# Global, regional, and national incidence, prevalence, and years lived with disability for 328 diseases and injuries for 195 countries, 1990–2016: a systematic analysis for the Global Burden of Disease Study 2016

**DOI:** 10.1016/S0140-6736(17)32154-2

**Published:** 2017-09-16

**Authors:** GBD 2016 Disease and Injury Incidence and Prevalence Collaborators, Amanuel Alemu Abajobir, Kalkidan Hassen Abate, Cristiana Abbafati, Kaja M Abbas, Foad Abd-Allah, Rizwan Suliankatchi Abdulkader, Abdishakur M Abdulle, Teshome Abuka Abebo, Semaw Ferede Abera, Victor Aboyans, Laith J Abu-Raddad, Ilana N Ackerman, Abdu Abdullahi Adamu, Olatunji Adetokunboh, Mohsen Afarideh, Ashkan Afshin, Sanjay Kumar Agarwal, Rakesh Aggarwal, Anurag Agrawal, Sutapa Agrawal, Hamid Ahmadieh, Muktar Beshir Ahmed, Miloud Taki Eddine Aichour, Amani Nidhal Aichour, Ibtihel Aichour, Sneha Aiyar, Rufus Olusola Akinyemi, Nadia Akseer, Faris Hasan Al Lami, Fares Alahdab, Ziyad Al-Aly, Khurshid Alam, Noore Alam, Tahiya Alam, Deena Alasfoor, Kefyalew Addis Alene, Raghib Ali, Reza Alizadeh-Navaei, Ala'a Alkerwi, François Alla, Peter Allebeck, Christine Allen, Fatma Al-Maskari, Rajaa Al-Raddadi, Ubai Alsharif, Shirina Alsowaidi, Khalid A Altirkawi, Azmeraw T Amare, Erfan Amini, Walid Ammar, Yaw Ampem Amoako, Hjalte H Andersen, Carl Abelardo T Antonio, Palwasha Anwari, Johan Ärnlöv, Al Artaman, Krishna Kumar Aryal, Hamid Asayesh, Solomon W Asgedom, Reza Assadi, Tesfay Mehari Atey, Niguse Tadele Atnafu, Sachin R Atre, Leticia Avila-Burgos, Euripide Frinel G Arthur Avokphako, Ashish Awasthi, Umar Bacha, Alaa Badawi, Kalpana Balakrishnan, Amitava Banerjee, Marlena S Bannick, Aleksandra Barac, Ryan M Barber, Suzanne L Barker-Collo, Till Bärnighausen, Simon Barquera, Lars Barregard, Lope H Barrero, Sanjay Basu, Bob Battista, Katherine E Battle, Bernhard T Baune, Shahrzad Bazargan-Hejazi, Justin Beardsley, Neeraj Bedi, Ettore Beghi, Yannick Béjot, Bayu Begashaw Bekele, Michelle L Bell, Derrick A Bennett, Isabela M Bensenor, Jennifer Benson, Adugnaw Berhane, Derbew Fikadu Berhe, Eduardo Bernabé, Balem Demtsu Betsu, Mircea Beuran, Addisu Shunu Beyene, Neeraj Bhala, Anil Bhansali, Samir Bhatt, Zulfiqar A Bhutta, Sibhatu Biadgilign, Burcu Kucuk Bicer, Kelly Bienhoff, Boris Bikbov, Charles Birungi, Stan Biryukov, Donal Bisanzio, Habtamu Mellie Bizuayehu, Dube Jara Boneya, Soufiane Boufous, Rupert R A Bourne, Alexandra Brazinova, Traolach S Brugha, Rachelle Buchbinder, Lemma Negesa Bulto Bulto, Blair R Bumgarner, Zahid A Butt, Lucero Cahuana-Hurtado, Ewan Cameron, Mate Car, Hélène Carabin, Jonathan R Carapetis, Rosario Cárdenas, David O Carpenter, Juan Jesus Carrero, Austin Carter, Felix Carvalho, Daniel C Casey, Valeria Caso, Carlos A Castañeda-Orjuela, Chris D Castle, Ferrán Catalá-López, Hsing-Yi Chang, Jung-Chen Chang, Fiona J Charlson, Honglei Chen, Mirriam Chibalabala, Chioma Ezinne Chibueze, Vesper Hichilombwe Chisumpa, Abdulaal A Chitheer, Devasahayam Jesudas Christopher, Liliana G Ciobanu, Massimo Cirillo, Danny Colombara, Cyrus Cooper, Paolo Angelo Cortesi, Michael H Criqui, John A Crump, Abel Fekadu Dadi, Koustuv Dalal, Lalit Dandona, Rakhi Dandona, José das Neves, Dragos V Davitoiu, Barbora de Courten, Diego De De Leo, Barthelemy Kuate Defo, Louisa Degenhardt, Selina Deiparine, Robert P Dellavalle, Kebede Deribe, Don C Des Jarlais, Subhojit Dey, Samath D Dharmaratne, Preet Kaur Dhillon, Daniel Dicker, Eric L Ding, Shirin Djalalinia, Huyen Phuc Do, E Ray Dorsey, Kadine Priscila Bender dos Santos, Dirk Douwes-Schultz, Kerrie E Doyle, Tim R Driscoll, Manisha Dubey, Bruce Bartholow Duncan, Ziad Ziad El-Khatib, Jerisha Ellerstrand, Ahmadali Enayati, Aman Yesuf Endries, Sergey Petrovich Ermakov, Holly E Erskine, Babak Eshrati, Sharareh Eskandarieh, Alireza Esteghamati, Kara Estep, Fanuel Belayneh Bekele Fanuel, Carla Sofia E Sa Farinha, André Faro, Farshad Farzadfar, Mir Sohail Fazeli, Valery L Feigin, Seyed-Mohammad Fereshtehnejad, João C Fernandes, Alize J Ferrari, Tesfaye Regassa Feyissa, Irina Filip, Florian Fischer, Christina Fitzmaurice, Abraham D Flaxman, Luisa Sorio Flor, Nataliya Foigt, Kyle J Foreman, Richard C Franklin, Nancy Fullman, Thomas Fürst, Joao M Furtado, Neal D Futran, Emmanuela Gakidou, Morsaleh Ganji, Alberto L Garcia-Basteiro, Teshome Gebre, Tsegaye Tewelde Gebrehiwot, Ayele Geleto, Bikila Lencha Gemechu, Hailay Abrha Gesesew, Peter W Gething, Alireza Ghajar, Katherine B Gibney, Paramjit Singh Gill, Richard F Gillum, Ibrahim Abdelmageem Mohamed Ginawi, Ababi Zergaw Giref, Melkamu Dedefo Gishu, Giorgia Giussani, William W Godwin, Audra L Gold, Ellen M Goldberg, Philimon N Gona, Amador Goodridge, Sameer Vali Gopalani, Atsushi Goto, Alessandra Carvalho Goulart, Max Griswold, Harish Chander Gugnani, Rahul Gupta, Rajeev Gupta, Tanush Gupta, Vipin Gupta, Nima Hafezi-Nejad, Gessessew Bugssa Hailu, Alemayehu Desalegne Hailu, Randah Ribhi Hamadeh, Samer Hamidi, Alexis J Handal, Graeme J Hankey, Sarah Wulf Hanson, Yuantao Hao, Hilda L Harb, Habtamu Abera Hareri, Josep Maria Haro, James Harvey, Mohammad Sadegh Hassanvand, Rasmus Havmoeller, Caitlin Hawley, Simon I Hay, Roderick J Hay, Nathaniel J Henry, Ileana Beatriz Heredia-Pi, Julio Montañez Hernandez, Pouria Heydarpour, Hans W Hoek, Howard J Hoffman, Nobuyuki Horita, H Dean Hosgood, Sorin Hostiuc, Peter J Hotez, Damian G Hoy, Aung Soe Htet, Guoqing Hu, Hsiang Huang, Chantal Huynh, Kim Moesgaard Iburg, Ehimario Uche Igumbor, Chad Ikeda, Caleb Mackay Salpeter Irvine, Kathryn H Jacobsen, Nader Jahanmehr, Mihajlo B Jakovljevic, Simerjot K Jassal, Mehdi Javanbakht, Sudha P Jayaraman, Panniyammakal Jeemon, Paul N Jensen, Vivekanand Jha, Guohong Jiang, Denny John, Sarah Charlotte Johnson, Catherine O Johnson, Jost B Jonas, Mikk Jürisson, Zubair Kabir, Rajendra Kadel, Amaha Kahsay, Ritul Kamal, Haidong Kan, Nadim E Karam, André Karch, Corine Kakizi Karema, Amir Kasaeian, Getachew Mullu Kassa, Nigussie Assefa Kassaw, Nicholas J Kassebaum, Anshul Kastor, Srinivasa Vittal Katikireddi, Anil Kaul, Norito Kawakami, Peter Njenga Keiyoro, Andre Pascal Kengne, Andre Keren, Yousef Saleh Khader, Ibrahim A Khalil, Ejaz Ahmad Khan, Young-Ho Khang, Ardeshir Khosravi, Jagdish Khubchandani, Aliasghar Ahmad Kiadaliri, Christian Kieling, Yun Jin Kim, Daniel Kim, Pauline Kim, Ruth W Kimokoti, Yohannes Kinfu, Adnan Kisa, Katarzyna A Kissimova-Skarbek, Mika Kivimaki, Ann Kristin Knudsen, Yoshihiro Kokubo, Dhaval Kolte, Jacek A Kopec, Soewarta Kosen, Parvaiz A Koul, Ai Koyanagi, Michael Kravchenko, Sanjay Krishnaswami, Kristopher J Krohn, G Anil Kumar, Pushpendra Kumar, Sanjiv Kumar, Hmwe H Kyu, Dharmesh Kumar Lal, Ratilal Lalloo, Nkurunziza Lambert, Qing Lan, Anders Larsson, Pablo M Lavados, Janet L Leasher, Paul H Lee, Jong-Tae Lee, James Leigh, Cheru Tesema Leshargie, Janni Leung, Ricky Leung, Miriam Levi, Yichong Li, Yongmei Li, Darya Li Kappe, Xiaofeng Liang, Misgan Legesse Liben, Stephen S Lim, Shai Linn, Patrick Y Liu, Angela Liu, Shiwei Liu, Yang Liu, Rakesh Lodha, Giancarlo Logroscino, Stephanie J London, Katharine J Looker, Alan D Lopez, Stefan Lorkowski, Paulo A Lotufo, Nicola Low, Rafael Lozano, Timothy C D Lucas, Erlyn Rachelle King Macarayan, Hassan Magdy Abd El Razek, Mohammed Magdy Abd El Razek, Mahdi Mahdavi, Marek Majdan, Reza Majdzadeh, Azeem Majeed, Reza Malekzadeh, Rajesh Malhotra, Deborah Carvalho Malta, Abdullah A Mamun, Helena Manguerra, Treh Manhertz, Ana Mantilla, Lorenzo G Mantovani, Chabila C Mapoma, Laurie B Marczak, Jose Martinez-Raga, Francisco Rogerlândio Martins-Melo, Ira Martopullo, Winfried März, Manu Raj Mathur, Mohsen Mazidi, Colm McAlinden, Madeline McGaughey, John J McGrath, Martin McKee, Claire McNellan, Suresh Mehata, Man Mohan Mehndiratta, Tefera Chane Mekonnen, Peter Memiah, Ziad A Memish, Walter Mendoza, Mubarek Abera Mengistie, Desalegn Tadese Mengistu, George A Mensah, Tuomo J Meretoja, Atte Meretoja, Haftay Berhane Mezgebe, Renata Micha, Anoushka Millear, Ted R Miller, Edward J Mills, Mojde Mirarefin, Erkin M Mirrakhimov, Awoke Misganaw, Shiva Raj Mishra, Philip B Mitchell, Karzan Abdulmuhsin Mohammad, Alireza Mohammadi, Kedir Endris Mohammed, Shafiu Mohammed, Sanjay K Mohanty, Ali H Mokdad, Sarah K Mollenkopf, Lorenzo Monasta, Marcella Montico, Maziar Moradi-Lakeh, Paula Moraga, Rintaro Mori, Chloe Morozoff, Shane D Morrison, Mark Moses, Cliff Mountjoy-Venning, Kalayu Birhane Mruts, Ulrich O Mueller, Kate Muller, Michele E Murdoch, Christopher J L Murray, Gudlavalleti Venkata Satyanarayana Murthy, Kamarul Imran Musa, Jean B Nachega, Gabriele Nagel, Mohsen Naghavi, Aliya Naheed, Kovin S Naidoo, Luigi Naldi, Vinay Nangia, Gopalakrishnan Natarajan, Dumessa Edessa Negasa, Ruxandra Irina Negoi, Ionut Negoi, Charles R Newton, Josephine Wanjiku Ngunjiri, Trang Huyen Nguyen, Quyen Le Nguyen, Cuong Tat Nguyen, Grant Nguyen, Minh Nguyen, Emma Nichols, Dina Nur Anggraini Ningrum, Sandra Nolte, Vuong Minh Nong, Bo Norrving, Jean Jacques N Noubiap, Martin J O'Donnell, Felix Akpojene Ogbo, In-Hwan Oh, Anselm Okoro, Olanrewaju Oladimeji, Tinuke Oluwasefunmi Olagunju, Andrew Toyin Olagunju, Helen E Olsen, Bolajoko Olubukunola Olusanya, Jacob Olusegun Olusanya, Kanyin Ong, John Nelson Opio, Eyal Oren, Alberto Ortiz, Aaron Osgood-Zimmerman, Majdi Osman, Mayowa O Owolabi, Mahesh PA, Rosana E Pacella, Adrian Pana, Basant Kumar Panda, Christina Papachristou, Eun-Kee Park, Charles D Parry, Mahboubeh Parsaeian, Scott B Patten, George C Patton, Katherine Paulson, Neil Pearce, David M Pereira, Norberto Perico, Konrad Pesudovs, Carrie Beth Peterson, Max Petzold, Michael Robert Phillips, David M Pigott, Julian David Pillay, Christine Pinho, Dietrich Plass, Martin A Pletcher, Svetlana Popova, Richie G Poulton, Farshad Pourmalek, Dorairaj Prabhakaran, Noela M Prasad, Narayan Prasad, Carrie Purcell, Mostafa Qorbani, Reginald Quansah, Beatriz Paulina Ayala Quintanilla, Rynaz H S Rabiee, Amir Radfar, Anwar Rafay, Kazem Rahimi, Afarin Rahimi-Movaghar, Vafa Rahimi-Movaghar, Mohammad Hifz Ur Rahman, Mahfuzar Rahman, Rajesh Kumar Rai, Sasa Rajsic, Usha Ram, Chhabi Lal Ranabhat, Zane Rankin, Puja C Rao, Paturi Vishnupriya Rao, Salman Rawaf, Sarah E Ray, Robert C Reiner, Nikolas Reinig, Marissa B Reitsma, Giuseppe Remuzzi, Andre M N Renzaho, Serge Resnikoff, Satar Rezaei, Antonio L Ribeiro, Luca Ronfani, Gholamreza Roshandel, Gregory A Roth, Ambuj Roy, Enrico Rubagotti, George Mugambage Ruhago, Soheil Saadat, Nafis Sadat, Mahdi Safdarian, Sare Safi, Saeid Safiri, Rajesh Sagar, Ramesh Sahathevan, Joseph Salama, Huda Omer Ba Saleem, Joshua A Salomon, Sundeep Santosh Salvi, Abdallah M Samy, Juan R Sanabria, Damian Santomauro, Itamar S Santos, João Vasco Santos, Milena M Santric Milicevic, Benn Sartorius, Maheswar Satpathy, Monika Sawhney, Sonia Saxena, Maria Inês Schmidt, Ione J C Schneider, Ben Schöttker, David C Schwebel, Falk Schwendicke, Soraya Seedat, Sadaf G Sepanlou, Edson E Servan-Mori, Tesfaye Setegn, Katya Anne Shackelford, Amira Shaheen, Masood Ali Shaikh, Mansour Shamsipour, Sheikh Mohammed Shariful Islam, Jayendra Sharma, Rajesh Sharma, Jun She, Peilin Shi, Chloe Shields, Girma Temam Shifa, Mika Shigematsu, Yukito Shinohara, Rahman Shiri, Reza Shirkoohi, Shreya Shirude, Kawkab Shishani, Mark G Shrime, Abla Mehio Sibai, Inga Dora Sigfusdottir, Diego Augusto Santos Silva, João Pedro Silva, Dayane Gabriele Alves Silveira, Jasvinder A Singh, Narinder Pal Singh, Dhirendra Narain Sinha, Eirini Skiadaresi, Vegard Skirbekk, Erica Leigh Slepak, Amber Sligar, David L Smith, Mari Smith, Badr H A Sobaih, Eugene Sobngwi, Reed J D Sorensen, Tatiane Cristina Moraes Sousa, Luciano A Sposato, Chandrashekhar T Sreeramareddy, Vinay Srinivasan, Jeffrey D Stanaway, Vasiliki Stathopoulou, Nicholas Steel, Murray B Stein, Dan J Stein, Timothy J Steiner, Caitlyn Steiner, Sabine Steinke, Mark Andrew Stokes, Lars Jacob Stovner, Bryan Strub, Michelle Subart, Muawiyyah Babale Sufiyan, Bruno F Sunguya, Patrick J Sur, Soumya Swaminathan, Bryan L Sykes, Dillon O Sylte, Rafael Tabarés-Seisdedos, Getachew Redae Taffere, Jukka S Takala, Nikhil Tandon, Mohammad Tavakkoli, Nuno Taveira, Hugh R Taylor, Arash Tehrani-Banihashemi, Tesfalidet Tekelab, Abdullah Sulieman Terkawi, Dawit Jember Tesfaye, Belay Tesssema, Ornwipa Thamsuwan, Katie E Thomas, Amanda G Thrift, Tenaw Yimer Tiruye, Ruoyan Tobe-Gai, Mette C Tollanes, Marcello Tonelli, Roman Topor-Madry, Miguel Tortajada, Mathilde Touvier, Bach Xuan Tran, Suryakant Tripathi, Christopher Troeger, Thomas Truelsen, Derrick Tsoi, Kald Beshir Tuem, Emin Murat Tuzcu, Stefanos Tyrovolas, Kingsley N Ukwaja, Eduardo A Undurraga, Chigozie Jesse Uneke, Rachel Updike, Olalekan A Uthman, Benjamin S Chudi Uzochukwu, Job F M van Boven, Santosh Varughese, Tommi Vasankari, S Venkatesh, Narayanaswamy Venketasubramanian, Ramesh Vidavalur, Francesco S Violante, Sergey K Vladimirov, Vasiliy Victorovich Vlassov, Stein Emil Vollset, Theo Vos, Fiseha Wadilo, Tolassa Wakayo, Yuan-Pang Wang, Marcia Weaver, Scott Weichenthal, Elisabete Weiderpass, Robert G Weintraub, Andrea Werdecker, Ronny Westerman, Harvey A Whiteford, Tissa Wijeratne, Charles Shey Wiysonge, Charles D A Wolfe, Rachel Woodbrook, Anthony D Woolf, Abdulhalik Workicho, Denis Xavier, Gelin Xu, Simon Yadgir, Mohsen Yaghoubi, Bereket Yakob, Lijing L Yan, Yuichiro Yano, Pengpeng Ye, Hassen Hamid Yimam, Paul Yip, Naohiro Yonemoto, Seok-Jun Yoon, Marcel Yotebieng, Mustafa Z Younis, Zoubida Zaidi, Maysaa El Sayed Zaki, Elias Asfaw Zegeye, Zerihun Menlkalew Zenebe, Xueying Zhang, Maigeng Zhou, Ben Zipkin, Sanjay Zodpey, Liesl Joanna Zuhlke

## Abstract

**Background:**

As mortality rates decline, life expectancy increases, and populations age, non-fatal outcomes of diseases and injuries are becoming a larger component of the global burden of disease. The Global Burden of Diseases, Injuries, and Risk Factors Study 2016 (GBD 2016) provides a comprehensive assessment of prevalence, incidence, and years lived with disability (YLDs) for 328 causes in 195 countries and territories from 1990 to 2016.

**Methods:**

We estimated prevalence and incidence for 328 diseases and injuries and 2982 sequelae, their non-fatal consequences. We used DisMod-MR 2.1, a Bayesian meta-regression tool, as the main method of estimation, ensuring consistency between incidence, prevalence, remission, and cause of death rates for each condition. For some causes, we used alternative modelling strategies if incidence or prevalence needed to be derived from other data. YLDs were estimated as the product of prevalence and a disability weight for all mutually exclusive sequelae, corrected for comorbidity and aggregated to cause level. We updated the Socio-demographic Index (SDI), a summary indicator of income per capita, years of schooling, and total fertility rate. GBD 2016 complies with the Guidelines for Accurate and Transparent Health Estimates Reporting (GATHER).

**Findings:**

Globally, low back pain, migraine, age-related and other hearing loss, iron-deficiency anaemia, and major depressive disorder were the five leading causes of YLDs in 2016, contributing 57·6 million (95% uncertainty interval [UI] 40·8–75·9 million [7·2%, 6·0–8·3]), 45·1 million (29·0–62·8 million [5·6%, 4·0–7·2]), 36·3 million (25·3–50·9 million [4·5%, 3·8–5·3]), 34·7 million (23·0–49·6 million [4·3%, 3·5–5·2]), and 34·1 million (23·5–46·0 million [4·2%, 3·2–5·3]) of total YLDs, respectively. Age-standardised rates of YLDs for all causes combined decreased between 1990 and 2016 by 2·7% (95% UI 2·3–3·1). Despite mostly stagnant age-standardised rates, the absolute number of YLDs from non-communicable diseases has been growing rapidly across all SDI quintiles, partly because of population growth, but also the ageing of populations. The largest absolute increases in total numbers of YLDs globally were between the ages of 40 and 69 years. Age-standardised YLD rates for all conditions combined were 10·4% (95% UI 9·0–11·8) higher in women than in men. Iron-deficiency anaemia, migraine, Alzheimer's disease and other dementias, major depressive disorder, anxiety, and all musculoskeletal disorders apart from gout were the main conditions contributing to higher YLD rates in women. Men had higher age-standardised rates of substance use disorders, diabetes, cardiovascular diseases, cancers, and all injuries apart from sexual violence. Globally, we noted much less geographical variation in disability than has been documented for premature mortality. In 2016, there was a less than two times difference in age-standardised YLD rates for all causes between the location with the lowest rate (China, 9201 YLDs per 100 000, 95% UI 6862–11943) and highest rate (Yemen, 14 774 YLDs per 100 000, 11 018–19 228).

**Interpretation:**

The decrease in death rates since 1990 for most causes has not been matched by a similar decline in age-standardised YLD rates. For many large causes, YLD rates have either been stagnant or have increased for some causes, such as diabetes. As populations are ageing, and the prevalence of disabling disease generally increases steeply with age, health systems will face increasing demand for services that are generally costlier than the interventions that have led to declines in mortality in childhood or for the major causes of mortality in adults. Up-to-date information about the trends of disease and how this varies between countries is essential to plan for an adequate health-system response.

**Funding:**

Bill & Melinda Gates Foundation, and the National Institute on Aging and the National Institute of Mental Health of the National Institutes of Health.

**Research in Context**

**Evidence before this study**

The Global Burden of Diseases, Injuries, and Risk Factors Study (GBD) produces the only assessment of prevalence, incidence, and years lived with disability (YLDs) for a comprehensive list of diseases and injuries, and for all countries from 1990 to the present. The World Health Organization has published YLD estimates for the years 2000–15 largely based on GBD 2015 results apart from ad hoc changes applied to selected disability weights and the prevalence of a small subset of causes. GBD 2016 is a reassessment of the burden of disease due to non-fatal diseases and injuries and updates the GBD 2015 study results.

**Added value of this study**

This study adds new knowledge about YLD rates globally and improves upon prior iterations of the GBD study in seven ways. First, new data were incorporated based on 56 356 unique data sources; these were composed mainly of peer-reviewed scientific literature identified by systematic reviews, reports from statistical agencies or ministries of health, household surveys, administrative data systems, claims data, and hospital data. In 2016, we had 14 521 sources from the scientific literature compared with 10 478 such sources used in GBD 2015. Our network of collaborators provided 2598 data sources for GBD 2016 compared with 968 available for GBD 2015; furthermore, 3430 sources of survey data were used in GBD 2016. These counts reflect our updated counting criteria for GBD 2016. Large amounts of new data for the main causes of YLDs were identified through our collaboration with the Indian Council of Medical Research and the Public Health Foundation of India. For particular diseases, the volume of available data increased substantially. Examples include Rapid Assessment of Avoidable Blindness surveys and the detailed studies reported in the Global Atlas of Helminth Infection for schistosomiasis and lymphatic filariasis. Second, we substantially changed the modelling approach for some diseases, such as cancers and tuberculosis. For cancer we improved our analysis of mortality-to-incidence ratios, resulting in considerably higher ratios in lower Socio-demographic Index (SDI) locations and thus lower YLD estimates. We also applied mortality-to-incidence ratios in the analysis of tuberculosis to better predict the gap between true incidence and notified cases—ie, undetected cases. Third, estimation at the subnational level was newly developed for Indonesia, and estimates for England were disaggregated into those for 150 local government areas. Fourth, we have disaggregated several causes to separately estimate drug-sensitive, multidrug-resistant, and extensively drug-resistant tuberculosis; latent tuberculosis infection; alcoholic cardiomyopathy; urogenital, musculoskeletal, and digestive congenital anomalies; and self-harm by firearm, to provide more detail within the GBD cause hierarchy. Additionally, Guinea worm disease was estimated due to the fact that there is policy interest in eradication, which is feasible in the near future. Sexual violence was added as a non-fatal cause of YLDs because of its relevance to several of the new sustainable development goal (SDG) targets. Fifth, we were able to incorporate inpatient hospital data by cause for 222 more locations and a final total of 3557 location-years. A separate analysis of total hospital admissions per capita by country, year, age, and sex allowed the use of hospital data sources that previously had been rejected because of incomplete knowledge about catchment populations. We extended our analyses of US medical claims data to impute a ratio of any health service contact for a diagnosis to inpatient episodes for chronic diseases that we applied to hospital inpatient data from elsewhere to predict prevalence. Sixth, we have extended our analyses of GBD results by SDI with new ways of presenting and visualising changes over time and the relationship with development. Seventh, we extended the terminal age group used in our analyses of older than 80 years into 80–84, 85–89, 90–94, and older than 95 years.

**Implications of all the available evidence**

As countries confront the effects of the epidemiological transition, there will be an increased need for up-to-date assessment of non-fatal health outcomes and exploration of the implications of growing numbers of individuals in need of chronic care as populations age. The GBD study provides opportunities to identify important non-fatal health trends across various locations and levels of development, and to assess the strength of available estimates.

## Introduction

Assessment of death rates by cause has been an essential component of tracking progress in global health. The Millennium Development Goals (MDGs) emphasised child, maternal, and infectious disease mortality and spurred investments that contributed to a rapid decline in mortality from these sources, although these have not been universally achieved at the same level.^1^, ^2^ Progress in reducing the disabling outcomes of disease has been much slower.^3^ The Global Burden of Diseases, Injuries, and Risk Factors Study 2015 (GBD 2015) estimated a modest 2·1% reduction in the age-standardised rate of years lived with disability (YLDs) for all causes compared with a 22·7% reduction in age-standardised rates of years of life lost (YLLs) for all causes between 2005 and 2015.^3^ The slower progress in addressing non-fatal compared with fatal health outcomes and ageing of populations make YLDs an increasingly important component of global disability-adjusted life-years (DALYs). In some high-income countries with advanced ageing, YLDs already make up more than half of the total burden in DALYs.^4^ The GBD Study is the only global effort to quantify non-fatal outcomes using a metric that allows comparisons between fatal and non-fatal outcomes of a comprehensive list of diseases and injuries.

There are several challenges in standardising the estimation of YLDs. For example, case definitions vary; there are diverse data sources and study methods; accessible data sources are sparse for many diseases, with large parts of the world lacking adequate data; and data for severity of outcomes are limited and lack a standardised approach. This annual update of the GBD study provides an opportunity to incorporate new data and improved methods within a standardised framework to enhance the precision and accuracy of estimation.

The extensive GBD network of more than 2518 collaborators from 133 countries and three non-sovereign locations have provided invaluable critiques of methods and helped to identify new data sources. Most of the debates arising from GBD 2015 have come as direct communications from the collaborative network or have been published by collaborators and other researchers. For example, collaborators have addressed omissions in the GBD cause list,^5^, ^6^, ^7^, ^8^, ^9^, ^10^ challenged the GBD hierarchy of causes,^11^ or explored the continual debate regarding disability weights.^12^ There is also a growing scientific literature on secondary analyses of GBD results—eg, with a focus on chronic kidney disease,^13^ oral diseases,^14^ or cardiovascular disease among the poorest billion.^15^

The primary objective of the non-fatal component of GBD 2016 was to estimate prevalence, incidence, and YLDs for 328 GBD causes from 1990 to 2016. For each cycle of GBD, the entire time series is re-estimated to incorporate new data and methods; thus, these results supersede previous GBD results. We explore the patterns of non-fatal disease over time and in comparison to expected levels based on an index of sociodemographic development.

## Methods

### Overview

The GBD study provides a standardised analytical approach for estimating incidence, prevalence, and YLDs by age, sex, cause, year, and location. We aim to use all accessible information on disease occurrence, natural history, and severity that passes minimum inclusion criteria set disease-by-disease (appendix 1, p 33). Our approach is to optimise the comparability of data collected by varying methods or different case definitions; find a consistent set of estimates between data for prevalence, incidence, and causes of death; and predict estimates for locations with sparse or absent data by borrowing information from other locations and using covariates.

In this study, we use different methods to reflect the available data and specific epidemiology of each disease. Our main approach is to combine all sources of information for a disease using the Bayesian meta-regression tool DisMod-MR 2.1.^16^ Subsequently, we use data for severity, the occurrence of particular consequences of diseases, or sequelae, to establish the proportion of prevalent cases experiencing each sequela. Several broad classes of alternative approaches are used within the GBD study. First, for injuries, non-fatal estimates must account for the cause of injury (eg, a fall), the nature of injury (eg, a fracture or head injury), the amount of disability arising in the short term, and permanent disability for a subset of cases. Second, cancers were estimated by assessing the association between mortality and incidence, taking into account the effect on survival of access to, and quality of, treatment for the cancer site. Third, we combined the natural history model strategy for HIV/AIDS with the DisMod-MR 2.1 modelling approach for tuberculosis as HIV infection affects outcomes in patients who also have tuberculosis. Fourth, models for malaria, hepatitis, and varicella relied on data of the presence of circulating antibodies or parasites in the blood to predict the incidence of clinical episodes for which we estimate disability. Fifth, neonatal disorders were estimated from birth prevalence data and cohort studies on the risk of death in the first month and the probability of long-term disabling outcomes. Sixth, incidence of rabies, whooping cough, diphtheria, and tetanus was estimated from cause-specific mortality rates and data on the case fatality of acute episodes (appendix 1, p 33).

Below we describe these modelling efforts organised into eight sections; the supplementary methods (appendix 1, p 1) presents a single source for additional detail of inputs, analytical processes, outputs, and methods specific to each cause. This study complies with the Guidelines for Accurate and Transparent Health Estimates Reporting (GATHER) recommendations (appendix 1, p 723).^17^

### 1 Geographic units and time periods

The GBD 2016 study was based on a geographic hierarchy that includes 195 countries and territories grouped within 21 regions and seven GBD super-regions (appendix 1, p 726). For this publication, we present subnational estimates in figures and only for Brazil, China, India, and the USA. Details of subnational estimates will be reported in separate publications.

Cause-specific estimation in GBD 2016 was done for the years 1990, 1995, 2000, 2006, 2010, and 2016 and interpolated to get a full time series. In view of policy priorities, a subset of results focus on change over the time period 2006–16. Results from GBD 2016 by year and location can be explored further in dynamic data visualisations.

### 2 GBD cause list

In the GBD Study, causes and their sequelae are collectively exhaustive and mutually exclusive and are organised in a hierarchy with five levels. Level 1 contains three broad cause groups: communicable, maternal, neonatal, and nutritional diseases; non-communicable diseases; and injuries. These are broken down into 21 Level 2 causes with further disaggregation into 163 Level 3 causes and 271 Level 4 causes. Sequelae of these causes are represented at Level 5 of the hierarchy.

For GBD 2016, we expanded the list of causes of non-fatal outcomes from 310 to 328. This involved the refinement of certain Level 3 causes into new Level 4 causes, including disaggregation of tuberculosis and HIV- tuberculosis into drug-susceptible tuberculosis, multidrug-resistant tuberculosis, extensively drug-resistant tuberculosis, and latent tuberculosis infection. Cardiomyopathy and myocarditis were further refined as alcoholic cardiomyopathy, myocarditis, and other cardiomyopathy. Other leukaemia was added as an additional sub-cause at Level 4. Self-harm was separated into self-harm by firearm and self-harm by other means. The previously named cause grouping “collective violence and legal intervention” was divided into two Level 4 causes: executions and police conflict. New causes of non-fatal outcomes added to the GBD hierarchy for 2016 included Zika virus disease; musculoskeletal, urogenital, and digestive congenital anomalies; Guinea worm disease; and sexual violence. Medication overuse headache was removed as a cause and, instead, characterised as a sequela of migraine and tension-type headache.

### 3 Sources of data

The first step in non-fatal estimation was the compilation of data sources from systematic data and literature searches conducted by cause. This process resulted in 4043 published studies newly included in GBD 2016, leading to a total of 14 521. Our network of collaborators for GBD 2016 provided 2598 data sources and studies. These were systematically screened, together with sources suggested by country-level experts, surveys located in multinational survey data catalogues, and Ministry of Health and Central Statistical Office websites. We analysed 18 792 sources of epidemiological surveillance data (country-years of disease reporting), up from 14 081 in 2015. All counts reflect our updated counting criteria for GBD 2016. The supplementary methods provides details of data adjustments, correction factors, and standardisations employed in incorporating these different data types (appendix 1, p 18).

The number of location-years of hospital inpatient data by cause increased from 1176 in GBD 2015 to 3557 in GBD 2016. This increase can be attributed to the addition of new years of data for some locations, as well as newly incorporated data for 16 countries where we had previously lacked clear information about the population covered. To allow their use in GBD, we first collated information from surveys and hospital administrative records to estimate hospital admission rates per capita for all GBD locations by age and sex, from 1990 to 2016, using DisMod-MR 2.1 (appendix 1, p 7). We then used inpatient data by cause from locations with unclear denominators as cause fractions of the all-cause inpatient admission rates. Three adjustment factors were derived from USA health insurance claims data on more than 80 million person-years of coverage. The first factor corrected for multiple inpatient episodes for the same cause in an individual. The second adjustment was to include secondary diagnostic fields. The third adjustment was to include any mention of a cause in inpatient or outpatient episodes of care as opposed to inpatient episodes with a primary diagnosis only. This new method of predicting prevalence or incidence from inpatient data allowed us to use these sources for 16 more causes than in 2015. The supplementary methods provides a detailed description of our process for inpatient data (appendix 1, p 11).

To provide a summary view on data availability, the number of causes at the most detailed level for which we have any prevalence or incidence data from 1980 to 2016 by location is presented in the appendix (appendix 1, p 722). An online search tool is available to view all data sources that were used in the estimation process for each cause.

### 4 Non-fatal disease models

Non-fatal diseases were modelled using DisMod-MR 2.1, a statistical method that synthesises sparse and heterogeneous epidemiological data for non-fatal outcomes. Estimation occurred at five levels: global, super-region, region, country, and subnational locations, with results from a higher level providing guidance for the analysis at a lower geographical level (appendix 1, p 18).

Custom models were created where DisMod-MR 2.1 does not capture the complexity of the disease, or if incidence and prevalence needed to be calculated from other data. Further details of these custom models can be found in the supplementary methods (appendix 1, p 18). Prevalence was estimated for nine impairments, disorders that are sequelae of multiple diseases and for which there were better data available to estimate the overall occurrence than for each underlying cause: anaemia, intellectual disability, epilepsy, hearing loss, vision loss, heart failure, infertility, pelvic inflammatory disease, and Guillain-Barré syndrome.

The methods for estimating YLDs from a number of diseases changed substantially for GBD 2016. We improved our estimation of mortality-to-incidence ratios for cancers to better reflect lower survival probabilities in low-income and middle-income locations based on each location's Socio-demographic Index (SDI) value. As a consequence, our prevalence and YLD estimates were lower in those locations but did not change much for higher-SDI locations. We made major changes to our modelling of tuberculosis. First, we made explicit estimates of latent tuberculosis infection from tuberculin skin testing data and the risk of developing active tuberculosis by induration size. Second, we predicted mortality-to-incidence ratios in locations with high data-quality ratings (4-star or 5-star using a system developed for the GBD 2016 causes of death estimation)^18^ and SDI as a covariate. We anchored the lower end of the SDI scale with a datapoint from an untreated cohort of pulmonary tuberculosis cases in the 1960s, half of whom had died after five years.^18^, ^19^ Third, we estimated incidence from these mortality-to-incidence ratios in all locations except those with higher reported notifications. Fourth, we modelled these incidence estimates as well as the prevalence data from surveys in low-income and middle-income countries and cause-specific mortality rates among the proportion of the population with latent infection in DisMod-MR 2.1. Fifth, we estimated the proportions of tuberculosis cases with multidrug-resistant tuberculosis or extensively drug-resistant tuberculosis from notification and survey data and included an increased risk of multidrug-resistant tuberculosis in HIV/AIDS-infected patients with tuberculosis from a meta-analysis.^20^

In our measles estimation strategy, we included the coverage of measles-containing vaccine second-dose (MCV2) rather than just the coverage of the primary vaccine as a covariate. As relatively few countries in sub-Saharan Africa have introduced MCV2, the estimated incidence for those locations is notably higher compared with previous estimates.

### 5 Severity distributions and disability weights

For 214 causes at Level 4 of the GBD hierarchy, sequelae were defined in terms of severity, usually graded as mild, moderate, or severe outcomes. We followed the same approach as in GBD 2015. For Zika virus disease, we included sequelae for those with symptomatic acute infection, a small proportion with Guillain-Barré syndrome, and the number of neonates with congenital Zika virus disease as reported to the Pan American Health Organization (PAHO). For sexual violence, we estimated YLDs associated with concurrent physical injuries and the short-term psychological outcomes following sexual violence.

A more substantial change in estimating severity was applied to stroke. A systematic review was done to collect data on modified Rankin scores, a measure of neurological disability.^21^ Levels of Rankin score were analysed in DisMod-MR 2.1 and mapped to the existing GBD health state lay descriptions for mild, moderate, and severe motor impairment from stroke, and, separately, the proportion of stroke patients with moderate-to-severe motor impairment who also experienced cognitive impairment. For GBD 2016 we used the same disability weights as in GBD 2013 and GBD 2015; the supplementary methods provides a complete listing of lay descriptions of all 235 health states used in GBD 2016 (appendix 1, p 799).

### 6 Comorbidity

We estimated comorbidity by simulating 40 000 individuals in every location-age-sex-year combination as exposed to the independent probability, based on the prevalence of the sequelae included in GBD 2016. In simulants with two or more sequelae, we assumed a multiplicative function to combine disability weights and then distributed the reduced combined weight proportionately among all comorbid sequelae. Averaging these adjusted values across all simulants with a particular sequela gave the adjusted value of YLDs. There was no change in the approach compared with GBD 2015.

### 7 YLD computation

All computations in GBD were done 1000 times, every time drawing from the distribution of the sampling error of data inputs, the uncertainty of data corrections for measurement errors, the uncertainty in coefficients from model fit (eg, in DisMod-MR 2.1), and the uncertainty of severity distributions and disability weights. Uncertainty bounds for a quantity of interest were defined by the 25th and 975th value of the ordered 1000 estimate values. If there was a change in disease estimates between locations or over time that was in the same direction in more than 950 of the 1000 samples we report it as significant. Age-standardised prevalence YLD rates were calculated based on the GBD reference population.^22^

The GBD cause hierarchy is comprehensive and includes 35 residual disease categories to capture YLDs from conditions for which we do not currently make separate estimates. For 22 of these residual categories, we made explicit epidemiological estimates of prevalence and incidence, and define sequelae based on the most common diseases in the Level 2 or 3 cause group and severity distributions from the Medical Expenditure Panel Survey (MEPS).^23^ For 13 residual categories, we had no epidemiological data and estimated YLDs from the ratio of YLDs to YLLs from explicitly modelled diseases in the cause category, assuming that relative to each death, the number of YLDs was similar to that of other diseases at the same level of the GBD hierarchy (appendix 1, p 29).

### 8 SDI and epidemiological transition

SDI is a summary measure that places all GBD locations on a spectrum of socioeconomic development.^24^ The SDI was developed for GBD 2015 to provide a comparable metric of overall development. This was achieved by using an equal weighting of lag-distributed income per capita, average years of education in the population over age 15 years, and total fertility rate.^22^ For GBD 2016, we modified the estimation of SDI by taking into consideration that SDI scales were subject to change based on increasing geographic units and an extended time period of analysis, affecting the interpretability across GBD iterations. We redefined the values of zero and one for each component of the index: zero now represents the level below which we have not observed GDP per capita or educational attainment or above which we have not observed the total fertility rate in known datasets. Maximum scores for educational attainment and Lagged Distributed Income represent a plateau in the relationship between each of the two components and life expectancy or under-5 mortality rates, suggesting no additional benefit. Analogously, the maximum score for total fertility rate represents the minimum level at which the relationship with the selected health outcomes plateaued. An SDI value was generated for each location and year as the geometric mean of each component score. Five SDI quintiles, high, high-middle, middle, low-middle, and low, were selected based on 2016 values of SDI; additional details are available in the supplementary methods (appendix 1).^22^

A Gaussian process regression was used to evaluate the average relationship for each age-sex-cause group, for cause-specific YLD rates on SDI at Levels 1, 2, and 3 of the GBD cause hierarchy using data from 1990 to 2016. These rates were used as the expected values for cause-specific YLD rates at a given level of SDI. Additional detail on this analysis is available in the supplementary methods (appendix 1, p 30) and in previous GBD publications.^18^

### Role of the funding source

The funder of the study had no role in study design, data collection, data analysis, data interpretation, or the writing of the report. All authors had full access to the data in the study and had final responsibility for the decision to submit for publication.

## Results

### Global prevalence and incidence

Global prevalence, incidence, and YLDs for 328 causes and nine impairments, as well as percent change of YLDs and percent change of age-standardised YLD rates from 2006 to 2016 are listed in the table. Cause-specific estimates for each year of the GBD estimation period 1990–2016 by location, age, and sex are available through an online results tool.

### Prevalence

In 2016, the ten causes with the greatest prevalence were caries of permanent teeth (2·44 billion, 95% UI 2·29 billion to 2·59 billion), latent tuberculosis infection (1·91 billion, 1·79 billion to 2·03 billion), tension-type headache (1·89 billion, 1·71 billion to 2·10 billion), age-related and other hearing loss (1·27 billion, 1·21 billion to 1·34 billion), iron-deficiency anaemia (1·24 billion cases, 1·21 billion to 1·28 billion), migraine (1·04 billion, 1·00 billion to 1·09 billion), glucose-6-phosphate dehydrogenase deficiency (G6PD) trait (866 million, 852 million to 882 million), genital herpes (860 million, 748 million to 992 million), refraction and accommodation disorders (854 million, 822 million to 886 million), and ascariasis (800 million, 738 million to 872 million; table). The vast majority of cases for two of these causes are asymptomatic sequelae that have no YLDs associated with them: genital herpes with no active lesions, and G6PD trait that does not result in anaemia. Similarly, latent tuberculosis infection is a highly prevalent cause but without any associated disability. The leading ten causes of prevalence accounted for 17·5% (95% uncertainty interval [UI] 15·8–19·3) of YLDs globally in 2016.

**Table:** Global prevalence, incidence, and YLDs for 2016, percentage change of YLD counts, and percentage change of age-standardised YLD rates between 2006 and 2016 for all causes and nine impairments

					**Prevalence in thousands (95% UI)**	**Incidence in thousands (95% UI)**	**YLDs in thousands (95% UI)**		
					2016 counts	2016 counts	2016 counts	Percentage change in counts between 2006 and 2016	Percentage change in age-standardised rates between 2006 and 2016
**All causes**					**7 122 683 (7 115 742 to 7 129 084)**	44 245 182 (42 139 690 to 46 565 214)*	**805 393 (601 661 to 1 045 626)**	**16·4 (16·0 to 16·9)**†	**–0·9 (−1·2 to −0·5)**†
**Communicable, maternal, neonatal, and nutritional disorders**					**4 943 086 (4 920 138 to 4 967 418)**	25 266 823 (23 366 307 to 27 404 017)*	**101 472 (72 326 to 136 902)**	**4·9 (2·7 to 7·3)**†	**–5·1 (−7·2 to −2·9)**†
	**HIV/AIDS and tuberculosis**				**2 051 462 (1 904 878 to 2 137 104)**	10 884 (9882 to 12 069)*	**6871 (4846 to 9324)**	**–3·6 (−15·5 to 7·1)**	**–16·9 (−27·3 to −7·3)**†
		Tuberculosis			1 918 597 (1 797 774 to 2 043 357)	9019 (8052 to 10 157)*	2839 (1886 to 3843)	6·3 (4·3 to 8·3)†	–10·6 (−12·0 to −9·1)†
			Drug-susceptible tuberculosis		9036 (8134 to 10 108)	8705 (7755 to 9818)	2735 (1815 to 3 696)	6·4 (4·3 to 8·6)†	–10·5 (−12·0 to −8·9)†
			Multidrug-resistant tuberculosis without extensive drug resistance		313 (279 to 353)	296 (261 to 336)	98 (66 to 136)	–1·0 (−10·1 to 8·9)	–16·9 (−24·5 to −8·7)†
			Extensively drug-resistant tuberculosis		19 (17 to 22)	18 (16 to 21)	6 (4 to 9)	181·8 (147·5 to 218·2)†	135·8 (107·4 to 166·0)†
			Latent tuberculosis infection		1 909 229 (1 788 354 to 2 034 184)	··	··	··	··
		HIV/AIDS			36 369 (34 215 to 39 122)	1865 (1690 to 2129)‡	4032 (2785 to 5587)	–9·5 (−24·9 to 7·8)	–20·9 (−34·7 to −4·9)†
			Drug-susceptible HIV/AIDS - Tuberculosis		1076 (760 to 1467)	1365 (981 to 1847)	415 (250 to 634)	–20·8 (−24·7 to −17·4)†	–30·0 (−33·4 to −27·0)†
			Multidrug-resistant HIV/AIDS - Tuberculosis without extensive drug resistance		28 (18 to 41)	36 (24 to 52)	11 (7 to 19)	–27·8 (−39·8 to −15·0)†	–36·2 (−46·7 to −24·9)†
			Extensively drug-resistant HIV/AIDS - Tuberculosis		1 (1 to 2)	1 (1 to 2)	0 (0 to 1)	147·8 (107·8 to 195·2)†	119·0 (84·1 to 161·0)†
			HIV/AIDS resulting in other diseases		35 264 (33 138 to 38 026)	1865 (1690 to 2129)	3605 (2467 to 5059)	–8·0 (−25·3 to 12·6)	–19·7 (−35·1 to −0·7)†
	**Diarrhoea, lower respiratory infections, and other common infectious diseases**				**434 596 (422 500 to 442 782)**	23 240 453 (21 377 295 to 25 359 204)*	**20 656 (14 317 to 28 476)**	**7·2 (6·4 to 7·9)**†	**–3·4 (−4·0 to −2·7)**†
		Diarrhoeal diseases			66 972 (63 500 to 70 778)	4 480 401 (4 246 997 to 4 737 769)	7506 (5176 to 10 240)	7·5 (6·5 to 8·5)†	–3·6 (−4·4 to −2·7)†
		Intestinal infectious diseases			810 (730 to 906)	15 532 (13 599 to 17 777)*	125 (84 to 175)	–16·2 (−22·8 to −8·9)†	–22·1 (−28·2 to −15·4)†
				Typhoid fever	746 (641 to 863)	11 774 (10 227 to 13 622)	114 (75 to 159)	–16·2 (−23·4 to −8·4)†	–22·0 (−28·5 to −14·6)†
				Paratyphoid fever	165 (145 to 187)	3758 (3328 to 4257)	10 (7 to 15)	–10·9 (−17·7 to −3·6)†	–18·6 (−24·8 to −12·1)†
				Other intestinal infectious diseases	··	··	1 (1 to 2)	–41·0 (−48·3 to −32·8)†	–45·1 (−51·9 to −37·7)†
			Lower respiratory infections		8 030 (7 582 to 8 548)	336 462 (313 085 to 361 622)	482 (320 to 678)	22·3 (20·0 to 24·9)†	7·1 (5·3 to 9·1)†
			Upper respiratory infections		235 768 (211 381 to 262 904)	17 778 134 (15 853 332 to 19 905 351)	5864 (3475 to 9057)	9·3 (8·3 to 10·6)†	–1·2 (−1·9 to −0·5)†
			Otitis media		106 062 (95 502 to 117 009)	451 730 (365 179 to 564 104)	3137 (1948 to 4665)	2·0 (−0·5 to 4·5)	–5·8 (−8·1 to −3·6)†
			Meningitis		14 423 (12 441 to 16 722)	2821 (2464 to 3310)	1483 (1039 to 1962)	10·8 (8·3 to 15·3)†	0·5 (−2·0 to 5·6)
				Pneumococcal meningitis	6180 (5170 to 7365)	612 (498 to 756)	634 (445 to 840)	7·6 (3·9 to 11·8)†	–2·8 (−6·2 to 1·1)
				H influenzae type B meningitis	2012 (1592 to 2545)	397 (291 to 534)	248 (171 to 330)	–13·8 (−18·5 to −9·5)†	–21·1 (−25·5 to −17·0)†
				Meningococcal infection	1661 (1207 to 2270)	561 (442 to 707)	168 (113 to 236)	19·5 (14·1 to 57·7)†	8·8 (3·1 to 54·0)†
				Other meningitis	4571 (3446 to 5833)	1250 (1056 to 1487)	432 (296 to 585)	34·8 (30·3 to 39·7)†	21·7 (17·6 to 26·1)†
		Encephalitis			14 908 (9877 to 21 755)	6534 (5957 to 7165)	1651 (1181 to 2145)	6·0 (3·1 to 8·7)†	–6·5 (−9·1 to −4·2)†
		Diphtheria			0 (0 to 0)	4 (4 to 6)	0 (0 to 0)	–58·0 (−69·3 to −44·4)†	–61·0 (−71·5 to −47·9)†
		Whooping cough			1593 (1242 to 2001)	11 627 (9069 to 14 610)	79 (47 to 124)	–22·5 (−25·0 to −19·9)†	–25·1 (−27·6 to −22·6)†
		Tetanus			160 (157 to 163)	90 (51 to 121)	4 (2 to 6)	–13·0 (−25·8 to 0·5)	–21·3 (−32·8 to −9·3)†
		Measles			245 (89 to 532)	8955 (3251 to 19 426)	22 (7 to 51)	–62·4 (−66·9 to −57·6)†	–64·0 (−68·3 to −59·4)†
		Varicella and herpes zoster			7602 (6996 to 8221)	148 162 (144 145 to 152 728)	304 (187 to 468)	21·4 (18·5 to 24·4)†	1·6 (−0·3 to 3·7)
		**Neglected tropical diseases and malaria**			**1 821 783 (1 778 850 to 1 853 280)**	440 947 (378 282 to 512 509)*	**13 665 (9345 to 19 194)**	**–10·6 (−15·1 to −6·0)**†	**–20·0 (−24·2 to −15·8)**†
		Malaria			128 948 (113 638 to 145 601)	213 098 (172 037 to 265 778)	1741 (1289 to 2326)	–11·1 (−15·9 to −6·1)†	–17·2 (−21·9 to −12·5)†
		Chagas disease			7201 (6065 to 8514)	180 (150 to 214)	63 (41 to 90)	9·2 (5·6 to 12·7)†	–10·8 (−13·6 to −8·0)†
		Leishmaniasis			4835 (4147 to 5523)	799 (618 to 1021)	275 (178 to 401)	26·9 (15·7 to 40·3)†	10·9 (0·7 to 23·9)†
				Visceral leishmaniasis	30 (28 to 32)	120 (112 to 129)	2 (1 to 3)	–54·2 (−59·2 to −48·6)†	–59·0 (−63·4 to −54·2)†
				Cutaneous and mucocutaneous leishmaniasis	4320 (3650 to 5238)	678 (497 to 902)	273 (177 to 399)	28·6 (16·8 to 42·9)†	12·5 (1·7 to 26·1)†
		African trypanosomiasis			7 (4 to 12)	5 (4 to 6)	2 (1 to 4)	–72·5 (−79·1 to −63·6)†	–75·3 (−81·3 to −67·4)†
		Schistosomiasis			189 774 (179 771 to 200 083)	71 385 (67 342 to 76 073)	1496 (755 to 2815)	–24·5 (−26·0 to −22·5)†	–33·2 (−34·6 to −31·4)†
		Cysticercosis			2676 (2232 to 3149)	··	421 (274 to 581)	–4·6 (−11·0 to 1·4)	–19·9 (−25·3 to −15·1)†
		Cystic echinococcosis			974 (674 to 1426)	204 (151 to 304)	90 (52 to 146)	–38·3 (−59·3 to −1·7)†	–45·6 (−64·1 to −14·2)†
		Lymphatic filariasis			29 382 (24 770 to 36 117)	7604 (6561 to 8750)	1189 (588 to 2115)	–37·3 (−52·4 to −26·0)†	–45·3 (−58·4 to −35·5)†
		Onchocerciasis			14 650 (9474 to 24 168)	3839 (1747 to 7518)	962 (452 to 1672)	–24·0 (−41·6 to −6·5)†	–32·6 (−48·5 to −17·5)†
		Trachoma			3338 (2 439 to 4492)	··	245 (162 to 354)	–0·5 (−6·5 to 5·2)	–23·8 (−28·5 to −19·3)†
		Dengue			6046 (3292 to 10 244)	101 064 (61 458 to 153 334)	982 (433 to 1829)	74·7 (42·5 to 293·7)†	58·4 (29·3 to 256·7)†
		Yellow fever			3 (1 to 8)	112 (32 to 283)	0 (0 to 0)	–18·1 (−30·1 to −3·5)†	–24·2 (−35·0 to −10·9)†
		Rabies			0 (0 to 1)	13 (7 to 19)	0 (0 to 0)	–46·7 (−56·0 to −33·4)†	–52·8 (−61·1 to −40·9)†
		Intestinal nematode infections			1 507 853 (1 450 985 to 1 542 581)	··	2946 (1713 to 4804)	–14·4 (−18·0 to −10·3)†	–22·7 (−26·0 to −18·9)†
				Ascariasis	799 683 (737 609 to 872 087)	··	924 (498 to 1 550)	–26·7 (−33·0 to −19·8)†	–33·7 (−39·5 to −27·5)†
				Trichuriasis	435 095 (410 564 to 464 463)	··	337 (186 to 574)	–20·0 (−27·9 to −11·6)†	–27·8 (−34·9 to −20·2)†
				Hookworm disease	450 683 (424 863 to 479 224)	··	1685 (1002 to 2649)	–4·2 (−9·5 to 1·3)	–13·5 (−18·3 to −8·5)†
			Food-borne trematodiases		74 725 (70 800 to 78 800)	34 989 (23 062 to 45 803)	1771 (924 to 3158)	6·7 (1·3 to 15·7)†	–7·0 (−11·4 to 0·5)
			Leprosy		523 (499 to 547)	55 (53 to 58)	32 (21 to 44)	1·2 (−1·3 to 3·6)	–18·1 (−20·0 to −16·2)†
			Ebola virus disease		1 (0 to 4)	0 (0 to 0)	0 (0 to 1)	··	··
			Zika virus disease		129 (97 to 179)	7598 (5696 to 10 664)	4 (3 to 6)	··	··
			Guinea worm disease		0 (0 to 0)	0 (0 to 0)	0 (0 to 0)	–99·5 (−99·7 to −99·4)†	–99·6 (−99·7 to −99·5)†
			Other neglected tropical diseases		49 493 (47 574 to 51 781)	··	1 445 (943 to 2094)	6·0 (3·6 to 8·2)†	–0·6 (−2·9 to 1·4)
		**Maternal disorders**			**8730 (8105 to 9254)**	118 296 (104 358 to 137 177)*	**852 (589 to 1181)**	**–5·8 (−18·4 to 10·1)**	**–14·6 (−25·8 to −0·2)**†
			Maternal haemorrhage		1403 (1155 to 1706)	9626 (7835 to 11 884)	60 (39 to 87)	3·3 (−19·8 to 32·5)	–5·7 (−26·6 to 21·4)
			Maternal sepsis and other pregnancy related infections		1662 (1256 to 2201)	10 377 (8385 to 13 151)	46 (23 to 83)	2·7 (−46·0 to 98·1)	–5·6 (−50·1 to 83·4)
			Maternal hypertensive disorders		4449 (2947 to 6101)	20 811 (18 216 to 23 245)	216 (120 to 349)	7·8 (−32·0 to 71·7)	–0·9 (−37·4 to 57·6)
			Maternal obstructed labour and uterine rupture		1041 (868 to 1229)	7238 (5821 to 9042)	336 (218 to 472)	–13·7 (−20·1 to −7·4)†	–22·6 (−28·2 to −17·0)†
			Maternal abortion, miscarriage, and ectopic pregnancy		576 (371 to 820)	70 245 (56 320 to 88 126)	63 (35 to 102)	–4·4 (−40·1 to 49·9)	–12·8 (−45·4 to 37·1)
			Other maternal disorders		··	··	131 (90 to 181)	–10·4 (−22·4 to 4·8)	–18·6 (−29·3 to −4·8)†
		**Neonatal disorders**			**80 244 (73 591 to 86 593)**	**··**	**13 737 (9642 to 19 774)**	**21·4 (9·0 to 36·3)**†	**11·4 (0·0 to 25·1)**†
			Neonatal preterm birth complications		66 092 (55 818 to 78 007)	··	8328 (5727 to 11 880)	18·4 (−0·6 to 41·3)	8·5 (−9·0 to 29·6)
			Neonatal encephalopathy due to birth asphyxia and trauma		12 134 (7668 to 19 247)	··	1596 (594 to 3694)	30·9 (21·6 to 46·0)†	18·9 (11·0 to 31·4)†
			Neonatal sepsis and other neonatal infections		6653 (2712 to 15 058)	··	2647 (893 to 6765)	29·7 (22·6 to 39·5)†	19·5 (13·1 to 28·3)†
			Haemolytic disease and other neonatal jaundice		2093 (1861 to 2337)	··	654 (498 to 822)	19·9 (16·0 to 23·5)†	9·5 (6·0 to 12·9)†
			Other neonatal disorders		··	··	512 (351 to 767)	7·0 (−3·7 to 19·2)	2·7 (−7·6 to 14·4)
		**Nutritional deficiencies**			**1 673 391 (1 633 390 to 1 689 822)**	598 813 (516 074 to 717 722)*	**41 431 (27 722 to 58 560)**	**6·4 (5·2 to 7·7)**†	**–2·6 (−3·7 to −1·5)**†
			Protein-energy malnutrition		63 135 (62 102 to 64 196)	43 590 (42 935 to 44 253)	3205 (2067 to 4487)	8·3 (3·4 to 13·3)†	3·9 (−0·8 to 8·7)
			Iodine deficiency		119 214 (105 724 to 133 961)	3043 (2708 to 3411)	3138 (2126 to 4398)	–6·1 (−7·6 to −4·4)†	–16·9 (−18·4 to −15·4)†
			Vitamin A deficiency		508 801 (445 148 to 601 630)	552 180 (469 062 to 671 144)	252 (159 to 388)	12·0 (8·7 to 15·5)†	2·6 (−0·3 to 5·6)
			Iron-deficiency anaemia		1 244 781 (1 205 796 to 1 284 426)	··	34 727 (22 960 to 49 579)	7·5 (6·2 to 8·9)†	–1·8 (−3·0 to −0·5)†
			Other nutritional deficiencies		··	··	109 (70 to 152)	8·5 (3·4 to 13·8)†	3·8 (−1·0 to 8·9)
		**Other communicable, maternal, neonatal, and nutritional diseases**			**1 728 266 (1 680 548 to 1 764 134)**	**857 430 (713 393 to 1 035 256)**	**4166 (2686 to 6243)**	**10·9 (8·4 to 13·1)**†	**–0·6 (−2·7 to 1·3)**
			Sexually transmitted diseases excluding HIV		1 175 931 (1 131 754 to 1 217 694)	552 776 (499 026 to 612 824)	2546 (1628 to 3983)	15·7 (13·9 to 17·7)†	1·9 (0·3 to 3·7)†
				Syphilis	56 092 (44 184 to 70 172)	38 838 (29 431 to 50 913)	188 (133 to 249)	29·2 (25·5 to 32·6)†	3·5 (1·1 to 6·0)†
				Chlamydial infection	93 574 (75 600 to 114 406)	73 667 (59 690 to 90 358)	516 (324 to 804)	10·2 (8·1 to 12·1)†	–1·3 (−3·1 to 0·3)
				Gonococcal infection	57 124 (46 375 to 70 820)	190 766 (147 718 to 242 152)	548 (341 to 851)	23·5 (19·4 to 27·6)†	10·5 (6·7 to 14·2)†
				Trichomoniasis	171 779 (148 048 to 196 705)	148 908 (126 821 to 171 971)	198 (76 to 421)	15·9 (14·8 to 17·1)†	1·8 (0·9 to 2·7)†
				Genital herpes	859 580 (748 108 to 991 917)	57 156 (49 205 to 67 554)	221 (71 to 507)	17·8 (15·5 to 19·7)†	–0·2 (−1·6 to 1·5)
				Other sexually transmitted diseases	36 588 (31 753 to 40 490)	43 441 (37 364 to 50 632)	875 (576 to 1284)	11·5† (9·3 to 13·8)	–0·9 (−3·0 to 1·2)
			Hepatitis		640 226 (566 219 to 731 671)	304 644 (182 678 to 477 007)	280 (175 to 413)	–20·9 (−33·6 to −5·7)†	–29·2† (−40·2 to −16·3)
				Acute hepatitis A	12 270 (3 159 to 25 111)	159 516 (41 071 to 326 448)	72 (39 to 119)	–29·0 (−60·7 to 27·6)	–35·1 (−63·2 to 14·6)
				Hepatitis B	468 435 (396 824 to 554 418)	118 976 (92 788 to 152 775)	165 (99 to 261)	–16·3 (−28·6 to −2·3)†	–26·7 (−36·7 to −14·9)†
				Hepatitis C	158 055 (141 445 to 175 144)	7094 (6474 to 7724)	6 (3 to 12)	5·1 (−0·4 to 11·4)	–5·3 (−10·4 to 0·8)
				Acute hepatitis E	1466 (1308 to 1645)	19 058 (17 003 to 21 385)	36 (23 to 54)	–25·7 (−32·1 to −19·0)†	–30·8 (−36·6 to −24·7)†
			Other infectious diseases		44 426 (42 785 to 46 296)	9 (6 to 13)	1 340 (879 to 1946)	11·5 (9·5 to 13·7)†	3·5 (1·6 to 5·7)†
	**Non-communicable diseases**				**6 681 939 (6 668 902 to 6 695 003)**	18 358 708 (17 561 181 to 19 263 162)*	**648 563 (481 335 to 836 494)**	**17·9 (17·5 to 18·4)**†	**–0·4 (−0·7 to −0·1)**†
		**Neoplasms**			**42 986 (42 692 to 43 241)**	**17 228 (16 713 to 17 802)**	**5180 (3830 to 6697)**	**30·5 (28·1 to 33·9)**†	**1·2 (−0·6 to 3·8)**
			Lip and oral cavity cancer		1387 (1 346 to 1 425)	382 (371 to 392)	142 (103 to 186)	32·7 (27·8 to 37·4)†	3·6 (−0·3 to 7·0)
			Nasopharynx cancer		332 (314 to 352)	96 (91 to 101)	40 (28 to 53)	8·4 (0·5 to 15·9)†	–11·2 (−17·5 to −5·3)†
			Other pharynx cancer		478 (452 to 494)	170 (159 to 176)	58 (42 to 76)	36·7 (26·9 to 43·2)†	5·8 (−1·7 to 10·8)
			Oesophageal cancer		556 (539 to 577)	443 (433 to 456)	112 (79 to 144)	9·1 (5·4 to 13·7)†	–16·2 (−19·0 to −12·8)†
			Stomach cancer		2199 (2144 to 2255)	1157 (1134 to 1180)	300 (218 to 384)	17·9 (14·6 to 21·5)†	–9·1 (−11·7 to −6·4)†
			Colon and rectum cancer		6323 (6112 to 6632)	1716 (1658 to 1795)	599 (442 to 780)	34·0 (28·4 to 41·1)†	1·7 (−2·4 to 7·1)
			Liver cancer		1027 (974 to 1076)	1008 (953 to 1042)	228 (163 to 298)	42·9 (36·6 to 49·9)†	11·4 (6·7 to 16·8)†
				Liver cancer due to hepatitis B	599 (516 to 675)	436 (380 to 488)	98 (68 to 131)	41·0 (33·4 to 50·8)†	11·3 (5·3 to 19·0)†
				Liver cancer due to hepatitis C	261 (236 to 286)	189 (170 to 207)	43 (31 to 58)	41·7 (35·4 to 47·7)†	8·0 (3·3 to 12·6)†
				Liver cancer due to alcohol use	186 (157 to 216)	148 (125 to 171)	34 (23 to 46)	48·4 (37·0 to 62·2)†	13·9 (5·1 to 24·6)†
				Liver cancer due to other causes	326 (288 to 371)	235 (210 to 264)	54 (38 to 71)	44·0 (35·2 to 52·5)†	13·2 (6·4 to 19·8)†
			Gallbladder and biliary tract cancer		169 (152 to 179)	184 (168 to 193)	41 (29 to 54)	21·3 (16·2 to 26·8)†	–7·9 (−11·8 to −3·7)†
			Pancreatic cancer		364 (349 to 375)	418 (406 to 425)	86 (60 to 111)	33·8 (29·2 to 37·5)†	1·8 (−1·8 to 4·7)
			Larynx cancer		638 (627 to 653)	187 (184 to 191)	75 (55 to 98)	22·8 (19·3 to 26·5)†	–5·6 (−8·3 to −2·8)†
			Tracheal, bronchus, and lung cancer		2836 (2750 to 2920)	2008 (1958 to 2055)	474 (345 to 598)	30·2 (26·1 to 33·9)†	0·1 (−3·0 to 3·0)
			Malignant skin melanoma		1347 (1156 to 1506)	282 (243 to 314)	90 (63 to 123)	37·9 (31·7 to 42·7)†	8·8 (3·8 to 12·6)†
			Non-melanoma skin cancer		853 (682 to 1066)	1521 (1109 to 2008)	31 (17 to 52)	5·8 (−3·3 to 19·7)	–21·4 (−28·0 to −10·8)†
				Non-melanoma skin cancer (squamous-cell carcinoma)	690 (415 to 996)	635 (386 to 922)	30 (16 to 51)	5·2 (−4·1 to 19·5)	–21·8 (−28·6 to −11·1)†
				Non-melanoma skin cancer (basal-cell carcinoma)	103 (65 to 167)	886 (574 to 1 262)	1 (0 to 2)	23·8 (16·1 to 31·7)†	–5·6 (−11·5 to 0·4)
			Breast cancer		8151 (7808 to 8610)	1702 (1629 to 1801)	739 (535 to 982)	26·9 (20·6 to 34·5)†	–1·4 (−6·2 to 4·1)
			Cervical cancer		1939 (1544 to 2082)	511 (414 to 542)	186 (128 to 247)	9·4 (2·7 to 18·3)†	–11·3 (−16·7 to −4·3)†
			Uterine cancer		1959 (1882 to 2076)	417 (401 to 442)	150 (107 to 201)	39·8 (32·9 to 50·0)†	7·2 (2·1 to 15·0)†
			Ovarian cancer		786 (743 to 809)	254 (242 to 260)	116 (84 to 150)	24·1 (16·9 to 29·4)†	–2·7 (−8·3 to 1·3)
			Prostate cancer		5697 (5098 to 6714)	1436 (1293 to 1619)	533 (384 to 707)	39·0 (32·3 to 48·1)†	4·4 (−0·7 to 11·4)
			Testicular cancer		340 (328 to 355)	67 (64 to 70)	24 (17 to 32)	29·8 (24·0 to 35·9)†	12·8 (7·8 to 18·2)†
			Kidney cancer		1289 (1226 to 1329)	342 (331 to 350)	114 (83 to 150)	27·7 (23·5 to 31·1)†	0·0 (−3·3 to 2·6)
			Bladder cancer		1767 (1721 to 1813)	437 (427 to 448)	165 (121 to 217)	31·1 (26·9 to 35·1)†	–0·6 (−3·7 to 2·5)
			Brain and nervous system cancer		781 (693 to 818)	330 (299 to 349)	106 (75 to 139)	37·9 (33·1 to 43·3)†	12·7 (8·8 to 17·2)†
			Thyroid cancer		1234 (1187 to 1313)	238 (229 to 253)	80 (56 to 110)	48·4 (41·5 to 58·2)†	17·9 (12·6 to 25·7)†
			Mesothelioma		53 (49 to 56)	35 (32 to 36)	11 (8 to 15)	27·7 (21·6 to 32·5)†	–1·0 (−5·8 to 2·6)
			Hodgkin's lymphoma		291 (264 to 322)	73 (66 to 82)	29 (21 to 39)	10·2 (7·3 to 13·2)†	–6·8 (−9·3 to −4·1)†
			Non-Hodgkin lymphoma		1670 (1545 to 1746)	461 (428 to 482)	147 (107 to 193)	45·2 (38·0 to 49·0)†	13·8 (8·1 to 16·8)†
			Multiple myeloma		358 (305 to 398)	138 (121 to 156)	70 (49 to 91)	42·8 (36·8 to 49·4)†	8·6 (4·1 to 13·7)†
			Leukaemia		1675 (1511 to 1765)	467 (423 to 488)	214 (153 to 284)	25·3 (18·7 to 32·2)†	2·1 (−3·1 to 7·5)
				Acute lymphoid leukaemia	292 (256 to 310)	76 (66 to 80)	36 (25 to 49)	31·1 (12·6 to 41·6)†	16·6 (0·7 to 25·8)†
				Chronic lymphoid leukaemia	460 (430 to 496)	105 (98 to 113)	57 (41 to 74)	37·4 (31·6 to 44·6)†	4·3 (0·0 to 9·5)†
				Acute myeloid leukaemia	160 (142 to 169)	103 (91 to 108)	29 (20 to 39)	32·8 (24·5 to 37·6)†	8·8 (2·2 to 12·6)†
				Chronic myeloid leukaemia	92 (84 to 99)	32 (29 to 34)	12 (8 to 16)	6·5 (1·9 to 11·0)†	–15·4 (−18·9 to −12·2)†
				Other leukaemia	672 (565 to 722)	150 (127 to 161)	81 (57 to 107)	16·3 (8·5 to 25·5)†	–3·7 (−9·9 to 3·7)
			Other neoplasms		2632 (2377 to 2720)	750 (682 to 772)	221 (160 to 292)	51·8 (44·9 to 56·7)†	21·7 (16·1 to 25·7)†
		**Cardiovascular diseases**			**469 454 (462 582 to 474 872)**	54 120 (51 965 to 56 447)*	**33 482 (24 477 to 43 376)**	**29·4 (26·7 to 31·9)**†	**1·0 (−1·1 to 2·9)**
			Rheumatic heart disease		29 677 (26 737 to 32 927)	1196 (1095 to 1308)	1456 (931 to 2116)	17·3 (13·9 to 20·6)†	3·4 (0·4 to 6·4)†
			Ischaemic heart disease		153 533 (145 992 to 160 779)	20 731 (19 530 to 22 072)	6916 (4812 to 9385)	29·3 (28·5 to 30·1)†	0·5 (−0·1 to 1·1)
			Cerebrovascular disease		79 574 (77 057 to 82 118)	13 677 (12 714 to 14 692)§	14 452 (10 150 to 18 414)	33·5 (27·3 to 39·2)†	2·7 (−2·0 to 7·1)
				Ischaemic stroke	67 595 (60 812 to 74 572)	9556 (8655 to 10 513)§	11 802 (8246 to 15 151)	35·2 (27·8 to 42·2)†	3·7 (−2·1 to 9·0)
				Haemorrhagic stroke	15 310 (13 857 to 16 954)	4120 (3764 to 4508)§	2650 (1896 to 3381)	26·6 (20·1 to 32·9)†	–1·6 (−6·6 to 3·4)
			Hypertensive heart disease		17 190 (14 948 to 19 774)	··	1380 (959 to 1 894)	31·6 (29·7 to 33·5)†	0·4 (−0·8 to 1·6)
			Cardiomyopathy and myocarditis		6118 (5779 to 6416)	2483 (2217 to 2784)*	559 (384 to 786)	18·7 (16·8 to 20·7)†	–4·0 (−5·4 to −2·6)†
				Myocarditis	1786 (1607 to 1977)	2483 (2217 to 2784)	133 (89 to 188)	13·1 (11·1 to 15·1)†	–1·4 (−2·9 to 0·0)†
				Alcoholic cardiomyopathy	1165 (994 to 1359)	··	97 (66 to 138)	20·6 (15·6 to 25·7)†	–5·4 (−9·0 to −1·7)†
				Other cardiomyopathy	4005 (3534 to 4525)	··	329 (226 to 462)	20·6 (18·3 to 22·9)†	–4·5 (−6·1 to −2·9)†
			Atrial fibrillation and flutter		46 311 (41 441 to 52 014)	3841 (3379 to 4388)	3614 (2439 to 5024)	32·0 (30·9 to 33·0)†	0·3 (−0·4 to 1·0)
			Peripheral vascular disease		120 145 (105 640 to 137 704)	11 020 (9557 to 12 778)	520 (244 to 941)	25·5 (23·8 to 27·3)†	–5·9 (−6·8 to −5·0)†
			Endocarditis		500 (448 to 568)	1172 (1068 to 1280)	40 (27 to 55)	23·0 (20·5 to 25·6)†	–3·7 (−5·6 to −1·9)†
			Other cardiovascular and circulatory diseases		87 122 (76 311 to 98 866)	··	4546 (3102 to 6404)	20·6† (18·7 to 22·6)	–2·2† (−3·4 to −1·0)
		**Chronic respiratory diseases**			**571 426 (560 139 to 582 621)**	94 696 (90 108 to 99 441)*	**30 954 (25 068 to 37 362)**	**24·0 (22·3 to 26·1)**†	**3·0 (1·8 to 4·2)**†
			Chronic obstructive pulmonary disease		251 631 (241 958 to 261 129)	18 721 (18 004 to 19 456)	16 288 (14 342 to 17 878)	28·8 (26·2 to 31·4)†	1·4 (−0·6 to 3·5)
			Pneumoconiosis		1111 (1043 to 1184)	99 (93 to 105)	162 (111 to 224)	32·0 (29·0 to 35·1)†	1·5 (−0·8 to 3·9)
				Silicosis	421 (380 to 464)	44 (41 to 49)	60 (41 to 84)	31·8 (26·1 to 37·1)†	1·0 (−3·2 to 4·8)
				Asbestosis	152 (138 to 170)	12 (11 to 13)	23 (16 to 32)	26·9 (24·3 to 29·6)†	–1·3 (−3·3 to 0·9)
				Coal workers pneumoconiosis	294 (256 to 340)	21 (19 to 24)	42 (28 to 60)	34·5 (27·0 to 42·4)†	2·6 (−3·0 to 8·6)
				Other pneumoconiosis	243 (210 to 277)	21 (18 to 24)	36 (24 to 50)	32·5 (27·6 to 37·8)†	2·8 (−1·0 to 7·0)
			Asthma		339 440 (319 582 to 360 796)	75 496 (71 071 to 80 177)	13 221 (8726 to 18 839)	17·2 (16·0 to 18·4)†	3·6 (2·5 to 4·6)†
			Interstitial lung disease and pulmonary sarcoidosis		3978 (3632 to 4352)	379 (348 to 412)	416 (282 to 596)	28·9 (26·7 to 31·2)†	1·7 (0·3 to 3·1)†
			Other chronic respiratory diseases		··	··	866 (752 to 955)	49·7 (46·2 to 53·0)†	34·2 (31·1 to 37·3)†
		**Cirrhosis and other chronic liver diseases**			**45 783 (43 781 to 47 831)**	**1638 (1575 to 1708)**	**1574 (1094 to 2165)**	**21·0 (19·4 to 22·6)**†	**1·8 (0·5 to 3·0)**†
			Cirrhosis and other chronic liver diseases due to hepatitis B		12 482 (11 611 to 13 474)	457 (421 to 500)	394 (274 to 545)	26·6 (24·1 to 29·4)†	2·9 (1·0 to 4·9)†
			Cirrhosis and other chronic liver diseases due to hepatitis C		10 903 (9925 to 11 984)	387 (353 to 426)	314 (215 to 433)	26·4 (23·6 to 29·2)†	2·2 (0·1 to 4·3)†
			Cirrhosis and other chronic liver diseases due to alcohol use		11 392 (10 521 to 12 386)	404 (373 to 439)	314 (220 to 431)	25·8 (23·1 to 28·6)†	0·2 (−1·7 to 2·4)
			Cirrhosis and other chronic liver diseases due to other causes		11 007 (10 105 to 11 958)	391 (363 to 421)	552 (376 to 772)	12·3 (10·0 to 14·6)†	1·7 (−0·4 to 3·7)
		**Digestive diseases**			**262 283 (257 495 to 266 222)**	211 761 (201 555 to 223 065)*	**7287 (5169 to 9878)**	**27·4 (24·7 to 30·3)**†	**5·9 (4·0 to 8·0)**†
			Peptic ulcer disease		74 335 (69 108 to 80 393)	10 329 (9 391 to 11 368)	1364 (891 to 2024)	38·7 (34·1 to 43·4)†	14·1 (10·5 to 17·7)†
			Gastritis and duodenitis		123 619 (112 199 to 135 163)	77 140 (70 074 to 85 452)	1672 (1094 to 2419)	38·6 (34·5 to 42·9)†	14·0 (10·9 to 17·2)†
			Appendicitis		857 (796 to 922)	22 580 (20 993 to 24 258)	263 (176 to 360)	9·8 (6·5 to 13·2)†	0·1 (−2·8 to 2·9)
			Paralytic ileus and intestinal obstruction		170 (155 to 186)	4651 (4265 to 5080)	54 (36 to 74)	25·2 (23·1 to 27·2)†	4·9 (3·6 to 6·3)†
			Inguinal, femoral, and abdominal hernia		51 239 (46 088 to 56 543)	36 240 (31 967 to 40 531)	2152 (1471 to 2993)	15·3 (12·9 to 17·6)†	–3·1 (−4·8 to −1·7)†
			Inflammatory bowel disease		6007 (5622 to 6446)	3518 (3275 to 3779)	863 (596 to 1185)	26·8 (24·0 to 29·6)†	3·8 (1·8 to 5·9)†
			Vascular intestinal disorders		104 (93 to 115)	583 (527 to 644)	32 (21 to 43)	25·7 (21·5 to 30·0)†	0·2 (−3·3 to 3·5)
			Gallbladder and biliary diseases		17 269 (15 618 to 19 012)	51 509 (46 439 to 56 953)	231 (158 to 325)	29·2 (25·4 to 32·7)†	4·9 (2·4 to 7·4)†
			Pancreatitis		584 (532 to 639)	5210 (4769 to 5703)	73 (49 to 102)	29·3 (25·1 to 33·7)†	5·8 (2·4 to 9·1)†
			Other digestive diseases		··	··	584 (413 to 784)	32·4 (29·4 to 35·6)†	10·1 (8·2 to 12·2)†
		**Neurological disorders**			**2 595 647 (2 542 097 to 2 651 997)**	**1 183 726 (1 021 730 to 1 362 193)**	**69 426 (47 199 to 92 890)**	**16·3 (14·5 to 18·3)**†	**0·4 (−1·0 to 2·0)**
			Alzheimer's disease and other dementias		43 836 (37 756 to 51 028)	7779 (6620 to 9135)	6415 (4506 to 8491)	38·2 (36·4 to 39·9)†	0·7 (−0·3 to 1·6)
			Parkinson's disease		6063 (4972 to 7325)	696 (564 to 850)	706 (457 to 974)	36·3 (33·6 to 38·6)†	3·3 (1·6 to 4·8)†
			Epilepsy		23 962 (20 402 to 27 737)	2 761 (2 308 to 3 268)	7547 (5111 to 10 458)	8·8 (−2·1 to 22·0)	–2·6 (−12·3 to 9·2)
			Multiple sclerosis		2221 (2034 to 2437)	69 (63 to 76)	584 (411 to 764)	24·5 (21·9 to 26·9)†	3·0 (0·8 to 5·1)†
			Motor neuron disease		331 (300 to 367)	58 (52 to 63)	70 (49 to 93)	21·2 (19·5 to 23·1)†	1·1 (0·1 to 2·1)†
			Migraine		1 044 772 (999 535 to 1 087 969)	110 316 (105 270 to 115 834)	45 122 (29 046 to 62 827)	14·3 (13·7 to 14·9)†	0·1 (−0·2 to 0·5)
			Tension-type headache		1 890 670 (1 707 786 to 2 097 762)	1 061 998 (899 411 to 1 241 310)	7195 (4615 to 10 500)	15·4 (13·7 to 17·0)†	0·4 (−0·4 to 1·4)
			Other neurological disorders		24 (16 to 33)	48 (32 to 66)	1786 (1212 to 2436)	30·1 (18·7 to 43·7)†	17·4 (7·4 to 30·2)†
		**Mental and substance use disorders**			**1 111 147 (1 095 612 to 1 128 948)**	410 091 (385 617 to 437 586)*	**150 476 (109 498 to 194 542)**	**12·9 (12·4 to 13·5)**†	**–1·4 (−1·8 to −1·0)**†
			Schizophrenia		20 883 (18 497 to 23 422)	1139 (1006 to 1287)	13 414 (9859 to 16 714)	16·7 (15·5 to 18·0)†	–0·9 (−1·7 to −0·2)†
			Alcohol use disorders		100 389 (89 592 to 111 659)	50 432 (44 249 to 56 797)	10 031 (6 883 to 13 787)	9·7 (7·7 to 11·8)†	–4·8 (−6·7 to −3·1)†
			Drug use disorders		61 968 (60 342 to 63 579)	7460 (6810 to 8140)	14 607 (10 464 to 19 045)	15·5 (13·7 to 17·3)†	1·4 (−0·2 to 3·0)
				Opioid use disorders	26 834 (23 563 to 30 952)	2795 (2443 to 3226)	11 132 (7725 to 14 577)	18·0 (15·8 to 20·1)†	2·7 (0·7 to 4·6)†
				Cocaine use disorders	5840 (5322 to 6473)	347 (304 to 391)	797 (501 to 1141)	11·8 (10·0 to 13·6)†	–1·4 (−2·9 to 0·2)
				Amphetamine use disorders	4955 (3693 to 6490)	440 (330 to 577)	658 (386 to 1038)	3·7 (0·4 to 6·8)†	–4·0 (−7·3 to −0·9)†
				Cannabis use disorders	22 094 (18 965 to 25 856)	3183 (2706 to 3776)	647 (401 to 946)	3·7 (1·2 to 6·0)†	–4·2 (−5·9 to −2·4)†
				Other drug use disorders	3944 (3537 to 4362)	695 (624 to 770)	1373 (919 to 1912)	10·6 (8·2 to 13·1)†	–1·5 (−3·6 to 0·7)
			Depressive disorders		268 172 (260 480 to 274 698)	274 704 (251 855 to 300 436)	44 208 (30 573 to 59 878)	13·2 (12·2 to 14·4)†	–3·6 (−4·3 to −2·9)†
				Major depressive disorder	167 836 (153 908 to 183 754)	257 768 (234 546 to 284 665)	34 105 (23 470 to 46 039)	11·2 (10·1 to 12·3)†	–4·9 (−5·6 to −4·1)†
				Dysthymia	105 580 (92 588 to 120 440)	16 935 (14 981 to 19 313)	10 104 (6 861 to 14 612)	20·5 (18·3 to 23·2)†	1·0 (−0·5 to 2·7)
			Bipolar disorder		43 908 (38 383 to 50 196)	3777 (3313 to 4331)	8954 (5588 to 13 186)	14·9 (13·8 to 16·0)†	0·8 (0·2 to 1·4)†
			Anxiety disorders		274 615 (255 323 to 294 796)	42 407 (39 560 to 45 513)	26 417 (18 440 to 35 634)	13·1 (11·9 to 14·3)†	–0·7 (−1·7 to 0·2)
			Eating disorders		10 523 (9368 to 11 716)	5440 (4039 to 7186)	2148 (1386 to 3091)	17·3 (15·5 to 19·0)†	9·1 (7·9 to 10·4)†
				Anorexia nervosa	2606 (1932 to 3450)	897 (665 to 1183)	556 (338 to 830)	12·2 (9·7 to 14·7)†	6·2 (4·0 to 8·4)†
				Bulimia nervosa	7559 (5765 to 9931)	4543 (3169 to 6256)	1592 (972 to 2392)	19·2 (16·9 to 21·2)†	10·2 (8·7 to 11·7)†
			Autistic spectrum disorders		62 174 (59 625 to 64 557)	450 (307 to 624)	9026 (6119 to 12 681)	11·4 (10·8 to 12·0)†	0·3 (−0·2 to 0·8)
				Autism	18 302 (15 766 to 21 177)	51 (26 to 86)	4649 (2977 to 6699)	11·3 (10·3 to 12·3)†	–0·1 (−0·9 to 0·8)
				Asperger syndrome and other autistic spectrum disorders	43 798 (36 425 to 52 403)	399 (258 to 572)	4377 (2864 to 6393)	11·5 (10·9 to 12·1)†	0·6 (0·2 to 1·1)†
			Attention-deficit or hyperactivity disorder		62 624 (57 564 to 68 596)	2872 (2562 to 3294)	755 (452 to 1197)	6·1 (5·0 to 7·1)†	–0·5 (−1·4 to 0·4)
			Conduct disorder		49 005 (40 946 to 57 034)	16 887 (13 566 to 20 196)	5947 (3 702 to 8 998)	2·2 (0·9 to 3·4)† 2·3	(1·1 to 3·4)†
			Idiopathic developmental intellectual disability		114 797 (69 506 to 161 353)	··	4611 (2202 to 7932)	2·5 (−0·9 to 4·6)	–6·9 (−10·0 to −4·9)†
			Other mental and substance use disorders		139 505 (122 047 to 160 430)	4523 (4004 to 5122)	10 357 (7059 to 14 807)	17·8 (17·2 to 18·5)†	0·1 (−0·4 to 0·5)
		**Diabetes, urogenital, blood, and endocrine diseases**			**2 927 330 (2 909 185 to 2 949 337)**	811 323 (769 165 to 854 588)*	**62 287 (44 312 to 84 848)**	**21·1 (19·7 to 22·6)**†	**–0·3 (−1·4 to 0·8)**
			Diabetes mellitus		383 453 (352 588 to 414 576)	20 828 (19 160 to 22 734)	28 584 (19 534 to 39 575)	23·6 (20·9 to 26·5)†	–1·2 (−3·3 to 1·2)
			Acute glomerulonephritis		104 (90 to 117)	1530 (1357 to 1729)	5 (3 to 8)	4·5 (2·4 to 6·8)†	–10·6 (−12·1 to −9·1)†
			Chronic kidney disease		275 930 (252 442 to 300 414)	21 329 (19 100 to 23 599)	8772 (6600 to 11 104)	30·4 (28·2 to 32·7)†	3·8 (2·2 to 5·3)†
				Chronic kidney disease due to diabetes mellitus	115 356 (101 848 to 130 636)	8979 (7742 to 10 377)	3695 (2768 to 4722)	33·4 (30·7 to 36·0)†	4·8 (3·1 to 6·7)†
				Chronic kidney disease due to hypertension	50 097 (44 151 to 55 693)	3626 (3131 to 4163)	1680 (1200 to 2159)	36·6 (33·4 to 40·3)†	4·8 (2·6 to 7·3)†
				Chronic kidney disease due to glomerulonephritis	49 615 (43 695 to 55 597)	3893 (3261 to 4529)	1479 (1093 to 1927)	20·6 (17·6 to 23·8)†	–0·4 (−2·6 to 2·0)
				Chronic kidney disease due to other causes	60 862 (52 600 to 69 124)	4831 (4135 to 5526)	1918 (1411 to 2502)	28·0 (25·3 to 30·7)†	4·0 (2·0 to 6·0)†
			Urinary diseases and male infertility		146 155 (135 457 to 155 774)	449 242 (412 808 to 488 929)*	4140 (2727 to 5875)	25·6 (24·3 to 27·0)†	–1·4 (−2·2 to −0·5)†
				Interstitial nephritis and urinary tract infections	6913 (6201 to 7660)	360 816 (325 242 to 399 931)	228 (142 to 345)	14·1 (12·0 to 16·1)†	1·0 (−0·5 to 2·4)
				Urolithiasis	2839 (2556 to 3162)	75 218 (67 725 to 83 689)	208 (140 to 286)	21·5 (18·6 to 24·4)†	0·3 (−1·9 to 2·4)
				Benign prostatic hyperplasia	103 655 (91 930 to 115 236)	13 208 (11 393 to 15 199)	3384 (2178 to 4835)	28·9 (27·5 to 30·3)†	–1·3 (−2·2 to −0·3)†
				Male infertility	28 044 (22 703 to 35 076)	··	165 (66 to 327)	19·3 (15·7 to 22·6)†	7·9 (4·9 to 10·6)†
				Other urinary diseases	··	··	155 (103 to 216)	–3·3 (−4·9 to −1·7)†	–16·4 (−17·5 to −15·3)†
			Gynaecological diseases		837 557 (822 589 to 852 391)	300 826 (284 093 to 317 864)*	10 195 (6918 to 14 656)	11·9 (10·6 to 13·4)†	–2·2 (−3·3 to −1·1)†
				Uterine fibroids	91 012 (82 090 to 100 488)	9394 (8303 to 10 601)	1375 (812 to 2218)	19·0 (17·9 to 20·1)†	0·2 (−0·5 to 1·0)
				Polycystic ovarian syndrome	10 642 (8653 to 12 885)	7651 (5518 to 10 179)	94 (43 to 173)	7·8 (6·4 to 9·4)†	–2·1 (−3·4 to −0·7)†
				Female infertility	31 477 (19 472 to 49 304)	··	180 (66 to 399)	38·5 (29·4 to 49·5)†	25·3 (17·7 to 34·8)†
				Endometriosis	3554 (3197 to 3935)	9262 (7894 to 10 960)	332 (224 to 459)	7·8 (5·2 to 10·7)†	–3·9 (−6·2 to −1·5)†
				Genital prolapse	250 418 (222 236 to 278 690)	16 608 (14 433 to 19 185)	778 (382 to 1409)	14·8 (13·7 to 15·9)†	–9·4 (−10·2 to −8·6)†
				Premenstrual syndrome	454 296 (432 912 to 474 453)	162 435 (153 441 to 171 301)	3791 (2344 to 5813)	9·2 (7·7 to 10·6)†	–2·0 (−3·3 to −0·8)†
				Other gynaecological diseases	104 692 (93 190 to 116 476)	95 475 (82 017 to 109 553)	3645 (2430 to 5090)	11·2 (8·8 to 14·2)†	–2·4 (−4·5 to 0·3)
			Haemoglobinopathies and haemolytic anaemias		1 804 470 (1 782 496 to 1 828 051)	··	6572 (4372 to 9566)	12·8 (10·9 to 14·9)†	1·8 (0·1 to 3·7)†
				Thalassaemias	366 (343 to 394)	··	23 (15 to 34)	–4·3 (−8·1 to −0·4)†	–8·5 (−12·2 to −4·8)†
				Thalassaemias trait	287 107 (276 495 to 298 645)	··	3280 (2157 to 4824)	10·9 (8·0 to 13·9)†	–0·6 (−3·1 to 2·0)
				Sickle cell disorders	3888 (3572 to 4470)	··	317 (222 to 441)	21·4 (17·1 to 26·0)†	15·6 (11·5 to 19·9)†
				Sickle cell trait	461 124 (418 742 to 516 943)	··	1555 (1014 to 2283)	16·8 (14·8 to 18·8)†	7·3 (5·4 to 9·1)†
				G6PD deficiency	331 513 (319 093 to 346 326)	··	26 (18 to 36)	14·5 (11·5 to 17·6)†	3·1 (0·4 to 5·9)†
				G6PD trait	865 927 (851 718 to 882 248)	··	1 (0 to 1)	17·6 (14·9 to 20·7)†	4·9 (2·6 to 7·5)†
				Other haemoglobinopathies and haemolytic anaemias	61 625 (59 671 to 63 634)	··	1370 (912 to 1967)	11·6 (9·4 to 14·3)†	–0·7 (−2·5 to 1·5)
			Endocrine, metabolic, blood, and immune disorders		127 493 (119 899 to 135 715)	17 569 (15 815 to 19 457)	4020 (2721 to 5537)	19·1 (17·4 to 20·8)†	–0·5 (−1·5 to 0·6)
		**Musculoskeletal disorders**			**1 270 630 (1 248 968 to 1 294 805)**	**652 005 (591 746 to 719 811)**	**137 832 (100 146 to 179 438)**	**19·9 (18·7 to 21·0)**†	**–1·2 (−1·9 to −0·5)**†
			Rheumatoid arthritis		21 337 (19 466 to 23 612)	1174 (1066 to 1298)	4989 (3427 to 6604)	28·8 (26·7 to 31·0)†	3·7 (2·1 to 5·2)†
			Osteoarthritis		301 567 (285 064 to 318 844)	14 696 (13 728 to 15 682)	16 283 (11 486 to 22 047)	31·5 (30·7 to 32·2)†	2·4 (1·9 to 3·0)†
			Low back and neck pain		748 081 (721 756 to 766 729)	313 434 (281 503 to 347 952)	86 584 (61 335 to 113 628)	19·3 (17·7 to 20·7)†	–1·3 (−2·4 to −0·5)†
				Low back pain	511 048 (457 208 to 572 056)	250 277 (220 585 to 283 681)	57 648 (40 820 to 75 877)	18·0 (16·0 to 20·1)†	–2·0 (−3·6 to −0·9)†
				Neck pain	290 524 (254 815 to 332 009)	63 157 (55 205 to 74 075)	28 936 (19 578 to 40 543)	21·9 (19·9 to 24·0)†	0·1 (−1·3 to 1·4)
			Gout		33 977 (30 617 to 37 923)	6435 (5666 to 7248)	1071 (742 to 1455)	26·2 (24·3 to 27·9)†	–0·1 (−1·3 to 1·1)
			Other musculoskeletal disorders		337 859 (289 444 to 390 647)	316 267 (266 448 to 375 370)	28 904 (19 554 to 40 812)	14·4 (11·5 to 17·2)†	–3·5 (−5·7 to −1·6)†
		**Other non-communicable diseases**			**5 292 032 (5 257 502 to 5 326 591)**	14 922 120 (14 136 832 to 15 793 314)*	**150 066 (102 792 to 212 136)**	**16·7 (16·0 to 17·2)**†	**–0·6 (−1·0 to −0·2)**†
			Congenital anomalies		82 890 (78 027 to 87 071)	··	9723 (6979 to 12 887)	9·1 (7·5 to 10·6)†	–1·5 (−3·0 to −0·2)†
				Neural tube defects	5782 (5232 to 6393)	··	1723 (1220 to 2299)	14·6 (12·1 to 17·4)†	3·0 (0·8 to 5·6)†
				Congenital heart anomalies	15 377 (13 712 to 17 128)	··	755 (332 to 1284)	8·5 (6·3 to 10·4)†	–0·9 (−2·9 to 0·8)
				Orofacial clefts	3590 (3230 to 3967)	··	46 (30 to 67)	3·5 (0·7 to 6·3)†	–7·3 (−9·8 to −4·8)†
				Down's syndrome	2031 (1776 to 2314)	··	185 (124 to 258)	8·3 (5·9 to 10·7)†	–1·1 (−3·4 to 1·1)
				Turner syndrome	2719 (2378 to 3089)	··	47 (22 to 76)	6·9 (3·2 to 10·4)†	–1·0 (−4·3 to 2·1)
				Klinefelter syndrome	3043 (2641 to 3482)	··	17 (8 to 32)	9·0 (6·2 to 11·7)†	0·0 (−2·5 to 2·5)
				Other chromosomal abnormalities	4310 (3734 to 4977)	··	494 (345 to 665)	4·3 (2·2 to 6·4)†	1·5 (−0·6 to 3·6)
				Congenital musculoskeletal and limb anomalies	10 812 (9 960 to 11 711)	··	1536 (1056 to 2113)	13·2 (11·1 to 15·2)†	–0·1 (−1·9 to 1·7)
				Urogenital congenital anomalies	7172 (6493 to 7894)	··	189 (112 to 298)	1·8 (0·1 to 3·1)†	–3·5 (−4·8 to −2·4)†
				Digestive congenital anomalies	9711 (8741 to 10 731)	··	427 (280 to 610)	9·0 (6·1 to 11·9)†	–3·2 (−5·8 to −0·6)†
				Other congenital anomalies	34 984 (24 584 to 49 402)	··	4302 (2844 to 6310)	6·8 (4·4 to 9·6)†	–3·9 (−6·1 to −1·4)†
			Skin and subcutaneous diseases		2 266 315 (2 242 994 to 2 285 332)	5 074 605 (4 838 566 to 5 335 844)	54 635 (36 830 to 79 320)	10·7 (10·2 to 11·3)†	1·2 (0·9 to 1·5)†
				Dermatitis	306 359 (290 041 to 324 193)	430 376 (398 274 to 462 932)	11 210 (6 714 to 18 218)	11·6 (10·8 to 12·4)†	1·1 (0·3 to 1·8)†
				Psoriasis	65 135 (62 708 to 67 812)	8170 (7861 to 8478)	5643 (4040 to 7377)	21·7 (20·8 to 22·6)†	4·2 (3·6 to 5·0)†
				Cellulitis	3018 (2842 to 3210)	61 333 (58 280 to 64 556)	170 (112 to 244)	14·0 (12·2 to 16·0)†	–0·5 (−1·9 to 1·0)
				Pyoderma	21 020 (20 491 to 21 564)	474 384 (461 024 to 488 662)	117 (47 to 243)	22·8 (21·9 to 23·6)†	5·2 (4·4 to 5·8)†
				Scabies	146 785 (127 773 to 170 009)	454 671 (392 690 to 529 184)	3788 (2104 to 6029)	3·2 (1·8 to 4·6)†	–5·4 (−6·2 to −4·8)†
				Fungal skin diseases	626 700 (568 967 to 690 267)	2 098 743 (1 884 346 to 2 337 325)	3509 (1403 to 7271)	18·0 (17·0 to 19·1)†	2·5 (2·2 to 2·9)†
				Viral skin diseases	193 171 (184 773 to 201 619)	276 755 (263 471 to 290 018)	5915 (3674 to 8828)	9·0 (8·5 to 9·5)†	–0·1 (−0·5 to 0·2)
				Acne vulgaris	614 771 (560 634 to 672 878)	429 822 (361 790 to 521 114)	15 836 (10 644 to 22 843)	5·1 (4·3 to 5·8)†	2·1 (1·5 to 2·6)†
				Alopecia areata	15 416 (14 906 to 15 948)	27 247 (26 347 to 28 203)	504 (323 to 760)	12·8 (11·8 to 13·9)†	–1·3 (−2·2 to −0·5)†
				Pruritus	66 780 (59 262 to 74 868)	53 046 (47 751 to 59 503)	709 (330 to 1 299)	18·2 (16·7 to 19·8)†	1·3 (0·8 to 1·8)†
				Urticaria	67 060 (59 299 to 77 018)	119 403 (105 497 to 135 993)	4030 (2576 to 5745)	9·4 (8·3 to 10·5)†	–0·3 (−0·8 to 0·3)
				Decubitus ulcer	1836 (1660 to 2017)	6722 (6040 to 7456)	290 (203 to 388)	27·0 (24·2 to 29·6)†	–0·8 (−2·8 to 1·0)
				Other skin and subcutaneous diseases	532 790 (519 540 to 545 725)	633 932 (616 730 to 651 838)	2913 (1409 to 5325)	25·3 (24·8 to 25·7)†	4·5 (4·3 to 4·7)†
			Sense organ diseases		1 843 201 (1 825 393 to 1 861 436)	708 004 (667 844 to 753 886)*	66 702 (46 534 to 92 392)	21·7 (20·7 to 22·5)†	–1·9 (−2·6 to −1·3)†
				Glaucoma	4761 (3973 to 5573)	··	461 (311 to 642)	35·7 (33·7 to 38·0)†	2·1 (0·7 to 3·6)†
				Cataract	83 628 (73 800 to 93 905)	··	5789 (4 135 to 7 915)	31·0 (29·4 to 32·6)†	0·0 (−1·2 to 1·1)
				Macular degeneration	6147 (5111 to 7401)	··	409 (278 to 556)	37·5 (34·7 to 40·4)†	2·7 (0·7 to 4·7)†
				Refraction and accommodation disorders	854 299 (822 109 to 885 545)	··	14 972 (9 341 to 23 362)	14·9 (13·7 to 15·9)†	–4·9 (−5·8 to −4·3)†
				Age-related and other hearing loss	1 271 675 (1 210 237 to 1 337 731)	··	36 288 (25 342 to 50 894)	22·3 (20·4 to 24·0)†	–1·7 (−2·9 to −0·6)†
				Other vision loss	29 249 (25 873 to 32 927)	··	2241 (1578 to 3016)	26·1 (23·9 to 28·1)†	0·7 (−0·8 to 2·0)
				Other sense organ diseases	210 697 (195 303 to 225 565)	708 004 (667 844 to 753 886)	6542 (4132 to 9828)	23·8 (23·1 to 24·4)†	0·9 (0·6 to 1·3)†
			Oral disorders		3 583 600 (3 475 812 to 3 657 885)	9 139 511 (8 388 756 to 9 926 992)*	19 006 (11 587 to 29 630)	22·2† (21·2 to 23·3)	–0·2 (−0·7 to 0·2)
				Caries of deciduous teeth	486 095 (398 110 to 573 474)	1 762 264 (1 264 051 to 2 391 119)	127 (56 to 249)	6·8 (4·5 to 8·5)†	0·2 (−1·9 to 1·8)
				Caries of permanent teeth	2 435 850 (2 288 064 to 2 586 461)	7 263 992 (6 715 107 to 7 835 458)	1708 (760 to 3325)	8·7 (8·0 to 9·4)†	–4·2 (−4·8 to −3·6)†
				Periodontal disease	750 847 (633 975 to 874 077)	89 840 (74 128 to 106 716)	4898 (1947 to 10 209)	25·8 (24·7 to 26·8)†	2·3 (1·8 to 2·9)†
				Edentulism and severe tooth loss	302 342 (263 801 to 339 035)	23 414 (19 894 to 27 222)	8338 (5467 to 11 760)	27·2 (26·0 to 28·4)†	–0·9 (−1·6 to −0·3)†
				Other oral disorders	134 457 (128 968 to 140 248)	··	3935 (2427 to 5908)	15·2 (14·6 to 15·8)†	0·0 (−0·3 to 0·3)
**Injuries**					**1 492 784 (1 413 602 to 1 584 568)**	619 651 (592 467 to 648 150)*	**55 358 (37 181 to 78 689)**	**22·1 (21·0 to 23·2)**†	**2·0 (1·2 to 2·6)**†
		**Transport injuries**			**241 194 (225 137 to 258 446)**	**75 419 (68 548 to 82 688)**	**12 345 (8227 to 17 658)**	**25·6 (24·5 to 26·6)**†	**4·1 (3·3 to 4·8)**†
			Road injuries		194 515 (182 106 to 208 390)	65 536 (58 946 to 72 433)	9983 (6650 to 14 255)	28·1 (27·2 to 29·0)†	5·5 (4·9 to 6·1)†
				Pedestrian road injuries	43 124 (38 450 to 48 872)	11 342 (9709 to 13 303)	2261 (1511 to 3222)	28·7 (27·1 to 30·1)†	5·8 (4·9 to 6·7)†
				Cyclist road injuries	38 983 (33 088 to 45 660)	15 288 (12 715 to 17 993)	1873 (1238 to 2710)	31·0 (30·1 to 31·8)†	8·1 (7·5 to 8·6)†
				Motorcyclist road injuries	46 084 (39 635 to 55 732)	11 346 (9534 to 13 518)	2336 (1555 to 3342)	29·5 (28·3 to 30·6)†	7·3 (6·5 to 8·0)†
				Motor vehicle road injuries	51 784 (43 776 to 59 354)	20 993 (18 156 to 23 790)	2821 (1895 to 3991)	20·9 (20·0 to 21·8)†	–1·0 (−1·8 to −0·2)†
				Other road injuries	14 539 (12 463 to 17 017)	6566 (5217 to 8267)	693 (457 to 1 014)	48·2 (46·5 to 49·7)†	22·9 (21·9 to 23·8)†
				Other transport injuries	46 680 (39 471 to 54 858)	9884 (8552 to 11 480)	2362 (1595 to 3372)	15·8 (14·2 to 17·5)†	–1·5 (−2·8 to −0·3)†
		**Unintentional injuries**			**1 127 878 (1 053 751 to 1 211 481)**	**510 685 (484 967 to 539 046)**	**37 696 (25 530 to 53 091)**	**22·9 (21·8 to 23·8)**†	**2·3 (1·5 to 2·9)**†
			Falls		474 230 (419 318 to 528 012)	208 272 (189 203 to 229 948)	18 946 (13 288 to 26 043)	26·7 (25·8 to 27·6)†	3·4 (2·6 to 4·1)†
			Drowning		4684 (4129 to 5324)	846 (763 to 938)	233 (153 to 336)	4·9 (2·9 to 6·9)†	–12·3 (−13·7 to −11·0)†
			Fire, heat, and hot substances		102 124 (87 477 to 121 846)	10 997 (9148 to 12 675)	2455 (1601 to 3666)	18·6† (17·0 to 19·9)	1·0 (−0·1 to 1·8)
			Poisonings		3526 (2984 to 4133)	4697 (3858 to 5639)	298 (192 to 437)	25·8 (24·5 to 27·2)†	8·9 (7·8 to 10·1)†
			Exposure to mechanical forces		197 786 (168 555 to 241 945)	67 906 (59 032 to 76 818)	4412 (2854 to 6440)	24·4 (23·2 to 25·5)†	3·9 (3·2 to 4·5)†
				Unintentional firearm injuries	7091 (5867 to 8629)	1429 (1052 to 1893)	228 (146 to 341)	23·5† (22·3 to 24·4)	4·0 (3·3 to 4·5)†
				Unintentional suffocation	9395 (7248 to 12 049)	1804 (1347 to 2370)	282 (174 to 438)	11·8 (10·3 to 13·5)†	–2·8 (−3·7 to −1·8)†
				Other exposure to mechanical forces	181 300 (153 213 to 224 295)	64 673 (56 323 to 73 039)	3 901 (2 536 to 5 696)	25·5 (24·2 to 26·6)†	4·5 (3·7 to 5·1)†
		Adverse effects of medical treatment			2924 (2328 to 3567)	38 245 (34 405 to 42 385)	390 (246 to 580)	11·8 (10·2 to 13·5)†	–4·4 (−6·0 to −2·8)†
		Animal contact			53 654 (47 673 to 61 282)	65 550 (56 597 to 74 579)	1667 (1126 to 2315)	11·0 (9·7 to 12·2)†	–4·0 (−4·9 to −3·2)†
			Venomous animal contact		24 759 (20 818 to 29 310)	31 691 (25 886 to 38 600)	1204 (813 to 1684)	11·7 (10·4 to 13·1)†	–2·4 (−3·5 to −1·3)†
			Non-venomous animal contact		28 894 (24 541 to 34 730)	33 859 (27 953 to 40 468)	463 (303 to 708)	9·1 (7·2 to 10·8)†	–7·9 (−9·4 to −6·7)†
		Foreign body			54 797 (45 419 to 63 613)	35 172 (30 752 to 40 749)	2040 (1308 to 3022)	16·6 (14·9 to 18·5)†	0·8 (−0·4 to 2·1)
			Pulmonary aspiration and foreign body in airway		26 682 (20 450 to 35 018)	10 801 (9 348 to 12 717)	1124 (719 to 1683)	12·4 (10·8 to 14·1)†	–2·1 (−3·3 to −0·9)†
			Foreign body in eyes		3168 (1880 to 4640)	15 443 (11 951 to 20 505)	186 (100 to 310)	26·8 (24·1 to 30·1)†	8·2 (6·3 to 10·3)†
			Foreign body in other body part		24 947 (20 403 to 31 426)	8929 (7508 to 10 562)	730 (468 to 1 083)	21·2 (19·5 to 23·1)†	3·8 (2·6 to 5·0)†
		Environmental heat and cold exposure			74 104 (63 212 to 86 149)	19 591 (16 473 to 23 142)	2756 (1829 to 3963)	18·9 (17·1 to 20·9)†	0·7 (−0·8 to 2·1)
		Other unintentional injuries			160 048 (131 378 to 191 818)	59 409 (51 226 to 68 804)	4500 (2972 to 6571)	20·5 (19·6 to 21·3)†	1·5 (0·7 to 2·3)†
	**Self-harm and interpersonal violence**				**230 043 (210 093 to 252 561)**	32 470 (28 739 to 36 265)*	**3884 (2672 to 5491)**	**9·3 (8·3 to 10·4)**†	**–5·8 (−6·3 to −5·4)**†
		Self-harm			11 924 (10 160 to 13 744)	6893 (6130 to 7759)	528 (342 to 764)	10·0 (8·9 to 11·2)†	–9·4 (−10·1 to −8·8)†
			Self-harm by firearm		380 (326 to 439)	98 (61 to 149)	13 (8 to 20)	3·6 (1·9 to 5·4)†	–12·5 (−13·8 to −11·2)†
			Self-harm by other specified means		11 544 (9770 to 13 356)	6795 (6037 to 7607)	515 (333 to 744)	10·2 (9·1 to 11·3)†	–9·3 (−10·1 to −8·7)†
		Interpersonal violence			218 118 (197 726 to 240 339)	25 577 (22 086 to 29 230)*	3356 (2325 to 4709)	9·2 (8·1 to 10·4)†	–5·2 (−5·7 to −4·8)†
			Assault by firearm		2899 (2388 to 3485)	710 (560 to 897)	104 (66 to 156)	14·9 (13·9 to 16·0)†	–3·6 (−4·3 to −2·9)†
			Assault by sharp object		15 012 (12 272 to 17 936)	5603 (4397 to 6946)	412 (269 to 601)	10·5 (9·0 to 12·0)†	–6·6 (−7·6 to −5·7)†
			Sexual violence		160 421 (141 228 to 181 452)	··	1366 (917 to 1946)	3·5 (2·2 to 4·8)†	–5·5 (−6·2 to −4·9)†
			Assault by other means		39 786 (33 335 to 48 036)	19 263 (16 510 to 22 244)	1474 (988 to 2129)	14·3 (13·4 to 15·1)†	–4·7 (−5·4 to −4·2)†
	**Forces of nature, conflict and terrorism, and executions and police conflict**				**54 091 (34 969 to 76 753)**	1077 (759 to 1328)*	**1433 (510 to 3006)**	**12·5 (5·0 to 18·2)**†	**–1·1 (−7·3 to 3·8)**
			Exposure to forces of nature		7571 (4293 to 11 602)	377 (231 to 534)	260 (71 to 610)	32·2 (17·0 to 39·5)†	16·4 (3·5 to 23·0)†
			Conflict and terrorism		40 684 (24 541 to 61 725)	··	1100 (432 to 2206)	10·8 (3·5 to 18·0)†	–2·4 (−8·5 to 3·9)
			Executions and police conflict		5836 (1906 to 9042)	700 (410 to 883)	131 (28 to 344)	–6·7 (−11·2 to −2·3)†	–20·0 (−23·6 to −16·3)†
**Impairments**					**··**	**··**	**··**	**··**	**··**
		Anaemia			2 042 728 (1 979 003 to 2 110 037)	··	61 649 (41 483 to 86 424)	9·2 (6·4 to 11·2)†	–2·2 (−4·8 to −0·3)†
		Developmental intellectual disability			196,243 (150 264 to 242 144)	··	19 925 (14 584 to 26 434)	8·3 (6·5 to 10·7)†	–1·8 (−3·4 to 0·4)
		Epilepsy			45 926 (39 942 to 54 564)	··	15 392 (11 594 to 20 379)	10·5 (2·5 to 19·1)†	–0·6 (−7·7 to 7·0)
		Guillain-Barré syndrome			65 (52 to 82)	··	19 (12 to 28)	16·7 (14·1 to 19·5)†	0·5 (−0·4 to 1·2)
		Hearing loss			1 390 482 (1 329 902 to 1 455 762)	··	42 630 (30 019 to 59 264)	18·8 (17·0 to 20·4)†	–2·5 (−3·6 to −1·5)†
		Heart failure			63 609 (56 944 to 71 388)	··	7783 (5857 to 9788)	30·2 (28·8 to 31·6)†	–0·6 (−1·6 to 0·3)
		Infertility			117 207 (94 952 to 145 048)	··	719 (310 to 1400)	18·7 (15·4 to 21·6)†	7·0 (4·3 to 9·4)†
		Pelvic inflammatory disease			4099 (3533 to 4740)	··	535 (360 to 743)	7·8 (5·5 to 10·1)†	–5·5 (−7·5 to −3·6)†
		Vision loss			1 009 472 (969 235 to 1 047 829)	··	28 748 (20 342 to 40 240)	20·4 (19·1 to 22·0)†	–1·3 (−2·4 to 0·8)

We did not calculate incidence for the nine impairments, certain neglected tropical diseases, and causes with only birth prevalence. Blank cells mean that no estimate is available. Cells with zeros indicate an estimate smaller than 500 as the table presents prevalence and incidence counts in thousands. To download the data in this table, please visit the Global Health Data Exchange (GHDx) at: http://ghdx.healthdata.org/node/311084.

* Incidence for aggregate causes represents the sum of the incidence of the causes below the aggregate.

† Percentage changes that are statistically significant.

‡ Incidence of HIV/AIDS represents new infections of HIV only and does not include new infections of tuberculosis in HIV positive cases.

§ Incidence estimates for stroke represent first-ever stroke only. YLDs=years lived with disability.

### Incidence

In 2016, the ten causes with the highest incidence were upper respiratory infections (17·78 billion, 15·85 billion to 19·90 billion), caries of permanent teeth (7·26 billion, 6·72 billion to 7·84 billion), diarrhoeal diseases (4·48 billion, 4·25 billion to 4·74 billion), fungal skin diseases (2·10 billion, 1·88 billion to 2·34 billion), caries of deciduous teeth (1·76 billion, 1·26 billion to 2·39 billion), tension-type headache (1·06 billion, 899 million to 1·24 billion), other sense organ diseases (708 million, 668 million to 754 million), other skin and subcutaneous diseases (634 million, 617 million to 652 million), vitamin A deficiency (552 million, 469 million to 671 million), and pyoderma (474 million, 461 million to 489 million; table). These ten causes of incidence accounted for 4·4% (3·7–5·6) of all YLDs globally in 2016.

### Overall global numbers of YLDs and trends 2006–2016

Globally, communicable, maternal, neonatal, and nutritional deficiency diseases accounted for 12·6% (95% UI 11·3–14·0) of YLDs in 2016 (101·5 million, 72·3 million to 136·9 million), while NCDs accounted for 80·6% (78·2–82·5) or 648·6 million (481·3 million to 836·5 million) of YLDs and injuries accounted for 6·9% (5·6–8·4) or 55·4 million (37·2 million to 78·7 million) of YLDs (table). In 2016, the age-standardised YLD rate for all causes was lowest in China at 9201 YLDs per 100 000 (95% UI 6862–11 943 per 100 000); Yemen had the highest age-standardised YLD rate at 14 774 YLDs per 100 000 (11 018–19 228 per 100 000).

**Figure 1:**
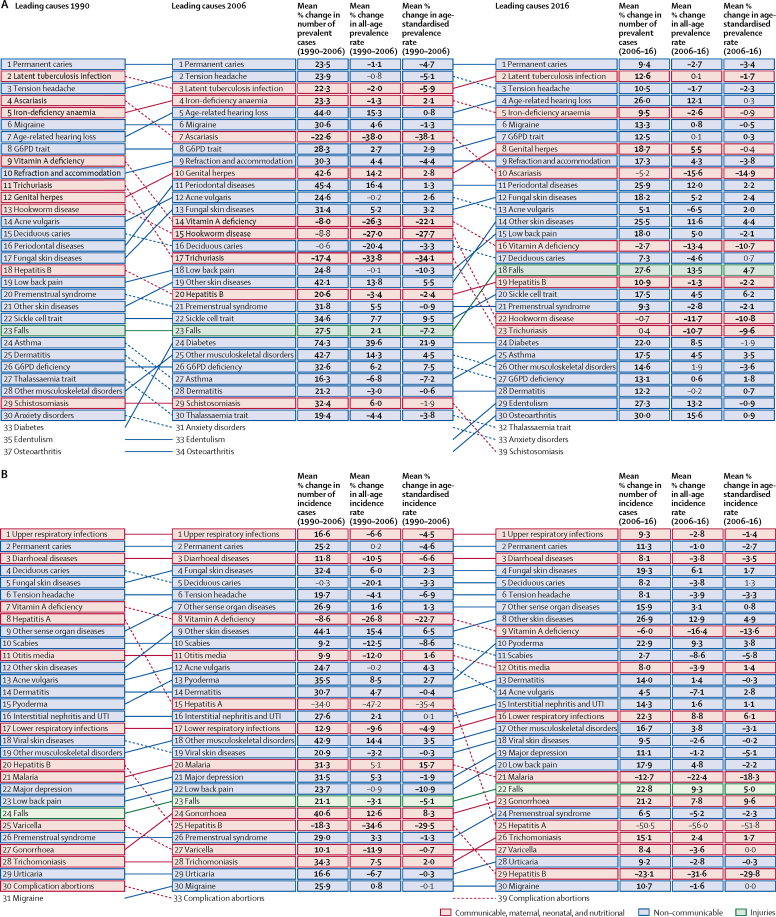

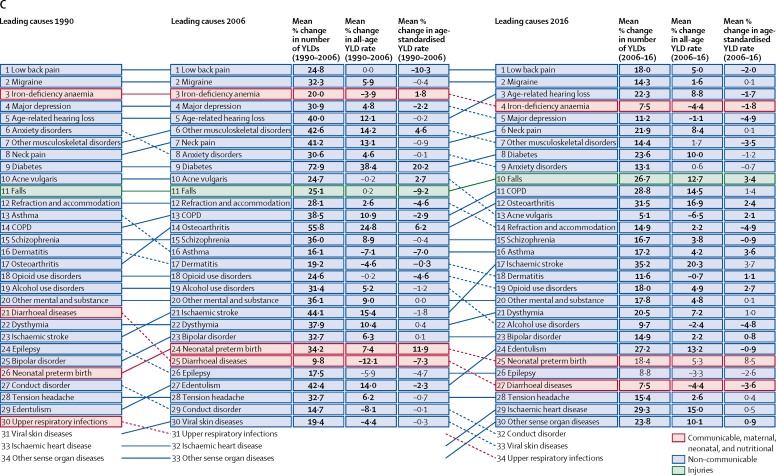
Leading 30 Level 4 causes of global prevalence (A), incidence (B), and YLDs (C), for 1990, 2006, and 2016, with mean percentage change of counts, and all-age and age-standardised rates Causes are connected by arrows between time periods; solid lines are increases and dashed lines are decreases. For the time period 1990–2006 and for 2006–16, three measures of change are shown: percentage change of counts, percentage change in the all-age rate, and percentage change in the age-standardised rate. Statistically significant changes are shown in bold. Solid lines are increases in rank and dashed lines are decreases in rank. YLDs=years lived with disability. G6PD=glucose-6-phosphate dehydrogenase deficiency. UTI=urinary tract infection. COPD=chronic obstructive pulmonary disease.

Figure 1C shows the change in global YLDs over time in three ways: mean percentage change in number of YLDs, mean percentage change in all-age rates, and mean percentage change in age-standardised YLD rates from 1990 to 2006 and from 2006 to 2016. The mean percentage change in the number of YLDs reflects the combined effects of population growth, population ageing, and epidemiological change. Population ageing and epidemiological change explain the mean percentage change in all-age rates. The mean percent change in age-standardised YLD rates reflects epidemiological change that is not due to ageing or population growth. All top-30 YLD causes increased in the number of YLDs between 1990 and 2016. Alcohol use disorder, major depressive disorder, and refraction and accommodation errors had the largest declines in age-standardised YLD rates from 2006 to 2016 but the change did not exceed 5%. Preterm birth complications had the largest increase in age-standardised YLD rates, 8·5%. Among the top 30 causes of YLDs, the difference between the change in all-age and age-standardised rates between 2006 and 2016 was 10% or greater for age-related and other hearing loss, diabetes, chronic obstructive pulmonary disease (COPD), osteoarthritis, ischaemic stroke, edentulism, and ischaemic heart disease, indicating that these diseases largely affect the elderly and therefore become more prominent causes of YLDs in an ageing global population.

Low back pain and migraine were the leading causes of YLDs in high-income, high-middle-income, and middle-SDI quintile countries, but iron-deficiency anaemia was the leading cause in low-middle-SDI and low-SDI quintiles in 2016 (appendix 2, p 14). Low back pain, migraine, and age-related and other hearing loss were in the top five causes of YLDs in all SDI quintiles. Major depressive disorders appeared in the top five in all quintiles but the middle, where it was displaced by diabetes. Neck pain was a top five cause of YLDs in high-income, high-middle-income, and middle-SDI quintiles. In 2016, 28 of the 30 leading causes of age-standardised YLD rates for high-SDI countries were NCDs, versus 23 out of 30 in low-SDI countries. Between 2006 and 2016, there were large drops in age-standardised rates of YLDs for malaria, HIV/AIDS, onchocerciasis, and schistosomiasis in the low-SDI quintile.

### Leading causes of YLDs by age-standardised rates

For men, the most common leading detailed cause of YLDs in 2016 was low back pain, resulting in the highest age-standardised rates of YLDs in 133 of 195 countries and territories, including every country in the high-income regions, central and eastern Europe, central Asia, Andean and Tropical Latin America, and eastern and central sub-Saharan Africa, as well as most countries in southeast Asia, north Africa and the Middle East, and western sub-Saharan Africa. Diabetes, the second most common leading cause of YLD rates for men, ranked first in 38 countries in Central Latin America, the Caribbean, Oceania, and North Africa and the Middle East. Iron-deficiency anaemia was the leading cause in India, Bhutan, Sudan, Yemen, and Mali. HIV/AIDS was the leading cause in South Africa, Lesotho, Swaziland, Namibia, and Botswana.

**Figure 2:**
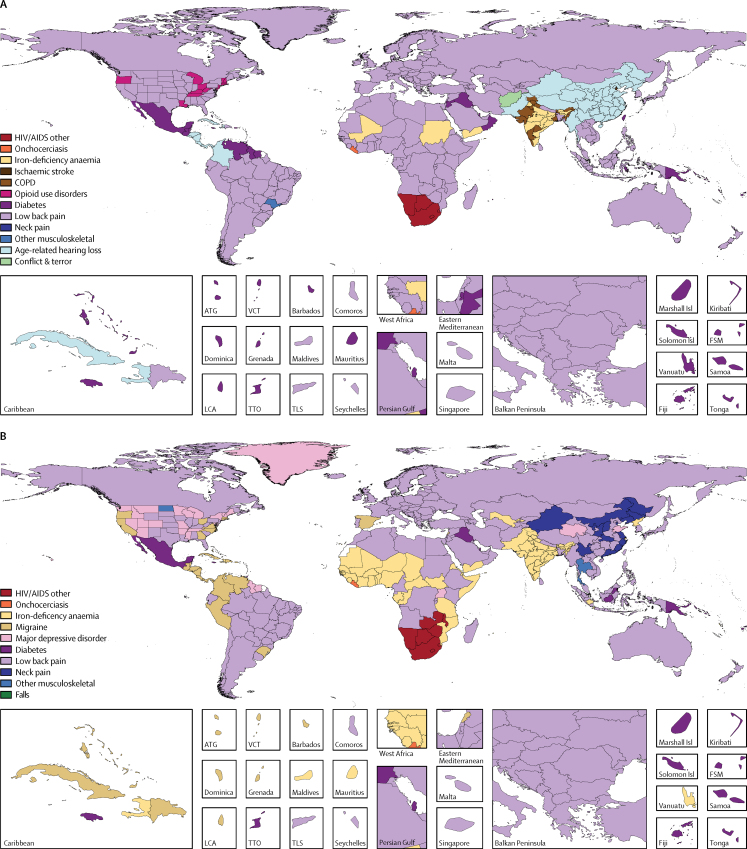
Leading Level 4 causes of age-standardised YLD rates by country for males (A) and females (B) in 2016 ATG=Antigua and Barbuda. COPD=chronic obstructive pulmonary disease. FSM=Federated States of Micronesia. LCA=Saint Lucia. Marshall Isl=Marshall Islands. Solomon Isl=Solomon Islands. TLS=Timor-Leste. TTO=Trinidad and Tobago. VCT=Saint Vincent and the Grenadines. YLDs=years lived with disability.

In 2016, low back pain was the leading cause of age-standardised YLD rates for women in 104 of the 195 countries and territories (figure 2B). It was the main cause of YLDs in almost all high-income, central Europe, eastern Europe, North Africa and the Middle East, and Andean and Tropical Latin American countries. Iron-deficiency anaemia was the leading cause for women (35 countries), followed by migraine and diabetes in 24 and 17 countries, respectively. HIV/AIDS was the leading cause in southern sub-Saharan Africa, Zambia, and Malawi.

### YLDs over time for countries classified into SDI quintiles in 2016

**Figure 3:**
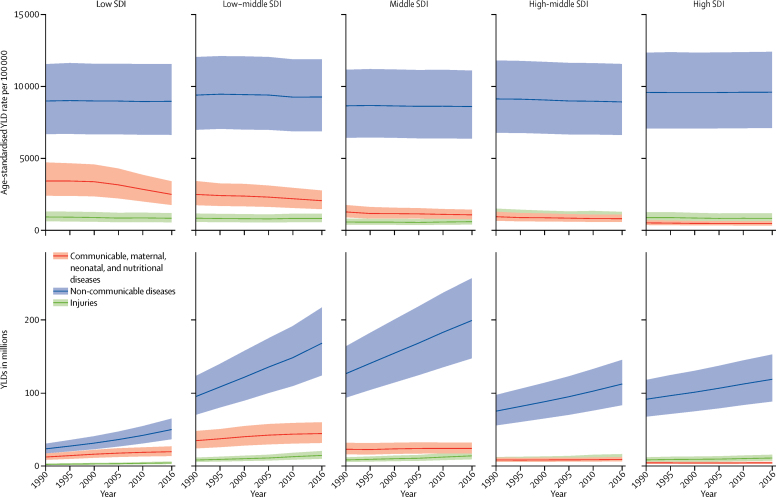
Trends of age-standardised YLD rates per 100 000 and total YLDs from 1990 to 2016, by GBD Level 1 causes, by SDI quintile SDI=Socio-demographic Index. YLDs=years lived with disability. Shaded areas show 95% uncertainty intervals.

Trends for the number of YLDs (in millions) and age-standardised YLD rates from 1990 to 2016 at Level 1 of the GBD cause hierarchy are shown by SDI quintile in figure 3. Generally, there was little or no change in the age-standardised rates of YLDs for NCDs, communicable, maternal, neonatal, and nutritional causes, or injuries, apart from a decrease in communicable, maternal, neonatal, and nutritional YLD rates between 2000 and 2016 in the low-SDI quintile. The large increase in total YLD numbers for NCDs reflects the combined effect of population growth, ageing, and epidemiological change. NCDs contributed the most YLDs at each SDI quintile, followed by communicable, maternal, neonatal, and nutritional causes and injuries, with exceptions for high and high-middle quintiles, where injuries contributed more YLDs than communicable, maternal, neonatal, and nutritional causes in 2016. This was particularly the case at the highest level of SDI, where YLDs from communicable, maternal, neonatal, and nutritional causes and injuries represented only a small fraction of overall YLDs.

### YLD pattern by age and sex

**Figure 4:**
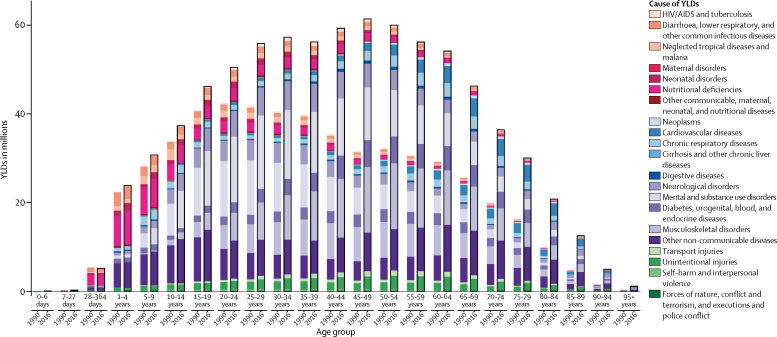
Global YLDs for 21 Level 2 causes by 23 GBD age groups for both sexes combined in 1990 and 2016 Composition of Level 2 causes of disability globally for males and females combined by age group showing difference in composition between 1990 and 2016. Number of total YLDs due to Level 2 causes is indicated by height of bar; causes are colour coded to highlight the relative number of total YLDs due to a specific cause. YLDs=years lived with disability.

Between 1990 and 2016 the number of YLDs for 21 Level 2 causes increased, particularly in the 40–69 year age range (figure 4). In childhood, other NCDs (skin diseases being the largest contributor in this category), nutritional deficiencies, infectious diseases, and mental and substance use disorders were the main causes of YLDs. After childhood, NCDs were the dominant source of YLDs. Mental and substance use disorders were the largest contributors to disability in young adults, while at older ages, other NCDs (with hearing loss and vision loss being the largest contributors to this category), musculoskeletal disorders, and cardiovascular diseases were the most important causes. YLDs from injuries were largest between ages 20 and 69 years.

**Figure 5:**
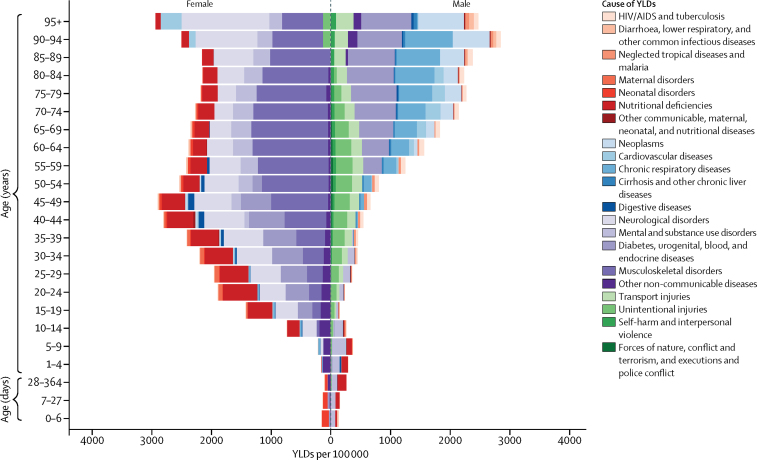
Sex difference in global YLD rates per 100 000 for 21 Level 2 causes by age, 2016 YLDs=years lived with disability.

YLD rates were higher in males than in females at ages under 10 years and between ages 75 and 94 years (figure 5). At all other ages, all-cause YLD rates were higher in females. Boys had higher YLD rates for mental disorders (particularly conduct disorder and autism), while girls had higher YLD rates for other non-communicable diseases (most of this difference is explained by higher YLD rates from dermatitis). At ages over 10 years, musculoskeletal disorders contributed most to higher YLD rates in females. The next two cause groups with higher YLD rates in females were neurological disorders (particularly migraine and, at older ages, Alzheimer's disease and other dementias) and nutritional deficiencies (mostly iron-deficiency anaemia). The sex differences in mental and substance use disorders were small, with slightly elevated rates in younger males, and somewhat higher rates in older females. The small overall sex difference masks much higher YLD rates from depressive disorder and anxiety in females and higher YLD rates from injuries, substance use disorders, and autism spectrum disorders in males. Adult males older than 55 years had higher YLD rates from diabetes, urogenital, blood, and endocrine disorders (diabetes and benign prostatic hyperplasia being the main drivers of differences), injuries, chronic respiratory diseases, and cancers.

### Rates of YLDs and change in rates of YLDs by cause

**Figure 6:**
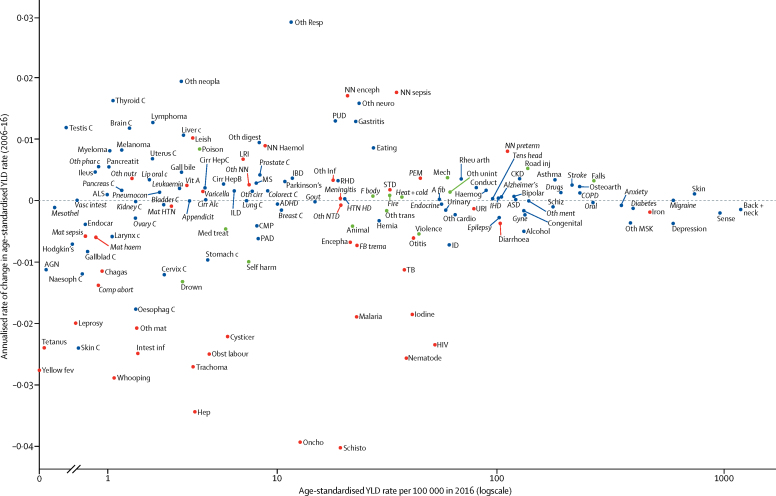
Relationship of global age-standardised YLD rates per 100 000 in 2016 and the annualised rate of change in age-standardised YLD rates for each Level 3 cause, 2006-2016 For both sexes combined. Age-standardized YLD rates are represented on a logarithmic scale on the x-axis. Causes for which the annualised rate of change is not significant are listed in italics. Level 3 causes related to shocks (forces of nature, conflict and terrorism, and executions and police conflict) are excluded. Not shown in figure (YLD, ARC): Diphtheria: (0.0003, −0.09), Measles: (0.3, −0.1), Afr Tryp: (0.03, −0.1), Echino: (1, −0.06), LF: (15.8, −0.06), Dengue: (13.2, 0.05), Rabies: (0.0009, −0.08), *Ebola:* (0.002, 0.3), Zika: (0.06, 0.6), GWD: (0.00001, −0.6). A Fib=Atrial fibrillation and flutter. ADHD=Attention-deficit/hyperactivity disorder. AGN=Acute glomerulonephritis. ALS=Motor neuron disease. ASD=Autistic spectrum disorders. Afr Tryp=African trypanosomiasis. Alcohol=Alcohol use disorders. Alzheimer's=Alzheimer's disease and other dementias. Animal=Animal contact. Anxiety=Anxiety disorders. Appendicit=Appendicitis. Asthma=Asthma. Back + neck=Low back and neck pain. Bipolar=Bipolar disorder. Bladder C=Bladder cancer. Brain C=Brain and nervous system cancer. Breast C=Breast cancer. CKD=Chronic kidney disease. CMP=Cardiomyopathy and myocarditis. COPD=Chronic obstructive pulmonary disease. Cervix C=Cervical cancer. Chagas=Chagas disease. Cirr Alc=Cirrhosis and other chronic liver diseases due to alcohol use. Cirr HepB=Cirrhosis and other chronic liver diseases due to hepatitis B. Cirr HepC=Cirrhosis and other chronic liver diseases due to hepatitis C. Colorect C=Colon and rectum cancer. Comp Abort=Maternal abortion, miscarriage, and ectopic pregnancy. Conduct=Conduct disorder. Congenital=Congenital birth defects. Cysticer=Cysticercosis. Dengue=Dengue. Depression=Depressive disorders. Diabetes=Diabetes mellitus. Diarrhoea=Diarrhoeal diseases. Diphtheria=Diphtheria. Drown=Drowning. Drugs=Drug use disorders. Eating=Eating disorders. Ebola=Ebola virus disease. Echino=Cystic echinococcosis. Encepha=Encephalitis. Endocar=Endocarditis. Endocrine=Endocrine, metabolic, blood, and immune disorders. Epilepsy=Epilepsy. F Body=Foreign body. FB Trema=Food-borne trematodiases. Falls=Falls. Fire=Fire, heat, and hot substances. GWD=Guinea worm disease. Gall Bile=Gallbladder and biliary diseases. Gallblad C=Gallbladder and biliary tract cancer. Gastritis=Gastritis and duodenitis. Gout=Gout. Gyne=Gynaecological diseases. HIV=HIV/AIDS. HTN HD=Hypertensive heart disease. Haemog=Haemoglobinopathies and haemolytic anaemias. Heat + cold=Environmental heat and cold exposure. Hep=Hepatitis. Hernia=Inguinal, femoral, and abdominal hernia. Hodgkin's=Hodgkin's lymphoma. IBD=Inflammatory bowel disease. ID=Idiopathic developmental intellectual disability. IHD=Ischaemic heart disease. ILD=Interstitial lung disease and pulmonary sarcoidosis. Ileus=Paralytic ileus and intestinal obstruction. Intest Inf=Intestinal infectious diseases. Iodine=Iodine deficiency. Iron=Iron-deficiency anaemia. Kidney C=Kidney cancer. LF=Lymphatic filariasis. LRI=Lower respiratory infections. Larynx C=Larynx cancer. Leish=Leishmaniasis. Leprosy=Leprosy. Leukaemia=Leukaemia. Lip Oral C=Lip and oral cavity cancer. Liver C=Liver cancer. Lung C=Tracheal, bronchus, and lung cancer. Lymphoma=Non-Hodgkin lymphoma. MS=Multiple sclerosis. Malaria=Malaria. Mat HTN=Maternal hypertensive disorders. Mat Haem=Maternal haemorrhage. Mat Sepsis=Maternal sepsis and other maternal infections. Measles=Measles. Mech=Exposure to mechanical forces. Med Treat=Adverse effects of medical treatment. Melanoma=Malignant skin melanoma. Meningitis=Meningitis. Mesothel=Mesothelioma. Migraine=Migraine. Myeloma=Multiple myeloma. NN Enceph=Neonatal encephalopathy due to birth asphyxia and trauma. NN Haemol=Haemolytic disease and other neonatal jaundice. NN Preterm=Neonatal preterm birth complications. NN Sepsis=Neonatal sepsis and other neonatal infections. Naesoph C=Naesopharynx cancer. Nematode=Intestinal nematode infections. Obst Labour=Maternal obstructed labour and uterine rupture. Oesophag C=Oesophageal cancer. Oncho=Onchocerciasis. Oral=Oral disorders. Osteoarth=Osteoarthritis. Oth Cardio=Other cardiovascular and circulatory diseases. Oth Cirr=Cirrhosis and other chronic liver diseases due to other causes. Oth Digest=Other digestive diseases. Oth Inf=Other infectious diseases. Oth MSK=Other musculoskeletal disorders. Oth Mat=Other maternal disorders. Oth Ment=Other mental and substance use disorders. Oth NN=Other neonatal disorders. Oth NTD=Other neglected tropical diseases. Oth Neopla=Other neoplasms. Oth Neuro=Other neurological disorders. Oth Nutr=Other nutritional deficiencies. Oth Phar C=Other pharynx cancer. Oth Resp=Other chronic respiratory diseases. Oth Trans=Other transport injuries. Oth Unint=Other unintentional injuries. Otitis=Otitis media. Ovary C=Ovarian cancer. PAD=Peripheral artery disease. PEM=Protein-energy malnutrition. PUD=Peptic ulcer disease. Pancreas C=Pancreatic cancer. Pancreatit=Pancreatitis. Parkinson's=Parkinson's disease. Pneumocon=Pneumoconiosis. Poison=Poisonings. Prostate C=Prostate cancer. RHD=Rheumatic heart disease. Rabies=Rabies. Rheu Arth=Rheumatoid arthritis. Road Inj=Road injuries. STD=Sexually transmitted diseases excluding HIV. Schisto=Schistosomiasis. Schiz=Schizophrenia. Self Harm=Self-harm. Sense=Sense organ diseases. Skin=Skin and subcutaneous diseases. Skin C=Non-melanoma skin cancer. Stomach C=Stomach cancer. Stroke=Cerebrovascular disease. TB=Tuberculosis. Tens Head=Tension-type headache. Testis C=Testicular cancer. Tetanus=Tetanus. Thyroid C=Thyroid cancer. Trachoma=Trachoma. URI=Upper respiratory infections. Urinary=Urinary diseases and male infertility. Uterus C=Uterine cancer. Varicella=Varicella and herpes zoster. Vasc Intest=Vascular intestinal disorders. Violence=Interpersonal violence. Vit A=Vitamin A deficiency. Whooping=Whooping cough. Yellow Fev=Yellow fever. YLD=years lived with disability. Zika=Zika virus disease.

Figure 6 shows the annualised rates of change in age-standardised YLD rates between 2006 and 2016, with Level 3 causes against age-standardised YLD rates presented on a log-scale from right to left. 15 causes significantly increased by more than 1%: six cancers (brain and nervous system, liver, non-Hodgkin lymphoma, testicular, thyroid, and other neoplasms), dengue, gastritis and duodenitis, leishmaniasis, neonatal encephalopathy due to birth asphyxia and trauma, neonatal sepsis and other neonatal infections, other chronic respiratory diseases, other neurological disorders, peptic ulcer disease, and Zika virus disease. Of 31 causes with declines of greater than 1·0% per year, 25 were communicable, maternal, neonatal, and nutritional causes and five were NCD causes. Road injuries was the only cause out of 30 causes with age-standardised rates of YLDs greater than 100 per 100 000 that significantly increased by more than 0·5% annually. There were no large causes with a greater than 0·5% decrease in age-standardised YLD rates.

### Leading causes of YLDs and deviations from expected levels based on SDI

Age-standardised YLD rates for the top ten most detailed causes are plotted by region and year against SDI in the supplementary results (appendix 2, p 4). The black solid line represents the expected value based on SDI. Two of the top ten causes, iron-deficiency anaemia and age-related and other hearing loss show a large decline in expected values with increasing SDI, while the regional estimates are relatively close to the expected line. The interpretation is that much of the variation in YLD rates for these two causes is linked to SDI. The expected lines for the other top ten conditions show a less clear pattern with SDI while regional estimates are dispersed widely around the line. These graphs show that most of the variation in YLD rates for these leading causes of YLDs depends on factors other than sociodemographic development, unlike the much clearer relationship between SDI and rates of YLLs for the leading causes of death.^18^

**Figure 7:**
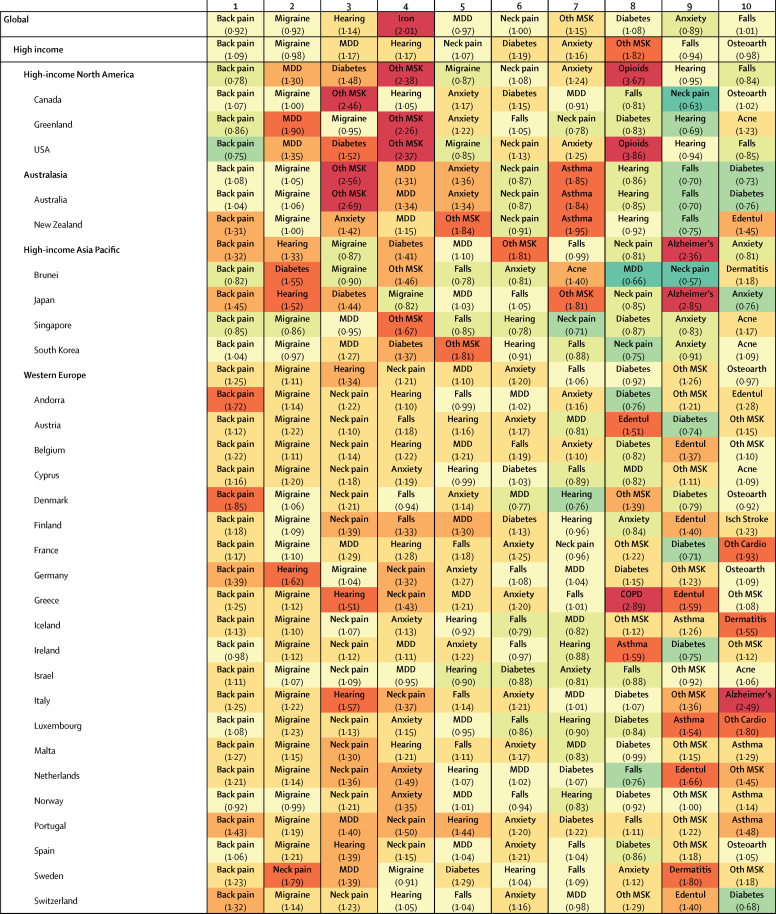

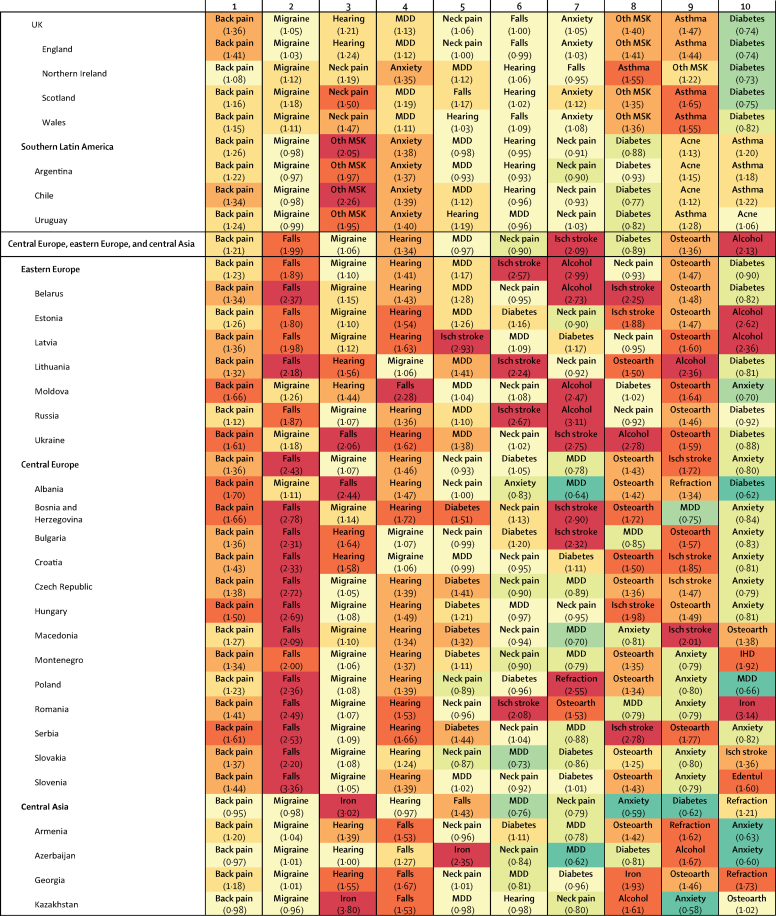

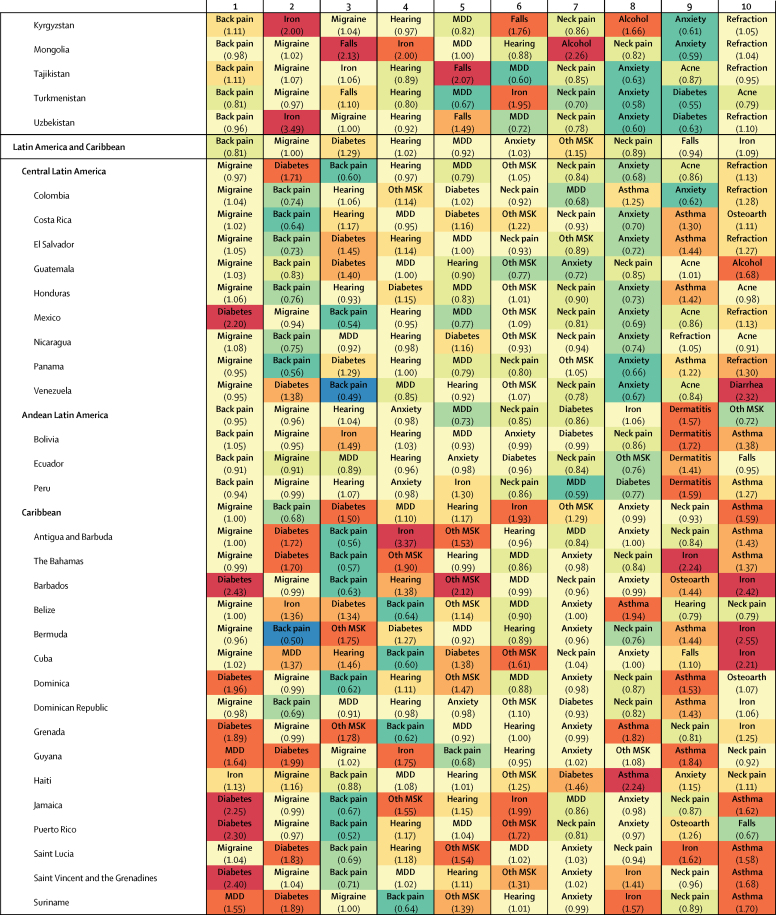

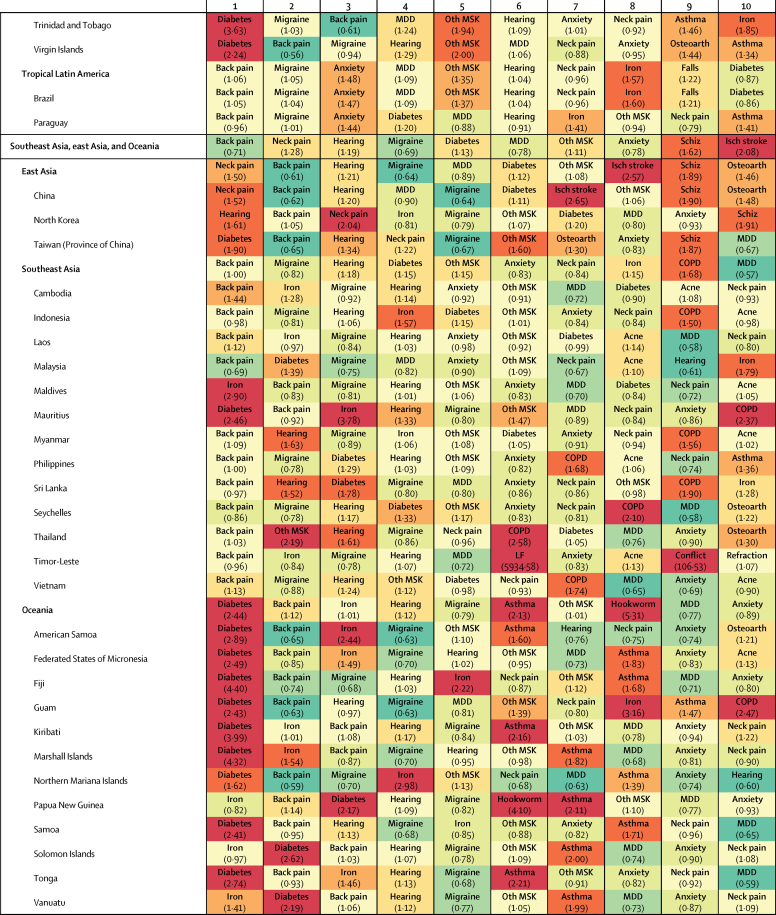

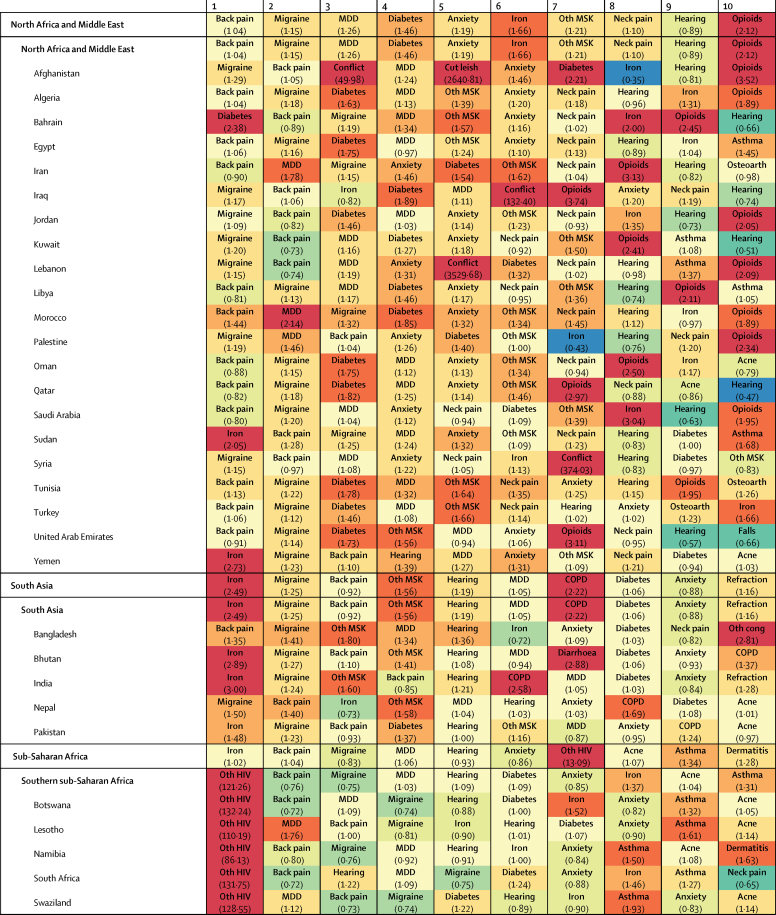

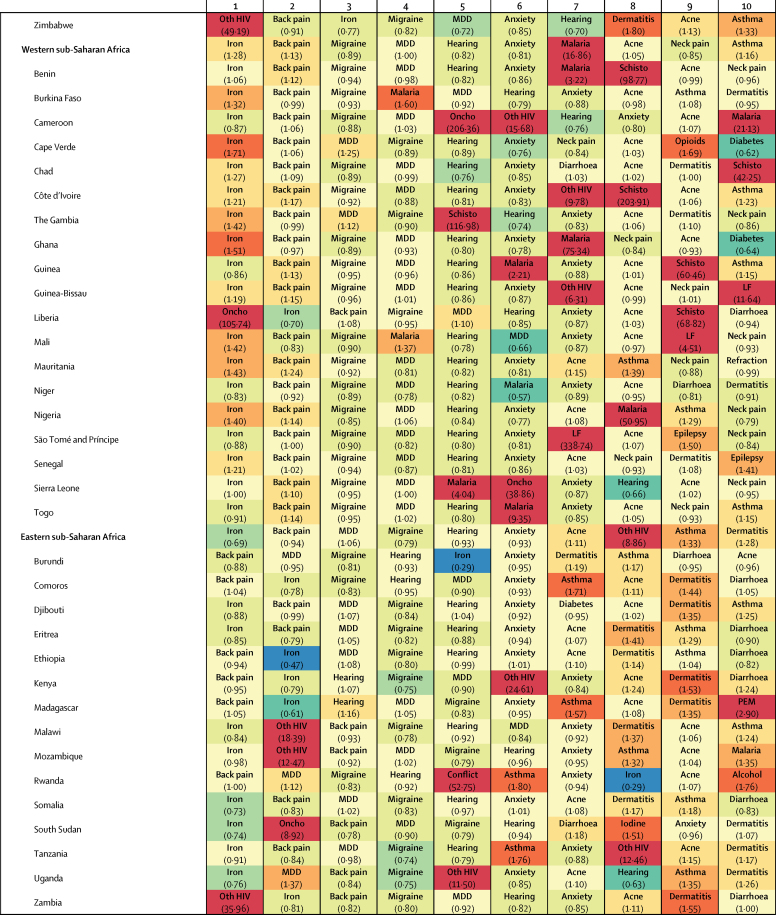

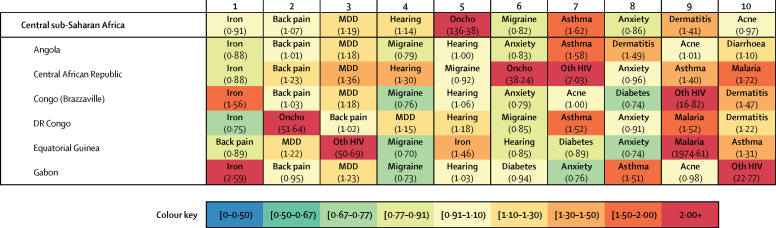
Leading ten causes of YLDs with ratio of observed YLDs to YLDs expected on the basis of SDI in 2016, by location Values shown in brackets represent the ratio of observed YLDs to predicted YLDs on the basis of SDI, rounded to two digits. Acne=Acne vulgaris. Alcohol=Alcohol use disorders. Alzheimer's=Alzheimer's disease and other dementias. Anxiety=Anxiety disorders. Asthma=Asthma. Back pain=Low back pain. COPD=Chronic obstructive pulmonary disease. Conflict=Conflict and terrorism. Cut Leish=Cutaneous and mucocutaneous leishmaniasis. Dermatitis=Dermatitis. Diabetes=Diabetes mellitus. Diarrhoea=Diarrhoeal diseases. Edentul=Edentulism and severe tooth loss. Epilepsy=Epilepsy. Falls=Falls. Hearing=Age-related and other hearing loss. Hookworm=Hookworm disease. IHD=Ischaemic heart disease. Iodine=Iodine deficiency. Iron=Iron-deficiency anaemia. Isch Stroke=Ischaemic stroke. LF=Lymphatic filariasis. MDD=Major depressive disorder. Malaria=Malaria. Migraine=Migraine. Neck Pain=Neck pain. Oncho=Onchocerciasis. Opioids=Opioid use disorders. Osteoarth=Osteoarthritis. Oth Cardio=Other cardiovascular and circulatory diseases. Oth Cong=Other congenital anomalies. Oth HIV=HIV/AIDS resulting in other diseases. Oth MSK=Other musculoskeletal disorders. PEM=Protein-energy malnutrition. Refraction=Refraction and accommodation disorders. SDI=Socio-demographic Index. Schisto=Schistosomiasis. Schiz=Schizophrenia. YLD=Years lived with disability.

Low back pain and migraine ranked in the top ten of YLDs in all 195 countries and territories in 2016. Adult-onset hearing loss was in the top ten in 193 countries and territories. Major depressive disorder ranked in the top ten in all but four countries. Anxiety disorders, iron-deficiency anaemia, neck pain, diabetes, and other musculoskeletal disorders ranked in the top ten of more than half of the countries and territories (figure 7).

Apart from showing the top ten causes of YLDs by country, figure 7 also shows for each top ten cause the variation from expected values based on SDI. The cells in dark red have at least twice the expected values and those in dark blue at least 50% lower values. In the high-income super-region, the USA had much higher than expected YLDs from opioid dependence. YLDs due to Alzheimer's disease and other dementia were more than double what was expected in Italy and Japan. Greece had much higher than expected rates of COPD. YLDs from other musculoskeletal disorders were more than twice the expected values in Australia, Canada, Chile, Greenland, and the USA. Many eastern and central European countries had much higher than expected YLDs from falls, alcohol use disorders, and ischaemic stroke. Four countries in central Asia had more than twice the expected rates of iron-deficiency anaemia. In Latin America, YLDs from diabetes were more than twice the expected rate in Barbados, Jamaica, Mexico, Puerto Rico, Saint Vincent and the Grenadines, Trinidad and Tobago, and the Virgin Islands. Venezuela had less than half the expected rate of YLDs from low back pain but more than double the expected rate of YLDs from diarrhoea. Haiti had higher than expected YLDs from asthma. YLDs from iron-deficiency anaemia were more than double the expected rate in five Caribbean nations. China had more than twice the expected YLD rate of ischaemic stroke, while North Korea had more than double the expected YLD rate for neck pain. In the southeast Asia region, YLD rates of iron-deficiency anaemia were more than twice the expected values in Maldives and Mauritius. Mauritius also had more than twice the expected YLD rates for diabetes and COPD. Thailand and the Seychelles had more than twice the expected rate of COPD. Timor-Leste was the only country in the region where lymphatic filariasis and long-term disability from conflict ranked among the top ten causes of YLDs. Diabetes ranked highly in all island nations of Oceania with much higher YLDs than expected based on SDI. In North Africa and the Middle East, more than twice the expected YLD rates from opioid dependence were found in Afghanistan, Bahrain, Iran, Iraq, Jordan, Kuwait, Lebanon, Libya, Palestine, Oman, Qatar, and the United Arab Emirates. Afghanistan and Bahrain had more than twice the expected YLD rates of diabetes. Long-term disability from injuries sustained during conflict ranked in the top ten for Afghanistan, Lebanon, Syria, and Iraq.

Iron-deficiency anaemia was much higher than expected in India and Bhutan. India had considerably higher than expected YLDs from COPD. Bangladesh was the only country in 2016 with YLDs from the residual category of other congenital anomalies in the top ten. Bhutan had more than double the expected YLDs from diarrhoea. HIV/AIDS was the leading cause of YLDs in southern sub-Saharan Africa. Malaria ranked in the top ten for YLDs in 14 sub-Saharan African countries. Three neglected tropical diseases, schistosomiasis, lymphatic filariasis, and onchocerciasis, ranked in the top ten of YLDs in six, three, and seven sub-Saharan African countries, respectively. In Madagascar, protein-energy malnutrition ranked in the top ten. Epilepsy was a top ten cause of YLDs in Senegal and São Tomé and Príncipe. Much lower than expected YLDs from iron-deficiency anaemia were found in Burundi, Ethiopia, and Rwanda. The long-term consequences of injuries sustained during the 1994 genocide in Rwanda still ranked among the top ten causes of YLDs in 2016.

## Discussion

### Summary of main findings

The largest Level 2 disease groups contributing to non-fatal burden in 2016 were mental and substance use disorders (18·7%, 95% UI 15·9–21·0), other NCDs (18·6%, 15·6–22·5), and musculoskeletal disorders (17·1%, 15·3–18·9), together covering more than half of global YLDs. Despite mostly stagnant age-standardised rates, the number of YLDs from NCDs grew rapidly in all the SDI quintiles, due to population growth and ageing. Across all causes of YLDs, age-standardised rates of YLDs decreased between 1990 and 2016 by 2·7% (95% UI 2·3–3·1) in contrast to the 39·1% (37·8–40·2) decrease in YLL rates over the same time period.^18^ Thus, the relative contribution of YLD to the overall burden of disease in DALYs increased from 21·7% (17·2–26·6) in 1990 to 33·5% (27·4–39·7) in 2016.

Age-standardised YLD rates for all conditions combined were 10·4% (9·0–11·8) higher in women compared to men. Iron-deficiency anaemia, migraine, Alzheimer's disease and other dementias, major depressive disorder, anxiety, and all musculoskeletal disorders apart from gout were the main conditions contributing to higher YLD rates in women. Men had higher age-standardised rates of substance use disorders, diabetes, cardiovascular diseases, cancers, and all injuries apart from sexual violence.

Globally, we found much less geographical variation in disability than has been documented for premature mortality. In 2016, there was a less than two-fold difference in age-standardised YLD rates for all causes between the location with the lowest (China, 9201 YLDs per 100 000, 95% UI 6862–11 943 per 100 000) and highest rates (Yemen, 14 774 YLDs per 100 000, 11 018–19 228 per 100 000). By contrast, there was a greater than tenfold range in age-standardised rates of YLLs between countries in GBD 2016.^18^ Two other countries with mainly Chinese populations, Singapore and Taiwan (Province of China), were ranked second and fourth lowest in terms of age-standardised YLD rates, respectively. The low YLD rates in China were largely determined by much lower prevalence of headaches, musculoskeletal disorders (in particular low back pain), major depressive disorder, iron-deficiency anaemia, falls, and anxiety. Low rates of reporting chronic pain and low prevalence of depressive disorder and anxiety in China and among Chinese immigrants elsewhere have frequently been reported, but these findings may partly be explained by standard diagnostic tools inadequately detecting cases in the Chinese and cultural differences in the perception and communication of pain.^25^, ^26^, ^27^, ^28^, ^29^

Trends across the causes of YLD were also different. For example, age-standardised YLD rates for NCDs were stagnant between 1990 and 2016 and showed little variation by SDI quintile; YLD rates for communicable, maternal, neonatal, and nutritional diseases were highest at lower SDI quintiles but with a steady drop; and injury YLD rates showed modest declines since 1990. Over the same period, age-standardised YLL rates of communicable, maternal, neonatal, and nutritional diseases rapidly declined, particularly in lower SDI quintiles. The decline in YLL rates from NCDs was slower than the decline in communicable, maternal, neonatal, and nutritional diseases but still substantial. The main causes of YLLs and YLDs were also notably different. Of the top 30 causes of YLLs, only seven appeared in the top 30 causes of YLDs (diabetes, falls, COPD, ischaemic stroke, neonatal preterm birth complication, diarrhoeal diseases, and ischaemic heart disease). At Level 4, 17 of the top 30 causes of YLDs were diseases for which we estimated no YLLs as they are not considered underlying causes of death.

### Cross-cutting themes

The availability and quality of epidemiological data the GBD Study draws upon to make non-fatal estimates vary enormously among diseases and by location. A multitude of study methods and preferred case definitions pose challenges to making comparable estimates. A large part of the effort in the GBD study in making non-fatal estimates is to identify and correct for known sources of measurement error. The main classes of data sources each have their own limitations. Surveys can be biased by low response rates or by exclusion of individuals who do not reside in a traditional household, and may be limited in the capacity to properly identify cases within the limited diagnostic means available during a survey. Administrative data on health service encounters include only those seeking and receiving health care and are likely biased toward more severe disease, but may offer more accurate diagnostic variables than available in surveys. With the development of electronic medical records and advancements in linkage between data sources, non-fatal population health assessments can benefit tremendously if survey and administrative records can be linked. This would help to overcome many of the limitations of individual data sources: administrative records can provide rich diagnostic information, while surveys can provide better information on cases that are not in contact with health services, exposure to risk factors, and health status measures that can provide information on severity. The challenges in low-income and middle-income countries will be to fund the data systems and infrastructure and development of technical expertise, and to provide access to generic rather than proprietary software to manage electronic medical record data and produce the linkages while preserving confidentiality.

In the GBD 2016 companion paper on causes of death^18^ we reported a faster decline in age-standardised YLL rates for the top ten causes of YLL (ischaemic heart disease, cerebrovascular disease, lower respiratory infections, diarrhoeal diseases, road injuries, neonatal preterm birth complications, malaria, COPD, HIV/AIDS, and neonatal encephalopathy) than for the rest of causes, globally and in each of the SDI quintiles. We hypothesised that a greater investment in large causes has led to greater improvements. We did not observe an equivalent rapid decline in the age-standardised YLD rates for these top ten causes of YLLs. YLD rates for ischaemic heart disease, stroke, LRI, COPD, and road injuries increased by less than 10% over the last ten years. YLDs for diarrhoeal diseases declined by less than 10%. The only top ten YLL causes with a greater decline in YLD rates were HIV/AIDS and malaria, though at a lesser pace than the change in YLL rates. Antiretroviral treatment greatly reduces the death rate in persons living with HIV but also improves the immune status of survivors. Thus, while there was a small increase in prevalence, the age-standardised YLD rate dropped by almost a fifth since 2006 as a larger proportion of cases were estimated to be in higher CD4 categories, for which we apply a lower disability weight. YLD rates from preterm birth complications and neonatal encephalopathy increased, while YLL rates dropped by almost a quarter. Improved survival through neonatal intensive care interventions predisposes survivors to the risk of long-term disabling outcomes. In terms of how we measure the burden of disease in DALYs, this means that large gains by prevention of YLLs can be accompanied by a smaller amount of health loss in terms of YLDs in new survivors. Similarly, health interventions that address mortality by reducing the case fatality among causes of disease or injury may in turn lead to an increase in non-fatal outcomes. For example, reducing cardiovascular risks in people with diabetes will increase the prevalence of those with sequelae such as neuropathy or vision loss. Similarly, preventing death from a myocardial infarction by revascularisation improves survival and thus exposes more people to heart failure as a complication.

The MDGs focused health policy on predominant infectious, neonatal, and maternal causes of death. The health-related SDGs have expanded into a number of non-communicable diseases and injuries. However, the focus remains on reducing mortality rather than the main causes of disability. The only goals that correspond with non-fatal outcomes are those related to substance use (goal 3.5) and physical and sexual violence (goals 5.2, 11.7, 16.1, and 16.2). Furthermore, the targets formulated on incidence of HIV, tuberculosis, malaria, and hepatitis were included because of the large number of deaths associated with these infectious diseases rather than presumably out of concern for their non-fatal outcomes.^1^ Diabetes and opioid use disorder are the only causes in the top 20 of age-standardised YLD rates at Level 4, globally, that are mentioned in the SDGs. The diabetes target, however, is formulated as an indicator of mortality only. The mental, musculoskeletal, sense organ, and neurological disorders that contribute to more than half of all YLDs are not considered. While this may partially be a bias toward assessing global health in terms of mortality, it may also reflect a perception that there is inadequate knowledge to address these major causes of disability. Indeed, the much smaller gap in YLD rates compared to the large differences in YLL rates between high-SDI and low-SDI countries suggests that the capacity to intervene in non-fatal outcomes is more limited.

Several threats exist that could lead to reversals in global health gains, such as widespread antimicrobial resistance,^30^, ^31^ conflict,^32^ climate change,^33^, ^34^ and obesity.^35^ Of these, obesity has the more immediately apparent health consequences and has a large effect on non-fatal outcomes such as diabetes, low back pain, and osteoarthritis. The increase in diabetes incidence due to obesity and the improved survival in people with diabetes, largely through prevention of deaths from cardiovascular complications, both contribute to increases in prevalence and a need for intensive long-term management to prevent fatal and disabling complications. Recent conflicts such as those in Libya, South Sudan, Syria, and Yemen are major health threats not only in terms of casualties but also because they lead to long-term physical and mental consequences directly related to the violence as well as the broader health consequences of disrupted health services and declining economic status.^36^

### Important changes in GBD 2016 compared with GBD 2015

The intensive collaborations with countries for which we present subnational estimates and the expanding network of GBD collaborators have contributed to a 36·2% increase in data sources for non-fatal outcome estimates compared to GBD 2015, as well as greater scrutiny of our methods and results. In particular, the close collaboration with the Indian Council for Medical Research and the Public Health Foundation of India, and 14 disease expert groups convened by these partners, has greatly enhanced the quality of the estimates for India. There was a 49·9% increase in non-fatal data sources in India, and disease experts provided access to state-level data for causes, while in GBD 2015 we had only access to national aggregates.

Increasingly, the GBD Study is making use of administrative data based on health care encounters. Public access to these data sources at the level of detail required for GBD analyses remains limited, and enormous scope remains to expand access to these valuable sources of health information in many more countries. By estimating total admission rates from both administrative and survey sources, we were able to increase the number of countries with available inpatient data from 26 in 2015 to 41 in 2016. To predict the level of disease under any type of care for other countries with inpatient data available, we applied the ratio of people with a diagnosis during inpatient and outpatient episodes to that of inpatient diagnoses only in US medical claims data. In our analyses for most conditions, the administrative data sources were adjusted to the level of a reference case set for the disease of interest. Thus, we make use of the detailed information on age and sex in these data sources while adjusting for a potential systematic bias compared to data sources with measurements according to the reference case. For instance, our reference case for diabetes prevalence was the proportion of a population with fasting plasma glucose greater than 7 mmol/L or on diabetes treatment in a representative survey. The adjustment of administrative data reflects the proportion of people with diabetes who may be unaware of their condition or who for other reasons have not contacted health services in the year of interest.

To estimate indicators of sexual violence for the SDGs, we added sexual violence as a subcause of interpersonal violence in the GBD hierarchy. The focus of the effort in GBD 2016 has been to estimate the prevalence of people who report sexual violence by intimate partners and other perpetrators in the last 12 months. For non-fatal outcomes, we quantified the immediate concurrent physical injuries and the more immediate mental health consequences. We realise this is a limited scope for estimating all relevant health loss from sexual violence. In future iterations of GBD we intend to include sexual violence as a risk factor for longer-term outcomes such as major depressive disorder, anxiety disorder, and substance use disorders. Going forward, we aim to become more comprehensive in our estimation of violence to include the wider health consequences of physical violence to children and adults as well as bullying.

Although efforts were undertaken to update data for all causes of YLDs in GBD 2016, a number of sources of new data were especially influential in improving estimates for selected causes. A large number of rapid-assessment vision loss surveys^37^ contributed to an increase in the number of sources to measure vision loss and its underlying causes from 397 in 2015 to 768 in 2016, with data available in 75 low-SDI and middle-SDI countries. Likewise, the Global Atlas for Helminth Infections Project provided a large amount of new data for schistosomiasis and lymphatic filariasis.^38^

A new approach to fitting ensembles of distributions improved the estimation of anaemia by better representing the tail end of the distributions of haemoglobin that most concerns our estimation of disability than can be derived from a single parametric distribution. This is important because for many surveys for which we rely on academic papers and survey reports we only have data for means and standard deviations.

The change in modelling strategy for tuberculosis was substantial. The new estimates of the prevalence of latent tuberculosis infection is of policy interest, as there are intervention strategies that target treatment of latent tuberculosis in high-risk individuals.^39^ It allowed us to model active disease among those with latent infection to improve the search for a consistent fit between cause of death rates, prevalence surveys, and notification of incident disease, the three main sources of epidemiological data on tuberculosis. Next, we estimated the association between SDI and the ratio of mortality to incidence, assuming this measure of case fatality is dependent on development as a proxy for quality of case finding and management. This provided greater alignment of mortality and non-fatal estimates of tuberculosis than we have been able to achieve in past GBD studies.

A substantial new effort on the severity of stroke was the analysis of 18 studies providing Rankin score data on the severity of disability in stroke survivors. Thus, we were able to make estimates by age, sex, year, and location to capture greater nuances in outcomes – for example, reflecting differences in access to rehabilitative services.

### Disease-specific considerations

The GBD study is the only source of comprehensive quantification of the disabling outcomes of diseases and injuries. For a number of diseases there are efforts to estimate global disease prevalence or incidence. Where appropriate, in the following sections on major sources of YLDs, comparisons with other global estimates are made; further detail can also be found in the supplementary methods (appendix 1, p 30).

### Mental and substance use disorders

GBD 2016 confirms that mental and substance use disorders, led by depressive disorders, are a major cause of non-fatal burden. With age-standardised prevalence and YLD rates for mental and substance use disorders showing less than 10% change between 1990 and 2016, apart from bulimia YLD rates which increased by almost 20%, our findings show no sizeable improvement in population mental health over time at the global level. Interventions can decrease the severity or increase remission for certain disorders and can be effectively administered in low-income, middle-income, and high-income settings.^40^ However, these have yet to be brought up to scale in most countries. Because there are no data measuring the severity of mental disorders in a consistent manner, we have been unable to capture treatment effects that alter severity of disease. By imposing severity distributions from two high-income countries only, we are probably underestimating the burden of mental disorders in countries with less access to quality care.

### Musculoskeletal disorders

Low back pain and neck pain are the two largest causes of musculoskeletal disability; their measurement is fully dependent on self-report measures. In GBD, we adjust for variations in recall period, anatomical location, minimum duration of episodes, and whether activity-limiting or not. Although less than 40% of low back pain can be attributed to occupational risks or increased BMI, there are no risks in GBD that are linked to neck pain. The lack of predictive covariates raises concern that we might be assigning measurement error as spatial variation and that the two to two-and-a-half-times difference in age-standardised prevalence between locations is overestimating true differences in disease occurrence of these two conditions.

### Diabetes

Several factors have contributed to the increase in the prevalence of diabetes. Ageing of populations along with greater exposure to lifestyle-related risk factors, most importantly high BMI, has increased the incidence of diabetes in almost all countries.^41^ At the same time, improvements in treatment of diabetes have increased the life expectancy among people with diabetes. The large increase in prevalence imposes a substantial economic burden on health-care systems. In the USA, diabetes was responsible for the largest health-care spending and the greatest increase over the past two decades among 155 health conditions.^42^ This highlights the importance of development and implementation of more effective population-level strategies to prevent diabetes.

### Dementia

Age-standardised prevalence of dementia varied four-times between countries. Dementia surveys generally have a two-step approach to identifying cases, with an initial screening phase followed by more intensive diagnostic procedures in those who screened positive. We scrutinised all sources of dementia and identified a wide range of screening and diagnostic tools, varying thresholds on these methods, and diagnoses made according to different classification systems. This made it impossible to identify a reference study method or case definition. Thus, it is likely that we are overestimating the variation in prevalence.

### Headaches

We changed our strategy to include medication overuse headache as a sequela of migraine and tension-type headache. This led to an increase in their prevalence and YLD estimates, particularly for migraine as more than two-thirds of medication overuse headache occurs in people with migraine as the primary headache. Thus, migraine has become the second largest cause of disability in 2016. As limited surveys are available that report on the frequency and duration of headache episodes, we have not been able to quantify any effect of treatment.

### Malaria

Globally, malaria case incidence trends estimated in GBD 2016 followed a very similar pattern to GBD 2015, where there was a gradual rise in cases until 2005 followed by a steady decline. For all years, however, the updated estimates are approximately one-quarter lower than those from GBD 2015, reflecting mainly lower estimates outside Africa and in particular for India. Outside Africa, and for lower-burden countries within Africa, 2016 estimates were produced using a spatiotemporal geostatistical model. Globally, this improved approach led to a reduction in estimated cases of around 75 million (about 26% reduction) of which most (roughly 60 million fewer cases) were in India, with other notable reductions in Myanmar (roughly 11 million fewer), Indonesia (roughly 3 million fewer), and Pakistan (roughly 1 million fewer). In high-burden countries in sub-Saharan Africa, where the methodology remained similar to GBD 2015, changes were relatively modest and reflected the inclusion of newly available cross-sectional parasite rate surveys or updates to data on malaria intervention coverage in recent years.

### Tuberculosis

For the first time, we have estimated the prevalence of latent tuberculosis infection. Globally, this disease affects 1·91 billion people (95% UI 1·79 billion to 2·03 billion) who are at risk of developing active disease when the immune system weakens through old age, HIV infection, diabetes, malnutrition, or excessive alcohol use. Globally, we estimate there were 10·4 million (9·37 million to 11·7 million) incident cases of tuberculosis in 2016, which is the same as the 10·4 million cases estimated by WHO for 2015.^43^ The separate estimation of 331 000 cases (95% UI 293 000–373 000) of multidrug-resistant and 19 800 incident cases (17 300–22 600) of extensively drug-resistant tuberculosis with or without HIV infections has great policy relevance, as the resources required for treatment of resistant cases are many times greater given that treatment needs to be applied for a longer period and with more expensive drugs. Drug resistance also requires more expensive detection and monitoring methods.^44^

### HIV/AIDS

A major change in methods for estimating burden of HIV/AIDS for GBD 2016 was the distribution of antiretroviral therapy coverage by age, sex, and CD4 count. We used two AIDS Indicator Surveys^45^, ^46^ to predict the age-sex-CD4 distribution of antiretroviral therapy coverage. This shifted the coverage distribution to groups with higher CD4 counts and gave a better fit to the data. UNAIDS produces periodic updates to global, regional, and national estimates of HIV/AIDS incidence and prevalence. In their latest assessment there were 36·7 million (30·8 million to 42·9 million) people living with HIV/AIDS in 2016, compared with 36·4 million (34·2 million to 39·1 million) estimated by GBD 2016.^47^ Comparisons of prevalence estimates at the country level in 2005 (the estimated peak of the HIV/AIDS epidemic globally) and 2016 show a high level of concordance between GBD 2016 and UNAIDS, with an average intra-class correlation coefficient of 0·992. For estimates of annual new infections, UNAIDS and GBD follow similar patterns, with UNAIDS having slightly lower estimates for years between 1996 and 2002. The estimates are similar between the two for most of the 2000s, with UNAIDS estimates showing a slightly faster rate of decline in annual new infections from 2008 to 2016 at the global level. GBD 2016 estimates about 1·9 million new infections globally in 2016, while UNAIDS estimates about 1.8 million for the same year.^47^

### Cancer

Country-specific cancer statistics are published by the International Agency for Research on Cancer for their GLOBOCAN project.^48^ Their most recent estimate of incidence for all cancers combined is 14·1 million cases in 2012, compared with GBD estimates of 14·8 million cases in 2010 and 17·2 million cases in 2016. The appendix provides a comparison of incidence by cancer type between GBD and GLOBOCAN (appendix 1, p 255).

### Zika virus disease

The large outbreak of Zika virus disease in Latin America in 2016 led WHO to declare it a public health emergency of international concern.^49^ There were 7·6 million new infections in 2016, 7·4 million of which occurred in Latin America and the Caribbean. Most of the health loss associated with Zika virus disease was from non-fatal outcomes, including fever and malaise during acute infection and congenital anomalies and Guillain-Barré syndrome as longer-term consequences.

### Guinea worm

Guinea worm disease was included as a cause of non-fatal health loss because eradication of the disease is achievable in the near future.^50^Although not having received much attention in general global health policy debates, a remarkable reduction from an estimated 3·5 million cases in the mid-1980s to just 15 cases in 2016 has been achieved. Guinea worm eradication interventions include the distribution of filter cloths and pipe filters for drinking water, treatment of water sources with larvacide, case detection, containment to prevent further transmission, and health education.^50^

### Limitations

The annual updates of GBD allow incremental improvements to methods. However, several measurement challenges in non-fatal estimation remain. The foremost concern is how best to disentangle measurement error from true variation in disease occurrence. We correct for known bias from non-reference methods or case definitions, but often have to rely on sparse information to make those adjustments. Lack of consensus among researchers on how to measure a disease can make it difficult to define a manageable set of alternative methods for which we can make adjustments. An extreme example is dementia: among 234 data sources we identified 228 different methods of screening, diagnostic tools, and diagnostic criteria. When possible, we use predictive covariates in our non-fatal models. For diseases with a lot of information on what drives differences in disease, covariates help to estimate true variation and minimise residual measurement error. However, many of the leading causes of YLDs lack strong predictors as indicated by our risk assessment, which assigned less than 20% of YLDs from mental, musculoskeletal, sense organ, and neurological disorders to the 79 risks evaluated in the GBD study.^51^

Second, many non-fatal models continue to rely on sparse data in some regions or super-regions despite the steady increase in data sources. With the addition of some new data sources, this can lead to large variation with previous GBD estimates. Alcohol dependence is a good example. Our estimate of the global prevalence of alcohol dependence increased by 70% after adding nine new data sources for Brazil, 12 for India, seven for sub-Saharan Africa, and four in eastern and central Europe. Until there is better coverage of data, non-fatal disease models will continue to be prone to estimates varying between GBD iterations.

Third, the adjustments to our hospital admission data for multiple admissions and secondary diagnosis fields reflect all health-care episodes rather than inpatient episodes only. Adjustments derived from a large, though non-representative, source of medical claims data in the USA are assumed to be generalisable. The generalisability of claims data, the validity of assuming diagnoses based on primary diagnosis alone or all diagnostic fields, and the validity of trends in claims data have been challenged.^52^, ^53^, ^54^, ^55^, ^56^ Also, there may be considerable inter-country variation in how diseases are treated between inpatient and outpatient settings. Our models attempt to adjust for such potential biases by using a covariate on claims and hospital admission data to correct for systematic error, although in a relatively crude manner, this assumes no geographical variation in the error. Gaining access to claims data from other countries, particularly in low-income and middle-income countries, is a crucial endeavour for future GBD iterations.

Fourth, our estimates of severity distributions for most diseases are based on sparse studies from the literature for diseases with a more established consensus on a measure of severity, or our analyses of three high-income surveys which combine diagnostic information with a general health status measure, the Short Form-12 (SF-12).^40^, ^57^, ^58^ Disease-specific researchers can provide better data with more routine use of a single established measure of severity in surveys and patient populations. Another potential improvement can be expected from countries that are able to link survey data with a general health assessment instrument like SF-12 and administrative diagnostic information. The latter approach has the advantage that it would allow adjustments for comorbidity, which can affect measures aiming to capture the severity of a single disease.

Fifth, apart from COPD, stroke, and epilepsy, where we have epidemiological data to estimate the geographical variation in the distribution of sequelae, for most diseases we are unable to capture differences in severity that occur due to treatment. As almost all our data sources on severity are from high-income countries, this would underestimate YLDs in low-income and middle-income countries with poorer access to and quality of treatment. If greater geographical information on severity of disease becomes available, we can incorporate a more explicit gradient based on our health-system access and quality index.^58^

Sixth, we have not yet been able to incorporate dependent comorbidity into our simulation methods. We aim for this to take place in GBD 2017 and will require substantial developmental work to make the computational code more efficient.

Seventh, most of the uncertainty in our YLD estimates comes from the disability weights. We have not conducted any new surveys since GBD 2013. With further data collection, we may be able to reduce some of the uncertainty by increasing the volume of data and by removing some of the ambiguities in lay descriptions, but large uncertainty is inherent to health state valuations that rely on perceptions by respondents of brief descriptions of complex health problems.

Eighth, the separate estimation of non-fatal models in DisMod-MR 2.1 for 1990, 1995, 2000, 2006, 2010, and 2016 implies uncertainty of estimates over time is independent. Also, compositional bias can lead to spurious time trends being estimated. DisMod-AT will allow for the more appealing simultaneous estimation over age and time and thus resolve the current limitations. However, as it is a more complex modelling tool, extensive testing and understanding what sensible settings are will need to take place before we can replace the current DisMod-MR approach.

Ninth, scrutiny of the multitude of results generated by GBD is a challenge. Increasingly, we generate visualisation tools to query results and intermediate steps in our analyses to better vet our results. We share our data and modelling tools with our expanding network of over 2500 collaborators who frequently query data sources and our measurements for particular diseases or locations. Also, by publishing all code and describing the methods used disease-by-disease we encourage scrutiny of every aspect of the GBD study.

Tenth, as our GBD collaborators have raised, the argument of including Guinea worm disease to document pending eradication can be used to justify the inclusion of poliomyelitis in the next round of GBD. There are also a number of large residual categories, particularly other musculoskeletal disorders and other cardiovascular disease, where we could make more detailed estimates in future rounds of GBD.

Eleventh, despite adding estimates of the prevalence of several asymptomatic states of disease that are precursors of disease that can be targeted with preventive interventions (such as latent tuberculosis infection, chronic hepatitis, or stage III chronic kidney disease without anaemia), we are not comprehensive in doing so. As diagnostic capacity increases, more asymptomatic precursor states can be identified and, in GBD, we will aim to include these if they can be measured at the population level and become relevant to policy.

### Future directions

We have introduced a quality grading system for cause of death sources for GBD 2016. We want to introduce a similar grading system of non-fatal data sources for GBD 2017. Important elements to capture in a grading system of non-fatal data sources will include the proportion of data sources using the reference method and case definition for a cause, the proportion of data sources representative of a location, and the amount of detail in reporting of age groups, sex, locations, and time periods covered. Unlike the location-specific grading of cause of death data sources, a grading system of non-fatal data sources will need to be location-specific and cause-specific, making it a much larger endeavour that will likely take several years to complete.

A prototype of a new version of a new analytical tool, DisMod-AT, is currently being tested. It has the advantage over DisMod-MR 2.1 in that it solves for the differential equations that govern the relationship between incidence, prevalence, remission, and mortality simultaneously over age and time. This is of great relevance for causes that vary substantially over time, and overcomes the assumption of a steady disease state that underlies previous versions of DisMod. We expect for GBD 2017 to run both the current tool and DisMod-AT in parallel for a number of time-sensitive causes with a view of DisMod-AT becoming the tool of choice from GBD 2018 onward.

We plan to reinstate a web survey facility that will allow derivation of disability weights for new health states; examination of the effect of changes in the wording of lay descriptions of health states on valuations provided by respondents; and creation of health states for combined impairments for which we currently estimate disability weights multiplicatively.

## Conclusion

Diverging trends in YLLs and YLDs raise the importance of non-fatal health outcomes as contributing to the burden of disease. Even if changes in trends for many causes are not large, more attention should be paid to assessing the burden of non-fatal health outcomes, as populations age and countries progress through the epidemiological transition, causing growing numbers of people to be in need of chronic care. More research is needed to identify risks of major disabling diseases, better methods of intervening by prevention, and treatment or improving severity of disability. As countries progress through the epidemiological transition, more attention should be paid to assessing the burden of non-fatal health outcomes, particularly in light of growing numbers of people in need of chronic care as populations age. More research is needed to identify risks of major disabling diseases, better methods of intervening by prevention, and treatment or improving severity of disability.
