# Supplementary material for: Global, regional, and national incidence, prevalence, and years lived with disability for 328 diseases and injuries for 195 countries, 1990–2016: a systematic analysis for the Global Burden of Disease Study 2016
Source: Lancet. 2017 Sep 16;390(10100):1211–59. doi: 10.1016/S0140-6736(17)32154-2 (PMC5605509; doi:10.1016/S0140-6736(17)32154-2)
Supplement: Supplementary appendix 1 [file mmc1.pdf]

THE LANCET

Supplementary appendix 1

This appendix formed part of the original submission and has been peer reviewed. We post it as supplied by the authors.

Supplement to: GBD 2016 Disease and Injury Incidence and Prevalence Collaborators. Global, regional, and national incidence, prevalence, and years lived with disability for 328 diseases and injuries for 195 countries, 1990–2016: a systematic analysis for the Global Burden of Disease Study 2016. *Lancet* 2017; **390:** 1211–59.

Methods appendix to “Global, regional, and national incidence, prevalence, and years lived with disability for 328 diseases and injuries for 195 countries, 1990–2016: a systematic analysis for the Global Burden of Disease Study 2016”

Preamble

This appendix provides further methodological detail for “Global, regional, and national incidence, prevalence, and years lived with disability for 328 diseases and injuries for 195 countries, 1990–2016: a systematic analysis for the Global Burden of Disease Study 2016” This study complies with the Guidelines for Accurate and Transparent Health Estimates Reporting (GATHER) recommendations. It includes detailed tables and information on data in an effort to maximize transparency in our estimation processes and provide a comprehensive description of analytical steps. We intend this appendix to be a living document, to be updated with each iteration of the Global Burden of Disease Study.

Table of Contents

Methods appendix to "Global, regional, and national incidence, prevalence, and years lived with disability for 328 diseases and injuries for 195 countries, 1990–2016: a systematic analysis for the Global Burden of Disease Study 2016"

Authors' Contributions i

Section 1. GBD overview 1

1.1 GATHER statement 1

1.2 Locations of the analysis 1

1.3 Time period of the analysis 1

1.4 GBD Cause List 1

1.5 GBD results 2

1.6 Data input sources 2

1.7 List of Acronyms 3

1.8 Funding Sources 5

Section 2. Nonfatal outcome estimation 6

2.1 Data sources, identification and extraction 6

2.1.1 Systematic reviews 6

2.1.2 Survey data preparation 6

2.1.3 Disease Registers 7

2.1.4 Estimation of Hospital Envelope 7

2.1.5 Claims, inpatient hospital, and outpatient data 11

2.1.6 Case notifications 17

2.2 Data adjustment 18

2.3 DisMod-MR 2.1 Estimation 18

2.4 Impairment and Underlying Cause Estimation 21

2.4.1 Impairment squeeze 22

2.5 Severity Distribution 22

2.6 Disability weights 25

2.7 Comorbidity correction (COMO) 28

2.8 YLD Computation, Uncertainty & Residual YLDs 29

2.9 Socio-Demographic Index (SDI) analysis & Epidemiological Transition 30

Section 3. Nonfatal cause-specific estimation process 33

Tuberculosis 34

HIV AIDS 43

Diarrheal diseases 54

Typhoid and Paratyphoid fever 62

Other intestinal infectious diseases 65

Lower respiratory infections 66
Upper respiratory infections 74
Otitis media 77
Meningitis 80
Encephalitis 87
Diphtheria 91
Whooping cough 93
Tetanus 95
Measles 97
Varicella and herpes zoster 99
Malaria 101
Chagas disease 106
Visceral leishmaniasis 109
Cutaneous and mucocutaneous leishmaniasis 111
African trypanosomiasis 114
Schistosomiasis 119
Cysticercosis 122
Cystic echinococcosis 126
Lymphatic filariasis 130
Onchocerciasis 132
Dengue 139
Yellow fever 141
Rabies 143
Ascariasis 145
Trichuriasis 150
Hookworm disease 155
Foodborne trematodiases 160
Leprosy 167
Ebola 169
Zika 175
Guinea worm 177
Other neglected tropical diseases 196
Maternal disorders 197
Obstetric Fistula 205
Preterm birth complications 208

Neonatal encephalopathy due to birth asphyxia and trauma 215
Neonatal sepsis and other neonatal infections 219
Hemolytic disease and other neonatal jaundice 223
Protein-energy malnutrition 227
Iodine deficiency 230
Vitamin A deficiency 233
Other nutritional deficiencies 237
Sexually transmitted diseases excluding HIV 238
Acute hepatitis A 247
Acute hepatitis B 249
Acute hepatitis C 251
Acute hepatitis E 253
Neoplasms (Cancer) 255
Rheumatic heart disease 271
Ischemic heart disease 277
Cerebrovascular disease 283
Acute Myocarditis 291
Atrial fibrillation and flutter 293
Peripheral arterial disease 297
Endocarditis 301
Other cardiovascular and circulatory diseases 304
Hemoglobinopathies and hemolytic anemias 306
COPD 312
Pneumoconiosis 371
Asthma 322
Interstitial lung disease 326
Other chronic respiratory diseases 329
Cirrhosis 330
Peptic ulcer disease 334
Gastritis and duodenitis 337
Appendicitis 341
Paralytic ileus and intestinal obstruction 344
Inguinal femoral and abdominal hernia 347
Inflammatory bowel disease 350
Vascular intestinal disorders 354

Gallbladder and biliary diseases 357
Pancreatitis 360
Other digestive diseases 363
Alzheimer's disease and other dementias 364
Parkinson's disease 369
Multiple sclerosis 376
Migraine 383
Tension-type headache 388
Motor neuron disease 393
Other neurological disorders 399
Schizophrenia 400
Alcohol dependence 404
Fetal alcohol syndrome 408
Opioid dependence 411
Cocaine dependence.. 415
Amphetamine dependence.. 419
Cannabis dependence.. 423
Other drug use disorders 427
Major depressive disorder 430
Dysthymia 436
Bipolar disorder 440
Anxiety disorders 445
Anorexia nervosa 449
Bulimia nervosa 452
Autism 455
Asperger syndrome 459
ADHD 462
Conduct disorder 466
Other mental and substance use disorders 470
Diabetes mellitus 473
Acute glomerulonephritis 485
Chronic kidney disease 488
Interstitial nephritis and urinary tract infections 493
Urolithiasis 497
BPH 500

Other urinary diseases ... 503
Uterine fibroids ... 504
Polycystic ovarian syndrome ... 507
Endometriosis ... 510
Genital prolapse ... 513
Premenstrual syndrome ... 516
Other gynecological diseases ... 519
EMBI disorders ... 521
Rheumatoid arthritis ... 524
Osteoarthritis ... 526
Low back pain ... 532
Neck pain ... 537
Gout ... 541
Other musculoskeletal disorders ... 544
Congenital anomalies ... 548
Dermatitis ... 583
Psoriasis ... 588
Cellulitis ... 591
Pyoderma ... 594
Scabies ... 597
Fungal skin diseases ... 600
Viral skin diseases ... 603
Acne vulgaris ... 607
Alopecia areata ... 610
Pruritus ... 613
Urticaria ... 616
Decubitus ulcer ... 619
Other skin and subcutaneous diseases ... 622
Other sense organ diseases ... 625
Deciduous caries ... 628
Permanent caries ... 633
Periodontal diseases ... 638
Edentulism and severe tooth loss ... 642
Other oral disorders ... 645
Injuries ... 646

Sexual Violence 657
Anemia 663
Epilepsy 674
Infertility 680
Intellectual disability 683
GBS 687
Pelvic inflammatory disease 691
Heart failure 694
Vision impairment 701
Hearing impairment 713
Section 4. Appendix: Tables and Figures 717
Appendix Figure 1a: Overview analytical flowchart for DisMod-MR 2.1 modelling strategies and injuries, GBD 2016 718
Appendix Figure 1b: Analytical flowchart for modelling strategies other than DisMod-MR 2.1 and injuries for selected nonfatal cause groups, GBD 2016 719
Appendix Figure 2: DisMod-MR 2.1 analytical cascade 720
Appendix Figure 3: Map of GBD 2016 Regions 721
Appendix Figure 4: Number of most detailed GBD causes with any incidence or prevalence data by location, 1980-2016 722
Appendix Table 1: GATHER checklist of information that should be included in reports of global health estimates, with description of compliance and location of information for GBD 2016. Global, regional, and national incidence, prevalence, and years lived with disability for 328 diseases and injuries for 195 countries, 1990-2016: a systematic analysis for the Global Burden of Disease Study 2016 723
Appendix Table 2: GBD 2016 location hierarchy with levels 726
Appendix Table 3: GBD 2016 cause and sequela hierarchy 743
Appendix Table 4: ICD-9 and ICD-10 codes by GBD 2016 nonfatal causes, impairments and nature of injury categories 791
Appendix Table 5: GBD 2016 sequelae, health states, health state lay descriptions, and disability weights 799
Appendix Table 6: GBD 2016 methods of estimating years lived with disability (YLDs) for 35 residual categories 850
Appendix Table 7: Socio-demographic Index R-squared values with lags up to 10 years 853
Appendix Table 8: Socio-demographic Index groupings by location, based on 2016 values 854
Appendix Table 9: Socio-demographic Index values for all estimated GBD locations, 1980 - 2016 870
Section 5. References 884

Authors’ Contributions

Managing the estimation process

Ashkan Afshin, Tahiya Alam, Christina Fitzmaurice, Emmanuela Gakidou, Caitie Hawley, Simon Hay, Nick Kassebaum, Stephen Lim, Christopher J L Murray, Mohsen Naghavi, Helen Elizabeth Olsen, Puja Rao, Greg Roth, Joseph Salama, Katya Shackelford, Chloe Shields, Shreya Shirude, Mari Smith, Jeffrey Stanaway, Caitlyn Steiner, and Theo Vos.

Writing the first draft of the manuscript

Audra Gold, Laurie Marczak, Christopher J L Murray, and Theo Vos.

Providing data or critical feedback on data sources

Cristiana Abbafati, Semaw Ferede Abera, Habtamu Abera Hareri, Laith J Abu-Raddad, Ilana Ackerman, Olatunji Adetokunboh, Rakesh Aggarwal, Anurag Agrawal, Aliasghar Ahmad Kiadaliri, Muktar Ahmed, Amani N Aichour, Ibtihel Aichour, Miloud TE Aichour, Nadia Akseer, Deena Al Asfoor, Fares Alahdab, Khurshid Alam, Kefyalew Addis Alene, Raghib Ali, Ala'a Alkerwi, François Alla, Peter Allebeck, Fatma Al-Maskari, Ubai Alsharif, Khalid Altirkawi, Dayane Gabriele Alves Silveira, Azmeraw T Amare, Erfan Amini, Walid Ammar, Palwasha Anwari, Johan Ärnlöv, Al Artaman, Krishna Kumar Aryal, Hamid Asayesh, Solomon W Asgedom, Reza Assadi, Ashish Awasthi, Umar Bacha, Kalpana Balakrishnan, Amitava Banerjee, Aleksandra Barac, Till Bärnighausen, Simon Barquera, Lars BarregardV, Lope Barrero, Basu Basu, Bernhard T Baune, Justin Beardsley, Neeraj Bedi, Yannick Béjot, Bayu Begashaw Bekele, Derrick Bennett, Isabela Bensenor, Eduardo Bernabe, Addisu Shunu Beyene, Neeraj Bhala, Anil Bhansali, Zulfiqar A Bhutta, Boris Bikbov, Charles Birungi, Donal Bisanzio, Soufiane Boufous, Gessessew Bugssa, Mate Car, Hélène Carabin, Jonathan Carapetis, David Carpenter, Juan-Jesus Carrero, Felix Carvalho, Valeria Caso, Carlos Castañeda-Orjuela, Chris Castle, Ferrán Catalá-López, Tefera Chane, Hsing-Yi Chang, Fiona Charlson, Honglei Chen, Mirriam Chibalabala, Abdulaal Chitheer, Massimo Cirillo, Danny Colombara, Michael Criqui, John Crump, Lalit Dandona, Rakhi Dandona, José das Neves, Louisa Degenhardt, Kebede Deribe, Christopher Devasahayam, Subhojit Dey, Samath Dharmaratne, Preet Dhillon, Balem Dimtsu, E Ray Dorsey, Dirk Douwes-Schultz, Kerrie Doyle, Tim Driscoll, Manisha Dubey, Bruce Duncan, Maysaa El Sayed Zaki, Jerisha Ellerstrand, Aman Endries, Sergey Ermakov, Holly Erskine, Sharareh Eskandarieh, Alireza Esteghamati, Carla Farinha, Andre Faro, Farshad Farzadfar, Mir Sohail Fazeli, Valery Feigin, Seyed-Mohammad Fereshtehnejad, João Fernandes, Alize Ferrari, Tesfaye R Feyissa, Florian Fischer, Luisa Flor, Richard Franklin, Thomas Fürst, João M Furtado, Neal Futran, Teshome Gebre, Ayele Geleto, Hailay Gesesew, Peter Gething, Alireza Ghajar, Katherine Gibney, Paramjit Gill, Ibrahim Ginawi, Melkamu Dedefo Gishu, Audra Gold, Philimon Gona, Atsushi Goto, Max Griswold, Harish Gugnani, Tanush Gupta, Nima Hafezi-Nejad, Alemayehu Hailu, Hilda Harb, Josep Maria Haro, James Harvey, Mohammad Sadegh Hassanvand, Hamid Yimam Hassen, Rasmus Havmoeller, Roderick Hay, Nathaniel Henry, Ileana B Heredia-Pi, Pouria Heydarpour, Hans Hoek, Howard J Hoffman, Nobuyuki Horita, H Dean Hosgood, Peter Hotez, Damian Hoy, Aung Soe Htet, Guoqing Hu, Kim Moesgaard Iburg, Ehimario Igumbor, Sheikh Mohammed Shariful Islam, Kathryn H Jacobsen, Nader Jahanmehr, Mihajlo Jakovljevic, Dube Jara, Simerjot Jassal, Mehdi Javanbakht, Panniyammakal Jeemon, Paul Jensen, Vivekanand Jha, Catherine Johnson, Jost B Jonas, Mikk Jürisson, Zubair Kabir, Rajendra Kadel, Ritul Kamal, Haidong Kan, André Karch, Corine Karema, Amir Kasaeian, Srinivasa Vittal Katikireddi, Anil Kaul, Norito Kawakami, Andre Keren, Yousef Khader, Ibrahim Khalil, Young-Ho Khang, Ardeshir Khosravi, Christian Kieling, Yohannes Kinfu, Ann Kristin Knudsen, Soewarta Kosen, Michael Kravchenko, Barthelemy Kuate Defo,

Burcu Kucuk Bicer, G Anil Kumar, Pushpendra Kumar, Basant Kumar Panda, Hmwe Kyu, Dharmesh Lal, Nkurunziza Lambert, Qing Lan, Janet Leasher, Jong-Tae Lee, Misgan Legesse Liben, James Leigh, Cheru T Leshargie, Janni Leung, Ricky Leung, Miriam Levi, Yichong Li, Xiaofeng Liang, Shiwei Liu, Katharine Looker, Alan Lopez, Stefan Lorkowski, Paulo Lotufo, Mohammed Magdy Abd El Razek, Marek Majdan, Azeem Majeed, Reza Malekzadeh, Abdullah Mamun, Lorenzo Mantovani, Ira Martopullo, Winfried März, Manu Mathur, Mohsen Mazidi, Colm McAlinden, John McGrath, Suresh Mehata, Man Mohan Mehndiratta, Ziad Memish, Walter Mendoza, Atte Meretoja, Haftay Berhane Mezgebe, Renata Micha, Ted Miller, Edward Mills, Vuong Minh Nong, Shiva Raj Mishra, Shafiu Mohammed, Sanjay K Mohanty, Ali Mokdad, Lorenzo Monasta, Julio Montañez, Maziar Moradi-Lakeh, Rintaro Mori, Ulrich Mueller, GVS Murthy, Kamarul Imran Musa, Jean Nachega, Gabriele Nagel, Kovin Naidoo, luigi Naldi, Lemma Negesa, Ionut Negoi, Charles R Newton, Minh Nguyen, Trang Nguyen Huyen, Quyen Nguyen Le, Cuong Nguyen Tat, Bo Norrving, Felix Ogbo, Olanrewaju Oladimeji, Andrew T Olagunju, Bolajoko Olusanya, Jacob Olusanya, Kanyin Ong, Alberto Ortiz, Majdi Osman, Mayowa Owolabi, Mahesh PA, Adrian Pana, Christina Papachristou, Scott Patten, Neil Pearce, David Pereira, Norberto Perico, Konrad Pesudovs, Max Petzold, Huyen Phuc Do, David Pigott, Dietrich Plass, Martin Pletcher, Farshad Pourmalek, Dorairaj Prabhakaran, Narayan Prasad, Noela Prasad, Mostafa Qorbani, Rynaz Rabiee, Anwa Rafay, Afarin Rahimi-Movaghar, Vafa Rahimi-Movaghar, Rajesh Kumar Rai, Rajesh Kumar Rai, Sasa Rajsic, Usha Ram, Chhabi Lal Ranabhat, Salman Rawaf, Giuseppe Remuzzi, Antonio Luiz Ribeiro, Luca Ronfani, Gholamreza Roshandel, Gregory Roth, Soheil Saadat, Saeid Safiri, Rajesh Sagar, Joshua A Salomon, Sundeep Salvi, Abdallah M Samy, Damian Santomauro, Itamar Santos, Milena Santric Milicevic, Maheswar Satpathy, Monika Sawhney, Sonia Saxena, Maria Inês Schmidt, David C Schwebel, Falk Schwendicke, Soraya Seedat, Sadaf Sepanlou, Edson Servan-Mori, Masood Ali Shaikh, Jayendra Sharma, Girma Temam Shifa, Reza Shirkoohi, Kawkab Shishani, Mark Shrime, Abla Mehio Sibai, Inga Dora Sigfusdottir, Diego Augusto Santos Silva, Jasvinder Singh, Narinder Pal Singh, Eirini Skiadaresi, Erica Leigh Slepak, Luciano Sposato, Chandrashekhar T Sreeramareddy, Vasiliki Stathopoulou, Timothy Steiner, Mark Stokes, Lars Jacob Stovner, Muawiyyah Babale Sufiyan, Rizwan Suliankatchi Abdulkader, Soumya Swaminathan, Bryan Sykes, Dillon Sylte, Rafael Tabarés-Seisdedos, Desalegn Tadese, Jukka Takala, Mohammad Tavakkoli, Nuno Taveira, Hugh Taylor, Abdullah Terkawi, Ornwipa Thamsuwan, Ruoyan Tobe-Gai, Marcello Tonelli, Bach Tran Xuan, Christopher Troeger, Derrick Tsoi, Stefanos Tyrovolas, Kingsley Nnanna Ukwaja, Eduardo Undurraga, Chigozie Uneke, Olalekan A Uthman, Job FM van Boven, Tommi Vasankari, Narayanaswamy Venketasubramanian, Ramesh Vidavalur, Francesco S Violante, Vasiliy Vlassov, Stein Emil Vollset, Theo Vos, Tolassa Wakayo, Marcia R Weaver, Elisabete Weiderpass, Robert Weintraub, Andrea Werdecker, Ronny Westerman, Harvey Whiteford, Tissa Wijeratne, Charles Shey Wiysonge, Charles Wolfe, Rachel Woodbrook, Anthony Woolf, Abdulhalik Workicho, Sarah Wulf Hanson, Denis Xavier, Gelin Xu, Simon Yadgir, Bereket Yakob, Lijing Yan, Pengpeng Ye, Naohiro Yonemoto, Marcel Yotebieng, Mustafa Younis, Zoubida Zaidi, Xueying Zhang, Maigeng Zhou, Ben Zipkin, and Liesl Zuhlke.

Developing methods or computational machinery

Peter Allebeck, Christine Allen, Solomon W Asgedom, Marlena Bannick, Katherine Battle, Samir Bhatt, Zulfiqar A Bhutta, Charles Birungi, Stan Biryukov, Donal Bisanzio, Ewan Cameron, Austin Carter, Daniel Casey, Chris Castle, Fiona Charlson, Danny Colombara, Christopher Devasahayam, Daniel Dicker, Holly Erskine, Mir Sohail Fazeli, Alize Ferrari, Christina Fitzmaurice, Kyle Foreman, Ayele Geleto, Peter Gething, Ellen Goldberg, Max Griswold, James Harvey, Catherine Johnson, Sarah Johnson, Nicholas J Kassebaum, James Leigh, Janni Leung, Angela Liu, Tim Lucas, Treh Manhertz, Ira Martopullo, Madeline McGaughey, Claire McNellan, Cliff Mountjoy-Venning, Lemma Negesa, Grant Nguyen, Emma Nichols, Kanyin Ong, Aaron Osgood-Zimmerman, Charles Parry, Katherine Paulson, Christine Pinho, Zane Rankin, Nikolas Reinig, Gregory Roth, Enrico Rubagotti, Joshua A Salomon, Abdallah M Samy, Damian Santomauro,

Maheswar Satpathy, David Smith, Chandrashekhar T Sreeramareddy, Vinay Srinivasan, Jeffrey Stanaway, Bryan Sykes, Dillon Sylte, Ornwipa Thamsuwan, Katie Thomas, Derrick Tsoi, Rachel L Updike, Theo Vos, Marcia R Weaver, Ronny Westerman, Tissa Wijeratne, and Gelin Xu.

Applying analytical methods to produce estimates

Fares Alahdab, Peter Allebeck, Christine Allen, Azmeraw T Amare, Marlena Bannick, Katherine Battle, Jennifer Benson, Adugnaw Berhane, Samir Bhatt, Zulfiqar A Bhutta, Charles Birungi, Stan Biryukov, Donal Bisanzio, Ewan Cameron, Jonathan Carapetis, Austin Carter, Daniel Casey, Fiona Charlson, Danny Colombara, Christopher Devasahayam, Daniel Dicker, Dirk Douwes-Schultz, Aman Endries, Holly Erskine, Mir Sohail Fazeli, João Fernandes, Alize Ferrari, Christina Fitzmaurice, Ayele Geleto, Peter Gething, Melkamu Dedefo Gishu, William Godwin, Ellen Goldberg, Max Griswold, Hamid Yimam Hassen, Nathaniel Henry, Caleb Irvine, Dube Jara, Catherine Johnson, Nicholas J Kassebaum, Jagdish Khubchandani, Yun Jin Kim, Hmwe Kyu, Misgan Legesse Liben, Janni Leung, Angela Liu, Patrick Liu, Tim Lucas, Helena Manguerra, Treh Manhertz, Ira Martopullo, Madeline McGaughey, Claire McNellan, Ali Mokdad, Cliff Mountjoy-Venning, Lemma Negesa, Grant Nguyen, Minh Nguyen, Emma Nichols, Helen Elizabeth Olsen, Jacob Olusanya, Kanyin Ong, Katherine Paulson, David Pigott, Martin Pletcher, Caroline Purcell, Zane Rankin, Nikolas Reinig, Marissa Reitsma, Gregory Roth, Joshua A Salomon, Damian Santomauro, Maheswar Satpathy, Masood Ali Shaikh, Vinay Srinivasan, Jeffrey Stanaway, Bryan Strub, Ornwipa Thamsuwan, Katie Thomas, Christopher Troeger, Derrick Tsoi, Rachel L Updike, Theo Vos, Tolassa Wakayo, Ronny Westerman, Tissa Wijeratne, Gelin Xu, Simon Yadgir, and Ben Zipkin.

Providing critical feedback on methods or results

Amanuel Abajobir, Cristiana Abbafati, Kaja Abbas, Abdishakur Abdulle, Teshome Abebo, Mubarek Abera, Semaw Ferede Abera, Habtamu Abera Hareri, Victor Aboyans, Laith J Abu-Raddad, Ilana Ackerman, Abdu Adamu, Olatunji Adetokunboh, Mohsen Afarideh, Anurag Agrawal, Aliasghar Ahmad Kiadaliri, Hamid Ahmadieh, Muktar Ahmed, Amani N Aichour, Ibtihel Aichour, Miloud TE Aichour, Rufus Akinyemi, Nadia Akseer, Deena Al Asfoor, Fares Alahdab, Ziyad Al-Aly, Khurshid Alam, Noore Alam, Kefyalew Addis Alene, Raghib Ali, Peter Allebeck, Reza Alizadeh-Navaei, Ala'a Alkerwi, Fatma Al-Maskari, Rajaa Al-Raddadi, Ubai Alsharif, Shirina Alsowaidi, Khalid Altirkawi, Dayane Gabriele Alves Silveira, Azmeraw T Amare, Erfan Amini, Yaw Ampem Amoako, Carl Abelardo Antonio, Palwasha Anwari, Johan Ärnlöv, Al Artaman, Krishna Kumar Aryal, Hamid Asayesh, Solomon W Asgedom, Nigussie Assefa, Tesfay Mehari Atey, Sachin Atre, Leticia Avila-Burgos, Euripide Avokpaho, Ashish Awasthi, Beatriz Paulina Ayala Quintanilla, Umar Bacha, Alaa Badawi, Aleksandra Barac, Suzanne Barker-Collo, Till Bärnighausen, Simon Barquera, Lars Barregard, Lope Barrero, Huda Basaleem, Basu Basu, Katherine Battle, Bernhard T Baune, Shahrzad Bazargan-Hejazi, Justin Beardsley, Neeraj Bedi, Ettore Beghi, Bayu Begashaw Bekele, Fanuel Belayneh, Michelle Bell, Derrick Bennett, Isabela Bensenor, Adugnaw Berhane, Derbew FikaduBe Berhe, Eduardo Bernabe, Mircea Beuran, Addisu Shunu Beyene, Neeraj Bhala, Samir Bhatt, Zulfiqar A Bhutta, Sibhatu Biadgilign, Boris Bikbov, Charles Birungi, Donal Bisanzio, Habtamu Mellie Bizuayehu, Soufiane Boufous, Alexandra Brazinova, Rachelle Buchbinder, Gessessew Bugssa, Zahid Butt, Lucero Cahuana-Hurtado, Ewan Cameron, Mate Car, Jonathan Carapetis, Rosario Cárdenas, Deborah Carvalho, Felix Carvalho, Carlos Castañeda-Orjuela, Ferrán Catalá-López, Tefera Chane, Jung-Chen Chang, Fiona Charlson, Vesper Chisumpa, Liliana G Ciobanu, Massimo Cirillo, Michael Criqui, John Crump, Koustuv Dalal, Lalit Dandona, Rakhi Dandona, José das Neves, Barbora de Courten, Diego de Leo, Louisa Degenhardt, Robert P Dellavalle, Kebede Deribe, Christopher Devasahayam, Subhojit Dey, Samath Dharmaratne, Preet Dhillon, Daniel Dicker, Balem Dimtsu, Eric L Ding, Dirk Douwes-Schultz, Kerrie Doyle, Tim Driscoll, Manisha Dubey, Dumessa Edessa, Maysaa El Sayed Zaki, Ziad El-Khatib, Ahmadali Enayati, Aman Endries, Holly Erskine, Babak Eshrati, Sharareh Eskandarieh, Alireza Esteghamati, Carla Farinha,

Andre Faro, Farshad Farzadfar, Mir Sohail Fazeli, Seyed-Mohammad Fereshtehnejad, João Fernandes, Alize Ferrari, Tesfaye R Feyissa, Irina Filip, Florian Fischer, Christina Fitzmaurice, Luisa Flor, Nataliya Foigt, Kyle Foreman, Richard Franklin, Thomas Fürst, João M Furtado, Neal Futran, Morsaleh Ganji, Teshome Gebre, Tsegaye Gebrehiwot, Ayele Geleto, Hailay Gesesew, Peter Gething, Katherine Gibney, Paramjit Gill, Melkamu Dedefo Gishu, Giorgia Giussani, Audra Gold, Ellen Goldberg, Philimon Gona, Amador Goodridge, Sameer Gopalani, Atsushi Goto, Alessandra c Goulart, Max Griswold, Harish Gugnani, Rahul Gupta, Rajeev Gupta, Tanush Gupta, Vipin Gupta, Nima Hafezi-Nejad, Alemayehu Hailu, Randah Hamadeh, Samer Hamidi, Yuantao Hao, Hamid Yimam Hassen, Rasmus Havmoeller, Roderick Hay, Simon Hay, Ileana B Heredia-Pi, Nobuyuki Horita, H Dean Hosgood, Sorin Hostiuc, Peter Hotez, Damian Hoy, Guoqing Hu, Hsiang Huang, Ehimario Igumbor, Sheikh Mohammed Shariful Islam, Kathryn H Jacobsen, Nader Jahanmehr, Mihajlo Jakovljevic, Dube Jara, Mehdi Javanbakht, Sudha Jayaraman, Panniyammakal Jeemon, Paul Jensen, Vivekanand Jha, Guohong Jiang, Denny John, Catherine Johnson, Sarah Johnson, Jost B Jonas, Mikk Jürisson, Rajendra Kadel, Amaha Kahsay, Ritul Kamal, Nadim Karam, André Karch, Corine Karema, Amir Kasaeian, Nicholas J Kassebaum, Anshul Kastor, Peter Keiyoro, Yousef Khader, Young-Ho Khang, Jagdish Khubchandani, Christian Kieling, Daniel Kim, Pauline Kim, Yun Jin Kim, Yohannes Kinfu, Adnan Kisa, Katarzyna Kissimova-Skarbek, Mika Kivimaki, Ann Kristin Knudsen, Dhaval Kolte, Jacek Kopec, Ai Koyanagi, Michael Kravchenko, Sanjay Krishnaswami, Kristopher Krohn, Barthelemy Kuate Defo, Burcu Kucuk Bicer, G Anil Kumar, Pushpendra Kumar, Basant Kumar Panda, Hmwe Kyu, Dharmesh Lal, Ratilal Lalloo, Nkurunziza Lambert, Faris Lami, Qing Lan, Anders Larsson, Pablo Lavados, Janet Leasher, Paul Lee, Misgan Legesse Liben, James Leigh, Bikila Lencha, Cheru T Leshargie, Janni Leung, Miriam Levi, Yongmei Li, Xiaofeng Liang, Shai Linn, Yang Liu, Rakesh Lodha, Katharine Looker, Alan Lopez, Stefan Lorkowski, Erlyn Rachelle Macarayan, Mohammed Magdy Abd El Razek, Mahdi Mahdavi, Marek Majdan, Reza Majdzadeh, Azeem Majeed, Reza Malekzadeh, Rajesh Malhotra, Deborah Malta, Abdullah Mamun, Treh Manhertz, Chabila Mapoma, Jose Martinez-Raga, Francisco Rogerlândio Martins-Melo, Ira Martopullo, Manu Mathur, Mohsen Mazidi, Colm McAlinden, John McGrath, Martin McKee, Suresh Mehata, Peter Memiah, Ziad Memish, Walter Mendoza, Zerihun Menlkalew, George Mensah, Tuomo Meretoja, Haftay Berhane Mezgebe, Haftay Berhane Mezgebe, Renata Micha, Ted Miller, Edward Mills, Vuong Minh Nong, Mojde Mirarefin, Erkin Mirrakhimov, Shiva Raj Mishra, Philip B Mitchell, Alireza Mohammadi, Kedir Mohammed, Shafiu Mohammed, Ali Mokdad, Lorenzo Monasta, Marcella Montico, Maziar Moradi-Lakeh, Cliff Mountjoy-Venning, Kalayu Mruts, Ulrich Mueller, GVS Murthy, Kamarul Imran Musa, Jean Nachega, Gabriele Nagel, Aliya Naheed, Vinay Nangia, Gopalakrishnan Natarajan, Lemma Negesa, Ionut Negoi, Ruxandra Irina Negoi, Josephine Ngunjiri, Grant Nguyen, Trang Nguyen Huyen, Quyen Nguyen Le, Cuong Nguyen Tat, Sandra Nolte, Bo Norrving, Jean Jacques Noubiap, Dina Nur Anggraini Ningrum, Martin O'Donnell, Felix Ogbo, Anselm Okoro, Olanrewaju Oladimeji, Andrew T Olagunju, Helen Elizabeth Olsen, Bolajoko Olusanya, Jacob Olusanya, Kanyin Ong, John Nelson Opio, Eyal Oren, Alberto Ortiz, Mayowa Owolabi, Mahesh PA, Rosana Pacella, Adrian Pana, Eun-Kee Park, Mahboubeh Parsaeian, Scott Patten, George Patton, Katherine Paulson, David Pereira, Norberto Perico, Konrad Pesudovs, Carrie Peterson, Michael R Phillips, Huyen Phuc Do, David Pigott, Julian Pillay, Dietrich Plass, Svetlana Popova, Richie Poulton, Farshad Pourmalek, Dorairaj Prabhakaran, Narayan Prasad, Mostafa Qorbani, Reginald Quansah, Amir Radfar, Kazem Rahimi, Afarin Rahimi-Movaghar, Vafa Rahimi-Movaghar, Mahfuzar Rahman, Mohammad Hifz Ur Rahman, Rajesh Kumar Rai, Sasa Rajsic, Usha Ram, Chhabi Lal Ranabhat, Puja Rao, Salman Rawaf, Sarah E Ray, Marissa Reitsma, Giuseppe Remuzzi, Andre Renzaho, Serge Resnikoff, Satar Rezaei, Antonio Luiz Ribeiro, Nikolas Reinig, Gholamreza Roshandel, Gregory Roth, Ambuj Roy, George Ruhago, Mahdi Safdarian, Sare Safi, Saeid Safiri, Rajesh Sagar, Ramesh Sahathevan, Joshua A Salomon, Sundeep Salvi, Abdallah M Samy, Damian Santomauro, Itamar Santos, Milena Santric Milicevic, Benn Sartorius, Maheswar Satpathy, Monika Sawhney, Sonia Saxena, Ben Schöttker, David C Schwebel, Falk Schwendicke, Soraya Seedat, Sadaf Sepanlou, Tesfaye Setegn, Amira Shaheen, Masood Ali Shaikh, Mansour Shamsipour, Rajesh Sharma, Jun

She, Chloe Shields, Girma Temam Shifa, Mika Shigematsu, Rahman Shiri, Reza Shirkoohi, Mark Shrime, Inga Dora Sigfusdottir, Diego Augusto Santos Silva, Jasvinder Singh, Eirini Skiadaresi, Amber Sligar, Mari Smith, Badr Sobaih, Eugene Sobngwi, Reed Sorensen, Tatiane Sousa, Luciano Sposato, Chandrashekhar T Sreeramareddy, Jeffrey Stanaway, Vasiliki Stathopoulou, Nicholas Steel, Murray Stein, Timothy Steiner, Sabine Steinke, Mark Stokes, Muawiyyah Babale Sufiyan, Rizwan Suliankatchi Abdulkader, Bruno Sunguya, Soumya Swaminathan, Bryan Sykes, Rafael Tabarés-Seisdedos, Niguse Tadele, Desalegn Tadese, Getachew Taffere, Jukka Takala, Nikhil Tandon, Hugh Taylor, Tesfalidet Tekelab, Abdullah Terkawi, Dawit Tesfaye, Belay Tessema, Ornwipa Thamsuwan, Amanda Thrift, Ruoyan Tobe-Gai, Marcello Tonelli, Roman Topor-Madry, Miguel Tortajada-Girbés, Mathilde Touvier, Bach Tran Xuan, Christopher Troeger, Derrick Tsoi, Kald Beshir Tuem, Kingsley Nnanna Ukwaja, Eduardo Undurraga, Chigozie Uneke, Olalekan A Uthman, Benjamin Uzochukwu, Job FM van Boven, Santosh Varughese, Tommi Vasankari, S Venkatesh, Narayanaswamy Venketasubramanian, Francesco S Violante, Paturi Vishnupriya Rao, Sergey Vladimirov, Vasiliy Vlassov, Stein Emil Vollset, Theo Vos, Fiseha Wadilo, Tolassa Wakayo, Yuan-Pang Wang, Marcia R Weaver, Scott Weichenthal, Elisabete Weiderpass, Robert Weintraub, Andrea Werdecker, Ronny Westerman, Harvey Whiteford, Tissa Wijeratne, Charles Shey Wiysonge, Anthony Woolf, Abdulhalik Workicho, Denis Xavier, Gelin Xu, Mohsen Yaghoubi, Bereket Yakob, Lijing Yan, Yuichiro Yano, Tenaw Yimer, Paul Siu Fai Yip, Naohiro Yonemoto, Marcel Yotebieng, Mustafa Younis, Zoubida Zaidi, Elias Asfaw Zegeye, Sanjay Zodpey, and Liesl Zuhlke.

Drafting the work or revising is critically for important intellectual content

Amanuel Abajobir, Cristiana Abbafati, Foad Abd-Allah, Abdishakur Abdulle, Mubarek Abera, Semaw Ferede Abera, Ilana Ackerman, Olatunji Adetokunboh, Mohsen Afarideh, Sanjay Agarwal, Sutapa Agrawal, Aliasghar Ahmad Kiadaliri, Muktar Ahmed, Rufus Akinyemi, Fares Alahdab, Khurshid Alam, Noore Alam, Tahiya Alam, Raghib Ali, Ala'a Alkerwi, Peter Allebeck, Fatma Al-Maskari, Shirina Alsowaidi, Azmeraw T Amare, Erfan Amini, Walid Ammar, Yaw Ampem Amoako, Hjalte H Andersen, Carl Abelardo Antonio, Palwasha Anwari, Johan Ärnlöv, Hamid Asayesh, Solomon W Asgedom, Tesfay Mehari Atey, Leticia Avila-Burgos, Euripide Avokpaho, Ashish Awasthi, Alaa Badawi, Amitava Banerjee, Aleksandra Barac, Suzanne Barker-Collo, Till Bärnighausen, Lope Barrero, Basu Basu, Bernhard T Baune, Shahrzad Bazargan-Hejazi, Neeraj Bedi, Michelle Bell, Derrick Bennett, Isabela Bensenor, Adugnaw Berhane, Eduardo Bernabe, Mircea Beuran, Addisu Shunu Beyene, Neeraj Bhala, Sibhatu Biadgilign, Zulfiqar A Bhutta, Gessessew Bugssa, Zahid Butt, Lucero Cahuana-Hurtado, Juan-Jesus Carrero, Carlos Castañeda-Orjuela, Chris Castle, Ferrán Catalá-López, Fiona Charlson, Chioma Chibueze, Liliana G Ciobanu, Danny Colombara, Cyrus Cooper, Paolo Angelo Cortesi, Michael Criqui, John Crump, Abel Dadi, Koustuv Dalal, Lalit Dandona, Rakhi Dandona, José das Neves, Dragos Davitoiu, Barbora de Courten, Louisa Degenhardt, Selina Deiparine, Robert P Dellavalle, Kebede Deribe, Don Des Jarlais, Christopher Devasahayam, Subhojit Dey, Samath Dharmaratne, Balem Dimtsu, Eric L Ding, E Ray Dorsey, Manisha Dubey, Bruce Duncan, Maysaa El Sayed Zaki, Aman Endries, Holly Erskine, Alireza Esteghamati, Andre Faro, Mir Sohail Fazeli, Seyed-Mohammad Fereshtehnejad, João Fernandes, Alize Ferrari, Tesfaye R Feyissa, Irina Filip, Florian Fischer, Nataliya Foigt, Thomas Fürst, João M Furtado, Morsaleh Ganji, Alberto Garcia-Basteiro, Teshome Gebre, Tsegaye Gebrehiwot, Ayele Geleto, Hailay Gesesew, Peter Gething, Alireza Ghajar, Katherine Gibney, Paramjit Gill, Richard Gillum, Melkamu Dedefo Gishu, Audra Gold, Philimon Gona, Amador Goodridge, Sameer Gopalani, Alessandra C Goulart, Rajeev Gupta, Tanush Gupta, Nima Hafezi-Nejad, Alemayehu Hailu, Randah Hamadeh, Alexis J Handal, Graeme Hankey, Hilda Harb, Josep Maria Haro, Hamid Yimam Hassen, Rasmus Havmoeller, Caitlin Hawley, Simon Hay, Ileana B Heredia-Pi, Nobuyuki Horita, H Dean Hosgood, Sorin Hostiuc, Peter Hotez, Damian Hoy, Guoqing Hu, Hsiang Huang, Ehimario Igumbor, Chad Ikeda, Sheikh Mohammed Shariful Islam, Kathryn H Jacobsen, Mihajlo Jakovljevic, Dube Jara, Sudha Jayaraman, Panniyammakal Jeemon, Paul Jensen, Vivekanand Jha,

Vivekanand Jha, Denny John, Catherine Johnson, Jost B Jonas, Mikk Jürisson, Rajendra Kadel, Ritul Kamal, André Karch, Amir Kasaeian, Nicholas J Kassebaum, Norito Kawakami, Peter Keiyoro, Andre Pascal Kengne, Yousef Khader, Ejaz Khan, Young-Ho Khang, Jagdish Khubchandani, Christian Kieling, Daniel Kim, Pauline Kim, Yun Jin Kim, Mika Kivimaki, Ann Kristin Knudsen, Yoshihiro Kokubo, Dhaval Kolte, Jacek Kopec, Parvaiz Koul, Ai Koyanagi, Kristopher Krohn, Barthelemy Kuate Defo, Burcu Kucuk Bicer, G Anil Kumar, Santhosh Kumar, Basant Kumar Panda, Hmwe Kyu, Dharmesh Lal, Anders Larsson, Pablo Lavados, Janet Leasher, Misgan Legesse Liben, Janni Leung, Miriam Levi, Giancarlo Logroscino, Stephanie J London, Alan Lopez, Paulo Lotufo, Mohammed Magdy Abd El Razek, Marek Majdan, Reza Majdzadeh, Azeem Majeed, Abdullah Mamun, Treh Manhertz, Francisco Rogerlândio Martins-Melo, Mohsen Mazidi, Colm McAlinden, Ziad Memish, Walter Mendoza, Zerihun Menlkalew, George Mensah, Atte Meretoja, Tuomo Meretoja, Haftay Berhane Mezgebe, Haftay Berhane Mezgebe, Ted Miller, Edward Mills, Vuong Minh Nong, Shiva Raj Mishra, Philip B Mitchell, Karzan Mohammad, Shafiu Mohammed, Ali Mokdad, Maziar Moradi-Lakeh, Paula Moraga, Shane Morrison, Cliff Mountjoy-Venning, Ulrich Mueller, Kamarul Imran Musa, Jean Nachega, Kovin Naidoo, Lemma Negesa, Ionut Negoi, Ruxandra Irina Negoi, Charles R Newton, Josephine Ngunjiri, Trang Nguyen Huyen, Quyen Nguyen Le, Cuong Nguyen Tat, Bo Norrving, Jean Jacques Noubiap, Felix Ogbo, In-Hwan Oh, Anselm Okoro, Olanrewaju Oladimeji, Andrew T Olagunju, Tinuke O Olagunju, Helen Elizabeth Olsen, Bolajoko Olusanya, Jacob Olusanya, Kanyin Ong, Eyal Oren, Alberto Ortiz, Mayowa Owolabi, Mahesh PA, Rosana Pacella, Christina Papachristou, George Patton, David Pereira, Konrad Pesudovs, Carrie Peterson, Huyen Phuc Do, Dietrich Plass, Richie Poulton, Mostafa Qorbani, Reginald Quansah, Amir Radfar, Anwa Rafay, Vafa Rahimi-Movaghar, Mahfuzar Rahman, Rajesh Kumar Rai, Salman Rawaf, Sarah E Ray, Nikolas Reinig, Andre Renzaho, Serge Resnikoff, Satar Rezaei, Antonio Luiz Ribeiro, Luca Ronfani, Gholamreza Roshandel, Gregory Roth, Ambuj Roy, George Ruhago, Mahdi Safdarian, Saeid Safiri, Rajesh Sagar, Joshua A Salomon, Abdallah M Samy, Damian Santomauro, Itamar Santos, João Vasco Santos, Milena Santric Milicevic, Maheswar Satpathy, Monika Sawhney, Maria Inês Schmidt, Ione Schneider, David C Schwebel, Falk Schwendicke, Sadaf Sepanlou, Masood Ali Shaikh, Mika Shigematsu, Rahman Shiri, Mark Shrime, Inga Dora Sigfusdottir, Diego Augusto Santos Silva, João Pedro Silva, Jasvinder Singh, Narinder Pal Singh, Dhirendra N Sinha, Eirini Skiadaresi, Vegard Skirbekk, Chandrashekhar T Sreeramareddy, Jeffrey Stanaway, Dan Stein, Sabine Steinke, Muawiyyah Babale Sufiyan, Bryan Sykes, Dillon Sylte, Desalegn Tadese, Arash Tehrani-Banihashemi, Belay Tessema, Ornwipa Thamsuwan, Amanda Thrift, Marcello Tonelli, Roman Topor-Madry, Miguel Tortajada-Girbés, Bach Tran Xuan, Kald Beshir Tuem, Stefanos Tyrovolas, Kingsley Nnanna Ukwaja, Eduardo Undurraga, Olalekan A Uthman, Job FM van Boven, Tommi Vasankari, Narayanaswamy Venketasubramanian, Vasiliy Vlassov, Stein Emil Vollset, Theo Vos, Fiseha Wadilo, Tolassa Wakayo, Yuan-Pang Wang, Elisabete Weiderpass, Robert Weintraub, Andrea Werdecker, Harvey Whiteford, Tissa Wijeratne, Charles Shey Wiysonge, Charles Wolfe, Denis Xavier, Gelin Xu, Bereket Yakob, Yuichiro Yano, Naohiro Yonemoto, Seok-Jun Yoon, Zoubida Zaidi, and Liesl Zuhlke.

Extracting, cleaning, or cataloging data; designing or coding figures and tables

Olatunji Adetokunboh, Muktar Ahmed, Sneha Aiyar, Tahiya Alam, Christine Allen, Reza Assadi, Robert Battista, Kadine Priscila Bender dos Santos, Jennifer Benson, Adugnaw Berhane, Donal Bisanzio, Daniel Casey, Chris Castle, Fiona Charlson, Danny Colombara, Christopher Devasahayam, Dirk Douwes-Schultz, Jerisha Ellerstrand, Sergey Ermakov, Holly Erskine, Alireza Esteghamati, Alize Ferrari, Luisa Flor, Ayele Geleto, Melkamu Dedefo Gishu, Ellen Goldberg, Max Griswold, Nathaniel Henry, Howard J Hoffman, Chad Ikeda, Dube Jara, Catherine Johnson, Sarah Johnson, Amir Kasaeian, Kristopher Krohn, Barthelemy Kuate Defo, Qing Lan, Janet Leasher, Misgan Legesse Liben, Janni Leung, Xiaofeng Liang, Angela Liu, Patrick Liu, Reza Malekzadeh, Helena Manguerra, Treh Manhertz, Ana Mantilla, Ira Martopullo, Mohsen

Mazidi, Claire McNellan, Alireza Mohammadi, Cliff Mountjoy-Venning, Lemma Negesa, Emma Nichols, Olanrewaju Oladimeji, Kanyin Ong, Mahesh PA, Katherine Paulson, David Pigott, Caroline Purcell, Mostafa Qorbani, Zane Rankin, Nikolas Reinig, Enrico Rubagotti, Abdallah M Samy, Damian Santomauro, Maheswar Satpathy, Peilin Shi, Erica Leigh Slepak, Jeffrey Stanaway, Bryan Strub, Michelle Subart, Dillon Sylte, Mohammad Tavakkoli, Ornwipa Thamsuwan, Katie Thomas, Derrick Tsoi, Rachel L Updike, Theo Vos, Tissa Wijeratne, Rachel Woodbrook, and Sarah Wulf Hanson.

Managing the overall research enterprise

Tahiya Alam, Peter Allebeck, Zulfiqar A Bhutta, Peter Gething, Audra Gold, Caitlin Hawley, Simon Hay, Chantal Huynh, Xiaofeng Liang, Chloe Morozoff, Helen Elizabeth Olsen, Puja Rao, Gregory Roth, Joseph Salama, Katya Shackelford, Chloe Shields, Shreya Shirude, Amber Sligar, Mari Smith, and Theo Vos.

Did not provide authorship contribution information

Traolach S Brugha, Ruth W Kimokoti, and Juan R Sanabria.

Section 1. GBD overview

1.1 GATHER statement

This study complies with the Guidelines for Accurate and Transparent Health Estimates Reporting (GATHER) recommendations. We have documented the steps involved in our analytical procedures and detailed the data sources used in compliance with the GATHER. For additional GATHER reporting, please refer to Appendix Table 1 in Section 4.

1.2 Locations of the analysis

The locations included in GBD 2016 have been arranged into a hierarchy of seven super-regions and a further nested set of 21 regions containing 195 countries and territories. These countries and territories for which GBD estimated global, regional, and national cause-specific mortality and years of life lost (YLLs) have not changed since GBD 2015. New subnational locations estimated for GBD 2016 were the local government areas of England and provinces of Indonesia. Subnational assessments for GBD 2016 included 26 states and one federal district for Brazil, 33 provinces and municipalities for China, nine regions and 150 local government areas for England, 31 states and union territories by urbanicity for India, 34 provinces for Indonesia, 47 prefectures for Japan, 47 counties for Kenya, 31 states and one federal district for Mexico, two areas for Sweden, nine provinces for South Africa, 13 states for the Kingdom of Saudi Arabia, and 50 states and one federal district for the United States. Combined, there were 335 locations at the first subnational unit level. Subnational Level 2 only applies to India (urban and rural populations by state) and England (the upper tier local health authorities). For this paper we present data at the national and territory level. Please refer to Appendix table 2 for more information.

1.3 Time period of the analysis

A complete set of cause-specific incidence, prevalence, and YLD numbers and rates were computed for the years 1990, 1995, 2000, 2005, 2010, and 2016. All GBD 2016 results and online data visualisations are available at http://vizhub.healthdata.org/gbd-compare[1] with access to results for all GBD metrics.

1.4 GBD Cause List

The GBD cause and sequelae list is organised hierarchically (see Appendix Table 3) to accommodate different purposes and needs of various users.

The first two levels aggregate causes into general grouping. At Level 1 there are three cause groups: communicable, maternal, neonatal, and nutritional diseases (CMNNDs); non-communicable diseases (NCDs); and injuries. These Level 1 aggregates are subdivided at Level 2 of the hierarchy into 21 cause groupings (e.g., neonatal disorders, neurological disorders, and transport injuries). The disaggregation into Levels 3 and 4 contains the finest level of detail for causes captured in GBD 2016. The greatest detail available for some causes, such as anxiety disorders or rheumatoid arthritis, is at Level 3 of the hierarchy while other specific causes are at Level 4 of the hierarchy with an aggregate category at Level 3 (for example, depressive disorders at Level 3 which encompasses major depressive disorders and dysthymia at Level 4). Sequelae of diseases and injuries are organised at Levels 5 and 6 of the hierarchy. In GBD, sequelae are defined as distinct, mutually exclusive, categories of health consequences that can be directly attributed to a cause. For example, both neuropathy and blindness due to diabetic retinopathy

are sequelae of diabetes; stroke and ischaemic heart disease are not, as these consequences cannot be categorically ascribed to diabetes in an individual despite good evidence for increased risk of these outcomes. The finest detail for all sequelae estimated in GBD is at Level 6 and is aggregated into summary sequelae categories (Level 5) for causes with large numbers of sequelae. Examples include the grouping of the infectious disease episodes and long-term sequelae of meningitis, and the grouping of 47 injury sequelae into seven summary categories (for example all fractures, spinal cord lesions, and head injuries). Sequelae in GBD are mutually exclusive and collectively exhaustive and thus our YLD estimates at each level of the hierarchy sum to the total of the level above. Prevalence aggregation is estimated at the level of individuals who may have more than one sequela or disease, and therefore are not additive.

The GBD cause list continues to evolve to reflect the policy relevance, public health, and medical care importance of the causes of major losses of health. The cause and sequelae list expanded following feedback from GBD 2015 and input from GBD 2016 collaborators. (Appendix Table 3). The incorporation of these changes expanded the cause list from the 310 causes with nonfatal estimates examined in GBD 2015 to 328 causes with nonfatal estimates and from 2,337 to 2,498 unique sequelae at Level 6 of the hierarchy. As in GBD 2015, we made no estimates of YLDs for just five causes, either because no disability is possible, as is the case with sudden infant death syndrome; because disability may occur rarely but at levels too low for accurate estimation given the data, as for aortic aneurysm; or because the disability is captured by the complicating causes that led to that cause of death, as for indirect maternal deaths, late maternal deaths, and maternal deaths aggravated by HIV/AIDS. Appendix Table 4 provides a list of International Classification of Diseases version 9 (ICD-9) and International Classification of Diseases version 10 (ICD-10) codes for all GBD causes and the nature of injury categories.

1.5 GBD results

Results from the Global Burden of Disease Study (GBD 2016) are now measured in terabytes. Results are available in an interactive data downloading tool on the Global Health Data exchange (GHDx). The tool contains the complete set of results from all summary papers; however, specialised tables from the papers are available as separate entries in the GHDx as were made available for GBD 2015.

The current version of the data download tool is available in the GHDx and contains core summary results for the Global Burden of Disease Study 2016 (GBD 2016): http://ghdx.healthdata.org/gbd-results-tool. The core summary results include deaths, YLLs, YLDs, and DALYs. It includes data for causes, risks, cause-risk attribution, aetiologies, and impairments.

In the GBD 2016 version, the tool also contains measures such as prevalence and incidence as well as rate of change data. Data above a certain size cannot be viewed online but can be downloaded. Depending on the size of the download, users may need to enter an email address and a download location will be sent to them when the files are prepared.

1.6 Data input sources

GBD 2016 incorporated a large number and wide variety of input sources to estimate mortality, causes of death and illness, and risk factors for 195 countries and territories from 1990 to 2016 and interpolated. These input sources are accessible through an interactive citation tool available in IHME’s GHDx.

Users can retrieve citations for a specific GBD component, cause or risk, and location by choosing from the available selection boxes. They can then view and access GHDx records for input sources and export a CSV file that includes the GHDx metadata, citations, and information about where the data were used in GBD. Additional metadata for each input source are available through the citation tool, as required by the GATHER statement.

The citation tool is accessible through the GHDx at http://ghdx.healthdata.org/global-burden-disease-study-2016

1.7 List of Acronyms

Adol: adolescent

Air poll mort: mortality attributable to air pollution

ANC: Antenatal care

ART: antiretroviral therapy

BTL: basic tabulation list

CDR: crude death rates

CHERG: Child Health Epidemiology Research Group

CKD: chronic kidney disease

CKD-DM: chronic kidney disease deaths attributable to diabetes

COD: causes of death

CODEm: cause of death ensemble modelling

COPD: chronic obstructive pulmonary disease

CR: cancer registry

CVD: cardiovascular disease

DAH: development assistance for health

DALY: disability-adjusted life-year

DHS: Demographic and Health Survey

DPT: diphtheria-pertussis-tetanus

DSP: Disease Surveillance Points

ELISA: enzyme-linked immunosorbent assay

EPEC: enteropathogenic E. coli

EPP: Estimation and Projection Package

ETEC: enterotoxigenic E. coli

GATHER: Guidelines for Accurate and Transparent Health Estimates Reporting

GBD: Global Burden of Diseases, Injuries, and Risk Factors Study

GEMS: Global Enteric Multicenter Study

GHDx: Global Health Data Exchange

GPRM: Global Price Reporting Mechanism

HAQ: Healthcare Access and Quality

HH air poll: household air pollution

Hib: Haemophilus influenzae type B

HIV CDR: Crude death rate due to HIV/AIDS

IAEG-SDGs: Inter-Agency and Expert Group on Sustainable Development Goal Indicators
IARC: International Agency for Research on Cancer
ICD: International Classification of Disease
IHD: ischemic heart disease
IHR: International Health Regulations
IOTF: International Obesity Task Force
IPV: intimate partner violence
ISIC: International Standard Industrial Classification
JMP: Joint Monitoring Programme
LDI: lag distributed income per capita
LRI: lower respiratory infection
MAP: Malaria Atlas Project
MCCD: Medical Certification of Causes of Death
MCEE: Maternal and Child Epidemiology Estimation group
MDG: Millennium Development Goal
MI: mortality/incidence ratio
MM: maternal mortality
MMR: maternal mortality ratio
MMS: Maternal Mortality Surveillance
Mort: mortality
NCD: non-communicable disease
NN mort: neonatal mortality
NTDs: neglected tropical diseases
OECD: Organisation for Economic Co-operation and Development
Occ risk burden: burden attributable to occupational risks
ODA: Official development assistance
PAF: population-attributable fraction
PCV3: Three-dose pneumococcal conjugate vaccine
PHE: population health equivalence
PM2.5: particulate matter <2.5μm in diameter
RMSE: root mean square error
RSV: respiratory syncytial virus
SBA: skilled birth attendance
SBH: summary birth history
SCD(R): Survey of Causes of Death (Rural)
SD: standard deviation
SDG: Sustainable Development Goals
SDI: Socio-demographic Index
SDSN: Sustainable Development Solutions Network
SEER: Surveillance, Epidemiology, and End Results Program
SEV: summary exposure variable
SRS: Sample Registration System

ST-GPR: spatiotemporal Gaussian process regression
TAC: TaqMan Array Card
TB: tuberculosis
TRIPS: Agreement on Trade-Related Aspects of Intellectual Property Rights
UHC: universal health coverage
UIs: Uncertainty intervals
UN: United Nations
UNICEF: United Nations Children's Fund
VA: verbal autopsy
VR: vital registration
WaSH: water, sanitation, and hygiene
WHO: World Health Organization
YLD: years lived with disability
YLL: years of life lost

1.8 Funding Sources

Funding for this research was provided by the Bill & Melinda Gates Foundation.

Section 2. Nonfatal outcome estimation

The GBD 2016 nonfatal estimation process is visually represented in Appendix Figures 1a and 1b illustrating the steps necessary to estimate incidence, prevalence, and YLDs for disease and injury sequelae in GBD 2016. Appendix Figure 1a outlines the general process of nonfatal outcome estimation from data inputs to finalization of YLD burden results; steps 3b and 3c of that process identify alternative modelling approaches employed for certain causes and injuries. Alternative approaches are illustrated in greater detail in Appendix Figure 1b. Conceptually, the estimation effort is divided into eight major components: (1) compiling data sources through data identification and extraction; (2) data adjustment; (3) estimation of prevalence and incidence by cause and sequelae using DisMod-MR 2.1 or alternative modelling strategies for selected cause groups; (4) estimation by impairment; (5) severity distributions; (6) incorporation of disability weights; (7) comorbidity adjustment; and (8) the estimation of YLDs by sequelae and causes. Appendix Section 3 contains additional detail specific to each disease, impairment and injury and their sequelae. Nonfatal modelling strategies vary significantly between causes.

2.1 Data sources, identification and extraction

2.1.1 Systematic reviews

For GBD 2015, updated systematic reviews were conducted for 101 causes and sequelae using data available through January of 2015. For GBD 2016 we conducted literature reviews for 116 causes and 4 impairments. For other diseases, only a small fraction of the existing data appears in the published literature and other sources predominate such as survey data, disease registers, notification data, hospital inpatient data, or claims data. Data were systematically screened from household surveys archived in the Global Health Data Exchange (ghdx.healthdata.org), including Demographic and Health Surveys, Multiple Indicator Cluster Surveys, Living Standards Measurement Surveys, and Reproductive Health Surveys. Other national health surveys were identified based on survey series that had yielded usable data for past rounds of GBD, sources suggested to us by in-country collaborators, and surveys identified in major multinational survey data catalogs, such as the International Household Survey Network and the World Health Organization (WHO) Central Data Catalog, as well as through country Ministry of Health and Central Statistical Office websites. Case notifications reported to the WHO were updated through 2016. Citations for all data sources used for nonfatal estimation in GBD 2016 are provided in searchable form through a web-tool (http://ghdx.healthdata.org/). A description of the search terms employed for cause-specific systematic reviews are detailed by cause in Appendix Section 3.

2.1.2 Survey data preparation

For GBD 2016, survey data for which we had access to the unit record data constituted a substantial part of the underlying data used in the estimation process. During extraction, we concentrated on demographic variables (such as location, sex, age), survey design variables (such as sampling strategy and sampling weights), and the variables used to define the population estimate (such as prevalence or a proportion) and a measure of uncertainty (standard error, confidence interval or sample size and number of cases).

2.1.3 Disease Registers

For GBD 2016 nonfatal estimation, disease registries were an important source for a select number of conditions such as cancers, end-stage renal disease, and congenital disorders. The GHDx source tool (http://ghdx.healthdata.org/data-type/disease-registry)[2] provides a comprehensive list of registry data used in GBD estimation processes.

2.1.4 Estimation of Hospital Envelope

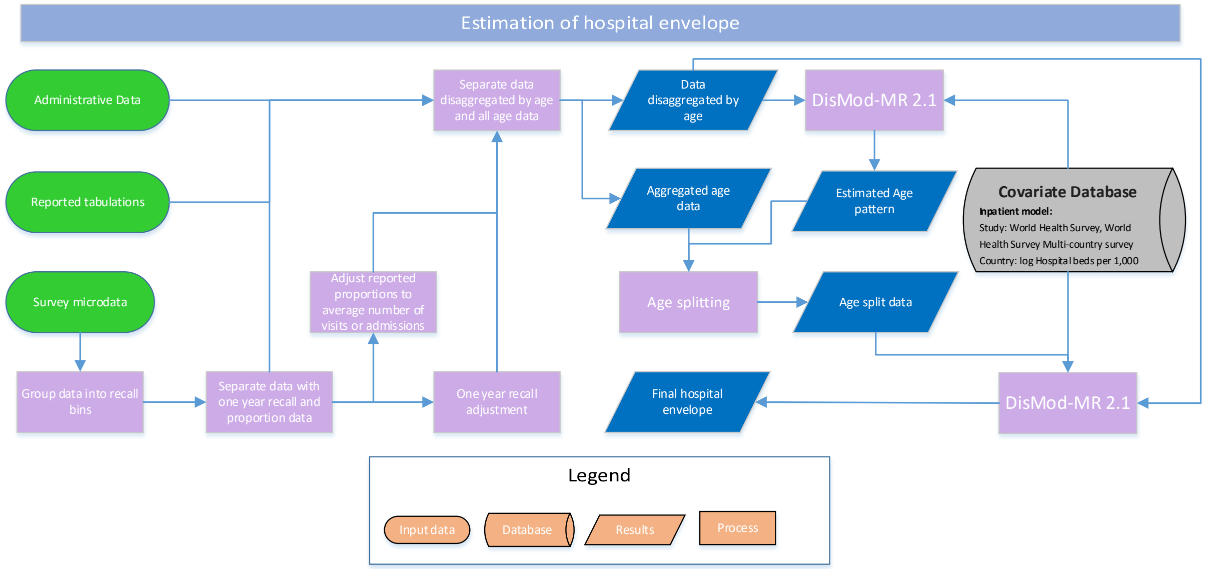

Input data and methodological summary

Case definition

We defined a hospital admission as the overnight admission into a formal healthcare facility but excluded admissions to long-term care (>120 days), nursing care facilities, and traditional or spiritual healers.

Input Data

We searched the GHDx for population surveys, administrative records, and censuses from January 1990 to September 2016. We applied five secondary data filters: “discharge”, “health facility”, “nationally representative”, “household”, or “outpatient.” We also applied ten keyword filters: “healthcare access”, “health care costs”, “healthcare economics”, “healthcare expenditure”, “healthcare services”, “healthcare use”, “outpatient facilities”, “patient counts”, “hospitals”, or “length of stay”. We applied no language restrictions to our search and required all returned records to either contain microdata or tabulated reports. We searched the returned records’ metadata looking for measures of inpatient care. For inclusion, we required all measures to be nationally or subnationally representative. Additionally, we consulted with experts and GBD collaborators to gather data sources that were not within the GHDx. In total, we accepted data sources from 2,855 location-years (1,560 from administrative records and 1,295 from population surveys).

Modelling Strategy

Data adjustment

We classified each of the accepted data sources into four data types: (a) proportion of survey respondents who were admitted into the hospital in the past 30 days; (b) proportion of survey respondents who were admitted to the hospital in the past year; (c) average number of admissions (utilization rate) reported by survey respondents in the past year; and (d) average number of visits reported by annual administrative records. We assigned measures reported by annual administrative records as our reference group as these data types were free from recall bias and most closely matched our case definition. In data sources where microdata was available, we extracted and binned the data based on gender and age groups of under-1, 1-4, 4-9, 10-14, through till 95+ years of age.

We crosswalked each of the three non-reference (survey) data types to the reference (administrative record) data type through the use of penalized spline regressions to account for non-systematic differences between the data types. For each non-reference data type and each sex, we looked for overlap between the non-reference data type and reference data type on the basis of location, year, age group, and sex. With the overlapping data, we calculated the ratio of the point estimate from the reference data type, μ_{ref}, to the non-reference data type, μ_s. We fit these ratios with a penalized spline regression shown below

$$\ln\left(\frac{\mu_{ref,i}}{\mu_{s,i}}\right) = h(age_i) + \varepsilon_i \ (1)$$

Where i denotes a given matched observation, $h(age_i)$ represents a basis function which estimated a cross-validated penalized spline over the population weighted mean age of the age group, and ε represents the residual. In the below figures, for each non-reference data type, we plot the ratio of μ_{ref} and μ_s across age and by sex and the predictions from the penalized spline regressions

Figure. Global age-sex specific crosswalks to equate each non-reference data type top the reference data type. For each non-reference data type and each sex, we plot the ratio of reference data points to non-reference data points, which were matched based on location, age group, year, and sex. Using a penalized spline regression, we estimated the crosswalk between each non-reference data type and the reference type. We plot the crosswalk and the associated prediction error below.

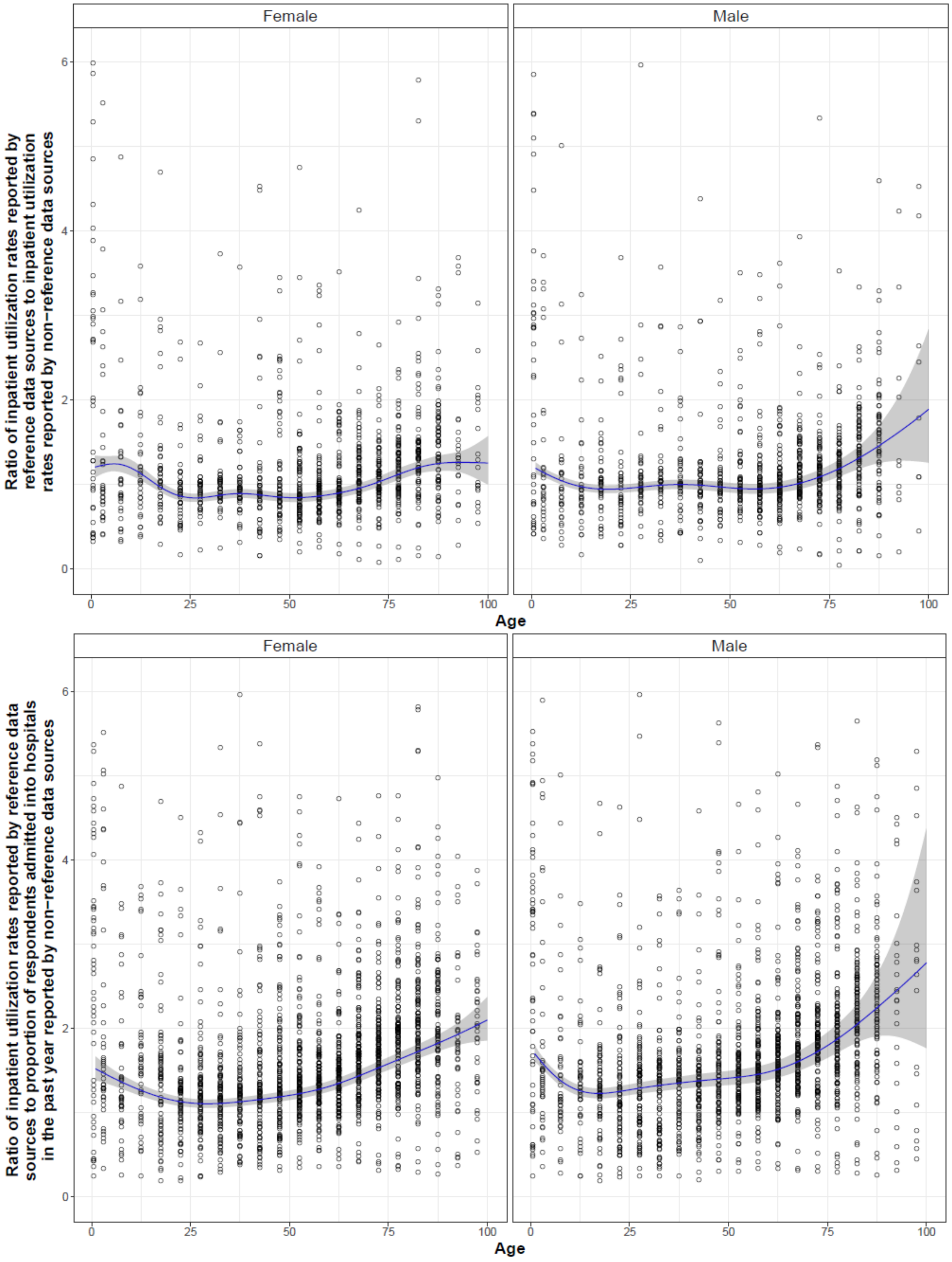

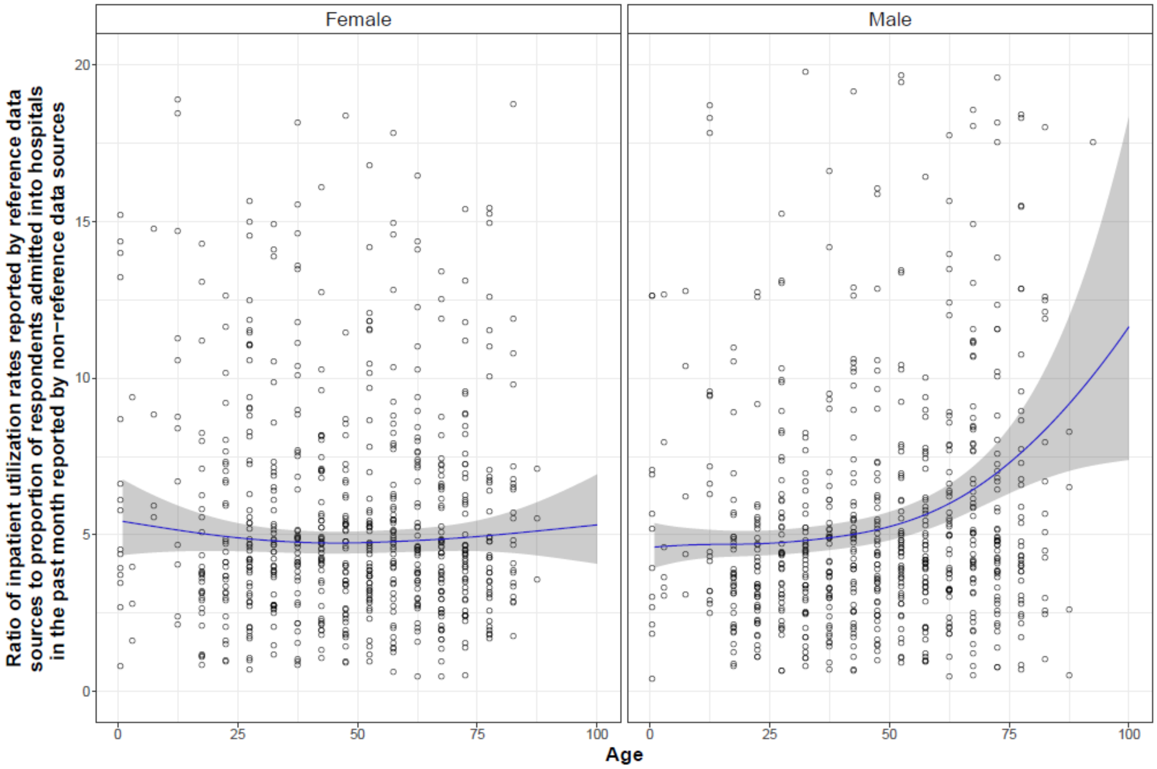

To crosswalk non-reference data types to reference data types, we multiplied non-reference data types by the exponentiated predictions from respective penalized spline regressions. Uncertainty from the adjustments were accounted for using the following equation

$$se_a = \sqrt{se_m^2 \cdot se_s^2 + se_m^2 \cdot \mu_s^2 + se_s^2 \cdot \mu_m^2} \quad (2)$$

Where se_a, se_m, and se_s are the standard errors of the adjusted non-reference data point, the exponentiated crosswalk prediction, and the non-reference data point, respectively. μ_s and μ_m are the means of the non-reference data point and the exponentiated crosswalk predictions from the penalized spline regressions.

Age-sex splitting

DisMod-MR 2.1 is capable of conducting age-integration but its performance degrades while integrating across wide age categories (e.g. all ages). To remedy this issue, we ran a DisMod-MR 2.1 model with data that was disaggregated by age to estimate countries' age-pattern and then applied the estimated age-pattern to split aggregated all age data. This procedure was operationalized by calculating a constant, k, which was the ratio of the aggregated all age data point, $\mu_{all\ age}$, to the all age estimated utilization rate from the DisMod-MR 2.1 model, $\widehat{\mu_d}$

$$k = \frac{\mu_{all\ age}}{\widehat{\mu_d}} \quad (3)$$

The constant, k, was then multiplied by age specific utilization rates from the DisMod-MR 2.1 model. The uncertainty from the data and the age-pattern were propagated following equation 2. The split data were then incorporated into the final DisMod-MR 2.1 model.

DisMod Modelling

To help explain variation in locations with little to no data, we used the country level covariate of natural log of hospital beds per 1,000. Study level covariates were used to denote World Health Surveys and World Health Organization's Multi-Country Survey Study on Health System Responsiveness. The country-level covariate of hospital beds per 1,000 was estimated using ST-GPR on data sourced from the World Bank. The study-level covariates were used as these two survey series were systematically higher than data points from other sources in the same locations and time period. Coefficients for the covariates are presented in below table.

Table. Estimated coefficients of the hospital envelope model.
Study-level covariates denote global dichotomous covariates that serve to adjust corresponding data points

Covariate	type	Coefficient	Exponentiated coefficient
World Health Survey	Study-level	0.44<br>(0.39 — 0.48)	1.55<br>(1.48 — 1.61)
World Health Organization Multi-country Survey Study on Health and Responsiveness	Study-level	0.49<br>(0.42 — 0.55)	1.63<br>(1.52 — 1.73)
log Hospital beds per 1,000	Country-level	0.17<br>(0.16 — 0.19)	1.19<br>(1.17 — 1.20)

2.1.5 Claims, inpatient hospital, and outpatient data

For GBD 2016, claims (linkage) data, inpatient hospital, and outpatient data played a key role in the nonfatal estimation process of many GBD causes.

Claims data

For GBD 2016, we accessed aggregate data derived from claims information in a database of US private health insurance and public insurance schemes of Medicaid and Medicare, for the years 2000, 2010, and 2012, commonly referred to as Marketscan. The population covered in each year was 3.3 million in 2000, 40.4 million in 2010, and 40.8 million in 2012. For each of these individuals, information on every health service encounter was collected and all episodes of care were linked to individuals by unique identifiers, which allowed us to aggregate data in multiple ways, including creating counts of claims and individuals for inpatient and outpatient care. Marketscan has fifteen diagnosis columns for both inpatient and outpatient episodes. We mapped all ICD-9 four- or five-digit-coded diagnoses to GBD causes (see Appendix Table 4). GBD conditions were categorized as "long-term" or "short-term" depending on cause duration. In a given year, for each individual in the claims data, a long-term case was defined as any mention in any diagnostic field associated with any claim, including inpatient and outpatient encounters. A short-term case was defined the same way, but assumed that claims within a condition-specific duration were the same case. In this way, an individual could have multiple short-term conditions in a given year, while avoiding double-counting cases with multiple claims from a single illness episode.

In GBD 2015, a subset of available facility types were used for short-term causes in outpatient claims data. In GBD 2016, we added more facility types, including the “office visit” facility type, which accounts for more than 50% of all outpatient data.

GBD 2016 Marketscan/Claims data extraction process

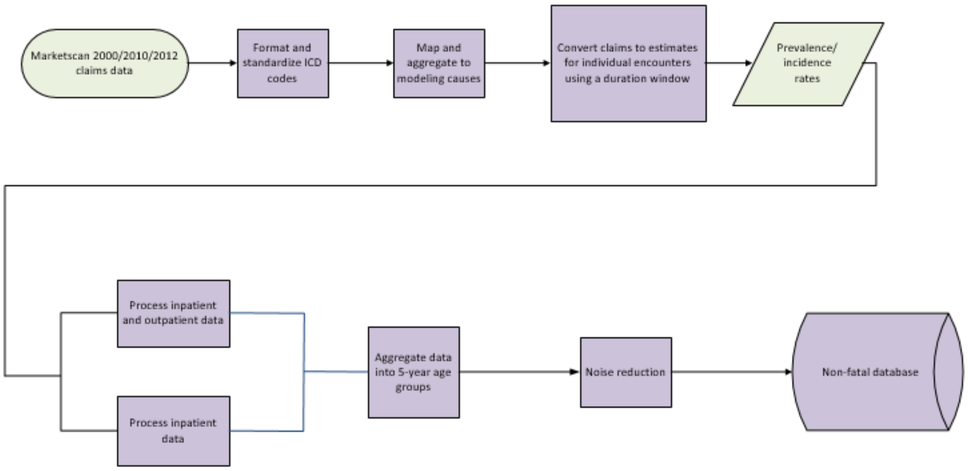

Inpatient hospital admissions

Inpatient hospital data were extracted from 3557 location-years in 41 countries. ICD coding was standardized across sources, and versions of ICD (Appendix Table 4).

For GBD 2015, one limitation of our use of hospital data in non-fatal disease estimation was the challenge of accessing accurate information on coverage populations for any given data source. Section 2.1.4 of the appendix describes the modelling strategy for the hospital envelope, an estimate of hospital utilization, i.e. the rate of inpatient episodes per capita by age, sex, year and location. For GBD 2016, we used this hospital utilization envelope in place of information on coverage population. We calculated demographic-specific (by age, sex, year, and location) cause fractions in each inpatient hospital data source and multiplied these by the hospital utilization envelope to produce a rate of incidence/prevalence in inpatient admissions. For countries with completed registration of all inpatient episodes, this method makes little difference compared to the previous approach of dividing inpatient episode numbers by population as the all-cause inpatient rates per population from these sources were inputs to the utilization model. For countries with incomplete coverage, where in the past we had to make assumptions on the proportion of the population covered or reject the source because of unknown catchment population, this method is an improvement.

Using the Marketscan claims data described above (with the exception of Marketscan 2000 data, due to the low coverage compared to two later years), we generated three scalars that were applied to the product of cause fractions generated by the inpatient hospital data and the utilization envelope on a

cause-by-cause basis. The scalars account for bias in inpatient hospital data from sources which were aggregated by ICD code and by primary diagnosis only. First, we corrected to account for multiple admissions for an individual. Second, we adjusted for non-primary diagnoses. Third, we corrected to account for inpatient and outpatient care. Combined with the uncorrected version (no scalar applied), this resulted in four types of incidence and prevalence estimates from inpatient hospital data: 1) (un-corrected) inpatient admissions by episode, primary diagnosis, 2) inpatient admissions by individual, primary diagnosis only, 3) inpatient hospital admissions, accounting for all diagnoses, 4) an estimate of any inpatient admission or outpatient visit by an individual, accounting for all diagnoses. These data were reviewed in conjunction with data from all other sources for each model that utilizes hospital data to determine which type of data adjustment was most appropriate as an input to non-fatal disease estimation.

The equations we used for each of the three scalars can be found below:

a) Adjustment to account for multiple admissions which gave us inpatient admissions by individual, primary diagnosis only
    a. $inpatient^{1°}_{episode} * \left(\frac{MS\ inpatient^{1°}_{indiv}}{MS\ inpatient^{1°}_{episode}}\right) = inpatient^{1°}_{indiv}$
b) Adjustment for non-primary diagnoses which gave us inpatient admissions by individual, all diagnoses
    a. $inpatient^{1°}_{episode} * \left(\frac{MS\ inpatient^{all}_{indiv}}{MS\ inpatient^{1°}_{episode}}\right) = inpatient^{all}_{indiv}$
c) Adjustment to account for inpatient and outpatient care which gave us inpatient admissions and outpatient visits by individual, all diagnoses
    a. $inpatient^{1°}_{episode} * \left(\frac{MS\ inpatient^{all}_{indiv} \cup MS\ outpatient^{all}_{indiv}}{MS\ inpatient^{1°}_{episode}}\right) = inpatient|outpatient^{all}_{indiv}$

For maternal causes, a new bundle was created that had every maternity-related ICD code mapped to it. At the end of the process, each maternal cause rate was divided by the rate of this all maternity bundle in order to adjust the denominators from population to live births.

Congenital and neonatal causes with similar age patterns were aggregated to create correction factors because the data in individual causes were too sparse to make them reasonable, and would leave the age pattern mostly flat. Injuries used a separate correction factor as well.

These scalars were then smoothed by fitting a Loess curve to the observed data for each combination of cause and sex. The span of the Loess smoothing varied depending on how many observable data points there were. For cause/sex subsets with 5 or fewer observations a span (fraction of number of points to consider when fitting a curve) of 1 was set. For 6 to 10 observations, a span of 0.75 was set, and for 11 or more observations, a span of 0.5 was set.

In cases where the third scalar, accounting for inpatient and outpatient care, was greater than 50, we determined it to be unstable and did not apply this scalar to the hospital data. Exceptions to this rule

were for congenital and neonatal causes (preterm births, neonatal hemoloytic disease, encephalopathy, sepsis), where babies hospitalized with these conditions often have comorbid states that make it very likely that the given code would not be listed as primary, as well as peripheral arterial disease and cirrhosis. For these causes, the exception is 100.

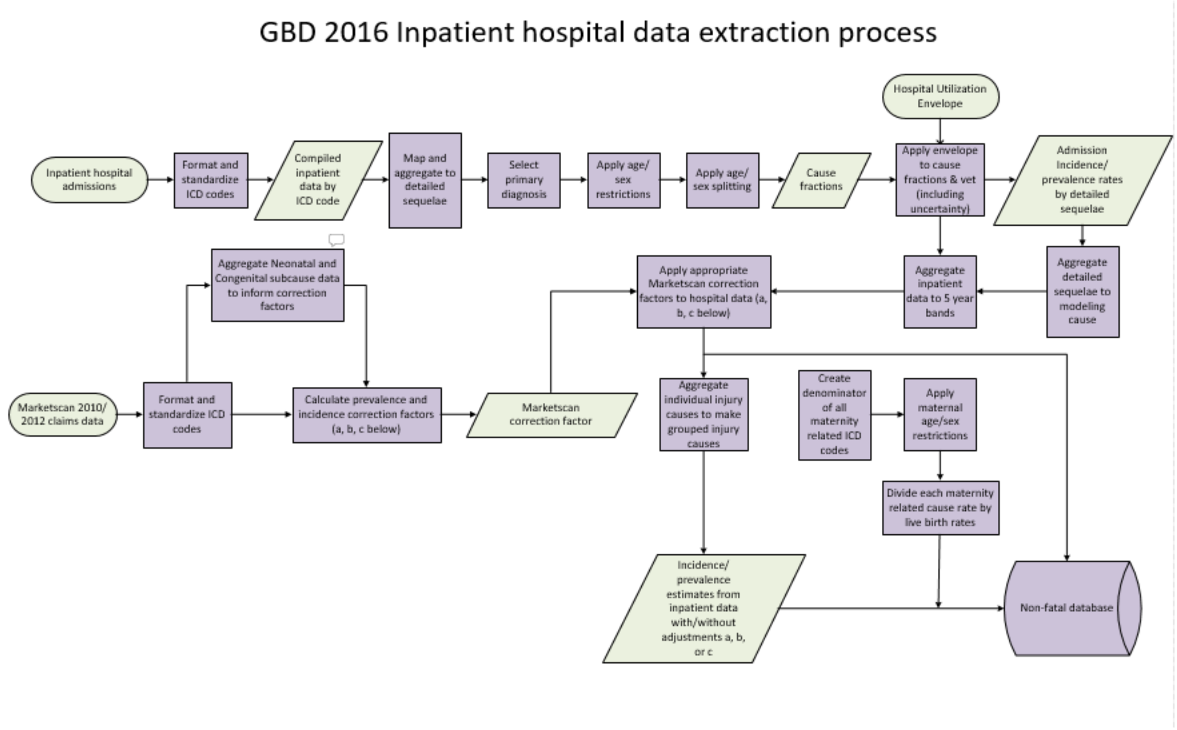

Outpatient

Outpatient encounter data were available from the US and Sweden for 68 location-years. In GBD 2016, Brazil and Mexico were included, but we dropped them this year due to lack of reliability (biases related to types of hospitals that were included in the datasets, etc). No changes were made in the processing of outpatient data from GBD 2015, aside from updates to the ICD mappings to GBD cause.

Similar to the inpatient hospital data, a scalar was calculated using Marketscan claims data to adjust for multiple visits per individual within one year (for long-term conditions), and within a cause-specific duration (for short-term causes). However, for the outpatient correction factor, we kept only 3 outpatient facility types (office, outpatient hospital, and outpatient NEC).

Table: Facility types used in outpatient Marketscan data

Outpatient Facility Name	Used in GBD 2015 for Incidence	Used in GBD 2016 for Incidence	Used in Outpatient Correction Factor
Office	no	yes	yes
Outpatient Hospital	yes	yes	yes
Independent Laboratory	yes	no	no
Emergency Room - Hospital	yes	yes	no
Patient Home	yes	yes	no
Inpatient Hospital	no	yes	no
Ambulatory Surgical Center	no	yes	no
End-Stage Renal Disease Facil	no	yes	no
Other Unlisted Facility	no	yes	no
Outpatient (NEC)	no	yes	yes
Skilled Nursing Facility	no	yes	no
Urgent Care Facility	no	yes	no
Ambulance (land)	no	yes	no
Independent Clinic	no	yes	no
Comprehensive Outpt Rehab Fac	no	yes	no
Nursing Facility	no	yes	no
Hospice	no	yes	no
Pharmacy (1)	no	no	no
Rural Health Clinic	no	yes	no
State/Local Public Health Clin	no	yes	no
Birthing Center	no	yes	no
Community Mental Health Center	no	yes	no
Federally Qualified Health Ctr	no	yes	no
Psych Facility Partial Hosp	no	yes	no
Residential Subst Abuse Facil	no	yes	no
Mass Immunization Center	no	yes	no
Custodial Care Facility	no	yes	no
Comprehensive Inpt Rehab Fac	no	yes	no
Psych Residential Treatmnt Ctr	no	yes	no
Inpatient Long-Term Care (NEC)	no	yes	no
Mobile Unit	no	yes	no
Assisted Living Facility	no	yes	no
School	no	yes	no
Inpatient Psychiatric Facility	no	yes	no
Walk-in Retail Health Clinic	no	yes	no
Military Treatment Facility	no	yes	no
Pharmacy (2)	no	no	no

Other Inpatient Care (NEC)	no	yes	no
Ambulance (air or water)	no	yes	no
Non-resident Subst Abuse Facil	no	yes	no
Intermed Care/Mental Retarded	no	yes	no
Group Home	no	yes	no
Adult Living Care Facility	no	yes	no
Temporary Lodging	no	yes	no
Homeless Shelter	no	yes	no
MISSING	no	yes	no

Table: Durations of causes

Duration in Days	GBD Cause
28	diarrheal diseases, clostridium difficile, pelvic inflammatory disease, acute otitis media, myocardial infarction due to ischemic heart disease, first ever acute hemorrhagic stroke, first ever acute ischemic stroke, upper respiratory infections, peptic ulcer disease, symptomatic episodes, gastritis and duodenitis, symptomatic episodes, appendicitis, vascular intestinal disorders, paralytic ileus and intestinal obstruction, gallbladder and biliary diseases, symptomatic episodes, pancreatitis cases, interstitial nephritis and urinary tract infections, acute urolithiasis, acute myocarditis, pelvic inflammatory disease due to gonococcal infection, cerebrovascular disease acute, pelvic inflammatory disease due to chlamydial infection
30	influenza
60	acute other sense organ diseases, lower respiratory infections
90	typhoid fever, paratyphoid fever, abscess and other bacterial skin diseases, other meningitis -- viral, gonococcal infection, trichomoniasis infection, adverse effects of medical treatment, foreign body in eyes, acute glomerulonephritis, cellulitis, impetigo, foreign body in other body part, pulmonary aspiration and foreign body in airway, other transport injuries, cyclist road injuries, motor vehicle road injuries, motorcyclist road injuries, other road injuries, pedestrian road injuries by road vehicle, poisonings, falls, fire, heat, and hot substances, environmental heat and cold exposure , other unintentional injuries, venomous animal contact, non-venomous animal contact, exposure to forces of nature, drowning, unintentional suffocation, other exposure to mechanical forces, unintentional firearm injuries, self-harm by other specified means, self-harm by firearm, sexual violence, assault by other means, assault by firearm, assault by sharp object, executions and police conflict, conflict and terrorism, typhoid and paratyphoid fever
120	acute encephalitis
180	endocarditis, early syphilis infection, chlamydial infection, maternal abortive outcome
365	diphtheria remission, whooping cough, meningitis nonfatal overall, mild impairment due to neonatal tetanus, varicella seroprevalence, measles, visceral leishmaniasis, cutaneous and mucocutaneous leishmaniasis, symptomatic cystic echinococcosis, disfigurement due to basal cell carcinoma, cutaneous squamous

	cell carcinoma, Guillain-Barré syndrome, ectopic pregnancy incidence ratio, maternal hemorrhage, hypertensive disorders of pregnancy, other maternal infections, obstructed labor, acute event, puerperal sepsis, decubitus ulcer, meningococcal meningitis incidence proportion, other meningitis incidence proportion, H influenza type b meningitis incidence proportion, pneumococcal meningitis incidence proportion, severe pre-eclampsia, eclampsia

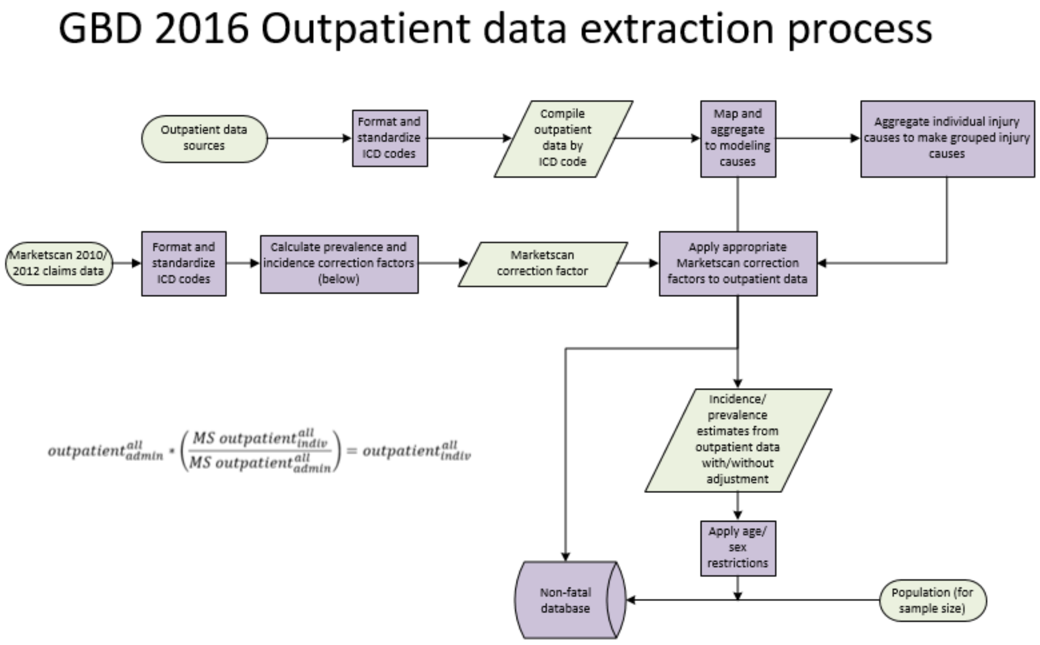

2.1.6 Case notifications

Case notifications, active screening, intervention coverage studies, and surveillance contributed to estimates of infectious diseases. If available, we extracted data from survey and administrative microdata; otherwise, data were extracted from published literature and reports. For many infectious diseases and neglected tropical diseases (NTDs), we made use of cases notified by countries to the World Health Organization (WHO) and other global monitoring entities. The causes for which we use WHO case notification data included tuberculosis (TB), measles, yellow fever, rabies, dengue, cholera, whooping cough, human African trypanosomiasis (HAT), meningitis, all sexually transmitted infections (STIs), and other infectious and NTDs, such as Ebola.

2.2 Data adjustment

In addition to the corrections applied to claims and hospital data, a number of other adjustments were applied to extracted nonfatal sources in order to make the data more consistent and suitable for modelling. In this second step of nonfatal estimation, commonly applied adjustments included age-sex splitting, adding study-level covariates, bias correction, adjustments for underreporting of notification data, and computing expected values of excess mortality.

Age-sex splitting was commonly applied to literature data reported by age or sex but not by age and sex. For GBD2016, we split all data reported in age groups with a width greater than 20 years, using age patterns from available survey microdata or regional patterns derived from an initial run of main modelling tool, DisMod-MR 2.1. We relied on the meta-regression component of DisMod-MR 2.1 for many of the bias correction of data for variations in study attributes such as case definitions and measurement method. DisMod-MR 2.1 calculates a single adjustment that is applied regardless of age, sex, or location. If enough data were available to differentiate these adjustments by age, sex, or location, or if detailed survey data were available to make more precise adjustments between different thresholds on a biochemical measure, we applied bias corrections to the data before entry into DisMod-MR 2.1. For instance, we crosswalked between 15 different case definitions with different thresholds of fasting plasma glucose or glycated hemoglobin levels for diabetes mellitus based on available survey data with individual records of the actual measurements. In another example, we corrected data reporting on one-year prevalence instead of point prevalence of alcohol dependence by age using studies reporting on both measures, as the average duration of alcohol dependence is greater in middle-aged and older individuals compared to young adults. The correction of notification data for underreporting relied on studies that had examined the gap between true incidence and notified cases.

In GBD 2016, we estimated expected values of excess mortality from prevalence or incidence and cause-specific mortality rate (CSMR) data for every cause for which deaths were estimated with the exception of a few causes with very low mortality rates such as uterine fibroids. We matched every prevalence data point (or incidence data for short-duration conditions) with the CSMR value corresponding to the age range, sex, year, and location of the data point. We restricted this to data points reporting age-groups spanning 20 years or less. The ratio of CSMR to prevalence (or incidence times a short duration) is conceptually equivalent to an excess mortality rate. To reflect a gradient in excess mortality, we added in all relevant models the log of lag distributed income (LDI) or the health access and quality index (HAQI) as a country covariate for excess mortality, with a strong prior that as LDI increases, excess mortality declines.

2.3 DisMod-MR 2.1 Estimation

a. Estimation of sequelae and causes

The most extensively used estimation method was the Bayesian meta-regression method DisMod-MR 2.1. For some causes such as HIV/AIDS or hepatitis B and C, disease-specific natural history models have been used where the underlying three state model in DisMod-MR 2.1 (susceptible, cases, dead) is insufficient to capture the complexity of a disease process. For some diseases with a range of sequelae differentiated

by severity, such as chronic obstructive pulmonary disease (COPD) or diabetes mellitus, DisMod-MR 2.1 was used to meta-analyze the data on overall prevalence with separate DisMod-MR 2.1 models of the proportions of cases with different severity levels or sequelae. Likewise, DisMod-MR 2.1 was used to meta-analyze data on the proportions of liver cancer and cirrhosis due to underlying etiologies such as hepatitis B, hepatitis C, and alcohol use.

b. DisMod-MR 2.1 description

Until GBD 2010, nonfatal estimates in burden of disease assessments were based on a single data source on prevalence, incidence, remission or a mortality risk selected by the researcher as most relevant to a particular location and time. For GBD 2010, we set a more ambitious goal: to evaluate all available information on a disease that passed a minimum quality standard. That required a different analytical tool that would be able to pool disparate information presented in varying age groupings and from data sources using different methods. The DisMod-MR 1.0 tool used in GBD 2010 evaluated and pooled all available data, adjusted data for systematic bias associated with methods that varied from the reference and produced estimates by world regions with uncertainty intervals using Bayesian statistical methods. For GBD 2013, the improved DisMod-MR 2.0 had increased computational speed allowing computations that were consistent between all disease parameters at the country rather than region level. The hundred-fold increase in speed of DisMod-MR 2.0 was partly due to a more efficient rewrite of the code in C++ but also by changing to a model specification using log rates rather than a negative binomial model used in DisMod-MR 1.0. In cross-validation tests, the log rates specification worked as well or better than the negative binomial specification.[3] For GBD2015, we rewrote the 'wrapper' code that organises the flow of data and settings at each level of the analytical cascade. The sequence of estimation occurs at five levels: global, super-region, region, country and, where applicable, subnational location. The super-region priors are generated at the global level with mixed-effects, nonlinear regression using all available data; the super-region fit, in turn, informs the region fit, and so on down the cascade. The wrapper gives analysts the choice to branch the cascade in terms of time and sex at different levels depending on data density. The default used in most models is to branch by sex after the global fit but to retain all years of data until the lowest level in the cascade. Appendix Figure 2 below summarizes the Dismod-MR process.

In updating the 'wrapper,' we consolidated the code base into a single language, Python, to make the code more transparent and efficient and to better deal with subnational estimation. The computational engine is limited to three levels of random effects; we differentiate estimates at the super-region, region and country level. In GBD 2013, the subnational units of China, the UK and Mexico were treated as 'countries' such that a random effect was estimated for every location with contributing data. However, the lack of a hierarchy between country and subnational units meant that the fit to country data contributed as much to the estimation of a subnational unit as the fits for all other countries in the region. We found inconsistency between the country fit and the aggregation of subnational estimates when the country's epidemiology varied from the average of the region. Adding an additional level of random effects required a prohibitively comprehensive rewrite of the underlying DisMod-MR engine. Instead, we added a fifth layer to the cascade, with subnational estimation informed by the country fit and country covariates, plus an adjustment based on the average of the residuals between the

subnational location's available data and its prior. This mimicked the impact of a random effect on estimates between subnationals.

In GBD 2015, we also improved how country covariates differentiate nonfatal estimates for diseases with sparse data. The coefficients for country covariates were re-estimated at each level of the cascade. For a given location, country coefficients were calculated using both data and prior information available for that location. In the absence of data, the coefficient of its parent location was used, in order to utilize the predictive power of our covariates in data sparse situations.

For GBD 2016, the computational engine (DisMod-MR 2.1) remained substantively unchanged from GBD 2015. We changed the prediction year set to generate fits for the years 1990, 1995, 2000, 2005, 2010, and 2016. We updated the age prediction sets to include age groups 80-84, 85-89, 90-94, and 95+, to comply with changes across all functional areas of the GBD. We also expanded the set of locations where subnational units are modeled; the set now includes: Brazil, China, England, India, Indonesia, Japan, Kenya, Mexico, Saudi Arabia, South Africa, Sweden, and the United States.

c. DisMod-MR 2.1 likelihood estimation

Analysts have the choice of using a Gaussian, log-Gaussian, Laplace or Log-Laplace likelihood function in DisMod-MR 2.1. The default log-Gaussian equation for the data likelihood is:

$$-log\left[p\left(y_j|\Phi\right)\right] = \log\left(\sqrt{2\pi}\right) + \log\left(\delta_j + s_j\right) + \frac{1}{2}\left(\frac{\log\left(a_j + \eta_j\right) - \log\left(m_j + \eta_j\right)}{\delta_j + s_j}\right)^2$$

where, y_j is a 'measurement value' (i.e., data point); Φ denotes all model random variables; η_j is the offset value, eta, for a particular 'integrand' (prevalence, incidence, remission, excess mortality rate, with-condition mortality rate, cause-specific mortality rate, relative risk or standardized mortality ratio) and a_j is the adjusted measurement for data point j, defined by:

$$a_j = e^{(-u_j - c_j)} y_j$$

where u_j is the total 'area effect' (i.e., the sum of the random effects at three levels of the cascade: super-region, region and country) and c_j is the total covariate effect (i.e., the mean combined fixed effects for sex, study level and country level covariates), defined by:

$$c_j = \sum_{k=0}^{K[I(j)]-1} \beta_{I(j),k} \hat{X}_{k,j}$$

with standard deviation

$$s_j = \sum_{l=0}^{L[I(j)]-1} \zeta_{I(j),l} \hat{Z}_{k,j}$$

where k denotes the mean value of each data point in relation to a covariate (also called x-covariate); I(j) denotes a data point for a particular integrand, j; $\beta_{I(j),k}$ is the multiplier of the kth x-covariate for the ith integrand; $\hat{X}_{k,j}$ is the covariate value corresponding to the data point j for covariate k; l denotes the standard deviation of each data point in relation to a covariate (also called z-covariate); $\zeta_{I(j),k}$ is the multiplier of the lth z-covariate for the ith integrand; and δ_j is the standard deviation for adjusted

measurement j, defined by:

$$\delta_j = log\left[y_j + e^{(-u_j-c_j)}\eta_j + c_j\right] - log\left[y_j + e^{(-u_j-c_j)}\eta_j\right]$$

Where m_j denotes the model for the jth measurement, not counting effects or measurement noise and defined by:

$$m_j = \frac{1}{B(j)-A(j)} \int_{A(j)}^{B(j)} I_j(\mathrm{a})\,\mathrm{da}$$

where A(j) is the lower bound of the age range for a data point; B(j) is the upper bound of the age range for a data point; and I_j denotes the function of age corresponding to the integrand for data point j.

2.4 Impairment and Underlying Cause Estimation

For GBD 2016, as in GBD 2015, we estimated the country-age-sex-year prevalence of nine impairments – step 4 of Appendix Figure 1a. Impairments in GBD are conditions or specific domains of functional health loss which are spread across many GBD causes as sequelae and for which there are better data to estimate the occurrence of the overall impairment than for each sequela based on the underlying cause. These impairments included: anaemia, epilepsy, hearing loss, heart failure, developmental intellectual disability, infertility, vision loss, Guillain-Barré syndrome, and pelvic inflammatory disease. Overall impairment prevalence was estimated using DisMod-MR 2.1. We constrained cause-specific estimates of impairments, as in the 19 causes of blindness, to sum to the total prevalence estimated for that impairment. Anaemia, epilepsy, hearing loss, heart failure, and developmental intellectual disability were estimated at different levels of severity. Estimates were made separately for primary infertility (those unable to conceive), secondary infertility (those having trouble conceiving again), and whether the impairment affected men and/or women. In the case of epilepsy, we determined the proportions with idiopathic and secondary epilepsy as well as the proportions with severe and less severe epilepsy using mixed effects regressions. The sparse data for the proportion of seizure-free, treated epilepsy were pooled in a random effects meta-analysis. DisMod-MR 2.1 models produced country-, age-, sex-, and year-specific severity levels of hearing loss and vision loss. Due to limited information on the severity levels of developmental intellectual disability, we assumed a similar distribution of severity globally, based on random effects meta-analysis of IQ-specific data for the overall impairment. This was supplemented by cause-specific severity distributions for chromosomal causes and iodine deficiency; the severity of developmental intellectual disability included in the long-term sequelae of causes including neonatal disorders, meningitis, encephalitis, neonatal tetanus, and malaria was estimated in combined health states of multiple impairments such as motor impairment, blindness, and/or seizures.[14] For GBD 2015, we changed the name of the intellectual disability impairment to specify that estimates reflect cases arising during the developmental period which we have defined as ages below 20. The severity of heart failure was derived from our Medical Expenditure Panel Surveys (MEPS) analysis and therefore was not specific for country, year, age, or sex.

A detailed description of the methods of each impairment can be found at the end of Section 3 of this appendix.

2.4.1 Impairment squeeze

For impairments like epilepsy, developmental intellectual disability, and blindness, mentioned above in Step 4, we often have better information regarding the total prevalence of the impairment rather than the prevalence of said impairment due to its various causes. For example, we have more data and a better idea of the total number of blind individuals (which we refer to herein as the blindness "envelope") in the world than we do for the number of individuals who are blind due to a specific cause like retinopathy of prematurity or cataract. We achieve this consistency by either "squeezing" or inflating the individual sequela prevalence values so that their sums fit into each appropriate envelope. Blindness, epilepsy and/or developmental intellectual disability appear in various combinations with motor impairment levels as sequelae for a number of neonatal disorders and infectious diseases like malaria and neonatal tetanus ("Moderate motor impairment with blindness and epilepsy due to neonatal tetanus," for example). This present an extra challenge as any squeeze or inflation of one of the impairments making up a sequela will affect the others. We set some rules on how to do these adjustments sequentially. First, when the envelope of an impairment is smaller than the sum of all contributing causes, we redistribute the 'excess' prevalent cases of combined impairment sequelae onto the sequelae that only have motor impairment (at mild, moderate or severe level) within the same cause grouping. Second, we apply the adjustments in a particular order such that we always fit at least one of the envelopes exactly where the other one or two envelopes may be exceeded by some amount. We first enforce a fit to the developmental intellectual disability impairment envelope, then epilepsy and, lastly, blindness. Thus, the developmental intellectual disability envelope will always match exactly, whereas the epilepsy and blindness envelopes may occasionally be exceeded on a draw-by-draw basis.

2.5 Severity Distribution

Sequelae were defined in terms of severity for 199 causes at Level 4 of the hierarchy (Appendix Table 3). We generally followed the same approach for estimating the distribution of severity as in GBD 2015. For Zika, we included sequelae for those with symptomatic acute infection, a small proportion with Guillain-Barré syndrome and the number of neonates with congenital Zika as reported to Pan American Health Organization (PAHO). For sexual violence, we estimated concurrent physical injuries and the more immediate psychological outcomes following sexual violence. For the added causes that were split from broader cause categories, the differentiation between drug-sensitive and drug-resistant tuberculosis, the creation of other leukemia, alcoholic and other cardiomyopathy, and self-harm by firearm or other means each follow the same pattern of assigning sequelae as for their parent causes. In cases in which severity was related to a particular impairment, such as mild, moderate, and severe heart failure due to ischemic heart disease, the analysis was driven by impairment estimation methods. Severity levels for conditions such as chronic kidney disease and COPD were modelled using DisMod-MR 2.1, while we performed meta-analyses to estimate the allocation of severity for causes such as rheumatoid arthritis, dementia, and multiple sclerosis.

For many causes we had inadequate data on severity from surveys or the epidemiological literature. For those diseases, we made use of three population surveys: the US Medical Expenditure Panel Survey (MEPS) 2000–2014, the [US] National Epidemiological Survey on Alcohol and Related Conditions (NESARC)

2000–2001 and 2004–2005, and the Australian National Survey of Mental Health and Wellbeing of Adults (NSMHWB) 1997.[20–22] Each dataset contained individual-level measurements of functional health status using the SF-12 Health Survey as well as diagnostic information on the conditions affecting each individual.

In order to use the data collected using SF-12 for measuring the distribution of severity, the individual SF-12 summary scores were mapped to an equivalent disability weight. A convenience sample of respondents was asked to complete SF-12 for the hypothetical individual living in a health state described using a selection of 60 of the 235 health states with their lay descriptions from the GBD Disability Weights (DW) surveys, reflecting the full range of severity. Each of these health states has a measured disability weight associated with it on a zero to one scale. In total, we collected 1,980 usable responses. To deal with heterogeneity in responses, we excluded from the statistical analysis responses that were more than two median absolute deviations from the median for each health state. After correcting for outliers, the rank order correlation between SF-12 scores for the hypothetical individuals in each health state characterized by the lay description with the measured disability weight was -0.815. The health states served as random effect groups, such that the composite score would be equal to the intercept plus the random effect estimated for that health state, or

$$DW_i = \alpha + U_{health\ state}.$$

The final relationship between SF-12 score and disability weight is shown below:

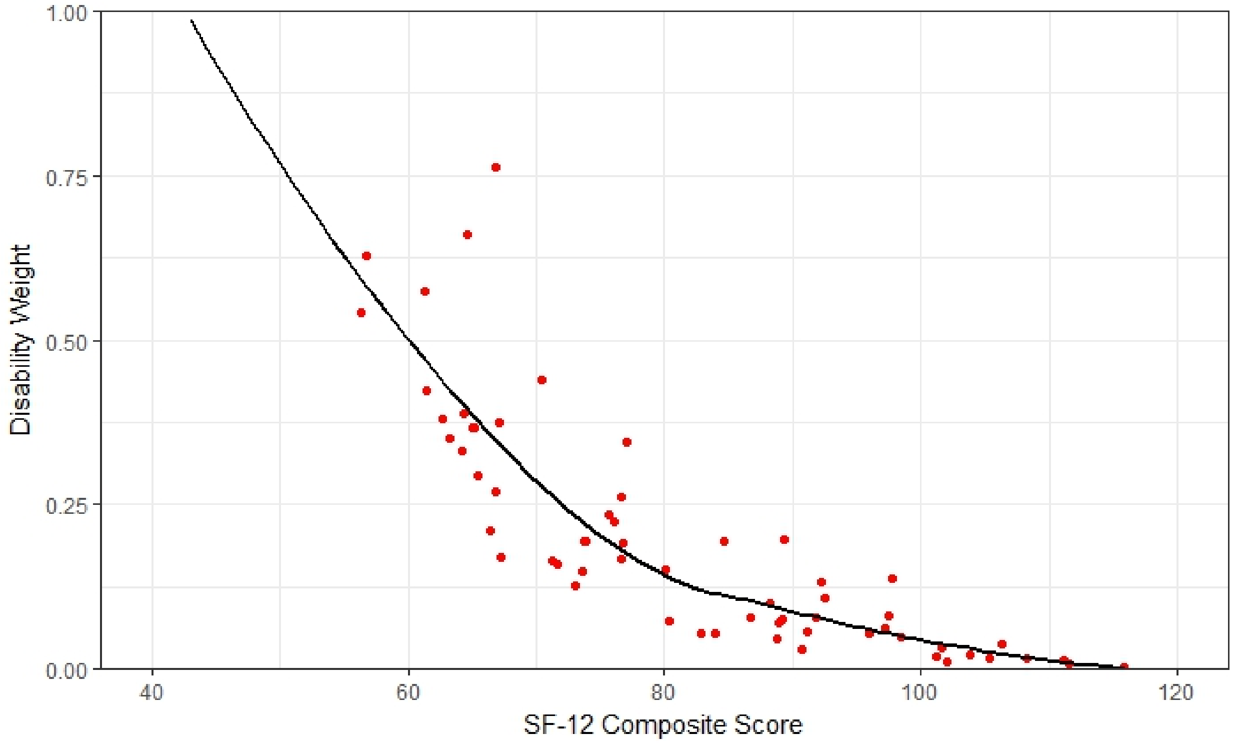

To generate a smooth mapping from SF-12 combined scores to the GBD disability weight space, we used LOESS regression on the random effects for each health state. Because disability weights are defined in the range from 0 to 1, we truncated the function at a combined SF-12 score of 116.36 (any combined score above this level was set to 0) and truncated the function at 42.7 so that any combined score less than that value was set to 1. All SF-12 survey data were thus transformed into disability weight space.

The second stage of the analysis was to build models predicting the transformed SF-12 scores as a function of the number of conditions suffered by each individual. First, variable selection was performed using LASSO regression to penalize the regression coefficients of highly correlated conditions. The tuning parameter, λ, controls the strength of the least-squares penalty. When λ=0, LASSO regression returns the same results as ordinary least-squares regression. Higher values of λ impose a stronger penalty and constrain a greater number of model parameters to 0. A 10-fold cross-validation was used to find the value of the λ that minimised the mean cross-validated error. This process resulted in a λ value of 0.0013 and eliminated 10 conditions from the analysis. Transformed SF-12 scores into the disability weight scale for the remaining 190 conditions were then modeled for each measure *m* of each individual *i* over *n* total conditions in the survey, as follows:

$$logit(DW)_{im} = \beta_0 + \beta_1 Condition1_{im} + \cdots + \beta_n Condition\, n_{im}$$

This equation effectively assumes that comorbid conditions act to change SF-12 scores in a multiplicative fashion rather than an additive fashion.

To estimate the comorbidity-corrected effect of each condition (i.e., in isolation) on total disability, we compared the predicted disability weight without the condition of interest (“counterfactual DW”) with the predicted disability weight including the condition of interest. Following the multiplicative comorbidity equation, the joint effect can be written:

$$Condition\ specificDW = 1 - \frac{1 - predictedDW_m}{1 - counterfactualDW_m}$$

The mean of this condition-specific effect over all observations is the population marginal effect of a condition.

Using the model above, we estimate a counterfactual disability weight – the total individual disability weight excluding the effect of the condition of interest. We compared the observed distribution of functional health status with this counterfactual distribution to determine the marginal effect of the condition of interest. In other words, we estimate the health state for each individual and for each condition as the cumulative individual weight minus the effects of all comorbid conditions.

$$Health\ state\ DW = 1 - \frac{1 - individual\ cumulativeDW_m}{1 - counterfactualDW_m}$$

The estimation strategy for health state-specific severity distributions where there are multiple severity categories involved binning individuals' weights into severity cutoffs (e.g., mild, moderate, and severe) for which disability weights were derived. These bins were defined using results from the GBD Disability Weights Studies[23] for conditions which had multiple health states defined. Cutoffs were taken as the midpoints between levels of health state and cases distributed into severity bins accordingly. Cases were considered asymptomatic if the counterfactual weight was equal to or exceeded the individual cumulative weight.

2.6 Disability weights

To compute YLDs for a particular health outcome in a given population, the number of people living with that outcome was multiplied by a disability weight that represents the magnitude of health loss associated with the outcome. Disability weights are measured on a scale from 0 to 1, with 0 implying a state that is equivalent to full health and 1 a state equivalent to death.

Disability weights used in GBD studies prior to GBD 2010 have been criticized for the method used (person trade-off), the small elite panel of international public health experts who determined the weights and the lack of consistency over time as the GBD cause list expanded and additional disability weights from a study in the Netherlands[24] were added or others derived by ad-hoc methods.

GBD 2010 Disability Weights Measurement Study

For GBD 2010, a primary data collection effort focused on measuring health loss rather than welfare loss using a standardized approach of simple comparison questions directed to the general public across diverse communities.

Household surveys were conducted between Oct 28, 2009 and June 23, 2010 in five countries (Bangladesh, Indonesia, Peru, Tanzania and the USA) selected to provide diversity across culture, language and socioeconomic status.

Personal face-to-face computer-assisted interviews were conducted for all household surveys with the exception of the survey in the USA which was conducted as computer-assisted telephone interviews. Households were randomly selected using a multistage stratified sampling design where the probability of selection was proportional to the population size. In all cases, samples were designed to be representative for a given geographical area with national representation in the case of the USA.

For every contacted household, an adult respondent aged 18 years or older was randomly selected by the survey programme using the Kish approach. For face-to-face interviews, up to three visits were made to selected households to establish contact. When a respondent was identified, up to three return visits were made in order to do the survey at a time when the respondent was available. For the US telephone surveys, repeat calls were made up to seven times.

A web based survey was posted at a dedicated URL between July 26, 2010 and May 16, 2011. The survey was initially available in English with subsequent availability in Spanish and Mandarin. Recruitment of respondents occurred through several channels, such as news items and editorials in scientific journals,

announcements at scientific meetings, postings on websites of institutions participating in the GBD, social networking and communication mobilization channels as well as direct contact with individuals and groups with known global health interests by tapping into the professional networks of the study investigators and their colleagues. Participants in the web based survey were required to be aged 18 or older. Household surveys obtained oral informed consent from all participants; written informed consent was obtained from participants in the web survey. Ethical review board approval was obtained from each household survey site and at the University of Washington, Seattle, WA.

Standardized survey instruments were developed to obtain comparative assessments of the full array of disease and injury sequelae, parsimoniously captured in 220 unique health states. Lay descriptions of health states formed the basis for all comparisons. These descriptions used simple, non-clinical vocabulary that emphasized the major functional consequences and symptoms associated with each health state. Development of these descriptions involved an iterative process of detailed consultation with experts participating in the GBD 2010 study with the goal of both capturing the most relevant details of each health state while avoiding ambiguity and ensuring consistency. Where possible, health states were grounded in standard clinical classifications systems, for example, the Canadian Cardiovascular Society grading scale was referenced for descriptions of stages of angina,[25] while the New York Heart Association functional classification was referenced for severity of heart failure.[26] Pilot testing indicated that the lay descriptions in face-to-face interviews should not exceed 30 words.

A paired comparison question formed the basis of all surveys. The questions in the survey were framed with the following statement, "A person's health may limit how well parts of his body or mind work. As a result, some people are not able to do all of the things in life that others may do, and some people are more severely limited than others. I am going to ask you a series of questions about different health problems. In each question, I will describe two different people…" Descriptions of two hypothetical people, each with a particular health state, were presented to respondents who were then asked which person they regarded as "the healthier". Health pairs in all surveys were selected by a randomizing computer algorithm. In the five household surveys, paired comparisons were presented for a subset of 108 health states pertaining to chronic conditions. The framing of chronic and acute conditions is different as they were presented as causing life-long or temporary health loss. We chose to only field health states that could be framed as lasting a lifetime in the household surveys as we hypothesized that presenting differently framed comparisons would be difficult to convey in face-to-face interviews. In the web survey we considered this more feasible as respondents could read and refer to the framing of the question for each pair-wise comparison. All 220 health states were thus evaluated in the web survey.

In addition, the web survey included "population health equivalence" (PHE) questions relating to population health and health programmes specifically – such as "Imagine two different health programmes. The first programme prevented 1,000 people from getting an illness that causes rapid death. The second programme prevented 2,000 people from getting an illness that is not fatal but causes lifelong health problems resulting in varying examples of health states at moderate to severe disability. Which programme would you say produced the greater overall health benefits?" This information was used to anchor the results from the pair-wise comparisons on the 0–1 disability weight scale.

GBD 2013 European Disability Weights Measurement Study

The GBD 2010 disability weights were critically dependent on the ways that outcomes were described to survey respondents. Descriptions for health states were designed to balance validity and parsimony and this necessarily meant that some details of different health states had to be omitted. As lay descriptions were developed collaboratively through individual expert groups organised around a particular set of health issues – some amount of variability in language and detail inevitably occurred. Criticisms and suggestions for improvement came from a number of commentators on the GBD 2010 disability weights measurement study.[27–29]

The GBD 2013 Study expanded the list of disease and injury causes and sequelae which were mapped to 235 unique health states. Additional data for the European Disability Weights Measurement Study were collected between September 23, 2013 and November 11, 2013 in Hungary, Italy, the Netherlands and Sweden. The initiation of these surveys was connected to a project sponsored by the European Centre for Disease Prevention and Control (the Burden of Communicable Diseases in Europe project).[30] The four selected countries were chosen to be representative of the four regions of Europe (east, south, middle, and north) in terms of age, sex and education of the respondents. Respondents were recruited from standing internet panels in each country on the basis of quota sampling with reference to age, sex and education in such a way as to maintain population representativeness of these characteristics. Eligible participants were aged 18–65 and were preselected in the case of the Netherlands, where age, sex and education of respondents were already known, or in the case of the other three countries, invited to participate via a web-link and then selected on the basis of their individual characteristics.

The protocol for the European disability weights measurement study followed the protocol that was developed and implemented in the GBD 2010 disability weights measurement study. Lay descriptions for some health states that lacked mention of an important symptom or for which consistency of wording across different levels of severity had been noted were reworded. The European disability weights measurement study included 255 health states, of which 183 were used in the analyses of GBD 2013. Those 183 consisted of 135 of the 220 health states that were included in the European disability weights measurement study with unmodified lay descriptions; 30 from GBD 2010 for which alternative lay descriptions were included. Disability weights were estimated for additional sequelae that were incorporated into GBD 2013 but had not been included in GBD 2010.

Finding high correlation in resulting disability weight values between the country surveys and the web survey, we analyzed the results of all surveys together. We ran probit regression analyses on the answers to the pair-wise comparison questions, with dummies for each health state with a value of 1 for the first state in a pair, –1 for the second of a pair being chosen, and 0 for all states other than the pair being considered. This method formalizes the intuition that if two health states in a pair produce similar health loss, the answers are likely to be evenly split; a pair of health states with very different health loss, will get many more responses favoring one over the other. The statistical methods infer the distances between values attached to different health states based on the frequencies of responses to the paired comparisons. A second analytic step was needed to anchor the resulting estimates onto the 0–1 disability

weights scale. We anchored results from the probit regression analysis onto the 0–1 scale using population health equivalence data from the GBD 2010 web survey using a linear regression of the probit coefficients from the analysis of paired comparisons on the logit-transformed disability weight estimates derived from interval regression of the population health equivalence responses. Using numerical integration, we then estimated mean values for disability weights on the natural 0–1 scale. Uncertainty was estimated by bootstrapping with 1000 samples.

A complete listing of the lay descriptions and values for the 235 health states used in GBD 2016 is provided in Appendix Table 5.

2.7 Comorbidity correction (COMO)

The final stage in the estimation of YLDs is a micro-simulation, which adjusts for comorbidity. We refer to this micro-simulation process as "COMO". For GBD 2016, we estimated the co-occurrence of different diseases by simulating 40,000 individuals in each location-age-sex-year combination as exposed to the independent probability of having any of the sequelae included in GBD 2016 based on disease prevalence. We tested the contribution of dependent and independent comorbidity in the US MEPS data, and found that independent comorbidity was the dominant factor even though there are well-known examples of dependent comorbidity, i.e., clustering of conditions such as diabetes and stroke or anxiety and alcohol use disorders. Age was the main predictor of comorbidity such that age-specific microsimulations accommodated most of the required comorbidity correction.[31]

The two components necessary for the computation of YLDs, prevalence of each disease sequelae and disability weights, are the two inputs into COMO. The prevalence values are primarily produced using DisMod-MR 2.1. The disability weights have been described above.

The micro-simulation, as performed for each age-sex-location-year, can best be represented as a four-step process. First, simulants are exposed to independent probabilities of having each sequela, where the probability is equal to the prevalence estimate. For each simulant, the probability of having a disease sequela is equal to the estimated prevalence from that draw from the uncertainty distribution. Each simulant is determined to have or not have the disease sequelae based on a draw from a binomial distribution. From this simulation, simulants end up having from none to multiple disease sequelae. Second, the disability weight for each simulant is estimated based on the disease sequelae that they have acquired. The formula for the cumulative disability weight for a simulant is one minus the multiplicative sum of one minus each disability weight present:

$$Simulant\ DW_l = 1 - \prod_{k=i}^{j}(1 - DW_k)$$

where the DW_k is the disability weight for the kth disease sequela that the simulant *l* has acquired. Once the simulant disability weight is computed, the disability weight attributable to each sequela for the simulant is calculated using the following formula:

$$ADW_{lk} = \frac{DW_k}{\sum_{k=i}^{k=j} DW_k} * Simulant\ DW_l$$

where ADW_{lk} is the attributable DW for disease sequela *k* in simulant *l*; DW_k is the disability weight for disease sequela *k*, and simulant DW_l is the disability weight for simulant *l* from the combination of all sequelae that they have acquired. This formula apportions the overall simulant disability weight to each condition in proportion to the disability weight of each condition in isolation.

Finally, YLDs per capita in an age-sex-country-year are computed by taking the sum of the attributable disability weights for a disease sequela across simulants.

$$YLD\ Rate_k = \frac{\sum_{l=1}^{n} ADW_{lk}}{n}$$

The actual number of YLDs from disease sequela *k* in an age-sex-location-year is then computed as the YLD rate *k* times the appropriate age-sex-location-year population.

By repeating the simulation process for each age-sex-country-year 1,000 times, the uncertainty in the prevalence of each disease sequela and the disability weight is propagated into the final comorbidity corrected YLD results. We selected 40,000 simulants for each age-sex- location-year group on the basis of simulation testing, which has shown that results were stable for YLDs at this number of simulants even in the younger age groups when prevalence is relatively low. For any given location-year-age-sex group, sequelae with prevalence of less than one in 20,000 were excluded from the microsimulation.

2.8 YLD Computation, Uncertainty & Residual YLDs

For GBD 2016, we computed YLDs by sequela as prevalence multiplied by the disability weight for the health state associated with that sequela. The uncertainty ranges reported around YLDs incorporates uncertainty in prevalence and uncertainty in the disability weight. To do this, we take the 1,000 samples of comorbidity-corrected YLDs and 1,000 samples of the disability weight to generate 1,000 samples of the YLD distribution. We assume no correlation in the uncertainty in prevalence and disability weights. The 95% uncertainty interval is reported as the 25th and 975th values of the distribution. Uncertainty intervals for YLDs at different points in time (1990, 1995, 2000, 2005, 2010, and 2016) for a given disease or sequela are correlated because of the shared uncertainty in the disability weight. For this reason, changes in YLDs over time can be significant even if the uncertainty intervals of the two estimates of YLDs largely overlap as significance is determined by the uncertainty around the prevalence estimates.

Residual YLDs

Despite expanding our list of causes and sequelae in successive GBD iterations, many diseases remain for which we do not explicitly estimate disease prevalence and YLDs. Less common diseases and their sequelae were included in 35 residual categories (Appendix Table 6). For 22 of these residual categories, epidemiological data on incidence or prevalence were available and these were modelled accordingly. For

13 residual categories, epidemiological data on incidence and prevalence were not available but sufficient cause of death data allowed for cause of death estimates. For these residual categories, we estimated YLDs by multiplying the residual YLL estimates by the ratio of YLDs to YLL from the estimates of level 3 causes in the same disease category that were explicitly modelled. This scaling was undertaken for each country-sex-year. This approach made the simplifying assumption that the residual diseases caused disability proportionate to the ratio of disability to mortality in explicitly modelled diseases. We did not include causes with large disability but no or little mortality in estimating these ratios. For example, we estimated the YLDs from other neurological disorders from the YLD to YLL ratios for dementia, multiple sclerosis, and Parkinson's disease, but did not include the YLDs from headaches and epilepsy in the ratio.

2.9 Socio-Demographic Index (SDI) analysis & Epidemiological Transition

a. Development of revised SDI indicator

The Socio-demographic Index (SDI) is a composite indicator of development status constructed for GBD 2015 whose components are strongly correlated with health outcomes. It is the geometric mean of 0 to 1 indices of total fertility rate, mean education for those aged 15 and older, and lag distributed income per capita.

SDI was calculated using the Human Development Index (HDI) methodology, wherein an index value was determined for each of the covariate inputs (log LDI, mean educational attainment over age 15, and TFR). For GBD 2015 these indices were computed on the basis of a relative scale, in which the upper and lower bounds were established by the maximum and minimum observed values, respectively, for each input over the entire estimation period of 1980–2015.
Prompted by the observations that the scales (and by extension SDI) were sensitive to the addition of new subnational locations as GBD becomes more granular and to the length of the time period over which SDI is computed, for GBD 2016 we implemented fixed scales in determining individual indices. Thus, an index score of 0 now represents the minimum level of each covariate input past which selected health outcomes can get no worse. An index score of 1 represents the maximum level of each covariate input past which selected health outcomes cease to improve. As a composite, a location with an SDI of 0 would have a theoretical minimum level of development relevant to these health outcomes, while a location with an SDI of 1 would have a theoretical maximum level of development relevant to these health outcomes.

We selected the minima and maxima of the scales by examining the relationships each of the inputs had with life expectancy at birth and under-5 mortality and identifying points of limiting returns at both high and low values, if they occurred prior to theoretical limits (eg, a TFR of 0). The final scales are summarised in the table below.

Input	Lower bound	Upper bound
TFR	1·5[a]	8
LDI per capita	250 USD (5·52 log USD)[b]	60,000 USD (11·00 log USD)
Mean educational attainment for ages 15 and older	0 years	17 years

[a] The low point of limiting returns for TFR was identified at 1 during GBD 2015; however, incorporating feedback with regard to accounting for a pattern of TFR rebound in highly developed countries, we instead set the lower limit of TFR at 1·5.

[b] The minimum for the LDI scale was originally set at the theoretical limit of 0 USD, as we did not observe an asymptotic relationship between log(LDI) and E_0 or 5q0 at lower values of log(LDI). Empirically, however, we also did not observe an LDI below 350 USD (5·86 log USD) for the estimation period 1970–2016. In log-space, this meant that approximately half of our scale was not being utilised, compressing the observed variation in LDI and diminishing its meaningful contribution to SDI. Accordingly, we set the lower limit on LDI to 250 USD (5·52 log USD) to ensure we were fully utilising the range of the scale to capture its variation across space and time, as is the case with the other two inputs.

Using the limits on the scales described above, we computed the index scores underlying SDI analogously to GBD 2015 as follows:

$$I_{Cly} = {(C_{ly} - C_{low})}\Big/{(C_{high} - C_{low})}$$

Where I_{cly} – the index for covariate C, location l, and year y – is equal to the difference between the value of that covariate in that location-year and the lower bound of the covariate divided by the difference between the upper and lower bounds for that covariate. If the values of input covariates fell outside the upper or lower bounds (eg, LDI per capita greater than 60,000 USD), they were mapped to the respective upper or lower bounds. We also note that the index value for TFR was computed as $1 - I_{TFRly}$, as lower TFRs correspond to higher levels of development, and thus higher index scores. For GBD 2016 we expanded the computation of SDI to 755 national and subnational locations spanning the time period 1970–2016.
The composite Socio-demographic Index is the geometric mean of these three indices for a given location-year. The cutoff values used to determine quintiles for analysis were then computed using country-level estimates of SDI for the year 2016, excluding countries with populations less than 1 million.

Example calculation

Below we present the calculation of SDI for Mexico in the year 2010:

$$TFR = 2.43;\ Mean\ educ\ yrs\ pc = 9.23;\ lnLDI = 9.58$$

$$I_{TFR} = 1 - \frac{2.43 - 1.5}{8 - 1.5} = .855$$

$$I_{Educ} = \frac{9.23 - 0}{17 - 0} = .543$$

$$I_{lnLDI} = \frac{9.58 - 5.52}{11.00 - 5.52} = .741$$

$$SDI = \sqrt[3]{I_{TFR} * I_{Educ} * I_{lnLDI}} = \sqrt[3]{.855 * .543 * .741} = .701$$

b. Age-sex-specific relationships between SDI and YLD rates

In order to evaluate the relationship between SDI and morbidity, we fit a Gaussian process regression using a linear prior to the mean function within a stochastic partial differential equation (SPDE) framework.

We first assume the following:

$$\ln(Y_{iasc}) \sim N(\mu_i, \sigma^2)$$

Where $Y_{,iasc}$ is the cause-specific YLD rate for a given level of SDI (*i*), age group (*a*), sex (*s*), and cause (*c*). We then specify a linear prior to the mean $\mu_{i,}$:

$$\mu_i = \alpha + \beta(SDI) + z_i$$

Where

$$z_i \sim GP(0, \Sigma_M)$$

GP refers to a Gaussian processs, and Σ_M refers to the Matern covariance function.

For causes where the relationship between SDI and YLD rates was markedly non-linear (e.g., many neglected tropical diseases), we instead specified a continuous linear piecewise prior to the mean with either one or two knots depending on the nature of the distinct changes in direction in the data.

Using SPDE, we specified additional priors on the range, variance, and precision of the mean function, as well as selected the number of underlying bases. These hyperparameters were chosen empirically and were identical for all age-sex-cause combinations. Values for the selected hyperparameters are displayed in the table below.

Hyperparmeter	Value
Range	0.2
Variance	1
Precision	1×10^{10}
Number of bases	2 (mesh points at 0.3, 0.7)

Regressions were run separately by age-sex-cause, using observed cause-specific YLD rates from all years 1990-2016 to produce predictions per level of SDI from 0 to 1 in increments of .005. We fit models on observations from all countries estimated in GBD and included state and province level estimates in lieu of national estimates for Brazil, China, and India due to their large populations (> 200 million) and small number of state-level units modelled in GBD (BRA – 27, IND – 31, CHN – 33) relative to population. Though the United States and Indonesia also fall under the designation of large-population (> 200 million), we fit models on national-level observations instead of state/province-level observations for these two countries as a result of the undue influence from the relatively large number of state-level units modelled in GBD relative to population (USA – 51, IDN – 34). Country and region dummy variables used in GBD 2015 were no longer included in this analysis. All models were fit using the INLA package in R.

Due to the more reliable estimation at more aggregate levels of cause specificity, we imposed a top-down hierarchical scaling scheme, in which the Level 1 causes were scaled to the predictions for all-cause morbidity, then Level 2 causes were scaled to their scaled Level 1 parents, and so on.

Having a complete set of age specific YLD rates, we were then able to produce a full set of age-standardised rates for every SDI level. In order to produce other age-aggregates of our results, we used the same modelling framework as above. In this case, however, we regarded the logit of the share of population in each age group as the dependent variable to estimate a smoothed relationship between population age-structure and SDI. Predictions for each age group at each level of SDI were rescaled to sum to 100%.

Section 3. Nonfatal cause-specific estimation process

Tuberculosis

Flowchart

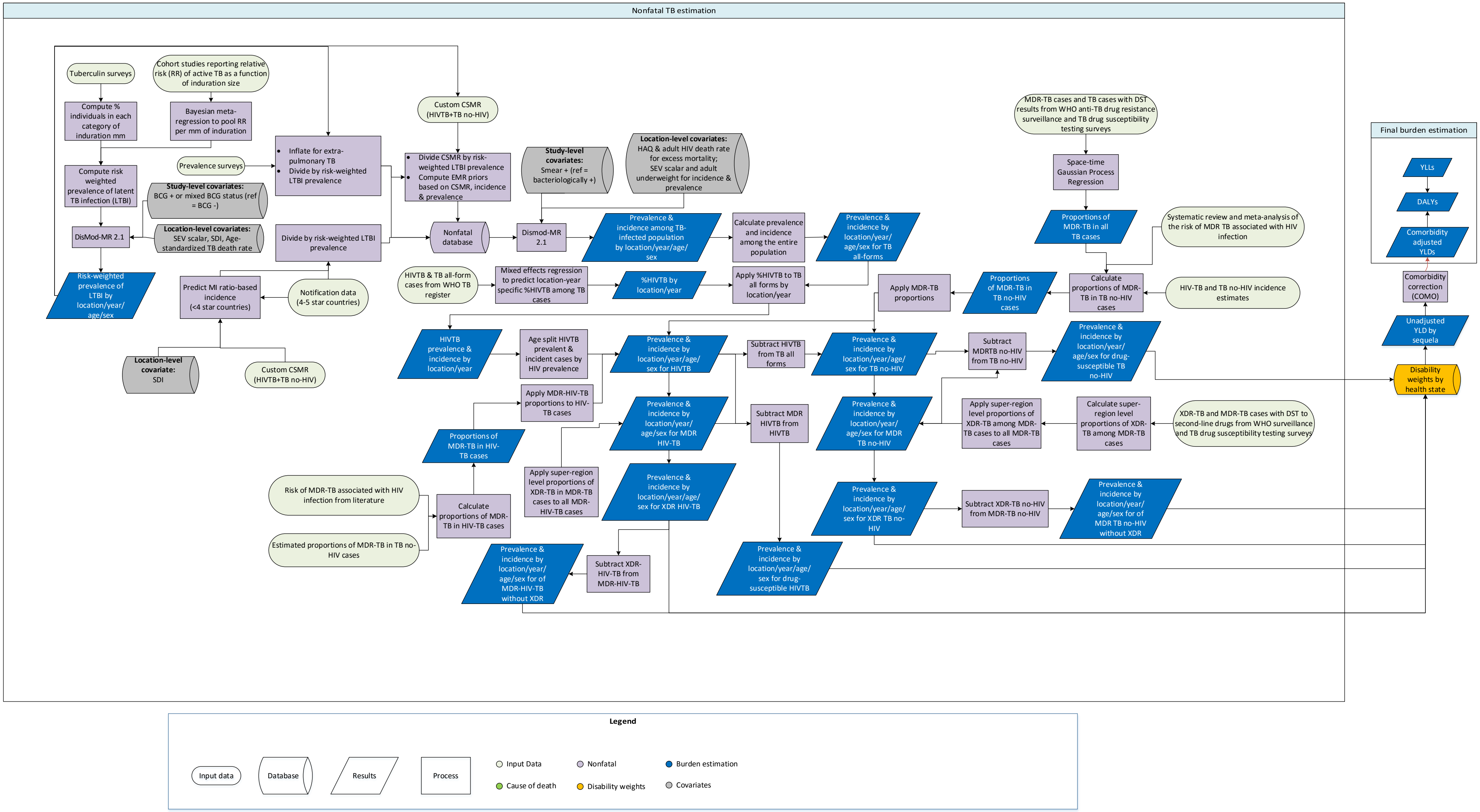

Case Definition

Tuberculosis (TB) is an infectious disease caused by Mycobacterium tuberculosis. The case definition includes all forms of TB including pulmonary TB and extrapulmonary TB which are bacteriologically confirmed or clinically diagnosed. For TB, the ICD 10 codes are A10-A19.9, B90-B90.9, K67.3, K93.0, M49.0, P37.0, and ICD 9 codes are 010-019.9, 137-137.9, 138.0, 138.9, 139.9, 320.4, 730.4-730.6. For HIV-TB, the ICD 10 code is B20.0.

Latent TB infection (a new sequela added for GBD 2016) is defined as an infection with Mycobacterium tuberculosis, without any symptoms or signs of active TB disease.

We have separately estimated the incidence and prevalence of multidrug-resistant tuberculosis and extensively drug-resistant tuberculosis by HIV status in GBD 2016. The case definitions of the new causes are shown below.

(1) Multidrug-resistant TB without extensive drug resistance: a form of TB (among HIV-negative individuals) that is resistant to the two most effective first-line anti-tuberculosis drugs (isoniazid and rifampicin), but is not resistant to any fluoroquinolone and any second-line injectable drugs (amikacin, kanamycin, or capreomycin).

(2) Extensively drug-resistant TB: a form of TB (among HIV-negative individuals) that is resistant to isoniazid and rifampicin, plus any fluoroquinolone and any second-line injectable drugs.

(3) Drug-susceptible TB: TB (among HIV-negative individuals) that is susceptible to isoniazid and rifampicin

(4) Multidrug-resistant HIV-TB without extensive drug resistance: a form of TB (among HIV-positive individuals) that is resistant to the two most effective first-line anti-tuberculosis drugs (isoniazid and rifampicin), but is not resistant to any fluoroquinolone and any second-line injectable drugs (amikacin, kanamycin, or capreomycin).

(5) Extensively drug-resistant HIV-TB: a form of TB (among HIV-positive individuals) that is resistant to isoniazid and rifampicin, plus any fluoroquinolone and any second-line injectable drugs

(6) Drug-susceptible HIV-TB: TB (among HIV-positive individuals) that is susceptible to isoniazid and rifampicin

Input data

Model Inputs

Input data for TB include annual case notifications, data from prevalence surveys, and estimated cause-specific mortality (CSMR) of TB among HIV-positive and HIV-negative individuals. From these inputs, we calculated 'priors' (expected values) on excess mortality to give more guidance to the model. An updated systematic review was done for GBD 2016 (the search terms are shown in the table below).

Input data for latent TB infection (LTBI) include: (1) population-based tuberculin surveys, and (2) cohort studies examining the risk of developing active TB disease as a function of induration size. We searched PubMed and Google Scholar, and also manually searched the reference list of relevant studies to aid

identification of additional studies. The search terms, number of studies identified, and number of studies included are shown in the table below.

Outcome	Search Terms	Total number of studies identified	Number of studies included
Tuberculosis*	PubMed search terms: ("tuberculosis"[MeSH] OR tuberculosis[Title/Abstract]) OR TB[Title/Abstract] OR Mycobacterium tuberculosis[Title/Abstract] AND prevalence[Title/Abstract] AND ("2015/01/01"[PDAT] : "2016/11/02"[PDAT]) NOT (animals[MESH] NOT humans[MESH])	1061	3
LTBI (tuberculin surveys)	PubMed search terms: ("tuberculin survey"[tiab] OR (("risk"[MeSH Terms] OR "risk"[tiab] OR "risk of"[tiab]) AND ("tuberculosis"[MeSH Terms] OR "tuberculosis"[tiab] OR "tuberculous"[tiab]) AND ("infection"[MeSH Terms] OR "infection"[tiab])) OR (("risk"[MeSH Terms] OR "risk"[tiab] OR "risk of"[tiab]) AND TB[tiab] AND ("infection"[MeSH Terms] OR "infection"[tiab])) OR "latent tuberculosis infection"[tiab] OR "latent TB infection"[tiab] OR "latent tuberculosis"[MESH]) AND ("survey"[tiab] OR “surveys”[tiab]) NOT (animals[MESH] NOT humans[MESH])<br><br>Google Scholar search terms: ("tuberculin survey") OR (("risk of tuberculous infection" OR "risk of tuberculosis infection" OR "risk of TB infection" OR "latent tuberculosis infection" OR "latent TB infection") AND “survey”)	9029	108
LTBI (cohort studies)	PubMed search terms: ("tuberculin"[tiab] OR ("tuberculin"[tiab] AND "positive"[tiab]) OR "Mantoux"[tiab] OR ("Mantoux"[tiab] AND "positive"[tiab]) OR "induration"[tiab]) AND (active[tiab] AND ("tuberculosis"[MeSH] OR "tuberculosis"[tiab])) AND ("risk"[MeSH] OR "risk"[tiab]) AND ("prospective"[tiab] OR "follow up"[tiab] OR "longitudinal"[tiab])<br><br>Google Scholar search terms: (("tuberculin" OR "Mantoux" OR “tuberculin reactivity”) AND (“risk of tuberculosis” OR “tuberculosis risk”)) -autopsy -autopsies -nosocomial -qualitative -prison -cancer -malignant -homeless -smoking	3624	27

* Updated systematic review, covering the period from 2015/01/01 to 2016/11/02

Input data for multidrug-resistant TB (MDR-TB) and extensively drug-resistant TB (XDR-TB) include: (i) the number of drug-resistant cases by type [MDR-TB, XDR-TB, TB cases with a drug sensitivity testing (DST) result for isoniazid and rifampicin, and MDR-TB cases with DST for second-line drugs] from routine

surveillance and surveys reported to the World Health Organization, and (ii) the risk of MDR-TB associated with HIV infection from the literature.[1]

Modeling Strategy

Overview

We made major changes to our modelling of TB. First, we estimated risk-weighted prevalence of LTBI by location, year, age and sex using data from population-based tuberculin surveys and cohort studies reporting on the risk of developing active TB disease as a function of induration size. Next, we divided the inputs on prevalence (from surveys in low and middle income countries), incidence (notification data from countries with a four or five-star rating, and estimated incidence for countries with a less than four-star rating), and CSMR by the risk-weighted LTBI prevalence in order to model TB among those at risk in each country. To generate incidence estimates, we first ran a regression using MI ratios (logit transformed) from locations with a 4 or 5-star rating on causes of death with SDI as a covariate anchoring the lower end of the SDI scale with a data point from the Bangalore study[2] reporting that 49.2% of 126 untreated new pulmonary TB cases were dead at the end of the 5-year follow up period, to predict age-sex specific MI ratios for all locations and years. We then estimated age-sex specific incidence using the predicted MI ratios and CSMR estimates. We used DisMod-MR 2.1, the GBD Bayesian meta-regression tool to generate consistent trends in all parameters. We then multiplied the DisMod-MR 2.1 outputs by the risk-weighted prevalence of LTBI to get population-level estimates of incidence and prevalence. Because the output from DisMod-MR 2.1 are for all forms of TB, we split them into MDR-TB and XDR-TB by HIV status. To do so, we estimated the proportions of TB cases with MDR-TB for all locations and years, using data from notifications and survey data. We then estimated the proportions of MDR-TB among HIV-negative individuals and MDR-TB among HIV-positive individuals based on the risk of MDR-TB associated with HIV infection from a meta-analysis[1]. To split MDR-TB into MDR-TB with and without extensive drug resistance, we pooled the limited notification and survey data on the proportion of MDR-TB cases who are extremely drug resistant by super-region, and applied these proportions to MDR-TB cases among HIV-negative and HIV-positive individuals respectively.

Modeling risk-weighted latent TB infection prevalence

Input data for modeling risk-weighted LTBI prevalence were from two sources: (i) population based tuberculin skin test (TST) surveys, and (ii) cohort studies examining the risk of developing active TB disease as a function of induration size. First, we extracted the prevalence of tuberculin skin testing results by induration size using the most detailed induration categories reported by studies. Second, from cohort studies reporting on the relative risk of developing active TB disease as a function of induration size, we pooled the risk of developing active TB by induration size in millimeters using the DisMod Ode computational engine. Third, we multiplied the LTBI prevalence by induration in millimeters ranging from 0-20+ with the relative risk of developing active TB at each induration size, and summed them up to derive risk-weighted LTBI prevalence for each age group.

Available evidence[3] suggests that people with very advanced HIV infection (CD4 counts <200 cells/mm^3) may have a false-negative TST (0mm induration) due to profound immune suppression, but still have very high risk for TB. For those who are HIV-positive, but with higher CD4 counts, the risk for active TB increases with greater induration size as in HIV-negative individuals (i.e., the shape of the tuberculin response curve is similar to that for the general population). To take into account the false-negative TST response in HIV cases with profound immune suppression, we first computed the proportion of HIV-positive individuals with CD4 counts <200 cells/mm for the 0mm induration group using our HIV prevalence estimates for that particular category. We then multiplied that proportion by the relative risk of developing active TB disease in the 0mm induration group compared with the 20+ mm induration group among HIV positive individuals. The relative risk was computed using data from a prospective, multicenter cohort study of HIV-positive people in the United States.[3]

Using the risk-weighted LTBI prevalence (adjusting for a false-negative TST among people with advanced HIV infection) as input data, we ran a DisMod MR 2.1 model with three location-level covariates, namely, Socio-demographic Index (SDI), Summary Exposure Variable (SEV) scalar for TB (a summary variable of the exposure levels of TB risk factors weighted by relative risk), and age-standardized TB mortality rate, to generate risk-weighted LTBI prevalence by location, year, age and sex. We included two study covariates (BCG positive, and mixed BCG status) where the reference category is BCG negative. We found no statistically significant difference between studies using different dosages of tuberculin purified protein derivative (PPD). We therefore did not include different PPD dosages as study covariates but added more uncertainty to data points from studies that used dosages larger or smaller than the standard dose of 5 tuberculin units per test dose of 0.1 ml, by entering them as z-covariates in Dismod.

Modeling TB incidence

Incidence inputs were from two different sources: (1) incidence from notification data for countries with a four or five-star rating on their cause of death data[4] as a proxy for the quality of health-related administrative data systems, and (2) estimated incidence for countries with a less than four-star rating. We used the age and sex-specific notifications (all new and relapse cases combined) in our analysis. Prior to 2013, notification data were available by case type (new pulmonary smear-positive, new pulmonary smear-negative, and new extra-pulmonary) and there were missing age data especially for younger age-groups in some countries. We imputed the missing age-groups for the three forms of TB notifications. Smear-positive age-specific notifications were inflated with the proportion smear-unknown and relapsed cases only reported at the country-year level. Some countries reported only pulmonary smear-positive cases for selected years. Missing smear-negative and extra-pulmonary cases were predicted from the adjusted smear-positive cases using a seemingly unrelated regression. All three types of notifications were added together to represent TB-all form incidence for countries with a four or five-star rating.

To generate incidence estimates for locations with a less than four-star rating, we ran a regression using MI ratios (logit transformed) from locations with a 4 or 5-star rating on causes of death as input data with SDI as a covariate anchoring the lower end of the SDI scale with a data point from a cohort study in the 1960s[2] reporting that 49.2% of 126 untreated new pulmonary TB cases were dead at the end of the 5-year follow up period, in order to predict age-sex specific MI ratios for all locations and years. We then

used the predicted MI ratios and cause specific mortality estimates to compute age-sex specific incidence estimates for locations with a less than four-star rating. For South Africa, a country with large inequality, we decided that the Health Care Access and Quality (HAQ) index would be a better health-related index than SDI for TB, a health outcome that differentially affects the poor. We therefore used the HAQ index instead, to predict incidence for South Africa. While the MI-ratios predicted using the SDI covariate were within a reasonable range for most countries with a less than four-star rating, there were some outliers with very high MI ratios. We replaced those MI ratios with the MI ratios computed based on notifications and CSMR for 2010. For outliers in other years, we assumed a similar proportional difference between predicted MI ratios and notifications-based MI ratios as in 2010 and adjusted the predicted MI ratios accordingly, which were then used to predict incidence.

We computed the age-sex specific incidence of TB among the latent TB-infected population, using TB incidence as the numerator and our estimated risk-weighted latent TB infection prevalence as the denominator. We included location-level covariates, namely, the age-standardized adult underweight prevalence, and the log-transformed age-standardized SEV scalar for TB to help inform variation over year and geography. We set bounds of 0.75 to 1.25 on the SEV scalar covariate where a value in log space of 1 would reflect perfect agreement with our risk factor estimates.

Modeling TB prevalence

Data from prevalence surveys reporting on pulmonary smear-positive TB and bacteriologically positive TB were included. Because incidence data are for all forms of TB, we adjusted prevalence surveys to account for extra-pulmonary cases. We ran a spatiotemporal Gaussian process regression to predict location-year-age-sex specific proportions of extra-pulmonary TB among all TB cases using data on the three forms of TB from the incidence data above. We then computed the extra-pulmonary inflation factor as *1+(proportion of extrapulmonary TB /(1- proportion of extrapulmonary TB))*, and applied it to data from prevalence surveys. We then computed the prevalence of TB among the TB-infected population, using TB prevalence as the numerator and our estimated risk-weighted LTBI prevalence as the denominator. We included a study covariate indicating whether it was bacteriologically positive TB (reference category) or smear-positive TB. We found no systematic bias between studies that used both symptoms and chest X-ray as screening methods and studies that used only one of the methods. We therefore did not adjust them for systematic bias but added more uncertainty to data points from studies that used only one of the screening methods (by using it as a z-covariate in Dismod). We also added more uncertainty to data points from sub-national surveys. We included the SEV scalar country-level covariate with priors that as the SEV scalar increases, prevalence increases.

Modeling TB excess mortality

We matched each prevalence data point and TB CSMR (TB and HIV-TB combined) by location, year, age, and sex to calculate excess mortality rate (EMR) as *EMR=CSMR/prevalence*. We also matched each incidence data point and TB CSMR by location, year, age, and sex to calculate EMR for countries with a four or five-star rating on their cause of death data. To reflect a gradient in EMR, we added the HAQ index, and adult HIV death rates as country-level covariates.

DisMod-MR 2.1

For each location, we included the following as input in the DisMod model: case notifications for locations with a four or five-star rating, predicted MI-ratio-based incidence for locations with a less than four-star rating, prevalence survey data where available, excess mortality estimates, and CSMR (TB and HIV-TB combined) by age and sex.

The output from the DisMod model was for all forms of TB in TB-infected population including both HIV-negative and HIV-positive individuals. We computed the incidence and prevalence of TB among the entire population, by multiplying the prevalence of LTBI with the DisMod model estimates.

Betas and exponentiated values from the DisMod model are shown in the table below.

Covariate	Parameter	Beta (95% CI)	Exponentiated beta (95% CI)
Smear positive TB	Prevalence	-0.75	0.47 (0.47 — 0.47)
Sex (male)	Prevalence	0.51	1.66 (1.55 — 1.79)
Sex (male)	Incidence	0.13	1.14 (1.14 — 1.14)
Age-standardized proportion adult underweight	Incidence	2.23	9.35 (8.73 — 9.72)
Age-standardized proportion adult underweight	Prevalence	2.95	19.13 (17.32 — 20.07)
Age-standardized SEV scalar (log-transformed)	Prevalence	0.78	2.19 (2.12 — 2.39)
Age-standardized SEV scalar (log-transformed)	Incidence	0.75	2.12 (2.12 — 2.12)
HAQ (log-transformed)	Excess mortality	-1.58	0.21 (0.19 — 0.22)
Adult HIV death rate	Excess mortality	0.96	2.61 (1.04 — 7.02)

HIV-TB incidence and prevalence

To distinguish HIV-TB from all forms of TB, we first estimated the proportions of HIV-TB cases among all TB cases using data on the number of TB cases recorded as HIV-positive and the number of TB cases with an HIV test result recorded in the WHO TB notifications register. We ran a mixed effects regression using the adult HIV death rate as a covariate to predict location-year specific HIV-TB proportions, which were then applied to TB incident and prevalent cases from DisMod, to generate HIV-TB incident and prevalent cases by location and year. These cases were then age-sex split based on the age-sex pattern of estimated HIV prevalence by location-year to generate location-year-age-sex specific HIV-TB incident and prevalent cases.

Multidrug-resistant TB, extensively drug-resistant TB and drug-susceptible TB

We ran a spatiotemporal Gaussian process regression to predict the proportions of MDR-TB cases among all TB cases for all locations and years. The input data for this regression (i.e., weighted average of the proportions of new and previously treated cases with MDR-TB) were based on the number of MDR-TB cases among new TB cases, MDR-TB cases among previously treated TB cases, and the number of new and previously treated TB cases with drug sensitivity testing for isoniazid and rifampicin from routine surveillance and surveys reported to the World Health Organization. We then used the predicted proportions to MDR-TB cases among all TB cases, along with the HIV-TB and TB no-HIV incidence estimates, and the relative risk of MDR-TB associated with HIV infection from the literature[1] to compute the proportions of MDR-TB cases among HIV negative TB cases $\left(PnoHIV_{c,y,a,s}\right)$ by location, year, age, and sex using the following formula:

$$PnoHIV_{c,y,a,s} = \frac{MDR_{c,y}}{\left(1 + \left(RR\frac{HIVTB_{c,y,a,s}}{TBnoHIV_{c,y,a,s}}\right)\right) TBnoHIV_{c,y,a,s}}$$

where $MDR_{c,y}$ is the number of all MDR-TB cases among HIV-positive and HIV-negative individuals by location and year, RR is the relative risk of MDR-TB associated with HIV infection, $HIVTB_{c,y,a,s}$ is the number of HIV-TB incident cases by location, year, age, and sex, and $TBnoHIV_{c,y,a,s}$ is the number of TB no-HIV incident cases by location, year, age, and sex.

We then applied the predicted proportions of MDR-TB cases among HIV negative TB cases to our predicted HIV-negative TB incident and prevalent cases to generate MDR-TB incident and prevalent cases by location, year, age, and sex. Next, we subtracted MDR-TB cases from all HIV-negative TB cases to generate drug-susceptible TB cases by location, year, age, and sex. To distinguish XDR-TB from MDR-TB, we aggregated the XDR-TB cases and MDR-TB cases (with drug sensitivity testing for second-line drugs) up to the super-region level and calculated the super-region level proportions of XDR-TB among MDR-TB cases, which were then applied to MDR-TB cases in corresponding countries within the super-regions to produce XDR-TB cases by location, year, age, and sex. We linearly extrapolated XDR-TB prevalence and incidence back assuming the rates were zero in 1992, one year before 1993 when XDR-TB was first recorded in USA surveillance data.[5] Finally, we subtracted XDR-TB cases from MDR-TB cases to generate MDR-TB (without XDR) cases by location, year, age, and sex.

Multidrug-resistant HIV-TB, extensively drug-resistant HIV-TB and drug-susceptible HIV-TB

To split HIV-TB into MDR-HIV-TB and drug-susceptible HIV-TB, we first calculated the proportions of MDR-HIV-TB among all HIV-TB cases ($PHIV_{c,y,a,s}$) for each location, year, age, and sex using the following formula:

$$PHIV_{c,y,a,s} = PnoHIV_{c,y,a,s}RR$$

where $PnoHIV_{c,y,a,s}$ is the proportions of MDR-TB among all HIV-negative TB cases for each location, year, age, and sex and RR is the relative risk of MDR-TB associated with HIV infection. We then applied

the predicted proportions of MDR-TB cases among HIV-TB cases to all HIV-TB case estimates to generate MDR-HIV-TB cases by location, year, age, and sex. Next, we subtracted MDR-HIV-TB cases from all HIV-TB cases to generate drug-susceptible HIV-TB cases by location, year, age, and sex. To separate out XDR-HIV-TB from MDR-HIV-TB, we applied the super-region level proportions of XDR-TB among MDR-TB cases, to MDR-HIV-TB cases in corresponding countries within the super-regions to produce XDR-HIV-TB cases by location, year, age, and sex. We linearly extrapolated XDR-HIV-TB prevalence and incidence back assuming the rates were zero in 1992, one year before 1993 when XDR-TB was first recorded in USA surveillance data.[5] Finally, we subtracted XDR-HIV-TB cases from MDR-HIV-TB cases to generate MDR-HIV-TB (without extensive drug resistance) cases by location, year, age, and sex.

Disability weights

The lay descriptions and disability weights for severity levels derived from the GBD Disability Weights study are shown below.

Health state Name	Lay description	Disability Weights (95% CI)
Tuberculosis, not HIV infected	has a persistent cough and fever, is short of breath, feels weak, and has lost a lot of weight	0.333 (0.224-0.454)
Tuberculosis, HIV infected	has a persistent cough and fever, shortness of breath, night sweats, weakness and fatigue and severe weight loss	0.408 (0.274-0.549)

For drug-susceptible TB, MDR-TB without extensive drug resistance, and XDR-TB, we used the same disability weight [0.333 (0.224-0.454)] as in non-HIV-infected TB. For drug-susceptible HIV-TB, MDR-HIV-TB without extensive drug resistance, and XDR-HIV-TB, we used the same disability weight [0.408 (0.274-0.549))] as in HIV-infected TB.

Reference

1. Mesfin YM, Hailemariam D, Biadglign S, Kibret KT. Association between HIV/AIDS and multi-drug resistance tuberculosis: a systematic review and meta-analysis. *PLoS One*. 2014 Jan 8;9(1):e82235.
2. Institute NT. Tuberculosis in a rural population of South India: a five-year epidemiological study. *Bulletin of the World Health Organization*. 1974;51(5):473.
3. Markowitz N, Hansen NI, Hopewell PC, Glassroth J, Kvale PA, Mangura BT, Wilcosky TC, Wallace JM, Rosen MJ, Reichman LB. Incidence of tuberculosis in the United States among HIV-infected persons. *Annals of internal medicine*. 1997 Jan 15;126(2):123-32.
4. GBD 2016 Mortality and Causes of Death Collaborators. Global, regional, and national age-sex specific mortality for 264 causes of death, 1980–2016: a systematic analysis for the Global Burden of Disease Study 2016. *The Lancet* (under review)
5. Centers for Disease Control and Prevention (CDC). Extensively Drug-Resistant Tuberculosis --- United States, 1993—2006. *MMWR*. 2007; 56(11);250-253.

HIV/AIDS

Flowchart

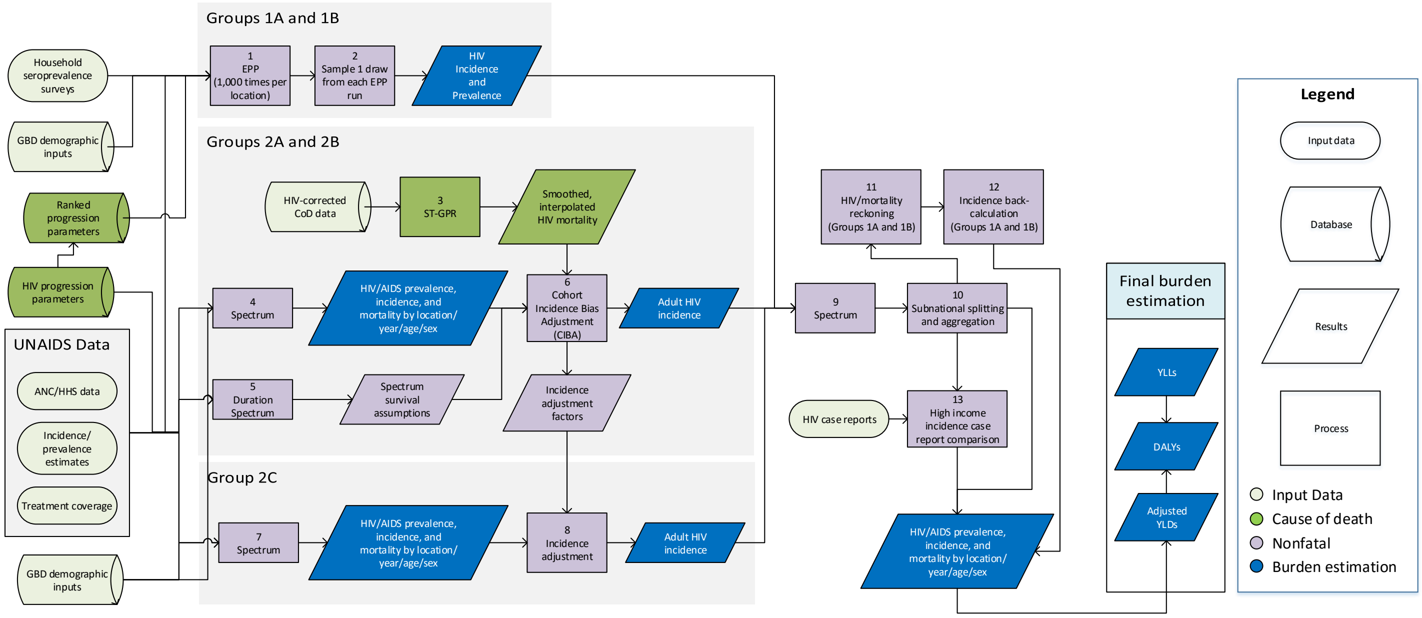

Case definition

Infection with the human immunodeficiency virus (HIV) causes influenza-like symptoms during the acute period following infection and can lead to acquired immunodeficiency syndrome (AIDS) if untreated. HIV attacks the immune system of its host, leaving infected individuals more susceptible to opportunistic infections like tuberculosis. Although there are two different subtypes of HIV, HIV-1 and HIV-2, no distinction is made in our estimation process or presentation of results. For HIV, ICD 10 codes are B20-B24, C46-C469, D84.9; ICD 9 codes are 042-044, 112-118 (after 1980), 130 (after 1980), 136.3-136.8 (after 1980), 176.0-176.9 (after 1980), 279 (after 1980); and ICD9 BTL codes are B184-B185.

Input data

Model inputs

Household seroprevalence surveys

Geographically representative HIV seroprevalence survey results were used as inputs to the model for countries with generalized HIV epidemics where available.

GBD demographic inputs

Location-specific population, fertility, and HIV-free survival rates from GBD 2016 and migration data from UNAIDS were used as inputs in modeling all locations.

UNAIDS data

The files compiled by UNAIDS for their HIV/AIDS estimation process were our main source of data for producing estimates of HIV burden. These files are typically country-specific and contain both demographic data (population, fertility, migration, and HIV-free survival rates) and HIV-specific information. In all cases except migration, we substituted in our own, internally consistent demographic estimates. The HIV-specific information includes what is needed to run both the Spectrum and Estimation and Projection Package (EPP) models. Spectrum requires data on AIDS mortality among people living with HIV with and without ART, CD4 progression among people living with HIV not on ART, ART coverage among adults and children, coverage of breastfeeding among women living with HIV, prevention of mother-to-child transmission coverage, and CD4 thresholds for treatment eligibility. EPP uses many of the same assumptions as Spectrum but fits a simpler model to HIV prevalence data from surveillance sites and large household surveys. Antenatal care, incidence, prevalence, and treatment coverage data from UNAIDS were used in modeling for all locations. We extracted all of these data from UNAIDS' proprietary formats.

For GBD 2016, we received national-level files for 81 countries and subnational-level files for 6 countries. For many of the GBD locations not covered by these files, we had UNAIDS files from an earlier year of estimation, which we used again. After combining, we were left with a set of 42 countries for which we have never received a UNAIDS file, many of them countries with small populations and/or low HIV prevalence. In those places, we generated regional averages of all needed inputs. This enabled us to run Spectrum for every GBD location.

In several cases, we have modified the structure or data in the UNAIDS files. In South Africa, which we have estimated at the province level since GBD 2015, we split the national-level UNAIDS file into nine provincial datasets. We used GBD 2016 demographic inputs for the provinces. These provinces are already fit as separate subpopulations in EPP, so we extracted the prevalence data for the individual provinces and assumed national rates for all other Spectrum inputs. In some locations that are estimated only at the national level in GBD 2016, we received subnational files from UNAIDS. In these cases, we split GBD 2016 demographic input data using the subnational relative relationships found in the UNAIDS files. Additionally, we identified that the ratio of fertility in HIV-positive women to HIV-negative women was negative in Indonesia. We used linear extrapolation to replace this value.

Vital registration data

We used all available sources of vital registration and sample registration data from the GBD Causes of Death database after garbage code redistribution and HIV/AIDS mis-coding correction, except in Group 1A countries as described below.[1,2] There are two different cause of death data sources for HIV/AIDS in China: the Disease Surveillance Point (DSP) system and the Notifiable Infectious Disease Reporting (NIDR) system. Both systems are administered by the Chinese Center for Disease Control and Prevention, but the reported number of deaths due to HIV is significantly lower in DSP. Therefore, we have used the provincial-level ratio of deaths due to HIV/AIDS from NIDR to those from DSP, choosing the larger ratio between years 2013 and 2014, and scaled the reported deaths in the DSP system, which is in turn used in the Space-Time Gaussian Process Regression (ST-GPR) process.

On-ART literature data

Data were identified by using search terms "HIV," "mortality," and "antiretroviral therapy" in PubMed searches across the literature. To be included, studies must include only HIV-positive people who receive antiretroviral therapy (ART) but who were ART-naïve prior to the study. In addition, studies must report either a duration-specific mortality proportion or a hazard ratio across age or sex, and must not include children.

For duration-specific survival data, studies must report uncertainty on mortality estimates or provide stratum-specific sample sizes and must include duration-specific data to allow for calculation of 0-6, 7-12, or 13-24 month conditional mortality. In addition, studies must either report separate mortality and loss-to-follow-up (LTFU) curves, be corrected for LTFU using vital registration data, or be conducted in a high-income setting. Finally, studies must report the percent of participants who are male, the median age of participants, and either data with specific data on the number of CD4 T lymphocytes (CD4 counts) or the median CD4 count used for the data.

Hazard ratio data for ages or sexes can only be used if the hazard ratios are controlled for other variables of interest (age, sex, and CD4 category).

In GBD 2013, we identified 102 papers for extraction. For GBD 2015, we included 13 additional studies informing the duration-specific mortality estimation process and 26 studies informing the age and sex hazard ratio estimation process (some studies were used and counted in both). We also added one study to our LTFU analysis. For GBD 2016, we included 12 additional studies informing the duration-specific mortality estimation process and 11 studies informing the age and sex hazard ratio estimation process (some studies were used and counted in both).

Off-ART literature data

In GBD 2013, to characterize uncertainty in the progression and death rates, we systematically reviewed the literature on mortality without ART. We searched terms related to pre-ART or ART-naive survival since seroconversion.[3] After screening, we identified 13 cohort studies that included the cohorts used by UNAIDS from which we extracted survival at each one-year point after infection. Screening for additional, recently published studies in GBD 2015 and GBD 2016 identified no new cohort studies for inclusion in this analysis.

Severity splits & disability weights

The basis of the GBD disability weight survey assessments are lay descriptions of sequelae highlighting major functional consequences and symptoms. The lay descriptions and disability weights for HIV/AIDS severity levels are shown below.

Severity level	Lay description	DW (95% CI)
Symptomatic HIV	has weight loss, fatigue, and frequent infections.	0.274 (0.184-0.377)
AIDS with antiretroviral treatment	has occasional fevers and infections. The person takes daily medication that sometimes causes diarrhea.	0.078 (0.052-0.111)
AIDS without antiretroviral treatment	has severe weight loss, weakness, fatigue, cough and fever, and frequent infections, skin rashes, and diarrhea.	0.582 (0.406-0.743)

The proportion of people living with HIV/AIDS who are being treated with anti-retroviral therapy is an output of Spectrum, the compartmental model used to make consistent incidence, prevalence, and mortality estimates described below.

Modeling strategy

In GBD 2016, our general modeling strategy for estimating HIV incidence, prevalence, and mortality is very similar to the strategy used in GBD 2015. We continue to use the Spectrum program rewritten in Python for GBD 2013 to facilitate faster and more flexible execution necessary for our more intensive computational needs. We made several changes to Spectrum's assumptions comparing to the Spectrum software used by UNAIDS. We also again ran EPP using an open-source computer program in R written by Jeffrey Eaton.[4] We ran EPP for all Group 1 countries in order to produce incidence and prevalence estimates that were consistent with the demographic and epidemiological assumptions used in GBD 2016.

On-ART

First, we corrected reported probabilities of death for loss to follow-up using an update of the approach developed by Verguet and colleagues.[5] Verguet and colleagues used tracing and follow-up studies to empirically estimate the relationship between death in LTFU and the rate of LTFU.

To create estimates of age-specific hazard ratios, we synthesized hazard ratio data in five broad age groups: 15-25, 25-35, 35-45, 45-55, 55-100, and modeled the data using DisMod-MR 2.0.

To create estimates of sex-specific hazard ratios, we use the *metan* function in Stata to create estimates of relative risks separately by region, using female age groups as the reference group.

The age and sex hazard ratios were applied to the study level mortality rates, accounting for the distribution of ages and sexes in the mortality data. We then subtracted HIV-free mortality from the model life table process to calculate study level age-sex HIV-specific mortality.

We used DisMod-MR 2.0 to synthesize the age-sex split study level data into estimates of conditional probability of death over initial CD4 count.[3] We modeled the data separately by duration, age, and sex and added a fixed effect on whether the study was conducted prior to 2002. We estimated all three regions together using a fixed effect for each region.

Changes for GBD 2016

In GBD 2016, we chose to age-sex split the data at the study level so that we could consider study-specific age-sex distributions, whereas previous GBD iterations relied upon region-specific distributions. Another change was a switch to estimating all regions together with fixed effects for each region. This allowed us to impart a CD4 trend in sub-Saharan Africa and other developing country estimates that led to more realistic estimates in the high CD4 categories where little data was available from those regions.

Off-ART

Following UNAIDS assumptions, no-ART mortality is modeled as shown in the figure below.[3]

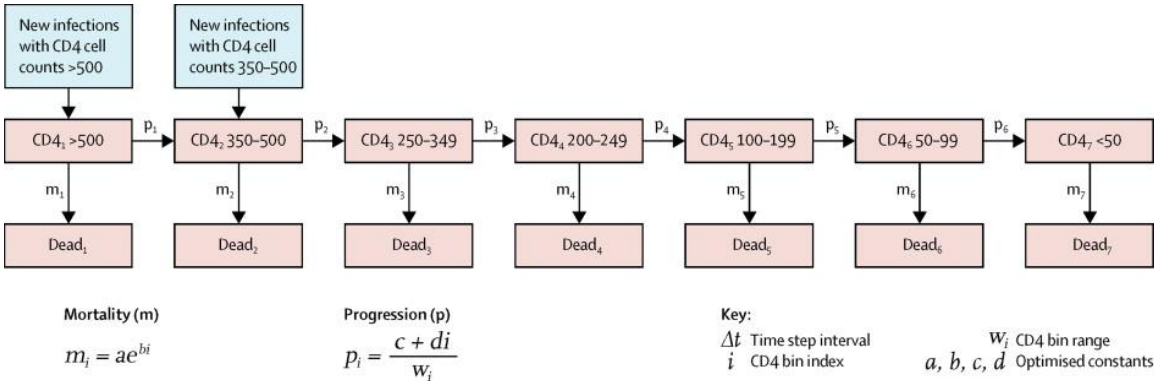

The death and progression rates between CD4 categories vary by age according to four age groups: 15–24 years, 25–34 years, 35–44 years, and 45 years or older. We modeled the logit of the conditional probability of death between years in these studies using the following formula:

$$logit\ (m_{ijk}) = \beta_0 + \sum_{i=1}^{4} \beta_{1i}\, a_i + \sum_{j=1}^{12} \beta_{2j} t_j + u_k + \varepsilon_{ijk}$$

In the formula, m is conditional probability of death from year t_j to t_{j+1}, a_i is an indicator variable for age group at seroconversion (15–24 years, 25–34 years, 35–44 years, and 45 years or older), t_j is an indicator variable of year since seroconversion, and u_K is a study-level random effect.

By sampling the variance-covariance matrix of the regression coefficients and the study-level random effect, we generated 1,000 survival curves for each age group that capture the systematic variation in survival across the available studies. For each of the 1,000 survival curves, we used a framework modeled after the UNAIDS optimization framework in which we find a set of progression and death rates that minimizes the sum of the squared errors for the fit to the survival curve.[6,7]

Burden estimation overview

UNAIDS uses two key analytical components in their epidemiological estimation. EPP is used to estimate incidence trajectories that are consistent with prevalence surveys and other prevalence measurements such as antenatal clinic serosurveillance. Spectrum is a compartmental HIV progression model used to generate age-specific incidence, prevalence, and death rates from the EPP incidence curves and assumptions about intervention scale-up and local variation in epidemiology.

For GBD 2013, we created an exact replica of Spectrum in Python. This enabled us to run thousands of iterations of the model at once on our computing cluster and allowed for more flexible input data structures. Additionally, in order to generate estimates with more realistic ranges of uncertainty than those in UNAIDS 2012, we adjusted all input data by uniformly sampled factors between 0.9 and 1.1. These changes, along with our new estimation of with- and without-ART mortality and CD4 progression parameters, persist into GBD 2016.

Due to the substantial differences in the quality and types of data available across different countries, we used three different methodologies to produce year-, age-, and sex-specific estimates of HIV incidence, prevalence, and mortality.

Countries with seroprevalence surveys and antenatal clinic data (Groups 1A and 1B)

We identified 50 countries – as well as subnational locations in India, Kenya, and South Africa – with at least 0.5% adult HIV prevalence and at least one geographically representative HIV seroprevalence survey or available antenatal care clinic (ANC) data. In order to ensure that our estimates of incidence and prevalence in these places were consistent with our estimates of HIV progression, we used a version of EPP written in R and C++ by Jeffrey Eaton to create new fits to the available prevalence data. The version of EPP used in GBD 2016 was an updated release from Jeffrey Eaton since completion of GBD 2015. In this new version, an ANC prevalence adjustment was included and incorporated with the 2016 lookup database and an additional parameter to estimate ANC variance inflation was included as well. In the ANC bias adjustment, instead of using the default universal assumption of the prior mean and standard deviation (SD) of the distribution that the adjustment follows, we selected the parameters based on each sub-population (general population and high risk population) in each location. For sub-populations with prevalence survey data, we used the default assumption with mean=0.15 and SD=1. For subpopulations without prevalence survey data, we chose the region/epidemic specific mean and SD based on the median probit difference and probit difference SD in Table 1 of Marsh et al.[8]

India's HIV epidemic is classified as concentrated in specific subpopulations rather than generalized to the full population, and only one prevalence survey, the 2005-2006 National Family Health Survey (NFHS-3), was available, so we used modified parameters for Indian states in EPP. We first calculated the mean of the median probit difference between men and women for "Countries with concentrated epidemics" in Table 1 of March et al as mentioned above, which was 0.245. Then we derived empirical parameters based on the difference between the ANC data and the NFHS-3 survey data in probit space to use for the general population. Specifically, we calculated the probit difference by taking the median of all raw ANC prevalence in years 2004 through 2006 and comparing to the 2005 prevalence survey data in probit space for three states with large HIV epidemics: Andhra Pradesh, Karnataka, and Maharashtra. From this empirical parameter derivation, we got the mean and SD value based on the three states as 0.124 and 0.051, respectively. We then used linear interpolation between the prevalence with a prior of 0.245 and the new prior of 0.124 to recalculate the mean and keep the SD the same as the empirical estimates. The final assumption of the prior mean and SD were 0.182 and 0.051, respectively. We did not make any adjustments for high risk populations.

In the new version of EPP, in addition to the equilibrium prior assumption of the force of infection in projection, a random walk approach is available as an alternative method. For locations with two or more prevalence surveys and a declining trend between the mean of the most recent two surveys, the random walk approach was chosen to project the force of infection. We assumed the change of the log scaled force of infection was following a normal distribution with mean equal to the median of the change of the modeled force of infection among the years having ART implemented or prevalence data, and the SD was equal to the default setting as the mean SD of the change of the modeled force of infections among the years having prevalence data. The projection year was chosen from the most recent year between the year with the lowest model force of infection and the year of the second latest survey data.

For Indian states, we used the equally weighted draw-level estimates of the equilibrium prior and random walk assumptions since we had no further information to support either assumption for each state. Here, the projection year of the random walk was the year with the lowest modeled force of infection because no locations had more than one prevalence survey, and the assumption of increasing ART coverage was supported by the data available to us.

In the new EPP code, an optimization step was added into IMIS function to speed up the parameter sampling step based on Raftery and Bao.[9] Two optimization methods have been introduced. The main algorithm is Broyden–Fletcher–Goldfarb–Shanno (BFGS) optimization. If BFGS fails, Nelder-Mead optimum is used instead. In our 2016 EPP model, by substituting in our own assumptions about HIV progression rates and on/off ART mortality, we were able to ensure that the implied relationship between incidence and mortality/prevalence in EPP is similar to that in Spectrum.

In Group 1 locations, we expect estimates of HIV burden to exhibit substantial uncertainty. To reflect this, we induced a perfect correlation between the previously independent draws of HIV mortality with and without ART and CD4 progression. We paired the draws of the three parameter sets internally and with each other in the following way: we sorted without-ART mortality and CD4 progression internally by age (not CD4), meaning the highest draw of HIV mortality without ART for age a_i and CD4 category c_l will be paired with the highest draw of HIV mortality without ART for age a_k and CD4 category c_l. In the same way, we sorted with-ART mortality internally by age, sex, CD4 count at treatment initiation, and duration on treatment. After this sorting process, the lowest indexed draw of each parameter has the highest values and vice versa. This means that we will use the most extreme possible parameter sets in EPP and Spectrum and should see a commensurate expansion in the range of the uncertainty.

To ensure that this expanded uncertainty is replicated in EPP, we fit the model once for every set of paired draws of the progression parameters for every location. This means that the first iteration of EPP for Uganda sees the highest draws of all three sets of progression parameters. Such a procedure is necessary because EPP currently has no mechanism for incorporating uncertainty in any inputs except prevalence data. This process (Process 1 in the HIV/AIDS Estimation Flowchart), produced 1,000 sets of EPP output for each of the locations that make up the 48 countries in the group. Every set of EPP outputs contains 500 consistent draws of HIV incidence and prevalence in adults aged 15-49. In many cases, the algorithm used to fit EPP, incremental mixture importance sampling, failed, resulting in fewer than 1,000 sets of EPP results.

For every location in the group, we sampled one of the 500 incidence/prevalence draws from each of the sets of EPP results (Process 2 in the HIV/AIDS Estimation Flowchart). By sampling one draw from each set, we ensured that the distribution of progression parameters dictating the relationship between incidence and prevalence was exactly the same as the distribution of the sorted parameters generated in the previous step. In locations where not all 1,000 iterations of EPP fit successfully, we sampled one draw from every iteration that did succeed and then resampled with replacement from that set of draws. To maintain the link between the input progression draws and the resulting incidence and prevalence draws from EPP, we replaced any parameter draw associated with a failed run of EPP with the parameter draw that that failed draw was replaced with. At the end of this process, for every location in the set of 48 countries, we were left with 1,000 linked draws of adult incidence and prevalence and the exact progression parameters that generated those draws.

We then ran these results, along with the previously described demographic and HIV-specific inputs, through Spectrum to produce location-, year-, age-, and sex-specific estimates of HIV incidence, prevalence, and mortality (Process 9 in the HIV/AIDS Estimation Flowchart).

The HIV/mortality reckoning process (Process 11 on the HIV/AIDS Estimation Flowchart) is intended as a method of reconciling separate estimates of HIV mortality (and its resulting effect on estimates of HIV-free and all-cause mortality) in Group 1 countries by averaging estimates of HIV mortality from the model

life table process and EPP-Spectrum. Additional details on the reckoning can be found in the GBD 2016 mortality manuscript.[10]

Since Spectrum produces HIV incidence, prevalence, and deaths that are consistent with one another over time, the reckoning process results in death numbers that are no longer consistent with the incidence and prevalence produced in Spectrum. In order to recreate this consistency, we recalculated incidence for all Group 1 locations using reckoned deaths and prevalence produced by Spectrum (Process 12 on the HIV/AIDS Estimation Flowchart). The updated incidence is calculated by aggregating counts of people living with HIV (PLWH), new infections, and deaths (among PLWH from HIV and other causes) at the year-sex level and calculating the following ratio for each sex:

$$r_t = \frac{[(PLWH_t - PLWH_{t-1}) + (Deaths_{hiv,t} + Deaths_{background,t})]}{NewHIV_t}$$

Age-specific counts of new infections are then scaled by their corresponding sex-year ratios.

Countries with vital registration data (Group 2A and 2B)

Vital registration is one of the highest-quality sources of data on HIV burden in many countries, so generating estimates that are consistent with these data, with necessary adjustment to account for any potential underreporting, is critical. We identified 114 countries – as well as 440 subnational locations from Brazil, China, Japan, Indonesia, Mexico, Sweden, the United Kingdom, and the United States – with at least two usable points of vital registration data, verbal autopsy (VA) data, or sample registration system (SRS) data. In India and Indonesia, we used SRS and VA data, respectively, as input mortality for CIBA. For India we extracted the resulting age-sex distribution of incidence, but scaled the level to match the adult incidence rate estimated from EPP for each state.

We imputed missing years of data to generate a complete time series for HIV from the estimated start year of the epidemic using ST-GPR. We analyzed mortality trends using ST-GPR starting in 1981, the year that HIV was first identified in the United States.[11] For ST-GPR, we adjusted the lambda (time weight) and GPR scale according to the completeness of vital registration data, with 4- and 5-star quality VR using parameters designed to follow the data more closely. We produced separate splines by country/age group, up to the peak year of death rate. We then ran a linear regression with random effects on region, age, and sex. Following this, we ran space-time residual smoothing, in which time, age, and space weights are used to inform smoothing of the residuals between data points and the linear regression estimate. From this process, we generated space-time estimates with the applied weights, along with the median absolute deviation (MAD) of the space-time estimates from the data. The MAD was calculated at various levels of the location hierarchy (e.g., subnational and national), and was added into the data variance term. The data variance and space-time estimates were then analyzed using Gaussian Process Regression to return a final estimate of mortality along with uncertainty.

Although Spectrum produces HIV mortality estimates that are within the realm of possibility in most countries using the incidence curves provided in the UNAIDS country files, it is a deterministic model that has not yet been integrated into an optimizable framework. Therefore, in order to “fit” it to vital registration data, we need to adjust input incidence.

To improve the fit of this process, in GBD 2015, we restructured Spectrum to add compartments that identify groups of people living with HIV by year of infection (Process 5 in the HIV/AIDS Estimation Flowchart). With this version of Spectrum we can output, among many other metrics, HIV deaths by year, age, sex, and infection cohort. This enables us to adjust incidence to fit to death much more precisely and without making any rigid assumptions about the time from HIV infection to HIV death.

We have incorporated these improvements into a cohort incidence bias adjustment (CIBA) process. First, we ran Spectrum normally to produce 1,000 draws of incidence, prevalence and mortality (Process 4 in the HIV/AIDS Estimation Flowchart). Then, by year, age, and sex, we took the ratio of VR deaths to Spectrum deaths to quantify the amount of bias in Spectrum. Using draw-level duration data from the new version of Spectrum, for every year-, age-, and sex-specific infection cohort, we calculated the share of all HIV deaths observed over the course of the projection period in that cohort that would occur in each year after the year of infection. For example, projecting from 1970 through 2016, we identified the cohort of men infected in 1992 at the age of 16, calculated the total number of HIV deaths in that cohort in all subsequent years through the end of 2016, and divided the annual number of deaths by that total. This showed us the distribution of deaths among that cohort over the projection period. In the most extreme case (infections in 2015), we could only produce one point of that distribution (2016), so that single value is exactly 1·0; 100% of the deaths observed in that cohort occurred in 2016.

We then used these distributions of death to weigh the ratio of VR deaths to Spectrum deaths, meaning that ratios in the years where we expect the largest share of deaths were weighed most heavily. We then multiplied the initial size of that cohort from the normal run of Spectrum by the sum of the combined ratios to get a new estimate of new cases in that year/age/sex combination.

We can write this method mathematically in the following way:

$$r_t = \frac{VR_t}{D_t}$$

$$\rho_t^{t-i} = \frac{d_t^{t-i}}{\sum_{k=t-i+1}^{n} d_k^{t-i}}$$

$$\alpha^{t-i} = \sum_{k=t-i+1}^{n} r_k * \rho_k^{t-i}$$

$$n_{\text{adjusted}}^{t-i} = \alpha^{t-i} * n^{t-i}$$

VR_t is the number of HIV/AIDS deaths in year t from ST-GPR, and D_t is the number of HIV/AIDS deaths from the first run of Spectrum. In the second equation, d_t^{t-i} is the number of HIV/AIDS deaths among members of infection cohort $t-i$ in year t, with $i \geq 1$, from the new, duration-tracking version of Spectrum, and n is final year of the projection. Therefore, ρ_t^{t-i} is the share of observed deaths in cohort $t-i$ that we expect to occur in year t. It follows that α^{t-i} is the weighted adjustment ratio described above, which we multiply by the estimated initial size of infection cohort $t-i$ as calculated in the first-stage Spectrum run to get the adjusted number of new cases, $n_{\text{adjusted}}^{t-i}$. This process is run separately for every sex, single-age, and draw.

CIBA (Process 6 in the HIV/AIDS Estimation Flowchart) allows ratios in each year after a given infection year to influence the final adjustment to incidence. The size of that influence is determined by the relative importance of that year in the cohort-year's distribution of deaths over time. The result is a new set of 1,000 draws of incidence and a set of 1,000 ratios of post-adjustment incidence to pre-adjustment incidence. We perform this adjustment using mean durations from the new version of Spectrum in order to try to shift the mean of the regular distribution of deaths.

Finally, to produce location-, year-, age-, and sex-specific estimates of HIV incidence, prevalence, and mortality, we ran the new estimates of incidence and all previously input data through Spectrum (Process 9 in the HIV/AIDS Estimation Flowchart).

Countries without survey data and vital registration data (Group 2C)

The remaining 31 countries – as well as 14 subnational locations from China and Saudi Arabia – had neither geographically representative seroprevalence surveys nor reliable vital registration systems. To produce estimates of HIV burden in these countries, we assumed that Spectrum is similarly biased as in other Group 2 countries. This involved running Spectrum (Process 7 in the HIV/AIDS Estimation Flowchart), adjusting incidence using 1,000 adjustment ratios randomly sampled from the entire set of CIBA results (Process 8), and rerunning Spectrum using the new draws of adjusted incidence (Process 9). As above, the estimates of incidence, prevalence, and mortality were incorporated into the rest of the machinery via the reckoning process.

Subnational splitting and aggregation

Spectrum results for India, Kenya, and UK subnational locations are modeled at higher levels of geography than our GBD locations. Spectrum results for India are produced at the state level, while GBD 2016 estimates were produced at the state urban-rural level; Spectrum models Kenya provinces, while we compute Kenyan estimates for 47 counties. Indonesia and the United Kingdom have Spectrum results at the national level, while GBD 2016 estimates Indonesian provinces and Upper Tier Local Authorities in the UK. To split the Spectrum results into more granular results for processing, we assign each GBD subnational unit to a Spectrum modeling unit. From this, we generate age/sex/year-specific proportions for population, HIV-specific death, and HIV-free mortality.

In Cote d'Ivoire, Haiti, Moldova, Mozambique, and Zimbabwe, the country files that we received from UNAIDS contained only subnational data without national-level aggregates. In these locations, we generated GBD 2016 demographic inputs for the provided subnational units using the proportions present in the UNAIDS files and ran the locations through EPP and Spectrum at the subnational level before aggregating to generate final national level GBD 2016 estimates. These aggregation and splitting steps are shown as Process 10 in the HIV/AIDS Estimation Flowchart.

HIV/AIDS resulting in other diseases

There are two Level 4 causes under the HIV/AIDS Level 3 cause in the GBD 2016 cause hierarchy. The modeling process for HIV/AIDS-tuberculosis is detailed in another part of this appendix. We computed the number of people living with HIV resulting in other diseases by subtracting the number of people living with HIV/AIDS-tuberculosis from all people living with HIV/AIDS at the 1,000 draw level.

References

1 Global, regional, and national age–sex specific all-cause and cause-specific mortality for 240 causes of death, 1990–2013: a systematic analysis for the Global Burden of Disease Study 2013. *The Lancet* 2015; **385**: 117–71.

2 Birnbaum JK, Murray CJ, Lozano R. Exposing misclassified HIV/AIDS deaths in South Africa. *Bull World Health Organ* 2011; **89**: 278–85.

3 Murray CJL, Ortblad KF, Guinovart C, *et al.* Global, regional, and national incidence and mortality for HIV, tuberculosis, and malaria during 1990–2013: a systematic analysis for the Global Burden of Disease Study 2013. *The Lancet* 2014; **384**: 1005–70.

4 jeffeaton/epp. GitHub. https://github.com/jeffeaton/epp (accessed April 21, 2016).

5 Verguet S, Lim SS, Murray CJL, Gakidou E, Salomon JA. Incorporating Loss to Follow-up in Estimates of Survival Among HIV-Infected Individuals in Sub-Saharan Africa Enrolled in Antiretroviral Therapy Programs. *J Infect Dis* 2013; **207**: 72–9.

6 Ghys PD, Zaba B, Prins M. Survival and mortality of people infected with HIV in low and middle income countries: results from the extended ALPHA network. *AIDS Lond Engl* 2007; **21 Suppl 6**: S1–4.

7 Hallett TB, Zaba B, Todd J, *et al.*, ALPHA Network. Estimating incidence from prevalence in generalised HIV epidemics: methods and validation. *PLoS Med* 2008; **5**: e80.

8 Marsh K, Mahy M, Salomon JA, Hogan DR. Assessing and adjusting for differences between HIV prevalence estimates derived from national population-based surveys and antenatal care surveillance, with applications for Spectrum 2013. *AIDS Lond Engl* 2014; **28 Suppl 4**: S497–505.

9 Raftery AE, Bao L. Estimating and Projecting Trends in HIV/AIDS Generalized Epidemics Using Incremental Mixture Importance Sampling. *Biometrics* 2010; **66**: 1162–73.

10 Wang H, Murray CJ, Carter A, He F. Global, regional, and national under-5 mortality, adult mortality, age-specific mortality, and life expectancy, 1970-2016: a systematic analysis for the Global Burden of Disease Study 2016. *The Lancet* 2017.

11 CDC. Pneumocystis Pneumonia --- Los Angeles. MMWR Wkly. 1981; published online June 5. http://www.cdc.gov/mmwr/preview/mmwrhtml/june_5.htm (accessed April 21, 2016).

Diarrheal Diseases

Flowchart

Diarrheal diseases

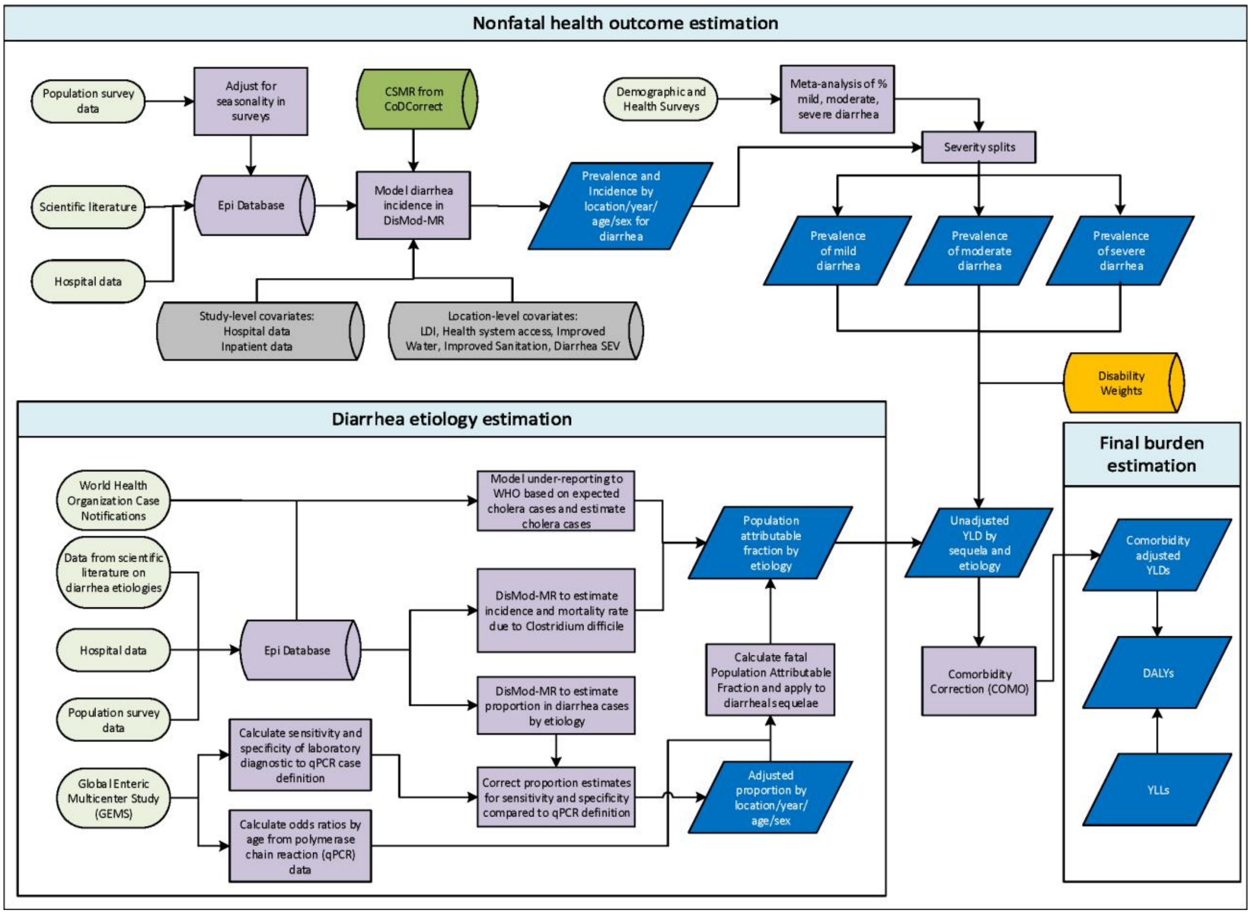

Case definition

We defined diarrheal disease episodes as three or more loose stools in a 24-hour period. In the diarrhea models, self-reported prevalence is the reference category for all data adjustments. Hospital input data use ICD9 codes 001-009.9 and ICD10 codes A00-A09. We also split diarrhea episodes into three severity levels: mild, moderate, and severe.

Input data

Model inputs

We used two main types of data in the diarrhea non-fatal burden estimation and the attribution of diarrheal etiologies. Moreover, we included all data sources used in GBD 2015 and conducted new reviews of scientific literature, surveys, and hospitalization data. We presented a summary of the data sources in Table Data.

The first type of data is the incidence and prevalence of diarrhea in community and hospital settings. Hospital data and healthcare utilization data were identified using the ICD9 codes 001-009.9 and ICD10 codes A00-A09. To be consistent with the survey data, we transformed the hospital and healthcare data from incidence to prevalence. A summary of the data sources is found in **Table 1**. We used data from population-representative surveys, such as the Demographic and Health Surveys and the Multiple Indicator Cluster Surveys. We converted the prevalence of maternal-reported two-week period from surveys to point prevalence in one-year age increments this equation.

$$Point\ Prevalence = Period\ Prevalence * \frac{Duration}{(Recall\ Period + Duration - 1)}$$

Survey data were adjusted for seasonality. An inclusion criterion for scientific literature is a study duration longer than 1 year to avoid bias in the seasonal timing of diarrhea. Surveys are frequently conducted over several months. To account for seasonal variation in diarrhea prevalence, we fit a simple sine/cosine model for each GBD region. The model is mixed-effects with random effects on each site. The model accounts for the year of the survey. The percent difference between the monthly model fit diarrhea prevalence and the mean fitted diarrheal prevalence is a scalar to adjust survey data by month and location.

Diarrhea duration was updated based on a systematic review with spatial variation (**Table 2**).[1]

The second type of data describes diarrhea etiologies. We extracted data on all etiologies except *C. difficile* from scientific literature that reported the proportion of diarrhea cases that tested positive for each pathogen. We completed a systematic literature review covering the years May 2015 to May 2016 for diarrhea prevalence, incidence, and all diarrhea etiologies. Inclusion criteria included diarrhea as the case definition, studies with a sample size of at least 100, and studies with at least one year of follow-up. We excluded studies that reported on diarrheal outbreaks exclusively and those that used acute gastroenteritis with or without diarrhea.

We pulled all articles using a PubMed search term that combined non-specific and etiology-specific diarrhea on May 24, 2016 (Search string*:* ((((((diarrhoea[title] OR diarrhoea[MeSH Terms] OR diarrhea[title] OR diarrhea[MeSH Terms]) NOT ((colitis[title/abstract] OR enterocolitis[title/abstract] OR inflammatory bowel[title/abstract] OR irritable[title/abstract]OR Crohn*[title/abstract] OR HIV[title] OR treatment[title] OR therapy[title])) NOT ((appendicitis[title/abstract] OR esophag*[title/abstract] OR surger*[title/abstract] OR gastritis[title/abstract] OR liver[title/abstract] OR case report[title] OR case-report[title] OR therapy[title] OR treatment[title])) AND ((2015/04/01:2016/12/31[PDat]) AND Humans[Mesh])))) OR (((diarrhoea[title] OR diarrhoea[MeSH Terms] OR diarrhea[title] OR diarrhea[MeSH Terms]) AND (salmonella[title/abstract] OR salmonella[MeSH Terms] aeromonas[title/abstract] OR aeromonas[MeSH Terms] OR shigell*[title/abstract] OR shigell*[MeSH Terms] OR enteropathogenic e coli [title/abstract] OR enteropathogenic e coli[MeSH Terms] OR enterotoxigenic e coli[title/abstract] OR enterotoxigenic e coli[MeSH Terms] OR campylobacter[title/abstract] OR campylobacter[MeSH Terms] OR amoebiasis[title/abstract] OR entamoeb*[title/abstract] OR amoebiasis[MeSH Terms] OR entamoeb*[MeSH Terms] OR cryptosporidi*[title/abstract] OR cryptosporidi*[MeSH Terms] OR rotavirus[title/abstract] OR rotavirus[MeSH Terms] OR norovirus[title/abstract] OR norovirus[MeSH Terms] OR adenovirus[title/abstract] OR adenovirus[MeSH Terms]) AND ((etiolog*[title/abstract] OR etiology[MeSH

Terms] OR cause[title/abstract] OR pathogen[title/abstract])) NOT ((colitis[title/abstract] OR enterocolitis[title/abstract] OR inflammatory bowel[title/abstract] OR irritable[title/abstract]OR Crohn*[title/abstract] OR HIV[title] OR treatment[title] OR therapy[title])) NOT ((appendicitis[title/abstract] OR esophag*[title/abstract] OR surger*[title/abstract] OR gastritis[title/abstract] OR liver[title/abstract] OR case report[title] OR case-report[title] OR therapy[title] OR treatment[title])) AND ((2015/04/01:2016/12/31[PDat]) AND Humans[Mesh]))))).

We identified 442 studies, of which 36 met our inclusion criteria. We extracted data points for location, sex, year, and age. For the data that describe proportion of episodes positive for a given pathogen, we assigned an age range based on the prevalence-weighted mean age of diarrhea in the appropriate year/sex/location if the age of the study participants was not reported.

We used the Global Enteric Multicenter Study (GEMS), a seven-site, case-control study of moderate-to-severe diarrhea in children under 5 years,[2] to calculate odds ratios for the diarrheal pathogens. We analyzed raw data for a systematic reanalysis, representative of the distribution of cases and controls by age and site, of roughly half of the 22,000 original GEMS samples that were tested for the presence of pathogen using quantitative polymerase chain reaction (qPCR).[3]

Severity split inputs

Diarrheal diseases have three severity levels: mild, moderate, and severe. The proportion of diarrhea cases that are assigned to each comes from a systematic review of diarrhea severity.[1] Mild cases are the proportion of diarrhea cases that did not seek medical care (64.8%); moderate cases are the proportion that sought medical care but did not have severe dehydration or bloody stool (28.9%); and severe cases are the proportion that sought medical care with severe dehydration or bloody stool (6.9%). These proportions are based on the frequency of dehydration and bloody stool among community-based studies reported in the systematic review.

Modeling strategy

The non-fatal diarrheal disease burden is modeled in DisMod-MR, a Bayesian meta-regression modeling framework. DisMod-MR produces estimates of the incidence, prevalence, and remission of diarrhea for each age, sex, location, and year. We defined remission, or the time to recovery, as five days average. Diarrhoeal disease episodes are characterized as three or more loose stools in a 24 hour period. The reference category for our input data is community based diarrhoea episodes such as data from population-representative surveys or community cohorts. Input data that are from a different population, such as hospital outpatient or inpatient groups, are adjusted by study-level covariates so that they are consistent with the reference category. This step occurs in DisMod.

Country-level covariates also inform the model. These include the proportion of the population that have access to improved sanitation, access to improved water sources, health system access, income per capita, and the SEV for diarrhea (**Table 3**).

We estimated diarrheal disease etiologies separately from overall diarrhea mortality using a counterfactual strategy for enteric adenovirus, *Aeromonas*, *Entamoeba histolytica* (amoebiasis), *Campylobacter enteritis*, *Cryptosporidium*, typical enteropathogenic *Escherichia coli* (t-EPEC),

enterotoxigenic *Escherichia coli* (ETEC), norovirus, non-typhoidal salmonella infections, rotavirus, and *Shigella*. *Vibrio cholerae* and *Clostridium difficile* were modeled separately.

Diarrheal etiologies are attributed to diarrheal deaths using a counter-factual approach. We calculated a population attributable fraction (PAF) from the proportion of severe diarrhea cases that are positive for each etiology. The PAF represents the relative reduction in diarrhea mortality if there was no exposure to a given etiology. As diarrhea can be caused by multiple pathogens and the pathogens may co-infect, PAFs can overlap and add up to more than 100%. We calculated the PAF from the proportion of severe diarrhea cases that are positive for each etiology. We assumed that hospitalized diarrhea cases are a proxy of severe and fatal cases. We used the following formula to estimate PAF:[4]

$$PAF = Proportion * (1 - \frac{1}{OR})$$

Where *Proportion* is the proportion of diarrhea cases positive for an etiology and *OR* is the odds ratio of diarrhea given the presence of the pathogen.

We dichotomized the continuous qPCR test result using the value of the cycle threshold (Ct) that most accurately discriminated between cases and controls. The Ct values range from 0 to 35 cycles representing the relative concentration of the target gene in the stool sample. A low value indicates a higher concentration of the pathogen while a value of 35 indicates the absence of the target in the sample. We used the lower Ct value when we had multiple Ct values for the cutpoint. The case definition for each pathogen is a Ct value that is below the established cutoff point.

We used a mixed effects conditional logistic regression model to calculate the odds ratio for under 1 year and 1-4 years old for each of our pathogens. The odds ratio for 1-4 years was applied to all GBD age groups over 5 years. There were three pathogen-age odds ratios that were not statistically significant: Aeromonas and Amoebiasis in under 1 year and Campylobacter in 1-4 years. The mean value of the odds ratio was above 1 in all three cases so we transformed the odds ratios for these three exceptions only in log-space such that exponentiated values could not be below 1. The transformation was:

Odds ratio = exp(log(or) – 1)) + 1

We modeled the proportion data using the meta-regression tool DisMod-MR to estimate the proportion of positive diarrhea cases for each separate etiology by location/year/age/sex and to adjust for the covariates.

We used the estimated sensitivity and specificity of the laboratory diagnostic technique used in the GEMS study compared to the qPCR case definition to adjust our proportion before we computed the PAF:[5]

$$Proportion_{True} = \frac{(Proportion_{Observed} + Specificity - 1)}{(Sensitivity + Specificity - 1)}$$

We used this correction to account for the fact that the proportions we used are based on a new test that is not consistent with the laboratory-based case definition (qPCR versus GEMS conventional laboratory testing for pathogens).[15]

Our literature review extracted the proportion of any enteropathogenic *Escherichia coli* (EPEC) without differentiating between typical (tEPEC) and atypical (aEPEC). In order to be consistent with the odds ratios

that we obtained, we adjusted our proportion estimates of any EPEC to typical EPEC only. This adjustment was informed by a subset of our literature review that reported both atypical and typical EPEC. We estimated a ratio by super-region of tEPEC to any EPEC and adjusted our proportion estimates accordingly. We found that the majority of EPEC diarrhea cases were positive for atypical EPEC, consistent with other published work.[3]

For *Vibrio cholerae* (cholera), we used the literature review to estimate expected number of cholera cases for each country-year using the incidence of diarrhoea, estimated using DisMod-MR, and the proportion of diarrhoea cases that are positive for cholera. We assigned cholera PAF using odds ratios from the qPCR results to estimate a number of cholera-attributable cases. We compared this expected number of cholera cases to the number reported to the World Health Organization at the country-year level.[6] We modeled the underreporting fraction to correct the cholera case notification data for all countries using health system access and the diarrhoea SEV scalar to predict total cholera cases. We used the age-specific proportion of positive cholera samples in DisMod and our incidence estimates to predict the number of cholera cases for each age/sex/year/location. Finally, we modeled the case fatality ratio of cholera using DisMod-MR and to estimate the number of cholera deaths.

For *C. difficile*, we modeled incidence and mortality in DisMod-MR for each age, sex, year, location. DisMod-MR is a Bayesian meta-regression tool that uses spatio-temporal information as priors to estimate prevalence, incidence, remission, and mortality for *C. difficile* infection. DisMod-MR uses a compartmental model to relate prevalence, incidence, remission, and mortality. We set remission in our model to 1 month.

There are several key updates to the approach to diarrhea etiology estimation in GBD 2016 compared to GBD 2015. There are new data used in the diarrhea DisMod-MR models and in the diarrhea proportion DisMod-MR models. We changed the age groups used in the odds ratio estimation. In GBD 2015 and before, odds ratios were estimated for each age group defined by GEMS: 0-1 year, 1-2 years, and 2-5 years. For GBD 2016, we have collapsed the last two age groups so that our odds are representative for under-1 and over-1 years. We have updated the survey data extraction methodology so that we account for the seasonality of diarrhea in each GBD region. We have changed the duration of diarrhea from an average of 4.2 days used in GBD 2015 and prior and updated the duration so that there is variation in space and uncertainty in the duration estimate. Last, we have updated the proportion severity splits based on a literature review.

Table 1. Nonfatal input data used in diarrhea modeling.

Type of data	Data points (#)
Facility - inpatient	14,913
Facility - other/unknown	6,540
Survey - cross-sectional	6,474
Other	2,231
**Total**	**30,158**

Figure 1. The number of data points used in diarrhea non-fatal modeling for each country is shown.

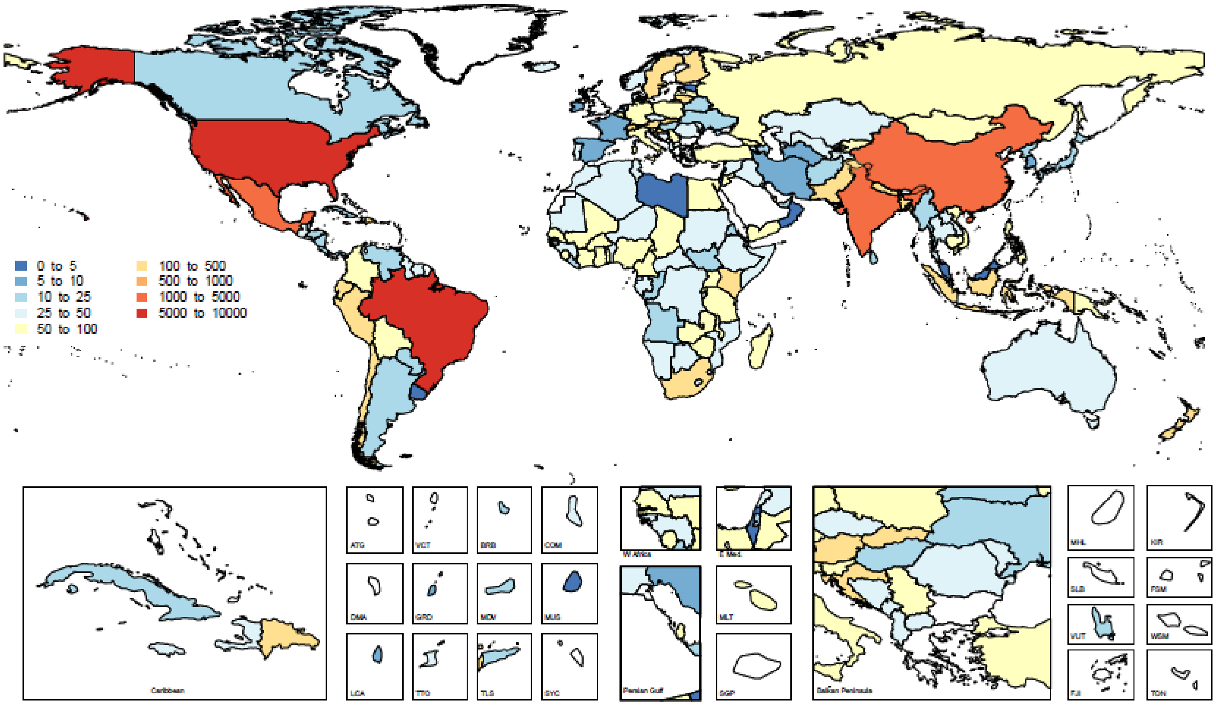

Table 2. Duration of diarrhea. Values are from a systematic review of diarrhea duration.[1]

	Mean	Lower	Upper
Global	4.3 days	4.2 days	4.4 days

Table 3. Severity splits, details on the severity levels for diarrhea in GBD 2015 and the associated disability weight (DW) with that severity.

Severity level	Lay description	DW (95% CI)
Mild	Has diarrhea defined as 3 or more loose stools in a 24-hour period with no dehydration	0.074 (0.049-0.104)
Moderate	Has diarrhea defined as 3 or more loose stools in a 24-hour period with painful cramps and feeling thirsty and any dehydration	0.188 (0.125-0.264)
Severe	Has diarrhea defined as 3 or more loose stools in a 24-hour period with painful cramps and is very thirsty or feels nauseated or tired and/or severely dehydrated	0.247 (0.164-0.348)

Table 4. Covariates. Summary of covariates used in the diarrhea DisMod-MR meta-regression model

Covariate	Type	Parameter	Exponentiated beta (95% Uncertainty Interval)
Low income hospital	Study-level		0.62 (0.24-1.08)
Inpatient population	Study-level		0.009 (0.005-0.02)
Marketscan data	Study-level		0.044 (0.028-0.092)
Diarrhea SEV	Country-level	Prevalence	0.87 (0.84-0.91)
Unsafe sanitation SEV	Country-level	Prevalence	2.36 (2.01-2.64)
Rotavirus vaccine coverage	Country-level	Prevalence	0.76 (0.74-0.79)
Sex	Country-level	Prevalence	0.95 (0.93-0.97)
Healthcare access and quality index	Country-level	Excess mortality	0.93 (0.93-0.93)
Sex	Country-level	Excess mortality	1.21 (1.18-1.23)

References

1 Lamberti LM, Fischer Walker CL, Black RE. Systematic review of diarrhea duration and severity in children and adults in low- and middle-income countries. *BMC Public Health* 2012; **12**: 276.

2 Kotloff KL, Nataro JP, Blackwelder WC, *et al.* Burden and aetiology of diarrhoeal disease in infants and young children in developing countries (the Global Enteric Multicenter Study, GEMS): a prospective, case-control study. *Lancet Lond Engl* 2013; **382**: 209–22.

3 Liu J, Gratz J, Amour C, *et al.* A laboratory-developed TaqMan Array Card for simultaneous detection of 19 enteropathogens. *J Clin Microbiol* 2013; **51**: 472–80.

4 Miettinen OS. Proportion of disease caused or prevented by a given exposure, trait or intervention. *Am J Epidemiol* 1974; **99**: 325–32.

5 Reiczigel J, Földi J, Ozsvári L. Exact confidence limits for prevalence of a disease with an imperfect diagnostic test. *Epidemiol Infect* 2010; **138**: 1674–8.

6 Platts-Mills JA, Operario DJ, Houpt ER. Molecular diagnosis of diarrhea: current status and future potential. *Curr Infect Dis Rep* 2012; **14**: 41–6.

7 Ochoa TJ, Barletta F, Contreras C, Mercado E. New insights into the epidemiology of enteropathogenic Escherichia coli infection. *Trans R Soc Trop Med Hyg* 2008; **102**: 852–6.

8 World Health Organization. Global Health Observatory data repository: Cholera. 2016. http://apps.who.int/gho/data/node.main.174?lang=en (accessed Aug 25, 2016).

9 Global Health Observatory data repository: Cholera. World Health Organ. http://apps.who.int/gho/data/node.main.174?lang=en (accessed April 20, 2016).

Typhoid and Paratyphoid Fever

Flowchart

Typhoid and paratyphoid fever

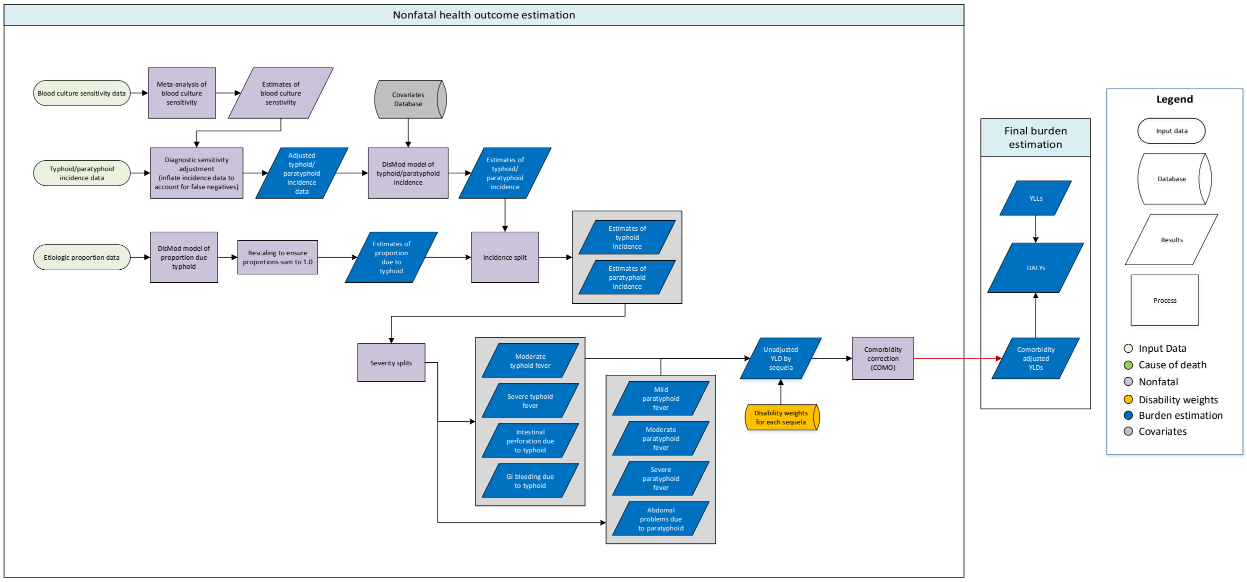

Case definition

Typhoid and paratyphoid are acute bacterial infections that most commonly cause febrile illness and gastrointestinal symptoms. Severe cases are associated with intestinal bleeding and perforation, altered mental state and, in some cases, death. We define a confirmed case as one for which there has been a positive blood culture test for either *Salmonella enterica typhi* or *paratyphi*. Diagnostic criteria do not typically accompany national surveillance reports; however, with blood culture being the standard diagnostic, we treat reported cases as confirmed. Given the poor sensitivity of blood culture, however, we estimated case definition as simply febrile illness resulting from an infection with *Salmonella enterica typhi* or *paratyphi*. This is effectively a counterfactual definition in which we attempt to estimate the number of true infections regardless of test result. These causes include all ICD-10 codes under the heading A01 (Typhoid and paratyphoid fevers).

Input data

Model inputs

Our incidence dataset included a combination of data from prospective cohort studies and national surveillance systems. Similarly, data on proportions due to typhoid and paratyphoid included a combination of prospective cohort studies and national surveillance systems.

Level	Incidence	Proportion
Data points	864	414

Studies	89	56
Locations	59	54
Regions	14	11

Severity splits

For GBD 2016, we derived severity splits based on a published review of enteric fever outcomes from (Azmatullah A, Qamar FN, Thaver D, et al. 2005).

Paratyphoid is split into four sequelae: mild (28.5% [15.6–44.2]), moderate (52.25% [27.2–77.7]), severe (14.25% [8.2–21.8]), and abdominal pain and distention (5.0% [2.8–7.6]):

Sequela	Description	Disability weight
Mild	Has a low fever and mild discomfort , but no difficulty with daily activities.	0.006 (0.002–0.012)
Moderate	Has a fever and aches, and feels weak, which causes some difficulty with daily activities.	0.051 (0.032–0.074)
Severe	Has a high fever and pain, and feels very weak, which causes great difficulty with daily activities.	0.133 (0.088–0.19)
Abdominal pain & distention due to paratyphoid	Has pain in the belly and feels nauseated. The person has difficulties with daily activities.	0.114 (0.078–0.159)

Similarly, typhoid is split into four sequelae: moderate (35.0% [26.0–44.3]), severe (47.75% [38.0–57.4]), severe abdominal pain and distention (17.0% [10.0–25.7]), and intestinal bleeding (0.25% [0–2.0]):

Sequela	Description	Disability weight
Moderate	Has a fever and aches, and feels weak, which causes some difficulty with daily activities.	0.051 (0.032–0.074)
Severe	Has a high fever and pain, and feels very weak, which causes great difficulty with daily activities.	0.133 (0.088–0.19)
Gastrointestinal bleeding	Vomits blood and feels nauseated.	0.325 (0.209–0.462)
Abdominal pain and distention (includes intestinal perforation)	Has severe pain in the belly and feels nauseated. The person is anxious and unable to carry out daily activities.	0.324 (0.22–0.442)

Modelling strategy

We first model total incidence of typhoid and paratyphoid combined. Second, we model the proportion of this total due to typhoid and the proportion due to paratyphoid. Finally, we split the case estimates into sequelae representing different major symptoms and levels of severity.

Total incidence was modelled using DisMod-MR, using the proportion of the population with access to clean water, and the proportion of the population living in the Indian Ocean monsoon belt as covariates. We performed a crosswalk using a study-level covariate indicating sources that were based on passive versus active surveillance, with active surveillance as the reference. This adjusts for incomplete case capture by passive surveillance. Incidence data were inflated to account for poor diagnostic sensitivity, based on an internal meta-analysis of the sensitivity of blood culture, the most common diagnostic used for typhoid. Similarly, we used two DisMod models to estimate etiologic proportions: one for the proportion of total incidence due to typhoid, and one for the proportion due to paratyphoid.

Typhoid cases are split between four sequelae: moderate typhoid fever, severe typhoid fever, severe typhoid fever with intestinal bleeding, and typhoid fever with abdominal complications. Paratyphoid cases are split between four sequelae: mild paratyphoid fever, moderate paratyphoid fever, severe paratyphoid fever, and paratyphoid fever with abdominal complications.

Changes from GBD 2015 to GBD 2016

We made no major changes in our methods between GBD 2015 and 2016.

Other intestinal infectious diseases

In addition to the intestinal infectious diseases described above, there are many diverse types of intestinal infectious diseases. Because these intestinal infectious diseases are diverse in their underlying causes and risk factors as well as in their associated health outcomes, modelling them together in a DisMod-MR model would not produce reliable estimates of prevalence or excess mortality. Instead, we calculated the YLDs caused by intestinal infectious diseases directly using a YLD/YLL ratio.

We calculated the ratio of YLDs to YLLs across the specified intestinal infectious diseases for which nonfatal outcomes were modelled, using YLL estimates from the GBD 2016 cause of death (CoD) analysis. We then multiplied this YLD/YLL ratio by the YLL estimates for other intestinal infectious diseases from the GBD 2016 CoD analysis, providing us with an estimate of the YLDs associated with other intestinal infectious diseases.

Lower respiratory infections (LRI)

Flowchart

Lower respiratory infections

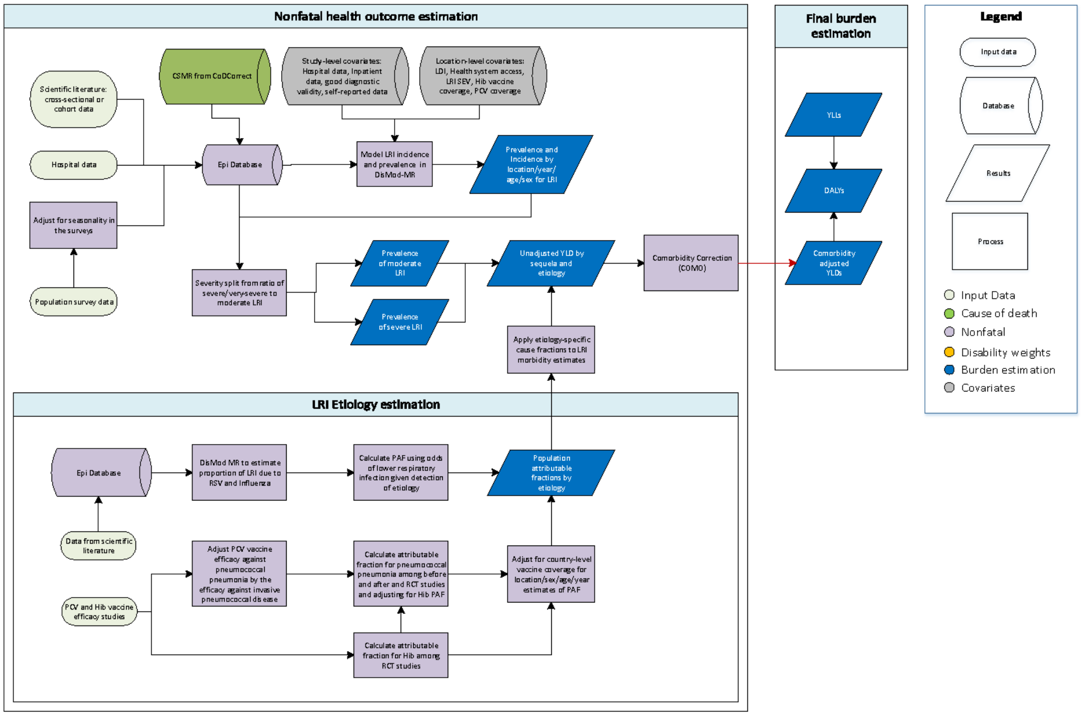

Case definition

We used clinician-diagnosed pneumonia or bronchiolitis as our case definition for lower respiratory infections (LRI). We included ICD9 codes 073.0-073.6, 079.82, 466-469, 480-489, 513.0, and 770.0 and ICD10 codes A48.1, J09-J22, J85.1, P23-P23.9, and U04. LRI etiologies are modeled separately from overall LRI incidence and prevalence. The etiologies include influenza, respiratory syncytial virus, *Streptococcus pneumoniae,* and *Haemophilus influenzae* type b and are episodes of LRI where the etiology is the causal pathogen in the infection.

Input data

Model inputs

Input data included all data used in GBD 2015. We used two primary types of input data for lower respiratory infections. The first is lower respiratory infection incidence and prevalence data. These data come from a systematic literature review, hospital inpatient and outpatient data, claims data from the US, and population-representative surveys (Table 1). The second type of data is on the etiologies of LRI. Influenza and respiratory syncytial virus (RSV) population attributable fractions were informed by a systematic literature review of the proportion of LRI cases that are positive for each pathogen.

Haemophilus influenzae type B (Hib) and *Streptococcus pneumoniae* (pneumococcal pneumonia) are informed by a systematic review of vaccine efficacy and effectiveness studies.

To estimate the non-fatal burden of lower respiratory infections (LRI), we conducted a systematic review of scientific literature for LRI incidence and prevalence (Search String: ('lower respiratory' [title/abstract] OR pneumonia[title/abstract] AND ('2015/05/01'[PDat] : '2016/6/31'[PDat])AND Humans[MeSH Terms] NOT(autoimmune[title/abstract] OR COPD [title/abstract] OR 'cystic fibrosis'[title/abstract]). Our inclusion criteria were studies that were published between May 2015 and June 2016, had a sample size of at least 100, were at least one year in duration, and included lower respiratory infections, pneumonia, or bronchiolitis in the case definition. Our literature review identified 631 articles, of which 48 were included and extracted. We also included studies referred by experts and identified through scientific literature references.

We also used self-reported prevalence of LRI symptoms from population-representative surveys, such as the Demographic and Health Survey and the Multiple Indicator Cluster Survey. When possible, we extracted survey data by 1-year age group and by sex. We converted these data from two-week period prevalence to point prevalence. The equation for this adjustment is

$$1) \quad Point\ Prevalence = \frac{Period\ Prevalence * Duration}{(Recall\ Period + Duration - 1)}$$

We performed a systematic review of the duration of symptoms of LRI. We sought consistency with our case definition of LRI and defined our duration as the time between the onset of symptoms to the resolution of increased work of breathing. Although crucial, there were very limited data on spatial, temporal, or age-specific duration which may vary based on severity, etiology, and treatment. We identified 485 titles from PubMed and extracted 6 studies which were used in a meta-analysis (mean duration 7.79 days, 6.2-9.64 days).

Survey data were adjusted for seasonality. An inclusion criterion for scientific literature is a study duration longer than 1 year to avoid bias in the seasonal timing of LRI. Surveys are frequently conducted over several months. To account for seasonal variation in LRI symptom prevalence, we fit a simple sine/cosine model for each GBD region. The model is mixed-effects with random effects on each site. The model accounts for the year of the survey and the case definition used. The percent difference between the monthly model fit diarrhea prevalence and the mean fitted diarrheal prevalence is a scalar to adjust survey data by month and location.

In addition to survey data, hospital inpatient, outpatient data, and US claims data were included in the LRI modeling. To make the data more consistent in the modeling process, we converted all incidence data to prevalence.

We updated our systematic review of scientific literature for the proportion of LRI that tested positive for influenza and respiratory syncytial virus (RSV) to include all data from GBD 2015 and from studies published between May 2015 and May 2016. Inclusion criteria were studies that had a sample size of at least 100, studies that were at least one year in duration, and studies describing lower respiratory infections, pneumonia, or bronchiolitis as the case definition. During our literature review we identified 209 studies, of which 7 met our inclusion criteria and were extracted. We excluded studies that described pandemic H1N1 influenza solely and studies that used influenza-like illness as the case definition. We

assigned an age range based on the prevalence-weighted mean age of LRI in the appropriate year/sex/location if the ages of the study participants were not reported.

We also conducted a systematic literature review of studies on the Hib vaccine and PCV effectiveness studies against X-ray-confirmed pneumonia and against pneumococcal and Hib disease until May 2016. For PCV studies, we extracted, if available, the distribution of pneumococcal pneumonia serotypes and the serotypes included in the PCV used in the study. No new studies were identified for GBD 2016. We excluded observational and case-control studies due to implausibly high vaccine efficacy estimates. Hib trial data were exclusively from children <5 years so we did not include the effect of Hib on ages over 5 years of age. PCV trial data are also frequently limited to younger age populations. To understand the contribution of pneumococcal pneumonia in older populations, we also included PCV efficacy studies that used before-after approaches.

Table 1: Data Inputs

Type of data	Data points (#)
Facility - inpatient	15,258
Facility - other/unknown	6,411
Survey - cross-sectional	7,772
Other	909
**Total**	**30,350**

Figure 1. The number of data points used in the LRI non-fatal modeling by country is shown.

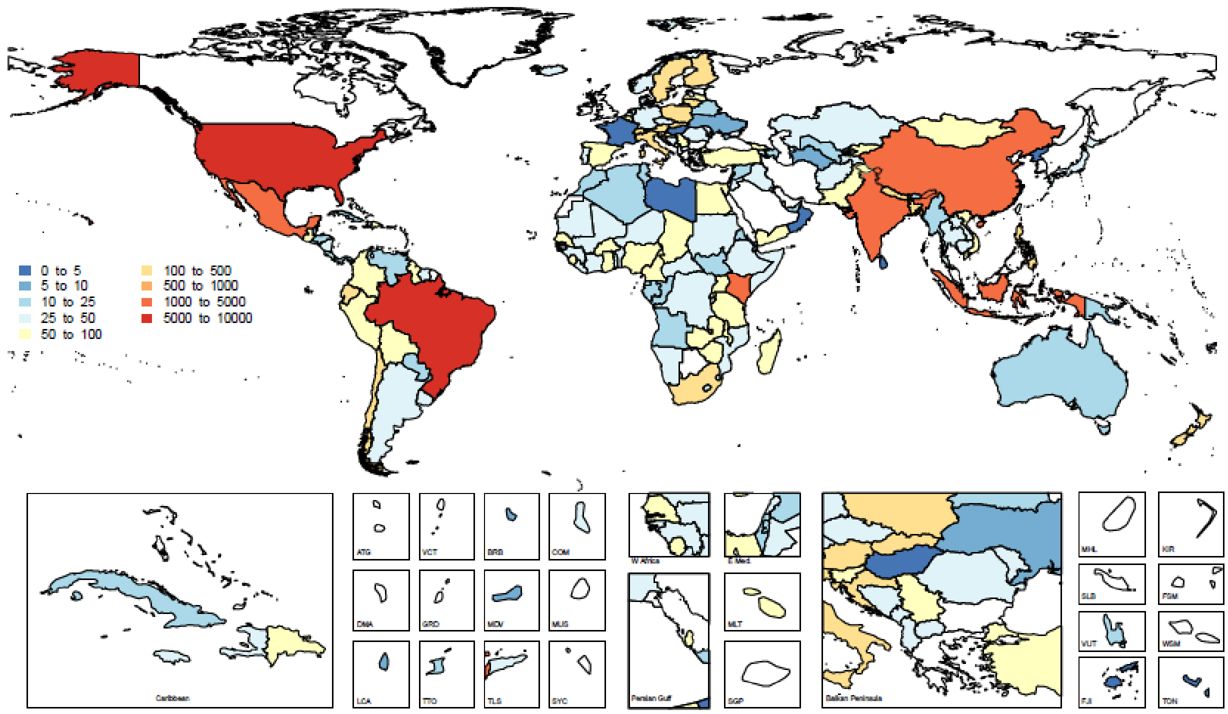

Severity splits

The distribution of moderate (85%) and severe (15%) lower respiratory infections is determined by a meta-analysis of the ratio of severe to all LRI from studies that report the incidence of moderate and severe lower respiratory infections.

We used the health states of acute infectious disease episode, moderate and severe, with the lay descriptions and disability weight values shown in table below:

Table 2: Severity Splits

Severity level	Lay description	DW (95% CI)
Moderate	Has a fever and aches and feels weak which causes some difficulty with daily activities.	0.051 (0.032-0.074)
Severe	Has a high fever and pain and feels very weak, which causes great difficulty with daily activities.	0.133 (0.088-0.19)

Modeling strategy

The non-fatal lower respiratory infection burden is modeled in DisMod-MR, a Bayesian meta-regression modeling framework. DisMod-MR produces estimates of the incidence, prevalence, and remission of LRI for each age, sex, location, and year. We defined the time to recovery as an average of 10

days (9-12 days) which corresponds with a remission 36.5. The models are informed by study-level covariates and by country-level covariates.

Input data are adjusted to our standard case definition. Data are adjusted by study-level binary covariates which describe if the source is a hospital or inpatient sample and if the data come from a self-reported survey (**Table 3**). Self-reported prevalence of LRI symptoms from population-representative surveys such as the Demographic and Health Survey (DHS) and the Multiple Indicator Cluster Survey (MICS) is used. Our case definition from symptom-based prevalence estimates is children in the last 2-weeks with fever and cough with difficulty breathing and symptoms located in the chest and/or chest and nose. This is consistent with the WHO Integrated Management of Childhood Illness guidelines definition of pneumonia and with the DHS and MICS definition of acute respiratory infection (ARI).[1] We extracted the prevalence of children under 5 years old that had fever and cough with difficulty breathing and included an indicator for this less-specific definition. Some surveys did not include the prevalence of LRI symptoms and fever so we adjusted survey prevalence estimates that did not include fever by studies that did based on a logistic regression.

Table 3: Model covariates

Study covariate	Type	Parameter	Exponentiated beta
Hospital inpatient population	Study-level	-	0.25 (0.23-0.27)
Hospital data from middle- or low-income country	Study-level	-	0.17 (0.16-0.19)
Self-reported	Study-level	-	4.05 (3.81-4.27)
Poor diagnostic validity	Study-level	-	1.72 (1.63-1.81)
Marketscan	Study-level	-	1.95 (1.77-2.12)
Hib vaccine coverage	Country-level	Prevalence	0.73 (0.70-0.76)
LRI SEV	Country-level	Prevalence	0.98 (0.91-1.07)
Socio-demographic index	Country-level	Prevalence	0.77 (0.59-1.06)
Healthcare access and quality index	Country-level	Excess mortality	0.96 (0.96-0.96)

Etiologies

We estimated LRI etiologies separately from overall LRI mortality using two distinct counterfactual modeling strategies to estimate population attributable fractions (PAFs), described in detail below. The PAF represents the relative reduction in LRI mortality if there was no exposure to a given etiology. As LRIs can be caused by multiple pathogens and the pathogens may co-infect, PAFs can overlap and add up to more than 100%. Separate strategies were used for viral- influenza and respiratory syncytial virus (RSV)- and bacterial- Streptococcus pneumoniae and Haemophilus influenzae type B- etiologies. We did not attribute etiologies to neonatal pneumonia deaths due to a dearth of reliable data in this age group. We calculated uncertainty of our PAF estimates from 1,000 draws of each parameter using normal distributions in log space.

Influenza and RSV. We calculated the population attributable fraction (PAF) from the proportion of severe LRI cases positive for influenza and RSV. We assumed that hospitalized LRI cases are a proxy of severe cases. We used the following formula to estimate PAF:[2]

$$PAF = Proportion * (1-1/OR)$$

Where Proportion is the proportion of LRI cases that test positive for influenza or RSV and OR is the odds ratio of LRI given the presence of the pathogen. We used an odds ratio of 5.1 (3.19 – 8.14) for influenza and 9.79 (4.98 – 19.27) for RSV from a recently published meta-analysis.[3] These odds ratios are marginally different from those used in GBD 2013.

We modeled the proportion data using the meta-regression tool DisMod-MR to estimate the proportion of LRI cases that are positive for influenza and RSV, separately, by location/year/age/sex. We accounted for study-level covariates in our models such as PCR as the diagnostic technique, studies that investigated RSV or influenza exclusively, and studies from inpatient populations.

As the case-fatality of viral causes of pneumonia is lower than for bacterial causes, we adjusted for differential case-fatality by determining the etiological fractions for mortality attributable to RSV and influenza (Table 2). We measured the etiologic fractions by applying a relative case-fatality adjustment based on in-hospital case-fatality, which we coded to specific pneumonia etiologies. Hospital admissions data of this type were limited to data from the USA, Austria, Brazil, and Mexico. We generated the pooled estimate of the case-fatality differential between bacterial (pneumococcus, Hib) and viral etiologies (RSV, influenza) using DisMod-MR.

Pneumococcal pneumonia and Hib. For Streptococcus pneumoniae (pneumococcal pneumonia) and Haemophilus influenzae type B (Hib), we calculated the population attributable fraction using a vaccine probe design.[4,5] The ratio of vaccine effectiveness against nonspecific pneumonia to pathogen-specific disease represents the fraction of pneumonia cases attributable to each pathogen.

To estimate the PAF for Hib and pneumococcal pneumonia, we calculated the ratio of vaccine effectiveness against nonspecific pneumonia to pathogen-specific pneumonia (Equations 1 and 3). We estimated a study-level estimate of PAF from a meta-analysis of these ratios. To estimate the PAF for Hib, we only used randomized controlled trials because of implausibly high values of vaccine efficacy in case-control studies. To estimate the PAF for pneumococcal pneumonia, we included RCTs and before and after vaccine introduction longitudinal studies.

We adjusted the study-level PAF estimate by vaccine coverage and expected vaccine performance to estimate country- and year-specific PAF values. For pneumococcal pneumonia, we adjusted the PAF by the final Hib PAF estimate and by vaccine serotype coverage. Finally, we used an age distribution of PAF modeled in DisMod to determine the PAF by age. Because of an absence of data describing vaccine efficacy against Hib in children older than two years, we did not attribute Hib to episodes of LRI in ages five years and older.

We used a vaccine probe design to estimate the PAF for pneumococcal pneumonia and (Hib) by first calculating the ratio of vaccine effectiveness against nonspecific pneumonia to pathogen-specific pneumonia at the study level (Equations 1 and 2).[4–6] We then adjusted this estimate by vaccine coverage and expected vaccine performance to estimate country- and year-specific PAF values (Equations 3 and 4).

1) $HibPAF_{Base} = 1 - \frac{VE_{Pneumonia}}{VE_{Hib}}$

2) $PneumoPAF_{Base} = 1 - \frac{VE_{Pneumonia} *(1-PAF_{Hib}*VE_{Hib\ Optimal})}{VE_{Streptococcus}*Cov_{Serotype}}$

3) $PAF_{Hib} = PAF_{Base} * \frac{(1-Cov_{Hib}*VE_{Hib\ Optimal})}{(1-PAF_{Base}*Cov_{Hib}*VE_{Hib\ Optimal})}$

4) $PAF_{Pneumo} = \frac{PAF_{Base}*(1-Cov_{PCV}*VE_{PCV\ Optimal})}{(1-PAF_{Hib}*Cov_{Hib}*VE_{Hib\ Optimal})*\left(1-\frac{PAF_{Base}Cov_{PCV}*VE_{PCV\ Optimal}}{(1-PAF_{Hib}*Cov_{Hib}*VE_{Hib\ Optimal})}\right)}$

Where $VE_{Pneumonia}$ is the vaccine efficacy against nonspecific pneumonia, VE_{Hib} is the vaccine efficacy against invasive Hib disease, $VE_{Streptococcus}$ is the vaccine efficacy against serotype-specific pneumococcal pneumonia, $Cov_{serotype}$ is the serotype-specific vaccine coverage for PCV,[7] $VE_{Hib\ Optimal}$ is the Hib effectiveness in the community (0.8)[8], PAF_{Hib} is the final PAF for Hib, Cov_{PCV} is the PCV coverage, Cov_{Hib} is the Hib coverage by country, and $VE_{PCV\ Optimal}$ is the vaccine effectiveness in the community (0.8).[9]

For Hib, we assumed that the vaccine efficacy against invasive Hib disease is the same against Hib pneumonia. For pneumococcal pneumonia, a recent study in adults [10] found that the vaccine efficacy against invasive pneumococcal disease may be significantly higher than against pneumococcal pneumonia. We used this ratio to adjust estimates of vaccine efficacy against invasive pneumococcal disease from other studies. However, recognizing that the study is unique in that it uses a urine antigen test among adults, we added uncertainty around our adjustment using a wide uniform distribution (median 0.65, 0.3-1.0).

There are several important differences in the approach to LRI etiology estimation in GBD 2016 compared to GBD 2015. We have added an adjustment for the seasonality of LRI symptom prevalence from population-representative surveys. We have performed an update to the average duration of LRI symptoms which decreased the mean duration of illness from 10 to 7.79 days. We have changed some of the covariates used in the modeling process and updated the input data for the LRI envelope and for its etiologies.

References

1 World Health Organization: Department of Child and Adolescent Health and Development. Handbook Integrated Management of Childhood Illness. 2005.

2 Miettinen OS. Proportion of disease caused or prevented by a given exposure, trait or intervention. *Am J Epidemiol* 1974; **99**: 325–32.

3 Shi T, McLean K, Campbell H, Nair H. Aetiological role of common respiratory viruses in acute lower respiratory infections in children under five years: A systematic review and meta-analysis. *J Glob Health* 2015; **5**: 10408.

4 Feikin DR, Scott JAG, Gessner BD. Use of vaccines as probes to define disease burden. *Lancet Lond Engl* 2014; **383**: 1762–70.

5 O'Brien KL, Wolfson LJ, Watt JP, *et al.* Burden of disease caused by Streptococcus pneumoniae in children younger than 5 years: global estimates. *Lancet Lond Engl* 2009; **374**: 893–902.

6 Watt JP, Wolfson LJ, O'Brien KL, *et al.* Burden of disease caused by Haemophilus influenzae type b in children younger than 5 years: global estimates. *Lancet Lond Engl* 2009; **374**: 903–11.

7 Johnson HL, Deloria-Knoll M, Levine OS, *et al.* Systematic evaluation of serotypes causing invasive pneumococcal disease among children under five: the pneumococcal global serotype project. *PLoS Med* 2010; **7**. DOI:10.1371/journal.pmed.1000348.

8 Swingler G, Fransman D, Hussey G. Conjugate vaccines for preventing Haemophilus influenzae type B infections. *Cochrane Database Syst Rev* 2007; : CD001729.

9 Lucero MG, Dulalia VE, Nillos LT, *et al.* Pneumococcal conjugate vaccines for preventing vaccine-type invasive pneumococcal disease and X-ray defined pneumonia in children less than two years of age. *Cochrane Database Syst Rev* 2009; : CD004977.

10 Bonten MJM, Huijts SM, Bolkenbaas M, *et al.* Polysaccharide conjugate vaccine against pneumococcal pneumonia in adults. *N Engl J Med* 2015; **372**: 1114–25.

Upper respiratory infections

Flowchart

Upper respiratory infections (URI)

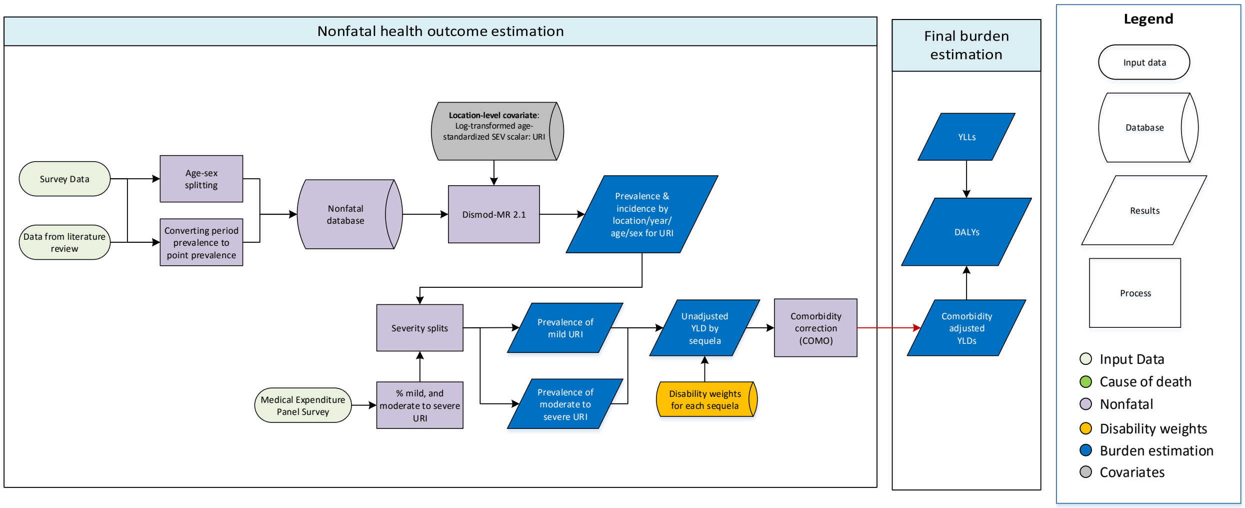

Case Definition

Upper respiratory infections (URI) include acute nasopharyngitis, sinusitis, pharyngitis, tonsillitis, laryngitis/tracheitis and epiglottitis. For URI, ICD 10 codes are J00-J02,J02.8-J03,J03.8-J06.9,J36,J36.0, and ICD 9 codes are 460-465.9,475-475.9,476.9.

Input data

Model Inputs

For GBD 2013, a systematic review of the prevalence of URI was conducted. The PubMed search terms were: ((upper respiratory infection[Title/Abstract] or rhinitis[Title/Abstract] or rhinosinusitis[Title/Abstract] or sinusitis[Title/Abstract] or nasopharyngitis[Title/Abstract] or rhinopharyngitis[Title/Abstract] or common cold[Title/Abstract] or pharyngitis[Title/Abstract] or tonsillitis[Title/Abstract] or epiglottitis[Title/Abstract] or supraglottitis[Title/Abstract] or laryngitis[Title/Abstract] or laryngotracheitis[Title/Abstract] or tracheitis[Title/Abstract]) AND (prevalence[Title/Abstract] OR incidence[Title/Abstract]) NOT (allergies or allergy or allergic rhinitis or asthma) AND (“2009”[Date – Publication] : “2013”[Date – Publication]))

The exclusion criteria were:

1. Studies that were not population-based, eg, hospital or clinic-based studies
2. Studies that did not provide primary data on epidemiological parameters, eg, a commentary piece
3. Studies with a sample size of less than 150
4. Reviews

Updates to systematic reviews are performed on an ongoing schedule across all GBD causes: an update for upper respiratory infections will be performed in the next one to two iterations. The table below shows the number of literature studies included in GBD 2016, as well as the number of countries or subnational units and GBD world regions represented.

	Prevalence	Incidence	Mortality risk
Studies	11	-	-
Countries/subnationals	10	-	-
GBD world regions	8	-	-

In addition, data from nationally representative surveys including United States National Health Interview Surveys, Demographic and Health Surveys, and Russia Longitudinal Monitoring Surveys were included.

Severity Splits

The table below shows the severity distributions based on the data from Medical Expenditure Panel Surveys where we categorized “acute nasopharyngitis or acute uri multi sites/nos” as mild URI and “acute sinusitis, acute pharyngitis, acute tonsillitis, and acute laryngitis/tracheitis and epiglottitis” as moderate URI.

**Age**	**Mild URI (proportion)**	**Moderate URI (proportion)**	**Standard error**
0-4	0.8475367	0.1524633	0.0235054
5-9	0.7957522	0.2042478	0.0214788
10-14	0.7510127	0.2489873	0.0219692
15-19	0.7800903	0.2199097	0.0254533
20-24	0.8277971	0.1722029	0.0340625
25-29	0.866052	0.133948	0.0372739
30-34	0.8716526	0.1283474	0.0385933
35-39	0.8711911	0.1288089	0.0404143
40-44	0.8723335	0.1276665	0.0413539
45-49	0.8835218	0.1164782	0.0450838
50-54	0.8862297	0.1137703	0.0483388
55-59	0.8882668	0.1117332	0.0532085
60-64	0.8837538	0.1162462	0.0618551
65-69	0.8844019	0.1155981	0.0741072
70-74	0.8912954	0.1087046	0.0867568
75-79	0.8846442	0.1153558	0.0937314
80-84	0.9130687	0.0869313	0.104148

The lay descriptions and disability weights for severity levels derived from the GBD Disability Weights study are shown below.

Severity level	Lay description	DW (95% CI)
Mild upper respiratory infections	has a low fever and mild discomfort , but no difficulty with daily activities.	0.006 (0.002–0.012)
Moderate/severe upper respiratory infections	has a fever and aches, and feels weak, which causes some difficulty with daily activities.	0.051 (0.032–0.074)

Modelling Strategy

URI was modeled using a standard DisMod MR2.2 model. We used the log-transformed age-standardized SEV scalar for URI as a location-level covariate.

Betas and exponentiated values are shown in the table below:

Covariate	Parameter	beta	Exponentiated beta
Log-transformed age-standardized SEV scalar for URI	Prevalence	4.69	108.32 (45.02 — 147.82)
Sex	Prevalence	-0.029	0.97 (0.95–0.99)

Otitis media

Flowchart

Otitis media (OM)

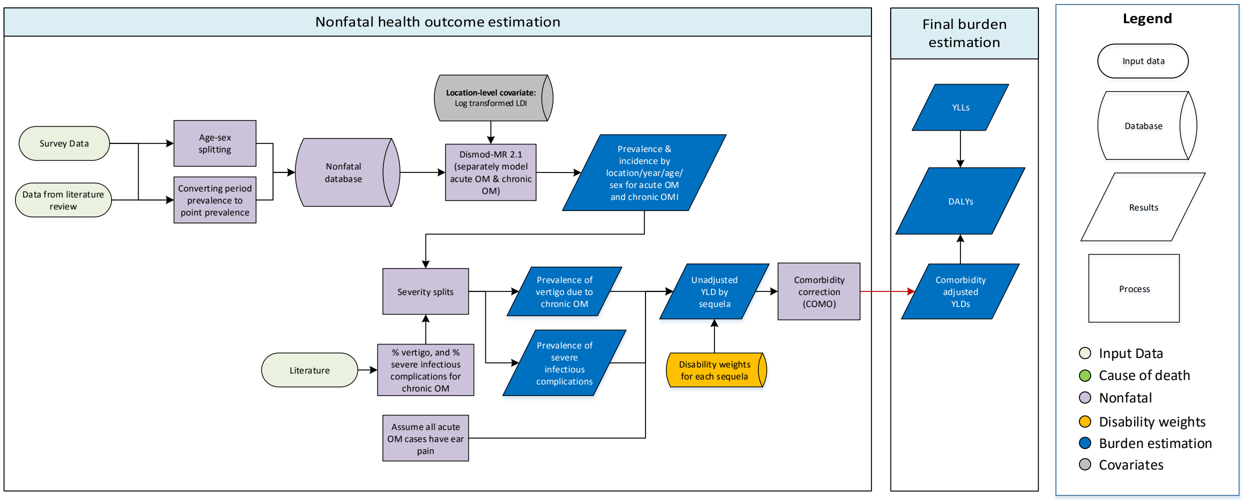

Case Definition

Otitis media is an infection of the middle ear space. We included acute otitis media, chronic otitis media, and hearing loss due to otitis media in the GBD non-fatal outcome modelling. (The hearing loss estimation is included in the hearing loss report provided separately.) The ICD 10 codes are H65-H75.83, and ICD 9 codes are 381-384.9.

Input data

Model Inputs

A systematic review of the prevalence of otitis media was conducted for GBD 2013. The PubMed search terms were: (((otitis media[Title/Abstract] AND (incidence[Title/Abstract] OR prevalence[Title/Abstract])) AND (“2009”[Date – Publication] : “2013”[Date – Publication])).

The exclusion criteria were:

5. Studies that were not population-based, eg, hospital or clinic-based studies
6. Studies that did not provide primary data on epidemiological parameters, eg commentaries
7. Studies with a sample size of less than 150
8. Reviews
9. Case series

Updates to systematic reviews are performed on an ongoing schedule across all GBD causes, and an update for otitis media will be performed in the next one to two iterations. The table below shows the number of literature studies included in GBD 2016, as well as the number of countries or subnational units and GBD world regions represented.

	Prevalence	Incidence	Remission
Studies	29	12	5
Countries/subnationals	20	8	4
GBD world regions	11	5	4

In addition, data from the United States Medical Expenditure Panel Surveys and Australia National Health Surveys were included.

Severity Splits

We assume that all acute otitis media cases would experience ear pain. The severity distributions for chronic otitis media based on the study by Lin and colleagues (2009) were as follows: (i) vertigo (2.9%, 95% CI: 2.4 to 3.6%), and (ii) severe infectious complications (0.05%, 95% CI: 0.01 to 0.2%). We considered the remaining 97% of chronic otitis media cases as asymptomatic. The lay descriptions and disability weights for severity levels derived from the GBD Disability Weights study are shown below.

Severity level	Lay description	DW (95% CI)
Ear pain	has an earache that causes some difficulty with daily activities	0.013 (0.007–0.024)
Vertigo due to chronic otitis media*		
Severe infectious complications due to chronic otitis media	has an earache that causes some difficulty with daily activities	0.013 (0.007–0.024)

* See the hearing loss report for the lay descriptions and disability weights for different severity levels.

Modelling Strategy

We modelled acute and chronic otitis media as separate non-fatal health outcomes using DisMod-MR 2.1. We assumed that the incidence of acute otitis media decreases after the age of 5 years. Log-transformed LDI covariate was used as a location-level covariate to model chronic otitis media.

Betas and exponentiated values are shown in the table below:

Acute otitis media DisMod model

Covariate	Parameter	Beta (95% CI)	Exponentiated beta (95% CI)
Sex	Prevalence	0.17	1.18 (1.01 – 1.38)
Sex	Incidence	-0.20	0.82 (0.76 – 0.88)

Chronic otitis media DisMod model

Covariate	Parameter	Beta (95% CI)	Exponentiated beta (95% CI)
Log LDI	Prevalence	-0.41	0.67 (0.61 – 0.76)
Sex	Prevalence	0.046	1.05 (0.75 – 1.46)

Reference

Lin, Y. S., Lin, L. C., Lee, F. P., & Lee, K. J. (2009). The prevalence of chronic otitis media and its complication rates in teenagers and adult patients. *Otolaryngology-Head and Neck Surgery*, *140*(2), 165-170.

Meningitis

Flowchart

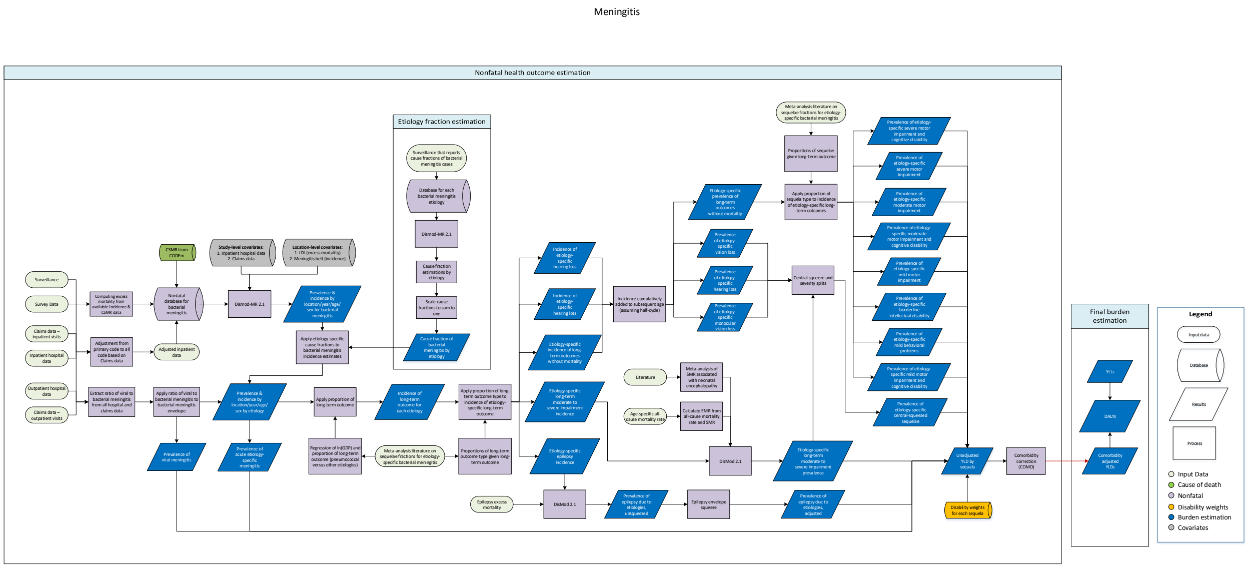

Case definition

Meningitis is a disease caused by inflammation of the meninges, the protective membrane surrounding the brain and spinal cord, and is typically caused by an infection in the cerebrospinal fluid. Symptoms include headache, fever, stiff neck, and sometimes seizures. Included in the GBD modeling were cases meeting ICD-10 diagnostic criteria for meningitis due to bacteria, viruses, or other causes (A39-A39.9, A87-A87.9, D86.81, G00.0-G00.8, G03-G03.8, Z20.811, and Z22.31) (1). In GBD 2016, meningitis encompasses viral meningitis and four bacterial etiologies: pneumococcal, haemophilus influenza type B (HiB), meningococcal, and other.

Input data

Model inputs

In the GBD 2010 study, a systematic review of literature was conducted to capture studies of incidence and excess mortality rate for all bacterial meningitis cases. For each of the four etiologies, literature included excess mortality rate, incidence, proportion, remission, and standardized mortality ratio. The inclusion criteria stipulated that: (1) the publication year must be between 1980 and 2010; (2) “caseness” was based on diagnoses by antigen test, blood test, cerebrospinal fluid test, polymerase chain reaction test, or latex agglutination test; (3) sufficient information must be provided on study method and sample characteristics to assess the quality of the study; and (4) study samples must be representative of the general population. No limitation was set on the language of publication. For GBD 2013, the search strategy was replicated to capture epidemiological studies published between 2010 and 2013. The search strategy was repeated in 2015 only to capture excess mortality – updates to systematic reviews are

performed on an ongoing schedule across all GBD causes, and a complete update for meningitis will be performed in the GBD 2017 iteration.

Additional sources we included in the acute bacterial meningitis model were inpatient-only hospital data and US claims data from 2000, 2010, and 2012, primary diagnosis and inpatient only. Sequelae and severity splits were informed by a meta-analysis, Edmond et al (2), while an internal meta-analysis informed mortality estimates for long-term moderate to severe impairments (3).

Data were outliered or excluded if we found them unreasonable when compared to regional, super-regional, and global rates.

For GBD 2016, we also included VR proportions for mortality due to the different etiologies of meningitis to inform the proportional splits of the meningitis parent model for GBD Cause of Death modeling.

The tables below show the number of studies included in GBD 2016, as well as the number of countries or subnational units and GBD world regions represented for the bacterial meningitis model and each model that informs the etiology split.

Table 1a. Acute bacterial meningitis

	Prevalence	Incidence	Mortality risk
Studies	0	70	70
Countries/subnationals	0	65/304	44/0
GBD world regions	0	20	17

Table 1b. Pneumococcal meningitis incidence proportion

	Proportion
Studies	67
Countries/subnationals	42/2
GBD world regions	18

Table 1c. Meningococcal meningitis incidence proportion

	Proportion
Studies	62
Countries/subnationals	39/2
GBD world regions	17

Table 1d. H influenza type B meningitis incidence proportion

	Proportion
Studies	68
Countries/subnationals	42/2
GBD world regions	18

Table 1e. Other bacterial meningitis incidence proportion

	Proportion
Studies	60

Countries/subnationals	37/2
GBD world regions	16

Modeling strategy

Non-fatal outcomes were modeled using a combination of custom models and DisMod-MR 2.1, with minor changes from the GBD 2015 modeling process. First, the overall incidence and prevalence of bacterial meningitis was modeled to estimate the short-term morbidity due to acute infection. This DisMod model had a set duration (1/remission) of 4 weeks with a range ±2 weeks. Literature and surveillance/notification data were flagged with covariates, as were US claims data with year-specific covariates to be crosswalked to the reference data, which was GBD 2016 inpatient-only hospital data. We used the function in DisMod-MR 2.1 to pull in cause-specific mortality rate (CSMR) data from our CODEm and CODcorrect analyses and matched with prevalence data points for the same location. We calculated excess mortality rate to estimate priors by dividing CSMR by prevalence, calculated from remission and incidence. To help inform trends where we lack data, we applied a country-level covariate for proportion of the population at the subnational and country levels that lives within the meningitis belt in sub-Saharan Africa. We forced a positive relationship, with a lower bound of 0 and an upper bound of 2. We also applied a lag-distributed income covariate to excess mortality, log transformed and forced negative with an upper bound of 0 and a lower bound of -1. Betas and exponentiated values (which can be interpreted as an odds ratio) are shown in the tables below for study-level covariates and country-level covariates.

Table 2. Study covariates

Study covariate	Parameter	Beta	Exponentiated beta
Surveillance/notification data	Incidence	-1.23 (-1.58 to -0.89)	0.29 (0.21 – 0.41)
Literature	Incidence	-0.24 (-0.32 to -0.15)	0.79 (0.73 – 0.4286)
Claims data – 2000	Incidence	-0.29 (-0.35 to -0.23)	0.75 (0.71 – 0.79)
Claims data – 2010	Incidence	-0.32 (-0.37 to -0.27)	0.73 (0.69 – 0.76)
Claims data – 2012	Incidence	-0.36 (-0.42 to -0.31)	0.69 (0.66 – 0.73)

Table 3. Country-level covariates

Country-level covariate	Parameter	Beta	Exponentiated beta
Meningitis belt (proportion of population)	Incidence	1.52 (0.95 – 1.97)	4.56 (2.59 – 7.19)
LDI (log transformed)	Excess mortality	-0.28 (-0.29 to -0.26)	0.76 (0.74 – 0.77)

Incidence of bacterial meningitis were split into four etiologies (pneumococcal, meningococcal, *H. influenzae* type B, and other bacterial meningitis) using four incidence proportion models run in DisMod-MR 2.1. Results from these models were squeezed to sum to 1 at the draw level for each location, year, age, and sex. We applied a Hib3 vaccine coverage covariate to the *H. influenzae* type B proportion model, the proportion of the population living in the meningitis belt covariate to the meningococcal meningitis

proportion model, a PCV3 coverage covariate to the pneumococcal meningitis model, and the aforementioned 3 covariates to the other meningitis incidence model.

Data for viral meningitis were only available from hospitals or US claims data, and not from population studies, so incidence and prevalence of viral meningitis were extrapolated from bacterial meningitis incidence by applying age- and sex-specific ratios between bacterial and viral cases from a combination of hospital data and US claims data. In addition to short-term sequelae as a result of acute bacterial and viral meningitis, we also modeled the long-term outcomes from bacterial meningitis infection.

Sequelae splits

We first split the long-term sequelae among survivors of acute infection. We calculated the acute-phase survivors by applying the excess mortality (calculated by the acute meningitis parent DisMod model) to the incidence of each etiology (excess mortality was converted to case fatality rate by $e^{(\text{-excess mortality x 1/(excess mortality + remission))}}$). The survivors were then subject for long-term sequelae by applying the post-discharge proportions of health consequences calculated by a meta-analysis by Edmond et al (2). We calculated the ratio of acute meningitis survivors that experience major long-term impairments for all etiologies, and the ratio of minor impairments to major impairments for pneumococcal meningitis versus all other etiologies (because pneumococcal meningitis showed significantly higher risk of morbidity than other etiologies). This ratio was based off a regression of log-transformed GDP and ratio values from Edmonds et al. The regression is shown below:

$$y = -0.33590 \ln(GDP) + 1.15230$$

We used these two ratios to calculate the proportions of survivors who contract a long-term minor impairment and those who contract a long-term major impairment. The proportion with major impairments were further split (again using pooled proportions from Edmond et al) into specific major impairments, which were grouped into vision loss, hearing loss, moderate-to-severe cognitive impairments, and epilepsy.

The calculated incidence of long-term sequelae was then converted to prevalence by two different approaches. For the sequelae not associated with excess mortality, which were vision loss, hearing loss, intellectual disability, motor impairment, and behavioral problems, the incidence of each age was cumulatively added up to the subsequent age (assuming half-cycle) to construct prevalence at each age. If the sequela is associated with excess mortality (epilepsy and moderate-to-severe cognitive impairments), the calculated incidence was uploaded into DisMod together with the corresponding mortality parameters (excess mortality data from the epilepsy envelope DisMod model, and standardized mortality ratio data from a neonatal encephalopathy meta-analysis, converted to excess mortality using all-cause mortality estimates) to estimate the prevalence. Vision loss, hearing loss, and epilepsy estimates were squeezed and severity split centrally.

Disability weights

The basis of the GBD disability weight survey assessments are lay descriptions of sequelae highlighting major functional consequences and symptoms. The lay descriptions and disability weights for sequelae associated with each etiology are shown below.

Table 5. Severity splits, lay descriptions, DWs

Severity split	Lay description	DW (95% CI)
Mild behavior problems	This person is hyperactive and has difficulty concentrating, remembering things, and completing tasks.	0.045 (0.028-0.066)
Mild hearing loss	This person has great difficulty hearing and understanding another person talking in a noisy place (for example, on an urban street).	0.01 (0.004-0.019)
Mild hearing loss with ringing	This person is unable to hear and understand another person talking, even in a quiet place, is unable to take part in a phone conversation. Difficulties with communicating and relating to others cause emotional impact at times (for example worry or depression).	0.021 (0.012-0.036)
Moderate hearing loss	This person has great difficulty hearing and understanding another person talking in a noisy place (for example, on an urban street), and sometimes has annoying ringing in the ears.	0.027 (0.015-0.042)
Moderate hearing loss with ringing	This person is unable to hear and understand another person talking, even in a quiet place, is unable to take part in a phone conversation. Difficulties with communicating and relating to others often cause worry, depression, or loneliness.	0.074 (0.048-0.107)
Moderately severe hearing loss	Custom DW from hearing loss impairment envelope	
Severe hearing loss	This person is unable to hear and understand another person talking, even in a quiet place, and unable to take part in a phone conversation. Difficulties with communicating and relating to others cause emotional impact at times (for example worry or depression).	0.158 (0.105-0.227)
Profound hearing loss	This person is unable to hear and understand another person talking, even in a quiet place, is unable to take part in a phone conversation, and has great difficulty hearing anything in any other situation. Difficulties with communicating and relating to others often cause worry, depression, or loneliness.	0.204 (0.134-0.288)
Complete hearing loss	This person cannot hear at all in any situation, including even the loudest sounds, and cannot communicate verbally or use a phone. Difficulties with communicating and relating to others often cause worry, depression, or loneliness.	0.215 (0.144-0.307)
Severe hearing loss with ringing	This person is unable to hear and understand another person talking, even in a quiet place, is unable to take part in a phone conversation, and has annoying ringing in the ears for more than 5 minutes	0.261 (0.175-0.36)

	at a time, almost every day. Difficulties with communicating and relating to others cause emotional impact at times (for example worry or depression),	
Profound hearing loss with ringing	This person is unable to hear and understand another person, even in a quiet place, is unable to take part in a phone conversation, has great difficulty hearing anything in any other situation, and has annoying ringing in the ears for more than 5 minutes at a time, several times a day. Difficulties with communicating and relating to others often cause worry, depression, or loneliness,	0.277 (0.182-0.387)
Complete hearing loss with ringing	This person cannot hear at all in any situation, including even the loudest sounds, and cannot communicate verbally or use a phone, and has very annoying ringing in the ears for more than half of the day. Difficulties with communicating and relating to others often cause worry, depression, or loneliness,	0.316 (0.212-0.435)
Moderate motor impairment	This person has some difficulty in moving around, and difficulty in lifting and holding objects, dressing and sitting upright, but is able to walk without help.	0.061 (0.04-0.089)
Moderate motor plus cognitive impairments	This person has some difficulty in moving around, holding objects, dressing and sitting upright, but can walk without help. This person has low intelligence and is slow in learning to speak and to do simple tasks.	0.203 (0.134-0.29)
Long-term mild motor impairment	This person has some difficulty in moving around but is able to walk without help.	0.01 (0.005-0.02)
Borderline intellectual disability	This person is slow in learning at school. As an adult, the person has some difficulty doing complex or unfamiliar tasks but otherwise functions independently.	0.011 (0.005-0.02)
Severe motor impairment	This person is unable to move around without help, and is not able to lift or hold objects, get dressed or sit upright.	0.402 (0.268-0.545)
Epilepsy	(combined DW)	NA
Blindness	Is completely blind, which causes great difficulty in some daily activities, worry and anxiety, and great difficulty going outside the home without assistance.	0.187 (0.124-0.26)
Severe acute episode of infectious disease	This person has a high fever and pain, and feels very weak, which causes great difficulty with daily activities.	0.133 (0.088-0.19)
Mild intellectual disability	This person has low intelligence and is slow in learning at school. As an adult, the person can live independently, but often needs help to raise children and can only work at simple supervised jobs.	0.043 (0.026-0.065)

Monocular distance vision loss	This person is blind in one eye and has difficulty judging distances	0.017 (0.009-0.029)
Mild motor plus cognitive impairments	This person has some difficulty in moving around but is able to walk without help. The person is slow in learning at school. As an adult, the person has some difficulty doing complex or unfamiliar tasks but otherwise functions independently.	0.031 (0.018-0.05)
Severe motor plus cognitive impairments	This person cannot move around without help, and cannot lift or hold objects, get dressed or sit upright. The person also has very low intelligence, speaks few words, and needs constant supervision and help with all daily activities.	0.542 (0.37-0.702)

Encephalitis

Flowchart

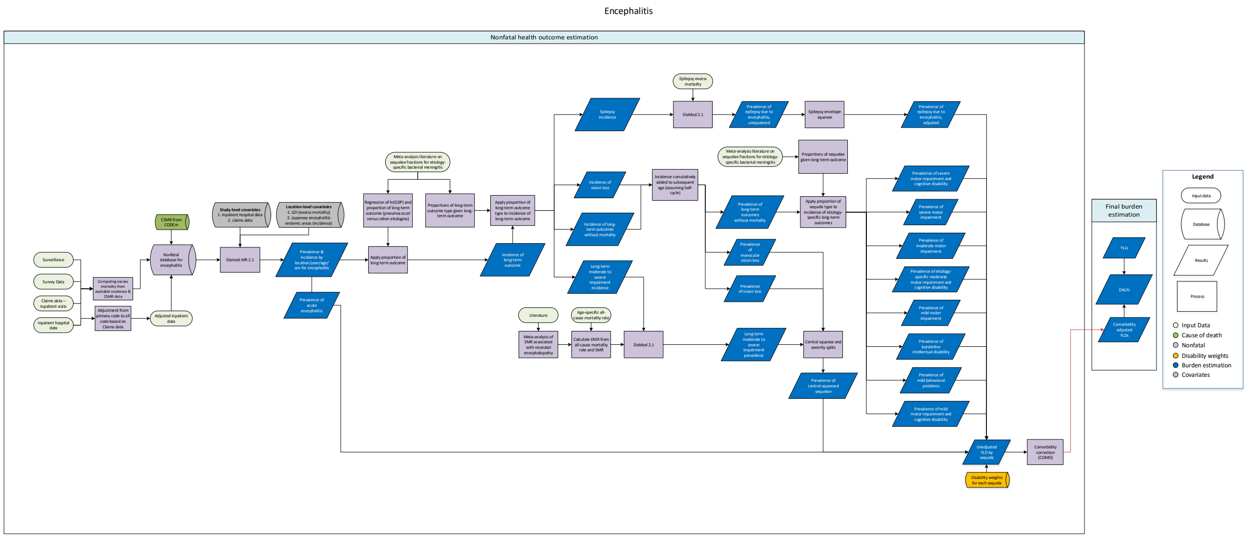

Case definition

Encephalitis is a disease caused by an acute inflammation of the brain. Symptoms of encephalitis can include flu-like symptoms like headache, fever, drowsiness, and fatigue, and at times, seizures, hallucinations, or stroke. Included in the GBD modeling were cases meeting ICD-10 diagnostic criteria for encephalitis (A83-A86.4, B94.1, F07.1, G04-G05.8) (1).

Input data

Model inputs

In the GBD 2010 study, a systematic review of literature was conducted to capture studies of incidence, excess mortality rate, remission, and standardized mortality ratio for encephalitis. These data sources included hospital data and literature. The inclusion criteria stipulated that: (1) the publication year must be between 1980 and 2010; (2) sufficient information must be provided on study method and sample characteristics to assess the quality of the study; and (3) study samples must be representative of the general population. No limitation was set on the language of publication. For GBD 2013, the GBD 2010 search strategy was replicated to capture epidemiological studies published between 2010 and 2013. We did not do a literature review for GBD 2016.

Additional sources we included in the acute bacterial meningitis model were inpatient-only hospital data and US claims data from 2000, 2010, and 2012, primary diagnosis and inpatient only. Sequelae and severity splits were informed by a meta-analysis, Edmond et al (2), while an internal meta-analysis informed mortality estimates for long-term moderate-to-severe impairments (3).

Data were outliered or excluded if we found they differed significantly when compared to regional, super-regional, and global rates.

The tables below show the number studies included in GBD 2016, as well as the number of countries or subnational units and GBD world regions represented for the encephalitis.

Table 1. Acute encephalitis

	Prevalence	Incidence
Studies	0	26
Countries/subnationals	0	37/291
GBD world regions	0	16

Modeling strategy

Non-fatal outcomes were modeled using a combination of custom models and DisMod-MR 2.1, with minor changes from the GBD 2015 modeling process. First, the overall incidence and prevalence of encephalitis was modeled to estimate the short-term morbidity due to acute infection. This DisMod model had a set duration (1/remission) between 2.9 and 3.1 weeks. We also imposed caps on excess mortality for ages 10-50, and a cap on incidence from ages 10-100. US claims data and literature data were flagged with year-specific covariates to be crosswalked to the reference data, which were inpatient-only, primary diagnosis hospital data. We used the function in DisMod-MR 2.1 to pull in cause-specific mortality rate (CSMR) data from our CODEm and CODcorrect analyses and match with incidence data points for the same location. We calculated excess mortality rate to estimate priors by dividing CSMR by prevalence, calculated from remission and incidence. To help inform trends where we lack data, we applied a binary country-level covariate at the subnational and country level that indicates if the location is in a Japanese Encephalitis endemic area (4). We forced a positive relationship, with a lower bound of 0 and an upper bound of 0.1. We also applied a lag-distributed income covariate to excess mortality, log transformed and forced negative with an upper bound of 0 and a lower bound of -1. Betas and exponentiated values (which can be interpreted as an odds ratio) are shown in the tables below for study-level covariates and country-level covariates.

Table 2. Study covariates

Study covariate	Parameter	beta	Exponentiated beta
Claims data – 2000	Incidence	-0.81 (-0.84 to – 0.78)	0.44 (0.43 – 0.46)
Claims data – 2010	Incidence	-0.56 (-0.59 to – 0.54)	0.57 (0.55 – 0.58)
Claims data – 2012	Incidence	-0.45 (-0.47 to -0.42)	0.64 (0.62 – 0.65)
Literature	Incidence	-1.23 (-1.36 to -1.13)	0.29 (0.26 – 0.32)

Table 3. Country-level covariates

Country-level covariate	Parameter	beta	Exponentiated beta
Japanese Encephalitis endemic area (binary)	Incidence	0.068 (0.030 – 0.097)	1.07 (1.03 – 1.10)

LDI (log transformed)	Excess mortality	-0.015 (-0.055 to -0.0002)	0.99 (0.95 – 1.00)

In addition to short-term sequelae as a result of acute encephalitis, we also modeled the long-term outcomes from encephalitis.

Sequelae Splits

We first split the long-term sequelae among survivors of acute infection. We calculated the acute phase survivors by applying the excess mortality (calculated by the acute meningitis DisMod model) to the incidence of each etiology (excess mortality was converted to case fatality rate by $e^{(\text{-excess mortality} \times 1/(\text{excess mortality + remission}))}$). The survivors were then subject to long-term sequelae by applying the post-discharge proportions of health consequences calculated by a meta-analysis by Edmond et al (2). We calculated the ratio of acute encephalitis survivors that result in a major long-term impairment, and the ratio of minor impairments to major impairments, based off a regression of log-transformed GDP and ratio values from Edmonds et al. The regression is shown below:

$$y = -0.33590\ln(GDP) + 1.15230$$

We assumed a similar pattern of health outcomes for encephalitis infection survivors as with other bacterial meningitis survivors (except hearing loss, as we could not find evidence of hearing loss as a consequence of encephalitis infection). We used these two ratios to calculate the proportions of survivors who contract a long-term minor impairment and those who contract a long-term major impairment. The proportion with major impairments were further split (again using pooled proportions from Edmond et al) into specific major impairments, which were grouped into vision loss, moderate to severe cognitive impairments, and epilepsy.

The calculated incidence of long-term sequelae was then converted to prevalence by two different approaches. For the sequelae not associated with excess mortality, which were vision loss, intellectual disability, motor impairment, and behavioral problems, the incidence of each age was cumulatively added up to the subsequent age (assuming half-cycle) to construct prevalence at each age. If the sequela is associated with excess mortality (epilepsy and moderate-to-severe cognitive impairments), the calculated incidence was uploaded into DisMod together with the corresponding mortality parameters (excess mortality data from the epilepsy envelope DisMod model, and standardized mortality ratio data from a neonatal encephalopathy meta-analysis, converted to excess mortality using all-cause mortality estimates) to estimate the prevalence. Vision loss and epilepsy estimates were squeezed and severity split centrally.

Disability weights

The basis of the GBD disability weight survey assessments is lay descriptions of sequelae highlighting major functional consequences and symptoms. The lay descriptions and disability weights for sequelae associated with encephalitis are shown below.

Table 4. Severity splits, lay descriptions, and DWs

Severity split	Lay description	DW (95% CI)

Mild behavior problems	This person is hyperactive and has difficulty concentrating, remembering things, and completing tasks.	0.045 (0.028-0.066)
Moderate motor impairment	This person has some difficulty in moving around, and difficulty in lifting and holding objects, dressing and sitting upright, but is able to walk without help.	0.061 (0.04-0.089)
Moderate motor plus cognitive impairments	This person has some difficulty in moving around, holding objects, dressing and sitting upright, but can walk without help. This person has low intelligence and is slow in learning to speak and to do simple tasks.	0.203 (0.134-0.29)
Long- term mild motor impairment	This person has some difficulty in moving around but is able to walk without help.	0.01 (0.005-0.02)
Borderline intellectual disability	This person is slow in learning at school. As an adult, the person has some difficulty doing complex or unfamiliar tasks but otherwise functions independently.	0.011 (0.005-0.02)
Severe motor impairment	This person is unable to move around without help, and is not able to lift or hold objects, get dressed, or sit upright.	0.402 (0.268-0.545)
Epilepsy	(combined DW)	NA
Blindness	Is completely blind, which causes great difficulty in some daily activities, worry and anxiety, and great difficulty going outside the home without assistance.	0.187 (0.124-0.26)
Acute encephalitis	This person has a high fever and pain, and feels very weak, which causes great difficulty with daily activities.	0.133 (0.088-0.19)
Mild intellectual disability	This person has low intelligence and is slow in learning at school. As an adult, the person can live independently but often needs help to raise children and can only work at simple supervised jobs.	0.043 (0.026-0.065)
Monocular distance vision loss	This person is blind in one eye and has difficulty judging distances	0.017 (0.009-0.029)
Mild motor plus cognitive impairments	This person has some difficulty in moving around but is able to walk without help. The person is slow in learning at school. As an adult, the person has some difficulty doing complex or unfamiliar tasks but otherwise functions independently.	0.031 (0.018-0.05)
Severe motor plus cognitive impairments	This person cannot move around without help, and cannot lift or hold objects, get dressed or sit upright. The person also has very low intelligence, speaks few words, and needs constant supervision and help with all daily activities.	0.542 (0.37-0.702)

No other significant changes were made to the modeling process for GBD 2016.

Diphtheria

Flowchart

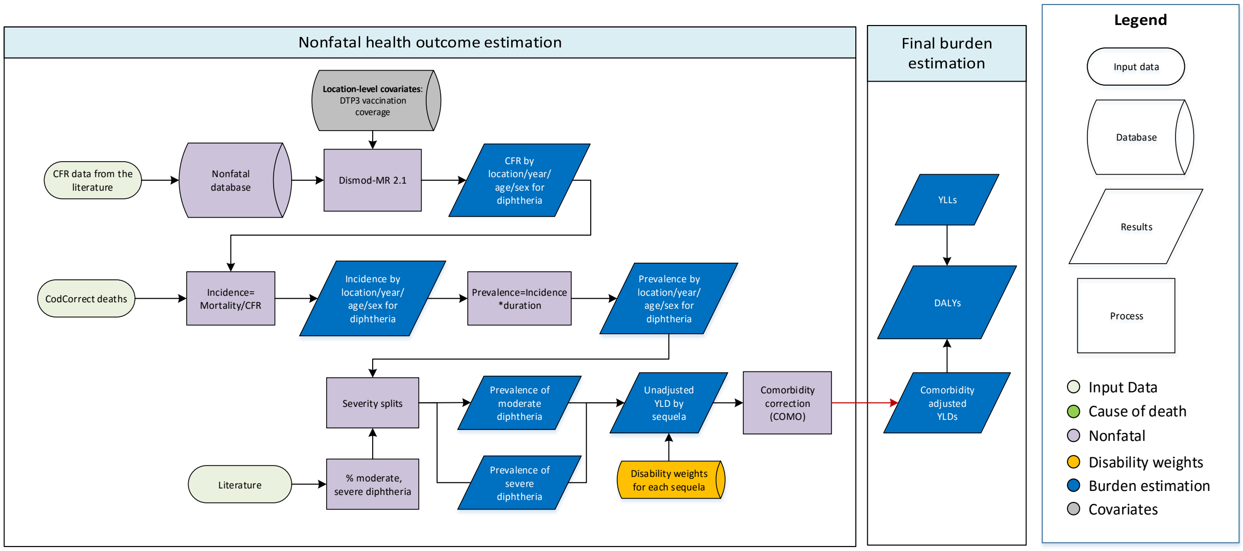

Case definition

Diphtheria is a bacterial infection caused by *Corynebacterium diphtheriae*. For diphtheria, ICD 10 codes are A36-A36.9, Z22.2, Z23.6, and ICD9 codes are 032-032.9, V02.4, V03.5, and V74.3.

Input data

Model inputs

For GBD 2016, input data included case fatality rate data from a systematic review of the literature and diphtheria mortality estimates from a negative binomial regression model.

A systematic review of diphtheria case fatality was conducted for GBD 2016 to add to the literature used in GBD 2013. The PubMed search terms were: ((diphtheria[Title/Abstract] AND case fatality[Title/Abstract])) AND ("2013"[Date - Publication]: "2016"[Date - Publication]).

Severity split & disability weights

We draw primarily on the literature, as well as patterns in data observed in South Asia and Central Asia, to assign the following severity distributions for diphtheria: 70% (95% CI:66.5–73.5%) moderate and 30% (95% CI: 26.5–33.5%) severe cases. The lay descriptions and disability weights for diphtheria severity levels derived from the GBD Disability Weights study are shown below.

Table 1. Severity splits, lay descriptions, and disability weights (DW)

Severity level	Lay description	DW (95% CI)
Moderate diphtheria	Has a fever and aches, and feels weak, which causes some difficulty with daily activities.	0.051 (0.032–0.074)
Severe diphtheria	Has a high fever and pain, and feels very weak, which causes great difficulty with daily activities.	0.133 (0.088–0.19)

Modelling strategy

We used DisMod-MR 2.1 as a meta-regression tool to pool the case fatality data and generate location-year-age-sex-specific case fatality rate estimates. We used lag-distributed income and the IHME health care access and quality index as location-level covariates. Diphtheria mortality was modelled using a negative binomial regression and data from the cause of death database with the DTP3 coverage covariate and age dummy variables. Incidence was then calculated as mortality rate divided by case fatality rate. Prevalence was calculated by multiplying incidence and duration, which was estimated to be a mean of 27.5 days, based on a meta-analysis of duration data from the literature.

The table below shows model covariate coefficients and exponentiated values (from the DisMod case-fatality model), which can be interpreted as odds ratios.

Table 2. Beta and exponentiated beta values

Covariate	Parameter	Coefficient (95% CI)	Exponentiated coefficient (95% CI)
DTP3 coverage (proportion)	Case fatality	-0.18 (-0.66, -0.01)	0.84 (0.52, 0.99)
Sex	Case fatality	0.15 (-0.58, 0.90)	1.16 (0.56, 2.46)

No other significant changes were made to the modelling strategy for GBD 2016.

Pertussis (whooping cough)

Flowchart

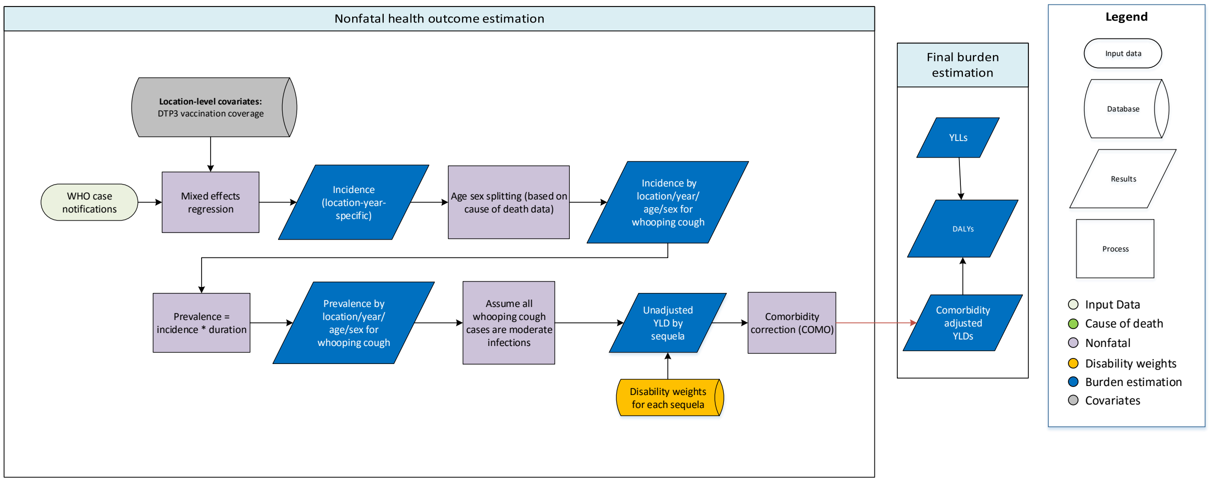

Case definition

Pertussis (whooping cough), is a contagious respiratory disease caused by the bacterium *Bordetella pertussis*. For pertussis, ICD 10 codes are A37-A37.91, Z23.7, and ICD 9 codes are 033-033.9, 484.3, V03.6.

Input data

Model inputs

For the GBD 2016 nonfatal estimation process, the primary source of input data was pertussis case notifications from the World Health Organization (WHO). We also used historical case notifications for the UK back to 1940 to inform the whooping cough natural history model.

Severity splits

For GBD 2015, we assumed all pertussis cases were moderate episodes of acute infectious disease because of associated symptoms and MEPS data. The lay description and disability weight derived from the GBD Disability Weights study are shown below.

Table 1. Severity splits, lay descriptions, and disability weights

Severity level	Lay description	DW (95% CI)
Moderate	Has a fever and aches and feels weak, which causes some difficulty with daily activities.	0.051 (0.032–0.074)

Modelling strategy

We modelled log-transformed incidence with a mixed-effects linear regression of case notifications from WHO (1985–2015) on diphtheria-tetanus-pertussis dose 3 (DTP3) vaccination coverage. Historical data of United Kingdom (UK) pertussis cases and UK DTP3 coverage rates (both back to 1940) were also used to inform the incidence model. The random effect by country allowed for registration completeness to vary by country. The results of this model were then used to predict incidence as a function of vaccine coverage. To correct for underreporting in case notifications, we used a value of the random effect that matched the highest random effect in a high income region – Switzerland (which has a pertussis monitoring system which captures a high percentage of cases) – to get an implied attack rate assumed to be the same for all unvaccinated populations. Uncertainty was estimated by taking 1,000 iterations of the predictions based on the variance-covariance matrix. Prevalence was calculated by multiplying incidence by an average duration assumed to be 50 days.

Tetanus

Flowchart

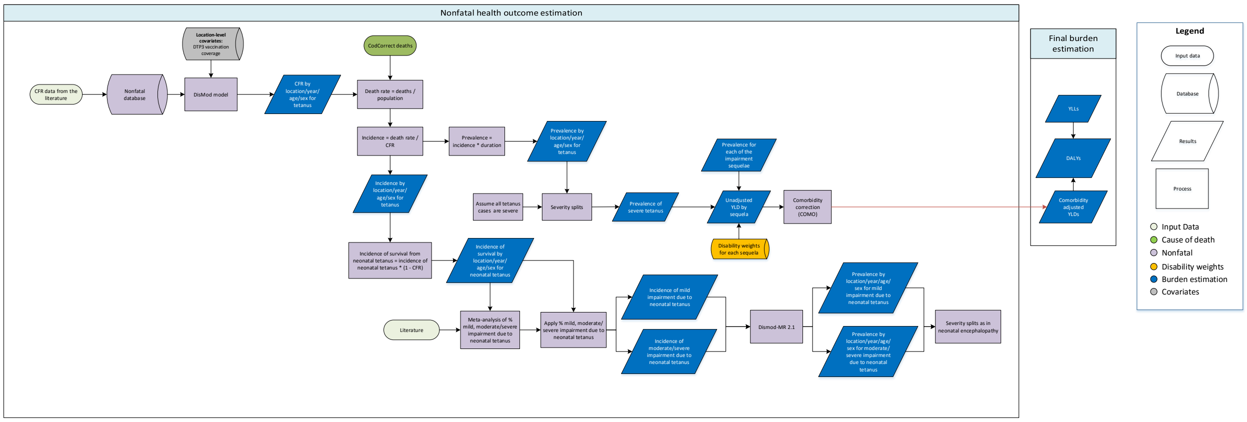

Case definition

Tetanus is a serious bacterial disease caused by the bacterium *Clostridium tetani.* For tetanus, the ICD 10 codes are A33-A35.0, Z23.5, and ICD 9 codes are 037-037.9, 771.3, V03.7.

Input data

Model inputs

For GBD 2016, input data for the estimation of tetanus included case fatality rate data extracted from a systematic review the literature and IHME tetanus mortality estimates calculated with CODEm.

A systematic review was conducted for GBD 2016. The PubMed search terms were: (tetanus[Title/Abstract]) AND (case fatality[Title/Abstract]) AND ("2013"[Date - Publication]: "2016"[Date - Publication]).

Severity split & disability weights

We assume that all tetanus cases are severe episodes of acute infectious diseases. The lay descriptions and disability weights for tetanus derived from the GBD Disability Weights study are shown below.

Table 1. Severity splits, lay descriptions, and disability weights (DW)

Severity level	Lay description	DW (95% CI)
Severe	Has a high fever and pain, and feels very weak, which causes great difficulty with daily activities.	0.133 (0.088–0.19)

Regarding the severity level of impairment due to neonatal tetanus, we assume the same distribution as in neonatal encephalopathy.

Modelling strategy

We used DisMod-MR 2.0 as a meta-regression tool to pool the case fatality data and generate location-year-age-sex-specific case fatality rate estimates. We used DTP3 coverage as a location-level covariate. Mortality was modelled using the standard CODEm tool on neonatal tetanus (ages 0-0.1) and non-neonatal tetanus (ages 1-80) separately for males and females. Incidence was then calculated as:

Incidence = mortality rate / case fatality rate

Prevalence was then computed based on the estimated incidence and duration draws derived from literature review.

To estimate mild and moderate impairment due to neonatal tetanus, we first computed the incidence of survival from neonatal tetanus as:

Incidence of survival = incidence * (1 - CFR)

We then conducted a meta-analysis of published studies to estimate the proportion of mild impairment due to neonatal tetanus and moderate-to-severe impairment due to neonatal tetanus. We applied these proportions to the estimated incidence of survival, to generate incidence of mild impairment due to neonatal tetanus and moderate-to-severe impairment due to neonatal tetanus, which were used as input data in DisMod 2.0. We ran two separate DisMod models (one for mild impairment due to neonatal tetanus, and one for moderate-to-severe impairment due to neonatal tetanus) to generate age-sex-year-country-specific estimates.

The table below shows betas and exponentiated values for the covariates used in the estimation process (from the DisMod case-fatality model), which can be interpreted as an odds ratio.

Table 3. Beta and exponentiated beta values

Covariate	Parameter	Beta (95% CI)	Exponentiated beta (95% CI)
DTP3 coverage (proportion)	Case fatality	0.52 (-0.061 — 1.32)	1.68 (0.94 — 3.76)
Sex	Case fatality	-0.12 (-0.35 — 0.10)	0.89 (0.71 — 1.11)

No other significant changes were made to the modelling strategy for GBD 2016.

Measles

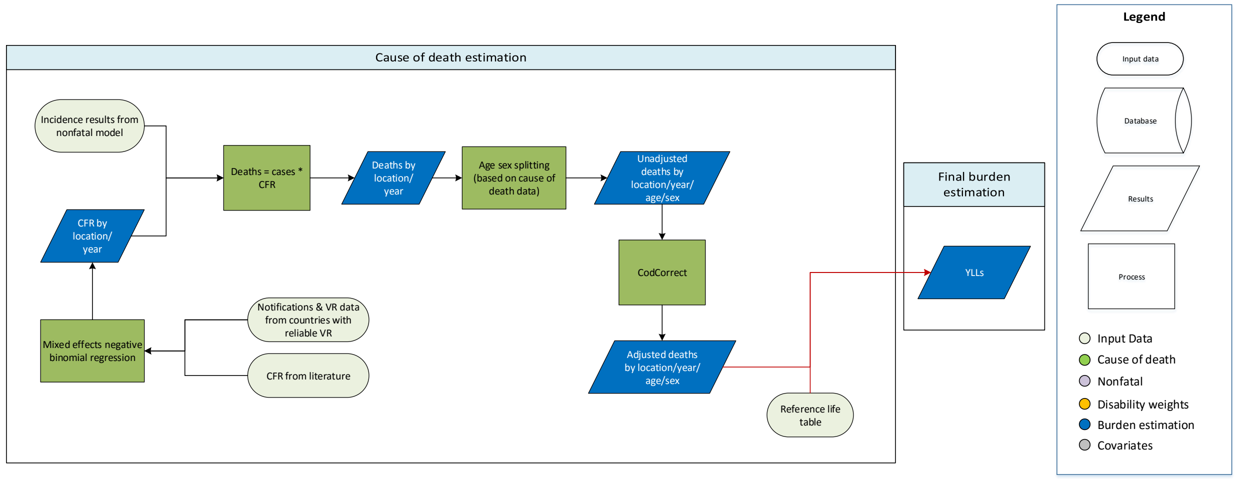

Input data

Vital registration data from the cause of death database were used for data-rich countries. To inform the natural history model, we used data from the following sources: World Health Organization (WHO) case notifications from 1995 to 2015; case notifications identified by collaborators; vital registration (VR) data in countries in the following three super-regions: high-income, Central Europe/Eastern Europe/Central Asia, and Latin America and Caribbean; and case fatality data identified through systematic literature reviews for GBD 2010, GBD 2013, and GBD 2016. The PubMed search query for GBD 2016 was: (measles [Title/Abstract]) AND (case fatality [Title/Abstract]) AND ("2013"[Date - Publication]: "2016"[Date - Publication]). Studies were included if they reported case fatality rate, number of deaths, and number of cases. Studies were excluded if they included non-representative samples only.

Modelling strategy – data-rich countries

Mortality was modelled separately for data-rich and other countries. For data-rich countries (ie, countries with vital registration more than 95% complete for more than 25 years), we used a general CODEm strategy to model VR data with measles-containing vaccination dose one (MCV1) coverage, childhood malnutrition, lagged distributed income, the Healthcare Access and Quality Index, and education as country-level covariates. We made estimations for the age range post-neonatal to 59 years.

Modelling strategy – other countries

Measles mortality in the remaining countries was modelled using a natural-history-based model. First, we modelled measles incidence with a mixed-effects linear regression of case notifications from WHO (1995–2015) on routine measles vaccination rates and supplementary immunization activities (SIAs). More precisely, log-transformed incidence rates were regressed on the log of the proportion unvaccinated with first- and second-dose measles-containing vaccine, and additional SIA coverage

lagged by one, two, three, four, and five years, with super-region, region, and country-level random effects. The results of this mixed-effects regression model were then used to predict location-year-specific incidence as a function of routine vaccine coverage and SIAs. To correct for underreporting in case notifications, we added the effect of a 95% attack rate, assumed to be the same across all unvaccinated populations. Uncertainty was estimated by taking 1,000 iterations of the predictions based on the variance-covariance matrix. For locations in three super-regions – high-income, Central Europe/Eastern Europe/Central Asia, and Latin America and Caribbean – we used reported measles cases as incident cases.

Second, the case fatality rate was modelled using a mixed-effects negative binomial regression with the child malnutrition covariate and study-level indicators (hospital-based or not; outbreak or not; and rural or urban/mixed), with country random effects. Uncertainty was estimated by taking 1,000 iterations of the predictions based on the variance-covariance matrix and uncertainty in country random effects. The fit of the model was evaluated using diagnostic plots of predicted versus observed values. Finally, estimated deaths were calculated at the 1,000-draw level as

$$deaths = incidence * CFR.$$

We estimated overall number of deaths and then assigned an age-sex distribution based on the age- and sex-specific patterns found in the cause of death data. We made estimations for the age range post-neonatal to 59 years.

Varicella and herpes zoster

Flowchart

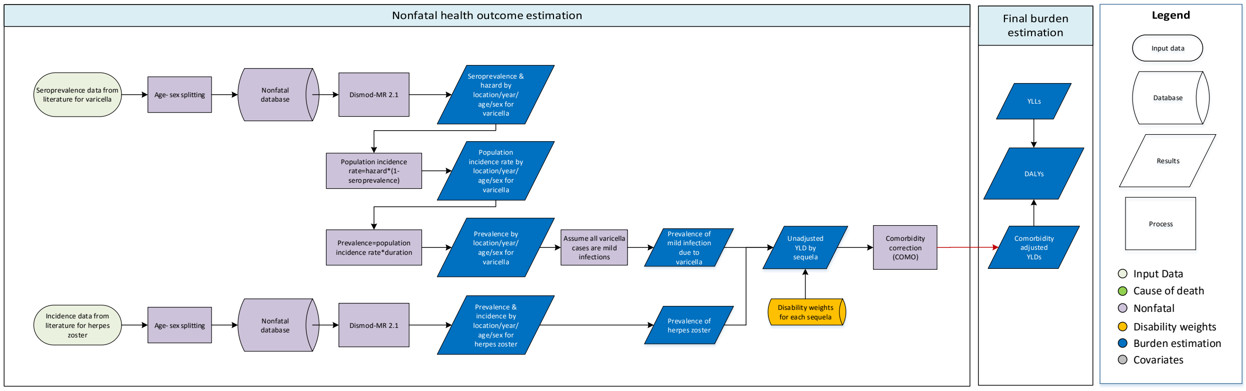

Case definition

Varicella (also known as chicken pox) is an acute infectious disease caused by varicella zoster virus. Herpes zoster (also known as shingles), is caused by the reactivation of the same virus that causes varicella. For varicella and herpes zoster, the ICD 10 codes are B01-B02.9, P35.8, Z20.820, and ICD 9 codes are 052-053.9, V01.71, V01.79, V05.4.

Input data

Model inputs

Input data for varicella were from published seroprevalence studies, and that for herpes zoster were from published incidence studies.

A systematic review was done for both varicella and herpes zoster for GBD 2016. The PubMed search query for varicella was as follows: (varicella[Title/Abstract] AND seroprevalence[Title/Abstract]) AND (incidence[Title/Abstract] OR prevalence[Title/Abstract]) NOT (herpes zoster[Title/Abstract] OR shingles[Title/Abstract]) AND ("2013"[Date - Publication] : "2016"[Date - Publication]).

The PubMed search query for herpes zoster was as follows: ((herpes zoster[Title/Abstract] OR shingles[Title/Abstract]) AND (incidence[Title/Abstract])) NOT (varicella[Title/Abstract] OR chicken pox[Title/Abstract]) AND ("2013"[Date - Publication] : "2016"[Date - Publication])

The exclusion criteria were:

10. Studies that were not population-based, eg, hospital or clinic-based studies
11. Studies that did not provide primary data on epidemiological parameters, eg, commentaries
12. Review articles
13. Case series
14. Self-reported cases

Severity splits & disability weights

We assume all varicella cases are mild episodes of acute infectious disease. Herpes zoster was a sequela studied in the GBD disability weight study. The lay descriptions and corresponding disability weights are presented in the table below.

Table 1. Severity level, lay description, and disability weights (DW)

Severity level	Lay description	DW (95% CI)
Mild acute infectious disease	Has a low fever and mild discomfort but no difficulty with daily activities.	0.006 (0.002–0.012)
Herpes zoster	Has a blistering skin rash that causes pain, with some burning and itching.	0.058 (0.035–0.09)

Modelling strategy

The modelling strategies for varicella and herpes zoster are outlined below:

I. Varicella seroprevalence data were first run through DisMod-MR 2.0. Detailed steps in the estimation of incidence and prevalence are shown below:

1. Model varicella seroprevalence data as prevalence in DisMod, after specifying zero remission and no excess mortality
2. Pick up incidence draws (which are actually hazards) from DisMod
3. Calculate population incidence rate = hazard *(1-prevalence) at the draw level
4. Calculate prevalence as prevalence = population incidence rate*duration (assumed to be 7 days)

II. Herpes Zoster morbidity was modeled using a standard DisMod model. We assume no excess mortality associated with herpes zoster.

Betas and exponentiated values (which can be interpreted as an odds ratio) are shown in the tables below.

Table 3a. Varicella DisMod model

Covariate	Parameter	Beta (95% CI)	Exponentiated beta (95% CI)
Sex (male)	Seroprevalence	-0.075 (-0.85 — 0.75)	0.93 (0.43 — 2.12)

Table 3b. Herpes zoster DisMod model

Covariate	Parameter	Beta (95% CI)	Exponentiated beta (95% CI)
Sex	Incidence	-0.14 (-0.28 — -0.0049)	0.87 (0.76 — 1.00)

No other significant changes were made to the GBD 2016 modelling strategy.

Malaria

Flowchart

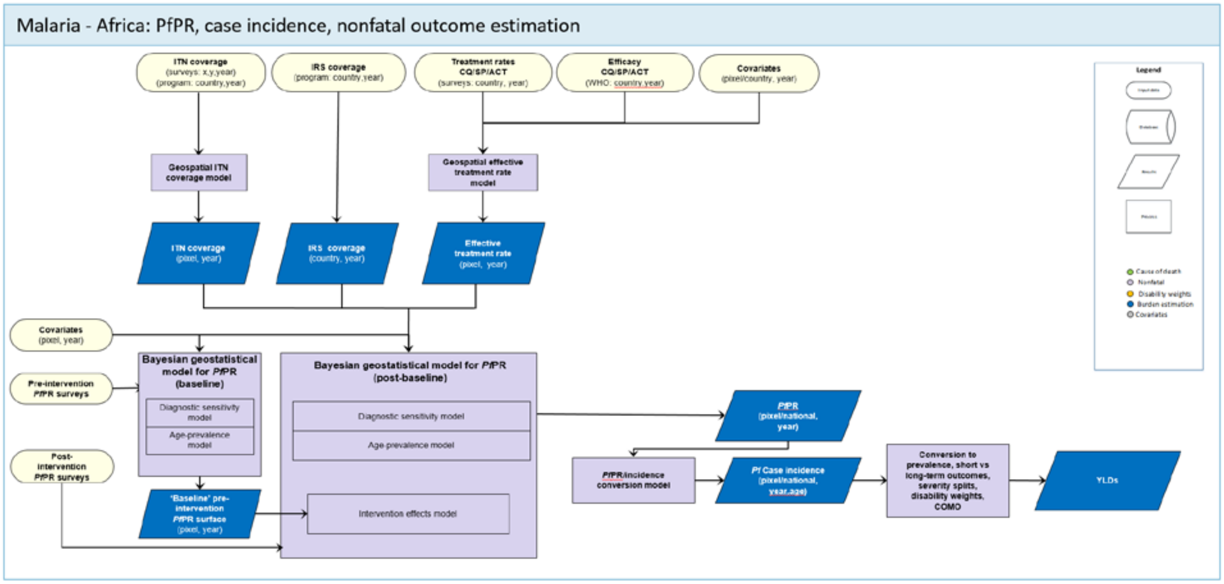

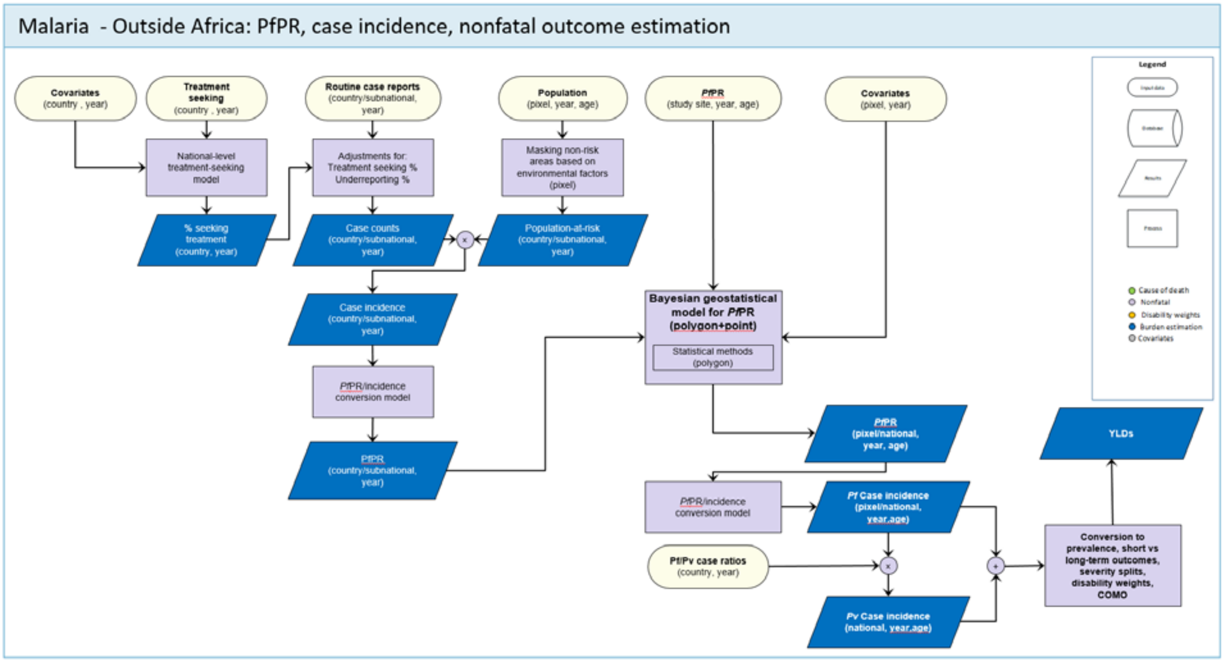

Case definition

Malaria is an acute parasitic mosquito-borne disease. An individual with uncomplicated malaria experiences one to two weeks of persistent fever, chills/shivering, sweating, joint pains and headache. The individual will likely be lethargic and feverish, causing loss of daily function during the attack. Individuals with an untreated *P. falciparum* infection may develop severe malaria, which includes the symptoms of uncomplicated malaria plus potentially swelling, difficulty breathing, unconsciousness, and death. Rapid diagnostic test or microscopy are considered the gold-standard diagnostic approaches for the purposes of GBD. The relevant ICD-10 codes are B50-B54.

Data input

Primary data inputs were:

(i) Routine malaria case reports from national routine surveillance systems. These were obtained at national level from the WHO World Malaria Report and at the subnational administrative level, wherever possible, via an exhaustive search of published and grey literature sources along with online data portals hosted by national ministries of health. Each retained record consisted of an annual count of malaria cases along with breakdown by whether confirmed/unconfirmed and by malaria parasite species.

(ii) Cross-sectional geolocated community-representative observations of infection prevalence for *Plasmodium falciparum* (referred to hereafter as *P. falciparum* parasite rate, *Pf*PR).

These malaria epidemiological metrics were augmented in the modelling by:

(iii) Malaria Atlas Project (MAP) modelled estimates of malaria control intervention population coverage (ITNs, IRS, antimalarials) resolved to 5 km x 5 km pixel-year level (for Africa) and country-year level (outside Africa).

(iv) A large suite of environmental, sociodemographic, and economic covariates resolved to 5 km x 5 km pixel-year level (for Africa) and country-year level (outside Africa).

Modelling strategy

The suitability, availability, and quality of *Pf*PR and routine case reporting data, as well as detailed intervention coverage information, differ markedly inside versus outside Africa. This meant we developed separate modelling strategy for countries inside Africa versus those outside. The exceptions were Algeria, Egypt, Morocco, Comoros, Mauritius, Cape Verde, Sao Tome, Principe, Rwanda, Botswana, Namibia, Eritrea, Djibouti, and South Africa which, despite being part of Africa, have epidemiologies and data availability/quality more akin to non-African settings.

*Pf*PR and case incidence modelling: Africa

Modelling was conducted in the following steps:

(i) The large assembly of geolocated *Pf*PR surveys maintained by MAP was used in a Bayesian spatiotemporal geostatistical model to predict *Pf*PR for every pixel-year in sub-Saharan Africa, representing an update to earlier work (Bhatt et al Nature, Gething et al NEJM). The model took into account (i) *Pf*PR survey participant age ranges and diagnostic type; (ii) coverage of ITNs, IRS, and effective antimalarial drug coverage and how these changed through time at

each data and prediction location; (iii) environmental conditions at each data and prediction location (including density of vegetation, temperature, humidity, rainfall, elevation, proximity to populated areas). The outcome was a predicted space-time “cube” of *Pf*PR, standardized to the 2-10 age range, for each year 1980–2016.

(ii) The *Pf*PR cube was then converted into an equivalent cube of the predicted incidence rate of clinical malaria. This conversion was achieved using an established model (Cameron et al Nature Communications) and allowed estimates stratified into three broad age bins (0-5; 5-15; <15).

*Pf*PR and case incidence modelling: Outside Africa

Malaria endemic countries outside Africa tend to have less *Pf*PR data than those inside, in part because prevalence is generally lower and thus *Pf*PR becomes an inefficient way to measure malaria risk. Conversely, routine surveillance systems outside Africa are generally stronger, meaning that reports of malaria cases from health systems are more reliable and provide some insight into the total malaria burden in the community. Modelling outside Africa was carried out in the following steps:

(i) National and subnational case reports were first subject to adjustments to identify and minimize bias. Bias in reported case numbers arises from various sources. First, a fraction of cases in the community will either seek no care or attend only a private or informal health care provider who would not provide a record of that case to the routine surveillance system. We adjusted for this by modelling the fraction of cases seeking care from different provider categories based on data from nationally representative cross-sectional household surveys (primarily from the Demographic and Health Survey (DHS) program and the Multiple Indicator Cluster Survey program). Second, cases reaching formal clinics may not be subject to a confirmatory diagnostic test. We adjusted for this by assuming the fraction of unconfirmed cases that were truly malaria would equal the fraction of positives among all those tested. Third, many routine surveillance systems fail to capture all case reports, with certain facilities/regions missing from the national totals in a given year. We adjusted for this based on reporting completeness statistics published nationally by WHO.

(ii) These adjusted routine case reports were georeferenced using digitized administrative boundary data using a large library of such boundaries maintained by MAP.

(iii) Each case report was converted to an estimate of clinical incidence rate by dividing over the estimated population at risk in each unit, with the latter quantity derived by combing high-resolution gridded population data with MAP models that exclude malaria risk based on aridity or temperature ranges not conducive to transmission.

(iv) The incidence rate for each unit was then converted to an inferred *Pf*PR value using the same model described earlier (Cameron et al). This allowed us to then combine these data with the true *Pf*PR survey data that existed, albeit sparsely, in many countries outside Africa.

(v) The combined *Pf*PR survey point data and (pseudo) *Pf*PR administrative unit data were then used in a Bayesian spatiotemporal geostatistical model to predict *Pf*PR at pixel-year level across all countries. As for the Africa model, *Pf*PR was standardized by age and diagnostic type and informed by a wide suite of covariates. An additional mechanism was developed to allow polygon (ie, administrative unit) and point (ie, survey) data to be used jointly to infer the predicted space-time surfaces.

(vi) As in Africa, the predicted *Pf*PR cube was then converted into an equivalent cube of the predicted incidence rate of clinical malaria.

Total malaria cases by country, year, sex

The pixel-level predictions of clinical incidence rate (both inside and outside Africa) were combined with high-resolution gridded population data to estimate total cases per pixel-year. These were then aggregated to GBD national/subnational geographies. For countries endemic for *P. vivax* and *P. falciparum*, we calculated the number of cases due to *P. vivax* applying the fraction of *P. vivax* and *P. falciparum* obtained from WHO and literature review. Total cases estimated in the MAP age bins were then redistributed to standard GBD age bins using the age pattern learned during the mortality/CoD estimation process (discussed in more detail in the GBD 2016 CoD paper).

Determining YLDs for malaria

As in GBD 2015, we use a two-step process for determining malaria severity. For acute cases, severity splits for mild, moderate, and severe malaria were produced by analysis of MEPS data. These sequelae and their associated disability weights are presented below.

Table 1. Severity level, lay description, and DW

Severity level	Lay description	DW (95% CI)
Mild	Has a low fever and mild discomfort but no difficulty with daily activities.	0.006 (0.002–0.012)
Moderate	Has a fever and aches and feels weak, which causes some difficulty with daily activities.	0.051 (0.032–0.074)
Severe	Has a high fever and pain and feels very weak, which causes great difficulty with daily activities.	0.133 (0.088–0.19)

To determine long-term neurological burden due to malaria, we use the work by Roca-Felter et al. (2008) that examined the number of uncomplicated cases that led to longer-term impairment. Analytically, this means multiplying incidence estimates (described in the section below) for persons under 20 by 0.00029 (0.000077–0.00057). This subset is then combined with excess mortality rates derived from all-cause mortality and standardized mortality ratios for neonatal encephalopathy (NE) in a DisMod model to produce prevalence estimates for all estimation years. Implicit in this process is an assumption that the disability and trend of impairment due to severe malaria follow NE. The subsequent severity splitting follows NE as well. Once the incidence estimation procedures were completed, the results were combined and converted to prevalence by matching each draw with a draw of duration. Consistent with GBD 2015, we use a uniform distribution between 14 and 28 days for duration.

References

Bhatt S, Weiss DJ, Cameron E, et al. The effect of malaria control on Plasmodium falciparum in Africa between 2000 and 2015. *Nature* 2015; 526: 207–11.

Cameron, E., K.E. Battle, S. Bhatt, D.J. Weiss, D. Bisanzio, B. Mappin, U. Dalrymple, S.I. Hay, D.L. Smith, J.T. Griffin, E.A. Wenger, P.A. Eckhoff, T.A. Smith, M.A. Penny, and P.W. Gething. 2015. Defining the relationship between infection prevalence and clinical incidence of Plasmodium falciparum malaria. *Nature Communications* 6:8170.

Gething, P.W., D.C. Casey, D.J. Weiss, D. Bisanzio, S. Bhatt, E. Cameron, K.E. Battle, U. Dalrymple, J. Rozier, P.C. Rao, M.J. Kutz, R.M. Barber, C. Huynh, K.A. Shackelford, M.M. Coates, G. Nguyen, M.S. Fraser, R. Kulikoff, H. Wang, M. Naghavi, D.L. Smith, C.J.L. Murray, S.I. Hay, and S.S. Lim. 2016. "Mapping Plasmodium falciparum Mortality in Africa between 1990 and 2015." New England Journal of Medicine 375 (25):2435-2445.

Chagas disease

Flowchart

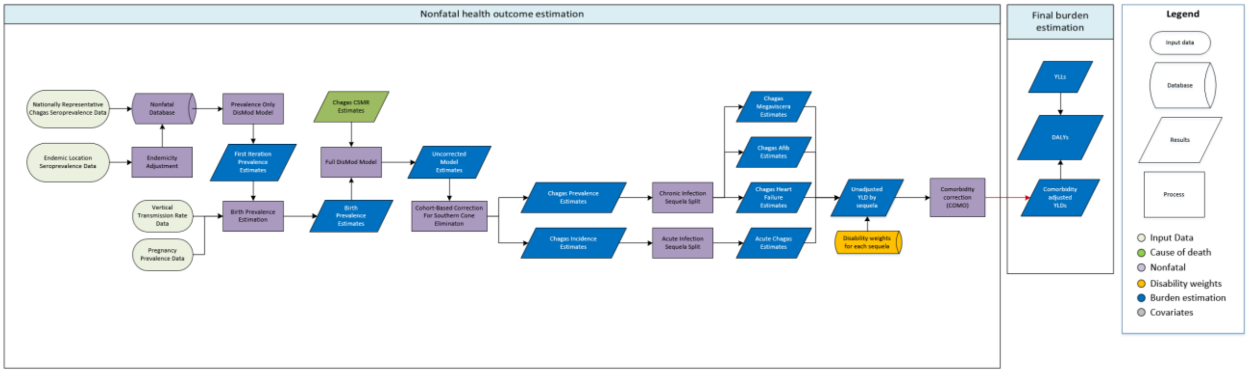

Case definition

Chagas disease is defined by infection with the protozoa *Trypanosoma cruzi,* which is transmitted by *Triatominae* insect vectors (most common), blood transfusion, organ transplant, and congenital transmission. It includes an acute phase corresponding with the time of infection, and is typically asymptomatic. Chronic infection may be latent (ie, asymptomatic), or result in cardiovascular or digestive sequelae. It includes all ICD-10 codes under the heading B57 (Chagas disease), with codes B57.0-B75.1 corresponding to the acute phase, B57.2 corresponding to chronic cardiovascular sequelae, and B57.3 corresponding to chronic digestive sequelae.

Input data

Model inputs

For GBD 2016 estimation, we used seroprevalence data to model Chagas. The table below illustrates the geographic distribution of model input data for the estimation process.

Table 1. Geographies

Level	Prevalence
Data points	407
Studies	56
Locations	20
Regions	4

We also use CSMR estimates in the modeling process, which will be addressed in further detail below.

Modelling strategy

We modeled Chagas disease using a full DisMod-MR 2.1 Bayesian meta-regression model incorporating seroprevalence data, as above, and CSMR estimates. We assume no remission. We eliminate all new infections, except those via vertical transmission, in Chile and Uruguay for years after the interruption of vector-based transmission (Abad-Franch F, Diotaiuti L, Gurgel-Gonçalves R, Gürtler RE. Certifying the interruption of Chagas disease transmission by native vectors: cui bono? Mem Inst Oswaldo Cruz 2013;108:251–4.; Coura JR. Chagas disease: control, elimination and eradication. Is it possible? Mem Inst Oswaldo Cruz 2013;108:962–7.). For non-endemic countries, we estimate the prevalence of imported chronic infections based on migration. For each non-endemic country, we estimate the total number of people infected with Chagas as the sum of the number of immigrants from each endemic country multiplied by the corresponding prevalence of Chagas in that endemic country.

We estimate five sequelae: symptomatic acute infection from incidence; and megaviscera, heart failure, atrial fibrillation, and chronic asymptomatic infection from prevalence. We assume that 5% of acute infections will be symptomatic (Teixeira AR, Nitz N, Guimaro MC, Gomes C, Santos-Buch CA. Chagas disease. Postgrad Med J 2006;82:788–98.). The proportion of chronic infections resulting in a given sequela varies by sex and age: the prevalence of megaviscera among those infected with Chagas ranges from 0% in children to nearly 10% among older adults (Coura JR, Naranjo MA, Willcox HP. Chagas' disease in the Brazilian Amazon: II. A serological survey. Rev Inst Med Trop São Paulo 1995; 37:103–7.); the prevalence of atrial fibrillation attributable to Chagas ranges from 0% among children to approximately 10% in men over 80 years of age (Ribeiro AL, Marcolino MS, Prineas RJ, Lima-Costa MF. Electrocardiographic abnormalities in elderly Chagas disease patients: 10-year follow-up of the Bambuí Cohort Study of Aging. J Am Heart Assoc 2014;3:e000632.); and the prevalence of heart failure attributable to Chagas among those who are infected ranges from 0% among young children, to a maximum of 23% among men over 80 years of age (Sabino EC, Ribeiro AL, Salemi VM, et al., for the National Heart, Lung, and Blood Institute Retrovirus Epidemiology Donor Study-II (REDS-II), International Component. Ten-year incidence of Chagas cardiomyopathy among asymptomatic Trypanosoma cruzi-seropositive former blood donors. Circulation 2013;127:1105–15.).

Severity splits and disability weights

The table below illustrates the sequelae, lay descriptions, and DWs for Chagas disease.

Table 2. Sequelae, lay description and DWs

Sequelae	Description	Disability Weight
Atrial fibrillation and flutter due to Chagas disease	Has periods of rapid and irregular heartbeats and occasional fainting.	0.224 (0.151–0.312)

Mild heart failure due to Chagas disease	Is short of breath and easily tires with moderate physical activity, such as walking uphill or more than a quarter-mile on level ground. The person feels comfortable at rest or during activities requiring less effort.	0.041 (0.026–0.062)
Moderate heart failure due to Chagas disease	Is short of breath and easily tires with minimal physical activity, such as walking only a short distance. The person feels comfortable at rest but avoids moderate activity.	0.072 (0.047–0.103)
Severe heart failure due to Chagas disease	Is short of breath and feels tired when at rest. The person avoids any physical activity, for fear of worsening the breathing problems.	0.179 (0.122–0.251)
Mild chronic digestive disease due to Chagas disease	Has some pain in the belly that causes nausea but does not interfere with daily activities.	0.011 (0.005–0.021)
Moderate chronic digestive disease due to Chagas disease	Has pain in the belly and feels nauseated. The person has difficulties with daily activities.	0.114 (0.078–0.159)
Acute Chagas disease	Has a fever and aches, and feels weak, which causes some difficulty with daily activities.	0.051 (0.032–0.074)
Asymptomatic Chagas disease	Latent Chagas infection (ie, chronic infection with no apparent symptoms)	NA

Changes from GBD 2015 to GBD 2016

We have made no substantive changes in the modelling strategy for endemic countries from GBD 2015 to GBD 2016.

Visceral Leishmaniasis

Flowchart

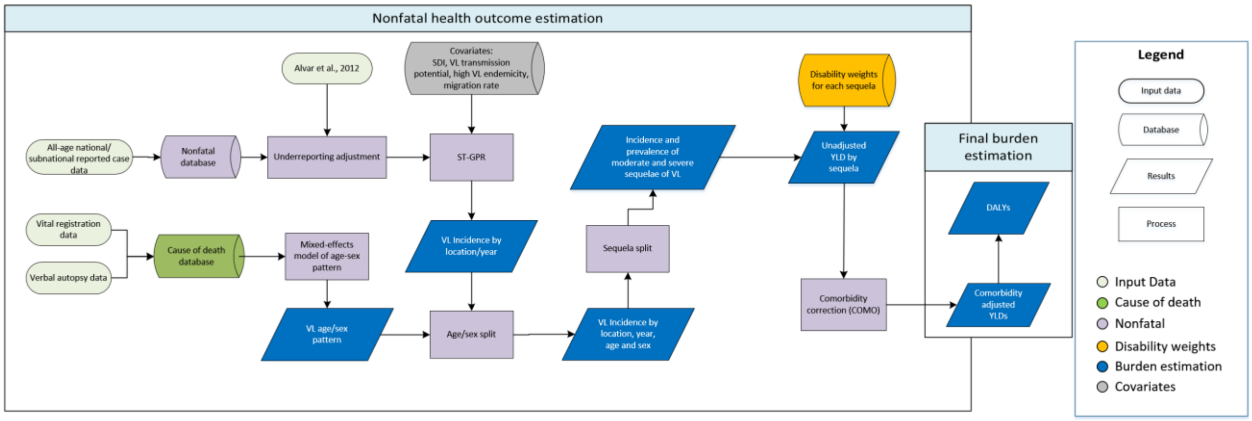

Input Data and Methodological Summary

Case Definition

Visceral leishmaniasis (VL) is the most serious manifestation of disease caused by the *Leishmania* parasite, transmitted through the bite of phlebotomine sand flies. Those infected typically present with fever, weight loss, anaemia, leukopenia, thrombocytopenia, and enlargement of the spleen and liver. If left untreated, it can be fatal. Transmission varies by geographic region, as approximately 70 animal species have been identified as potential reservoir hosts of the parasite. The ICD9 code related to visceral leishmaniasis is 085.0, and the ICD10 code is B55.0.

Input data

No systematic review of literature was done for VL for GBD 2016; however, WHO country profile datasets were updated to include the most recent case reports, and subnational reporting data from India, Brazil and Mexico were added. The table below outlines the number of location-years of reporting data, and the number of locations (countries or subnational units) and GBD world regions represented.

	incidence
Location-years	2,034
Locations	188
GBD world regions	14

Modelling strategy

We inflated VL case reports to account for underreporting, based on underreporting factors that were published by Alvar et al.(1) After adjusting for underreporting, we modelled all-age VL incidence using Space-Time Gaussian Process Regression (ST-GPR), with four covariates: socio-demographic index, leishmaniasis transmission potential (binary), high leishmaniasis endemicity (binary), and migration rate. Given that the vast majorty of location-years of case reports are for all ages and both sexes combined, mortality data offered the richest source of data on the age/sex distribution of VL. We modelled age-sex patterns using a mixed effects model of the logit-transformed proportion of cases that occur in each age/sex category, with fixed effects on age (indicator variables for each GBD age group) and sex, and hierarchical random effects region nested within super-region, and random slopes on age category within each level of the geographic hierarchy. Finally, we applied the age/sex patterns from the age-specific model to the total incidence estimates from the all-age ST-GPR model to estimate incidence by location, year, age and sex. Resultant incidence draws are then assumed to have a duration of three months, from which prevalence is calculated. Of those three months, three weeks are assumed to be spend with severe infection, and nine with moderate infection.

Changes from GBD 2015 to GBD 2016

We have substantially revised this model for GBD 2016. Whereas GBD 2015 incidence estimates were derived from a DisMod model, we now estimate all-age incidence via ST-GPR, and age/patterns via a custom mixed-effects model. This approach has the advantage of being able to estimate trends with more detail and accuracy, and of being able to more effectively utilize all-age data, which represent the majority of location-years of incidence data for VL.

Cutaneous & Mucocutaneous Leishmaniasis

Flowchart

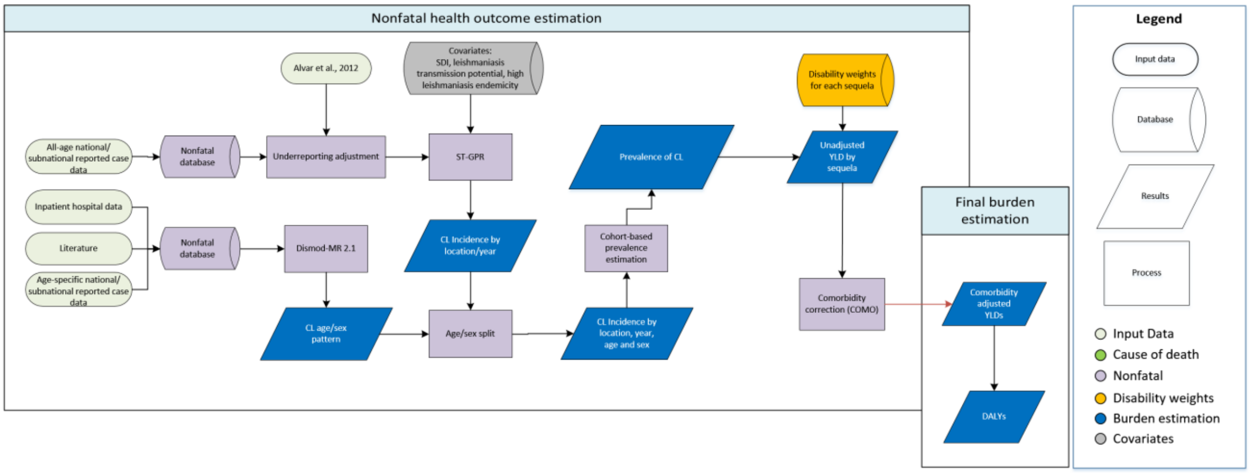

Input Data and Methodological Summary

Case Definition

Cutaneous leishmaniasis (CL) is the most common manifestation of disease caused by the *Leishmania* parasite, transmitted through the bite of phlebotomine sand flies. It causes the appearance of skin lesions, often beginning as papules or nodules and developing into ulcers, on parts of the body exposed to the bite of the sand fly. Mucocutaneous leishmaniasis (MCL) is a much more exceptional – and severe – presentation. Primarily isolated to Latin America, MCL infections can result in degradation of the mucous membranes, typically following an ulcerative sore from CL infection. Transmission varies by geographic region, as approximately 70 animal species have been identified as potential reservoir hosts of the parasite.

Input data

No systematic review of literature was done for Cutaneous and Mucocutaneous Leishmaniasis for GBD 2016; however, WHO country profile datasets were updated to include the most recent case reports, and subnational reporting data from India, Brazil and Mexico were added. The table below outlines the number of location-years of reporting data, and the number of locations (countries or subnational units) and GBD world regions represented.

	incidence
Location-years	1,368
Locations	132
GBD world regions	16

Modelling strategy

We first compiled two CL datasets: 1) a dataset of all-age annual CL case reports, and 2) a dataset of age/sex specific incidence data. Where countries reported cases by age or sex, those age/sex-specific case reports were collapsed to all-age/both sex totals for inclusion the all-age database. All-age/both-sex case reports were then inflated to account for underreporting, based on underreporting factors that were published by Alvar et al.(1)

After adjusting for underreporting, we modelled all-age CL incidence using Space-Time Gaussian Process Regression (ST-GPR). Age-sex patterns were modelled using DisMod-MR and the age/sex-specific dataset. Finally, we applied the age/sex patterns from the age-specific DisModel model to the total incidence estimates from the all-age ST-GPR model to estimate incidence by location, year, age and sex.

We estimated total prevalence as the sum of the prevalence of acute symptoms and long-term sequelae. The prevalence of acute symptoms was calculated as the product of incidence and duration, with duration equal to six-months.(2) We estimated the prevalence of long-term sequelae based on the proportion of cases that would result in lasting facial scars. To do this, we first obtained the average proportion of sores that occur on the face based on a sample-weighted average of the proportion from four studies conducted in North Africa/Middle East (the region with the most data and a fairly high incidence of CL).(3–6) The average proportion of facial sores was 0.476. Of these people, we only assigned long-term to sequelae to those who did not have appropriate access to health care, which we estimated based on a normalized health system access covariate. We multiplied CL incidence times the proportion of people with facial sores (46%), times the proportion of people without health system access in each location-year to obtain incidence of people with long-term sequelae. Assuming no remission or excess mortality, we stream those with long-term sequelae through cohort space to estimate prevalence.

Changes from GBD 2015 to GBD 2016

We have substantially revised this model for GBD 2016. Whereas GBD 2015 incidence estimates were derived from a DisMod model, we now estimate all-age incidence via ST-GPR, and age/patterns via a DisMod model. This approach has the advantage of being able to estimate trends with more detail and accuracy, and of being able to more effectively utilize all-age data, which represent the majority of location-years of incidence data for CL. Moreover, previous iterations of GBD estimated prevalence from incidence by carrying cases across age groups within a GBD estimation year, rather than carrying cases across cohort space. The previous approach offers a good approximation under relatively stable conditions (i.e. relatively stable incidence and health system access), but it performs poorly where there are non-trivial trends in incidence. We have improved our approach here, and now estimate prevalence in cohort space.

1. Alvar J, Vélez ID, Bern C, Herrero M, Desjeux P, Cano J, et al. Leishmaniasis Worldwide and Global Estimates of Its Incidence. PLOS ONE. 2012 May 31;7(5):e35671.

2. Reithinger R, Dujardin J-C, Louzir H, Pirmez C, Alexander B, Brooker S. Cutaneous leishmaniasis. Lancet Infect Dis. 2007 Sep 1;7(9):581–96.

3. CLINICAL PICTURE OF CUTANEOUS LEISHMANIASIS IN ISFAHAN, IRAN - MOMENI - 1994 - International Journal of Dermatology - Wiley Online Library [Internet]. [cited 2017 Jul 10]. Available from: http://onlinelibrary.wiley.com/doi/10.1111/j.1365-4362.1994.tb01039.x/abstract

4. Gurel MS, Ulukanligil M, Ozbilge H. Cutaneous leishmaniasis in Sanliurfa: epidemiologic and clinical features of the last four years (1997–2000). Int J Dermatol. 2002 Jan 1;41(1):32–7.

5. Sharifi I, Fekri AR, Aflatonian MR, Nadim A, Nikian Y, Kamesipour A. Cutaneous leishmaniasis in primary school children in the south-eastern Iranian city of Bam, 1994-95. Bull World Health Organ. 1998;76(3):289–93.

6. Mujtaba G, Khalid M. Cutaneous leishmaniasis in Multan, Pakistan. Int J Dermatol. 1998 Nov 1;37(11):843–5.

Human African Trypanosomiasis (HAT)

Flowchart

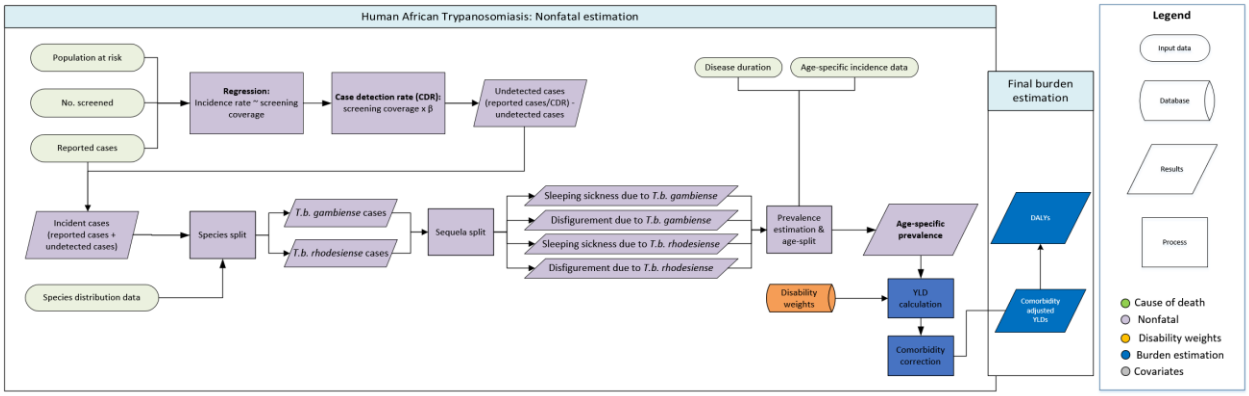

Input Data & Methodological Summary

Case Definition

Human African trypanosomiasis (HAT), also known as sleeping sickness, is a vector-borne disease which is transmitted by the bite of the tsetse fly. It is caused by the parasite *Trypanosoma brucei* with two subspecies, namely *T.b. rhodesience* (makes up less than 5% of total HAT cases) and *T.b. gambiense*. Cases are diagnosed through laboratory methods which rest on finding the parasite in body fluid or tissue by microscopy. In highly endemic or epidemic areas where the likelihood of false positives in serological tests is deemed lower, a seropositive individual is considered affected even in the absence of parasitological confirmation. The ICD-10 codes for HAT are B56.0, B56.1 and B56.9.

Input data

Model inputs

A literature search was done for GBD 2013 and for GBD 2015. The GBD 2015 search was conducted between 1/1/2013 and 8/10/2015, and the number of initial hits was 87. Of these, five sources were extracted for data. The literature search was updated by reviewing all publications from 2016; of the 138 sources reviewed, one additional source was identified to describe the age/sex distribution of HAT infection.

Additional input data used to estimate mortality due to HAT included a) population at risk estimates from GBD 2010 ArcGIS analysis using geocoded case notifications for 2000 to 2009(1) and population Count Grid estimates from Gridded Population of the World 3,(2,3) b) population screened from 1997 to 2004,(4) c) historical data from GBD 2010 on total number of HAT cases reported(1,4,5) and d) cases reported annually to the WHO(6) – for Kenya, a study on cases reported subnationally(7) was used to split the national cases into five counties (HomaBay, Migori, Busia, Bungoma, Kakamega). In addition,

age-specific incidence data from active screening undertaken in the Democratic Republic of Congo(8) and Uganda(9) were used to inform age pattern for deaths.

Data on the number of cases identified through active screening were obtained from a Weekly Epidemiological Record report (4) for *T.b. gambiense* HAT reported from 1997-2004 which reported active case finding data for the following countries: The Gambia, Ghana, Guinea Bissau, Liberia, Niger, Sierra Leone, and Senegal. No active case detection screening coverage data are currently available for *T.b. rhodesiense* HAT-endemic countries.

National case data from 1990-2015 were obtained from the World Health Organization's official surveillance data (available via the Global Health Observatory).

The table below shows the number of location-years of reporting data, and the number of locations (countries or subnational units) and GBD world regions represented.

	incidence
Location-years	805
Locations	29
GBD world regions	4

Based on available historical data post-1980, the following countries (years) were included in the estimation: Botswana (1983), Ethiopia (1980-1983), Guinea-Bissau (1980-1983, 1985-1987), Rwanda (1980, 1982-1988), and Sierra Leone (1981-1982). Five subnational locations (out of 49) for Kenya were also added in the estimation for GBD 2016.

Severity splits/sequelae

The basis of the GBD disability weight (DW) survey assessments are lay descriptions of sequelae highlighting major functional consequences and symptoms. The lay descriptions and disability weights for sequelae due to HAT are shown below.

Sequela	Lay description	DW (95% CI)
Skin disfigurement, level 1	Has a slight, visible physical deformity that is sometimes sore or itchy. Others notice the deformity, which causes some worry and discomfort.	0.027 (0.015–0.042)
Motor plus cognitive impairments, severe	Cannot move around without help, and cannot lift or hold objects, get dressed or sit upright. The person also has very low intelligence, speaks few words, and needs constant supervision and help with all daily activities	0.542 (0.37–0.702)

Modelling strategy

The nonfatal model for HAT is implemented as follows:

1. The incidence of reported HAT cases among the population at risk was calculated as the total number of reported cases divided by the population at risk.

2. To estimate the number of cases that were likely undetected by country and year, a multi-level mixed-effects linear regression of natural log-transformed incidence rate (ratio of reported HAT cases to population at risk) on natural log-transformed screening coverage (ratio of number screened for HAT to population at risk), with country random effects, was performed. Gaps were then filled using exponential interpolation between years and extrapolation from 2015 to 2016 for reported cases. This model generates a beta-coefficient which is used to estimate the case detection rate (see step 3).

   For country-years in which no screening coverage data were reported:

   - Among countries with data reported, 1997-2004, the proportion of the at risk population screened from 1997 was used retrospectively for the period 1990-1996 and the screening coverage from 2004 was carried forward from 2005-2016.
   - For countries with no screening data reported, the mean screening coverage for the region was used to impute a value over time.

3. Using the mean and variance-covariance matrix from the regression as parameters, a multivariate normal distribution was used to generate 1,000 draws of case detection rate (CDR), given the expected screening coverage. Undetected deaths were then estimated as the difference between the ratio of reported cases to CDR and reported cases (reported cases/CDR – reported cases). Estimates of incidence were obtained by adding the reported cases to the undetected cases.

4. Total HAT cases were split between *T.b. gambiense* and *T.b. rhodesiense*. We assigned all cases to *T.b. gambiense* for Angola, Benin, Burkina Faso, Cameroon, Central African Republic, Chad, Congo, Cote d'Ivoire, Democratic Republic of the Congo, Equatorial Guinea, Gabon, Ghana, Guinea, Guinea-Bissau, Mali, Nigeria, Sierra Leone, South Sudan, and Togo. We assigned all cases to *T.b. rhodesiense* for Botswana, Ethiopia, Kenya, Malawi, Mozambique, Rwanda, Tanzania, Zambia, and Zimbabwe. Cases in Uganda were split by species based on species-specific case reports.

5. Assuming the same proportion in treated and untreated cases, the incidence estimates were then split into the two sequelae, skin disfigurement and sleeping disorder. This was done by generating 1,000 draws of the splitting proportion for the sequelae (70%–74% with sleeping disorder) based on a study that reported presence of symptoms at admission of patients in treatment centers [14] – draws were generated from a beta distribution with alpha parameter = 1884 and beta parameter = 649.

6. To compute prevalence of HAT, we generate 1,000 draws of total duration of symptoms in untreated cases with a mean duration of 3 years (1.8 – 4.1) for *T.b. gambiense* and 0.5 years (0.46 – 0.54) for *T.b. rhodesiense*, and an estimated duration of six months for all treated cases (both species) (10,11). Prevalence of treated and untreated cases were summed.

7. For untreated cases, we assumed that half the duration is spent with sleeping disorder (severe motor and cognitive impairment) and disfigurement.(12) Treated (ie, reported) cases are assumed to have been prevalent for 0.5 years, and for the fraction of treated cases that present with sleeping disorder, it was assumed that this is present for half the total duration and that the rest of the duration is spent suffering from disfiguring skin disease. Treated cases that don't present with sleeping disorder were assigned disfigurement for the entire duration.

8. An age-pattern was applied to the all-age incidence estimates using the incidence studies from DRC and Uganda.(8,9,13) The age-pattern in GBD 2016 employed a cubic spline to account for the higher risk of infection among working age adults (as opposed to GBD 2015 in which a step-function at age 20 years was employed to differentiate risk). Without information on sex-specific incidence, we assumed equal incidence rates between both sexes.

Changes from GBD 2015 to GBD 2016

We have made two changes to our modelling strategy between GBD 2015 to GBD 2016. First, we now split nonfatal cases between *T.b. gambiense* and *T.b. rhodesiense*, and apply different durations to the two. Second, we have improved our method for estimating the age-pattern of cases and now use cubic spline based regression model rather the previous step function.

References

1. Simarro PP, Cecchi G, Paone M, Franco JR, Diarra A, Ruiz JA, et al. The Atlas of human African trypanosomiasis: a contribution to global mapping of neglected tropical diseases. Int J Health Geogr. 2010;9:57.

2. Gridded Population of the World (GPW), Version 3: Population Count Grid. Palisades, NY: Socioeconomic Data and Applications Center (SEDAC); Center for International Earth Science Information Network (CIESIN) at Columbia University; Centro Internacional de Agricultura Tropical (CIAT); 2005.

3. Gridded Population of the World (GPW), Version 3: Population Count Grid, Future Estimates. Palisades, NY: Socioeconomic Data and Applications Center (SEDAC); Center for International Earth Science Information Network (CIESIN) at Columbia University; United Nations Food and Agriculture Programme (FAO); Centro Internacional de Agricultura Tropical (CIAT); 2005.

4. Weekly Epidemiological Record (WER). World Health Organization; 2006 Feb p. 69–80. Report No.: 81, 8.

5. World Health Organization, Department of Communicable Disease Surveillance and Response (CDS). WHO Report on Global Surveillance of Epidemic-prone Infectious Diseases. World Health Organization; 2000.

6. World Health Organization. Human African Trypanosomiasis [Internet]. Global Health Observatory data repository. [cited 2016 Apr 21]. Available from: http://apps.who.int/gho/data/node.main.A1635?lang=en

7. Rutto JJ, Karuga JW. Temporal and spatial epidemiology of sleeping sickness and use of geographical information system (GIS) in Kenya. J Vector Borne Dis. 2009 Mar;46(1):18–25.

8. Lutumba P, Makieya E, Shaw A, Meheus F, Boelaert M. Human African trypanosomiasis in a rural community, Democratic Republic of Congo. Emerg Infect Dis. 2007 Feb;13(2):248–54.

9. Fèvre EM, Odiit M, Coleman PG, Woolhouse MEJ, Welburn SC. Estimating the burden of rhodesiense sleeping sickness during an outbreak in Serere, eastern Uganda. BMC Public Health. 2008;8:96.

10. Odiit M, Kansiime F, Enyaru JC. Duration of symptoms and case fatality of sleeping sickness caused by Trypanosoma brucei rhodesiense in Tororo, Uganda. East Afr Med J. 1997 Dec;74(12):792–5.

11. Checchi F, Filipe JA, Haydon DT, Chandramohan D, Chappuis F. Estimates of the duration of the early and late stage of gambiense sleeping sickness. BMC Infect Dis. 2008 Feb 8;8:16.

12. Blum J, Schmid C, Burri C. Clinical aspects of 2541 patients with second stage human African trypanosomiasis. Acta Trop. 2006 Jan;97(1):55–64.

13. Kato CD, Nanteza A, Mugasa C, Edyelu A, Matovu E, Alibu VP. Clinical Profiles, Disease Outcome and Co-Morbidities among T. b. rhodesiense Sleeping Sickness Patients in Uganda. PLOS ONE. 2015 Feb 26;10(2):e0118370.

Schistosomiasis

Flowchart

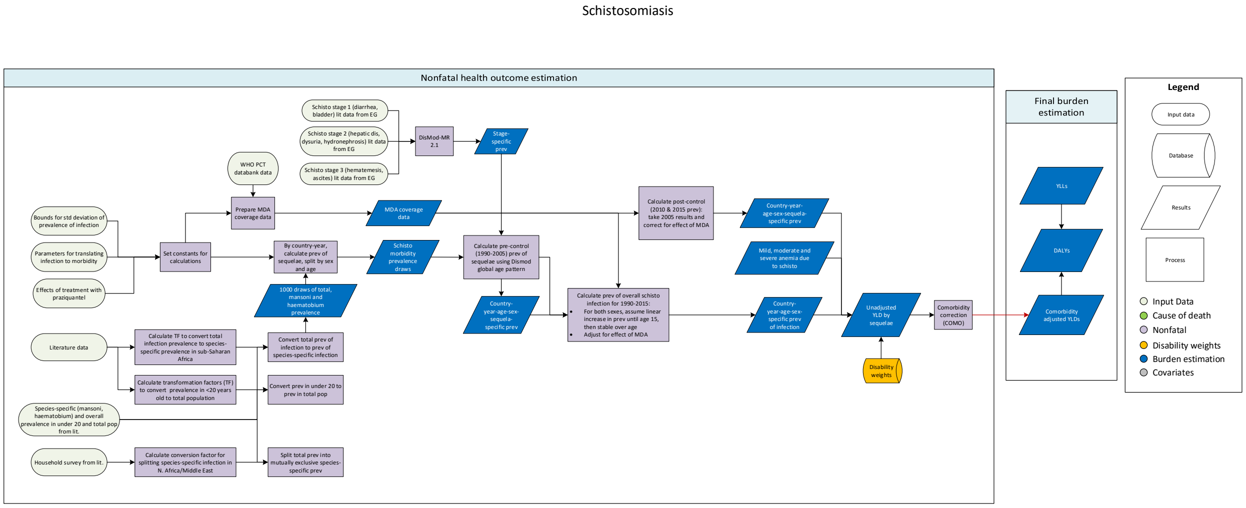

Case definition

Schistosomiasis, also known as bilharzia or "snail fever," is a helminth disease caused by infection with five species of the parasite *Schistosoma,* namely, *S. mansoni, S. japonicum, S. haematobium, S. mekongi, and S. intercalatuma*. It is considered a neglected tropical disease (NTD). The first three species cause the most infection and the last two rarely cause disease. Diagnosis is made by microscopic exam of stool or urine for parasite eggs. For less advanced infections, serologic techniques are used. The ICD-10 codes for schistosomiasis are B65-B65.9.

Input data

Model inputs

To model nonfatal outcomes due to schistosomiasis, we conducted a systematic literature review, extracting prevalence data from 1980 to 2016 for the five species of schistosomiasis listed above. The search string used in the systematic review is (schistosom*[Title/Abstract] OR bilharzia*[Title/Abstract] OR "snail fever"[Title/Abstract]) AND ("1990"[Date - Publication] : "3000"[Date - Publication]) AND (epidemiolog* OR inciden* OR prevalen* OR seroprevalen*) NOT (animals[mesh] NOT humans[mesh]). Additionally, we used data compiled by the Global Atlas of Helminth Infections (GAHI), which includes grey literature and unpublished data.

Population at risk/mass drug administration data

Population at risk estimates and MDA data were taken from the WHO PCT Databank [1].

Severity splits/sequelae

The table below shows the list of clinical sequelae (including mild, moderate, and severe anaemia) due to schistosomiasis, their lay descriptions, and the associated disease stages and disability weights. Using literature [1], a list of eight possible clinical sequelae and anaemia sequelae were defined (mild infection, mild diarrhoea, haematemesis (vomiting blood), hepatomegaly, ascites (buildup of fluid in the peritoneal cavity), dysuria (painful urination), bladder pathology, hydronephrosis (swelling of kidney due to buildup of urine in the kidney), mild anaemia, moderate anaemia, and severe anaemia).

Table 2. Clinical sequela, lay descriptions, disease stages, and DWs

Clinical sequela	Lay description	Disease stage	Disability weights (DWs)
Mild infection	has a low fever and mild discomfort , but no difficulty with daily activities	1	0.006 (0.002–0.012)
Mild diarrhoea		1	0.056
Hepatomegaly	has some pain in the belly that causes nausea but does not interfere with daily activities	2	0.011 (0.005–0.021)
Dysuria	has some pain in the belly that causes nausea but does not interfere with daily activities	2	0.011 (0.005–0.021)
Hydronephrosis	has some pain in the belly that causes nausea but does not interfere with daily activities	2	0.011 (0.005–0.021)
Haematemesis	vomits blood and feels nauseated	3	0.325 (0.209–0.463)
Ascites	has pain in the belly and feels nauseated. The person has difficulties with daily activities	3	0.114 (0.078–0.159)
Bladder pathology	has some pain in the belly that causes nausea but does not interfere with daily activities	3	0.011 (0.005–0.021)
Mild anaemia	feels slightly tired and weak at times, but this does not interfere with normal daily activities	NA	0.004 (0.001–0.008)
Moderate anaemia	feels moderate fatigue, weakness, and shortness of breath after exercise, making daily activities more difficult	NA	0.052 (0.034–0.076)
Severe anaemia	feels very weak, tired, and short of breath, and has problems with activities that require physical effort or deep concentration	NA	0.149 (0.101–0.210)

Modelling strategy

The morbidity model for schistosomiasis involved a multi-step process. First, we ran a single-parameter prevalence model in DisMod-MR 2.0 using the prevalence data extracted in the systematic review and from the GAHI database. We make the assumption that all of our data are measured within a population at risk – therefore, the estimates from the DisMod model represent prevalence estimates among the population at risk for schistosomiasis. Additionally, we included the MDA treatment data from the WHO as a country-level covariate in the DisMod model. Second, we then scaled the prevalence estimates to the population at risk estimates from the WHO PCT Databank to get age/sex/location/year all-schistosomiasis prevalence envelopes. 3) We ran a generalized linear model to get species-specific proportional prevalence on data from literature that reported both *S. haematobium* and *S. mansoni* infection, and 4) literature-informed parameters (a, b, c) for translating infection (x) to morbidity (y): y =

(a + bx^c)/(1 + bx^c) – a [2-4]. We used the species-specific conversion factors calculated in step (3) to split the all-schistosomiasis envelope into species-specific schistosomiasis. We then used the parameters determined in step (4) to translate infection into morbidity to get age/sex/year/location-specific prevalence of sequelae. The burden of anaemia due to schistosomiasis was estimated (see anaemia documentation for details).

Model evaluation was done by separately assessing the fit of the single-parameter DisMod models and checking the final estimates produced after age-sex splits. Plots of time trends of prevalence across locations and age were used to evaluate the results. In addition, maps of the global distribution of total schistosomiasis prevalence and prevalence of sequelae due to schistosomiasis were also assessed across time.

Changes from GBD 2015 to GBD 2016

The main change made from GBD 2015 was the systematic review and using extracted data in a DisMod model to estimate prevalence within the population at risk. In addition, newly updated data from the WHO PCT databank were downloaded and used in the model, and geographic restrictions were updated.

References

1. World Health Organization (WHO). WHO PCT Databank - Schistosomiasis. Geneva, Switzerland: World Health Organization (WHO).
2. van der Werf MJ, de Vlas SJ, Brooker S, et al. Quantification of clinical morbidity associated with schistosome infection in sub-Saharan Africa. Acta Trop. 2003;86(2-3):125-39
3. van der Werf MJ, de Vlas SJ, Looman CW, Nagelkerke NJ, Habbema JD, Engels D. Associating community prevalence of Schistosoma mansoni infection with prevalence of signs and symptoms. Acta Trop. 2002;82(2):127-37
4. van der Werf MJ, de Vlas SJ. Diagnosis of urinary schistosomiasis: A novel approach to compare bladder pathology measured by ultrasound and three methods for hematuria detection. Am. J. Trop. Med. Hyg. 2004;82:98-106

Cysticercosis

Flowchart

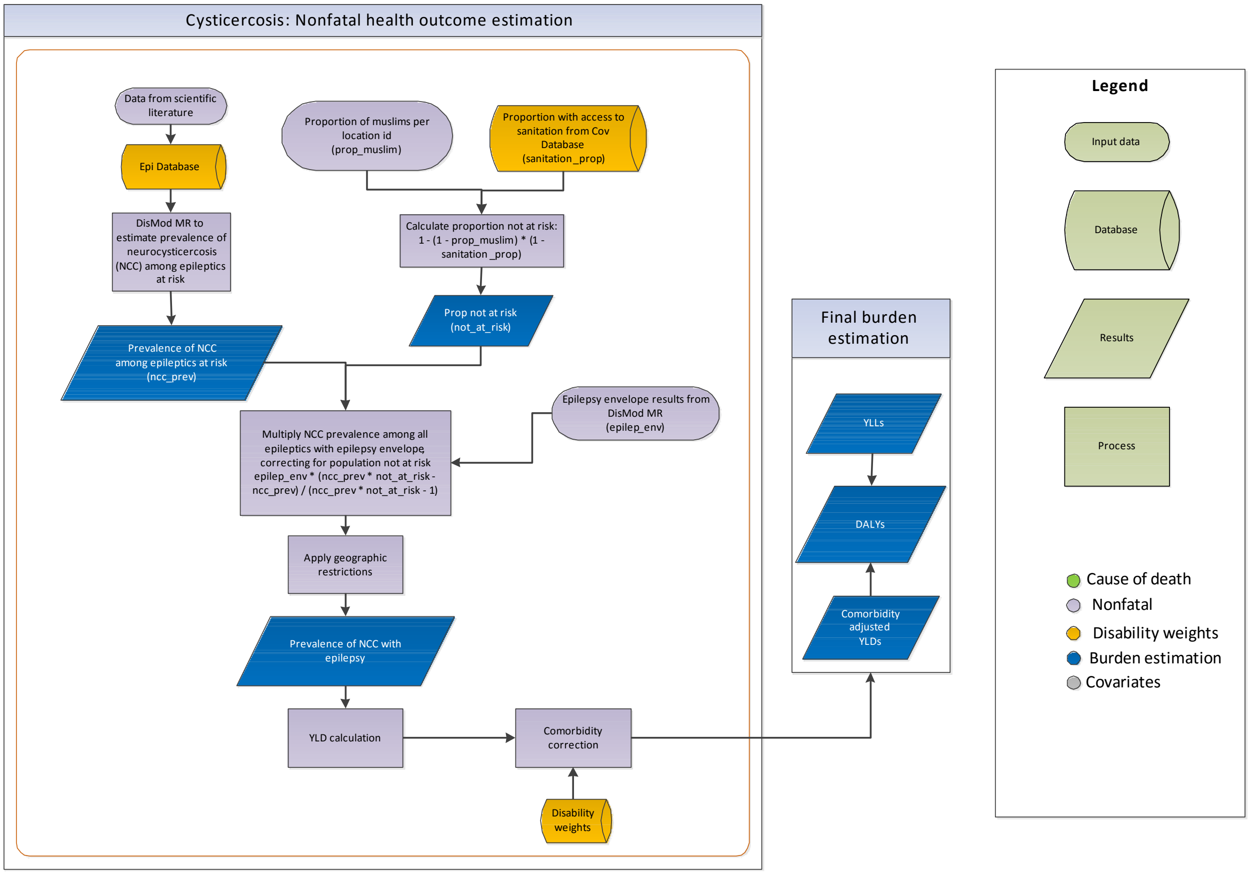

Input Data & Methodological Summary

Case Definition

Cysticercosis, or Neurocysticercosis (NCC), is a parasitic disease caused by the pig tapeworm, *Taenia solium.* It is transmitted via ingestion of eggs or gravid proglottids shed by a human or non-human host with an intestinal infection of the same helminth known as Taeniasis. In rare cases, auto-infection is also possible among people with intestinal infections. Diagnosis is made by magnetic resonance imaging (MRI) or computerized tomography (CT) brain scans to identify cysts. The ICD-10 codes for Cysticercosis are B69-B69.9.

Input data

Systematic Literature Review

The nonfatal estimation for cysticercosis focused on estimating prevalence of NCC among epileptics at risk as well as the prevalence of NCC with epilepsy. A systematic review of literature was conducted in PubMed for GBD 2015 using the following search string:

> ("cysticercosis"[Title/Abstract] OR "neurocysticercosis"[Title/Abstract] OR "cysticerciasis"[Title/Abstract] OR "Taenia solium"[Title/Abstract]) AND ("1990"[Date – Publication] : "2015"[Date – Publication]) AND (epidemiology OR prevalence)).

This yielded 1,038 studies of which 166 were included during the title/abstract screening. Following the full-text screening, 17 studies were included and extracted – studies were excluded because of one or more of the following reasons:

15. study not in epileptics
16. study not population-based
17. study does not have primary data on prevalence of NCC among epileptics at risk
18. study not in humans (some studies were on cysticercosis in pigs)
19. study on comorbidities with NCC (other than epilepsy)
20. study on sub-population, eg, patients with neurological disorders
21. review study

We combined the newly extracted studies with studies extracted during GBD 2013. The table below shows the number of studies finally included, and the number of countries or subnational units and GBD world regions represented.

	prevalence
Studies	31
Countries/subnationals	23
GBD world regions	8

A study-level covariate was also created in GBD 2015 to indicate the type of diagnosis for each study, ie, definitive or probable. Of the 77 rows of country-year-age-sex data, there were 15 rows with definitive diagnosis and 62 rows with probable diagnosis.

Covariates

Data was ascertained from the PEW Research Center [1] on the proportion of the population that is Muslim and incorporated as a continuous covariate with a range between 0 and 1.

Epilepsy Envelope

The modelling process incorporates 100 draws of epilepsy envelope prevalence from the GBD 2016 epilepsy DisMod MR model – details on this modeling process can be found elsewhere.

Geographic Restrictions

We conducted a literature review to determine the geographic extent of the disease and classify locations based on whether the disease is absent or present in each year. Locations that were

geographically restricted in any given year did not have estimates made for them but could have imported cases attributed to them at a later stage. Of note, we did not attempt a complete systematic review, since a single high-quality source could offer sufficient evidence of presence. Evidence of absence or presence was not available for every location for each year and so assumptions were made for missing years by taking into consideration the epidemiological characteristics of the disease. If evidence indicated disease presence for two non-consecutive years, we assumed presence for all years between the two. If evidence indicated disease absence for two non-consecutive years, we assumed absence for all years between the two. If evidence indicated a change in status (i.e. from absent to present, or present to absent) between two non-consecutive years than we conducted targeted searches to ascertain the relevant year of introduction or elimination for that location. In the cases where presence or absence information was missing for the start or end years of our study interval (1990-2016) without evidence of any introduction or elimination events within the interval, we applied the status of the first and last presence/absence observations respectively to all years between the interval bound and the observation year. For cysticercosis, we performed targeted searches to classify location-years in PubMed and Google Scholar. Our map was populated by 21 peer-reviewed articles and meta-analyses and WHO reports.

Modelling strategy

DisMod MR was used to model the prevalence of NCC among epileptics at risk. In the model, pigs per capita and religion (binary, >50% Muslim) were used as country-level covariates. In addition, the prevalence of "definitive diagnosis" was transformed to that of "probable and definitive diagnosis" so as to not underestimate overall prevalence.

After running DisMod, we adjusted the fraction of people with epilepsy attributable to cysticercosis in endemic countries for the population at risk based on the proportion of the population without access to sanitation and the proportion of the population that is Muslim. Predicted NCC prevalence among epileptics at risk was calculated such that Prevalence=P×(NM-N)/(NM-1), where P = prevalence of all-cause epilepsy in total population, N = proportion of NCC among epileptics at risk (non-Muslims without access to sanitation), and M = proportion of population not at risk of contracting NCC. It was assumed that the prevalence of epilepsy due to causes other than NCC is the same regardless of whether a population is at risk or not. It was also assumed that Muslims and non-Muslims have equal access to sanitation.

Geographic restrictions were applied to set prevalence to zero in non-endemic locations.

Model evaluation was done by separately assessing the fit of the DisMod MR model and checking the estimates produced after estimating prevalence of NCC with epilepsy. Plots of time trends of prevalence across locations and age were used to evaluate the results. In addition, maps of the global distribution of prevalence of NCC among epileptics at risk and prevalence of NCC with epilepsy were also assessed across time.

Only minor changes were made to the GBD2015 modeling strategy including slight changes to the model parameters in Dismod MR, a change in covariates, new geographic restrictions and an updating of the proportion of population with Muslim data by filling in subnational locations with national proportions – this was done due to lack of data on this covariate at the subnational level.

References:

5. "Table: Muslim Population by Country Pew Research Center, Washington, D.C." (July 7, 2017). http://www.pewforum.org/2011/01/27/table-muslim-population-by-country/

Cystic Echinococcosis

Flowchart

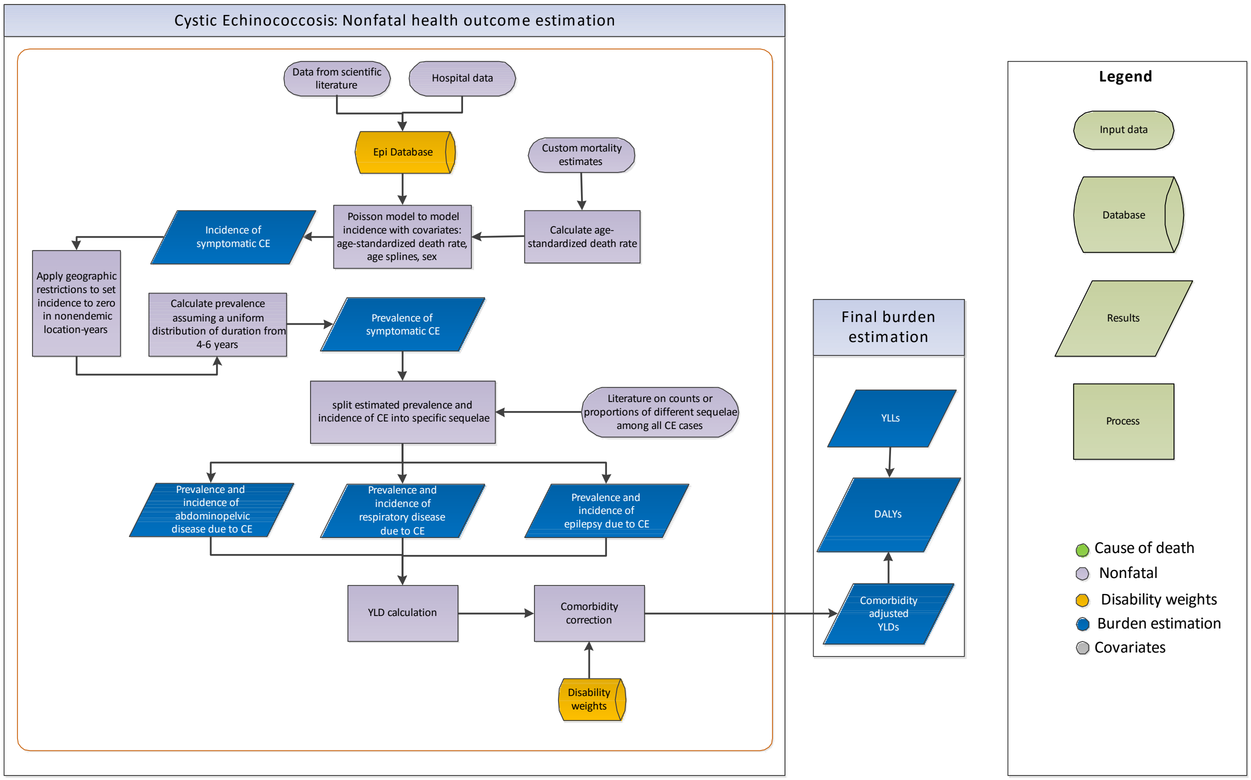

Input Data & Methodological Summary

Case definition

Cystic echinococcosis is a parasitic disease caused by infection with the *Echinococcus granulosis* tapeworm. It is a natural parasite of canines, with sheep being the most common intermediate host in the two-stage lifecycle, but can be spread to humans through ingestion of soil, water, or food contaminated with the fecal matter of an infected dog containing infective eggs. Diagnosis is made by clinical findings, imaging, serology, and tissue pathology. The ICD-9 and ICD-10 codes for echinococcosis are 122.0-122.9 and B67-B67.9, respectively.

Input data

Systematic Literature Review

The nonfatal estimation for cystic echinococcosis (CE) focused on estimating incidence and prevalence of CE and its sequelae. A systematic review of literature was conducted in PubMed for GBD 2015 using the following search string:

("echinococcosis"[Title/Abstract] OR "hydatid disease"[Title/Abstract] OR "hydatidosis"[Title/Abstract] OR "echinococcal disease"[Title/Abstract] OR "Echinococcus granulosus infection"[Title/Abstract]) AND (“1990”[Date – Publication] : “2015”[Date – Publication]) AND (epidemiology OR incidence OR prevalence).

This yielded 1,619 studies of which 279 were included during the title/abstract screening. Following the full-text screening, 77 studies (32 incidence, 43 prevalence and 2 both) were included and extracted – studies were excluded because of one or more of the following reasons:

22. study not population-based
23. study does not have primary data on prevalence and/or incidence
24. study not in humans
25. study on sub-populations
26. review study

Since we were interested in modelling symptomatic CE cases, we only used data on incidence of patients diagnosed by imaging techniques (mainly ultrasonography). Therefore, we excluded prevalence data which were mostly from serological studies.

Data from these extracted studies were combined with data from studies extracted during GBD 2013.

The table below shows the number of studies finally included, and the number of countries or subnational units and GBD world regions represented.

	incidence
Studies	84
Countries/subnationals	137

Hospital Data

Hospital data prepared by the GBD team. This data was adjusted to account for multiple hospital episodes of a single case and non-primary diagnoses.

	incidence
Data Sources	59
Countries/subnationals	319

Geographic Restrictions

We conducted a literature review to determine the geographic extent of the disease and classify locations based on whether the disease is absent or present in each year. Locations that were geographically restricted in any given year did not have estimates made for them but could have imported cases attributed to them at a later stage. Of note, we did not attempt a complete systematic review, since a single high-quality source could offer sufficient evidence of presence. Evidence of absence or presence was not available for every location for each year and so assumptions were made for missing years by taking into consideration the epidemiological characteristics of the disease. If evidence indicated disease presence for two non-consecutive years, we assumed presence for all years between the two. If evidence indicated disease absence for two non-consecutive years, we assumed

absence for all years between the two. If evidence indicated a change in status (i.e. from absent to present, or present to absent) between two non-consecutive years than we conducted targeted searches to ascertain the relevant year of introduction or elimination for that location. In the cases where presence or absence information was missing for the start or end years of our study interval (1990-2016) without evidence of any introduction or elimination events within the interval, we applied the status of the first and last presence/absence observations respectively to all years between the interval bound and the observation year. For cystic echinococcosis, we performed targeted searches to classify location-years in PubMed and Google Scholar. Our map was populated by 23 peer-reviewed articles and meta-analyses.

Sequelae due to cystic echinoccocosis

The table below shows the sequelae due to echinococcosis and their associated disability weights.

Sequela	Lay description	DW (95% CI)
Chronic respiratory disease	has cough and shortness of breath after heavy physical activity, but is able to walk long distances and climb stairs.	0.019 (0.011–0.033)
Abdominal problems	has pain in the belly and feels nauseated. The person has difficulties with daily activities.	0.114 (0.078–0.159)
Epilepsy	(Combined DW)	NA

Modelling strategy

A Poisson model was used to model cystic echinococcosis incidence. Mortality estimates from the custom mortality model were age-standardized and used as a covariate in the model along with age splines and sex. One thousand draws were generated from the predicted mean and predicted standard error.

Geographic restrictions were applied to set incidence to zero in location-years where the disease was not endemic.

These incidence draws were used to calculate prevalence at the draw level by drawing a value of duration of clinical disease from a uniform distribution between four and six years and assuming that each incident case, on average, gets infected in the middle of a five-year age group. In the youngest age groups where individuals are less than one year old, prevalence was equal to have of the incidence. For all individuals over one year old, half of the incidence-years in each age group were assigned to the prevalence in that age bin and the remaining incidence was assigned to the age bin following it.

After producing all-case prevalence draws, a thousand draws of proportions for abdominal, respiratory, and epileptic symptoms among echinococcosis cases adding up to 1 were generated. Uncertainty in the splitting proportions was captured by drawing them from a Dirichlet distribution, informed by published data on cysts localization [1]. On average, the proportions of abdominal, respiratory, and epileptic symptoms due to echinococcosis were 0.8, 0.19, and 0.01, respectively. These proportions were used to split the prevalence and incidence from DisMod into the three sequelae.

Model evaluation was done by separately assessing the fit of the DisMod MR model and checking the estimates produced after estimating incidence and prevalence of sequelae due to cystic echinococcosis.

Plots of time trends of incidence and prevalence across locations and age were used to evaluate the results. In addition, maps of the global distribution of incidence and prevalence were assessed across time.

References

6. Eckert J, Deplazes P. Biological, Epidemiological, and Clinical Aspects of Echinococcosis, a Zoonosis of Increasing Concern. *Clin Microbiol Rev*, 2004; 17(1): 107-35

Lymphatic Filariasis

Flowchart

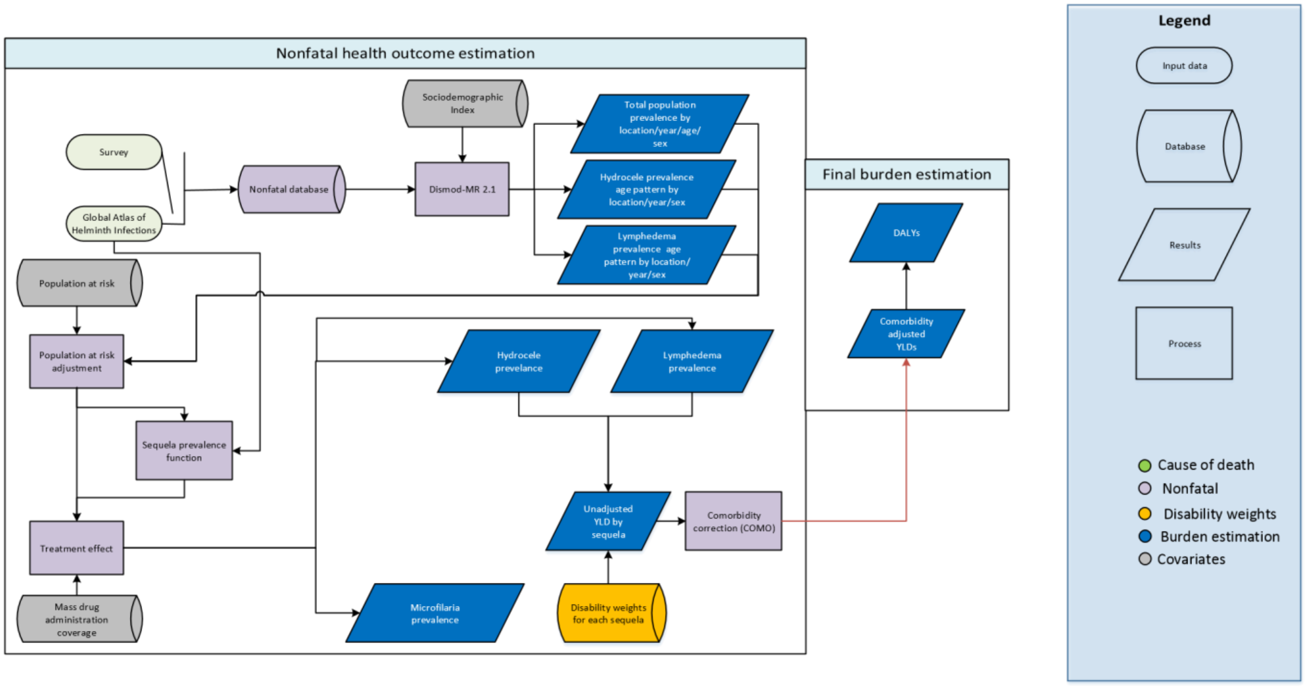

Input Data and Methodological Summary

Case Definition

Lymphatic filariasis (LF) is a neglected tropical disease spread in which threadlike nematodes invade the lymphatic system. The worms responsible – *Wuchereria bancrofti*, *Brugia malayi,* and *Brugia timori* – are spread from human to human via mosquitoes. The most prominent clinical manifestations of LF are lymphedema (a swelling of the legs, also known in its more extreme manifestation as elephantiasis) and hydrocele (a collection of fluid in the sac around the testicles).

Input data

A systematic review of literature for GBD 2016 in the PubMed database was done on October 14, 2016, for prevalence and incidence data using the search (Lymphatic filariasis AND prevalence) OR (Lymphatic filariasis AND (prevalence OR incidence OR "mass drug administration" OR MDA OR coverage)) OR (Lymphedema, hydrocele) OR (Transmission Assessment Survey (TAS)) OR (Lymphatic filariasis AND mapping).

Population at risk and MDA coverage data come from the WHO PCT Databank [1].

Modelling strategy

Data on prevalence of microfilaria is modelled using Dismod-MR 2.1. Due to the focal nature of lymphatic filariasis, we make the assumption that data collected are from endemic locations unless specifically specified in literature or survey methods. If the data are nationally representative, we adjust the data points by multiplying by the inverse of the proportion of the population at risk. Due to the fact that data is collected in endemic locations or we adjust it so that it is within the population at risk, we then scaled the DisMod-MR 2.1 estimates according to at-risk population in order to attain nationally representative values. We developed a new MDA location-level covariate that is used in the DisMod model based off WHO PCT Databank data, informing prevalence estimates.

For lymphedema and hydrocele, we incorporate survey data from the Global LF Atlas in a non-linear error-in-variables regression that determines the prevalence of lymphedema and hydrocele as functions of microfilaria prevalence, which is then applied to the total microfilaria DisMod model in order to attain an envelope of cases by location-year. Separately, all available prevalence data for these conditions is modeled in DisMod in order to determine an age-sex pattern.

In the estimation of lymphedema and hydrocele prevalence, we perform the same population at-risk correction that is done on microfilaria prevalence. For hydrocele prevalence after treatment, we take the value before MDA rollout in 2000 and reduce that by the same treatment efficacy function described for microfilaria prevalence, using dosage-reduction data specific to hydrocele along with the location-year specific MDA coverage. For lymphedema, we assume no new cases appear among treated individuals. As such, we reduce lymphedema prevalence in post-treatment years in accordance with MDA coverage.

Sequela	Data points	Regions	Countries	Subnational units
Prevalence of detectable microfilaria	1,552	10	40	28
Lymphedema due to lymphatic filariasis	511	10	25	15
Hydrocele due to lymphatic filariasis	265	8	22	12

Changes from GBD 2015 to GBD 2016

We conducted a new literature review, and utilized data from recent years and the MDA covariate to predict the time trend rather than last year's non-linear regression to estimate the reduction of microfilaria as a function of treatments per person.

Onchocerciasis

Flowchart

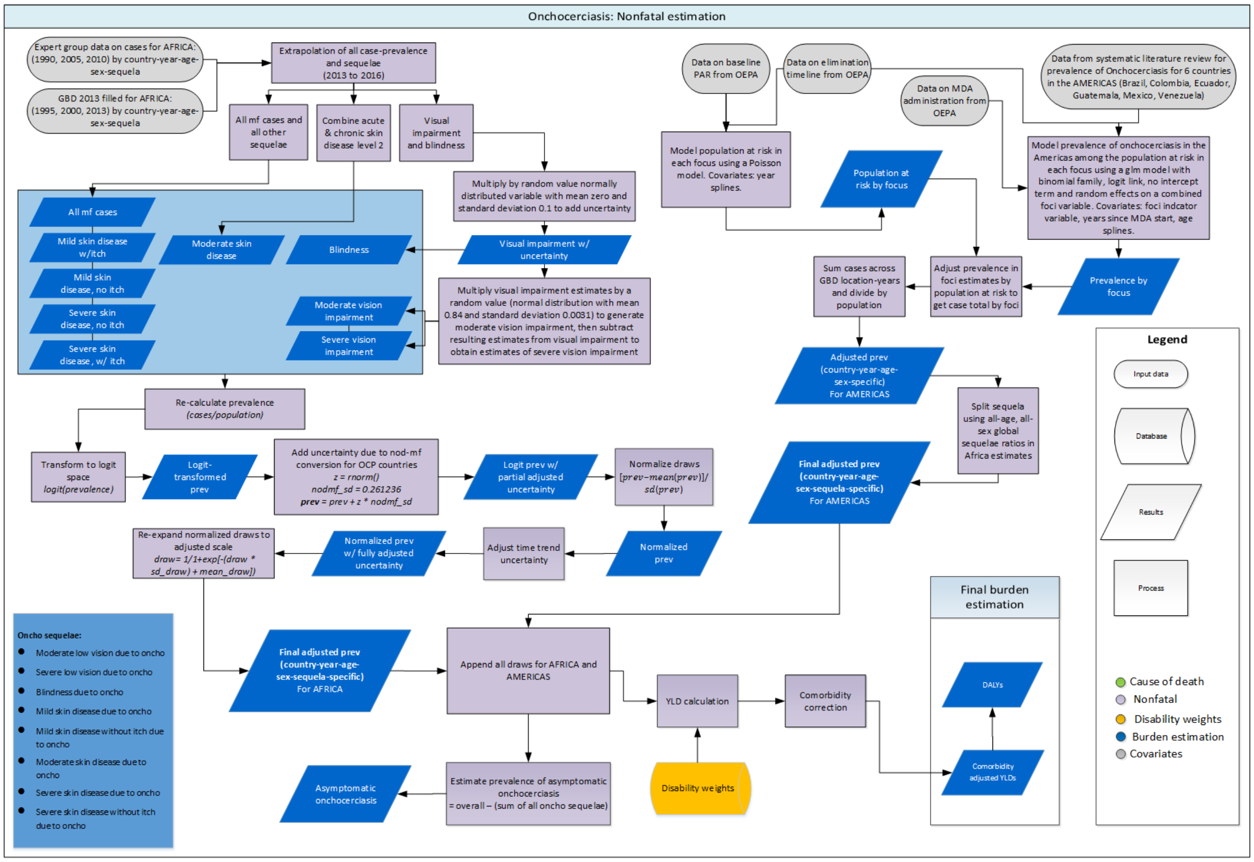

Input data & methodological summary

Case definition

Onchocerciasis, also known as river blindness, is a parasitic disease caused by the helminth *Onchocerca volvulus*. It is transmitted via the bite of one of several species of *Similium* blackflies that have historically bred in fast-moving freshwater rivers and tributaries throughout sub-Saharan Africa, Central America, and South America. Diagnosis can be made by skin snip biopsy to identify larvae, surgical removal of nodules and exam for adult worms, slit lamp exam of anterior part of the eye where larvae or lesions caused by them are visible, and antibody tests (mostly useful to visitors to areas with parasites). The ICD-10 code for onchocerciasis is B73.

Input data

Model inputs

Prevalence data prepared by the GBD 2010 expert group (EG) was used for modelling the nonfatal outcomes resulting from onchocerciasis in Africa. This included 1,000 draws of infection and morbidity

(visual impairment, blindness, and skin conditions) cases with confidence intervals categorized by country, age, and sex for years 1990, 1995, 2000, 2005, and 2010. Details about the materials and methods used by the EG to generate these draws can be found elsewhere [1-5]. These data represented all African countries included in the African Programme for Onchocerciasis Control (APOC) and the Onchocerciasis Control Programme (OCP) for which initial Rapid Epidemiological Mapping of Onchocerciasis (REMO) assessments demonstrated a need for Community-Directed Treatment with Ivermectin (CDTI) (defined as having a prevalence of skin nodules greater than 20%). Four countries – Rwanda, Mozambique, Kenya and Gabon – were designated as hypo-endemic countries after initial REMO assessments and not included due to sparsity of cases and paucity of data. Estimates for Sudan from GBD 2010 were reassigned to South Sudan in GBD 2013 after its independence in 2011 since REMO assessments indicated that the vast majority of cases occurred in that area of the former Sudan. The tables below show the countries included in each program and the number of corresponding GBD locations they represent.

	**APOC Countries**	**OCP Countries**
Countries included	Angola, Burundi, Cameroon, Central African Republic, Chad, Congo, Democratic Republic of Congo, Ethiopia, Equatorial Guinea, Liberia, Malawi, Nigeria, South Sudan, Tanzania, and Uganda	Benin, Burkina Faso, Côte d'Ivoire, Ghana, Guinea Bissau, Guinea, Mali, Niger, Senegal, Sierra Leone, and Togo
Hypo-endemic countries not included	Rwanda, Mozambique, Kenya, Gabon, Sudan	
GBD countries & subnationals	15	11
GBD world regions	3	1

Prevalence data for modelling non-fatal outcomes resulting from onchocerciasis in the Americas was extracted via a systematic literature review. Web of Science, Scopus, and PubMed were searched with the following search strings:

**Database**	**Search string**	**Yield**
PubMed	(oncho*[Title/Abstract] OR "river blindness"[Title/Abstract] OR "O. volvulus"[Title/Abstract] OR "robles disease"[Title/Abstract] OR "blinding filariasis"[Title/Abstract] OR "coast erysipelas"[Title/Abstract] OR “sowda” [Title/Abstract] OR “nodding syndrome”[Title/Abstract]) AND (“1980”[Date – Publication] : “2016”[Date – Publication]) AND (epidemiology[Title/Abstract] OR prevalence[Title/Abstract] OR incidence[Title/Abstract] OR surveillance[Title/Abstract] OR”MDA”[Title/Abstract] OR “Mass Drug Administration”[Title/Abstract] OR “Community-directed treatment with ivermectin”[Title/Abstract] OR “CDTI”[Title/Abstract] OR “mass treatment”[Title/Abstract] OR “multiple ivermectin treatments”[Title/Abstract] OR “monthly doses of ivermectin”[Title/Abstract] OR “large scale treatment”[Title/Abstract] OR REMO[Title/Abstract] OR “Rapid epidemiological mapping of onchocerciasis”[Title/Abstract] OR APOC[Title/Abstract] OR “African Programme for Onchocerciasis Control”[Title/Abstract] OR OCP[Title/Abstract] OR “Onchocerciasis Control Programme”[Title/Abstract]) NOT(Animals[MeSH] NOT Humans[MeSH])	986
Web of Science	TS=(oncho* OR "river blindness" OR "O. volvulus" OR "robles disease" OR "blinding filariasis" OR "coast erysipelas" OR sowda OR “nodding syndrome”) AND TS=(epidemiology	1,144

	OR prevalence OR incidence OR surveillance OR MDA OR “Mass Drug Administration” OR “Community-directed treatment with ivermectin” OR CDTI OR “mass treatment” OR “multiple ivermectin treatments” OR “monthly doses of ivermectin” OR “large scale treatment” OR REMO OR “Rapid epidemiological mapping of onchocerciasis” OR APOC OR “African Programme for Onchocerciasis Control” OR OCP OR “Onchocerciasis Control Programme”) NOT TS=((Animals NOT Humans))	
SCOPUS	(TITLE-ABS-KEY(oncho* OR "river blindness" OR "O. volvulus" OR "robles disease" OR "blinding filariasis" OR "coast erysipelas")) AND TITLE-ABS-KEY(epidemiology OR prevalence OR incidence OR surveillance OR MDA OR "Mass Drug Administration" OR "Community-directed treatment with ivermectin" OR CDTI OR "mass treatment" OR "multiple ivermectin treatments" OR "monthly doses of ivermectin" OR "large scale treatment" OR REMO OR "Rapid epidemiological mapping of onchocerciasis" OR APOC OR "African Programme for Onchocerciasis Control" OR OCP OR "Onchocerciasis Control Programme") AND NOT KEY(Animals NOT Humans) AND PUBYEAR > 1979	2,000

This yielded 4,130 results in total which was reduced to 2,502 after removing duplicates. The title and abstracts were screened for inclusion or exclusion with the following criteria:

Exclusion Criteria:

- Pre-1980
- Non-original source
- Non-representative population
  - Vulnerable populations (eg, slum-dwellers, prisoners, orphans, high-risk jobs, etc.)
  - Hospital-based samples (including saved stool samples)
  - Non-native peoples (eg, migrants, expats, nomads, etc.)
  - Immunosuppression/illness (eg, HIV, TB, CA, RA, asthma, malaria, handicap, etc.)
- Non-human population
- Does not meet case definition
- Case-control study

Sixty-one articles were identified for full text screening and extraction from the historically endemic American countries: Guatemala, Brazil, Ecuador, Venezuela, Mexico, and Colombia.

Severity splits/sequelae

The table below shows the list of common clinical manifestations of onchocerciasis and the sequelae to which they have been mapped along with the lay description and the associated disability weight (DW) of each sequela.

Clinical manifestation	Sequela name	Lay description	DW
Uveitis; Punctate keratitis; Optic neuritis; Torpid Iritis; Onchochorioretinitis	Moderate vision impairment	has vision problems that make it difficult to recognize faces or objects across a room	0.031 (0.019–0.049)
Sclerosing keratitis; Optic neuropathy; Optic atrophy;	Severe vision impairment	has severe vision loss, which causes difficulty in daily activities, some emotional impact (for	0.184 (0.125–0.258)

Choroidoretinopathy; Cataracts		example worry), and some difficulty going outside the home without assistance	
Blindness	Blindness	is completely blind, which causes great difficulty in some daily activities, worry and anxiety, and great difficulty going outside the home without assistance	0.187 (0.124–0.260)
Acute papular onchodermatitis; Onchocercomata (subcutaneous nodules)	Mild skin disease	has a slight, visible physical deformity that is sometimes sore or itchy. Others notice the deformity, which causes some worry and discomfort	0.027 (0.015–0.042)
Chronic papular onchodermatitis; Lichenified obchodermatitis (“sowda”); Lymphadenopathy	Mild skin disease without itch	has a slight, visible physical deformity that others notice, which causes some worry and discomfort	0.011 (0.005–0.021)
Skin atrophy; Depigmentation (“leopard skin”)	Moderate skin disease	has a visible physical deformity that is sore and itchy. Other people stare and comment, which causes the person to worry. The person has trouble sleeping and concentrating	0.188 (0.124–0.267)
Hanging groin; Lymphoedema	Severe skin disease without itch	has an obvious physical deformity that makes others uncomfortable, which causes the person to avoid social contact, feel worried, sleep poorly, and think about suicide	0.405 (0.275–0.546)
	Asymptomatic onchocerciasis	NA	NA

Modelling strategy

The nonfatal modelling for onchocerciasis included four major steps. In the first step, GBD 2010 prevalence was extrapolated to obtain GBD 2016 estimates. Acute skin disease level 2 and chronic skin disease level 2 were summed to create the moderate skin disease sequela. Uncertainty was quantified and provided by the EG for all estimates except those of visual impairment and blindness. In these cases, each of the OCP draws the number of cases were multiplied by a random value (the exponent of a normally distributed variable with mean zero and standard deviation 0.1) in order to add uncertainty. Within each draw, the same randomly drawn value was applied to all country-year-age-sex estimates. Visual impairment was then split into moderate and severe vision impairment by first multiplying the visual impairment estimates by a random value (from a normal distribution with mean 0.84 and standard deviation 0.0031) to generate moderate vision impairment, and then subtracting the resulting estimates from visual impairment to obtain estimates of severe vision impairment. Prevalence of sequelae was calculated by dividing the cases by the population.

The second step in modelling morbidity due to onchocerciasis was the adjustment of uncertainty in the conversion of nodule prevalence to microfilaria (mf) prevalence and in the effects of mass drug administration (MDA). To adjust for uncertainty in translation of nodule prevalence to mf prevalence,

the final OCP draws from the first step were logit transformed and uncertainty was added from a random value drawn from a normal distribution to the transformed estimates. The resulting estimates were then normalized and scaled using estimates published elsewhere [1]. To adjust for uncertainty due to MDA, the year when MDA with Ivermectin started was set according to the table below.

Country	MDA start year
Angola, Burundi, South Sudan	2005
Congo, Ethiopia, DRC	2001
Cameroon, Central African Republic, Equatorial Guinea, Liberia, Nigeria, Uganda	1999
Chad, Niger, Tanzania	1998
Malawi	1997
All others	1990

The uncertainty in the time trend was then multiplied by the normalized prevalence estimates and the final prevalence was obtained by re-expanding the scaled normalized draws and adjusting the scale back from logit scale.

In the third step, prevalence of onchocerciasis in the Americas was modelled separately and combined with the Africa model. Onchocerciasis is known to have occurred in six countries of Central and Southern America: Mexico, Guatemala, Colombia, Ecuador, Brazil and Venezuela. The epidemiology of onchocerciasis is very different in these countries than in Africa because it has only occurred in relatively small, well defined foci. These foci have been mapped and thoroughly monitored since the early 1990s with the formation of the Onchocerciasis Elimination Program of the Americas (OEPA) and all of the prevalence surveys conducted are only representative of these areas. Additionally, certain foci are geographically continuous across national boundaries. Therefore, we modelled onchocerciasis in these countries at the foci level among the population at risk in each foci instead of at the national level. Population at risk for each focus was modelled using data from OEPA on baseline population at risk [6] and data from OEPA and peer reviewed studies on dates of elimination in each focus [6-19]. This was done with a Poisson model using year splines as a covariate and 1,000 draws of the population are risk were drawn from the predicted mean and standard error. The prevalence of disease among the population at risk was subsequently modeled using a generalized linear model with a binomial family, logit link, no intercept term and random effects on a combined-foci variable created by grouping foci by geographic contiguity and nearness when data was sparse. Covariates included an indicator term on the foci, the number of years since MDA began and splines on age. One thousand draws of prevalence were calculated form one thousand draws of beta values from the variance-covariance matrix and adjusted by the estimated population at risk in each foci-year to determine the number of cases. The cases were then summed by GBD location and year and divided by national population to find the national prevalence. While the model predicted case values very close to zero in the countries where elimination has occurred, these were overwritten to zero values for all years after certified elimination. The ratio of global all-age, all-sex prevalence of each sequelae to the all-cases prevalence from the Africa estimates was applied to all-cases prevalence from the Americas to calculate prevalence of each sequelae.

Lastly, to estimate the prevalence of asymptomatic onchocerciasis, the prevalence of morbidity (vision loss, blindness and skin conditions) was subtracted from the overall onchocerciasis prevalence.

Moderate vision impairment, severe vision impairment and blindness estimates were each multiplied by a factor of 8/33 before subtraction to account for cases that have concurring symptoms.

Model evaluation was done by separately assessing plots of time trends of prevalence across locations and age for each sequela. In addition, maps of the global distribution of total onchocerciasis prevalence and prevalence of sequelae due to onchocerciasis were also assessed across time.

Changes from GBD 2015 to GBD 2016

Prevalence of onchocerciasis in foci in the Americas was not previously included but is now being modelled.

References

7. Zouré HG, Noma M, Tekle AH, Amazigo UV, Diggle PJ, Giorgi E, Remme JH. The geographic distribution of onchocerciasis in the 20 participating countries of the African Programme for Onchocerciasis Control: (2) pre-control endemicity levels and estimated number infected. Parasites & Vectors. 2014. 7-326.
8. Coffeng L, Stolk W, Hoerauf A, Habbema D, Bakker R, Hopkins A, de Vlas S. Elimination of African onchocerciasis: modeling the impact of increasing the frequency of ivermectin mass treatment. PLoS One. 2014. 9(12):e115886.
9. Coffeng LE, Stolk WA, Zouré HG, Veerman JL, Agblewonu KB, Murdoch ME, Noma M, Fobi G, Richardus JH, Bundy DA, Habbema D, de Vlas SJ, Amazigo UV. African Programme For Onchocerciasis Control 1995-2015: model-estimated health impact and cost. PLoS Negl Trop Dis. 2013; 7(1): e2032.
10. Murdoch ME, Asuzu MC, Hagan M, Makunde WH, Ngoumou P, Ogbuagu KF, Okello D, Ozoh G, Remme J. Onchocerciasis: the clinical and epidemiological burden of skin disease in Africa. Ann Trop Med Parasitol. 2002; 96(3): 283-296.
11. Brieger WR, Awedoba AK, Eneanya CI, Hagan M, Ogbuagu KF, Okello DO, Ososanya OO, Ovuga EB, Noma M, Kale OO, Burnham GM, Remme JH. The effects of ivermectin on onchocercal skin disease and severe itching: results of a multicentre trial. Trop Med Int Health. 1998; 3(12): 951-61.
12. México. http://www.oepa.net/Mexico.htm (accessed July 7, 2017).
13. Guatemala. http://www.oepa.net/guatemala.html (accessed July 7, 2017).
14. Venezuela. http://www.oepa.net/venezuela.html (accessed July 7, 2017).
15. Colombia. http://www.oepa.net/colombia.html (accessed July 7, 2017).
16. Ecuador. http://www.oepa.net/ecuador.html (accessed July 7, 2017).
17. Rodríguez-Pérez MA, Unnasch TR, Domínguez-Vázquez A, *et al.* Lack of Active Onchocerca volvulus Transmission in the Northern Chiapas Focus of Mexico. *The American Journal of Tropical Medicine and Hygiene* 2010; **83**: 15–20.
18. Rodríguez-Pérez MA, Domínguez-Vázquez A, Unnasch TR, *et al.* Interruption of Transmission of Onchocerca volvulus in the Southern Chiapas Focus, México. *PLOS Neglected Tropical Diseases* 2013; **7**: e2133.
19. Rodríguez-Pérez MA, Unnasch TR, Domínguez-Vázquez A, *et al.* Interruption of Transmission of Onchocerca volvulus in the Oaxaca Focus, Mexico. *The American Journal of Tropical Medicine and Hygiene* 2010; **83**: 21–7.

20. Cruz-Ortiz N, Gonzalez RJ, Lindblade KA, *et al.* Elimination of Onchocerca volvulus Transmission in the Huehuetenango Focus of Guatemala. Journal of Parasitology Research. 2012. https://www.hindawi.com/journals/jpr/2012/638429/abs/ (accessed July 7, 2017).
21. Jr FR, Rizzo N, Espinoza CED, *et al.* One Hundred Years After Its Discovery in Guatemala by Rodolfo Robles, Onchocerca volvulus Transmission Has Been Eliminated from the Central Endemic Zone. *The American Journal of Tropical Medicine and Hygiene* 2015; **93**: 1295–304.
22. Gonzalez RJ, Cruz-Ortiz N, Rizzo N, *et al.* Successful interruption of transmission of Onchocerca volvulus in the Escuintla-Guatemala focus, Guatemala. *PLoS Negl Trop Dis* 2009; **3**: e404.
23. Lindblade KA, Arana B, Zea-Flores G, *et al.* Elimination of Onchocercia volvulus transmission in the Santa Rosa focus of Guatemala. *Am J Trop Med Hyg* 2007; **77**: 334–41.
24. Convit J, Schuler H, Borges R, *et al.* Interruption of Onchocerca volvulus transmission in Northern Venezuela. *Parasites & Vectors* 2013; **6**: 289.
25. WHO | WHO declares Ecuador free of onchocerciasis (river blindness). WHO. http://www.who.int/neglected_diseases/ecuador_free_from_onchocerciasis/en/ (accessed July 7, 2017).

Dengue

Flowchart

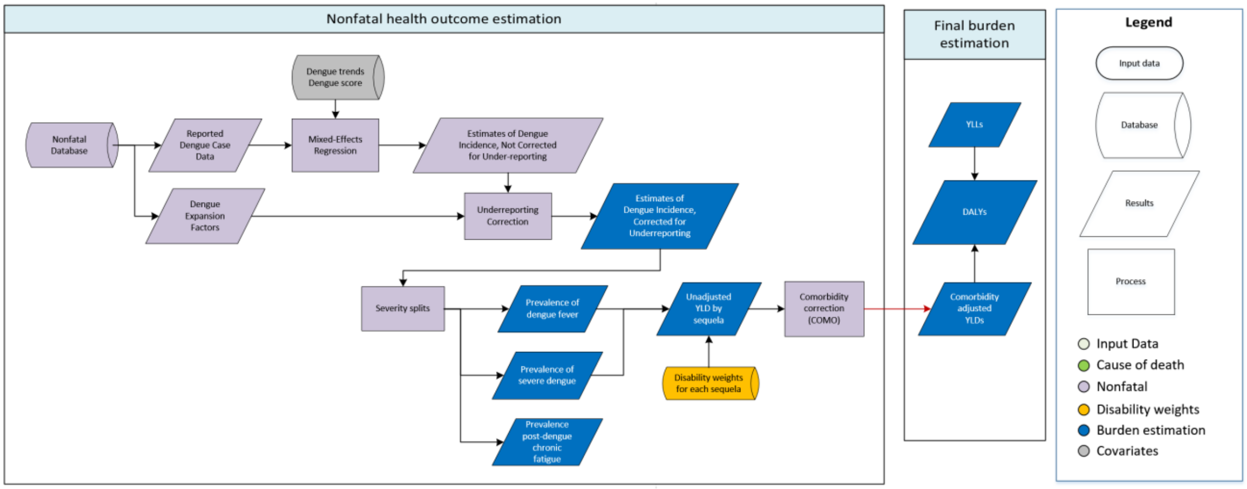

Case definition

Dengue is mosquito-borne viral infection that causes febrile illness and, in severe cases, jaundice, hemorrhage, and death. It includes all ICD-10 codes under the heading A90 (Dengue fever [classical dengue]) and A91 (Dengue hemorrhagic fever).

Input data

Model inputs

For GBD 2016, we modelled dengue incidence based on officially reported cases. The table below illustrates the geographic distribution of data points used in our analysis.

Table 1. Geographies

Level	Incidence
Data points	2,920
Studies	70
Locations	201
Regions	15

While no systematic update was conducted, we did incorporate new expansion factor data that were provided by collaborators and have updated to the latest available case reports for GBD 2016.

Modelling strategy

The methods used to model dengue incidence remain unchanged from GBD 2015, and are an improved variant of the methods used for GBD 2013 that were described by Stanaway et al. Briefly, we derive two dengue-specific covariates: first a variable to define the expected spatial distribution of the disease based on principal components analysis of dengue CSMR estimates and dengue transmission probability and, second, a variable to define the country-specific trends, based on a mixed-effects model of reported cases. We then estimate a mixed-effects negative binomial model with number of reported cases as the dependent variable, fixed effects on the aforementioned spatial and temporal covariates, and random effects on location. These random effects are assumed to correspond to deviations in reporting completeness and, calibrating against published expansion factor data (ie, estimates of the degree of underreporting), they are inflated to adjust for underreporting estimates. The resulting incidence estimates are split into moderate (94.5%) and severe (5.5%) sequelae, based on the proportion of reported cases that were severe. We assume that 8.4% of symptomatic infections will produce post-acute chronic fatigue lasting an average of six months (Teixeira L de AS, Lopes JSM, Martins AG da C, Campos FAB, Miranzi S de SC, Nascentes GAN. Persistence of dengue symptoms in patients in Uberaba, Minas Gerais State, Brazil. *Cad Saúde Pública* 2010; **26**: 624–30.).

Severity splits and disability weights

Table 2. Sequelae, lay descriptions, and DWs

Sequela	Lay description	Disability Weight (DW)
Moderate	Has a fever and aches, and feels weak, which causes some difficulty with daily activities.	0.051 (0.032–0.074)
Severe	Has a high fever and pain, and feels very weak, which causes great difficulty with daily activities.	0.133 (0.088–0.19)
Asymptomatic	Infection with no apparent illness.	NA

Changes from GBD 2015 to GBD 2016

We have made no substantive changes in the modelling strategy from GBD 2015 to GBD 2016.

References

1. Stanaway JD, Shepard DS, Undurraga EA, Halasa YA, Coffeng LE, Brady OJ, et al. The global burden of dengue: an analysis from the Global Burden of Disease Study 2013. *The Lancet Infectious Diseases* [Internet]. 2016 Feb [cited 2016 May 23].

2. Bhatt S, Gething PW, Brady OJ, Messina JP, Farlow AW, Moyes CL, et al. The global distribution and burden of dengue. *Nature*. 2013 Apr 25;496(7446):504–7.

Yellow Fever

Flowchart

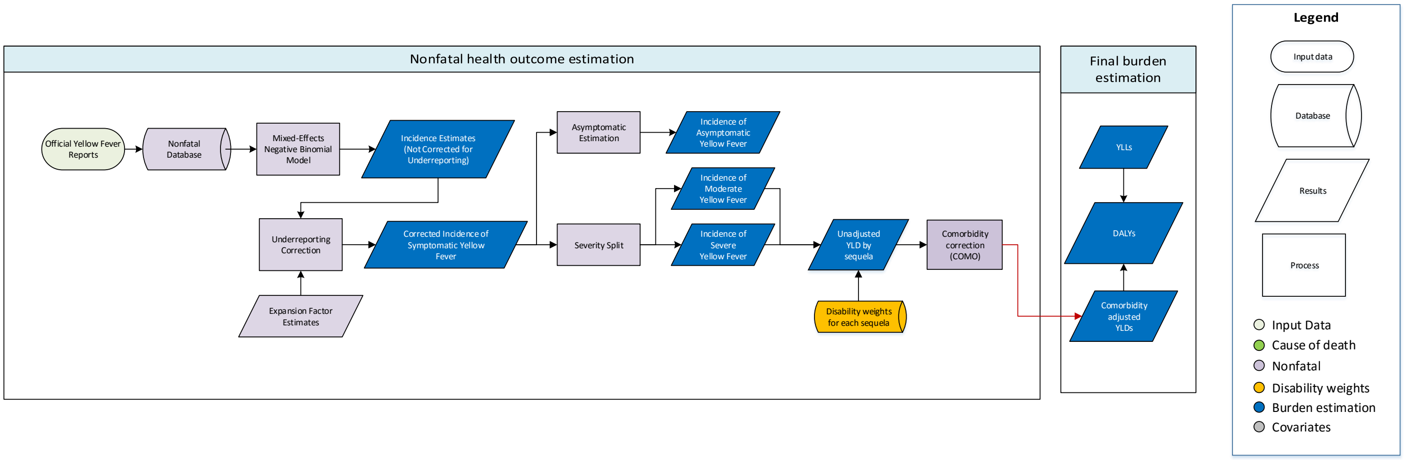

Case definition

Yellow fever is mosquito-borne viral infection that causes febrile illness and, in severe cases, jaundice, haemorrhage, and death. It is considered a neglected tropical disease (NTD). It includes all ICD-10 codes under the heading A95 (yellow fever).

Input data

Model inputs

Case data for the yellow fever estimate process comes from official case reports filed with the World Health Organization. The table below shows the distribution of said data geographically for the GBD 2016 estimation process.

Table 1. Data spread

Level	Incidence
Data points	909
Studies	19
Locations	47
Regions	9

We have updated to the latest available case reports for GBD 2016.

Severity splits

Yellow fever is split into three levels of severity: moderate (33% [13–52]), severe (12% [5–26]), and asymptomatic (55% [37–74]). The table below illustrates this breakdown.

Table 2. Sequela, description, and disability weight (DW)

Sequela	Description	Disability weight (DW)
Moderate	Has a fever and aches, and feels weak, which causes some difficulty with daily activities.	0.051 (0.032–0.074)
Severe	Has a high fever and pain, and feels very weak, which causes great difficulty with daily activities.	0.133 (0.088–0.19)
Asymptomatic	Infection with no apparent illness.	NA

Modelling strategy

We modelled reported cases of yellow fever using a mixed-effects negative binomial model, with fixed effects for year and random effects for super-region, region, and country. We assume that yellow fever cases are underreported, and that this underreporting mirrors that for dengue (a disease for which we have better data on underreporting). With that, we estimate symptomatic cases as the product of our base case estimates and dengue expansion factors (ie, the factor by which you must multiply reported cases to derive true cases). Based on published estimates from Johansson et al (2014), we assume that 27% of symptomatic cases will be severe.

Changes from GBD 2015 to GBD 2016

We have made no substantive changes in the modelling strategy for endemic countries from GBD 2015 to GBD 2016.

Rabies

Flowchart

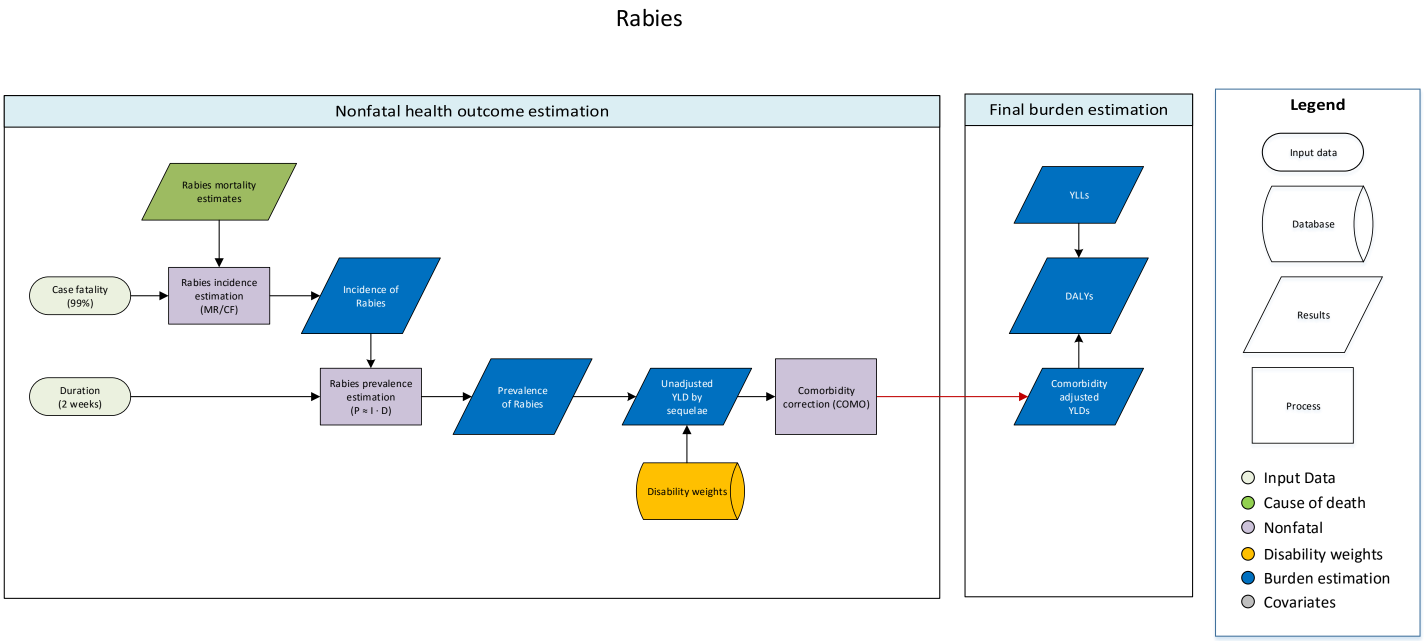

Input data and methodological summary

Case definition

Rabies is a fatal viral infection, transmitted by animal bites. Without prophylactic vaccination the disease is almost universally fatal. The disease has a long incubation period (1-3 months), and early intervention with prophylactic vaccination is nearly 100% effective in preventing symptomatic disease. It is considered a neglected tropical disease (NTD). We model symptomatic infections, not including those infections in which intervention prevented the onset of symptomatic disease, corresponding to the ICD10 code A82.

Input data

Model inputs

As we derive our estimate of cases from our estimate of deaths, no incidence data are used in the model. For GBD 2016, we modelled rabies mortality using all available data in the cause of death database. Data points were outliered if they reported an improbable number of rabies deaths (eg, zero rabies deaths in a hyperendemic country) or if their inclusion in the model yielded distorted trends. In some cases multiple data sources for the same location differed dramatically both in their quality and reported rabies mortality (eg, a verbal autopsy and vital registration source). In these cases the lower-quality data source was outliered.

Modelling strategy

We derive estimates of the number of symptomatic rabies infections (ie, those not averted through prophylactic vaccination) based on rabies mortality estimates, assuming 99% case fatality. All cases are assumed to be severe.

We modelled rabies mortality using a two-model hybrid approach 1) a global CODEm model of all locations, using all data in the CoD database; and 2) a CODEm model restricted to data-rich countries.

Sequela description and DW

There is only one sequela and associated disability weight for rabies, which is severe. The lay description is included in the table below.

Table 2. Sequela, description, and DW

Sequela	Description	Disability Weight (95% CI)
Severe	Has a high fever and pain, and feels very weak, which causes great difficulty with daily activities.	0.133 (0.088–0.19)

Changes from GBD 2015 to GBD 2016

We have made no substantive changes in the modelling strategy for rabies from GBD 2015.

Ascariasis

Flowchart

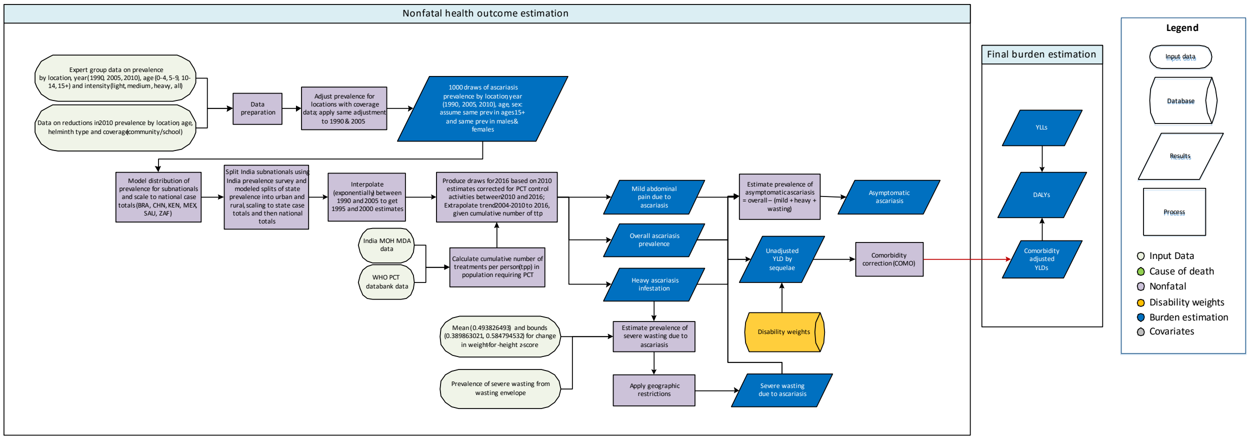

Input data and methodological summary

Case definition

Ascariasis is a helminthic disease caused by the parasitic roundworm *Ascaris lumbricoides*. It is one of the three intestinal nematode infections (INI), or soil-transmitted helminthiasis (STH), that are modelled in GBD. Diagnosis is made by examination of stool by microscope or PCR, with or without concentration procedures. The ICD-10 codes for ascariasis are B77-B77.9.

Input data

Expert Group Data

Input data for this model was compiled from various sources. Prevalence data prepared by the GBD expert group (EG) during GBD 2010 [1, 2] contained mean prevalence with confidence intervals, stratified by location, year (1990, 2005, 2010), age group (0-4, 5-9, 10-14, 15+ years) and intensity of infection (light, medium, heavy, all). For the model, light infestation was not attributed any disability. The expert group also provided data on reduction of prevalence in 2010, stratified by location, age group and Mass Drug Administration (MDA) coverage strategy (community/school). The table below shows the number of countries or subnationals units and GBD world regions represented in the data.

Table 1a. Geographic Spread of Expert Group Prevalence Data

	prevalence
Countries/subnationals	160
GBD world regions	17

Mass Drug Administration

Data on national MDA program coverage was downloaded from the WHO PCT Databank source site [3]. This data spanned 119 GBD locations over 13 years beginning in 2003.

India Ministry of Health

Collaborators from the Indian Ministry of Health provided supplementary prevalence data [4] and MDA coverage data for India [5]. This data was available for all Indian states but was not available at the most detailed GBD location specification for the country: urban and rural. Data on MDA coverage was available for the years 2013-2016 while prevalence data was only available for 2015.

Severe Wasting Estimates

To inform the wasting model, 1,000 draws of severe wasting prevalence among children under 5 years were ascertained from GBD 2016 estimates – the methods used to generate estimates of wasting prevalence are detailed elsewhere (part of risk factors documentation) [6].

Geographic Restrictions

We conducted a literature review to determine the geographic extent of the disease and classify locations based on whether the disease is absent or present in each year. Locations that were geographically restricted in any given year did not have estimates made for them but could have imported cases attributed to them at a later stage. Of note, we did not attempt a complete systematic review, since a single high-quality source could offer sufficient evidence of presence. Evidence of absence or presence was not available for every location for each year and so assumptions were made for missing years by taking into consideration the epidemiological characteristics of the disease. If evidence indicated disease presence for two non-consecutive years, we assumed presence for all years between the two. If evidence indicated disease absence for two non-consecutive years, we assumed absence for all years between the two. If evidence indicated a change in status (i.e. from absent to present, or present to absent) between two non-consecutive years than we conducted targeted searches to ascertain the relevant year of introduction or elimination for that location. In the cases where presence or absence information was missing for the start or end years of our study interval (1990-2016) without evidence of any introduction or elimination events within the interval, we applied the status of the first and last presence/absence observations respectively to all years between the interval bound and the observation year. Our search was done in conjunction with the title/abstract screening portion of a systematic literature review for prevalence data. The search strings and yield can be viewed in the table below for each of the databases queried.

Table 1b. Geographic Restriction Search Strings

Database	Search String	Yield
PubMed	(Ascariasis[Title/Abstract] OR Ascaris[Title/Abstract] OR "A. lumbricoides"[Title/Abstract] OR Ascaris[MeSH] OR Trichuris[Title/Abstract] OR Trichuriasis[Title/Abstract] OR "Whip Worm"[Title/Abstract] OR "T. trichura"[Title/Abstract] OR Trichuris[MeSH] OR Hookworm[Title/Abstract] OR "A. duodenale"[Title/Abstract] OR "Ancylostoma duodenale"[Title/Abstract] OR ancylostomiasis[Title/Abstract] OR "N. americanus"[Title/Abstract] OR "Necator americanus"[Title/Abstract] OR necatoriasis[Title/Abstract] OR Ancylostoma [MeSH] OR Necator[MeSH]) AND (prevalence[Title/Abstract] OR incidence[Title/Abstract] OR epidemiology[Title/Abstract] OR surveillance[Title/Abstract]) NOT(Animals[MeSH] NOT Humans[MeSH])	2376
Web of Science	(Ascariasis OR Ascaris OR A. lumbricoides OR Trichuris OR Trichuriasis OR Whip Worm OR T. trichura OR Hookworm OR A. duodenale OR Ancylostoma duodenale OR anclyostomiasis OR N. americanus OR Necator americanus OR	2266

	necatoriasis) AND TOPIC:(prevalence OR incidence OR epidemiology OR surveillance) NOTTOPIC: ((Animals NOT Humans))<br>Timespan: 1980-2016. Indexes: SCI-EXPANDED, SSCI, A&HCI, ESCI.	
SCOPUS	TITLE-ABS_KEY (ascariasis OR ascaris OR a. lumbricoides OR trichuris OR trichuriasis OR whip worm OR t. trichura OR hookworm OR a. duodenale OR ancylostoma duodenale OR anclyostomiasis OR n. americanus OR necator americanus OR necatoriasis) AND PUBYEAR>1979	29

These papers were used to classify location-years for all locations and years present in the literature. Additionally, systematic literature reviews, meta-analyses, national health statistics publications and collaborator input were used to classify location-years not present in the literature review wherever possible.

Severity splits/sequelae

The table below shows the list of sequelae due to ascariasis and the associated disability weights (DW). Prevalence of medium infection and heavy infection were mapped to *mild abdominopelvic problems* and *heavy infestation of ascariasis* respectively. Light infection was not attributed any disability.

Table 2. Sequelae, lay descriptions, and disability weights (DWs)

Sequela	Lay description	DW
Mild abdominopelvic problems	has some pain in the belly that causes nausea but does not interfere with daily activities	0.011 (0.005–0.021)
Heavy infestation	has cramping pain and a bloated feeling in the belly	0.027 (0.015–0.043)
Severe wasting	is extremely skinny and has no energy	0.128 (0.082–0.183)
Asymptomatic ascariasis	N/A	N/A

Modelling strategy

In the estimation of morbidity due to ascariasis, the EG data were first prepared by formatting the location names to be consistent with the GBD 2016 location names and applying the 2010 prevalence to 1990 and 2005 for sub-Saharan Africa countries – estimates for these two years were missing. This was followed by using the data on reductions in 2010 prevalence to adjust the prevalence for locations with coverage data. After this adjustment, only data for medium infection, heavy infection, and all infection were retained.

Using the mean prevalence and the upper and lower bounds of the mean provided by the EG, 1,000 draws of prevalence were generated. This was done by multiplying the mean estimates by the exponent of random draws from a normal distribution with mean = 0 and standard deviation = sd, where sd = abs(abs(ln(upper)-ln(lower))/(invnormal(0.975)*2). These draws were created for all GBD age-groups, assuming the same prevalence in ages 15+ and same prevalence in males and females.

Since the draws were only at the national level, subnational locations were modelled for Brazil, China, Indonesia, Kenya, Mexico, Saudi Arabia, and South Africa. This was done using a generalized linear model with a binomial family, logit link and robust standard errors. The predicted cases for all subnational locations were summed and scaled to the national case total. India subnationals were separately derived by applying the proportion of all national cases in each state from the Indian Ministry of Health data [4] to

the national total. We then applied the modelled proportion of cases in urban or rural locations such that the sum of urban and rural cases in a state equals the state total and the sum of all state cases equals the national total.

To get 1995 and 2000 estimates, exponential interpolation of estimates between 1990 and 2005 was performed. The draws for 2016 were produced based on 2010 estimates corrected for PCT control activities between 2010 and 2016 – this was done by extrapolating the 2004–2010 trend to 2016, given cumulative number of treatments per person calculated using data from the WHO PCT Databank [3] and the Indian Ministry of Health MDA coverage data[5]. The 2004-2010 trend was applied to all intensities of infection. Prevalence was assumed to be zero for the countries with missing input data and also in children younger than 28 days. The resulting estimates were 1,000 draws of ascariasis prevalence by GBD location, year, age, sex, and intensity level (mild, heavy, overall infection). To estimate the prevalence of asymptomatic ascariasis, prevalence of mild and heavy infestation was subtracted from the overall ascariasis prevalence.

The final step in the modelling process was to estimate the prevalence of severe wasting due to ascariasis in age groups 28–364 days and 1–4 years. This was done separately using 1,000 draws of prevalence of heavy infestation due to ascariasis and the wasting envelope prevalence. The initial step in determining prevalence of severe wasting due to ascariasis was generating 1,000 draws of change in weight-for-height z-score per heavy prevalent case from a random normal distribution with mean = 0.493826493 and standard deviation = 0.04972834 (calculated from upper and lower bounds of the mean estimate). The mean, upper, and lower bounds were provided by a GBD collaborator who calculated them based on a published article [6]. The prevalence of severe wasting due to ascariasis was then obtained as a function of change in weight-for-height z-score (z_change) such that prevalence = p_wasting_env – Phi(Phi_inv(p_wasting_env) – z_change*p), where p_wasting_env = wasting envelope prevalence, Phi_inv is the inverse standard normal cumulative distribution function (cdf), and p = prevalence of heavy ascariasis infestation. Finally, geographic restrictions were applied to set prevalence to zero in countries with wasting that are not endemic for ascariasis.

Model evaluation was done by plotting prevalence of overall ascariasis and that of each sequela against year for each location and age group. Maps of the global distribution of total ascariasis prevalence and prevalence of sequelae due to ascariasis were also assessed across time and age.

Only minor changes were made to the GBD 2015 modelling strategy including the incorporation of updated data from the WHO PCT databank [3] and data from the Indian Ministry of Health, new modelling of subnational prevalence distribution and new geographic restrictions.

References

26. Brooker S, Pullan R, Smith J, and Hotez P. Chapter: Intestinal nematodes. Cluster D: Communicable Diseases, Neglected Tropical Diseases Group. Global Burden of Diseases, Injuries, and Risk Factors Study. 2011 (4 July). 1-24.
27. Brooker S & Smith JL. Impact of hookworm infection and deworming on anaemia in non-pregnant populations: a systematic review. *Tropical Medicine and International Health*. 2010. 15,7,776-795.

28. WHO | PCT databank. WHO. http://www.who.int/neglected_diseases/preventive_chemotherapy/sth/en/ (accessed July 7, 2017).
29. Prevalence data. GBD India Collaborators. Personal Communication.
30. MDA Coverage data. GBD India Collaborators. Personal Communication.
31. Hall A, Hewitt G, Tuffrey V, de Silva N. A review and meta-analysis of the impact of intestinal worms on child growth and nutrition. *Maternal and Child Nutrition*. 2008. 4. 118-236.

Trichuriasis

Flowchart

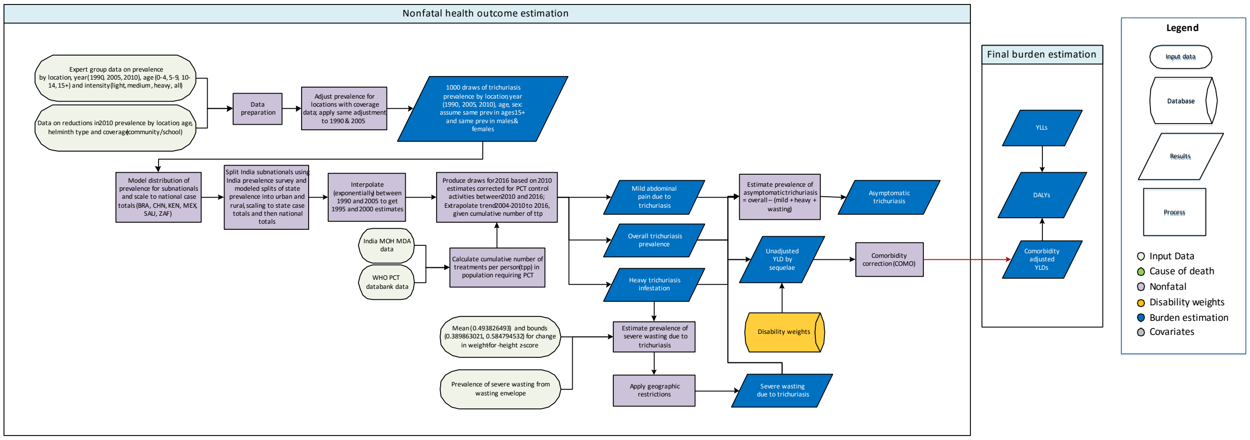

Input data and methodological summary

Case definition

Trichuriasis is a helminth diseases caused by the parasitic roundworm *Trichuris trichiura*. It is one of the three intestinal nematode infections (INI), or soil-transmitted helminthiasis (STH), that we model in GBD. Diagnosis is made by examination of stool by microscope or PCR, with or without concentration procedures. The ICD-10 code for trichuriasis is B79.

Input data

Expert Group Data

Input data for this model was compiled from various sources. Prevalence data prepared by the GBD expert group (EG) during GBD 2010 [1, 2] contained mean prevalence with confidence intervals, stratified by location, year (1990, 2005, 2010), age group (0-4, 5-9, 10-14, 15+ years) and intensity of infection (light, medium, heavy, all). For the model, light infestation was not attributed any disability. The expert group also provided data on reduction of prevalence in 2010, stratified by location, age group and Mass Drug Administration (MDA) coverage strategy (community/school). The table below shows the number of countries or subnational units and GBD world regions represented in the data.

Table 1a. Geographic Spread of Expert Group Prevalence Data

	prevalence
Countries/subnationals	160
GBD world regions	17

Mass Drug Administration

Data on national MDA program coverage was downloaded from the WHO PCT Databank source site [3]. This data spanned 119 GBD locations over 13 years beginning in 2003.

India Ministry of Health

Collaborators from the Indian Ministry of Health provided supplementary prevalence data [4] and MDA coverage data for India [5]. This data was available for all Indian states but was not available at the most detailed GBD location specification for the country: urban and rural. Data on MDA coverage was available for the years 2013-2016 while prevalence data was only available for 2015.

Severe Wasting Estimates

To inform the wasting model, 1,000 draws of severe wasting prevalence among children under 5 years were ascertained from GBD 2016 estimates – the methods used to generate estimates of wasting prevalence are detailed elsewhere (part of risk factors documentation) [6].

Geographic Restrictions

We conducted a literature review to determine the geographic extent of the disease and classify locations based on whether the disease is absent or present in each year. Locations that were geographically restricted in any given year did not have estimates made for them but could have imported cases attributed to them at a later stage. Of note, we did not attempt a complete systematic review, since a single high-quality source could offer sufficient evidence of presence. Evidence of absence or presence was not available for every location for each year and so assumptions were made for missing years by taking into consideration the epidemiological characteristics of the disease. If evidence indicated disease presence for two non-consecutive years, we assumed presence for all years between the two. If evidence indicated disease absence for two non-consecutive years, we assumed absence for all years between the two. If evidence indicated a change in status (i.e. from absent to present, or present to absent) between two non-consecutive years than we conducted targeted searches to ascertain the relevant year of introduction or elimination for that location. In the cases where presence or absence information was missing for the start or end years of our study interval (1990-2016) without evidence of any introduction or elimination events within the interval, we applied the status of the first and last presence/absence observations respectively to all years between the interval bound and the observation year. Our search was done in conjunction with the title/abstract screening portion of a systematic literature review for prevalence data. The search strings and yield can be viewed in the table below for each of the databases queried.

Table 1b. Geographic Restriction Search Strings

Database	Search String	Yield
PubMed	(Ascariasis[Title/Abstract] OR Ascaris[Title/Abstract] OR "A. lumbricoides"[Title/Abstract] OR Ascaris[MeSH] OR Trichuris[Title/Abstract] OR Trichuriasis[Title/Abstract] OR "Whip Worm"[Title/Abstract] OR "T. trichura"[Title/Abstract] OR Trichuris[MeSH] OR Hookworm[Title/Abstract] OR "A. duodenale"[Title/Abstract] OR "Ancylostoma duodenale"[Title/Abstract] OR ancylostomiasis[Title/Abstract] OR "N. americanus"[Title/Abstract] OR "Necator americanus"[Title/Abstract] OR necatoriasis[Title/Abstract] OR Ancylostoma [MeSH] OR Necator[MeSH]) AND (prevalence[Title/Abstract] OR	2376

	incidence[Title/Abstract] OR epidemiology[Title/Abstract] OR surveillance[Title/Abstract]) NOT(Animals[MeSH] NOT Humans[MeSH])	
Web of Science	(Ascariasis OR Ascaris OR A. lumbricoides OR Trichuris OR Trichuriasis OR Whip Worm OR T. trichura OR Hookworm OR A. duodenale OR Ancylostoma duodenale OR anclyostomiasis OR N. americanus OR Necator americanus OR necatoriasis) AND TOPIC:(prevalence OR incidence OR epidemiology OR surveillance) NOTTOPIC: ((Animals NOT Humans))<br>Timespan: 1980-2016. Indexes: SCI-EXPANDED, SSCI, A&HCI, ESCI.	2266
SCOPUS	TITLE-ABS_KEY (ascariasis OR ascaris OR a. lumbricoides OR trichuris OR trichuriasis OR whip worm OR t. trichura OR hookworm OR a. duodenale OR ancylostoma duodenale OR anclyostomiasis OR n. americanus OR necator americanus OR necatoriasis) AND PUBYEAR>1979	29

These papers were used to classify location-years for all locations and years present in the literature. Additionally, systematic literature reviews, meta-analyses, national health statistics publications and collaborator input were used to classify location-years not present in the literature review wherever possible.

Severity splits/sequelae

The table below shows the list of sequelae due to trichuriasis and the associated disability weights (DW). Prevalence of medium infection and heavy infection were mapped to *mild abdominopelvic problems* and *heavy infestation of trichuriasis* respectively. Light infection was not attributed any disability.

Sequela	Lay description	DW (95% CI)
Mild abdominopelvic problems	has some pain in the belly that causes nausea but does not interfere with daily activities	0.011 (0.005–0.021)
Heavy infestation	has cramping pain and a bloated feeling in the belly	0.027 (0.015–0.044)
Severe wasting	is extremely skinny and has no energy	0.128 (0.082–0.183)
Asymptomatic trichuriasis	N/A	N/A

In the estimation of morbidity due to trichuriasis, the EG data were first prepared by formatting the location names to be consistent with the GBD 2016 location names and applying the 2010 prevalence to 1990 and 2005 for sub-Saharan Africa countries – estimates for these two years were missing. This was followed by using the data on reductions in 2010 prevalence to adjust the prevalence for locations with coverage data. After this adjustment, only data for medium infection, heavy infection, and all infection were retained.

Using the mean prevalence and the upper and lower bounds of the mean provided by the EG, 1,000 draws of prevalence were generated. This was done by multiplying the mean estimates by the exponent of random draws from a normal distribution with mean = 0 and standard deviation = sd, where sd = abs(abs(ln(upper)-ln(lower))/(invnormal(0.975)*2). These draws were created for all GBD age-groups, assuming the same prevalence in ages 15+ and same prevalence in males and females.

Since the draws were only at the national level, subnational locations were modelled for Brazil, China, Indonesia, Kenya, Mexico, Saudi Arabia, and South Africa. This was done using a generalized linear model with a binomial family, logit link and robust standard errors. The predicted cases for all subnational

locations were summed and scaled to the national case total. India subnationals were separately derived by applying the proportion of all national cases in each state from the Indian Ministry of Health data [4] to the national total. We then applied the modelled proportion of cases in urban or rural locations such that the sum of urban and rural cases in a state equals the state total and the sum of all state cases equals the national total.

To get 1995 and 2000 estimates, exponential interpolation of estimates between 1990 and 2005 was performed. The draws for 2016 were produced based on 2010 estimates corrected for PCT control activities between 2010 and 2016 – this was done by extrapolating the 2004–2010 trend to 2016, given cumulative number of treatments per person calculated using data from the WHO PCT Databank [3] and the Indian Ministry of Health MDA coverage data[5]. The 2004-2010 trend was applied to all intensities of infection. Prevalence was assumed to be zero for the countries with missing input data and also in children younger than 28 days. The resulting estimates were 1,000 draws of trichuriasis prevalence by GBD location, year, age, sex, and intensity level (mild, heavy, overall infection). To estimate the prevalence of asymptomatic trichuriasis, prevalence of mild and heavy infestation was subtracted from the overall trichuriasis prevalence.

Erroneous outliers in the original prevalence data in several countries were dealt with by replacing national prevalence with the associated regional prevalence and re-assigning all heavy infection cases to mild infection in countries with consistently operating MDA programs. This applied to countries where original prevalence data produced more than 100 YLDs per 100,000 people including Kiribati, Marshall Islands, Malaysia, Philippines, Swaziland and South Africa. These were significant outliers and not thought plausible according to available literature.

The final step in the modelling process was to estimate the prevalence of severe wasting due to trichuriasis in age groups 28–364 days and 1–4 years. This was done separately using 1,000 draws of prevalence of heavy infestation due to trichuriasis and the wasting envelope prevalence. The initial step in determining prevalence of severe wasting due to trichuriasis was generating 1,000 draws of change in weight-for-height z-score per heavy prevalent case from a random normal distribution with mean = 0.493826493 and standard deviation = 0.04972834 (calculated from upper and lower bounds of the mean estimate). The mean, upper, and lower bounds were provided by a GBD collaborator who calculated them based on a published article [6]. The prevalence of severe wasting due to trichuriasis was then obtained as a function of change in weight-for-height z-score (z_change) such that prevalence = p_wasting_env – Phi(Phi_inv(p_wasting_env) – z_change*p), where p_wasting_env = wasting envelope prevalence, Phi_inv is the inverse standard normal cumulative distribution function (cdf), and p = prevalence of heavy trichuriasis infestation. Finally, geographic restrictions were applied to set prevalence to zero in countries with wasting that are not endemic for trichuriasis.

Model evaluation was done by plotting prevalence of overall trichuriasis and that of each sequela against year for each location and age group. Maps of the global distribution of total trichuriasis prevalence and prevalence of sequelae due to trichuriasis were also assessed across time and age.

Only minor changes were made to the GBD 2015 modelling strategy including the incorporation of updated data from the WHO PCT databank [3] and data from the Indian Ministry of Health, new modelling of subnational prevalence distribution and new geographic restrictions.

References

32. Brooker S, Pullan R, Smith J, and Hotez P. Chapter: Intestinal nematodes. Cluster D: Communicable Diseases, Neglected Tropical Diseases Group. Global Burden of Diseases, Injuries, and Risk Factors Study. 2011 (4 July). 1-24.
33. Brooker S & Smith JL. Impact of hookworm infection and deworming on anaemia in non-pregnant populations: a systematic review. *Tropical Medicine and International Health*. 2010. 15,7,776-795.
34. WHO | PCT databank. WHO. http://www.who.int/neglected_diseases/preventive_chemotherapy/sth/en/ (accessed July 7, 2017).
35. Prevalence data. GBD India Collaborators. Personal Communication.
36. MDA Coverage data. GBD India Collaborators. Personal Communication.
37. Hall A, Hewitt G, Tuffrey V, de Silva N. A review and meta-analysis of the impact of intestinal worms on child growth and nutrition. *Maternal and Child Nutrition*. 2008. 4. 118-236.

Hookworm Disease

Flowchart

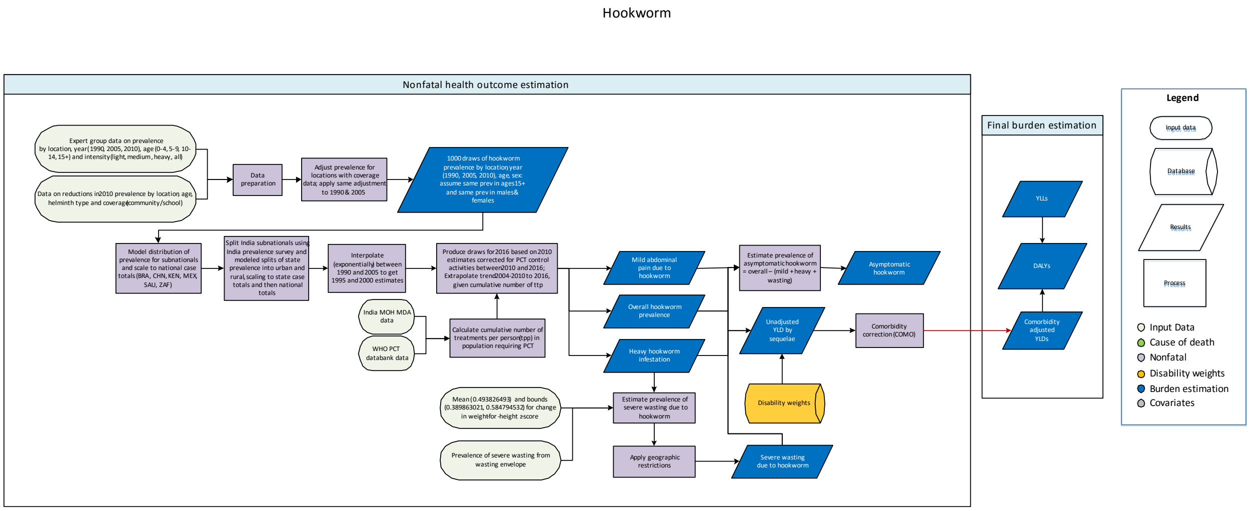

Input data and methodological summary

Case Definition

Hookworm disease is a helminthic disease caused by the parasitic roundworms, *Ancylostoma duodenale* and *Necator americanus*. It is one of the three intestinal nematode infections (INI), or soil-transmitted helminthiasis (STH), that we model in GBD. Diagnosis is made by examination of stool by microscope or PCR, with or without concentration procedures. The ICD-10 codes for hookworm disease are B76-B76.9.

Input data

Expert Group Data

Input data for this model was compiled from various sources. Prevalence data prepared by the GBD expert group (EG) during GBD 2010 [1, 2] contained mean prevalence with confidence intervals, stratified by location, year (1990, 2005, 2010), age group (0-4, 5-9, 10-14, 15+ years) and intensity of infection (light, medium, heavy, all). For the model, light infestation was not attributed any disability. The expert group also provided data on reduction of prevalence in 2010, stratified by location, age group and Mass Drug Administration (MDA) coverage strategy (community/school). The table below shows the number of countries or subnational units and GBD world regions represented in the data.

Table 1a. Geographic Spread of Expert Group Prevalence Data

	prevalence
Countries/subnationals	160
GBD world regions	17

Mass Drug Administration

Data on national MDA program coverage was downloaded from the WHO PCT Databank source site [3]. This data spanned 119 GBD locations over 13 years beginning in 2003.

India Ministry of Health

Collaborators from the Indian Ministry of Health provided supplementary prevalence data [4] and MDA coverage data for India [5]. This data was available for all Indian states but was not available at the most detailed GBD location specification for the country: urban and rural. Data on MDA coverage was available for the years 2013-2016 while prevalence data was only available for 2015.

Severe Wasting Estimates

To inform the wasting model, 1,000 draws of severe wasting prevalence among children under 5 years were ascertained from GBD 2016 estimates – the methods used to generate estimates of wasting prevalence are detailed elsewhere (part of risk factors documentation) [6].

Geographic Restrictions

We conducted a literature review to determine the geographic extent of the disease and classify locations based on whether the disease is absent or present in each year. Locations that were geographically restricted in any given year did not have estimates made for them but could have imported cases attributed to them at a later stage. Of note, we did not attempt a complete systematic review, since a single high-quality source could offer sufficient evidence of presence. Evidence of absence or presence was not available for every location for each year and so assumptions were made for missing years by taking into consideration the epidemiological characteristics of the disease. If evidence indicated disease presence for two non-consecutive years, we assumed presence for all years between the two. If evidence indicated disease absence for two non-consecutive years, we assumed absence for all years between the two. If evidence indicated a change in status (i.e. from absent to present, or present to absent) between two non-consecutive years than we conducted targeted searches to ascertain the relevant year of introduction or elimination for that location. In the cases where presence or absence information was missing for the start or end years of our study interval (1990-2016) without evidence of any introduction or elimination events within the interval, we applied the status of the first and last presence/absence observations respectively to all years between the interval bound and the observation year. Our search was done in conjunction with the title/abstract screening portion of a systematic literature review for prevalence data. The search strings and yield can be viewed in the table below for each of the databases queried.

Table 1b. Geographic Restriction Search Strings

Database	Search String	Yield
PubMed	(Ascariasis[Title/Abstract] OR Ascaris[Title/Abstract] OR "A. lumbricoides"[Title/Abstract] OR Ascaris[MeSH] OR Trichuris[Title/Abstract] OR Trichuriasis[Title/Abstract] OR "Whip Worm"[Title/Abstract] OR "T. trichura"[Title/Abstract] OR Trichuris[MeSH] OR Hookworm[Title/Abstract] OR "A. duodenale"[Title/Abstract] OR "Ancylostoma duodenale"[Title/Abstract] OR ancylostomiasis[Title/Abstract] OR "N. americanus"[Title/Abstract] OR "Necator americanus"[Title/Abstract] OR necatoriasis[Title/Abstract] OR Ancylostoma [MeSH] OR Necator[MeSH]) AND (prevalence[Title/Abstract] OR	2376

	incidence[Title/Abstract] OR epidemiology[Title/Abstract] OR surveillance[Title/Abstract]) NOT(Animals[MeSH] NOT Humans[MeSH])	
Web of Science	(Ascariasis OR Ascaris OR A. lumbricoides OR Trichuris OR Trichuriasis OR Whip Worm OR T. trichura OR Hookworm OR A. duodenale OR Ancylostoma duodenale OR anclyostomiasis OR N. americanus OR Necator americanus OR necatoriasis) AND TOPIC:(prevalence OR incidence OR epidemiology OR surveillance) NOTTOPIC: ((Animals NOT Humans))<br>Timespan: 1980-2016. Indexes: SCI-EXPANDED, SSCI, A&HCI, ESCI.	2266
SCOPUS	TITLE-ABS_KEY (ascariasis OR ascaris OR a. lumbricoides OR trichuris OR trichuriasis OR whip worm OR t. trichura OR hookworm OR a. duodenale OR ancylostoma duodenale OR anclyostomiasis OR n. americanus OR necator americanus OR necatoriasis) AND PUBYEAR>1979	29

These papers were used to classify location-years for all locations and years present in the literature. Additionally, systematic literature reviews, meta-analyses, national health statistics publications and collaborator input were used to classify location-years not present in the literature review wherever possible.

Severity splits/sequelae

The table below shows the list of sequelae due to hookworm and the associated disability weights (DW). Prevalence of medium infection and heavy infection were mapped to *mild abdominopelvic problems* and *heavy infestation of hookworm* respectively. Light infection was not attributed any disability.

Table 2. Sequelae, lay descriptions, and disability weights (DWs)

Sequela	Lay description	DW
Mild abdominopelvic problems	has some pain in the belly that causes nausea but does not interfere with daily activities	0.011 (0.005–0.021)
Heavy infestation	has cramping pain and a bloated feeling in the belly	0.027 (0.015–0.044)
Severe wasting	is extremely skinny and has no energy	0.128 (0.082–0.183)
Asymptomatic hookworm disease	NA	NA
Mild anaemia	feels slightly tired and weak at times, but this does not interfere with normal daily activities	0.004 (0.001–0.008)
Moderate anaemia	feels moderate fatigue, weakness, and shortness of breath after exercise, making daily activities more difficult	0.052 (0.034–0.076)
Severe anaemia	feels very weak, tired and short of breath, and has problems with activities that require physical effort or deep concentration	0.149 (0.101–0.210)

Modelling strategy

In the estimation of morbidity due to hookworm disease, the EG data were first prepared by formatting the location names to be consistent with the GBD 2016 location names and applying the 2010 prevalence

to 1990 and 2005 for sub-Saharan Africa countries – estimates for these two years were missing. This was followed by using the data on reductions in 2010 prevalence to adjust the prevalence for locations with coverage data. After this adjustment, only data for medium infection, heavy infection, and all infection was retained.

Using the mean prevalence and the upper and lower bounds of the mean provided by the EG, 1,000 draws of prevalence were generated. This was done by multiplying the mean estimates by the exponent of random draws from a normal distribution with mean = 0 and standard deviation = sd, where sd = abs(abs(ln(upper)-ln(lower))/(invnormal(0.975)*2). These draws were created for all GBD age groups, assuming the same prevalence in ages 15+ and same prevalence in males and females.

Since the draws were only at the national level, subnational locations were modelled for Brazil, China, Indonesia, Kenya, Mexico, Saudi Arabia, and South Africa. This was done using a generalized linear model with a binomial family, logit link and robust standard errors. The predicted cases for all subnational locations were summed and scaled to the national case total. India subnationals were separately derived by applying the proportion of all national cases in each state from the Indian Ministry of Health data [4] to the national total. We then applied the modelled proportion of cases in urban or rural locations such that the sum of urban and rural cases in a state equals the state total and the sum of all state cases equals the national total.

To get 1995 and 2000 estimates, exponential interpolation of estimates between 1990 and 2005 was performed. The draws for 2016 were produced based on 2010 estimates corrected for PCT control activities between 2010 and 2016 – this was done by extrapolating the 2004–2010 trend to 2016, given cumulative number of treatments per person calculated using data from the WHO PCT Databank [3] and the Indian Ministry of Health MDA coverage data[5]. The 2004-2010 trend was applied to all intensities of infection. Prevalence was assumed to be zero for the countries with missing input data and also in children younger than 28 days. The resulting estimates were 1,000 draws of hookworm prevalence by GBD location, year, age, sex, and intensity level (mild, heavy, overall infection). To estimate the prevalence of asymptomatic hookworm, prevalence of mild and heavy infestation was subtracted from the overall hookworm prevalence.

The final step in the modelling process was to estimate the prevalence of severe wasting due to hookworm in age groups 28–364 days and 1–4 years. This was done separately using 1,000 draws of prevalence of heavy infestation due to hookworm and the wasting envelope prevalence. The initial step in determining prevalence of severe wasting due to hookworm was generating 1,000 draws of change in weight-for-height z-score per heavy prevalent case from a random normal distribution with mean = 0.493826493 and standard deviation = 0.04972834 (calculated from upper and lower bounds of the mean estimate). The mean, upper, and lower bounds were provided by a GBD collaborator who calculated them based on a published article [6]. The prevalence of severe wasting due to hookworm was then obtained as a function of change in weight-for-height z-score (z_change) such that prevalence = p_wasting_env – Phi(Phi_inv(p_wasting_env) – z_change*p), where p_wasting_env = wasting envelope prevalence, Phi_inv is the inverse standard normal cumulative distribution function (cdf), and p = prevalence of heavy hookworm infestation. Finally, geographic restrictions were applied to set prevalence to zero in countries with wasting that are not endemic for hookworm.

The burden of anemia due to hookworm disease was estimated seperately (see anemia documentation for details).

Model evaluation was done by plotting prevalence of overall hookworm disease and that of each sequelae against year for each location and age group. Maps of the global distribution of total hookworm disease prevalence and prevalence of sequelae due to hookworm disease were also assessed across time and age.

Only minor changes were made to the GBD 2015 modelling strategy including the incorporation of updated data from the WHO PCT databank [3] and data from the Indian Ministry of Health, new modelling of subnational prevalence distribution and new geographic restrictions.

References

38. Brooker S, Pullan R, Smith J, and Hotez P. Chapter: Intestinal nematodes. Cluster D: Communicable Diseases, Neglected Tropical Diseases Group. Global Burden of Diseases, Injuries, and Risk Factors Study. 2011 (4 July). 1-24.
39. Brooker S & Smith JL. Impact of hookworm infection and deworming on anaemia in non-pregnant populations: a systematic review. *Tropical Medicine and International Health*. 2010. 15,7,776-795.
40. WHO | PCT databank. WHO. http://www.who.int/neglected_diseases/preventive_chemotherapy/sth/en/ (accessed July 7, 2017).
41. Prevalence data. GBD India Collaborators. Personal Communication.
42. MDA Coverage data. GBD India Collaborators. Personal Communication.
43. Hall A, Hewitt G, Tuffrey V, de Silva N. A review and meta-analysis of the impact of intestinal worms on child growth and nutrition. *Maternal and Child Nutrition*. 2008. 4. 118-236.

Foodborne Trematodiases

Flowcharts

Clonorchiasis

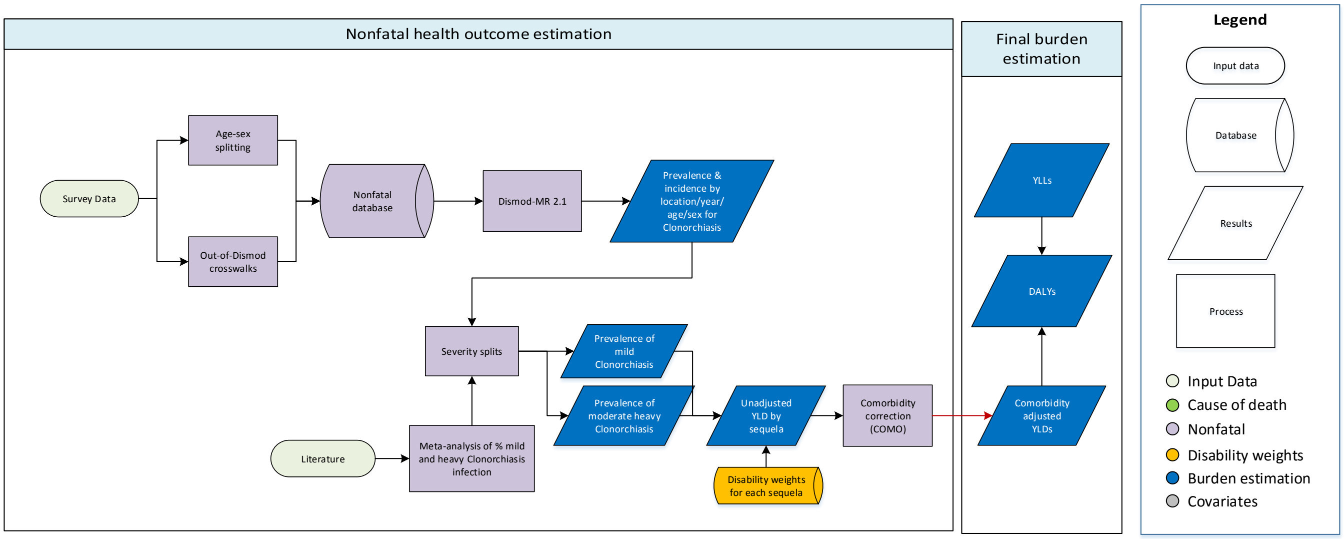

Fascioliasis

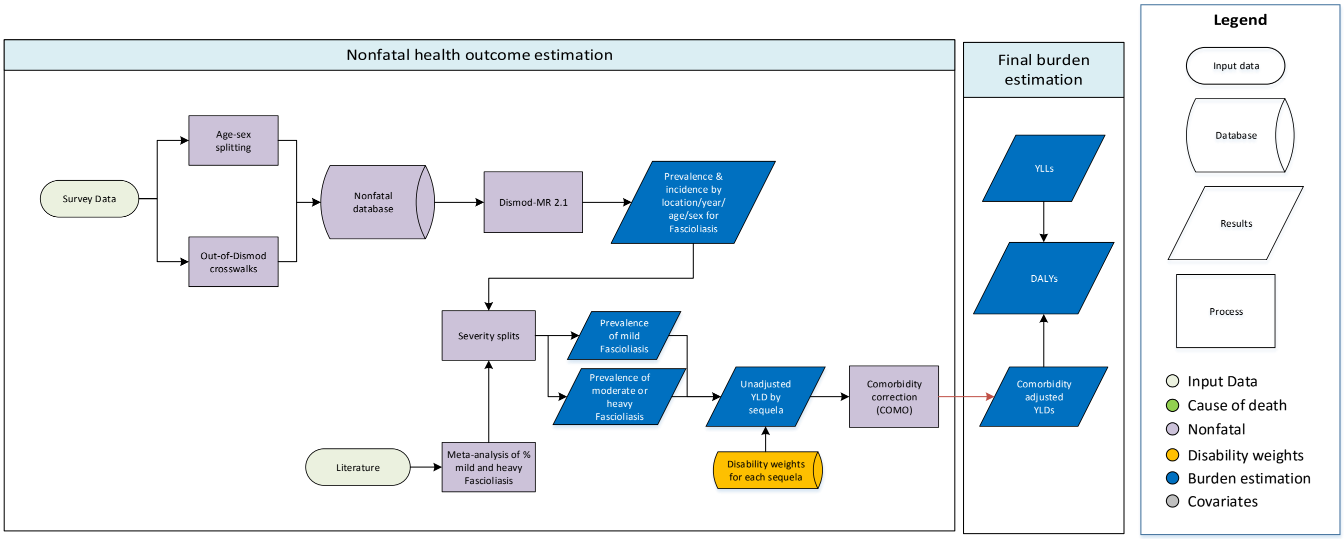

Intestinal fluke

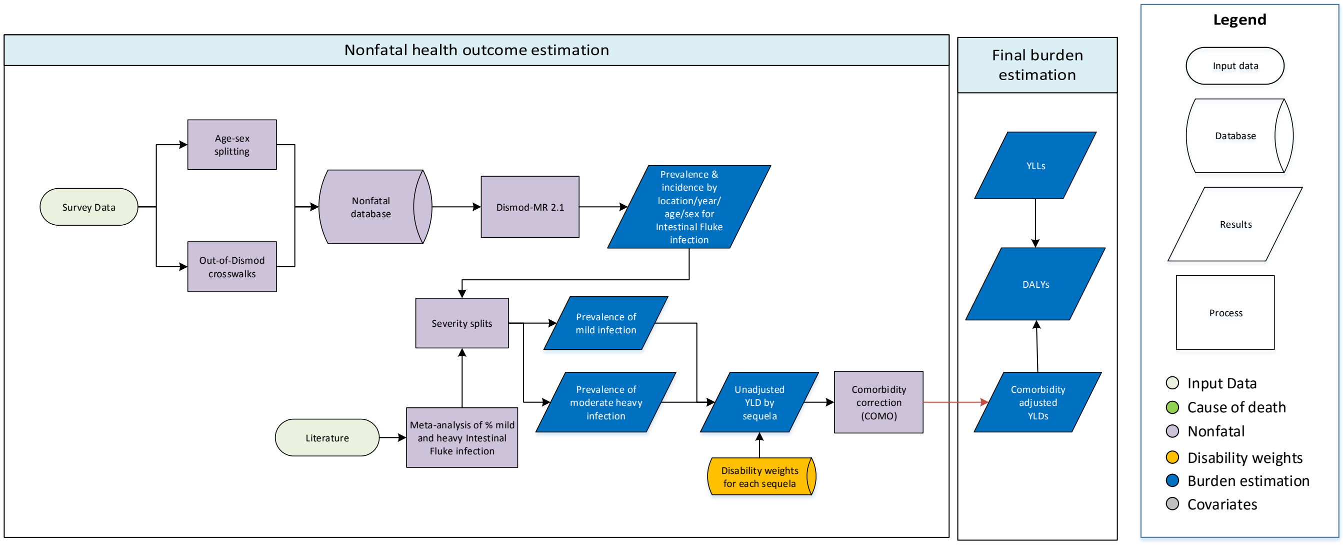

Opisthorchiasis

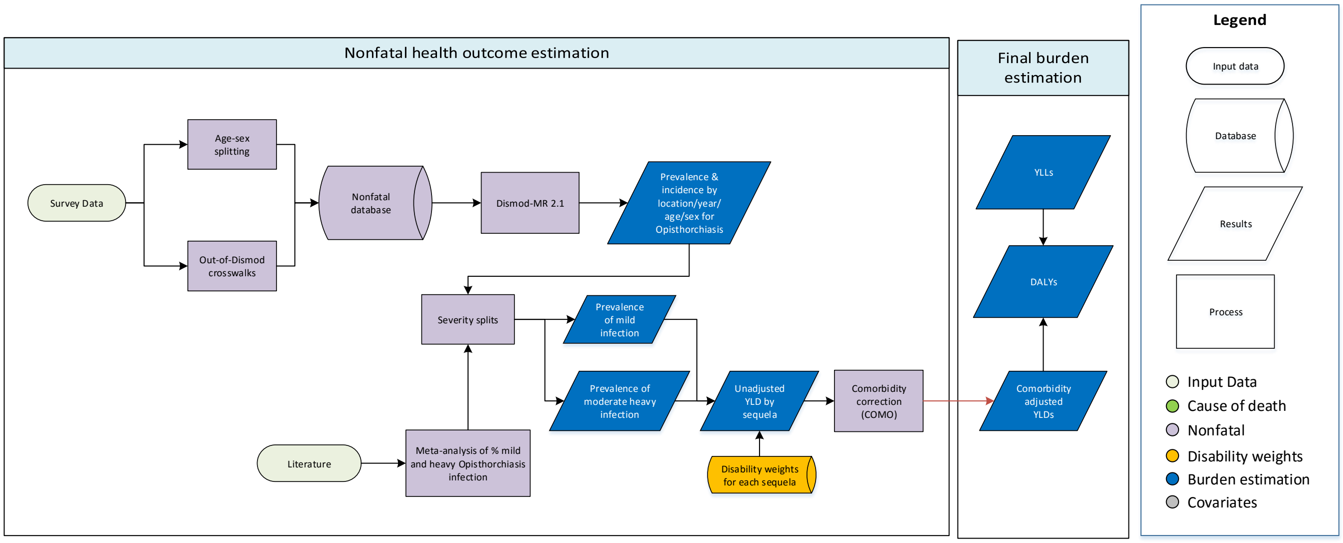

Paragonimiasis

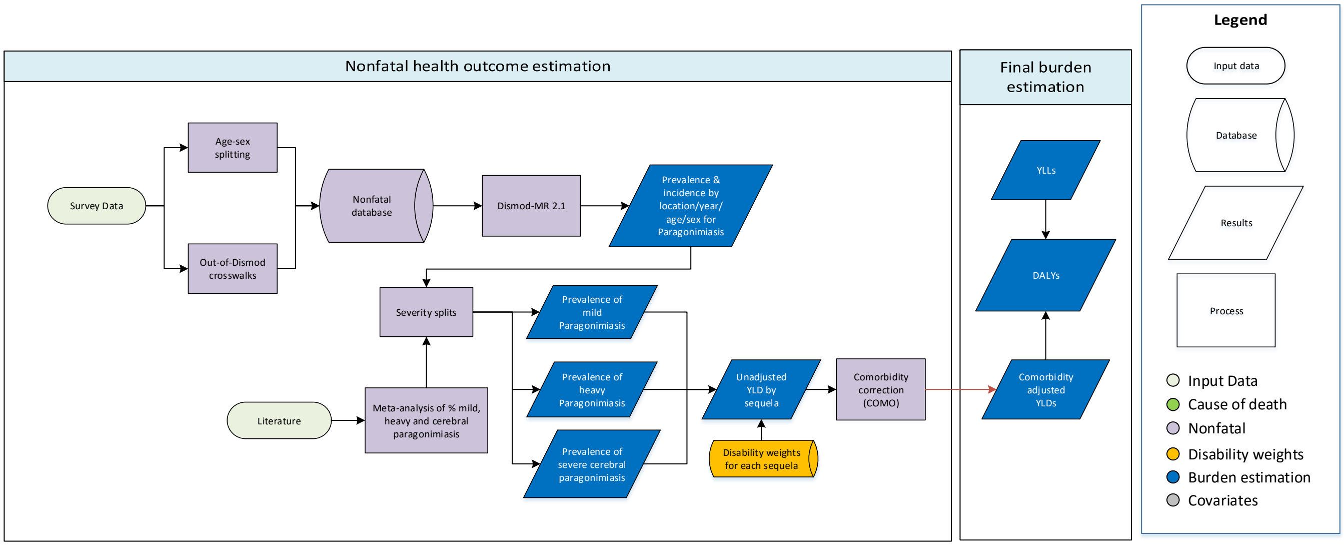

Input Data & Methodological Summary

Case definition

Human foodborne trematodiases (FBT) is defined as the infection with parasitic worms of the class trematoda, which are also known as flukes. Trematodes are transmitted via contaminated food and infection is highly related to food habits. Definitive hosts, including humans, become infected when ingesting viable metacercariae by consuming contaminated aquatic products (eg, watercress). In the ICD-10, FBT are listed under code B66 [1].

FBT is subdivided into six types of FBT (see Table 1):

- Clonorchiasis
- Fascioliasis
- Intestinal fluke
- Opisthorchiasis
- Paragonimiasis (normal and cerebral infections)

Table 1. Subtypes of FBT

	Species of FBT	Also known as:	Carcinogen
1	Chlonorchiasis	(Chinese) Liver fluke	Associated with choliangiocarcinoma
2	Opisthorchiasis<br>(*O viverrini* & *O felineus*)	Liver fluke	Associated with choliangiocarcinoma<br>(*O viverrini*)
3	Fascioliasis	Liver fluke	No available evidence
4	Intenstinal fluke	Liver fluke	No available evidence
5	Paragonimiasis	Lung fluke	

Thresholds for heavy infection and duration by species of FBT

The majority of people infected with FBTs are asymptomatic. When symptoms do occur they are often non-specific. Among the clinical symptomatic group, severity is associated with worm burden, typically measured by fecal egg counts, and the duration of infection. The thresholds for heavy infection and duration by species of FBT are shown in Table 2. The clinical presentation of FBT depends on the target organs (liver, lung, or intestines). Clonorchiasis and opisthorchiasis patients may suffer from loss of appetite, fullness, indigestion, diarrhoea, pain in the right upper quadrant, lassitude, weight loss, ascites, and oedema.[2, 3] Cholangitis, obstructive jaundice, intra-abdominal mass, cholecystitis, and gallbladder or intrahepatic stones may occur as complications.[3, 4]

Table 2. Thresholds for heavy infection and duration by species of FBT

	Species of FBT	Case thresholds for heavy infection	Duration
1	Chlonorchiasis	10,000 eggs per g of feces	lifelong
2	Opisthorchiasis	10,000 eggs per g of feces	lifelong
3	Fascioliasis	1,000 eggs per g of faces	lifelong
4	Intenstinal fluke	1,000 eggs per g of faces	lifelong
5	Paragonimiasis	100 eggs per 5 ml sputum	lifelong
6	Cerebral paragonimiasis	Any infection of the brain with flukes and/or eggs of *Paragonimus* spp.	lifelong

Input data

Model inputs

For GBD 2010, the data came from the expert group and is the result of their analysis. The expert group analysis used the results of a systematic literature review performed by Furst et al. as a starting point for the analysis.[5] Furst et al. searched PubMed, WHOLIS, FAOBIB, Embase, CAB Abstracts, Literatura Latino Americana e do Caribe em Ciências de Saùde (LILACS), ISI Web of Science, BIOSIS preview, Science Direct, African Journals OnLine (AJOL), and the System for Information on Grey Literature in Europe (SIGLE), period Jan 1, 1980, to

Dec 31, 2008. The initial number of studies identified through the literature review was ~34,000 references. The literature review included extracted data from 181 studies. For GBD 2013 and GBD 2015 the search strategy was replicated to capture epidemiological studies published between 2008 and 2015. Due to the cyclical nature of systematic review for GBD causes, no data collection was scheduled for GBD 2016. As such, foodborne trematodiases will be a priority for the next iteration of the study.

Input data for the assessment of the total national number of infected people

Only studies that used countrywide surveys to estimate the national prevalence rates were included (or for China, province-wide surveys). Reason for choosing only national studies is that FBT shows a highly focal spatial distribution and local cross-sectional surveys would profoundly under- or overestimate true

national prevalences. We decided not to model national and subnational together and get a coefficient on subnational, because there is not a one-fits-all relationship across the world. Infection is highly related to food habits and there are highly varying differences between national and subnational prevalence rates. The final GBD 2016 dataset contained 29 prevalence studies from 17 countries. We used raw data from the selected studies as input for DisMod.

Prevalence intestinal fluke infection

Intestinal fluke is different from the other types of FBT, because there are several pathogens that fall under intestinal fluke infection. It can be caused by pathogens, such as Metagonimus spp., Echinostoma spp., Neodiplostomatidae.[6] When assessing the prevalence of intestinal fluke infection, we added the identified prevalence for each parasite species in order to obtain the overall prevalence of intestinal fluke infections. This approach may lead to a certain overestimation of the true prevalence, because people may be co-infected with more than one intestinal fluke species. There is no sufficient evidence about the proportion of co-infections, but the resulting overestimation of the true prevalence may be more than offset by the assumptions made in our previous modelling approach and the many challenges in generating the underlying epidemiological parameters (eg, diagnostic inaccuracy in the detection of infections with the more than 50 intestinal fluke species). Also of note: the transmission source of intestinal fluke infections are species-specific and therefore vary. For instance, *Fasciolopsis buski* is usually transmitted by eating raw water plants with the infective parasite stage attached to the water plants, whereas Neodiplostomatidae are transmitted by eating undercooked and infested frogs, snakes, and tadpoles. Because of these different transmission pathways, the rate of co-infection might in fact be smaller than expected.

Input data to differentiate between asymptomatic and heavy infections

We estimated the proportion of heavily infected among all infected in all available national and regional cross-sectional surveys. It is expected that heavy infection increases with age and there are data available on heavy infection by age group. We therefore decided to include age-dependent rates of heavy infection for clonorchiasis, opisthorchiasis, and intenstinal fluke infection. For (cerebral) paragonimiasis and fascioliasis there were not sufficient age-dependent data on high intensity FBT infection.

Modelling strategy

We used a three-step process for the disease modelling of FBT. In the first step we used DisMod-MR 2.0 to estimate assess the prevalence of FBT by age, sex, year, and country. In the second we differentiated between asymptomatic and heavy infections. MetaXL (a meta-analysis add in for Microsoft Excel) was used to estimate the proportion of heavy infected among all infected by age group for clonorchiasis, opisthorchiasis, and intenstinal fluke infection (see Table 3 and 4). These proportions were used to estimate the prevalence of heavy FBT infection.
The third step consisted of deselecting countries that have no autochtonous case reports of FBT (input 34,000 references from literature review).

Table 3. Percentage of high intensity infection by age group and type of FBT (based on eight FBT prevalence studies)

Age category	Clonorchiasis			Opisthorchiasis			Intestinal fluke infection		
	Mean	Low	High	Mean	Low	High	Mean	Low	High

0-9	30%	17%	44%	10%	0%	29%	8%	3%	14%
10-19	15%	0%	43%	15%	0%	69%	11%	8%	14%
20-29	18%	10%	29%	16%	0%	52%	18%	15%	21%
30-39	17%	5%	34%	21%	0%	56%	22%	17%	28%
40-49	22%	13%	32%	28%	1%	68%	22%	13%	32%
50-59	18%	0%	49%	29%	0%	75%	17%	9%	28%
60+	32%	18%	47%	25%	0%	64%	15%	8%	23%

Table 4. Percentage of high-intensity infection by type of FBT (based on 4 FBT prevalence studies)

Type of FBT	Mean	Low	High
Paragonimiasis	23%	0%	59%
Fascioliasis	19%	3%	41%

Cerebral paragonimiasis

It was assumed that 0.8% of paragonimiasis cases have cerebral involvement. This proportion was used to estimate the prevalence of cerebral paragonimiasis. This proportion is based on one study. The data are from Oh SJ. The rate of cerebral involvement in paragonimiasis: an epidemiologic study. *Jpn J Parasitol* 1969;18:211-14. The study was performed in Paju, South Korea. This is an area with 6,738 inhabitants and according to the survey, it was estimated that 29.6% of all individuals would react to intradermal test (= an immunological reaction indicating previous or current contact to the parasite). 25% of all “positive reactors” may have eggs in their sputum (= active infection with the parasite currently present in the human host). If these rates are applied to the community as a whole, the number of patients with active paragonimiasis would be at least 498 (=6,738*0.296*0.250). Furthermore, four cases of cerebral paragonimiasis were found in this community. Therefore, four out of 498 individuals with active paragonimus infection suffered from cerebral infection (=0.80%; 95% confidence interval 0.019%-1.587%).

Severity splits and disability weights

For GBD 2016, FBT was not split into health states with different severities. The table below shows the GBD 2016 disability weights that were used to calculate the burden of FBT in YLDs.

Table 5. Disability weights that were used to calculate FBT YLDs

Sequelae	Severity description	Health state name	Disability weight
Asymptomatic clonorchiasis	Clonorchiasis, currently without symptoms	N/A	0.000 (0.000–0.000)
Heavy clonorchiasis	Abdominal pain and nausea reported as moderate	Abdominopelvic problem, moderate	0.114 (0.078–0.159)
Asymptomatic opisthorchiasis	Opisthorchiasis, currently without symptoms	N/A	0.000 (0.000–0.000)

Heavy opisthorchiasis	Abdominal pain and nausea reported as moderate	Abdominopelvic problem, moderate	0.114 (0.078–0.159)
Asymptomatic fascioliasis	Fascioliasis, currently without symptoms	N/A	0.000 (0.000–0.000)
Heavy fascioliasis	Abdominal pain and nausea reported as moderate	Abdominopelvic problem, moderate	0.114 (0.078–0.159)
Asymptomatic intestinal fluke infection	Intestinal fluke infection, currently without symptoms	N/A	0.000 (0.000–0.000)
Heavy intestinal fluke infection	Abdominal pain and nausea reported as moderate	Abdominopelvic problem, moderate	0.114 (0.078–0.159)
Asymptomatic paragonimiasis	Paragonimiasis, currently without symptoms	N/A	0.000 (0.000–0.000)
Heavy paragonimiasis	Cough, fever, and weight loss	Tuberculosis, not HIV-infected	0.333 (0.224–0.454)
Cerebral paragonimiasis	Epilepsy due to cerebral paragonimiasis	Epilepsy, less severe (seizures < once per month)	0.263 (0.173–0.367)
		Epilepsy, severe (seizures >= once per month	0.552 (0.375–0.710)

Note. N/A: not applicable

Changes from GBD 2015 to GBD 2016

We have made no substantive changes in the modelling strategy from GBD 2015 to GBD 2016.

References

1. WHO. *International Statistical Classification of Diseases and Related Health Problems. 10th Revision. Version for 2007.* 2007 [cited 2009 October 14, 2009]; Available from: http://apps.who.int/classifications/apps/icd/icd10online/.
2. Rim, H.J., *Clonorchiasis: an update.* J Helminthol, 2005. **79**(3): p. 269-81.
3. Pungpak, S., et al., *Clinical features in severe opisthorchiasis viverrini.* Southeast Asian J Trop Med Public Health, 1985. **16**(3): p. 405-9.
4. Rim, H.J., *The current pathobiology and chemotherapy of clonorchiasis.* Korean J Parasitol, 1986. **24**(Suppl.): p. 1-141.
5. Furst, T., J. Keiser, and J. Utzinger, *Global burden of human food-borne trematodiasis: a systematic review and meta-analysis.* Lancet Infect Dis, 2012. **12**(3): p. 210-21.
6. Furst, T., et al., *Manifestation, diagnosis, and management of foodborne trematodiasis.* BMJ, 2012. **344**: p. e4093.

Leprosy

Flowchart

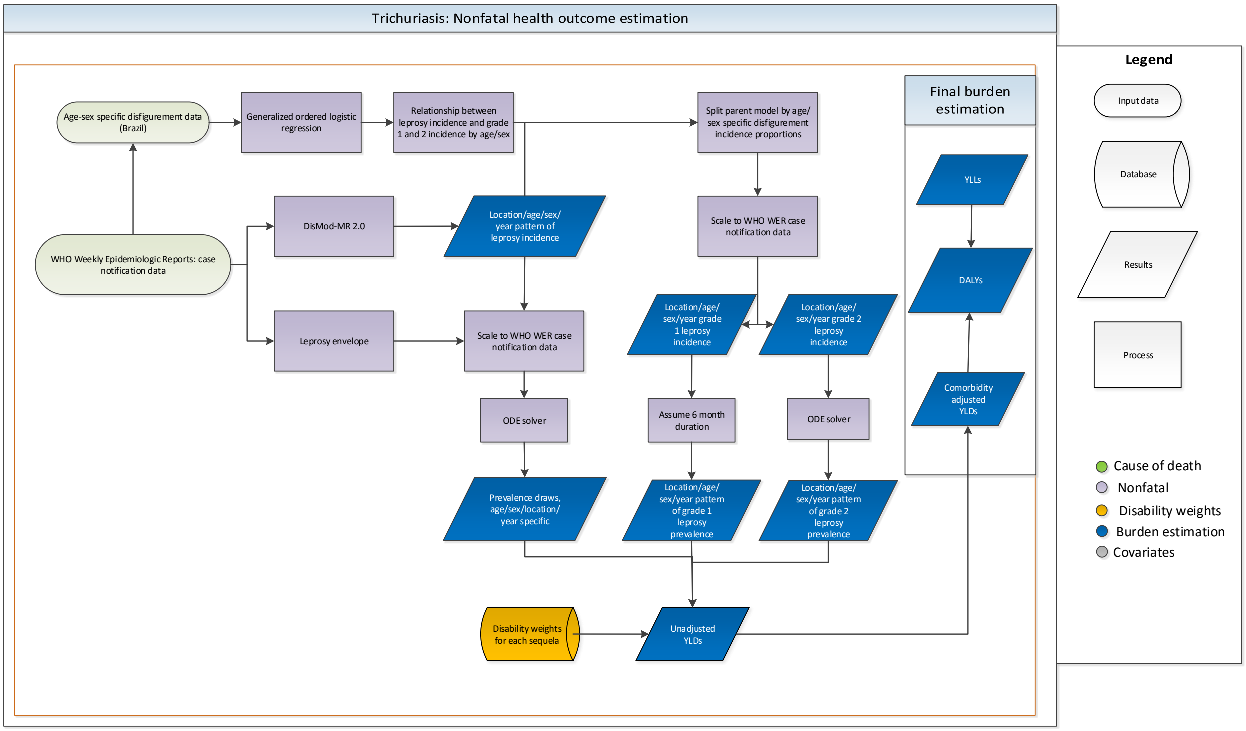

Input Data and Methodological Summary

Case definition

Leprosy is a chronic bacterial infection caused by *Mycobacterium leprae*, primarily affecting the nervous system, skin, respiratory tract, and eyes. Transmission is facilitated through contact with fluid from the nose and mouth of an infected individual. The ICD-10 codes for leprosy are A30.9.

Input data

To model nonfatal outcomes due to leprosy, WHO Weekly Epidemiological Record (WER) case notification data were used from 1987 to 2012 to capture incident cases of leprosy. This is the same database that was used to model GBD 2015 estimates, and due to the cyclical nature of systematic reviews for GBD causes, no data collection was scheduled for GBD 2016. As such, leprosy will be a priority for the next iteration of the study. Stage-specific incidence data for grade 1 and grade 2 leprosy that are used to define age-sex patterns came from Brazil case notification data.

Modelling strategy

We used a multi-step process for the disease modeling of leprosy. In the first step, we ran a single-parameter model using DisMod-MR 2.0 to estimate the leprosy incidence age pattern by age, sex, year, and country. Then, we scaled the incidence outputs to the WHO WER cases, and used the ordinary differential equations (ODE) solver to calculate prevalence from the scaled DisMod-MR 2.0 incidence outputs.

Severity data were prepared by running a generalized ordered logistic regression using Brazil case notification data to get the relationship between leprosy incidence and grade 1 and grade 2 incidence by age and sex. We then used this relationship to split the parent DisMod-MR 2.0 model, and again scaled to WHO WER severity-specific cases. For disfigurement grade 1, we apply a duration of six months to get prevalence estimates. For disfigurement grade 2, we again use the ODE solver to get prevalence estimates.

Model evaluation was done by separately assessing the fit of the parent DisMod model and checking the final estimates produced after age-sex splits. Plots of time trends of prevalence across locations and age were used to evaluate the results. In addition, maps of the global distribution of leprosy prevalence and prevalence of sequelae due to leprosy were also assessed across time.

Changes from GBD 2015 to GBD 2016

We have made no substantive changes in the modelling strategy from GBD 2015 to GBD 2016.

Ebola

Flowchart

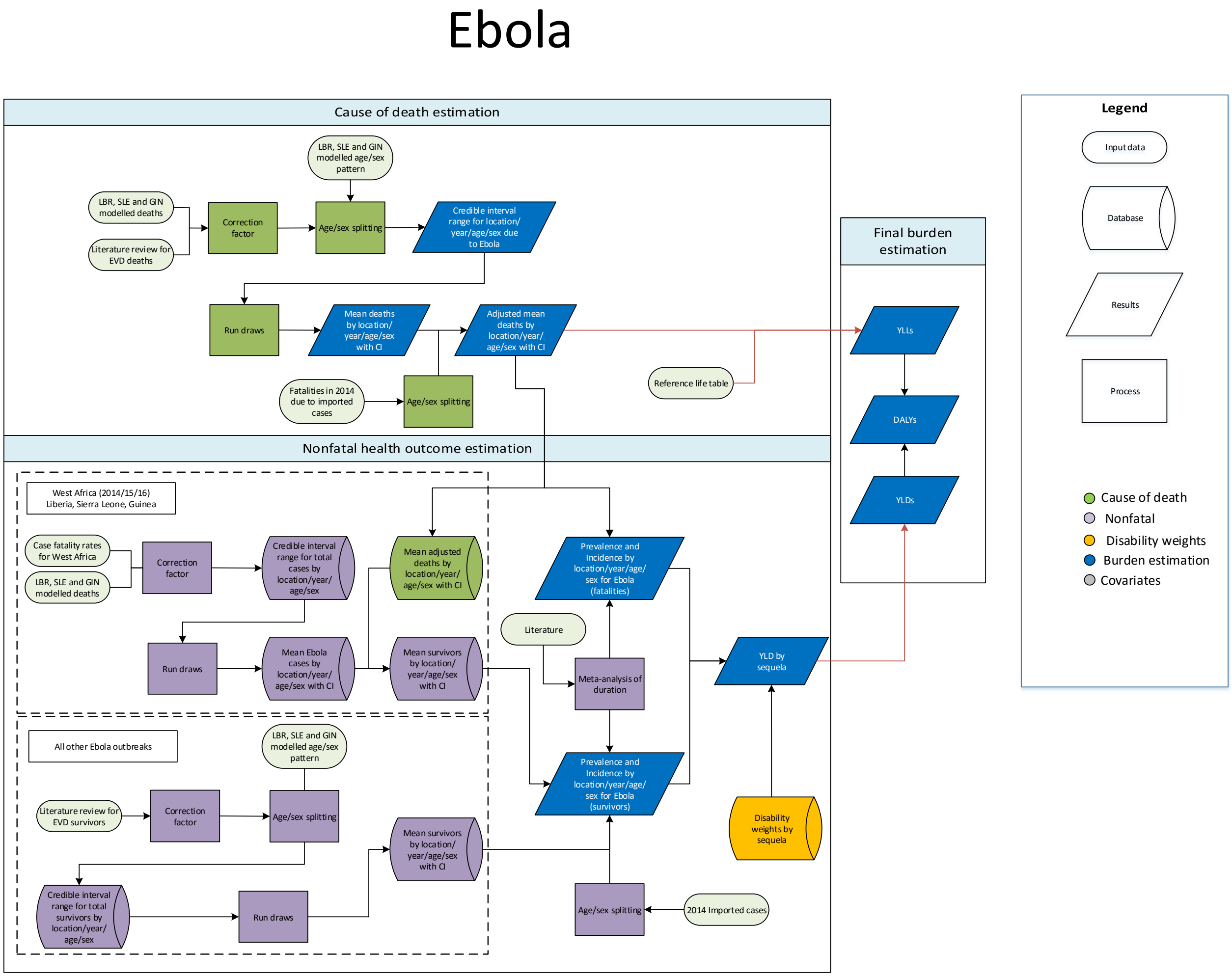

Input data and methodological summary

Background and case definition

Ebola virus disease is a relatively rare viral pathogen linked with high case fatality rates in both humans and non-human primates. The disease is zoonotic, and while bats have been implicated as reservoirs, definitive host species are yet to be identified. Once a human becomes infected after viral transmission from animal sources either directly or indirectly, secondary human-to-human transmission is possible, primarily through exchange of infectious bodily fluids and secretions. Clinical cases typically present initially as a febrile illness, similar to a number of different pathogens, subsequently followed by haemorrhagic complications, and often death. Historically there have been a number of outbreaks,

usually no more than a few hundred cases and typically constrained to one country, focused in Central Africa. The West African outbreak, however, which started in Guinea in 2013, has claimed more lives than all previous outbreaks combined, and spread across the region seeding additional outbreaks. There is an ICD code for Ebola, A98.4, but no data used in the modelling reference that coding (ie, all the data are from literature extractions). Data for Ebola virus disease were only included if the case was identified as either "probable" or "confirmed" as per WHO definitions [http://www.who.int/csr/resources/publications/ebola/ebola-case-definition-contact-en.pdf]. A confirmed case is any suspected or probable case with a positive laboratory result through either detection of virus RNA via reverse transcriptase-polymerase chain reaction, or by detection of IgM antibodies directed against Ebola. A probable case is any suspected case evaluated by a clinician or any deceased suspected case with an epidemiological link to a confirmed case.

Input data

Model inputs

Two distinct sequelae were assigned to Ebola virus disease (EVD) to be incorporated into the YLD estimation process: (i) sequela associated with the initial symptomatic phase of the infection (associated with all cases of Ebola virus disease) and (ii) sequela characterizing the long-term post-EVD consequences of infection. As such, data were required both to ascertain the number of deaths as well as those surviving from each outbreak.

Data on fatal cases were inherited from the GBD 2016 mortality estimation process and were converted into incidence of cases of Ebola (with fatal outcomes) by cross-referencing locational annualized population estimates.

In order to calculate the numbers of survivors from each outbreak, two data sources were referenced, one based upon modelled estimates of the main three countries in the West African Ebola outbreak (namely Sierra Leone, Liberia, and Guinea), supplemented by WHO Situation Reports covering the clusters of 2016 cases and literature references covering all other subsequent outbreaks.

Researchers from Imperial College London (UK), as part of the WHO Ebola response team, provided modelled estimates for the number of fatalities that result from a given number of reported cases (provided by line lists from WHO). This method was used in a variety of papers to generate baseline estimates of case fatality rates and other key epidemiological measures while correcting for the lag period between initially reporting a case and the final outcome of that case (whether it be death or survival). The full data cleaning and methodology are reported elsewhere.[1,2] Bespoke estimates were provided for GBD for Liberia, Sierra Leone, and Guinea and were stratified by age, sex, and year. Death data from Guinea ranged from February 28, 2014, until September 27, 2015, with data from Liberia ranging from March 20, 2014, to May 4, 2015, and data from Sierra Leone ranging from May 21 until September 28, 2015. To these estimates, calculated case fatality ratios[1,2] were applied in order to generate an estimate of the total number of cases stratified by age, sex, and country.

For all other outbreaks, numbers of survivors were directly evaluated based upon numbers published in a previous review[3,4] and consulting original documents describing these outbreaks. This initial review was also updated to include the outbreak that occurred in the Democratic Republic of the Congo in 2014,[5] and

cases in 2016. This resulted in datasets describing each outbreak with variable degrees of detail: some fully describing the age and sex breakdown of all survivors [eg, Rosello et al.[6]] and others simply providing the final total. Only confirmed or probable cases were included as per the case definition. Outbreaks that spanned multiple years, in the absence of sufficient data providing an accurate breakdown, were apportioned between the years by evenly assigning a uniform number of survivors to each month of the outbreak's duration. An additional search was conducted to identify imported cases from the West African outbreak during 2014 and 2015.

Table 1. Sequelae and disability weights (DWs) associated with Ebola

Sequelae	Description	Disability weight
Infectious disease, acute episode, severe	Has a high fever and pain and feels very weak, which causes great difficulty with daily activities	0.133 (0.088–0.19)
Infectious disease, post-acute consequences (fatigue, emotional lability, insomnia)	Is always tired and easily upset. The person feels pain all over the body and is depressed	0.219 (0.148–0.308)

It was not possible to create bespoke disability weights for the more specific sequelae often associated with Ebola virus disease (eg, haemorrhaging or ocular complications in survivors), so existing disability weights were co-opted. General high fevers and weakness characterize the majority of presenting cases[7] with long-term complications generally related to weakness and arthralgia.[8]

Modelling strategy

Data on cases (both survivors and fatalities) resulting from imported cases from 2014 and 2015 were used as specific count data as it was assumed to be an accurate representation of the cases and outbreaks in these countries, all of which were on high alert for importation of cases.[9,10]

The other input data were processed prior to inclusion in GBD to account for any potential underreporting of deaths. A meta-analysis of existing underreporting studies from the literature was performed, using a random effects model with a DerSimonian-Laird estimator. A variety of sources were included, capturing a number of different estimation processes, all identified by literature review. The figure below shows the different effect sizes of the different studies, as well as the resulting GBD 2016 correction factor, with the GBD 2015 correction factor for reference. The correction factor ranged from 1.5147 to 2.5720 with a mean of 2.0433.

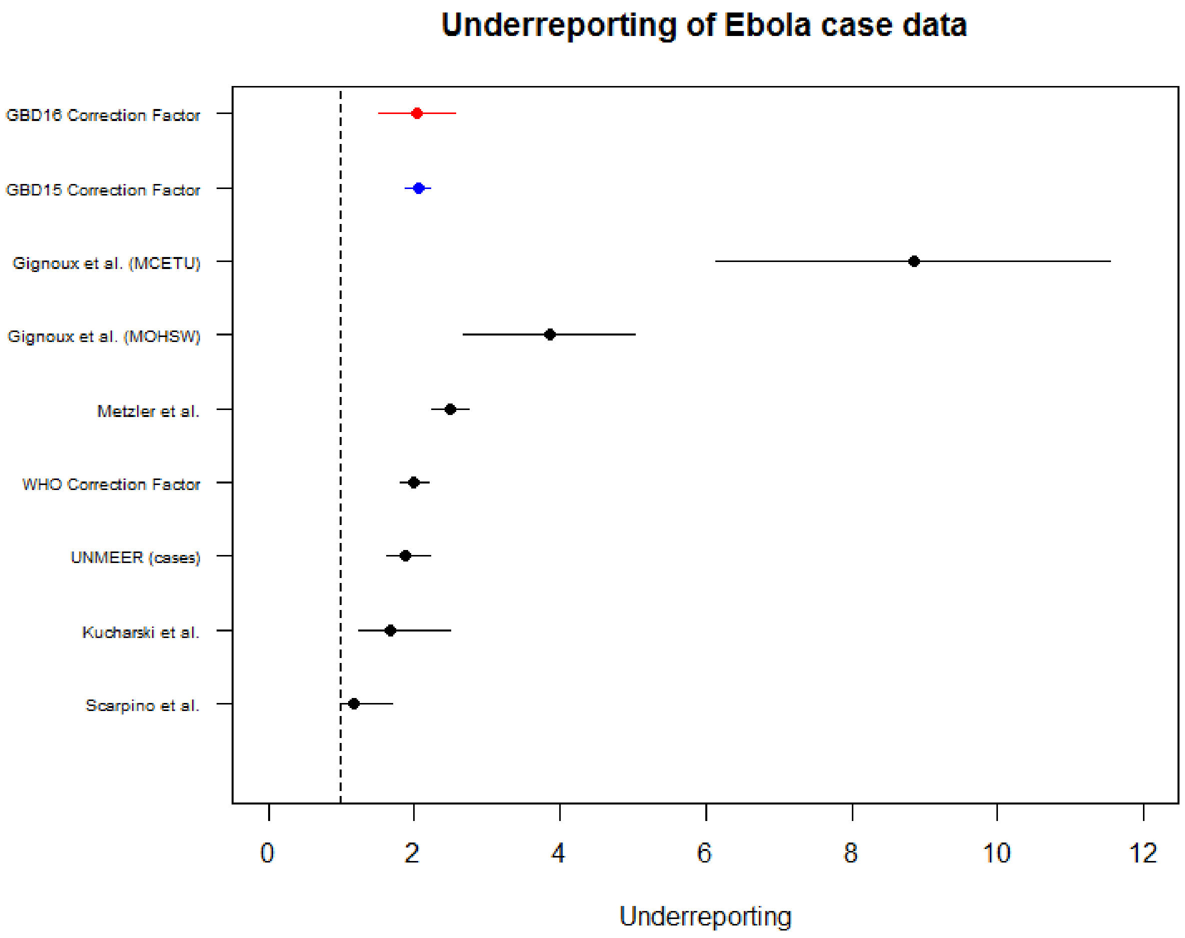

In order to capture this potential variation, all input data were multiplied by the lower and upper limit of this estimated correction factor; these numbers then provided the lower and upper bounds from which draw values were taken. For outbreaks where no data were supplied for age and/or sex, the pattern observed in the West African outbreak (for which there were the most comprehensive data) was used to apportion these total values.

One thousand draws were taken from a normal distribution fitted between these lower and upper bound values, which generated mean estimates stratified by age, sex, location, and year along with credible intervals for these numbers. For the West African outbreak, this generated total case numbers, from which the estimated number of deaths was subtracted in order to provide an estimate for the total number of survivors. For all other outbreaks, this data processing directly estimated the total number of survivors from each outbreak. These count data were converted into prevalence estimates by cross-referencing estimates of population size.

In order to estimate the duration of the sequelae categories, previous modelled assessments of the West African outbreak were consulted.[1,2] The duration of initial infection for patients was calculated as the total time period between onset of symptoms to death or to discharge from hospital (8.2 days [7.9–8.4] and 15.1 [14.6–15.6], respectively). These time periods were assumed to be appropriate for

characterizing all other outbreaks. This time period was then assigned a disability weight corresponding to "infectious disease, acute episode, severe."

For long-term sequelae estimation, the proportion of survivors still suffering post-acute consequences was modelled using an exponential function with proportions of survivors still reporting poor health states (derived from a number of survivor studies[8,12–21]) reported over different time periods. The average duration of post-Ebola sequelae was then calculated as 0.9042 years (0.3673–1.4268).

The final combination of YLDs associated with prevalent initial onset of disease and prevalent post-EVD consequences was then calculated to provide an overall YLD estimate stratified by age, sex, location, and year. Estimates were provided for the years 1990, 1995, 2000, 2005, 2010, 2015, and 2016 as per non-fatal GBD estimation protocols.

Potential limitations

Data on Ebola outbreaks prior to 2014 are sparse, and as a result many values derived from the West African outbreak were assumed to be valid for historic outbreaks as well. This may mask significant differences that exist between these outbreaks, some of which were caused by different species of Ebola virus. In order to minimize this problem we chose to implement a data-driven approach – for those outbreaks where sufficiently detailed historical data could be obtained, this was used in preference to any assumed age/sex breakdown.

Haemorrhagic manifestations are currently not considered as an explicit health state for disability weighting, and as a result, the current classification (of infectious disease, acute episode, severe) may be an underestimate. In contrast, the post-Ebola disease sequelae disability weighting may overestimate this burden, particularly when applied over a long period of time. In both instances, however, these disability weightings represent the most relevant linkages in the absence of bespoke values being generated.

Due to so few historical survivors of Ebola virus disease, only a handful of studies have tracked the long-term sequelae among cohorts of survivors beyond a two-year period. Given the large number of survivors from the West African outbreak, it is likely that future of parameterization of this component will become much better data-driven. The current log-linear regression model extends for a period of 20 years and therefore could prove to be an overestimate of duration. In addition, ocular manifestations are not currently considered within the sequelae envelope – future iterations will consider health states such as "Distance vision, severe impairment: has severe vision loss, which causes difficulty in daily activities, some emotional impact (for example worry), and some difficulty going outside the home without assistance."

References

1 Agua-Agum J, Ariyarajah A, Aylward B, *et al.* West African Ebola Epidemic after One Year — Slowing but Not Yet under Control. *N Engl J Med* 2015; **372**: 584–7.

2 Ebola Virus Disease in West Africa - The First 9 Months of the Epidemic and Forward Projections. *N Engl J Med* 2014; **371**: 1481–95.

3 Pigott DM, Golding N, Mylne A, *et al.* Mapping the zoonotic niche of Ebola virus disease in Africa. *Elife* 2014; **3**: e04395.

4 Mylne A, Brady OJ, Huang Z, *et al.* A comprehensive database of the geographic spread of past human Ebola outbreaks. *Sci Data* 2014; **1**: 140042.

5 Maganga GD, Kapetshi J, Berthet N, *et al.* Ebola virus disease in the Democratic Republic of Congo. *N Engl J Med* 2014; **371**: 2083–91.

6 Rosello A, Mossoko M, Flasche S, *et al.* Ebola virus disease in the Democratic Republic of the Congo, 1976-2014. *Elife* 2015; **4**. DOI:10.7554/eLife.09015.

7 Schieffelin JS, Shaffer JG, Goba A, *et al.* Clinical Illness and Outcomes in Patients with Ebola in Sierra Leone. *N Engl J Med* 2014; **371**: 2092–100.

8 Tiffany A, Vetter P, Mattia J, *et al.* Ebola Virus Disease Complications as Experienced by Survivors in Sierra Leone. *Clin Infect Dis* 2016; **62**: 1360–6.

9 Fasina FO, Shittu A, Lazarus D, *et al.* Transmission dynamics and control of Ebola virus disease outbreak in Nigeria, July to September 2014. *Euro Surveill* 2014; **19**: 20920.

10 Althaus CL, Low N, Musa EO, Shuaib F, Gsteiger S. Ebola virus disease outbreak in Nigeria: Transmission dynamics and rapid control. *Epidemics* 2015; **11**: 80–4.

11 UNMEER. Sierra Leone: Ebola emergency Weekly Situation Report No. 7. 2014 https://www.humanitarianresponse.info/system/files/documents/files/UNMEER_NERC_SitRep_07Dec.pdf.

12 Clark D V, Kibuuka H, Millard M, *et al.* Long-term sequelae after Ebola virus disease in Bundibugyo, Uganda: a retrospective cohort study. *Lancet Infect Dis* 2015; **15**: 905–12.

13 Qureshi AI, Chughtai M, Loua TO, *et al.* Study of Ebola Virus Disease Survivors in Guinea. *Clin Infect Dis* 2015; **61**: 1035–42.

14 Rowe AK, Bertolli J, Khan AS, *et al.* Clinical, virologic, and immunologic follow-up of convalescent Ebola hemorrhagic fever patients and their household contacts, Kikwit, Democratic Republic of the Congo. Commission de Lutte contre les Epidémies à Kikwit. *J Infect Dis* 1999; **179 Suppl**: S28-35.

15 Bwaka MA, Bonnet MJ, Calain P, *et al.* Ebola hemorrhagic fever in Kikwit, Democratic Republic of the Congo: clinical observations in 103 patients. *J Infect Dis* 1999; **179 Suppl**: S1-7.

16 Mohammed H, Vandy AO, Stretch R, *et al.* Sequelae and Other Conditions in Ebola Virus Disease Survivors, Sierra Leone, 2015. *Emerg Infect Dis* 2017; **23**: 66–73.

17 Nanyonga M, Saidu J, Ramsay A, Shindo N, Bausch DG. Sequelae of Ebola Virus Disease, Kenema District, Sierra Leone. *Clin Infect Dis* 2016; **62**: 125–6.

18 Mattia JG, Vandy MJ, Chang JC, *et al.* Early clinical sequelae of Ebola virus disease in Sierra Leone: a cross-sectional study. *Lancet Infect Dis* 2016; **16**: 331–8.

19 Epstein L, Wong KK, Kallen AJ, Uyeki TM. Post-Ebola Signs and Symptoms in U.S. Survivors. *N Engl J Med* 2015; **373**: 2484–6.

20 Etard J-F, Sow MS, Leroy S, *et al.* Multidisciplinary assessment of post-Ebola sequelae in Guinea (Postebogui): an observational cohort study. *Lancet Infect Dis* 2017. DOI:10.1016/S1473-3099(16)30516-3.

21 Scott JT, Sesay FR, Massaquoi TA, Idriss BR, Sahr F, Semple MG. Post-Ebola Syndrome, Sierra Leone. *Emerg Infect Dis* 2016; **22**: 641–6.

Zika

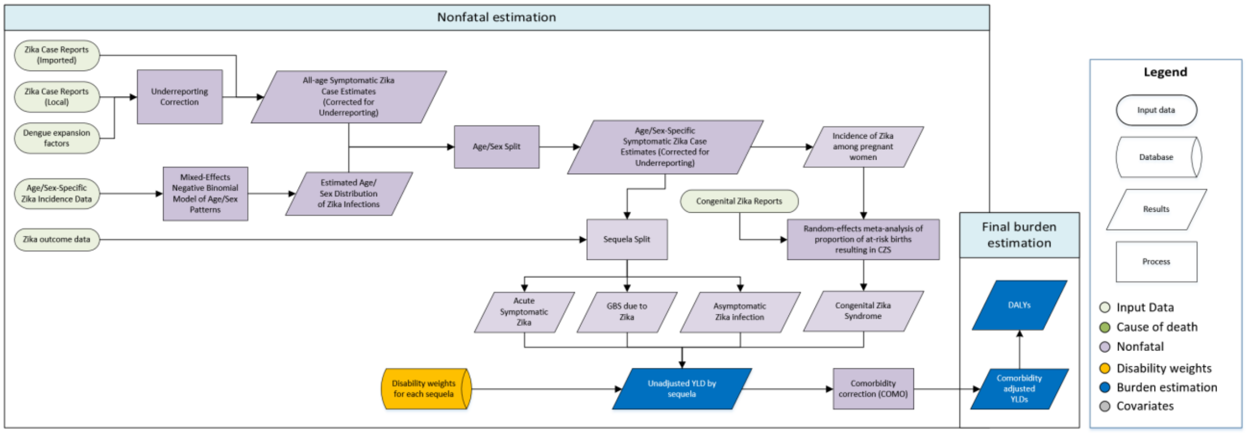

Input data

Data on cases of acute Zika and Congenital Zika Syndrome (CZS) come from official reports, primarily from PAHO.

Modeling strategy

We estimate the all-age incidence of symptomatic Zika as the product of reported Zika cases and country-specific expansion factors that adjust for underreporting. Those expansion factors are derived from our dengue model and the methods used for their estimation are detailed in the dengue model documentation and by Stanaway et al.(1) A subset of incidence data were age/sex-specific, and we used a mixed-effects negative binomial model with cubic splines on age and interaction terms with sex to estimate the age/sex distribution of cases. We then split total incidence based on the age/sex-distribution model to estimate the incidence of symptomatic Zika by location, year, age, and sex.

We conducted a meta-analysis of three studies(2–4) to estimate the proportion of all Zika infections that are symptomatic. We estimate that 41% of Zika infections are symptomatic (14 – 68%), with 59% being asymptomatic. We then estimated incidence of asymptomatic infections as,

$$I_{asymp} = \frac{I_{symp}}{Pr_{symp}} - I_{symp}$$

Where I_{asymp} is the incidence of asymptomatic infections, I_{symp} is the incidence of symptomatic Zika, and Pr_{symp} is the proportion of infections that are symptomatic (i.e. 41%).

We assume that the incidence of Zika among pregnant women equals the incidence of Zika among all women, within a given location, year, and age group. We then estimate the number of pregnant women infected with Zika as the product of incidence of Zika and the number of pregnant women in every location, year, and age group. Finally, we used an intercept only, mixed-effects Poisson regression model, with random effects on location, the number of at-risk births as the exposure term, and the

number of reported CZS cases as the outcome to estimate proportion of at-risk births (i.e. those in which the mother was infected with Zika during pregnancy) resulting in CZS.

Changes from GBD 2015 to GBD 2016

Zika is a new cause for GBD 2016.

References

1. Stanaway JD, Shepard DS, Undurraga EA, Halasa YA, Coffeng LE, Brady OJ, et al. The global burden of dengue: an analysis from the Global Burden of Disease Study 2013. Lancet Infect Dis [Internet]. 2016 Feb [cited 2016 May 23]; Available from: http://linkinghub.elsevier.com/retrieve/pii/S1473309916000268

2. Gallian P, Cabié A, Richard P, Paturel L, Charrel RN, Pastorino B, et al. Zika virus in asymptomatic blood donors in Martinique. Blood. 2017 Jan 12;129(2):263–6.

3. Duffy MR, Chen T-H, Hancock WT, Powers AM, Kool JL, Lanciotti RS, et al. Zika virus outbreak on Yap Island, Federated States of Micronesia. N Engl J Med. 2009 Jun 11;360(24):2536–43.

4. Aubry M, Teissier A, Huart M, Merceron S, Vanhomwegen J, Roche C, et al. Zika Virus Seroprevalence, French Polynesia, 2014–2015. Emerg Infect Dis. 2017 Apr;23(4):669–72.

Dracunculiasis (Guinea worm) Appendix

Guinea Worm

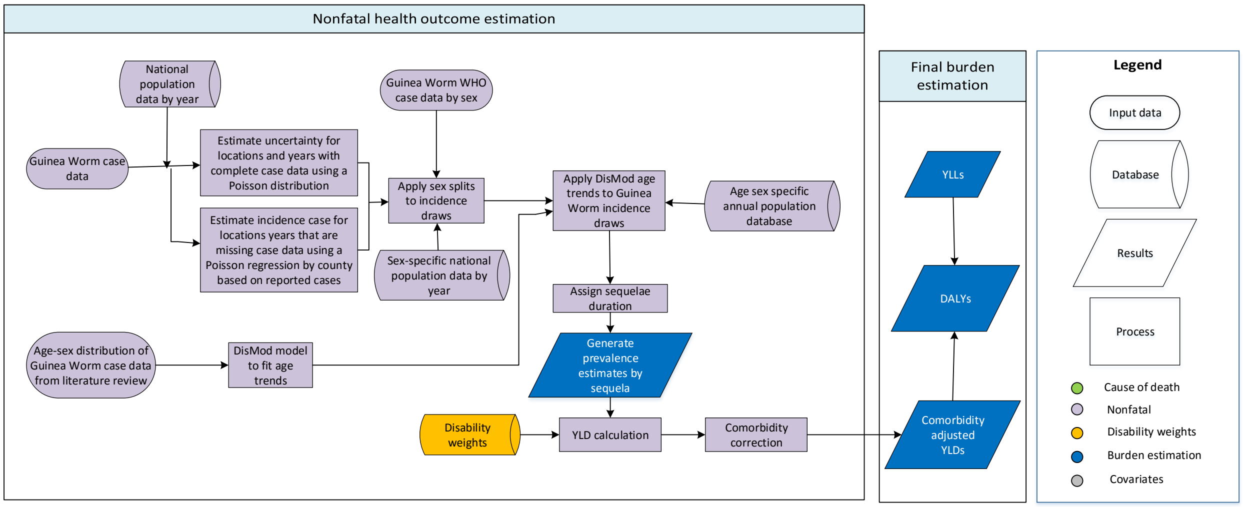

Background

Guinea-worm disease is caused by the parasitic worm *Dracunculus medinensis*. The transmission cycle begins when Guinea worm larvae are released in stagnant water (e.g., ponds, lakes, open wells) where they are ingested by freshwater copepods (small crustaceans sometimes called water fleas) of the genus *Cyclops* [1]. When a person consumes water containing *Cyclops*, the copepods are dissolved by gastric acids and intestinal enzymes and the larvae are released. Larvae then migrate through the intestinal wall and travel to the connective tissues. The larvae mature and mate 60–90 days after infection; shortly thereafter, the male dies and the pregnant female worm continues to move through the victim's connective tissues. Approximately 10–14 months post-infection, the adult worm creates a painful burning blister on the skin that develops and enlarges over several days, usually from the feet or lower limbs. Blister formation may be preceded by a slight fever, itchy rash, nausea, vomiting, and diarrhea. To relieve the pain associated with the worm's emergence, infected persons immerse the infected part of their body in local stagnant water sources, such as ponds. Upon entering the water, the female worm will expel her larvae and the cycle can begin again [1-4].

The global campaign to eradicate Guinea worm began in 1980, when the U.S. Centers for Disease Control and Prevention (CDC) suggested that Guinea worm eradication would be an ideal indicator of the success of the International Drinking Water Supply and Sanitation Decade of 1981–1990; in 1981, Guinea worm eradication was adopted as a sub-goal of this United Nations advocacy effort [1, 5]. In 1986, the World Health Assembly adopted a resolution to eliminate Guinea worm disease and since then The Carter Center has led a coalition that includes ministries of health of endemic countries, CDC, the World Health Organization (WHO), the United Nations Children's Fund (UNICEF), thousands of village volunteers and supervisory staff supported by numerous donors [5].

To break the cycle of transmission, ministries of health in endemic countries implement a suite of interventions: case detection and containment; provision of safe water sources; distribution of filter cloths and pipe filters; water source treatment with Abate® (a larvacide) and health education.

By design, the Guinea worm eradication programmatic infrastructure covers the entire at-risk population in endemic countries. Since case containment[6] is a key intervention designed to not only interrupt transmission but also monitor progress towards eradication, incident cases of guinea worm disease are nationally representative. To implement case containment as an intervention, all cases of Guinea worm disease are identified. Containment is defined as: detection within 24 hours of the worm's emergence; the patient did not contaminate any water source; the patient received proper wound care and health education on not entering any water source; a supervisor verified the case as dracunculiasis within 7 days; and Abate® is used if there is any uncertainty about contamination of water sources or known contamination of water sources [7]. Case reporting occurs at the village level on a monthly basis; case data are then aggregated within the national Guinea Worm Eradication Program and reported to the World Health Organization. In settings where annual case reports are low (suggesting no transmission) or transmission has been interrupted, cash rewards are promoted to enhance surveillance activities.

Input Data & Methodological Summary

Case Definition

A Guinea worm case is defined as an individual with Guinea worm disease. A person is counted as a case only once in a calendar year, i.e., when the first Guinea worm emerged from that person, although an individual may have more than one worm emerge at a time and/or more than one worm emerge during the year. These cases are confirmed through the Guinea worm eradication program infrastructure by clinical exam and verification by local supervisors. All specimens from all case-patients are sent to the U.S. Centers for Disease Control and Prevention (CDC) for laboratory evaluation and confirmation [7].

Input data

Model inputs

Geographic restrictions

Only the following countries were identified as guinea-worm endemic as of 1990[8]: Benin, Burkina Faso, Cameroon, Central African Republic, Chad, Cote d'Ivoire, Ethiopia, Ghana, India, Kenya, Mali, Mauritania, Niger, Nigeria, Pakistan, Senegal, Sudan, South Sudan, Togo, Uganda, and Yemen[8]. Any country not reporting Guinea worm in 1990 is not included in the GBD model.

Geographic restrictions by year were also implemented to account for the period post-transmission to reflect the accomplishments of the Guinea worm eradication campaign. Geographic restriction for countries that were endemic in 1990 was defined based on data reported post-interruption of transmission. In the GBD analysis, Guinea worm disease was no longer modeled for the year that followed the last reported case (imported or indigenous) provided that the subsequent years through 2015 also had no case reports. To ensure that cases were attributed to burden in the country in which the case was detected, both indigenous and imported cases were included. For example, Kenya reported its last (imported) case in 2005, and as no other cases were reported through 2015, incidence

from 2006 onwards is zero. For Chad, a country that had years during which no cases were reported, the model covers the entire period 1990-2015.

Data sources

1) Case data by location, by year
2) Literature review of age/sex distribution
3) Literature review for sequelae (type, duration and proportion)

Case data: Annual case data were reported by the WHO in the Weekly Epidemiological Record. For years or locations for which WER reports were not published, the following sources were also used to extract case counts:

1) CDC's MMWR reports
2) 1990-1999 total country reports from Hopkins *et al*[8]
3) India subnational estimates: India MOH report (1984-1999)
4) The Carter Center's Guinea worm wrap-up: disaggregation of case totals for Sudan and South Sudan pre-2011 (independence) to ensure case totals from 1990-2010 are consistent with current national boundaries; 2016 provisional case data.

The number of cases annually was compared to official total numbers published in WER 2016 to ensure accuracy of data entry.

Subnational data

India: Subnational data for India was obtained from the Ministry of Health for the period 1984-1999; cases were reported by year and state: http://www.ncdc.gov.in/index2.asp?slid=329&sublinkid=216

Kenya: Subnational data from Kenya was requested from the MOH but not obtained. To split cases by subnational unit, the Carter Center Guinea Worm Wrap Up was reviewed to identify districts with endemic villages. A national survey conducted 1993/1994 found cases in Turkana and West Pokot counties, but case totals were not reported by county. Indigenous transmission was interrupted in 1995, with imported cases reported until 2005. WER reports from 1999-2006 document that all imported cases from 1998-2005 occurred in Turkana County. All cases in Kenya are currently analyzed in GBD as occurring in Turkana County as we are unable to disaggregate the data.

Accounting for possible under-reporting

Once national eradication programs were initiated, national case searches were conducted to improve the accuracy of national case estimates. These searches were designed to enumerate prevalent Guinea worm disease cases and identify endemic villages to direct intervention and surveillance activities. For the majority of years included in the GBD analysis, the total number of Guinea worm cases reported is equivalent to a national census, as all cases are identified and reported. Nevertheless, not all endemic countries were able to initiate full national surveillance as of 1990. National case searches began as follows: India (1980); Pakistan (1987); Cameroon and Nigeria (1988); Ghana (1989); Benin, Burkina Faso, Central African Republic, Côte d'Ivoire and Togo (1990); Niger, Mauritania, Mali, Senegal, Uganda and Yemen (1991); Ethiopia and Sudan (1992); and Chad and Kenya (1993). By 1995, all endemic countries had begun implementing case-containment strategies[2].

The model does not account for the possibility that cases occurred in communities that were not included in routine surveillance or did not achieve 100% reporting coverage over time. However, any cases that may have been undetected would likely not have been a significant increase over annual totals given the comprehensive nature of Guinea worm disease surveillance activities. Nevertheless, there are years for which the annual case data is inconsistent with preceding/following annual case totals and could not be accounted for in our model. For example, Niger reported 500 cases in 1992, despite reporting 32,829 cases in 1991 and 25,346 cases in 1993. In those instances, the following data points were identified as outliers and excluded from analysis as follows:

Table 1. List of reported case data outliered in the analysis to account for possible under-reporting

Country	Year	Reported Cases
Central African Republic	1996	9
Central African Republic	1997	5
Ethiopia	1992	303
Kenya (Turkana County)	1990	6
Uganda	1990	4,704
Uganda*	1992	126,369
Benin	1991	4,006
Benin	1992	4,315
Chad	1992	156
Cote d'Ivoire	1990	1,360
Mali	1990	884
Mauritania	1992	1,557
Niger	1992	500
Senegal	1990	38
Togo	1990	3,042
Togo	1991	5,118
South Sudan*	1996	116,844
Sudan	1994	132

*For these two data points, we do not dispute that over 100,000 cases of Guinea worm likely occurred. However, given the amount of missing data in the early time series for these two countries, inclusion of these resulted in implausibly high case predictions (over 1 million cases in Uganda in 1990 and over 1.5 million for South Sudan from 1990-1995).

Age/Sex distribution

Generally, the risk of Guinea worm infection varies according to sex or age-specific differences in access to safe drinking water. A study in Ethiopia found women were more likely to experience Guinea worm disease than men; in India, men experienced greater risk of infection [1]. Exposure to unsafe water sources varies largely on mobility patterns and type of water sources: communities in which infected

water is carried in for consumption are more likely to see more Guinea worm disease in children and older adults [9]. Once interventions to control the spread of Guinea worm infection are implemented, the age and sex distribution likely changes to reflect variation in coverage and uptake of eradication interventions, such as larvacide of water sources and case-containment rates; age/sex case data are currently not available.

The evidence base available to describe risk of infection by age is as follows:

1) Studies from Nigeria:
    a. Adeyeba *et al* [10]: Guinea worm disease not common among children <1 year of age; increase in risk by age
    b. Kale *et al* [11]: More boys ages 5-9 years than girls were infected (11.9% v. 6.8%); Women ages 20-29 years had higher prevalence of infection than men (13.4% v. 4.7%); Overall, the prevalence in both men and women was highest in ages 10-14 years and 30 years or older.
    c. Greenwood *et al* [12]: The mean age of male cases was 25.8 years (95% CI: 23.9, 27.7) and 26.9 years for females (95% CI: 23.7, 30.1).
2) Other countries:
    a. Sudan [13]: No significant age trend among lower-endemicity villages, higher endemicity villages (n=4) had higher prevalence in children and older adults. This study attributes the difference in age trends to community-level water source.
    b. Ghana [14]: The trend in age of first infection reported was similar for males and females, with more females experiencing first infection between 15-19 years and males between 20-24 years of age. The proportion of men with Guinea worm disease was much higher than women among ages 25-54 years of age. Adults >15 years of age were more likely to be infected than children.

The evidence base available to describe the risk of infection by gender is as follows:

1) Studies from Nigeria:
    a. Adeyeba *et al* [10]: No difference among males and female
    b. Kale *et al* [11]: No overall gender difference comparing total males infected to total females infected, although gender differences for certain age groups (see notes above).
    c. Greenwood *et al* [12]: Two-thirds of cases reported among 47 villages from 1971-1974 were male.

WHO Weekly Epidemiological Record (WER) age reports: Age and sex data were reported by country for 2009 onwards; these data capture the age distribution for Chad, Ethiopia, Ghana, Mali, and South Sudan. We excluded these data as the age/sex distribution is only described for children <15 years or adults, which does not permit fitting an age trend across multiple categories.

WER sex-specific data: Sex-specific differences in the burden of Guinea worm disease could reflect differing levels of access to eradication program interventions, in addition to risk factors associated with local transmission dynamics. Since the data reported from 2009-2015 are the only available data nationally representative, we used the overall sex difference to generate sex-specific incidence and prevalence, with females experiencing a slightly higher risk (53%) compared to males (47%):

Table 2. WHO Weekly Epidemiological Record total worm burden by gender, by year

Year	Female	Male	Total	% Fem	% Male
**2009**	1699	1490	3189	53%	47%
**2010**	976	821	1797	54%	46%
**2011**	524	534	1058	50%	50%
**2012**	273	269	542	50%	50%
**2013**	79	69	148	53%	47%
**2014**	63	63	126	50%	50%
**2015**	9	13	22	41%	59%
**Total**	3623	3259	6882	53%	47%

There is limited evidence to suggest that risk varies jointly by sex and age; however, evidence for this modification also suggests that such age and sex specific risks may vary by endemic community within a given geography (in some settings, women at higher risk, in others men, but not for all age strata). Without additional data sources in which cases are disaggregated by age and sex, this joint relationship is not modeled.

To model age-specific variation, we used data from seven studies with age-specific case data to generate an age-trend in a Dismod model. We further assumed no Guinea worm disease occurred in infants less than 1 year of age.

Severity splits/sequelae

Sequelae associated with Guinea worm relate to the wound at the site of the worm's emergence, which can include abscesses and chronic ulcerations. Joint and tissue damage can occur, as well as secondary infection in connective tissues [15]. During the worm's emergence, which takes approximately one month to exit the body, the ulcer is painful and itchy [1]. The wound is subject to secondary infection and scarring. Possible long-term consequences of Guinea worm infection include arthritis or other permanent damage to connective tissues; however, data on this is limited. In the Greenwood study, 41.7% of all cases experienced infection at the site of emergence and the annual proportion of cases with definite arthritis ranged from 1.6% to 7.3% of all cases.

While an individual experiences Guinea worm disease, they are generally unable to work and have limited mobility at the time prior and during emergence and in the subsequent period in which they are healing. Although most worms emerge in the feet and lower legs, there are reports of worms exiting at other sites [15], which could cause other disability not accounted for here. A study in Nigeria found that 98% of worms emerged in the lower limbs[16]. The Greenwood study also observed that 88.4% emerged in the lower limbs. Therefore, for the purposes of estimating the burden of Guinea worm disease in the GBD, all disability associated with Guinea worm disease is attributed to lower limb conditions, pain and lack of mobility. Due to limited data, we cannot account for differential disability based on number of worms emerging at the same time.

The following evidence base was reviewed to determine the proportion of cases attributed to each sequela, as well as duration of sequelae.

Duration of disability and type of disability:

Studies from Nigeria:

1) Adeyeba *et al* [10]: 93.4% incapacitated for an average of 26 days
2) Smith *et al* [17]: Average disability duration 12.7 weeks; 58% unable to leave the home for a mean duration of 4.2 weeks; duration of disability greater among those older than 50 years compared to those younger than 50 years
3) Okoye *et al* [16]: 21% of cases were totally incapacitated due to their infection (not permanently disabled)
4) Kate *et al* [11]: A survey of 17 villages from 1971-1975 found that duration of disability was approximately 100 days
5) Greenwood *et al* [12]: Weekly visits to 47 villages from 1971-1974 reported mean duration of illness ranging from 4.2 weeks to 7.2 weeks. 17.4% of cases had an active infection which persisted for 10 weeks or more.

Other countries:

6) Benin [18]: From two villages in highly endemic areas, estimated that 39-59 days of disability experienced after worm emergence.
7) Ghana [19]: 28.2% experienced pain 12-18 months post emergence; 5% unable to carry out at least one daily activity, 0.5% permanently impaired (ligament damage to thumb)
8) Ghana [14]: Complete disability experienced among males with Guinea worm disease lasted approximately 5 weeks among those untreated. Among cases provided supportive care (wound management), the duration of disability was 2.5 weeks.

For all cases, we assume each experiences pain and disfigurement (level 2), and musculoskeletal problems lower limb (moderate) for a period of one month, followed by two months of pain and disfigurement (mild). We then assume that 30% of all cases will then experience disfigurement level 1 with itch/pain for an additional 9 months (approximately a year of disability) to account for longer-term disability associated with recovery.

Table 3. Sequela associated with Guinea worm disease in Global Burden of Disease study

Sequela	Lay description	DW (95% CI)
Disfigurement, level 2, with itch/pain	Has a visible physical deformity that is sore and itchy. Other people stare and comment, which causes the person to worry. The person has trouble sleeping and concentrating.	0.188 (0.125-0.267)
Disfigurement, level 1, with itch/pain	Has a slight, visible physical deformity that is sometimes sore or itchy. Others notice the deformity, which causes some worry and discomfort.	0.027 (0.015-0.042)
Musculoskeletal problems, lower limbs, moderate	Has moderate pain in the leg, which makes the person limp, and causes some difficulty walking, standing, lifting and carrying heavy things, getting up and down and sleeping.	0.079 (0.054-0.11)

Modeling strategy

Total incidence

The incidence of Guinea worm disease is modeled in GBD using two approaches: for years and locations for which case data were reported, 1,000 draws of incidence were estimated using a beta distribution of cases and total population minus cases. For years and locations for which case data were missing (largely the early 1990s) a Poisson regression of all case data was implemented per country, using the total population as the offset. The predicted incidence and standard error was used to generate a random distribution of 1,000 incidence draws. Incidence is multiplied by duration of sequelae to calculate prevalence. Country-level incidence predictions are shown in the following figures.

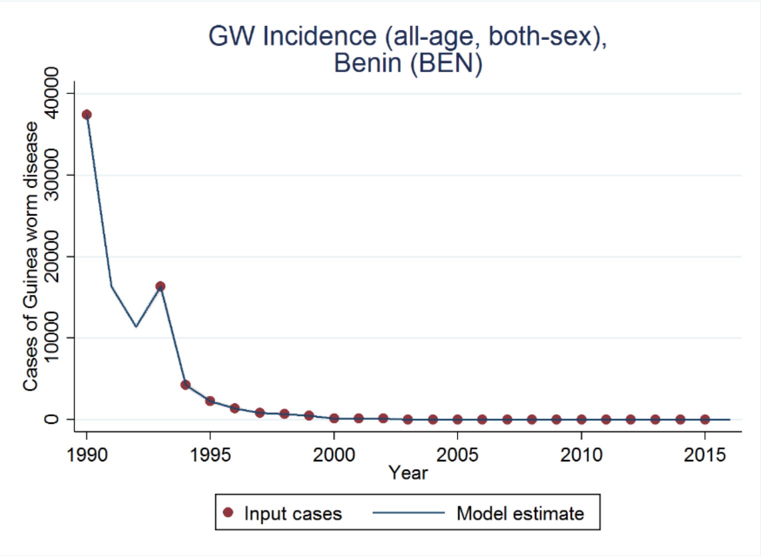
GW Incidence (all-age, both-sex),
Benin (BEN)
Cases of Guinea worm disease
0
10000
20000
30000
40000
1990
1995
2000
2005
2010
2015
Year
Input cases
Model estimate

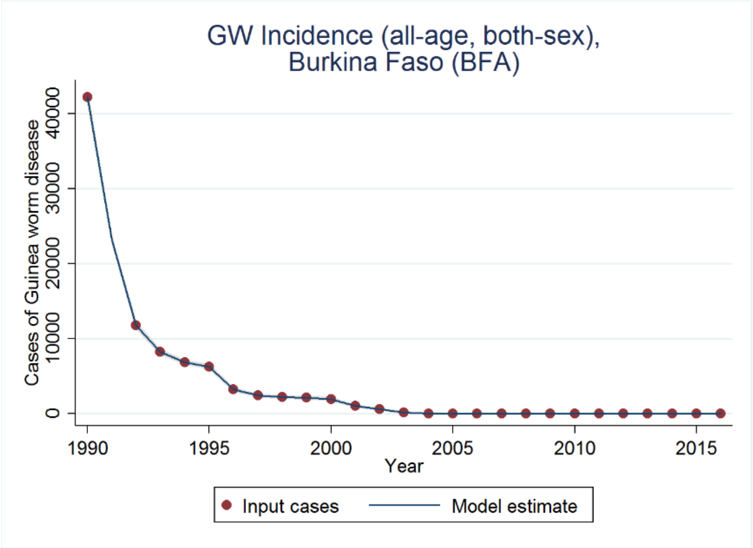
GW Incidence (all-age, both-sex),
Burkina Faso (BFA)
Cases of Guinea worm disease
0
10000
20000
30000
40000
1990
1995
2000
2005
2010
2015
Year
Input cases
Model estimate

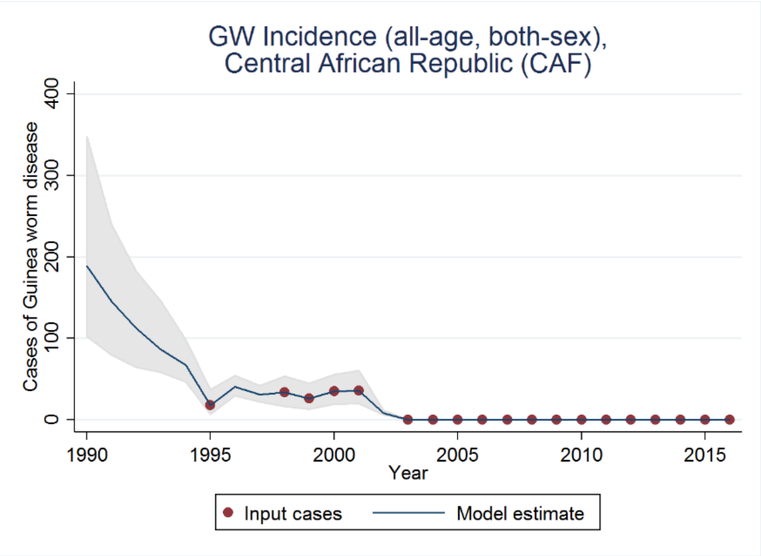
GW Incidence (all-age, both-sex),
Central African Republic (CAF)
Cases of Guinea worm disease
0
100
200
300
400
1990
1995
2000
2005
2010
2015
Year
Input cases
Model estimate

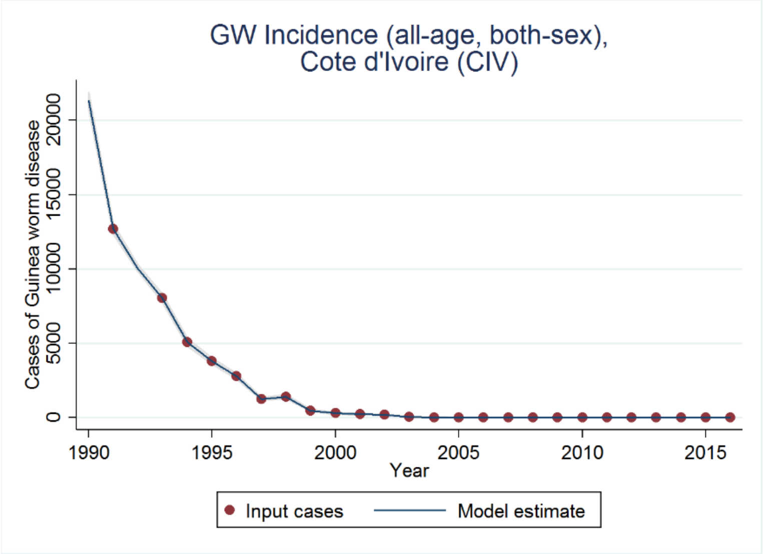
GW Incidence (all-age, both-sex),
Cote d'Ivoire (CIV)
Cases of Guinea worm disease
0
5000
10000
15000
20000
1990
1995
2000
2005
2010
2015
Year
Input cases
Model estimate

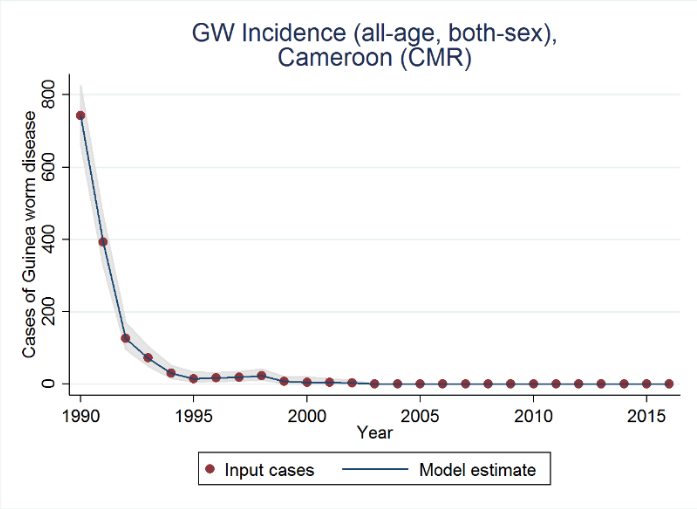
GW Incidence (all-age, both-sex),
Cameroon (CMR)
Cases of Guinea worm disease
0
200
400
600
800
1990
1995
2000
2005
2010
2015
Year
Input cases
Model estimate

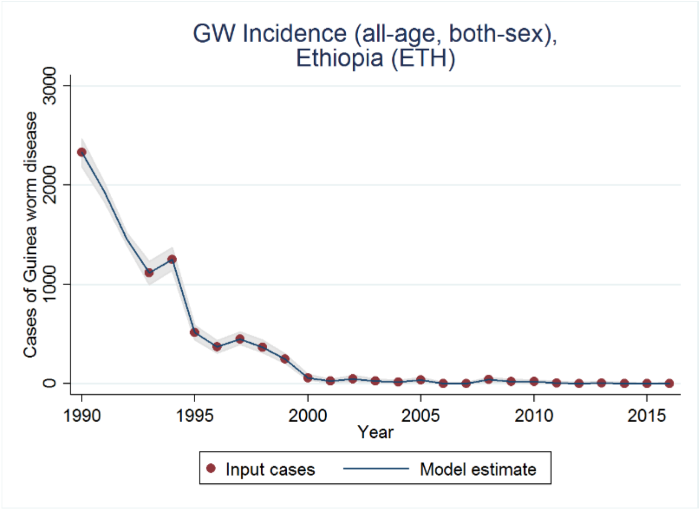
GW Incidence (all-age, both-sex),
Ethiopia (ETH)
Cases of Guinea worm disease
0
1000
2000
3000
1990
1995
2000
2005
2010
2015
Year
Input cases
Model estimate

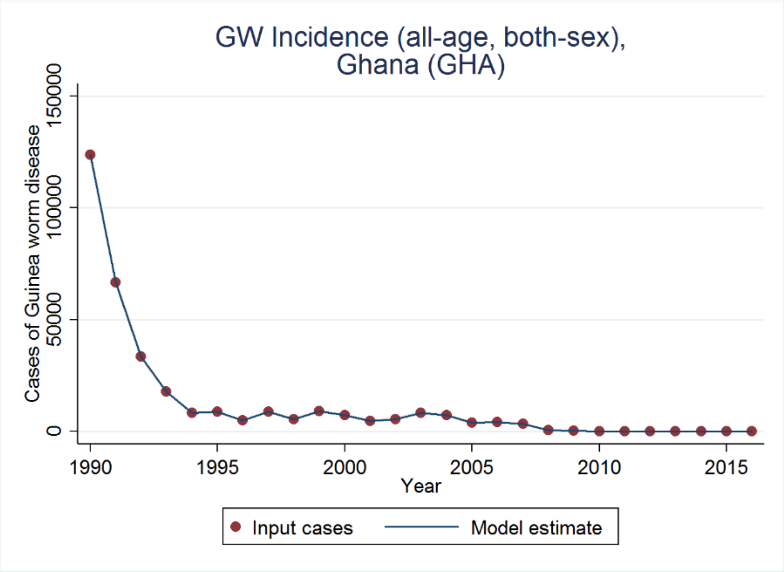
GW Incidence (all-age, both-sex),
Ghana (GHA)
Cases of Guinea worm disease
0
50000
100000
150000
1990
1995
2000
2005
2010
2015
Year
Input cases
Model estimate

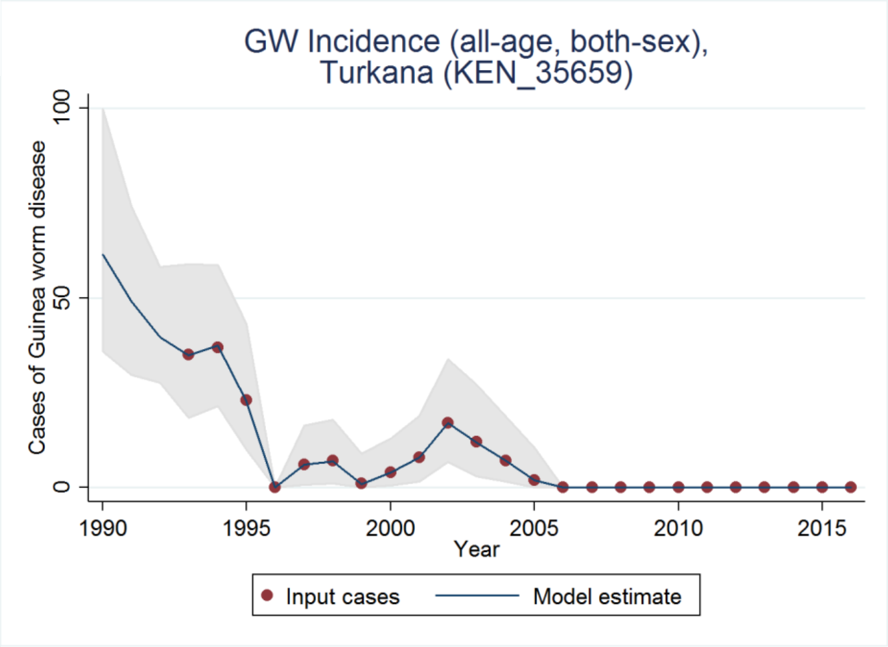
GW Incidence (all-age, both-sex),
Turkana (KEN_35659)
Cases of Guinea worm disease
0
50
100
1990
1995
2000
2005
2010
2015
Year
Input cases
Model estimate

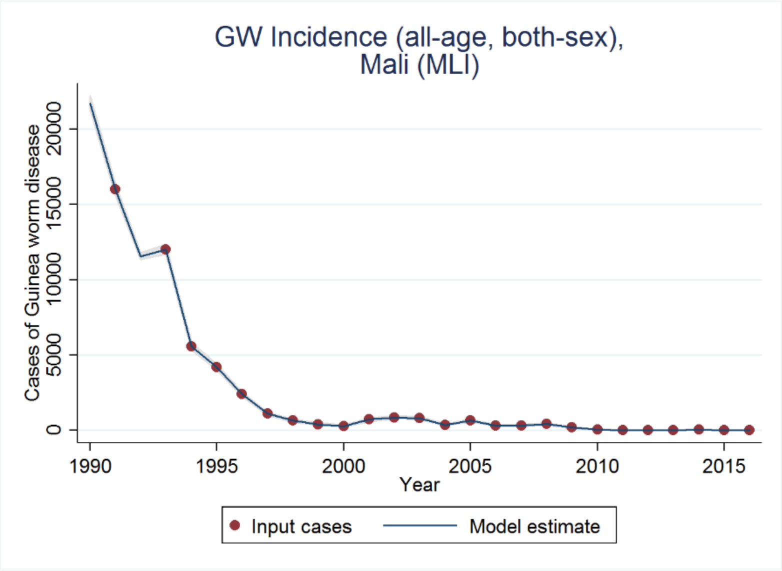

GW Incidence (all-age, both-sex),
Mali (MLI)
Cases of Guinea worm disease
0
5000
10000
15000
20000
1990
1995
2000
2005
2010
2015
Year
Input cases
Model estimate

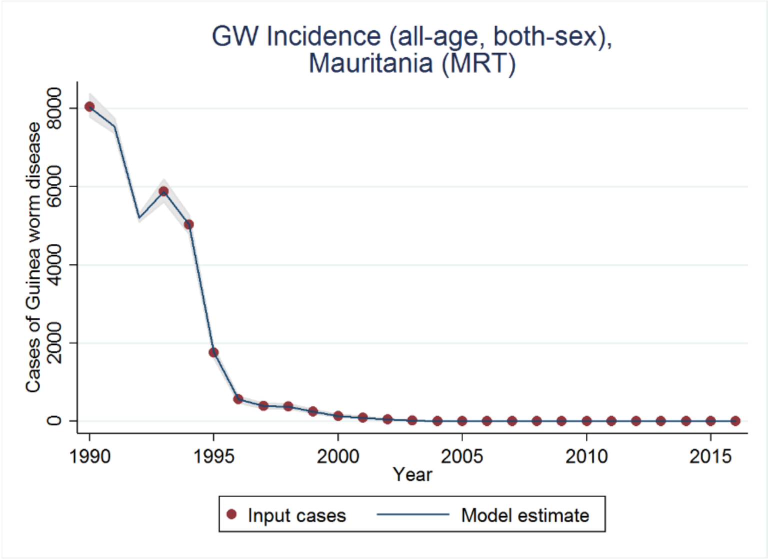

GW Incidence (all-age, both-sex),
Mauritania (MRT)
Cases of Guinea worm disease
0
2000
4000
6000
8000
1990
1995
2000
2005
2010
2015
Year
Input cases
Model estimate

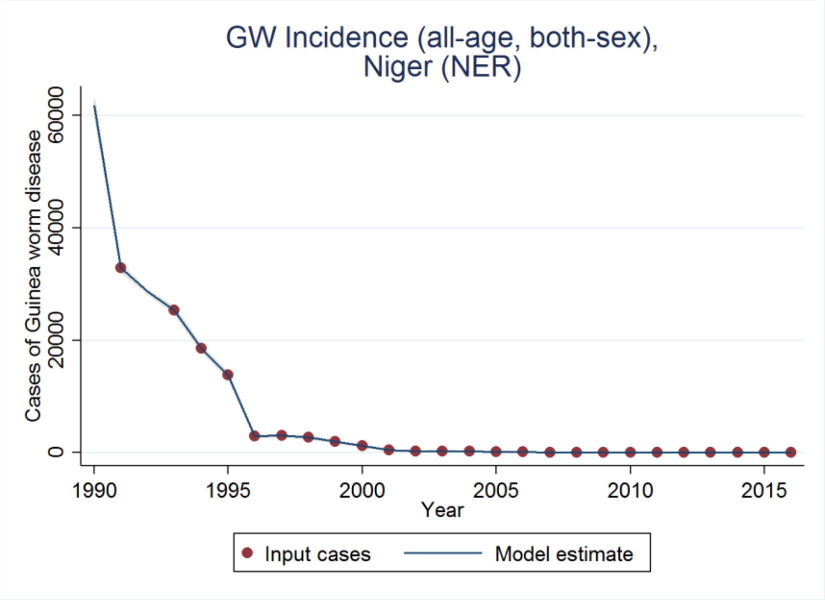
GW Incidence (all-age, both-sex),
Niger (NER)
Cases of Guinea worm disease
0
20000
40000
60000
1990
1995
2000
2005
2010
2015
Year
Input cases
Model estimate

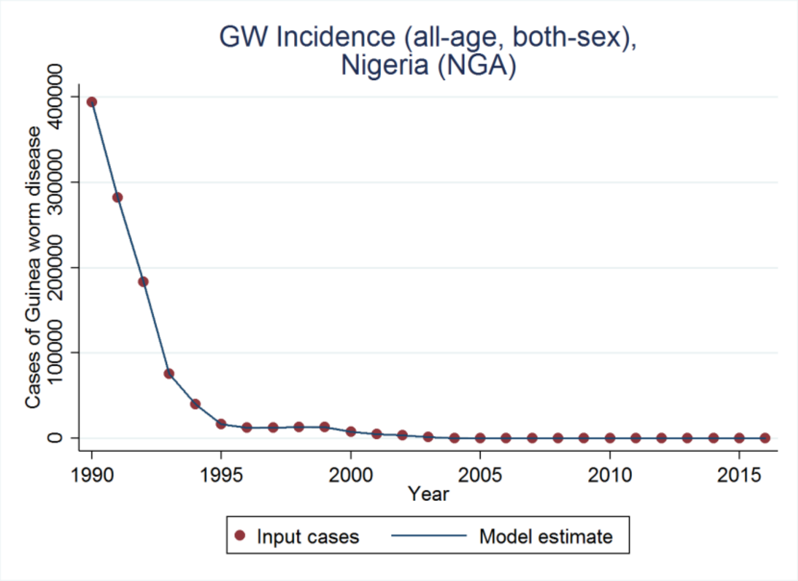
GW Incidence (all-age, both-sex),
Nigeria (NGA)
Cases of Guinea worm disease
0
100000
200000
300000
400000
1990
1995
2000
2005
2010
2015
Year
Input cases
Model estimate

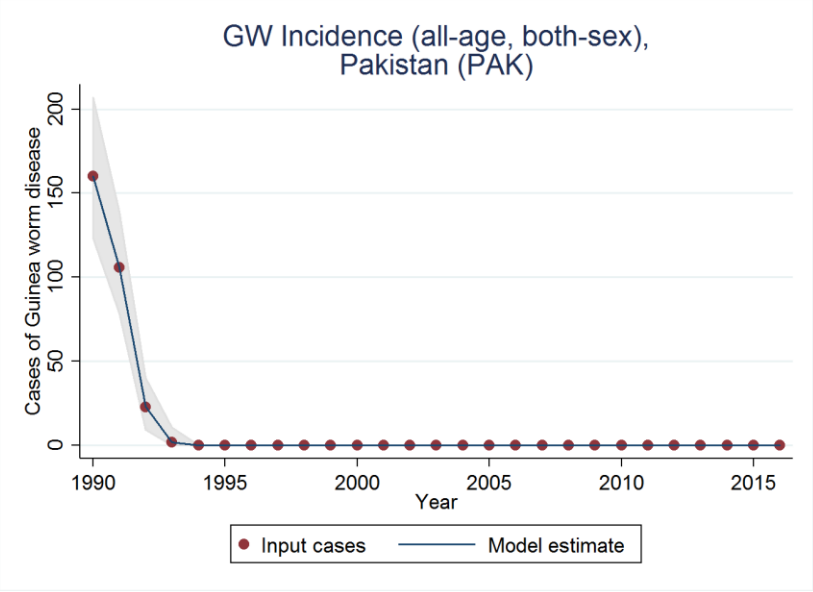
GW Incidence (all-age, both-sex),
Pakistan (PAK)
Cases of Guinea worm disease
0
50
100
150
200
1990
1995
2000
2005
2010
2015
Year
Input cases
Model estimate

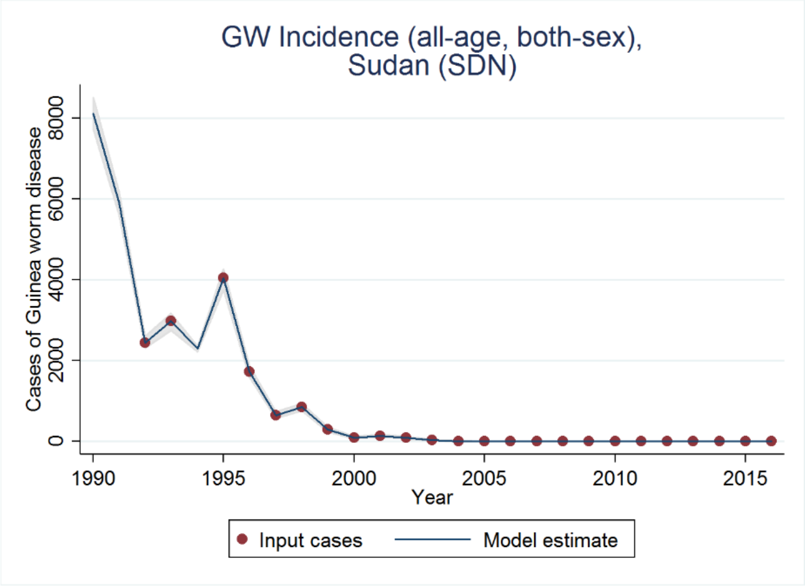
GW Incidence (all-age, both-sex),
Sudan (SDN)
Cases of Guinea worm disease
0
2000
4000
6000
8000
1990
1995
2000
2005
2010
2015
Year
Input cases
Model estimate

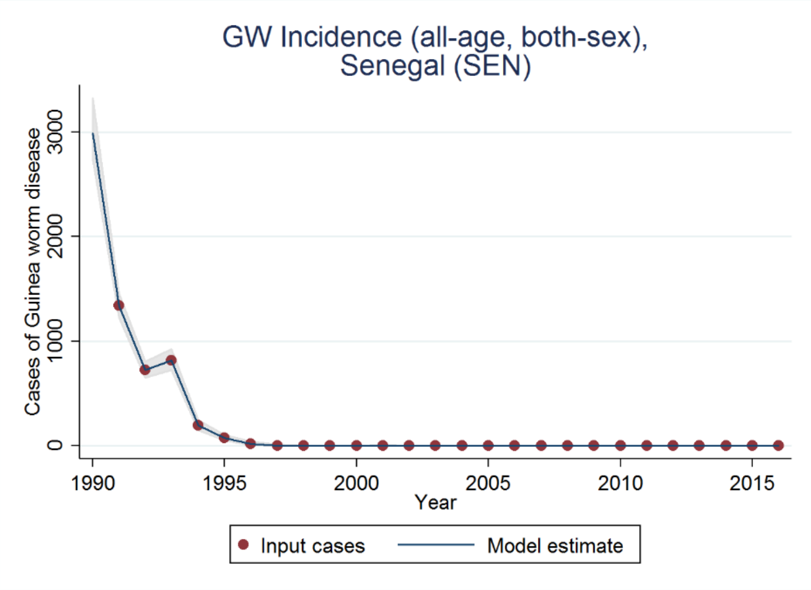
GW Incidence (all-age, both-sex),
Senegal (SEN)
Cases of Guinea worm disease
0
1000
2000
3000
1990
1995
2000
2005
2010
2015
Year
Input cases
Model estimate

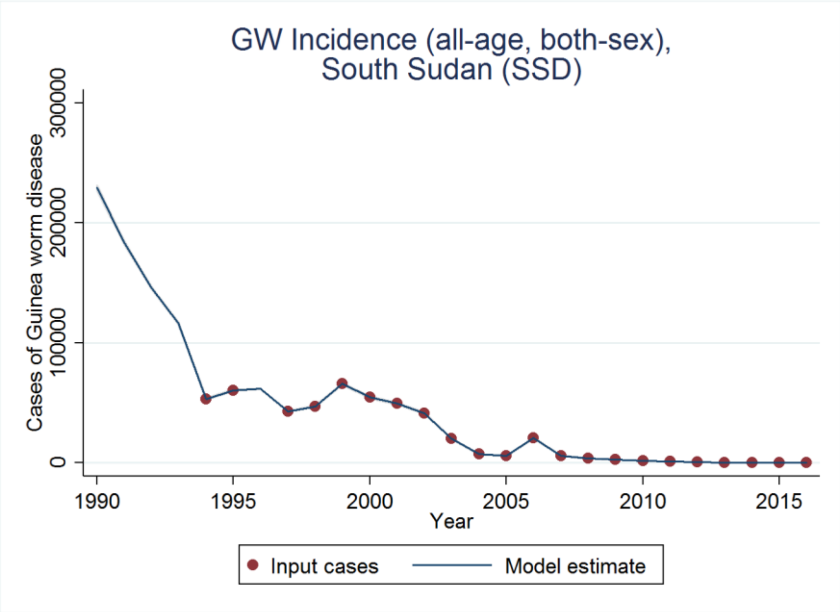
GW Incidence (all-age, both-sex),
South Sudan (SSD)
Cases of Guinea worm disease
0
100000
200000
300000
1990
1995
2000
2005
2010
2015
Year
Input cases
Model estimate

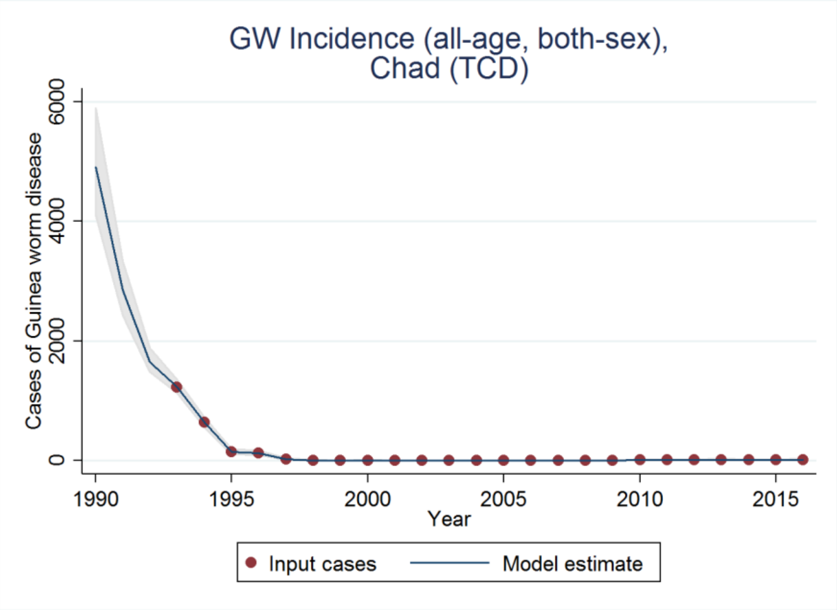
GW Incidence (all-age, both-sex),
Chad (TCD)
Cases of Guinea worm disease
0
2000
4000
6000
1990
1995
2000
2005
2010
2015
Year
Input cases
Model estimate

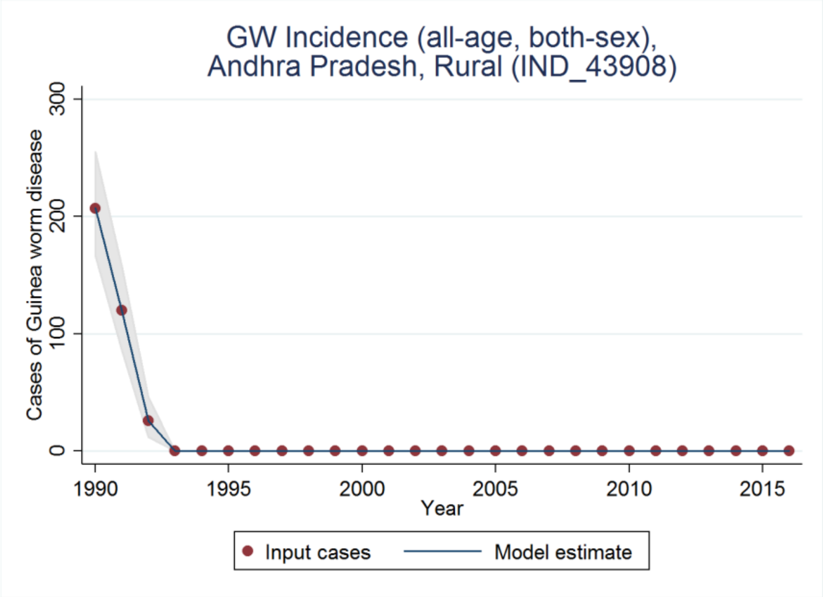
GW Incidence (all-age, both-sex),
Andhra Pradesh, Rural (IND_43908)
Cases of Guinea worm disease
0
100
200
300
1990
1995
2000
2005
2010
2015
Year
Input cases
Model estimate

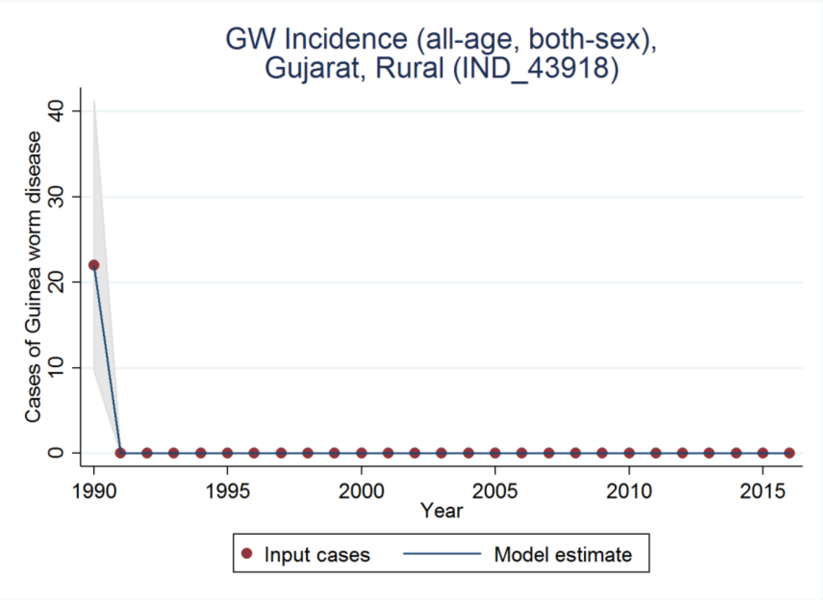
GW Incidence (all-age, both-sex),
Gujarat, Rural (IND_43918)
Cases of Guinea worm disease
0
10
20
30
40
1990
1995
2000
2005
2010
2015
Year
Input cases
Model estimate

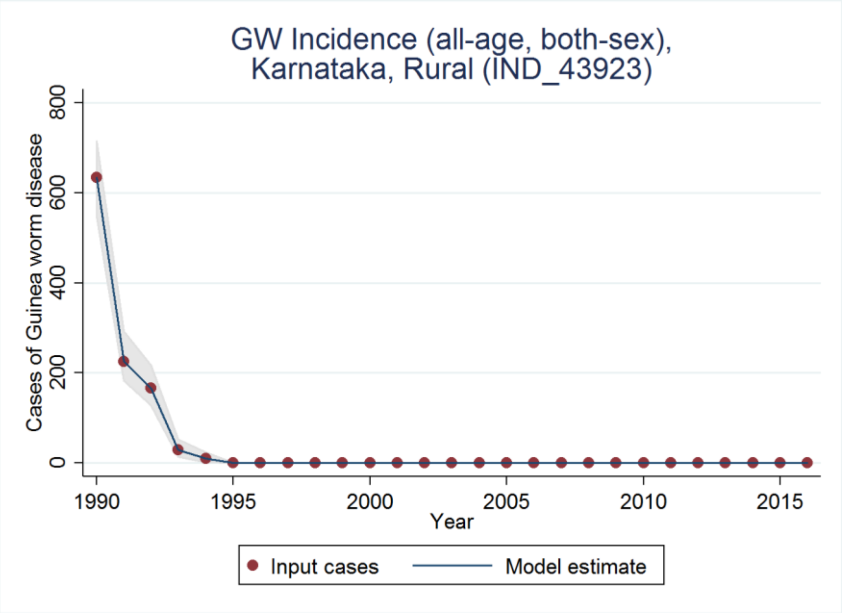
GW Incidence (all-age, both-sex),
Karnataka, Rural (IND_43923)
Cases of Guinea worm disease
0
200
400
600
800
1990
1995
2000
2005
2010
2015
Year
Input cases
Model estimate

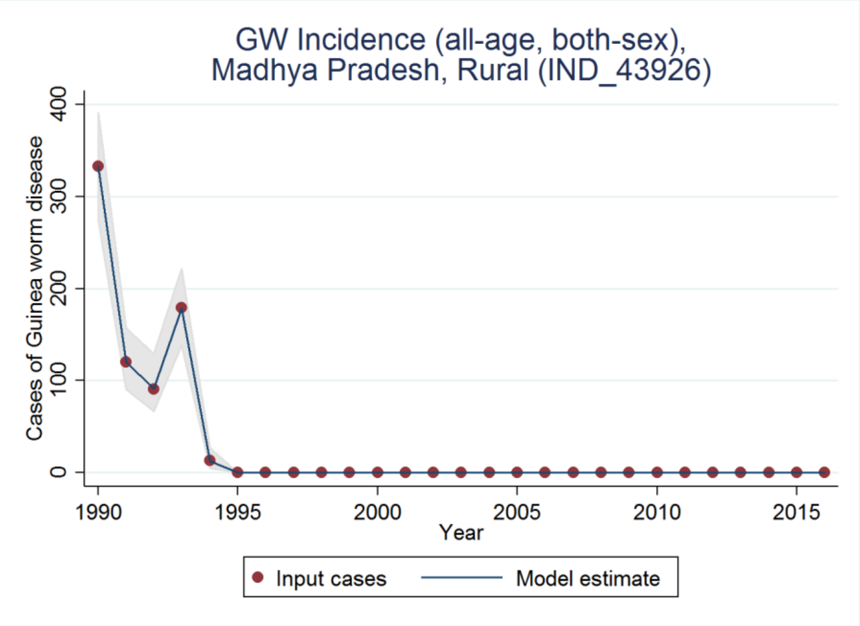
GW Incidence (all-age, both-sex),
Madhya Pradesh, Rural (IND_43926)
Cases of Guinea worm disease
0
100
200
300
400
1990
1995
2000
2005
2010
2015
Year
Input cases
Model estimate

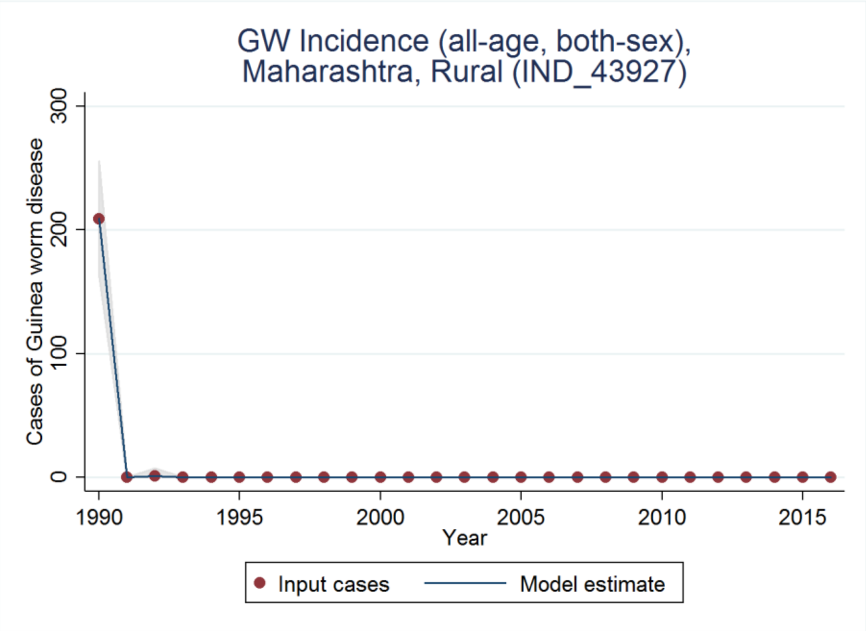

GW Incidence (all-age, both-sex),
Maharashtra, Rural (IND_43927)
Cases of Guinea worm disease
0
100
200
300
1990
1995
2000
2005
2010
2015
Year
Input cases
Model estimate

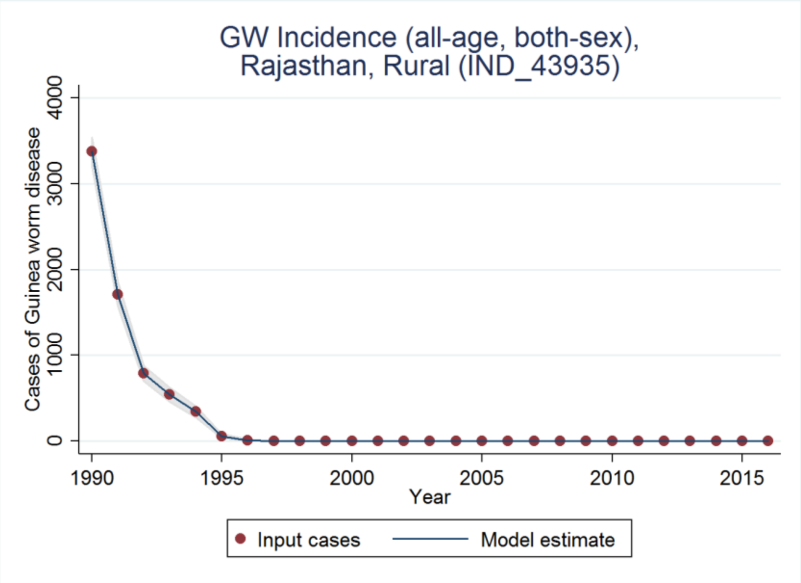

GW Incidence (all-age, both-sex),
Rajasthan, Rural (IND_43935)
Cases of Guinea worm disease
0
1000
2000
3000
4000
1990
1995
2000
2005
2010
2015
Year
Input cases
Model estimate

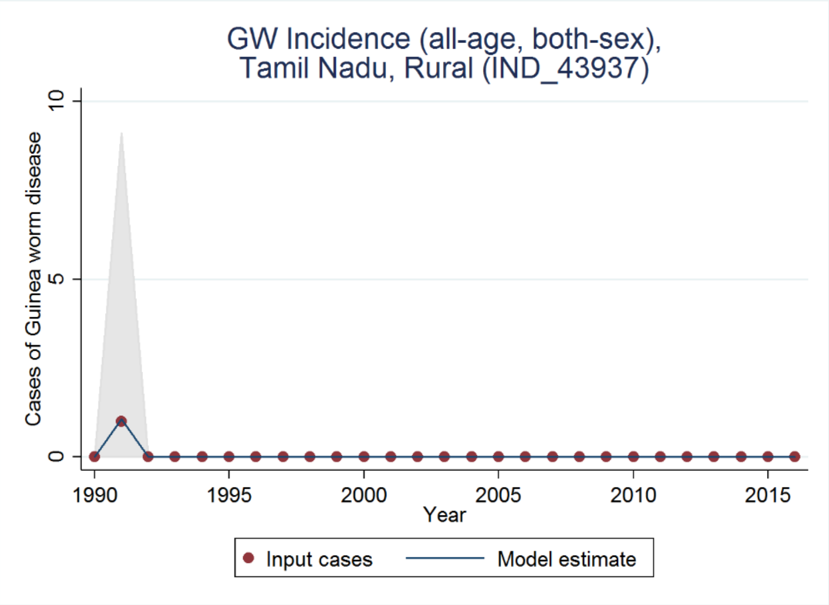

GW Incidence (all-age, both-sex),
Tamil Nadu, Rural (IND_43937)
Cases of Guinea worm disease
0
5
10
1990
1995
2000
2005
2010
2015
Year
Input cases
Model estimate

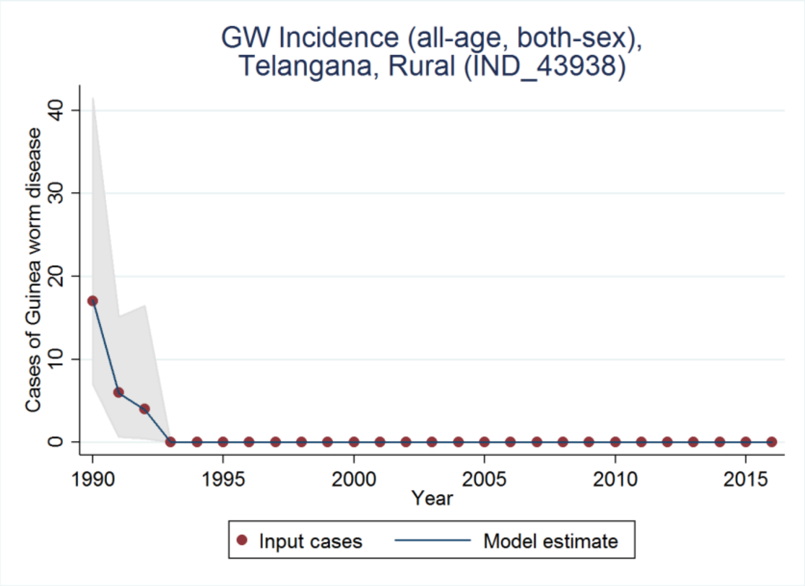

GW Incidence (all-age, both-sex),
Telangana, Rural (IND_43938)
Cases of Guinea worm disease
0
10
20
30
40
1990
1995
2000
2005
2010
2015
Year
Input cases
Model estimate

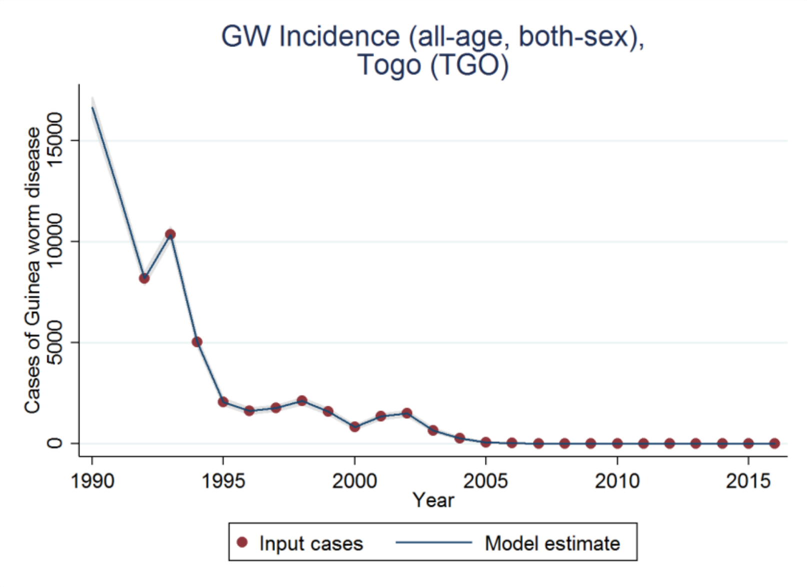
GW Incidence (all-age, both-sex),
Togo (TGO)
Cases of Guinea worm disease
0
5000
10000
15000
1990
1995
2000
2005
2010
2015
Year
Input cases
Model estimate

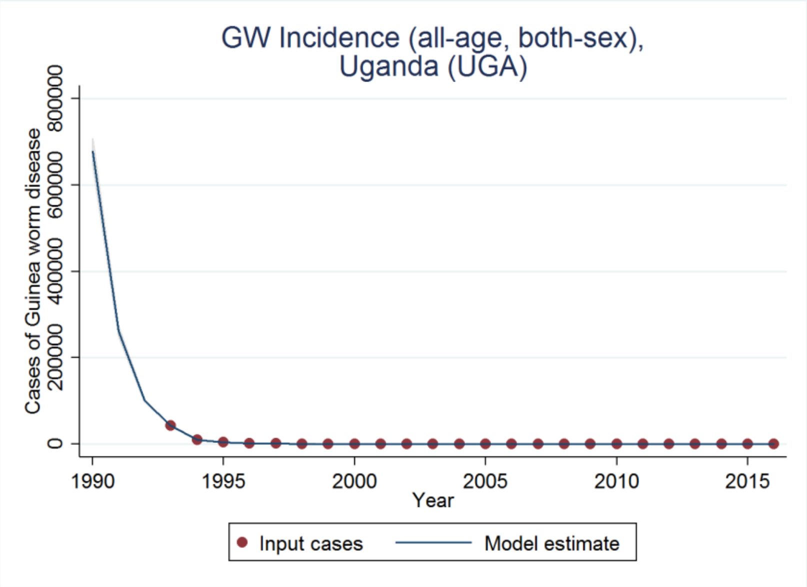
GW Incidence (all-age, both-sex),
Uganda (UGA)
Cases of Guinea worm disease
0
200000
400000
600000
800000
1990
1995
2000
2005
2010
2015
Year
Input cases
Model estimate

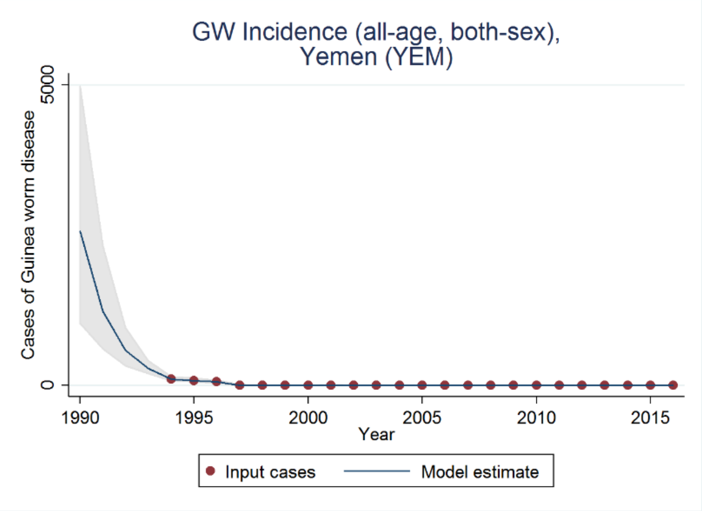
GW Incidence (all-age, both-sex),
Yemen (YEM)
Cases of Guinea worm disease
0
5000
1990
1995
2000
2005
2010
2015
Year
Input cases
Model estimate

Figure 1. Overall comparison of model versus reported cases (excluding outliers)

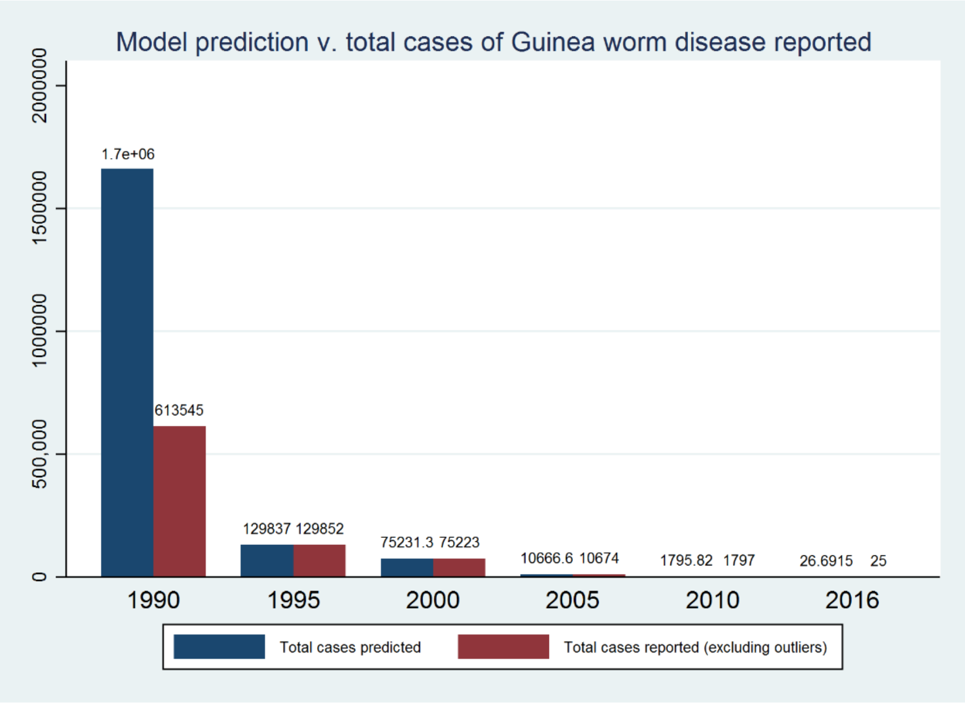

Sex-specific incidence

To account for the proportion of cases in females compared to males (53% to 47%), the incidence draws were multiplied by the sex proportion and the total population (to estimate number of cases by sex), then divided by the sex-specific total population for that year to calculate sex-specific incidence.

Age-specific incidence

In order to generate age-specific incidence, a literature search was conducted to identify national and subnational data sources in which age-specific prevalence was reported. The only nationally representative data available were WER reports from 2009 onwards; however, age was only reported as less than 15 years of age or older than 15 years of age. In order to generate a trend over the life course, eight subnational data sources were identified. The prevalence of guinea worm disease was extracted by age category reported in the original paper. An age trend was then fit using DisMod 2.0, with the following model settings:

Age mesh points: 0 0.01 5 10 15 20 25 30 35 40 45 50 55 60 65 70 75 80 85 90 100

Drill year: 2000; Drill location: Global; no birth prevalence; 30 year time window

The age data were used to generate one single age trend that we assumed applied to all locations, and all estimation periods from 1990-2016.

Figure 2. Age-specific prevalence model generated by DisMod

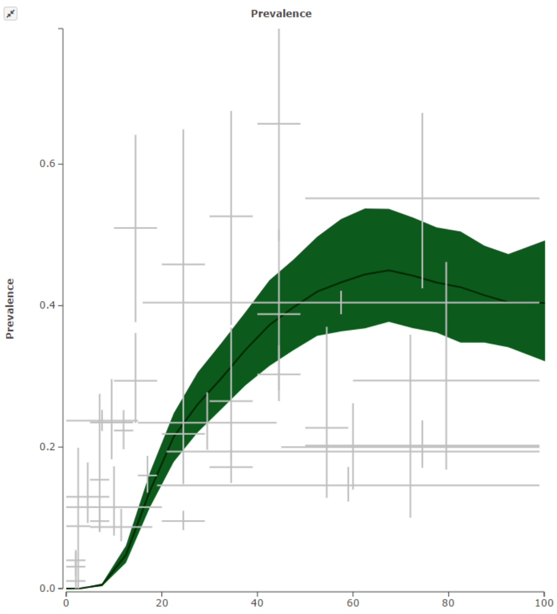

To apply this age prevalence curve to the sex-split incidence draws, 1,000 draws of output were downloaded from DisMod and applied to the incidence data as follows:

j indexes the age strata

i indexes the draw (1 to 1,000)

sex cases draw is the total number of cases for the sex stratum (all ages)

$$age\ cases_j = DisMod\ Draw_{i,j} * age\ population_j$$

$$age\ incidence\ draw_i = \frac{age\ cases_j \left(\frac{sex\ cases\ draw_i}{total\ cases}\right)}{age\ population_j}$$

Under the assumption that Guinea worm disease occurs approximately 1 year post-infection, incidence among children aged less than one year was set to zero.

Sequelae splits

Prevalence of the sequelae listed in Table 3 was calculated by multiplying the age and sex-specific incidence draw by the duration of the health state (in years).

1) Guinea worm pain associated with worm emergence (Level 2): all cases, 1 month
2) Guinea worm pain associated with worm emergence (Level 1): all cases, 2 months plus 30% of cases for an additional 9 months

3) Lower limb musculoskeletal problems: all cases, 1 month

Estimates were produced for 1990, 1995, 2000, 2005, 2010, and 2016.

References

1. Cairncross S, Muller R, Zagaria N. Dracunculiasis (Guinea worm disease) and the eradication initiative. Clin Microbiol Rev. 2002;15(2):223-46.
2. Biswas G, Sankara DP, Agua-Agum J, Maiga A. Dracunculiasis (guinea worm disease): eradication without a drug or a vaccine. Philos Trans R Soc Lond B Biol Sci. 2013;368(1623):20120146.
3. Ruiz-Tiben E, Hopkins DR. Dracunculiasis (Guinea worm disease) eradication. Adv Parasitol. 2006;61:275-309.
4. Greenaway C. Dracunculiasis (guinea worm disease). CMAJ. 2004;170(4):495-500.
5. Hopkins DR, Ruiz-Tiben E, Downs P, Withers PC, Jr., Roy S. Dracunculiasis eradication: neglected no longer. Am J Trop Med Hyg. 2008;79(4):474-9.
6. Kappus KD, Hopkins DR, Ruiz-Tiben E, Imtiaz R, Andersen J, Azam M, et al. A strategy to speed the eradication of dracunculiasis. World Health Forum. 1991;12(2):220-5.
7. Prevention CfDCa. Guinea worm wrap-up Atlanta, GA: WHO Collborating center for Research, Training and Eradication of Dracunculiasis, CDC; 2015.
8. Hopkins DR, Ruiz-Tiben E, Diallo N, Withers PC, Jr., Maguire JH. Dracunculiasis eradication: and now, Sudan. Am J Trop Med Hyg. 2002;67(4):415-22.
9. Watts SJ, Brieger WR, Yacoob M. Guinea worm: an in-depth study of what happens to mothers, families and communities. Soc Sci Med. 1989;29(9):1043-9.
10. Adeyeba OA, Kale OO. Epidemiology of dracunculiasis and its socio-economic impact in a village in south-west Nigeria. West Afr J Med. 1991;10(3-4):208-15.
11. Kale OO. The clinico-epidemiological profile of guinea worm in the Ibadan district of Nigeria. Am J Trop Med Hyg. 1977;26(2):208-14.
12. Greenwood B, Greenwood A, Bradley A. Guinea worm infection in northern Nigeria: reflections on a disease approaching eradication. Trop Med Int Health. 2017.
13. Tayeh A, Cairncross S. The impact of dracunculiasis on the nutritional status of children in South Kordofan, Sudan. Ann Trop Paediatr. 1996;16(3):221-6.
14. Belcher DW, Wurapa FK, Ward WB, Lourie IM. Guinea worm in southern Ghana: its epidemiology and impact on agricultural productivity. Am J Trop Med Hyg. 1975;24(2):243-9.
15. Muller R. Guinea worm disease: epidemiology, control, and treatment. Bull World Health Organ. 1979;57(5):683-9.
16. Okoye SN, Onwuliri CO, Anosike JC. A survey of predilection sites and degree of disability associated with guineaworm (Dracunculus medinensis). Int J Parasitol. 1995;25(9):1127-9.
17. Smith GS, Blum D, Huttly SR, Okeke N, Kirkwood BR, Feachem RG. Disability from dracunculiasis: effect on mobility. Ann Trop Med Parasitol. 1989;83(2):151-8.
18. Chippaux JP, Banzou A, Agbede K. [Social and economic impact of dracunculosis: a longitudinal study carried out in 2 villages in Benin]. Bull World Health Organ. 1992;70(1):73-8.
19. Hours M, Cairncross S. Long-term disability due to guinea worm disease. Trans R Soc Trop Med Hyg. 1994;88(5):559-60.

Other neglected tropical diseases

In addition to the neglected tropical diseases described above, there are many diverse types of neglected tropical diseases, which are encompassed by the following ICD 10 codes: A68-A68.9, A69.2-A69.5, A75-A75.9, A77-A79.9, A92-A94.0, A96-A96.9, A98-A98.8, B58, B59-B60.8, B68-B68.9, B70-B72.0, B74.3-B75, B78-B78.9, B80-B81.8, B83-B83.8, P37.1.

Because these neglected tropical diseases are diverse in their underlying causes and risk factors as well as in their associated health outcomes, modelling them together in a DisMod-MR model would not produce reliable estimates of prevalence or excess mortality. Instead, we calculated the YLDs caused by neglected tropical diseases directly using a YLD/YLL ratio.

We calculated the ratio of YLDs to YLLs across the specified neglected tropical diseases for which nonfatal outcomes were modelled, using YLL estimates from the GBD 2015 cause of death (CoD) analysis. We then multiplied this YLD/YLL ratio by the YLL estimates for other neglected tropical diseases from the GBD 2015 CoD analysis, providing us with an estimate of the YLDs associated with other neglected tropical diseases.

Changes from GBD 2015 to GBD 2016

We have made no substantive changes in the modelling strategy from GBD 2015 to GBD 2016.

Maternal Disorders

1) Abortion, ectopic pregnancy, and miscarriage; 2) Obstructed labor and uterine rupture; 3) Maternal hemorrhage; 4) Maternal sepsis and other maternal infections; 5) Maternal hypertensive disorders; 6) Other maternal disorders

Flowchart

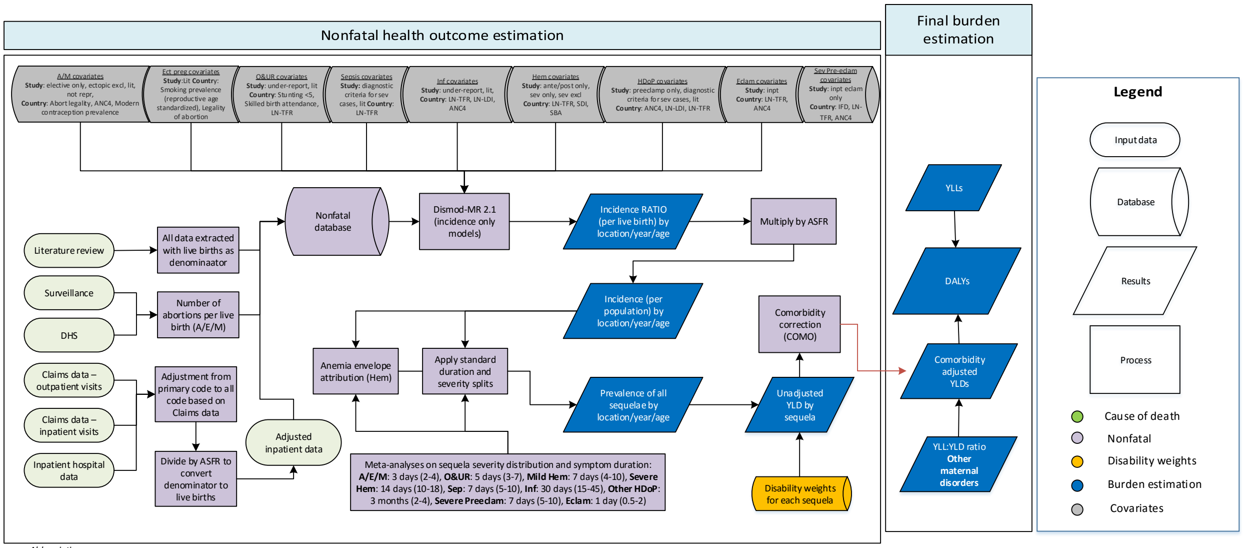

Abbreviations
A/M: Abortion and miscarriage; Ect Preg: Ectopic pregnancy; O&UR: Obstructed labor and uterine rupture; Hem: Maternal hemorrhage; Sep: Maternal sepsis; Inf: Other maternal infections; HDoP: Maternal hypertensive disorders; Eclam: Eclampsia; Sev pre-eclam: Severe pre-eclampsia; ASFR: Age-specific fertility rate; Stunting <5: Stunting (proportion <2SD height for age, <5 years); MS: Marketscan; ANC4: coverage of 4 visits of antenatal care; SDI: Socio-Demographic Index; lit: Literature data; inpt: Inpatient data; not repr: Not representative; LN: Natural log; TFR: Total fertility rate; LDI: Lag-distributed income per capita; SBA: Skilled birth attendance (proportion), IFD: In facility delivery

Input data and methodological summary

Case definition

Maternal disorders are those complications occurring during pregnancy, childbirth, and the postpartum period. Seven different statistical models were completed for GBD 2016. These included 1) abortion, ectopic pregnancy, miscarriage, 2) obstructed labor and uterine rupture, 3) maternal hemorrhage (including placental disorders), 4) maternal sepsis, 5) other maternal infections, 6) hypertensive disorders of pregnancy, 7) eclampsia. Other direct maternal disorders were estimated using a YLD-to-YLL ratio approach used for multiple different GBD causes. Late maternal death and indirect maternal disorders, including HIV-related maternal death, were not assigned any disability as the disability associated with them was assumed to be included in estimates for the underlying conditions. ICD-9 and ICD-10 codes for each of the statistical models are contained in the table below.

Table 1. ICD codes for maternal disorders

International classification of diseases codes for maternal disorders in GBD 2015 non-fatal analysis		
Model	ICD10 code	ICD9 code

Abortion, miscarriage	O01-O08, O36.4	631, 634-639
Ectopic pregnancy	O00	633-633.91
Maternal hemorrhage	O20, O43.2, O44-O46, O62.2, O67, O72	640-641, 661.0, 666
Eclampsia	O15	642.6
Hypertensive disorders of pregnancy	O11-O16	642.3, 642.4, 642.5, 642.6, 642.7, 642.9
Severe Pre-eclampsia	O14.1-O14.13	642.5-642.54
Obstructed labor and uterine rupture	O64-O66, O71, O83	659-660, 662, 665, 669.5, 669.6
Other maternal infections	O23, O41, O75.2-3, O86, O91	646.5, 646.6, 659.2, 672.0, 674.1, 674.2, 674.3, 675
Maternal sepsis	O85	659.3, 670

Input data

Systematic literature reviews were completed for GBD 2010, GBD 2013 and GBD 2015. These were updated on August 31st 2016 using the combined search string below. In addition, we searched ministry of health websites for pregnancy complication data and used from Confidential Enquiry and other sources used in our maternal mortality analyses when they presented data on pregnancy complications. We also performed snowball searches for abortion reporting and surveillance data systems, finding multiple such systems throughout high income countries and several locations in Central and Eastern Europe. Inpatient and outpatient data were used, as was claims data from MarketScan in the United States. This data was extracted and processed as described in the section on hospital data, including use of primary-to-any inpatient ratio to correct for under-reporting of pregnancy complications in hospital datasets that rely only on primary discharge codes. All data was extracted in standard fashion, uploaded and stored on a centralized SQL database. The final dataset contents for each of the models is shown below as well.

Maternal Disorders

((("Postpartum Hemorrhage" OR "Uterine Hemorrhage") OR (maternal[Title/Abstract] OR pregnant[Title/Abstract] OR pregnancy[Title/Abstract] OR mothers) AND (haemorrhag*[Title/Abstract] OR hemorrhag*[Title/Abstract]) NOT "case report"[All fields]) AND (2015/04/30[PDat] : 2016/12/31[PDat]) AND humans[MeSH Terms]) OR ((("induced abortion" OR "Therapeutic abortion" OR "legal Abortion" OR "medical abortion" OR "miscarriage" OR "Abortion, Induced"[Mesh] OR "Abortion, Therapeutic"[Mesh] OR "Abortion, Legal"[Mesh] OR "ectopic Pregnancy") NOT ("case report"[Title/Abstract] OR "birth defect"[Title/Abstract] OR congenital[Title/Abstract])) AND (2015/04/30[PDat] : 2016/12/31[PDat]) AND humans[MeSH Terms]) OR (("obstructed labour" OR "obstructed labor" OR "labour dystocia" OR "labor dystocia" OR dystocia OR "cephalopelvic disproportion" OR "cephalo-pelvic disproportion") AND (2015/03/15[PDAT] : 2016/12/31[PDAT]) AND (Humans[MeSH Terms])) OR ((("obstetric fistula" OR "vesicovaginal fistula") OR

"rectovaginal fistula") AND (2015/03/15[PDAT] : 2016/12/31[PDAT]) AND humans[MeSH Terms]) OR (("Puerperal Infection"[Mesh] OR "Puerperal Infection" OR ((maternal[Title/Abstract] OR pregnant[Title/Abstract] OR pregnancy[Title/Abstract]) AND (Sepsis OR infection[Title/Abstract]))) NOT "case report" AND (2015/07/31[PDat] : 2016/12/31[PDat]) AND humans[MeSH Terms]) OR ((Pre-Eclampsia[Title/Abstract] OR preeclampsia[Title/Abstract] OR Eclampsia[Title/Abstract] OR Pre-Eclampsia[Mesh] OR Eclampsia[Mesh] OR "Hypertension, Pregnancy-Induced"[Mesh] OR "pregnancy induced hypertension"[Title/Abstract] OR "gestational hypertension"[Title/Abstract] OR "Hypertensive disorders of pregnancy"[Title/Abstract]) NOT ("case report" OR "kidney don*"[Title/Abstract] OR polymorphism*[Title/Abstract] OR endotheli*[Title/Abstract]) AND (2015/07/31[PDat] : 2016/12/31[PDat]) AND "humans"[MeSH Terms]) **2519 hits, 22 new sources extracted.**

	Incidence
Studies	22
Countries/subnationals	21/3
GBD world regions	10

Modeling strategy

We estimated the incidence ratio of each category of pregnancy complications using DisMod-MR 2.1, with the exception of other maternal disorders which we estimated using a YLD-to-YLL ratio approach used in multiple causes across the GBD 2016. The reason is that most literature and surveillance data is expressed in terms of number of events per live birth rather than per population. Hospital and claims data, which was centrally processed for all GBD 2016 causes to have population as the denominator, was transformed to have live births as the denominator by dividing by age-specific fertility rate (ASFR; live births per population).

We used the datasets described above to estimate incidence ratio for each age-sex-location-year in the GBD 2016 location hierarchy using DisMod-MR 2.1. A number of study-level covariates were used to crosswalk from non-standard sub-populations or case definitions. For most conditions, Marketscan claims data or literature data were considered to be the closest approximation of the true incidence of complications so were identified as the reference category. A series of country covariates were then chosen to help drive the magnitude of estimates in areas of sparse or absent data. As fertility rate is partially related to the number of cases of a given complication, the natural log of total fertility rate (TFR) from our demographics analysis was used in most of the models. No specific age or slope priors were used. All models were run with a time window of five years. The quantitative results of study-level and country-level covariates for each condition are shown below.

Abortion and miscarriage

**Study-level covariate**	**Parameter**	**Location level**	**beta**	**Exponentiated beta**
Not representative	Incidence	Global	-1.2e-03 (-2.6e-03 — -2.6e-04)	1.00 (1.00 — 1.00)

Hospital Inpatient	Incidence	Global	-7.9e-04 (-2.4e-03 — -3.8e-04)	1.00 (1.00 — 1.00)
Elective pregnancy terminations only	Incidence	Global	-9.7e-03 (-0.054 — -3.8e-05)	0.99 (0.95 — 1.00)
Marketscan, year 2000	Incidence	Global	-1 (-1.99 — -0.03)	0.37 (0.14 — 0.97)
Marketscan, year 2010	Incidence	Global	-1.02 (-2 — 0)	0.36 (0.14 — 1.00)
Marketscan, year 2012	Incidence	Global	-1.01 (-2 — -0.051)	0.36 (0.14 — 0.95)

**Country-level covariate**	**Parameter**	**Location level**	**beta**	**Exponentiated beta**
Legality of Abortion	Incidence	Global	0.020 (0.019 — 0.021)	1.02 (1.02 — 1.02)
Antenatal Care (4 visits) Coverage (proportion)	Incidence	Global	-3.8e-04 (-1.6e-03 — -6.0e-06)	1.00 (1.00 — 1.00)
Contraception (Modern) Prevalence (proportion)	Incidence	Global	-0.53 (-0.6 — -0.33)	0.59 (0.55 — 0.72)

Ectopic pregnancy

Study-level covariate	Parameter	Location level	beta	Exponentiated beta
Hospital Inpatient	Incidence	Global	2.50 (2.50 — 2.50)	12.18 (12.17 — 12.18)

Country-level covariate	Parameter	Location level	beta	Exponentiated beta
Legality of Abortion	Incidence	Global	-0.012 (-0.013 — -0.011)	0.99 (0.99 — 0.99)

Maternal hemorrhage

**Study-level covariate**	**Parameter**	**Location level**	**beta**	**Exponentiated beta**
Postpartum hemorrhage only	Incidence	Global	-6.6e-03 (-0.022 — -3.9 e-04)	0.99 (0.98 — 1.00)
Hospital Inpatient	Incidence	Global	1.00 (1.00 — 1.00)	2.72 (2.71 — 2.72)
Antepartum hemorrhage only	Incidence	Global	-0.63 (-0.99 — -0.28)	0.53 (0.37 — 0.76)
Severe hemorrhage excluded	Incidence	Global	-0.027 (-0.1 — -7.3e-04)	0.97 (0.90 — 1.00)
Severe hemorrhage only	Incidence	Global	-0.12 (-0.28 — -7.9e-03)	0.89 (0.76 — 0.99)

Country-level covariate	Parameter	Location level	beta	Exponentiated beta
Skilled Birth Attendance (proportion)	Incidence	Global	-3.5e-03 (-0.013 — -3.3e-04)	1.00 (0.99 — 1.00)
Sociodemographic Index	Incidence	Global	1.8e-03 (5.1e-05 — 8.3e-03)	1.00 (1.00 — 1.01)
Total Fertility Rate	Incidence	Global	-0.1 (-0.11 — -0.1)	0.90 (0.90 — 0.90)

Hypertensive disorders of pregnancy

Study-level covariate	Parameter	Location level	beta	Exponentiated beta
Hospital Inpatient	Incidence	Global	0.40 (0.37 — 0.46)	1.50 (1.45 — 1.58)
Literature	Incidence	Global	-0.3 (-0.3 — -0.28)	0.74 (0.74 — 0.76)
Diagnostic criteria for severe cases	Incidence	Global	-1.84 (-1.88 — -1.78)	0.16 (0.15 — 0.17)
Includes only preeclampsia data (not other gestational hypertension	Incidence	Global	-1.16 (-1.17 — -1.13)	0.31 (0.31 — 0.32)

Country-level covariate	Parameter	Location level	beta	Exponentiated beta
Antenatal Care (4 visits) Coverage (proportion)	Incidence	Global	-1.5e-04 (-1.5e-04 — -1.3e-05)	1.00 (1.00 — 1.00)
LDI (I$ per capita)	Incidence	Global	-3.6e-0.5(-1.4e-04 — -9.9e-06)	1.00 (1.00 — 1.00)
Total Fertility Rate	Incidence	Global	-1.5 (-1.5 — -1.5)	0.22 (0.22 — 0.22)

Eclampsia

Study-level covariate	Parameter	Location level	beta	Exponentiated beta
Hospital Inpatient	Incidence	Global	1.99 (1.85 — 2.00)	7.28 (6.39 — 7.39)
Literature	Incidence	Global	-1.34 (-1.54 — -1.25)	0.26 (0.21 — 0.29)

Country-level covariate	Parameter	Location level	beta	Exponentiated beta

Antenatal Care (4 visits) Coverage (proportion)	Incidence	Global	-4.11 (-6.08 — -3.14	2.3e-03 — 0.043)
Total Fertility Rate	Incidence	Global	-0.98 (-1.06 — -0.94)	0.37 (0.35 — 0.39)

Severe pre-eclampsia

Study-level covariate	Parameter	Location level	beta	Exponentiated beta
Hospital Inpatient	Incidence	Global	2.00 (2.00 — 2.00)	7.39 (7.38 — 7.39)

Country-level covariate	Parameter	Location level	beta	Exponentiated beta
Antenatal Care (4 visits) Coverage (proportion)	Incidence	Global	-1.6e-03 (-5.2e-03 – -5.3e-05)	1.00 (0.99 — 1.00)
Total Fertility Rate	Incidence	Global	-1.5 (-1.5 — -1.49)	0.22 (0.22 — 0.23)
LDI (I$ per capita)	Incidence	Global	-3.3e-04(-1.6e-03 – -1.8e-05)	1.00 (1.00 — 1.00)

Obstructed labor and uterine rupture

Study-level covariate	Parameter	Location level	beta	Exponentiated beta
Under Reported	Incidence	Global	-8.8e-03 (-0.04 - -1.8e-04)	0.99 (0.96 — 1.00)
Hospital Inpatient	Incidence	Global	2.00 (2.00 — 2.00)	7.38 (7.37 — 7.39)
Marketscan, year 2000	Incidence	Global	1.99 (1.95 — 2.00)	7.29 (7.04 — 7.39)
Marketscan, year 2010	Incidence	Global	1.88 (1.80 — 1.95)	6.52 (6.05 — 7.02)
Marketscan, year 2012	Incidence	Global	1.85 (1.77 — 1.91)	6.33 (5.88 — 6.77)

Country-level covariate	Parameter	Location level	beta	Exponentiated beta
Stunting (proportion <2SD height for age, <5 years)	Incidence	Global	0.11 (0.0061 — 0.36)	1.11 (1.01 — 1.44)
Skilled Birth Attendance (proportion)	Incidence	Global	-0.015 (-0.059 — -5.8e-04)	0.99 (0.94 — 1.00)

Maternal sepsis

Study-level covariate	Parameter	Location level	beta	Exponentiated beta
Hospital Inpatient	Incidence	Global	3.00 (3.00 — 3.00)	20.08 (20.07 — 20.09)
Diagnostic criteria for severe cases	Incidence	Global	-7.1e-03 (-0.026 - -4.7e-04)	0.99 (0.97 — 1.00)
Marketscan, year 2000	Incidence	Global	1.99 (1.97 — 2.00)	7.31 (7.14 — 7.39)
Marketscan, year 2010	Incidence	Global	1.99 (1.96 — 2.00)	7.32 (7.12 — 7.39)
Marketscan, year 2012	Incidence	Global	1.98 (1.93 — 2.00)	7.21 (6.88 — 7.38)

Other maternal infections:

Study-level covariate	Parameter	Location level	beta	Exponentiated beta
Hospital Inpatient	Incidence	Global	0.50 (0.50 — 0.50)	1.65 (1.65 — 1.65)
Under Reported	Incidence	Global	-0.52 (-1.18 — -0.046)	0.59 (0.31 — 0.96)
Marketscan, year 2000	Incidence	Global	1.66 (1.43 — 1.79)	5.25 (4.16 — 5.99)
Marketscan, year 2010	Incidence	Global	1.85 (1.62 — 1.98)	6.37 (5.07 — 7.21)
Marketscan, year 2012	Incidence	Global	1.91 (1.68 — 2.00)	6.72 (5.34 — 7.37)

Country-level covariate	Parameter	Location level	beta	Exponentiated beta
Socio-demographic Index	Incidence	Global	-7.4e-03 (-0.024 - -1.7e-04)	0.99 (0.98 — 1.00)

Severity splits

After completion of DisMod-MR 2.1 models, all age-specific ratios were then converted to population rates by multiplying by ASFR. Maternal hemorrhage was split between moderate (500-<1000ml blood loss) and severe (>1000 blood loss) on the basis of a meta-analysis of 19 studies[1]. Data on the average duration of acute symptoms were not available so, after consultation with clinician collaborators, we assigned a duration of 7 days (+/-3) for moderate hemorrhage and 14 days (+/- 4) for severe hemorrhage. The total maternal hemorrhage incidence served as input to the causal attribution process of the overall anemia envelope, which is described separately. This is a change from GBD 2013, when only the prevalence of maternal hemorrhage was considered in anemia causal attribution which was inappropriate because the disability from hemorrhage-induced anemia is longer-lasting than the acute hemorrhagic event, a situation that was not previously reflected. Acute disability was calculated assuming incident cases of abortion, ectopic pregnancy, and miscarriage persist for an average of 3 days (+/-1) and obstructed labor was assigned a duration of 5 days (+/-2). Again, these determinations were based on clinical expert determination as we could not identify any data to inform this.

Hypertensive disorders of pregnancy (HDoP) and maternal sepsis and other maternal infections were estimated in two models each. HDoP YLD estimates included severe preeclampsia and other HDoP which were derived from two models of total HDoP and severe pre-eclampsia. The duration of severe preeclampsia was assigned to be 7 days (+/-2) and other HDoP was assigned a duration of 3 months (2-4). Eclampsia was a separate model, assigned a duration of 1 day (+/-1). A large number of those with severe preeclampsia go on to have long term sequelae of the condition[2], as do those with eclampsia[3,4], both of which were included in the estimates of HDoP and were a bulk of the YLD for those conditions. Maternal sepsis was assigned a duration of 5 days (+/-2) and, based on the same data identified in our review of pelvic inflammatory disease (PID; described separately), a proportion of incident cases were estimated to continue on to have secondary infertility due to maternal sepsis. Other maternal infections were assigned a wide potential duration of 15 to 45 days (mean 30).

Uncertainty and model selection

For all maternal disorders, uncertainty bounds include uncertainty due to input data, crosswalks from non-reference definitions, uncertainty in numerical solutions (posteriors) of each DisMod-MR 2.1 model, duration of symptoms, and proportion of all persons with each type of symptom.

In consultation with GBD researchers and collaborators, final models were selected on a combination of qualitative and quantitative goodness of fit to input data, plausibility of geographic and temporal trends, consistency of age pattern, and, when available, comparison with other published studies on the epidemiology of pregnancy complications. Directionality, magnitude, and plausibility of study-level and country-level covariates was also considered in the process of model development. Of note, due to the nature of statistical modeling, final results do not always cover the values reported in input data.

Obstetric fistula

Flowchart

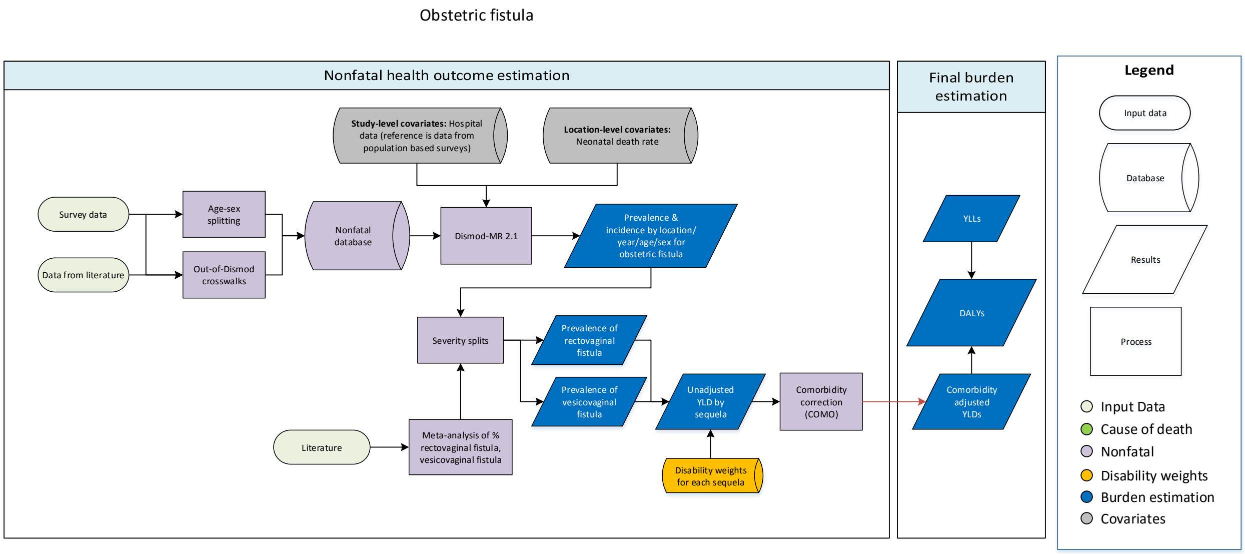

Case Definition

Obstetric fistula is a severe long-term complication of prolonged obstructed labor in which a fistula (hole) develops between the birth canal and the bladder and/or rectum. The ICD codes include:

Input data

Model Inputs

A systematic review was conducted for GBD 2015. The PubMed search terms were: (('obstetric fistula'[All Fields] OR 'vesicovaginal fistula'[All Fields]) OR 'rectovaginal fistula'[All Fields]) AND ('2013'[PDAT] : '2015'[PDAT]) AND 'humans'[MeSH Terms].

The exclusion criteria were:

27. Studies that did not provide primary data on epidemiological parameters, e.g. commentaries
28. Case series
29. Reviews

The table below shows the number of literature studies included in GBD2015, as well as the number of countries or subnational units and GBD world regions represented.

	Prevalence	Incidence	Mortality risk
Studies	8	3	-
Countries/subnationals	8	3	-

GBD world regions	3	1	-

In addition to using data from published studies, we also included data from UNFPA reports, and nationally representative Demographic and Health Surveys and Multiple Indicator Cluster Surveys.

Severity Splits

The following severity distributions were assigned based a meta-analysis of published studies[1-4] and Pakistan Demographic and Health survey (2006-2007): vesicovaginal fistula (90.8%, 95% CI: 85.0 to 95.4%); rectovaginal fistula (9.2%, 95% CI: 4.6 to 15.0%. The lay descriptions and disability weights for severity levels derived from the GBD Disability Weights study are shown below.

Severity level	Lay description	DW (95% CI)
vesicovaginal fistula	has an abnormal opening between the bladder and the vagina, which makes her unable to control urinating. The woman is anxious and depressed.	0.342 (0.227-0.478)
rectovaginal fistula	has an abnormal opening between her vagina and rectum causing flatulence and feces to escape through the vagina. The person gets infections in her vagina, and has pain when urinating.	0.501 (0.339-0.657)

Modeling Strategy

Obstetric fistula was modeled using DisMod-MR 2.1. We used neonatal mortality rate as a country-level covariate. We also included a study-level covariate indicating whether it was a hospital-based or community-based study. Remission was calculated, using the cure data from 11 Demographic and Health surveys, by dividing the number of cured obstetric fistula cases by total person years of follow up of all cases (cured, uncured and untreated). The person year of follow up for uncured or untreated fistula cases was calculated as the time interval (in year) between the last birth and the date of interview. For cured cases, we assumed that the person year of follow up was half the time interval (in year) between the last birth and the date of interview.

Betas and exponentiated values (which can be interpreted as an odds ratio) are shown in the table below:

Covariate	Parameter	Beta (95% CI)	Exponentiated beta (95% CI)
Neonatal mortality rate	Prevalence	1.96 (1.85 — 2.00)	7.09 (6.35 — 7.38)
Neonatal mortality rate	Incidence	0.54 (0.026 — 1.14)	1.72 (1.03 — 3.13)
Neonatal mortality rate	Remission	-0.-73 (-0.99 — -0.28)	0.48 (0.37 — 0.76)
Hospital data	Prevalence	-1.75 (-1.99 — -1.27)	0.17 (0.14 — 0.28)
Hospital data	Incidence	-0.65 (-1.86 — 0.85)	0.52 (0.16 — 2.35)

References

1. Danso KA, Martey J, Wall LL, Elkins TE. The epidemiology of genitourinary fistulae in Kumasi, Ghana, 1977–1992. International Urogynecology Journal. 1996;7(3):117-120.

2. Wall LL, Karshima JA, Kirschner C, Arrowsmith SD. The obstetric vesicovaginal fistula: characteristics of 899 patients from Jos, Nigeria. Am. J. Obstet. Gynecol. 2004;190(4):1011-1016.

3. Das R, Sengupta S. Vesico-vaginal fistula of obstetric origin. J. Obstet. Gynaecol. India. 1969;19:383-389.

4. Mahfouz NP. Urinary and Faecal Fistulae. BJOG. 1938;45(3):405-424.

Preterm Birth Complications

Flowchart

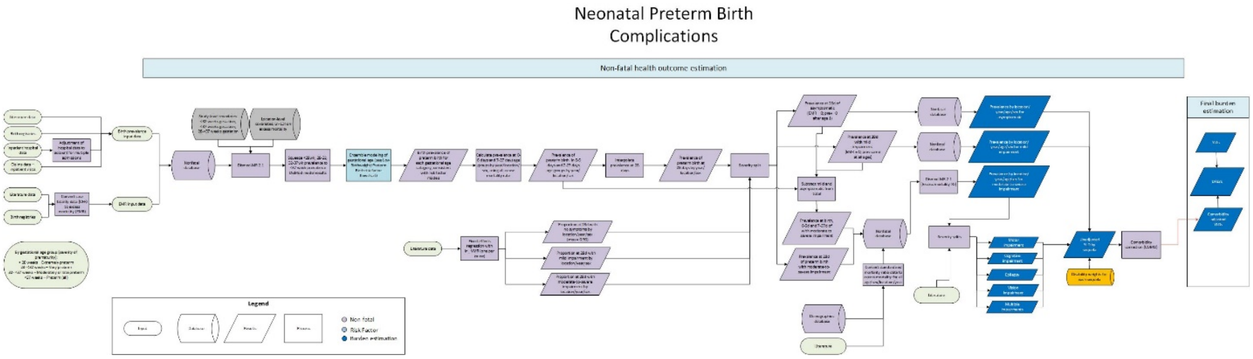

Input data and methodological summary

Case definition

Preterm birth is defined as live birth before 37 completed weeks of gestation. In our analysis, we further break down this cause into three sub-categories of preterm birth, based on gestational age: extremely preterm (<28 weeks), very preterm (28 to <32 weeks), and moderate to late preterm (32 to <37 weeks). These categories are based on the World Health Organization (WHO) definition of preterm birth.[1]

Input data

Model inputs

A systematic review was completed for GBD 2010, and for GBD 2013 and GBD 2016, a review was conducted on literature published since the previous addition. The PubMed database was searched using the following search string: ((("Infant, Premature"[Mesh] OR ("infant"[All Fields] AND "premature"[All Fields]) OR "premature infant"[All Fields] OR ("preterm"[All Fields] AND "infant"[All Fields]) OR "preterm infant"[All Fields] OR ("infant, newborn"[MeSH Terms] OR ("infant"[All Fields] AND "newborn"[All Fields]) OR "newborn infant"[All Fields] OR ("newborn"[All Fields] AND "infant"[All Fields])) AND (premature[All Fields] OR preterm[All Fields]) OR "premature birth"[MeSH Terms] OR ("premature"[All Fields] AND "birth"[All Fields]) OR "premature birth"[All Fields] OR ("preterm"[All Fields] AND "birth"[All Fields]) OR "preterm birth"[All Fields]) ((("Infant, Premature"[Mesh] OR ("infant"[All Fields] AND "premature"[All Fields]) OR "premature infant"[All Fields] OR ("preterm"[All Fields] AND "infant"[All Fields]) OR "preterm infant"[All Fields] OR ("infant, newborn"[MeSH Terms] OR ("infant"[All Fields] AND "newborn"[All Fields]) OR "newborn infant"[All Fields] OR ("newborn"[All Fields] AND "infant"[All Fields])) AND (premature[All Fields] OR preterm[All Fields]) OR "premature birth"[MeSH Terms] OR ("premature"[All Fields] AND "birth"[All Fields]) OR "premature birth"[All Fields] OR ("preterm"[All Fields] AND "birth"[All Fields]) OR "preterm birth"[All Fields]) AND ("2012"[PDAT] : "3000"[PDAT]) AND "humans"[MeSH Terms].

The exclusion criteria were:

[1] http://www.who.int/mediacentre/factsheets/fs363/en/

1. Studies that did not provide primary data on epidemiological parameters, e.g., a commentary piece
2. Non-representative studies (e.g., only high-risk pregnancies)
3. Reviews

The table below shows the number of literature studies included in the estimates for GBD 2016, as well as the number of countries or subnational units and GBD world regions represented.

Table 1a-c. Literature Data geographies

Preterm Birth <28 Weeks

	Birth prevalence	Case fatality
Studies	219	125
Geographic locations	281	99
GBD world regions	16	18

Preterm Birth 28-<32 Weeks

	Birth prevalence	Case fatality
Studies	227	117
Geographic locations	290	97
GBD world regions	18	17

Preterm Birth 32-<37 Weeks

	Birth prevalence	Case fatality
Studies	86	93
Geographic locations	244	82
GBD world regions	14	10

In addition to literature data, data from US claims data for 2010 and 2012 by US state and hospital data from 98 additional national or subnational locations were considered to inform the estimates of the three gestational age preterm categories. Only inpatient data was included from these datasets, as we believed it would be more representative of the true prevalence of preterm birth than outpatient data: preterm infants in the countries from which hospital data was available are almost sure to be admitted to the hospital, whereas outpatient data is more likely to capture repeated visits by the same child as they grow.

In accordance with methodology from other global estimates of preterm birth[2], we have outliered sources reporting preterm births (<37 weeks) as less than 3% of all births, as this was determined to be biologically implausible.

Severity splits and disability weights

The basis of the GBD disability weight (DW) survey assessments are lay descriptions of sequelae highlighting major functional consequences of symptoms. The lay descriptions and disability weights for

[2] Blencowe H, Cousens S, Oestergaard MZ, Chou D, Moller A, Narwal R, Adler A, Vera Garcia C, Rohde S, Say L, Lawn J. National, regional, and worldwide estimates of preterm birth rates in the year 2010 with time trends since 1990 for selected countries: a systematic analysis and implications. *Lancet* 2012; 379: 2162–72

preterm birth are shown below. Further severity levels are calculated by combining several of these disability weights, e.g., moderate motor plus cognitive impairment with blindness.

Table 2. Severity, lay description, and DWs

Severity level	Lay description
Motor plus cognitive impairments, mild	has some difficulty in moving around but is able to walk without help. The person is slow in learning at school. As an adult, the person has some difficulty doing complex or unfamiliar tasks but otherwise functions independently.
Motor impairment, mild	has some difficulty in moving around but is able to walk without help.
Motor impairment, moderate	has some difficulty in moving around, and difficulty in lifting and holding objects, dressing and sitting upright, but is able to walk without help.
Motor impairment, severe	is unable to move around without help, and is not able to lift or hold objects, get dressed or sit upright.
Distance vision, mild impairment	has some difficulty with distance vision, for example reading signs, but no other problems with eyesight.
Distance vision, moderate impairment	has vision problems that make it difficult to recognize faces or objects across a room.
Distance vision, severe impairment	has severe vision loss, which causes difficulty in daily activities, some emotional impact (for example worry), and some difficulty going outside the home without assistance.
Distance vision blindness	is completely blind, which causes great difficulty in some daily activities, worry and anxiety, and great difficulty going outside the home without assistance.
Motor plus cognitive impairments, mild	Combined DW
Motor impairment with blindness, moderate	Combined DW
Motor impairment with epilepsy, moderate	Combined DW
Motor impairment with blindness and epilepsy, moderate	Combined DW
Motor plus cognitive impairment with blindness, moderate	Combined DW
Motor plus cognitive impairment with epilepsy, moderate	Combined DW
Motor plus cognitive impairment with blindness and epilepsy, moderate	Combined DW
Motor impairment with blindness, severe	Combined DW
Motor impairment with epilepsy, severe	Combined DW
Motor impairment with blindness and epilepsy, severe	Combined DW
Motor impairment with blindness and epilepsy, severe	Combined DW
Motor plus cognitive impairment with epilepsy, severe	Combined DW
Motor plus cognitive impairment with blindness and epilepsy, severe	Combined DW

To determine the proportion of people within each of these severity levels, one study informed moderate-to-severe impairment splits, and for mild impairments cases were divided equally into both categories.[3]

Modeling strategy

Burden from each of the gestational age categories of preterm birth (extreme, very, and moderate to late) is modeled separately, using similar methods.

For a given gestational age group, an initial DisMod MR 2.2 model is run using prevalence and case fatality data. An initial DisMod MR 2.2 model is also run for all preterm births (<37 weeks). Prior to input into DisMod, case fatality ratio (CFR) data is transformed into excess mortality rate (EMR) space using the formula:

$$EMR = -\frac{\ln(1-CFR)}{\frac{28}{365.25}}$$

which is analogous to the transformation of cumulative incidence to incidence rate. The denominator in this equation is derived from the definition of our CFR parameter, which is death in the first 28 days of life. The output from this first-step DisMod model is prevalence in the first two neonatal age groups. For this model, remission and incidence are both set to zero, as no one can be born prematurely after birth, and no one can cease to have been born premature after the fact. Study covariates were created for hospital data, US claims data, and literature sources that use non-standard gestational age categories.

Preterm Birth <28 Weeks

Study-level covariate	Parameter	Geography level	beta	Exponentiated beta
Sex	Prevalence	Global	0.068 (0.047 — 0.090)	1.07 (1.05 — 1.09)
Hospital data	Prevalence	Global	0.010 (-2 — 1.98)	1.01 (0.14 — 7.24)
Birth prevalence of preterm birth, gestational age < 32 weeks	Prevalence	Global	1.04 (1.01 — 1.05)	2.82 (2.76 — 2.86)
inpatient-only Marketscan, year 2010	Prevalence	Global	-2.18 (-3.89 — 0.95)	0.11 (0.020 — 2.58)
inpatient-only Marketscan, year 2012	Prevalence	Global	-1.01 (-3.95 — 1.90)	0.36 (0.019 — 6.71)
Sex	Excess Mortality	Global	0.11 (0.079 — 0.15)	1.12 (1.08 — 1.16)

[3] Hagberg et al. Acta Paediatrica 1996, 85:954-60

Country-level covariate	Parameter	beta	Exponentiated beta
Healthcare Access & Quality Index	Prevalence	-0.012 (-0.014 — -0.01)	0.99 (0.99 — 0.99)
LDI (I$ per capita)	Excess Mortality Rate	-0.11 (-0.16 — -0.07)	0.90 (0.85 — 0.93)
Healthcare Access & Quality Index	Excess Mortality Rate	-0.013 (-0.016 — -0.0099)	0.99 (0.98 — 0.99)

Preterm Birth 28-<32 Weeks

Study-level covariate	Parameter	Geography level	beta	Exponentiated beta
Sex	Prevalence	Global	0.067 (0.051 — 0.079)	1.07 (1.05 — 1.08)
Hospital data	Prevalence	Global	-0.02 (-2 — 1.93)	0.98 (0.14 — 6.87)
Birth prevalence of preterm birth, gestational age < 32 weeks	Prevalence	Global	0.45 (0.43 — 0.46)	1.57 (1.54 — 1.59)
Birth prevalence of preterm birth, gestational age 28-37 weeks	Prevalence	Global	2.20 (2.19 — 2.22)	9.06 (8.92 — 9.21)
inpatient-only Marketscan, year 2010	Prevalence	Global	1.30 (0.36 — 1.97)	3.65 (1.43 — 7.14)
inpatient-only Marketscan, year 2012	Prevalence	Global	- -1 (-4 — 1.99)	0.37 (0.018 — 7.32)
Sex	Excess Mortality	Global	0.21 (0.16 — 0.27)	1.24 (1.17 — 1.31)

Country-level covariate	Parameter	beta	Exponentiated beta
Healthcare Access & Quality Index	Prevalence	-0.0079 (-0.0097 — -0.0065)	0.99 (0.99 — 0.99)
LDI (I$ per capita)	Excess Mortality Rate	-0.66 (-0.78 — -0.59)	0.51 (0.46 — 0.56)
Healthcare Access & Quality Index	Excess Mortality Rate	-0.0089 (-0.014 — -0.0026)	0.99 (0.99 — 1.00)

Preterm Birth <37 Weeks

Study-level covariate	Parameter	Geography level	beta	Exponentiated beta
Sex	Prevalence	Global	0.066 (0.056 — 0.076)	1.07 (1.06 — 1.08)
Hospital data	Prevalence	Global	-0.013 (-2 — 1.97)	0.99 (0.14 — 7.17)

Birth prevalence of preterm birth, gestational age 28-37 weeks	Prevalence	Global	0.12 (0.11 — 0.13)	1.13 (1.12 — 1.14)
inpatient-only Marketscan, year 2010	Prevalence	Global	-0.9 (-3.6 — 1.53)	0.41 (0.027 — 4.60)
inpatient-only Marketscan, year 2012	Prevalence	Global	-0.99 (-3.94 — 1.96)	0.37 (0.019 — 7.13)
Sex	Excess Mortality	Global	0.13 (0.084 — 0.18)	1.14 (1.09 — 1.19)

Country-level covariate	Parameter	beta	Exponentiated beta
Healthcare Access & Quality Index	Prevalence	-0.0048 (-0.0061 — -0.0035)	1.00 (0.99 — 1.00)
LDI (I$ per capita)	Excess Mortality Rate	-0.47 (-0.55 — -0.41)	0.62 (0.58 — 0.66)
Healthcare Access & Quality Index	Excess Mortality Rate	-0.0051 (-0.0091 — -0.00036)	0.99 (0.99 — 1.00)

Using prevalence in the first two neonatal age groups, prevalence at 0-6 days, 7-27 days, and 28 days is calculated via interpolation. Functionally, this is birth prevalence minus all those who have died in the first 28 days, our case fatality parameter.

Next, using mild impairment proportion and moderate-to-severe impairment proportion data, we ran either mixed-effect hierarchical regressions or meta-analyses to generate country-year-sex-specific estimates of both parameters (we used a meta-analysis when there was not sufficient data to support a regression model). We then calculated the birth prevalence of each severity level. As mild impairment and moderate-severe impairment were calculated separately, it was possible that they could sum to a value greater than one. To address this, we checked the sum of the two values in any of the 1,000 iterations of the uncertainty analysis, and if greater than 0.9, proportionately rescaled both estimates to sum to 0.9 in any of the 1,000 iterations of the uncertainty analysis (we picked 0.9 rather than 1 to allow at least some probability of a child having no impairment). We then proceeded with the calculation of the birth prevalence of impairment:

Mild impairment birth prevalence = prevalence at 28 days * proportion mild impairment

Moderate/severe impairment birth prevalence = prevalence at 28 days * proportion mod-severe impairment

For moderate/severe impairment, results of these latter calculations were then combined with excess mortality estimates derived from the standard mortality ratios (SMR) of cerebral palsy and used as inputs into a second DisMod MR 2.2 model. For this model, remission and incidence were also set to zero. For

mild impairment, we assumed no excess mortality and no remission, and as such simply applied the birth prevalence of mild impairment to every age group.

No other significant changes were made to the modeling strategy for GBD 2016.

Neonatal encephalopathy due to birth asphyxia and birth trauma

Flowchart

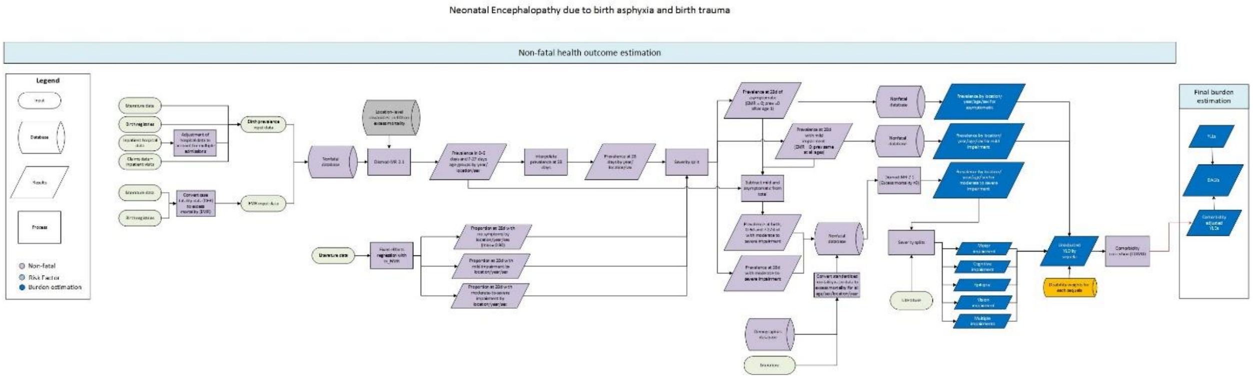

Input data and methodological summary

Case definition

Neonatal encephalopathy (NE) due to birth asphyxia and birth trauma, also called hypoxic-ischemic encephalopathy, is defined as injury to the brain in the first few moments or days of life in an infant born at term. NE has multiple etiologies, and is defined more by its symptoms – abnormal neurological function, including reduced level of consciousness, seizures, depression of tone and reflexes, or difficulty maintaining respiration – than its origin. NE can occur when an infant is deprived of oxygen during delivery or sustains physical trauma to the head, among other causes.

Input data

Model inputs

A systematic review was completed for GBD 2010, and for GBD 2013 and GBD 2015, a review was conducted on literature published since the previous addition. The PubMed database was searched using the following search string**:** ((("infant"[Title/Abstract] OR "newborn"[Title/Abstract] OR "newborn infant"[Title/Abstract]) AND ("encephalopathy"[Title/Abstract] OR "neonatal encephalopathy"[Title/Abstract] OR "perinatal asphyxia"[Title/Abstract] OR "asphyxia neonatorum"[Title/Abstract] OR "newborn encephalopathy"[Title/Abstract] OR "hypoxic ischaemic encephalopathy"[Title/Abstract] OR ("birth trauma"[Title/Abstract] AND "birth asphyxia"[Title/Abstract]))) AND ("2012"[PDAT] : "3000"[PDAT]) AND "humans"[MeSH Terms])

The exclusion criteria were:

1. Studies that did not provide primary data on epidemiological parameters, e.g., a commentary piece
2. Non-representative studies (e.g., only high-risk pregnancies)
3. Reviews

The table below shows the number of literature studies included in GBD 2016, as well as the number of countries or subnational units and GBD world regions represented. No additional literature searches were done for GBD 2016.

Table 1. Geographic representation

	Birth prevalence	Case fatality
Studies	38	36
Locations	27	25
GBD world regions	13	12

In addition to literature data, data from US claims data for 2000, 2010, and 2012 by US state were included. Only inpatient data was included from these datasets, because we believed it would be more representative of the true prevalence of neonatal encephalopathy than outpatient data: infants with neonatal encephalopathy in the countries from which hospital data was available are almost sure to be admitted to the hospital, whereas outpatient data is more likely to capture repeated visits by the same child as they grow.

Severity splits

The basis of the GBD disability weight (DW) survey assessments are lay descriptions of sequelae highlighting major functional consequences of symptoms. The lay descriptions and disability weights for neonatal encephalopathy birth are shown below.

Table 2. Severity level, lay description and DWs

Severity level	Lay description
Motor plus cognitive impairments, mild	has some difficulty in moving around but is able to walk without help.The person is slow in learning at school. As an adult, the person has some difficulty doing complex or unfamiliar tasks but otherwise functions independently.
Motor impairment, mild	has some difficulty in moving around but is able to walk without help.
Motor impairment, moderate	has some difficulty in moving around, and difficulty in lifting and holding objects, dressing and sitting upright, but is able to walk without help.
Motor impairment, severe	is unable to move around without help, and is not able to lift or hold objects, get dressed or sit upright.
Distance vision blindness	is completely blind, which causes great difficulty in some daily activities, worry and anxiety, and great difficulty going outside the home without assistance.
Motor impairment with blindness, moderate	(combined DW)
Motor impairment with epilepsy, moderate	(combined DW)
Motor impairment with blindness and epilepsy, moderate	(combined DW)
Motor plus cognitive impairment with blindness, moderate	(combined DW)
motor plus cognitive impairment with epilepsy, moderate	(combined DW)

motor plus cognitive impairment with blindness and epilepsy	(combined DW)
Motor impairment with blindness, severe	(combined DW)
Motor impairment with epilepsy, severe	(combined DW)
Motor impairment with blindness and epilepsy, severe	(combined DW)
Motor plus cognitive impairment with blindness, severe	(combined DW)
Motor plus cognitive impairment with epilepsy, severe	(combined DW)

**For disability weights and combined disability weights, please refer to the disability weights table in the appendix.

To determine the proportion of people within each of these severity levels, one study informed moderate-to-severe impairment splits, and mild impairments cases were divided equally into both categories.[3]

Modeling strategy

An initial DisMod MR 2.2 model is run using prevalence and case fatality data. Prior to input into DisMod, case fatality ratio (CFR) data is transformed into excess mortality rate (EMR) space using the formula $EMR = -\frac{\ln(1-CFR)}{\frac{28}{365.25}}$, which is analogous to the transformation of cumulative incidence to incidence rate. The denominator in this equation is derived from the definition of our CFR parameter, which is death in the first 28 days of life. The output from this first-step DisMod model is prevalence in the first two neonatal age groups. For this model, remission and incidence are both set to zero, as no one can be born with encephalopathy after birth, and no one can cease to have been born with encephalopathy after the fact. Study covariates were created for hospital data and US claims data.

Table 3

Study-level covariate	Parameter	Location level	beta	Exponentiated beta
Sex	Prevalence	Global	-0.036 (-0.11 — 0.047)	0.96 (0.89 — 1.05)
Inpatient-only Marketscan, year 2000	Prevalence	Global	1.64 (1.44 — 1.85)	5.18 (4.20 — 6.39)
Inpatient-only Marketscan, year 2010	Prevalence	Global	1.91 (1.76 — 2.00)	6.78 (5.84 — 7.36)
Inpatient-only Marketscan, year 2012	Prevalence	Global	1.96 (1.85 — 2.00)	7.07 (6.39 — 7.38)

[3] Badawi et al Developmental Medicine & Child Neurology, 2005, 47:293-8

Hospital data – Central Europe, Eastern Europe, & Central Asia	Prevalence	Global	-1.23 (-1.43 — -1.03)	0.29 (0.24 — 0.36)
Sex	Excess Mortality Rate	Global	0.16 (-0.58 — 0.93)	1.18 (0.56 — 2.53)

Country-level covariate	Parameter	beta	Exponentiated beta
Healthcare Access & Quality Index	Prevalence	-0.0084 (-0.016 — -0.0012)	0.99 (0.98 — 1.00)
In-Facility Delivery (proportion)	Prevalence	-0.34 (-0.87 — -0.02)	0.71 (0.42 — 0.98)
Skilled Birth Attendance (proportion)	Prevalence	-0.63 (-1.47 — -0.041)	0.53 (0.23 — 0.96)
LDI (I$ per capita)	Excess Mortality Rate	-0.23 (-0.37 — -0.086)	0.79 (0.69 — 0.92)

Using prevalence in the first two neonatal age groups, prevalence at 28 days is calculated via interpolation. Functionally, this is birth prevalence minus all those who have died in the first 28 days, our case fatality parameter.

Next, using mild impairment proportion and moderate-to-severe impairment proportion data, we ran either mixed-effect hierarchical regressions or meta-analyses to generate country-year-sex-specific estimates of both parameters (we used a meta-analysis when there was not sufficient data to support a regression model). We then calculated the birth prevalence of each severity level. As mild impairment and moderate-severe impairment were calculated separately, it was possible that they could sum to a value greater than one. To address this, we checked the sum of the two values in any of the 1,000 iterations of the uncertainty analysis, and if greater than 0.9, proportionately rescaled both estimates to sum to 0.9 in any of the 1,000 iterations of the uncertainty analysis (we picked 0.9 rather than 1 to allow at least some probability of a child having no impairment). We then proceeded with the calculation of the birth prevalence of impairment:

Mild impairment birth prevalence = prevalence at 28 days * proportion mild impairment

Moderate/severe impairment birth prevalence = prevalence at 28 days * proportion mod-severe impairment

For moderate/severe impairment, results of these latter calculations were then combined with excess mortality estimates derived from the standard mortality ratios (SMR) of cerebral palsy and used as inputs into a second DIsMod MR 2.1 model. For this model, remission and incidence were also set to zero. For mild impairment, we assumed no excess mortality and no remission, and as such simply applied the birth prevalence of mild impairment to every age group.

No other significant changes were made to the GBD 2016 modeling process.

Neonatal sepsis and other neonatal infections

Flowchart

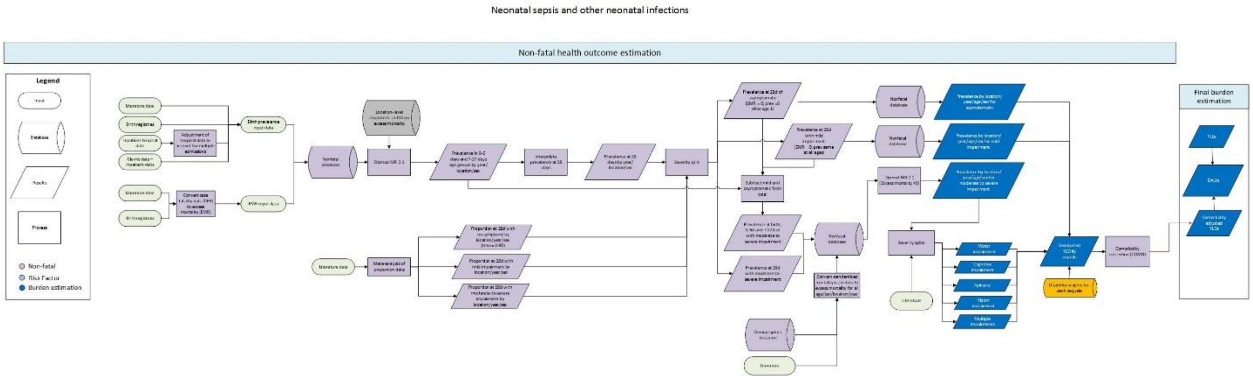

Input data and methodological summary

Case definition

Neonatal sepsis and other neonatal infections are infections during the neonatal period that advance to a systemic blood stream infection, the underlying cause of which can be meningitis, gastroenteritis, or other etiologies. Neonatal pneumonia, however, is not included – it is captured in our modeling of pneumonia as a separate entity. ICD codes associated with these causes include:

Input data

Model inputs

A systematic review was completed for GBD 2010, and for GBD 2013 and GBD 2015, a review was conducted on literature published since the previous addition. The PubMed database was searched using the following search string: (("infant"[Title/Abstract] OR "newborn"[Title/Abstract] OR "newborn infant"[Title/Abstract]) AND ("neonatal sepsis"[All Fields] OR "neonatal septicaemia"[All Fields] OR "neonatal meningitis"[All Fields] OR "early sepsis"[All Fields] OR "early septicaemia"[All Fields] OR "tetanus"[All Fields] OR "meningitis"[All Fields] OR "sepsis"[All Fields])) AND ("2012"[PDAT] : "3000"[PDAT]) AND "humans"[MeSH Terms]

The exclusion criteria were:

1. Studies that did not provide primary data on epidemiological parameters, e.g., a commentary piece
2. Non-representative studies (e.g., only high-risk pregnancies)
3. Reviews

The table below shows the number of literature studies included in GBD 2016, as well as the number of countries or subnational units and GBD world regions represented. Please note – no literature review was conducted for GBD 2016.

Table 1. Geographic representation

	Birth prevalence	Case fatality
Studies	2	15
Locations	2	15
GBD world regions	2	10

In addition to literature data, data from US claims data for 2000, 2010, and 2012 by US state were included. Only inpatient data was included from these datasets, because we believed it would be more representative of the true prevalence of neonatal sepsis than outpatient data: infants with neonatal sepsis in the countries from which hospital data was available are almost sure to be admitted to the hospital, whereas outpatient data is more likely to capture repeated visits by the same child as they grow. Remission was set to 26, as a cause of neonatal sepsis is assumed to last two weeks. Incidence is set to 0 after 27 days, as by definition, neonatal sepsis must occur within the neonatal period (0-27 days).

Severity splits

The basis of the GBD disability weight (DW) survey assessments are lay descriptions of sequelae highlighting major functional consequences of symptoms. The lay descriptions and disability weights for neonatal sepsis birth are shown below.

Table 2. Severity level, lay description and DWs

Severity level	Lay description
Motor plus cognitive impairments, mild	has some difficulty in moving around but is able to walk without help. The person is slow in learning at school. As an adult, the person has some difficulty doing complex or unfamiliar tasks but otherwise functions independently.
Motor impairment, mild	has some difficulty in moving around but is able to walk without help.
Motor impairment, moderate	has some difficulty in moving around, and difficulty in lifting and holding objects, dressing and sitting upright, but is able to walk without help.
Motor impairment, severe	is unable to move around without help, and is not able to lift or hold objects, get dressed or sit upright.
Distance vision blindness	is completely blind, which causes great difficulty in some daily activities, worry and anxiety, and great difficulty going outside the home without assistance.
Motor impairment with blindness, moderate	Combined DW
Motor impairment with epilepsy, moderate	Combined DW
Motor impairment with blindness, moderate	Combined DW
Motor plus cognitive impairment with blindness, moderate	Combined DW
Motor plus cognitive impairment with epilepsy, moderate	Combined DW
Motor plus cognitive impairment with blindness and epilepsy, moderate	Combined DW
Motor impairment with blindness, severe	Combined DW
Motor impairment with epilepsy, severe	Combined DW
Motor impairment with blindness, severe	Combined DW

Motor plus cognitive impairment, severe	Combined DW
Motor plus cognitive impairment with epilepsy, severe	Combined DW
Motor plus cognitive impairment with blindness and epilepsy, severe	Combined DW
Infection due to neonatal sepsis, severe	Combined DW

**For disability weights and combined disability weights, please refer to the disability weights table in the appendix.

To determine the proportion of people within each of these severity levels, one study informed moderate-to-severe impairment splits, and for mild impairments cases were divided equally into both categories.[4]

Modeling strategy

For GBD 2016, we used the same modeling strategy employed for GBD 2015. The methodology is outlined below.

An initial DisMod MR 2.1 model is run using prevalence and case fatality data. Prior to input into DisMod, case fatality ratio (CFR) data is transformed into excess mortality rate (EMR) space using the formula:

$$EMR = -\frac{\ln(1 - CFR)}{\frac{28}{365.25}}$$

which is analogous to the transformation of cumulative incidence to incidence rate. The denominator in this equation is derived from the definition of our CFR parameter, which is death in the first 28 days of life. The output from this first-step DisMod model is prevalence in the first two neonatal age groups. A study covariate were created literature.

Study-level covariate	Parameter	Location level	beta	Exponentiated beta
Sex	Prevalence	Global	0.12 (0.068 — 0.17)	1.12 (1.07 — 1.18)
Literature	Prevalence	Global	1.70 (1.18 — 1.99)	5.47 (3.24 — 7.30)

Country-level covariate	Parameter	beta	Exponentiated beta
Healthcare Access & Quality Index	Prevalence	0.0051 (-0.0097 — 0.020)	1.01 (0.99 — 1.02)

[4] Badawi et al Developmental Medicine & Child Neurology, 2005, 47:293-8

LDI (I$ per capita)	Excess Mortality Rate	-0.2 (-0.49 — -0.0097)	0.82 (0.61 — 0.99)
Healthcare Access & Quality Index	Excess Mortality Rate	-0.017 (-0.04 — -0.00089)	0.98 (0.96 — 1.00)
SEV Unsafe Water	Prevalence	0.12 (0.0060 — 0.39)	1.13 (1.01 — 1.47)
SEV Unsafe Sanitation	Prevalence	0.12 (0.0018 — 0.45)	1.13 (1.00 — 1.57)

Using prevalence in the first two neonatal age groups, prevalence at 28 days is calculated via interpolation. Functionally, this is birth prevalence minus all those who have died in the first 28 days, our case fatality parameter.

Next, using mild impairment proportion and moderate-to-severe impairment proportion data, we ran either mixed-effect hierarchical regressions or meta-analyses to generate country-year-sex-specific estimates of both parameters (we used a meta-analysis when there was not sufficient data to support a regression model). We then calculated the birth prevalence of each severity level. As mild impairment and moderate-severe impairment were calculated separately, it was possible that they could sum to a value greater than one. To address this, we checked the sum of the two values in any of the 1,000 iterations of the uncertainty analysis, and if greater than 0.9, proportionately rescaled both estimates to sum to 0.9 in any of the 1,000 iterations of the uncertainty analysis (we picked 0.9 rather than 1 to allow at least some probability of a child having no impairment). We then proceeded with the calculation of the birth prevalence of impairment:

Mild impairment birth prevalence = prevalence at 28 days * proportion mild impairment

Moderate/severe impairment birth prevalence = prevalence at 28 days * proportion mod-severe impairment

For moderate/severe impairment, results of these latter calculations were then combined with excess mortality estimates derived from the standard mortality ratios (SMR) of cerebral palsy and used as inputs into a second DisMod MR 2.1 model. For this model, remission and incidence were also set to zero. For mild impairment, we assumed no excess mortality and no remission, and as such simply applied the birth prevalence of mild impairment to every age group.

Hemolytic disease and other neonatal jaundice

Flowchart

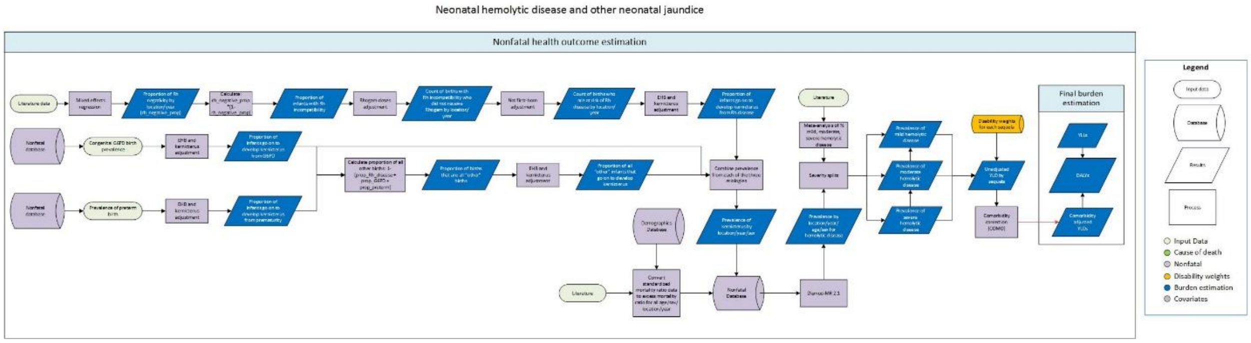

Case definition

Hemolytic disease of the newborn and other neonatal jaundice refers to several etiologies by which an infant develops extreme hyperbilirunemia (EHB) and can then go on to develop kernicterus. The etiologies that we model for GBD are EHB from Rhesus (Rh) disease, preterm birth, glucose-6-phosphate dehydrogenase deficiency (G6PD), and other causes.

Input data

Model inputs

A systematic review was completed for GBD 2010, and for GBD 2013 and GBD 2015, a review was conducted on literature published since the previous addition. The PubMed database was searched using the following search string: (("infant"[Title/Abstract] OR "newborn"[Title/Abstract] OR "newborn infant"[Title/Abstract]) AND ("haemolytic"[All Fields] OR "hyperbilirubinemia"[All Fields] OR "jaundice"[All Fields] OR "glucose-6-phosphate dehydrogenase deficiency"[All Fields] OR "G6PD deficiency"[All Fields] OR "hyperbilirubinemia"[All Fields] OR "EHB"[All Fields] OR "phototherapy"[All Fields] OR "ABO incompatibility"[All Fields] OR "RH incompatibility"[All Fields] OR "rh blood group system"[All Fields] OR "Rhesus"[All Fields] OR "Rhesus disease"[All Fields] OR "erythroblastosis fetalis"[All Fields] OR "kernicterus"[All Fields])) AND ("2012"[PDAT] : "3000"[PDAT]) Sort by: PublicationDate

The exclusion criteria were:

1. Studies that did not provide primary data on epidemiological parameters, e.g., a commentary piece
2. Non-representative studies (e.g., only high-risk pregnancies)
3. Reviews

For hemolytic disease, much of the input data comes from other GBD models, which are described in detail in the "modeling strategy" section. However, the modeling process for EHB from Rh disease involves literature data for several parameters, the breadth of which are described in the tables below:

Table 1a. Rh negativity

	Prevalence
Studies	48
Countries/subnationals	47
GBD world regions	13

Table 1b. Children who are not first-born

	Prevalence
Studies	82
Countries/subnationals	81
GBD world regions	14

Table 1c. Proportion of Preterm Infants who go on to have ROP

	Prevalence
Studies	32
Countries/subnationals	28
GBD world regions	12

**Please note that US claims data and hospital data were not included in the hemolytic disease modeling process because they are not coded separately by etiology. They could not be slotted into the existing modeling framework.

Severity splits & disability weights

The basis of the GBD disability weight (DW) survey assessments are lay descriptions of sequelae highlighting major functional consequences and symptoms. The lay descriptions and disability weights for hemolytic disease and other neonatal jaundice levels are shown below.

Table 2. Severity, lay description and DWs

Severity level	Lay description	DW (95% CI)
Motor impairment, moderate	has some difficulty in moving around, and difficulty in lifting and holding objects, dressing and sitting upright, but is able to walk without help.	0.061 (0.04-0.089)
Motor impairment, severe	is unable to move around without help, and is not able to lift or hold objects, get dressed or sit upright.	0.402 (0.268-0.545)
Distance vision blindness	is completely blind, which causes great difficulty in some daily activities, worry and anxiety, and great difficulty going outside the home without assistance.	0.187 (0.124-0.26)

To determine the proportion of people within each of these severity levels, one study informed moderate-to-severe impairment splits, and for mild impairments cases were divided equally into both categories.[5]

Modeling strategy

The modeling strategy can be described as two main steps. For each of the four etiologies (Rh disease, G6PD deficiency, preterm birth, and other causes) we estimated the prevalence of extreme hyperbilirubinemia (EHB or bilirubin>25 or exchange transfusion). Then, we multiplied this prevalence by an estimated proportion of EHB cases who go on to develop kernicterus. We used development of kernicterus as our criterion for incidence of long-term moderate/severe impairment.

Rh Disease

We began with data on the prevalence of Rh negativity in the population, the number of Rhogam (Rh_0 Immune Globulin) doses distributed to countries in 2010, and the proportion of children who are not firstborn. Mixed effect regressions were run on Rh negativity and birth order greater than one to generate estimates of these values for every country-year. We made the assumptions that the proportion of Rhogam doses to Rh-incompatible children stayed constant over time, and that countries with NMR<5 had complete Rhogam coverage. This yields the following equation for the prevalence of EHB in countries with NMR>5:

EHB Prevalence = Rh negative prevalence * (1 – Rh negative prevalence) * (2010 Rhogam doses / 2010 Rh incompatible babies) * (not-firstborn prevalence) * 0.15

The 0.15 multiplier represents results of previous calculations[5] showing the proportion of women developing Rh isoimmunization with a risk for anti-Rh antibodies complicating subsequent pregnancies.

Finally, to generate estimates of kernicterus prevalence, we multiplied the prevalence of EHB by 0.0072 (0.0038, 0.112) -- the proportion of children with EHB who develop kernicterus (extracted from previously published values).[6,7]

G6PD, Preterm, and Other

The other three pathways of kernicterus modeling simply involve multiplication by scalars. For each of these, given a complete set of prevalence estimates from other GBD models, we multiplied by a scalar representing the proportion of children who are expected to develop EHB (see the table below for the values of these scalars). We then adjusted that estimate upward by a factor of 2.45 (1.44, 4.16) for countries in which the NMR is greater than 15, a value utilized in previous publications[4] to reflect heightened risk in those countries where access to phototherapy for prevention of EHB is not standard. Finally, these EHB prevalence values were multiplied by a set of NMR-dependent scalars representing the proportion of EHB cases that go on to develop kernicterus. See table below, for those values.

[5] Badawi et al. Developmental Medicine & Child Neurology, 2005, 47:293-8

Table 3. EHB proportion and CIs

Cause	Source of birth prevalence estimates	EHB proportion	95% CI
G6PD	Congenital DisMod model, GBD 2016.	0.0013	(0.00085, 0.002)
Preterm birth complications	Custom modeling for preterm conditions, discussed elsewhere in this document (summed over all gestational ages).	0.00045	(0.00029, 0.0007)
Other	Global births – (Rh disease birth prevalence + preterm complications birth prevalence + G6PD birth prevalence)	0.00038	(0.00033, 0.00163)

Table 4. EHB proportions used in G6PD, preterm, and other estimates.

NMR	Kernicterus proportion	95% CI
<5	0.23	(0.099, 0.361)
5-15	0.35	(0.12, 0.58)
>=15	0.438	(0.255, 0.621)

Table 5: NMR-dependent kernicterus proportions applied to G6PD, preterm, and other EHB estimates.

Final Kernicterus Prevalence

We estimated preterm kernicterus in order to arrive at a proper value for the number of "other" children at risk of kernicterus but we do not actually include our preterm estimates in our measures of kernicterus since we assume that its disability is captured in our preterm models. Thus, we generate our final birth prevalence of kernicterus by:

Kernicterus birth prevalence = (kernicterus prevalence from Rh disease) + (kernicterus prevalence from G6PD) + (kernicterus prevalence from other disorders).

These estimates, along with estimates of excess mortality associated with kernicterus, are then used as inputs into our disease modeling tool (DisMod) to generate estimates of moderate to severe impairment for all ages.

No other significant changes were made to the GBD 2016 estimation process.

Protein-energy malnutrition

Flowchart

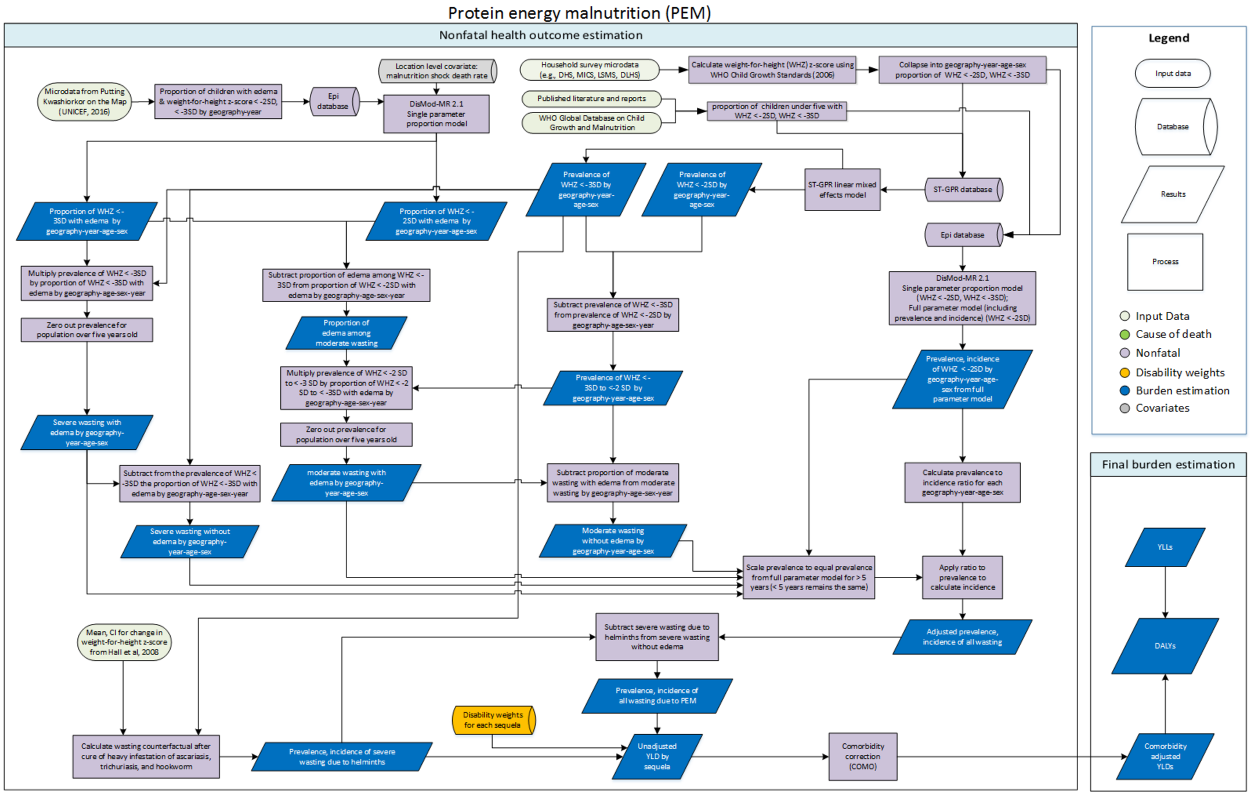

Input data and methodological summary

Case Definition

For protein energy malnutrition (PEM), ICD 10 codes are E40-E46.9, E64.0, and ICD 9 codes are 260-263.9. Our assessment of nonfatal PEM includes the quantification of nonfatal health loss associated with moderate and severe acute malnutrition, commonly referred to as "wasting," and was defined in terms of weight-for-height Z-scores (WHZ) on the WHO 2006 growth standard for children. We quantified nonfatal PEM burden in four mutually-exclusive and collectively exhaustive categories, reflecting distinct gradations of disability that can occur: moderate wasting without edema (WHZ < -2SD to < -3 SD), moderate wasting ***with edema*** (WHZ < -2SD to < -3 SD), severe wasting without edema (WHZ < -3SD), and severe wasting ***with edema*** (WHZ < -3SD). The aggregate of categories that include "edema" can be considered equivalent to the disease state commonly referred to as "kwashiorkor" and severe wasting can likewise be considered equivalent to "marasmus."

This classification reflects a moderate shift from GBD 2015, when moderate wasting without edema was not included in our nonfatal estimates, and by definition is associated with higher prevalence estimates than previously published by GBD. The other GBD 2015 categories—kwashiorkor, marasmus, and severe wasting—have unchanged case definitions, but have been renamed for clarity and consistency. This revised GBD 2016 case definition more closely aligns with other and allows for better application to the international nutrition community's programming and estimates related to non-fatal PEM.

Input data

The input data for this model come in three primary streams. First, we used individual-level and tabulated child anthropometry data from health surveys, literature, national reports, and centralized to inform the prevalence of WHZ decrement in each category corresponding to our case definitions. Second, to inform the proportion of children under 5 years who have signs of organ failure manifested as edema (i.e. kwashiorkor), we used a compiled dataset of surveys conducted using Standardized Monitoring and Assessment of Relief and Transitions (SMART) methods. Third, we used the cause-specific mortality rate (CSMR) results of our causes-of-death GBD analyses to inform spatiotemporal and age patterns in PEM mortality, which helped also inform patterns of PEM morbidity. These data sources and modeling process are described in other GBD publications on cause-specific mortality. All data was extracted with the most detailed standard demographic identifiers available, including age, sex, country, year, and subnational location if available.

Modelling Strategy

We used five parallel models to inform our estimates, the first two of which were completed in ST-GPR and the second three of which were completed in DisMod-MR 2.1: 1) Prevalence of WHZ <-2 in children under 5 years, 2) Prevalence of WHZ <-3 in children under 5 years, 3) Proportion of those with WHZ <-2 who have edema in under 5 years, 4) Proportion of those with WHZ <-3 who have edema in under 5 years, and 5) Prevalence, incidence, and excess-mortality of WHZ <-2 in all ages. As a final step, we subtracted a number of cases of PEM where the underlying etiology is severe worm infestation.

The results of the first four models were used for children under 5 years. Arithmetic transformations were performed to ensure that the final results fit into the mutually-exclusive, collectively-exhaustive categories described in the first section above. We assumed zero prevalence of edema in people over 5 years old. The results of the final model were used for all age groups 5 years and older and the proportion of moderate versus severe wasting in each of those age groups was derived from the first set of models.

The final model represents the first attempt we have made in GBD to estimate incidence for PEM, for which there is comparatively little empirical data. Using available information from scientific publications, which suggest the mean duration of illness is 9 months, and conversations with collaborators and nutrition experts, we applied what we consider a plausible set of remission rate bounds of 0.25-1.25 (# of remitted cases of PEM per person-year of illness) to the final of the five models. These bounds allowed DisMod-MR 2.1 to mathematically derive an internally-consistent solution for incidence, prevalence, remission, excess mortality, and cause-specific mortality using all available data. This could only be done for the aggregate PEM definition (prevalence of WHZ <-2) to ensure that the case definition for prevalence matched that of the mortality results. The incidence-to-prevalence ratio derived from the final model was applied equally across all the categories of nonfatal PEM. Future work in systematically

evaluating longitudinal datasets on nutrition and growth failure will allow us to improving the empirical basis for PEM incidence estimates, including improved resolution for the component categories.

We applied disability weights from the GBD disability weight survey to the prevalence of the above sequela according to their corresponding health state and severity level. The sequela, along with their lay descriptions and disability weights for health states derived from the GBD Disability Weights study are shown below. We assumed that those with moderate wasting, but no edema, did not have any direct disability due to this condition.

Table 1. Sequela, severity, lay description, and DWs

Sequela	Health State Name	Lay description	DW (95% CI)
Moderate Wasting without Edema	Asymptomatic	--	--
Moderate Wasting with Edema	Kwashiorkor	Is very tired and irritable and has diarrhea.	0.051 (0.031-0.079)
Severe Wasting without Edema	Severe wasting	Is extremely skinny and has no energy.	0.128 (0.082-0.183)
Severe Wasting with Edema	Kwashiorkor + Severe wasting	Is very tired and irritable and has diarrhea.	0.051 (0.031-0.079)
		Is extremely skinny and has no energy.	0.128 (0.082-0.183)

Because both worms and PEM can cause wasting, we needed to divide out the wasting envelope to attribute wasting to both PEM and worms. We determined the amount of wasting attributable to worms by referencing Hall et al. 2008[6] to determine the mean and confidence interval estimates of the z-score shift. We then calculated the counterfactual wasting prevalence given no worms, according to the z-score shift. From this, we calculated the fraction of wasting that is attributable to worms, and assigned the remainder of wasting to PEM. We assumed no edema due to worms and the same prevalence-to-incidence ratio as in each of the other models.

Following the assignment of disability weights to the various sequelae, the resulting disability adjusted life years (YLDs) go through the comorbidity simulator, which accounts for any comorbidity and corrects accordingly. The final outputs are comorbidity adjusted YLDs, which are combined with years of life lost (YLLs) for final disability-adjusted life years (DALYs).

[6] Hall A, Hewitt G, Tuffrey V, de Silva N. A review and meta-analysis of the impact of intestinal worms on child growth and nutrition. Maternal and Child Nutrition. 2008. 4. 118-236.

Iodine deficiency

Flowchart

Iodine deficiency

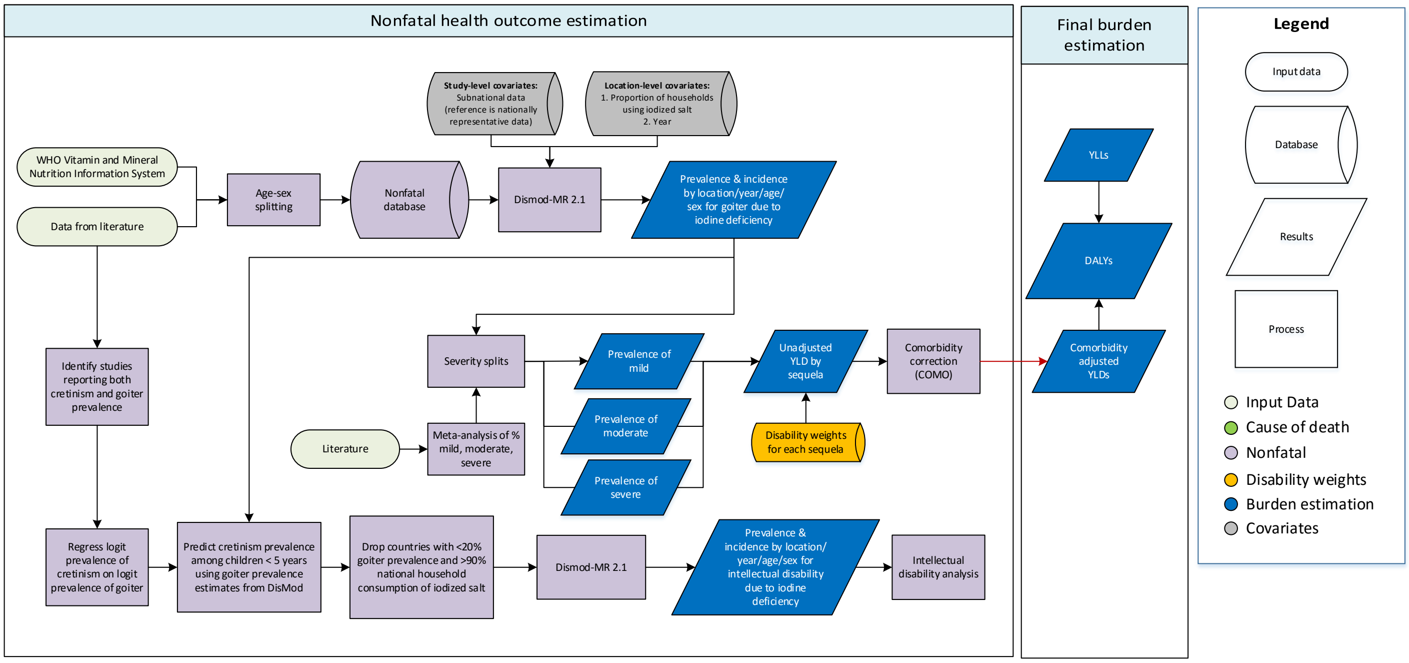

Input data and methodological summary

Case definition

Our assessment of the nonfatal burden of iodine deficiency includes estimates of only the subset of iodine deficiency associated with visible goiter (grade 2) and its associated sequelae, including thyroid dysfunction, heart failure, and intellectual disability (historically referred to as "cretinism"). It does not include estimates of sub-clinical iodine deficiency or non-visible goiter (grade 1) induced by iodine deficiency. Expanding to include all forms of subclinical iodine deficiency is a goal of future iterations of GBD analyses. The corresponding ICD-10 codes from the causes of death analysis for iodine deficiency are E00-E02. Further details of mortality modeling can be found in the GBD 2016 cause-specific mortality publication.

Input data

For GBD 2016, data from the WHO Vitamin and Mineral Nutrition Information System and published studies were used. This extraction and an accompanying systematic review were last conducted for GBD 2013. The PubMed search terms were: ((iodine deficiency[Title/Abstract] AND prevalence[Title/Abstract]) AND ("2009"[Date – Publication] : "2013"[Date – Publication]))

The exclusion criteria were:

1. Studies that were not population-based, eg, hospital or clinic-based studies
2. Studies that did not provide primary data on epidemiological parameters, eg, commentaries
3. Review articles
4. Case series
5. Self-reported cases

Updates to systematic reviews are performed on an ongoing schedule across all GBD causes; an update for iodine deficiency will be performed in the next one to two iterations. The table below shows the number of literature studies included in GBD 2016, as well as the number of countries or subnational units and GBD world regions represented.

Table 1. Geographic representation

	Prevalence	Incidence	Mortality risk
Studies	25	-	-
Countries/subnationals	8	-	-
GBD world regions	5	-	-

Modelling strategy

For GBD 2016, we modelled the prevalence of grade 2 goiter in DisMod-MR 2.1. We chose to model grade 2 goiter over grade 1 goiter because of the greater reliability and consistency of the clinical diagnosis of grade 2 goiter worldwide. We used a study-level covariate to indicate national and subnational observations, where nationally representative studies were set as the reference category. We used household iodized salt consumption proportion as a country-level covariate.

We estimated the prevalence of intellectual disability due to iodine deficiency (cretinism) by regressing data points from studies reporting both cretinism and goiter prevalence in the same population. To do so, we first transformed cretinism prevalence and goiter prevalence into logit space, regressed the logit prevalence of cretinism on the logit prevalence of goiter, and predicted for all locations using the goiter estimates from the DisMod model above. We dropped locations with total goiter prevalence less than 20% and locations with household iodized salt consumption greater than 90%. We kept observations in children younger than 5 years and use these data as incidence input in DisMod to generate location-year-age-sex-specific estimates. We assumed zero remission as the disease is a lifelong condition, and zero incidence after age 5. We repeated the dropout criteria of total goiter prevalence and iodized salt consumption on the DisMod output. The severity split for intellectual disability due to iodine deficiency is presented separately in the section for intellectual disability.

Heart failure attributable to iodine deficiency was modelled separately, and the methods for this outcome are presented separately in the section for heart failure and its etiologies.

Betas and exponentiated values (which can be interpreted as an odds ratio) for the grade 2 goiter DisMod model are shown in the table below.

Table 2. Beta and exponentiated beta values

Covariate	Parameter	beta	Exponentiated beta
Proportions of households using iodized salts	Prevalence	-0.55 (-0.60, -0.41)	0.58 (0.55, 0.66)
Subnational	Prevalence	0.50 (0.48, 0.50)	1.64 (1.62, 1.65)
Sex	Prevalence	-0.63 (-0.85, -0.42)	0.53 (0.43, 0.66)

Severity splits & disability weights

Our approach to apportioning cases of grade 2 goiter was modified in GBD 2016 to improve internal consistency of YLD estimates. The primary change was that in GBD 2015, each of the types of symptoms (heart failure, thyroid dysfunction, intellectual disability) were considered to be mutually-exclusive, but recognizing they are not, we constructed independent joint probabilities for each. Initial severity proportions have not changed since GBD 2010: visible goiter without symptoms of thyroid dysfunction (proportion=0.915, 95% confidence interval (CI): 0.904-0.926); goiter with symptoms of thyroid dysfunction (proportion=0.085, 95% confidence interval (CI): 0.084-0.086). The regression results for intellectual disability (ID) were applied to the total estimates of iodine deficiency. All of those with ID were assumed to have symptoms of thyroid dysfunction. Heart failure due to iodine deficiency, which was estimated separately, was assumed to only occur in the subset of persons with profound ID.

The lay descriptions and disability weights for severity levels derived from the GBD Disability Weights study are shown below.

Table 3. Severity, health state, lay description, and DWs for sequelae specific to iodine deficiency

Severity level	Health state	Lay description	DW (95% CI)
Visible goiter without symptoms	Disfigurement, level 2	Has a visible physical deformity that causes others to stare and comment. As a result, the person is worried and has trouble sleeping and concentrating.	0.067 (0.044–0.096)
Visible goiter with symptoms	Goiter	Has a large mass in the front of the neck. The person sometimes has weakness and fatigue, constipation and weight gain.	0.199 (0.133–0.276)

No other significant changes were made to the modelling strategy for GBD 2016.

Vitamin A deficiency

Flowchart

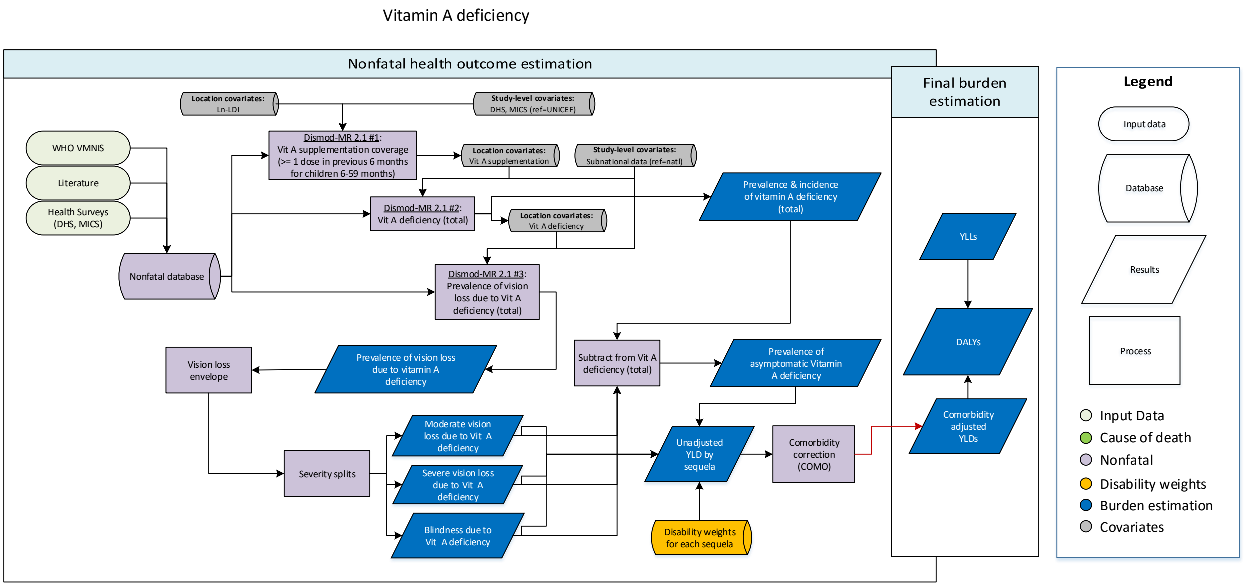

Input data and methodological summary

Case definition

For GBD 2016, the case assessment of vitamin A deficiency involves the quantification of total Vitamin A deficiency (serum retinol < 0.7 μmol/L) as well as blindness and vision loss due to Vitamin A deficiency, which are associated with corneal ulcerations and corneal scars. ICD 10 codes are E50-E50.9, E64.1, and ICD 9 codes are 264-264.9.

To ensure we were using as much information as possible, and therefore maximize the data basis of our estimates, we modeled Vitamin A deficiency sequentially. The first step was to estimate the coverage of Vitamin A supplementation. Although the typical metric on which supplementation is tracked is 2+ doses of Vitamin A in the previous 12 months for children under 5 years, most existing health surveys do not routinely provide sufficient information to calculate it. Our case definition for the supplementation model was therefore the proportion of children 6-59 months of age who received at least one dose of Vitamin A in the previous 6 months. Supplementation estimates were then used as a location-level covariate to guide incidence and prevalence models of overall Vitamin A deficiency, which was subsequently used as a location-level covariate to guide prevalence estimates of vision loss due to Vitamin A deficiency. The difference between total Vitamin A deficiency and vision loss due to Vitamin A deficiency is considered asymptomatic. Total Vitamin A deficiency is separately considered as a risk factor in the GBD 2016 comparative risk assessment analysis.

Input data

For GBD 2016, we used data from the WHO Vitamin and Mineral Nutrition Information System, health surveys such as DHS and MICS, and studies identified through literature review. A systematic review was last conducted for GBD 2013. The PubMed search terms were: ((vitamin A deficiency[Title/Abstract]

AND prevalence[Title/Abstract]) AND ("2009"[Date – Publication] : "2013"[Date – Publication])). The table below shows the number of data points included in the final datasets. Exclusion criteria were:

1. Studies that were not population-based, e.g., hospital or clinic-based studies
2. Studies that did not provide primary data on epidemiological parameters, e.g., commentaries
3. Review articles
4. Case series
5. Self-reported cases

Table 1. Geographic representation of datasets used for three stages of Vitamin A deficiency nonfatal burden estimation (number of data points per location)

Location	Supplementation (proportion)	Deficiency (prevalence)	Vision Loss (Relative risk)	Vision Loss (prevalence)
Global	900	365	1	81
East Asia	12	10		
Southeast Asia	102	45		21
Oceania	24	18		
Central Asia	51	31		
Central Europe	2	1		
Australasia		1		
Southern Latin America		1		
High-income North America		4		
Caribbean	17	12		1
Andean Latin America	25	10		
Central Latin America	33	54		1
Tropical Latin America	1	2		2
North Africa and Middle East	49	37		18
South Asia	61	21		21
Central Sub-Saharan Africa	60	9		1
Eastern Sub-Saharan Africa	182	63		10
Southern Sub-Saharan Africa	49	15		1
Western Sub-Saharan Africa	232	31		5

Modelling strategy

All Vitamin A deficiency estimates were made using DisMod-MR 2.1. As described above, we first estimated Vitamin A supplementation coverage. Although all data was from ages 6-59 months, we assumed no difference in age pattern of supplementation coverage and used the natural log of lag-distributed income per capita (LN-LDI) as a location-level covariate to inform estimates where data was absent. DHS and MICS data was cross-walked to the reference data source, which came from UNICEF (http://data.worldbank.org/indicator/SN.ITK.VITA.ZS).

Table 2: Covariate effects for Vitamin A supplementation model

Measure	Covariate	Type	Value	Exponentiated
Prevalence	MICS	Study-level	-0.59	0.56

			(-0.73 — -0.42)	(0.48 — 0.65)
**Prevalence**	DHS	Study-level	-0.092 (-0.22 — 0.038)	0.91 (0.80 — 1.04)
**Prevalence**	LDI (I$ per capita)	Country-level	0.0094 (0.00061 — 0.039)	1.01 (1.00 — 1.04)

Second, we estimated the age- and sex-specific prevalence of Vitamin A deficiency (serum retinol < 0.7 µmol/L). WHO VMNIS was the primary data source for this model and was supplemented with data from DHS and other health surveys where testing was performed. We assumed the following in our model: no excess mortality, birth prevalence is possible, and that incidence and remission are both decreasing after age 5. Incidence estimates were therefore derived from the changing age pattern of prevalence with some allowance for remission. Data from subnational locations was crosswalked to the reference data sources of nationally-representative data. Females were found to have 1.09 times higher Vitamin A deficiency, although the uncertainty in that ratio ranged from 0.97 to 1.24. Location-level covariates were used for Vitamin A supplementation coverage from the above model as well as GBD 2016 Socio-demographic Index (SDI) numbers.

Table 3: Covariate effects for Vitamin A deficiency model

Measure	Covariate	Type	Value	Exponentiated
**Prevalence**	Sex	Study-level	0.086 (-0.027 — 0.21)	1.09 (0.97 — 1.24)
**Prevalence**	Subnational	Study-level	0.00074 (-0.15 — 0.17)	1.00 (0.86 — 1.19)
**Prevalence**	Vit A suppl. coverage	Country-level	-0.38 (-0.71 — -0.099)	0.68 (0.49 — 0.91)
**Prevalence**	SDI	Country-level	-2.25 (-2.87 — -1.36)	0.10 (0.057 — 0.26)

Third, for models of prevalence of blindness and vision loss due to vitamin A deficiency, this was run as a single-parameter meta-regression on prevalence, so incidence estimates were not generated. Data from subnational locations was crosswalked to the reference data sources of nationally-representative data. Vitamin A deficiency prevalence was used as a location-level covariate. Two covariates that were used in GBD 2015 were removed in the GBD 2016 models. These were 1) proportion of children who are underweight and 2) year. The first was removed to eliminate a modeling circularity where, because of timing of processes, the only available results for this covariate at time of modeling Vitamin A deficiency were from the previous round of GBD estimation. The second was removed because it functionally assumes a fixed time trend in the prevalence of vision loss, which may not be the case everywhere.

Table 4: Covariate effects for Vision loss due to Vitamin A deficiency model

Measure	Covariate	Type	Value	Exponentiated
**Prevalence**	Sex	Study-level	0.040 (-1.44 — 1.53)	1.04 (0.24 — 4.60)
**Prevalence**	Subnational	Study-level	0.19	1.20

			(0.0069 — 0.46)	(1.01 — 1.59)
**Prevalence**	Vit A Def Prev (age-stdized)	Country-level	1.10 (0.054 — 1.96)	3.00 (1.06 — 7.08)

Severity splits and disability weights

Our GBD 2016 results include explicit estimates of total Vitamin A deficiency, although those without vision loss are assumed to be asymptomatic. Description of how our estimates of total vision loss described above are parsed into moderate vision loss, severe vision loss, and blindness can be found in the modeling description for the "vision loss impairment". Sequelae and corresponding disability weights for each of the health states associated with Vitamin A deficiency are shown in the table below.

Table 5. Severity, lay description and Disability Weight (DW)

Severity level	Lay description	DW (95% CI)
**Asymptomatic Vitamin A deficiency**		0
**Moderate vision loss**	Has vision problems that make it difficult to recognize faces or objects across a room.	0.031 (0.019-0.049)
**Severe vision loss**	Has severe vision loss, which causes difficulty in daily activities, some emotional impact (for example worry), and some difficulty going outside the home without assistance.	0.184 (0.125-0.259)
**Blindness**	Is completely blind, which causes great difficulty in some daily activities, worry and anxiety, and great difficulty going outside the home without assistance.	0.187 (0.124-0.26)

Other nutritional deficiencies

Other nutritional deficiencies encompass a wide variety of causes of morbidity, ranging from vitamin deficiencies to other nutritional anemias. GBD 2016 treats these causes as a single category, given their relatively limited burden, diversity in underlying causes and risk factors, and data availability. Instead of modeling them in a traditional modeling format, we calculate the YLDs associated with other nutritional deficiencies using a YLD/YLL ratio.

The first input for this non-fatal portion of other nutritional deficiencies burden is the YLL estimates from the GBD 2016 causes of death (CoD) analysis. The causes and their associated ICD-10 codes that constitute other nutritional deficiencies for CoD are listed below. Additionally, CoD includes specific models for Protein Energy Malnutrition, another nutritional cause of morbidity and mortality; as Protein Energy Malnutrition has a specific non fatal model that results in YLDs, we can calculate the YLD/YLL ratio for Protein Energy Malnutrition. We multiply the YLL estimates for other nutritional deficiencies from CoD by the YLD/YLL ratio for PEM, providing us with an estimate of the YLDs associated with other nutritional deficiencies.

GBD cause	ICD-10 code
Other nutritional deficiencies	D51-D52.0 (vitamin B12 deficiency anemia and folate deficiency anemia)
Other nutritional deficiencies	D52.8-D53.9 (other nutritional anemias)
Other nutritional deficiencies	D64.3 (other sideroblastic anemias)
Other nutritional deficiencies	E51-E61.9 (thiamine, niacin, other B group vitamins, ascorbic acid, vitamin D, other vitamin, dietary calcium, dietary selenium, dietary zinc, and other nutrient element deficiencies)
Other nutritional deficiencies	E63-E64.0 (other nutritional deficiencies and sequelae of protein-calorie malnutrition)
Other nutritional deficiencies	E64.2-E64.9 (sequelae of vitamin C deficiency, rickets, other nutritional deficiencies, and unspecified nutritional deficiencies)
Other nutritional deficiencies	M12.1-M12.19 (Kaschin-Beck disease)

Sexually transmitted infections (STIs), excluding HIV:
Chlamydia, gonorrhea, trichomonas, genital herpes due to HSV-2, syphilis (early infection and adult tertiary syphilis), and other STIs

Flowchart

Sexually-transmitted infections excluding HIV:
Chlamydia, Gonorrhea, Trichomonas, Genital herpes due to HSV-2, Syphilis (early and adult tertiary)

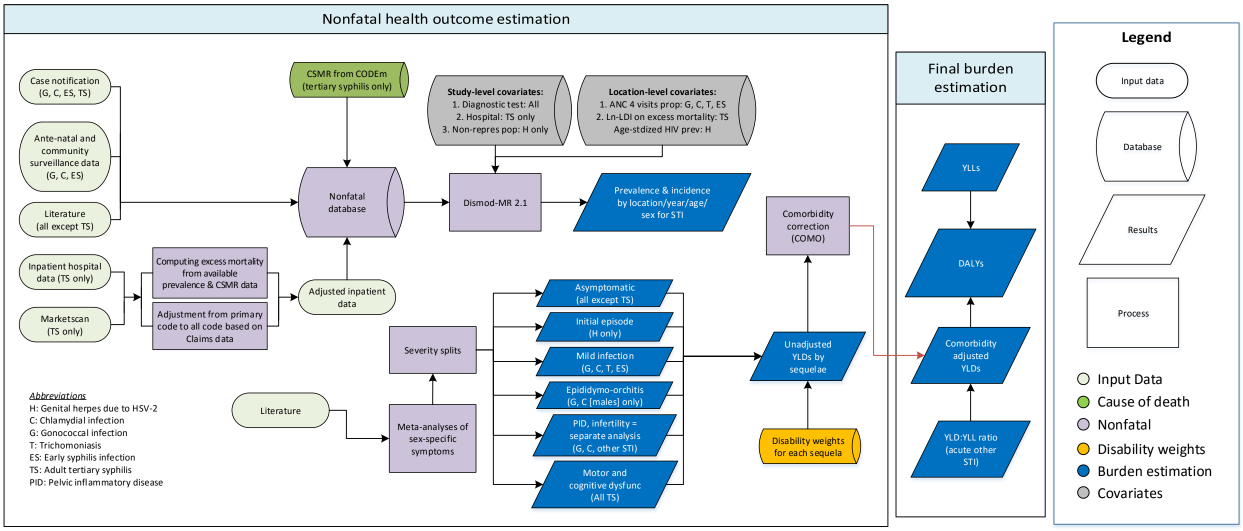

Input data and methodological summary

Case definition

For GBD 2016, we estimated the prevalence and incidence of genital and reproductive tract infection with several common sexually transmitted infections, including *Chlamydia trachomatis*, *Neisseria gonorrhea*, *Trichomonas vaginalis*, *Treponema pallidum* (syphilis), and genital herpes associated with seroprevalent HSV-2. Separate estimates were completed for early syphilis and adult tertiary syphilis. ICD-9 and ICD-10 codes associated with each cause in the GBD 2016 cause-specific mortality analyses are listed below. Of note, mortality was assumed to be zero in trichomoniasis and genital herpes infection, and YLDs due to congenital syphilis were not estimated.

Table 1. International classification of diseases codes for sexually transmitted infections in GBD 2015 cause of death analysis

Condition	ICD10 code	ICD9 code
Sexually transmitted infections (STI) excl HIV	A50-A60, A63-A64, I98.0, K67.0-K67.2, M73.0-M73.1	090-099,131,614
Syphilis	A50-A53, I98.0, K67.0-K67.2, M73.0-M73.1	090-097
Congenital syphilis	A50	090
Early syphilis	A51	091

Adult tertiary syphilis	A52, I98.0	093-095
Chlamydial infection	A55-A56,K67.0	099.41,099.5
Gonococcal infection	A54,K67.1	098
Trichomoniasis	A59,K67.0	131
Genital herpes	A60	054.1
Other STI	A57-A58, A63-A64, M73.0	099 (except 099.41, 099.5)

Input data

Model inputs

Systematic literature reviews were completed on April 17, 2015, for chlamydia, gonorrhea, trichomonas, genital herpes, and early syphilis. Three related search strings were used as many studies report on multiple infections. With the exception of the early syphilis literature review, which was completed in GBD 2015, these were the same search strings and strategies as were employed in GBD 2013.

462 initial hits; 54 sources selected for full text review and data extraction: (((chlamydia[Title/Abstract] OR chlamydia tracomatis[Title/Abstract] OR trachoma[Title/Abstract]) AND prevalence[Title/Abstract]) AND ('2013'[Date - Publication] : '2015'[Date - Publication])) /// ((gonorrhea[Title/Abstract] OR Neisseria[Title/Abstract] OR gonococcal[Title/Abstract]) AND prevalence[Title/Abstract]) AND ("2013"[PDAT] : "2015"[PDAT]) /// ((trichomonal[Title/Abstract] OR trichomonas[Title/Abstract]) AND prevalence[Title/Abstract]) AND ('2013'[PDAT] : '2015'[PDAT])

1265 initial hits; 178 sources selected for full text review and data extraction: ("syphilis"[MeSH] OR "Treponema pallidum"[MeSH]) NOT "Yaws"[MeSH] AND "prevalence"[MeSH] AND "1990"[PDAT] : "2015"[PDAT] AND "humans"[MeSH] **///** ("syphilis"[MeSH] OR "Treponema pallidum"[Mesh]) NOT "Yaws"[MeSH] AND ("incidence"[MeSH]) AND ("1990"[PDAT] : "2015"[PDAT]) AND "humans"[MeSH])

13 initial hits; 1 selected for full text review and data extraction: herpes"[Title/Abstract] OR "Herpesvirus 2, Human"[Mesh]) AND ("Prevalence"[Title/Abstract] OR "Incidence"[Title/Abstract] AND ("2015"[PDAT] : "2015"[PDAT])

The genital herpes dataset was supplemented by those sources contained in recent published estimates completed by Looker and colleagues.[1,2] For all other STI, most notably early syphilis, we supplemented our datasets with manual search of national ministry of health websites, antenatal clinic surveillance reports, and case-notification data from locations where centralized reporting is mandatory.

To be included, a study must report on laboratory-confirmed diagnosis of an STI. For each STI, the reference category for diagnostic modality was a DNA-based test (eg, PCR or other nucleic acid amplification test) and data using any other diagnostic test were quantitatively crosswalked to the reference category using binary study-level covariates in DisMod-MR 2.1 For genital herpes, any sources that did not use nucleic acid amplification were excluded.

For early syphilis, crosswalks were performed in a country-specific manner because non-treponemal false-positive rate is dependent on several epidemiological factors, including age, sex, and endemicity of other infections. Given the potential variability in immunological reasons for false positives in each of the other STI categories, we also did not perform any adjustment for published sensitivity and specificity of

tests, as these results are often drawn from particular populations that may not be representative of the global experience for each infection.

For all STI, including genital herpes, sources were excluded if the sample population was drawn exclusively from a high-risk group (eg, HIV-positive, men who have sex with men [MSM], or sex workers).

All surveillance data were considered prevalence when modeling chlamydia and gonorrhea, and all data were considered prevalence when modeling early syphilis.

Data were outliered or excluded if we found them unreasonable when compared to regional, super-regional, and global rates. These data included European surveillance data, much of which was unreasonably low.

Composition of final datasets are shown below for each of the different STI models.

Gonococcal infection

	Prevalence	Incidence
Studies	177	5
Countries/subnationals	75/240	3
GBD world regions	20	3

Chlamydial infection

	Prevalence	Incidence
Studies	203	2
Countries/subnationals	93/221	1/0
GBD world regions	21	1

Early syphilis

	Prevalence
Studies	409
Countries/subnationals	167/155
GBD world regions	21

Adult tertiary syphilis

	Prevalence
Studies	34
Countries/subnationals	17/127
GBD world regions	7

Trichomoniasis infection

	Prevalence
Studies	115

Countries/subnationals	62/18
GBD world regions	20

Genital herpes

	Prevalence	Incidence
Studies	269	20
Countries/subnationals	72/38	12/8
GBD world regions	20	7

Modelling strategy

Overall, we have made no substantive changes to the estimation strategy since GBD 2015. We estimated the nonfatal burden of STI in three parts. For the first part we estimated the prevalence, incidence, remission, and case fatality from acute infection associated with gonococcus, chlamydia, trichomoniasis, genital herpes (from herpes simplex virus 2), early syphilis, and adult tertiary syphilis using the data above and DisMod-MR 2.1. Not all cases of STI are symptomatic, so we used literature review to guide splitting prevalent cases into asymptomatic and symptomatic health states.[3,4] Specific modelling considerations in DisMod-MR 2.1 for each STI are described below.

The second part, which is estimation of prevalence, incidence, remission and case fatality from pelvic inflammatory disease (PID) and PID-induced primary and secondary infertility, is described in a separate section on those conditions. Briefly, for PID we used ICD-9 and ICD-10 coded discharge datasets, Marketscan claims database, and systematic literature review to develop a dataset that was subsequently modelled for all locations using DisMod-MR 2.1. Processing steps for discharge and claims data are described separately. PID was then split into underlying ideologies (chlamydia, gonorrhea, and other) using results of supplemental literature review and DisMod-MR 2.1 models of the proportion due to each etiology. PID-induced primary and secondary infertility assuming only a fixed subset of incident PID cases develop infertility and that there is no remission in these cases.

The third and final part involved finding the ratio of YLD to YLL ratio for all STI (excluding other STI) and then applying that same ratio to other STI YLLs.

Gonococcal infection

Prevalence data were the primary input from literature, case notification systems, and surveillance. Incidence was restricted to occur only between ages 10 and 69. EMR was set to have a maximum value of 0.0001. Remission rate bounds were set to be between 2 and 5 (translating to a duration of 10 to 26 weeks), and are the same as we used in GBD 2015, GBD 2013, and GBD 2010.[3,4] Study covariates included crosswalks for data where case diagnosis was based on culture or other non-nucleic acid amplification tests (PCR is the gold-standard diagnostic method). Surveillance/notification data were also crosswalked against the reference definition. Female-to-male ratio of prevalence and incidence was restricted to not exceed 2.01:1, an approach that was used to account for relatively sparse data from males. Coverage proportion of four visits of antenatal care coverage (ANC4) was the only country covariate and was assigned to prevalence.

We assigned the proportion of persons who developed symptoms of infection and/or epididymo-orchitis. Both were unchanged from GBD 2010 and are the same as used in WHO analyses. Males were assigned a fixed proportion of each of the following health states. Epididymo-orchitis differed for locations with long time series of high-quality vital registration data ("data-rich") compared to locations with poor data ("all others"). This split was performed for developed versus developing countries in GBD 2013 and GBD 2010. Data-rich proportion was 0.03 (0.015–0.045) and all others was 0.0975 (0.0483–0.143). A proportion of the remainder, 0.5875 (0.5288–0.6463), were assigned a health state of mild, acute infectious disease. The remainder were assumed to be asymptomatic. Females were estimated to have a proportion with mild, acute infectious disease of 0.34 (0.306–0.374) and the remainder asymptomatic.[3,4] Females have fewer symptoms of gonorrhea than males, which results in higher YLDs among males, despite a lower number of cases.

Gonococcal infection

Study-level covariate	Parameter	Location level	beta	Exponentiated beta
Sex	prevalence	Global	0.37 (0.30 – 0.70)	1.45 (1.29 – 2.01)
Culture-positive	prevalence	Global	-0.0054 (-0.039 to – 0.000071)	0.99 (0.96 – 1.00)
Diagnosis other	prevalence	Global	-0.0063 (-0.051 to - 0.00019)	0.99 (0.95 – 1.00)

Country-level covariate	Parameter	beta	Exponentiated beta
Antenatal Care (4 visits) Coverage (proportion)	prevalence	-0.021 (-0.098 to - 0.0000033)	0.98 (0.91 – 1.00)

Chlamydial infection

Chlamydia estimation methods were the same as used for gonococcal infection described above, with a few exceptions. Remission bounds were set between 0.5 and 0.9599, and the female-to-male sex ratio of prevalence and incidence was restricted to not exceed 1.35:1. The proportion of antenatal care coverage (4 visits) was applied as a country-level covariate.

The approach to estimating sequelae was the same as for gonorrhea, although the proportions in each state were different. Data- rich proportion was 0.02 (0.01–0.03) and all others was 0.0625 (0.0325–0.0975). A proportion of the remainder, 0.505 (0.4545–0.5555), were assigned a health state of mild, acute infectious disease. The remainder were assumed to be asymptomatic. Females were estimated to have proportion with mild, acute infectious disease of 0.17 (0.153–0.187) and the remainder asymptomatic.[3,4] Again, despite a higher number of cases among females, the YLD-per case among males was higher.

Chlamydial infection

Study-level covariate	Parameter	Location level	beta	Exponentiated beta
Diagnosis other	prevalence	Global	-0.031 (-0.18 to - 0.00029)	0.97 (0.83 – 1.00)

Culture-positive	prevalence	Global	-0.024 (-0.12 to -0.00058)	0.98 (0.89 – 1.00)

Country-level covariate	Parameter	beta	Exponentiated beta
Antenatal Care (4 visits) Coverage (proportion)	prevalence	-0.024 (-0.093 to -0.00013)	0.98 (0.91 – 1.00)

Trichomoniasis

Trichomoniasis estimation methods were similar to those used for gonococcal and chlamydia infection. However, the female-to-male sex ratio was unbounded, and excess mortality rate was assumed to be zero. Remission was bounded from 0.7199 and 0.89. Surveillance and case-notification data for trichomonas was considered to be unreliable and not used. The reference definition for diagnostic was PCR, and wet mount and culture positive were crosswalked against the reference definition.

Males are assumed to be 100% asymptomatic with trichomoniasis, while 0.34 (0.306–0.374) of prevalent females were assigned a health state of mild, acute infectious disease.[3,4]

Trichomonas infection

Study-level covariate	Parameter	Location level	beta	Exponentiated beta
Diagnostic wet mount	prevalence	Global	-0.41 (-0.5 to -0.24)	0.66 (0.61 – 0.79)
Culture-positive	prevalence	Global	-0.15 (-0.38 to -0.01)	0.86 (0.68 – 0.99)

Country-level covariate	Parameter	beta	Exponentiated beta
Antenatal care (4 visits) coverage (proportion)	prevalence	-0.052 (-0.098 to -0.003)	0.95 (0.91 – 1.00)

Genital herpes due to HSV-2

Genital herpes estimation assumed remission and mortality are both zero. Incidence estimates were thus based on mathematical integration of age-specific prevalence data. Incidence was restricted to occur between ages 10 and 59. After careful analysis, the modeling strategy for genital herpes has evolved to exclude high-risk populations. High-risk groups that were excluded included MSM, HIV-positive, prisoners, sex workers, drug users, and STI clinic samples. However, blood donor samples were included, and crosswalked to the general population assuming the prevalence is lower than that of the general population. Data on pregnant populations were also crosswalked, without a priori knowledge of direction. Crosswalks were performed at the regional level to allow for variation in the quantitative relationship. A single country covariate, age-standardized HIV prevalence, was used to guide estimates in locations with sparse data in recognition of the strong relationship between HSV-2 and HIV transmission.

Many with initial genital herpes infection have a painful rash that, while not as severe as that accompanying zoster reactivation, is more severe than that associated with recurrent genital herpes episodes. A systematic literature review revealed a few studies that informed our estimation that 0.175 (0.10–0.25) of initial herpes cases have symptoms of moderate, acute infectious disease lasting 3 (2–4) weeks and 0.189 have prevalent persons have 6 (5–7) recurrent episodes per year each lasting 2 (1–3) weeks.[5–7]

Genital herpes

Study-level covariate	Parameter	Location level	beta	Exponentiated beta
Study Population Blood Donors	Prevalence	Region	-0.38 (-0.62 to -0.16)	0.68 (0.54 – 0.86)
Study Population Pregnant	Prevalence	Region	-0.11 (-0.25 to -0.034)	0.90 (0.78 – 1.03)

Country-level covariate	Parameter	beta	Exponentiated beta
HIV age-standardized prevalence	Prevalence	0.057 (0.0038 – 0.098)	1.06 (1.00 – 1.10)

Early syphilis

For early syphilis estimation methods, data were assumed to be prevalence measures. Age range was restricted to be from 10 to 64 years. Excess mortality was assumed to be 0, and incidence rates were assumed to be less than 0.3. Remission bounds were between 0.5 and 2. Blood donor samples consistently had prevalence values that were lower than that of the general population, and were crosswalked accordingly. Data from studies using only treponemal tests, or only non-treponemal tests, were additionally crosswalked to the reference definition of both treponemal and non-treponemal testing. Crosswalks were performed in a country-specific manner to reflect the variability in false positive rate in non-treponemal tests as a function of age, sex, and endemicity of other non-STI infectious conditions. The proportion of antenatal care coverage was applied as a country covariate on prevalence.

Our review of literature on proportion of early syphilis cases with symptoms led to assignment of a 0.686 (0.627–0.745) of prevalent cases to have a combined duration of primary and secondary symptoms lasting 23 (13–44) days.[8–11] The remainder were considered asymptomatic.

Early syphilis infection

Study-level covariate	Parameter	Location level	beta	Exponentiated beta
Study Population Blood Donors	prevalence	Country	-0.2 (-0.2 to -0.19)	0.82 (0.82 – 0.82)
Non-trep only	prevalence	Country	0.0021 (0.00008 – 0.0094)	1.00 (1.00 – 1.01)

Country-level covariate	Parameter	beta	Exponentiated beta

Antenatal Care (4 visits) Coverage (proportion)	prevalence	-0.2 (-0.2 to -0.2)	0.82 (0.82 to 0.82)

Adult tertiary syphilis

Prevalence data were the primary input. Incidence was assumed to not occur until age 15. Excess mortality rate was capped at 0.1, which equates to minimum duration of five years, and no remission was assumed. Cause-specific mortality rate (CSMR) results from GBD 2016 mortality and causes of death analysis were included in the model, which was also used to back-calculate excess mortality rate (EMR) data to match each input prevalence datum.[12] CSMR priors were not passed on through the cascade so as to restrict the utility of these data to back-calculating prevalence at the country level. Study-level covariates were used to crosswalk surveillance data to the reference source of hospital data. Natural log of lag-distributed income (LN-LDI) was used as a country-level covariate on EMR. Non transformed syphilis prevalence was used as used as a country-level covariate on prevalence. An increasing slope prior was applied to incidence from ages 30 to 100.

Two notable differences exist between GBD 2016 and previous estimates of adult tertiary syphilis. First, in GBD 2015 all cases of adult tertiary syphilis were assumed to be symptomatic and were assigned the same disability that describes a health state of moderate motor and cognitive dysfunction. For GBD 2016, there were 8 new sequelae, including asymptomatic adult tertiary syphilis. Second, the reference source was hospital data, which was adjusted for the likelihood of tertiary syphilis to appear as an inpatient or outpatient case.

Adult tertiary syphilis

Study-level covariate	Parameter	Location level	beta	Exponentiated beta
Surveillance/notification data	prevalence	Global	-1.1 (-1.23 to -0.98)	0.33 (0.29 – 0.38)

Country-level covariate	Parameter	beta	Exponentiated beta
LDI (I$ per capita)	excess mortality rate	-0.24 (-0.29 to -0.2)	0.79 (0.75 – 0.82)
Syphilis prevalence (proportion)	Prevalence	0.098 (0.0021 – 0.41)	1.10 (1.00 – 1.51)

Other sexually transmitted infections

To calculate YLDs due to acute infection with other STI, we calculated the YLD to YLL ratio for all STI (excluding other STI) and then applied that same ratio to other STI YLLs. YLDs were also estimated to other STI as a result of the proportion of PID and PID-induced infertility that was not due to gonorrhea or chlamydia.

Uncertainty and model selection

For all STI estimates, uncertainty bounds include uncertainty due to input data, including CSMR from GBD 2016 mortality and causes of death analysis, crosswalks from non-reference definitions, uncertainty in numerical solutions (posteriors) of each DisMod-MR 2.1 model, and proportion of all infections with each type of symptom.

In consultation with GBD researchers and collaborators, final models were selected on a combination of qualitative and quantitative goodness of fit to input data, plausibility of geographic and temporal trends, consistency of age pattern, and, when available, comparison with other published studies on STI epidemiology. Directionality, magnitude, and plausibility of study-level and country-level covariates was also considered in the process of model development. Of note, due to the nature of statistical modelling, final results do not always cover the values reported in input data.

No other changes were made to the modelling strategy for GBD 2016.

Acute hepatitis A

Flowchart

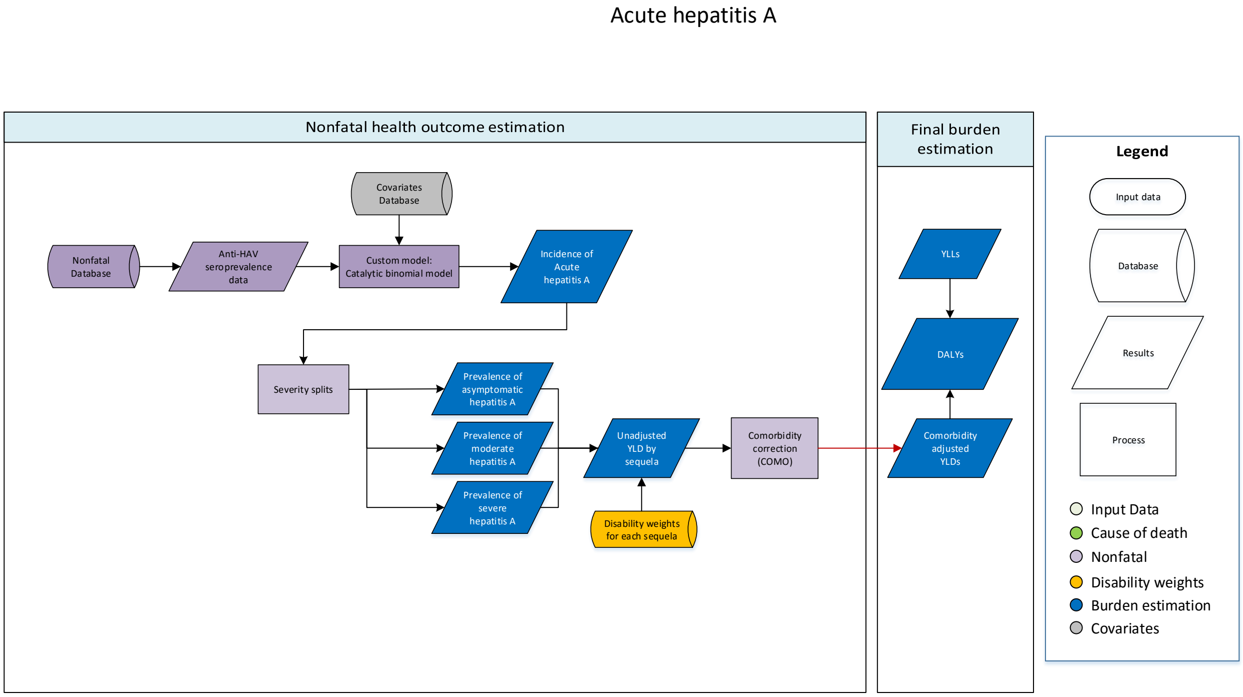

Case definition

We define acute hepatitis A as an infection with the hepatitis A virus resulting in anti-HAV IgG seroconversion, regardless of symptoms. It includes all ICD-10 codes under the heading B15 (Acute hepatitis A).

Input data

Model inputs

We use anti-HAV seroprevalence data from population-based studies and surveys for the incidence model.

Level	Prevalence
Data points	3668
Studies	469
Locations	175
Regions	21

Updates to systematic reviews are performed on an ongoing schedule across all GBD causes, an update for hepatitis A will be performed in the next 1-2 iterations.

Severity splits & disability weights

Based on information published by Armstrong et al, (Armstrong GL, Bell BP. Hepatitis A Virus Infections in the United States: Model-Based Estimates and Implications for Childhood Immunization. Pediatrics. 2002 May 1;109(5):839–45.) we assume that the probability of symptomatic infection increases with age from ~1% in the first year of life to ~85% in adulthood.

The table below illustrates the sequelae associated with Acute Hep A, as well as the lay descriptions and associated disability weights.

Sequela	Description	Disability Weight
Moderate	Has a fever and aches, and feels weak, which causes some difficulty with daily activities.	0.051 (0.032-0.074)
Severe	Has a high fever and pain, and feels very weak, which causes great difficulty with daily activities.	0.133 (0.088-0.19)
Asymptomatic	Infection with no apparent illness of	NA

Modelling strategy

Given its reasonably stable force of infection among susceptible people across age groups, we used a catalytic binomial model to estimate incidence of acute hepatitis A based on anti-HAV seroprevalence. The catalytic binomial model is a binomial generalized linear model with a complementary log-log link, and an offset term for log-age. Since anti-HAV is a lifetime marker of past infection, and a given individual can only be infected once, seroprevalence at age *t* is equal to the cumulative incidence (CI) over *t* years. Assuming constant force of infection, we can estimate the incidence rate (IR) as,

$$CI = 1 - e^{-IR \cdot t}$$

We can rearrange this equation to solve for the log-IR:

$$\ln(IR) = \frac{\ln(-\ln(1 - CI))}{\ln(t)}$$

Thus, by using the complimentary log-log link for CI (i.e. ln(-ln(1-CI)) with an offset for log-age, we are able to model the incidence rate of infection from seroprevalence data. To inform the model in the absence of data we use a predictive covariate derived from principal components analysis of lag-distributed income (LDI) and the proportion of the population with access to improved water. We use a mixed effects model with fixed effects on the aforementioned PCA-derived covariate, and nested hierarchical random effects on super-region, region, country, and sub-national locations.

Changes from GBD 2015 to GBD 2016

Our overall approach has not changed from that used in GBD 2016

Acute hepatitis B

Flowchart

Acute hepatitis B

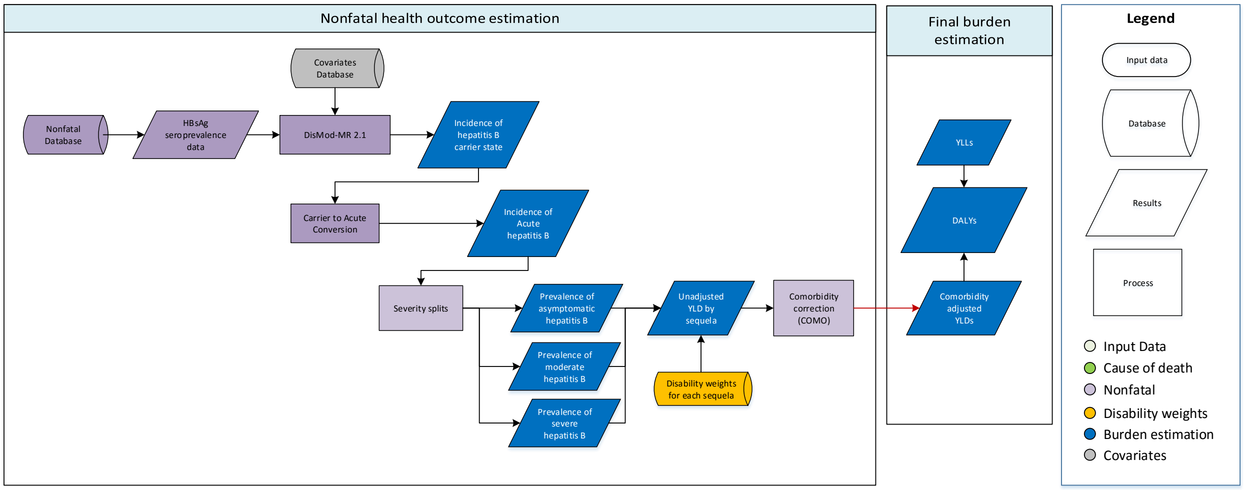

Case definition

We define acute hepatitis B as the period corresponding to initial infection with the hepatitis B virus, regardless of symptoms. It includes all ICD-10 codes under the heading B16 (Acute hepatitis B).

Input data

Model inputs

We use hepatitis B surface antigen (HBsAg) seroprevalence data from population-based studies and surveys for the incidence model.

Level	Prevalence
Data points	2987
Studies	312
Locations	145
Regions	19

Updates to systematic reviews are performed on an ongoing schedule across all GBD causes, an update for hepatitis A will be performed in the next 1-2 iterations.

Modelling strategy

We model the incidence of chronic HBsAg carriage using a full DisMod model of HBsAg seroprevalence. We then convert incidence of chronic carriage to total incidence of hepatitis B infection by dividing age-specific estimates of the incidence of chronic carriage by age-specific estimates of the probability of infection resulting in carriage based on Edmunds et al (Edmunds WJ, Medley GF, Nokes DJ, Hall AJ, Whittle HC. The influence of age on the development of the hepatitis B carrier state. Proc Biol Sci. 1993 Aug 23;253(1337):197–201):

$$P(carrier \mid age \leq 6\ months) = 0.885$$

$$P(carrier \mid 6\ months \leq age < 25\ years) = e^{-0.645 \times age^{0.455}}$$

$$P(carrier \mid age \geq 25\ years) = e^{-0.645 \times 25^{0.455}} = 0.061$$

We then split symptomatic cases into moderate (73%) and severe (27%) severities based on data from McMahon et al (McMahon BJ, Alward WL, Hall DB, Heyward WL, Bender TR, Francis DP, et al. Acute hepatitis B virus infection: relation of age to the clinical expression of disease and subsequent development of the carrier state. J Infect Dis. 1985 Apr;151(4):599–603).

Sequela	Description	Disability Weight
Moderate	Has a fever and aches, and feels weak, which causes some difficulty with daily activities.	0.051 (0.032-0.074)
Severe	Has a high fever and pain, and feels very weak, which causes great difficulty with daily activities.	0.133 (0.088-0.19)
Asymptomatic	Infection with no apparent illness of	NA

Changes from GBD 2015 to GBD 2016

We have updated the severity splits, but the modeling strategy remains otherwise unchanged from GBD 2013.

Acute hepatitis C

Flowchart

Acute hepatitis C

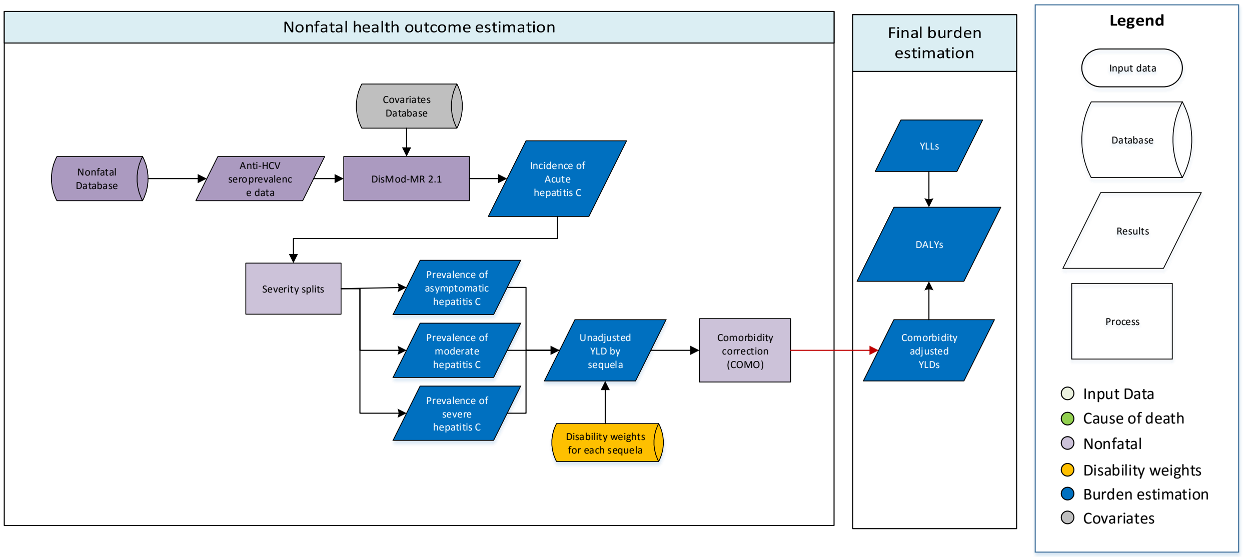

Case definition

We define acute hepatitis C as the period corresponding to initial infection with the hepatitis C virus, resulting in anti-HCV IgG seroconversion, regardless of symptoms. It includes all ICD-10 codes under the heading B17.1 (Acute hepatitis C).

Input data

Model inputs

To estimate morbidity for HepC, we use anti-HCV seroprevalence data from population-based studies and surveys to estimate incidence and prevalence of hepatitis C infection:

Level	Prevalence
Data points	5242
Studies	239
Locations	521
Regions	21

Model

We model the incidence and prevalence of hepatitis C infection using a full DisMod model of anti-HCV seroprevalence data. We divide incident infections into asymptomatic (75%), moderate (24%), and severe (1%) states. Based on a meta-analysis, we estimate that 75% of anti-HCV positive people are chronically infected.

Sequela	Description	Disability Weight
Moderate	Has a fever and aches, and feels weak, which causes some difficulty with daily activities.	0.051 (0.032-0.074)
Severe	Has a high fever and pain, and feels very weak, which causes great difficulty with daily activities.	0.133 (0.088-0.19)
Asymptomatic	Infection with no apparent illness of	NA

Changes from GBD 2015 to GBD 2016

We have made no substantive changes in the modeling strategy from GBD 2015.

Acute hepatitis E

Flowchart

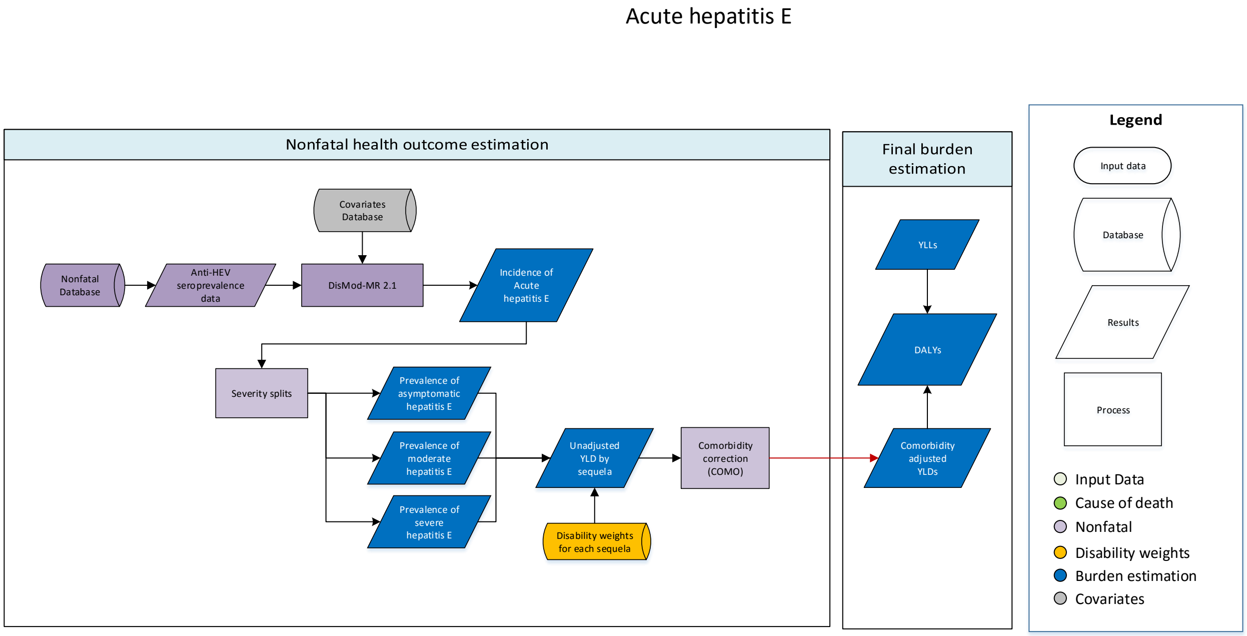

Case definition

For GBD 2016, we define acute hepatitis E as an infection with the hepatitis E virus resulting in anti-HEV IgG seroconversion, regardless of symptoms. It includes all ICD-10 codes under the heading B17.2 (Acute hepatitis E).

Input data

Model inputs

For GBD 2016 estimation, we used anti-HEV seroprevalence data from population-based studies and surveys to estimate incidence of infection. The table below indicates the number of data points included in terms of prevalence by location hierarchy:

Level	Prevalence
Data points	433
Studies	85
Locations	56
Regions	19

Modeling Strategy

The GBD 2016 estimation process for HepE uses a DisMod MR 2.1 model, which is a Bayesian meta-regression. We model the incidence of Hepatitis E using a full DisMod MR 2.1 model of anti-HEV seroprevalence, assuming no remission. Based on information published by Rein et al[1] we assume that the probability of symptomatic infection increases with age from ~1% in the first year of life to ~60% in adulthood.

The table below illustrates the sequelae associated with Acute HepE, along with their descriptions and disability weights.

Sequela	Description	Disability Weight
Moderate	Has a fever and aches, and feels weak, which causes some difficulty with daily activities.	0.051 (0.032-0.074)
Severe	Has a high fever and pain, and feels very weak, which causes great difficulty with daily activities.	0.133 (0.088-0.19)
Asymptomatic	Infection with no apparent illness of	NA

Changes from GBD 2015 to GBD 2016

We have made no substantive changes in the modeling strategy from GBD 2015.

REFERENCES

1. Rein DB, Stevens GA, Theaker J, Wittenborn JS, Wiersma ST. The global burden of hepatitis E virus genotypes 1 and 2 in 2005. Hepatology. 2012 Apr 1;55(4):988–97.

Neoplasms (Cancer)

All cancers except for non-melanoma skin cancer (basal cell carcinoma and squamous cell carcinoma)

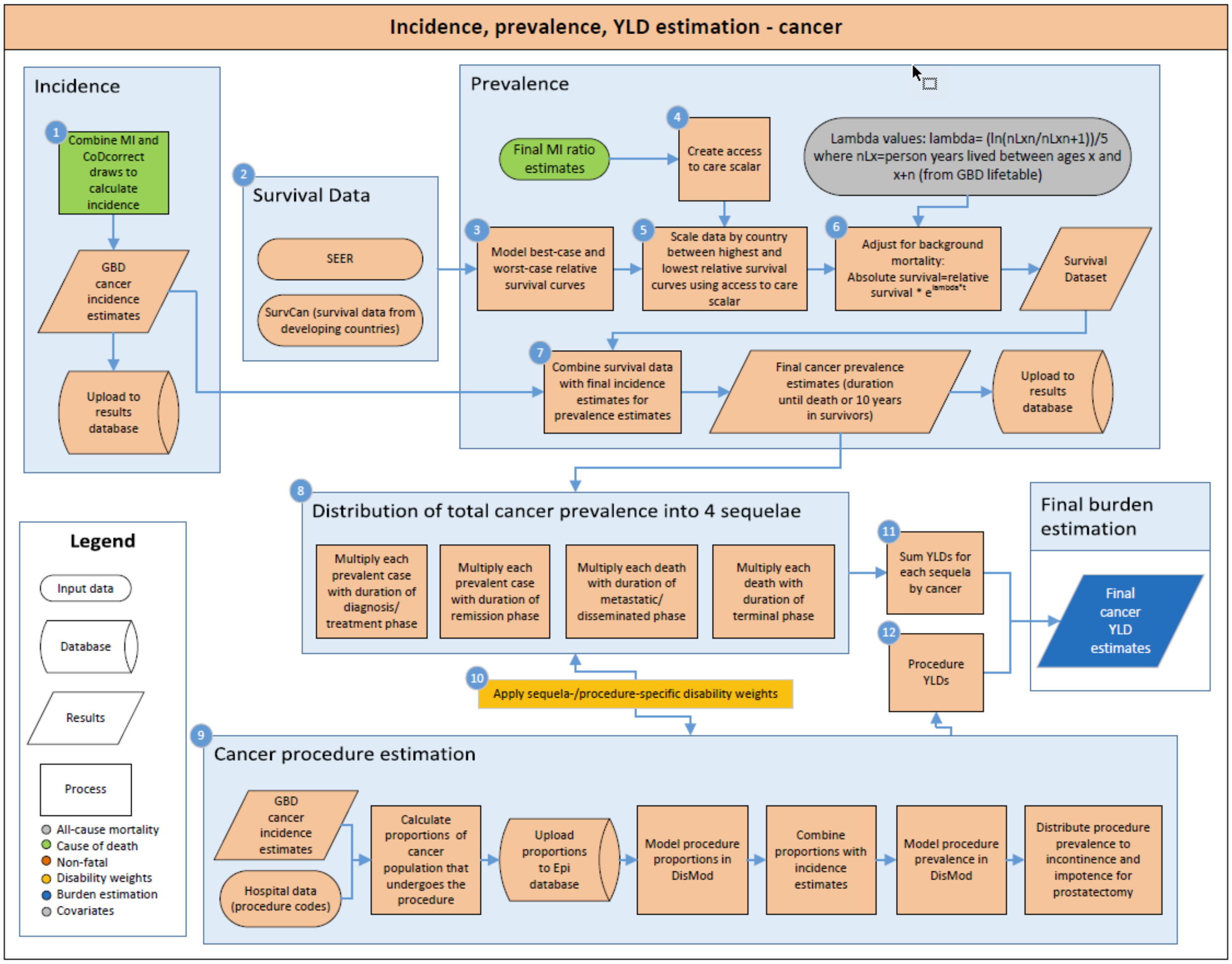

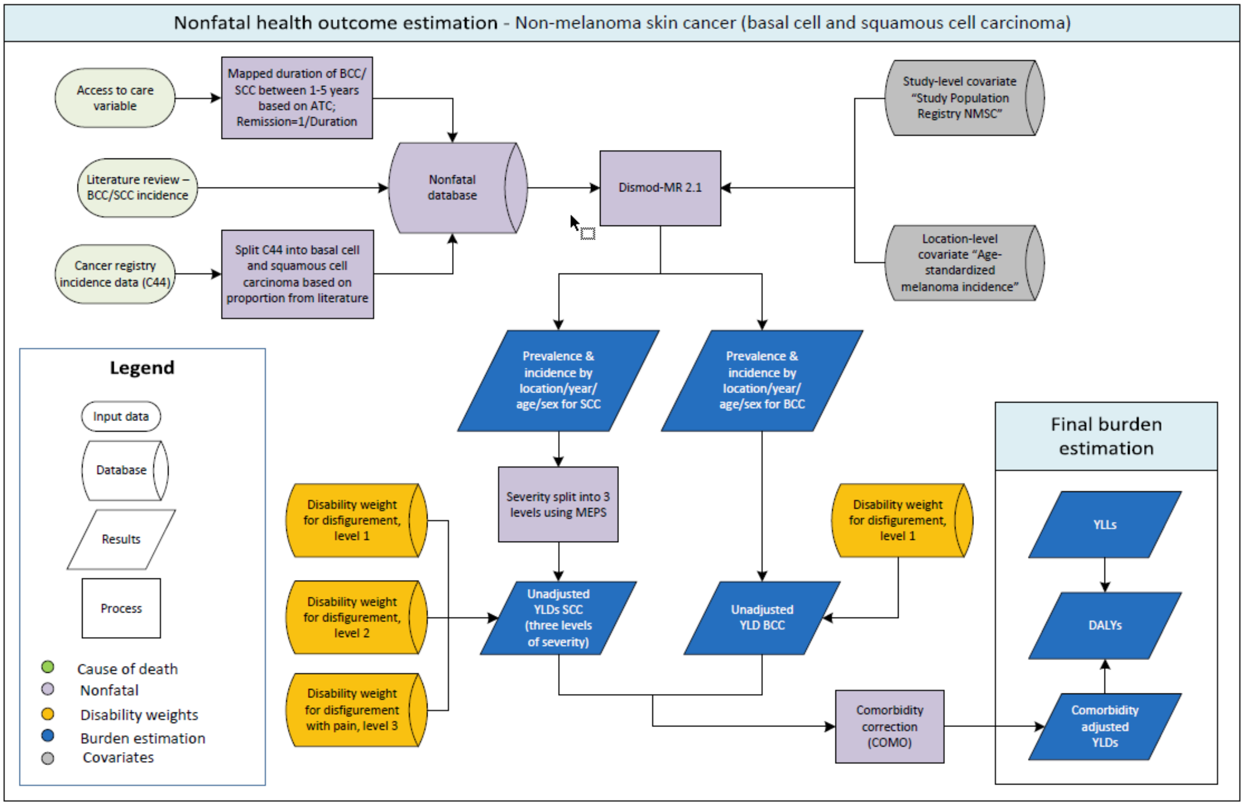

Input data and methodological appendix

Case definition

For GBD 2016, incidence, prevalence, and disability are estimated for all cancers as defined in ICD-10 (C00-C96). Prevalence for all cancers is estimated for a maximum of 10 years after incidence as in GBD 2013 and GBD 2015. Prevalence extending beyond the 10 year period is only estimated for permanent sequelae from procedures.

To estimate disability for each cancer, total prevalence is split into four sequelae: 1. diagnosis and primary therapy; 2. controlled phase; 3. metastatic phase; and 4. terminal phase. Diagnosis and primary therapy are defined as the time from symptoms onset to end of treatment. Controlled phase is defined as the time after finishing primary treatment and either cure (defined as survival after 10 years) or metastatic phase. Metastatic phase is defined as the time period of intensive treatment for metastatic disease, terminal phase is defined as the one month period prior to death. Each of these sequelae has a separate disability weight (**Error! Reference source not found.**). Additional disability is estimated for breast cancer (disability due to mastectomy), larynx cancer (disability due to laryngectomy), colon and rectum cancer (disability due to stoma), bladder cancer (disability due to incontinence), and prostatectomy (disability due to incontinence and impotence). The associated ICD codes for neoplasms estimated for GBD 2016 are listed below, as well as in Appendix Table 4.

Table 1. GBD cancer causes with respective ICD codes

GBD cause	ICD9	ICD10
Bladder cancer	188.0-188.9	C67-C67.9
Brain and nervous system cancer	191.0-192.9	C70-C72.9
Breast cancer	174.0-175.9	C50-C50.929
Cervical cancer	180.0-180.9	C53-C53.9, D26.0
Colon and rectum cancer	153.0-154.9, 209.1-209.17	C18-C21.9
Esophageal cancer	150.0-150.9	C15-C15.9
Gallbladder and biliary tract cancer	156.0-156.9, 209.25-209.27	C23-C24.9
Hodgkin disease	201.0-201.98	C81-C81.99
Kidney cancer	189.0-189.1, 209.24	C64-C65.9
Larynx cancer	161.0-161.9	C32-C32.9
Leukemia	208.0-208.92	C94.1, C94.7-C95.92
Acute lymphoid leukemia ALL	204.0-204.02	C91.0-C91.02
Acute myeloid leukemia AML	205.0-205.02, 205.3-205.32, 206.0-206.02, 207.0	C92.0-C92.02, C92.3-C92.62, C93.0-C93.02, C94.0-C94.02, C94.2-C94.22, C94.4-C94.5
Chronic lymphoid leukemia CLL	204.1-204.12	C91.1-C91.12
Chronic myeloid leukemia CML	205.1-205.12, 206.1-206.12, 207.1	C92.1-C92.12
Other leukemia	204, 204.2, 204.5, 204.8, 204.9, 205, 205.2, 205.8, 205.9, 206, 206.2, 206.8, 206.9, 207, 207.0-207.2, 207.8, 207.9, 208, 208.0-208.2, 208.4, 208.7, 208.8, 208.9	C91, C91.2, C91.3-C91.9, C92, C92.2, C92.7-C92.9, C93, C93.1-C93.3, C93.5, C93.7-C93.9, C94, C94.1, C94.3, C94.6-C94.8, C95, C95.1-C95.2, C95.4, C95.6, C95.7, C95.9
Liver cancer	155.0-155.9	C22-C22.9
Lung, bronchus, and trachea cancer	162.0-162.9, 209.21	C33-C34.92
Non-Hodgkin lymphoma	200.0-200.9, 202.0-202.98	C82-C86.6, C96-C96.9
Malignant skin melanoma	172.0-172.9	C43-C43.9
Mesothelioma	158.9, 163.0-163.9	C45-C45.9
Oral and Lip Cancer	140.0-145.9	C0-C08.9
Multiple myeloma and immunoproliferative diseases	203.0-203.9	C88-C90.9
Nasopharynx cancer	147.0-147.9	C11-C11.9
Non-melanoma skin cancer (Basal cell carcinoma)	173.0-173.01, 173.09-173.11, 173.19-173.21, 173.29-173.31,	C44.0-C44.01, C44.09-C44.119, C44.19-C44.219, C44.29-C44.319,

	173.39-173.41, 173.49-173.51, 173.59-173.61, 173.69-173.71, 173.79-173.81, 173.89-173.91, 173.99, 216.0-216.9, 232.0-232.9, 238.2	C44.39-C44.41, C44.49-C44.519, C44.59-C44.619, C44.69-C44.719, C44.79-C44.80, C44.82-C44.91, C44.99
Non-melanoma skin cancer (Squamous-cell carcinoma)	173.02, 173.12, 173.22, 173.32, 173.42, 173.52, 173.62, 173.72, 173.82, 173.92	C44.02, C44.12-C44.129, C44.22-C44.229, C44.32-C44.329, C44.42, C44.52-C44.529, C44.62-C44.629, C44.72-C44.729, C44.81, C44.92, D04-D04.9, D49.2
Other neoplasms	158.0-158.8, 209.4-209.57, 209.61, 209.63-209.67, 210.0-211.8, 212.0-212.8, 213.0-215.9, 217.0-221.8, 222.0-222.8, 223.0-223.89, 224.0-229.0, 229.8, 230.1-230.8, 231.0-231.2, 233.0-233.2, 233.31-233.32, 233.4-233.5, 233.7, 234.0-234.8, 235.0, 235.4, 235.6-235.8, 236.1-236.2, 236.4-236.5, 236.7, 236.91-237.3, 237.5-238.1, 238.3-238.5, 239.2-239.4, 239.6	D00.00-D00.2, D01.0-D01.3, D02.0-D02.3, D03-D03.9, D05-D06.9, D07.0-D07.2, D07.4-D07.5, D09.0, D09.2-D09.3, D09.8, D10.0-D10.7, D11-D12.9, D13.0-D13.7, D14.0-D14.32, D15-D24.9, D27-D27.9, D28.0-D28.7, D29.0-D29.8, D30.0-D30.8, D31-D36.7, D37.01-D37.5, D38.0-D38.5, D39.1-D39.2, D39.8, D40.0-D40.8, D41.0-D41.8, D42-D43.9, D44.0-D44.8, D45-D45.9, D47-D47.0, D47.2-D47.9, D48.0-D48.7, D49.3-D49.4, D49.6
Other cancers	152.0-152.9, 160.0-160.9, 164.0-164.9, 170.0-171.9, 181.0-181.9, 182.9, 183.2-183.8, 184.0-184.4, 184.8, 187.1-187.8, 189.2-189.8, 190.0-190.9, 194.0-194.8, 209.0-209.03,	C17-C17.9, C3-C31.9, C37-C38.8, C4-C41.9, C47-C5, C51-C52.9, C57-C57.8, C58-C58.0, C60-C60.9, C63-C63.8, C66-C66.9, C68.0-C68.8, C69-C7, C74-C75.8, D49.81

	209.22, 209.31-209.36	
Other pharynx cancer	146.0-146.9, 148.0-148.9	C09-C10.9, C12-C13.9
Ovarian cancer	183.0	C56-C56.9
Pancreatic cancer	157.0-157.9	C25-C25.9
Prostate cancer	185.0-185.9	C61-C61.9
Stomach cancer	151.0-151.9, 209.23	C16-C16.9
Testicular cancer	186.0-186.9	C62-C62.92
Thyroid cancer	193.0-193.9	C73-C73.9
Uterine cancer	182.0-182.8	C54-C54.9
Garbage code	149.0-149.9, 159.0-159.9, 165.0-165.9, 169.0, 173.0, 176.0-179.9, 183.9-184.0, 184.5, 184.9, 187.0, 187.9, 189.0, 189.9, 194.9-199.9, 209.0, 209.2, 209.29-209.3, 209.6, 209.62, 209.69-210.0, 211.0, 211.9-212.0, 212.9, 221.0, 221.9-222.0, 222.9-223.0, 223.9, 229.0-229.1, 229.9-230.0, 230.9-231.0, 231.8-231.9, 233.0, 233.3, 233.39, 233.6, 233.9-234.0, 234.9-235.3, 235.5, 235.9-236.0, 236.3, 236.6, 236.9, 237.4, 238.0, 238.6-239.1, 239.5, 239.7-239.9	C14-C14.9, C26-C29, C35-C36, C39-C39.9, C42, C44, C46-C46.9, C55-C55.9, C57.9, C59-C6, C63.9, C68, C68.9, C75.9-C80.9, C87, C97-D00.0, D01, D01.4-D02, D02.4-D02.9, D07, D07.3-D07.39, D07.6-D09, D09.1-D09.19, D09.7, D09.9-D10, D10.9, D13, D13.9-D14, D14.4, D28, D28.9-D29, D29.9-D30, D30.9, D36.9-D37.0, D37.6-D38, D38.6-D39.0, D39.7, D39.9-D40, D40.9-D41, D41.9, D44, D44.9, D46-D46.9, D47.1, D48, D48.9-D49.1, D49.5, D49.7-D49.8, D49.89-D49.9

Input data

Cancer incidence is directly estimated from cancer mortality using mortality to incidence ratios (MIR). Data sources for cancer mortality are described in detail elsewhere.[1] Data sources to scale countries between a hypothetical best- and a hypothetical worst case survival remained the same for the worst case survival as in GBD 2015 where we used a combination of the 1950 US Mortality Files with "Cancer Survival in Africa, Asia, the Caribbean and Central America" (SurvCan) data.[2,3] For mesothelioma, gallbladder cancer, and the leukemia subtypes SEER 1973 survival data for the lower boundary was used since these cancers are not included in the US Mortality Files from 1950. We updated the hypothetical

best case survival using 5-year survival data from the SEER Cancer Statistics Review (CSR), 1975-2013.[4] To estimate the proportion of cancer patients undergoing procedures we used SEER data form 1983 to 2008[5] and Mexico Hospital Data from 2001 to 2009[7]. Data sources used to adjust procedure sequelae will be listed below.

Modeling strategy

Estimation of cancer mortality and MIR estimation has been described in the GBD 2016 Mortality and Causes of Death capstone paper. The final GBD cancer mortality estimates are being transformed to incidence estimate by using separately estimated MIR. To summarize the MIR estimation process, incidence and mortality data from cancer registries were matched by cancer, age, sex, year, and location to generate MI ratios. Compared to GBD 2015 we used more narrow inclusion criteria to select the input for the MIR model. Only sources were included that reported both, incidence and mortality. We dropped MIR if cases were less than 25. Compared to the outliering process for GBD 2015, which relied on the binary classification of “developed” versus “developing” countries, we used SDI quintiles for GBD 2016 for dropping unrealistic data in the input dataset. MI ratios from SDI quintiles 1-4 were dropped if they were below the median MI ratio of quintile 5. This was done under the assumption that these MI ratios reflect incomplete death ascertainment. To not artificially increase the predicted MI ratio, MI ratios from SDI quintiles 1-4 were dropped if they were above the third interquartile range (IQR) x 1.5 IQR. MI ratios above 2 were dropped. To avoid MIR predictions for all age groups and all years above 1, the MIR input data was divided by upper caps. Upper caps were generated using the 95th percentile of MIR in the cleaned input dataset for all cancers combined. To avoid too low MIR predictions especially in young age groups with little data, the MIR data input was divided by lower caps, which were the 5th percentile of the cleaned input dataset by cancer.

A fixed effect logistic regression was used to fit the model input using the Socio-demographic Index (SDI) as a covariate with age and sex as categorical variables. The model was run separately by cancer and results were rescaled using the lower and upper caps.

$$\text{logit}\left(MI\ ratio_{c,a,s,t}\right) = \alpha + \beta_1 SDI_{c,t} + \sum_{a}^{A} \beta_2 I_a + \beta_3 I_s + \epsilon_{c,a,s,t}$$

c: country, a: age group, t: time (years); s: sex
SDI: Socio-demographic index (index using log lag dependent income per capita (LDI), average educational attainment in the population over age 15, and total fertility rate (TFR))
I: indicator variable
$\epsilon_{c,a,s,t}$: error term

Data points were outliered manually if they clearly influenced the model in an unrealistic way. For example, a data point was marked as an outlier if it created a single-year, single age group spike in model predictions. Results from the final linear model were used as input for space-time smoothing and a Gaussian Process Regression (ST-GPR) (Appendix Section 2.9). Compared to GBD 2013 and 2015 the MI ratio estimation continues to be revised since the modeling strategy used for GBD 2015 yielded MI ratios for low- and low-middle SDI that were very close to MI ratios from high-SDI countries for certain cancers.

Final MI ratio estimates at the 1000 draw level were combined with final mortality estimates (as well at the 1000 draw level) to generate incidence estimates. It was assumed that uncertainty in the MI ratio is independent of uncertainty in the estimated mortality.

After transforming the final GBD cancer mortality estimates to incidence estimates (step 1 in the flowchart), incidence was combined with the relative yearly survival estimates up to 10 years (step 8 in the flowchart). To estimate cancer prevalence, relative cancer survival was estimated by scaling cancer specific survival between the "best case" and "worst case" survival, using the survival data sources listed above (step 2, 3, and 5 in the flowchart). To transform relative to absolute survival (adjusting for background mortality), GBD 2016 lifetables were used (step 6 and 7 in the flowchart) to calculate lambda values: lambda= (ln(nLxn/nLxn+1))/5 where nLx=person years lived between ages x and x+n (from GBD lifetable). Absolute survival was then calculated using an exponential survival function (absolute survival = relative survival * $e^{lambda*t}$).

The access to cancer care variable to scale countries between the best and worst case survival was estimated using the same method as for GBD 2013 and GBD 2015 (step 4 in the flowchart)[8]:

$$Access\ to\ care = 1 - \frac{Age\ standardized\ MIR_{cys} - Age\ standardized\ MIR_{min}}{Age\ standardized\ MIR_{max} - Age\ standardized\ MIR_{min}}$$

c=country; y=year; s=sex; Age-standardized MI $ratio_{min}$=lowest MIR for all countries and years; Age standardized MIR_{max}=highest MIR for all countries and years

Survivors beyond 10 years were considered cured. The survivor population prevalence was divided into two sequelae (1. diagnosis and primary therapy; 2. controlled phase). The yearly prevalence of the population that did not survive beyond 10 years was then divided into the four sequelae by assigning the fixed durations for the diagnosis and primary therapy phase, metastatic phase, and terminal phase and assigning the remaining prevalence to the controlled phase (step 9 in the flowchart). Duration of the treatment sequelae (1. diagnosis and primary therapy; 2. controlled phases; 3. metastatic phase; 4. terminal phase) remained the same as for GBD 2013 and GBD 2015.[8] Table 3 lists the duration including sources used.

Table 3. Duration of four prevalence sequelae by cancer

	Diagnosis/ Treatment (months)	Remission	Disseminated/metastatic (months)	Note	Terminal (months)
Esophageal cancer	5[9]	Calculated based on remainder of time after attributing other sequelae.	4.6[10]	SEER Summary Stage 1997 (Distant site/node involved) 1995-2000	1 months
Stomach cancer	5.2[9]		3.88[10]	SEER Summary Stage 1997 (Distant site/node involved) 1995-2000	
Liver cancer	4		2.51[10]	SEER Summary Stage 1997 (Distant site/node involved) 1995-2000	
Larynx cancer	5.3[9]		8.84[10]	SEER Stage IVc	

Lung cancer	3.3[11]	4.51[10]	SEER Summary Stage 1997 (Distant site/node involved) 1995-2000
Breast cancer	3[11]	17.7[10]	SEER Summary Stage 1997 (Distant site/node involved) 1995-2000
Cervical cancer	4.8[9]	9.21[10]	SEER Summary Stage 1997 (Distant site/node involved) 1995-2000
Uterine cancer	4.6[9]	11.6[10]	SEER Summary Stage 1997 (Distant site/node involved) 1995-2000
Prostate cancer	4[11]	30.35[10]	SEER Summary Stage 1997 (Distant site/node involved) 1995-2000
Colorectal cancer	4[11]	9.69[10]	SEER Summary Stage 1997 (Distant site/node involved) 1995-2000
Oral cancer	5.3[9]	9.33[10]	SEER Stage IVc
Nasopharyngeal cancer	5.3[9]	13.19[10]	SEER Stage IVc
Cancer of other part of pharynx	5.3[9]	7.91[10]	SEER Stage IVc
Gallbladder cancer	4	3.47[10]	SEER Summary Stage 1997 (Distant site/node involved) 1995-2000
Pancreas cancer	4.1[9]	2.54[10]	SEER Summary Stage 1997 (Distant site/node involved) 1995-2000
Melanoma	2.9[12]	7.18[10]	SEER Summary Stage 1997 (Distant site/node involved) 1995-2000
Ovarian cancer	3.2[11]	25.6[10]	SEER Summary Stage 1997 (Distant site/node involved) 1995-2000
Testicular cancer	3.7[9]	19.47[10]	SEER Stage III
Kidney cancer	5.3[9]	5.38[10]	SEER Summary Stage 1997 (Distant site/node involved) 1995-2000
Bladder cancer	5.1[9]	5.8[10]	SEER Summary Stage 1997 (Distant site/node involved) 1995-2000
Brain cancer	5	6.93[10]	SEER Median age standardized survival all patients, all years

Thyroid cancer	3		19.39[10]	SEER Stage IVc	
Mesothelioma	4		7.75[10]	SEER Summary Stage 1997 (Distant site/node involved) 1995-2000	
Hodgkin lymphoma	3.7[11]		26[14]		
Non Hodgkin lymphoma	3.7[11]		7.7[14]		
Multiple myeloma	7[9]		36.82[10]	SEER Median age standardized survival all patients, all years	
Leukemia[9]	5		43.67[10]	SEER Median age standardized survival all patients, all years	
ALL	12		7.02[10]	SEER Median age standardized survival all patients, all years	
AML	6		4.6[10]	SEER Median age standardized survival all patients, all years	
CLL	6		48[15]	SEER Median age standardized survival all patients, all years	
CML	6		4.6[10]	SEER Median age standardized survival for AML (patients with CML die in blast crisis, which is treated like AML) all patients, all years	
Leukemia other	6		48[15]	SEER Median age standardized survival all patients, all years	
Other	4.4 (mean of other cancer durations)		15.81[10]	SEER Median age standardized survival all patients, all years	

For cancer specific procedure sequelae hospital data were used to estimate the number of cancer patients undergoing mastectomy, laryngectomy, stoma, prostatectomy, and cystectomy (step 10 in the flowchart). These proportions remained the same as in GBD 2013 and GBD 2015.[8] Proportions were generated by dividing the rate of procedures generated from the diagnostic codes in the hospital dataset and the coverage population by the GBD age-, and sex-specific disease incidence rates for that country. Diagnostic codes used are listed in table 4:

Table 4. Procedure codes used to estimate cancer procedure proportions		
Procedure	Cancer	Procedure code (ICD-9_CM)
Mastectomy	Breast cancer	854, 8541, 8542, 8543, 8544, 8545,<br>8546, 8547, 8548
Laryngectomy	Larynx cancer	301, 303, 304, 3029
Stoma	Colon and rectum cancer	461, 4610, 4611, 4613, 4862
Cystectomy	Bladder cancer	5771, 5779
Prostatectomy	Prostate	603, 604, 605, 606, 6062

To estimate procedure-related disability for certain cancers, the procedure proportions (proportion of cancer population that undergoes procedures) from hospital data was used as input for a proportion model in Dismod-MR 2.1 in order to estimate the proportions for all locations, by age, and by sex.

Since colostomy or ileostomy procedures are done for reasons other than cancer a literature review was done to determine the proportion of ostomies due to colorectal cancer. The “all cause” colostomy proportions were multiplied by 0.58 based on the results of the literature review showing that on average 58% of ostomies are done for colorectal cancer.[16–18]

The final procedure proportions were applied to the incidence cases of the respective cancers and multiplied with the proportion of the incidence population surviving for 10 years to determine the incident cases of the cancer population that underwent procedures. These incident cases were used again as an input for DisMod-MR 2.1 with a remission specification of zero and an excess mortality rate prior of 0 to 0.1. This approach was different compared to GBD 2015 where we used the cause-specific mortality of the specific cancer to obtain prevalence of the sequela. The approach was changed since the mortality for the population undergoing disease specific procedures (e.g. mastectomy for breast cancer) is likely closer to the general population after they have survived for a period of time compared to the cause-specific mortality of the underlying disease (e.g. breast cancer).

Since disability associated with prostatectomy comes from impotence and incontinence and not from the prostatectomy itself 18% of the prostatectomy prevalence was assumed to be incontinent and 55% was assumed to be impotent based on a literature review done for GBD 2013.[19–26]

Since all sequelae for a cause need to be mutually exclusive, the controlled phase for the cancers with additional procedure related disability was adjusted to only include the population without procedure related disability (= controlled phases prevalence of the total population – controlled phase prevalence of the proportion that experienced procedure related disability) (step 11 in the flowchart). The disability weight for the prevalence of the population that experiences additional disability was adjusted to reflect the combined disability of the controlled phase as well as the procedure.

Lastly, the procedure sequelae prevalence and general sequelae prevalence were multiplied with disability weights (Table 5) for the procedures to obtain the number of YLDs (steps 11, 12, 13 in the flowchart). The sum of these YLDs is the final YLD estimate associated with each cancer.

Table 5. Lay description and disability weights				
Health state	Lay description	Estimate	Uncertainty interval	
Cancer, diagnosis and primary therapy (cancer_diagnosis)	This person has pain, nausea, fatigue, weight loss and high anxiety.	0.288	0.193	0.399
Cancer, controlled phase (generic_medication)	This person has a chronic disease that requires medication every day and causes some worry but minimal interference with daily activities.	0.049	0.031	0.072
Cancer, metastatic (cancer_metastatic)	This person has severe pain, extreme fatigue, weight loss and high anxiety.	0.451	0.307	0.600
Terminal phase, with medication (cancer_terminal_treat)	This person has lost a lot of weight and regularly uses strong medication to avoid constant pain. The person has no appetite, feels nauseous, and needs to spend most of the day in bed.	0.540	0.377	0.687
Mastectomy (cancer_mastectomy)	This person had one of her breasts removed and sometimes has pain or swelling in the arms.	0.036	0.020	0.057
Stoma (cancer_stoma)	This person has a pouch attached to an opening in the belly to collect and empty stools.	0.095	0.063	0.131
Laryngectomy (speech_problems)	This person has difficulty speaking, and others find it difficult to understand.	0.051	0.032	0.078
Urinary incontinence (incontinence)	This person cannot control urinating.	0.139	0.094	0.198
Impotence (impotence)	This person has difficulty in obtaining or maintaining an erection.	0.017	0.009	0.030

Estimating non-melanoma skin cancer (squamous and basal cell carcinoma)

Mortality due to squamous cell skin cancer was estimated in the same way that all other cancers were estimated using the same methods as in GBD 2015 with the exception that only vital registration system data (and not cancer registry data) was the sources for squamous cell cancer mortality. CODEm models were run to generate estimates for all countries, years, and age groups by sex. As in GBD 2015 for basal cell carcinoma of the skin (BCC) we did not estimate any mortality given that this is a very rare event.

We estimated squamous cell and basal cell skin cancer incidence by using cancer registry as well as primary literature data for incidence. Only cancer registries that were listed in CI5 VIII as registering squamous cell carcinoma or basal cell carcinoma respectively, were included in the analysis.[27] For cancer registry data reported at the three digit level (C44: Other and unspecified malignant neoplasm of skin), proportions from Karagas et al were used to split C44 into squamous cell carcinoma and basal cell carcinoma.[28] We did not add any new data compared to GBD 2015. DisMod-MR 2.1 was used to model incidence and prevalence. Prevalence was calculated as function of two extreme scenarios (duration 1 versus 5 years). Country, age, sex and year specific duration was estimated using a country-age-sex-year specific relative access-to-care-score.

The access to care score was based on the melanoma mortality to incidence ratio:

$$Access\ to\ care = 1 - \frac{Age\ standardized\ MIR_{cys} - Age\ standardized\ MIR_{min}}{Age\ standardized\ MIR_{max} - Age\ standardized\ MIR_{min}}$$

c=country; y=year; s=sex; Age-standardized MI $ratio_{min}$=lowest MIR for all countries and years; Age standardized MIR_{max}=highest MIR for all countries and years

Remission was calculated as the inverse of the duration estimates and used as additional input for DisMod-MR 2.1.

To reflect differing degrees of disability due to squamous cell carcinoma we used three levels of severity that were derived from MEPS (more detail on severity splits is found in Appendix Section 2.5). Prevalence was multiplied by distinct disability weights (Table 6) to generated YLDs.

Table 6. Lay description and disability weights			
Cause	Health state		Estimate with uncertainty interval
Cutaneous squamous cell carcinoma, mild	Disfigurement, level 1	has a slight, visible physical deformity that others notice, which causes some worry and discomfort.	0.011 (0.005-0.021)
Cutaneous squamous cell carcinoma, moderate	Disfigurement, level 2	has a visible physical deformity that causes others to stare and comment. As a result, the person is worried and has trouble sleeping and concentrating.	0.067 (0.044-0.096)
Cutaneous squamous cell carcinoma, severe	Disfigurement, level 3, with itch/pain	has an obvious physical deformity that is very painful and itchy. The physical deformity makes others uncomfortable, which causes the person to avoid social contact, feel worried, sleep poorly, and think about suicide.	0.576 (0.401-0.731)

Disfigurement due to basal cell carcinoma	Disfigurement, level 1	has a slight, visible physical deformity that others notice, which causes some worry and discomfort.	0.011 (0.005-0.021)

SR Table 15 contains a comparison of GBD 2016 and GLOBOCAN estimated cancer incidence by cause and sex for 2012.

There are no other significant changes to the GBD 2016 neoplasms modeling process.

References

1 GBD 2016 Causes of Death Collaborators. Global, regional, and national age-sex specific mortality for 264 causes of death, 1980–2016: a systematic analysis for the Global Burden of Disease Study 2016. *The Lancet* 2017.

2 Sankaranarayanan R, Swaminathan R, World Health Organization, International Agency for Research on Cancer, editors. Cancer survival in Africa, Asia, the Caribbean and Central America. Lyon, France: International Agency for Research on Cancer, World Health Organization, 2011.

3 National Center for Health Statistics, Centers for Disease Control and, Prevention. US Mortality Files. 61-Year Trends in U.S. Cancer Death Rates. http://seer.cancer.gov/archive/csr/1975_2010/results_merged/topic_historical_mort_trends.pdf.

4 Howlader N, Noone A, Krapcho M, *et al.* SEER Cancer Statistics Review, 1975-2013. 2015; published online Nov. http://seer.cancer.gov/csr/1975_2013/.

5 National Cancer Institute (United States). United States SEER Cancer Data 1973-2010. Bethesda, United States: National Cancer Institute (United States). .

6 Canadian Institute for Health Information (CIHI). Canada Discharge Abstract Database 1994-2009. Ottawa, Canada: Canadian Institute for Health Information (CIHI). .

7 Ministry of Health (Mexico). Mexico Ministry of Health Hospital Discharges 2000-2012. Mexico City, México: Ministry of Health (Mexico). .

8 Fitzmaurice C, Dicker D, Pain A, *et al.* The Global Burden of Cancer 2013. *JAMA Oncol* 2015; published online May 28. DOI:10.1001/jamaoncol.2015.0735.

9 Neal RD, Din NU, Hamilton W, *et al.* Comparison of cancer diagnostic intervals before and after implementation of NICE guidelines: analysis of data from the UK General Practice Research Database. *Br J Cancer* 2014; **110**: 584–92.

10 Surveillance, Epidemiology, and End Results (SEER) Program (www.seer.cancer.gov) SEER*Stat Database: Incidence - SEER 18 Regs Research Data + Hurricane Katrina Impacted Louisiana Cases, Nov 2012 Sub (1973-2010 varying) - Linked To County Attributes - Total U.S., 1969-2011 Counties, National Cancer Institute, DCCPS, Surveillance Research Program, Surveillance Systems Branch, released April 2013, based on the November 2012 submission. .

11 Allgar VL, Neal RD. Delays in the diagnosis of six cancers: analysis of data from the National Survey of NHS Patients: Cancer. *Br J Cancer* 2005; **92**: 1959–70.

12 Neal RD, Cannings-John R, Hood K, *et al.* Excision of malignant melanomas in North Wales: effect of location and surgeon on time to diagnosis and quality of excision. *Fam Pract* 2008; **25**: 221–7.

13 Nolan RC, Chan MT-L, Heenan PJ. A clinicopathologic review of lethal nonmelanoma skin cancers in Western Australia. *J Am Acad Dermatol* 2005; **52**: 101–8.

14 Kewalramani T, Nimer SD, Zelenetz AD, *et al.* Progressive disease following autologous transplantation in patients with chemosensitive relapsed or primary refractory Hodgkin's disease or aggressive non-Hodgkin's lymphoma. *Bone Marrow Transplant* 2003; **32**: 673–9.

15 Esteban D, Tovar N, Jiménez R, *et al.* Patients with relapsed/refractory chronic lymphocytic leukaemia may benefit from inclusion in clinical trials irrespective of the therapy received: a case-control retrospective analsysis. *Blood Cancer J* 2015; **5**: e356.

16 Canova C, Giorato E, Roveron G, Turrini P, Zanotti R. Validation of a stoma-specific quality of life questionnaire in a sample of patients with colostomy or ileostomy. *Colorectal Dis Off J Assoc Coloproctology G B Irel* 2013; **15**: e692-698.

17 Caricato M, Ausania F, Ripetti V, Bartolozzi F, Campoli G, Coppola R. Retrospective analysis of long-term defunctioning stoma complications after colorectal surgery. *Colorectal Dis Off J Assoc Coloproctology G B Irel* 2007; **9**: 559–61.

18 Erwin-Toth P, Thompson SJ, Davis JS. Factors impacting the quality of life of people with an ostomy in North America: results from the Dialogue Study. *J Wound Ostomy Cont Nurs Off Publ Wound Ostomy Cont Nurses Soc WOCN* 2012; **39**: 417-422; quiz 423-424.

19 Catalona WJ, Carvalhal GF, Mager DE, Smith DS. Potency, continence and complication rates in 1,870 consecutive radical retropubic prostatectomies. *J Urol* 1999; **162**: 433–8.

20 Donnellan SM, Duncan HJ, MacGregor RJ, Russell JM. Prospective assessment of incontinence after radical retropubic prostatectomy: objective and subjective analysis. *Urology* 1997; **49**: 225–30.

21 Eastham JA, Kattan MW, Rogers E, *et al.* Risk factors for urinary incontinence after radical prostatectomy. *J Urol* 1996; **156**: 1707–13.

22 Kundu SD, Roehl KA, Eggener SE, Antenor JAV, Han M, Catalona WJ. Potency, continence and complications in 3,477 consecutive radical retropubic prostatectomies. *J Urol* 2004; **172**: 2227–31.

23 Potosky AL, Davis WW, Hoffman RM, *et al.* Five-Year Outcomes After Prostatectomy or Radiotherapy for Prostate Cancer: The Prostate Cancer Outcomes Study. *JNCI J Natl Cancer Inst* 2004; **96**: 1358–67.

24 Sacco E, Prayer-Galetti T, Pinto F, *et al.* Urinary incontinence after radical prostatectomy: incidence by definition, risk factors and temporal trend in a large series with a long-term follow-up. *BJU Int* 2006; **97**: 1234–41.

25 Stanford JL, Feng Z, Hamilton AS, *et al.* Urinary and sexual function after radical prostatectomy for clinically localized prostate cancer: the Prostate Cancer Outcomes Study. *JAMA* 2000; **283**: 354–60.

26 Walsh PC, Marschke P, Ricker D, Burnett AL. Patient-reported urinary continence and sexual function after anatomic radical prostatectomy. *Urology* 2000; **55**: 58–61.

27 Parkin D, Whelan S, Ferlay J, Teppo L, Thomas D. Cancer Incidence in Five Continents VIII. Lyon: IARC, 2002.

28 Karagas MR, Greenberg ER, Spencer SK, Stukel TA, Mott LA. Increase in incidence rates of basal cell and squamous cell skin cancer in New Hampshire, USA. New Hampshire Skin Cancer Study Group. *Int J Cancer J Int Cancer* 1999; **81**: 555–9.

Rheumatic Heart Disease

Flowchart

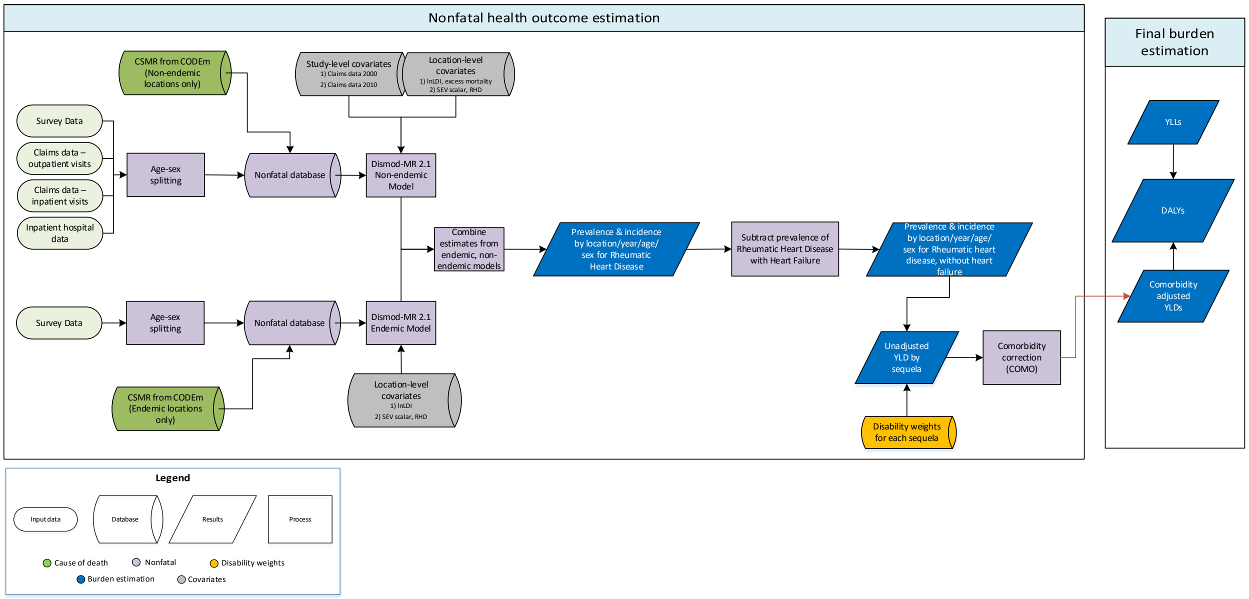

Input data and methodological appendix

Case definition

Rheumatic heart disease (RHD) was defined as a clinical diagnosis by a physician with or without confirmation using echocardiography. This case definition for echocardiographic confirmation of RHD follows the World Heart Federation criteria for echocardiographic diagnosis of rheumatic heart disease (1).

Criterion	Definition
1. Echocardiography	Prevalent rheumatic heart disease based on echocardiographic assessment and clinical confirmation
2. Clinical diagnosis	Prevalent rheumatic heart disease based on physician diagnosis

ICD codes for data included from hospital records can be found in Methods Appendix Table 4.

Input data

Model inputs

We did not perform a systematic review for GBD 2016. A systematic review was performed for GBD 2013 and updated for GBD 2015. The GBD 2015 search information encompassed the following:

- Search terms: ('rheumatic heart disease' AND epidemiology[MeSH Subheading]) OR ('acute rheumatic fever' AND epidemiology[MeSH Subheading]) OR ('rheumatic fever' AND

epidemiology[MeSH Subheading]) OR (RHD AND epidemiology[MeSH Subheading]) OR ('valvular heart disease' AND epidemiology[MeSH Subheading]) OR (((streptococcus OR streptococci) AND heart) AND epidemiology[MeSH Subheading]) OR (heart AND valve AND disease AND epidemiology[MeSH Subheading]) OR ('mitral valve stenosis' AND epidemiology[MeSH Subheading]) OR (('rheumatic heart disease' OR 'rheumatic fever') AND prevalence) OR (('rheumatic heart disease' OR 'rheumatic fever') AND incidence) OR (('rheumatic heart disease' OR 'rheumatic fever') AND ('standardized mortality ratio' OR SMR)) OR ('rheumatic heart disease' OR 'rheumatic fever' AND 'case fatality')
- Dates included in search: 1/1/2013 – 3/16/2015
- Number of initial hits: 2,045
- Number of sources included: 17

These differed from the GBD 2013 search terms:

- (hasabstract[text] AND Humans[Mesh] AND middle age[MeSH])) OR 21) AND ((rheumatic heart disease/epidemiology[Mesh] OR rheumatic heart disease/mortality[Mesh]) AND (prevalence[Title/Abstract] OR incidence[Title/Abstract]) AND ("2010"[Date - Publication] : "3000"[Date - Publication]) AND (hasabstract[text] AND Humans[Mesh] AND middle age[MeSH]))

The table below illustrates the number of literature studies included in GBD 2016, as well as the number of countries or subnational units and GBD world regions represented.

Endemic model			
	Prevalence	Incidence	Mortality risk
Studies	54	0	0
Countries/subnationals	29	0	0
GBD world regions	9	0	0

Non-endemic model			
	Prevalence	Incidence	Mortality risk
Studies	8	0	0
Countries/subnationals	5	0	0
GBD world regions	5	0	0

We did not include any non-literature-based data types other than the hospital and claims data described elsewhere. Hospital and claims data were available only for the non-endemic country model. We used uncorrected inpatient hospital data as the correction factors generated using US claims data were exceptionally high (~100X) and could not be validated using data sources from other geographies. As patterns of disease and access to care vary widely, using uncorrected hospital data avoids inappropriately scaling inpatient data in other countries based on the very high use of RHD codes in the US in all-position diagnosis and outpatient claims data. We excluded all outpatient data, as they were implausibly low when compared with inpatient data from the same locations and claims data.

For the non-endemic country model, we included study-level covariates for inpatient hospital data and claims data from 2000 and 2010 to adjust these data points, using as reference the data obtained from literature and claims data from 2012.

Severity splits and disability weights

Severity level	Lay description	DW (95% CI)
Rheumatic heart disease, not including heart failure	Has a chronic disease that requires medication every day and causes some worry but minimal interference with daily activities.	0.049 (0.031–0.072)

Modelling strategy

For GBD 2016 estimation, we ran two models – one for non-endemic countries and one for endemic countries. We defined endemicity based on GBD 2016 RHD mortality estimates and the Socio-demographic Index estimates (SDI) for the year 2016. For mortality, we used a threshold of 0.15/100,000 deaths in children aged 5-9; for SDI, we used a cutpoint of 0.6. These thresholds were selected using as priors expert opinion on locations where RHD is known to be endemic. Locations above the threshold for deaths or below the threshold for SDI were categorised as endemic; all other locations were categorised as non-endemic. For GBD 2015, these decisions were made at the country level. For GBD 2016, they were made at the national level for countries modelled nationally, and at the subnational level for the subset of countries modelled subnationally.

Non-endemic model: We included hospital data, claims data, and limited literature data on prevalence. We also included CSMR from our mortality estimates of RHD for non-endemic locations only. A prior of no remission was set, and excess mortality was capped at 0.1 for all ages. We included study-level covariates for claims data from 2000 and 2010, cross-walking them to data from the literature and claims data in 2012. We also included the log-transformed age-standardised SEV scalar for RHD and the natural log of lagged distributed income (lnLDI, I$ per capita) as a country-level covariates for prevalence and excess mortality, respectively.

Endemic model: We included prevalence data from surveys published in the literature. As with the high-income model, we included CSMR from our mortality estimates of RHD for endemic locations only. A prior of no remission was set for all ages, and excess mortality was capped at 0.07, the highest observed mean excess mortality rate data point observed in this model. We also set priors of 0 on incidence for ages 0 to 1 and 50 to 100 to account for patterns of incidence in endemic countries. We used lnLDI as fixed-effect country-level covariates on prevalence and excess mortality, enforcing an inverse relationship for both. The log-transformed, age-standardized SEV scalar was also used as a fixed-effect country-level covariate on prevalence.

We combined estimates from the endemic and non-endemic models, selecting estimates for the locations identified as non-endemic from the non-endemic model and estimates for the locations identified as endemic from the endemic model. Estimates of heart failure due to RHD were then subtracted from the estimates for RHD, giving the overall prevalence of RHD without heart failure. A description of the modelling strategy for heart failure due to RHD can be found in the Heart Failure appendix. We evaluated models based on comparing estimates with input data as well as estimates from previous rounds of GBD.

The table below shows the country covariates, parameters, betas, and exponentiated betas:

Covariate	Parameter	beta	Exponentiated beta
Endemic model			
Log-transformed age-standardized SEV scalar: RHD	Prevalence	0.93 (0.76 – 1.22)	2.55 (2.13 – 3.37)
LDI (I$ per capita)	Excess mortality rate	-0.28 (-0.48 – -0.11)	0.76 (0.62 – 0.90)
Non-endemic model			
All Marketscan, year 2000	Study-level	0.19 (0.090 – 0.30)	1.21 (1.09 – 1.34)
All Marketscan, year 2010	Study-level`	0.44 (0.36 – 0.52)	1.55 (1.43 – 1.68)
Log-transformed age-standardized SEV scalar: RHD	Prevalence	0.78 (0.75 – 0.89)	2.19 (2.12 – 2.43)
LDI (I$ per capita)	Excess mortality rate	-0.54 (-0.57 – -0.5)	0.58 (0.57 – 0.61)

We changed the process of selecting locations for the endemic and non-endemic models. In previous rounds, this decision had been based on country development status and income level, and the models were referred to as low-income and high-income. However, because these models are trying to capture differences between locations where RHD is endemic and locations where the disease is extremely rare, we are now using death data and the Socio-demographic Index to identify which model should be used for estimates for each location. In addition, we are now making this determination at the subnational level for the relevant countries.

Endemic Locations: North Korea, Cambodia, Laos, the Maldives, Myanmar, the Philippines, Timor-Leste, Fiji, Kiribati, Marshall Islands, Federated States of Micronesia, Papua New Guinea, Samoa, Solomon Islands, Tonga, Vanuatu, Azerbaijan, Georgia, Tajikistan, Turkmenistan, Uzbekistan, Albania, Belize, Dominica, Dominican Republic, Grenada, Guyana, Haiti, Jamaica, Saint Lucia, Saint Vincent and the Grenadines, Suriname, Bolivia, Ecuador, Guatemala, Honduras, Nicaragua, Algeria, Egypt, Iran, Iraq, Libya, Morocco, Palestine, Syria, Yemen, Afghanistan, Bangladesh, Bhutan, Nepal, Pakistan, Angola, the Central African Republic, Congo (Brazzaville), Democratic Republic of the Congo, Burundi, Comoros, Djibouti, Eritrea, Ethiopia, Madagascar, Malawi, Mauritius, Mozambique, Rwanda, Somalia, Tanzania, Uganda, Zambia, Lesotho, Namibia, Swaziland, Zimbabwe, Benin, Burkina Faso, Cameroon, Cape Verde, Chad, Cote d'Ivoire, The Gambia, Ghana, Guinea, Guinea-Bissau, Liberia, Mali, Mauritania, Niger, Nigeria, Sao Tome and Principe, Senegal, Sierra Leone, Togo, American Samoa, Guam, South Sudan, Eastern Cape, Free State, Gauteng, KwaZulu-Natal, Limpopo, Mpumalanga, North-West, Northern Cape, Western Cape, Chongqing, Gansu, Guizhou, Hainan, Inner Mongolia, Qinghai, Tibet, Xinjiang, Yunnan, Sudan, Sumatera Barat, Bengkulu, Kalimantan Utara, Jawa Tengah, Bali, Nusa Tenggara Barat, Nusa Tenggara Timur, Kalimantan Barat, Kalimantan Tengah, Kalimantan Selatan, Kalimantan Timur, Sulawesi Utara, Sulawesi Tengah, Sulawesi Tenggara, Gorontalo, Sulawesi Barat, Maluku, Maluku Utara, Papua Barat, Papua, Acre, Amapá, Bahia,
Goiás, Maranhão, Pará, Pernambuco, Piaui, Sergipe, Tocantins, Baringo, Bomet, Bungoma, Busia, Elgeyo-Marakwet, Embu, Garissa, HomaBay, Isiolo, Kajiado, Kakamega, Kericho, Kilifi, Kirinyaga, Kisii, Kisumu, Kitui, Kwale, Laikipia, Lamu, Machakos, Makueni, Mandera, Marsabit, Meru, Migori, Mombasa, Murang'a, Nairobi, Nakuru, Nandi, Narok, Nyamira, Nyandarua, Nyeri, Samburu, Siaya, TaitaTaveta, TanaRiver, TharakaNithi, TransNzoia, Turkana, UasinGishu, Vihiga, Wajir, WestPokot, Andhra Pradesh, Urban, Assam, Urban, Bihar, Urban, Chhattisgarh, Urban, Delhi, Urban, Goa, Urban, Gujarat, Urban, Haryana, Urban, Himachal Pradesh, Urban, Jammu and Kashmir, Urban, Jharkhand, Urban, Karnataka, Urban, Kerala, Urban, Madhya Pradesh, Urban, Maharashtra, Urban, Manipur, Urban, Meghalaya, Urban, Odisha, Urban, Punjab, Urban, Rajasthan, Urban, Tamil Nadu, Urban, Telangana, Urban, Tripura, Urban, Uttar Pradesh,

Urban, Uttarakhand, Urban, West Bengal, Urban, Andhra Pradesh, Rural, Arunachal Pradesh, Rural, Assam, Rural, Bihar, Rural, Chhattisgarh, Rural, Delhi, Rural, Gujarat, Rural, Haryana, Rural, Himachal Pradesh, Rural, Jammu and Kashmir, Rural, Jharkhand, Rural, Karnataka, Rural, Kerala, Rural, Madhya Pradesh, Rural, Maharashtra, Rural, Manipur, Rural, Meghalaya, Rural, Nagaland, Rural, Odisha, Rural Punjab, Rural, Rajasthan, Rural, Sikkim, Rural, Tamil Nadu, Rural, Telangana, Rural, Tripura, Rural, Uttar Pradesh, Rural, Uttarakhand, Rural, West Bengal, Rural, The Six Minor Territories, Rural, The Six Minor Territories, Urban

Non-endemic Locations: Taiwan (Province of China), Malaysia, Sri Lanka, Thailand, Vietnam, Armenia, Kazakhstan, Kyrgyzstan, Mongolia, Bosnia and Herzegovina, Bulgaria, Croatia, Czech Republic, Hungary, Macedonia, Montenegro, Poland, Romania, Serbia, Slovakia, Slovenia, Belarus, Estonia, Latvia, Lithuania, Moldova, Russia, Ukraine, Brunei, South Korea, Singapore, Australia, New Zealand, Andorra, Austria, Belgium, Cyprus, Denmark, Finland, France, Germany, Greece, Iceland, Ireland, Israel, Italy, Luxembourg, Malta, Netherlands, Norway, Portugal, Spain, Switzerland, Argentina, Chile, Uruguay, Canada, Antigua and Barbuda, The Bahamas, Barbados, Cuba, Trinidad and Tobago, Peru, Colombia, Costa Rica, El Salvador, Panama, Venezuela, Paraguay, Bahrain, Jordan, Kuwait, Lebanon, Oman, Qatar, Tunisia, Turkey, United Arab Emirates, Equatorial Guinea, Gabon, Seychelles, Botswana, Bermuda, Greenland, Hong Kong Special Administrative Region of China, Macao Special Administrative Region of China, Northern Mariana Islands, Puerto Rico, Virgin Islands, U.S., Northern Ireland, Scotland, Anhui, Beijing, Fujian, Guangdong, Guangxi, Hebei, Heilongjiang, Henan, Hubei, Hunan, Jiangsu, Jiangxi, Jilin, Liaoning, Ningxia, Shaanxi, Shandong, Shanghai, Shanxi, Sichuan, Tianjin, Zhejiang, Alabama, Alaska, Arizona, Arkansas, California, Colorado, Connecticut, Delaware, District of Columbia, Florida, Georgia, Hawaii, Idaho, Illinois, Indiana, Iowa, Kansas, Kentucky, Louisiana, Maine, Maryland, Massachusetts, Michigan, Minnesota, Mississippi, Missouri, Montana, Nebraska, Nevada, New Hampshire, New Jersey, New Mexico, New York, North Carolina, North Dakota, Ohio, Oklahoma, Oregon, Pennsylvania, Rhode Island, South Carolina, South Dakota, Tennessee, Texas, Utah, Vermont, Virginia, Washington, West Virginia, Wisconsin, Wyoming, Wales, Aguascalientes, Baja California, Baja California Sur, Campeche, Coahuila, Colima, Chiapas, Chihuahua, Distrito Federal, Durango, Guanajuato, Guerrero, Hidalgo, Jalisco, México, Michoacán de Ocampo, Morelos, Nayarit, Nuevo León, Oaxaca, Puebla, Querétaro, Quintana Roo, San Luis Potosí, Sinaloa, Sonora, Tabasco, Tamaulipas, Tlaxcala, Veracruz de Ignacio de la Llave, Yucatán, Zacatecas, Aceh, Sumatera Utara, Riau, Jambi, Sumatera Selatan, Lampung, Bangka Belitung, Kepulauan Riau, Jakarta, Jawa Barat, Yogyakarta, Jawa Timur, Banten, Sulawesi Selatan, Alagoas, Amazonas, Ceará, Distrito Federal, Espírito Santo, Minas Gerais, Mato Grosso do Sul, Mato Grosso, Paraíba, Paraná, Rio de Janeiro, Rio Grande do Norte, Rondônia, Roraima, Rio Grande do Sul, Santa Catarina, São Paulo, Sweden except Stockholm, Stockholm, Hokkaidō, Aomori, Iwate, Miyagi, Akita, Yamagata, Fukushima, Ibaraki, Tochigi, Gunma, Saitama, Chiba, Tōkyō, Kanagawa, Niigata, Toyama, Ishikawa, Fukui, Yamanashi, Nagano, Gifu, Shizuoka, Aichi, Mie, Shiga, Kyōto, Ōsaka, Hyōgo, Nara, Wakayama, Tottori, Shimane, Okayama, Hiroshima, Yamaguchi, Tokushima, Kagawa, Ehime, Kōchi, Fukuoka, Saga, Nagasaki, Kumamoto, Ôita, Miyazaki, Kagoshima, Okinawa, Kiambu, Arunachal Pradesh, Urban, Mizoram, Urban, Nagaland, Urban, Sikkim, Urban, Goa, Rural, Mizoram, Rural, Ha'il, Qassim, Riyadh, Tabuk, Madinah, Makkah, Bahah, Northern Borders, Jawf, Jizan, 'Asir, Najran, Eastern Province, Darlington, Northumberland, Stockton-on-Tees, Newcastle upon Tyne, North Tyneside, Redcar and Cleveland, County Durham, Gateshead, Middlesbrough, South Tyneside, Sunderland, Hartlepool, Cheshire East, Stockport, Trafford, Cheshire, West and Chester, Sefton, Lancashire, Cumbria, Bolton, Wirral, Bury, St Helens, Warrington, Oldham, Rochdale, Wigan, Halton, Liverpool, Tameside, Salford, Blackburn with Darwen, Knowsley, Blackpool, Manchester, North Yorkshire, East Riding of Yorkshire, York, North East Lincolnshire, Calderdale, North Lincolnshire, Bradford, Kirklees, Leeds, Sheffield, Wakefield, Rotherham, Doncaster, Kingston upon Hull, City of Barnsley, Northamptonshire, Leicestershire, Lincolnshire, Rutland, Derby, Derbyshire,

Nottinghamshire, Nottingham, Leicester, Warwickshire, Herefordshire, County of, Solihull, Shropshire, Worcestershire, Staffordshire, Dudley, Coventry, Telford and Wrekin, Stoke-on-Trent, Walsall, Wolverhampton, Birmingham, Sandwell, Bedford, Central Bedfordshire, Suffolk, Hertfordshire, Essex, Cambridgeshire, Thurrock, Norfolk, Southend-on-Sea, Peterborough, Luton, Richmond upon Thames, Kensington and Chelsea, Barnet, Westminster, Bromley, Bexley, Redbridge, Merton, Brent, Hillingdon, Havering, Kingston upon Thames, Sutton, Harrow, Enfield, Croydon, Hammersmith and Fulham, Ealing, Greenwich, Wandsworth, Waltham Forest, Camden, Lambeth, Lewisham, Hounslow, Southwark, Newham, Barking and Dagenham, Haringey, Hackney, Islington, Tower Hamlets, Wokingham, Buckinghamshire, Surrey, Windsor and Maidenhead, West Berkshire, Hampshire, Bracknell Forest, West Sussex, Oxfordshire, Reading, Kent, Brighton and Hove, Medway, East Sussex, Portsmouth, Isle of Wight, Milton Keynes, Southampton, Slough, South Gloucestershire, Dorset, Wiltshire, North Somerset, Devon, Poole, Bath and North East Somerset, Gloucestershire, Somerset, Swindon, Torbay, Bristol, City of Bournemouth, Cornwall, Plymouth

Other than this update to model assignment, there were no substantive changes in the modelling strategy from GBD 2015.

1. Reményi, B. et al. Nat. Rev. Cardiol. 9, 297–309 (2012); published online 28 February 2012

Ischemic Heart Disease

Flowchart

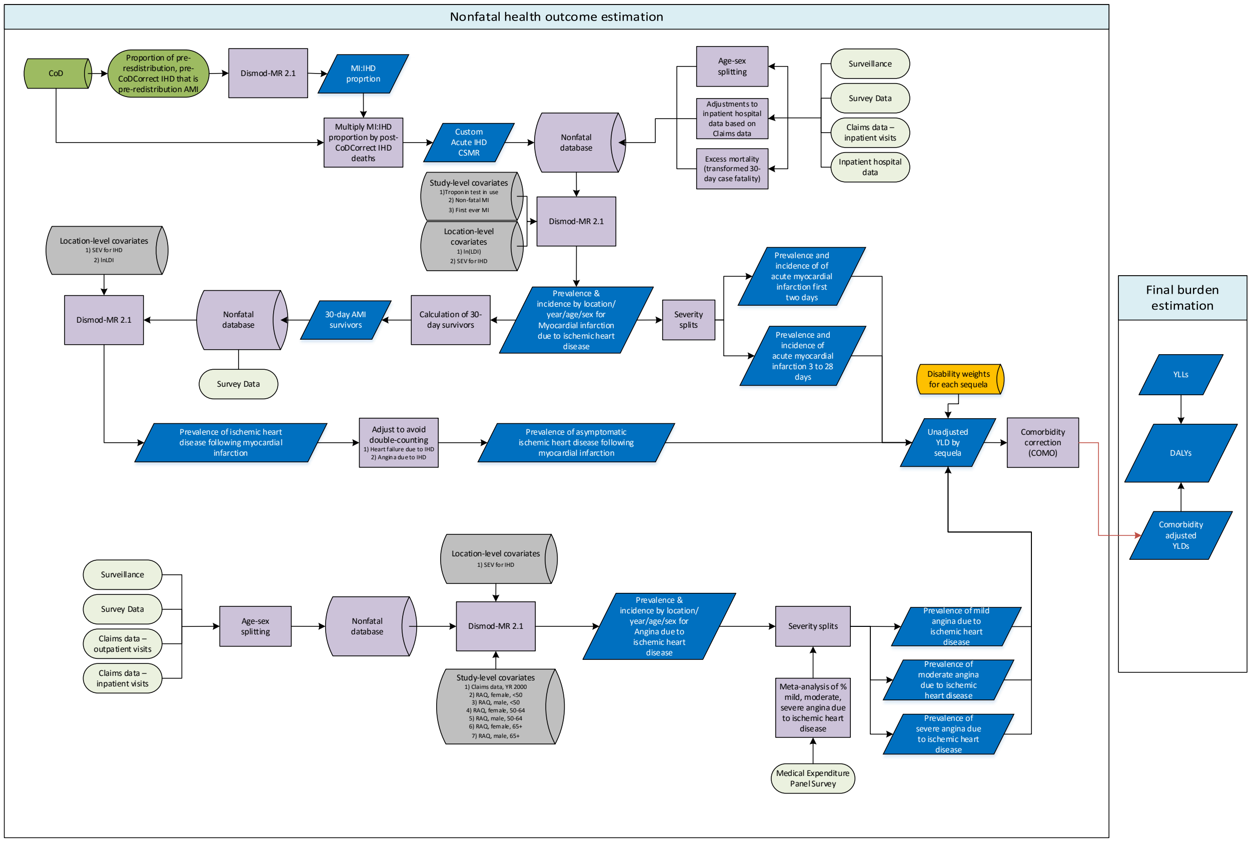

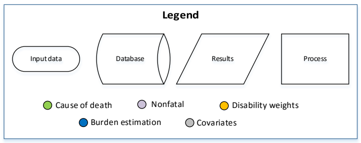

Input data and methodological summary

Case definition

<u>Case definitions:</u>

1) Acute myocardial infarction (MI): Definite and possible MI according to the third universal definition of myocardial infarction:
    a. When there is clinical evidence of myocardial necrosis in a clinical setting consistent with myocardial ischemia or
    b. Detection of a rise and/or fall of cardiac biomarker values and with at least one of the following: i) symptoms of ischaemia, ii) new or presumed new ST-segment-T wave changes or new left bundle branch block, iii) development of pathological Q waves in the

ECG, iv) imaging evidence of new loss of viable myocardium or new regional wall motion abnormality, or v) identification of an intracoronary thrombus by angiography or autopsy.

c. Sudden (abrupt) unexplained cardiac death, involving cardiac arrest or no evidence of a noncoronary cause of death
d. Prevalent MI is considered to last from the onset of the event to 28 days after the event and is divided into an acute phase (0–2 days) and subacute (3–28 days).

2) Chronic IHD
   a. Angina; clinically diagnosed stable exertional angina pectoris or definite angina pectoris according to the Rose Angina Questionnaire, physician diagnosis, or taking nitrate medication for the relief of chest pain.
   b. Asymptomatic ischemic heart disease following myocardial infarction; survival to 28 days following incident MI. The GBD study does not use estimates based on ECG evidence for prior MI, due to its limited specificity and sensitivity (1).

ICD codes used for inclusion of hospital and claims data for MI and angina can be found in Methods Appendix Table 4.

Input data

Model inputs

Myocardial infarction

A systematic review for myocardial infarction was not performed for GBD 2016. Updates to systematic reviews are performed on an ongoing schedule across all GBD causes; an update for myocardial infarction will be performed in the next one to two iterations.

A systematic review was done for myocardial infarction for GBD 2015. The search strings used were extensive; a full list will be provided on request.

The dates of the search were 1/1/2009 – 2/3/2015. 38,522 studies were returned; 194 were extracted (this number includes extractions that were done for STEMI/NSTEMI models and revascularization models that are not currently part of the MI modelling process but may be in the future).

A systematic review for myocardial infarction was also done for GBD 2013. The extensive search terms for that review can be found here will be provided on request.

	Prevalence	Incidence	Mortality risk
Studies	0	93	61
Countries/subnationals	0	39	32
GBD world regions	0	8	10

Apart from inpatient hospital and inpatient claims data, we did not include any data from sources other than the literature for myocardial infarction. We excluded data with broad age ranges where it was impossible to obtain more granular data, as these data caused the known age pattern for increased risk of myocardial infarction to be masked in the estimates generated from DisMod.

We corrected inpatient hospital data outside of DisMod to account for the fact that these data sources do not capture the out-of-hospital cardiac arrest deaths which are part of the universal definition of MI. Using as a model the adjustment factors developed to translate tobacco consumption prevalence to tobacco consumption frequency, we matched administrative hospital data to population-based literature data based on age group, sex, and super region (1). For the adjustment factor, we developed the following generalized additive model on matched data

$$\ln(\mu_{ref,i}) = \beta_0 + \beta_1 \ln(\mu_{b,i}) + s(age_i) + \varepsilon_i$$

Where i represents a given matched observation, age signifies the data point's age group, $s(x)$ represents a penalized spline where the smoothing parameter is chosen through cross validation and μ_{ref} and μ_b denote the mean of the data point from literature and the mean of the inpatient hospital data point, respectively. Predictions from the model were then taken as the adjusted data points. The standard error of each corrected data point was adjusted to account for the uncertainty due to the correction.

We included a study-level covariate to correct for the change in diagnostic criteria to include troponin measurements within DisMod. This adjustment was applied to data collected before 2000. We also included a study-level covariate to adjust data points within DisMod that captured only first-ever MI, using studies where all events were included as the reference. We also adjusted estimates within DisMod from studies that only included non-fatal cases using study-level covariates with sources that included fatal and non-fatal cases as reference.

Angina

A systematic review was not performed for GBD 2016. Updates to systematic reviews are performed on an ongoing schedule across all GBD causes; an update for angina will be performed in the next one to two iterations.

A systematic review for angina was not done for GBD 2015, but was for GBD 2013. The search terms for that are here: (Angina Pectoris/epidemiology[Mesh] OR Angina Pectoris/mortality[Mesh]) AND (prevalence[Title/Abstract] OR incidence[Title/Abstract]) AND ("2010"[Date - Publication] : "3000"[Date - Publication])

Literature data included: Angina

	Prevalence	Incidence	Mortality risk
Studies	72	0	7
Countries/subnationals	73	0	7
GBD world regions	20	0	5

We included survey data (including NHANES and World Health Study questionnaires) which included the RAQ items. Prevalence of angina was calculated using the standard algorithm to determine whether the RAQ was positive or negative.

We excluded data with broad age ranges where it was impossible to obtain more granular data, as these data caused the known age pattern for increased risk of angina to be masked in the estimates generated from DisMod.

We included sex- and age-group-specific covariates to adjust prevalence data points obtained from the RAQ using the claims data as the reference since the RAQ has been shown to be neither sensitive nor specific.

We also included US claims data, but did not include inpatient hospital data from any locations. Stable angina (unstable angina is modeled as part of MI) is expected to be rare in inpatient, but common in outpatient data as it is a condition usually managed on an outpatient basis, except for specific surgical interventions. This discrepancy leads to implausible correction factors based on inpatient/outpatient information from claims data (~150X); thus adjusted data cannot be used. Including uncorrected data in the model is likely to lead to incorrect estimates as hospitalization and procedure rates are likely to vary between geographies based on access to and patterns of care. All outpatient data was excluded as it was implausibly low for all locations when compared with literature and claims data.

Severity split inputs

Acute myocardial infarction was split into two severity levels by length of time since the event – days 1 and 2 versus days 3 through 28. Disability weights were established for these two severities using the standard approach for GBD 2015.

Angina was split into mild, moderate, and severe groups using information from MEPS. Disability weights were established for these severities using the standard approach for GBD 2015.

Acute myocardial infarction

Severity level	Lay description	DW (95% CI)
Acute myocardial infarction, days 1-2	Has severe chest pain that becomes worse with any physical activity. The person feels nauseated, short of breath, and very anxious.	0.432 (0.288–0.579)
Acute myocardial infarction, days 3-28	Gets short of breath after heavy physical activity, and tires easily, but has no problems when at rest. The person has to take medication every day and has some anxiety.	0.074 (0.049–0.105)

Angina pectoris

Severity level	Lay description	DW (95% CI)
Mild angina	Has chest pain that occurs with strenuous physical activity, such as running or lifting heavy objects. After a brief rest, the pain goes away.	0.033 (0.02 – 0.052)
Moderate angina	Has chest pain that occurs with moderate physical activity, such as walking uphill or more than half a kilometer (around a	0.08 (0.052 – 0.113)

	quarter-mile) on level ground. After a brief rest, the pain goes away.	
Severe angina	Has chest pain that occurs with minimal physical activity, such as walking only a short distance. After a brief rest, the pain goes away. The person avoids most physical activities because of the pain.	0.167 (0.11 – 0.24)

Modelling strategy

Myocardial infarction

- We first calculated custom cause-specific mortality estimates using cause of death data prior to garbage code redistribution, generating age-sex-country-specific proportions of IHD deaths that were due to MI (acute IHD) vs those due to other causes of IHD (chronic IHD). Estimates of this proportion for all locations were then generated using a DisMod proportion-only model. Due to a high degree of variability in pre-redistribution coding practices by location, we used the global age-, sex-, and year-specific proportions of acute deaths in subsequent calculations. The global proportions were multiplied by post-CodCorrect (final GBD estimates) IHD deaths to generate CSMR estimates for MI, even though GBD reports only deaths for all IHD taken together. These data, along with incidence and excess mortality data, informed a DisMod model to estimate the prevalence and incidence of myocardial infarction due to ischemic heart disease.
- These estimates were split into prevalence and incidence estimates for days 1-2 and days 3-28 post-event. Disability weights were assigned to each of these two groupings.
- We set a value prior of one month for remission (11/13) from the MI health state. We also set a value prior for the maximum excess mortality rate of 10 for all ages. We included lnLDI as a fixed-effect country-level covariate on excess mortality, forcing an inverse relationship.

Study covariate	Parameter	Beta	Exponentiated beta
Diagnostic blood sample (troponin)	Incidence	-0.45 (-0.47 – -0.44)	0.64 (0.62 - 0.64)
LDI (I$ per capita)	Excess mortality rate	-0.1 (-0.1 – -0.1)	0.9 (0.9 – 0.9)
First ever MI	Incidence	-0.66 (-0.67 – -0.65)	0.52 (0.51 – 0.52)
Non-fatal MI	Incidence	-0.40 (-0.41 – -0.40)	0.67 (0.66 – 0.67)
Log-transformed age-standardized SEV scalar: IHD	Incidence	1.24 (1.21 – 1.25)	3.44 (3.34 – 3.49)

Asymptomatic ischaemic heart disease

- Excess mortality estimates from the myocardial infarction model were used to generate data of the incidence of surviving 28 days post-event.
- We used these data, along with the estimates of CSMR due to chronic IHD (the other part of the proportion described in step 1) and excess mortality data in a DisMod model to estimate the prevalence of persons with IHD following myocardial infarction. This estimate included subjects with angina and heart failure; a proportion of this prevalence was removed in order to avoid double-counting based on evidence from the literature (2). The result of this step generates estimates of asymptomatic ischaemic heart disease following myocardial infarction.
- We set a value prior of 0 for remission for all ages.
- No study- or country-level covariates were included for the model.

Angina

- We used prevalence data from the literature and USA claims databases, along with data on mortality risk to estimate the prevalence and incidence of angina for all locations.
- The proportion of mild, moderate, and severe angina was determined by the standard approach for severity splitting for GBD 2015.
- We included a value prior of 0 for remission for all ages. We also included a value prior of 1 for excess mortality for all ages.
- We included age- and sex-specific study-level covariates to adjust data points based on RAQ, using data points from the claims database as the reference.
- We also included the log-transformed, age-standardized SEV scalar for IHD as a fixed effect country-level covariate.

Study covariate	Parameter	Beta	Exponentiated beta
RAQ, female, less than 50	Prevalence	2.33 (2.30 – 2.40)	10.28 (9.98 – 11.06)
RAQ, male, less than 50	Prevalence	0.94 (0.92 – 0.95)	2.57 (2.51 – 2.59)
RAQ, female, 50 to 64	Prevalence	1.44 (1.35 – 1.50)	4.22 (3.87 – 4.47)
RAQ, male, 50 to 64	Prevalence	0.83 (0.80 – 0.90)	2.30 (2.23 – 2.46)
RAQ, female, 65 plus	Prevalence	0.29 (0.27 – 0.30)	1.34 (1.31 – 1.35)
RAQ, male, 65 plus	Prevalence	0.19 (0.11 – 0.29)	1.21 (1.11 – 1.33)
Log-transformed age-standardized SEV scalar: IHD	Prevalence	1.25 (1.23 – 1.25)	3.47 (3.43 – 3.49)
LDI (I$ per capita)	Excess mortality rate	-0.55 (-1 – -0.1)	0.58 (0.37 – 0.90)

There have been no substantive changes in the modelling strategy for myocardial infarction, asymptomatic ischemic heart disease following myocardial infarction, and angina from GBD 2015.

1. Reitsma, Marissa B., et al. "Smoking prevalence and attributable disease burden in 195 countries and territories, 1990–2015: a systematic analysis from the Global Burden of Disease Study 2015." The Lancet.

Ischaemic Stroke & Haemorrhagic Stroke

Flowchart

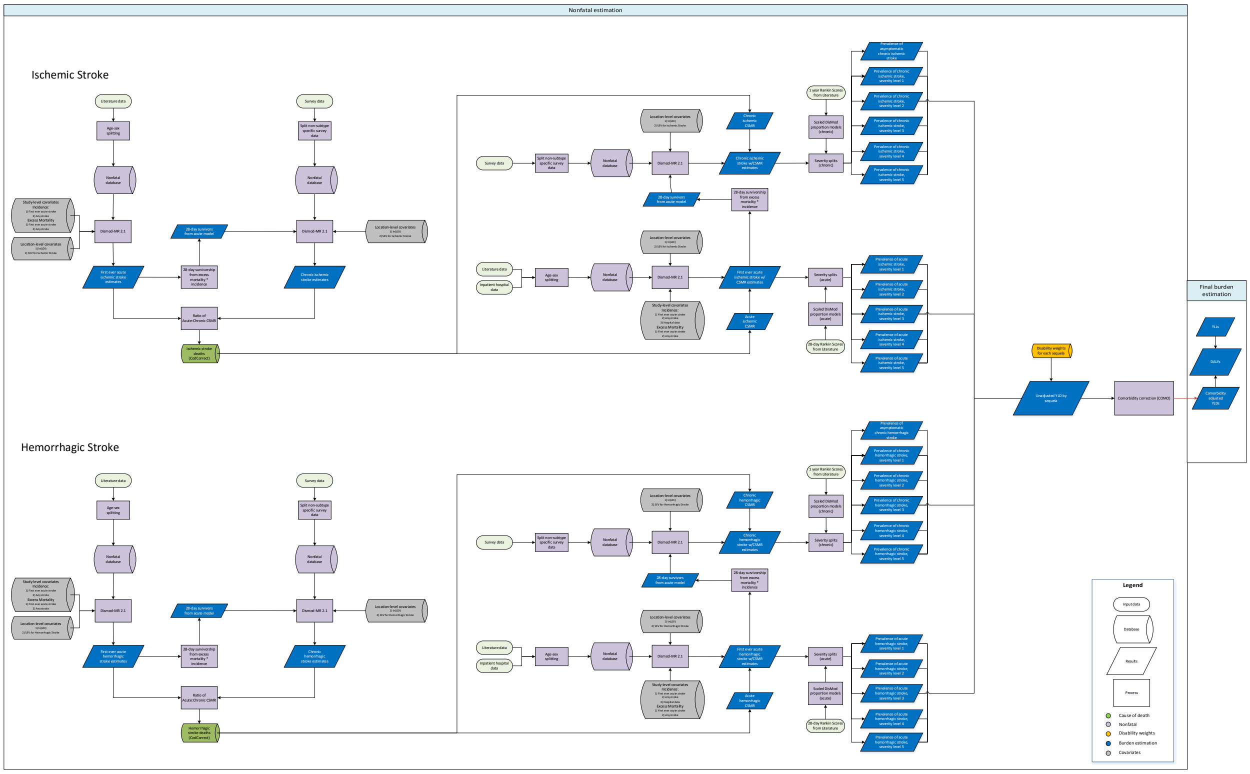

Input data and methodological summary

Case definition

Stroke was defined according to WHO criteria – rapidly developing clinical signs of focal (at times global) disturbance of cerebral function lasting more than 24 hours or leading to death with no apparent cause other than that of vascular origin(1). Data on transient ischaemic attack (TIA) were not included.

Acute stroke: Stroke cases are considered acute from the day of incidence of a first-ever stroke through day 28 following the event.

Chronic stroke: Stroke cases are considered chronic beginning 28 days following the occurrence of an event. Chronic stroke includes the sequelae of an acute stroke AND all recurrent stroke events. GBD 2015 adopts this broader definition of chronic stroke than was used in prior iterations in order to model acute strokes using only first-ever incident events.

Ischaemic stroke: Incident ischaemic stroke is defined as the occurrence of first-ever ischaemic stroke, based on clinical diagnosis by a physician using diagnostic imaging. Ischaemic strokes are considered to include all vascular events leading to limited blood flow to brain tissue, with

resulting infarction, including atherosclerotic and thromboembolic strokes but excluding strokes in which the underlying cause is intracranial haemorrhage.

Haemorrhagic or other strokes: This cause includes all non-ischaemic strokes of a vascular cause including subarachnoid and stroke due to intracranial haemorrhage.

ICD codes used for inclusion of hospital and claims data can be found in Appendix Table 4

Input data

Model inputs

A systematic review was not performed for GBD 2016. Updates to systematic reviews are performed on an ongoing schedule across all GBD causes; an update for cerebrovascular disease will be performed in the next iteration.

A systematic review of the literature was performed in GBD 2013. The search terms used were: (stroke[Mesh]) AND (prevalence[Title/Abstract] OR incidence[Title/Abstract]) AND ("2010"[Date - Publication] : "3000"[Date - Publication]) AND (hasabstract[text] AND Humans[Mesh] AND middle age[MeSH])) (hasabstract[text] AND Humans[Mesh] AND middle age[MeSH])) OR 21) AND ((hemorrhagic stroke/epidemiology[Mesh] OR hemorrhagic stroke/mortality[Mesh]) AND (prevalence[Title/Abstract] OR incidence[Title/Abstract]) AND ("2010"[Date - Publication] : "3000"[Date - Publication]) AND (hasabstract[text] AND Humans[Mesh] AND middle age[MeSH]))

The tables below indicate the number of literature studies included in GBD 2016, as well as the number of countries or subnational units and GBD world regions represented.

Acute Ischaemic stroke

	Prevalence	Incidence	Mortality risk
Studies	0	73	14
Countries/subnationals	0	55	12
GBD world regions	0	14	5

Acute Haemorrhagic or other stroke

	Prevalence	Incidence	Mortality risk
Studies	0	73	10
Countries/subnationals	0	51	11
GBD world regions	0	13	4

Chronic Ischaemic stroke

	Prevalence	Incidence	Mortality risk
Studies	53	0	8
Countries/subnationals	50	0	4
GBD world regions	14	0	2

Chronic Haemorrhagic or other stroke

	Prevalence	Incidence	Mortality risk
Studies	53	0	8
Countries/subnationals	50	0	4
GBD world regions	14	0	2

We included inpatient hospital data, adjusted for readmission and primary to any diagnosis using correction factors estimated from US claims data. We excluded data for locations where the data points were implausibly low (Vietnam, Philippines, India). In addition, we included unpublished stroke registry data for acute ischaemic and acute haemorrhagic strokes. We also included survey data for chronic cerebrovascular disease. These surveys were identified based on expert opinion and review of major survey series focused on world health that included questions regarding self-reported history of stroke.

As with many models in GBD, the diversity of data sources available means that we needed to adjust available data to our preferred or reference case definition (2). For the first ever acute stroke models we used DisMod to estimate the statistical association between measurements taken using different case definitions and then used these estimates to adjust the non-referent datapoints. We included study-level covariates to adjust data points for first and recurrent strokes combined, using data for first strokes only as reference. We also included study-level covariates to adjust ischaemic and haemorrhagic strokes combined (all stroke), using as reference studies with subtype-specific information.

Severity split inputs

The table below illustrates the severity level, lay description, and disability weights for GBD 2016. In previous iterations of the GBD, severity splits for stroke were based on the standard approach described elsewhere (3). For GBD 2016, we undertook a review to identify epidemiologic literature which reported the degree of disability at 28 days (for acute stroke) or one year (for chronic stroke) using the modified Rankin scale (mRS) and the Mini-mental State Examination (MMSE) or the Montreal Cognitive Assessment (MoCA). The mRS assesses functional capabilities, while the MMSE and MoCA tests provide evaluations of cognitive functioning. We then mapped these measures to the existing GBD categories as indicated below. This appproach allowed us to include location-specific information and can be updated as more data on functional or cognitive status become available.

Acute stroke severity splits

Severity level	Lay description	Modified Rankin Score	Cognitive Status	DW (95% CI)
Stroke, mild	Has some difficulty in moving around and some weakness in one hand, but is able to walk without help.	1	N/A	0.019 (0.01–0.032)
Stroke, moderate	Has some difficulty in moving around, and in using the hands for lifting and holding things, dressing, and grooming.	2, 3	MoCA>=24 or MMSE>=26	0.07 (0.046–0.099)

Stroke, moderate plus cognition problems	Has some difficulty in moving around, in using the hands for lifting and holding things, dressing and grooming, and in speaking. The person is often forgetful and confused.	2, 3	MoCA<24 or MMSE<26	0.316 (0.206–0.437)
Stroke, severe	Is confined to bed or a wheelchair, has difficulty speaking, and depends on others for feeding, toileting, and dressing.	4, 5	MoCA>=24 or MMSE>=26	0.552 (0.377–0.707)
Stroke, severe plus cognition problems	Is confined to bed or a wheelchair, depends on others for feeding, toileting, and dressing, and has difficulty speaking, thinking clearly, and remembering things.		MoCA<24 or MMSE<26	0.588 (0.411–0.744)

Chronic stroke severity splits

Severity level	Lay description	Modified Rankin Score	Cognitive Status	DW (95% CI)
Stroke, asymptomatic		0	N/A	N/A
Stroke, long-term consequences, mild	Has some difficulty in moving around and some weakness in one hand, but is able to walk without help.	1	N/A	0.019 (0.01–0.032)
Stroke, long-term consequences, moderate	Has some difficulty in moving around, and in using the hands for lifting and holding things, dressing, and grooming.	2, 3	MoCA>=24 or MMSE>=26	0.07 (0.046–0.099)
Stroke, long-term consequences, moderate plus cognition problems	Has some difficulty in moving around, in using the hands for lifting and holding things, dressing and grooming, and in speaking. The person is often forgetful and confused.	2, 3	MoCA<24 or MMSE<26	0.316 (0.206–0.437)
Stroke, long-term consequences, severe	Is confined to bed or a wheelchair, has difficulty speaking, and depends on others for feeding, toileting, and dressing.	4, 5	MoCA>=24 or MMSE>=26	0.552 (0.377–0.707)
Stroke, long-term consequences, severe plus cognition problems	Is confined to bed or a wheelchair, depends on others for feeding, toileting, and dressing, and has difficulty speaking, thinking clearly, and remembering things.	4, 5	MoCA<24 or MMSE<26	0.588 (0.411–0.744)

Severity split literature data availability

	Acute Proportion	Chronic Proportion
Studies	6	13
Countries/subnationals	5	11
GBD world regions	5	5

We used DisMod MR 2.1, a Bayesian meta-regression tool, to model the six severity levels, with an independent proportion model for each. Reports which grouped mRS scores differently than our mapping (e.g. 0-2) were adjusted in DisMod by estimating the association between these alternate groupings and our preferred mappings. These statistical associations were used to adjust data points to the referent category as necessary. The six models were scaled such that the sum of the proportions for all levels equaled 1.

Modelling strategy

Three general approaches were employed for all of the components of the stroke modelling process, detailed in the table below.

- Data points were adjusted from nonstandard to standard case deinitions using estimates from statistical models generated by DisMod for the acute models. Coefficients for these crosswalks can be found in the tables for fixed effects located below.
- The GBD summary exposure value, which is the relative risk-weighted prevalence of exposure, for ischaemic or haemorrhagic stroke as appropriate and a covariate for country income were used as country-level covariates for all models (4). Coefficients for these covariates can be found in the tables for fixed effects located below.
- Two versions of each stroke model were run, referred to as step 1 and step 2 models. First, we ran the step 1 DisMod-MR models for acute and chronic subtype-specific stroke using only incidence, prevalence, and case fatality data as inputs. We then used the ratio of acute:chronic cause-specific mortality estimated by these models to divide GBD stroke deaths into acute and chronic stroke deaths, using the global average for the proportion of acute:chronic stroke mortality. The acute and chronic models were then run (step 2) using the same incidence, prevalence, and case fatality data as well as the custom cause-specific mortality rates as input data.

Step 1

- We generated estimates for first-ever acute ischaemic and first-ever acute haemorrhagic stroke using DisMod-MR 2.1 with data collected on stroke incidence and excess mortality. We set value priors of 11 to 13 on remission for all ages to establish a one-month duration for these acute sequelae.
- We then calculated the rate of surviving until 28 days after an acute event for both ischaemic and haemorrhagic stroke using the modelled estimates of excess mortality and incidence.
- These survivor data were then used in the chronic ischaemic and chronic haemorrhagic stroke models as incidence inputs.
- We then ran the chronic stroke models, using the survivor incidence data and excess mortality data. Non-subtype-specific prevalence data were split into ischaemic and haemorrhagic components using the ratio of 28-day survivors from the first stage acute models. We set a value prior of 0 on remission for all ages.

- Implausible or extreme outliers in input data were dropped from these estimation results.
- From these four models, we generated the proportions of deaths due to acute ischaemic, chronic ischaemic, acute haemorrhagic, and chronic haemorrhagic stroke, and split the post-CoDCorrect stroke deaths generated from the GBD mortality estimates into these four parts, by multiplying the location-, sex-, age- and year-specific CSMR results by the global proportions estimated from the DisMod models. Thus, the mortality rates due to acute ischaemic, chronic ischaemic, acute haemorrhagic, and chronic haemorrhagic stroke are driven by all available data on incidence, prevalence, and excess mortality data for stroke. These CSMR estimates were then uploaded into the non-fatal database and used as inputs for models in Step 2.

Step 2

- We re-ran the first-ever acute ischaemic and first-ever acute haemorrhagic models with CSMR as derived from CoDCorrect and epidemiologic data as described above. Twenty-eight-day survivorship was recalculated from these models and uploaded into the chronic ischaemic and chronic haemorrhagic stroke with CSMR models. These chronic models also use CSMR as derived from CoDCorrect and epidemiologic data as described above.
- Implausible or extreme outliers were dropped from these estimation results.

Models were evaluated based on expert opinion, comparison with previous iterations, and model fit.

Changes in the modelling of stroke for GBD 2016

Several changes were made to the modelling strategy for stroke for the GBD 2016 study. In GBD 2015 and prior, chronic stroke was modelled for both subtypes (ischaemic and haemorrhagic or other) together to estimate the total prevalence of chronic stroke. For the GBD 2016 study, each stroke subtype was modelled independently, resulting in separate acute and chronic stroke models for each subtype. This change was made in order to simplify the stroke modeling process and to ensure that both subtypes were estimated correctly. In the GBD2015 and prior studies, severity splits were based on estimates derived from standard GBD analysis of the U.S. Medical Expenditure Panel Survey. For the GBD2016 study, a review of studies reporting modified Rankin scores following stroke was performed and disability weights were applied using a model of modified Rankin level by age and sex as described above.

1) Hatano S. Experience from a mulicentre stroke register: a preliminary report. Bull WHO 54, 541-553. 1976.
2) GBD 2015 Disease and Injury Incidence and Prevalence Collaborators. Global, regional, and national incidence, prevalence, and years lived with disability for 310 diseases and injuries, 1990-2015: a systematic analysis for the Global Burden of Disease Study 2015. Lancet. 2016 Oct 8;388(10053):1545-1602. doi: 10.1016/S0140-6736(16)31678-6.
3) Burstein et al. Estimating distributions of health state severity for the global burden of disease study. Population Health Metrics (2015) 13:31
4) GBD 2015 Risk Factors Collaborators. Global, regional, and national comparative risk assessment of 79 behavioural, environmental and occupational, and metabolic risks or clusters of risks, 1990-2015: a systematic analysis for the Global Burden of Disease Study 2015. Lancet. 2016 Oct 8;388(10053):1659-1724. doi: 10.1016/S0140-6736(16)31679-8.

The tables below indicate the covariates used by cause in the estimation process, as well as the beta and exponentiated beta values.

Step 1:

Cause	Variable name	Measure	beta	Exponentiated beta
Chronic ischaemic stroke	Log-transformed SEV scalar: Isch Stroke	Prevalence	0.83 (0.75 — 1.03)	2.29 (2.12 — 2.80)
Chronic ischaemic stroke	LDI (I$ per capita)	Excess mortality rate	-0.16 (-0.29 — -0.1)	0.85 (0.75 — 0.90)
Chronic haemorrhagic stroke	Log-transformed SEV scalar: Hem Stroke	Prevalence	0.79 (0.75 — 0.92)	2.21 (2.12 — 2.50)
Chronic haemorrhagic stroke	LDI (I$ per capita)	Excess mortality rate	-0.12 (-0.16 — -0.1)	0.89 (0.85 — 0.90)
First ever acute haemorrhagic stroke	Hospital data	Incidence	0.54 (0.54 – 0.54)	1.71 (1.71 – 1.72)
First ever acute haemorrhagic stroke	Any stroke	Incidence	1.27 (1.27 – 1.28)	3.57 (3.56 – 3.59)
First ever acute haemorrhagic stroke	First-ever acute stroke, ischemic or hemorrhagic	Incidence	0.52 (0.52 – 0.53)	1.69 (1.68 – 1.71)
First ever acute haemorrhagic stroke	Log-transformed age-standardized SEV scalar: hemorrhagic stroke	Incidence	0.77 (0.75 – 0.82)	2.17 (2.12 – 2.27)
First ever acute haemorrhagic stroke	Any stroke	Excess mortality rate	-0.48 (-0.66 – -0.32)	0.62 (0.52 – 0.73)
First ever acute haemorrhagic stroke	First-ever acute stroke, ischemic or hemorrhagic	Excess mortality rate	-0.081 (-0.3 – 0.16)	0.62 (0.52 – 0.73)
First ever acute ischaemic stroke	Hospital data	Incidence	0.38 (0.37 – 0.38)	1.46 (1.45 – 1.46)
First ever acute ischaemic stroke	Any stroke	Incidence	0.31 (0.29 – 0.33)	1.37 (1.34 – 1.39)
First ever acute ischaemic stroke	First-ever acute stroke, ischemic or hemorrhagic	Incidence	0.37 (0.36 – 0.38)	1.44 (1.43 – 1.46)
First ever acute ischaemic stroke	Log-transformed age-standardized SEV scalar: ischemic stroke	Incidence	1.16 (1.09 – 1.22)	3.21 (2.99 – 3.39)

Step 2:

Cause	Variable name	Measure	beta	Exponentiated beta
Chronic ischemic stroke with CSMR	Log-transformed SEV scalar: Ischaemic stroke	Prevalence	0.89 (0.75 – 1.19)	2.44 (2.13 – 3.27)
Chronic ischemic stroke with CSMR	LDI (I$ per capita)	Excess mortality rate	-0.49 (-0.5 – -0.46)	0.61 (0.61 – 0.63)
Chronic haemorrhagic stroke with CSMR	Log-transformed SEV scalar: Haemorrhagic stroke	Prevalence	0.88 (0.75 – 1.15)	2.40 (2.13 – 3.17)
Chronic haemorrhagic stroke with CSMR	LDI (I$ per capita)	Excess mortality rate	-0.48 (-0.5 – -0.44)	0.62 (0.61 – 0.64)
First-ever acute haemorrhagic stroke with CSMR	Any stroke	Incidence	1.27 (1.27 – 1.29)	3.58 (3.56 – 3.62)
First-ever acute haemorrhagic stroke with CSMR	First-ever acute stroke, ischemic or hemorrhagic	Incidence	0.52 (0.52 – 0.54)	1.69 (1.68 – 1.71)
First-ever acute haemorrhagic stroke with CSMR	Log-transformed SEV scalar: Hem stroke	Incidence	1.11 (1.01 – 1.20)	3.03 (2.74 – 3.33)
First-ever acute haemorrhagic stroke with CSMR	Any stroke	Excess mortality rate	-0.37 (-0.49 – -0.27)	0.69 (0.62 – 0.77)
First-ever acute haemorrhagic stroke with CSMR	First-ever acute stroke, ischemic or hemorrhagic	Excess mortality rate	0.023 (-0.2 – 0.23)	1.02 (0.82 – 1.25)
First-ever acute ischaemic stroke with CSMR	Any stroke	Incidence	0.32 (0.30 – 0.33)	1.38 (1.35 – 1.39)
First-ever acute ischaemic stroke with CSMR	First-ever acute stroke, ischemic or hemorrhagic	Incidence	0.37 (0.36 – 0.38)	1.44 (1.43 – 1.46)
First-ever acute ischaemic stroke with CSMR	Log-transformed age-standardized SEV scalar: Ischemic stroke	Incidence	1.11 (1.05 – 1.18)	3.04 (2.86 – 3.26)
First-ever acute ischaemic stroke with CSMR	Any stroke	Excess mortality rate	-0.34 (-0.45 – -0.24)	0.71 (0.64 – 0.79)
First-ever acute ischaemic stroke with CSMR	First-ever acute stroke, ischemic or hemorrhagic	Excess mortality rate	-0.69 (-0.82 – -0.56)	0.50 (0.44 – 0.57)

Acute Myocarditis

Flowchart

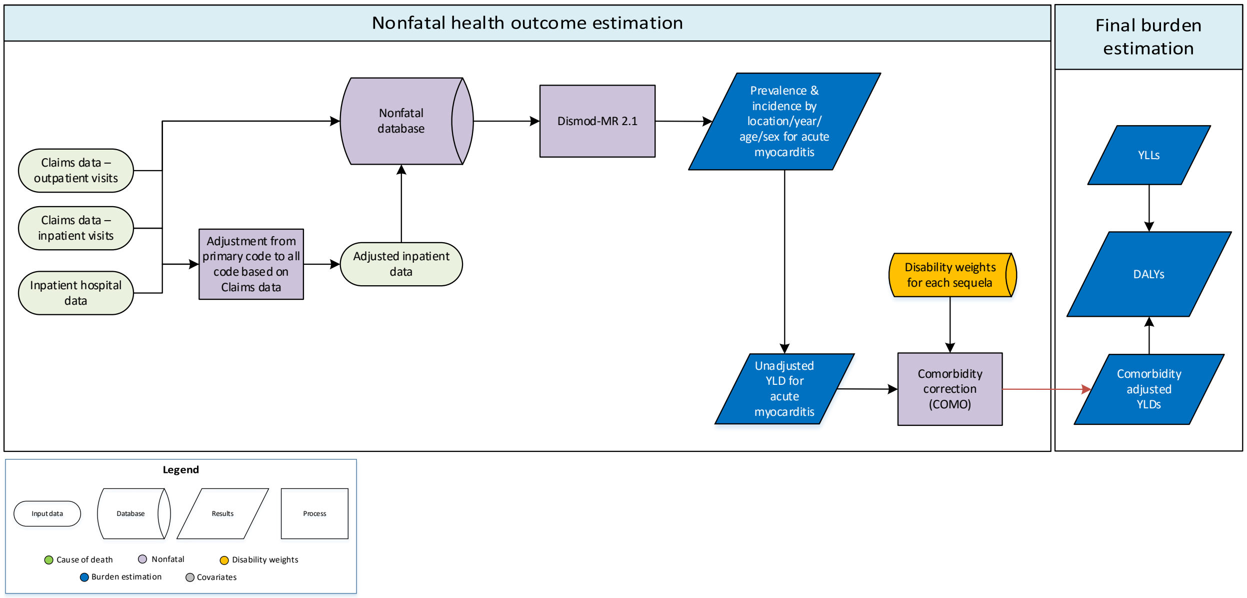

Input data and methodological summary

Case definition

Myocarditis refers to a heterogenous group of diseases with variable clinical and pathological features. Acute myocarditis was defined for GBD as the acute and time-limited symptoms of myocarditis separate from its chronic heart failure-related sequelae. Heart failure due to myocarditis is estimated separately in GBD (see methods for heart failure). Symptoms of acute myocarditis are nonspecific and include a flu-like or gastrointestinal syndrome, followed by anginal-type chest pain, arrhythmias, syncope, or heart failure.

A list of the ICD codes included can be found in Methods Appendix Table 4.

Input data

Model inputs

The preferred data sources for acute myocarditis was hospital admission data and other health facility data identifying cases of acute myocarditis.

A systematic review was performed for GBD 2013 and updated for GBD 2015. A systematic review was not performed for GBD 2016.

The GBD 2015 search terms included: (cardiomyopathy AND epidemiology[MeSH Subheading]) OR (myocarditis AND epidemiology[MeSH Subheading]) OR (cardiomyopathy AND (incidence OR prevalence OR “case fatality”)) OR (myocarditis AND (incidence OR prevalence OR “case fatality”))

- Dates included in search: 1/1/2013 – 3/16/2015
- Number of initial hits: 3,598
- Number of sources included: 0

The GBD 2013 search terms included: (hasabstract[text] AND Humans[Mesh] AND middle age[MeSH])) OR 21) AND ((cardiomyopathy/epidemiology[Mesh] OR cardiomyopathy/mortality[Mesh]) AND (prevalence[Title/Abstract] OR incidence[Title/Abstract]) AND ("2010"[Date - Publication] : "3000"[Date - Publication]) AND (hasabstract[text] AND Humans[Mesh] AND middle age[MeSH]))

We did not include any non-literature-based data, apart from the hospital and claims data described elsewhere. We used inpatient hospital data adjusted for readmission, primary to any diagnosis, and inpatient to outpatient utilization based on correction factors generated using US claims data. We excluded all outpatient data, as they were implausibly low when compared with inpatient data from the same locations and with claims data. Inpatient hospital data points which were more than 5-fold different from the mean value for High-income North America, Central Europe and Western Europe for that age-sex group were excluded.

We included study-level covariates for inpatient hospital data and claims data from 2000 and 2010 to adjust these data points, using as reference the claims data from 2012.

Severity splits and disability weights

Severity level	Lay description	DW (95% CI)
Acute myocarditis	Has a fever and aches, and feels weak, which causes some difficulty with daily activities.	0.051 (0.032–0.074)

Modelling strategy

For GBD 2016, we estimated myocarditis using a DisMod MR-2.1 Bayesian meta-regression model, setting a minimum of 3 and maximum of 5 as value priors on remission to establish an average duration of three months. We set a value prior of 0 for all ages on excess mortality. We included study-level covariates on incidence for claims data from 2000 and 2010. Country-level covariates used included the cardiomyopathy and myocarditis summary exposure variable (SEV) on incidence and lag distributed income (LDI) per capita (I$) on excess mortality.

The table below gives the parameters, betas, and exponentiated betas for study-level and country-level covariates used in the model

Study covariate	Parameter	beta	Exponentiated beta
LDI (I$ per capita)	Excess mortality rate	-0.3 (-0.49 – -0.11)	0.74 (0.61 – 0.90)
All MarketScan, year 2000	Incidence	0.0036 (-0.072 – 0.082)	1.00 (0.93 – 1.09)
All MarketScan, year 2010	Incidence	-0.046 (-0.12 – 0.020)	0.96 (0.89 – 1.02)
Log-transformed age-standardized SEV scalar: CMP	Incidence	0.89 (0.75 – 1.18)	2.44 (2.13 – 3.24)

No substantive changes were made to the modelling approach for GBD 2016.

Atrial Fibrillation and Flutter

Flowchart

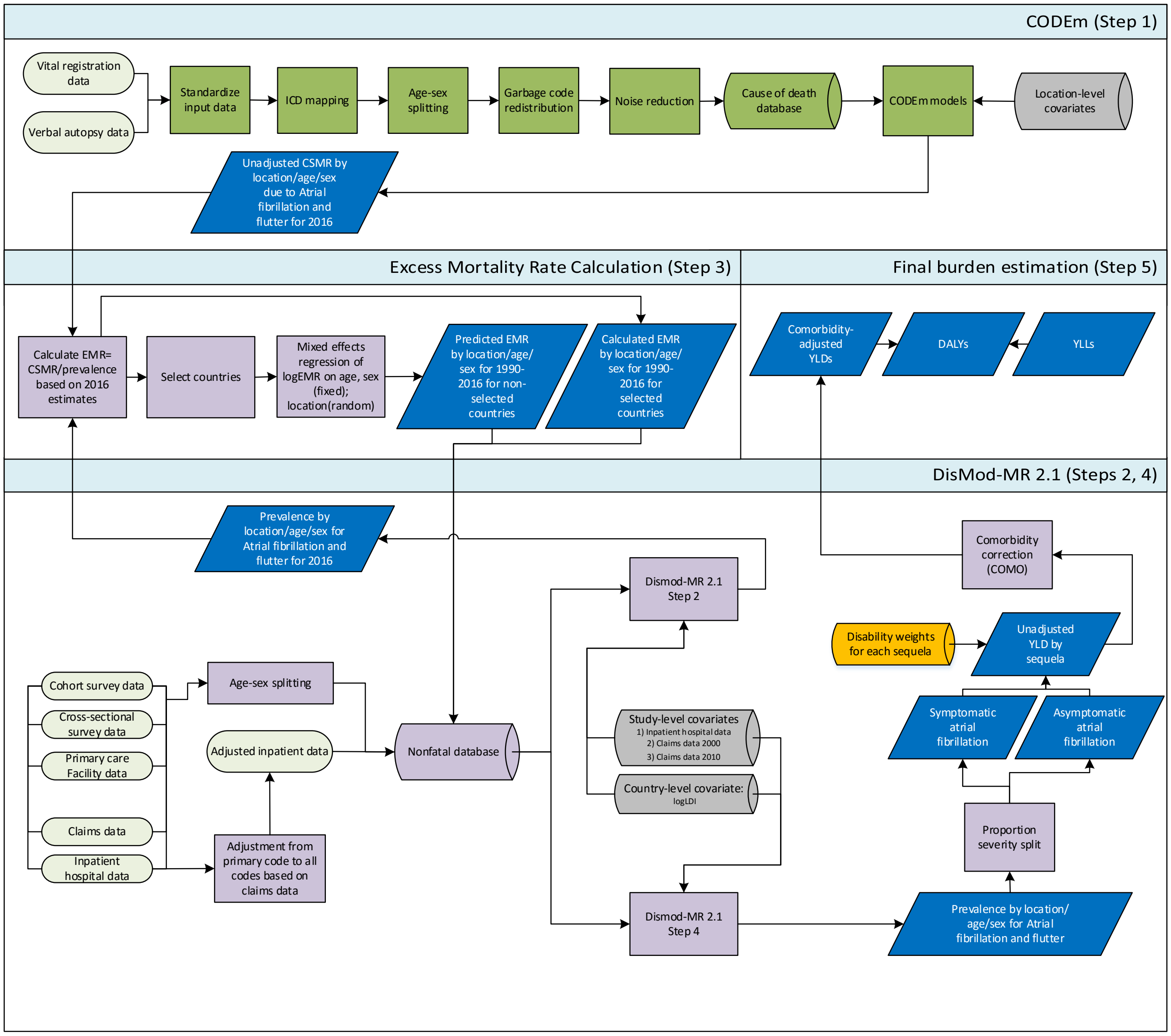

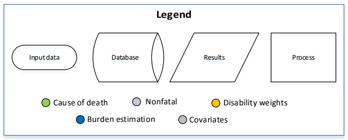

Input data and methodological summary

Case definition

Atrial fibrillation was defined as a diagnosis with atrial fibrillation or atrial flutter by ECG findings. ICD codes used for inclusion of hospital and claims data can be found in Methods Appendix Table 4.

Input data

Model inputs

We did not perform a systematic review for GBD 2016. A systematic review was performed for GBD 2015 with the following search terms: (“atrial fibrillation” AND epidemiology[MeSH Subheading]) OR (“atrial flutter” AND epidemiology[MeSH Subheading]) OR (“atrial fibrillation” AND (prevalence OR incidence OR “case fatality”)) OR (“atrial flutter” AND (prevalence OR incidence OR “case fatality”)) OR (“heart atrium fibrillation” AND epidemiology[MeSH Subheading]) OR (“heart atrium fibrillation” AND (prevalence OR incidence OR “case fatality”))

The dates of the search were 1/1/2013 – 3/15/2016. There were 5,630 studies returned and, of those, 27 were extracted.

A systematic review was also performed for GBD 2013, with the search terms: (hasabstract[text] AND Humans[Mesh] AND middle age[MeSH])) OR 21) AND ((atrial fibrillation/epidemiology[Mesh] OR atrial fibrillation/mortality[Mesh]) AND (prevalence[Title/Abstract] OR incidence[Title/Abstract]) AND ("2010"[Date - Publication] : "3000"[Date - Publication]) AND (hasabstract[text] AND Humans[Mesh] AND middle age[MeSH]))

The table below shows the data inputs:

	Prevalence	Incidence	Mortality risk
Studies	71	24	15
Countries/subnationals	42	17	12
GBD world regions	8	3	6

Apart from hospital and claims data points on prevalence, no non-literature-based data were included. We included hospital data corrected for readmission, primary to any diagnosis, and inpatient to outpatient utilization ratios using adjustment factors calculated from US claims data. We excluded hospital data in certain geographies (e.g. Philippines, China, India) where the data were implausibly low, as well as any year groupings with where the corrected prevalence value exceeded 0.3 for any age group. This threshold was based on the highest reported prevalence in the available literature. We excluded all outpatient administrative data as the values for all locations were implausibly low. We included study-level covariates to adjust the inpatient hospital data and the claims data from 2000 and 2010 within DisMod, using as reference literature data and the claims data from 2012.

Severity splits & disability weights

Atrial fibrillation is split into symptomatic and asymptomatic based on standard GBD proportion information. The table below includes lay descriptions and disability weights for the severity levels of atrial fibrillation:

Severity level	Lay description	DW (95% CI)
Asymptomatic	No symptoms	N/A
Symptomatic	Has periods of rapid and irregular heartbeats and occasional fainting	0.224 (0.151–0.312)

Modelling strategy

In order to address changes in coding practices for atrial fibrillation that resulted in an implausible trend of increasing death certificate-based mortality rates, we used a prevalence-based modeling approach that combined DisMod-MR and CODEm models to generate estimates for atrial fibrillation and flutter. This approach, first used in GBD 2015, allowed us to more generate more accurate estimates, using observed prevalence and incidence rates along with modeled excess mortality rates generated from prevalence and cause-specific mortality estimates.

- In Step 1, we estimated deaths for atrial fibrillation using a standard CODEm approach.
- In Step 2, we estimated prevalence rates in DisMod-MR using data from published reports of cross-sectional and cohort surveys and primary care facility data. We also used claims data covering inpatient and outpatient visits for the United States along with inpatient hospital data from 22 countries. As the inpatient hospital data only included information from the primary code for each visit, prevalence rates for these data were adjusted based on the age- and sex-specific proportions of atrial fibrillation in the primary codes versus secondary codes in the US claims data.
- In Step 3, we calculated the excess mortality rate (EMR) for 2016 (defined as the cause-specific mortality rate (CSMR) estimated from CODEm divided by the prevalence rate from DisMod-MR). We then selected 27 countries based on four conditions: 1) ranking of 4 or 5 stars on the newly developed system for assessing the quality of VR data; 2) prevalence data available from the literature was included in the DisMod-MR 2.1 estimation; 3) prevalence rate ≥ 0.005; and, 4) CSMR ≥ 0.00002. These conditions were set to ensure that the VR data were of sufficient quality to generate reasonable estimates, that prevalence estimates for that location were informed directly by representative data for that population, and that cause-specific mortality and prevalence estimates were high enough to ensure reasonable results from the regression. Using information from these countries as input data, we ran a linear mixed-effects regression of logEMR on sex, age, and location. Sex and age were treated as fixed effects for the regression, while location was considered a random effect. We then predicted age- and sex-specific EMR using the results of this regression for all non-selected countries. Countries included in the regression were assigned their directly calculated values. These EMR data points were assigned to the time period 1990–2016 and uploaded into the Nonfatal database.
- In Step 4, we re-ran DisMod-MR including the EMR estimated in Step 3 and using log-transformed lagged distributed income (LDI) as a country-level covariate. Based on information from other regressions, we set the bounds at -1.5 to -0.25. We also included study-level covariates to cross-walk the inpatient hospital and claims data from 2000 and 2010 to the reference data, which included literature data and claims data from 2012. We included a value prior of 0 for remission for all ages. We also set a value prior of 0 for excess mortality for ages 0-30.

The prevalence from the DisMod-MR model in Step 4 was used as the finalized output for upload to COMO and further processing into YLDs and DALYs. Models were evaluated based on expert opinion, comparison with results from previous rounds of GBD, and model fit.

The tables below includes the study covariates, parameters, betas, and exponentiated betas.

DisMod Covariates – Step 2

Study covariate	Parameter	Beta	Exponentiated beta
Hospital data	Prevalence	-0.000086 (-0.19 – 0.097)	1.0 (0.82 – 1.10)
All MarketScan, year 2000	Prevalence	-0.47 (-0.5 – -0.44)	0.63 (0.61 – 0.64)
All MarketScan, year 2010	Prevalence	-0.003 (-0.024 – -0.014)	1.0 (0.98 – 1.01)
Log-transformed age-standardized SEV scalar: A Fib	Prevalence	0.75 (0.75 – 0.75)	2.12 (2.12 – 2.12)
LDI (I$ per capita)	Excess mortality rate	-0.48 (-0.5 – -0.43)	0.62 (0.61 – 0.65)

DisMod Covariates – Step 4

Study covariate	Parameter	Beta	Exponentiated beta
All MarketScan, year 2000	Prevalence	-0.46 (-0.49 – -0.43)	0.63 (0.62 – 0.65)
All MarketScan, year 2010	Prevalence	-0.0021 (-0.025 – -0.021)	1.0 (0.98 – 1.02)
Log-transformed age-standardized SEV scalar: A Fib	Prevalence	0.75 (0.75 – 0.75)	2.12 (2.12 – 2.12)
LDI (I$ per capita)	Excess mortality rate	-0.1 (-0.1 – -0.1)	0.9 (0.9 – 0.9)

No substantative changes were made to the modelling strategy for GBD 2016.

Peripheral Arterial Disease

Flowchart

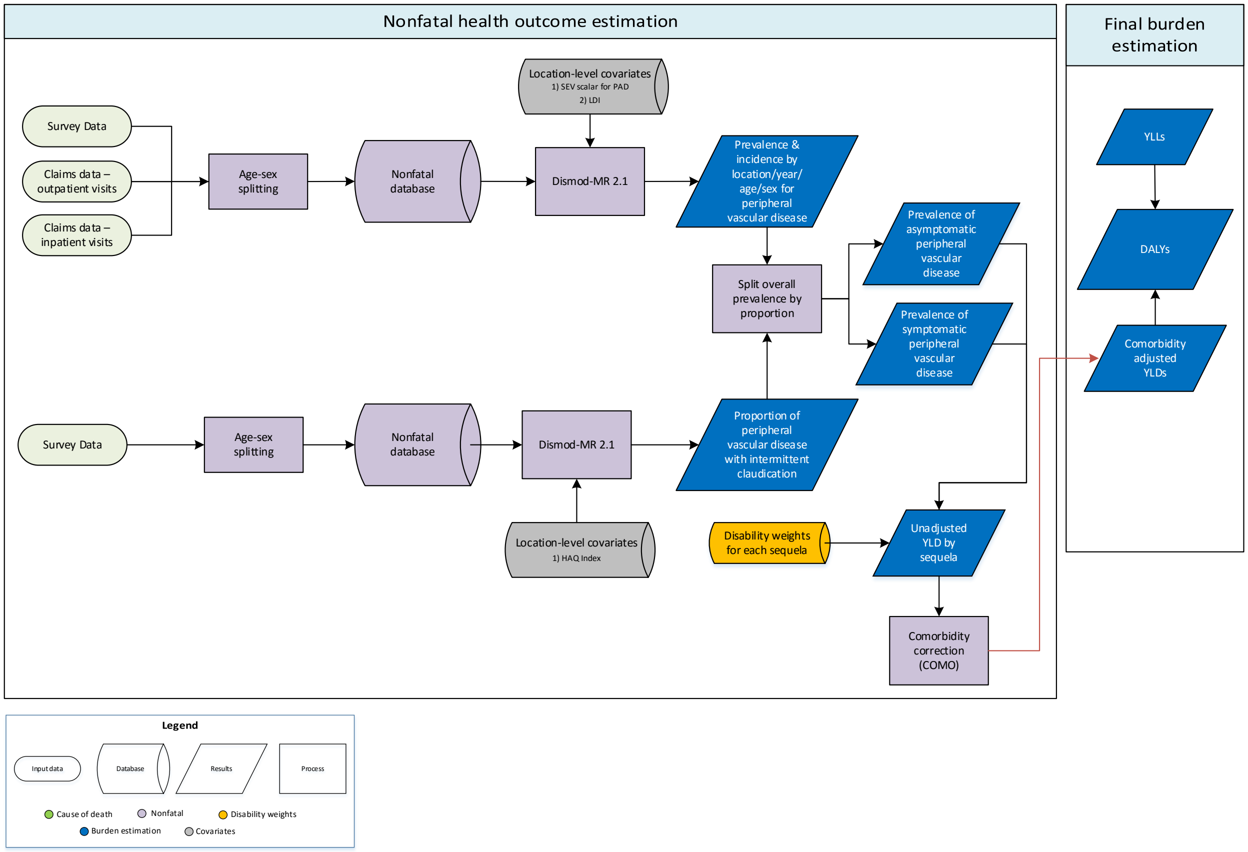

Input data and methodological appendix

Case definition

For GBD 2016, peripheral arterial disease was defined as having an ankle-brachial index (ABI) <0.9. Intermittent claudication was defined clinically.

Specific ICD codes for claims data included can be found in Methods Appendix Table 4.

Input data

Model inputs

We did not perform a systematic review for GBD 2016.

A systematic review was performed for peripheral arterial disease and intermittent claudication for GBD 2015. The search terms were: ('peripheral vascular disease'[TIAB] AND 'epidemiology'[Subheading]) OR ('peripheral arterial disease'[TIAB] AND 'epidemiology'[Subheading]) OR ('peripheral artery disease'[TIAB] AND 'epidemiology'[Subheading]) OR ('intermittent claudication'[TIAB] AND 'epidemiology'[Subheading]) OR ('ankle-brachial index'[TIAB] AND 'epidemiology'[Subheading]) OR ('ankle brachial index'[TIAB] AND

'epidemiology'[Subheading]) OR ('peripheral artery occlusive disease'[TIAB] AND 'epidemiology'[Subheading]) OR ('peripheral obliterative arteriopathy'[TIAB] AND 'epidemiology'[Subheading]) OR ('peripheral vascular disease'[TIAB] AND 'prevalence'[MeSH Terms]) OR ('peripheral vascular disease'[TIAB] AND 'incidence'[MeSH Terms]) OR ('peripheral vascular disease'[TIAB] AND 'case fatality'[All Fields]) OR ('symptomatic claudication'[TIAB] AND (proportion[All Fields] OR percent[All Fields]))

The search was conducted from 1/1/13 to 3/16/2015. 1,658 results were returned, of which six were extracted.

A systematic review was also performed for peripheral arterial disease and intermittent claudication for GBD 2013. Search terms can be provided upon request.

The table below shows the number of literature studies included in GBD 2015, as well as the number of countries or subnational units and GBD world regions represented.

Peripheral arterial disease

	Prevalence	Incidence	Mortality risk
Studies	16	2	1
Countries/subnationals	11	2	1
GBD world regions	7	2	1

Proportion with intermittent claudication

	Proportion
Studies	9
Countries/subnationals	4
GBD world regions	3

Apart from the claims data, we did not include any non-literature-based data types. We did not use inpatient hospital data, as peripheral arterial disease is expected to be rare in inpatient data, but common in outpatient data as it is a condition usually managed on an outpatient basis, except for specific surgical interventions. This discrepancy leads to implausible correction factors based on inpatient/outpatient information from claims data (~150X); thus adjusted data cannot be used. Including uncorrected data in the model is likely to lead to incorrect estimates as hospitalization and procedure rates are likely to vary between geographies based on access to and patterns of care.

We corrected US claims data outside of DisMod, using as reference literature reports of prevalence. Using as a model the adjustment factors developed to translate tobacco consumption prevalence to tobacco consumption frequency, we matched administrative claims data to population-based literature data based on age group, sex, and super region (1). For the adjustment factor, we developed the following generalized additive model on matched data

$$\ln(\mu_{ref,i}) = \beta_0 + \beta_1 \ln(\mu_{b,i}) + s(age_i) + \varepsilon_i$$

Where i represents a given matched observation, age signifies the data point's age group, $s(x)$ represents a penalized spline where the smoothing parameter is chosen through cross validation and μ_{ref} and μ_b denote the mean of the data point from literature and the mean of the claims data point,

respectively. Predictions from the model were then taken as the adjusted data points. The standard error of each corrected data point was adjusted to account for the uncertainty due to the correction.

Outpatient data were not included as the rates were implausibly low for all locations.

Severity splits and disability weights

We used the proportion of intermittent claudication to split the overall prevalence of peripheral arterial disease into symptomatic and asymptomatic peripheral vascular disease. The table below illustrates these values:

Severity level	Lay description	DW (95% CI)
Asymptomatic	No symptoms	No DW assigned
Symptomatic	Has cramping pains in the legs after walking a medium distance. The pain goes away after a short rest.	0.014 (0.007–0.025)

Modelling strategy

For GBD 2016, we used DisMod MR 2.1 to model the overall prevalence of peripheral arterial disease using prevalence data from literature studies and claims data. Claims data were adjusted outside of DisMod using the ratio of prevalence reported in literature studies to the prevalence figures in the claims data by age and sex. We also included the log-transformed, age-standardized SEV scalar for PAD and log-transformed LDI as fixed-effect, country-level covariates. We set value priors of 0 for incidence from ages 0 to 30. We also set a value prior of 0 for remission for all ages. Finally, we set a value prior of a maximum value of 0.25 on excess mortality for all ages.

The table below illustrates the study covariates, parameters, beta, and exponentiated beta values for the overall peripheral vascular disease model.

Covariate	Parameter	Beta	Exponentiated beta
Log-transformed age-standardized SEV scalar: PVD	Prevalence	1.22 (1.17 – 1.25)	3.40 (3.21 – 3.49)
LDI (I$ per capita)	Excess mortality rate	-0.30 (-0.50 – -0.1)	0.74 (0.61 – 0.90)

We used DisMod MR-2.1 to model the proportion of peripheral vascular disease with intermittent claudication. We set a value prior of 0 for proportion for ages 0 to 40.

Covariate	Parameter	Beta	Exponentiated beta
Healthcare access and quality index	Proportion	-0.013 (-0.04 – -0.00034)	0.99 (0.96 – 1.00)

To obtain final estimates for the sequelae of interest, we multiplied the prevalence model by the proportion model at the draw level to generate the prevalence of symptomatic and asymptomatic peripheral vascular disease.

Models were evaluated based on expert review, comparisons with estimates from prior rounds of GBD, and assessing model fit.

We corrected an error in the mapping of ICD-coded data for peripheral arterial disease – in previous iterations of GBD, unspecified atherosclerosis was assigned to peripheral arterial disease. As this code likely represents a mix of central and peripheral atherosclerosis, we excluded these data to ensure specificity of diagnosis. Other this correction, there have been no substantive changes from GBD 2015 in terms of modelling strategy for peripheral arterial disease.

1. Reitsma, Marissa B., et al. "Smoking prevalence and attributable disease burden in 195 countries and territories, 1990–2015: a systematic analysis from the Global Burden of Disease Study 2015." The Lancet.

Acute Endocarditis

Flowchart

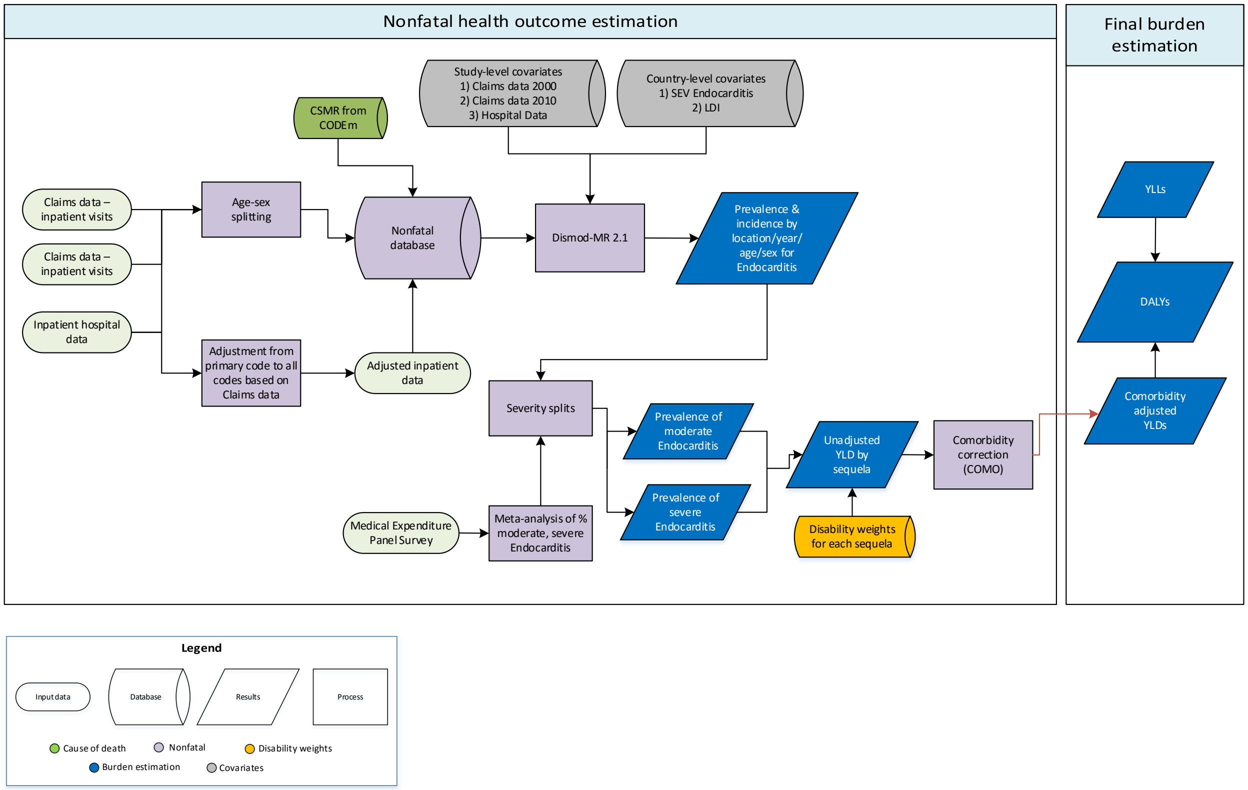

Input data and methodological appendix

Case definition

Our case definition for acute endocarditis was a clinical diagnosis of infective endocarditis. The ICD codes included can be found in Methods Appendix Table 4.

Input data

Model inputs

A systematic review was performed for GBD 2013 and updated for GBD 2015. We did not perform a systematic review for GBD 2016. The following search terms were used: ((‘endocarditis’[MeSH Terms] OR ‘endocarditis’[All Fields]) AND ‘epidemiology’[Subheading]) OR ((‘endocarditis’[MeSH Terms] OR ‘endocarditis’[All Fields]) AND ((‘epidemiology’[Subheading] OR ‘epidemiology’[All Fields] OR ‘incidence’[All Fields] OR ‘incidence’[MeSH Terms]) OR (‘epidemiology’[Subheading] OR ‘epidemiology’[All Fields] OR ‘prevalence’[All Fields] OR ‘prevalence’[MeSH Terms]) OR ‘case fatality’[All Fields])) OR ((‘endocardium’[MeSH Terms] OR ‘endocardium’[All Fields]) AND inflammation[TIAB] AND ‘epidemiology’[Subheading]) OR ((‘endocardium’[MeSH Terms] OR ‘endocardium’[All Fields]) AND inflammation[TIAB] AND ((‘epidemiology’[Subheading] OR ‘epidemiology’[All Fields] OR ‘incidence’[All

Fields] OR 'incidence'[MeSH Terms]) OR ('epidemiology'[Subheading] OR 'epidemiology'[All Fields] OR 'prevalence'[All Fields] OR 'prevalence'[MeSH Terms]) OR 'case fatality'[All Fields]))

- Dates included in search: 1/1/2013 – 3/16/2015
- Number of initial hits: 1,246
- Number of sources included: 6

The table below shows the number of literature studies included in GBD 2016, as well as the number of countries or subnational units and GBD world regions represented.

	Prevalence	Incidence	Mortality risk
Studies	0	14	1
Countries/subnationals	0	7	1
GBD world regions	0	3	1

We did not include any non-literature-based data types, apart from the hospital and claims data described elsewhere. We excluded all outpatient data, as they were implausibly low when compared with inpatient data from the same locations and claims data. We used hospital data corrected for readmission and primary to any diagnosis based on the correction factors generated using US claims data. We excluded any inpatient hospital data points which were more than 5-fold different from the mean value for High-income North America, Central Europe and Western Europe for that age-sex group.

We included study-level covariates for inpatient hospital data and claims data from 2000 and 2010 to adjust these data points, using as reference the data obtained from literature and claims data from 2012.

Severity split inputs

We used the standard GBD approach, which utilizes MEPS data to split overall estimates of endocarditis into moderate and severe categories. The table below includes the severity level, lay descriptions, and DWs associated with acute endocarditis.

Severity level	Lay description	DW (95% CI)
Moderate	Has a fever and aches, and feels weak, which causes some difficulty with daily activities.	0.051 (0.032–0.074)
Severe	Has a high fever and pain, and feels very weak, which causes great difficulty with daily activities.	0.133 (0.088–0.19)

Modelling strategy

For GBD 2016, we estimated endocarditis using a DisMod MR-2.1 Bayesian meta-regression model, setting a minimum of 11 and maximum of 13 as value priors on remission to establish an average duration of one month. We included study-level covariates on incidence for inpatient hospital data and claims data from 2000 and 2010. Country-level covariates used included the endocarditis summary exposure variable (SEV) on incidence and lag distributed income (LDI) per capita (I$) on excess mortality.

We evaluated models by comparing model fits with the data and with results from previous GBD estimation cycles. In prior iterations of GBD, we used modelled data for remission; based on information from the literature and expert review, we have revised this to use an average duration of one month for all locations. For GBD 2015, we had restricted claims and inpatient hospital data to primary diagnosis position only; as mentioned above, we are now using data with a diagnosis of endocarditis in any position.

The table below gives the parameters, betas, and exponentiated betas for study-level and country-level covariates used in the model

Covariate	Parameter	Beta	Exponentiated beta
Hospital data	Incidence	0.70 (0.66 – 0.72)	2.01 (1.93 – 2.06)
Inpatient-only Marketscan, year 2000	Incidence	-0.34 (-0.39 – -0.29)	0.71 (0.68 – 0.75)
Inpatient-only Marketscan, year 2010	Incidence	-0.18 (-0.22 – -0.13)	0.84 (0.80 – 0.88)
LDI (I$per capita)	Excess mortality rate	-0.14 (-0.16 – -0.13)	0.87 (0.85 – 0.88)
Log-transformed age-standardized SEV scalar: endocarditis	Incidence	0.89 (0.75 – 0.86)	2.20 (2.12 – 2.37)

No other significant changes were made to the modelling strategy from GBD 2015.

Other cardiovascular disease

Flowchart

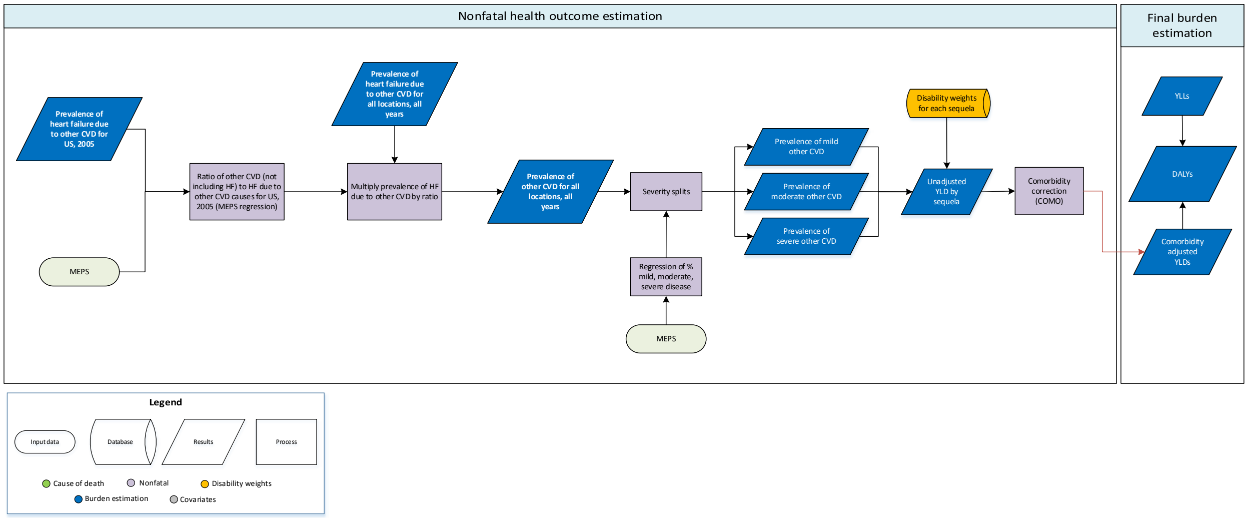

Case definition

Other cardiovascular disease is a residual category resulting from the GBD approach of estimating the total burden of all causes. Prevalence estimates are produced in order to provide YLDs consistent with the estimated YLLs from the death modeling process and to enable the calculation of DALYs.

Conditions included in this cause, based on ICD codes used for both fatal and nonfatal modeling, are: Other diseases of pulmonary vessels, Acute pericarditis, Other diseases of pericardium, Pericarditis in diseases classified elsewhere, Nonrheumatic mitral valve disorders, Nonrheumatic aortic valve disorders, Nonrheumatic tricuspid valve disorders, Nonrheumatic pulmonary valve disorders, Paroxysmal tachycardia, Cardiac septal defect, acquired, Rupture of chordae tendineae, not elsewhere classified, Rupture of papillary muscle, not elsewhere classified, Intracardiac thrombosis, not elsewhere classified, Cerebral amyloid angiopathy, Other aneurysm, Other disorders of arteries and arterioles, Diseases of capillaries, Disorders of arteries, arterioles and capillaries in diseases classified elsewhere, Phlebitis and thrombophlebitis, Portal vein thrombosis, Other venous embolism and thrombosis, Varicose veins of lower extremities, Varicose veins of other sites, Other disorders of veins, Nonspecific lymphadenitis, Other noninfective disorders of lymphatic vessels and lymph nodes, Other disorders of circulatory system in diseases classified elsewhere

Input data

As this is a residual category, we used data from the Medical Expenditure Panel Survey and modeled estimates from heart failure due to other cardiovascular disease to estimate prevalence of other cardiovascular disease.

Severity split inputs

The table below includes lay descriptions and disability weights for the severity levels of other cardiovascular disease for GBD 2016.

Severity level	Lay description	DW (95% CI)
Mild	Is short of breath and easily tires with moderate physical activity, such as walking uphill or more than a quarter-mile on level ground. The person feels comfortable at rest or during activities requiring less effort.	0.041 (0.026–0.062)
Moderate	Is short of breath and easily tires with minimal physical activity, such as walking only a short distance. The person feels comfortable at rest but avoids moderate activity.	0.072 (0.047–0.103)
Severe	Is short of breath and feels tired when at rest. The person avoids any physical activity, for fear of worsening the breathing problems.	0.179 (0.122–0.251)

Modelling strategy

To obtain prevalence estimates of other cardiovascular disease, we used MEPS data combined with prevalence estimates of heart failure due to other CVD for the US in 2005 to estimate the ratio of the prevalence of HF due to other CVD causes to to the prevalence of other CVD causes. We then applied this ratio to the age-, sex-, and year-specific prevalence estimates for heart failure due to other CVD causes for all locations to generate prevalence estimates of other cardiovascular disease.

No significant changes were made to the modelling strategy for GBD 2016.

Hemoglobinopathies and Hemolytic Anaemias

Sickle cell disorders, thalassemias, glucose-6-phosphate dehydrogenase (G6PD) deficiency, sickle cell trait, thalassemia trait, hemizygous G6PD deficiency, other hemoglobinopathies and hemolytic anaemias

Flowchart

Hemoglobinopathies and hemolytic anemias: Sickle cell disorders, Thalassemias, Glucose-6-phosphate dehydrogenase (G6PD) deficiency, Sickle cell trait, Thalassemia trait, Hemizygous G6PD deficiency, Other hemoglobinopathies and hemolytic anemias

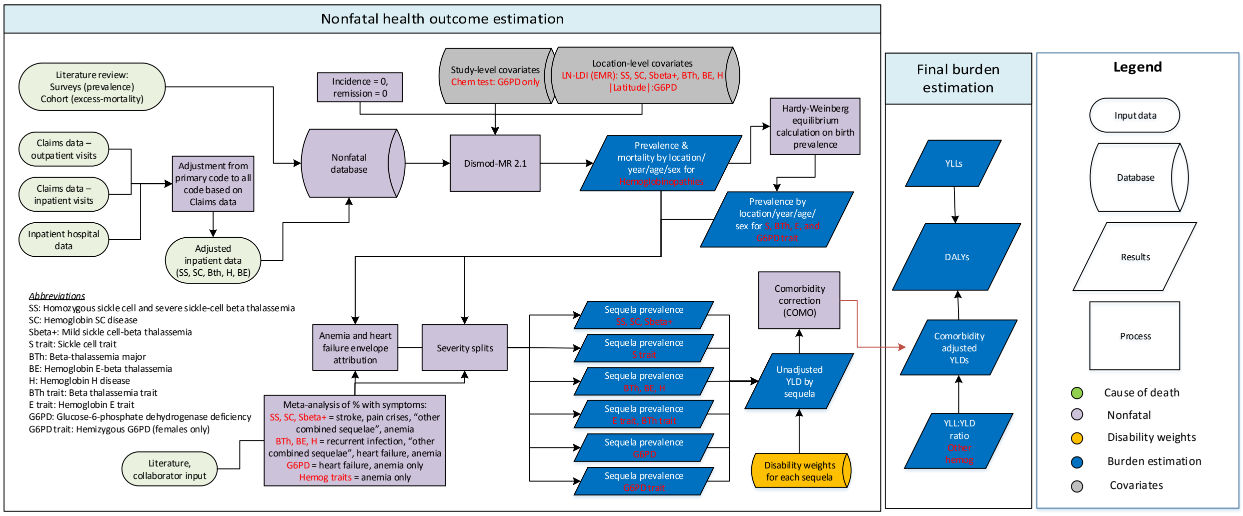

Input data and methodological summary

Case definition

Hemoglobinopathies and hemolytic anaemias span four GBD causes: thalassemias, sickle cell disorders, glucose-6-phosphate dehydrogenase (G6PD) deficiency, and other hemoglobinopathies. ICD-9 and ICD-10 codes for each are contained in Table 1. Within each category, several unique combinations of genetic mutations lead to distinct phenotypes with different natural history, which has led us to estimate several distinct subtypes of thalassemias and sickle cell disorders. The three thalassemia models included 1) beta-thalassemia major, 2) hemoglobin E/beta-thalassemia, and 3) hemoglobin H disease. Sickle cell models included 1) homozygous sickle cell and severe sickle cell/beta-thalassemia, 2) hemoglobin SC disease, and 3) "mild" sickle cell-beta thalassemia. We also estimated the burden of glucose-6-phosphate dehydrogenase (G6PD) deficiency. Finally, we estimated prevalence and YLD due to other hemoglobinopathies and hemolytic anemias assuming the YLD-to-YLL ratio for each age, sex, location, and year was similar to that of the aggregate of sickle cell, thalassemias, and G6PD deficiency. This approach was used in multiple causes across GBD 2015.

TABLE 1. International classification of diseases codes for hemoglobinopathies and hemolytic anaemias in GBD 2015 cause of death analysis		
Condition	ICD-10 code	ICD-9 code
Total	D55-D59	282.0-282.1, 282.7-285.8, 282.2-282.3, 282.5-282.6, 282.4
Thalassemias	D56	282.4
Sickle cell disorders	D57	282.5-282.6
G6PD deficiency	D55	282.2-282.3
Other hemoglobinopathies and hemolytic anemias	D58-D64.8	282.0-282.1, 282.7-285.8

Input data

Model inputs

Systematic literature reviews were completed for GBD 2010 and GBD 2013. These were updated on May 1, 2015, using the following search strings:

(thalassemias[Title/Abstract] AND (prevalence[Title/Abstract] OR survival[Title/Abstract] OR mortality[Title/Abstract]) AND ("2008"[PDAT] : "2013"[PDAT])) AND "humans"[MeSH Terms]

(sickle cell[Title/Abstract] AND (mortality[Title/Abstract] OR survival[Title/Abstract] OR prevalence[Title/Abstract]) AND ("2008"[PDAT] : "2013"[PDAT])) AND "humans"[MeSH Terms]

(G6PD[Title/Abstract] OR G6PD deficiency[Title/Abstract] OR glucose-6 phosphate dehydrogenase[Title/Abstract] OR glucose-6-phosphate dehydrogenase deficiency[Title/Abstract] AND (survival[Title/Abstract] OR mortality[Title/Abstract]) AND ("2008"[PDAT] : "2013"[PDAT])) AND "humans"[MeSH Terms]

Of note, upon the recommendation from multiple GBD collaborators, we identified and re-extracted all primary data that had been used in GBD 2013. In some situations, this process identified cases of sickle cell or thalassemia that had been assigned to incorrect subtypes, but mostly the data were correct and verified. We excluded any data where the results presented in a study were themselves the result of modelling exercises. The most significant change as a result of re-extraction was when we identified that much of the literature data used in GBD 2013 from females with G6PD deficiency actually did not correspond to our case definition of homozygous disease, but rather included combined case counts for homozygotes and hemizygotes. We only included homozygous disease in our datasets for GBD 2015, which has led to much lower estimates of G6PD deficiency in females.

We extracted prevalence data from population-level and community surveys as well as with-condition mortality and excess-mortality data from cohort studies. Age-specific survival proportions were converted to with-condition mortality rates as needed. We also included data from hospital and claims data for a subset of hemoglobinopathy models, including beta-thalassemia major, hemoglobin E/beta-thalassemia, homozygous sickle cell and severe sickle cell/beta-thalassemia, hemoglobin SC disease, and mild sickle cell/beta-thalassemia. The extraction and processing of hospital and claims data is described separately. Composition of final datasets are shown below for each of the different hemoglobinopathies and hemolytic anaemias models.

Data availability

Homozygous sickle cell and severe sickle cell/beta-thalassemia (2097):

	Prevalence	Mortality risk
Studies	110	25
Countries/subnationals	83/44	11/15
GBD world regions	13	8

Hemoglobin SC disease (2100):

	Prevalence	Mortality risk
Studies	63	12
Countries/subnationals	42/21	4/7
GBD world regions	12	4

Mild sickle cell/beta-thalassemia (2103):

	Prevalence	Mortality risk
Studies	20	9
Countries/subnationals	57/7	22/9
GBD world regions	13	3

Beta-thalassemia major (2085):

	Prevalence	Mortality risk
Studies	27	6
Countries/subnationals	86/33	6/1
GBD world regions	18	3

Hemoglobin E/beta-thalassemia (2087):

	Prevalence	Mortality risk
Studies	7	1
Countries/subnationals	23/20	1/1
GBD world regions	3	1

Hemoglobin H disease (2089):

	Prevalence
Studies	11
Countries/subnationals	19/23
GBD world regions	9

G6PD deficiency (2112):

	Prevalence

Studies	278
Countries/subnationals	89/39
GBD world regions	18

Modelling strategy

Besides data re-extraction and addition of hospital and claims data, we have made no substantive changes to the estimation strategy since 2013. We estimated the nonfatal burden of hemoglobinopathies in three parts.

First, we used the datasets described above to estimate prevalence for each age-sex-location-year in the GBD 2015 location hierarchy using DisMod-MR 2.1. For mild sickle cell/beta-thalassemia models, study-level covariates were used to identify and crosswalk those data from Marketscan in the year 2000, which were systematically lower than later years, to the years 2010 and 2012. The magnitude of prevalence in later years of claims data for this model, and in all years for other models, was similar to that of literature data from the same locations, so they were considered equivalent and no additional crosswalks were performed. In all sickle cell models and beta-thalassemia major, the natural log of lag-distributed income per capita (LN-LDI) was used as a sole country covariate on excess mortality, meant to reflect the profound impact that health system financial resources can have on survival from these conditions. For G6PD deficiency, data where diagnosis was made only on the basis of chemical or reagent testing was crosswalked to the reference definition of genetic G6PD deficiency; absolute value of latitude was the sole country covariate for this model.

Second, we calculated prevalence of hemoglobinopathy traits (sickle cell trait, hemoglobin E trait, hemoglobin beta trait, G6PD trait) by back-calculating from birth prevalence estimates from corresponding DisMod-MR 2.1 models, assuming Hardy-Weinberg equilibrium, and no excess mortality. Third, age-specific prevalence for all subtypes of hemoglobinopathies were paired with estimated sequelae distributions from a series of cohort studies and clinical data in GBD 2010 and GBD 2013. This included consideration of the burden of anaemia associated with homozygous and heterozygous persons and ensuring the estimates of hemoglobinopathy-induced anaemia were internally consistent with overall estimates for each condition, including prevention of double counting. The anaemia estimation process is described separately.

Third, and finally, we found the ratio of YLD to YLL ratio for all hemoglobinopathies and then applied it to YLLs estimated for other hemoglobinopathies and hemolytic anaemias in our cause-specific mortality analysis. Quantitative crosswalk results for each model are shown below.

Covariate, parameter, beta, and exponentiated beta values

Homozygous sickle cell and severe sickle cell/beta-thalassemia (2097):

Study-level covariate	Parameter	Location level	beta	Exponentiated beta
LDI (I$ per capita)	Excess mortality rate	Global	-0.14	0.87<br>(0.86 - 0.88)

Hemoglobin SC disease (2100):

Study-level covariate	Parameter	Location level	beta	Exponentiated beta
LDI (I$ per capita)	Excess mortality rate	Global	-0.03	0.97<br>(0.95 – 1.00)

Mild sickle cell/beta-thalassemia (2103):

Study-level covariate	Parameter	Location level	beta	Exponentiated beta
LDI (I$ per capita)	Excess mortality rate	Global	-0.19	0.83<br>(0.74 - 0.98)
All MarketScan, year 2000	Prevalence	Global	-0.48	.6199<br>(0.54 - 0.71)

Beta-thalassemia major (2085):

Study-level covariate	Parameter	Location level	beta	Exponentiated beta
LDI (I$ per capita)	Excess mortality rate	Global	-0.58	0.56<br>(0.55 - 0.58)

Hemoglobin E/beta-thalassemia (2087):

Study-level covariate	Parameter	Location level	beta	Exponentiated beta
Year	Prevalence	Global	0.02<br>(0.00 - 0.02)	1.02<br>(1.00 - 1.02)

Hemoglobin H disease (2089):

Study-level covariate	Parameter	Location level	beta	Exponentiated beta
Year	Prevalence	Global	-0.01	0.99<br>(0.98 - 1.02)

G6PD deficiency (2112):

Study-level covariate	Parameter	Location level	beta	Exponentiated beta
Absolute value of average latitude	Prevalence	Super region	-0.01	0.99<br>(0.98 - 1.00)

Diagnostic modality based on chemical/ reagent testing	Prevalence	Super region	0.322	1.38 (1.16 - 1.69)
Year	Prevalence	Super region	-0.01	0.99 (0.98 - 0.99)

Sequelae

With the exception of anaemia, only homozygous individuals were considered to experience disability. Estimated sequelae of thalassemias included anaemia (described separately), heart failure (described separately), and periodic severe infection. Another series of common, but not universal, sequelae also occur in those with thalassemias, including splenomegaly, skeletal deformity, delayed growth/puberty, diabetes, hypothyroidism, and leg ulcers. Given sparse data on the occurrence of these sequelae, they were approximated with a health state named "other combined sequelae of thalassemia," for which we used the disability weight corresponding to a health state of "generic uncomplicated disease, anxiety about diagnosis and daily medication" which, of note, was also used to approximate the disability for those with cancer in remission. For sickle cell disorders, we similarly estimated YLDs for anaemia (described separately), stroke, and pain crises separately and approximated the myriad additional complications of sickle cell disease with the health state "other combined sequelae of sickle cell disease." The only sequelae estimated for G6PD deficiency were anaemia (described separately) and heart failure (described separately). Notably, however, G6PD deficiency is considered to be asymptomatic for a vast majority of those with the condition, with only a very small subset of around 1 in 1,000,000 having chronic hemolysis (Class I disease) and approximately 1% having periodic hemolytic episodes (Class II disease) with exposure to environmental, pharmaceutical, or food products. Females heterozygous for G6PD deficiency exhibit chimerism, as one X chromosome becomes dominant in each of the red blood cells, so we estimated half as many heterozygous females will be symptomatic as homozygous females.

Uncertainty and model selection

For all hemoglobinopathies estimates, uncertainty bounds include uncertainty due to input data, crosswalks from non-reference definitions in study covariates above, uncertainty in numerical solutions (posteriors) of each DisMod-MR 2.1 model, and proportion of all persons with each type of symptom.

In consultation with GBD researchers and collaborators, final models were selected on a combination of qualitative and quantitative goodness of fit to input data, plausibility of geographic and temporal trends, consistency of age pattern, and, when available, comparison with other published studies on hemoglobinopathy epidemiology. Directionality, magnitude, and plausibility of study-level and country-level covariates was also considered in the process of model development. Of note, due to the nature of statistical modelling, final results do not always cover the values reported in input data.

No other significant changes were made to the GBD 2015 modelling strategy.

Chronic Obstructive Pulmonary Disease (COPD)

Flowchart

Chronic Obstructive Pulmonary Disease (COPD)

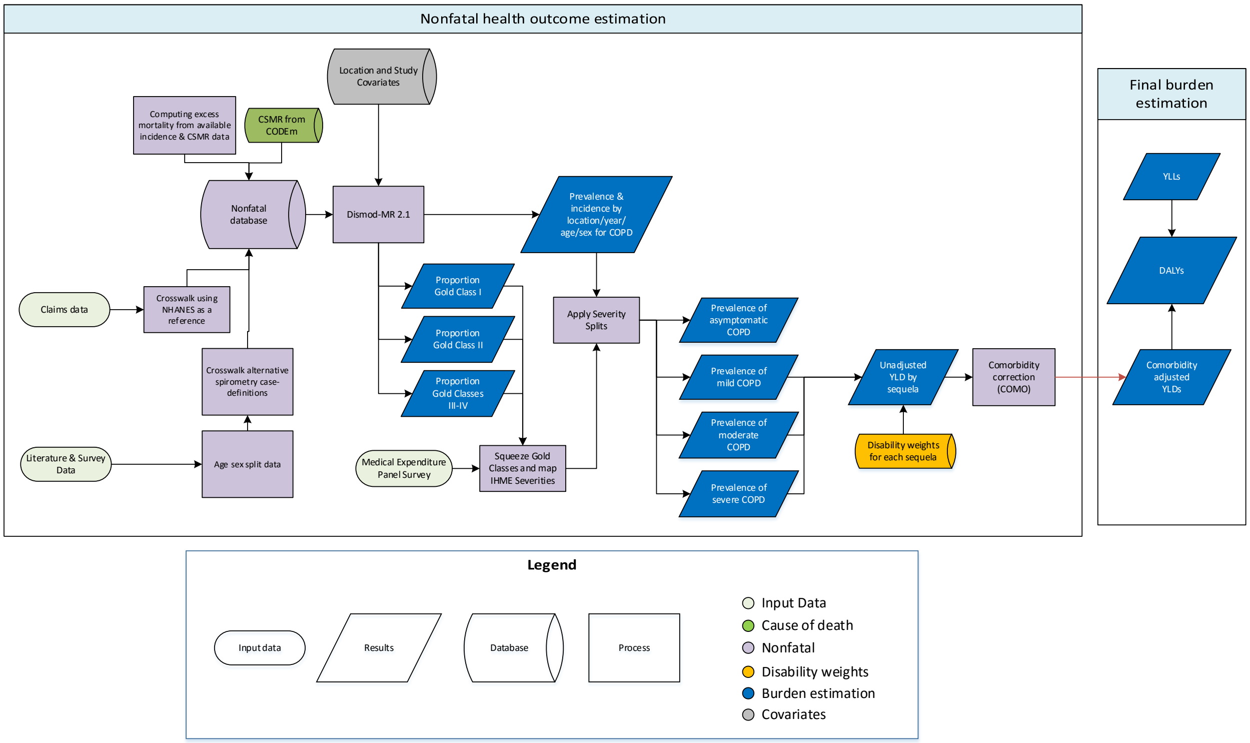

Input data and methodological summary

Case definition

COPD is defined as in the Global Initiative for Chronic Obstructive Lung Disease (GOLD) classification: a measurement of <0.7 FEV_1/FVC (one second of forceful exhalation/total forced expiration) on spirometry after bronchodilation. It should be noted that this is the same reference definition as was used for GBD 2015, but it is different from GBD 2013 where the "Lower Limit of Normal (LLN)," ie, relative to an age- and sex-specific norm for the FEV_1/FVC ratio, was the reference. We made this decision because the severity grading of COPD follows the GOLD Class definition rather than the LLN concept. The definitions of the severity classes in the GOLD classification are provided below.

GOLD CLASS	FEV_1 Score
I: Mild	>=80% of normal
II: Moderate	50-79% of normal
IV: Severe	<50% of normal

ICD-10 codes associated with COPD include J40, J41, J42, J43, J44, and J47. The corresponding ICD-9 codes are 490-492, 494, and 496.

Input data

For GBD 2016, we updated the systematic review from previous iterations. The full search term was:

(chronic obstructive pulmonary disease[Title/Abstract] AND (prevalence[Title/Abstract] or incidence [Title/Abstract] or mortality [Title/Abstract] or death [Title/Abstract]) AND "Cross-Sectional Studies"[MeSH Terms]) Filters: Publication date from 04/01/2015 to 11/01/2016; Humans

Twenty-three new sources were extracted. Studies excluding smokers were excluded from the review.

In addition, we include survey data with spirometry measurements, including the National Health and Nutrition Examination Study series in the United States. The Study of Aging and Global Health (SAGE) series was examined for GBD 2015 but ultimately excluded as the spirometry data had implausible FEV_1/FVC values (eg, over 1).

Data using alternative case-definitions of COPD prevalence (i.e. LLN) were crosswalked to the reference case-definition with age-specific ratios derived from studies reporting prevalence using both the alternative and reference case-definitions.

Furthermore, claims data for the United States were included. Additional information on the claims data collection and pre-corrections are provided elsewhere. Briefly, we determined US national and state-level estimates of COPD prevalence from a database of individual level ICD-coded health service encounters. Persons with any claim associated with COPD were marked as a prevalent case for that year.

For GBD 2016, an additional correction was made for COPD US claims data. Under the assumption that NHANES estimates are more accurate than claims data estimates because they use spirometry measurements, we derived an age-specific crosswalk to adjust US claims data according to the ratio between NHANES and the national-level US claims estimates.

A table describing the density and distribution of the available data informing the COPD estimation process is provided below.

	Proportion by GOLD Class	Prevalence	Incidence
Studies	15	80	4
Countries or subnational locations	27	119	4
Regions	15	16	3

Modelling strategy

As described above, the estimation of COPD burden occurs in three main steps. The first is the estimation of prevalence and incidence using a DisMod-MR 2.1 model. The second is the separate estimation of the proportions by three GOLD class groupings in DisMod-MR 2.1. The third is the combination of these two processes to derive prevalence by severity.

Step 1: Main COPD model

Prior settings include remission of 0 and an incidence ceiling of 0.0002 before age 20. The latter was necessary to avoid a kick-up of estimates in childhood at an age range with few or no primary data.

Similar to other causes, we include estimates of cause-specific mortality rate (CSMR) and derived estimates of excess mortality rate (EMR) by dividing every prevalence data point by the CSMR value for the corresponding location, age, sex, and year. We did not estimate EMR for data points with an age range greater than 20 years.

To assist estimation, each model includes a series of country-level covariates that describe spatiotemporal patterns. Where available, we use the COPD standardized exposure variables (SEV), which aggregate multiple risk factors into a single variable. We also use the log of LDI on EMR to capture country-level variation of EMR, assuming a negative coefficient (ie, lower mortality with rising GDP).

Step 2: GOLD class models

The GOLD class models use data from surveys that specified prevalence by GOLD class after expressing the values as a proportion of all COPD cases. We use fixed effects from the SEV scalar and the log of lag-distributed income (LDI) per capita to assist estimation.

Table of model coefficients for COPD and GOLD class models

Cause	Variable_name	Measure	Beta	Exponentiated
COPD	LDI (I$ per capita)	excess mortality rate	-0.5 (-0.5 — -0.5)	0.61 (0.60 — 0.61)
COPD	Log age-standardized SEV scalar: COPD	prevalence	0.75 (0.75 — 0.76)	2.12 (2.12 — 2.15)
GOLD I proportion	Socio-demographic Index	proportion	0.93 (-0.96 — 1.97)	2.54 (0.38 — 7.18)
GOLD I proportion	Log age-standardized SEV scalar: COPD	proportion	-0.17 (-0.62 — 0.33)	0.84 (0.54 — 1.39)
GOLD II proportion	Socio-demographic Index	proportion	0.93 (-0.96 — 1.97)	2.54 (0.38 — 7.18)
GOLD II proportion	Log age-standardized SEV scalar: COPD	proportion	-0.17 (-0.62 — 0.33)	0.84 (0.54 — 1.39)
GOLD III+IV proportion	Socio-demographic Index	proportion	0.35 (-1.66 — 1.88)	1.42 (0.19 - 6.57)
GOLD III+IV proportion	Log age-standardized SEV scalar: COPD	proportion	0.018 (-0.8 — 0.78)	1.02 (0.45 — 2.17)

Severity

The three GOLD class groupings reflect a grading based on a physiological measurement rather than a direct measurement of disease severity. In order to map the epidemiological findings by GOLD Class into the three COPD health states for which we have disability weights (DW), we used the 2001–2011 Medical Expenditure Panel Survey (MEPS) data from the United States. Specifically, we convert the GOLD class designations estimated for the USA in 2005 (the midpoint of MEPS years of analyses) into GBD classifications of asymptomatic, mild, moderate, and severe COPD.

The table below shows the three health states of COPD and the corresponding lay descriptions and disability weights. The graph shows the average proportion by GOLD Class (after scaling to 100%) across all ages for USA in 2005. We also show the proportion of MEPS respondents reporting any health service contact in the past year for COPD with a DW value attributable to COPD of 0, mild range (0 to midpoint between DWs for mild and moderate), moderate range (midpoint of DW values mild and moderate to midpoint of DW values for moderate and severe) and severe range (midpoint between DW values moderate and severe or higher). The DW value for COPD was derived from a regression with indicator variables for all health states reported by MEPS respondents and their reported overall level of disability derived from a conversion of 12-Item Short Form Surveys (SF-12) answers to GBD DW values. This analysis gave the severity distribution for each GBD cause reported in MEPS after correcting for any comorbid causes individual respondents reported during a year.

Health state	Lay description	DW (95% CI)
Mild COPD	This person has cough and shortness of breath after heavy physical activity, but is able to walk long distances and climb stairs.	0.019 (0.011–0.033)
Moderate COPD	This person has cough, wheezing, and shortness of breath, even after light physical activity. The person feels tired and can walk only short distances or climb only a few stairs.	0.225 (0.153–0.31)
Severe COPD	This person has cough, wheezing, and shortness of breath all the time. The person has great difficulty walking even short distances or climbing any stairs, feels tired when at rest, and is anxious.	0.408 (0.273–0.556)

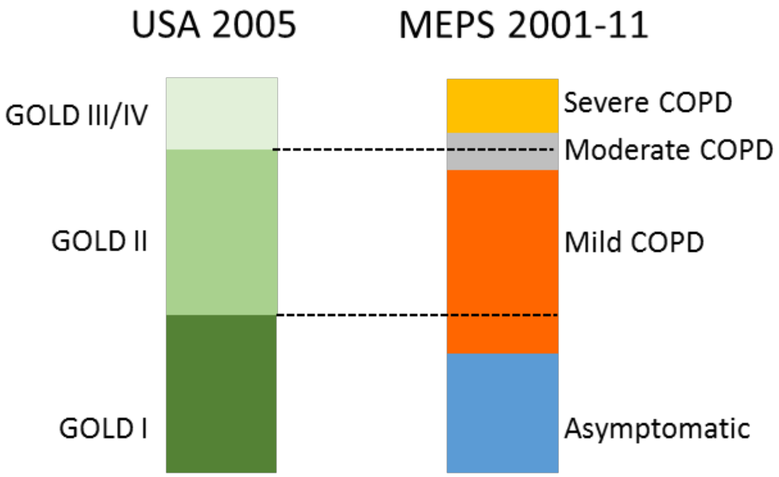

The algorithm to translate GOLD Class to COPD DW categories first assigns GOLD III&IV to severe COPD and what remains to moderate. Next, GOLD class I is assigned to the asymptomatic category first and what remains goes to mild COPD. This algorithm is repeated for each age and sex category and for all 1,000 draws from the DisMod models of GOLD classes and the MEPS analyses. We end up with proportions of each of the GOLD class categories that map onto GBD COPD health states with uncertainty bounds determined by the 25th and 975th values of the 1,000 draws. These values are then applied to the estimates of the proportion of cases by GOLD class category, after scaling to 100%, by location, year, age, and sex. This assumes that the relationship between GOLD class and GBD COPD health states in the United States applies everywhere.

Pneumoconiosis

Coal Worker's Pneumoconiosis, Asbestosis, Silicosis, and Other Pneumoconiosis

Flowchart

Pneumoconiosis:
Coal Worker's, Asbestosis, Silicosis, Other

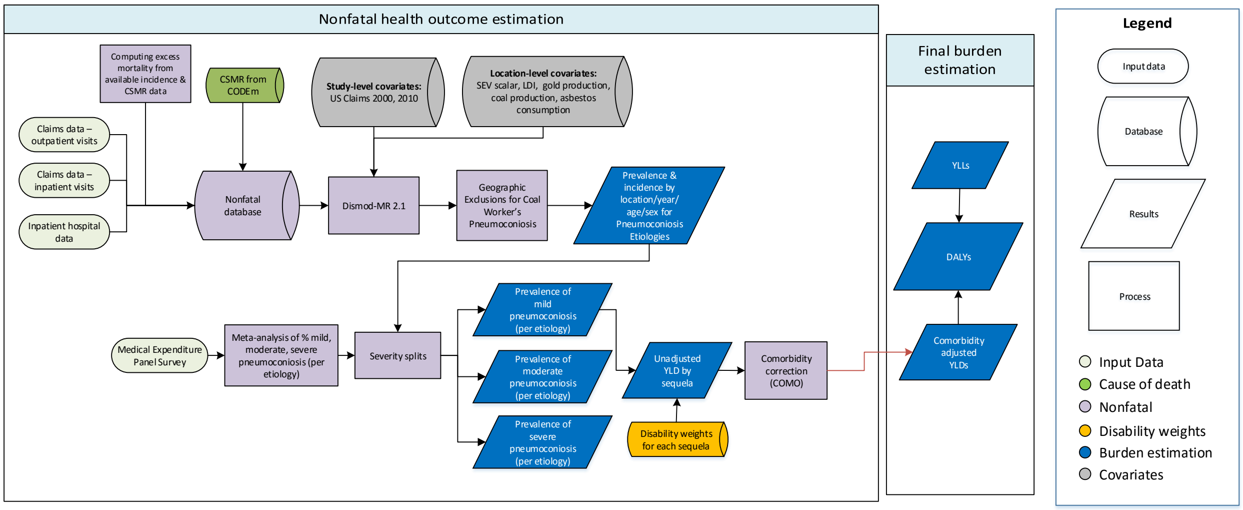

Input data and methodological appendix

Case definition

Pneumoconiosis is a chronic lung disease typified by lung scarring and other interstitial damage caused by exposure to dust and other containments – usually through occupational exposure. For GBD, we model pneumoconiosis by exposure type: coal, asbestos, silica, and other.

Input data

A systematic review of the literature was conducted for pneumoconiosis using the following search string in PubMed:

(Pneumoconiosis[Title/Abstract] AND prevalence[Title/Abstract])

The search was restricted to humans and publications between January 2012 and November 2016. The search produced 31 hits, although no sources included useable data. Many studies were excluded on the basis of being from non-representative populations (eg, studies of prevalence of pneumoconiosis among groups of coal workers).

Data used to make estimates of pneumoconiosis are predominantly from four main sources. The first is literature data from previous systematic reviews – usually from smaller-scale studies or surveys of prevalence. The second are occupational exposure reports and registries produced by governmental

agencies. The third data format we use are collated hospital inpatient reports. The fourth main category of data are claims data – particularly for the United States. Greater detail on the preparation of the collated inpatient data and claims data is provided elsewhere. An overview of the data density and distribution by measure and location is present below for the four pneumoconiosis variants.

**Silicosis**	Prevalence	Incidence	Mortality risk
Studies	48	3	0
Locations	214	3	0
Regions	13	3	0

**Coal worker's**	Prevalence	Incidence	Mortality risk
Studies	45	4	0
Locations	239	4	0
Regions	12	3	0

**Asbestosis**	Prevalence	Incidence	Mortality risk
Studies	45	3	0
Locations	263	3	0
Regions	12	3	0

**Other**	Prevalence	Incidence	Mortality risk
Studies	58	0	0
Locations	299	0	0
Regions	15	0	0

For all etiologies, we use a sex-specific correction factor of the hospital inpatient data where numbers are adjusted upward by the ratio of primary diagnosis to secondary diagnosis present in the claims data.

Severity split inputs

Data to inform estimates of the severity gradient due to pneumoconiosis etiologies are derived from previous analyses of the Medical Expenditure Panel Survey (MEPS). The disability weights are also shared.

Severity level	Lay description	DW (95% CI)
Mild	Has cough and shortness of breath after heavy physical activity, but is able to walk long distances and climb stairs.	0.019 (0.011–0.033)
Moderate	Has cough, wheezing, and shortness of breath, even after light physical activity. The person feels tired and can walk only short distances or climb only a few stairs.	0.225 (0.153–0.312)
Severe	Has cough, wheezing, and shortness of breath all the time. The person has great difficulty walking even short distances or climbing any stairs, feels tired when at rest, and is anxious.	0.408 (0.273–0.556)

Modelling strategy

Estimates for the pneumoconiosis etiologies are produced using a standard DisMod-MR 2.1 approach.

For all etiologies, we use prior settings of zero remission. Additionally, we assume no incidence and prevalence before the age of 10.

Claims data for 2000 and 2010 are adjusted via study covariates to account for systematically low estimates relative to the 2012 claims data. Implicit in this adjustment is the assumption that variation between years of claims data is a function of data collection inconsistencies and noise. As pneumoconiosis is a chronic disease largely caused by long-term occupational exposure and there is no evidence of rapid occupational shifts during the period of study, we ascribe observed differences to collection noise/error.

Similar to other causes, we include estimates of cause-specific mortality rate (CSMR) and Excess Mortality Rate (EMR) in this. The source and estimation of these rates are discussed elsewhere.

To assist estimation, each model includes a series of country-level covariates that describe spatiotemporal patterns.

For the most part, the same covariates from GBD 2015 were used. Adjustments were also made to the coal and asbestos covariates as follows.

The coal production covariate was improved to include subnational data for the United States and India. United States state-level data for 2001-2015 came from the U.S. Energy Information Administration. India state-level data for 2005-2014 came from the Ministry of Coal in India. We scaled these figures to the national estimates from the BP Satistical Review of World Energy 2016. For years with missing state-level data we split the national-level data according to the proportions by state in the closest year for which we did have state-level data.

We also created a covariate for asbestos consumption per capita with a 30-year lag, and used that instead of the GBD 2015 asbestos production covariate. This change is based on the idea that asbestos production may be too limited in scope, given that asbestosis may occur in locations where asbestos is used and handled but not necessarily mined. To create the asbestos consumption covariate we used data from the United States Geological Survey to run a model in DisMod 2.1. A 30-year lag was placed on this model to account for the delay between asbestos consumption and occurrence of disease.

Where available, we use standardized exposure variables (SEV) which aggregates multiple risk factors into a single variable. A full accounting is below:

Cause	Measure	Variable_Name	Beta	Exponentiated
Asbestosis	excess mortality rate	LDI (I$ per capita)	-0.055 (-0.14 — -0.012)	0.95 (0.87 — 0.99)
Asbestosis	prevalence	All MarketScan, year 2010	-0.00057 (-0.0013 — -0.00013)	1.00 (1.00 — 1.00)

Asbestosis	prevalence	All MarketScan, year 2000	-0.00019 (-0.00087 — -0.000033)	1.00 (1.00 — 1.00)
Asbestosis	Prevalence	Asbestos Consumption (per capita)	0.0091 (0.00051 — 0.023)	1.01 (1.00 — 1.02)
Coal worker's	prevalence	All MarketScan, year 2000	-0.0074 (-0.025 — -0.00035)	0.99 (0.98 — 1.00)
Coal worker's	prevalence	Log-transformed age-standardized SEV scalar: Coal W	1.00 (0.76 — 1.24)	2.72 (2.14 — 3.46)
Coal worker's	prevalence	Coal Production (per capita)	1.19 (0.086 — 1.97)	3.28 (1.09 — 7.19)
Coal worker's	prevalence	All MarketScan, year 2010	-0.011 (-0.042 — -0.00023))	0.99 (0.96 — 1.00)
Coal worker's	excess mortality rate	LDI (I$ per capita)	-1 (-1 — -1)	0.37 (0.37 — 0.37)
Other pneumoconiosis	prevalence	All MarketScan, year 2010	-0.0097 (-0.035 — -0.000015)	0.99 (0.97 — 1.00))
Other pneumoconiosis	prevalence	Log-transformed SEV scalar: Oth Pneum	0.78 (0.75 — 0.87)	2.19 (2.12 — 2.39)
Other pneumoconiosis	excess mortality rate	LDI (I$ per capita)	-1 (-1 — -1)	0.37 (0.37 — 0.37)
Other pneumoconiosis	prevalence	All MarketScan, year 2000	-0.0095 (-0.034 — -0.0002)	0.99 (0.97 — 1.00)
Silicosis	prevalence	All MarketScan, year 2000	-0.0069 (-0.021 — -0.00032)	0.99 (0.98 — 1.00)
Silicosis	excess mortality rate	LDI (I$ per capita)	-0.5 (-0.5 — -0.5)	0.61 (0.61 — 0.61)
Silicosis	prevalence	All MarketScan, year 2010	-0.0076 (-0.027 — -0.00037)	0.99 (0.97 — 1.00)
Silicosis	prevalence	Log-transformed age-standardized SEV scalar: Silicosis	1.24 (1.21 — 1.25)	3.45 (3.36 — 3.49)
Silicosis	Prevalence	Gold Production (per capita)	2.75 (0.17 — 4.91)	15.71 (1.18 — 135.10)

To account for country level differences in excess mortality (perhaps as a function of available medical care) we use ln(lag distributed income) as a proxy measure.

For GBD 2015 prevalence and incidence of coal worker’s pneumoconiosis and asbestosis were set to zero in locations without a history of coal mining or asbestosis production given the causal and necessary relationship between respective occupational exposure and disease. For GBD 2016 we removed the geographical exclusions for asbestosis, given our move to a consumption covariate, as mentioned above.

Asthma

Flowchart

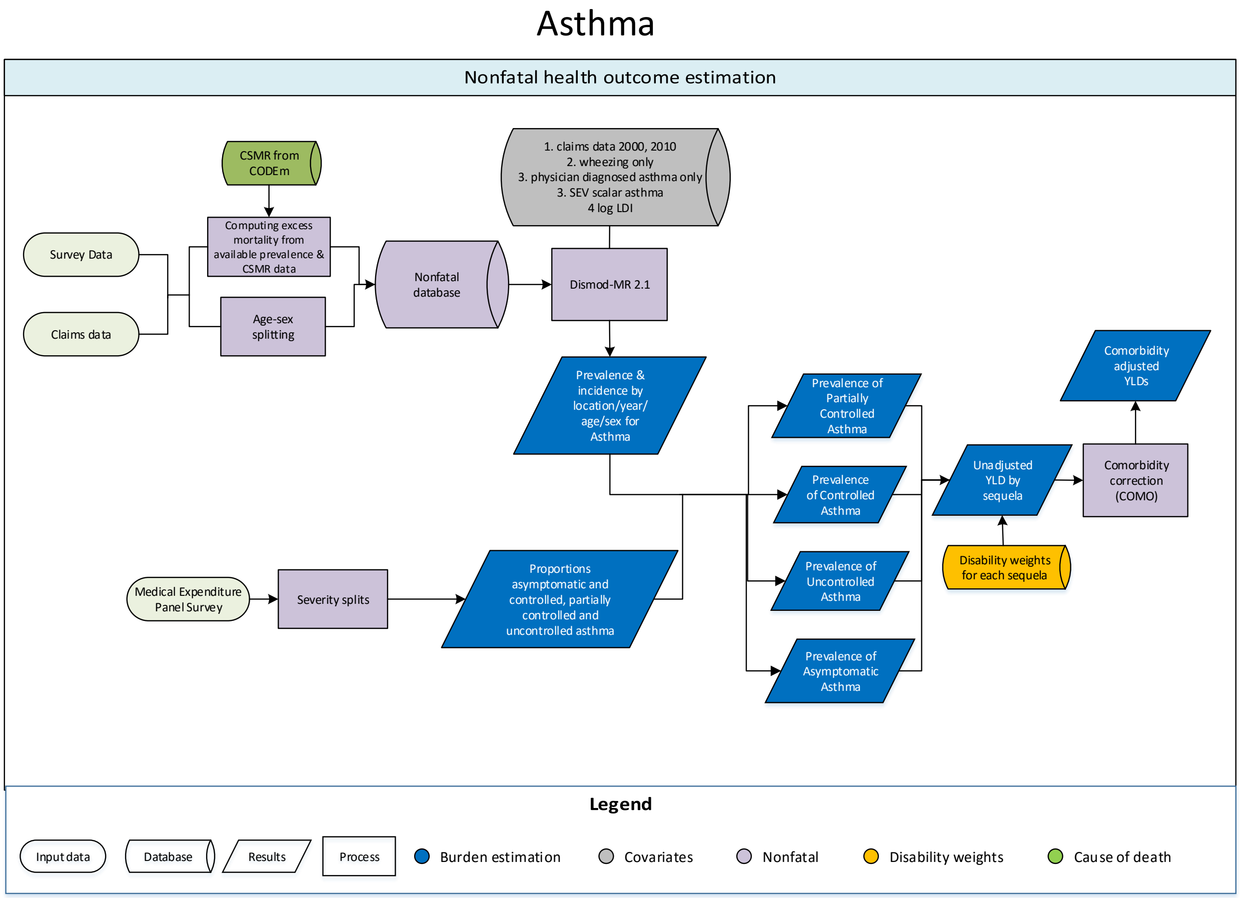

Case definition

Asthma is a chronic lung disease marked by spasms in the bronchi usually resulting from an allergic reaction or hypersensitivity and causing difficulty in breathing. We define asthma as a doctor's diagnosis and wheezing in the past year. The relevant ICD-10 codes are J45 and J46. ICD-9 code is 493.

Input data

For GBD 2016, we did a full systematic review of the literature on asthma. We used the following search string in PubMed and filtered by studies of humans published between January 2012 and November 2016.

(Asthma[Title/Abstract] AND prevalence[Title/Abstract] AND "Cross-Sectional Studies"[MeSH Terms])

From this search, we had 533 hits. Of these, 47 sources were extracted after full-text screening and incorporated into the GBD 2016 model.

Additional survey data added for GBD 2016 were mainly from India: International Study of Asthma and Allergies in Childhood (ISAAC); National Family Health Survey; WHO Study on global AGEIng and adult health (SAGE); and Indian Study on Epidemiology of Asthma, Respiratory Symptoms and Chronic Bronchitis (INSEARCH).

Surveys carried out as part of the ISAAC collaboration are the most important source of prevalence data in children.

The following table provides a description of the data density and distribution by location and epidemiological measure (including the claims data discussed below).

	Prevalence	Incidence	Mortality risk
Studies	302	7	6
Countries/subnational locations	354	5	3
Regions	21	1	1

In addition to literature and survey data, we use claims data from the United States from 2000, 2010, and 2012. Information on the source and preparation of these data are provided in detail elsewhere. Briefly, we determined US national and state level estimates of asthma prevalence from a database of individual-level ICD-coded health service encounters for three years. Persons with any claim associated with asthma were marked as a prevalent case for that year. Aggregated estimates were then adjusted using a noise-reduction algorithm. These corrected data were then used in the modelling process.

Modelling strategy

We use DisMod-MR 2.1 as the main modelling tool for asthma. Prior settings include a maximum remission of 0.3 (reflecting the upper bound of the highest observed data) and no incidence between the ages of 0 and 0.5 year, as a diagnosis cannot be made in young infants.

Data points from the ISAAC studies were reported for both sexes combined. We sex-split before modelling using the ratios derived from the 2012 US claims data (1).

Data that describe wheezing in the past year, but do not report presence/absence of an accompanying diagnosis are crosswalked to the reference category using a study-level covariate in DisMod. As the table below shows, studies that only report wheezing are systematically higher than reference data points and are adjusted down -- dividing by the exponentiated coefficient. Data that describe prevalence of lifetime diagnosis of asthma but not accompanying wheezing in the past year are also crosswalked to the reference category using a study-level covariate.

To account for country-level differences in excess mortality as a function of available medical care we use log lag-distributed income (LDI) as a covariate and assume a negative coefficient. The effect size is shown below.

Claims data for 2000 and 2010 are adjusted via study covariates to account for systematically lower estimates relative to the 2012 claims data. Implicit in this adjustment is the assumption that variation between years of claims data is a function of data-collection inconsistencies.

Similar to other causes, we include estimates of cause-specific mortality rate (CSMR) and excess mortality rate (EMR) derived as a matched value for each prevalence data point dividing CSMR by prevalence. We restrict these EMR calculations to data points of 20-year age span or less.

To assist estimation, the model includes a series of country-level covariates that describe spatiotemporal patterns. Specifically, we use log LDI and the asthma standardized exposure variable (SEV), a scalar that combines exposure of all GBD risks that influence asthma. A full covariate list, including the study-level covariates, described above are presented in the following table with their associated effects:

Variable_name	Measure	Beta	Exponentiated
Wheezing only	prevalence	0.46<br>(0.43 - 0.50)	1.59<br>(1.53 — 1.65)
Physician diagnosed asthma only	prevalence	-0.091<br>(-0.14 — -0.038)	0.91<br>(0.87 — 0.96)
Claims data 2000	prevalence	-0.53<br>(-0.56 - -0.5)	0.59<br>(0.57 — 0.61)
Claims data 2010	prevalence	-0.13<br>(-0.15 — -0.1)	0.88<br>(0.86 — 0.90)
Log SEV scalar: asthma	prevalence	1.24<br>(1.20 — 1.25)	3.44<br>(3.33 — 3.49)
Log LDI (I$ per capita)	excess mortality rate	-0.5<br>(-0.5 — -0.5)	0.61<br>(0.61 — 0.61)

Severity split inputs

Lay descriptions and disability weights for the asthma health states are shown in the table below. The distribution between the three health states is derived from an analysis of the US Medical Expenditure Panel Surveys (MEPS). The methods are described in full in a separate section of this appendix. Briefly, MEPS is an ongoing survey of health service encounters with as its main objective to collect data on health expenditure. Panels are recruited every year and followed up for a period of two years. Diagnostic information provided by respondents on the reasons for any health care contact are coded into three-digit ICD-9 codes by professional coders.

Twice over the two-year follow-up period respondents are asked to fill in 12-Item Short Form Surveys (SF-12). From convenience samples asking respondents to fill in SF-12 for 60 of the GBD health states, IHME has created a mapping from SF-12 scores to GBD Disability Weights (DW). We perform a regression with indicator variables for all GBD causes that we can identify from the ICD codes in MEPS to derive for each individual with a diagnosis the amount of disability that can be attributed to that condition after

controlling for any comorbid conditions. Anyone with a diagnosis of asthma in whom the disability assigned to asthma is negative or zero we assume is asymptomatic (at the time of asking SF-12 question relating to their health status in the past four weeks). Non-zero values we bin into the three health states assuming a split between these at the midpoint between DW values. The table below gives the proportions in MEPS in each of the health states and an asymptomatic state.

Severity level	Lay description	DW (95% CI)	Severity distribution
Asymptomatic			36.2% (35.0–37.3%)
Controlled	This person has wheezing and cough once a month, which does not cause difficulty with daily activities.	0.015 (0.007–0.026)	19.9% (13.6–27.8%)
Partially controlled	This person has wheezing and cough once a week, which causes some difficulty with daily activities.	0.036 (0.022–0.055)	20.6% (15.1–25.8%)
Uncontrolled	This person has wheezing, cough, and shortness of breath more than twice a week, which causes difficulty with daily activities and sometimes wakes the person at night.	0.133 (0.086–0.192)	23.3% (18.7–30.3%)

There were no significant changes in modelling approach from GBD 2015.

Interstitial Lung Disease and Pulmonary Sarcoidosis (ILD)

Flowchart

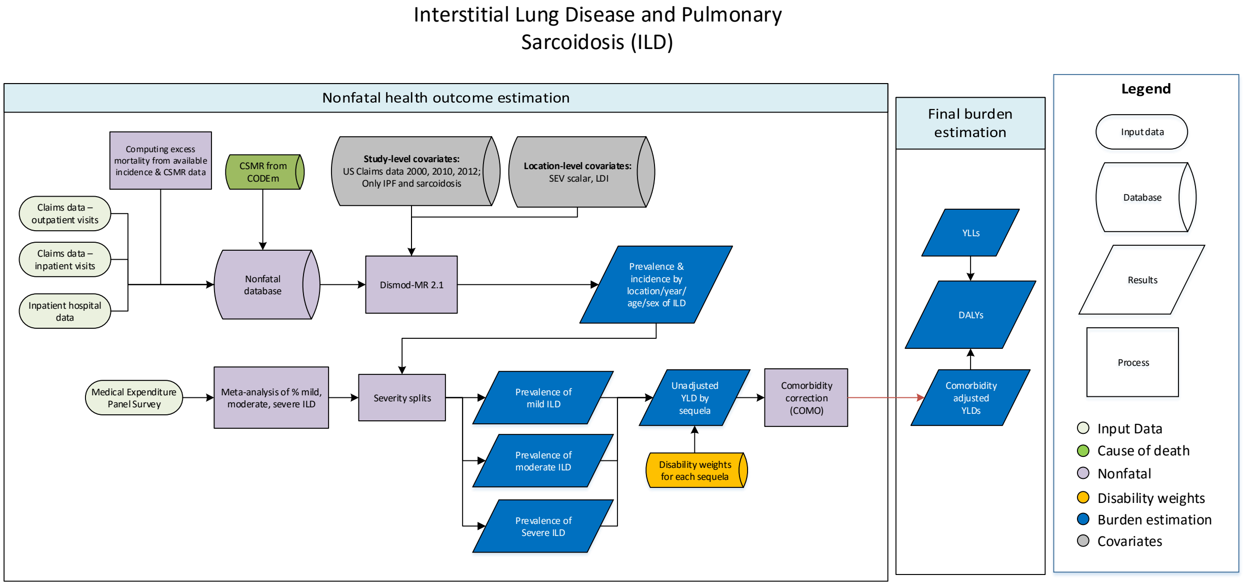

Case definition

Interstitial lung diseases and pulmonary sarcoidosis are a collection of chronic respiratory diseases that impair lung function and oxygen uptake through scarring and/or inflammation. The relevant ICD codes are D86 and J84. For interstitial lung disease, we use the American Thoracic Society as the gold standard definition.

Input data

Model Inputs

No systematic review of the literature was conducted for ILD for this iteration of the Global Burden of Disease. These reviews done on a rotating basis and updates will be made for a future iteration.

Data used to make estimates of ILD are predominantly from three main sources. The first is literature data from previous systematic reviews – usually from smaller-scale studies of prevalence. The second main data type is claims data for the United States. The source and preparation of these data is described elsewhere. The third main data type is adjusted hospital inpatient records. Because these records only report primary diagnosis, we a priori adjust the numbers by a sex-specific factor based on patterns observed in the US claims data – that is, the ratio of primary to secondary diagnoses.

The following table provides a picture of the number of available studies along with their distribution globally and by epidemiological profile. In short, the ILD data landscape is rather sparse. The available data are largely skewed toward high-income countries like the United States or the member countries of

the European Union. The relatively high number of subnational units with data is largely a function of claims data in the United States and hospital data from Mexico and Brazil.

	Prevalence	Incidence	Mortality risk
Studies	51	19	0
Locations	261	15	0
Regions	11	6	0

Severity splits

Data to inform estimates of the severity gradient due to ILD are derived from previously analyses of the Medical Expenditure Panel Survey (MEPS). The table below illustrates the lay descriptions and disability weights associated with different levels of severity of interstitial lung disease.

Severity level	Lay description	DW (95% CI)
Mild	Has cough and shortness of breath after heavy physical activity, but is able to walk long distances and climb stairs.	0.019 (0.011–0.033)
Moderate	Has cough, wheezing, and shortness of breath, even after light physical activity. The person feels tired and can walk only short distances or climb only a few stairs.	0.225 (0.153–0.312)
Severe	Has cough, wheezing, and shortness of breath all the time. The person has great difficulty walking even short distances or climbing any stairs, feels tired when at rest, and is anxious.	0.408 (0.273–0.556)

Modelling strategy

Estimates for ILD are produced using a standard DisMod-MR 2.1 approach. We use prior settings of zero remission and we constrain the super-region random effects to -0.5 to 0.5 to ensure model stability.

As described above, we use an a priori adjustment of hospital inpatient data to correct for secondary diagnoses available in the claims data.

Similar to other causes, we include estimates of cause-specific mortality rate (CSMR) and Excess Mortality Rate (EMR). The source and estimation of these rates are discussed elsewhere.

The GBD ethic is to use all available data sources where reasonable. Because ILD consists of many smaller etiologies not broken out here, we make crosswalks to account for measurement, case definition, and study design differences. The full list is below:

Variable_Name	Measure	Beta	Exponentiated
Only idiopathic pulmonary fibrosis and sarcoidosis	prevalence	-1.49 (-1.96 — -0.88)	0.22 (0.14 — 0.41)
All MarketScan, year 2000	prevalence	1.15 (1.10 — 1.19)	3.15 (3.00 — 3.29)

All MarketScan, year 2010	prevalence	1.40<br>(1.37 — 1.44)	4.07<br>(3.93 — 4.24)
All MarketScan, year 2012	prevalence	1.40<br>(1.35 — 1.43)	4.04<br>(3.88 — 4.19)
Only idiopathic pulmonary fibrosis and sarcoidosis	incidence	-0.87<br>(-1.53 — -0.19)	0.42<br>(0.22 — 0.83)
LDI (I$ per capita)	excess mortality rate	-0.2<br>(-0.2 — -0.2)	0.82<br>(0.82 — 0.82)

To account for country level differences in excess mortality (perhaps as a function of available medical care) we use ln(lag distributed income) as a proxy measure. The effect size is shown above.

Other chronic respiratory diseases

In addition to the chronic respiratory diseases described above, there are many diverse types of chronic respiratory diseases with a range of severities and associated sequelae. Because these chronic respiratory diseases are diverse in their underlying causes and risk factors as well as in their associated health outcomes, modelling them together in a DisMod-MR model would not produce reliable estimates of prevalence or excess mortality. Instead, we calculated the YLDs caused by other chronic respiratory diseases directly using a YLD/YLL ratio.

We calculated the ratio of YLDs to YLLs across the specified chronic respiratory diseases for which nonfatal outcomes were modelled, using YLL estimates from the GBD 2016 cause of death (CoD) analysis. We then multiplied this YLD/YLL ratio by the YLL estimates for other chronic respiratory diseases from the GBD 2016 CoD analysis, providing us with an estimate of the YLDs associated with other chronic respiratory diseases.

Cirrhosis

Flowchart

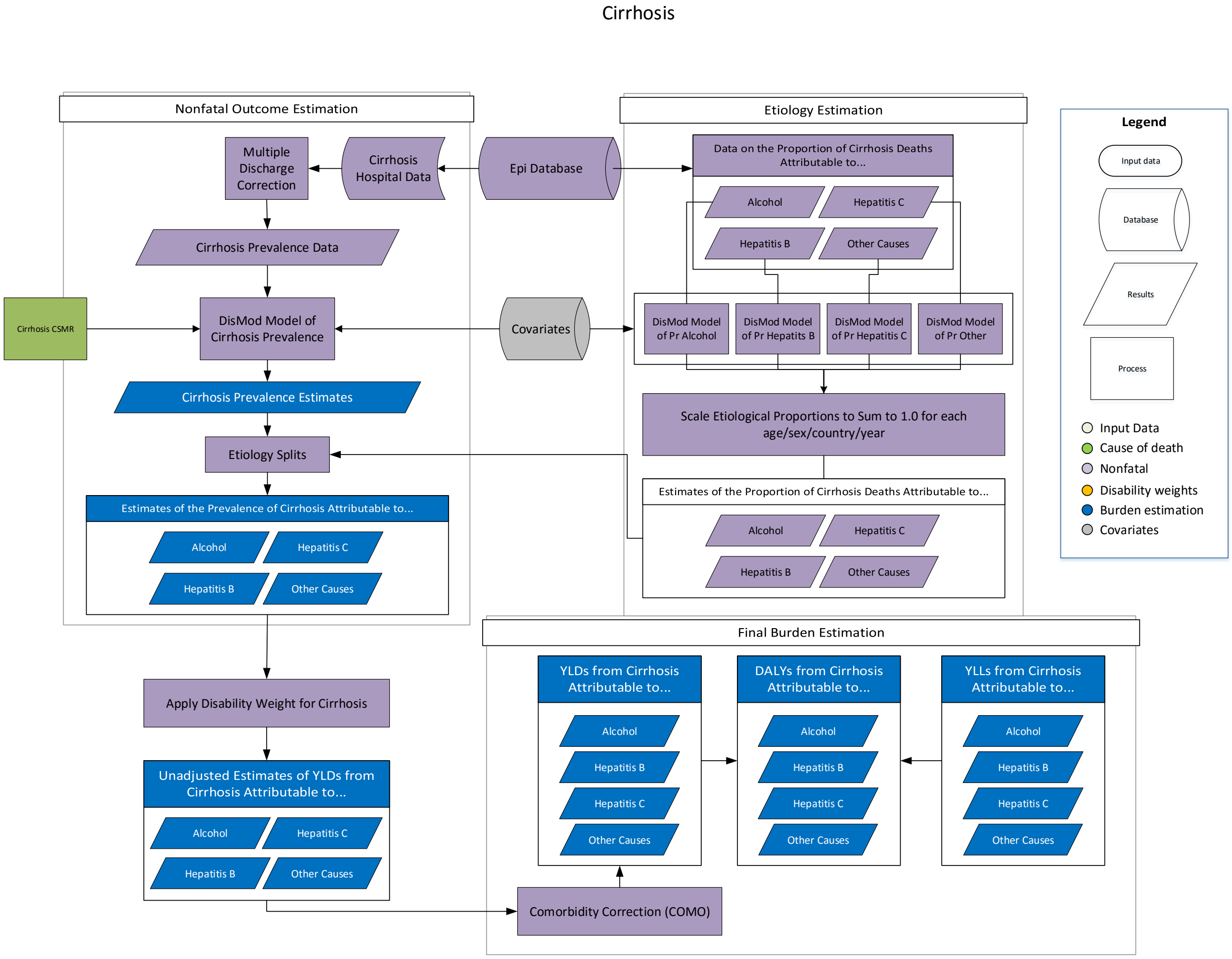

Input data and methodological summary

Case definition

Cirrhosis is a chronic liver disease most often caused by alcohol use or chronic infection with hepatitis B or C. Early disease is typically asymptomatic as the liver's resilience compensates for cirrhotic damage. Decompensated cirrhosis occurs when the disease progresses beyond the capacity of the liver to compensate for the damage, and is marked by profound symptoms, health loss and, often, death. We model decompensated cirrhosis, defined by cirrhosis (or a closely related diagnosis code) as the primary diagnosis in hospital data. We model total cirrhosis (compensated plus decompensated) when cirrhosis is a secondary diagnosis in hospital data. This includes ICD1-0 codes K70-K77, I85, P78.81.

Input data

Model inputs

For GBD 2016, we modelled cirrhosis prevalence based on hospital data, which have been updated to reflect the new estimation cycle. This year, we added data from MarketScan to our prevalence estimation process. The table below indicates the number of data points, as well as the location and regional breakdown of non-hospital and non-MarketScan data. Decompensated cirrhosis only used inpatient MarketScan data, whereas compensated used inpatient and outpatient.

Table 1. Data inputs for the cirrhosis parent model (not including hospital inpatient data)

	Prevalence	Incidence
Studies	0	1
Countries/subnationals	0	1
Countries	0	1
GBD world regions	0	1
GBD super-regions	0	1

We model etiologic proportions based on published estimates of the proportion of cirrhosis due to alcohol use, hepatitis B, hepatitis C, and other causes:

Table 2. Data inputs for the etiologic proportion models

	Alcohol	Hepatitis B	Hepatitis C	Other
Studies	54	80	88	41
Countries/subnationals	27	41	46	21
Countries	25	35	39	20
GBD world regions	14	17	18	11
GBD super-regions	7	7	7	7

For GBD 2016, we conducted a systematic review of the literature to capture studies of the proportion of cirrhosis attributable to alcohol, hepatitis B, hepatitis C, and other causes. We searched the peer-reviewed literature via PubMed and solicited sources from GBD collaborators.

The inclusion criteria stipulated that: 1) the publication year must be from 1980 onward; 2) the sample had to be a representative sample of those with decompensated cirrhosis (eg, studies of patients with both HCC and cirrhosis were excluded); 3) sufficient information must be provided on study method and sample characteristics to assess the quality of the study; 4) hepatitis B and C were confirmed via HBsAg, in the case of hepatitis B, and anti-HCV IgG, in the case of hepatitis C.

Data were outliered or excluded if we found them unreasonable when compared to regional, super-regional, and global rates.

Severity splits

The basis of the GBD disability weight survey assessments are lay descriptions of sequelae highlighting major functional consequences and symptoms.

Table 3. Severity level and lay descriptions

Sequela name	Health state description	Disability weight (95% CI)
Cirrhosis of the liver due to alcohol, decompensated	Has a swollen belly and swollen legs. The person feels weakness, fatigue and loss of appetite.	0.178 (0.122-0.25)
Cirrhosis of the liver due to hepatitis B, decompensated	Has a swollen belly and swollen legs. The person feels weakness, fatigue and loss of appetite.	0.178 (0.123-0.25)
Cirrhosis of the liver due to hepatitis C, decompensated	Has a swollen belly and swollen legs. The person feels weakness, fatigue and loss of appetite.	0.178 (0.122-0.25)
Cirrhosis of the liver due to other cause, decompensated	Has a swollen belly and swollen legs. The person feels weakness, fatigue and loss of appetite.	0.178 (0.122-0.25)

Modelling strategy

We modelled cirrhosis prevalence using hospital data and CSMR estimates, assuming no remission. For GBD 2016 we modeled compensated cirrhosis. Compensated cirrhosis was estimated by subtracting year, sex, age, and location specific draws of compensated cirrhosis from the corresponding draws of total cirrhosis. To estimate the prevalence of cirrhosis due to alcohol, cirrhosis due to hepatitis B, cirrhosis due to hepatitis C, and cirrhosis due to other causes, we developed aetiological proportion models using DisMod, and used the results of these models to split the compensated and decompensated cirrhosis prevalence estimates.

Given the similar etiologies for liver cancer and cirrhosis we integrated the etiology models for these two causes. We have more data for liver cancer etiologies than we do for cirrhosis. Therefore, we first developed four single-parameter DisMod models, each to estimate the proportion of liver cancer due to a given cause (ie, alcohol, hepatitis B, hepatitis C, and other). These models included as covariates alcohol consumption (litres per capita), hepatitis B surface antigen (HBsAg) seroprevalence, and hepatitis C (anti-HCV IgG) seroprevalence. Moreover, the model for the proportion due to alcohol included a binary covariate indicating countries with a predominantly Muslim population (thought to be associated with very low alcohol consumption). Estimates from these liver cancer models were then used as covariates (along with alcohol, HBsAg, and anti-HCV) in the four corresponding cirrhosis aetiology models. Estimates from these cirrhosis models were then similarly used as covariates in the corresponding liver cancer models. Proportions from the four etiology models were then rescaled to sum to one at the draw level, and used to split the parent compensated and decompensated cirrhosis estimates.

Study level covariates were used to adjusted prevalence data from hospital inpatient data and US claims data (2000 and 2010) toward the level of other prevalence data points, which were more representative of the general population. The healthcare quality and access index was used as a location-level covariate on excess mortality to guide estimates for countries with few or no data.

Table 4. Beta and exponentiated values for total cirrhosis

Study covariate	Parameter	Beta	Exp(beta)
Hospital Inpatient	Prevalence	1.40 (1.40 - 1.40)	4.05 (4.05 - 4.06)
Claims data - 2000	Prevalence	-0.70 (-0.74 - -0.66)	0.50 (0.47 - 0.52)

Claims data - 2010	Prevalence	-0.03 (-0.07 - -0.00)	0.97 (0.93 - 1.00)

Table 5. Location-level beta and exponentiated values for total cirrhosis

Country-level covariate	Parameter	Beta	Exp(beta)
Healthcare access and quality index	Excess mortality rate	-0.08 (-0.09 - -0.07)	0.93 (0.91 - 0.93)

Table 6. Beta and exponentiated values for decompensated cirrhosis

Study covariate	Parameter	Beta	Exp(beta)
Inpatient only claims data – 2000	Incidence	-0.9 (-0.94 — -0.87)	0.40 (0.39 — 0.42)
Inpatient only claims data – 2010	Incidence	-0.011 (-0.031 — -0.00031)	0.99 (0.97 – 1.00)
Hospital inpatient data	Incidence	-2.5 (-2.5 — -2.5)	0.082 (0.082 – 0.082)

Table 7. Location-level beta and exponentiated values for decompensated cirrhosis

Country-level covariate	Parameter	Beta	Exp(beta)
Healthcare access and quality index	Excess mortality rate	-0.02 (-0.021 — -0.019)	0.98 (0.98 — 0.98)

Changes from GBD 2015 to GBD 2016

We added the MarketScan database to our input data and modeled compensated cirrhosis for the first time.

Peptic Ulcer Disease

Flowchart

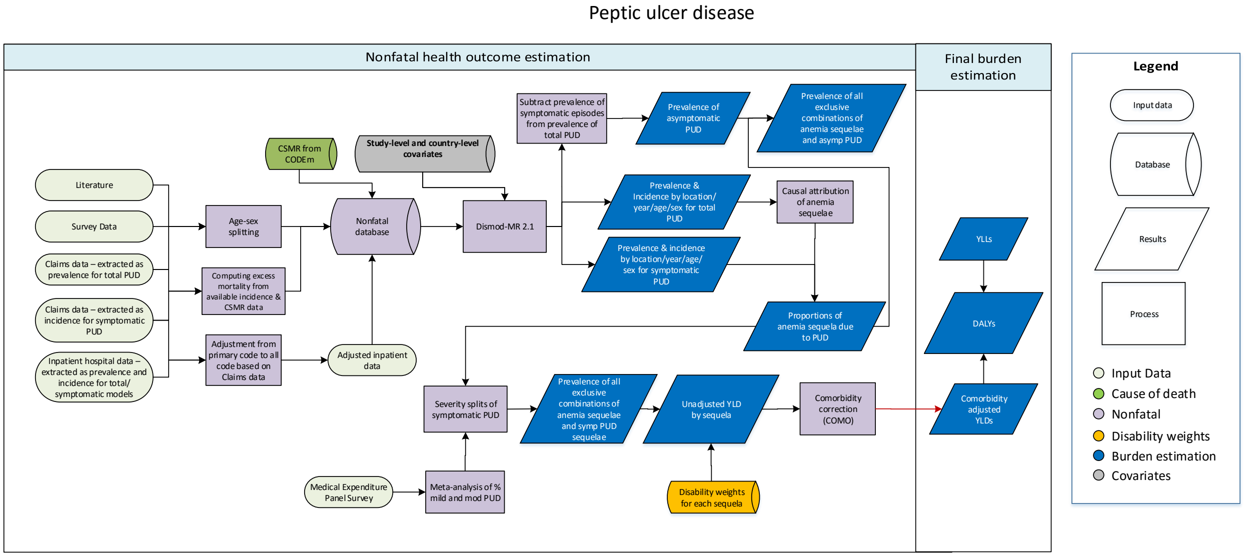

Case definition

Peptic ulcer disease is a digestive disorder involving ulcers in the lining of the stomach (gastric ulcers) or the duodenum (duodenal ulcers), diagnosed by endoscopy. Peptic ulcer disease is often asymptomatic with periodic symptomatic episodes of heartburn, bloating, nausea, or vomiting, and in severe cases, bleeding. ICD codes included are K25, K26, K27, K28, and K31.

Input data

Model inputs

In GBD 2016, chronic peptic ulcer disease was renamed to total peptic ulcer disease, symptomatic and asymptomatic (total peptic ulcer). Data inputs were separate for the total peptic ulcer and symptomatic episode models. A systematic review of literature was conducted to capture studies of prevalence, incidence associated with peptic ulcer disease. For GBD 2016, the search was conducted in peer-reviewed literature via PubMed. The inclusion criteria stipulated that (1) "caseness" must be based on clinical threshold as established by the ICD; (2) sufficient information must be provided on study methods and sample characteristics to assess the quality of the study; and (3) study samples must be representative of the general population (ie, samples of patients prescribed gastroscopies due to gastric pain or populations with *H. pylori* bacteria were excluded).

For the total peptic ulcer dataset we also included hospital inpatient and US claims data from 2000, 2010, and 2012 at the US state level, extracted as prevalence. The claims data were extracted using all diagnoses, and the hospital data were adjusted for only recording primary diagnoses, using a correction factor from claims data. For GBD 2016, we also extracted national surveys for doctor-confirmed, self-reported diagnoses of peptic ulcer disease and adjusted the data with a study-level covariate.

For the symptomatic episode dataset, we also considered literature sources that specifically referenced bleeding, perforations, and hospital visits, and extracted hospital inpatient and US claims data from 2000, 2010, and 2012 at the US state level, extracted as incidence to capture individual episodes. These data were similarly extracted and adjusted to estimate all diagnoses. Data were outliered or excluded if we found them unreasonable when compared to regional, super-regional, and global rates.

The tables below show the number of studies included in GBD 2016, as well as the number of countries or subnational units and GBD world regions represented.

Total peptic ulcer disease, symptomatic and asymptomatic

	Prevalence	Incidence
Studies	31	1
Countries	41	1
Countries/subnationals	324	1
GBD world regions	6	1

Symptomatic episodes of peptic ulcer disease

	Prevalence	Incidence
Studies	0	7
Countries	0	17
Countries/subnationals	0	119
GBD world regions	0	3

Severity split & disability weight

The basis of the GBD disability weight survey assessments are lay descriptions of sequelae highlighting major functional consequences and symptoms. For GBD 2016, we added a mild sequela to symptomatic episodes of peptic ulcer disease with proportions of cases derived from the MEPS. The lay descriptions and disability weights for peptic ulcer disease are shown below:

Severity level	Lay description	DW (95% CI)
Mild peptic ulcer disease, symptomatic episodes	This person has some pain in the belly that causes nausea but does not interfere with daily activities.	0.011 (0.005-0.021)
Moderate peptic ulcer disease, symptomatic episodes	This person has pain in the belly and feels nauseous. The person has difficulties with daily activities.	0.114 (0.080-0.159)

*The numerous sequelae generated from exclusive combinations of anemia and peptic ulcer disease each contain custom disability weights. More information can be found in the appendix detailing disability weights.

Modelling strategy

The DisMod model for total peptic ulcer disease included bounding remission from 0.1 to 0.5 (a duration of two to ten years), zero incidence from 0 to 5 years of age. Reference data were from literature and our most complete claims data set (2012). We marked inpatient, other US claims years data, and national survey data with study-level covariates. We used the function in DisMod-MR 2.1 to pull in cause-specific mortality rate (CSMR) data from our CODEm and CODcorrect analyses and match with prevalence data points for the same location. We calculated excess mortality rate to estimate priors by dividing CSMR by prevalence. We also applied a lag-distributed income and a healthcare access and quality index country-covariate to excess mortality, forced negative. Betas and exponentiated values (which can be interpreted as an odds ratio) are shown in the tables below for study-level covariates and country-level covariates.

Covariate type	Covariate	Parameter	beta	Exponentiated beta
Study-level covariate	Hospital Inpatient	Prevalence	0.73 (0.67 - 0.79)	2.08 (1.96 - 2.20)
Study-level covariate	Self-reported	Prevalence	0.60 (0.52 - 0.67)	1.82 (1.69 - 1.95)
Study-level covariate	Claims data - 2000	Prevalence	-0.27 (-0.31 - -0.22)	0.77 (0.73 - 0.80)
Study-level covariate	Claims data - 2010	Prevalence	0.05 (0.01 - 0.09)	1.05 (1.01 - 1.10)
Country covariate	LDI (I$ per capita)	Excess mortality rate	-0.47 (-0.49 - -0.45)	0.63 (0.61 - 0.64)
Country covariate	Healthcare access and quality index	Excess mortality rate	-0.01 (-0.01 - -0.01)	0.99 (0.99 - 0.99)

The symptomatic episodes of peptic ulcer disease DisMod model bounded remission from 16.5 to 17.5 (a duration of about three weeks). We also assumed no incidence from 0 to 5 years old, and excess mortality capped at 0.1 for all ages. The reference data were US claims data from 2012, and we marked other US claims years data, inpatient hospital data with study-level covariates. We used the function in DisMod-MR 2.1 to pull in cause-specific mortality rate (CSMR) data from our CODEm and CODcorrect analyses and match with prevalence data points for the same location. We calculated excess mortality rate to estimate priors by dividing CSMR by prevalence. We also applied a lag-distributed income covariate to excess mortality, log transformed and forced negative with an upper bound of -0.1 and a lower bound of -1, and a log-transformed age-standardized death rate for stomach cancer to incidence. Betas and exponentiated values (which can be interpreted as an odds ratio) are shown in the tables below for study-level covariates and country-level covariates.

Covariate type	Covariate	Parameter	beta	Exponentiated beta
Study-level covariate	Hospital Inpatient	Incidence	0.77 (0.71 - 0.83)	2.16 (2.03 - 2.30)
Study-level covariate	Claims data - 2000	Incidence	0.04 (-0.01 - 0.09)	1.04 (0.99 - 1.09)
Study-level covariate	Claims data - 2010	Incidence	-0.08 (-0.12 - -0.03)	0.93 (0.89 - 0.97)
Country covariate	LDI (I$ per capita)	Excess mortality rate	-0.61 (-0.63 - -0.58)	0.55 (0.53 - 0.56)

To calculate prevalence asymptomatic peptic ulcer disease, we took the estimated prevalence of symptomatic episodes, and subtracted it from our estimated prevalence of total peptic ulcer disease. Methods for causal attribution of anemia due to peptic ulcer can be found elsewhere in the appendix detailing strategies for impairments. Each final combination of sequela of anemia and peptic ulcer disease underwent exclusivity adjustments to prevent double counting.

Gastritis and duodenitis

Flowchart

Gastritis and duodenitis

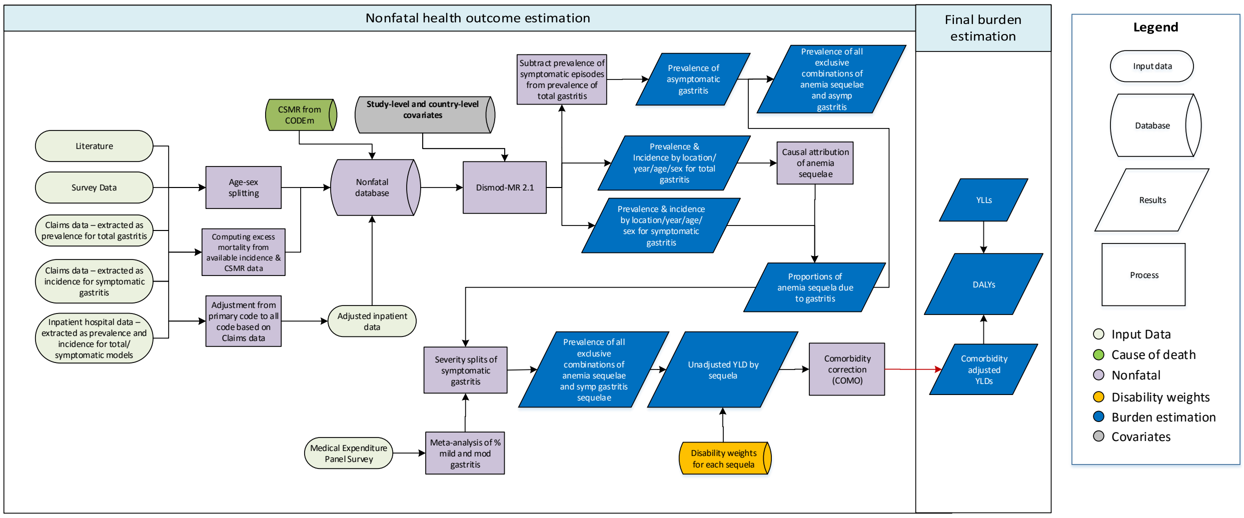

Case definition

Gastritis and duodenitis are digestive disorders involving inflammation of the stomach lining (gastritis) or the duodenum (duodenitis). Gastritis and duodenitis can often be asymptomatic with periodic symptomatic episodes of nausea, vomiting, indigestion, stomach pain, and in severe cases, internal bleeding. ICD code included is K29.

Input data

Model inputs

In GBD 2016, chronic gastritis and duodenitis was renamed to total gastritis and duodenitis, symptomatic and asymptomatic (total gastritis). Data inputs were separate for the total gastritis and symptomatic episode models. A systematic review of literature was conducted to capture studies of prevalence, and incidence associated with gastritis and duodenitis. The inclusion criteria were studies that are representative of the national population (ie. excluded populations of patients with *H. pylori*), and studies with sufficient information methods and sample characteristics. Reviews were excluded from the search results.

In addition to literature data, we included hospital inpatient and US claims data from 2000, 2010, and 2012 at the US state level, extracted as prevalence for the total gastritis dataset. The claims data were extracted using all diagnoses, and the hospital data were adjusted for only recording primary diagnoses, using a correction factor from claims data.

For the symptomatic episode dataset, we used extracted hospital inpatient and US claims data from 2000, 2010, and 2012 at the US state level, extracted as incidence to capture individual episodes. These

data were similarly extracted and adjusted to estimate all diagnoses. Data were outliered or excluded if we found them unreasonable when compared to regional, super-regional, and global rates.

The tables below show the number of studies included in GBD 2016, as well as the number of countries or subnational units and GBD world regions represented.

Total gastritis and duodenitis, symptomatic and asymptomatic

	Prevalence	Incidence
Studies	82	1
Countries		
Countries/subnationals	164	1
GBD world regions	16	1

Symptomatic episodes of gastritis and duodenitis

	Prevalence	Incidence
Studies	0	49
Countries		
Countries/subnationals	0	143
GBD world regions	0	8

Disability weights

The basis of the GBD disability weight survey assessments are lay descriptions of sequelae highlighting major functional consequences and symptoms. For GBD 2016, we added the mild sequela for symptomatic episodes of gastritis with proportions of cases derived from the MEPS. The lay descriptions and disability weights for gastritis and duodenitis are shown below:

Severity split	Lay description	DW (95% CI)
Mild gastritis and duodenitis, symptomatic episodes	This person has some pain in the belly that causes nausea but does not interfere with daily activities.	0.011 (0.005-0.021)
Moderate gastritis and duodenitis, symptomatic episodes	This person has pain in the belly and feels nauseated. The person has difficulties with daily activities.	0.114 (0.078 — 0.159)

*The numerous sequelae generated from exclusive combinations of anemia and gastritis each contain custom disability weights. More information can be found in the appendix detailing disability weights.

Modelling strategy

The DisMod model for total gastritis and duodenitis included bounding remission from 0 to 1 (a minimum duration of one year). Reference data were from literature and our most complete US claims dataset (2012). We marked inpatient and other US claims years data with study-level covariates. We used the function in DisMod-MR 2.1 to pull in cause-specific mortality rate (CSMR) data from our CODEm and CODcorrect analyses and match with prevalence data points for the same geography. We calculated excess mortality rate to estimate priors by dividing CSMR by prevalence. We also applied a country-level

covariate for alcohol consumption to prevalence, which we forced positive with a lower bound of 0 and an upper bound of 2. Additionally, we applied lag-distributed income and healthcare access and quality index covariates to excess mortality, forced negative with an upper bound of 0 and a lower bound of -1. Betas and exponentiated values (which can be interpreted as an odds ratio) are shown in the tables below for study-level covariates and country-level covariates.

Covariate type	Covariate	Parameter	beta	Exponentiated beta
Study-level covariate	Hospital Inpatient	Prevalence	-0.81 (-0.86 - -0.70)	0.44 (0.42 - 0.50)
Study-level covariate	Outpatient	Prevalence	-0.28 (-1.23 - 0.79)	0.76 (0.29 - 2.20)
Study-level covariate	Claims data - 2000	Prevalence	0.05 (0.01 - 0.09)	1.05 (1.01 - 1.09)
Study-level covariate	Claims data - 2010	Prevalence	0.23 (0.20 - 0.27)	1.26 (1.22 - 1.31)
Country covariate	Alcohol (liters per capita)	Prevalence	0.00 (0.00 - 0.00)	1.00 (1.00 - 1.00)
Country covariate	LDI (I$ per capita)	Excess mortality rate	-0.14 (-0.17 - -0.12)	0.87 (0.85 - 0.89)
Country covariate	Healthcare access and quality index	Excess mortality rate	-0.07 (-0.07 - -0.07)	0.93 (0.93 - 0.93)

The symptomatic episodes of gastritis and duodenitis DisMod models bounded remission from 52 to 54 (a duration of about one week). We also assumed no incidence from 0 to 2 years old. The reference data were US claims data from 2012, and we marked other US claims years data and inpatient data with specific study-level covariates. We used the function in DisMod-MR 2.1 to pull in cause-specific mortality rate (CSMR) data from our CODEm and CODcorrect analyses and match with incidence data points for the same geography. We calculated excess mortality rate to estimate priors by dividing CSMR by incidence/remission. We also applied a country-level covariate for alcohol consumption to incidence, which we forced positive with a lower bound of 0 and an upper bound of 2. Additionally, we applied lag-distributed income and healthcare access and quality index covariates to excess mortality, forced negative with an upper bound of 0 and a lower bound of -1. Betas and exponentiated values (which can be interpreted as an odds ratio) are shown in the tables below for study-level covariates and country-level covariates.

Covariate type	Covariate	Parameter	beta	Exponentiated beta
Study-level covariate	Hospital Inpatient	Incidence	-1.00 (-1.00 - -0.99)	0.37 (0.37 - 0.37)
Study-level covariate	Claims data - 2000	Incidence	0.01 (-0.03 - 0.05)	1.01 (0.97 - 1.05)
Study-level covariate	Claims data - 2010	Incidence	0.19 (0.16 - 0.22)	1.21 (1.17 - 1.25)
Country covariate	Alcohol (liters per capita)	Incidence	0.00 (0.00 - 0.01)	1.00 (1.00 - 1.01)
Country covariate	LDI (I$ per capita)	Excess mortality rate	-0.14 (-0.16 - -0.12)	0.87 (0.85 - 0.89)
Country covariate	Healthcare access and quality index	Excess mortality rate	-0.04 (-0.04 - -0.03)	0.96 (0.96 - 0.97)

To calculate prevalence of asymptomatic gastritis and duodenitis, we took the estimated prevalence of symptomatic episodes, and subtracted it from our estimated prevalence of total gastritis and duodenitis. Methods for causal attribution of anemia due to gastritis can be found elsewhere in the appendix

detailing strategies for impairments. Each final combination of sequela of anemia and gastritis underwent exclusivity adjustments to prevent double counting.

Appendicitis

Flowchart

Appendicitis

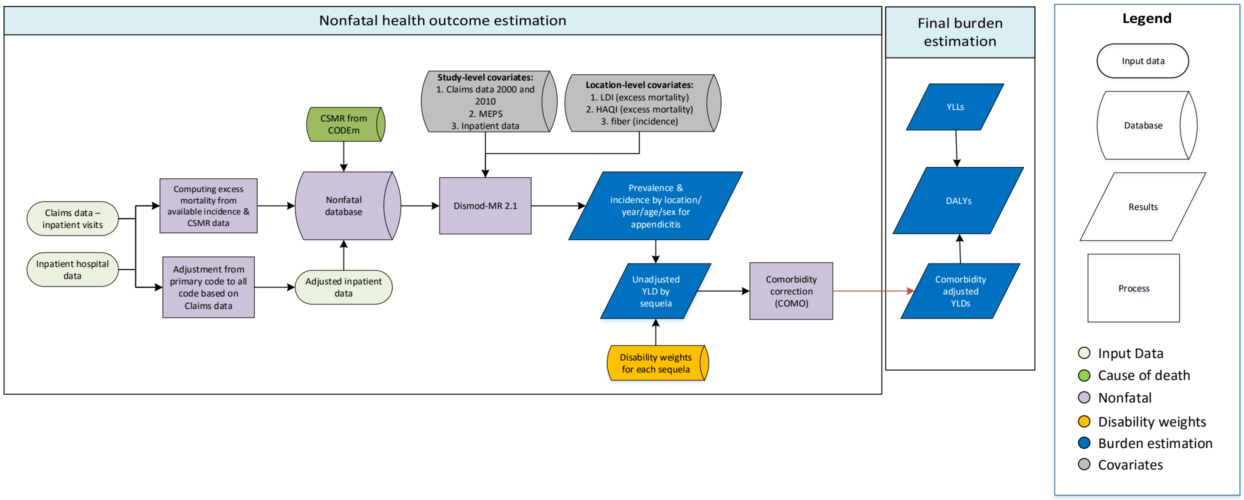

Case definition

Appendicitis is an inflammation of the appendix that causes nausea, vomiting, and sharp pain in the right lower abdomen. Appendicitis requires surgery, or septic shock may set in and the patient will be at risk for severe complications, including sepsis and death. ICD-10 codes included are K35-K35.3, K35.8, K35.80, K35.89, K35.9, K36, K36.0, K37, K37.0, K37.9, and K38.3.

Input data

Model inputs

Since GBD 2010, the data used for appendicitis are hospital inpatient data across 103 separate locations and US claims data for 2000, 2010, and 2012 by US state, primary diagnoses only. Descriptions of search strategies for hospital and claims data are included elsewhere in the appendix. The agreed-upon approach for appendicitis was to rely primarily on these data sources and not conduct a formal literature review. Data were outliered or excluded if we found them unreasonable when compared to regional, super-regional, and global rates.

Table 1. Data inputs (not including hospital inpatient data)

	Prevalence	Incidence
Studies	0	3
Countries/subnationals	0	319
Countries	0	40
GBD world regions	0	15
GBD super-regions	0	7

Severity splits

The basis of the GBD disability weight survey assessments are lay descriptions of sequelae highlighting major functional consequences and symptoms.

Table 2. Severity level and lay description.

Severity split	Lay description	DW (95% CI)
Appendicitis, severe	This person has severe pain in the belly and feels nauseated. The person is anxious and unable to carry out daily activities.	0.324 (0.219–0.442)

Modelling strategy

DisMod-MR 2.1, a Bayesian meta-regression tool, was used to estimate appendicitis prevalence by age, sex, year, and geography (subnational [select countries], country, region, super-region). Prior settings in the DisMod model included bounding remission from 25 to 27 (a duration of about two weeks) for all age groups and capping excess mortality at 0.31. We used study-level covariates to adjust incidence derived from MEPS, and US claims data for 2000 and 2010 toward the level of other incidence data points, which were more representative of the general population.

We used the function in DisMod-MR 2.1 to pull in cause-specific mortality rate (CSMR) data from our CODEm and CODcorrect analyses and match with prevalence data points for the same geography. We calculated excess mortality rate to estimate priors by dividing CSMR by prevalence. We also applied a fiber (g per day) country-level covariate to incidence, forcing a positive relationship with a lower bound of 0. A lag-distributed income (LDI) covariate was applied to excess mortality, log-transformed and forced negative with an upper bound of -0.1 and a lower bound of 0. Similarly, a health care access and quality index (HAQI) covariate was also forced negative (-2, 0) on excess mortality.

Betas and exponentiated values (which can be interpreted as an odds ratio) are shown in the tables below for study-level covariates and country-level covariates:

Table 3. Study-level beta and exponentiated values

Covariate	Parameter	beta	Exponentiated beta
MEPS	Incidence	0.43 (0.14 - 0.72)	1.53 (1.15 - 2.06)
inpatient-only Marketscan, year 2000	Incidence	-0.13 (-0.18 - -0.10)	0.88 (0.84 - 0.91)
inpatient-only Marketscan, year 2010	Incidence	-0.06 (-0.10 - -0.02)	0.94 (0.91 - 0.98)

Table 4. Location-level beta and exponentiated values

Covariate	Parameter	beta	Exponentiated beta
fiber adjusted(g)	Incidence	-0.04 (-0.05 - -0.04)	0.96 (0.95 - 0.96)
LDI (I$ per capita)	Excess mortality rate	-0.00 (-0.00 - -0.00)	1.00 (1.00 - 1.00)

Healthcare access and quality index	Excess mortality rate	-0.00 (-0.00 - -0.00)	1.00 (1.00 - 1.00)

Paralytic Ileus and Intestinal Obstruction

Flowchart

Paralytic ileus and intestinal obstruction (ileus)

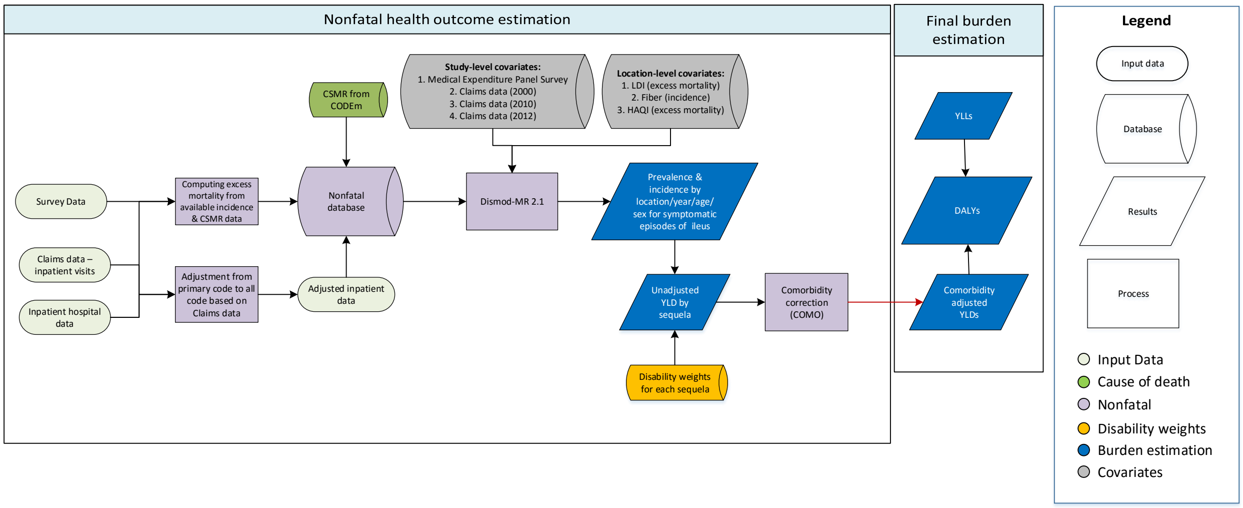

Case definition

Paralytic ileus and intestinal obstruction is a lack of digestive propulsion caused by failed peristalsis, typically requiring surgery. ICD code for paralytic ileus and intestinal obstruction is K56.

Input data

Model inputs

Since GBD 2010, the data used for paralytic ileus and intestinal obstruction have been hospital inpatient data and US claims data for 2000, 2010, and 2012 by US state, primary diagnoses only. Descriptions of search strategies for hospital and claims data are included elsewhere in the appendix. The agreed-upon approach for paralytic ileus and intestinal obstruction was to only use these data sources, and not conduct a literature review. Data were outliered or excluded if we found them unreasonable when compared to regional, super-regional, and global rates.

Table 1. Model inputs (not including hospital inpatient data)

	Prevalence	Incidence
Studies	0	4
Countries/subnationals	0	347
Countries	0	42
GBD world regions	0	16
GBD super-regions	0	7

Severity splits

The basis of the GBD disability weight survey assessments is lay descriptions of sequelae highlighting major functional consequences and symptoms.

Table 2. Severity level and lay description.

Severity level	Lay description	DW (95% CI)
Paralytic ileus and intestinal obstruction, severe	This person has severe pain in the belly and feels nauseated. The person is anxious and unable to carry out daily activities.	0.324 (0.219–0.442)

Modelling strategy

DisMod-MR 2.1, a Bayesian meta-regression tool, was used to estimate paralytic ileus and intestinal obstruction prevalence by age, sex, year, and geography (subnational [select countries], country, region, super-region).

Paralytic ileus and intestinal obstruction was modelled with remission set between 25 and 26 for all age groups, implying a duration of approximately two weeks. We also set a prior for the maximum incidence of 0.002 for ages 0 to 5. The reference data were hospital inpatient data, primary diagnosis only. Study-level covariates were used to adjust incidence derived from the Medical Expenditure Panel Survey (MEPS) and US claims data for 2000, 2010, and 2012 toward the levels in hospital inpatient data.

We used the function in DisMod-MR 2.1 to pull in cause-specific mortality rate (CSMR) data from our CODEm and CODcorrect analyses and match with incidence data points for the same geography. We calculated the excess mortality rate to estimate priors by dividing CSMR by incidence/remission. We also applied location-level covariates: fibre consumption to incidence with an upper bound of 0 and a lower bound of -2, a lag-distributed income covariate to excess mortality, log-transformed and forced negative with an upper bound of 0 and a lower bound of -1, and a health care access and quality index (HAQI) covariate with an upper bound of 0 and lower bound of -2.

Betas and exponentiated values (which can be interpreted as an odds ratio) are shown in the tables below for study-level covariates and country-level covariates:

Table 3. Study-level beta and exponentiated values

Covariate	Parameter	beta	Exponentiated beta
MEPS	Incidence	0.05 (-0.18 - 0.28)	1.05 (0.84 - 1.32)
inpatient-only Marketscan, year 2000	Incidence	0.27 (0.22 - 0.31)	1.30 (1.25 - 1.36)
inpatient-only Marketscan, year 2010	Incidence	0.63 (0.59 - 0.66)	1.88 (1.81 - 1.94)
inpatient-only Marketscan, year 2012	Incidence	0.60 (0.56 - 0.64)	1.82 (1.76 - 1.89)

Table 4. Location-level beta and exponentiated values

Covariate	Parameter	beta	Exponentiated beta
fiber adjusted(g)	Incidence	-0.01 (-0.03 - -0.00)	0.99 (0.97 - 1.00)
LDI (I$ per capita)	Excess mortality rate	-0.23 (-0.25 - -0.20)	0.80 (0.78 - 0.82)

Healthcare access and quality index	Excess mortality rate	-0.02 (-0.02 - -0.02)	0.98 (0.98 - 0.98)

Inguinal, femoral and abdominal hernias

Flowchart

Inguinal, femoral and abdominal hernias

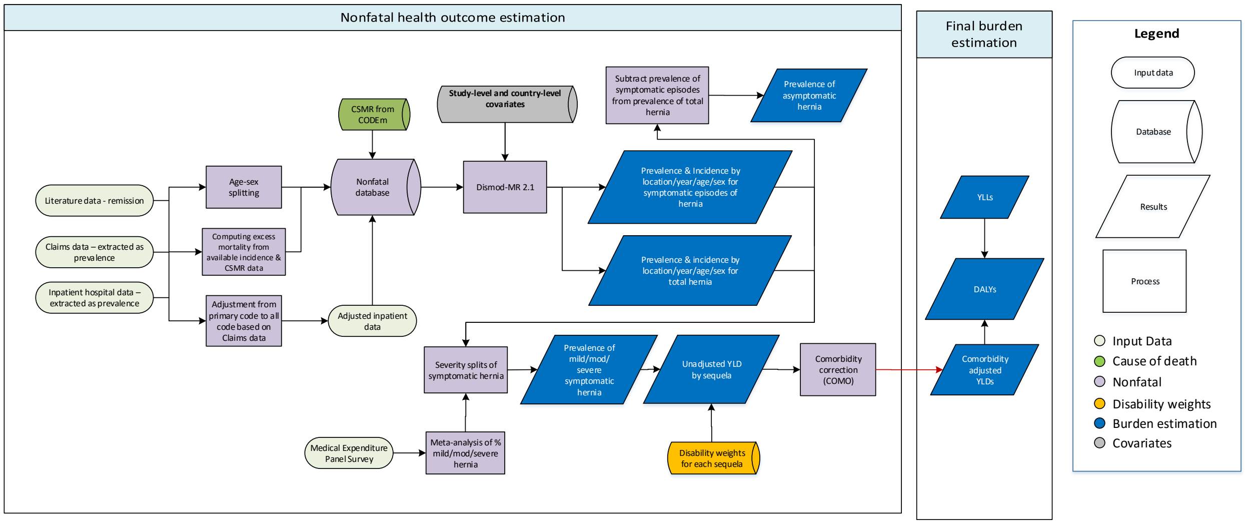

Flowchart

Case definition

A hernia is a digestive disorder that occurs when an internal organ protrudes through an opening in the tissue that holds it in place. Hernias most commonly occur in the inner and outer groin, and in the abdomen, and require surgical intervention. However, it can take several months before surgery occurs, resulting in a chronic condition. ICD codes used are K40, K41, K42, K44, K45, and K46.

Input data

Model inputs

For GBD 2010 and 2013, the data used for hernias were hospital inpatient data extracted as prevalence and remission data calculated based off a regression from a paper describing mean wait times for elective surgery in OECD countries. For GBD 2016, we use the same data, but only included regression results for OECD countries. We also included US claims data for 2000, 2010, and 2012 by US state. The agreed-upon approach for hernias was to use only these data sources, and not conduct a literature review. For GBD 2016, we renamed chronic inguinal, femoral, and abdominal hernias to total inguinal, femoral, and abdominal hernias.

In GBD 2016 we created a new database and model for inguinal, femoral and abdominal hernias, symptomatic episodes, which models prevalence and incidence of symptomatic episodes within inpatient settings as a primary diagnosis. The input data for the symptomatic database include the hospital inpatient data (primary diagnoses only) extracted as prevalence, US claims data (inpatient only) for 2000, 2010, and 2012, and the remission data on elective surgery wait times described above.

Data were outliered or excluded if we found them unreasonable when compared to regional, super-regional, and global rates.

The tables below show the number of studies included in GBD 2016, as well as the number of countries or subnational units and GBD world regions represented. These numbers reflect both the total and symptomatic databases.

Total inguinal, femoral, and abdominal hernias

	Prevalence	Incidence	Remission
Studies	0	0	1
Countries	39	0	33
Countries/subnationals	329	0	33
GBD world regions	7	0	4

Inguinal, femoral, and abdominal hernias, symptomatic episodes

	Prevalence	Incidence	Remission
Studies	0	0	1
Countries	40	0	33
Countries/subnationals	332	0	33
GBD world regions	7	0	4

Disability weights

The basis of the GBD disability weight survey assessments are lay descriptions of sequelae highlighting major functional consequences and symptoms. For GBD 2016, we added moderate and severe sequela to prevalent cases of symptomatic hernias . The lay descriptions and disability weights for inguinal, abdominal, and femoral hernias are shown below:

Severity split	Lay description	DW (95% CI)
Mild Inguinal, abdominal, and femoral hernia, symptomatic episodes	This person has some pain in the belly that causes nausea but does not interfere with daily activities.	0.011 (0.005-0.021)
Moderate Inguinal, abdominal, and femoral hernia, symptomatic episodes	This person has pain in the belly and feels nauseous. The person has difficulties with daily activities.	0.114 (0.080-0.159)
Severe Inguinal, abdominal, and femoral hernia, symptomatic episodes	This person has severe pain in the belly and feels nauseated. The person is anxious and unable to carry out daily activities.	0.324 (0.219-0.442)

Modelling strategy

We modelled total hernia and symptomatic hernia using separate DisMod models, informed by prevalence and incidence data from hospital and US claims data, paired with the calculated remission from the regression described in Siciliani et al[1].. Priors were set with a maximum incidence of 0.02 for ages 0 to 4, and bounds on remission from 0 to 5 (a minimum duration of about ten weeks) – we also assumed prevalence at birth due to umbilical hernias. Reference data were US claims data from 2012, and we marked inpatient hospital data and the remaining years of US claims data with separate study-level covariates. We used the function in DisMod-MR 2.1 to pull in cause-specific mortality rate (CSMR) data from our CODEm and CoDcorrect analyses and match with incidence data points for the same geography. We calculated excess mortality rate to estimate priors by dividing CSMR by incidence/remission. We also applied a lag-distributed income covariate to both remission and excess mortality, log transformed. We forced this covariate negative for excess mortality (bounds from -1 to 0), and positive for remission (bounds from 0.5 to 2). Betas and exponentiated values (which can be interpreted as an odds ratio) are shown in the tables below for study-level covariates and country-level covariates.

Total inguinal, femoral, and abdominal hernias

Covariate type	Covariate	Parameter	beta	Exponentiated beta
Study-level covariate	Hospital Inpatient	Prevalence	-2.02 (-2.06 - -2.00)	0.13 (0.13 - 0.14)
Study-level covariate	Claims data - 2000	Prevalence	-0.37 (-0.41 - -0.33)	0.69 (0.67 - 0.72)
Study-level covariate	Claims data - 2010	Prevalence	-0.06 (-0.08 - -0.03)	0.95 (0.92 - 0.97)
Country covariate	LDI (I$ per capita)	Excess mortality rate	1.01 (0.90 - 1.11)	2.74 (2.45 - 3.04)
Country covariate	LDI (I$ per capita)	Excess mortality rate	-0.00 (-0.00 - -0.00)	1.00 (1.00 - 1.00)

Inguinal, femoral, and abdominal hernias, symptomatic episodes

Covariate type	Covariate	Parameter	beta	Exponentiated beta
Study-level covariate	Hospital Inpatient	Prevalence	-1.28 (-1.30 - -1.25)	0.28 (0.27 - 0.29)
Study-level covariate	Claims data - 2000	Prevalence	-0.68 (-0.70 - -0.65)	0.51 (0.50 - 0.52)
Country covariate	LDI (I$ per capita)	Excess mortality rate	0.85 (0.51 - 0.98)	2.34 (1.66 - 2.66)
Country covariate	LDI (I$ per capita)	Excess mortality rate	-0.00 (-0.00 - -0.00)	1.00 (1.00 - 1.00)

We calculated prevalence of asymptomatic hernias by subtracting the estimated prevalence of symptomatic hernias from our estimated prevalence of total hernias.

Inflammatory Bowel Disease

Flowchart

Inflammatory Bowel Disease (IBD)

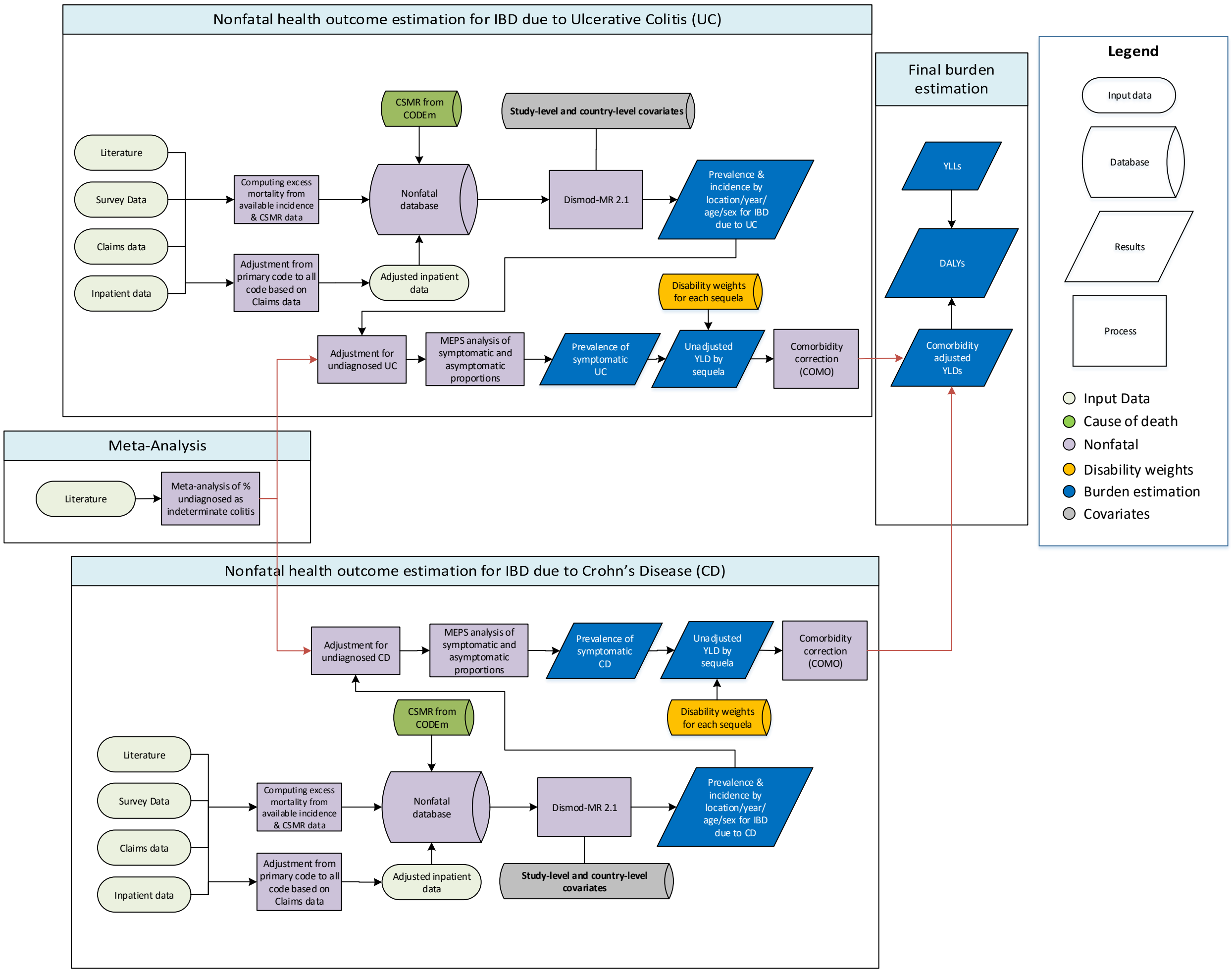

Case definition

Inflammatory bowel disease is a type of digestive disorder involving inflammation of the colon and gastrointestinal tract, most commonly classified as Crohn's disease (inflammation of the small and large intestine) and ulcerative colitis (inflammation of the colon and rectum). In a significant proportion of cases of inflammatory bowel disease, neither Crohn's disease nor ulcerative colitis is definitively the diagnosis, and a diagnosis of indeterminate colitis is applied. ICD codes are K50 for Crohn's disease, K51 for ulcerative colitis, and K52 for indeterminate colitis.

Input data

Model inputs

Data inputs were separate for ulcerative colitis and Crohn's disease, but a single systematic review of literature was conducted to capture studies of prevalence and incidence for all inflammatory bowel diseases for GBD 2016. Studies were excluded if they were not representative of the national population, or if they had insufficient study and sampling methods. Reviews were excluded from the search results. In addition to literature data, we included US claims data from 2000, 2010, and 2012 at the US state level, extracted as prevalence. For GBD 2016 we included inpatient hospital data as model inputs. Data were outliered or excluded if we found them unreasonable when compared to regional, super-regional, and global rates.

The tables below show the number of studies included in GBD 2016, as well as the number of countries or subnational units and GBD world regions represented.

Noninfective inflammatory bowel disease due to ulcerative colitis

	Prevalence	Incidence
Studies	15	98
Countries	41	52
Countries/subnationals	286	61
GBD world regions	7	6

Noninfective inflammatory bowel disease due to Crohn's disease

	Prevalence	Incidence
Studies	17	84
Countries	46	47
Countries/subnationals	292	55
GBD world regions	7	5

Disability weights

The basis of the GBD disability weight survey assessments are lay descriptions of sequelae highlighting major functional consequences and symptoms. For GBD 2016, we used the MEPS to find the proportion of asymptomatic prevalence for both ulcerative colitis and crohn's disease. The lay descriptions and disability weights for sequelae associated with inflammatory bowel disease are shown below, and are applied to symptomatic cases only:

Severity split	Lay description	DW (95% CI)
Crohn's disease, symptomatic	This person has cramping abdominal pain, has diarrhea several times a day, and feels very tired for two months every year. When the person does not have symptoms, there is anxiety about them returning.	0.231 (0.156-0.32)

Ulcerative colitis, symptomatic	This person has cramping abdominal pain, has diarrhea several times a day, and feels very tired for two months every year. When the person does not have symptoms, there is anxiety about them returning.	0.231 (0.156-0.32)

Modeling strategy

The modelling strategy for all inflammatory bowel disease encompasses separate DisMod models for ulcerative colitis and Crohn's disease, which are then adjusted to account for inflammatory bowel disease due to indeterminate colitis.

The DisMod model for ulcerative colitis included setting remission to 0 for all age groups and setting incidence to 0 for ages 0 to 1. Reference data were US claims data from 2012, and we marked hospital inpatient, literature, and the remaining years of US claims data with separate study-level covariates. We used lag-distributed income (log-transformed) and healthcare access and quality index country level covariates on excess morality (forced negative) to further inform the model. We applied a country-level covariate of absolute value of average latitude to prevalence, and a ln-ASDR (age standardized death rate) fixed effect on prevalence. The ASDR data are taken from our CODEm and CODcorrect analyses for all inflammatory bowel disease. Betas and exponentiated values (which can be interpreted as an odds ratio) are shown in the tables below for study-level covariates and country-level covariates.

**Covariate type**	**Covariate**	**Parameter**	**beta**	**Exponentiated beta**
Study-level covariate	Hospital Inpatient	Prevalence	0.40 (0.40 - 0.40)	1.49 (1.49 - 1.49)
Study-level covariate	MEPS	Prevalence	-0.01 (-0.02 - -0.00)	0.99 (0.98 - 1.00)
Study-level covariate	Literature	Prevalence	-0.00 (-0.01 - -0.00)	1.00 (0.99 - 1.00)
Study-level covariate	Claims data - 2000	Prevalence	-0.00 (-0.01 - -0.00)	1.00 (0.99 - 1.00)
Study-level covariate	Claims data - 2010	Prevalence	-0.00 (-0.00 - -0.00)	1.00 (1.00 - 1.00)
Country covariate	Absolute value of average latitude	Prevalence	0.05 (0.05 - 0.06)	1.06 (1.06 - 1.06)
Country covariate	LDI (I$ per capita)	Excess mortality rate	-0.50 (-1.00 - -0.02)	0.61 (0.37 - 0.98)
Country covariate	Healthcare access and quality index	Excess mortality rate	-0.50 (-1.00 - 0.00)	0.61 (0.37 - 1.00)

The DisMod model for Crohn's disease included setting remission to 0 for all age groups and setting incidence to 0 for ages 0 to 2. Reference data were US claims data from 2012 and inpatient data. We marked literature, MEPS, and the remaining years of US claims data with specific study-level covariates. We used lag-distributed income (log-transformed) and healthcare access and quality index country level covariates on excess morality (forced negative) to further inform the model. We applied a country-level ln-ASDR (age standardized death rate) fixed effect on prevalence. The ASDR data are taken from our CODEm and COD correct analyses for all inflammatory bowel disease. Betas and exponentiated values (which can be interpreted as an odds ratio) are shown in the tables below for study-level covariates and country-level covariates.

Covariate type	Covariate	Parameter	beta	Exponentiated beta
Study-level covariate	MEPS	Prevalence	-0.01 (-0.04 - -0.00)	0.99 (0.96 - 1.00)
Study-level covariate	Literature	Prevalence	-0.00 (-0.01 - -0.00)	1.00 (0.99 - 1.00)
Study-level covariate	Claims data - 2000	Prevalence	-0.00 (-0.00 - -0.00)	1.00 (1.00 - 1.00)
Study-level covariate	Claims data - 2010	Prevalence	-0.00 (-0.00 - -0.00)	1.00 (1.00 - 1.00)
Country covariate	LDI (I$ per capita)	Excess mortality rate	-0.47 (-0.97 - -0.02)	0.62 (0.38 - 0.98)
Country covariate	Healthcare access and quality index	Excess mortality rate	-0.17 (-0.23 - -0.10)	0.84 (0.79 - 0.91)

We conducted a meta-analysis to calculate the ratio of inflammatory bowel disease cases that are categorized as indeterminate colitis to cases that are characterized as either Crohn's disease or ulcerative colitis. The results of this meta-analysis were a mean ratio of 0.059 (0.047-0.071). We then took the prevalence results of the DisMod models for ulcerative colitis and Crohn's disease and adjusted them by multiplying by a normal distribution of draws from 1.059 (1.047-1.071) to encompass all inflammatory bowel disease cases.

Vascular Intestinal Disorders

Flowchart

Vascular Intestinal Disorders

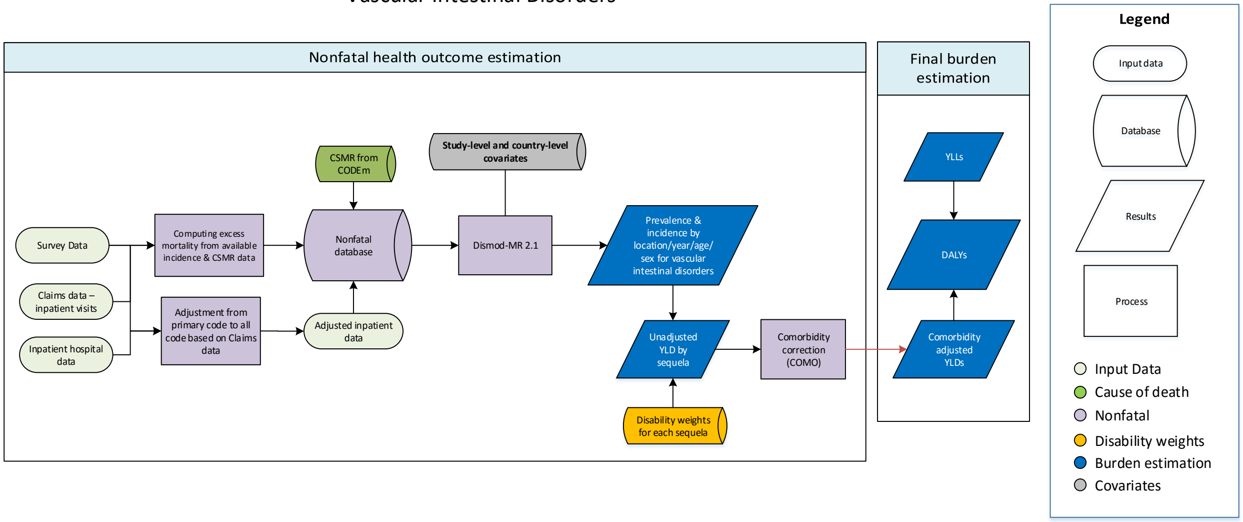

Input data and methodological summary

Case definition

Vascular intestinal disorder, also known as intestinal ischemia, occurs when there is decreased blood supply to the gastrointestinal tract causing injury to the bowel. Vascular intestinal disorders typically require surgery. The ICD code for vascular intestinal disorders is K55.

Input data

Model inputs

For GBD 2016, the data used for vascular intestinal disorders are hospital inpatient data and US claims data for 2000, 2010, and 2012 by US state, primary diagnoses only. Descriptions of search strategies for hospital and claims data are included elsewhere in the appendix. The agreed-upon approach for vascular intestinal disorders was to use only these data sources and not conduct a literature review. Data were outliered or excluded if we found them unreasonable when compared to regional, super-regional, and global rates.

The table below shows the number of literature studies included in GBD 2016, as well as the number of countries or subnational units and GBD world regions represented.

Table 1. Data inputs

	Incidence
Studies	0
Countries	11
Countries/subnationals	97
GBD world regions	5

Disability weights

The basis of the GBD disability weight survey assessments are lay descriptions of sequelae highlighting major functional consequences and symptoms. The lay descriptions and disability weights for vascular intestinal disorders are shown below:

Table 2. Severity level and lay descriptions

Severity split	Lay description	DW (95% CI)
Vascular intestinal disorders, severe	This person has severe pain in the belly and feels nauseated. The person is anxious and unable to carry out daily activities.	0.324 (0.219-0.442)

Modelling strategy

Prior settings in the DisMod model included bounding remission from 2 to 12 (a duration from about four weeks to half a year) for all age groups and capping excess mortality at 10. The reference data were US claims data from year 2012, primary diagnosis only, and we marked inpatient US claims data years 2000 and 2010, and hospital inpatient data with separate study-level covariates.

We used the function in DisMod-MR 2.1 to pull in cause-specific mortality rate (CSMR) data from our CODEm and CoDcorrect analyses and match with incidence data points for the same geography. We calculated excess mortality rate to estimate priors by dividing CSMR by incidence/remission. We also applied a lag-distributed income covariate (log transformed) and healthcare access and quality index covariate to excess mortality, forced negative with an upper bound of 0 and a lower bound of -1.

Betas and exponentiated values (which can be interpreted as an odds ratio) are shown in the tables below for study-level covariates and country-level covariates:

Table 3. Study covariate

**Covariate type**	**Covariate**	**Parameter**	**beta**	**Exponentiated beta**
Study-level covariate	Hospital Inpatient	Incidence	0.29 (0.21 - 0.34)	1.33 (1.24 - 1.40)
Study-level covariate	inpatient-only Marketscan, year 2000	Incidence	-0.56 (-0.61 - -0.51)	0.57 (0.54 - 0.60)

Study-level covariate	inpatient-only Marketscan, year 2010	Incidence	0.01 (-0.04 - 0.05)	1.01 (0.96 - 1.05)
Country covariate	LDI (I$ per capita)	Excess mortality rate	-0.43 (-0.51 - -0.35)	0.65 (0.60 - 0.70)
Country covariate	Healthcare access and quality index	Excess mortality rate	-0.03 (-0.04 - -0.03)	0.97 (0.96 - 0.97)

Gallbladder and Biliary Diseases

Flowchart

Gallbladder and biliary diseases

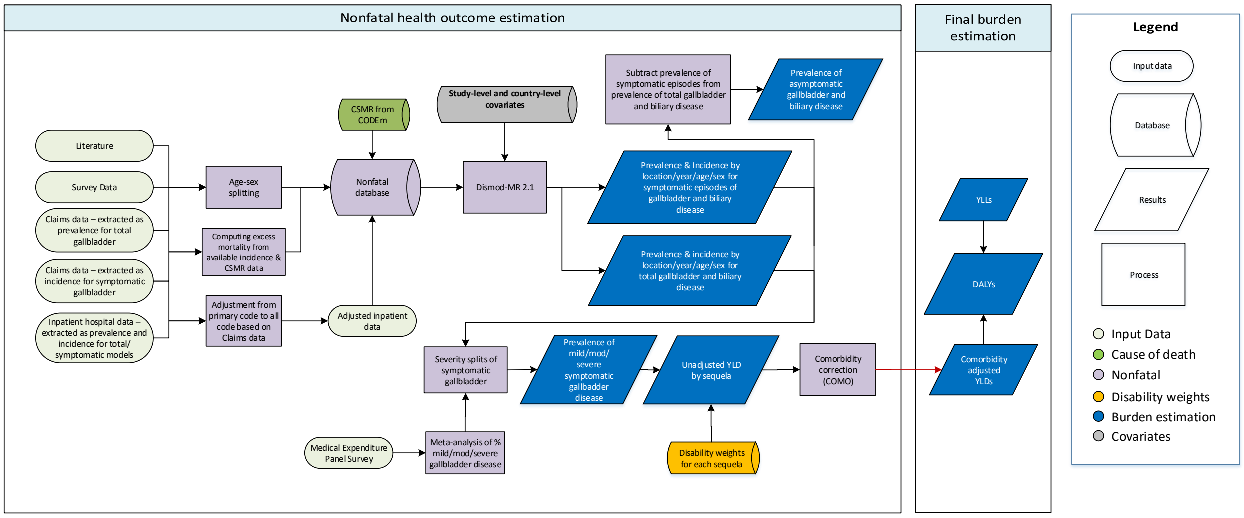

Input data and methodological summary

Case definition

Gallbladder and biliary diseases are digestive disorders including gallstones, cholecystitis, cholangitis, and other diseases of the gallbladder and biliary tract. Cholecystitis is an inflammation of the gallbladder, and cholangitis is an infection or inflammation of the bile duct, the result of a bacterial infection – both are often the result of gallstones. Gallbladder and biliary diseases, especially the presence of gallstones, can often be asymptomatic with periodic symptomatic episodes of severe abdominal pain, nausea, vomiting, and at times fever. ICD codes included are K80, K81, K82, and K83.

Input data

Model inputs

For GBD 2016, we renamed chronic gallbladder and biliary diseases to total gallbladder and biliary diseases (total gallbladder disease). Data inputs were separate for the total gallbladder disease and symptomatic episode models. A systematic review of literature was conducted to capture studies of prevalence and incidence of gallbladder and biliary diseases. For GBD 2016, we performed a systematic literature search using PubMed. Studies not representative of the national population (ie. *H. Pylori* cohorts, patients presenting with pain), studies without sufficient information on study and sampling methods, and reviews were excluded. In addition to literature data, we included hospital inpatient and US claims data from 2000, 2010, and 2012 at the US state level, extracted as prevalence for the total gallbladder model, and extracted an incident case for the symptomatic gallbladder model. Data were

outliered or excluded if we found them unreasonable when compared to regional, super-regional, and global rates.

The tables below show the number of studies included in GBD 2016, as well as the number of countries or subnational units and GBD world regions represented.

Total gallbladder and biliary diseases

	Prevalence	Incidence
Studies	28	2
Countries	45	2
Countries/subnationals	307	2
GBD world regions	7	2

Symptomatic episodes of gallbladder and biliary diseases

	Prevalence	Incidence
Studies	1	1
Countries	1	38
Countries/subnationals	1	320
GBD world regions	1	6

Disability weights

The basis of the GBD disability weight survey assessments are lay descriptions of sequelae highlighting major functional consequences and symptoms. For GBD 2016, we included the mild and severe sequela and applied the disability weights to prevalent cases in the symptomatic gallbladder model. The lay descriptions and disability weights for gallbladder and biliary disease are shown below:

Severity split	Lay description	DW (95% CI)
Mild gallbladder and biliary disease, symptomatic episodes	This person has some pain in the belly that causes nausea but does not interfere with daily activities.	0.011 (0.005-0.021)
Moderate gallbladder and biliary disease, symptomatic episodes	This person has pain in the belly and feels nauseous. The person has difficulties with daily activities.	0.114 (0.080-0.159)
Severe gallbladder and biliary disease, symptomatic episodes	This person has severe pain in the belly and feels nauseated. The person is anxious and unable to carry out daily activities.	0.324 (0.219-0.442)

Modelling strategy

The DisMod model for total gallbladder and biliary diseases included bounding remission from 0 to 1 (a minimum duration of one year). Reference data were from literature and US claims data year 2012, and we marked inpatient hospital and US claims data years 2000 and 2010 with separate year-specific study-

level covariates. We used the function in DisMod-MR 2.1 to pull in cause-specific mortality rate (CSMR) data from our CODEm and CODcorrect analyses and match with prevalence data points for the same geography. We calculated excess mortality rate to estimate priors by dividing CSMR by prevalence. We also applied a lag-distributed income covariate to excess mortality, log transformed and forced negative with an upper bound of 0 and a lower bound of -1. Betas and exponentiated values (which can be interpreted as an odds ratio) are shown in the tables below for study-level covariates and country-level covariates.

**Covariate type**	**Covariate**	**Parameter**	**beta**	**Exponentiated beta**
Study-level covariate	Hospital Inpatient	Prevalence	0.60 (0.59 - 0.60)	1.82 (1.81 - 1.82)
Study-level covariate	Claims data - 2000	Prevalence	0.19 (0.15 - 0.23)	1.21 (1.16 - 1.26)
Study-level covariate	Claims data - 2010	Prevalence	-0.02 (-0.05 - 0.02)	0.98 (0.95 - 1.02)
Country covariate	LDI (I$ per capita)	Excess mortality rate	-0.56 (-0.57 - -0.54)	0.57 (0.56 - 0.58)

The symptomatic episodes of gallbladder and biliary diseases DisMod model bounded remission from 9 to 26 (a duration of about two to six weeks). The reference data were US claims data from 2012, and we marked other US claims years data, inpatient data, and literature with specific study-level covariates. We used the function in DisMod-MR 2.1 to pull in cause-specific mortality rate (CSMR) data from our CODEm and CODcorrect analyses and match with incidence data points for the same geography. We calculated excess mortality rate to estimate priors by dividing CSMR by incidence/remission. We also applied a lag-distributed income covariate to excess mortality, log transformed and forced negative with an upper bound of 0 and a lower bound of -1. Betas and exponentiated values (which can be interpreted as an odds ratio) are shown in the tables below for study-level covariates and country-level covariates.

**Covariate type**	**Covariate**	**Parameter**	**beta**	**Exponentiated beta**
Study-level covariate	Hospital Inpatient	Incidence	0.70 (0.70 - 0.70)	2.01 (2.01 - 2.01)
Study-level covariate	Literature	Incidence	-0.87 (-1.13 - -0.60)	0.42 (0.32 - 0.55)
Study-level covariate	Claims data - 2000	Incidence	-0.34 (-0.37 - -0.30)	0.71 (0.69 - 0.74)
Study-level covariate	Claims data - 2010	Incidence	-0.00 (-0.01 - -0.00)	1.00 (0.99 - 1.00)
Country covariate	LDI (I$ per capita)	Excess mortality rate	-0.66 (-0.67 - -0.65)	0.51 (0.51 - 0.52)

To calculate prevalence of asymptomatic gallbladder and biliary diseases, we took the estimated prevalence of symptomatic episodes and subtracted it from our estimated prevalence of total gallbladder and biliary diseases.

Pancreatitis

Flowchart

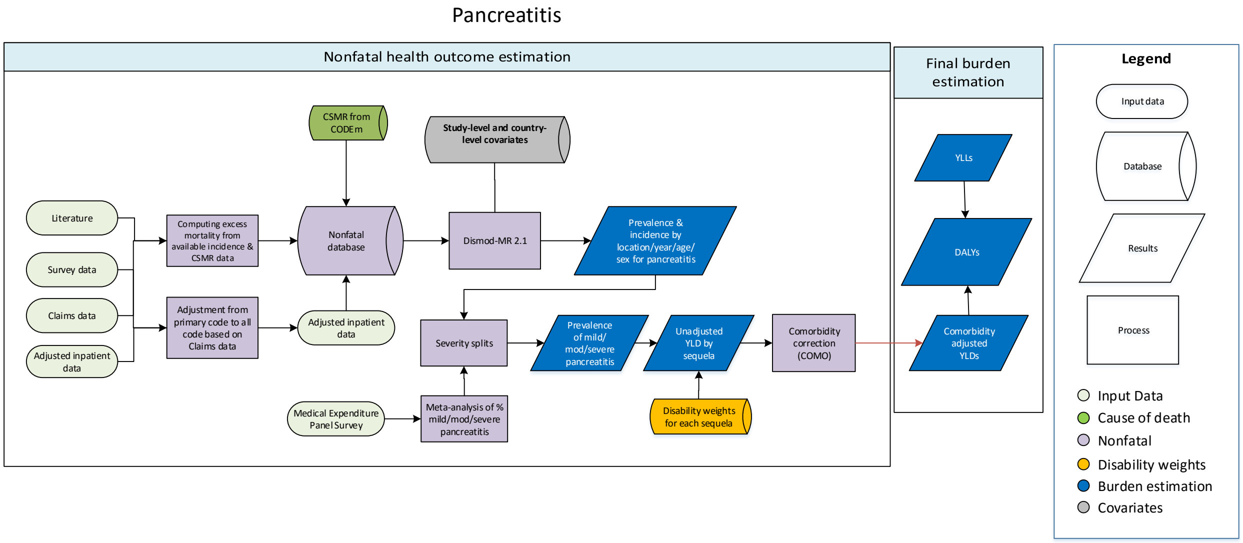

Input data and methodological summary

Case definition

Pancreatitis is the inflammation of the pancreas, and often results in nausea, stomach pain, and vomiting. We model acute and chronic pancreatitis together, using ICD codes K85 and K86.

Input data

Model inputs

For GBD 2016, a systematic review of the prevalence and incidence of pancreatitis throughout the world was conducted. The exclusion criteria were studies clearly not representative of the national population (ie. alcoholics or smokers), self-reported data, and reviews. In addition to literature, we included hospital inpatient and US claims data for 2000, 2010, and 2012 by US state. Data were outliered or excluded if we found them unreasonable when compared to regional, super-regional, and global rates.

The table below shows the number of literature studies included in GBD 2016, as well as the number of countries or subnational units and GBD world regions represented.

	Prevalence	Incidence
Studies	5	19
Countries	4	40
Countries/subnationals	4	318
GBD world regions	2	6

Disability weights

The basis of the GBD disability weight survey assessments are lay descriptions of sequelae highlighting major functional consequences and symptoms. The lay descriptions and disability weights for pancreatitis are shown below:

Severity split	Lay description	DW (95% CI)
Pancreatitis cases, mild	This person has some pain in the belly that causes nausea but does not interfere with daily activities.	0.011 (0.005-0.021)
Pancreatitis cases, moderate	This person has pain in the belly and feels nauseous. The person has difficulties with daily activities.	0.114 (0.080-0.159)
Pancreatitis cases, severe	This person has severe pain in the belly and feels nauseated. The person is anxious and unable to carry out daily activities.	0.324 (0.219-0.442)

Modelling strategy

Prior settings in the DisMod model included bounding remission from 8 to 9 (a duration from about six weeks) for all ages. The reference data were literature and US claims data from 2012. We marked hospital inpatient and other US claims years data with a separate year-specific study-level covariate. Additionally, we marked literature that reported acute pancreatitis only, with an acute study-level covariate.

We used the function in DisMod-MR 2.1 to pull in cause-specific mortality rate (CSMR) data from our CODEm and CODcorrect analyses and match with incidence and prevalence data points for the same geography. We calculated excess mortality rate to estimate priors by dividing CSMR by incidence/remission (or prevalence, if applicable). We also applied a country-level log-transformed age-standardized SEV scalar covariate for pancreatitis to incidence with bounds of 0.75 to 1.25, and a lag-distributed income covariate to excess mortality, log-transformed and forced negative with an upper bound of 0 and a lower bound of -1.

Betas and exponentiated values (which can be interpreted as an odds ratio) are shown in the tables below for study-level covariates and country-level covariates:

**Covariate type**	**Covariate**	**Parameter**	**beta**	**Exponentiated beta**
Study-level covariate	Acute	Incidence	-0.92 (-1.06 - -0.78)	0.40 (0.35 - 0.46)
Study-level covariate	Hospital Inpatient	Incidence	0.90 (0.90 - 0.90)	2.46 (2.46 - 2.46)
Study-level covariate	Claims data - 2000	Incidence	-0.44 (-0.48 - -0.40)	0.64 (0.62 - 0.67)

Study-level covariate	Claims data -2010	Incidence	0.07 (0.03 - 0.10)	1.07 (1.03 - 1.10)
Country covariate	SEV scalar, Pancreatitis	Incidence	1.24 (1.21 - 1.25)	3.45 (3.35 - 3.49)
Country covariate	LDI (I$ per capita)	Excess mortality rate	-1.00 (-1.00 - -1.00)	0.37 (0.37 - 0.37)

Other digestive diseases

In addition to the digestive diseases described above, there are many diverse types of digestive diseases with a range of severities and associated sequelae. Because these digestive diseases are diverse in their underlying causes and risk factors as well as in their associated health outcomes, modelling them together in a DisMod-MR model would not produce reliable estimates of prevalence or excess mortality. Instead, we calculated the YLDs caused by digestive diseases directly using a YLD/YLL ratio.

We calculated the ratio of YLDs to YLLs across the specified digestive diseases for which nonfatal outcomes were modelled, using YLL estimates from the GBD 2016 cause of death (CoD) analysis. We then multiplied this YLD/YLL ratio by the YLL estimates for other digestive diseases from the GBD 2016 CoD analysis, providing us with an estimate of the YLDs associated with other digestive diseases.

Alzheimer’s Disease and Other Dementias

Flowchart

Alzheimer's Disease and Other Dementias

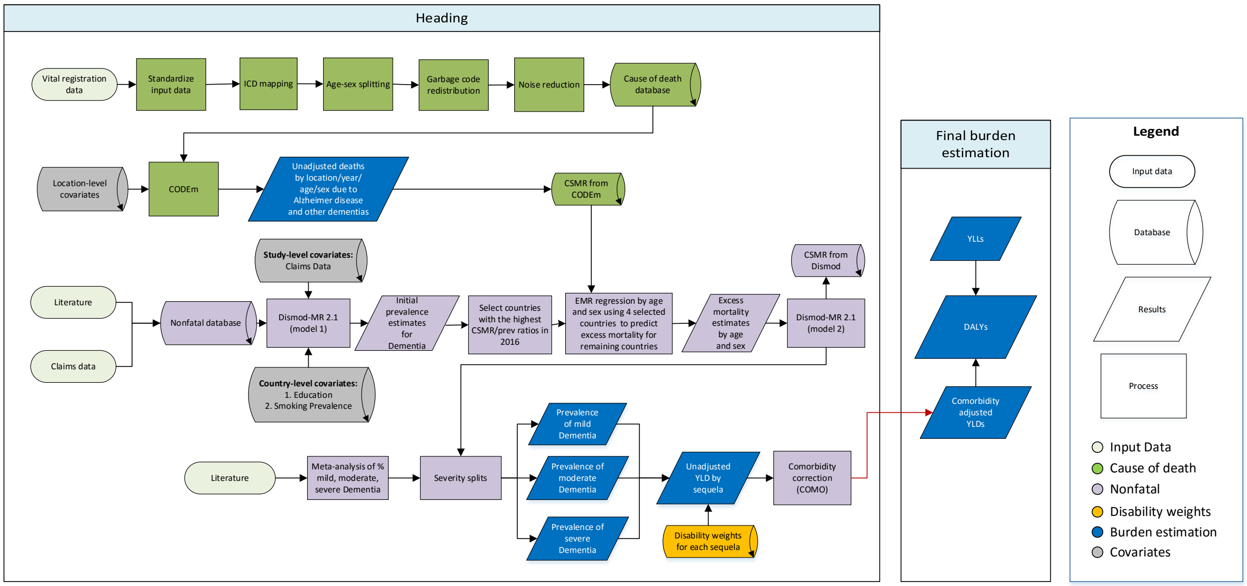

Input data and methodological summary

Case definition

Dementia is a progressive, degenerative, and chronic neurological disorder typified by memory impairment and other neurological dysfunctions. For the purposes of GBD 2016, we use the Diagnostic and Statistical Manual of Mental Disorders III, IV or V, or ICD case definitions as the reference. A wide array of diagnostic and screening instruments exists, including Clinical Dementia Rating scale (CDR), Mini Mental State Examination (MMSE), and the Geriatric Mental State (GMS). For severity rating purposes we use the CDR as the reference. The relevant ICD-10 codes for dementia are F00, F01, F02, F03, G30, and G31. The ICD-9 codes are 290, 291.2, 291.8, 294 and 331.

Unlike most causes in the Global Burden of Disease project, dementia mortality and morbidity estimates are modelled jointly. This is because of marked discrepancies between prevalence data and cause of death data. Specifically, prevalence data suggest little to no variation over time (eg, 1990–2016) whereas age-standardized mortality rates in vital registrations in high-income countries have increased multiple times over this same period. Additionally, prevalence variation between countries is much smaller than the variation in death rates assigned to dementia in vital registration. We attribute these discrepancies to changing coding practices rather than epidemiological change.

Because of this joint procedure, descriptions of the mortality estimation process are included where relevant.

Input data

Model inputs

To inform our estimates of burden due to dementia, we use mortality data from vital registration systems, as well as prevalence data from surveys, and administrative data such as claims sources.

An updated systematic review was conducted covering January 2015 to October 2016, and search terms[1] were set to capture studies for all dementia, including its sub-types. The search yielded 1,208 initial hits and 27 were marked for extraction. Inclusion criteria comprised studies that reported prevalence, incidence, remission rate, excess mortality rate, relative risk of mortality, standardised mortality ratio, or with-condition mortality rate. Studies with no clearly defined sample were excluded. A flow chart documenting this review is displayed below.

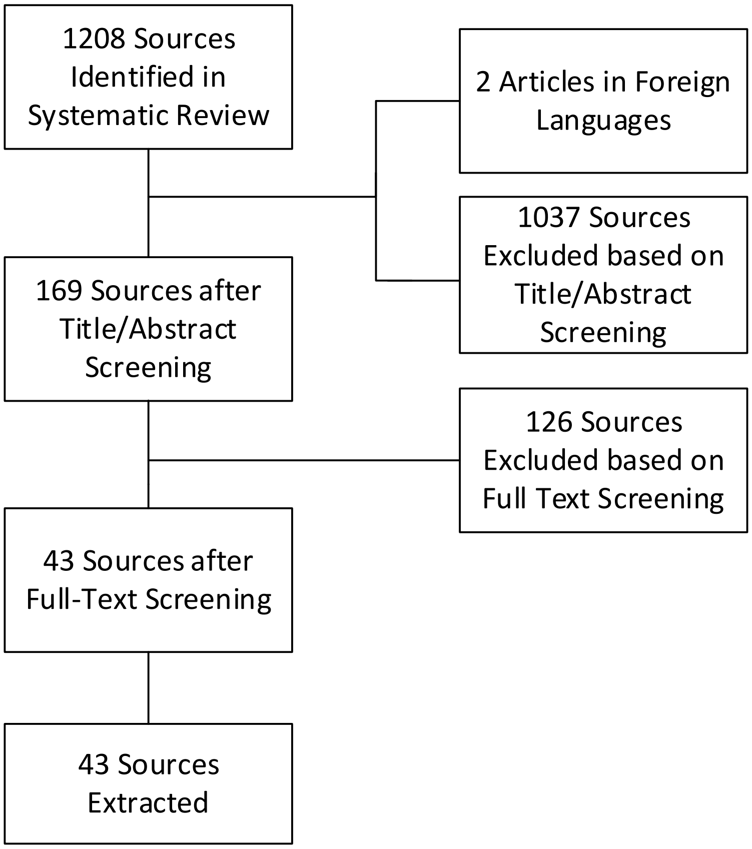

[1] ((dementia[Title/Abstract]) AND ((incidence[Title/Abstract]) or (prevalence[Title/Abstract])) AND ("2015/01/23"[Date - Publication] : "3000"[Date - Publication])

Additionally, a table describing the density and distribution of the epidemiological data available for GBD 2016 is presented below:

	Prevalence	Incidence	Mortality risk	Severity
Studies	174	60	15	17
Countries/subnationals	131	37	19	15
GBD world regions	17	10	9	8

Severity splits

In GBD 2013 (and still used in GBD 2016), we extracted data from studies reporting on mild, moderate, and severe dementia. As the data indicate an age pattern with greater proportions with more severe disease in the very old, we restricted our analyses to studies reporting on severity <70, 70-79, and 80+ ages. Most of these studies reported severity based on the CDR scale: CDR=1 as mild, CDR=2 as moderate, and CDR=3 as severe dementia. Other studies report staging of dementia according to MMSE; the Functional capacity scale; the Cambridge Mental Disorders of the Elderly Examination (CAMDEX); the scale of Hughes and the Geriatric Mental State (GMS). This year we excluded all studies that used the DSM III criteria, as we found that these sources reported systematically higher severities. We used a random effects meta-analysis to pool the data by severity level.

We multiplied estimations of prevalence (country-year-sex-age-specific) by the fractions of mild, moderate, and severe dementia and estimated 95% uncertainty intervals at the 1,000-draw level.

Severity level	Lay description	DW (95% CI)	Severity distribution
Mild	The person has some trouble remembering recent events and finds it hard to concentrate and make decisions and plans.	0.069 (0.046–0.099)	<70: 79% (65–86%)<br>70-79: 71% (51–84%)<br>80+: 61% (44–76%)
Moderate	The person has memory problems and confusion, feels disoriented, at times hears voices that are not real, and needs help with some daily activities.	0.377 (0.252–0.508)	<70: 17% (5–24%)<br>70-79: 19% (8–37%)<br>80+: 26% (16–34%)
Severe	The person has complete memory loss, no longer recognizes close family members, and requires help with all daily activities.	0.449 (0.304–0.595)	<70: 4% (2–42%)<br>70-79: 9% (8–17%)<br>80+: 12% (9–24%)

Modelling strategy

As mentioned above, the estimation of morbidity due to dementia occurs in conjunction with the mortality estimation.

First, we ran a CODEm model for dementia and extracted the mortality rates by age, sex, and geography for 2016.

Second, we ran a DisMod-MR 2.1 model with all data on incidence, prevalence, and mortality risk (relative risk, standardized mortality ratio, or with-condition mortality rates) and a setting of zero remission and

extracted 2016 prevalence by age, sex, and geography. To account for potential systematic differences between claims and survey data, we crosswalked for each year of claims data.

Third, we selected countries where the sum of cause-specific mortality rate to prevalence ratio for males and females exceeded 0.4 (excluding small island nations and those without vital registration). This resulted in choosing the United States, Sweden, Finland and Puerto Rico. The choice to pick fewer countries for this regression compared to GBD 2015, which used 30 countries in the EMR regression, was motivated by a desire to reduce the spread in EMR values, as countries used in the regression retain their original EMR values.

Fourth, we used a linear effects regression with dummies on age group and sex to predict excess mortality (ie, the ratio of cause-specific mortality rate and prevalence) by age and sex, the results of which are found in the Tables below.

Table: Fixed effect coefficients of EMR regression. Outcome: ln(EMR)

Independent variables	Coef	Std. error	P value	95% Confidence Interval	
Male	0.047	0.054	0.394	-0.06	0.153
Age 40-59	-3.299	0.115	0.000	-3.524	-3.073
Age 60-64	-2.627	0.115	0.000	-2.852	-2.401
Age 65-69	-2.395	0.115	0.000	-2.62	-2.169
Age 70-74	-2.068	0.115	0.000	-2.293	-1.842
Age 75- 79	-1.686	0.115	0.000	-1.911	-1.461
Age 80-84	-1.362	0.115	0.000	-1.587	-1.137
Age 85-89	-0.949	0.115	0.000	-1.175	-0.724
Age 90-94	-0.426	0.115	0.001	-0.651	-0.2
Constant	-1.539	0.086	0.000	-1.707	-1.371

Table: Predicted EMR values by age and sex (95% CI)

	Male	Female
Age 40-59	0.008 (0.007 - 0.01)	0.008 (0.007 - 0.009)
Age 60-64	0.016 (0.014 - 0.019)	0.016 (0.013 - 0.018)
Age 65-69	0.021 (0.017 - 0.024)	0.02 (0.017 - 0.023)
Age 70-74	0.029 (0.024 - 0.034)	0.027 (0.023 - 0.032)
Age 75- 80	0.042 (0.035 - 0.049)	0.04 (0.034 - 0.047)
Age 80-84	0.058 (0.049 - 0.068)	0.055 (0.047 - 0.064)
Age 85-89	0.088 (0.074 - 0.104)	0.084 (0.07 - 0.098)
Age 90-94	0.147 (0.124 - 0.174)	0.14 (0.118 - 0.165)
Age 95+	0.226 (0.19 - 0.268)	0.216 (0.183 - 0.254)

Fifth, these estimates were added to a second DisMod-MR 2.1 model as pertaining to the full 1990–2016 estimation period. For the four countries included in the regression, we retained their age- and sex-specific ratios and entered those also as pertaining to the full 1990–2016 estimation period. Thus, the model reflects the cause-specific mortality rate if all countries over time would have had the average propensity to code to dementia as an underlying cause of death similar to the selected four countries in 2016.

In this model, we assumed zero remission as well as zero excess mortality and incidence until age 40. Because of lack of consistency between prevalence and incidence data in locations where the underlying data, we excluded incidence data from the final model. In a few locations we found good consistency between prevalence and incidence and these were locations where incidence and prevalence were collected as part of the same study. In other locations (Beijing, Australia, Italy, Canada, various states in the US, Mexico, and Nigeria) we noted that DisMod-MR 2.1 was pushing the fit above the available prevalence data and below incidence – "averaging the difference." In all cases the incidence and prevalence data were collected by different studies. We decided to drop the incidence estimates as measuring incidence of dementia when symptoms are still mild is more prone to measurement bias than measuring prevalence when the diagnosis has become more obvious over time.

The table below provides additional information on the country covariates used in this model, as well as beta and exponentiated beta values.

Variable	Measure	Beta	Exponentiated Beta Value (CI)
Mean years of education, age-standardized	prevalence	-0.053	0.95 (0.85–1.00)
Smoking prevalence, age-standardized both sex	Prevalence	0.11	1.11 (1.06–1.17)
US claims data 2000	prevalence	-0.69	0.50 (0.48–0 .55)
US claims data 2010	prevalence	-0.25	0.78 (0.75–0.86)
US claims data 2012	prevalence	-0.25	0.78 (0.75–0.86)

As described above, we used crosswalks to standardize the claims data relative to existing literature data. Age-standardized education was used as a proxy for general brain health/use that may be protective of dementia – specifically Alzheimer's disease. Smoking prevalence (age-standardised, both sexes) was also used as a covariate to guide estimates, as the literature has shown a positive relationship between smoking and dementia.

Parkinson's Disease

Flowchart

Parkinson’s Disease

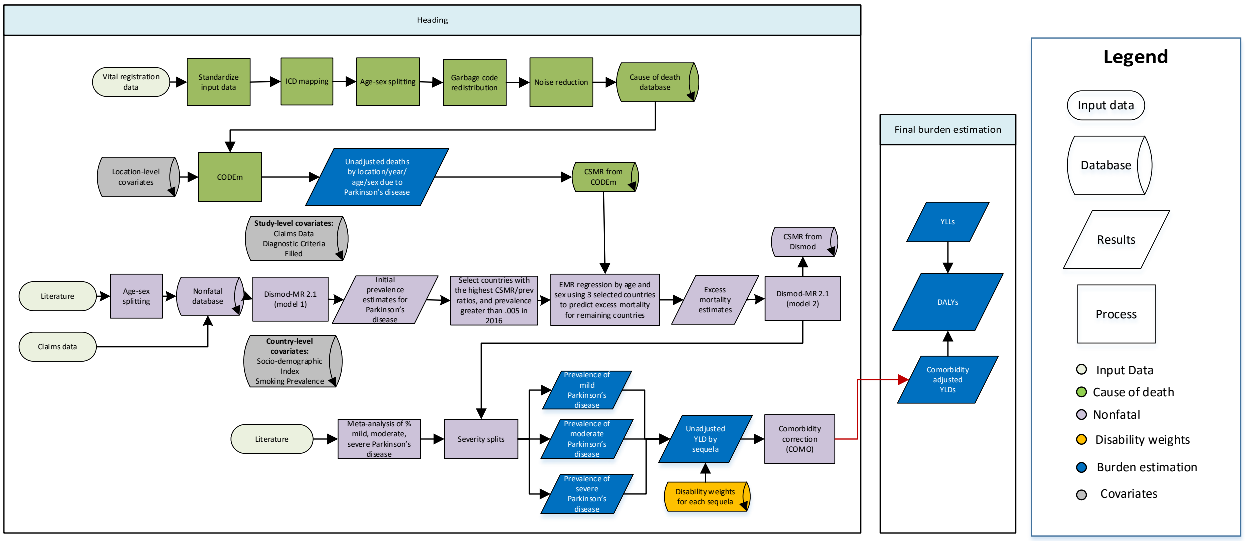

Case definition

Parkinson’s disease is a chronic, degenerative, and progressive neurological condition typified by the loss of motor mobility and control – most notably tremors. The corresponding ICD-10 codes are G20, G21, and G22. Our case definition for GBD is the presence of at least two of the four primary symptoms: (1) tremors/trembling, (2) bradykinesia, (3) stiffness of limbs and torso, and (4) posture instability.

Unlike most causes in the Global Burden of Disease project, Parkinson’s disease mortality and morbidity estimates are modelled jointly. This is because of marked discrepancies between prevalence data and cause of death data. Specifically, prevalence data suggest little to no variation over time (eg, 1990–2016) whereas age-standardized mortality rates in vital registrations in high-income countries have increased multiple times over this same period. Additionally, prevalence variation between countries is much smaller than the variation in death rates assigned to Parkinson’s disease in vital registration. We attribute these discrepancies to changing coding practices rather than epidemiological change.

Because of this joint procedure, descriptions of the mortality estimation process are included where relevant.

Input data

Model inputs

To inform our estimates of burden due to Parkinson’s disease, we use mortality data from vital registration systems, as well as prevalence data from surveys, and administrative data such as claims sources.

For GBD 2015, a systematic review was conducted from January 2011 to December 2015, and the search terms were set to capture studies for Parkinson's disease.[1] This search term resulted in 1,433 initial hits with 17 sources marked for extraction. Studies with no clearly defined sample or that drew from specific clinic/patient organizations were excluded.

The following table provides a description of the density and distribution of literature data informing the Parkinson's estimates:

	Prevalence	Incidence	Mortality risk
Studies	94	34	10
Countries/subnationals	134	31	10
Regions	16	9	3

Beyond the exclusion of studies using non-representative populations, there are no substantial adjustment or outliering criteria for the Parksinson's model. Certain studies have been outliered on a case-by-case basis due to subsequent review and exclusion due to inappropriateness of the study design, and overly broad age and sex groups that conflict with existing gold-standard age-sex-specific data – where possible.

Severity splits

As in GBD 2013, we use Hoehn and Yahr stages to determine severity. However, for GBD 2016, the cutpoints were updated in order to more accurately correspond with the lay descriptions of severities. Specifically, a Hoehn and Yahr stage 4 now corresponds to a designation of severe, where before it was classified as moderate.

Severity	Stage
Mild	≤2.0
Moderate	2.5-3.5
Severe	≥4

For GBD 2016, a literature review was completed to update the severity splits meta-analysis. The systematic review covered 1/1/2008 to 11/10/2016 and the search terms were set to capture studies reporting prevalence of Parkinson's by Hoehn and Yahr stage.[2] The search term resulted in 234 hits with 21 marked for extraction.

The following figures show the results of the meta-analysis on Hoehn and Yahr stage:

[1] (((((Parkinson disease AND epidemiology) AND ("2011/01/01"[PDat] : "2015/12/31"[PDat]))) AND ((Parkinson disease AND epidemiology))))

[2] (("1/1/2008"[Date - Publication] : "2016"[Date - Publication])) AND (parkinson disease[MeSH Terms] OR parkinson disease) AND (epidemiology OR prevalence OR incidence) AND (Hoehn) AND (Yahr) AND (stage)

Figure 1. Percentage of mild cases of Parkinson's disease in population-based studies

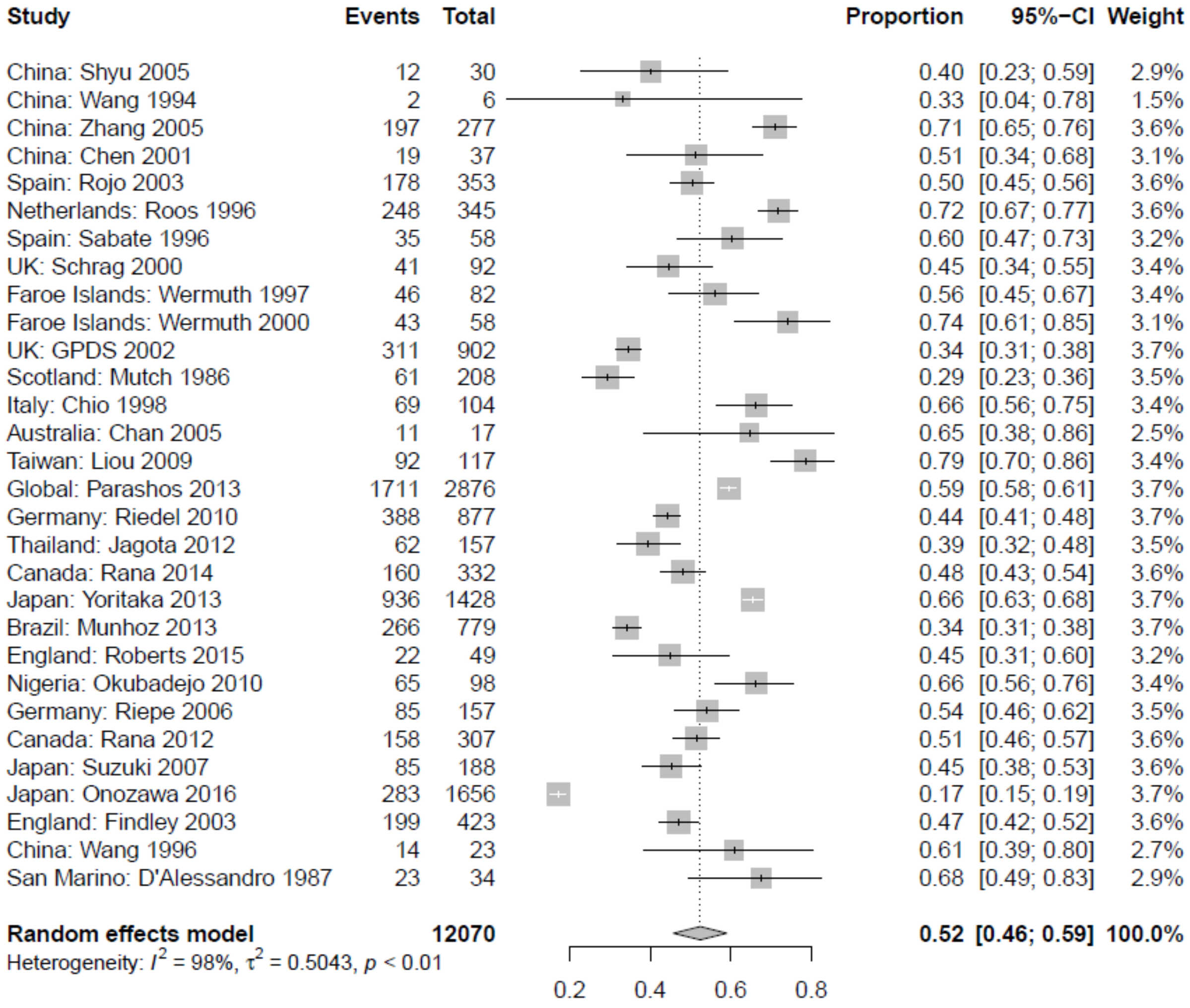

Figure 2. Percentage of moderate cases of Parkinson's disease in population-based studies

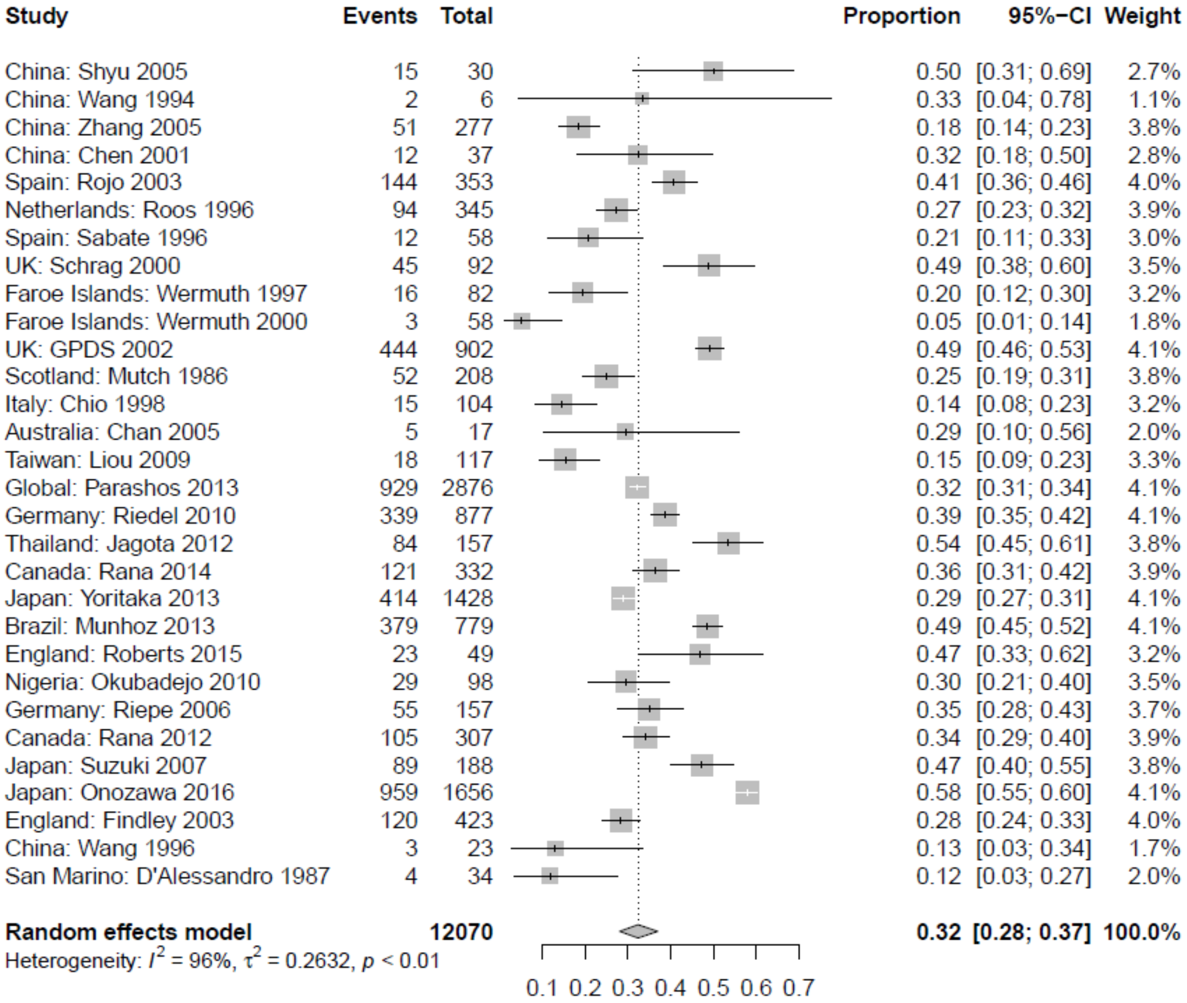

Figure 3. Percentage of severe cases of Parkinson's disease in population-based studies

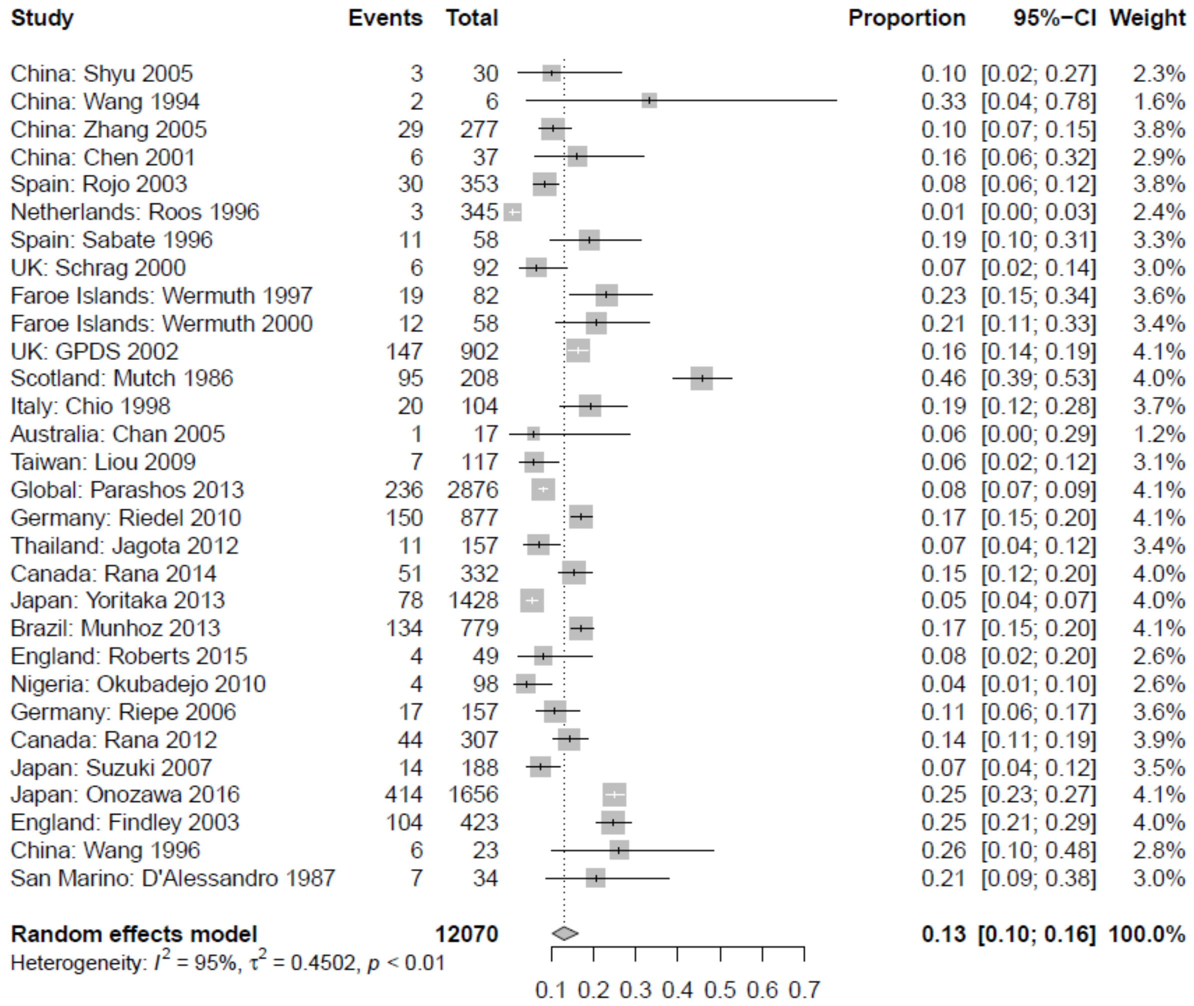

Severity estimates were generated by multiplying estimates of prevalence (country-year-sex-age-specific) by the fractions of mild, moderate, and severe PD and estimated 95% confidence intervals by taking 1,000 draws.

The following table provides the lay description and disability weights associated with Parkinson's disease.

Severity level	Lay description	DW (95% CI)
Mild	Has mild tremors and moves a little slowly, but is able to walk and do daily activities without assistance.	0.01 (0.005–0.019)
Moderate	Has moderate tremors and moves slowly, which causes some difficulty in walking and daily activities. The person has some trouble swallowing, talking, sleeping, and remembering things.	0.267 (0.181–0.372)

Severe	Has severe tremors and moves very slowly, which causes great difficulty in walking and daily activities. The person falls easily and has a lot of difficulty talking, swallowing, sleeping, and remembering things.	0.575 (0.396–0.73)

Modelling strategy

First, we ran a CODEm model for Parkinson disease and extracted the mortality rates by age, sex, and geography for 2016.

Second, we ran a DisMod-MR 2.1 model with all data on incidence, prevalence, and mortality risk (relative risk, standardized mortality ratio, or with-condition mortality rates) and a setting of zero remission and extracted 2016 prevalence by age, sex, and geography. To account for potential systematic differences between claims and survey data, we crosswalked for each year of claims data.

Third, we selected the three countries (United States, Finland, and Austria) with the highest cause-specific mortality rate (from step 1) to prevalence (from step two) ratio, which also had prevalence rates above 0.0005, and a population greater than 1 million.

Fourth, we used a linear effects regression with dummies on age group and sex to predict excess mortality (ie, the ratio of cause-specific mortality rate and prevalence) by age and sex, the results of which are found in the tables below.

Table: Fixed-effect coefficients of EMR regression. Outcome: ln(EMR)					
Independent variables	Coef	Std. error	P value	95% Confidence Interval	
Male	0.214	0.074	0.006	0.069	0.359
Age 40-59	-3.522	0.157	0.000	-3.829	-3.214
Age 60-64	-2.716	0.157	0.000	-3.024	-2.409
Age 65-69	-2.236	0.157	0.000	-2.544	-1.929
Age 70-74	-1.686	0.157	0.000	-1.993	-1.378
Age 75- 80	-1.194	0.157	0.000	-1.502	-0.887
Age 80-84	-0.779	0.157	0.000	-1.087	-0.471
Age 85-89	-0.493	0.157	0.003	-0.800	-0.185
Age 90-94	-0.203	0.157	0.202	-0.511	0.104
Constant	-2.097	0.117	0.000	-2.326	-1.867

Table: Predicted EMR values by age and sex (95% CI)		
	Male	Female
Age 40-59	0.005 (0.004 - 0.006)	0.004 (0.003 - 0.005)
Age 60-64	0.01 (0.008 - 0.013)	0.008 (0.006 - 0.01)
Age 65-69	0.016 (0.013 - 0.02)	0.013 (0.01 - 0.016)
Age 70-74	0.028 (0.023 - 0.035)	0.023 (0.018 - 0.029)
Age 75- 80	0.047 (0.037 - 0.059)	0.037 (0.029 - 0.046)
Age 80-84	0.071 (0.055 - 0.089)	0.057 (0.045 - 0.07)
Age 85-89	0.093 (0.073 - 0.117)	0.076 (0.06 - 0.093)

Age 90-94	0.126 (0.099 - 0.155)	0.101 (0.08 - 0.127)
Age 95+	0.154 (0.122 - 0.191)	0.123 (0.097 - 0.153)

Fifth, these estimates were added to a second DisMod-MR 2.1 model as pertaining to the full 1990–2016 estimation period. For the three countries included in the regression, we retained their age- and sex-specific ratios and entered those also as pertaining to the full 1990–2016 estimation period. Thus, the model reflects the cause-specific mortality rate if all countries over time would have had the average propensity to code to dementia as an underlying cause of death similar to the selected three countries in 2016.

In this model, we assumed zero remission among all ages, with no incidence or excess mortality for ages zero to 20 years old. We ignore data on incidence, relative risk, standardized mortality ratio, and with-condition mortality as these were shown to be inconsistent with prevalence estimates. We also constrain the super-region random effects for prevalence and incidence to -0.25 and 0.25 to account for spurious inflation of regional differences.

We make one study-level crosswalk: Diagnostic Criteria. Studies that do not use the gold-standard case definition of presence of at least two of the four main symptoms are crosswalked to meet this gold standard definition. The table below shows the effect of this crosswalk, which results in a downward adjustment of non-standard data points. For GBD 2015, an additional adjustment was made for Case Ascertainment, or studies that ascertain cases on clinical record review rather than using live diagnostic processes. However, this adjustment was not significant for GBD 2016 and was therefore changed to a z-cov, which effects the uncertainty of the estimates.

Additionally, claims data for 2000, 2010, and 2012 are adjusted via study covariates to account for systematic differences between the claims data and the literature. We use a country-level crosswalk to assist DisMod in estimating global patterns. We use Socio-demographic Index as a proxy to capture possible social and cultural risk factors or modifiers of Parkinson's prevalence.

The following table provides an overview of the study-level and country covariates used in the Parkinson's model.

Covariate	Measure	Beta	Exponentiated
Socio-demographic Index	prevalence	1.44<br>(1.26 – 1.63)	4.22<br>(3.53 – 5.08)
All MarketScan, year 2012	prevalence	-0.0043<br>(-0.016 - -0.00051)	1.00<br>(0.98 – 1.00)
All MarketScan, year 2010	prevalence	-0.0083<br>(-0.025 - -0.00021)	0.99<br>(0.97 – 1.00)
All MarketScan, year 2000	prevalence	-0.0086<br>(-0.022 - -0.00044)	0.99<br>(0.98 – 1.00)
(Un)Filled diagnostic criteria	prevalence	0.20<br>(0.13 - 0.27)	1.22<br>(1.14 – 1.31)

Multiple Sclerosis (MS)

Flowchart

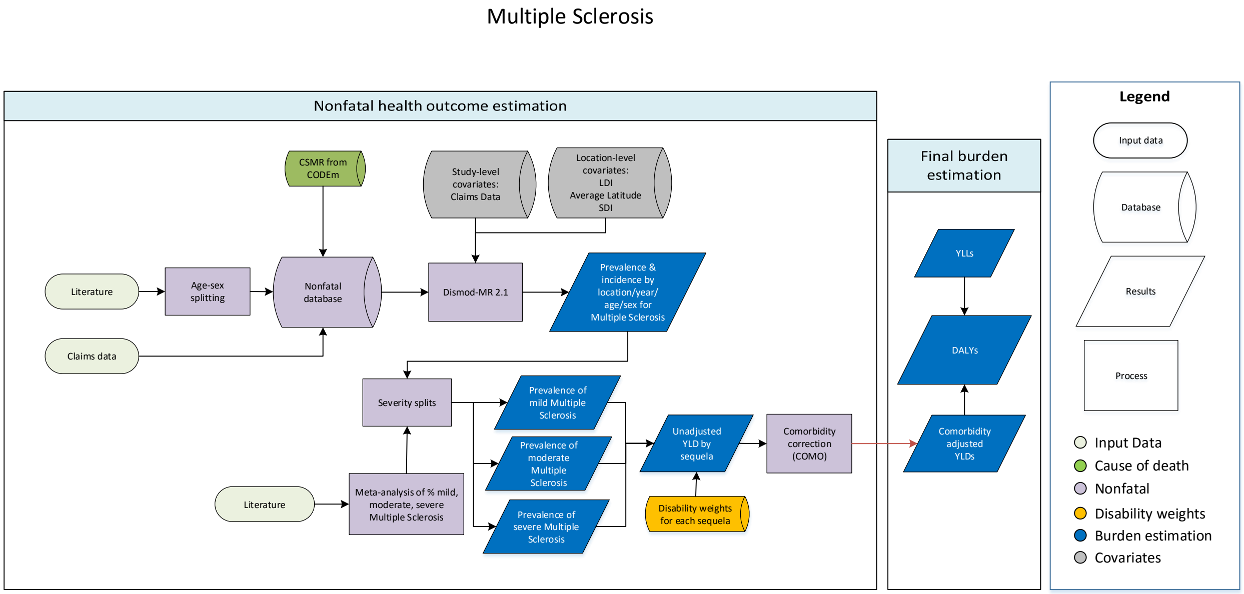

Input data and methodological summary

Case definition

Multiple sclerosis is a chronic, degenerative, and progressive neurological condition typified by the damaging of the myelin sheaths. For GBD, the McDonald's criteria for diagnosis are considered the gold standard, but other definitions such as Poser Committee's criteria and self-report of a doctor's diagnosis are also included. The ICD-10 code for MS is G35.

Input data

A systematic review was conducted for MS for GBD 2015. The search using (multiple sclerosis AND epidemiology AND ("2011/01/01"[PDat] : "2015/12/31"[PDat])) from 1/1/2011-7/15/15 yielded 1756 hits with 28 sources marked for extraction.

The data underpinning estimates of burden due to MS are generally of two types. The first are representative, population-based surveys. This includes retrospective case/hospital report analysis, nationally representative health studies and the like. Studies with no clearly defined sample or that draw from specific clinic/patient organizations were excluded during the systematic review phase. The second type are claims data from the United States from 2000, 2010, and 2012. Additional information on the source and preparation of these data is provided elsewhere.

The following table provides a description of the density and distribution of literature data informing the MS estimates:

	Prevalence	Incidence	Mortality risk
Studies	132	65	14
Countries/subnationals	105	26	10
GBD world regions	11	8	3

Beyond the exclusion of studies using non-representative populations, there are no substantial adjustment or outliering criteria for the MS model. Certain studies have been outliered on a case-by-case basis due to: (1) subsequent review and exclusion due to inappropriate of the study design, and overly broad age and sex groups that conflict with existing gold standard age-sex specific data – where possible.

Severity splits

For GBD 2016, we updated the meta-analysis of all eligible studies that reported EDSS. The search using (("2008"[Date - Publication] : "2016"[Date - Publication])) AND (multiple sclerosis[MeSH Terms] OR multiple sclerosis) AND (epidemiology OR prevalence OR incidence) AND ("Kurtzke's Expanded Disability Status Scale") from 1/1/2008 to 11/14/2016 yielded 355 hits, with 10 marked for extraction.

As in GBD 2013, we use Kurtzke's Expanded Disability Status Scale (EDSS) to determine severity splits for MS. However, for GBD 2016 we added a category for asymptomatic multiple sclerosis in order to capture the initial stages of relapse-remitting multiple sclerosis which has no disability associated. The EDSS scores corresponding to each severity are as follows:

Asymptomatic: EDSS = 0
Mild: 0 < EDSS ≤ 3.5
Moderate: 3.5 < EDSS ≤ 6.5
Severe: 6.5 < EDSS ≤ 9.5

The table below illustrates severity levels, lay descriptions, and DWs.

Severity level	Lay description	DW (95% CI)
Asymptomatic	-	0<br>(0-0)
Mild	Has mild loss of feeling in one hand, is a little unsteady while walking, has slight loss of vision in one eye, and often needs to urinate urgently.	0.183<br>(0.124–0.253)
Moderate	Needs help walking, has difficulty with writing and arm coordination, has loss of vision in one eye and cannot control urinating.	0.463<br>(0.313–0.613)
Severe	Has slurred speech and difficulty swallowing. The person has weak arms and hands, very limited and stiff leg movement, has loss of vision in both eyes and cannot control urinating.	0.719<br>(0.534–0.858)

Because not all sources had information on the number of cases with EDSS stage 0, instead reporting on a mild category, we implemented a two-step meta-analysis strategy. First we subsetted the studies to those that reported on the number of cases with EDSS stage 0, and did meta-analyses on the proportion of asymptomatic and mild cases. Then, we conducted meta-analyses on the full dataset to get the proportion mild, moderate and severe and we squeezed the asymptomatic and mild categories from the previous meta-analyses into the mild category established by the meta-analysis on the full dataset.

The following figures provide the result of the first meta-analysis on the asymptomatic and mild categories.

Figure 1. Asymptomatic cases of MS

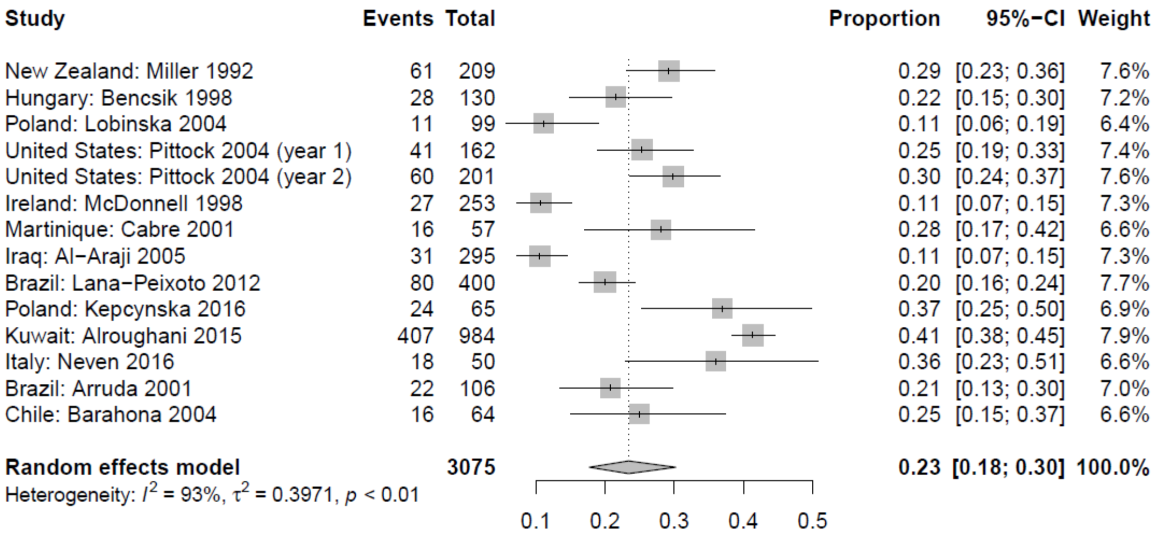

Figure 2. Mild Cases of MS

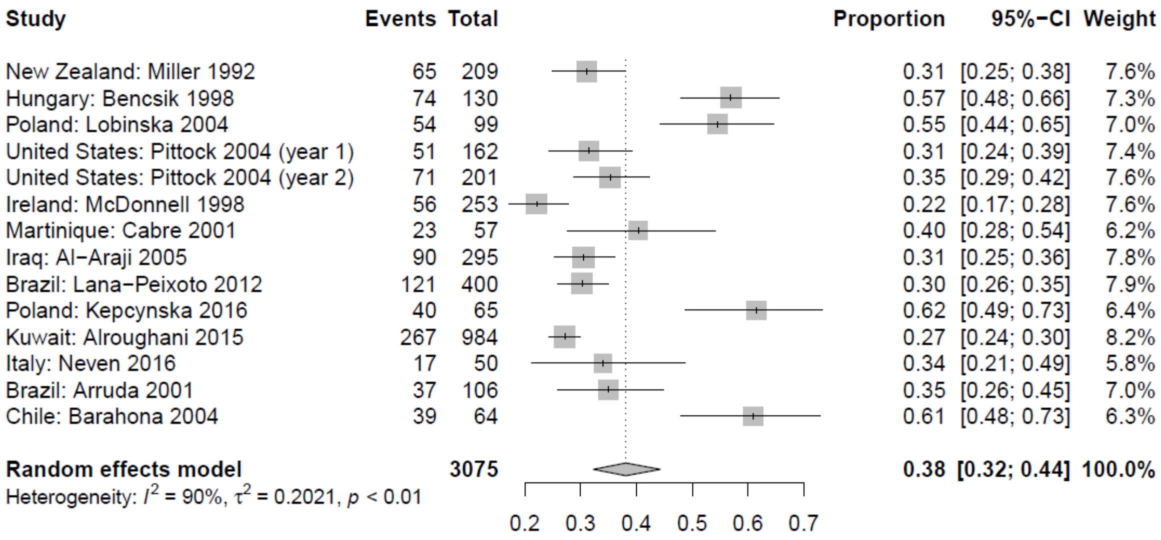

The following figures provide the result of the second meta-analysis on the mild, moderate and severe categories.

Figure 3. Mild Cases of MS (Including both Asymptomatic and Mild Categories)

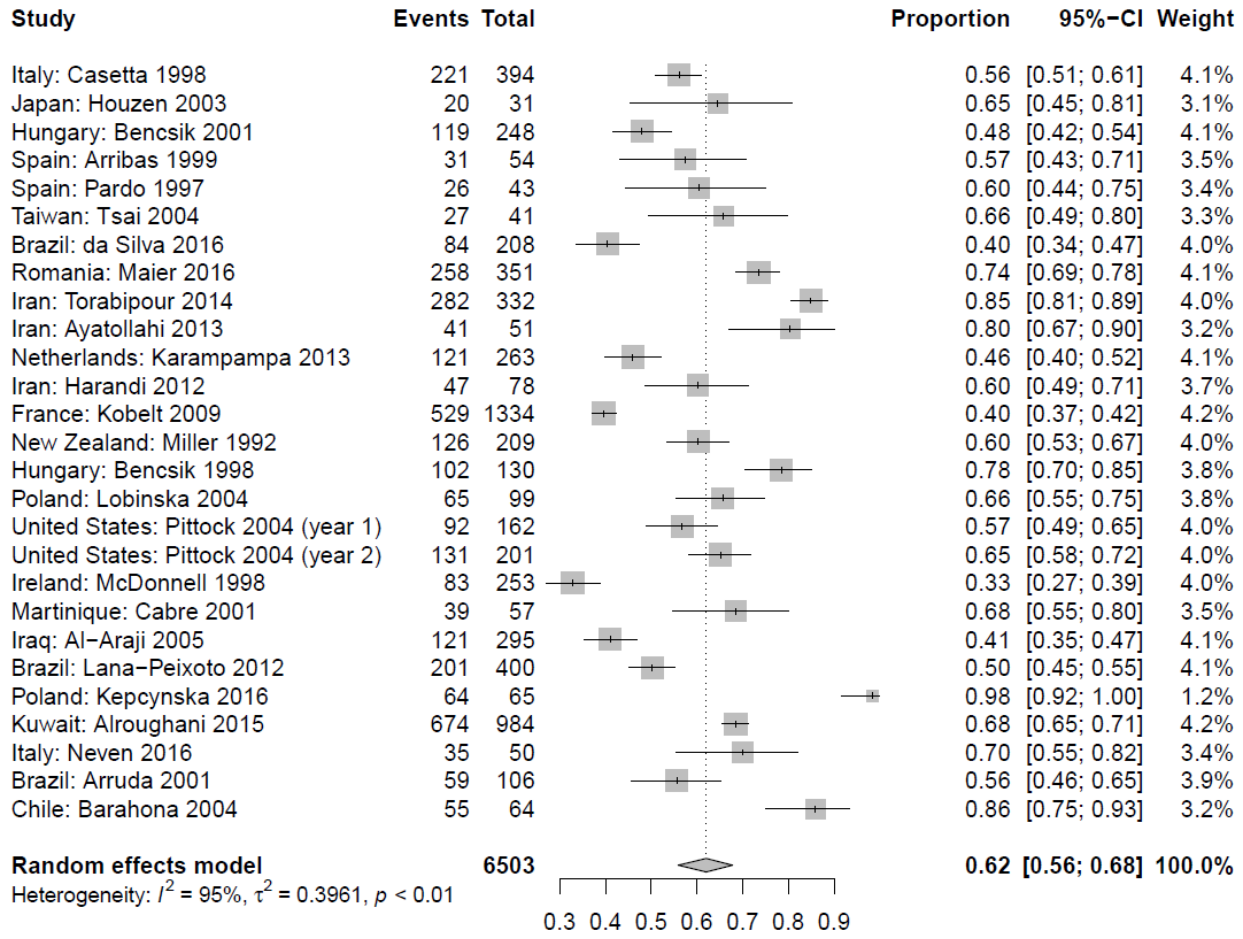

Figure 4. Moderate Cases of MS

Study	Events	Total	Proportion	95%−CI	Weight
Italy: Casetta 1998	69	394	0.18	[0.14; 0.22]	4.1%
Japan: Houzen 2003	4	31	0.13	[0.04; 0.30]	2.3%
Hungary: Bencsik 2001	87	248	0.35	[0.29; 0.41]	4.1%
Spain: Arribas 1999	16	54	0.30	[0.18; 0.44]	3.4%
Spain: Pardo 1997	9	43	0.21	[0.10; 0.36]	3.0%
Taiwan: Tsai 2004	10	41	0.24	[0.12; 0.40]	3.1%
Brazil: da Silva 2016	91	208	0.44	[0.37; 0.51]	4.0%
Romania: Maier 2016	86	351	0.25	[0.20; 0.29]	4.1%
Iran: Torabipour 2014	33	332	0.10	[0.07; 0.14]	3.9%
Iran: Ayatollahi 2013	7	51	0.14	[0.06; 0.26]	2.9%
Netherlands: Karampampa 2013	113	263	0.43	[0.37; 0.49]	4.1%
Iran: Harandi 2012	12	78	0.15	[0.08; 0.25]	3.3%
France: Kobelt 2009	669	1334	0.50	[0.47; 0.53]	4.2%
New Zealand: Miller 1992	41	209	0.20	[0.14; 0.26]	3.9%
Hungary: Bencsik 1998	21	130	0.16	[0.10; 0.24]	3.7%
Poland: Lobinska 2004	22	99	0.22	[0.14; 0.32]	3.7%
United States: Pittock 2004 (year 1)	54	162	0.33	[0.26; 0.41]	4.0%
United States: Pittock 2004 (year 2)	56	201	0.28	[0.22; 0.35]	4.0%
Ireland: McDonnell 1998	121	253	0.48	[0.42; 0.54]	4.1%
Martinique: Cabre 2001	18	57	0.32	[0.20; 0.45]	3.5%
Iraq: Al−Araji 2005	110	295	0.37	[0.32; 0.43]	4.1%
Brazil: Lana−Peixoto 2012	150	400	0.38	[0.33; 0.42]	4.1%
Poland: Kepcynska 2016	22	65	0.34	[0.23; 0.47]	3.6%
Kuwait: Alroughani 2015	244	984	0.25	[0.22; 0.28]	4.2%
Italy: Neven 2016	15	50	0.30	[0.18; 0.45]	3.3%
Brazil: Arruda 2001	34	106	0.32	[0.23; 0.42]	3.8%
Chile: Barahona 2004	33	64	0.52	[0.39; 0.64]	3.6%
**Random effects model**		**6503**	**0.28**	**[0.24; 0.33]**	**100.0%**

Heterogeneity: $I^2 = 94\%$, $\tau^2 = 0.3479$, $p < 0.01$

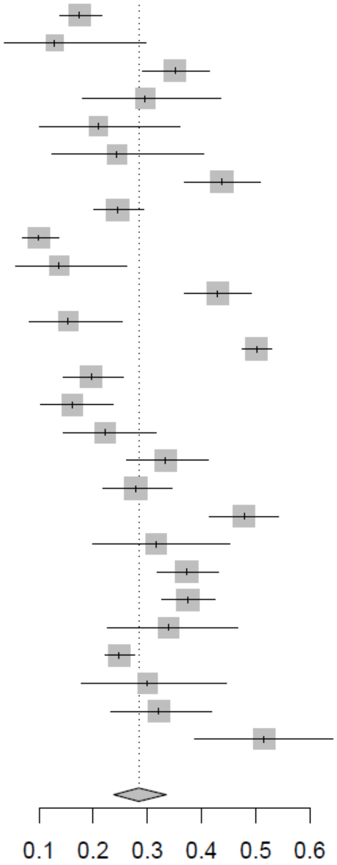

Figure 5. Severe Cases of MS

Study	Events	Total	Proportion	95%-CI	Weight
Italy: Casetta 1998	104	394	0.26	[0.22; 0.31]	4.6%
Japan: Houzen 2003	7	31	0.23	[0.10; 0.41]	3.2%
Hungary: Bencsik 2001	42	248	0.17	[0.12; 0.22]	4.4%
Spain: Arribas 1999	7	54	0.13	[0.05; 0.25]	3.3%
Spain: Pardo 1997	8	43	0.19	[0.08; 0.33]	3.4%
Taiwan: Tsai 2004	5	41	0.12	[0.04; 0.26]	3.0%
Brazil: da Silva 2016	33	208	0.16	[0.11; 0.22]	4.3%
Romania: Maier 2016	7	351	0.02	[0.01; 0.04]	3.4%
Iran: Torabipour 2014	17	332	0.05	[0.03; 0.08]	4.1%
Iran: Ayatollahi 2013	3	51	0.06	[0.01; 0.16]	2.5%
Netherlands: Karampampa 2013	29	263	0.11	[0.08; 0.15]	4.3%
Iran: Harandi 2012	19	78	0.24	[0.15; 0.35]	4.0%
France: Kobelt 2009	136	1334	0.10	[0.09; 0.12]	4.7%
New Zealand: Miller 1992	42	209	0.20	[0.15; 0.26]	4.4%
Hungary: Bencsik 1998	7	130	0.05	[0.02; 0.11]	3.4%
Poland: Lobinska 2004	12	99	0.12	[0.06; 0.20]	3.8%
United States: Pittock 2004 (year 1)	46	162	0.28	[0.22; 0.36]	4.4%
United States: Pittock 2004 (year 2)	53	201	0.26	[0.20; 0.33]	4.5%
Ireland: McDonnell 1998	49	253	0.19	[0.15; 0.25]	4.5%
Martinique: Cabre 2001	1	57	0.02	[0.00; 0.09]	1.3%
Iraq: Al−Araji 2005	65	295	0.22	[0.17; 0.27]	4.5%
Brazil: Lana−Peixoto 2012	50	400	0.12	[0.09; 0.16]	4.5%
Poland: Kepcynska 2016	3	65	0.05	[0.01; 0.13]	2.5%
Kuwait: Alroughani 2015	66	984	0.07	[0.05; 0.08]	4.6%
Italy: Neven 2016	0	50	0.00	[0.00; 0.07]	0.8%
Brazil: Arruda 2001	13	106	0.12	[0.07; 0.20]	3.9%
Chile: Barahona 2004	13	64	0.20	[0.11; 0.32]	3.8%
**Random effects model**		**6503**	**0.13**	**[0.10; 0.16]**	**100.0%**

Heterogeneity: $I^2 = 90\%$, $\tau^2 = 0.3794$, $p < 0.01$

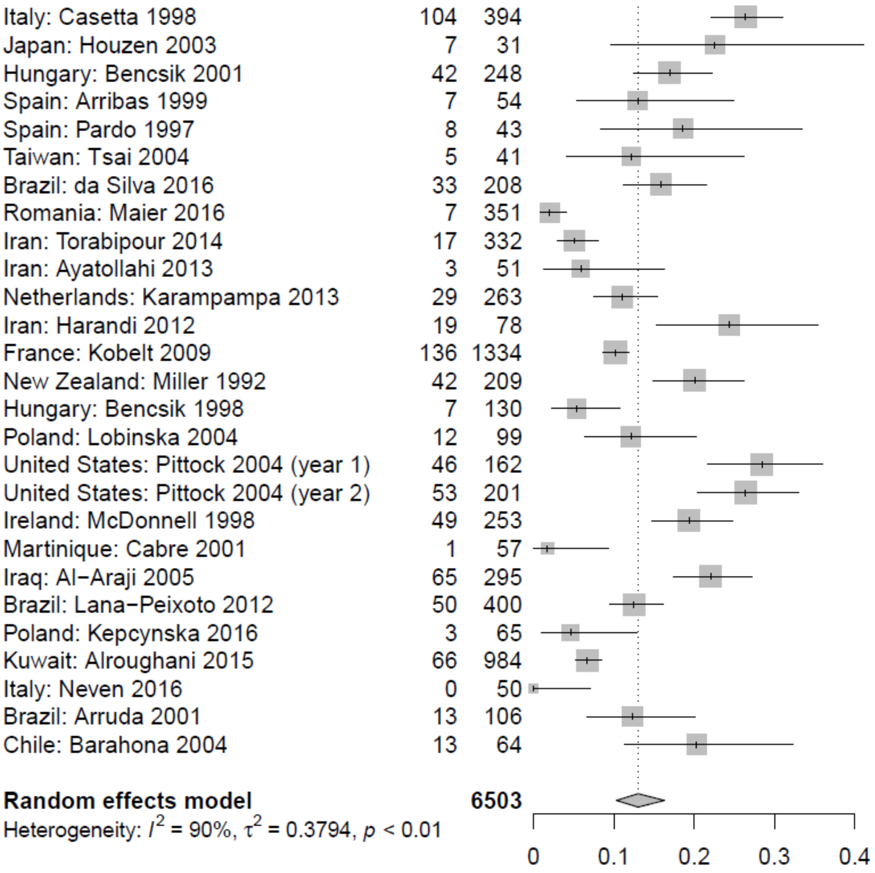

Modelling strategy

We use DisMod 2.1 as the main analytical tool for the MS estimation process. Prior settings include zero remission for all ages, and no incidence or excess mortality for persons under 4 years old. We also constrain the super-region random effects for prevalence and incidence to -0.5 and 0.5 to account for spurious inflation of regional differences.

Claims data for 2000 and 2010 are adjusted via study covariates to account for systematically low estimates relative to the 2012 claims data. Implicit in this adjustment is the assumption that variation between years of claims data is a function of data collection inconsistencies and noise.

Similar to other cases we use GBD estimates of cause-specific mortality rate (CSMR) and Excess Mortality Rate (EMR) in this model.

To assist the estimation process, we use a several country-level covariates. These effects plus those of the study covariates are presented below.

Covariate	Measure	Beta	Exponentiated	Parameter Type
Absolute value of average latitude	prevalence	.029 (.026 - .040)	1.03 (1.03 - 1.04)	Country-level
Absolute value of average latitude	incidence	.055 (.0095 - .061)	1.06 (1.01 - 1.06)	Country-level
All MarketScan, year 2000	prevalence	-.26 (-.29 - -.22)	.77 (.75 - .80)	x-cov
All MarketScan, year 2010	prevalence	-.0011 (-.0034 - -.0017)	.99 (.97 – 1.00)	x-cov
Healthcare access and quality index	excess mortality rate	-.048 (-.21 - -.035)	.95 (.81 - .97)	Country-level
SDI	prevalence	1.98 (1.96- 2.00)	7.24 (7.06- 7.38)	Country-level

As described in the literature, extreme latitude is associated with higher prevalence and incidence of MS. While the pathway that affects MS is not fully understood, our results suggest a sizable relationship. Our operationalization of latitude is created by a population-weighted average of latitude by country and taking the absolute value. The underlying population distribution rasters are part of the Gridded Population of the World dataset.

Although there are no known cures for MS, we expect disease management to differ globally – largely as a function of available resources. To capture this, we use the healthcare access and quality index covariate to capture this relationship in the estimation of excess mortality.

To capture possible social and cultural risk factors or modifiers of MS prevalence, we include SDI as a covariate.

Migraine

Flowchart

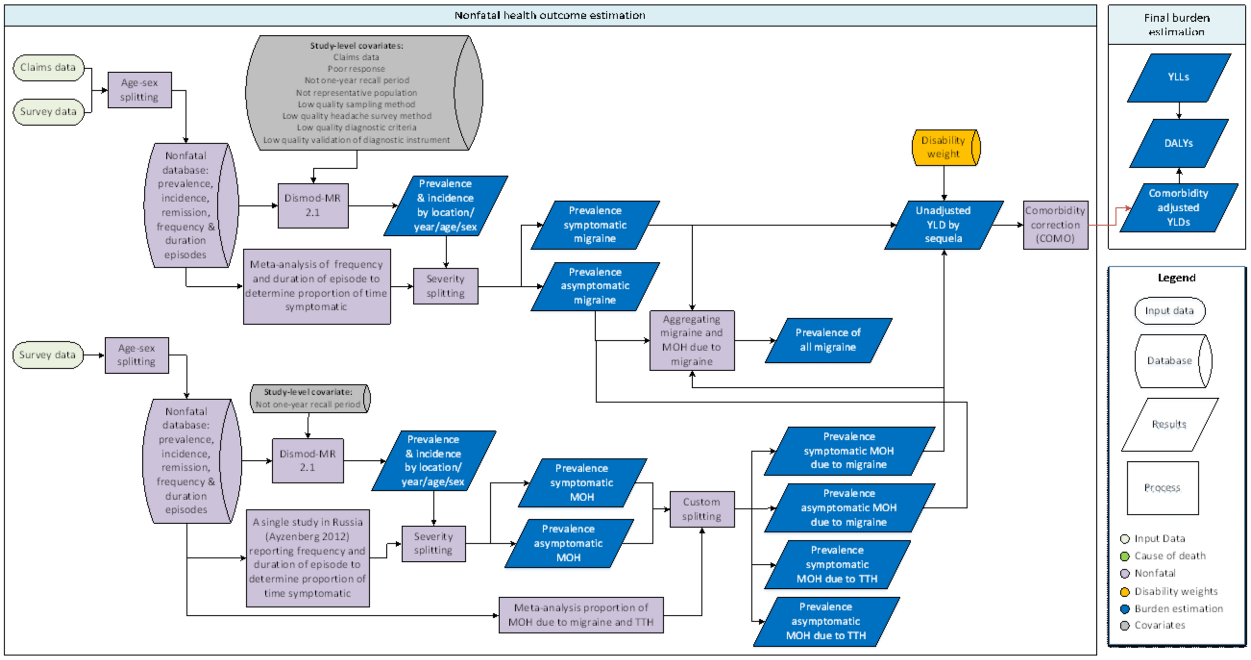

Case definition

Migraine is a class of disabling primary headache disorders, characterized by recurrent unilateral pulsatile headaches. The two major subtypes are common migraine (without aura) and classic migraine (with aura or neurological symptoms). In GBD we do not distinguish subtypes as most epidemiological studies report on overall migraine only. The ICD-10 code for migraine is G43 and ICD-9 code is 346.

Migraine is due to medication overuse headache (MOH) when the additional International Classification of Headache Disorders (ICHD) diagnostic criteria for MOH are met. The criteria are:

A. Headache present on ≥15 days/month fulfilling criteria C and D

B. Regular overuse (ie, >2 days per week) for ≥3 months of one or more drugs that can be taken for acute and/or symptomatic treatment of headache

C. Headache has developed or markedly worsened during medication overuse

D. Headache resolves or reverts to its previous pattern within 2 months after discontinuation of overused medication

It also explicitly states that if a person qualifies for chronic migraine or chronic tension-type headache (TTH) as well as MOH, both diagnoses should be given. However, our GBD headache collaborators, Steiner and Stovner, say that in survey practice, a screening question on chronic headache is used first,

followed by questions to determine if medication overuse headache is probable (ie, fitting all criteria but criterion D).

Only one study used in GBD was able to meet criterion D making a final diagnosis after a trial of detoxification. Of 25 cases with probably MOH, 15 were confirmed as MOH.

Input data

Model inputs

A systematic reviews of migraine and MOH were conducted for GBD 2010 and updated for GBD 2013. In GBD 2015, three new representative surveys conducted by GBD collaborators in Norway; Karnataka, India; and Nepal were added. In GBD 2016, four new representative surveys conducted by GBD collaborators in Ethiopia, Germany, Denmark, and Sweden were included for migraine, and two surveys in Norway and Ethiopia were included for MOH.

Inclusion criteria of the systematic reviews were:

- Representative, population-based surveys
- Reporting of prevalence of migraine headache

In addition, US claims data for 2000, 2010, and 2012 by US state were used.

The table below illustrates the geographic distribution of migraine data.

	Prevalence	Incidence	Remission	Frequency and duration episodes
Studies	123	4	1	16
Countries/subnational locations	113	4	1	16
GBD world regions	16	2	1	9

The table below shows the geographic distribution of MOH data.

	Prevalence	Incidence	Remission
Studies	26	0	7
Countries/subnational locations	20	0	5
GBD world regions	8	0	1

Severity splits

To determine the proportion of time over a year spent in an episode of migraine headache, 16 studies providing data on the frequency of episodes and the average duration of episodes were meta-analyzed. As these studies reported frequency and duration of episodes by disparate categories, an assumption was made that the mean represented each category. For each study the estimated proportion of time symptomatic is 0.085 (0.058–0.112).

The basis of GBD disability weight (DW) survey assessments are lay descriptions of sequelae highlighting major functional consequences and symptoms. The lay description and DW for migraine are shown below.

Severity level	Lay description	DW (95% CI)
Mild	This person has severe, throbbing head pain, and nausea that cause great difficulty in daily activities and sometimes confine the person to bed. Moving around, light, and noise make it worse.	0.434 (0.285–0.603)

Modelling strategy

We used a list of binary covariates which are a modified version of quality indicators of epidemiological studies on headache (Steiner TJ, Stovner LJ et al [2013]. Improving quality in population surveys of headache prevalence, burden, and cost: key methodological considerations. J Headache Pain, 14: 87) and shown in the table below.

Study covariate	Notation	
	Less desirable (1)	Reference (zero)
Other than one-year recall period	Point prevalence	One-year prevalence
Not representative	Selected population	General population or community-based sample from whole country OR general population or community-based sample from defined region within a country, or school-based (for children)
Low-quality sampling method	Not stated OR no (or failed) attempt to secure representativeness	Total defined population, or random sample corrected for population demographics OR random sample uncorrected for population demographics
Poor response	Not stated, or <70%	70–100%
Low-quality survey method and type of interviewer	Not stated OR self-administered (unsupervised) questionnaire OR telephone or face-to-face interview by untrained or unspecified interviewer(s)	Face-to-face interview with headache expert
Low-quality validation of diagnostic instrument	Instrument not specified or not validated OR validated, but sensitivity and/or specificity <70% OR validated only in screen-positive sub-sample, or in clinic or unspecified sample, but sensitivity and specificity ≥70%	Validated in target population or similar, and sensitivity and specificity ≥70%, or all diagnoses made in face-to-face or telephone interviews by headache expert
Low-quality diagnostic criteria	Not stated OR stated, other than ICHD OR ICHD (or reasonable modification)	ICHD (or reasonable modification)

We added separate covariates for lifetime recall and three years of claims data from MarketScan (2000, 2010, and 2012).

Prior settings in the DisMod model included setting incidence to 0 before age 5 based on expert advice. We also assume no excess mortality due to migraine. Remission rate is set to be between 0 and 0.1.

All study covariates were initially evaluated as x-cov (which means that data points are adjusted to the reference value if a systematic bias is detected); those that did not have a significant coefficient, were entered as z-cov (which means that a multiplier is applied to the standard error of such data points to indicate they are less certain values because they did not meet the reference criteria for study quality). The table below shows the fixed effect values of the x-covs which are in log space (as DisMod uses an offset lognormal model) as well as the exponentiated values which for an x-cov can be interpreted as an odds ratio.

Only the covariate for low quality survey method and type of interviewer, poor response and three years of claim data remain as x-covs.

The covariates for other than one-year recall period, low-quality sampling method, low-quality diagnostic criteria, low-quality validation of diagnostic instrument, and not representative studies had non-significant coefficients as a x-cov and were subsequently used as z-covs.

Study covariate	Parameter	beta	Exponentiated beta
Low quality survey method and type of interviewer	Prevalence	0.09	1.09 (0.99 – 1.19)
Poor response	Prevalence	- 0.25	0.78 (0.71 – 0.85)
Claims data US 2000	Prevalence	- 2.45	0.086 (0.082 – 0.095)
Claims data US 2010	Prevalence	- 2.07	0.13 (0.12 – 0.14)
Claims data US 2012	Prevalence	- 1.97	0.14 (0.13 – 0.15)

MOH is initially modelled separately in DisMod, then we include MOH prevalence due to migraine as a sequela of migraine. Prior settings in the DisMod model included setting incidence to 0 before age 5 based on expert advice. We also assume no excess mortality due to MOH. Based on the seven literatures on remission (listed in references below), we set bounds of the remission to be between 0 and 0.4

All study covariates for MOH are evaluated using the same strategy as modelling for migraine.

The study with recall period other than one year was the only covariate used as a x-covariate; however, its coefficient was insignificant: beta = -0.19 and exponentiated beta = 0.83 (0.66 – 1.04). The others were subsequently used as z-covariates.

Study covariate	Parameter	beta	Exponentiated beta
Low-quality diagnostic criteria	Prevalence	0.24	1.27 (1.05–1.59)
Poor response	Prevalence	-0.64	0.53 (0.42–0.65)

In GBD 2015, to the prevalence output from DisMod, we first applied the finding from da Silva (2010) that 60% (40.8–79.2%) of "probable" cases were confirmed cases of MOH. However, headache collaborators have argued that this would leave the 40% unaccounted for, as surveys first ask about chronic headache and those on medication before applying criteria for those with migraine and TTH. Thus, in GBD 2016, we

no longer multiplied the 60% (40.8–79.2%) to the prevalence from DisMod; ie, consider all the "probable" cases as MOH cases. For the severity split, we estimate the proportion of time "symptomatic," ie, with headache, from the Ayzenberg (2012) estimate of 23.1 days a month with headache and multiply estimates by 75.9% (72.9–78.8%).

In addition, we multiplied 73% (64–82%) to the symptomatic MOH prevalence to be symptomatic MOH due to migraine and the rest of the prevalent cases are symptomatic MOH due to TTH.

References

- Colas R, Munoz P, Temprano R, Gomez C, Pascual J. Chronic daily headache with analgesic overuse: Epidemiology and impact on quality of life. Neurology. 2004; 62(8): 1338-42.
- Katsarava Z, Muessig M, Dzagnidze A, Fritsche G, Diener HC, Limmroth V. Medication overuse headache: rates and predictors for relapse in a 4-year prospective study. Cephalalgia. 2005; 25(1): 12-5.
- Jonsson P, Hedenrud T, Linde M. Epidemiology of medication overuse headache in the general Swedish population. Cephalalgia. 2011; 31(9): 1015-22.

New studies on remission from medication overuse headache

- Fontanillas N, Colás R, Muñoz P, Oterino A, Pascual J. Long-term evolution of chronic daily headache with medication overuse in the general population. Headache. 2010; 50(6): 981-8.
- Pini LA, Cicero AF, Sandrini M. Long-term follow-up of patients treated for chronic headache with analgesic overuse. Cephalalgia. 2001; 21(9): 878-83.
- Suhr B, Evers S, Bauer B, Gralow I, Grotemeyer KH, Husstedt IW. Drug-induced headache: long-term results of stationary versus ambulatory withdrawal therapy. Cephalalgia. 1999; 19(1): 44-9.
- Katsarava Z, Muessig M, Dzagnidze A, Fritsche G, Diener HC, Limmroth V. Medication overuse headache: rates and predictors for relapse in a 4-year prospective study. Cephalalgia. 2005; 25(1): 12-5.
- Schnider P, Aull S, Baumgartner C, Marterer A, Wöber C, Zeiler K, Wessely P. Long-term outcome of patients with headache and drug abuse after inpatient withdrawal: five-year follow-up. Cephalalgia. 1996; 16(7): 481-5.
- Tribl GG, Schnider P, Wöber C, Aull S, Auterith A, Zeiler K, Wessely P. Are there predictive factors for long-term outcome after withdrawal in drug-induced chronic daily headache?. Cephalalgia. 2001; 21(6): 691-6.
- Hagen K, Albretsen C, Vilming ST, Salvesen R, Grønning M, Helde G, Gravdahl G, Zwart JA, Stovner LJ. A 4-year follow-up of patients with medication-overuse headache previously included in a randomized multicentre study. J Headache Pain. 2011; 12(3): 315-22.

Tension-type Headache

Flowchart

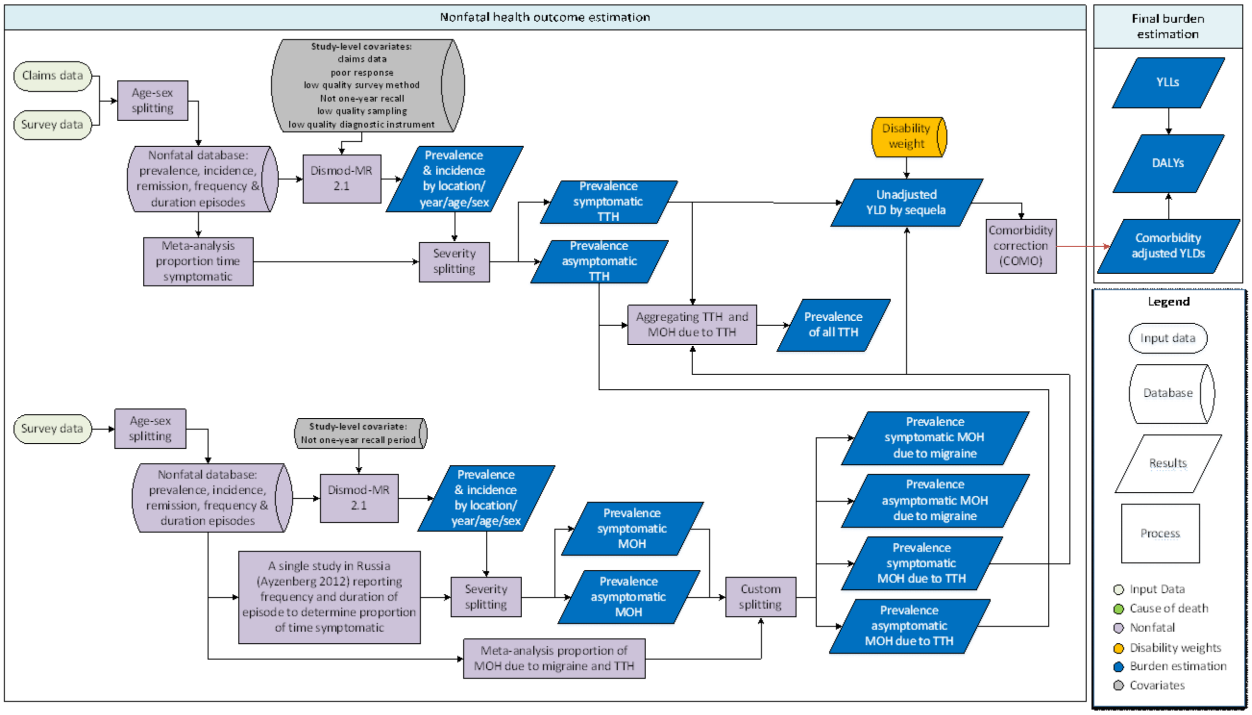

Case definition

Tension-type headache (TTH) is characterized by a dull, non-pulsatile, diffuse, band-like (or vice-like) pain of mild to moderate intensity in the head, scalp, or neck. The ICD-10 code for migraine is G44.2 and the ICD-9 code is 339.1.

TTH is due to medication overuse headache (MOH) when the additional International Classification of Headache Disorders (ICHD) diagnostic criteria for MOH are met. The criteria are:

E. Headache present on ≥15 days/month fulfilling criteria C and D

F. Regular overuse (ie, >2 days per week) for ≥3 months of one or more drugs that can be taken for acute and/or symptomatic treatment of headache

G. Headache has developed or markedly worsened during medication overuse

H. Headache resolves or reverts to its previous pattern within 2 months after discontinuation of overused medication

It also explicitly states that if a person qualifies for chronic migraine or chronic TTH as well as MOH, both diagnoses should be given. However, our headache GBD collaborators, Steiner and Stovner, say that in

survey practice, a screening question on chronic headache is used first, followed by questions to determine if medication overuse headache is probable (ie, fitting all criteria but criterion D).

Only one study used in GBD was able to meet criterion D making a final diagnosis after a trial of detoxification. Of 25 cases with probable MOH, 15 were confirmed as MOH.

Input data

Model Inputs

A systematic review of TTH was conducted for GBD 2010 and updated for GBD 2013. In GBD 2015 three new representative surveys conducted by GBD collaborators in Norway; Karnataka, India; and Nepal were added. In GBD 2016, two new representative surveys conducted by GBD collaborators in Ethiopia and Germany were added. Inclusion criteria of the systematic reviews were:

- Representative, population-based surveys
- Reporting of prevalence of TTH headache

In addition, US claims data for 2000, 2010, and 2012 by US state were included.

The table below illustrates the geographic distribution of TTH data.

	Prevalence	Incidence	Remission	Frequency and duration episodes
Studies	86	1	0	9
Countries/subnational locations	103	1	0	7
GBD world regions	15	1	0	6

The table below shows the geographic distribution of MOH data.

	Prevalence	Incidence	Remission
Studies	26	0	7
Countries/subnational locations	20	0	5
GBD world regions	8	0	1

Severity splits

To determine the proportion of time over a year spent in an episode of TTH headache, nine studies providing data on the frequency of episodes and the average duration of episodes were meta-analyzed. As these studies reported frequency and duration of episodes by disparate categories, an assumption was made that the mean represented each category. The estimated proportion of time symptomatic is 0.058 (0.023–0.092).

The basis of GBD disability weight (DW) survey assessments are lay descriptions of sequelae highlighting major functional consequences and symptoms. The lay description and DW for TTH are shown below.

Severity level	Lay description	DW (95% CI)
Mild	This person has a moderate headache that also affects the neck, which causes difficulty in daily activities	0.036 (0.023–0.053)

Modelling strategy

We used a list of binary covariates which are a modified version of quality indicators of epidemiological studies on headache (Steiner TJ, Stovner LJ et al (2013). Improving quality in population surveys of headache prevalence, burden and cost: key methodological considerations. *J Headache Pain*, 14: 87) and shown in the table below.

**Study covariate**	**Notation**	
	**Less desirable (1)**	**Reference (zero)**
**Other than one-year recall period**	Point prevalence	One-year prevalence
**Not representative**	selected population	general population or community-based sample from whole country OR general population or community-based sample from defined region within a country, or school-based (for children)
**Low-quality sampling method**	not stated OR no (or failed) attempt to secure representativeness	total defined population, or random sample corrected for population demographics OR random sample uncorrected for population demographics
**Poor response**	not stated, or <70%	70–100%
**Low-quality survey method and type of interviewer**	not stated OR self-administered (unsupervised) questionnaire OR telephone or face-to-face interview by untrained or unspecified interviewer(s)	face-to-face interview with headache expert
**Low-quality validation of diagnostic instrument**	instrument not specified or not validated OR validated, but sensitivity and/or specificity <70% OR validated only in screen-positive sub-sample, or in clinic or unspecified sample, but sensitivity and specificity ≥70%	validated in target population or similar, and sensitivity and specificity ≥70%, or all diagnoses made in face-to-face or telephone interviews by headache expert
**Low-quality diagnostic criteria**	not stated OR stated, other than ICHD OR ICHD (or reasonable modification)	ICHD (or reasonable modification)

We added separate covariates for chronic headache, lifetime recall and the three years of claims data from MarketScan (2000, 2010, and 2012).

Prior settings in the DisMod model included setting incidence to 0 before age 5 based on expert advice. We also assume no excess mortality due to TTH. In the absence of any data on remission we set bounds between 0 and 0.5, ie, ensuring an average duration of at least two years.

All study covariates were initially evaluated as x-cov (which means that data points are adjusted to the reference value if a systematic bias is detected); those that did not have a significant coefficient were entered as z-cov (which means that a multiplier is applied to the standard error of such data points to indicate they are less certain values because they did not meet the reference criteria for study quality). The table below shows the fixed-effect values of the x-covs which are in log space (as DisMod uses an offset lognormal model) as well as the exponentiated values which for a x-cov can be interpreted as an odds ratio.

The covariate for other than one-year recall period and not representative had non-significant coefficients as a x-cov and were subsequently used as a z-cov.

Study covariate	Parameter	beta	Exponentiated beta
Chronic headache	Prevalence	-0.37	0.69 (0.41 – 0.98)
Recall lifetime	Prevalence	0.74	2.09 (1.36 – 2.69)
Claims data US 2000	Prevalence	- 4.49	0.011 (0.011 – 0.011)
Claims data US 2010	Prevalence	- 4.12	0.016 (0.016 – 0.017)
Claims data US 2012	Prevalence	- 4.00	0.018 (0.018 – 0.019)

The very low coefficients in claims data mean that few cases of TTH are included in claims data. Data points were crosswalked up by a factor 50 or more. We decided to include the data with such large crosswalks as we had no other data for the states of the USA and the crosswalks estimated by DisMod were within range of the data from three US studies in Massachusetts, Maryland, and Kentucky.

MOH is initially modelled separately in DisMod, then we include the MOH prevalence due to TTH as a sequela of TTH. Prior settings in the DisMod model included setting incidence to 0 before age 5 based on expert advice. We also assume no excess mortality due to MOH. Based on seven literature sources on remission (listed in references below), we set bounds of the remission to be between 0 and 0.4.

All study covariates for MOH are evaluated using the same strategy as modelling for TTH.

The study with recall period other than one year was the only covariate used as a x-covariate; however, its coefficient was insignificant: beta = -0.19 and exponentiated beta = 0.83 (0.66 – 1.04). The others were subsequently used as z-covariates.

In GBD 2015, to the prevalence output from DisMod, we first applied the finding from da Silva (2010) that 60% (40.8–79.2) of "probable" cases were confirmed cases of MOH. However, headache collaborators have argued that this would leave the 40% unaccounted for, as surveys first ask about chronic headache and those on medication before applying criteria for those with migraine and TTH. Thus, in GBD 2016, we no longer multiplied the 60% (40.8–79.2) to the prevalence from DisMod; ie, consider all the "probable" cases as MOH cases. For the severity split, we estimate the proportion of time "symptomatic," ie, with headache from the Ayzenberg (2012) estimate of 23.1 days a month with headache and multiply estimates by 75.9% (72.9–78.8).

In addition, we multiplied 73% (64–82) to the symptomatic MOH prevalence to be symptomatic MOH due to migraine and the rest of the prevalent cases are symptomatic MOH due to TTH.

References

- Colas R, Munoz P, Temprano R, Gomez C, Pascual J. Chronic daily headache with analgesic overuse: Epidemiology and impact on quality of life. *Neurology*. 2004; 62(8): 1338-1342.
- Katsarava Z, Muessig M, Dzagnidze A, Fritsche G, Diener HC, Limmroth V. Medication overuse headache: rates and predictors for relapse in a 4-year prospective study. *Cephalalgia*. 2005; 25(1): 12-15.
- Jonsson P, Hedenrud T, Linde M. Epidemiology of medication overuse headache in the general Swedish population. *Cephalalgia*. 2011; 31(9): 1015-1022.

New studies on remission from medication overuse headache

- Fontanillas N, Colás R, Muñoz P, Oterino A, Pascual J. Long-term evolution of chronic daily headache with medication overuse in the general population. Headache. 2010; 50(6): 981-8.
- Pini LA, Cicero AF, Sandrini M. Long-term follow-up of patients treated for chronic headache with analgesic overuse. Cephalalgia. 2001; 21(9): 878-83.
- Suhr B, Evers S, Bauer B, Gralow I, Grotemeyer KH, Husstedt IW. Drug-induced headache: long-term results of stationary versus ambulatory withdrawal therapy. Cephalalgia. 1999; 19(1): 44-9.
- Katsarava Z, Muessig M, Dzagnidze A, Fritsche G, Diener HC, Limmroth V. Medication overuse headache: rates and predictors for relapse in a 4-year prospective study. Cephalalgia. 2005; 25(1): 12-5.
- Schnider P, Aull S, Baumgartner C, Marterer A, Wöber C, Zeiler K, Wessely P. Long-term outcome of patients with headache and drug abuse after inpatient withdrawal: five-year follow-up. Cephalalgia. 1996; 16(7): 481-5.
- Tribl GG, Schnider P, Wöber C, Aull S, Auterith A, Zeiler K, Wessely P. Are there predictive factors for long-term outcome after withdrawal in drug-induced chronic daily headache?. Cephalalgia. 2001; 21(6): 691-6.
- Hagen K, Albretsen C, Vilming ST, Salvesen R, Grønning M, Helde G, Gravdahl G, Zwart JA, Stovner LJ. A 4-year follow-up of patients with medication-overuse headache previously included in a randomized multicentre study. J Headache Pain. 2011; 12(3): 315-22.

Motor Neuron Diseases

Flowchart

Motor neuron disease

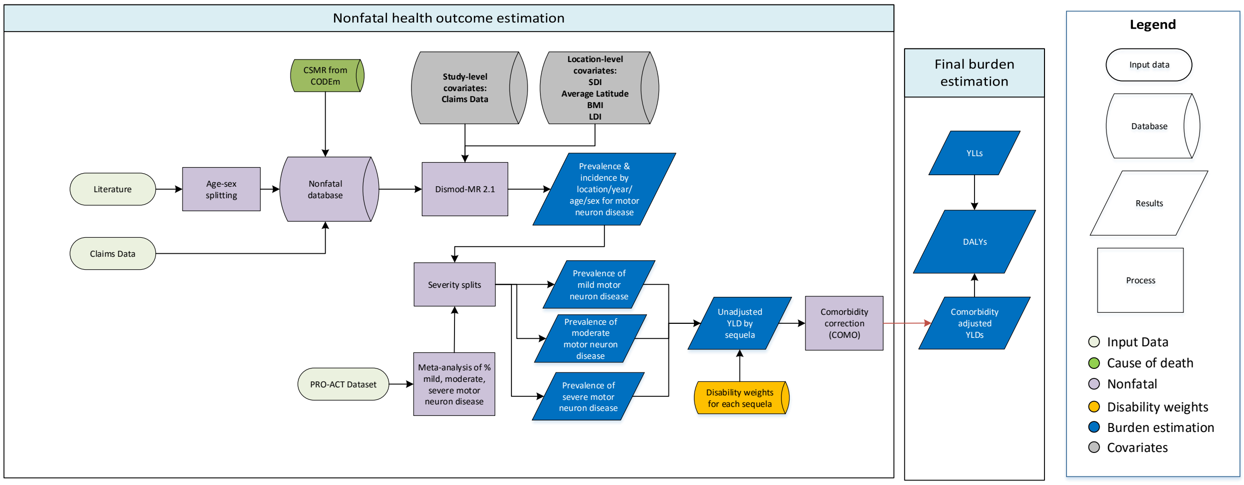

Case definition

Motor neuron diseases (MND) are a set of chronic, degenerative, and progressive neurological conditions typified by the destruction of motor neurons and the subsequent deterioration of voluntary muscle activity. The most common MND is amyotrophic lateral sclerosis. The ICD-10 code corresponding to motor neuron diseases is G12. Our gold standard diagnostic criteria are the El Escorial Criteria, with other similar criteria (eg, the original set from World Federation of Neurology) if necessary.

Input data

A full systematic review was conducted for GBD 2015. The following search string guided our search, which resulted in 3,146 hits with 58 sources meeting extraction criteria: (1) the study is a representative population-based study, (2) reports on prevalence, incidence, remission, excess mortality, relative risk of mortality, standardized mortality ratio, or with-condition mortality rate. Studies with no clearly defined sample were excluded.

(('motor neuron disease'[MeSH Terms] OR ('motor'[All Fields] AND 'neuron'[All Fields] AND 'disease'[All Fields]) OR 'motor neuron disease'[All Fields] OR ('motor'[All Fields] AND 'neuron'[All Fields] AND 'diseases'[All Fields]) OR 'motor neuron diseases'[All Fields]) OR ('amyotrophic lateral sclerosis'[MeSH Terms] OR ('amyotrophic'[All Fields] AND 'lateral'[All Fields] AND 'sclerosis'[All Fields]) OR 'amyotrophic lateral sclerosis'[All Fields]) OR ALS[All Fields] OR ('motor neuron disease'[MeSH Terms] OR ('motor'[All Fields] AND 'neuron'[All Fields] AND 'disease'[All Fields]) OR 'motor neuron disease'[All Fields] OR ('primary'[All Fields] AND 'lateral'[All Fields] AND 'sclerosis'[All Fields]) OR 'primary lateral sclerosis'[All Fields]) OR ('Politics Life Sci'[Journal] OR 'pls'[All Fields]) OR ('muscular atrophy, spinal'[MeSH Terms] OR ('muscular'[All Fields] AND 'atrophy'[All Fields] AND 'spinal'[All Fields]) OR 'spinal muscular atrophy'[All Fields] OR ('progressive'[All Fields] AND 'muscular'[All Fields] AND 'atrophy'[All Fields]) OR 'progressive muscular atrophy'[All Fields]) OR PBP[All Fields] OR ('pseudobulbar palsy'[MeSH Terms] OR

('pseudobulbar'[All Fields] AND 'palsy'[All Fields]) OR 'pseudobulbar palsy'[All Fields])) AND (('epidemiology'[Subheading] OR 'epidemiology'[All Fields] OR 'epidemiology'[MeSH Terms]) OR population-based[All Fields])

The following table provides an overview of the density and distribution of the data used for GBD 2016.

	Prevalence	Incidence	Mortality risk
Studies	11	47	3
Countries/subnational units	57	46	3
Regions	5	7	2

Beyond the literature data, we also make use of claims data from the United States for 2000, 2010, and 2012. Descriptions of the source and preparation of this data are provided elsewhere.

Except for excluding studies using non-representative populations, there are no substantial adjustments or outliering criteria for the MND model. Certain studies have been outliered on a case-by-case basis due to subsequent review and exclusion due to inappropriateness of the study design and case definition.

Severity splits

To calculate severity and disability due to MND we analyzed a dataset from Pooled Resource Open-access ALS Clinical Trials (PRO-ACT). This dataset contains the largest ALS clinical trials dataset, with a total of 8,635 ALS patient records from multiple completed clinical trials. Among these, we conducted the final analysis with n=4838 (56%) of the patients with complete ALS Function Rating Score (ALSFRS) with average follow-up time of 184 days (min: -22, max: 648), in which 2,999 (62%) received experimental (medication) treatments and 1,301 (27%) received placebo (in these trials, the medications tested were found to be no better than placebo with respect to their effects on ALS progressions).

The ALSFRS is an instrument for evaluating the functional status of patients with Amyotrophic Lateral Sclerosis. It can be used to monitor functional changes in a patient over time. It measures (1) speech, (2) salivation, (3) swallowing, (4) handwriting, (5) cutting food and handling utensils (with or without gastrostomy), (6) dressing and hygiene, (7) turning in bed and adjusting bed clothes, (8) walking, (9) climbing stairs, and (10) breathing. Each task is rated on a 5-point scale from 0 = can't do, to 4 = normal ability. Individual item scores are summed to produce a reported total score of between 0 and 40 (worst to best). ALSFRS has been revised to ALSFRS-R, which includes 12 questions (ALSFRS Q10 changes to (10) Dyspnea, (11) Orthopnea, and (12) Respiratory insufficiency), with individual item scores summed to a score between 0 and 48.

In order to eliminate any bias from the treatment effects on the ALSFRS, only the first observation at the time of trial is selected. If the first observation is missing at the time of trial (or prior), the next non-missing observation is selected to be included in the final analysis.

We subsequently mapped ALSFRS scores into GBD severities, and sequelae into different combinations of speech problems, chronic obstructive pulmonary disease, and motor impairment using the following logic:

Motor impairment

The ALSFRS assess motor function of the legs through questions on walking (Q8) and stair climbing (Q9).

Combined score	Severity level
8	None
5-7	Mild
2-4	Moderate
0-1	Severe

The ALSFRS also assesses motor impairment through questions on handwriting (Q4), cutting food and handling utensils (Q5), and dressing and hygiene (Q6).

Combined score	Severity level
12	None
9-11	Mild
3-8	Moderate
0-2	Severe

After determining case severity on these two separate metrics, we aggregate by taking the most severe ranking (eg, severe + mild = a severe case).

Respiratory problems:

Question 10 of the ALSFRS describes breathing difficulty as a function of MND.

ALSFRS score	Description	Severity level
4	Normal	None
3	Shortness of breath with minimal exertion	Mild
2	Shortness of breath at rest	Moderate
0-1	Intermittent ventilator assistance required/ventilator-dependent	Severe

Speech problems

Speech impairment due to MND is derived from ALSFRS question 1, which describes speech impediments. A score of 4 on this question denotes no impairment, while all other values suggest some impairment.

Creating sequelae

After determining the severity status of each case for the three symptom umbrellas, we subsequently estimated the relative proportion of each combination of symptom class and their respective severities. Those without any symptoms (eg, no severity) were categorized as having worry about the diagnosis for disability estimation. The following table displays the various sequelae and their associated proportions.

Sequela	Proportion (Mean)	Proportion (Lower)	Proportion (Upper)
Mild motor impairment, mild respiratory problems and speech problems due to motor neuron disease	0.01779	0.01658	0.01909
Mild motor impairment, moderate respiratory problems and speech problems due to motor neuron disease	0.00270	0.00225	0.00324
Mild motor impairment, severe respiratory problems and speech problems due to motor neuron disease	0.00082	0.00059	0.00113
Mild motor impairment, and speech problems due to motor neuron disease	0.02052	0.01922	0.02190
Moderate motor impairment, mild respiratory problems and speech problems due to motor neuron disease	0.03377	0.03210	0.03552
Moderate motor impairment, moderate respiratory problems and speech problems due to motor neuron disease	0.00715	0.00640	0.00799
Moderate motor impairment, severe respiratory problems and speech problems due to motor neuron disease	0.00286	0.00240	0.00342
Moderate motor impairment, and speech problems due to motor neuron disease	0.03041	0.02883	0.03208
Severe motor impairment, mild respiratory problems and speech problems due to motor neuron disease	0.05242	0.05035	0.05457
Severe motor impairment, moderate respiratory problems and speech problems due to motor neuron disease	0.02247	0.02111	0.02392
Severe motor impairment, severe respiratory problems and speech problems due to motor neuron disease	0.01365	0.01259	0.01479
Severe motor impairment and speech problems due to motor neuron disease	0.04765	0.04567	0.04970
Mild respiratory problems and speech problems due to motor neuron disease	0.01157	0.01060	0.01263
Moderate respiratory problems and speech problems due to motor neuron disease	0.00142	0.00111	0.00182
Severe respiratory problems and speech problems due to motor neuron disease	0.00023	0.00013	0.00043
Speech problems due to motor neuron disease	0.02457	0.02315	0.02608
Mild motor impairment and mild respiratory problems due to motor neuron disease	0.02245	0.02109	0.02389
Mild motor impairment and moderate respiratory problems due to motor neuron disease	0.00275	0.00230	0.00329
Mild motor impairment and severe respiratory problems due to motor neuron disease	0.00068	0.00047	0.00097
Mild motor impairment due to motor neuron disease	0.10388	0.10103	0.10681
Moderate motor impairment and mild respiratory problems due to motor neuron disease	0.06744	0.06511	0.06985
Moderate motor impairment and moderate respiratory problems due to motor neuron disease	0.01302	0.01199	0.01413
Moderate motor impairment and severe respiratory problems due to motor neuron disease	0.00412	0.00356	0.00477
Moderate motor impairment due to motor neuron disease	0.20136	0.19760	0.20518

Severe motor impairment and mild respiratory problems due to motor neuron disease	0.06902	0.06666	0.07146
Severe motor impairment and moderate respiratory problems due to motor neuron disease	0.02000	0.01872	0.02137
Severe motor impairment and severe respiratory problems due to motor neuron disease	0.01062	0.00969	0.01163
Severe motor impairment due to motor neuron disease	0.15037	0.14702	0.15378
Mild respiratory problems due to motor neuron disease	0.00643	0.00571	0.00723
Moderate respiratory problems due to motor neuron disease	0.00044	0.00028	0.00069
Severe respiratory problems due to motor neuron disease	0.00005	0.00001	0.00017
Asymptomatic, but worry about diagnosis due to motor neuron disease	0.03738	0.03562	0.03921

To determine disability due to these sequelae, we use the standard multiplicative aggregation formula as described in the main text. The following table provides description and disability weight assigned to the sequelae as appropriate.

Symptom group	Severity level	Lay description	DW (95%)
Respiratory problems	Asymptomatic		
Respiratory problems	Mild	Has cough and shortness of breath after heavy physical activity, but is able to walk long distances and climb stairs.	0.019 (0.011–0.033)
Respiratory problems	Moderate	Has cough, wheezing, and shortness of breath, even after light physical activity. The person feels tired and can walk only short distances or climb only a few stairs.	0.225 (0.153–0.31)
Respiratory problems	Severe	Has cough, wheezing, and shortness of breath all the time. The person has great difficulty walking even short distances or climbing any stairs, feels tired when at rest, and is anxious.	0.408 (0.273–0.556)
Motor impairment	Asymptomatic		
Motor impairment	Mild	Has some difficulty in moving around but is able to walk without help.	0.01 (0.005–0.019)
Motor impairment	Moderate	Has some difficulty in moving around and difficulty in lifting and holding objects, dressing, and sitting upright, but is able to walk without help.	0.061 (0.04–0.089)
Motor impairment	Severe	Is unable to move around without help, and is not able to lift or hold objects, get dressed, or sit upright.	0.402 (0.268–0.545)

Speech problems	No		
Speech problems	Yes	Has difficulty speaking, and others find it difficult to understand.	0.051 (0.032–0.078)
Asymptomatic, but worry	Yes	Has a disease diagnosis that causes some worry but minimal interference with daily activities.	0.012 (0.006–0.023)

Modelling strategy

We use DisMod 2.1 as the main analytical tool for MND estimation. Prior settings are limited to 0 remission at all ages. We also constrain the super-region random effects for prevalence and incidence to -0.5 and 0.5 to account for spurious inflation of regional differences.

Claims data for 2000 and 2010 are adjusted via study covariates to account for systematically low estimates relative to the 2012 claims data. Implicit in this adjustment is the assumption that variation between years of claims data is a function of data collection inconsistencies and noise.

Similar to other cases we use GBD estimates of cause-specific mortality rate (CSMR) and Excess Mortality Rate (EMR) in this model. The source and estimation of these rates are discussed elsewhere.

To assist the estimation process we use several country-level covariates.

Covariate	Measure	Beta	Exponentiated
Absolute value of average latitude	prevalence	.014 (.012 - .016)	1.01 (1.01 - 1.02)
LDI (I$ per capita)	excess mortality rate	-.5 (-.5 - -.5)	.61 (.61 - .61)
All MarketScan, year 2010	prevalence	-.017 (-.038 - -.0014)	.98 (.96 – 1.00)
All MarketScan, year 2000	prevalence	-.026 (-0.054 - -.0037)	.97 (.95 - 1.00)

Although there are no known cures for MND, we expect disease management to differ globally – largely as a function of available resources. To capture this, we use the natural log of lagged distributed income per capita as a proxy to capture this relationship in the estimation of excess mortality.

As described in the literature, extreme latitude may be associated with higher prevalence and incidence of motor neuron disease. While the pathway that affects motor neuron disease is not fully understood, our results suggest a relationship. Our operationalization of latitude is created by a population-weighted average of latitude by country and taking the absolute value. The underlying population distribution rasters are part of the Gridded Population of the World dataset.

Other neurological disorders

In addition to the neurological disorders described above, there are many diverse types of neurological disorders with a range of severities and associated sequelae. Because these neurological disorders are diverse in their underlying causes and risk factors as well as in their associated health outcomes, modelling them together in a DisMod-MR model would not produce reliable estimates of prevalence or excess mortality. Instead, we calculated the YLDs caused by neurological disorders directly using a YLD/YLL ratio.

We calculated the ratio of YLDs to YLLs across the specified neurological disorders for which nonfatal outcomes were modelled, using YLL estimates from the GBD 2016 cause of death (CoD) analysis. We then multiplied this YLD/YLL ratio by the YLL estimates for other neurological disorders from the GBD 2016 CoD analysis, providing us with an estimate of the YLDs associated with other neurological disorders.

Schizophrenia

Flowchart

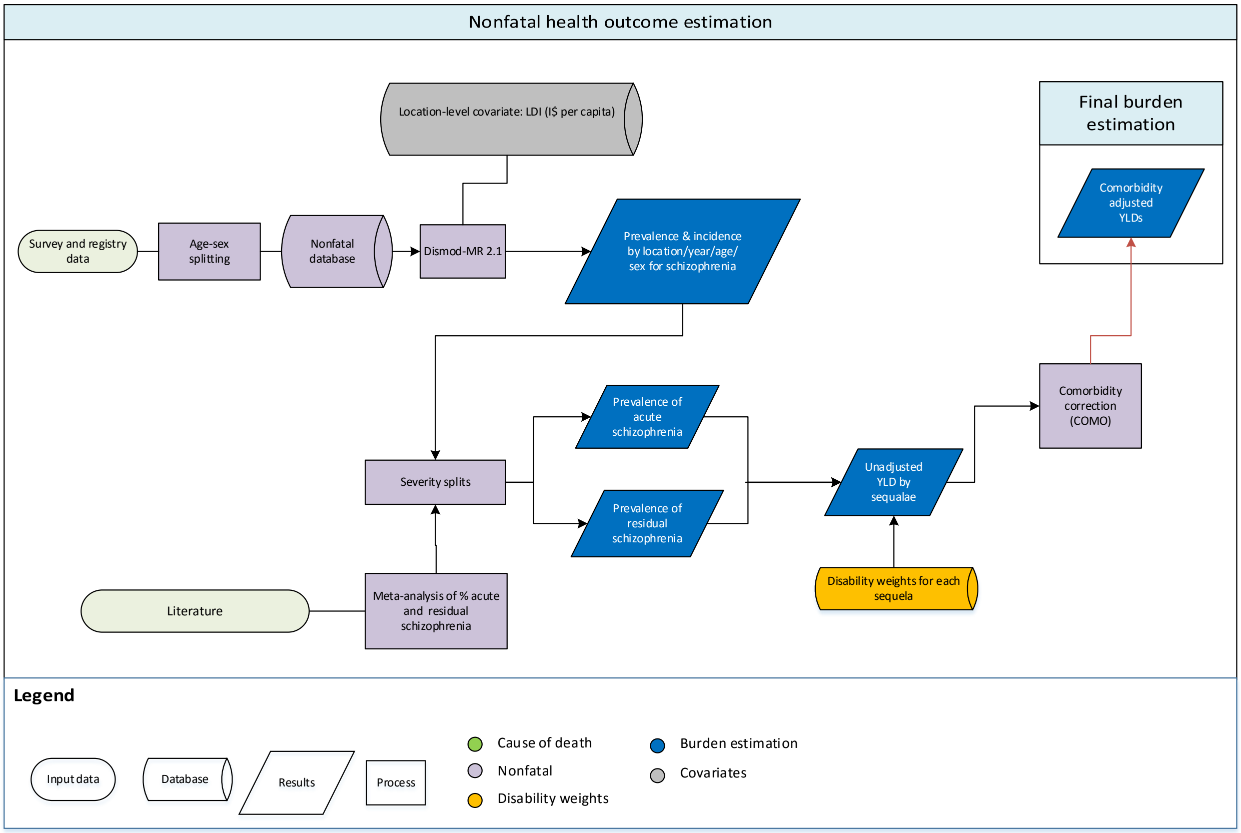

Case Definition

Schizophrenia is a chronic psychotic disorder which involves the experience of positive symptoms (eg, delusions, hallucinations, thought disorder) and negative symptoms (eg, flat affect, loss of interest, and emotional withdrawal). Included in the GBD disease modelling were cases meeting the Diagnostic and Statistical Manual of Mental Disorders (DSM-IV-TR) or the International Classification of Diseases (ICD-10) diagnostic criteria for schizophrenia (DSM: 295.10-295.30, 295.60, 295.90; ICD: F20.9).[1,2] Diagnostic criteria are:

A. Two (or more) of the following, each present for a significant portion of time during a one-month period (or less if successfully treated):

1. Delusions
2. Hallucinations
3. Disorganized speech
4. Grossly disorganized or catatonic behavior
5. Negative symptoms

B. Social/occupational dysfunction

C. Continuous signs of the disturbance persist for at least 6 months.

D. Exclusions must be met for schizoaffective and mood disorders, substance and general medical conditions, and a relationship to a pervasive development disorder.

Input data

Model Inputs

For GBD 2015 a systematic review of literature was conducted to capture studies of prevalence, incidence, remission, duration, and excess mortality associated with schizophrenia. In summary, the search was conducted in three stages involving searches of the peer-reviewed literature (via Medline, Embase and PubMed), the grey literature, and expert consultation. The inclusion criteria stipulated that: (1) the publication year must be from 1980 onward; (2) "caseness" must be based on clinical threshold as established by the DSM or ICD; (3) sufficient information must be provided on study method and sample characteristics to assess the quality of the study; and (4) study samples must be representative of the general population (ie, inpatient or pharmacological treatment samples, case studies, veterans or refugee samples were excluded). No limitation was set on the language of publication. In GBD 2016, a complete review of data sources logged in the Global Health Index (GHDx) was undertaken. This led to the inclusion of data from an additional 23 data sources.

The table below shows the number of studies included in GBD 2016, as well as the number of countries or subnational units and GBD world regions represented.

	Prevalence	Incidence	Duration	Excess mortality
Studies	64	30	5	37
Countries/subnational geographies	52	27	17	35
GBD world regions	12	7	8	11

Age and sex splitting

In GBD 2016 reported estimates of prevalence were split by age and sex where possible. Firstly, if studies reported prevalence for broad age groups by sex (eg, prevalence in 15 to 65 year old males and females separately), and also by specific age groups but for both sexes combined (eg, prevalence in 15 to 30 year olds, then in 31 to 65 year olds, for males and females combined); age-specific estimates were split by sex using the reported sex ratio and bounds of uncertainty. Secondly, where studies reported estimates across age groups spanning 20 years or more, these were split into five-year age groups using the prevalence age pattern estimated by DisMod-MR.

Severity splits

The basis of the GBD disability weight survey assessments are lay descriptions of sequelae highlighting major functional consequences and symptoms. The lay descriptions and disability weights for schizophrenia severity levels are shown below.

Severity level	Lay description	DW (95% CI)
acute state	Hears and sees things that are not real and is afraid, confused, and sometimes violent. The person has great difficulty with communication and daily activities, and sometimes wants to harm or kill himself (or herself).	0.778 (0.606–0.9)
residual state	Hears and sees things that are not real and has trouble communicating. The person can be forgetful, has difficulty with daily activities, and thinks about hurting himself (or herself).	0.588 (0.411–0.754)

Severity splits used in GBD 2016 were consistent with those used in GBD 2013 for schizophrenia. Information on the distribution of acute and residual states of schizophrenia was obtained from a separate systematic review of the literature.[4] Meta-XL (a Microsoft Excel add-in for meta-analysis) was used to pool estimates across all studies to calculate the overall proportion of schizophrenia cases in each health state – acute 63% (29%–91%) and residual state 37% (9%–71%).

Modelling strategy

The GBD 2016 epidemiological modelling strategy for schizophrenia made use of DisMod-MR 2.1 to estimate prevalence by age, sex, year, and country. Data across all epidemiological parameters were included in the modelling process. We assumed no incidence and prevalence before age 10 and after age 80. This minimum age of onset was corroborated with expert feedback and existing literature on schizophrenia. Remission was also restricted to a maximum of 0.04 as guided by data available in the dataset.

Study-level covariates can be used in DisMod-MR 2.1 to accommodate for between-study variability in the raw prevalence data. In GBD 2016 tested covariates failed to demonstrate significance resulting in a model without the inclusion of any covariates.

A location-level covariate, LDI, was also included. This covariate represents a moving average of gross domestic product (GDP) over time. LDI was also applied to excess mortality data with a negative relationship assumed. The table below illustrates the covariate, parameter, beta and exponentiated beta values for the model.

Location-level covariate	Parameter	beta	Exponentiated beta
LDI	Excess mortality rate	-0.55 (-1 — -0.1)	0.58 (0.37 — 0.90)

Citations

1. American Psychiatric Association. Diagnostic and Statistical Manual of Mental Disorders (DSM-IV-TR). 4th, Text Revision ed. Washington DC: American Psychiatric Association; 2000.

2. World Health Organization. The ICD-10 Classification of Mental and Behavioural Disorders. Clinical descriptions and diagnostic guidelines. Geneva: World Health Organization; 1992.

3. Ferrari AJ, Norman RE, Freedman G, et al. The burden attributable to mental and substance use disorders as risk factors for suicide: findings from the Global Burden of Disease Study 2010. *PloS one* 2014; **9**(4): e91936.

4. Ferrari AJ, Saha S, McGrath JJ, et al. Health states for schizophrenia and bipolar disorder within the Global Burden of Disease 2010 Study. *Population Health Metrics* 2012; **10**(1): 16.

Alcohol dependence

Flowchart

Alcohol use disorders

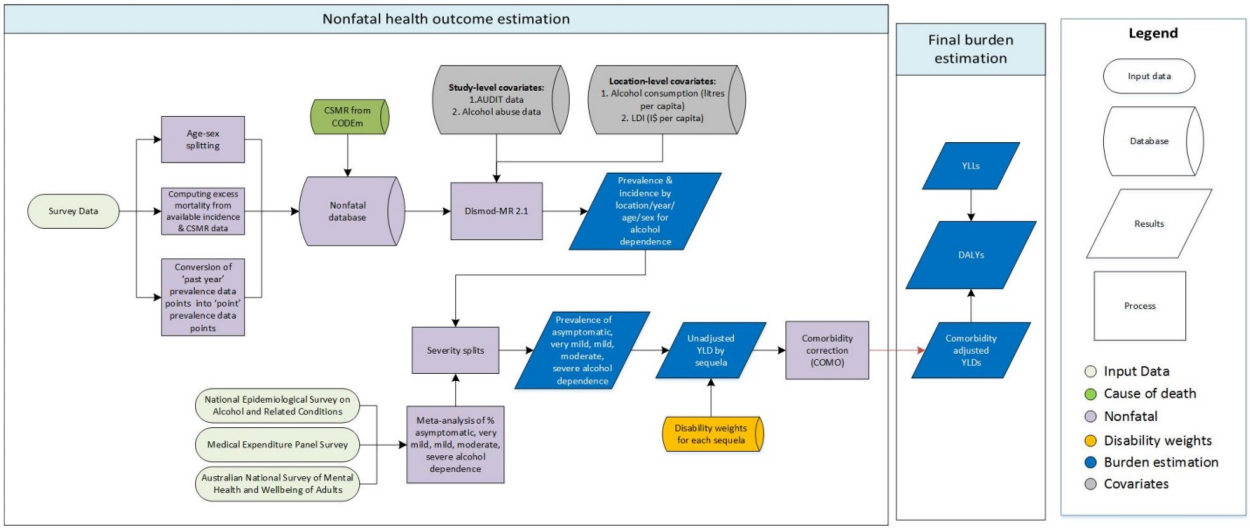

Case definition

Alcohol dependence is a substance-related disorder involving a dysfunctional pattern of alcohol use. According to the Diagnostic and Statistical Manual of Mental Disorders (DSM-IV) criteria for alcohol dependence, at least three out of seven of the following criteria must be manifested during a 12-month period:

- Tolerance
- Withdrawal symptoms or clinically defined alcohol withdrawal syndrome
- Use in larger amounts or for longer periods than intended
- Persistent desire or unsuccessful efforts to cut down on alcohol use
- Time is spent obtaining alcohol or recovering from effects
- Social, occupational, and recreational pursuits are given up or reduced because of alcohol use
- Use is continued despite knowledge of alcohol-related harm (physical or psychological)

The DSM-IV codes for alcohol dependence is 303.90, and the corresponding International Classification of Diseases (ICD-10) codes are F10.1 and F10.2.[1,2]

Input data

Model inputs

In GBD 2013 and GBD 2016, systematic reviews of literature were conducted to capture studies of prevalence, incidence, remission, duration, and excess mortality associated with alcohol dependence. In summary, the search was conducted in three stages involving searches of the peer-reviewed literature (via Medline, Embase, and PubMed), the grey literature, and expert consultation.

The inclusion criteria stipulated that (1) "caseness" must be based on clinical threshold as established by the DSM and ICD; (2) sufficient information must be provided on study method and sample characteristics to assess the quality of the study; and (3) study samples must be representative of the general population (ie, inpatient or pharmacological treatment samples (accepted for estimates of mortality), case studies, veterans or refugee samples were excluded).

An adjustment was made outside of DisMod-MR 2.1 to adjust past-year prevalence estimates of alcohol dependence toward the level they would have been had the study measured point prevalence, as the latter is less susceptible to recall bias. Given that remission from alcohol dependence (and hence, average disease duration) vary considerably with age, we also applied an age pattern to this adjustment that cannot be replicated within DisMod-MR 2.1 by use of covariates. The first step was to estimate the average duration by taking the inverse of remission. Next, we applied an adjustment factor from one-year to point prevalence using the following formula where average duration is expressed in years:

$$\text{adjustment factor} = \frac{\text{average duration}}{\text{average duration} + 1}$$

Age-specific adjustment factors were applied to all one-year prevalence estimates propagating sampling uncertainty around the prevalence and remission input data through to the final adjusted prevalence estimates.

Prevalence estimates were split by age and sex where possible outside of DisMod-MR 2.1. Firstly if studies reported prevalence for broad age groups by sex (eg, prevalence in 15 to 65 year old males and females separately), and also by specific age groups but for both sexes combined (eg, prevalence in 15 to 30 year olds, then in 31 to 65 year olds, for males and females combined); age-specific estimates were split by sex using the reported sex ratio and bounds of uncertainty. Secondly, where studies reported estimates across age groups spanning 20 years or more, these were split into five-year age groups using the regional prevalence age pattern estimated by DisMod-MR 2.1.

The final dataset for GBD 2016 included 5,140 prevalence estimates, 25 incidence estimates, 14 remission estimates, and 87 excess mortality estimates. The table below shows the number of studies for each parameter as well as the number of countries/subnationals and GBD world regions covered by the available data.

	Prevalence	Incidence	Remission	Mortality
Studies	185	3	4	38
Countries/subnationals	202	3	4	23
GBD world regions	19	3	3	6

Severity splits & disability weights

The basis of the GBD disability weight survey assessments are lay descriptions of sequelae highlighting major functional consequences and symptoms. The lay descriptions and disability weights for alcohol dependence severity levels are shown below.

Severity level	Lay description	DW (95% CI)
Very mild	Drinks alcohol daily and has difficulty controlling the urge to drink. When sober, the person functions normally.	0.123 (0.082–0.177)
Mild	Drinks a lot of alcohol and sometimes has difficulty controlling the urge to drink. While intoxicated, the person has difficulty performing daily activities.	0.235 (0.16–0.327)
Moderate	Drinks a lot, gets drunk almost every week and has great difficulty controlling the urge to drink. Drinking and recovering cause great difficulty in daily activities, sleep loss, and fatigue.	0.373 (0.248–0.508)
Severe	Gets drunk almost every day and is unable to control the urge to drink. Drinking and recovering replace most daily activities. The person has difficulty thinking, remembering and communicating, and feels constant pain and fatigue.	0.57 (0.396–0.732)

**asymptomatic cases carried no disability weight*

Severity splits used in GBD 2016 were consistent with those used in GBD 2015. The United States' Medical Expenditure Panel Survey (MEPS, conducted in annual waves since 1996)[3], the US National Epidemiological Survey on Alcohol and Related Conditions (NESARC, conducted in two waves from 2001–2002 and 2004–2005)[4], and the Australian National Survey of Mental Health and Wellbeing of Adults (NSMHWB, conducted in 1997)[5] were used to estimate the proportion of alcohol dependence cases in the asymptomatic 40.9% (38.4%–43.3%); very mild 46.9% (43.7%–50.0%); mild 4.0% (1.8%–5.8%); moderate 3.4% (2.3%–4.5%); and severe 4.8% (3.0%–7.0%) disease categories.

Modelling strategy

The GBD 2016 epidemiological modelling strategy for alcohol dependence made use of DisMod-MR 2.1 to estimate prevalence by age, sex, year, and location. Standardized mortality ratio and relative risk data were excluded in the modelling process. Instead we pulled in cause-specific mortality rate (CSMR) data from our CODEm and CODcorrect analyses and matched it with prevalence data points for the same geography and study year to estimate priors on excess mortality rates (by dividing CSMR by prevalence). We assumed no incidence and mortality before age 10. An upper limit of 0.6 was placed on remission (in line with data from the US National Epidemiological Survey on Alcohol and Related Conditions (NESARC) as well as a declining trend with age to restrict DisMod-MR 2.1 from straying too far from the data inputs.

Within DisMod-MR 2.1, study-level covariates were used to accommodate for other sources of between-study variability in the raw prevalence data. Combined abuse and dependence prevalence estimates were crosswalked down toward dependence-only estimates. Similarly, prevalence estimates using AUDIT were crosswalked down toward prevalence estimates from diagnostic (non-AUDIT) measures.

Country-level covariates were also included. The LDI covariate represents a moving average of gross domestic product (GDP) over time. LDI was also applied to excess mortality data with a negative relationship assumed. Alcohol consumption was also represented by a covariate representing this in terms of litres of alcohol per capita.

Study/country covariate	Parameter	beta	Exponentiated beta
Aabuse and dependence	Prevalence	0.78 (0.66 — 0.92)	2.19 (1.93 — 2.50)
AUDIT	Prevalence	1.33 (1.24 — 1.41)	3.77 (3.47 — 4.08)
Alcohol (litres per capita)	Prevalence	0.50 (0 — 1.00)	1.65 (1.00 — 2.72)
LDI (I$ per capita)	Excess mortality	-0.11 (-0.14 — -0.1)	0.89 (0.87 — 0.90)

References

1. American Psychiatric Association. Diagnostic and Statistical Manual of Mental Disorders (DSM-IV-TR). 4th, Text Revision ed. Washington DC: American Psychiatric Association; 2000.
2. World Health Organization. The ICD-10 Classification of Mental and Behavioural Disorders. Clinical descriptions and diagnostic guidelines. Geneva: World Health Organization; 1992.
3. Agency for Healthcare Research and Quality. United States Medical Expenditure Panel Survey. Rockville, United States: Agency for Healthcare Research and Quality.
4. Introduction to the National Epidemiologic Survey on Alcohol and Related Conditions [http://pubs.niaaa.nih.gov/publications/arh29-2/74-78.htm]. Access date 1 December 2014.
5. Australian Bureau of Statistics. National Survey of Mental Health and Wellbeing of Adults 1997. Canberra: Australian Bureau of Statistics.

Fetal alcohol syndrome

Flowchart

Fetal alcohol syndrome (FAS)

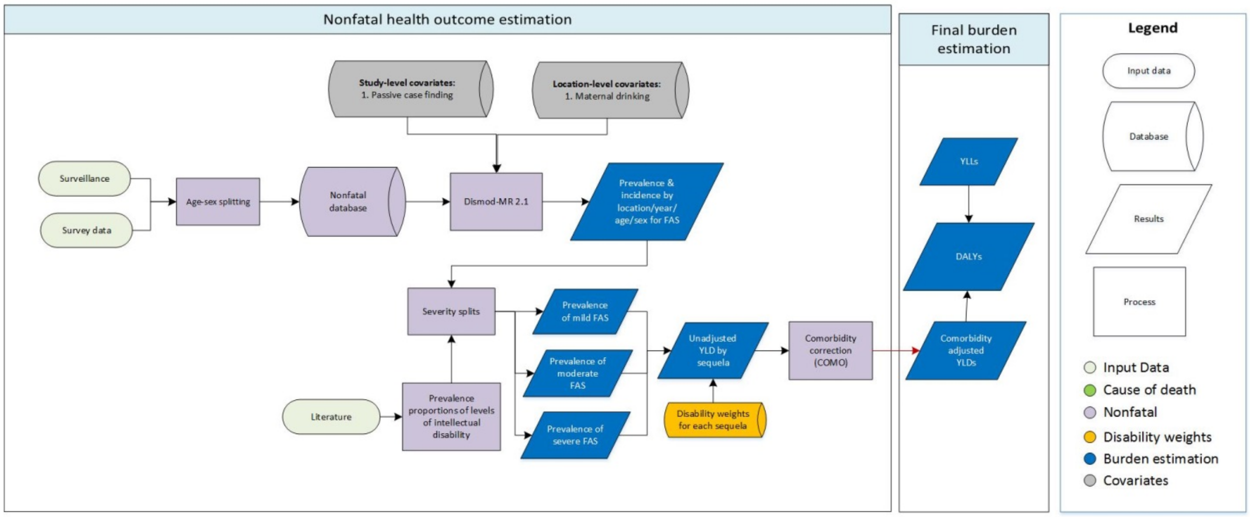

Input data and methodological summary

Case definition

Fetal alcohol syndrome (FAS; ICD-10: Q86.0) is a disorder caused by maternal drinking during pregnancy and is the most severe form of fetal alcohol spectrum disorder (FASD). In GBD, only FAS cases were included in the model. Other manifestations of FASD including partial fetal alcohol syndrome, alcohol-related neurodevelopmental disorder, and alcohol-related birth defects were not included. FAS is characterized by maternal alcohol exposure which results in certain patterns of facial anomalies such as short palpebral fissures and abnormalities in the premaxillary zone (eg, flat upper lip, flattened philtrum, and flat midface), growth retardation (eg, decelerating weight over time not due to nutrition), and central nervous system neurodevelopmental abnormalities (eg, decreased cranial size at birth) in the offspring.[1] Cases were defined according to diagnostic guidelines set by the US institute of Medicine, the British Pediatric Association, and other recognized bodies in the area.

Input data

Model inputs

A series of systematic literature reviews were conducted to capture studies reporting the prevalence, incidence, remission, and excess mortality of FAS. The reviews incorporated searches of peer-reviewed literature via electronic databases and consultation with experts. In order for a study to be included, it must use recognized classifications of FAS (eg, the US Institute of Medicine) and provide sufficient details on study methodology and sample characteristics to determine study quality. No limitation was set on the language of publication. Data from the European Surveillance of Congenital Anomalies (EUROCAT) were also included and updated where relevant. This methodology was utilized in GBD 2015. Updates to

systematic reviews are performed on an ongoing schedule across all GBD causes, an update for FAS will be performed in the next 1-2 iterations. The final dataset for GBD 2016 included 175 prevalence estimates and 13 excess mortality estimates (from studies of individuals with intellectual disability).

	Prevalence	Mortality
Studies	81	5
Countries/subnationals	53	4
GBD world regions	10	3

Severity split inputs

There were no data available which gave prevalence of FAS by severity. As such, severity splits for FAS were calculated by matching FAS severity to categories of IQ in children for which prevalence data are available. Severe FAS was matched to an IQ of less than 50, moderate FAS to an IQ of 50 to 69, mild FAS to an IQ of 74 to 84, and asymptomatic FAS to an IQ of 85 or higher. Prevalence data for these IQ levels were then used to calculate severity splits for FAS.

Severity level	Lay description	DW (95% CI)
Mild	Is a little slow in developing physically and mentally, which causes some difficulty in learning but no other difficulties in daily activities.	0.016 (0.008–0.03)
Moderate	Is slow in developing physically and mentally, which causes some difficulty in daily activities.	0.056 (0.035–0.083)
Severe	Is very slow in developing physically and mentally, which causes great difficulty in daily activities.	0.179 (0.119–0.257)

Modelling strategy

Prevalence was set to begin from birth. Incidence was set to zero given cases cannot manifest after birth (despite the fact they may not be diagnosed immediately at birth). Remission was also set to zero. A covariate was included in the model which addressed the heterogeneity introduced by different case-finding methods, ie, active versus passive case-finding. Estimates from known high-drinking populations (eg, indigenous populations) were not considered representative of the general population and were excluded. A country-level covariate was included for GBD 2016 representing the log proportion of pregnant women who drink during their pregnancy, estimated from a meta-analysis.[2]

The table below illustrates the covariate, parameter, beta and exponentiated beta values for the model.

Study/country covariate	Parameter	beta	Exponentiated beta
Passive case finding	Prevalence	0.39 (0.0046–1.14)	1.48 (1.00–3.14)
Maternal drinking	Prevalence	0.66 (0.014–1.45)	1.93 (1.01–4.25)

References

1. Stratton K, Howe C, Battaglia F, editors. Fetal alcohol syndrome. Diagnosis, epidemiology, prevention, and treatment. Washington DC: National Academy Press; 1996.
2. Popova S, Lange S, Probst C, Gmel G, Rehm J. Estimation of national, regional, and global prevalence of alcohol use during pregnancy and fetal alcohol syndrome: a systematic review and meta-analysis. *The Lancet Global Health* 2017.

Opioid dependence

Flowchart

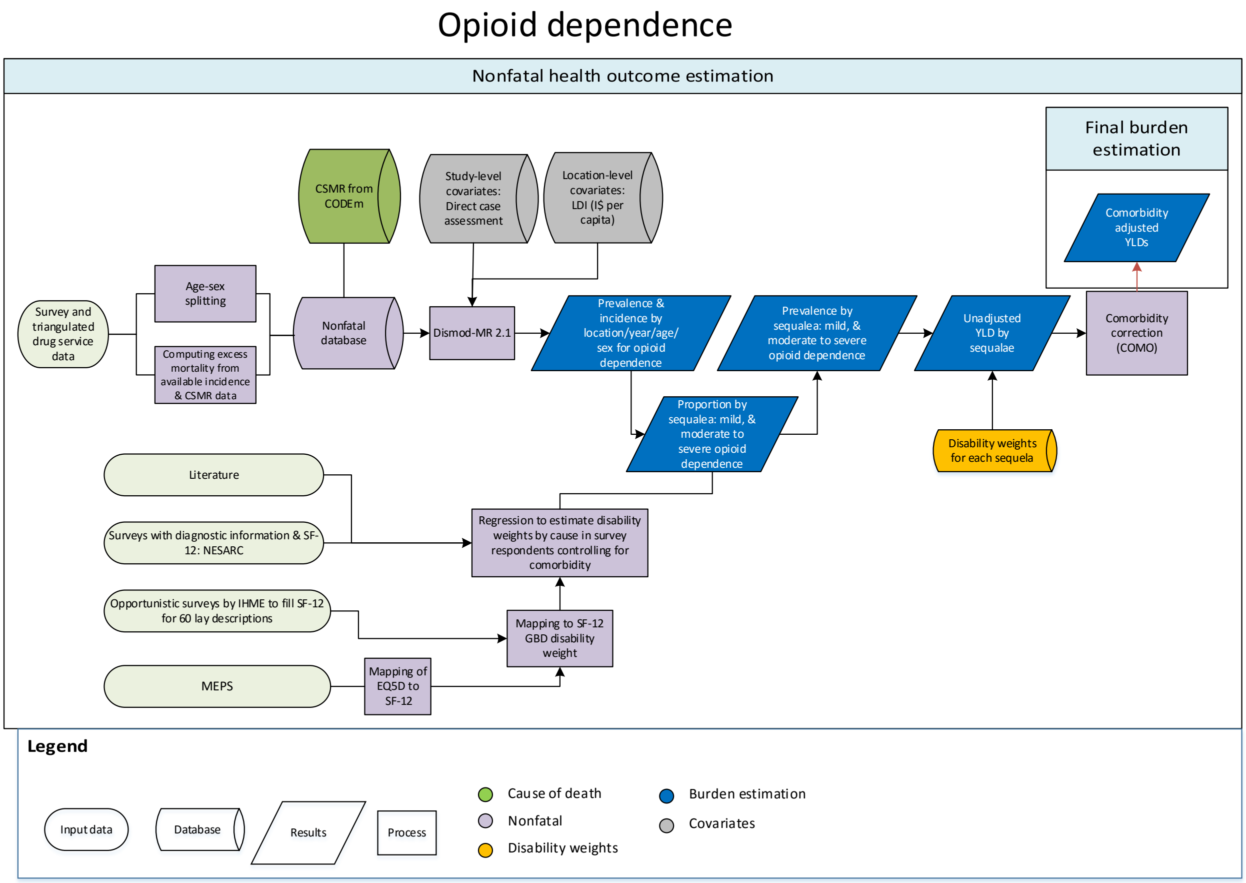

Case Definition

Opioid dependence is a substance-related disorder involving a dysfunctional pattern of opioid use. Included in the GBD disease modelling were cases meeting the Diagnostic and Statistical Manual of Mental Disorders (DSM-IV-TR) or the International Classification of Diseases (ICD-10) diagnostic criteria for opioid dependence (DSM: 304.00; ICD: F11.2), excluding those cases due to a general medical condition.[1,2] According to DSM-IV TR criteria, dependence involves a maladaptive pattern of substance use leading to clinically significant impairment or distress. At least three of the following symptoms must be experienced within the same 12-month period:

- Tolerance, characterized by either
  - a need for increased amounts of the substance to achieve intoxication; or
  - markedly diminished effect with continued use of the same amount of the substance;
- Withdrawal, characterized by either
  - Withdrawal symptoms characteristic to dependence; or
  - the same (or similar) substance is taken to avoid withdrawal symptoms;
- Substance taken in progressively larger amounts or for longer period;

- Persistent desire or unsuccessful efforts to reduce substance use;
- Disproportionate time dedicated to obtaining the substance;
- Other important activities are given up because of the substance use; and
- Substance use is continued despite knowledge of physical or psychological problems occurring as a result of the substance.

Input data

Model inputs

A systematic review of the literature was conducted to capture studies of prevalence, incidence, remission, and excess mortality associated with opioid dependence. In summary, the search was conducted in three stages involving searches of the peer-reviewed literature (via Medline, Embase, and Pubmed), the grey literature and, expert consultation. The agreed-upon approach for mental and substance use disorders was to conduct electronic database searches on a rolling basis. All three stages of GBD 2010's literature review were repeated for GBD 2013 to capture additional data published up to 2013. For GBD 2015, stages 2 and 3 of the literature review were updated, and in GBD 2016, the peer-reviewed database search (stage 1) was conducted via Medline, Embase, and Psycinfo to capture studies published from 2013 to 2016.

The inclusion criteria stipulated that 1) the publication year must be from 1980 onward; 2) "caseness" must be based on clinical threshold as established by the DSM or ICD; 3) sufficient information must be provided on study method and sample characteristics to assess the quality of the study; and (4) study samples must be representative of the general population (ie, inpatient or pharmacological treatment samples, case studies, veterans or refugee samples were excluded). No limitation was set on the language of publication. Methods used for this systematic review have been reported in greater detail elsewhere.[3,4] The table below shows the number of studies included, as well as the number of countries or subnational units and GBD world regions represented.

	Prevalence	Remission	Mortality rate
Studies	66	8	41
Countries/subnational geographies	191	25	22
GBD world regions	10	5	6

Age and sex splitting

In GBD 2016, reported estimates of prevalence were split by age and sex where possible. First, if studies reported prevalence for broad age groups by sex (eg, prevalence in 15 to 65 year old males and females separately), and also by specific age groups but for both sexes combined (eg, prevalence in 15 to 30 year olds, then in 31 to 65 year olds, for males and females combined); age-specific estimates were split by sex using the reported sex ratio and bounds of uncertainty. Second, where studies reported estimates across age groups spanning 20 years or more, these were split into five-year age groups using the prevalence age pattern estimated by DisMod MR.

Severity splits

The basis of the GBD disability weight survey assessments are lay descriptions of sequelae highlighting major functional consequences and symptoms. The lay descriptions and disability weights for opioid dependence severity levels are shown below.

Severity level	Lay description	DW (95% CI)
Mild	Uses heroin (or methadone) daily and has difficulty controlling the habit. When not using, the person functions normally.	0.335 (0.221–0.473)
Moderate to severe	Uses heroin daily and has difficulty controlling the habit. When the effects wear off, the person feels severe nausea, agitation, vomiting, and fever. The person has a lot of difficulty in daily activities.	0.697 (0.510–0.843)

The proportion of people with opioid dependence within each of the severity levels was determined based on available data from US National Epidemiological Survey on Alcohol and Related Conditions (NESARC), conducted in two waves from 2001–2002 and 2004–2005,[5] and the Comorbidity and Trauma study conducted in 2005-2008.[6,7] The estimated distribution of opioid dependent cases by severity were asymptomatic (16%, 13%–19%), mild (37%, 20%–55%), and moderate/severe (47%, 29%–64%).

Modelling Strategy

We ran a DisMod-MR model to produce estimates by age, sex, year, and country. We assumed no incidence and excess mortality before age 15. This minimum age of onset was corroborated with expert feedback and existing literature on opioid dependence. We also assumed no incidence after age 64 as supported by data from various sources including the European Monitoring Centre for Drugs and Drug Addiction.[8] An upper limit of 0.2 was placed on remission consistent with limits in the dataset. Cause-specific mortality rates (CSMR) from the GBD 2016 cause of death model for opioid use disorders were included as data points in the DisMod-MR model.

The prevalence dataset included data points using “direct” or “indirect” survey methods. “Direct” methods of measuring opioid dependence predominantly involve surveys of the general population that ask if respondents use or are dependent on opioid. Surveys tend to underestimate the prevalence of the most harmful and stigmatized forms of illicit drug use in ways that probably vary between countries and cultures.[9] “Indirect” methods are considered superior; they use different sources of data to indirectly estimate the total number of drug users (methods include “multiplier methods,” back-projection and capture-recapture methods). Due to insufficient data on dependence from indirect survey methods (considered to be the gold standard for GBD purposes), estimates derived from direct survey methods were included in the modelling. The cv_direct covariate was then used to adjust for whether a direct or indirect survey method was used. A crosswalk was estimated to convert all dependence estimates obtained via direct methods in the dataset, into its equivalent value if the study had measured dependence estimates obtained via indirect methods. A direct:indirect dependence ratio of 0.39 (0.22–0.78) was calculated by DisMod-MR based on comparable direct and indirect dependence estimates in the dataset.

Betas and exponentiated values (which can be interpreted as an odds ratio) for each study-level covariate are shown in the table below:

Study covariate	Parameter	beta	Exponentiated beta
cv_direct	Prevalence	-0.95 (-0.95 – -0.95)	0.39 (0.39–0.39)

Citations

1. American Psychiatric Association. Diagnostic and Statistical Manual of Mental Disorders (DSM-IV-TR). 4th, Text Revision ed Washington DC: American Psychiatric Association; 2000.
2. World Health Organization. The ICD-10 Classification of Mental and Behavioural Disorders. Clinical descriptions and diagnostic guidelines Geneva: World Health Organization; 1992.
3. Degenhardt L, Bucello C, Calabria B, Nelson P, Roberts A, Hall W, et al. What data are available on the extent of illicit drug use and dependence globally? Results of four systematic reviews. Drug and alcohol dependence. 2011.
4. Calabria B, Degenhardt L, Briegleb C, Vos T, Hall W, Lynskey M, et al. Systematic review of prospective studies investigating "remission" from amphetamine, cannabis, cocaine or opioid dependence. Addictive Behaviors. 2010.
5. Grant BF, Dawson DA. National Institute on Alcohol Abuse and Alcoholism. Alcohol Health & Research World. 2006; 29(2): p. 74.
6. Shand FL, Slade T, Degenhardt L, Baillie A, Nelson EC. Opioid dependence latent structure: two classes with differing severity? Addiction. 2011; 106(3): p. 590-8.
7. Shand FL, Degenhardt L, Slade T, Nelson EC. Sex differences amongst dependent heroin users: Histories, clinical characteristics and predictors of other substance dependence. Addictive behaviors. 2011; 36(1): p. 27-36.
8. European Montioring Centre for Drugs and Drug Addiction. Lisbon, Portugal; 2014.
9. Reuter P, Trautmann F. A Report on Global Illicit Drugs Markets 1998-2007. Utrecht; 2009.

Cocaine dependence

Flowchart

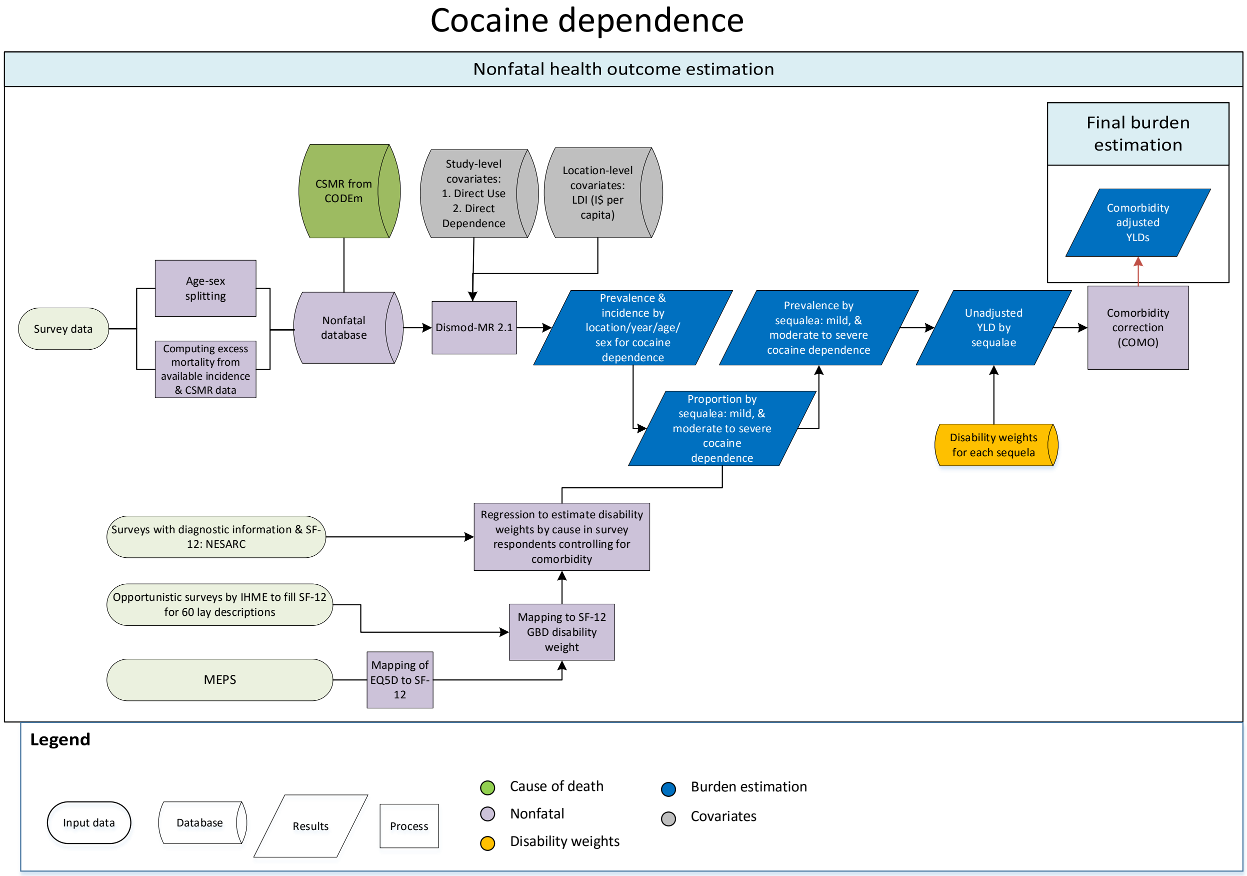

Case Definition

Cocaine dependence is a substance-related disorder involving a dysfunctional pattern of cocaine use. Included in the GBD disease modelling were cases meeting the Diagnostic and Statistical Manual of Mental Disorders (DSM-IV-TR) or the International Classification of Diseases (ICD-10) diagnostic criteria for cocaine dependence (DSM: 304.20; ICD: F14.2), excluding those cases due to a general medical condition.[1,2] According to DSM-IV TR criteria, dependence involves a maladaptive pattern of substance use leading to clinically significant impairment or distress. At least three of the following symptoms must be experienced within the same 12-month period:

- Tolerance, characterized by either
  - a need for increased amounts of the substance to achieve intoxication; or
  - markedly diminished effect with continued use of the same amount of the substance;
- Withdrawal, characterized by either
  - Withdrawal symptoms characteristic to dependence; or
  - the same (or similar) substance is taken to avoid withdrawal symptoms;
- Substance taken in progressively larger amounts or for longer period;

- Persistent desire or unsuccessful efforts to reduce substance use;
- Disproportionate time dedicated to obtaining the substance;
- Other important activities are given up because of the substance use; and
- Substance use is continued despite knowledge of physical or psychological problems occurring as a result of the substance.

Input data

Model inputs

A systematic review of the literature was conducted to capture studies of prevalence, incidence, remission, and excess mortality associated with cocaine dependence. In summary, the search was conducted in three stages involving searches of the peer-reviewed literature (via Medline, Embase, and Pubmed), the grey literature and, expert consultation. The agreed-upon approach for mental and substance use disorders was to conduct electronic database searches on a rolling basis. All three stages of GBD 2010's literature review were repeated for GBD 2013 to capture additional data published up to 2013. For GBD 2015, stages 2 and 3 of the literature review were updated and in GBD 2016, the peer-reviewed database search (stage 1) was conducted via Medline, Embase, and Psycinfo to capture studies published from 2013 to 2016.

The inclusion criteria stipulated that 1) the publication year must be from 1980 onward; 2) "caseness" must be based on clinical threshold as established by the DSM or ICD; 3) sufficient information must be provided on study method and sample characteristics to assess the quality of the study; and (4) study samples must be representative of the general population (ie, inpatient or pharmacological treatment samples, case studies, veterans or refugee samples were excluded). No limitation was set on the language of publication. Methods used for this systematic review have been reported in greater detail elsewhere.[3,4] The table below shows the number of studies included, as well as the number of countries or subnational units and GBD world regions represented.

	**Prevalence**	**Remission**	**Standardized mortality ratio, with-condition mortality rate**
Studies	118	3	7
Countries/subnational geographies	274	3	7
GBD world regions	15	2	3

Age and sex splitting

In GBD 2016, reported estimates of prevalence were split by age and sex where possible. First, if studies reported prevalence for broad age groups by sex (eg, prevalence in 15 to 65 year old males and females separately), and also by specific age groups but for both sexes combined (eg, prevalence in 15 to 30 year olds, then in 31 to 65 year olds, for males and females combined); age-specific estimates were split by sex using the reported sex ratio and bounds of uncertainty. Second, where studies reported estimates across

age groups spanning 20 years or more, these were split into five-year age groups using the prevalence age pattern estimated by DisMod-MR.

Severity splits

The basis of the GBD disability weight survey assessments are lay descriptions of sequelae highlighting major functional consequences and symptoms. The lay descriptions and disability weights for cocaine dependence severity levels are shown below.

Severity level	Lay description	DW (95% CI)
Mild	Uses cocaine at least once a week and has some difficulty controlling the habit. When not using, the person functions normally.	0.116 (0.074–0.165)
Moderate to severe	Uses cocaine and has difficulty controlling the habit. The person sometimes has mood swings, anxiety, paranoia, hallucinations and sleep problems, and has some difficulty in daily activities.	0.479 (0.324–0.634)

The proportion of people with cocaine dependence within each of the severity levels were determined based on available data from US National Epidemiological Survey on Alcohol and Related Conditions (NESARC), conducted in two waved from 2001-2002 and 2004-2005 [5]. The estimated distribution of cocaine dependent cases by severity were asymptomatic (50%, 37%—64%), mild (25%, 18%—33%), and moderate/severe (25%, 17%—33%).

Modelling Strategy

We ran a DisMod-MR model to produce estimates by age, sex, year, and country. We assumed no incidence, remission, and excess mortality before age 15, and an upper limit of 0.2 on remission. The minimum age of onset was corroborated with expert feedback and existing literature from various sources including the European Monitoring Centre for Drugs and Drug Addiction.[6] Cause-specific mortality rates (CSMR) from the GBD 2016 cause of death model for cocaine use disorders was included as data points in the DisMod-MR model.

The prevalence dataset included data points of both use and dependence estimated using “direct” or “indirect” survey methods. “Direct” methods of measuring amphetamine dependence predominantly involve surveys of the general population that ask if respondents use or are dependent on amphetamine. Surveys tend to underestimate the prevalence of the most harmful and stigmatized forms of illicit drug use in ways that probably vary between countries and cultures.[7] “Indirect” methods are considered superior; they use different sources of data to indirectly estimate the total number of drug users (methods include “multiplier methods,” back-projection and capture-recapture methods). Due to the lack of data available on cocaine dependence from indirect survey methods (considered to be the gold standard for GBD purposes), estimates of use and/or estimates from direct survey methods were included in the modelling. Study-level covariates were then used to accommodate for between-study variability in the raw prevalence data. The cv_direct use covariate was used to adjust for whether direct or indirect survey methods were used. This converted all use estimates obtained via direct methods in the dataset, into its equivalent value if the study had measured dependence estimates obtained via indirect methods. A ratio of direct use:indirect dependence was calculated by comparing similar direct

use and indirect dependence estimates in the dataset. To allow for meaningful comparisons, paired direct use and indirect dependence estimates needed to be similar in terms of the country they were from, year, age group, sex, and prevalence type. To maximize the number of data points available for this ratio paired estimates for psychostimulants (ie, both cocaine and amphetamine) were used. Once a dataset was set up with paired direct use and indirect dependence estimates, MetaXL (a meta-analysis add-in for Microsoft Excel) was utilized to estimate a ratio of direct use:indirect dependence, whereby direct use estimates were found to be 3.6 (2.6–5.2) times higher than indirect dependence estimates. This ratio was used in DisMod-MR to adjust all use estimates in the dataset downward, toward the level they would have been had the study reported indirect dependence. A similar method was used to adjust prevalence estimates of cocaine dependence obtained via direct methods toward the level they would have been had the study measured cocaine dependence using indirect methods. The estimated ratio of direct dependence: indirect dependence was 0.5 (0.2–1.1).

Betas and exponentiated values (which can be interpreted as an odds ratio) for each study-level covariate are shown in the table below:

Study covariate	Parameter	beta	Exponentiated beta
cv_direct use	Prevalence	1.29 (1.29–1.29)	3.63 (3.63–3.63)
cv_direct dependence	Prevalence	-0.68 (-0.68 – -0.68)	0.51 (0.51–0.51)

Citations

1. American Psychiatric Association. Diagnostic and Statistical Manual of Mental Disorders (DSM-IV-TR). 4th, Text Revision ed Washington DC: American Psychiatric Association. 2000.

2. World Health Organization. The ICD-10 Classification of Mental and Behavioural Disorders. Clinical descriptions and diagnostic guidelines Geneva: World Health Organization. 1992.

3. Degenhardt L, Bucello C, Calabria B, Nelson P, Roberts A, Hall W, et al. What data are available on the extent of illicit drug use and dependence globally? Results of four systematic reviews. Drug and alcohol dependence. 2011.

4. Calabria B, Degenhardt L, Briegleb C, Vos T, Hall W, Lynskey M, et al. Systematic review of prospective studies investigating “remission” from amphetamine, cannabis, cocaine or opioid dependence. Addictive Behaviors. 2010.

5. Grant BF, Dawson DA. National Institute on Alcohol Abuse and Alcoholism. Alcohol Health & Research World. 2006; 29(2): p. 74.

6. European Montioring Centre for Drugs and Drug Addiction. Lisbon, Portugal 2014.

7. Reuter P, Trautmann F. A Report on Global Illicit Drugs Markets 1998-2007. Utrecht. 2009.

Amphetamine dependence

Flowchart

Amphetamine dependence

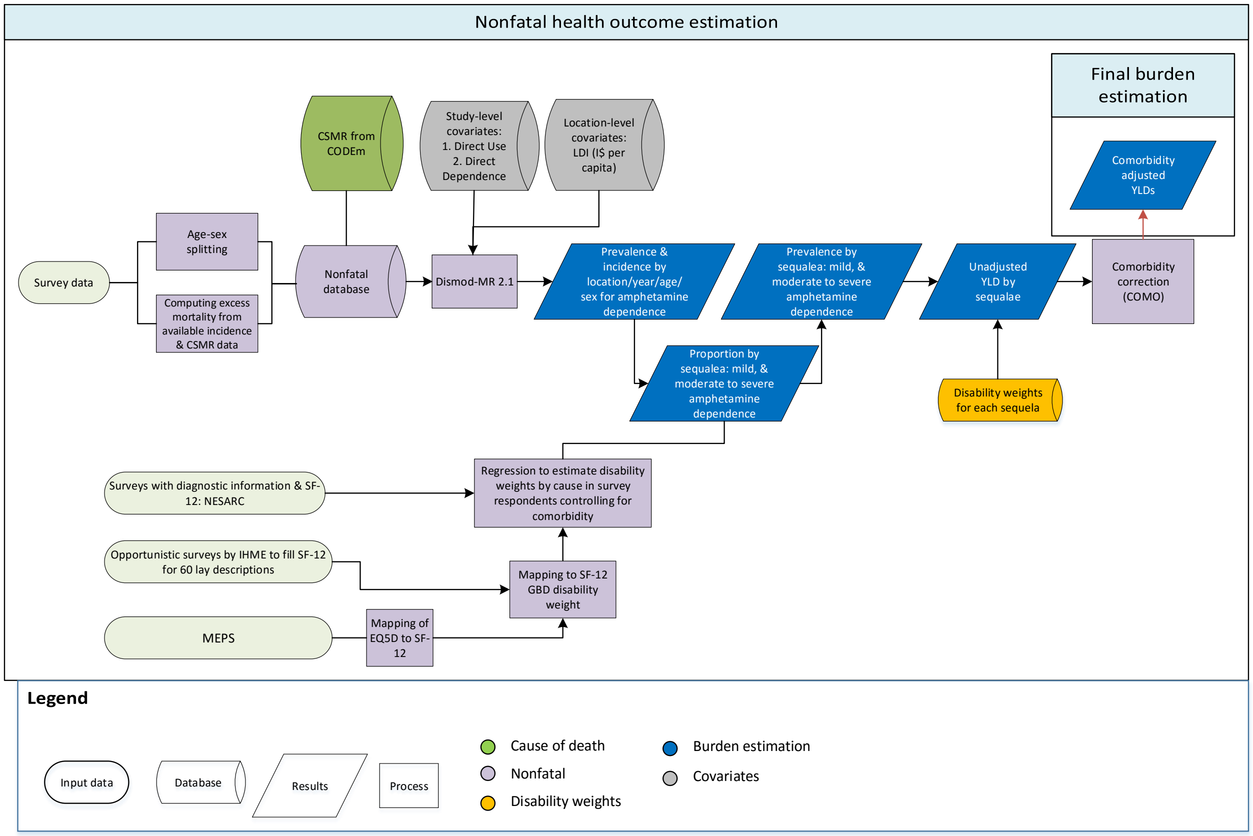

Case definition

Amphetamine dependence is a substance-related disorder involving a dysfunctional pattern of amphetamine use. Included in the GBD disease modelling were cases meeting the Diagnostic and Statistical Manual of Mental Disorders (DSM-IV-TR) or the International Classification of Diseases (ICD-10) diagnostic criteria for amphetamine dependence (DSM: 304.40; ICD: F15.2), excluding those cases due to a general medical condition.[1,2] According to DSM-IV TR criteria, dependence involves a maladaptive pattern of substance use, leading to clinically significant impairment or distress. At least three of the following symptoms must be experienced within the same 12-month period:

- Tolerance, characterized by either
  - a need for increased amounts of the substance to achieve intoxication; or
  - markedly diminished effect with continued use of the same amount of the substance;
- Withdrawal, characterized by either

- Withdrawal symptoms characteristic to dependence; or
    - the same (or similar) substance is taken to avoid withdrawal symptoms;
- Substance taken in progressively larger amounts or for longer period;
- Persistent desire or unsuccessful efforts to reduce substance use;
- Disproportionate time dedicated to obtaining the substance;
- Other important activities are given up because of the substance use; and
- Substance use is continued despite knowledge of physical or psychological problems occurring as a result of the substance.

Input data

Model inputs

A systematic review of the literature was conducted to capture studies of prevalence, incidence, remission, and excess mortality associated with amphetamine dependence. In summary, the search was conducted in three stages involving searches of the peer-reviewed literature (via Medline, Embase and Pubmed), the grey literature and, expert consultation. The agreed-upon approach for mental and substance use disorders was to conduct electronic database searches on a rolling basis. All three stages of GBD 2010's literature review were repeated for GBD 2013 to capture additional data published up to 2013. For GBD 2015, stages 2 and 3 of the literature review were updated and in GBD 2016, the peer-reviewed database search (stage 1) was conducted via Medline, Embase and Psycinfo to capture studies published from 2013 to 2016.

The inclusion criteria stipulated that: 1) the publication year must be from 1980 onward; 2) "caseness" must be based on clinical threshold as established by the DSM or ICD; 3) sufficient information must be provided on study method and sample characteristics to assess the quality of the study; and (4) study samples must be representative of the general population (ie, inpatient or pharmacological treatment samples, case studies, veterans, or refugee samples were excluded). No limitation was set on the language of publication. Methods used for this systematic review have been reported in greater detail elsewhere.[3,4] The table below shows the number of studies included, as well as the number of countries or subnational units and GBD world regions represented.

	**Prevalence**	**Remission**	**Mortality rate**
Studies	88	2	6
Countries/subnational geographies	274	3	7
GBD world regions	13	2	3

Age and sex splitting

In GBD 2016, reported estimates of prevalence were split by age and sex where possible. First, if studies reported prevalence for broad age groups by sex (eg, prevalence in 15 to 65 year old males and females separately), and also by specific age groups but for both sexes combined (eg, prevalence in 15 to 30 year olds, then in 31 to 65 year olds, for males and females combined); age-specific estimates were split by sex using the reported sex ratio and bounds of uncertainty. Second, where studies reported estimates across age groups spanning 20 years or more, these were split into five-year age groups using the prevalence age pattern estimated by DisMod MR.

Severity splits

The basis of the GBD disability weight survey assessments are lay descriptions of sequelae highlighting major functional consequences and symptoms. The lay descriptions and disability weights for amphetamine dependence severity levels are shown below.

Severity level	Lay description	DW (95% CI)
Mild	Uses stimulants (drugs) at least once a week and has some difficulty controlling the habit. When not using, the person functions normally.	0.079 (0.051–0.114)
Moderate to severe	Uses stimulants (drugs) and has difficulty controlling the habit. The person sometimes has depression, hallucinations, and mood swings, and has difficulty in daily activities.	0.486 (0.329–0.637)

The proportion of people with amphetamine dependence within each of the severity levels was determined based on available data from US National Epidemiological Survey on Alcohol and Related Conditions (NESARC), conducted in two waves from 2001–2002 and 2004–2005.[5] The estimated distribution of amphetamine dependent cases by severity were asymptomatic (55%, 40%–71%), mild (19%, 12%–27%), and moderate/severe (26%, 16%–35%).

Modelling Strategy

We ran a DisMod-MR model to produce estimates by age, sex, year, and country. We assumed no incidence, remission, and excess mortality before age 15, and an upper limit of 0.35 on remission. The minimum age of onset was corroborated with expert feedback and existing literature from various sources including the European Monitoring Centre for Drugs and Drug Addiction.[6] Cause-specific mortality rates (CSMR) from the GBD 2016 cause of death model for amphetamine use disorders was included as data-points in the DisMod-MR model.

The prevalence dataset included data-points of both use and dependence estimated using “direct” or “indirect” survey methods. “Direct” methods of measuring amphetamine dependence predominantly involve surveys of the general population that ask if respondents use or are dependent on amphetamine. Surveys tend to underestimate the prevalence of the most harmful and stigmatized forms of illicit drug use in ways that probably vary between countries and cultures.[7] “Indirect” methods are considered superior; they use different sources of data to indirectly estimate the total number of drug users (methods include “multiplier methods,” back-projection and capture-recapture methods). Due to the lack of data available on amphetamine dependence from indirect survey methods (considered to be the gold standard for GBD purposes), estimates of use and/or estimates from direct survey methods were included in the modelling. Study-level covariates were then used to accommodate for between-study variability in the raw prevalence data. The cv_direct use covariate was used to adjust for whether direct or indirect survey methods were used. This converted all use estimates obtained via direct methods in the dataset, into its equivalent value if the study had measured dependence estimates obtained via indirect methods. A ratio of direct use:indirect dependence was calculated by comparing similar direct use and indirect dependence estimates in the dataset. To allow for meaningful comparisons, paired direct use and indirect dependence estimates needed to be similar in terms of the country they were from,

year, age group, sex and, prevalence type. To maximize the number of data points available for this ratio paired estimates for psychostimulants (ie, both cocaine and amphetamine) were used. Once a dataset was set up with paired direct use and indirect dependence estimates, MetaXL (a meta-analysis add-in for Microsoft Excel) was utilized to estimate a ratio of direct use:indirect dependence, whereby direct use estimates were found to be 3.6 (2.6–5.2) times higher than indirect dependence estimates. This ratio was used in DisMod-MR to adjust all use estimates in the dataset downward, toward the level they would have been had the study reported indirect dependence. A similar method was used to adjust prevalence estimates of amphetamine dependence obtained via direct methods towards the level they would have been had the study measured amphetamine dependence using indirect methods. The estimated ratio of direct dependence: indirect dependence was 0.5 (0.2–1.1).

Betas and exponentiated values (which can be interpreted as an odds ratio) for each study level covariate are shown in the table below:

Study covariate	Parameter	beta	Exponentiated beta
cv_direct use	Prevalence	1.29 (1.29–1.29)	3.63 (3.63–3.63)
cv_direct dependence	Prevalence	-0.68 (-0.68 – -0.68)	0.51 (0.51–0.51)

Citations

1. American Psychiatric Association. Diagnostic and Statistical Manual of Mental Disorders (DSM-IV-TR). 4th, Text Revision ed Washington DC: American Psychiatric Association. 2000.
2. World Health Organization. The ICD-10 Classification of Mental and Behavioural Disorders. Clinical descriptions and diagnostic guidelines Geneva: World Health Organization. 1992.
3. Degenhardt L, Bucello C, Calabria B, Nelson P, Roberts A, Hall W, et al. What data are available on the extent of illicit drug use and dependence globally? Results of four systematic reviews. Drug and alcohol dependence. 2011.
4. Calabria B, Degenhardt L, Briegleb C, Vos T, Hall W, Lynskey M, et al. Systematic review of prospective studies investigating “remission” from amphetamine, cannabis, cocaine or opioid dependence. Addictive Behaviors. 2010.
5. Grant BF, Dawson DA. National Institute on Alcohol Abuse and Alcoholism. Alcohol Health & Research World. 2006; 29(2): p. 74.
6. European Montioring Centre for Drugs and Drug Addiction. Lisbon, Portugal 2014.
7. Reuter P, Trautmann F. A Report on Global Illicit Drugs Markets 1998-2007. Utrecht. 2009.

Cannabis Dependence

Flowchart

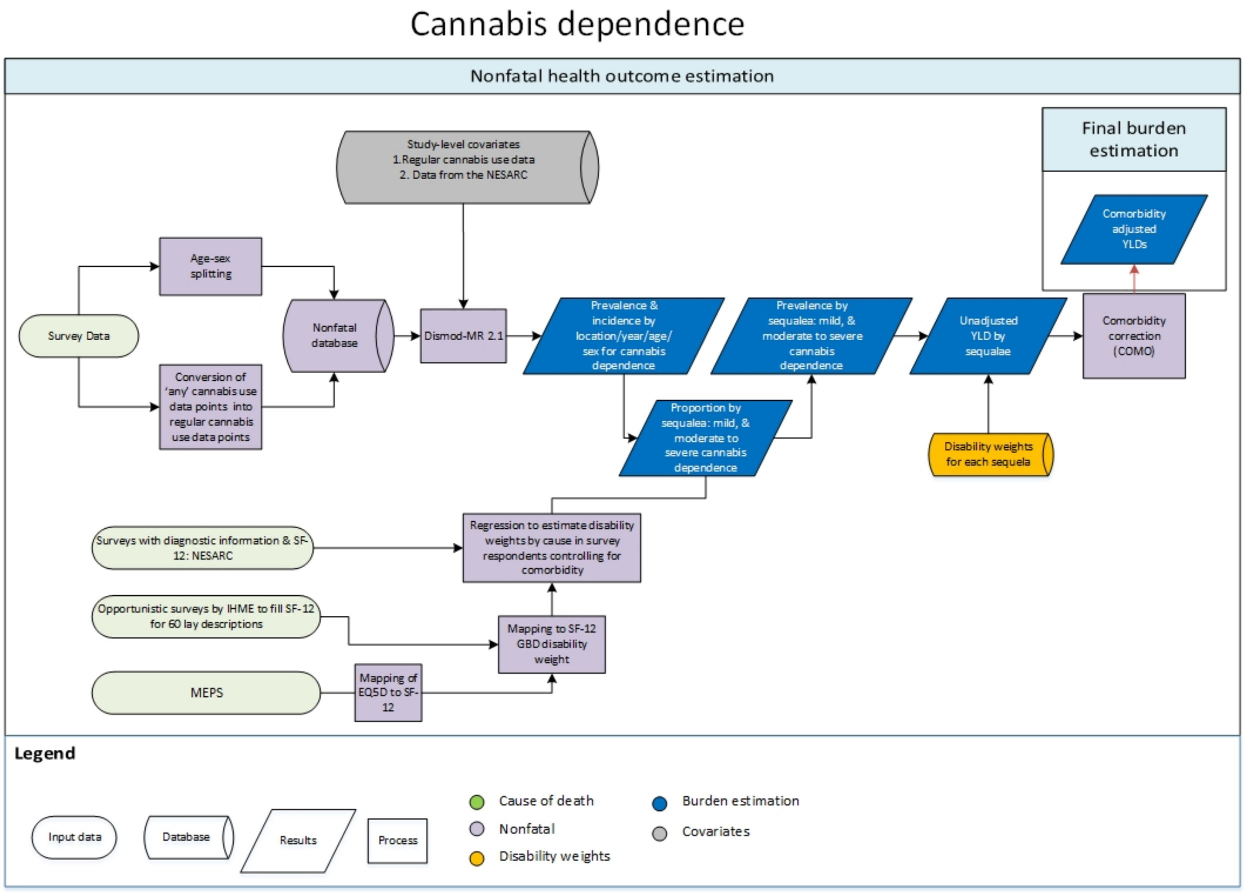

Case Definition

Cannabis dependence is a substance-related disorder involving a dysfunctional pattern of cannabis use. Included in GBD disease modelling were cases meeting diagnostic criteria for cannabis dependence according to the Diagnostic and Statistical Manual of Mental Disorders (DSM), or the equivalent diagnosis in the International Classification of Diseases (ICD). These were identified by the following codes: DSM:304.30, ICD:F12.2; excluding those cases due to a general medical condition.[1,2]

According to DSM-IV-TR criteria, cannabis dependence involves a maladaptive pattern of cannabis use, leading to clinically significant impairment or distress. At least three of the following symptoms must be experienced within the same 12-month period:

- Tolerance, characterized by either
    - a need for increased amounts of the substance to achieve intoxication; or
    - markedly diminished effect with continued use of the same amount of the substance;
- Withdrawal, characterized by either

   - Withdrawal symptoms characteristic to cannabis dependence; or
   - the same (or similar) substance is taken to avoid withdrawal symptoms;
- substance taken in progressively larger amounts or for longer period;
- persistent desire or unsuccessful efforts to reduce substance use;
- disproportionate time dedicated to obtaining the substance;
- other important activities are given up because of the substance use; and
- substance use is continued despite knowledge of physical or psychological problems occurring as a result of the substance.

Input data

Model inputs

For GBD 2010, a systematic review of the literature was conducted to capture studies of prevalence, incidence, remission, and excess mortality associated with cannabis dependence. In summary, the search was conducted in three stages involving electronic searches of the peer-reviewed literature (via Medline, Embase, and PubMed), the grey literature and, expert consultation. The agreed-upon approach for mental and substance use disorders was to conduct electronic database searches on a rolling basis. All three stages of GBD 2010's literature review were repeated for GBD 2013 to capture additional data published up to 2013. For GBD 2015, stages 2 and 3 of the literature review were updated and in GBD 2016, the peer-reviewed database search (stage 1) was conducted via Medline, Embase, and Psycinfo to capture studies published from 2013 to 2016.

The inclusion criteria stipulated that: (1) the publication year must be from 1980 onward; (2) "caseness" must be based on clinical threshold as established by the DSM or ICD; (3) sufficient information must be provided on study method and sample characteristics to assess the quality of the study; and (4) study samples must be representative of the general population (ie, inpatient or pharmacological treatment samples, case studies, veterans or refugee samples were excluded). No limitation was set on the language of publication. Methods used for this systematic review have been reported in greater detail elsewhere.[3-6] The table below shows the number of studies included, as well as the number of countries or subnational units and GBD world regions represented.

	Prevalence	Remission	Mortality
Studies	236	3	-
Countries/subnational geographies	315	3	-
GBD world regions	20	3	-

Age and sex splitting

In GBD 2016, reported estimates of prevalence were split by age and sex where possible. First, if studies reported prevalence for broad age groups by sex (eg, prevalence in 15 to 65 year old males and females separately), and also by specific age groups but for both sexes combined (eg, prevalence in 15 to 30 year olds, then in 31 to 65 year olds, for males and females combined); age-specific estimates were split by sex using the reported sex ratio and bounds of uncertainty. Second, where studies reported estimates across age groups spanning 20 years or more, these were split into five-year age groups using the prevalence age pattern estimated by DisMod MR.

Severity splits

The basis of the GBD disability weight survey assessments are lay descriptions of sequelae highlighting major functional consequences and symptoms. The lay descriptions and disability weights for cannabis dependence severity levels are shown below.

Severity level	Lay description	DW (95% CI)
Mild	Uses marijuana at least once a week and has some difficulty controlling the habit. When not using, the person functions normally.	0.039 (0.024–0.06)
Moderate to severe	Uses marijuana daily and has difficulty controlling the habit. The person sometimes has mood swings, anxiety, and hallucinations, and has some difficulty in daily activities.	0.266 (0.178–0.364)

The US National Epidemiological Survey on Alcohol and Related Conditions (NESARC, conducted in two waves from 2001-2002 and 2004-2005)[7] was used to estimate the proportion of cannabis dependence cases asymptomatic (58%, 51%–63%), mild (36%, 31%–42%) and moderate to severe (6%, 4%–8%).

Modelling Strategy

The epidemiological modelling strategy for cannabis dependence made use of DisMod-MR. Due to insufficient data, estimates of any cannabis use and regular (ie, weekly) cannabis use were included in the disease modelling of cannabis dependence in a two-step process. At step 1, a crosswalk was estimated to convert estimates of any use in the dataset into its equivalent value if the study had measured regular use. To do this a ratio of use:regular use was calculated by comparing similar regular use and use estimates in the dataset. To allow for meaningful comparisons, paired regular use and use estimates needed to be similar in terms of the country they were from, year, age group, sex, and prevalence type. Once a dataset was set up with paired regular use and use estimates, MetaXL (a meta-analysis add-in for Microsoft Excel) was used to estimate a ratio of use: regular use whereby use estimates were found to be 2.9 (2.5–3.3) times higher than regular use estimates. This ratio was used to adjust all use estimates in the dataset downwards, toward the level they would have been had the study reported regular cannabis use. Step 2 involved the DisMod-MR modelling of the regular cannabis use (from step 1) and cannabis dependence data. This cannabis regular use/dependence dataset was modelled using a study-level covariate which adjusted estimates of regular cannabis use toward the desirable which were estimates of cannabis dependence.

Study-level covariates were used to accommodate for between-study variability in the raw prevalence data. As mentioned previously, a cv_regular use covariate adjusted all regular use estimates toward the level they would have been if the study had measured cannabis dependence. This covariate was informed by a cannabis regular use: dependence ratio (4.1, 3.9–4.6) estimated outside of DisMod-MR using the same methodology outlined above for the use:regular use ratio. Based on expert advice, a cv_nesarc covariate adjusted all estimates derived from the NESARC toward the level they would have been if they had been derived by other surveys. Drug use disorders are not well-captured in household surveys. This is especially an issue in NESARC as the sampling strategy used was biased toward less severe cases of drug use disorders. We also tested a cv_school survey covariate adjusting estimates derived from school

surveys to the level they would have been had the study conducted a fully representative population survey; however, this did not have a statistically significant effect on prevalence. Betas and exponentiated values (which can be interpreted as an odds ratio) for each study-level covariate are shown in the table below.

Study covariate	Parameter	beta	Exponentiated beta
cv_regular use	Prevalence	1.40 (1.40–1.40)	4.06 (4.06–4.06)
cv_nesarc	Prevalence	-0.709 (-0.86 – -0.54)	0.50 (0.42–0.58)
cv_school survey	Prevalence	0.03 (-0.05 – 0.12)	1.03 (0.956–1.12)

References

1. American Psychiatric Association. Diagnostic and Statistical Manual of Mental Disorders (DSM-IV-TR). 4th, Text Revision ed. Washington DC: American Psychiatric Association; 2000.
2. World Health Organization. The ICD-10 Classification of Mental and Behavioural Disorders. Clinical descriptions and diagnostic guidelines. Geneva: World Health Organization; 1992.
3. Calabria B, Degenhardt L, Briegleb C, et al. Systematic review of prospective studies investigating "remission" from amphetamine, cannabis, cocaine or opioid dependence. *Addictive Behaviors* 2010; **35**(8): 741-9.
4. Calabria B, Degenhardt L, Hall W, Lynskey M. Does cannabis use increase the risk of death? Systematic review of epidemiological evidence on adverse effects of cannabis use. *Drug Alcohol Rev* 2010; **29**(3): 318-30.
5. Calabria B, Degenhardt L, Nelson P, et al. What do we know about the extent of cannabis use and dependence? Results of a global systematic review. Sydney: National Drug and Alcohol Research Centre, University of NSW, 2010.
6. Degenhardt L, Ferrari AJ, Calabria B, et al. The global epidemiology and contribution of cannabis use and dependence to the global burden of disease: results from the GBD 2010 study. *PloS one* 2013; **8**(10): e76635.
7. Introduction to the National Epidemiologic Survey on Alcohol and Related Conditions [http://pubs.niaaa.nih.gov/publications/arh29-2/74-78.htm]. Access date 1 December 2014.

Other drug use disorders

Flowchart

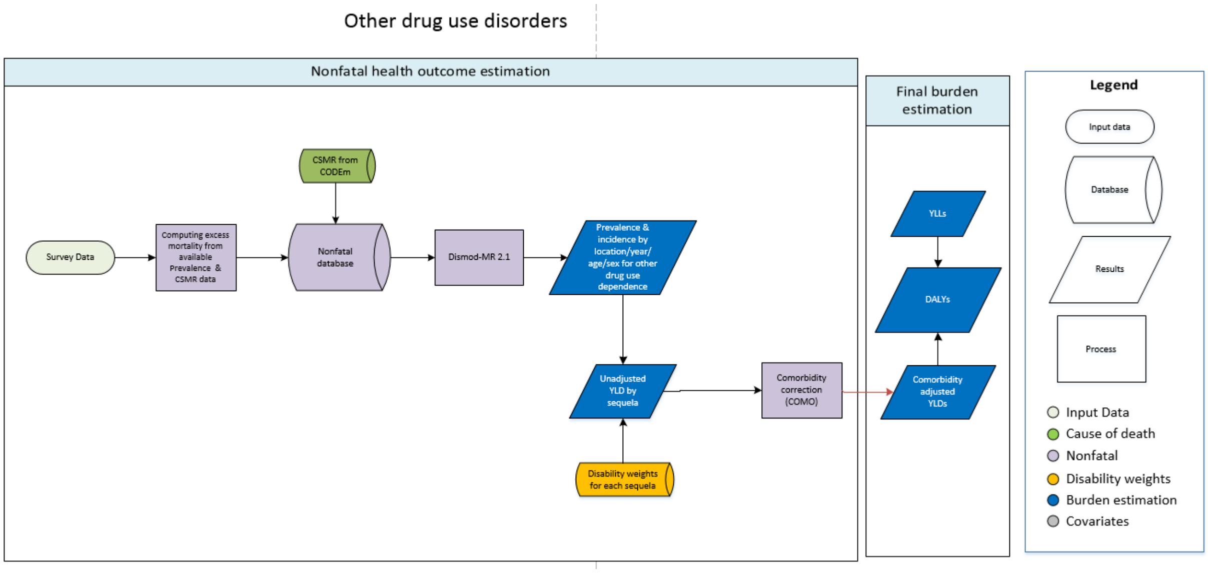

Case definition

In addition to the four drug use disorders for which we specifically estimate non-fatal burden (opioid, cocaine, amphetamine, and cannabis dependence), we also estimate the burden attributable to a residual cause of "other drug use disorders." This is made up of an aggregate group of other forms of drug dependence. Included in the GBD disease modelling were cases meeting the Diagnostic and Statistical Manual of Mental Disorders (DSM-IV-TR)[1] or the International Classification of Diseases (ICD-10)[2] diagnostic criteria for:

- Hallucinogen dependence
- Inhalant or solvent dependence
- Sedative dependence
- Tranquilizer dependence
- Other medicines, drugs, substance dependence

According to DSM-IV TR criteria, dependence involves a maladaptive pattern of substance use, leading to clinically significant impairment or distress. At least three of the following symptoms must be experienced within the same 12-month period:

- Tolerance, characterized by either
    - a need for increased amounts of the substance to achieve intoxication; or
    - markedly diminished effect with continued use of the same amount of the substance;
- Withdrawal, characterized by either

- Withdrawal symptoms characteristic to dependence; or
    - the same (or similar) substance is taken to avoid withdrawal symptoms;
- Substance taken in progressively larger amounts or for longer period;
- Persistent desire or unsuccessful efforts to reduce substance use;
- Disproportionate time dedicated to obtaining the substance;
- Other important activities are given up because of the substance use; and
- Substance use is continued despite knowledge of physical or psychological problems occurring as a result of the substance.

Input data

Model inputs

Prevalence estimates were obtained from the Australian National Survey of Mental Health and Wellbeing (NSMHWB) conducted in 1997[3],and the US National Epidemiological Survey on Alcohol and Related Conditions (NESARC), conducted in two waves in 2001–2002[4] and 2004–2005.[5] Given that other forms of drug dependence often co-occur with the four types of drug dependence for which we estimate non-fatal burden (opioid, cocaine, amphetamine, and cannabis dependence), an adjustment for co-morbidity is important so as not to overestimate the overall burden attributable to drug dependence. Participants meeting criteria for any other form of drug dependence from each of the surveys used were counted as a prevalent case only if they did not simultaneously meet criteria for opioid, cocaine, amphetamine, or cannabis dependence.

Severity splits

The basis of the GBD disability weight survey assessments are lay descriptions of sequelae highlighting major functional consequences and symptoms. The average disability weight estimated for cocaine and amphetamine dependence was applied to all cases in this residual group of other drug use disorders. The cocaine and amphetamine lay descriptions and disability weights are shown below:

Severity level	Lay description	DW (95% CI)
**Amphetamine dependence**		
Mild	Uses stimulants (drugs) at least once a week and has some difficulty controlling the habit. When not using, the person functions normally.	0.079 (0.051–0.114)
Moderate to severe	Uses stimulants (drugs) and has difficulty controlling the habit. The person sometimes has depression, hallucinations, and mood swings, and has difficulty in daily activities.	0.486 (0.329–0.637)
**Cocaine dependence**		
Mild	Uses cocaine at least once a week and has some difficulty controlling the habit. When not using, the person functions normally.	0.116 (0.074–0.165)
Moderate to severe	Uses cocaine and has difficulty controlling the habit. The person sometimes has mood swings, anxiety, paranoia, hallucinations, and sleep problems, and has some difficulty in daily activities.	0.479 (0.324–0.634)

Modelling Strategy

The GBD 2016 epidemiological modelling strategy made use of DisMod-MR. A number of additional expert priors were used in order to run a full parameter model. We assumed no incidence before age 14, a maximum of 0.0004 on incidence from the age of 60 years onward, and a maximum remission of 0.2. These priors were corroborated with expert feedback and existing literature on drug use disorders including the European Monitoring Centre for Drugs and Drug Addiction.[6] Finally, cause-specific mortality rates (CSMR) from the GBD 2016 cause of death model for other drug use disorders were included as data-points in the DisMod-MR model.

References

1. American Psychiatric Association. Diagnostic and Statistical Manual of Mental Disorders (DSM-IV-TR). 4th, Text Revision ed. Washington DC: 2000.
2. World Health Organization. The ICD-10 Classification of Mental and Behavioural Disorders. Clinical descriptions and diagnostic guidelines. Geneva: 1992.
3. Australia Bureau of Statistics. Australia National Survey of Mental Health and Wellbeing 1997. Canberra: 1997.
4. National Institute on Alcohol Abuse and Alcoholism (NIAAA), National Institutes of Health (NIH). United States National Epidemiologic Survey on Alcohol and Related Conditions 2001-2002. 2002.
5. National Institute on Alcohol Abuse and Alcoholism (NIAAA), National Institutes of Health (NIH). United States National Epidemiologic Survey on Alcohol and Related Conditions 2004-2005. 2005.
6. European Monitoring Centre for Drugs and Drug Addiction. Lisbon, Portugal: 2014.

Major Depressive Disorder

Flowchart

Major depressive disorder (MDD)

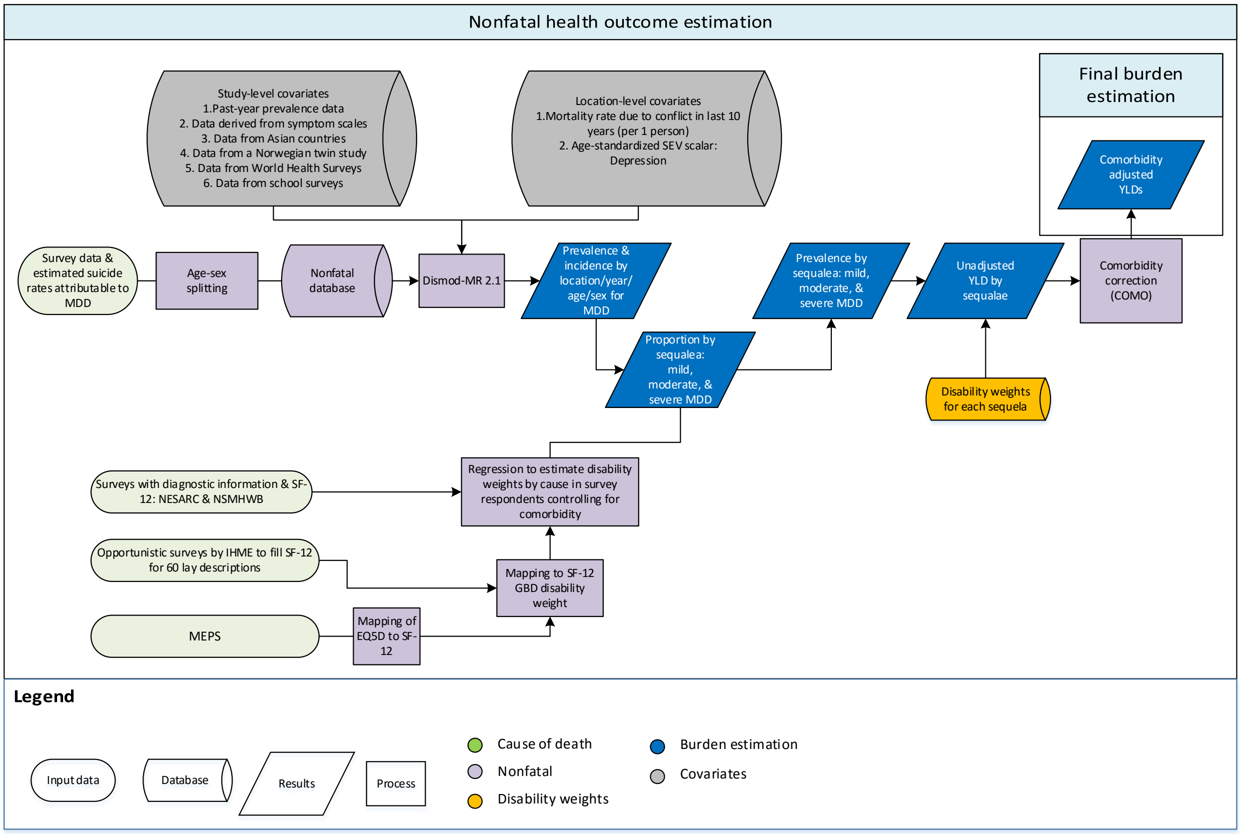

Case Definition

Major depressive disorder (MDD) is an episodic mood disorder involving the experience of one or more major depressive episode(s). Included in GBD disease modelling were cases meeting diagnostic criteria for MDD according to the Diagnostic and Statistical Manual of Mental Disorders (DSM) or the equivalent diagnosis of recurrent depression in the International Classification of Diseases (ICD). These were identified by the following codes: DSM-IV-TR: 296.21–24, 296.31–34; ICD-10: F32.0–9, F33.0–9; excluding those cases due to a general medical condition or substance induced cases.[1,2]

According to DSM-IV-TR criteria, MDD involves the presence of at least one major depressive episode, which is the experience of depressed mood almost all day, every day, for at least two weeks. Mood must represent a change from the person's baseline and impaired functioning must be observed across social, occupational, and educational domains. Additionally, a total of five out nine criteria must be met to make a diagnosis and at least one of the five criteria should either be:

- "depressed mood" for most of every day; or

- “loss of interest in nearly all activities” for most of every day.

The other seven criteria are:

- change in eating, appetite or weight;
- excessive sleeping or insomnia;
- agitated or slow motor activity;
- fatigue;
- feeling worthless or inappropriately guilty;
- trouble concentrating; and
- repeated thoughts about death

MDD was modelled as an episodic disorder with the average length of a major depressive episode (ie, duration) specified. This was consistent with previously proposed methodology for the modelling of MDD for burden of disease purposes.[3-5]

Input data

Model Inputs

For GBD 2010, a systematic review of the literature was conducted to capture studies of prevalence, incidence, duration, and excess mortality associated with MDD. In summary, the search was conducted in three stages involving electronic searches of the peer-reviewed literature (via PsycInfo, Embase and PubMed), the grey literature and, expert consultation. The agreed-upon approach for mental and substance use disorders was to conduct electronic database searches on a rolling basis. All three stages of GBD 2010’s literature review were repeated for GBD 2013 to capture additional data published up to 2013. In GBD 2015, stages two and three of the literature review were conducted, and in GBD 2016, stage 1 was repeated to update our electronic database search to capture data sources published between January 2013 and September 2016.

The inclusion criteria stipulated that: (1) the publication year must be from 1980 onward; (2) “caseness” must be based on clinical threshold as established by the DSM or ICD; (3) sufficient information must be provided on study method and sample characteristics to assess the quality of the study; and (4) study samples must be representative of the general population (ie, inpatient or pharmacological treatment samples, case studies, veterans or refugee samples were excluded). No limitation was set on the language of publication. Methods used for this systematic review have been reported in greater detail elsewhere.[6,7] The table below shows the number of studies included in GBD 2016, as well as the number of countries or subnational units and GBD world regions represented.

	Prevalence	Incidence	Duration	Excess mortality
Studies	344	3	5	22
Countries/subnational geographies	249	3	2	18
GBD world regions	20	2	2	5

Age- and sex-splitting

In GBD 2016, reported estimates of prevalence were split by age and sex where possible. First, if studies reported prevalence for broad age groups by sex (eg, prevalence in 15 to 65 year old males and females separately), and also by specific age groups but for both sexes combined (eg, prevalence in 15 to 30 year olds, then in 31 to 65 year olds, for males and females combined); age-specific estimates were split by sex using the reported sex ratio and bounds of uncertainty. Second, where studies reported estimates across age groups spanning 20 years or more, these were split into five-year age groups using the prevalence age pattern estimated by DisMod MR 2.1.

Attributable suicide estimates

Given that MDD is an established risk factor for suicide,[8] in GBD 2016 we supplemented the available data on excess mortality with estimated suicide rates (by age, sex, year, and location) attributable to MDD. These were estimated using GBD's comparative risk assessment methodology whereby the current health status was compared with a theoretical-minimum-risk exposure defined as the counterfactual status of the absence of MDD in the population. Population attributable fractions (PAFs) were estimated using this established formula:

$$PAF = \frac{p\,(RR-1)}{p\,(RR-1)+1}$$

P referred to the exposure distribution, which in this case was the DisMod-MR 2.1 prevalence rates of MDD by age, sex, location and year. RR referred to the pooled relative-risk of suicide due to MDD obtained from an existing systematic review and meta-analysis.[8] Age, sex, year, and location-specific PAFs were multiplied by their corresponding GBD suicide rate to estimate the proportion of suicide cases attributable to MDD. These were entered as cause-specific mortality rates in our epidemiological model for MDD.

Severity splits

The basis of the GBD disability weight survey assessments are lay descriptions of sequelae highlighting major functional consequences and symptoms. The lay descriptions and disability weights for MDD severity levels are shown below.

Severity level	Lay description	DW (95% CI)
Mild	Feels persistent sadness and has lost interest in usual activities. The person sometimes sleeps badly, feels tired, or has trouble concentrating but still manages to function in daily life with extra effort.	0.145 (0.099–0.209)
Moderate	Has constant sadness and has lost interest in usual activities. The person has some difficulty in daily life, sleeps badly, has trouble concentrating, and sometimes thinks about harming himself (or herself).	0.396 (0.267-0.531)
Severe	Has overwhelming, constant sadness and cannot function in daily life. The person sometimes loses touch with reality and wants to harm or kill himself (or herself).	0.658 (0.477-0.807)

To determine the proportion of people with MDD within each of the severity levels, the US National Epidemiological Survey on Alcohol and Related Conditions (NESARC, conducted in two waves from 2001 to 2002 and 2004 to 2005)[9] and the Australian National Survey of Mental Health and Wellbeing of Adults (NSMHWB, conducted in 1997)[10] were used to estimate the proportion of MDD cases asymptomatic (13%, 10%–17%), mild (59%, 49%–69%), moderate (17%, 13%–22%), and severe (10%, 3%–20%).

Modelling Strategy

The GBD 2016 epidemiological modelling strategy for MDD made use of DisMod-MR 2.1. Data across all epidemiological parameters were initially included in the modelling process. However, given that the few incidence data points available typically excluded cases of MDD at baseline, new major depressive episodes in people with previous episodes were not counted and incidence was underestimated. For this reason, we chose to exclude all raw incidence data in the final model and instead, allowed Dismod-MR 2.1 to calculate incidence based on data from other parameters. We assumed no incidence and prevalence before age 3. This minimum age of onset was corroborated with expert feedback and existing MDD literature.[6] An average remission rate for a major depressive episode of 1.45 (1.3–1.6) was used. This was derived from the four longitudinal studies[11-14] fitting a lognormal curve with least squared differences to data on the proportion of incident cases still fulfilling the case definition for major depression at intervals over a one-year period. As data were only available for a follow-up of one year, a decision had to be made about the maximum allowable duration of an episode. Setting this at 40 years, the average duration implied by the lognormal fit was 0.65 (0.59–0.70) of a year.[15]

Study-level covariates were used to accommodate between-study variability in the raw prevalence data. A cv_past year recall covariate adjusted all data points derived from past year prevalence toward the level they would have been if the study had captured point/past-month prevalence. The latter prevalence period is less affected by recall bias. A cv_symptom scale covariate adjusted all data points derived using a symptom scale towards the level they would have been if the scale had strictly adhered to DSM or ICD thresholds for MDD. A cv_asian data points covariate was used to adjust all estimates from East Asia, Southeast Asia and Asia Pacific high-income using a ratio based on a study in China. Phillips and collaborators[16] reported that the prevalence of MDD in China was 2.07% while the prevalence of mood disorders not otherwise specified (NOS) was 2.06%. Of the 808 individuals diagnosed with mood disorders NOS, 467 (58%) met criteria for minor depression (defined by DSM-IV-TR as two to four of nine symptoms of depression lasting for ≥2 weeks). There is evidence to suggest that these reported cases of minor depression are likely misdiagnosed cases MDD as DSM/ICD diagnostic criteria are not sensitive to cross-cultural presentations of MDD in Asia.[16-19] Based on this, a ratio of MDD + minor depression: MDD only (1.53, 1.45–1.63) was derived from data presented by Phillips and collaborators and used to adjust prevalence estimates from Asia in the model. The aim of this adjustment was not to capture sub-syndromal depression but instead, to pick up on diagnoses of MDD where there is evidence to suggest that the use of Western-based criteria have underestimated prevalence. A cv_school survey covariate adjusted estimates derived from school surveys downwards, to the level they would have been had the study conducted a fully representative population survey. A cv_World Health Survey covariate was used to adjust all World Health Survey data downwards. The World Health Surveys are surveys conducted by the World Health Organization in close to 70 countries. While these surveys capture useful information on the prevalence of depression, they make use of a symptom scale which does not fully meet DSM and ICD criteria for MDD. This covariate works in

essentially the same way as the symptom scale covariate in adjusting World Health Survey estimates downwards towards the level they would have been had the study strictly adhered to DSM or ICD thresholds for MDD.

Location-level covariates were also included in the MDD model. A covariate identifying for each GBD location, the mean mortality rate in the previous 10 years due to war and terrorism informed the estimation of prevalence given existing evidence to show a positive association between conflict status and the prevalence of MDD.[20,21] An age-standardised SEV scalar was also included. This made use of the fraction of MDD burden caused by its relevant risk factors combined to inform the estimation of prevalence. Intimate partner violence and childhood sexual violence are the two established risk factors of MDD for which attributable burden is estimated in GBD studies. Betas and exponentiated values (which can be interpreted as an odds ratio) for each study- and country-level covariate are shown in the table below:

Study/Country covariate	Parameter	beta	Exponentiated beta
cv_asian datapoints	Prevalence	-0.43 (-0.48 — -0.37)	0.65 (0.62 — 0.69)
cv_past year recall	Prevalence	0.63 (0.59 — 0.67)	1.88 (1.80 — 1.96)
cv_symptom scale	Prevalence	1.00 (0.94 — 1.06)	2.72 (2.57 — 2.90)
cv_school survey	Prevalence	0.29 (0.16 — 0.42)	1.33 (1.17 — 1.52)
cv_world health survey	Prevalence	0.78 (0.71 — 0.85)	2.18 (2.04 — 2.35)
Mean war mortality rate in the previous ten years	Prevalence	0.49 (0.022 — 0.98)	1.63 (1.02 — 2.65)
Age-standardised SEV scalar: Depression	Prevalence	0.77 (0.75 — 0.82)	2.17 (2.12 — 2.27)

Citations

1. American Psychiatric Association. Diagnostic and Statistical Manual of Mental Disorders (DSM). Washington: American Psychiatric Association, 1952.
2. World Health Organization. The ICD-10 Classification of Mental and Behavioural Disorders. Clinical descriptions and diagnostic guidelines. Geneva: World Health Organization; 1992.
3. Vos T, Mathers C, Herrman H, et al. The burden of mental disorders in Victoria, 1996. *Soc Psychiatry Psychiatr Epidemiol* 2001; **36**(2): 53-62.
4. Vos T, Mathers CD. The burden of mental disorders: a comparison of methods between the Australian burden of disease studies and the Global Burden of Disease study. *Bull World Health Organ* 2000; **78**(4): 427-38.
5. Ustun TB, Kessler RC. Global burden of depressive disorders: the issue of duration. *Br J Psychiatry* 2002; **181**: 181-3.
6. Ferrari AJ, Somerville AJ, Baxter AJ, et al. Global variation in the prevalence and incidence of major depressive disorder: a systematic review of the epidemiological literature. *Psychol Med* 2013; **43**(3): 471-81.
7. Ferrari AJ, Charlson FJ, Norman RE, et al. Burden of depressive disorders by country, sex, age, and year: findings from the global burden of disease study 2010. *PLoS Med* 2013; **10**(11): e1001547.
8. Ferrari AJ, Norman RE, Freedman G, et al. The burden attributable to mental and substance use disorders as risk factors for suicide: findings from the Global Burden of Disease Study 2010. *PloS one* 2014; **9**(4): e91936.

9. Introduction to the National Epidemiologic Survey on Alcohol and Related Conditions [http://pubs.niaaa.nih.gov/publications/arh29-2/74-78.htm]. Access date 1 December 2014.
10. Australian Bureau of Statistics. National Survey of Mental Health and Wellbeing of Adults 1997. Canberra: Australian Bureau of Statistics.
11. Kendler KS, Walters EE, Kessler RC. The prediction of length of major depressive episodes: results from an epidemiological sample of female twins. *Psychol Med* 1997; **27**(1): 107-17.
12. Lewinsohn PM, Clarke GN, Seeley JR, Rohde P. Major depression in community adolescents: age at onset, episode duration, and time to recurrence. *J Am Acad Child Adolesc Psychiatry* 1994; **33**(6): 809-18.
13. Spijker J, de Graaf R, Bijl RV, Beekman AT, Ormel J, Nolen WA. Duration of major depressive episodes in the general population: results from The Netherlands Mental Health Survey and Incidence Study (NEMESIS). *Br J Psychiatry* 2002; **181**: 208-13.
14. McLeod JD, Kessler RC, Landis KR. Speed of recovery from major depressive episodes in a community sample of married men and women. *J Abnorm Psychol* 1992; **101**(2): 277-86.
15. Vos T, Haby MM, Barendregt JJ, Kruijshaar M, Corry J, Andrews G. The burden of major depression avoidable by longer-term treatment strategies. *Archives of General Psychiatry* 2004; **61**(11): 1097-103.
16. Phillips MR, Zhang J, Shi Q, et al. Prevalence, treatment, and associated disability of mental disorders in four provinces in China during 2001–05: an epidemiological survey. *The Lancet* 2009; **373**: 2041–53.
17. Bromet E, Andrade LH, Hwang I, et al. Cross-national epidemiology of DSM-IV major depressive episode. *BMC medicine* 2011; **9**: 90.
18. Wang PS, Aguilar-Gaxiola S, Alonso J, et al. Use of mental health services for anxiety, mood, and substance disorders in 17 countries in the WHO world mental health surveys. *The Lancet* 2007; **370**(9590): 841-50.
19. Simon GE, Goldberg D, Von Korff M, Ustun T. Understanding cross-national differences in depression prevalence. *Psychological Medicine* 2002; **32**(4): 585–94.
20. Karam E, Bou GM. Psychosocial consequences of war among civilian populations. *Current Opinion in Psychiatry* 2013; **16**(413–419).
21. Steel Z, Chey T, Silove D, Marnane C, Bryant RA, van Ommeren M. Association of torture and other potentially traumatic events with mental health outcomes among populations exposed to mass conflict and displacement: a systematic review and meta-analysis. *JAMA* 2009; **302**(5): 537-49.

Dysthymia

Flowchart

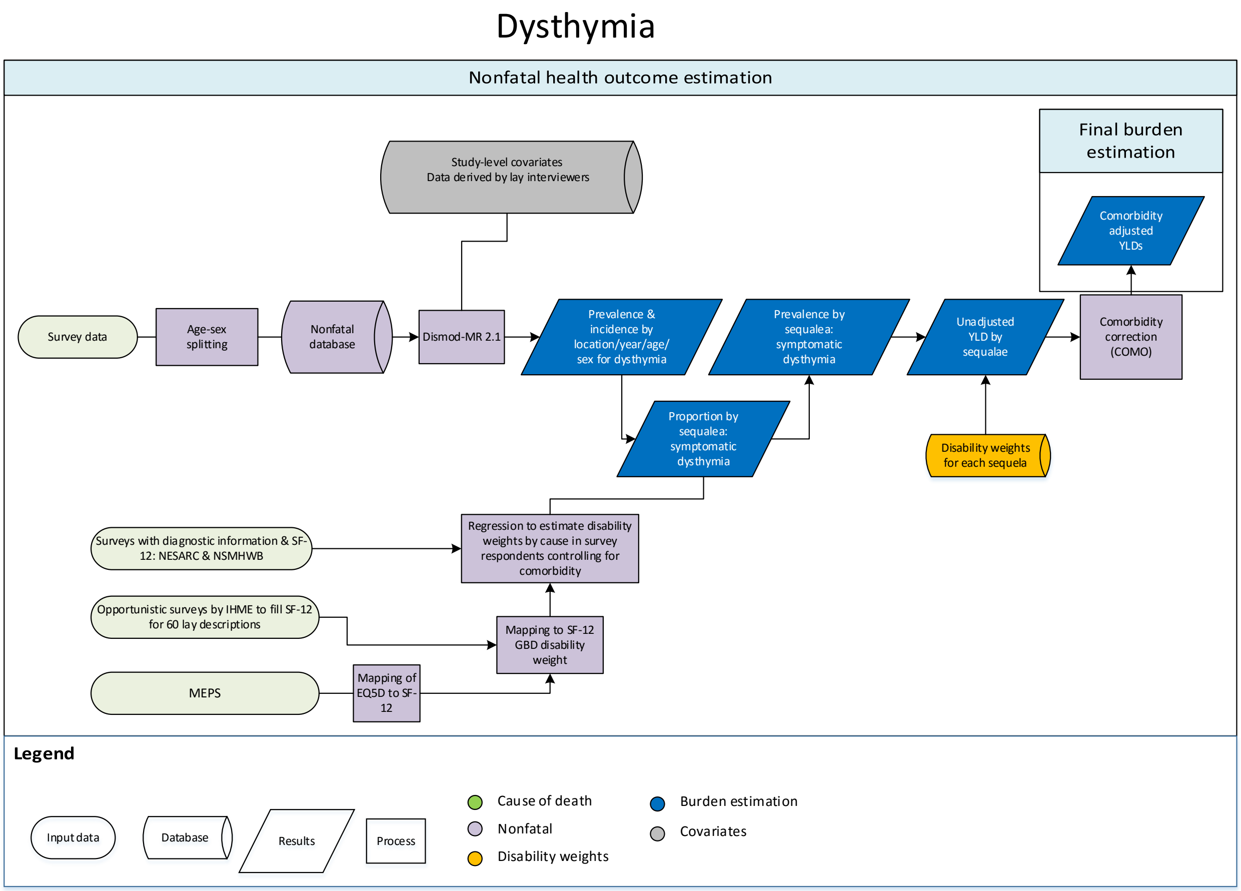

Case Definition

Dysthymia is a mood disorder consisting of chronic depression, demonstrating less severe but longer-lasting symptoms than major depressive disorder. Included in GBD disease modelling were cases meeting diagnostic criteria for dysthymia according to the Diagnostic and Statistical Manual of Mental Disorders (DSM), or the equivalent diagnosis in the International Classification of Diseases (ICD). These were identified by the following codes: DSM-IV-TR: 300.4, ICD-10: F34.1; excluding those cases due to a general medical condition or substance-induced cases.[1,2]

According to DSM-IV TR criteria, dysthymia involves the experience of chronically depressed mood for most of the day, most days that not, for at least two year (or at least one year in children and adolescents). During this period, at least two of the following symptoms must also be experienced:

- poor appetite or overeating;
- insomnia or hypersomnia;
- low energy or fatigue;
- low self-esteem;

- poor concentration or indecisiveness; and
- feelings of hopelessness

Input data

Model Inputs

For GBD 2010, a systematic review of the literature was conducted to capture studies of prevalence, incidence, remission, and excess mortality associated with dysthymia. In summary, the search was conducted in three stages involving electronic searches of the peer-reviewed literature (via Medline, Embase, and PubMed), the grey literature and, expert consultation. The agreed-upon approach for mental and substance use disorders was to conduct electronic database searches on a rolling basis. All three stages of GBD 2010's literature review were repeated for GBD 2013 to capture additional data published up to 2013. In GBD 2015, stages two and three of the literature review were conducted, and in GBD 2016, stage 1 was repeated to update our electronic database search to capture data sources published between January 2013 and September 2016.

The inclusion criteria stipulated that: (1) the publication year must be from 1980 onward; (2) "caseness" must be based on clinical threshold as established by the DSM or ICD; (3) sufficient information must be provided on study method and sample characteristics to assess the quality of the study; and (4) study samples must be representative of the general population (ie, inpatient or pharmacological treatment samples, case studies, veterans, or refugee samples were excluded). No limitation was set on the language of publication. Methods used for this systematic review have been reported in greater detail elsewhere.[3,4] The table below shows the number of studies included in GBD 2016, as well as the number of countries or subnational units and GBD world regions represented.

	Prevalence	Incidence	Remission	Excess mortality
Studies	94	2	2	-
Countries/subnational geographies	65	2	2	-
GBD world regions	15	1	2	-

Age- and sex-splitting

In GBD 2016, reported estimates of prevalence were split by age and sex where possible. First, if studies reported prevalence for broad age groups by sex (eg, prevalence in 15 to 65 year old males and females separately), and also by specific age groups but for both sexes combined (eg, prevalence in 15 to 30 year olds, then in 31 to 65 year olds, for males and females combined); age-specific estimates were split by sex using the reported sex ratio and bounds of uncertainty. Second, where studies reported estimates across age groups spanning 20 years or more, these were split into five-year age groups using the prevalence age pattern estimated by DisMod-MR 2.1.

Severity splits

The basis of the GBD disability weight survey assessments are lay descriptions of sequelae highlighting major functional consequences and symptoms. The lay description and disability weight for a

symptomatic state of dysthymia are shown below. Given the milder and more stable presentation of dysthymia, it was assigned the same disability weight as that for mild major depressive disorder.

Severity level	Lay description	DW (95% CI)
Symptomatic dysthymia	Feels persistent sadness and has lost interest in usual activities. The person sometimes sleeps badly, feels tired, or has trouble concentrating but still manages to function in daily life with extra effort.	0.145 (0.099–0.209)

To determine the proportion of people with symptomatic and asymptomatic dysthymia, the US National Epidemiological Survey on Alcohol and Related Conditions (NESARC, conducted in two waves from 2001 to 2002 and 2004 to 2005)[5] and the Australian National Survey of Mental Health and Wellbeing of Adults (NSMHWB, conducted in 1997)[6] were used to estimate the proportion of dysthymia cases asymptomatic (29%, 23%–36%) and symptomatic (71%, 64%–77%).

Modelling Strategy

The GBD 2016 epidemiological modelling strategy for dysthymia made use of DisMod-MR 2.1. Data across all epidemiological parameters were initially included in the modelling process. The incidence studies reported estimates which were very low relative to the prevalence data. As prevalence studies contributed much greater world coverage than incidence studies, we excluded the incidence data, relying instead on data from the other parameters. We assumed no incidence and prevalence before age 3. This minimum age of onset was corroborated with expert feedback and was consistent with the available data. Excess-mortality was set to 0 as there is no epidemiological evidence to suggest that dysthymia is associated with a statistically significant risk of mortality.[3,4]

Study-level covariates were used to accommodate between-study variability in the raw prevalence data. A cv_lay interviewer covariate created a crosswalk between prevalence derived from clinically trained interviewers (desirable) and prevalence derived from lay-interviewers. A cv_past year prevalence covariate was originally included to adjust all data points derived from past year prevalence toward the level they would have been if the study had captured point/past-month prevalence. As the effect of this covariate was not statistically significant, it was excluded from the final model. Given that dysthymia is being modelled as a chronic disorder with a long duration of between six and 10 years, it was not surprising that we did not detect significant variation between point and past-year prevalence.

Betas and exponentiated values (which can be interpreted as an odds ratio) for each study level covariate are shown in the table below:

Study covariate	Parameter	beta	Exponentiated beta
cv_lay interviewer	Prevalence	-0.36 (-0.50 — -0.23)	0.70 (0.61 — 0.79)

Given that there was an overall paucity of epidemiological data available for dysthymia, and the data available were very heterogeneous given differences in the data collection methodology used between studies, we applied a restriction on location random-effects of -0.3 to 0.3 to further guide the estimation of prevalence.

Citations

1. American Psychiatric Association. Diagnostic and Statistical Manual of Mental Disorders (DSM-IV-TR). 4th, Text Revision ed. Washington DC: American Psychiatric Association; 2000.
2. World Health Organization. The ICD-10 Classification of Mental and Behavioural Disorders. Clinical descriptions and diagnostic guidelines. Geneva: World Health Organization; 1992.
3. Charlson FJ, Ferrari AJ, Flaxman AD, Whiteford HA. The epidemiological modelling of dysthymia: application for the Global Burden of Disease Study 2010. *J Affect Disord* 2013; **151**(1): 111-20.
4. Ferrari AJ, Charlson FJ, Norman RE, et al. Burden of depressive disorders by country, sex, age, and year: findings from the global burden of disease study 2010. *PLoS Med* 2013; **10**(11): e1001547.
5. Introduction to the National Epidemiologic Survey on Alcohol and Related Conditions [http://pubs.niaaa.nih.gov/publications/arh29-2/74-78.htm]. Access date 1 December 2014.
6. Australian Bureau of Statistics. National Survey of Mental Health and Wellbeing of Adults 1997. Canberra: Australian Bureau of Statistics.

Bipolar disorder

Flowchart

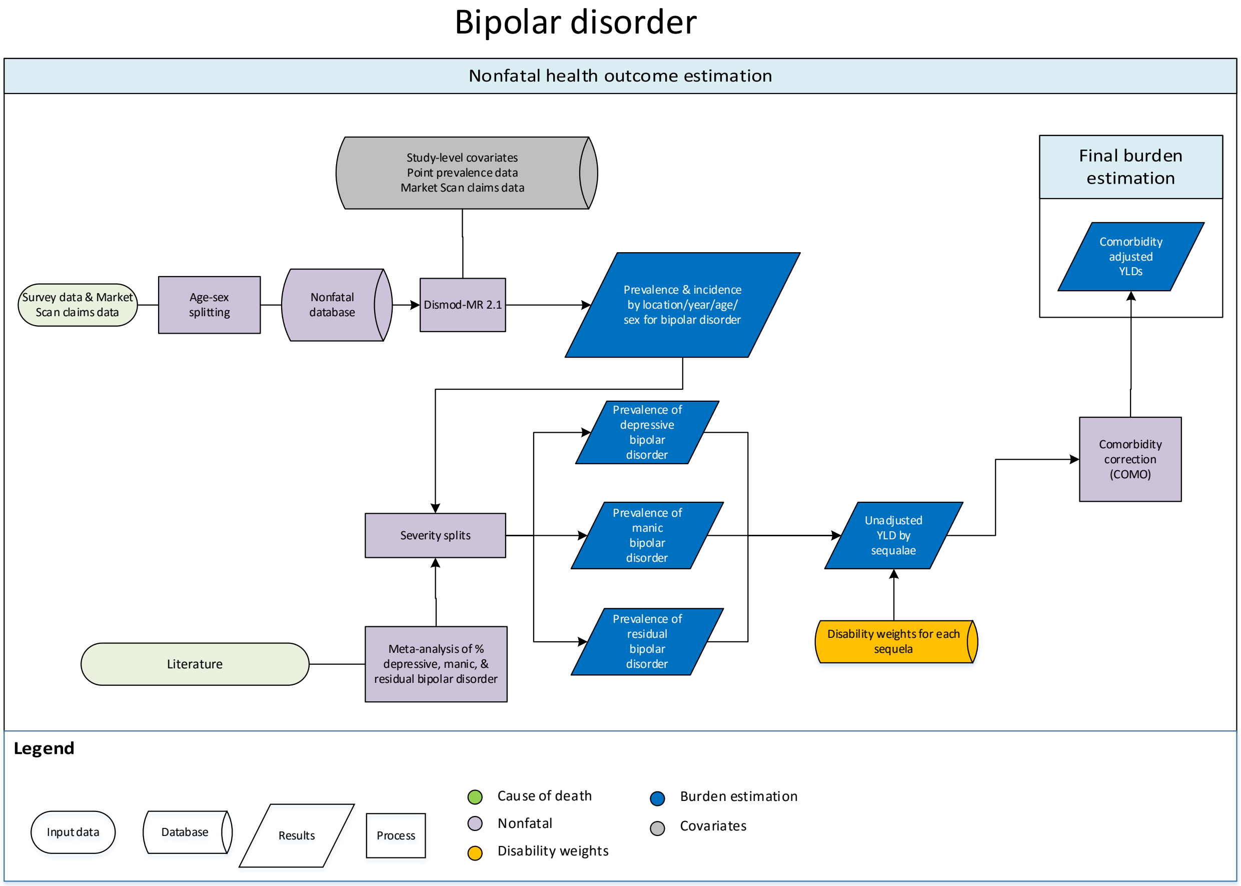

Case Definition

Bipolar disorder is a chronic mood disorder with little or no complete remission. Included in GBD disease modelling were cases meeting diagnostic criteria for bipolar disorder according to the Diagnostic and Statistical Manual of Mental Disorders (DSM), or the equivalent diagnosis in the International Classification of Diseases (ICD).[1,2] These were identified by the following codes: DSM-IV-TR: 296.0–296.8, 296.89, 301.13; ICD-10: F31.0–F31.6, F31.8–F31.9, F34.0–F34.1, excluding those cases due to a general medical condition or substance induced cases. A diagnosis of bipolar disorder involves the experience of one or more manic or hypomanic episode(s), which can be accompanied by a major depressive episode.

According to DSM-IV-TR a manic episode involves the experience of elevated, expansive, or irritable mood lasting for at least one week. During this period, at least three (or four if mood is only irritable) of the following symptoms must also be experienced;

- inflated self-esteem or grandiosity;
- decreased need for sleep;
- more talkative;

- flight of ideas or experience that thoughts are racing;
- distractibility;
- increase in goal-directed activity; and
- excessive involvement in pleasurable activities with high potential for painful consequences.

A hypomanic episode involves the experience of elevated, expansive, or irritable mood lasting for at least four days. During this period, at least three (or four if mood is only irritable) of the symptoms previously listed for a manic episode must also be experienced.

A major depressive episode involves the experience of depressed mood almost all day, every day, for at least two weeks. A total of five of nine criteria must be met to make a diagnosis and at least one of the five criteria should either be:

- "depressed mood" for most of every day; or
- "loss of interest in nearly all activities" for most of every day.

The other seven criteria are:

- change in eating, appetite, or weight;
- excessive sleeping or insomnia;
- agitated or slow motor activity;
- fatigue;
- feeling worthless or inappropriately guilty;
- trouble concentrating; and
- repeated thoughts about death.

Different subtypes of bipolar disorder can be diagnosed depending on the combination of symptoms experienced. Bipolar I is characterized by at least one manic episode, which can also alternate with a major depressive episode. Bipolar II is characterized by hypomanic episodes alternating with major depressive episodes. Cyclothymia is characterized by subsyndromal hypomanic and major depressive episode episodes. Bipolar disorder not otherwise specified is characterized by clinically significant symptoms of bipolar disorder which do not meet criteria for the other diagnoses.[1,2] In GBD 2016 we estimated burden for the entire spectrum of bipolar disorder simultaneously, rather than individually for each subtype of the disorder. At a minimum, epidemiological studies needed to report on bipolar I and bipolar II combined to be included in analyses.

Input data

Model inputs

For GBD 2010, a systematic review of the literature was conducted to capture studies of prevalence, incidence, remission, and excess mortality associated with bipolar disorder. In summary, the search was conducted in three stages involving electronic searches of the peer-reviewed literature (via Medline, Embase and PubMed), the grey literature and, expert consultation. The agreed-upon approach for mental and substance use disorders was to conduct electronic database searches on a rolling basis. All three stages of GBD 2010's literature review were repeated for GBD 2013 to capture additional data published up to 2013. In GBD 2015, stages two and three of the literature review were conducted, and

in GBD 2016, stage 1 was repeated to update our electronic database search to capture data sources published between January 2013 and September 2016.

The inclusion criteria stipulated that: (1) the publication year must be from 1980 onward; (2) "caseness" must be based on clinical threshold as established by the DSM or ICD; (3) sufficient information must be provided on study method and sample characteristics to assess the quality of the study; and (4) study samples must be representative of the general population (ie, inpatient or pharmacological treatment samples, case studies, veterans or refugee samples were excluded). No limitation was set on the language of publication. Methods used for this systematic review have been reported in greater detail elsewhere.[3] As previously explained, burden was estimated for the entire spectrum of bipolar disorder simultaneously. Combined estimates of all subtypes of bipolar disorders were required. Studies reporting separate estimates for bipolar I, bipolar II, cyclothymia, and/or bipolar not otherwise specified were accepted if sufficient information was available to sum the disorder-specific estimates. At a minimum, studies needed to report on bipolar I and bipolar II. The table below shows the number of studies included in GBD 2016, as well as the number of countries or subnational units and GBD world regions represented.

	Prevalence	Incidence	Remission	Excess mortality
Studies	58	3	-	13
Countries/subnational geographies	96	2	-	12
GBD world regions	14	2	-	4

Age- and sex-splitting

In GBD 2016, reported estimates of prevalence were split by age and sex where possible. First, if studies reported prevalence for broad age groups by sex (eg, prevalence in 15 to 65 year old males and females separately) and by specific age groups but for both sexes combined (eg, prevalence in 15 to 30 year olds, then in 31 to 65 year olds, for males and females combined), age-specific estimates were split by sex using the reported sex ratio and bounds of uncertainty. Second, where studies reported estimates across age groups spanning 20 years or more, these were split into five-year age groups using the prevalence age pattern estimated by DisMod-MR 2.1.

MarketScan data

In GBD 2016, we made use of United States (US) MarketScan data in our prevalence dataset for the years 2010 and 2012. These were prevalence data for bipolar disorder derived from claims information in a database of private and public insurance schemes. Given the sparseness of the bipolar disorder prevalence dataset, this allowed us to incorporate detailed prevalence estimates by state, sex, and age in our modelling. Evaluation of the age-pattern of MarketScan data revealed that it was consistent to what can be observed in population-representative survey estimates; however, given that this data source only captures a subset of the population, the actual levels of prevalence and the sex difference in prevalence were not and had to be adjusted accordingly. These adjustments are discussed further in the section on "Modelling strategy."

Severity splits inputs

The basis of the GBD disability weight survey assessments are lay descriptions of sequelae highlighting major functional consequences and symptoms. The lay descriptions and disability weights for bipolar disorder severity levels are shown below.

Severity level	Lay description	DW (95% CI)
Manic	Is hyperactive, hears and believes things that are not real, and engages in impulsive and aggressive behavior that endanger the person and others.	0.492 (0.341–0.646)
Depressive*	Has constant sadness and has lost interest in usual activities. The person has some difficulty in daily life, sleeps badly, has trouble concentrating, and sometimes thinks about harming himself (or herself).	0.396 (0.267–0.531)
Residual	Has mild mood swings, irritability, and some difficulty with daily activities.	0.032 (0.018–0.051)

*Note.*Equivalent to the disability weight estimated for moderate major depressive disorder*

Information on the distribution of manic, depressive, and residual states of bipolar disorder was obtained from a separate systematic review of the literature.[5] Meta-XL (a Microsoft Excel add-in for meta-analysis) was used to pool estimates across all studies to calculate the overall proportion of bipolar cases in each health state. Six studies provided information on the proportion of bipolar disorder cases in a manic (21%, 12%–33%), depressive (23%, 10%–39%) or residual state (52%, 28%–77%).

Modelling strategy

The GBD 2016 epidemiological modelling strategy for bipolar disorder made use of DisMod-MR 2.1. Data across all epidemiological parameters were initially included in the modelling process. The two studies on incidence reported 0% and 0.1% incidence of bipolar disorder and were low relative to the prevalence data. They were excluded from the final model where incidence was estimated using data from other parameters. We assumed no incidence and prevalence before age 10. Remission was set to a maximum of 0.05 in agreement with literature and expert advice suggesting no or very little complete remission from bipolar disorder.[6,7]

Study-level covariates were used to accommodate for between-study variability in the raw prevalence data. A cv_point recall covariate adjusted all data points derived from point/past-month prevalence toward the level they would have been if the study had captured 12-month prevalence. We set 12-month prevalence as the desirable level due to the episodic nature of bipolar disorder. Estimates of point prevalence surveying symptoms experienced in the past 30 days or less may fail to diagnose cases of bipolar disorder in a residual state, thereby underestimating prevalence. Two cv_market scan covariates for the years 2010 and 2012 adjusted MarketScan prevalence estimates upward toward the level they would have been had they been representative of the general population. The ratio used for this upward adjustment was estimated outside of DisMod-MR 2.1 by comparing modelled prevalence estimates for the US before MarketScan data were included in the model and corresponding MarketScan data points. MetaXL (a meta-analysis add-in for Microsoft Excel) was used to estimate the

adjustment ratio whereby Market scan estimates were 1.42 (1.28–1.58) times lower than prevalence estimated from survey data representative of the general US population.

The corresponding beta and exponentiated value (which can be interpreted as an odds ratio) is shown in the table below:

Study covariate	Parameter	beta	Exponentiated beta
cv_point recall	Prevalence	-0.60 (-0.90 — -0.42)	0.55 (0.41 — 0.66)
cv_market scan 2010	Prevalence	-0.35 (-0.44 — -0.28)	0.71 (0.64 — 0.76)
Cv_market scan 2012	Prevalence	-0.32 (-0.42 — -0.25)	0.73 (0.66 — 0.78)

An analysis of the sex pattern in MarketScan data also showed that the male: female prevalence ratio was 0.68 (0.62–0.76) compared to 0.88 (0.80–0.97) in population-representative survey data. It is likely that females are overrepresented in claims data for bipolar disorder as they are more likely to make use of such services. So as not to bias our sex pattern in prevalence, the estimated sex ratio in population-representative survey data was used as a setting to guide the sex pattern in our estimation of prevalence.

Given that there was an overall paucity in epidemiological data available for bipolar disorder, and the data available were very heterogeneous given differences in the data-collection methodology used between studies, we applied a restriction on location random-effects of -0.3 to 0.3 to further guide the estimation of prevalence.

Citations

1. American Psychiatric Association. Diagnostic and Statistical Manual of Mental Disorders (DSM). Washington: American Psychiatric Association, 1952.
2. World Health Organization. The ICD-10 Classification of Mental and Behavioural Disorders. Clinical descriptions and diagnostic guidelines. Geneva: World Health Organization; 1992.
3. Ferrari AJ, Baxter AJ, Whiteford HA. A systematic review of the global distribution and availability of prevalence data for bipolar disorder. *J Affect Disord* 2011; **134**(1-3): 1-13.
4. Ferrari AJ, Norman RE, Freedman G, et al. The burden attributable to mental and substance use disorders as risk factors for suicide: findings from the Global Burden of Disease Study 2010. *PLoS One* 2014; **9**(4): e91936.
5. Ferrari AJ, Saha S, McGrath JJ, et al. Health states for schizophrenia and bipolar disorder within the Global Burden of Disease 2010 Study. *Population health metrics* 2012; **10**(1): 16.
6. Angst J, Sellaro R. Historical perspectives and natural history of bipolar disorder. *Biological Psychiatry* 2000; **48**(6): 445–57.
7. Colom F, Vieta E. The road to DSM-V. Bipolar disorder episode and course specifiers. *Psychopathology* 2009; **42**(4): 209–18.

Anxiety Disorders

Flowchart

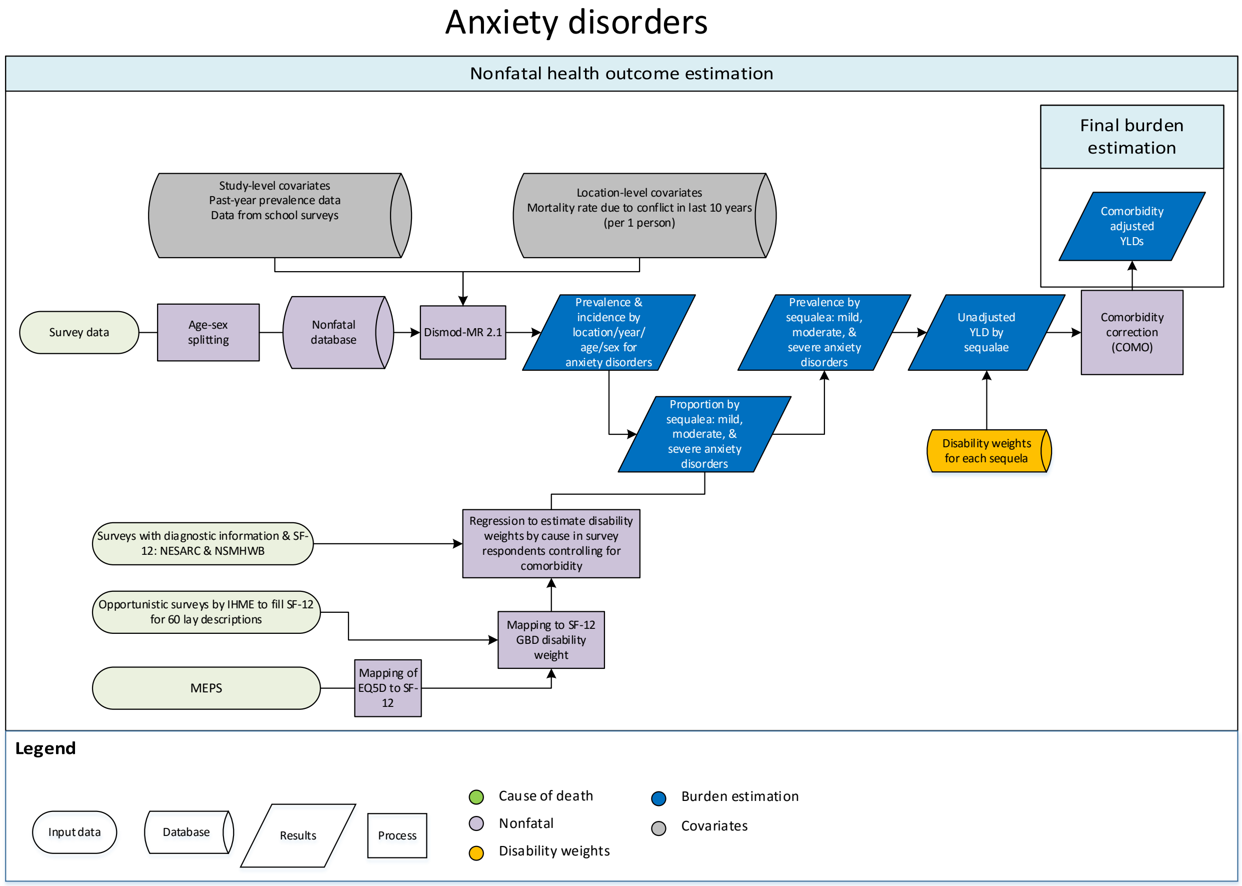

Case Definition

Anxiety disorders are characterized by experiences of intense of fear and distress, typically in combination with other physiological symptoms. We aimed to capture all cases of anxiety disorders reaching diagnostic threshold defined by the Diagnostic and Statistical Manual of Mental Disorders (DSM) or the WHO International Classification of Diseases (ICD-10).[1,2] Included disorders are listed below and can be identified by the DSM-IV-TR and ICD-10 coding systems as: DSM IV TR: 299.8, 300.0-300.3, 309.21, 309.81 and ICD-10: F40-42, F43.1, F93.0-93.2, F93.8. Excluded were anxiety disorders due to a general medical condition and substance-induced anxiety disorder.

- panic disorder;
- agoraphobia;
- specific phobia;
- social phobia;
- obsessive-compulsive disorder;
- post-traumatic stress disorder;

- acute stress disorder;
- generalized anxiety disorder;
- separation anxiety disorder; and
- anxiety disorder not otherwise specified

As specific anxiety disorders frequently co-occur, anxiety disorders were modeled as a single cause for "any" anxiety disorder in GBD 2016 to avoid the double-counting of individuals meeting criteria for more than one anxiety disorder. Epidemiological estimates reporting an outcome for "any" or "total" anxiety disorders were included in analyses.

Input data

Model inputs

For GBD 2010, a systematic review of the literature was conducted to capture studies of prevalence, incidence, remission, and excess mortality associated with anxiety disorders. In summary, the search was conducted in three stages involving electronic searches of the peer-reviewed literature (via Medline, Embase, and PubMed), the grey literature and, expert consultation. The agreed-upon approach for mental and substance use disorders was to conduct electronic database searches on a rolling basis. All three stages of GBD 2010's literature review were repeated for GBD 2013 to capture additional data published up to 2013. In GBD 2015, stages two and three of the literature review were conducted, and in GBD 2016, stage 1 was repeated to update our electronic database search to capture data sources published between January 2013 and August 2016.

The inclusion criteria stipulated that: (1) the publication year must be from 1980 onward; (2) "caseness" must be based on clinical threshold as established by the DSM or ICD; (3) sufficient information must be provided on study method and sample characteristics to assess the quality of the study; and (4) study samples must be representative of the general population (ie, inpatient or pharmacological treatment samples, case studies, veterans, or refugee samples were excluded). No limitation was set on the language of publication. Methods used for this systematic review have been reported in greater detail elsewhere.[3-5] The table below shows the number of studies included in GBD 2016, as well as the number of countries or subnational units and GBD world regions represented.

	Prevalence	Incidence	Remission
Studies	132	3	3
Countries/subnational geographies	103	2	3
GBD world regions	18	2	2

Age- and sex-splitting

In GBD 2016, reported estimates of prevalence were split by age and sex where possible. First, if studies reported prevalence for broad age groups by sex (eg, prevalence in 15 to 65 year old males and females separately), and also by specific age groups but for both sexes combined (eg, prevalence in 15 to 30 year olds, then in 31 to 65 year olds, for males and females combined); age-specific estimates were split by sex using the reported sex ratio and bounds of uncertainty. Second, where studies reported estimates across age groups spanning 20 years or more, these were split into five-year age groups using the prevalence age pattern estimated by DisMod-MR 2.1.

Severity splits

The basis of the GBD disability weight survey assessments are lay descriptions of sequelae highlighting major functional consequences and symptoms. The lay descriptions and disability weights for anxiety disorder severity levels are shown below.

Severity level	Lay description	DW (95% CI)
Mild	Feels mildly anxious and worried, which makes it slightly difficult to concentrate, remember things, and sleep. The person tires easily but is able to perform daily activities.	0.03 (0.018–0.046)
Moderate	Feels anxious and worried, which makes it difficult to concentrate, remember things, and sleep. The person tires easily and finds it difficult to perform daily activities.	0.133 (0.091–0.186)
Severe	Constantly feels very anxious and worried, which makes it difficult to concentrate, remember things, and sleep. The person has lost pleasure in life and thinks about suicide.	0.523 (0.362–0.677)

To determine the proportion of people with anxiety disorders within each of the severity levels, the United States' Medical Expenditure Panel Survey (MEPS, conducted in annual waves since 1996)[6], the US National Epidemiological Survey on Alcohol and Related Conditions (NESARC, conducted in two waves from 2001 to 2002 and 2004 to 2005)[7], and the Australian National Survey of Mental Health and Wellbeing of Adults (NSMHWB, conducted in 1997)[8] were used to estimate the proportion of anxiety disorder cases asymptomatic (28.8%, 27.5%–30.1%), mild (39.3%, 34.2%–44.2%), moderate (19.1%, 15.8%–22.7%) and severe (12.7%, 9.2%–16.7%).

Modelling strategy

The GBD 2016 epidemiological modelling strategy for anxiety disorders made use of DisMod-MR 2.1. Data across all epidemiological parameters were initially included in the modelling process. The incidence studies reported estimates which were very low relative to the prevalence data. As prevalence studies contributed much greater world coverage than incidence studies, we excluded the incidence data, relying instead on data from the other parameters. We assumed no incidence and prevalence before age 2 and after age 95. This minimum age of onset was corroborated with expert feedback and existing literature on anxiety disorders. Remission was set to a maximum of 0.2, consistent with the data points available.

Study-level covariates were used to accommodate for between-study variability in the raw prevalence data. A cv_past year recall covariate adjusted all data points derived from past year prevalence toward the level they would have been if the study had captured point/past-month prevalence. The latter prevalence period is less affected by recall bias. A cv_school survey covariate adjusted estimates derived from school surveys downward to the level they would have been had the study conducted a fully

representative population survey. A country-level covariate identifying for each GBD location the mean mortality rate in the previous 10 years due to war and terrorism was also included in the anxiety disorders model. This informed the estimation of prevalence given existing evidence to show a positive association between conflict status and the prevalence for anxiety disorders.[9,10] Betas and exponentiated values (which can be interpreted as an odds ratio) for each study-level covariate are shown in the table below:

Study/country covariate	Parameter	beta	Exponentiated beta
cv_past year recall	Prevalence	0.36 (0.30 — 0.42)	1.43 (1.36 — 1.53)
cv_school survey	Prevalence	0.30 (0.15 — 0.46)	1.35 (1.17 — 1.58)
Mean war mortality rate in the previous 10 years	Prevalence	0.50 (0.030 — 0.97)	1.66 (1.03 — 2.65)

Citations

1. American Psychiatric Association. Diagnostic and Statistical Manual of Mental Disorders (DSM-IV-TR). Fourth Edition, Text Revision ed. Washington DC: American Psychiatric Association; 2000.
2. World Health Organization. The ICD-10 Classification of Mental and Behavioural Disorders. Clinical descriptions and diagnostic guidelines. Geneva: World Health Organization; 1992.
3. Baxter AJ, Scott KM, Vos T, Whiteford HA. Global prevalence of anxiety disorders: a systematic review and meta-regression. *Psychological Medicine* 2013; **43**(05): 897-910.
4. Baxter AJ, Vos T, Scott KM, Ferrari AJ, Whiteford HA. The global burden of anxiety disorders in 2010. *Psychological Medicine* 2014; **44**(11): 2363-74.
5. Ferrari AJ, Norman RE, Freedman G, et al. The burden attributable to mental and substance use disorders as risk factors for suicide: findings from the Global Burden of Disease Study 2010. *PLoS One* 2014; **9**(4): e91936.
6. Agency for Healthcare Research and Quality. United States Medical Expenditure Panel Survey. Rockville, United States: Agency for Healthcare Research and Quality.
7. Introduction to the National Epidemiologic Survey on Alcohol and Related Conditions [http://pubs.niaaa.nih.gov/publications/arh29-2/74-78.htm]. Access date 1 December 2014.
8. Australian Bureau of Statistics. National Survey of Mental Health and Wellbeing of Adults 1997. Canberra: Australian Bureau of Statistics.
9. Karam E, Bou GM. Psychosocial consequences of war among civilian populations. *Current Opinion in Psychiatry* 2013; **16**(413–419).
10. Steel Z, Chey T, Silove D, Marnane C, Bryant RA, van Ommeren M. Association of torture and other potentially traumatic events with mental health outcomes among populations exposed to mass conflict and displacement: a systematic review and meta-analysis. *JAMA* 2009; **302**(5): 537-49.

Anorexia Nervosa

Flowchart

Anorexia nervosa

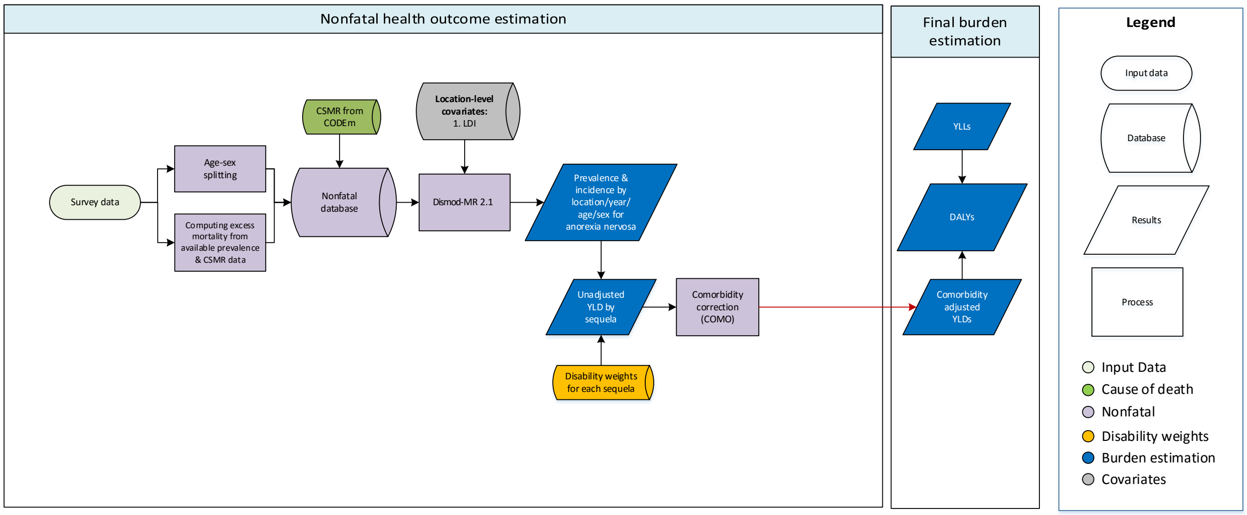

Case definition

According to the Diagnostic and Statistical Manual of Mental Disorders fourth edition, text revision (DSM-IV-TR),[1] anorexia nervosa (AN) is an eating disorder characterized by:

a) Refusal to maintain body weight at or above a minimally normal weight for age and height (eg, weight loss leading to maintenance of body weight less than 85% of that expected; or failure to make expected weight gain during period of growth, leading to body weight less than 85% of that expected).
b) Intense fear of gaining weight or becoming fat, even though underweight (expanded to include any behavior that interferes with weight gain in DSM-5[2]).
c) Disturbance in the way in which one's body weight or shape is experienced, undue influence of body weight or shape on self-evaluation, or denial of the seriousness of the current low body weight.
d) In postmenarcheal females, amenorrhea, ie, the absence of at least three consecutive menstrual cycles (this criterion was removed in DSM-5[2]).

Included in GBD were cases meeting diagnostic criteria according to DSM[1] or the International Classification of Diseases (ICD).[3] These were identified by the following codes: 307.1 (DSM-IV-TR) and F50.0 (ICD-10). Different versions of DSM (DSM-III, DSM-III-R, DSM-IV, DSM-IV-TR, and DSM-5) and ICD (ICD-9 and ICD-10) were accepted.

Input data

Model inputs

A series of systematic literature reviews were conducted to capture studies reporting the prevalence, incidence, remission, and excess mortality of AN. The reviews incorporated searches of peer-reviewed literature via electronic databases, investigations of grey literature, and consultation with experts. In order for a study to be included, it must have been published during or after 1980, use DSM or ICD criteria to define cases, provide sufficient details on study methodology and sample characteristics to determine study quality, and be representative of the general population rather than a special population, eg, prison inmates. No limitation was set on the language of publication. Detailed descriptions of this methodology have been published elsewhere.[4] This methodology was utilized in GBD 2010, GBD 2013, and GBD 2015. The systematic review will be updated again for GBD 2017.

The final dataset for GBD 2016 included 110 prevalence estimates, 40 incidence estimates, 20 remission estimates, and 28 excess mortality estimates. The table below shows the number of studies for each parameter as well as the number of countries/subnationals and GBD world regions covered by the available data.

	Prevalence	Incidence	Remission	Mortality
Studies	49	6	19	22
Countries/subnationals	35	6	14	15
GBD world regions	10	2	4	3

Disability weight

No severity splits were applied to AN. The lay description and disability weight for AN are shown in the table below.

Lay description	DW (95% CI)
Feels an overwhelming need to starve and exercises excessively to lose weight. The person is very thin, weak, and anxious.	0.224 (0.150–0.312)

Modelling strategy

We assumed no incidence prior to age 5 or from 50 years onward. These settings are in line with those placed on the corresponding cause of death model for anorexia nervosa. A cap of 0.6 was placed on remission in order to obtain a more plausible fit of the model. We used the function in DisMod-MR 2.1 to pull in cause-specific mortality rate (CSMR) data from our CODEm and CODcorrect analyses. As such, other mortality data (standardized mortality ratios and relative risks) were excluded. We also used these CSMR data to estimate priors on excess mortality rates (EMR) by matching them with prevalence data points for the same geography and study year and dividing CSMR by prevalence. A country-level covariate, lagged distributed income (LDI), was included. This covariate represents a moving average of gross domestic product (GDP) over time. The limits placed on this covariate meant that prevalence was assumed to increase with rising GDP. LDI was also applied to excess mortality data in order to better inform regional distribution. The table below illustrates the covariates, parameters, beta and exponentiated beta values for AN.

Covariate	Parameter	beta	Exponentiated beta
LDI ($ per capita)	Prevalence	0.43 (0.28–0.50)	1.54 (1.32–1.64)
LDI ($ per capita)	Excess mortality	-0.36 (-0.49 – -0.13)	0.70 (0.61–0.88)

References

1. American Psychiatric Association. Diagnostic and Statistical Manual of Mental Disorders (DSM-IV-TR). 4th, Text Revision ed. Washington DC: American Psychiatric Association; 2000.

2. American Psychiatric Association. Diagnostic and Statistical Manual of Mental Disorders, Fifth Edition. Arlington, VA: American Psychiatric Association; 2013.

3. World Health Organisation. ICD-10 Classification of Mental and Behavioural Disorders: Clinical descriptions and diagnostic guidelines. Geneva: World Health Organisation; 1992.

4. Whiteford HA, Degenhardt L, Rehm J, et al. Global burden of disease attributable to mental and substance use disorders: findings from the Global Burden of Disease Study 2010. *The Lancet* 2013; **382**(9904): 1575-86.

Bulimia nervosa

Flowchart

Bulimia nervosa

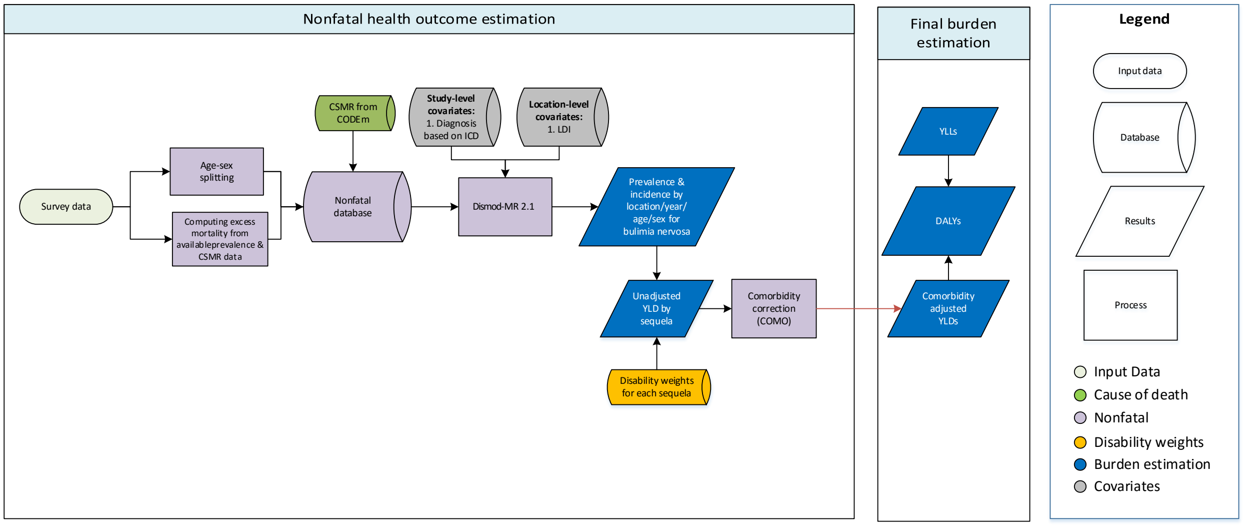

Case definition

According to the Diagnostic and Statistical Manual of Mental Disorders fourth edition, text revision (DSM-IV-TR),[1] bulimia nervosa (BN) is an eating disorder characterized by:

a) Recurrent episodes of binge eating. An episode of binge eating is characterized by both of the following:
   1) eating, in a discrete period of time (eg, within any two-hour period), an amount of food that is definitely larger than most people would eat during a similar period of time and under similar circumstances
   2) a sense of lack of control over eating during the episode (eg, a feeling that one cannot stop eating or control what or how much one is eating)
b) Recurrent inappropriate compensatory behavior in order to prevent weight gain, such as self-induced vomiting; misuse of laxatives, diuretics, enemas, or other medications; fasting; or excessive exercise.
c) The binge eating and inappropriate compensatory behaviors both occur, on average, at least twice a week for three months (changed to once a week for three months in DSM-5[2]).
d) Self-evaluation is unduly influenced by body shape and weight.
e) The disturbance does not occur exclusively during episodes of anorexia nervosa.

Included in GBD were cases meeting diagnostic criteria according to DSM[1] or the International Classification of Diseases (ICD).[3] These were identified by the following codes: 307.51 (DSM-IV-TR) and F50.1 (ICD-10). Different versions of DSM (DSM-III, DSM-III-R, DSM-IV, DSM-IV-TR, and DSM-5) and ICD (ICD-9 and ICD-10) were accepted.

Input data

Model inputs

A series of systematic literature reviews were conducted to capture studies reporting the prevalence, incidence, remission, and excess mortality of BN. The reviews incorporated searches of peer-reviewed literature via electronic databases, investigations of grey literature, and consultation with experts. In order for a study to be included, it must have been published during or after 1980, use DSM or ICD criteria to define cases, provide sufficient details on study methodology and sample characteristics to determine study quality, and be representative of the general population rather than a special population, eg, prison inmates. No limitation was set on the language of publication. Detailed descriptions of this methodology have been published elsewhere.[4] This methodology was utilized in GBD 2010, GBD 2013, and GBD 2015. The systematic review will be updated again for GBD 2017.

The final dataset for GBD 2016 included 148 prevalence estimates, 24 incidence estimates, 14 remission estimates, and 11 excess mortality estimates. The table below shows the number of studies for each parameter as well as the number of countries/subnationals and GBD world regions covered by the available data.

	Prevalence	Incidence	Remission	Mortality
Studies	49	4	14	11
Countries/subnationals	36	4	11	9
GBD world regions	11	1	3	2

Disability weight

No severity splits were applied to BN. The lay description and disability weight for BN is shown in the table below.

Lay description	DW (95% CI)
Has uncontrolled overeating followed by guilt, starving, and vomiting to lose weight.	0.223 (0.149–0.311)

Modelling strategy

We assumed no incidence prior to 10 years of age or onward from 40 years of age. We used the function in DisMod-MR 2.1 to pull in cause-specific mortality rate (CSMR) data from our CODEm and CODcorrect analyses. As such, other mortality data (standardized mortality ratios and relative risks) were excluded. We also used CSMR data to estimate priors on excess mortality rates (EMR) by matching them with prevalence data points for the same geography and study year and dividing CSMR by prevalence. A study-level covariate was applied which adjusted estimates based on ICD criteria toward those based on DSM criteria. A country-level covariate, lagged distributed income (LDI), was also included. This covariate represents a moving average of gross domestic product (GDP) over time. The limits placed on this covariate meant that prevalence was assumed to increase with rising GDP. LDI was also applied to excess mortality data in order to better inform regional distribution. The table below illustrates the covariates, parameters, beta and exponentiated beta values for BN.

Covariate	Parameter	beta	Exponentiated beta
ICD classification	Prevalence	0.66 (0.015–1.34)	1.93 (1.01–3.80)

LDI	Prevalence	0.43 (0.26–0.50)	1.53 (1.30–1.65)
LDI	Excess mortality	-0.33 (-0.49 – -0.12)	0.72 (0.61–0.89)

References

1. American Psychiatric Association. Diagnostic and Statistical Manual of Mental Disorders (DSM-IV-TR). 4th, Text Revision ed. Washington DC: American Psychiatric Association; 2000.
2. American Psychiatric Association. Diagnostic and Statistical Manual of Mental Disorders, Fifth Edition. Arlington, VA: American Psychiatric Association; 2013.
3. World Health Organisation. ICD-10 Classification of Mental and Behavioural Disorders: Clinical descriptions and diagnostic guidelines. Geneva: World Health Organisation; 1992.
4. Whiteford HA, Degenhardt L, Rehm J, et al. Global burden of disease attributable to mental and substance use disorders: findings from the Global Burden of Disease Study 2010. *The Lancet* 2013; **382**(9904): 1575-86.

Autism

Flowchart

Autism

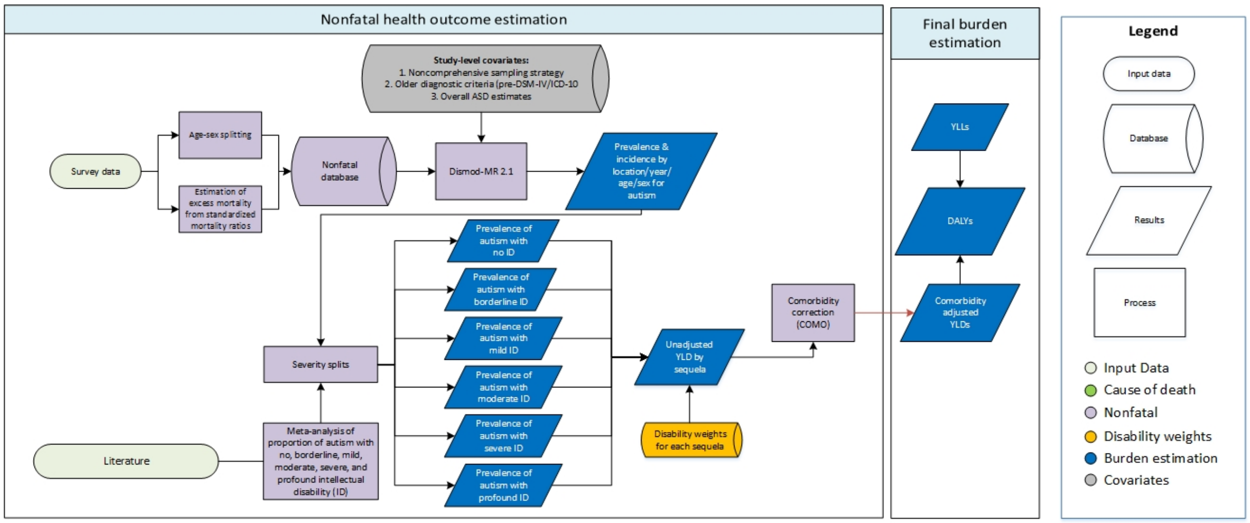

Case definition

Autism (also known as autistic disorder or childhood autism) is an autistic spectrum disorder (ASD) with onset occurring in early childhood. It is characterized by severe and pervasive impairment in several areas of development, including social interaction and communication skills, along with restricted and repetitive patterns of behaviors and/or interests. As per criteria set by the Diagnostic and Statistical Manual of Mental Disorders fourth edition, text revision (DSM-IV-TR),[1] diagnosis requires a total of six (or more) symptoms, with at least two symptoms of qualitative impairment in social interaction and at least one symptom of both qualitative impairment in communication and restricted, repetitive, stereotyped behavior. The recognized symptoms include:

Qualitative impairment in social interaction

a) marked impairment in the use of multiple nonverbal behaviors such as eye-to-eye gaze, facial expression, body postures, and gestures to regulate social interaction
b) failure to develop peer relationships appropriate to developmental level
c) a lack of spontaneous seeking to share enjoyment, interests, or achievements with other people
d) lack of social or emotional reciprocity

Qualitative impairments in communication

a) delay in, or total lack of, the development of spoken language (not accompanied by an attempt to compensate through alternative modes of communication such as gesture)
b) in individuals with adequate speech, marked impairment in the ability to initiate or sustain a conversation with others
c) stereotyped and repetitive use of language or idiosyncratic language
d) lack of varied, spontaneous make-believe play or social imitative play appropriate to developmental level

Restricted repetitive and stereotyped patterns of behavior, interests, and activities

a) encompassing preoccupation with one or more stereotyped and restricted patterns of interest that is abnormal either in intensity or focus
b) apparently inflexible adherence to specific, nonfunctional routines or rituals
c) stereotyped and repetitive motor mannerisms
d) persistent preoccupation with parts of objects

Delays or abnormal functioning with onset prior to three years of age in at social interaction, language interaction, or symbolic or imaginate play is also required. Included in GBD were cases meeting diagnostic criteria according to DSM[1] or the International Classification of Diseases (ICD).[2] These were identified by the following codes: 299.00 (DSM-IV-TR) and F84 (ICD-10). Different versions of DSM (DSM-III, DSM-III-R, DSM-IV, DSM-IV-TR, and DSM-5) and ICD (ICD-9 and ICD-10) were accepted.

Input data

Model inputs

A series of systematic literature reviews were conducted to capture studies reporting the prevalence, incidence, remission, and excess mortality of autism. The reviews incorporated searches of peer-reviewed literature via electronic databases, investigations of grey literature, and consultation with experts. In order for a study to be included, it must have been published during or after 1980, use DSM or ICD criteria to define cases, provide sufficient details on study methodology and sample characteristics to determine study quality, and be representative of the general population rather than a special population eg, prison inmates. No limitation was set on the language of publication. Detailed descriptions of this methodology have been published elsewhere.[3] This methodology was utilized in GBD 2010, GBD 2013, and GBD 2016.

Prevalence estimates were split by age and sex where possible outside of DisMod-MR 2.1. Firstly if studies reported prevalence for broad age groups by sex (eg, prevalence in 15 to 65 year old males and females separately), and also by specific age groups but for both sexes combined (eg, prevalence in 15 to 30 year olds, then in 31 to 65 year olds, for males and females combined); age-specific estimates were split by sex using the reported sex ratio and bounds of uncertainty. Secondly, where studies reported estimates across age groups spanning 20 years or more, these were split into five-year age groups using the regional prevalence age pattern estimated by DisMod-MR 2.1.

The final dataset for GBD 2016 included 174 prevalence estimates, 24 incidence estimates, 5 remission estimates, and 11 standardized mortality ratio estimates. The table below shows the number of studies for each parameter as well as the number of countries/subnationals and GBD world regions covered by the available data.

	Prevalence	Incidence	Remission	Mortality
Studies	67	3	5	3
Countries/subnationals	50	3	4	3
GBD world regions	10	3	2	2

Severity split inputs

Autism is one of the causes that contributes to the intellectual disability (ID) envelope. As such, a gradation of autism by level of severity was needed. Meta-analyses were conducted using data from six studies reporting information on the IQ level in those with autism in order to calculate the severity splits

by six sequelae: autism with 1) no ID, 2) borderline ID, 3) mild ID, 4) moderate ID, 5) severe ID, and 6) profound ID. The lay descriptions and disability weights for autism and each level of intellectual disability are shown in the table below.

Health state	Lay description	DW (95% CI)
Autism	Has severe problems interacting with others and difficulty understanding simple questions or directions. The person has great difficulty with basic daily activities and becomes distressed by any change in routine.	0.262 (0.176–0.365)
ID, borderline	Is slow in learning at school. As an adult, the person has some difficulty doing complex or unfamiliar tasks but otherwise functions independently.	0.011 (0.005–0.024)
ID, mild	Has low intelligence and is slow in learning at school. As an adult, the person can live independently, but often needs help to raise children and can only work at simple supervised jobs.	0.043 (0.028–0.067)
ID, moderate	Has low intelligence, and is slow in learning to speak and to do even simple tasks. As an adult, the person requires a lot of support to live independently and raise children. The person can only work at the simplest supervised jobs.	0.098 (0.064–0.142)
ID, severe	Has very low intelligence and cannot speak more than a few words, needs constant supervision and help with most daily activities, and can do only the simplest tasks.	0.157 (0.104–0.219)
ID, profound	Has very low intelligence, has almost no language, and does not understand even the most basic requests or instructions. The person requires constant supervision and help for all activities.	0.196 (0.126–0.272)

Modelling strategy

We assumed no incidence from 15 years of age onward. A small setting was placed on excess mortality whereby only minimal excess mortality was allowed over the lifespan. Remission was set to 0 after expert consultation revealed we would not expect remission for autism. Settings for excess mortality and remission differ from settings used in GBD 2015 where excess mortality was originally set to 0 and a small setting was placed on remission whereby only minimal remission was allowed over the lifespan. The mortality data in the autism dataset consist of standardized mortality ratio estimates from high-income locations. DisMod MR 2.1 produced good global fit for these data; however, the region fit did not follow the data, leading to high estimates of standardised mortality ratio in high-income countries and low estimates of standardised mortality ratio in low- and middle-income countries. Excess mortality estimates by age, sex, and year were therefore calculated by pulling global estimates of standardised mortality ratio from DisMod MR 2.1 and applying the following formula:

$$\text{excess mortality rate} = (\text{standardized mortality ratio} - 1) \times \text{all cause mortality rate}$$

Three study-level covariates were applied which: 1) adjusted estimates using a limited sampling strategy towards those using comprehensive sampling strategies (eg, those including private households and mainstream schools as well as healthcare and remedial therapy facilities), and 2) adjusted estimates based on older diagnostic criteria (prior to DSM-IV and ICD-10) toward estimates made using more

current criteria, and 3) adjusted estimates of ASD overall (ie, studies that did not report on the individual disorders) toward estimates representing autism. The third covariate is an addition in GBD 2016.

Study covariate	Parameter	beta	Exponentiated beta
Non-comprehensive sampling strategy	Prevalence	-0.32 (-0.6 – -0.041)	0.73 (0.55–0.96)
Identifies data classified by older criteria (ie, before DSM-IV and ICD-10)	Prevalence	-0.54 (-0.9 – -0.18)	0.58 (0.41–0.83)
Overall ASD estimates	Prevalence	1.25 (0.9–1.60)	3.47 (2.45–4.94)

References

1. American Psychiatric Association. Diagnostic and Statistical Manual of Mental Disorders (DSM-IV-TR). 4th, Text Revision ed. Washington DC: American Psychiatric Association; 2000.
2. World Health Organisation. ICD-10 Classification of Mental and Behavioural Disorders: Clinical descriptions and diagnostic guidelines. Geneva: World Health Organisation; 1992.
3. Baxter AJ, Brugha TS, Erskine HE, Scheurer RW, Vos T, Scott JG. The epidemiology and global burden of autism spectrum disorders. *Psychological Medicine* 2014; **45**(3): 601-13.

Asperger's syndrome & other autistic spectrum disorders

Flowchart

Asperger's syndrome and other autistic spectrum disorders

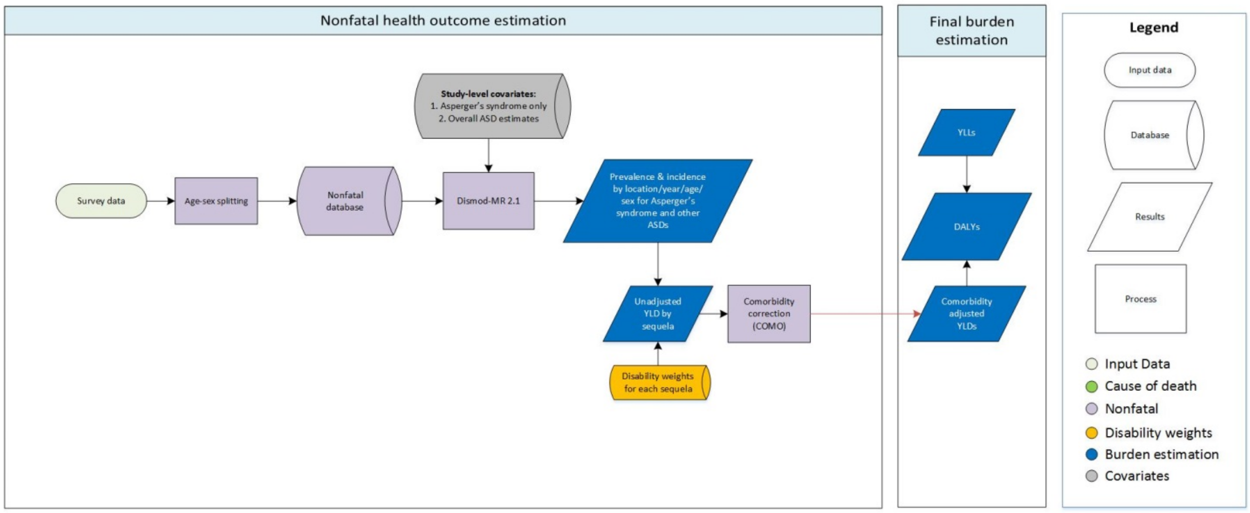

Case definition

Asperger's syndrome is an autistic spectrum disorder (ASD) characterized by severe and sustained impairment in social interaction skills along with restricted and repetitive patterns of behavior or interests. As per criteria set by the Diagnostic and Statistical Manual of Mental Disorders fourth edition, text revision (DSM-IV-TR),[1] diagnosis requires at least two symptoms of qualitative impairment in social interaction and at least one symptom of restricted, repetitive, stereotyped behavior. The recognized symptoms include:

Qualitative impairment in social interaction

a) marked impairment in the use of multiple nonverbal behaviors such as eye-to-eye gaze, facial expression, body postures, and gestures to regulate social interaction
b) failure to develop peer relationships appropriate to developmental level
c) a lack of spontaneous seeking to share enjoyment, interests, or achievements with other people
d) lack of social or emotional reciprocity

Restricted repetitive and stereotyped patterns of behavior, interests, and activities

a) encompassing preoccupation with one or more stereotyped and restricted patterns of interest that is abnormal either in intensity or focus
b) apparently inflexible adherence to specific, nonfunctional routines or rituals
c) stereotyped and repetitive motor mannerisms
d) persistent preoccupation with parts of objects

Unlike autism, there is no clinically significant delay in language acquisition or cognitive development. Included in GBD were cases meeting diagnostic criteria according to DSM[1] or the International Classification of Diseases (ICD).[2] These were identified by the following codes: 299.8 (DSM-IV-TR) and

F84.5 (ICD-10). Different versions of DSM (DSM-III, DSM-III-R, DSM-IV, DSM-IV-TR, and DSM-5) and ICD (ICD-9 and ICD-10) were accepted. Estimates of other ASDs were also included such as Rett's disorder (DSM-IV-TR: 299.8, ICD-10: F84.2), childhood disintegrative disorder (DSM-IV-TR: 299.1, ICD-10: F84.3), atypical autism (ICD-10: F84.1), overactive disorder associated with mental retardation and stereotyped movements (ICD-10: F84.4), and pervasive disorder not otherwise specified (DSM-IV-TR: 299.8, ICD-10: F84.8-F84.9).

Input data

Model inputs

A series of systematic literature reviews were conducted to capture studies reporting the prevalence, incidence, remission, and excess mortality of Asperger's syndrome and other ASDs. The reviews incorporated searches of peer-reviewed literature via electronic databases, investigations of grey literature, and consultation with experts. In order for a study to be included, it must have been published during or after 1980, use DSM or ICD criteria to define cases, provide sufficient details on study methodology and sample characteristics to determine study quality, and be representative of the general population rather than a special population, eg, prison inmates. No limitation was set on the language of publication. Detailed descriptions of this methodology have been published elsewhere.[3] This methodology was utilized in GBD 2010, GBD 2013, and GBD 2016.

Prevalence estimates were split by age and sex where possible outside of DisMod-MR 2.1. Firstly if studies reported prevalence for broad age groups by sex (eg, prevalence in 15 to 65 year old males and females separately), and also by specific age groups but for both sexes combined (eg, prevalence in 15 to 30 year olds, then in 31 to 65 year olds, for males and females combined); age-specific estimates were split by sex using the reported sex ratio and bounds of uncertainty. Secondly, where studies reported estimates across age groups spanning 20 years or more, these were split into five-year age groups using the regional prevalence age pattern estimated by DisMod-MR 2.1.

The final dataset for GBD 2016 included 78 prevalence estimates, 14 incidence estimates, 2 remission estimates, and 1 excess mortality estimate. The table below shows the number of studies for each parameter as well as the number of countries/subnationals and GBD world regions covered by the available data.

	Prevalence	Incidence	Remission	Mortality
Studies	31	2	2	1
Countries/subnationals	32	2	2	1
GBD world regions	8	2	1	1

Disability weight

No severity splits were applied to autism. The lay description and disability weight for autism are shown in the table below.

Lay description	DW (95% CI)
Has difficulty interacting with other people and is slow to understand or respond to questions. The person is often preoccupied with one thing and has some difficulty with basic daily activities.	0.104 (0.071–0.147)

Modelling strategy

We assumed no incidence from 15 years of age onward. This was changed from GBD 2015 where no incidence was assumed from 20 years of age onward. A small setting was placed on remission whereby only minimal remission was allowed over the lifespan. Excess mortality was set to 0 given the limited data demonstrating an association between Asperger's syndrome and other ASDs and an increased risk of death. Only one study reported the excess mortality associated with Asperger's syndrome and other ASDs and found no significantly increased risk of death. Two study-level covariates were applied. The first adjusted estimates of Asperger's syndrome only toward those including both Asperger's syndrome and other ASDs. The second adjusted estimates of ASD overall (ie, studies that did not report on the individual disorders) toward estimates representing Asperger's syndrome and other ASDs. This approach was largely consistent with that of GBD 2015 with the exception of the addition of the latter covariate and the earlier age in which we assume no incidence onward.

Study covariate	Parameter	beta	Exponentiated beta
Asperger's syndrome only	Prevalence	-0.39 (-0.88 – -0.034)	0.67 (0.42–0.97)
Overall ASD estimates	Prevalence	0.97 (0.53–1.17)	2.65 (1.84–3.67)

References

1. American Psychiatric Association. Diagnostic and Statistical Manual of Mental Disorders (DSM-IV-TR). 4th, Text Revision ed. Washington DC: American Psychiatric Association; 2000.
2. World Health Organisation. ICD-10 Classification of Mental and Behavioural Disorders: Clinical descriptions and diagnostic guidelines. Geneva: World Health Organisation; 1992.
3. Baxter AJ, Brugha TS, Erskine HE, Scheurer RW, Vos T, Scott JG. The epidemiology and global burden of autism spectrum disorders. *Psychological Medicine* 2014; **45**(3): 601-13.

Attention-deficit/hyperactivity disorder (ADHD)

Flowchart

Attention-deficit/hyperactivity disorder

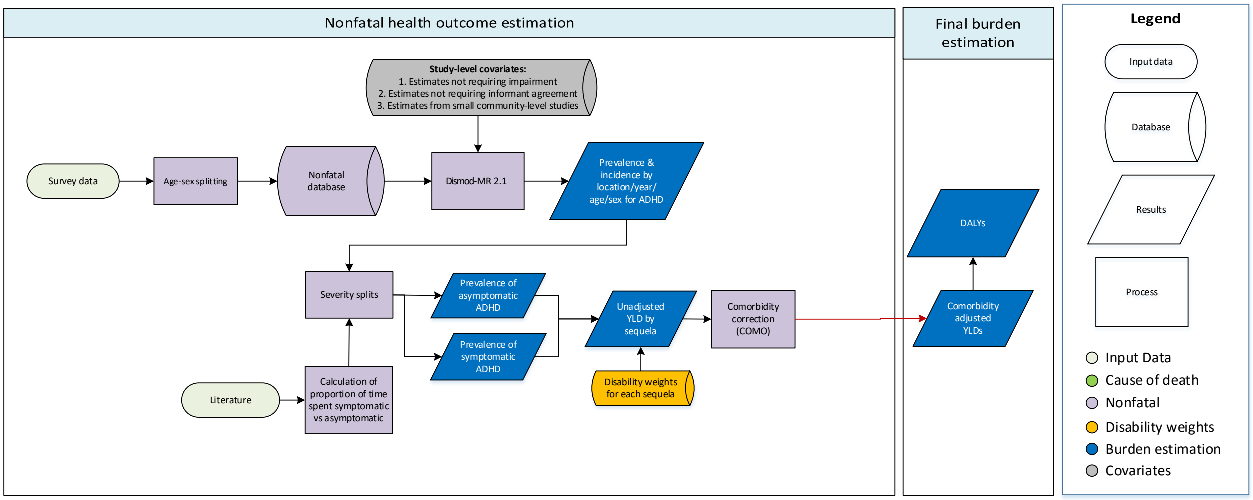

Case definition

Attention-deficit/hyperactivity disorder (ADHD) is an externalizing behavior disorder characterized by persistent inattention and/or hyperactivity-impulsivity. As per criteria set by the Diagnostic and Statistical Manual of Mental Disorders fourth edition, text revision (DSM-IV-TR)[1], diagnosis requires six or more symptoms of inattention or hyperactivity-impulsivity to have persisted for at least six months in two or more settings causing significant impairment to functioning, with at least some impairing symptoms being present prior to 7 years of age (12 years of age in DSM-5[2]). Recognized symptoms include:

Inattention:

- often fails to give close attention to details or makes careless mistakes in schoolwork, work, or other activities
- often has difficulty sustaining attention in tasks or play activities
- often does not seem to listen when spoken to directly
- often does not follow through on instructions and fails to finish school work, chores, or duties in the workplace (not due to oppositional behavior or failure to understand instructions)
- often has difficulty organizing tasks and activities
- often avoids, dislikes, or is reluctant to engage in tasks that require sustained mental effort (such as schoolwork or homework)
- often loses things necessary for tasks or activities (eg, toys, school assignments, pencils, books, or tools)
- is often easily distracted by extraneous stimuli
- is often forgetful in daily activities

Hyperactivity

- often fidgets with hands or feet or squirms in seat
- often leaves seat in classroom or in other situations in which remaining seated is expected
- often runs about or climbs excessively in situations in which it is inappropriate (in adolescents or adults, may be limited to subjective feelings of restlessness)
- often has difficulty playing or engaging in leisure activities quietly
- is often "on the go" or often acts as if "driven by a motor"
- often talks excessively

Impulsivity

- often blurts out answers before questions have been completed
- often has difficulty awaiting turn
- often interrupts or intrudes on others (eg, butts into conversations or games)

Included in GBD were cases meeting diagnostic criteria according to DSM[1] or the International Classification of Diseases (ICD)[3] (called "hyperkinetic disorder" in ICD). These were identified by the following codes: 314.0, 314.01 (DSM-IV-TR) and F90 (ICD-10). Different versions of DSM (DSM-III, DSM-III-R, DSM-IV, DSM-IV-TR, and DSM-5) and ICD (ICD-9 and ICD-10) were accepted.

Input data

Model inputs

A series of systematic literature reviews were conducted to capture studies reporting the prevalence, incidence, remission, and excess mortality of ADHD. The reviews incorporated searches of peer-reviewed literature via electronic databases, investigations of grey literature, and consultation with experts. In order for a study to be included, it must have been published during or after 1980, use DSM or ICD criteria to define cases, provide sufficient details on study methodology and sample characteristics to determine study quality, and be representative of the general population rather than a special population, eg, prison inmates. No limitation was set on the language of publication. Detailed descriptions of this methodology have been published elsewhere.[4] This methodology was utilized in GBD 2010 and GBD 2013. GBD 2015 included additional sources identified by GBD experts and microdata where available. The systematic review methodology used in GBD 2010 and 2013 was replicated to update the dataset for GBD 2016.

The final dataset for GBD 2016 included 290 prevalence estimates, five incidence estimates, 20 remission estimates, and three excess mortality estimates. The table below shows the number of studies for each parameter as well as the number of countries/subnationals and GBD world regions covered by the available data.

	Prevalence	Incidence	Remission	Mortality
Studies	131	2	14	2
Countries/subnationals	76	2	12	2
GBD world regions	16	1	3	2

Reported estimates of prevalence were split by age and sex where possible. If studies reported prevalence for broad age groups by sex (eg, prevalence in 5-18 year old males and females separately) and by specific age groups but for both sexes combined (eg, prevalence for 5-12 year olds and 13-18 year olds, for males and females combined), age-specific estimates were split by sex using the reported sex

ratio and bounds of uncertainty. Also, where studies reported estimates across age groups spanning 20 years or more, these were split into five-year age groups using the prevalence age pattern estimated by DisMod-MR 2.1.

Severity split inputs

A severity split for the proportion of time spent symptomatic versus asymptomatic was based on data from the Great Smoky Mountains Study which assessed the levels of disability found in children and adolescents with mental disorders.[5] Of those with ADHD, 48% reported disability while 20% of individuals with no diagnosis reported disability at the time of survey. Using these as estimates of the proportion of time with disability in the "average case," the proportion of disability in children without a diagnosis was subtracted from the proportion with disability for ADHD, giving an adjusted proportion of 28%. Detailed descriptions of this methodology have been published elsewhere.[6] The lay description and disability weight for ADHD is shown in the table below.

Lay description	DW (95% CI)
Is hyperactive and has difficulty concentrating, remembering things, and completing tasks	0.045 (0.028–0.066)

Modelling strategy

We assumed no incidence prior to 3 years of age or onward from 12 years of age. The minimum age of onset was set in consultation with experts and based on current literature, while the upper age limit on incidence was set in line with the latest DSM-5 criteria. Remission was set to zero prior to 12 years, in line with the restriction on incidence. Excess mortality was set to zero given only three estimates were found for this parameter. Three covariates were included in the model. The first covariate was an informant covariate which adjusted estimates not requiring agreement between informants (eg, diagnosis made if either a teacher or parent indicates ADHD) toward estimates which required informant agreement. The second covariate adjusted estimates not requiring impairment (or those not specifying whether impairment was required) for diagnosis toward those which required impairment. The third covariate adjusted studies using small, community samples toward studies representative of entire regions or countries. Bounds for these covariates were calculated from the epidemiological data and applied in DisMod-MR 2.1.

Study covariate	Parameter	beta	Exponentiated beta
No informant agreement	Prevalence	0.53 (0.45–0.72)	1.71 (1.57–2.05)
No impairment	Prevalence	0.068 (0.0043–0.21)	1.07 (1.00–1.24)
Small, community-level studies	Prevalence	0.53 (0.31–0.75)	1.69 (1.36–2.12)

References

1. American Psychiatric Association. Diagnostic and Statistical Manual of Mental Disorders (DSM-IV-TR). 4th, Text Revision ed. Washington DC: American Psychiatric Association; 2000.

2. American Psychiatric Association. Diagnostic and Statistical Manual of Mental Disorders, Fifth Edition. Arlington, VA: American Psychiatric Association; 2013.

3. World Health Organisation. ICD-10 Classification of Mental and Behavioural Disorders: Clinical descriptions and diagnostic guidelines. Geneva: World Health Organisation; 1992.
4. Erskine HE, Ferrari AJ, Nelson P, et al. Research Review: Epidemiological modelling of attention-deficit/hyperactivity disorder and conduct disorder for the Global Burden of Disease Study 2010. *Journal of Child Psychology and Psychiatry* 2013; **54**(12): 1263-74.
5. Ezpeleta L, Keeler G, Erkanli A, Costello EJ, Angold A. Epidemiology of Psychiatric Disability in Childhood and Adolescence. *J Child Psychol Psychiatry* 2001; **42**(7): 901-14.
6. Erskine HE, Ferrari AJ, Polanczyk GV, et al. The global burden of conduct disorder and attention-deficit/hyperactivity disorder in 2010. *J Child Psychol Psychiatry* 2014; **55**(4): 328-36.

Conduct Disorder

Flowchart

Conduct disorder

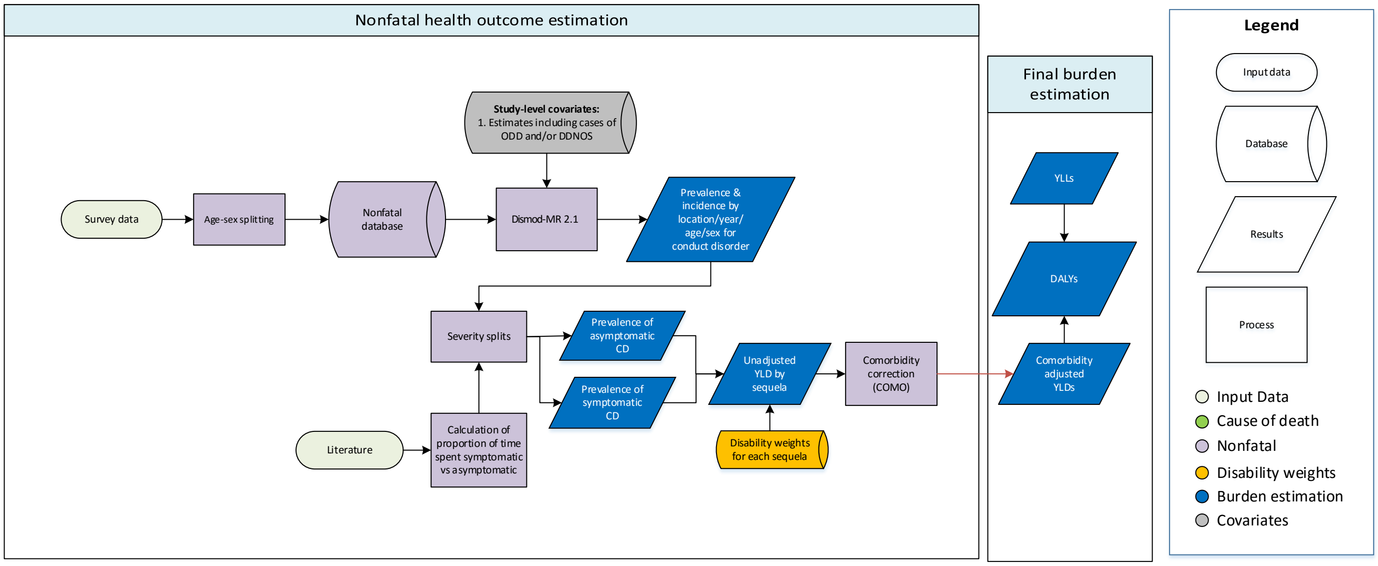

Case definition

Conduct disorder (CD) is an externalizing behavior disorder characterized by a pattern of antisocial behavior that violates the basic rights of others or major age-appropriate societal norms. As per criteria set by the Diagnostic and Statistical Manual of Mental Disorders fourth edition, text revision (DSM-IV-TR),[1] diagnosis requires three or more of the following symptoms to be present in the past 12 months (with at least one present in the last six months) and cause significant impairment in functioning. Symptoms include:

Aggression to people and animals

- often bullies, threatens, or intimidates others
- often initiates physical fights
- has used a weapon that can cause serious physical harm to others (eg, a bat, brick, broken bottle, knife, gun)
- has been physically cruel to people
- has been physically cruel to animals
- has stolen while confronting a victim (eg, mugging, purse snatching, extortion, armed robbery)
- has forced someone into sexual activity

Destruction of property

- has deliberately engaged in fire setting with the intention of causing serious damage
- has deliberately destroyed others' property (other than by fire setting)

Deceitfulness or theft

- has broken into someone else's house, building, or car
- often lies to obtain goods or favors or to avoid obligations (ie, "cons" others)
- has stolen items of nontrivial value without confronting a victim (eg, shoplifting, but without breaking and entering; forgery)

Serious violations of rules

- often stays out at night despite parental prohibitions, beginning before age 13 years
- has run away from home overnight at least twice while living in parental or parental surrogate home (or once without returning for a lengthy period)
- is often truant from school, beginning before age 13 years

CD is considered a disorder of childhood but can be diagnosed in adults who display such behaviors yet do not meet the criteria for antisocial personality disorder. However, there are almost no studies measuring adult CD as existing studies in this area tend to measure adult antisocial behavior rather than adult CD.[2] As such, only childhood CD (ie, cases prior to 18 years of age) was modeled in GBD.

Included in GBD were cases meeting diagnostic criteria according to DSM[1] or the International Classification of Diseases (ICD).[3] These were identified by the following codes: 312 (DSM-IV-TR) and F91 (ICD-10). Different versions of DSM (DSM-III, DSM-III-R, DSM-IV, DSM-IV-TR, and DSM-5) and ICD (ICD-9 and ICD-10) were accepted. Estimates also including oppositional defiant disorder (ODD; DSM-IV-TR: 313.81, ICD-10: F91.3) or disruptive behavior disorder not otherwise specified (DDNOS, DSM-IV-TR: 312.9, ICD-10: 91.9) were accepted and adjusted with a covariate during the modelling process.

Input data

Model inputs

A series of systematic literature reviews were conducted to capture studies reporting the prevalence, incidence, remission, and excess mortality of CD. The reviews incorporated searches of peer-reviewed literature via electronic databases, investigations of grey literature, and consultation with experts. In order for a study to be included, it must have been published during or after 1980, use DSM or ICD criteria to define cases, provide sufficient details on study methodology and sample characteristics to determine study quality, and be representative of the general population rather than a special population, eg, prison inmates. No limitation was set on the language of publication. Detailed descriptions of this methodology have been published elsewhere.[2] This methodology was utilized in GBD 2010 and GBD 2013. GBD 2015 included additional sources identified by GBD experts and microdata where available. The systematic review methodology used in GBD 2010 and 2013 was replicated to update the dataset for GBD 2016.

The final dataset for GBD 2016 included 176 prevalence estimates, 12 incidence estimates, and 11 remission estimates. No estimates of excess mortality were found for CD. The table below shows the number of studies for each parameter as well as the number of countries/subnationals and GBD world regions covered by the available data.

	Prevalence	Incidence	Remission	Mortality
Studies	65	4	5	0
Countries/subnationals	50	6	6	0

GBD world regions	14	2	2	0

Reported estimates of prevalence were split by age and sex where possible. If studies reported prevalence for broad age groups by sex (eg, prevalence in 5-18 year old males and females separately) and by specific age groups but for both sexes combined (eg, prevalence for 5-12 year olds and 13-18 year olds, for males and females combined), age-specific estimates were split by sex using the reported sex ratio and bounds of uncertainty.

Severity split inputs

A severity split for the proportion of time spent symptomatic versus asymptomatic was based on data from the Great Smoky Mountains Study which assessed the levels of disability found in children and adolescents with mental disorders.[4] Of those with CD, 72% reported disability while 20% of individuals with no diagnosis reported disability at the time of survey. Using these as estimates of the proportion of time with disability in the "average case," the proportion of disability in children without a diagnosis was subtracted from the proportion with disability for CD, giving an adjusted proportion of 52%. Detailed descriptions of this methodology have been published elsewhere.[5] The lay description and disability weight for CD is shown in the table below.

Lay description	DW (95% CI)
Has frequent behavior problems, which are sometimes violent. The person often has difficulty interacting with other people and feels irritable	0.241 (0.159–0.341)

Modelling strategy

We assumed no incidence or prevalence prior to 5 years of age or after 18 years of age. The minimum age of onset was set in consultation with experts while the upper age limit was set in line with DSM criteria. Excess mortality was set to zero given the absence of data demonstrating an association between CD and an increased risk of death. Remission and incidence were capped between ages 4 and 17 years in order to gain more plausible output. A covariate was used to adjust any prevalence estimates which also included cases of oppositional defiant disorder and/or disruptive behavior disorder not otherwise specified towards those including CD only.

Covariate	Parameter	beta	Exponentiated beta
Identifies estimates also containing ODD &/or DDNOS cases	Prevalence	0.65 (0.41 — 0.89)	1.91 (1.51 — 2.43)

References

1. American Psychiatric Association. Diagnostic and Statistical Manual of Mental Disorders (DSM-IV-TR). 4th, Text Revision ed. Washington DC: American Psychiatric Association; 2000.

2. Erskine HE, Ferrari AJ, Nelson P, et al. Research Review: Epidemiological modelling of attention-deficit/hyperactivity disorder and conduct disorder for the Global Burden of Disease Study 2010. *Journal of Child Psychology and Psychiatry* 2013; **54**(12): 1263-74.

3. World Health Organisation. ICD-10 Classification of Mental and Behavioural Disorders: Clinical descriptions and diagnostic guidelines. Geneva: World Health Organisation; 1992.
4. Ezpeleta L, Keeler G, Erkanli A, Costello EJ, Angold A. Epidemiology of Psychiatric Disability in Childhood and Adolescence. *J Child Psychol Psychiatry* 2001; **42**(7): 901-14.
5. Erskine HE, Ferrari AJ, Polanczyk GV, et al. The global burden of conduct disorder and attention-deficit/hyperactivity disorder in 2010. *Journal of Child Psychology and Psychiatry* 2014; **55**(4): 328-36.

Other mental disorders

Flow chart

Other mental disorders: Personality disorders

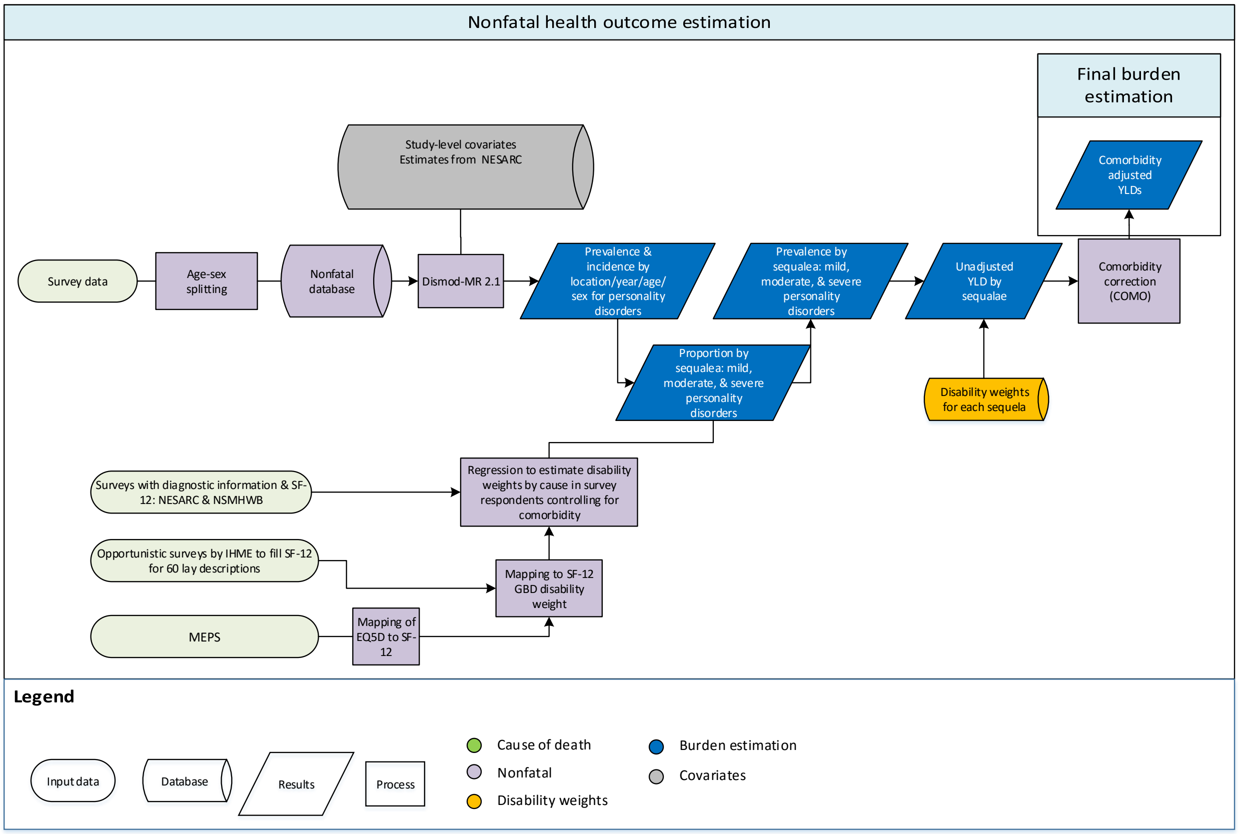

Case Definition

In addition to the individual mental disorders for which we estimate burden, we also estimate the non-fatal burden attributable to a residual cause of "other mental disorders." This is made up of an aggregate group of personality disorders. Personality disorders are characterized by pervasive, inflexible and maladaptive patterns of behaviour and inner experience which are markedly different from what is considered to be acceptable in the individual's culture. These disorders tend to be chronic and are associated with significant distress or disability. Included in GBD 2016 were cases meeting diagnostic criteria for personality disorders according to the Diagnostic and Statistical Manual of Mental Disorders (DSM), or the equivalent diagnosis in the International Classification of Diseases (ICD).[1,2] The aggregated group of personality disorders used in GBD 2016 captured any of the following;

- Paranoid personality disorder
- Schizoid personality disorder

- Schizotypal personality disorder
- Antisocial personality disorder
- Borderline personality disorder
- Histrionic personality disorder
- Narcissistic personality disorder
- Avoidant personality disorder
- Dependent personality disorder
- Obsessive-compulsive personality disorder
- Personality disorder not otherwise specified

Input data

Model inputs

Prevalence estimates for the above personality disorders were obtained from the US National Epidemiological Survey on Alcohol and Related Conditions (NESARC, conducted in two waves from 2001–2002 and 2004–2005)[3] and the Australian National Survey of Mental Health and Wellbeing of Adults (NSMHWB, conducted in 1997).[4] Given that personality disorders often co-occur with other mental and substance use disorders, an adjustment for comorbidity is important so as not to overestimate the overall burden attributable to mental and substance use disorders. Participants meeting criteria for any type of personality disorders from the NESARC and NSMHWB surveys were counted as a prevalent case only if they did not simultaneously meet criteria for another mental and substance use disorder featured in GBD 2016.

Severity splits

The basis of the GBD disability weight survey assessments are lay descriptions of sequelae highlighting major functional consequences and symptoms. The lay descriptions and disability weights applied to the personality disorders within this residual group are shown below and were those estimated for anxiety disorders.

Severity level	Lay description	DW (95% CI)
Mild	Feels mildly anxious and worried, which makes it slightly difficult to concentrate, remember things, and sleep. The person tires easily but is able to perform daily activities.	0.03 (0.018–0.046)
Moderate	Feels anxious and worried, which makes it difficult to concentrate, remember things, and sleep. The person tires easily and finds it difficult to perform daily activities.	0.133 (0.091–0.186)
Severe	Constantly feels very anxious and worried, which makes it difficult to concentrate, remember things, and sleep. The person has lost pleasure in life and thinks about suicide.	0.523 (0.362–0.677)

To determine the proportion of people with personality disorders within each of the severity levels, the NSMHWB survey was used to estimate the proportion of cases asymptomatic (30%, 28%–32%), mild (41%, 33%–47%), moderate (15%, 11%–20%) and severe (14%, 10%–18%).

Modelling Strategy

The GBD 2016 epidemiological modelling strategy made use of DisMod-MR 2.1. As we only had prevalence data available, a number of expert priors were used in order to run a full-parameter model. We assumed no incidence and prevalence before age 14. This minimum age of onset was corroborated with expert feedback and DSM criteria highlighting the fact that personality disorders typically become recognizable during adolescence and early adulthood. Remission was set to a maximum of 0.01, given that these are understood to be chronic disorders with little or no complete remission. Excess mortality was set to 0 in this model, in the absence of mortality data required for DisMod-MR 2.1 modelling purposes.

Study-level covariates were used to accommodate for between-study variability in the raw prevalence data. A cv_NESARC covariate adjusted all data points derived from NESARC toward the level of data points from estimates from the NSMHWB. The latter survey was made up of a more representative list of personality disorders and produced estimates along the levels of what we would expect for personality disorders. The corresponding beta and exponentiated value (which can be interpreted as an odds ratio) is shown in the table below:

Study covariate	Parameter	beta	Exponentiated beta
cv_NESARC	Prevalence	0.67 (0.35–0.96)	1.95 (1.42–2.60)

In this model, global prevalence was exclusively estimated using prevalence estimates from two surveys from the United States and Australia where we had unit record data available to estimate the prevalence of personality disorders, excluding those not simultaneously meeting criteria for another mental or substance use disorder. Given the sparsity of data, we applied a restriction on location random-effects of -0.1 to 0.1 to further guide prevalence estimation. We are currently undertaking a literature review of population-survey data on the epidemiology of personality disorders across low-, middle-, and high-income countries with the aim of providing more robust and globally representative burden estimates for personality disorders in future GBD studies.

References

1. American Psychiatric Association. Diagnostic and Statistical Manual of Mental Disorders (DSM). Washington: American Psychiatric Association, 1952.
2. World Health Organization. The ICD-10 Classification of Mental and Behavioural Disorders. Clinical descriptions and diagnostic guidelines. Geneva: World Health Organization; 1992.
3. Introduction to the National Epidemiologic Survey on Alcohol and Related Conditions [http://pubs.niaaa.nih.gov/publications/arh29-2/74-78.htm]. Access date 1 December 2014.
4. Australian Bureau of Statistics. National Survey of Mental Health and Wellbeing of Adults 1997. Canberra: Australian Bureau of Statistics.

Diabetes Mellitus

Flowchart

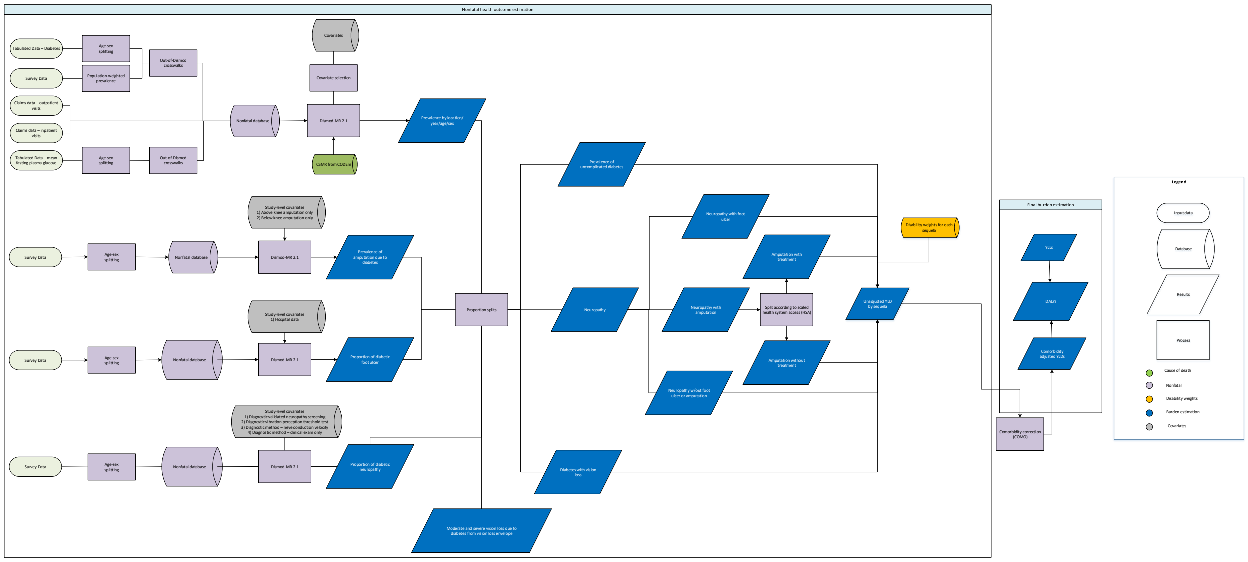

Case definition

The case definitions and diagnostic criteria are presented in the table below. For full accounting of associated ICD 9 and ICD 10 codes, please refer to **Appendix Table 4.**

Criterion	Definition
1. Diabetes mellitus parent	Diabetes mellitus (DM) was defined as fasting plasma glucose (FPG) > 126 mg/dL (7 mmol/L) or being on treatment for diabetes.
2. Uncomplicated diabetes mellitus	Cases of DM that do not have any of the following complications: neuropathy, foot ulcer, leg amputation, or vision loss
3. Diabetic neuropathy	Cases of DM that experience diagnosable neuropathy
4. Diabetic foot due to neuropathy	Cases of DM that currently have a foot ulcer
5. Diabetic neuropathy and amputation with treatment	Cases of DM that have had a leg amputation above or below the knee, with treatment consisting of a prosthetic limb
6. Diabetic neuropathy and amputation without treatment	Cases of DM that have had a leg amputation above or below the knee, with no prosthetic limb

7. Moderate vision impairment due to diabetes mellitus	Cases of DM that have moderate vision loss due to diabetic retinopathy
8. Severe vision impairment due to diabetes mellitus	Cases of DM that have severe vision loss due to diabetic retinopathy
9. Blindness due to diabetes mellitus	Cases of DM that have blindness due to diabetic retinopathy

Diabetes mellitus parent:

Data seeking

1. A systematic review of the literature was done for GBD 2016 with the following search terms:

Diabetes mellitus search string: (diabetes[TI] AND (prevalence[TIAB] OR incidence[TIAB])) OR ('diabetes mellitus'[MeSH Terms] AND 'epidemiology'[MeSH Terms]) OR (diabetes[TI] AND 'epidemiology'[MeSH Terms]) NOT gestational[All Fields] NOT ('neoplasms'[MeSH Terms] OR 'neoplasms'[All Fields] OR 'cancer'[All Fields]) NOT ('mice'[MeSH Terms] OR 'mice'[All Fields]) NOT ('schizophrenia'[MeSH Terms] OR 'schizophrenia'[All Fields]) NOT ('emigrants and immigrants'[MeSH Terms] OR ('emigrants'[All Fields] AND 'immigrants'[All Fields]) OR 'emigrants and immigrants'[All Fields] OR 'immigrants'[All Fields]) NOT ('pregnancy'[MeSH Terms] OR 'pregnancy'[All Fields] OR 'gestation'[All Fields]) NOT ('rats'[MeSH Terms] OR 'rats'[All Fields] OR 'rat'[All Fields]) NOT ('kidney'[MeSH Terms] OR 'kidney'[All Fields]) NOT renal[All Fields] NOT ('vitamins'[Pharmacological Action] OR 'vitamins'[MeSH Terms] OR 'vitamins'[All Fields] OR 'vitamin'[All Fields])

And

FPG search string: ((“glucose”[Mesh] OR “hyperglycemia”[Mesh] OR “prediabetic state”[Mesh]) AND "Geographic Locations"[Mesh] NOT "United States"[Mesh]) AND ("humans"[Mesh] AND "adult"[MeSH]) AND ("Data Collection"[Mesh] OR "Health Services Research"[Mesh] OR "Population Surveillance"[Mesh] OR "Vital statistics"[Mesh] OR "Population"[Mesh] OR "Epidemiology"[Mesh] OR "surve*"[TiAb]) NOT Comment[ptyp] NOT Case Reports[ptyp]) NOT "hospital"[TiAb]

Search date: January 5, 2017

The search took place for the following dates: 1/1/2016– 12/31/2016. The number of studies returned was 1,976, and the number of studies extracted was 26.

2. We systematically searched the Global Health Data Exchange (GHDx) for multi-country survey programs, national surveys, and longitudinal studies that was tagged with either fasting plasma glucose (FPG) or Diabetes mellitus. Each data source we found was tagged with whether the file contained microdata and whether the file contained data on FPG (biomarker). In the interest of time, we prioritized the data sources we reviewed and extracted based on whether the source was tagged with microdata and biomarker information.

3. To capture any remaining sources not identified in the GHDx or in PubMed, we looked to other leaders in the field to ensure our datasets were as comprehensive as possible. These included data sources used by other research groups that report on the global burden of diabetes[3,4], microdata from not-yet published national studies, and publications that were not captured in the PubMed search string.

Figure 1: PRISMA diagram of data sources used in GBD 2016 Diabetes mellitus model

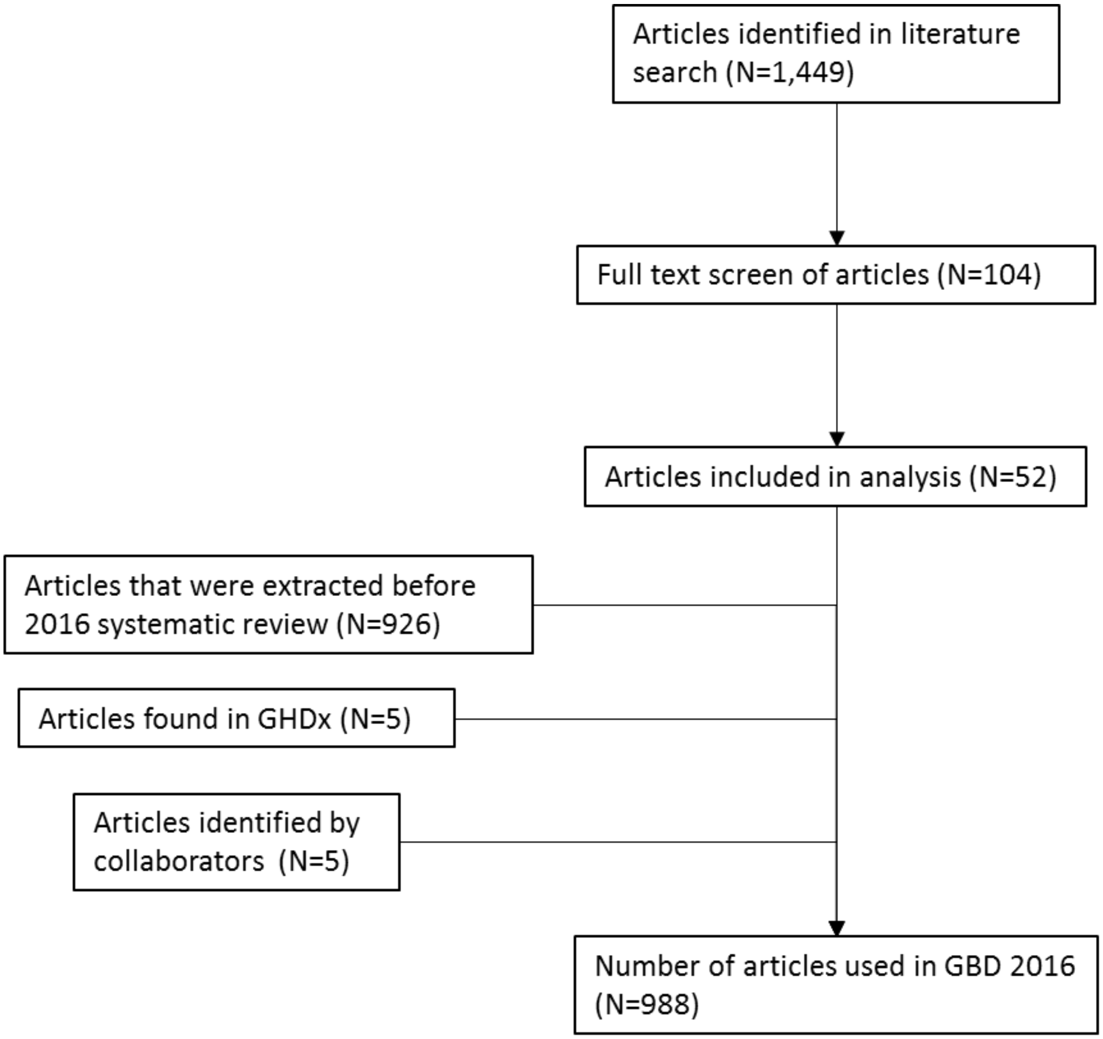

The table below illustrates the number of data sources used for the GBD 2016 estimation of diabetes mellitus parent:

	Prevalence	Incidence	Mortality risk	Relative risk
Studies	806	132	8	1

Data inputs

Purpose:
To incorporate all available data related to population-representative estimates of diabetes, we accepted other measures of blood sugar (hemoglobin A1c, oral glucose tolerance test, post prandial glucose test) to define diabetes and mean fasting plasma glucose (FPG) in a population when data on diabetes was not available as data inputs.

Data:
1. Data inputs come from 4 sources:
   - Estimates of diabetes in a representative population
   - Estimates of mean FPG in a representative population
   - Individual-level data of fasting plasma glucose measured from surveys
   - MarketScan, which are insurance claims data from the United States

When a study reported both mean fasting plasma glucose (FPG) and prevalence of diabetes, we used the prevalence of diabetes. Where possible, individual-level data from a cohort superseded any data described in a study. Individual-level data was collapsed and aggregated to produce estimates for each age group, sex, location, and year a survey is conducted.

2. Covariates

To inform our estimates in data-sparse countries, we systematically tested a range of covariates and selected two covariates based on AIC and adjusted R^2. These included prevalence of obesity per location and lag-distributed income per capita (LDI).

Data processing

We perform several processing steps to the data in order to address sampling and measurement inconsistencies that will ensure the data are comparable across data sources and between high fasting plasma glucose modeling efforts.

1. *Small sample size:* Estimates in a sex and age group with a sample size <30 persons was considered a small sample size. In order to avoid small sample size problems that may bias estimates, data were collapsed into the next age group in the same study till the sample size reached at least 30 persons. The intent of collapsing the data is to preserve as much granularity

between age groups as possible which determined whether the collapse occurred with a younger or older age group. If the entire study sample consisted of <30 persons and did not include a population-weight, the study was excluded from the modeling process. The estimates were re-calculated if case count and sample size were available or the population-weighted estimate was calculated when only sample size was available.

2. *Time, Age, and Sex Splitting*

   a. Time: Prior to modeling in DisMod, any study period that spanned more than 5 years was duplicated.
   b. Age: Prior to modeling in DisMod, data provided in age groups wider than the GBD 5-year age groups were split using the global age pattern of diabetes mellitus from data that were in age groups less than 20-year age groups. Uncertainty was propagated by multiplying the standard error of the data performed by the square root of the number of splits performed.
   c. Sex: Prior to modeling in DisMod, data that does not differentiate gender is split into male and female according to the global male to female ratio from data with sex-specific data. Uncertainty was propagated by multiplying the standard error of the data performed by the square root of the number of splits performed.

3. *Mean FPG processing:* For more details on how datapoints on mean FPG was processed, please see the High Fasting Plasma Glucose capstone appendix in the GBD 2016 Risk Factors Paper.

4. *Crosswalks*
   1. Case-definition

   We performed adjustments (crosswalks) to datapoints to standardize data to a reference definition: fasting plasma glucose (FPG) >126 mg/dL (7 mmol/L) or on treatment.

      a. Prevalence
         i. Single-component

         Single component case definitions consisted of diabetes defined based on the level of only one biomarker (e.g., FPG, HbA1c).

            1. FPG

            We used an ensemble distribution to standardized the case definition of diabetes in surveys by estimating the prevalence of diabetes under different thresholds of FPG. We used individual-level measures of FPG in surveys of a representative population. This allowed us to capture the non-systematic change in the proportion of population above different levels of FPG. We adjusted the datapoint by applying the ratio between FPG above 126 mg/dL and the case-definition used in the study. For more details on the approach used in the ensemble distribution, please see the GBD 2016 Risk Factors Paper.

            2. HbA1c

            We assumed that HbA1c >6.5% was equivalent to FPG >126 mg/dL.

         ii. Multi-component

Multi-component case definition consisted of studies where more than 1 glucose test was used in the study to identify different segments of the population (e.g. FPG and PPG).

1. Multi-component that includes FPG >126 mg/dL
Multi-component case definitions that consisted of FPG >126 mg/dL were assumed to be equivalent to the reference case definition FPG >126 mg/dL or Treatment.

2. Multi-component that does not include FPG >126 mg/dL
Case definitions that did not include FPG >126 mg/dL were excluded from the model.

b. Non-prevalence measure
Data from studies with non-prevalence measures (e.g., incidence, relative risk, excess mortality) were marked with the case definition and adjusted to the reference case definition within DisMod.

2. Marketscan
Data from MarketScan were included in the model and a study-level covariate was included in the model to adjust them. These datapoints were adjusted to the reference case definition within DisMod.

3. Estimate prevalence of diabetes from mean FPG
We also used the ensemble distribution to estimate the prevalence of diabetes based on mean FPG in locations where data on prevalence of diabetes were not available. For more details on the approach used in the ensemble distribution, please see the GBD 2016 Risk Factors Paper.

Amputation due to diabetes mellitus

Data seeking

A systematic review of the literature was performed for GBD 2016 with the following search terms:

(‘diabetes mellitus’[MeSH Terms] OR (‘diabetes’[All Fields] AND ‘mellitus’[All Fields]) OR ‘diabetes mellitus’[All Fields]) AND ‘amputation’[All Fields] AND (proportion OR prevalence OR incidence) NOT gestational NOT cancer NOT mice NOT schizophrenia NOT immigrants NOT gestation NOT rat NOT kidney NOT renal NOT vitamin

- Dates of search: 1/1/16-12/31/16
- Number of studies returned: 16
- Number of studies extracted: 1

The table below indicates the data inputs for the GBD 2016 estimation of amputation due to diabetes mellitus.

	Prevalence	Incidence	Mortality risk
Studies	12	32	5

Diabetic neuropathy

Data seeking

A systematic review of the literature was performed for GBD 2016 with the following search terms:

(“diabetes mellitus”[MeSH Terms] OR (“diabetes”[All Fields] AND “mellitus”[All Fields]) OR “diabetes mellitus”[All Fields]) AND neuropathy[All Fields] AND (proportion OR prevalence OR incidence) NOT gestational NOT cancer NOT mice NOT schizophrenia NOT immigrants NOT gestation NOT rat NOT kidney NOT renal NOT vitamin

- Dates: 1/1/16-12/31/16
- Number of studies returned: 170
- Number of studies extracted: 1

The table below illustrates the model inputs for the GBD 2016 estimation process:

	Proportion
Studies	89

Diabetic foot ulcer

Data seeking

A systematic review of the literature was performed for GBD 2016 with the following search terms:

((("diabetes mellitus"[MeSH Terms] OR ("diabetes"[All Fields] AND "mellitus"[All Fields]) OR "diabetes mellitus"[All Fields] OR "diabetes"[All Fields]) AND ("foot"[MeSH Terms] OR "foot"[All Fields]) AND ("ulcer"[MeSH Terms] OR "ulcer"[All Fields])) NOT ("neoplasms"[MeSH Terms] OR "neoplasms"[All Fields] OR "cancer"[All Fields]) NOT ("mice"[MeSH Terms] OR "mice"[All Fields]) NOT ("emigrants and immigrants"[MeSH Terms] OR ("emigrants"[All Fields] AND "immigrants"[All Fields]) OR "emigrants and immigrants"[All Fields] OR "immigrants"[All Fields]) NOT ("pregnancy"[MeSH Terms] OR "pregnancy"[All Fields] OR "gestation"[All Fields]) NOT ("vitamins"[Pharmacological Action] OR "vitamins"[MeSH Terms]

OR "vitamins"[All Fields] OR "vitamin"[All Fields]) NOT renal[All Fields] NOT ("kidney"[MeSH Terms] OR "kidney"[All Fields]) AND (proportion[All Fields] OR "incidence"[All Fields] OR "prevalence"[All Fields]) NOT ("schizophrenia"[MeSH Terms] OR "schizophrenia"[All Fields]) NOT ("rats"[MeSH Terms] OR "rats"[All Fields] OR "rat"[All Fields]))

- Dates: 1/1/16-12/31/16
- Number of studies returned: 48
- Number of studies extracted: 0

The table below illustrates the data inputs used for the GBD 2016 modeling process.

	Proportion
Studies	43

Modeling strategy

DisMod Model

For GBD 2016, we estimated the overall prevalence of diabetes using DisMod MR-2.1, a Bayesian metaregression. DisMod-MR produces estimates of the prevalence of diabetes for each age, sex, geographic location, and year. We also estimated amputation due to diabetes mellitus, diabetic neuropathy, and diabetic foot using DisMod. We then multiply all proportion draws from neuropathy/foot/amputation models by the parent diabetes model so that all estimates are in the same population-space.

Next, we squeeze (neuropathy + moderate vision loss + severe vision loss) to (90% of parent diabetes) prevalence if sum exceeds that 90%. This is to ensure that at least 10% of diabetes cases are uncomplicated for all draws. We then squeeze (amputation + foot ulcer) to (90% of neuropathy) prevalence if sum exceeds 90%. This is to ensure that at least 10% of diabetic neuropathy cases do not have foot ulcer or amputation for all draws. This treats foot ulcer and amputation as mutually exclusive categories by assuming a patient won’t have both simultaneously.

From here, we calculate uncomplicated diabetes as the remainder of diabetes cases exclusive of neuropathy and vision loss. In addition, we estimate the prevalence of amputation due to diabetes is split into with and without treatment using scaled HSA values. For diabetic amputation, we calculated a distribution of treated versus untreated amputation, defined as receiving a prosthetic or not. We first rescaled the IHME health system access estimates to be between 0 and 0.9, under the assumption that 10% of amputees will not receive a prosthetic, even in high income countries. We based this assumption on the retrospective study by Moore et al, which found that about 80% of patients following major lower extremity amputation were fitted with prostheses in the authors’ institutions from 1978 to 1986 in the USA. We then performed a population-weighted average of this country-specific value to obtain a proxy for the proportion of amputees that receive a prosthetic by super region. Because these are rough

estimates based on large assumptions, we applied confidence intervals of +/- 50% of the value to reflect our uncertainty.

Diabetes mellitus

- We set a value prior of 0 for remission for ages 0 to 14
- We set a value prior of a maximum value of 0.01 for remission for ages 15 to 100
- We set a value prior of a maximum value of 0.15 for excess mortality for all ages
- We set a value prior of 0 for incidence for ages 0 to 1
- We set a value prior of a maximum value of 0.1 for incidence for ages 1 to 100

Study covariate	Parameter	beta	Exponentiated beta
Sex	With-condition mortality rate	0.27 (-0.9 — 1.49)	1.31 (0.41 — 4.45)
LDI (I$ per capita)	Excess mortality rate	-0.24 (-0.25 — -0.22)	0.79 (0.78 — 0.80)
All MarketScan, year 2000	Prevalence	-0.48 (-0.53 — -0.43)	0.62 (0.59 — 0.65)
All MarketScan, year 2010	Prevalence	-0.17 (-0.21 — -0.11)	0.85 (0.81 — 0.90)
All MarketScan, year 2012	Prevalence	-0.15 (-0.2 — -0.091)	0.86 (0.82 — 0.91)
Obesity	Prevalence	2.76 (2.46 — 3.07)	15.79 (11.66 — 21.57)
Sex	Prevalence	0.17 (0.15 — 0.19)	1.18 (1.16 — 1.21)
Sex	Incidence	0.035 (-0.042 — 0.11)	1.04 (0.96 — 1.12)
Sex	Excess mortality rate	0.18 (0.15 — 0.20)	1.19 (1.16 — 1.23)
Sex	Cause-specific mortality rate	0.00030 (-0.0058 — 0.0059)	1.00 (0.99 — 1.01)

Our estimate of the age-standardized global prevalence of diabetes is slightly lower than the estimates reported previously by the NCD Risk Factor Collaboration (NCD-RisC) and International Diabetes Federation (IDF). IDF reported a prevalence for the year 2013 of 8.3% (7.2–11.3) at ages 20 to 80, compared to our estimate for 2016 of 6.0% (5.1–7.0) for the same age range and using the IDF method of age-standardization (NCD-Risc: http://www.sciencedirect.com/science/article/pii/S0140673616006188 IDF: https://www.idf.org/component/attachments/attachments.html?id=1093&task=download.) The NCD-RisC estimates of prevalence for ages over 18 for the year 2014 were 9.0% (7.2–11.1) in males and 7.9% (6.7–9.7) in females, compared to our 2016 estimates of 5.3% (4.5–6.2) and 4.9% (4.1–5.7), respectively. Several factors can explain the difference in estimates. We include a greater number of data sources but

exclude surveys with self-reported diagnosis of diabetes unlike NCD-RisC. We also define the whole distribution of fasting plasma glucose (FPG) and thus have a more accurate way of including surveys that report on FPG only in our diabetes disease model.

Amputation due to diabetes

- We set a value prior of 0 for incidence for ages 0 to 15
- We set a value prior of 0 for remission for all ages
- We crosswalked the incidence of either above or below knee amputation only to the incidence of all amputations

DisMod Model

Study covariate	Parameter	beta	Exponentiated beta
Above knee amputation only	Incidence	-0.32 (-0.6 — -0.034)	0.72 (0.55 — 0.97)
Below knee amputation only	Incidence	-0.44 (-0.72 — -0.18)	0.64 (0.49 — 0.83)

Diabetic neuropathy

- We set a value prior on the proportion of 0 from ages 0 to 1
- We crosswalked data from studies using alternate diagnostic criteria using as reference studies which used the monofilament test as their diagnostic criteria

DisMod Model

Study covariate	Parameter	Beta	Exponentiated beta
Diagnostic vibration perception threshold test	Proportion	-0.13 (-0.33 — 0.11)	0.88 (0.72 — 1.12)
Diagnostic method - nerve conduction velocity	Proportion	-0.25 (-0.5 — 0.029)	0.78 (0.61 — 1.03)
Diagnostic method - clinical exam only	Proportion	-0.044 (-0.27 — 0.21)	0.96 (0.76 — 1.24)
Diagnostic validated neuropathy scoring	Proportion	-0.021 (-0.23 — 0.20)	0.98 (0.79 — 1.23)

Diabetic foot ulcer

- We set a value prior on the proportion of 0 from ages 0 to 10.
- We crosswalked data from studies investigating hospitalized patients only using as reference studies which captured all diabetic foot ulcers.

DisMod Model

Study covariate	Parameter	beta	Exponentiated beta
Hospital data	Proportion	0.52 (0.13 — 0.87)	1.68 (1.14 — 2.38)

Severity split inputs

Severity splits and disability weights were determined by the GBD disability weight survey assessment for diabetes mellitus. The table below illustrates the severity levels, lay descriptions, and associated disability weights:

Severity level	Lay description	DW (95% CI)
Uncomplicated diabetes mellitus	Has a chronic disease that requires medication every day and causes some worry, but minimal interference with daily activities	0.049 (0.031 – 0.072)
Diabetic neuropathy	Has pain, tingling, and numbness in the arms, legs, hands, and feet. The person sometimes gets cramps and muscle weakness.	0.133 (0.089 – 0.187)
Diabetic neuropathy with diabetic foot	Has a sore on the foot that is swollen and causes some difficulty in walking.	[a]
Diabetic neuropathy with treated amputation	Has lost part of one leg, leaving pain and tingling in the stump. The person has an artificial leg that helps in moving around.	[a]
Diabetic neuropathy with untreated amputation	Has lost part of one leg, leaving pain and tingling in the stump. The person does not have an artificial leg, has frequent sores, and uses crutches.	[a]
Moderate vision loss due to diabetes mellitus	Has vision problems that make it difficult to recognize faces or objects across a room.	0.031 (0.019 – 0.049)
Severe vision loss due to diabetes mellitus	Has severe vision loss, which causes difficulty in daily activities, some emotional impact (for example worry), and	0.184 (0.125 – 0.259)

	some difficulty going outside the home without assistance.	
Blindness due to diabetes mellitus	Is completely blind, which causes great difficulty in some daily activities, worry and anxiety, and great difficulty going outside the home without assistance.	0.187 (0.124 – 0.26)

[a] *The disability weights are produced from a combination of two health states: neuropathy and diabetic foot/amputation*

Moore TJ, Barron J, Hutchinson F 3rd, Golden C, Ellis C, Humphries D. **Prosthetic usage following major lower extremity amputation.** *Clin Orthop Relat Res.* 1989 Jan;(238):219-24.

Acute Glomerulonephritis

Flowchart

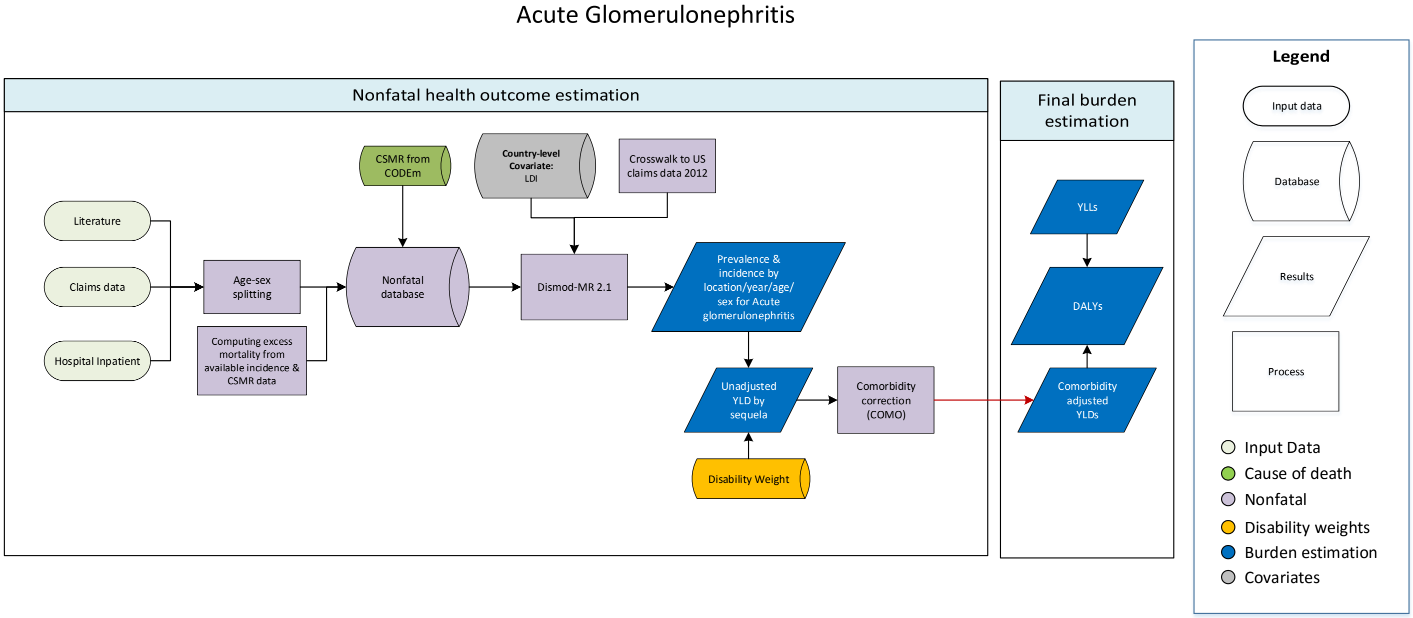

Case Definition

Acute glomerulonephritis (AG) (or post-infectious glomerulonephritis) is an acute episode of hematuria, edema, hypertension, and acute kidney injury that typically follows infection with specific strains of group A beta-hemolytic streptococcus. As used in the GBD study, this term is synonymous with post-streptococcal or post-staphylococcal glomerulonephritis. This disease is typically seen in children but can demonstrate a bimodal distribution as early life immunity wanes within older years. ICD codes include N00, N00.0, N00.1, N00.2, N00.3, N00.4, N00.5, N00.6, N00.7, N00.8, N00.9, N01, N01.1, N01.2, N01.3, N01.4, N01.5, N01.6, N01.7, N01.8, and N01.9.

Input data

Model inputs

For GBD 2010, a systematic review of the prevalence of AG throughout the world was conducted. This search was updated for GBD 2013 and GBD 2015. For GBD 2016 a PubMed search was conducted using the following search terms: (Acute Glomerulonephritis[Title/Abstract] OR GN [Title/Abstract]) AND (Prevalen*[Title/Abstract] OR Inciden*[Title/Abstract]) AND ("2015/01/01"[PDAT] : "3000"[PDAT]) AND "humans"[MeSH Terms].

The exclusion criteria were:

35. Studies clearly not representative of the national population
36. Studies that did not provide primary data on epidemiological parameters, eg, a commentary piece

37. Studies that describe non-infectious glomerulonephritis epidemiology

Twenty-eight studies were identified though this search. Two studies were identified for full text screening. Neither of these studies was determined to meet inclusion criteria after full-text screening. The most recent literature source dates from 2013. Twenty-one articles have been included in total.

Data from US claims data for 2000, 2010, and 2012 by US state were included. Hospital inpatient data was also included. Inpatient data points that exceeded the maximum observed age-standardized rate in the US inpatient data by a margin of 25% or greater were excluded from analysis.

The table below shows the number of studies included in GBD 2016, as well as the number of countries and GBD world regions represented by either studies or hospital data.

	Incidence
Studies	73
Geographies	360
Regions	15

Severity split & disability weight

The basis of the GBD disability weight assessment is lay descriptions of sequelae highlighting major functional consequences and symptoms. Disability weighting (DW) for AG associates with systemic symptoms of fever, aches, weakness, and some difficulty with daily activities. The lay description and disability weight for acute glomerulonephritis are shown below.

Cause	Lay description	DW (95% CI)
Acute glomerulonephritis	Has a fever and aches, and feels weak, which causes some difficulty with daily activities.	0.051 (0.032–0.074)

Modelling strategy

We ran a DisMod-MR 2.1 model to produce estimates by age, sex, year, and country. Prior settings in the DisMod model included setting remission of three to four weeks. It was assumed that no one was born with AG.

We applied a crosswalk to all US claims data to adjust to 2012 US claims data. Inpatient data was adjusted to account for multiple admissions and multiple diagnoses, based on MarketScan data, and was adjusted to 2012 US claims data in DisMod. We used the function in DisMod-MR 2.1 to pull in cause-specific mortality rate (CSMR) data from our CODEm and CODcorrect analyses and match them with incidence data points for the same geography and study year to estimate priors on excess mortality rates (by dividing CSMR by incidence).

We included the covariate Lagged Distributed Income (LDI) as a country-level covariate to inform excess mortality, with bounds of -0.5, -0.1.

Betas and exponentiated values (which can be interpreted as an odds ratio) are shown in the table below:

Covariate	Parameter	beta	Exponentiated beta
Claims data - 2000	Incidence	-0.2 (-0.24 — -0.16)	0.82 (0.79 — 0.86)
Claims data - 2010	Incidence	-0.11 (-0.15 — -0.063)	0.89 (0.86 — 0.94)
Hospital data	Incidence	-0.9 (-0.9 — -0.9)	0.41 (0.41 — 0.41)
LDI (I$ per capita)	Excess mortality rate	-0.1 (-0.1 — -0.1)	0.90 (0.90 — 0.90)

Compared to GBD 2015, major changes include inclusion of inpatient data from a greater number of geographies.

Chronic Kidney Disease

Flowchart

Chronic Kidney Disease (CKD)

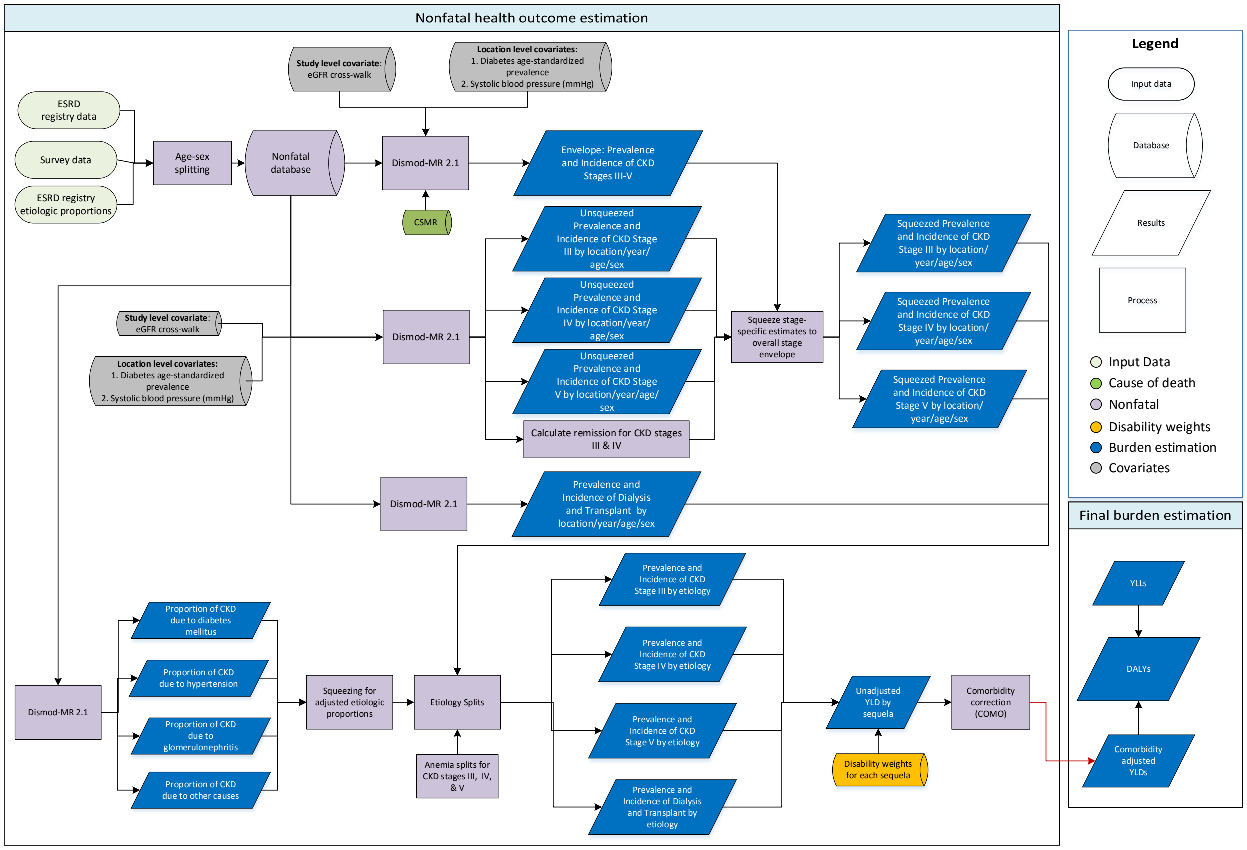

Case definition

Chronic kidney disease (CKD) is defined as a permanent loss of renal function as indicated by estimated glomerular filtration rate (eGFR). The GBD study considers five stages of CKD as defined by degree of loss of renal function or receipt of renal replacement therapy: CKD Stage III (eGFR 30-60ml/min/1.73m^2), CKD Stage IV (eGFR 15-30ml/min/1.73m^2), CKD Stage V (eGFR <15ml/min/1.73m^2, not on renal replacement therapy), maintenance dialysis, and renal transplantation.[1] The ICD-10 codes associated with CKD include N18.1-N18.9.

Input data

Model inputs

For GBD 2010, a systematic review of the prevalence of CKD throughout the world was conducted. This search was updated for GBD 2013 and GBD 2015. For GBD 2016 this literature search was repeated using PubMed search terms: ((((("chronic kidney disease"[Title/Abstract]) AND prevalen*[Title/Abstract]) AND ("2015/1/1"[Date - Publication] : "3000"[Date - Publication])) NOT ((animals[MeSH] NOT humans[MeSH])))).

The exclusion criteria were:

1. Studies clearly not representative of the national population
2. Studies that did not provide primary data on epidemiological parameters, eg, a commentary piece
3. Studies of a specific etiology of CKD
4. Studies not reporting on CKD by stage

This literature search was augmented by identification of population-based surveys that measured renal function. For maintenance dialysis and renal transplantation, data were largely obtained from registry reports.

The next planned PubMed search will be conducted for GBD 2017.

Disease	Number of sources
CKD Stage III	112
CKD Stage IV	94
CKD Stage V	92
Maintenance dialysis	534
Renal transplantation	430

Severity splits & disability weights

Estimates of prevalence and incidence are split using CKD etiology proportion models, resulting in CKD estimates by stage and etiology. Then a portion of each etiology split for CKD stages III, IV, and V is attributed a disability weight associated with mild, moderate, or severe anemia.[2]

Severity level	Lay description	Disability weight (95% CI)
CKD stage III without anemia	Asymptomatic	--
CKD stage III with mild anemia	Feels slightly tired and weak at times, but this does not interfere with normal daily activities.	0.004 (0.001–0.008)
CKD stage III with moderate anemia	Feels moderate fatigue, weakness, and shortness of breath after exercise, making daily activities more difficult.	0.052 (0.034–0.076)
CKD stage III with severe anemia	Feels very weak, tired, and short of breath, and has problems with activities that require physical effort or deep concentration.	0.149 (0.101–0.21)

CKD stage IV without anemia	Tires easily, has nausea, reduced appetite, and difficulty sleeping.	0.104 (0.07–0.147)
CKD stage IV with mild anemia		0.108 (0.072–0.151)
CKD stage IV with moderate anemia		0.15 (0.103–0.207)
CKD stage IV with severe anemia		0.237 (0.165–0.324)
CKD stage V without anemia	Has lost a lot of weight and has constant pain. The person has no appetite, feels nauseated, and needs to spend most of the day in bed.	0.569 (0.389–0.727)
CKD stage V with mild anemia		0.570 (0.391–0.727)
CKD stage V with moderate anemia		0.591 (0.414–0.743)
CKD stage V with severe anemia		0.631 (0.456–0.782)
End-stage renal disease, on dialysis	Is tired and has itching, cramps, headache, joint pains, and shortness of breath. The person needs intensive medical care every other day lasting about half a day.	0.571 (0.397–0.725)
End-stage renal disease, with kidney transplant	Sometimes feels tired and down, and has some difficulty with daily activities.	0.024 (0.014–0.039)

Etiology proportion models are informed by renal registry etiology proportion data. These proportions are applied to the CKD stages as described above. Etiologies included in the GBD study include diabetes mellitus, hypertension, glomerulonephritis, and “other.” “Other” excludes urologic diseases and toxins/poisons as well as cases of unknown etiology.

Modelling strategy

We ran a DisMod-MR 2.1 model to produce estimates by age, sex, year, and country for each stage of CKD. To account for progression of individuals from stage III to stage IV and stage IV to stage V, we informed remission for stages III and IV. Remission data for stage IV was calculated as the ratio of the incidence of stage V and prevalence of stage IV at the gender, age, and country-matched level. Remission data for stage III were calculated as the ratio of resulting stage IV incidence and stage III prevalence at the gender, age, and country-matched level. Remission was set to 0 for stage V and the excess mortality parameter was used to account for progression to end-stage renal disease and mortality due to CKD stage V. Bounds on excess mortality were informed using a meta-analysis of survival analyses of individuals with untreated CKD stage V.

Data from sources reporting the prevalence of stage III, IV, and V CKD was aggregated to represent the prevalence of stage III-V CKD. We ran a DisMod-MR 2.1 model to produce estimates by age, sex, year, and country for aggregate stage III-V CKD. We used the function in DisMod-MR 2.1 to pull in cause-specific mortality rate (CSMR) data from our chronic kidney disease CODEm and CODcorrect analyses and match them with incidence data points for the same geography and study year to estimate priors on excess mortality rates (by dividing CSMR by incidence). In order to enforce more consistency between stage

models, prevalence of CKD stage III, IV, and V were then scaled to sum to the prevalence and incidence of the stage III-V CKD model, at the gender, age, and country-matched level.

A full description of priors and covariates included in each model can be found in the table below:

	Priors (min,max)	Study-level covariate	Country-level covariate
CKD stage III	Remission (0, 0.75)<br>Excess mortality (0, 0.05)	Adjust for estimating equation	Diabetes age-standardised prevalence
			Mean Systolic Blood Pressure
CKD stage IV	Remission (0, 0.75)<br>Excess mortality (0, 0.05)	Adjust for estimating equation	Diabetes age-standardised prevalence
			Mean Systolic Blood Pressure
			Age-standardised prevalence of CKD stage III
CKD stage V	Remission (0, 0)<br>Incidence (0, 0.001), age 0-20<br>Excess mortality (0.29, 0.54)	Adjust for estimating equation	Diabetes age-standardised prevalence
			Mean Systolic Blood Pressure
			Age-standardised prevalence of CKD stage III
CKD stage III-V	Remission (0, 0)<br>Excess mortality (0, 0.54)	Adjust for estimating equation	Diabetes age-standardised prevalence
			Mean Systolic Blood Pressure

We crosswalked data reporting glomerular filtration rate (GFR) estimated with the Modification of Diet in Renal Disease (MDRD) equation to data reported using the CKD-Epi equation as our baseline. GFR reported for children was estimated using the Schwartz equation as the gold standard among the pediatric population. Bounds on the MDRD-CKD-Epi cross walk were informed using a meta-analysis of studies reporting prevalence of CKD by stage using both the MDRD and CKD-Epi equations. Betas and exponentiated values for this crosswalk are shown in the table below:

	Study covariate	Parameter	beta	Exponentiated beta
Stage III	eGFR calculated with MDRD Equation	Prevalence	0.27 (0.26 — 0.28)	1.31 (1.30 — 1.32)
Stage IV	eGFR calculated with MDRD Equation	Prevalence	-0.0085 (-0.026 — 0.012)	0.99 (0.97 — 1.01)
Stage V	eGFR calculated with MDRD Equation	Prevalence	-0.093 (-0.14 — -0.035)	0.91 (0.87 — 0.97)
Stage III-V	eGFR calculated with MDRD Equation	Prevalence	0.24 (0.23 — 0.25)	1.27 (1.26 — 1.28)

The maintenance dialysis and renal transplant models include bounds on location random effects for East Asia and High-income Asia Pacific. As Taiwan (Province of China) and Japan have rates of renal replacement therapy far out of proportion to other countries in their regions, these bounds prevent renal replacement therapy estimates for surrounding countries lacking data from overinflating. Remission data

for dialysis was calculated as the ratio of the incidence of renal transplantation to prevalence of dialysis at the gender, age, and country-matched level.

Major changes for GBD 2016 include attribution of anemia to stage V CKD, the switch from the MDRD equation to the CKD-Epi equation as our reference, and the use of a CKD envelope to scale individual stage estimates.

Interstitial Nephritis

Flowchart

Interstitial Nephritis (IN)

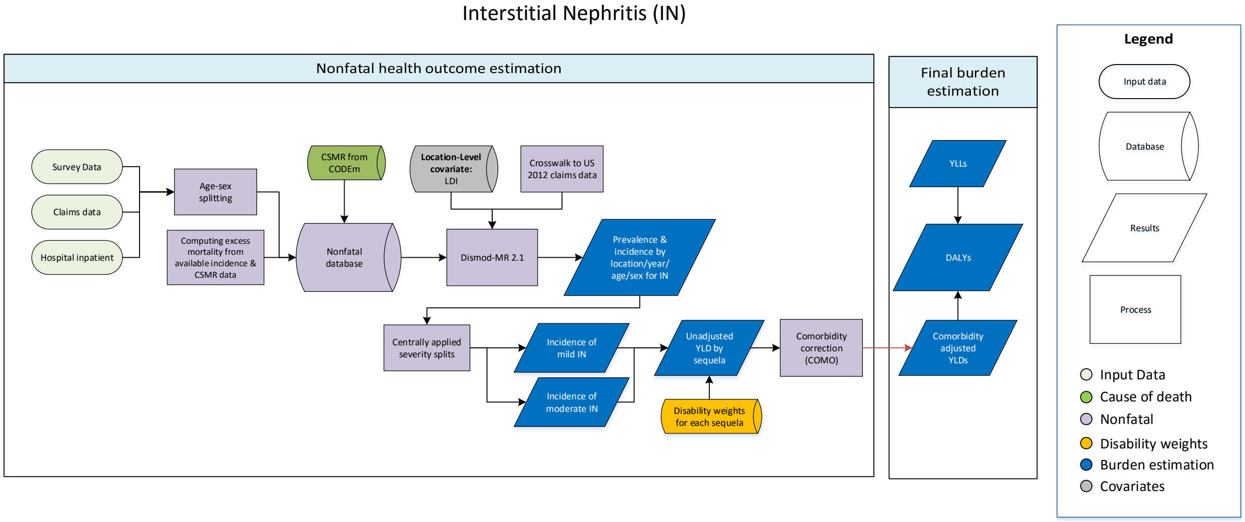

Case definition

Interstitial nephritis (IN) is defined as a kidney infection that can lead to systemic symptoms such as fever and weakness and can cause discomfort and difficulty with daily activities.[1] ICD codes include N10, N10.0, N10.9, N11, N11.0, N11.1, N11.8, N11.9, N12, N12.0, and N12.9.

Input data

Model inputs

The interstitial nephritis model is informed by survey data, US state-level claims data, and global hospital inpatient and outpatient data. For GBD 2016, a systematic review of the prevalence of IN throughout the world was conducted using the following search terms: (((Interstitial Nephritis[Title/Abstract]) OR (Urinary Tract Infection[Title/Abstract]))AND (Inciden*[Title/Abstract])) AND ("2013/01/01"[Date - Publication] : "3000"[Date - Publication]) NOT (animals[MeSH] NOT humans[MeSH]).

The exclusion criteria were:

42. Studies clearly not representative of the national population
43. Studies that did not provide primary data on epidemiological parameters, eg, a commentary piece
44. Studies of a specific type of interstitial nephritis

The table below shows the number of countries and GBD world regions represented.

	Incidence
Countries/subnationals	330
GBD world regions	15

Data from US claims data for 2000, 2010, and 2012 by US state were included. Hospital inpatient data was also included. Inpatient data points that exceeded the maximum observed age-standardized rate in the US claims data by a margin of 25% or greater were excluded from analysis.

Severity splits & disability weights

The basis of the GBD disability weight survey assessments is lay descriptions of sequelae highlighting major functional consequences and symptoms. Interstitial nephritis is split into mild and moderate severity. Mild severity is associated with a disability weight that correlates with low fever, mild discomfort, but no difficulty with daily activities. Moderate discomfort is associated with a disability weight that correlates with systemic symptoms of fever, aches, weakness, and some difficulty with daily activities. The lay descriptions and disability weights for IN are shown below.

Severity level	Lay description	DW (95% CI)
Interstitial nephritis and urinary tract infection, mild	Has a low fever and mild discomfort, but no difficulty with daily activities.	0.006 (0.002, 0.012)
Interstitial nephritis and urinary tract infection, moderate	Has a fever and aches, and feels weak, which causes some difficulty with daily activities.	0.051 (0.032, 0.074)

The severity distribution of interstitial nephritis was derived from analysis of the Medical Expenditure Panel Surveys (MEPS). MEPS is an overlapping panel survey of the non-institutionalized US population that collects data on respondents' health service interactions. Panels are initiated every year. Each panel is two years long and consists of five rounds. In 2000, MEPS began using 12-Item Short Form Surveys (SF-12) to collect data on functional health status. The SF-12 survey is administered twice per panel (about once per year).

In order to translate SF-12 scores into GBD disability weights, 62 lay descriptions for conditions representing the full range of disability weight values (from most mild to most severe) were selected. A convenience sample of respondents was then asked to complete an SF-12 form for an individual with the health state described in the lay descriptions of these conditions. Composite mental and physical SF-12 score was regressed on GBD disability weight to derive the relationship between disability weight and SF-12 score. Individual respondent scores were then regressed on reported conditions to obtain a comorbidity-corrected condition-specific disability weight. The distribution of these condition-specific weights was used derive the proportion of individuals with the conditions that fall within each GBD severity category.

Severity	Distribution
Mild Interstitial Nephritis and UTI	0.362 (0.258, 0.478)
Moderate Interstitial Nephritis and UTI	0.638 (0.522, 0.742)

Modelling strategy

We ran a DisMod MR-2.1 model to produce estimates by age, sex, year, and location. Prior settings in the IN DisMod 2.1 model included remission after one week between ages 0 and 100. We used the function in DisMod-MR 2.1 to pull in cause-specific mortality rate (CSMR) data from our CODEm and CODcorrect

analyses and match them with incidence data points for the same geography and study year to estimate priors on excess mortality rates (by dividing CSMR by incidence).

We applied a crosswalk to all US claims data to adjust to 2012 US claims data. Inpatient data was adjusted to account for multiple admissions and multiple diagnoses, based on MarketScan data, and an age-sex-specific crosswalk was applied to adjust inpatient hospital data to 2012 US claims data. This crosswalk was applied by comparing the ratio of prevalence indicated by 2012 US claims data to that indicated by US inpatient data and applying these age- and sex-specific adjustment factors to global inpatient hospital data. Adjustment factors and their associated standard errors are presented in the table below.

Sex	Age start	Age end	Correction Factor	Standard Error
Female	0	0.999	8.927	0.117
Female	1	4	9.460	0.168
Female	5	9	17.699	0.331
Female	10	14	21.029	0.345
Female	15	19	29.738	0.350
Female	20	24	31.087	0.327
Female	25	29	31.140	0.324
Female	30	34	30.919	0.318
Female	35	39	24.843	0.299
Female	40	44	18.959	0.254
Female	45	49	16.083	0.230
Female	50	54	13.398	0.199
Female	55	59	12.035	0.215
Female	60	64	10.162	0.180
Female	65	69	9.014	0.167
Female	70	74	7.441	0.136
Female	75	79	6.268	0.135
Female	80	84	5.391	0.117
Female	85	89	4.628	0.097
Female	90	94	4.289	0.094
Female	95	99	3.877	0.100

Sex	Age start	Age end	Correction Factor	Standard Error
Male	0	0.999	3.8295	0.0848
Male	1	4	6.8094	0.1377
Male	5	9	12.6029	0.2488
Male	10	14	14.6992	0.2576
Male	15	19	23.5695	0.2911
Male	20	24	24.8601	0.3068
Male	25	29	17.6689	0.2530
Male	30	34	15.5750	0.2220
Male	35	39	13.9135	0.2181
Male	40	44	11.2504	0.1873
Male	45	49	9.5610	0.1669
Male	50	54	7.0199	0.1374
Male	55	59	5.7911	0.1390
Male	60	64	5.7911	0.1310
Male	65	69	5.2306	0.1233
Male	70	74	4.7013	0.1039
Male	75	79	4.1767	0.0968
Male	80	84	3.7635	0.0823
Male	85	89	3.6148	0.0736
Male	90	94	3.3424	0.0752
Male	95	99	3.4442	0.0960

We included the covariate Lagged Distributed Income (LDI) as a country-level covariate to inform excess mortality, with bounds of -0.5, -0.1. We used this covariate based on the assumption of a higher likelihood of mortality based on the developmental status of a country.

Betas and exponentiated values (which can be interpreted as an odds ratio) are shown in the table below:

Study covariate	Parameter	beta	Exponentiated beta
Claims data – 2000	Incidence	-0.042 (-0.072 — -0.01)	0.96 (0.93 — 0.99)
Claims data –2010	Incidence	-0.00074 (-0.0039 — -0.000091)	1.00 (1.00 — 1.00)

Compared to GBD 2015, major changes include inpatient data from a greater number of geographies and an age-sex-specific adjustment of inpatient hospital data to US claims data.

Acute Urolithiasis

Flowchart

Acute Urolithiasis

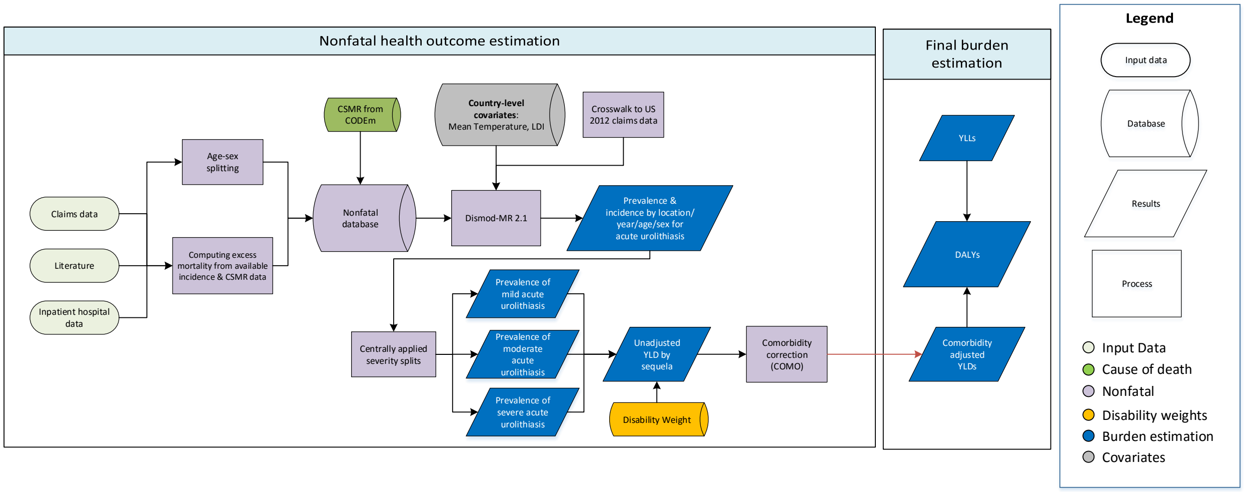

Case definition

Acute urolithiasis (AU) is an acute and usually symptomatic episode of urolithiasis, defined as stone formation located anywhere along the genitourinary tract.[1] Associated ICD codes include N20, N20.0, N20.1, N20.2, N20.9, N21, N21.1, N21.8, N21.9, N22, N22.0, N22.8, N23, and N23.0.

Input data

Model inputs

For GBD 2010, a systematic review of the prevalence of AU throughout the world was conducted. This search was updated for GBD 2013 and again for GBD 2016. A PubMed search was conducted using the following search terms: (Urolithiasis[Title/Abstract] OR Kidney Stones[Title/Abstract]) AND (Prevalence[Title/Abstract] OR Incidence[Title/Abstract]) AND ("2013/01/01"[PDAT] : "3000"[PDAT]) AND "humans"[MeSH Terms].

The exclusion criteria were:

45. Studies clearly not representative of the national population
46. Studies that did not provide primary data on epidemiological parameters, eg, a commentary piece
47. Studies of a specific type of urolithiasis

Four new studies were added based on this systematic review. The below table indicates the number of studies included and number of countries/regions represented by either study or hospital data:

	Prevalence	Incidence
Sources	5	70
Countries/subnationals	5	329

GBD world regions	4	14

Data from US claims data for 2000, 2010, and 2012 by US state were included. Hospital inpatient and outpatient data were also included. Inpatient data points that exceeded the maximum observed age-standardized rate in the US claims data by a margin of 25% or greater were excluded from analysis.

Severity splits & disability weights

The basis of the GBD disability weight survey assessments is lay descriptions of sequelae highlighting major functional consequences and symptoms. Urolithiasis is split into mild, moderate, and severe categories. The lay descriptions and disability weights for urolithiasis are shown below.

Severity level	Lay description	DW (95% CI)
Mild acute urolithiasis	Has some pain in the belly that causes nausea but does not interfere with daily activities.	0.011 (0.005, 0.021)
Moderate acute urolithiasis	Has pain in the belly and feels nauseous. The person has difficulties with daily activities.	0.114 (0.078, 0.159)
Severe acute urolithiasis	Has severe pain in the belly and feels nauseous. The person is anxious and unable to carry out daily activities.	0.324 (0.220, 0.442)

The severity distribution of urolithiasis was derived from analysis of the Medical Expenditure Panel Surveys (MEPS). MEPS is an overlapping panel survey of the non-institutionalized US population that collects data on respondents' health service interactions. Panels are initiated every year. Each panel is two years long and consists of five rounds. In 2000, MEPS began using 12-Item Short Form Surveys (SF-12) to collect data on functional health status. The SF-12 survey is administered twice per panel (about once per year).

In order to translate SF-12 scores into GBD disability weights, 62 lay descriptions for conditions representing the full range of disability weight values (from most mild to most severe) were selected. A convenience sample of respondents was then asked to complete an SF-12 form for an individual with the health state described in the lay descriptions of these conditions. Composite mental and physical SF-12 score was regressed on GBD disability weight to derive the relationship between disability weight and SF-12 score. Individual respondent scores were then regressed on reported conditions to obtain a comorbidity-corrected condition-specific disability weight. The distribution of these condition-specific weights was used derive the proportion of individuals with the conditions that fall within each GBD severity category.

Severity	Distribution
Mild acute urolithiasis	0.642 (0.536, 0.734)
Moderate acute urolithiasis	0.217 (0.149, 0.296)
Severe acute urolithiasis	0.141 (0.108, 0.178)

Modelling strategy

For GBD 2016, we modeled AU using DisMod MR 2.1, a Bayesian meta-regression modelling program. Prior settings in the DisMod model included setting remission of two weeks. We applied a crosswalk to all US claims data and hospital inpatient data to adjust to 2012 US claims data. We used the function in

DisMod-MR 2.1 to pull in cause-specific mortality rate (CSMR) data from our CODEm and CODcorrect analyses and match them with incidence data points for the same geography and study year to estimate priors on excess mortality rates (by dividing CSMR by incidence). We set bounds on location random effects for incidence due to sparse data.

Betas and exponentiated values (which can be interpreted as an odds ratio) are shown in the table below:

Study covariate	Parameter	beta	Exponentiated beta
Claims data - 2000	Incidence	-0.51 (-0.54 — -0.47)	0.60 (0.58 — 0.62)
Claims data - 2010	Incidence	-0.0022 (-0.0065 — -0.00017)	1.00 (0.99 — 1.00)
Hospital, inpatient	Incidence	-1.76 (-1.8 — -1.74)	0.17 (0.17 — 0.18)

For GBD 2016 we modelled only acute urolithiasis. Chronic urolithiasis estimates were directly derived from acute urolithiasis data in previous GBD iterations. Applying this technique for this GBD iteration would have required being able to identify acute urolithiasis episodes that repeated within an individual, which is a level of detail we currently do not estimate.

Compared to GBD 2015, major changes include inclusion of inpatient data from a greater number of geographies, estimation of the severity distribution of acute urolithiasis, and exclusion of chronic cases of urolithiasis.

Benign Prostatic Hypertrophy (BPH)

Flowchart

Benign Prostatic Hypertrophy (BPH)

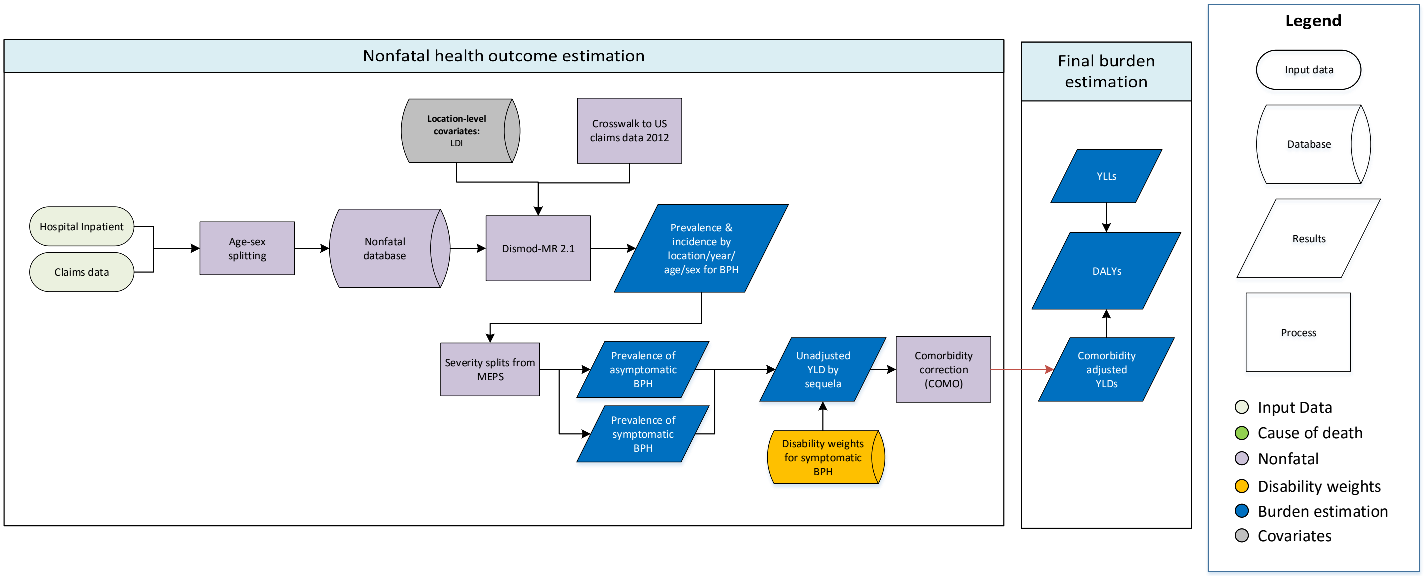

Case definition

Benign prostatic hypertrophy (BPH) is defined as a benign proliferation of prostatic tissue, often leading to symptoms such as urinary retention, bladder outlet obstruction, or urinary tract infection.[1,2] The ICD codes for BPH include N40, N40.0, N40.1, N40.2, N40.3, and N40.9.

Input data

Model inputs

The BPH model is informed by US state-level claims data and global hospital inpatient data. US claims data from years 2000, 2010, and 2012 were included. Inpatient data that exceeded the maximum observed age-standardized rate in the US inpatient data by a margin of 25% or greater were excluded from analysis.

	Prevalence
Sources	62
Countries/subnationals	355
GBD world regions	15

Severity splits

The basis of the GBD disability weight survey assessments are lay descriptions of sequelae highlighting major functional consequences and symptoms of a given cause. BPH is split into symptomatic and asymptomatic types. There is no disability weight (DW) assigned to asymptomatic cases of BPH. The DW associated with symptomatic BPH regards urinary frequency that is sometimes associated with pain – as seen in the table below, which offers further information.

Severity level	Lay description	DW (95% CI)
Asymptomatic	N/A	0
Symptomatic	Feels the urge to urinate frequently, but when passing urine it comes out slowly and sometimes is painful.	0.067 (0.043–0.097)

The proportions symptomatic and asymptomatic were derived from analysis of the Medical Expenditure Panel Surveys (MEPS). MEPS is an overlapping panel survey of the non-institutionalized US population that collects data on respondents' health service interactions. Panels are initiated every year. Each panel is two years long and consists of five rounds. In 2000, MEPS began using 12-Item Short Form Surveys (SF-12) to collect data on functional health status. The SF-12 survey is administered twice per panel (about once per year).

In order to translate SF-12 scores into GBD disability weights, 62 lay descriptions for conditions representing the full range of disability weight values (from most mild to most severe) were selected. A convenience sample of respondents was then asked to complete an SF-12 form for an individual with the health state described in the lay descriptions of these conditions. Composite mental and physical SF-12 score was regressed on GBD disability weight to derive the relationship between disability weight and SF-12 score. Individual respondent scores were then regressed on reported conditions to obtain a comorbidity-corrected condition-specific disability weight. The distribution of these condition-specific weights was used derive the proportion of individuals with the conditions that fall within each GBD severity category.

Severity	Distribution
Asymptomatic BPH	0.472 (0.459–0.487)
Symptomatic BPH	0.528 (0.513–0.541)

Modelling strategy

We ran a DisMod 2.1 model to produce estimates by age, sex, year, and location. Prior settings in the BPH DisMod 2.1 model include setting incidence and remission prior to age 40 years to 0. We set an upper bound on remission after age 40 to 0.1, corresponding to a maximum duration of 10 years. We also determined that there was no excess mortality related to BPH.

We applied a crosswalk to all US claims data to adjust to 2012 US claims data. Inpatient data was adjusted to account for multiple admissions and multiple diagnoses, based on MarketScan data, and an age-specific crosswalk was applied to adjust inpatient hospital data to 2012 US claims data. This crosswalk was

applied by comparing the ratio of prevalence indicated by 2012 US claims data to that indicated by US inpatient data and applying these age-specific adjustment factors to global inpatient hospital data. Adjustment factors and their associated standard errors are presented in the table below.

Age start	Age end	Adjustment factor	Standard error
40	44	21.528	0.402
45	49	23.031	0.350
50	54	10.461	0.244
55	59	6.718	0.232
60	64	5.402	0.195
65	69	3.951	0.166
70	74	3.407	0.132
75	79	3.172	0.119
80	84	2.942	0.098
85	89	3.004	0.085
90	94	3.874	0.092
95	99	2.601	0.127

Betas and exponentiated values (which can be interpreted as an odds ratio) are shown in the table below:

Study covariate	Parameter	beta	Exponentiated beta
Claims data – 2000	Prevalence	-0.016 (-0.051 — -0.0014)	0.98 (0.95 — 1.00)
Claims data – 2010	Prevalence	-0.0047 (-0.018 — -0.00016)	1.00 (0.98 — 1.00)
Mean BMI	Prevalence	-0.04 (-0.051 — -0.028)	0.96 (0.95 — 0.97)

Compared to GBD 2015, major changes include exclusion of literature data on BPH due to issues of representativeness and inconsistent case definitions, inclusion of inpatient data from a greater number of geographies, and an age-specific adjustment of inpatient hospital data to US claims data.

Other urinary diseases

In addition to the urinary diseases described above, there are many diverse types of urinary diseases, with a range of severities and associated sequelae. Because these urinary diseases are diverse in their underlying causes and risk factors as well as in their associated health outcomes, modelling them together in a DisMod-MR model would not produce reliable estimates of prevalence or excess mortality. Instead, we calculated the YLDs caused by other congenital disorders directly using a YLD/YLL ratio.

We calculated the ratio of YLDs to YLLs across the specified urinary diseases for which nonfatal outcomes were modelled, using YLL estimates from the GBD 2016 cause of death (CoD) analysis. We then multiplied this YLD/YLL ratio by the YLL estimates for other urinary diseases from the GBD 2016 CoD analysis, providing us with an estimate of the YLDs association with other urinary diseases.

Uterine Fibroids

Flowchart

Uterine fibroids

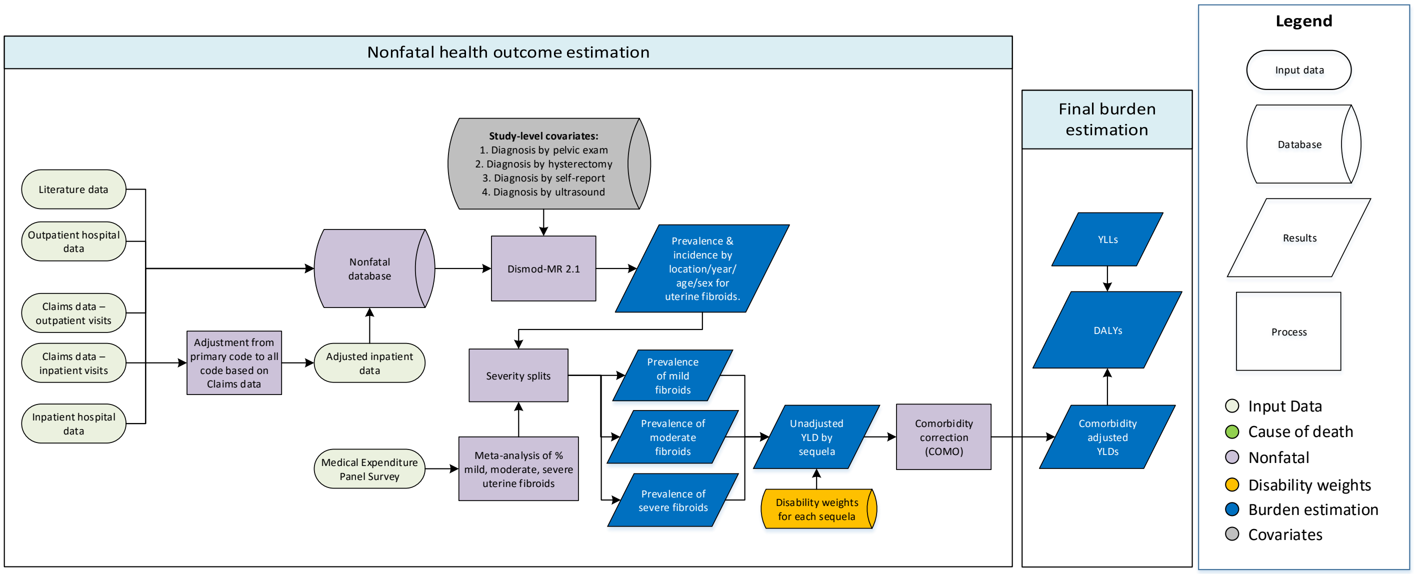

Case definition

Uterine fibroids, also called uterine myomas or leiomyomas, are non-cancerous, compact tumors that occur in the uterus. Fibroids can be diagnosed in a number of ways, including pelvic exam, ultrasound, and hysterectomy. Our reference definition is diagnosis by pelvic exam or ultrasound because it is the most common. However, we incorporate studies that include diagnosis by self-report, pelvic exam only, ultrasound only, hysterectomy only, and all combinations of the three. Refer to the Appendix for ICD codes.

Input data

Model inputs

For GBD 2010, a systematic review of uterine fibroids throughout the world was conducted. Ovid MEDLINE, EMBASE, CINAHL, CAB abstracts, WHOLIS, and ISGLE database were searched. The agreed approach for uterine fibroids was to conduct a PubMed literature search every three years. A PubMed search was conducted as part of the initial review in 2010 and is next due for GBD 2017. Exclusion criteria for the initial systematic review were:

1. Studies that did not provide primary data on epidemiological parameters, e.g., a commentary piece
2. Reviews
3. Clearly non-representative studies (e.g., only high-risk pregnant women)

The table below shows the number of literature studies included in GBD 2016, as well as the number of countries or subnational units and GBD world regions represented.

	Prevalence	Incidence
Studies	12	4
Countries/subnationals	16	17
GBD world regions	7	3

In addition, US claims data for 2000, 2012, and 2012 by US state were included. Inpatient and outpatient hospital data were also included.

Severity splits

The basis of the GBD disability weight (DW) survey assessment are lay descriptions of sequelae highlighting major functional consequences and symptoms. The lay descriptions and disability weights for uterine fibroids are shown below. Further severity levels are calculated by combining several of these disability weights, e.g., mild abdominal pain with moderate anemia. It should be noted that anemia alone is not ascribed to fibroids, but only in conjunction with mild abdominal pain. The disability weights are listed for reference.

Severity	Lay description	DW (95% CI)
Abdominopelvic problem, mild	has some pain in the belly that causes nausea but does not interfere with daily activities.	0.011 (0.005-0.021)
Anemia, mild	feels slightly tired and weak at times, but this does not interfere with normal daily activities.	0.004 (0.001-0.008)
Anemia, moderate	feels moderate fatigue, weakness, and shortness of breath after exercise, making daily activities more difficult.	0.052 (0.034-0.076)
Anemia, severe	feels very weak, tired and short of breath, and has problems with activities that require physical effort or deep concentration.	0.149 (0.101-0.21)

To determine the proportion of women with uterine fibroids who fall into each severity level, data from the Medical Expenditure Panel Survey (MEPS) is used.

Modeling strategy

The amount of data included in our model increased significantly with GBD 2015 due to the addition of US claims data and hospital data. Our DisMod MR 2.2 settings remained the same as those used in GBD 2015.

As in previous GBD iterations, incidence was set to zero prior to 15 years of age and after 51. We assume no excess mortality from uterine fibroids.

Diagnosis by either ultrasound or pelvic exam was set as the reference category. Study-level covariates for diagnosis by hysterectomy only and self-report were included.

Study covariate	Measure	Parameter	beta	Exponentiated beta
Hysterectomy only	incidence	x-cov	-1.48 (-1.59 — -1.37)	0.23 (0.20 — 0.25)
All MarketScan, year 2000	Prevalence	x-cov	-1.02 (-1.03 — -1.01)	0.36 (0.36 — 0.36)
All MarketScan, year 2012	Prevalence	x-cov	-0.73 (-0.77 — -0.69)	0.48 (0.46 — 0.50)

All MarketScan, year 2010	Prevalence	x-cov	-0.76 (-0.79 — -0.75)	0.47 (0.46 — 0.47)
Self-reported	prevalence	x-cov	-0.23 (-0.26 — -0.22)	0.79 (0.77 — 0.80)

No other significant changes were made to the GBD 2015 modeling strategy.

Polycystic Ovarian Syndrome (PCOS)

Flowchart

Polycystic ovarian syndrome

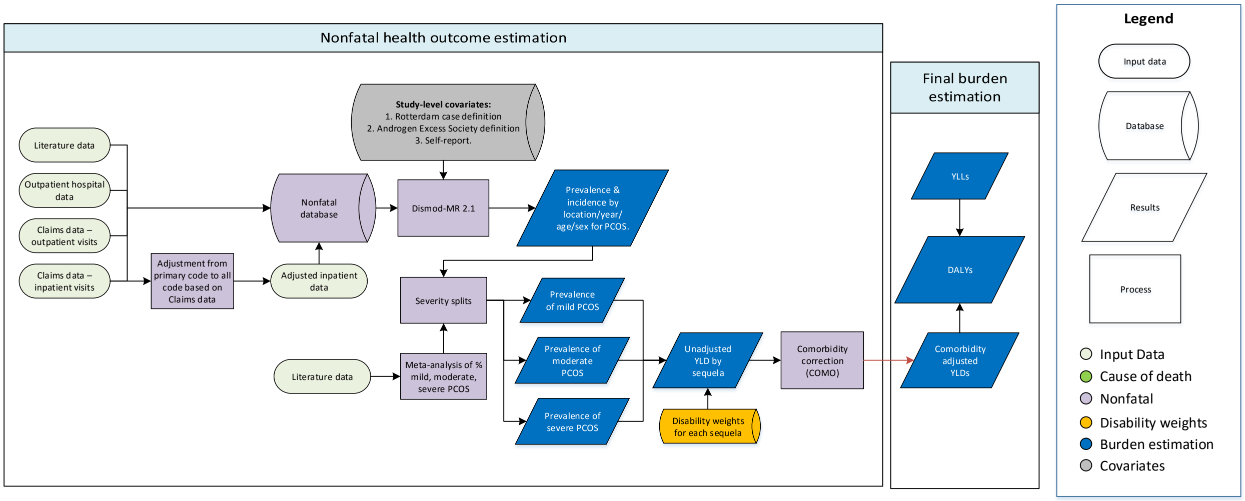

Case definition

Polycystic ovarian syndrome (PCOS) is a condition that affects women's ovaries and can lead to a variety of symptoms. Women with PCOS often have enlarged ovaries that contain pockets of fluid, and symptoms include infrequent menstruation, excess hair growth, acne, and obesity.

Input data

Model inputs

For GBD 2010, a systematic review of PCOS throughout the world was conducted. Ovid MEDLINE, EMBASE, CINAHL, CAB abstracts, WHOLIS, and ISGLE database were searched. The agreed approach for PCOS was to conduct a PubMed literature search every three years. A PubMed search was conducted as part of the initial review in 2010 and is next due for GBD 2017. Exclusion criteria for the initial systematic review were:

1. Studies that did not provide primary data on epidemiological parameters, e.g., a commentary piece
2. Reviews
3. Clearly non-representative studies (e.g., only high-risk pregnant women)

The table below shows the number of literature studies included in GBD 2016, as well as the number of countries or subnational units and GBD world regions represented.

	Prevalence
Studies	21
Countries/subnationals	17
GBD world regions	10

In addition, US claims data for 2000, 2012 and 2012 by US state were included. Inpatient and outpatient hospital data were also included.

Severity splits

The basis of the GBD disability weight (DW) survey assessment are lay descriptions of sequelae highlighting major functional consequences and symptoms. The lay descriptions and disability weights for premenstrual syndrome are shown below. Further severity levels are calculated by combining several of these disability weights, e.g., mild disfigurement and primary infertility.

Severity	Lay description	DW (95% CI)
Disfigurement, level 1	has a slight, visible physical deformity that others notice, which causes some worry and discomfort.	0.011 (0.005-0.021)
Infertility, primary	wants to have a child and has a fertile partner, but the couple cannot conceive.	0.008 (0.003-0.015)
Infertility, secondary	has at least one child, and wants to have more children. The person has a fertile partner, but the couple cannot conceive.	0.005 (0.002-0.011)

To determine the proportion of people within each of these severity levels, one study was consulted.[10] Tehrani et al. included information on the proportion of women who experience primary infertility and hyperandrogenism. Percentages were combined to calculate the proportion of women who fall into both hyperandrogenism and infertility categories.

Modeling strategy

The amount of data included in our model increased significantly with GBD 2016 due to the addition of US claims data and inpatient and outpatient hospital data.

The table below illustrates the study covariates, measures, parameters, beta, and exponentiated beta values.

Study covariate	Measure	parameter	beta	Exponentiated beta
All MarketScan, year 2000	Prevalence	x-cov	-2.51 (-2.55 — -2.5)	0.081 (0.078 — 0.082)
All MarketScan, year 2010	Prevalence	x-cov	-1.41 (-1.44 — -1.4)	0.24 (0.24 — 0.25)
All MarketScan, year 2012	Prevalence	x-cov	-1.41 (-1.44 — -1.4)	0.25 (0.24 — 0.25)

[10] Tehrani FR, Simbar M, Tohidi M, Hosseinpanah F, Azizi F. The prevalence of polycystic ovary syndrome in a community sample of Iranian population: Iranian PCOS prevalence study. <i>Reprod Biol Endocrinol</i>. 2011; 9: 39.

Androgen Excess Society case definition	Prevalence	z-cov	0.47 (0.033 — 0.96)	1.59 (1.03 — 2.61)
Rotterdam case definition	Prevalence	x-cov	0.31 (0.21 — 0.40)	1.36 (1.23 — 1.49)
Self-report	prevalence	z-cov	0.48 (0.11 — 0.96)	1.62 (1.12 — 2.62)
Hospital Inpatient	Prevalence	x-cov	-0.99 (-1.13 — -0.86)	0.37 (0.32 — 0.42)
Healthcare access and quality index	Excess mortality rate	Country-level	-0.043 (-0.05 — -0.034)	0.96 (0.95 — 0.97)

As in previous GBD iterations, incidence was set to zero prior to 15 years of age and after 50. This is because a woman must enter puberty before she can get PCOS, and the condition spontaneously goes into remission with the onset of menopause.

Case definitions for PCOS vary widely, including varying rosters of symptoms over various time periods. We use as our reference definition the NIH/NICHD criteria, for which three signs must be present: clinical or biochemical evidence of hyperandrogenism, oligomenorrhea, and the exclusion of other disorders. We include study-level covariates for other common case definitions, including the Rotterdam and Androgen Excess Society (AES) definitions, as well as self-report.

No significant changes took place for GBD 2016.

Endometriosis

Flowchart

Endometriosis

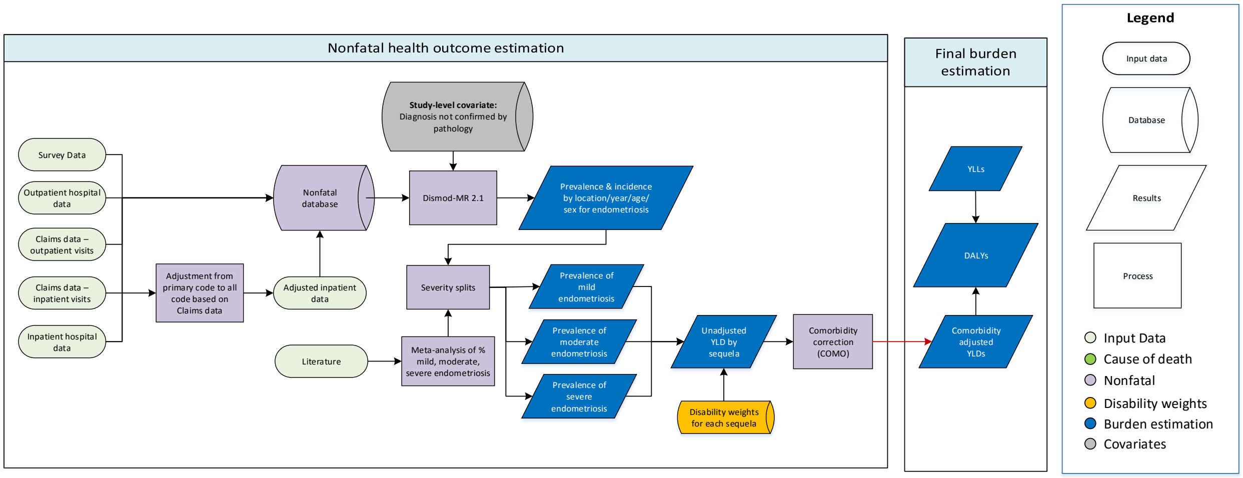

Case definition

Endometriosis is defined as growth of tissue that usually lies inside the uterus outside of it. Common symptoms include chronic pain and infertility. Our reference case definition of endometriosis is diagnosis accompanied by pathological confirmation.

Input data

Model inputs

For GBD 2010, a systematic review of endometriosis throughout the world was conducted. Ovid MEDLINE, EMBASE, CINAHL, CAB abstracts, WHOLIS, and ISGLE database were searched. The agreed approach for endometriosis was to conduct a PubMed literature search every three years. A PubMed search was conducted as part of the initial review in 2010. Exclusion criteria for the initial systematic review were:

1. Studies that did not provide primary data on epidemiological parameters, e.g., a commentary piece
2. Reviews
3. Clearly non-representative studies (e.g., only high-risk pregnant women)

The table below shows the number of literature studies included in GBD 2016, as well as the number of countries or subnational units and GBD world regions represented.

	Prevalence	Incidence
Studies	7	10
Countries/subnationals	7	8
GBD world regions	4	2

In addition, US claims data for 2000, 2012 and 2012 by US state were included. Inpatient and outpatient hospital data were also included.

Severity splits & disability weights

The basis of the GBD disability weight (DW) survey assessment are lay descriptions of sequelae highlighting major functional consequences and symptoms. The lay descriptions and disability weights for endometriosis are shown below. Further severity levels are calculated by combining several of these disability weights, e.g., moderate abdominal pain and secondary infertility.

Severity	Lay description	DW (95% CI)
Abdominopelvic problem, mild	has some pain in the belly that causes nausea but does not interfere with daily activities.	0.011 (0.005-0.021)
Abdominopelvic problem, moderate	has pain in the belly and feels nauseous. The person has difficulties with daily activities.	0.114 (0.078-0.159)
Abdominopelvic problem, severe	has severe pain in the belly and feels nauseous. The person is anxious and unable to carry out daily activities.	0.324 (0.219-0.442)
Infertility, primary	wants to have a child and has a fertile partner, but the couple cannot conceive.	0.008 (0.003-0.015)
Infertility, secondary	has at least one child, and wants to have more children. The person has a fertile partner, but the couple cannot conceive.	0.005 (0.002-0.011)

To determine the proportion of people within each of these severity levels, three studies were consulted (ALWHS YEAR; Sinaii et al. 2002 & 2008). One addressed the proportion of women who become infertile, one addressed the proportion with chronic abdominal pain, and the last one severity of abdominal pain. Estimates were combined across studies to calculate the proportion of women who fall into both abdominal pain and infertility categories.

Modeling strategy

We use DisMod-MR 2.1, a Bayesian meta-regression epidemiological model, to generate estimates for Endometriosis by age, sex, year, and country. The amount of data included in our model increased significantly with GBD 2015 due to the addition of US claims data and hospital data.

Diagnosis confirmed by pathology was set as the reference definition. In addition to study-level covariate on diagnosis not confirmed by pathology, study-level covariates were added for each year of US claims data as well as inpatient and outpatient hospital data. The table below illustrates covariates, measures, parameters, beta, and exponentiated beta values.

Study covariate	Measure	Parameter	beta	Exponentiated beta
Endometriosis not confirmed by pathology	incidence	x-cov	-0.56 (-0.9 — -0.23)	0.57 (0.41 — 0.80)
All MarketScan, year 2000	prevalence	x-cov	-1.5 (-1.96 — -0.96)	0.21 (0.14 — 0.37)
All MarketScan, year 2010	prevalence	x-cov	-1.54 (-1.98 — -0.99)	0.92 (0.85 — 0.99)

All MarketScan, year 2012	prevalence	x-cov	. -1.55 (-1.99 — -0.99)	0.21 (0.14 — 0.37)
Hospital inpatient	prevalence	x-cov	-1.13 (-1.61 — -0.58)	0.32 (0.20 — 0.56)
Healthcare access and quality index	Excess mortality rate	Country-level	-0.02 (-0.02 — -0.02)	0.98 (0.98 — 0.98)

As in previous GBD iterations, incidence was set to zero prior to 15 years of age and after 51. This is because a woman must enter puberty before she can get endometriosis, and the condition spontaneously goes into remission with the onset of menopause.

There have been no additional significant changes to the modeling strategy for GBD 2016.

Genital Prolapse

Flowchart

Genital prolapse

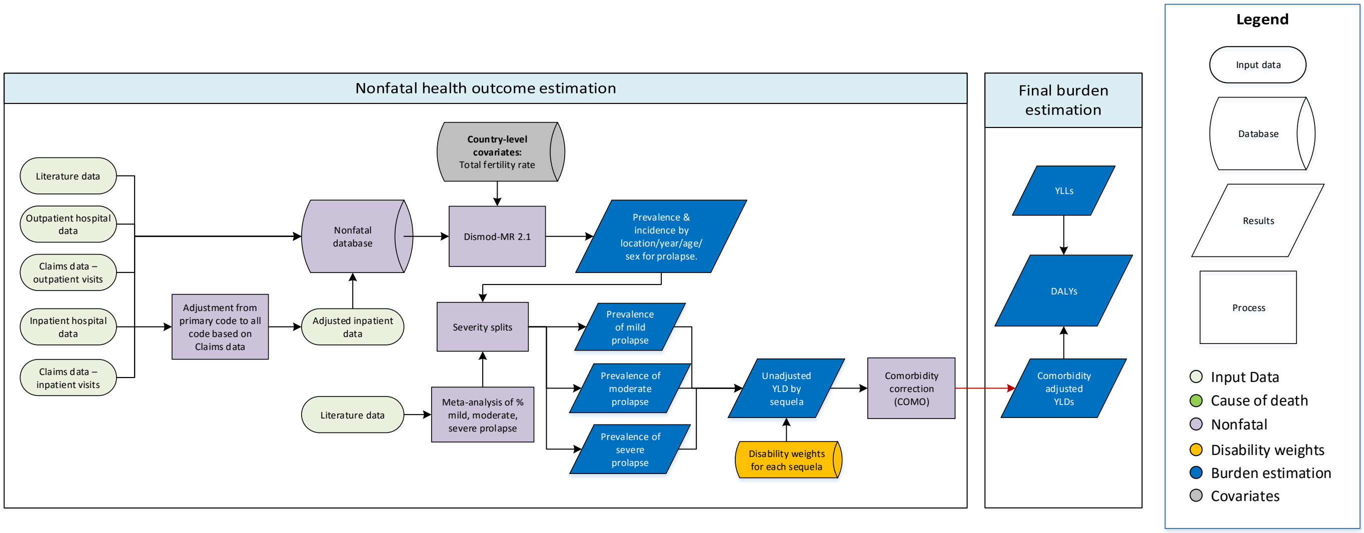

Case definition

Genital prolapse, also called female pelvic organ prolapse, is the clinically relevant descent of one of more of the pelvic structures, including the uterus, bladder, rectum, small or large bowl, or vagina. Risk of prolapse increases with age, and can be exacerbated by vaginal childbirth or physical strain. ICD codes associated with genital prolapse include: N81.

Input data

Model inputs

For GBD 2010, a systematic review of genital prolapse throughout the world was conducted. Ovid MEDLINE, EMBASE, CINAHL, CAB abstracts, WHOLIS, and ISGLE database were searched. The agreed approach for genital prolapse was to conduct a PubMed literature search every three years. A PubMed search was conducted as part of the initial review in 2010 and is next due for GBD 2017. Exclusion criteria for the initial systematic review were:

1. Studies that did not provide primary data on epidemiological parameters, e.g., a commentary piece
2. Reviews
3. Clearly non-representative studies (e.g., only high-risk pregnant women)

The table below shows the number of literature studies included in GBD 2016, as well as the number of countries or subnational units and GBD world regions represented.

	Prevalence

Studies	13
Countries/subnationals	12
GBD world regions	8

In addition, US claims data for 2000, 2010 and 2012 by US state were included. Outpatient and inpatient hospital data were also included.

Severity splits

The basis of the GBD disability weight (DW) survey assessment are lay descriptions of sequelae highlighting major functional consequences and symptoms. The lay descriptions and disability weights for genital prolapse are shown below. Further severity levels are calculated by combining several of these disability weights, e.g., mild abdominal pain and stress incontinence.

Severity	Lay description	DW (95% CI)
Stress incontinence	loses small amounts of urine without meaning to when coughing, sneezing, laughing or during physical exercise.	0.02 (0.011-0.035)
Abdominopelvic problem, mild	has some pain in the belly that causes nausea but does not interfere with daily activities.	0.011 (0.005-0.021)

To determine the proportion of people within each of these severity levels, two studies were consulted.[11],[12] Scherf and Slieker-Ten Hove included information on the proportion of women with prolapse who experience a bulging sensation as well as stress incontinence. Percentages were combined to calculate the proportion of women who fall into both stress incontinence and bulging sensation categories.

Modeling strategy

The amount of data included in our model increased significantly with GBD 2015 due to the addition of US claims data. Our DisMod MR 2.2 settings remained the same as GBD 2015.

As in previous GBD iterations, incidence was set to zero prior to 15 years of age. This is because it is highly unlikely a woman would experience genital prolapse before entering her childbearing years.

This year we have added covariates for each year of the US claims data as well as the inpatient hospital data. The table below illustrates covariates, measures, parameters, beta, and exponentiated beta values.

**Study covariate**	**Measure**	**Parameter**	**beta**	**Exponentiated beta**
All MarketScan, year 2000	prevalence	x-cov	-1.99 (-2 — -1.99)	0.14 (0.14 — 0.14)
All MarketScan, year 2010	prevalence	x-cov	-1.99 (-2 — -1.97)	0.14 (0.14 — 0.14)
All MarketScan, year 2012	prevalence	x-cov	-1.99 (-2 — -1.96)	0.14 (0.14 — 0.14)

[11] Epidemiology of pelvic organ prolapse in rural Gambia, West Africa. Scherf, 2002.
[12] Symptomatic pelvic organ prolapse and possible risk factors in a general population. Slieker-Ten Howe, 2009.

Hospital Inpatient	Prevalence	x-cov	-2.17 (-2.33 — -2.08)	0.11 (0.097 — 0.13)
Total fertility rate	prevalence	Country-level covariate	0.99 (0.97 — 1.00)	2.69 (2.63 — 2.72)

No other significant changes to modeling strategy were made for GBD 2016.

Premenstrual Syndrome (PMS)

Flowchart

Premenstrual syndrome

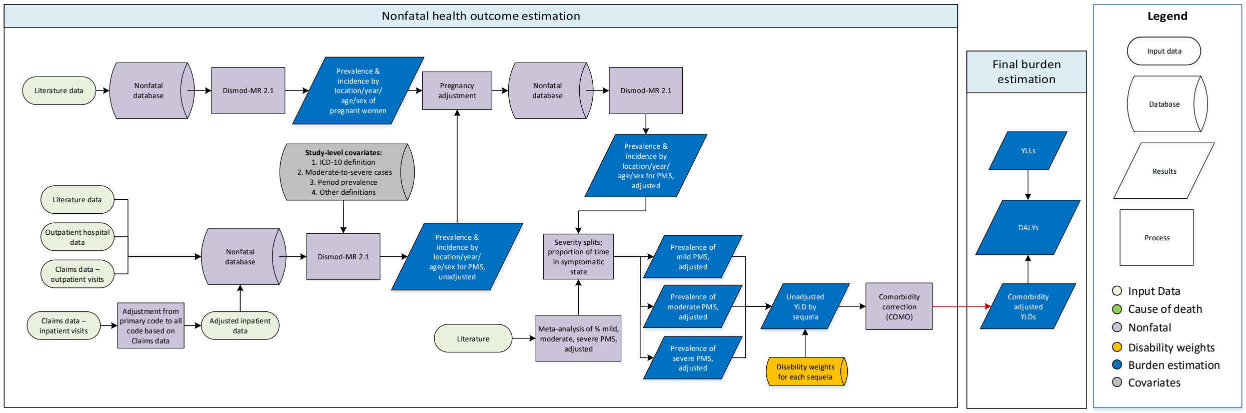

Case definition

Premenstrual syndrome refers to psychological and physical symptoms that occur in the weeks leading up to a woman's period in her menstrual cycle. Symptoms are extremely varied in nature and severity, but include tenderness, bloating, irritability, fatigue, abdominal pain, and altered mental states. Symptoms cease when a woman is pregnant and once she reaches menopause.

Input data

Model inputs

For GBD 2010, a systematic review of premenstrual syndrome throughout the world was conducted. Ovid MEDLINE, EMBASE, CINAHL, CAB abstracts, WHOLIS, and ISGLE database were searched. The agreed approach for premenstrual syndrome was to conduct a PubMed literature search every three years. A PubMed search was conducted as part of the initial review in 2010 and is next due for GBD 2017. Exclusion criteria for the initial systematic review were:

1. Studies that did not provide primary data on epidemiological parameters, e.g., a commentary piece
2. Reviews
3. Clearly non-representative studies (e.g., only high-risk pregnant women)

The table below shows the number of literature studies included in GBD 2015, as well as the number of countries or subnational units and GBD world regions represented. Note that data on the proportion of women who are pregnant is used during the pregnancy adjustment, described in detail in the modeling strategy section.

PMS

	Prevalence

Studies	50
Countries/subnationals	43
GBD world regions	11

Women who are pregnant

	Prevalence
Studies	2
Countries/subnationals	188
GBD world regions	21

Inpatient hospital data was not incorporated, as we believed that inpatient data on PMS would fluctuate wildly with across geographies and different coding practices, and would not represent the true prevalence of PMS in the population.

Severity splits and disability weights

The basis of the GBD disability weight (DW) survey assessment are lay descriptions of sequelae highlighting major functional consequences and symptoms. The lay descriptions and disability weights for premenstrual syndrome are shown below. Further severity levels are calculated by combining several of these disability weights, e.g., abdominal pain and depression due to premenstrual syndrome.

Severity	Lay description	DW (95% CI)
Major depressive disorder, mild episode	feels persistent sadness and has lost interest in usual activities. The person sometimes sleeps badly, feels tired, or has trouble concentrating but still manages to function in daily life with extra effort.	0.145 (0.099-0.209)
Abdominopelvic problem, mild	has some pain in the belly that causes nausea but does not interfere with daily activities.	0.011 (0.005-0.021)

To determine the proportion of people within each of these severity levels, five studies were consulted. Three studies addressed the proportion of women with PMS who experience depression, and two other studies addressed the proportion of women with PMS who experience abdominal pain. Estimates were pooled across studies to get final proportions, and were combined across studies to calculate the proportion of women who fall into both abdominal pain and depression categories.

Modeling strategy

Our DisMod MR 2.2 settings remained the same as those used in GBD 2015 and, no new data was added for GBD 2016.

As in previous GBD iterations, incidence was set to zero prior to 15 years of age and after 50. This is because a woman must enter puberty before she can get premenstrual syndrome, and the condition spontaneously goes into remission with the onset of menopause. We assume no excess mortality from PMS.

Case definitions for PMS vary widely, including varying rosters of symptoms over various time periods. We use as our reference definition the American College of Obstetricians and Gynecologists (ACOG) criteria, which states that the patient reports at least one of each of the following affective and somatic symptoms during the five days before their menses and appear in three consecutive cycles: depression, angry outbursts, irritability, anxiety, confusion, social withdrawal; breast tenderness, abdominal bloating, headache, or swelling of extremities. We include study-level covariates for other common case definitions, including the ICD-10 definition, and the Premenstrual Symptoms Screening Tool (PSST) definition. We also include covariates for studies that only examine moderate to severe PMS, or period prevalence of PMS.

Other gynaecological conditions

Flowchart

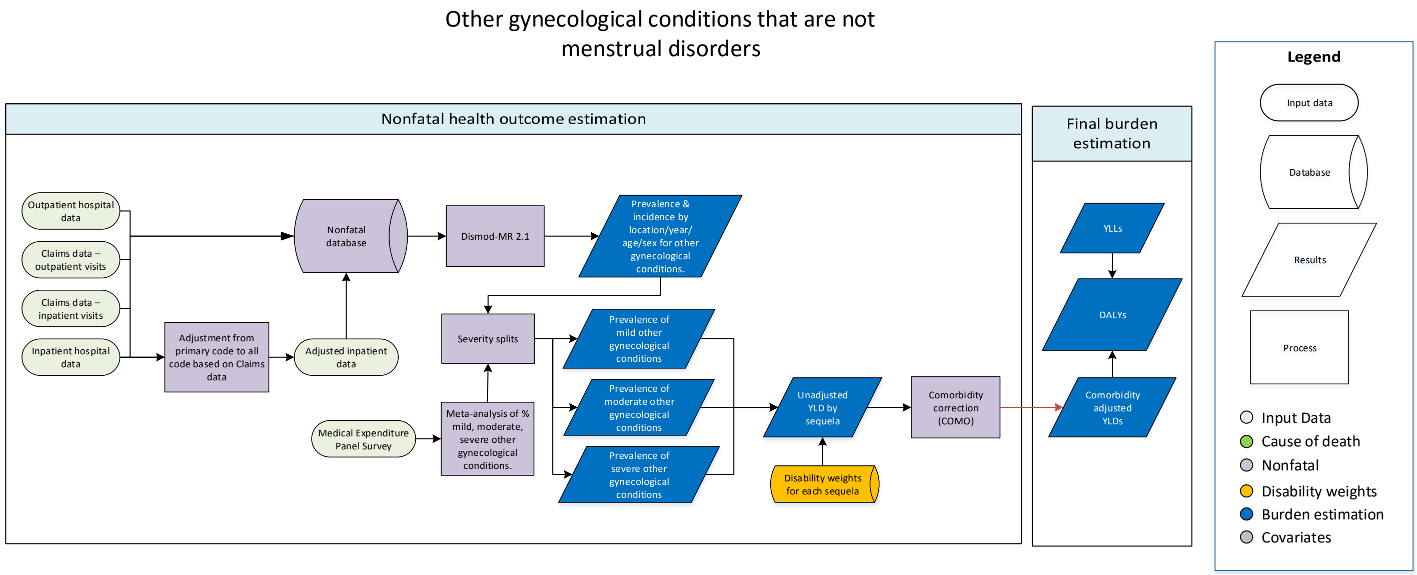

Case definition

Other gynaecological conditions encompasses all disorders that are not menstruation- or bleeding-related that do not fall under the heading of any of the other gynaecological causes. They only affect women.

Input data

Model inputs

No literature data are used to inform models of other gynaecological conditions. Previously, only inpatient and outpatient hospital data were used. For GBD 2015, US claims data for 2000, 2012 and 2012 by US state were added.

Severity splits & disability weights

The basis of the GBD disability weight (DW) survey assessment are lay descriptions of sequelae highlighting major functional consequences and symptoms. The lay descriptions and disability weights for other gynaecological conditions are shown below.

Severity	Lay description	DW (95% CI)
Abdominopelvic problem, mild	has some pain in the belly that causes nausea but does not interfere with daily activities.	0.011 (0.005–0.021)
Abdominopelvic problem, moderate	has pain in the belly and feels nauseated. The person has difficulties with daily activities.	0.114 (0.078–0.159)
Abdominopelvic problem, severe	has severe pain in the belly and feels nauseated. The person is anxious and unable to carry out daily activities.	0.324 (0.219–0.442)
Anaemia, mild	feels slightly tired and weak at times, but this does not interfere with normal daily activities.	0.004 (0.001–0.008)

Anaemia, moderate	feels moderate fatigue, weakness, and shortness of breath after exercise, making daily activities more difficult.	0.052 (0.034–0.076)
Anaemia, severe	feels very weak, tired and short of breath, and has problems with activities that require physical effort or deep concentration.	0.149 (0.101–0.21)

To determine the proportion of women with endometriosis who fall into each severity level, data from the Medical Expenditure Panel Survey (MEPS) are used.

Modelling strategy

We ran a DisMod MR 2.1 model to estimate the global burden of gynaecological diseases. The amount of data included in our model increased significantly with GBD 2015 due to the addition of US claims data. Many of our DisMod MR 2.1 settings remained the same, but new study-level covariates were added to accommodate the new data. These covariates are shown in the table below, along with measures, parameters, beta, and exponentiated beta values.

Study covariate	Measure	Parameter	beta	Exponentiated beta
All MarketScan, year 2000	Prevalence	x-cov	4.00 (4.00 — 4.00)	54.52 (54.43 — 54.60)
All MarketScan, year 2010	prevalence	x-cov	3.99 (3.98 — 4.00)	54.30 (53.57 — 54.60)
All MarketScan, year 2012	Prevalence	x-cov	3.99 (3.97 — 4.00)	53.86 (52.83 — 54.54)
Hospital inpatient	prevalence	x-cov	-1.96 (-2.17 — -1.76)	0.14 (0.11 — 0.17)

As in previous GBD iterations, incidence was set to zero prior to 15 years of age. We assume no excess mortality from other gynecological conditions over the same age range.

No other significant changes were made to the GBD 2016 estimation process.

Endocrine, Metabolic, Blood and Immune Disorders

Flowchart

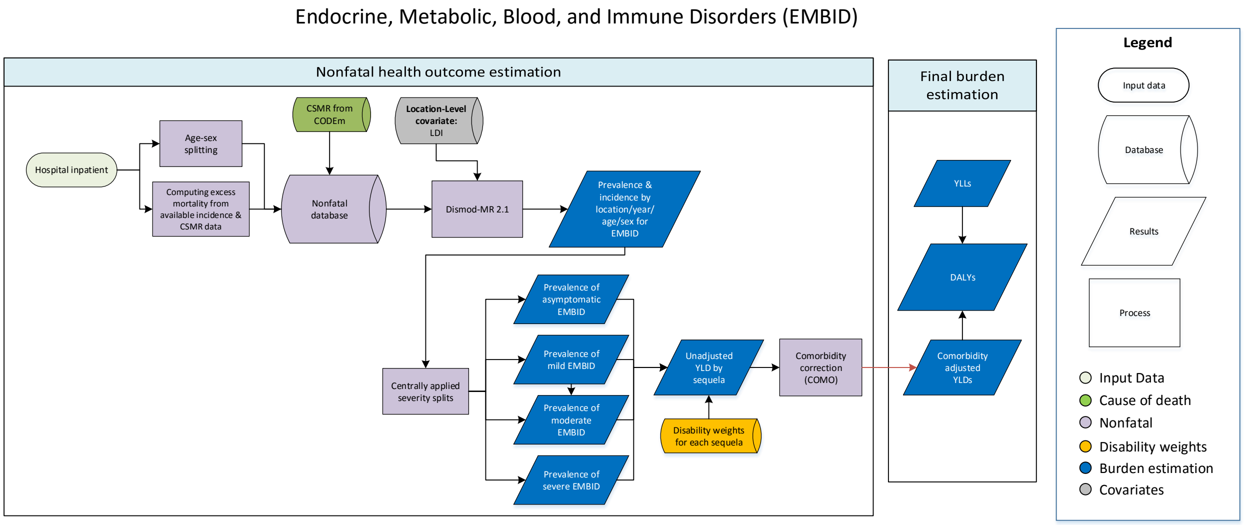

Case definition

Endocrine, metabolic, blood, and immune disorders (EMBID) is a residual cause consisting of conditions that do not map to other causes within the diabetes, urogenital, blood and endocrine disease hierarchy. This residual group consists mainly of thyroid disorders, rare metabolic and immune disorders, and blood disorders not resulting in anaemia . From the ICD chapter on Endocrine, Metabolic, and Immune Disorders (the E chapter) GBD's definition of EMBID excludes the codes for nutritional deficiencies, diabetes and anaemia which are modelled as separate causes; as well as those for obesity and hypercholesterolemia which are modeled as risks, not causes.

ICD 10 codes for EMBID include: D64.4, D64.8, D68-D68.6, D68.8-D68.9, D69-D69.4, D69.6, D69.8, D70-D70.4, D70.8-D70.9, D72-D72.1, D72.8-D72.9, D73- D73.5, D73.8-D73.9, D74.0, D74.8-D74.9, D75-D75.2, D75.8-D75.9, D76-D76.3, D80-D80.9, D81-D81.9, D82-D82.4, D82.8-D82.9, D83-D83.2, D83.8-D83.9, D84-D84.1, D84.8-D84.9, D86.8, D89-D89.3, D89.8-D89.9, E03-E03.1, E03.3-E03.5, E03.8-E03.9, E04-E04.2, E04.8-E04.9, E05-E05.5, E05.8-E05.9, E06-E06.3, E06.5, E06.9, E07-E07.1, E07.8-E07.9, E16.1-E16.4, E16.8-E16.9, E20-E20.1, E20.8-E20.9, E21-E21.5, E22-E22.2, E22.8-E22.9, E23.0, E23.2-E23.3, E23.6-E23.7, E24-E24.1, E24.3-E24.4, E24.8-E24.9, E25.0, E25.8-E25.9, E26-E26.1, E26.8-E26.9, E27-E27.2, E27.4-E27.5, E27.8-E27.9, E28-E28.1, E28.3, E28.8-E28.9, E29-E29.1, E29.8-E29.9, E30-E30.1, E30.8-E30.9, E31-E31.2, E31.8-E31.9, E32-E32.1, E32.8-E32.9, E34-E34.5, E34.8-E34.9, E67-E67.3, E67.8, E70-E70.5, E70.8-E70.9, E71-E71.5, E72-E72.5, E72.8-E72.9, E73-E73.1, E73.8-E73.9, E74-E74.4, E74.8-E74.9, E75-E75.6, E76-E76.3, E76.8-E76.9, E77-E77.1, E77.8-E77.9, E79-E79.2, E79.8-E79.9, E80-E80.7, E83-E83.9, E84-E84.9, E85-E85.9, E88-E88.9.

Input data

Model inputs

The EBMID model is informed by global hospital inpatient data. Global inpatient data points that exceeded the maximum observed age-standardized rate in the US inpatient data by a margin of 25% or greater were excluded from analysis.

The table below shows the number of countries and GBD world regions represented.

	Prevalence
Countries/subnationals	125
GBD world regions	14

Severity splits & disability weights

The basis of the GBD disability weight survey assessments is lay descriptions of sequelae highlighting major functional consequences and symptoms. EMBID is split into asymptomatic, mild, moderate, and severe categories. The lay descriptions and disability weights for EMBID are shown below.

Severity level	Lay description	DW (95% CI)
Asymptomatic endocrine, metabolic, blood, and immune disorders	--	--
Mild endocrine, metabolic, blood, and immune disorders	Has low energy and feels cold.	0.019 (0.01–0.032)
Moderate endocrine, metabolic, blood, and immune disorders	Feels nervous, has palpitations, sweats a lot, and has difficulty sleeping.	0.145 (0.096–0.202)
Severe endocrine, metabolic, blood, and immune disorders	Easily bruises and sometimes bleeds from the gums and nose; feels weak and has some difficulty with daily activities.	0.159 (0.106–0.226)

The severity distribution of EMBID was derived from analysis of the Medical Expenditure Panel Surveys (MEPS). MEPS is an overlapping panel survey of the non-institutionalized US population that collects data on respondents' health service interactions. Panels are initiated every year. Each panel is two years long and consists of five rounds. In 2000, MEPS began using 12-Item Short Form Surveys (SF-12) to collect data on functional health status. The SF-12 survey is administered twice per panel (about once per year).

In order to translate SF-12 scores into GBD disability weights, 62 lay descriptions for conditions representing the full range of disability weight values (from most mild to most severe) were selected. A convenience sample of respondents was then asked to complete an SF-12 form for an individual with the health state described in the lay descriptions of these conditions. Composite mental and physical SF-12 score was regressed on GBD disability weight to derive the relationship between disability weight and SF-12 score. Individual respondent scores were then regressed on reported conditions to obtain a comorbidity-corrected condition-specific disability weight. The distribution of these condition-specific

weights was used derive the proportion of individuals with the conditions that fall within each GBD severity category.

Severity	Distribution
Asymptomatic endocrine, metabolic, blood, and immune disorders	0.410 (0.398, 0.423)
Mild endocrine, metabolic, blood, and immune disorders	0.387 (0.328, 0.430)
Moderate endocrine, metabolic, blood, and immune disorders	0.061 (0.042, 0.060)
Severe endocrine, metabolic, blood, and immune disorders	0.142 (0.115, 0.173)

Modelling strategy

We ran a DisMod MR-2.1 model to produce estimates by age, sex, year, and location. Prior settings in the EMBID DisMod model include an upper bound on remission of 0.25, corresponding to a maximum duration of four years. We used the function in DisMod-MR 2.1 to pull in cause-specific mortality rate (CSMR) data from our CODEm and CODcorrect analyses and match them with incidence data points for the same geography and study year to estimate priors on excess mortality rates (by dividing CSMR by incidence).

We included the covariate Lagged Distributed Income (LDI) as a country-level covariate to inform excess mortality, with bounds of -0.5, -0.1.

Compared to GBD 2015, major changes include inclusion of inpatient data from a greater number of geographies and placing bounds on remission to reflect the chronic nature of many conditions within the endocrine, metabolic, blood, and immune disorders cause grouping.

Rheumatoid arthritis

Flowchart

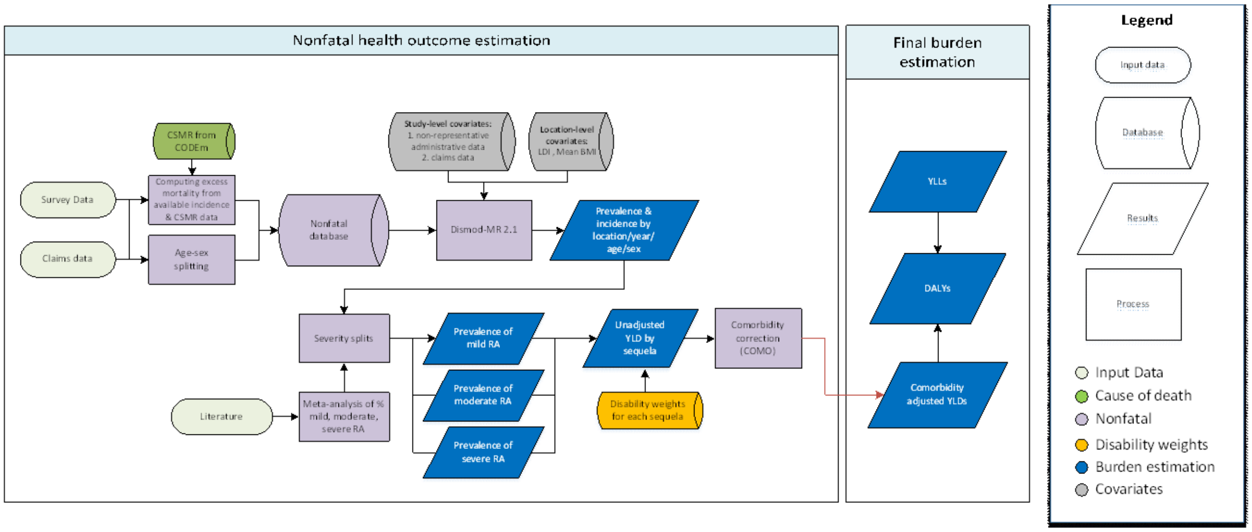

Case definition

Rheumatoid arthritis (RA) is a systemic autoimmune disorder that causes pain and swelling of the joints. While RA is known to affect internal organs in addition to the joints, these extra-articular effects are not factored into the disability weights (DW) used in GBD. The reference case definition for rheumatoid arthritis is based on the 1987 criteria by the American College of Rheumatology (ACR 1987)[1] which stipulate seven diagnostic criteria, of which four need to be satisfied for a diagnosis. Criteria 1 through 4 must have been present for at least six weeks (see table below). For RA, ICD-10 codes are M05, M06, and M08 and ICD-9 codes are 714.0–714.9.

Input data

Model inputs

For GBD 2010, a systematic review of the prevalence of RA throughout the world was conducted. Ovid MEDLINE, EMBASE, CINAHL, CAB abstracts, WHOLIS, and SIGLE databases were searched using the following search terms: (rheumatoid arthritis OR rheumatic disease* OR rheumatism) AND (prevalen* OR inciden* OR cross-sectional OR cross sectional OR epidemiol* OR survey OR population-based OR population based OR population study OR population sample OR cohort OR follow-up OR follow up OR longitudinal OR regist* OR data collection).

The exclusion criteria were:

48. Studies clearly not representative of the national population
49. Studies that were not population-based, eg, hospital or clinic-based studies
50. Studies that did not provide primary data on epidemiological parameters, eg, a commentary piece

51. Studies of a specific type of RA, eg, seropositive RA
52. Studies with a sample size of less than 150
53. Reviews

The most recent PubMed search was conducted in GBD 2013 using the above search terms. Opportunistically, we added scientific literatures and population surveys encountered for GBD 2015 and GBD 2016. The table below shows the number of literature studies included in GBD 2016, as well as the number of countries or subnational units and GBD world regions represented.

	Prevalence	Incidence	Mortality risk
Studies	68	25	25
Countries/subnationals	91	16	16
GBD world regions	16	6	3

In addition, data from US claims data for 2000, 2010, and 2012 by US state were included. We decided not to use hospital inpatient data as we considered they would not be representative of true prevalence and that variation between countries in the proportion of true prevalent cases captured in hospital inpatient data systems would likely vary more than can be captured by a single crosswalk in DisMod-MR 2.1. We compared the rates of RA in the outpatient data from Norway, Sweden, Canada, and the USA and found implausibly large differences with the rates from the claims data. The US outpatient rates were half the value of the claims data and those for the other countries much lower still. For those reasons we decided not to use the outpatient data.

Severity splits

The basis of the GBD disability weight survey assessments are lay descriptions of sequelae highlighting major functional consequences and symptoms. The lay descriptions and disability weights for RA severity levels are shown below.

Severity level	Lay description	DW (95% CI)
Mild	This person has moderate pain and stiffness in the arms and hands which causes difficulty lifting, carrying, and holding things, and trouble sleeping because of the pain.	0.117 (0.080–0.163)
Moderate	This person has pain and deformity in most joints, causing difficulty moving around, getting up and down, and using the hands for lifting and carrying. The person often feels fatigue.	0.317 (0.216–0.440)
Severe	This person has severe, constant pain, and deformity in most joints, causing difficulty moving around, getting up and down, eating, dressing, lifting, carrying, and using the hands. The person often feels sadness, anxiety, and extreme fatigue.	0.581 (0.403–0.739)

To determine the proportion of people with RA within each of the severity levels, seven studies from three regions provided information on the severity of RA. Severity was classified according to Health Assessment Questionnaire scores, with the cutoff scores for each severity level: <1 mild; 1–1.875

moderate; and ≥2 severe. Estimates were pooled across studies. We used a random effects meta-analysis model. The pooled percentages were mild 48.8% (37.9–59.6), moderate 37.6% (29.3–46.2) and severe 12.2% (7.8–17.4). After streaming out 1,000 draws assuming a binomial distribution, percentages were scaled to sum to 1 at each draw.

Modelling strategy

Prior settings in the DisMod model included setting remission to 0.009-0.021, and it was assumed that there was no incidence or prevalence of RA before the age of 5 years.

Data from all sources were re-extracted to better reflect the range of case definitions. We set the American College of Rheumatology (ACR) 1987 criteria[1] as the reference. We marked studies using the Rome 1961,[2] American Rheumatology Association (ARA) 1958,[3] or European League against Rheumatism (EULAR)[4] criteria with a single study covariate "non-ACR_1987" as there were inadequate studies with each alternative classification system to do separate crosswalks.

Additional study covariates were created for studies using administrative health system data sources; for studies covering regional rather than (sub)-nationally representative populations; and for claims data.

We used the function in DisMod-MR 2.1 to pull in cause-specific mortality rate (CSMR) data from our CODEm and CODcorrect analyses and match it with prevalence data points for the same geography and study year to estimate priors on excess mortality rates (by dividing CSMR by prevalence). In GBD 2016, CSMR data include the age groups 80-84, 85-89, 90-94, and 95+ years.

We retained just two RA diagnosis criteria and three years of claims data as x-cov (ie, based on a significant coefficient indicating evidence of a systematic bias). Betas and exponentiated values (which can be interpreted as an odds ratio) for these covariates are shown in the table below:

Study covariate	Parameter	beta	Exponentiated beta
RA criteria other than ACR 1987	Prevalence	-0.81	0.45 (0.39 – 0.52)
RA diagnosis from admin data	Prevalence	-0.041	0.96 (0.92 – 1.00)
Claims data - 2000	Prevalence	-0.26	0.77 (0.75 – 0.80)
Claims data - 2010	Prevalence	-0.03	0.97 (0.95 – 0.99)
Claims data - 2012	Prevalence	-0.0028	1.00 (0.99 – 1.00)

The non-representative covariate were used as z-cov, meaning that DisMod estimates a value that gets added to the standard deviation of data points to reflect that these were not estimated according to our reference case definition/study method.

References

1 Arnett FC, Edworthy SM, Bloch DA, *et al.* The American Rheumatism Association 1987 revised criteria for the classification of rheumatoid arthritis. *Arthritis Rheum* 1988; **31**: 315–24.

2 Kellgren JH. Diagnostic criteria for population studies. *Bull Rheum Dis* 1962; **13**: 291–2.

3 Bennett GA, Cobb S, Jacox R, Jessar RA, Ropes MW. Proposed diagnostic criteria for rheumatoid arthritis. *Bull Rheum Dis* 1956; **7**: 121–4.

4 Vonkeman HE, van de Laar MAFJ. The new European League Against Rheumatism/American College of Rheumatology diagnostic criteria for rheumatoid arthritis: how are they performing? *Curr Opin Rheumatol* 2013; **25**: 354–9.

Osteoarthritis

Flowchart

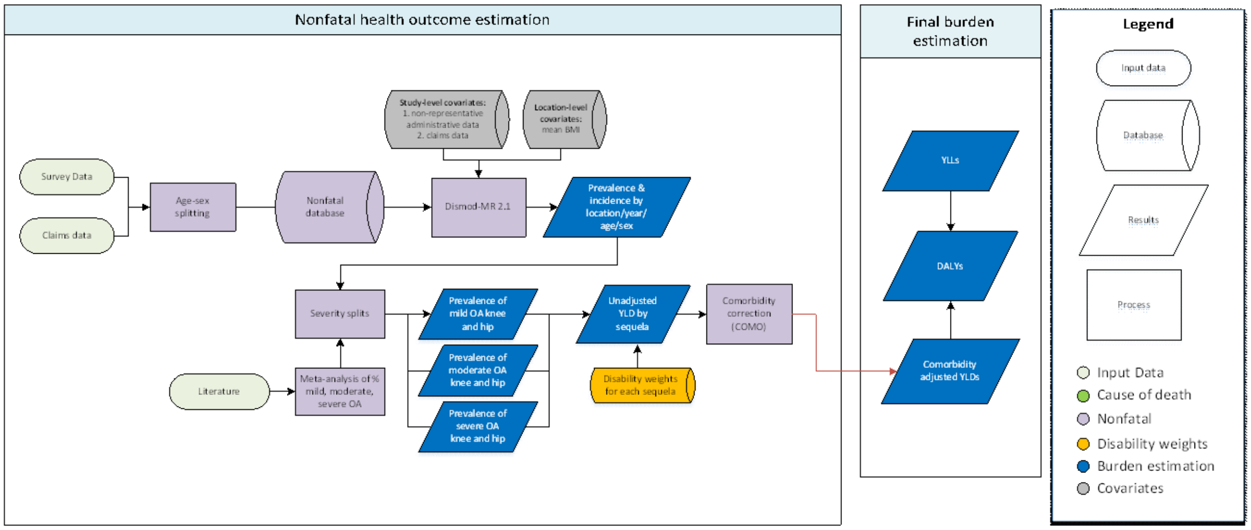

Case definition

The osteoarthritis (OA) reference case definition is symptomatic osteoarthritis of the hip or knee radiologically confirmed as Kellgren-Lawrence grade 2-4. Grade 2 symptomatic requires one defined osteophyte in hip or knee and pain for at least one month out of the last 12. Grade 3-4 symptomatic requires osteophytes and joint space narrowing in hip or knee with deformity also present for grade 4, and pain for at least one month out of the last 12 months.

OA is the most common form of arthritis, involving inflammation and breakdown of joints. For the purposes of OA estimates for this GBD study, only hip and knee sites were reviewed. The hip and knee are the common sites of OA in the larger joints and are considered to produce the greatest disability. Failure of these joints can lead to need for joint replacement surgery, if available, and thus contributes to a significant proportion of the high direct health care costs attributable to arthritis. OA of the spine is also common; however, it was considered that any symptoms and disability related to the cervical and/or lumbar spine would be captured in the estimates of low back pain and neck pain. Hand OA involving the fingers and thumbs is another common site for OA, but as it often overlaps with knee OA and could also be captured in the "Other Musculoskeletal disorders" category, it was not considered as a separate entity in these GBD OA estimates.

ICD-10 codes for OA of the hip and knee are M16 and M17, respectively. The ICD-9 code for OA is 715, without specific codes for hip and knee sites.

Input data

Model inputs

A systematic review of the prevalence, incidence and mortality of OA was performed for the years 1980 to 2009 on MEDLINE, EMBASE, CINAHL, CAB Abstracts, WHO Library (WHOLIS) and OpenSIGLE for GBD2010. For prevalence and incidence, the following search terms were used: (osteoarth* OR gonarthr*) AND (prevalen* OR inciden* OR cross-sectional OR cross sectional OR epidemiol* OR survey OR population-based OR population based OR population study OR population sample OR cohort OR follow-up OR follow up OR longitudinal OR regist*) AND (list of names of all GBD countries). For mortality, the following search terms were used: (osteoarth* OR gonarthr*) AND (Mortality OR death OR standardised mortality ratio OR standardized mortality ratio OR case fatality OR cross-sectional OR cross sectional OR epidemiol* OR survey OR population-based OR population based OR population study OR population sample OR cohort OR follow-up OR follow up OR longitudinal OR regist*) AND (list of names of all GBD countries).

Exclusion criteria were:

54. Sub-populations clearly not representative of the national population
55. Not a population-based study
56. Low sample size (less than 150)
57. Review rather than original studies

The most recent PubMed search was conducted in GBD 2013 using the above search terms. Opportunistically, we added scientific literatures and population surveys encountered during data review for GBD 2015 and GBD 2016. The table below shows the number of literature studies included in GBD 2016, as well as the number of countries or subnational units and GBD world regions represented.

	Prevalence		Incidence		Mortality risk	Severity
	OA hip	OA knee	OA hip	OA knee		
Studies	56	82	5	14	3	4
Countries/subnationals	90	100	5	14	2	3
GBD world regions	9	10	2	3	2	3

In addition, data from US claims data for 2000, 2010, and 2012 by US state were included. We decided not to use hospital inpatient data as we considered it would not be representative of true prevalence, and that variation between countries in the proportion of true prevalent cases captured in hospital inpatient data system would likely vary more than can be captured by a single crosswalk in DisMod-MR 2.1.

Severity splits

The basis of the GBD disability weight survey assessments are lay descriptions of sequelae highlighting major functional consequences and symptoms. The lay descriptions and disability weights for OA severity levels are shown below.

Severity level	Lay description	DW (95% CI)

Mild	This person has pain in the leg, which causes some difficulty running, walking long distances, and getting up and down.	0.023 (0.013–0.037)
Moderate	This person has moderate pain in the leg, which makes the person limp, and causes some difficulty walking, standing, lifting and carrying heavy things, getting up and down, and sleeping.	0.079 (0.054–0.110)
Severe	This person has severe pain in the leg, which makes the person limp and causes a lot of difficulty walking, standing, lifting and carrying heavy things, getting up and down, and sleeping.	0.165 (0.112–0.232)

To determine the proportion of people with OA within each of the severity levels, four studies from three regions provided information on the severity of OA. Severity was classified based on the Western Ontario and McMaster Universities Arthritis Index (WOMAC) with scores 0-5 taken as mild, 6-13 as moderate, and 14 and higher as severe. Estimates were pooled across studies using a random effects meta-analysis model. The pooled percentages were mild 47.0% (42.2–51.9), moderate 35.9% (31.3–40.7), and severe 17.1% (12.9–21.6) pooled between patient and physician ratings in a study from Bangladesh which we apply to low- and middle-income countries. The pooled proportions from three high-income countries were mild 74.3% (64.8–82.7), moderate 24.3% (16.4–33.1), and severe 1.1% (0.6–1.7). After streaming out 1,000 draws assuming a binomial distribution, percentages were scaled to sum to 1 at each draw.

Modelling strategy

Prior settings in the DisMod model included setting remission to 0, and it was assumed that there was no incidence or prevalence of OA before the age of 30 years. We assumed in the final model that excess mortality is zero. While there are some data on excess mortality risk, the values of hazard ratios or standardised mortality ratios are close to one, with some studies reporting mean estimates less than one.

We used study covariates for studies that reported on X-rays only, self-reported OA with pain, reporting a physician diagnosis of OA, and OA with symptoms but no X-ray diagnosis. For each of these covariates we estimated the crosswalk prior to DisMod comparing like geographies which had data according to the reference case definition and the alternative. In DisMod we set bounds with an upper and lower limit. We added covariates for each of three years of claims data in the USA. Betas and exponentiated values (which can be interpreted as an odds ratio) for these covariates are shown in the table below:

Study covariate	Parameter	OA hip		OA knee	
		beta	Exponentiated beta	beta	Exponentiated beta
Self-reported OA with pain	Prevalence	1.25	3.48 (3.03 – 3.96)	0.37	1.45 (1.14 – 1.69)
Reported physician diagnosis OA	Prevalence	0.97	2.64 (2.45 – 2.72)	0.092	1.10 (0.85 – 1.30)
Radiography only	Prevalence	0.95	2.59 (2.33 – 2.71)	0.12	1.13 (0.88 – 1.32)
OA with symptoms but no radiography confirmation	Prevalence	1.18	3.26 (3.01 – 3.77)	0.68	1.97 (1.44 – 2.42)
Claims data - 2000	Prevalence	-0.53	0.59 (0.58 – 0.60)	-1.39	0.40 (0.19 – 0.29)
Claims data - 2010	Prevalence		not significant	-0.91	0.40 (0.29 – 0.47)

Claims data - 2012	Prevalence		not significant	-0.84	0.43 (0.32 – 0.51)
Reported physician diagnosis OA	Incidence	0.91	2.49 (2.24 — 2.71)	0.64	1.89 (0.89 – 2.69)
Radiography only	Incidence	0.91	2.47 (2.24 — 2.71)	0.77	2.17 (1.33 – 2.69)

We added mean body mass index (BMI) and the summary exposure variable (SEV) scalar for OA (ie, a composite measure of the exposure to risks in GBD that affect OA: BMI) as country-level covariates of prevalence measure, and the SEV-OA as a country-level covariate of incidence measure. The coefficient for BMI in OA hip model was 0.029 (exponentiated 1.03; 1.01–1.05) meaning that for every unit increase in mean BMI in the population OA prevalence increases by 3%.

Low back pain (LBP)

Flowchart

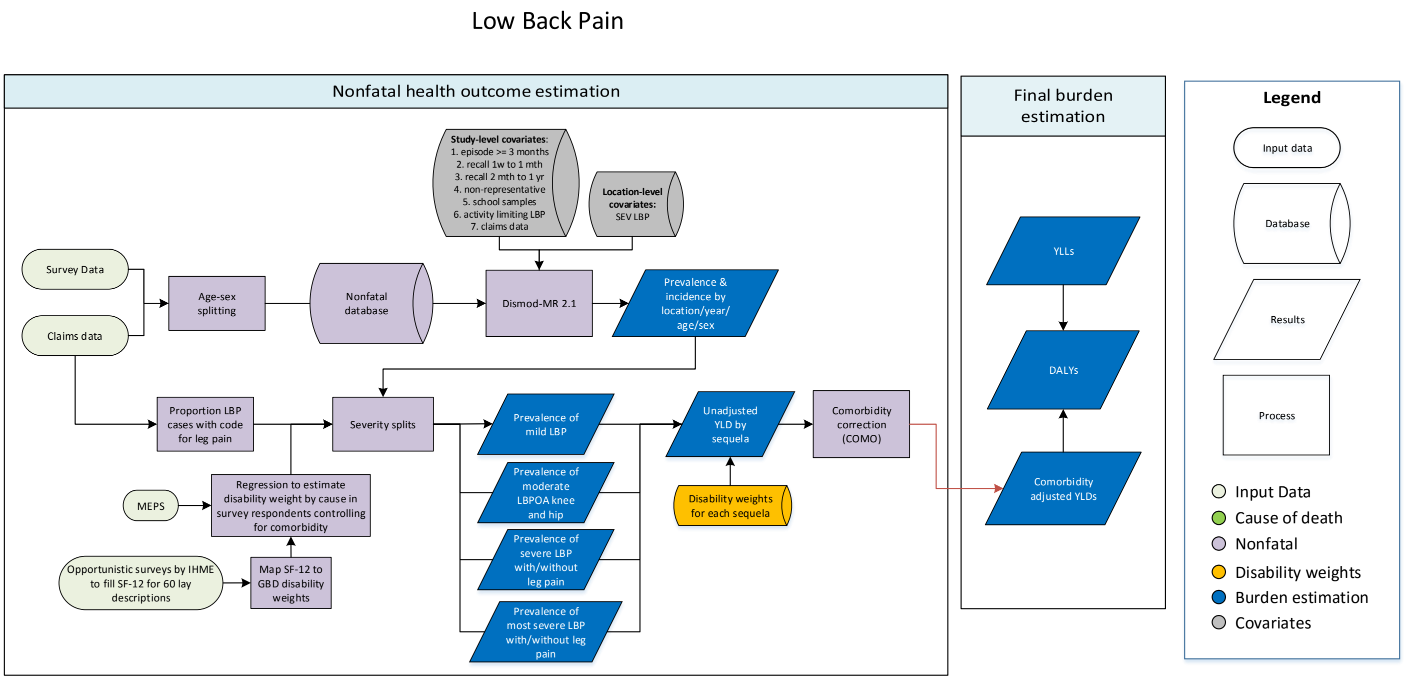

Case definition

Low back pain (LBP) is defined as low back pain (with or without pain referred into one or both lower limbs) that lasts for at least one day. The "low back" is defined as the area on the posterior aspect of the body from the lower margin of the twelfth ribs to the lower gluteal folds.

ICD-10 codes for LBP are M54.3, M54.4 and M54.5. The ICD-9 code is 724.

Input data

Model inputs

Ovid Medline, EMBase, and CINAHL electronic databases were searched for GBD2010. There were no age, sex, or language restrictions. The terms "back pain," "lumbar pain," "back ache," "backache," and "lumbago" were used individually and combined with each of the following: "prevalence," "incidence," "cross-sectional," and "epidemiology." The search was updated for GBD 2015 in PUBMED through to August 2015.

Exclusion criteria were:

58. Sub-populations clearly not representative of the national population

59. Not a population-based study
60. Low sample size (less than 150)
61. Review rather than original studies

Additional information was derived from unit record data of surveys in the GHDx, GBD's repository of population health data including the World Health surveys and national health surveys. The table below shows the number of studies and surveys included in GBD 2016, as well as the number of countries or subnational units and GBD world regions represented.

	Prevalence	Incidence	Remission
Studies/surveys	253	4	4
Countries/subnationals	172	3	2
GBD world regions	20	1	1

In addition, data from US claims data for 2000 and 2010 by US state were included.

Severity splits

The basis of the GBD disability weight survey assessments are lay descriptions of sequelae highlighting major functional consequences and symptoms. The lay descriptions and disability weights for LBP severity levels are shown below.

Severity level	Lay description	DW (95% CI)
Low back pain, mild	This person has mild back pain, which causes some difficulty dressing, standing, and lifting things.	0.020 (0.011–0.035)
Low back pain, moderate	This person has moderate back pain, which causes difficulty dressing, sitting, standing, walking, and lifting things.	0.054 (0.035–0.079)
Low back pain, severe without leg pain	This person has severe back pain, which causes difficulty dressing, sitting, standing, walking, and lifting things. The person sleeps poorly and feels worried.	0.272 (0.182–0.373)
Low back pain, severe with leg pain	This person has severe back and leg pain, which causes difficulty dressing, sitting, standing, walking, and lifting things. The person sleeps poorly and feels worried.	0.325 0.219–0.446)
Low back pain, most severe without leg pain	This person has constant back pain, which causes difficulty dressing, sitting, standing, walking, and lifting things. The person sleeps poorly, is worried, and has lost some enjoyment in life.	0.372 (0.250–0.506)
Low back pain, most severe with leg pain	This person has constant back and leg pain, which causes difficulty dressing, sitting, standing, walking, and lifting things. The person sleeps poorly, is worried, and has lost some enjoyment in life.	0.384 (0.256–0.518)

The severity distributions are derived from an analysis of the Medical Expenditure Panel Surveys (MEPS) in the US. MEPS is an overlapping continuous panel survey of the United States non-institutionalized population whose primary purpose is to collect information on the use and cost of health care. Panels are two years long and are conducted in five rounds, which are conducted every five to six months. A new panel begins annually, while the last panel is in its second year

(http://www.meps.ahrq.gov/survey_comp/hc_data_collection.jsp). Each panel typically contains about 30,000 to 35,000 individual respondents.

MEPS was initiated in 1996 but only began collecting health status data in the form of SF-12 responses in 2000. For GBD 2016 we used data from 2000–2014. Respondents self-administer the SF-12 twice per panel, at rounds 2 and 4, typically about a year apart. Only adults 18 years and older completed the SF-12. MEPS also usually collects information on diagnoses based on self-report of reasons for encounters with health services. In addition, diagnoses are derived through additional questions on "problems that bother you" or conditions that led to "disability days," ie, days out of role due to illness. Professional coders translate the verbatim text into three digit ICD-9 codes. The main reason for LBP being measured in MEPS relates to health care contact. From MEPS, the severity distribution for LBP without leg pain and with leg pain were derived as shown in the below table.

Severity level	Distribution without leg pain	Distribution with leg pain
Low back pain, mild	0.41 (0.31–0.53)	0.27 (0.19–0.37)
Low back pain, moderate	0.35 (0.25–0.44)	0.36 (0.28–0.43)
Low back pain, severe	0.10 (0.08–0.12)	0.14 (0.10–0.16)
Low back pain, most severe	0.14 (0.09–0.20)	0.23 (0.15–0.32)

We used US claims data (2012) to derive the proportion of cases with low back pain who report leg pain. The proportions were different by age group as shown in Figure 1. The proportion in each severity level in each age group is calculated by multiplying the proportion in the severity level and the proportion with or without leg pain.

Figure 1: Proportion of LBP with leg pain

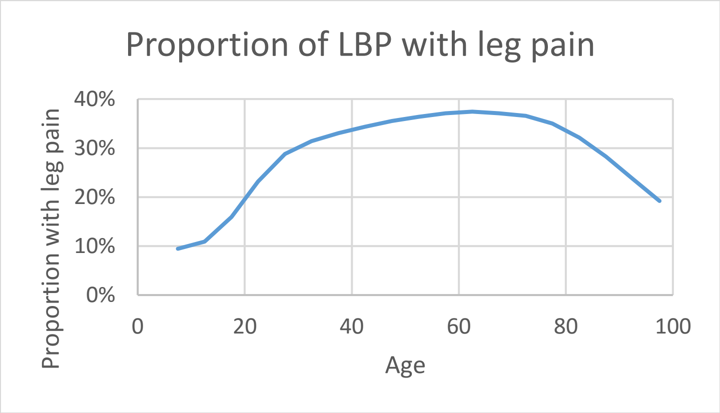

Age (years)	Proportion with leg pain
5–9	9.4 (9.1–9.8) %
10–14	10.9 (10.7–11.1) %
15–19	15.9 (15.8–16.1) %

20–24	23.2 (23.0–23.4) %
25–29	28.8 (28.6–28.9) %
30–34	31.4 (31.3–31.6) %
35–39	33.1 (32.9–33.2) %
40–44	34.3 (34.2–34.4) %
45–49	35.5 (35.4–35.6) %
50–54	36.4 (36.3–36.5) %
55–59	37.1 (37.0–37.2) %
60–64	37.4 (37.3–37.5) %
65–69	37.1 (36.9–37.3) %
70–74	36.5 (36.4–36.7) %
75–79	35.0 (34.8–35.2) %
80–84	32.1 (31.9–32.4) %
85–89	28.3 (28.0–28.5) %
90–94	23.7 (23.2–24.2) %
95–100	19.2 (18.2–20.2) %

Modelling Strategy

Prior settings in the DisMod model included setting excess mortality to 0, and it was assumed that there was no incidence or prevalence of low back pain before the age of 5 years.

We used study covariates for studies that reported a too broad anatomical region, episode duration of greater than three months, recall periods of one week to one month, recall periods between two months and one year, activity-limiting LBP, and studies conducted among schoolchildren or otherwise non-representative samples as studies covering regional rather than (sub)-nationally representative populations. The mean and standard error for the coefficient of the covariate of studies among schoolchildren population were calculated and used for a constraint in DisMod. The other covariates have their coefficients constraint by reasonable ranges in the direction whether they increased or decreased the estimates. We added covariates for each of three years of claims data in the USA. Betas and exponentiated values (which can be interpreted as an odds ratio) for these two covariates are shown in the table below:

Study covariate	Parameter	beta	Exponentiated beta[1]
Anatomical region too broad	Prevalence	0.00049	1.00 (1.00 — 1.00)
episode duration >= 3 months	Prevalence	-0.58	0.56 (0.53 — 0.59)
recall periods of 1 week to 1 month	Prevalence	0.67	1.96 (1.90 – 2.02)
recall periods between 2 months and one year	Prevalence	0.67	1.95 (1.89 – 2.01)
Not representative	Prevalence	0.17	1.19 (1.14 — 1.25)
studies among school children	Prevalence	1.00	2.72 (2.72 – 2.72)
Activity-limiting LBP	Prevalence	-0.82	0.44 (0.42 – 0.46)
Claims data - 2000	Prevalence	-0.55	0.57 (0.56 — 0.59)

Claims data - 2010	Prevalence	-0.098	0.91 (0.89 – 0.92)

[1] interpretation examples: in DisMod-MR 2.1 activity limiting LBP data points showed a systematic bias downward and were adjusted up by dividing by 0.72, while data points of recall greater than two months were adjusted downward dividing by 2.36.

We dropped the covariate for claims data 2012 as it had a non-significant coefficient close to zero. We included the SEV scalar for low back pain as a country covariate. This combines the exposure measures for risks estimated to impinge on LBP in GBD: occupational ergonomic exposure and increased BMI. We set bounds of 0.75 to 1.25 as the SEV is constructed in a way that if our risk estimates are accurate the value should be 1.

Neck pain (NP)

Flowchart

Neck Pain

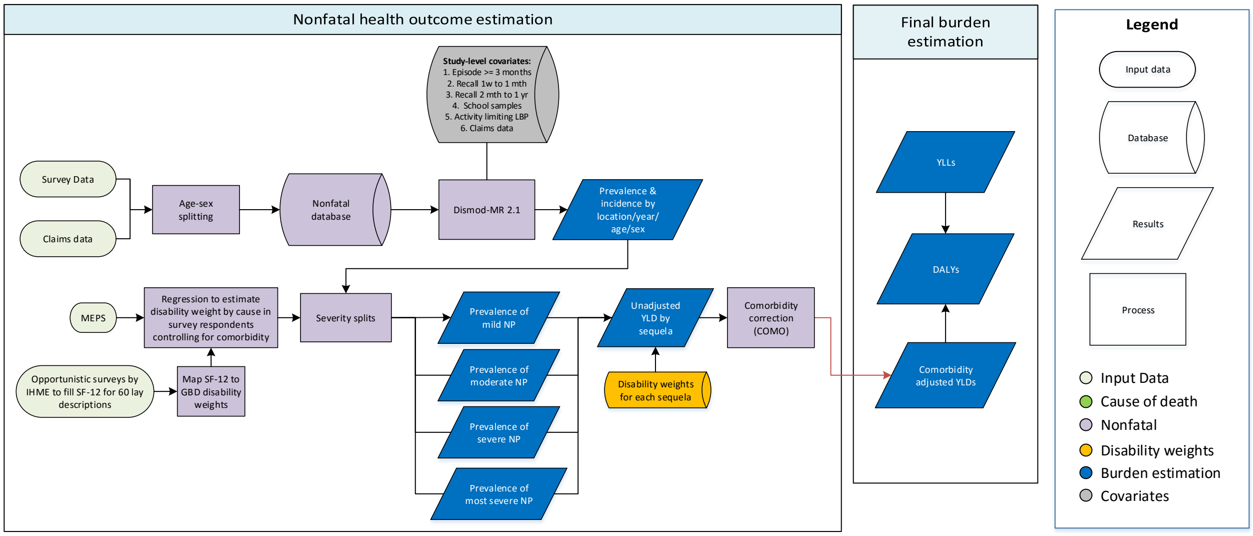

Case definition

Neck pain (NP) was defined as*: neck pain (+/- pain referred into the upper limb(s)) that lasts for at least one day*.

ICD-10 code for neck pain is M54.2. The ICD-9 code is 723.1.

Input data

Model inputs

Ovid MEDLINE, EMBASE, CINAHL, CAB abstracts, WHOLIS, and SIGLE databases were searched for GBD 2010. There were no age, sex, or language restrictions. The terms *neck pain, neck ache, neckache, and cervical pain* individually and combined with each of the following terms*: prevalen*, inciden*, cross-sectional, cross sectional, epidemiol*, survey, population-based, population based, population study, population sample*. The search was updated for GBD 2013 and GBD 2015 in PUBMED through to August 2015.

Exclusion criteria were:

62. Sub-populations clearly not representative of the national population
63. Not a population-based study

64. Studies on a specific type of neck pain (eg, following neck fracture)
65. Low sample size (less than 150)
66. Review rather than original studies

Additional information was derived from unit record data of surveys in GHDx, GBD's repository of population health data including NHANES and NHIS in the USA. The table below shows the number of studies and surveys included in GBD 2016, as well as the number of countries or subnational units and GBD world regions represented.

	Prevalence	Incidence	Remission
Studies/surveys	81	2	1
Countries/subnationals	91	1	1
GBD world regions	11	1	1

In addition, data from US claims data for 2000, 2010, and 2012 by US state were included.

Severity splits

The basis of the GBD disability weight survey assessments are lay descriptions of sequelae highlighting major functional consequences and symptoms. The lay descriptions and disability weights for neck pain severity levels are shown below.

Severity level	Lay description	DW (95% CI)	Proportions
Neck pain, mild	This person has neck pain, and has difficulty turning the head and lifting things	0.052 (0.036–0.074)	0.67 (0.57–0.75)
Neck pain, moderate	This person has constant neck pain, and has difficulty turning the head, holding arms up, and lifting things	0.112 (0.079–0.162)	0.12 (0.08–0.19)
Neck pain, severe	This person has severe neck pain, and difficulty turning the head and lifting things. The person gets headaches and arm pain, sleeps poorly, and feels tired and worried	0.226 (0.147–0.323)	0.06 (0.05–0.07)
Neck pain, most severe	This person has constant neck pain and arm pain, and difficulty turning the head, holding arms up, and lifting things. The person gets headaches, sleeps poorly, and feels tired and worried	0.300 0.199–0.434)	0.15 (0.11–0.20)

The severity distributions are derived from an analysis of the Medical Expenditure Panel Surveys (MEPS) in the US. MEPS is an overlapping continuous panel survey of the United States non-institutionalized population whose primary purpose is to collect information on the use and cost of health care. Panels are two years long and are conducted in five rounds, which are conducted every five to six months. A new panel begins annually, while the last panel is in its second year (http://www.meps.ahrq.gov/survey_comp/hc_data_collection.jsp). Each panel typically contains about 30,000 to 35,000 individual respondents.

MEPS was initiated in 1996 but only began collecting health status data in the form of SF-12 responses in 2000. For GBD 2016 we used data from 2000–2014. Respondents self-administer the SF-12 twice per panel, at rounds two and four, typically about a year apart. Only adults 18 years and older completed the

SF-12. MEPS also usually collects information on diagnoses based on self-report of reasons for encounters with health services. In addition, diagnoses are derived through additional questions on "problems that bother you" or conditions that led to "disability days," ie, days out of role due to illness. Professional coders translate the verbatim text into three-digit ICD-9 codes. The main reason for neck pain being measured in MEPS relates to health care contact.

In order to derive a crosswalk of SF-12 values into a scale comparable with that used by the GBD disability weights, small studies on convenience samples were conducted asking respondents to fill in SF-12 to reflect 62 lay descriptions of diverse severity that were used to derive the GBD disability weights. From these responses a relationship between SF-12 summary score and the GBD DWs was derived. With regression methods, average disability weights were calculated for each of 156 conditions for which there were corresponding diagnoses in MEPS, while controlling for any co-morbid other condition by adding dummy variables for each condition. As our case definition is for point prevalence of neck pain, we ignored the proportion of MEPS respondents with a neck pain diagnosis for whom in our regression we found no disability attributable to neck pain. For the remaining cases we binned the amount of DW attributed to neck pain across the four health states assuming thresholds at the midpoints between DW values.

Modeling strategy

Prior settings in the DisMod model included setting excess mortality to 0, and it was assumed that there was no incidence or prevalence of neck pain before the age of 5 years.

We used study covariates for studies that reported a too broad anatomical region, episode duration of greater than three months, recall periods of one week to one month, recall periods between two months and one year, activity-limiting neck pain, and studies conducted among schoolchildren. We added covariates for each of three years of claims data in the USA.

With exceptions for broad anatomical region, studies among schoolchildren population and activity limiting neck pain whose coefficients were consistent with their reasonable ranges, the means and the upper and lower bounds for the covariates were calculated by crosswalking with NHANES data as a baseline. The table below shows the prior values for those covariates' coefficients.

Study covariate	Lower bound	Mean	Upper bound
Anatomical region too broad	0		2
episode duration >= 3 months	-0.81	-0.74	-0.67
recall periods of 1 week to 1 month	0.67	0.77	0.87
recall periods between 2 months and one year	0.77	0.87	0.97
studies among school children	0		3
Activity-limiting neck pain	-3		0
Claims data - 2000	-2.20	-2.08	-1.96
Claims data - 2010	-1.73	-1.57	-1.41
Claims data - 2012	-1.65	-1.50	-1.35

Betas and exponentiated values (which can be interpreted as an odds ratio) for these two covariates are shown in the table below:

Study covariate	Parameter	beta	Exponentiated beta[1]
Anatomical region too broad	Prevalence	0.52	1.68 (1.53 – 1.84)
episode duration >= 3 months	Prevalence	-0.68	0.51 (0.49 – 0.51)
recall periods of 1 week to 1 month	Prevalence	0.86	2.37 (2.32 – 2.39)
recall periods between 2 months and one year	Prevalence	0.79	2.21 (2.16 – 2.29)
studies among school children	Prevalence	2.53	12.54 (10.64 – 14.94)
Activity-limiting neck pain	Prevalence	-1.42	0.24 (0.19 – 0.31)
Claims data - 2000	Prevalence	-2.19	0.11 (0.11 – 0.11)
Claims data - 2010	Prevalence	-1.60	0.20 (0.20 – 0.21)
Claims data - 2012	Prevalence	-1.51	0.22 (0.22 – 0.23)

[1] interpretation examples: in DisMod-MR 2.1 activity-limiting NP data points showed a systematic bias downward and were adjusted up by dividing by 0.34 while data points of recall greater than 2 months were adjusted downward dividing by 4.63.

Gout

Flowchart

Gout

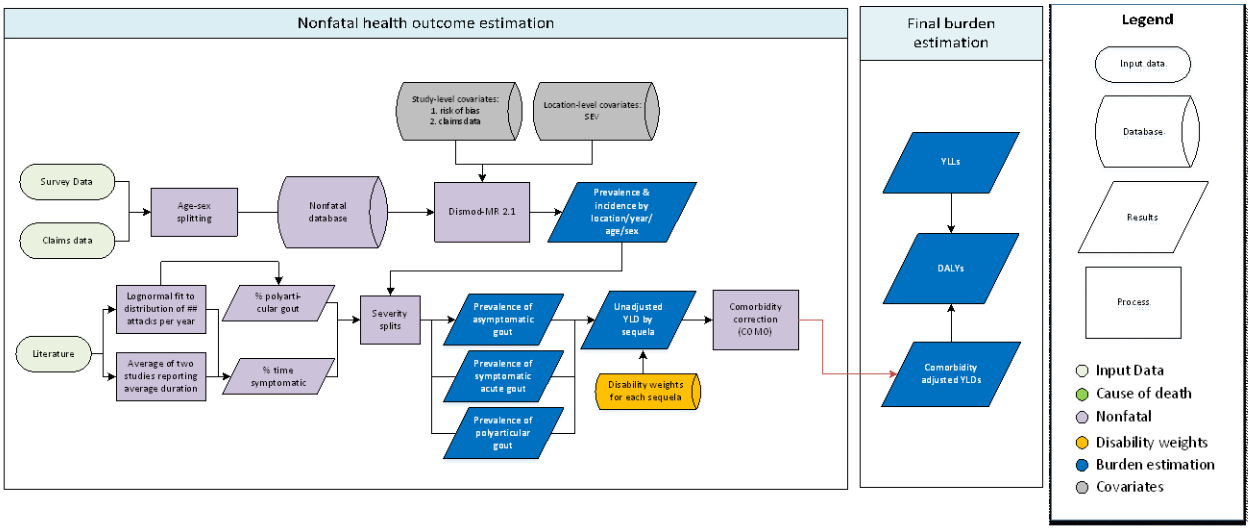

Case definition

Gout is a rheumatic disease that is characterized by formation of monosodium urate (MSU) crystals in the synovial fluid of joints and in other tissues causing inflammation. The crystal formation is caused by elevated urate levels in extracellular fluids. It is more common in men. GBD uses the case definition of primary gout given by the American College of Rheumatology, generally referred to as ARA 1977 survey criteria requiring the presence of MSU crystals in joint fluid or the presence of a tophus proven to contain MSU crystals and at least six of 12 gout symptoms or findings (>1 attack of acute arthritis, development of maximal inflammation within a day, attack of monarticular arthritis, observation of joint erythema, pain or swelling in the first MTP joint, unilaterally attack involving the first MTP joint, unilateral attack involving tarsal joint, suspected tophus, hyperuricemia, asymmetrical swelling within a joint on X-ray and negative culture of joint fluid for microorganisms during attack of joint inflammation) to make a diagnosis. The ICD-10 code for gout is M10 and the ICD9 code is 274.

Input data

Model Inputs

For GBD 2010 literature searches were performed for years 1980 to 2009 on MEDLINE, EMBASE, CINAHL, CAB Abstracts, WHO Library (WHOLIS), and OpenSIGLE. For prevalence and incidence, the following search terms were used: (gout* OR hyperuricemia) AND (prevalen* OR inciden* OR cross-sectional OR cross sectional OR epidemiol* OR survey OR population-based OR population based OR population study OR population sample OR cohort OR follow-up OR follow up OR longitudinal OR regist*) AND (list of names of all GBD countries). For mortality, the following search terms were used: (gout* OR

hyperuricemia) AND (Mortality OR death OR standardised mortality ratio OR standardized mortality ratio OR case fatality OR cross-sectional OR cross sectional OR epidemiol* OR survey OR population-based OR population based OR population study OR population sample OR cohort OR follow-up OR follow up OR longitudinal OR regist*) AND (list of names of all GBD countries).

Exclusion criteria were:

- Sub-populations clearly not representative of the national population
- Not a population-based study
- Low sample size (less than 150)
- Review

The most recent PubMed search was conducted in GBD 2013 using the above search terms. Opportunistically, additional studies encountered during data review were added for GBD 2015. Since we did not add new literature in GBD 2016, the most recent literature source dates from 2014. The table below shows the number of literature studies included in GBD 2016, as well as the number of countries or subnational units and GBD world regions represented.

	Prevalence	Incidence	Mortality risk
Studies	73	11	3
Countries/subnationals	90	7	2
GBD world regions	12	4	1

In addition, data from US claims data for 2000, 2010, and 2012 by state were included.

Severity splits

The basis of the GBD disability weight survey assessments are lay descriptions of sequelae highlighting major functional consequences and symptoms. The lay descriptions and disability weights for gout severity levels are shown below.

Severity level	Lay description	DW (95% CI)
Gout, acute	This person has severe pain and swelling in the leg, making it very difficult to get up and down, stand, walk, lift, and carry heavy things. The person has trouble sleeping because of the pain.	0.295 (0.196–0.409)
Polyarticular gout (same as for severe RA)	This person has severe, constant pain and deformity in most joints, causing difficulty moving around, getting up and down, eating, dressing, lifting, carrying, and using the hands. The person often feels sadness, anxiety, and extreme fatigue.	0.581 (0.403–0.739)

We used three studies on the distribution of the number of gout attacks per year and fitted a lognormal curve using a least squared differences method. In the absence of data on the proportion of gout cases who have chronic polyarticular gout, we assumed the proportion is equal to those who would have 52 attacks a year (ie, weekly) or more as implied by the lognormal curve.

The average number of attacks was estimated from the lognormal fit: 5.66 (5.14–6.18). From two studies we derived an average duration of attacks of 6.1 (5.4–6.8) days by simple averaging. The resulting proportion of time symptomatic for acute gout was taken as the multiplication of these two estimates divided by the number of days in a year: 9.4% (8.0–10.9%).

Figure 1: Distribution of cases by frequency

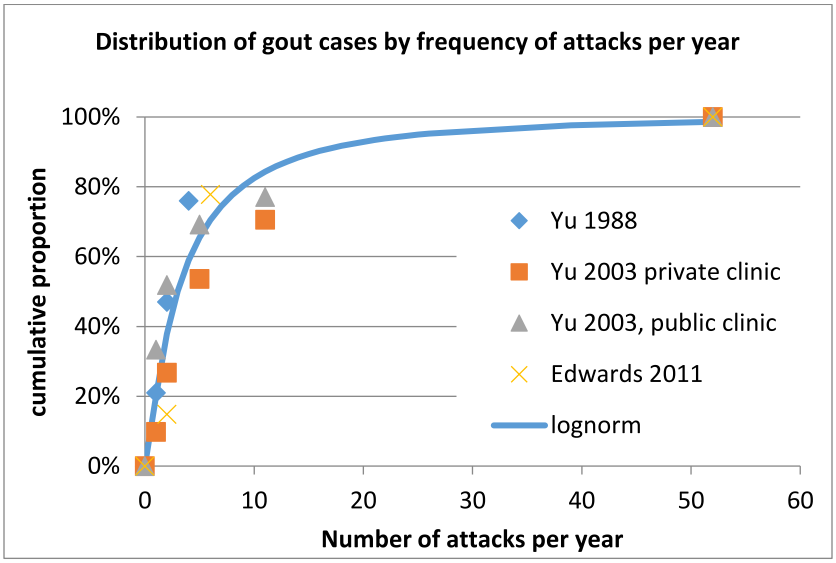

Modelling strategy

Initially we set remission to zero but found this made incidence and prevalence inconsistent. As the ratio of prevalence and incidence in similar locations was in the order of 10:1 we decided to allow remission to range between 0 and 0.2 and that made incidence and prevalence more consistent. We assumed that there was no incidence or prevalence of gout before the age of 15 years.

We used a covariate for the 2000 US claims data but assumed the latter two years reflect the reference case definition. For studies relying on self-reported diagnoses or not stating diagnostic criteria were flagged with a covariate for a risk of bias. However, the risk of bias covariate was found to be insignificant; thus, it was used as a covariate that affects only uncertainty but not mean estimates.

Betas and exponentiated values (which can be interpreted as an odds ratio) for the claims covariate are shown in the table below:

Study covariate	Parameter	beta	Exponentiated beta
Claims data – 2000	Prevalence	-0.66	0.52 (0.50–0.53)

We added the Summary Exposure Variable (SEV) scalar for gout which summarizes exposure to risks estimated in GBD to impinge on gout, ie, low glomerular filtration rate as a country covariate. We set bounds of 0.75 to 1.25 as the SEV is constructed in a way that if our risk estimates are accurate the value should be 1.

Other musculoskeletal disorders (MSK)

Flowchart

Other musculoskeletal disorders

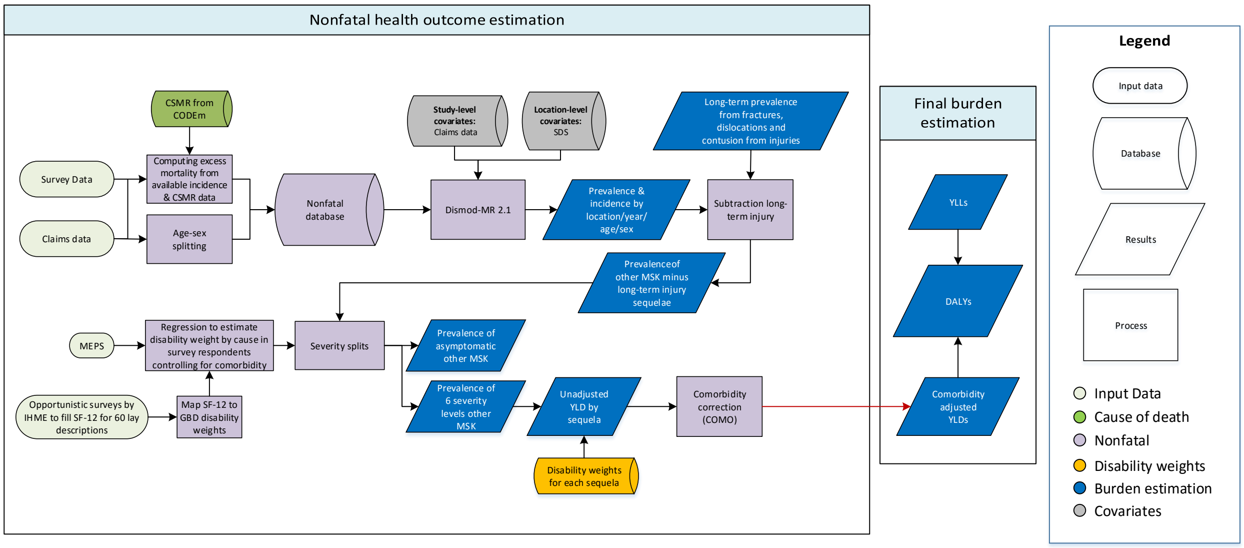

Case definition

Other musculoskeletal (MSK) disorders is a heterogeneous rest category comprising a wide range of disorders of muscles, bones, and ligaments that are not included in the five GBD defined musculoskeletal diseases rheumatoid arthritis, osteoarthritis, low back and neck pain, and gout, and are not captured as long-term sequelae of injuries.

The table below provides detail of the ICD-10 and ICD-9 codes included in this category.

ICD-10 codes	ICD-9 codes
L93—Lupus erythematosus	710.0
M00-M02—Infectious arthropathies	711
M08, M11-M13—Inflammatory polyarthropathies	712–713
M20-M25—Other joint disorders	716–719
M30-M35—Systemic connective tissue disorders	710.1-710.9
M40-M43—Deforming dorsopathies	737
M45-M46—Spondylopathies	720–721
M60 -M63—Disorders of muscles	725
M65-M68—Disorders of synovium and tendon	726–728
M70- M73, M75-M79—Other soft tissue disorders	729
M80-M85—Disorders of bone density and structure	733.0-2
M86—Osteomyelitis	730.1-730.3, 730.7-9
M87-M90—Other osteopathies	731, 733.3-9

M91-M94—Chondropathies<br>M95-M99—Other disorders of the MSK system and connective tissue	732<br>734–736, 738–739

Input data

Model Inputs

The above ICD codes were used to extract other MSK prevalence from US claims data for 2000, 2010, and 2012 by US state. The systematic review concentrated on finding health surveys that measured an overall amount of musculoskeletal disorders and complaints and reported information to distinguish a rest category that was not OA, RA, gout, or low back or neck pain. These data sources are based on self-reported musculoskeletal conditions or symptoms and not on the listed ICD codes.

The table below shows the number of studies and surveys included in GBD 2016, as well as the number of countries or subnational units and GBD world regions represented.

	Prevalence	Incidence	Remission
Studies/surveys	45	2	1
Countries/subnationals	70	1	1
GBD world regions	11	1	1

Severity splits

The basis of the GBD disability weight survey assessments are lay descriptions of sequelae highlighting major functional consequences and symptoms. The lay descriptions and disability weights for other MSK severity levels are shown below. They include the three levels of health states that are used for osteoarthritis and rheumatoid arthritis, each.

Severity level	Lay description	DW (95% CI)	Proportions
Asymptomatic			0.28 (0.27–0.29)
Musculoskeletal problems, lower limbs, mild	This person has pain in the leg, which causes some difficulty running, walking long distances, and getting up and down.	0.023 (0.013–0.040)	0.22 (0.15–0.30)
Musculoskeletal problems, upper limbs, mild	This person has mild pain and stiffness in the arms and hands. The person has some difficulty lifting, carrying, and holding things.	0.028 (0.017–0.046)	0.20 (0.15–0.29)
Musculoskeletal problems, upper limbs, moderate	This person has moderate pain and stiffness in the arms and hands, which causes difficulty lifting, carrying, and holding things, and trouble sleeping because of the pain.	0.115 (0.079–0.163)	0.10 (0.06–0.15)

Musculoskeletal problems, lower limbs, severe	This person has severe pain in the leg, which makes the person limp and causes a lot of difficulty walking, standing, lifting and carrying heavy things, getting up and down, and sleeping.	0.163 0.109–0.224	0.06 (0.04–0.07)
Musculoskeletal problems, generalized, moderate	This person has pain and deformity in most joints, causing difficulty moving around, getting up and down, and using the hands for lifting and carrying. The person often feels fatigue.	0.312 (0.201–0.438)	0.07 (0.06–0.08)
Musculoskeletal problems, generalized, severe	This person has severe, constant pain and deformity in most joints, causing difficulty moving around, getting up and down, eating, dressing, lifting, carrying, and using the hands. The person often feels sadness, anxiety, and extreme fatigue.	0.572 (0.370–0.758)	0.07 (0.07–0.08)

The severity distributions are derived from an analysis of the Medical Expenditure Panel Surveys (MEPS) in the US. MEPS is an overlapping continuous panel survey of the United States non-institutionalized population whose primary purpose is to collect information on the use and cost of health care. Panels are two years long and are conducted in five rounds, which are conducted every five to six months. A new panel begins annually, while the last panel is in its second year (http://www.meps.ahrq.gov/survey_comp/hc_data_collection.jsp). Each panel typically contains about 30,000 to 35,000 individual respondents.

MEPS was initiated in 1996, but only began collecting health status data in the form of 12-Item Short Form Survey (SF-12) responses in 2000. For GBD 2016 we used data from 2000–2014. Respondents self-administer the SF-12 twice per panel, at rounds two and four, typically about a year apart. Only adults 18 years and older completed the SF-12. MEPS also usually collects information on diagnoses based on self-report of reasons for encounters with health services. In addition, diagnoses are derived through additional questions on “problems that bother you” or conditions that led to “disability days,” ie, days out of role due to illness. Professional coders translate the verbatim text into three-digit ICD-9 codes. The main reason for other MSK being measured in MEPS relates to health care contact.

In order to derive a crosswalk of SF-12 values into a scale comparable with that used by the GBD disability weights, small studies on convenience samples were conducted asking respondents to fill in SF-12 to reflect 62 lay descriptions of diverse severity that were used to derive the GBD disability weights. From these responses a relationship between SF-12 summary score and the GBD DWs was derived. With regression methods, average disability weights were calculated for each of 156 conditions for which there were corresponding diagnoses in MEPS, while controlling for any comorbid other condition by adding dummy variables for each condition. We binned the amount of DW attributed to neck pain across the seven health states assuming thresholds at the midpoints between DW values.

Modeling Strategy

Prior settings in the DisMod model included setting excess mortality to 0, and it was assumed that there was no incidence or prevalence of other MSK before the age of 10 years. In the absence of any meaningful data on incidence and remission for such a heterogeneous category of disorders, we made a rather arbitrary decision of remission 0.5–1, ie, an average duration of 1-2 years. We included cause-specific mortality rate (CSMR) data for other MSK and estimated priors on excess mortality rate by dividing all prevalence data points by the corresponding CSMR. In GBD 2016, new CSMR data include older age group, ie, 80-85, 85-90, 90-95, and 95+ years, in addition to 80+ years in GBD 2015.

We used study covariates for each of the three years of claims data in the USA. Betas and exponentiated values (which can be interpreted as an odds ratio) for these two covariates are shown in the table below:

Study covariate	Parameter	beta	Exponentiated beta[1]
Claims data - 2000	Prevalence	0.54	1.72 (1.66 — 1.81)
Claims data - 2010	Prevalence	0.87	2.40 (2.31 — 2.53)
Claims data - 2012	Prevalence	0.91	2.49 (2.40 — 2.62)

[1] interpretation examples: in DisMod-MR 2.1 2012 claims data are reduced by a factor 2.71.

We allow positive coefficients on claims data as all our other data sources are based on other MSK disorders in the absence of low back pain, neck pain, OA, RA, and gout, while claims data reflect the one-year prevalence of having an ICD-coded other MSK condition mentioned. As there are multiple other MSK conditions that last less than a year, it is not surprising to find higher one-year prevalence in claims data then the point prevalence estimates derived from surveys.

We use the GBD sociodemographic scalar variable as a covariate with a small coefficient of 0.17 estimated by DisMod-MR 2.1.

In order to avoid double counting, we subtract the long-term sequelae of fractures, dislocations, and contusions due to injuries from other MSK, as the surveys from which we derive prevalence estimates make no distinction between cases with other MSK problems that are or are not due to injuries.

Congenital Anomalies

This write-up covers the following causes: congenital heart defects, neural tube defects, cleft lip and cleft palate, congenital anomalies of the urogenital system, congenital anomalies of the gastrointestinal tract, musculoskeletal congenital anomalies, Down Syndrome, Turner Syndrome, Klinefelter Syndrome, and other chromosomal abnormalities, genetic syndromes and micro-deletions

Flowchart

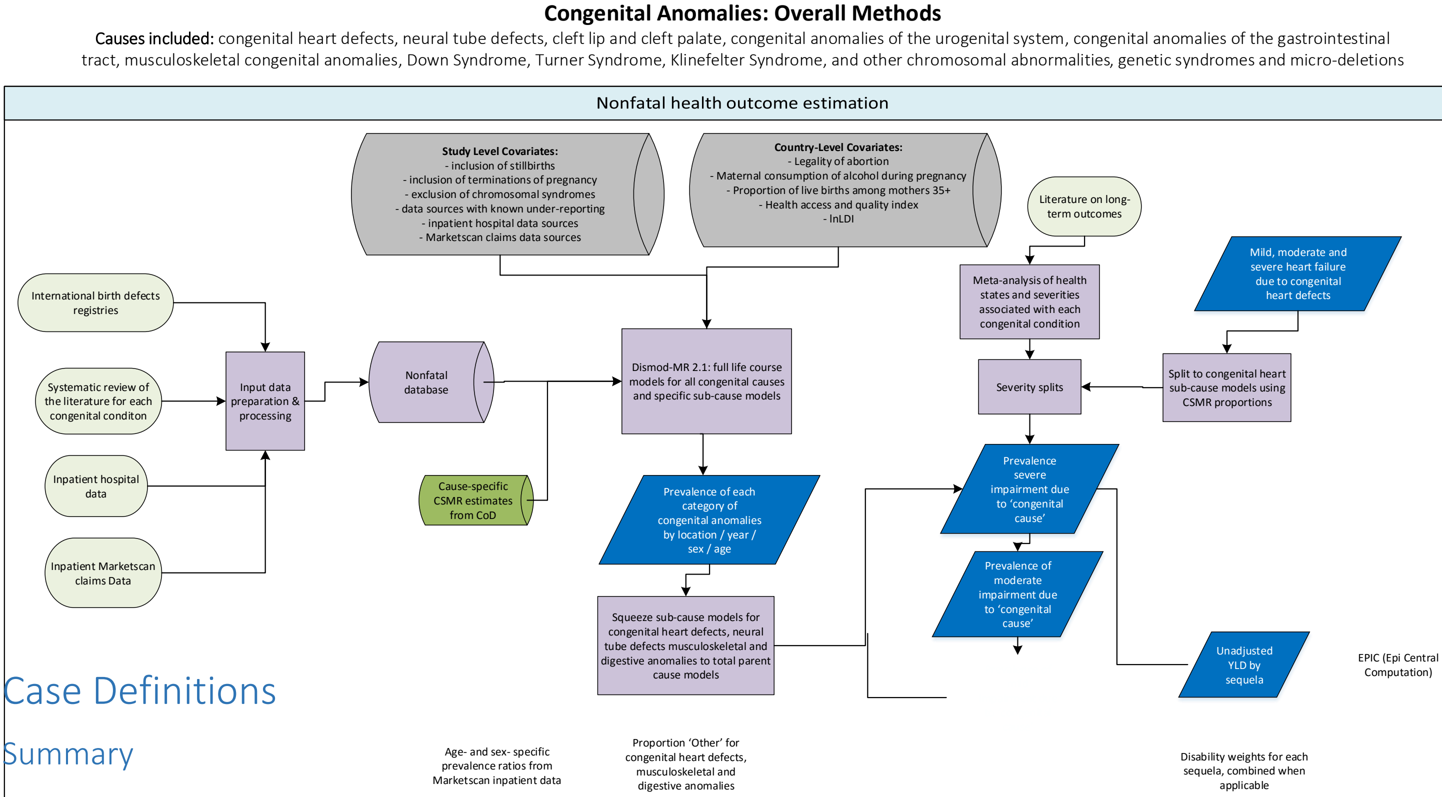

Case Definitions

Summary

The GBD case definition of congenital anomalies includes any condition present at birth that is a result of abnormalities of embryonic development, excluding those that are directly the result of infections or substance abuse (e.g. fetal alcohol syndrome, congenital syphilis) and excludes minor anomalies as they are defined by EUROCAT. Further, our GBD case definition includes only live births and excludes all terminations of pregnancy following prenatal diagnosis and stillbirths.

We have estimated the prevalence and associated disability of the following categories of congenital birth defects

1. Neural tube defects
   a. Anencephaly
   b. Encephalocele
   c. Spina bifida
2. Congenital heart defects
   a. Single ventricle and single ventricle pathway defects

b. Severe congenital heart defects excluding single ventricle and single ventricle pathway defects
c. Critical malformations of great vessels, congenital valvular heart disease and patent ductus arteriosis
d. Ventricular septal defect and atrial septal defect
e. Other congenital cardiovascular anomalies

3. **Orofacial clefts: Cleft lip and cleft palate**
4. **Down Syndrome**
5. **Turner Syndrome**
6. **Klinefelter Syndrome**
7. **Other chromosomal abnormalities, genetic syndromes, and micro-deletions**
    a. Edwards Syndrome and Patau Syndrome
8. **Congenital anomalies of the urogenital system**
    a. Congenital urinary anomalies
    b. Congenital genital anomalies
9. **Congenital anomalies of the digestive system**
    a. Congenital diaphragmatic hernia
    b. Congenital malformations of the abdominal wall
    c. Congenital atresia and/or stenosis of the gastrointestinal tract
    d. Other congenital malformations of the gastrointestinal tract
10. **Musculoskeletal congenital anomalies**
    a. Polydactyly and syndactyly
    b. Limb reduction defects
    c. Other musculoskeletal congenital anomalies
11. **Other congenital anomalies: all birth defects (excluding minor anomalies) not contained in the other categories.**

Urogenital, musculoskeletal, and gastrointestinal are all new causes estimated for the first time in GBD 2016. Previously all were included in the "other congenital anomalies" category.

This appendix will first describe the input data sources and aspects of the modeling strategy that are common to all sub-types of congenital anomalies. We will then provide a description of the case definitions, ICD-10 codes, and health states associated with each of the component congenital causes, as well as the specific modeling strategies employed in each congenital cause, including the model settings, study-level and country-level covariates, and other modeling decisions made.

Input Data Sources

Several types of data sources are used in the estimation of congenital anomalies: literature prevalence, with-condition mortality and excess mortality data, birth prevalence and neonatal with-condition mortality data from a number of international birth defects registries and surveillance systems, inpatient hospital and Marketscan claims data prepared internally by the GBD research team, and cause-specific mortality estimates produced by the GBD 2016 causes of death analysis.

We conducted a systematic review of the available literature for all types of congenital anomalies by constructing search strings designed to capture information on the prevalence, associated mortality and long-term health outcomes associated with each sub-category of congenital anomalies. All results were screened – first abstracts, then full-text screenings – to ensure the availability of required information and the representativeness of the reported population, and the exclusion of duplicate data also reported as part of the birth registry data inputs.

We extracted data from a number of international birth defects registries. The International Clearinghouse for Birth Defects Surveillance and Research (ICBDSR) reports birth prevalence from a number of international member registries. The World Atlas Report also published birth prevalence estimates from these international registries prior to the publication of ICBDSR reports. The European Surveillance of Congenital Anomalies (EUROCAT) reports the birth prevalence of anomalies as for a variety of locations in Western Europe as reported by participating member registries. China's Maternal and Child Health Surveillance survey (MCHS) reports birth prevalence and early neonatal mortality data for all subnational locations of China. The National Birth Defects Prevention Network (NBDPN) reports birth prevalence estimates as compiled by a number of subnational registries within the United States. The Birth Defects Registry of India (BDRI) reports congenital anomalies from participating hospitals within India.

Inpatient hospital and Marketscan claims data for all congenital anomalies causes and sub-cause models was prepped centrally by the GBD research team. The inpatient hospital data was adjusted for multiple inpatient visits by individuals and for the correction of primary diagnoses to all diagnoses including the congenital ICD codes. For more information on the preparation of these data sources, see elsewhere in this appendix.

Modeling Strategy: Overview

All available input data was utilized in a series DisMod-MR models in order to estimate the prevalence of each category of congenital anomalies across the full life course for each location/age/sex combination. Incidence was set to 0 for all congenital models, as congenital conditions occur at the time of birth and by definition there are no incident cases after birth. Remission was allowed only in the models of a select subset of causes for which surgical intervention or spontaneous remission can completely eliminate the disability due to that congenital condition: namely, cleft lip and/or palate, polydactyly and syndactyly, and ventricular septal defect and atrial septal defect. Cause-specific priors and slope priors were used to guide biologically plausible DisMod-MR estimates of excess mortality and remission where applicable. For a subset of conditions, a decreasing slope prior on excess mortality rate was applied to capture the highest risk of mortality from congenital conditions in the neonatal age groups and a subsequent decreasing risk of mortality from congenital conditions later in life. Excess mortality was set to zero after 70 years of age in all models, in keeping with the GBD cause of death estimates for all congenital causes.

For each of congenital heart, musculoskeletal and gastrointestinal anomalies, we used as DisMod-MR model to estimate the total prevalence of all conditions within that cause category. We then squeezed the sum of the specific sub-cause prevalence estimates to these total prevalence estimates in order to ensure internal consistency of our cause-level and sub-cause estimates. We used age- and sex-specific prevalence ratios derived from the Marketscan inpatient claims data to split off a proportion of other heart, musculoskeletal, and gastrointestinal anomalies from the total estimates for each cause.

Study-level Covariates

A number of the input data sources used for the estimation of congenital birth defects are known to have biases leading to under-reporting or over-reporting relative to the true prevalence of congenital anomalies among live births and all subsequent age groups. We used study-level covariates in the each of the DisMod-MR models to adjust for these under-reporting and over-reporting biases.

Where necessary, we used a study-level covariate to adjust for the inclusion of stillbirths in the reported birth prevalence estimates in literature and registry data sources, as stillbirths are not included in our

case definition of prevalence among live births. We also used a study-level covariate to adjust for data sources that included terminations of pregnancy in their birth prevalence estimates. In all models except for the chromosomal conditions, we also used a study-level covariate to adjust for under-reporting in data sources that were extracted to exclude co-occurring chromosomal conditions from the reported prevalence estimates.

For a subset of congenital causes, particularly the congenital heart defects, we noted substantial differences in the lists of case definitions being reported to the various congenital registries. Across all types of congenital heart defects, the National Birth Defects Prevention Network (NBDPN) had the most complete list of reported case definitions – ie, the highest case ascertainment – and was considered the gold standard among all birth registry data sources. We used registry-specific crosswalks to adjust all other birth defects registries to match the case ascertainment seen in the NBDPN.

We also included a series of study-level covariates to adjust for under-reporting in the inpatient hospital data and Marketscan claims data. This included one study-level covariate used specifically to adjust the 2010 inpatient Marketscan data and another used specifically to adjust the 2012 Marketscan data to the reference literature and registry data sources.

Country-level Covariates

Country-level covariates were used in each of the congenital DisMod-MR models based on literature information about the risk factors for these birth defects. Folic acid availability was used as a covariate on prevalence for all neural tube defects models and a subset of the congenital musculoskeletal anomalies models. The legality of abortion was used as a covariate on prevalence for conditions in which prenatal diagnosis is commonly available and the prognosis is severe enough to cause high rate of termination of pregnancy following prenatal diagnosis: these include all chromosomal conditions and a subset of the congenital heart defects. Maternal consumption of alcohol during pregnancy, as a proportion of all pregnancies, was used as a covariate on prevalence for all congenital heart defects. The proportion of live births by mothers age 35+ was used as a covariate on all chromosomal models.

Across many of the congenital models, the Health Access and Quality Index covariate was used to guide the global pattern of with-condition mortality and excess mortality, as was the natural log of the lag-distributed income per capita (lnLDI). For most of the severe congenital conditions, the mortality associated with the condition is highly dependent on access to adequate surgical interventions and other medical care during the first hours, weeks, and years of life.

Assigning health states and sequelae for long-term outcomes

To determine the distribution of health outcomes associated with the congenital causes, we performed a review of available literature on the long-term health outcomes of survivors in cohorts born with each type of congenital malformation. For conditions requiring surgical intervention shortly after birth to ensure survival, the health states included in the disability weight calculations correspond to the post-surgery outcomes reported in cohorts of individuals born with these life-threatening congenital conditions. Where data was available on from multiple cohorts, we pooled these cohorts together to calculate the proportion of individuals with each health state. Where data on the joint distribution of the long-term health outcomes was not available, we assumed independence of each long-term health outcome. Combined disability weights were calculated for all necessary combinations of existing disability weights.

Case definitions and modeling strategy specifics

Neural tube defects

Case definitions

Neural tube defects occur when neural tube fails to close completely during development. The GBD 2016 case definition includes spina bifida, in which part of the spinal cord and/or meninges are uncovered by skin; encephalocele, a congenital defect characterized by sac-like protrusions of the brain and meninges through openings in the skull; and anencephaly, the absence of a major portion of the brain, skull, and scalp. Spina bifida occulta, a much less severe form of spina bifida where the defect in vertebral column remains covered by skin, is excluded from the GBD case definition of spina bifida. All infants born with anencephaly die during the first few weeks of life, as there is no remission and no cure for this condition. Infants born with spina bifida or encephalocele typically require surgical intervention during the first few weeks of life, and thereafter may experience a range of neural and motor complications. Our case definitions of spina bifida and encephalocele do not consider surgical intervention for either condition as remission. In GBD 2016, spina bifida, encephalocele, and anencephaly are each modeled separately and then fit to a total model of all neural tube defects. Spina bifida corresponds to the ICD-10 codes Q05.0, Q05.4,Q05.6, Q05.7, Q05.8, and Q05.9. Encephalocele corresponds to the ICD-10 codes Q01.2, Q01.8, and Q01.9. Anencephaly corresponds to the ICD-10 codes Q00.0 and Q00.2.

Health states associated with neural tube defects

All infants with anencephaly are assigned the health state of severe motor and cognitive impairment. Cases of spina bifida and encephalocele are split into every combination of mild, moderate and severe motor impairment, all severities of intellectual disability, and urinary incontinence. These proportions were calculated using a pooled analysis of available literature on the long-term outcomes in cohorts of individuals born with each sub-type of neural tube defects. The distribution of health states associated with encephalocele (references: NIDs 292406, 292320, and 292322) was derived separately from the distribution of health states associated with spina bifida (references: NIDs 292324 and 292327), although these two categories of neural tube defects are associated with the same list of long-term outcome sequela.

Neural tube defects overall modeling strategy and model settings

In order to ensure internal consistency of the estimates of each sub-type of neural tube defects, we developed a model of the total prevalence of neural tube defects and used these location, year, sex and age-specific prevalence estimates to scale the estimates of anencepaly, encephalocele and spina bifida prevalence. This modeling strategy allowed us to incorporate the cause-specific mortality estimates form the GBD 2016 Cause of Death analysis and also allowed us to use literature data where the prevalence and mortality estimates were reported for the total of all neural tube defects only.

The DisMod-MR model of total neural tube defects used cause-specific mortality (CSMR) estimates from the GBD cause of death analysis for neural tube defects. This model had a minimum excess mortality of 3.0 for the first week of age and a decreasing slope prior on excess mortality rate for all ages as the risk of death due to neural tube defects is greatest shortly after birth. The model also used an increased smoothness (maximum xi=1) on excess mortality rate in order to allow high excess mortality in the early neonatal age group. Random effects on prevalence were limited to +- 0.5 in order to limit geographic variation in the estimated birth prevalence.

Anencephaly modeling strategy and model settings

The life expectancy for infants born with anencephaly is on the order of hours or days; none of these infants survive past the neonatal age period. Because of the extremely high excess mortality associated with this condition and the short age range over which the prevalence varies, we used a custom modeling process to estimate the prevalence of anencephaly. We first used DisMod-MR to model the prevalence of anencephaly at birth for every location, year, age and sex combination. We then used literature data on outcomes largest available cohort of infants born with anencephaly (references: NID 294812 and NID 296668)), using the precise time of death information from this cohort to create a life table that applied the high excess mortality rates to all cases of anencephaly at birth.

We applied these mortality rates to both sex and all locations, generating the time lived by infants with anencephaly during the early and late neonatal age groups by location, year and sex. We then used GBB 2016 mortality estimates to calculate the time lived by all infants during the early and late neonatal age groups by location, year and sex, and used these two values to calculate the prevalence of anencephaly in the early and late neonatal age groups; after one month of age, all available literature indicates that no infants born with anencephaly are still alive.

The DisMod-MR model for the birth prevalence of anencephaly has random effects on prevalence limited to +- 1. As this model was designed to estimate only the prevalence at birth, incidence, remission and excess mortality were set to zero for all ages, and the only age mesh points were 0 and 100 years of age.

Encephalocele modeling strategy and model settings

The DisMod-MR model for encephalocele had a minimum excess mortality of 5.0 for the first week of age and a decreasing slope prior on excess mortality rate for all ages. The model also used an increased smoothness on excess mortality rate (maximum xi=1). Random effects on prevalence were limited to +- 0.5 as we expect limited geographic variation in the birth prevalence of encephalocele.

Spina bifida modeling strategy and model settings

The DisMod-MR model for spina bifida had a minimum excess mortality of 3.0 for the first week of age, a decreasing slope prior on excess mortality rate for all ages, and a maximum smoothness on excess mortality rate of xi=1. Random effects on prevalence were also limited to +- 0.5.

Covariates used in the DisMod-MR total neural tube defects model

Covariate name	Type	Measure	Beta value	Exponentiated value
Folic acid unadjusted (ug)	Country covariate	Prevalence	-0.000 (-0.000 - -0.000)	1.000 (1.000 - 1.000)
LDI (I$ per capita)	Country covariate	Excess mortality rate	-0.101 (-0.104 - -0.100)	0.904 (0.901 - 0.905)
All MarketScan, year 2010	Study-level x-covariate	Prevalence	-1.040 (-1.108 - -0.975)	0.353 (0.330 - 0.377)

All MarketScan, year 2012	Study-level x-covariate	Prevalence	-1.023 (-1.096 - -0.958)	0.359 (0.334 - 0.384)
Chromosomal diagnoses excluded	Study-level x-covariate	Prevalence	-0.298 (-0.300 - -0.292)	0.742 (0.741 - 0.746)
Hospital data for ages over 1 year only	Study-level x-covariate	Prevalence	-0.252 (-0.256 - -0.250)	0.778 (0.774 - 0.779)
Hospital data for the under-1 year age group	Study-level x-covariate	Prevalence	0.249 (0.247 - 0.250)	1.283 (1.280 - 1.284)
Stillbirths included as cases	Study-level x-covariate	Prevalence	0.298 (0.296 - 0.300)	1.348 (1.345 - 1.349)
Terminations of pregnancy included as cases	Study-level x-covariate	Prevalence	0.008 (0.000 - 0.028)	1.008 (1.000 - 1.028)
Under Reported	Study-level x-covariate	Prevalence	-0.003 (-0.013 - -0.000)	0.997 (0.987 - 1.000)

- Covariates used in the DisMod-MR birth prevalence model of anencephaly

**Covariate name**	**Type**	**Measure**	**Beta value**	**Exponentiated value**
Folic acid unadjusted (ug)	Country covariate	Prevalence	-0.004 (-0.048 - -0.000)	0.996 (0.954 - 1.000)
Legality of Abortion	Country covariate	Prevalence	-0.014 (-0.018 - -0.000)	0.986 (0.982 - 1.000)
Chromosomal diagnoses excluded	Study-level x-covariate	Prevalence	-0.156 (-0.254 - -0.000)	0.855 (0.776 - 1.000)
Hospital Inpatient	Study-level x-covariate	Prevalence	-0.420 (-0.583 - -0.001)	0.657 (0.558 - 0.999)
Stillbirths included as cases	Study-level x-covariate	Prevalence	0.797 (0.251 - 0.942)	2.220 (1.286 - 2.564)
Terminations of pregnancy included as cases	Study-level x-covariate	Prevalence	0.184 (0.001 - 0.378)	1.203 (1.001 - 1.459)
Folic acid unadjusted (ug)	Country covariate	Prevalence	-0.004 (-0.048 - -0.000)	0.996 (0.954 - 1.000)

Covariates used in the DisMod-MR model of encephalocele

**Covariate name**	**Type**	**Measure**	**Beta value**	**Exponentiated value**
Folic acid unadjusted (ug)	Country covariate	Prevalence	-0.002 (-0.037 - -0.000)	0.998 (0.963 - 1.000)
Healthcare access and quality index	Country covariate	With-condition mortality rate	-0.005 (-0.018 - -0.000)	0.995 (0.982 - 1.000)
LDI (I$ per capita)	Country covariate	Excess mortality rate	-0.626 (-0.996 - -0.250)	0.535 (0.370 - 0.779)

Legality of Abortion	Country covariate	Prevalence	-0.008 (-0.011 - -0.000)	0.992 (0.989 - 1.000)
All MarketScan, year 2010	Study-level x-covariate	Prevalence	-0.712 (-0.877 - -0.130)	0.491 (0.416 - 0.878)
All MarketScan, year 2012	Study-level x-covariate	Prevalence	-0.681 (-0.844 - -0.118)	0.506 (0.430 - 0.888)
Chromosomal diagnoses excluded	Study-level x-covariate	Prevalence	-0.284 (-0.300 - -0.004)	0.752 (0.741 - 0.996)
Hospital data for ages over 1 year only	Study-level x-covariate	Prevalence	-0.089 (-0.166 - -0.002)	0.915 (0.847 - 0.998)
Hospital data for the under-1 year age group	Study-level x-covariate	Prevalence	-0.024 (-0.285 - -0.000)	0.976 (0.752 - 1.000)
Stillbirths included as cases	Study-level x-covariate	Prevalence	0.267 (0.005 - 0.299)	1.306 (1.005 - 1.349)
Terminations of pregnancy included as cases	Study-level x-covariate	Prevalence	0.110 (0.005 - 0.246)	1.116 (1.005 - 1.279)

Covariates used in the DisMod-MR model of spina bifida

Covariate name	Type	Measure	Beta value	Exponentiated value
Folic acid unadjusted (ug)	Country covariate	Prevalence	-0.002 (-0.029 - -0.000)	0.998 (0.971 - 1.000)
Healthcare access and quality index	Country covariate	With-condition mortality rate	-0.013 (-0.059 - -0.001)	0.987 (0.942 - 0.999)
LDI (I$ per capita)	Country covariate	Excess mortality rate	-0.625 (-1.000 - -0.250)	0.535 (0.368 - 0.779)
Legality of Abortion	Country covariate	Prevalence	-0.007 (-0.009 - -0.000)	0.993 (0.991 - 1.000)
All MarketScan, year 2010	Study-level x-covariate	Prevalence	-1.114 (-1.249 - -0.880)	0.328 (0.287 - 0.415)
All MarketScan, year 2012	Study-level x-covariate	Prevalence	-1.081 (-1.211 - -0.869)	0.339 (0.298 - 0.419)
Chromosomal diagnoses excluded	Study-level x-covariate	Prevalence	-0.287 (-0.300 - -0.000)	0.750 (0.741 - 1.000)
Hospital data for ages over 1 year only	Study-level x-covariate	Prevalence	-0.006 (-0.019 - -0.000)	0.994 (0.981 - 1.000)
Hospital data for the under-1 year age group	Study-level x-covariate	Prevalence	-0.004 (-0.012 - -0.000)	0.996 (0.988 - 1.000)
Stillbirths included as cases	Study-level x-covariate	Prevalence	0.011 (0.001 - 0.039)	1.011 (1.001 - 1.040)

-

Congenital heart anomalies

Case definitions

There are many distinct types of congenital heart anomalies with a range of anatomical patterns, severities, and requirements for medical treatment. For the purposes of estimating nonfatal outcomes, in GBD 2016 congenital heart anomalies were split into five-sub categories based on both the anatomical characteristics and the treatment requirements of each condition.

The first sub-cause category, single ventricle and single ventricle pathway defects include tricuspid atresia, hypoplastic left heart syndrome, mitral valve atresia, single left ventricle, double outlet right ventricle, and pulmonary atresia; the corresponding ICD-10 codes are Q20.1, Q20.2, Q20.4, Q22.4, Q22.6 and Q23.4. Each of the single ventricle and single ventricle pathway conditions requires surgical intervention shortly after birth to ensure infant survival.

The second sub-cause category, severe congenital heart defects excluding single ventricle and single ventricle pathway defects, includes common arterial trunk, common truncus, discordant ventriculoaterial connection, transposition of great vessels, atrioventricular septal defect, endocardial cushion defect, Tetralogy of fallot, aortopulmonary septal defect, pulmonary valve atresia, congenital stenosis of aortic valve, and total anomalous pulmonary venous connection. This category of severe congenital heart defects includes ICD-10 codes Q20.0; Q20.3; Q21.2; Q21.3; Q21.4; Q22.0; Q23.0 and Q26.2.

The third sub-cause category is critical malformations of great vessels, congenital valvular heart disease and patent ductus arteriosis. The malformations of vessels and valves in this sub-cause category include Ebstein's anomaly, congenital pulmonary valve stenosis, pulmonary valve insufficiency, other malformations of the pulmonary valve, malformations of the tricuspid valve, tricuspid atresia or stenosis, insufficiency of the aortic valve, mitral stenosis or insufficiency, and other malformations of aortic and mitral valves. Patent ductus arteriosis cases are only included among infants of >37 weeks gestational age, as premature infants often have minor patent ductus arteriosis that closes shortly after birth. The ICD-10 codes corresponding to the critical malformations of great vessels category include Q22.1, Q22.2, Q22.3, Q22.5, Q22.8, Q22.9, Q23.1, Q23.2, Q23.3, Q23.8, Q23, Q25.1, Q25.2, Q25.3, Q25.4, Q25.5, and Q25.0. The majority of these conditions require medical attention shortly within the first few weeks of life.

The fourth sub-cause category, ventricular septal defects and atrial septal defects, includes holes in the walls separating the chambers of the heart. Many of these septal defects close spontaneously, while other require surgical care. The ICD-10 codes corresponding to ventricular septal defect and atrial septal defect are Q21.0 and Q21.1, respectively.

The fifth and final sub-cause category of congenital heart defects is other congenital cardiovascular anomalies, which correspond to ICD-10 codes Q27, Q27.1, Q27.2, Q27.3, Q27.30, Q27.31, Q27.32, Q27.33, Q27.34, Q27.39, Q27.4, Q27.8, Q27.9, Q28, Q28.0, Q28.1, Q28.2, Q28.3, Q28.8 and Q28.9.

Health states associated with congenital heart anomalies

Every case of congenital heart defects was associated with a health state of congenital heart disease, except for a proportion of ventricular and atrial septal defects which are considered asymptomatic. All congenital heart defects cases were split into a proportion without intellectual disability and a proportion with every severity from borderline to profound intellectual disability. The proportion of congenital heart anomalies cases experiencing each severity of intellectual disability were calculated using available literature sources on the prevalence and severity of intellectual disability in congenital heart defect populations (references: NIDs 292230, 292237 and 292240). The proportion of VSD/ASD cases attributed to the asymptomatic category was derived from literature sources on the long-term outcomes of patients

diagnosed with septal defects at birth (references: NIDs 261403, 292273 and 292275). GBD estimates of congenital heart failure were assigned to the congenital heart defect categories according to the proportion of total congenital heart cause-specific mortality assigned to each category of congenital heart defects.

Modeling strategy and model settings

In order to ensure internally consistent estimates of the prevalence of congenital heart anomalies, we developed a model of the total prevalence of congenital heart anomalies and fit the estimates of each sub-type of congenital heart anomalies proportionally to these total envelope estimates. The prevalence estimates of other congenital heart anomalies were derived as proportions of the total congenital heart estimates, using age- and sex-specific proportions from Marketscan claims data.

The National Birth Defects Prevention Network (NBDPN), the United States state-level birth defects reporting system, includes the most comprehensive case reporting of any registry data source. As reported in the tables below, the congenital heart models each used registry-specific study-level covariate crosswalks to adjust input data from all other birth registries upward according to the composition of cases included in the NBDPN. All hospital and Marketscan data were also adjusted using study-level covariates with the NBDPN and literature prevalence values as the reference data.

Total congenital heart anomalies model settings

In the DisMod model of total congenital heart anomalies, random effects on prevalence were limited to +- 0.5 in order to limit geographic variation in the estimates of birth prevalence. The model included a decreasing slope prior on excess mortality rate for all ages, as the risk of death due to congenital heart anomalies is greatest shortly after birth. The minimum excess mortality rate for the neonatal age range was set to 5.0. The smoothness on excess mortality rate was increased to Xi=5.0 in order to allow high excess mortality in the neonatal age groups and lower excess mortality rates in older ages.

During model development, all registry prevalence values below the threshold of 3 per 1,000 were marked with a study-level covariate to indicate under-reporting, regardless of whether a comprehensive list of ICD codes or cases was included in the input data. These under-reported data sources were then adjusted upwards to the reference data.

Single ventricle and single ventricle pathway defects model settings

In the DisMod model of single ventricle and single ventricle pathway heart defects, random effects on prevalence were limited to +- 0.5 in order to limit the estimated geographic variation in birth prevalence. A minimum excess mortality rate of 10.0 was set for the early neonatal period in order to capture the high mortality risk, based on expert priors and a review available literature on the mortality risk among infants born with single ventricle and single ventricle pathway heart defects. The smoothness on excess mortality rate was set to 5.0 in order to fit steep changes in the excess mortality rate during the first weeks of life, and a decreasing slope prior on excess mortality rate was applied to all ages, as the risk of death due to these congenital heart anomalies is greatest shortly after birth and diminishes over the life course.

Severe congenital heart defects excluding single ventricle and single ventricle pathway defects model settings

In the DisMod model of congenital heart defects excluding single ventricle and single ventricle pathway defects, random effects on prevalence were limited to +- 0.5. A minimum excess mortality rate of 5.0 for the early neonatal period was enforced in order to capture the high risk of mortality associated with these conditions, and a decreasing slope prior on excess mortality rate was applied for all ages. The smoothness on excess mortality rate was set to Xi = 1.0 in order to allow the model to fit steep changes in the mortality rate of these conditions in the neonatal age period.

Critical malformations of great vessels, congenital valvular heart disease and patent ductus arteriosis model settings

In the DisMod model of critical malformations of great vessels, congenital valvular heart disease and patent ductus arteriosis, random effects on prevalence were limited to +- 0.5. A minimum excess mortality rate of 5.0 was set for the early neonatal period in order to capture the high mortality risk associated with these conditions. A decreasing slope prior was applied to excess mortality rate for all ages, as the risk of death due to congenital heart anomalies is highest shortly after birth. The smoothness on excess mortality was increased to Xi = 3.0 in order to fit steep changes in the mortality associated with these conditions during and after the neonatal period.

Ventricular septal defects and atrial septal defects model settings

In the DisMod model of ventricular septal defects and atrial septal defects (VSD/ASD), remission was set to zero for all ages. Cases of septal defects that spontaneously close over time were considered as part of the asymptomatic proportion of VSD/ASD rather than remitted cases. Random effects on prevalence were limited to +- 0.3 in order to limit the random geographic variation in the estimated birth prevalence. No minimum excess mortality rate was set in this model, as VSD/ASD cases are not associated with excess mortality rates as high as the other subtypes of congenital heart defects. The smoothness on excess mortality rate was set to Xi=1.0, and a decreasing slope prior was set on excess mortality rate for all ages.

Covariates used in the DisMod-MR model of total congenital heart anomalies

Covariate name	Type	Measure	Beta value	Exponentiated value
Healthcare access and quality index	Country covariate	Prevalence	0.005 (0.003 - 0.011)	1.005 (1.003 - 1.011)
LDI (I$ per capita)	Country covariate	Excess mortality rate	-0.999 (-1.250 - -0.752)	0.368 (0.287 - 0.472)
Maternal alcohol consumption during pregnancy (proportion)	Country covariate	Prevalence	0.479 (0.398 - 0.499)	1.614 (1.489 - 1.647)
Chromosomal diagnoses excluded	Study-level x-covariate	Prevalence	-0.009 (-0.021 - -0.001)	0.991 (0.979 - 0.999)
Hospital data for ages over 1 year only	Study-level x-covariate	Prevalence	-0.000 (-0.002 - -0.000)	1.000 (0.998 - 1.000)
Hospital data for the under-1 year age group	Study-level x-covariate	Prevalence	-0.080 (-0.133 - -0.034)	0.923 (0.876 - 0.967)
ICDBSR to NBDPN registry case composition adjustment	Study-level x-covariate	Prevalence	-1.168 (-1.212 - -1.122)	0.311 (0.298 - 0.326)
Stillbirths included as cases	Study-level x-covariate	Prevalence	0.001 (0.000 - 0.003)	1.001 (1.000 - 1.003)
Terminations of pregnancy included as cases	Study-level x-covariate	Prevalence	0.000 (0.000 - 0.002)	1.000 (1.000 - 1.002)
Under Reported	Study-level x-covariate	Prevalence	-0.910 (-0.942 - -0.873)	0.403 (0.390 - 0.418)
World Atlas to NBDPN registry case composition adjustment	Study-level x-covariate	Prevalence	-1.458 (-1.512 - -1.404)	0.233 (0.220 - 0.246)
Healthcare access and quality index	Country covariate	Prevalence	0.005 (0.003 - 0.011)	1.005 (1.003 - 1.011)
LDI (I$ per capita)	Country covariate	Excess mortality rate	-0.999 (-1.250 - -0.752)	0.368 (0.287 - 0.472)
Maternal alcohol consumption during pregnancy (proportion)	Country covariate	Prevalence	0.479 (0.398 - 0.499)	1.614 (1.489 - 1.647)

Covariates used in the DisMod-MR model of single ventricle and single ventricle pathway defects

Covariate name	Type	Measure	Beta value	Exponentiated value
LDI (I$ per capita)	Country covariate	Excess mortality rate	-0.750 (-1.000 - -0.507)	0.472 (0.368 - 0.602)
Maternal alcohol consumption during pregnancy (proportion)	Country covariate	Prevalence	0.061 (0.001 - 0.204)	1.063 (1.001 - 1.227)

All MarketScan, year 2010	Study-level x-covariate	Prevalence	-0.025 (-0.093 - -0.001)	0.975 (0.912 - 0.999)
All MarketScan, year 2012	Study-level x-covariate	Prevalence	-0.023 (-0.079 - -0.001)	0.977 (0.924 - 0.999)
Chromosomal diagnoses excluded	Study-level x-covariate	Prevalence	-0.084 (-0.191 - -0.002)	0.919 (0.826 - 0.998)
EUROCAT to NBDPN registry case composition adjustment	Study-level x-covariate	Prevalence	-0.989 (-1.000 - -0.953)	0.372 (0.368 - 0.386)
Hospital data for ages over 1 year only	Study-level x-covariate	Prevalence	-0.001 (-0.004 - -0.000)	0.999 (0.996 - 1.000)
Hospital data for the under-1 year age group	Study-level x-covariate	Prevalence	-0.449 (-0.725 - -0.316)	0.638 (0.484 - 0.729)
Stillbirths included as cases	Study-level x-covariate	Prevalence	0.004 (0.000 - 0.012)	1.004 (1.000 - 1.012)
Terminations of pregnancy included as cases	Study-level x-covariate	Prevalence	0.058 (0.003 - 0.146)	1.060 (1.003 - 1.157)
Under Reported	Study-level x-covariate	Prevalence	-0.994 (-1.000 - -0.979)	0.370 (0.368 - 0.376)
Under Reported	Study-level z-covariate	With-condition mortality rate	1.530 (1.256 - 1.856)	4.620 (3.511 - 6.398)

Covariates used in the DisMod-MR model of severe congenital heart defects excluding single ventricle and single ventricle pathway defects

Covariate name	Type	Measure	Beta value	Exponentiated value
Healthcare access and quality index	Country covariate	With-condition mortality rate	-0.019 (-0.056 - -0.001)	0.981 (0.946 - 0.999)
LDI (I$ per capita)	Country covariate	Excess mortality rate	-0.628 (-0.993 - -0.255)	0.534 (0.370 - 0.775)
Maternal alcohol consumption during pregnancy (proportion)	Country covariate	Prevalence	0.121 (0.004 - 0.341)	1.128 (1.004 - 1.406)
Chromosomal diagnoses excluded	Study-level x-covariate	Prevalence	-0.270 (-0.299 - -0.192)	0.764 (0.741 - 0.826)
EUROCAT to NBDPN registry case composition adjustment	Study-level x-covariate	Prevalence	-0.995 (-1.000 - -0.979)	0.370 (0.368 - 0.376)
Hospital data for ages over 1 year only	Study-level x-covariate	Prevalence	-0.001 (-0.006 - -0.000)	0.999 (0.994 - 1.000)
Hospital data for the under-1 year age group	Study-level x-covariate	Prevalence	-0.018 (-0.089 - -0.000)	0.982 (0.915 - 1.000)

ICDBSR to NBDPN registry case composition adjustment	Study-level x-covariate	Prevalence	-0.885 (-0.993 - -0.782)	0.413 (0.371 - 0.458)
Stillbirths included as cases	Study-level x-covariate	Prevalence	0.003 (0.000 - 0.012)	1.003 (1.000 - 1.012)
Terminations of pregnancy included as cases	Study-level x-covariate	Prevalence	0.012 (0.000 - 0.042)	1.012 (1.000 - 1.043)
Under Reported	Study-level x-covariate	Prevalence	-0.844 (-0.962 - -0.749)	0.430 (0.382 - 0.473)
World Atlas to NBDPN registry case composition adjustment	Study-level x-covariate	Prevalence	-0.994 (-1.000 - -0.973)	0.370 (0.368 - 0.378)

Covariates used in the DisMod-MR model of critical malformations of great vessels, congenital valvular heart disease and patent ductus arteriosis

Covariate name	Type	Measure	Beta value	Exponentiated value
Healthcare access and quality index	Country covariate	With-condition mortality rate	-0.035 (-0.075 - -0.002)	0.965 (0.928 - 0.998)
LDI (I$ per capita)	Country covariate	Excess mortality rate	-0.273 (-0.500 - -0.055)	0.761 (0.607 - 0.946)
Maternal alcohol consumption during pregnancy (proportion)	Country covariate	Prevalence	0.310 (0.002 - 0.493)	1.364 (1.002 - 1.637)
All MarketScan, year 2010	Study-level x-covariate	Prevalence	-0.003 (-0.010 - -0.000)	0.997 (0.990 - 1.000)
All MarketScan, year 2012	Study-level x-covariate	Prevalence	-0.006 (-0.030 - -0.000)	0.994 (0.971 - 1.000)
Chromosomal diagnoses excluded	Study-level x-covariate	Prevalence	-0.226 (-0.297 - -0.058)	0.797 (0.743 - 0.944)
EUROCAT to NBDPN registry case composition adjustment	Study-level x-covariate	Prevalence	-0.908 (-0.999 - -0.748)	0.403 (0.368 - 0.473)
Hospital data for ages over 1 year only	Study-level x-covariate	Prevalence	-0.004 (-0.019 - -0.000)	0.996 (0.981 - 1.000)
Hospital data for the under-1 year age group	Study-level x-covariate	Prevalence	-0.075 (-0.625 - -0.001)	0.928 (0.535 - 0.999)
ICDBSR to NBDPN registry case composition adjustment	Study-level x-covariate	Prevalence	-0.997 (-1.000 - -0.989)	0.369 (0.368 - 0.372)
Stillbirths included as cases	Study-level x-covariate	Prevalence	0.002 (0.000 - 0.007)	1.002 (1.000 - 1.007)
Terminations of pregnancy included as cases	Study-level x-covariate	Prevalence	0.039 (0.001 - 0.129)	1.040 (1.001 - 1.138)

Under Reported	Study-level x-covariate	Prevalence	-0.985 (-0.999 - -0.944)	0.373 (0.368 - 0.389)
World Atlas to NBDPN registry case composition adjustment	Study-level x-covariate	Prevalence	-0.993 (-1.000 - -0.970)	0.371 (0.368 - 0.379)
Under Reported	Study-level z-covariate	With-condition mortality rate	1.400 (1.145 - 1.706)	4.056 (3.142 - 5.507)

Covariates used in the DisMod-MR model of ventricular septal defects and atrial septal defects

**Covariate name**	**Type**	**Measure**	**Beta value**	**Exponentiated value**
Healthcare access and quality index	Country covariate	With-condition mortality rate	-0.002 (-0.009 - -0.000)	0.998 (0.991 - 1.000)
LDI (I$ per capita)	Country covariate	Excess mortality rate	-0.025 (-0.050 - 0.000)	0.975 (0.952 - 1.000)
Maternal alcohol consumption during pregnancy (proportion)	Country covariate	Prevalence	0.014 (0.001 - 0.051)	1.014 (1.001 - 1.052)
All MarketScan, year 2010	Study-level x-covariate	Prevalence	-0.065 (-0.115 - -0.019)	0.937 (0.892 - 0.982)
All MarketScan, year 2012	Study-level x-covariate	Prevalence	-0.014 (-0.045 - -0.001)	0.986 (0.956 - 0.999)
Hospital data for ages over 1 year only	Study-level x-covariate	Prevalence	-0.004 (-0.014 - -0.000)	0.996 (0.986 - 1.000)
Hospital data for the under-1 year age group	Study-level x-covariate	Prevalence	-0.005 (-0.017 - -0.000)	0.995 (0.983 - 1.000)
Stillbirths included as cases	Study-level x-covariate	Prevalence	0.017 (0.001 - 0.062)	1.017 (1.001 - 1.064)
Terminations of pregnancy included as cases	Study-level x-covariate	Prevalence	0.012 (0.001 - 0.049)	1.012 (1.001 - 1.050)
Under Reported	Study-level x-covariate	Prevalence	-0.529 (-0.707 - -0.323)	0.589 (0.493 - 0.724)
Healthcare access and quality index	Country covariate	With-condition mortality rate	-0.002 (-0.009 - -0.000)	0.998 (0.991 - 1.000)

Cleft lip & cleft palate (orofacial clefts)

Case definition and associated health states

Orofacial clefts include isolated cleft lip, isolated cleft palate, and combined cleft lip and cleft palate. Cleft lip is an opening in the upper lip that may extend into the nose, and with cleft palate, the roof of the mouth contains an opening into the nose. Both conditions are the result of the tissues of the face not joining properly during development. These conditions can be successfully treated by surgery, which is typically done during the first few months or years of life but may occasionally be completed later in life. The sequelae associated with orofacial clefts are disfigurement level 1, disfigurement level 2, and disfigurement level 2 with speech problems. Additionally, a proportion of the population with orofacial clefts is considered to be asymptomatic. The proportion of cleft cases with associated speech problems was calculated following a review of available literature on orofacial cleft health outcomes (need source(s) from Nick?).

The GBD 2016 case definition of orofacial clefts includes isolated cleft palate, which corresponds to ICD-10 codes Q35.2, Q35.3, Q35.5, Q35.6, Q35.7, Q35.8, and Q35.9, and cleft palate with or without cleft lip, which corresponds to ICD-10 codes Q36.0, Q36.1, Q36.9, Q37.1, Q37.5, Q37.8, and Q37.9.

Modeling strategy and model settings

The DisMod-MR model of orofacial clefts had random effects on prevalence limited to +- 0.5, as we expected limited variation in birth prevalence of orofacial clefts. The model settings allow increased smoothness on both excess mortality rate and remission (maximum Xi = 5.0) in order to fit steep changes in the rates mortality and remission during the first few years of life.

Incidence was set to zero for all ages. Remission was set to zero for the first three months of life, as cleft lip and/or palate are rarely corrected in the first few months of life. A maximum remission of 0.8 was set for ages three months to two years, the age range in which cleft repair is most commonly performed, allowing up to 75% of cleft cases to be repaired between three months and 2 years of age. Remission was bounded from 0 to 0.07 for ages 2 to 5 years, 0 to 0.004 for ages 5 to 20 years, then bounded from 0 to 0.002 for ages 20 to 50 years, and set at 0 for ages 50 years +. These limits on remission reflect our priors that up to 20% of remaining cleft cases are repaired between 2 and 5 years of age, another 5% may be repaired between 5 and 20 years of age, and a maximum 5% of remaining cases are surgically repaired between ages 20 and 50 years.

Priors on excess mortality rate were set at a maximum of 2.5 for the early neonatal period, 0.9 for the late neonatal period, 0.24 for the rest of the first year of life, 0.05 for ages 1 to 5 years, and was set to 0 for ages after 5 years. These limits on excess mortality reflect our priors that up to 5% of individuals with orofacial clefts die in the first week of life, up to 5% die in the following three weeks, up to 20% die in the next 11 months, another maximum of 20% before 5 years of ages, and a maximum of 5% of the remaining individuals die between ages 5 and 10 years.

During model development, all birth registry prevalence values below 2 per 10,000 were excluded as outliers, as these data are considered low enough to indicate severe under-reporting in the input data.

Covariates used in the DisMod-MR model of orofacial clefts

Covariate name	Type	Measure	Beta value	Exponentiated value

Folic acid unadjusted (ug)	Country covariate	Prevalence	-0.000 (-0.000 - -0.000)	1.000 (1.000 - 1.000)
Healthcare access and quality index	Country covariate	Prevalence	-0.006 (-0.008 - -0.005)	0.994 (0.992 - 0.995)
LDI (I$ per capita)	Country covariate	Excess mortality rate	-0.350 (-0.350 - -0.349)	0.705 (0.705 - 0.705)
Chromosomal diagnoses excluded	Study-level x-covariate	Prevalence	-0.202 (-0.237 - -0.167)	0.817 (0.789 - 0.846)
Hospital data for ages over 1 year only	Study-level x-covariate	Prevalence	0.557 (0.505 - 0.607)	1.746 (1.656 - 1.835)
Hospital data for the under-1 year age group	Study-level x-covariate	Prevalence	-0.192 (-0.242 - -0.144)	0.825 (0.785 - 0.866)
Stillbirths included as cases	Study-level x-covariate	Prevalence	0.176 (0.142 - 0.211)	1.192 (1.153 - 1.236)
Under Reported	Study-level x-covariate	Prevalence	-0.519 (-0.592 - -0.454)	0.595 (0.553 - 0.635)

Down Syndrome

Case definition and associated health states

Down syndrome, also known as Trisomy 21, is the presence of a third copy of chromosome 21, typically caused by nondisjunction during the production of gametes. Down syndrome is associated with several specific physical characteristics, including decreased muscle tone, flat facial features, an upward slant to the eyes, abnormally shaped ears, a single deep crease across the center of the palm, folded skin on the inner corners of the eyes, and ability to extend joints beyond the usual, among others. The GBD 2016 case definition of Down syndrome includes ICD-10 codes Q90.0, Q90.1, Q90.2, and Q90.9.

Individuals with Down syndrome may have several combinations of sequelae: those included in the GBD sequelae list are intellectual disability, congenital heart disease, and dementia. The joint distribution of intellectual disability, congenital heart disease, and dementia associated with cases of Down Syndrome was derived from a review of literature on long-term outcomes in cohorts of Down Syndrome individuals. To calculate the severity distribution of intellectual disability due to Down Syndrome, we used literature values for the IQ distribution of individuals with Down Syndrome (reference: NID 149859) and calculated the area under the curve. We obtained age-specific proportions of individuals with Down Syndrome and dementia, and thus global age patterns were modeled to calculate the proportion of the population with each combination of sequelae for each of the following age ranges: 0-44 years, 45-49 years, 50-54 years, 55-69 years, 70-79 years, and 80+ years.

Modeling strategy and model settings

The DisMod-MR model of Down Syndrome excluded all data with a prevalence of zero as outliers, as we expect that these low values are indicative of under-reporting in the data sources. The DisMod model used cause-specific mortality rate data from the corresponding Down Syndrome model in the GBD 2016 Cause of Death analysis, and converted these data to excess mortality rate estimates where matching prevalence data is available. Random effects on prevalence were limited to +- 0.5 and random effects on excess mortality rate are limited to +- 1.0 in order to limit the geographic variation in birth prevalence allowed in the model. The maximum smoothness on excess mortality rate was increased to x= 2.0 in order to fit the observed steep decline in the mortality risk associated with Down Syndrome after the neonatal age range.

Of note, the use of cause-specific mortality data in the nonfatal model of Down Syndrome is a substantial change in the modeling strategy as compared to the previous iterations of the GBD, and results in much better-informed excess mortality estimates driving the Down Syndrome prevalence estimates across the life course.

Covariates used in the DisMod-MR model of Down Syndrome

Covariate name	Type	Measure	Beta value	Exponentiated value
LDI (I$ per capita)	Country covariate	Excess mortality rate	-0.337 (-0.368 - -0.304)	0.714 (0.692 - 0.738)
Legality of Abortion	Country covariate	Prevalence	-0.001 (-0.002 - -0.000)	0.999 (0.998 - 1.000)
Live Births 35+ (proportion)	Country covariate	Prevalence	0.008 (0.001 - 0.025)	1.008 (1.001 - 1.025)

Hospital data for ages over 1 year only	Study-level x-covariate	Prevalence	0.140 (0.107 - 0.182)	1.151 (1.113 - 1.199)
Hospital data for the under-1 year age group	Study-level x-covariate	Prevalence	0.499 (0.497 - 0.500)	1.647 (1.644 - 1.649)
Stillbirths included as cases	Study-level x-covariate	Prevalence	-0.039 (-0.049 - -0.011)	0.962 (0.952 - 0.989)
Terminations of pregnancy included as cases	Study-level x-covariate	Prevalence	-0.033 (-0.050 - 0.006)	0.968 (0.952 - 1.006)
LDI (I$ per capita)	Country covariate	Excess mortality rate	-0.337 (-0.368 - -0.304)	0.714 (0.692 - 0.738)
Legality of Abortion	Country covariate	Prevalence	-0.001 (-0.002 - -0.000)	0.999 (0.998 - 1.000)

Turner Syndrome

Case definitions and associated health states

Turner syndrome, also known as 45 XO, is a condition in which a female is partly or completely missing an X chromosome. Turner syndrome can lead to a variety of medical and developmental problems, including short height, failure to start puberty, infertility, heart defects, learning disabilities, and difficulty with social adjustment. The GBD case definition of Turner syndrome includes ICD-10 codes Q96.0, Q96.3, and Q96.9. The sequelae associated with Turner syndrome are congenital heart disease, infertility, and the combination of both congenital heart disease and infertility; additionally, a subset of individuals with Turner syndrome are asymptomatic. The distribution of these sequelae was determined by a review of existing literature on the long-term health consequences of Turner Syndrome.

Modeling strategy and model settings

One of the known limitations to the use of birth prevalence data on Turner Syndrome is that individuals with Turner Syndrome are commonly diagnosed later in life rather than prenatally or at birth. Thus, we implemented a correction factor to account for under-diagnosis in all birth registry data sources, using available literature on the trends in age pattern of Turner Syndrome diagnosis over time (source: NID 283283); although improvements in diagnoses have occurred over time, only between 15% and 30% of all diagnosed Turner Syndrome cases are diagnosed before one year of age. Additionally, the reported denominators from all birth registries – the number of live births in each registry catchment area – were adjusted to include only female births using the GBD fertility estimates of the age, year, and location-specific proportion of total live births that are female. Furthermore, all prevalence data with values of zero were excluded as outliers, as these low values indicate severe under-reporting in the input data. These modeling strategy changes address known causes of under-reporting of Turner Syndrome in the previous iterations of the GBD and resulted in higher estimates of Turner Syndrome than were reported previously.

The DisMod-MR model of Turner Syndrome had an excess mortality rate capped at 0.1 (slightly higher than the highest available literature estimate of excess mortality rate?). The model did not have a slope prior set on excess mortality rate as the risk of mortality associated with Turner Syndrome is not specific to the neonatal ages. This model also allows an increased maximum smoothness on excess mortality rate and random effects on prevalence limited to +- 0.5 in order to limit random geographic variation in the estimated birth prevalence of Turner Syndrome.

Covariates used in the DisMod-MR model of Turner Syndrome

Covariate name	Type	Measure	Beta value	Exponentiated value
Healthcare access and quality index	Country covariate	Excess mortality rate	-0.122 (-0.250 - 0.000)	0.885 (0.779 - 1.000)
LDI (I$ per capita)	Country covariate	Excess mortality rate	-0.149 (-0.298 - 0.000)	0.861 (0.742 - 1.000)
Live Births 35+ (proportion)	Country covariate	Prevalence	-0.245 (-0.297 - -0.119)	0.782 (0.743 - 0.887)
All MarketScan, year 2010	Study-level x-covariate	Prevalence	-0.958 (-1.061 - -0.856)	0.384 (0.346 - 0.425)

All MarketScan, year 2012	Study-level x-covariate	Prevalence	-0.969 (-1.070 - -0.865)	0.380 (0.343 - 0.421)

Klinefelter Syndrome

Case definitions and associated health states

Klinefelter syndrome, also known as 47 XXY, is a condition in which a male is born with an extra X chromosome in all or some of his cells. We also include other genotypes with supranumary X chromosomes, e.g. XXXY, XXXXY, etc. The primary feature of Klinefelter syndrome is sterility, but it can cause a variety of other conditions, including weaker muscles, increased height, poor coordination abilities, smaller genitals, breast growth, and reduced sexual drive as a result of lower testosterone levels. The GBD 2016 case definition of Klinefelter syndrome includes ICD-10 codes Q98.0, Q98.5, and Q99.8. The sequelae associated with Klinefelter syndrome are borderline intellectual disability, mild intellectual disability, primary infertility, the combination of borderline intellectual disability and infertility, and the combination of mild intellectual disability and infertility. In addition, a subset of individuals with Klinefelter syndrome are asymptomatic. The distribution of these sequelae was determined by a review of existing literature on the long-term health consequences of Turner Syndrome.

Modeling strategy and model settings

As discussed above for Turner Syndrome, one limitation to the use of birth registry data for the estimation of Klinefelter Syndrome is that many individuals with Klinefelter Syndrome are not diagnosed prenatally or at birth. To correct this systematic under-reporting in the birth registry data, we applied a correction factor to all birth registry input data using available literature on the age pattern of Klinefelter Syndrome diagnosis (source: NID 283326). We also adjusted the both-sex live birth denominators provided in registry data using location, age, and year-specific proportions of all live births that were male. Furthermore, all prevalence data with values of zero were excluded as outliers, as these low values indicate severe under-reporting in the input data. These modeling strategy changes address known causes of under-reporting in the previous iterations of the GBD and resulted in higher estimates of Klinefelter Syndrome than were reported previously.

The DisMod-MR model of Klinefelter Syndrome had an excess mortality rate maximum limit of 0.075, allowing the model to fit estimates of excess mortality up to slightly higher than the highest reported literature values. The model did not have a slope prior set on excess mortality and allowed an increased smoothness on excess mortality rate. As with several of the other models of chromosomal conditions, random effects on prevalence were limited to +- 0.5 in order to limit random geographic variation in the estimates of birth prevalence.

Covariates used in the DisMod-MR model of Klinefelter Syndrome

Covariate name	Type	Measure	Beta value	Exponentiated value
LDI (I$ per capita)	Country covariate	Excess mortality rate	-0.150 (-0.300 - 0.000)	0.860 (0.741 - 1.000)

Legality of Abortion	Country covariate	Prevalence	-0.000 (-0.000 - -0.000)	1.000 (1.000 - 1.000)
Live Births 35+ (proportion)	Country covariate	Prevalence	0.266 (0.164 - 0.299)	1.304 (1.178 - 1.349)
All MarketScan, year 2010	Study-level x-covariate	Prevalence	-2.879 (-3.044 - -2.752)	0.056 (0.048 - 0.064)
All MarketScan, year 2012	Study-level x-covariate	Prevalence	-2.848 (-3.010 - -2.727)	0.058 (0.049 - 0.065)
Hospital data for ages over 1 year only	Study-level x-covariate	Prevalence	-1.722 (-1.801 - -1.642)	0.179 (0.165 - 0.194)

Other chromosomal abnormalities, genetic syndromes, and microdeletions

Case definitions and associated health states

Unbalanced chromosomal rearrangements are genetic anomalies that typically occur due to meiotic nondisjunction, when homologous chromosomes do not separate normally in nuclear division during gamete formation. The GBD case definition of other chromosomal rearrangements includes 47,XXX (Triple X syndrome), other meiotic nondisjunction events, other female sex chromosome abnormalities, and other unspecified chromosomal abnormalities. The GBD 2016 case definition corresponds to the ICD-10 codes Q92.0, Q97.0, Q97.8, and Q99.9. Excluded from this definition are the chromosomal abnormalities of Down syndrome, Turner syndrome, Klinefelter syndrome, Edward syndrome and Patau syndrome, which are each modeled separately. The sequelae associated with other chromosomal rearrangements include intellectual disability, intellectual disability with dementia, intellectual disability with congenital heart disease and dementia, and intellectual disability with congenital heart disease. Additionally, a proportion of the individuals with unbalanced chromosomal rearrangements are asymptomatic. In the absence of available literature on the long-term health outcomes among individuals with other chromosomal conditions, the severity distributions associated with Down Syndrome were used for the sequela associated with other chromosomal anomalies.

Edwards Syndrome, also known as Trisomy 18, is the condition in which infants are born with a third copy of chromosome 18. Patau syndrome, also known as Trisomy 13, is the condition in which infants are born with a third copy of chromosome 13. The GBD estimates the combined prevalence of these two conditions in a single model as they present similarly and are associated with similar rates of excess mortality. Infants with Edwards syndrome typically have low birthweights and a range of associated conditions including a small head and jaw, limb abnormalities, and severe intellectual disability. Infants with Patau syndrome have a range of associated defects including musculoskeletal anomalies, developmental abnormalities of the nervous system such as microcephaly, congenital heart defects and severe intellectual disability. The ICD-10 code for Edwards syndrome is Q91.3 and the ICD-10 code for Patau syndrome is Q91.7. In the GBD 2016, all cases of Edwards and Patau syndrome are assigned the sequela of severe motor and cognitive impairment, and a proportion of these cases are also associated with congenital heart disease. The proportion of cases with associated congenital heart disease was 0.775, derived by pooling estimates from available literature on the health states associated with the two trisomies (references: NID 292226 and NID 292228).

Modeling strategy and model settings

The DisMod-MR model of other chromosomal abnormalities, genetic syndromes, and microdeletions was used to estimate the prevalence and mortality among infants with chromosomal conditions that are not explicitly included in any other congenital models. This model used cause-specific mortality estimates from the corresponding model of other chromosomal anomalies in the GBD 2016 Cause of Death analysis; these cause-specific mortality estimates were then converted to excess mortality where overlapping prevalence data were available. Random effects on prevalence were limited to +- 0.5 as limited variation in the birth prevalence of chromosomal anomalies is expected across geographies. The maximum allowed smoothness on excess mortality rate was set to Xi=2.0 in order to fit high mortality rates in the early age groups.

In the DisMod-MR model of Edwards Syndrome and Patau Syndrome, random effects on prevalence were limited to +- 0.5, reflecting the expectation of limited geographic variation in the birth prevalence of Edwards Syndrome and Patau Syndrome. An increasing slope prior was set on excess mortality rate

for all ages, as individuals with these trisomies generally die within the first few years of life. The model allowed a maximum smoothness of Xi = 2.0 in order to fit high excess mortality in the early age groups.

All input data with birth prevalence values of zero were excluded as outliers, as these values represent under-reporting and low case ascertainment in the input data rather than a true lack of these chromosomal conditions in the corresponding locations.

Covariates used in the DisMod-MR model of other chromosomal abnormalities

**Covariate name**	**Type**	**Measure**	**Beta value**	**Exponentiated value**
Healthcare access and quality index	Country covariate	Excess mortality rate	-0.000 (-0.000 - -0.000)	1.000 (1.000 - 1.000)
Legality of Abortion	Country covariate	Prevalence	-0.006 (-0.007 - -0.005)	0.994 (0.993 - 0.995)
Live Births 35+ (proportion)	Country covariate	Prevalence	0.293 (0.279 - 0.299)	1.340 (1.321 - 1.349)
All MarketScan, year 2010	Study-level x-covariate	Prevalence	-0.388 (-0.455 - -0.320)	0.679 (0.635 - 0.726)
All MarketScan, year 2012	Study-level x-covariate	Prevalence	-0.278 (-0.353 - -0.206)	0.757 (0.703 - 0.814)
Hospital data for ages over 1 year only	Study-level x-covariate	Prevalence	-0.528 (-0.561 - -0.490)	0.590 (0.571 - 0.613)
Hospital data for the under-1 year age group	Study-level x-covariate	Prevalence	-0.002 (-0.006 - -0.000)	0.998 (0.994 - 1.000)
Healthcare access and quality index	Country covariate	Excess mortality rate	-0.000 (-0.000 - -0.000)	1.000 (1.000 - 1.000)
Legality of Abortion	Country covariate	Prevalence	-0.006 (-0.007 - -0.005)	0.994 (0.993 - 0.995)
Live Births 35+ (proportion)	Country covariate	Prevalence	0.293 (0.279 - 0.299)	1.340 (1.321 - 1.349)

Covariates used in the DisMod model of Edwards Syndrome and Patau Syndrome

**Covariate name**	**Type**	**Measure**	**Beta value**	**Exponentiated value**
Healthcare access and quality index	Country covariate	Excess mortality rate	-0.124 (-0.250 - -0.004)	0.883 (0.779 - 0.996)
LDI (I$ per capita)	Country covariate	Excess mortality rate	-0.025 (-0.050 - -0.000)	0.975 (0.951 - 1.000)
Legality of Abortion	Country covariate	Prevalence	-0.002 (-0.004 - -0.000)	0.998 (0.996 - 1.000)
Live Births 35+ (proportion)	Country covariate	Prevalence	0.213 (0.034 - 0.298)	1.238 (1.034 - 1.348)
All MarketScan, year 2010	Study-level x-covariate	Prevalence	-2.087 (-2.288 - -1.871)	0.124 (0.101 - 0.154)

All MarketScan, year 2012	Study-level x-covariate	Prevalence	-2.022 (-2.233 - -1.815)	0.132 (0.107 - 0.163)
Hospital data	Study-level x-covariate	Prevalence	-0.003 (-0.010 - -0.001)	0.997 (0.990 - 0.999)
Stillbirths included as cases	Study-level x-covariate	Prevalence	0.012 (0.000 - 0.046)	1.012 (1.000 - 1.047)

Musculoskeletal congenital anomalies

Case definitions and associated health states

The GBD 2016 definition of musculoskeletal congenital anomalies includes any anomalies of the muscles or skeletal system present at birth that are not caused by a defined chromosomal syndrome. Within the range of congenital musculoskeletal anomalies, we explicitly model three sub-categories: polydactyly and syndactyly, limb reduction defects, and all other congenital musculoskeletal anomalies.

Polydactyly is the condition of being born with at least one extra digit on either the hand or the foot, while syndactyly is absence of at least one digit. Our case definition of polydactyly corresponds to ICD-10 code Q69, and syndactyly corresponds to Q70. The sequela associated with all cases of polydactyly and syndactyly is level 1 disfigurement. Limb reduction defects are the condition where a part or all of the arm or limb of a fetus fails to form during development, so that the limb is either reduced from its normal size or missing entirely. The GBD case definition of limb reduction defects corresponds with ICD-10 codes Q71 (all three-digit codes under Q71), Q72 (all three-digit codes), Q73.0, Q73.1 and Q73.8.

The other congenital musculoskeletal anomalies included within the total estimate of congenital musculoskeletal anomalies includes clubfoot, skeletal dysplasias, congenital deformities of the spine, congenital dysplasia of the hip, and other congenital musculoskeletal anomalies. This "other" category corresponds to ICD-10 codes Q65, Q65.0, Q65.00, Q65.01, Q65.02, Q65.1; Q65.2; Q65.8; Q65.81; Q65.82; Q65.89; Q65.9; Q66; Q66.0; Q66.1; Q68; Q68.1; Q68.2; Q68.6; Q68.8; Q74; Q74.1; Q74.2; Q74.3; Q74.9; Q75; Q75.0; Q75.5; Q75.9; Q79.8; Q79.9, Q76; Q76.1; Q76.2; Q76.3; Q76.4; Q76.41; Q76.411; Q76.412; Q76.413; Q76.414; Q76.415; Q76.419; Q76.42; Q76.425; Q76.426; Q76.427; Q76.428; Q76.429; Q76.49; Q76.8; Q76.9, Q77; Q77.0; Q77.1; Q77.2; Q77.3; Q77.4; Q77.5; Q77.6; Q77.7; Q77.8; Q77.9; Q78; Q78.0; Q78.1; Q78.2; Q78.3; Q78.4; Q78.5; Q78.6; Q78.8, and Q78.9.

All cases of polydactyly and syndactyly are assigned the health state of level 1 disfigurement. Remission is allowed in the model of polydactyly and syndactyly, as individuals born with these conditions may have them surgically corrected and are then no longer considered within our case definition. However, remission is not included in the models of limb reduction defects or other congenital musculoskeletal defects, as these conditions typically cannot be fully surgically corrected. All cases of limb reduction defects are associated with level 2 disfigurement. A proportion of limb reduction defect cases are associated with no motor impairment, mild motor impairment with and without pain, and moderate motor impairment with and without pain. The distribution of health states associated with congenital limb reduction was derived from an analysis of available literature on the long-term outcomes among individuals with congenital limb reductions (references: NIDs 292277 and 292279).

In the absence comprehensive literature on the long-term outcomes associated with the category of other congenital musculoskeletal anomalies, prevalence estimates of other congenital musculoskeletal anomalies were assigned health states using the proportions derived for limb reduction defects.

Modeling strategy and model settings

As with other categories of congenital anomalies, a model of total musculoskeletal anomalies was used as an envelope model and the sub-categories of congenital musculoskeletal anomalies were squeezed proportionally to these total estimates. The prevalence of other congenital musculoskeletal anomalies was derived from Marketscan claims data as the age- and sex-specific proportion of total musculoskeletal congenital anomaly cases that are not included in the polydactyly and syndactyly or limb reduction defects categories.

The DisMod model of total musculoskeletal anomalies used cause-specific mortality estimates from the corresponding model in the GBD Causes of Death analysis, and converted these data to excess mortality estimates where corresponding prevalence data were available. Random effects on prevalence were limited to +- 0.5 in order to limit geographic variation in the birth prevalence of congenital musculoskeletal anomalies. Smoothness on excess mortality rate was increased to Xi= 2.0 to allow the model to fit a steep decrease in excess mortality rate after the earliest age groups. The model also included a decreasing slope prior on excess mortality rate for all ages, as the risk of mortality from congenital musculoskeletal anomalies is greatest shortly after birth and decreases over age.

In the DisMod model of limb reduction defects, random effects on prevalence were limited to +- 0.3 in order to limit geographic variation in the estimated birth prevalence. The excess mortality rate was set to a maximum of 0.5 before 70 years of age in to reflect the relatively low mortality risk of congenital limb anomalies, and a decreasing slope prior on excess mortality rate was set for all ages as the risk of mortality is highest in the earliest age groups.

The DisMod model of polydactly and syndactyly limited random effects on prevalence to +- 0.5, as we expected limited geographic variation in the birth prevalence estimates. Excess mortality was set to 0 for all ages. The remission rate was bounded from 0 to 0.02 for the first three months of life, as surgical correction of polydactyly or syndactyly rarely occurs in the first few months of life. Remission was bounded between 0 and 0.5 for ages 2 to 5 years, the ages during which surgical correction is most likely to occur, then set to a maximum of 0.02 after 5 years of age. The smoothness on remission was set to Xi= 1.5 in order to facilitate steep changes in remission rates during the first few years of life.

Covariates used in the DisMod-MR model of total musculoskeletal anomalies

Covariate name	Type	Measure	Beta value	Exponentiated value
Healthcare access and quality index	Country covariate	With-condition mortality rate	-0.250 (-0.500 - -0.003)	0.779 (0.607 - 0.997)
LDI (I$ per capita)	Country covariate	Excess mortality rate	-0.325 (-0.500 - -0.243)	0.722 (0.607 - 0.784)
Legality of Abortion	Country covariate	Prevalence	-0.000 (-0.001 - -0.000)	1.000 (0.999 - 1.000)
All MarketScan, year 2010	Study-level x-covariate	Prevalence	-0.003 (-0.009 - -0.001)	0.997 (0.991 - 0.999)
All MarketScan, year 2012	Study-level x-covariate	Prevalence	-0.004 (-0.010 - -0.000)	0.996 (0.990 - 1.000)

Chromosomal diagnoses excluded	Study-level x-covariate	Prevalence	-0.007 (-0.025 - -0.000)	0.993 (0.975 - 1.000)
Hospital data for ages over 1 year only	Study-level x-covariate	Prevalence	0.456 (0.399 - 0.500)	1.577 (1.490 - 1.648)
Hospital data for the under-1 year age group	Study-level x-covariate	Prevalence	0.496 (0.487 - 0.499)	1.642 (1.627 - 1.648)
Stillbirths included as cases	Study-level x-covariate	Prevalence	0.001 (0.000 - 0.004)	1.001 (1.000 - 1.004)

Covariates used in the DisMod-MR model of polydactyly and syndactyly

Covariate name	Type	Measure	Beta value	Exponentiated value
LDI (I$ per capita)	Country covariate	Remission	0.999 (0.500 - 1.500)	2.717 (1.649 - 4.482)
All MarketScan, year 2010	Study-level x-covariate	Prevalence	-0.774 (-0.899 - -0.646)	0.461 (0.407 - 0.524)
All MarketScan, year 2012	Study-level x-covariate	Prevalence	-0.713 (-0.842 - -0.590)	0.490 (0.431 - 0.555)
Chromosomal diagnoses excluded	Study-level x-covariate	Prevalence	-0.019 (-0.049 - -0.001)	0.981 (0.952 - 0.999)
Hospital data for ages over 1 year only	Study-level x-covariate	Prevalence	0.996 (0.985 - 1.000)	2.709 (2.677 - 2.718)
Stillbirths included as cases	Study-level x-covariate	Prevalence	0.003 (0.000 - 0.008)	1.003 (1.000 - 1.008)
Terminations of pregnancy included as cases	Study-level x-covariate	Prevalence	0.010 (0.000 - 0.034)	1.010 (1.000 - 1.035)
Under Reported	Study-level x-covariate	Prevalence	-0.995 (-1.000 - -0.978)	0.370 (0.368 - 0.376)
LDI (I$ per capita)	Country covariate	Remission	0.999 (0.500 - 1.500)	2.717 (1.649 - 4.482)

Covariates used in the DisMod-MR model of limb reduction defects

Covariate name	Type	Measure	Beta value	Exponentiated value
LDI (I$ per capita)	Country covariate	Excess mortality rate	-0.050 (-0.100 - 0.000)	0.952 (0.905 - 1.000)
Legality of Abortion	Country covariate	Prevalence	-0.002 (-0.004 - -0.000)	0.998 (0.996 - 1.000)
All MarketScan, year 2010	Study-level x-covariate	Prevalence	-0.017 (-0.062 - -0.000)	0.983 (0.940 - 1.000)
All MarketScan, year 2012	Study-level x-covariate	Prevalence	-0.086 (-0.282 - -0.001)	0.917 (0.755 - 0.999)
Chromosomal diagnoses excluded	Study-level x-covariate	Prevalence	-0.075 (-0.114 - -0.038)	0.928 (0.893 - 0.963)

Hospital data for ages over 1 year only	Study-level x-covariate	Prevalence	0.536 (0.431 - 0.641)	1.709 (1.539 - 1.898)
Hospital data for the under-1 year age group	Study-level x-covariate	Prevalence	0.543 (0.385 - 0.703)	1.721 (1.469 - 2.020)
Stillbirths included as cases	Study-level x-covariate	Prevalence	0.126 (0.072 - 0.171)	1.134 (1.075 - 1.186)
Terminations of pregnancy included as cases	Study-level x-covariate	Prevalence	0.015 (0.000 - 0.051)	1.015 (1.000 - 1.053)
Under Reported	Study-level x-covariate	Prevalence	-0.073 (-0.125 - -0.027)	0.930 (0.883 - 0.973)

Urogenital congenital anomalies

Case definitions and associated health states

The GBD 2016 case definition of urogenital congenital anomalies include anomalies of the genitals and the urinary system that are present at birth. While some types of urogenital congenital anomalies encompass both the urinary and genital systems, we have assigned each congenital condition as a malformation of either the urinary or the genital system and model anomalies of the urinary and genital systems separately. Urinary anomalies include congenital malformation of the collecting system, ureter, bladder, and kidney, as well as bladder exstrophy and epispadias. The ICD-10 codes included in the category of urinary anomalies are Q64.0, Q64.1, Q60-Q61 and Q62-Q63. Genital anomalies include hypospadias, ambiguous or indeterminate sex, other congenital abnormalities of the male genitalia, and a variety of female genital malformations. ICD-10 codes Q50-Q52, Q54, Q56, and Q55 (excluding Q55.20-Q55.21) are included in the case definition of congenital genital anomalies. Undescended testicles are excluded from the case definition of genital anomalies, as this is not considered a severe condition. Remission is not permitted in the models or either urinary or genital anomalies, as individuals who receive surgical corrections of these conditions after birth typically continue to experience health consequences as a result of these congenital conditions.

The total prevalence of congenital urinary anomalies was split into proportions with and without each of the following health states: urinary incontinence, impotence, recurrent urinary tract infections and other recurring abdominal issues, and atypical genitalia (corresponding to disfigurement, level 1 in the GBD Disability Weights Study). Cases of congenital genital anomalies was split into proportions with and without primary infertility, impotence, recurrent urinary tract infections and other recurring abdominal issues, and atypical genitalia. Estimates were produced for the prevalence of every possible combination of those long-term sequela, assuming independence between the outcomes.

The distribution of these long-term outcomes was derived from a review of available literature on the long-term outcomes experienced cohorts of individuals born with a range of congenital urogenital anomalies (references: NIDs 292284, 292293, 292295, 292297, 292299, 292316, and 292318).

Modeling strategy and model settings

Congenital urogenital anomalies were modeled as two distinct categories, with distinct model specifications: urinary congenital anomalies and genital congenital anomalies.In the DisMod model of congenital urinary anomalies, random effects on random effects on prevalence were limited to +- 0.5 and random effects on with-condition mortality were limited to +- 1.0. The maximum excess mortality rate was set to 5.0 for the first year of life and 2.0 for ages 1 year to 70 years. The excess mortality rate set to 0 for ages 70+ years, consistent with the GBD 2016 Cause of Death models which exclude congenital urogenital anomalies as a cause of death after 70 years of age. The smoothness on excess mortality rate was set to Xi = 0.6 in order to fit changes in the excess mortality rate during the neonatal period.

In the DisMod model of congenital genital anomalies, random effects on prevalence were limited to +- 0.5 in order to limit random geographic variation in the estimates of birth prevalence, and random effects on with-condition mortality were limited to +- 1.0. The maximum excess mortality rate was set to 0.5 for all ages < 70 years, according to maximum observed excess mortality rates in available literature data on congenital genital anomalies. The excess mortality rate set to 0 for ages 70+ years, consistent with the GBD 2016 Cause of Death models which exclude congenital urogenital anomalies as a cause of death after 70 years of age.

Covariates used in the DisMod-MR model of urinary congenital anomalies

Covariate name	Type	Measure	Beta value	Exponentiated value
LDI (I$ per capita)	Country covariate	Excess mortality rate	-0.498 (-0.750 - -0.256)	0.608 (0.472 - 0.774)
All MarketScan, year 2010	Study-level x-covariate	Prevalence	-0.018 (-0.065 - -0.000)	0.983 (0.937 - 1.000)
All MarketScan, year 2012	Study-level x-covariate	Prevalence	-0.013 (-0.054 - -0.001)	0.987 (0.947 - 0.999)
Chromosomal diagnoses excluded	Study-level x-covariate	Prevalence	-0.206 (-0.297 - -0.132)	0.814 (0.743 - 0.876)
Hospital data for ages over 1 year only	Study-level x-covariate	Prevalence	-0.001 (-0.002 - -0.000)	0.999 (0.998 - 1.000)
Hospital data for the under-1 year age group	Study-level x-covariate	Prevalence	-0.013 (-0.143 - -0.000)	0.987 (0.867 - 1.000)
Stillbirths included as cases	Study-level x-covariate	Prevalence	0.002 (0.000 - 0.005)	1.002 (1.000 - 1.005)
Terminations of pregnancy included as cases	Study-level x-covariate	Prevalence	0.230 (0.142 - 0.294)	1.258 (1.152 - 1.342)

Covariates used in the DisMod-MR model of congenital genital anomalies

Covariate name	Type	Measure	Beta value	Exponentiated value
LDI (I$ per capita)	Country covariate	Remission	0.556 (0.100 - 0.977)	1.743 (1.105 - 2.655)
All MarketScan, year 2010	Study-level x-covariate	Prevalence	-0.309 (-0.436 - -0.181)	0.734 (0.647 - 0.835)
All MarketScan, year 2012	Study-level x-covariate	Prevalence	-0.333 (-0.445 - -0.207)	0.717 (0.641 - 0.813)
Chromosomal diagnoses excluded	Study-level x-covariate	Prevalence	-0.030 (-0.076 - -0.001)	0.971 (0.927 - 0.999)
EUROCAT to NBDPN registry case composition adjustment	Study-level x-covariate	Prevalence	-0.383 (-0.494 - -0.273)	0.682 (0.610 - 0.761)
Hospital data for ages over 1 year only	Study-level x-covariate	Prevalence	0.256 (0.122 - 0.374)	1.291 (1.129 - 1.453)
Hospital data for the under-1 year age group	Study-level x-covariate	Prevalence	-0.485 (-0.500 - -0.444)	0.616 (0.607 - 0.641)
ICDBSR to NBDPN registry case composition adjustment	Study-level x-covariate	Prevalence	-0.519 (-0.628 - -0.422)	0.595 (0.534 - 0.656)

Congenital anomalies of the digestive system

Case definitions

Congenital anomalies of the digestive system include any anomalies of the gastrointestinal tract present at birth as the result of abnormal embryonic development. As with the other congenital causes, this variety of digestive system abnormalities is split into four sub-cause categories. Congenital diaphragmatic hernia, a life-threatening malformation of the diaphragm that allows the abdominal organs to push into the chest cavity and obstructs proper formation of the lungs, is modeled separately from all other congenital malformations of the digestive system. Congenital diaphragmatic hernia corresponds to ICD-10 code Q79.0.

All congenital malformations of the abdominal wall are modeled together as a distinct sub-category. The primary diagnoses in this category are gastroschisis, omphalocele, and prune belly syndrome, corresponding to ICD-10 codes Q79.3, Q79.2, and Q79.4, respectively. All variations of atresia and/or stenosis of the digestive tract are modeled together as the third distinct sub-category of digestive congenital anomalies. This includes biliary atresia, esophageal atresia and/or stenosis with and without tracheoesophageal fistula, and atresia and stenosis of the small intestine, large intestine, rectum and anus. The ICD-10 codes included in the atresia and stenosis sub-cause category are Q42.0; Q42.1; Q42.2; Q42.3; Q42.4; Q42.8; Q42.9, Q42.8; Q42.9, Q42.0; Q42.1; Q42.2; Q42.3; Q42.4; Q41 (Q41.0; Q41.1; Q41.2; Q41.8; Q41.9;), Q44.2, Q39.0; Q39.1 and Q39.2. The final category of digestive congenital anomalies estimated in the GBD is other congenital malformations and diseases of the digestive system. This includes ICD-10 codes Q38 (Q38.0; Q38.3; Q38.4; Q38.6; Q38.7; Q38.8); Q39(Q39.3; Q39.4; Q39.5; Q39.6; Q39.8; Q39.9); Q40(Q40.0; Q40.1; Q40.2; Q40.3; Q40.8; Q40.9); Q43(Q43.1; Q43.2; Q43.3; Q43.4; Q43.5; Q43.6; Q43.7; Q43.8; Q43.9); Q44(Q44.0; Q44.1; Q44.3; Q44.4; Q44.5; Q44.6; Q44.7); Q45(Q45.0; Q45.1; Q45.2; Q45.3; Q45.8; Q45.9); Q79.1, and Q79.5(Q79.51; Q79.59). Inguinal hernias present at birth are excluded from the case definition of gastrointestinal congenital anomalies and are modeled separately as part of the estimation of inguinal hernias.

Most congenital anomalies of the gastrointestinal tract require surgical correction early in life in order to ensure infant survival. Following surgical intervention, individuals born with these congenital anomalies may experience a range of long-term outcomes as a result of their congenital conditions. Thus, we do not permit remission in the models of any congenital digestive anomalies and the health states associated with the various types of digestive anomalies reflect the long-term outcomes experienced by individuals with surgically corrected congenital malformations.

Health states associated with congenital anomalies of the digestive system

The health outcomes associated with congenital diaphragmatic hernia include every combination of disfigurement, chronic abdominal pain, mild chronic respiratory problems and breathlessness, mild intellectual disability, and a proportion of patients who are asymptomatic. The distribution of these long-term health outcomes was derived from a pooled analysis of available literature on the long-term outcomes in surviving patients born with congenital diaphragmatic hernias (references: NIDs 281144, 292221 and 292224).

The health outcomes associated with congenital malformations of the abdominal wall include every combination of constipation, chronic abdominal pain, and disfigurement and concern about scars. The distribution of these outcomes was calculated from a pooled analysis of literature sources on the long-term outcomes among surviving individuals born with congenital malformations of the abdominal wall (references: NIDs 292217 and 292235). Similarly, the outcomes associated with congenital atresia and/or stenosis of the abdominal tract include every combination of dysphagia, acid reflux, chronic abdominal

pain and/or nausea, and chronic respiratory problems; the distribution of these long-term outcomes was also derived from available long-term follow-up studies (references: NIDs 292215 and 292242).

The distribution of health outcomes associated with other congenital anomalies of the gastrointestinal tract was considered to be the same as the health outcomes associated with atresia and/or stenosis of the abdominal tract.

Modeling strategy and model settings

In order to ensure internal consistency in the estimates of each sub-type of congenital digestive anomalies, we generated a model to estimate the total prevalence and associated mortality due to all congenital digestive anomalies, then fit the estimates of each sub-type of congenital digestive anomalies proportionally to the envelope of this total model. The prevalence of other congenital anomalies of the digestive tract was calculated as a proportion of the total digestive anomalies estimates, using age- and sex-specific proportions derived from Marketscan claims data, which has the most detailed ICD-9 and ICD-10 coding information available of any input data sources. This modeling strategy allowed us to utilize the GBD 2016 Cause of Death estimates as input to the total congenital digestive anomalies estimates and also allowed us to incorporate literature data that reported only the total prevalence of all digestive anomalies.

The DisMod model of total congenital digestive anomalies used cause-specific mortality estimates from the corresponding GBD 2016 Cause of Death model of congenital digestive anomalies, and these data were converted to excess mortality estimates where corresponding cause-specific mortality estimates were available. The model had random effects on prevalence limited to +- 0.5 and a decreasing slope prior on excess mortality rate was set for all ages. The smoothness on excess mortality rate was increased to Xi = 1.0 in order to fit steep changes in excess mortality rate during the neonatal age period.

In the DisMod model of congenital diaphragmatic hernia, random effects on prevalence were set to +- 0.5 and random effects on with-condition mortality were set to +- 1.0. The minimum excess mortality for the early neonatal age period was set to 2.0. A decreasing slope prior on excess mortality rate was set for all ages, as the risk of mortality due to congenital diaphragmatic hernia is highest shortly after birth and diminishes over the life course following surgical correction of the condition. Smoothness on excess mortality rate was increased to Xi= 1.0 in order to fit steep changes in excess mortality rate during the first weeks of life.

The DisMod model of congenital malformations of the abdominal wall had random effects on prevalence limited to +- 0.5 and random effects on with-condition mortality were limited to +- 1.0. The minimum excess mortality rate was set to 3.0 in the early neonatal period according to the range of excess mortality estimates observed in literature data. A decreasing slope prior on excess mortality rate was set for all ages, and the smoothness on excess mortality rate was set to Xi = 0.8, allowing the model to fit a steep decrease in the excess mortality rate after the neonatal age period.

In the DisMod model of congenital diaphragmatic hernia, random effects on prevalence were set to +- 0.5 and random effects on with-condition mortality were set to +- 1.0. A decreasing slope prior on excess mortality rate was set for all ages, as the risk of mortality due to these congenital digestive anomalies highest shortly after birth. The smoothness on excess mortality rate was increased to Xi = 1.0 in order to fit steep changes in excess mortality rate during the first weeks of life.

Covariates used in the DisMod-MR model of total congenital digestive anomalies

Covariate name	Type	Measure	Beta value	Exponentiated value
LDI (I$ per capita)	Country covariate	Excess mortality rate	-0.158 (-0.195 - -0.124)	0.854 (0.823 - 0.883)
Chromosomal diagnoses excluded	Study-level x-covariate	Prevalence	-0.047 (-0.086 - -0.007)	0.954 (0.917 - 0.993)
Hospital data for ages over 1 year only	Study-level x-covariate	Prevalence	-0.237 (-0.276 - -0.193)	0.789 (0.759 - 0.824)
Hospital data for the under-1 year age group	Study-level x-covariate	Prevalence	0.381 (0.318 - 0.440)	1.464 (1.375 - 1.552)
Registry data	Study-level x-covariate	Prevalence	-0.199 (-0.240 - -0.162)	0.819 (0.787 - 0.850)
Stillbirths included as cases	Study-level x-covariate	Prevalence	0.003 (0.000 - 0.015)	1.003 (1.000 - 1.015)
Terminations of pregnancy included as cases	Study-level x-covariate	Prevalence	0.048 (0.006 - 0.114)	1.049 (1.006 - 1.121)
Under Reported	Study-level x-covariate	Prevalence	-0.910 (-0.968 - -0.852)	0.403 (0.380 - 0.427)
Under Reported	Study-level z-covariate	With-condition mortality rate	1.657 (1.207 - 1.975)	5.246 (3.343 - 7.207)

Covariates used in the DisMod-MR model of congenital diaphragmatic hernia

Covariate name	Type	Measure	Beta value	Exponentiated value
Healthcare access and quality index	Country covariate	With-condition mortality rate	-0.252 (-0.498 - 0.000)	0.777 (0.608 - 1.000)
LDI (I$ per capita)	Country covariate	Excess mortality rate	-0.627 (-1.000 - -0.250)	0.534 (0.368 - 0.779)
All MarketScan, year 2010	Study-level x-covariate	Prevalence	-1.571 (-1.692 - -1.445)	0.208 (0.184 - 0.236)
All MarketScan, year 2012	Study-level x-covariate	Prevalence	-1.566 (-1.688 - -1.447)	0.209 (0.185 - 0.235)
Chromosomal diagnoses excluded	Study-level x-covariate	Prevalence	-0.115 (-0.183 - -0.043)	0.892 (0.833 - 0.958)
Hospital data	Study-level x-covariate	Prevalence	-0.003 (-0.007 - -0.000)	0.997 (0.993 - 1.000)
Stillbirths included as cases	Study-level x-covariate	Prevalence	0.008 (0.000 - 0.024)	1.008 (1.000 - 1.025)

Covariates used in the DisMod-MR model of congenital malformations of the abdominal wall

Covariate name	Type	Measure	Beta value	Exponentiated value
Healthcare access and quality index	Country covariate	With-condition mortality rate	-0.025 (-0.049 - -0.006)	0.975 (0.952 - 0.994)
LDI (I$ per capita)	Country covariate	Excess mortality rate	-0.425 (-0.750 - -0.102)	0.654 (0.472 - 0.903)
Chromosomal diagnoses excluded	Study-level x-covariate	Prevalence	-0.019 (-0.057 - -0.000)	0.981 (0.945 - 1.000)
Hospital data for ages over 1 year only	Study-level x-covariate	Prevalence	-0.007 (-0.021 - -0.000)	0.993 (0.979 - 1.000)
Hospital data for the under-1 year age group	Study-level x-covariate	Prevalence	-0.002 (-0.008 - -0.000)	0.998 (0.992 - 1.000)
Stillbirths included as cases	Study-level x-covariate	Prevalence	0.027 (0.001 - 0.077)	1.027 (1.001 - 1.080)
Terminations of pregnancy included as cases	Study-level x-covariate	Prevalence	0.102 (0.033 - 0.180)	1.107 (1.034 - 1.198)
Under Reported	Study-level x-covariate	Prevalence	-0.705 (-0.785 - -0.625)	0.494 (0.456 - 0.535)

Covariates used in the DisMod-MR model of congenital atresia and/or stenosis of the digestive tract

Covariate name	Type	Measure	Beta value	Exponentiated value
Healthcare access and quality index	Country covariate	Excess mortality rate	-0.250 (-0.496 - 0.000)	0.779 (0.609 - 1.000)
LDI (I$ per capita)	Country covariate	Excess mortality rate	-0.050 (-0.100 - -0.000)	0.951 (0.905 - 1.000)
Chromosomal diagnoses excluded	Study-level x-covariate	Prevalence	-0.274 (-0.300 - -0.222)	0.760 (0.741 - 0.801)
Hospital data for ages over 1 year only	Study-level x-covariate	Prevalence	-0.003 (-0.008 - -0.000)	0.997 (0.992 - 1.000)
Hospital data for the under-1 year age group	Study-level x-covariate	Prevalence	-0.001 (-0.003 - -0.000)	0.999 (0.997 - 1.000)
Stillbirths included as cases	Study-level x-covariate	Prevalence	0.002 (0.000 - 0.005)	1.002 (1.000 - 1.005)
Terminations of pregnancy included as cases	Study-level x-covariate	Prevalence	0.018 (0.001 - 0.064)	1.019 (1.001 - 1.066)
Under Reported	Study-level x-covariate	Prevalence	-0.992 (-1.000 - -0.968)	0.371 (0.368 - 0.380)
Under Reported	Study-level z-covariate	With-condition mortality rate	1.544 (1.203 - 1.968)	4.683 (3.330 - 7.156)

Other congenital anomalies

In addition of the specific types of congenital anomalies outlined in the preceding pages, there are a number of other types of defects that may be present at birth. These other congenital defects include anomalies of the ears, eyes, face and neck, respiratory malformation and diseases, skin disorders, phakomatoses and other neurological disorders that are not included in the case definition of neural tube defects. Estimates of the YLDs attributable to these other congenital anomalies are derived from a YLL:YLD ratio using YLLs from the cause of death (COD) estimates for other congenital anomalies.

Dermatitis

Flowcharts for Atopic Dermatitis, Contact Dermatitis, & Seborrheic Dermatitis

Atopic Dermatitis

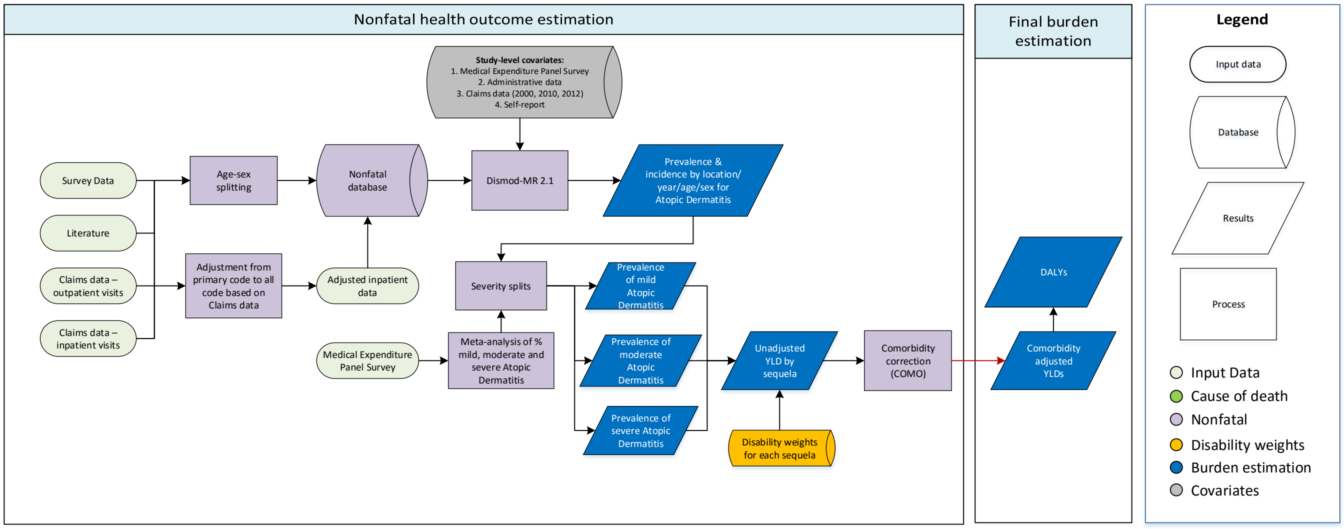

Contact Dermatitis

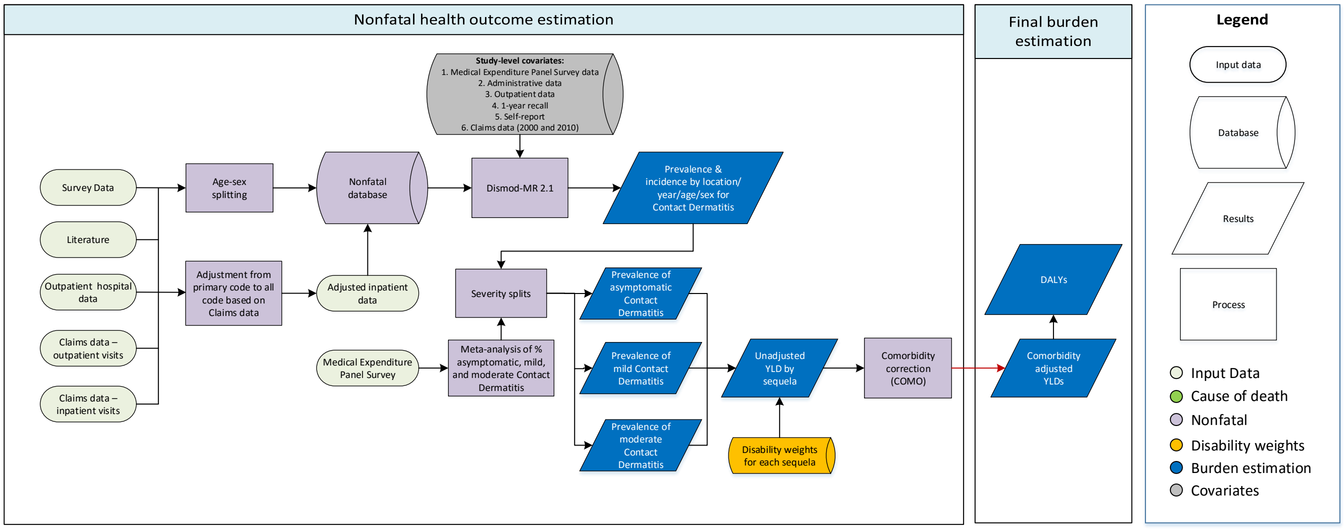

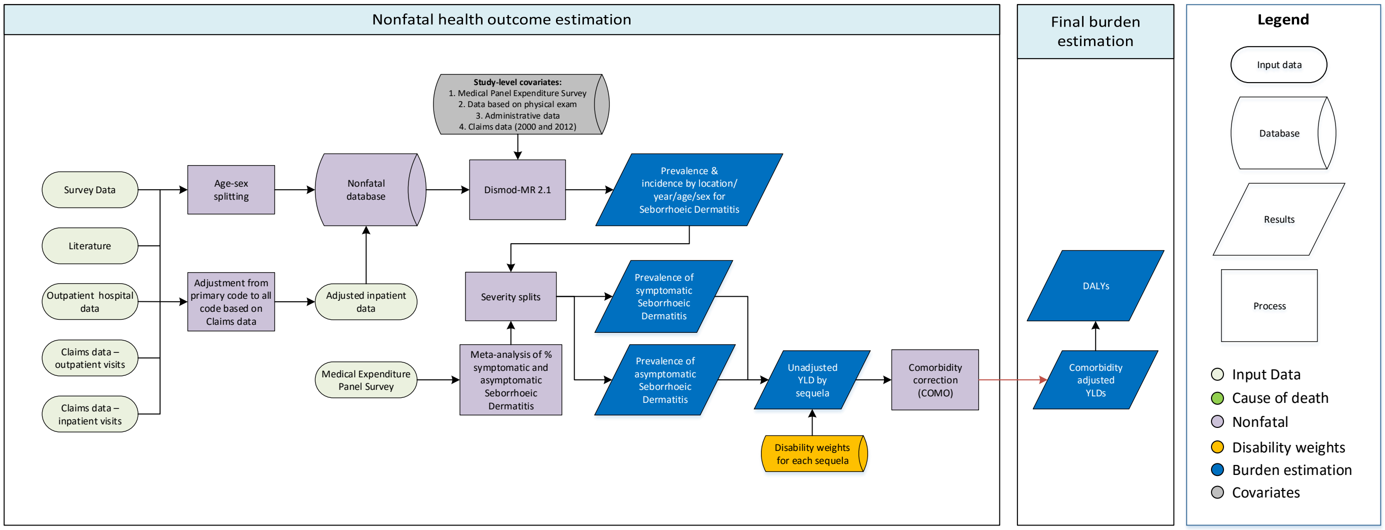

Case definition

Dermatitis was included in the GBD 2016 cause group of skin and subcutaneous conditions and consists of atopic dermatitis, contact dermatitis, and seborrheic dermatitis. Dermatitis, or eczema, refers to an inflammation of the skin. Atopic dermatitis is an inflammatory, relapsing, and non-contagious skin disorder characterized by an itchy rash (ICD-10: L20) (1). Contact dermatitis is a localized rash or irritation of the skin caused by allergens or irritants (ICD: 10: L22-26) (1). Seborrhoeic dermatitis is an inflammatory skin disorder affecting the sebaceous-gland-rich areas of skin, generally the scalp, face, and torso (ICD-10: L21) (1). It is characterized by scaly, flaky, itchy, and red skin on the affected area. We estimated burden separately for atopic dermatitis, contact dermatitis, and seborrheic dermatitis in order to better accommodate differences in the epidemiology and burden between the subtypes of dermatitis.

Input data

Model inputs

In the GBD 2010, a literature-based dataset was provided by the skin conditions expert group. Data from the Medical Expenditure Panel Survey (MEPS) in the United States in 2000–2009 (2) were also included to inform the age pattern of the prevalence output. Data from the NHANES study and the NHIS study (both from the US) were not extracted, as questions regarding dermatitis were too broad (ie, asked whether a respondent had experienced eczema or any other rash). Dermatitis (known as “eczema”) was modelled as a single disorder. For GBD 2013, the literature was updated and dermatitis was disaggregated into eczema, contact dermatitis, and seborrheic dermatitis. The agreed-upon approach for dermatitis diseases was to undertake a literature review every two years. Therefore, we undertook a new literature review for GBD 2016. The data for dermatitis were expanded based on recommendations of research articles and reviews by the skin expert group. Additionally, hospital outpatient and US claims data for 2000, 2010, and 2012 were used, where appropriate. Data were outliered or excluded if we found them unreasonable when compared to regional, super-regional, and global rates. See descriptions of individual modelling approaches for more information.

Table 1. Model inputs

		Prevalence	Incidence
Atopic dermatitis	Studies	197	0
	Countries/subnationals	227	0
	Countries	96	0
	GBD world regions	19	0
	GBD super-regions	7	0
Contact dermatitis	Studies	24	1
	Countries/subnationals	68	1
	Countries	14	1
	GBD world regions	8	1
	GBD super-regions	6	1
Seborrheic dermatitis	Studies	24	1
	Countries/subnationals	71	1
	Countries	19	1
	GBD world regions	10	1
	GBD super-regions	7	1

Severity splits and disability weights

The basis of the GBD disability weight survey assessments are lay descriptions of sequelae highlighting major functional consequences and symptoms. Severity was split into three levels of disfigurement with pain/itch. The severity splits and disability weights applied in GBD 2015 were also used for GBD 2016. See below for a lay descriptions of the severity levels.

Table 2. Severity level and lay description.

Sequela	Severity level	Lay description	DW (95% CI)
Mild atopic dermatitis	Disfigurement, level 1 with itch/pain	The person has a slight, visible physical deformity that is sometimes sore or itchy. Others notice the deformity, which causes some worry and discomfort.	0.027 (0.015–0.042)
Moderate atopic dermatitis	Disfigurement, level 2, with itch/pain	The person has a visible physical deformity that is sore and itchy. Other people stare and comment, which causes the person to worry. The person has trouble sleeping and concentrating.	0.188 (0.124–0.267)

Severe atopic dermatitis	Disfigurement, level 3, with itch/pain	The person has an obvious physical deformity that is very painful and itchy. The physical deformity makes others uncomfortable, which causes the person to avoid social contact, feel worried, sleep poorly, and think about suicide.	0.576 (0.401–0.731)
Mild contact dermatitis	Disfigurement, level 1 with itch/pain	The person has a slight, visible physical deformity that is sometimes sore or itchy. Others notice the deformity, which causes some worry and discomfort.	0.027 (0.015–0.042)
Moderate contact dermatitis	Disfigurement, level 2, with itch/pain	The person has a visible physical deformity that is sore and itchy. Other people stare and comment, which causes the person to worry. The person has trouble sleeping and concentrating.	0.188 (0.124–0.267)
Symptomatic seborrheic dermatitis	Disfigurement, level 1 with itch/pain	The person has a slight, visible physical deformity that is sometimes sore or itchy. Others notice the deformity, which causes some worry and discomfort.	0.027 (0.015–0.042)

Modelling strategy

DisMod-MR 2.1 was used to estimate prevalence by age, sex, year, and geography (subnational [select countries], country, region, super-region) for atopic dermatitis, contact dermatitis, and seborrheic dermatitis. Separate models were run for each cause.

Atopic dermatitis: Since our available data mostly contained information on prevalence, we specified additional expert priors to further inform analyses. Excess mortality was set to zero while a setting of 0-0.2 (equivalent to five years to life time duration) was placed on remission. Study-level covariates were used to adjust prevalence estimates from the Medical Expenditure Panel Survey (MEPS), US claims data for 2000, 2010, and 2012, self-reported data, and administrative data toward data based on clinical examination. To improve regional and global estimates, the minimum coefficient of variation was set at 0.4 and location random effects for Paraguay, Sweden, and England were restricted to [-0.25, 0.25], [-

0.25, 0.25], and [-0.5, 0.5], respectively. A time window of 10 years was used to determine which data points were used for a particular year of fit.

Contact dermatitis: Similar to atopic dermatitis, mostly prevalence data were available for contact dermatitis. Per expert advice, the remission parameter was set from 0.1 to 4, excess mortality was set to zero, and incidence was set to zero prior to age 6. Study-level covariates were used to adjust data from the Medical Expenditure Panel Survey (MEPS), outpatient data, administrative data, self-reported data, data based on a one-year recall, and claims data for 2000 and 2010 toward the values in the 2012 claims data. In order to improve model estimates, location random effects were added for Sweden [-2, 2], Norway [-2, 2], Denmark [0, 0.5], Italy [-2, 2], Germany [-2, 2], and France [-0.5, 0]. "A time window of 25 years was used to determine which data points were used for a particular year of fit.

Seborrheic dermatitis: As with contact dermatitis, the available data were mostly prevalence estimates. Per expert advice, settings were placed on incidence as follows: 0-4 years = 0-0.1, and 60-100 = 0-0.01. Excess mortality was set to zero while a setting of 0.1-12 was placed on remission, implying a duration of one month to 10 years. Study-level covariates were used to adjust prevalence estimates from US claims data for 2000 and 2010, MEPS, administrative data, and clinical exam data toward other data points.

Table 3. Study-level beta and exponentiated values

	Study covariate	Parameter	Beta	Exp(beta)
Atopic dermatitis	MEPS	Prevalence	-1.08 (-1.23 - -0.95)	0.34 (0.29 - 0.39)
	Self-reported	Prevalence	0.39 (0.34 - 0.44)	1.48 (1.41 - 1.56)
	Administrative data	Prevalence	-0.33 (-0.54 - -0.12)	0.72 (0.58 - 0.89)
	Claims data - 2000	Prevalence	-1.90 (-1.99 - -1.83)	0.15 (0.14 - 0.16)
	Claims data - 2010	Prevalence	-1.51 (-1.60 - -1.43)	0.22 (0.20 - 0.24)
	Claims data - 2012	Prevalence	-1.47 (-1.56 - -1.40)	0.23 (0.21 - 0.25)
Contact dermatitis	MEPS	Prevalence	-0.36 (-0.79 - -0.03)	0.69 (0.46 - 0.97)
	Outpatient	Prevalence	-0.90 (-1.82 - -0.05)	0.41 (0.16 - 0.95)
	Recall 1 year	Prevalence	-0.18 (-1.02 - 0.86)	0.83 (0.36 - 2.37)
	Self-reported	Prevalence	1.65 (0.98 - 1.99)	5.23 (2.68 - 7.30)
	Administrative data	Prevalence	1.20 (-0.14 - 1.97)	3.32 (0.87 - 7.16)
	Claims data - 2000	Prevalence	0.32 (0.24 - 0.39)	1.37 (1.27 - 1.48)
	Claims data - 2010	Prevalence	0.53 (0.47 - 0.60)	1.70 (1.59 - 1.81)
Seborrhoeic dermatitis	Diagnosis Physical Exam	Prevalence	1.72 (1.03 - 2.00)	5.61 (2.82 - 7.39)
	MEPS	Prevalence	-1.96 (-2.00 - -1.86)	0.14 (0.14 - 0.16)
	Administrative data	Prevalence	1.83 (1.30 - 2.00)	6.22 (3.65 - 7.39)
	Claims data - 2000	Prevalence	-0.31 (-0.36 - -0.20)	0.73 (0.69 - 0.82)
	Claims data - 2010	Prevalence	0.14 (0.10 - 0.25)	1.15 (1.10 - 1.28)

Psoriasis

Flowchart

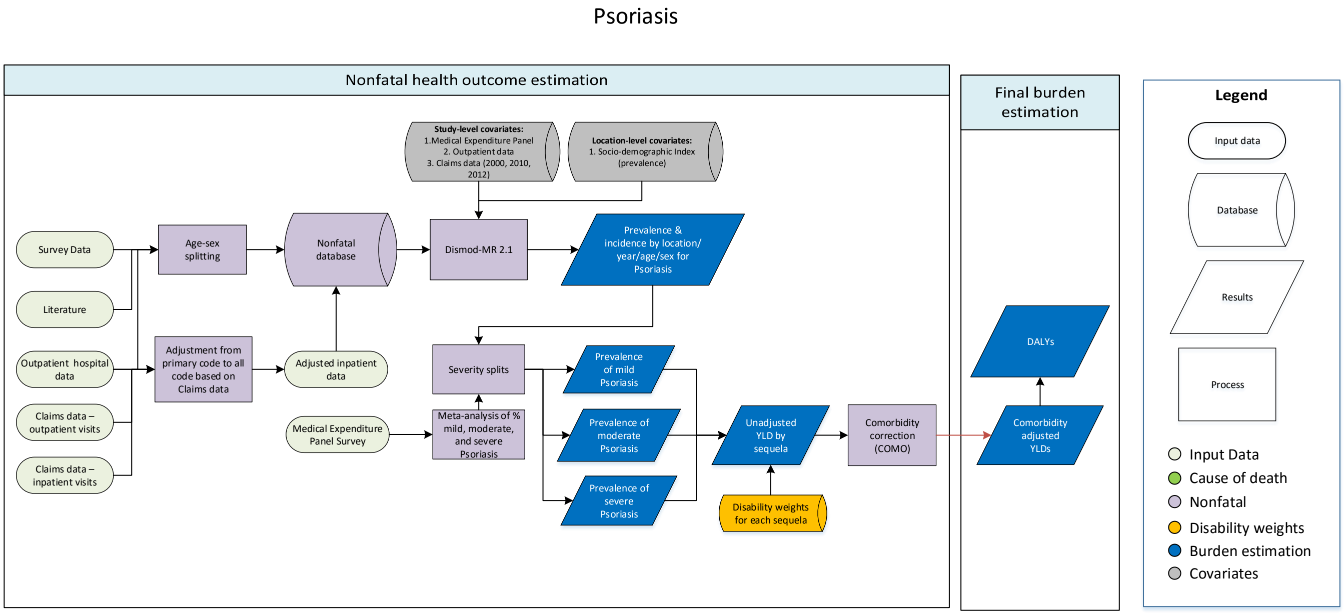

Case definition

Psoriasis was included in the GBD 2016 cause group of skin and subcutaneous conditions. According to the International Classification of Diseases (ICD-10), it is a skin disease marked by itchy or sore patches of thick, red skin with silvery scales (ICD-10: L40, L41) (1,2).

Input data

Model inputs

In the GBD 2010 study, a systematic review of the literature was conducted using PubMed and Google Scholar to capture epidemiological data for psoriasis. The literature search also included any relevant data from the Medical Expenditure Panel Survey (MEPS) in the United States for 2000–2009, the Australian National Health Survey 1995–1996, 2001, 2004–2005, 2007–2008, and the US National Health and Nutrition Examination Survey (NHANES) in 2002 and 2005.

The inclusion criteria stipulated that studies (1) must be published between 1980 and 2012; (2) must provide data on the incidence or prevalence of psoriasis; (3) must use samples representative of the general population (ie, samples derived from the experimental arm of clinical trials or based in dermatology clinics were excluded); (4) must use a sample size larger than 100; and (5) must provide sufficient information on study method and sample characteristics to assess the quality of the study. For GBD 2013, the GBD 2010 search strategy was replicated to capture epidemiological studies published between 2012 and 2014. The agreed-upon approach for psoriasis was to undertake a literature review every two years. Therefore, we conducted an updated literature review through October 1, 2016, for GBD

2016. Hospital outpatient and US claims data from 2000, 2010, and 2012 were also included in GBD 2016. Data were outliered or excluded if we found them unreasonable when compared to regional, super-regional, and global rates.

Table 1. Data inputs

	Prevalence	Incidence
Studies	72	6
Countries/subnationals	92	2
Countries	30	3
GBD world regions	13	2
GBD super-regions	6	1

Severity splits

As was the case in GBD 2010, GBD 2013, and GBD 2015, disability weights were estimated for disfigurement with itch/pain, levels 1, 2, and 3. The disability weights used for GBD 2015 were also used in 2016.

Table 2. Severity level and lay description.

Sequela	Severity level	Lay description	DW (95% CI)
Mild psoriasis	Disfigurement, level 1 with itch/pain	The individual has a slight, visible physical deformity that is sometimes sore or itchy. Others notice the deformity, which causes some worry and discomfort.	0.027 (0.015–0.042)
Moderate psoriasis	Disfigurement, level 2, with itch/pain	The individual has a visible physical deformity that is sore and itchy. Other people stare and comment, which causes the person to worry. The person has trouble sleeping and concentrating.	0.188 (0.124–0.267)
Severe psoriasis	Disfigurement, level 3, with itch/pain	The individual has an obvious physical deformity that is very painful and itchy. The physical deformity makes others uncomfortable, which causes the person to avoid social contact, feel worried, sleep poorly, and think about suicide.	0.576 (0.401–0.731)

Modelling strategy

DisMod-MR 2.1, a Bayesian meta-regression tool, was used to estimate prevalence by age, sex, year, and geography (subnational [select countries], country, region, super-region) for psoriasis.

Psoriasis was modelled with remission set between 0.05 and 0.15, implying a duration between 6.6 and 20 years. This was in line with the available epidemiological data, expert opinion, and previous GBD work. Excess mortality was assumed to be zero. The datasets for psoriasis were sufficiently large to make use of a relatively short time window of 10 years to determine which data points were used for a particular year of fit.

Study-level covariates were used to adjust incidence derived from the Medical Expenditure Panel Survey (MEPS) and US claims data for 2000, 2010, and 2012, and outpatient data toward the level of other prevalence and incidence data points, which were more representative of the general population. Additionally, the data were extremely heterogeneous. Therefore, the random effects were constrained to (-0.25, 0.25) except in China (0, 0.5) and Taiwan (Province of China) (-0.5, 0). SDI was used as a location-level covariate to guide estimates for countries with few or no data.

Table 3. Study-level beta and exponentiated values

Covariate	Parameter	Beta	Exp(beta)
MEPS	Prevalence	-0.98 (-1.15 - -0.88)	0.37 (0.32 - 0.42)
Outpatient	Prevalence	-0.57 (-0.62 - -0.52)	0.56 (0.54 - 0.59)
Claims data - 2000	Prevalence	-1.21 (-1.25 - -1.17)	0.30 (0.29 - 0.31)
Claims data - 2010	Prevalence	-0.96 (-0.98 - -0.92)	0.38 (0.38 - 0.40)
Claims data - 2012	Prevalence	-0.88 (-0.90 - -0.84)	0.42 (0.41 - 0.43)

Table 4. Location-level beta and exponentiated values

Covariate	Parameter	Beta	Exp(beta)
Socio-demographic Index	Prevalence	1.91 (1.77 — 1.99)	6.73 (5.90 — 7.34)

Cellulitis

Flowchart

Cellulitis

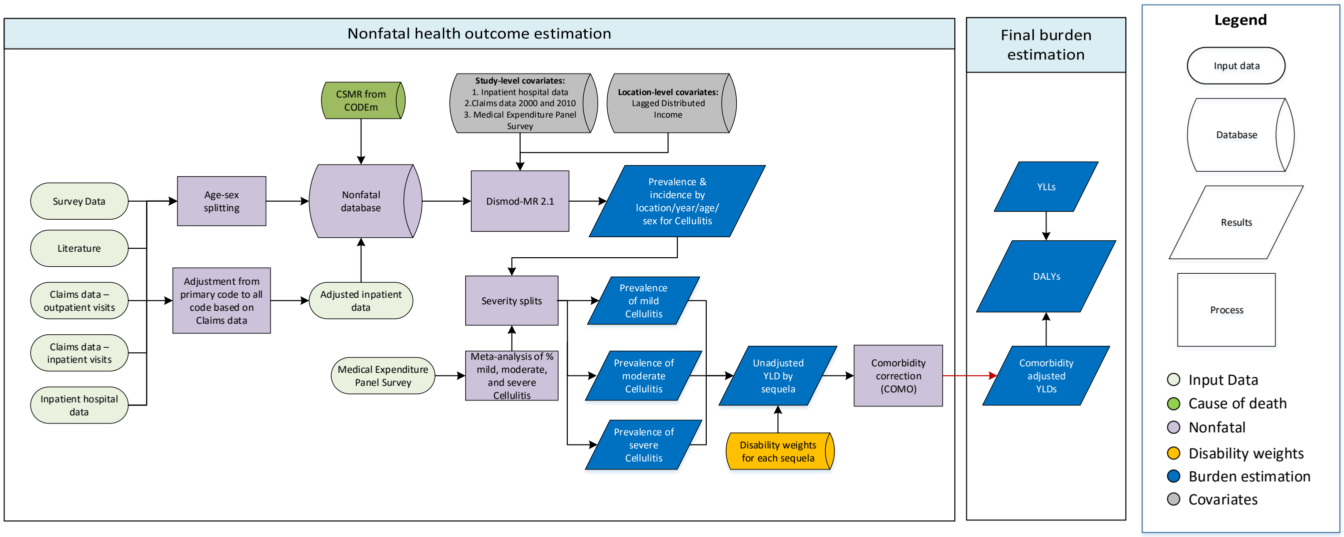

Case definition

Cellulitis was included in the GBD 2016 cause group of skin and subcutaneous conditions. Cellulitis is a skin disease marked by a bacterial infection that affects and spreads through the skin and soft tissues. Symptoms of cellulitis include pain, tenderness, and reddening in the affected area, fever, chills, and lymphadenopathy (ICD-10: L03) (1).

Input data

Model inputs

In the GBD 2010 study, a systematic review of the literature was conducted using PubMed and Google Scholar to capture epidemiological data for cellulitis. Due to lack of published data on the epidemiology of cellulitis, the literature search also included relevant incidence data from national inpatient or outpatient records in Europe, North America, and Latin America. When years in the national data from the hospital records overlapped, inpatient and outpatient data were summed together in an effort to better estimate the population incidence of cellulitis. The final dataset also included survey data from the Medical Expenditure Panel Survey (MEPS), US claims data, and cause-specific mortality rates for cellulitis estimated by CODEm.

The inclusion criteria stipulated that studies (1) must be published between 1980 and 2012; (2) must provide data on the incidence or prevalence of cellulitis; (3) must use samples representative of the general population (ie, samples derived from the experimental arm of clinical trials or based in dermatology clinics were excluded); (4) must use a sample size larger than 100; and (5) must provide sufficient information on study method and sample characteristics to assess the quality of the study. For GBD 2013, the GBD 2010 search strategy was replicated to capture epidemiological studies published between 2012 and 2014. In addition, hospital inpatient data and data from US claims for 2000, 2010, and 2012 by US state were included in GBD 2016. Data were outliered or excluded if we found them unreasonable when compared to regional, super-regional, and global rates.

Table 1. Model inputs (not including hospital inpatient data)

	Prevalence	Incidence
Studies	0	3
Countries/subnationals	0	318
Countries	0	38
GBD world regions	0	15
GBD super-regions	0	7

Severity splits

The basis of the GBD disability weight survey assessments are lay descriptions of sequelae highlighting major functional consequences and symptoms. Severity splits for cellultis were calculated via the Medical Expenditure Panel Survey (MEPS) regression and outlined in the table below.

Table 2. Severity level and lay description.

Sequela	Severity level	Lay description	DW (95% CI)
Mild cellulitis	Infectious disease, acute episode, mild	This person has a low fever and mild discomfort, but no difficulty with daily activities.	0.006 (0.002–0.012)
Moderate cellulitis	Infectious disease, acute episode, moderate	This person has a fever and aches, and feels weak, which causes some difficulty with daily activities.	0.051 (0.032–0.074)
Severe cellulitis	Infectious disease, acute episode, severe	This person has a high fever and pain, and feels	0.133 (0.088–0.19)

		very weak, which causes great difficulty with daily activities.	

Modelling strategy

DisMod-MR 2.1, a Bayesian meta-regression tool, was used to estimate cellulitis prevalence by age, sex, year, and geography (subnational [select countries], country, region, super-region). Cellulitis was modeled with remission set between 12 and 30, implying a duration of 12 days to one month. This was in line with the available epidemiological data, expert opinion, and previous GBD work. The cellulitis dataset was sufficiently large to make use of a relatively short time window of five years to determine which data points were used for a particular year of fit.

Study-level covariates were used to adjust incidence derived from MEPS, hospital inpatient data, and US claims data for 2000 and 2010 toward the level of other incidence data points, which were more representative of the general population. US claims data for 2000 and 2010 were set at a maximum of zero, whereas MEPS and inpatient data were set with a maximum of two. Log-transformed lagged distributed income (LDI) was used as a location-level covariates to guide estimates for locations with little or no data. LDI was restricted to a range of -0.5 to -0.1.

Table 3. Study-level beta and exponentiated values

Covariate	Parameter	Beta	Exp(beta)
MEPS	Incidence	-1.97 (-2.00 - -1.91)	0.14 (0.14 - 0.15)
Claims data - 2000	Incidence	-0.52 (-0.55 - -0.49)	0.60 (0.58 - 0.61)
Claims data - 2010	Incidence	-0.06 (-0.09 - -0.04)	0.94 (0.92 - 0.97)

Table 4. Location-level beta and exponentiated values

Covariate	Parameter	Beta	Exp(beta)
Log (LDI)	Excess mortality rate	-0.50 (-0.50 — -0.50)	0.61 (0.61 — 0.61)

Pyoderma

Flowchart

Pyoderma

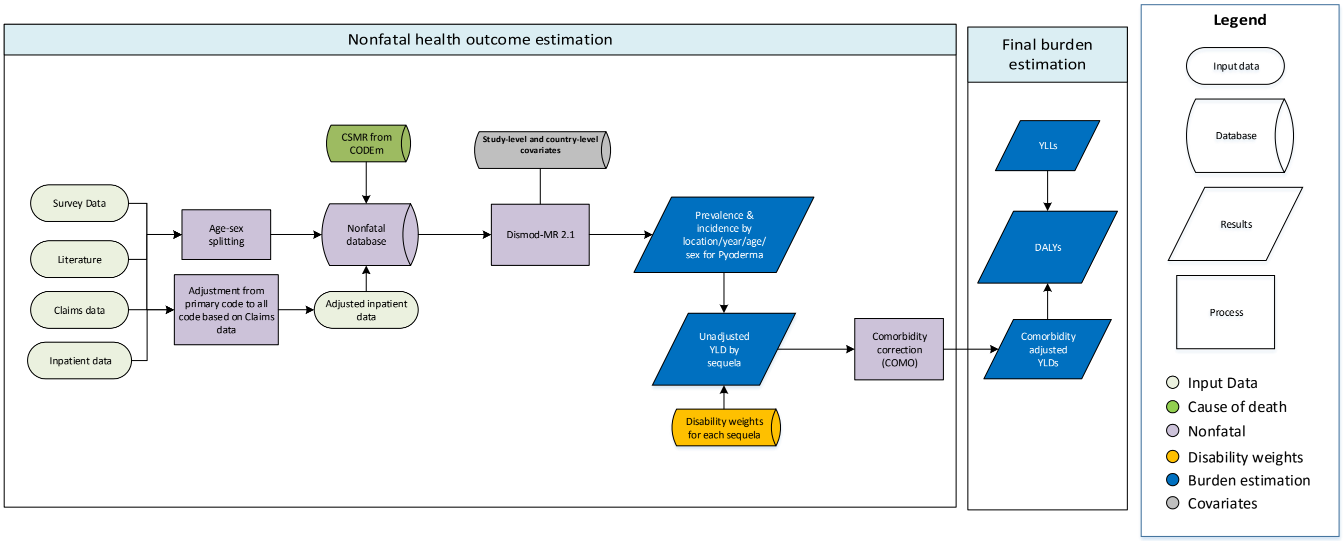

Case definition

Pyoderma refers to any skin disease that is pyogenic, ie, involves the development of pus. These include superficial bacterial conditions such as impetigo, furuncles, ulcers, and abscesses. For GBD 2016, pyoderma was modelled as two separate groups: impetigo, and abscess and other bacterial skin diseases. Impetigo is a highly contagious bacterial skin infection often characterized by red sores, which eventually leak pus or fluid (ICD-10: L01). An abscess is a collection of pus that builds up within the tissue of the body, with carbuncles and furuncles being examples of specific types of abscess. The abscess and other bacterial skin diseases group included all bacterial skin diseases except impetigo (ICD-10: L00, L02, L04, L05, L08).

Input data

Model inputs

For both impetigo and abscess and other bacterial skin diseases in GBD 2010, a literature review was conducted using PubMed and Google Scholar. The inclusion criteria were studies which were published between 1980 and 2010 and provided data on relevant disease incidence or prevalence. Exclusion criteria were studies with no incidence or prevalence data provided, not community- or population-based, outside of year range, sample size smaller than 100, experimental arm of clinical trial, papers that provided estimates rather than data, and studies that were based in dermatology clinics. The agreed approach for pyoderma disease was to undertake a literature review every two years. Therefore, no literature review was undertaken for GBD 2015. For GBD 2016, the GBD 2013 search strategy was replicated to capture epidemiological studies published between 2014 and 2016. Hospital inpatient data were used as model inputs for abscesses and other bacterial skin diseases, but were omitted for

impetigo, as the adjustment factor from primary diagnoses codes to all diagnoses codes were found to be implausible.

Table 1. Data inputs

		Prevalence	Incidence
Impetigo	Studies	8	2
	Countries	6	3
	Countries/subnationals	6	53
	GBD world regions	4	1
Abscess and other bacterial skin diseases	Studies	1	0
	Countries	2	38
	Countries/subnationals	2	322
	GBD world regions	1	7

Severity splits and disability weights

Information on the distribution of cases of impetigo and abscess and other bacterial skin diseases, asymptomatic, and within disfigurement levels 1 and 2, were obtained from the MEPS. The symptomatic cases were assigned the disability weight of a mild acute infectious disease case.

Table 2. Severity level and lay description

Sequela	Severity level	Lay description	DW (95% CI)
Impetigo	Infectious disease, acute episode, mild	The person has a low fever and mild discomfort, but no difficulty with daily activities.	0.006 (0.002–0.012)
Abscesses and other bacterial skin diseases	Infectious disease, acute episode, mild	The person has a low fever and mild discomfort, but no difficulty with daily activities.	0.006 (0.002–0.012)

Modelling strategy

DisMod-MR 2.1 was used to estimate prevalence by age, sex, year, and geography (country, region, super-region) for impetigo and abscess and other bacterial skin diseases. Separate models were run for each disease.

Impetigo: Per expert advice, we assumed a remission of 17 to 20, equating to a duration between approximately two and three weeks. A value prior was also placed on incidence, restricting the range between zero and one. In addition, study-level covariates were placed on incidence to adjust US claims

data for 2000 and 2010 toward the reference literature data and US 2012 claims data. A country-level covariate, log transformed lagged distributed income (I$ per capita), which represents a moving average of gross domestic product (GDP) over time, was also included to inform prevalence and excess mortality estimates. We also used the cause-specific mortality rates for pyoderma estimated using CODEm. We used a time window of 5 years to determine which data points were used for a particular year of fit.

Abscess and other bacterial skin diseases: Per expert advice, a remission setting of 17 to 30 was applied, which equated to a duration of two to six weeks. Study-level covariates adjusted incidence estimates from US claims data for 2000 and 2010 and hospital inpatient data toward the reference literature data and US 2012 claims data. We also used the cause-specific mortality rates for pyoderma estimated using CODEm. In addition, we used a log transformed lagged distributed income (I$ per capita) country covariates on excess mortality. We used a time window of 5 years to determine which data points were used for a particular year of fit.

Table 3. Beta and exponentiated values

Impetigo

**Covariate type**	**Covariate**	**Parameter**	**beta**	**Exponentiated beta**
Study-level covariate	Claims data - 2000	Incidence	-0.30 (-0.32 - -0.28)	0.74 (0.72 - 0.75)
Study-level covariate	Claims data - 2010	Incidence	-0.09 (-0.10 - -0.07)	0.92 (0.90 - 0.93)
Country covariate	LDI (I$ per capita)	Prevalence	0.09 (0.00 - 0.33)	1.09 (1.00 - 1.40)
Country covariate	LDI (I$ per capita)	Excess mortality rate	-0.20 (-0.20 - -0.20)	0.82 (0.82 - 0.82)

Abscesses and other bacterial skin diseases

**Covariate type**	**Covariate**	**Parameter**	**beta**	**Exponentiated beta**
Study-level covariate	Hospital Inpatient	Incidence	-2.40 (-2.40 - -2.39)	0.09 (0.09 - 0.09)
Study-level covariate	Claims data - 2000	Incidence	-0.32 (-0.35 - -0.28)	0.73 (0.70 - 0.76)
Study-level covariate	Claims data - 2010	Incidence	0.02 (-0.01 - 0.05)	1.02 (0.99 - 1.05)
Country covariate	LDI (I$ per capita)	Excess mortality rate	-0.18 (-0.20 - -0.17)	0.83 (0.82 - 0.85)

Scabies

Flowchart

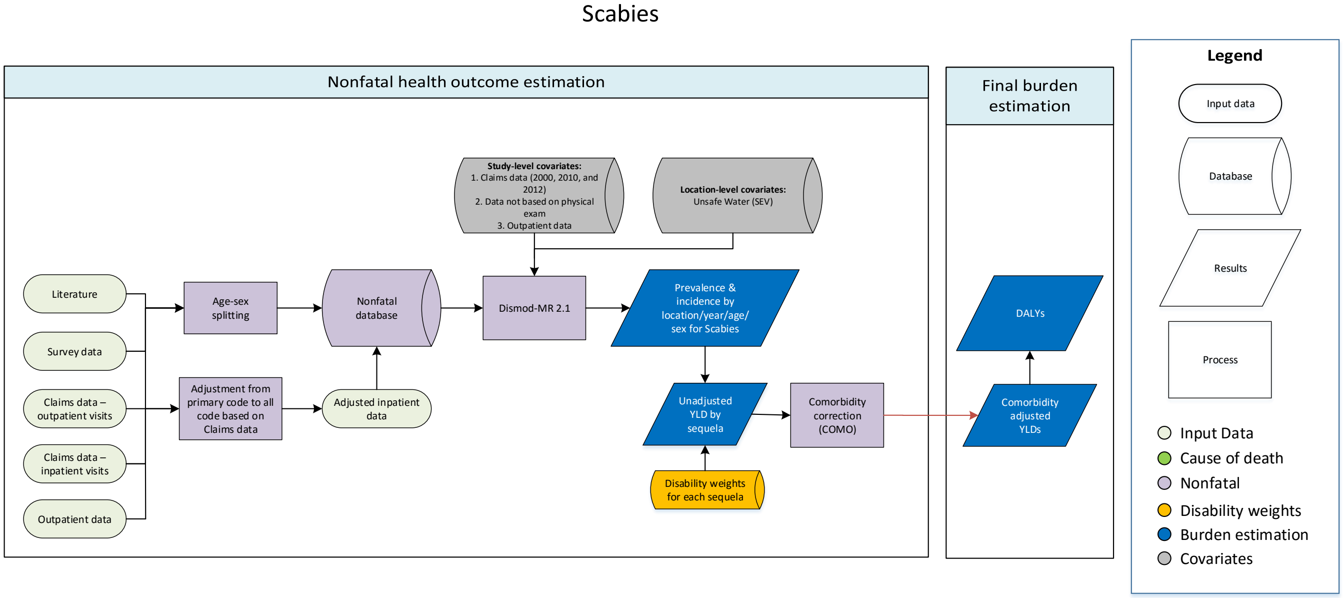

Case definition

Scabies was included in the GBD 2016 cause group of skin and subcutaneous conditions. According to the International Classification of Diseases (ICD-10), scabies is a skin disease caused by the microscopic mite *Sarcoptes scabiei*. The main symptom is an itchy, pimple-like rash (ICD-10: B86) (1).

Input data

Model inputs

In the GBD 2010 study, a systematic review of the literature was conducted using PubMed and Google Scholar to capture epidemiological data for scabies. The inclusion criteria stipulated that studies (1) must be published between 1980 and 2012; (2) must provide data on the incidence or prevalence of scabies; (3) must use samples representative of the general population (ie, samples derived from the experimental arm of clinical trials or based in dermatology clinics were excluded); (4) must use a sample size larger than 100; and (5) must provide sufficient information on study method and sample characteristics to assess the quality of the study. For GBD 2013, the GBD 2010 search strategy was replicated to capture epidemiological studies published between 2012 and 2014. The agreed-upon approach for scabies was to undertake a literature review every two years. Therefore, we updated the systematic review through October 6, 2016, for GBD 2016. Additionally, US claims data from 2000, 2010, and 2012 were included in GBD 2016. Data were outliered or excluded if we found them unreasonable when compared to regional, super-regional, and global rates.

Table 1. Data inputs

	Prevalence	Incidence
Studies	68	20
Countries/subnationals	85	6
Countries	33	5
GBD world regions	17	4
GBD super-regions	7	3

Severity splits

Scabies was assigned the disability weight for disfigurement level 1. The disability weights used for GBD 2015 were also used for GBD 2016.

Table 2. Severity level and lay descriptions

Severity level	Lay description	DW (95% CI)
Disfigurement, level 1 with itch/pain	The individual has a slight, visible physical deformity that is sometimes sore or itchy. Others notice the deformity, which causes some worry and discomfort.	0.027 (0.015–0.042)

Modelling strategy

DisMod-MR 2.1, a Bayesian meta-regression tool, was used to estimate scabies prevalence by age, sex, year, and geography (subnational [select countries], country, region, super-region).

Scabies was modelled with remission set between 2.5 and 3.5, implying four to five months of duration, and excess mortality was assumed to be zero. This was in line with the available epidemiological data, expert opinion, and previous GBD work.

The datasets for scabies were sufficiently large to make use of a relatively short time window of five years to determine which data points were used for a particular year of fit. Additionally, to improve estimation across all regions, we restricted location random effects to (-0.25, 0.25) in Cambodia, Mali, Nepal, Fiji, Timor-Leste, Vanuatu, the Oceania, Southeast Asia, and East Asia GBD regions, and the corresponding super-region. We also restricted the random effect in Kenya (0, 0.5). We used the unsafe water SEV (summary exposure value) as a location-level covariate and set the minimum coefficient of variation at 0.4.

Study-level covariates were used to adjust prevalence derived from outpatient data, US claims data for 2000, 2010, and 2012, and data not based on clinical exams toward the level of other prevalence and incidence data points, which were more representative of the general population.

Table 3. Study-level beta and exponentiated values

Covariate	Parameter	Beta	Exp(beta)
Claims data - 2000	Prevalence	-1.91 (-2.00 — -1.82)	0.15 (0.14 — 0.16)
Claims data - 2010	Prevalence	-1.51 (-1.62 — -1.43)	0.22 (0.20 — 0.24)
Claims data - 2012	Prevalence	-1.29 (-1.39 — -1.20)	0.27 (0.25 — 0.30)
Data was not based on physical examinations	Prevalence	-1.36 (-1.72 — -0.98)	0.26 (0.18 — 0.37)
Outpatient	Prevalence	1.83 (1.70 — 1.97)	6.26 (5.45 — 7.14)

Table 4. Location-level beta and exponentiated values

Covariate	Parameter	Beta	Exp(beta)
Unsafe water (SEV)	Prevalence	1.98 (1.94 — 2.00)	7.24 (6.92 — 7.38)

Fungal Skin Diseases

Flowchart

Tinea Capitis

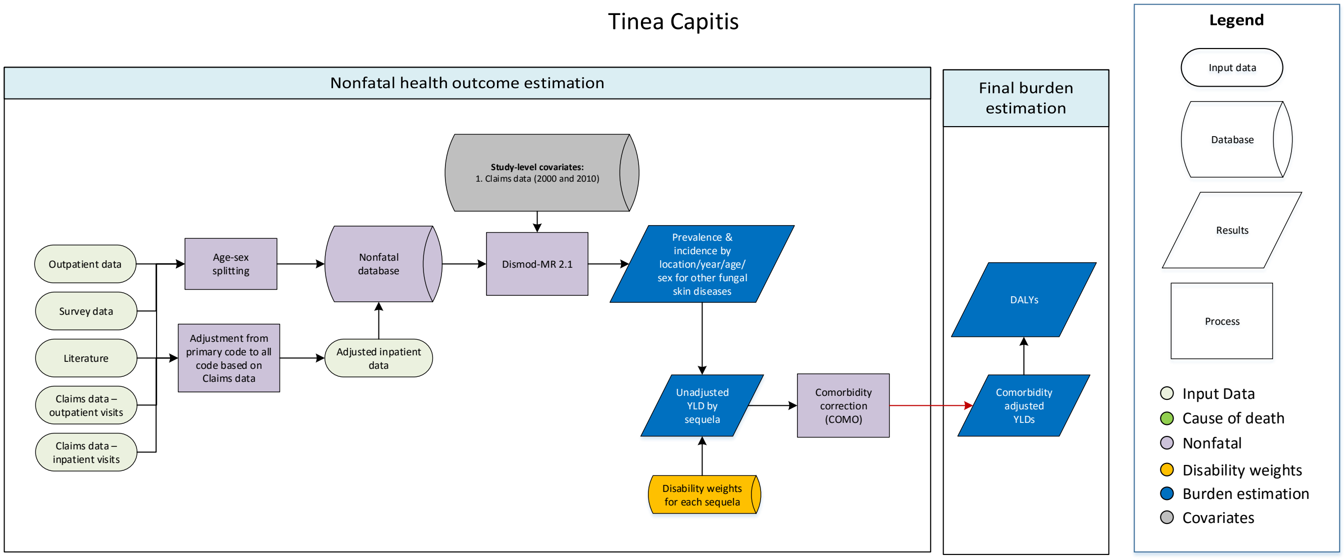

Other Fungal Skin Diseases

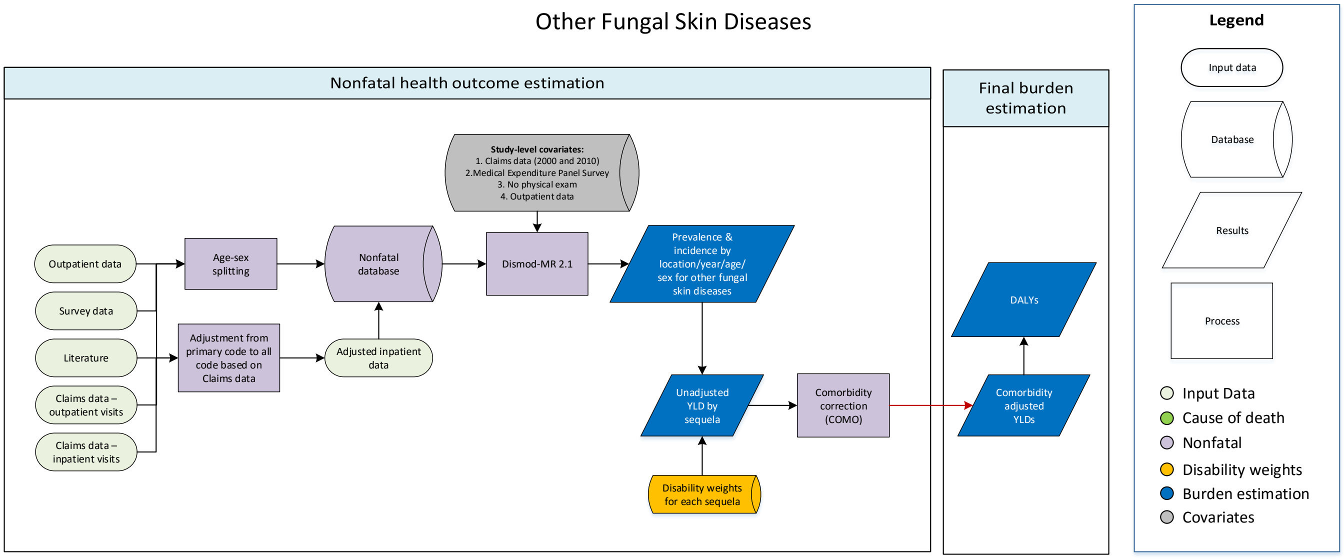

Case definition

Fungal diseases were included in the GBD 2016 cause group of skin and subcutaneous conditions and consisted of tinea capitis and a residual group of "any" other fungal disease. Similar to GBD 2015, tinea capitis was modelled separately from the other fungal skin diseases. This was done to better accommodate differences in burden between tinea capitis and other subtypes of fungal skin diseases.

Tinea capitis is a fungal infection of the scalp and associated hair. It is characterized by the appearance of thickened scaly swellings or as expanding raised red rings (ringworm), mainly caused by species of *Microsporum*, *Trichophyton,* and *Epidermophyton* (ICD-10: B35.0) (1).

The residual group of "any" other fungal skin disease included any fungal skin disease that was specifically not tinea capitis or onychomycosis (ie, fungal nail infection). The ICD-10 (1) list of other fungal skin diseases includes tinea manuum (ICD-10: B35.2), or hand ringworm; tinea pedis (ICD-10: B35.3), or athlete's foot; tinea corporis (ICD-10:B35.4), or ringworm of the body; tinea imbricata (ICD-10:B35.5), a superficial fungal infection limited to parts of Asia and Central America; tinea cruris (ICD-10:B35.6), also known as dhobi itch, groin ringworm, or jock itch. In GBD 2016, we added dermatophytosis (ICD-10:B35.9).

Input data

Model inputs

For GBD 2010, a systematic review of the literature using PubMed and Google Scholar was conducted to capture epidemiological data for fungal skin diseases. The literature search also included any relevant data from the Medical Expenditure Panel Survey (MEPS) in the United States in 2000–2009. The inclusion criteria stipulated that studies (1) must be published between 1980 and 2012; (2) must provide data on the incidence or prevalence of fungal skin diseases; (3) must use samples representative of the general population (ie, samples derived from the experimental arm of clinical trials or based in dermatology clinics were excluded); (4) must use a sample size larger than 100; and (5) must provide sufficient information on study method and sample characteristics to assess the quality of the study. For GBD 2013, the GBD 2010 search strategy was replicated to capture epidemiological studies published between 2012 and 2014. Data were outliered or excluded if we found them unreasonable when compared to regional, super-regional, and global rates.

Table 1. Data inputs

		Prevalence	Incidence
Tinea capitis	Studies	23	0
	Countries/subnationals	68	0
	Countries	17	0
	GBD world regions	7	0
	GBD super-regions	4	0
Other fungal skin diseases	Studies	30	0
	Countries/subnationals	74	0
	Countries	22	0
	GBD world regions	11	0
	GBD super-regions	6	0

In addition, data from US claims for 2000, 2010, and 2012 by US state were included for both tinea capitis and other fungal skin diseases. We also used hospital outpatient data for other fungal skin diseases but decided not to use it for tinea capitis because we decided it would not be representative of true prevalence, and variation between countries in the proportion of true prevalent cases captured in hospital inpatient and outpatient data would likely vary more than can be captured by a single crosswalk

in DisMod-MR 2.1. For tinea capitis, we compared the rates in the outpatient data from Norway, Sweden, Canada, and the USA and found implausibly large differences with the rates from the claims data.

Severity splits

The basis of the GBD disability weight survey assessments are lay descriptions of sequelae highlighting major functional consequences and symptoms. The same disability weight was used for both tinea capitis and other fungal skin diseases.

Table 2. Severity level and lay description

Severity level	Lay description	DW (95% CI)
Infectious disease, acute episode, mild	The person has a low fever and mild discomfort but no difficulty with daily activities.	0.006 (0.002–0.012)

Modelling strategy

DisMod-MR 2.1, a Bayesian meta-regression tool, was used to estimate tinea capitis and other fungal skin diseases prevalence by age, sex, year, and geography (subnational [select countries], country, region, super-region). Separate models were run for tinea capitis and other fungal skin diseases.

Tinea capitis. To help inform the distribution of tinea capitis across the lifespan, excess mortality was set at zero, remission was set at 0.5 to 4, and incidence was set to zero between 20 and 100 years. This was in agreement with the available prevalence data and expert advice. We made use of a relatively long time window of 20 years to determine which data points were used for a particular year of fit. This means that for the year 2000, for instance, DisMod-MR 2.1 incorporated all data points ranging from 1980 to present to estimate prevalence. Study-level covariates in the final model included adjustments for hospital outpatient data and US claims data for 2000 and 2010. In addition, we limited random effects for sub-Saharan Africa (-2,3) and Western Europe (-0.1, 1) to improve model estimates.

Other fungal skin diseases. The modelling strategy was similar to that for tinea capitis with remission set between 0.33 and 4. As Medical Expenditure Panel Survey (MEPS) data points were included in this model, we used a study-level covariate to adjust estimates derived from MEPS, outpatient data, and US claims data for 2000 and 2010 toward the level of prevalence observed in US claims data from 2012 and the literature. We also included a covariate to add uncertainty for data that did not arise from physical examination.

Table 3. Study-level beta and exponentiated values

Cause	Study covariate	Parameter	Beta	Exp(beta)
Tinea capitis	Claims data - 2000	Prevalence	-0.13 (-0.18 - -0.08)	0.88 (0.84 - 0.92)
	Claims data - 2010	Prevalence	-0.10 (-0.14 - -0.06)	0.90 (0.87 - 0.94)
Other fungal skin diseases	MEPS	Prevalence	-0.98 (-1.11 - -0.85)	0.37 (0.33 - 0.43)
	Outpatient	Prevalence	-0.99 (-1.99 - 0.00)	0.37 (0.14 - 1.00)
	Claims data - 2000	Prevalence	-0.20 (-0.23 - -0.18)	0.81 (0.80 - 0.83)
	Claims data - 2010	Prevalence	-0.05 (-0.07 - -0.03)	0.95 (0.94 - 0.97)
	Data was not based on physical examinations*	Prevalence	1.45 (0.66 - 1.98)	4.28 (1.94 - 7.24)

** Covariate on the variance.*

Viral skin diseases

Flowchart

Viral Warts

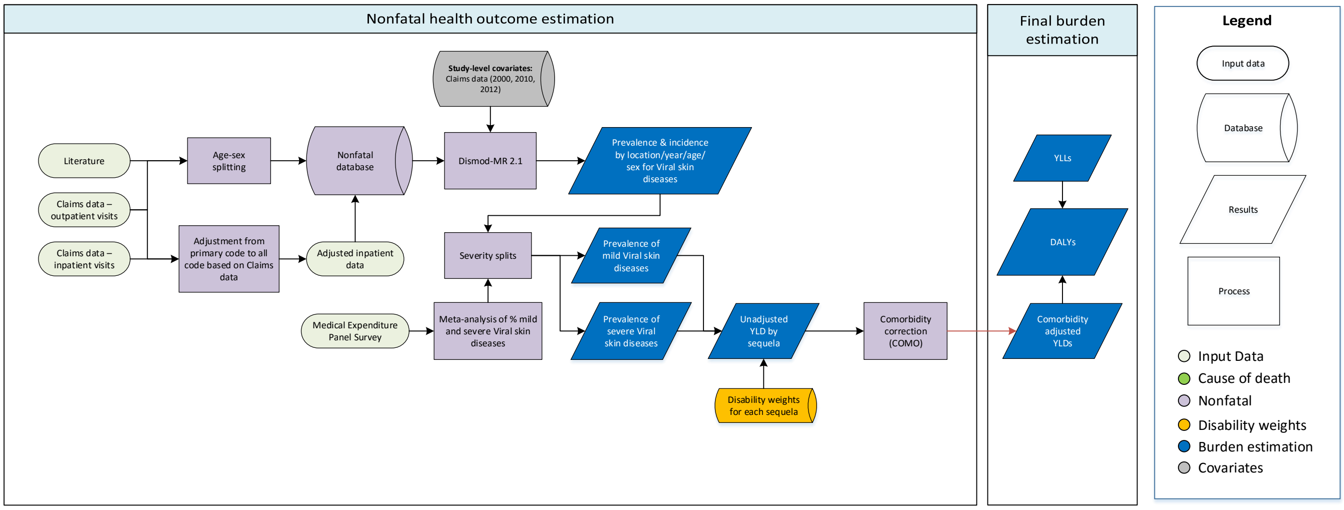

Molluscum Contagiosum

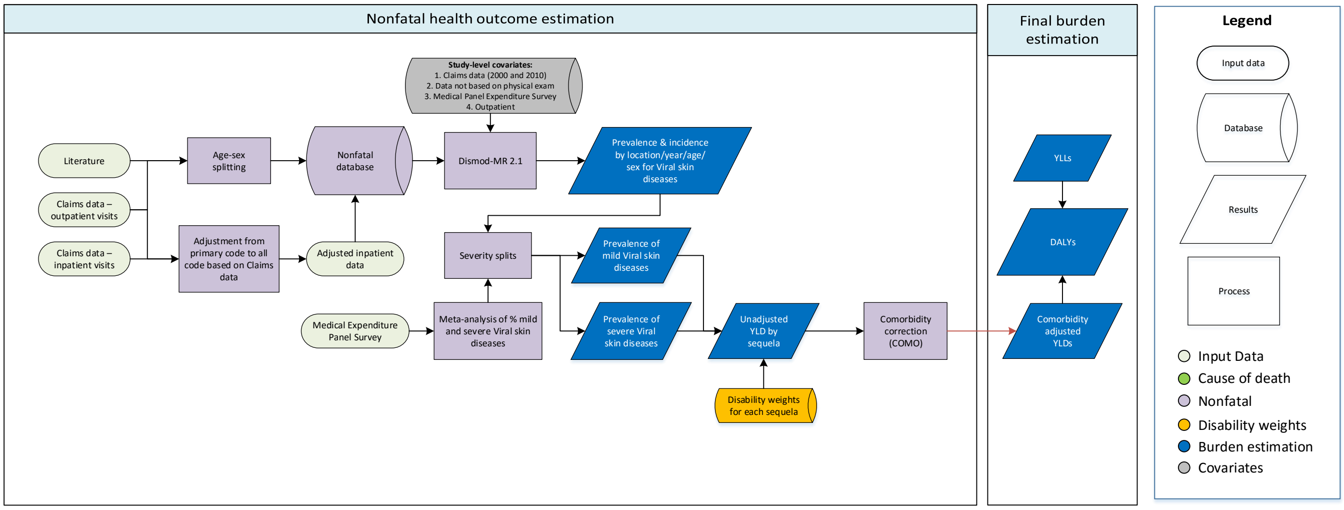

Case definition

Viral skin diseases consist of viral warts and molluscum contagiosum. Viral warts are raised growths on the surface of the skin caused by an infection with the human papillomavirus (ICD-10: B07). Molluscum contagiosum is a viral infection of the skin or occasionally of the mucous membranes characterized by the appearance of waxy, dome-shaped nodules. It is caused by a DNA poxvirus called the molluscum contagiosum virus (ICD-10: B08.1) (1). In GBD 2016, we modelled viral warts and molluscum contagiosum

separately in order to better accommodate differences in burden between the subtypes of viral skin diseases.

Input data

Model inputs

In the GBD 2010 study, a systematic review of the literature was conducted using PubMed and Google Scholar, to capture epidemiological data for viral skin diseases. Due to lack of published data on the epidemiology of viral skin diseases, the literature search also included relevant incidence data from national inpatient or outpatient records in the USA. The inclusion criteria stipulated that studies (1) must be published between 1980 and 2012; (2) must provide data on the incidence or prevalence of viral warts or molluscum contagiosum; (3) must use samples representative of the general population (ie, samples derived from the experimental arm of clinical trials or based in dermatology clinics were excluded); (4) must use a sample size larger than 100; and (5) must provide sufficient information on study method and sample characteristics to assess the quality of the study. For GBD 2013, the GBD 2010 search strategy was replicated to capture epidemiological studies published between 2012 and 2014. Data were outliered or excluded if we found them unreasonable when compared to regional, super-regional, and global rates.

Table 1. Input data

Cause		Prevalence	Incidence
Viral warts	Studies	24	1
	Countries/subnationals	71	1
	Countries	20	1
	GBD world regions	12	1
	GBD super-regions	7	1
Molluscum contagiosum	Studies	11	3
	Countries/subnationals	60	3
	Countries	10	2
	GBD world regions	6	1
	GBD super-regions	4	1

Outpatient data and data from US claims for 2000, 2010, and 2012 by US state were included in GBD 2016, where appropriate. See descriptions of individual modelling approaches for more information.

Severity splits

In GBD 2016, cases of both disorders were allocated a distribution between mild acute infectious disease and disfigurement level 2. The severity splits and disability weights used in GBD 2015 were also applied in GBD 2016.

Table 2. Sequela and disability weight

Sequela	Severity level	Lay description	DW (95% CI)

Mild viral warts	Infectious disease, acute episode, mild	The person has a low fever and mild discomfort, but no difficulty with daily activities.	0.006 (0.002–0.012)
Severe viral warts	Disfigurement, level 2	The person has a visible physical deformity that causes others to stare and comment. As a result, the person is worried and has trouble sleeping and concentrating.	0.067 (0.044–0.096)
Mild molluscum contagiosum	Infectious disease, acute episode, mild	The person has a low fever and mild discomfort but no difficulty with daily activities.	0.006 (0.002–0.012)
Severe molluscum contagiosum	Disfigurement, level 2	The person has a visible physical deformity that causes others to stare and comment. As a result, the person is worried and has trouble sleeping and concentrating.	0.067 (0.044–0.096)

Modelling strategy

For GBD 2016, DisMod-MR 2.1 was used to estimate prevalence, by age, sex, year, and geography (subnational [select countries], country, region, super-region) for viral warts and molluscum contagiosum. Separate models were run for each disease, as illustrated throughout this cause write-up.

Viral warts. Viral warts were modelled with excess mortality set to 0 and remission set between 0.25 and 2, implying a duration of 0.5 to 4 years. This was in line with the levels of prevalence and incidence data, as well as expert opinion. A number of additional settings were used to ensure that DisMod-MR 2.1 sufficiently followed available data points. Incidence was restricted to a maximum of 0.1, and we made use of a relatively long time window of 25 years to determine which data points were used for a particular year of fit. We limited the prevalence random effects for Andean Latin America (-0.2, 0.2) in order to improve model fit. Study-level covariates were used to adjust US claims data for 2000, 2010, and 2012 toward other data points.

Molluscum contagiosum. As available data only contained information on prevalence and incidence, we specified additional expert priors to further inform analyses. Molluscum contagiosum was modelled with excess mortality set to 0 and remission set between 0.5 and 2, implying a duration of 0.5 to 2 years. This

was in line with the available epidemiological data, expert opinion, and previous GBD work. We used a time window of 25 years to determine which data points to include for a particular year of fit. Due to data heterogeneity, we restricted the location random effects to between -0.5 and 0.5 for the Netherlands and select GBD regions and super-regions (Southern Latin America, sub-Saharan Africa, high-income, South Asia, and Southeast Asia, East Asia, and Oceania). Study-level covariates were used to adjust data not based on examination, MEPS, outpatient, and US claims data for 2000 and 2010 toward other data points.

Table 3. Study-level beta and exponentiated values

	Covariate	Parameter	Beta	Exp(beta)
Viral warts	Claims data - 2000	Prevalence	-0.31 (-0.34 — -0.26)	0.73 (0.71 — 0.77)
	Claims data - 2010	Prevalence	-0.19 (-0.22 — -0.14)	0.83 (0.80 — 0.87)
	Claims data - 2012	Prevalence	-0.16 (-0.19 — -0.11)	0.85 (0.83 — 0.89)
Molluscum contagiosum	Claims data - 2000	Prevalence	-1.78 (-1.96 - -1.52)	0.17 (0.14 - 0.22)
	Claims data - 2010	Prevalence	-1.20 (-1.38 - -0.96)	0.30 (0.25 - 0.38)
	Claims data - 2012	Prevalence	-1.06 (-1.23 - -0.82)	0.35 (0.29 - 0.44)

Acne Vulgaris

Flowchart

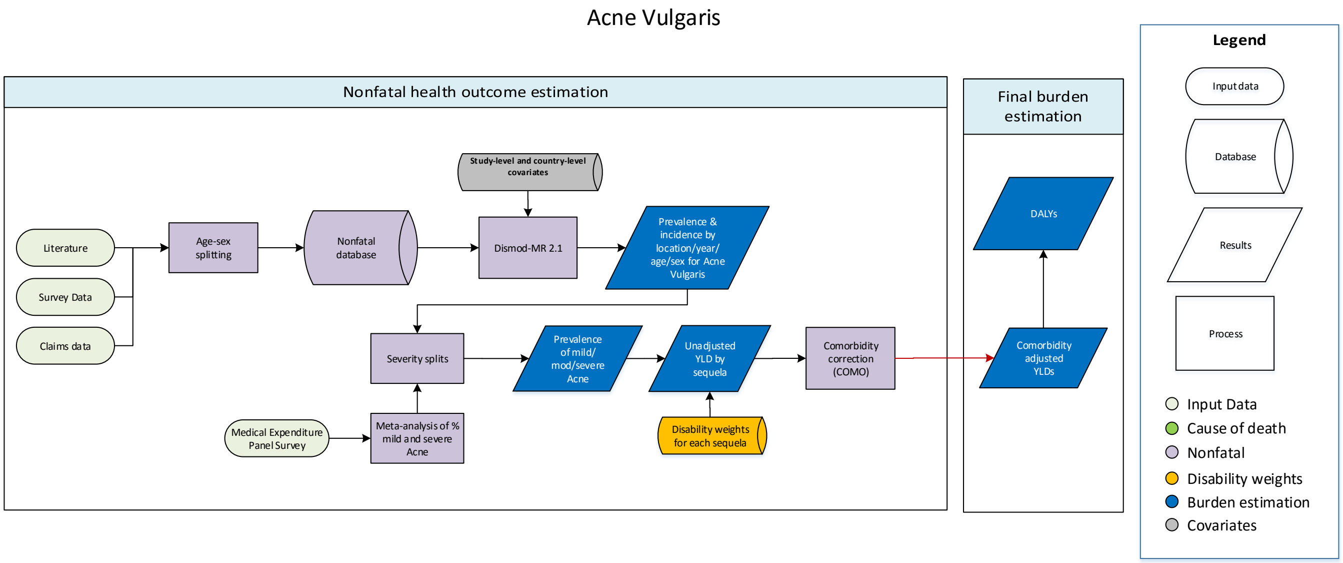

Case definition

Acne vulgaris was included in the GBD 2016 cause group of skin and subcutaneous conditions. Acne vulgaris (or acne) is a chronic inflammatory disease of the pilosebaceous unit associated with an increase in sebum secretion. Included in the GBD 2016 modelling were cases meeting ICD-10 diagnostic criteria for acne vulgaris (ICD-10: l70**).

Input data

Model inputs

In the GBD 2010 study, a systematic review of the literature was conducted using PubMed and Google Scholar to capture epidemiological data for acne vulgaris. The inclusion criteria stipulated that studies (1) must be published between 1980 and 2012; (2) must provide data on the incidence or prevalence of acne vulgaris; (3) must use samples representative of the general population (ie, samples derived from the experimental arm of clinical trials or based in dermatology clinics were excluded); (4) must use a sample size larger than 100; and (5) must provide sufficient information on study method and sample characteristics to assess the quality of the study. The agreed-upon approach for acne vulgaris was to undertake a literature review every two years. For GBD 2016, the GBD 2010 search strategy was replicated in PubMed to capture epidemiological studies published between 2013 and 2016. Additionally, US claims data from 2000, 2010, and 2012 were included.

Table 1. Input data

	Prevalence
Studies	59
Countries	33
Countries/subnationals	86
GBD world regions	7

Severity splits

The table below illustrates the severity level, lay description, and disability weight for acne. In GBD 2016, we added two additional severity levels – disfigurement 2 and disfigurement 3. The disability weight of each severity of acne was applied across forty percent of the total prevalence cases to account for biases in outpatient utilization. The remaining sixty percent of prevalence cases were considered mild cases (disfigurement level 1). These proportions were generated using the ratio of patients seeking care captured from claims data, to all individuals from captured in literature surveying the general population.

Table 2. Severity level and lay description

Sequela	Severity level	Lay description	DW (95% CI)
Mild acne vulgaris	Disfigurement, level 1	The individual has a slight, visible physical deformity that others notice, which causes some worry and discomfort.	0.011 (0.005–0.021)
Moderate acne vulgaris	Disfigurement, level 2	The individual has a visible physical deformity that causes others to stare and comment. As a result, the person is worried and has trouble sleeping and concentrating.	0.067 (0.044–0.096)
Severe acne vulgaris	Disfigurement, level 3	The individual has an obvious physical deformity that makes others uncomfortable, which causes the person to avoid social contact, feel worried, sleep poorly, and think about suicide.	0.405 (0.275–0.546)

Modelling strategy

DisMod-MR 2.1 was used to estimate prevalence by age, sex, year, and country for acne vulgaris.

Since our available data only contained information on prevalence, we specified additional expert priors to further inform analyses. We assumed zero excess mortality and remission from 0.38 to 0.6, implying a duration of approximately two to three years. This was in line with the available epidemiological data, expert opinion, and previous GBD work. A value prior of zero was set for incidence between the ages of 0 and 6, and 61 and 100. We used a time window of five years to determine which data points were used for a particular year of fit.

Study-level covariates were used to adjust prevalence from US claims data for 2000, 2010, and 2012 toward the level of other prevalence data points, which were more representative of the general population. A study-level covariate was applied to data that were not based on physical examinations.

Furthermore, since the data were extremely heterogeneous, the random effects were constrained to (-0.3, 0.3) for regions such as East Asia. In addition, sociodemographic status and sugar consumption were used as country-level covariates to guide estimates for countries with few or no data.

The table below indicates the study covariates, parameters, beta, and exponentiated beta values used in GBD 2016.

Table 3. Beta and exponentiated values

**Covariate type**	**Covariate**	**Parameter**	**beta**	**Exponentiated beta**
Study-level covariate	Data was not based on physical examinations	Prevalence	0.23 (-0.24 - 0.68)	1.26 (0.79 - 1.97)
Study-level covariate	Claims data – 2000	Prevalence	-1.83 (-1.99 - -1.62)	0.16 (0.14 - 0.20)
Study-level covariate	Claims data – 2010	Prevalence	-1.69 (-1.85 - -1.49)	0.18 (0.16 - 0.23)
Study-level covariate	Claims data – 2012	Prevalence	-1.63 (-1.80 - -1.43)	0.20 (0.17 - 0.24)
Country covariate	Socio-demographic Index	Prevalence	0.95 (0.84 - 1.00)	2.60 (2.32 - 2.72)
Country covariate	sugar adjusted(g)	Prevalence	0.00 (-0.00 - 0.00)	1.00 (1.00 - 1.00)

Alopecia Areata

Flowchart

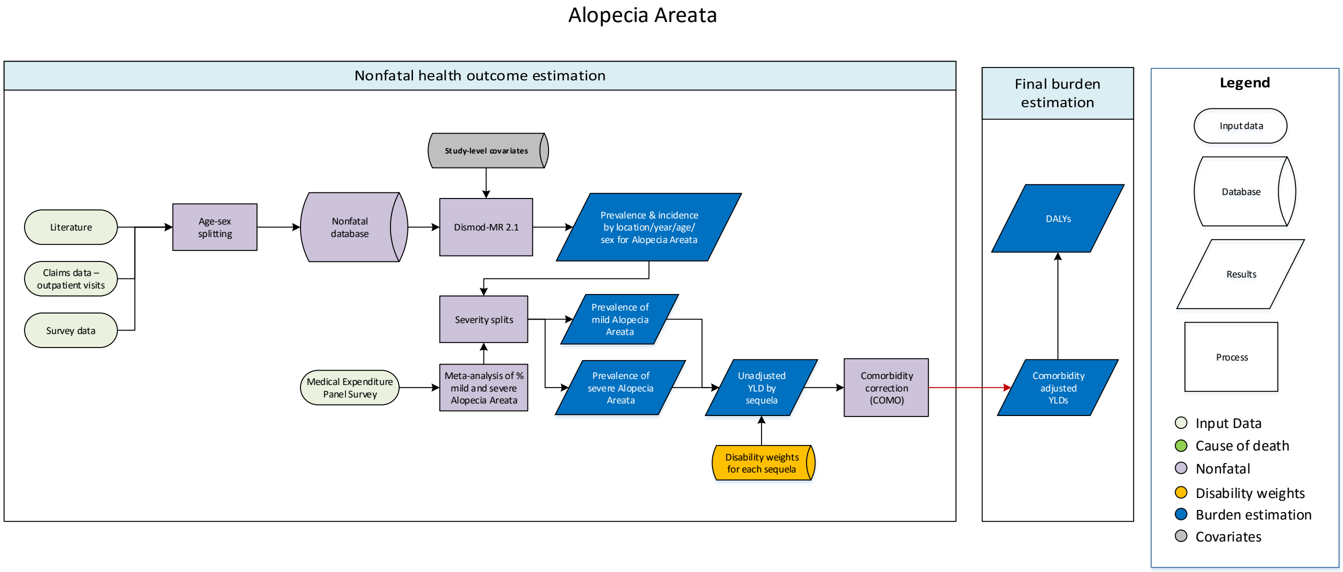

Case definition

Alopecia areata was included in the GBD 2016 cause group of skin and subcutaneous conditions. Alopecia areata is an autoimmune disease that results in hair loss on the scalp and other parts of the body. Included in the GBD disease modelling were cases meeting ICD-10 diagnostic criteria for alopecia (ICD-10: L63).

Input data

Model inputs

In the GBD 2016 study, a systematic review of the literature was conducted using PubMed to expand the GBD dataset (1980–2014) with new epidemiological data for Alopecia areata between 2014 and 2016. The inclusion criteria stipulated that studies (1) must provide data on the incidence or prevalence of alopecia areata; (2) must use samples representative of the general population (ie, samples derived from the experimental arm of clinical trials or based in dermatology clinics were excluded); (3) must use a sample size larger than 100; and (4) must provide sufficient information on study method and sample characteristics to assess the quality of the study. The agreed-upon approach for alopecia areata was to undertake a literature review every two years. Additionally, US claims data from 2000, 2010, and 2012 were included.

Table 1. Model inputs

	Prevalence	Incidence
Studies	18	1
Countries	14	1

Countries/subnationals	64	1
GBD world regions	6	1

Severity splits & disability weights

The table below illustrates the sequela, severity level, lay description, and disability weights associated with Alopecia areata.

Table 2. Severity level and lay description

Sequela	Severity level	Lay description	DW (95% CI)
Mild alopecia areata	Disfigurement, level 1	The individual has a slight, visible physical deformity that others notice, which causes some worry and discomfort.	0.011 (0.005–0.021)
Severe alopecia areata	Disfigurement, level 2	The individual has a visible physical deformity that causes others to stare and comment. As a result, the person is worried and has trouble sleeping and concentrating.	0.067 (0.044–0.096)

Modelling strategy

DisMod-MR 2.1 was used to estimate prevalence by age, sex, year, and country for alopecia areata. We assumed zero excess mortality and remission priors implying a minimum duration of seven months. This was in line with the available epidemiological data, expert opinion, and previous GBD work. We used a time window of 20 years to determine which data points were used for a particular year of fit.

Study-level covariates were used to adjust prevalence derived from US claims data for 2000 and 2010 toward the level of other prevalence data, which were more representative of the general population. To improve estimation across all regions, the minimum global coefficient of variation was set at 0.1. In addition, significant sex differences were observed in the US claims data, resulting in a higher prevalence in females compared to males, likely due to more females seeking health consultations for alopecia areata compared to males. To minimize this effect, we set the sex covariate to zero, but this had minimal impact on the global estimates.

Table 3. Beta and exponentiated values

**Covariate type**	**Covariate**	**Parameter**	**beta**	**Exponentiated beta**
Study-level covariate	Claims data - 2000	Prevalence	-0.69 (-0.74 - -0.64)	0.50 (0.48 - 0.53)

Study-level covariate	Claims data - 2010	Prevalence	-0.07 (-0.11 - -0.02)	0.93 (0.89 - 0.98)

Pruritus

Flowchart

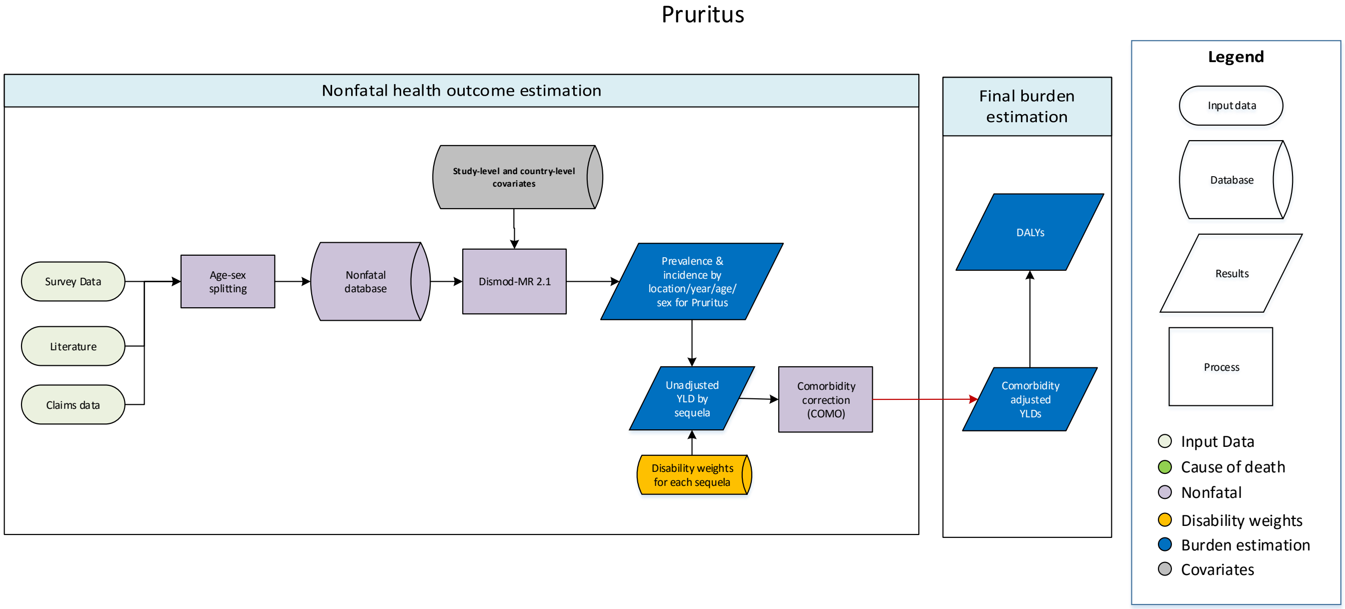

Case definition

Pruritus was included in the GBD 2016 cause group of skin and subcutaneous conditions. Pruritus (or itching) can be a symptom of a condition or disease. Included in the GBD disease modelling were cases meeting ICD-10 diagnostic criteria for pruritus (ICD-10: L29).

Input data

Model inputs

In the GBD 2010 study, a systematic review of the literature was conducted using PubMed and Google Scholar to capture epidemiological data for pruritus. The inclusion criteria stipulated that studies (1) must be published between 1980 and 2012; (2) must provide data on the incidence or prevalence of pruritus; (3) must use samples representative of the general population (ie, samples derived from the experimental arm of clinical trials or based in dermatology clinics were excluded); (4) must use a sample size larger than 100; and (5) must provide sufficient information on study method and sample characteristics to assess the quality of the study. The agreed-upon approach for pruritus was to undertake a literature review every two years. For GBD 2016, the GBD 2010 search strategy was replicated in PubMed to capture epidemiological studies published between 2013 and 2016. Additionally, US claims data from 2000, 2010, and 2012 were included.

Table 1. Data inputs

		Prevalence
Pruritus	Studies	5
	Countries	5
	Countries/subnationals	56
	GBD world regions	2

Severity splits

The table below illustrates the severity level, lay description, and disability weight for pruritus.

Table 2. Severity level and lay description

Sequela	Severity level	Lay description	DW (95% CI)
Pruritus	Disfigurement, level 1	The individual has a slight, visible physical deformity that others notice, which causes some worry and discomfort.	0.011 (0.005–0.021)

Modelling strategy

DisMod-MR 2.1 was used to estimate prevalence by age, sex, year, and country for pruritus.

Per expert advice, remission was set from 0.2 to 1, implying a duration of three months to one years. We used a time window of 25 years to determine which data points were used for a particular year of fit.

Study-level covariates were used to adjust prevalence derived from self-reported literature, MEPS, and US claims data for 2000 and 2010 toward the level of US claims data 2012, which were more representative of the general population.

The data were extremely heterogeneous. Therefore, the random effects were constrained to (-0.2, 0.2) and lagged distributed income was used as a country-level covariate to guide estimates for countries with few or no data.

Table 3. Beta and exponentiated values

**Covariate type**	**Covariate**	**Parameter**	**beta**	**Exponentiated beta**
Study-level covariate	MEPS	Prevalence	-1.33 (-1.65 - -1.07)	0.26 (0.19 - 0.34)
Study-level covariate	Self-reported	Prevalence	1.61 (0.95 - 1.99)	5.02 (2.59 - 7.32)
Study-level covariate	Claims data – 2000	Prevalence	-0.74 (-0.78 - -0.71)	0.48 (0.46 - 0.49)

Study-level covariate	Claims data – 2010	Prevalence	-0.16 (-0.18 - -0.14)	0.85 (0.84 - 0.87)
Country covariate	LDI (I$ per capita)	Prevalence	0.06 (-0.11 - 0.19)	1.06 (0.90 - 1.21)

Urticaria

Flowchart

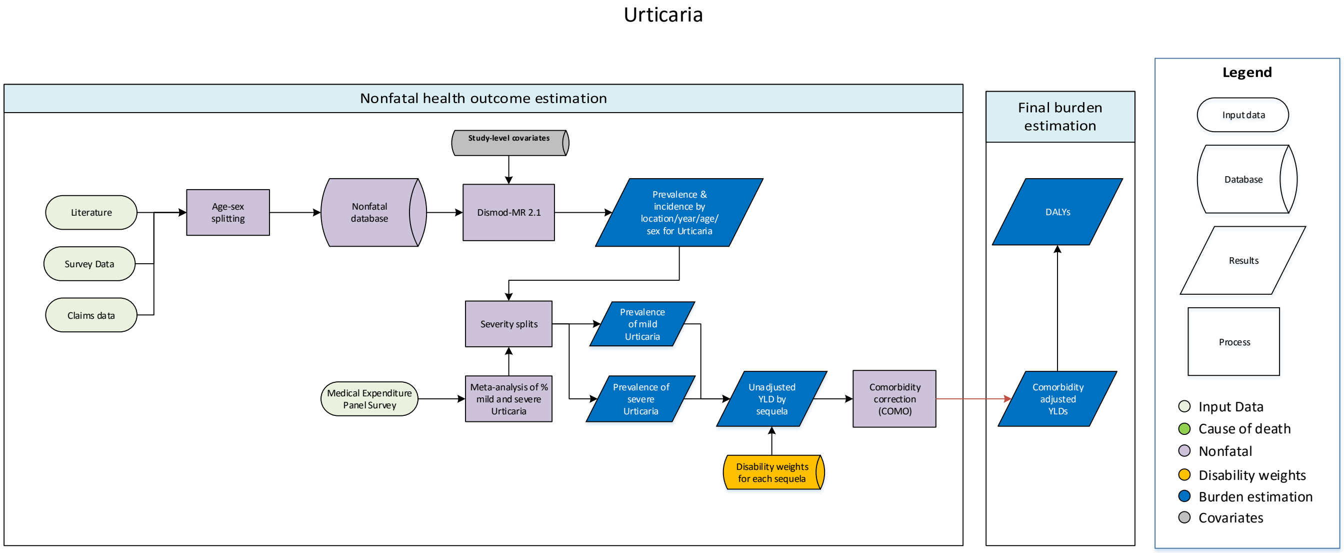

Case definition

Urticaria was included in the GBD 2016 cause group of skin and subcutaneous conditions. Urticaria (hives) refers to a skin reaction that causes itchy, raised bumps. Included in the GBD disease modelling were cases meeting ICD-10 diagnostic criteria for urticaria (ICD-10: L50).

Input data

Model inputs

In the GBD 2010 study, a systematic review of the literature was conducted using PubMed and Google Scholar to capture epidemiological data for urticaria. The inclusion criteria stipulated that studies (1) must be published between 1980 and 2012; (2) must provide data on the incidence or prevalence of urticaria; (3) must use samples representative of the general population (ie, samples derived from the experimental arm of clinical trials or based in dermatology clinics were excluded); (4) must use a sample size larger than 100; and (5) must provide sufficient information on study method and sample characteristics to assess the quality of the study.

The agreed-upon approach for urticaria was to undertake a literature review every two years. For GBD 2016, the GBD 2010 search strategy was replicated to capture epidemiological studies published between the previous 2013 and 2016. Additionally, US claims data from 2000, 2010, and 2012 were included in the data used for GBD 2016.

The table below illustrates the data inputs used in GBD 2016 by number of studies, geographic location, and prevalence/incidence.

Table 1. Data inputs

	Prevalence
Studies	22
Countries	21
Countries/subnationals	72
GBD world regions	6

Severity splits & disability weights

The table below illustrates the severity level, lay description, and disability weight for urticaria.

Table 2. Severity level and lay descriptions

Sequela	Severity level	Lay description	DW (95% CI)
Mild urticaria	Disfigurement, level 1 with itch/pain	The person has a slight, visible physical deformity that is sometimes sore or itchy. Others notice the deformity, which causes some worry and discomfort.	0.027 (0.015–0.042)
Severe urticaria	Disfigurement, level 2, with itch/pain	The person has a visible physical deformity that is sore and itchy. Other people stare and comment, which causes the person to worry. The person has trouble sleeping and concentrating.	0.188 (0.124–0.267)

Modelling strategy

DisMod-MR 2.1 was used to estimate prevalence by age, sex, year, and country for urticaria.

The available data were mainly composed of prevalence estimates with a few incidence data points. For GBD 2016, we made both prevalence and incidence estimates. We used a time window set to 25 years. We set excess mortality to zero and remission between 0.5 to 2, implying a duration between ½ and 2 years. In addition, location random effects were constrained to (-0.3, 0.3). US claims data from year 2000 and 2010 were adjusted towards the most complete US 2012 set. Specific data points were outliered if they were overestimates or underestimates in comparison to country, regional and global patterns.

The table below illustrates the covariate used in the modelling process, as well as associated parameters, beta, and exponentiated beta (confidence interval) values for GBD 2016.

Table 3. Study covariate

Covariate type	Covariate	Parameter	beta	Exponentiated beta
Study-level covariate	Claims data - 2000	Prevalence	-0.23 (-0.25 - -0.22)	0.79 (0.78 - 0.80)
Study-level covariate	Claims data - 2010	Prevalence	-0.06 (-0.08 - -0.05)	0.94 (0.92 - 0.95)

Decubitus Ulcer

Flowchart

Decubitus Ulcer

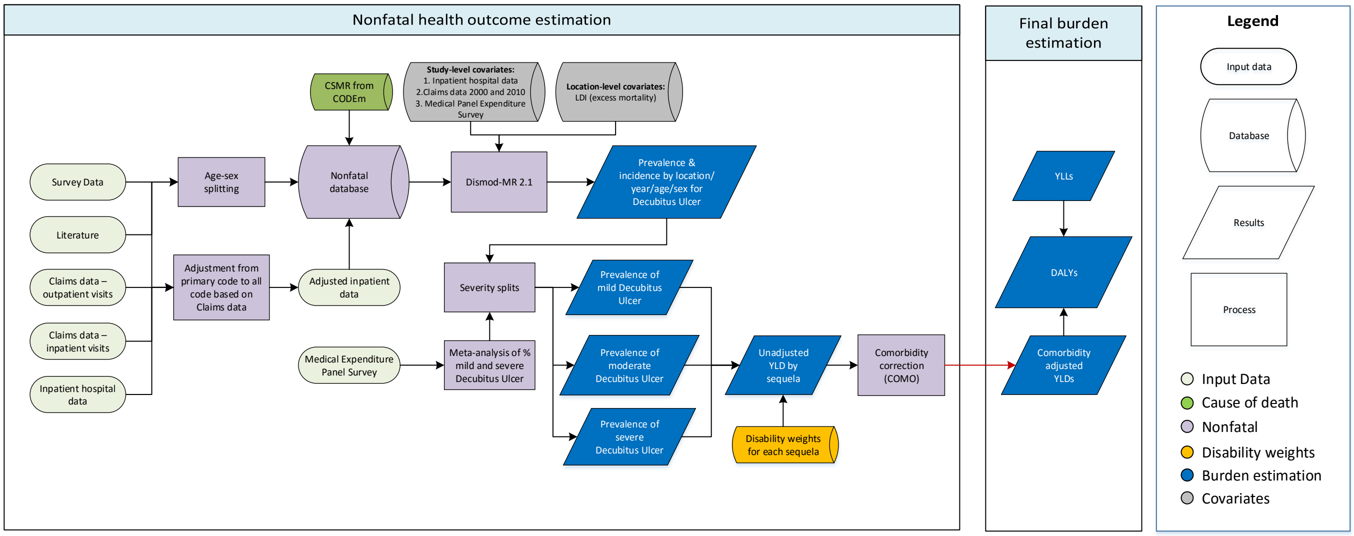

Case definition

Decubitus ulcer was included in the GBD 2016 cause group of skin and subcutaneous conditions. Decubitus ulcer, also known as pressure ulcer/sore, is an injury to the skin and underlying tissue resulting from an obstruction of blood flow due to pressure on the skin. Included in the GBD modelling were cases meeting ICD-10 criteria for decubitus ulcer (ICD-10: L89) (1).

Input data

Model inputs

In the GBD 2010 study, a systematic review of the literature was conducted using PubMed and Google Scholar to capture epidemiological data for decubitus ulcer. The inclusion criteria stipulated that studies (1) must be published between 1980 and 2012; (2) must provide data on the incidence or prevalence of decubitus ulcer; (3) must use samples representative of the general population (ie, samples derived from the experimental arm of clinical trials or based in dermatology clinics were excluded); (4) must use a sample size larger than 100; and (5) must provide sufficient information on study method and sample characteristics to assess the quality of the study. The data from literature were sparse but contained both prevalence and incidence estimates. For GBD 2013, the GBD 2010 search strategy was replicated to capture epidemiological studies published between 2012 and 2014. The available data were from high-income countries. Hospital inpatient and US claims data from 2000, 2010, and 2012 were also used for GBD 2016. The final dataset also included cause-specific mortality rates for decubitus ulcer estimated by

CODEm. Data were outliered or excluded if we found them unreasonable when compared to regional, super-regional, and global rates.

Table 1. Data inputs (not including hospital inpatient data)

	Prevalence	Incidence
Studies	0	2
Countries/subnationals	0	308
Countries	0	34
GBD world regions	0	15
GBD super-regions	0	6

Severity splits

For GBD 2016, decubitus ulcer was assigned the disability weight, disfigurement with itch/pain, levels 1, 2, and 3. The disability weights used for GBD 2015 were based on disfigurement only.

Table 2. Severity level and lay descriptions

Sequela	Severity level	Lay description	DW (95% CI)
Mild decubitus ulcer	Disfigurement, level 1 with itch/pain	The person has a slight, visible physical deformity that is sometimes sore or itchy. Others notice the deformity, which causes some worry and discomfort.	0.027 (0.015–0.042)
Moderate decubitus ulcer	Disfigurement, level 2, with itch/pain	The person has a visible physical deformity that is sore and itchy. Other people stare and comment, which causes the person to worry. The person has trouble sleeping and concentrating.	0.188 (0.124–0.267)
Severe decubitus ulcer	Disfigurement, level 3, with itch/pain	The person has an obvious physical deformity that is very painful and itchy. The physical deformity makes others uncomfortable, which causes the person to avoid social contact, feel worried, sleep poorly, and think about suicide.	0.576 (0.401–0.731)

Modelling strategy

DisMod-MR 2.1 was used to estimate prevalence by age, sex, year, and geography (subnational [select countries], country, region, super-region) for decubitus ulcer. Per expert advice, remission was set from 3 to 4, implying a duration of three to four months. This was based on the assumption that remission does not change with treatment. These values were also in line with the available epidemiological data, expert opinion, and previous GBD work. The decubitus ulcer dataset was sufficiently large to make use of a relatively short time window of five years to determine which data points were used for a particular year of fit.

Study-level covariates were used to adjust incidence derived from hospital inpatient data, MEPS, and US claims data for 2000 and 2010 toward the level of other incidence data points, which were more representative of the general population. We excluded estimates less than 10E-6. Log-transformed lagged distributed income (LDI) was used as a location-level covariate on excess mortality to guide estimates for countries with few or no data.

Table 3. Beta and exponentiated values

Study covariate	Parameter	Beta	Exp(beta)
Hospital Inpatient	Incidence	-0.00 (-0.00 - -0.00)	1.00 (1.00 - 1.00)
MEPS	Incidence	-0.08 (-1.12 - 0.85)	0.93 (0.33 - 2.35)
Claims data - 2000	Incidence	-0.52 (-0.56 - -0.47)	0.60 (0.57 - 0.62)
Claims data - 2010	Incidence	-0.30 (-0.34 - -0.26)	0.74 (0.71 - 0.77)

Table 4. Location-level beta and exponentiated values

Country-level covariate	Parameter	Beta	Exp(beta)
LDI (log-transformed)	Excess mortality rate	-0.50 (-0.50 — -0.49)	0.61 (0.61 — 0.61)

Other skin and subcutaneous diseases

Flowchart

Other Skin and Subcutaneous Diseases (OSSD)

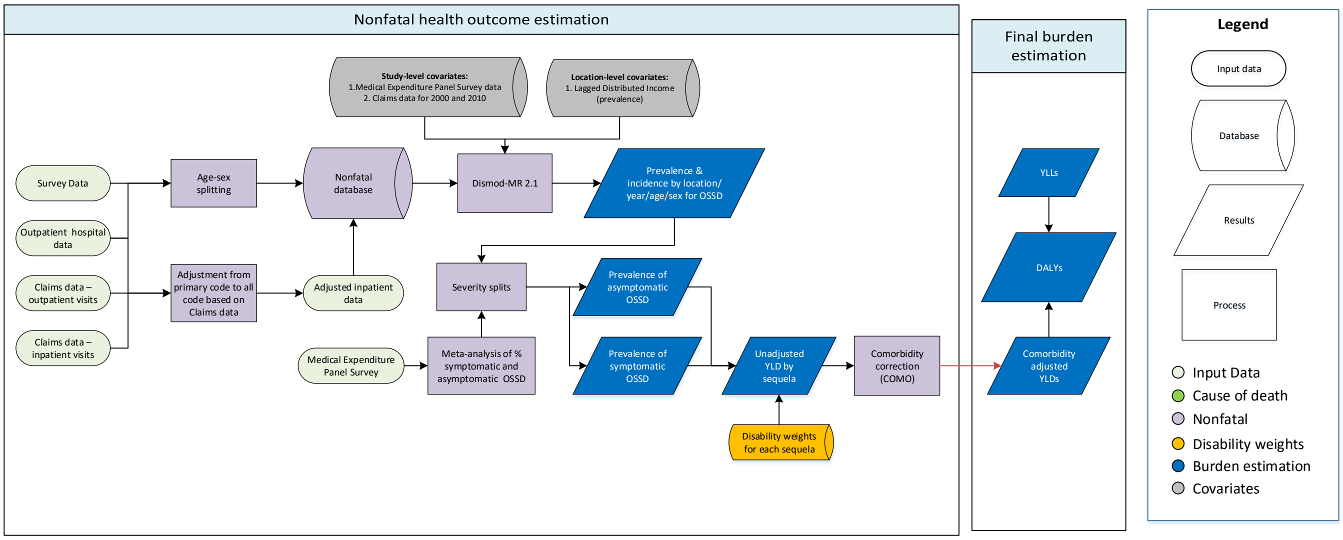

Case definition

The other skin and subcutaneous diseases category encompassed a large group of skin conditions not captured in the other skin categories. We included cases meeting the following ICD-10 diagnostic criteria: other viral infections characterized by skin and mucous membrane lesions, not elsewhere classified (B08), unspecified viral infection characterized by skin and mucous membrane lesions (B09), pediculosis and phthiriasis (B85), myiasis (B87), other infestations (B88), sarcoidosis of skin (D86.3), porphyria cutanea tarda (E80.1), other and unspecified porphyria (E80.2), pemphigus (L10), other acantholytic disorders (L11), pemphigoid (L12), other bullous disorders (L13), bullous disorders in diseases classified elsewhere (L14), lichen simplex chronicus and prurigo (L28), pityriasis rosea (L42), lichen planus (L43), other papulosquamous disorders (L44), papulosquamous disorders in diseases classified elsewhere (L45), exfoliation due to erythematous conditions according to extent of body surface involved (L49), erythema multiforme (L51), erythema nodosum (L52), other erythematous conditions (L53), erythema in diseases classified elsewhere (L54), other acute skin changes due to ultraviolet radiation (L56), skin changes due to chronic exposure to nonionizing radiation (L57), other disorders of skin and subcutaneous tissue related to radiation (L59), nail disorders (L60), nail disorders in diseases classified elsewhere (L62), androgenic alopecia (L64), other nonscarring hair loss (L65), cicatricial alopecia [scarring hair loss] (L66), hair color and hair shaft abnormalities (L67), hypertrichosis (L68), rosacea (L71), follicular cysts of skin and subcutaneous tissue (L72), other follicular disorders (L73), eccrine sweat disorders (L74), apocrine sweat disorders (L75), vitiligo (L80), other disorders of pigmentation (L81), seborrheic keratosis (L82), acanthosis

nigricans (L83), corns and callosities (L84), other epidermal thickening (L85), keratoderma in diseases classified elsewhere (L86), transepidermal elimination disorders (L87), atrophic disorders of skin (L90), hypertrophic disorders of skin (L91), granulomatous disorders of skin and subcutaneous tissue (L92), other localized connective tissue disorders (L94), vasculitis limited to skin, not elsewhere classified (L95), and other disorders of skin and subcutaneous tissue in diseases classified elsewhere (L99).

Input data

Model inputs

In the GBD 2010 study, a systematic review of the literature was conducted using PubMed and Google Scholar to capture epidemiological data for skin diseases not captured in the other skin categories. The inclusion criteria stipulated that studies (1) must be published between 1980 and 2012; (2) must provide data on the incidence or prevalence; (3) must use samples representative of the general population (ie, samples derived from the experimental arm of clinical trials or based in dermatology clinics were excluded); (4) must use a sample size larger than 100; and (5) must provide sufficient information on study method and sample characteristics to assess the quality of the study. For GBD 2013, the GBD 2010 search strategy was replicated to capture epidemiological studies published between 2012 and 2014. Hospital outpatient data and data from US claims for 2000, 2010, and 2012 by US state were included in GBD 2016. Data were outliered or excluded if we found them unreasonable when compared to regional, super-regional, and global rates.

Table 1. Data inputs (not including hospital inpatient data)

	Prevalence
Studies	1
Countries/subnationals	52
Countries	1
GBD world regions	1
GBD super-regions	1

Severity split & disability weight

Skin and other subcutaneous diseases were assigned the disability weight for disfigurement level 1.

Table 2. Severity level and lay description

Sequela	Severity level	Lay description	DW (95% CI)
Symptomatic other skin and subcutaneous diseases	Disfigurement, level 1	The person has a slight, visible physical deformity that others notice, which causes some worry and discomfort.	0.011 (0.005–0.021)

Modelling strategy

DisMod-MR 2.1, a Bayesian meta-regression tool, was used to estimate prevalence by age, sex, year, and geography (subnational [select countries], country, region, super-region) for skin and other subcutaneous diseases.

We assumed remission of one, implying a duration of 12 months. Similar to GBD 2015, we used a time window of 25 years to determine which data points were used for a particular year of fit.

Study-level covariates, which were used to adjust prevalence, were derived from the Medical Expenditure Panel Survey (MEPS) and US claims data for 2000 and 2010 toward the level of other prevalence data, which were more representative of the general population. In addition, log-transformed lagged distributed income was used as a location-level covariate to guide estimates for countries with few or no data.

Table 3. Study-level beta and exponentiated values

Covariate	Parameter	Beta	Exp(beta)
Claims data – 2000	Prevalence	-0.28 (-0.29 — -0.27)	0.76 (0.75 — 0.77)
Claims data – 2000	Prevalence	-0.06 (-0.07 — -0.05)	0.94 (0.93 — 0.95)
MEPS	Prevalence	-0.64 (-0.73 — -0.54)	0.53 (0.48 — 0.58)

Table 4. Location-level beta and exponentiated values

Covariate	Parameter	Beta	Exp(beta)
LDI (log-transformed)	Prevalence	0.28 (0.27 — 0.30)	1.33 (1.31 — 1.35)

Other sense organ diseases

Flowchart

Other sense organ diseases

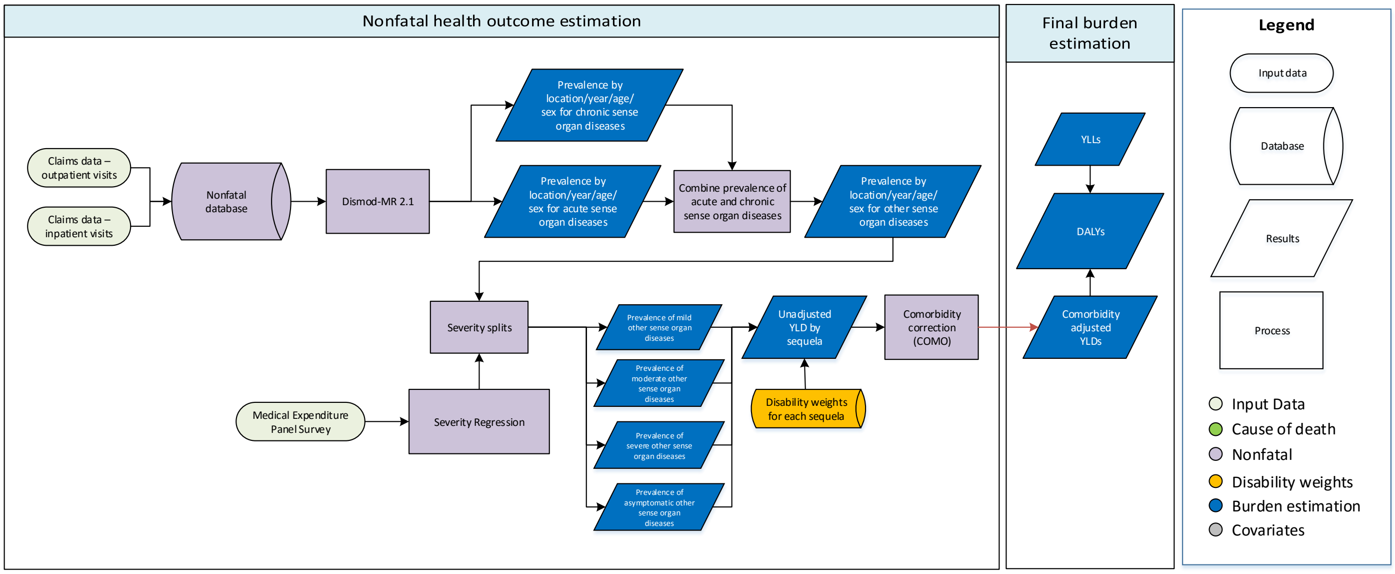

Case definition

Other sense organ disease is a residual cause capturing both acute and chronic conditions that do not map to other causes, but lead to non-trivial morbidity. These include the following ICD codes: 077, 360, 364, 370-77, 379, 380, 386, and 388, which encompass a plethora of eye and ear disorders and conditions.

077	Other diseases of conjunctiva due to viruses and chlamydiae
360	Disorders of the globe
364	Disorders of iris and ciliary body
370-77	Keratits, Corneal opacity and other disorders of cornea, Disorders of conjunctiva, Inflammation of eyelids, Other disorders of eyelids, Disorders of lacrimal system, Disorders of the orbit, Disorders of optic nerve and visual pathways
379	Other disorders of eye
380	Disorders of external ear
386	Vertiginous syndromes and other disorders of vestibular system
388	Other disorders of ear

Input data

Model inputs

For GBD 2016, we used claims data from US claims data to model other sense organ diseases, since these conditions would not appear in inpatient hospital data. ICD-9 codes were assigned at the five-digit level to either acute or chronic conditions as listed elsewhere in the Appendix table 4.

Severity splits & disability weights

The basis of the GBD disability weight survey assessments are lay descriptions of sequelae highlighting major functional consequences and symptoms. Severity splits for other sense organ diseases were calculated via the Medical Expenditure Panel Survey (MEPS) regression and outlined in the table below.

<u>Chronic:</u>

Severity	Proportion	Health state	Disability weight
Severe	0.21 (0.15–0.28)	Vertigo	
Moderate	0.37 (0.30–0.42)	This person has slight physical deformity which causes some worry and discomfort	0.011 (0.005–0.021)
Asymptomatic	0.42 (0.41–0.44)	Asymptomatic	N/A

<u>Acute</u>

Severity	Proportion	Health state	Disability weight
Mild	0.30 (0.23–0.37)	This person has low fever and mild discomfort but no difficulty with daily activities	0.006 (0.002–0.012)
Moderate	0.25 (0.18–0.32)	This person has slight physical deformity which causes some worry and discomfort	0.011 (0.005–0.021)
Asymptomatic	0.45 (0.43–0.46)	Asymptomatic	N/A

Modelling strategy

For GBD 2016, hospital data were extracted separately for the chronic and acute conditions included in other sense organ diseases. The chronic data were extracted as prevalence, and acute as incidence. We then ran two separate DisMod-MR 2.1 models. The chronic model, with prevalence data, was run as a prevalence-only model. The acute model was run as a full model with incidence data, assuming zero excess mortality and duration of one week (remission 52). In both models, to correct for systematically lower data from 2000 MarketScan claims, we used a study-level covariate to crosswalk the 2000 data. Since the only data source is from the United States, we did not use any country-level covariates in this model.

We then aggregated chronic and acute prevalence outputs, resulting in the prevalence of other sense organ diseases by country, age, year, and sex.

Results from GBD 2016 are higher for other sense organ diseases because in GBD 2013 we used MEPS self-reported claims data, whereas in GBD 2015 and GBD 2016 MarketScan more accurately captures the

occurrence of these conditions. In GBD 2016 we were able to separately model acute and chronic conditions.

Caries of Deciduous Teeth

Flowchart

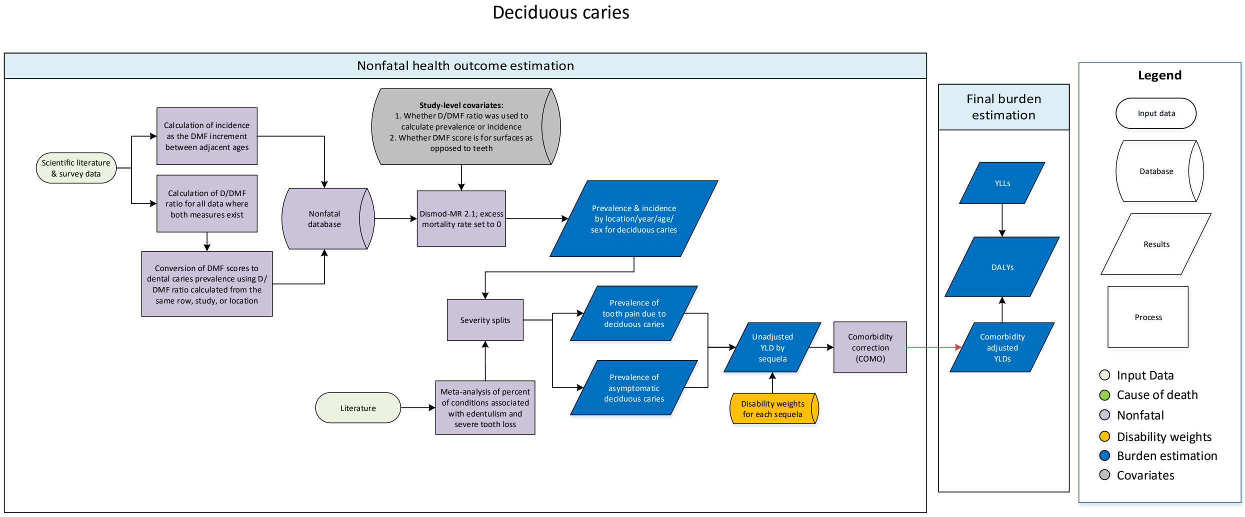

Case definition

The case definition for dental caries is “teeth with unmistakable coronal cavity at dentin level, root cavity in cementum that feels soft or leathery to probing, temporary or permanent restorations, or missing teeth extracted due to a caries lesion.” This definition corresponds to an ICD-9 code of 521.0 and an ICD-10 code of K02.3 – K02.9. Most caries are subclinical in the sense that they do not cause symptoms a majority of the time. Once a carious lesion develops, it will occasionally recede without intervention. Generally, however, it worsens with time and eventually requires either filling or extraction. The major sequela associated with the condition is symptomatic caries, which is defined as “a toothache which causes some difficulty eating.”

Deciduous teeth are colloquially known by several names throughout the world, including “baby,” “milk,” or “fall” teeth. They start erupting in infants around 6 months of age and generally finish by the end of year three. Exfoliation of deciduous teeth begins around age 5-6 and usually is complete by age 12-14, when only permanent teeth remain. The name of this cause for GBD 2016 has been changes from ‘deciduous caries’ to ‘caries of deciduous teeth’.

Dental caries and the DMFT index

Public health dentists commonly measure dental caries using the dmft/DMFT index, which is an incremental measure of the proportion of unhealthy teeth and is also a measure of an individual’s lifetime prevalence of caries. Lowercase letters (dmft) are used for deciduous dentition and uppercase letters (DMFT) for permanent dentition. D is for decayed, M for missing, F for filled, and T for teeth. The maximum dmft score is 20 and the maximum DMFT score is 32. Furthermore, some dentists prefer to measure dental caries in terms of tooth surfaces, rather than number of teeth, and report their results using an analogous dmfs/DMFS index. The maximum dmfs score is 88, and the maximum DMFS score is 128 or 148 depending on whether the third molars are counted.

The DMFT index is easy to measure and inter-rater reliability is high. However, the primary shortcoming of the DMFT is that it does not discriminate well between current and past caries. Strategies we employed to maximally utilize dmf/DMF data for estimating the prevalence of burden due to caries of deciduous teeth are described below.

Input data

Model inputs

Literature reviews

A literature review was conducted by the expert group for GBD 2010, and an additional systematic review was performed for GBD 2013. The search terms used in the GBD 2013 literature review for deciduous caries were (Deciduous caries[Title/Abstract]) OR (milk caries[Title/Abstract]) OR (baby caries[Title/Abstract]) OR (caries[Title/Abstract]) OR (dental health[Title/Abstract]) OR (oral health[Title/Abstract]) AND (prevalence[Title/Abstract]) AND ("2010"[Date - Publication] : "2013"[Date - Publication]). Updates to systematic reviews are performed on an ongoing schedule across all GBD causes; an update for deciduous and permanent caries will be performed in the next one to two iterations.

We eliminated many data points to avoid repetition in the dataset, while striving to maintain as much data detail as possible. Redundancy tended to arise in three data descriptors: age, gender, and urbanicity. Our order of preference for maintaining detail was age, followed by gender, then urbanicity. Additionally, many of the studies presented dmft or DMFT scores, which represent lifetime prevalence and were often described as "caries experience." For the purposes of measuring the burden of disability from dental caries, we considered only data on current prevalence to be relevant, and thus converted lifetime prevalence data to current prevalence and incidence where possible (see below).

Conversion of dmf/DMF scores to prevalence and incidence

Caries of deciduous teeth

Many of the studies that reported lifetime prevalence of deciduous caries (dmf scores) provided detail on the component breakdown of these scores. We used these data to calculate a d/dmf ratio and convert the lifetime prevalence value into one that reflected current prevalence. For example, if the lifetime prevalence was reported as 0.8 with d = 1.5 and dmft = 2 (d/dmf ratio = 0.75), we adjusted the prevalence value to 0.6. When possible, we used within-study d/dmf ratios to convert lifetime prevalence to current prevalence. Otherwise, we converted to current prevalence using a weighted average d/dmf ratio at the country, region, super-region, or global level, in that order of preference.

For studies reporting dmf scores for successive age intervals, the increment in the dmf values between examinations was considered to be equivalent to the caries incidence over the study duration. We extrapolated incidence data from two types of studies. First, for longitudinal or cohort studies, we calculated the caries increment over successive ages and time periods as the difference between the DMF scores at each time point. Narrow age and time intervals were preferred; most were of three years or less. We did not extrapolate incidence data if the age or time interval was greater than 10 years. Secondly, if a study only performed a single cross-sectional examination, but reported data in age intervals of three years or less, we extrapolated incidence data in the same manner. Narrow age ranges

were considered necessary for this incidence extrapolation because the dental health of population cohorts has been observed to change in just a few years when preventive measures are instituted.

Data availability for deciduous caries:

	Prevalence	Continuous DMF score converted to prevalence	Continuous DMF score converted to incidence
Studies	188	119	64
Countries/subnationals	72/51	57/21	34/167
GBD world regions	19	19	16

Modeling strategy

Separate estimates for caries of deciduous and permanent teeth

The natural histories of caries of deciduous teeth and caries of permanent teeth share many similarities, but they also share some important differences. Age patterns of decay in permanent and deciduous dentition are distinct, and duration of a carious lesion in deciduous teeth also tends to be shorter than an untreated episode of permanent caries. Sugar consumption and feeding with formula are both associated with development of deciduous carries, but not with permanent caries. Finally, it is unclear whether the gender patterns and regional differences are the same for both deciduous and permanent caries. For all of these reasons, we elected to model deciduous caries and permanent caries as separate entities and then add the estimates together for an overall estimation of the global burden of dental caries. This modeling approach was also taken in GBD 2010 and GBD 2013.

DisMod model development: caries of deciduous teeth

Serious health consequences of deciduous caries were assumed to be uncommon and death from permanent caries very rare. For purposes of modeling, we therefore assigned excess mortality to be zero from age 0 to 100. We fixed incidence and prevalence at zero after age 12 when exfoliation is presumed to be complete. This age was chosen because 11 was the oldest age of a non-zero prevalence data point. We additionally assigned incidence and prevalence to be zero before age 6 months to indicate that this condition never begins at birth and is absent in the neonatal and post-neonatal periods. Incidence bounds of 0 to 4.0 for ages 1 to 10 years were chosen based on examining the dataset and adding a comfortable margin to the highest reported value. An upper remission bound of 1.0 for ages 0 to 5 years was chosen in order to fit the sharp increase in prevalence over this age range. As prevalence was assigned to be zero after age 14 anyway, we elected to not include a lower remission bounds.

No country-level covariates were included. We used a study-level covariate to indicate whether a given prevalence data point was of “true” current prevalence or calculated from lifetime prevalence using the d/dmf ratio. For both incidence and prevalence, we also used study-level covariates to indicate whether the dmf scores were for surfaces as opposed to teeth.

Because no “true” unconverted incidence values were present in the dataset, we could not crosswalk the extrapolated incidence values to reference incidence values for deciduous caries. Instead, we used higher heterogeneity settings for incidence values than for prevalence (0.7 for incidence, 0.2 for prevalence). We

calculated age mesh points at ages 0, 0.5, 1, 2, 3, 4, 5, 6, 7, 8, 9, 10, 11, 12, 13, 14, 15, and 100 years. High smoothness settings were used for both incidence and prevalence to allow for dynamic age trends.

Covariate name	Type	Measure	Beta value	Exponentiated value
LDI (I$ per capita)	Country covariate	Prevalence	-0.101 (-0.142 - -0.067)	0.904 (0.868 - 0.935)
sugar unadjusted(g)	Country covariate	Incidence	0.002 (-0.001 - 0.004)	1.002 (0.999 - 1.004)
DMF score is for surfaces	Study-level x-covariate	Prevalence	0.266 (0.193 - 0.340)	1.305 (1.213 - 1.405)
DMF score is for surfaces	Study-level x-covariate	Incidence	1.970 (0.000 - 3.896)	7.173 (1.000 - 49.182)
d/dmf ratio used to convert lifetime prevalence	Study-level x-covariate	Prevalence	0.246 (0.193 - 0.302)	1.279 (1.213 - 1.353)

Models were vetted based on the biological plausibility of the results, the extent to which estimates fit the data, and the plausibility of the range of estimates across location hierarchies.

Correction for edentulism

One systematic source of bias in the literature was the exclusion of edentate individuals form the study populations, which leads to systematic overestimation of caries prevalence when modeled over the entire population. To account for this bias, we used our GBD estimates of edentulism and severe tooth prevalence to adjust YLD estimates for dental caries. Using the final DisMod estimates for prevalence of edentulousness, we calculated the mean prevalence for each age and sex and averaged the 1990 and 2015 values. We then calculated a population-weighted mean prevalence for each region and each GBD super-region. The resulting super-regional averages were used to adjust the DisMod estimates for prevalence of permanent caries in calculating years lost due to disability (YLDs).

Disability weights

As described above, the GBD definition of disability associated with symptomatic dental caries is “this person has a toothache, which causes some difficulty eating.” The disability weight associated with this condition is 0.01 (0.005 – 0.019), as derived from the GBD Disability Weights Study.

Not all those with dental caries experience this disability all the time. We considered only those with active dentinal decay to experience symptomatic tooth pain. Those with deciduous caries who had undergone exfoliation or had their cavities filled were considered to have no disability. Likewise, those with permanent caries who had received fillings, had their cavities extracted, or lost a carious tooth altogether were considered to have no disability. Thus, two additional pieces of information are required to complete the calculation of years of life lived with disability (YLDs): proportion with symptoms and duration of disability.

To determine duration, we adapted the method employed by the Australian Burden of Disease (AusBoD) Study in 1996. For total duration, we used the posterior estimates of duration from final DisMod models. For those with symptoms, we split this total duration into two distinct phases of caries disability. The “initial” phase is characterized by *periodic* pain that we assigned to occur an average of one hour per day. The “terminal” phase is a period of *constant* symptoms at the end of an episode. The length of the

terminal phase was determined by literature review as described by the AusBoD group. For deciduous caries we used a study by Mason, et al. of children in the UK presenting to a casualty ward with tooth pain [1]. The length of time each child had been experiencing tooth pain was recorded. Based on the distribution of time courses, a log-normal distribution was plotted that approximated the average duration of *constant* symptoms at 27.6 days leading up to seeking care. For permanent caries, a similar study of the tooth pain experience of adults in New Zealand who presented to hospital dental departments and an emergency clinic resulted in an estimated 55.2 days spent in the terminal phase of caries. For those with severe disease, the length of time spent in the terminal phase was subtracted from the total duration to determine the amount of time spent in the initial phase. For those with mild disease, we considered the entire duration to be spent in the initial phase.

To determine proportion with symptoms, we completed a supplemental literature review of tooth pain and caries. We identified a total of 21 studies with data about the prevalence of pain. The studies were grouped according to the type of dentition studied (deciduous or permanent) and the location of the study group (high-income or low- and middle-income countries). We extracted data on the proportion in each group that described symptoms of pain related to their caries as well as a subset who described their symptoms as being severe. The proportions in each group were weighted according to sample size to give estimates of the relative sizes of three groups: asymptomatic, mild, and severe.

We considered asymptomatic individuals to experience no disability. Those with mild disease spent the entire duration in the initial phase of disease (one hour of pain per day). Those with severe disease spent a majority of the duration in the initial phase followed by a period of time in the terminal phase (constant pain). YLDs were calculated by multiplying the prevalence, duration, proportion, and disability weight for each age, country, sex, and year.

No other significant changes were made to the modeling strategy from GBD 2015 to GBD 2016.

Caries of Permanent Teeth

Flowchart

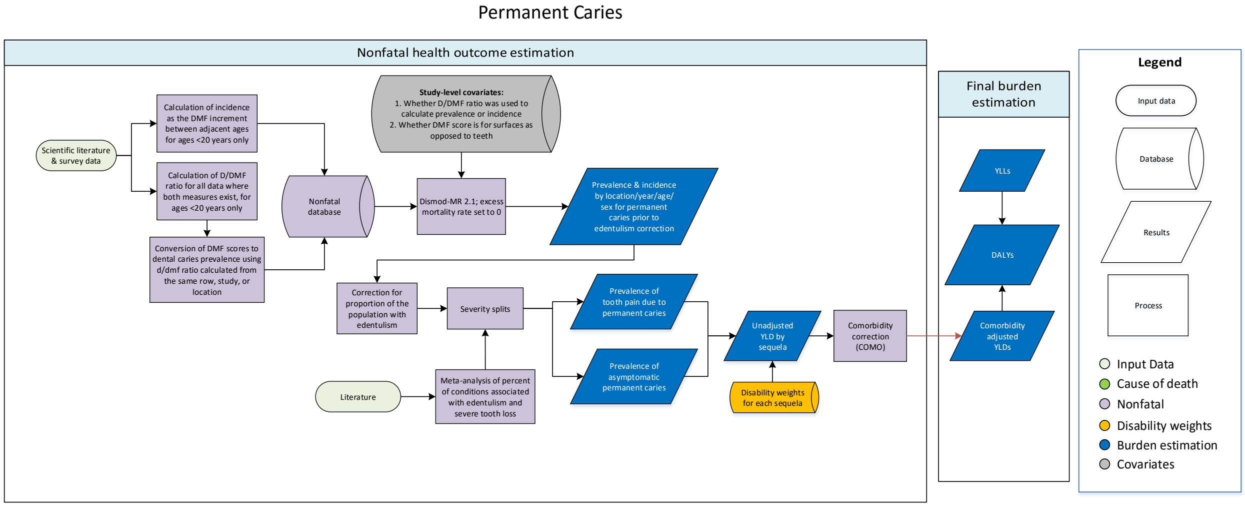

Case definition

The case definition for dental caries is “teeth with unmistakable coronal cavity at dentin level, root cavity in cementum that feel soft or leathery to probing, temporary or permanent restorations, or missing teeth extracted due to a caries lesion.” This definition corresponds to an ICD-9 code of 521.0 and an ICD-10 code of K02.3 – K02.9. Most caries are subclinical in the sense that they do not cause symptoms a majority of the time. Once a carious lesion develops, it will occasionally recede without intervention. Generally, however, it worsens with time and eventually requires either filling or extraction. The major sequela associated with the condition is symptomatic caries, which is defined as “a toothache, which causes some difficulty eating.”

Dental caries and the DMFT index

Public health dentists commonly measure dental caries using the dmft/DMFT index, which is an incremental measure of the proportion of unhealthy teeth and is also a measure of an individual’s lifetime prevalence of caries. Lowercase letters (dmft) are used for deciduous dentition and uppercase letters (DMFT) for permanent dentition. D is for decayed, M for missing, F for filled, and T for teeth. The maximum dmft score is 20 and the maximum DMFT score is 32. Furthermore, some dentists prefer to measure dental caries in terms of tooth surfaces, rather than number of teeth, and report their results using an analogous dmfs/DMFS index. The maximum dmfs score is 88, and the maximum DMFS score is 128 or 148 depending on whether the third molars are counted.

The DMFT index is easy to measure and inter-rater reliability is high. However, the primary shortcoming of the DMFT is that it does not discriminate well between current and past caries. Strategies we employed to maximally utilize dmf/DMF data for estimating the prevalence of burden due to permanent caries are described below.

Input data

Literature reviews

A literature review was conducted by the expert group for GBD 2010, and an additional systematic review was performed for GBD 2013. The search terms used in the GBD 2013 literature review for permanent caries were (Permanent caries[Title/Abstract]) OR (caries prevalence[Title/Abstract]) OR (dental health[Title/Abstract]) OR (oral health[Title/Abstract]) AND (prevalence[Title/Abstract]) AND ("2010"[Date - Publication] : "2013"[Date - Publication]). Updates to systematic reviews are performed on an ongoing schedule across all GBD causes, an update for deciduous and permanent caries will be performed in the next one to two iterations.

We eliminated many data points to avoid repetition in the dataset, while striving to maintain as much data detail as possible. Redundancy tended to arise in three data descriptors: age, gender, and urbanicity. Our order of preference for maintaining detail was age, followed by gender, then urbanicity. Additionally, many of the studies presented dmft or DMFT scores, which represent lifetime prevalence and were often described as "caries experience." For the purposes of measuring the burden of disability from dental caries, we considered only data on current prevalence to be relevant, and thus converted lifetime prevalence data to current prevalence and incidence where possible (see below).

Conversion of dmf/DMF scores to prevalence and incidence

Caries of Permanent Teeth

Whereas in the deciduous dentition, a vast majority of the dmf index is accounted for by caries, tooth loss is a major contributor to the DMF index for the permanent dentition. Caries of permanent teeth may not necessarily be the primary driver of this tooth loss, as other factors such as periodontal disease and trauma may contribute significantly. Thus, we performed the conversions of DMF scores to prevalence and incidence values as described above for permanent caries only in individuals ages 20 years or less.

Data availability for caries of permanent teeth:

	Prevalence	Incidence	Continuous DMF score converted to prevalence	Continuous DMF score converted to incidence
Studies	160	4	89	31
Countries/subnationals	158/68	4/2	46/19	24/11
GBD world regions	19	3	19	14

Modeling strategy

Separate estimates of caries of deciduous teeth and caries of permanent teeth

The natural history of deciduous and permanent caries share many similarities, but they also share some important differences. Age patterns of decay in permanent and deciduous dentition are distinct, and duration of a carious lesion in deciduous teeth also tends to be shorter than an untreated episode of permanent caries. Sugar consumption and feeding with formula are both associated with development of

deciduous carries, but not with permanent caries. Finally, it is unclear whether the gender patterns and regional differences are the same for both deciduous and permanent caries. For all of these reasons, we elected to model deciduous caries and permanent caries as separate entities and then add the estimates together for an overall estimation of the global burden of dental caries. This is the modeling approach which was also taken in GBD 2010 and GBD 2013.

DisMod model development: caries of permanent teeth

Serious health consequences of permanent caries were also assumed to be uncommon and death from permanent caries very rare. We therefore assigned excess mortality to be zero from age 0 to 100. The dataset suggested that permanent caries are sometimes incident in 5-year-olds, so we fixed incidence and prevalence at 0 for ages 0 to 4. Incidence bounds were again chosen based on examining the dataset and adding a margin to the highest reported value. In this case, incidence bounds were 0 – 2. Lower bounds for remission were set at 0.2 and upper bounds were set at 3.

As with permanent caries, no country-level covariates were included. We used a study-level covariate to indicate whether a given prevalence data point was of “true” current prevalence or calculated from lifetime prevalence using the D/DMF ratio, and another study-level covariate to indicate whether a given incidence values was extrapolated from DMF scores. For both incidence and prevalence, we also used study-level covariates to indicate whether the DMF scores were for surfaces as opposed to teeth.

We calculated age mesh points at ages 0, 4, 5, 8, 10, 12, 15, 19, 20, 25, 29, 30, 35, 40, 45, 50, 55, 60, 70, 80, 90, and 100 years. Heterogeneity was set to 0.5 for both incidence and prevalence. High smoothness settings were used for both incidence and prevalence to allow for dynamic age trends.

Covariate name	Type	Measure	Beta value	Exponentiated value
LDI (I$ per capita)	Country covariate	Prevalence	-0.345 (-0.433 - -0.259)	0.708 (0.648 - 0.772)
sugar unadjusted(g)	Country covariate	Incidence	-0.003 (-0.004 - -0.001)	0.997 (0.996 - 0.999)
DMF score is for surfaces	Study-level x-covariate	Incidence	1.000 (0.000 - 2.000)	2.717 (1.000 - 7.389)
DMF score is for surfaces	Study-level x-covariate	Prevalence	0.324 (0.185 - 0.453)	1.383 (1.203 - 1.573)
DMF score used to calculate incidence	Study-level x-covariate		0.266 (0.160 - 0.299)	1.304 (1.174 - 1.348)
d/dmf ratio used to convert lifetime prevalence	Study-level x-covariate	Prevalence	-0.204 (-0.285 - -0.120)	0.815 (0.752 - 0.887)

Although studies were screened carefully during data extraction to ensure that they specified whether they were measuring permanent or deciduous caries, some data points were marked as outliers during modeling due to their high prevalence values in young ages, as it was deemed likely that some of these studies were reporting deciduous in addition to permanent caries.

As with deciduous caries, models for permanent caries were vetted based on the biological plausibility of the results, the extent to which estimates fit the data, and the plausibility of the range of estimates across location hierarchies.

Correction for edentulism

One systematic source of bias in the literature was the exclusion of edentate individuals from the study populations, which leads to systematic overestimation of caries prevalence when modeled over the entire population. To account for this bias, we used our GBD estimates of edentulism and severe tooth prevalence to adjust YLD estimates for dental caries. Using the final DisMod estimates for prevalence of edentulousness, we calculated the mean prevalence for each age and sex and averaged the 1990 and 2015 values. We then calculated a population-weighted mean prevalence for each region and each GBD super-region. The resulting super-regional averages were used to adjust the DisMod estimates for prevalence of permanent caries in calculating years lost due to disability (YLDs).

Disability weights

As described above, the GBD definition of disability associated with symptomatic dental caries is "this person has a toothache, which causes some difficulty eating." The disability weight associated with this condition is 0.01 (0.005 – 0.019), as derived from the GBD Disability Weights study.

Not all those with dental caries experience this disability all the time. We considered only those with active dentinal decay to experience symptomatic tooth pain. Those with deciduous caries who had undergone exfoliation or had their cavities filled were considered to have no disability. Likewise, those with permanent caries who had received fillings, had their cavities extracted, or lost a carious tooth altogether were considered to have no disability. Thus, two additional pieces of information are required to complete the calculation of years of life lived with disability (YLDs): proportion with symptoms and duration of disability.

To determine duration, we adapted the method employed by the Australian Burden of Disease (AusBoD) Study in 1996. For total duration, we used the posterior estimates of duration from final DisMod models. For those with symptoms, we split this total duration into two distinct phases of caries disability. The "initial" phase is characterized by *periodic* pain that we assigned to occur an average of one hour per day. The "terminal" phase is a period of *constant* symptoms at the end of an episode. The length of the terminal phase was determined by literature review as described by the AusBoD group. For deciduous caries we used a study by Mason, et al. of children in the UK presenting to a casualty ward with tooth pain [1]. The length of time each child had been experiencing tooth pain was recorded. Based on the distribution of time courses, a log-normal distribution was plotted that approximated the average duration of *constant* symptoms at 27.6 days leading up to seeking care. For permanent caries, a similar study of the tooth pain experience of adults in New Zealand who presented to hospital dental departments and an emergency clinic [2] resulted in an estimated 55.2 days spent in the terminal phase of caries. For those with severe disease, the length of time spent in the terminal phase was subtracted from the total duration to determine the amount of time spent in the initial phase. For those with mild disease, we considered the entire duration to be spent in the initial phase.

To determine proportion with symptoms, we completed a supplemental literature review of tooth pain and caries. We identified a total of 21 studies with data about the prevalence of pain. The studies were grouped according to the type of dentition studied (deciduous or permanent) and the location of the study group (high-income or low- and middle-income countries). We extracted data on the proportion in each group that described symptoms of pain related to their caries as well as a subset who described their symptoms as being severe. The proportions in each group were weighted according to sample size to give estimates of the relative sizes of three groups: asymptomatic, mild, and severe.

We considered asymptomatic individuals to experience no disability. Those with mild disease spent the entire duration in the initial phase of disease (one hour of pain per day). Those with severe disease spent a majority of the duration in the initial phase followed by a period of time in the terminal phase (constant pain). YLDs were calculated by multiplying the prevalence, duration, proportion, and disability weight for each age, country, sex, and year.

No additional changes were made to this modeling strategy.

Chronic Periodontal Disease

Flowchart

Periodontal diseases

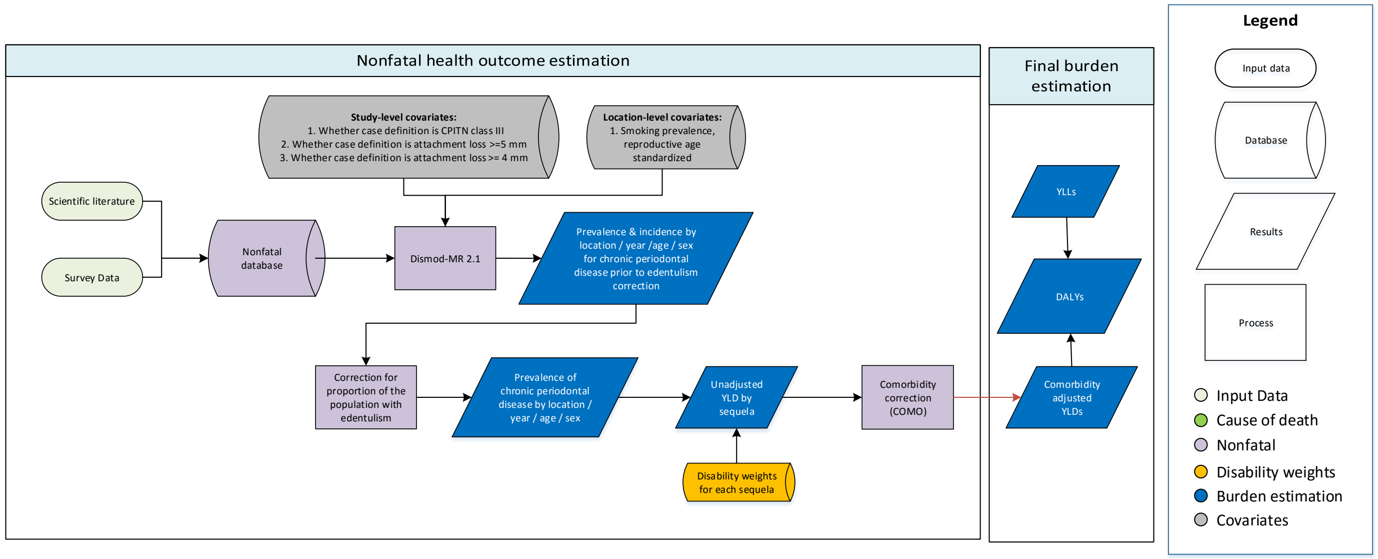

Case definition

Chronic periodontal disease is caused by chronic bacterial infection around the teeth. Symptoms of gingivitis, the mildest form of the disease, include swelling, redness, and propensity of the gums to bleed when perturbed. If the infection is not treated appropriately, it will eventually spread below the gum line leading to a chronic inflammatory state of the periodontal tissues. Over time, there will be loss of gingival tissue and alveolar bone destruction. Teeth will become loose and may need to be extracted.

The GBD definition of disability associated with symptomatic severe periodontal disease is “bad breath, a bad taste in the mouth, and gums that bleed a little from time to time, but which does not interfere with daily activities.” The ICD-10 codes for periodontal disease are K05.0 – K05.6, and the ICD-9 codes are 523.0 – 523.9.

Defining periodontal disease in a meaningful, reproducible manner has been an ongoing challenge for public health dentists. Attachment loss (AL) and pocket depth (PD) have emerged as the most common metrics of periodontal health measurement. Attachment loss (AL) is measured as the difference between the distance from the gingival margin to the bottom of the pocket and the distance from the cemento-enamel junction to the bottom of the pocket.

The Community Periodontal Index of Treatment Needs (CPITN) is a classification system that was developed by the WHO as a standardized method of periodontal health measurement [1]. CPITN classification is based on quantifying the probing depth between teeth and gums. The mouth is divided into 6 sections, called sextants. Sextants with fewer than two teeth are excluded. Multiple teeth in each sextant are examined. A standard-sized probe is used with depth markings from 3.5 to 5.5 mm. The probe

is inserted into the sulcus between a tooth and the gingiva until it meets resistance. The surrounding area is then explored with the probe to determine the maximum depth of the pocket. Multiple areas around each tooth are probed. Scores range from 0 to 4 in order of increasing severity. When the CPITN method was employed, we considered those with Class 4 only. We excluded studies in which the study population was reported as the number of sextants rather than the number of individuals surveyed.

In 2007, a new CDC proposal for gold standard diagnosis of severe, chronic periodontitis was published. This standard specified that a more strict definition of the condition should be implemented. This more exclusive definition of chronic periodontal disease includes $\geq$ 2 interproximal sites with AL $\geq$ 6 mm ***AND*** $\geq$ 1 interproximal site with PD $\geq$ 5 mm [2].

We included the following definitions of severe periodontal disease commonly found in the literature:

1. Community Periodontal Index of Treatment Needs (CPITN) – Class 4 only
2. Clinical Attachment Loss (AL) > 6mm
3. Clinical Attachment Loss (AL) > 5mm
4. Clinical Attachment Loss (AL) > 4mm
5. Gingival Pocket Depth (PD) > 5mm

If more than one type of data was included in a study, our first preference was for CPITN = 4, followed by AL >6 mm, with PD >5 considered the least accurate representation of the GBD case definition. AL > 6mm was preferred over AL > 5mm, followed by AL > 4mm. All definitions were extracted for each datum as available this time, and a series of study covariates were used to crosswalk non-standard definitions to the reference standard of CPITN stage 4 (see below).

Input data

Model inputs

For GBD 2010, a review of the literature on periodontal disease prevalence was conducted by the Expert Group. A new systematic review was conducted for GBD 2013. The GBD 2013 literature review used the following search terms: (Peridontal disease[Title/Abstract]) OR (periodontitis[Title/Abstract]) OR (periodontal[Title/Abstract]) AND (prevalence[Title/Abstract]) AND ("2010"[Date - Publication] : "2013"[Date - Publication]).

In both systematic reviews, there was a hierarchical preference for case definitions: if more than one type of data was included in a study, our first preference was AL followed by PD, with CPITN considered the least accurate representation of the GBD case definition. Updates to systematic reviews are performed on an ongoing schedule across all GBD causes; an update for chronic periodontal disease will be performed in the next one to two iterations.

	**Prevalence**	**Mortality**
Studies	97	4
Countries/subnationals	48/15	3/1
GBD world regions	18	2

Modeling strategy

Overview

Evidence for chronic periodontal disease being a direct, proximate cause of death is lacking. As such, it was not included in overall causes of death analysis. However, there is a developing body of literature to suggest that those with chronic periodontal disease may be at increased risk of death from other causes. Relative risk data were, therefore, included in modeling of morbidity, but overall years of life lost (YLLs) were estimated to be zero. Models of disease burden due to chronic periodontal disease instead focused on estimating morbidity (YLDs) associated with the condition, and chronic periodontal disease was not included in risk factor analysis of any other condition.

Correction for edentulism

Bias in the dataset was felt to be limited, but some systematic bias was present in the definition of the study populations. In virtually all studies, edentate persons were excluded from evaluation. This exclusion is justified in the context of periodontal disease surveillance because advanced periodontal disease is not common in those who are toothless. To account for the systematic bias inherent in excluding those with severe tooth loss from the denominator, we discounted the prevalence numbers estimated by DisMod MR 2.1. For example, if 40% of 70-74 year old females were estimated to be edentate in a certain region, the corresponding estimates for advanced periodontal disease prevalence were reduced to 60% of the original value.

DisMod model development

Mortality was fixed to zero and relative risk was fixed to 1.0 before age 30, as any excess cardiovascular events that occur in those with severe tooth loss would not be expected at young ages. Incidence and prevalence were assigned to be zero until age 8 as periodontal disease is largely considered to be a disease of adulthood. Incidence was allowed to rise beginning at age 9, based on the youngest age at which there was a non-zero point estimate for prevalence in the dataset.

Bounds were assigned for remission and excess mortality to improve plausibility in the DisMod estimates. Remission was bounded 0 to 0.05 and excess mortality rate was bounded to 0.0001. We considered both bounds to be within reasonable ranges for the observed natural history of the disease.

Study-level covariates were created for whether the data use a case definition of CPITN class III periodontal disease as opposed to the reference definition of class IV, and for whether the data use a case definition of attachment loss >=5 mm as opposed to the reference definition of >= 6mm. We did not identify any studies for which the only case definition reported was attachment loss >= 4mm. Reproductive age-standardized smoking prevalence was used as a country-level covariate.

Models were vetted based on the biological plausibility of the results, the extent to which estimates fit the data, and the plausibility of the range of estimates across location hierarchies.

Covariate name	Type	Measure	Beta value	Exponentiated value
Smoking Prevalence (Reproductive Age Standardized)	Country covariate	Prevalence	0.097 (0.004 - 0.194)	1.102 (1.004 - 1.215)
Data corresponds to those with CPITN class III periodontal disease (class IV is reference)	Study-level x-covariate	Prevalence	0.211 (0.008 - 0.575)	1.235 (1.008 - 1.777)
Data corresponds to those with attachment loss >=5 mm (reference is >=6 mm)	Study-level x-covariate	Prevalence	0.903 (0.657 - 1.126)	2.468 (1.928 - 3.083)

Disability calculation and YLDs

Because those who are edentate cannot have advanced periodontal disease, we corrected for toothlessness as described above. Using the DisMod estimates for prevalence of edentulism, we calculated the mean prevalence for each age and sex and averaged the 1990 and 2015 values. We then calculated a population-weighted mean prevalence for each region followed by the same for each GBD super-region. The resulting super-regional averages were used to adjust the estimates for prevalence of advanced periodontal disease in calculating years lost due to disability (YLDs).

We considered all estimated prevalent cases of chronic periodontal disease to experience the disability described by “bad breath, a bad taste in the mouth, and gums that bleed a little from time to time, but this does not interfere with daily activities.” The GBD Disability Survey differentiated between those who experience pain and those who do not, but the calculated disability weight was the same for both forms of the condition, 0.007 (0.003 – 0.014).

No other changes were made for GBD 2016.

Edentulism & Severe Tooth Loss

Flowchart

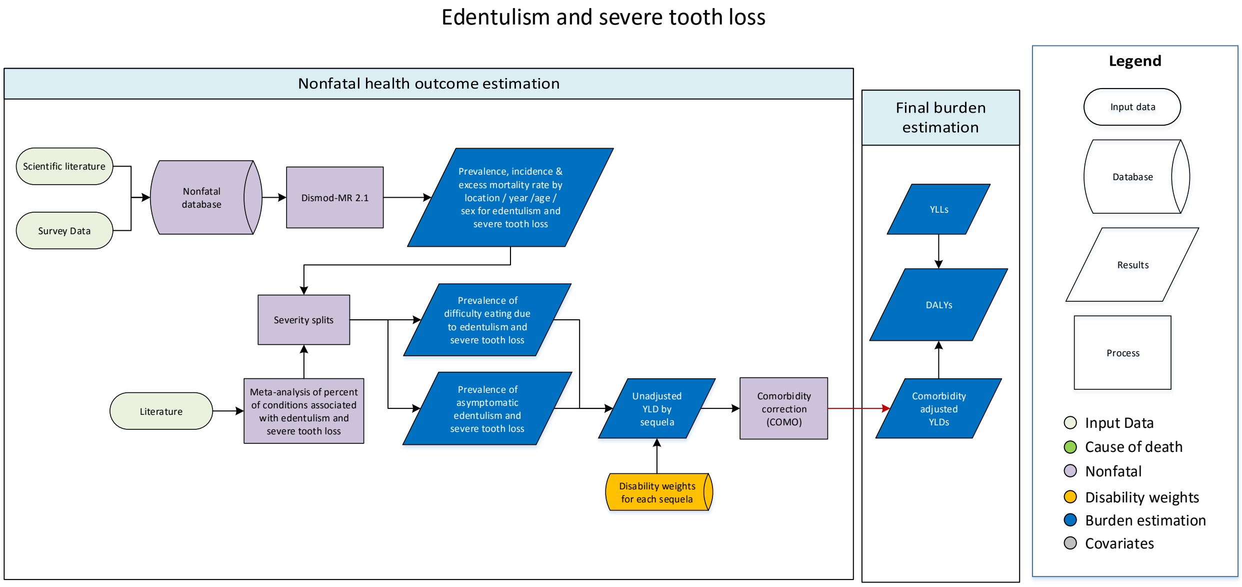

Case definition

The case definition of edentulism and severe tooth loss includes any individual with fewer than nine remaining permanent teeth; toothlessness of infancy is not included. The assessment of this disease includes quantification of the prevalence of the disease as well as estimation of the major sequelae: asymptomatic toothlessness and symptomatic toothlessness leading to "great difficulty in eating meat, fruits, and vegetables." A small body of evidence has begun to emerge that implicates edentulousness as predisposing individuals to increased risk for ischemic cardiovascular events including myocardial infarction and stroke. These data are sparse but have been included in models estimating burden of major tooth loss. However, edentulism was not included in the risk factor analysis for ischemic cardiovascular diseases.

Input data

Model inputs

An initial literature review was done by the Expert Group for GBD 2010, including published articles as well as the results of national and subnational reports. A new systematic review was completed for GBD 2013. The search terms for this systematic review included: (Edentulism[Title/Abstract]) OR (edentulous[Title/Abstract]) OR (endentulousness[Title/Abstract]) OR (severe tooth loss[Title/Abstract]) OR (total tooth loss[Title/Abstract]) OR (complete tooth loss[Title/Abstract]) AND (prevalence[Title/Abstract]) AND ("2010"[Date - Publication] : "2013"[Date - Publication]).

While an additional literature review was not performed for GBD 2015, new World Health Survey data were added for 47 countries. Updates to systematic reviews are performed on an ongoing schedule

across all GBD causes; an update for edentulism and severe tooth loss will be performed in the next one to two iterations.

Bias in the dataset was considered to be negligible. Diagnostic criteria for this condition are very clear (< 9 teeth). Additionally, all included studies are considered representative of the study population. Thus, covariates to account for excess variability were not deemed necessary. Few data points were marked as outliers during the modeling process.

	Prevalence	Incidence	Mortality
Sources	151	11	8
Countries/subnationals	75/11	4/5	5/4
GBD world regions	20	4	3

Modeling strategy

Overview

First, estimates for the prevalence of edentulism and severe toothlessness were calculated for each location/year/sex/age using DisMod-MR 2.1. Then, estimates of the proportion of the population with access to dentures were generated for each location, and the disability weight for "great difficulty in eating meat, fruits, and vegetables" was applied to the proportion of the population with edentulism and no access to dentures.

DisMod model development

As would be expected for an irreversible condition, remission was fixed at zero for all ages. Mortality and relative risk were both fixed at zero before age 30, as any excess cardiovascular events resulting from severe tooth loss would not be expected at younger ages. We also assigned incidence and prevalence to be zero during childhood. Incidence was allowed to rise beginning at age 15, which was chosen based on the age at which the permanent dentition is expected to have fully formed in all individuals. The random effect limits for all locations were bounded at +/- 1.

As mentioned above, the criteria for diagnosis of edentulism are straightforward, and bias in the dataset was considered negligible. Thus, no study-level covariates were used in modeling the prevalence of edentulism. We included lnLDI as a country-level covariate, with a minimum beta value of 0.02; see results in the table below.

Covariate name	Type	Measure	Beta value	Exponentiated value
LDI (I$ per capita)	Country covariate	Prevalence	0.026 (0.020 - 0.043)	1.027 (1.021 - 1.044)

Models were vetted based on the biological plausibility of the results, the extent to which estimates fit the data, and the plausibility of the range of estimates across location hierarchies. We have made no substantive changes in the modeling strategy from GBD 2013.

Disability weights

The disability weight used for symptomatic toothlessness leading to "great difficulty in eating meats, fruits, and vegetables" is 0.067 (0.045 – 0.095) as determined by the GBD Disability Survey. We considered all those with severe tooth loss and no access to dentures to experience this disability. However, the proportion of those with edentulism and severe tooth loss who have dentures has not been studied extensively.

In order to estimate the proportion of edentulous individuals with no access to dentures, we completed a supplemental literature review of dentures prevalence. Only six systematic surveys of dentures prevalence were identified, all in high- and middle-income countries. All were completed since 2000. After extracting the data from the studies, we performed linear regressions of denture presence and denture absence against health system access (HSA), a standardized covariate of treatment availability used in many disease estimation models. From the results of the regression, the prevalence of no dentures was calculated for all super-regions. We then completed a population-weighted average of all countries in the super-region based on 2003 populations, the average year of the dentures studies. Uncertainties for the prevalence of dentures were calculated by finding the standard deviation and standard error of the calculated prevalence values.

The estimated prevalence of dentures in each location was used to calculate the proportion of individuals with asymptomatic edentulism and severe tooth loss (e.g., those who have access to dentures) and difficulty eating due to edentulism and severe tooth loss (e.g., those without access to dentures). This latter sequela was included as a cause of years lost due to disability (YLDs).

Other Oral Disorders

Other oral disorders encompass a wide variety of dental, tongue, and jaw disorders and malformations, including all oral disorders that are not included in the case definitions of permanent or deciduous dental caries, periodontal disease, or edentulism and severe tooth loss. All data on the prevalence of other oral disorders were obtained from the United States Medical Expenditure Panel Surveys, a nationally representative survey conducted yearly from 1996 to 2011 by the US Agency for Healthcare Research and Quality.

These data were modelled in DisMod-MR 2.1 using a prevalence-only model with age mesh points set at 0, 0.5, 1, 5, 10, 20, 30, 40, 50, 60, 70, 80, 90, and 100 years of age. Heterogeneity for prevalence was set to the default of 0.5, and smoothness for prevalence was set to the default of 0.3. No study-level or country-level covariates were used in this model other than the study-level covariate for sex, which was fixed at the super-region level. This model provided us with estimates of the prevalence of other oral disorders for every location/age/sex combination.

Injuries

Flowchart

Nonfatal Injuries Flow Chart

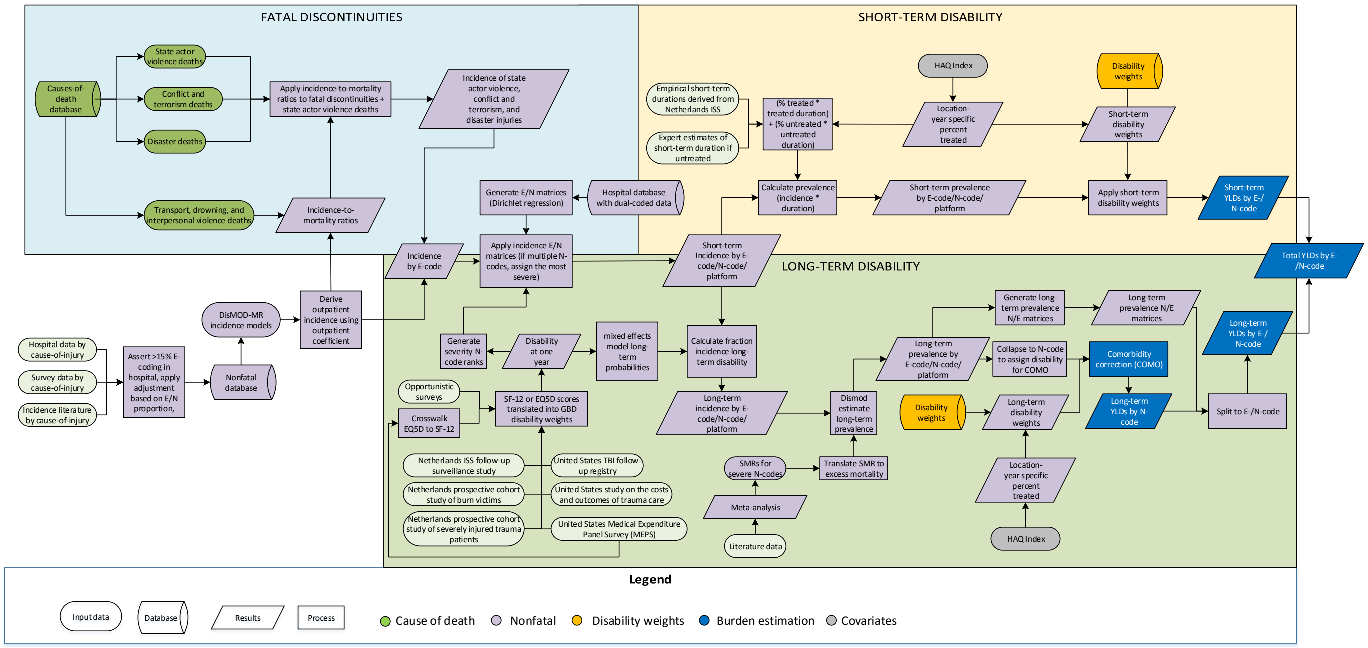

Case definition

For GBD 2016, the Injuries estimation process for non-fatal health outcomes encompasses a range of 29 causes, including transport injuries, falls, drowning, self-harm, interpersonal violence, and animal contact. Injury incidence is defined using ICD-9 codes E000-E999 and ICD-10 chapters V to Y. For non-fatal estimation, Chapters S and T in ICD-10 and codes 800-999 in ICD9 are used to estimate morbidity. Each of these 29 causes can result in a variety of physical injury sequelae (i.e. traumatic brain injury), which we call the "nature of injury." Though the initial DisMod models are the "cause of injury" level (ie, drowning), each cause of injury is broken out into cause-nature pairs. For the first time in GBD 2016, we report incidence, prevalence, and YLDs due to injuries at the cause-nature pair level rather than the cause of injury level in aggregate.

We make additional distinctions between inpatient and outpatient injuries, and between short-term and long-term injuries. Inpatient injuries are defined as injuries that led to hospitalization, whereas outpatient injuries are ones that occurred in outpatient settings or emergency care. We define short-term injuries as injuries lasting less than one year, and long-term injuries as those lasting longer than one year, at which point we assume lifelong disability.

Input data

Model inputs

For GBD 2016, to estimate morbidity from injuries, we used data from hospital records, emergency department records, and surveys to produce years lost to disability (YLDs) by country, year, sex, age, external cause-of-injury, and nature of injury category.

Unfortunately, quite a few countries report their data using a mix of cause of injury and nature of injury codes. In order to retain as much of the data as possible, we included all datasets that had at least 15% of cases coded to the cause of injury. Previously, we chose 45% as the threshold, but in order to include as much data as possible for GBD 2016, we lowered the threshold to 15%. We made this distinction after assessing the proportions of major injury causes (road injury and falls) in each of the data sources. We concluded that there were no obvious differences between country data with 15%–45% coverage of external cause codes and those with more than 45% coverage. Below the 15% threshold, the cause of nature coding became more disproportionate when compared to sources with higher cause of nature coding. (We assessed the raw hospital data to make sure that there was no disproportionate coding to certain causes in the 15%–45% cause of injury coding range. We increased the cause-specific injury cases from these datasets proportionately to sum to the total number of injury cases.

Conflict, war and executions and police conflict data were obtained from the Uppsala Conflict Data Program [17], the International Institute for Strategic Studies [18], the Armed Conflict Location and Event Dataset [TBD] and the Social Conflict Analysis Database [TBD], vital registration systems. Disaster data were obtained from the International Disaster Database from the Center for Research on the Epidemiology of Disasters [19].

Data searches

For GBD 2016, hospital and emergency department records were supplemented with more recent and available site-years. Additionally, we reviewed a list of sources, which was composed primarily of reports and surveys that could be incorporated into non-fatal estimates of injuries prior to estimation. Hospital utilization envelopes that gave reliable denominators for hospital data allowed for the use of more data sources. We applied correction factors to account for repeat hospital visits within a three-month time window (derived from US claims data) to the incidence estimates to avoid double-counting multiple health service contacts for the same injury.

Infrequently, data points were marked as outliers. Reasons for this were that the data point did not follow the age or time pattern as expected and/or if the incidence rate of people sustaining an injury from a certain cause of injury was not plausible.

Modelling strategy

As in previous GBD iterations, two categories of injury severity were separately modelled: injuries warranting inpatient care and injuries warranting other health care. Injuries warranting inpatient care refer to injury cases of sufficient severity to require inpatient care, if there are no restrictions in access to health care. Injuries warranting other health care refer to injury cases of sufficient severity to require health care attention but not hospitalisation. This category includes emergency department visits. In order to best measure the burden of injuries, the GBD 2016 estimates excluded trivial injuries by

restricting morbidity analysis to cases warranting some form of health care in a system with full access to health care. We intended to include cases with injuries that did not receive care in areas with restricted access to health care, but that would have warranted some type of health care in a system with full access to health care. In some surveys, after asking about recall of injuries in the past month or year respondents were further probed whether they sought care and if they did not what the reasons were. This allowed us to include cases who cited financial or geographical barriers as reasons for not seeking care.

Cause-of-injury incidence

The list of unique (ie, not counting aggregate categories like road injuries or interpersonal violence) cause-of-injury categories has increased to 29 for GBD 2015 following the addition of "self-harm by firearm," and "self-harm by other specified means." The fatal discontinuities cause-of-injury categories now only include "conflict and terrorism" and "exposure to forces of nature, disaster" rather than "collective violence and legal intervention," which encompassed both in GBD 2015. Included previously in "collective violence and legal intervention" were injuries due to "executions and police conflict." For GBD 2016, we are treating executions and police conflict as a typical cause of injury that has a steady state over time rather than as a fatal discontinuity.

The majority of incidence data exist at the external cause-of-injury level. Thus, incidence for cause-of-injury categories was modelled using DisMod-MR 2.0. DisMod-MR 2.0 is a descriptive epidemiological meta-regression tool that produces simultaneous estimates of disease incidence, prevalence, remission, and mortality. Multiple datasets from hospital, emergency/outpatient departments, and survey datasets were fed into these incidence models. We separately estimated two categories of injury severity: inpatient and outpatient injuries. For GBD 2016 we used two covariates in each DisMod-MR model as a multiplier from inpatient to outpatient incidence, namely covariates "outpatient," "in- and outpatient."

Due to the sporadic nature of the incidence of injuries and a lack of time-trend that results from fatal discontinuities, DisMod-MR 2.0 was not used to model incidence due to fatal discontinuities, including exposure to forces of nature (ie, natural disaster) and conflict and terrorism. For GBD 2015, to estimate incidence that would be expected to occur when there are fatal discontinuities, the mortality rate for these cause-of-injury categories was multiplied by the average country-year-age-sex-specific incidence-to-mortality ratio within several cause-of-injury categories that likely exhibit similar case-fatality ratios. This method resulted in some outliers driven by patterns in the cause-of-injury categories used to compute the incidence-mortality ratios that did not reflect accurate patterns in the fatal discontinuities. For GBD 2016, the incidence-to-mortality ratios were averaged over super-region, year, and sex to limit the variability in the ratios applied to fatal discontinuities. For disaster incidence, the incidence-to-mortality ratio was calculated as an average of the road injuries cause, and drowning if there was a water-related natural disaster in that specific country-year noted in the International Disaster Database [19]. For conflict and terrorism, the incidence-to-mortality ratio was calculated as an average of the road injuries and interpersonal violence causes. We treated executions and police conflict as similar to the fatal discontinuities in that we imputed the incidence using the incidence-to-mortality ratio of interpersonal violence.

Follow-up studies

Similar to GBD 2015, we used follow-up data obtained from a pooled dataset of six follow-up studies from China, the Netherlands, and the US, which followed up patients for at least one year after the injury and the Medical Expenditure Panel Survey (MEPS) [20-27].

Details of injury follow-up surveys used in GBD 2016

Dataset	Year	Type of data collected	Type of patients	Setting	Sample size* and response
Guangdong follow up survey, China[#]	2006–2007	Follow up survey among sample of ISS patients	Patients (15+ years) who were hospitalized that had been injured by road traffic injury, fall, blunt or penetrating trauma	Based on three national injury surveillance hospitals in Zhuhai, Guangdong Province in China	998 (response 87%)
LIS follow up survey, Netherlands[1]	2001–2002	Follow-up survey among stratified sample of ISS patients (oversampling less common, severe injuries)	Patients (15+ years) who visited the Emergency Department of a hospital and were discharged to the home environment and patients who were admitted to hospital	Based on 17 public hospitals in the Netherlands	8,564 (response 37%)
LIS follow-up survey, Netherlands[2]	2007–2008	Follow-up survey among stratified sample of ISS patients (oversampling less common, severe injuries)	Patients (15+ years) who visited the Emergency Department of a hospital and were discharged to the home environment and patients who were admitted to hospital	Based on 15 public hospitals in the Netherlands	8,057 (response 36%)
NSCOT – National study on Costs and Outcomes of Trauma, USA[4]	2001–2002	A prospective cohort study was conducted among a sample of adult trauma patients treated at Level I trauma centers and non-trauma center hospitals	Patients treated for a moderate to severe injury (as defined by at least one injury of an Abbreviated Injury Scale (AIS) score of 3 or greater	Based on 69 hospitals in 12 states in the US	5,191 (response 61%)
SCTBIFR – South Carolina Traumatic Brain injury Follow-up Registry, USA[5]	1999–2002	A prospective cohort study was conducted among injured in-patients with a traumatic brain injury-related injury	Patients (15+ years) who were admitted to hospitals and met the CDC case definition of TBI – trauma to the head associated with altered consciousness, amnesia, neurological abnormalities, skull fracture, intracranial lesion, or death	Discharged from all nonfederal in-state acute care hospitals	7,613 (response 28%)

Dataset	Year	Type of data collected	Type of patients	Setting	Sample size* and response
Burns outcome study, Netherlands[6]	2003–2006	A multicenter prospective cohort was conducted among adult (severe) burn patients	Injury patients who sustained severe burns	Three public hospitals with specialized burn units.	311 (response 78%)

*number of patients that met the inclusion criteria; response rate = percentage of patients who responded to the follow-up survey (in case of multiple follow-up times the response rate of the first follow-up moment is reported).

data from CDC China, jointly analyzed by study authors from IHME and China CDC

MEPS is a large-scale overlapping continuous panel survey of the US non-institutionalized population that collects information on use and cost of health care and SF12 responses [28]. SF-12 responses are elicited twice over the two-year period that any individual is part of the study. Thus, MEPS offered the benefit of including health state measures of non-injured and destined to be injured and the benefit of having pre-injury and post-injury SF-12 responses. We pooled all available MEPS data over a 19-year span.

The follow-up studies used different patient reported outcome measures to assess health status, namely the SF-36, Version 1 SF-12, and the EQ-5D [29-31]. To enable comparison across the six datasets, it was necessary to analyse the data in a standardised patient-reported outcome measure. First, we mapped all patient-reported outcome measures to Version 2 SF-12 (SF-12v2). Second, we normalised the health status measurements by mapping the SF-12 scores to a corresponding disability weight based on several opportunistic surveys asking respondents to score SF-12 based on the lay descriptions for a selection of 60 GBD health states [12]. We ran a regression of logit-transformed disability weight on nature-of-injury category and age group and never-injured status. The pooled dataset informed both the nature-of-injury category hierarchy and the long-term probability of injuries, discussed below.

Nature-of-injury category hierarchy

Multiple injuries can occur in one individual. For GBD 2016, a nature-of-injuries severity hierarchy was developed to establish a one-to-one relationship between cause-of-injury and nature-of-injury category. This means that in case of multiple injury the nature-of-injury category that was likely to be responsible for the largest burden was selected. To construct the hierarchy, we used data from the pooled dataset of follow-up studies. The output of the regression of logit-transformed disability weight on nature-of-injury category and individual characteristics of the follow-up studies were used to calculate the mean long-term disability attributable each nature-of-injury category. The ranking of nature-of-injury categories by their long-term disability weights formed the basis of our severity hierarchy. Hierarchies were developed separately, for injuries warranting inpatient care and injuries warranting other health care.

Nature of injury hierarchies: combination of empirical hierarchies estimated from pooled follow-up study and expert adjustments, for inpatient and outpatient injuries

Rank	Inpatient Hierarchy	Outpatient Hierarchy
1	Spinal cord lesion below neck level	Fracture of pelvis
2	Amputation of lower limbs, bilateral	Fracture of patella, tibia or fibula, or ankle

3	Amputation of upper limbs, bilateral	Fracture of hip
4	Spinal cord lesion at neck level	Fracture of skull
5	Fracture of hip	Amputation of thumb
6	Fracture of femur, other than femoral neck	Fracture of vertebral column
7	Amputation of upper limb, unilateral	Multiple fractures, dislocations, crashes, wounds, sprains, and strains
8	Amputation of lower limb, unilateral	Internal hemorrhage in abdomen and pelvis
9	Multiple fractures, dislocations, crashes, wounds, sprains, and strains	Fracture of femur, other than femoral neck
10	Effect of different environmental factors	Dislocation of hip
11	Fracture of patella, tibia or fibula, or ankle	Amputation of toe/toes
12	Moderate-Severe traumatic brain injury	Fracture of hand (wrist and other distal part of hand)
13	Fracture of foot bones except ankle	Amputation of fingers (excluding thumb)
14	Internal hemorrhage in abdomen and pelvis	Burns, <20% of total burned surface area without lower airway burns
15	Crush injury	Dislocation of knee
16	Minor traumatic brain injury	Contusion in any part of the body
17	Fracture of pelvis	Minor traumatic brain injury
18	Nerve injury	Foreign body in respiratory system
19	Severe chest injury	Severe chest injury
20	Dislocation of hip	Drowning and nonfatal submersion
21	Burns, >= 20% total burned surface area or >= 10% burned surface are if head/neck or hands/wrist involved w/o lower airway burns	Asphyxiation
22	Lower airway burns	Poisoning requiring urgent care
23	Fracture of skull	Effect of different environmental factors
24	Amputation of thumb	Foreign body in GI and urogenital system
25	Fracture of hand (wrist and other distal part of hand)	Fracture of sternum and/or fracture of one or more ribs
26	Fracture of vertebral column	Nerve injury
27	Contusion in any part of the body	Fracture of face bones
28	Open wound(s)	Dislocation of shoulder
29	Amputation of toe/toes	Injury to eyes
30	Dislocation of knee	Fracture of clavicle, scapula, or humerus
31	Amputation of fingers (excluding thumb)	Fracture of radius and/or ulna
32	Drowning and nonfatal submersion	Fracture of foot bones except ankle
33	Asphyxiation	Foreign body in ear
34	Burns, <20% total burned surface area without lower airway burns	Muscle and tendon injuries, including sprains and strains lesser dislocations
35	Muscle and tendon injuries, including sprains and strains lesser dislocations	Superficial injury of any part of the body
36	Fracture of face bones	Open wound(s)
37	Foreign body in respiratory system	Complications following therapeutic procedures
38	Poisoning requiring urgent care	
39	Foreign body in GI and urogenital system	
40	Fracture of sternum and/or fracture of one or more ribs	
41	Dislocation of shoulder	

42	Injury to eyes	
43	Fracture of clavicle, scapula, or humerus	
44	Fracture of radius and/or ulna	
45	Foreign body in ear	
46	Superficial injury of any part of the body	
47	Complications following therapeutic procedures	

Cause-nature matrices

Due to the fact that injury disability is linked more to nature-of-injury than to cause-of-injury, matrices were generated to map the proportion of each cause-of-injury category that results in a particular nature-of-injury category. These matrices are based on a collection of dual-coded (eg, both cause-of-injury and nature-of-injury coded) hospital and emergency department datasets. The data for this step came from inpatient, outpatient, and emergency room discharge data from Argentina, Bulgaria, China, Colombia, Cyprus, the Czech Republic, Denmark, Egypt, Estonia, Hungary, Iceland, Iran, Italy, Latvia, Malta, Mauritius, Mexico, Mozambique, the Netherlands, Norway, Portugal, Slovenia, Spain, Sweden, Macedonia, Uganda, US, and Zambia. We applied our nature-of-injury severity hierarchy above to assert that every observation had one cause-of-injury and one nature-of-injury.

In GBD 2015, negative binomial models were used to estimate the probability of each nature-of-injury category resulting from each cause-of-injury category. For GBD 2016, Dirichlet models were used instead in order to estimate all of the nature-of-injury category proportions for one cause-of-injury simultaneously. These models allow for more consistent borrowing of information across age, sex, inpatient/outpatient, and high/low-income countries, and assert that the nature-of-injury proportions within a cause-of-injury category must add up to 1. One cause-nature matrix was created for each combination of injury warranting hospital admission versus injury warranting other health care, high/low-income countries, male/female, and age category. Applying these matrices to our cause-of-injury incidence from DisMod-MR, we produced cases of injury warranting hospital admission and incidence of injury warranting other health care by cause and nature of injury.

Probability of permanent health loss

Disability due to injury was assumed to affect all cases in the short term with a proportion having long-term (permanent) outcomes. The probability of long-term outcomes was needed to estimate the incidence and subsequently the prevalence of cases with permanent health loss. In our conceptual model, individuals who suffer a non-fatal injury will, in the long-term, return to either full or partial health. If one-year post-injury patients return to a health status with more disability than their pre-injury health status, injury patients are assumed to have permanent disability from their injury. The difference between the pre-injury health states and health status one year after injury is assumed to be their permanent level of injury-related disability. We assessed the probability of developing permanent health loss using the pooled dataset of follow-up studies and the Medical Expenditure Panel Survey (MEPS) that were also used to generate the nature-of-injury hierarchy. To assess the probability of permanent health loss we estimated the effects using a logit-linear mixed effects regression:

$$\begin{aligned} Logit(DW)_{im} = {} & \alpha + \beta(injuries_{im}) + \beta(never\ injured_i) + \beta(never\ injured_i * age_i) \\ & + \beta(fracture\ of\ pelvis_i) + \beta(fracture\ of\ pelvis_i * age_i) + \beta(poisoning_i * age_i) \\ & + \beta(moderate\ to\ severe\ TBI_i * age_i) + RE_c + RE_i \end{aligned}$$

where we included dummies for all the nature-of-injury categories $(injuries_{im})$, with the reference category being no injury (from MEPS dataset. We also include a dummy for never injured prior to the current injury, age, interactions between age and never injured status, and interactions with three long-term nature-of-injury categories that were found to significantly vary with age: pelvis fractures, poisonings, and moderate/severe traumatic brain injuries. In notation, subscript m refers to patient-reported outcome measure, i refers to individual and $c$refers to country. Random effects (RE) were included to control for variation between countries and individuals.

After predicting overall disability at one-year follow-up, we estimated a counterfactual by setting all observations to "no injury," the reference group for $\beta(injuries_{im})$ in our model. The disability attributable to the nature-of-injury at one year was assumed to be the difference between our counterfactual of no injury and predicted disability with injury. The probability of treated long-term outcomes was estimated via the ratio of this attributable disability relative to the long-term disability weight for that injury.

$$Probability\ of\ long - term\ disability = \frac{with\ injury\ disability_{im} - counterfactual\ disability_{im}}{DW_m}$$

We developed estimates of the probability of permanent health loss by nature-of-injury category, injury severity level (injuries warranting inpatient admission and injuries warranting other health care) and age. These probabilities are shown in Figure X for three selected age groups (25-30, 50-55, 75-80), and selected nature-of-injury categories by inpatient and outpatient. Moderate-severe TBI and spinal cord lesions only have inpatient injury long-term probabilities, and nerve injury, open wounds, and severe chest injury have long-term probabilities of 0 for outpatient cases.

Figure X. Long-term probabilities derived from the MEPS data for selected nature of injuries and age groups

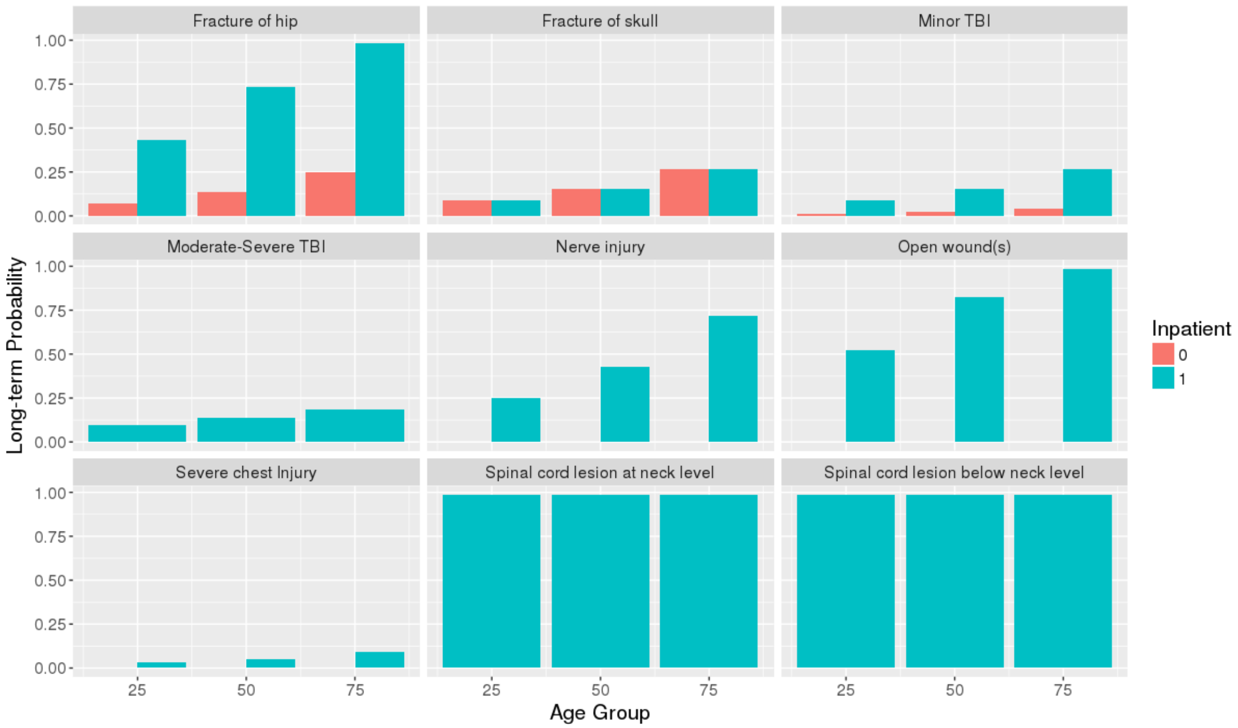

<u>Disability associated with treated and untreated cases</u>

For many nature-of-injury categories GBD 2016 has a separate disability weight for treated and for untreated cases [14]. To estimate the percent treated for injuries in a given location-year, we used the HAQ Index with the same strategy described for the probability of permanent health loss. In GBD 2015, we used a proxy covariate that defined health system access (HSA) based on a combination of vaccination rates, proportion of deliveries by a skilled birth attendant, in-facility birth, and antenatal care. We used this to estimate the ratio of treated to untreated injuries for each country-year grouping and assigned a location-year-specific disability weight equal to a weighted average of the treated and untreated disability weights for each nature-of-injury category/severity-level grouping. We assumed that all locations had at least 10% of all injuries treated and capped the maximum HSA value at the lowest HSA value for an OECD country in a given year. This OECD HSA value for 2015 was used to scale all other location-years between 10% and 100% treated based on their HSA value.

In GBD 2016, we changed this method significantly. Instead of using the HSA covariate, we use the Healthcare Access and Quality (HAQ) Index. The HAQ Index better reflects access to care related to injury treatment rather than to maternal and child healthcare. We did not assert that all OECD countries had the same value, but rather chose a reasonable cutoff for the HAQ Index (75 on a scale of 0 – 100) as the threshold at and above which 100% of injuries were treated. This value captured most OECD countries for all years back to 1980, rather than just the most recent year. We then scaled all remaining location-years between 10% and 100% treated based on their HAQ Index value and used that as the percent treated in a given location-year. This was done at the draw level to propagate uncertainty. Similar to GBD 2015, we made the decision to ignore any long-term disability from outpatient injuries from open wound, poisoning, and contusion because of implausibly high estimates of long-term disability.

Duration of short-term health loss

To determine the duration for treated cases of short-term injury we analyzed patient responses of two Dutch Injury Surveillance System follow-up studies of 2001–2003 and 2007–2009 [20, 21]. These studies collected data at 2.5, 5, 9, and 12 months post-injury on whether injury patients were still experiencing problems due to their injury [20, 21]. If not, the patients were asked how many days they had experienced problems. The injury patients that still reported having problems one year after the injury were assumed to be captured in our analysis of permanent disability. The duration for treated cases of short-term injury was estimated for injuries warranting inpatient admission and injuries warranting other health care separately. The estimates were supplemented by expert-driven estimates of short-term duration for nature of injury categories that did not appear in the Dutch dataset and untreated injuries.

Calculation of prevalence from incidence data – short-term injury

For short-term injury outcomes, which were assumed to be less than one year in duration, the prevalence for each cause-of-injury/nature-of-injury/severity-level grouping was approximated by the incidence for that grouping multiplied by the associated nature-of-injury/severity-level-specific duration.

Calculation of prevalence from incidence data – permanent health loss

For permanent health loss, we assumed no remission and thus integrated incidence over time to arrive at prevalence estimates. We used DisMod ODE (ie, the “engine” of DisMod-MR 2.1) to carry out this integration for each combination of cause of injury and nature of injury by country year and sex. For this step we used random effects meta-analysis to pool data on standardized mortality ratios derived from literature reviews for spinal cord injury, burns covering more than 20% of the body, moderate to severe traumatic brain injury, hip fracture, and multiple significant injuries (see for more detail [12]). Here we include examples of these meta-analyses: hip fractures and traumatic brain injuries.

Figure XX. Meta-analyses of standardized mortality ratios derived from literature reviews: hip fractures and traumatic brain injury.

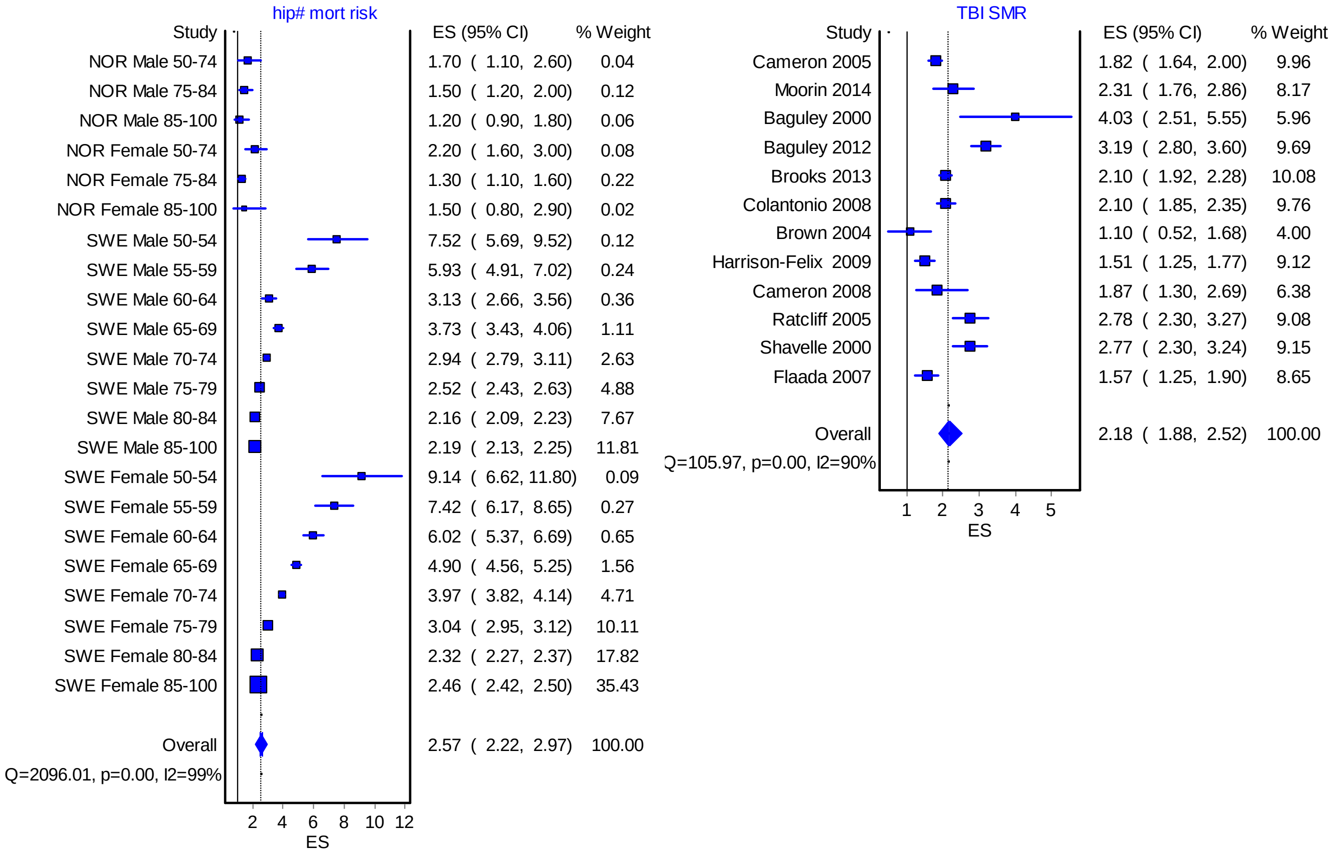

For all other nature-of-injury categories, we assumed no long-term excess mortality. For the incidence estimates derived from fatal discontinuities – "exposure to forces of nature," and "conflict and terrorism" – we did not use DisMod as discontinuities by definition violate the assumption of a steady state in DisMod to estimate prevalence from incidence. For these two cause-of-injury categories, we coded the differential equations from DisMod ODE that determine the relationship between incidence, remission, mortality risk, and prevalence into Python and streamed out the prevalence from the incidence in the years of war or disaster by integrating over one year at a time.

No other significant changes were made to the injuries modelling process for GBD 2016.

Sexual Violence

Flowchart

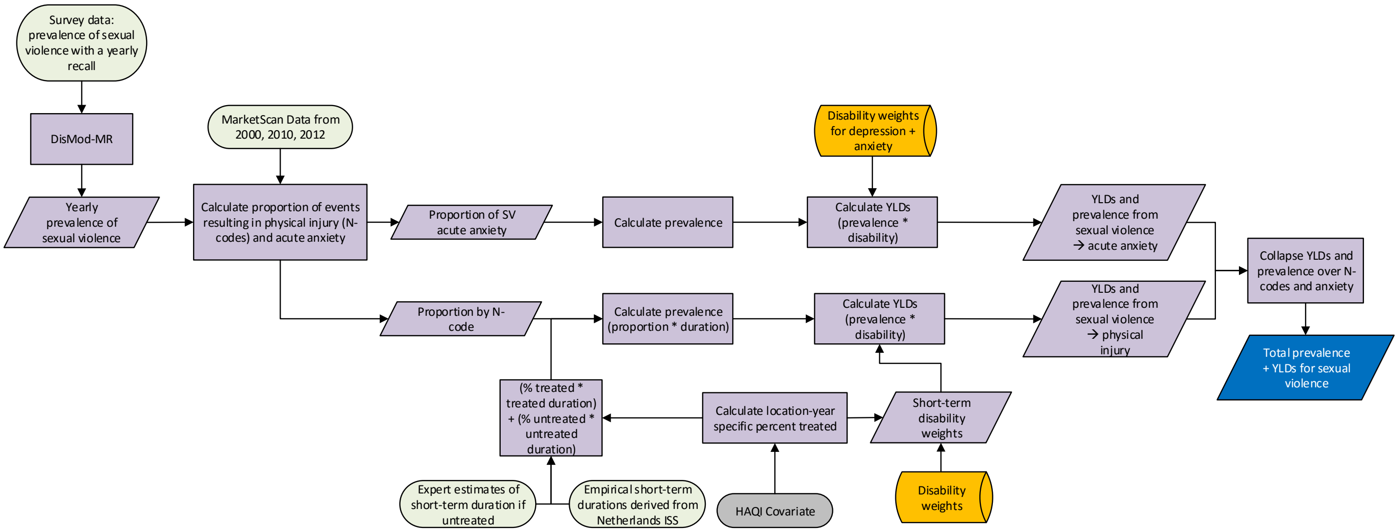

Case definition

Sexual violence is a new cause for GBD 2016. We estimate the yearly prevalence of sexual violence, ie, the proportion of the population that experienced at least one event of sexual violence in the last year. We define sexual violence as any sexual assault, including both penetrative sexual violence (rape) and non-penetrative sexual violence (other forms of unwanted sexual touching).

Input data

Model inputs

The majority of the data for sexual violence comes from various health and demographic surveys. We include many Demographic and Health Surveys (DHS) and Reproductive Health Surveys (RHS). Other survey series include the US Behavioral Risk Factor Surveillance Survey (BRFSS), the British Crime Surveys. Table 1 shows the number of unique location-sources for prevalence of sexual violence with a yearly recall that we used in the DisMod models.

Table 1. Unique location-sources for data on sexual violence yearly prevalence used in the DisMod model by Global Burden of Disease super-region.

Super-region name	Unique number of sources
Southeast Asia, East Asia, and Oceania	10
Central Europe, Eastern Europe, and Central Asia	23
High-income	55
Latin America and Caribbean	26
North Africa and Middle East	7
South Asia	91
Sub-Saharan Africa	46

Many other non-survey data sources exist for sexual violence. We explored the use of the United Nations Office on Drugs and Crime (UNODC) Statistics [TBD] that covers a wide range of geographies from 2003 to 2014. However, these estimates are based only on police reports, and their incidence is about 20 times lower than the incidence seen in the same location-years from survey data. Although we could include a covariate in our models to adjust for this underreporting, we deemed the source unusable because of the magnitude of the difference between the police reports and survey data. Survey data typically range between 1% and 10% of individuals experiencing sexual violence in the last year. Figure 1 shows the incidence estimates from the UNODC data, where most of the estimates are below about 0.05%. The geographic pattern is the opposite of what we see in survey data, with higher-income countries having higher estimates in the UNODC data. Additionally, the reports were not age-sex-specific, and the definition for what constitutes sexual violence varies across countries.

We also chose not to include the Centers for Disease Control non-fatal injury reports of sexual violence. Although this data source includes age- and sex-specific estimates for sexual violence in the United States, only sexual violence cases which resulted in physical injury are reported. These estimates are also systematically lower than the survey data, to the degree at which any adjustment with covariates would be unreliable. Lastly, we excluded a source from the United States Federal Bureau of Investigation: The Uniform Crime Reporting (UCR) program. The FBI estimates are produced at the state level for the United States, and are meant to be comparable across states. However, police report data for sexual violence are systematically lower, similar in magnitude to the UNODC data, so we chose to exclude it.

Figure 1. United Nations Office of Drugs and Crime Statistics: estimates of sexual violence (incidence per person), color by Global Burden of Disease super-regions.

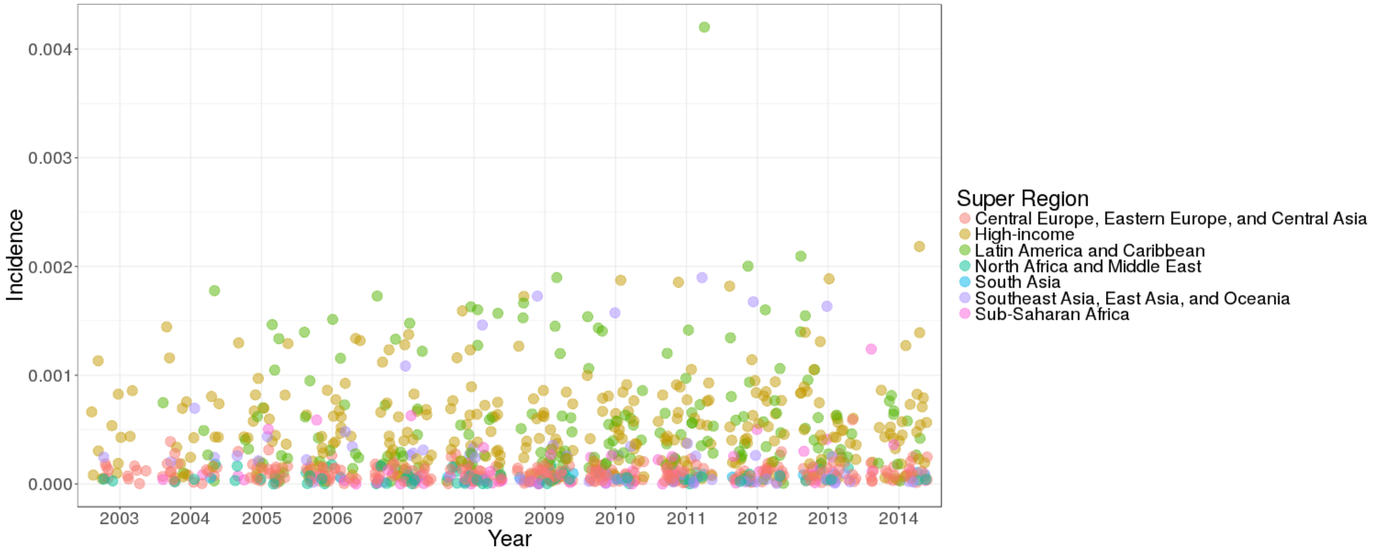

Data searches

To find large data sources for sexual violence, we searched through the Global Health Data Exchange (GHDx) to identify survey series with relevant questions, and reviewed surveys that were being used for intimate partner violence (IPV) already. We identified 107 sources with relevant data that were being

used for IPV, and 33 additional surveys with sexual violence questions. We excluded sources that only asked about lifetime prevalence of sexual violence because our case definition is specific to the past year. We extracted data on the perpetrator of sexual violence where possible (partner versus non-partner).

We completed a systematic review in PubMed to identify additional sources. We identified 415 sources and excluded 314 based on title/abstract screening. Of the 111 sources remaining after title/abstract screening, 84 did not have usable data, were of non-representative samples, or referenced data that was already being captured from the GHDx. Samples were non-representative if they were only taken among high-risk populations (war-afflicted, sex workers, intravenous drug users, etc.), sexually abused individuals, or women suffering intimate partner violence. We also excluded studies that only asked about sexual violence in the context of alcohol. After full-text screening, 18 sources remained, only two of which had yearly recall prevalence.

Modelling strategy

Prevalence of sexual violence

To produce estimates of the yearly prevalence of sexual violence, we used DisMod-MR 2.0. DisMod-MR 2.0 is a descriptive epidemiological meta-regression tool that uses the integrative systems modelling approach to produce simultaneous estimates of disease incidence, prevalence, remission, and mortality. To preserve variation between male- and female-specific estimates, we have separate models for men and women.

We make various assumptions within DisMod-MR 2.0 including no excess mortality due to sexual violence and no incidence between 0–2 and 98–100 years of age. Because of the different ways that questions about sexual violence in the last year can be asked, we include multiple study-level covariates (for coefficient estimates, see Table 2). We bounded the covariates at logical values to minimize the effect of collinearity between the covariates, ie, we expect studies that ask about penetrative sexual violence only to have lower estimates of sexual violence overall, so that covariate has an upper bound of 1.

Table 2. Study- and country-level covariates for DisMod-MR 2.0 yearly recall prevalence models for sexual violence.

Covariate	Covariate Bounds	Exponentiated Value
Study-level covariates		
Physically forced sexual violence only	Upper: 1	0.94 (0.82 – 1.00)
Ever-partnered people only	None	1.49 (1.16 – 2.01)
Ever-married people only	None	2.19 (1.53 – 2.97)
Specifies specifically degrading or humiliating sex acts	Upper: 1	0.96 (0.87 – 1.00)
Ever had sex	None	1.23 (1.09 – 1.42)
Ever married or lived with a partner	None	1.44 (1.15 – 1.93)
Does not include coerced or feared sex in definition	Upper: 1	0.94 (0.84 – 1.00)
Penetrative sexual violence only	Upper: 1	0.66 (0.56 – 0.75)

Does not include non-partner non-penetrative	Upper: 1	0.99 (0.97 – 1.00)
Only includes partner sexual violence	Upper: 1	0.85 (0.65 – 0.99)
Includes attempted sexual violence	Lower: 1	1.36 (1.04 – 1.97)
Country-level covariates		
Alcohol (liters per capita)		1.00 (0.37 – 2.72)
Socio-demographic Index		0.12 (0.088 – 0.16)

Years lived with disability (YLDs) due to sexual violence

In our calculations of years lived with disability due to sexual violence, for GBD 2016 we are only considering the short-term physical and psychological effects of sexual violence. In future GBD iterations, we will be including sexual violence as a risk factor including both sexual violence in the last year and lifetime exposure to sexual violence (independent from, and in interaction with, intimate partner violence). Including sexual violence as a risk will allow us to capture the long-term mental health consequences of sexual violence, which we are unable to capture just considering past yearly recall of sexual violence and its consequences for up to one year.

To calculate the years lived with disability (YLDs) due to having experienced sexual violence in the past year, we utilize claims data from the United States from the years 2000, 2010, and 2012 to assess sexual violence sequelae. We search through the claims database for the following ICD9 diagnosis codes: 995.53 (child sexual abuse), 995.83 (adult sexual abuse), and E960.1 (rape). We considered sequelae relating to both physical injuries and mental health consequences, in the short-term.

Physical injury

For the physical injury sequelae, we looked for any nature-of-injury ICD9 code on the same date of contact with medical service providers for a sexual violence ICD9 code (above), and categorized the nature-of-injury codes as we do for the general injuries nonfatal modeling process (see appendix: nonfatal injuries). We calculate the proportion of individuals with any sexual violence code that result in each of the physical injuries categories. This strategy is similar to the strategy that we use for the cause-nature of injury matrices in the general injuries modeling process, but we have an additional category for no physical injury result, since the majority of sexual violence incidents do not result in physical injury in the claims database. Additionally, since we only have one data source, we do not model these proportions with Dirichlet regression like we do for the injuries cause-nature of injury matrices, but just compute them directly from the claims data. To estimate the physical injuries component of YLDs, we multiply the DisMod estimates of yearly prevalence of sexual violence by these proportions and then multiply by each physical injuries' respective short-term duration and disability weight that we use in the general injuries process (see appendix: nonfatal injuries).

Acute anxiety and/or reaction to stress

For the mental and psychological sequelae of sexual violence, we searched an individual being coded to any of the following ICD9 codes at any point *after* a sexual violence incident was noted. The codes are meant to reflect conditions relating to an "acute anxiety and/or reaction to stress" condition following a traumatic incident, displayed in Table 2.

Table 2. ICD9 codes included in the "acute anxiety and/or reaction to stress" condition as a sequela for sexual violence.

ICD9 Code	Condition Description
**308**	Acute reaction to stress
**308**	Predominant disturbance of emotions
**308.1**	Predominant disturbance of consciousness
**308.2**	Predominant psychomotor disturbance
**308.3**	Other acute reactions to stress
**308.4**	Mixed disorders as reaction to stress
**308.9**	Unspecified acute reaction to stress
**309**	Adjustment reaction
**309**	Adjustment disorder with depressed mood
**309.1**	Prolonged depressive reaction
**309.2**	Adjustment reaction with predominant disturbance of other emotions
**309.21**	Separation anxiety disorder
**309.22**	Emancipation disorder of adolescence and early adult life
**309.23**	Specific academic or work inhibition
**309.24**	Adjustment disorder with anxiety
**309.28**	Adjustment disorder with mixed anxiety and depressed mood
**309.29**	Other adjustment reactions with predominant disturbance of other emotions
**309.3**	Adjustment disorder with disturbance of conduct
**309.4**	Adjustment disorder with mixed disturbance of emotions and conduct
**309.8**	Other specified adjustment reactions
**309.81**	Posttraumatic stress disorder
**309.82**	Adjustment reaction with physical symptoms
**309.83**	Adjustment reaction with withdrawal
**309.89**	Other specified adjustment reactions
**309.9**	Unspecified adjustment reaction

It is possible that the appearance of one of these ICD9 codes is entirely unrelated to the sexual violence incident. Additionally, the appearance of one of these codes could be related instead to underlying depression and anxiety. To control for these confounding factors, we also searched for these ICD9 codes among individuals that were not victims of sexual violence in the past year. We used Poisson regression with robust standard errors to model the relative risk of the "acute anxiety and/or reaction to stress" comparing individuals with and without sexual violence within the year, controlling for underlying diagnoses of depression and anxiety constant:

$$\log(\lambda) = \beta_0 + \beta_1(sexual\ violence) + \beta_2(depression) + \beta_3(anxiety) + \beta_4(female) + \beta_5(age)$$

where λ is the risk of "acute anxiety and/or reaction to stress," and e^{β_1} is the relative risk of "acute anxiety and/or reaction to stress" comparing those experiencing at least one sexual violence incident to those with no sexual violence incidence, holding underlying depression, anxiety, sex, and age constant.

We can approximate the risk of “acute anxiety and/or reaction to stress” for each age and sex experiencing sexual violence by:

$$\lambda_{age,sex} = e^{\beta_1} * (e^{\beta_0} * e^{sex*\beta_4+age*\beta_5}) - (e^{\beta_0} * e^{sex*\beta_4+age*\beta_5})$$

The claims data had n = 70,6707,63 observations (n = 8,331 sexual violence cases). Using the equation above, the transformed coefficients and transformed robust standard errors (transformations were performed with the Delta method) are shown in Table 3.

Table 3. Estimates of the risk of “acute anxiety and/or reaction to stress” ($\lambda_{age,sex}$) among people experiencing sexual violence over a year time-period, specific to age and sex.

Age	Male		Female	
	Estimate	*Standard error*	*Estimate*	*Standard error*
**0**	0.0967	0.0023	0.1205	0.0028
**5**	0.0933	0.0021	0.1162	0.0027
**10**	0.0899	0.0021	0.1120	0.0026
**15**	0.0867	0.0020	0.1080	0.0025
**20**	0.0836	0.0020	0.1042	0.0024
**25**	0.0806	0.0019	0.1004	0.0024
**30**	0.0777	0.0018	0.0968	0.0023
**35**	0.0749	0.0018	0.0934	0.0022
**40**	0.0722	0.0017	0.0900	0.0021
**45**	0.0697	0.0016	0.0868	0.0020
**50**	0.0672	0.0016	0.0837	0.0020
**55**	0.0648	0.0015	0.0807	0.0019
**60**	0.0624	0.0015	0.0778	0.0018
**65**	0.0602	0.0014	0.0750	0.0018
**70**	0.0581	0.0014	0.0723	0.0017
**75**	0.0560	0.0013	0.0697	0.0016
**80**	0.0540	0.0013	0.0672	0.0016
**85**	0.0520	0.0012	0.0648	0.0015
**90**	0.0502	0.0012	0.0625	0.0015
**95**	0.0484	0.0011	0.0603	0.0014

We multiplied the prevalence of yearly sexual violence by $\lambda_{age,sex}$ to get the prevalence of “acute anxiety and/or reaction to stress” due exclusively to sexual violence. To estimate YLDs for this sexual violence sequela, we used the average of the disability weights for mild depression and anxiety. For simplicity, we assume a duration of one year, thus the YLDs for the mental and psychological stress component of sexual violence is the product of the residual probability of “acute anxiety and/or reaction to stress” and the disability weight.

Anemia Impairment

Flowchart

Anemia Impairment Estimation

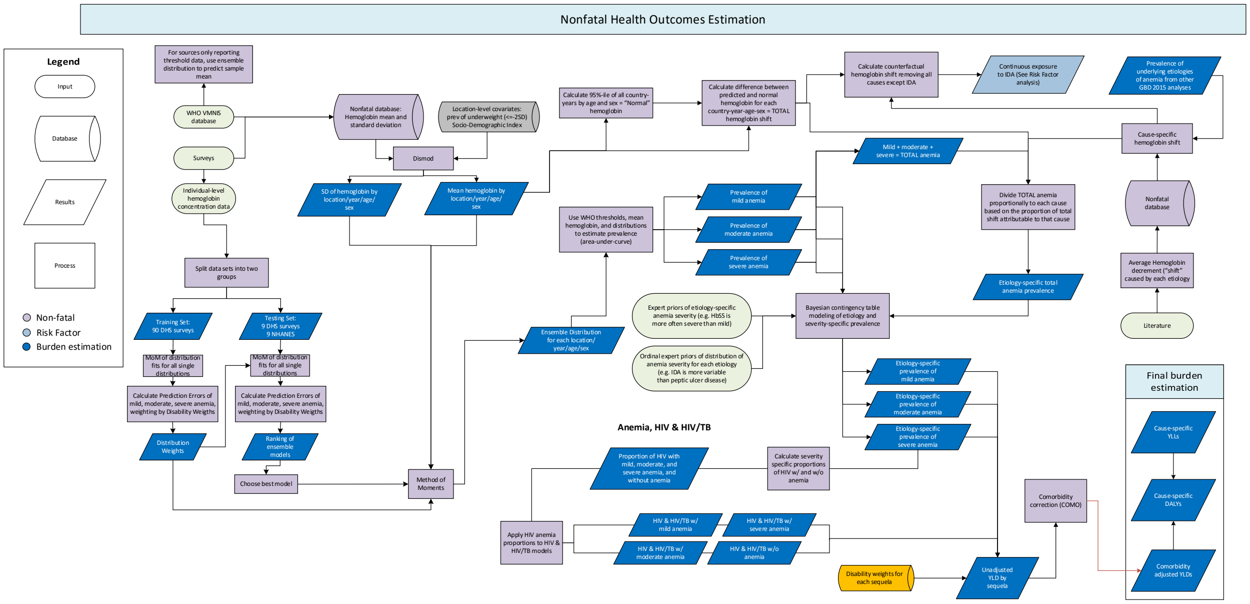

Input data and methodological summary

Case definition

The prevalence of anemia is defined by the following WHO thresholds for hemoglobin in g/L.

Severity definitions used to calculate GBD 2016 anemia envelope			
	Severity of anemia		
	Mild	Moderate	Severe
Age < 5 years			
Males	100 - 109 g/L	70 - 99 g/L	< 70 g/L
Females	100 - 109 g/L	70 - 99 g/L	< 70 g/L
Age 5 - 14 years			
Males	110 - 114 g/L	70 - 99 g/L	< 70 g/L
Females	110 - 114 g/L	70 - 99 g/L	< 70 g/L
Age 15+ years			
Males	110 – 129 g/L	80 - 109 g/L	< 80 g/L
Females, non-pregnant	110 - 119 g/L	80 - 109 g/L	< 80 g/L
Females, pregnant	100 – 109 g/L	70 - 99 g/L	< 70 g/L

Modelling Strategy Summary:

As with GBD 2015, the anemia model has two main steps: estimation of the anemia envelope and causal attribution. The envelope approach utilizes the main source of anemia data – population mean hemoglobin and prevalence of all-cause anemia – to estimate the overall prevalence of anemia impairment. The causal attribution assigns all cases of anemia to a specific cause and severity in an internally consistent method.

Input data

Model inputs

The envelope approach to the anemia impairment utilizes data from a variety of sources. Population-based surveys of hemoglobin concentration were the primary input to our analytic dataset. Examples include the Demographic and Health Survey (DHS) and Multiple Indicator Cluster Survey (MICS) series, along with other national and subnational surveys that completed hemoglobin testing. We supplemented with pertinent sources downloaded from the WHO Vitamin and Mineral Nutrition Information System (VMNIS) available at http://www.who.int/vmnis/database/anaemia/countries/en/. A full source list is available elsewhere in this appendix. Most used a HemoCue test, adjusted for altitude, and excluded those with terminal or acute medical conditions. Inclusion, exclusion and diagnostic criteria for other studies were similar and can be found in each study.

Modelling strategy

Anemia Impairment Envelope

1) Estimation of population mean and standard deviation of hemoglobin

We ran two Dismod-MR models – one for mean hemoglobin and one for standard deviation of hemoglobin. In both models, we included fixed effects on pregnancy status, underweight (proportion of children under 5 <2SD weight for age), and sociodemographic index (SDI). A z-cov was used for studies not representative of the location modelled. Mean-hemoglobin was used as a fixed effect in the standard deviation model.

2) Estimation of prevalence of anemia by severity

We modelled the full distribution of hemoglobin for each population (location/age/year/sex), from which we applied the WHO thresholds to calculate prevalence of each severity of anemia. In GBD 2015, a Weibull distribution was fit using shape and scale parameters estimated from mean hemoglobin. For GBD 2016, we combine multiple two-parameter distributions to create a more precise and unbiased ensemble distribution.

First, we created a training and testing set of individual-level hemoglobin measurements. The training set consisted of 90 DHS surveys, providing 290 group-specific samples of microdata from children <5, males 15-45, pregnant females 15-45, and non-pregnant females 15-45 (not all groups were sampled in each DHS). A set of two-parameter distributions (gamma, mirror gamma, Weibull, mirror lognormal, and mirror gumbel) were fit to the sample's hemoglobin mean and variance. These distributions were combined using weights optimized by a loss function of severity-specific prediction error weighted by the ratio of the severity's disability weight (DW) to mild anemia DW. Weights were constrained to be positive

and sum to 1, so that the resultant ensemble distribution is a proper probability density function. All permutations of the 5 distributions were tested (ie, we optimized weights for both a mix of all 5 distributions as well as a gamma-weibull two-way combination).

The loss function is

$$\sum_{i=1}^{n_i}\sum_{j=1}^{n_j}\sum_{k=1}^{n_k} r_j|p_{ijk} - \hat{p}_{ijk}|$$

Where

$$\hat{p}_{ijk} = \sum_{z=1}^{n_z} w_z \int_{t1_{jk}}^{t2_{jk}} PDF_{ijz}$$

n_i is a list of surveys (in either the training or testing set)

n_j is the list of groups: children <5, males 15-45, pregnant females 15-45, non-pregnant females 15-45, males >45, and females >45

n_k is the list of severities (mild, moderate, severe)

n_z is the list of distributions (gamma, mirror gamma, Weibull, mirror lognormal, and mirror gumbel)

r is the ratio of the severity *j* disability weight to that of mild anemia

r_k = 13 for moderate and r_k = 40 for severe

PDF is a probability density function fit to the sample mean and variance

t1 and t2 are the lower and upper bounds to the WHO anemia definition for the group

w is the set of distribution weights (each constrained to be positive) such that

$\sum_{z=1}^{n_z} w_z = 1$ and all $w_z > 0$

Therefore $\sum_{z=1}^{n_z} w_z * PDF_z$ describes the ensemble probability density function that can be integrated to calculate prevalence for any severity

The testing set consisted of 9 NHANES and 9 DHS surveys not included in the training data. Inclusion of NHANES as half the testing set ensured out of sample predictive validity by challenging the global weights, as it provided the ensemble distribution with high-income data (DHS is from LMIC countries) and data from adults >45 (DHS did not take blood tests from the elderly). We selected the combination of distributions (including all individual component distributions) that minimized the loss function.

With a set of component distributions and global weights, we then modelled the distribution of hemoglobin in each location/year/age/sex by fitting each component distribution using modelled mean and standard deviation, then weighting to create the ensemble distribution $\sum_{z=1}^{n_z} w_z * PDF_z$. We

integrated area under the curve for each group-specific WHO threshold to calculate prevalence of anemia by severity.

Because anemia thresholds depend on pregnancy, we separately modelled the distribution of pregnant and non-pregnant females. The method for fitting the ensemble distribution to pregnant women was identical to that of non-pregnant, but used the mean and variance from the two Dismod models adjusted by the estimated beta on the pregnancy status fixed effect. The prevalence of anemia in pregnant and non-pregnant women were weighted by the pregnancy rate and combined to estimate population prevalence of anemia. The pregnancy rate for each age is estimated as

$$pregnancy = \quad (ASFR + SB) * 46/52$$

Where

ASFR is the location- and age-specific fertility rate

SB is the location-specific stillbirth rate

Below are some examples of the hemoglobin distributions for various locations and groups, combining the sample distribution (histogram), individual distributions, and ensemble distribution.

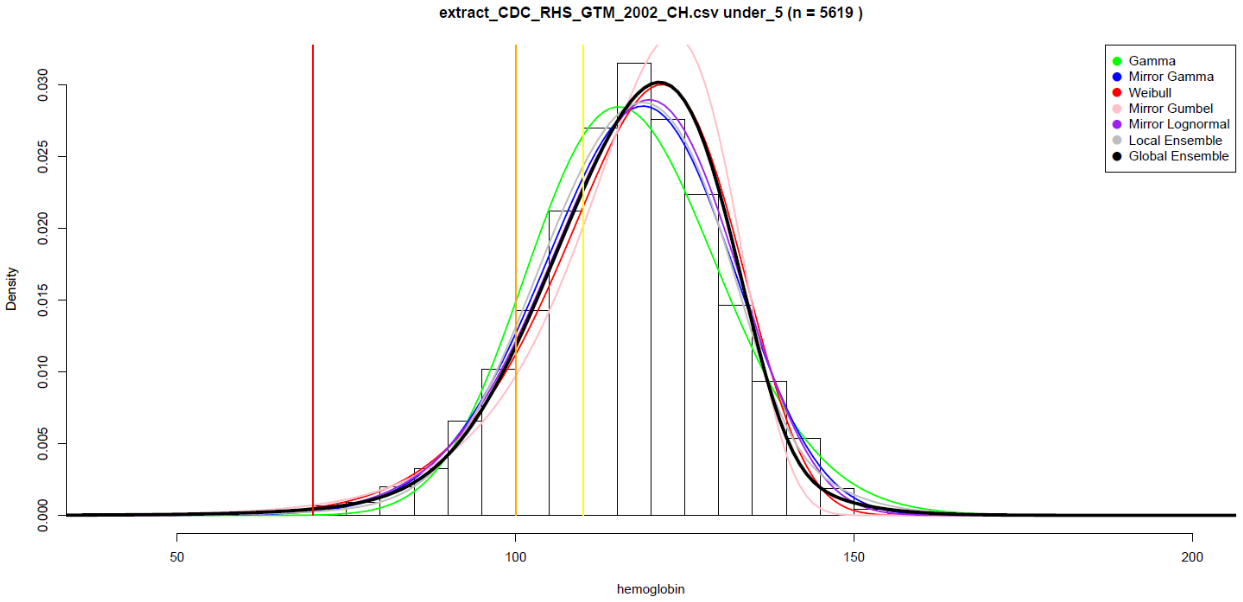

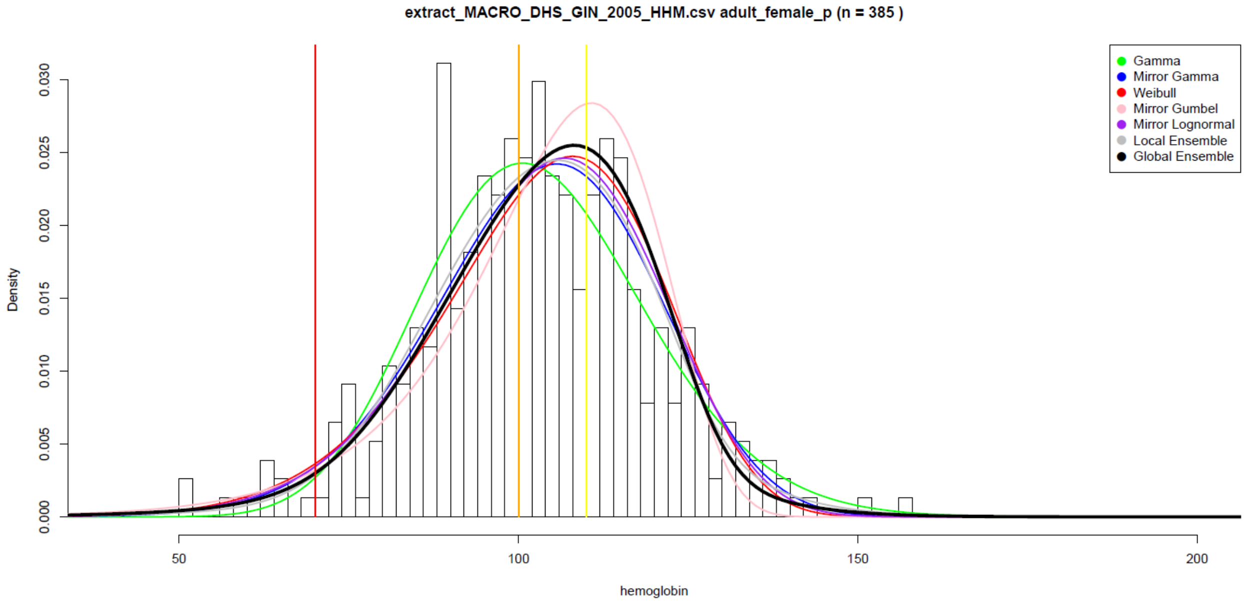
extract_MACRO_DHS_GIN_2005_HHM.csv adult_female_p (n = 385)
Gamma
Mirror Gamma
Weibull
Mirror Gumbel
Mirror Lognormal
Local Ensemble
Global Ensemble
Density
hemoglobin

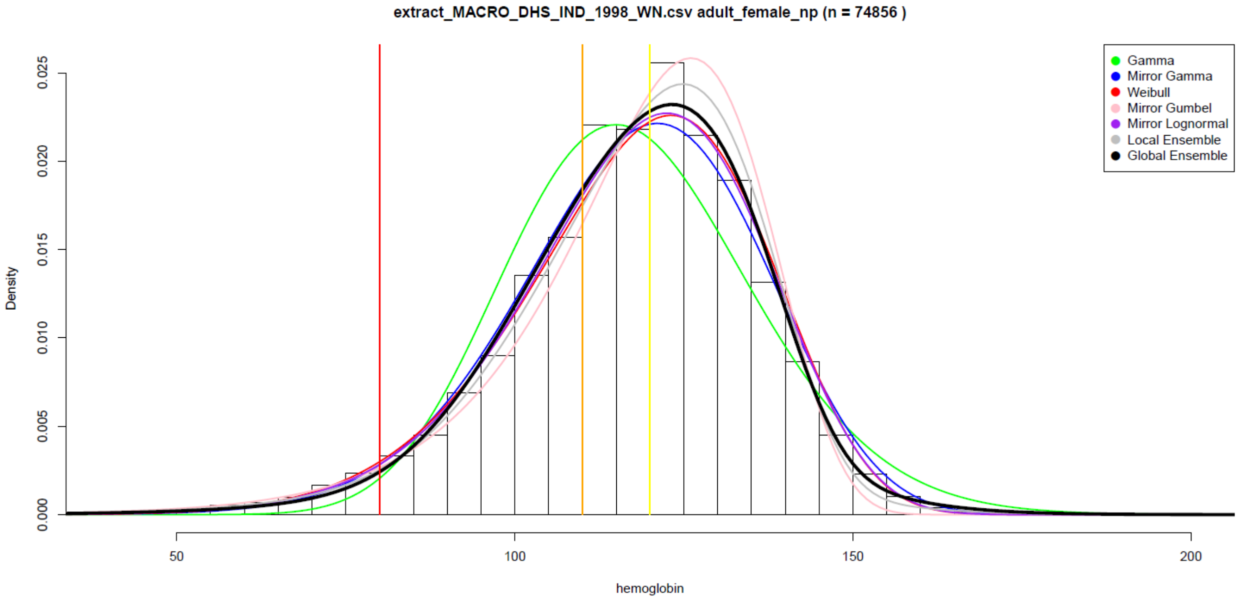
extract_MACRO_DHS_IND_1998_WN.csv adult_female_np (n = 74856)
Gamma
Mirror Gamma
Weibull
Mirror Gumbel
Mirror Lognormal
Local Ensemble
Global Ensemble
Density
hemoglobin

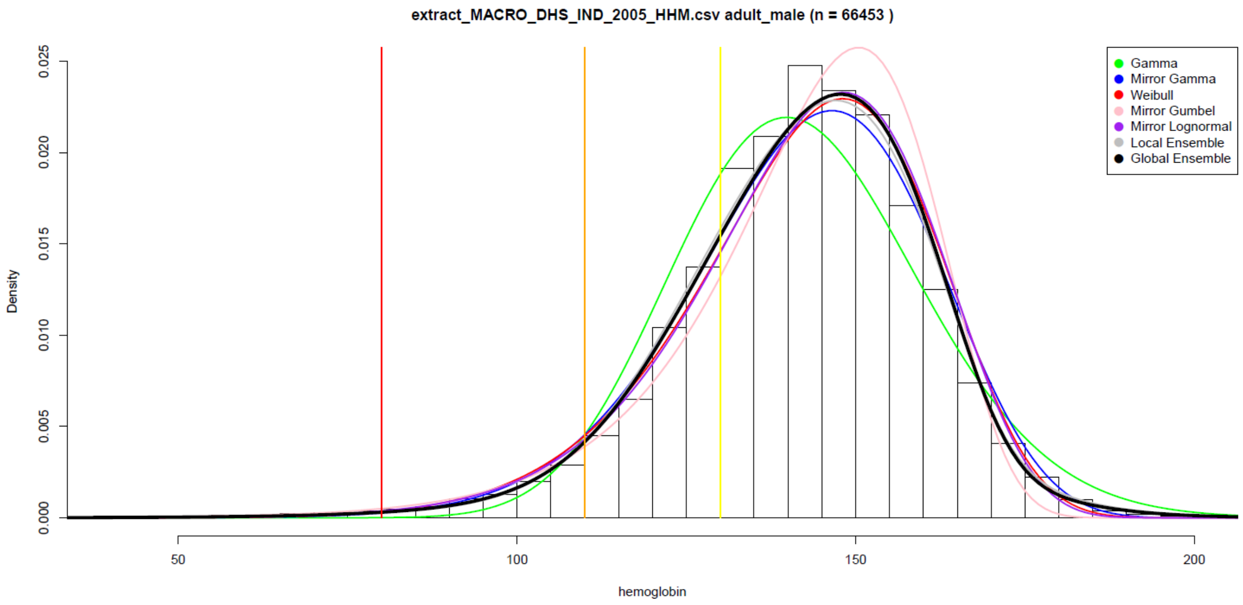
extract_MACRO_DHS_IND_2005_HHM.csv adult_male (n = 66453)
Gamma
Mirror Gamma
Weibull
Mirror Gumbel
Mirror Lognormal
Local Ensemble
Global Ensemble
Density
0.000
0.005
0.010
0.015
0.020
0.025
50
100
150
200
hemoglobin

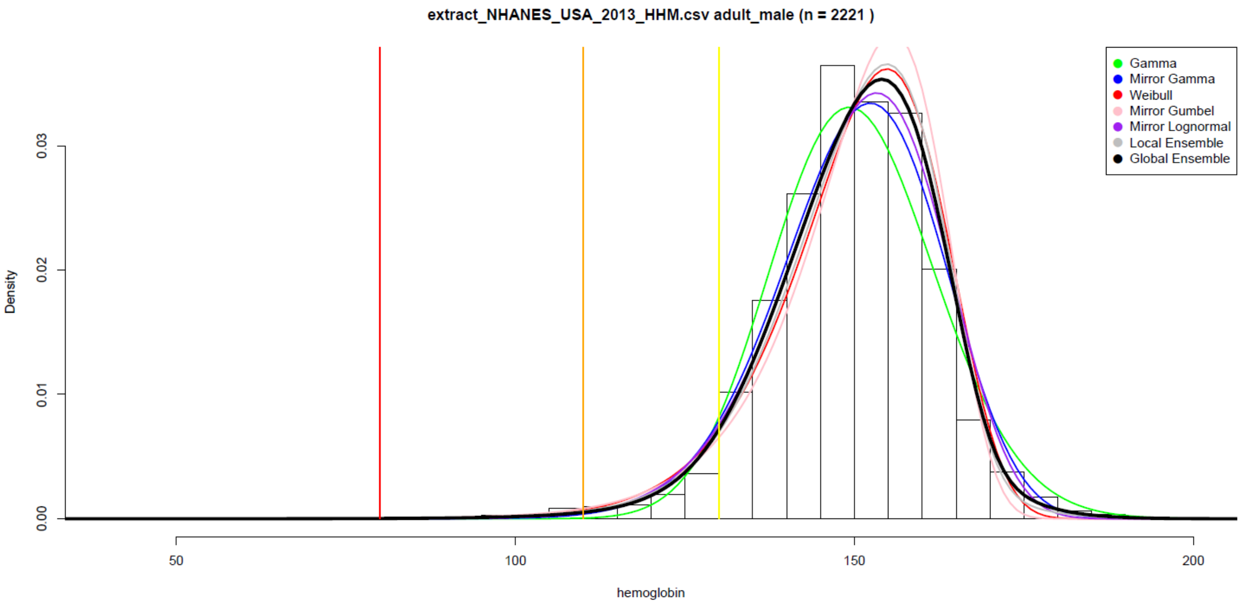
extract_NHANES_USA_2013_HHM.csv adult_male (n = 2221)
Gamma
Mirror Gamma
Weibull
Mirror Gumbel
Mirror Lognormal
Local Ensemble
Global Ensemble
Density
0.00
0.01
0.02
0.03
50
100
150
200
hemoglobin

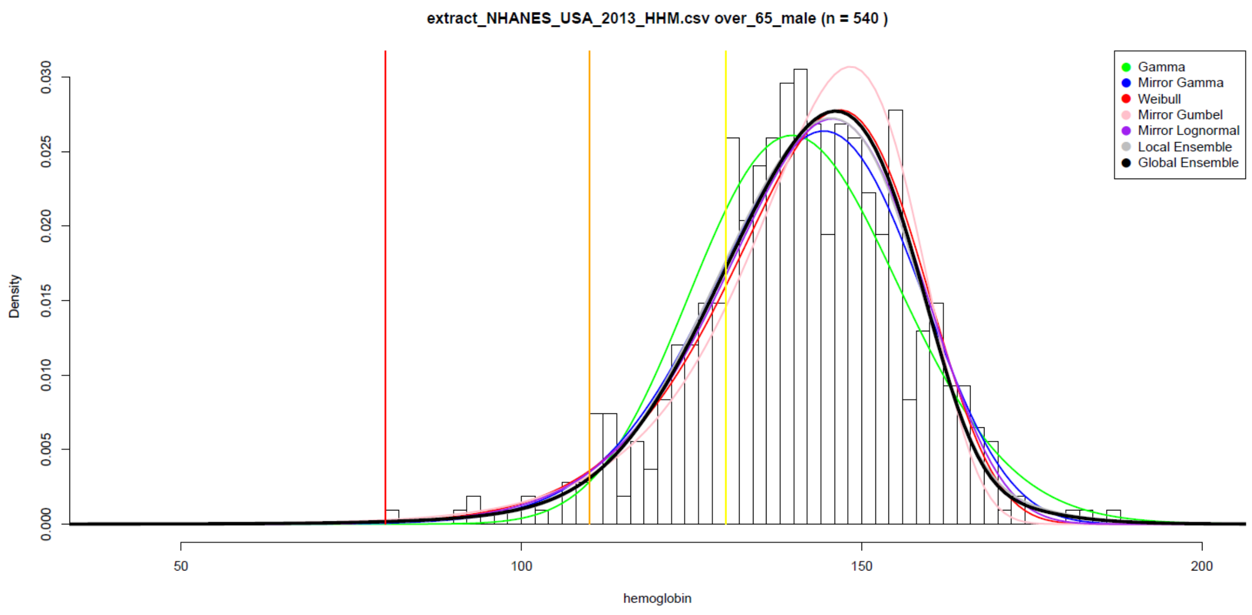

Causal Attribution

We performed cause-specific attribution on the anemia envelope using information on cause-specific prevalence and hemoglobin shift, which uses the same overall method as in the GBD 2010 with the addition of a number of causes and updates to hemoglobin shifts for inputs to causal attribution.

Total "hemoglobin shift" was determined as the difference between the normal and predicted mean hemoglobin levels for each population group. We denoted the normal hemoglobin level as the global 95th percentile of the distribution of mean-hemoglobin within each age- group, sex, and year. We then determined a total shift for each country in the corresponding age-group, sex, and year by finding the difference between the global "normal" and the country-specific predicted mean hemoglobin. Our model of attribution followed that, because the shift is a disease state experienced by 100% of the population, then the sum of cause-specific hemoglobin shifts times the prevalence of each contributing cause should add up to the total. We summed shift times prevalence estimates from all causes, compared to the total predicted hemoglobin shift, and proportionally assigned. We distributed the residual envelope among seven remaining causes.

Of note, our iron-deficiency anemia (IDA) estimates include acute and chronic hemorrhagic states for which supplementation may be helpful, but poor nutritional intake is not the only underlying problem. A few causes in this category – hookworm, schistosomiasis, upper gastrointestinal bleeding, and gynecologic diseases – were considered separately from IDA because there was enough data from GBD prevalence estimation processes to do so. Distribution of anemia burden to IDA only after assignment to "known" causes avoided double counting of these cases. Most other causes of anemia not specifically considered were included in the "other" categories.

For all causes with specific population-specific prevalence estimates, we enforced a condition where the sum of mild, moderate, and severe anemia would not exceed the total prevalence within each population. Additionally, because inherent in our method of determining "normal" hemoglobin is the fact that 5% of population groups will have zero, or negative, total shift, we assigned a minimum of 10% of all

anemia to be assigned to residual causes based on review of findings from National Health and Nutrition Examination Survey (NHANES) in the United states[5,6].

We again used Bayesian contingency table modelling methods developed for GBD 2013 to disaggregate marginal estimates of anemia severity and aetiology into a complete set of prevalence estimates for aetiology/severity pairs. Marginal estimates of column sums (total anaemia prevalence by severity [mild, moderate, severe]) and row sums (total aetiology prevalence for each cause) were paired with priors on the etiology-specific hemoglobin shifts (the same as were used for overall etiologic attribution) and rank order of variation of severity (e.g. malaria-induced anaemia severity is highly variable while that due to homozygous sickle cell disease is less so). Nonlinear optimization methods were then used to populate a complete matrix of aetiology-severity estimates from the marginal estimates and distribution priors. We found the maximum a posteriori (MAP) point estimate for 5 samples from estimated posterior distributions independently for each population group, then scaled the results to ensure row sums were non-zero and column sums matched the original draws. We then took the mean of the scaled posteriors for each population group. To estimate uncertainty for each scaled posterior mean, we first calculated the ratio of each draw to the mean of all draws for the anemia envelope. For non-residual causes, we also calculated the ratio of each draws to the mean of all draws. We then multiplied the scaled posterior mean by these ratios.

The anemia causal attribution process produces estimates for mild, moderate, and severe anemia due to HIV. Using these estimates, we calculated proportions of HIV with mild, moderate, severe, and no anemia for each demographic group. GBD produces estimates for seven HIV sub-causes: early HIV, symptomatic HIV, AIDS with antiretroviral treatment, AIDS without antiretroviral treatment, drug-sensitive HIV/AIDS – Tuberculosis, multidrug-resistant HIV/AIDS – Tuberculosis, and extensively drug-resistant HIV/AIDS – Tuberculosis. We assumed the anemia severity proportions were equivalent across the seven sub-causes, and estimated the anemia severity levels for each by multiplying the HIV sub-causes by the anemia proportions.

Priors: Largest*

Anemia Subtype	Expected Largest
Malaria (PFPR)	0
Schistosomiasis	0
Hookworm	0
Other NTDs	0
Maternal Hem.	1
Iron Deficiency	0
Other Infectious Diseases	0
Peptic Ulcer Disease (PUD)	2
Gastritis	2
CKD due to Diabetes, Stage 3	1
CKD due to Diabetes, Stage 4	1

CKD due to Diabetes, Stage 5	2
CKD due to Hypertension, Stage 3	1
CKD due to Hypertension, Stage 4	1
CKD due to Hypertension, Stage 5	2
ckd_glomerulo3	1
ckd_glomerulo4	1
ckd_glomerulo5	2
CKD due to Other, Stage 3	1
CKD due to Other, Stage 4	1
CKD due to Other, Stage 5	2
Uterine Fibroids	0
Other Gyne	0
Other Hemoglobinopathies	1
Endocrine, Metabolic & Other Blood Disorders	0
hemog_g6pd	1
hemog_g6pd_hemi	1
hemog_thalass_btm	2
hemog_thalass_btt	1
hemog_thalass_ett	0
hemog_thalass_hebt	2
hemog_thalass_hhd	2
hemog_sickle_hscbt	2
hemog_sickle_hscd	2
hemog_sickle_scbt	2
hemog_sickle_sct	0
HIV	0

*0 = mild anemia; 1 = moderate anemia; 2 = severe anemia

Priors: Severity

Anemia Subtype	Expected Variation
Malaria	1
Gastritis	2
Peptic Ulcer Disease (PUD)	3
Maternal Hem.	4
Endocrine	5
Other Gyne	6
Other Infectious	7
Other NTDs	8
ckd_other3	9
ckd_htn3	10
ckd_diabetes3	11
ckd_glomerulo3	12
ckd_other4	13
ckd_htn4	14
ckd_diabetes4	15
ckd_glomerulo4	16
ckd_other5	17
ckd_htn5	18
ckd_diabetes5	19
ckd_glomerulo5	20
hemog_thalass_ett	21
hemog_sickle_sct	22
hemog_thalass_btt	23
hemog_g6pd	24
hemog_g6pd_hemi	25
Iron Deficiency	26
Uterine Fibroids	27
hemog_sickle_hscd	28
Schistosomiasis	29
hemog_thalass_hhd	30
Other Hemoglobinopathies	31
hemog_sickle_scbt	32
Hookworm	33
hemog_thalass_hebt	34
hemog_sickle_hscbt	35
hemog_thalass_btm	36
HIV	37

Causes for which allocation of residual anemia envelope was based on fixed proportion redistribution methods*:

Iron-deficiency anemia (IDA)
Uterine fibroids
Other infectious diseases
Other gynecological disorders
Other neglected tropical diseases
Other endocrine, nutrition, blood and immune disorders
Other hemoglobinopathies and hemolytic anemias

* A minimum of 10% of all anemia was assigned to residual categories based on analysis of NHANES-III data from the United States

References

1. Kassebaum NJ. The Global Burden of Anemia. *Hematology/Oncology Clinics* 2016; **30**: 247–308.
2. Kassebaum NJ, Jasrasaria R, Naghavi M, *et al.* A systematic analysis of global anemia burden from 1990 to 2010. *Blood* 2014; **123**: 615–24.
3. WHO | Haemoglobin concentrations for the diagnosis of anaemia and assessment of severity. Geneva: World Health Organization, 2011 http://www.who.int/vmnis/indicators/haemoglobin.pdf.
4. Kates EH, Kates JS. Anemia and polycythemia in the newborn. *Pediatr Rev* 2007; **28**: 33–4.
5. Centers for Disease Control and Prevention (CDC). Iron deficiency--United States, 1999-2000. *MMWR Morb Mortal Wkly Rep* 2002; **51**: 897–9.
6. Looker AC, Dallman PR, Carroll MD, Gunter EW, Johnson CL. PRevalence of iron deficiency in the united states. *JAMA* 1997; **277**: 973–6.

Epilepsy Impairment

Flowchart

Epilepsy

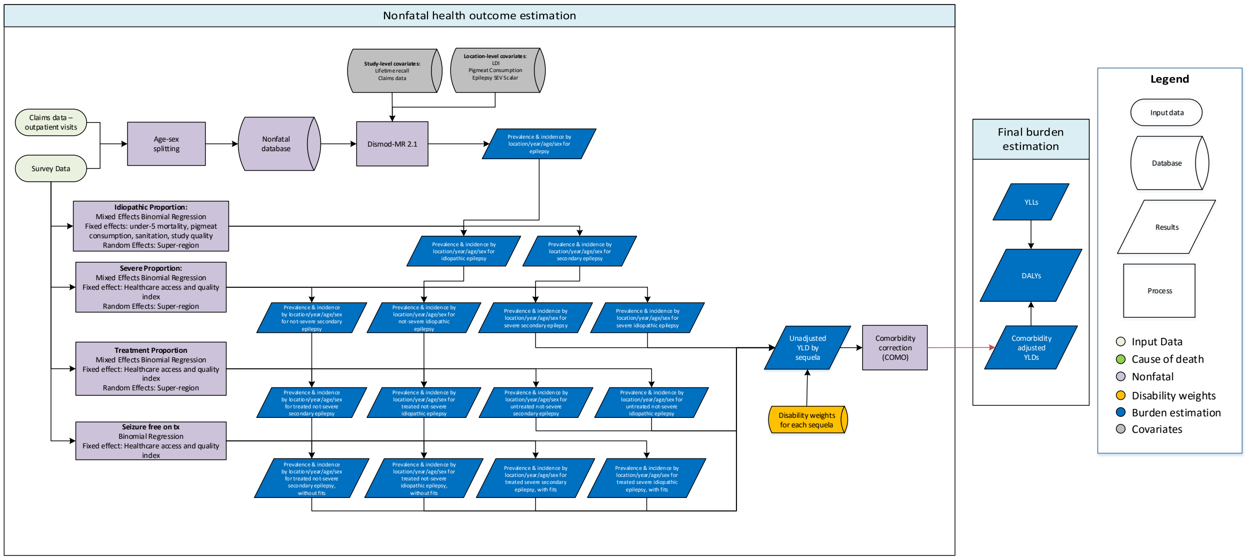

Case definition

Since GBD 2013, we have used the following definitions from the “Guidelines for Epidemiologic Studies on Epilepsy”: 1) Epilepsy: a condition characterized by recurrent (two or more) epileptic seizures, unprovoked by any immediate identified cause, and 2) “Active” epilepsy: a prevalent case of active epilepsy is defined as a person with epilepsy who has had at least one epileptic seizure in the previous five years, regardless of antiepileptic drug (AED) treatment. We also use the following ICD-10 codes for epilepsy: G40 (Neuro, epilepsy, total) and G41 (Neuro, epilepsy, status epilepticus). We defined severe epilepsy as having seizures one or more times per month.

Input data

Model inputs

For GBD 2016, we conducted a systematic review covering 10/1/2014 to 10/7/2016 using the following search string:

("2014/10/01"[PDAT] : "2016"[PDAT]) AND ("epilepsy"[MeSH Terms] OR "epilepsy, partial, motor"[MeSH Terms] OR "epilepsy, benign neonatal"[MeSH Terms] OR "epilepsy, reflex"[MeSH Terms] OR "myoclonic epilepsy, juvenile"[MeSH Terms] OR "epilepsy, frontal lobe"[MeSH Terms] OR "epilepsy, complex partial"[MeSH Terms] OR "epilepsy, post-traumatic"[MeSH Terms] OR "epilepsy, temporal lobe"[MeSH Terms] OR "epilepsy, absence"[MeSH Terms] OR "epilepsy, tonic-clonic"[MeSH Terms] OR "epilepsies, myoclonic"[MeSH Terms] OR "epilepsies, partial"[MeSH Terms] OR epilepsy[Title/Abstract]) AND (incidence[Title/Abstract] OR prevalence[Title/Abstract]) NOT(animals[MeSH] NOT humans[MeSH]).

Of 952 hits, 32 were marked for extraction. Additional data seeking efforts also led to the addition of 12 more sources on epilepsy prevalence in India subnationals. A flow chart documenting the review is displayed below.

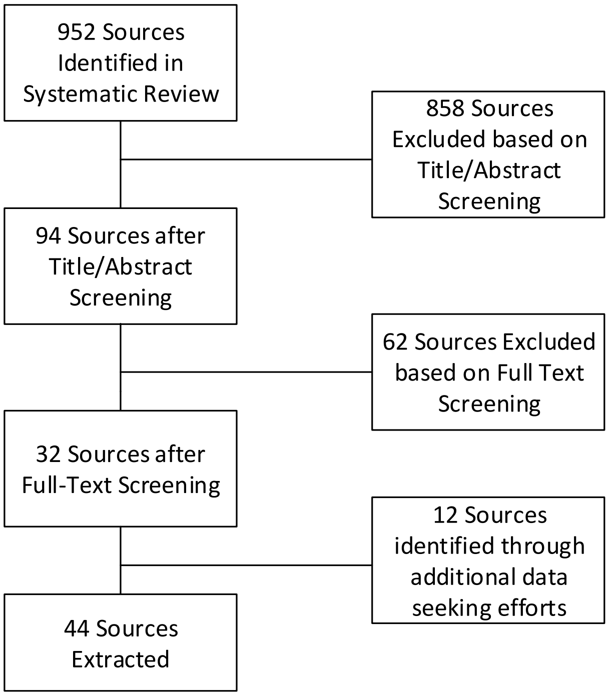

We included representative, population-based surveys that reported of prevalence, incidence, remission rate, excess mortality rate, relative risk of mortality, standardized mortality ratio, or with-condition mortality rate. We excluded studies with no clearly defined sample (eg, among clinic attenders or patient organization members with non-specific or non-representative catchment area). The table below details the model inputs used to estimate the epilepsy impairment.

	Prevalence	Incidence	Mortality risk
Studies	317	81	23
Countries/subnationals	192	51	23
GBD world regions	20	15	10

The inputs for the regressions used to split the epilepsy impairment envelope were also updated for GBD 2016. These regressions are used to determine the proportion of epilepsy that is primary or idiopathic, the proportion of epilepsy that is severe (one or more fits per month), the proportion of epilepsy that is untreated (the treatment gap), and the proportion of treated epilepsy that is treated without fits (no fits reported in the preceding year).

For GBD 2016, a new systematic review was conducted covering 1/1/2006 to 10/17/2016 using the search term:

("2006"[PDAT] : "2016"[PDAT]) AND ("epilepsy"[MeSH Terms] OR "epilepsy, partial, motor"[MeSH Terms] OR "epilepsy, benign neonatal"[MeSH Terms] OR "epilepsy, reflex"[MeSH Terms] OR "myoclonic epilepsy, juvenile"[MeSH Terms] OR "epilepsy, frontal lobe"[MeSH Terms] OR "epilepsy, complex partial"[MeSH Terms] OR "epilepsy, post-traumatic"[MeSH Terms] OR "epilepsy, temporal lobe"[MeSH Terms] OR

"epilepsy, absence"[MeSH Terms] OR "epilepsy, tonic-clonic"[MeSH Terms] OR "epilepsies, myoclonic"[MeSH Terms] OR "epilepsies, partial"[MeSH Terms] OR epilepsy[Title/Abstract]) AND (incidence[Title/Abstract] OR prevalence[Title/Abstract] OR epidemiology[Title/Abstract]) AND (sever*[Title/Abstract] OR treated[Title/Abstract] OR “drug resistant epilepsy”[MeSH] OR “treatment resistant”[Title/Abstract] OR proportion[Title/Abstract] OR “clinical characteristics”[Title/Abstracts] OR “treatment gap”[Title/Abstract]) NOT(animals[MeSH] NOT humans[MeSH])

Of 1,234 hits, 37 were marked for extraction. Additional data seeking efforts also led to the addition of five more sources on the treatment gap in India subnationals. A flow chart documenting the review is displayed below.

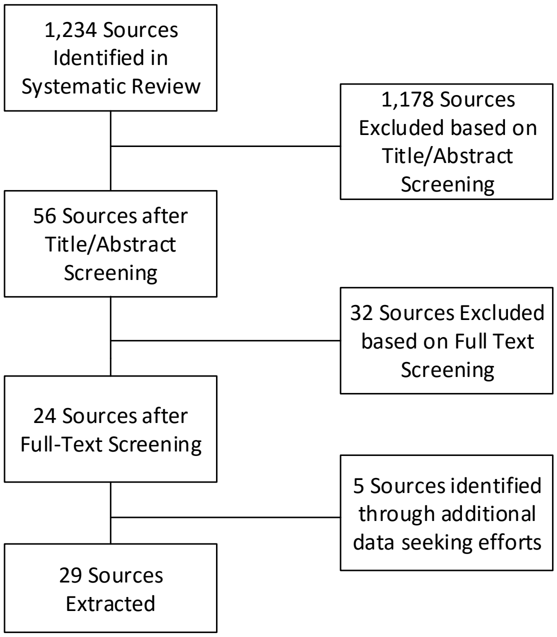

Severity splits & disability weights

The table below illustrates the severity levels, descriptions, and disability weights associated with epilepsy. These are calculated using regressions from literature (ie, frequency of seizures).

Severity level	Lay description	Disability weights (95% CI)
severe (seizures >= once per month)	This person has sudden seizures one or more times each month, with violent muscle contractions and stiffness, loss of consciousness, and loss of urine or bowel control. Between seizures the person has memory loss and difficulty concentrating.	0.552 (0.375–0.71)
less severe (seizures < once per month)	This person has sudden seizures two to five times a year, with	0.263 (0.173–0.367)

	violent muscle contractions and stiffness, loss of consciousness, and loss of urine or bowel control.	
Treated without fits	This person has a chronic disease that requires medication every day and causes some worry but minimal interference with daily activities.	0.049 (0.031–0.072)

Modelling strategy

We modelled the prevalence of epilepsy in two steps: first, we created an epilepsy impairment envelope. Second, we split the envelope into primary (or idiopathic) and secondary epilepsies. Each of these were subdivided into "severe" (on average one or more fits per month) and "non-severe." Non-severe cases were subdivided into "treated" and "un-treated." Finally, "treated" cases were divided into "treated cases with fits" (between one and 11 fits on average in the preceding year) and "treated cases without fits" (no fits reported in the preceding year).

In the first step, we used the DisMod-MR tool for the epilepsy impairment envelope to model a consistent fit between incidence, prevalence, remission, and standardized mortality ratio data while using meta-regression to correct data points with non-reference study quality characteristics. We found no systematic bias for the covariate "non-standard case definition" indicating studies that did not define "active epilepsy" and additionally the covariate was not significant as a "z-cov", which acts as a multiplier applied to the standard error and thus results in these data points being given less weight in the analysis than the "reference" data points. Therefore, we excluded this covariate from the model. We also included data on lifetime prevalence and therefore added a covariate on lifetime prevalence data points. We also included country-level covariates on prevalence for the SEV epilepsy scalar, which summarizes the epilepsy risk exposure level for each country, and pig meat consumption per capita, which is used as a proxy for the level of neurocysticercosis, a common cause of secondary epilepsy. We included cause-specific mortality rate (CSMR) results from the epilepsy mortality model as input data to the DisMod model. Where age-specific prevalence data was available, we calculated excess mortality rate (EMR) from prevalence and CSMR. We included the log of the lag distributed income (LDI) as a covariate on EMR to account for lower mortality in developed countries. We included Bayesian priors on remission to account for the scarcity of remission data. We set bounds on remission from 0 to 0.25 from age 0-60 and 0 to 0.05 from age 61-100. The table below indicates the covariates used in the estimation process, as well as parameters, betas, and exponentiated betas.

Measure	Variable Name	Beta	Exponentiated
prevalence	Recall Lifetime	0.18 (0.15 – 0.22)	1.20 (1.17–1.24)
prevalence	All MarketScan, year 2000	-0.89 (-0.94 – -0.83)	0.41 (0.39–0.43)
prevalence	All MarketScan, year 2010	-0.43 (-0.47 – -0.37)	0.65 (0.62–0.69)

prevalence	All MarketScan, year 2012	-0.35 (-0.41 – -0.30)	0.70 (0.67–0.74)
prevalence	Pig Meat Consumption (kg per capita)	0.0054 (0.00012 – 0.015)	1.01 (1.00–1.02)
Prevalence	Log-transformed age-standardized SEV scalar for epilepsy	0.79 (0.75-0.90)	2.21 (2.12-2.46)
excess mortality rate	LDI (I$ per capita)	-0.55 (-1 – -0.1)	0.58 (1.37–0.90)

In the second step, we used a mixed-effects generalized linear models (binomial family) to predict the proportion of idiopathic epilepsy, the proportion of severe epilepsy, the proportion of treated epilepsy and the proportion of epilepsy that is treated without fits.

Because not all of the data on the proportion of idiopathic epilepsy uses optimal case finding methods (using CT scans or MRI's in addition to EEG's in order to diagnose secondary epilepsy), for GBD 2016 we decided to do add a covariate to crosswalk studies with non-optimal case finding methods to those with adequate methods. The regression for the proportion of epilepsy that is idiopathic therefore has fixed effects on this study quality covariate as well as the under-5 mortality rate, the log of pig meat consumption (per capita), and the proportion of a country with access to proper sanitation, as well as a random effect on super-region.

We used similar models to predict the proportion of severe epilepsy and treatment gap based on the reported proportions extracted from the systematic review. To predict the proportion of severe epilepsy and the treatment gap, we used mixed-effects models with a fixed effect on the log of HAQ Index and a random effect on super-region.

For GBD 2015, a meta-analysis was used to generate two different pooled estimates for proportion of treated epilepsy that is seizure-free in developing and developed countries, as there was not enough data to run a regression. However, for GBD 2016 the expanded dataset allowed for the implementation of a generalized linear model (binomial family) to generate predictions for the proportion of treated epilepsy that is seizure-free. We used a fixed effect on the log of HAQ Index.

We tested a fixed effect on Socio-demographic Index (SDI) and random effects on region and country in different models, but they did not improve the model. We generated 1,000 draws of country-specific estimates for each year between 1980 and 2016 for each of the models. The table below shows the betas from these regressions.

Regression	covariate	beta	SE
Idiopathic	Under-5 Mortality	-65.82	7.50
Idiopathic	Pig meat consumption	-0.12	0.02
Idiopathic	Sanitation	0.45	0.16
Idiopathic	Study Quality	0.88	0.07
Severe	HAQ Index	-2.15	0.24

Treatment Gap	HAQ Index	-3.17	0.18
Treated without fits	HAQ Index	3.65	0.21

Infertility Impairment

Flowchart

Infertility

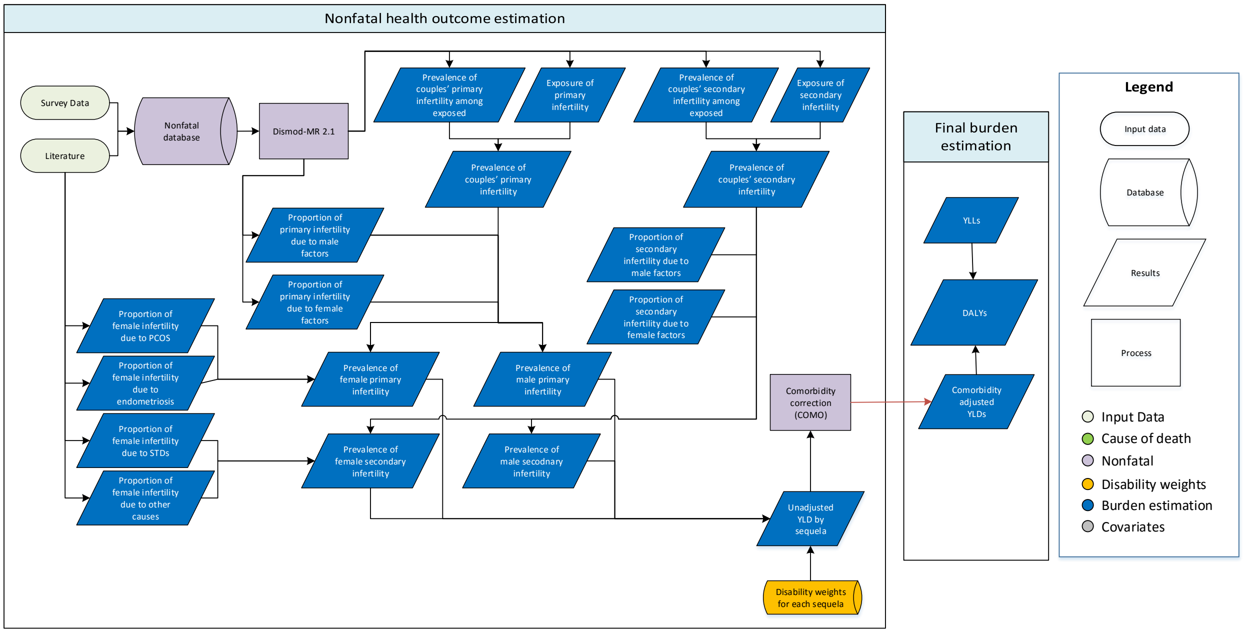

Case definition

For GBD 2016, the following case definitions were used for infertility:

1. Primary infertility is defined as a couple who have not had a live birth, who wish to have a child, and have been in a union for more than five years without using contraceptives.

2. Secondary infertility is defined in a couple who wish to have a child and have been in a union for more than five years without using contraceptives since the last live birth.

Input data

Model inputs

We included data for women in five-year age groups between ages 15 and 49 from population-based surveys including the Demographic and Health Surveys (DHS), World Fertility Surveys (WFS), Reproductive Health Surveys (RHS), Family and Fertility Survey (FFS), and others (EUR, NSF, PCD, PFM). Although these surveys only interviewed women, the resultant estimate of prevalence is an indicator of couples' infertility, as it is not possible to determine in a survey which partner is the cause of the infertility. Because some surveys ask questions of only ever-married women, we separately estimated the prevalence of four parameters:

Table 1: Infertility parameters modeled in DisMod 3 for estimation of couples' infertility			
**Infertility Type**	**Parameter**	**Numerator**	**Denominator**
Primary	Prevalence of primary infertility among exposed women	Married 5+ years; no contraception for 5+ years prior to survey; no previous births; desires a child	Numerator OR: Married 5+ years; 1 or more birth
Primary	Prevalence of exposure to primary infertility	Married 5+ years; no contraception for 5+ years prior to survey	All women
Secondary	Prevalence of secondary infertility among exposed women	Married 5+ years; no contraception for 5+ years prior to survey; last birth 5+ years ago; desires a child	Numerator OR: Married 5+ years; first birth 5+ years ago; last birth <5 years ago
Secondary	Prevalence of exposure to secondary infertility	Married 5+ years; no contraception for 5+ years prior to survey; one or more child	All women

As described below, infertility will be estimated by multiplying prevalence among exposed by the exposure. The table below illustrates the extent of data coverage for the infertility impairment for GBD 2016.

Table 2: Data coverage

Model	Sources	Subnational	Country	Region	Super-region
primary infertility impairment	314	1	113	20	7
secondary infertility impairment	315	1	112	19	7
primary infertility exposure	266	1	101	17	7
secondary infertility exposure	267	1	101	17	7
proportion male primary infertility	18	2	15	8	6
proportion female primary infertility	18	2	15	8	6
proportion male secondary infertility	18	2	15	8	6
proportion female secondary infertility	18	2	15	8	6

Sequelae/disability weights

Health state name	Health state description	Disability weight
Infertility, primary	This person wants to have a child and has a fertile partner, but the couple cannot conceive.	0.008 (0.003-0.015)
Infertility, secondary	This person has at least one child, and wants to have more children. The person has a fertile partner, but the couple cannot conceive.	0.005 (0.002-0.011)

Modelling strategy

For GBD 2016, we estimated the prevalence of primary and secondary infertility by sex and cause in three steps.

1) Estimation of couples' infertility

To estimate the prevalence of infertility among couples, we first ran four DisMod-MR 2.1 models to estimate the four parameters detailed above. For prevalence of infertility, we tried using the natural log of the age-standardized death rate (lnASDR) of STDs, but it was not statistically significant so we did not use it in the final model.

Next, we estimated primary and secondary infertility from DisMod-MR 2.0 models by multiplying the estimates for prevalence of infertility among exposed women by the prevalence of exposure to infertility to obtain prevalence of infertility among all women and all men.

2) Estimation of infertility by sex

We ran four DisMod models to estimate the proportion of primary and secondary infertility due to male or female causes. Because infertility in some couples is attributable to both partners rather than just one, the sum of the proportion of couples' prevalence due to male factor infertility and due to female factor infertility is greater than 1. Again, we tried using lnASDR of STDs as a covariate, but it was not statistically significant so we did not use it in the final model. We multiplied our prevalence of primary and secondary infertility derived in step 1 by the proportion due to male and female factors to estimate primary and secondary infertility by sex.

3) Causal attribution

There are seven identified causes of female infertility in the GBD 2013 cause list: chlamydia, gonorrhea, other sexually transmitted diseases, maternal sepsis, polycystic ovarian syndrome, endometriosis, and Turner syndrome. For each of these diseases, we determined the prevalence of infertility by a literature review of the probability of becoming infertile due to that disease. For sexually transmitted diseases we applied a proportion with infertility derived from Westrom et al. (1992, Sex Transm Dis) to incident cases of pelvic inflammatory disease and streamed out prevalence over the fertile age range using DisMod-MR 2.0.12. We added all the disease-specific estimates of prevalence and assigned the remaining proportion to categories of "female primary infertility due to other causes" and "female secondary infertility due to other causes." We assumed all infertility from Turner syndrome is primary infertility and all infertility following maternal sepsis is secondary infertility. The only recognized cause of male infertility in the GBD 2013 cause list is Klinefelter syndrome. We assigned all other male infertility to "male infertility due to other causes."

We have made no substantive changes in the modelling strategy from GBD 2015.

Developmental Intellectual Disability Impairment

Flowchart

Case definition

Developmental intellectual disability is a condition of below-average intelligence or mental ability. Consistent with the American Association on Intellectual and Developmental Disabilities, we define developmental intellectual disability as a condition originating before age 18 (thus, does not include impairment due to stroke, Alzheimer's, etc.). We model the following severities, as measured by intelligence quotient (IQ) tests:

Type of intellectual disability	IQ
Profound	0 to 19
Severe	20 to 34
Moderate	35 to 49
Mild	50 to 69
Borderline	70 to 85

Input data

Model inputs

1) Prevalence data: Prevalence of intellectual disability (IQ <70) came from a systematic review starting 1/1/1990 using the following search string:

(((intellectual disability[MeSH Terms]) AND prevalence[Title/Abstract]) AND ('1990'[Date - Publication] : '3000'[Date - Publication]))

For GBD 2015, this search had 2,115 hits, of which 13 were extracted that had not been previously included in GBD. We included studies that estimate the general population prevalence of intellectual disability. We excluded studies that do not use a case definition based on intelligence quotient (IQ) or investigated non-representative groups, like hospital patients or people of a specific ethnicity.

2) IQ mean and standard deviation: Data for mean and SD of IQ came from three source types:
    a. IQ instruments: We conducted a systematic review for IQ scores from standardized tests designed to measure intelligence. Given the vast number of instruments that are used to measure IQ, we used instrument-specific search strings. In total, our search had 85,000 hits, of which 69 were extracted. We excluded non-general populations or non-school populations (eg, gifted or impaired populations). We extracted the mean and SD of IQ, in addition to whether or not a norming procedure had been applied to the data. Where available, we extracted the raw mean and SD as well as the normalized mean and SD.
    b. International Educational Attainment (IEA) surveys: Scores from standardized mathematics, reading, and/or science exams were extracted from school-based surveys, given that IEA tests measure a closely related concept to IQ in school-age populations.

c.

Survey	Country-Years
Programme for International Student Assessment (PISA)	73 country-years
Progress in International Reading Literacy Study (PIRLS)	212 country-years
Trends in International Mathematics and Science Study (TIMSS)	156 country-years

d. Cognitive function surveys: Scores from cognitive function surveys were used where IQ and IEA data were unavailable, particularly for older populations and infants.

Survey	Country-Years
Survey of Health, Ageing, and Retirement in Europe (SHARE)	131 country-years
WHO Study on Global AGEing and Adult Health (SAGE)	12 country-years
Longitudinal Study of Ageing (LSA)	TBD
Health and Retirement Survey (HRS)	TBD
Bayley Scales of Infant and Toddler Development	TBD; from literature

Severity split/disability weight

Health state	Description	Disability weight
Borderline intellectual functioning	This person is slow in learning at school. As an adult, the person has some difficulty doing complex or unfamiliar tasks but otherwise functions independently.	0.011 (0.005–0.02)
Intellectual disability/mental retardation, mild	This person has low intelligence and is slow in learning at school. As an adult, the person can live independently, but often needs help to raise children and can only work at simple supervised jobs.	0.043 (0.026–0.064)
Intellectual disability/mental retardation, moderate	This person has low intelligence, and is slow in learning to speak and to do even simple tasks. As an adult, the person requires a lot of support to live independently and raise children. The person can only work at the simplest supervised jobs.	0.1 (0.066–0.142)
Intellectual disability/mental retardation, severe	This person has very low intelligence and cannot speak more than a few words, needs constant supervision and help with most daily activities, and can do only the simplest tasks.	0.16 (0.107–0.226)
Intellectual disability/mental retardation, profound	This person has very low intelligence, has almost no language, and does not understand even the most basic requests or instructions. The person requires constant supervision and help for all activities.	0.2 (0.133–0.283)

Modelling strategy

We modelled the prevalence estimates of intellectual disability (ID), both aetiology-specific IDs and idiopathic ID, over multiple steps. For GBD 2016, our modelling strategy changed significantly. We additionally used IQ distribution data to inform the estimates of ID prevalence, as follows.

1) *Estimate Intellectual Disability Prevalence (IQ <70)*

First, we ran a DisMod-MR 2.1 model to estimate the total prevalence of intellectual disability of level IQ <70. We included lag-distributed income and education in the model.

2) *Estimate population mean and SD of Intelligence Quotient*

Second, we crosswalked International Educational Attainment data and cognitive function surveys to one standard IQ measure. We crosswalked by matching country/age/year sources for which we had two or more types of data. Using the IQ data and the data converted to IQ as inputs, we ran a single-parameter, continuous DisMod-MR 2.1 model estimating the mean and SE of IQ using standardized measure. In this model, we included log-transformed LDI (per capita), proportion underweight, and log-transformed education (age-standardized) as country-level covariates in this model. We additionally included as a covariate an indicator of instrument-type. These instrument-types were determined based upon similarity of theoretical constructs and concurrent validity.

3) *Fit parametric distribution to mean and SD of IQ and prevalence of intellectual disability*

We fit a distribution using the mean and SD of IQ, and the prevalence of ID using an ensemble approach. The ensemble approach takes a weighted average of multiple distributions, rather than a single distribution. The distribution is fit via a Method of Moments, where each potential distribution is weighted based upon in-sample fit. This allows us to adapt to different skew over time and geographies, and is a highly generalizable approach that does not specify the underlying distribution.

4) *Severity-specific intellectual disability*

We split the total prevalence of idiopathic ID into four severity levels: mild (IQ 50-69), moderate (IQ 35-49), severe (IQ 20-34), and profound (IQ below 20).

5) *Causal attribution*

We estimated prevalence of each etiology-specific ID by models from the following parent causes. Since we are modelling only developmental intellectual disability, causes such as stroke and Alzheimer's are not included in the causal attribution process.

Neonatal preterm birth complications (<28w, 28-32w, 32-36w)
Neonatal encephalopathy due to birth asphyxia and trauma
Hemolytic disease and other neonatal jaundice
Meningitis (pneumococcal, H influenza type B, meningococcal, other bacterial)
Encephalitis
Malaria
Neonatal tetanus
Iodine deficiency
African trypanosomiasis
Down syndrome
Klinefelter syndrome
Chromosomal unbalanced rearrangements

Neural tube defects
Hypertensive disorders of pregnancy (eclampsia, preeclampsia)
Autism
Fetal alcohol syndrome

For autism, we identified six studies reporting severity of intellectual disability. We conducted a meta-analysis to produce the following severity distribution which we applied to the prevalence of autism to produce severity-specific ID due to autism.

ID severity	Mean	SE
None	0.161	0.034
Borderline	0.161	0.034
Mild	0.375	0.037
Moderate	0.190	0.031
Severe	0.090	0.177
Profound	0.024	0.134

We calculated prevalence of idiopathic ID by subtracting all severity- and aetiology-specific IDs from the severity-specific envelope ID assuming the residuals to represent idiopathic. If the residual was less than 5% of the severity-specific envelope, the prevalence of all aetiology-specific IDs were proportionally squeezed to fit within 95% of the envelope, leaving 5% for idiopathic ID.

As we estimated the prevalence of individual aetiology-specific IDs by models from the respective parent causes, the squeezing may result in a distorted balance of prevalence estimates within their parent causes. With the aim to maintain consistencies of prevalence within each of the parent causes, we added the difference between the original and the squeezed prevalence estimates to the "motor impairment" sequela if the squeezed sequela represented "motor and cognitive impairment." For autism, we obtained the fraction of cases that result in ID from literature (0.29; 0.27–0.30 95% CI) and applied to the subtraction and squeezing processes. We assume all ID cases due to iodine deficiency (cretinism) to result in either severe or profound level, and Klinefelter syndrome cases that result in ID will have either borderline or mild level.

In GBD 2013, all aetiology-specific models were squeezed into the overall (IQ <70) envelope. In 2015, we squeeze each model into its discrete severity envelope. GBD 2016 methods are still in development and will be finalized later this summer.

Guillain–Barré Syndrome (GBS) Impairment

Flowchart

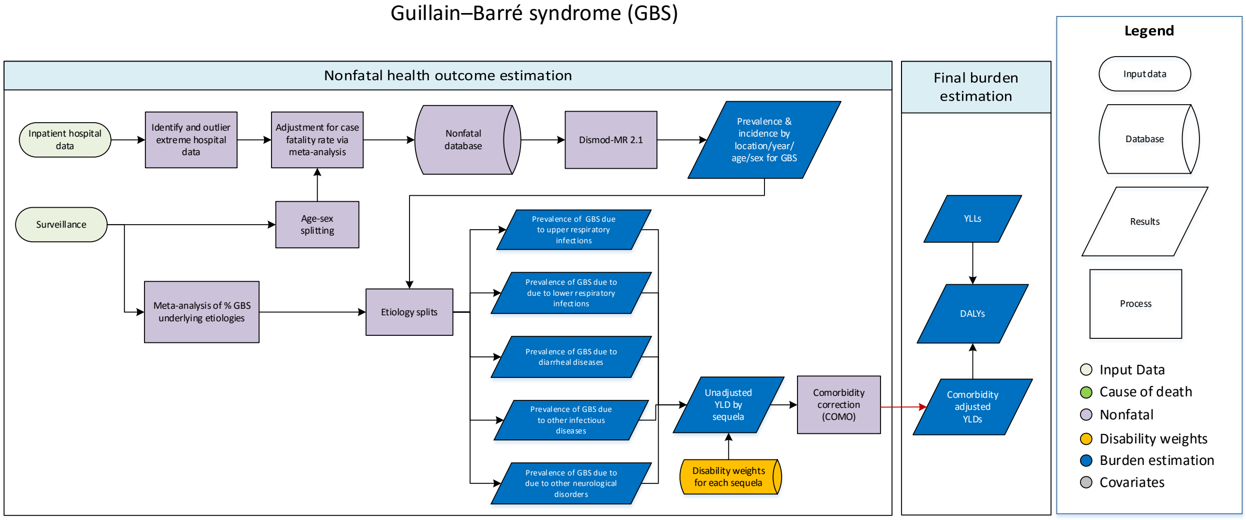

Case definition

Guillain-Barré syndrome is a rare condition that usually occurs as a complication of respiratory or gastrointestinal infection. It is considered an immune-mediated nerve dysfunction with rapid onset of weakness in feet and hands ascending toward the trunk. In the acute phase about a quarter of cases required mechanical ventilation for survival. The majority of cases fully recover within months to a year. The following ICD codes are used G61.0 (GBS) and 357.0 (Acute infective polyneuritis). These codes are also referenced in Methods Appendix Table 4.

Input data

Model inputs

A systematic search was performed in Pubmed on 28.01.2010 which produced a systematic review by McGrogan A et al. (2009). The search covered 1980-2008 using keywords "Guillain-Barré Syndrome" or "polyradiculoneuropathy" and "incidence" or "epidemiology." This review included 63 relevant studies (published between 1980 and 2008) which reported on Guillain-Barré incidence rate and provided the estimated incidence rates for 25 countries in nine GBD regions). There were 35 studies from our systematic review that provided information on the underlying cause: 31 mentioning all of the identified underlying infectious diseases; 26 providing a proportion for upper respiratory infections, three for influenza, 25 for diarrheal disease, and 14 for other diseases.

An additional search was undertaken for more information on mortality, remission, and duration of GBS. As the mortality, remission, and case-fatality rate did not vary significantly across the studies and there were time constraints, instead of doing a systematic search for GBS mortality and remission, a general search was carried out. We extracted case fatality rate from five studies, and remission from four studies.

measure	Data sources	Country/Subnational Coverage	GBD World Regions
incidence	61	111	9

Updates to systematic reviews are performed on an ongoing schedule across all GBD causes, and an update for Guillain-Barré syndrome will be performed in the next one to two iterations. Inpatient hospital data were extracted using the ICD codes listed above. Only primary diagnoses were considered, with the reasoning that Guillain-Barré syndrome should appear as a primary diagnosis and we do not wish to include follow-up visits that may be listed as secondary or tertiary codes.

We still had some locations with systematically high or low hospital data. In order to systematically outlier hospital data that was implausible, we outliered all data from any sex-specific country-years where the age-standardized rates were higher than 125% of the second highest sex-specific country-year of US hospital data or 75% of the second lowest sex-specific country-year of US hospital data. This assumes US hospital data to be the gold standard of hospital data.

Etiology splits

Information on etiology splits come from a systematic review and meta-analysis of the literature completed for GBD 2010. This review searched for articles providing information on the proportion of Guillain-Barré cases with any described etiological cause, the proportion of Guillain-Barré cases attributed to influenza, the proportion of Guillain-Barré cases attributed to upper respiratory infections, the proportion of Guillain-Barré cases attributed to diarrheal diseases and the proportion of Guillain-Barré cases attributed to other infections. This review yielded 35 articles; a breakdown of how many articles inform each proportion contributing to the split is provided below:

Split	Number of Sources
All specified etiologies	31
Influenza	3
Upper respiratory infections	26
Diarrheal diseases	25
Other infectious diseases	14

First the envelope for Guillain-Barré cases due to all specified etiologies is established by doing a meta-analysis on the proportions reported in the studies included. Then, the proportions for each of the other splits are squeezed to fit the envelope created in the all specified meta-analysis. Finally, the difference between all specified and 100% is attributed to other neurological disorders. The final results of these etiology splits are shown below:

Etiology	Mean	Lower	Upper
Other neurological disorders	0.382	0.331	0.669
Influenza	0.119	0.071	0.192
Upper respiratory infections	0.319	0.27	0.372
Diarrheal diseases	0.109	0.086	0.135
Other infectious diseases	0.071	0.054	0.093

Disability weights

One health state was used for all Guillain-Barré cases. It is described as paralyzed from the waist down, cannot feel or move the legs, and has difficulties with urine and bowel control. The person uses a wheelchair to move around. The disability weight is 0.296 (0.198–0.414).

Modelling strategy

All data, from both the literature review and hospital extraction, were corrected for the survival rate. A random effects meta-analysis calculated a 95% case fatality rate (95% CI 93–98%). A forest plot showing the results of this meta-analysis is displayed below. As mortality mainly occurs during the acute phase of the disease (usually within four weeks of onset), the pooled survival rate was used to get the incidence of the people surviving after the acute phase of the GBS. Where surveillance data were reported at age groups of over 20 years, we applied the age pattern derived from US Marketscan claims data to age-split the data.

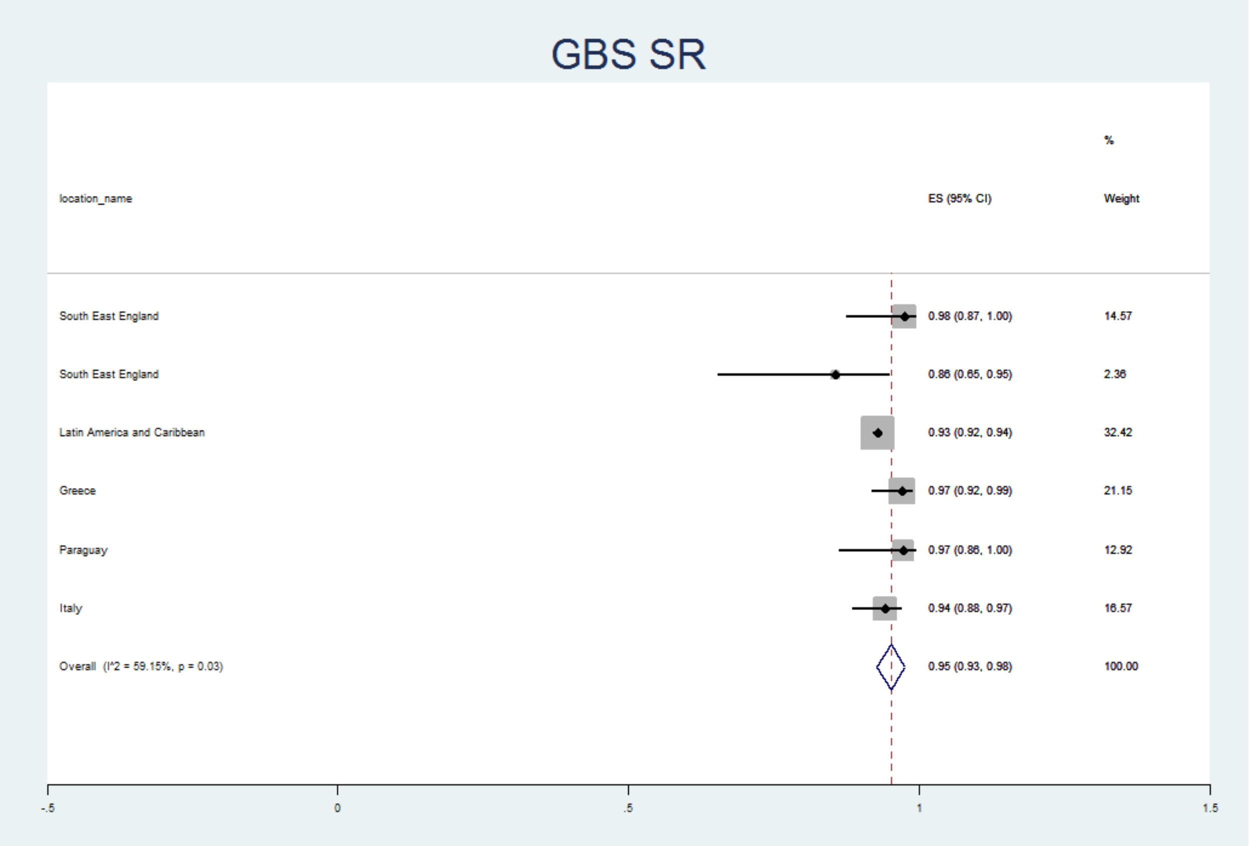

Dismod MR was used to estimate prevalence of Guillain-Barré syndrome for every location, year, age, and sex. We included a study-level covariate on hospital data in order to adjust for a potential systematic bias in this type of data. The exponentiated beta for this covariate was 1.30 (1.26-1.33). We then split the overall prevalence of the impairment by underlying etiology (upper respiratory infections, influenza, diarrheal diseases, other infections, and other neurological causes). We used random effects meta-analysis to pool these proportions. We squeeze the proportions for influenza, diarrheal diseases, upper

respiratory infections, and other infectious diseases to add to the proportion for all identified infectious underlying diseases. We assigned the complement to one of the proportion with any underlying infectious disease to a rest category of “idiopathic Guillain-Barre syndrome” that is classified under neurological disorders.

There are no substantive modelling changes from GBD 2015.

Pelvic Inflammatory Disease (PID) Impairment

Flowchart

Pelvic Inflammatory Disease

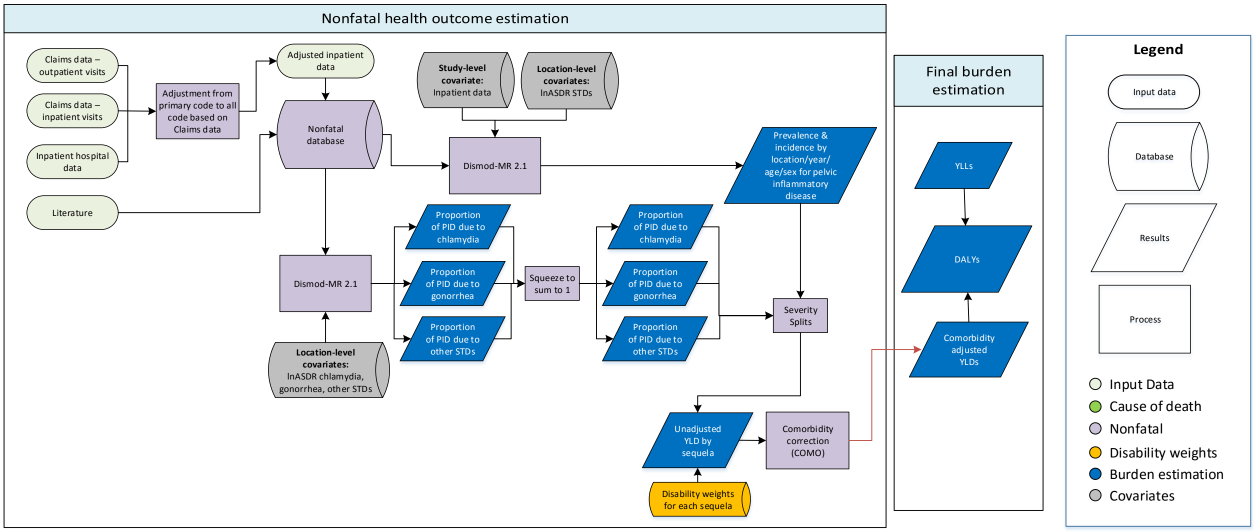

Case definition

Pelvic inflammatory disease (PID) is an infection of the female reproductive organs presenting as a complication of infection by a sexually transmitted disease. It can irreversibly damage the uterus, fallopian tubes, or other parts of the female reproductive system, leading to infertility.

Input data

Model inputs

A systematic review was completed for GBD 2013 on October 28, 2013, using the following search terms:

- (("pelvic inflammatory disease"[Title/Abstract] OR "salpingitis"[Title/Abstract]) AND (“1994”[Date – Publication] : “2013”[Date – Publication]))

In addition, we included studies with confirmed clinical diagnosis in general population.

No systematic review was conducted for GBD 2015. Updates to systematic reviews are performed on an ongoing schedule across all GBD causes, and an update for PID will be performed in the next one to two iterations.

Inpatient outpatient MarketScan data were extracted as incidence rates, as per the ICD codes above. We also extracted inpatient hospital data and employed two crosswalks derived from MarketScan data. First, we adjusted hospital inpatient data using a ratio of primary diagnoses for PID to all diagnoses, since the sexually transmitted disease (rather than PID, the consequent syndrome) may appear as the primary

diagnosis. Second, we adjusted using a ratio of inpatient/outpatient diagnoses, since not all PID must be treated in an inpatient facility. The result is an estimate of total PID incidence.

PID incidence input data

The table below illustrates the data sources used in GBD 2016:

Measure	Data sources	Subnational coverage	Country coverage	Region coverage	Super-region coverage
Incidence	37	86	22	6	3

A subset of the studies from the systematic review reported the underlying etiology of PID, allowing us to estimate the proportion of PID due to chlamydia, gonorrhea, and other sexually transmitted diseases.

Proportion of PID due to	data_sources	subnational_coverage	country_coverage	region_coverage	super_region_coverage
chlamydia	15	5	8	6	3
gonorrhea	10	5	6	4	2
other STDs	15	5	8	6	3

Health states and disability weights

Health state name	Health state description	Disability weight
Abdominopelvic problem, severe	This person has severe pain in the belly and feels nauseated. The person is anxious and unable to carry out daily activities.	0.324 (0.219–0.442)
Abdominopelvic problem, moderate	This person has pain in the belly and feels nauseated. The person has difficulties with daily activities.	0.114 (0.078–0.159)

Modelling strategy

First, we estimated the total incidence and prevalence of pelvic inflammatory disease using a Dismod-MR 2.1. We used a study-level covariate on hospital inpatient data. This has the effect of crosswalking inpatient incidence (from hospital inpatient facilities) to total PID incidence (derived from MarketScan inpatient and outpatient claims data). We used the natural log of the age-standardised death rate (lnASDR) of sexually transmitted diseases (excluding HIV) as a location fixed effect, since these diseases cause PID and thus their mortality is correlated to PID incidence. We used Bayesian priors on remission (13–17) and excess mortality rate (0–0.15).

Covariate	Parameter	beta	Exponentiated beta
Hospital inpatient (study-level)	Incidence	0.65 (-0.77 – -0.59)	0.52 (0.46–0.56)
lnASDR STDs (location)	Incidence	-0.65(-0.77 – -0.59)	0.52 (0.46–0.56)

Second, we ran three separate DisMod models for the proportion of PID due to the following three causes: chlamydia, gonorrhea, and other STDs. For each model, we used the lnASDR of the underlying STD.

Covariate	Parameter	Exponentiated beta
lnASDR chlamydia	Proportion	1.07 (1.00–1.22)
lnASDR gonorrhea	Proportion	1.19 (1.02–1.34)
lnASDR STDs	Proportion	1.00 (1.00–1.01)

DisMod estimate for the proportions due to each etiology were proportionally squeezed to sum to 1.

We extracted MarketScan claims data for the first time in GBD 2015. As described in detail above, this allowed us to make two crosswalks (from primary diagnosis to all diagnoses, and from inpatient to all diagnoses) that we had not previously. In GBD 2013, we only were able to use a ratio of inpatient:outpatient diagnoses from one literature source (Rein et al.). The combined effect of these two crosswalks is that we estimate substantially more PID cases than estimated in GBD 2013.

No additional changes were made to the estimation process for GBD 2016.

References

1. Rein DB, Kassler WJ, Irwin KL, et al. Direct medical cost of pelvic inflammatory disease and its sequelae: decreasing, but still substantial1. *Obstetrics & Gynecology*. 2000;95(3):397–402.

Heart failure impairment envelope and other cardiovascular disease

Flowcharts

Overall modelling strategy

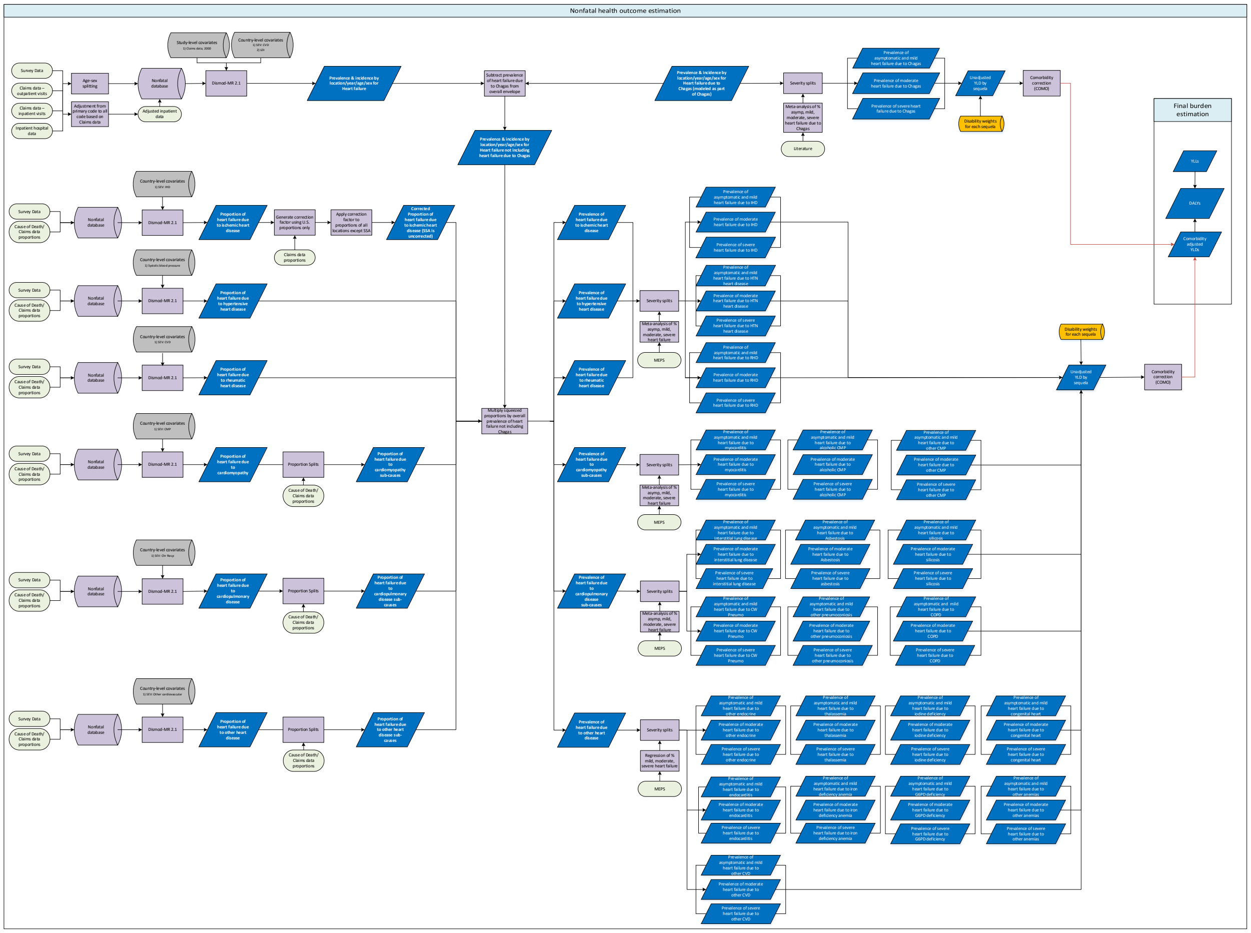

Proportion splits and correction factor estimation

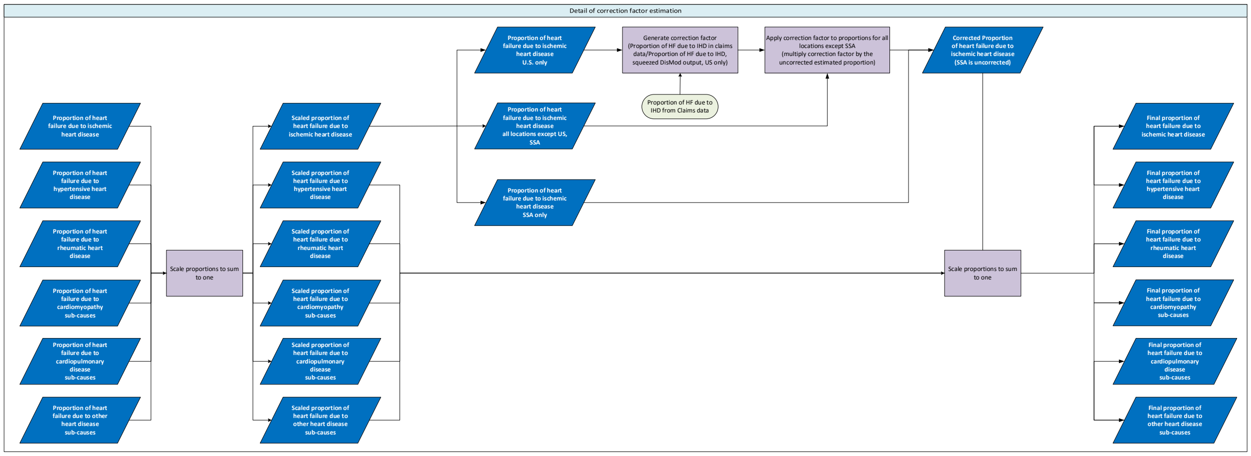

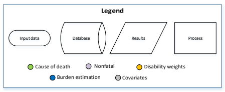

Case definition

Heart failure was diagnosed clinically using structured criteria such as the Framingham or European Society of Cardiology criteria. Previous iterations of GBD modelled symptomatic (i.e., NYHA Class II and above) episodes of HF only. Beginning in GBD 2016, we used ACC/AHA Stage C and above to capture both persons who are currently symptomatic and those who have been diagnosed with heart failure, but are currently asymptomatic.

Framingham Criteria (1): Must fulfill two major criteria or one major and two minor criteria.
Major criteria: Paroxysmal nocturnal dyspnoea, neck vein distention, rales, radiographic cardiomegaly, acute pulmonary oedema, S3 gallop, increased central venous pressure (>16 cm H_2O at right atrium), hepatojugular reflux; weight loss >4.5 kg in 5 days in response to treatment
Minor criteria: bilateral ankle oedema, nocturnal cough, dyspnoea on ordinary exertion, hepatomegaly, pleural effusion, decrease in vital capacity by one-third from maximum recorded, tachycardia (heart rate>120 beats/min).

European Society of Cardiology (2):
Typical signs (elevated jugular venous pressure, pulmonary crackles and peripheral oedema) and symptoms (e.g. breathlessness, ankle swelling and fatigue) caused by a structural and/or functional cardiac abnormality, resulting in a reduced cardiac output and/or elevated intracardiac pressures at rest or during stress.

Input data

Model inputs

A systematic review was performed for GBD 2016. The search terms used were: "heart failure"[TIAB] AND (epidemiology[MeSH Terms] OR prevalence[TIAB] OR incidence[TIAB] OR mortality[TIAB]) AND ("1990/01/01"[PDAT] : "2016/09/02"[PDAT]) NOT "animal model" NOT rat NOT mice NOT diabetes[TIAB] NOT "renal transplant"[TIAB]. The dates of the search were 01/01/1990 through 09/02/2016. 37,891 initial hits were returned, and 57 sources were added. An unstructured review of the literature yielded an additional 30 sources, of which six were extracted.

A systematic review was not performed for GBD 2015 or GBD 2013; however, the GBD 2010 study had conducted a systematic literature review for heart failure.

Overall heart failure prevalence

	Prevalence	Incidence	Mortality risk
Studies	22	24	53
Countries/subnationals	18	20	35
GBD world regions	7	6	9

Heart failure due to ischaemic heart disease

	Proportion
Studies	34
Countries/subnationals	51

GBD world regions	9

Heart failure due to hypertensive heart disease

	Proportion
Studies	35
Countries/subnationals	38
GBD world regions	9

Heart failure due to rheumatic heart disease

	Proportion
Studies	28
Countries/subnationals	49
GBD world regions	11

Heart failure due to cardiomyopathy

	Proportion
Studies	32
Countries/subnationals	51
GBD world regions	10

Heart failure due to cardiopulmonary disease

	Proportion
Studies	12
Countries/subnationals	10
GBD world regions	5

Heart failure due to other cardiovascular and circulatory diseases

	Proportion
Studies	19
Countries/subnationals	22
GBD world regions	6

Non-literature data included claims data and GBD 2016 death estimates.

Severity split inputs

The table below includes lay descriptions and disability weights for the severity levels of heart failure for GBD 2016.

Severity level	Lay description	DW (95% CI)
Mild	Is short of breath and easily tires with moderate physical activity, such as walking uphill or more than a quarter-mile on level ground. The person feels comfortable at rest or during activities requiring less effort.	0.041 (0.026–0.062)
Moderate	Is short of breath and easily tires with minimal physical activity, such as walking only a short distance. The person feels comfortable at rest but avoids moderate activity.	0.072 (0.047–0.103)
Severe	Is short of breath and feels tired when at rest. The person avoids any physical activity, for fear of worsening the breathing problems.	0.179 (0.122–0.251)

Modelling strategy

The general analytical strategy included estimating the overall prevalence of heart failure using DisMod MR 2.1, followed by a multi-step analysis of published literature, claims data, and cause of death data to estimate the aetiological fraction for each cause of heart failure. The latter process includes an initial assessment of the fraction of heart failure cases attributable to each of the high-level parent cause groupings, followed by further division into the detailed causes within each of these groupings, and finally a correction factor applied to adjust these proportions. The selection for aetiological causes was based on a review of the literature and expert opinion regarding diseases that lead to congestive heart failure.

We first estimated an overall prevalence of AHA/ACC stage C or D heart failure using literature data, hospital data, and claims data. Hospital data were adjusted outside of DisMod, using as reference data points reporting prevalence from the literature. Using as a model the adjustment factors developed to translate tobacco consumption prevalence to tobacco consumption frequency, we matched administrative claims data to population-based literature data based on age group, sex, and super region (3). For the adjustment factor, we developed the following generalized additive model on matched data

$$\ln(\mu_{ref,i}) = \beta_0 + \beta_1 \ln(\mu_{b,i}) + s(age_i) + \varepsilon_i$$

Where i represents a given matched observation, age signifies the data point's age group, $s(x)$ represents a penalized spline where the smoothing parameter is chosen through cross validation and μ_{ref} and μ_b denote the mean of the data point from literature and the mean of the claims data point, respectively. Predictions from the model were then taken as the adjusted data points. The standard error of each corrected data point was adjusted to account for the uncertainty due to the correction.

We set a prior of no remission and capped excess mortality at 1.

Heart failure due to Chagas was modelled separately as part of the Chagas modelling strategy. We subtracted the prevalence of heart failure due to Chagas from the overall heart failure prevalence to give an adjusted prevalence of heart failure due to all other aetiologies.

Our estimation of the aetiological causes of heart failure makes several assumptions and has several limitations. First, we assume that each case of heart failure only has one cause. Second, we rely on claims data from the United States, the only country where detailed person-level claims data were available, to assess the association between heart failure and underlying aetiologies. Third, we rely on mortality estimates due to these underlying causes (regardless of heart failure) to estimate their contribution to heart failure deaths in countries where detailed person-level claims data is not yet available. Fourth, we utilize the association between claims data and death certificate data in the United States to adjust for the differences in coding for ischemic heart disease, the most common cause of heart failure globally, in claims data versus death certificates. This approach allows us to produce estimates for all locations and can be updated to include more detailed health record and claims data from additional locations as they become available.

To estimate the aetiological fractions for each cause, we calculated the proportion of people with a diagnosis of one of the underlying causes of heart failure who also had a diagnosis of heart failure listed using claims data from the United States. These proportions were then multiplied by age-, sex-, and location-specific deaths (post CoDCorrect) to yield an overall number for each underlying cause of heart failure. We then divided these cause-specific totals by the sum of deaths for all aetiologies in order to yield a proportion of deaths due to heart failure for each cause. These proportions, along with literature data, were used to inform DisMod MR 2.1 models for the six broadest and mutually exclusive and collectively exhaustive cause groupings: ischaemic heart disease, hypertensive heart disease, cardiomyopathy and myocarditis, rheumatic heart disease, cardiopulmonary disease, and other cardiovascular and circulatory diseases. An exception to this approach was made for Sub-Saharan Africa where we excluded the proportion estimates generated from claims and death data, relying instead on published literature to determine the proportions of heart failure etiologies. This decision was based on expert opinion that local patterns differed significantly from what would have been determined from claims and death data. The THESUS-HF study, a large-scale, prospective, echocardiographic study of heart failure aetiologies in multiple African countries, provided these proportions (3). The results of these six proportion models were scaled to sum to one.

For heart failure due to cardiopulmonary disease, heart failure due to cardiomyopathy and myocarditis, and heart failure due to other causes, we calculated the proportion for each sub-cause according to the proportion of that cause within each larger aggregate group.

After the initial splitting and scaling steps, we compared the estimated proportions for the United States with the proportions originally calculated from the US claims data. The estimated proportion of HF due to IHD was much higher than the proportion of HF due to IHD in the claims data. This difference was due to the large number of IHD deaths not related to heart failure. In order to correct this over-estimation, we generated a correction factor for HF due to IHD by taking the proportion of HF due to IHD from the claims data and dividing it by the proportion of HF due to IHD from the scaled DisMod results. We then multiplied the proportion of HF due to IHD from the scaled DisMod results by this correction factor for all locations except Sub-Saharan Africa. Since echocardiographic evidence rather than claims data were used to model HF etiology proportions in Sub-Saharan Africa, we did not adjust the estimated proportion of HF due to IHD for this super region. In the next step, after the proportion of HF due to IHD has been corrected, the proportions were all rescaled to sum to one. These final scaled proportions were then multiplied by the overall HF envelope (after Chagas adjustment) to yield prevalence estimates of HF due to all aetiologies.

These estimates were then split into asymptomatic and mild, moderate, and severe heart failure based on an analysis of MEPS data.

Models were evaluated based on expert opinion, comparison of results with other rounds of GBD, and model fit.

Overall heart failure impairment envelope

Study covariate	Parameter	beta	Exponentiated beta
All MarketScan, year 2000	Prevalence	-0.3 (-0.32 – -0.28)	0.74 (0.72 – 0.76)
Log-transformed age-standardised SEV scalar: CVD	Prevalence	1.06 (0.98 – 1.12)	2.88 (2.67 – 3.07)
LDI (I$ per capita)	Excess mortality rate	-0.3 (-0.49 – -0.1)	0.74 (0.61 – 0.90)

Six main sub-cause proportion envelopes

Sub-cause	Covariate	Parameter	beta	Exponentiated beta
Heart failure due to cardiomyopathy impairment envelope	Log-transformed age-standardised SEV scalar: CMP	Proportion	0.75 (0.75 – 0.76)	2.12 (2.12 – 2.14)
Heart failure due to cardiopulmonary disease impairment envelope	Log-transformed age-standardised SEV scalar: Chr Resp	Proportion	0.81 (0.79 – 0.83)	2.25 (2.20 – 2.30)
Heart failure due to hypertensive heart disease impairment envelope	Systolic blood pressure (mmHg)	Proportion	0.000010 (0.0000023 – 0.000015)	1.00 (1.00 – 1.00)
Heart failure due to ischaemic heart disease impairment envelope	Log-transformed age-standardised SEV scalar: IHD	Proportion	0.75 (0.75 - 0.75)	2.12 (2.12 – 2.12)
Heart failure due to other causes impairment envelope	Log-transformed SEV scalar: Oth Cardio	Proportion	0.75 (0.75 – 0.75)	2.12 (2.12 – 2.12)
Heart failure due to valvular heart disease impairment envelope	Log-transformed age-standardised SEV scalar: CVD	Proportion	0.75 (0.75 – 0.75)	2.12 (2.12 – 2.12)

No other significant changes were made to the modelling strategy for GBD 2016.

1) http://www.framinghamheartstudy.org/share/protocols/soe0_03s_protocol.pdf
2) 2016 ESC Guidelines for the diagnosis and treatment of acute and chronic heart failure: The Task Force for the diagnosis and treatment of acute and chronic heart failure of the European Society of Cardiology

(ESC) Developed with the special contribution of the Heart Failure Association (HFA) of the ESC. Eur Heart J 2016; 37 (27): 2129-2200.
3) Reitsma, Marissa B., et al. "Smoking prevalence and attributable disease burden in 195 countries and territories, 1990–2015: a systematic analysis from the Global Burden of Disease Study 2015." The Lancet.
4) Damasceno A, Mayosi BM, Sani M, Ogah OS, Mondo C, Ojji D, Dzudie A, Kouam CK, Suliman A, Schrueder N, Yonga G, Ba SA, Maru F, Alemayehu B, Edwards C, Davison BA, Cotter G, Sliwa K. The Causes, Treatment, and Outcome of Acute Heart Failure in 1006 Africans From 9 Countries. Results of the Sub-Saharan Africa Survey of Heart Failure. Arch Intern Med. 2012;172(18):1386-1394.

Vision Impairment

Flowcharts

Vision Impairment

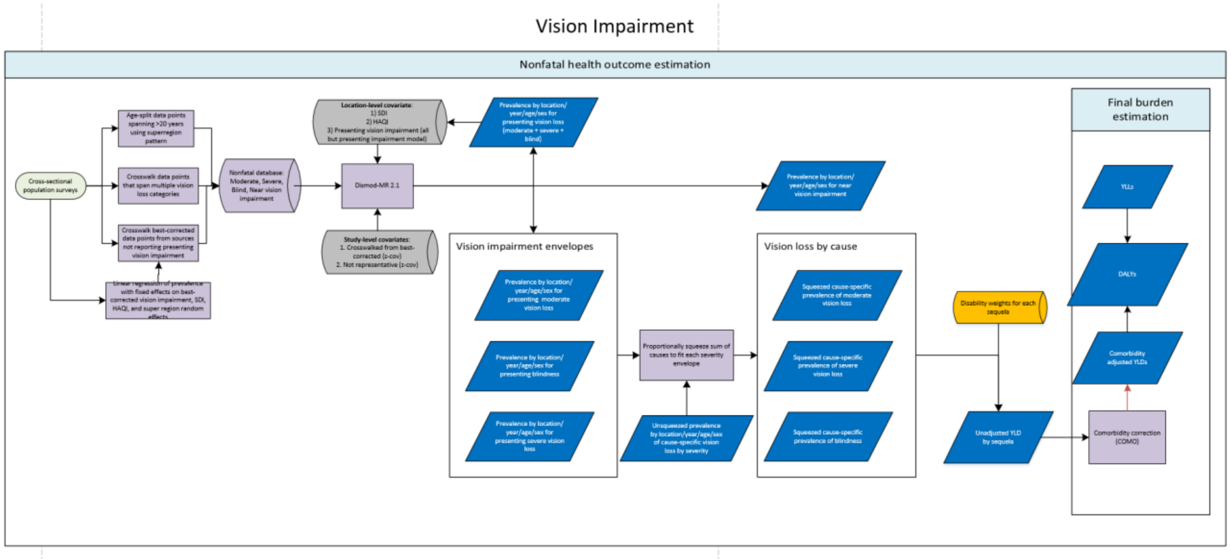

Diabetic Retinopathy

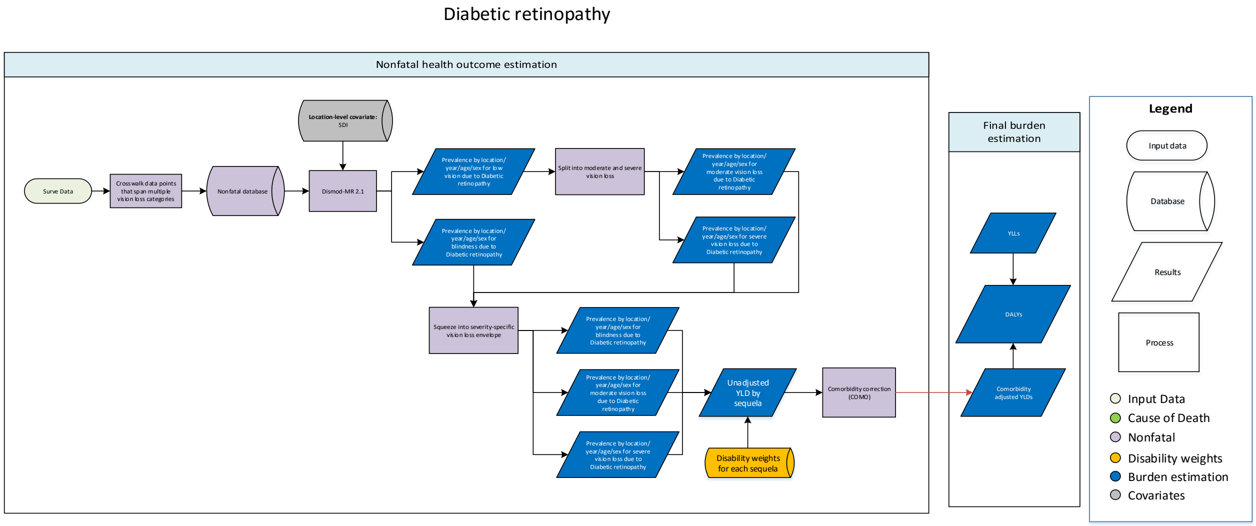

Macular Degeneration

Macular degeneration

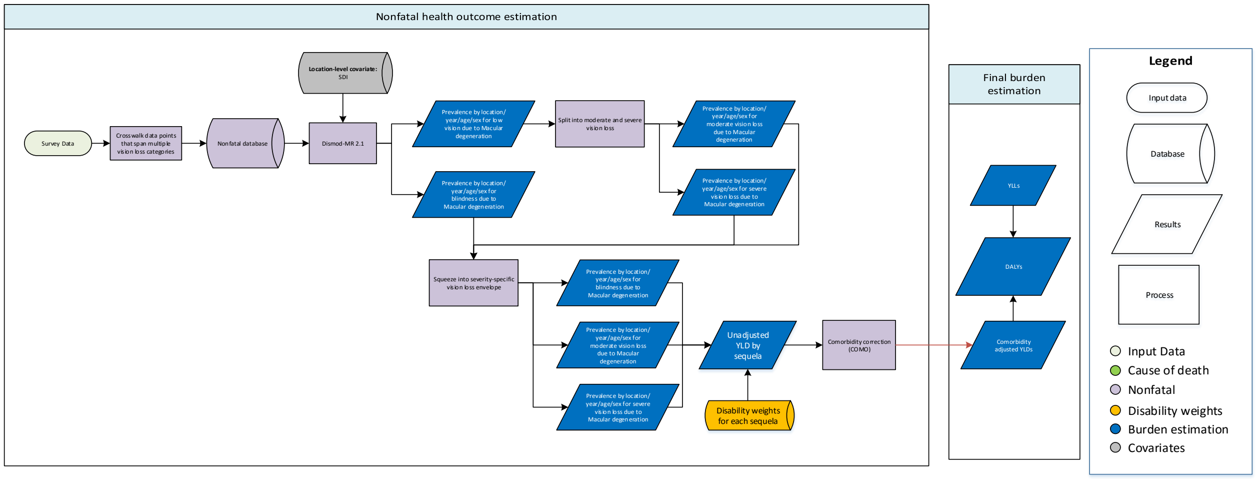

Cataract

Cataract

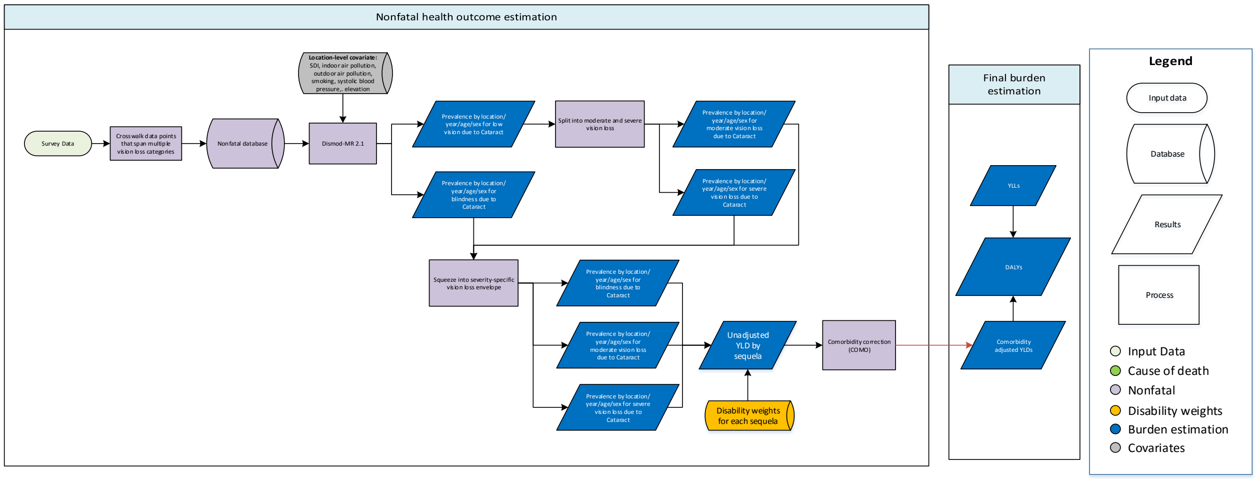

Glaucoma

Glaucoma

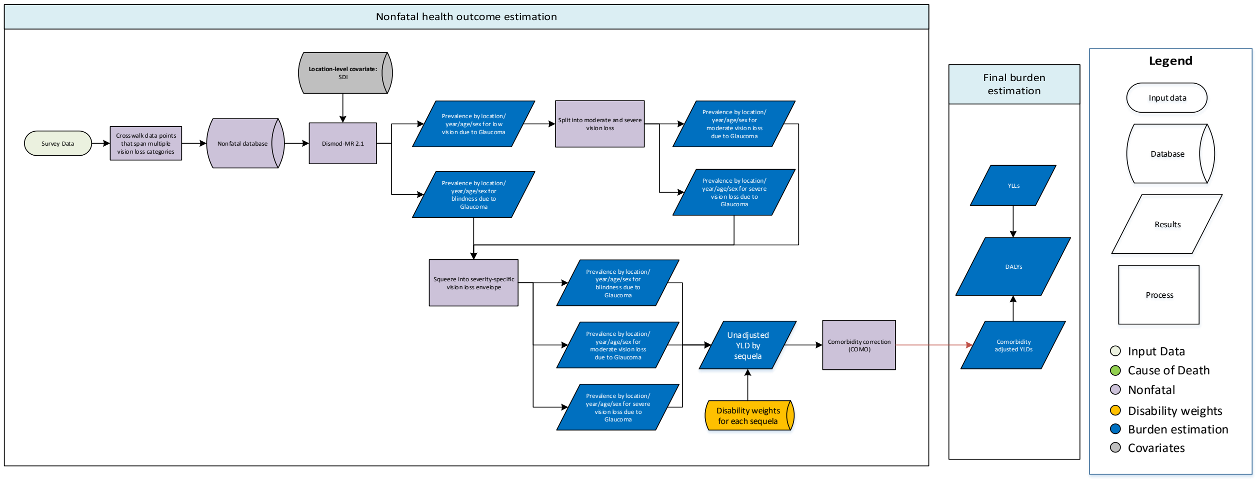

Trachoma

Trachoma

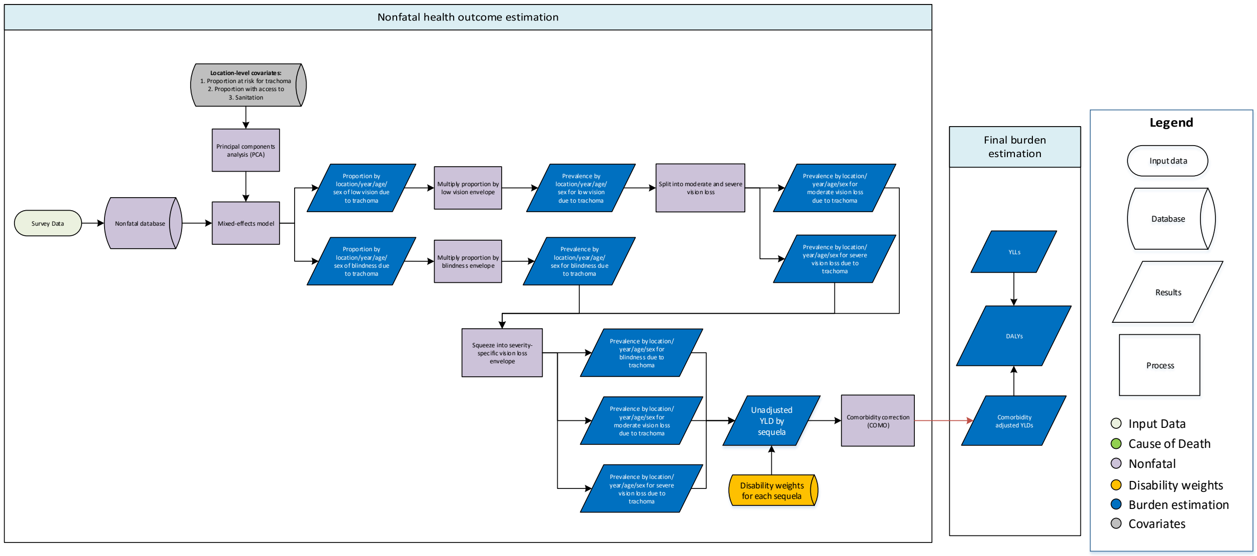

Other vision loss

Other vision loss

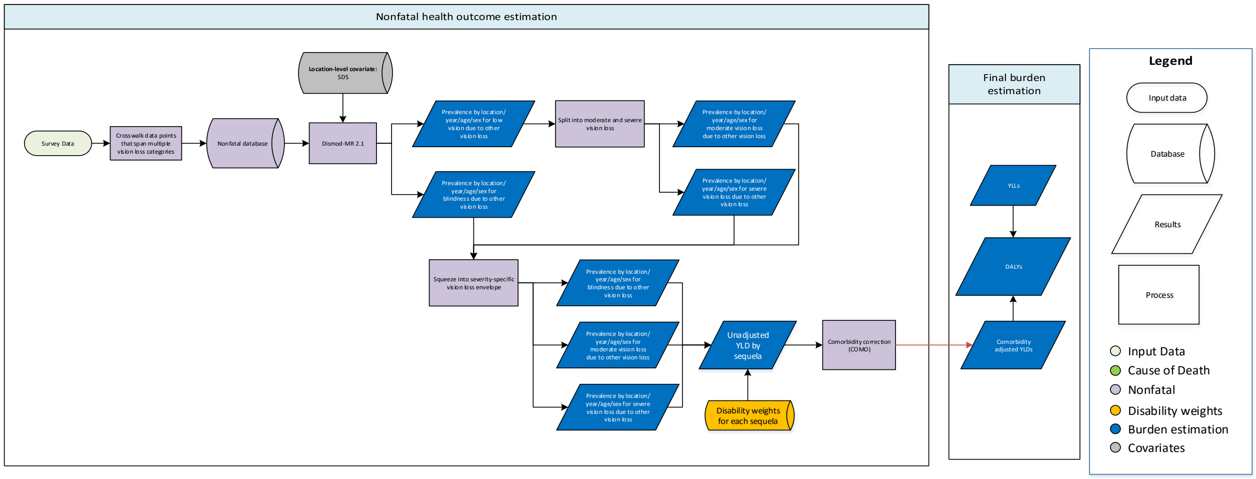

Case definition

We model vision impairment as visual acuity <6/18 according to the Snellen chart. The following impairments are modeled:

Condition	Case definition
Blindness	Visual acuity of <3/60 or <10% visual field around central fixation
Severe vision impairment	≥3/60 and <6/60
Moderate vision impairment	≥6/60 and <6/18
Near vision impairment envelope	Near visual acuity of <6/18 distance equivalent

Near vision impairment describes the progressive inability to focus on near objects as individuals age, and is also called presbyopia. This impairs the ability to read. The majority of presbyopia can be corrected by the use of reading glasses, contact lenses, or refractive surgery.

We model vision impairment due to the following causes: uncorrected refractive error, cataract, glaucoma, macular degeneration, diabetic retinopathy, trachoma, Vitamin A deficiency, retinopathy of prematurity, meningitis, encephalitis, onchocerciasis, and other vision loss. Vision loss due to vitamin A deficiency, retinopathy of prematurity, meningitis, encephalitis, and onchocerciasis are modelled as part of their underlying cause as described in their respective sections.

Refractive error is blurry vision due to the lens's inability to focus. The blurriness caused by refractive error can be addressed through the use of contact lenses, glasses, or refractive surgery. Cataract is

clouding of the lens of the eye due to protein buildup that impairs vision. Glaucoma is a condition with increased intraocular pressure which can lead to damage of the optic nerve. Macular degeneration is a deterioration of the macula, leading to central vision loss. Diabetic retinopathy is damage to the retina caused by damaged blood vessels that can leak blood into the retina and cause scarring of the retina. Trachoma results from a conjunctival bacterial infection (*Chlamydia trachomatis*) that produces inflammation and scarring which leads to an inversion of the eyelids and eyelashes scratching the cornea, which eventually leads to scarring of the cornea and vision impairment or blindness.

Input data

Model inputs

Data on overall vision impairment come from surveys measuring visual acuity in representative population-based studies, either from publications in peer-reviewed and grey literature or surveys for which we had the unit record data. Data were excluded if no test was used of visual acuity that can be converted to the Snellen scale, and if a study did not assess "presenting" or "best-corrected" vision. A subset of these studies that reported vision loss by cause were used to estimate the prevalence of vision loss due to cataract, glaucoma, macular degeneration, diabetic retinopathy, and other causes.

For GBD 2015, we conducted a systematic review for new sources since GBD 2013 (covering 1/1/2013 – 5/20/2015), using the following search string:

((((glaucoma[Title/Abstract] OR cataract[Title/Abstract] OR macular[Title/Abstract] OR 'refractive error'[Title/Abstract] OR presbyopia[Title/Abstract]) OR (('blindness'[MeSH Terms] OR 'blindness'[All Fields]) OR 'vision, low'[MeSH Terms])) AND ('2013'[PDAT] : '3000'[PDAT])) AND 'humans'[MeSH Terms]) AND (prevalence[Title/Abstract] OR incidence[Title/Abstract] OR epidemiology[Title/Abstract])

This yielded 1,169 results, of which we extracted 20 sources. Furthermore, we extracted from the following nationally representative surveys measuring visual acuity: the WHO Studies on Global Ageing and Adult Health (SAGE) and the United States National Health and Examination Surveys (NHANES).

For GBD 2016, we did a comprehensive extraction of the Rapid Assessment of Avoidable Blindness (RAAB) repository (http://raabdata.info/), a database of vision impairment studies in developing settings across the world. There are 266 site-years of data, the majority of which have publicly available reports or publications of the data. A standardized methodology was used by all sources in the repository, allowing inclusion of all available reports. In addition, we added two state-level national surveys from India.

Due to the sparse literature reporting measured near-vision visual acuity, we also extracted data from the following nationally representative studies measuring self-reported near vision loss: SAGE; NHANES; the Surveys of Health, Ageing, and Retirement in Europe (SHARE); the Multi-Country Survey Study on Health and Responsiveness (MCSS); and the World Health Surveys (WHS).

Several adjustments were made to raw data.

1) Where studies reported visual acuity spanning multiple thresholds (eg, <6/60, rather than separate severe and blind estimates), we crosswalked using ratios predicted by a linear regression on age, using data from studies reporting vision loss by each severity.

2) Some studies reported best-corrected vision impairment, but not presenting vision impairment (PVI). We crosswalked these data points using a linear regression of logit-transformed PVI prevalence with fixed effects on best-corrected VI, healthcare quality and access index (HAQI) and Socio-demographic Index (SDI) and super-region random effects. This gave us a predicted PVI data points for these studies not explicitly reporting PVI. These crosswalked data points were flagged with a study-level covariate that increased standard error in DisMod.
3) Where data points spanned more than 20 years of age, we age-split using an algorithm that applies the age-pattern of the super-region to split the data to five-year age groups.

Whereas other vision impairment aetiologies are modelled based on prevalence data, vision impairment due to trachoma is modelled as a proportion of the overall vision impairment envelope, a strategy that was chosen based on the nature of available data.

Health states and disability weights

Health state name	Health state description	Disability weight
Distance vision, severe impairment	This person has severe vision loss, which causes difficulty in daily activities, some emotional impact (for example, worry), and some difficulty going outside the home without assistance.	0.184 (0.125–0.259)
Distance vision, moderate impairment	This person has vision problems that make it difficult to recognize faces or objects across a room.	0.031 (0.019–0.049)
Distance vision blindness	This person is completely blind, which causes great difficulty in some daily activities, worry and anxiety, and great difficulty going outside the home without assistance.	0.187 (0.124–0.26)
Presbyopia	This person has difficulty seeing things that are nearer than 3 feet, but has no difficulty with seeing things at a distance.	0.011 (0.005–0.02)

Modelling strategy

We modelled the prevalence of vision loss in two steps. In the first step, we estimated the total prevalence estimates of presenting vision loss: moderate vision impairment, severe vision impairment, blindness, and near vision impairment (presbyopia). We directly derived prevalence of near vision impairment from this step, whereas the remaining three models that reflect different severity levels of distance vision loss continued to the next step.

1) Estimate severity-specific vision impairment (the "envelopes")

First, we ran five DisMod-MR 2.1 models to estimate the total prevalence estimates of presenting vision loss: moderate vision impairment, severe vision impairment, blindness, near vision impairment (presbyopia), and presenting vision impairment (moderate + severe + blindness). The presenting vision impairment model was used as a covariate in the severity-specific models to improve consistency across severities.

Betas and exponentiated values, which can be interpreted as an odds ratio, are shown in the table below for each covariate. The best-corrected covariate indicates whether the test measures visual acuity with the level of correction the patient presents with (best_corrected = 0) or the ophthalmologist provides additional correction via pinhole (best_corrected = 1). Rapid-assessment corrects for potential biases in cause-specific vision loss from studies using expedited visual acuity measurement. Socio-demographic

Index (SDI) and healthcare access and quality index (HAQI) are used as location covariates as a proxy measure of access to eye care such as cataract surgery. Non-representative studies are those not representative at the level they are used to model (eg, a state-level survey assigned to a country), including a z-cov adjusts for potential bias. Data points that were crosswalked from best-corrected visual acuity are flagged with a z-cov to adjust uncertainty in the crosswalk process. Non-standard severity definition is used to crosswalk between the self-report questionnaire of SHARE (nonstandard) and the other surveys, including SAGE and NHANES, which are crosswalked to examination data using the self-reported covariate.

Model	Covariate name	Type	Measure	Beta value	Exponentiated value
Vision impairment due to glaucoma unsqueezed	Socio-demographic Index	Country covariate	Prevalence	-0.235 (-0.690 - -0.008)	0.791 (0.502 - 0.992)
Vision impairment due to glaucoma unsqueezed	diagnostic rapid assessment of loss	Study-level x-covariate	Prevalence	0.012 (0.002 - 0.033)	1.012 (1.002 - 1.033)
Vision impairment due to glaucoma unsqueezed	Not representative	Study-level z-covariate	Prevalence	0.022 (0.003 - 0.067)	1.023 (1.003 - 1.069)
Blindness due to glaucoma unsqueezed	Socio-demographic Index	Country covariate	Prevalence	-0.256 (-0.690 - -0.010)	0.775 (0.501 - 0.990)
Blindness due to glaucoma unsqueezed	diagnostic rapid assessment of loss	Study-level x-covariate	Prevalence	0.010 (0.000 - 0.030)	1.010 (1.000 - 1.030)
Blindness due to glaucoma unsqueezed	Not representative	Study-level z-covariate	Prevalence	0.024 (0.000 - 0.085)	1.025 (1.000 - 1.089)
Vision impairment due to cataract unsqueezed	Elevation Over 1500m (proportion)	Country covariate	Prevalence	0.119 (0.006 - 0.319)	1.127 (1.006 - 1.376)
Vision impairment due to cataract unsqueezed	Indoor Air Pollution (All Cooking Fuels)	Country covariate	Prevalence	0.031 (0.000 - 0.111)	1.032 (1.000 - 1.118)
Vision impairment due to cataract unsqueezed	Outdoor Air Pollution (PM2.5)	Country covariate	Prevalence	0.008 (0.003 - 0.014)	1.008 (1.003 - 1.014)
Vision impairment due to cataract unsqueezed	Smoking Prevalence (Age-standardized, both sexes)	Country covariate	Prevalence	0.776 (0.035 - 1.675)	2.174 (1.036 - 5.340)
Vision impairment due to cataract unsqueezed	Socio-demographic Index	Country covariate	Prevalence	-0.612 (-0.962 - -0.157)	0.542 (0.382 - 0.855)
Vision impairment due to cataract unsqueezed	Systolic Blood Pressure (mmHg)	Country covariate	Prevalence	0.002 (0.000 - 0.007)	1.002 (1.000 - 1.007)

Vision impairment due to cataract unsqueezed	diagnostic rapid assessment of loss	Study-level x-covariate	Prevalence	0.031 (0.009 - 0.063)	1.031 (1.009 - 1.065)
Vision impairment due to cataract unsqueezed	Not representative	Study-level z-covariate	Prevalence	0.014 (0.003 - 0.039)	1.014 (1.003 - 1.039)
Blindness due to cataract unsqueezed	Elevation Over 1500m (proportion)	Country covariate	Prevalence	0.641 (0.420 - 0.868)	1.898 (1.522 - 2.382)
Blindness due to cataract unsqueezed	Indoor Air Pollution (All Cooking Fuels)	Country covariate	Prevalence	0.408 (0.143 - 0.660)	1.504 (1.153 - 1.936)
Blindness due to cataract unsqueezed	Outdoor Air Pollution (PM2.5)	Country covariate	Prevalence	0.000 (0.000 - 0.001)	1.000 (1.000 - 1.001)
Blindness due to cataract unsqueezed	Smoking Prevalence (Age-standardized, both sexes)	Country covariate	Prevalence	0.757 (0.036 - 1.723)	2.132 (1.036 - 5.601)
Blindness due to cataract unsqueezed	Socio-demographic Index	Country covariate	Prevalence	-0.965 (-1.000 - -0.864)	0.381 (0.368 - 0.421)
Blindness due to cataract unsqueezed	Systolic Blood Pressure (mmHg)	Country covariate	Prevalence	0.002 (0.000 - 0.008)	1.002 (1.000 - 1.009)
Blindness due to cataract unsqueezed	diagnostic rapid assessment of loss	Study-level x-covariate	Prevalence	0.002 (0.000 - 0.009)	1.002 (1.000 - 1.009)
Blindness due to cataract unsqueezed	Not representative	Study-level z-covariate	Prevalence	0.004 (0.000 - 0.010)	1.004 (1.000 - 1.010)
Vision impairment due to macular degeneration unsqueezed	Socio-demographic Index	Country covariate	Prevalence	0.350 (-0.432 - 0.921)	1.419 (0.650 - 2.512)
Vision impairment due to macular degeneration unsqueezed	diagnostic rapid assessment of loss	Study-level x-covariate	Prevalence	0.047 (0.005 - 0.126)	1.049 (1.005 - 1.134)
Vision impairment due to macular degeneration unsqueezed	Not representative	Study-level z-covariate	Prevalence	0.058 (0.008 - 0.150)	1.060 (1.008 - 1.162)
Blindness due to macular degeneration unsqueezed	Socio-demographic Index	Country covariate	Prevalence	0.328 (-0.563 - 0.959)	1.389 (0.570 - 2.609)
Blindness due to macular degeneration unsqueezed	diagnostic rapid assessment of loss	Study-level x-covariate	Prevalence	0.016 (0.002 - 0.050)	1.016 (1.002 - 1.052)
Blindness due to macular degeneration unsqueezed	Not representative	Study-level z-covariate	Prevalence	0.014 (0.001 - 0.047)	1.014 (1.001 - 1.048)

Near vision impairment due to presbyopia due to uncorrected refractive error	Socio-demographic Index	Country covariate	Prevalence	-1.803 (-1.999 - -1.451)	0.165 (0.135 - 0.234)
Near vision impairment due to presbyopia due to uncorrected refractive error	Non-standard severity definition	Study-level x-covariate	Prevalence	-0.195 (-0.200 - -0.186)	0.822 (0.819 - 0.830)
Near vision impairment due to presbyopia due to uncorrected refractive error	Self-reported	Study-level x-covariate	Prevalence	-0.102 (-0.120 - -0.089)	0.903 (0.886 - 0.915)
Vision impairment due to other vision loss unsqueezed	Socio-demographic Index	Country covariate	Prevalence	-0.113 (-0.351 - -0.006)	0.893 (0.704 - 0.994)
Vision impairment due to other vision loss unsqueezed	diagnostic rapid assessment of loss	Study-level x-covariate	Prevalence	0.054 (0.012 - 0.103)	1.056 (1.012 - 1.109)
Vision impairment due to other vision loss unsqueezed	Not representative	Study-level z-covariate	Prevalence	0.186 (0.126 - 0.239)	1.205 (1.135 - 1.270)
Blindness due to other vision loss unsqueezed	Socio-demographic Index	Country covariate	Prevalence	-0.179 (-0.472 - -0.005)	0.836 (0.624 - 0.995)
Blindness due to other vision loss unsqueezed	diagnostic rapid assessment of loss	Study-level x-covariate	Prevalence	0.164 (0.123 - 0.211)	1.178 (1.131 - 1.235)
Blindness due to other vision loss unsqueezed	Not representative	Study-level z-covariate	Prevalence	0.057 (0.019 - 0.107)	1.059 (1.020 - 1.113)
Vision impairment envelope	Socio-demographic Index	Country covariate	Prevalence	-1.899 (-1.997 - -1.605)	0.150 (0.136 - 0.201)
Blindness impairment envelope	Healthcare access and quality index	Country covariate	Prevalence	-0.020 (-0.024 - -0.013)	0.980 (0.976 - 0.987)
Blindness impairment envelope	Presenting vision impairment	Country covariate	Prevalence	0.506 (0.291 - 0.743)	1.659 (1.337 - 2.102)
Blindness impairment envelope	Socio-demographic Index	Country covariate	Prevalence	-0.115 (-0.345 - -0.002)	0.891 (0.708 - 0.998)
Blindness impairment envelope	Not representative	Study-level z-covariate	Prevalence	0.000 (0.000 - 0.002)	1.000 (1.000 - 1.002)
Blindness impairment envelope	best-corrected crosswalk	Study-level z-covariate	Prevalence	0.002 (0.000 - 0.007)	1.002 (1.000 - 1.007)
Moderate vision impairment envelope	Presenting vision impairment	Country covariate	Prevalence	0.775 (0.668 - 0.868)	2.170 (1.951 - 2.383)
Moderate vision impairment envelope	Socio-demographic Index	Country covariate	Prevalence	-0.041 (-0.170 - -0.000)	0.960 (0.844 - 1.000)

Moderate vision impairment envelope	Not representative	Study-level z-covariate	Prevalence	0.000 (0.000 - 0.002)	1.000 (1.000 - 1.002)
Moderate vision impairment envelope	best-corrected crosswalk	Study-level z-covariate	Prevalence	0.160 (0.130 - 0.194)	1.174 (1.139 - 1.214)
Severe vision impairment envelope	Presenting vision impairment	Country covariate	Prevalence	0.509 (0.383 - 0.636)	1.664 (1.466 - 1.889)
Severe vision impairment envelope	Socio-demographic Index	Country covariate	Prevalence	-0.018 (-0.056 - -0.001)	0.983 (0.945 - 0.999)
Severe vision impairment envelope	Not representative	Study-level z-covariate	Prevalence	0.001 (0.000 - 0.003)	1.001 (1.000 - 1.003)
Severe vision impairment envelope	best-corrected crosswalk	Study-level z-covariate	Prevalence	0.034 (0.011 - 0.062)	1.035 (1.011 - 1.064)
Vision impairment due to diabetes mellitus	Diabetes Age-Standardized Prevalence (proportion)	Country covariate	Prevalence	1.465 (0.673 - 2.255)	4.328 (1.961 - 9.535)
Vision impairment due to diabetes mellitus	Socio-demographic Index	Country covariate	Prevalence	-1.192 (-1.957 - -0.132)	0.304 (0.141 - 0.876)
Vision impairment due to diabetes mellitus	diagnostic rapid assessment of loss	Study-level x-covariate	Prevalence	0.011 (0.000 - 0.036)	1.011 (1.000 - 1.036)
Vision impairment due to diabetes mellitus	Not representative	Study-level z-covariate	Prevalence	0.089 (0.005 - 0.239)	1.093 (1.005 - 1.270)
Blindness due to diabetes mellitus unsqueezed	Diabetes Age-Standardized Prevalence (proportion)	Country covariate	Prevalence	3.805 (3.352 - 3.993)	44.905 (28.560 - 54.217)
Blindness due to diabetes mellitus unsqueezed	Socio-demographic Index	Country covariate	Prevalence	-1.594 (-1.989 - -0.597)	0.203 (0.137 - 0.550)
Blindness due to diabetes mellitus unsqueezed	diagnostic rapid assessment of loss	Study-level x-covariate	Prevalence	0.070 (0.002 - 0.230)	1.072 (1.002 - 1.259)
Blindness due to diabetes mellitus unsqueezed	Not representative	Study-level z-covariate	Prevalence	0.373 (0.193 - 0.586)	1.453 (1.213 - 1.796)
Moderate vision impairment due to uncorrected refractive error unsqueezed	Socio-demographic Index	Country covariate	Prevalence	-0.955 (-0.998 - -0.830)	0.385 (0.369 - 0.436)
Severe vision impairment due to uncorrected refractive error unsqueezed	Socio-demographic Index	Country covariate	Prevalence	-0.899 (-0.996 - -0.621)	0.407 (0.369 - 0.538)

Blindness due to uncorrected refractive error unsqueezed	Socio-demographic Index	Country covariate	Prevalence	-0.968 (-1.000 - -0.870)	0.380 (0.368 - 0.419)

2) Estimate cause-specific vision impairment

In the second step, we estimated the prevalence of vision loss due to multiple causes: refractive error, cataract, glaucoma, macular degeneration, diabetic retinopathy, retinopathy due to prematurity, trachoma, vitamin A deficiency, onchocerciasis, meningitis, and other causes not classified elsewhere. The vision loss due to retinopathy of prematurity, vitamin A deficiency, onchocerciasis, meningitis, tetanus, and neonatal conditions was modeled as part of these underlying causes. Vision loss due to trachoma is modelled as a proportion of the envelope, with separate proportion models for vision impairment and blindness. For each of cataract, glaucoma, macular degeneration, diabetic retinopathy, and other vision loss, we ran two DisMod-MR 2.1 models: one for the combined category of moderate and severe vision loss due to the cause, and one for blindness due to the cause. Moderate and severe vision loss were modelled together because input data were mostly available for the aggregate. Refractive error was modelled in three models, one for each severity. We used the following age restrictions:

Cause	Minimum age
Cataracts	20
Glaucoma	45
Macular degeneration	45
Diabetic retinopathy	20
Trachoma	15
Other vision loss	0

For the cataract model, we used known risk factors – hypertension, smoking, air pollution, and elevation.

For cataract and refractive error, we used presenting vision impairment as a covariate, as these are the main causes of vision impairment and are treatable and thus should have greater covariance with overall vision impairment than less common causes such as glaucoma or macular degeneration.

We estimated the proportions of low vision and blindness due to trachoma using custom mixed-effects models. For consistency, the two models (blindness and low vision) were parameterized identically and differ only in their input data. Our model included fixed effects on age (using cubic splines with knots at 0, 40, and 100 years of age), sex, and a covariate derived from a principal components analysis of the proportion of the population at risk for trachoma and the proportion of the population with access to sanitation. We included nested random effects on super-region, region, and country. Finally, we applied geographic and age restrictions to ensure that we estimate zero proportions in non-endemic locations and among those younger than 15 year of age (as scarring of the cornea due to trachoma takes decades to develop). The prevalence of trachoma at each severity level was calculated by multiplying the proportion of vision loss (vision impairment or blindness) due to trachoma by the corresponding best-corrected vision loss envelope.

We split the moderate plus severe vision loss estimates for each cause into moderate and severe using the ratio of best-corrected moderate and severe vision loss envelopes. As exceptions, onchocerciasis and retinopathy of prematurity were modelled for moderate and severe vision loss as part of the estimation process of these causes.

We scaled the cause-specific vision loss prevalence to the total prevalence of the best-corrected vision loss envelopes for each of the three severity levels. The final result is prevalence of vision loss due to each cause by severity.

The following changes have been implemented since GBD 2015:

- DisMod is not designed to handle wide-age data points – by age-splitting the input data we improve model fits.
- In the severity-specific vision impairment models, we use overall presenting vision impairment as a covariate, ensuring greater consistency between severities.
- In GBD 2013 vision impairment models, best-corrected vision data were crosswalked within DisMod using a single beta for all ages and locations. By crosswalking the input data, we allow the ratio between presenting and best-corrected vision impairment to vary with age and location.
- In GBD 2013, we estimated the ratio of vision impairment due to refractive error. In 2016, we are estimating the prevalence of refractive error, as it shows greater covariance with predictors such as SDI and HAQI. This allows the second step (squeezing causes to the envelopes) to include refractive error as an input.

Hearing Impairment

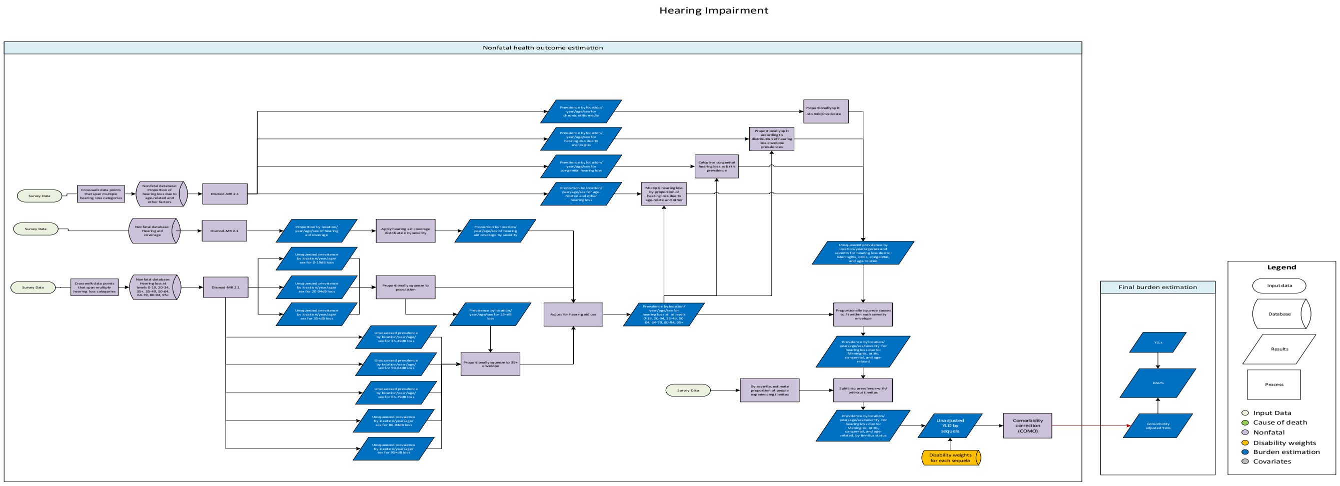

Case definition

For GBD 2016, hearing impairment modeled the following severities of hearing loss:

Severity thresholds of interest for hearing loss	
Severity	Threshold (in decibels)
None	0–19
Mild	20–34
Moderate	35–49
Moderately severe	50–64
Severe	65–79
Profound	80–94
Complete	95+

We model the following causes of hearing loss: congenital, meningitis, otitis, and age-related and other hearing loss. Hearing loss due to meningitis and otitis are modelled as part of their underlying cause as described in their respective sections. Congenital hearing loss is defined as hearing loss present at birth. Age-related and other hearing loss includes causes not identified as meningitis, otitis, or congenital. This includes presbycusis, the gradual increase in hearing loss over age frequently caused by the natural breakdown of neurons in the inner ear. For all causes, we estimate hearing loss with and without tinnitus, the perception of noise or ringing in the ears.

Input data

Model inputs

For the estimation of the severity-specific envelopes, we used a series of systematic reviews and survey extraction. Data sources up to 2008 were identified by a published systematic review (http://www.ncbi.nlm.nih.gov/pubmed/19444763). A systematic review covering 2008–2013 was conducted with the following search terms:

(hearing impairment[Title/Abstract] OR deafness[Title/Abstract] OR hearing loss[Title/Abstract]) AND (prevalence[Title/Abstract]) AND ("2008"[PDAT] : "3000"[PDAT]) AND (cross sectional OR survey)

In addition, we extracted hearing loss measurement from the United States National Health and Examination Surveys (NHANES). Self-reported data, from both the literature and surveys, were excluded. This includes censuses in the Integrated Public Use Microdata Series (IPUMS), the WHO Studies on Global Ageing and Adult Health (SAGE), and the WHO Multi-Country Survey Study on Health and Responsiveness (MCSS). Systematic reviews and self-reported survey data (including MCSS, SAGE, and NHANES) were used to estimate hearing aid coverage.

For GBD 2016, we conducted a systematic review on November 30, 2016, using the following search terms:

(hearing impairment[Title/Abstract] OR deafness[Title/Abstract] OR hearing loss[Title/Abstract] OR audiometry[Title/Abstract]) AND (prevalence[Title/Abstract]) AND ("2008/11/26"[PDAT] : "3000"[PDAT]) AND (cross sectional OR survey)

This returned 239 results, of which 17 were accepted.

Where studies reported hearing loss spanning multiple thresholds (eg, 80+, rather than 80-94 and 95+), we crosswalked using ratios predicted by a linear regression on age, using NHANES microdata. Where studies reported severity categories that did not align with GBD thresholds, we crosswalked using NHANES microdata to the nearest GBD severity category, as long as the upper and lower thresholds were not more than 10dB different.

Health states and disability weights

Health state name	Health state description	Disability weight
Hearing loss, mild	This person has great difficulty hearing and understanding another person talking in a noisy place (for example, on an urban street).	0.01 (0.004–0.019)
Hearing loss, mild, with ringing	This person is unable to hear and understand another person talking, even in a quiet place, is unable to take part in a phone conversation. Difficulties with communicating and relating to others cause emotional impact at times (for example worry or depression).	0.021 (0.012–0.036)
Hearing loss, moderate	This person has great difficulty hearing and understanding another person talking in a noisy place (for example, on an urban street), and sometimes has annoying ringing in the ears.	0.027 (0.015–0.042)
Hearing loss, moderate, with ringing	This person is unable to hear and understand another person talking, even in a quiet place, and has great difficulty hearing anything in any other situation. Difficulties with communicating and relating to others often cause worry, depression or loneliness.	0.074 (0.048–0.107)
Hearing loss, moderately severe	(custom DW from hearing loss impairment envelope)	0.092 (0.064–0.129)
Hearing loss, moderately severe, with ringing	(custom DW from hearing loss impairment envelope)	0.167 (0.114–0.231)

Hearing loss, severe	This person is unable to hear and understand another person talking in a noisy place (for example, on an urban street), and has difficulty hearing another person talking even in a quiet place or on the phone.	0.158 (0.104–0.227)
Hearing loss, severe, with ringing	This person is unable to hear and understand another person talking, even in a quiet place, has great difficulty hearing anything in any other situation, Difficulties with communicating and relating to others often cause worry, depression, or loneliness.	0.261 (0.174–0.361)
Hearing loss, profound	This person is unable to hear and understand another person talking in a noisy place, has difficulty hearing another person talking even in a quiet place or on the phone, and has annoying ringing in the ears for 5 minutes at a time, almost every day.	0.204 (0.134–0.288)
Hearing loss, profound, with ringing	This person cannot hear at all in any situation, including even the loudest sounds, and cannot communicate verbally or use a phone. Difficulties with communicating and relating to others often cause worry, depression or loneliness.	0.277 (0.182–0.388)
Hearing loss, complete	This person is unable to hear and understand another person talking, even in a quiet place, and unable to take part in a phone conversation. Difficulties with communicating and relating to others cause emotional impact at times (for example worry or depression).	0.215 (0.143–0.307)
Hearing loss, complete, with ringing	This person cannot hear at all in any situation, including even the loudest sounds, and cannot communicate verbally or use a phone. Difficulties with communicating and relating to others often cause worry, depression or loneliness.	0.316 (0.211–0.436)

Modelling strategy

We modelled the prevalence of hearing loss over five steps. First, we ran three DisMod-MR 2.1 models to estimate the total prevalence estimates of hearing loss: normal hearing (0–19dB), mild hearing loss (20–34dB), and moderate hearing loss and above (35+ dB). We squeezed the prevalence estimates from these DisMod-MR 2.0 models to fit within the entire population of each country. We estimated prevalence of normal hearing for this squeezing purpose only, and hence did not form part of further analysis. Betas and exponentiated values, which can be interpreted as an odds ratio, are shown in the table below for each covariate.

Model	Covariate name	Type	Measure	Beta value	Exponentiated value
Hearing loss impairment at 35+ dB	Socio-demographic Index	Country covariate	Prevalence	-1.451 (-1.984 - -0.486)	0.234 (0.138 - 0.615)
Hearing loss impairment at 95+ dB	Socio-demographic Index	Country covariate	Prevalence	-0.584 (-1.595 - -0.024)	0.557 (0.203 - 0.976)
Hearing aids (proportion of total hearing loss)	LDI (I$ per capita)	Country covariate	Prevalence	0.726 (0.498 - 0.979)	2.066 (1.646 - 2.662)
Hearing loss impairment at 0-19 dB	Socio-demographic Index	Country covariate	Prevalence	0.058 (0.001 - 0.182)	1.059 (1.001 - 1.200)

Second, we ran five additional DisMod-MR 2.1 models for each severity levels of hearing loss above mild: moderate (35–49dB), moderately severe (50–64dB), severe (65–79dB), profound (80–94dB), and complete (95+). We then squeezed the prevalence estimates from these models to fit within the prevalence that were estimated for 35+dB in the first step. By the end of the second step, we had estimated prevalence of six severity levels of hearing loss, including mild (20–34dB). We also ran a DisMod-MR 2.0 model for the coverage of hearing aids, using (logged) lag distributed income (LDI) as a covariate.

Third, we adjusted the prevalence of each severity level by accounting for hearing aids. We assumed the use of hearing aids reduced the severity by one level. Data obtained from a survey in Norway provided detailed information on people with hearing aids, which was used to estimate the proportion of hearing

aids for each severity level. We ran a log-linear regression on age with binary indicator for severity levels. We calculated country-specific hearing aid coverage by multiplying the severity-specific coverage in Norway by the ratio of hearing aid coverage in a given country to that of Norway for each age-sex. We shifted the identified fraction of people in each severity level a level below, except for complete hearing loss, which we assumed was not correctable by hearing aids. This provided the adjusted prevalence of six severity levels of all-cause hearing loss.

Fourth, we estimated the prevalence of hearing loss due to multiple causes: otitis media, congenital, meningitis (pneumococcal, H influenza type B meningitis, meningococcal, and other bacterial), and age-related and other causes not classified elsewhere. For congenital hearing loss, we assumed that all hearing losses occurring at the time of birth are of congenital nature. We assumed that all hearing loss due to otitis media is at the mild or moderate level. We implemented proportional squeezes to scale cause-specific hearing loss prevalence to the total prevalence of each severity level.

Finally, we estimated the percent of people experiencing tinnitus for at least five minutes per day by severity level using data from the NHANES and two datasets from the United Kingdom. We calculated confidence intervals assuming a binomial distribution. We assumed the same distribution of tinnitus across all types of hearing loss.

Section 4. Appendix: Tables and Figures

Appendix Figure 1a: Overview analytical flowchart for DisMod-MR 2.1 modelling strategies and injuries, GBD 2016

Appendix Figure 1b: Analytical flowchart for modelling strategies other than DisMod-MR 2.1 and injuries for selected nonfatal cause groups, GBD 2016

Appendix Figure 2: DisMod-MR 2.1 analytical cascade

Appendix Figure 3: Map of GBD 2016 Regions

Appendix Figure 4: Number of most detailed GBD causes with any incidence or prevalence data by location, 1980-2016

Appendix Table 1: GATHER checklist of information that should be included in reports of global health estimates, with description of compliance and location of information for GBD 2016. Global, regional, and national incidence, prevalence, and years lived with disability for 328 diseases and injuries for 195 countries, 1990-2016: a systematic analysis for the Global Burden of Disease Study 2016

Appendix Table 2: GBD 2016 location hierarchy with levels

Appendix Table 3: GBD 2016 cause and sequela hierarchy

Appendix Table 4: ICD-9 and ICD-10 codes by GBD 2016 nonfatal causes, impairments and nature of injury categories

Appendix Table 5: GBD 2016 sequelae, health states, health state lay descriptions, and disability weights

Appendix Table 6: GBD 2016 methods of estimating years lived with disability (YLDs) for 35 residual categories

Appendix Table 7: Socio-demographic Index R-squared values with lags up to 10 years

Appendix Table 8: Socio-demographic Index groupings by location, based on 2016 values

Appendix Table 9: Socio-demographic Index values for all estimated GBD locations, 1980 - 2016

Appendix Figure 1a: Overview analytical flowchart for DisMod-MR 2.1 modelling strategies and injuries, GBD 2016

Ovals represent data inputs, square boxes represent analytical steps, cylinders represent databases, and parallelograms represent intermediate and final results. The flowchart is color-coded by major estimation component: raw data sources, in pink; data adjustments, in yellow; DisMod-MR 2.1 estimation, in purple; alternative modelling strategies, in light green; injury modeling strategy, in dark green; estimation of impairments and underlying causes, in brown; severity distributions and comorbidity correction, in blue; disability weights in orange; and cause of death and demographic inputs, in grey. GBD = Global Burden of Disease; TB=tuberculosis; HIV =human immunodeficiency virus; AIDS=acquired immunodeficiency syndrome; SF-12=Short Form 12 questions; MEPS=Medical Expenditure Panel Surveys; CSMR=cause-specific mortality rate; SMR=standardized mortality ratio; YLDs=years lived with disability; YLLs=years of life lost.

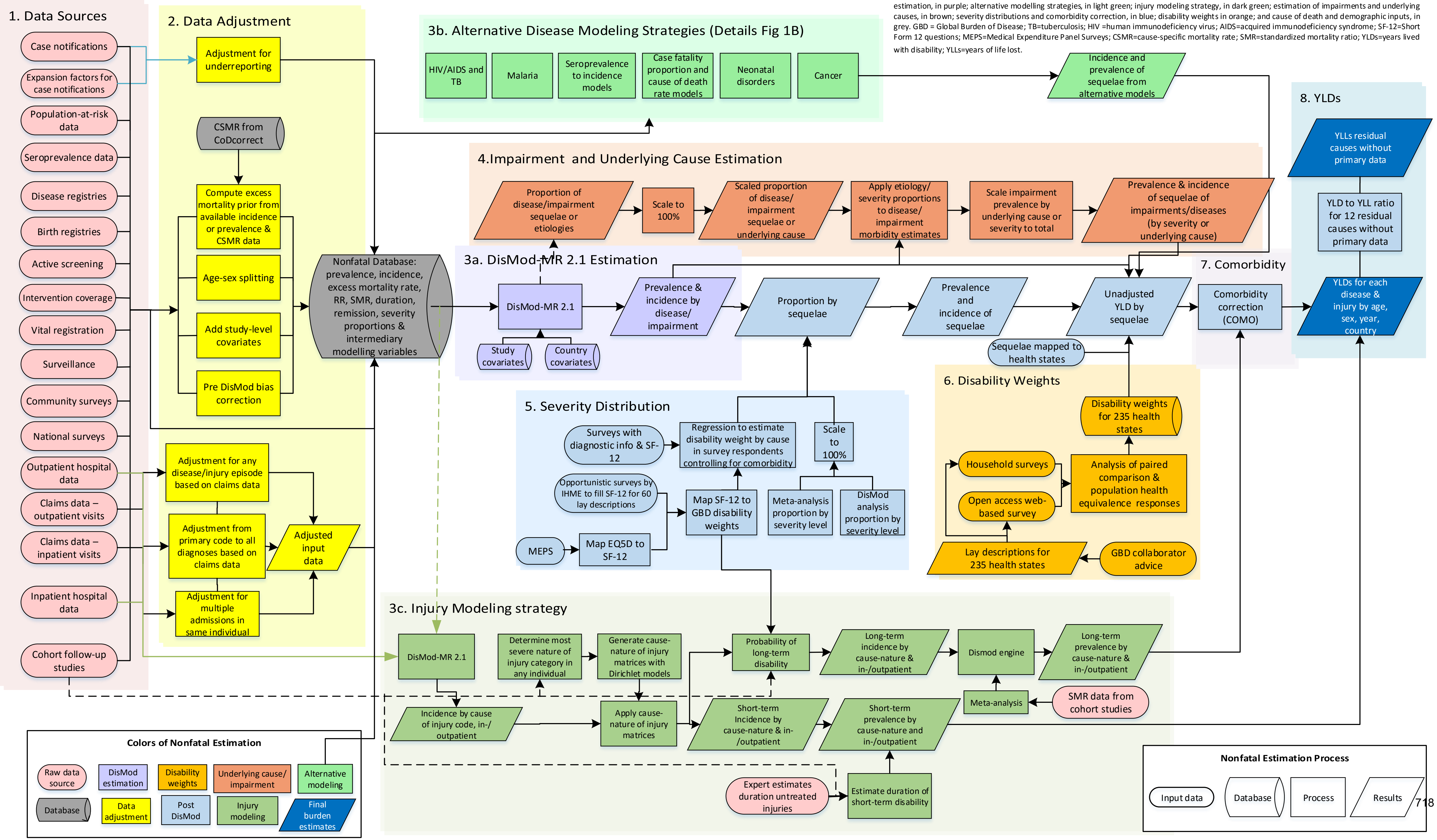

Appendix Figure 1b: Analytical flowchart for modelling strategies other than DisMod-MR 2.1 and injuries for selected nonfatal cause groups, GBD 2016

Ovals represent data inputs, square boxes represent analytical steps, cylinders represent databases, and parallelograms represent intermediate and final results. The flowchart is color-coded by major estimation component: raw data sources, in pink; data adjustments, in yellow; DisMod-MR 2.1 estimation, in purple; alternative modelling strategies, in light green; injury modeling strategy, in dark green; estimation of impairments and underlying causes, in brown; severity distributions and comorbidity correction, in blue; disability weights in orange; and cause of death and demographic inputs, in grey. GBD = Global Burden of Disease; TB=tuberculosis; HIV =human immunodeficiency virus; AIDS=acquired immunodeficiency syndrome; SF-12=Short Form 12 questions; MEPS=Medical Expenditure Panel Surveys; CSMR=cause-specific mortality rate; SMR=standardized mortality ratio; YLDs=years lived with disability; YLLs=years of life lost.

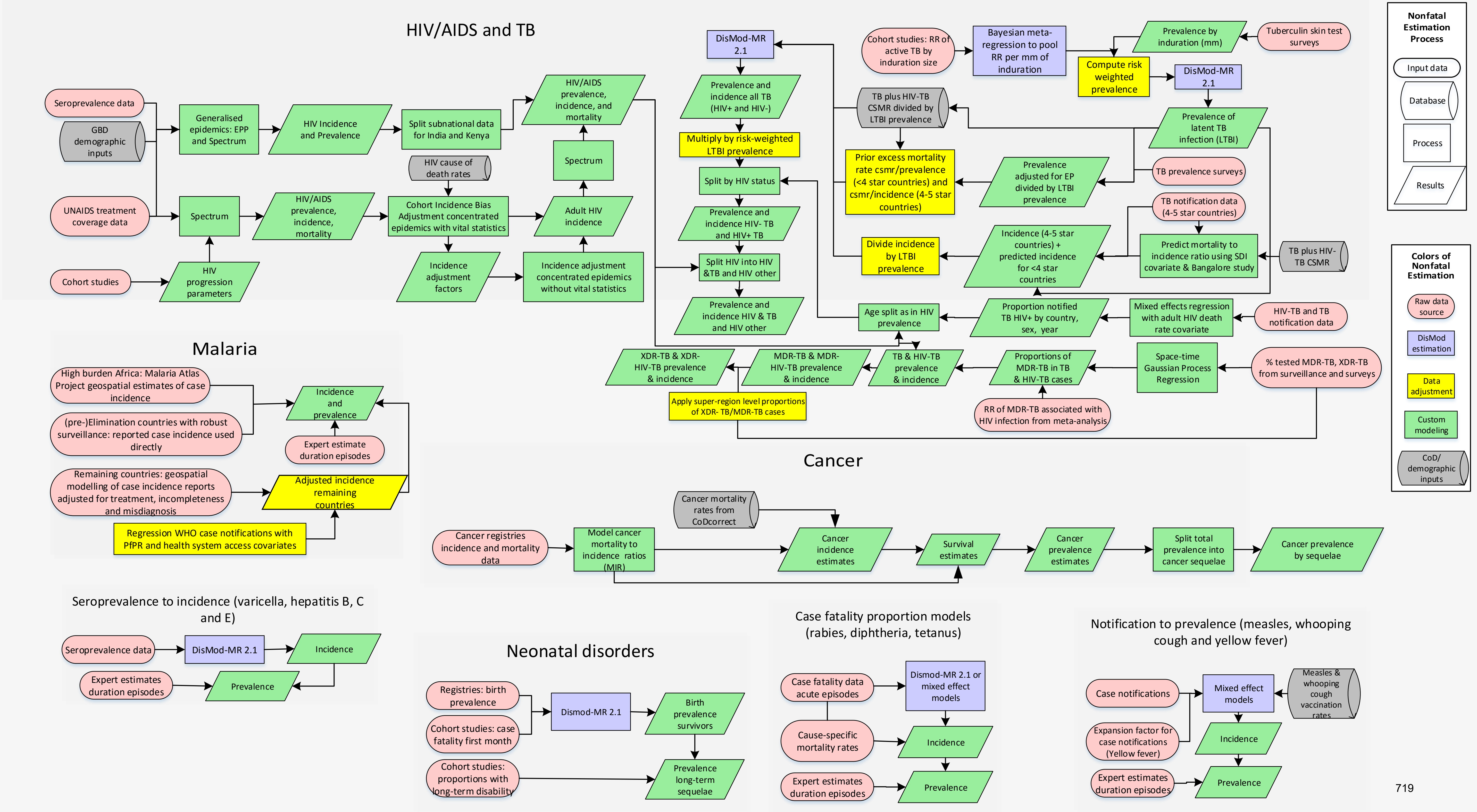

Appendix Figure 2: DisMod-MR 2.1 analytical cascade

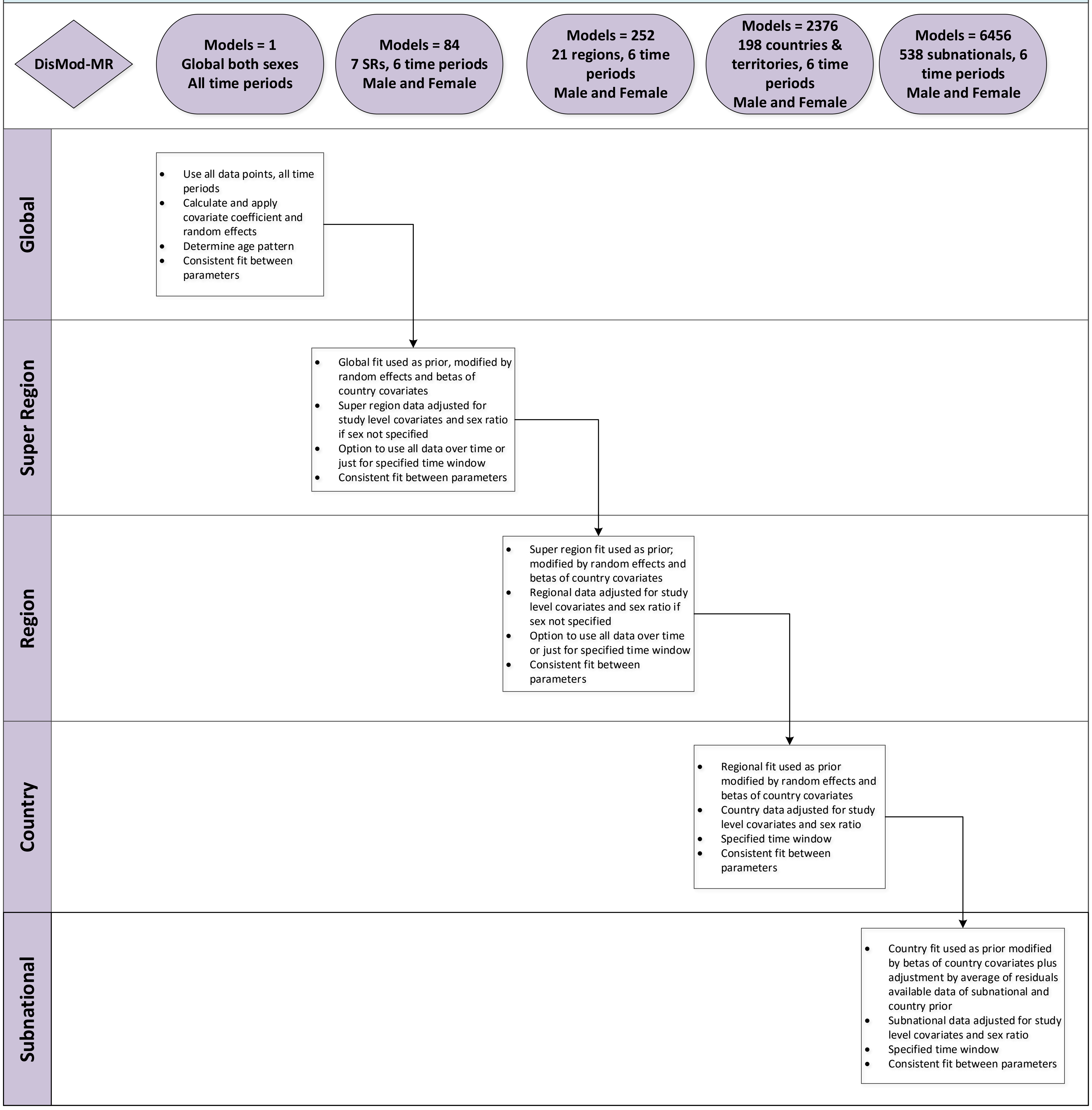

Appendix Figure 3: Map of GBD 2016 Regions

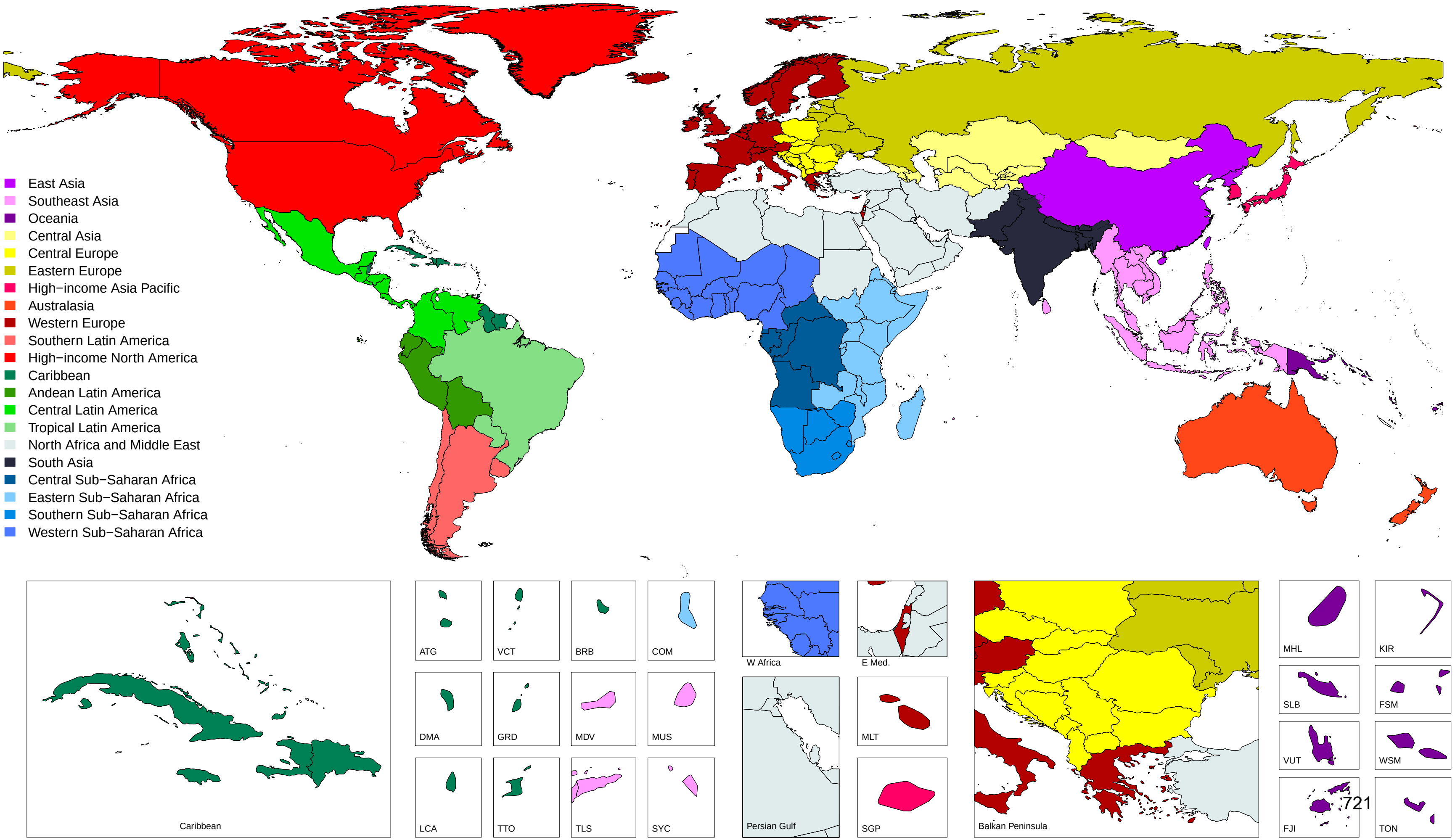

Appendix Figure 3: Number of most detailed GBD causes with any incidence or prevalence data by location, 1980 to 2016

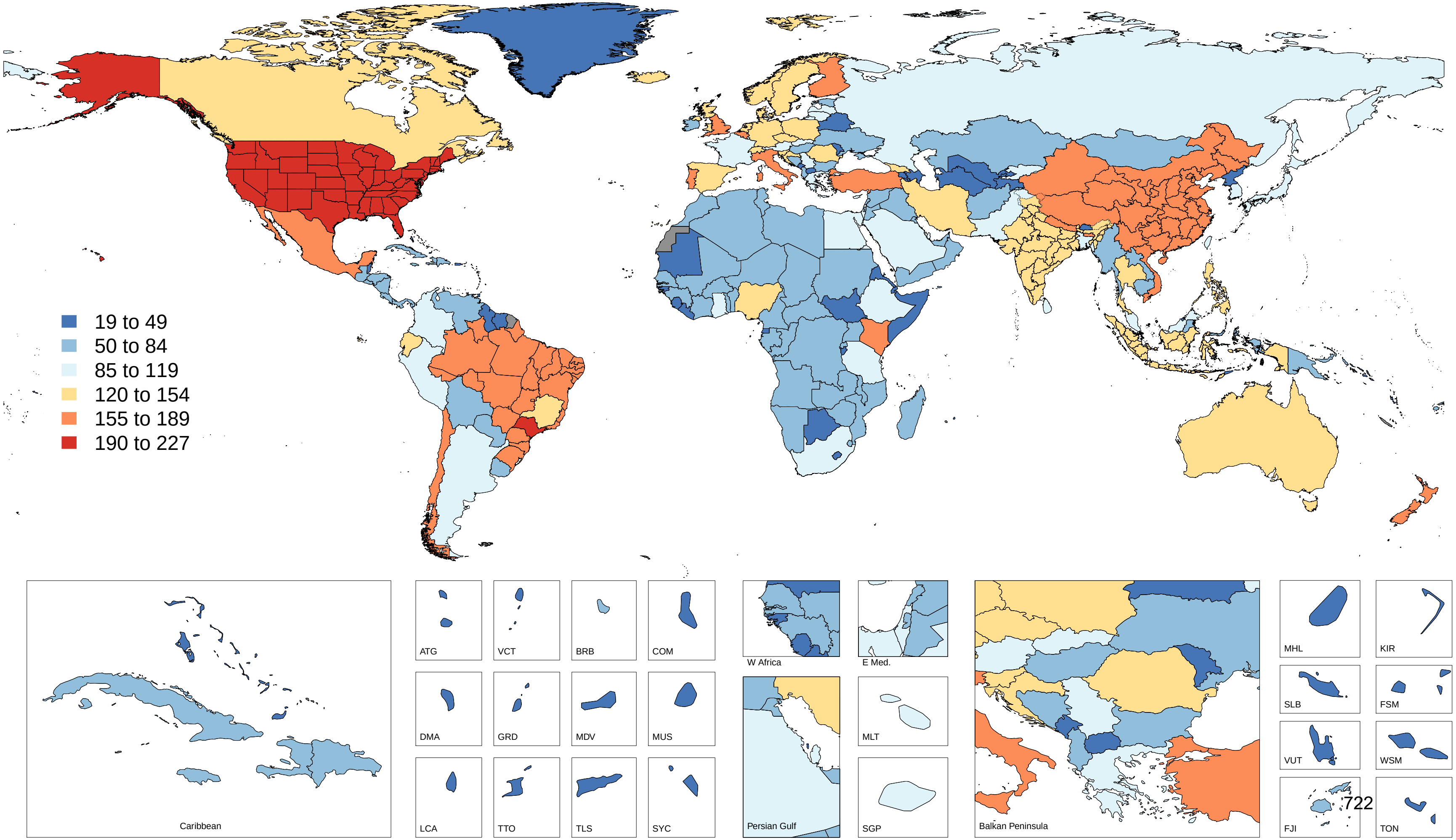

Appendix Table 1: GATHER checklist of information that should be included in reports of global health estimates, with description of compliance and location of information for GBD 2016. Global, regional, and national incidence, prevalence, and years lived with disability for 328 diseases and injuries for 195 countries, 1990-2016: a systematic analysis for the Global Burden of Disease Study 2016			
**#**	**GATHER checklist item**	**Description of compliance**	**Reference**
**Objectives and funding**			
1	Define the indicators, populations, and time periods for which estimates were made.	Narrative provided in paper and methods appendix describing indicators, definitions, and populations	Manuscript (Methods—Overview, Geographic units and time periods) and methods appendix
2	List the funding sources for the work.	Funding sources listed in paper	Summary (Funding)
**Data Inputs**			
For all data inputs from multiple sources that are synthesized as part of the study:			
3	Describe how the data were identified and how the data were accessed.	Narrative description of data seeking methods provided	Manuscript (Methods) and methods appendix Section 2
4	Specify the inclusion and exclusion criteria. Identify all ad-hoc exclusions.	Narrative about inclusion and exclusion criteria by data type provided; ad-hoc exclusions in cause-specific write ups	Methods appendix Section 2 and Section 3
5	Provide information on all included data sources and their main characteristics. For each data source used, report reference information or contact name/institution, population represented, data collection method, year(s) of data collection, sex and age range, diagnostic criteria or measurement method, and sample size, as relevant.	An interactive, online data source tool that provides metadata for data sources by component, geography, cause, risk, or impairment has been developed	Online data citation tool http://ghdx.healthdata.org/global-burden-disease-study-2016
6	Identify and describe any categories of input data that have potentially important biases (e.g., based on characteristics listed in item 5).	Summary of known biases by cause included in methods appendix	Methods appendix Section 2
For data inputs that contribute to the analysis but were not synthesized as part of the study:			
7	Describe and give sources for any other data inputs.	Included in online data source tool	http://ghdx.healthdata.org/global-burden-disease-study-2016
For all data inputs:			

8	Provide all data inputs in a file format from which data can be efficiently extracted (e.g., a spreadsheet as opposed to a PDF), including all relevant meta-data listed in item 5. For any data inputs that cannot be shared due to ethical or legal reasons, such as third-party ownership, provide a contact name or the name of the institution that retains the right to the data.	Downloads of input data available through online tools, including data visualization tools and data query tools; input data not available in tools will be made available upon request	Online data visualization tools, data query tools, and the Global Health Data Exchange
**Data analysis**			
9	Provide a conceptual overview of the data analysis method. A diagram may be helpful.	Flow diagrams of the overall methodological processes, as well as cause-specific modeling processes, have been provided	Manuscript (Methods) and methods appendix (appendix Figure 2)
10	Provide a detailed description of all steps of the analysis, including mathematical formulae. This description should cover, as relevant, data cleaning, data pre-processing, data adjustments and weighting of data sources, and mathematical or statistical model(s).	Flow diagrams and corresponding methodological write-ups for each cause, as well as the databases and modeling processes, have been provided	Manuscript (Methods) and methods appendix (Appendix Figure 1, Appendix Section 3)
11	Describe how candidate models were evaluated and how the final model(s) were selected.	Provided in the methodological write-ups	Appendix section 3
12	Provide the results of an evaluation of model performance, if done, as well as the results of any relevant sensitivity analysis.	Provided in the methodological write-ups	Appendix section 3
13	Describe methods for calculating uncertainty of the estimates. State which sources of uncertainty were, and were not, accounted for in the uncertainty analysis.	Provided in the methodological write-ups	Appendix section 3
14	State how analytic or statistical source code used to generate estimates can be accessed.	Access statement provided	Code is provided in an online repository
**Results and Discussion**			
15	Provide published estimates in a file format from which data can be efficiently extracted.	GBD 2016 results are available through online data visualization tools, the Global Health Data Exchange, and the online data query tool	Manuscript, supplementary results, and online data tools (data visualization tools, data query tools, and the Global Health Data Exchange)

16	Report a quantitative measure of the uncertainty of the estimates (e.g. uncertainty intervals).	Uncertainty intervals are provided with all results	Manuscript, supplementary results, and online data tools (data visualization tools, data query tools, and the Global Health Data Exchange); [illegible]
17	Interpret results in light of existing evidence. If updating a previous set of estimates, describe the reasons for changes in estimates.	Discussion of methodological changes between GBD rounds provided in the narrative of the manuscript and methods appendix	Manuscript (Methods and Discussion) and methods appendix section 3
18	Discuss limitations of the estimates. Include a discussion of any modeling assumptions or data limitations that affect interpretation of the estimates.	Discussion of limitations provided in the narrative of the manuscript, as well as in the methodological write-ups in the methods appendix	Manuscript (Limitations) and methods appendix

Appendix Table 2: GBD 2016 location hierarchy with levels

Location	Level
Global	0
Southeast Asia, East Asia, and Oceania	1
East Asia	2
China	3
Anhui	4
Beijing	4
Chongqing	4
Fujian	4
Gansu	4
Guangdong	4
Guangxi	4
Guizhou	4
Hainan	4
Hebei	4
Heilongjiang	4
Henan	4
Hong Kong Special Administrative Region of China	4
Hubei	4
Hunan	4
Inner Mongolia	4
Jiangsu	4
Jiangxi	4
Jilin	4
Liaoning	4
Macao Special Administrative Region of China	4
Ningxia	4
Qinghai	4
Shaanxi	4
Shandong	4
Shanghai	4
Shanxi	4
Sichuan	4
Tianjin	4
Tibet	4
Xinjiang	4
Yunnan	4
Zhejiang	4
North Korea	3
Taiwan (Province of China)	3
Southeast Asia	2
Cambodia	3
Indonesia	3
Aceh	4
Bali	4
Bangka Belitung	4
Banten	4

Appendix Table 2: GBD 2016 location hierarchy with levels

Location	Level
Bengkulu	4
Gorontalo	4
Jakarta	4
Jambi	4
Cental Java	4
West Java	4
East Java	4
West Kalimantan	4
South Kalimantan	4
Central Kalimantan	4
East Kalimantan	4
North Kalimantan	4
Riau Islands	4
Lampung	4
Maluku	4
North Maluku	4
Nusa Tenggara Barat	4
East Nusa Tenggara	4
Papua	4
West Papua	4
Riau	4
West Sulawesi	4
South Sumatera	4
Central Sulawesi	4
Southeast Sulawesi	4
North Sulawesi	4
West Sumatera	4
South Sumatera	4
North Sumatera	4
Yogyakarta	4
Laos	3
Malaysia	3
Maldives	3
Mauritius	3
Myanmar	3
Philippines	3
Sri Lanka	3
Seychelles	3
Thailand	3
Timor-Leste	3
Vietnam	3
Oceania	2
American Samoa	3
Federated States of Micronesia	3
Fiji	3
Guam	3

Appendix Table 2: GBD 2016 location hierarchy with levels	
**Location**	**Level**
Kiribati	3
Marshall Islands	3
Northern Mariana Islands	3
Papua New Guinea	3
Samoa	3
Solomon Islands	3
Tonga	3
Vanuatu	3
Central Europe, Eastern Europe, and Central Asia	1
Central Asia	2
Armenia	3
Azerbaijan	3
Georgia	4
Georgia	3
Kazakhstan	3
Kyrgyzstan	3
Mongolia	3
Tajikistan	3
Turkmenistan	3
Uzbekistan	3
Central Europe	2
Albania	3
Bosnia and Herzegovina	3
Bulgaria	3
Croatia	3
Czech Republic	3
Hungary	3
Macedonia	3
Montenegro	3
Poland	3
Romania	3
Serbia	3
Slovakia	3
Slovenia	3
Eastern Europe	2
Belarus	3
Estonia	3
Latvia	3
Lithuania	3
Moldova	3
Russia	3
Ukraine	3
High-income	1
High-income Asia Pacific	2
Brunei	3
Japan	3

Appendix Table 2: GBD 2016 location hierarchy with levels

Location	Level
Aichi	4
Akita	4
Aomori	4
Chiba	4
Ehime	4
Fukui	4
Fukuoka	4
Fukushima	4
Gifu	4
Gunma	4
Hiroshima	4
Hokkaidō	4
Hyōgo	4
Ibaraki	4
Ishikawa	4
Iwate	4
Kagawa	4
Kagoshima	4
Kanagawa	4
Kōchi	4
Kumamoto	4
Kyōto	4
Mie	4
Miyagi	4
Miyazaki	4
Nagano	4
Nagasaki	4
Nara	4
Niigata	4
Ôita	4
Okayama	4
Okinawa	4
Ōsaka	4
Saga	4
Saitama	4
Shiga	4
Shimane	4
Shizuoka	4
Tochigi	4
Tokushima	4
Tōkyō	4
Tottori	4
Toyama	4
Wakayama	4
Yamagata	4
Yamaguchi	4

Appendix Table 2: GBD 2016 location hierarchy with levels

Location	Level
Yamanashi	4
South Korea	3
Singapore	3
Australasia	2
Australia	3
New Zealand	3
Western Europe	2
Andorra	3
Austria	3
Belgium	3
Cyprus	3
Denmark	3
Finland	3
France	3
Germany	3
Greece	3
Iceland	3
Ireland	3
Israel	3
Italy	3
Luxembourg	3
Malta	3
Netherlands	3
Norway	3
Portugal	3
Spain	3
Sweden	3
Stockholm	4
Sweden except Stockholm	4
Switzerland	3
United Kingdom	3
England	4
East Midlands	5
Derby	6
Derbyshire	6
Leicester	6
Leicestershire	6
Lincolnshire	6
Northamptonshire	6
Nottingham	6
Nottinghamshire	6
Rutland	6
East of England	5
Bedford	6
Cambridgeshire	6
Central Bedfordshire	6

Appendix Table 2: GBD 2016 location hierarchy with levels	
**Location**	**Level**
Essex	6
Hertfordshire	6
Luton	6
Norfolk	6
Peterborough	6
Southend-on-Sea	6
Suffolk	6
Thurrock	6
Greater London	5
Barking and Dagenham	6
Barnet	6
Bexley	6
Brent	6
Bromley	6
Camden	6
Croydon	6
Ealing	6
Enfield	6
Greenwich	6
Hackney	6
Hammersmith and Fulham	6
Haringey	6
Harrow	6
Havering	6
Hillingdon	6
Hounslow	6
Islington	6
Kensington and Chelsea	6
Kingston upon Thames	6
Lambeth	6
Lewisham	6
Merton	6
Newham	6
Redbridge	6
Richmond upon Thames	6
Southwark	6
Sutton	6
Tower Hamlets	6
Waltham Forest	6
Wandsworth	6
Westminster	6
North East England	5
County Durham	6
Darlington	6
Gateshead	6
Hartlepool	6

Appendix Table 2: GBD 2016 location hierarchy with levels	
**Location**	**Level**
Middlesbrough	6
Newcastle upon Tyne	6
North Tyneside	6
Northumberland	6
Redcar and Cleveland	6
South Tyneside	6
Stockton-on-Tees	6
Sunderland	6
North West England	5
Blackburn with Darwen	6
Blackpool	6
Bolton	6
Bury	6
Cheshire East	6
Cheshire West and Chester	6
Cumbria	6
Halton	6
Knowsley	6
Lancashire	6
Liverpool	6
Manchester	6
Oldham	6
Rochdale	6
Salford	6
Sefton	6
St Helens	6
Stockport	6
Tameside	6
Trafford	6
Warrington	6
Wigan	6
Wirral	6
South East England	5
Bracknell Forest	6
Brighton and Hove	6
Buckinghamshire	6
East Sussex	6
Hampshire	6
Isle of Wight	6
Kent	6
Medway	6
Milton Keynes	6
Oxfordshire	6
Portsmouth	6
Reading	6
Slough	6

Appendix Table 2: GBD 2016 location hierarchy with levels

Location	Level
Southampton	6
Surrey	6
West Berkshire	6
West Sussex	6
Windsor and Maidenhead	6
Wokingham	6
South West England	5
Bath and North East Somerset	6
Bournemouth	6
Bristol, City of	6
Cornwall	6
Devon	6
Dorset	6
Gloucestershire	6
North Somerset	6
Plymouth	6
Poole	6
Somerset	6
South Gloucestershire	6
Swindon	6
Torbay	6
Wiltshire	6
West Midlands	5
Birmingham	6
Coventry	6
Dudley	6
Herefordshire, County of	6
Sandwell	6
Shropshire	6
Solihull	6
Staffordshire	6
Stoke-on-Trent	6
Telford and Wrekin	6
Walsall	6
Warwickshire	6
Wolverhampton	6
Worcestershire	6
Yorkshire and the Humber	5
Barnsley	6
Bradford	6
Calderdale	6
Doncaster	6
East Riding of Yorkshire	6
Kingston upon Hull, City of	6
Kirklees	6
Leeds	6

Appendix Table 2: GBD 2016 location hierarchy with levels

Location	Level
North East Lincolnshire	6
North Lincolnshire	6
North Yorkshire	6
Rotherham	6
Sheffield	6
Wakefield	6
York	6
Northern Ireland	4
Scotland	4
Wales	4
Southern Latin America	2
Argentina	3
Chile	3
Uruguay	3
High-income North America	2
Canada	3
Greenland	3
USA	3
Alabama	4
Alaska	4
Arizona	4
Arkansas	4
California	4
Colorado	4
Connecticut	4
Delaware	4
District of Columbia	4
Florida	4
Georgia	3
Georgia	4
Hawaii	4
Idaho	4
Illinois	4
Indiana	4
Iowa	4
Kansas	4
Kentucky	4
Louisiana	4
Maine	4
Maryland	4
Massachusetts	4
Michigan	4
Minnesota	4
Mississippi	4
Missouri	4
Montana	4

Appendix Table 2: GBD 2016 location hierarchy with levels	
**Location**	**Level**
Nebraska	4
Nevada	4
New Hampshire	4
New Jersey	4
New Mexico	4
New York	4
North Carolina	4
North Dakota	4
Ohio	4
Oklahoma	4
Oregon	4
Pennsylvania	4
Rhode Island	4
South Carolina	4
South Dakota	4
Tennessee	4
Texas	4
Utah	4
Vermont	4
Virginia	4
Washington	4
West Virginia	4
Wisconsin	4
Wyoming	4
Latin America and Caribbean	1
Caribbean	2
Antigua and Barbuda	3
The Bahamas	3
Barbados	3
Belize	3
Bermuda	3
Cuba	3
Dominica	3
Dominican Republic	3
Grenada	3
Guyana	3
Haiti	3
Jamaica	3
Puerto Rico	3
Saint Lucia	3
Saint Vincent and the Grenadines	3
Suriname	3
Trinidad and Tobago	3
Virgin Islands, U.S.	3
Andean Latin America	2
Bolivia	3

Appendix Table 2: GBD 2016 location hierarchy with levels

Location	Level
Ecuador	3
Peru	3
Central Latin America	2
Colombia	3
Costa Rica	3
El Salvador	3
Guatemala	3
Honduras	3
Mexico	3
Aguascalientes	4
Baja California	4
Baja California Sur	4
Campeche	4
Chiapas	4
Chihuahua	4
Coahuila	4
Colima	4
Distrito Federal	4
Distrito Federal	4
Durango	4
Guanajuato	4
Guerrero	4
Hidalgo	4
Jalisco	4
México	4
Michoacán de Ocampo	4
Morelos	4
Nayarit	4
Nuevo León	4
Oaxaca	4
Puebla	4
Querétaro	4
Quintana Roo	4
San Luis Potosí	4
Sinaloa	4
Sonora	4
Tabasco	4
Tamaulipas	4
Tlaxcala	4
Veracruz de Ignacio de la Llave	4
Yucatán	4
Zacatecas	4
Nicaragua	3
Panama	3
Venezuela	3
Tropical Latin America	2

Appendix Table 2: GBD 2016 location hierarchy with levels

Location	Level
Brazil	3
Acre	4
Alagoas	4
Amapá	4
Amazonas	4
Bahia	4
Ceará	4
Distrito Federal	4
Distrito Federal	4
Espírito Santo	4
Goiás	4
Maranhão	4
Mato Grosso	4
Mato Grosso do Sul	4
Minas Gerais	4
Pará	4
Paraíba	4
Paraná	4
Pernambuco	4
Piaui	4
Rio de Janeiro	4
Rio Grande do Norte	4
Rio Grande do Sul	4
Rondônia	4
Roraima	4
Santa Catarina	4
São Paulo	4
Sergipe	4
Tocantins	4
Paraguay	3
North Africa and Middle East	1
North Africa and Middle East	2
Afghanistan	3
Algeria	3
Bahrain	3
Egypt	3
Iran	3
Iraq	3
Jordan	3
Kuwait	3
Lebanon	3
Libya	3
Morocco	3
Palestine	3
Oman	3
Qatar	3

Appendix Table 2: GBD 2016 location hierarchy with levels	
**Location**	**Level**
Saudi Arabia	3
'Asir	4
Bahah	4
Eastern Province	4
Ha'il	4
Jawf	4
Jizan	4
Madinah	4
Makkah	4
Najran	4
Northern Borders	4
Qassim	4
Riyadh	4
Tabuk	4
Sudan	3
Syria	3
Tunisia	3
Turkey	3
United Arab Emirates	3
Yemen	3
South Asia	1
South Asia	2
Bangladesh	3
Bhutan	3
India	3
Andhra Pradesh	4
Andhra Pradesh, Rural	5
Andhra Pradesh, Urban	5
Arunāchal Pradesh	4
Arunāchal Pradesh, Rural	5
Arunāchal Pradesh, Urban	5
Assam	4
Assam, Rural	5
Assam, Urban	5
Bihār	4
Bihār, Rural	5
Bihār, Urban	5
Chhattīsgarh	4
Chhattīsgarh, Rural	5
Chhattīsgarh, Urban	5
Delhi	4
Delhi, Rural	5
Delhi, Urban	5
Goa	4
Goa, Rural	5
Goa, Urban	5

Appendix Table 2: GBD 2016 location hierarchy with levels	
**Location**	**Level**
Gujarāt	4
Gujarāt, Rural	5
Gujarāt, Urban	5
Haryāna	4
Haryāna, Rural	5
Haryāna, Urban	5
Himachal Pradesh	4
Himachal Pradesh, Rural	5
Himachal Pradesh, Urban	5
Jammu and Kashmīr	4
Jammu and Kashmīr, Rural	5
Jammu and Kashmīr, Urban	5
Jharkhand	4
Jharkhand, Rural	5
Jharkhand, Urban	5
Karnātaka	4
Karnātaka, Rural	5
Karnātaka, Urban	5
Kerala	4
Kerala, Rural	5
Kerala, Urban	5
Madhya Pradesh	4
Madhya Pradesh, Rural	5
Madhya Pradesh, Urban	5
Mahārāshtra	4
Mahārāshtra, Rural	5
Mahārāshtra, Urban	5
Manipur	4
Manipur, Rural	5
Manipur, Urban	5
Meghālaya	4
Meghālaya, Rural	5
Meghālaya, Urban	5
Mizoram	4
Mizoram, Rural	5
Mizoram, Urban	5
Nāgāland	4
Nāgāland, Rural	5
Nāgāland, Urban	5
Orissa	4
Orissa, Rural	5
Orissa, Urban	5
Punjab	4
Punjab, Rural	5
Punjab, Urban	5
Rājasthān	4

Appendix Table 2: GBD 2016 location hierarchy with levels

Location	Level
Rājasthān, Rural	5
Rājasthān, Urban	5
Sikkim	4
Sikkim, Rural	5
Sikkim, Urban	5
Tamil Nādu	4
Tamil Nādu, Rural	5
Tamil Nādu, Urban	5
Telangana	4
Telangana, Rural	5
Telangana, Urban	5
Tripura	4
Tripura, Rural	5
Tripura, Urban	5
Uttar Pradesh	4
Uttar Pradesh, Rural	5
Uttar Pradesh, Urban	5
Uttarakhand	4
Uttarakhand, Rural	5
Uttarakhand, Urban	5
West Bengal	4
West Bengal, Rural	5
West Bengal, Urban	5
The Six Minor Territories	4
The Six Minor Territories, Rural	5
The Six Minor Territories, Urban	5
Nepal	3
Pakistan	3
Sub-Saharan Africa	1
Central Sub-Saharan Africa	2
Angola	3
the Central African Republic	3
Congo (Brazzaville)	3
Democratic Republic of the Congo	3
Equatorial Guinea	3
Gabon	3
Eastern Sub-Saharan Africa	2
Burundi	3
Comoros	3
Djibouti	3
Eritrea	3
Ethiopia	3
Kenya	3
Baringo	4
Bomet	4
Bungoma	4

Appendix Table 2: GBD 2016 location hierarchy with levels	
**Location**	**Level**
Busia	4
Elgeyo-Marakwet	4
Embu	4
Garissa	4
HomaBay	4
Isiolo	4
Kajiado	4
Kakamega	4
Kericho	4
Kiambu	4
Kilifi	4
Kirinyaga	4
Kisii	4
Kisumu	4
Kitui	4
Kwale	4
Laikipia	4
Lamu	4
Machakos	4
Makueni	4
Mandera	4
Marsabit	4
Meru	4
Migori	4
Mombasa	4
Murang’a	4
Nairobi	4
Nakuru	4
Nandi	4
Narok	4
Nyamira	4
Nyandarua	4
Nyeri	4
Samburu	4
Siaya	4
TaitaTaveta	4
TanaRiver	4
TharakaNithi	4
TransNzoia	4
Turkana	4
UasinGishu	4
Vihiga	4
Wajir	4
WestPokot	4
Madagascar	3
Malawi	3

Appendix Table 2: GBD 2016 location hierarchy with levels

Location	Level
Mozambique	3
Rwanda	3
Somalia	3
South Sudan	3
Tanzania	3
Uganda	3
Zambia	3
Southern Sub-Saharan Africa	2
Botswana	3
Lesotho	3
Namibia	3
South Africa	3
Eastern Cape	4
Free State	4
Gauteng	4
KwaZulu-Natal	4
Limpopo	4
Mpumalanga	4
North-West	4
Northern Cape	4
Western Cape	4
Swaziland	3
Zimbabwe	3
Western Sub-Saharan Africa	2
Benin	3
Burkina Faso	3
Cameroon	3
Cape Verde	3
Chad	3
Cote d'Ivoire	3
The Gambia	3
Ghana	3
Guinea	3
Guinea-Bissau	3
Liberia	3
Mali	3
Mauritania	3
Niger	3
Nigeria	3
Sao Tome and Principe	3
Senegal	3
Sierra Leone	3
Togo	3

Appendix Table 3: GBD 2016 cause and sequela hierarchy
**Causes and sequelae**
All causes
Communicable, maternal, neonatal, and nutritional diseases
HIV/AIDS and tuberculosis
Tuberculosis
Drug-susceptible tuberculosis
Multidrug-resistant tuberculosis without extensive drug resistance
Extensively drug-resistant tuberculosis
Latent tuberculosis infection
HIV/AIDS
Drug-susceptible HIV/AIDS - Tuberculosis
Drug-susceptible HIV/AIDS - Tuberculosis with mild anemia
Drug-susceptible HIV/AIDS - Tuberculosis with moderate anemia
Drug-susceptible HIV/AIDS - Tuberculosis with severe anemia
Drug-susceptible HIV/AIDS - Tuberculosis without anemia
Multidrug-resistant HIV/AIDS - Tuberculosis without extensive drug resistance
Multidrug-resistant HIV/AIDS - Tuberculosis without extensive drug resistance with mild anemia
Multidrug-resistant HIV/AIDS - Tuberculosis without extensive drug resistance with moderate anemia
Multidrug-resistant HIV/AIDS - Tuberculosis without extensive drug resistance with severe anemia
Multidrug-resistant HIV/AIDS - Tuberculosis without extensive drug resistance without anemia
Extensively drug-resistant HIV/AIDS - Tuberculosis
Extensively drug-resistant HIV/AIDS - Tuberculosis with mild anemia
Extensively drug-resistant HIV/AIDS - Tuberculosis with moderate anemia
Extensively drug-resistant HIV/AIDS - Tuberculosis with severe anemia
Extensively drug-resistant HIV/AIDS - Tuberculosis without anemia
HIV/AIDS resulting in other diseases
AIDS with antiretroviral treatment with mild anemia
AIDS with antiretroviral treatment with moderate anemia
AIDS with antiretroviral treatment with severe anemia
AIDS with antiretroviral treatment without anemia
AIDS without antiretroviral treatment with mild anemia
AIDS without antiretroviral treatment with moderate anemia
AIDS without antiretroviral treatment with severe anemia
AIDS without antiretroviral treatment without anemia
Early HIV with mild anemia
Early HIV with moderate anemia
Early HIV with severe anemia
Early HIV without anemia
Symptomatic HIV with mild anemia
Symptomatic HIV with moderate anemia
Symptomatic HIV with severe anemia
Symptomatic HIV without anemia

Diarrhea, lower respiratory, and other common infectious diseases
Diarrheal diseases
Guillain-Barré syndrome due to diarrheal diseases
Mild diarrheal diseases
Moderate diarrheal diseases
Severe diarrheal diseases
Intestinal infectious diseases
Typhoid fever
Acute typhoid infection
Gastrointestinal bleeding due to typhoid
Intestinal perforation due to typhoid
Severe typhoid fever
Paratyphoid fever
Acute paratyphoid infection
Intestinal perforation due to paratyphoid
Moderate paratyphoid fever
Severe paratyphoid fever
Other intestinal infectious diseases
Lower respiratory infections
Guillain-Barré syndrome due to lower respiratory infections
Moderate lower respiratory infections
Severe lower respiratory infections
Upper respiratory infections
Guillain-Barré syndrome due to upper respiratory infections
Mild upper respiratory infections
Moderate upper respiratory infections
Otitis media
Acute otitis media
Mild hearing loss due to chronic otitis media
Mild hearing loss with ringing due to chronic otitis media
Moderate hearing loss due to chronic otitis media
Moderate hearing loss with ringing due to chronic otitis media
Severe infectious complications due to chronic otitis media
Vertigo with mild hearing loss and ringing due to chronic otitis media
Vertigo with mild hearing loss due to chronic otitis media
Vertigo with moderate hearing loss and ringing due to chronic otitis media
Vertigo with moderate hearing loss due to chronic otitis media
Meningitis
Pneumococcal meningitis
Acute pneumococcal meningitis
Blindness due to pneumococcal meningitis
Borderline intellectual disability due to pneumococcal meningitis
Complete hearing loss due to pneumococcal meningitis

Complete hearing loss with ringing due to pneumococcal meningitis
Epilepsy due to pneumococcal meningitis
Mild behavioral problems due to pneumococcal meningitis
Mild hearing loss due to pneumococcal meningitis
Mild hearing loss with ringing due to pneumococcal meningitis
Mild intellectual disability due to pneumococcal meningitis
Mild motor impairment due to long term due to pneumococcal meningitis
Mild motor plus cognitive impairments due to pneumococcal meningitis
Moderate hearing loss due to pneumococcal meningitis
Moderate hearing loss with ringing due to pneumococcal meningitis
Moderate motor impairment due to pneumococcal meningitis
Moderate motor plus cognitive impairments due to pneumococcal meningitis
Moderate vision impairment due to pneumococcol meningitis
Moderately severe hearing loss due to pneumococcal meningitis
Moderately severe hearing loss with ringing due to pneumococcal meningitis
Monocular distance vision loss due to pneumococcal meningitis
Profound hearing loss due to pneumococcal meningitis
Profound hearing loss with ringing due to pneumococcal meningitis
Severe hearing loss due to pneumococcal meningitis
Severe hearing loss with ringing due to pneumococcal meningitis
Severe motor impairment due to pneumococcal meningitis
Severe motor plus cognitive impairments due to pneumococcal meningitis
Severe vision impairment due to pneumococcol meningitis
H influenzae type B meningitis
Acute H influenzae type B meningitis
Blindness due to H influenzae type B meningitis
Borderline intellectual disability due to H influenzae type B meningitis
Complete hearing loss due to H influenzae type B meningitis
Complete hearing loss with ringing due to H influenzae type B meningitis
Epilepsy due to H influenzae type B meningitis
Mild behavioral problems due to H influenzae type B meningitis
Mild hearing loss due to H influenzae type B meningitis
Mild hearing loss with ringing due to H influenzae type B meningitis
Mild intellectual disability due to H influenzae type B meningitis
Mild motor impairment due to long term due to H influenzae type B meningitis
Mild motor plus cognitive impairments due to H influenzae type B meningitis
Moderate hearing loss due to H influenzae type B meningitis
Moderate hearing loss with ringing due to H influenzae type B meningitis
Moderate motor impairment due to H influenzae type B meningitis
Moderate motor plus cognitive impairments due to H influenzae type B meningitis
Moderate vision impairment due to H influenza type B meningitis
Moderately severe hearing loss due to H influenzae type B meningitis
Moderately severe hearing loss with ringing due to H influenzae type B meningitis

Monocular distance vision loss due to H influenzae type B meningitis
Profound hearing loss due to H influenzae type B meningitis
Profound hearing loss with ringing due to H influenzae type B meningitis
Severe hearing loss due to H influenzae type B meningitis
Severe hearing loss with ringing due to H influenzae type B meningitis
Severe motor impairment due to H influenzae type B meningitis
Severe motor plus cognitive impairments due to H influenzae type B meningitis
Severe vision impairment due to H influenza type B meningitis
Meningococcal meningitis
Acute meningococcal meningitis
Blindness due to meningococcal meningitis
Borderline intellectual disability due to meningococcal meningitis
Complete hearing loss due to meningococcal meningitis
Complete hearing loss with ringing due to meningococcal meningitis
Epilepsy due to meningococcal meningitis
Mild behavioral problems due to meningococcal meningitis
Mild hearing loss due to meningococcal meningitis
Mild hearing loss with ringing due to meningococcal meningitis
Mild intellectual disability due to meningococcal meningitis
Mild motor impairment due to long term due to meningococcal meningitis
Mild motor plus cognitive impairments due to meningococcal meningitis
Moderate hearing loss due to meningococcal meningitis
Moderate hearing loss with ringing due to meningococcal meningitis
Moderate motor impairment due to meningococcal meningitis
Moderate motor plus cognitive impairments due to meningococcal meningitis
Moderate vision impairment due to meningococcal meningitis
Moderately severe hearing loss due to meningococcal meningitis
Moderately severe hearing loss with ringing due to meningococcal meningitis
Monocular distance vision loss due to meningococcal meningitis
Profound hearing loss due to meningococcal meningitis
Profound hearing loss with ringing due to meningococcal meningitis
Severe hearing loss due to meningococcal meningitis
Severe hearing loss with ringing due to meningococcal meningitis
Severe motor impairment due to meningococcal meningitis
Severe motor plus cognitive impairments due to meningococcal meningitis
Severe vision impairment due to meningococcal meningitis
Other meningitis
Acute viral meningitis
Blindness due to other bacterial meningitis
Borderline intellectual disability due to other bacterial meningitis
Complete hearing loss due to other bacterial meningitis
Complete hearing loss with ringing due to other bacterial meningitis
Epilepsy due to other meningitis

Mild behavioral problems due to other bacterial meningitis
Mild hearing loss due to other bacterial meningitis
Mild hearing loss due with ringing to other bacterial meningitis
Mild intellectual disability due to other bacterial meningitis
Mild motor impairment due to long term due to other bacterial meningitis
Mild motor plus cognitive impairments due to other bacterial meningitis
Moderate hearing loss due to other bacterial meningitis
Moderate hearing loss with ringing due to other bacterial meningitis
Moderate motor impairment due to other bacterial meningitis
Moderate motor plus cognitive impairments due to other bacterial meningitis
Moderate vision impairment due to other bacterial meningitis
Moderately severe hearing loss due to other bacterial meningitis
Moderately severe hearing loss with ringing due to other bacterial meningitis
Monocular distance vision loss due to other bacterial meningitis
Other acute bacterial meningitis
Profound hearing loss due to other bacterial meningitis
Profound hearing loss with ringing due to other bacterial meningitis
Severe hearing loss due to other bacterial meningitis
Severe hearing loss with ringing due to other bacterial meningitis
Severe motor impairment due to other bacterial meningitis
Severe motor plus cognitive impairments due to other bacterial meningitis
Severe vision impairment due to other bacterial meningitis
Encephalitis
Acute encephalitis
Blindness due to encephalitis
Borderline intellectual disability due to encephalitis
Epilepsy due to encephalitis
Mild behavioral problems due to encephalitis
Mild intellectual disability due to encephalitis
Mild motor impairment due to long term due to encephalitis
Mild motor plus cognitive impairments due to encephalitis
Moderate motor impairment due to encephalitis
Moderate motor plus cognitive impairments due to encephalitis
Moderate vision impairment due to encephalitis
Monocular distance vision loss due to encephalitis
Severe motor impairment due to encephalitis
Severe motor plus cognitive impairments due to encephalitis
Severe vision impairment due to encephalitis
Diphtheria
Moderate diphtheria
Severe diphtheria
Whooping cough
Tetanus

Mild motor impairment due to neonatal tetanus
Mild motor plus cognitive impairments due to neonatal tetanus
Moderate motor impairment due to neonatal tetanus
Moderate motor impairment with blindness and epilepsy due to neonatal tetanus
Moderate motor impairment with blindness due to neonatal tetanus
Moderate motor impairment with epilepsy due to neonatal tetanus
Moderate motor plus cognitive impairment with blindness and epilepsy due to neonatal tetanus
Moderate motor plus cognitive impairment with blindness due to neonatal tetanus
Moderate motor plus cognitive impairment with epilepsy due to neonatal tetanus
Severe motor impairment due to neonatal tetanus
Severe motor impairment with blindness and epilepsy due to neonatal tetanus
Severe motor impairment with blindness due to neonatal tetanus
Severe motor impairment with epilepsy due to neonatal tetanus
Severe motor plus cognitive impairment with blindness and epilepsy due to neonatal tetanus
Severe motor plus cognitive impairment with blindness due to neonatal tetanus
Severe motor plus cognitive impairment with epilepsy due to neonatal tetanus
Severe tetanus
Measles
Moderate measles
Severe measles
Varicella and herpes zoster
Chickenpox
Herpes zoster
Neglected tropical diseases and malaria
Malaria
Asymptomatic malaria parasitemia (PfPR)
Mild anemia due to malaria parasitemia (PfPR)
Mild malaria
Mild malaria with mild anemia
Mild malaria with moderate anemia
Mild malaria with severe anemia
Moderate anemia due to malaria parasitemia (PfPR)
Moderate malaria
Moderate malaria with mild anemia
Moderate malaria with moderate anemia
Moderate malaria with severe anemia
Moderate motor impairment due to malaria
Moderate motor impairment with blindness and epilepsy due to malaria
Moderate motor impairment with blindness due to malaria
Moderate motor impairment with epilepsy due to malaria
Moderate motor plus cognitive impairment with blindness and epilepsy due to malaria
Moderate motor plus cognitive impairment with blindness due to malaria
Moderate motor plus cognitive impairment with epilepsy due to malaria

Severe anemia due to malaria parasitemia (PfPR)
Severe malaria
Severe malaria with mild anemia
Severe malaria with moderate anemia
Severe malaria with severe anemia
Severe motor impairment due to malaria
Severe motor impairment with blindness and epilepsy due to malaria
Severe motor impairment with blindness due to malaria
Severe motor impairment with epilepsy due to malaria
Severe motor plus cognitive impairment with blindness and epilepsy due to malaria
Severe motor plus cognitive impairment with blindness due to malaria
Severe motor plus cognitive impairment with epilepsy due to malaria
Chagas disease
Acute Chagas disease
Asymptomatic Chagas disease
Asymptomatic and mild heart failure due to Chagas disease
Atrial fibrillation and flutter due to Chagas disease
Mild chronic digestive disease due to Chagas disease
Moderate chronic digestive disease due to Chagas disease
Moderate heart failure due to Chagas disease
Severe heart failure due to Chagas disease
Leishmaniasis
Visceral leishmaniasis
Moderate visceral leishmaniasis
Severe visceral leishmaniasis
Cutaneous and mucocutaneous leishmaniasis
African trypanosomiasis
Skin disfigurement due to Trypanosoma brucei gambiense
Skin disfigurement due to Trypanosoma brucei rhodesiense
Sleeping sickness due to Trypanosoma brucei gambiense
Sleeping sickness due to Trypanosoma brucei rhodesiense
Schistosomiasis
Ascites due to schistosomiasis
Bladder pathology due to schistosomiasis
Dysuria due to schistosomiasis
Hematemesis due to schistosomiasis
Hepatomegaly due to schistosomiasis
Hydronephrosis due to schistosomiasis
Mild anemia due to schistosomiasis
Mild diarrhea due to schistosomiasis
Mild schistosomiasis
Moderate anemia due to schistosomiasis
Severe anemia due to schistosomiasis

Cysticercosis
Neurocysticercosis with epilepsy
Cystic echinococcosis
Abdominal problems due to cystic echinococcosis
Chronic respiratory disease due to cystic echinococcosis
Epilepsy due to echinococcosis
Lymphatic filariasis
Acute adenolymphangitis due to lymphatic filariasis
Hydrocele due to lymphatic filariasis
Lymphedema due to lymphatic filariasis
Prevalence of detectable microfiliaria due to lymphatic filariasis
Onchocerciasis
Asymptomatic onchocerciasis
Blindness due to onchocerciasis
Mild skin disease due to onchocerciasis
Mild skin disease without itch due to onchocerciasis
Moderate skin disease due to onchocerciasis
Moderate vision impairment due to onchocerciasis
Severe skin disease due to onchocerciasis
Severe skin disease without itch due to onchocerciasis
Severe vision impairment due to onchocerciasis
Trachoma
Blindness due to trachoma
Moderate vision impairment due to trachoma
Severe vision impairment due to trachoma
Dengue
Moderate dengue
Post-dengue chronic fatigue syndrome
Severe dengue
Yellow fever
Asymptomatic yellow fever
Moderate yellow fever
Severe yellow fever
Rabies
Intestinal nematode infections
Ascariasis
Asymptomatic ascariasis
Heavy infestation of ascariasis
Mild abdominopelvic problems due to ascariasis
Severe wasting due to ascariasis
Trichuriasis
Asymptomatic trichuriasis
Heavy infestation of trichuriasis

Mild abdominopelvic problems due to trichuriasis
Severe wasting due to trichuriasis
Hookworm disease
Asymptomatic hookworm disease
Heavy infestation of hookworm
Mild abdominopelvic problems due to hookworm disease
Mild anemia due to hookworm disease
Moderate anemia due to hookworm disease
Severe anemia due to hookworm disease
Severe wasting due to hookworm disease
Food-borne trematodiases
Asymptomatic clonorchiasis
Asymptomatic fascioliasis
Asymptomatic intestinal fluke infection
Asymptomatic opisthorchiasis
Asymptomatic paragonimiasis
Cerebral paragonimiasis
Heavy clonorchiasis due to food-borne trematodiases
Heavy fascioliasis due to food-borne trematodiases
Heavy intestinal fluke infection due to food-borne trematodiases
Heavy opisthorchiasis due to food-borne trematodiases
Mild paragonimiasis due to food-borne trematodiases
Moderate paragonimiasis due to food-borne trematodiases
Severe paragonimiasis due to food-borne trematodiases
Leprosy
Disfigurement level 1 due to leprosy
Disfigurement level 2 due to leprosy
Ebola
Ebola cases
Post-Ebola chronic fatigue syndrome
Zika virus
Acute Zika infection
Asymptomatic Zika infection
Congenital Zika syndrome
Guillain–Barré syndrome due to Zika infection
Guinea worm disease
Mild pain due to Guinea worm emergence
Moderate pain due to Guinea worm emergence
Moderate reduced mobility due to Guinea worm emergence
Other neglected tropical diseases
Acute infection due to other neglected tropical diseases
Mild anemia due to other neglected tropical diseases
Moderate anemia due to other neglected tropical diseases

Severe anemia due to other neglected tropical diseases
Maternal disorders
Maternal hemorrhage
Maternal hemorrhage (< 1L blood lost)
Maternal hemorrhage (> 1L blood lost)
Mild anemia due to maternal hemorrhage
Moderate anemia due to maternal hemorrhage
Severe anemia due to maternal hemorrhage
Maternal sepsis and other maternal infections
Infertility due to puerperal sepsis
Other maternal infections
Puerperal sepsis
Maternal hypertensive disorders
Eclampsia
Long term sequelae of eclampsia
Long term sequelae of severe pre-eclampsia
Other hypertensive disorders of pregnancy
Severe pre-eclampsia
Maternal obstructed labor and uterine rupture
Obstructed labor, acute event
Rectovaginal fistula
Vesicovaginal fistula
Maternal abortion, miscarriage, and ectopic pregnancy
Ectopic Pregnancy
Maternal abortive outcome
Indirect maternal deaths
Late maternal deaths
Maternal deaths aggravated by HIV/AIDS
Other maternal disorders
Neonatal disorders
Neonatal preterm birth complications
Asymptomatic neonatal preterm birth 28-<32 wks
Asymptomatic neonatal preterm birth 32-<37wks
Asymptomatic neonatla preterm birth <28wks
Asymptomatic retinopathy of prematurity
Blindness due to retinopathy of prematurity
Mild motor impairment due to neonatal preterm birth complications 28-32wks
Mild motor impairment due to neonatal preterm birth complications 32-36wks
Mild motor impairment due to neonatal preterm birth complications <28wks
Mild motor plus cognitive impairments due to neonatal preterm birth complications 28-32wks
Mild motor plus cognitive impairments due to neonatal preterm birth complications 32-36wks
Mild motor plus cognitive impairments due to neonatal preterm birth complications <28wks
Mild vision impairment due to retinopathy of prematurity

	Moderate motor impairment due to neonatal preterm birth complications 28-32wks
	Moderate motor impairment due to neonatal preterm birth complications 32-36wks
	Moderate motor impairment due to neonatal preterm birth complications <28wks
	Moderate motor impairment with blindness and epilepsy due to neonatal preterm birth complications 28-32wks
	Moderate motor impairment with blindness and epilepsy due to neonatal preterm birth complications 32-36wks
	Moderate motor impairment with blindness and epilepsy due to neonatal preterm birth complications <28wks
	Moderate motor impairment with blindness due to neonatal preterm birth complications 28-32wks
	Moderate motor impairment with blindness due to neonatal preterm birth complications 32-36wks
	Moderate motor impairment with blindness due to neonatal preterm birth complications <28wks
	Moderate motor impairment with epilepsy due to neonatal preterm birth complications 28-32wks
	Moderate motor impairment with epilepsy due to neonatal preterm birth complications 32-36wks
	Moderate motor impairment with epilepsy due to neonatal preterm birth complications <28wks
	Moderate motor plus cognitive impairment with blindness and epilepsy due to neonatal preterm birth complications 28-32wks
	Moderate motor plus cognitive impairment with blindness and epilepsy due to neonatal preterm birth complications 32-36wks
	Moderate motor plus cognitive impairment with blindness and epilepsy due to neonatal preterm birth complications <28wks
	Moderate motor plus cognitive impairment with blindness due to neonatal preterm birth complications 28-32wks
	Moderate motor plus cognitive impairment with blindness due to neonatal preterm birth complications 32-36wks
	Moderate motor plus cognitive impairment with blindness due to neonatal preterm birth complications <28wks
	Moderate motor plus cognitive impairment with epilepsy due to neonatal preterm birth complications 28-32wks
	Moderate motor plus cognitive impairment with epilepsy due to neonatal preterm birth complications 32-36wks
	Moderate motor plus cognitive impairment with epilepsy due to neonatal preterm birth complications <28wks
	Moderate vision impairment due to retinopathy of prematurity
	Severe motor impairment due to neonatal preterm birth complications 28-32wks
	Severe motor impairment due to neonatal preterm birth complications 32-36wks
	Severe motor impairment due to neonatal preterm birth complications <28wks
	Severe motor impairment with blindness and epilepsy due to neonatal preterm birth complications 28-32wks
	Severe motor impairment with blindness and epilepsy due to neonatal preterm birth complications 32-36wks
	Severe motor impairment with blindness and epilepsy due to neonatal preterm birth complications <28wks
	Severe motor impairment with blindness due to neonatal preterm birth complications 28-32wks
	Severe motor impairment with blindness due to neonatal preterm birth complications 32-36wks
	Severe motor impairment with blindness due to neonatal preterm birth complications <28wks
	Severe motor impairment with epilepsy due to neonatal preterm birth complications 28-32wks
	Severe motor impairment with epilepsy due to neonatal preterm birth complications 32-36wks
	Severe motor impairment with epilepsy due to neonatal preterm birth complications <28wks
	Severe motor plus cognitive impairment with blindness and epilepsy due to neonatal preterm birth complications 28-32wks
	Severe motor plus cognitive impairment with blindness and epilepsy due to neonatal preterm birth complications 32-36wks
	Severe motor plus cognitive impairment with blindness and epilepsy due to neonatal preterm birth complications <28wks
	Severe motor plus cognitive impairment with blindness due to neonatal preterm birth complications 28-32wks
	Severe motor plus cognitive impairment with blindness due to neonatal preterm birth complications 32-36wks
	Severe motor plus cognitive impairment with blindness due to neonatal preterm birth complications <28wks

Severe motor plus cognitive impairment with epilepsy due to neonatal preterm birth complications 28-32wks
Severe motor plus cognitive impairment with epilepsy due to neonatal preterm birth complications 32-36wks
Severe motor plus cognitive impairment with epilepsy due to neonatal preterm birth complications <28wks
Severe vision impairment due to retinopathy of prematurity
Neonatal encephalopathy due to birth asphyxia and trauma
Asymptomatic neonatal encephalopathy due to birth asphyxia and trauma
Mild motor impairment due to neonatal encephalopathy due to birth asphyxia and trauma
Mild motor plus cognitive impairments due to neonatal encephalopathy due to birth asphyxia and trauma
Moderate motor impairment due to neonatal encephalopathy due to birth asphyxia and trauma
Moderate motor impairment with blindness and epilepsy due to neonatal encephalopathy due to birth asphyxia and trauma
Moderate motor impairment with blindness due to neonatal encephalopathy due to birth asphyxia and trauma
Moderate motor impairment with epilepsy due to neonatal encephalopathy due to birth asphyxia and trauma
Moderate motor plus cognitive impairment with blindness and epilepsy due to neonatal encephalopathy due to birth asphyxia and trauma
Moderate motor plus cognitive impairment with blindness due to neonatal encephalopathy due to birth asphyxia and trauma
Moderate motor plus cognitive impairment with epilepsy due to neonatal encephalopathy due to birth asphyxia and trauma
Severe motor impairment due to neonatal encephalopathy due to birth asphyxia and trauma
Severe motor impairment with blindness and epilepsy due to neonatal encephalopathy due to birth asphyxia and trauma
Severe motor impairment with blindness due to neonatal encephalopathy due to birth asphyxia and trauma
Severe motor impairment with epilepsy due to neonatal encephalopathy due to birth asphyxia and trauma
Severe motor plus cognitive impairment with blindness and epilepsy due to neonatal encephalopathy due to birth asphyxia and trauma
Severe motor plus cognitive impairment with blindness due to neonatal encephalopathy due to birth asphyxia and trauma
Severe motor plus cognitive impairment with epilepsy due to neonatal encephalopathy due to birth asphyxia and trauma
Neonatal sepsis and other neonatal infections
Asymptomatic neonatal sepsis and other neonatal infections
Mild motor impairment due to neonatal sepsis and other neonatal infections
Mild motor plus cognitive impairments due to neonatal sepsis and other neonatal infections
Moderate motor impairment due to neonatal sepsis and other neonatal infections
Moderate motor impairment with blindness and epilepsy due to neonatal sepsis and other neonatal infections
Moderate motor impairment with blindness due to neonatal sepsis and other neonatal infections
Moderate motor impairment with epilepsy due to neonatal sepsis and other neonatal infections
Moderate motor plus cognitive impairment with blindness and epilepsy due to neonatal sepsis and other neonatal infections
Moderate motor plus cognitive impairment with blindness due to neonatal sepsis and other neonatal infections
Moderate motor plus cognitive impairment with epilepsy due to neonatal sepsis and other neonatal infections
Severe infection due to neonatal sepsis and other neonatal infections
Severe motor impairment due to neonatal sepsis and other neonatal infections
Severe motor impairment with blindness and epilepsy due to neonatal sepsis and other neonatal infections
Severe motor impairment with blindness due to neonatal sepsis and other neonatal infections
Severe motor impairment with epilepsy due to neonatal sepsis and other neonatal infections

Severe motor plus cognitive impairment with blindness and epilepsy due to neonatal sepsis and other neonatal infections
Severe motor plus cognitive impairment with blindness due to neonatal sepsis and other neonatal infections
Severe motor plus cognitive impairment with epilepsy due to neonatal sepsis and other neonatal infections
Hemolytic disease and other neonatal jaundice
Moderate motor impairment due to hemolytic disease and other neonatal jaundice
Moderate motor impairment with blindness and epilepsy due to hemolytic disease and other neonatal jaundice
Moderate motor impairment with blindness due to hemolytic disease and other neonatal jaundice
Moderate motor impairment with epilepsy due to hemolytic disease and other neonatal jaundice
Moderate motor plus cognitive impairment with blindness and epilepsy due to hemolytic disease and other neonatal jaundice
Moderate motor plus cognitive impairment with blindness due to hemolytic disease and other neonatal jaundice
Moderate motor plus cognitive impairment with epilepsy due to hemolytic disease and other neonatal jaundice
Severe motor impairment severe due to hemolytic disease and other neonatal jaundice
Severe motor impairment with blindness and epilepsy due to hemolytic disease and other neonatal jaundice
Severe motor impairment with blindness due to hemolytic disease and other neonatal jaundice
Severe motor impairment with epilepsy due to hemolytic disease and other neonatal jaundice
Severe motor plus cognitive impairment with blindness and epilepsy due to hemolytic disease and other neonatal jaundice
Severe motor plus cognitive impairment with blindness due to hemolytic disease and other neonatal jaundice
Severe motor plus cognitive impairment with epilepsy due to hemolytic disease and other neonatal jaundice
Other neonatal disorders
Nutritional deficiencies
Protein-energy malnutrition
Moderate wasting with edema
Moderate wasting without edema
Severe wasting with edema
Severe wasting without edema
Iodine deficiency
Visible goiter with profound intellectual disability and mild heart failure due to iodine deficiency
Visible goiter with profound intellectual disability and moderate heart failure due to iodine deficiency
Visible goiter with profound intellectual disability due to iodine deficiency
Visible goiter with profound intellectual disability with severe heart failure due to iodine deficiency
Visible goiter with severe intellectual disability due to iodine deficiency
Visible goiter with symptoms without intellectual disability or heart failure
Visible goiter without symptoms
Vitamin A deficiency
Asymptomatic vitamin A deficiency
Blindness due to vitamin A deficiency
Moderate vision impairment loss due to vitamin A deficiency
Severe vision impairment loss due to vitamin A deficiency
Iron-deficiency anemia
Asymptomatic and mild heart failure due to iron-deficiency anemia
Mild iron-deficiency anemia

Moderate heart failure due to iron-deficiency anemia
Moderate iron-deficiency anemia
Severe heart failure due to iron-deficiency anemia
Severe iron-deficiency anemia
Other nutritional deficiencies
Other communicable, maternal, neonatal, and nutritional diseases
Sexually transmitted diseases excluding HIV
Syphilis
Asymptomatic adult tertiary syphilis
Asymptomatic early syphilis infection
Cardiovascular complications due to adult tertiary syphilis
Mild early syphilis infection
Neurological problems and cardiovascular complications due to adult tertiary syphilis
Neurological problems due to adult tertiary syphilis
Severe disfigurement and cardiovascular complications due to adult tertiary syphilis
Severe disfigurement and neurological problems due to adult tertiary syphilis
Severe disfigurement due to adult tertiary syphilis
Severe disfigurement, neurological problems, and cardiovascular complications due to adult tertiary syphilis
Chlamydial infection
Asymptomatic chlamydial infection
Epididymo-orchitis due to chlamydial infection
Mild chlamydial infection
Moderate pelvic inflammatory diseases due to chlamydial infection
Primary infertility due to chlamydial infection
Secondary infertility due to chlamydial infection
Severe pelvic inflammatory diseases due to chlamydial infection
Gonococcal infection
Asymptomatic gonococcal infection
Epididymo-orchitis due to gonococcal infection
Mild gonococcal infection
Moderate pelvic inflammatory diseases due to gonococcal infection
Primary infertility due to gonococcal infection
Secondary infertility due to gonococcal infection
Severe pelvic inflammatory diseases due to gonococcal infection
Trichomoniasis
Acute trichomoniasis infection
Asymptomatic trichomoniasis infection
Genital herpes
Asymptomatic genital herpes
Moderate infection due to initial genital herpes episode
Symptomatic genital herpes
Other sexually transmitted diseases
Moderate pelvic inflammatory diseases due to other sexually transmitted diseases

Primary infertility due to other sexually transmitted diseases
Secondary infertility due to other sexually transmitted diseases
Severe pelvic inflammatory diseases due to other sexually transmitted diseases
Hepatitis
Acute hepatitis A
Asymptomatic acute hepatitis A
Moderate acute hepatitis A
Severe acute hepatitis A
Hepatitis B
Asymptomatic acute hepatitis B
Chronic hepatitis B
Moderate acute hepatitis B
Severe acute hepatitis B
Hepatitis C
Asymptomatic acute hepatitis C
Chronic hepatitis C
Moderate acute hepatitis C
Severe acute hepatitis C
Acute hepatitis E
Asymptomatic acute hepatitis E
Moderate acute hepatitis E
Severe acute hepatitis E
Other infectious diseases
Guillain-Barré syndrome due to other infectious diseases
Mild anemia due to other infectious diseases
Moderate anemia due to other infectious diseases
Severe anemia due to other infectious diseases
Non-communicable diseases
Neoplasms
Lip and oral cavity cancer
Controlled phase of mouth cancer
Diagnosis and primary therapy phase of mouth cancer
Metastatic phase of mouth cancer
Terminal phase of mouth cancer
Nasopharynx cancer
Controlled phase of nasopharynx cancer
Diagnosis and primary therapy phase of nasopharynx cancer
Metastatic phase of nasopharynx cancer
Terminal phase of nasopharynx cancer
Other pharynx cancer
Controlled phase of other pharynx cancer
Diagnosis and primary therapy phase of other pharynx cancer
Metastatic phase of other pharynx cancer

Terminal phase of other pharynx cancer
Esophageal cancer
Controlled phase of esophageal cancer
Diagnosis and primary therapy phase of esophageal cancer
Metastatic phase of esophageal cancer
Terminal phase of esophageal cancer
Stomach cancer
Controlled phase of stomach cancer
Diagnosis and primary therapy phase of stomach cancer
Metastatic phase of stomach cancer
Terminal phase of stomach cancer
Colon and rectum cancer
Controlled phase of colon and rectum cancers
Diagnosis and primary therapy phase of colon and rectum cancers
Metastatic phase of colon and rectum cancers
Stoma due to colon and rectum cancer
Terminal phase of colon and rectum cancers
Liver cancer
Liver cancer due to hepatitis B
Controlled phase of liver cancer due to hepatitis B
Diagnosis and primary therapy phase of liver cancer due to hepatitis B
Metastatic phase of liver cancer due to hepatitis B
Terminal phase of liver cancer due to hepatitis B
Liver cancer due to hepatitis C
Controlled phase of liver cancer due to hepatitis C
Diagnosis and primary therapy phase of liver cancer due to hepatitis C
Metastatic phase of liver cancer due to hepatitis C
Terminal phase of liver cancer due to hepatitis C
Liver cancer due to alcohol use
Controlled phase of liver cancer due to alcohol use
Diagnosis and primary therapy phase of liver cancer due to alcohol use
Metastatic phase of liver cancer due to alcohol use
Terminal phase of liver cancer due to alcohol use
Liver cancer due to other causes
Controlled phase of liver cancer due to other causes
Diagnosis and primary therapy phase of liver cancer due to other causes
Metastatic phase of liver cancer due to other causes
Terminal phase of liver cancer due to other causes
Gallbladder and biliary tract cancer
Controlled phase of gallbladder and biliary tract cancer
Diagnosis and primary therapy phase of gallbladder and biliary tract cancer
Metastatic phase of gallbladder and biliary tract cancer
Terminal phase of gallbladder and biliary tract cancer

Pancreatic cancer
Controlled phase of pancreatic cancer
Diagnosis and primary therapy phase of pancreatic cancer
Metastatic phase of pancreatic cancer
Terminal phase of pancreatic cancer
Larynx cancer
Controlled phase of larynx cancer
Diagnosis and primary therapy phase of larynx cancer
Laryngectomy due to larynx cancer
Metastatic phase of larynx cancer
Terminal phase of larynx cancer
Tracheal, bronchus, and lung cancer
Controlled phase of lung, bronchus, and trachea cancer
Diagnosis and primary therapy phase of lung, bronchus, and trachea cancer
Metastatic phase of lung, bronchus, and trachea cancer
Terminal phase of lung, bronchus, and trachea cancer
Malignant skin melanoma
Controlled phase of malignant skin melanoma
Diagnosis and primary therapy phase of malignant skin melanoma
Metastatic phase of malignant skin melanoma
Terminal phase of malignant skin melanoma
Non-melanoma skin cancer
Non-melanoma skin cancer (squamous-cell carcinoma)
Mild disfigurement due to squamous cell carcinoma
Moderate disfigurement due to squamous cell carcinoma
Severe disfigurement due to squamous cell carcinoma
Non-melanoma skin cancer (basal-cell carcinoma)
Disfigurement due to basal cell carcinoma
Breast cancer
Controlled phase of breast cancer
Diagnosis and primary therapy phase of breast cancer
Mastectomy due to breast cancer
Metastatic phase of breast cancer
Terminal phase of breast cancer
Cervical cancer
Controlled phase of cervical cancer
Diagnosis and primary therapy phase of cervical cancer
Metastatic phase of cervical cancer
Terminal phase of cervical cancer
Uterine cancer
Controlled phase of uterine cancer
Diagnosis and primary therapy phase of uterine cancer
Metastatic phase of uterine cancer

Terminal phase of uterine cancer
Ovarian cancer
Controlled phase of ovarian cancer
Diagnosis and primary therapy phase of ovarian cancer
Metastatic phase of ovarian cancer
Terminal phase of ovarian cancer
Prostate cancer
Controlled phase of prostate cancer
Diagnosis and primary therapy phase of prostate cancer
Impotence due to prostate cancer
Incontinence due to prostate cancer
Metastatic phase of prostate cancer
Terminal phase of prostate cancer
Testicular cancer
Controlled phase of testicular cancer
Diagnosis and primary therapy phase of testicular cancer
Metastatic phase of testicular cancer
Terminal phase of testicular cancer
Kidney cancer
Controlled phase of kidney cancer
Diagnosis and primary therapy phase of kidney cancer
Metastatic phase of kidney cancer
Terminal phase of kidney cancer
Bladder cancer
Controlled phase of bladder cancer
Diagnosis and primary therapy phase of bladder cancer
Metastatic phase of bladder cancer
Terminal phase of bladder cancer
Urinary incontinence due to bladder cancer
Brain and nervous system cancer
Controlled phase of brain and nervous system cancers
Diagnosis and primary therapy phase of brain and nervous system cancers
Metastatic phase of brain and nervous system cancers
Terminal phase of brain and nervous system cancers
Thyroid cancer
Controlled phase of thyroid cancer
Diagnosis and primary therapy phase of thyroid cancer
Metastatic phase of thyroid cancer
Terminal phase of thyroid cancer
Mesothelioma
Controlled phase of mesothelioma
Diagnosis and primary therapy phase of mesothelioma
Metastatic phase of mesothelioma

Terminal phase of mesothelioma
Hodgkin lymphoma
Controlled phase of Hodgkin disease
Diagnosis and primary therapy phase of Hodgkin disease
Metastatic phase of Hodgkin disease
Terminal phase of Hodgkin disease
Non-Hodgkin lymphoma
Controlled phase of non-Hodgkin lymphoma
Diagnosis and primary therapy phase of non-Hodgkin lymphoma
Metastatic phase of non-Hodgkin lymphoma
Terminal phase of non-Hodgkin lymphoma
Multiple myeloma
Controlled phase of multiple myeloma
Diagnosis and primary therapy phase of multiple myeloma
Metastatic phase of multiple myeloma
Terminal phase of multiple myeloma
Leukemia
Acute lymphoid leukemia
Controlled phase of acute lymphoid leukemia
Diagnosis and primary therapy phase of acute lymphoid leukemia
Metastatic phase of acute lymphoid leukemia
Terminal phase of acute lymphoid leukemia
Chronic lymphoid leukemia
Controlled phase of chronic lymphoid leukemia
Diagnosis and primary therapy phase of chronic lymphoid leukemia
Metastatic phase of chronic lymphoid leukemia
Terminal phase of chronic lymphoid leukemia
Acute myeloid leukemia
Controlled phase of acute myeloid leukemia
Diagnosis and primary therapy phase of acute myeloid leukemia
Metastatic phase of acute myeloid leukemia
Terminal phase of acute myeloid leukemia
Chronic myeloid leukemia
Controlled phase of chronic myeloid leukemia
Diagnosis and primary therapy phase of chronic myeloid leukemia
Metastatic phase of chronic myeloid leukemia
Terminal phase of chronic myeloid leukemia
Other leukemia
Controlled phase of other leukemia
Diagnosis and primary therapy phase of other leukemia
Metastatic phase of other leukemia
Terminal phase of other leukemia
Other neoplasms

Controlled phase of other neoplasms
Diagnosis and primary therapy phase of other neoplasms
Metastatic phase of other neoplasms
Terminal phase of other neoplasms
Cardiovascular diseases
Rheumatic heart disease
Asymptomatic and mild heart failure due to rheumatic heart disease
Moderate heart failure due to rheumatic heart disease
Rheumatic heart disease, without heart failure
Severe heart failure due to rheumatic heart disease
Ischemic heart disease
Acute myocardial infarction 3 to 28 days
Acute myocardial infarction first 2 days
Asymptomatic and mild heart failure due to ischemic heart disease
Asymptomatic angina due to ischemic heart disease
Asymptomatic ischemic heart disease following myocardial infarction
Mild angina due to ischemic heart disease
Moderate angina due to ischemic heart disease
Moderate heart failure due to ischemic heart disease
Severe angina due to ischemic heart disease
Severe heart failure due to ischemic heart disease
Cerebrovascular disease
Ischemic stroke
Acute ischemic stroke severity level 1
Acute ischemic stroke severity level 2
Acute ischemic stroke severity level 3
Acute ischemic stroke severity level 4
Acute ischemic stroke severity level 5
Asymptomatic chronic ischemic stroke
Chronic ischemic stroke severity level 1
Chronic ischemic stroke severity level 2
Chronic ischemic stroke severity level 3
Chronic ischemic stroke severity level 4
Chronic ischemic stroke severity level 5
Hemorrhagic stroke
Acute hemorrhagic stroke severity level 1
Acute hemorrhagic stroke severity level 2
Acute hemorrhagic stroke severity level 3
Acute hemorrhagic stroke severity level 4
Acute hemorrhagic stroke severity level 5
Asymptomatic chronic hemorrhagic stroke
Chronic hemorrhagic stroke severity level 1
Chronic hemorrhagic stroke severity level 2

Chronic hemorrhagic stroke severity level 3
Chronic hemorrhagic stroke severity level 4
Chronic hemorrhagic stroke severity level 5
Hypertensive heart disease
Asymptomatic and mild heart failure due to hypertensive heart disease
Moderate heart failure due to hypertensive heart disease
Severe heart failure due to hypertensive heart disease
Cardiomyopathy and myocarditis
Myocarditis
Acute myocarditis
Asymptomatic and mild heart failure due to myocarditis
Moderate heart failure due to myocarditis
Severe heart failure due to myocarditis
Alcoholic cardiomyopathy
Asymptomatic and mild heart failure due to alcoholic cardiomyopathy
Moderate heart failure due to alcoholic cardiomyopathy
Severe heart failure due to alcoholic cardiomyopathy
Other cardiomyopathy
Asymptomatic and mild heart failure due to other cardiomyopathy
Moderate heart failure due to other cardiomyopathy
Severe heart failure due to other cardiomyopathy
Atrial fibrillation and flutter
Asymptomatic atrial fibrillation and flutter
Symptomatic atrial fibrillation and flutter
Aortic aneurysm
Peripheral artery disease
Asymptomatic peripheral vascular disease
Symptomatic claudication due to peripheral vascular disease
Endocarditis
Asymptomatic and mild heart failure due to endocarditis
Moderate endocarditis
Moderate heart failure due to endocarditis
Severe endocarditis
Severe heart failure due to endocarditis
Other cardiovascular and circulatory diseases
Asymptomatic and mild heart failure due to other cardiovascular diseases
Asymptomatic other cardiovascular diseases
Mild other cardiovascular diseases
Moderate heart failure due to other cardiovascular diseases
Moderate other cardiovascular diseases
Severe heart failure due to other cardiovascular diseases
Severe other cardiovascular diseases
Chronic respiratory diseases

Chronic obstructive pulmonary disease
Asymptomatic and mild heart failure due to severe chronic obstructive pulmonary disease
Asymptomatic chronic obstructive pulmonary disease
Mild chronic obstructive pulmonary disease
Moderate chronic obstructive pulmonary disease
Moderate heart failure due to severe chronic obstructive pulmonary disease
Severe chronic obstructive pulmonary disease without heart failure
Severe heart failure due to severe chronic obstructive pulmonary disease
Pneumoconiosis
Silicosis
Asymptomatic and mild heart failure due to severe silicosis
Asymptomatic silicosis
Mild silicosis
Moderate heart failure due to severe silicosis
Moderate silicosis
Severe heart failure due to severe silicosis
Severe silicosis without heart failure
Asbestosis
Asymptomatic and mild heart failure due to severe asbestosis
Asymptomatic asbestosis
Mild asbestosis
Moderate asbestosis
Moderate heart failure due to severe asbestosis
Severe asbestosis without heart failure
Severe heart failure due to severe asbestosis
Coal workers pneumoconiosis
Asymptomatic and mild heart failure due to severe coal workers pneumoconiosis
Asymptomatic coal workers pneumoconiosis
Mild coal workers pneumoconiosis
Moderate coal workers pneumoconiosis
Moderate heart failure due to severe coal workers pneumoconiosis
Severe coal workers pneumoconiosis without heart failure
Severe heart failure due to severe coal workers pneumoconiosis
Other pneumoconiosis
Asymptomatic and mild heart failure due to severe other pneumoconiosis
Asymptomatic other pneumoconiosis
Mild other pneumoconiosis
Moderate heart failure due to severe other pneumoconiosis
Moderate other pneumoconiosis
Severe heart failure due to severe other pneumoconiosis
Severe other pneumoconiosis without heart failure
Asthma
Asymptomatic asthma

Controlled asthma
Partially controlled asthma
Uncontrolled asthma
Interstitial lung disease and pulmonary sarcoidosis
Asymptomatic and mild heart failure due to severe interstitial lung disease and pulmonary sarcoidosis
Asymptomatic interstitial lung disease and pulmonary sarcoidosis
Mild interstitial lung disease and pulmonary sarcoidosis
Moderate heart failure due to severe interstitial lung disease and pulmonary sarcoidosis
Moderate interstitial lung disease and pulmonary sarcoidosis
Severe heart failure due to severe interstitial lung disease and pulmonary sarcoidosis
Severe interstitial lung disease and pulmonary sarcoidosis without heart failure
Other chronic respiratory diseases
Cirrhosis and other chronic liver diseases
Cirrhosis and other chronic liver diseases due to hepatitis B
Cirrhosis and other chronic liver diseases due to hepatitis B, compensated
Cirrhosis and other chronic liver diseases due to hepatitis B, decompensated
Cirrhosis and other chronic liver diseases due to hepatitis C
Cirrhosis and other chronic liver diseases due to hepatitis C, compensated
Cirrhosis and other chronic liver diseases due to hepatitis C, decompensated
Cirrhosis and other chronic liver diseases due to alcohol use
Cirrhosis and other chronic liver diseases due to alcohol, compensated
Cirrhosis and other chronic liver diseases due to alcohol, decompensated
Cirrhosis and other chronic liver diseases due to other causes
Cirrhosis and other chronic liver diseases due to other cause, compensated
Cirrhosis and other chronic liver diseases due to other cause, decompensated
Digestive diseases
Peptic ulcer disease
Asymptomatic peptic ulcer disease with mild anemia
Asymptomatic peptic ulcer disease with moderate anemia
Asymptomatic peptic ulcer disease with severe anemia
Asymptomatic peptic ulcer disease without anemia
Mild anemia due to peptic ulcer disease
Mild symptomatic peptic ulcer disease with mild anemia
Mild symptomatic peptic ulcer disease with moderate anemia
Mild symptomatic peptic ulcer disease with severe anemia
Mild symptomatic peptic ulcer disease without anemia
Moderate anemia due to peptic ulcer disease
Moderate symptomatic peptic ulcer disease with mild anemia
Moderate symptomatic peptic ulcer disease with moderate anemia
Moderate symptomatic peptic ulcer disease with severe anemia
Moderate symptomatic peptic ulcer disease without anemia
Severe anemia due to peptic ulcer disease
Gastritis and duodenitis

Asymptomatic gastritis and duodenitis with mild anemia
Asymptomatic gastritis and duodenitis with moderate anemia
Asymptomatic gastritis and duodenitis with severe anemia
Asymptomatic gastritis and duodenitis without anemia
Mild anemia due to gastritis and duodenitis
Mild symptomatic gastritis and duodenitis with mild anemia
Mild symptomatic gastritis and duodenitis with moderate anemia
Mild symptomatic gastritis and duodenitis with severe anemia
Mild symptomatic gastritis and duodenitis without anemia
Moderate anemia due to gastritis and duodenitis
Moderate symptomatic gastritis and duodenitis with mild anemia
Moderate symptomatic gastritis and duodenitis with moderate anemia
Moderate symptomatic gastritis and duodenitis with severe anemia
Moderate symptomatic gastritis and duodenitis without anemia
Severe anemia due to gastritis and duodenitis
Appendicitis
Paralytic ileus and intestinal obstruction
Inguinal, femoral, and abdominal hernia
Asymptomatic inguinal, femoral, and abdominal hernia
Mild symptomatic inguinal, femoral and abdominal hernia
Moderate symptomatic inguinal, femoral and abdominal hernia
Severe symptomatic inguinal, femoral and abdominal hernia
Inflammatory bowel disease
Asymptomatic Crohn's disease
Asymptomatic ulcerative colitis
Crohn's disease
Ulcerative colitis
Vascular intestinal disorders
Gallbladder and biliary diseases
Asymptomatic gallbladder and biliary diseases
Mild symptomatic episodes gallbladder and biliary diseases
Moderate symptomatic episodes gallbladder and biliary diseases
Severe symptomatic episodes gallbladder and biliary diseases
Pancreatitis
Mild pancreatitis episodes
Moderate pancreatitis episodes
Severe pancreatitis episodes
Other digestive diseases
Neurological disorders
Alzheimer disease and other dementias
Mild Alzheimer disease and other dementias
Moderate Alzheimer disease and other dementias
Severe Alzheimer disease and other dementias

Parkinson disease
Mild Parkinson disease
Moderate Parkinson disease
Severe Parkinson disease
Epilepsy
Less severe epilepsy
Seizure-free, treated epilepsy
Severe epilepsy
Multiple sclerosis
Asymptomatic multiple sclerosis
Mild multiple sclerosis
Moderate multiple sclerosis
Severe multiple sclerosis
Motor neuron disease
Diagnosis of motor neuron disease
Mild motor impairment and mild respiratory problems due to motor neuron disease
Mild motor impairment and severe respiratory problems due to motor neuron disease
Mild motor impairment and speech problems due to motor neuron disease
Mild motor impairment due to motor neuron disease
Mild motor impairment, mild respiratory problems, and speech problems due to motor neuron disease
Mild motor impairment, moderate respiratory problems, and speech problems due to motor neuron disease
Mild motor impairment, severe respiratory problems and speech problems due to motor neuron disease
Mild respiratory problems and speech problems due to motor neuron disease
Mild respiratory problems due to motor neuron disease
Moderate motor impairment and mild respiratory problems due to motor neuron disease
Moderate motor impairment and moderate respiratory problems due to motor neuron disease
Moderate motor impairment and severe respiratory problems due to motor neuron disease
Moderate motor impairment and speech problems due to motor neuron disease
Moderate motor impairment due to motor neuron disease
Moderate motor impairment, mild respiratory problems, and speech problems due to motor neuron disease
Moderate motor impairment, moderate respiratory problems, and speech problems due to motor neuron disease
Moderate motor impairment, severe respiratory problems, and speech problems due to motor neuron disease
Moderate respiratory problems and speech problems due to motor neuron disease
Moderate respiratory problems due to motor neuron disease
Severe motor impairment and mild respiratory problems due to motor neuron disease
Severe motor impairment and moderate respiratory problems due to motor neuron disease
Severe motor impairment and severe respiratory problems due to motor neuron disease
Severe motor impairment and speech problems due to motor neuron disease
Severe motor impairment due to motor neuron disease
Severe motor impairment, mild respiratory problems, and speech problems due to motor neuron disease
Severe motor impairment, moderate respiratory problems, and speech problems due to motor neuron disease
Severe motor impairment, severe respiratory problems, and speech problems due to motor neuron disease
Severe respiratory problems and speech problems due to motor neuron disease

Severe respiratory problems due to motor neuron disease
Speech problems due to motor neuron disease
Migraine
Asymptomatic medication overuse headache due to migraine
Asymptomatic migraine
Symptomatic medication overuse headache due to migraine
Symptomatic migraine
Tension-type headache
Asymptomatic medication overuse headache due to tension-type headache
Asymptomatic tension-type headache
Symptomatic medication overuse headache due to tension-type headache
Symptomatic tension-type headache
Other neurological disorders
Guillain-Barré syndrome due to other neurological disorders
Mental and substance use disorders
Schizophrenia
Schizophrenia acute state
Schizophrenia residual state
Alcohol use disorders
Asymptomatic alcohol dependence
Asymptomatic fetal alcohol syndrome
Mild alcohol dependence
Mild fetal alcohol syndrome
Moderate alcohol dependence
Moderate fetal alcohol syndrome
Severe alcohol dependence
Severe fetal alcohol syndrome
Very mild alcohol dependence
Drug use disorders
Opioid use disorders
Asymptomatic opioid dependence
Mild opioid dependence
Severe opioid dependence
Cocaine use disorders
Asymptomatic cocaine dependence
Mild cocaine dependence
Severe cocaine dependence
Amphetamine use disorders
Asymptomatic amphetamine dependence
Mild amphetamine dependence
Severe amphetamine dependence
Cannabis use disorders
Asymptomatic cannabis dependence

Mild cannabis dependence
Severe cannabis dependence
Other drug use disorders
Depressive disorders
Major depressive disorder
Major depressive disorder, currently without symptoms
Mild major depressive disorder
Moderate major depressive disorder
Severe major depressive disorder
Dysthymia
Dysthymia, currently without symptoms
Symptomatic dysthymia
Bipolar disorder
Bipolar disorder depressive state
Bipolar disorder manic state
Bipolar disorder residual state
Anxiety disorders
Anxiety disorders, currently without symptoms
Mild anxiety disorders
Moderate anxiety disorders
Severe anxiety disorders
Eating disorders
Anorexia nervosa
Bulimia nervosa
Autistic spectrum disorders
Autism
Autism with borderline intellectual disability
Autism with mild intellectual disability
Autism with moderate intellectual disability
Autism with profound intellectual disability
Autism with severe intellectual disability
Autism without intellectual disability
Asperger syndrome and other autistic spectrum disorders
Attention-deficit/hyperactivity disorder
Attention-deficit/hyperactivity disorder, currently without symptoms
Symptomatic attention-deficit/hyperactivity disorder
Conduct disorder
Conduct disorder, currently without symptoms
Symptomatic conduct disorder
Idiopathic developmental intellectual disability
Borderline idiopathic developmental intellectual disability
Mild idiopathic developmental intellectual disability
Moderate idiopathic developmental intellectual disability

Profound idiopathic developmental intellectual disability
Severe idiopathic developmental intellectual disability
Other mental and substance use disorders
Mild other mental disorders
Moderate other mental disorders
Other mental disorders, currently without symptoms
Severe other mental disorders
Diabetes, urogenital, blood, and endocrine diseases
Diabetes mellitus
Blindness due to diabetes mellitus
Diabetic foot due to neuropathy
Diabetic neuropathy
Diabetic neuropathy and amputation with treatment
Diabetic neuropathy and amputation without treatment
Moderate vision impairment due to diabetes mellitus
Severe vision impairment due to diabetes mellitus
Uncomplicated diabetes mellitus
Acute glomerulonephritis
Chronic kidney disease
Chronic kidney disease due to diabetes mellitus
End-stage renal disease after transplant due to diabetes mellitus
End-stage renal disease on dialysis due to diabetes mellitus
Stage III chronic kidney disease and mild anemia due to diabetes mellitus
Stage III chronic kidney disease and moderate anemia due to diabetes mellitus
Stage III chronic kidney disease and severe anemia due to diabetes mellitus
Stage III chronic kidney disease without anemia due to diabetes mellitus
Stage IV chronic kidney disease untreated and mild anemia due to diabetes mellitus
Stage IV chronic kidney disease untreated and moderate anemia due to diabetes mellitus
Stage IV chronic kidney disease untreated and severe anemia due to diabetes mellitus
Stage IV chronic kidney disease untreated without anemia due to diabetes mellitus
Stage V chronic kidney disease untreated and mild anemia due to diabetes mellitus
Stage V chronic kidney disease untreated and moderate anemia due to diabetes mellitus
Stage V chronic kidney disease untreated and severe anemia due to diabetes mellitus
Stage V chronic kidney disease untreated without anemia due to diabetes mellitus
Chronic kidney disease due to hypertension
End-stage renal disease after transplant due to hypertension
End-stage renal disease on dialysis due to hypertension
Stage III chronic kidney disease and mild anemia due to hypertension
Stage III chronic kidney disease and moderate anemia due to hypertension
Stage III chronic kidney disease and severe anemia due to hypertension
Stage III chronic kidney disease without anemia due to hypertension
Stage IV chronic kidney disease untreated and mild anemia due to hypertension
Stage IV chronic kidney disease untreated and moderate anemia due to hypertension

Stage IV chronic kidney disease untreated and severe anemia due to hypertension
Stage IV chronic kidney disease untreated without anemia due to hypertension
Stage V chronic kidney disease untreated and mild anemia due to hypertension
Stage V chronic kidney disease untreated and moderate anemia due to hypertension
Stage V chronic kidney disease untreated and severe anemia due to hypertension
Stage V chronic kidney disease untreated without anemia due to hypertension
Chronic kidney disease due to glomerulonephritis
End-stage renal disease after transplant due to glomerulonephritis
End-stage renal disease on dialysis due to glomerulonephritis
Stage III chronic kidney disease and mild anemia due to glomerulonephritis
Stage III chronic kidney disease and moderate anemia due to glomerulonephritis
Stage III chronic kidney disease and severe anemia due to glomerulonephritis
Stage III chronic kidney disease without anemia due to glomerulonephritis
Stage IV chronic kidney disease untreated and mild anemia due to glomerulonephritis
Stage IV chronic kidney disease untreated and moderate anemia due to glomerulonephritis
Stage IV chronic kidney disease untreated and severe anemia due to glomerulonephritis
Stage IV chronic kidney disease untreated without anemia due to glomerulonephritis
Stage V chronic kidney disease untreated and mild anemia due to glomerulonephritis
Stage V chronic kidney disease untreated and moderate anemia due to glomerulonephritis
Stage V chronic kidney disease untreated and severe anemia due to glomerulonephritis
Stage V chronic kidney disease untreated without anemia due to glomerulonephritis
Chronic kidney disease due to other causes
End-stage renal disease after transplant due to other causes
End-stage renal disease on dialysis due to other causes
Stage III chronic kidney disease and mild anemia due to other causes
Stage III chronic kidney disease and moderate anemia due to other causes
Stage III chronic kidney disease and severe anemia due to other causes
Stage III chronic kidney disease without anemia due to other causes
Stage IV chronic kidney disease untreated and mild anemia due to other causes
Stage IV chronic kidney disease untreated and moderate anemia due to other causes
Stage IV chronic kidney disease untreated and severe anemia due to other causes
Stage IV chronic kidney disease untreated without anemia due to other causes
Stage V chronic kidney disease untreated and mild anemia due to other causes
Stage V chronic kidney disease untreated and moderate anemia due to other causes
Stage V chronic kidney disease untreated and severe anemia due to other causes
Stage V chronic kidney disease untreated without anemia due to other causes
Urinary diseases and male infertility
Interstitial nephritis and urinary tract infections
Mild interstitial nephritis and urinary tract infections
Moderate interstitial nephritis and urinary tract infections
Urolithiasis
Mild urolithiasis episodes
Moderate urolithiasis episodes

Severe urolithiasis episodes
Benign prostatic hyperplasia
Asymptomatic benign prostatic hyperplasia
Symptomatic benign prostatic hyperplasia
Male infertility
Primary male infertility
Secondary male infertility
Other urinary diseases
Gynecological diseases
Uterine fibroids
Asymptomatic uterine fibroids
Mild abdominal pain due to uterine fibroids, with mild anemia
Mild abdominal pain due to uterine fibroids, with moderate anemia
Mild abdominal pain due to uterine fibroids, with severe anemia
Mild abdominal pain due to uterine fibroids, without anemia
Polycystic ovarian syndrome
Asymptomatic polycystic ovarian syndrome
Hirsutism and primary infertility due to polycystic ovarian syndrome
Hirsutism and secondary infertility due to polycystic ovarian syndrome
Hirsutism due to polycystic ovarian syndrome
Primary infertility due to polycystic ovarian syndrome
Secondary infertility due to polycystic ovarian syndrome
Female infertility
Idiopathic primary female infertility
Idiopathic secondary female infertility
Endometriosis
Asymptomatic endometriosis
Mild abdominal pain and primary infertility due to endometriosis
Mild abdominal pain and secondary infertility due to endometriosis
Mild abdominal pain due to endometriosis
Moderate abdominal pain and primary infertility due to endometriosis
Moderate abdominal pain and secondary infertility due to endometriosis
Moderate abdominal pain due to endometriosis
Primary infertility due to endometriosis
Secondary infertility due to endometriosis
Severe abdominal pain and primary infertility due to endometriosis
Severe abdominal pain and secondary infertility due to endometriosis
Severe endometriosis
Genital prolapse
Abdominal pain and stress incontinence due to genital prolapse
Abdominal pain due to genital prolapse
Asymptomatic genital prolapse
Stress incontinence due to genital prolapse

Premenstrual syndrome
Abdominal pain and depression due to premenstrual syndrome
Abdominal pain due to premenstrual syndrome
Asymptomatic premenstrual syndrome
Depression due to premenstrual syndrome
Other gynecological diseases
Asymptomatic other gynecological disorders
Mild anemia due to other gynecological diseases
Mild other gynecological disorders
Moderate anemia due to other gynecological diseases
Moderate other gynecological disorders
Severe anemia due to other gynecological diseases
Severe other gynecological disorders
Hemoglobinopathies and hemolytic anemias
Thalassemias
Asymptomatic and mild heart failure due to thalassemias
Beta-thalassemia major, severe infection with severe anemia
Beta-thalassemia major, with mild anemia
Beta-thalassemia major, with moderate anemia
Beta-thalassemia major, with severe anemia
Beta-thalassemia major, without anemia
Hemoglobin E/Beta-thalassemia, without anemia
Hemoglobin E/beta-thalassemia, severe infection with severe anemia
Hemoglobin E/beta-thalassemia, with mild anemia
Hemoglobin E/beta-thalassemia, with moderate anemia
Hemoglobin E/beta-thalassemia, with severe anemia
Hemoglobin H disease, severe infection with severe anemia
Hemoglobin H disease, with mild anemia
Hemoglobin H disease, with moderate anemia
Hemoglobin H disease, with severe anemia
Hemoglobin H disease, without anemia
Moderate heart failure due to thalassemias
Severe heart failure due to thalassemias
Thalassemias trait
Asymptomatic B-thalassemia trait
Asymptomatic hemoglobin E trait
Mild anemia due to B-thalassemia trait
Mild anemia due to hemoglobin E trait
Moderate anemia due to B-thalassemia trait
Moderate anemia due to hemoglobin E trait
Severe anemia due to B-thalassemia trait
Severe anemia due to hemoglobin E trait
Sickle cell disorders

Hemoglobin SC disease, with mild anemia
Hemoglobin SC disease, with moderate anemia
Hemoglobin SC disease, with severe anemia
Hemoglobin SC disease, with stroke and severe anemia
Hemoglobin SC disease, with stroke, without anemia
Hemoglobin SC disease, with vaso-occlusive crisis and severe anemia
Hemoglobin SC disease, with vaso-occlusive crisis and stroke, without anemia
Hemoglobin SC disease, with vaso-occlusive crisis, stroke, and severe anemia
Hemoglobin SC disease, with vaso-occlusive crisis, without anemia
Hemoglobin SC disease, without anemia
Homozygous sickle cell and severe sickle cell/beta-thalassemia, with mild anemia
Homozygous sickle cell and severe sickle cell/beta-thalassemia, with moderate anemia
Homozygous sickle cell and severe sickle cell/beta-thalassemia, with severe anemia
Homozygous sickle cell and severe sickle cell/beta-thalassemia, with stroke and severe anemia
Homozygous sickle cell and severe sickle cell/beta-thalassemia, with stroke, without anemia
Homozygous sickle cell and severe sickle cell/beta-thalassemia, with vaso-occlusive crisis and severe anemia
Homozygous sickle cell and severe sickle cell/beta-thalassemia, with vaso-occlusive crisis and stroke, without anemia
Homozygous sickle cell and severe sickle cell/beta-thalassemia, with vaso-occlusive crisis, stroke, and severe anemia
Homozygous sickle cell and severe sickle cell/beta-thalassemia, with vaso-occlusive crisis, without anemia
Homozygous sickle cell and severe sickle cell/beta-thalassemia, without anemia
Mild sickle cell/beta-thalassemia, with mild anemia
Mild sickle cell/beta-thalassemia, with moderate anemia
Mild sickle cell/beta-thalassemia, with severe anemia
Mild sickle cell/beta-thalassemia, with stroke and severe anemia
Mild sickle cell/beta-thalassemia, with stroke, without anemia
Mild sickle cell/beta-thalassemia, with vaso-occlusive crisis and severe anemia
Mild sickle cell/beta-thalassemia, with vaso-occlusive crisis and stroke, without anemia
Mild sickle cell/beta-thalassemia, with vaso-occlusive crisis, stroke, and severe anemia
Mild sickle cell/beta-thalassemia, with vaso-occlusive crisis, without anemia
Mild sickle cell/beta-thalassemia, without anemia
Sickle cell trait
Asymptomatic sickle cell trait
Mild anemia due to sickle cell trait
Moderate anemia due to sickle cell trait
Severe anemia due to sickle cell trait
G6PD deficiency
Asymptomatic G6PD deficiency
Asymptomatic and mild heart failure due to G6PD deficiency
Mild anemia due to G6PD deficiency
Moderate anemia due to G6PD deficiency
Moderate heart failure due to G6PD deficiency
Severe anemia due to G6PD deficiency

Severe heart failure due to G6PD deficiency
G6PD trait
Asymptomatic hemizygous G6PD deficiency
Mild anemia due to hemizygous G6PD deficiency
Moderate anemia due to hemizygous G6PD deficiency
Severe anemia due to hemizygous G6PD deficiency
Other hemoglobinopathies and hemolytic anemias
Asymptomatic and mild heart failure due to other hemoglobinopathies and hemolytic anemias
Mild anemia due to other hemoglobinopathies and hemolytic anemias
Moderate anemia due to other hemoglobinopathies and hemolytic anemias
Moderate heart failure due to other hemoglobinopathies and hemolytic anemias
Severe anemia due to other hemoglobinopathies and hemolytic anemias
Severe heart failure due to other hemoglobinopathies and hemolytic anemias
Endocrine, metabolic, blood, and immune disorders
Asymptomatic and mild heart failure due to endocrine, metabolic, blood, and immune disorders
Asymptomatic endocrine, metabolic, blood, and immune disorders
Mild anemia due to endocrine, metabolic, blood, and immune disorders
Mild endocrine, metabolic, blood, and immune disorders
Moderate anemia due to endocrine, metabolic, blood, and immune disorders
Moderate endocrine, metabolic, blood, and immune disorders
Moderate heart failure due to endocrine, metabolic, blood, and immune disorders
Severe anemia due to endocrine, metabolic, blood, and immune disorders
Severe endocrine, metabolic, blood, and immune disorders
Severe heart failure due to endocrine, metabolic, blood, and immune disorders
Musculoskeletal disorders
Rheumatoid arthritis
Mild rheumatoid arthritis
Moderate rheumatoid arthritis
Severe rheumatoid arthritis
Osteoarthritis
Mild osteoarthritis of the hip
Mild osteoarthritis of the knee
Moderate osteoarthritis of the hip
Moderate osteoarthritis of the knee
Severe osteoarthritis of the hip
Severe osteoarthritis of the knee
Low back and neck pain
Low back pain
Mild low back pain with leg pain
Mild low back pain without leg pain
Moderate low back pain with leg pain
Moderate low back pain without leg pain
Most severe low back pain with leg pain

Most severe low back pain without leg pain
Severe low back pain with leg pain
Severe low back pain without leg pain
Neck pain
Mild neck pain
Moderate neck pain
Most severe neck pain
Severe neck pain
Gout
Asymptomatic gout
Polyarticular gout
Symptomatic episodes of gout
Other musculoskeletal disorders
Asymptomatic other musculoskeletal disorders
Other musculoskeletal disorders severity level 1
Other musculoskeletal disorders severity level 2
Other musculoskeletal disorders severity level 3
Other musculoskeletal disorders severity level 4
Other musculoskeletal disorders severity level 5
Other musculoskeletal disorders severity level 6
Other non-communicable diseases
Congenital birth defects
Neural tube defects
Asymptomatic encephalocele following treatment
Borderline intellectual disability and incontinence due to encephalocele
Borderline intellectual disability due to encephalocele
Incontinence due to encephalocele
Mild intellectual disability and incontinence due to encephalocele
Mild intellectual disability due to encephalocele
Mild motor impairment and borderline intellectual disability due to encephalocele
Mild motor impairment and borderline intellectual disability due to spina bifida
Mild motor impairment and incontinence due to encephalocele
Mild motor impairment and incontinence due to spina bifida
Mild motor impairment and mild intellectual disability due to encephalocele
Mild motor impairment and mild intellectual disability due to spina bifida
Mild motor impairment and moderate intellectual disability due to encephalocele
Mild motor impairment and moderate intellectual disability due to spina bifida
Mild motor impairment and profound intellectual disability due to encephalocele
Mild motor impairment and profound intellectual disability due to spina bifida
Mild motor impairment and severe intellectual disability due to encephalocele
Mild motor impairment and severe intellectual disability due to spina bifida
Mild motor impairment due to encephalocele
Mild motor impairment due to spina bifida

	Mild motor impairment, borderline intellectual disability and incontinence due to encephalocele
	Mild motor impairment, borderline intellectual disability and incontinence due to spina bifida
	Mild motor impairment, mild intellectual disability and incontinence due to encephalocele
	Mild motor impairment, mild intellectual disability and incontinence due to spina bifida
	Mild motor impairment, moderate intellectual disability and incontinence due to encephalocele
	Mild motor impairment, moderate intellectual disability and incontinence due to spina bifida
	Mild motor impairment, profound intellectual disability and incontinence due to encephalocele
	Mild motor impairment, profound intellectual disability and incontinence due to spina bifida
	Mild motor impairment, severe intellectual disability and incontinence due to encephalocele
	Mild motor impairment, severe intellectual disability and incontinence due to spina bifida
	Moderate intellectual disability and incontinence due to encephalocele
	Moderate intellectual disability due to encephalocele
	Moderate motor impairment and borderline intellectual disability due to encephalocele
	Moderate motor impairment and borderline intellectual disability due to spina bifida
	Moderate motor impairment and incontinence due to encephalocele
	Moderate motor impairment and incontinence due to spina bifida
	Moderate motor impairment and mild intellectual disability due to encephalocele
	Moderate motor impairment and mild intellectual disability due to spina bifida
	Moderate motor impairment and moderate intellectual disability due to encephalocele
	Moderate motor impairment and moderate intellectual disability due to spina bifida
	Moderate motor impairment and profound intellectual disability due to encephalocele
	Moderate motor impairment and profound intellectual disability due to spina bifida
	Moderate motor impairment and severe intellectual disability due to encephalocele
	Moderate motor impairment and severe intellectual disability due to spina bifida
	Moderate motor impairment due to encephalocele
	Moderate motor impairment due to spina bifida
	Moderate motor impairment, borderline intellectual disability and incontinence due to encephalocele
	Moderate motor impairment, borderline intellectual disability and incontinence due to spina bifida
	Moderate motor impairment, mild intellectual disability and incontinence due to encephalocele
	Moderate motor impairment, mild intellectual disability and incontinence due to spina bifida
	Moderate motor impairment, moderate intellectual disability and incontinence due to encephalocele
	Moderate motor impairment, moderate intellectual disability and incontinence due to spina bifida
	Moderate motor impairment, profound intellectual disability and incontinence due to encephalocele
	Moderate motor impairment, profound intellectual disability and incontinence due to spina bifida
	Moderate motor impairment, severe intellectual disability and incontinence due to encephalocele
	Moderate motor impairment, severe intellectual disability and incontinence due to spina bifida
	Profound intellectual disability and incontinence due to encephalocele
	Profound intellectual disability due to encephalocele
	Severe intellectual disability and incontinence due to encephalocele
	Severe intellectual disability due to encephalocele
	Severe motor and cognitive impairment due to anencephaly
	Severe motor impairment and borderline intellectual disability due to encephalocele
	Severe motor impairment and borderline intellectual disability due to spina bifida

Severe motor impairment and incontinence due to encephalocele
Severe motor impairment and incontinence due to spina bifida
Severe motor impairment and mild intellectual disability due to encephalocele
Severe motor impairment and mild intellectual disability due to spina bifida
Severe motor impairment and moderate intellectual disability due to encephalocele
Severe motor impairment and moderate intellectual disability due to spina bifida
Severe motor impairment and profound intellectual disability due to encephalocele
Severe motor impairment and profound intellectual disability due to spina bifida
Severe motor impairment and severe intellectual disability due to encephalocele
Severe motor impairment and severe intellectual disability due to spina bifida
Severe motor impairment due to encephalocele
Severe motor impairment due to spina bifida
Severe motor impairment, borderline intellectual disability and incontinence due to encephalocele
Severe motor impairment, borderline intellectual disability and incontinence due to spina bifida
Severe motor impairment, mild intellectual disability and incontinence due to encephalocele
Severe motor impairment, mild intellectual disability and incontinence due to spina bifida
Severe motor impairment, moderate intellectual disability and incontinence due to encephalocele
Severe motor impairment, moderate intellectual disability and incontinence due to spina bifida
Severe motor impairment, profound intellectual disability and incontinence due to encephalocele
Severe motor impairment, severe intellectual disability and incontinence due to encephalocele
Severe motor impairment, severe intellectual disability and incontinence due to spina bifida
profound motor impairment, profound intellectual disability and incontinence due to spina bifida
Congenital heart anomalies
Asymptomatic ventricular septal defect and atrial septal defect
Congenital heart disease and borderline intellectual disability without heart failure due to critical malformations of great vessels, congenital valvular heart disease and patent ductus arteriosus
Congenital heart disease and borderline intellectual disability without heart failure due to other congenital cardiovascular anomalies
Congenital heart disease and borderline intellectual disability without heart failure due to severe congenital heart anomalies excluding single ventricle heart defects
Congenital heart disease and borderline intellectual disability without heart failure due to single ventricle and single ventricle pathway heart defects
Congenital heart disease and borderline intellectual disability without heart failure due to ventricular septal defect and atrial septal defect
Congenital heart disease and mild heart failure without intellectual disability due to critical malformations of great vessels, congenital valvular heart disease and patent ductus arteriosus
Congenital heart disease and mild heart failure without intellectual disability due to severe congenital heart anomalies excluding single ventricle heart defects
Congenital heart disease and mild heart failure without intellectual disability due to single ventricle and single ventricle pathway heart defects
Congenital heart disease and mild heart failure without intellectual disability due to ventricular septal defect and atrial septal defect
Congenital heart disease and mild intellectual disability without heart failure due to severe congenital heart anomalies excluding single ventricle heart defects
Congenital heart disease and mild intellectual disability without heart failure due to single ventricle and single ventricle pathway heart defects
Congenital heart disease and mild intellectual disability without heart failure due to critical malformations of great vessels, congenital valvular heart disease and patent ductus arteriosus
Congenital heart disease and mild intellectual disability without heart failure due to other congenital cardiovascular anomalies

Congenital heart disease and mild intellectual disability without heart failure due to ventricular septal defect and atrial septal defect
Congenital heart disease and moderate heart failure without intellectual disability due to critical malformations of great vessels, congenital valvular heart disease and patent ductus arteriosus
Congenital heart disease and moderate heart failure without intellectual disability due to severe congenital heart anomalies excluding single ventricle heart defects
Congenital heart disease and moderate heart failure without intellectual disability due to single ventricle and single ventricle pathway heart defects
Congenital heart disease and moderate heart failure without intellectual disability due to ventricular septal defect and atrial septal defect
Congenital heart disease and moderate intellectual disability without heart failure due to severe congenital heart anomalies excluding single ventricle heart defects
Congenital heart disease and moderate intellectual disability without heart failure due to single ventricle and single ventricle pathway heart defects
Congenital heart disease and moderate intellectual disability without heart failure due to critical malformations of great vessels, congenital valvular heart disease and patent ductus arteriosus
Congenital heart disease and moderate intellectual disability without heart failure due to other congenital cardiovascular anomalies
Congenital heart disease and moderate intellectual disability without heart failure due to ventricular septal defect and atrial septal defect
Congenital heart disease and profound intellectual disability without heart failure due to critical malformations of great vessels, congenital valvular heart disease and patent ductus arteriosus
Congenital heart disease and profound intellectual disability without heart failure due to other congenital cardiovascular anomalies
Congenital heart disease and profound intellectual disability without heart failure due to severe congenital heart anomalies excluding single ventricle heart defects
Congenital heart disease and profound intellectual disability without heart failure due to single ventricle and single ventricle pathway heart defects
Congenital heart disease and profound intellectual disability without heart failure due to ventricular septal defect and atrial septal defect
Congenital heart disease and severe heart failure without intellectual disability due to critical malformations of great vessels, congenital valvular heart disease and patent ductus arteriosus
Congenital heart disease and severe heart failure without intellectual disability due to severe congenital heart anomalies excluding single ventricle heart defects
Congenital heart disease and severe heart failure without intellectual disability due to single ventricle and single ventricle pathway heart defects
Congenital heart disease and severe heart without intellectual disability due to ventricular septal defect and atrial septal defect
Congenital heart disease and severe intellectual disability without heart failure due to severe congenital heart anomalies excluding single ventricle heart defects
Congenital heart disease and severe intellectual disability without heart failure due to critical malformations of great vessels, congenital valvular heart disease and patent ductus arteriosus
Congenital heart disease and severe intellectual disability without heart failure due to other congenital cardiovascular anomalies
Congenital heart disease and severe intellectual disability without heart failure due to single ventricle and single ventricle pathway heart defects
Congenital heart disease and severe intellectual disability without heart failure due to ventricular septal defect and atrial septal defect
Congenital heart disease without heart failure or intellectual disability due to critical malformations of great vessels, congenital valvular heart disease and patent ductus arteriosus
Congenital heart disease without heart failure or intellectual disability due to ventricular septal defect and atrial septal defect
Congenital heart disease without intellectual disability or heart failure due to other congenital cardiovascular anomalies
Congenital heart disease without intellectual disability or heart failure due to severe congenital heart anomalies excluding single ventricle heart defects
Congenital heart disease without intellectual disability or heart failure due to single ventricle and single ventricle pathway heart defects
Congenital heart disease, boderline intellectual disability and mild heart failure due to critical malformations of great vessels, congenital valvular heart disease and patent ductus arteriosus

Congenital heart disease, boderline intellectual disability and mild heart failure due to severe congenital heart anomalies excluding single ventricle heart defects
Congenital heart disease, boderline intellectual disability and mild heart failure due to single ventricle and single ventricle pathway heart defects
Congenital heart disease, boderline intellectual disability and mild heart failure due to ventricular septal defect and atrial septal defect
Congenital heart disease, borderline intellectual disability and moderate heart failure due to critical malformations of great vessels, congenital valvular heart disease and patent ductus arteriosus
Congenital heart disease, borderline intellectual disability and moderate heart failure due to severe congenital heart anomalies excluding single ventricle heart defects
Congenital heart disease, borderline intellectual disability and moderate heart failure due to single ventricle and single ventricle pathway heart defects
Congenital heart disease, borderline intellectual disability and moderate heart failure due to ventricular septal defect and atrial septal defect
Congenital heart disease, borderline intellectual disability and severe heart failure due to critical malformations of great vessels, congenital valvular heart disease and patent ductus arteriosus
Congenital heart disease, borderline intellectual disability and severe heart failure due to severe congenital heart anomalies excluding single ventricle heart defects
Congenital heart disease, borderline intellectual disability and severe heart failure due to single ventricle and single ventricle pathway heart defects
Congenital heart disease, borderline intellectual disability and severe heart failure due to ventricular septal defect and atrial septal defect
Congenital heart disease, mild intellectual disability and mild heart failure due to critical malformations of great vessels, congenital valvular heart disease and patent ductus arteriosus
Congenital heart disease, mild intellectual disability and mild heart failure due to severe congenital heart anomalies excluding single ventricle heart defects
Congenital heart disease, mild intellectual disability and mild heart failure due to single ventricle and single ventricle pathway heart defects
Congenital heart disease, mild intellectual disability and mild heart failure due to ventricular septal defect and atrial septal defect
Congenital heart disease, mild intellectual disability and moderate heart failure due to critical malformations of great vessels, congenital valvular heart disease and patent ductus arteriosus
Congenital heart disease, mild intellectual disability and moderate heart failure due to severe congenital heart anomalies excluding single ventricle heart defects
Congenital heart disease, mild intellectual disability and moderate heart failure due to single ventricle and single ventricle pathway heart defects
Congenital heart disease, mild intellectual disability and moderate heart failure due to ventricular septal defect and atrial septal defect
Congenital heart disease, mild intellectual disability and severe heart failure due to critical malformations of great vessels, congenital valvular heart disease and patent ductus arteriosus
Congenital heart disease, mild intellectual disability and severe heart failure due to severe congenital heart anomalies excluding single ventricle heart defects
Congenital heart disease, mild intellectual disability and severe heart failure due to single ventricle and single ventricle pathway heart defects
Congenital heart disease, mild intellectual disability and severe heart failure due to ventricular septal defect and atrial septal defect
Congenital heart disease, moderate intellectual disability and mild heart failure due to critical malformations of great vessels, congenital valvular heart disease and patent ductus arteriosus
Congenital heart disease, moderate intellectual disability and mild heart failure due to severe congenital heart anomalies excluding single ventricle heart defects
Congenital heart disease, moderate intellectual disability and mild heart failure due to single ventricle and single ventricle pathway heart defects
Congenital heart disease, moderate intellectual disability and mild heart failure due to ventricular septal defect and atrial septal defect
Congenital heart disease, moderate intellectual disability and moderate heart failure due to critical malformations of great vessels, congenital valvular heart disease and patent ductus arteriosus
Congenital heart disease, moderate intellectual disability and moderate heart failure due to severe congenital heart anomalies excluding single ventricle heart defects
Congenital heart disease, moderate intellectual disability and moderate heart failure due to single ventricle and single ventricle pathway heart defects

Congenital heart disease, moderate intellectual disability and moderate heart failure due to ventricular septal defect and atrial septal defect
Congenital heart disease, moderate intellectual disability and severe heart failure due to critical malformations of great vessels, congenital valvular heart disease and patent ductus arteriosus
Congenital heart disease, moderate intellectual disability and severe heart failure due to severe congenital heart anomalies excluding single ventricle heart defects
Congenital heart disease, moderate intellectual disability and severe heart failure due to single ventricle and single ventricle pathway heart defects
Congenital heart disease, moderate intellectual disability and severe heart failure due to ventricular septal defect and atrial septal defect
Congenital heart disease, profound intellectual disability and mild heart failure due to critical malformations of great vessels, congenital valvular heart disease and patent ductus arteriosus
Congenital heart disease, profound intellectual disability and mild heart failure due to severe congenital heart anomalies excluding single ventricle heart defects
Congenital heart disease, profound intellectual disability and mild heart failure due to single ventricle and single ventricle pathway heart defects
Congenital heart disease, profound intellectual disability and mild heart failure due to ventricular septal defect and atrial septal defect
Congenital heart disease, profound intellectual disability and moderate heart failure due to critical malformations of great vessels, congenital valvular heart disease and patent ductus arteriosus
Congenital heart disease, profound intellectual disability and moderate heart failure due to severe congenital heart anomalies excluding single ventricle heart defects
Congenital heart disease, profound intellectual disability and moderate heart failure due to single ventricle and single ventricle pathway heart defects
Congenital heart disease, profound intellectual disability and moderate heart failure due to ventricular septal defect and atrial septal defect
Congenital heart disease, profound intellectual disability and severe heart failure due to critical malformations of great vessels, congenital valvular heart disease and patent ductus arteriosus
Congenital heart disease, profound intellectual disability and severe heart failure due to severe congenital heart anomalies excluding single ventricle heart defects
Congenital heart disease, profound intellectual disability and severe heart failure due to single ventricle and single ventricle pathway heart defects
Congenital heart disease, profound intellectual disability and severe heart failure due to ventricular septal defect and atrial septal defect
Congenital heart disease, severe intellectual disability and mild heart failure due to critical malformations of great vessels, congenital valvular heart disease and patent ductus arteriosus
Congenital heart disease, severe intellectual disability and mild heart failure due to severe congenital heart anomalies excluding single ventricle heart defects
Congenital heart disease, severe intellectual disability and mild heart failure due to single ventricle and single ventricle pathway heart defects
Congenital heart disease, severe intellectual disability and mild heart failure due to ventricular septal defect and atrial septal defect
Congenital heart disease, severe intellectual disability and moderate heart failure due to critical malformations of great vessels, congenital valvular heart disease and patent ductus arteriosus
Congenital heart disease, severe intellectual disability and moderate heart failure due to severe congenital heart anomalies excluding single ventricle heart defects
Congenital heart disease, severe intellectual disability and moderate heart failure due to single ventricle and single ventricle pathway heart defects
Congenital heart disease, severe intellectual disability and moderate heart failure due to ventricular septal defect and atrial septal defect
Congenital heart disease, severe intellectual disability and severe heart failure due to critical malformations of great vessels, congenital valvular heart disease and patent ductus arteriosus
Congenital heart disease, severe intellectual disability and severe heart failure due to severe congenital heart anomalies excluding single ventricle heart defects
Congenital heart disease, severe intellectual disability and severe heart failure due to single ventricle and single ventricle pathway heart defects
Congenital heart disease, severe intellectual disability and severe heart failure due to ventricular septal defect and atrial septal defect
Orofacial clefts

Asymptomatic orofacial clefts
Disfigurement level 1 due to orofacial clefts
Disfigurement level 2 and speech problems due to orofacial clefts
Disfigurement level 2 due to orofacial clefts
Down syndrome
Asymptomatic Down syndrome
Borderline intellectual disability and mild dementia due to Down syndrome
Borderline intellectual disability and moderate dementia due to Down syndrome
Borderline intellectual disability and severe dementia due to Down syndrome
Borderline intellectual disability due to Down syndrome
Borderline intellectual disability with congenital heart disease due to Down syndrome
Borderline intellectual disability, mild dementia, and congenital heart disease due to Down syndrome
Borderline intellectual disability, moderate dementia, and congenital heart disease due to Down syndrome
Borderline intellectual disability, severe dementia, and congenital heart disease due to Down syndrome
Congenital heart disease and mild dementia due to Down syndrome
Congenital heart disease and moderate dementia due to Down syndrome
Congenital heart disease and severe dementia due to Down syndrome
Isolated congenital heart disease due to Down syndrome
Mild dementia due to Down syndrome
Mild intellectual disability and mild dementia due to Down syndrome
Mild intellectual disability and moderate dementia due to Down syndrome
Mild intellectual disability and severe dementia due to Down syndrome
Mild intellectual disability due to Down syndrome
Mild intellectual disability with congenital heart disease due to Down syndrome
Mild intellectual disability, mild dementia, and congenital heart disease due to Down syndrome
Mild intellectual disability, moderate dementia, and congenital heart disease due to Down syndrome
Mild intellectual disability, severe dementia, and congenital heart disease due to Down syndrome
Moderate dementia due to Down syndrome
Moderate intellectual disability and mild dementia due to Down syndrome
Moderate intellectual disability and moderate dementia due to Down syndrome
Moderate intellectual disability and severe dementia due to Down syndrome
Moderate intellectual disability due to Down syndrome
Moderate intellectual disability with congenital heart disease due to Down syndrome
Moderate intellectual disability, mild dementia, and congenital heart disease due to Down syndrome
Moderate intellectual disability, moderate dementia, and congenital heart disease due to Down syndrome
Moderate intellectual disability, severe dementia, and congenital heart disease due to Down syndrome
Profound intellectual disability and mild dementia due to Down syndrome
Profound intellectual disability and moderate dementia due to Down syndrome
Profound intellectual disability and severe dementia due to Down syndrome
Profound intellectual disability due to Down syndrome
Profound intellectual disability with congenital heart disease due to Down syndrome
Profound intellectual disability, mild dementia, and congenital heart disease due to Down syndrome
Profound intellectual disability, moderate dementia, and congenital heart disease due to Down syndrome

Profound intellectual disability, severe dementia, and congenital heart disease due to Down syndrome
Severe dementia due to Down syndrome
Severe intellectual disability and mild dementia due to Down syndrome
Severe intellectual disability and moderate dementia due to Down syndrome
Severe intellectual disability and severe dementia due to Down syndrome
Severe intellectual disability due to Down syndrome
Severe intellectual disability with congenital heart disease due to Down syndrome
Severe intellectual disability, mild dementia, and congenital heart disease due to Down syndrome
Severe intellectual disability, moderate dementia, and congenital heart disease due to Down syndrome
Severe intellectual disability, severe dementia, and congenital heart disease due to Down syndrome
Turner syndrome
Asymptomatic Turner syndrome
Congenital heart disease due to Turner syndrome
Congenital heart disease with infertility due to Turner syndrome
Primary infertility due to Turner syndrome
Klinefelter syndrome
Asymptomatic Klinefelter syndrome
Borderline intellectual disability due to Klinefelter syndrome
Borderline intellectual disability with infertility due to Klinefelter syndrome
Mild intellectual disability due to Klinefelter syndrome
Mild intellectual disability with infertility due to Klinefelter syndrome
Primary infertility due to Klinefelter syndrome
Other chromosomal abnormalities
Asymptomatic other chromosomal abnormalities
Borderline intellectual disability and mild dementia due to other chromosomal abnormalities
Borderline intellectual disability and moderate dementia due to other chromosomal abnormalities
Borderline intellectual disability and severe dementia due to other chromosomal abnormalities
Borderline intellectual disability due to other chromosomal abnormalities
Borderline intellectual disability with congenital heart disease due to other chromosomal abnormalities
Borderline intellectual disability, mild dementia, and congenital heart disease due to other chromosomal abnormalities
Borderline intellectual disability, moderate dementia, and congenital heart disease due to other chromosomal abnormalities
Borderline intellectual disability, severe dementia, and congenital heart disease due to other chromosomal abnormalities
Congenital heart disease and mild dementia due to other chromosomal abnormalities
Congenital heart disease and moderate dementia due to other chromosomal abnormalities
Congenital heart disease and severe dementia due to other chromosomal abnormalities
Isolated congenital heart disease due to other chromosomal abnormalities
Mild dementia due to other chromosomal abnormalities
Mild intellectual disability and mild dementia due to other chromosomal abnormalities
Mild intellectual disability and moderate dementia due to other chromosomal abnormalities
Mild intellectual disability and severe dementia due to other chromosomal abnormalities
Mild intellectual disability due to other chromosomal abnormalities

Mild intellectual disability with congenital heart disease due to other chromosomal abnormalities
Mild intellectual disability, mild dementia, and congenital heart disease due to other chromosomal abnormalities
Mild intellectual disability, moderate dementia, and congenital heart disease due to other chromosomal abnormalities
Mild intellectual disability, severe dementia, and congenital heart disease due to other chromosomal abnormalities
Moderate dementia due to other chromosomal abnormalities
Moderate intellectual disability and mild dementia due to other chromosomal abnormalities
Moderate intellectual disability and moderate dementia due to other chromosomal abnormalities
Moderate intellectual disability and severe dementia due to other chromosomal abnormalities
Moderate intellectual disability due to other chromosomal abnormalities
Moderate intellectual disability with congenital heart disease due to other chromosomal abnormalities
Moderate intellectual disability, mild dementia, and congenital heart disease due to other chromosomal abnormalities
Moderate intellectual disability, moderate dementia, and congenital heart disease due to other chromosomal abnormalities
Moderate intellectual disability, severe dementia, and congenital heart disease due to other chromosomal abnormalities
Profound intellectual disability and mild dementia due to other chromosomal abnormalities
Profound intellectual disability and moderate dementia due to other chromosomal abnormalities
Profound intellectual disability and severe dementia due to other chromosomal abnormalities
Profound intellectual disability due to other chromosomal abnormalities
Profound intellectual disability with congenital heart disease due to other chromosomal abnormalities
Profound intellectual disability, mild dementia, and congenital heart disease due to other chromosomal abnormalities
Profound intellectual disability, moderate dementia, and congenital heart disease due to other chromosomal abnormalities
Profound intellectual disability, severe dementia, and congenital heart disease due to other chromosomal abnormalities
Severe dementia due to other chromosomal abnormalities
Severe intellectual disability and mild dementia due to other chromosomal abnormalities
Severe intellectual disability and moderate dementia due to other chromosomal abnormalities
Severe intellectual disability and severe dementia due to other chromosomal abnormalities
Severe intellectual disability due to other chromosomal abnormalities
Severe intellectual disability with congenital heart disease due to other chromosomal abnormalities
Severe intellectual disability, mild dementia, and congenital heart disease due to other chromosomal abnormalities
Severe intellectual disability, moderate dementia, and congenital heart disease due to other chromosomal abnormalities
Severe intellectual disability, severe dementia, and congenital heart disease due to other chromosomal abnormalities
Severe motor and cognitive impairment due to Edward Syndrome or Patau Syndrome
Severe motor and cognitive impairment with congenital heart disease due to Edward Syndrome or Patau Syndrome
Congenital musculoskeletal and limb anomalies
Disfigurement level 1 due to polydactyly and syndactyly
Disfigurement level 2 and mild motor impairment due to congenital limb deficiency
Disfigurement level 2 and mild motor impairment due to other congenital musculoskeletal anomalies
Disfigurement level 2 and moderate motor impairment due to congenital limb deficiency
Disfigurement level 2 and moderate motor impairment due to other congenital musculoskeletal anomalies
Disfigurement level 2 due to congenital limb deficiency
Disfigurement level 2 due to other congenital musculoskeletal anomalies

Disfigurement level 2 with pain and mild motor impairment due to congenital limb deficiency
Disfigurement level 2 with pain and mild motor impairment due to other congenital musculoskeletal anomalies
Disfigurement level 2 with pain and moderate motor impairment due to congenital limb deficiency
Disfigurement level 2 with pain and moderate motor impairment due to other congenital musculoskeletal anomalies
Disfigurement level 2 with pain due to congenital limb deficiency
Disfigurement level 2 with pain due to other congenital musculoskeletal anomalies
Urogenital congenital anomalies
Asymptomatic congenital anomalies of the urinary tract
Asymptomatic congenital genital anomalies
Atypical genital and recurrent urinary tract infections and other abdominal issues due to congenital anomalies of the urinary tract
Atypical genital and recurrent urinary tract infections and other abdominal issues due to congenital genital anomalies
Atypical genitalia and impotence due to congenital anomalies of the urinary tract
Atypical genitalia and impotence due to congenital genital anomalies
Atypical genitalia and incontinence due to congenital anomalies of the urinary tract
Atypical genitalia and primary infertility due to congenital genital anomalies
Atypical genitalia due to congenital anomalies of the urinary tract
Atypical genitalia due to congenital genital anomalies
Atypical genitalia, incontinence and impotence due to congenital anomalies of the urinary tract
Atypical genitalia, incontinence, impotence, and recurrent urinary tract infections or other abdominal issues and impotence due to congenital anomalies of the urinary tract
Atypical genitalia, infertility and impotence due to congenital genital anomalies
Atypical genitalia, infertility, impotence, and recurrent urinary tract infections or other abdominal issues and impotence due to congenital genital anomalies
Atypical genitalia, recurrent urinary tract infections or other abdominal issues and impotence due to congenital anomalies of the urinary tract
Atypical genitalia, recurrent urinary tract infections or other abdominal issues and impotence due to congenital genital anomalies
Atypical genitalia, recurrent urinary tract infections or other abdominal issues and incontinence due to congenital anomalies of the urinary tract
Atypical genitalia, recurrent urinary tract infections or other abdominal issues and infertility due to congenital genital anomalies
Impotence and recurrent urinary tract infections or other abdominal issues due to congenital anomalies of the urinary tract
Impotence and recurrent urinary tract infections or other abdominal issues due to congenital genital anomalies
Impotence due to congenital anomalies of the urinary tract
Impotence due to congenital genital anomalies
Incontinence and impotence due to congenital anomalies of the urinary tract
Incontinence and recurrent urinary tract infections or other abdominal issues due to congenital anomalies of the urinary tract
Incontinence due to congenital anomalies of the urinary tract
Incontinence, impotence, and recurrent urinary tract infections or other abdominal issues and impotence due to congenital anomalies of the urinary tract
Infertility and impotence due to congenital genital anomalies
Infertility, impotence, and recurrent urinary tract infections or other abdominal issues and impotence due to congenital genital anomalies
Primary infertility and recurrent urinary tract infections or other abdominal issues due to congenital genital anomalies
Primary infertility due to congenital genital anomalies
Recurrent urinary tract infections or other abdominal issues due to congenital anomalies of the urinary tract

Recurrent urinary tract infections or other abdominal issues due to congenital genital anomalies
Digestive congenital anomalies
Mild chronic respiratory problems and developmental delay due to congenital diaphragmatic hernia
Acid reflux, dyspahgia, and/or constipation due to other congenital malformations of the digestive tract
Asymptomatic congenital atresia and/or stenosis of the digestive tract
Asymptomatic congenital diaphragmatic hernia
Asymptomatic congenital malformations of the abdominal wall after treatment
Asymptomatic other congenital malformations of the digestive tract
Chronic abdominal pain and concern about scars due to congenital malformations of the abdominal wall
Chronic abdominal pain and developmental delay due to congenital diaphragmatic hernia
Chronic abdominal pain and disfigurement due to congenital diaphragmatic hernia
Chronic abdominal pain and mild chronic respiratory problems due to congenital diaphragmatic hernia
Chronic abdominal pain and/or nausea due to other congenital malformations of the digestive tract
Chronic abdominal pain and/or nausea with acid reflux, dyspahgia, and/or constipation due to other congenital malformations of the digestive tract
Chronic abdominal pain due to congenital atresia and/or stenosis of the digestive tract
Chronic abdominal pain due to congenital diaphragmatic hernia
Chronic abdominal pain due to congenital malformations of the abdominal wall
Chronic abdominal pain, chronic respiratory problems and developmental delay due to congenital diaphragmatic hernia
Chronic abdominal pain, disfigurement and chronic respiratory problems due to congenital diaphragmatic hernia
Chronic abdominal pain, disfigurement and developmental delay due to congenital diaphragmatic hernia
Chronic abdominal pain, disfigurement, developmental delay and chronic respiratory problems due to congenital diaphragmatic hernia
Chronic respiratory problems and abdominal pain due to congenital atresia and/or stenosis of the digestive tract
Chronic respiratory problems and dysphagia or acid reflux due to congenital atresia and/or stenosis of the digestive tract
Chronic respiratory problems including difficulty breaking and recurrent upper respiratory infections due to atresia and/or stenosis of the digestive tract
Constipation and chronic abdominal pain due to congenital malformations of the abdominal wall
Constipation and concern about scars due to congenital malformations of the abdominal wall
Constipation due to congenital malformations of the abdominal wall
Constipation, chronic abdominal pain and concern about scars due to congenital malformations of the abdominal wall
Developmental delay or mild intellectual disability due to congenital diaphragmatic hernia
Disfigurement and developmental delay due to congenital diaphragmatic hernia
Disfigurement and mild chronic respiratory problems due to congenital diaphragmatic hernia
Disfigurement due to congenital diaphragmatic hernia
Disfigurement from scars following treatment for congenital malformations of the abdominal wall
Disfigurement, chronic respiratory problems and developmental delay due to congenital diaphragmatic hernia
Dysphagia or acid reflux and chronic abdominal pain due to congenital atresia and/or stenosis of the digestive tract
Dysphagia or acid reflux due to congenital atresia and/or stenosis of the digestive tract
Dysphagia or acid reflux, chronic abdominal pain and chronic respiratory problems due to congenital atresia and/or stenosis of the digestive tract
Mild chronic respiratory problems and breathlessness due to congenital diaphragmatic hernia
Other congenital birth defects
Complete hearing loss due to other congenital anomalies

Complete hearing loss with ringing due to other congenital anomalies
Mild hearing loss due to other congenital anomalies
Mild hearing loss with ringing due to other congenital anomalies
Moderate hearing loss due to other congenital anomalies
Moderate hearing loss with ringing due to other congenital anomalies
Moderately severe hearing loss due to other congenital anomalies
Moderately severe hearing loss with ringing due to other congenital anomalies
Profound hearing loss due to other congenital anomalies
Profound hearing loss with ringing due to other congenital anomalies
Severe hearing loss due to other congenital anomalies
Severe hearing loss with ringing due to other congenital anomalies
Skin and subcutaneous diseases
Dermatitis
Asymptomatic contact dermatitis
Asymptomatic seborrhoeic dermatitis
Mild atopic dermatitis
Mild contact dermatitis
Moderate atopic dermatitis
Moderate contact dermatitis
Severe atopic dermatitis
Symptomatic seborrhoeic dermatitis
Psoriasis
Mild psoriasis
Moderate psoriasis
Severe psoriasis
Cellulitis
Mild cellulitis
Moderate cellulitis
Severe cellulitis
Pyoderma
Abscess and other bacterial skin diseases
Impetigo
Scabies
Fungal skin diseases
Other fungal skin diseases
Tinea capitis
Viral skin diseases
Mild molluscum contagiosum
Mild viral warts
Severe molluscum contagiosum
Severe viral warts
Acne vulgaris
Mild acne vulgaris

Moderate acne vulgaris
Severe acne vulgaris
Alopecia areata
Mild alopecia areata
Severe alopecia areata
Pruritus
Urticaria
Mild urticaria
Severe urticaria
Decubitus ulcer
Mild decubitus ulcer
Moderate decubitus ulcer
Severe decubitus ulcer
Other skin and subcutaneous diseases
Asymptomatic other skin and subcutaneous diseases
Symptomatic other skin and subcutaneous diseases
Sense organ diseases
Glaucoma
Blindness due to glaucoma
Moderate vision impairment due to glaucoma
Severe vision impairment due to glaucoma
Cataract
Blindness due to cataract
Moderate vision impairment due to cataract
Severe vision impairment due to cataract
Macular degeneration
Blindness due to macular degeneration
Moderate vision impairment due to macular degeneration
Severe vision impairment due to macular degeneration
Refraction and accommodation disorders
Blindness due to uncorrected refractive error
Moderate vision impairment due to uncorrected refractive error
Near vision impairment
Severe vision impairment due to uncorrected refractive error
Age-related and other hearing loss
Complete hearing loss due to age-related and other hearing loss
Complete hearing loss with ringing due to age-related and other hearing loss
Mild hearing loss due to age-related and other hearing loss
Mild hearing loss with ringing due to age-related and other hearing loss
Moderate hearing loss due to age-related and other hearing loss
Moderate hearing loss with ringing due to age-related and other hearing loss
Moderately severe hearing loss due to age-related and other hearing loss
Moderately severe hearing loss with ringing due to age-related and other hearing loss

Profound hearing loss due to age-related and other hearing loss
Profound hearing loss with ringing due to age-related and other hearing loss
Severe hearing loss due to age-related and other hearing loss
Severe hearing loss with ringing due to age-related and other hearing loss
Other vision loss
Blindness due to other vision loss
Moderate vision impairment due to other vision loss
Severe vision impairment due to other vision loss
Other sense organ diseases
Asymptomatic acute sense organ diseases
Asymptomatic chronic sense organ diseases
Mild acute sense organ diseases
Moderate acute sense organ diseases
Moderate chronic sense organ diseases
Severe chronic sense organ diseases
Oral disorders
Caries of deciduous teeth
Asymptomatic caries of deciduous teeth
Pain due to caries of deciduous teeth
Caries of permanent teeth
Asymptomatic caries of permanent teeth
Pain due to caries of permanent teeth
Periodontal diseases
Chronic periodontal diseases
Edentulism and severe tooth loss
Asymptomatic edentulism and severe tooth loss
Difficulty eating due to edentulism and severe tooth loss
Other oral disorders
Mild other oral disorders
Severe other oral disorders
Sudden infant death syndrome
Injuries
Transport injuries
Road injuries
Pedestrian road injuries
Cyclist road injuries
Motorcyclist road injuries
Motor vehicle road injuries
Other road injuries
Other transport injuries
Unintentional injuries
Falls
Drowning

Fire, heat, and hot substances
Poisonings
Exposure to mechanical forces
Unintentional firearm injuries
Unintentional suffocation
Other exposure to mechanical forces
Adverse effects of medical treatment
Animal contact
Venomous animal contact
Non-venomous animal contact
Foreign body
Pulmonary aspiration and foreign body in airway
Foreign body in eyes
Foreign body in other body part
Environmental heat and cold exposure
Other unintentional injuries
Self-harm and interpersonal violence
Self-harm
Self-harm by firearm
Self-harm by other specified means
Interpersonal violence
Physical violence by firearm
Physical violence by sharp object
Sexual violence
Physical violence by other means
Forces of nature, conflict and terrorism, and executions and police conflict
Exposure to forces of nature
Conflict and terrorism
Executions and police conflict

Appendix Table 4: ICD-9 and ICD-10 codes by GBD 2016 nonfatal causes, impairments, and nature of injury categories				
**Cause**	**ICD10**	**ICD10 Used in Hospital/Claims Analyses**	**ICD9**	**ICD9 Used in Hospital/Claims Analyses**
Tuberculosis	A10-A14, A15-A18.89, A19-A19.9, B90-B90.9, K67.3, K93.0, M49.0, N74.0-N74.1, P37.0, U84.3, Z03.0, Z11.1, Z20.1, Z23.2		010-019.9, 137-137.9, 320.4, 730.4-730.6, V01.1, V03.2, V12.01, V74.1	
Drug-susceptible tuberculosis	A10-A14, A15-A18.89, A19-A19.9, B90-B90.9, K67.3, K93.0, M49.0, N74.0-N74.1, P37.0		010-019.9, 137-137.9, 320.4, 730.4-730.6	
Multidrug-resistant tuberculosis without extensive drug resistance	U84.3			
Extensively drug-resistant tuberculosis				
Latent tuberculosis infection				
HIV/AIDS	B20-B23.8, B24-B24.0, B97.81, C46-C46.52, C46.7-C46.9, O98.7-O98.73, Z11.4, Z20.6, Z21, Z22.6, Z83.0		042-044.9, 176-176.9	
Drug-susceptible HIV/AIDS-Tuberculosis	B20.0			
Multidrug-resistant HIV/AIDS-Tuberculosis without extensive drug resistance				
Extensively drug-resistant HIV/AIDS-Tuberculosis				
HIV/AIDS resulting in other diseases	B20.1-B23.8, B24-B24.0, B97.81, C46-C46.52, C46.7-C46.9		176-176.9	
Diarrheal diseases	A00-A00.9, A02-A07, A07.2-A07.4, A08-A08.8, A09, Z22.1, Z23.0	A00-A00.9, A02-A09	001-001.9, 003-009.9, V01.0, V01.83, V02.0, V02.2-V02.3, V03.0, V74.0	001-001.9, 003-009.9
Intestinal infectious diseases	A01-A01.4, A07.0-A07.1, A07.8-A07.9, Z11.0, Z20.0-Z20.09		002-002.9	
Typhoid fever	A01.0-A01.09		002.0	
Paratyphoid fever	A01.1-A01.4		002.1-002.9	
Other intestinal infectious diseases	A07.0-A07.1, A07.8-A07.9			
Lower respiratory infections	A48.1, A70, B96.0-B96.1, B97.21, B97.4-B97.6, J09-J18.2, J18.8-J18.9, J19.6-J22.9, J85.1, P23-P23.9, U04-U04.9, Z25.1	A48.1, A70, B96.0-B97.6, J09-J22.9, J85.1, P23-P23.9, U04-U04.9	079.82, 466-469, 470.0, 480-484, 484.1-489, 510-511.9, 513.0-513.9, 770.0, V01.82, V04.7, V04.81	079.82, 466-470.0, 480-484, 484.1-489, 510-513.9, 770.0
Upper respiratory infections	J00-J06.9, J36-J36.0		460-465.9, 475-475.9	
Otitis media	H65-H70.93		381-383.9	
Meningitis	A39-A39.9, A87-A87.9, D86.81, G00-G03.9, G06-G09.9, Z20.811, Z22.31	A39-A39.9, A87-A87.9, D86.81, G00-G03.9	036-036.9, 047-049.9, 054.72, 320-320.3, 320.5-322.9, 324-326.9, V01.84	036-036.9, 047-049.9, 054.72, 320-320.3, 320.5-322.9
Pneumococcal meningitis	G00.1		320.1	
H influenzae type B meningitis	G00.0		320.0	
Meningococcal meningitis	A39-A39.9		036-036.9, 320.5	
Other meningitis	A87-A87.9, D86.81, G03-G03.9		047-049.9, 054.72, 321-322.9	
Encephalitis	A83-A85.2, A85.8-A86.0, B94.1, F07.1, G04-G05.8, Z24.1	A83-A86.0, B94.1, F07.1, G04-G05.8	062-064.9, 310.89, 323-323.9, V05.0-V05.1	062-064.9, 310.89, 323-323.9
Diphtheria	A36-A36.9, Z22.2, Z23.6		032-032.9, V02.4, V03.5, V74.3	
Whooping cough	A37-A37.91, Z23.7		033-033.9, V03.6	
Tetanus	A33-A35.0, Z23.5		037-037.9, 771.3, V03.7	
Measles	B05-B05.9, Z24.4		055-055.9, 484.0, V04.2, V73.2	
Varicella and herpes zoster	B01-B02.9, Z20.820		052-053.9, V01.71-V01.79, V05.4	
Malaria	B50-B50.0, B50.8-B52.0, B52.8-B53.1, B53.8-B54.0, P37.3-P37.4		084-084.9	
Chagas disease	B57-B57.5, K93.1		086-086.2, 425.6	
Leishmaniasis	B55-B55.9, Z26.0		085-085.9, V05.2	
Visceral leishmaniasis	B55.0	B55.0	085.0	085.1-085.5
Cutaneous and mucocutaneous leishmaniasis	B55.1-B55.2	B55.1-B55.2	085.1-085.5	085.0
African trypanosomiasis	B56-B56.9		086.3-086.9	
Schistosomiasis	B65-B65.9		120-120.9	
Cysticercosis	B69-B69.9		123.1	
Cystic echinococcosis	B67-B67.4, B67.8-B67.99	B67-B67.99	122-122.4, 122.8-122.9	122-122.9
Lymphatic filariasis	B74-B74.2		125.0-125.2	
Onchocerciasis	B73-B73.1		125.3	
Trachoma	A71-A71.9, A74.0, B94.0		076-076.9	
Dengue	A90-A91.0		061-061.8	
Yellow fever	A95-A95.9, Z24.3		060-060.9, V04.4, V73.4	
Rabies	A82-A82.9, Z20.3, Z24.2		071-071.9, V01.5, V04.5	
Intestinal nematode infections	B76-B77.9, B79, Z11.6		126-126.9, 127.0, 127.3, V75.7	
Ascariasis	B77-B77.9		127.0	
Trichuriasis	B79		127.3	
Hookworm disease	B76-B76.9		126-126.9	
Food-borne trematodiases	B66-B66.9, B72.0		121-121.9	
Leprosy	A30-A30.9, B92		030-030.9	
Ebola	A98.4			
Zika virus	U06-U06.9		066.3	
Guinea worm disease	B72			
Other neglected tropical diseases	A68-A68.9, A69.2-A69.29, A69.8-A69.9, A75-A75.9, A77-A79.9, A92-A94.0, A96-A96.9, A98-A98.3, A98.5-A99.0, B33.0-B33.1, B60-B60.8, B64, B67.5-B67.7, B68-B68.9, B70-B71.9, B74.3-B75, B78-B78.9, B80-B83.9, B89, P37.1		065-066.2, 066.4-066.9, 080-083.9, 087-088.9, 122.5-122.7, 123-123.0, 123.2-125, 125.4-125.9, 127, 127.1-127.2, 127.4-129.0	

Appendix Table 4: ICD-9 and ICD-10 codes by GBD 2016 nonfatal causes, impairments, and nature of injury categories				
**Cause**	**ICD10**	**ICD10 Used in Hospital/Claims Analyses**	**ICD9**	**ICD9 Used in Hospital/Claims Analyses**
Maternal hemorrhage	O20-O20.9, O43.2-O43.239, O44-O44.00, O44.03-O46.93, O62.2, O67-O67.9, O72-O72.3	O20-O20.9, O43.2-O43.239, O44-O44.00, O44.03-O46.93, O62.2-O62.2, O67-O67.9, O72-O72.3	640-641.93, 661.2-661.23, 666-666.9	640-641.93, 661.2-661.23, 666-666.9
Maternal sepsis and other maternal infections	O23-O23.93, O41.1-O41.93, O85-O86.89, O91-O91.23	O23-O23.93, O41.1-O41.93, O85-O86.89, O91-O91.23	646.5-646.64, 658.4-658.93, 659.3-659.33, 670-670.9, 675-675.94	646.5-646.64, 658.4-658.93, 659.2-659.33, 670-670.9, 672-672.04, 674.1-674.34, 675-675.94
Maternal hypertensive disorders	O10-O16.9	O10-O16.9	642-642.94	642-642, 642.3-642.94
Maternal obstructed labor and uterine rupture	O64-O66.9, O70-O71.9, O83-O84.9	O64-O66.9, O70-O71.9, O83-O84.9	652.7-652.73, 653-653.93, 659.0-659.13, 660-660.93, 664-665.94, 669.5-669.61	652.7-652.73, 653-653.93, 659.0-659.13, 660-660.93, 664-665.94, 669.5-669.61
Maternal abortion, miscarriage, and ectopic pregnancy	N96, O00-O08.9, O36.7-O36.73	N96-N96, O00-O08.9, O36.7-O36.73	630-639.9, 656.4-656.43	630-639.9, 656.4-656.43
Other maternal disorders	F53-F54, O09-O09.93, O18.0, O21-O22.93, O24.4-O24.439, O25-O26.93, O28-O36.63, O36.8-O36.93, O38.4, O40-O41.03, O42-O43.199, O43.8-O43.93, O44.01-O44.02, O47-O48.1, O60-O62.1, O62.3-O63.9, O68-O69.9, O73-O77.9, O80-O82.9, O87-O90.9, O92-O92.79, O94-O95, O96-O97.9, Z03.7-Z03.79, Z32-Z37.9, Z39-Z39.2, Z64.0-Z64.3, Z87.5-Z87.6		643-646.44, 646.7-646.93, 648.1-648.14, 649.7-652.63, 652.8-652.93, 654-655.23, 655.7-656.33, 656.5-658.33, 659, 659.4-659.93, 661-661.13, 661.3-663.93, 667-669.44, 669.7-669.94, 671-674.94, 676-679.14, V13.1-V13.21, V15.21-V15.22, V22-V24.2, V27-V28.9, V72.4-V72.42, V82.4, V91-V91.99	
Neonatal preterm birth complications	P07.2-P07.39, P22-P22.9, P25-P28.9, P61.2, P77-P77.9	P07.2-P07.39, P77-P77.9	765.21-765.9, 769-770, 770.2-770.9, 776.6, 777.5-777.53	764-765.9, 777.5-777.53
Neonatal encephalopathy due to birth asphyxia and trauma	P02-P03.9, P10-P15.9, P20-P21.9, P24-P24.9, P90-P91.9	P02-P03.9, P10-P21.9, P24-P24.9, P90-P91.9	761.7-763.9, 767-768, 768.2-768.9, 779.0-779.2	761.7-763.9, 767.0-768.9, 772.1-772.2, 779.0-779.2
Neonatal sepsis and other neonatal infections	P36-P36.9, P38-P39.9	P36-P36.9, P38-P39.9	771, 771.4-771.89	771, 771.4-771.89
Hemolytic disease and other neonatal jaundice	P55-P59.9		773-774.9	
Other neonatal disorders	P00-P01.9, P04-P05.9, P07-P07.18, P08-P09, P19-P19.9, P29-P29.9, P50-P54.9, P60-P61.1, P61.3-P61.9, P70-P72.9, P74-P74.9, P75.0-P76.9, P78-P78.9, P80-P81.9, P83-P84, P92-P92.9, P94-P94.9, P96, P96.3-P96.9, P99.9		655.3-655.63, 760-761.6, 764-765.20, 766-766.9, 771.9-772.9, 775-775.1, 775.4-776.5, 776.7-777.4, 777.6-779, 779.3-779.5, 779.7-779.9	
Protein-energy malnutrition	E40-E46.9, E64.0		260-263.9	
Iodine deficiency	E00-E02		244.2	
Vitamin A deficiency	E50-E50.9, E64.1		264-264.9	
Iron-deficiency anemia	D50-D50.9		280-280.9	
Other nutritional deficiencies	D51-D53.9, E51-E61.9, E63-E64, E64.2-E64.9		265-269.9, 273-273.9, 281-281.2	
Sexually transmitted diseases excluding HIV	A50-A60.9, A63-A64.0, B63, I98.0, K67.0-K67.2, M73.0-M73.8, N70-N71.9, N73-N74, N74.2-N74.8, Z11.3, Z20.2, Z22.4		054.1, 054.11-054.19, 090-099.9, 131-131.9, 613-615.9, V01.6, V02.7-V02.9, V73.8, V73.88, V73.9-V73.98, V74.5-V74.6	
Syphilis	A50-A53.9, I98.0, K67.2, M73.1-M73.8	A51-A52.9, I98.0	090-097.9	091-092.9, 093.2
Chlamydial infection	A55-A56.8, K67.0, N74.4		099.41, 099.5	
Gonococcal infection	A54-A54.9, K67.1, M73.0, N74.3		098-098.9	
Trichomoniasis	A59-A59.9		131-131.9	
Genital herpes	A60-A60.9		054.1, 054.11-054.19	
Other sexually transmitted diseases	A57-A58, A63-A64.0, B63, N70-N71.9, N73-N74, N74.2, N74.8		099-099.40, 099.49, 099.50-099.9, 613-615.9	
Hepatitis	B15-B19.9, B94.2, K71.2, K71.6, P35.3, Z20.5, Z22.5-Z22.59, Z24.6		070-070.9, V02.6-V02.69, V05.3	
Acute hepatitis A	B15-B15.9		070.0-070.1	
Hepatitis B	B16-B16.9, B17.0, B18.0-B18.1, B19.1-B19.11, P35.3		070.2-070.31, 070.42, 070.52	
Hepatitis C	B17.1-B17.11, B18.2, B19.2-B19.21		070.41, 070.44, 070.51, 070.7-070.71	
Acute hepatitis E	B17.2		070.43, 070.53	
Other infectious diseases	A20-A28.9, A31-A32.9, A38-A38.9, A42-A44.9, A48-A48.0, A48.2-A49.9, A65-A65.0, A69-A69.1, A74, A74.8-A74.9, A80-A81.9, A88-A89.0, B00-B00.9, B03-B04, B06-B06.9, B10-B10.89, B25-B27.99, B33, B33.3-B34.9, B37-B37.2, B37.5-B47.1, B47.9-B49, B58-B59, B91, B94, B94.8-B96, B96.2-B97.2, B97.29-B97.39, B97.7-B97.8, B97.89, B99-B99.9, G14-G14.6, I00, I02, I02.9, I96-I96.9, I98.1, J85-J85.0, J85.2-J85.3, J86-J86.9, K75.0, K75.3, K76.3, M49.1, M89.6-M89.69, P35-P35.2, P35.8-P35.9, P37, P37.2, P37.5-P37.9, R02-R02.9, U82-U84, U85-U89, Z11, Z11.2, Z11.5-Z11.59, Z11.8-Z11.9, Z16-Z16.39, Z20, Z20.4, Z20.7-Z20.810, Z20.818-Z20.82, Z20.828-Z20.9, Z22-Z22.0, Z22.3, Z22.32-Z22.39, Z22.8-Z22.9, Z23.3-Z23.4, Z24.0, Z24.5, Z25.0, Z83.1		020-029, 031-031.9, 034-034.9, 039-039.4, 039.8-040, 040.1-041.9, 045-046.9, 050-051.9, 054-054.0, 054.10, 054.2-054.71, 054.73-054.9, 056-059.9, 072-074.1, 074.20, 074.3-075.9, 078, 078.2-079.81, 079.83-079.99, 100-101.6, 104-104.9, 112-112.0, 112.3-118.9, 130-130.9, 136-136.0, 136.2-136.9, 138-139.9, 390-390.9, 392, 392.9, 572.0-572.1, 771.0-771.2, V01, V01.2-V01.4, V01.7, V01.8-V01.81, V01.89-V02, V02.1, V02.5-V02.59, V03.1, V03.3-V03.4, V03.8, V03.9-V04.1, V04.3, V04.6, V04.8, V04.89-V05, V05.8-V06, V07-V07.3, V09-V09.91, V12.00, V12.02-V12.09, V18.8, V58.62, V73.0-V73.1, V73.3, V73.81, V73.89, V73.99, V74.8-V74.9, V75.0-V75.4, V75.9	
Lip and oral cavity cancer	C00-C07, C08-C08.9, Z85.81-Z85.810		140-145.9, V76.42	
Nasopharynx cancer	C11-C11.9		147-147.9	
Other pharynx cancer	C09-C10.9, C12-C13.9		146-146.9, 148-148.9	
Esophageal cancer	C15-C15.9, Z85.01		150-150.9	
Stomach cancer	C16-C16.9, Z12.0, Z85.02-Z85.028		151-151.9, 209.23, V10.04	
Colon and rectum cancer	C18-C19.0, C20, C21-C21.8, Z12.1-Z12.13, Z85.03-Z85.048, Z86.010		153-154.9, 209.1-209.17, V10.05-V10.06, V76.41, V76.5-V76.52	
Liver cancer	C22-C22.4, C22.7-C22.9, Z85.05		155-155.9, V10.07	

Appendix Table 4: ICD-9 and ICD-10 codes by GBD 2016 nonfatal causes, impairments, and nature of injury categories				
Cause	ICD10	ICD10 Used in Hospital/Claims Analyses	ICD9	ICD9 Used in Hospital/Claims Analyses
Liver cancer due to hepatitis B				
Liver cancer due to hepatitis C				
Liver cancer due to alcohol use				
Liver cancer due to other causes				
Gallbladder and biliary tract cancer	C23, C24-C24.9		156-156.9	
Pancreatic cancer	C25-C25.9, Z85.07		157-157.9	
Larynx cancer	C32-C32.9, Z85.21		161-161.9, V10.21	
Tracheal, bronchus, and lung cancer	C33, C34-C34.92, Z12.2, Z80.1-Z80.2, Z85.1-Z85.20		162-162.9, 209.21, V10.1-V10.20, V16.1-V16.2, V16.4-V16.40	
Malignant skin melanoma	C43-C43.9, Z85.82-Z85.828		172-172.9	
Non-melanoma skin cancer	C44-C44.99		173-173.99, 216-216.9, 232-232.9	
Non-melanoma skin cancer (squamous-cell carcinoma)	C44.02, C44.12-C44.129, C44.22-C44.229, C44.32-C44.329, C44.42, C44.52-C44.529, C44.62-C44.629, C44.72-C44.729, C44.82, C44.92	C44.02, C44.12-C44.129, C44.22-C44.229, C44.32-C44.329, C44.42, C44.52-C44.529, C44.62-C44.629, C44.72-C44.729, C44.82, C44.92	173.02, 173.12, 173.22, 173.32, 173.42, 173.52, 173.62, 173.72, 173.82, 173.92	173.02, 173.12, 173.22, 173.32, 173.42, 173.52, 173.62, 173.72, 173.82, 173.92
Non-melanoma skin cancer (basal-cell carcinoma)	C44.01, C44.11-C44.119, C44.21-C44.219, C44.31-C44.319, C44.41, C44.51-C44.519, C44.61-C44.619, C44.71-C44.719, C44.81, C44.91		173.01, 173.11, 173.21, 173.31, 173.41, 173.51, 173.60-173.61, 173.71, 173.81, 173.91	
Breast cancer	C50-C50.629, C50.8-C50.929, Z12.3-Z12.39, Z80.3, Z85.3, Z86.000		174-175.9, V10.3, V16.3	
Cervical cancer	C53-C53.9, Z12.4, Z85.41		180-180.9, V10.41, V72.32	
Uterine cancer	C54-C54.3, C54.8-C54.9, Z85.42, Z86.001		182-182.9	
Ovarian cancer	C56-C56.2, C56.9, Z80.41, Z85.43		183-183.0, 183.8-183.9, V10.43, V16.41	
Prostate cancer	C61-C61.9, Z12.5, Z80.42, Z85.46		185-185.9, V10.46, V16.42, V76.44	
Testicular cancer	C62-C62.92, Z80.43, Z85.47-Z85.48		186-186.9, V10.47-V10.48, V16.43	
Kidney cancer	C64-C64.2, C64.9-C65.9, Z80.51, Z85.52-Z85.54		189-189.1, 189.5-189.6, 209.24	
Bladder cancer	C67-C67.9, Z12.6-Z12.79, Z80.52, Z85.51		188-188.9, V10.51, V16.52, V76.3	
Brain and nervous system cancer	C70-C70.1, C70.9-C72.9, Z85.841-Z85.848, Z86.011		191-191.9	
Thyroid cancer	C73, Z85.850		193-193.9	
Mesothelioma	C45-C45.2, C45.7, C45.9			
Hodgkin lymphoma	C81-C81.49, C81.7-C81.79, C81.9-C81.99, Z85.71-Z85.72		201-201.98, V10.72	
Non-Hodgkin lymphoma	C82-C85.29, C85.7-C86.6, C96-C96.9		200-200.9, 202-202.98	
Multiple myeloma	C88-C90.32		203-203.9	
Leukemia	C91-C93.7, C93.9-C95.2, C95.7-C95.92, Z80.6, Z85.6		204-208.92, V10.59-V10.69, V16.6	
Acute lymphoid leukemia	C91.0-C91.02		204.0-204.02	
Chronic lymphoid leukemia	C91.1-C91.12		204.1-204.12	
Acute myeloid leukemia	C92.0-C92.02, C92.3-C92.62, C93.0-C93.02, C94.0-C94.02, C94.2-C94.22, C94.4-C94.5		205.0-205.02, 205.3-205.32, 206.0-206.02, 207.0	
Chronic myeloid leukemia	C92.1-C92.12		205.1-205.12, 206.1-206.12, 207.1	
Other leukemia				
Other neoplasms	C17-C17.9, C30-C30.1, C31-C31.9, C37-C37.0, C38-C38.8, C40-C41.4, C41.8-C41.9, C47-C4A, C51-C52, C57-C57.8, C58-C58.0, C60-C60.9, C63-C63.8, C66-C66.9, C68.0-C68.8, C69-C69.92, C74-C75.5, C75.8		152-152.9, 158-158.9, 160-160.9, 163-164.9, 170-171.9, 181-181.9, 183.2-183.5, 184-184.9, 187-187.9, 189.2-189.4, 189.8-190.9, 192-192.9, 194-194.8, 209-209.03, 209.22, 209.25-209.27, 209.31-209.36	
Rheumatic heart disease	I01-I01.9, I02.0, I05-I09.9	I01-I09.9	391-391.9, 392.0, 393-398.99	391-398.99
Ischemic heart disease	I20-I21.6, I21.9-I25.9, Z82.4-Z82.49	I20-I25.9	410-414.9, V17.3	410-414.9
Cerebrovascular disease	G45-G46.8, I60-I64, I64.1, I65-I69.998, Z82.3		430-439.6, V12.54, V17.1	
Ischemic stroke	G45-G46.8, I63-I63.9, I65-I66.9, I67.2-I67.848, I69.3-I69.4	I63-I63.9, I67.2-I67.848, I69.3-I69.4	433-435.9, 437.0-437.2, 437.4-437.9	434-434.91, 437.0-437.2, 437.4-437.9
Hemorrhagic stroke	I60-I62.9, I67.0-I67.1, I69.0-I69.298	I60-I62.9, I67.0-I67.1, I69.0-I69.298	430-432.9, 437.3	430-432.9
Hypertensive heart disease	I11-I11.2, I11.9		402-402.91	
Cardiomyopathy and myocarditis	B33.2-B33.20, B33.22-B33.24, D86.85, I40-I41.8, I42-I43.8, I51.4-I51.6		074.2, 074.23, 422-422.99, 425-425.5, 425.7-425.9, 429.0-429.1	
Myocarditis	B33.2-B33.20, B33.22-B33.24, D86.85, I40-I41.8, I51.4-I51.6	B33.2-B33.20, B33.22-B33.24, D86.85, I40-I41.8, I51.4-I51.6	074.2, 074.23, 422-422.99, 429.0-429.1	074.2, 074.23, 422-422.99, 429.0-429.1
Alcoholic cardiomyopathy	I42.6		425.5	
Other cardiomyopathy	I42.0-I42.5, I42.7-I43.8		425.0-425.4, 425.7-425.9	
Atrial fibrillation and flutter	I48-I48.92	I48-I48.92	427.3-427.32	427.3-427.32
Peripheral artery disease	I70.2-I70.92, I73-I73.9	I70.2-I73.9	440.2-440.29, 440.4-440.9, 443-443.9	440.2-440.29, 440.4-443.9
Endocarditis	B33.21, I33-I33.9, I38-I38.0, I39-I39.9	B33.21, I33-I39.9	074.22, 421-421.9, 424, 424.4-424.99	074.22, 421-421.9, 424-424.99
Other cardiovascular and circulatory diseases	I30-I32.8, I34-I37.9, I51-I51.3, I51.7-I52.8, I72-I72.9, I77-I83.93, I86-I89.0, I89.9, I95.0-I95.1, I98, I98.8-I99.9, K75.1		074.21, 417-417.9, 420-420.99, 423-423.9, 424.0-424.3, 429, 429.2-429.9, 442-442.9, 447-454.9, 456, 456.3-457, 457.1, 457.8-458.1, 459-459.9	
Chronic obstructive pulmonary disease	J41-J42.4, J43-J44.9, J47-J47.9	J41-J44.9, J47-J47.9	490-492.9, 494-494.9, 496-499	491-492.9, 494-499
Pneumoconiosis	J60-J65.0, J92.0		500-505.9	
Silicosis	J62-J62.9	J62-J62.9	502-502.9	502-502.9
Asbestosis	J61-J61.0, J92.0	J61-J61.0, J92.0	501-501.9	501-501.9
Coal workers pneumoconiosis	J60-J60.0	J60-J60.0	500-500.9	500-500.9

Appendix Table 4: ICD-9 and ICD-10 codes by GBD 2016 nonfatal causes, impairments, and nature of injury categories				
**Cause**	**ICD10**	**ICD10 Used in Hospital/Claims Analyses**	**ICD9**	**ICD9 Used in Hospital/Claims Analyses**
Other pneumoconiosis	J63-J65.0	J63-J65.0	503-505.9	503-505.9
Asthma	J45-J46.0, Z82.5	J45-J46.0	493-493.92, V17.5	493-493.92
Interstitial lung disease and pulmonary sarcoidosis	D86-D86.2, D86.89-D86.9, J84-J84.9	D86-D86.2, D86.89-D86.9, J84-J84.9	135-135.9, 515, 515.9-516.9	135-135.9, 515-516.9
Other chronic respiratory diseases	G47.3-G47.39, J30-J35.9, J37-J39.9, J66-J68.9, J70.8-J70.9, J82, J90-J90.0, J91-J92, J92.9-J93.12, J93.8-J94.9, J96.1-J96.8, J98-J99.8		278.03, 327.2-327.29, 470, 470.9-474.9, 476-479, 495-495.9, 506-508.9, 512-513, 514-514.9, 515.0, 517-518.4, 518.7-519.0, 519.11-519.9	
Cirrhosis and other chronic liver diseases	I85-I85.9, I98.2, K70-K71.11, K71.3-K71.51, K71.7-K75, K75.2, K75.4-K76.2, K76.4-K77.8, Z52.6, Z94.4	I85-I85.9, I98.2, K70-K77.8	456.0-456.21, 570-572, 572.2-573.9, V42.7, V59.6	456.0-456.21, 570-573.9
Cirrhosis and other chronic liver diseases due to hepatitis B				
Cirrhosis and other chronic liver diseases due to hepatitis C				
Cirrhosis and other chronic liver diseases due to alcohol use				
Cirrhosis and other chronic liver diseases due to other causes				
Peptic ulcer disease	K25-K27.7, K28-K28.9	K25-K28.9	531-533.8, 534-534.91	531-534.91
Gastritis and duodenitis	K29-K29.91	K29-K29.91	535-535.9	535-535.9
Appendicitis	K35-K37.9	K35-K37.9	540-542.9	540-542.9
Paralytic ileus and intestinal obstruction	K56-K56.9	K56-K56.9	560-560.39, 560.8-560.9, 569.87	560-560.9, 569.87
Inguinal, femoral, and abdominal hernia	K40-K42.9, K44-K46.9	K40-K42.9, K44-K46.9	550-551.1, 551.3-552.1, 552.3-553.1, 553.3-553.9	550-551.1, 551.3-552.1, 552.3-553.1, 553.3-553.9
Inflammatory bowel disease	K50-K51.319, K51.5-K52.9, K58	K50-K51.919, K58	555-556.9, 558-558.9, 564.1, 569.5	555-556.9, 564.1
Vascular intestinal disorders	K55-K55.9	K55.0-K55.1	557-557.9, 569.84-569.86	557.0-557.9
Gallbladder and biliary diseases	K80-K80.81, K81-K83.9, K87-K87.1	K80-K83.9, K87-K87.1	574-576.9	574-576.9
Pancreatitis	K85-K86.9	K85-K86.9	577-577.9	577-577.9
Other digestive diseases	I84-I84.9, K20-K23.8, K30-K31.9, K38-K38.9, K51.4-K51.419, K57-K57.93, K58.0-K62.6, K62.8-K67, K67.8-K68.1, K68.12-K68.9, K90-K90.9, K92-K92.9, K93.8		455-455.9, 530-530.86, 530.89-530.9, 536-536.3, 536.8-538, 543-543.9, 562-562.13, 564-564.09, 564.5-569.49, 569.81-569.83, 569.89-569.9, 578-579.9	
Alzheimer disease and other dementias	F00-F03.91, F06.2, G30-G31.1, G31.8-G32.89	F00-F06.2, G30-G31.1, G31.8-G32.89	290-290.9, 294.0-294.9, 331-331.2, 331.6-331.7, 331.82, 331.89-331.9	290-290.9, 294.0-294.9, 331-331.7, 331.82-331.9
Parkinson disease	G20-G20.9	G20-G20.9	332-332.9	332-332.9
Epilepsy	G40-G41.9, Z82.0		345-345.91	
Multiple sclerosis	G35-G35.0	G35-G35.0	340-340.9	340-340.9
Motor neuron disease				335-335.9
Migraine	G43-G43.919	G43-G43.919	346-346.93	346-346.93
Tension-type headache	G44-G44.89	G44.2-G44.41	307.81, 339-339.89	307.81, 339.1-339.3
Other neurological disorders	G10-G10.0, G11-G13.8, G21-G21.0, G21.2-G24, G24.1-G25.0, G25.2-G25.3, G25.5, G25.8-G26.0, G36-G37.9, G50-G54.1, G54.5-G62, G62.1-G65.2, G70-G72, G72.1-G73.7, G80-G83.9, G89-G93.6, G93.8-G95.29, G95.8-G96, G96.1, G96.12-G96.9, G98-G99.8, M33-M33.99, M60-M60.19, M60.8-M60.9, M79.7		307.8-307.80, 307.89, 330-330.9, 331.3-331.5, 331.8, 331.83, 333-333.91, 333.93-338.4, 342-344.9, 348-348.9, 350-353.0, 353.5-357.5, 357.7-359.23, 359.29-359.9, 710.3-710.4, 725-725.9, 728-728.85, 728.87-728.9, 775.2, 780.96	
Schizophrenia	F20-F20.9, F25-F25.9		295-295.35, 295.5-295.8	
Alcohol use disorders	F10-F10.99, G31.2, R78.0, X45-X45.9, X65-X65.9, Y15-Y15.9, Z81.1		291-291.9, 303-303.93, 305-305.03, 790.3, E860-E860.19, V11.3	
Drug use disorders	F11-F19.99, P96.1, R78.1-R78.9, Z81.2-Z81.4		292-292.9, 304-304.93, 305.1-305.93, E850.0-E850.29, V15.8-V15.86	
Opioid use disorders	F11-F11.99, R78.1		304.0-304.03, 305.5-305.53, E850.0-E850.29	
Cocaine use disorders	F14-F14.99, R78.2		304.2-304.23, 305.2-305.23, 305.6-305.63	
Amphetamine use disorders	F15-F15.99		304.4-304.43, 305.7-305.73	
Cannabis use disorders	F12-F12.99		304.3-304.33	
Other drug use disorders	F13-F13.99, F16-F19.99, P96.1, R78.3-R78.9		292-292.9, 304, 304.1-304.13, 304.5-304.93, 305.1-305.13, 305.3-305.43, 305.8-305.93	
Depressive disorders	F32-F33.9, F34.1		296.2-296.36, 300.4, 311-311.9	
Major depressive disorder	F32-F33.9		296.2-296.36, 311-311.9	
Dysthymia	F34.1		300.4	
Bipolar disorder	F30-F31.9, F34.0	F30-F31.9, F34.0	296-296.16, 296.4-296.81	296-296.16, 296.4-296.81
Anxiety disorders	F40-F44.9, F93-F93.2		300-300.3, 308-309.9	
Eating disorders	F50-F50.9		307.1, 307.5-307.59	
Anorexia nervosa	F50.0-F50.1		307.1	
Bulimia nervosa	F50.2-F50.5		307.51, 307.54	
Autistic spectrum disorders	F84-F84.9		299-299.91	
Autism	F84.0-F84.4		299.0-299.11	
Asperger syndrome and other autistic spectrum disorders	F84, F84.5-F84.9		299, 299.8-299.91	
Attention-deficit/hyperactivity disorder	F90-F90.9		314-314.9	
Conduct disorder	F91-F92.9		312-312.9	
Idiopathic developmental intellectual disability	F70-F79.9, Z81.0		317-319.9, V18.4	

Appendix Table 4: ICD-9 and ICD-10 codes by GBD 2016 nonfatal causes, impairments, and nature of injury categories				
**Cause**	**ICD10**	**ICD10 Used in Hospital/Claims Analyses**	**ICD9**	**ICD9 Used in Hospital/Claims Analyses**
Other mental and substance use disorders	F04-F06.1, F06.3-F07.0, F08-F09.9, F21-F24, F26-F29.9, F34, F34.8-F34.9, F38-F39, F45-F49, F51-F52.9, F55-F55.8, F56-F69.0, F80-F83, F85-F89.0, F93.3-F99.0, G47-G47.29, G47.4-G47.9, R40-R40.4, R45-R55.0, Z03.2, Z04.6-Z04.72, Z13.4, Z64, Z81, Z81.8, Z86.5-Z86.59		293-294, 295.4-295.45, 295.80-295.95, 296.82-298.9, 300.5-302.9, 306-307.0, 307.2-307.49, 307.6-307.7, 307.9, 310-310.1, 313-313.9, 316-316.9, 327-327.19, 327.3-327.8, 347-347.9, 780-780.2, 780.93, 780.97, 797-797.9, 799.2-799.29, V11.0-V11.2, V11.4-V12.0, V17-V17.0, V40-V41.9, V79-V79.9	
Diabetes mellitus	E08-E08.11, E08.3-E08.9, E10-E10.11, E10.3-E11.1, E11.3-E12.1, E12.3-E13.11, E13.3-E14.1, E14.3-E14.9, R73-R73.9	E08-E08.11, E08.3-E08.9, E10-E10.11, E10.3-E11.1, E11.3-E12.1, E12.3-E13.11, E13.3-E14.1, E14.3-E14.9	249-249.31, 249.5-250.39, 250.5-250.99, 790.2-790.29	249-249.31, 249.5-250.39, 250.5-250.99
Acute glomerulonephritis	N00-N01.9	N00-N01.9	580-580.9	580-580.9
Chronic kidney disease	D63.1, E08.2-E08.29, E10.2-E10.29, E11.2-E11.29, E12.2, E13.2-E13.29, E14.2, I12-I13.9, N02-N08.8, N18-N19, Q62-Q62.8, Q64.2-Q64.9, Z49-Z49.32, Z52.4, Z99.2		249.4-249.41, 250.4-250.49, 285.21, 403-404.93, 581-587.9, 753.2-753.29, 753.4, 753.6-753.9, V13.03-V13.09, V18.6, V18.69, V42.0, V45.1-V45.12, V45.73, V56-V56.8, V59.4, V81.5-V81.6	
Chronic kidney disease due to diabetes mellitus	E08.2-E08.29, E10.2-E10.29, E11.2-E11.29, E12.2, E13.2-E13.29, E14.2		249.4-249.41, 250.4-250.49	
Chronic kidney disease due to hypertension	I12-I13.9		403-404.93	
Chronic kidney disease due to glomerulonephritis	N03-N06.9, N08-N08.8		581-583.9	
Chronic kidney disease due to other causes	N02-N02.9, N07-N07.9, Q62-Q62.8, Q64.2-Q64.9		753.2-753.29, 753.4, 753.6-753.9	
Urinary diseases and male infertility	N10-N12.9, N15-N16.8, N20-N23.0, N25-N29, N29.1-N37.8, N39-N46.02, N46.022-N46.12, N46.122-N49.1, N49.8-N52.1, N52.8-N53.9, Z43.7		588-596.8, 596.89-598.1, 598.8-609, 788.3-788.91, V26.5, V26.52	
Interstitial nephritis and urinary tract infections	N10-N12.9, N15-N16.8, N30-N30.91, N34-N34.3, N39.0	N10-N12.9, N15-N16.8, N30-N39.0	590-590.9, 595-595.9, 597-597.9, 599.0	590-590.9, 595-595.9, 597-597.9, 599.0
Urolithiasis	N20-N23.0	N20-N23.0	592-592.9, 594-594.9	592-594.9
Benign prostatic hyperplasia	N40-N40.9	N40-N40.9	600-600.91	600-600.91
Male infertility	N46-N46.02, N46.022-N46.12, N46.122-N46.9, Z43.7		606-606.9, V26.5, V26.52	
Other urinary diseases	N25-N29, N29.1-N29.8, N31-N33.8, N35-N37.8, N39, N39.1-N39.9, N41-N45.9, N47-N49.1, N49.8-N52.1, N52.8-N53.9		588-589.9, 593-593.9, 596-596.8, 596.89-596.9, 598-598.1, 598.8-599, 599.1-599.9, 601-605.9, 607-609, 788.3-788.91	
Gynecological diseases	B37.3-B37.49, D25-D28.2, E28.2, N60-N64.9, N72-N72.0, N75-N77.8, N80-N95.9, N97-N98.9, Z31-Z31.9		112.1-112.2, 218-218.9, 256.4, 610-611.9, 616-629.9, V26-V26.49, V26.51, V26.8-V26.9, V59.7-V59.74	
Uterine fibroids	D25-D28.2	D25-D28.2	218-218.9	218-218.9
Polycystic ovarian syndrome	E28.2	E28.2	256.4	256.4
Female infertility	N97-N98.9		628-628.9	
Endometriosis	N80-N80.9	N80-N80.9	617-617.9	617-617.9
Genital prolapse	N81-N81.9	N81-N81.9	618-618.9	618-618.9
Premenstrual syndrome	N85.0-N85.1, N92-N93.9, N94.3, N95.0		621.2-621.3, 621.31-621.32, 621.34, 625.4, 626, 626.2-626.9, 627.0-627.1	
Other gynecological diseases	B37.3-B37.49, N60-N64.9, N72-N72.0, N75-N77.8, N83-N85, N85.2-N91.5, N94-N94.2, N94.4-N95, N95.1-N95.9, Z31-Z31.9	B37.3-B37.49, N60-N64.9, N72-N72.0, N75-N77.8, N83-N94.2, N94.4-N95.9	112.1-112.2, 610-611.9, 616-616.9, 620-621.1, 621.30, 621.33, 621.35-625.3, 625.5-625.9, 626.0-626.1, 627, 627.2-627.9, 629-629.9, V26-V26.49, V26.51, V26.8-V26.9, V59.7-V59.74	112.1-112.2, 610-611.9, 616-616.9, 620-625.3, 625.5-629.9
Hemoglobinopathies and hemolytic anemias	D55-D61.9		282-284.9	
Thalassemias	D56-D56.3, D56.5-D56.9, D57.4-D57.419		282.4-282.49, 282.6-282.62	
Thalassemias trait				
Sickle cell disorders	D57-D57.3, D57.8-D57.819		282.5, 282.63-282.69	
Sickle cell trait				
G6PD deficiency	D55-D55.2		282.3	
G6PD trait				
Other hemoglobinopathies and hemolytic anemias	D55.3-D55.9, D56.4, D58-D61.9		282-282.2, 282.7-284.9	
Endocrine, metabolic, blood, and immune disorders	D66-D69.49, D69.6-D70.0, D70.2-D77, D80-D84.9, D86.8, D86.82-D86.84, D86.86-D86.87, D89-D89.9, E03-E03.1, E03.3-E06.3, E06.5-E07.9, E15-E16, E16.1-E16.9, E20-E23.0, E23.2-E24.1, E24.3-E27.2, E27.4-E28.1, E28.3-E32.9, E34-E35.8, E65-E66.09, E66.2-E68, E70-E80.09, E80.3-E88.9, E90-E998, Z83.4-Z83.49	D66-D69.49, D69.6-D70.0, D70.2-D77, D80-D84.9, D86.8, D86.82-D86.84, D86.86-D86.87, D89-D89.9, E03-E03.1, E03.3-E06.3, E06.5-E07.9, E15-E16, E16.1-E23.0, E23.2-E24.1, E24.3-E27.2, E27.4-E28.1, E28.3-E35.8, E67-E80.09, E80.3-E88.9, E90-E998	240-244, 244.8-246.9, 251-256.39, 256.8-259.9, 270-272.9, 275-278.02, 278.1-279.9, 286-289.9, 775.3, V12.2-V12.49, V18.1-V18.19, V45.86, V77.0, V77.3-V77.8, V77.91, V85-V85.45, V85.51, V85.53-V85.54	240-244, 244.8-246.9, 251-256.39, 256.8-259.9, 270-271.9, 275-279.9, 285-289.9, 775.3
Rheumatoid arthritis	M05-M06.9	M05-M06.9	714-714.9	714-714.9
Osteoarthritis	M16-M17.9	M16-M17.9	715-715.9	715-715.9
Low back and neck pain	G54.2-G54.4, M47-M49, M49.2-M51.9, M53-M54.9, M99.0-M99.04, M99.1-M99.14, M99.2-M99.24, M99.3-M99.34, M99.4-M99.44, M99.5-M99.54, M99.6-M99.64, M99.7-M99.74, M99.8-M99.84		353.1-353.4, 721-724.9	

Appendix Table 4: ICD-9 and ICD-10 codes by GBD 2016 nonfatal causes, impairments, and nature of injury categories				
Cause	ICD10	ICD10 Used in Hospital/Claims Analyses	ICD9	ICD9 Used in Hospital/Claims Analyses
Low back pain	G54.4, M47.015-M47.019, M47.15-M47.18, M47.25-M47.28, M47.815-M47.818, M47.896-M47.899, M48.05-M48.08, M48.16-M48.19, M48.25-M48.27, M48.35-M48.38, M48.45-M48.48, M48.55-M48.58, M49.85-M49.88, M51.05-M51.07, M51.15-M51.17, M51.25-M51.27, M51.35-M51.37, M51.45-M51.47, M51.85-M51.87, M53.3, M53.85-M53.88, M54.05-M54.09, M54.15-M54.18, M54.3-M54.5, M99.03-M99.04, M99.13-M99.14, M99.23-M99.24, M99.33-M99.34, M99.43-M99.44, M99.53-M99.54, M99.63-M99.64, M99.73-M99.74, M99.83-M99.84	G54.4, M47.015-M47.019, M47.15-M47.18, M47.25-M47.28, M47.815-M47.818, M47.896-M47.899, M48.05-M48.08, M48.16-M48.19, M48.25-M48.27, M48.35-M48.38, M48.45-M48.48, M48.55-M48.58, M49.85-M49.88, M51.05-M51.87, M53.3, M53.85-M53.88, M54.05-M54.09, M54.15-M54.18, M54.3-M54.5, M99.03-M99.04, M99.13-M99.14, M99.23-M99.24, M99.33-M99.34, M99.43-M99.44, M99.53-M99.54, M99.63-M99.64, M99.73-M99.74, M99.83-M99.84	353.1, 353.4, 721.3, 721.42, 722.10, 722.32, 722.52, 722.73, 722.83, 722.93, 724.02-724.03, 724.2-724.3, 724.6-724.79	353.1, 353.4, 721.3-721.42, 722.10-722.52, 722.73, 722.83, 722.93-724.79
Neck pain	G54.2, M47.011-M47.013, M47.11-M47.13, M47.21-M47.23, M47.811-M47.813, M47.892-M47.894, M48.01-M48.03, M48.12-M48.14, M48.21-M48.23, M48.31-M48.33, M48.41-M48.43, M48.51-M48.53, M49.81-M49.83, M50-M50.93, M53.0-M53.1, M53.81-M53.83, M54.01-M54.03, M54.11-M54.13, M54.2, M54.81, M99.01, M99.11, M99.21, M99.31, M99.41, M99.51, M99.61, M99.71, M99.81	G54.2, M47.011-M47.013, M47.11-M47.13, M47.21-M47.23, M47.811-M47.813, M47.892-M47.894, M48.01-M48.03, M48.12-M48.14, M48.21-M48.23, M48.31-M48.33, M48.41-M48.43, M48.51-M48.53, M49.81-M49.83, M50-M50.93, M53.0-M53.1, M53.81-M53.83, M54.01-M54.03, M54.11-M54.13, M54.2, M99.01, M99.11, M99.21, M99.31, M99.41, M99.51, M99.61, M99.71, M99.81	353.2, 721.0-721.1, 722.0, 722.71, 722.81, 722.91, 723-723.9	353.2, 721.0-721.1, 722.0, 722.71, 722.81, 722.91
Gout	M10-M10.19, M10.3-M10.9	M10-M10.19, M10.3-M10.9	274-274.9, 712.0-712.09	274-274.9, 712.0-712.09
Other musculoskeletal disorders	I27.1, L93-L93.2, M00-M03.6, M07-M09.8, M11-M15.9, M18-M25.9, M30-M32.9, M34-M36.8, M40-M43.9, M45-M46.99, M61-M63.89, M65-M68.8, M70-M72.4, M72.8-M73, M75-M77.9, M79-M79.676, M79.8-M87.09, M87.2-M89.59, M89.7-M95.9, M99, M99.05-M99.09, M99.15-M99.19, M99.25-M99.29, M99.35-M99.39, M99.45-M99.49, M99.55-M99.59, M99.65-M99.69, M99.75-M99.79, M99.85-M99.9	I27.1-I27.1, L93-L93.2, M00-M03.6, M07-M09.8, M11-M15.9, M18-M25.9, M30-M46.99, M61-M72.4, M72.8-M87.09, M87.2-M95.9, M99, M99.05-M99.09, M99.13-M99.19, M99.25-M99.29, M99.35-M99.39, M99.45-M99.49, M99.55-M99.59, M99.65-M99.69, M99.75-M99.79, M99.85-M99.9	416.1, 446-446.9, 710-710.2, 710.5-712, 712.1-713.8, 715-720.9, 726-727.9, 729-730.39, 730.7-739.9	416.1-416.1, 446-446.9, 710-712, 712.1-713.8, 715-720.9, 726-730.39, 730.7-739.9
Congenital birth defects	P96.0, Q00-Q07.9, Q10-Q18.9, Q20-Q28.9, Q30-Q45.9, Q50-Q61.9, Q63-Q64.19, Q65-Q87.89, Q89-Q89.8, Q90-Q93.9, Q95-Q99.9, Z13.7-Z13.79, Z14-Z15.89, Z82.7-Z82.79, Z87.7-Z87.798		237.7-237.79, 740-753.19, 753.3, 753.5, 754.3-759.9, V13.6-V13.69, V18.61, V18.9, V19.5-V19.8, V82.3	
Neural tube defects	Q00-Q01.9, Q05-Q05.9, Q07.01, Q07.03	Q00-Q07.03	740-741.93, 742.0	740-742.0
Congenital heart anomalies	Q20-Q27, Q27.1-Q28.9	Q20-Q28.9	745-747.9	745-747.9
Orofacial clefts	Q35-Q37.9	Q35-Q37.9	749-749.9	749-749.9
Down syndrome	Q90-Q90.9	Q90-Q90.9	758.0	758.0
Turner syndrome	Q96-Q96.9	Q96-Q96.9	758.6	758.6
Klinefelter syndrome	Q98-Q98.9	Q98-Q98.9	758.7	758.7
Other chromosomal abnormalities	Q91-Q93.9, Q95, Q95.2-Q95.9, Q97-Q97.9, Q99-Q99.9	Q91-Q95.9, Q97-Q97.9, Q99-Q99.9	758, 758.1-758.2, 758.4-758.5, 758.8-758.9	758, 758.1-758.5, 758.8-759.89
Congenital musculoskeletal and limb anomalies	Q65-Q65.2, Q65.8-Q66.1, Q68, Q68.1-Q68.2, Q68.6-Q74, Q74.1-Q75.1, Q75.4-Q76, Q76.1-Q76.49, Q76.8-Q79, Q79.6-Q79.9, Q87-Q87.89	Q65-Q79, Q79.6-Q87.89	754.3-756.0, 758.3-758.39, 759.7-759.89	754-756.59, 756.8-756.9
Urogenital congenital anomalies	P96.0, Q50-Q52.2, Q52.4, Q52.6-Q52.9, Q54-Q55.2, Q55.22-Q61.9, Q63-Q63.2, Q63.8-Q64.19	Q50-Q61.9, Q63-Q64.19	752.0-752.9, 753.0-753.19, 753.3, 753.5	752.0-753.9
Digestive congenital anomalies	Q38-Q38.0, Q38.3-Q38.4, Q38.6-Q43, Q43.1-Q45.8, Q79.0-Q79.59	Q38-Q45.8, Q79.0-Q79.59	750-751, 751.1-751.9, 756.6-756.79	750-751.9, 756.6-756.79
Other congenital birth defects	Q02-Q04.9, Q06-Q07.00, Q07.02, Q07.8-Q07.9, Q10-Q18.9, Q27.0, Q30-Q34.9, Q38.1-Q38.2, Q38.5, Q43.0, Q45.9, Q52.3, Q52.5, Q53-Q53.9, Q55.20-Q55.21, Q63.3, Q65.3-Q65.6, Q66.2-Q67.8, Q68.0, Q68.3-Q68.5, Q74.0, Q75.2-Q75.3, Q76.0, Q76.5-Q76.7, Q80-Q86.8, Q89-Q89.8, Q95.0-Q95.1		237.7-237.79, 742, 742.1-744.9, 748-748.9, 751.0, 752, 753, 756.2-756.3, 757-757.9, 759-759.6, 759.9	
Skin and subcutaneous diseases	A46-A46.0, A66-A67.3, A67.9, B07-B09, B35-B36.9, B85-B88.9, D86.3, E80.1-E80.29, I89.1-I89.8, L00-L05.92, L08-L08.9, L10-L14.0, L20-L23.2, L23.4-L27.0, L27.2-L30.9, L40-L45, L49-L54.0, L56, L56.2-L57.9, L59-L60.9, L62-L64, L64.8-L68.9, L70-L75.9, L80-L92.9, L94-L95.9, L97-L99.8, M72.5-M72.6, N49.2-N49.3		035-035.9, 040.0, 078.0-078.19, 102-103.9, 110-111.9, 132-134.9, 136.1, 457.2-457.3, 680-709.3, 709.8-709.9, 728.86, 785.4	
Dermatitis	L20-L23.2, L23.4-L27.0, L27.2-L27.9, L30-L30.9	L20-L23.2, L23.4-L25.9	690-692.7, 692.79-692.9	690-692.7, 692.79-692.9
Psoriasis	L40-L41.9	L40-L41.9	696-696.9	696-696.9
Cellulitis	L03-L03.91, M72.5-M72.6	L03-L03.91, M72.5-M72.6	681-682.9, 728.86	
Pyoderma	A46-A46.0, A66-A67.3, A67.9, I89.1-I89.8, L00-L02.93, L04-L05.92, L08-L08.9, L88, L97-L98.499, N49.2-N49.3	A46-A46.0, A66-A67.9, I89.1-I89.8, L00-L02.93, L04-L08.9, L88, L97-L98.499, N49.2-N49.3	035-035.9, 040.0, 102-103.9, 457.2-457.3, 680-680.9, 683-689, 785.4	035-035.9, 040.0, 102-103.9, 457.2-457.3, 680-689, 785.4
Scabies	B86	B86	133-133.6	133-133.6
Fungal skin diseases	B35-B36.9	B35-B36.9	110-111.9	110-111.9
Viral skin diseases	B07-B07.9, B08.1	B07-B07.9, B08.1	078.0-078.19	078.0-078.19

Appendix Table 4: ICD-9 and ICD-10 codes by GBD 2016 nonfatal causes, impairments, and nature of injury categories				
Cause	ICD10	ICD10 Used in Hospital/Claims Analyses	ICD9	ICD9 Used in Hospital/Claims Analyses
Acne vulgaris	L70-L70.9	L70-L70.9	706.0-706.1	706.0-706.1
Alopecia areata	L63-L63.9	L63-L63.9	704.0-704.09	704.0-704.09
Pruritus	L29-L29.9	L29-L29.9	698-699	698-699
Urticaria	L50-L50.9	L50-L50.9	708-708.9	708-708.9
Decubitus ulcer	L89-L89.95	L89-L89.95	707-707.09, 707.2-707.7	707-707.09, 707.2-707.7
Other skin and subcutaneous diseases	B08-B08.09, B08.2-B09, B85-B85.4, B87-B88.9, D86.3, E80.1-E80.29, L10-L14.0, L28-L28.2, L42-L45, L49-L49.9, L51-L54.0, L56, L56.2-L57.9, L59-L60.9, L62-L62.8, L64, L64.8-L68.9, L71-L75.9, L80-L87.9, L90-L92.9, L94-L95.9, L98.5-L99.8	B08-B08.09, B08.2-B09, B85-B85.4, B87-B88.9, D86.3, E80.1-E80.29, L10-L14.0, L28-L28.2, L42-L49.9, L51-L54.0, L56, L56.2-L57.9, L59-L62.8, L64, L64.8-L68.9, L71-L75.9, L80-L87.9, L90-L92.9, L94-L95.9, L98.5-L99.8	132-132.9, 133.8-134.9, 136.1, 692.70-692.77, 693-695.9, 697-697.9, 700-704, 704.1-706, 706.2-706.9, 707.1-707.19, 707.8-707.9, 709-709.3, 709.8-709.9	132-132.9, 133.8-134.9, 136.1, 692.70-692.77, 693-695.9, 697-697.9, 700-704, 704.1-706, 706.2-706.9, 707.1-707.19, 707.8-707.9, 709-709.3, 709.8-709.9
Sense organ diseases	B30-B30.9, H00-H02.8, H02.82-H02.9, H03.0-H06.3, H10-H11.9, H13-H13.8, H15-H22.8, H25-H28.8, H30-H36.8, H40-H40.9, H42-H44.539, H44.8-H55.89, H57-H58.9, H60-H62.8, H71-H75.83, H80-H83.93, H90-H91, H91.1-H94.83, R43-R44.9, Z01.0-Z01.12, Z13.5, Z41.3, Z52.5, Z82.1-Z82.2, Z83.5-Z83.6, Z94.7, Z97.3-Z97.4		077-077.99, 360-360.44, 360.8-374.85, 374.87-376.52, 376.8-379.59, 379.8-380.9, 384-389.9, V19.0-V19.3, V42.5, V43.0-V43.1, V45.6-V45.69, V45.78, V48.4-V48.5, V50.3, V58.71, V59.5, V74.4, V80-V80.0, V80.09-V80.3	
Glaucoma	H40-H40.9, H42-H42.8		365-365.9	
Cataract	H25-H26.9, H28-H28.8		366-366.9	
Macular degeneration	H35.3-H35.389		362.5-362.57	
Refraction and accommodation disorders	H52-H52.7		367-367.9	
Age-related and other hearing loss	H71-H75.83, H80-H80.93, H83-H83.93, H90-H91, H91.1-H91.93, H94-H94.83		384-385.9, 387-387.9, 388.1-388.2, 389-389.9	
Other vision loss	H27-H27.9, H31-H35.23, H35.4-H36.8, H46-H51.9, H53-H54.9		360.8-362.43, 362.6-363.9, 368-369.9, 377-378.9	
Other sense organ diseases	B30-B30.9, H00-H02.8, H02.82-H02.9, H03.0-H06.3, H10-H11.9, H13-H13.8, H15-H22.8, H30-H30.93, H43-H44.539, H44.8-H45.8, H55-H55.89, H57-H58.9, H60-H62.8, H81-H82.9, H92-H93.93, R43-R44.9	B30-B30.9, H00-H02.8, H02.82-H22.8, H30-H30.93, H43-H44.539, H44.8-H45.8, H55-H58.9, H60-H62.8, H81-H82.9, H92-H93.93	077-077.99, 360-360.44, 364-364.9, 370-374.85, 374.87-376.52, 376.8-376.9, 379-379.59, 379.8-380.9, 386-386.9, 388-388.02, 388.3-388.9	380-380.9, 386-388.02, 388.3-388.9
Oral disorders	K00-K08.499, K08.8-K14.9, M26-M27.9		520-525.54, 525.8-526.61, 526.69-529.9	
Caries of deciduous teeth				
Caries of permanent teeth	K02-K02.9		521.0-521.09	
Periodontal diseases	K05-K06.9		523-523.9	
Edentulism and severe tooth loss	K08.0-K08.499		525.0-525.19, 525.4-525.54	
Other oral disorders	K00-K01.1, K03-K04.99, K07-K08, K08.8-K14.9, M26-M27.9		520-521, 521.1-522.9, 524-525, 525.2-525.3, 525.8-526.61, 526.69-529.9	
Road injuries	V01-V04.99, V06-V80.929, V82-V82.9, V87.2-V87.3	V01-V04.99, V06-V80.929, V82-V82.9, V87.2-V87.3	E800.3, E801.3, E802.3, E803.3, E804.3, E805.3, E806.3, E807.3-E810.6, E811.0-E820.6, E821.0-E821.6, E822.0-E826.1, E826.3-E827.0, E827.3-E828.0, E828.4-E829.4	E800.3, E801.3, E802.3, E803.3, E804.3, E805.3, E806.3, E807.3-E810.6, E811.0-E820.6, E821.0-E821.6, E822.0-E826.1, E826.3-E827.0, E827.3-E828.0, E828.4-E829.4
Pedestrian road injuries	V01-V04.99, V06-V09.9	V01-V04.99, V06-V09.9	E811.7, E812.7, E813.7, E814.7, E815.7, E816.7, E817.7, E818.7, E819.7, E822.7, E823.7, E824.7, E825.7-E826.0, E827.0, E828.0, E829.0	E811.7, E812.7, E813.7, E814.7, E815.7, E816.7, E817.7, E818.7, E819.7, E822.7, E823.7, E824.7, E825.7-E826.0, E827.0, E828.0, E829.0
Cyclist road injuries	V10-V19.9	V10-V19.9	E800.3, E801.3, E802.3, E803.3, E804.3, E805.3, E806.3, E807.3, E810.6, E811.6, E812.6, E813.6, E814.6, E815.6, E816.6, E817.6, E818.6, E819.6, E820.6, E821.6, E822.6, E823.6, E824.6, E825.6, E826.1	E800.3, E801.3, E802.3, E803.3, E804.3, E805.3, E806.3, E807.3, E810.6, E811.6, E812.6, E813.6, E814.6, E815.6, E816.6, E817.6, E818.6, E819.6, E820.6, E821.6, E822.6, E823.6, E824.6, E825.6, E826.1
Motorcyclist road injuries	V20-V29.9	V20-V29.9	E810.2-E810.3, E811.2-E811.3, E812.2-E812.3, E813.2-E813.3, E814.2-E814.3, E815.2-E815.3, E816.2-E816.3, E817.2-E817.3, E818.2-E818.3, E819.2-E819.3, E820.2-E820.3, E821.2-E821.3, E822.2-E822.3, E823.2-E823.3, E824.2-E824.3, E825.2-E825.3	E810.2-E810.3, E811.2-E811.3, E812.2-E812.3, E813.2-E813.3, E814.2-E814.3, E815.2-E815.3, E816.2-E816.3, E817.2-E817.3, E818.2-E818.3, E819.2-E819.3, E820.2-E820.3, E821.2-E821.3, E822.2-E822.3, E823.2-E823.3, E824.2-E824.3, E825.2-E825.3
Motor vehicle road injuries	V30-V79.9, V87.2-V87.3	V30-V79.9, V87.2-V87.3	E810.0-E810.1, E811.0-E811.1, E812.0-E812.1, E813.0-E813.1, E814.0-E814.1, E815.0-E815.1, E816.0-E816.1, E817.0-E817.1, E818.0-E818.1, E819.0-E819.1, E820.0-E820.1, E821.0-E821.1, E822.0-E822.1, E823.0-E823.1, E824.0-E824.1, E825.0-E825.1	E810.0-E810.1, E811.0-E811.1, E812.0-E812.1, E813.0-E813.1, E814.0-E814.1, E815.0-E815.1, E816.0-E816.1, E817.0-E817.1, E818.0-E818.1, E819.0-E819.1, E820.0-E820.1, E821.0-E821.1, E822.0-E822.1, E823.0-E823.1, E824.0-E824.1, E825.0-E825.1
Other road injuries	V80-V80.929, V82-V82.9	V80-V80.929, V82-V82.9	E810.4-E810.5, E811.4-E811.5, E812.4-E812.5, E813.4-E813.5, E814.4-E814.5, E815.4-E815.5, E816.4-E816.5, E817.4-E817.5, E818.4-E818.5, E819.4-E819.5, E820.4-E820.5, E821.4-E821.5, E822.4-E822.5, E823.4-E823.5, E824.4-E824.5, E825.4-E825.5, E826.3-E826.4, E827.3-E827.4, E828.4, E829.4	E810.4-E810.5, E811.4-E811.5, E812.4-E812.5, E813.4-E813.5, E814.4-E814.5, E815.4-E815.5, E816.4-E816.5, E817.4-E817.5, E818.4-E818.5, E819.4-E819.5, E820.4-E820.5, E821.4-E821.5, E822.4-E822.5, E823.4-E823.5, E824.4-E824.5, E825.4-E825.5, E826.3-E826.4, E827.3-E827.4, E828.4, E829.4
Other transport injuries	V00-V00.898, V05-V05.99, V81-V81.9, V83-V86.99, V88.2-V98.8	V00-V00.898, V05-V05.99, V81-V81.9, V83-V86.99, V88.2-V98.8	E800-E800.2, E801-E801.2, E802-E802.2, E803-E803.2, E804-E804.2, E805-E805.2, E806-E806.2, E807-E807.2, E810.7, E820.7, E821.7, E826.2, E827.2, E828.2, E830-E849.9, E929.1	E800-E800.2, E801-E801.2, E802-E802.2, E803-E803.2, E804-E804.2, E805-E805.2, E806-E806.2, E807-E807.2, E810.7, E820.7, E821.7, E826.2, E827.2, E828.2, E830-E849.9, E929.1
Falls	W00-W19.9	W00-W19.9	E880-E888.9, E929.3	E880-E888.9, E929.3
Drowning	W65-W70.9, W73-W74.9	W65-W74.9	E910-E910.99	E910-E910.99
Fire, heat, and hot substances	X00-X06.9, X08-X19.9	X00-X19.9	E890-E899.09, E924-E924.99, E929.4	E890-E899.09, E924-E924.99, E929.4

Appendix Table 4: ICD-9 and ICD-10 codes by GBD 2016 nonfatal causes, impairments, and nature of injury categories

Cause	ICD10	ICD10 Used in Hospital/Claims Analyses	ICD9	ICD9 Used in Hospital/Claims Analyses
Poisonings	X48-X48.9	X48-X48.9	E863-E863.99	E850.3-E869.99, E929.2
Exposure to mechanical forces	W20-W38.9, W40-W43.9, W45.0-W45.2, W46-W46.2, W49-W52, W75-W76.9	W20-W38.9, W40-W43.9, W45.0-W45.2, W46-W52, W75-W76.9	E913-E913.19, E916-E922.99, E928.1-E928.7	E913-E913.19, E916-E922.99, E928.1-E928.7
Unintentional firearm injuries	W32-W34.9	W32-W34.9	E922-E922.99, E928.7	E922-E922.99, E928.7
Unintentional suffocation	W75-W76.9	W75-W76.9	E913-E913.19	E913-E913.19
Other exposure to mechanical forces	W20-W31.9, W35-W38.9, W40-W43.9, W45.0-W45.2, W46-W46.2, W49-W52	W20-W31.9, W35-W38.9, W40-W43.9, W45.0-W45.2, W46-W52	E916-E921.99, E928.1-E928.6	E916-E921.99, E928.1-E928.6
Adverse effects of medical treatment	D69.5-D69.59, D70.1, D78-D78.89, E03.2, E06.4, E09-E09.9, E16.0, E23.1, E24.2, E27.3, E36-E36.8, E66.1, E89-E89.9, G21.1-G21.19, G24.0-G24.09, G25.1, G25.4, G25.6-G25.79, G62.0, G72.0, G93.7, G96.0, G96.11, G97-G97.9, H59-H59.89, H91.0-H91.09, H95-H95.9, I95.2-I95.81, I97-I97.9, J70-J70.5, J95-J95.9, K08.5-K08.59, K43-K43.9, K62.7, K68.11, K91-K91.9, K94-K95.89, L23.3, L27.1, L56.0-L56.1, L64.0, L76-L76.82, M10.2-M10.29, M87.1-M87.19, M96-M96.9, N14-N14.4, N46.021, N46.121, N52.2-N52.39, N65-N65.1, N99-N99.9, P93-P93.8, P96.2, X46-X47.9, Y40-Y84.9, Y88-Y88.3, Z21.0, Z42-Z43.0, Z43.8-Z43.9, Z48-Z48.9, Z51-Z51.9, Z88-Z88.9, Z92-Z94.0, Z94.6, Z94.8-Z94.9, Z96-Z96.49, Z96.6-Z97.2, Z97.8-Z99.12, Z99.3-Z99.9	D69.5-D69.59, D70.1, D78-D78.89, E03.2, E06.4, E09-E09.9, E16.0, E23.1, E24.2, E27.3, E36-E36.8, E66.1, E89-E89.9, G21.1-G25.79, G62.0-G97.9, H59-H59.89, H91.0-H91.09, H95-H95.9, I95.2-I97.9, J70-J70.5, J95-J95.9, K08.5-K08.59, K43-K43.9, K62.7, K68.11, K91-K91.9, K94-K95.89, L23.3, L27.1, L56.0-L56.1, L64.0, L76-L76.82, M10.2-M10.29, M87.1-M87.19, M96-M96.9, N14-N14.4, N46.021, N46.121, N52.2-N52.39, N65-N65.1, N99-N99.9, P93-P96.2, X46-X47.9, Y40-Y84.9, Y88-Y88.3, Z21.0, Z42-Z51.9, Z88-Z99.9	244.0-244.1, 244.3, 331.81, 333.92, 349-349.9, 357.6, 359.24, 379.6-379.63, 440.3-440.32, 457.0, 458.2-458.29, 518.6, 519.00-519.1, 525.6-525.79, 526.62-526.63, 530.87, 536.4-536.49, 539-539.9, 551.2-551.29, 552.2-552.29, 553.2-553.29, 564.2-564.4, 569.6-569.8, 596.81-596.83, 598.2, 612-612.1, 780.66, 995.89, E862-E862.99, E867-E879.9, E930-E949.9, V44-V45, V45.2-V45.4, V45.7, V45.77, V45.79-V45.8, V45.87-V45.89	244.0-244.1, 244.3, 331.81, 333.92, 349-349.9, 357.6-359.24, 379.6-379.63, 440.3-440.32, 457.0, 458.2-458.29, 518.6-519.1, 525.6-525.79, 526.62-526.63, 530.87, 536.4-539.9, 551.2-551.29, 552.2-552.29, 553.2-553.29, 564.2-569.8, 596.81-596.83, 598.2, 612-612.1, 780.66, 995.89, E870-E879.9, E930-E949.9, V44-V45.89
Animal contact	W52.0-W62.9, W64-W64.9, X20-X29.9	W52.0-W64.9, X20-X29.9	E905-E906.99	E905-E906.99
Venomous animal contact				E905-E905.99
Non-venomous animal contact	W52.0-W62.9, W64-W64.9, X20-X29.9	W52.0-W64.9, X20-X29.9	E905-E906.99	E906-E906.99
Foreign body	H02.81-H02.819, H44.6-H44.799, M60.2-M60.28, W44-W45, W45.3-W45.9, W78-W80.9, W83-W84.9		360.5-360.69, 374.86, 376.6, 709.4, 770.1-770.18, E911-E912.09, E913.8-E915.09	
Pulmonary aspiration and foreign body in airway	W78-W80.9, W83-W84.9	W78-W80.9, W83-W84.9	770.1-770.18, E911-E912.09, E913.8-E913.99	770.1-770.18, E911-E912.09, E913.8-E913.99
Foreign body in eyes	H02.81-H02.819, H44.6-H44.799	H02.81-H02.819, H44.6-H44.799	360.5-360.69, 374.86, 376.6, E914-E914.09	360.5-360.69, 374.86, 376.6, E914-E914.09
Foreign body in other body part	M60.2-M60.28, W44-W45, W45.3-W45.9	M60.2-M60.28, W44-W45, W45.3-W45.9	709.4, E915-E915.09	709.4, E915-E915.09
Environmental heat and cold exposure	L55-L55.9, L58-L58.9, W88-W94.9, W97.9, W99-W99.9, X30-X32.9, X39-X39.9	L55-L55.9, L58-L58.9, W88-W99.9, X30-X32.9, X39-X39.9	E900-E902.99, E926-E926.99, E929.5	E900-E902.99, E926-E926.99, E929.5
Other unintentional injuries	W39-W39.9, W77-W77.9, W81-W81.9, X50-X54.9, X57-X58.9	W39-W39.9, W77-W77.9, W81-W81.9, X50-X58.9	E903-E904.99, E913.2-E913.39, E923-E923.99, E927-E928.09, E928.8-E928.89	E903-E904.99, E913.2-E913.39, E923-E923.99, E927-E928.09, E928.8-E928.89
Self-harm	X60-X64.9, X66-X84.9, Y87.0	X60-X64.9, X66-X84.9, Y87.0	E950-E959	E950-E959
Self-harm by firearm	X72-X74.9	X72-X74.9	E955-E955.9	E955-E955.9
Self-harm by other specified means	X60-X64.9, X66-X67.9, X69-X71.9, X75-X75.9, X77-X84.9, Y87.0	X60-X64.9, X66-X67.9, X69-X71.9, X75-X75.9, X77-X84.9, Y87.0	E950-E954, E956-E958.0, E958.2-E959	E950-E954, E956-E958.0, E958.2-E959
Interpersonal violence	X85-Y08.9, Y87.1-Y87.2	X85-Y08.9, Y87.1-Y87.2	E960-E969	E960-E969
Physical violence by firearm	X93-X95.9	X93-X95.9	E965-E965.4	E965-E965.4
Physical violence by sharp object	X99-X99.9	X99-X99.9	E966	E966
Sexual violence	Y05-Y05.9		E960-E960.1	
Physical violence by other means	X85-X92.9, X96-X98.9, Y00-Y04.9, Y06-Y08.9, Y87.1-Y87.2	X85-X92.9, X96-X98.9, Y00-Y04.9, Y06-Y08.9, Y87.1-Y87.2	E961-E964, E965.5-E965.9, E967-E969	E961-E964, E965.5-E965.9, E967-E969
Exposure to forces of nature	X33-X38.9		E907-E909.9	
Conflict and terrorism	U00-U03, Y36-Y38.9, Y89.1		E979-E999.1	
Executions and police conflict	Y35-Y35.93, Y89.0		E970-E978	

NOTE: This is a comprehensive mapping of ICD codes to GBD categories for nonfatal estimation. Not all causes use hospital data. Detailed case definitions disease by disease are provided in the disease and injury specific write-ups. A small number of causes have no corresponding ICD codes.

Appendix Table 5. GBD 2016 sequelae, health states, health state lay descriptions, and disability weights			
**Sequela**	**Health state name**	**Health state lay description**	**Disability weight**
Drug-susceptible tuberculosis	Tuberculosis, not HIV infected	has a persistent cough and fever, is short of breath, feels weak, and has lost a lot of weight.	0.333 (0.224–0.454)
Multidrug-resistant tuberculosis without extensive drug resistance	Tuberculosis, not HIV infected	has a persistent cough and fever, is short of breath, feels weak, and has lost a lot of weight.	0.333 (0.224–0.454)
Extensively drug-resistant tuberculosis	Tuberculosis, not HIV infected	has a persistent cough and fever, is short of breath, feels weak, and has lost a lot of weight.	0.333 (0.224–0.454)
Latent tuberculosis infection	Asymptomatic	--	--
Drug-susceptible HIV/AIDS - Tuberculosis with severe anemia	Tuberculosis, HIV infected and anemia, severe	(combined DW)	0.495 (0.353–0.64)
Drug-susceptible HIV/AIDS - Tuberculosis with moderate anemia	Tuberculosis, HIV infected and anemia, moderate	(combined DW)	0.439 (0.307–0.577)
Drug-susceptible HIV/AIDS - Tuberculosis with mild anemia	Tuberculosis, HIV infected and anemia, mild	(combined DW)	0.411 (0.278–0.551)
Drug-susceptible HIV/AIDS - Tuberculosis without anemia	Tuberculosis, HIV infected	has a persistent cough and fever, shortness of breath, night sweats, weakness and fatigue and severe weight loss.	0.408 (0.274–0.549)
Multidrug-resistant HIV/AIDS - Tuberculosis without extensive drug resistance with severe anemia	Tuberculosis, HIV infected and anemia, severe	(combined DW)	0.495 (0.353–0.64)
Multidrug-resistant HIV/AIDS - Tuberculosis without extensive drug resistance without anemia	Tuberculosis, HIV infected	has a persistent cough and fever, shortness of breath, night sweats, weakness and fatigue and severe weight loss.	0.408 (0.274–0.549)
Multidrug-resistant HIV/AIDS - Tuberculosis without extensive drug resistance with moderate anemia	Tuberculosis, HIV infected and anemia, moderate	(combined DW)	0.439 (0.307–0.577)
Multidrug-resistant HIV/AIDS - Tuberculosis without extensive drug resistance with mild anemia	Tuberculosis, HIV infected and anemia, mild	(combined DW)	0.411 (0.278–0.551)
Extensively drug-resistant HIV/AIDS - Tuberculosis with mild anemia	Tuberculosis, HIV infected and anemia, mild	(combined DW)	0.411 (0.278–0.551)
Extensively drug-resistant HIV/AIDS - Tuberculosis with severe anemia	Tuberculosis, HIV infected and anemia, severe	(combined DW)	0.495 (0.353–0.64)
Extensively drug-resistant HIV/AIDS - Tuberculosis without anemia	Tuberculosis, HIV infected	has a persistent cough and fever, shortness of breath, night sweats, weakness and fatigue and severe weight loss.	0.408 (0.274–0.549)
Extensively drug-resistant HIV/AIDS - Tuberculosis with moderate anemia	Tuberculosis, HIV infected and anemia, moderate	(combined DW)	0.439 (0.307–0.577)
Early HIV with severe anemia	Anemia, severe; Generic uncomplicated disease anxiety	--	--
Symptomatic HIV without anemia	HIV cases, symptomatic, pre-AIDS	has weight loss, fatigue, and frequent infections.	0.274 (0.184–0.377)
Early HIV with mild anemia	Anemia, mild; Generic uncomplicated disease anxiety	--	--
AIDS without antiretroviral treatment without anemia	AIDS cases, not receiving ARV treatment	has severe weight loss, weakness, fatigue, cough and fever, and frequent infections, skin rashes and diarrhea.	0.582 (0.406–0.743)
AIDS with antiretroviral treatment without anemia	HIV/AIDS cases, receiving ARV treatment	has occasional fevers and infections. The person takes daily medication that sometimes causes diarrhea.	0.078 (0.052–0.111)
Early HIV with moderate anemia	Anemia, moderate; Generic uncomplicated disease anxiety	--	--
Early HIV without anemia	Generic uncomplicated disease: anxiety about diagnosis	has a disease diagnosis that causes some worry but minimal interference with daily activities.	0.012 (0.006–0.023)
Symptomatic HIV with moderate anemia	HIV cases, symptomatic, pre-AIDS and anemia, moderate	(combined DW)	0.312 (0.217–0.418)
Symptomatic HIV with severe anemia	HIV cases, symptomatic, pre-AIDS and anemia, severe	(combined DW)	0.381 (0.269–0.505)
AIDS with antiretroviral treatment with mild anemia	HIV/AIDS cases, receiving ARV treatment and anemia, mild	(combined DW)	0.081 (0.054–0.116)
AIDS with antiretroviral treatment with moderate anemia	HIV/AIDS cases, receiving ARV treatment and anemia, moderate	(combined DW)	0.125 (0.085–0.176)
AIDS with antiretroviral treatment with severe anemia	HIV/AIDS cases, receiving ARV treatment and anemia, severe	(combined DW)	0.215 (0.148–0.295)
AIDS without antiretroviral treatment with mild anemia	AIDS cases, not receiving ARV treatment and anemia, mild	(combined DW)	0.583 (0.409–0.743)
AIDS without antiretroviral treatment with moderate anemia	AIDS cases, not receiving ARV treatment and anemia, moderate	(combined DW)	0.603 (0.43–0.758)
AIDS without antiretroviral treatment with severe anemia	AIDS cases, not receiving ARV treatment and anemia, severe	(combined DW)	0.642 (0.47–0.792)
Symptomatic HIV with mild anemia	HIV cases, symptomatic, pre-AIDS and anemia, mild	(combined DW)	0.277 (0.189–0.379)
Mild diarrheal diseases	Diarrhea, mild	has diarrhea three or more times a day with occasional discomfort in the belly.	0.074 (0.049–0.104)
Guillain-Barré syndrome due to diarrheal diseases	Spinal cord lesion below neck level (treated)	is paralyzed from the waist down, cannot feel or move the legs and has difficulties with urine and bowel control. The person uses a wheelchair to move around.	0.296 (0.198–0.414)
Severe diarrheal diseases	Diarrhea, severe	has diarrhea three or more times a day with severe belly cramps. The person is very thirsty and feels nauseous and tired.	0.247 (0.164–0.348)
Moderate diarrheal diseases	Diarrhea, moderate	has diarrhea three or more times a day, with painful cramps in the belly and feeling thirsty	0.188 (0.125–0.264)
Intestinal perforation due to typhoid	Abdominopelvic problem, severe	has severe pain in the belly and feels nauseous. The person is anxious and unable to carry out daily activities.	0.324 (0.22–0.442)
Severe typhoid fever	Infectious disease, acute episode, severe	has a high fever and pain, and feels very weak, which causes great difficulty with daily activities.	0.133 (0.088–0.19)
Gastrointestinal bleeding due to typhoid	Gastric bleeding	vomits blood and feels nauseous.	0.325 (0.209–0.462)

Appendix Table 5. GBD 2016 sequelae, health states, health state lay descriptions, and disability weights			
**Sequela**	**Health state name**	**Health state lay description**	**Disability weight**
Acute typhoid infection	Infectious disease, acute episode, moderate	has a fever and aches, and feels weak, which causes some difficulty with daily activities.	0.051 (0.032–0.074)
Acute paratyphoid infection	Infectious disease, acute episode, mild	has a low fever and mild discomfort , but no difficulty with daily activities.	0.006 (0.002–0.012)
Moderate paratyphoid fever	Infectious disease, acute episode, moderate	has a fever and aches, and feels weak, which causes some difficulty with daily activities.	0.051 (0.032–0.074)
Intestinal perforation due to paratyphoid	Abdominopelvic problem, moderate	has pain in the belly and feels nauseous. The person has difficulties with daily activities.	0.114 (0.078–0.159)
Severe paratyphoid fever	Infectious disease, acute episode, severe	has a high fever and pain, and feels very weak, which causes great difficulty with daily activities.	0.133 (0.088–0.19)
Other intestinal infectious diseases	Post-COMO calculation for residuals (YLL/YLD ratio, other methods)	--	--
Guillain-Barré syndrome due to lower respiratory infections	Spinal cord lesion below neck level (treated)	is paralyzed from the waist down, cannot feel or move the legs and has difficulties with urine and bowel control. The person uses a wheelchair to move around.	0.296 (0.198–0.414)
Severe lower respiratory infections	Infectious disease, acute episode, severe	has a high fever and pain, and feels very weak, which causes great difficulty with daily activities.	0.133 (0.088–0.19)
Moderate lower respiratory infections	Infectious disease, acute episode, moderate	has a fever and aches, and feels weak, which causes some difficulty with daily activities.	0.051 (0.032–0.074)
Mild upper respiratory infections	Infectious disease, acute episode, mild	has a low fever and mild discomfort , but no difficulty with daily activities.	0.006 (0.002–0.012)
Moderate upper respiratory infections	Infectious disease, acute episode, moderate	has a fever and aches, and feels weak, which causes some difficulty with daily activities.	0.051 (0.032–0.074)
Guillain-Barré syndrome due to upper respiratory infections	Spinal cord lesion below neck level (treated)	is paralyzed from the waist down, cannot feel or move the legs and has difficulties with urine and bowel control. The person uses a wheelchair to move around.	0.296 (0.198–0.414)
Vertigo with moderate hearing loss and ringing due to chronic otitis media	Vertigo with moderate hearing loss and ringing	--	--
Vertigo with moderate hearing loss due to chronic otitis media	Vertigo with moderate hearing loss	--	--
Severe infectious complications due to chronic otitis media	Ear pain	has an ear-ache that causes some difficulty with daily activities.	0.013 (0.007–0.024)
Acute otitis media	Ear pain	has an ear-ache that causes some difficulty with daily activities.	0.013 (0.007–0.024)
Vertigo with mild hearing loss and ringing due to chronic otitis media	Vertigo with mild hearing loss and ringing	--	--
Vertigo with mild hearing loss due to chronic otitis media	Vertigo with mild hearing loss	--	--
Moderate hearing loss due to chronic otitis media	Hearing loss, moderate	is unable to hear and understand another person talking in a noisy place (for example, on an urban street), and has difficulty hearing another person talking even in a quiet place or on the phone.	0.027 (0.015–0.042)
Mild hearing loss due to chronic otitis media	Hearing loss, mild	has great difficulty hearing and understanding another person talking in a noisy place (for example, on an urban street).	0.01 (0.004–0.019)
Moderate hearing loss with ringing due to chronic otitis media	Hearing loss, moderate, with ringing	is unable to hear and understand another person talking in a noisy place (for example, on an urban street), and has difficulty hearing another person talking even in a quiet place or on the phone, and has annoying ringing in the ears for more than 5 minutes at a time, almost everyday.	0.074 (0.049–0.107)
Mild hearing loss with ringing due to chronic otitis media	Hearing loss, mild, with ringing	has great difficulty hearing and understanding another person talking in a noisy place (for example, on an urban street), and sometimes has annoying ringing in the ears.	0.021 (0.012–0.036)
Epilepsy due to pneumococcal meningitis	Epilepsy	(combined DW)	--
Profound hearing loss due to pneumococcal meningitis	Hearing loss, profound	is unable to hear and understand another person talking, even in a quiet place, is unable to take part in a phone conversation, and has great difficulty hearing anything in any other situation. Difficulties with communicating and relating to othersoften cause worry, depression, and loneliness.	0.204 (0.134–0.288)
Acute pneumococcal meningitis	Infectious disease, acute episode, severe	has a high fever and pain, and feels very weak, which causes great difficulty with daily activities.	0.133 (0.088–0.19)
Severe hearing loss with ringing due to pneumococcal meningitis	Hearing loss, severe, with ringing	is unable to hear and understand another person talking, even in a quiet place, and unable to take part in a phone conversation, and has annoying ringing in the ears for more than 5 minutes at a time, almost everyday. Difficulties with communicating and relating to others cause emotional impact at times (for example worry or depression).	0.261 (0.175–0.36)
Moderately severe hearing loss due to pneumococcal meningitis	Hearing loss, moderately severe	(custom DW from hearing loss impairment envelope)	--
Borderline intellectual disability due to pneumococcal meningitis	Borderline intellectual functioning	is slow in learning at school. As an adult, the person has some difficulty doing complex or unfamiliar tasks but otherwise functions independently.	0.011 (0.005–0.02)
Moderate hearing loss with ringing due to pneumococcal meningitis	Hearing loss, moderate, with ringing	is unable to hear and understand another person talking in a noisy place (for example, on an urban street), and has difficulty hearing another person talking even in a quiet place or on the phone, and has annoying ringing in the ears for more than 5 minutes at a time, almost everyday.	0.074 (0.049–0.107)
Profound hearing loss with ringing due to pneumococcal meningitis	Hearing loss, profound, with ringing	[illegible] in a quiet place, is unable to take part in a phone conversation, has great difficulty hearing anything in any other situation, and has annoying ringing in the ears for more than 5 minutes at a time, several times a day. Difficulties with communicating and relating to others often cause worry, [illegible]	0.277 (0.182–0.387)

Appendix Table 5. GBD 2016 sequelae, health states, health state lay descriptions, and disability weights			
**Sequela**	**Health state name**	**Health state lay description**	**Disability weight**
Severe hearing loss due to pneumococcal meningitis	Hearing loss, severe	is unable to hear and understand another person talking, even in a quiet place, and unable to take part in a phone conversation. Difficulties with communicating and relating to others cause emotional impact at times (for example worry or depression).	0.158 (0.105–0.227)
Mild behavioral problems due to pneumococcal meningitis	Attention deficit hyperactivity disorder	is hyperactive and has difficulty concentrating, remembering things, and completing tasks.	0.045 (0.028–0.066)
Severe motor plus cognitive impairments due to pneumococcal meningitis	Motor plus cognitive impairments, severe	cannot move around without help, and cannot lift or hold objects, get dressed or sit upright. The person also has very low intelligence, speaks few words, and needs constant supervision and help with all daily activities.	0.542 (0.374–0.702)
Mild motor impairment due to long term due to pneumococcal meningitis	Motor impairment, mild	has some difficulty in moving around but is able to walk without help.	0.01 (0.005–0.019)
Monocular distance vision loss due to pneumococcal meningitis	Distance vision, monocular	is blind in one eye and has difficulty judging distances	0.017 (0.009–0.029)
Severe vision impairment due to pneumococcol meningitis	Distance vision, severe impairment	has severe vision loss, which causes difficulty in daily activities, some emotional impact (for example worry), and some difficulty going outside the home without assistance.	0.184 (0.125–0.258)
Moderately severe hearing loss with ringing due to pneumococcal meningitis	Hearing loss, moderately severe, with ringing	(custom DW from hearing loss impairment envelope)	--
Moderate motor impairment due to pneumococcal meningitis	Motor impairment, moderate	has some difficulty in moving around, and difficulty in lifting and holding objects, dressing and sitting upright, but is able to walk without help.	0.061 (0.04–0.089)
Mild hearing loss with ringing due to pneumococcal meningitis	Hearing loss, mild, with ringing	has great difficulty hearing and understanding another person talking in a noisy place (for example, on an urban street), and sometimes has annoying ringing in the ears.	0.021 (0.012–0.036)
Moderate motor plus cognitive impairments due to pneumococcal meningitis	Motor plus cognitive impairments, moderate	has some difficulty in moving around, holding objects, dressing and sitting upright, but can walk without help. The person has low intelligence and is slow in learning to speak and to do simple tasks.	0.203 (0.134–0.29)
Mild intellectual disability due to pneumococcal meningitis	Intellectual disability / mental retardation, mild	has low intelligence and is slow in learning at school. As an adult, the person can live independently, but often needs help to raise children and can only work at simple supervised jobs.	0.043 (0.026–0.064)
Complete hearing loss with ringing due to pneumococcal meningitis	Hearing loss, complete, with ringing	cannot hear at all in any situation, including even the loudest sounds, and cannot communicate verbally or use a phone, and has very annoying ringing in the ears for more than half of the day. Difficulties with communicating and relating to others often cause worry, depression or loneliness.	0.316 (0.212–0.435)
Mild motor plus cognitive impairments due to pneumococcal meningitis	Motor plus cognitive impairments, mild	has some difficulty in moving around but is able to walk without help. The person is slow in learning at school. As an adult, the person has some difficulty doing complex or unfamiliar tasks but otherwise functions independently.	0.031 (0.018–0.05)
Moderate hearing loss due to pneumococcal meningitis	Hearing loss, moderate	is unable to hear and understand another person talking in a noisy place (for example, on an urban street), and has difficulty hearing another person talking even in a quiet place or on the phone.	0.027 (0.015–0.042)
Moderate vision impairment due to pneumococcol meningitis	Distance vision, moderate impairment	has vision problems that make it difficult to recognize faces or objects across a room.	0.031 (0.019–0.049)
Mild hearing loss due to pneumococcal meningitis	Hearing loss, mild	has great difficulty hearing and understanding another person talking in a noisy place (for example, on an urban street).	0.01 (0.004–0.019)
Severe motor impairment due to pneumococcal meningitis	Motor impairment, severe	is unable to move around without help, and is not able to lift or hold objects, get dressed or sit upright.	0.402 (0.268–0.545)
Blindness due to pneumococcal meningitis	Distance vision blindness	is completely blind, which causes great difficulty in some daily activities, worry and anxiety, and great difficulty going outside the home without assistance.	0.187 (0.124–0.26)
Complete hearing loss due to pneumococcal meningitis	Hearing loss, complete	cannot hear at all in any situation, including even the loudest sounds, and cannot communicate verbally or use a phone. Difficulties with communicating and relating to others often cause worry, depression or loneliness.	0.215 (0.144–0.307)
Monocular distance vision loss due to H influenzae type B meningitis	Distance vision, monocular	is blind in one eye and has difficulty judging distances	0.017 (0.009–0.029)
Profound hearing loss due to H influenzae type B meningitis	Hearing loss, profound	is unable to hear and understand another person talking, even in a quiet place, is unable to take part in a phone conversation, and has great difficulty hearing anything in any other situation. Difficulties with communicating and relating to othersoften cause worry, depression, and loneliness.	0.204 (0.134–0.288)
Complete hearing loss due to H influenzae type B meningitis	Hearing loss, complete	cannot hear at all in any situation, including even the loudest sounds, and cannot communicate verbally or use a phone. Difficulties with communicating and relating to others often cause worry, depression or loneliness.	0.215 (0.144–0.307)
Blindness due to H influenzae type B meningitis	Distance vision blindness	is completely blind, which causes great difficulty in some daily activities, worry and anxiety, and great difficulty going outside the home without assistance.	0.187 (0.124–0.26)
Complete hearing loss with ringing due to H influenzae type B meningitis	Hearing loss, complete, with ringing	cannot hear at all in any situation, including even the loudest sounds, and cannot communicate verbally or use a phone, and has very annoying ringing in the ears for more than half of the day. Difficulties with communicating and relating to others often cause worry, depression or loneliness.	0.316 (0.212–0.435)

Appendix Table 5. GBD 2016 sequelae, health states, health state lay descriptions, and disability weights			
**Sequela**	**Health state name**	**Health state lay description**	**Disability weight**
Severe hearing loss with ringing due to H influenzae type B meningitis	Hearing loss, severe, with ringing	is unable to hear and understand another person talking, even in a quiet place, and unable to take part in a phone conversation, and has annoying ringing in the ears for more than 5 minutes at a time, almost everyday. Difficulties with communicating and relating to others cause emotional impact at times (for example worry or depression).	0.261 (0.175–0.36)
Profound hearing loss with ringing due to H influenzae type B meningitis	Hearing loss, profound, with ringing	is unable to hear and understand another person talking, even in a quiet place, is unable to take part in a phone conversation, has great difficulty hearing anything in any other situation, and has annoying ringing in the ears for more than 5 minutes at a time, several times a day. Difficulties with communicating and relating to others often cause worry, depression, or loneliness.	0.277 (0.182–0.387)
Severe vision impairment due to H influenza type B meningitis	Distance vision, severe impairment	has severe vision loss, which causes difficulty in daily activities, some emotional impact (for example worry), and some difficulty going outside the home without assistance.	0.184 (0.125–0.258)
Moderate vision impairment due to H influenza type B meningitis	Distance vision, moderate impairment	has vision problems that make it difficult to recognize faces or objects across a room.	0.031 (0.019–0.049)
Mild hearing loss with ringing due to H influenzae type B meningitis	Hearing loss, mild, with ringing	has great difficulty hearing and understanding another person talking in a noisy place (for example, on an urban street), and sometimes has annoying ringing in the ears.	0.021 (0.012–0.036)
Moderate hearing loss with ringing due to H influenzae type B meningitis	Hearing loss, moderate, with ringing	is unable to hear and understand another person talking in a noisy place (for example, on an urban street), and has difficulty hearing another person talking even in a quiet place or on the phone, and has annoying ringing in the ears for more than 5 minutes at a time, almost everyday.	0.074 (0.049–0.107)
Severe hearing loss due to H influenzae type B meningitis	Hearing loss, severe	is unable to hear and understand another person talking, even in a quiet place, and unable to take part in a phone conversation. Difficulties with communicating and relating to others cause emotional impact at times (for example worry or depression).	0.158 (0.105–0.227)
Moderate motor plus cognitive impairments due to H influenzae type B meningitis	Motor plus cognitive impairments, moderate	has some difficulty in moving around, holding objects, dressing and sitting upright, but can walk without help. The person has low intelligence and is slow in learning to speak and to do simple tasks.	0.203 (0.134–0.29)
Mild intellectual disability due to H influenzae type B meningitis	Intellectual disability / mental retardation, mild	has low intelligence and is slow in learning at school. As an adult, the person can live independently, but often needs help to raise children and can only work at simple supervised jobs.	0.043 (0.026–0.064)
Mild motor plus cognitive impairments due to H influenzae type B meningitis	Motor plus cognitive impairments, mild	has some difficulty in moving around but is able to walk without help. The person is slow in learning at school. As an adult, the person has some difficulty doing complex or unfamiliar tasks but otherwise functions independently.	0.031 (0.018–0.05)
Severe motor impairment due to H influenzae type B meningitis	Motor impairment, severe	is unable to move around without help, and is not able to lift or hold objects, get dressed or sit upright.	0.402 (0.268–0.545)
Mild hearing loss due to H influenzae type B meningitis	Hearing loss, mild	has great difficulty hearing and understanding another person talking in a noisy place (for example, on an urban street).	0.01 (0.004–0.019)
Severe motor plus cognitive impairments due to H influenzae type B meningitis	Motor plus cognitive impairments, severe	cannot move around without help, and cannot lift or hold objects, get dressed or sit upright. The person also has very low intelligence, speaks few words, and needs constant supervision and help with all daily activities.	0.542 (0.374–0.702)
Moderately severe hearing loss due to H influenzae type B meningitis	Hearing loss, moderately severe	(custom DW from hearing loss impairment envelope)	--
Moderate hearing loss due to H influenzae type B meningitis	Hearing loss, moderate	is unable to hear and understand another person talking in a noisy place (for example, on an urban street), and has difficulty hearing another person talking even in a quiet place or on the phone.	0.027 (0.015–0.042)
Mild behavioral problems due to H influenzae type B meningitis	Attention deficit hyperactivity disorder	is hyperactive and has difficulty concentrating, remembering things, and completing tasks.	0.045 (0.028–0.066)
Acute H influenzae type B meningitis	Infectious disease, acute episode, severe	has a high fever and pain, and feels very weak, which causes great difficulty with daily activities.	0.133 (0.088–0.19)
Mild motor impairment due to long term due to H influenzae type B meningitis	Motor impairment, mild	has some difficulty in moving around but is able to walk without help.	0.01 (0.005–0.019)
Epilepsy due to H influenzae type B meningitis	Epilepsy	(combined DW)	--
Moderately severe hearing loss with ringing due to H influenzae type B meningitis	Hearing loss, moderately severe, with ringing	(custom DW from hearing loss impairment envelope)	--
Moderate motor impairment due to H influenzae type B meningitis	Motor impairment, moderate	has some difficulty in moving around, and difficulty in lifting and holding objects, dressing and sitting upright, but is able to walk without help.	0.061 (0.04–0.089)
Borderline intellectual disability due to H influenzae type B meningitis	Borderline intellectual functioning	is slow in learning at school. As an adult, the person has some difficulty doing complex or unfamiliar tasks but otherwise functions independently.	0.011 (0.005–0.02)
Mild behavioral problems due to meningococcal meningitis	Attention deficit hyperactivity disorder	is hyperactive and has difficulty concentrating, remembering things, and completing tasks.	0.045 (0.028–0.066)
Mild hearing loss with ringing due to meningococcal meningitis	Hearing loss, mild, with ringing	has great difficulty hearing and understanding another person talking in a noisy place (for example, on an urban street), and sometimes has annoying ringing in the ears.	0.021 (0.012–0.036)
Complete hearing loss due to meningococcal meningitis	Hearing loss, complete	cannot hear at all in any situation, including even the loudest sounds, and cannot communicate verbally or use a phone. Difficulties with communicating and relating to others often cause worry, depression or loneliness.	0.215 (0.144–0.307)

Appendix Table 5. GBD 2016 sequelae, health states, health state lay descriptions, and disability weights

Sequela	Health state name	Health state lay description	Disability weight
Severe hearing loss due to meningococcal meningitis	Hearing loss, severe	is unable to hear and understand another person talking, even in a quiet place, and unable to take part in a phone conversation. Difficulties with communicating and relating to others cause emotional impact at times (for example worry or depression).	0.158 (0.105–0.227)
Mild intellectual disability due to meningococcal meningitis	Intellectual disability / mental retardation, mild	has low intelligence and is slow in learning at school. As an adult, the person can live independently, but often needs help to raise children and can only work at simple supervised jobs.	0.043 (0.026–0.064)
Mild hearing loss due to meningococcal meningitis	Hearing loss, mild	has great difficulty hearing and understanding another person talking in a noisy place (for example, on an urban street).	0.01 (0.004–0.019)
Moderate hearing loss due to meningococcal meningitis	Hearing loss, moderate	is unable to hear and understand another person talking in a noisy place (for example, on an urban street), and has difficulty hearing another person talking even in a quiet place or on the phone.	0.027 (0.015–0.042)
Profound hearing loss due to meningococcal meningitis	Hearing loss, profound	is unable to hear and understand another person talking, even in a quiet place, is unable to take part in a phone conversation, and has great difficulty hearing anything in any other situation. Difficulties with communicating and relating to othersoften cause worry, depression, and loneliness.	0.204 (0.134–0.288)
Borderline intellectual disability due to meningococcal meningitis	Borderline intellectual functioning	is slow in learning at school. As an adult, the person has some difficulty doing complex or unfamiliar tasks but otherwise functions independently.	0.011 (0.005–0.02)
Moderately severe hearing loss due to meningococcal meningitis	Hearing loss, moderately severe	(custom DW from hearing loss impairment envelope)	--
Moderate vision impairment due to meningococcal meningitis	Distance vision, moderate impairment	has vision problems that make it difficult to recognize faces or objects across a room.	0.031 (0.019–0.049)
Blindness due to meningococcal meningitis	Distance vision blindness	is completely blind, which causes great difficulty in some daily activities, worry and anxiety, and great difficulty going outside the home without assistance.	0.187 (0.124–0.26)
Mild motor plus cognitive impairments due to meningococcal meningitis	Motor plus cognitive impairments, mild	has some difficulty in moving around but is able to walk without help. The person is slow in learning at school. As an adult, the person has some difficulty doing complex or unfamiliar tasks but otherwise functions independently.	0.031 (0.018–0.05)
Epilepsy due to meningococcal meningitis	Epilepsy	(combined DW)	--
Moderate motor impairment due to meningococcal meningitis	Motor impairment, moderate	has some difficulty in moving around, and difficulty in lifting and holding objects, dressing and sitting upright, but is able to walk without help.	0.061 (0.04–0.089)
Severe vision impairment due to meningococcal meningitis	Distance vision, severe impairment	has severe vision loss, which causes difficulty in daily activities, some emotional impact (for example worry), and some difficulty going outside the home without assistance.	0.184 (0.125–0.258)
Severe motor impairment due to meningococcal meningitis	Motor impairment, severe	is unable to move around without help, and is not able to lift or hold objects, get dressed or sit upright.	0.402 (0.268–0.545)
Moderate motor plus cognitive impairments due to meningococcal meningitis	Motor plus cognitive impairments, moderate	has some difficulty in moving around, holding objects, dressing and sitting upright, but can walk without help. The person has low intelligence and is slow in learning to speak and to do simple tasks.	0.203 (0.134–0.29)
Moderate hearing loss with ringing due to meningococcal meningitis	Hearing loss, moderate, with ringing	is unable to hear and understand another person talking in a noisy place (for example, on an urban street), and has difficulty hearing another person talking even in a quiet place or on the phone, and has annoying ringing in the ears for more than 5 minutes at a time, almost everyday.	0.074 (0.049–0.107)
Severe motor plus cognitive impairments due to meningococcal meningitis	Motor plus cognitive impairments, severe	cannot move around without help, and cannot lift or hold objects, get dressed or sit upright. The person also has very low intelligence, speaks few words, and needs constant supervision and help with all daily activities.	0.542 (0.374–0.702)
Monocular distance vision loss due to meningococcal meningitis	Distance vision, monocular	is blind in one eye and has difficulty judging distances	0.017 (0.009–0.029)
Profound hearing loss with ringing due to meningococcal meningitis	Hearing loss, profound, with ringing	is unable to hear and understand another person talking, even in a quiet place, is unable to take part in a phone conversation, has great difficulty hearing anything in any other situation, and has annoying ringing in the ears for more than 5 minutes at a time, several times a day. Difficulties with communicating and relating to others often cause worry, [illegible]	0.277 (0.182–0.387)
Mild motor impairment due to long term due to meningococcal meningitis	Motor impairment, mild	has some difficulty in moving around but is able to walk without help.	0.01 (0.005–0.019)
Acute meningococcal meningitis	Infectious disease, acute episode, severe	has a high fever and pain, and feels very weak, which causes great difficulty with daily activities.	0.133 (0.088–0.19)
Severe hearing loss with ringing due to meningococcal meningitis	Hearing loss, severe, with ringing	is unable to hear and understand another person talking, even in a quiet place, and unable to take part in a phone conversation, and has annoying ringing in the ears for more than 5 minutes at a time, almost everyday. Difficulties with communicating and relating to others cause emotional impact at times (for example worry or depression).	0.261 (0.175–0.36)
Complete hearing loss with ringing due to meningococcal meningitis	Hearing loss, complete, with ringing	cannot hear at all in any situation, including even the loudest sounds, and cannot communicate verbally or use a phone, and has very annoying ringing in the ears for more than half of the day. Difficulties with communicating and relating to others often cause worry, depression or loneliness.	0.316 (0.212–0.435)
Moderately severe hearing loss with ringing due to meningococcal meningitis	Hearing loss, moderately severe, with ringing	(custom DW from hearing loss impairment envelope)	--

Appendix Table 5. GBD 2016 sequelae, health states, health state lay descriptions, and disability weights			
**Sequela**	**Health state name**	**Health state lay description**	**Disability weight**
Mild intellectual disability due to other bacterial meningitis	Intellectual disability / mental retardation, mild	has low intelligence and is slow in learning at school. As an adult, the person can live independently, but often needs help to raise children and can only work at simple supervised jobs.	0.043 (0.026–0.064)
Moderate hearing loss with ringing due to other bacterial meningitis	Hearing loss, moderate, with ringing	is unable to hear and understand another person talking in a noisy place (for example, on an urban street), and has difficulty hearing another person talking even in a quiet place or on the phone, and has annoying ringing in the ears for more than 5 minutes at a time, almost everyday.	0.074 (0.049–0.107)
Mild motor plus cognitive impairments due to other bacterial meningitis	Motor plus cognitive impairments, mild	has some difficulty in moving around but is able to walk without help. The person is slow in learning at school. As an adult, the person has some difficulty doing complex or unfamiliar tasks but otherwise functions independently.	0.031 (0.018–0.05)
Borderline intellectual disability due to other bacterial meningitis	Borderline intellectual functioning	is slow in learning at school. As an adult, the person has some difficulty doing complex or unfamiliar tasks but otherwise functions independently.	0.011 (0.005–0.02)
Moderate motor plus cognitive impairments due to other bacterial meningitis	Motor plus cognitive impairments, moderate	has some difficulty in moving around, holding objects, dressing and sitting upright, but can walk without help. The person has low intelligence and is slow in learning to speak and to do simple tasks.	0.203 (0.134–0.29)
Mild behavioral problems due to other bacterial meningitis	Attention deficit hyperactivity disorder	is hyperactive and has difficulty concentrating, remembering things, and completing tasks.	0.045 (0.028–0.066)
Severe motor plus cognitive impairments due to other bacterial meningitis	Motor plus cognitive impairments, severe	cannot move around without help, and cannot lift or hold objects, get dressed or sit upright. The person also has very low intelligence, speaks few words, and needs constant supervision and help with all daily activities.	0.542 (0.374–0.702)
Severe motor impairment due to other bacterial meningitis	Motor impairment, severe	is unable to move around without help, and is not able to lift or hold objects, get dressed or sit upright.	0.402 (0.268–0.545)
Moderately severe hearing loss due to other bacterial meningitis	Hearing loss, moderately severe	(custom DW from hearing loss impairment envelope)	--
Monocular distance vision loss due to other bacterial meningitis	Distance vision, monocular	is blind in one eye and has difficulty judging distances	0.017 (0.009–0.029)
Mild hearing loss due to other bacterial meningitis	Hearing loss, mild	has great difficulty hearing and understanding another person talking in a noisy place (for example, on an urban street).	0.01 (0.004–0.019)
Moderate hearing loss due to other bacterial meningitis	Hearing loss, moderate	is unable to hear and understand another person talking in a noisy place (for example, on an urban street), and has difficulty hearing another person talking even in a quiet place or on the phone.	0.027 (0.015–0.042)
Epilepsy due to other meningitis	Epilepsy	(combined DW)	--
Moderately severe hearing loss with ringing due to other bacterial meningitis	Hearing loss, moderately severe, with ringing	(custom DW from hearing loss impairment envelope)	--
Severe hearing loss due to other bacterial meningitis	Hearing loss, severe	is unable to hear and understand another person talking, even in a quiet place, and unable to take part in a phone conversation. Difficulties with communicating and relating to others cause emotional impact at times (for example worry or depression).	0.158 (0.105–0.227)
Blindness due to other bacterial meningitis	Distance vision blindness	is completely blind, which causes great difficulty in some daily activities, worry and anxiety, and great difficulty going outside the home without assistance.	0.187 (0.124–0.26)
Profound hearing loss due to other bacterial meningitis	Hearing loss, profound	is unable to hear and understand another person talking, even in a quiet place, is unable to take part in a phone conversation, and has great difficulty hearing anything in any other situation. Difficulties with communicating and relating to othersoften cause worry, depression, and loneliness.	0.204 (0.134–0.288)
Moderate vision impairment due to other bacterial meningitis	Distance vision, moderate impairment	has vision problems that make it difficult to recognize faces or objects across a room.	0.031 (0.019–0.049)
Complete hearing loss with ringing due to other bacterial meningitis	Hearing loss, complete, with ringing	cannot hear at all in any situation, including even the loudest sounds, and cannot communicate verbally or use a phone, and has very annoying ringing in the ears for more than half of the day. Difficulties with communicating and relating to others often cause worry, depression or loneliness.	0.316 (0.212–0.435)
Complete hearing loss due to other bacterial meningitis	Hearing loss, complete	cannot hear at all in any situation, including even the loudest sounds, and cannot communicate verbally or use a phone. Difficulties with communicating and relating to others often cause worry, depression or loneliness.	0.215 (0.144–0.307)
Profound hearing loss with ringing due to other bacterial meningitis	Hearing loss, profound, with ringing	is unable to hear and understand another person talking, even in a quiet place, is unable to take part in a phone conversation, has great difficulty hearing anything in any other situation, and has annoying ringing in the ears for more than 5 minutes at a time, several times a day. Difficulties with communicating and relating to others often cause worry, [illegible]	0.277 (0.182–0.387)
Severe hearing loss with ringing due to other bacterial meningitis	Hearing loss, severe, with ringing	is unable to hear and understand another person talking, even in a quiet place, and unable to take part in a phone conversation, and has annoying ringing in the ears for more than 5 minutes at a time, almost everyday. Difficulties with communicating and relating to others cause emotional impact at times (for example worry or depression).	0.261 (0.175–0.36)
Mild hearing loss due with ringing to other bacterial meningitis	Hearing loss, mild, with ringing	has great difficulty hearing and understanding another person talking in a noisy place (for example, on an urban street), and sometimes has annoying ringing in the ears.	0.021 (0.012–0.036)

Appendix Table 5. GBD 2016 sequelae, health states, health state lay descriptions, and disability weights			
**Sequela**	**Health state name**	**Health state lay description**	**Disability weight**
Moderate motor impairment due to other bacterial meningitis	Motor impairment, moderate	has some difficulty in moving around, and difficulty in lifting and holding objects, dressing and sitting upright, but is able to walk without help.	0.061 (0.04–0.089)
Severe vision impairment due to other bacterial meningitis	Distance vision, severe impairment	has severe vision loss, which causes difficulty in daily activities, some emotional impact (for example worry), and some difficulty going outside the home without assistance.	0.184 (0.125–0.258)
Mild motor impairment due to long term due to other bacterial meningitis	Motor impairment, mild	has some difficulty in moving around but is able to walk without help.	0.01 (0.005–0.019)
Other acute bacterial meningitis	Infectious disease, acute episode, severe	has a high fever and pain, and feels very weak, which causes great difficulty with daily activities.	0.133 (0.088–0.19)
Acute viral meningitis	Infectious disease, acute episode, severe	has a high fever and pain, and feels very weak, which causes great difficulty with daily activities.	0.133 (0.088–0.19)
Blindness due to encephalitis	Distance vision blindness	is completely blind, which causes great difficulty in some daily activities, worry and anxiety, and great difficulty going outside the home without assistance.	0.187 (0.124–0.26)
Epilepsy due to encephalitis	Epilepsy	(combined DW)	--
Monocular distance vision loss due to encephalitis	Distance vision, monocular	is blind in one eye and has difficulty judging distances	0.017 (0.009–0.029)
Severe motor plus cognitive impairments due to encephalitis	Motor plus cognitive impairments, severe	cannot move around without help, and cannot lift or hold objects, get dressed or sit upright. The person also has very low intelligence, speaks few words, and needs constant supervision and help with all daily activities.	0.542 (0.374–0.702)
Moderate motor plus cognitive impairments due to encephalitis	Motor plus cognitive impairments, moderate	has some difficulty in moving around, holding objects, dressing and sitting upright, but can walk without help. The person has low intelligence and is slow in learning to speak and to do simple tasks.	0.203 (0.134–0.29)
Mild motor plus cognitive impairments due to encephalitis	Motor plus cognitive impairments, mild	has some difficulty in moving around but is able to walk without help. The person is slow in learning at school. As an adult, the person has some difficulty doing complex or unfamiliar tasks but otherwise functions independently.	0.031 (0.018–0.05)
Mild behavioral problems due to encephalitis	Attention deficit hyperactivity disorder	is hyperactive and has difficulty concentrating, remembering things, and completing tasks.	0.045 (0.028–0.066)
Severe motor impairment due to encephalitis	Motor impairment, severe	is unable to move around without help, and is not able to lift or hold objects, get dressed or sit upright.	0.402 (0.268–0.545)
Acute encephalitis	Infectious disease, acute episode, severe	has a high fever and pain, and feels very weak, which causes great difficulty with daily activities.	0.133 (0.088–0.19)
Borderline intellectual disability due to encephalitis	Borderline intellectual functioning	is slow in learning at school. As an adult, the person has some difficulty doing complex or unfamiliar tasks but otherwise functions independently.	0.011 (0.005–0.02)
Moderate motor impairment due to encephalitis	Motor impairment, moderate	has some difficulty in moving around, and difficulty in lifting and holding objects, dressing and sitting upright, but is able to walk without help.	0.061 (0.04–0.089)
Mild intellectual disability due to encephalitis	Intellectual disability / mental retardation, mild	has low intelligence and is slow in learning at school. As an adult, the person can live independently, but often needs help to raise children and can only work at simple supervised jobs.	0.043 (0.026–0.064)
Mild motor impairment due to long term due to encephalitis	Motor impairment, mild	has some difficulty in moving around but is able to walk without help.	0.01 (0.005–0.019)
Moderate vision impairment due to encephalitis	Distance vision, moderate impairment	has vision problems that make it difficult to recognize faces or objects across a room.	0.031 (0.019–0.049)
Severe vision impairment due to encephalitis	Distance vision, severe impairment	has severe vision loss, which causes difficulty in daily activities, some emotional impact (for example worry), and some difficulty going outside the home without assistance.	0.184 (0.125–0.258)
Severe diphtheria	Infectious disease, acute episode, severe	has a high fever and pain, and feels very weak, which causes great difficulty with daily activities.	0.133 (0.088–0.19)
Moderate diphtheria	Infectious disease, acute episode, moderate	has a fever and aches, and feels weak, which causes some difficulty with daily activities.	0.051 (0.032–0.074)
Whooping cough	Infectious disease, acute episode, moderate	has a fever and aches, and feels weak, which causes some difficulty with daily activities.	0.051 (0.032–0.074)
Severe motor plus cognitive impairment with blindness due to neonatal tetanus	Severe motor plus cognitive impairment with blindness	(combined DW)	--
Severe motor impairment with blindness and epilepsy due to neonatal tetanus	Severe motor impairment with blindness and epilepsy	(combined DW)	--
Severe motor impairment with epilepsy due to neonatal tetanus	Severe motor impairment with epilepsy	(combined DW)	--
Severe motor impairment with blindness due to neonatal tetanus	Severe motor impairment with blindness	(combined DW)	--
Moderate motor plus cognitive impairment with blindness and epilepsy due to neonatal tetanus	Moderate motor plus cognitive impairment with blindness and epilepsy	(combined DW)	--
Moderate motor plus cognitive impairment with epilepsy due to neonatal tetanus	Moderate motor plus cognitive impairment with epilepsy	(combined DW)	--
Moderate motor impairment with epilepsy due to neonatal tetanus	Moderate motor impairment with epilepsy	(combined DW)	--
Moderate motor impairment with blindness and epilepsy due to neonatal tetanus	Moderate motor impairment with blindness and epilepsy	(combined DW)	--
Severe motor impairment due to neonatal tetanus	Motor impairment, severe	is unable to move around without help, and is not able to lift or hold objects, get dressed or sit upright.	0.402 (0.268–0.545)
Mild motor impairment due to neonatal tetanus	Motor impairment, mild	has some difficulty in moving around but is able to walk without help.	0.01 (0.005–0.019)
Moderate motor impairment due to neonatal tetanus	Motor impairment, moderate	has some difficulty in moving around, and difficulty in lifting and holding objects, dressing and sitting upright, but is able to walk without help.	0.061 (0.04–0.089)

Appendix Table 5. GBD 2016 sequelae, health states, health state lay descriptions, and disability weights

Sequela	Health state name	Health state lay description	Disability weight
Severe motor plus cognitive impairment with epilepsy due to neonatal tetanus	Severe motor plus cognitive impairment with epilepsy	(combined DW)	--
Severe tetanus	Infectious disease, acute episode, severe	has a high fever and pain, and feels very weak, which causes great difficulty with daily activities.	0.133 (0.088–0.19)
Mild motor plus cognitive impairments due to neonatal tetanus	Motor plus cognitive impairments, mild	has some difficulty in moving around but is able to walk without help. The person is slow in learning at school. As an adult, the person has some difficulty doing complex or unfamiliar tasks but otherwise functions independently.	0.031 (0.018–0.05)
Moderate motor plus cognitive impairment with blindness due to neonatal tetanus	Moderate motor plus cognitive impairment with blindness	(combined DW)	--
Severe motor plus cognitive impairment with blindness and epilepsy due to neonatal tetanus	Severe motor plus cognitive impairment with blindness and epilepsy	(combined DW)	--
Moderate motor impairment with blindness due to neonatal tetanus	Moderate motor impairment with blindness	(combined DW)	--
Moderate measles	Infectious disease, acute episode, moderate	has a fever and aches, and feels weak, which causes some difficulty with daily activities.	0.051 (0.032–0.074)
Severe measles	Infectious disease, acute episode, severe	has a high fever and pain, and feels very weak, which causes great difficulty with daily activities.	0.133 (0.088–0.19)
Chickenpox	Infectious disease, acute episode, mild	has a low fever and mild discomfort , but no difficulty with daily activities.	0.006 (0.002–0.012)
Herpes zoster	Herpes zoster	has a blistering skin rash that causes pain, with some burning and itching.	0.058 (0.035–0.09)
Moderate motor impairment with epilepsy due to malaria	Moderate motor impairment with epilepsy	(combined DW)	--
Moderate malaria with moderate anemia	Infectious disease, acute episode, moderate, with moderate anemia	(combined DW)	0.099 (0.065–0.142)
Moderate malaria with severe anemia	Infectious disease, acute episode, moderate, with severe anemia	(combined DW)	0.192 (0.133–0.263)
Moderate motor plus cognitive impairment with epilepsy due to malaria	Moderate motor plus cognitive impairment with epilepsy	(combined DW)	--
Severe malaria	Infectious disease, acute episode, severe	has a high fever and pain, and feels very weak, which causes great difficulty with daily activities.	0.133 (0.088–0.19)
Severe motor plus cognitive impairment with blindness and epilepsy due to malaria	Severe motor plus cognitive impairment with blindness and epilepsy	(combined DW)	--
Severe motor impairment due to malaria	Motor impairment, severe	is unable to move around without help, and is not able to lift or hold objects, get dressed or sit upright.	0.402 (0.268–0.545)
Moderate motor impairment due to malaria	Motor impairment, moderate	has some difficulty in moving around, and difficulty in lifting and holding objects, dressing and sitting upright, but is able to walk without help.	0.061 (0.04–0.089)
Mild malaria	Infectious disease, acute episode, mild	has a low fever and mild discomfort , but no difficulty with daily activities.	0.006 (0.002–0.012)
Moderate malaria with mild anemia	Infectious disease, acute episode, moderate, with mild anemia	(combined DW)	0.054 (0.034–0.079)
Severe motor plus cognitive impairment with epilepsy due to malaria	Severe motor plus cognitive impairment with epilepsy	(combined DW)	--
Moderate malaria	Infectious disease, acute episode, moderate	has a fever and aches, and feels weak, which causes some difficulty with daily activities.	0.051 (0.032–0.074)
Mild anemia due to malaria parasitemia (PfPR)	Anemia, mild	feels slightly tired and weak at times, but this does not interfere with normal daily activities.	0.004 (0.001–0.008)
Moderate anemia due to malaria parasitemia (PfPR)	Anemia, moderate	feels moderate fatigue, weakness, and shortness of breath after exercise, making daily activities more difficult.	0.052 (0.034–0.076)
Severe motor plus cognitive impairment with blindness due to malaria	Severe motor plus cognitive impairment with blindness	(combined DW)	--
Severe motor impairment with blindness due to malaria	Severe motor impairment with blindness	(combined DW)	--
Severe anemia due to malaria parasitemia (PfPR)	Anemia, severe	feels very weak, tired and short of breath, and has problems with activities that require physical effort or deep concentration.	0.149 (0.101–0.209)
Severe motor impairment with blindness and epilepsy due to malaria	Severe motor impairment with blindness and epilepsy	(combined DW)	--
Moderate motor plus cognitive impairment with blindness and epilepsy due to malaria	Moderate motor plus cognitive impairment with blindness and epilepsy	(combined DW)	--
Mild malaria with severe anemia	Infectious disease, acute episode, mild, with severe anemia	(combined DW)	0.154 (0.105–0.214)
Severe motor impairment with epilepsy due to malaria	Severe motor impairment with epilepsy	(combined DW)	--
Severe malaria with severe anemia	Infectious disease, acute episode, severe, with severe anemia	(combined DW)	0.262 (0.184–0.359)
Severe malaria with moderate anemia	Infectious disease, acute episode, severe, with moderate anemia	(combined DW)	0.178 (0.122–0.247)
Moderate motor impairment with blindness and epilepsy due to malaria	Moderate motor impairment with blindness and epilepsy	(combined DW)	--
Moderate motor impairment with blindness due to malaria	Moderate motor impairment with blindness	(combined DW)	--
Mild malaria with mild anemia	Infectious disease, acute episode, mild, with mild anemia	(combined DW)	0.009 (0.004–0.02)
Severe malaria with mild anemia	Infectious disease, acute episode, severe, with mild anemia	(combined DW)	0.137 (0.091–0.192)
Asymptomatic malaria parasitemia (PfPR)	Asymptomatic	--	--
Mild malaria with moderate anemia	Infectious disease, acute episode, mild, with moderate anemia	(combined DW)	0.057 (0.037–0.085)
Moderate motor plus cognitive impairment with blindness due to malaria	Moderate motor plus cognitive impairment with blindness	(combined DW)	--

Appendix Table 5. GBD 2016 sequelae, health states, health state lay descriptions, and disability weights			
**Sequela**	**Health state name**	**Health state lay description**	**Disability weight**
Moderate heart failure due to Chagas disease	Heart failure, moderate	is short of breath and easily tires with minimal physical activity, such as walking only a short distance. The person feels comfortable at rest but avoids moderate activity.	0.072 (0.047–0.103)
Atrial fibrillation and flutter due to Chagas disease	Cardiac conduction disorders and cardiac dysrhythmias	has periods of rapid and irregular heartbeats and occasional fainting.	0.224 (0.151–0.312)
Asymptomatic and mild heart failure due to Chagas disease	Heart failure, mild	is short of breath and easily tires with moderate physical activity, such as walking uphill or more than a quarter-mile on level ground. The person feels comfortable at rest or during activities requiring less effort.	0.041 (0.026–0.062)
Mild chronic digestive disease due to Chagas disease	Abdominopelvic problem, mild	has some pain in the belly that causes nausea but does not interfere with daily activities.	0.011 (0.005–0.021)
Acute Chagas disease	Infectious disease, acute episode, moderate	has a fever and aches, and feels weak, which causes some difficulty with daily activities.	0.051 (0.032–0.074)
Severe heart failure due to Chagas disease	Heart failure, severe	is short of breath and feels tired when at rest. The person avoids any physical activity, for fear of worsening the breathing problems.	0.179 (0.122–0.251)
Asymptomatic Chagas disease	Asymptomatic	--	--
Moderate chronic digestive disease due to Chagas disease	Abdominopelvic problem, moderate	has pain in the belly and feels nauseous. The person has difficulties with daily activities.	0.114 (0.078–0.159)
Moderate visceral leishmaniasis	Infectious disease, acute episode, moderate	has a fever and aches, and feels weak, which causes some difficulty with daily activities.	0.051 (0.032–0.074)
Severe visceral leishmaniasis	Infectious disease, acute episode, severe	has a high fever and pain, and feels very weak, which causes great difficulty with daily activities.	0.133 (0.088–0.19)
Cutaneous and mucocutaneous leishmaniasis	Disfigurement, level 2	has a visible physical deformity that causes others to stare and comment. As a result, the person is worried and has trouble sleeping and concentrating.	0.067 (0.044–0.096)
Sleeping sickness due to Trypanosoma brucei rhodesiense	Disfigurement, level 1 with itch/pain	has a slight, visible physical deformity that is sometimes sore or itchy. Others notice the deformity, which causes some worry and discomfort.	0.027 (0.015–0.042)
Skin disfigurement due to Trypanosoma brucei gambiense	Disfigurement, level 1 with itch/pain	has a slight, visible physical deformity that is sometimes sore or itchy. Others notice the deformity, which causes some worry and discomfort.	0.027 (0.015–0.042)
Skin disfigurement due to Trypanosoma brucei rhodesiense	Motor plus cognitive impairments, severe	cannot move around without help, and cannot lift or hold objects, get dressed or sit upright. The person also has very low intelligence, speaks few words, and needs constant supervision and help with all daily activities.	0.542 (0.374–0.702)
Sleeping sickness due to Trypanosoma brucei gambiense	Motor plus cognitive impairments, severe	cannot move around without help, and cannot lift or hold objects, get dressed or sit upright. The person also has very low intelligence, speaks few words, and needs constant supervision and help with all daily activities.	0.542 (0.374–0.702)
Mild diarrhea due to schistosomiasis	Diarrhea, mild	has diarrhea three or more times a day with occasional discomfort in the belly.	0.074 (0.049–0.104)
Severe anemia due to schistosomiasis	Anemia, severe	feels very weak, tired and short of breath, and has problems with activities that require physical effort or deep concentration.	0.149 (0.101–0.209)
Hematemesis due to schistosomiasis	Gastric bleeding	vomits blood and feels nauseous.	0.325 (0.209–0.462)
Hepatomegaly due to schistosomiasis	Abdominopelvic problem, mild	has some pain in the belly that causes nausea but does not interfere with daily activities.	0.011 (0.005–0.021)
Moderate anemia due to schistosomiasis	Anemia, moderate	feels moderate fatigue, weakness, and shortness of breath after exercise, making daily activities more difficult.	0.052 (0.034–0.076)
Mild anemia due to schistosomiasis	Anemia, mild	feels slightly tired and weak at times, but this does not interfere with normal daily activities.	0.004 (0.001–0.008)
Hydronephrosis due to schistosomiasis	Abdominopelvic problem, mild	has some pain in the belly that causes nausea but does not interfere with daily activities.	0.011 (0.005–0.021)
Ascites due to schistosomiasis	Abdominopelvic problem, moderate	has pain in the belly and feels nauseous. The person has difficulties with daily activities.	0.114 (0.078–0.159)
Mild schistosomiasis	Infectious disease, acute episode, mild	has a low fever and mild discomfort , but no difficulty with daily activities.	0.006 (0.002–0.012)
Dysuria due to schistosomiasis	Abdominopelvic problem, mild	has some pain in the belly that causes nausea but does not interfere with daily activities.	0.011 (0.005–0.021)
Bladder pathology due to schistosomiasis	Abdominopelvic problem, mild	has some pain in the belly that causes nausea but does not interfere with daily activities.	0.011 (0.005–0.021)
Neurocysticercosis with epilepsy	Epilepsy	(combined DW)	--
Abdominal problems due to cystic echinococcosis	Abdominopelvic problem, moderate	has pain in the belly and feels nauseous. The person has difficulties with daily activities.	0.114 (0.078–0.159)
Epilepsy due to echinococcosis	Epilepsy	(combined DW)	--
Chronic respiratory disease due to cystic echinococcosis	COPD and other chronic respiratory problems, mild	has cough and shortness of breath after heavy physical activity, but is able to walk long distances and climb stairs.	0.019 (0.011–0.033)
Lymphedema due to lymphatic filariasis	Lymphatic filariasis, symptomatic	has swollen legs with hard and thick skin, which causes difficulty in moving around.	0.109 (0.073–0.154)
Hydrocele due to lymphatic filariasis	Epididymo-orchitis	has swelling and tenderness in the testicles and pain during urination.	0.128 (0.086–0.18)
Acute adenolymphangitis due to lymphatic filariasis	Infectious disease, acute episode, moderate	has a fever and aches, and feels weak, which causes some difficulty with daily activities.	0.051 (0.032–0.074)
Prevalence of detectable microfiliaria due to lymphatic filariasis	Asymptomatic	--	--
Mild skin disease due to onchocerciasis	Disfigurement, level 1 with itch/pain	has a slight, visible physical deformity that is sometimes sore or itchy. Others notice the deformity, which causes some worry and discomfort.	0.027 (0.015–0.042)

Appendix Table 5. GBD 2016 sequelae, health states, health state lay descriptions, and disability weights			
**Sequela**	**Health state name**	**Health state lay description**	**Disability weight**
Severe vision impairment due to onchocerciasis	Distance vision, severe impairment	has severe vision loss, which causes difficulty in daily activities, some emotional impact (for example worry), and some difficulty going outside the home without assistance.	0.184 (0.125–0.258)
Moderate vision impairment due to onchocerciasis	Distance vision, moderate impairment	has vision problems that make it difficult to recognize faces or objects across a room.	0.031 (0.019–0.049)
Asymptomatic onchocerciasis	Asymptomatic	--	--
Severe skin disease without itch due to onchocerciasis	Disfigurement, level 3	has an obvious physical deformity that makes others uncomfortable, which causes the person to avoid social contact, feel worried, sleep poorly, and think about suicide.	0.405 (0.275–0.546)
Severe skin disease due to onchocerciasis	Disfigurement, level 2, with itch/pain	has a visible physical deformity that is sore and itchy. Other people stare and comment, which causes the person to worry. The person has trouble sleeping and concentrating.	0.188 (0.125–0.267)
Blindness due to onchocerciasis	Distance vision blindness	is completely blind, which causes great difficulty in some daily activities, worry and anxiety, and great difficulty going outside the home without assistance.	0.187 (0.124–0.26)
Mild skin disease without itch due to onchocerciasis	Disfigurement, level 1	has a slight, visible physical deformity that others notice, which causes some worry and discomfort.	0.011 (0.005–0.021)
Moderate skin disease due to onchocerciasis	Disfigurement, level 2, with itch/pain	has a visible physical deformity that is sore and itchy. Other people stare and comment, which causes the person to worry. The person has trouble sleeping and concentrating.	0.188 (0.125–0.267)
Blindness due to trachoma	Distance vision blindness	is completely blind, which causes great difficulty in some daily activities, worry and anxiety, and great difficulty going outside the home without assistance.	0.187 (0.124–0.26)
Severe vision impairment due to trachoma	Distance vision, severe impairment	has severe vision loss, which causes difficulty in daily activities, some emotional impact (for example worry), and some difficulty going outside the home without assistance.	0.184 (0.125–0.258)
Moderate vision impairment due to trachoma	Distance vision, moderate impairment	has vision problems that make it difficult to recognize faces or objects across a room.	0.031 (0.019–0.049)
Severe dengue	Infectious disease, acute episode, severe	has a high fever and pain, and feels very weak, which causes great difficulty with daily activities.	0.133 (0.088–0.19)
Post-dengue chronic fatigue syndrome	Infectious disease, post-acute consequences (fatigue, emotional lability, insomnia)	is always tired and easily upset. The person feels pain all over the body and is depressed.	0.219 (0.148–0.308)
Moderate dengue	Infectious disease, acute episode, moderate	has a fever and aches, and feels weak, which causes some difficulty with daily activities.	0.051 (0.032–0.074)
Asymptomatic yellow fever	Asymptomatic	--	--
Moderate yellow fever	Infectious disease, acute episode, moderate	has a fever and aches, and feels weak, which causes some difficulty with daily activities.	0.051 (0.032–0.074)
Severe yellow fever	Infectious disease, acute episode, severe	has a high fever and pain, and feels very weak, which causes great difficulty with daily activities.	0.133 (0.088–0.19)
Rabies	Infectious disease, acute episode, severe	has a high fever and pain, and feels very weak, which causes great difficulty with daily activities.	0.133 (0.088–0.19)
Severe wasting due to ascariasis	Severe wasting	is extremely skinny and has no energy.	0.128 (0.082–0.183)
Asymptomatic ascariasis	Asymptomatic	--	--
Heavy infestation of ascariasis	Intestinal nematode infections, symptomatic	has cramping pain and a bloated feeling in the belly.	0.027 (0.015–0.043)
Mild abdominopelvic problems due to ascariasis	Abdominopelvic problem, mild	has some pain in the belly that causes nausea but does not interfere with daily activities.	0.011 (0.005–0.021)
Heavy infestation of trichuriasis	Intestinal nematode infections, symptomatic	has cramping pain and a bloated feeling in the belly.	0.027 (0.015–0.043)
Severe wasting due to trichuriasis	Severe wasting	is extremely skinny and has no energy.	0.128 (0.082–0.183)
Mild abdominopelvic problems due to trichuriasis	Abdominopelvic problem, mild	has some pain in the belly that causes nausea but does not interfere with daily activities.	0.011 (0.005–0.021)
Asymptomatic trichuriasis	Asymptomatic	--	--
Mild anemia due to hookworm disease	Anemia, mild	feels slightly tired and weak at times, but this does not interfere with normal daily activities.	0.004 (0.001–0.008)
Severe anemia due to hookworm disease	Anemia, severe	feels very weak, tired and short of breath, and has problems with activities that require physical effort or deep concentration.	0.149 (0.101–0.209)
Asymptomatic hookworm disease	Asymptomatic	--	--
Mild abdominopelvic problems due to hookworm disease	Abdominopelvic problem, mild	has some pain in the belly that causes nausea but does not interfere with daily activities.	0.011 (0.005–0.021)
Severe wasting due to hookworm disease	Severe wasting	is extremely skinny and has no energy.	0.128 (0.082–0.183)
Heavy infestation of hookworm	Intestinal nematode infections, symptomatic	has cramping pain and a bloated feeling in the belly.	0.027 (0.015–0.043)
Moderate anemia due to hookworm disease	Anemia, moderate	feels moderate fatigue, weakness, and shortness of breath after exercise, making daily activities more difficult.	0.052 (0.034–0.076)
Asymptomatic fascioliasis	Asymptomatic	--	--
Heavy clonorchiasis due to food-borne trematodiases	Abdominopelvic problem, moderate	has pain in the belly and feels nauseous. The person has difficulties with daily activities.	0.114 (0.078–0.159)
Heavy fascioliasis due to food-borne trematodiases	Abdominopelvic problem, moderate	has pain in the belly and feels nauseous. The person has difficulties with daily activities.	0.114 (0.078–0.159)
Heavy intestinal fluke infection due to food-borne trematodiases	Abdominopelvic problem, moderate	has pain in the belly and feels nauseous. The person has difficulties with daily activities.	0.114 (0.078–0.159)
Asymptomatic opisthorchiasis	Asymptomatic	--	--

Appendix Table 5. GBD 2016 sequelae, health states, health state lay descriptions, and disability weights

Sequela	Health state name	Health state lay description	Disability weight
Asymptomatic intestinal fluke infection	Asymptomatic	--	--
Cerebral paragonimiasis	Epilepsy	(combined DW)	--
Asymptomatic paragonimiasis	Asymptomatic	--	--
Moderate paragonimiasis due to food-borne trematodiases	COPD and other chronic respiratory problems, moderate	has cough, wheezing and shortness of breath, even after light physical activity. The person feels tired and can walk only short distances or climb only a few stairs.	0.225 (0.153–0.31)
Severe paragonimiasis due to food-borne trematodiases	COPD and other chronic respiratory problems, severe	has cough, wheezing and shortness of breath all the time. The person has great difficulty walking even short distances or climbing any stairs, feels tired when at rest, and is	0.408 (0.273–0.556)
Mild paragonimiasis due to food-borne trematodiases	COPD and other chronic respiratory problems, mild	has cough and shortness of breath after heavy physical activity, but is able to walk long distances and climb stairs.	0.019 (0.011–0.033)
Heavy opisthorchiasis due to food-borne trematodiases	Abdominopelvic problem, moderate	has pain in the belly and feels nauseous. The person has difficulties with daily activities.	0.114 (0.078–0.159)
Asymptomatic clonorchiasis	Asymptomatic	--	--
Disfigurement level 2 due to leprosy	Disfigurement, level 2	has a visible physical deformity that causes others to stare and comment. As a result, the person is worried and has trouble sleeping and concentrating.	0.067 (0.044–0.096)
Disfigurement level 1 due to leprosy	Disfigurement, level 1	has a slight, visible physical deformity that others notice, which causes some worry and discomfort.	0.011 (0.005–0.021)
Post-Ebola chronic fatigue syndrome	Infectious disease, post-acute consequences (fatigue, emotional lability, insomnia)	is always tired and easily upset. The person feels pain all over the body and is depressed.	0.219 (0.148–0.308)
Ebola cases	Infectious disease, acute episode, severe	has a high fever and pain, and feels very weak, which causes great difficulty with daily activities.	0.133 (0.088–0.19)
Guillain–Barré syndrome due to Zika infection	Spinal cord lesion below neck level (treated)	is paralyzed from the waist down, cannot feel or move the legs and has difficulties with urine and bowel control. The person uses a wheelchair to move around.	0.296 (0.198–0.414)
Asymptomatic Zika infection	Asymptomatic	--	--
Acute Zika infection	Infectious disease, acute episode, moderate	has a fever and aches, and feels weak, which causes some difficulty with daily activities.	0.051 (0.032–0.074)
Congenital Zika syndrome	Severe motor plus cognitive impairment with epilepsy	(combined DW)	--
Mild pain due to Guinea worm emergence	Disfigurement, level 1 with itch/pain	has a slight, visible physical deformity that is sometimes sore or itchy. Others notice the deformity, which causes some worry and discomfort.	0.027 (0.015–0.042)
Moderate pain due to Guinea worm emergence	Disfigurement, level 2, with itch/pain	has a visible physical deformity that is sore and itchy. Other people stare and comment, which causes the person to worry. The person has trouble sleeping and concentrating.	0.188 (0.125–0.267)
Moderate reduced mobility due to Guinea worm emergence	Musculoskeletal problems, lower limbs, moderate	has moderate pain in the leg, which makes the person limp, and causes some difficulty walking, standing, lifting and carrying heavy things, getting up and down and sleeping.	0.079 (0.054–0.11)
Mild anemia due to other neglected tropical diseases	Anemia, mild	feels slightly tired and weak at times, but this does not interfere with normal daily activities.	0.004 (0.001–0.008)
Moderate anemia due to other neglected tropical diseases	Anemia, moderate	feels moderate fatigue, weakness, and shortness of breath after exercise, making daily activities more difficult.	0.052 (0.034–0.076)
Acute infection due to other neglected tropical diseases	Post-COMO calculation for residuals (YLL/YLD ratio, other methods)	--	--
Severe anemia due to other neglected tropical diseases	Anemia, severe	feels very weak, tired and short of breath, and has problems with activities that require physical effort or deep concentration.	0.149 (0.101–0.209)
Mild anemia due to maternal hemorrhage	Anemia, mild	feels slightly tired and weak at times, but this does not interfere with normal daily activities.	0.004 (0.001–0.008)
Moderate anemia due to maternal hemorrhage	Anemia, moderate	feels moderate fatigue, weakness, and shortness of breath after exercise, making daily activities more difficult.	0.052 (0.034–0.076)
Severe anemia due to maternal hemorrhage	Anemia, severe	feels very weak, tired and short of breath, and has problems with activities that require physical effort or deep concentration.	0.149 (0.101–0.209)
Maternal hemorrhage (> 1L blood lost)	Abdominopelvic problem, severe	has severe pain in the belly and feels nauseous. The person is anxious and unable to carry out daily activities.	0.324 (0.22–0.442)
Maternal hemorrhage (< 1L blood lost)	Abdominopelvic problem, moderate	has pain in the belly and feels nauseous. The person has difficulties with daily activities.	0.114 (0.078–0.159)
Puerperal sepsis	Infectious disease, acute episode, severe	has a high fever and pain, and feels very weak, which causes great difficulty with daily activities.	0.133 (0.088–0.19)
Other maternal infections	Infectious disease, acute episode, moderate	has a fever and aches, and feels weak, which causes some difficulty with daily activities.	0.051 (0.032–0.074)
Infertility due to puerperal sepsis	Infertility, secondary	has at least one child, and wants to have more children. The person has a fertile partner, but the couple cannot conceive.	0.005 (0.002–0.011)
Other hypertensive disorders of pregnancy	Generic uncomplicated disease: worry and daily medication	has a chronic disease that requires medication every day and causes some worry but minimal interference with daily activities.	0.049 (0.031–0.072)
Long term sequelae of severe pre-eclampsia	Tension-type headaches, mild motor plus cognitive impairment	(combined DW)	--
Long term sequelae of eclampsia	Tension-type headaches, mild motor plus cognitive impairment	(combined DW)	--
Severe pre-eclampsia	Moderate abdominal pain, tension-type headaches, mild motor plus cognitive impairment	(combined DW)	--
Eclampsia	Moderate abdominal pain and severe epilepsy	(combined DW)	--
Obstructed labor, acute event	Abdominopelvic problem, severe	has severe pain in the belly and feels nauseous. The person is anxious and unable to carry out daily activities.	0.324 (0.22–0.442)

Appendix Table 5. GBD 2016 sequelae, health states, health state lay descriptions, and disability weights			
**Sequela**	**Health state name**	**Health state lay description**	**Disability weight**
Vesicovaginal fistula	Vesicovaginal fistula	has an abnormal opening between the bladder and the vagina, which makes her unable to control urinating. The woman is anxious and depressed.	0.342 (0.227–0.478)
Rectovaginal fistula	Rectovaginal fistula	has an abnormal opening between her vagina and rectum causing flatulence and feces to escape through the vagina. The person gets infections in her vagina, and has pain when urinating.	0.501 (0.339–0.657)
Ectopic Pregnancy	Abdominopelvic problem, moderate	has pain in the belly and feels nauseous. The person has difficulties with daily activities.	0.114 (0.078–0.159)
Maternal abortive outcome	Abdominopelvic problem, moderate	has pain in the belly and feels nauseous. The person has difficulties with daily activities.	0.114 (0.078–0.159)
Other maternal disorders	Post-COMO calculation for residuals (YLL/YLD ratio, other methods)	--	--
Severe motor plus cognitive impairment with epilepsy due to neonatal preterm birth complications <28wks	Severe motor plus cognitive impairment with epilepsy	(combined DW)	--
Severe motor plus cognitive impairment with epilepsy due to neonatal preterm birth complications 32-36wks	Severe motor plus cognitive impairment with epilepsy	(combined DW)	--
Severe motor plus cognitive impairment with epilepsy due to neonatal preterm birth complications 28-32wks	Severe motor plus cognitive impairment with epilepsy	(combined DW)	--
Mild motor plus cognitive impairments due to neonatal preterm birth complications <28wks	Motor plus cognitive impairments, mild	has some difficulty in moving around but is able to walk without help. The person is slow in learning at school. As an adult, the person has some difficulty doing complex or unfamiliar tasks but otherwise functions independently.	0.031 (0.018–0.05)
Severe motor plus cognitive impairment with blindness and epilepsy due to neonatal preterm birth complications <28wks	Severe motor plus cognitive impairment with blindness and epilepsy	(combined DW)	--
Mild motor plus cognitive impairments due to neonatal preterm birth complications 32-36wks	Motor plus cognitive impairments, mild	has some difficulty in moving around but is able to walk without help. The person is slow in learning at school. As an adult, the person has some difficulty doing complex or unfamiliar tasks but otherwise functions independently.	0.031 (0.018–0.05)
Severe motor plus cognitive impairment with blindness due to neonatal preterm birth complications <28wks	Severe motor plus cognitive impairment with blindness	(combined DW)	--
Moderate motor plus cognitive impairment with blindness and epilepsy due to neonatal preterm birth complications <28wks	Moderate motor plus cognitive impairment with blindness and epilepsy	(combined DW)	--
Moderate motor plus cognitive impairment with blindness and epilepsy due to neonatal preterm birth complications 32-36wks	Moderate motor plus cognitive impairment with blindness and epilepsy	(combined DW)	--
Severe motor plus cognitive impairment with blindness and epilepsy due to neonatal preterm birth complications 28-32wks	Severe motor plus cognitive impairment with blindness and epilepsy	(combined DW)	--
Mild motor plus cognitive impairments due to neonatal preterm birth complications 28-32wks	Motor plus cognitive impairments, mild	has some difficulty in moving around but is able to walk without help. The person is slow in learning at school. As an adult, the person has some difficulty doing complex or unfamiliar tasks but otherwise functions independently.	0.031 (0.018–0.05)
Severe motor impairment with epilepsy due to neonatal preterm birth complications 32-36wks	Severe motor impairment with epilepsy	(combined DW)	--
Severe motor impairment due to neonatal preterm birth complications 32-36wks	Motor impairment, severe	is unable to move around without help, and is not able to lift or hold objects, get dressed or sit upright.	0.402 (0.268–0.545)
Severe motor plus cognitive impairment with blindness due to neonatal preterm birth complications 32-36wks	Severe motor plus cognitive impairment with blindness	(combined DW)	--
Severe motor impairment with epilepsy due to neonatal preterm birth complications <28wks	Severe motor impairment with epilepsy	(combined DW)	--
Severe motor impairment with blindness due to neonatal preterm birth complications 32-36wks	Severe motor impairment with blindness	(combined DW)	--
Severe motor impairment with epilepsy due to neonatal preterm birth complications 28-32wks	Severe motor impairment with epilepsy	(combined DW)	--
Mild motor impairment due to neonatal preterm birth complications <28wks	Motor impairment, mild	has some difficulty in moving around but is able to walk without help.	0.01 (0.005–0.019)
Mild motor impairment due to neonatal preterm birth complications 28-32wks	Motor impairment, mild	has some difficulty in moving around but is able to walk without help.	0.01 (0.005–0.019)
Mild motor impairment due to neonatal preterm birth complications 32-36wks	Motor impairment, mild	has some difficulty in moving around but is able to walk without help.	0.01 (0.005–0.019)
Severe motor impairment with blindness due to neonatal preterm birth complications <28wks	Severe motor impairment with blindness	(combined DW)	--
Severe motor impairment due to neonatal preterm birth complications 28-32wks	Motor impairment, severe	is unable to move around without help, and is not able to lift or hold objects, get dressed or sit upright.	0.402 (0.268–0.545)
Severe motor impairment with blindness and epilepsy due to neonatal preterm birth complications <28wks	Severe motor impairment with blindness and epilepsy	(combined DW)	--
Moderate motor impairment due to neonatal preterm birth complications 28-32wks	Motor impairment, moderate	has some difficulty in moving around, and difficulty in lifting and holding objects, dressing and sitting upright, but is able to walk without help.	0.061 (0.04–0.089)
Moderate motor impairment due to neonatal preterm birth complications <28wks	Motor impairment, moderate	has some difficulty in moving around, and difficulty in lifting and holding objects, dressing and sitting upright, but is able to walk without help.	0.061 (0.04–0.089)
Moderate motor impairment due to neonatal preterm birth complications 32-36wks	Motor impairment, moderate	has some difficulty in moving around, and difficulty in lifting and holding objects, dressing and sitting upright, but is able to walk without help.	0.061 (0.04–0.089)
Severe motor impairment with blindness due to neonatal preterm birth complications 28-32wks	Severe motor impairment with blindness	(combined DW)	--
Severe motor impairment with blindness and epilepsy due to neonatal preterm birth complications 32-36wks	Severe motor impairment with blindness and epilepsy	(combined DW)	--
Severe motor plus cognitive impairment with blindness due to neonatal preterm birth complications 28-32wks	Severe motor plus cognitive impairment with blindness	(combined DW)	--

Appendix Table 5. GBD 2016 sequelae, health states, health state lay descriptions, and disability weights			
**Sequela**	**Health state name**	**Health state lay description**	**Disability weight**
Severe motor impairment due to neonatal preterm birth complications <28wks	Motor impairment, severe	is unable to move around without help, and is not able to lift or hold objects, get dressed or sit upright.	0.402 (0.268–0.545)
Severe motor impairment with blindness and epilepsy due to neonatal preterm birth complications 28-32wks	Severe motor impairment with blindness and epilepsy	(combined DW)	--
Moderate motor plus cognitive impairment with epilepsy due to neonatal preterm birth complications 28-32wks	Moderate motor plus cognitive impairment with epilepsy	(combined DW)	--
Blindness due to retinopathy of prematurity	Distance vision blindness	is completely blind, which causes great difficulty in some daily activities, worry and anxiety, and great difficulty going outside the home without assistance.	0.187 (0.124–0.26)
Severe motor plus cognitive impairment with blindness and epilepsy due to neonatal preterm birth complications 32-36wks	Severe motor plus cognitive impairment with blindness and epilepsy	(combined DW)	--
Moderate motor impairment with blindness and epilepsy due to neonatal preterm birth complications 32-36wks	Moderate motor impairment with blindness and epilepsy	(combined DW)	--
Moderate motor impairment with blindness and epilepsy due to neonatal preterm birth complications 28-32wks	Moderate motor impairment with blindness and epilepsy	(combined DW)	--
Moderate motor impairment with epilepsy due to neonatal preterm birth complications <28wks	Moderate motor impairment with epilepsy	(combined DW)	--
Moderate motor impairment with epilepsy due to neonatal preterm birth complications 28-32wks	Moderate motor impairment with epilepsy	(combined DW)	--
Moderate motor impairment with blindness and epilepsy due to neonatal preterm birth complications <28wks	Moderate motor impairment with blindness and epilepsy	(combined DW)	--
Moderate motor impairment with epilepsy due to neonatal preterm birth complications 32-36wks	Moderate motor impairment with epilepsy	(combined DW)	--
Moderate motor plus cognitive impairment with blindness due to neonatal preterm birth complications 28-32wks	Moderate motor plus cognitive impairment with blindness	(combined DW)	--
Moderate motor impairment with blindness due to neonatal preterm birth complications 32-36wks	Moderate motor impairment with blindness	(combined DW)	--
Moderate motor impairment with blindness due to neonatal preterm birth complications 28-32wks	Moderate motor impairment with blindness	(combined DW)	--
Moderate motor impairment with blindness due to neonatal preterm birth complications <28wks	Moderate motor impairment with blindness	(combined DW)	--
Moderate motor plus cognitive impairment with blindness due to neonatal preterm birth complications <28wks	Moderate motor plus cognitive impairment with blindness	(combined DW)	--
Moderate motor plus cognitive impairment with blindness due to neonatal preterm birth complications 32-36wks	Moderate motor plus cognitive impairment with blindness	(combined DW)	--
Asymptomatic neonatal preterm birth 32-<37wks	Asymptomatic	--	--
Moderate motor plus cognitive impairment with blindness and epilepsy due to neonatal preterm birth complications 28-32wks	Moderate motor plus cognitive impairment with blindness and epilepsy	(combined DW)	--
Severe vision impairment due to retinopathy of prematurity	Distance vision, severe impairment	has severe vision loss, which causes difficulty in daily activities, some emotional impact (for example worry), and some difficulty going outside the home without assistance.	0.184 (0.125–0.258)
Moderate motor plus cognitive impairment with epilepsy due to neonatal preterm birth complications <28wks	Moderate motor plus cognitive impairment with epilepsy	(combined DW)	--
Mild vision impairment due to retinopathy of prematurity	Distance vision, mild impairment	has some difficulty with distance vision, for example reading signs, but no other problems with eyesight.	0.003 (0.001–0.007)
Moderate vision impairment due to retinopathy of prematurity	Distance vision, moderate impairment	has vision problems that make it difficult to recognize faces or objects across a room.	0.031 (0.019–0.049)
Moderate motor plus cognitive impairment with epilepsy due to neonatal preterm birth complications 32-36wks	Moderate motor plus cognitive impairment with epilepsy	(combined DW)	--
Asymptomatic neonatla preterm birth <28wks	Asymptomatic	--	--
Asymptomatic neonatal preterm birth 28-<32 wks	Asymptomatic	--	--
Asymptomatic retinopathy of prematurity	Asymptomatic	--	--
Severe motor impairment due to neonatal encephalopathy due to birth asphyxia and trauma	Motor impairment, severe	is unable to move around without help, and is not able to lift or hold objects, get dressed or sit upright.	0.402 (0.268–0.545)
Moderate motor impairment due to neonatal encephalopathy due to birth asphyxia and trauma	Motor impairment, moderate	has some difficulty in moving around, and difficulty in lifting and holding objects, dressing and sitting upright, but is able to walk without help.	0.061 (0.04–0.089)
Moderate motor impairment with blindness due to neonatal encephalopathy due to birth asphyxia and trauma	Moderate motor impairment with blindness	(combined DW)	--
Severe motor plus cognitive impairment with epilepsy due to neonatal encephalopathy due to birth asphyxia and trauma	Severe motor plus cognitive impairment with epilepsy	(combined DW)	--
Moderate motor impairment with epilepsy due to neonatal encephalopathy due to birth asphyxia and trauma	Moderate motor impairment with epilepsy	(combined DW)	--
Moderate motor plus cognitive impairment with blindness and epilepsy due to neonatal encephalopathy due to birth asphyxia and [illegible]	Moderate motor plus cognitive impairment with blindness and epilepsy	(combined DW)	--
Mild motor impairment due to neonatal encephalopathy due to birth asphyxia and trauma	Motor impairment, mild	has some difficulty in moving around but is able to walk without help.	0.01 (0.005–0.019)
Moderate motor plus cognitive impairment with epilepsy due to neonatal encephalopathy due to birth asphyxia and trauma	Moderate motor plus cognitive impairment with epilepsy	(combined DW)	--
Severe motor impairment with blindness and epilepsy due to neonatal encephalopathy due to birth asphyxia and trauma	Severe motor impairment with blindness and epilepsy	(combined DW)	--
Mild motor plus cognitive impairments due to neonatal encephalopathy due to birth asphyxia and trauma	Motor plus cognitive impairments, mild	has some difficulty in moving around but is able to walk without help. The person is slow in learning at school. As an adult, the person has some difficulty doing complex or unfamiliar tasks but otherwise functions independently.	0.031 (0.018–0.05)
Severe motor plus cognitive impairment with blindness and epilepsy due to neonatal encephalopathy due to birth asphyxia and trauma	Severe motor plus cognitive impairment with blindness and epilepsy	(combined DW)	--

Appendix Table 5. GBD 2016 sequelae, health states, health state lay descriptions, and disability weights			
**Sequela**	**Health state name**	**Health state lay description**	**Disability weight**
Moderate motor impairment with blindness and epilepsy due to neonatal encephalopathy due to birth asphyxia and trauma	Moderate motor impairment with blindness and epilepsy	(combined DW)	--
Asymptomatic neonatal encephalopathy due to birth asphyxia and trauma	Asymptomatic	--	--
Moderate motor plus cognitive impairment with blindness due to neonatal encephalopathy due to birth asphyxia and trauma	Moderate motor plus cognitive impairment with blindness	(combined DW)	--
Severe motor impairment with epilepsy due to neonatal encephalopathy due to birth asphyxia and trauma	Severe motor impairment with epilepsy	(combined DW)	--
Severe motor plus cognitive impairment with blindness due to neonatal encephalopathy due to birth asphyxia and trauma	Severe motor plus cognitive impairment with blindness	(combined DW)	--
Severe motor impairment with blindness due to neonatal encephalopathy due to birth asphyxia and trauma	Severe motor impairment with blindness	(combined DW)	--
Moderate motor impairment with epilepsy due to neonatal sepsis and other neonatal infections	Moderate motor impairment with epilepsy	(combined DW)	--
Moderate motor plus cognitive impairment with epilepsy due to neonatal sepsis and other neonatal infections	Moderate motor plus cognitive impairment with epilepsy	(combined DW)	--
Severe motor impairment with blindness due to neonatal sepsis and other neonatal infections	Severe motor impairment with blindness	(combined DW)	--
Severe motor plus cognitive impairment with blindness and epilepsy due to neonatal sepsis and other neonatal infections	Severe motor plus cognitive impairment with blindness and epilepsy	(combined DW)	--
Mild motor impairment due to neonatal sepsis and other neonatal infections	Motor impairment, mild	has some difficulty in moving around but is able to walk without help.	0.01 (0.005–0.019)
Severe motor impairment with epilepsy due to neonatal sepsis and other neonatal infections	Severe motor impairment with epilepsy	(combined DW)	--
Asymptomatic neonatal sepsis and other neonatal infections	Asymptomatic	--	--
Moderate motor impairment with blindness due to neonatal sepsis and other neonatal infections	Moderate motor impairment with blindness	(combined DW)	--
Severe motor impairment due to neonatal sepsis and other neonatal infections	Motor impairment, severe	is unable to move around without help, and is not able to lift or hold objects, get dressed or sit upright.	0.402 (0.268–0.545)
Severe infection due to neonatal sepsis and other neonatal infections	Infectious disease, acute episode, severe	has a high fever and pain, and feels very weak, which causes great difficulty with daily activities.	0.133 (0.088–0.19)
Moderate motor plus cognitive impairment with blindness and epilepsy due to neonatal sepsis and other neonatal infections	Moderate motor plus cognitive impairment with blindness and epilepsy	(combined DW)	--
Moderate motor impairment due to neonatal sepsis and other neonatal infections	Motor impairment, moderate	has some difficulty in moving around, and difficulty in lifting and holding objects, dressing and sitting upright, but is able to walk without help.	0.061 (0.04–0.089)
Severe motor plus cognitive impairment with epilepsy due to neonatal sepsis and other neonatal infections	Severe motor plus cognitive impairment with epilepsy	(combined DW)	--
Severe motor impairment with blindness and epilepsy due to neonatal sepsis and other neonatal infections	Severe motor impairment with blindness and epilepsy	(combined DW)	--
Mild motor plus cognitive impairments due to neonatal sepsis and other neonatal infections	Motor plus cognitive impairments, mild	has some difficulty in moving around but is able to walk without help. The person is slow in learning at school. As an adult, the person has some difficulty doing complex or unfamiliar tasks but otherwise functions independently.	0.031 (0.018–0.05)
Severe motor plus cognitive impairment with blindness due to neonatal sepsis and other neonatal infections	Severe motor plus cognitive impairment with blindness	(combined DW)	--
Moderate motor plus cognitive impairment with blindness due to neonatal sepsis and other neonatal infections	Moderate motor plus cognitive impairment with blindness	(combined DW)	--
Moderate motor impairment with blindness and epilepsy due to neonatal sepsis and other neonatal infections	Moderate motor impairment with blindness and epilepsy	(combined DW)	--
Severe motor impairment with blindness and epilepsy due to hemolytic disease and other neonatal jaundice	Severe motor impairment with blindness and epilepsy	(combined DW)	--
Severe motor impairment with epilepsy due to hemolytic disease and other neonatal jaundice	Severe motor impairment with epilepsy	(combined DW)	--
Severe motor plus cognitive impairment with blindness and epilepsy due to hemolytic disease and other neonatal jaundice	Severe motor plus cognitive impairment with blindness and epilepsy	(combined DW)	--
Severe motor plus cognitive impairment with blindness due to hemolytic disease and other neonatal jaundice	Severe motor plus cognitive impairment with blindness	(combined DW)	--
Severe motor plus cognitive impairment with epilepsy due to hemolytic disease and other neonatal jaundice	Severe motor plus cognitive impairment with epilepsy	(combined DW)	--
Moderate motor impairment with blindness and epilepsy due to hemolytic disease and other neonatal jaundice	Moderate motor impairment with blindness and epilepsy	(combined DW)	--
Moderate motor plus cognitive impairment with epilepsy due to hemolytic disease and other neonatal jaundice	Moderate motor plus cognitive impairment with epilepsy	(combined DW)	--
Moderate motor impairment with epilepsy due to hemolytic disease and other neonatal jaundice	Moderate motor impairment with epilepsy	(combined DW)	--
Moderate motor plus cognitive impairment with blindness due to hemolytic disease and other neonatal jaundice	Moderate motor plus cognitive impairment with blindness	(combined DW)	--
Moderate motor impairment with blindness due to hemolytic disease and other neonatal jaundice	Moderate motor impairment with blindness	(combined DW)	--
Severe motor impairment severe due to hemolytic disease and other neonatal jaundice	Motor impairment, severe	is unable to move around without help, and is not able to lift or hold objects, get dressed or sit upright.	0.402 (0.268–0.545)
Moderate motor plus cognitive impairment with blindness and epilepsy due to hemolytic disease and other neonatal jaundice	Moderate motor plus cognitive impairment with blindness and epilepsy	(combined DW)	--
Moderate motor impairment due to hemolytic disease and other neonatal jaundice	Motor impairment, moderate	has some difficulty in moving around, and difficulty in lifting and holding objects, dressing and sitting upright, but is able to walk without help.	0.061 (0.04–0.089)
Severe motor impairment with blindness due to hemolytic disease and other neonatal jaundice	Severe motor impairment with blindness	(combined DW)	--

Appendix Table 5. GBD 2016 sequelae, health states, health state lay descriptions, and disability weights

Sequela	Health state name	Health state lay description	Disability weight
Other neonatal disorders	Post-COMO calculation for residuals (YLL/YLD ratio, other methods)	--	--
Moderate wasting without edema	Asymptomatic	--	--
Moderate wasting with edema	Kwashiorkor	is very tired and irritable and has diarrhea.	0.051 (0.031–0.079)
Severe wasting without edema	Severe wasting	is extremely skinny and has no energy.	0.128 (0.082–0.183)
Severe wasting with edema	Kwashiorkor and severe wasting	(combined DW)	0.172 (0.115–0.238)
Visible goiter with profound intellectual disability due to iodine deficiency	Intellectual disability / mental retardation, profound and Iodine-deficiency goiter	(combined DW)	0.358 (0.252–0.475)
Visible goiter with severe intellectual disability due to iodine deficiency	Intellectual disability / mental retardation, severe and Iodine-deficiency goiter	(combined DW)	0.326 (0.233–0.438)
Visible goiter with profound intellectual disability and mild heart failure due to iodine deficiency	Intellectual disability / mental retardation, profound and Iodine-deficiency goiter and heart failure, mild	(combined DW)	0.384 (0.276–0.502)
Visible goiter without symptoms	Disfigurement, level 1	has a slight, visible physical deformity that others notice, which causes some worry and discomfort.	0.011 (0.005–0.021)
Visible goiter with profound intellectual disability and moderate heart failure due to iodine deficiency	Intellectual disability / mental retardation, profound and Iodine-deficiency goiter and heart failure, moderate	(combined DW)	0.403 (0.293–0.524)
Visible goiter with profound intellectual disability with severe heart failure due to iodine deficiency	Intellectual disability / mental retardation, profound and Iodine-deficiency goiter and heart failure, severe	(combined DW)	0.471 (0.344–0.602)
Visible goiter with symptoms without intellectual disability or heart failure	Iodine-deficiency goiter	has a large mass in the front of the neck. The person sometimes has weakness and fatigue, constipation and weight gain.	0.199 (0.133–0.276)
Moderate vision impairment loss due to vitamin A deficiency	Distance vision, moderate impairment	has vision problems that make it difficult to recognize faces or objects across a room.	0.031 (0.019–0.049)
Blindness due to vitamin A deficiency	Distance vision blindness	is completely blind, which causes great difficulty in some daily activities, worry and anxiety, and great difficulty going outside the home without assistance.	0.187 (0.124–0.26)
Severe vision impairment loss due to vitamin A deficiency	Distance vision, severe impairment	has severe vision loss, which causes difficulty in daily activities, some emotional impact (for example worry), and some difficulty going outside the home without assistance.	0.184 (0.125–0.258)
Asymptomatic vitamin A deficiency	Asymptomatic	--	--
Moderate heart failure due to iron-deficiency anemia	Heart failure, moderate	is short of breath and easily tires with minimal physical activity, such as walking only a short distance. The person feels comfortable at rest but avoids moderate activity.	0.072 (0.047–0.103)
Mild iron-deficiency anemia	Anemia, mild	feels slightly tired and weak at times, but this does not interfere with normal daily activities.	0.004 (0.001–0.008)
Asymptomatic and mild heart failure due to iron-deficiency anemia	Heart failure, mild	is short of breath and easily tires with moderate physical activity, such as walking uphill or more than a quarter-mile on level ground. The person feels comfortable at rest or during activities requiring less effort.	0.041 (0.026–0.062)
Severe iron-deficiency anemia	Anemia, severe	feels very weak, tired and short of breath, and has problems with activities that require physical effort or deep concentration.	0.149 (0.101–0.209)
Severe heart failure due to iron-deficiency anemia	Heart failure, severe	is short of breath and feels tired when at rest. The person avoids any physical activity, for fear of worsening the breathing problems.	0.179 (0.122–0.251)
Moderate iron-deficiency anemia	Anemia, moderate	feels moderate fatigue, weakness, and shortness of breath after exercise, making daily activities more difficult.	0.052 (0.034–0.076)
Other nutritional deficiencies	Post-COMO calculation for residuals (YLL/YLD ratio, other methods)	--	--
Cardiovascular complications due to adult tertiary syphilis	Infectious disease, acute episode, moderate	has a fever and aches, and feels weak, which causes some difficulty with daily activities.	0.051 (0.032–0.074)
Severe disfigurement, neurological problems, and cardiovascular complications due to adult tertiary syphilis	Level 3 disfigurement, moderate motor plus cognitive impairments, and moderate infectious disease, acute episode	(combined DW)	0.547 (0.402–0.691)
Severe disfigurement and cardiovascular complications due to adult tertiary syphilis	Level 3 disfigurement and moderate infectious disease, acute episode	(combined DW)	0.435 (0.306–0.571)
Neurological problems due to adult tertiary syphilis	Motor plus cognitive impairments, moderate	has some difficulty in moving around, holding objects, dressing and sitting upright, but can walk without help. The person has low intelligence and is slow in learning to speak and to do simple tasks.	0.203 (0.134–0.29)
Severe disfigurement due to adult tertiary syphilis	Disfigurement, level 3	has an obvious physical deformity that makes others uncomfortable, which causes the person to avoid social contact, feel worried, sleep poorly, and think about suicide.	0.405 (0.275–0.546)
Asymptomatic early syphilis infection	Asymptomatic	--	--
Mild early syphilis infection	Infectious disease, acute episode, mild	has a low fever and mild discomfort , but no difficulty with daily activities.	0.006 (0.002–0.012)
Neurological problems and cardiovascular complications due to adult tertiary syphilis	Moderate motor plus cognitive impairments and moderate infectious disease, acute episode	(combined DW)	0.243 (0.168–0.333)
Asymptomatic adult tertiary syphilis	Asymptomatic	--	--
Severe disfigurement and neurological problems due to adult tertiary syphilis	Level 3 disfigurement and moderate motor plus cognitive impairments	(combined DW)	0.523 (0.378–0.669)
Secondary infertility due to chlamydial infection	Infertility, secondary	has at least one child, and wants to have more children. The person has a fertile partner, but the couple cannot conceive.	0.005 (0.002–0.011)

Appendix Table 5. GBD 2016 sequelae, health states, health state lay descriptions, and disability weights			
**Sequela**	**Health state name**	**Health state lay description**	**Disability weight**
Asymptomatic chlamydial infection	Asymptomatic	--	--
Moderate pelvic inflammatory diseases due to chlamydial infection	Abdominopelvic problem, moderate	has pain in the belly and feels nauseous. The person has difficulties with daily activities.	0.114 (0.078–0.159)
Severe pelvic inflammatory diseases due to chlamydial infection	Abdominopelvic problem, severe	has severe pain in the belly and feels nauseous. The person is anxious and unable to carry out daily activities.	0.324 (0.22–0.442)
Epididymo-orchitis due to chlamydial infection	Epididymo-orchitis	has swelling and tenderness in the testicles and pain during urination.	0.128 (0.086–0.18)
Primary infertility due to chlamydial infection	Infertility, primary	wants to have a child and has a fertile partner, but the couple cannot conceive.	0.008 (0.003–0.015)
Mild chlamydial infection	Infectious disease, acute episode, mild	has a low fever and mild discomfort , but no difficulty with daily activities.	0.006 (0.002–0.012)
Primary infertility due to gonococcal infection	Infertility, primary	wants to have a child and has a fertile partner, but the couple cannot conceive.	0.008 (0.003–0.015)
Epididymo-orchitis due to gonococcal infection	Epididymo-orchitis	has swelling and tenderness in the testicles and pain during urination.	0.128 (0.086–0.18)
Severe pelvic inflammatory diseases due to gonococcal infection	Abdominopelvic problem, severe	has severe pain in the belly and feels nauseous. The person is anxious and unable to carry out daily activities.	0.324 (0.22–0.442)
Asymptomatic gonococcal infection	Asymptomatic	--	--
Mild gonococcal infection	Infectious disease, acute episode, mild	has a low fever and mild discomfort , but no difficulty with daily activities.	0.006 (0.002–0.012)
Secondary infertility due to gonococcal infection	Infertility, secondary	has at least one child, and wants to have more children. The person has a fertile partner, but the couple cannot conceive.	0.005 (0.002–0.011)
Moderate pelvic inflammatory diseases due to gonococcal infection	Abdominopelvic problem, moderate	has pain in the belly and feels nauseous. The person has difficulties with daily activities.	0.114 (0.078–0.159)
Asymptomatic trichomoniasis infection	Asymptomatic	--	--
Acute trichomoniasis infection	Infectious disease, acute episode, mild	has a low fever and mild discomfort , but no difficulty with daily activities.	0.006 (0.002–0.012)
Moderate infection due to initial genital herpes episode	Infectious disease, acute episode, moderate	has a fever and aches, and feels weak, which causes some difficulty with daily activities.	0.051 (0.032–0.074)
Asymptomatic genital herpes	Asymptomatic	--	--
Symptomatic genital herpes	Infectious disease, acute episode, mild	has a low fever and mild discomfort , but no difficulty with daily activities.	0.006 (0.002–0.012)
Primary infertility due to other sexually transmitted diseases	Infertility, primary	wants to have a child and has a fertile partner, but the couple cannot conceive.	0.008 (0.003–0.015)
Severe pelvic inflammatory diseases due to other sexually transmitted diseases	Abdominopelvic problem, severe	has severe pain in the belly and feels nauseous. The person is anxious and unable to carry out daily activities.	0.324 (0.22–0.442)
Other sexually transmitted diseases	Post-COMO calculation for residuals (YLL/YLD ratio, other methods)	--	--
Secondary infertility due to other sexually transmitted diseases	Infertility, secondary	has at least one child, and wants to have more children. The person has a fertile partner, but the couple cannot conceive.	0.005 (0.002–0.011)
Moderate pelvic inflammatory diseases due to other sexually transmitted diseases	Abdominopelvic problem, moderate	has pain in the belly and feels nauseous. The person has difficulties with daily activities.	0.114 (0.078–0.159)
Asymptomatic acute hepatitis A	Asymptomatic	--	--
Severe acute hepatitis A	Infectious disease, acute episode, severe	has a high fever and pain, and feels very weak, which causes great difficulty with daily activities.	0.133 (0.088–0.19)
Moderate acute hepatitis A	Infectious disease, acute episode, moderate	has a fever and aches, and feels weak, which causes some difficulty with daily activities.	0.051 (0.032–0.074)
Severe acute hepatitis B	Infectious disease, acute episode, severe	has a high fever and pain, and feels very weak, which causes great difficulty with daily activities.	0.133 (0.088–0.19)
Moderate acute hepatitis B	Infectious disease, acute episode, moderate	has a fever and aches, and feels weak, which causes some difficulty with daily activities.	0.051 (0.032–0.074)
Chronic hepatitis B	Asymptomatic	--	--
Asymptomatic acute hepatitis B	Asymptomatic	--	--
Moderate acute hepatitis C	Infectious disease, acute episode, moderate	has a fever and aches, and feels weak, which causes some difficulty with daily activities.	0.051 (0.032–0.074)
Severe acute hepatitis C	Infectious disease, acute episode, severe	has a high fever and pain, and feels very weak, which causes great difficulty with daily activities.	0.133 (0.088–0.19)
Chronic hepatitis C	Asymptomatic	--	--
Asymptomatic acute hepatitis C	Asymptomatic	--	--
Asymptomatic acute hepatitis E	Asymptomatic	--	--
Moderate acute hepatitis E	Infectious disease, acute episode, moderate	has a fever and aches, and feels weak, which causes some difficulty with daily activities.	0.051 (0.032–0.074)
Severe acute hepatitis E	Infectious disease, acute episode, severe	has a high fever and pain, and feels very weak, which causes great difficulty with daily activities.	0.133 (0.088–0.19)
Guillain-Barré syndrome due to other infectious diseases	Spinal cord lesion below neck level (treated)	is paralyzed from the waist down, cannot feel or move the legs and has difficulties with urine and bowel control. The person uses a wheelchair to move around.	0.296 (0.198–0.414)
Mild anemia due to other infectious diseases	Anemia, mild	feels slightly tired and weak at times, but this does not interfere with normal daily activities.	0.004 (0.001–0.008)
Other infectious diseases	Post-COMO calculation for residuals (YLL/YLD ratio, other methods)	--	--
Moderate anemia due to other infectious diseases	Anemia, moderate	feels moderate fatigue, weakness, and shortness of breath after exercise, making daily activities more difficult.	0.052 (0.034–0.076)
Severe anemia due to other infectious diseases	Anemia, severe	feels very weak, tired and short of breath, and has problems with activities that require physical effort or deep concentration.	0.149 (0.101–0.209)

Appendix Table 5. GBD 2016 sequelae, health states, health state lay descriptions, and disability weights			
**Sequela**	**Health state name**	**Health state lay description**	**Disability weight**
Controlled phase of mouth cancer	Generic uncomplicated disease: worry and daily medication	has a chronic disease that requires medication every day and causes some worry but minimal interference with daily activities.	0.049 (0.031–0.072)
Metastatic phase of mouth cancer	Cancer, metastatic	has severe pain, extreme fatigue, weight loss and high anxiety.	0.451 (0.307–0.6)
Diagnosis and primary therapy phase of mouth cancer	Cancer, diagnosis and primary therapy	has pain, nausea, fatigue, weight loss and high anxiety.	0.288 (0.193–0.399)
Terminal phase of mouth cancer	Terminal phase, with medication (for cancers, end-stage kidney/liver disease)	has lost a lot of weight and regularly uses strong medication to avoid constant pain. The person has no appetite, feels nauseous, and needs to spend most of the day in bed.	0.54 (0.377–0.687)
Terminal phase of nasopharynx cancer	Terminal phase, with medication (for cancers, end-stage kidney/liver disease)	has lost a lot of weight and regularly uses strong medication to avoid constant pain. The person has no appetite, feels nauseous, and needs to spend most of the day in bed.	0.54 (0.377–0.687)
Controlled phase of nasopharynx cancer	Generic uncomplicated disease: worry and daily medication	has a chronic disease that requires medication every day and causes some worry but minimal interference with daily activities.	0.049 (0.031–0.072)
Metastatic phase of nasopharynx cancer	Cancer, metastatic	has severe pain, extreme fatigue, weight loss and high anxiety.	0.451 (0.307–0.6)
Diagnosis and primary therapy phase of nasopharynx cancer	Cancer, diagnosis and primary therapy	has pain, nausea, fatigue, weight loss and high anxiety.	0.288 (0.193–0.399)
Controlled phase of other pharynx cancer	Generic uncomplicated disease: worry and daily medication	has a chronic disease that requires medication every day and causes some worry but minimal interference with daily activities.	0.049 (0.031–0.072)
Metastatic phase of other pharynx cancer	Cancer, metastatic	has severe pain, extreme fatigue, weight loss and high anxiety.	0.451 (0.307–0.6)
Terminal phase of other pharynx cancer	Terminal phase, with medication (for cancers, end-stage kidney/liver disease)	has lost a lot of weight and regularly uses strong medication to avoid constant pain. The person has no appetite, feels nauseous, and needs to spend most of the day in bed.	0.54 (0.377–0.687)
Diagnosis and primary therapy phase of other pharynx cancer	Cancer, diagnosis and primary therapy	has pain, nausea, fatigue, weight loss and high anxiety.	0.288 (0.193–0.399)
Controlled phase of esophageal cancer	Generic uncomplicated disease: worry and daily medication	has a chronic disease that requires medication every day and causes some worry but minimal interference with daily activities.	0.049 (0.031–0.072)
Terminal phase of esophageal cancer	Terminal phase, with medication (for cancers, end-stage kidney/liver disease)	has lost a lot of weight and regularly uses strong medication to avoid constant pain. The person has no appetite, feels nauseous, and needs to spend most of the day in bed.	0.54 (0.377–0.687)
Metastatic phase of esophageal cancer	Cancer, metastatic	has severe pain, extreme fatigue, weight loss and high anxiety.	0.451 (0.307–0.6)
Diagnosis and primary therapy phase of esophageal cancer	Cancer, diagnosis and primary therapy	has pain, nausea, fatigue, weight loss and high anxiety.	0.288 (0.193–0.399)
Diagnosis and primary therapy phase of stomach cancer	Cancer, diagnosis and primary therapy	has pain, nausea, fatigue, weight loss and high anxiety.	0.288 (0.193–0.399)
Metastatic phase of stomach cancer	Cancer, metastatic	has severe pain, extreme fatigue, weight loss and high anxiety.	0.451 (0.307–0.6)
Terminal phase of stomach cancer	Terminal phase, with medication (for cancers, end-stage kidney/liver disease)	has lost a lot of weight and regularly uses strong medication to avoid constant pain. The person has no appetite, feels nauseous, and needs to spend most of the day in bed.	0.54 (0.377–0.687)
Controlled phase of stomach cancer	Generic uncomplicated disease: worry and daily medication	has a chronic disease that requires medication every day and causes some worry but minimal interference with daily activities.	0.049 (0.031–0.072)
Metastatic phase of colon and rectum cancers	Cancer, metastatic	has severe pain, extreme fatigue, weight loss and high anxiety.	0.451 (0.307–0.6)
Terminal phase of colon and rectum cancers	Terminal phase, with medication (for cancers, end-stage kidney/liver disease)	has lost a lot of weight and regularly uses strong medication to avoid constant pain. The person has no appetite, feels nauseous, and needs to spend most of the day in bed.	0.54 (0.377–0.687)
Stoma due to colon and rectum cancer	Stoma and generic medication	(combined DW)	0.139 (0.094–0.192)
Diagnosis and primary therapy phase of colon and rectum cancers	Cancer, diagnosis and primary therapy	has pain, nausea, fatigue, weight loss and high anxiety.	0.288 (0.193–0.399)
Controlled phase of colon and rectum cancers	Generic uncomplicated disease: worry and daily medication	has a chronic disease that requires medication every day and causes some worry but minimal interference with daily activities.	0.049 (0.031–0.072)
Controlled phase of liver cancer due to hepatitis B	Generic uncomplicated disease: worry and daily medication	has a chronic disease that requires medication every day and causes some worry but minimal interference with daily activities.	0.049 (0.031–0.072)
Terminal phase of liver cancer due to hepatitis B	Terminal phase, with medication (for cancers, end-stage kidney/liver disease)	has lost a lot of weight and regularly uses strong medication to avoid constant pain. The person has no appetite, feels nauseous, and needs to spend most of the day in bed.	0.54 (0.377–0.687)
Diagnosis and primary therapy phase of liver cancer due to hepatitis B	Cancer, diagnosis and primary therapy	has pain, nausea, fatigue, weight loss and high anxiety.	0.288 (0.193–0.399)
Metastatic phase of liver cancer due to hepatitis B	Cancer, metastatic	has severe pain, extreme fatigue, weight loss and high anxiety.	0.451 (0.307–0.6)
Controlled phase of liver cancer due to hepatitis C	Generic uncomplicated disease: worry and daily medication	has a chronic disease that requires medication every day and causes some worry but minimal interference with daily activities.	0.049 (0.031–0.072)
Metastatic phase of liver cancer due to hepatitis C	Cancer, metastatic	has severe pain, extreme fatigue, weight loss and high anxiety.	0.451 (0.307–0.6)
Diagnosis and primary therapy phase of liver cancer due to hepatitis C	Cancer, diagnosis and primary therapy	has pain, nausea, fatigue, weight loss and high anxiety.	0.288 (0.193–0.399)

Appendix Table 5. GBD 2016 sequelae, health states, health state lay descriptions, and disability weights			
**Sequela**	**Health state name**	**Health state lay description**	**Disability weight**
Terminal phase of liver cancer due to hepatitis C	Terminal phase, with medication (for cancers, end-stage kidney/liver disease)	has lost a lot of weight and regularly uses strong medication to avoid constant pain. The person has no appetite, feels nauseous, and needs to spend most of the day in bed.	0.54 (0.377–0.687)
Diagnosis and primary therapy phase of liver cancer due to alcohol use	Cancer, diagnosis and primary therapy	has pain, nausea, fatigue, weight loss and high anxiety.	0.288 (0.193–0.399)
Terminal phase of liver cancer due to alcohol use	Terminal phase, with medication (for cancers, end-stage kidney/liver disease)	has lost a lot of weight and regularly uses strong medication to avoid constant pain. The person has no appetite, feels nauseous, and needs to spend most of the day in bed.	0.54 (0.377–0.687)
Metastatic phase of liver cancer due to alcohol use	Cancer, metastatic	has severe pain, extreme fatigue, weight loss and high anxiety.	0.451 (0.307–0.6)
Controlled phase of liver cancer due to alcohol use	Generic uncomplicated disease: worry and daily medication	has a chronic disease that requires medication every day and causes some worry but minimal interference with daily activities.	0.049 (0.031–0.072)
Controlled phase of liver cancer due to other causes	Generic uncomplicated disease: worry and daily medication	has a chronic disease that requires medication every day and causes some worry but minimal interference with daily activities.	0.049 (0.031–0.072)
Metastatic phase of liver cancer due to other causes	Cancer, metastatic	has severe pain, extreme fatigue, weight loss and high anxiety.	0.451 (0.307–0.6)
Diagnosis and primary therapy phase of liver cancer due to other causes	Cancer, diagnosis and primary therapy	has pain, nausea, fatigue, weight loss and high anxiety.	0.288 (0.193–0.399)
Terminal phase of liver cancer due to other causes	Terminal phase, with medication (for cancers, end-stage kidney/liver disease)	has lost a lot of weight and regularly uses strong medication to avoid constant pain. The person has no appetite, feels nauseous, and needs to spend most of the day in bed.	0.54 (0.377–0.687)
Diagnosis and primary therapy phase of gallbladder and biliary tract cancer	Cancer, diagnosis and primary therapy	has pain, nausea, fatigue, weight loss and high anxiety.	0.288 (0.193–0.399)
Terminal phase of gallbladder and biliary tract cancer	Terminal phase, with medication (for cancers, end-stage kidney/liver disease)	has lost a lot of weight and regularly uses strong medication to avoid constant pain. The person has no appetite, feels nauseous, and needs to spend most of the day in bed.	0.54 (0.377–0.687)
Metastatic phase of gallbladder and biliary tract cancer	Cancer, metastatic	has severe pain, extreme fatigue, weight loss and high anxiety.	0.451 (0.307–0.6)
Controlled phase of gallbladder and biliary tract cancer	Generic uncomplicated disease: worry and daily medication	has a chronic disease that requires medication every day and causes some worry but minimal interference with daily activities.	0.049 (0.031–0.072)
Metastatic phase of pancreatic cancer	Cancer, metastatic	has severe pain, extreme fatigue, weight loss and high anxiety.	0.451 (0.307–0.6)
Terminal phase of pancreatic cancer	Terminal phase, with medication (for cancers, end-stage kidney/liver disease)	has lost a lot of weight and regularly uses strong medication to avoid constant pain. The person has no appetite, feels nauseous, and needs to spend most of the day in bed.	0.54 (0.377–0.687)
Controlled phase of pancreatic cancer	Generic uncomplicated disease: worry and daily medication	has a chronic disease that requires medication every day and causes some worry but minimal interference with daily activities.	0.049 (0.031–0.072)
Diagnosis and primary therapy phase of pancreatic cancer	Cancer, diagnosis and primary therapy	has pain, nausea, fatigue, weight loss and high anxiety.	0.288 (0.193–0.399)
Metastatic phase of larynx cancer	Cancer, metastatic	has severe pain, extreme fatigue, weight loss and high anxiety.	0.451 (0.307–0.6)
Laryngectomy due to larynx cancer	Speech problems and generic medication	(combined DW)	0.098 (0.063–0.145)
Controlled phase of larynx cancer	Generic uncomplicated disease: worry and daily medication	has a chronic disease that requires medication every day and causes some worry but minimal interference with daily activities.	0.049 (0.031–0.072)
Diagnosis and primary therapy phase of larynx cancer	Cancer, diagnosis and primary therapy	has pain, nausea, fatigue, weight loss and high anxiety.	0.288 (0.193–0.399)
Terminal phase of larynx cancer	Terminal phase, with medication (for cancers, end-stage kidney/liver disease)	has lost a lot of weight and regularly uses strong medication to avoid constant pain. The person has no appetite, feels nauseous, and needs to spend most of the day in bed.	0.54 (0.377–0.687)
Metastatic phase of lung, bronchus, and trachea cancer	Cancer, metastatic	has severe pain, extreme fatigue, weight loss and high anxiety.	0.451 (0.307–0.6)
Diagnosis and primary therapy phase of lung, bronchus, and trachea cancer	Cancer, diagnosis and primary therapy	has pain, nausea, fatigue, weight loss and high anxiety.	0.288 (0.193–0.399)
Controlled phase of lung, bronchus, and trachea cancer	Generic uncomplicated disease: worry and daily medication	has a chronic disease that requires medication every day and causes some worry but minimal interference with daily activities.	0.049 (0.031–0.072)
Terminal phase of lung, bronchus, and trachea cancer	Terminal phase, with medication (for cancers, end-stage kidney/liver disease)	has lost a lot of weight and regularly uses strong medication to avoid constant pain. The person has no appetite, feels nauseous, and needs to spend most of the day in bed.	0.54 (0.377–0.687)
Controlled phase of malignant skin melanoma	Generic uncomplicated disease: worry and daily medication	has a chronic disease that requires medication every day and causes some worry but minimal interference with daily activities.	0.049 (0.031–0.072)
Metastatic phase of malignant skin melanoma	Cancer, metastatic	has severe pain, extreme fatigue, weight loss and high anxiety.	0.451 (0.307–0.6)
Terminal phase of malignant skin melanoma	Terminal phase, with medication (for cancers, end-stage kidney/liver disease)	has lost a lot of weight and regularly uses strong medication to avoid constant pain. The person has no appetite, feels nauseous, and needs to spend most of the day in bed.	0.54 (0.377–0.687)
Diagnosis and primary therapy phase of malignant skin melanoma	Cancer, diagnosis and primary therapy	has pain, nausea, fatigue, weight loss and high anxiety.	0.288 (0.193–0.399)
Severe disfigurement due to squamous cell carcinoma	Disfigurement, level 3, with itch/pain	has an obvious physical deformity that is very painful and itchy. The physical deformity makes others uncomfortable, which causes the person to avoid social contact, feel worried, sleep poorly, and think about suicide.	0.576 (0.401–0.731)

Appendix Table 5. GBD 2016 sequelae, health states, health state lay descriptions, and disability weights			
**Sequela**	**Health state name**	**Health state lay description**	**Disability weight**
Moderate disfigurement due to squamous cell carcinoma	Disfigurement, level 2	has a visible physical deformity that causes others to stare and comment. As a result, the person is worried and has trouble sleeping and concentrating.	0.067 (0.044–0.096)
Mild disfigurement due to squamous cell carcinoma	Disfigurement, level 1	has a slight, visible physical deformity that others notice, which causes some worry and discomfort.	0.011 (0.005–0.021)
Disfigurement due to basal cell carcinoma	Disfigurement, level 1	has a slight, visible physical deformity that others notice, which causes some worry and discomfort.	0.011 (0.005–0.021)
Diagnosis and primary therapy phase of breast cancer	Cancer, diagnosis and primary therapy	has pain, nausea, fatigue, weight loss and high anxiety.	0.288 (0.193–0.399)
Mastectomy due to breast cancer	Mastectomy and generic medication	(combined DW)	0.083 (0.052–0.124)
Controlled phase of breast cancer	Generic uncomplicated disease: worry and daily medication	has a chronic disease that requires medication every day and causes some worry but minimal interference with daily activities.	0.049 (0.031–0.072)
Metastatic phase of breast cancer	Cancer, metastatic	has severe pain, extreme fatigue, weight loss and high anxiety.	0.451 (0.307–0.6)
Terminal phase of breast cancer	Terminal phase, with medication (for cancers, end-stage kidney/liver disease)	has lost a lot of weight and regularly uses strong medication to avoid constant pain. The person has no appetite, feels nauseous, and needs to spend most of the day in bed.	0.54 (0.377–0.687)
Terminal phase of cervical cancer	Terminal phase, with medication (for cancers, end-stage kidney/liver disease)	has lost a lot of weight and regularly uses strong medication to avoid constant pain. The person has no appetite, feels nauseous, and needs to spend most of the day in bed.	0.54 (0.377–0.687)
Diagnosis and primary therapy phase of cervical cancer	Cancer, diagnosis and primary therapy	has pain, nausea, fatigue, weight loss and high anxiety.	0.288 (0.193–0.399)
Controlled phase of cervical cancer	Generic uncomplicated disease: worry and daily medication	has a chronic disease that requires medication every day and causes some worry but minimal interference with daily activities.	0.049 (0.031–0.072)
Metastatic phase of cervical cancer	Cancer, metastatic	has severe pain, extreme fatigue, weight loss and high anxiety.	0.451 (0.307–0.6)
Controlled phase of uterine cancer	Generic uncomplicated disease: worry and daily medication	has a chronic disease that requires medication every day and causes some worry but minimal interference with daily activities.	0.049 (0.031–0.072)
Terminal phase of uterine cancer	Terminal phase, with medication (for cancers, end-stage kidney/liver disease)	has lost a lot of weight and regularly uses strong medication to avoid constant pain. The person has no appetite, feels nauseous, and needs to spend most of the day in bed.	0.54 (0.377–0.687)
Diagnosis and primary therapy phase of uterine cancer	Cancer, diagnosis and primary therapy	has pain, nausea, fatigue, weight loss and high anxiety.	0.288 (0.193–0.399)
Metastatic phase of uterine cancer	Cancer, metastatic	has severe pain, extreme fatigue, weight loss and high anxiety.	0.451 (0.307–0.6)
Diagnosis and primary therapy phase of ovarian cancer	Cancer, diagnosis and primary therapy	has pain, nausea, fatigue, weight loss and high anxiety.	0.288 (0.193–0.399)
Controlled phase of ovarian cancer	Generic uncomplicated disease: worry and daily medication	has a chronic disease that requires medication every day and causes some worry but minimal interference with daily activities.	0.049 (0.031–0.072)
Metastatic phase of ovarian cancer	Cancer, metastatic	has severe pain, extreme fatigue, weight loss and high anxiety.	0.451 (0.307–0.6)
Terminal phase of ovarian cancer	Terminal phase, with medication (for cancers, end-stage kidney/liver disease)	has lost a lot of weight and regularly uses strong medication to avoid constant pain. The person has no appetite, feels nauseous, and needs to spend most of the day in bed.	0.54 (0.377–0.687)
Diagnosis and primary therapy phase of prostate cancer	Cancer, diagnosis and primary therapy	has pain, nausea, fatigue, weight loss and high anxiety.	0.288 (0.193–0.399)
Impotence due to prostate cancer	Impotence and generic medication	(combined DW)	0.065 (0.04–0.1)
Terminal phase of prostate cancer	Terminal phase, with medication (for cancers, end-stage kidney/liver disease)	has lost a lot of weight and regularly uses strong medication to avoid constant pain. The person has no appetite, feels nauseous, and needs to spend most of the day in bed.	0.54 (0.377–0.687)
Controlled phase of prostate cancer	Generic uncomplicated disease: worry and daily medication	has a chronic disease that requires medication every day and causes some worry but minimal interference with daily activities.	0.049 (0.031–0.072)
Metastatic phase of prostate cancer	Cancer, metastatic	has severe pain, extreme fatigue, weight loss and high anxiety.	0.451 (0.307–0.6)
Incontinence due to prostate cancer	Incontinence and generic medication	(combined DW)	0.181 (0.124–0.248)
Terminal phase of testicular cancer	Terminal phase, with medication (for cancers, end-stage kidney/liver disease)	has lost a lot of weight and regularly uses strong medication to avoid constant pain. The person has no appetite, feels nauseous, and needs to spend most of the day in bed.	0.54 (0.377–0.687)
Metastatic phase of testicular cancer	Cancer, metastatic	has severe pain, extreme fatigue, weight loss and high anxiety.	0.451 (0.307–0.6)
Diagnosis and primary therapy phase of testicular cancer	Cancer, diagnosis and primary therapy	has pain, nausea, fatigue, weight loss and high anxiety.	0.288 (0.193–0.399)
Controlled phase of testicular cancer	Generic uncomplicated disease: worry and daily medication	has a chronic disease that requires medication every day and causes some worry but minimal interference with daily activities.	0.049 (0.031–0.072)
Diagnosis and primary therapy phase of kidney cancer	Cancer, diagnosis and primary therapy	has pain, nausea, fatigue, weight loss and high anxiety.	0.288 (0.193–0.399)
Metastatic phase of kidney cancer	Cancer, metastatic	has severe pain, extreme fatigue, weight loss and high anxiety.	0.451 (0.307–0.6)
Terminal phase of kidney cancer	Terminal phase, with medication (for cancers, end-stage kidney/liver disease)	has lost a lot of weight and regularly uses strong medication to avoid constant pain. The person has no appetite, feels nauseous, and needs to spend most of the day in bed.	0.54 (0.377–0.687)

Appendix Table 5. GBD 2016 sequelae, health states, health state lay descriptions, and disability weights			
**Sequela**	**Health state name**	**Health state lay description**	**Disability weight**
Controlled phase of kidney cancer	Generic uncomplicated disease: worry and daily medication	has a chronic disease that requires medication every day and causes some worry but minimal interference with daily activities.	0.049 (0.031–0.072)
Controlled phase of bladder cancer	Generic uncomplicated disease: worry and daily medication	has a chronic disease that requires medication every day and causes some worry but minimal interference with daily activities.	0.049 (0.031–0.072)
Urinary incontinence due to bladder cancer	Incontinence and generic medication	(combined DW)	0.181 (0.124–0.248)
Terminal phase of bladder cancer	Terminal phase, with medication (for cancers, end-stage kidney/liver disease)	has lost a lot of weight and regularly uses strong medication to avoid constant pain. The person has no appetite, feels nauseous, and needs to spend most of the day in bed.	0.54 (0.377–0.687)
Diagnosis and primary therapy phase of bladder cancer	Cancer, diagnosis and primary therapy	has pain, nausea, fatigue, weight loss and high anxiety.	0.288 (0.193–0.399)
Metastatic phase of bladder cancer	Cancer, metastatic	has severe pain, extreme fatigue, weight loss and high anxiety.	0.451 (0.307–0.6)
Diagnosis and primary therapy phase of brain and nervous system cancers	Cancer, diagnosis and primary therapy	has pain, nausea, fatigue, weight loss and high anxiety.	0.288 (0.193–0.399)
Terminal phase of brain and nervous system cancers	Terminal phase, with medication (for cancers, end-stage kidney/liver disease)	has lost a lot of weight and regularly uses strong medication to avoid constant pain. The person has no appetite, feels nauseous, and needs to spend most of the day in bed.	0.54 (0.377–0.687)
Metastatic phase of brain and nervous system cancers	Cancer, metastatic	has severe pain, extreme fatigue, weight loss and high anxiety.	0.451 (0.307–0.6)
Controlled phase of brain and nervous system cancers	Generic uncomplicated disease: worry and daily medication	has a chronic disease that requires medication every day and causes some worry but minimal interference with daily activities.	0.049 (0.031–0.072)
Terminal phase of thyroid cancer	Terminal phase, with medication (for cancers, end-stage kidney/liver disease)	has lost a lot of weight and regularly uses strong medication to avoid constant pain. The person has no appetite, feels nauseous, and needs to spend most of the day in bed.	0.54 (0.377–0.687)
Controlled phase of thyroid cancer	Generic uncomplicated disease: worry and daily medication	has a chronic disease that requires medication every day and causes some worry but minimal interference with daily activities.	0.049 (0.031–0.072)
Metastatic phase of thyroid cancer	Cancer, metastatic	has severe pain, extreme fatigue, weight loss and high anxiety.	0.451 (0.307–0.6)
Diagnosis and primary therapy phase of thyroid cancer	Cancer, diagnosis and primary therapy	has pain, nausea, fatigue, weight loss and high anxiety.	0.288 (0.193–0.399)
Terminal phase of mesothelioma	Terminal phase, with medication (for cancers, end-stage kidney/liver disease)	has lost a lot of weight and regularly uses strong medication to avoid constant pain. The person has no appetite, feels nauseous, and needs to spend most of the day in bed.	0.54 (0.377–0.687)
Controlled phase of mesothelioma	Generic uncomplicated disease: worry and daily medication	has a chronic disease that requires medication every day and causes some worry but minimal interference with daily activities.	0.049 (0.031–0.072)
Diagnosis and primary therapy phase of mesothelioma	Cancer, diagnosis and primary therapy	has pain, nausea, fatigue, weight loss and high anxiety.	0.288 (0.193–0.399)
Metastatic phase of mesothelioma	Cancer, metastatic	has severe pain, extreme fatigue, weight loss and high anxiety.	0.451 (0.307–0.6)
Controlled phase of Hodgkin disease	Generic uncomplicated disease: worry and daily medication	has a chronic disease that requires medication every day and causes some worry but minimal interference with daily activities.	0.049 (0.031–0.072)
Metastatic phase of Hodgkin disease	Cancer, metastatic	has severe pain, extreme fatigue, weight loss and high anxiety.	0.451 (0.307–0.6)
Diagnosis and primary therapy phase of Hodgkin disease	Cancer, diagnosis and primary therapy	has pain, nausea, fatigue, weight loss and high anxiety.	0.288 (0.193–0.399)
Terminal phase of Hodgkin disease	Terminal phase, with medication (for cancers, end-stage kidney/liver disease)	has lost a lot of weight and regularly uses strong medication to avoid constant pain. The person has no appetite, feels nauseous, and needs to spend most of the day in bed.	0.54 (0.377–0.687)
Metastatic phase of non-Hodgkin lymphoma	Cancer, metastatic	has severe pain, extreme fatigue, weight loss and high anxiety.	0.451 (0.307–0.6)
Terminal phase of non-Hodgkin lymphoma	Terminal phase, with medication (for cancers, end-stage kidney/liver disease)	has lost a lot of weight and regularly uses strong medication to avoid constant pain. The person has no appetite, feels nauseous, and needs to spend most of the day in bed.	0.54 (0.377–0.687)
Controlled phase of non-Hodgkin lymphoma	Generic uncomplicated disease: worry and daily medication	has a chronic disease that requires medication every day and causes some worry but minimal interference with daily activities.	0.049 (0.031–0.072)
Diagnosis and primary therapy phase of non-Hodgkin lymphoma	Cancer, diagnosis and primary therapy	has pain, nausea, fatigue, weight loss and high anxiety.	0.288 (0.193–0.399)
Terminal phase of multiple myeloma	Terminal phase, with medication (for cancers, end-stage kidney/liver disease)	has lost a lot of weight and regularly uses strong medication to avoid constant pain. The person has no appetite, feels nauseous, and needs to spend most of the day in bed.	0.54 (0.377–0.687)
Diagnosis and primary therapy phase of multiple myeloma	Cancer, diagnosis and primary therapy	has pain, nausea, fatigue, weight loss and high anxiety.	0.288 (0.193–0.399)
Metastatic phase of multiple myeloma	Cancer, metastatic	has severe pain, extreme fatigue, weight loss and high anxiety.	0.451 (0.307–0.6)
Controlled phase of multiple myeloma	Generic uncomplicated disease: worry and daily medication	has a chronic disease that requires medication every day and causes some worry but minimal interference with daily activities.	0.049 (0.031–0.072)
Controlled phase of acute lymphoid leukemia	Generic uncomplicated disease: worry and daily medication	has a chronic disease that requires medication every day and causes some worry but minimal interference with daily activities.	0.049 (0.031–0.072)
Diagnosis and primary therapy phase of acute lymphoid leukemia	Cancer, diagnosis and primary therapy	has pain, nausea, fatigue, weight loss and high anxiety.	0.288 (0.193–0.399)

Appendix Table 5. GBD 2016 sequelae, health states, health state lay descriptions, and disability weights			
**Sequela**	**Health state name**	**Health state lay description**	**Disability weight**
Metastatic phase of acute lymphoid leukemia	Cancer, metastatic	has severe pain, extreme fatigue, weight loss and high anxiety.	0.451 (0.307–0.6)
Terminal phase of acute lymphoid leukemia	Terminal phase, with medication (for cancers, end-stage kidney/liver disease)	has lost a lot of weight and regularly uses strong medication to avoid constant pain. The person has no appetite, feels nauseous, and needs to spend most of the day in bed.	0.54 (0.377–0.687)
Metastatic phase of chronic lymphoid leukemia	Cancer, metastatic	has severe pain, extreme fatigue, weight loss and high anxiety.	0.451 (0.307–0.6)
Terminal phase of chronic lymphoid leukemia	Terminal phase, with medication (for cancers, end-stage kidney/liver disease)	has lost a lot of weight and regularly uses strong medication to avoid constant pain. The person has no appetite, feels nauseous, and needs to spend most of the day in bed.	0.54 (0.377–0.687)
Diagnosis and primary therapy phase of chronic lymphoid leukemia	Cancer, diagnosis and primary therapy	has pain, nausea, fatigue, weight loss and high anxiety.	0.288 (0.193–0.399)
Controlled phase of chronic lymphoid leukemia	Generic uncomplicated disease: worry and daily medication	has a chronic disease that requires medication every day and causes some worry but minimal interference with daily activities.	0.049 (0.031–0.072)
Diagnosis and primary therapy phase of acute myeloid leukemia	Cancer, diagnosis and primary therapy	has pain, nausea, fatigue, weight loss and high anxiety.	0.288 (0.193–0.399)
Controlled phase of acute myeloid leukemia	Generic uncomplicated disease: worry and daily medication	has a chronic disease that requires medication every day and causes some worry but minimal interference with daily activities.	0.049 (0.031–0.072)
Terminal phase of acute myeloid leukemia	Terminal phase, with medication (for cancers, end-stage kidney/liver disease)	has lost a lot of weight and regularly uses strong medication to avoid constant pain. The person has no appetite, feels nauseous, and needs to spend most of the day in bed.	0.54 (0.377–0.687)
Metastatic phase of acute myeloid leukemia	Cancer, metastatic	has severe pain, extreme fatigue, weight loss and high anxiety.	0.451 (0.307–0.6)
Diagnosis and primary therapy phase of chronic myeloid leukemia	Cancer, diagnosis and primary therapy	has pain, nausea, fatigue, weight loss and high anxiety.	0.288 (0.193–0.399)
Metastatic phase of chronic myeloid leukemia	Cancer, metastatic	has severe pain, extreme fatigue, weight loss and high anxiety.	0.451 (0.307–0.6)
Terminal phase of chronic myeloid leukemia	Terminal phase, with medication (for cancers, end-stage kidney/liver disease)	has lost a lot of weight and regularly uses strong medication to avoid constant pain. The person has no appetite, feels nauseous, and needs to spend most of the day in bed.	0.54 (0.377–0.687)
Controlled phase of chronic myeloid leukemia	Generic uncomplicated disease: worry and daily medication	has a chronic disease that requires medication every day and causes some worry but minimal interference with daily activities.	0.049 (0.031–0.072)
Metastatic phase of other leukemia	Cancer, metastatic	has severe pain, extreme fatigue, weight loss and high anxiety.	0.451 (0.307–0.6)
Controlled phase of other leukemia	Generic uncomplicated disease: worry and daily medication	has a chronic disease that requires medication every day and causes some worry but minimal interference with daily activities.	0.049 (0.031–0.072)
Diagnosis and primary therapy phase of other leukemia	Cancer, diagnosis and primary therapy	has pain, nausea, fatigue, weight loss and high anxiety.	0.288 (0.193–0.399)
Terminal phase of other leukemia	Terminal phase, with medication (for cancers, end-stage kidney/liver disease)	has lost a lot of weight and regularly uses strong medication to avoid constant pain. The person has no appetite, feels nauseous, and needs to spend most of the day in bed.	0.54 (0.377–0.687)
Terminal phase of other neoplasms	Terminal phase, with medication (for cancers, end-stage kidney/liver disease)	has lost a lot of weight and regularly uses strong medication to avoid constant pain. The person has no appetite, feels nauseous, and needs to spend most of the day in bed.	0.54 (0.377–0.687)
Diagnosis and primary therapy phase of other neoplasms	Cancer, diagnosis and primary therapy	has pain, nausea, fatigue, weight loss and high anxiety.	0.288 (0.193–0.399)
Metastatic phase of other neoplasms	Cancer, metastatic	has severe pain, extreme fatigue, weight loss and high anxiety.	0.451 (0.307–0.6)
Controlled phase of other neoplasms	Generic uncomplicated disease: worry and daily medication	has a chronic disease that requires medication every day and causes some worry but minimal interference with daily activities.	0.049 (0.031–0.072)
Severe heart failure due to rheumatic heart disease	Heart failure, severe	is short of breath and feels tired when at rest. The person avoids any physical activity, for fear of worsening the breathing problems.	0.179 (0.122–0.251)
Rheumatic heart disease, without heart failure	Generic uncomplicated disease: worry and daily medication	has a chronic disease that requires medication every day and causes some worry but minimal interference with daily activities.	0.049 (0.031–0.072)
Moderate heart failure due to rheumatic heart disease	Heart failure, moderate	is short of breath and easily tires with minimal physical activity, such as walking only a short distance. The person feels comfortable at rest but avoids moderate activity.	0.072 (0.047–0.103)
Asymptomatic and mild heart failure due to rheumatic heart disease	Heart failure, mild	is short of breath and easily tires with moderate physical activity, such as walking uphill or more than a quarter-mile on level ground. The person feels comfortable at rest or during activities requiring less effort.	0.041 (0.026–0.062)
Acute myocardial infarction first 2 days	Acute myocardial infarction, days 1-2	has severe chest pain that becomes worse with any physical activity,. The person feels nauseous, short of breath, and very anxious.	0.432 (0.288–0.579)
Asymptomatic and mild heart failure due to ischemic heart disease	Heart failure, mild	is short of breath and easily tires with moderate physical activity, such as walking uphill or more than a quarter-mile on level ground. The person feels comfortable at rest or during activities requiring less effort.	0.041 (0.026–0.062)
Asymptomatic angina due to ischemic heart disease	Asymptomatic	--	--
Severe heart failure due to ischemic heart disease	Heart failure, severe	is short of breath and feels tired when at rest. The person avoids any physical activity, for fear of worsening the breathing problems.	0.179 (0.122–0.251)

Appendix Table 5. GBD 2016 sequelae, health states, health state lay descriptions, and disability weights			
**Sequela**	**Health state name**	**Health state lay description**	**Disability weight**
Acute myocardial infarction 3 to 28 days	Acute myocardial infarction, days 3-28	gets short of breath after heavy physical activity, and tires easily, but has no problems when at rest. The person has to take medication every day and has some anxiety.	0.074 (0.049–0.105)
Moderate angina due to ischemic heart disease	Angina pectoris, moderate	has chest pain that occurs with moderate physical activity, such as walking uphill or more than half a kilometer (around a quarter-mile) on level ground. After a brief rest, the pain goes away.	0.08 (0.052–0.113)
Asymptomatic ischemic heart disease following myocardial infarction	Asymptomatic	--	--
Severe angina due to ischemic heart disease	Angina pectoris, severe	has chest pain that occurs with minimal physical activity, such as walking only a short distance. After a brief rest, the pain goes away. The person avoids most physical activities because of the pain.	0.167 (0.11–0.24)
Moderate heart failure due to ischemic heart disease	Heart failure, moderate	is short of breath and easily tires with minimal physical activity, such as walking only a short distance. The person feels comfortable at rest but avoids moderate activity.	0.072 (0.047–0.103)
Mild angina due to ischemic heart disease	Angina pectoris, mild	has chest pain that occurs with strenuous physical activity, such as running or lifting heavy objects. After a brief rest, the pain goes away.	0.033 (0.02–0.052)
Acute ischemic stroke severity level 1	Stroke, long-term consequences, mild	has some difficulty in moving around and some weakness in one hand, but is able to walk without help.	0.019 (0.01–0.032)
Chronic ischemic stroke severity level 1	Stroke, long-term consequences, mild	has some difficulty in moving around and some weakness in one hand, but is able to walk without help.	0.019 (0.01–0.032)
Acute ischemic stroke severity level 2	Stroke, long-term consequences, moderate	has some difficulty in moving around, and in using the hands for lifting and holding things, dressing and grooming.	0.07 (0.046–0.099)
Chronic ischemic stroke severity level 5	Stroke, long-term consequences, severe plus cognition problems	is confined to bed or a wheelchair, depends on others for feeding, toileting and dressing, and has difficulty speaking, thinking clearly and remembering things.	0.588 (0.411–0.744)
Chronic ischemic stroke severity level 2	Stroke, long-term consequences, moderate	has some difficulty in moving around, and in using the hands for lifting and holding things, dressing and grooming.	0.07 (0.046–0.099)
Acute ischemic stroke severity level 5	Stroke, long-term consequences, severe plus cognition problems	is confined to bed or a wheelchair, depends on others for feeding, toileting and dressing, and has difficulty speaking, thinking clearly and remembering things.	0.588 (0.411–0.744)
Acute ischemic stroke severity level 3	Stroke, long-term consequences, moderate plus cognition problems	has some difficulty in moving around, in using the hands for lifting and holding things, dressing and grooming, and in speaking. The person is often forgetful and confused.	0.316 (0.206–0.437)
Chronic ischemic stroke severity level 4	Stroke, long-term consequences, severe	is confined to bed or a wheelchair, has difficulty speaking and depends on others for feeding, toileting and dressing.	0.552 (0.377–0.707)
Chronic ischemic stroke severity level 3	Stroke, long-term consequences, moderate plus cognition problems	has some difficulty in moving around, in using the hands for lifting and holding things, dressing and grooming, and in speaking. The person is often forgetful and confused.	0.316 (0.206–0.437)
Acute ischemic stroke severity level 4	Stroke, long-term consequences, severe	is confined to bed or a wheelchair, has difficulty speaking and depends on others for feeding, toileting and dressing.	0.552 (0.377–0.707)
Asymptomatic chronic ischemic stroke	Asymptomatic	--	--
Chronic hemorrhagic stroke severity level 1	Stroke, long-term consequences, mild	has some difficulty in moving around and some weakness in one hand, but is able to walk without help.	0.019 (0.01–0.032)
Acute hemorrhagic stroke severity level 5	Stroke, long-term consequences, severe plus cognition problems	is confined to bed or a wheelchair, depends on others for feeding, toileting and dressing, and has difficulty speaking, thinking clearly and remembering things.	0.588 (0.411–0.744)
Chronic hemorrhagic stroke severity level 5	Stroke, long-term consequences, severe plus cognition problems	is confined to bed or a wheelchair, depends on others for feeding, toileting and dressing, and has difficulty speaking, thinking clearly and remembering things.	0.588 (0.411–0.744)
Acute hemorrhagic stroke severity level 1	Stroke, long-term consequences, mild	has some difficulty in moving around and some weakness in one hand, but is able to walk without help.	0.019 (0.01–0.032)
Acute hemorrhagic stroke severity level 2	Stroke, long-term consequences, moderate	has some difficulty in moving around, and in using the hands for lifting and holding things, dressing and grooming.	0.07 (0.046–0.099)
Chronic hemorrhagic stroke severity level 2	Stroke, long-term consequences, moderate	has some difficulty in moving around, and in using the hands for lifting and holding things, dressing and grooming.	0.07 (0.046–0.099)
Chronic hemorrhagic stroke severity level 4	Stroke, long-term consequences, severe	is confined to bed or a wheelchair, has difficulty speaking and depends on others for feeding, toileting and dressing.	0.552 (0.377–0.707)
Acute hemorrhagic stroke severity level 3	Stroke, long-term consequences, moderate plus cognition problems	has some difficulty in moving around, in using the hands for lifting and holding things, dressing and grooming, and in speaking. The person is often forgetful and confused.	0.316 (0.206–0.437)
Chronic hemorrhagic stroke severity level 3	Stroke, long-term consequences, moderate plus cognition problems	has some difficulty in moving around, in using the hands for lifting and holding things, dressing and grooming, and in speaking. The person is often forgetful and confused.	0.316 (0.206–0.437)
Acute hemorrhagic stroke severity level 4	Stroke, long-term consequences, severe	is confined to bed or a wheelchair, has difficulty speaking and depends on others for feeding, toileting and dressing.	0.552 (0.377–0.707)
Asymptomatic chronic hemorrhagic stroke	Asymptomatic	--	--
Asymptomatic and mild heart failure due to hypertensive heart disease	Heart failure, mild	is short of breath and easily tires with moderate physical activity, such as walking uphill or more than a quarter-mile on level ground. The person feels comfortable at rest or during activities requiring less effort.	0.041 (0.026–0.062)
Severe heart failure due to hypertensive heart disease	Heart failure, severe	is short of breath and feels tired when at rest. The person avoids any physical activity, for fear of worsening the breathing problems.	0.179 (0.122–0.251)
Moderate heart failure due to hypertensive heart disease	Heart failure, moderate	is short of breath and easily tires with minimal physical activity, such as walking only a short distance. The person feels comfortable at rest but avoids moderate activity.	0.072 (0.047–0.103)

Appendix Table 5. GBD 2016 sequelae, health states, health state lay descriptions, and disability weights			
**Sequela**	**Health state name**	**Health state lay description**	**Disability weight**
Asymptomatic and mild heart failure due to myocarditis	Heart failure, mild	is short of breath and easily tires with moderate physical activity, such as walking uphill or more than a quarter-mile on level ground. The person feels comfortable at rest or during activities requiring less effort.	0.041 (0.026–0.062)
Acute myocarditis	Infectious disease, acute episode, moderate	has a fever and aches, and feels weak, which causes some difficulty with daily activities.	0.051 (0.032–0.074)
Severe heart failure due to myocarditis	Heart failure, severe	is short of breath and feels tired when at rest. The person avoids any physical activity, for fear of worsening the breathing problems.	0.179 (0.122–0.251)
Moderate heart failure due to myocarditis	Heart failure, moderate	is short of breath and easily tires with minimal physical activity, such as walking only a short distance. The person feels comfortable at rest but avoids moderate activity.	0.072 (0.047–0.103)
Asymptomatic and mild heart failure due to alcoholic cardiomyopathy	Heart failure, mild	is short of breath and easily tires with moderate physical activity, such as walking uphill or more than a quarter-mile on level ground. The person feels comfortable at rest or during activities requiring less effort.	0.041 (0.026–0.062)
Moderate heart failure due to alcoholic cardiomyopathy	Heart failure, moderate	is short of breath and easily tires with minimal physical activity, such as walking only a short distance. The person feels comfortable at rest but avoids moderate activity.	0.072 (0.047–0.103)
Severe heart failure due to alcoholic cardiomyopathy	Heart failure, severe	is short of breath and feels tired when at rest. The person avoids any physical activity, for fear of worsening the breathing problems.	0.179 (0.122–0.251)
Moderate heart failure due to other cardiomyopathy	Heart failure, moderate	is short of breath and easily tires with minimal physical activity, such as walking only a short distance. The person feels comfortable at rest but avoids moderate activity.	0.072 (0.047–0.103)
Severe heart failure due to other cardiomyopathy	Heart failure, severe	is short of breath and feels tired when at rest. The person avoids any physical activity, for fear of worsening the breathing problems.	0.179 (0.122–0.251)
Asymptomatic and mild heart failure due to other cardiomyopathy	Heart failure, mild	is short of breath and easily tires with moderate physical activity, such as walking uphill or more than a quarter-mile on level ground. The person feels comfortable at rest or during activities requiring less effort.	0.041 (0.026–0.062)
Asymptomatic atrial fibrillation and flutter	Asymptomatic	--	--
Symptomatic atrial fibrillation and flutter	Cardiac conduction disorders and cardiac dysrhythmias	has periods of rapid and irregular heartbeats and occasional fainting.	0.224 (0.151–0.312)
Asymptomatic peripheral vascular disease	Asymptomatic	--	--
Symptomatic claudication due to peripheral vascular disease	Claudication	has cramping pains in the legs after walking a medium distance. The pain goes away after a short rest.	0.014 (0.007–0.025)
Moderate endocarditis	Infectious disease, acute episode, moderate	has a fever and aches, and feels weak, which causes some difficulty with daily activities.	0.051 (0.032–0.074)
Asymptomatic and mild heart failure due to endocarditis	Heart failure, mild	is short of breath and easily tires with moderate physical activity, such as walking uphill or more than a quarter-mile on level ground. The person feels comfortable at rest or during activities requiring less effort.	0.041 (0.026–0.062)
Severe endocarditis	Infectious disease, acute episode, severe	has a high fever and pain, and feels very weak, which causes great difficulty with daily activities.	0.133 (0.088–0.19)
Severe heart failure due to endocarditis	Heart failure, severe	is short of breath and feels tired when at rest. The person avoids any physical activity, for fear of worsening the breathing problems.	0.179 (0.122–0.251)
Moderate heart failure due to endocarditis	Heart failure, moderate	is short of breath and easily tires with minimal physical activity, such as walking only a short distance. The person feels comfortable at rest but avoids moderate activity.	0.072 (0.047–0.103)
Moderate other cardiovascular diseases	Heart failure, moderate	is short of breath and easily tires with minimal physical activity, such as walking only a short distance. The person feels comfortable at rest but avoids moderate activity.	0.072 (0.047–0.103)
Severe heart failure due to other cardiovascular diseases	Heart failure, severe	is short of breath and feels tired when at rest. The person avoids any physical activity, for fear of worsening the breathing problems.	0.179 (0.122–0.251)
Asymptomatic other cardiovascular diseases	Asymptomatic	--	--
Mild other cardiovascular diseases	Heart failure, mild	is short of breath and easily tires with moderate physical activity, such as walking uphill or more than a quarter-mile on level ground. The person feels comfortable at rest or during activities requiring less effort.	0.041 (0.026–0.062)
Asymptomatic and mild heart failure due to other cardiovascular diseases	Heart failure, mild	is short of breath and easily tires with moderate physical activity, such as walking uphill or more than a quarter-mile on level ground. The person feels comfortable at rest or during activities requiring less effort.	0.041 (0.026–0.062)
Severe other cardiovascular diseases	Heart failure, severe	is short of breath and feels tired when at rest. The person avoids any physical activity, for fear of worsening the breathing problems.	0.179 (0.122–0.251)
Moderate heart failure due to other cardiovascular diseases	Heart failure, moderate	is short of breath and easily tires with minimal physical activity, such as walking only a short distance. The person feels comfortable at rest but avoids moderate activity.	0.072 (0.047–0.103)
Severe heart failure due to severe chronic obstructive pulmonary disease	Severe COPD and other chronic respiratory, with severe heart failure	--	--
Asymptomatic and mild heart failure due to severe chronic obstructive pulmonary disease	Severe COPD and other chronic respiratory, with mild heart failure	--	--

Appendix Table 5. GBD 2016 sequelae, health states, health state lay descriptions, and disability weights			
**Sequela**	**Health state name**	**Health state lay description**	**Disability weight**
Moderate chronic obstructive pulmonary disease	COPD and other chronic respiratory problems, moderate	has cough, wheezing and shortness of breath, even after light physical activity. The person feels tired and can walk only short distances or climb only a few stairs.	0.225 (0.153–0.31)
Moderate heart failure due to severe chronic obstructive pulmonary disease	Severe COPD and other chronic respiratory, with moderate heart failure	--	--
Asymptomatic chronic obstructive pulmonary disease	Asymptomatic	--	--
Mild chronic obstructive pulmonary disease	COPD and other chronic respiratory problems, mild	has cough and shortness of breath after heavy physical activity, but is able to walk long distances and climb stairs.	0.019 (0.011–0.033)
Severe chronic obstructive pulmonary disease without heart failure	COPD and other chronic respiratory problems, severe	has cough, wheezing and shortness of breath all the time. The person has great difficulty walking even short distances or climbing any stairs, feels tired when at rest, and is	0.408 (0.273–0.556)
Moderate heart failure due to severe silicosis	Severe COPD and other chronic respiratory, with moderate heart failure	--	--
Mild silicosis	COPD and other chronic respiratory problems, mild	has cough and shortness of breath after heavy physical activity, but is able to walk long distances and climb stairs.	0.019 (0.011–0.033)
Moderate silicosis	COPD and other chronic respiratory problems, moderate	has cough, wheezing and shortness of breath, even after light physical activity. The person feels tired and can walk only short distances or climb only a few stairs.	0.225 (0.153–0.31)
Asymptomatic and mild heart failure due to severe silicosis	Severe COPD and other chronic respiratory, with mild heart failure	--	--
Severe silicosis without heart failure	COPD and other chronic respiratory problems, severe	has cough, wheezing and shortness of breath all the time. The person has great difficulty walking even short distances or climbing any stairs, feels tired when at rest, and is	0.408 (0.273–0.556)
Asymptomatic silicosis	Asymptomatic	--	--
Severe heart failure due to severe silicosis	Severe COPD and other chronic respiratory, with severe heart failure	--	--
Asymptomatic and mild heart failure due to severe asbestosis	Severe COPD and other chronic respiratory, with mild heart failure	--	--
Moderate heart failure due to severe asbestosis	Severe COPD and other chronic respiratory, with moderate heart failure	--	--
Mild asbestosis	COPD and other chronic respiratory problems, mild	has cough and shortness of breath after heavy physical activity, but is able to walk long distances and climb stairs.	0.019 (0.011–0.033)
Severe asbestosis without heart failure	COPD and other chronic respiratory problems, severe	has cough, wheezing and shortness of breath all the time. The person has great difficulty walking even short distances or climbing any stairs, feels tired when at rest, and is	0.408 (0.273–0.556)
Severe heart failure due to severe asbestosis	Severe COPD and other chronic respiratory, with severe heart failure	--	--
Moderate asbestosis	COPD and other chronic respiratory problems, moderate	has cough, wheezing and shortness of breath, even after light physical activity. The person feels tired and can walk only short distances or climb only a few stairs.	0.225 (0.153–0.31)
Asymptomatic asbestosis	Asymptomatic	--	--
Severe coal workers pneumoconiosis without heart failure	COPD and other chronic respiratory problems, severe	has cough, wheezing and shortness of breath all the time. The person has great difficulty walking even short distances or climbing any stairs, feels tired when at rest, and is	0.408 (0.273–0.556)
Mild coal workers pneumoconiosis	COPD and other chronic respiratory problems, mild	has cough and shortness of breath after heavy physical activity, but is able to walk long distances and climb stairs.	0.019 (0.011–0.033)
Severe heart failure due to severe coal workers pneumoconiosis	Severe COPD and other chronic respiratory, with severe heart failure	--	--
Asymptomatic and mild heart failure due to severe coal workers pneumoconiosis	Severe COPD and other chronic respiratory, with mild heart failure	--	--
Asymptomatic coal workers pneumoconiosis	Asymptomatic	--	--
Moderate heart failure due to severe coal workers pneumoconiosis	Severe COPD and other chronic respiratory, with moderate heart failure	--	--
Moderate coal workers pneumoconiosis	COPD and other chronic respiratory problems, moderate	has cough, wheezing and shortness of breath, even after light physical activity. The person feels tired and can walk only short distances or climb only a few stairs.	0.225 (0.153–0.31)
Moderate other pneumoconiosis	COPD and other chronic respiratory problems, moderate	has cough, wheezing and shortness of breath, even after light physical activity. The person feels tired and can walk only short distances or climb only a few stairs.	0.225 (0.153–0.31)
Asymptomatic other pneumoconiosis	Asymptomatic	--	--
Mild other pneumoconiosis	COPD and other chronic respiratory problems, mild	has cough and shortness of breath after heavy physical activity, but is able to walk long distances and climb stairs.	0.019 (0.011–0.033)
Severe heart failure due to severe other pneumoconiosis	Severe COPD and other chronic respiratory, with severe heart failure	--	--
Severe other pneumoconiosis without heart failure	COPD and other chronic respiratory problems, severe	has cough, wheezing and shortness of breath all the time. The person has great difficulty walking even short distances or climbing any stairs, feels tired when at rest, and is	0.408 (0.273–0.556)
Moderate heart failure due to severe other pneumoconiosis	Severe COPD and other chronic respiratory, with moderate heart failure	--	--
Asymptomatic and mild heart failure due to severe other pneumoconiosis	Severe COPD and other chronic respiratory, with mild heart failure	--	--
Controlled asthma	Asthma, controlled	has wheezing and cough once a month, which does not cause difficulty with daily activities.	0.015 (0.007–0.026)
Uncontrolled asthma	Asthma, uncontrolled	has wheezing, cough and shortness of breath more than twice a week, which causes difficulty with daily activities and sometimes wakes the person at night.	0.133 (0.086–0.192)
Asymptomatic asthma	Asymptomatic	--	--

Appendix Table 5. GBD 2016 sequelae, health states, health state lay descriptions, and disability weights			
**Sequela**	**Health state name**	**Health state lay description**	**Disability weight**
Partially controlled asthma	Asthma, partially controlled	has wheezing and cough once a week, which causes some difficulty with daily activities.	0.036 (0.022–0.055)
Severe heart failure due to severe interstitial lung disease and pulmonary sarcoidosis	Severe COPD and other chronic respiratory, with severe heart failure	--	--
Severe interstitial lung disease and pulmonary sarcoidosis without heart failure	COPD and other chronic respiratory problems, severe	has cough, wheezing and shortness of breath all the time. The person has great difficulty walking even short distances or climbing any stairs, feels tired when at rest, and is	0.408 (0.273–0.556)
Moderate heart failure due to severe interstitial lung disease and pulmonary sarcoidosis	Severe COPD and other chronic respiratory, with moderate heart failure	--	--
Mild interstitial lung disease and pulmonary sarcoidosis	COPD and other chronic respiratory problems, mild	has cough and shortness of breath after heavy physical activity, but is able to walk long distances and climb stairs.	0.019 (0.011–0.033)
Moderate interstitial lung disease and pulmonary sarcoidosis	COPD and other chronic respiratory problems, moderate	has cough, wheezing and shortness of breath, even after light physical activity. The person feels tired and can walk only short distances or climb only a few stairs.	0.225 (0.153–0.31)
Asymptomatic interstitial lung disease and pulmonary sarcoidosis	Asymptomatic	--	--
Asymptomatic and mild heart failure due to severe interstitial lung disease and pulmonary sarcoidosis	Severe COPD and other chronic respiratory, with mild heart failure	--	--
Other chronic respiratory diseases	Post-COMO calculation for residuals (YLL/YLD ratio, other methods)	--	--
Cirrhosis and other chronic liver diseases due to hepatitis B, compensated	Asymptomatic	--	--
Cirrhosis and other chronic liver diseases due to hepatitis B, decompensated	Decompensated cirrhosis of the liver	has a swollen belly and swollen legs. The person feels weakness, fatigue and loss of appetite.	0.178 (0.123–0.25)
Cirrhosis and other chronic liver diseases due to hepatitis C, decompensated	Decompensated cirrhosis of the liver	has a swollen belly and swollen legs. The person feels weakness, fatigue and loss of appetite.	0.178 (0.123–0.25)
Cirrhosis and other chronic liver diseases due to hepatitis C, compensated	Asymptomatic	--	--
Cirrhosis and other chronic liver diseases due to alcohol, compensated	Asymptomatic	--	--
Cirrhosis and other chronic liver diseases due to alcohol, decompensated	Decompensated cirrhosis of the liver	has a swollen belly and swollen legs. The person feels weakness, fatigue and loss of appetite.	0.178 (0.123–0.25)
Cirrhosis and other chronic liver diseases due to other cause, compensated	Asymptomatic	--	--
Cirrhosis and other chronic liver diseases due to other cause, decompensated	Decompensated cirrhosis of the liver	has a swollen belly and swollen legs. The person feels weakness, fatigue and loss of appetite.	0.178 (0.123–0.25)
Severe anemia due to peptic ulcer disease	Anemia, severe	feels very weak, tired and short of breath, and has problems with activities that require physical effort or deep concentration.	0.149 (0.101–0.209)
Moderate symptomatic peptic ulcer disease with mild anemia	Moderate abdominal pain with mild anemia	(combined DW)	0.118 (0.081–0.163)
Moderate symptomatic peptic ulcer disease with moderate anemia	Moderate abdominal pain with moderate anemia	(combined DW)	0.16 (0.109–0.22)
Moderate symptomatic peptic ulcer disease without anemia	Abdominopelvic problem, moderate	has pain in the belly and feels nauseous. The person has difficulties with daily activities.	0.114 (0.078–0.159)
Mild anemia due to peptic ulcer disease	Anemia, mild	feels slightly tired and weak at times, but this does not interfere with normal daily activities.	0.004 (0.001–0.008)
Asymptomatic peptic ulcer disease without anemia	Asymptomatic	--	--
Mild symptomatic peptic ulcer disease with severe anemia	Mild abdominal pain with severe anemia	(combined DW)	--
Moderate anemia due to peptic ulcer disease	Anemia, moderate	feels moderate fatigue, weakness, and shortness of breath after exercise, making daily activities more difficult.	0.052 (0.034–0.076)
Moderate symptomatic peptic ulcer disease with severe anemia	Mild abdominal pain with severe anemia	(combined DW)	--
Asymptomatic peptic ulcer disease with moderate anemia	Anemia, moderate	feels moderate fatigue, weakness, and shortness of breath after exercise, making daily activities more difficult.	0.052 (0.034–0.076)
Asymptomatic peptic ulcer disease with severe anemia	Anemia, severe	feels very weak, tired and short of breath, and has problems with activities that require physical effort or deep concentration.	0.149 (0.101–0.209)
Mild symptomatic peptic ulcer disease without anemia	Abdominopelvic problem, mild	has some pain in the belly that causes nausea but does not interfere with daily activities.	0.011 (0.005–0.021)
Mild symptomatic peptic ulcer disease with moderate anemia	Mild abdominal pain with moderate anemia	(combined DW)	--
Asymptomatic peptic ulcer disease with mild anemia	Anemia, mild	feels slightly tired and weak at times, but this does not interfere with normal daily activities.	0.004 (0.001–0.008)
Mild symptomatic peptic ulcer disease with mild anemia	Mild abdominal pain with mild anemia	(combined DW)	--
Mild anemia due to gastritis and duodenitis	Anemia, mild	feels slightly tired and weak at times, but this does not interfere with normal daily activities.	0.004 (0.001–0.008)
Mild symptomatic gastritis and duodenitis with moderate anemia	Mild abdominal pain with moderate anemia	(combined DW)	--
Asymptomatic gastritis and duodenitis with moderate anemia	Anemia, moderate	feels moderate fatigue, weakness, and shortness of breath after exercise, making daily activities more difficult.	0.052 (0.034–0.076)
Moderate symptomatic gastritis and duodenitis with severe anemia	Moderate abdominal pain with severe anemia	(combined DW)	0.246 (0.171–0.334)
Moderate anemia due to gastritis and duodenitis	Anemia, moderate	feels moderate fatigue, weakness, and shortness of breath after exercise, making daily activities more difficult.	0.052 (0.034–0.076)
Asymptomatic gastritis and duodenitis without anemia	Asymptomatic	--	--
Moderate symptomatic gastritis and duodenitis with moderate anemia	Moderate abdominal pain with moderate anemia	(combined DW)	0.16 (0.109–0.22)
Asymptomatic gastritis and duodenitis with mild anemia	Anemia, mild	feels slightly tired and weak at times, but this does not interfere with normal daily activities.	0.004 (0.001–0.008)

Appendix Table 5. GBD 2016 sequelae, health states, health state lay descriptions, and disability weights			
**Sequela**	**Health state name**	**Health state lay description**	**Disability weight**
Asymptomatic gastritis and duodenitis with severe anemia	Anemia, severe	feels very weak, tired and short of breath, and has problems with activities that require physical effort or deep concentration.	0.149 (0.101–0.209)
Mild symptomatic gastritis and duodenitis without anemia	Abdominopelvic problem, mild	has some pain in the belly that causes nausea but does not interfere with daily activities.	0.011 (0.005–0.021)
Mild symptomatic gastritis and duodenitis with severe anemia	Mild abdominal pain with severe anemia	(combined DW)	--
Severe anemia due to gastritis and duodenitis	Anemia, severe	feels very weak, tired and short of breath, and has problems with activities that require physical effort or deep concentration.	0.149 (0.101–0.209)
Mild symptomatic gastritis and duodenitis with mild anemia	Mild abdominal pain with mild anemia	(combined DW)	--
Moderate symptomatic gastritis and duodenitis with mild anemia	Moderate abdominal pain with mild anemia	(combined DW)	0.118 (0.081–0.163)
Moderate symptomatic gastritis and duodenitis without anemia	Abdominopelvic problem, moderate	has pain in the belly and feels nauseous. The person has difficulties with daily activities.	0.114 (0.078–0.159)
Appendicitis	Abdominopelvic problem, severe	has severe pain in the belly and feels nauseous. The person is anxious and unable to carry out daily activities.	0.324 (0.22–0.442)
Paralytic ileus and intestinal obstruction	Abdominopelvic problem, severe	has severe pain in the belly and feels nauseous. The person is anxious and unable to carry out daily activities.	0.324 (0.22–0.442)
Asymptomatic inguinal, femoral, and abdominal hernia	Asymptomatic	--	--
Mild symptomatic inguinal, femoral and abdominal hernia	Abdominopelvic problem, mild	has some pain in the belly that causes nausea but does not interfere with daily activities.	0.011 (0.005–0.021)
Moderate symptomatic inguinal, femoral and abdominal hernia	Abdominopelvic problem, moderate	has pain in the belly and feels nauseous. The person has difficulties with daily activities.	0.114 (0.078–0.159)
Severe symptomatic inguinal, femoral and abdominal hernia	Abdominopelvic problem, severe	has severe pain in the belly and feels nauseous. The person is anxious and unable to carry out daily activities.	0.324 (0.22–0.442)
Asymptomatic Crohn's disease	Asymptomatic	--	--
Asymptomatic ulcerative colitis	Asymptomatic	--	--
Ulcerative colitis	Crohn disease or ulcerative colitis	has cramping abdominal pain, has diarrhea several times a day, and feels very tired for two months every year. When the person does not have symptoms, there is anxiety about them returning.	0.231 (0.156–0.32)
Crohn's disease	Crohn disease or ulcerative colitis	has cramping abdominal pain, has diarrhea several times a day, and feels very tired for two months every year. When the person does not have symptoms, there is anxiety about them returning.	0.231 (0.156–0.32)
Vascular intestinal disorders	Abdominopelvic problem, severe	has severe pain in the belly and feels nauseous. The person is anxious and unable to carry out daily activities.	0.324 (0.22–0.442)
Asymptomatic gallbladder and biliary diseases	Asymptomatic	--	--
Severe symptomatic episodes gallbladder and biliary diseases	Abdominopelvic problem, severe	has severe pain in the belly and feels nauseous. The person is anxious and unable to carry out daily activities.	0.324 (0.22–0.442)
Mild symptomatic episodes gallbladder and biliary diseases	Abdominopelvic problem, mild	has some pain in the belly that causes nausea but does not interfere with daily activities.	0.011 (0.005–0.021)
Moderate symptomatic episodes gallbladder and biliary diseases	Abdominopelvic problem, moderate	has pain in the belly and feels nauseous. The person has difficulties with daily activities.	0.114 (0.078–0.159)
Moderate pancreatitis episodes	Abdominopelvic problem, moderate	has pain in the belly and feels nauseous. The person has difficulties with daily activities.	0.114 (0.078–0.159)
Mild pancreatitis episodes	Abdominopelvic problem, mild	has some pain in the belly that causes nausea but does not interfere with daily activities.	0.011 (0.005–0.021)
Severe pancreatitis episodes	Abdominopelvic problem, severe	has severe pain in the belly and feels nauseous. The person is anxious and unable to carry out daily activities.	0.324 (0.22–0.442)
Other digestive diseases	Post-COMO calculation for residuals (YLL/YLD ratio, other methods)	--	--
Severe Alzheimer disease and other dementias	Dementia, severe	has complete memory loss; no longer recognizes close family members; and requires help with all daily activities.	0.449 (0.304–0.595)
Mild Alzheimer disease and other dementias	Dementia, mild	has some trouble remembering recent events, and finds it hard to concentrate and make decisions and plans.	0.069 (0.046–0.099)
Moderate Alzheimer disease and other dementias	Dementia, moderate	has memory problems and confusion, feels disoriented, at times hears voices that are not real, and needs help with some daily activities.	0.377 (0.252–0.508)
Mild Parkinson disease	Parkinson disease, mild	has mild tremors and moves a little slowly, but is able to walk and do daily activities without assistance.	0.01 (0.005–0.019)
Moderate Parkinson disease	Parkinson disease, moderate	has moderate tremors and moves slowly, which causes some difficulty in walking and daily activities. The person has some trouble swallowing, talking, sleeping, and	0.267 (0.181–0.372)
Severe Parkinson disease	Parkinson disease, severe	has severe tremors and moves very slowly, which causes great difficulty in walking and daily activities. The person falls easily and has a lot of difficulty talking, swallowing, sleeping, and remembering things.	0.575 (0.396–0.73)
Severe epilepsy	Epilepsy, seizures >= once a month	has sudden seizures one or more times each month, with violent muscle contractions and stiffness, loss of consciousness, and loss of urine or bowel control. Between seizures the person has memory loss and difficulty concentrating.	0.552 (0.375–0.71)
Less severe epilepsy	Epilepsy, seizures 1-11 per year	has sudden seizures two to five times a year, with violent muscle contractions and stiffness, loss of consciousness, and loss of urine or bowel control.	0.263 (0.173–0.367)

Appendix Table 5. GBD 2016 sequelae, health states, health state lay descriptions, and disability weights			
**Sequela**	**Health state name**	**Health state lay description**	**Disability weight**
Seizure-free, treated epilepsy	Generic uncomplicated disease: worry and daily medication	has a chronic disease that requires medication every day and causes some worry but minimal interference with daily activities.	0.049 (0.031–0.072)
Mild multiple sclerosis	Multiple sclerosis, mild	has mild loss of feeling in one hand, is a little unsteady while walking, has slight loss of vision in one eye, and often needs to urinate urgently.	0.183 (0.124–0.253)
Severe multiple sclerosis	Multiple sclerosis, severe	has slurred speech and difficulty swallowing. The person has weak arms and hands, very limited and stiff leg movement, has loss of vision in both eyes and cannot control urinating.	0.719 (0.534–0.858)
Moderate multiple sclerosis	Multiple sclerosis, moderate	needs help walking, has difficulty with writing and arm coordination, has loss of vision in one eye and cannot control urinating.	0.463 (0.313–0.613)
Asymptomatic multiple sclerosis	Asymptomatic	--	--
Severe respiratory problems and speech problems due to motor neuron disease	Severe respiratory and speech problems	(combined DW)	0.438 (0.304–0.581)
Moderate respiratory problems and speech problems due to motor neuron disease	Moderate respiratory and speech problems	(combined DW)	0.265 (0.184–0.36)
Mild respiratory problems and speech problems due to motor neuron disease	Mild respiratory and speech problems	(combined DW)	0.069 (0.043–0.106)
Severe motor impairment and speech problems due to motor neuron disease	Severe motor impairment and speech problems	(combined DW)	0.432 (0.306–0.572)
Moderate motor impairment and speech problems due to motor neuron disease	Moderate motor impairment and speech problems	(combined DW)	0.109 (0.071–0.158)
Mild motor impairment and speech problems due to motor neuron disease	Mild motor impairment and speech problems	(combined DW)	0.061 (0.038–0.094)
Severe motor impairment and severe respiratory problems due to motor neuron disease	Severe motor impairment and severe respiratory problems	(combined DW)	0.641 (0.47–0.796)
Severe motor impairment and mild respiratory problems due to motor neuron disease	Severe motor impairment and mild respiratory problems	(combined DW)	0.413 (0.286–0.553)
Moderate motor impairment, severe respiratory problems, and speech problems due to motor neuron disease	Moderate motor impairment with severe respiratory problems and speech problems	(combined DW)	0.472 (0.339–0.611)
Moderate motor impairment and severe respiratory problems due to motor neuron disease	Moderate motor impairment and severe respiratory problems	(combined DW)	0.414 (0.281–0.559)
Moderate motor impairment and moderate respiratory problems due to motor neuron disease	Moderate motor impairment and moderate respiratory problems	(combined DW)	0.233 (0.16–0.322)
Moderate motor impairment and mild respiratory problems due to motor neuron disease	Moderate motor impairment and mild respiratory problems	(combined DW)	0.029 (0.015–0.051)
Mild motor impairment and severe respiratory problems due to motor neuron disease	Mild motor impairment and severe respiratory problems	(combined DW)	0.443 (0.311–0.587)
Mild motor impairment and mild respiratory problems due to motor neuron disease	Mild motor impairment and mild respiratory problems	(combined DW)	0.079 (0.05–0.117)
Severe motor impairment, severe respiratory problems, and speech problems due to motor neuron disease	Severe motor impairment with severe respiratory problems and speech problems	(combined DW)	0.659 (0.495–0.809)
Severe motor impairment, moderate respiratory problems, and speech problems due to motor neuron disease	Severe motor impairment with moderate respiratory problems and speech problems	(combined DW)	0.557 (0.412–0.705)
Severe motor impairment and moderate respiratory problems due to motor neuron disease	Severe motor impairment and moderate respiratory problems	(combined DW)	0.534 (0.382–0.685)
Moderate motor impairment due to motor neuron disease	Motor impairment, moderate	has some difficulty in moving around, and difficulty in lifting and holding objects, dressing and sitting upright, but is able to walk without help.	0.061 (0.04–0.089)
Moderate motor impairment, mild respiratory problems, and speech problems due to motor neuron disease	Moderate motor impairment with mild respiratory problems and speech problems	(combined DW)	0.126 (0.081–0.183)
Mild respiratory problems due to motor neuron disease	COPD and other chronic respiratory problems, mild	has cough and shortness of breath after heavy physical activity, but is able to walk long distances and climb stairs.	0.019 (0.011–0.033)
Severe respiratory problems due to motor neuron disease	COPD and other chronic respiratory problems, severe	has cough, wheezing and shortness of breath all the time. The person has great difficulty walking even short distances or climbing any stairs, feels tired when at rest, and is	0.408 (0.273–0.556)
Moderate respiratory problems due to motor neuron disease	COPD and other chronic respiratory problems, moderate	has cough, wheezing and shortness of breath, even after light physical activity. The person feels tired and can walk only short distances or climb only a few stairs.	0.225 (0.153–0.31)
Mild motor impairment due to motor neuron disease	Motor impairment, mild	has some difficulty in moving around but is able to walk without help.	0.01 (0.005–0.019)
Mild motor impairment, mild respiratory problems, and speech problems due to motor neuron disease	Mild motor impairment with mild respiratory problems and speech problems	(combined dw)	0.079 (0.049–0.123)
Mild motor impairment, moderate respiratory problems, and speech problems due to motor neuron disease	Mild motor impairment with moderate respiratory problems and speech problems	(combined dw)	0.272 (0.191–0.369)
Speech problems due to motor neuron disease	Speech problems	has difficulty speaking, and others find it difficult to understand.	0.051 (0.032–0.078)
Severe motor impairment due to motor neuron disease	Motor impairment, severe	is unable to move around without help, and is not able to lift or hold objects, get dressed or sit upright.	0.402 (0.268–0.545)
Diagnosis of motor neuron disease	Generic uncomplicated disease: anxiety about diagnosis	has a disease diagnosis that causes some worry but minimal interference with daily activities.	0.012 (0.006–0.023)
Severe motor impairment, mild respiratory problems, and speech problems due to motor neuron disease	Severe motor impairment with mild respiratory problems and speech problems	(combined DW)	0.443 (0.316–0.58)
Moderate motor impairment, moderate respiratory problems, and speech problems due to motor neuron disease	Moderate motor impairment with moderate respiratory problems and speech problems	(combined DW)	0.309 (0.221–0.414)
Mild motor impairment, severe respiratory problems and speech problems due to motor neuron disease	Mild motor impairment with severe respiratory problems and speech problems	(combined DW)	0.444 (0.311–0.585)
Asymptomatic medication overuse headache due to migraine	Asymptomatic	--	--

Appendix Table 5. GBD 2016 sequelae, health states, health state lay descriptions, and disability weights			
**Sequela**	**Health state name**	**Health state lay description**	**Disability weight**
Symptomatic migraine	Headache, migraine	has severe, throbbing head pain and nausea that cause great difficulty in daily activities and sometimes confine the person to bed. Moving around, light, and noise make it worse.	0.441 (0.294–0.588)
Symptomatic medication overuse headache due to migraine	Headache, medication overuse	has daily headaches, felt as dull pain and often lasting all day, with poor sleep, nausea and fatigue. The person takes medicine for the headaches, which provides little relief but is needed to avoid having worse symptoms.	0.223 (0.146–0.313)
Asymptomatic migraine	Asymptomatic	--	--
Symptomatic medication overuse headache due to tension-type headache	Headache, medication overuse	has daily headaches, felt as dull pain and often lasting all day, with poor sleep, nausea and fatigue. The person takes medicine for the headaches, which provides little relief but is needed to avoid having worse symptoms.	0.223 (0.146–0.313)
Asymptomatic medication overuse headache due to tension-type headache	Asymptomatic	--	--
Asymptomatic tension-type headache	Asymptomatic	--	--
Symptomatic tension-type headache	Headache, tension-type	has a moderate headache that also affects the neck, which causes difficulty in daily activities.	0.037 (0.022–0.057)
Other neurological disorders	Post-COMO calculation for residuals (YLL/YLD ratio, other methods)	--	--
Guillain-Barré syndrome due to other neurological disorders	Spinal cord lesion below neck level (treated)	is paralyzed from the waist down, cannot feel or move the legs and has difficulties with urine and bowel control. The person uses a wheelchair to move around.	0.296 (0.198–0.414)
Schizophrenia acute state	Schizophrenia, acute state	hears and sees things that are not real and is afraid, confused, and sometimes violent. The person has great difficulty with communication and daily activities, and sometimes wants to harm or kill himself (or herself).	0.778 (0.606–0.9)
Schizophrenia residual state	Schizophrenia, residual state	hears and sees things that are not real and has trouble communicating. The person can be forgetful, has difficulty with daily activities, and thinks about hurting himself (or	0.588 (0.411–0.754)
Very mild alcohol dependence	Alcohol use disorder, very mild	drinks alcohol daily and has difficulty controlling the urge to drink. When sober, the person functions normally.	0.123 (0.082–0.177)
Severe fetal alcohol syndrome	Fetal alcohol syndrome, severe	is very slow in developing physically and mentally, which causes great difficulty in daily activities.	0.179 (0.119–0.257)
Mild alcohol dependence	Alcohol use disorder, mild	drinks a lot of alcohol and sometimes has difficulty controlling the urge to drink. While intoxicated, the person has difficulty performing daily activities.	0.235 (0.16–0.327)
Moderate fetal alcohol syndrome	Fetal alcohol syndrome, moderate	is slow in developing physically and mentally, which causes some difficulty in daily activities.	0.056 (0.035–0.083)
Asymptomatic fetal alcohol syndrome	Asymptomatic	--	--
Asymptomatic alcohol dependence	Asymptomatic	--	--
Mild fetal alcohol syndrome	Fetal alcohol syndrome, mild	is a little slow in developing physically and mentally, which causes some difficulty in learning but no other difficulties in daily activities.	0.016 (0.008–0.03)
Severe alcohol dependence	Alcohol use disorder, severe	gets drunk almost every day and is unable to control the urge to drink. Drinking and recovering replace most daily activities. The person has difficulty thinking, remembering and communicating, and feels constant pain and fatigue.	0.57 (0.396–0.732)
Moderate alcohol dependence	Alcohol use disorder, moderate	drinks a lot, gets drunk almost every week and has great difficulty controlling the urge to drink. Drinking and recovering cause great difficulty in daily activities, sleep loss,	0.373 (0.248–0.508)
Asymptomatic opioid dependence	Asymptomatic	--	--
Severe opioid dependence	Heroin and other opioid dependence	uses heroin daily and has difficulty controlling the habit. When the effects wear off, the person feels severe nausea, agitation, vomiting and fever. The person has a lot of difficulty in daily activities.	0.697 (0.51–0.843)
Mild opioid dependence	Heroin and other opioid dependence, mild	uses heroin (or methadone)Â daily and has difficulty controlling the habit. When not using, the person functions normally.	0.335 (0.221–0.473)
Mild cocaine dependence	Cocaine dependence, mild	uses cocaine at least once a week and has some difficulty controlling the habit. When not using, the person functions normally.	0.116 (0.074–0.165)
Asymptomatic cocaine dependence	Asymptomatic	--	--
Severe cocaine dependence	Cocaine dependence	uses cocaine and has difficulty controlling the habit. The person sometimes has mood swings, anxiety, paranoia, hallucinations and sleep problems, and has some difficulty in daily activities.	0.479 (0.324–0.634)
Severe amphetamine dependence	Amphetamine dependence	uses stimulants (drugs) and has difficulty controlling the habit. The person sometimes has depression, hallucinations and mood swings, and has difficulty in daily activities.	0.486 (0.329–0.637)
Mild amphetamine dependence	Amphetamine dependence, mild	uses stimulants (drugs) at least once a week and has some difficulty controlling the habit. When not using, the person functions normally.	0.079 (0.051–0.114)
Asymptomatic amphetamine dependence	Asymptomatic	--	--
Asymptomatic cannabis dependence	Asymptomatic	--	--
Mild cannabis dependence	Cannabis dependence, mild	uses marijuana at least once a week and has some difficulty controlling the habit. When not using, the person functions normally.	0.039 (0.024–0.06)

Appendix Table 5. GBD 2016 sequelae, health states, health state lay descriptions, and disability weights			
**Sequela**	**Health state name**	**Health state lay description**	**Disability weight**
Severe cannabis dependence	Cannabis dependence	uses marijuana daily and has difficulty controlling the habit. The person sometimes has mood swings, anxiety and hallucinations, and has some difficulty in daily activities.	0.266 (0.178–0.364)
Other drug use disorders	Cocaine dependence, mild	uses cocaine at least once a week and has some difficulty controlling the habit. When not using, the person functions normally.	0.116 (0.074–0.165)
Severe major depressive disorder	Major depressive disorder, severe episode	has overwhelming, constant sadness and cannot function in daily life. The person sometimes loses touch with reality and wants to harm or kill himself (or herself).	0.658 (0.477–0.807)
Moderate major depressive disorder	Major depressive disorder, moderate episode	has constant sadness and has lost interest in usual activities. The person has some difficulty in daily life, sleeps badly, has trouble concentrating, and sometimes thinks about harming himself (or herself).	0.396 (0.267–0.531)
Major depressive disorder, currently without symptoms	Asymptomatic	--	--
Mild major depressive disorder	Major depressive disorder, mild episode	feels persistent sadness and has lost interest in usual activities. The person sometimes sleeps badly, feels tired, or has trouble concentrating but still manages to function in daily life with extra effort.	0.145 (0.099–0.209)
Dysthymia, currently without symptoms	Asymptomatic	--	--
Symptomatic dysthymia	Major depressive disorder, mild episode	feels persistent sadness and has lost interest in usual activities. The person sometimes sleeps badly, feels tired, or has trouble concentrating but still manages to function in daily life with extra effort.	0.145 (0.099–0.209)
Bipolar disorder manic state	Bipolar disorder, manic episode	is hyperactive, hears and believes things that are not real, and engages in impulsive and aggressive behavior that endanger the person and others.	0.492 (0.341–0.646)
Bipolar disorder residual state	Bipolar disorder, residual state	has mild mood swings, irritability and some difficulty with daily activities.	0.032 (0.018–0.051)
Bipolar disorder depressive state	Major depressive disorder, moderate episode	has constant sadness and has lost interest in usual activities. The person has some difficulty in daily life, sleeps badly, has trouble concentrating, and sometimes thinks about harming himself (or herself).	0.396 (0.267–0.531)
Anxiety disorders, currently without symptoms	Asymptomatic	--	--
Moderate anxiety disorders	Anxiety disorders, moderate	feels anxious and worried, which makes it difficult to concentrate, remember things, and sleep. The person tires easily and finds it difficult to perform daily activities.	0.133 (0.091–0.186)
Severe anxiety disorders	Anxiety disorders, severe	constantly feels very anxious and worried, which makes it difficult to concentrate, remember things and sleep. The person has lost pleasure in life and thinks about suicide.	0.523 (0.362–0.677)
Mild anxiety disorders	Anxiety disorders, mild	feels mildly anxious and worried, which makes it slightly difficult to concentrate, remember things, and sleep. The person tires easily but is able to perform daily activities.	0.03 (0.018–0.046)
Anorexia nervosa	Anorexia nervosa	feels an overwhelming need to starve and exercises excessively to lose weight. The person is very thin, weak and anxious.	0.224 (0.15–0.312)
Bulimia nervosa	Bulimia nervosa	has uncontrolled overeating followed by guilt, starving, and vomiting to lose weight.	0.223 (0.149–0.311)
Autism with profound intellectual disability	Autism with profound intellectual disability	(combined DW)	--
Autism with moderate intellectual disability	Autism with moderate intellectual disability	(combined DW)	--
Autism with mild intellectual disability	Autism with mild intellectual disability	(combined DW)	--
Autism with borderline intellectual disability	Autism with borderline intellectual disability	(combined DW)	--
Autism without intellectual disability	Autism without intellectual disability	(combined DW)	--
Autism with severe intellectual disability	Autism with severe intellectual disability	(combined DW)	--
Asperger syndrome and other autistic spectrum disorders	Asperger syndrome	has difficulty interacting with other people, and is slow to understand or respond to questions. The person is often preoccupied with one thing and has some difficulty with basic daily activities.	0.104 (0.071–0.147)
Symptomatic attention-deficit/hyperactivity disorder	Attention deficit hyperactivity disorder	is hyperactive and has difficulty concentrating, remembering things, and completing tasks.	0.045 (0.028–0.066)
Attention-deficit/hyperactivity disorder, currently without symptoms	Asymptomatic	--	--
Symptomatic conduct disorder	Conduct disorder	has frequent behavior problems, which are sometimes violent. The person often has difficulty interacting with other people and feels irritable.	0.241 (0.159–0.341)
Conduct disorder, currently without symptoms	Asymptomatic	--	--
Mild idiopathic developmental intellectual disability	Intellectual disability / mental retardation, mild	has low intelligence and is slow in learning at school. As an adult, the person can live independently, but often needs help to raise children and can only work at simple supervised jobs.	0.043 (0.026–0.064)
Borderline idiopathic developmental intellectual disability	Borderline intellectual functioning	is slow in learning at school. As an adult, the person has some difficulty doing complex or unfamiliar tasks but otherwise functions independently.	0.011 (0.005–0.02)
Profound idiopathic developmental intellectual disability	Intellectual disability / mental retardation, profound	has very low intelligence, has almost no language, and does not understand even the most basic requests or instructions. The person requires constant supervision and help for all activities.	0.2 (0.133–0.283)

Appendix Table 5. GBD 2016 sequelae, health states, health state lay descriptions, and disability weights			
**Sequela**	**Health state name**	**Health state lay description**	**Disability weight**
Moderate idiopathic developmental intellectual disability	Intellectual disability / mental retardation, moderate	has low intelligence, and is slow in learning to speak and to do even simple tasks. As an adult, the person requires a lot of support to live independently and raise children. The person can only work at the simplest supervised jobs.	0.1 (0.066–0.142)
Severe idiopathic developmental intellectual disability	Intellectual disability / mental retardation, severe	has very low intelligence and cannot speak more than a few words, needs constant supervision and help with most daily activities, and can do only the simplest tasks.	0.16 (0.107–0.226)
Other mental disorders, currently without symptoms	Asymptomatic	--	--
Moderate other mental disorders	Anxiety disorders, moderate	feels anxious and worried, which makes it difficult to concentrate, remember things, and sleep. The person tires easily and finds it difficult to perform daily activities.	0.133 (0.091–0.186)
Severe other mental disorders	Anxiety disorders, severe	constantly feels very anxious and worried, which makes it difficult to concentrate, remember things and sleep. The person has lost pleasure in life and thinks about suicide.	0.523 (0.362–0.677)
Mild other mental disorders	Anxiety disorders, mild	feels mildly anxious and worried, which makes it slightly difficult to concentrate, remember things, and sleep. The person tires easily but is able to perform daily activities.	0.03 (0.018–0.046)
Moderate vision impairment due to diabetes mellitus	Distance vision, moderate impairment	has vision problems that make it difficult to recognize faces or objects across a room.	0.031 (0.019–0.049)
Diabetic foot due to neuropathy	Diabetic neuropathy with diabetic foot	--	--
Severe vision impairment due to diabetes mellitus	Distance vision, severe impairment	has severe vision loss, which causes difficulty in daily activities, some emotional impact (for example worry), and some difficulty going outside the home without assistance.	0.184 (0.125–0.258)
Diabetic neuropathy	Diabetic neuropathy	has pain, tingling and numbness in the arms, legs, hands and feet. The person sometimes gets cramps and muscle weakness.	0.133 (0.089–0.187)
Uncomplicated diabetes mellitus	Generic uncomplicated disease: worry and daily medication	has a chronic disease that requires medication every day and causes some worry but minimal interference with daily activities.	0.049 (0.031–0.072)
Diabetic neuropathy and amputation with treatment	Diabetic neuropathy with treated amputation	--	--
Diabetic neuropathy and amputation without treatment	Diabetic neuropathy with untreated amputation	--	--
Blindness due to diabetes mellitus	Distance vision blindness	is completely blind, which causes great difficulty in some daily activities, worry and anxiety, and great difficulty going outside the home without assistance.	0.187 (0.124–0.26)
Acute glomerulonephritis	Infectious disease, acute episode, moderate	has a fever and aches, and feels weak, which causes some difficulty with daily activities.	0.051 (0.032–0.074)
Stage V chronic kidney disease untreated and severe anemia due to diabetes mellitus	Severe anemia and terminal phase, without medication (for cancers, end-stage kidney/liver disease)	(combined DW)	0.631 (0.456–0.782)
End-stage renal disease after transplant due to diabetes mellitus	End-stage renal disease, with kidney transplant	sometimes feels tired and down, and has some difficulty with daily activities.	0.024 (0.014–0.039)
Stage IV chronic kidney disease untreated without anemia due to diabetes mellitus	Chronic kidney disease (stage IV)	tires easily, has nausea, reduced appetite and difficulty sleeping.	0.104 (0.07–0.147)
Stage III chronic kidney disease and severe anemia due to diabetes mellitus	Anemia, severe	feels very weak, tired and short of breath, and has problems with activities that require physical effort or deep concentration.	0.149 (0.101–0.209)
Stage III chronic kidney disease and mild anemia due to diabetes mellitus	Anemia, mild	feels slightly tired and weak at times, but this does not interfere with normal daily activities.	0.004 (0.001–0.008)
Stage IV chronic kidney disease untreated and mild anemia due to diabetes mellitus	Mild anemia with Stage IV CKD	--	--
Stage III chronic kidney disease without anemia due to diabetes mellitus	Asymptomatic	--	--
Stage IV chronic kidney disease untreated and moderate anemia due to diabetes mellitus	Moderate anemia with Stage IV CKD	--	--
Stage V chronic kidney disease untreated without anemia due to diabetes mellitus	Terminal phase, without medication (for cancers, end-stage kidney/liver disease)	has lost a lot of weight and has constant pain. The person has no appetite, feels nauseous, and needs to spend most of the day in bed.	0.569 (0.389–0.727)
Stage IV chronic kidney disease untreated and severe anemia due to diabetes mellitus	Severe anemia with Stage IV CKD	--	--
Stage V chronic kidney disease untreated and moderate anemia due to diabetes mellitus	Moderate anemia and terminal phase, without medication (for cancers, end-stage kidney/liver disease)	(combined DW)	0.591 (0.414–0.743)
End-stage renal disease on dialysis due to diabetes mellitus	End-stage renal disease, on dialysis	is tired and has itching, cramps, headache, joint pains and shortness of breath. The person needs intensive medical care every other day lasting about half a day.	0.571 (0.398–0.725)
Stage V chronic kidney disease untreated and mild anemia due to diabetes mellitus	Mild anemia and terminal phase, without medication (for cancers, end-stage kidney/liver disease)	(combined DW)	0.57 (0.391–0.727)
Stage III chronic kidney disease and moderate anemia due to diabetes mellitus	Anemia, moderate	feels moderate fatigue, weakness, and shortness of breath after exercise, making daily activities more difficult.	0.052 (0.034–0.076)
Stage IV chronic kidney disease untreated and moderate anemia due to hypertension	Moderate anemia with Stage IV CKD	--	--
Stage III chronic kidney disease without anemia due to hypertension	Asymptomatic	--	--
Stage V chronic kidney disease untreated and moderate anemia due to hypertension	Moderate anemia and terminal phase, without medication (for cancers, end-stage kidney/liver disease)	(combined DW)	0.591 (0.414–0.743)

Appendix Table 5. GBD 2016 sequelae, health states, health state lay descriptions, and disability weights			
**Sequela**	**Health state name**	**Health state lay description**	**Disability weight**
Stage V chronic kidney disease untreated and severe anemia due to hypertension	Severe anemia and terminal phase, without medication (for cancers, end-stage kidney/liver disease)	(combined DW)	0.631 (0.456–0.782)
End-stage renal disease on dialysis due to hypertension	End-stage renal disease, on dialysis	is tired and has itching, cramps, headache, joint pains and shortness of breath. The person needs intensive medical care every other day lasting about half a day.	0.571 (0.398–0.725)
Stage V chronic kidney disease untreated and mild anemia due to hypertension	Mild anemia and terminal phase, without medication (for cancers, end-stage kidney/liver disease)	(combined DW)	0.57 (0.391–0.727)
Stage III chronic kidney disease and mild anemia due to hypertension	Anemia, mild	feels slightly tired and weak at times, but this does not interfere with normal daily activities.	0.004 (0.001–0.008)
Stage IV chronic kidney disease untreated and severe anemia due to hypertension	Severe anemia with Stage IV CKD	--	--
Stage IV chronic kidney disease untreated without anemia due to hypertension	Chronic kidney disease (stage IV)	tires easily, has nausea, reduced appetite and difficulty sleeping.	0.104 (0.07–0.147)
Stage V chronic kidney disease untreated without anemia due to hypertension	Terminal phase, without medication (for cancers, end-stage kidney/liver disease)	has lost a lot of weight and has constant pain. The person has no appetite, feels nauseous, and needs to spend most of the day in bed.	0.569 (0.389–0.727)
Stage III chronic kidney disease and severe anemia due to hypertension	Anemia, severe	feels very weak, tired and short of breath, and has problems with activities that require physical effort or deep concentration.	0.149 (0.101–0.209)
Stage IV chronic kidney disease untreated and mild anemia due to hypertension	Mild anemia with Stage IV CKD	--	--
End-stage renal disease after transplant due to hypertension	End-stage renal disease, with kidney transplant	sometimes feels tired and down, and has some difficulty with daily activities.	0.024 (0.014–0.039)
Stage III chronic kidney disease and moderate anemia due to hypertension	Anemia, moderate	feels moderate fatigue, weakness, and shortness of breath after exercise, making daily activities more difficult.	0.052 (0.034–0.076)
End-stage renal disease after transplant due to glomerulonephritis	End-stage renal disease, with kidney transplant	sometimes feels tired and down, and has some difficulty with daily activities.	0.024 (0.014–0.039)
Stage IV chronic kidney disease untreated without anemia due to glomerulonephritis	Chronic kidney disease (stage IV)	tires easily, has nausea, reduced appetite and difficulty sleeping.	0.104 (0.07–0.147)
Stage V chronic kidney disease untreated and moderate anemia due to glomerulonephritis	Moderate anemia and terminal phase, without medication (for cancers, end-stage kidney/liver disease)	(combined DW)	0.591 (0.414–0.743)
Stage III chronic kidney disease and moderate anemia due to glomerulonephritis	Anemia, moderate	feels moderate fatigue, weakness, and shortness of breath after exercise, making daily activities more difficult.	0.052 (0.034–0.076)
Stage III chronic kidney disease and severe anemia due to glomerulonephritis	Anemia, severe	feels very weak, tired and short of breath, and has problems with activities that require physical effort or deep concentration.	0.149 (0.101–0.209)
Stage III chronic kidney disease and mild anemia due to glomerulonephritis	Anemia, mild	feels slightly tired and weak at times, but this does not interfere with normal daily activities.	0.004 (0.001–0.008)
Stage III chronic kidney disease without anemia due to glomerulonephritis	Asymptomatic	--	--
Stage V chronic kidney disease untreated and mild anemia due to glomerulonephritis	Mild anemia and terminal phase, without medication (for cancers, end-stage kidney/liver disease)	(combined DW)	0.57 (0.391–0.727)
Stage IV chronic kidney disease untreated and severe anemia due to glomerulonephritis	Severe anemia with Stage IV CKD	--	--
Stage IV chronic kidney disease untreated and moderate anemia due to glomerulonephritis	Moderate anemia with Stage IV CKD	--	--
Stage V chronic kidney disease untreated and severe anemia due to glomerulonephritis	Severe anemia and terminal phase, without medication (for cancers, end-stage kidney/liver disease)	(combined DW)	0.631 (0.456–0.782)
Stage V chronic kidney disease untreated without anemia due to glomerulonephritis	Terminal phase, without medication (for cancers, end-stage kidney/liver disease)	has lost a lot of weight and has constant pain. The person has no appetite, feels nauseous, and needs to spend most of the day in bed.	0.569 (0.389–0.727)
Stage IV chronic kidney disease untreated and mild anemia due to glomerulonephritis	Mild anemia with Stage IV CKD	--	--
End-stage renal disease on dialysis due to glomerulonephritis	End-stage renal disease, on dialysis	is tired and has itching, cramps, headache, joint pains and shortness of breath. The person needs intensive medical care every other day lasting about half a day.	0.571 (0.398–0.725)
Stage V chronic kidney disease untreated without anemia due to other causes	Terminal phase, without medication (for cancers, end-stage kidney/liver disease)	has lost a lot of weight and has constant pain. The person has no appetite, feels nauseous, and needs to spend most of the day in bed.	0.569 (0.389–0.727)
Stage III chronic kidney disease without anemia due to other causes	Asymptomatic	--	--
Stage IV chronic kidney disease untreated without anemia due to other causes	Chronic kidney disease (stage IV)	tires easily, has nausea, reduced appetite and difficulty sleeping.	0.104 (0.07–0.147)
Stage III chronic kidney disease and mild anemia due to other causes	Anemia, mild	feels slightly tired and weak at times, but this does not interfere with normal daily activities.	0.004 (0.001–0.008)
Stage IV chronic kidney disease untreated and mild anemia due to other causes	Mild anemia with Stage IV CKD	--	--
Stage III chronic kidney disease and moderate anemia due to other causes	Anemia, moderate	feels moderate fatigue, weakness, and shortness of breath after exercise, making daily activities more difficult.	0.052 (0.034–0.076)
Stage IV chronic kidney disease untreated and moderate anemia due to other causes	Moderate anemia with Stage IV CKD	--	--
Stage V chronic kidney disease untreated and moderate anemia due to other causes	Moderate anemia and terminal phase, without medication (for cancers, end-stage kidney/liver disease)	(combined DW)	0.591 (0.414–0.743)

Appendix Table 5. GBD 2016 sequelae, health states, health state lay descriptions, and disability weights			
**Sequela**	**Health state name**	**Health state lay description**	**Disability weight**
Stage III chronic kidney disease and severe anemia due to other causes	Anemia, severe	feels very weak, tired and short of breath, and has problems with activities that require physical effort or deep concentration.	0.149 (0.101–0.209)
Stage IV chronic kidney disease untreated and severe anemia due to other causes	Severe anemia with Stage IV CKD	--	--
End-stage renal disease after transplant due to other causes	End-stage renal disease, with kidney transplant	sometimes feels tired and down, and has some difficulty with daily activities.	0.024 (0.014–0.039)
Stage V chronic kidney disease untreated and severe anemia due to other causes	Severe anemia and terminal phase, without medication (for cancers, end-stage kidney/liver disease)	(combined DW)	0.631 (0.456–0.782)
Stage V chronic kidney disease untreated and mild anemia due to other causes	Mild anemia and terminal phase, without medication (for cancers, end-stage kidney/liver disease)	(combined DW)	0.57 (0.391–0.727)
End-stage renal disease on dialysis due to other causes	End-stage renal disease, on dialysis	is tired and has itching, cramps, headache, joint pains and shortness of breath. The person needs intensive medical care every other day lasting about half a day.	0.571 (0.398–0.725)
Mild interstitial nephritis and urinary tract infections	Infectious disease, acute episode, mild	has a low fever and mild discomfort , but no difficulty with daily activities.	0.006 (0.002–0.012)
Moderate interstitial nephritis and urinary tract infections	Infectious disease, acute episode, moderate	has a fever and aches, and feels weak, which causes some difficulty with daily activities.	0.051 (0.032–0.074)
Moderate urolithiasis episodes	Abdominopelvic problem, moderate	has pain in the belly and feels nauseous. The person has difficulties with daily activities.	0.114 (0.078–0.159)
Severe urolithiasis episodes	Abdominopelvic problem, severe	has severe pain in the belly and feels nauseous. The person is anxious and unable to carry out daily activities.	0.324 (0.22–0.442)
Mild urolithiasis episodes	Abdominopelvic problem, mild	has some pain in the belly that causes nausea but does not interfere with daily activities.	0.011 (0.005–0.021)
Symptomatic benign prostatic hyperplasia	Benign prostatic hypertrophy, symptomatic cases	feels the urge to urinate frequently, but when passing urine it comes out slowly and sometimes is painful.	0.067 (0.043–0.097)
Asymptomatic benign prostatic hyperplasia	Asymptomatic	--	--
Primary male infertility	Infertility, primary	wants to have a child and has a fertile partner, but the couple cannot conceive.	0.008 (0.003–0.015)
Secondary male infertility	Infertility, secondary	has at least one child, and wants to have more children. The person has a fertile partner, but the couple cannot conceive.	0.005 (0.002–0.011)
Other urinary diseases	Post-COMO calculation for residuals (YLL/YLD ratio, other methods)	--	--
Asymptomatic uterine fibroids	Asymptomatic	--	--
Mild abdominal pain due to uterine fibroids, with severe anemia	Mild abdominal pain with severe anemia	(combined DW)	--
Mild abdominal pain due to uterine fibroids, with moderate anemia	Mild abdominal pain with moderate anemia	(combined DW)	--
Mild abdominal pain due to uterine fibroids, without anemia	Abdominopelvic problem, mild	has some pain in the belly that causes nausea but does not interfere with daily activities.	0.011 (0.005–0.021)
Mild abdominal pain due to uterine fibroids, with mild anemia	Mild abdominal pain with mild anemia	(combined DW)	--
Secondary infertility due to polycystic ovarian syndrome	Disfigurement level 1 and secondary infertility	(combined DW)	--
Hirsutism due to polycystic ovarian syndrome	Disfigurement, level 1	has a slight, visible physical deformity that others notice, which causes some worry and discomfort.	0.011 (0.005–0.021)
Hirsutism and secondary infertility due to polycystic ovarian syndrome	Infertility, secondary	has at least one child, and wants to have more children. The person has a fertile partner, but the couple cannot conceive.	0.005 (0.002–0.011)
Asymptomatic polycystic ovarian syndrome	Asymptomatic	--	--
Hirsutism and primary infertility due to polycystic ovarian syndrome	Disfigurement level 1 and primary infertility	(combined DW)	--
Primary infertility due to polycystic ovarian syndrome	Infertility, primary	wants to have a child and has a fertile partner, but the couple cannot conceive.	0.008 (0.003–0.015)
Idiopathic secondary female infertility	Infertility, secondary	has at least one child, and wants to have more children. The person has a fertile partner, but the couple cannot conceive.	0.005 (0.002–0.011)
Idiopathic primary female infertility	Infertility, primary	wants to have a child and has a fertile partner, but the couple cannot conceive.	0.008 (0.003–0.015)
Mild abdominal pain and secondary infertility due to endometriosis	Mild abdominal pain and secondary infertility	(combined DW)	--
Moderate abdominal pain and secondary infertility due to endometriosis	Moderate abdominal pain and secondary infertility	(combined DW)	--
Severe abdominal pain and secondary infertility due to endometriosis	Severe abdominal pain and secondary infertility	(combined DW)	--
Severe endometriosis	Abdominopelvic problem, severe	has severe pain in the belly and feels nauseous. The person is anxious and unable to carry out daily activities.	0.324 (0.22–0.442)
Primary infertility due to endometriosis	Infertility, primary	wants to have a child and has a fertile partner, but the couple cannot conceive.	0.008 (0.003–0.015)
Secondary infertility due to endometriosis	Infertility, secondary	has at least one child, and wants to have more children. The person has a fertile partner, but the couple cannot conceive.	0.005 (0.002–0.011)
Moderate abdominal pain due to endometriosis	Abdominopelvic problem, moderate	has pain in the belly and feels nauseous. The person has difficulties with daily activities.	0.114 (0.078–0.159)
Asymptomatic endometriosis	Asymptomatic	--	--
Severe abdominal pain and primary infertility due to endometriosis	Severe abdominal pain and primary infertility	(combined DW)	--
Mild abdominal pain due to endometriosis	Abdominopelvic problem, mild	has some pain in the belly that causes nausea but does not interfere with daily activities.	0.011 (0.005–0.021)
Moderate abdominal pain and primary infertility due to endometriosis	Moderate abdominal pain and primary infertility	(combined DW)	--
Mild abdominal pain and primary infertility due to endometriosis	Mild abdominal pain and primary infertility	(combined DW)	--
Abdominal pain due to genital prolapse	Abdominopelvic problem, mild	has some pain in the belly that causes nausea but does not interfere with daily activities.	0.011 (0.005–0.021)

Appendix Table 5. GBD 2016 sequelae, health states, health state lay descriptions, and disability weights			
**Sequela**	**Health state name**	**Health state lay description**	**Disability weight**
Asymptomatic genital prolapse	Asymptomatic	--	--
Abdominal pain and stress incontinence due to genital prolapse	Mild abdominal pain and stress incontinence	(combined DW)	--
Stress incontinence due to genital prolapse	Stress incontinence	loses small amounts of urine without meaning to when coughing, sneezing, laughing or during physical exercise.	0.02 (0.011–0.035)
Abdominal pain due to premenstrual syndrome	Abdominopelvic problem, mild	has some pain in the belly that causes nausea but does not interfere with daily activities.	0.011 (0.005–0.021)
Asymptomatic premenstrual syndrome	Asymptomatic	--	--
Depression due to premenstrual syndrome	Major depressive disorder, mild episode	feels persistent sadness and has lost interest in usual activities. The person sometimes sleeps badly, feels tired, or has trouble concentrating but still manages to function in daily life with extra effort.	0.145 (0.099–0.209)
Abdominal pain and depression due to premenstrual syndrome	Mild abdominal pain and mild depression	(combined DW)	--
Severe other gynecological disorders	Abdominopelvic problem, severe	has severe pain in the belly and feels nauseous. The person is anxious and unable to carry out daily activities.	0.324 (0.22–0.442)
Mild other gynecological disorders	Abdominopelvic problem, mild	has some pain in the belly that causes nausea but does not interfere with daily activities.	0.011 (0.005–0.021)
Moderate anemia due to other gynecological diseases	Anemia, moderate	feels moderate fatigue, weakness, and shortness of breath after exercise, making daily activities more difficult.	0.052 (0.034–0.076)
Moderate other gynecological disorders	Abdominopelvic problem, moderate	has pain in the belly and feels nauseous. The person has difficulties with daily activities.	0.114 (0.078–0.159)
Severe anemia due to other gynecological diseases	Anemia, severe	feels very weak, tired and short of breath, and has problems with activities that require physical effort or deep concentration.	0.149 (0.101–0.209)
Asymptomatic other gynecological disorders	Asymptomatic	--	--
Mild anemia due to other gynecological diseases	Anemia, mild	feels slightly tired and weak at times, but this does not interfere with normal daily activities.	0.004 (0.001–0.008)
Beta-thalassemia major, with moderate anemia	Anemia, moderate; Generic uncomplicated disease anxiety	--	--
Asymptomatic and mild heart failure due to thalassemias	Heart failure, mild	is short of breath and easily tires with moderate physical activity, such as walking uphill or more than a quarter-mile on level ground. The person feels comfortable at rest or during activities requiring less effort.	0.041 (0.026–0.062)
Moderate heart failure due to thalassemias	Heart failure, moderate	is short of breath and easily tires with minimal physical activity, such as walking only a short distance. The person feels comfortable at rest but avoids moderate activity.	0.072 (0.047–0.103)
Hemoglobin E/beta-thalassemia, with moderate anemia	Anemia, moderate; Generic uncomplicated disease anxiety	--	--
Hemoglobin H disease, with moderate anemia	Anemia, moderate; Generic uncomplicated disease anxiety	--	--
Hemoglobin H disease, severe infection with severe anemia	Infectious disease, acute episode, severe; Generic uncomplicated disease anxiety; Anemia, severe	--	--
Beta-thalassemia major, with severe anemia	Anemia, severe; Generic uncomplicated disease anxiety	--	--
Hemoglobin E/beta-thalassemia, with severe anemia	Anemia, severe; Generic uncomplicated disease anxiety	--	--
Beta-thalassemia major, severe infection with severe anemia	Infectious disease, acute episode, severe; Generic uncomplicated disease anxiety; Anemia, severe	--	--
Hemoglobin H disease, with mild anemia	Anemia, mild; Generic uncomplicated disease anxiety	--	--
Hemoglobin E/beta-thalassemia, severe infection with severe anemia	Infectious disease, acute episode, severe; Generic uncomplicated disease anxiety; Anemia, severe	--	--
Hemoglobin E/Beta-thalassemia, without anemia	Generic uncomplicated disease: anxiety about diagnosis	has a disease diagnosis that causes some worry but minimal interference with daily activities.	0.012 (0.006–0.023)
Hemoglobin E/beta-thalassemia, with mild anemia	Anemia, mild; Generic uncomplicated disease anxiety	--	--
Beta-thalassemia major, with mild anemia	Anemia, mild; Generic uncomplicated disease anxiety	--	--
Beta-thalassemia major, without anemia	Generic uncomplicated disease: anxiety about diagnosis	has a disease diagnosis that causes some worry but minimal interference with daily activities.	0.012 (0.006–0.023)
Hemoglobin H disease, without anemia	Generic uncomplicated disease: anxiety about diagnosis	has a disease diagnosis that causes some worry but minimal interference with daily activities.	0.012 (0.006–0.023)
Severe heart failure due to thalassemias	Heart failure, severe	is short of breath and feels tired when at rest. The person avoids any physical activity, for fear of worsening the breathing problems.	0.179 (0.122–0.251)
Hemoglobin H disease, with severe anemia	Anemia, severe; Generic uncomplicated disease anxiety	--	--
Moderate anemia due to B-thalassemia trait	Anemia, moderate	feels moderate fatigue, weakness, and shortness of breath after exercise, making daily activities more difficult.	0.052 (0.034–0.076)
Asymptomatic B-thalassemia trait	Asymptomatic	--	--
Asymptomatic hemoglobin E trait	Asymptomatic	--	--
Moderate anemia due to hemoglobin E trait	Anemia, moderate	feels moderate fatigue, weakness, and shortness of breath after exercise, making daily activities more difficult.	0.052 (0.034–0.076)
Mild anemia due to B-thalassemia trait	Anemia, mild	feels slightly tired and weak at times, but this does not interfere with normal daily activities.	0.004 (0.001–0.008)
Mild anemia due to hemoglobin E trait	Anemia, mild	feels slightly tired and weak at times, but this does not interfere with normal daily activities.	0.004 (0.001–0.008)

Appendix Table 5. GBD 2016 sequelae, health states, health state lay descriptions, and disability weights			
**Sequela**	**Health state name**	**Health state lay description**	**Disability weight**
Severe anemia due to hemoglobin E trait	Anemia, severe	feels very weak, tired and short of breath, and has problems with activities that require physical effort or deep concentration.	0.149 (0.101–0.209)
Severe anemia due to B-thalassemia trait	Anemia, severe	feels very weak, tired and short of breath, and has problems with activities that require physical effort or deep concentration.	0.149 (0.101–0.209)
Homozygous sickle cell and severe sickle cell/beta-thalassemia, without anemia	Generic uncomplicated disease: anxiety about diagnosis	has a disease diagnosis that causes some worry but minimal interference with daily activities.	0.012 (0.006–0.023)
Hemoglobin SC disease, without anemia	Generic uncomplicated disease: anxiety about diagnosis	has a disease diagnosis that causes some worry but minimal interference with daily activities.	0.012 (0.006–0.023)
Mild sickle cell/beta-thalassemia, without anemia	Generic uncomplicated disease: anxiety about diagnosis	has a disease diagnosis that causes some worry but minimal interference with daily activities.	0.012 (0.006–0.023)
Hemoglobin SC disease, with vaso-occlusive crisis, stroke, and severe anemia	Generic uncomplicated disease anxiety; long-term consequences due to stroke; severe abdominopelvic problem; Anemia, severe	--	--
Mild sickle cell/beta-thalassemia, with vaso-occlusive crisis, stroke, and severe anemia	Generic uncomplicated disease anxiety; long-term consequences due to stroke; severe abdominopelvic problem; Anemia, severe	--	--
Mild sickle cell/beta-thalassemia, with stroke, without anemia	Generic uncomplicated disease anxiety; long-term consequences due to stroke	--	--
Homozygous sickle cell and severe sickle cell/beta-thalassemia, with vaso-occlusive crisis and stroke, without anemia	Generic uncomplicated disease anxiety; long-term consequences due to stroke; severe abdominopelvic problem	--	--
Hemoglobin SC disease, with vaso-occlusive crisis and stroke, without anemia	Generic uncomplicated disease anxiety; long-term consequences due to stroke; severe abdominopelvic problem	--	--
Mild sickle cell/beta-thalassemia, with vaso-occlusive crisis and stroke, without anemia	Generic uncomplicated disease anxiety; long-term consequences due to stroke; severe abdominopelvic problem	--	--
Homozygous sickle cell and severe sickle cell/beta-thalassemia, with vaso-occlusive crisis and severe anemia	Moderate abdominal pain; Anemia, severe; Generic uncomplicated disease anxiety	--	--
Homozygous sickle cell and severe sickle cell/beta-thalassemia, with mild anemia	Anemia, mild; Generic uncomplicated disease anxiety	--	--
Hemoglobin SC disease, with stroke, without anemia	Generic uncomplicated disease anxiety; long-term consequences due to stroke	--	--
Homozygous sickle cell and severe sickle cell/beta-thalassemia, with stroke, without anemia	Generic uncomplicated disease anxiety; long-term consequences due to stroke	--	--
Mild sickle cell/beta-thalassemia, with vaso-occlusive crisis, without anemia	Generic uncomplicated disease anxiety and severe abdominopelvic problem	--	--
Hemoglobin SC disease, with vaso-occlusive crisis, without anemia	Generic uncomplicated disease anxiety and severe abdominopelvic problem	--	--
Hemoglobin SC disease, with vaso-occlusive crisis and severe anemia	Moderate abdominal pain; Anemia, severe; Generic uncomplicated disease anxiety	--	--
Homozygous sickle cell and severe sickle cell/beta-thalassemia, with severe anemia	Anemia, severe; Generic uncomplicated disease anxiety	--	--
Hemoglobin SC disease, with severe anemia	Anemia, severe; Generic uncomplicated disease anxiety	--	--
Homozygous sickle cell and severe sickle cell/beta-thalassemia, with stroke and severe anemia	Generic uncomplicated disease anxiety; Long-term consequences due to stroke; Anemia, severe	--	--
Hemoglobin SC disease, with stroke and severe anemia	Generic uncomplicated disease anxiety; Long-term consequences due to stroke; Anemia, severe	--	--
Hemoglobin SC disease, with mild anemia	Anemia, mild; Generic uncomplicated disease anxiety	--	--
Mild sickle cell/beta-thalassemia, with vaso-occlusive crisis and severe anemia	Moderate abdominal pain; Anemia, severe; Generic uncomplicated disease anxiety	--	--
Mild sickle cell/beta-thalassemia, with mild anemia	Anemia, mild; Generic uncomplicated disease anxiety	--	--
Mild sickle cell/beta-thalassemia, with severe anemia	Anemia, severe; Generic uncomplicated disease anxiety	--	--
Mild sickle cell/beta-thalassemia, with moderate anemia	Anemia, moderate; Generic uncomplicated disease anxiety	--	--
Homozygous sickle cell and severe sickle cell/beta-thalassemia, with vaso-occlusive crisis, stroke, and severe anemia	Generic uncomplicated disease anxiety; long-term consequences due to stroke; severe abdominopelvic problem; Anemia, severe	--	--
Hemoglobin SC disease, with moderate anemia	Anemia, moderate; Generic uncomplicated disease anxiety	--	--
Homozygous sickle cell and severe sickle cell/beta-thalassemia, with moderate anemia	Anemia, moderate; Generic uncomplicated disease anxiety	--	--
Homozygous sickle cell and severe sickle cell/beta-thalassemia, with vaso-occlusive crisis, without anemia	Generic uncomplicated disease anxiety and severe abdominopelvic problem	--	--
Mild sickle cell/beta-thalassemia, with stroke and severe anemia	Generic uncomplicated disease anxiety; Long-term consequences due to stroke; Anemia, severe	--	--
Asymptomatic sickle cell trait	Asymptomatic	--	--
Moderate anemia due to sickle cell trait	Anemia, moderate	feels moderate fatigue, weakness, and shortness of breath after exercise, making daily activities more difficult.	0.052 (0.034–0.076)
Mild anemia due to sickle cell trait	Anemia, mild	feels slightly tired and weak at times, but this does not interfere with normal daily activities.	0.004 (0.001–0.008)
Severe anemia due to sickle cell trait	Anemia, severe	feels very weak, tired and short of breath, and has problems with activities that require physical effort or deep concentration.	0.149 (0.101–0.209)

Appendix Table 5. GBD 2016 sequelae, health states, health state lay descriptions, and disability weights			
**Sequela**	**Health state name**	**Health state lay description**	**Disability weight**
Asymptomatic and mild heart failure due to G6PD deficiency	Heart failure, mild	is short of breath and easily tires with moderate physical activity, such as walking uphill or more than a quarter-mile on level ground. The person feels comfortable at rest or during activities requiring less effort.	0.041 (0.026–0.062)
Mild anemia due to G6PD deficiency	Anemia, mild	feels slightly tired and weak at times, but this does not interfere with normal daily activities.	0.004 (0.001–0.008)
Severe heart failure due to G6PD deficiency	Heart failure, severe	is short of breath and feels tired when at rest. The person avoids any physical activity, for fear of worsening the breathing problems.	0.179 (0.122–0.251)
Severe anemia due to G6PD deficiency	Anemia, severe	feels very weak, tired and short of breath, and has problems with activities that require physical effort or deep concentration.	0.149 (0.101–0.209)
Asymptomatic G6PD deficiency	Asymptomatic	--	--
Moderate heart failure due to G6PD deficiency	Heart failure, moderate	is short of breath and easily tires with minimal physical activity, such as walking only a short distance. The person feels comfortable at rest but avoids moderate activity.	0.072 (0.047–0.103)
Moderate anemia due to G6PD deficiency	Anemia, moderate	feels moderate fatigue, weakness, and shortness of breath after exercise, making daily activities more difficult.	0.052 (0.034–0.076)
Mild anemia due to hemizygous G6PD deficiency	Anemia, mild	feels slightly tired and weak at times, but this does not interfere with normal daily activities.	0.004 (0.001–0.008)
Severe anemia due to hemizygous G6PD deficiency	Anemia, severe	feels very weak, tired and short of breath, and has problems with activities that require physical effort or deep concentration.	0.149 (0.101–0.209)
Moderate anemia due to hemizygous G6PD deficiency	Anemia, moderate	feels moderate fatigue, weakness, and shortness of breath after exercise, making daily activities more difficult.	0.052 (0.034–0.076)
Asymptomatic hemizygous G6PD deficiency	Asymptomatic	--	--
Mild anemia due to other hemoglobinopathies and hemolytic anemias	Anemia, mild	feels slightly tired and weak at times, but this does not interfere with normal daily activities.	0.004 (0.001–0.008)
Other hemoglobinopathies and hemolytic anemias	Post-COMO calculation for residuals (YLL/YLD ratio, other methods)	--	--
Severe heart failure due to other hemoglobinopathies and hemolytic anemias	Heart failure, severe	is short of breath and feels tired when at rest. The person avoids any physical activity, for fear of worsening the breathing problems.	0.179 (0.122–0.251)
Asymptomatic and mild heart failure due to other hemoglobinopathies and hemolytic anemias	Heart failure, mild	is short of breath and easily tires with moderate physical activity, such as walking uphill or more than a quarter-mile on level ground. The person feels comfortable at rest or during activities requiring less effort.	0.041 (0.026–0.062)
Moderate heart failure due to other hemoglobinopathies and hemolytic anemias	Heart failure, moderate	is short of breath and easily tires with minimal physical activity, such as walking only a short distance. The person feels comfortable at rest but avoids moderate activity.	0.072 (0.047–0.103)
Moderate anemia due to other hemoglobinopathies and hemolytic anemias	Anemia, moderate	feels moderate fatigue, weakness, and shortness of breath after exercise, making daily activities more difficult.	0.052 (0.034–0.076)
Severe anemia due to other hemoglobinopathies and hemolytic anemias	Anemia, severe	feels very weak, tired and short of breath, and has problems with activities that require physical effort or deep concentration.	0.149 (0.101–0.209)
Severe anemia due to endocrine, metabolic, blood, and immune disorders	Anemia, severe	feels very weak, tired and short of breath, and has problems with activities that require physical effort or deep concentration.	0.149 (0.101–0.209)
Asymptomatic endocrine, metabolic, blood, and immune disorders	Asymptomatic	--	--
Severe endocrine, metabolic, blood, and immune disorders	Thrombocytopenic purpura	easily bruises and sometimes bleeds from the gums and nose; feels weak and has some difficulty with daily activities.	0.159 (0.106–0.226)
Moderate heart failure due to endocrine, metabolic, blood, and immune disorders	Heart failure, moderate	is short of breath and easily tires with minimal physical activity, such as walking only a short distance. The person feels comfortable at rest but avoids moderate activity.	0.072 (0.047–0.103)
Asymptomatic and mild heart failure due to endocrine, metabolic, blood, and immune disorders	Heart failure, mild	is short of breath and easily tires with moderate physical activity, such as walking uphill or more than a quarter-mile on level ground. The person feels comfortable at rest or during activities requiring less effort.	0.041 (0.026–0.062)
Mild anemia due to endocrine, metabolic, blood, and immune disorders	Anemia, mild	feels slightly tired and weak at times, but this does not interfere with normal daily activities.	0.004 (0.001–0.008)
Mild endocrine, metabolic, blood, and immune disorders	Hypothyroidism	has low energy and feels cold.	0.019 (0.01–0.032)
Moderate anemia due to endocrine, metabolic, blood, and immune disorders	Anemia, moderate	feels moderate fatigue, weakness, and shortness of breath after exercise, making daily activities more difficult.	0.052 (0.034–0.076)
Severe heart failure due to endocrine, metabolic, blood, and immune disorders	Heart failure, severe	is short of breath and feels tired when at rest. The person avoids any physical activity, for fear of worsening the breathing problems.	0.179 (0.122–0.251)
Moderate endocrine, metabolic, blood, and immune disorders	Hyperthyroidism	feels nervous, has palpitations, sweats a lot and has difficulty sleeping.	0.145 (0.096–0.202)
Moderate rheumatoid arthritis	Musculoskeletal problems, generalized, moderate	has pain and deformity in most joints, causing difficulty moving around, getting up and down, and using the hands for lifting and carrying. The person often feels fatigue.	0.317 (0.216–0.44)
Mild rheumatoid arthritis	Musculoskeletal problems, upper limbs, moderate	has moderate pain and stiffness in the arms and hands, which causes difficulty lifting, carrying, and holding things, and trouble sleeping because of the pain.	0.117 (0.08–0.163)
Severe rheumatoid arthritis	Musculoskeletal problems, generalized, severe	has severe, constant pain and deformity in most joints, causing difficulty moving around, getting up and down, eating, dressing, lifting, carrying and using the hands. The person often feels sadness, anxiety and extreme fatigue.	0.581 (0.403–0.739)

Appendix Table 5. GBD 2016 sequelae, health states, health state lay descriptions, and disability weights			
**Sequela**	**Health state name**	**Health state lay description**	**Disability weight**
Moderate osteoarthritis of the knee	Musculoskeletal problems, lower limbs, moderate	has moderate pain in the leg, which makes the person limp, and causes some difficulty walking, standing, lifting and carrying heavy things, getting up and down and sleeping.	0.079 (0.054–0.11)
Severe osteoarthritis of the hip	Musculoskeletal problems, lower limbs, severe	has severe pain in the leg, which makes the person limp and causes a lot of difficulty walking, standing, lifting and carrying heavy things, getting up and down, and sleeping.	0.165 (0.112–0.232)
Severe osteoarthritis of the knee	Musculoskeletal problems, lower limbs, severe	has severe pain in the leg, which makes the person limp and causes a lot of difficulty walking, standing, lifting and carrying heavy things, getting up and down, and sleeping.	0.165 (0.112–0.232)
Mild osteoarthritis of the knee	Musculoskeletal problems, lower limbs, mild	has pain in the leg, which causes some difficulty running, walking long distances, and getting up and down.	0.023 (0.013–0.037)
Moderate osteoarthritis of the hip	Musculoskeletal problems, lower limbs, moderate	has moderate pain in the leg, which makes the person limp, and causes some difficulty walking, standing, lifting and carrying heavy things, getting up and down and sleeping.	0.079 (0.054–0.11)
Mild osteoarthritis of the hip	Musculoskeletal problems, lower limbs, mild	has pain in the leg, which causes some difficulty running, walking long distances, and getting up and down.	0.023 (0.013–0.037)
Mild low back pain without leg pain	Low back pain, mild	has mild back pain, which causes some difficulty dressing, standing, and lifting things.	0.02 (0.011–0.035)
Moderate low back pain without leg pain	Low back pain, moderate	has moderate back pain, which causes difficulty dressing, sitting, standing, walking, and lifting things.	0.054 (0.035–0.079)
Most severe low back pain with leg pain	Back pain, most severe, with leg pain	has constant back and leg pain, which causes difficulty dressing, sitting, standing, walking, and lifting things. The person sleeps poorly, is worried, and has lost some enjoyment in life.	0.384 (0.256–0.518)
Most severe low back pain without leg pain	Back pain, most severe, without leg pain	has constant back pain, which causes difficulty dressing, sitting, standing, walking, and lifting things. The person sleeps poorly, is worried, and has lost some enjoyment in life.	0.372 (0.25–0.506)
Severe low back pain with leg pain	Back pain, severe, with leg pain	has severe back and leg pain, which causes difficulty dressing, sitting, standing, walking, and lifting things. The person sleeps poorly and feels worried.	0.325 (0.219–0.446)
Severe low back pain without leg pain	Back pain, severe, without leg pain	has severe back pain, which causes difficulty dressing, sitting, standing, walking, and lifting things. The person sleeps poorly and feels worried.	0.272 (0.182–0.373)
Mild low back pain with leg pain	Mild low back pain with leg pain	(combined DW)	0.02 (0.011–0.035)
Moderate low back pain with leg pain	Moderate low back pain with leg pain	(combined DW)	0.054 (0.035–0.079)
Severe neck pain	Neck pain, severe	has severe neck pain, and difficulty turning the head and lifting things. The person gets headaches and arm pain, sleeps poorly, and feels tired and worried.	0.229 (0.153–0.317)
Most severe neck pain	Neck pain, most severe	has constant neck pain and arm pain, and difficulty turning the head, holding arms up, and lifting things. The person gets headaches, sleeps poorly, and feels tired and worried.	0.304 (0.202–0.415)
Moderate neck pain	Neck pain, moderate	has constant neck pain, and has difficulty turning the head, holding arms up, and lifting things	0.114 (0.075–0.162)
Mild neck pain	Neck pain, mild	has neck pain, and has difficulty turning the head and lifting things.	0.053 (0.034–0.078)
Asymptomatic gout	Asymptomatic	--	--
Polyarticular gout	Musculoskeletal problems, generalized, severe	has severe, constant pain and deformity in most joints, causing difficulty moving around, getting up and down, eating, dressing, lifting, carrying and using the hands. The person often feels sadness, anxiety and extreme fatigue.	0.581 (0.403–0.739)
Symptomatic episodes of gout	Gout, acute	has severe pain and swelling in the leg, making it very difficult to get up and down, stand, walk, lift, and carry heavy things. The person has trouble sleeping because of the	0.295 (0.196–0.409)
Other musculoskeletal disorders severity level 5	Musculoskeletal problems, generalized, moderate	has pain and deformity in most joints, causing difficulty moving around, getting up and down, and using the hands for lifting and carrying. The person often feels fatigue.	0.317 (0.216–0.44)
Other musculoskeletal disorders severity level 4	Musculoskeletal problems, lower limbs, severe	has severe pain in the leg, which makes the person limp and causes a lot of difficulty walking, standing, lifting and carrying heavy things, getting up and down, and sleeping.	0.165 (0.112–0.232)
Other musculoskeletal disorders severity level 6	Musculoskeletal problems, generalized, severe	has severe, constant pain and deformity in most joints, causing difficulty moving around, getting up and down, eating, dressing, lifting, carrying and using the hands. The person often feels sadness, anxiety and extreme fatigue.	0.581 (0.403–0.739)
Asymptomatic other musculoskeletal disorders	Asymptomatic	--	--
Other musculoskeletal disorders severity level 3	Musculoskeletal problems, upper limbs, moderate	has moderate pain and stiffness in the arms and hands, which causes difficulty lifting, carrying, and holding things, and trouble sleeping because of the pain.	0.117 (0.08–0.163)
Other musculoskeletal disorders severity level 2	Musculoskeletal problems, upper limbs, mild	has mild pain and stiffness in the arms and hands. The person has some difficulty lifting, carrying and holding things.	0.028 (0.017–0.045)
Other musculoskeletal disorders severity level 1	Musculoskeletal problems, lower limbs, mild	has pain in the leg, which causes some difficulty running, walking long distances, and getting up and down.	0.023 (0.013–0.037)
Severe motor impairment, severe intellectual disability and incontinence due to spina bifida	Severe motor impairment with severe intellectual disability and incontinence	(combined DW)	0.564 (0.418–0.71)
Severe motor impairment, moderate intellectual disability and incontinence due to spina bifida	Severe motor impairment with moderate intellectual disability and incontinence	(combined DW)	0.534 (0.391–0.675)
Severe motor impairment, moderate intellectual disability and incontinence due to encephalocele	Severe motor impairment with moderate intellectual disability and incontinence	(combined DW)	0.534 (0.391–0.675)

Appendix Table 5. GBD 2016 sequelae, health states, health state lay descriptions, and disability weights			
**Sequela**	**Health state name**	**Health state lay description**	**Disability weight**
Severe motor impairment, borderline intellectual disability and incontinence due to encephalocele	Severe motor impairment, borderline intellectual functioning, and urinary incontinence	(combined DW)	0.489 (0.353–0.632)
Severe motor impairment, profound intellectual disability and incontinence due to encephalocele	Severe motor impairment with severe intellectual disability and incontinence	(combined DW)	0.564 (0.418–0.71)
Severe motor impairment, mild intellectual disability and incontinence due to encephalocele	Severe motor impairment with mild intellectual disability and incontinence	(combined DW)	0.505 (0.367–0.647)
profound motor impairment, profound intellectual disability and incontinence due to spina bifida	Severe motor impairment with profound intellectual disability and incontinence	(combined DW)	0.584 (0.435–0.73)
Severe motor impairment and mild intellectual disability due to spina bifida	Severe motor impairment with mild intellectual disability	(combined DW)	0.427 (0.3–0.567)
Severe motor impairment and mild intellectual disability due to encephalocele	Severe motor impairment with mild intellectual disability	(combined DW)	0.427 (0.3–0.567)
Severe motor impairment, severe intellectual disability and incontinence due to encephalocele	Severe motor impairment with severe intellectual disability and incontinence	(combined DW)	0.564 (0.418–0.71)
Severe motor impairment, mild intellectual disability and incontinence due to spina bifida	Severe motor impairment with mild intellectual disability and incontinence	(combined DW)	0.505 (0.367–0.647)
Mild motor impairment and incontinence due to spina bifida	Mild motor impairment and urinary incontinence	(combined DW)	0.148 (0.1–0.207)
Mild motor impairment, severe intellectual disability and incontinence due to encephalocele	Mild motor impairment, servere intellectual functioning, and urinary incontinence	(combined DW)	0.284 (0.201–0.385)
Moderate motor impairment, severe intellectual disability and incontinence due to spina bifida	Moderate motor impairment, servere intellectual functioning, and urinary incontinence	(combined DW)	0.32 (0.228–0.429)
Moderate motor impairment, severe intellectual disability and incontinence due to encephalocele	Moderate motor impairment, servere intellectual functioning, and urinary incontinence	(combined DW)	0.32 (0.228–0.429)
Severe motor impairment and moderate intellectual disability due to spina bifida	Severe motor impairment with moderate intellectual disability	(combined DW)	0.461 (0.324–0.603)
Mild motor impairment and incontinence due to encephalocele	Mild motor impairment and urinary incontinence	(combined DW)	0.148 (0.1–0.207)
Moderate motor impairment and incontinence due to spina bifida	Moderate motor impairment and urinary incontinence	(combined DW)	0.191 (0.132–0.263)
Moderate motor impairment and incontinence due to encephalocele	Moderate motor impairment and urinary incontinence	(combined DW)	0.191 (0.132–0.263)
Mild motor impairment, profound intellectual disability and incontinence due to spina bifida	Mild motor impairment, profound intellectual functioning, and urinary incontinence	(combined DW)	0.318 (0.224–0.426)
Moderate motor impairment, profound intellectual disability and incontinence due to encephalocele	Mild motor impairment, profound intellectual functioning, and urinary incontinence	(combined DW)	0.318 (0.224–0.426)
Mild motor impairment and profound intellectual disability due to spina bifida	Mild motor impairment with profound intellectual disability	(combined DW)	0.208 (0.142–0.289)
Mild motor impairment, severe intellectual disability and incontinence due to spina bifida	Mild motor impairment, servere intellectual functioning, and urinary incontinence	(combined DW)	0.284 (0.201–0.385)
Severe motor impairment and moderate intellectual disability due to encephalocele	Severe motor impairment with moderate intellectual disability	(combined DW)	0.461 (0.324–0.603)
Severe motor impairment and profound intellectual disability due to spina bifida	Severe motor impairment with profound intellectual disability	(combined DW)	0.519 (0.37–0.668)
Severe motor impairment and severe intellectual disability due to encephalocele	Severe motor impairment with severe intellectual disability	(combined DW)	0.496 (0.355–0.641)
Severe motor impairment, borderline intellectual disability and incontinence due to spina bifida	Severe motor impairment, borderline intellectual functioning, and urinary incontinence	(combined DW)	0.489 (0.353–0.632)
Mild motor impairment, mild intellectual disability and incontinence due to encephalocele	Mild motor impairment, mild intellectual functioning, and urinary incontinence	(combined DW)	0.184 (0.128–0.253)
Moderate motor impairment, borderline intellectual disability and incontinence due to encephalocele	Moderate motor impairment, borderline intellectual functioning, and urinary incontinence	(combined DW)	0.2 (0.139–0.273)
Moderate motor impairment, borderline intellectual disability and incontinence due to spina bifida	Moderate motor impairment, borderline intellectual functioning, and urinary incontinence	(combined DW)	0.2 (0.139–0.273)
Mild motor impairment, borderline intellectual disability and incontinence due to encephalocele	Mild motor impairment, borderline intellectual functioning, and urinary incontinence	(combined DW)	0.157 (0.108–0.218)
Mild motor impairment, borderline intellectual disability and incontinence due to spina bifida	Mild motor impairment, borderline intellectual functioning, and urinary incontinence	(combined DW)	0.157 (0.108–0.218)
Moderate motor impairment and severe intellectual disability due to encephalocele	Moderate motor impairment and severe intellectual functioning	(combined DW)	0.211 (0.145–0.293)
Moderate motor impairment and severe intellectual disability due to spina bifida	Moderate motor impairment and severe intellectual functioning	(combined DW)	0.211 (0.145–0.293)
Moderate motor impairment, mild intellectual disability and incontinence due to spina bifida	Moderate motor impairment, mild intellectual functioning, and urinary incontinence	(combined DW)	0.272 (0.191–0.364)
Mild motor impairment and severe intellectual disability due to spina bifida	Mild motor impairment and severe intellectual functioning	(combined DW)	0.169 (0.113–0.237)
Mild motor impairment and moderate intellectual disability due to encephalocele	Mild motor impairment and moderate intellectual functioning	(combined DW)	0.109 (0.073–0.154)
Mild motor impairment and moderate intellectual disability due to spina bifida	Mild motor impairment and moderate intellectual functioning	(combined DW)	0.109 (0.073–0.154)
Moderate motor impairment and mild intellectual disability due to encephalocele	Moderate motor impairment and mild intellectual functioning	(combined DW)	0.101 (0.066–0.146)
Moderate motor impairment, mild intellectual disability and incontinence due to encephalocele	Moderate motor impairment, mild intellectual functioning, and urinary incontinence	(combined DW)	0.272 (0.191–0.364)
Mild motor impairment, moderate intellectual disability and incontinence due to spina bifida	Mild motor impairment, moderate intellectual functioning, and urinary incontinence	(combined DW)	0.233 (0.161–0.314)
Mild motor impairment, moderate intellectual disability and incontinence due to encephalocele	Mild motor impairment, moderate intellectual functioning, and urinary incontinence	(combined DW)	0.233 (0.161–0.314)
Moderate motor impairment and mild intellectual disability due to spina bifida	Moderate motor impairment and mild intellectual functioning	(combined DW)	0.101 (0.066–0.146)

Appendix Table 5. GBD 2016 sequelae, health states, health state lay descriptions, and disability weights			
**Sequela**	**Health state name**	**Health state lay description**	**Disability weight**
Mild motor impairment, mild intellectual disability and incontinence due to spina bifida	Mild motor impairment, mild intellectual functioning, and urinary incontinence	(combined DW)	0.184 (0.128–0.253)
Borderline intellectual disability and incontinence due to encephalocele	Borderline intellectual functioning and urinary incontinence	(combined DW)	0.148 (0.101–0.206)
Moderate motor impairment, moderate intellectual disability and incontinence due to encephalocele	Moderate motor impairment, moderate intellectual functioning, and urinary incontinence	(combined DW)	0.272 (0.191–0.364)
Moderate motor impairment, moderate intellectual disability and incontinence due to spina bifida	Moderate motor impairment, moderate intellectual functioning, and urinary incontinence	(combined DW)	0.272 (0.191–0.364)
Mild intellectual disability and incontinence due to encephalocele	Mild intellectual disability and urinary incontinence	(combined DW)	0.176 (0.12–0.242)
Moderate intellectual disability and incontinence due to encephalocele	Moderate intellectual disability and urinary incontinence	(combined DW)	0.225 (0.156–0.304)
Severe motor impairment and severe intellectual disability due to spina bifida	Severe motor impairment with severe intellectual disability	(combined DW)	0.496 (0.355–0.641)
Severe intellectual disability and incontinence due to encephalocele	Severe intellectual disability and urinary incontinence	(combined DW)	0.276 (0.194–0.376)
Mild motor impairment and borderline intellectual disability due to spina bifida	Mild motor impairment and borderline intellectual functioning	(combined DW)	0.021 (0.01–0.039)
Mild motor impairment and borderline intellectual disability due to encephalocele	Mild motor impairment and borderline intellectual functioning	(combined DW)	0.021 (0.01–0.039)
Moderate motor impairment and borderline intellectual disability due to spina bifida	Moderate motor impairment and borderline intellectual functioning	(combined DW)	0.071 (0.045–0.106)
Moderate motor impairment and borderline intellectual disability due to encephalocele	Moderate motor impairment and borderline intellectual functioning	(combined DW)	0.071 (0.045–0.106)
Severe motor impairment and borderline intellectual disability due to spina bifida	Severe motor impairment and borderline intellectual functioning	(combined DW)	0.408 (0.279–0.55)
Severe motor impairment and borderline intellectual disability due to encephalocele	Severe motor impairment and borderline intellectual functioning	(combined DW)	0.408 (0.279–0.55)
Profound intellectual disability and incontinence due to encephalocele	Profound intellectual disability and urinary incontinence	(combined DW)	0.311 (0.217–0.418)
Mild motor impairment and severe intellectual disability due to encephalocele	Mild motor impairment and severe intellectual functioning	(combined DW)	0.169 (0.113–0.237)
Moderate motor impairment, profound intellectual disability and incontinence due to spina bifida	Moderate motor impairment with profound intellectual disability and incontinence	(combined DW)	0.352 (0.254–0.465)
Moderate intellectual disability due to encephalocele	Intellectual disability / mental retardation, moderate	has low intelligence, and is slow in learning to speak and to do even simple tasks. As an adult, the person requires a lot of support to live independently and raise children. The person can only work at the simplest supervised jobs.	0.1 (0.066–0.142)
Incontinence due to encephalocele	Urinary incontinence	cannot control urinating.	0.139 (0.094–0.198)
Moderate motor impairment due to spina bifida	Motor impairment, moderate	has some difficulty in moving around, and difficulty in lifting and holding objects, dressing and sitting upright, but is able to walk without help.	0.061 (0.04–0.089)
Moderate motor impairment due to encephalocele	Motor impairment, moderate	has some difficulty in moving around, and difficulty in lifting and holding objects, dressing and sitting upright, but is able to walk without help.	0.061 (0.04–0.089)
Borderline intellectual disability due to encephalocele	Borderline intellectual functioning	is slow in learning at school. As an adult, the person has some difficulty doing complex or unfamiliar tasks but otherwise functions independently.	0.011 (0.005–0.02)
Severe motor impairment and incontinence due to spina bifida	Severe motor impairment with incontinence	(combined DW)	--
Severe motor impairment and incontinence due to encephalocele	Severe motor impairment with incontinence	(combined DW)	--
Mild motor impairment and profound intellectual disability due to encephalocele	Severe motor impairment with incontinence	(combined DW)	--
Moderate motor impairment and profound intellectual disability due to encephalocele	Severe motor impairment with incontinence	(combined DW)	--
Severe motor and cognitive impairment due to anencephaly	Motor plus cognitive impairments, severe	cannot move around without help, and cannot lift or hold objects, get dressed or sit upright. The person also has very low intelligence, speaks few words, and needs constant supervision and help with all daily activities.	0.542 (0.374–0.702)
Severe motor impairment and profound intellectual disability due to encephalocele	Severe motor impairment with incontinence	(combined DW)	--
Mild intellectual disability due to encephalocele	Intellectual disability / mental retardation, mild	has low intelligence and is slow in learning at school. As an adult, the person can live independently, but often needs help to raise children and can only work at simple supervised jobs.	0.043 (0.026–0.064)
Moderate motor impairment and moderate intellectual disability due to encephalocele	Motor plus cognitive impairments, moderate	has some difficulty in moving around, holding objects, dressing and sitting upright, but can walk without help. The person has low intelligence and is slow in learning to speak and to do simple tasks.	0.203 (0.134–0.29)
Mild motor impairment and mild intellectual disability due to encephalocele	Motor plus cognitive impairments, mild	has some difficulty in moving around but is able to walk without help. The person is slow in learning at school. As an adult, the person has some difficulty doing complex or unfamiliar tasks but otherwise functions independently.	0.031 (0.018–0.05)
Mild motor impairment and mild intellectual disability due to spina bifida	Motor plus cognitive impairments, mild	has some difficulty in moving around but is able to walk without help. The person is slow in learning at school. As an adult, the person has some difficulty doing complex or unfamiliar tasks but otherwise functions independently.	0.031 (0.018–0.05)
Severe motor impairment due to encephalocele	Motor impairment, severe	is unable to move around without help, and is not able to lift or hold objects, get dressed or sit upright.	0.402 (0.268–0.545)

Appendix Table 5. GBD 2016 sequelae, health states, health state lay descriptions, and disability weights

Sequela	Health state name	Health state lay description	Disability weight
Severe motor impairment due to spina bifida	Motor impairment, severe	is unable to move around without help, and is not able to lift or hold objects, get dressed or sit upright.	0.402 (0.268–0.545)
Profound intellectual disability due to encephalocele	Intellectual disability / mental retardation, profound	has very low intelligence, has almost no language, and does not understand even the most basic requests or instructions. The person requires constant supervision and help for all activities.	0.2 (0.133–0.283)
Moderate motor impairment and profound intellectual disability due to spina bifida	Moderate motor impairment with profound intellectual disability	(combined DW)	0.249 (0.174–0.338)
Severe intellectual disability due to encephalocele	Intellectual disability / mental retardation, severe	has very low intelligence and cannot speak more than a few words, needs constant supervision and help with most daily activities, and can do only the simplest tasks.	0.16 (0.107–0.226)
Mild motor impairment, profound intellectual disability and incontinence due to encephalocele	Severe motor impairment with incontinence	(combined DW)	--
Mild motor impairment due to spina bifida	Motor impairment, mild	has some difficulty in moving around but is able to walk without help.	0.01 (0.005–0.019)
Asymptomatic encephalocele following treatment	Asymptomatic	--	--
Mild motor impairment due to encephalocele	Motor impairment, mild	has some difficulty in moving around but is able to walk without help.	0.01 (0.005–0.019)
Moderate motor impairment and moderate intellectual disability due to spina bifida	Motor plus cognitive impairments, moderate	has some difficulty in moving around, holding objects, dressing and sitting upright, but can walk without help. The person has low intelligence and is slow in learning to speak and to do simple tasks.	0.203 (0.134–0.29)
Congenital heart disease, boderline intellectual disability and mild heart failure due to severe congenital heart anomalies excluding single ventricle heart defects	Congenital heart disease, borderline intellectual functioning, and mild heart failure	(combined DW)	0.052 (0.032–0.081)
Congenital heart disease, boderline intellectual disability and mild heart failure due to critical malformations of great vessels, congenital valvular heart disease and patent ductus arteriosus	Congenital heart disease, borderline intellectual functioning, and mild heart failure	(combined DW)	0.052 (0.032–0.081)
Congenital heart disease, boderline intellectual disability and mild heart failure due to ventricular septal defect and atrial septal defect	Congenital heart disease, borderline intellectual functioning, and mild heart failure	(combined DW)	0.052 (0.032–0.081)
Congenital heart disease, severe intellectual disability and moderate heart failure due to severe congenital heart anomalies excluding single ventricle heart defects	Congenital heart disease, severe intellectual disability, and moderate heart failure	(combined DW)	0.22 (0.151–0.302)
Congenital heart disease, borderline intellectual disability and moderate heart failure due to severe congenital heart anomalies excluding single ventricle heart defects	Congenital heart disease, borderline intellectual functioning, and moderate heart failure	(combined DW)	0.082 (0.053–0.12)
Congenital heart disease, borderline intellectual disability and moderate heart failure due to critical malformations of great vessels, congenital valvular heart disease and patent ductus arteriosus	Congenital heart disease, borderline intellectual functioning, and moderate heart failure	(combined DW)	0.082 (0.053–0.12)
Congenital heart disease, borderline intellectual disability and moderate heart failure due to ventricular septal defect and atrial septal defect	Congenital heart disease, borderline intellectual functioning, and moderate heart failure	(combined DW)	0.082 (0.053–0.12)
Congenital heart disease, boderline intellectual disability and mild heart failure due to single ventricle and single ventricle pathway heart defects	Congenital heart disease, borderline intellectual functioning, and mild heart failure	(combined DW)	0.052 (0.032–0.081)
Congenital heart disease, borderline intellectual disability and severe heart failure due to severe congenital heart anomalies excluding single ventricle heart defects	Congenital heart disease,borderline intellectual functioning, and severe heart failure	(combined DW)	0.188 (0.13–0.258)
Congenital heart disease, borderline intellectual disability and severe heart failure due to critical malformations of great vessels, congenital valvular heart disease and patent ductus arteriosus	Congenital heart disease,borderline intellectual functioning, and severe heart failure	(combined DW)	0.188 (0.13–0.258)
Congenital heart disease, borderline intellectual disability and severe heart failure due to ventricular septal defect and atrial septal defect	Congenital heart disease,borderline intellectual functioning, and severe heart failure	(combined DW)	0.188 (0.13–0.258)
Congenital heart disease, mild intellectual disability and mild heart failure due to single ventricle and single ventricle pathway heart defects	Congenital heart disease, mild intellectual disability, and mild heart failure	(combined DW)	0.082 (0.052–0.121)
Congenital heart disease, mild intellectual disability and mild heart failure due to severe congenital heart anomalies excluding single ventricle heart defects	Congenital heart disease, mild intellectual disability, and mild heart failure	(combined DW)	0.082 (0.052–0.121)
Congenital heart disease, borderline intellectual disability and moderate heart failure due to single ventricle and single ventricle pathway heart defects	Congenital heart disease, borderline intellectual functioning, and moderate heart failure	(combined DW)	0.082 (0.053–0.12)
Congenital heart disease, borderline intellectual disability and severe heart failure due to single ventricle and single ventricle pathway heart defects	Congenital heart disease,borderline intellectual functioning, and severe heart failure	(combined DW)	0.188 (0.13–0.258)
Congenital heart disease and severe heart failure without intellectual disability due to severe congenital heart anomalies excluding single ventricle heart defects	Congenital heart disease and severe heart failure	(combined DW)	0.179 (0.122–0.251)
Congenital heart disease and severe heart failure without intellectual disability due to critical malformations of great vessels, congenital valvular heart disease and patent ductus arteriosus	Congenital heart disease and severe heart failure	(combined DW)	0.179 (0.122–0.251)
Congenital heart disease, severe intellectual disability and moderate heart failure due to single ventricle and single ventricle pathway heart defects	Congenital heart disease, severe intellectual disability, and moderate heart failure	(combined DW)	0.22 (0.151–0.302)
Congenital heart disease, profound intellectual disability and severe heart failure due to ventricular septal defect and atrial septal defect	Congenital heart disease, profound intellectual disability, and severe heart failure	(combined DW)	0.342 (0.239–0.457)
Congenital heart disease, profound intellectual disability and severe heart failure due to critical malformations of great vessels, congenital valvular heart disease and patent ductus arteriosus	Congenital heart disease, profound intellectual disability, and severe heart failure	(combined DW)	0.342 (0.239–0.457)
Congenital heart disease, profound intellectual disability and severe heart failure due to severe congenital heart anomalies excluding single ventricle heart defects	Congenital heart disease, profound intellectual disability, and severe heart failure	(combined DW)	0.342 (0.239–0.457)

Appendix Table 5. GBD 2016 sequelae, health states, health state lay descriptions, and disability weights			
**Sequela**	**Health state name**	**Health state lay description**	**Disability weight**
Congenital heart disease, profound intellectual disability and severe heart failure due to single ventricle and single ventricle pathway heart defects	Congenital heart disease, profound intellectual disability, and severe heart failure	(combined DW)	0.342 (0.239–0.457)
Congenital heart disease, profound intellectual disability and moderate heart failure due to ventricular septal defect and atrial septal defect	Congenital heart disease, profound intellectual disability, and moderate heart failure	(combined DW)	0.257 (0.181–0.351)
Congenital heart disease, profound intellectual disability and moderate heart failure due to critical malformations of great vessels, congenital valvular heart disease and patent ductus arteriosus	Congenital heart disease, profound intellectual disability, and moderate heart failure	(combined DW)	0.257 (0.181–0.351)
Congenital heart disease, profound intellectual disability and moderate heart failure due to severe congenital heart anomalies excluding single ventricle heart defects	Congenital heart disease, profound intellectual disability, and moderate heart failure	(combined DW)	0.257 (0.181–0.351)
Congenital heart disease, profound intellectual disability and moderate heart failure due to single ventricle and single ventricle pathway heart defects	Congenital heart disease, profound intellectual disability, and moderate heart failure	(combined DW)	0.257 (0.181–0.351)
Congenital heart disease, profound intellectual disability and mild heart failure due to ventricular septal defect and atrial septal defect	Congenital heart disease, profound intellectual disability, and mild heart failure	(combined DW)	0.233 (0.162–0.317)
Congenital heart disease, profound intellectual disability and mild heart failure due to critical malformations of great vessels, congenital valvular heart disease and patent ductus arteriosus	Congenital heart disease, profound intellectual disability, and mild heart failure	(combined DW)	0.233 (0.162–0.317)
Congenital heart disease, profound intellectual disability and mild heart failure due to severe congenital heart anomalies excluding single ventricle heart defects	Congenital heart disease, profound intellectual disability, and mild heart failure	(combined DW)	0.233 (0.162–0.317)
Congenital heart disease, profound intellectual disability and mild heart failure due to single ventricle and single ventricle pathway heart defects	Congenital heart disease, profound intellectual disability, and mild heart failure	(combined DW)	0.233 (0.162–0.317)
Congenital heart disease, severe intellectual disability and severe heart failure due to ventricular septal defect and atrial septal defect	Congenital heart disease, severe intellectual disability, and severe heart failure	(combined DW)	0.31 (0.22–0.419)
Congenital heart disease, severe intellectual disability and severe heart failure due to critical malformations of great vessels, congenital valvular heart disease and patent ductus arteriosus	Congenital heart disease, severe intellectual disability, and severe heart failure	(combined DW)	0.31 (0.22–0.419)
Congenital heart disease, severe intellectual disability and severe heart failure due to severe congenital heart anomalies excluding single ventricle heart defects	Congenital heart disease, severe intellectual disability, and severe heart failure	(combined DW)	0.31 (0.22–0.419)
Congenital heart disease, severe intellectual disability and severe heart failure due to single ventricle and single ventricle pathway heart defects	Congenital heart disease, severe intellectual disability, and severe heart failure	(combined DW)	0.31 (0.22–0.419)
Congenital heart disease, severe intellectual disability and moderate heart failure due to ventricular septal defect and atrial septal defect	Congenital heart disease, severe intellectual disability, and moderate heart failure	(combined DW)	0.22 (0.151–0.302)
Congenital heart disease, severe intellectual disability and moderate heart failure due to critical malformations of great vessels, congenital valvular heart disease and patent ductus arteriosus	Congenital heart disease, severe intellectual disability, and moderate heart failure	(combined DW)	0.22 (0.151–0.302)
Congenital heart disease and mild heart failure without intellectual disability due to single ventricle and single ventricle pathway heart defects	Congenital heart disease and mild heart failure	(combined DW)	0.041 (0.026–0.062)
Congenital heart disease and mild heart failure without intellectual disability due to severe congenital heart anomalies excluding single ventricle heart defects	Congenital heart disease and mild heart failure	(combined DW)	0.041 (0.026–0.062)
Congenital heart disease and mild heart failure without intellectual disability due to critical malformations of great vessels, congenital valvular heart disease and patent ductus arteriosus	Congenital heart disease and mild heart failure	(combined DW)	0.041 (0.026–0.062)
Congenital heart disease and mild heart failure without intellectual disability due to ventricular septal defect and atrial septal defect	Congenital heart disease and mild heart failure	(combined DW)	0.041 (0.026–0.062)
Congenital heart disease and moderate heart failure without intellectual disability due to single ventricle and single ventricle pathway heart defects	Congenital heart disease and moderate heart failure	(combined DW)	0.072 (0.047–0.103)
Congenital heart disease and moderate heart failure without intellectual disability due to severe congenital heart anomalies excluding single ventricle heart defects	Congenital heart disease and moderate heart failure	(combined DW)	0.072 (0.047–0.103)
Congenital heart disease and moderate heart failure without intellectual disability due to critical malformations of great vessels, congenital valvular heart disease and patent ductus arteriosus	Congenital heart disease and moderate heart failure	(combined DW)	0.072 (0.047–0.103)
Congenital heart disease and moderate heart failure without intellectual disability due to ventricular septal defect and atrial septal defect	Congenital heart disease and moderate heart failure	(combined DW)	0.072 (0.047–0.103)
Congenital heart disease and severe heart failure without intellectual disability due to single ventricle and single ventricle pathway heart defects	Congenital heart disease and severe heart failure	(combined DW)	0.179 (0.122–0.251)
Congenital heart disease, mild intellectual disability and mild heart failure due to critical malformations of great vessels, congenital valvular heart disease and patent ductus arteriosus	Congenital heart disease, mild intellectual disability, and mild heart failure	(combined DW)	0.082 (0.052–0.121)
[truncated: 367,836 more chars]
