# Supplementary material for: Global, regional, and national incidence, prevalence, and years lived with disability for 328 diseases and injuries for 195 countries, 1990–2016: a systematic analysis for the Global Burden of Disease Study 2016
Source: Lancet. 2017 Sep 16;390(10100):1211–59. doi: 10.1016/S0140-6736(17)32154-2 (PMC5605509; doi:10.1016/S0140-6736(17)32154-2)
Supplement: Supplementary appendix 2 [file mmc2.pdf]

THE LANCET

Supplementary appendix 2

This appendix formed part of the original submission and has been peer reviewed. We post it as supplied by the authors.

Supplement to: GBD 2016 Disease and Injury Incidence and Prevalence Collaborators. Global, regional, and national incidence, prevalence, and years lived with disability for 328 diseases and injuries for 195 countries, 1990–2016: a systematic analysis for the Global Burden of Disease Study 2016. *Lancet* 2017; **390:** 1211–59.

Supplementary Results to “Global, regional, and national incidence, prevalence, and years lived with disability for 328 diseases and injuries for 195 countries, 1990–2016: a systematic analysis for the Global Burden of Disease Study 2016”

This component of the appendix provides supplementary results for incidence, prevalence, and years of life lived with disability (YLDs).

Supplementary Results: Tables & Figures

SR Figure 1: All-cause years lived with disability (YLD) rate per capita by location and sex, 1990, 1995, 2000, 2005, 2010 and 2016, pg. 1

SR Figure 2: Co-evolution of age-standardised YLD rates per 100,000 for top-ten level 4 causes and the socio-demographic index (SDI), by GBD region, pg. 4

Figure 3: Leading 30 Level 4 causes of YLDs, by SDI quintile for 1990, 2006, and 2016, with mean percentage change in number of YLDs, and all-age and age-standardised YLD rates, pg. 14

SR Table 1: Global prevalence and incidence for 2016, percentage change in prevalence and incidence counts and age-standardized prevalence and incidence rates between 2006 and 2016 for all causes and nine impairments, pg. 19

SR Table 2: Global sequela-level prevalence, incidence and YLDs, all ages, 2006, 2016, pg. 25

SR Table 3: Global prevalence and years lived with disability (YLDs) for 2016, percentage change of YLD counts and percentage change of age-standardised YLD rates between 2006 and 2016 of anemia by cause, all ages and both sexes combined, pg. 64

SR Table 4: Global prevalence and years lived with disability (YLDs) for 2016, percentage change of YLD counts and percentage change of age-standardised YLD rates between 2006 and 2016 of developmental intellectual disability by cause, all ages and both sexes combined, pg. 65

SR Table 5: Global prevalence and years lived with disability (YLDs) for 2016, percentage change of YLD counts and percentage change of age-standardised YLD rates between 2006 and 2016 of epilepsy by cause, all ages and both sexes combined, pg. 66

SR Table 6: Global prevalence and years lived with disability (YLDs) for 2016, percent change in counts, and percent change in age-standardised rates between 2005 and 2016 of Guillain-Barré syndrome by cause, all ages and both sexes, combined, pg. 67

SR Table 7: Global prevalence and years lived with disability (YLDs) for 2016, percentage change of YLD counts and percentage change of age-standardised YLD rates between 2006 and 2016 of hearing loss by cause, all ages and both sexes combined, pg. 68

SR Table 8: Global prevalence and years lived with disability (YLDs) for 2016, percentage change of YLD counts and percentage change of age-standardised YLD rates between 2006 and 2016 of heart failure by cause, all ages and both sexes combined, pg. 69

SR Table 9: Global prevalence and years lived with disability (YLDs) for 2016, percentage change of YLD counts and percentage change of age-standardised YLD rates between 2006 and 2016 of infertility by cause, all ages and both sexes combined, pg. 70

SR Table 10: Global prevalence and years lived with disability (YLDs) for 2016, percentage change of YLD counts and percentage change of age-standardised YLD rates between 2006 and 2016 of pelvic inflammatory disease by cause, all ages and both sexes combined, pg. 71

SR Table 11: Global prevalence and years lived with disability (YLDs) for 2016, percentage change of YLD counts and percentage change of age-standardised YLD rates between 2006 and 2016 of vision loss by cause, all ages and both sexes combined, pg. 72

SR Table 12: Global prevalence, incidence, and YLDs for 2016, percentage change of YLD counts and percentage change of YLD rates between 2006 and 2016 by cause, for each age group, separately, pg. 73

SR Table 13a-i: Global prevalence and YLDs for 2016, percentage change of YLD counts and percentage change of YLD rates between 2006 and 2016 by impairment and for each age group, separately, pg. 234

SR Table 14: Prevalence, incidence and YLDs for 2016, percentage change of YLD counts and percentage change of age-standardised YLD rates between 2006 and 2016 by cause and impairments, for all ages, for each location, separately, pg. 435

SR Table 15: Comparison of GBD 2016 and GLOBOCAN estimated cancer incidence by cause and sex, 2012, pg. 1800

SR Figure 1: Age-standardised all-cause years lived with disability (YLD) rate per capita by location and sex, 1990, 1995, 2000, 2005, 2010, and 2016	Males						Females					
	1990	1995	2000	2005	2010	2016	1990	1995	2000	2005	2010	2016
**Global**	10.8%	10.8%	10.8%	10.7%	10.6%	10.5%	12.0%	11.9%	11.8%	11.7%	11.7%	11.6%
High SDI	10.4%	10.4%	10.3%	10.3%	10.3%	10.3%	11.6%	11.5%	11.5%	11.5%	11.5%	11.5%
High-middle SDI	10.7%	10.5%	10.4%	10.3%	10.2%	10.1%	11.6%	11.4%	11.3%	11.2%	11.2%	11.1%
Middle SDI	9.9%	9.8%	9.8%	9.7%	9.7%	9.7%	11.1%	11.0%	10.9%	10.9%	10.9%	10.8%
Low-middle SDI	12.0%	12.0%	12.0%	11.9%	11.6%	11.5%	13.4%	13.4%	13.2%	13.1%	12.9%	12.8%
Low SDI	12.9%	13.0%	12.9%	12.6%	12.3%	11.9%	13.7%	13.7%	13.6%	13.3%	13.0%	12.7%
**High-income**	10.3%	10.3%	10.3%	10.2%	10.2%	10.2%	11.6%	11.6%	11.5%	11.5%	11.5%	11.5%
**High-income North America**	10.9%	11.0%	11.0%	10.9%	10.9%	10.9%	12.2%	12.2%	12.2%	12.2%	12.3%	12.3%
Canada	10.0%	10.0%	10.0%	9.8%	9.9%	10.0%	11.3%	11.3%	11.3%	11.2%	11.3%	11.2%
Greenland	11.2%	11.1%	11.0%	10.9%	10.9%	10.8%	12.7%	12.7%	12.7%	12.7%	12.6%	12.4%
USA	11.0%	11.1%	11.1%	11.0%	11.1%	11.0%	12.3%	12.3%	12.3%	12.4%	12.4%	12.4%
**Australasia**	10.5%	10.7%	10.4%	10.3%	10.3%	10.3%	11.7%	11.7%	11.6%	11.6%	11.6%	11.5%
Australia	10.6%	10.7%	10.4%	10.3%	10.4%	10.3%	11.7%	11.7%	11.6%	11.6%	11.7%	11.5%
New Zealand	10.4%	10.3%	10.3%	10.2%	10.2%	10.2%	11.7%	11.6%	11.7%	11.6%	11.5%	11.5%
**High-income Asia Pacific**	9.6%	9.6%	9.5%	9.5%	9.5%	9.5%	10.6%	10.6%	10.5%	10.5%	10.6%	10.6%
Brunei	10.3%	10.2%	10.0%	10.0%	9.9%	9.9%	11.2%	11.0%	10.9%	10.8%	10.7%	10.6%
Japan	9.3%	9.4%	9.4%	9.4%	9.4%	9.5%	10.5%	10.4%	10.4%	10.4%	10.5%	10.6%
Singapore	9.4%	9.2%	9.1%	8.9%	8.9%	8.9%	10.5%	10.3%	10.2%	9.9%	9.9%	9.9%
South Korea	10.3%	10.1%	9.9%	9.9%	9.9%	9.7%	11.1%	10.9%	10.8%	10.8%	10.8%	10.7%
**Western Europe**	10.2%	10.2%	10.0%	10.0%	9.9%	9.9%	11.6%	11.6%	11.4%	11.4%	11.4%	11.4%
Andorra	10.5%	10.4%	10.3%	10.3%	10.3%	10.3%	12.0%	11.9%	11.9%	11.8%	11.9%	11.8%
Austria	10.1%	10.2%	10.1%	10.0%	10.0%	9.9%	11.4%	11.4%	11.2%	11.1%	11.2%	11.2%
Belgium	10.2%	10.2%	10.3%	10.3%	10.2%	10.1%	11.5%	11.6%	11.6%	11.7%	11.6%	11.5%
Cyprus	9.9%	9.9%	9.9%	9.8%	9.8%	9.7%	11.6%	11.5%	11.4%	11.3%	11.3%	11.2%
Denmark	10.3%	10.5%	10.3%	10.4%	10.5%	10.4%	11.8%	11.9%	11.7%	11.8%	11.8%	11.9%
Finland	11.0%	10.9%	10.9%	10.9%	10.7%	10.5%	12.1%	12.1%	11.9%	11.9%	11.7%	11.7%
France	9.8%	9.7%	9.6%	9.5%	9.7%	9.5%	11.5%	11.3%	11.2%	11.1%	11.2%	11.2%
Germany	10.4%	10.4%	10.3%	10.2%	10.2%	10.1%	11.8%	11.8%	11.6%	11.6%	11.5%	11.5%
Greece	9.8%	9.7%	9.6%	9.6%	9.7%	9.7%	11.4%	11.3%	11.3%	11.3%	11.3%	11.2%
Iceland	10.1%	10.0%	9.9%	9.9%	9.9%	9.8%	11.7%	11.7%	11.6%	11.5%	11.5%	11.4%
Ireland	10.3%	10.3%	10.2%	10.2%	10.2%	10.2%	11.7%	11.7%	11.6%	11.6%	11.6%	11.5%
Israel	9.9%	9.9%	9.9%	9.8%	9.8%	9.8%	11.3%	11.3%	11.2%	11.2%	11.2%	11.1%
Italy	10.0%	10.0%	9.8%	9.7%	9.6%	9.6%	11.5%	11.3%	11.1%	11.1%	11.1%	11.1%
Luxembourg	10.8%	10.8%	10.7%	10.6%	10.4%	10.2%	12.1%	12.1%	12.0%	11.9%	11.8%	11.7%
Malta	10.0%	9.9%	9.9%	9.9%	9.9%	9.7%	11.5%	11.4%	11.3%	11.3%	11.3%	11.3%
Netherlands	10.2%	10.3%	10.3%	10.2%	10.1%	10.0%	11.9%	12.0%	12.0%	11.9%	11.8%	11.7%
Norway	10.2%	10.2%	10.2%	10.1%	10.0%	9.9%	11.2%	11.3%	11.1%	11.0%	11.0%	10.9%
Portugal	10.2%	10.2%	10.0%	9.9%	9.9%	9.8%	11.7%	11.6%	11.5%	11.4%	11.5%	11.4%
Spain	9.6%	9.6%	9.4%	9.4%	9.3%	9.2%	11.1%	11.0%	10.8%	10.7%	10.8%	10.8%
Sweden	10.2%	10.3%	10.1%	10.1%	10.2%	10.2%	11.9%	12.0%	11.9%	11.9%	11.8%	11.8%
Switzerland	10.5%	10.6%	10.7%	10.5%	10.4%	10.0%	12.0%	12.2%	12.4%	12.3%	12.2%	11.7%
UK	10.6%	10.6%	10.5%	10.4%	10.4%	10.3%	12.0%	11.9%	11.8%	11.8%	11.7%	11.7%
England	10.6%	10.6%	10.5%	10.5%	10.4%	10.3%	12.0%	12.0%	11.8%	11.8%	11.8%	11.8%
Northern Ireland	10.2%	10.2%	10.2%	10.2%	10.2%	10.1%	11.7%	11.7%	11.7%	11.6%	11.6%	11.5%
Scotland	10.6%	10.6%	10.5%	10.4%	10.5%	10.4%	11.8%	11.8%	11.6%	11.6%	11.6%	11.7%
Wales	10.3%	10.3%	10.2%	10.2%	10.2%	10.2%	11.7%	11.6%	11.5%	11.4%	11.4%	11.5%
**Southern Latin America**	10.2%	10.2%	10.2%	10.1%	10.1%	10.1%	11.3%	11.2%	11.2%	11.2%	11.2%	11.1%
Argentina	10.2%	10.2%	10.2%	10.1%	10.1%	10.0%	11.2%	11.1%	11.1%	11.1%	11.1%	11.1%
Chile	10.4%	10.3%	10.4%	10.3%	10.3%	10.2%	11.7%	11.5%	11.7%	11.5%	11.5%	11.3%
Uruguay	9.7%	9.7%	9.6%	9.6%	9.7%	9.7%	10.8%	10.8%	10.7%	10.7%	10.8%	10.8%
**Central Europe, Eastern Europe, and Central Asia**	11.4%	11.4%	11.3%	11.2%	11.1%	11.0%	11.6%	11.5%	11.4%	11.3%	11.3%	11.2%
**Eastern Europe**	11.6%	11.6%	11.7%	11.4%	11.3%	11.1%	11.7%	11.7%	11.6%	11.5%	11.4%	11.4%
Belarus	11.1%	11.1%	11.1%	11.1%	11.2%	11.0%	11.6%	11.5%	11.5%	11.4%	11.5%	11.4%
Estonia	11.6%	11.5%	11.3%	11.1%	11.1%	10.9%	12.1%	11.9%	11.8%	11.6%	11.5%	11.4%
Latvia	11.8%	11.5%	11.4%	11.1%	11.2%	10.9%	12.0%	11.9%	11.7%	11.5%	11.5%	11.4%
Lithuania	11.5%	11.3%	11.2%	11.1%	11.2%	11.0%	11.8%	11.7%	11.6%	11.5%	11.5%	11.3%
Moldova	11.3%	11.1%	10.8%	10.7%	10.7%	10.6%	12.1%	11.9%	11.7%	11.5%	11.5%	11.3%
Russia	11.7%	11.8%	11.9%	11.6%	11.5%	11.2%	11.7%	11.7%	11.7%	11.5%	11.4%	11.4%
Ukraine	11.3%	11.2%	11.2%	11.1%	11.0%	10.9%	11.7%	11.6%	11.5%	11.4%	11.4%	11.3%
**Central Europe**	11.3%	11.1%	11.0%	10.9%	11.0%	10.9%	11.3%	11.2%	11.0%	10.9%	10.9%	10.8%
Albania	10.5%	10.4%	10.4%	10.3%	10.2%	10.2%	10.8%	10.7%	10.7%	10.5%	10.5%	10.5%
Bosnia and Herzegovina	10.9%	11.0%	10.9%	10.9%	11.0%	11.0%	11.2%	11.1%	10.8%	10.8%	10.9%	10.9%
Bulgaria	11.2%	11.0%	10.8%	10.7%	10.7%	10.7%	11.0%	10.9%	10.8%	10.7%	10.7%	10.7%
Croatia	11.0%	10.8%	10.7%	10.7%	10.7%	10.6%	10.9%	10.7%	10.6%	10.6%	10.6%	10.5%
Czech Republic	11.4%	11.2%	11.1%	11.2%	11.3%	11.2%	11.6%	11.3%	11.1%	11.2%	11.3%	11.2%
Hungary	11.6%	11.4%	11.2%	11.0%	11.0%	10.9%	11.7%	11.5%	11.2%	11.1%	11.1%	11.1%
Macedonia	10.6%	10.5%	10.4%	10.4%	10.5%	10.5%	10.9%	10.8%	10.7%	10.7%	10.7%	10.7%
Montenegro	10.8%	10.7%	10.7%	10.6%	10.6%	10.6%	10.8%	10.7%	10.7%	10.7%	10.7%	10.7%
Poland	11.3%	11.1%	11.0%	11.0%	11.0%	10.9%	11.2%	11.0%	10.8%	10.8%	10.8%	10.7%
Romania	11.5%	11.3%	11.1%	11.0%	10.9%	10.9%	11.4%	11.4%	11.2%	11.1%	11.1%	11.0%
Serbia	10.9%	10.8%	10.8%	10.8%	10.9%	10.8%	11.1%	11.1%	11.1%	11.0%	11.0%	10.9%
Slovakia	11.4%	11.2%	11.2%	11.1%	11.1%	11.0%	11.1%	11.0%	10.9%	10.8%	10.9%	10.8%
Slovenia	11.5%	11.3%	11.3%	11.4%	11.4%	11.1%	11.5%	11.3%	11.2%	11.3%	11.2%	11.2%
**Central Asia**	10.8%	10.7%	10.6%	10.5%	10.5%	10.5%	11.4%	11.3%	11.2%	11.2%	11.2%	11.1%
Armenia	10.7%	10.5%	10.4%	10.3%	10.3%	10.2%	10.9%	10.7%	10.6%	10.7%	10.6%	10.5%
Azerbaijan	10.3%	10.3%	10.1%	10.1%	10.1%	10.1%	10.9%	10.8%	10.7%	10.7%	10.7%	10.6%
Georgia	10.3%	10.1%	9.9%	9.9%	10.1%	10.2%	10.5%	10.3%	10.2%	10.3%	10.4%	10.4%
Kazakhstan	11.2%	11.3%	11.2%	11.2%	11.2%	11.1%	11.5%	11.5%	11.4%	11.4%	11.4%	11.3%

Kyrgyzstan	11.2%	11.1%	11.0%	10.7%	10.6%	10.6%	11.6%	11.5%	11.4%	11.3%	11.3%	11.2%
Mongolia	10.7%	10.7%	10.8%	10.9%	11.1%	11.1%	11.3%	11.2%	11.1%	11.1%	11.2%	11.1%
Tajikistan	10.6%	10.6%	10.5%	10.4%	10.3%	10.2%	11.2%	11.1%	11.1%	11.0%	10.9%	10.7%
Turkmenistan	10.3%	10.2%	10.2%	10.1%	10.1%	9.9%	11.1%	10.9%	10.7%	10.6%	10.6%	10.5%
Uzbekistan	10.7%	10.6%	10.5%	10.4%	10.4%	10.3%	11.9%	11.8%	11.6%	11.5%	11.5%	11.3%
**Latin America and Caribbean**	10.3%	10.2%	10.2%	10.1%	10.0%	10.0%	11.2%	11.1%	11.1%	11.0%	10.9%	10.8%
**Central Latin America**	9.8%	9.7%	9.6%	9.5%	9.5%	9.5%	10.5%	10.4%	10.3%	10.2%	10.2%	10.2%
Colombia	9.5%	9.4%	9.4%	9.3%	9.2%	9.1%	10.3%	10.2%	10.1%	10.1%	10.0%	9.8%
Costa Rica	9.2%	9.2%	9.2%	9.1%	9.1%	9.1%	10.1%	10.1%	10.0%	10.0%	10.0%	9.9%
El Salvador	10.1%	9.9%	9.8%	9.8%	9.7%	9.6%	10.5%	10.3%	10.2%	10.2%	10.2%	10.1%
Guatemala	10.8%	10.7%	10.7%	10.5%	10.3%	10.1%	11.4%	11.2%	11.2%	11.1%	10.9%	10.7%
Honduras	9.8%	9.8%	9.7%	9.6%	9.6%	9.6%	10.9%	10.8%	10.8%	10.7%	10.7%	10.6%
Mexico	10.0%	9.8%	9.7%	9.6%	9.6%	9.7%	10.6%	10.5%	10.3%	10.2%	10.3%	10.3%
Nicaragua	9.6%	9.4%	9.4%	9.3%	9.3%	9.2%	10.3%	10.2%	10.1%	10.0%	10.1%	10.0%
Panama	9.3%	9.2%	9.2%	9.2%	9.2%	9.3%	10.2%	10.0%	10.0%	9.9%	10.0%	10.0%
Venezuela	9.5%	9.4%	9.4%	9.4%	9.4%	9.5%	10.3%	10.2%	10.1%	10.0%	10.0%	10.0%
**Andean Latin America**	10.4%	10.2%	10.1%	10.0%	9.9%	9.8%	11.2%	11.0%	10.8%	10.7%	10.6%	10.6%
Bolivia	11.3%	11.1%	10.8%	10.7%	10.6%	10.5%	12.1%	11.9%	11.6%	11.5%	11.4%	11.3%
Ecuador	10.1%	10.1%	10.0%	9.9%	9.8%	9.7%	10.8%	10.7%	10.5%	10.4%	10.4%	10.4%
Peru	10.2%	10.0%	9.9%	9.8%	9.7%	9.7%	11.0%	10.8%	10.7%	10.6%	10.5%	10.5%
**Caribbean**	10.4%	10.5%	10.5%	10.5%	10.6%	10.3%	11.6%	11.5%	11.4%	11.4%	11.5%	11.3%
Antigua and Barbuda	10.5%	10.5%	10.5%	10.5%	10.4%	10.4%	11.0%	11.1%	11.0%	11.0%	11.0%	11.0%
The Bahamas	10.3%	10.3%	10.2%	10.1%	10.0%	10.1%	11.2%	11.1%	11.1%	11.1%	11.0%	11.1%
Barbados	10.1%	10.2%	10.2%	10.1%	10.1%	10.1%	11.1%	11.1%	11.1%	11.1%	11.2%	11.2%
Belize	10.2%	10.3%	10.5%	10.5%	10.5%	10.4%	11.4%	11.3%	11.4%	11.4%	11.5%	11.4%
Bermuda	10.4%	10.3%	10.1%	10.0%	9.9%	9.9%	11.1%	11.0%	10.9%	10.8%	10.7%	10.6%
Cuba	9.9%	9.8%	9.7%	9.7%	9.7%	9.8%	11.3%	11.1%	10.9%	10.7%	10.7%	10.7%
Dominica	10.3%	10.3%	10.3%	10.3%	10.3%	10.4%	10.9%	10.9%	10.8%	10.8%	10.9%	10.9%
Dominican Republic	9.8%	9.8%	10.0%	10.0%	9.9%	9.8%	10.8%	10.9%	10.8%	10.8%	10.9%	10.7%
Grenada	11.0%	10.6%	10.6%	10.6%	10.7%	10.7%	12.0%	11.6%	11.5%	11.5%	11.5%	11.5%
Guyana	11.3%	11.4%	11.5%	11.5%	11.4%	11.2%	12.1%	12.2%	12.3%	12.3%	12.3%	12.1%
Haiti	12.1%	12.1%	12.1%	11.9%	12.5%	11.4%	13.1%	13.1%	13.1%	13.0%	13.2%	12.5%
Jamaica	10.2%	10.3%	10.3%	10.3%	10.3%	10.4%	11.5%	11.5%	11.5%	11.5%	11.5%	11.5%
Puerto Rico	10.3%	10.6%	10.7%	10.7%	10.5%	10.4%	10.8%	10.8%	10.9%	10.9%	10.9%	10.8%
Saint Lucia	11.3%	10.7%	10.6%	10.5%	10.4%	10.4%	12.2%	11.6%	11.3%	11.2%	11.3%	11.2%
Saint Vincent and the Grenadines	10.6%	10.6%	10.7%	10.7%	10.8%	10.8%	11.5%	11.5%	11.6%	11.5%	11.5%	11.4%
Suriname	10.5%	10.6%	10.6%	10.7%	10.8%	10.8%	11.6%	11.7%	11.7%	11.8%	11.8%	11.7%
Trinidad and Tobago	10.7%	10.8%	10.8%	11.0%	11.0%	11.0%	11.7%	11.7%	11.7%	11.6%	11.6%	11.5%
Virgin Islands	10.1%	10.1%	10.1%	10.1%	10.2%	10.2%	10.7%	10.7%	10.7%	10.7%	10.8%	10.8%
**Tropical Latin America**	10.7%	10.6%	10.7%	10.7%	10.6%	10.5%	12.0%	11.9%	12.0%	11.9%	11.6%	11.5%
Brazil	10.7%	10.6%	10.7%	10.7%	10.6%	10.5%	12.0%	11.9%	12.0%	11.9%	11.6%	11.5%
Paraguay	10.1%	10.1%	10.3%	10.4%	10.3%	10.4%	11.6%	11.6%	11.5%	11.6%	11.7%	11.7%
**Southeast Asia, East Asia, and Oceania**	9.4%	9.2%	9.2%	9.1%	9.1%	9.1%	10.7%	10.5%	10.4%	10.3%	10.3%	10.2%
**East Asia**	8.9%	8.8%	8.7%	8.6%	8.6%	8.6%	10.4%	10.2%	10.0%	9.9%	10.0%	9.9%
China	8.9%	8.8%	8.7%	8.6%	8.6%	8.6%	10.4%	10.2%	10.0%	9.9%	9.9%	9.8%
North Korea	8.7%	9.0%	8.9%	8.9%	8.9%	9.0%	10.5%	11.2%	11.5%	11.1%	10.9%	10.5%
Taiwan (Province of China)	9.3%	9.0%	8.9%	8.8%	8.8%	8.9%	10.8%	10.5%	10.4%	10.3%	10.3%	10.2%
**Southeast Asia**	10.6%	10.5%	10.5%	10.3%	10.2%	10.1%	11.3%	11.2%	11.1%	11.0%	10.9%	10.8%
Cambodia	12.2%	12.1%	12.2%	11.9%	11.4%	11.0%	13.1%	13.0%	13.0%	12.5%	12.0%	11.6%
Indonesia	10.3%	10.2%	10.2%	10.2%	10.2%	10.1%	11.2%	11.1%	11.1%	10.9%	11.0%	11.0%
Laos	11.3%	11.2%	11.1%	10.9%	10.7%	10.5%	12.3%	12.1%	11.9%	11.7%	11.4%	11.2%
Malaysia	10.0%	10.0%	9.9%	9.9%	10.0%	10.0%	11.0%	10.8%	10.6%	10.6%	10.7%	10.6%
Maldives	11.3%	11.1%	10.7%	10.3%	10.2%	10.0%	12.6%	12.3%	11.9%	11.5%	11.2%	11.0%
Mauritius	10.9%	10.7%	10.8%	10.8%	10.8%	10.8%	11.8%	11.6%	11.4%	11.5%	11.5%	11.5%
Myanmar	11.7%	11.5%	11.3%	11.1%	11.0%	10.6%	12.3%	11.9%	11.7%	11.5%	11.3%	10.9%
Philippines	11.3%	11.2%	11.3%	11.1%	10.8%	10.6%	11.3%	11.2%	11.4%	11.4%	11.1%	10.9%
Sri Lanka	10.5%	10.5%	10.4%	10.3%	10.3%	10.1%	11.0%	10.9%	10.8%	10.7%	10.7%	10.5%
Seychelles	10.1%	10.1%	10.1%	10.0%	10.0%	10.0%	10.5%	10.4%	10.4%	10.5%	10.4%	10.4%
Thailand	10.3%	10.3%	10.3%	10.1%	9.9%	9.9%	11.1%	11.0%	10.9%	10.7%	10.6%	10.5%
Timor-Leste	12.2%	12.0%	12.1%	11.8%	11.6%	11.2%	12.2%	11.8%	11.6%	11.5%	11.3%	10.9%
Vietnam	10.1%	10.0%	9.9%	9.8%	9.6%	9.4%	11.1%	10.9%	10.8%	10.6%	10.3%	10.1%
**Oceania**	11.6%	11.6%	11.6%	11.6%	11.7%	11.6%	12.9%	12.8%	12.9%	12.9%	12.9%	12.9%
American Samoa	11.5%	11.5%	11.5%	11.4%	11.4%	11.4%	12.0%	11.9%	12.1%	12.1%	12.0%	12.1%
Federated States of Micronesia	11.1%	11.1%	11.1%	11.1%	10.9%	11.2%	12.2%	12.1%	12.1%	12.1%	12.0%	12.1%
Fiji	11.6%	11.7%	11.8%	11.5%	11.5%	11.2%	12.3%	12.4%	12.5%	12.5%	12.4%	12.3%
Guam	9.8%	9.8%	9.8%	10.0%	10.2%	10.3%	11.2%	11.1%	11.1%	11.2%	11.2%	11.5%
Kiribati	11.3%	11.5%	11.7%	11.8%	11.8%	11.7%	12.8%	12.7%	12.7%	12.7%	12.6%	12.6%
Marshall Islands	11.5%	11.6%	11.7%	11.9%	11.9%	12.0%	12.6%	12.7%	13.0%	13.1%	13.1%	13.0%
Northern Mariana Islands	10.1%	10.0%	10.0%	10.1%	10.2%	10.3%	11.3%	11.1%	11.1%	11.1%	11.2%	11.4%
Papua New Guinea	11.8%	11.8%	11.8%	11.8%	11.8%	11.8%	13.1%	13.1%	13.1%	13.2%	13.1%	13.1%
Samoa	10.9%	10.9%	10.7%	10.6%	10.6%	10.5%	11.5%	11.5%	11.5%	11.6%	11.6%	11.6%
Solomon Islands	11.1%	11.2%	11.1%	11.3%	11.2%	11.3%	12.7%	12.7%	12.7%	12.9%	12.8%	12.7%
Tonga	10.8%	10.7%	10.8%	10.8%	10.9%	11.0%	12.4%	12.2%	12.3%	12.3%	12.3%	12.3%
Vanuatu	11.2%	11.1%	11.1%	11.0%	11.2%	11.0%	12.9%	12.7%	12.7%	12.8%	12.9%	12.9%
**North Africa and Middle East**	12.4%	12.2%	12.0%	11.9%	11.8%	11.8%	14.0%	13.8%	13.7%	13.6%	13.5%	13.4%
**North Africa and Middle East**	12.4%	12.2%	12.0%	11.9%	11.8%	11.8%	14.0%	13.8%	13.7%	13.6%	13.5%	13.4%
Afghanistan	14.9%	14.7%	14.5%	14.1%	13.9%	13.6%	16.5%	16.3%	16.2%	15.8%	15.6%	15.4%
Algeria	11.9%	11.7%	11.5%	11.3%	11.3%	11.3%	13.7%	13.4%	13.2%	13.1%	13.1%	13.0%
Bahrain	12.2%	12.2%	12.2%	12.0%	11.8%	11.7%	14.4%	14.5%	14.4%	14.2%	14.0%	13.9%
Egypt	12.2%	12.0%	11.8%	11.8%	11.7%	11.5%	13.8%	13.6%	13.4%	13.5%	13.4%	13.1%
Iran	12.3%	12.2%	12.0%	11.8%	11.8%	11.8%	13.6%	13.4%	13.4%	13.3%	13.3%	13.2%

Iraq	13.8%	13.7%	13.4%	13.3%	13.3%	13.2%	14.9%	14.8%	14.5%	14.3%	14.4%	14.3%
Jordan	12.2%	11.9%	11.7%	11.6%	11.6%	11.5%	14.0%	13.7%	13.5%	13.4%	13.3%	13.1%
Kuwait	11.2%	11.2%	11.2%	11.2%	11.2%	11.0%	13.0%	13.0%	13.0%	12.9%	12.9%	12.7%
Lebanon	12.6%	12.2%	11.7%	11.5%	11.4%	11.3%	13.6%	13.3%	13.1%	13.0%	12.9%	12.8%
Libya	11.6%	11.5%	11.4%	11.4%	11.5%	11.6%	13.3%	13.2%	13.1%	13.1%	13.1%	13.1%
Morocco	12.3%	12.1%	12.0%	11.9%	11.8%	11.7%	14.2%	13.8%	13.7%	13.6%	13.7%	13.4%
Palestine	11.9%	11.8%	11.8%	11.8%	11.9%	11.9%	13.3%	13.1%	13.0%	13.0%	13.2%	13.2%
Oman	12.1%	11.7%	11.5%	11.3%	11.4%	11.3%	13.6%	13.3%	12.9%	12.7%	12.6%	12.6%
Qatar	11.9%	11.8%	11.8%	11.6%	11.5%	11.3%	13.4%	13.4%	13.3%	13.2%	13.1%	12.9%
Saudi Arabia	11.6%	11.4%	11.2%	11.0%	11.0%	10.9%	14.1%	13.8%	13.7%	13.4%	13.2%	13.0%
Sudan	13.4%	13.2%	13.0%	12.8%	12.7%	12.5%	15.0%	14.8%	14.6%	14.3%	14.2%	14.0%
Syria	11.7%	11.5%	11.3%	11.1%	11.1%	11.4%	13.5%	13.3%	13.0%	12.9%	12.8%	12.8%
Tunisia	11.4%	11.2%	11.2%	11.2%	11.1%	11.0%	12.8%	12.7%	12.4%	12.3%	12.4%	12.3%
Turkey	12.0%	11.6%	11.4%	11.2%	11.1%	10.9%	13.6%	13.2%	13.0%	12.8%	12.7%	12.7%
United Arab Emirates	12.1%	11.9%	11.7%	11.6%	11.5%	11.5%	13.7%	13.3%	13.2%	13.0%	13.0%	12.8%
Yemen	14.9%	14.7%	14.4%	14.1%	14.0%	13.7%	17.1%	16.9%	16.7%	16.3%	16.2%	15.8%
**South Asia**	12.3%	12.3%	12.3%	12.2%	11.9%	11.8%	14.0%	13.9%	13.6%	13.5%	13.3%	13.3%
**South Asia**	12.3%	12.3%	12.3%	12.2%	11.9%	11.8%	14.0%	13.9%	13.6%	13.5%	13.3%	13.3%
Bangladesh	12.4%	12.1%	11.8%	11.7%	11.3%	11.0%	13.9%	13.7%	13.5%	13.2%	12.7%	12.4%
Bhutan	12.4%	12.1%	12.0%	11.8%	11.7%	11.6%	14.3%	14.0%	13.8%	13.5%	13.4%	13.2%
India	12.4%	12.4%	12.5%	12.3%	12.0%	12.0%	14.1%	14.0%	13.8%	13.7%	13.5%	13.5%
Nepal	12.4%	12.1%	12.0%	11.8%	11.5%	11.3%	13.2%	13.0%	12.9%	12.8%	12.5%	12.3%
Pakistan	11.5%	11.3%	11.2%	11.4%	11.4%	11.3%	13.1%	13.0%	12.9%	13.0%	12.9%	12.7%
**Sub-Saharan Africa**	12.5%	12.6%	12.7%	12.5%	12.1%	11.7%	13.0%	13.1%	13.1%	13.0%	12.7%	12.2%
**Southern Sub-Saharan Africa**	11.8%	12.2%	13.0%	13.2%	13.0%	12.5%	12.0%	12.3%	13.2%	13.7%	13.5%	12.9%
Botswana	11.9%	13.2%	14.4%	14.3%	13.5%	12.6%	11.9%	12.9%	14.2%	14.5%	13.5%	12.8%
Lesotho	12.0%	12.5%	13.5%	14.3%	14.0%	13.6%	12.6%	12.9%	13.9%	14.8%	14.5%	14.1%
Namibia	11.8%	12.2%	12.8%	13.0%	12.4%	12.0%	11.9%	12.2%	12.7%	13.1%	12.6%	12.0%
South Africa	11.8%	12.1%	12.9%	13.2%	13.1%	12.7%	12.1%	12.2%	13.0%	13.6%	13.6%	13.0%
Swaziland	11.9%	12.6%	14.1%	14.9%	14.8%	13.7%	12.0%	12.6%	14.1%	15.1%	14.9%	13.7%
Zimbabwe	11.6%	12.8%	13.4%	13.2%	12.5%	11.6%	11.7%	12.7%	13.4%	13.6%	13.0%	12.1%
**Western Sub-Saharan Africa**	12.6%	12.5%	12.5%	12.3%	12.0%	11.5%	13.4%	13.3%	13.3%	13.1%	12.8%	12.3%
Benin	13.2%	12.9%	12.6%	12.3%	12.1%	11.7%	14.0%	13.7%	13.5%	13.2%	13.0%	12.6%
Burkina Faso	14.4%	14.2%	13.8%	13.0%	12.3%	11.7%	14.5%	14.4%	14.0%	13.6%	13.1%	12.7%
Cameroon	12.7%	12.8%	12.9%	12.8%	12.3%	11.7%	13.5%	13.6%	13.6%	13.5%	13.0%	12.5%
Cape Verde	11.5%	11.5%	11.5%	11.3%	11.2%	10.9%	12.1%	12.0%	11.9%	11.7%	11.6%	11.4%
Chad	12.7%	12.8%	13.0%	13.0%	12.6%	12.4%	13.6%	13.6%	13.8%	13.7%	13.4%	13.1%
Cote d'Ivoire	13.2%	13.1%	12.9%	12.5%	12.3%	12.0%	14.1%	13.8%	13.6%	13.3%	13.1%	12.8%
The Gambia	11.9%	11.9%	12.0%	12.0%	12.0%	11.7%	13.5%	13.4%	13.3%	13.3%	13.1%	12.9%
Ghana	11.8%	11.6%	11.5%	11.4%	11.3%	10.9%	12.8%	12.5%	12.4%	12.2%	12.1%	11.6%
Guinea	12.3%	12.1%	12.0%	11.8%	11.7%	11.6%	13.6%	13.3%	13.2%	13.0%	12.9%	12.7%
Guinea-Bissau	12.5%	12.5%	12.4%	12.4%	12.3%	12.1%	13.3%	13.2%	13.1%	13.1%	13.0%	12.7%
Liberia	14.2%	14.6%	14.1%	13.9%	13.5%	12.8%	14.8%	15.0%	14.6%	14.5%	14.1%	13.6%
Mali	13.4%	13.3%	13.0%	12.8%	12.3%	11.8%	13.8%	13.6%	13.4%	13.1%	12.8%	12.5%
Mauritania	12.1%	11.9%	11.8%	11.6%	11.3%	11.2%	13.1%	12.8%	12.5%	12.3%	12.2%	12.0%
Niger	11.9%	11.8%	11.7%	11.6%	11.3%	11.1%	12.7%	12.7%	12.6%	12.5%	12.2%	12.0%
Nigeria	12.5%	12.5%	12.6%	12.3%	12.0%	11.5%	13.3%	13.3%	13.4%	13.2%	12.9%	12.3%
Sao Tome and Principe	11.7%	11.7%	11.7%	11.6%	11.5%	11.3%	11.9%	11.9%	11.8%	11.7%	11.6%	11.4%
Senegal	11.8%	11.8%	11.6%	11.6%	11.6%	11.3%	12.7%	12.6%	12.4%	12.4%	12.3%	12.0%
Sierra Leone	12.7%	12.8%	13.0%	12.8%	12.2%	11.7%	13.0%	13.1%	13.3%	13.2%	12.9%	12.4%
Togo	12.0%	12.1%	12.3%	12.1%	11.7%	11.2%	13.1%	13.1%	13.2%	13.1%	12.7%	12.1%
**Eastern Sub-Saharan Africa**	12.2%	12.4%	12.4%	12.1%	11.7%	11.3%	12.5%	12.6%	12.7%	12.4%	12.0%	11.6%
Burundi	11.8%	12.1%	12.0%	11.7%	11.2%	10.9%	12.0%	12.2%	12.2%	11.9%	11.3%	11.0%
Comoros	12.1%	11.9%	11.9%	11.4%	11.1%	11.0%	12.2%	12.0%	11.9%	11.6%	11.4%	11.3%
Djibouti	11.1%	11.1%	11.2%	11.2%	11.1%	11.0%	11.5%	11.4%	11.6%	11.6%	11.5%	11.3%
Eritrea	11.9%	11.7%	11.5%	11.4%	11.3%	11.1%	12.5%	12.1%	11.8%	11.6%	11.6%	11.4%
Ethiopia	12.1%	11.9%	11.8%	11.5%	11.2%	11.0%	12.5%	12.2%	12.1%	11.8%	11.4%	11.1%
Kenya	11.0%	11.6%	11.9%	11.6%	11.3%	11.2%	11.3%	11.7%	12.0%	11.8%	11.5%	11.4%
Madagascar	11.9%	11.8%	11.7%	11.7%	11.4%	11.2%	12.3%	12.1%	12.0%	11.9%	11.7%	11.3%
Malawi	12.7%	13.4%	13.6%	13.4%	12.6%	11.8%	12.5%	13.1%	13.5%	13.4%	12.7%	12.0%
Mozambique	12.7%	12.9%	13.1%	13.1%	13.1%	12.5%	13.0%	13.1%	13.2%	13.4%	13.3%	12.7%
Rwanda	11.8%	13.0%	12.6%	11.9%	11.3%	11.0%	12.2%	13.1%	12.7%	12.1%	11.4%	11.1%
Somalia	11.7%	11.6%	11.8%	11.6%	11.7%	11.4%	12.5%	12.4%	12.5%	12.3%	12.3%	12.1%
South Sudan	14.7%	14.9%	14.3%	13.9%	13.2%	12.7%	15.3%	15.3%	14.9%	14.4%	13.6%	13.2%
Tanzania	12.1%	12.4%	12.3%	12.0%	11.6%	11.0%	12.5%	12.8%	12.8%	12.6%	12.2%	11.6%
Uganda	13.8%	14.1%	13.7%	13.1%	12.4%	11.8%	13.8%	13.9%	13.9%	13.4%	12.9%	12.2%
Zambia	11.7%	12.6%	13.1%	12.9%	12.4%	12.0%	12.1%	12.9%	13.4%	13.3%	12.8%	12.3%
**Central Sub-Saharan Africa**	14.0%	14.0%	13.9%	13.6%	13.2%	12.7%	14.2%	14.2%	14.2%	13.9%	13.5%	13.0%
Angola	12.9%	12.8%	12.6%	12.4%	12.2%	12.0%	13.3%	13.2%	13.1%	12.8%	12.6%	12.3%
Central African Republic	14.4%	14.6%	14.6%	14.4%	13.8%	13.5%	14.3%	14.4%	14.6%	14.4%	14.0%	13.7%
Congo (Brazzaville)	13.5%	13.5%	13.4%	12.9%	12.6%	12.1%	13.9%	14.0%	14.0%	13.7%	13.4%	13.0%
DR Congo	14.4%	14.3%	14.3%	14.0%	13.5%	12.9%	14.6%	14.5%	14.5%	14.3%	13.8%	13.2%
Equatorial Guinea	13.3%	13.1%	12.8%	12.6%	12.4%	12.2%	13.8%	13.6%	13.3%	13.2%	12.9%	12.6%
Gabon	12.6%	12.5%	12.5%	12.3%	12.1%	11.9%	13.2%	13.2%	13.4%	13.4%	13.3%	12.9%

SR Figure 2: Co−evolution of age−standardised YLD rates per 100,000 for top ten YLD Level 4 causes and the socio−demographic index (SDI), by GBD region

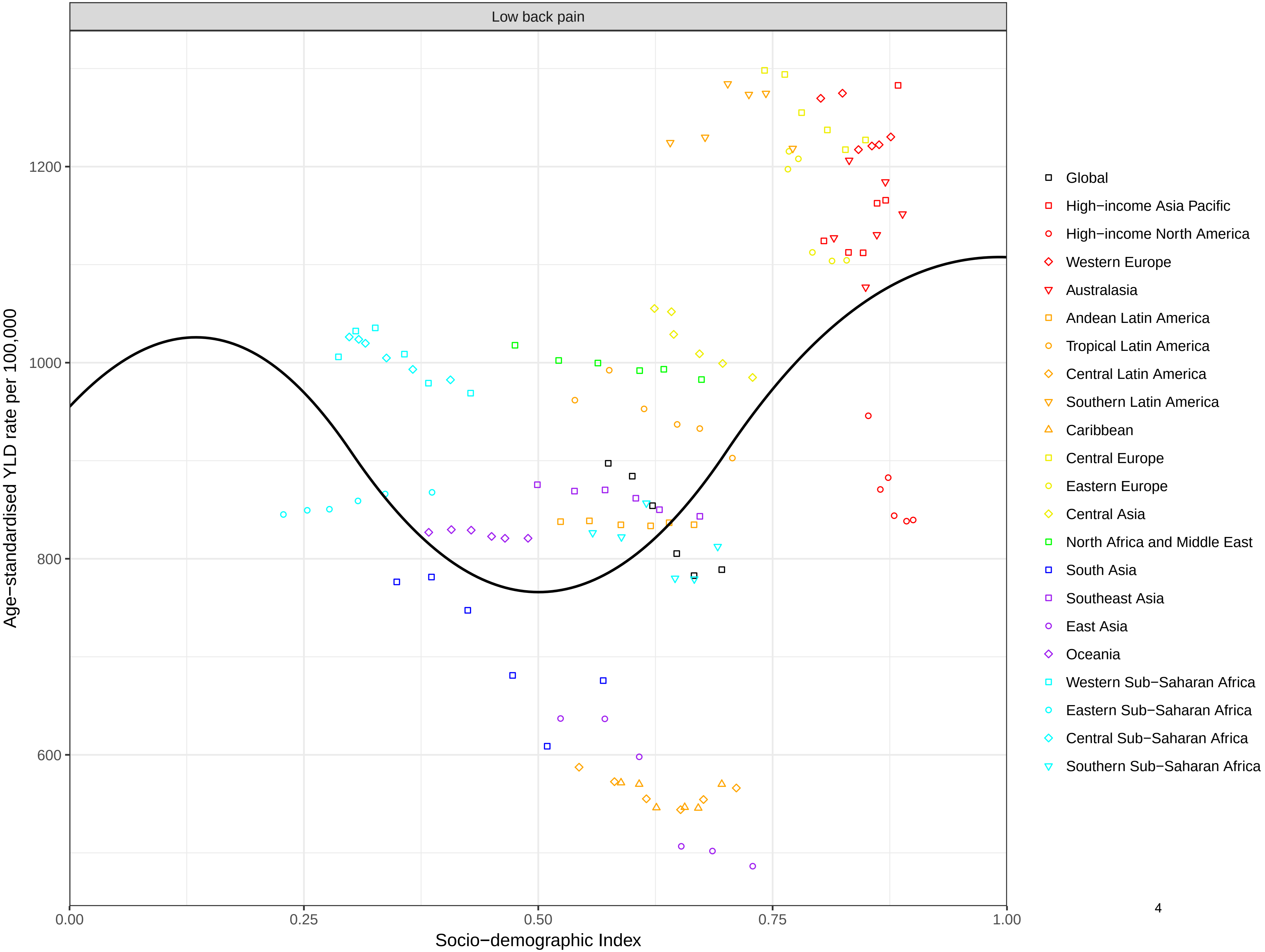

SR Figure 2: Co−evolution of age−standardised YLD rates per 100,000 for top ten YLD Level 4 causes and the socio−demographic index (SDI), by GBD region

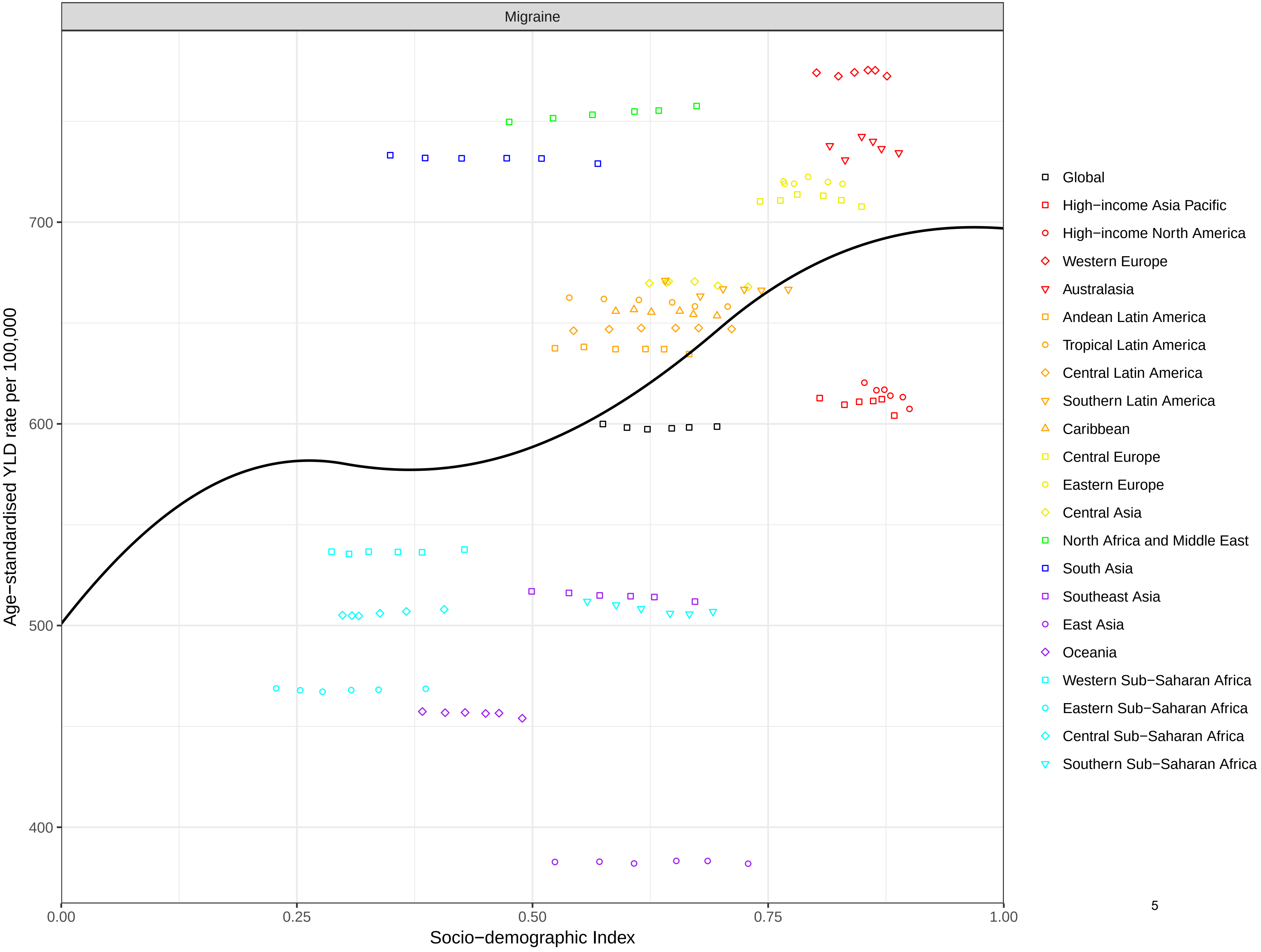

SR Figure 2: Co−evolution of age−standardised YLD rates per 100,000 for top ten YLD Level 4 causes and the socio−demographic index (SDI), by GBD region

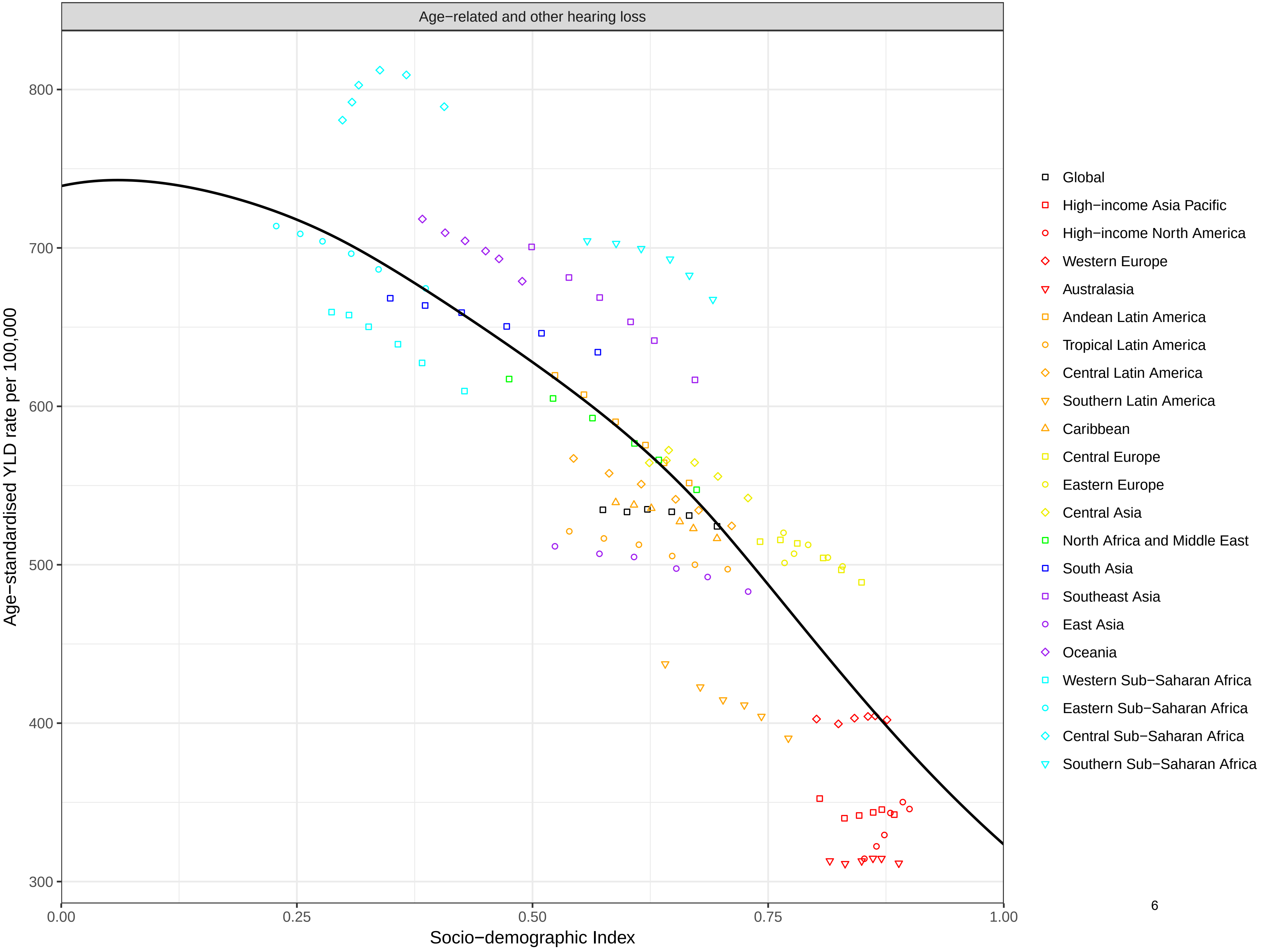

SR Figure 2: Co−evolution of age−standardised YLD rates per 100,000 for top ten YLD Level 4 causes and the socio−demographic index (SDI), by GBD region

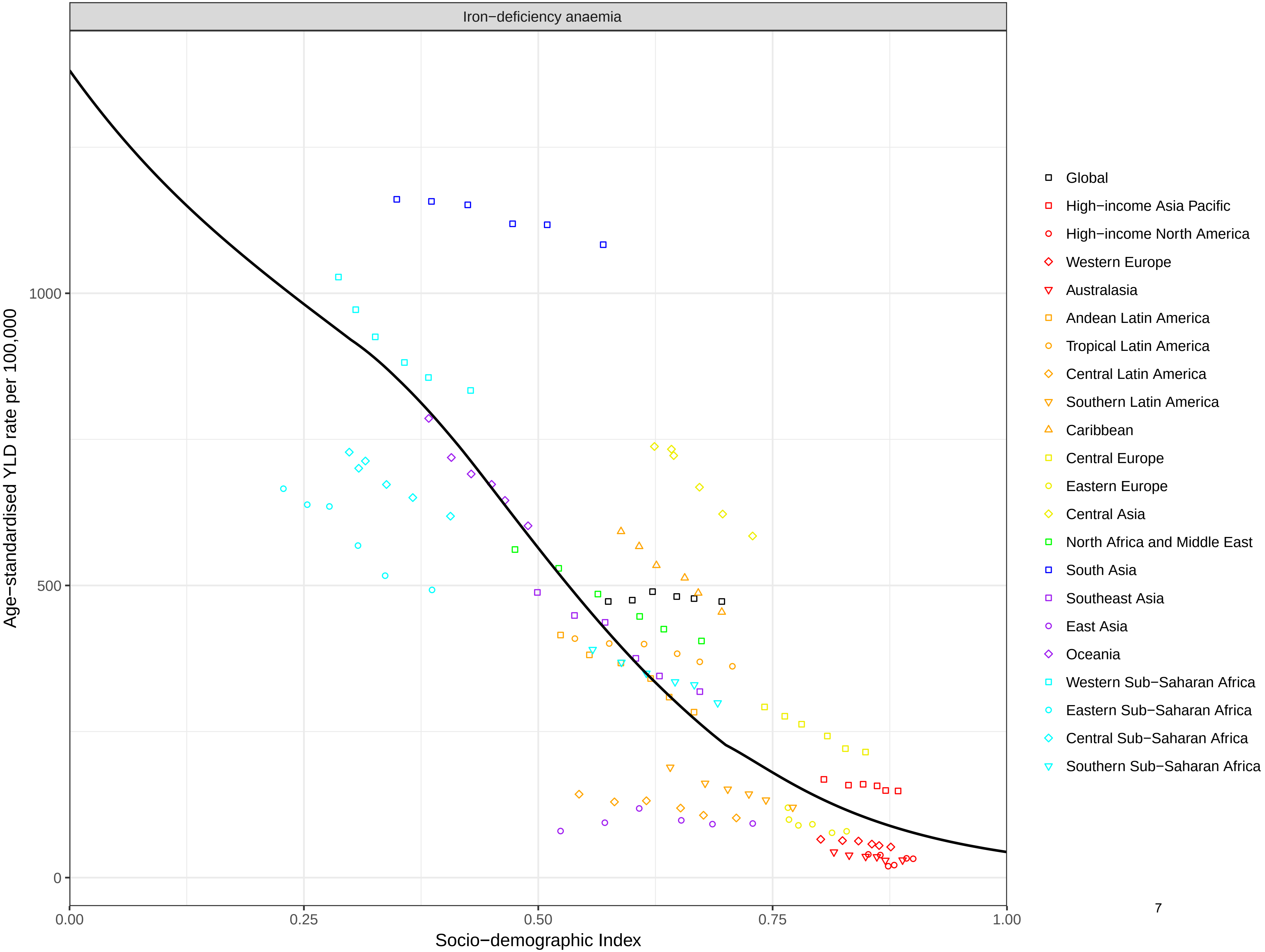

SR Figure 2: Co−evolution of age−standardised YLD rates per 100,000 for top ten YLD Level 4 causes and the socio−demographic index (SDI), by GBD region

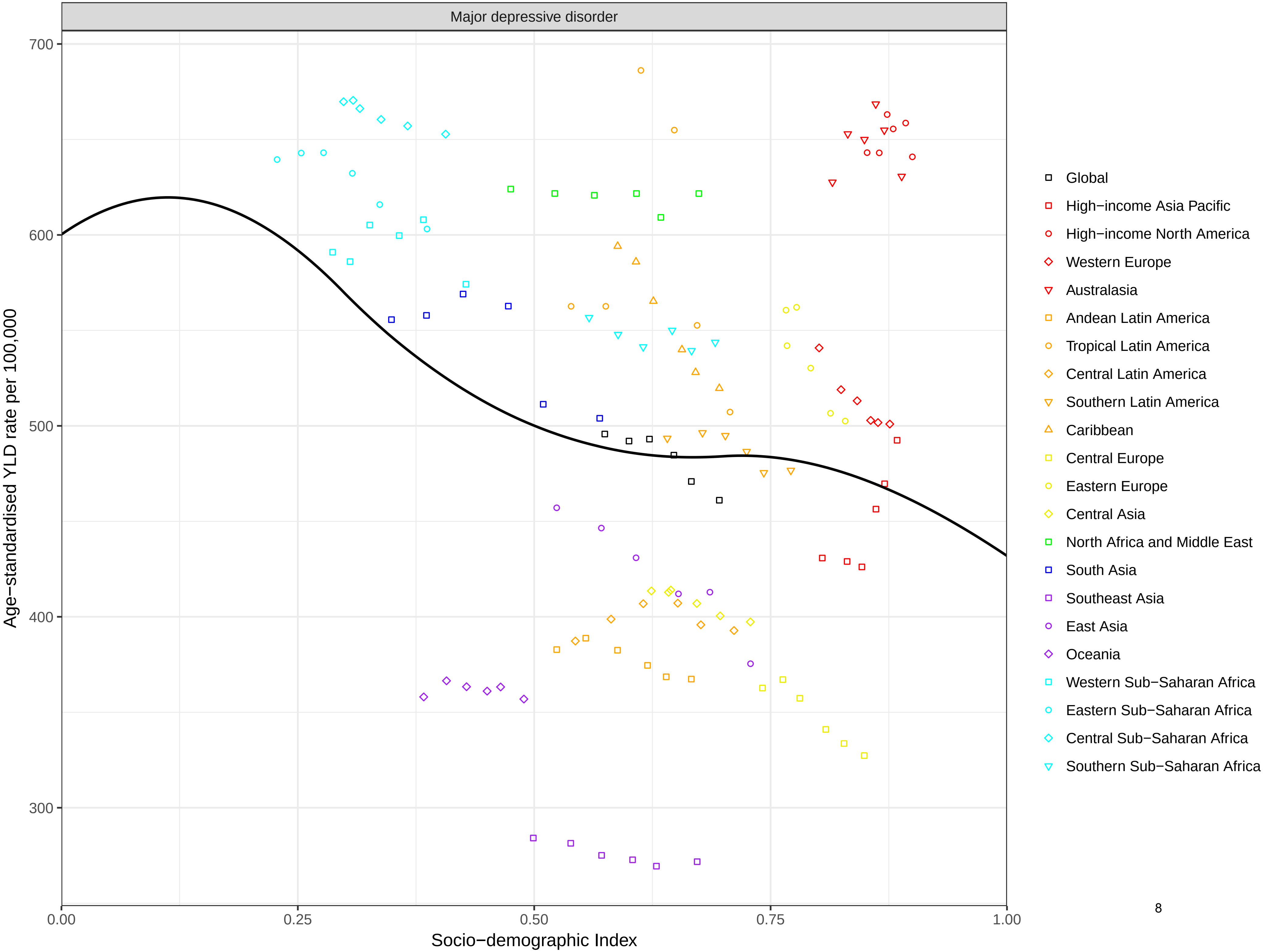

SR Figure 2: Co−evolution of age−standardised YLD rates per 100,000 for top ten YLD Level 4 causes and the socio−demographic index (SDI), by GBD region

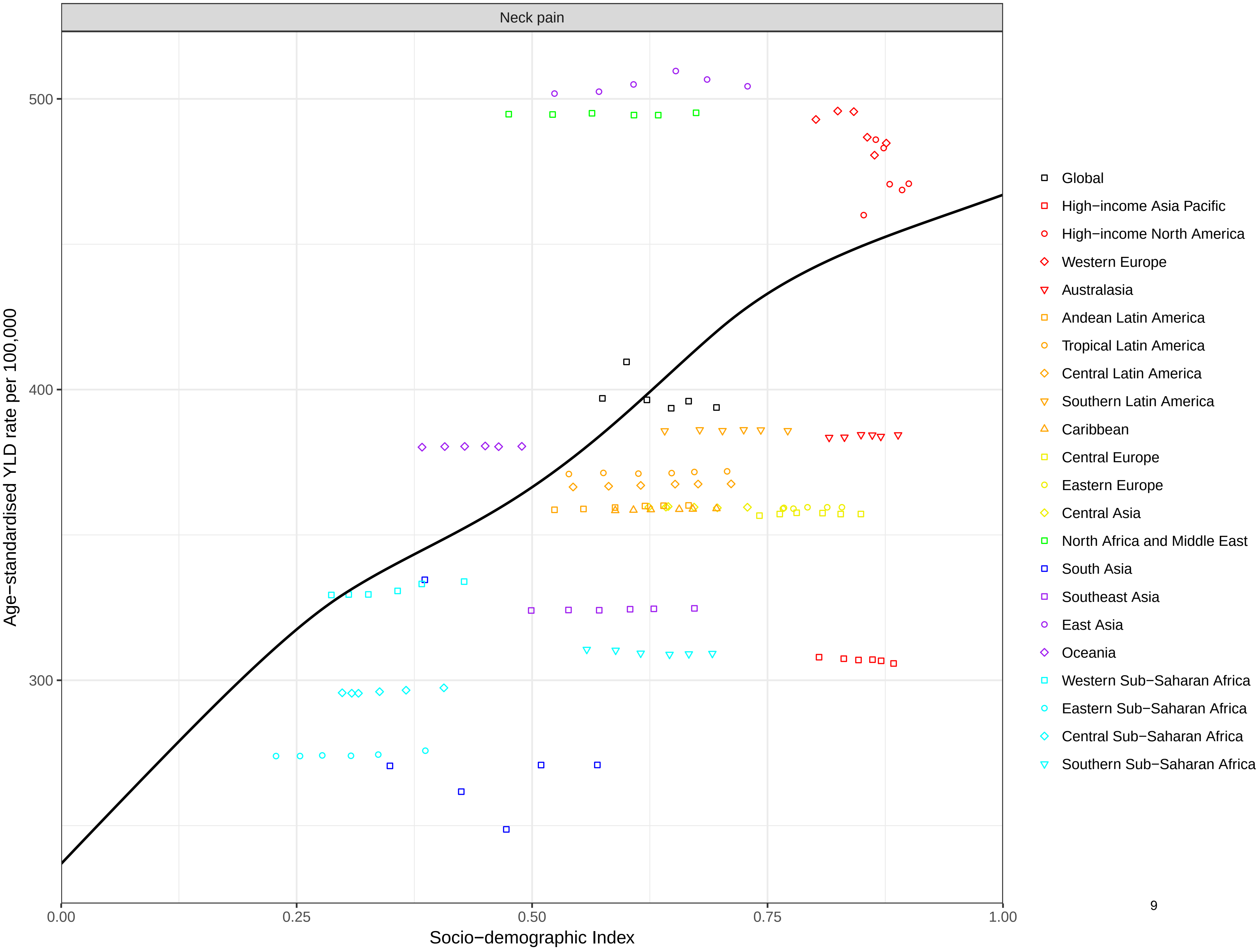

SR Figure 2: Co−evolution of age−standardised YLD rates per 100,000 for top ten YLD Level 4 causes and the socio−demographic index (SDI), by GBD region

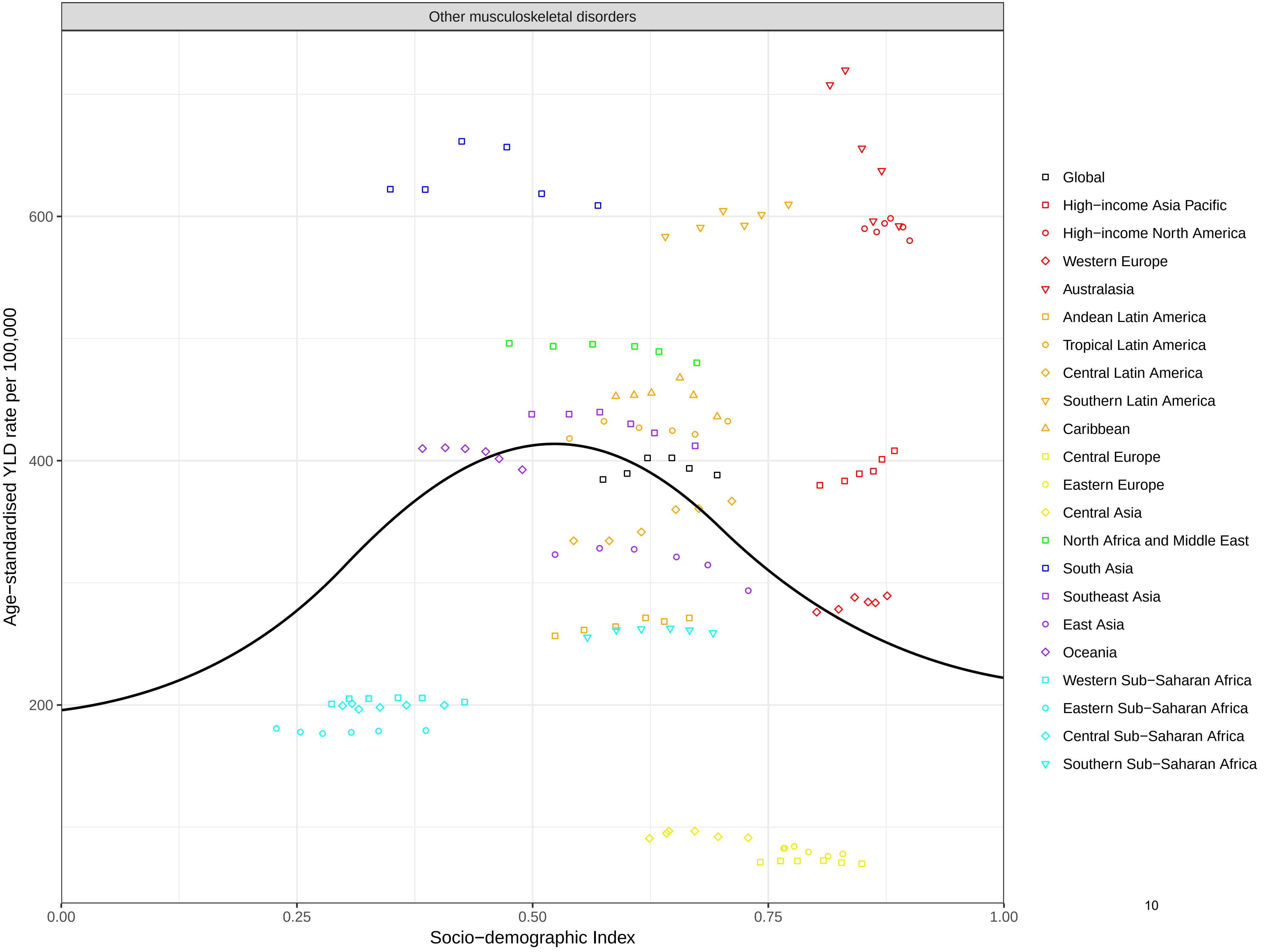

SR Figure 2: Co−evolution of age−standardised YLD rates per 100,000 for top ten YLD Level 4 causes and the socio−demographic index (SDI), by GBD region

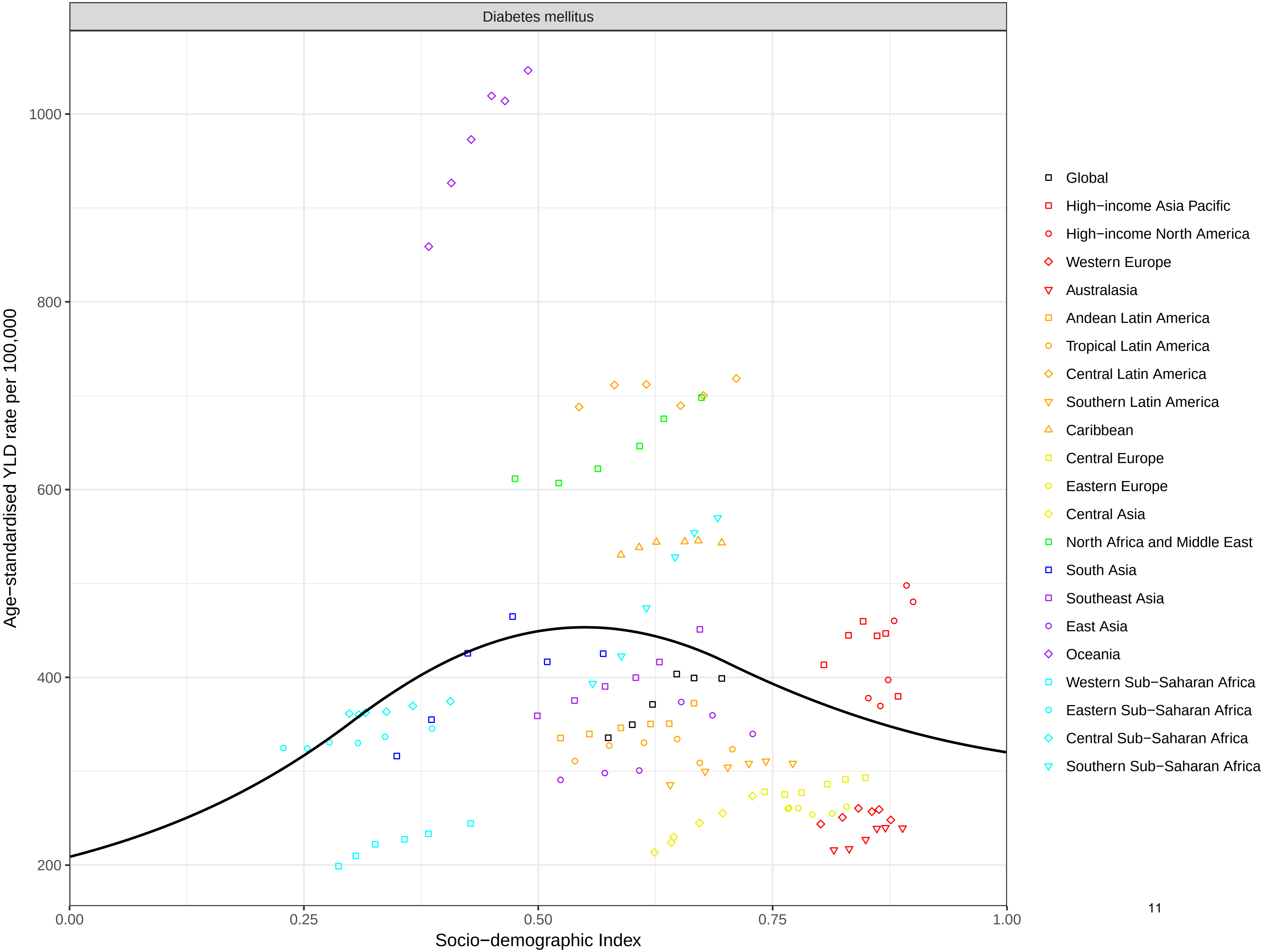

SR Figure 2: Co−evolution of age−standardised YLD rates per 100,000 for top ten YLD Level 4 causes and the socio−demographic index (SDI), by GBD region

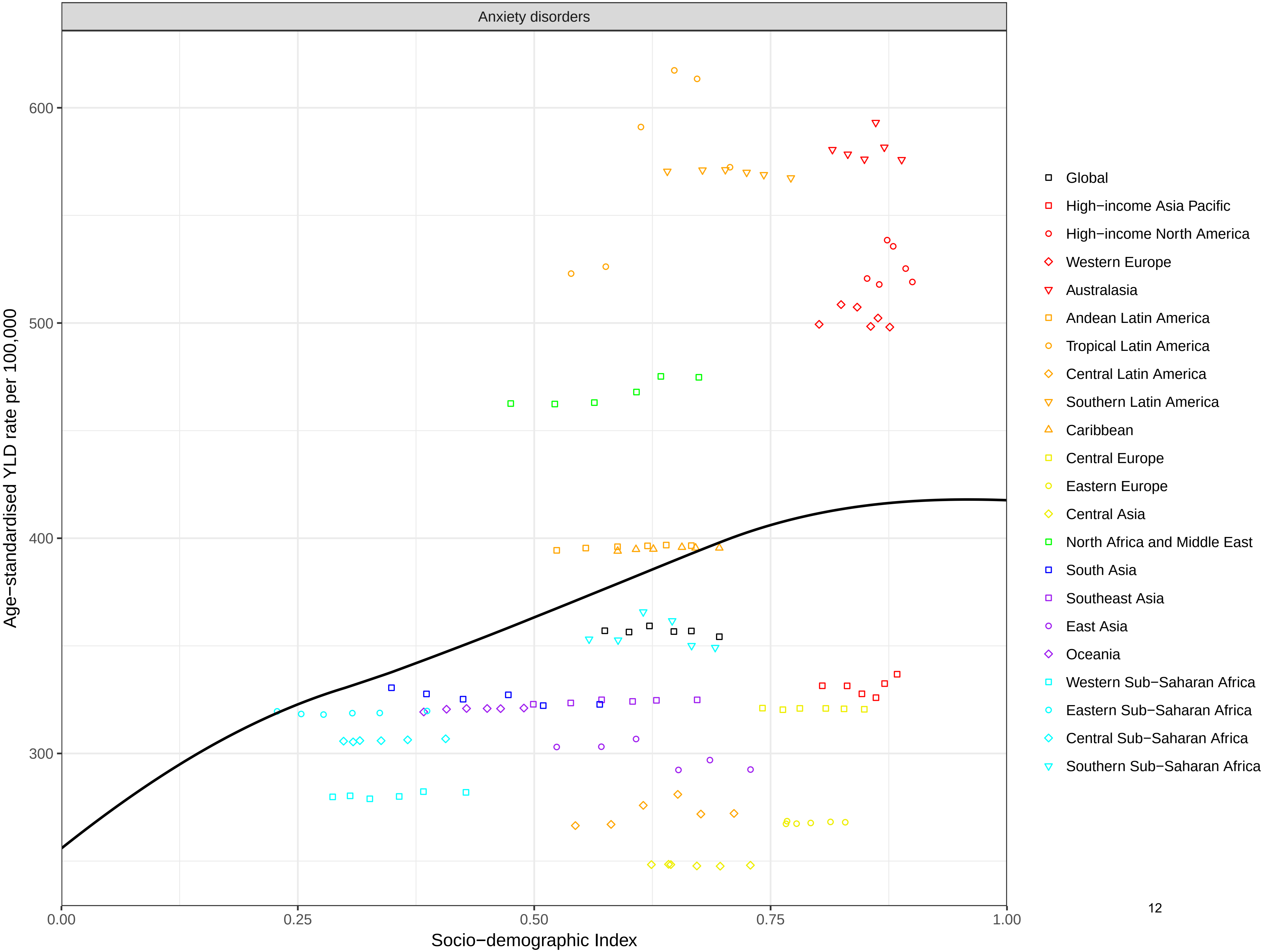

SR Figure 2: Co−evolution of age−standardised YLD rates per 100,000 for top ten YLD Level 4 causes and the socio−demographic index (SDI), by GBD region

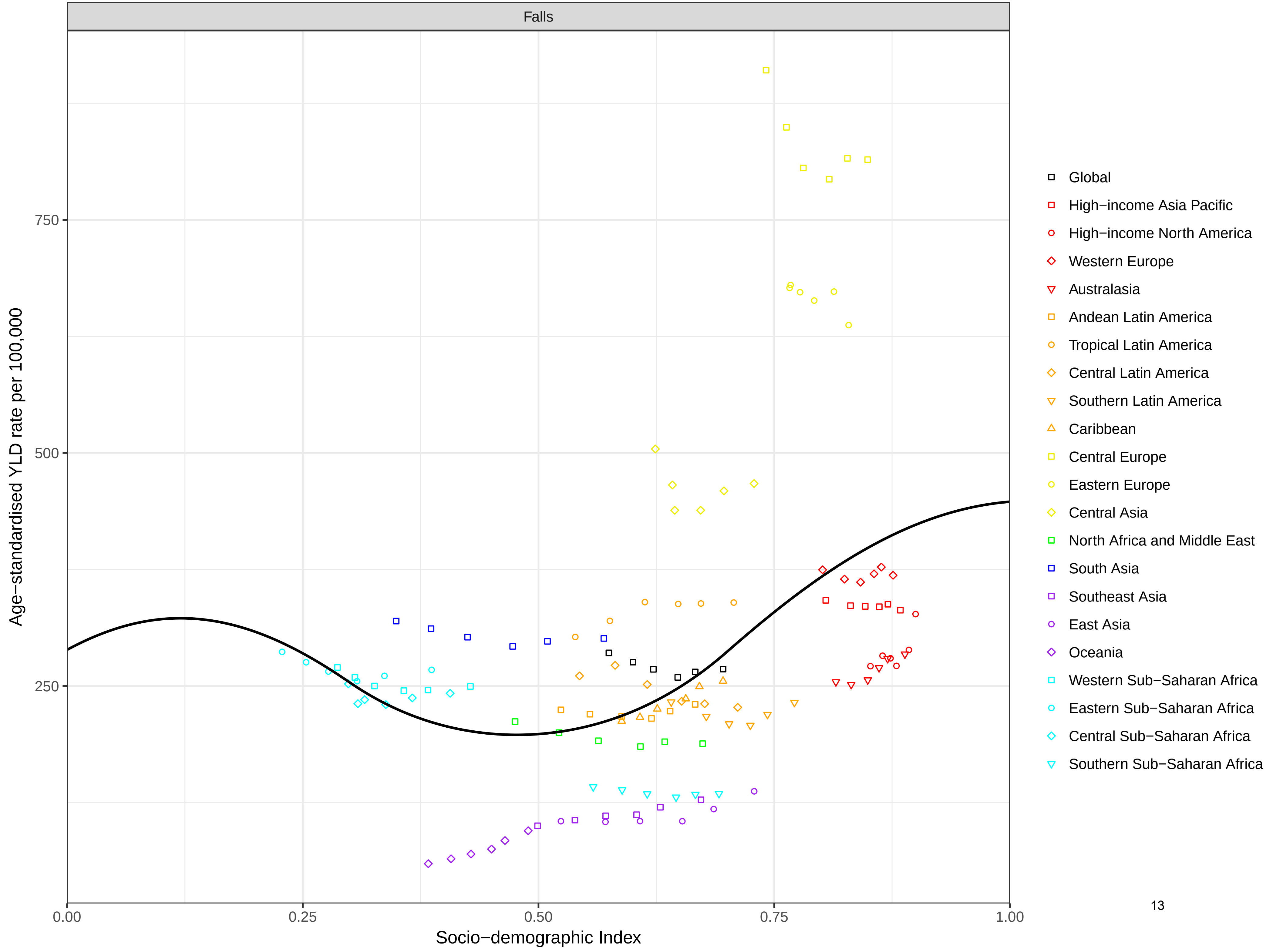

SR Figure 3: Leading 30 Level 4 causes of YLDs, high SDI quintile for 1990, 2006, and 2016, with mean percentage change in number of YLDs, and all-age and age-standardised YLD rates

Causes are connected by arrows between time periods; solid lines are increases and dashed lines are decreases. For the time period 1990 to 2006 and for 2006 to 2016, three measures of change are shown: percentage change in the number of YLDs, percentage change in the all-age YLD rate, and percentage change in the age-standardised YLD rate. Communicable, maternal, neonatal, and nutritional disorders are shown in red, non-communicable causes in blue, and injuries in green. Statistically significant changes are shown in bold. COPD=chronic obstructive pulmonary disease. YLDs=years lived with disability.

Leading causes 1990	Leading causes 2006	Mean % change number of YLDs 1990-2006	Mean % change all-age YLD rate 1990-2006	Mean % change age-standardised YLD rate 1990-2006	Leading causes 2016	Mean % change number of YLDs 2006-2016	Mean % change all-age YLD rate 2006-2016	Mean % change age-standardised YLD rate 2006-2016
1 Low back pain	1 Low back pain	**13.3**	3.1	**-4.8**	1 Low back pain	**11.3**	**5.6**	1.3
2 Migraine	2 Migraine	**10.4**	0.5	**-0.8**	2 Migraine	**2.8**	**-2.4**	**-0.9**
3 Major depression	3 Major depression	**12.0**	2.0	0.7	3 Age-related hearing loss	**19.6**	**13.6**	-0.6
4 Neck pain	4 Neck pain	**22.9**	**11.9**	0.2	4 Major depression	**5.4**	0.1	0.4
5 Anxiety disorders	5 Age-related hearing loss	**33.0**	**21.1**	1.5	5 Neck pain	**10.8**	**5.2**	-0.2
6 Age-related hearing loss	6 Anxiety disorders	**12.6**	**2.5**	1.4	6 Diabetes	**15.1**	**9.2**	-1.4
7 Other musculoskeletal	7 Diabetes	**43.7**	**30.8**	**12.4**	7 Falls	**22.2**	**16.0**	**5.5**
8 Falls	8 Other musculoskeletal	**21.2**	**10.3**	**4.9**	8 Anxiety disorders	**4.0**	-1.2	-0.3
9 Diabetes	9 Falls	**18.8**	**8.2**	**-4.7**	9 Other musculoskeletal	**7.5**	2.1	0.6
10 Acne vulgaris	10 Acne vulgaris	**3.4**	**-5.9**	**4.3**	10 Osteoarthritis	**19.6**	**13.5**	**1.1**
11 Asthma	11 Osteoarthritis	**37.5**	**25.2**	**5.3**	11 Acne vulgaris	**-2.9**	**-7.8**	-0.2
12 Dermatitis	12 Asthma	1.9	**-7.2**	**-6.7**	12 Opioid use disorders	**12.2**	**6.5**	**9.7**
13 Opioid use disorders	13 Opioid use disorders	**11.2**	1.3	**7.6**	13 Edentulism	**20.0**	**13.9**	0.7
14 Osteoarthritis	14 Dermatitis	**7.6**	**-2.1**	**2.0**	14 Ischemic stroke	**13.6**	**7.9**	**-6.8**
15 Schizophrenia	15 Ischemic stroke	**28.1**	**16.6**	**-5.6**	15 COPD	**15.6**	**9.7**	-2.4
16 Edentulism	16 Edentulism	**26.2**	**14.9**	**-2.6**	16 Asthma	**1.5**	**-3.6**	**-3.7**
17 Ischemic stroke	17 COPD	**36.2**	**24.0**	**5.3**	17 Alzheimer disease	**31.8**	**25.1**	0.1
18 COPD	18 Schizophrenia	**17.2**	**6.7**	**1.7**	18 Dermatitis	**4.7**	-0.6	**0.8**
19 Bipolar disorder	19 Alzheimer disease	**48.8**	**35.5**	0.7	19 Schizophrenia	**6.6**	1.2	0.7
20 Other mental & substance	20 Other mental & substance	**15.5**	**5.2**	0.5	20 Other mental & substance	**7.5**	**2.0**	0.4
21 Alcohol use disorders	21 Bipolar disorder	**11.7**	1.7	0.6	21 Bipolar disorder	**4.9**	-0.5	0.3
22 Other cardiovascular	22 Alcohol use disorders	4.7	-4.7	-2.8	22 Dysthymia	**9.1**	**3.6**	1.2
23 Ischemic heart disease	23 Dysthymia	**15.0**	**4.7**	-0.4	23 Alcohol use disorders	**3.5**	-1.7	0.5
24 Dysthymia	24 Refraction & accommodation	**14.0**	**3.8**	**-5.0**	24 Refraction & accommodation	**9.3**	**3.7**	**-3.4**
25 Refraction & accommodation	25 Other cardiovascular	-2.0	**-10.8**	**-24.1**	25 Other cardiovascular	**10.2**	**4.6**	**-7.0**
26 Alzheimer disease	26 Psoriasis	**20.1**	**9.3**	**4.8**	26 Psoriasis	**10.4**	**4.8**	**2.5**
27 Psoriasis	27 Ischemic heart disease	0.0	**-9.0**	**-24.8**	27 Ischemic heart disease	**12.3**	**6.6**	**-7.7**
28 Upper respiratory infect	28 Rheumatoid arthritis	**27.8**	**16.3**	**2.7**	28 Rheumatoid arthritis	**16.0**	**10.1**	**2.0**
29 Rheumatoid arthritis	29 Other sense organ	**24.9**	**13.7**	**0.8**	29 Other sense organ	**15.7**	**9.9**	0.5
30 Other sense organ	30 Atrial fibrillation	**32.3**	**20.4**	-3.1	30 Atrial fibrillation	**22.3**	**16.1**	**-1.2**
34 Atrial fibrillation	32 Upper respiratory infect				32 Upper respiratory infect			

Legend:
Communicable, maternal, neonatal, and nutritional
Non-communicable
Injuries

SR Figure 3: Leading 30 Level 4 causes of YLDs, high-middle SDI quintile for 1990, 2006, and 2016, with mean percentage change in number of YLDs, and all-age and age-standardised YLD rates

Causes are connected by arrows between time periods; solid lines are increases and dashed lines are decreases. For the time period 1990 to 2006 and for 2006 to 2016, three measures of change are shown: percentage change in the number of YLDs, percentage change in the all-age YLD rate, and percentage change in the age-standardised YLD rate. Communicable, maternal, neonatal, and nutritional disorders are shown in red, non-communicable causes in blue, and injuries in green. Statistically significant changes are shown in bold. COPD=chronic obstructive pulmonary disease. YLDs=years lived with disability.

Leading causes 1990	Leading causes 2006	Mean % change number of YLDs 1990-2006	Mean % change all-age YLD rate 1990-2006	Mean % change age-standardised YLD rate 1990-2006	Leading causes 2016	Mean % change number of YLDs 2006-2016	Mean % change all-age YLD rate 2006-2016	Mean % change age-standardised YLD rate 2006-2016
1 Low back pain	1 Low back pain	**18.2**	-0.5	**-11.9**	1 Low back pain	**15.4**	**3.9**	**-3.8**
2 Migraine	2 Migraine	**26.2**	**6.2**	**-2.3**	2 Migraine	**11.5**	0.3	**-1.2**
3 Major depression	3 Major depression	**27.9**	**7.7**	**-2.1**	3 Major depression	**9.1**	-1.8	**-5.0**
4 Age-related hearing loss	4 Age-related hearing loss	**32.7**	**11.7**	**-2.1**	4 Age-related hearing loss	**19.8**	**7.8**	**-3.7**
5 Falls	5 Neck pain	**41.5**	**19.1**	**2.8**	5 Neck pain	**22.3**	**10.1**	1.0
6 Neck pain	6 Anxiety disorders	**30.1**	**9.5**	**3.5**	6 Diabetes	**27.5**	**14.8**	2.1
7 Anxiety disorders	7 Falls	**13.1**	**-4.8**	**-14.2**	7 Falls	**14.5**	**3.1**	**-5.2**
8 Diabetes	8 Diabetes	**54.5**	**30.1**	**12.3**	8 Anxiety disorders	**11.9**	0.7	0.7
9 Other musculoskeletal	9 Other musculoskeletal	**44.3**	**21.5**	**9.1**	9 Other musculoskeletal	**21.0**	**8.9**	**3.3**
10 Iron-deficiency anemia	10 Osteoarthritis	**45.4**	**22.4**	**4.6**	10 Osteoarthritis	**30.0**	**17.0**	**1.8**
11 Acne vulgaris	11 Ischemic stroke	**31.6**	**10.8**	**-5.8**	11 Ischemic stroke	**30.9**	**17.8**	2.1
12 Ischemic stroke	12 Acne vulgaris	**22.8**	**3.4**	**5.0**	12 Schizophrenia	**21.6**	**9.4**	**3.3**
13 Alcohol use disorders	13 Alcohol use disorders	**20.4**	1.4	**-8.0**	13 Refraction & accommodation	**14.0**	**2.6**	**-5.1**
14 Osteoarthritis	14 Schizophrenia	**38.2**	**16.4**	**4.2**	14 Acne vulgaris	**-3.5**	**-13.1**	**3.2**
15 Asthma	15 Refraction & accommodation	**19.7**	0.8	**-6.8**	15 Alcohol use disorders	2.2	**-8.0**	**-11.1**
16 Refraction & accommodation	16 Iron-deficiency anemia	**-5.3**	**-20.3**	**-14.5**	16 Opioid use disorders	**10.4**	-0.6	-3.9
17 Schizophrenia	17 Opioid use disorders	**27.9**	**7.6**	0.5	17 Asthma	**12.8**	1.6	**2.3**
18 Opioid use disorders	18 Asthma	**6.3**	**-10.5**	**-8.9**	18 Iron-deficiency anemia	0.7	**-9.4**	**-6.9**
19 Dermatitis	19 Other mental & substance	**32.7**	**11.7**	0.2	19 Other mental & substance	**17.4**	**5.6**	0.1
20 Other mental & substance	20 Dysthymia	**33.4**	**12.3**	0.4	20 Edentulism	**24.2**	**11.8**	**-2.1**
21 Bipolar disorder	21 Edentulism	**34.7**	**13.4**	**-2.6**	21 Dysthymia	**19.9**	**7.9**	0.7
22 Dysthymia	22 Bipolar disorder	**27.6**	**7.4**	-1.0	22 COPD	**25.8**	**13.3**	0.3
23 Edentulism	23 Dermatitis	**10.7**	**-6.8**	0.3	23 Bipolar disorder	**10.4**	-0.7	-0.4
24 COPD	24 COPD	**29.4**	**8.9**	**-5.3**	24 Ischemic heart disease	**25.3**	**12.8**	**-1.3**
25 Ischemic heart disease	25 Ischemic heart disease	**30.6**	**9.9**	**-6.9**	25 Dermatitis	**9.8**	-1.2	1.1
26 Tension headache	26 Tension headache	**27.4**	**7.2**	**-2.0**	26 Tension headache	**13.3**	2.0	-0.5
27 Epilepsy	27 Epilepsy	8.2	-8.9	-6.4	27 Alzheimer disease	**38.3**	**24.5**	1.5
28 Upper respiratory infect	28 Other sense organ	**33.6**	**12.5**	**1.2**	28 Other sense organ	**22.9**	**10.6**	0.9
29 Neonatal preterm birth	29 Upper respiratory infect	**11.0**	**-6.6**	**-3.1**	29 Psoriasis	**21.0**	**8.9**	**5.2**
30 Conduct disorder	30 Alzheimer disease	**58.6**	**33.6**	**6.9**	30 Upper respiratory infect	**6.5**	**-4.1**	**-2.0**
34 Other sense organ	31 Neonatal preterm birth				32 Epilepsy			
35 Psoriasis	32 Psoriasis				34 Neonatal preterm birth			
38 Alzheimer disease	35 Conduct disorder				44 Conduct disorder			

Legend:
Communicable, maternal, neonatal, and nutritional
Non-communicable
Injuries

SR Figure 3: Leading 30 Level 4 causes of YLDs, middle SDI quintile for 1990, 2006, and 2016, with mean percentage change in number of YLDs, and all-age and age-standardised YLD rates

Causes are connected by arrows between time periods; solid lines are increases and dashed lines are decreases. For the time period 1990 to 2006 and for 2006 to 2016, three measures of change are shown: percentage change in the number of YLDs, percentage change in the all-age YLD rate, and percentage change in the age-standardised YLD rate. Communicable, maternal, neonatal, and nutritional disorders are shown in red, non-communicable causes in blue, and injuries in green. Statistically significant changes are shown in bold. COPD=chronic obstructive pulmonary disease. YLDs=years lived with disability.

Leading causes 1990	Leading causes 2006	Mean % change number of YLDs 1990-2006	Mean % change all-age YLD rate 1990-2006	Mean % change age-standardised YLD rate 1990-2006
1 Low back pain	1 Low back pain	**26.0**	**4.8**	**-12.1**
2 Migraine	2 Migraine	**35.5**	**12.7**	**2.3**
3 Major depression	3 Age-related hearing loss	**42.0**	**18.1**	**-3.4**
4 Age-related hearing loss	4 Major depression	**27.3**	**5.9**	**-4.5**
5 Other musculoskeletal	5 Neck pain	**49.3**	**24.1**	-0.9
6 Neck pain	6 Other musculoskeletal	**43.1**	**19.0**	**2.6**
7 Anxiety disorders	7 Diabetes	**84.5**	**53.4**	**21.3**
8 Iron-deficiency anemia	8 Anxiety disorders	**29.6**	**7.8**	0.7
9 Diabetes	9 Iron-deficiency anemia	**16.5**	**-3.1**	**3.4**
10 Acne vulgaris	10 Acne vulgaris	**17.7**	**-2.1**	**5.4**
11 Refraction & accommodation	11 Schizophrenia	**36.0**	**13.1**	**-2.2**
12 Schizophrenia	12 Refraction & accommodation	**27.9**	**6.4**	**-6.6**
13 Asthma	13 Osteoarthritis	**71.6**	**42.7**	**7.2**
14 COPD	14 COPD	**43.6**	**19.5**	**-5.8**
15 Osteoarthritis	15 Asthma	**11.9**	**-7.0**	**-4.3**
16 Dermatitis	16 Ischemic stroke	**63.7**	**36.1**	**6.9**
17 Opioid use disorders	17 Dysthymia	**43.3**	**19.1**	-0.2
18 Epilepsy	18 Other mental & substance	**39.0**	**15.6**	0.1
19 Other mental & substance	19 Dermatitis	**12.5**	**-6.5**	**1.1**
20 Dysthymia	20 Falls	**39.8**	**16.2**	-0.1
21 Falls	21 Opioid use disorders	**11.9**	**-6.9**	**-12.5**
22 Diarrheal diseases	22 Alcohol use disorders	**41.5**	**17.7**	**6.0**
23 Neonatal preterm birth	23 Epilepsy	13.4	-5.7	-3.8
24 Ascariasis	24 Neonatal preterm birth	**22.1**	1.6	**8.1**
25 Alcohol use disorders	25 Bipolar disorder	**36.5**	**13.5**	**4.0**
26 Ischemic stroke	26 Tension headache	**34.9**	**12.2**	0.9
27 Conduct disorder	27 Diarrheal diseases	**-3.9**	**-20.1**	**-13.4**
28 Scabies	28 Conduct disorder	**4.2**	**-13.4**	0.1
29 Bipolar disorder	29 Viral skin diseases	**12.6**	**-6.3**	0.2
30 Viral skin diseases	30 Edentulism	**61.5**	**34.3**	**2.4**
32 Tension headache	31 Other sense organ			
34 Other sense organ	32 Scabies			
40 Edentulism	34 Ischemic heart disease			
49 Ischemic heart disease	76 Ascariasis			

Leading causes 2016	Mean % change number of YLDs 2006-2016	Mean % change all-age YLD rate 2006-2016	Mean % change age-standardised YLD rate 2006-2016
1 Low back pain	**17.8**	**9.7**	**-1.2**
2 Migraine	**12.1**	**4.4**	**2.2**
3 Age-related hearing loss	**22.4**	**14.0**	**-3.0**
4 Neck pain	**20.8**	**12.6**	-0.8
5 Diabetes	**24.3**	**15.8**	-1.6
6 Major depression	**8.1**	0.7	**-6.6**
7 Other musculoskeletal	**11.6**	**4.0**	**-4.8**
8 Anxiety disorders	**9.8**	**2.3**	0.1
9 Iron-deficiency anemia	**3.3**	**-3.8**	**-1.7**
10 Osteoarthritis	**34.7**	**25.4**	**1.8**
11 COPD	**28.9**	**20.1**	-1.4
12 Schizophrenia	**12.3**	**4.6**	**-2.1**
13 Refraction & accommodation	**15.8**	**7.9**	**-5.0**
14 Acne vulgaris	**-4.5**	**-11.0**	**3.3**
15 Ischemic stroke	**50.1**	**39.8**	**12.2**
16 Falls	**38.8**	**29.3**	**15.4**
17 Asthma	**12.6**	**4.9**	**5.3**
18 Dysthymia	**20.0**	**11.8**	0.0
19 Other mental & substance	**15.3**	**7.4**	0.1
20 Opioid use disorders	**10.4**	2.8	-0.8
21 Dermatitis	**5.3**	**-1.9**	**1.3**
22 Alcohol use disorders	**8.3**	0.9	**-3.1**
23 Neonatal preterm birth	11.5	3.9	8.6
24 Bipolar disorder	**13.3**	**5.6**	**3.7**
25 Epilepsy	1.3	-5.6	-4.5
26 Edentulism	**34.1**	**25.0**	**1.2**
27 Tension headache	**13.0**	**5.3**	**1.9**
28 Other sense organ	**24.3**	**15.8**	**1.1**
29 Ischemic heart disease	**39.3**	**29.7**	**6.0**
30 Diarrheal diseases	**1.5**	**-5.5**	**-4.2**
33 Viral skin diseases			
35 Conduct disorder			
36 Scabies			
98 Ascariasis			

Legend:
Communicable, maternal, neonatal, and nutritional
Non-communicable
Injuries

SR Figure 3: Leading 30 Level 4 causes of YLDs, low-middle SDI quintile for 1990, 2006, and 2016, with mean percentage change in number of YLDs, and all-age and age-standardised YLD rates

Causes are connected by arrows between time periods; solid lines are increases and dashed lines are decreases. For the time period 1990 to 2006 and for 2006 to 2016, three measures of change are shown: percentage change in the number of YLDs, percentage change in the all-age YLD rate, and percentage change in the age-standardised YLD rate. Communicable, maternal, neonatal, and nutritional disorders are shown in red, non-communicable causes in blue, and injuries in green. Statistically significant changes are shown in bold. COPD=chronic obstructive pulmonary disease. YLDs=years lived with disability.

Leading causes 1990	Leading causes 2006	Mean % change number of YLDs 1990-2006	Mean % change all-age YLD rate 1990-2006	Mean % change age-standardised YLD rate 1990-2006	Leading causes 2016	Mean % change number of YLDs 2006-2016	Mean % change all-age YLD rate 2006-2016	Mean % change age-standardised YLD rate 2006-2016
1 Iron-deficiency anemia	1 Iron-deficiency anemia	**21.5**	**-10.6**	**-7.2**	1 Iron-deficiency anemia	**6.4**	**-8.8**	**-6.7**
2 Migraine	2 Migraine	**45.4**	**7.0**	0.1	2 Migraine	**20.7**	**3.5**	-0.1
3 Low back pain	3 Low back pain	**40.3**	**3.2**	**-7.3**	3 Low back pain	**23.3**	**5.7**	**-1.6**
4 Major depression	4 Major depression	**47.9**	**8.8**	1.5	4 Age-related hearing loss	**23.5**	**5.9**	**-3.1**
5 Age-related hearing loss	5 Other musculoskeletal	**54.9**	**14.0**	**4.3**	5 Major depression	**13.8**	-2.4	**-8.5**
6 Other musculoskeletal	6 Age-related hearing loss	**43.9**	**5.9**	**-3.2**	6 Other musculoskeletal	**16.4**	-0.2	**-6.7**
7 Anxiety disorders	7 Anxiety disorders	**43.6**	**5.6**	0.6	7 Anxiety disorders	**19.2**	**2.2**	-0.8
8 COPD	8 Diabetes	**109.9**	**54.5**	**37.4**	8 Diabetes	**24.8**	**7.0**	**-3.6**
9 Refraction & accommodation	9 COPD	**38.8**	2.1	**-9.2**	9 COPD	**35.2**	**16.0**	**2.7**
10 Neck pain	10 Neck pain	**48.0**	**8.9**	**-3.2**	10 Neck pain	**33.2**	**14.2**	**3.9**
11 Acne vulgaris	11 Refraction & accommodation	**34.1**	**-1.3**	**-8.5**	11 Refraction & accommodation	**14.4**	**-1.9**	**-10.0**
12 Diarrheal diseases	12 Acne vulgaris	**43.5**	**5.6**	**5.6**	12 Acne vulgaris	**16.8**	0.2	**5.1**
13 Diabetes	13 Falls	**37.4**	**1.1**	**-6.4**	13 Falls	**34.2**	**15.1**	**5.5**
14 Falls	14 Diarrheal diseases	**12.9**	**-17.0**	**-15.8**	14 Neonatal preterm birth	**28.8**	10.4	14.9
15 Iodine deficiency	15 Neonatal preterm birth	**73.7**	**27.8**	**34.9**	15 Osteoarthritis	**38.0**	**18.3**	**3.2**
16 Dermatitis	16 Dermatitis	**30.5**	**-4.0**	0.4	16 Asthma	**30.5**	**11.9**	**10.0**
17 Asthma	17 Asthma	**33.1**	**-2.1**	**-3.2**	17 Diarrheal diseases	**7.4**	**-7.9**	**-8.4**
18 Epilepsy	18 Osteoarthritis	**62.6**	**19.6**	**4.3**	18 Dermatitis	**16.0**	-0.5	**2.3**
19 Schizophrenia	19 Schizophrenia	**49.1**	**9.7**	-0.7	19 Schizophrenia	**25.1**	**7.2**	-0.4
20 Neonatal preterm birth	20 Alcohol use disorders	**49.7**	**10.2**	1.6	20 Opioid use disorders	**34.5**	**15.3**	**10.0**
21 Osteoarthritis	21 Opioid use disorders	**53.1**	**12.7**	**4.8**	21 Other mental & substance	**24.8**	**7.0**	0.3
22 Alcohol use disorders	22 Other mental & substance	**49.6**	**10.1**	0.2	22 Alcohol use disorders	**14.3**	-2.0	**-7.2**
23 Conduct disorder	23 Epilepsy	21.5	-10.6	-10.0	23 Dysthymia	**26.2**	**8.2**	1.0
24 Other mental & substance	24 Dysthymia	**49.8**	**10.3**	0.7	24 Cataract	**34.1**	**15.0**	-0.9
25 Intellectual disability	25 Conduct disorder	**29.2**	**-5.0**	0.5	25 Bipolar disorder	**22.0**	**4.6**	1.0
26 Opioid use disorders	26 Bipolar disorder	**47.8**	**8.8**	**1.3**	26 Epilepsy	14.7	-1.6	-1.5
27 Protein-energy malnutrition	27 Intellectual disability	**26.5**	**-6.9**	**-7.6**	27 Tension headache	**21.1**	**3.8**	-0.3
28 Other congenital	28 Cataract	**64.5**	**21.1**	**3.3**	28 Conduct disorder	**9.3**	**-6.3**	1.7
29 Dysthymia	29 Tension headache	**44.7**	**6.5**	-0.6	29 Intellectual disability	3.0	**-11.7**	**-11.2**
30 Bipolar disorder	30 Other congenital	**22.5**	**-9.8**	**-8.9**	30 Protein-energy malnutrition	**11.2**	-4.6	7.0
31 Tension headache	31 Protein-energy malnutrition				31 Other congenital			
33 Cataract	34 Iodine deficiency				48 Iodine deficiency			

Legend:
Communicable, maternal, neonatal, and nutritional
Non-communicable
Injuries

SR Figure 3: Leading 30 Level 4 causes of YLDs, low SDI quintile for 1990, 2006, and 2016, with mean percentage change in number of YLDs, and all-age and age-standardised YLD rates

Causes are connected by arrows between time periods; solid lines are increases and dashed lines are decreases. For the time period 1990 to 2006 and for 2006 to 2016, three measures of change are shown: percentage change in the number of YLDs, percentage change in the all-age YLD rate, and percentage change in the age-standardised YLD rate. Communicable, maternal, neonatal, and nutritional disorders are shown in red, non-communicable causes in blue, and injuries in green. Statistically significant changes are shown in bold. COPD=chronic obstructive pulmonary disease. YLDs=years lived with disability.

Leading causes 1990
1 Iron-deficiency anemia
2 Low back pain
3 Migraine
4 Major depression
5 Age-related hearing loss
6 Anxiety disorders
7 Onchocerciasis
8 Iodine deficiency
9 Diarrheal diseases
10 Asthma
11 Malaria
12 Refraction & accommodation
13 Acne vulgaris
14 Dermatitis
15 Other musculoskeletal
16 Schistosomiasis
17 Neck pain
18 Falls
19 Diabetes
20 Epilepsy
21 COPD
22 Conduct disorder
23 Intellectual disability
24 Alcohol use disorders
25 Other congenital
26 Bipolar disorder
27 Lymphatic filariasis
28 Protein-energy malnutrition
29 Other mental & substance
30 Viral skin diseases
32 HIV/AIDS other
33 Osteoarthritis
36 Opioid use disorders

Leading causes 2006	Mean % change number of YLDs 1990-2006	Mean % change all-age YLD rate 1990-2006	Mean % change age-standardised YLD rate 1990-2006
1 Iron-deficiency anemia	**42.5**	**-9.9**	**-9.6**
2 Low back pain	**53.5**	**-2.9**	**-2.7**
3 Migraine	**60.2**	**1.3**	0.5
4 Major depression	**57.7**	-0.3	-0.5
5 Age-related hearing loss	**55.5**	**-1.7**	-0.9
6 Anxiety disorders	**60.6**	1.6	0.4
7 Acne vulgaris	**72.5**	**9.1**	**5.6**
8 Diarrheal diseases	**48.9**	**-5.8**	**-3.9**
9 HIV/AIDS other	**255.7**	**125.0**	**129.5**
10 Asthma	**53.1**	**-3.2**	**-3.5**
11 Other musculoskeletal	**64.5**	**4.1**	**4.3**
12 Dermatitis	**57.8**	-0.2	0.2
13 Malaria	**51.3**	**-4.3**	**-3.2**
14 Refraction & accommodation	**50.9**	**-4.6**	**-3.0**
15 Diabetes	**74.6**	**10.4**	**12.3**
16 Neck pain	**55.8**	**-1.5**	-0.6
17 Schistosomiasis	**48.0**	**-6.4**	**-8.2**
18 Onchocerciasis	-5.5	**-40.2**	**-42.4**
19 Falls	**37.5**	**-13.0**	**-11.5**
20 Conduct disorder	**62.4**	**2.7**	0.5
21 Epilepsy	**48.8**	-5.9	-6.6
22 Iodine deficiency	**-27.2**	**-53.9**	**-54.0**
23 Intellectual disability	**62.0**	2.5	1.5
24 Alcohol use disorders	**57.9**	-0.1	-1.9
25 COPD	**41.3**	**-10.6**	**-9.5**
26 Bipolar disorder	**62.0**	**2.5**	0.8
27 Other congenital	**56.0**	-1.4	-1.3
28 Other mental & substance	**59.9**	1.1	0.5
29 Viral skin diseases	**59.4**	0.8	0.2
30 Protein-energy malnutrition	**52.0**	-3.9	1.4
31 Osteoarthritis			
33 Opioid use disorders			
34 Lymphatic filariasis			

Leading causes 2016	Mean % change number of YLDs 2006-2016	Mean % change all-age YLD rate 2006-2016	Mean % change age-standardised YLD rate 2006-2016
1 Iron-deficiency anemia	**21.2**	**-8.4**	**-7.7**
2 Low back pain	**36.2**	**3.0**	0.9
3 Migraine	**33.8**	1.2	-0.5
4 Major depression	**31.8**	-0.4	**-3.0**
5 Age-related hearing loss	**29.8**	**-1.9**	**-2.8**
6 Anxiety disorders	**34.2**	1.4	0.1
7 Acne vulgaris	**39.3**	**5.3**	**4.7**
8 Asthma	**39.6**	**5.5**	**6.0**
9 Other musculoskeletal	**31.1**	-0.9	**-3.4**
10 Dermatitis	**32.4**	0.0	1.0
11 Diarrheal diseases	**18.4**	**-10.5**	**-9.2**
12 Diabetes	**36.0**	2.8	1.1
13 Neck pain	**38.7**	**4.9**	**2.4**
14 Refraction & accommodation	**21.7**	**-8.0**	**-9.4**
15 Falls	**36.7**	**3.3**	**1.4**
16 HIV/AIDS other	-16.5	**-36.9**	**-40.0**
17 Malaria	**-14.0**	**-35.0**	**-34.5**
18 Epilepsy	35.2	2.2	2.0
19 Conduct disorder	**32.1**	-0.2	1.0
20 Alcohol use disorders	**37.7**	**4.1**	1.1
21 COPD	**36.1**	2.9	1.7
22 Schistosomiasis	**-17.3**	**-37.5**	**-38.5**
23 Intellectual disability	**18.8**	**-10.2**	**-10.5**
24 Bipolar disorder	**36.0**	**2.8**	0.9
25 Other mental & substance	**36.4**	**3.1**	0.5
26 Opioid use disorders	**44.5**	**9.2**	**6.2**
27 Osteoarthritis	**38.9**	**5.0**	**2.4**
28 Viral skin diseases	**31.6**	-0.5	0.4
29 Onchocerciasis	**-21.2**	**-40.4**	**-40.0**
30 Other congenital	**22.6**	**-7.3**	**-7.3**
35 Protein-energy malnutrition			
37 Iodine deficiency			
48 Lymphatic filariasis			

Legend:
Communicable, maternal, neonatal, and nutritional
Non-communicable
Injuries

SR Table 1: Global prevalence and incidence for 2016, percentage change in prevalence and incidence counts and age-standardized prevalance and incidence rates between 2006 and 2016 for all causes and nine impairments						
Cause	Prevalence in thousands (95% UI)			Incidence in thousands (95% UI)		
	2016 counts	Percentage change in counts between 2006 and 2016	Percentage change in age-standardised rates between 2006 and 2016	2016 counts	Percentage change in counts between 2006 and 2016	Percentage change in age-standardised rates between 2006 and 2016
**All causes**	**7,122,683<br>(7,115,742 to 7,129,084)**	**12.4<br>(12.2 to 12.6)**	**-0.2<br>(-0.4 to -0.1)**	**44,245,182†<br>(42,139,690 to 46,565,213)**	**9.8<br>(8.7 to 10.9)**	**-2.0<br>(-2.9 to -1.1)**
**Communicable, maternal, neonatal, and nutritional disorders**	**4,943,086<br>(4,920,138 to 4,967,417)**	**10.2<br>(9.7 to 10.7)**	**-2.1<br>(-2.6 to -1.7)**	**25,266,823†<br>(23,366,307 to 27,404,017)**	**7.5<br>(5.7 to 9.4)**	**-3.0<br>(-4.5 to -1.4)**
**HIV/AIDS and tuberculosis**	**2,051,462<br>(1,904,878 to 2,137,104)**	**12.4<br>(11.6 to 13.3)**	**-1.8<br>(-2.5 to -1.2)**	**10,885†<br>(9,882 to 12,069)**	**-3.5<br>(-6.3 to -0.1)**	**-15.7<br>(-18.1 to -12.8)**
Tuberculosis	1,918,597<br>(1,797,774 to 2,043,357)	12.6<br>(11.7 to 13.4)	-1.7<br>(-2.2 to -1.2)	9,019†<br>(8,052 to 10,157)	1.2<br>(-0.4 to 2.8)	-12.4<br>(-13.8 to -11.0)
Drug-susceptible tuberculosis	9,036<br>(8,134 to 10,108)	6.5<br>(4.7 to 8.5)	-10.7<br>(-12.1 to -9.3)	8,705<br>(7,755 to 9,818)	1.3<br>(-0.3 to 3.0)	-12.3<br>(-13.7 to -10.9)
Multidrug-resistant tuberculosis without extensive drug resistance	313<br>(279 to 353)	-1.4<br>(-10.3 to 7.7)	-17.6<br>(-25.0 to -10.2)	296<br>(261 to 336)	-5.5<br>(-13.2 to 2.0)	-18.5<br>(-25.1 to -11.9)
Extensively drug-resistant tuberculosis	19<br>(17 to 22)	182.1<br>(147.5 to 218.2)	136.1<br>(107.6 to 166.1)	18<br>(16 to 21)	155.6<br>(124.1 to 189.1)	119.5<br>(92.8 to 147.9)
Latent tuberculosis infection	1,909,229<br>(1,788,353 to 2,034,184)	12.6<br>(11.7 to 13.4)	-1.7<br>(-2.2 to -1.1)	--	--	--
HIV/AIDS	36,369<br>(34,215 to 39,122)	19.1<br>(16.5 to 22.0)	5.1<br>(2.9 to 7.5)	1,865^<br>(1,690 to 2,129)	-21.2<br>(-29.4 to -8.5)	-28.8<br>(-36.4 to -17.2)
Drug-susceptible HIV/AIDS - Tuberculosis	1,076<br>(760 to 1,467)	-21.4<br>(-24.9 to -18.3)	-30.5<br>(-33.7 to -27.8)	1,365<br>(981 to 1,847)	-24.4<br>(-27.8 to -21.5)	-33.2<br>(-36.3 to -30.7)
Multidrug-resistant HIV/AIDS - Tuberculosis without extensive drug resistance	28<br>(18 to 41)	-28.6<br>(-40.3 to -16.4)	-36.9<br>(-47.2 to -26.2)	36<br>(24 to 52)	-28.5<br>(-41.4 to -13.8)	-36.9<br>(-48.2 to -23.9)
Extensively drug-resistant HIV/AIDS - Tuberculosis	1<br>(1 to 2)	147.4<br>(107.7 to 195.0)	118.7<br>(83.6 to 160.7)	1<br>(1 to 2)	131.1<br>(94.2 to 171.3)	104.5<br>(72.0 to 139.8)
HIV/AIDS resulting in other diseases	35,264<br>(33,138 to 38,026)	21.0<br>(18.3 to 24.2)	6.8<br>(4.5 to 9.5)	1,865<br>(1,690 to 2,129)	-21.2<br>(-29.4 to -8.5)	-28.8<br>(-36.4 to -17.2)
**Diarrhoea, lower respiratory infections, and other common infectious diseases**	**434,596<br>(422,500 to 442,782)**	**7.7<br>(7.0 to 8.4)**	**-2.8<br>(-3.4 to -2.2)**	**23,240,453†<br>(21,377,295 to 25,359,204)**	**9.1<br>(8.2 to 10.1)**	**-1.7<br>(-2.2 to -1.2)**
Diarrhoeal diseases	66,972<br>(63,500 to 70,778)	7.7<br>(6.9 to 8.6)	-3.6<br>(-4.4 to -2.9)	4,480,401<br>(4,246,997 to 4,737,769)	8.1<br>(7.3 to 9.0)	-3.5<br>(-4.2 to -2.7)
Intestinal infectious diseases	810<br>(730 to 906)	-18.8<br>(-24.2 to -13.4)	-24.3<br>(-29.3 to -19.2)	15,532†<br>(13,599 to 17,777)	-15.6<br>(-17.1 to -13.7)	-21.7<br>(-23.1 to -20.0)
Typhoid fever	746<br>(641 to 863)	-16.8<br>(-22.6 to -10.0)	-22.4<br>(-27.9 to -16.0)	11,774<br>(10,227 to 13,622)	-16.9<br>(-18.5 to -14.9)	-22.6<br>(-24.2 to -20.8)
Paratyphoid fever	165<br>(145 to 187)	-11.1<br>(-16.2 to -5.5)	-18.7<br>(-23.2 to -13.7)	3,758<br>(3,328 to 4,257)	-11.0<br>(-14.4 to -7.6)	-18.6<br>(-21.8 to -15.6)
Other intestinal infectious diseases	--	--	--	--	--	--
Lower respiratory infections	8,030<br>(7,582 to 8,548)	23.1<br>(21.1 to 25.5)	7.4<br>(5.9 to 9.1)	336,462<br>(313,085 to 361,622)	22.3<br>(20.0 to 24.6)	6.1<br>(4.4 to 7.7)
Upper respiratory infections	235,768<br>(211,381 to 262,904)	9.5<br>(8.4 to 10.7)	-1.3<br>(-1.9 to -0.6)	17,778,134<br>(15,853,332 to 19,905,351)	9.3<br>(8.2 to 10.6)	-1.4<br>(-2.0 to -0.7)
Otitis media	106,062<br>(95,502 to 117,009)	4.0<br>(3.0 to 5.1)	-4.4<br>(-5.3 to -3.5)	451,730<br>(365,179 to 564,104)	8.0<br>(6.6 to 9.6)	1.4<br>(0.0 to 2.9)
Meningitis	14,423<br>(12,441 to 16,722)	5.8<br>(1.2 to 10.5)	-5.4<br>(-9.6 to -1.3)	2,821<br>(2,464 to 3,310)	12.0<br>(9.5 to 14.4)	4.8<br>(2.5 to 7.2)
Pneumococcal meningitis	6,180<br>(5,170 to 7,365)	-2.9<br>(-6.4 to 0.6)	-13.6<br>(-16.7 to -10.4)	612<br>(498 to 756)	-15.1<br>(-19.7 to -10.6)	-20.7<br>(-24.9 to -16.4)
H influenzae type B meningitis	2,012<br>(1,592 to 2,545)	-32.3<br>(-36.9 to -26.8)	-38.9<br>(-43.2 to -33.8)	397<br>(291 to 534)	-41.1<br>(-46.7 to -35.1)	-43.9<br>(-49.5 to -38.1)
Meningococcal infection	1,661<br>(1,207 to 2,270)	16.7<br>(11.0 to 40.0)	4.8<br>(-0.8 to 31.5)	561<br>(442 to 707)	19.8<br>(13.3 to 26.3)	12.5<br>(6.2 to 18.6)
Other meningitis	4,571<br>(3,446 to 5,833)	58.9<br>(51.1 to 66.5)	41.4<br>(34.6 to 48.0)	1,250<br>(1,056 to 1,487)	90.6<br>(80.3 to 101.1)	75.5<br>(66.2 to 85.3)
Encephalitis	14,908<br>(9,877 to 21,755)	6.3<br>(4.7 to 8.2)	-7.8<br>(-9.3 to -5.8)	6,534<br>(5,957 to 7,165)	12.4<br>(10.9 to 14.1)	0.0<br>(-1.0 to 1.1)
Diphtheria	0<br>(0 to 0)	-58.0<br>(-69.3 to -44.4)	-61.0<br>(-71.5 to -47.9)	4<br>(3 to 6)	-58.0<br>(-69.3 to -44.4)	-61.0<br>(-71.5 to -47.9)
Whooping cough	1,593<br>(1,242 to 2,001)	-22.6<br>(-24.4 to -20.8)	-25.2<br>(-27.0 to -23.5)	11,627<br>(9,069 to 14,610)	-22.6<br>(-24.4 to -20.8)	-25.2<br>(-27.0 to -23.5)
Tetanus	160<br>(157 to 163)	9.3<br>(7.3 to 12.1)	-1.1<br>(-3.1 to 1.3)	90<br>(51 to 121)	-60.0<br>(-66.0 to -52.5)	-63.2<br>(-68.7 to -56.5)
Measles	245<br>(89 to 532)	-62.6<br>(-66.6 to -58.2)	-64.3<br>(-68.1 to -60.0)	8,955<br>(3,251 to 19,426)	-62.6<br>(-66.6 to -58.2)	-64.3<br>(-68.1 to -60.0)
Varicella and herpes zoster	7,602<br>(6,996 to 8,221)	16.8<br>(14.9 to 18.8)	1.0<br>(-0.2 to 2.5)	148,162<br>(144,145 to 152,728)	8.4<br>(7.6 to 9.2)	0.0<br>(-0.4 to 0.4)
**Neglected tropical diseases and malaria**	**1,821,783<br>(1,778,850 to 1,853,279)**	**-0.4<br>(-2.6 to 1.8)**	**-10.6<br>(-12.6 to -8.7)**	**440,947†<br>(378,282 to 512,509)**	**3.8<br>(-4.9 to 14.3)**	**-5.2<br>(-13.0 to 4.2)**
Malaria	128,947<br>(113,638 to 145,601)	-23.3<br>(-28.8 to -17.0)	-28.8<br>(-34.1 to -22.8)	213,098<br>(172,037 to 265,778)	-12.7<br>(-23.9 to -3.1)	-18.3<br>(-28.7 to -9.2)
Chagas disease	7,201<br>(6,065 to 8,514)	1.3<br>(-1.9 to 4.1)	-13.3<br>(-15.9 to -10.9)	180<br>(150 to 214)	-7.5<br>(-10.3 to -4.5)	-15.2<br>(-17.7 to -12.5)
Leishmaniasis	4,835<br>(4,147 to 5,523)	22.9<br>(5.0 to 44.8)	6.9<br>(-9.0 to 26.9)	799<br>(618 to 1,021)	-19.8<br>(-38.1 to 3.5)	-28.1<br>(-44.6 to -7.2)
Visceral leishmaniasis	30<br>(28 to 32)	-54.5<br>(-58.5 to -50.4)	-59.4<br>(-62.9 to -55.8)	120<br>(112 to 129)	-54.5<br>(-58.5 to -50.4)	-59.4<br>(-62.9 to -55.8)
Cutaneous and mucocutaneous leishmaniasis	4,320<br>(3,650 to 5,238)	28.4<br>(17.2 to 42.7)	11.9<br>(1.8 to 25.4)	679<br>(497 to 902)	-7.3<br>(-32.8 to 25.7)	-16.3<br>(-39.3 to 13.4)
African trypanosomiasis	7<br>(4 to 12)	-74.4<br>(-79.9 to -67.4)	-77.1<br>(-82.1 to -70.5)	5<br>(4 to 6)	-80.1<br>(-82.7 to -76.8)	-82.2<br>(-84.7 to -79.3)
Schistosomiasis	189,774<br>(179,771 to 200,083)	-26.6<br>(-27.9 to -25.3)	-35.3<br>(-36.6 to -34.1)	71,385<br>(67,342 to 76,073)	-15.3<br>(-19.8 to -10.4)	-23.7<br>(-27.7 to -19.4)
Cysticercosis	2,675<br>(2,232 to 3,149)	1.5<br>(-4.8 to 7.6)	-15.1<br>(-20.4 to -9.9)	--	--	--
Cystic echinococcosis	974<br>(674 to 1,426)	-38.7<br>(-59.4 to -2.2)	-46.1<br>(-64.2 to -14.3)	204<br>(151 to 304)	-38.4<br>(-59.7 to -2.2)	-45.6<br>(-64.7 to -14.1)
Lymphatic filariasis	29,382<br>(24,770 to 36,117)	-42.4<br>(-47.4 to -39.5)	-48.8<br>(-53.3 to -46.3)	7,604<br>(6,561 to 8,750)	7.1<br>(-11.0 to 27.5)	-3.7<br>(-19.8 to 13.9)
Onchocerciasis	14,650<br>(9,474 to 24,168)	-26.9<br>(-38.6 to -15.7)	-34.6<br>(-45.1 to -24.6)	3,839<br>(1,747 to 7,518)	39.9<br>(-8.5 to 94.7)	27.1<br>(-16.7 to 76.7)
Trachoma	3,338<br>(2,439 to 4,492)	0.5<br>(-8.5 to 8.0)	-23.2<br>(-30.0 to -17.3)	--	--	--
Dengue	6,046<br>(3,292 to 10,244)	75.1<br>(43.7 to 294.2)	58.4<br>(30.0 to 256.6)	101,064<br>(61,458 to 153,334)	75.2<br>(43.8 to 290.0)	58.5<br>(30.1 to 252.9)
Yellow fever	3<br>(1 to 8)	-18.0<br>(-30.1 to -3.5)	-24.1<br>(-35.0 to -10.9)	112<br>(32 to 283)	-18.0<br>(-30.1 to -3.5)	-24.1<br>(-35.0 to -10.9)
Rabies	1<br>(0 to 1)	-46.7<br>(-56.0 to -33.4)	-52.8<br>(-61.1 to -40.9)	13<br>(7 to 19)	-46.7<br>(-56.0 to -33.4)	-52.8<br>(-61.1 to -40.9)
Intestinal nematode infections	1,507,853<br>(1,450,985 to 1,542,581)	2.2<br>(-0.8 to 5.2)	-8.2<br>(-10.9 to -5.5)	--	--	--
Ascariasis	799,683<br>(737,609 to 872,087)	-5.2<br>(-14.7 to 5.7)	-14.9<br>(-23.4 to -5.1)	--	--	--
Trichuriasis	435,095<br>(410,564 to 464,463)	0.4<br>(-7.6 to 9.2)	-9.6<br>(-16.9 to -1.6)	--	--	--
Hookworm disease	450,683<br>(424,863 to 479,224)	-0.7<br>(-8.3 to 7.9)	-10.8<br>(-17.7 to -3.1)	--	--	--
Food-borne trematodiases	74,725<br>(70,800 to 78,800)	6.1<br>(3.9 to 8.3)	-7.3<br>(-9.3 to -5.4)	34,989<br>(23,063 to 45,803)	29.8<br>(18.6 to 49.0)	3.7<br>(-4.3 to 20.6)
Leprosy	523<br>(499 to 547)	0.9<br>(0.6 to 1.2)	-18.4<br>(-18.7 to -17.9)	55<br>(53 to 58)	-3.7<br>(-4.4 to -3.1)	-18.9<br>(-19.3 to -18.4)
Ebola virus disease	1<br>(0 to 4)	--	--	0<br>(0 to 0)	--	--
Zika virus disease	129<br>(97 to 179)	--	--	7,598<br>(5,696 to 10,664)	--	--
Guinea worm disease	0<br>(0 to 0)	-99.6<br>(-99.7 to -99.4)	-99.6<br>(-99.7 to -99.5)	0<br>(0 to 0)	-99.6<br>(-99.7 to -99.4)	-99.6<br>(-99.7 to -99.5)

Other neglected tropical diseases	49,493<br>(47,574 to 51,781)	7.2<br>(5.9 to 8.5)	-0.8<br>(-2.0 to 0.4)	--	--	--
**Maternal disorders**	**8,730<br>(8,105 to 9,255)**	**1.8<br>(-7.3 to 12.3)**	**-7.3<br>(-15.6 to 2.0)**	**118,297†<br>(104,358 to 137,177)**	**-1.2<br>(-3.3 to 0.9)**	**-9.6<br>(-11.5 to -7.9)**
Maternal haemorrhage	1,403<br>(1,155 to 1,706)	6.1<br>(-2.5 to 16.1)	-3.3<br>(-11.1 to 5.7)	9,626<br>(7,835 to 11,884)	2.0<br>(-0.9 to 5.3)	-7.0<br>(-9.5 to -4.1)
Maternal sepsis and other pregnancy related infections	1,662<br>(1,256 to 2,201)	7.2<br>(-25.7 to 56.8)	-3.7<br>(-32.9 to 40.2)	10,377<br>(8,385 to 13,151)	0.6<br>(-2.1 to 3.5)	-7.1<br>(-9.3 to -4.9)
Maternal hypertensive disorders	4,449<br>(2,947 to 6,101)	7.7<br>(-32.1 to 75.0)	-1.0<br>(-37.5 to 60.4)	20,811<br>(18,217 to 23,245)	6.8<br>(3.7 to 9.4)	-1.8<br>(-4.4 to 0.3)
Maternal obstructed labour and uterine rupture	1,041<br>(867 to 1,229)	-14.0<br>(-19.0 to -8.9)	-22.9<br>(-27.2 to -18.3)	7,238<br>(5,821 to 9,042)	0.3<br>(-2.6 to 3.7)	-8.3<br>(-10.6 to -5.4)
Maternal abortion, miscarriage, and ectopic pregnancy	576<br>(371 to 820)	-4.6<br>(-40.1 to 49.0)	-13.0<br>(-45.5 to 36.6)	70,245<br>(56,320 to 88,126)	-4.1<br>(-7.0 to -1.2)	-12.5<br>(-14.8 to -10.2)
Other maternal disorders	--	--	--	--	--	--
**Neonatal disorders**	**80,244<br>(73,591 to 86,593)**	**13.1<br>(8.9 to 18.5)**	**3.4<br>(-0.3 to 8.4)**	**--**	**--**	**--**
Neonatal preterm birth complications	66,092<br>(55,818 to 78,007)	11.3<br>(2.5 to 21.9)	1.7<br>(-6.2 to 11.4)	--	--	--
Neonatal encephalopathy due to birth asphyxia and trauma	12,134<br>(7,668 to 19,247)	18.6<br>(8.7 to 27.6)	7.8<br>(-1.3 to 16.0)	--	--	--
Neonatal sepsis and other neonatal infections	6,653<br>(2,713 to 15,058)	28.9<br>(21.9 to 36.6)	18.6<br>(12.4 to 25.2)	--	--	--
Haemolytic disease and other neonatal jaundice	2,093<br>(1,861 to 2,337)	22.7<br>(20.1 to 25.5)	12.4<br>(10.0 to 15.0)	--	--	--
Other neonatal disorders	--	--	--	--	--	--
**Nutritional deficiencies**	**1,673,391<br>(1,633,390 to 1,689,822)**	**6.2<br>(5.5 to 6.9)**	**-3.7<br>(-4.4 to -3.1)**	**598,813†<br>(516,074 to 717,722)**	**-5.9<br>(-8.6 to -2.7)**	**-13.2<br>(-15.7 to -10.1)**
Protein-energy malnutrition	63,135<br>(62,102 to 64,197)	0.7<br>(-2.1 to 3.4)	-3.5<br>(-6.2 to -0.9)	43,590<br>(42,935 to 44,253)	-3.2<br>(-5.6 to -0.8)	-6.5<br>(-8.9 to -4.3)
Iodine deficiency	119,214<br>(105,725 to 133,961)	-5.9<br>(-7.0 to -4.6)	-17.0<br>(-17.9 to -15.9)	3,043<br>(2,708 to 3,411)	-18.3<br>(-19.3 to -17.0)	-21.8<br>(-22.8 to -20.6)
Vitamin A deficiency	508,801<br>(445,148 to 601,630)	-2.7<br>(-4.7 to -0.5)	-10.7<br>(-12.6 to -8.5)	552,180<br>(469,061 to 671,144)	-6.0<br>(-8.9 to -2.6)	-13.6<br>(-16.4 to -10.3)
Iron-deficiency anaemia	1,244,781<br>(1,205,796 to 1,284,426)	9.5<br>(8.6 to 10.6)	-0.9<br>(-1.7 to 0.0)	--	--	--
Other nutritional deficiencies	--	--	--	--	--	--
**Other communicable, maternal, neonatal, and nutritional diseases**	**1,728,266<br>(1,680,548 to 1,764,134)**	**14.1<br>(12.3 to 15.9)**	**-1.2<br>(-2.7 to 0.2)**	**857,430<br>(713,393 to 1,035,257)**	**-14.1<br>(-38.9 to 25.6)**	**-21.5<br>(-44.3 to 16.5)**
Sexually transmitted diseases excluding HIV	1,175,931<br>(1,131,754 to 1,217,694)	16.9<br>(15.8 to 18.0)	-0.3<br>(-1.2 to 0.6)	552,776<br>(499,026 to 612,824)	13.8<br>(11.0 to 16.5)	2.4<br>(0.0 to 5.0)
Syphilis	56,092<br>(44,184 to 70,172)	10.8<br>(6.7 to 14.7)	-1.5<br>(-5.4 to 1.7)	38,838<br>(29,431 to 50,913)	9.5<br>(4.4 to 13.7)	-1.8<br>(-6.5 to 1.8)
Chlamydial infection	93,574<br>(75,600 to 114,406)	7.1<br>(5.8 to 8.4)	-3.4<br>(-4.4 to -2.4)	73,667<br>(59,690 to 90,359)	5.0<br>(3.7 to 6.4)	-4.8<br>(-5.6 to -3.9)
Gonococcal infection	57,124<br>(46,375 to 70,820)	20.9<br>(14.0 to 27.4)	9.1<br>(2.7 to 14.7)	190,766<br>(147,718 to 242,152)	21.2<br>(13.3 to 27.5)	9.6<br>(2.6 to 15.3)
Trichomoniasis	171,779<br>(148,048 to 196,705)	15.8<br>(14.9 to 16.6)	1.4<br>(0.8 to 1.9)	148,908<br>(126,821 to 171,971)	15.1<br>(14.1 to 16.1)	1.7<br>(1.1 to 2.4)
Genital herpes	859,580<br>(748,108 to 991,917)	18.7<br>(16.9 to 20.5)	-0.4<br>(-1.8 to 1.1)	57,156<br>(49,205 to 67,554)	8.3<br>(6.1 to 10.3)	2.4<br>(0.6 to 4.2)
Other sexually transmitted diseases	36,588<br>(31,753 to 40,490)	10.8<br>(8.3 to 13.2)	-1.0<br>(-3.1 to 0.9)	43,441<br>(37,364 to 50,632)	6.7<br>(4.6 to 8.8)	-6.6<br>(-8.3 to -4.9)
Hepatitis	640,226<br>(566,219 to 731,671)	11.0<br>(5.7 to 16.4)	-2.3<br>(-6.8 to 2.0)	304,644<br>(182,678 to 477,007)	-40.6<br>(-71.0 to 44.0)	-43.7<br>(-72.9 to 38.5)
Acute hepatitis A	12,270<br>(3,159 to 25,111)	-50.5<br>(-88.9 to 205.7)	-51.8<br>(-89.3 to 197.0)	159,516<br>(41,071 to 326,448)	-50.5<br>(-88.9 to 205.7)	-51.8<br>(-89.3 to 197.0)
Hepatitis B	468,435<br>(396,824 to 554,418)	10.9<br>(9.7 to 12.2)	-2.2<br>(-3.1 to -1.2)	118,976<br>(92,788 to 152,775)	-23.1<br>(-32.7 to -11.9)	-29.8<br>(-37.9 to -20.2)
Hepatitis C	158,055<br>(141,445 to 175,144)	24.0<br>(23.3 to 24.6)	6.7<br>(6.5 to 7.0)	7,094<br>(6,474 to 7,724)	5.1<br>(3.7 to 6.6)	-5.2<br>(-6.9 to -3.6)
Acute hepatitis E	1,466<br>(1,308 to 1,645)	-33.5<br>(-35.6 to -31.4)	-37.8<br>(-39.7 to -36.0)	19,058<br>(17,003 to 21,385)	-33.5<br>(-35.6 to -31.4)	-37.8<br>(-39.7 to -36.0)
Other infectious diseases	44,426<br>(42,785 to 46,296)	7.4<br>(6.2 to 8.6)	-1.4<br>(-2.5 to -0.3)	9<br>(6 to 13)	16.7<br>(14.3 to 19.2)	0.7<br>(-0.3 to 1.6)
**Non-communicable diseases**	**6,681,939<br>(6,668,902 to 6,695,003)**	**13.1<br>(13.0 to 13.2)**	**0.0<br>(-0.1 to 0.1)**	**18,358,708†<br>(17,561,181 to 19,263,162)**	**12.8<br>(12.4 to 13.3)**	**-0.9<br>(-1.2 to -0.6)**
**Neoplasms**	**42,986<br>(42,692 to 43,241)**	**41.3<br>(40.3 to 42.4)**	**8.9<br>(8.1 to 9.7)**	**17,228<br>(16,713 to 17,803)**	**28.4<br>(26.3 to 31.3)**	**-0.8<br>(-2.5 to 1.3)**
Lip and oral cavity cancer	1,387<br>(1,346 to 1,425)	34.8<br>(30.2 to 38.7)	5.4<br>(1.9 to 8.3)	382<br>(371 to 392)	33.1<br>(28.3 to 36.9)	3.7<br>(0.1 to 6.5)
Nasopharynx cancer	332<br>(314 to 352)	8.4<br>(0.8 to 15.6)	-10.9<br>(-17.0 to -5.1)	96<br>(91 to 101)	8.4<br>(1.2 to 15.2)	-11.2<br>(-16.9 to -5.8)
Other pharynx cancer	478<br>(452 to 494)	42.4<br>(34.0 to 47.6)	10.3<br>(3.8 to 14.3)	170<br>(159 to 176)	36.5<br>(27.3 to 42.3)	5.6<br>(-1.4 to 10.2)
Oesophageal cancer	556<br>(539 to 577)	13.5<br>(10.2 to 18.0)	-12.7<br>(-15.2 to -9.2)	443<br>(433 to 456)	7.1<br>(4.7 to 10.6)	-17.8<br>(-19.7 to -15.2)
Stomach cancer	2,199<br>(2,144 to 2,255)	25.3<br>(21.9 to 28.5)	-3.5<br>(-6.1 to -1.0)	1,157<br>(1,134 to 1,180)	15.4<br>(12.9 to 17.8)	-11.3<br>(-13.2 to -9.4)
Colon and rectum cancer	6,323<br>(6,112 to 6,632)	38.0<br>(32.5 to 45.8)	5.1<br>(1.0 to 10.9)	1,716<br>(1,658 to 1,795)	33.8<br>(28.2 to 41.0)	1.4<br>(-2.7 to 6.7)
Liver cancer	1,027<br>(974 to 1,076)	86.5<br>(77.1 to 96.6)	45.1<br>(38.2 to 52.4)	1,008<br>(953 to 1,042)	37.6<br>(32.4 to 43.1)	7.2<br>(3.2 to 11.4)
Liver cancer due to hepatitis B	599<br>(516 to 675)	70.0<br>(60.1 to 81.5)	34.1<br>(26.5 to 42.9)	437<br>(380 to 489)	35.0<br>(29.6 to 42.1)	6.4<br>(2.3 to 11.8)
Liver cancer due to hepatitis C	261<br>(236 to 286)	60.8<br>(53.2 to 67.7)	22.8<br>(16.9 to 28.1)	189<br>(170 to 207)	37.7<br>(32.3 to 42.1)	4.8<br>(0.8 to 8.3)
Liver cancer due to alcohol use	186<br>(157 to 216)	70.2<br>(56.2 to 85.8)	31.0<br>(20.2 to 42.8)	148<br>(125 to 171)	44.3<br>(33.1 to 57.1)	10.8<br>(2.2 to 20.5)
Liver cancer due to other causes	326<br>(288 to 371)	71.7<br>(60.0 to 82.3)	34.9<br>(25.9 to 43.3)	235<br>(210 to 264)	38.3<br>(31.1 to 44.1)	8.6<br>(2.9 to 13.2)
Gallbladder and biliary tract cancer	169<br>(152 to 179)	24.4<br>(18.5 to 30.9)	-5.7<br>(-10.2 to -0.7)	184<br>(169 to 193)	20.7<br>(15.9 to 25.7)	-8.6<br>(-12.2 to -4.8)
Pancreatic cancer	364<br>(349 to 375)	38.7<br>(33.6 to 42.3)	5.5<br>(1.6 to 8.3)	418<br>(406 to 425)	33.6<br>(29.6 to 36.2)	1.4<br>(-1.7 to 3.5)
Larynx cancer	638<br>(627 to 653)	25.3<br>(22.4 to 28.9)	-3.5<br>(-5.7 to -0.8)	187<br>(184 to 191)	21.9<br>(19.1 to 25.4)	-6.4<br>(-8.5 to -3.8)
Tracheal, bronchus, and lung cancer	2,836<br>(2,750 to 2,920)	39.3<br>(35.7 to 42.9)	7.0<br>(4.3 to 9.8)	2,008<br>(1,958 to 2,055)	28.4<br>(25.3 to 31.5)	-1.4<br>(-3.7 to 0.9)
Malignant skin melanoma	1,347<br>(1,156 to 1,506)	39.7<br>(33.6 to 44.0)	10.6<br>(5.9 to 14.0)	282<br>(243 to 314)	38.7<br>(32.7 to 42.9)	9.3<br>(4.5 to 12.6)
Non-melanoma skin cancer	853<br>(682 to 1,066)	5.3<br>(-2.4 to 14.1)	-21.8<br>(-27.6 to -15.3)	1,521<br>(1,109 to 2,008)	12.4<br>(6.3 to 18.9)	-15.3<br>(-20.3 to -10.1)
Non-melanoma skin cancer (squamous-cell carcinoma)	690<br>(415 to 996)	4.2<br>(-4.8 to 19.3)	-22.8<br>(-29.3 to -11.8)	635<br>(386 to 922)	4.7<br>(-3.7 to 19.0)	-21.8<br>(-28.2 to -11.1)
Non-melanoma skin cancer (basal-cell carcinoma)	103<br>(65 to 167)	23.7<br>(17.1 to 31.1)	-5.9<br>(-10.6 to -0.5)	886<br>(574 to 1,262)	18.6<br>(13.2 to 23.2)	-9.7<br>(-13.8 to -6.1)
Breast cancer	8,151<br>(7,808 to 8,610)	30.6<br>(23.5 to 39.5)	1.9<br>(-3.4 to 8.6)	1,702<br>(1,629 to 1,801)	29.0<br>(21.8 to 38.0)	0.4<br>(-5.0 to 7.2)
Cervical cancer	1,939<br>(1,544 to 2,082)	10.6<br>(3.8 to 19.5)	-10.4<br>(-15.7 to -3.3)	511<br>(414 to 542)	8.9<br>(2.3 to 17.7)	-11.9<br>(-17.1 to -4.9)
Uterine cancer	1,959<br>(1,882 to 2,076)	41.8<br>(35.6 to 51.7)	9.0<br>(4.3 to 16.4)	417<br>(401 to 442)	40.4<br>(34.2 to 50.1)	7.6<br>(3.0 to 15.0)
Ovarian cancer	786<br>(743 to 809)	25.3<br>(19.0 to 29.1)	-1.9<br>(-6.6 to 1.1)	254<br>(242 to 260)	24.1<br>(18.0 to 27.9)	-3.0<br>(-7.6 to -0.1)
Prostate cancer	5,697<br>(5,098 to 6,714)	44.8<br>(37.5 to 55.6)	9.6<br>(4.1 to 18.1)	1,436<br>(1,293 to 1,619)	40.1<br>(34.2 to 49.2)	5.7<br>(1.2 to 12.9)
Testicular cancer	340<br>(328 to 355)	32.2<br>(27.2 to 38.2)	15.4<br>(11.0 to 20.7)	67<br>(64 to 70)	30.5<br>(25.6 to 36.3)	13.7<br>(9.5 to 18.7)
Kidney cancer	1,289<br>(1,226 to 1,329)	28.7<br>(24.8 to 32.1)	1.1<br>(-2.0 to 3.7)	342<br>(331 to 350)	27.7<br>(24.1 to 30.7)	-0.3<br>(-3.0 to 2.0)

Bladder cancer	1,767 (1,721 to 1,813)	33.7 (29.7 to 37.6)	1.6 (-1.4 to 4.6)	437 (427 to 448)	31.0 (27.1 to 34.8)	-0.9 (-3.9 to 1.9)
Brain and nervous system cancer	781 (693 to 818)	42.6 (37.2 to 47.3)	17.5 (13.1 to 21.3)	330 (299 to 349)	36.9 (32.3 to 42.2)	11.9 (8.1 to 16.2)
Thyroid cancer	1,234 (1,187 to 1,313)	51.2 (44.7 to 61.1)	20.9 (15.8 to 28.8)	238 (229 to 253)	49.7 (43.3 to 59.4)	19.4 (14.3 to 27.2)
Mesothelioma	53 (49 to 56)	28.9 (23.0 to 33.2)	0.1 (-4.5 to 3.4)	35 (33 to 36)	27.6 (21.7 to 31.9)	-1.1 (-5.7 to 2.1)
Hodgkin lymphoma	291 (264 to 322)	16.1 (13.6 to 18.5)	-1.2 (-3.3 to 0.8)	73 (66 to 82)	10.3 (8.0 to 12.6)	-6.3 (-8.4 to -4.5)
Non-Hodgkin's lymphoma	1,669 (1,545 to 1,746)	50.8 (43.6 to 54.2)	18.2 (12.6 to 20.8)	461 (428 to 482)	44.5 (37.8 to 47.5)	13.1 (7.8 to 15.5)
Multiple myeloma	358 (305 to 398)	48.1 (42.6 to 54.9)	12.7 (8.6 to 18.0)	139 (121 to 155)	38.0 (33.2 to 43.0)	5.0 (1.3 to 8.7)
Leukaemia	1,675 (1,511 to 1,765)	25.3 (18.0 to 33.2)	3.2 (-2.6 to 9.5)	467 (423 to 489)	26.0 (19.3 to 32.4)	2.9 (-2.4 to 7.9)
Acute lymphoid leukaemia	292 (256 to 310)	32.1 (12.1 to 43.4)	18.3 (0.8 to 28.3)	76 (66 to 80)	30.8 (12.6 to 40.8)	16.2 (0.4 to 25.1)
Chronic lymphoid leukaemia	460 (430 to 496)	38.6 (33.4 to 45.7)	6.0 (2.2 to 11.3)	105 (98 to 113)	38.1 (33.0 to 44.9)	5.0 (1.3 to 10.0)
Acute myeloid leukaemia	159 (141 to 169)	32.3 (20.2 to 39.4)	10.3 (0.9 to 15.7)	103 (91 to 108)	33.3 (25.4 to 37.9)	8.7 (2.6 to 12.3)
Chronic myeloid leukaemia	92 (84 to 99)	7.3 (2.7 to 11.8)	-14.6 (-18.2 to -11.3)	32 (29 to 34)	6.3 (1.9 to 10.9)	-15.7 (-19.0 to -12.5)
Other leukaemia	672 (565 to 723)	16.4 (8.1 to 26.4)	-2.5 (-9.0 to 5.5)	150 (127 to 161)	16.9 (9.0 to 26.4)	-2.8 (-9.0 to 4.8)
Other neoplasms	2,632 (2,377 to 2,719)	60.0 (53.0 to 64.7)	28.5 (22.8 to 32.3)	750 (682 to 772)	51.6 (45.1 to 55.7)	21.2 (15.8 to 24.5)
**Cardiovascular diseases**	**469,454 (462,582 to 474,872)**	**26.7 (26.0 to 27.3)**	**-0.8 (-1.3 to -0.3)**	**54,120† (51,965 to 56,447)**	**23.1 (21.8 to 24.5)**	**-5.1 (-6.0 to -4.0)**
Rheumatic heart disease	29,677 (26,737 to 32,927)	16.7 (12.9 to 20.1)	3.2 (-0.1 to 6.3)	1,196 (1,095 to 1,308)	8.7 (5.6 to 11.5)	-0.5 (-3.4 to 2.3)
Ischaemic heart disease	153,533 (145,992 to 160,779)	24.5 (22.2 to 26.7)	-3.8 (-5.6 to -2.0)	20,731 (19,530 to 22,073)	15.6 (14.0 to 17.2)	-11.7 (-13.0 to -10.5)
Cerebrovascular disease	79,574 (77,057 to 82,118)	32.3 (29.9 to 35.0)	2.0 (0.0 to 4.1)	13,677‡ (12,713 to 14,692)	31.9 (27.0 to 36.8)	0.7 (-2.9 to 4.5)
Ischaemic stroke	67,595 (60,812 to 74,572)	33.5 (26.3 to 40.2)	2.7 (-2.9 to 8.1)	9,556‡ (8,655 to 10,513)	37.5 (31.1 to 44.0)	4.6 (-0.2 to 9.7)
Haemorrhagic stroke	15,310 (13,857 to 16,954)	25.8 (19.5 to 32.2)	-1.7 (-6.7 to 3.3)	4,120‡ (3,764 to 4,508)	20.7 (14.7 to 26.7)	-7.4 (-11.9 to -2.9)
Hypertensive heart disease	17,190 (14,948 to 19,774)	31.7 (29.9 to 33.6)	0.3 (-0.7 to 1.4)	--	--	--
Cardiomyopathy and myocarditis	6,118 (5,779 to 6,416)	19.8 (17.8 to 21.9)	-4.7 (-6.5 to -3.0)	2,483† (2,217 to 2,784)	19.1 (17.7 to 20.8)	-0.7 (-1.2 to -0.3)
Myocarditis	1,786 (1,607 to 1,977)	14.1 (12.9 to 15.3)	-1.4 (-1.9 to -0.9)	2,483 (2,217 to 2,784)	19.1 (17.7 to 20.8)	-0.7 (-1.2 to -0.3)
Alcoholic cardiomyopathy	1,165 (994 to 1,359)	20.5 (16.7 to 24.3)	-5.5 (-8.3 to -2.8)	--	--	--
Other cardiomyopathy	4,005 (3,534 to 4,525)	21.0 (18.9 to 23.0)	-4.7 (-6.0 to -3.4)	--	--	--
Atrial fibrillation and flutter	46,311 (41,441 to 52,014)	32.0 (31.2 to 32.9)	0.2 (-0.4 to 0.7)	3,841 (3,379 to 4,388)	30.9 (29.7 to 32.0)	0.3 (-0.4 to 0.9)
Peripheral vascular disease	120,145 (105,640 to 137,704)	30.0 (29.0 to 31.0)	-1.6 (-2.3 to -0.9)	11,020 (9,557 to 12,778)	28.4 (26.9 to 30.1)	-2.0 (-2.7 to -1.4)
Endocarditis	500 (448 to 568)	23.2 (21.6 to 25.0)	-3.6 (-4.7 to -2.5)	1,172 (1,068 to 1,280)	24.3 (22.2 to 26.6)	3.5 (2.1 to 5.0)
Other cardiovascular and circulatory diseases	87,122 (76,311 to 98,866)	20.7 (18.9 to 22.6)	-2.4 (-3.5 to -1.3)	--	--	--
**Chronic respiratory diseases**	**571,426 (560,139 to 582,621)**	**20.9 (20.3 to 21.5)**	**1.7 (1.2 to 2.3)**	**94,696† (90,108 to 99,441)**	**19.6 (18.7 to 20.6)**	**2.9 (2.2 to 3.6)**
Chronic obstructive pulmonary disease	251,631 (241,958 to 261,129)	26.7 (25.6 to 27.9)	-0.6 (-1.6 to 0.3)	18,721 (18,004 to 19,457)	23.1 (21.8 to 24.5)	-3.1 (-4.0 to -2.1)
Pneumoconiosis	1,111 (1,043 to 1,184)	32.4 (31.1 to 33.7)	1.7 (0.8 to 2.6)	99 (93 to 105)	29.0 (27.6 to 30.6)	-1.4 (-2.3 to -0.4)
Silicosis	421 (380 to 464)	32.3 (30.2 to 34.9)	1.3 (-0.2 to 3.1)	45 (41 to 49)	28.8 (26.4 to 31.3)	-3.0 (-4.4 to -1.6)
Asbestosis	153 (138 to 170)	27.1 (25.8 to 28.6)	-1.1 (-2.1 to 0.0)	12 (11 to 13)	31.0 (29.4 to 32.9)	2.8 (1.5 to 4.1)
Coal workers pneumoconiosis	294 (256 to 340)	35.0 (32.0 to 38.1)	2.9 (0.7 to 5.2)	21 (19 to 25)	28.6 (25.2 to 31.8)	-1.4 (-3.7 to 1.2)
Other pneumoconiosis	243 (210 to 277)	32.6 (30.2 to 35.3)	2.8 (1.0 to 4.6)	21 (18 to 24)	28.8 (25.7 to 32.2)	-0.1 (-2.0 to 1.9)
Asthma	339,440 (319,582 to 360,796)	17.5 (16.3 to 18.6)	3.5 (2.5 to 4.5)	75,496 (71,071 to 80,177)	18.7 (17.5 to 19.8)	4.5 (3.6 to 5.4)
Interstitial lung disease and pulmonary sarcoidosis	3,978 (3,632 to 4,351)	29.6 (28.2 to 30.9)	2.0 (1.2 to 2.7)	379 (348 to 412)	34.7 (33.0 to 36.5)	4.6 (3.9 to 5.5)
Other chronic respiratory diseases	--	--	--	--	--	--
**Cirrhosis and other chronic liver diseases**	**45,783 (43,781 to 47,831)**	**27.8 (26.6 to 29.0)**	**5.0 (4.0 to 5.9)**	**1,638 (1,575 to 1,708)**	**19.6 (18.3 to 20.9)**	**-4.0 (-4.9 to -3.1)**
Cirrhosis and other chronic liver diseases due to hepatitis B	12,482 (11,611 to 13,474)	29.7 (28.1 to 31.4)	5.7 (4.6 to 6.8)	457 (421 to 500)	21.0 (19.3 to 22.6)	-3.9 (-5.2 to -2.8)
Cirrhosis and other chronic liver diseases due to hepatitis C	10,903 (9,925 to 11,984)	30.5 (28.8 to 32.2)	5.9 (4.7 to 7.1)	387 (353 to 426)	22.2 (20.6 to 23.9)	-3.7 (-4.9 to -2.5)
Cirrhosis and other chronic liver diseases due to alcohol use	11,392 (10,521 to 12,386)	29.5 (27.7 to 31.2)	4.2 (3.0 to 5.4)	404 (373 to 439)	21.0 (19.2 to 22.7)	-5.0 (-6.3 to -3.7)
Cirrhosis and other chronic liver diseases due to other causes	11,007 (10,105 to 11,958)	21.6 (20.0 to 23.2)	4.2 (3.3 to 5.2)	391 (363 to 421)	14.2 (13.0 to 15.7)	-3.2 (-4.1 to -2.3)
**Digestive diseases**	**262,283 (257,495 to 266,222)**	**29.1 (28.0 to 30.2)**	**6.6 (5.7 to 7.5)**	**211,761† (201,555 to 223,065)**	**26.5 (25.3 to 27.7)**	**4.9 (4.3 to 5.6)**
Peptic ulcer disease	74,335 (69,108 to 80,393)	34.1 (31.6 to 36.9)	9.9 (8.1 to 12.0)	10,329 (9,391 to 11,368)	13.5 (11.4 to 15.4)	-5.1 (-6.9 to -3.5)
Gastritis and duodenitis	123,619 (112,199 to 135,163)	34.0 (31.3 to 36.6)	9.7 (7.9 to 11.5)	77,140 (70,073 to 85,452)	27.1 (24.7 to 29.6)	4.8 (3.4 to 6.1)
Appendicitis	857 (796 to 922)	9.9 (9.1 to 10.8)	0.0 (-0.5 to 0.5)	22,580 (20,993 to 24,258)	9.9 (9.1 to 10.8)	0.0 (-0.5 to 0.5)
Paralytic ileus and intestinal obstruction	170 (155 to 186)	25.7 (23.9 to 27.6)	5.1 (4.1 to 6.1)	4,651 (4,265 to 5,080)	26.1 (24.3 to 27.9)	4.9 (3.8 to 5.8)
Inguinal, femoral, and abdominal hernia	51,239 (46,088 to 56,543)	17.0 (14.3 to 19.9)	-0.8 (-2.5 to 1.2)	36,241 (31,967 to 40,531)	41.6 (39.3 to 44.5)	14.4 (13.0 to 15.9)
Inflammatory bowel disease	6,007 (5,622 to 6,446)	27.4 (25.1 to 29.8)	4.0 (2.4 to 5.7)	3,518 (3,275 to 3,779)	9.0 (8.0 to 10.0)	-5.5 (-6.1 to -4.9)
Vascular intestinal disorders	104 (93 to 115)	26.0 (23.4 to 28.7)	-0.1 (-1.8 to 1.9)	583 (527 to 645)	23.9 (21.3 to 26.5)	-1.9 (-4.0 to 0.1)
Gallbladder and biliary diseases	17,269 (15,618 to 19,012)	25.4 (22.9 to 28.0)	3.6 (2.1 to 5.2)	51,509 (46,439 to 56,953)	28.7 (26.1 to 31.2)	4.1 (2.7 to 5.6)
Pancreatitis	584 (532 to 639)	30.5 (27.2 to 33.9)	6.6 (4.2 to 9.1)	5,210 (4,769 to 5,703)	29.9 (26.6 to 33.3)	6.1 (3.7 to 8.6)
Other digestive diseases	--	--	--	--	--	--
**Neurological disorders**	**2,595,647 (2,542,097 to 2,651,997)**	**12.0 (11.4 to 12.5)**	**-1.4 (-1.9 to -0.9)**	**1,183,726 (1,021,730 to 1,362,193)**	**8.5 (7.1 to 9.7)**	**-3.0 (-3.5 to -2.5)**
Alzheimer's disease and other dementias	43,836 (37,756 to 51,028)	37.5 (35.9 to 39.0)	0.7 (-0.3 to 1.5)	7,779 (6,620 to 9,135)	37.7 (35.4 to 40.3)	0.6 (-0.5 to 1.5)
Parkinson's disease	6,063 (4,971 to 7,325)	36.3 (34.2 to 38.2)	3.2 (1.8 to 4.4)	696 (564 to 850)	34.8 (32.9 to 37.0)	3.0 (1.7 to 4.1)
Epilepsy	23,962 (20,402 to 27,737)	15.1 (4.9 to 26.6)	2.3 (-6.6 to 12.5)	2,761 (2,308 to 3,268)	11.7 (1.4 to 23.1)	2.6 (-6.8 to 13.2)
Multiple sclerosis	2,221 (2,034 to 2,437)	24.8 (23.4 to 26.4)	3.1 (1.9 to 4.4)	69 (63 to 76)	16.8 (15.2 to 18.5)	4.5 (3.4 to 5.6)
Motor neuron disease	331 (300 to 367)	21.2 (19.5 to 23.1)	1.1 (0.2 to 2.2)	57 (53 to 63)	27.2 (25.3 to 29.1)	2.0 (0.8 to 3.1)

Migraine	1,044,771 (999,535 to 1,087,969)	13.3 (12.9 to 13.7)	-0.5 (-0.7 to -0.2)	110,317 (105,270 to 115,834)	10.7 (10.1 to 11.2)	0.0 (-0.2 to 0.3)
Tension-type headache	1,890,670 (1,707,786 to 2,097,762)	10.5 (9.5 to 11.5)	-2.3 (-2.7 to -1.9)	1,061,998 (899,411 to 1,241,310)	8.1 (6.5 to 9.4)	-3.3 (-3.9 to -2.8)
Other neurological disorders	24 (16 to 33)	16.8 (14.1 to 19.6)	0.5 (-0.4 to 1.2)	49 (32 to 66)	16.9 (14.5 to 19.6)	0.7 (-0.3 to 1.6)
**Mental and substance use disorders**	**1,111,147 (1,095,612 to 1,128,948)**	**12.1 (11.6 to 12.6)**	**-1.5 (-1.9 to -1.0)**	**410,091† (385,617 to 437,586)**	**10.8 (9.9 to 11.5)**	**-3.5 (-4.1 to -3.0)**
Schizophrenia	20,883 (18,497 to 23,422)	16.9 (16.0 to 18.0)	-0.9 (-1.5 to -0.4)	1,139 (1,006 to 1,287)	8.0 (6.0 to 9.8)	-2.4 (-3.2 to -1.8)
Alcohol use disorders	100,389 (89,592 to 111,659)	9.7 (7.8 to 11.8)	-4.9 (-6.7 to -3.2)	50,432 (44,249 to 56,797)	10.1 (7.8 to 12.4)	-3.5 (-5.4 to -1.7)
Drug use disorders	61,968 (60,342 to 63,579)	10.8 (9.5 to 11.9)	-0.9 (-2.0 to 0.1)	7,460 (6,810 to 8,140)	4.7 (3.2 to 6.1)	-1.6 (-2.6 to -0.7)
Opioid use disorders	26,834 (23,563 to 30,952)	18.1 (16.0 to 20.1)	2.6 (0.7 to 4.3)	2,795 (2,443 to 3,226)	12.1 (10.0 to 14.1)	1.0 (-0.6 to 2.5)
Cocaine use disorders	5,840 (5,322 to 6,473)	12.0 (11.0 to 13.0)	-1.4 (-2.4 to -0.5)	347 (304 to 391)	3.3 (2.0 to 4.5)	1.7 (0.7 to 2.7)
Amphetamine use disorders	4,955 (3,693 to 6,490)	3.6 (1.1 to 6.2)	-4.1 (-6.6 to -1.7)	440 (330 to 577)	-5.7 (-8.3 to -2.7)	-7.1 (-8.9 to -5.0)
Cannabis use disorders	22,095 (18,965 to 25,855)	3.6 (1.8 to 5.4)	-4.3 (-5.5 to -3.1)	3,183 (2,706 to 3,776)	-1.2 (-3.1 to 1.0)	-3.9 (-5.3 to -2.2)
Other drug use disorders	3,944 (3,537 to 4,362)	17.2 (15.9 to 18.5)	1.0 (-0.1 to 2.0)	695 (624 to 770)	14.3 (12.8 to 15.8)	0.6 (-0.3 to 1.4)
Depressive disorders	268,172 (260,480 to 274,697)	14.8 (13.9 to 15.8)	-2.7 (-3.5 to -2.0)	274,704 (251,855 to 300,436)	11.5 (10.4 to 12.5)	-4.8 (-5.5 to -4.1)
Major depressive disorder	167,836 (153,908 to 183,754)	11.4 (10.3 to 12.5)	-5.0 (-5.7 to -4.3)	257,768 (234,546 to 284,665)	11.1 (9.9 to 12.2)	-5.1 (-5.9 to -4.4)
Dysthymia	105,580 (92,588 to 120,440)	20.8 (18.5 to 23.3)	1.0 (-0.5 to 2.6)	16,935 (14,981 to 19,313)	17.9 (15.7 to 20.2)	0.9 (-0.5 to 2.3)
Bipolar disorder	43,908 (38,383 to 50,196)	15.0 (14.1 to 16.0)	0.6 (0.3 to 0.9)	3,777 (3,313 to 4,331)	10.5 (9.4 to 11.8)	2.2 (1.8 to 2.5)
Anxiety disorders	274,615 (255,323 to 294,796)	13.3 (12.2 to 14.5)	-0.8 (-1.7 to 0.1)	42,407 (39,560 to 45,513)	11.6 (10.5 to 12.7)	0.0 (-0.9 to 0.9)
Eating disorders	10,523 (9,368 to 11,716)	17.5 (14.7 to 20.1)	9.3 (6.7 to 11.7)	5,440 (4,039 to 7,186)	10.5 (8.7 to 12.3)	7.4 (6.8 to 8.1)
Anorexia nervosa	2,606 (1,932 to 3,450)	12.2 (10.7 to 13.8)	6.2 (4.9 to 7.5)	897 (665 to 1,183)	11.0 (9.5 to 12.4)	7.0 (6.0 to 7.9)
Bulimia nervosa	7,559 (5,765 to 9,931)	19.2 (17.2 to 20.8)	10.1 (9.1 to 11.1)	4,543 (3,169 to 6,257)	10.5 (8.4 to 12.6)	7.5 (6.8 to 8.3)
Autistic spectrum disorders	62,174 (59,625 to 64,557)	11.6 (10.1 to 13.1)	0.4 (-0.9 to 1.7)	450 (307 to 624)	5.0 (4.3 to 5.6)	-0.3 (-0.6 to 0.0)
Autism	18,302 (15,766 to 21,177)	11.5 (10.9 to 12.0)	-0.1 (-0.6 to 0.4)	51 (26 to 86)	6.1 (5.1 to 7.0)	0.9 (0.2 to 1.7)
Asperger syndrome and other autistic spectrum disorders	43,798 (36,425 to 52,403)	11.6 (11.3 to 11.9)	0.6 (0.5 to 0.7)	399 (258 to 572)	4.8 (4.1 to 5.5)	-0.4 (-0.8 to -0.1)
Attention-deficit/hyperactivity disorder	62,624 (57,564 to 68,597)	6.1 (5.4 to 6.8)	-0.5 (-1.0 to 0.1)	2,873 (2,562 to 3,294)	9.0 (8.1 to 9.8)	2.3 (1.4 to 3.0)
Conduct disorder	49,005 (40,946 to 57,034)	2.1 (1.1 to 3.1)	2.2 (1.3 to 3.2)	16,887 (13,566 to 20,196)	4.1 (2.9 to 5.4)	1.8 (0.9 to 2.8)
Idiopathic developmental intellectual disability	114,797 (69,506 to 161,353)	3.9 (1.9 to 5.2)	-5.2 (-7.0 to -4.0)	--	--	--
Other mental and substance use disorders	139,505 (122,047 to 160,430)	18.0 (17.7 to 18.4)	0.0 (-0.1 to 0.1)	4,523 (4,003 to 5,122)	5.6 (4.8 to 6.7)	0.3 (0.0 to 0.5)
**Diabetes, urogenital, blood, and endocrine diseases**	**2,927,330 (2,909,185 to 2,949,337)**	**15.1 (14.3 to 16.0)**	**0.2 (-0.6 to 0.9)**	**811,323† (769,165 to 854,588)**	**13.2 (12.3 to 14.1)**	**-0.6 (-1.2 to -0.1)**
Diabetes mellitus	383,453 (352,588 to 414,576)	22.0 (19.3 to 24.8)	-1.9 (-4.1 to 0.3)	20,828 (19,161 to 22,734)	18.5 (15.2 to 22.2)	-0.7 (-3.3 to 2.0)
Acute glomerulonephritis	104 (90 to 117)	4.4 (2.3 to 6.5)	-10.8 (-12.0 to -9.6)	1,530 (1,357 to 1,729)	5.2 (3.1 to 7.3)	-10.1 (-11.2 to -9.0)
Chronic kidney disease	275,930 (252,442 to 300,414)	30.8 (28.2 to 33.2)	2.1 (0.2 to 3.9)	21,329 (19,100 to 23,599)	30.8 (27.3 to 33.9)	2.8 (0.2 to 5.3)
Chronic kidney disease due to diabetes mellitus	115,356 (101,848 to 130,636)	34.4 (31.6 to 37.2)	4.0 (2.0 to 6.1)	8,979 (7,742 to 10,377)	35.3 (31.6 to 39.1)	4.5 (1.4 to 7.3)
Chronic kidney disease due to hypertension	50,097 (44,151 to 55,693)	27.5 (24.2 to 30.7)	-0.2 (-2.5 to 2.2)	3,626 (3,131 to 4,163)	17.5 (13.5 to 21.1)	-4.2 (-7.3 to -1.4)
Chronic kidney disease due to glomerulonephritis	49,615 (43,695 to 55,597)	27.8 (24.7 to 30.8)	-0.7 (-3.0 to 1.5)	3,893 (3,261 to 4,529)	37.4 (33.0 to 41.7)	4.4 (1.0 to 7.5)
Chronic kidney disease due to other causes	60,862 (52,601 to 69,124)	29.4 (26.4 to 32.2)	2.7 (0.5 to 4.8)	4,831 (4,135 to 5,526)	28.6 (24.9 to 32.0)	3.7 (0.9 to 6.2)
Urinary diseases and male infertility	146,155 (135,457 to 155,774)	26.1 (24.8 to 27.4)	0.5 (-0.5 to 1.6)	449,242† (412,808 to 488,929)	15.8 (14.5 to 17.1)	0.9 (0.3 to 1.5)
Interstitial nephritis and urinary tract infections	6,913 (6,201 to 7,660)	14.3 (12.8 to 15.8)	1.0 (0.2 to 1.7)	360,815 (325,242 to 399,931)	14.3 (12.8 to 15.8)	1.1 (0.3 to 1.8)
Urolithiasis	2,839 (2,556 to 3,162)	22.1 (20.5 to 23.7)	0.5 (-0.3 to 1.4)	75,218 (67,725 to 83,689)	22.0 (20.4 to 23.6)	0.5 (-0.3 to 1.4)
Benign prostatic hyperplasia	103,655 (91,930 to 115,236)	28.9 (27.6 to 30.3)	-1.3 (-2.2 to -0.3)	13,208 (11,393 to 15,199)	27.2 (25.7 to 28.6)	-1.5 (-2.4 to -0.5)
Male infertility	28,044 (22,703 to 35,076)	18.8 (15.4 to 22.1)	7.2 (4.4 to 9.8)	--	--	--
Other urinary diseases	--	--	--	--	--	--
Gynecological diseases	837,557 (822,589 to 852,391)	12.8 (12.1 to 13.4)	-3.4 (-4.0 to -2.8)	300,826† (284,093 to 317,864)	7.6 (6.4 to 9.0)	-3.1 (-4.3 to -1.9)
Uterine fibroids	91,012 (82,090 to 100,488)	20.9 (20.2 to 21.7)	0.1 (-0.2 to 0.4)	9,395 (8,303 to 10,601)	11.0 (9.6 to 12.5)	-2.3 (-3.0 to -1.7)
Polycystic ovarian syndrome	10,642 (8,653 to 12,885)	7.7 (6.7 to 8.6)	-2.3 (-3.1 to -1.4)	7,651 (5,518 to 10,179)	18.2 (16.0 to 20.2)	9.6 (8.0 to 11.2)
Female infertility	31,477 (19,472 to 49,304)	38.3 (29.3 to 50.4)	25.1 (17.2 to 35.5)	--	--	--
Endometriosis	3,554 (3,197 to 3,935)	8.0 (6.5 to 9.6)	-3.8 (-4.9 to -2.6)	9,262 (7,894 to 10,960)	-6.4 (-8.3 to -4.4)	-11.0 (-12.6 to -9.5)
Genital prolapse	250,418 (222,236 to 278,690)	14.8 (13.7 to 15.8)	-9.5 (-10.3 to -8.8)	16,608 (14,433 to 19,185)	6.6 (5.3 to 7.8)	-8.6 (-9.8 to -7.5)
Premenstrual syndrome	454,296 (432,912 to 474,453)	9.3 (7.9 to 10.5)	-2.1 (-3.3 to -1.0)	162,435 (153,441 to 171,301)	6.5 (5.0 to 7.9)	-2.3 (-3.6 to -1.3)
Other gynecological diseases	104,692 (93,190 to 116,476)	11.2 (8.9 to 14.2)	-2.4 (-4.3 to 0.2)	95,475 (82,017 to 109,553)	10.2 (7.2 to 14.2)	-3.7 (-6.1 to -0.1)
Haemoglobinopathies and haemolytic anaemias	1,804,470 (1,782,496 to 1,828,051)	13.3 (11.7 to 14.9)	1.3 (0.0 to 2.7)	--	--	--
Thalassemias	366 (343 to 394)	-3.4 (-5.0 to -1.7)	-7.6 (-9.2 to -6.0)	--	--	--
Thalassaemias trait	287,107 (276,495 to 298,645)	11.3 (10.6 to 12.1)	-1.1 (-1.8 to -0.4)	--	--	--
Sickle cell disorders	3,888 (3,571 to 4,469)	20.1 (17.2 to 23.2)	14.9 (12.1 to 17.8)	--	--	--
Sickle cell trait	461,124 (418,742 to 516,943)	17.5 (16.7 to 18.8)	6.2 (5.5 to 7.5)	--	--	--
G6PD deficiency	331,513 (319,093 to 346,326)	13.1 (12.6 to 13.7)	1.8 (1.3 to 2.3)	--	--	--
G6PD trait	865,927 (851,718 to 882,248)	12.5 (12.3 to 12.7)	0.3 (0.1 to 0.5)	--	--	--
Other haemoglobinopathies and hemolytic anaemias	61,625 (59,671 to 63,634)	12.0 (10.9 to 13.1)	-1.4 (-2.3 to -0.5)	--	--	--
Endocrine, metabolic, blood, and immune disorders	127,493 (119,899 to 135,715)	18.8 (17.5 to 20.1)	-0.6 (-1.4 to 0.3)	17,569 (15,815 to 19,457)	24.9 (22.9 to 26.9)	0.3 (-0.9 to 1.7)
**Musculoskeletal disorders**	**1,270,630 (1,248,968 to 1,294,805)**	**19.9 (19.2 to 20.6)**	**-1.2 (-1.7 to -0.7)**	**652,005 (591,746 to 719,811)**	**17.7 (16.3 to 19.3)**	**-2.3 (-3.3 to -1.3)**
Rheumatoid arthritis	21,337 (19,466 to 23,612)	29.2 (27.1 to 31.1)	3.7 (2.3 to 5.0)	1,174 (1,065 to 1,298)	25.2 (22.8 to 27.5)	4.3 (2.9 to 5.7)
Osteoarthritis	301,567 (285,064 to 318,844)	30.0 (29.5 to 30.5)	0.9 (0.6 to 1.2)	14,696 (13,728 to 15,682)	25.6 (24.7 to 26.5)	0.8 (0.4 to 1.1)

Low back and neck pain	748,081 (721,756 to 766,729)	19.3 (18.3 to 20.4)	-1.2 (-2.0 to -0.4)	313,434 (281,503 to 347,952)	18.2 (16.6 to 19.7)	-1.7 (-2.7 to -0.9)
Low back pain	511,048 (457,208 to 572,056)	18.0 (16.1 to 19.9)	-2.1 (-3.6 to -1.0)	250,277 (220,585 to 283,681)	17.9 (15.9 to 19.8)	-2.2 (-3.4 to -1.1)
Neck pain	290,524 (254,815 to 332,009)	22.1 (20.1 to 24.2)	0.0 (-1.4 to 1.3)	63,157 (55,205 to 74,075)	19.7 (17.5 to 21.9)	0.2 (-1.0 to 1.3)
Gout	33,977 (30,617 to 37,923)	26.5 (25.4 to 27.7)	-0.1 (-0.6 to 0.5)	6,435 (5,666 to 7,248)	26.0 (24.7 to 27.3)	0.2 (-0.4 to 0.8)
Other musculoskeletal disorders	337,859 (289,444 to 390,647)	14.6 (11.6 to 17.4)	-3.6 (-5.7 to -1.6)	316,267 (266,448 to 375,370)	16.7 (13.8 to 19.7)	-3.1 (-5.1 to -1.3)
**Other non-communicable diseases**	**5,292,032 (5,257,502 to 5,326,591)**	**13.4 (13.2 to 13.7)**	**-0.3 (-0.5 to -0.1)**	**14,922,120† (14,136,832 to 15,793,314)**	**12.8 (12.2 to 13.3)**	**-0.7 (-1.0 to -0.3)**
Congenital anomalies	82,890 (78,027 to 87,071)	7.9 (5.7 to 10.1)	-2.2 (-4.1 to -0.2)	--	--	--
Neural tube defects	5,782 (5,232 to 6,393)	14.5 (12.5 to 17.0)	2.9 (1.1 to 5.2)	--	--	--
Congenital heart anomalies	15,377 (13,711 to 17,128)	7.4 (5.9 to 8.8)	-1.4 (-2.8 to -0.1)	--	--	--
Orofacial clefts	3,590 (3,229 to 3,967)	10.9 (8.6 to 12.8)	-1.2 (-3.2 to 0.6)	--	--	--
Down's syndrome	2,031 (1,775 to 2,315)	7.2 (4.9 to 9.4)	-1.2 (-3.4 to 1.0)	--	--	--
Turner syndrome	2,719 (2,378 to 3,089)	6.5 (5.0 to 7.9)	-1.3 (-2.7 to 0.0)	--	--	--
Klinefelter syndrome	3,043 (2,641 to 3,482)	7.0 (5.7 to 8.4)	0.6 (-0.7 to 1.9)	--	--	--
Other chromosomal abnormalities	4,310 (3,734 to 4,977)	3.7 (1.8 to 5.4)	1.5 (-0.3 to 3.2)	--	--	--
Congenital musculoskeletal and limb anomalies	10,812 (9,960 to 11,711)	12.5 (10.6 to 14.3)	-0.6 (-2.3 to 1.1)	--	--	--
Urogenital congenital anomalies	7,172 (6,493 to 7,894)	4.0 (3.2 to 4.8)	-2.9 (-3.7 to -2.2)	--	--	--
Digestive congenital anomalies	9,711 (8,741 to 10,731)	9.5 (7.1 to 11.9)	-3.1 (-5.3 to -0.9)	--	--	--
Other congenital anomalies	34,984 (24,584 to 49,402)	7.9 (5.1 to 10.9)	-3.2 (-5.7 to -0.6)	--	--	--
Skin and subcutaneous diseases	2,266,315 (2,242,994 to 2,285,332)	13.6 (13.2 to 14.0)	1.5 (1.2 to 1.9)	5,074,605 (4,838,566 to 5,335,844)	16.0 (15.4 to 16.7)	1.3 (1.1 to 1.5)
Dermatitis	306,359 (290,041 to 324,193)	12.2 (11.6 to 12.8)	0.7 (0.1 to 1.2)	430,376 (398,274 to 462,932)	14.0 (13.2 to 14.8)	-0.3 (-0.5 to -0.2)
Psoriasis	65,135 (62,708 to 67,812)	22.0 (21.5 to 22.4)	4.1 (3.9 to 4.4)	8,170 (7,861 to 8,478)	21.3 (20.8 to 21.8)	5.1 (4.9 to 5.3)
Cellulitis	3,018 (2,842 to 3,210)	14.4 (13.6 to 15.1)	-0.5 (-0.8 to -0.1)	61,333 (58,280 to 64,556)	14.5 (13.8 to 15.2)	-1.5 (-1.8 to -1.1)
Pyoderma	21,020 (20,491 to 21,564)	22.9 (22.5 to 23.3)	5.0 (4.7 to 5.3)	474,385 (461,024 to 488,663)	22.9 (22.5 to 23.3)	3.8 (3.5 to 4.1)
Scabies	146,785 (127,773 to 170,009)	3.3 (1.9 to 4.7)	-5.4 (-6.1 to -4.9)	454,671 (392,690 to 529,184)	2.7 (1.3 to 4.2)	-5.8 (-6.5 to -5.2)
Fungal skin diseases	626,700 (568,967 to 690,267)	18.2 (17.2 to 19.3)	2.4 (2.1 to 2.7)	2,098,743 (1,884,346 to 2,337,325)	19.3 (18.0 to 20.6)	1.7 (1.4 to 1.9)
Viral skin diseases	193,171 (184,773 to 201,619)	9.1 (8.8 to 9.5)	-0.2 (-0.4 to 0.0)	276,755 (263,471 to 290,018)	9.5 (9.1 to 10.0)	-0.2 (-0.4 to 0.0)
Acne vulgaris	614,771 (560,634 to 672,878)	5.1 (4.5 to 5.8)	2.0 (1.6 to 2.5)	429,822 (361,790 to 521,114)	4.5 (3.5 to 5.7)	2.8 (1.7 to 4.1)
Alopecia areata	15,415 (14,906 to 15,948)	12.9 (12.6 to 13.2)	-1.3 (-1.5 to -1.2)	27,247 (26,347 to 28,203)	12.7 (12.4 to 13.0)	-1.3 (-1.5 to -1.2)
Pruritus	66,780 (59,262 to 74,868)	18.4 (16.9 to 19.9)	1.2 (0.9 to 1.6)	53,046 (47,751 to 59,503)	18.1 (16.6 to 19.7)	1.2 (0.9 to 1.5)
Urticaria	67,060 (59,299 to 77,018)	9.5 (8.6 to 10.5)	-0.3 (-0.4 to -0.2)	119,403 (105,497 to 135,993)	9.2 (8.3 to 10.2)	-0.3 (-0.5 to -0.2)
Decubitus ulcer	1,836 (1,660 to 2,017)	27.9 (25.7 to 30.0)	-0.9 (-2.3 to 0.4)	6,722 (6,040 to 7,456)	28.3 (25.9 to 30.4)	-0.8 (-2.2 to 0.5)
Other skin and subcutaneous diseases	532,790 (519,540 to 545,725)	25.5 (25.1 to 25.9)	4.4 (4.3 to 4.6)	633,932 (616,730 to 651,837)	26.9 (26.4 to 27.4)	4.9 (4.7 to 5.1)
Sense organ diseases	1,843,201 (1,825,393 to 1,861,436)	21.5 (21.1 to 21.9)	-0.7 (-1.0 to -0.4)	708,004† (667,844 to 753,886)	15.9 (15.5 to 16.3)	0.8 (0.7 to 1.0)
Glaucoma	4,761 (3,973 to 5,573)	34.4 (32.3 to 36.2)	1.2 (-0.2 to 2.4)	--	--	--
Cataract	83,628 (73,800 to 93,905)	32.3 (29.4 to 34.8)	0.8 (-1.3 to 2.6)	--	--	--
Macular degeneration	6,147 (5,111 to 7,401)	43.0 (39.9 to 45.8)	7.2 (5.0 to 9.3)	--	--	--
Refraction and accommodation disorders	854,299 (822,109 to 885,545)	17.3 (16.6 to 17.9)	-3.8 (-4.2 to -3.4)	--	--	--
Age-related and other hearing loss	1,271,675 (1,210,237 to 1,337,731)	26.0 (25.2 to 26.8)	0.3 (-0.1 to 0.7)	--	--	--
Other vision loss	29,249 (25,873 to 32,927)	26.0 (23.7 to 28.3)	0.8 (-0.5 to 2.1)	--	--	--
Other sense organ diseases	210,697 (195,303 to 225,565)	23.5 (23.1 to 24.0)	0.8 (0.6 to 1.0)	708,004 (667,844 to 753,886)	15.9 (15.5 to 16.3)	0.8 (0.7 to 1.0)
Oral disorders	3,583,600 (3,475,812 to 3,657,885)	12.2 (11.7 to 12.8)	-1.4 (-1.8 to -1.0)	9,139,511† (8,388,756 to 9,926,991)	10.8 (10.0 to 11.5)	-1.9 (-2.5 to -1.3)
Caries of deciduous teeth	486,095 (398,110 to 573,474)	7.3 (5.4 to 8.5)	0.7 (-1.1 to 1.8)	1,762,264 (1,264,051 to 2,391,119)	8.2 (5.3 to 10.0)	1.3 (-1.4 to 3.1)
Caries of permanent teeth	2,435,850 (2,288,064 to 2,586,461)	9.4 (8.7 to 10.1)	-3.4 (-3.8 to -3.0)	7,263,992 (6,715,107 to 7,835,458)	11.3 (10.4 to 12.2)	-2.7 (-3.1 to -2.3)
Periodontal disease	750,847 (633,975 to 874,077)	25.9 (24.8 to 26.9)	2.2 (1.7 to 2.8)	89,841 (74,128 to 106,716)	24.2 (22.1 to 25.8)	2.3 (1.6 to 2.9)
Edentulism and severe tooth loss	302,342 (263,801 to 339,035)	27.3 (26.2 to 28.5)	-0.9 (-1.6 to -0.3)	23,414 (19,894 to 27,222)	26.8 (24.8 to 29.0)	-1.2 (-2.0 to -0.4)
Other oral disorders	134,457 (128,968 to 140,248)	15.4 (14.8 to 15.9)	0.0 (-0.2 to 0.1)	--	--	--
**Injuries**	**1,492,784 (1,413,602 to 1,584,568)**	**24.7 (24.2 to 25.1)**	**3.7 (3.4 to 4.0)**	**619,651† (592,467 to 648,150)**	**17.5 (16.5 to 18.5)**	**2.9 (2.1 to 3.8)**
**Transport injuries**	**241,195 (225,137 to 258,446)**	**27.7 (26.9 to 28.5)**	**5.1 (4.7 to 5.6)**	**75,419 (68,548 to 82,688)**	**20.3 (17.5 to 23.5)**	**6.5 (4.0 to 9.2)**
Road injuries	194,515 (182,106 to 208,390)	29.1 (28.2 to 30.1)	5.7 (5.1 to 6.3)	65,536 (58,945 to 72,433)	21.6 (18.4 to 25.2)	7.5 (4.7 to 10.5)
Pedestrian road injuries	43,124 (38,450 to 48,872)	31.4 (29.5 to 33.3)	7.5 (6.5 to 8.5)	11,343 (9,709 to 13,303)	23.2 (18.7 to 27.8)	8.4 (4.6 to 12.1)
Cyclist road injuries	38,983 (33,088 to 45,660)	30.5 (28.4 to 32.7)	7.1 (5.9 to 8.2)	15,288 (12,715 to 17,993)	22.2 (17.9 to 27.1)	8.3 (4.7 to 12.1)
Motorcyclist road injuries	46,084 (39,635 to 55,732)	31.1 (29.9 to 32.7)	7.9 (7.2 to 8.8)	11,347 (9,534 to 13,518)	22.3 (17.6 to 27.1)	9.5 (5.7 to 13.3)
Motor vehicle road injuries	51,784 (43,776 to 59,354)	20.5 (18.9 to 22.2)	-2.1 (-3.1 to -1.1)	20,993 (18,157 to 23,790)	15.6 (11.4 to 19.8)	1.5 (-2.1 to 4.9)
Other road injuries	14,539 (12,463 to 17,017)	47.8 (45.1 to 50.3)	21.9 (20.5 to 23.3)	6,566 (5,217 to 8,267)	38.8 (33.3 to 44.7)	23.3 (19.7 to 27.5)
Other transport injuries	46,680 (39,471 to 54,858)	22.1 (21.4 to 22.9)	2.9 (2.3 to 3.4)	9,883 (8,552 to 11,480)	11.9 (10.1 to 14.2)	0.5 (-0.8 to 2.0)
**Unintentional injuries**	**1,127,878 (1,053,751 to 1,211,481)**	**24.7 (24.2 to 25.1)**	**3.7 (3.4 to 4.0)**	**510,685 (484,967 to 539,046)**	**18.1 (17.0 to 19.3)**	**3.2 (2.2 to 4.2)**
Falls	474,230 (419,318 to 528,012)	27.6 (26.6 to 28.5)	4.7 (4.2 to 5.1)	208,272 (189,203 to 229,948)	22.8 (20.4 to 25.6)	5.0 (2.9 to 7.0)
Drowning	4,684 (4,129 to 5,324)	12.4 (11.3 to 13.4)	-6.9 (-7.5 to -6.4)	846 (763 to 938)	2.9 (0.7 to 5.2)	-11.0 (-13.2 to -8.9)
Fire, heat, and hot substances	102,124 (87,477 to 121,846)	20.9 (19.6 to 22.1)	1.4 (0.3 to 2.4)	10,997 (9,148 to 12,675)	12.6 (9.7 to 15.2)	1.9 (-0.9 to 4.5)
Poisonings	3,526 (2,984 to 4,133)	28.3 (26.6 to 29.8)	9.0 (7.8 to 10.2)	4,697 (3,858 to 5,639)	21.4 (18.1 to 24.4)	9.5 (6.6 to 11.9)

Exposure to mechanical forces	197,786<br>(168,555 to 241,945)	26.0<br>(24.9 to 27.1)	4.7<br>(4.1 to 5.3)	67,906<br>(59,032 to 76,818)	19.1<br>(16.6 to 21.7)	6.4<br>(4.3 to 8.5)
Unintentional firearm injuries	7,091<br>(5,867 to 8,629)	24.0<br>(22.9 to 25.2)	2.9<br>(2.1 to 3.6)	1,429<br>(1,052 to 1,893)	17.7<br>(13.4 to 21.9)	6.7<br>(3.1 to 10.0)
Unintentional suffocation	9,395<br>(7,248 to 12,049)	12.8<br>(7.2 to 17.9)	-2.2<br>(-6.6 to 2.0)	1,804<br>(1,347 to 2,370)	7.6<br>(2.4 to 12.3)	-0.5<br>(-5.2 to 3.8)
Other exposure to mechanical forces	181,300<br>(153,213 to 224,295)	26.9<br>(25.7 to 27.9)	5.2<br>(4.6 to 5.7)	64,673<br>(56,323 to 73,039)	19.5<br>(16.9 to 22.2)	6.6<br>(4.5 to 8.7)
Adverse effects of medical treatment	2,924<br>(2,328 to 3,567)	11.8<br>(10.2 to 13.4)	-4.4<br>(-6.0 to -2.8)	38,245<br>(34,405 to 42,385)	11.8<br>(10.2 to 13.5)	-4.5<br>(-6.1 to -2.9)
Animal contact	53,654<br>(47,673 to 61,282)	15.8<br>(15.0 to 16.6)	-2.3<br>(-2.8 to -1.9)	65,550<br>(56,597 to 74,579)	10.0<br>(7.5 to 12.3)	-1.4<br>(-3.6 to 0.4)
Venomous animal contact	24,759<br>(20,818 to 29,310)	18.0<br>(17.1 to 19.0)	0.2<br>(-0.3 to 0.8)	31,691<br>(25,886 to 38,600)	10.9<br>(7.9 to 13.5)	-0.5<br>(-3.1 to 1.6)
Non-venomous animal contact	28,895<br>(24,541 to 34,730)	14.0<br>(13.1 to 14.9)	-4.4<br>(-5.0 to -3.8)	33,859<br>(27,953 to 40,468)	9.2<br>(6.8 to 11.3)	-2.2<br>(-4.2 to -0.4)
Foreign body	54,797<br>(45,419 to 63,613)	22.5<br>(20.7 to 24.7)	4.9<br>(3.6 to 6.5)	35,172<br>(30,752 to 40,749)	20.1<br>(18.2 to 22.6)	6.4<br>(4.8 to 7.9)
Pulmonary aspiration and foreign body in airway	26,682<br>(20,450 to 35,018)	18.5<br>(15.8 to 21.6)	2.2<br>(0.2 to 4.7)	10,801<br>(9,348 to 12,717)	13.6<br>(11.7 to 15.7)	0.4<br>(-1.5 to 2.5)
Foreign body in eyes	3,168<br>(1,880 to 4,640)	27.5<br>(24.8 to 31.4)	9.2<br>(7.4 to 11.1)	15,443<br>(11,951 to 20,505)	24.5<br>(20.9 to 29.5)	9.9<br>(7.0 to 12.8)
Foreign body in other body part	24,947<br>(20,403 to 31,426)	26.5<br>(24.7 to 27.9)	7.5<br>(6.4 to 8.7)	8,929<br>(7,508 to 10,562)	21.3<br>(19.5 to 23.2)	8.7<br>(7.2 to 10.4)
Environmental heat and cold exposure	74,104<br>(63,212 to 86,149)	24.3<br>(22.9 to 25.3)	5.1<br>(4.3 to 5.9)	19,591<br>(16,473 to 23,143)	15.5<br>(13.2 to 17.7)	2.6<br>(0.9 to 4.1)
Other unintentional injuries	160,048<br>(131,378 to 191,818)	21.9<br>(20.9 to 23.0)	2.7<br>(2.0 to 3.5)	59,409<br>(51,227 to 68,804)	15.4<br>(13.0 to 17.4)	2.3<br>(0.3 to 4.1)
**Self-harm and interpersonal violence**	**230,043<br>(210,093 to 252,561)**	**7.7<br>(6.6 to 8.7)**	**-5.1<br>(-5.5 to -4.7)**	**32,470†<br>(28,739 to 36,265)**	**6.0<br>(3.8 to 8.0)**	**-6.0<br>(-8.0 to -4.2)**
Self-harm	11,924<br>(10,160 to 13,744)	14.4<br>(13.7 to 15.0)	-7.5<br>(-8.3 to -6.6)	6,893<br>(6,130 to 7,759)	1.6<br>(-1.0 to 4.1)	-13.6<br>(-15.8 to -11.3)
Self-harm by firearm	380<br>(326 to 439)	11.1<br>(9.6 to 12.8)	-7.0<br>(-8.8 to -5.0)	98<br>(61 to 149)	-0.6<br>(-6.4 to 4.9)	-11.5<br>(-17.5 to -5.7)
Self-harm by other specified means	11,544<br>(9,770 to 13,356)	14.5<br>(13.8 to 15.1)	-7.5<br>(-8.3 to -6.6)	6,795<br>(6,037 to 7,607)	1.6<br>(-1.0 to 4.2)	-13.6<br>(-15.8 to -11.3)
Interpersonal violence	218,118<br>(197,726 to 240,339)	7.3<br>(6.2 to 8.4)	-4.9<br>(-5.4 to -4.6)	25,577†<br>(22,086 to 29,230)	7.2<br>(4.6 to 9.6)	-3.6<br>(-6.0 to -1.6)
Assault by firearm	2,899<br>(2,387 to 3,485)	15.7<br>(13.7 to 17.6)	-4.3<br>(-5.6 to -2.9)	711<br>(560 to 897)	8.8<br>(4.5 to 13.0)	-1.3<br>(-5.3 to 2.3)
Assault by sharp object	15,012<br>(12,272 to 17,936)	15.5<br>(14.3 to 16.7)	-3.7<br>(-4.7 to -2.7)	5,603<br>(4,397 to 6,946)	5.8<br>(2.0 to 9.3)	-3.8<br>(-7.3 to -0.8)
Sexual violence	160,421<br>(141,228 to 181,452)	4.4<br>(3.2 to 5.7)	-5.4<br>(-6.0 to -4.9)	--	--	--
Assault by other means	39,786<br>(33,335 to 48,036)	16.7<br>(16.0 to 17.3)	-3.5<br>(-4.0 to -3.1)	19,263<br>(16,510 to 22,244)	7.6<br>(5.0 to 10.2)	-3.6<br>(-6.0 to -1.4)
**Forces of nature, conflict and terrorism, and executions and police conflict**	**54,091<br>(34,969 to 76,753)**	**23.8<br>(18.5 to 30.1)**	**8.9<br>(3.9 to 14.8)**	**1,077†<br>(759 to 1,328)**	**-30.1<br>(-36.7 to -22.4)**	**-37.0<br>(-43.0 to -29.8)**
Exposure to forces of nature	7,571<br>(4,293 to 11,602)	37.8<br>(21.3 to 52.6)	21.3<br>(6.9 to 33.7)	377<br>(231 to 534)	-35.4<br>(-49.1 to -21.2)	-41.3<br>(-53.9 to -28.5)
Conflict and terrorism	40,684<br>(24,541 to 61,725)	24.7<br>(18.0 to 33.3)	10.2<br>(3.8 to 18.4)	--	--	--
Executions and police conflict	5,836<br>(1,906 to 9,042)	4.7<br>(2.1 to 18.5)	-10.5<br>(-13.1 to 1.8)	700<br>(411 to 883)	-26.9<br>(-33.9 to -17.5)	-34.4<br>(-40.5 to -26.2)
**Impairments**	**--**	**--**	**--**	**--**	**--**	**--**
Anaemia	2,042,728<br>(1,979,003 to 2,110,037)	12.6<br>(11.5 to 13.7)	0.5<br>(-0.4 to 1.4)	--	--	--
Developmental intellectual disability	196,243<br>(150,264 to 242,144)	6.5<br>(5.9 to 7.2)	-3.2<br>(-3.7 to -2.5)	--	--	--
Epilepsy	45,926<br>(39,942 to 54,564)	16.8<br>(10.4 to 23.6)	4.5<br>(-1.3 to 10.7)	--	--	--
Guillain-Barre syndrome	65<br>(52 to 82)	18.4<br>(15.6 to 21.4)	1.9<br>(1.2 to 2.7)	--	--	--
Hearing loss	1,390,482<br>(1,329,902 to 1,455,762)	23.8<br>(23.1 to 24.5)	-0.2<br>(-0.5 to 0.2)	--	--	--
Heart failure	63,609<br>(56,944 to 71,388)	29.3<br>(28.1 to 30.5)	-1.3<br>(-2.0 to -0.6)	--	--	--
Infertility	117,207<br>(94,952 to 145,048)	20.1<br>(16.6 to 23.5)	8.0<br>(5.1 to 10.6)	--	--	--
Pelvic inflammatory disease	4,099<br>(3,533 to 4,740)	7.7<br>(6.0 to 9.5)	-5.6<br>(-7.0 to -4.2)	--	--	--
Vision loss	1,009,472<br>(969,235 to 1,047,829)	18.8<br>(18.0 to 19.5)	-3.1<br>(-3.6 to -2.6)	--	--	--

We did not calculate incidence for the nine impairments, certain NTDs, and causes with only birth prevalence. YLDs for residual categories do not have prevalence estimates.

† Incidence for aggregate causes represents the sum of the incidence of the causes below the aggregate.

‡ Incidence estimates for stroke represent first-ever stroke only.

^ Incidence of HIV/AIDS represents new infections of HIV only and does not include new infections of Tuberculosis in HIV positive cases.

SR Table 2: Global sequela-level prevalence, incidence, and YLDs, all ages, 2006 and 2016

Cause	Sequela	Prevalent cases in thousands (95% uncertainty interval)		Incident cases in thousands (95% uncertainty interval)		YLDs in thousands (95% uncertainty interval)	
		2006	2016	2006	2016	2006	2016
Drug-susceptible tuberculosis	Drug-susceptible tuberculosis	8,482.5 (7,576.9 to 9,570.5)	9,035.7 (8,134.5 to 10,107.5)	8,593.3 (7,637.4 to 9,770.9)	8,705.2 (7,754.6 to 9,817.7)	2,570.0 (1,703.7 to 3,481.7)	2,735.0 (1,814.8 to 3,696.5)
Multidrug-resistant tuberculosis without extensive drug resistance	Multidrug-resistant tuberculosis without extensive drug resistance	317.4 (281.0 to 360.1)	312.9 (279.1 to 352.9)	312.8 (277.5 to 355.6)	295.6 (261.4 to 335.6)	98.8 (65.5 to 136.0)	97.8 (65.6 to 136.4)
Extensively drug-resistant tuberculosis	Extensively drug-resistant tuberculosis	6.8 (6.0 to 7.8)	19.2 (16.9 to 22.1)	7.2 (6.3 to 8.3)	18.5 (16.1 to 21.2)	2.3 (1.5 to 3.2)	6.4 (4.2 to 9.0)
Latent tuberculosis infection	Latent tuberculosis infection	1,695,755.7 (1,582,887.5 to 1,808,835.0)	1,909,229.2 (1,788,353.5 to 2,034,184.5)	--	--	< 0.05 (0.0 to < 0.05)	< 0.05 (0.0 to < 0.05)
Drug-susceptible HIV/AIDS - Tuberculosis	Drug-susceptible HIV/AIDS - Tuberculosis with severe anaemia	14.4 (9.9 to 20.5)	11.4 (7.9 to 16.4)	15.6 (11.1 to 22.0)	11.9 (8.3 to 16.7)	7.0 (4.2 to 10.8)	5.6 (3.4 to 8.6)
Drug-susceptible HIV/AIDS - Tuberculosis	Drug-susceptible HIV/AIDS - Tuberculosis without anaemia	1,097.5 (786.2 to 1,463.4)	835.4 (591.9 to 1,131.8)	1,444.1 (1,058.9 to 1,926.8)	1,056.7 (757.5 to 1,433.9)	415.8 (250.8 to 634.8)	317.9 (190.8 to 486.8)
Drug-susceptible HIV/AIDS - Tuberculosis	Drug-susceptible HIV/AIDS - Tuberculosis with moderate anaemia	101.7 (74.3 to 138.8)	85.6 (60.9 to 119.6)	133.2 (98.5 to 178.1)	105.2 (76.5 to 142.6)	42.4 (26.2 to 63.2)	36.0 (22.0 to 54.8)
Drug-susceptible HIV/AIDS - Tuberculosis	Drug-susceptible HIV/AIDS - Tuberculosis with mild anaemia	154.4 (112.6 to 207.6)	143.5 (101.6 to 198.4)	212.1 (156.7 to 281.4)	190.9 (138.0 to 260.7)	59.5 (36.2 to 90.0)	55.8 (33.5 to 85.2)
Multidrug-resistant HIV/AIDS - Tuberculosis without extensive drug resistance	Multidrug-resistant HIV/AIDS - Tuberculosis without extensive drug resistance with mild anaemia	4.6 (3.1 to 6.5)	4.0 (2.7 to 6.0)	6.1 (4.2 to 8.7)	5.5 (3.6 to 8.1)	1.9 (1.1 to 2.9)	1.7 (1.0 to 2.7)
Multidrug-resistant HIV/AIDS - Tuberculosis without extensive drug resistance	Multidrug-resistant HIV/AIDS - Tuberculosis without extensive drug resistance with severe anaemia	0.4 (0.2 to 0.6)	0.3 (0.2 to 0.5)	0.4 (0.3 to 0.6)	0.3 (0.2 to 0.5)	0.2 (0.1 to 0.3)	0.1 (0.1 to 0.2)
Multidrug-resistant HIV/AIDS - Tuberculosis without extensive drug resistance	Multidrug-resistant HIV/AIDS - Tuberculosis without extensive drug resistance without anaemia	31.1 (20.6 to 44.6)	21.1 (13.9 to 31.2)	39.9 (26.8 to 56.7)	27.1 (17.8 to 39.2)	12.4 (7.2 to 19.6)	8.5 (4.9 to 14.0)
Multidrug-resistant HIV/AIDS - Tuberculosis without extensive drug resistance	Multidrug-resistant HIV/AIDS - Tuberculosis without extensive drug resistance with moderate anaemia	2.9 (2.0 to 4.2)	2.4 (1.6 to 3.5)	3.7 (2.5 to 5.2)	2.9 (1.9 to 4.2)	1.3 (0.8 to 2.0)	1.0 (0.6 to 1.7)
Extensively drug-resistant HIV/AIDS - Tuberculosis	Extensively drug-resistant HIV/AIDS - Tuberculosis with moderate anaemia	< 0.05 (< 0.05 to 0.1)	0.1 (0.1 to 0.2)	< 0.05 (< 0.05 to 0.1)	0.1 (0.1 to 0.2)	< 0.05 (< 0.05 to < 0.05)	< 0.05 (< 0.05 to 0.1)
Extensively drug-resistant HIV/AIDS - Tuberculosis	Extensively drug-resistant HIV/AIDS - Tuberculosis with severe anaemia	< 0.05 (< 0.05 to < 0.05)	< 0.05 (< 0.05 to < 0.05)	< 0.05 (< 0.05 to < 0.05)	< 0.05 (< 0.05 to < 0.05)	< 0.05 (< 0.05 to < 0.05)	< 0.05 (< 0.05 to < 0.05)
Extensively drug-resistant HIV/AIDS - Tuberculosis	Extensively drug-resistant HIV/AIDS - Tuberculosis without anaemia	0.4 (0.2 to 0.6)	0.9 (0.5 to 1.3)	0.4 (0.3 to 0.7)	0.9 (0.6 to 1.4)	0.2 (0.1 to 0.3)	0.4 (0.2 to 0.6)
Extensively drug-resistant HIV/AIDS - Tuberculosis	Extensively drug-resistant HIV/AIDS - Tuberculosis with mild anaemia	0.1 (< 0.05 to 0.1)	0.2 (0.1 to 0.3)	0.1 (< 0.05 to 0.1)	0.3 (0.2 to 0.4)	< 0.05 (< 0.05 to < 0.05)	0.1 (< 0.05 to 0.1)
HIV/AIDS resulting in other diseases	Early HIV without anaemia	13,236.9 (11,643.8 to 14,306.3)	8,688.1 (8,118.7 to 9,206.3)	1,864.1 (1,528.4 to 2,299.3)	1,423.1 (1,276.2 to 1,634.9)	160.0 (80.6 to 289.5)	105.2 (52.0 to 190.7)
HIV/AIDS resulting in other diseases	AIDS without antiretroviral treatment with severe anaemia	16.0 (6.4 to 33.4)	9.5 (5.1 to 18.4)	--	--	9.7 (3.7 to 21.5)	6.1 (2.9 to 12.5)
HIV/AIDS resulting in other diseases	Symptomatic HIV without anaemia	4,628.9 (4,110.5 to 4,952.1)	2,982.5 (2,795.9 to 3,148.8)	--	--	1,183.0 (801.1 to 1,619.2)	766.5 (514.7 to 1,038.8)
HIV/AIDS resulting in other diseases	AIDS with antiretroviral treatment with severe anaemia	10.0 (7.3 to 13.8)	97.2 (88.5 to 108.0)	--	--	2.1 (1.3 to 3.4)	19.8 (13.6 to 27.0)
HIV/AIDS resulting in other diseases	Early HIV with severe anaemia	90.0 (81.6 to 98.2)	70.4 (62.1 to 81.1)	17.3 (15.3 to 19.8)	15.1 (12.6 to 18.2)	13.7 (9.5 to 18.9)	11.0 (7.3 to 15.4)
HIV/AIDS resulting in other diseases	Early HIV with moderate anaemia	1,235.6 (1,086.2 to 1,351.0)	858.7 (795.1 to 927.0)	187.1 (159.6 to 227.2)	146.4 (131.1 to 167.3)	74.8 (47.4 to 111.7)	52.3 (33.8 to 78.1)
HIV/AIDS resulting in other diseases	AIDS without antiretroviral treatment with mild anaemia	454.4 (184.6 to 964.7)	254.9 (131.1 to 518.3)	--	--	240.3 (89.7 to 548.9)	138.7 (61.8 to 308.6)
HIV/AIDS resulting in other diseases	AIDS with antiretroviral treatment with moderate anaemia	129.9 (99.0 to 168.5)	1,380.5 (1,283.7 to 1,490.4)	--	--	15.6 (9.4 to 23.5)	162.5 (109.1 to 225.9)
HIV/AIDS resulting in other diseases	Early HIV with mild anaemia	2,090.4 (1,827.7 to 2,282.4)	1,671.6 (1,549.5 to 1,808.9)	297.9 (243.9 to 371.3)	280.6 (254.0 to 316.6)	32.6 (15.6 to 61.7)	26.1 (12.4 to 49.5)
HIV/AIDS resulting in other diseases	AIDS with antiretroviral treatment without anaemia	2,518.6 (1,748.9 to 3,436.4)	14,045.0 (12,899.5 to 15,247.6)	--	--	187.1 (106.2 to 301.2)	1,036.1 (686.5 to 1,471.5)
HIV/AIDS resulting in other diseases	Symptomatic HIV with mild anaemia	731.7 (644.6 to 791.1)	574.0 (531.0 to 622.4)	--	--	189.1 (128.7 to 256.8)	149.8 (100.9 to 202.8)
HIV/AIDS resulting in other diseases	AIDS without antiretroviral treatment with moderate anaemia	255.3 (119.3 to 508.6)	136.3 (79.5 to 253.2)	--	--	140.7 (60.6 to 300.7)	77.8 (39.6 to 157.0)
HIV/AIDS resulting in other diseases	Symptomatic HIV with moderate anaemia	430.6 (380.9 to 467.0)	293.5 (272.7 to 317.8)	--	--	125.2 (87.1 to 166.4)	86.5 (59.7 to 114.9)
HIV/AIDS resulting in other diseases	Symptomatic HIV with severe anaemia	31.2 (28.4 to 34.0)	24.0 (21.2 to 27.6)	--	--	11.5 (8.0 to 15.3)	9.0 (6.3 to 12.2)
HIV/AIDS resulting in other diseases	AIDS without antiretroviral treatment without anaemia	2,840.5 (1,221.2 to 5,841.6)	1,384.8 (796.4 to 2,601.1)	--	--	1,497.9 (591.5 to 3,304.2)	742.3 (366.3 to 1,511.9)
HIV/AIDS resulting in other diseases	AIDS with antiretroviral treatment with mild anaemia	433.0 (301.1 to 588.4)	2,793.5 (2,542.1 to 3,103.4)	--	--	33.6 (18.7 to 53.4)	215.0 (141.4 to 303.6)
Diarrhoeal diseases	Guillain-Barre syndrome due to diarrhoeal diseases	6.0 (4.1 to 8.3)	7.0 (4.7 to 9.7)	12.3 (8.8 to 17.1)	14.4 (10.2 to 20.0)	1.8 (1.0 to 2.8)	2.1 (1.2 to 3.2)
Diarrhoeal diseases	Mild diarrhoeal diseases	39,904.9 (37,815.9 to 42,114.1)	42,991.8 (40,762.5 to 45,436.2)	2,660,079.9 (2,523,310.9 to 2,806,465.4)	2,876,408.0 (2,726,564.8 to 3,041,638.0)	2,807.2 (1,888.0 to 3,938.5)	3,018.2 (2,025.4 to 4,239.3)
Diarrhoeal diseases	Moderate diarrhoeal diseases	17,963.4 (17,023.1 to 18,957.9)	19,353.0 (18,349.5 to 20,453.4)	1,197,450.3 (1,135,882.9 to 1,263,346.6)	1,294,831.6 (1,227,378.9 to 1,369,210.9)	3,181.0 (2,148.5 to 4,405.0)	3,417.7 (2,315.6 to 4,729.2)
Diarrhoeal diseases	Severe diarrhoeal diseases	4,288.8 (4,064.3 to 4,526.3)	4,620.6 (4,381.0 to 4,883.3)	285,896.4 (271,197.0 to 301,629.5)	309,146.7 (293,042.0 to 326,905.0)	993.9 (676.3 to 1,396.3)	1,067.9 (721.7 to 1,484.4)
Typhoid fever	Intestinal perforation due to typhoid	184.3 (155.7 to 220.0)	153.5 (129.8 to 182.0)	2,403.9 (2,065.2 to 2,810.1)	1,998.8 (1,719.2 to 2,313.8)	58.3 (37.6 to 83.9)	49.0 (31.7 to 69.8)
Typhoid fever	Acute typhoid infection	190.2 (161.5 to 222.6)	157.9 (135.2 to 185.9)	4,962.5 (4,275.9 to 5,754.9)	4,117.4 (3,573.4 to 4,767.4)	9.5 (5.8 to 14.2)	7.9 (4.7 to 12.0)
Typhoid fever	Gastrointestinal bleeding due to typhoid	2.6 (1.6 to 4.1)	2.1 (1.3 to 3.6)	33.7 (21.1 to 53.6)	28.0 (17.2 to 45.7)	0.8 (0.4 to 1.5)	0.7 (0.4 to 1.3)
Typhoid fever	Severe typhoid fever	518.7 (440.5 to 614.7)	432.1 (366.2 to 501.6)	6,769.4 (5,835.8 to 7,879.8)	5,629.7 (4,890.0 to 6,539.4)	66.8 (42.2 to 98.5)	55.9 (36.2 to 82.4)
Paratyphoid fever	Intestinal perforation due to paratyphoid	8.3 (7.2 to 9.6)	7.4 (6.3 to 8.6)	215.7 (188.8 to 248.8)	192.1 (167.6 to 221.4)	0.9 (0.6 to 1.3)	0.8 (0.6 to 1.2)
Paratyphoid fever	Moderate paratyphoid fever	83.9 (72.8 to 97.0)	74.4 (65.0 to 86.0)	2,184.6 (1,914.3 to 2,480.9)	1,944.3 (1,712.6 to 2,205.9)	4.2 (2.6 to 6.4)	3.8 (2.3 to 5.7)

Paratyphoid fever	Acute paratyphoid infection	46.4 (40.2 to 53.9)	41.3 (35.8 to 48.1)	1,211.8 (1,066.3 to 1,391.3)	1,078.1 (945.6 to 1,233.2)	0.3 (0.1 to 0.6)	0.2 (0.1 to 0.5)
Paratyphoid fever	Severe paratyphoid fever	46.9 (40.6 to 54.5)	41.7 (36.1 to 49.0)	611.1 (535.8 to 701.0)	543.8 (477.3 to 624.3)	6.3 (4.0 to 9.2)	5.6 (3.7 to 8.2)
Other intestinal infectious diseases	Other intestinal infectious diseases	--	--	--	--	2.3 (1.5 to 3.3)	1.4 (0.9 to 1.9)
Lower respiratory infections	Severe lower respiratory infections	981.3 (871.5 to 1,107.6)	1,208.0 (1,077.2 to 1,355.6)	41,528.8 (36,409.6 to 46,915.4)	50,789.6 (44,807.7 to 57,044.2)	124.4 (80.2 to 175.9)	151.9 (98.1 to 215.2)
Lower respiratory infections	Moderate lower respiratory infections	5,535.2 (5,161.0 to 5,931.1)	6,814.1 (6,418.7 to 7,237.2)	233,571.1 (216,537.8 to 252,668.3)	285,656.0 (265,647.9 to 308,203.8)	267.5 (171.3 to 391.4)	327.4 (209.5 to 477.3)
Lower respiratory infections	Guillain-Barre syndrome due to lower respiratory infections	6.5 (3.5 to 10.0)	7.6 (4.1 to 11.7)	13.7 (7.4 to 21.7)	16.0 (8.6 to 25.3)	1.9 (0.9 to 3.3)	2.3 (1.1 to 3.9)
Upper respiratory infections	Moderate upper respiratory infections	95,773.5 (74,187.4 to 118,652.7)	104,829.0 (81,221.1 to 129,836.8)	7,184,285.8 (5,631,965.6 to 8,962,619.9)	7,850,917.3 (6,174,851.1 to 9,787,950.4)	4,680.9 (2,788.8 to 7,206.4)	5,118.0 (3,045.0 to 7,867.7)
Upper respiratory infections	Guillain-Barre syndrome due to upper respiratory infections	17.7 (12.8 to 23.8)	20.6 (14.9 to 27.7)	36.1 (26.8 to 48.5)	42.1 (31.2 to 56.7)	5.2 (3.1 to 8.2)	6.1 (3.6 to 9.5)
Upper respiratory infections	Mild upper respiratory infections	119,616.0 (96,309.7 to 145,441.9)	130,918.1 (105,505.3 to 158,738.8)	9,084,167.2 (7,436,092.8 to 11,062,677.4)	9,927,174.6 (8,087,532.6 to 12,032,598.1)	677.0 (268.1 to 1,379.1)	740.4 (293.6 to 1,505.7)
Otitis media	Vertigo with mild hearing loss due to chronic otitis media	3,400.8 (2,664.8 to 4,374.4)	3,405.3 (2,671.2 to 4,375.9)	< 0.05 (0.0 to < 0.05)	< 0.05 (0.0 to < 0.05)	398.7 (237.9 to 609.7)	398.9 (240.1 to 609.8)
Otitis media	Vertigo with moderate hearing loss due to chronic otitis media	3,387.1 (2,661.8 to 4,334.3)	3,393.8 (2,670.7 to 4,339.5)	< 0.05 (0.0 to < 0.05)	< 0.05 (0.0 to < 0.05)	445.3 (267.6 to 679.0)	445.9 (269.2 to 680.9)
Otitis media	Acute otitis media	22,624.9 (18,208.2 to 28,109.0)	24,489.7 (19,683.7 to 30,283.7)	418,083.8 (337,904.6 to 518,062.5)	451,706.3 (365,132.6 to 564,096.9)	296.5 (147.9 to 526.4)	320.9 (159.4 to 569.1)
Otitis media	Moderate hearing loss due to chronic otitis media	7,943.4 (6,452.8 to 9,613.5)	8,158.5 (6,573.0 to 9,835.4)	< 0.05 (0.0 to < 0.05)	< 0.05 (0.0 to < 0.05)	205.2 (112.2 to 342.0)	210.6 (118.8 to 351.4)
Otitis media	Vertigo with moderate hearing loss and ringing due to chronic otitis media	3,118.7 (2,528.0 to 3,809.1)	3,135.3 (2,540.5 to 3,820.7)	< 0.05 (0.0 to < 0.05)	< 0.05 (0.0 to < 0.05)	532.6 (340.5 to 779.2)	534.9 (344.8 to 778.3)
Otitis media	Mild hearing loss due to chronic otitis media	46,447.9 (40,904.2 to 52,906.1)	47,960.9 (42,269.8 to 54,406.7)	< 0.05 (0.0 to < 0.05)	< 0.05 (0.0 to < 0.05)	434.0 (204.2 to 839.0)	448.0 (210.1 to 860.1)
Otitis media	Vertigo with mild hearing loss and ringing due to chronic otitis media	3,398.5 (2,664.8 to 4,359.2)	3,403.8 (2,671.2 to 4,371.4)	< 0.05 (0.0 to < 0.05)	< 0.05 (0.0 to < 0.05)	432.2 (259.8 to 655.7)	432.6 (260.3 to 655.8)
Otitis media	Severe infectious complications due to chronic otitis media	54.2 (1.6 to 188.0)	54.2 (1.6 to 187.7)	22.9 (0.7 to 86.9)	23.2 (0.8 to 87.7)	0.7 (< 0.05 to 2.8)	0.7 (< 0.05 to 2.8)
Otitis media	Moderate hearing loss with ringing due to chronic otitis media	1,787.6 (1,144.4 to 2,509.4)	1,868.0 (1,180.2 to 2,600.1)	< 0.05 (0.0 to < 0.05)	< 0.05 (0.0 to < 0.05)	126.6 (67.8 to 208.1)	132.3 (71.7 to 223.1)
Otitis media	Mild hearing loss with ringing due to chronic otitis media	9,795.8 (8,322.4 to 11,398.2)	10,192.2 (8,623.7 to 11,780.3)	< 0.05 (0.0 to < 0.05)	< 0.05 (0.0 to < 0.05)	204.2 (110.7 to 338.2)	212.3 (115.7 to 353.4)
Pneumococcal meningitis	Mild hearing loss with ringing due to pneumococcal meningitis	124.8 (77.0 to 180.5)	111.6 (68.6 to 161.2)	--	--	2.7 (1.2 to 5.0)	2.4 (1.1 to 4.5)
Pneumococcal meningitis	Profound hearing loss with ringing due to pneumococcal meningitis	16.6 (1.3 to 26.7)	12.9 (1.1 to 21.1)	--	--	4.6 (0.4 to 8.0)	3.6 (0.3 to 6.3)
Pneumococcal meningitis	Profound hearing loss due to pneumococcal meningitis	9.3 (0.7 to 15.8)	7.3 (0.6 to 11.9)	--	--	1.9 (0.1 to 3.5)	1.5 (0.1 to 2.7)
Pneumococcal meningitis	Mild motor impairment due to long term due to pneumococcal meningitis	831.0 (507.2 to 1,304.7)	726.5 (438.1 to 1,148.4)	--	--	8.2 (3.4 to 17.5)	7.1 (3.0 to 15.4)
Pneumococcal meningitis	Blindness due to pneumococcal meningitis	9.4 (6.7 to 12.5)	8.1 (5.8 to 10.8)	--	--	1.8 (1.0 to 2.7)	1.5 (0.9 to 2.4)
Pneumococcal meningitis	Moderate hearing loss with ringing due to pneumococcal meningitis	91.6 (7.5 to 132.6)	78.4 (6.5 to 115.4)	--	--	6.7 (0.5 to 11.4)	5.8 (0.4 to 9.9)
Pneumococcal meningitis	Complete hearing loss due to pneumococcal meningitis	11.5 (0.9 to 17.7)	9.1 (0.7 to 14.2)	--	--	2.5 (0.2 to 4.4)	2.0 (0.2 to 3.4)
Pneumococcal meningitis	Epilepsy due to pneumococcal meningitis	241.5 (209.3 to 277.4)	262.4 (224.4 to 305.8)	--	--	45.2 (31.5 to 62.0)	46.7 (31.5 to 65.4)
Pneumococcal meningitis	Mild hearing loss due to pneumococcal meningitis	471.4 (291.9 to 678.1)	421.6 (256.7 to 610.6)	--	--	4.4 (1.7 to 9.3)	3.9 (1.5 to 8.3)
Pneumococcal meningitis	Severe hearing loss with ringing due to pneumococcal meningitis	20.2 (1.7 to 31.1)	16.0 (1.4 to 25.2)	--	--	5.3 (0.4 to 9.1)	4.2 (0.3 to 7.2)
Pneumococcal meningitis	Severe motor impairment due to pneumococcal meningitis	491.9 (381.7 to 631.7)	592.9 (462.2 to 763.4)	--	--	188.1 (115.5 to 271.4)	226.5 (142.4 to 329.2)
Pneumococcal meningitis	Severe motor plus cognitive impairments due to pneumococcal meningitis	217.3 (106.8 to 315.8)	226.0 (102.5 to 334.8)	--	--	115.5 (53.0 to 182.4)	120.6 (51.5 to 193.1)
Pneumococcal meningitis	Mild motor plus cognitive impairments due to pneumococcal meningitis	503.8 (290.8 to 780.3)	437.2 (246.9 to 680.9)	--	--	15.2 (7.2 to 27.8)	13.2 (6.2 to 24.2)
Pneumococcal meningitis	Mild intellectual disability due to pneumococcal meningitis	563.1 (430.5 to 706.6)	649.8 (493.2 to 818.5)	--	--	23.3 (13.4 to 36.9)	26.9 (15.5 to 42.4)
Pneumococcal meningitis	Severe vision impairment due to pneumococcol meningitis	17.2 (12.7 to 23.2)	15.5 (11.4 to 21.1)	--	--	3.2 (1.9 to 4.9)	2.9 (1.7 to 4.5)
Pneumococcal meningitis	Moderate motor impairment due to pneumococcal meningitis	390.5 (312.8 to 486.7)	467.9 (369.6 to 591.0)	--	--	23.3 (14.3 to 35.3)	27.9 (16.8 to 42.9)
Pneumococcal meningitis	Severe hearing loss due to pneumococcal meningitis	11.5 (0.9 to 18.1)	9.1 (0.7 to 14.7)	--	--	1.8 (0.1 to 3.2)	1.4 (0.1 to 2.5)
Pneumococcal meningitis	Acute pneumococcal meningitis	36.3 (30.4 to 43.0)	31.0 (25.7 to 37.1)	720.5 (591.5 to 888.5)	611.7 (498.3 to 755.5)	4.8 (3.0 to 7.2)	4.1 (2.6 to 6.1)
Pneumococcal meningitis	Moderately severe hearing loss with ringing due to pneumococcal meningitis	49.2 (4.1 to 72.7)	40.6 (3.4 to 59.7)	--	--	8.2 (0.7 to 13.7)	6.8 (0.5 to 11.4)
Pneumococcal meningitis	Borderline intellectual disability due to pneumococcal meningitis	620.3 (367.6 to 961.4)	540.5 (317.9 to 855.5)	--	--	6.5 (2.7 to 14.2)	5.7 (2.3 to 12.5)
Pneumococcal meningitis	Moderate motor plus cognitive impairments due to pneumococcal meningitis	370.5 (277.4 to 476.9)	420.3 (306.6 to 545.9)	--	--	72.5 (43.4 to 109.4)	82.3 (48.8 to 125.8)
Pneumococcal meningitis	Monocular distance vision loss due to pneumococcal meningitis	548.0 (323.4 to 868.3)	477.7 (281.0 to 769.4)	--	--	8.8 (3.8 to 16.8)	7.7 (3.3 to 14.8)
Pneumococcal meningitis	Complete hearing loss with ringing due to pneumococcal meningitis	13.9 (1.1 to 21.4)	11.0 (0.9 to 17.1)	--	--	4.4 (0.4 to 7.7)	3.5 (0.3 to 6.0)
Pneumococcal meningitis	Mild behavioral problems due to pneumococcal meningitis	305.0 (177.5 to 487.7)	265.9 (157.8 to 426.0)	--	--	13.2 (6.7 to 23.7)	11.6 (5.8 to 20.9)

Pneumococcal meningitis	Moderate hearing loss due to pneumococcal meningitis	211.4 (17.4 to 304.0)	181.2 (15.0 to 267.1)	--	--	5.5 (0.4 to 10.2)	4.7 (0.4 to 9.0)
Pneumococcal meningitis	Moderately severe hearing loss due to pneumococcal meningitis	102.1 (8.5 to 150.6)	84.5 (7.2 to 124.0)	--	--	9.3 (0.8 to 15.7)	7.7 (0.6 to 13.2)
Pneumococcal meningitis	Moderate vision impairment due to pneumococcol meningitis	87.3 (65.8 to 116.9)	75.5 (55.3 to 102.3)	--	--	2.7 (1.5 to 4.6)	2.3 (1.3 to 3.9)
H influenzae type B meningitis	Moderate motor impairment due to H influenzae type B meningitis	167.0 (128.0 to 213.6)	160.3 (121.2 to 208.7)	--	--	10.2 (6.1 to 16.0)	9.8 (5.8 to 15.3)
H influenzae type B meningitis	Severe hearing loss due to H influenzae type B meningitis	5.7 (0.5 to 9.2)	3.2 (0.3 to 5.3)	--	--	0.9 (0.1 to 1.6)	0.5 (< 0.05 to 1.0)
H influenzae type B meningitis	Severe motor impairment due to H influenzae type B meningitis	233.8 (177.4 to 303.4)	227.9 (171.3 to 293.2)	--	--	93.7 (55.3 to 138.2)	91.2 (53.9 to 135.1)
H influenzae type B meningitis	Borderline intellectual disability due to H influenzae type B meningitis	554.0 (234.3 to 910.8)	301.2 (123.3 to 506.1)	--	--	5.8 (1.9 to 13.1)	3.2 (1.0 to 7.4)
H influenzae type B meningitis	Mild hearing loss due to H influenzae type B meningitis	202.1 (112.2 to 295.0)	111.1 (59.5 to 166.1)	--	--	1.9 (0.7 to 4.1)	1.1 (0.4 to 2.4)
H influenzae type B meningitis	Mild hearing loss with ringing due to H influenzae type B meningitis	53.5 (29.7 to 78.0)	29.4 (15.7 to 44.0)	--	--	1.2 (0.5 to 2.3)	0.6 (0.3 to 1.3)
H influenzae type B meningitis	Moderately severe hearing loss with ringing due to H influenzae type B meningitis	22.7 (1.9 to 33.8)	11.9 (1.0 to 19.0)	--	--	3.8 (0.3 to 6.6)	2.0 (0.2 to 3.5)
H influenzae type B meningitis	Moderate hearing loss with ringing due to H influenzae type B meningitis	40.9 (3.4 to 60.6)	22.2 (1.8 to 34.5)	--	--	3.0 (0.2 to 5.4)	1.6 (0.1 to 3.0)
H influenzae type B meningitis	Complete hearing loss due to H influenzae type B meningitis	5.3 (0.4 to 8.4)	2.8 (0.2 to 4.6)	--	--	1.1 (0.1 to 2.0)	0.6 (< 0.05 to 1.1)
H influenzae type B meningitis	Severe motor plus cognitive impairments due to H influenzae type B meningitis	112.6 (54.7 to 167.6)	98.0 (43.3 to 147.6)	--	--	60.9 (27.7 to 97.6)	53.0 (23.1 to 85.6)
H influenzae type B meningitis	Profound hearing loss due to H influenzae type B meningitis	4.5 (0.4 to 7.4)	2.5 (0.2 to 4.2)	--	--	0.9 (0.1 to 1.7)	0.5 (< 0.05 to 1.0)
H influenzae type B meningitis	Mild motor impairment due to long term due to H influenzae type B meningitis	306.9 (135.4 to 506.7)	167.3 (75.5 to 285.4)	--	--	3.1 (1.0 to 6.9)	1.7 (0.5 to 3.8)
H influenzae type B meningitis	Monocular distance vision loss due to H influenzae type B meningitis	331.6 (140.3 to 552.0)	180.3 (76.5 to 320.2)	--	--	5.4 (1.9 to 10.7)	2.9 (1.0 to 6.2)
H influenzae type B meningitis	Complete hearing loss with ringing due to H influenzae type B meningitis	6.4 (0.5 to 9.9)	3.3 (0.3 to 5.4)	--	--	2.0 (0.2 to 3.5)	1.1 (0.1 to 1.9)
H influenzae type B meningitis	Severe hearing loss with ringing due to H influenzae type B meningitis	10.0 (0.8 to 15.9)	5.6 (0.5 to 9.4)	--	--	2.6 (0.2 to 4.6)	1.5 (0.1 to 2.7)
H influenzae type B meningitis	Profound hearing loss with ringing due to H influenzae type B meningitis	8.0 (0.6 to 13.2)	4.4 (0.3 to 7.5)	--	--	2.2 (0.2 to 4.1)	1.2 (0.1 to 2.4)
H influenzae type B meningitis	Mild behavioral problems due to H influenzae type B meningitis	83.1 (38.4 to 141.6)	45.2 (20.6 to 78.8)	--	--	3.7 (1.4 to 7.0)	2.0 (0.8 to 3.9)
H influenzae type B meningitis	Moderately severe hearing loss due to H influenzae type B meningitis	47.2 (4.0 to 70.3)	24.7 (2.1 to 39.0)	--	--	4.4 (0.4 to 7.5)	2.3 (0.2 to 4.0)
H influenzae type B meningitis	Mild intellectual disability due to H influenzae type B meningitis	142.1 (106.4 to 184.6)	133.2 (99.1 to 173.4)	--	--	6.0 (3.5 to 9.6)	5.7 (3.2 to 9.1)
H influenzae type B meningitis	Blindness due to H influenzae type B meningitis	0.6 (0.4 to 0.9)	0.3 (0.2 to 0.5)	--	--	0.1 (0.1 to 0.2)	0.1 (< 0.05 to 0.1)
H influenzae type B meningitis	Moderate vision impairment due to H influenza type B meningitis	6.3 (4.5 to 8.5)	3.5 (2.3 to 5.0)	--	--	0.2 (0.1 to 0.3)	0.1 (0.1 to 0.2)
H influenzae type B meningitis	Severe vision impairment due to H influenza type B meningitis	1.1 (0.8 to 1.5)	0.7 (0.4 to 1.0)	--	--	0.2 (0.1 to 0.3)	0.1 (0.1 to 0.2)
H influenzae type B meningitis	Moderate motor plus cognitive impairments due to H influenzae type B meningitis	197.1 (147.6 to 251.3)	180.3 (132.2 to 231.2)	--	--	39.8 (24.3 to 59.5)	36.3 (22.1 to 54.4)
H influenzae type B meningitis	Acute H influenzae type B meningitis	30.9 (24.8 to 38.1)	18.0 (13.5 to 23.6)	674.6 (528.4 to 853.3)	397.3 (291.1 to 533.7)	4.1 (2.6 to 6.3)	2.4 (1.5 to 3.7)
H influenzae type B meningitis	Epilepsy due to H influenzae type B meningitis	118.6 (98.4 to 141.4)	122.8 (102.3 to 148.5)	--	--	22.8 (15.3 to 32.0)	22.6 (14.9 to 32.4)
H influenzae type B meningitis	Mild motor plus cognitive impairments due to H influenzae type B meningitis	185.0 (83.1 to 314.4)	100.2 (42.6 to 173.0)	--	--	5.7 (2.2 to 11.2)	3.1 (1.2 to 6.1)
H influenzae type B meningitis	Moderate hearing loss due to H influenzae type B meningitis	94.4 (8.0 to 139.8)	51.2 (4.3 to 79.0)	--	--	2.5 (0.2 to 4.8)	1.4 (0.1 to 2.6)
Meningococcal infection	Mild hearing loss with ringing due to meningococcal meningitis	27.9 (16.4 to 41.3)	32.3 (18.5 to 48.2)	--	--	0.6 (0.3 to 1.1)	0.7 (0.3 to 1.3)
Meningococcal infection	Severe hearing loss due to meningococcal meningitis	2.7 (0.2 to 4.2)	2.9 (0.2 to 4.6)	--	--	0.4 (< 0.05 to 0.7)	0.5 (< 0.05 to 0.8)
Meningococcal infection	Epilepsy due to meningococcal meningitis	39.1 (32.0 to 48.2)	46.6 (38.3 to 57.3)	--	--	7.6 (5.0 to 10.6)	8.6 (5.5 to 12.3)
Meningococcal infection	Mild intellectual disability due to meningococcal meningitis	64.6 (47.6 to 85.4)	77.6 (56.2 to 107.1)	--	--	2.8 (1.6 to 4.5)	3.3 (1.8 to 5.6)
Meningococcal infection	Moderate hearing loss with ringing due to meningococcal meningitis	20.6 (1.8 to 29.8)	22.6 (1.9 to 33.2)	--	--	1.5 (0.1 to 2.6)	1.7 (0.1 to 2.8)
Meningococcal infection	Mild motor plus cognitive impairments due to meningococcal meningitis	73.4 (21.3 to 132.6)	83.6 (24.4 to 150.4)	--	--	2.3 (0.6 to 4.8)	2.6 (0.7 to 5.4)
Meningococcal infection	Moderately severe hearing loss due to meningococcal meningitis	23.8 (2.0 to 36.2)	25.9 (2.1 to 39.0)	--	--	2.2 (0.2 to 3.7)	2.4 (0.2 to 4.1)
Meningococcal infection	Acute meningococcal meningitis	22.4 (18.2 to 27.8)	26.8 (21.6 to 33.0)	468.4 (371.1 to 587.5)	561.4 (442.1 to 706.7)	3.0 (1.9 to 4.4)	3.5 (2.2 to 5.2)
Meningococcal infection	Profound hearing loss due to meningococcal meningitis	2.5 (0.2 to 4.2)	2.8 (0.2 to 4.6)	--	--	0.5 (< 0.05 to 1.0)	0.6 (< 0.05 to 1.1)
Meningococcal infection	Mild hearing loss due to meningococcal meningitis	105.5 (62.0 to 155.8)	122.0 (70.4 to 182.5)	--	--	1.0 (0.4 to 2.1)	1.2 (0.4 to 2.5)
Meningococcal infection	Mild behavioral problems due to meningococcal meningitis	32.7 (10.0 to 58.8)	37.2 (11.6 to 66.4)	--	--	1.4 (0.4 to 2.9)	1.6 (0.5 to 3.3)
Meningococcal infection	Profound hearing loss with ringing due to meningococcal meningitis	4.5 (0.4 to 7.3)	4.9 (0.4 to 8.0)	--	--	1.2 (0.1 to 2.3)	1.4 (0.1 to 2.5)
Meningococcal infection	Moderate hearing loss due to meningococcal meningitis	47.6 (4.0 to 69.3)	52.3 (4.4 to 76.7)	--	--	1.3 (0.1 to 2.3)	1.4 (0.1 to 2.6)

Meningococcal infection	Blindness due to meningococcal meningitis	11.1 (7.9 to 15.6)	12.5 (8.7 to 17.9)	--	--	2.1 (1.2 to 3.2)	2.3 (1.4 to 3.6)
Meningococcal infection	Moderately severe hearing loss with ringing due to meningococcal meningitis	11.4 (0.9 to 17.5)	12.5 (1.0 to 18.8)	--	--	1.9 (0.2 to 3.2)	2.1 (0.2 to 3.5)
Meningococcal infection	Complete hearing loss due to meningococcal meningitis	2.7 (0.2 to 4.3)	2.8 (0.2 to 4.5)	--	--	0.6 (< 0.05 to 1.0)	0.6 (< 0.05 to 1.1)
Meningococcal infection	Moderate vision impairment due to meningococcal meningitis	106.1 (76.8 to 143.7)	117.0 (83.1 to 158.7)	--	--	3.2 (1.8 to 5.4)	3.6 (2.0 to 6.0)
Meningococcal infection	Severe motor impairment due to meningococcal meningitis	105.4 (79.6 to 139.2)	138.7 (97.2 to 256.8)	--	--	42.3 (25.8 to 63.7)	55.0 (31.4 to 98.7)
Meningococcal infection	Mild motor impairment due to long term due to meningococcal meningitis	121.0 (36.6 to 213.9)	137.9 (43.8 to 249.4)	--	--	1.2 (0.3 to 2.9)	1.4 (0.3 to 3.3)
Meningococcal infection	Severe hearing loss with ringing due to meningococcal meningitis	4.7 (0.4 to 7.5)	5.0 (0.4 to 8.0)	--	--	1.2 (0.1 to 2.1)	1.3 (0.1 to 2.3)
Meningococcal infection	Monocular distance vision loss due to meningococcal meningitis	129.7 (39.1 to 238.1)	147.7 (44.9 to 270.8)	--	--	2.1 (0.5 to 4.6)	2.4 (0.7 to 5.2)
Meningococcal infection	Complete hearing loss with ringing due to meningococcal meningitis	3.3 (0.3 to 5.1)	3.4 (0.3 to 5.4)	--	--	1.0 (0.1 to 1.8)	1.1 (0.1 to 1.9)
Meningococcal infection	Borderline intellectual disability due to meningococcal meningitis	217.6 (65.5 to 388.7)	247.8 (74.7 to 449.9)	--	--	2.3 (0.6 to 5.2)	2.6 (0.6 to 5.9)
Meningococcal infection	Moderate motor plus cognitive impairments due to meningococcal meningitis	89.1 (66.5 to 118.8)	105.1 (76.6 to 142.5)	--	--	18.0 (10.8 to 27.7)	21.3 (12.5 to 33.9)
Meningococcal infection	Severe motor plus cognitive impairments due to meningococcal meningitis	54.9 (26.9 to 79.4)	61.1 (27.7 to 95.5)	--	--	29.7 (14.1 to 47.4)	33.0 (14.3 to 55.4)
Meningococcal infection	Severe vision impairment due to meningococcal meningitis	24.0 (16.9 to 32.8)	29.6 (21.2 to 40.4)	--	--	4.4 (2.6 to 6.8)	5.4 (3.3 to 8.4)
Meningococcal infection	Moderate motor impairment due to meningococcal meningitis	74.1 (55.6 to 96.0)	101.9 (67.5 to 257.0)	--	--	4.5 (2.6 to 7.0)	6.1 (3.2 to 13.8)
Other meningitis	Severe motor plus cognitive impairments due to other bacterial meningitis	123.8 (59.1 to 182.5)	142.9 (63.8 to 217.0)	--	--	67.0 (29.0 to 107.9)	77.2 (31.4 to 127.4)
Other meningitis	Borderline intellectual disability due to other bacterial meningitis	414.8 (165.2 to 692.8)	758.2 (297.7 to 1,268.2)	--	--	4.4 (1.4 to 9.5)	8.0 (2.6 to 17.2)
Other meningitis	Mild intellectual disability due to other bacterial meningitis	148.3 (108.3 to 191.3)	194.0 (141.9 to 248.6)	--	--	6.3 (3.5 to 10.1)	8.3 (4.6 to 13.1)
Other meningitis	Severe hearing loss with ringing due to other bacterial meningitis	7.7 (0.6 to 11.8)	13.6 (1.1 to 20.7)	--	--	2.0 (0.2 to 3.5)	3.5 (0.3 to 6.2)
Other meningitis	Severe motor impairment due to other bacterial meningitis	238.0 (179.8 to 310.3)	330.7 (253.1 to 424.7)	--	--	95.2 (57.3 to 143.9)	131.5 (79.3 to 197.5)
Other meningitis	Profound hearing loss with ringing due to other bacterial meningitis	5.6 (0.4 to 9.0)	10.7 (0.9 to 16.9)	--	--	1.5 (0.1 to 2.8)	3.0 (0.2 to 5.2)
Other meningitis	Mild hearing loss due with ringing to other bacterial meningitis	47.0 (28.8 to 68.2)	86.3 (52.9 to 123.4)	--	--	1.0 (0.5 to 1.9)	1.8 (0.9 to 3.5)
Other meningitis	Other acute bacterial meningitis	33.5 (28.4 to 39.6)	61.8 (53.1 to 72.8)	655.9 (547.7 to 781.0)	1,250.4 (1,055.7 to 1,486.8)	4.5 (2.9 to 6.5)	8.2 (5.4 to 11.9)
Other meningitis	Monocular distance vision loss due to other bacterial meningitis	248.3 (96.4 to 425.9)	453.9 (176.0 to 772.5)	--	--	4.0 (1.3 to 8.8)	7.3 (2.3 to 15.7)
Other meningitis	Profound hearing loss due to other bacterial meningitis	3.1 (0.2 to 5.1)	6.0 (0.5 to 9.8)	--	--	0.6 (< 0.05 to 1.2)	1.2 (0.1 to 2.3)
Other meningitis	Acute viral meningitis	235.7 (206.9 to 270.8)	262.9 (229.0 to 307.0)	--	--	31.0 (20.5 to 44.8)	34.5 (22.6 to 50.1)
Other meningitis	Blindness due to other bacterial meningitis	9.1 (6.6 to 12.1)	15.8 (11.5 to 21.0)	--	--	1.7 (1.0 to 2.6)	3.0 (1.8 to 4.6)
Other meningitis	Complete hearing loss due to other bacterial meningitis	4.0 (0.3 to 6.1)	7.0 (0.6 to 10.6)	--	--	0.9 (0.1 to 1.5)	1.5 (0.1 to 2.6)
Other meningitis	Epilepsy due to other meningitis	106.6 (91.3 to 122.6)	131.3 (111.5 to 152.5)	--	--	20.0 (13.5 to 27.6)	23.5 (15.6 to 33.1)
Other meningitis	Mild motor plus cognitive impairments due to other bacterial meningitis	138.6 (54.7 to 235.7)	253.3 (99.7 to 430.2)	--	--	4.3 (1.4 to 8.8)	7.8 (2.4 to 16.2)
Other meningitis	Moderate motor plus cognitive impairments due to other bacterial meningitis	204.3 (153.6 to 262.4)	262.3 (191.8 to 338.2)	--	--	41.3 (25.1 to 62.1)	52.9 (31.9 to 80.5)
Other meningitis	Moderate motor impairment due to other bacterial meningitis	169.1 (130.8 to 214.6)	227.8 (174.0 to 295.0)	--	--	10.3 (6.3 to 16.1)	13.8 (8.4 to 21.6)
Other meningitis	Mild behavioral problems due to other bacterial meningitis	62.7 (24.5 to 108.0)	114.6 (44.6 to 196.5)	--	--	2.8 (1.0 to 5.3)	5.1 (1.8 to 9.6)
Other meningitis	Mild motor impairment due to long term due to other bacterial meningitis	232.8 (92.4 to 394.0)	425.9 (171.3 to 717.6)	--	--	2.3 (0.6 to 5.4)	4.2 (1.1 to 9.8)
Other meningitis	Moderate hearing loss with ringing due to other bacterial meningitis	33.2 (2.8 to 47.4)	60.0 (5.0 to 86.1)	--	--	2.5 (0.2 to 4.2)	4.4 (0.3 to 7.5)
Other meningitis	Mild hearing loss due to other bacterial meningitis	177.5 (108.4 to 257.0)	325.6 (200.4 to 465.1)	--	--	1.7 (0.7 to 3.6)	3.1 (1.2 to 6.5)
Other meningitis	Moderate hearing loss due to other bacterial meningitis	76.8 (6.4 to 109.6)	138.8 (11.4 to 198.0)	--	--	2.0 (0.1 to 3.8)	3.6 (0.3 to 6.9)
Other meningitis	Moderately severe hearing loss with ringing due to other bacterial meningitis	17.3 (1.4 to 25.6)	31.4 (2.6 to 45.9)	--	--	2.9 (0.2 to 4.9)	5.2 (0.4 to 8.8)
Other meningitis	Moderately severe hearing loss due to other bacterial meningitis	36.0 (3.0 to 53.3)	65.3 (5.4 to 95.0)	--	--	3.3 (0.3 to 5.6)	6.0 (0.5 to 10.0)
Other meningitis	Moderate vision impairment due to other bacterial meningitis	79.6 (60.4 to 103.7)	145.7 (112.4 to 188.3)	--	--	2.4 (1.4 to 4.2)	4.5 (2.6 to 7.5)
Other meningitis	Complete hearing loss with ringing due to other bacterial meningitis	4.8 (0.4 to 7.3)	8.3 (0.7 to 12.8)	--	--	1.5 (0.1 to 2.6)	2.6 (0.2 to 4.4)
Other meningitis	Severe vision impairment due to other bacterial meningitis	13.7 (10.2 to 18.5)	29.2 (21.6 to 39.4)	--	--	2.5 (1.6 to 3.9)	5.4 (3.3 to 8.3)
Other meningitis	Severe hearing loss due to other bacterial meningitis	4.4 (0.4 to 6.7)	7.7 (0.6 to 11.8)	--	--	0.7 (0.1 to 1.2)	1.2 (0.1 to 2.1)
Encephalitis	Mild motor impairment due to long term due to encephalitis	1,462.9 (282.2 to 3,249.2)	1,535.0 (300.4 to 3,394.4)	--	--	14.2 (2.6 to 40.1)	14.9 (2.7 to 41.3)

Encephalitis	Mild behavioral problems due to encephalitis	392.5 (71.4 to 886.2)	410.6 (75.1 to 917.6)	--	--	16.9 (2.9 to 41.0)	17.7 (3.0 to 43.0)
Encephalitis	Severe motor impairment due to encephalitis	1,373.4 (1,030.1 to 1,795.7)	1,564.3 (1,172.1 to 2,035.0)	--	--	515.7 (317.7 to 755.0)	585.4 (357.0 to 859.9)
Encephalitis	Epilepsy due to encephalitis	804.5 (706.4 to 912.4)	871.7 (775.2 to 981.5)	--	--	137.4 (93.8 to 182.5)	140.0 (95.2 to 187.3)
Encephalitis	Monocular distance vision loss due to encephalitis	1,551.3 (307.8 to 3,458.6)	1,622.7 (322.1 to 3,617.8)	--	--	24.7 (4.1 to 63.2)	25.9 (4.3 to 66.3)
Encephalitis	Acute encephalitis	323.3 (291.8 to 358.5)	362.9 (329.8 to 399.4)	5,813.5 (5,248.8 to 6,427.7)	6,534.3 (5,956.6 to 7,165.2)	42.4 (28.2 to 60.4)	47.6 (32.1 to 67.6)
Encephalitis	Severe motor plus cognitive impairments due to encephalitis	735.8 (388.8 to 1,043.9)	708.7 (330.3 to 1,042.6)	--	--	370.7 (181.6 to 558.0)	357.1 (163.6 to 557.1)
Encephalitis	Mild intellectual disability due to encephalitis	860.5 (660.1 to 1,094.5)	924.2 (712.8 to 1,188.0)	--	--	35.5 (19.8 to 57.0)	38.1 (21.3 to 61.9)
Encephalitis	Blindness due to encephalitis	94.0 (72.8 to 117.9)	99.9 (77.8 to 125.1)	--	--	17.3 (10.7 to 25.0)	18.4 (11.5 to 26.5)
Encephalitis	Moderate motor impairment due to encephalitis	897.8 (694.5 to 1,145.7)	1,001.6 (764.4 to 1,299.1)	--	--	52.8 (32.4 to 80.1)	58.8 (35.4 to 90.2)
Encephalitis	Borderline intellectual disability due to encephalitis	2,588.8 (489.1 to 5,609.8)	2,707.3 (510.6 to 5,881.5)	--	--	27.0 (4.4 to 72.1)	28.2 (4.7 to 75.4)
Encephalitis	Moderate motor plus cognitive impairments due to encephalitis	1,218.9 (936.8 to 1,524.6)	1,282.4 (958.8 to 1,631.2)	--	--	233.4 (144.9 to 337.8)	244.9 (151.3 to 360.9)
Encephalitis	Moderate vision impairment due to encephalitis	729.4 (597.8 to 875.5)	779.6 (633.1 to 930.9)	--	--	21.6 (12.7 to 35.2)	23.1 (13.4 to 37.2)
Encephalitis	Mild motor plus cognitive impairments due to encephalitis	867.7 (162.5 to 1,908.6)	903.7 (170.4 to 1,975.7)	--	--	25.9 (4.4 to 61.9)	27.0 (4.7 to 64.0)
Encephalitis	Severe vision impairment due to encephalitis	121.4 (97.4 to 148.3)	133.8 (106.4 to 165.9)	--	--	21.4 (13.9 to 31.2)	23.8 (15.4 to 34.3)
Diphtheria	Moderate diphtheria	0.5 (0.4 to 0.7)	0.2 (0.2 to 0.3)	7.1 (5.6 to 9.4)	3.0 (2.4 to 3.9)	< 0.05 (< 0.05 to < 0.05)	< 0.05 (< 0.05 to < 0.05)
Diphtheria	Severe diphtheria	0.2 (0.2 to 0.3)	0.1 (0.1 to 0.1)	3.1 (2.4 to 4.1)	1.3 (1.0 to 1.6)	< 0.05 (< 0.05 to < 0.05)	< 0.05 (< 0.05 to < 0.05)
Whooping cough	Whooping cough	2,057.2 (1,591.1 to 2,596.1)	1,592.8 (1,242.4 to 2,001.4)	15,017.7 (11,615.3 to 18,951.6)	11,627.4 (9,069.2 to 14,610.5)	101.9 (60.3 to 158.8)	79.0 (46.9 to 124.5)
Tetanus	Moderate motor plus cognitive impairment with epilepsy due to neonatal tetanus	< 0.05 (< 0.05 to < 0.05)	< 0.05 (< 0.05 to < 0.05)	--	--	< 0.05 (< 0.05 to < 0.05)	< 0.05 (< 0.05 to < 0.05)
Tetanus	Severe tetanus	12.5 (7.1 to 16.2)	5.0 (2.9 to 6.8)	225.4 (130.4 to 281.5)	90.2 (51.3 to 120.8)	1.7 (0.9 to 2.5)	0.7 (0.3 to 1.1)
Tetanus	Moderate motor plus cognitive impairment with blindness and epilepsy due to neonatal tetanus	< 0.05 (< 0.05 to < 0.05)	< 0.05 (< 0.05 to < 0.05)	--	--	< 0.05 (< 0.05 to < 0.05)	< 0.05 (< 0.05 to < 0.05)
Tetanus	Moderate motor impairment due to neonatal tetanus	0.1 (0.1 to 0.1)	0.1 (0.1 to 0.1)	--	--	< 0.05 (< 0.05 to < 0.05)	< 0.05 (< 0.05 to < 0.05)
Tetanus	Severe motor plus cognitive impairment with blindness due to neonatal tetanus	< 0.05 (< 0.05 to < 0.05)	< 0.05 (< 0.05 to < 0.05)	--	--	< 0.05 (< 0.05 to < 0.05)	< 0.05 (< 0.05 to < 0.05)
Tetanus	Moderate motor plus cognitive impairment with blindness due to neonatal tetanus	< 0.05 (< 0.05 to < 0.05)	< 0.05 (< 0.05 to < 0.05)	--	--	< 0.05 (< 0.05 to < 0.05)	< 0.05 (< 0.05 to < 0.05)
Tetanus	Moderate motor impairment with blindness due to neonatal tetanus	< 0.05 (< 0.05 to < 0.05)	< 0.05 (< 0.05 to < 0.05)	--	--	< 0.05 (< 0.05 to < 0.05)	< 0.05 (< 0.05 to < 0.05)
Tetanus	Severe motor impairment with epilepsy due to neonatal tetanus	< 0.05 (< 0.05 to < 0.05)	< 0.05 (< 0.05 to < 0.05)	--	--	< 0.05 (< 0.05 to < 0.05)	< 0.05 (< 0.05 to < 0.05)
Tetanus	Severe motor plus cognitive impairment with epilepsy due to neonatal tetanus	< 0.05 (< 0.05 to < 0.05)	< 0.05 (< 0.05 to < 0.05)	--	--	< 0.05 (< 0.05 to < 0.05)	< 0.05 (< 0.05 to < 0.05)
Tetanus	Moderate motor impairment with blindness and epilepsy due to neonatal tetanus	< 0.05 (< 0.05 to < 0.05)	< 0.05 (< 0.05 to < 0.05)	--	--	< 0.05 (< 0.05 to < 0.05)	< 0.05 (< 0.05 to < 0.05)
Tetanus	Mild motor plus cognitive impairments due to neonatal tetanus	65.2 (41.4 to 88.8)	75.5 (47.8 to 102.7)	--	--	2.0 (1.0 to 3.5)	2.3 (1.2 to 4.0)
Tetanus	Severe motor impairment with blindness due to neonatal tetanus	< 0.05 (< 0.05 to < 0.05)	< 0.05 (< 0.05 to < 0.05)	--	--	< 0.05 (< 0.05 to < 0.05)	< 0.05 (< 0.05 to < 0.05)
Tetanus	Moderate motor impairment with epilepsy due to neonatal tetanus	< 0.05 (< 0.05 to < 0.05)	< 0.05 (< 0.05 to < 0.05)	--	--	< 0.05 (< 0.05 to < 0.05)	< 0.05 (< 0.05 to < 0.05)
Tetanus	Severe motor plus cognitive impairment with blindness and epilepsy due to neonatal tetanus	< 0.05 (< 0.05 to < 0.05)	< 0.05 (< 0.05 to < 0.05)	--	--	< 0.05 (< 0.05 to < 0.05)	< 0.05 (< 0.05 to < 0.05)
Tetanus	Severe motor impairment with blindness and epilepsy due to neonatal tetanus	< 0.05 (< 0.05 to < 0.05)	< 0.05 (< 0.05 to < 0.05)	--	--	< 0.05 (< 0.05 to < 0.05)	< 0.05 (< 0.05 to < 0.05)
Tetanus	Severe motor impairment due to neonatal tetanus	0.1 (0.1 to 0.1)	0.1 (0.1 to 0.1)	--	--	< 0.05 (< 0.05 to 0.1)	< 0.05 (< 0.05 to < 0.05)
Tetanus	Mild motor impairment due to neonatal tetanus	68.2 (45.2 to 92.0)	79.2 (52.5 to 106.7)	--	--	0.7 (0.3 to 1.4)	0.8 (0.3 to 1.6)
Measles	Severe measles	329.0 (116.8 to 734.9)	123.0 (44.8 to 274.4)	11,985.4 (4,179.5 to 26,690.7)	4,478.6 (1,587.5 to 9,921.2)	42.6 (13.9 to 101.2)	16.1 (5.2 to 37.4)
Measles	Moderate measles	327.6 (115.5 to 705.1)	122.4 (43.8 to 262.5)	11,981.7 (4,332.0 to 26,088.3)	4,476.7 (1,595.1 to 9,705.9)	16.2 (5.0 to 36.2)	6.1 (1.9 to 13.8)
Varicella and herpes zoster	Herpes zoster	4,358.1 (3,888.8 to 4,828.9)	5,338.1 (4,727.8 to 5,964.6)	24,472.4 (21,988.5 to 27,099.9)	30,196.2 (26,953.1 to 33,652.5)	237.8 (146.9 to 361.9)	290.5 (179.6 to 446.3)
Varicella and herpes zoster	Chickenpox	2,151.8 (2,101.7 to 2,207.8)	2,263.7 (2,215.9 to 2,322.8)	112,193.6 (109,629.2 to 114,909.1)	117,966.2 (115,537.8 to 120,901.8)	12.3 (5.0 to 25.8)	13.0 (5.2 to 27.3)
Malaria	Severe motor impairment with blindness due to malaria	2.8 (1.8 to 4.0)	3.2 (2.0 to 4.7)	--	--	1.4 (0.8 to 2.2)	1.6 (0.9 to 2.6)
Malaria	Asymptomatic malaria parasitemia (PfPR)	130,459.8 (121,272.2 to 142,301.2)	98,612.5 (85,935.1 to 112,039.6)	< 0.05 (0.0 to < 0.05)	< 0.05 (0.0 to < 0.05)	< 0.05 (0.0 to < 0.05)	< 0.05 (0.0 to < 0.05)
Malaria	Moderate motor plus cognitive impairment with blindness due to malaria	46.1 (39.2 to 53.6)	56.8 (47.8 to 66.5)	--	--	16.1 (10.7 to 22.2)	19.8 (13.2 to 27.5)
Malaria	Moderate motor impairment due to malaria	291.0 (252.5 to 333.3)	370.9 (318.4 to 431.2)	--	--	17.2 (11.1 to 25.0)	22.0 (14.0 to 32.3)
Malaria	Mild malaria	1,376.7 (617.6 to 2,560.5)	1,098.6 (339.7 to 2,653.0)	161,858.3 (139,089.0 to 192,317.7)	141,279.0 (113,842.8 to 176,265.2)	7.7 (2.2 to 19.0)	6.1 (1.4 to 17.4)

Malaria	Moderate malaria	689.3 (309.8 to 1,282.7)	550.0 (171.0 to 1,322.2)	81,072.3 (69,364.2 to 96,608.7)	70,764.1 (56,880.3 to 88,162.6)	33.7 (13.2 to 65.3)	26.9 (7.7 to 61.4)
Malaria	Severe anaemia due to malaria parasitemia (PfPR)	853.4 (634.6 to 1,078.5)	536.0 (350.6 to 737.8)	< 0.05 (0.0 to < 0.05)	< 0.05 (0.0 to < 0.05)	120.0 (75.2 to 178.2)	75.6 (44.0 to 119.9)
Malaria	Mild malaria with mild anaemia	3,115.2 (2,115.5 to 4,167.2)	3,076.7 (2,040.8 to 4,126.4)	< 0.05 (0.0 to < 0.05)	< 0.05 (0.0 to < 0.05)	28.8 (10.8 to 63.1)	28.4 (10.3 to 62.3)
Malaria	Moderate motor plus cognitive impairment with epilepsy due to malaria	260.4 (225.4 to 301.6)	320.3 (276.9 to 372.1)	--	--	89.6 (61.6 to 122.0)	108.4 (72.4 to 148.9)
Malaria	Severe malaria with severe anaemia	2.6 (0.3 to 8.1)	2.1 (0.2 to 6.2)	< 0.05 (0.0 to < 0.05)	< 0.05 (0.0 to < 0.05)	0.7 (0.1 to 2.0)	0.5 (0.1 to 1.6)
Malaria	Moderate motor plus cognitive impairment with blindness and epilepsy due to malaria	24.6 (20.7 to 29.2)	30.3 (25.3 to 36.0)	--	--	11.8 (8.3 to 15.7)	14.4 (10.0 to 19.2)
Malaria	Moderate motor impairment with epilepsy due to malaria	140.1 (121.9 to 161.1)	172.5 (149.2 to 197.9)	--	--	33.5 (24.1 to 44.9)	40.1 (28.2 to 54.3)
Malaria	Severe malaria with moderate anaemia	32.4 (3.6 to 99.3)	27.4 (3.0 to 83.3)	< 0.05 (0.0 to < 0.05)	< 0.05 (0.0 to < 0.05)	5.6 (0.6 to 16.6)	4.8 (0.5 to 14.5)
Malaria	Severe malaria with mild anaemia	22.9 (2.6 to 70.3)	22.6 (2.5 to 68.1)	< 0.05 (0.0 to < 0.05)	< 0.05 (0.0 to < 0.05)	3.1 (0.4 to 9.1)	3.1 (0.3 to 9.2)
Malaria	Mild malaria with severe anaemia	360.8 (240.7 to 494.6)	282.8 (184.7 to 398.7)	< 0.05 (0.0 to < 0.05)	< 0.05 (0.0 to < 0.05)	53.1 (30.5 to 80.9)	41.7 (23.9 to 64.5)
Malaria	Severe motor impairment with epilepsy due to malaria	46.8 (40.4 to 54.4)	57.6 (49.8 to 67.0)	--	--	24.1 (16.5 to 32.7)	29.4 (20.3 to 40.1)
Malaria	Severe malaria	10.2 (0.9 to 35.0)	8.0 (0.7 to 29.1)	1,210.4 (138.8 to 3,372.3)	1,055.0 (124.7 to 2,929.7)	1.3 (0.1 to 4.5)	1.1 (0.1 to 3.9)
Malaria	Moderate anaemia due to malaria parasitemia (PfPR)	11,874.1 (8,788.6 to 14,932.2)	8,217.1 (5,374.6 to 11,248.5)	< 0.05 (0.0 to < 0.05)	< 0.05 (0.0 to < 0.05)	590.7 (364.4 to 923.3)	410.4 (225.4 to 676.6)
Malaria	Moderate motor impairment with blindness and epilepsy due to malaria	13.1 (11.0 to 15.4)	16.1 (13.6 to 19.1)	--	--	5.1 (3.6 to 6.8)	6.2 (4.4 to 8.4)
Malaria	Severe motor plus cognitive impairment with blindness due to malaria	38.0 (22.8 to 49.6)	42.8 (24.2 to 58.2)	--	--	23.5 (13.4 to 33.2)	26.7 (14.2 to 39.1)
Malaria	Severe motor plus cognitive impairment with epilepsy due to malaria	215.4 (132.7 to 281.4)	243.0 (138.9 to 329.2)	--	--	127.9 (73.2 to 179.8)	144.0 (76.4 to 208.4)
Malaria	Moderate motor impairment with blindness due to malaria	8.3 (5.4 to 12.1)	9.5 (5.9 to 14.3)	--	--	2.0 (1.1 to 3.2)	2.3 (1.3 to 3.8)
Malaria	Severe motor impairment due to malaria	370.2 (290.7 to 489.7)	494.0 (381.5 to 646.3)	--	--	141.9 (87.8 to 208.1)	189.3 (116.4 to 275.7)
Malaria	Severe motor plus cognitive impairment with blindness and epilepsy due to malaria	20.2 (12.6 to 26.8)	22.8 (12.8 to 31.6)	--	--	14.1 (8.1 to 19.9)	15.8 (8.6 to 23.3)
Malaria	Moderate malaria with severe anaemia	180.6 (119.9 to 247.0)	141.6 (93.1 to 200.1)	< 0.05 (0.0 to < 0.05)	< 0.05 (0.0 to < 0.05)	33.2 (19.2 to 51.5)	26.2 (14.8 to 40.3)
Malaria	Mild anaemia due to malaria parasitemia (PfPR)	9,514.2 (7,571.5 to 11,530.0)	7,396.1 (5,180.6 to 9,721.1)	< 0.05 (0.0 to < 0.05)	< 0.05 (0.0 to < 0.05)	34.4 (11.8 to 78.4)	26.9 (8.9 to 63.1)
Malaria	Severe motor impairment with blindness and epilepsy due to malaria	4.4 (3.7 to 5.1)	5.4 (4.6 to 6.3)	--	--	2.7 (1.9 to 3.5)	3.3 (2.4 to 4.3)
Malaria	Moderate malaria with moderate anaemia	2,204.4 (1,481.6 to 3,038.1)	1,865.1 (1,238.4 to 2,614.2)	< 0.05 (0.0 to < 0.05)	< 0.05 (0.0 to < 0.05)	211.1 (117.6 to 338.6)	178.9 (100.5 to 282.9)
Malaria	Mild malaria with moderate anaemia	4,402.6 (2,968.0 to 6,065.4)	3,725.0 (2,461.8 to 5,213.6)	< 0.05 (0.0 to < 0.05)	< 0.05 (0.0 to < 0.05)	243.5 (132.5 to 400.4)	206.2 (112.9 to 329.9)
Malaria	Moderate malaria with mild anaemia	1,559.8 (1,061.3 to 2,089.4)	1,540.5 (1,026.4 to 2,066.5)	< 0.05 (0.0 to < 0.05)	< 0.05 (0.0 to < 0.05)	81.4 (44.8 to 131.5)	80.5 (44.8 to 129.6)
Chagas disease	Asymptomatic and mild heart failure due to Chagas disease	250.7 (162.4 to 365.9)	283.2 (185.1 to 408.6)	--	--	9.8 (5.1 to 16.9)	11.0 (5.8 to 18.8)
Chagas disease	Asymptomatic Chagas disease	6,137.5 (5,237.1 to 7,163.9)	6,154.6 (5,154.1 to 7,307.0)	--	--	< 0.05 (0.0 to < 0.05)	< 0.05 (0.0 to < 0.05)
Chagas disease	Moderate chronic digestive disease due to Chagas disease	248.2 (191.9 to 314.6)	257.0 (196.9 to 325.4)	--	--	26.8 (16.9 to 39.3)	27.7 (17.5 to 40.4)
Chagas disease	Severe heart failure due to Chagas disease	18.6 (12.1 to 27.2)	21.0 (13.7 to 30.3)	--	--	3.1 (1.7 to 5.1)	3.6 (2.0 to 5.7)
Chagas disease	Moderate heart failure due to Chagas disease	21.5 (13.9 to 31.4)	24.3 (15.9 to 35.1)	--	--	1.5 (0.8 to 2.4)	1.7 (0.9 to 2.8)
Chagas disease	Mild chronic digestive disease due to Chagas disease	372.3 (287.9 to 471.9)	385.5 (295.3 to 488.2)	--	--	4.0 (1.9 to 7.5)	4.1 (1.9 to 7.9)
Chagas disease	Atrial fibrillation and flutter due to Chagas disease	61.8 (46.0 to 81.3)	74.1 (54.7 to 97.8)	--	--	12.4 (7.8 to 18.2)	14.8 (9.4 to 21.9)
Chagas disease	Acute Chagas disease	1.1 (0.5 to 1.9)	1.0 (0.5 to 1.8)	194.5 (164.2 to 228.5)	179.9 (150.1 to 214.0)	0.1 (< 0.05 to 0.1)	0.1 (< 0.05 to 0.1)
Visceral leishmaniasis	Moderate visceral leishmaniasis	49.5 (46.7 to 52.6)	22.6 (21.0 to 24.1)	264.2 (248.9 to 280.7)	120.3 (111.9 to 128.6)	2.4 (1.5 to 3.5)	1.1 (0.7 to 1.6)
Visceral leishmaniasis	Severe visceral leishmaniasis	16.5 (15.6 to 17.5)	7.5 (7.0 to 8.0)	--	--	2.1 (1.4 to 3.0)	1.0 (0.6 to 1.4)
Cutaneous and mucocutaneous leishmaniasis	Cutaneous and mucocutaneous leishmaniasis	3,364.6 (2,612.2 to 4,371.5)	4,320.2 (3,650.3 to 5,237.6)	731.6 (636.3 to 842.6)	678.5 (496.7 to 902.1)	212.4 (131.6 to 329.3)	273.1 (177.2 to 398.8)
African trypanosomiasis	Skin disfigurement due to Trypanosoma brucei gambiense	14.1 (8.7 to 22.2)	3.6 (2.1 to 6.2)	5.9 (4.5 to 7.6)	1.2 (0.9 to 1.6)	0.4 (0.2 to 0.7)	0.1 (< 0.05 to 0.2)
African trypanosomiasis	Sleeping sickness due to Trypanosoma brucei gambiense	12.5 (7.0 to 20.6)	3.3 (1.7 to 5.9)	17.1 (13.4 to 22.0)	3.5 (2.8 to 4.5)	6.3 (3.0 to 11.1)	1.8 (0.8 to 3.4)
African trypanosomiasis	Sleeping sickness due to Trypanosoma brucei rhodesiense	0.4 (0.2 to 0.7)	< 0.05 (< 0.05 to 0.1)	1.5 (0.8 to 2.7)	0.2 (0.1 to 0.4)	< 0.05 (< 0.05 to < 0.05)	< 0.05 (< 0.05 to < 0.05)
African trypanosomiasis	Skin disfigurement due to Trypanosoma brucei rhodesiense	0.6 (0.3 to 1.1)	0.1 (< 0.05 to 0.2)	0.5 (0.3 to 0.9)	0.1 (< 0.05 to 0.1)	0.3 (0.2 to 0.6)	< 0.05 (< 0.05 to 0.1)
Schistosomiasis	Bladder pathology due to schistosomiasis	19,687.0 (17,857.2 to 21,670.7)	9,000.7 (7,732.6 to 10,388.0)	--	--	210.6 (104.1 to 399.6)	96.5 (46.9 to 184.5)
Schistosomiasis	Hepatomegaly due to schistosomiasis	13,500.9 (12,616.0 to 14,333.7)	17,849.7 (16,665.4 to 19,005.4)	--	--	144.3 (71.6 to 267.1)	191.2 (95.1 to 356.8)
Schistosomiasis	Hematemesis due to schistosomiasis	6.6 (6.3 to 6.9)	8.8 (8.4 to 9.2)	--	--	2.1 (1.4 to 3.1)	2.9 (1.9 to 4.1)

Schistosomiasis	Moderate anaemia due to schistosomiasis	5,641.9 (5,304.6 to 6,008.8)	4,117.0 (3,832.2 to 4,421.8)	--	--	277.5 (185.1 to 397.8)	203.3 (136.4 to 291.5)
Schistosomiasis	Severe anaemia due to schistosomiasis	440.6 (405.6 to 477.5)	345.9 (316.6 to 381.1)	--	--	61.0 (41.9 to 84.7)	48.1 (32.6 to 66.5)
Schistosomiasis	Dysuria due to schistosomiasis	151.4 (107.2 to 219.0)	60.3 (16.4 to 131.9)	--	--	1.6 (0.7 to 3.3)	0.6 (0.1 to 1.8)
Schistosomiasis	Mild anaemia due to schistosomiasis	10,170.9 (9,565.8 to 10,789.7)	8,904.4 (8,330.8 to 9,517.0)	--	--	36.4 (13.0 to 78.7)	31.9 (11.4 to 68.9)
Schistosomiasis	Ascites due to schistosomiasis	469.5 (449.8 to 489.1)	627.2 (599.6 to 653.6)	--	--	50.5 (34.8 to 69.8)	67.6 (46.8 to 93.6)
Schistosomiasis	Hydronephrosis due to schistosomiasis	5,357.1 (4,970.9 to 5,770.9)	3,224.3 (2,922.7 to 3,548.3)	--	--	57.2 (28.5 to 108.4)	34.5 (17.0 to 65.0)
Schistosomiasis	Mild diarrhea due to schistosomiasis	5.2 (3.6 to 7.0)	0.6 (0.3 to 0.9)	--	--	0.4 (0.2 to 0.6)	< 0.05 (< 0.05 to 0.1)
Schistosomiasis	Mild schistosomiasis	203,133.9 (195,079.5 to 210,972.7)	145,634.7 (138,464.7 to 152,882.3)	84,306.3 (81,061.0 to 87,841.8)	71,385.4 (67,341.7 to 76,073.3)	1,140.7 (458.7 to 2,393.0)	819.6 (329.8 to 1,697.7)
Cysticercosis	Neurocysticercosis with epilepsy	2,636.5 (2,273.0 to 3,027.4)	2,675.5 (2,231.9 to 3,148.7)	--	--	441.1 (299.6 to 598.5)	420.9 (274.1 to 580.8)
Cystic echinococcosis	Abdominal problems due to cystic echinococcosis	1,283.4 (899.5 to 1,864.9)	787.2 (542.5 to 1,169.3)	270.1 (206.3 to 376.9)	166.5 (122.0 to 247.2)	138.5 (80.0 to 221.2)	85.5 (48.4 to 137.0)
Cystic echinococcosis	Chronic respiratory disease due to cystic echinococcosis	290.9 (195.9 to 435.5)	178.4 (117.9 to 262.8)	61.2 (44.1 to 87.9)	37.7 (26.7 to 55.0)	5.5 (2.6 to 10.4)	3.4 (1.6 to 6.3)
Cystic echinococcosis	Epilepsy due to echinococcosis	13.2 (3.7 to 30.6)	8.1 (2.0 to 18.3)	--	--	2.7 (0.7 to 6.2)	1.6 (0.4 to 3.7)
Lymphatic filariasis	Hydrocele due to lymphatic filariasis	10,975.1 (3,483.3 to 22,742.3)	6,043.5 (2,050.7 to 12,663.9)	--	--	1,321.9 (402.0 to 2,841.2)	729.4 (235.5 to 1,621.1)
Lymphatic filariasis	Lymphedema due to lymphatic filariasis	5,443.5 (3,673.1 to 7,513.2)	4,366.5 (2,941.9 to 6,021.5)	7,102.1 (6,285.0 to 8,062.1)	7,604.4 (6,561.1 to 8,749.7)	556.6 (313.4 to 876.0)	446.2 (250.8 to 703.4)
Lymphatic filariasis	Prevalence of detectable microfiliaria due to lymphatic filariasis	34,211.0 (32,616.3 to 35,967.3)	18,697.7 (17,847.0 to 19,595.0)	--	--	< 0.05 (0.0 to < 0.05)	< 0.05 (0.0 to < 0.05)
Lymphatic filariasis	Acute adenolymphangitis due to lymphatic filariasis	396.1 (253.3 to 583.7)	274.6 (181.2 to 388.0)	--	--	19.2 (10.2 to 32.6)	13.3 (7.2 to 21.7)
Onchocerciasis	Mild skin disease without itch due to onchocerciasis	6,024.3 (3,326.8 to 9,346.3)	5,010.5 (1,990.2 to 9,600.0)	--	--	64.1 (25.1 to 134.9)	53.4 (15.1 to 127.9)
Onchocerciasis	Severe skin disease due to onchocerciasis	2,097.1 (766.3 to 3,564.9)	1,644.2 (365.9 to 3,224.7)	--	--	365.9 (129.6 to 676.2)	287.7 (61.7 to 603.4)
Onchocerciasis	Blindness due to onchocerciasis	91.6 (58.9 to 135.4)	73.0 (35.8 to 132.4)	--	--	15.4 (8.8 to 25.1)	12.3 (5.4 to 23.8)
Onchocerciasis	Asymptomatic onchocerciasis	5,489.2 (353.7 to 14,579.9)	2,741.5 (5.3 to 10,644.9)	--	--	< 0.05 (0.0 to < 0.05)	< 0.05 (0.0 to < 0.05)
Onchocerciasis	Severe vision impairment due to onchocerciasis	170.5 (109.2 to 251.6)	165.0 (77.3 to 300.8)	--	--	28.2 (16.1 to 43.1)	27.2 (12.2 to 51.1)
Onchocerciasis	Moderate vision impairment due to onchocerciasis	542.1 (322.3 to 829.0)	543.6 (230.3 to 1,021.1)	--	--	15.6 (7.4 to 28.0)	15.7 (5.4 to 34.0)
Onchocerciasis	Severe skin disease without itch due to onchocerciasis	52.0 (5.6 to 114.2)	42.4 (3.8 to 97.2)	--	--	18.7 (1.8 to 44.1)	15.4 (1.3 to 37.0)
Onchocerciasis	Mild skin disease due to onchocerciasis	1,456.8 (532.1 to 2,456.8)	1,496.4 (399.3 to 2,741.5)	2,743.0 (1,877.2 to 3,981.0)	3,838.6 (1,746.6 to 7,517.7)	37.3 (11.7 to 75.3)	38.5 (9.2 to 84.7)
Onchocerciasis	Moderate skin disease due to onchocerciasis	4,129.1 (2,070.4 to 6,714.0)	2,933.2 (997.4 to 5,634.6)	--	--	721.2 (332.8 to 1,258.9)	512.4 (172.7 to 1,038.6)
Trachoma	Blindness due to trachoma	566.3 (473.1 to 684.2)	534.3 (442.9 to 643.4)	--	--	93.4 (61.0 to 130.1)	88.2 (58.2 to 122.9)
Trachoma	Moderate vision impairment due to trachoma	2,173.2 (1,524.7 to 2,942.4)	2,202.8 (1,443.2 to 3,164.5)	--	--	60.8 (34.2 to 103.7)	61.7 (33.2 to 108.4)
Trachoma	Severe vision impairment due to trachoma	583.1 (420.2 to 764.0)	600.4 (415.7 to 813.6)	--	--	92.3 (56.5 to 137.6)	95.2 (57.0 to 144.2)
Dengue	Severe dengue	122.3 (47.4 to 220.9)	214.4 (116.4 to 368.3)	3,189.0 (1,236.4 to 5,760.5)	5,589.9 (3,036.0 to 9,602.4)	16.1 (5.6 to 30.2)	28.2 (13.4 to 51.8)
Dengue	Moderate dengue	895.9 (367.0 to 1,449.3)	1,569.4 (957.3 to 2,368.0)	54,499.3 (22,323.7 to 88,163.0)	95,474.3 (58,236.9 to 144,052.8)	43.8 (16.6 to 78.6)	76.6 (39.2 to 136.1)
Dengue	Post-dengue chronic fatigue syndrome	2,434.2 (890.8 to 4,684.0)	4,262.1 (1,990.2 to 7,793.6)	--	--	502.1 (173.8 to 1,012.1)	877.0 (365.9 to 1,662.0)
Yellow fever	Asymptomatic yellow fever	2.1 (0.5 to 6.0)	1.7 (0.4 to 4.8)	76.5 (19.8 to 212.0)	62.7 (16.0 to 174.7)	< 0.05 (0.0 to < 0.05)	< 0.05 (0.0 to < 0.05)
Yellow fever	Severe yellow fever	0.5 (0.1 to 1.4)	0.4 (0.1 to 1.1)	17.5 (3.9 to 50.0)	14.3 (3.2 to 40.2)	0.1 (< 0.05 to 0.2)	0.1 (< 0.05 to 0.2)
Yellow fever	Moderate yellow fever	1.2 (0.3 to 3.3)	1.0 (0.2 to 2.7)	43.2 (10.9 to 115.7)	35.4 (9.1 to 92.6)	0.1 (< 0.05 to 0.2)	< 0.05 (< 0.05 to 0.1)
Rabies	Rabies	1.0 (0.6 to 1.2)	0.5 (0.3 to 0.7)	25.0 (15.3 to 32.4)	13.3 (7.2 to 19.1)	0.1 (0.1 to 0.2)	0.1 (< 0.05 to 0.1)
Ascariasis	Mild abdominopelvic problems due to ascariasis	32,045.1 (30,426.5 to 33,867.0)	25,500.8 (23,968.4 to 27,345.6)	--	--	344.1 (170.3 to 647.9)	273.6 (132.0 to 521.4)
Ascariasis	Severe wasting due to ascariasis	94.7 (70.9 to 119.4)	76.8 (57.6 to 100.4)	--	--	11.7 (6.9 to 18.6)	9.5 (5.5 to 14.8)
Ascariasis	Asymptomatic ascariasis	776,030.7 (729,377.0 to 830,201.6)	749,226.7 (687,300.7 to 822,583.7)	--	--	< 0.05 (0.0 to < 0.05)	< 0.05 (0.0 to < 0.05)
Ascariasis	Heavy infestation of ascariasis	35,069.7 (32,120.7 to 38,158.0)	24,878.3 (22,945.2 to 27,006.2)	--	--	903.5 (501.8 to 1,455.0)	640.4 (345.3 to 1,047.1)
Trichuriasis	Asymptomatic trichuriasis	409,844.4 (386,534.6 to 433,735.6)	415,634.6 (390,790.5 to 444,430.7)	--	--	< 0.05 (0.0 to < 0.05)	< 0.05 (0.0 to < 0.05)
Trichuriasis	Heavy infestation of trichuriasis	11,013.3 (9,904.2 to 12,418.8)	8,407.4 (7,657.7 to 9,424.3)	--	--	283.8 (154.3 to 467.2)	216.7 (118.8 to 352.9)
Trichuriasis	Mild abdominopelvic problems due to trichuriasis	12,587.9 (11,910.4 to 13,318.4)	11,039.6 (10,412.6 to 11,781.1)	--	--	135.3 (66.7 to 254.6)	118.7 (58.0 to 226.7)
Trichuriasis	Severe wasting due to trichuriasis	19.4 (14.4 to 25.3)	13.0 (9.6 to 17.3)	--	--	2.4 (1.4 to 3.8)	1.6 (0.9 to 2.6)

Hookworm disease	Heavy infestation of hookworm	37,980.0 (35,745.6 to 40,406.5)	34,965.4 (32,862.0 to 37,060.8)	--	--	976.1 (536.7 to 1,554.2)	897.5 (503.7 to 1,446.3)
Hookworm disease	Severe anaemia due to hookworm disease	514.6 (465.1 to 570.5)	541.9 (487.8 to 609.1)	--	--	72.6 (49.2 to 99.2)	76.5 (50.9 to 108.1)
Hookworm disease	Mild anaemia due to hookworm disease	15,991.9 (14,980.4 to 17,102.5)	18,052.7 (16,831.0 to 19,359.3)	--	--	57.7 (20.3 to 128.3)	65.0 (22.9 to 142.3)
Hookworm disease	Mild abdominopelvic problems due to hookworm disease	19,233.8 (18,538.3 to 20,032.3)	18,892.0 (18,175.6 to 19,660.3)	--	--	206.1 (102.0 to 390.6)	202.4 (99.0 to 386.5)
Hookworm disease	Severe wasting due to hookworm disease	64.7 (49.3 to 83.1)	58.7 (44.1 to 77.6)	--	--	8.0 (4.7 to 12.6)	7.3 (4.3 to 11.7)
Hookworm disease	Moderate anaemia due to hookworm disease	8,822.1 (8,208.9 to 9,493.0)	8,776.0 (8,164.3 to 9,480.5)	--	--	439.2 (288.1 to 644.2)	436.7 (287.4 to 630.3)
Hookworm disease	Asymptomatic hookworm disease	371,194.8 (348,494.1 to 397,256.7)	369,396.1 (344,627.2 to 395,452.4)	--	--	< 0.05 (0.0 to < 0.05)	< 0.05 (0.0 to < 0.05)
Food-borne trematodiases	Heavy fascioliasis due to food-borne trematodiases	184.0 (45.1 to 398.3)	219.1 (52.9 to 475.5)	--	--	19.6 (4.7 to 45.0)	23.4 (5.5 to 53.6)
Food-borne trematodiases	Cerebral paragonimiasis	261.5 (76.8 to 543.5)	249.1 (75.4 to 504.4)	--	--	43.3 (11.4 to 95.4)	37.3 (10.5 to 81.7)
Food-borne trematodiases	Asymptomatic paragonimiasis	22,927.8 (12,974.8 to 30,013.1)	22,567.9 (12,833.2 to 29,094.2)	910.5 (513.6 to 1,231.9)	730.9 (411.9 to 972.7)	< 0.05 (0.0 to < 0.05)	< 0.05 (0.0 to < 0.05)
Food-borne trematodiases	Asymptomatic intestinal fluke infection	1,414.9 (1,169.8 to 1,681.7)	1,817.5 (1,490.1 to 2,182.5)	142.6 (120.9 to 167.9)	179.9 (150.5 to 214.9)	< 0.05 (0.0 to < 0.05)	< 0.05 (0.0 to < 0.05)
Food-borne trematodiases	Heavy intestinal fluke infection due to food-borne trematodiases	193.8 (110.2 to 313.4)	280.9 (153.5 to 465.7)	--	--	20.9 (10.4 to 36.6)	30.1 (14.4 to 54.0)
Food-borne trematodiases	Mild paragonimiasis due to food-borne trematodiases	4,775.3 (663.2 to 11,415.5)	4,701.4 (652.5 to 11,218.1)	189.7 (25.8 to 446.6)	152.3 (20.8 to 365.9)	89.2 (11.6 to 226.0)	87.6 (11.7 to 220.3)
Food-borne trematodiases	Asymptomatic fascioliasis	709.4 (500.8 to 933.9)	824.1 (599.8 to 1,043.7)	78.6 (55.2 to 101.8)	91.2 (65.9 to 115.2)	< 0.05 (0.0 to < 0.05)	< 0.05 (0.0 to < 0.05)
Food-borne trematodiases	Moderate paragonimiasis due to food-borne trematodiases	1,120.5 (153.4 to 2,664.2)	1,103.2 (151.2 to 2,599.2)	44.6 (6.4 to 104.8)	35.8 (4.9 to 84.5)	239.2 (32.5 to 606.8)	234.2 (31.2 to 587.8)
Food-borne trematodiases	Asymptomatic clonorchiasis	28,051.0 (25,725.4 to 30,842.3)	29,789.6 (27,379.7 to 32,815.6)	1,863.2 (1,665.1 to 2,102.8)	1,846.7 (1,647.6 to 2,086.4)	< 0.05 (0.0 to < 0.05)	< 0.05 (0.0 to < 0.05)
Food-borne trematodiases	Severe paragonimiasis due to food-borne trematodiases	1,360.3 (185.9 to 3,226.5)	1,339.3 (182.1 to 3,186.0)	54.0 (7.6 to 128.1)	43.4 (5.9 to 104.2)	522.4 (71.3 to 1,343.6)	511.1 (69.2 to 1,300.8)
Food-borne trematodiases	Heavy opisthorchiasis due to food-borne trematodiases	787.9 (202.4 to 1,717.0)	1,223.1 (289.7 to 2,668.0)	--	--	80.5 (20.7 to 182.6)	124.7 (29.8 to 288.8)
Food-borne trematodiases	Heavy clonorchiasis due to food-borne trematodiases	5,875.8 (5,117.4 to 6,681.8)	6,605.4 (5,697.8 to 7,603.4)	--	--	644.4 (420.4 to 903.7)	722.8 (467.4 to 1,003.1)
Food-borne trematodiases	Asymptomatic opisthorchiasis	2,766.5 (1,688.6 to 3,669.8)	4,004.0 (2,361.7 to 5,207.2)	23,683.3 (15,329.7 to 30,919.3)	31,909.0 (20,054.2 to 42,650.3)	< 0.05 (0.0 to < 0.05)	< 0.05 (0.0 to < 0.05)
Leprosy	Disfigurement level 1 due to leprosy	21.7 (20.5 to 23.1)	19.7 (18.7 to 21.0)	43.4 (41.0 to 46.2)	39.5 (37.3 to 41.9)	0.2 (0.1 to 0.4)	0.2 (0.1 to 0.4)
Leprosy	Disfigurement level 2 due to leprosy	496.9 (473.8 to 519.5)	503.5 (479.5 to 527.3)	14.0 (13.5 to 14.6)	15.8 (15.2 to 16.4)	31.1 (21.1 to 43.5)	31.4 (21.2 to 43.7)
Ebola virus disease	Ebola cases	< 0.05 (0.0 to < 0.05)	< 0.05 (< 0.05 to < 0.05)	< 0.05 (0.0 to < 0.05)	< 0.05 (< 0.05 to < 0.05)	< 0.05 (0.0 to < 0.05)	< 0.05 (< 0.05 to < 0.05)
Ebola virus disease	Post-Ebola chronic fatigue syndrome	< 0.05 (0.0 to < 0.05)	0.6 (< 0.05 to 4.0)	< 0.05 (0.0 to < 0.05)	< 0.05 (< 0.05 to < 0.05)	< 0.05 (0.0 to < 0.05)	0.1 (< 0.05 to 0.9)
Zika virus disease	Congenital Zika syndrome	< 0.05 (0.0 to < 0.05)	2.8 (1.9 to 5.4)	< 0.05 (0.0 to < 0.05)	2.4 (1.6 to 5.1)	< 0.05 (0.0 to < 0.05)	1.7 (1.0 to 2.8)
Zika virus disease	Acute Zika infection	< 0.05 (0.0 to < 0.05)	44.5 (34.1 to 62.0)	< 0.05 (0.0 to < 0.05)	2,704.2 (2,072.0 to 3,769.0)	< 0.05 (0.0 to < 0.05)	2.2 (1.3 to 3.5)
Zika virus disease	Asymptomatic Zika infection	< 0.05 (0.0 to < 0.05)	80.4 (57.9 to 116.5)	< 0.05 (0.0 to < 0.05)	4,891.5 (3,521.3 to 7,087.7)	< 0.05 (0.0 to < 0.05)	< 0.05 (0.0 to < 0.05)
Zika virus disease	Guillain-Barre syndrome due to Zika infection	< 0.05 (0.0 to < 0.05)	0.9 (0.7 to 1.3)	--	--	< 0.05 (0.0 to < 0.05)	0.3 (0.2 to 0.5)
Guinea worm disease	Mild pain due to Guinea worm emergence	2.4 (2.3 to 2.4)	< 0.05 (< 0.05 to < 0.05)	--	--	0.1 (< 0.05 to 0.1)	< 0.05 (< 0.05 to < 0.05)
Guinea worm disease	Moderate mobility reduced due to guinea worm emergence	0.5 (0.5 to 0.5)	< 0.05 (< 0.05 to < 0.05)	--	--	< 0.05 (< 0.05 to 0.1)	< 0.05 (< 0.05 to < 0.05)
Guinea worm disease	Moderate pain due to Guinea worm emergence	0.5 (0.5 to 0.5)	< 0.05 (< 0.05 to < 0.05)	6.0 (5.9 to 6.2)	< 0.05 (< 0.05 to < 0.05)	0.1 (0.1 to 0.1)	< 0.05 (< 0.05 to < 0.05)
Other neglected tropical diseases	Mild anaemia due to other neglected tropical diseases	23,341.7 (22,679.7 to 24,022.0)	25,517.3 (24,682.4 to 26,455.0)	--	--	84.7 (30.3 to 186.1)	92.5 (33.0 to 202.2)
Other neglected tropical diseases	Moderate anaemia due to other neglected tropical diseases	21,535.8 (20,331.6 to 22,836.0)	22,628.8 (21,281.6 to 24,159.5)	--	--	1,081.2 (704.4 to 1,582.2)	1,136.2 (736.0 to 1,649.4)
Other neglected tropical diseases	Acute infection due to other neglected tropical diseases	--	--	--	--	12.5 (5.5 to 21.7)	24.6 (13.0 to 42.5)
Other neglected tropical diseases	Severe anaemia due to other neglected tropical diseases	1,296.4 (1,118.3 to 1,528.1)	1,346.7 (1,131.1 to 1,697.9)	--	--	184.8 (120.1 to 257.0)	191.9 (125.1 to 273.6)
Maternal haemorrhage	Maternal haemorrhage (< 1L blood lost)	153.6 (86.4 to 232.1)	158.2 (93.5 to 247.5)	8,028.0 (6,381.9 to 10,096.2)	8,193.0 (6,612.1 to 10,132.8)	17.0 (8.8 to 29.8)	17.5 (9.1 to 30.5)
Maternal haemorrhage	Severe anaemia due to maternal haemorrhage	21.7 (17.3 to 26.8)	26.9 (21.5 to 33.5)	--	--	3.2 (2.0 to 4.6)	4.0 (2.5 to 5.8)
Maternal haemorrhage	Mild anaemia due to maternal haemorrhage	737.6 (581.3 to 924.6)	806.5 (654.9 to 993.2)	--	--	2.7 (0.9 to 5.8)	2.9 (1.0 to 6.4)
Maternal haemorrhage	Moderate anaemia due to maternal haemorrhage	354.9 (270.3 to 461.6)	356.4 (280.8 to 456.2)	--	--	17.7 (10.8 to 27.0)	17.8 (10.8 to 27.0)
Maternal haemorrhage	Maternal haemorrhage (> 1L blood lost)	54.1 (33.0 to 83.9)	55.2 (34.0 to 83.4)	1,408.7 (949.4 to 1,984.2)	1,432.7 (982.9 to 1,977.2)	17.3 (9.1 to 29.5)	17.7 (9.6 to 30.1)
Maternal sepsis and other pregnancy related infections	Infertility due to puerperal sepsis	712.5 (628.2 to 803.1)	812.2 (717.8 to 910.7)	35.5 (31.5 to 39.8)	39.5 (35.1 to 44.2)	3.6 (1.4 to 7.7)	4.1 (1.6 to 8.8)
Maternal sepsis and other pregnancy related infections	Puerperal sepsis	2.2 (1.4 to 3.3)	2.3 (1.4 to 3.4)	115.0 (87.0 to 144.3)	117.5 (89.8 to 147.7)	0.3 (0.2 to 0.5)	0.3 (0.2 to 0.5)
Maternal sepsis and other pregnancy related infections	Other maternal infections	834.9 (451.1 to 1,380.8)	847.4 (448.7 to 1,355.4)	10,169.4 (8,089.8 to 13,104.3)	10,219.7 (8,222.7 to 13,002.2)	40.7 (19.5 to 73.8)	41.4 (19.8 to 77.2)

Maternal hypertensive disorders	Eclampsia	5.4 (2.1 to 10.2)	5.3 (1.8 to 10.4)	1,987.7 (1,602.7 to 2,458.9)	1,951.4 (1,576.3 to 2,400.1)	3.3 (1.2 to 6.5)	3.2 (1.1 to 6.6)
Maternal hypertensive disorders	Long term sequelae of severe pre-eclampsia	21.8 (20.9 to 22.9)	24.7 (23.5 to 26.0)	325.4 (311.1 to 341.2)	369.0 (352.0 to 387.9)	1.5 (0.9 to 2.3)	1.6 (1.0 to 2.5)
Maternal hypertensive disorders	Long term sequelae of eclampsia	12.2 (11.5 to 13.0)	13.9 (13.1 to 14.7)	186.6 (176.8 to 197.0)	212.2 (201.4 to 224.2)	0.8 (0.5 to 1.3)	0.9 (0.6 to 1.4)
Maternal hypertensive disorders	Other hypertensive disorders of pregnancy	4,080.5 (2,740.1 to 5,719.5)	4,390.5 (2,886.0 to 6,042.8)	16,362.5 (14,198.2 to 18,458.2)	17,514.2 (14,981.7 to 19,982.7)	192.7 (110.3 to 313.0)	207.8 (113.8 to 340.0)
Maternal hypertensive disorders	Severe pre-eclampsia	11.7 (7.0 to 17.9)	14.3 (8.6 to 22.7)	632.6 (445.7 to 898.1)	764.2 (526.6 to 1,112.1)	2.0 (1.1 to 3.4)	2.5 (1.3 to 4.5)
Maternal obstructed labour and uterine rupture	Vesicovaginal fistula	1,060.2 (882.4 to 1,264.5)	897.9 (745.5 to 1,082.1)	196.2 (160.9 to 240.3)	189.3 (151.5 to 232.9)	333.9 (215.8 to 472.0)	283.8 (185.8 to 407.5)
Maternal obstructed labour and uterine rupture	Obstructed labor, acute event	95.9 (58.2 to 145.6)	97.1 (57.3 to 146.9)	7,007.0 (5,485.9 to 8,965.3)	7,039.4 (5,611.8 to 8,839.8)	30.1 (15.9 to 50.0)	30.5 (16.2 to 51.0)
Maternal obstructed labour and uterine rupture	Rectovaginal fistula	55.1 (14.8 to 112.3)	46.4 (12.7 to 92.6)	10.2 (2.8 to 21.2)	9.8 (2.8 to 19.5)	26.1 (6.8 to 56.4)	22.2 (5.9 to 46.1)
Maternal abortion, miscarriage, and ectopic pregnancy	Maternal abortive outcome	581.2 (370.3 to 842.6)	552.0 (343.7 to 795.1)	70,440.8 (55,263.7 to 89,943.9)	67,299.2 (53,502.3 to 85,164.8)	63.5 (34.3 to 102.6)	60.4 (33.3 to 98.2)
Maternal abortion, miscarriage, and ectopic pregnancy	Ectopic Pregnancy	23.0 (14.7 to 34.4)	24.2 (15.5 to 35.8)	2,805.4 (2,151.1 to 3,657.0)	2,945.5 (2,276.4 to 3,846.6)	2.6 (1.4 to 4.3)	2.8 (1.5 to 4.6)
Other maternal disorders	Other maternal disorders	--	--	--	--	170.9 (121.1 to 232.7)	145.0 (101.2 to 198.5)
Neonatal preterm birth complications	Severe motor plus cognitive impairment with blindness and epilepsy due to neonatal preterm birth complications 28-32wks	74.4 (31.7 to 110.6)	81.4 (32.1 to 131.0)	--	--	51.1 (21.8 to 79.7)	55.6 (22.1 to 92.2)
Neonatal preterm birth complications	Mild motor impairment due to neonatal preterm birth complications 32-36wks	3,410.9 (2,296.6 to 4,640.8)	3,394.6 (2,313.2 to 4,691.1)	--	--	33.7 (15.0 to 66.1)	33.4 (14.8 to 67.9)
Neonatal preterm birth complications	Moderate motor plus cognitive impairment with blindness due to neonatal preterm birth complications 32-36wks	128.6 (83.6 to 185.3)	160.0 (70.3 to 318.1)	--	--	45.1 (25.9 to 72.5)	56.1 (22.5 to 116.5)
Neonatal preterm birth complications	Severe motor impairment with epilepsy due to neonatal preterm birth complications 28-32wks	242.5 (186.4 to 309.6)	276.5 (187.8 to 405.1)	--	--	120.6 (79.5 to 169.6)	135.2 (80.6 to 210.3)
Neonatal preterm birth complications	Severe motor impairment due to neonatal preterm birth complications <28wks	1,802.8 (1,008.4 to 2,962.4)	2,283.1 (921.1 to 4,809.0)	--	--	686.4 (346.9 to 1,199.1)	865.7 (305.4 to 1,877.6)
Neonatal preterm birth complications	Severe motor impairment with blindness and epilepsy due to neonatal preterm birth complications <28wks	15.7 (9.5 to 24.6)	18.4 (8.2 to 35.1)	--	--	9.3 (5.2 to 15.2)	10.8 (4.5 to 21.8)
Neonatal preterm birth complications	Moderate motor impairment with epilepsy due to neonatal preterm birth complications <28wks	503.5 (299.6 to 776.5)	589.8 (262.0 to 1,118.4)	--	--	109.9 (59.9 to 175.8)	122.7 (50.7 to 241.1)
Neonatal preterm birth complications	Severe motor impairment due to neonatal preterm birth complications 28-32wks	2,779.0 (2,048.6 to 3,781.7)	3,453.1 (2,143.3 to 5,186.2)	--	--	1,058.4 (631.1 to 1,607.8)	1,310.9 (697.9 to 2,224.5)
Neonatal preterm birth complications	Moderate motor plus cognitive impairment with blindness due to neonatal preterm birth complications 28-32wks	223.3 (166.3 to 289.7)	267.8 (178.5 to 386.9)	--	--	77.1 (47.4 to 112.5)	91.7 (50.8 to 144.6)
Neonatal preterm birth complications	Moderate motor impairment with blindness due to neonatal preterm birth complications 28-32wks	85.3 (57.7 to 120.4)	103.1 (62.4 to 158.8)	--	--	20.1 (11.4 to 31.7)	24.2 (13.0 to 41.6)
Neonatal preterm birth complications	Asymptomatic neonatla preterm birth <28wks	143.9 (84.5 to 227.4)	156.3 (88.9 to 255.0)	--	--	< 0.05 (0.0 to < 0.05)	< 0.05 (0.0 to < 0.05)
Neonatal preterm birth complications	Blindness due to retinopathy of prematurity	1,107.2 (554.3 to 1,580.2)	1,269.6 (676.9 to 1,797.0)	--	--	195.3 (91.4 to 316.9)	223.3 (109.8 to 357.0)
Neonatal preterm birth complications	Severe motor impairment with blindness due to neonatal preterm birth complications <28wks	19.3 (11.3 to 30.7)	23.9 (10.8 to 45.8)	--	--	9.9 (5.6 to 17.1)	12.2 (5.1 to 25.4)
Neonatal preterm birth complications	Mild motor plus cognitive impairments due to neonatal preterm birth complications <28wks	1,237.4 (656.4 to 2,192.3)	1,393.0 (724.2 to 2,472.4)	--	--	37.3 (16.0 to 74.8)	42.0 (17.9 to 85.8)
Neonatal preterm birth complications	Moderate motor impairment with blindness and epilepsy due to neonatal preterm birth complications 32-36wks	41.2 (28.2 to 58.4)	48.8 (21.8 to 100.0)	--	--	15.1 (9.3 to 23.7)	17.5 (7.4 to 38.4)
Neonatal preterm birth complications	Severe motor impairment with blindness due to neonatal preterm birth complications 28-32wks	28.4 (19.2 to 41.2)	34.3 (21.0 to 52.8)	--	--	14.5 (8.7 to 22.5)	17.6 (9.6 to 29.6)
Neonatal preterm birth complications	Moderate motor impairment with blindness due to neonatal preterm birth complications <28wks	58.0 (34.0 to 93.6)	71.9 (33.0 to 138.9)	--	--	13.7 (7.3 to 24.7)	17.0 (7.0 to 34.8)
Neonatal preterm birth complications	Severe motor impairment due to neonatal preterm birth complications 32-36wks	1,606.3 (1,033.0 to 2,434.4)	2,106.3 (871.5 to 4,562.9)	--	--	612.5 (343.0 to 1,007.8)	802.5 (311.1 to 1,867.5)
Neonatal preterm birth complications	Moderate motor plus cognitive impairment with blindness and epilepsy due to neonatal preterm birth complications 28-32wks	119.0 (87.3 to 156.2)	142.6 (94.3 to 210.6)	--	--	54.9 (36.0 to 77.9)	64.7 (37.4 to 100.1)
Neonatal preterm birth complications	Severe motor plus cognitive impairment with epilepsy due to neonatal preterm birth complications 28-32wks	792.6 (336.7 to 1,153.3)	867.8 (334.5 to 1,366.9)	--	--	465.9 (185.4 to 728.6)	504.5 (201.1 to 834.7)
Neonatal preterm birth complications	Severe motor impairment with blindness and epilepsy due to neonatal preterm birth complications 32-36wks	13.7 (9.2 to 19.9)	16.3 (7.3 to 33.3)	--	--	8.2 (5.0 to 12.5)	9.6 (3.9 to 20.9)
Neonatal preterm birth complications	Asymptomatic retinopathy of prematurity	3,729.4 (2,606.9 to 5,188.9)	3,995.4 (2,776.4 to 5,566.6)	--	--	< 0.05 (0.0 to < 0.05)	< 0.05 (0.0 to < 0.05)
Neonatal preterm birth complications	Severe motor plus cognitive impairment with epilepsy due to neonatal preterm birth complications <28wks	502.2 (183.2 to 834.3)	559.7 (177.0 to 1,136.5)	--	--	300.4 (105.4 to 526.1)	330.9 (92.4 to 698.4)
Neonatal preterm birth complications	Moderate motor impairment with blindness and epilepsy due to neonatal preterm birth complications 28-32wks	68.9 (52.4 to 87.8)	73.6 (48.9 to 107.4)	--	--	25.3 (16.6 to 36.3)	26.4 (15.4 to 41.2)
Neonatal preterm birth complications	Severe motor impairment with epilepsy due to neonatal preterm birth complications 32-36wks	146.9 (99.8 to 208.1)	173.9 (76.1 to 349.9)	--	--	73.8 (45.2 to 115.8)	86.5 (35.6 to 180.0)
Neonatal preterm birth complications	Mild motor impairment due to neonatal preterm birth complications 28-32wks	6,738.1 (3,959.4 to 10,365.9)	7,309.2 (4,246.4 to 11,319.4)	--	--	66.6 (26.4 to 144.0)	72.2 (28.0 to 155.1)
Neonatal preterm birth complications	Moderate motor impairment with blindness due to neonatal preterm birth complications 32-36wks	50.6 (30.8 to 77.2)	64.1 (27.1 to 134.6)	--	--	12.0 (6.4 to 19.8)	15.2 (6.1 to 32.9)
Neonatal preterm birth complications	Moderate motor plus cognitive impairment with epilepsy due to neonatal preterm birth complications 32-36wks	726.1 (459.9 to 1,032.6)	904.4 (391.6 to 1,928.8)	--	--	236.9 (136.8 to 373.8)	286.6 (114.3 to 627.9)
Neonatal preterm birth complications	Mild motor impairment due to neonatal preterm birth complications <28wks	1,379.2 (739.7 to 2,345.4)	1,564.5 (805.4 to 2,673.6)	--	--	13.6 (5.1 to 30.0)	15.4 (5.6 to 33.6)
Neonatal preterm birth complications	Severe motor plus cognitive impairment with blindness due to neonatal preterm birth complications <28wks	88.6 (33.3 to 149.5)	98.8 (31.3 to 200.8)	--	--	55.3 (19.9 to 96.2)	61.6 (19.3 to 129.7)
Neonatal preterm birth complications	Mild motor plus cognitive impairments due to neonatal preterm birth complications 32-36wks	2,912.7 (1,921.7 to 4,102.4)	2,875.2 (1,810.3 to 4,043.7)	--	--	87.9 (44.5 to 154.1)	86.6 (43.0 to 150.7)
Neonatal preterm birth complications	Moderate motor impairment due to neonatal preterm birth complications 32-36wks	1,249.7 (813.5 to 1,826.3)	1,607.2 (670.6 to 3,554.1)	--	--	74.0 (41.0 to 124.3)	94.9 (36.2 to 232.2)

Neonatal preterm birth complications	Severe motor plus cognitive impairment with blindness due to neonatal preterm birth complications 28-32wks	139.8 (61.9 to 208.0)	153.1 (62.1 to 250.9)	--	--	87.3 (38.6 to 137.4)	95.6 (39.3 to 164.2)
Neonatal preterm birth complications	Moderate motor plus cognitive impairment with epilepsy due to neonatal preterm birth complications 28-32wks	1,259.5 (944.3 to 1,627.8)	1,509.8 (1,020.5 to 2,166.2)	--	--	407.1 (263.0 to 577.2)	473.1 (268.9 to 732.7)
Neonatal preterm birth complications	Moderate motor impairment with epilepsy due to neonatal preterm birth complications 32-36wks	440.4 (299.5 to 624.6)	521.2 (232.6 to 1,062.8)	--	--	96.1 (59.2 to 148.4)	108.5 (45.5 to 235.5)
Neonatal preterm birth complications	Mild motor plus cognitive impairments due to neonatal preterm birth complications 28-32wks	6,099.2 (3,592.3 to 9,223.0)	6,595.9 (3,821.1 to 10,055.1)	--	--	184.1 (84.2 to 332.0)	199.0 (89.9 to 361.9)
Neonatal preterm birth complications	Moderate motor impairment with epilepsy due to neonatal preterm birth complications 28-32wks	727.1 (553.1 to 940.3)	829.1 (555.7 to 1,188.5)	--	--	156.5 (104.6 to 223.8)	169.8 (101.5 to 267.2)
Neonatal preterm birth complications	Moderate motor plus cognitive impairment with blindness and epilepsy due to neonatal preterm birth complications 32-36wks	68.5 (43.9 to 100.0)	85.3 (37.2 to 171.1)	--	--	31.7 (19.0 to 49.7)	38.9 (15.7 to 82.7)
Neonatal preterm birth complications	Asymptomatic neonatal preterm birth 28-<32 wks	678.2 (453.2 to 953.5)	683.9 (447.4 to 983.9)	--	--	< 0.05 (0.0 to < 0.05)	< 0.05 (0.0 to < 0.05)
Neonatal preterm birth complications	Severe motor plus cognitive impairment with blindness and epilepsy due to neonatal preterm birth complications <28wks	47.1 (16.8 to 79.4)	52.5 (16.5 to 106.5)	--	--	32.4 (11.4 to 57.5)	35.9 (10.2 to 75.3)
Neonatal preterm birth complications	Severe motor plus cognitive impairment with blindness and epilepsy due to neonatal preterm birth complications 32-36wks	44.4 (17.8 to 70.2)	49.7 (16.0 to 106.7)	--	--	30.5 (11.7 to 50.8)	34.0 (10.6 to 74.7)
Neonatal preterm birth complications	Mild vision impairment due to retinopathy of prematurity	834.6 (280.5 to 1,797.9)	894.2 (300.6 to 1,938.0)	--	--	2.5 (0.6 to 7.8)	2.6 (0.6 to 8.4)
Neonatal preterm birth complications	Moderate motor impairment with blindness and epilepsy due to neonatal preterm birth complications <28wks	47.1 (28.2 to 72.5)	55.2 (23.6 to 106.5)	--	--	17.3 (9.4 to 27.8)	19.8 (7.9 to 39.7)
Neonatal preterm birth complications	Moderate motor plus cognitive impairment with epilepsy due to neonatal preterm birth complications <28wks	779.5 (446.6 to 1,219.7)	948.2 (396.6 to 1,953.6)	--	--	253.9 (130.9 to 427.3)	299.3 (118.6 to 622.3)
Neonatal preterm birth complications	Severe motor impairment with blindness and epilepsy due to neonatal preterm birth complications 28-32wks	22.7 (16.8 to 30.2)	25.9 (17.0 to 37.9)	--	--	13.5 (9.1 to 19.0)	15.2 (9.1 to 23.8)
Neonatal preterm birth complications	Severe motor plus cognitive impairment with blindness due to neonatal preterm birth complications 32-36wks	83.5 (34.4 to 131.3)	93.5 (29.8 to 197.5)	--	--	52.2 (19.5 to 88.7)	58.4 (18.0 to 123.5)
Neonatal preterm birth complications	Moderate motor impairment due to neonatal preterm birth complications <28wks	1,398.7 (812.6 to 2,189.0)	1,746.2 (708.2 to 3,687.3)	--	--	82.7 (43.2 to 145.9)	102.9 (37.1 to 234.5)
Neonatal preterm birth complications	Moderate motor impairment due to neonatal preterm birth complications 28-32wks	2,145.6 (1,577.3 to 2,847.0)	2,618.1 (1,628.4 to 3,998.5)	--	--	126.8 (75.4 to 195.2)	154.6 (80.4 to 260.8)
Neonatal preterm birth complications	Asymptomatic neonatal preterm birth 32-<37wks	6,682.1 (5,559.0 to 7,905.0)	6,219.8 (5,099.6 to 7,423.7)	--	--	< 0.05 (0.0 to < 0.05)	< 0.05 (0.0 to < 0.05)
Neonatal preterm birth complications	Severe motor impairment with blindness due to neonatal preterm birth complications 32-36wks	16.9 (10.3 to 25.4)	21.4 (9.1 to 43.6)	--	--	8.6 (4.9 to 14.2)	10.9 (4.5 to 23.4)
Neonatal preterm birth complications	Severe motor impairment with epilepsy due to neonatal preterm birth complications <28wks	168.0 (100.7 to 258.2)	196.7 (83.7 to 376.6)	--	--	84.5 (45.1 to 137.8)	97.6 (39.6 to 198.0)
Neonatal preterm birth complications	Severe vision impairment due to retinopathy of prematurity	1,864.2 (1,287.4 to 2,573.8)	2,269.4 (1,569.2 to 3,153.5)	--	--	323.7 (192.3 to 503.8)	393.5 (231.3 to 615.8)
Neonatal preterm birth complications	Moderate vision impairment due to retinopathy of prematurity	3,139.5 (1,694.4 to 4,773.4)	3,750.3 (2,019.1 to 5,753.7)	--	--	93.7 (42.3 to 171.6)	111.8 (50.0 to 205.1)
Neonatal preterm birth complications	Severe motor plus cognitive impairment with epilepsy due to neonatal preterm birth complications 32-36wks	473.3 (192.1 to 745.7)	530.1 (171.6 to 1,119.7)	--	--	282.8 (110.3 to 475.2)	313.7 (91.6 to 688.4)
Neonatal preterm birth complications	Moderate motor plus cognitive impairment with blindness and epilepsy due to neonatal preterm birth complications <28wks	73.6 (41.7 to 116.8)	89.5 (37.7 to 180.0)	--	--	34.1 (18.3 to 57.1)	40.7 (15.9 to 83.5)
Neonatal preterm birth complications	Moderate motor plus cognitive impairment with blindness due to neonatal preterm birth complications <28wks	138.2 (80.2 to 218.4)	168.1 (69.5 to 334.2)	--	--	48.5 (26.2 to 81.3)	58.9 (22.5 to 121.4)
Neonatal encephalopathy due to birth asphyxia and trauma	Asymptomatic neonatal encephalopathy due to birth asphyxia and trauma	477.6 (333.2 to 675.6)	497.8 (343.3 to 702.5)	--	--	< 0.05 (0.0 to < 0.05)	< 0.05 (0.0 to < 0.05)
Neonatal encephalopathy due to birth asphyxia and trauma	Severe motor impairment with blindness due to neonatal encephalopathy due to birth asphyxia and trauma	11.2 (3.8 to 25.6)	15.8 (6.0 to 33.9)	--	--	5.7 (1.8 to 13.5)	8.1 (2.8 to 18.0)
Neonatal encephalopathy due to birth asphyxia and trauma	Moderate motor plus cognitive impairment with blindness due to neonatal encephalopathy due to birth asphyxia and trauma	78.7 (22.1 to 196.3)	107.1 (31.7 to 257.7)	--	--	27.8 (6.8 to 71.1)	37.7 (9.7 to 91.1)
Neonatal encephalopathy due to birth asphyxia and trauma	Moderate motor plus cognitive impairment with epilepsy due to neonatal encephalopathy due to birth asphyxia and trauma	444.4 (124.8 to 1,124.1)	604.5 (178.0 to 1,477.9)	--	--	145.6 (36.7 to 394.0)	194.5 (53.0 to 500.3)
Neonatal encephalopathy due to birth asphyxia and trauma	Severe motor plus cognitive impairment with epilepsy due to neonatal encephalopathy due to birth asphyxia and trauma	273.1 (45.3 to 727.8)	357.7 (73.1 to 887.2)	--	--	164.7 (28.6 to 462.0)	214.1 (44.0 to 552.8)
Neonatal encephalopathy due to birth asphyxia and trauma	Moderate motor impairment due to neonatal encephalopathy due to birth asphyxia and trauma	996.3 (343.1 to 2,342.4)	1,326.5 (482.2 to 3,033.4)	--	--	59.2 (18.2 to 149.2)	78.7 (24.2 to 197.6)
Neonatal encephalopathy due to birth asphyxia and trauma	Moderate motor plus cognitive impairment with blindness and epilepsy due to neonatal encephalopathy due to birth asphyxia and trauma	41.9 (11.7 to 105.6)	57.0 (16.8 to 140.5)	--	--	19.3 (5.1 to 52.1)	26.1 (7.1 to 67.8)
Neonatal encephalopathy due to birth asphyxia and trauma	Severe motor plus cognitive impairment with blindness due to neonatal encephalopathy due to birth asphyxia and trauma	48.2 (7.8 to 125.6)	63.1 (12.8 to 152.5)	--	--	30.2 (4.9 to 79.5)	39.6 (7.7 to 98.0)
Neonatal encephalopathy due to birth asphyxia and trauma	Mild motor plus cognitive impairments due to neonatal encephalopathy due to birth asphyxia and trauma	2,851.2 (1,516.5 to 4,491.3)	3,135.0 (1,677.3 to 4,945.0)	--	--	86.0 (37.8 to 158.5)	94.7 (42.1 to 176.5)
Neonatal encephalopathy due to birth asphyxia and trauma	Mild motor impairment due to neonatal encephalopathy due to birth asphyxia and trauma	3,212.3 (1,928.7 to 4,802.7)	3,550.2 (2,072.7 to 5,287.7)	--	--	31.9 (12.5 to 64.5)	35.2 (14.0 to 72.4)
Neonatal encephalopathy due to birth asphyxia and trauma	Severe motor plus cognitive impairment with blindness and epilepsy due to neonatal encephalopathy due to birth asphyxia and trauma	25.6 (4.5 to 67.4)	33.5 (6.7 to 82.3)	--	--	17.6 (3.1 to 47.3)	22.9 (4.7 to 58.8)
Neonatal encephalopathy due to birth asphyxia and trauma	Moderate motor impairment with epilepsy due to neonatal encephalopathy due to birth asphyxia and trauma	334.8 (108.9 to 780.9)	447.3 (163.6 to 987.2)	--	--	71.1 (21.7 to 182.9)	92.5 (31.0 to 222.4)
Neonatal encephalopathy due to birth asphyxia and trauma	Moderate motor impairment with blindness due to neonatal encephalopathy due to birth asphyxia and trauma	32.5 (10.9 to 76.2)	46.2 (17.4 to 98.7)	--	--	7.7 (2.3 to 18.6)	10.9 (3.6 to 25.0)
Neonatal encephalopathy due to birth asphyxia and trauma	Severe motor impairment with blindness and epilepsy due to neonatal encephalopathy due to birth asphyxia and trauma	10.5 (3.5 to 26.1)	14.0 (5.2 to 32.5)	--	--	6.2 (2.0 to 15.8)	8.2 (2.9 to 20.1)
Neonatal encephalopathy due to birth asphyxia and trauma	Moderate motor impairment with blindness and epilepsy due to neonatal encephalopathy due to birth asphyxia and trauma	31.3 (10.5 to 72.9)	41.8 (15.6 to 90.8)	--	--	11.3 (3.6 to 28.5)	15.0 (5.2 to 35.3)
Neonatal encephalopathy due to birth asphyxia and trauma	Severe motor impairment due to neonatal encephalopathy due to birth asphyxia and trauma	1,249.6 (427.3 to 3,076.9)	1,687.4 (620.9 to 3,951.0)	--	--	479.9 (139.0 to 1,239.4)	645.1 (197.2 to 1,634.2)
Neonatal encephalopathy due to birth asphyxia and trauma	Severe motor impairment with epilepsy due to neonatal encephalopathy due to birth asphyxia and trauma	111.6 (36.8 to 262.3)	149.1 (54.4 to 331.4)	--	--	55.4 (17.6 to 138.6)	73.2 (25.4 to 174.7)
Neonatal sepsis and other neonatal infections	Severe motor impairment with blindness due to neonatal sepsis and other neonatal infections ?	37.5 (13.1 to 93.2)	48.9 (17.8 to 120.1)	--	--	19.2 (6.2 to 48.7)	25.0 (8.6 to 61.9)
Neonatal sepsis and other neonatal infections	Moderate motor impairment with blindness and epilepsy due to neonatal sepsis and other neonatal infections	60.3 (13.0 to 158.2)	78.7 (18.6 to 201.0)	--	--	22.4 (4.6 to 55.4)	28.8 (6.6 to 70.5)

Neonatal sepsis and other neonatal infections	Moderate motor impairment with blindness due to neonatal sepsis and other neonatal infections	54.5 (15.3 to 137.7)	73.7 (23.2 to 178.8)	--	--	12.9 (3.4 to 33.6)	17.4 (5.1 to 43.5)
Neonatal sepsis and other neonatal infections	Severe motor plus cognitive impairment with blindness and epilepsy due to neonatal sepsis and other neonatal infections	133.2 (45.4 to 322.0)	173.6 (62.7 to 408.6)	--	--	91.0 (29.3 to 221.7)	118.2 (39.7 to 287.9)
Neonatal sepsis and other neonatal infections	Severe infection due to neonatal sepsis and other neonatal infections	124.3 (67.2 to 215.9)	120.5 (65.3 to 208.9)	--	--	16.2 (7.6 to 30.5)	15.7 (7.4 to 29.4)
Neonatal sepsis and other neonatal infections	Severe motor impairment with blindness and epilepsy due to neonatal sepsis and other neonatal infections	1,522.5 (512.5 to 4,090.1)	2,016.4 (697.6 to 5,313.4)	--	--	855.9 (266.0 to 2,337.9)	1,123.6 (367.2 to 2,989.9)
Neonatal sepsis and other neonatal infections	Moderate motor plus cognitive impairment with blindness due to neonatal sepsis and other neonatal infections	32.0 (6.8 to 84.6)	41.8 (10.2 to 107.0)	--	--	11.3 (2.5 to 30.8)	14.7 (3.5 to 39.9)
Neonatal sepsis and other neonatal infections	Moderate motor plus cognitive impairment with blindness and epilepsy due to neonatal sepsis and other neonatal infections	1,188.0 (410.9 to 3,048.1)	1,549.2 (550.4 to 3,794.3)	--	--	525.8 (166.6 to 1,387.9)	676.1 (219.0 to 1,764.7)
Neonatal sepsis and other neonatal infections	Severe motor impairment due to neonatal sepsis and other neonatal infections	42.1 (14.5 to 100.7)	55.3 (20.9 to 126.3)	--	--	16.9 (5.5 to 42.0)	22.2 (7.9 to 54.2)
Neonatal sepsis and other neonatal infections	Severe motor plus cognitive impairment with epilepsy due to neonatal sepsis and other neonatal infections	12.5 (4.2 to 31.0)	16.3 (5.8 to 40.1)	--	--	7.8 (2.5 to 19.8)	10.1 (3.5 to 25.5)
Neonatal sepsis and other neonatal infections	Moderate motor plus cognitive impairment with epilepsy due to neonatal sepsis and other neonatal infections	340.0 (73.1 to 902.0)	444.1 (106.6 to 1,116.7)	--	--	114.7 (23.4 to 296.7)	146.3 (33.0 to 364.7)
Neonatal sepsis and other neonatal infections	Mild motor impairment due to neonatal sepsis and other neonatal infections	226.8 (106.7 to 428.5)	254.2 (120.8 to 473.2)	--	--	2.3 (0.7 to 5.9)	2.6 (0.8 to 6.5)
Neonatal sepsis and other neonatal infections	Moderate motor impairment with epilepsy due to neonatal sepsis and other neonatal infections	579.0 (163.8 to 1,395.4)	783.7 (242.8 to 1,931.6)	--	--	126.9 (31.7 to 323.6)	165.8 (44.9 to 412.7)
Neonatal sepsis and other neonatal infections	Severe motor plus cognitive impairment with blindness due to neonatal sepsis and other neonatal infections	14.0 (4.6 to 33.4)	18.4 (6.9 to 42.8)	--	--	8.7 (2.9 to 21.0)	11.5 (4.2 to 27.2)
Neonatal sepsis and other neonatal infections	Asymptomatic neonatal sepsis and other neonatal infections	89.4 (47.8 to 157.1)	92.5 (49.3 to 161.4)	--	--	< 0.05 (0.0 to < 0.05)	< 0.05 (0.0 to < 0.05)
Neonatal sepsis and other neonatal infections	Mild motor plus cognitive impairments due to neonatal sepsis and other neonatal infections	203.0 (92.7 to 380.0)	227.2 (103.1 to 416.2)	--	--	6.3 (2.3 to 14.2)	7.1 (2.7 to 15.7)
Neonatal sepsis and other neonatal infections	Moderate motor impairment due to neonatal sepsis and other neonatal infections	102.2 (30.3 to 244.2)	138.4 (44.6 to 332.3)	--	--	6.2 (1.6 to 15.9)	8.4 (2.3 to 21.8)
Neonatal sepsis and other neonatal infections	Severe motor impairment with epilepsy due to neonatal sepsis and other neonatal infections	399.0 (136.1 to 974.8)	520.1 (190.1 to 1,243.2)	--	--	196.6 (61.3 to 503.9)	253.3 (84.1 to 637.5)
Hemolytic disease and other neonatal jaundice	Severe motor plus cognitive impairment with blindness and epilepsy due to hemolytic disease and other neonatal jaundice	15.7 (6.6 to 22.7)	17.8 (6.9 to 26.0)	--	--	10.8 (4.4 to 16.6)	12.2 (4.6 to 19.0)
Hemolytic disease and other neonatal jaundice	Moderate motor impairment with epilepsy due to hemolytic disease and other neonatal jaundice	157.6 (133.4 to 183.9)	187.9 (158.4 to 219.7)	--	--	36.2 (25.7 to 49.2)	41.4 (29.2 to 56.7)
Hemolytic disease and other neonatal jaundice	Severe motor impairment severe due to hemolytic disease and other neonatal jaundice	530.5 (429.7 to 661.7)	673.3 (549.3 to 829.1)	--	--	206.3 (126.8 to 293.6)	260.5 (164.4 to 366.5)
Hemolytic disease and other neonatal jaundice	Moderate motor impairment with blindness and epilepsy due to hemolytic disease and other neonatal jaundice	14.8 (12.4 to 17.4)	17.6 (14.7 to 20.8)	--	--	5.5 (3.8 to 7.4)	6.4 (4.4 to 8.8)
Hemolytic disease and other neonatal jaundice	Moderate motor plus cognitive impairment with blindness due to hemolytic disease and other neonatal jaundice	44.1 (34.7 to 54.0)	53.6 (41.4 to 65.5)	--	--	15.5 (10.2 to 21.8)	18.8 (12.3 to 26.2)
Hemolytic disease and other neonatal jaundice	Severe motor impairment with blindness and epilepsy due to hemolytic disease and other neonatal jaundice	4.9 (4.1 to 5.8)	5.9 (4.9 to 6.9)	--	--	2.9 (2.1 to 3.9)	3.5 (2.5 to 4.5)
Hemolytic disease and other neonatal jaundice	Severe motor impairment with blindness due to hemolytic disease and other neonatal jaundice	6.0 (4.2 to 8.2)	7.6 (5.3 to 10.3)	--	--	3.0 (1.9 to 4.6)	3.9 (2.4 to 5.7)
Hemolytic disease and other neonatal jaundice	Moderate motor plus cognitive impairment with blindness and epilepsy due to hemolytic disease and other neonatal jaundice	23.5 (18.6 to 28.8)	28.6 (22.5 to 35.1)	--	--	11.0 (7.5 to 15.0)	13.2 (8.7 to 18.0)
Hemolytic disease and other neonatal jaundice	Severe motor plus cognitive impairment with epilepsy due to hemolytic disease and other neonatal jaundice	152.3 (65.2 to 214.9)	169.4 (67.0 to 246.7)	--	--	94.8 (37.7 to 144.8)	104.5 (38.6 to 165.4)
Hemolytic disease and other neonatal jaundice	Moderate motor impairment with blindness due to hemolytic disease and other neonatal jaundice	17.9 (12.7 to 24.5)	22.7 (16.0 to 30.9)	--	--	4.2 (2.5 to 6.5)	5.4 (3.3 to 8.1)
Hemolytic disease and other neonatal jaundice	Moderate motor impairment due to hemolytic disease and other neonatal jaundice	408.1 (338.5 to 488.5)	510.2 (423.4 to 618.6)	--	--	24.5 (15.2 to 37.2)	30.6 (19.2 to 46.0)
Hemolytic disease and other neonatal jaundice	Severe motor impairment with epilepsy due to hemolytic disease and other neonatal jaundice	52.6 (44.6 to 61.8)	62.7 (52.8 to 74.9)	--	--	26.7 (18.5 to 36.3)	31.4 (21.5 to 43.3)
Hemolytic disease and other neonatal jaundice	Severe motor plus cognitive impairment with blindness due to hemolytic disease and other neonatal jaundice	29.5 (13.1 to 42.1)	33.4 (13.1 to 48.5)	--	--	18.4 (7.5 to 27.8)	20.8 (8.4 to 32.5)
Hemolytic disease and other neonatal jaundice	Moderate motor plus cognitive impairment with epilepsy due to hemolytic disease and other neonatal jaundice	248.8 (198.6 to 299.4)	302.4 (234.6 to 361.9)	--	--	85.9 (57.5 to 120.5)	101.7 (66.2 to 142.6)
Other neonatal disorders	Other neonatal disorders	--	--	--	--	478.1 (345.4 to 685.2)	511.6 (350.7 to 766.7)
Protein-energy malnutrition	Severe wasting without edema	23,784.7 (22,829.2 to 24,775.3)	25,747.8 (25,228.3 to 26,347.4)	17,719.5 (17,083.9 to 18,392.9)	18,131.9 (17,810.7 to 18,489.2)	2,954.8 (1,911.3 to 4,167.7)	3,199.5 (2,062.9 to 4,480.2)
Protein-energy malnutrition	Moderate wasting without edema	38,871.3 (37,180.2 to 40,599.4)	37,349.0 (36,159.9 to 38,623.0)	27,281.6 (26,142.1 to 28,439.4)	25,434.9 (24,713.7 to 26,216.7)	< 0.05 (0.0 to < 0.05)	< 0.05 (0.0 to < 0.05)
Protein-energy malnutrition	Moderate wasting with edema	2.8 (0.3 to 14.2)	7.7 (0.8 to 25.3)	--	--	0.1 (< 0.05 to 0.7)	0.4 (< 0.05 to 1.3)
Protein-energy malnutrition	Severe wasting with edema	28.5 (17.9 to 45.9)	30.5 (18.1 to 51.4)	22.2 (14.3 to 34.4)	22.9 (13.8 to 37.6)	4.8 (2.5 to 8.2)	5.1 (2.5 to 8.7)
Iodine deficiency	Visible goiter with profound intellectual disability with severe heart failure due to iodine deficiency	< 0.05 (< 0.05 to 0.1)	0.1 (< 0.05 to 0.1)	--	--	< 0.05 (< 0.05 to < 0.05)	< 0.05 (< 0.05 to < 0.05)
Iodine deficiency	Visible goiter with severe intellectual disability due to iodine deficiency	1,204.8 (720.5 to 1,676.6)	1,098.2 (618.2 to 1,566.5)	--	--	366.7 (195.4 to 554.6)	334.2 (170.2 to 520.5)
Iodine deficiency	Visible goiter with profound intellectual disability and mild heart failure due to iodine deficiency	0.1 (0.1 to 0.1)	0.1 (0.1 to 0.1)	--	--	< 0.05 (< 0.05 to < 0.05)	< 0.05 (< 0.05 to 0.1)
Iodine deficiency	Visible goiter without symptoms	117,228.2 (103,899.1 to 131,946.1)	110,421.4 (97,966.5 to 124,262.9)	3,407.4 (3,038.3 to 3,820.6)	2,784.5 (2,477.6 to 3,124.2)	1,251.3 (580.3 to 2,291.8)	1,177.6 (547.1 to 2,163.1)
Iodine deficiency	Visible goiter with profound intellectual disability and moderate heart failure due to iodine deficiency	< 0.05 (< 0.05 to < 0.05)	< 0.05 (< 0.05 to < 0.05)	--	--	< 0.05 (< 0.05 to < 0.05)	< 0.05 (< 0.05 to < 0.05)
Iodine deficiency	Visible goiter with profound intellectual disability due to iodine deficiency	1,361.1 (710.1 to 1,848.5)	1,415.7 (728.7 to 1,916.0)	--	--	455.6 (215.7 to 704.8)	473.8 (221.7 to 733.1)
Iodine deficiency	Visible goiter with symptoms without intellectual disability or heart failure	6,891.5 (5,867.3 to 8,036.6)	6,278.7 (5,319.5 to 7,334.3)	316.4 (281.8 to 354.2)	258.5 (230.4 to 289.6)	1,267.6 (846.9 to 1,793.5)	1,152.4 (766.2 to 1,622.0)
Vitamin A deficiency	Blindness due to vitamin A deficiency	104.6 (62.8 to 164.7)	109.6 (67.4 to 168.8)	--	--	19.5 (9.9 to 34.4)	20.4 (10.7 to 35.7)

Vitamin A deficiency	Moderate vision impairment loss due to vitamin A deficiency	4,177.2<br>(3,103.3 to 5,652.2)	4,616.0<br>(3,435.6 to 6,208.1)	--	--	125.7<br>(68.5 to 211.6)	138.8<br>(77.2 to 230.8)
Vitamin A deficiency	Severe vision impairment loss due to vitamin A deficiency	453.4<br>(298.5 to 630.5)	527.3<br>(341.3 to 734.2)	--	--	80.2<br>(44.9 to 129.0)	93.2<br>(51.4 to 150.2)
Vitamin A deficiency	Asymptomatic vitamin A deficiency	518,107.4<br>(457,821.4 to 601,202.9)	503,548.6<br>(439,987.5 to 597,169.7)	587,473.7<br>(504,061.9 to 696,250.3)	552,179.9<br>(469,061.5 to 671,143.9)	< 0.05<br>(0.0 to < 0.05)	< 0.05<br>(0.0 to < 0.05)
Iron-deficiency anaemia	Severe heart failure due to iron-deficiency anaemia	1.4<br>(1.2 to 1.7)	1.9<br>(1.5 to 2.2)	--	--	0.2<br>(0.2 to 0.4)	0.3<br>(0.2 to 0.5)
Iron-deficiency anaemia	Severe iron-deficiency anaemia	35,441.6<br>(33,057.9 to 38,532.4)	37,673.4<br>(34,717.1 to 41,911.6)	--	--	4,978.0<br>(3,363.0 to 6,836.8)	5,282.4<br>(3,582.0 to 7,206.2)
Iron-deficiency anaemia	Moderate iron-deficiency anaemia	503,993.5<br>(484,869.6 to 524,071.9)	541,805.9<br>(521,064.0 to 565,349.5)	--	--	25,179.9<br>(16,571.5 to 36,565.2)	27,042.4<br>(17,848.8 to 38,997.1)
Iron-deficiency anaemia	Asymptomatic and mild heart failure due to iron-deficiency anaemia	2.4<br>(2.0 to 2.8)	3.2<br>(2.7 to 3.7)	--	--	0.1<br>(0.1 to 0.2)	0.1<br>(0.1 to 0.2)
Iron-deficiency anaemia	Moderate heart failure due to iron-deficiency anaemia	0.5<br>(0.4 to 0.7)	0.7<br>(0.5 to 0.9)	--	--	< 0.05<br>(< 0.05 to 0.1)	< 0.05<br>(< 0.05 to 0.1)
Iron-deficiency anaemia	Mild iron-deficiency anaemia	596,849.1<br>(581,476.3 to 612,526.3)	665,295.7<br>(646,870.7 to 684,901.7)	--	--	2,156.2<br>(761.1 to 4,704.9)	2,402.0<br>(846.8 to 5,243.8)
Other nutritional deficiencies	Other nutritional deficiencies	--	--	--	--	100.2<br>(64.9 to 141.4)	108.6<br>(70.1 to 152.1)
Syphilis	Neurological problems due to adult tertiary syphilis	23.0<br>(15.0 to 34.1)	30.2<br>(19.4 to 45.1)	1.3<br>(0.8 to 2.0)	1.7<br>(1.0 to 2.6)	4.7<br>(2.6 to 7.5)	6.1<br>(3.4 to 9.9)
Syphilis	Mild early syphilis infection	2,149.4<br>(984.0 to 3,935.5)	2,374.8<br>(1,062.3 to 4,365.6)	1,515.2<br>(694.7 to 2,616.9)	1,657.8<br>(758.3 to 2,858.2)	12.1<br>(3.4 to 30.2)	13.4<br>(3.7 to 34.1)
Syphilis	Asymptomatic early syphilis infection	47,578.7<br>(38,528.9 to 58,624.8)	52,572.4<br>(41,128.2 to 66,105.2)	33,899.5<br>(26,461.9 to 43,562.4)	37,115.1<br>(27,812.9 to 48,903.8)	< 0.05<br>(0.0 to < 0.05)	< 0.05<br>(0.0 to < 0.05)
Syphilis	Neurological problems and cardiovascular complications due to adult tertiary syphilis	39.9<br>(27.1 to 55.6)	52.3<br>(35.5 to 73.4)	2.4<br>(1.6 to 3.4)	3.1<br>(2.0 to 4.4)	9.5<br>(5.4 to 14.9)	12.5<br>(7.1 to 19.6)
Syphilis	Asymptomatic adult tertiary syphilis	469.3<br>(411.4 to 537.6)	614.6<br>(531.6 to 711.8)	27.3<br>(23.6 to 31.6)	34.5<br>(29.3 to 40.6)	< 0.05<br>(0.0 to < 0.05)	< 0.05<br>(0.0 to < 0.05)
Syphilis	Severe disfigurement and cardiovascular complications due to adult tertiary syphilis	73.8<br>(56.4 to 94.6)	96.8<br>(73.9 to 124.3)	4.4<br>(3.4 to 5.8)	5.7<br>(4.3 to 7.4)	30.7<br>(19.7 to 44.1)	40.2<br>(25.6 to 58.4)
Syphilis	Severe disfigurement due to adult tertiary syphilis	135.8<br>(112.7 to 162.4)	177.9<br>(147.1 to 214.4)	8.0<br>(6.5 to 9.6)	10.1<br>(8.1 to 12.3)	52.2<br>(34.4 to 72.6)	68.2<br>(44.0 to 93.9)
Syphilis	Cardiovascular complications due to adult tertiary syphilis	69.7<br>(51.7 to 90.3)	91.3<br>(67.7 to 118.0)	4.1<br>(3.1 to 5.3)	5.3<br>(3.9 to 6.8)	3.5<br>(2.0 to 5.3)	4.6<br>(2.6 to 7.1)
Syphilis	Severe disfigurement and neurological problems due to adult tertiary syphilis	46.6<br>(32.8 to 63.4)	61.1<br>(42.9 to 83.5)	2.8<br>(2.0 to 3.8)	3.6<br>(2.5 to 4.9)	23.8<br>(14.7 to 35.2)	31.2<br>(19.1 to 46.4)
Syphilis	Severe disfigurement, neurological problems, and cardiovascular complications due to adult tertiary syphilis	15.9<br>(8.1 to 27.1)	20.8<br>(10.4 to 35.1)	1.0<br>(0.4 to 1.6)	1.2<br>(0.6 to 2.0)	8.7<br>(4.0 to 15.4)	11.4<br>(5.2 to 20.4)
Chlamydial infection	Moderate pelvic inflammatory diseases due to chlamydial infection	744.3<br>(636.1 to 872.3)	816.9<br>(700.6 to 955.1)	11,337.3<br>(9,661.7 to 13,369.2)	12,414.6<br>(10,647.1 to 14,549.0)	80.6<br>(53.4 to 114.9)	88.6<br>(59.4 to 125.2)
Chlamydial infection	Asymptomatic chlamydial infection	52,850.4<br>(41,422.5 to 66,462.0)	55,472.1<br>(43,155.6 to 69,859.2)	40,351.6<br>(30,984.9 to 51,240.7)	41,643.0<br>(31,817.5 to 53,253.1)	< 0.05<br>(0.0 to < 0.05)	< 0.05<br>(0.0 to < 0.05)
Chlamydial infection	Severe pelvic inflammatory diseases due to chlamydial infection	92.4<br>(75.6 to 112.9)	101.4<br>(83.3 to 123.3)	1,399.3<br>(1,140.9 to 1,702.8)	1,532.3<br>(1,249.0 to 1,861.1)	29.5<br>(19.0 to 43.1)	32.4<br>(20.9 to 46.7)
Chlamydial infection	Secondary infertility due to chlamydial infection	8,485.6<br>(7,048.8 to 9,844.8)	9,946.1<br>(8,076.0 to 11,679.3)	--	--	42.6<br>(16.8 to 88.9)	50.0<br>(19.2 to 104.9)
Chlamydial infection	Mild chlamydial infection	21,242.8<br>(16,566.5 to 26,746.1)	22,745.0<br>(17,515.2 to 28,610.2)	16,035.5<br>(12,337.4 to 20,278.4)	16,927.6<br>(13,029.5 to 21,503.3)	120.7<br>(44.9 to 265.9)	129.2<br>(47.7 to 287.1)
Chlamydial infection	Epididymo-orchitis due to chlamydial infection	1,420.4<br>(984.9 to 1,965.2)	1,567.0<br>(1,092.9 to 2,161.4)	1,052.0<br>(739.4 to 1,440.8)	1,149.2<br>(808.4 to 1,572.4)	175.6<br>(104.5 to 280.9)	193.8<br>(114.8 to 312.9)
Chlamydial infection	Primary infertility due to chlamydial infection	2,574.0<br>(2,073.8 to 3,148.1)	2,925.4<br>(2,273.4 to 3,653.0)	--	--	19.0<br>(8.2 to 37.3)	21.6<br>(9.2 to 42.4)
Gonococcal infection	Moderate pelvic inflammatory diseases due to gonococcal infection	253.2<br>(211.3 to 303.0)	275.9<br>(231.2 to 329.2)	3,881.4<br>(3,228.7 to 4,684.2)	4,222.9<br>(3,517.1 to 5,066.0)	27.8<br>(17.9 to 40.0)	30.3<br>(19.9 to 43.4)
Gonococcal infection	Mild gonococcal infection	20,569.0<br>(17,017.1 to 24,997.5)	25,174.9<br>(19,891.9 to 31,718.4)	73,542.2<br>(58,693.3 to 90,189.4)	89,967.1<br>(68,842.7 to 114,298.8)	116.5<br>(45.0 to 247.0)	142.7<br>(55.0 to 309.6)
Gonococcal infection	Secondary infertility due to gonococcal infection	3,186.7<br>(2,692.5 to 3,694.3)	3,697.8<br>(3,046.2 to 4,397.1)	--	--	16.0<br>(6.2 to 32.7)	18.5<br>(7.1 to 38.9)
Gonococcal infection	Primary infertility due to gonococcal infection	906.5<br>(719.7 to 1,124.2)	1,024.6<br>(790.6 to 1,302.1)	--	--	6.7<br>(2.9 to 13.1)	7.6<br>(3.3 to 14.9)
Gonococcal infection	Asymptomatic gonococcal infection	20,097.1<br>(16,591.5 to 24,327.5)	24,147.3<br>(18,879.9 to 30,536.8)	71,544.9<br>(57,832.4 to 87,328.7)	86,005.1<br>(65,475.1 to 109,822.2)	< 0.05<br>(0.0 to < 0.05)	< 0.05<br>(0.0 to < 0.05)
Gonococcal infection	Epididymo-orchitis due to gonococcal infection	2,186.4<br>(1,575.4 to 2,951.8)	2,768.9<br>(1,959.2 to 3,794.9)	7,960.4<br>(5,480.4 to 10,988.5)	10,049.6<br>(6,785.0 to 13,940.1)	266.4<br>(160.6 to 407.2)	337.6<br>(202.7 to 521.9)
Gonococcal infection	Severe pelvic inflammatory diseases due to gonococcal infection	31.4<br>(25.3 to 39.2)	34.2<br>(27.6 to 42.5)	479.0<br>(384.8 to 591.3)	521.2<br>(418.2 to 640.9)	10.2<br>(6.4 to 15.0)	11.1<br>(6.9 to 16.3)
Trichomoniasis	Acute trichomoniasis infection	30,406.7<br>(25,509.3 to 35,419.9)	35,256.9<br>(29,495.7 to 41,083.0)	26,896.0<br>(22,274.5 to 31,834.7)	31,026.7<br>(25,631.0 to 36,816.5)	171.0<br>(65.1 to 362.0)	198.2<br>(75.9 to 420.8)
Trichomoniasis	Asymptomatic trichomoniasis infection	117,997.1<br>(101,525.9 to 135,055.9)	136,522.0<br>(117,931.9 to 156,948.4)	102,501.6<br>(87,738.6 to 118,057.7)	117,881.8<br>(100,416.7 to 135,904.8)	< 0.05<br>(0.0 to < 0.05)	< 0.05<br>(0.0 to < 0.05)
Genital herpes	Symptomatic genital herpes	31,429.6<br>(14,572.9 to 52,277.2)	37,294.5<br>(17,313.1 to 61,984.1)	1,149.5<br>(551.6 to 1,993.0)	1,244.8<br>(594.5 to 2,151.2)	174.6<br>(50.5 to 402.0)	207.0<br>(60.1 to 476.8)
Genital herpes	Asymptomatic genital herpes	692,666.9<br>(605,077.5 to 798,124.2)	821,996.9<br>(716,759.4 to 951,546.1)	25,239.0<br>(21,716.8 to 29,806.9)	27,333.3<br>(23,421.2 to 32,385.2)	< 0.05<br>(0.0 to < 0.05)	< 0.05<br>(0.0 to < 0.05)
Genital herpes	Moderate infection due to initial genital herpes episode	266.9<br>(60.4 to 619.2)	289.1<br>(65.4 to 674.3)	26,388.5<br>(22,822.9 to 30,915.2)	28,578.1<br>(24,602.3 to 33,776.9)	13.3<br>(2.9 to 33.2)	14.4<br>(3.1 to 35.7)
Other sexually transmitted diseases	Other sexually transmitted diseases	--	--	--	--	270.3<br>(170.0 to 419.9)	317.6<br>(200.3 to 497.3)
Other sexually transmitted diseases	Moderate pelvic inflammatory diseases due to other sexually transmitted diseases	2,387.7<br>(2,021.9 to 2,782.8)	2,554.1<br>(2,188.7 to 2,959.3)	36,237.8<br>(30,908.5 to 42,815.2)	38,668.6<br>(33,345.2 to 45,152.0)	258.2<br>(169.8 to 365.0)	276.3<br>(183.7 to 390.0)
Other sexually transmitted diseases	Secondary infertility due to other sexually transmitted diseases	24,427.6<br>(20,377.4 to 28,076.8)	27,150.3<br>(22,268.1 to 31,649.1)	--	--	122.7<br>(47.6 to 256.2)	136.3<br>(52.8 to 284.4)
Other sexually transmitted diseases	Primary infertility due to other sexually transmitted diseases	5,913.3<br>(4,684.9 to 7,344.9)	6,567.2<br>(5,072.8 to 8,306.6)	--	--	43.7<br>(18.5 to 84.5)	48.5<br>(20.6 to 95.4)

Other sexually transmitted diseases	Severe pelvic inflammatory diseases due to other sexually transmitted diseases	296.3 (240.5 to 361.4)	317.0 (260.1 to 386.3)	4,472.6 (3,653.1 to 5,465.5)	4,772.6 (3,915.1 to 5,784.8)	90.1 (58.2 to 128.5)	96.6 (62.4 to 137.5)
Acute hepatitis A	Moderate acute hepatitis A	2,031.7 (1,273.9 to 2,915.4)	1,440.0 (954.3 to 2,029.8)	26,412.1 (16,560.7 to 37,899.8)	18,720.3 (12,406.4 to 26,387.6)	99.2 (51.4 to 159.1)	70.4 (38.2 to 116.9)
Acute hepatitis A	Asymptomatic acute hepatitis A	22,760.8 (3,433.6 to 49,402.1)	10,820.0 (1,783.1 to 23,652.1)	295,890.7 (44,637.3 to 642,227.6)	140,660.6 (23,180.7 to 307,477.7)	< 0.05 (0.0 to < 0.05)	< 0.05 (0.0 to < 0.05)
Acute hepatitis A	Severe acute hepatitis A	14.6 (9.2 to 21.5)	10.4 (6.8 to 14.8)	190.0 (119.8 to 279.5)	134.9 (88.0 to 192.1)	2.0 (1.0 to 3.2)	1.4 (0.7 to 2.3)
Hepatitis B	Chronic hepatitis B	405,838.6 (343,919.9 to 481,407.4)	456,224.0 (385,512.4 to 541,076.2)	12,300.2 (9,806.9 to 15,582.2)	13,150.0 (10,458.7 to 16,557.3)	< 0.05 (0.0 to < 0.05)	< 0.05 (0.0 to < 0.05)
Hepatitis B	Moderate acute hepatitis B	2,075.9 (1,458.8 to 2,789.9)	1,740.8 (1,232.7 to 2,341.2)	17,991.3 (12,643.0 to 24,178.9)	15,086.8 (10,683.0 to 20,290.7)	100.4 (56.9 to 158.2)	83.9 (47.2 to 134.0)
Hepatitis B	Asymptomatic acute hepatitis B	13,583.6 (10,437.4 to 17,405.3)	9,822.7 (7,624.9 to 12,813.2)	117,724.1 (90,457.2 to 150,846.1)	85,130.4 (66,082.5 to 111,047.5)	< 0.05 (0.0 to < 0.05)	< 0.05 (0.0 to < 0.05)
Hepatitis B	Severe acute hepatitis B	769.1 (344.3 to 1,341.1)	647.2 (287.0 to 1,144.7)	6,665.7 (2,983.8 to 11,622.7)	5,609.2 (2,487.7 to 9,920.7)	97.2 (37.4 to 183.4)	81.5 (31.1 to 155.7)
Hepatitis C	Severe acute hepatitis C	4.7 (2.3 to 9.2)	4.9 (2.5 to 9.7)	40.8 (20.3 to 79.8)	42.9 (21.3 to 83.7)	0.6 (0.3 to 1.4)	0.7 (0.3 to 1.5)
Hepatitis C	Asymptomatic acute hepatitis C	328.8 (233.5 to 387.1)	345.7 (244.8 to 407.9)	2,849.7 (2,023.8 to 3,355.3)	2,995.9 (2,121.8 to 3,535.3)	< 0.05 (0.0 to < 0.05)	< 0.05 (0.0 to < 0.05)
Hepatitis C	Moderate acute hepatitis C	110.9 (59.8 to 201.9)	116.6 (61.9 to 212.5)	961.3 (518.1 to 1,749.4)	1,010.8 (536.8 to 1,841.8)	5.6 (2.6 to 10.7)	5.8 (2.7 to 11.4)
Hepatitis C	Chronic hepatitis C	127,058.6 (113,280.5 to 141,321.0)	157,587.7 (140,996.3 to 174,642.5)	2,895.8 (2,585.6 to 3,215.3)	3,044.6 (2,719.2 to 3,384.5)	< 0.05 (0.0 to < 0.05)	< 0.05 (0.0 to < 0.05)
Acute hepatitis E	Asymptomatic acute hepatitis E	1,262.3 (1,129.9 to 1,403.3)	765.2 (685.6 to 853.5)	16,410.5 (14,689.2 to 18,242.7)	9,947.8 (8,913.1 to 11,095.2)	< 0.05 (0.0 to < 0.05)	< 0.05 (0.0 to < 0.05)
Acute hepatitis E	Severe acute hepatitis E	31.4 (25.6 to 37.9)	23.4 (19.5 to 28.3)	408.1 (332.2 to 492.6)	304.2 (254.0 to 368.1)	4.2 (2.6 to 6.2)	3.1 (2.0 to 4.6)
Acute hepatitis E	Moderate acute hepatitis E	910.3 (785.2 to 1,051.7)	677.4 (579.4 to 783.8)	11,833.8 (10,207.5 to 13,671.5)	8,805.8 (7,532.4 to 10,189.5)	44.5 (27.7 to 66.8)	33.1 (20.8 to 50.1)
Other infectious diseases	Moderate anaemia due to other infectious diseases	16,519.0 (15,671.4 to 17,445.2)	18,379.3 (17,359.2 to 19,569.9)	--	--	828.1 (541.9 to 1,210.8)	921.6 (598.8 to 1,334.7)
Other infectious diseases	Severe anaemia due to other infectious diseases	923.0 (820.7 to 1,056.4)	1,109.3 (953.5 to 1,358.8)	--	--	130.9 (85.6 to 180.1)	157.2 (103.1 to 221.3)
Other infectious diseases	Guillain-Barre syndrome due to other infectious diseases	3.9 (2.6 to 5.6)	4.5 (3.0 to 6.6)	8.0 (5.2 to 11.2)	9.3 (6.1 to 13.2)	1.2 (0.7 to 1.8)	1.3 (0.8 to 2.2)
Other infectious diseases	Mild anaemia due to other infectious diseases	23,926.3 (23,248.7 to 24,597.5)	24,932.9 (24,149.3 to 25,753.0)	--	--	86.7 (30.8 to 190.5)	90.2 (32.0 to 197.4)
Other infectious diseases	Other infectious diseases	--	--	--	--	155.0 (94.3 to 237.2)	169.3 (103.1 to 260.3)
Lip and oral cavity cancer	Terminal phase of mouth cancer	11.6 (11.4 to 11.8)	15.1 (14.6 to 15.5)	--	--	6.3 (4.4 to 8.0)	8.1 (5.6 to 10.4)
Lip and oral cavity cancer	Diagnosis and primary therapy phase of mouth cancer	98.4 (97.1 to 99.9)	132.0 (128.2 to 135.6)	287.0 (283.3 to 291.1)	382.0 (370.7 to 392.1)	26.8 (17.9 to 36.7)	35.8 (24.3 to 49.1)
Lip and oral cavity cancer	Metastatic phase of mouth cancer	85.1 (83.7 to 86.3)	110.7 (107.2 to 113.9)	--	--	36.0 (24.5 to 47.2)	46.8 (31.7 to 61.6)
Lip and oral cavity cancer	Controlled phase of mouth cancer	833.8 (821.8 to 847.1)	1,129.0 (1,095.8 to 1,160.7)	--	--	38.0 (24.7 to 55.5)	51.4 (33.4 to 73.8)
Nasopharynx cancer	Controlled phase of nasopharynx cancer	240.9 (232.4 to 249.4)	261.1 (246.7 to 277.3)	--	--	11.5 (7.3 to 16.8)	12.4 (7.9 to 18.4)
Nasopharynx cancer	Terminal phase of nasopharynx cancer	3.7 (3.6 to 3.8)	4.0 (3.8 to 4.2)	--	--	2.0 (1.4 to 2.5)	2.2 (1.5 to 2.8)
Nasopharynx cancer	Diagnosis and primary therapy phase of nasopharynx cancer	28.0 (27.0 to 28.9)	30.3 (28.7 to 32.1)	88.2 (85.3 to 91.0)	95.6 (90.8 to 101.1)	8.0 (5.3 to 11.1)	8.7 (5.8 to 12.2)
Nasopharynx cancer	Metastatic phase of nasopharynx cancer	33.8 (32.8 to 34.8)	36.6 (34.9 to 38.6)	--	--	15.1 (10.3 to 20.2)	16.4 (11.0 to 22.1)
Other pharynx cancer	Terminal phase of other pharynx cancer	6.7 (6.5 to 6.9)	8.8 (8.2 to 9.2)	--	--	3.6 (2.5 to 4.6)	4.8 (3.3 to 6.2)
Other pharynx cancer	Diagnosis and primary therapy phase of other pharynx cancer	32.6 (32.0 to 33.3)	46.2 (43.6 to 47.7)	124.2 (121.7 to 127.0)	169.6 (159.4 to 175.5)	9.2 (6.2 to 12.8)	12.9 (8.7 to 18.0)
Other pharynx cancer	Controlled phase of other pharynx cancer	255.7 (250.8 to 261.4)	369.5 (349.2 to 381.4)	--	--	11.8 (7.6 to 16.9)	17.0 (10.9 to 25.0)
Other pharynx cancer	Metastatic phase of other pharynx cancer	40.9 (39.9 to 41.9)	54.0 (50.4 to 56.1)	--	--	17.7 (11.9 to 23.6)	23.2 (15.8 to 30.8)
Oesophageal cancer	Terminal phase of oesophageal cancer	31.6 (31.2 to 32.0)	33.4 (32.7 to 34.4)	--	--	16.3 (11.4 to 20.8)	17.5 (12.1 to 22.2)
Oesophageal cancer	Diagnosis and primary therapy phase of oesophageal cancer	53.9 (52.7 to 55.0)	61.1 (59.2 to 63.5)	413.1 (407.5 to 418.8)	442.6 (433.3 to 456.2)	14.7 (10.0 to 20.3)	16.7 (11.4 to 23.0)
Oesophageal cancer	Controlled phase of oesophageal cancer	259.4 (252.1 to 267.4)	307.9 (295.9 to 321.7)	--	--	11.9 (7.7 to 17.3)	14.1 (9.1 to 20.6)
Oesophageal cancer	Metastatic phase of oesophageal cancer	145.3 (143.3 to 147.2)	153.7 (150.6 to 158.4)	--	--	59.3 (41.0 to 77.6)	63.2 (43.4 to 83.5)
Stomach cancer	Metastatic phase of stomach cancer	266.0 (263.3 to 268.8)	297.3 (291.8 to 302.8)	--	--	106.9 (74.0 to 139.5)	119.6 (82.2 to 156.4)
Stomach cancer	Terminal phase of stomach cancer	68.6 (67.9 to 69.3)	76.6 (75.2 to 78.0)	--	--	34.7 (24.5 to 43.6)	38.9 (27.1 to 49.5)
Stomach cancer	Controlled phase of stomach cancer	1,194.2 (1,177.8 to 1,211.9)	1,549.1 (1,508.9 to 1,592.1)	--	--	54.2 (35.4 to 78.6)	70.1 (45.8 to 101.2)
Stomach cancer	Diagnosis and primary therapy phase of stomach cancer	226.3 (223.5 to 229.1)	275.7 (269.1 to 282.4)	1,002.5 (991.9 to 1,013.4)	1,156.6 (1,134.4 to 1,179.8)	58.9 (40.6 to 79.7)	71.6 (48.7 to 97.6)
Colon and rectum cancer	Metastatic phase of colon and rectum cancers	367.4 (363.1 to 372.0)	471.7 (455.7 to 491.7)	--	--	144.8 (99.7 to 187.1)	185.5 (128.1 to 243.2)
Colon and rectum cancer	Stoma due to colon and rectum cancer	268.0 (258.6 to 277.5)	341.5 (329.0 to 354.2)	57.8 (55.9 to 59.7)	73.2 (70.6 to 75.8)	33.8 (23.7 to 45.6)	43.0 (29.8 to 58.0)
Colon and rectum cancer	Controlled phase of colon and rectum cancers	3,574.8 (3,526.9 to 3,623.1)	5,006.4 (4,828.6 to 5,272.6)	--	--	161.2 (104.3 to 230.9)	225.8 (148.1 to 325.1)

Colon and rectum cancer	Diagnosis and primary therapy phase of colon and rectum cancers	319.3 (315.6 to 323.1)	438.3 (423.7 to 459.4)	1,224.5 (1,210.1 to 1,239.5)	1,642.3 (1,584.7 to 1,721.6)	81.7 (56.3 to 110.5)	111.9 (76.5 to 152.1)
Colon and rectum cancer	Terminal phase of colon and rectum cancers	51.0 (50.4 to 51.6)	64.8 (62.6 to 67.6)	--	--	25.9 (18.2 to 32.8)	33.0 (23.2 to 42.2)
Liver cancer due to hepatitis B	Metastatic phase of liver cancer due to hepatitis B	62.5 (55.5 to 69.3)	80.9 (70.5 to 90.4)	--	--	26.9 (17.9 to 35.9)	34.6 (22.4 to 46.9)
Liver cancer due to hepatitis B	Terminal phase of liver cancer due to hepatitis B	24.9 (22.1 to 27.6)	32.2 (28.1 to 36.0)	--	--	13.4 (8.9 to 17.5)	17.2 (11.4 to 22.7)
Liver cancer due to hepatitis B	Controlled phase of liver cancer due to hepatitis B	189.9 (168.0 to 211.7)	379.8 (326.9 to 429.6)	--	--	8.9 (5.6 to 13.0)	17.7 (11.1 to 26.0)
Liver cancer due to hepatitis B	Diagnosis and primary therapy phase of liver cancer due to hepatitis B	74.9 (66.5 to 82.7)	105.9 (91.8 to 118.6)	323.3 (287.1 to 357.7)	436.5 (380.1 to 488.5)	20.5 (13.5 to 28.3)	28.7 (18.8 to 39.8)
Liver cancer due to hepatitis C	Metastatic phase of liver cancer due to hepatitis C	26.1 (23.6 to 28.6)	34.7 (31.3 to 38.2)	--	--	11.3 (7.7 to 15.3)	15.1 (10.3 to 20.5)
Liver cancer due to hepatitis C	Terminal phase of liver cancer due to hepatitis C	10.4 (9.4 to 11.4)	13.8 (12.5 to 15.2)	--	--	5.6 (3.9 to 7.3)	7.4 (5.1 to 9.7)
Liver cancer due to hepatitis C	Diagnosis and primary therapy phase of liver cancer due to hepatitis C	32.2 (29.2 to 35.3)	45.7 (41.2 to 50.2)	137.1 (124.3 to 150.1)	188.7 (170.2 to 207.4)	8.9 (5.9 to 12.4)	12.5 (8.4 to 17.7)
Liver cancer due to hepatitis C	Controlled phase of liver cancer due to hepatitis C	93.9 (85.8 to 102.2)	167.0 (150.0 to 182.9)	--	--	4.3 (2.8 to 6.4)	7.7 (4.9 to 11.2)
Liver cancer due to alcohol use	Metastatic phase of liver cancer due to alcohol use	19.8 (16.6 to 23.3)	27.8 (23.4 to 32.3)	--	--	8.9 (5.9 to 12.2)	12.4 (8.3 to 17.0)
Liver cancer due to alcohol use	Controlled phase of liver cancer due to alcohol use	58.1 (48.8 to 67.9)	112.3 (94.2 to 131.1)	--	--	2.7 (1.7 to 4.1)	5.2 (3.3 to 7.8)
Liver cancer due to alcohol use	Diagnosis and primary therapy phase of liver cancer due to alcohol use	23.6 (19.8 to 27.7)	35.1 (29.6 to 40.7)	102.3 (85.8 to 120.2)	147.7 (124.6 to 171.3)	6.7 (4.3 to 9.5)	9.9 (6.6 to 14.3)
Liver cancer due to alcohol use	Terminal phase of liver cancer due to alcohol use	7.9 (6.6 to 9.3)	11.1 (9.3 to 12.9)	--	--	4.3 (2.9 to 5.6)	6.0 (4.1 to 7.9)
Liver cancer due to other causes	Diagnosis and primary therapy phase of liver cancer due to other causes	39.5 (35.4 to 44.3)	57.1 (51.1 to 64.3)	169.7 (152.3 to 190.4)	234.8 (210.3 to 264.1)	11.0 (7.3 to 15.2)	15.7 (10.4 to 21.5)
Liver cancer due to other causes	Terminal phase of liver cancer due to other causes	13.0 (11.7 to 14.6)	17.3 (15.5 to 19.4)	--	--	7.0 (4.8 to 9.2)	9.3 (6.3 to 12.2)
Liver cancer due to other causes	Metastatic phase of liver cancer due to other causes	32.7 (29.3 to 36.6)	43.4 (38.8 to 48.8)	--	--	14.3 (9.6 to 19.2)	18.9 (12.5 to 25.8)
Liver cancer due to other causes	Controlled phase of liver cancer due to other causes	104.8 (93.1 to 118.0)	208.4 (182.8 to 239.4)	--	--	4.9 (3.1 to 7.3)	9.7 (6.1 to 14.4)
Gallbladder and biliary tract cancer	Controlled phase of gallbladder and biliary tract cancer	57.7 (51.7 to 60.6)	74.6 (65.4 to 80.8)	--	--	2.7 (1.7 to 4.0)	3.5 (2.2 to 5.1)
Gallbladder and biliary tract cancer	Metastatic phase of gallbladder and biliary tract cancer	42.2 (39.1 to 44.2)	50.7 (46.5 to 53.3)	--	--	18.2 (12.5 to 24.0)	21.9 (14.8 to 29.5)
Gallbladder and biliary tract cancer	Terminal phase of gallbladder and biliary tract cancer	12.1 (11.3 to 12.7)	14.6 (13.4 to 15.4)	--	--	6.5 (4.5 to 8.3)	7.8 (5.4 to 10.1)
Gallbladder and biliary tract cancer	Diagnosis and primary therapy phase of gallbladder and biliary tract cancer	23.9 (22.2 to 24.9)	29.2 (26.6 to 30.7)	152.2 (141.5 to 159.5)	183.7 (168.5 to 193.2)	6.7 (4.5 to 9.2)	8.2 (5.4 to 11.4)
Pancreatic cancer	Metastatic phase of pancreatic cancer	63.8 (63.4 to 64.3)	85.0 (82.6 to 86.6)	--	--	27.0 (18.5 to 35.4)	35.7 (24.6 to 47.0)
Pancreatic cancer	Terminal phase of pancreatic cancer	25.1 (24.9 to 25.3)	33.5 (32.5 to 34.1)	--	--	13.4 (9.3 to 17.0)	17.7 (12.3 to 22.6)
Pancreatic cancer	Controlled phase of pancreatic cancer	102.9 (97.9 to 107.7)	150.5 (140.5 to 159.2)	--	--	4.7 (3.0 to 6.9)	6.9 (4.5 to 10.0)
Pancreatic cancer	Diagnosis and primary therapy phase of pancreatic cancer	70.3 (69.7 to 70.9)	94.6 (91.8 to 96.5)	312.5 (310.2 to 314.9)	417.7 (405.7 to 425.4)	18.9 (12.9 to 25.9)	25.4 (17.1 to 34.5)
Larynx cancer	Diagnosis and primary therapy phase of larynx cancer	50.6 (49.9 to 51.4)	62.8 (61.7 to 64.3)	138.5 (136.3 to 141.0)	168.4 (165.2 to 172.9)	13.9 (9.3 to 19.1)	17.4 (11.7 to 24.1)
Larynx cancer	Controlled phase of larynx cancer	325.6 (319.5 to 332.2)	412.5 (403.0 to 425.7)	--	--	15.0 (9.7 to 21.6)	18.9 (12.3 to 27.3)
Larynx cancer	Terminal phase of larynx cancer	7.1 (7.0 to 7.2)	8.3 (8.2 to 8.5)	--	--	3.8 (2.7 to 4.9)	4.5 (3.1 to 5.8)
Larynx cancer	Laryngectomy due to larynx cancer	75.2 (72.8 to 77.6)	94.9 (91.8 to 97.9)	14.9 (14.4 to 15.3)	18.6 (18.0 to 19.2)	7.0 (4.5 to 10.1)	8.8 (5.7 to 12.8)
Larynx cancer	Metastatic phase of larynx cancer	50.4 (49.6 to 51.2)	59.2 (58.2 to 60.6)	--	--	21.5 (14.8 to 28.5)	25.6 (17.7 to 33.8)
Tracheal, bronchus, and lung cancer	Controlled phase of lung, bronchus, and trachea cancer	1,222.0 (1,194.3 to 1,252.0)	1,790.1 (1,728.4 to 1,850.7)	--	--	55.2 (36.1 to 79.4)	80.7 (53.2 to 116.6)
Tracheal, bronchus, and lung cancer	Metastatic phase of lung, bronchus, and trachea cancer	529.0 (523.2 to 534.5)	666.9 (650.9 to 682.0)	--	--	208.4 (145.1 to 269.8)	261.9 (180.5 to 339.5)
Tracheal, bronchus, and lung cancer	Terminal phase of lung, bronchus, and trachea cancer	117.3 (116.0 to 118.5)	147.9 (144.3 to 151.2)	--	--	57.1 (40.3 to 72.0)	71.8 (50.7 to 90.3)
Tracheal, bronchus, and lung cancer	Diagnosis and primary therapy phase of lung, bronchus, and trachea cancer	168.3 (165.5 to 170.9)	231.1 (224.2 to 237.8)	1,563.3 (1,545.6 to 1,579.3)	2,007.8 (1,958.4 to 2,055.3)	43.6 (29.7 to 58.9)	59.7 (41.3 to 80.4)
Malignant skin melanoma	Diagnosis and primary therapy phase of malignant skin melanoma	45.5 (39.9 to 50.9)	63.4 (54.5 to 70.7)	203.0 (178.7 to 226.9)	281.5 (243.1 to 314.2)	12.6 (8.3 to 17.5)	17.3 (11.6 to 24.3)
Malignant skin melanoma	Terminal phase of malignant skin melanoma	3.5 (3.2 to 3.9)	4.7 (4.1 to 5.1)	--	--	1.9 (1.3 to 2.5)	2.5 (1.7 to 3.2)
Malignant skin melanoma	Controlled phase of malignant skin melanoma	893.1 (782.7 to 999.7)	1,249.3 (1,070.5 to 1,397.5)	--	--	41.0 (25.9 to 59.4)	57.2 (35.9 to 83.9)
Malignant skin melanoma	Metastatic phase of malignant skin melanoma	22.4 (20.2 to 24.7)	30.1 (26.4 to 32.9)	--	--	9.7 (6.5 to 13.1)	12.9 (8.6 to 17.2)
Non-melanoma skin cancer (squamous-cell carcinoma)	Mild disfigurement due to squamous cell carcinoma	529.5 (327.6 to 788.0)	551.5 (332.7 to 789.7)	485.7 (304.2 to 745.4)	508.4 (307.7 to 752.4)	5.4 (2.3 to 11.2)	5.6 (2.4 to 11.7)
Non-melanoma skin cancer (squamous-cell carcinoma)	Moderate disfigurement due to squamous cell carcinoma	98.9 (56.2 to 158.7)	103.0 (58.2 to 165.6)	90.8 (51.4 to 145.2)	95.0 (53.8 to 149.3)	6.0 (3.1 to 10.4)	6.3 (3.1 to 11.1)
Non-melanoma skin cancer (squamous-cell carcinoma)	Severe disfigurement due to squamous cell carcinoma	33.7 (16.3 to 62.6)	35.1 (16.7 to 64.1)	30.3 (14.5 to 53.7)	31.7 (15.3 to 55.1)	16.9 (7.9 to 31.4)	17.9 (8.1 to 33.5)
Non-melanoma skin cancer (basal-cell carcinoma)	Disfigurement due to basal cell carcinoma	82.9 (54.0 to 129.0)	102.6 (65.0 to 166.8)	747.2 (500.1 to 1,055.7)	886.1 (574.1 to 1,261.5)	0.9 (0.4 to 1.8)	1.1 (0.4 to 2.2)

Breast cancer	Terminal phase of breast cancer	26.0 (25.0 to 27.7)	31.5 (30.1 to 33.9)	--	--	13.7 (9.5 to 17.5)	16.6 (11.4 to 21.3)
Breast cancer	Metastatic phase of breast cancer	364.0 (350.9 to 386.8)	442.8 (423.7 to 474.5)	--	--	144.3 (100.5 to 187.7)	175.3 (119.3 to 228.1)
Breast cancer	Mastectomy due to breast cancer	3,093.2 (3,023.2 to 3,163.1)	3,780.2 (3,683.9 to 3,867.3)	529.4 (517.9 to 541.3)	642.3 (627.0 to 658.8)	235.0 (150.8 to 341.7)	286.9 (184.5 to 416.5)
Breast cancer	Controlled phase of breast cancer	2,469.5 (2,276.3 to 2,624.2)	3,518.8 (3,179.2 to 3,941.3)	--	--	113.9 (72.2 to 165.1)	162.1 (104.1 to 237.8)
Breast cancer	Diagnosis and primary therapy phase of breast cancer	289.9 (280.8 to 296.6)	377.8 (361.1 to 400.2)	789.9 (752.4 to 827.8)	1,059.9 (979.5 to 1,163.2)	75.3 (51.8 to 102.0)	98.0 (66.9 to 134.2)
Cervical cancer	Metastatic phase of cervical cancer	123.8 (103.8 to 132.1)	130.7 (107.9 to 138.7)	--	--	52.7 (35.7 to 69.7)	56.3 (37.4 to 74.6)
Cervical cancer	Diagnosis and primary therapy phase of cervical cancer	148.6 (122.2 to 156.2)	163.3 (130.7 to 175.0)	469.4 (388.9 to 492.1)	511.2 (414.0 to 542.3)	40.4 (26.8 to 56.4)	44.7 (29.4 to 62.7)
Cervical cancer	Terminal phase of cervical cancer	17.2 (14.4 to 18.4)	18.1 (15.0 to 19.2)	--	--	9.3 (6.4 to 11.9)	9.8 (6.7 to 12.6)
Cervical cancer	Controlled phase of cervical cancer	1,464.3 (1,192.8 to 1,543.3)	1,626.7 (1,289.4 to 1,751.9)	--	--	67.6 (42.1 to 99.2)	75.2 (46.2 to 110.2)
Uterine cancer	Controlled phase of uterine cancer	1,222.9 (1,194.3 to 1,250.6)	1,739.1 (1,670.4 to 1,843.6)	--	--	55.8 (36.4 to 81.8)	79.3 (51.3 to 116.0)
Uterine cancer	Metastatic phase of uterine cancer	49.6 (48.3 to 50.7)	66.1 (63.7 to 69.6)	--	--	20.9 (14.1 to 27.4)	27.6 (18.7 to 36.7)
Uterine cancer	Diagnosis and primary therapy phase of uterine cancer	103.5 (101.0 to 105.8)	146.1 (140.4 to 154.8)	297.3 (290.5 to 304.0)	417.4 (400.8 to 441.7)	27.4 (18.7 to 37.3)	38.6 (26.3 to 53.7)
Uterine cancer	Terminal phase of uterine cancer	5.6 (5.4 to 5.7)	7.4 (7.1 to 7.8)	--	--	3.0 (2.1 to 3.8)	4.0 (2.8 to 5.2)
Ovarian cancer	Diagnosis and primary therapy phase of ovarian cancer	31.1 (30.1 to 31.6)	39.0 (36.8 to 40.2)	204.3 (199.8 to 208.8)	253.5 (242.4 to 260.2)	8.9 (6.0 to 12.2)	11.1 (7.4 to 15.5)
Ovarian cancer	Controlled phase of ovarian cancer	443.4 (427.0 to 450.7)	558.2 (524.8 to 575.9)	--	--	20.3 (13.2 to 29.3)	25.5 (16.5 to 37.3)
Ovarian cancer	Terminal phase of ovarian cancer	10.4 (10.2 to 10.7)	12.8 (12.3 to 13.1)	--	--	5.6 (3.9 to 7.2)	6.9 (4.8 to 8.9)
Ovarian cancer	Metastatic phase of ovarian cancer	142.2 (139.3 to 145.8)	175.6 (168.5 to 180.0)	--	--	58.8 (40.9 to 77.1)	72.6 (49.5 to 95.4)
Prostate cancer	Metastatic phase of prostate cancer	360.2 (319.2 to 387.2)	471.9 (407.8 to 521.5)	--	--	136.4 (94.0 to 177.5)	178.5 (122.0 to 235.2)
Prostate cancer	Incontinence due to prostate cancer	195.7 (111.0 to 286.5)	255.7 (145.0 to 374.7)	39.8 (23.7 to 60.9)	51.8 (31.0 to 79.4)	31.3 (16.1 to 50.8)	40.8 (21.3 to 65.5)
Prostate cancer	Impotence due to prostate cancer	591.3 (495.5 to 688.8)	772.7 (646.4 to 903.0)	123.7 (103.6 to 143.3)	161.1 (134.7 to 187.4)	35.0 (21.8 to 51.9)	45.7 (28.5 to 68.2)
Prostate cancer	Terminal phase of prostate cancer	19.1 (16.8 to 20.6)	24.8 (21.3 to 27.1)	--	--	9.3 (6.4 to 11.8)	12.1 (8.2 to 15.5)
Prostate cancer	Diagnosis and primary therapy phase of prostate cancer	230.7 (212.3 to 260.3)	335.3 (302.3 to 396.5)	861.3 (794.4 to 962.0)	1,222.8 (1,092.5 to 1,412.2)	57.7 (38.1 to 78.1)	83.9 (55.9 to 115.4)
Prostate cancer	Controlled phase of prostate cancer	2,537.6 (2,251.6 to 2,971.6)	3,836.7 (3,346.0 to 4,749.5)	--	--	113.8 (71.2 to 169.4)	172.1 (107.4 to 259.2)
Testicular cancer	Diagnosis and primary therapy phase of testicular cancer	14.4 (14.0 to 14.7)	19.0 (18.3 to 19.9)	51.2 (50.1 to 52.4)	66.8 (64.5 to 69.7)	4.1 (2.8 to 5.7)	5.5 (3.6 to 7.6)
Testicular cancer	Metastatic phase of testicular cancer	5.8 (5.6 to 5.9)	6.7 (6.5 to 6.9)	--	--	2.6 (1.8 to 3.5)	3.0 (2.0 to 4.0)
Testicular cancer	Controlled phase of testicular cancer	236.4 (230.9 to 242.0)	313.6 (302.3 to 327.7)	--	--	11.3 (7.3 to 16.5)	15.0 (9.6 to 22.2)
Testicular cancer	Terminal phase of testicular cancer	0.5 (0.5 to 0.5)	0.6 (0.6 to 0.6)	--	--	0.3 (0.2 to 0.3)	0.3 (0.2 to 0.4)
Kidney cancer	Controlled phase of kidney cancer	856.5 (820.6 to 874.0)	1,104.5 (1,046.3 to 1,140.6)	--	--	39.3 (25.4 to 56.9)	50.6 (32.7 to 73.0)
Kidney cancer	Metastatic phase of kidney cancer	48.1 (47.1 to 49.3)	60.4 (59.1 to 61.9)	--	--	20.8 (14.0 to 27.6)	26.1 (17.7 to 34.4)
Kidney cancer	Diagnosis and primary therapy phase of kidney cancer	87.7 (84.5 to 89.4)	112.7 (107.4 to 116.1)	268.0 (263.9 to 271.7)	342.1 (330.8 to 349.9)	23.8 (16.0 to 32.3)	30.5 (20.4 to 41.9)
Kidney cancer	Terminal phase of kidney cancer	9.3 (9.2 to 9.6)	11.7 (11.5 to 12.0)	--	--	5.0 (3.5 to 6.4)	6.3 (4.4 to 8.1)
Bladder cancer	Metastatic phase of bladder cancer	59.1 (58.3 to 60.0)	74.0 (72.1 to 75.7)	--	--	24.3 (16.7 to 31.9)	30.4 (20.9 to 40.1)
Bladder cancer	Terminal phase of bladder cancer	10.9 (10.8 to 11.1)	13.6 (13.3 to 14.0)	--	--	5.8 (4.0 to 7.4)	7.2 (5.0 to 9.1)
Bladder cancer	Controlled phase of bladder cancer	1,014.7 (1,000.4 to 1,028.6)	1,367.8 (1,327.1 to 1,408.7)	--	--	45.6 (29.7 to 65.4)	61.5 (40.4 to 88.2)
Bladder cancer	Diagnosis and primary therapy phase of bladder cancer	115.0 (113.6 to 116.3)	152.9 (149.0 to 156.7)	307.6 (303.5 to 311.6)	403.4 (392.5 to 414.1)	29.9 (20.5 to 40.6)	39.6 (26.8 to 53.7)
Bladder cancer	Urinary incontinence due to bladder cancer	122.6 (118.6 to 126.6)	159.1 (153.9 to 164.4)	26.3 (25.5 to 27.1)	34.0 (32.9 to 35.1)	20.3 (14.2 to 27.2)	26.3 (18.3 to 35.5)
Brain and nervous system cancer	Controlled phase of brain and nervous system cancers	404.3 (356.5 to 420.3)	584.8 (515.0 to 613.7)	--	--	19.1 (11.9 to 28.1)	27.5 (17.3 to 40.4)
Brain and nervous system cancer	Diagnosis and primary therapy phase of brain and nervous system cancers	49.5 (44.0 to 51.4)	70.7 (62.7 to 74.1)	240.7 (218.3 to 250.1)	329.7 (298.9 to 348.8)	14.2 (9.4 to 20.0)	20.3 (13.6 to 28.4)
Brain and nervous system cancer	Metastatic phase of brain and nervous system cancers	79.5 (72.7 to 83.7)	106.6 (97.3 to 113.4)	--	--	35.7 (23.7 to 48.0)	47.8 (32.1 to 64.0)
Brain and nervous system cancer	Terminal phase of brain and nervous system cancers	14.3 (13.1 to 15.1)	19.1 (17.5 to 20.3)	--	--	7.7 (5.4 to 10.0)	10.3 (7.1 to 13.3)
Thyroid cancer	Diagnosis and primary therapy phase of thyroid cancer	37.3 (36.5 to 38.0)	56.3 (54.2 to 59.9)	159.2 (155.8 to 162.1)	238.2 (229.3 to 252.9)	10.7 (7.1 to 15.0)	16.2 (10.8 to 22.6)
Thyroid cancer	Metastatic phase of thyroid cancer	15.4 (15.0 to 15.7)	20.2 (19.6 to 21.1)	--	--	6.9 (4.7 to 9.1)	9.0 (6.1 to 12.1)
Thyroid cancer	Terminal phase of thyroid cancer	1.3 (1.3 to 1.3)	1.7 (1.6 to 1.7)	--	--	0.7 (0.5 to 0.9)	0.9 (0.6 to 1.2)

Thyroid cancer	Controlled phase of thyroid cancer	762.7<br>(746.2 to 776.4)	1,156.2<br>(1,111.8 to 1,230.0)	--	--	35.4<br>(23.0 to 51.3)	53.6<br>(34.6 to 78.1)
Mesothelioma	Terminal phase of mesothelioma	2.1<br>(2.0 to 2.2)	2.6<br>(2.4 to 2.7)	--	--	1.1<br>(0.8 to 1.4)	1.4<br>(1.0 to 1.8)
Mesothelioma	Controlled phase of mesothelioma	22.6<br>(21.7 to 23.8)	29.4<br>(27.5 to 30.9)	--	--	1.1<br>(0.7 to 1.6)	1.4<br>(0.9 to 2.1)
Mesothelioma	Diagnosis and primary therapy phase of mesothelioma	3.9<br>(3.7 to 4.1)	5.0<br>(4.7 to 5.3)	27.2<br>(26.1 to 28.6)	34.8<br>(32.5 to 36.5)	1.1<br>(0.7 to 1.6)	1.4<br>(1.0 to 2.0)
Mesothelioma	Metastatic phase of mesothelioma	12.4<br>(11.9 to 13.0)	15.8<br>(14.7 to 16.6)	--	--	5.5<br>(3.7 to 7.3)	7.0<br>(4.7 to 9.3)
Hodgkin lymphoma	Metastatic phase of Hodgkin disease	24.8<br>(22.1 to 28.6)	25.4<br>(22.7 to 29.3)	--	--	11.2<br>(7.5 to 15.3)	11.5<br>(7.7 to 15.5)
Hodgkin lymphoma	Controlled phase of Hodgkin disease	210.2<br>(190.9 to 228.7)	247.8<br>(225.0 to 273.0)	--	--	10.1<br>(6.2 to 14.6)	11.9<br>(7.5 to 17.4)
Hodgkin lymphoma	Diagnosis and primary therapy phase of Hodgkin disease	13.8<br>(12.5 to 15.0)	16.1<br>(14.6 to 17.8)	66.5<br>(59.6 to 73.8)	73.3<br>(66.0 to 82.3)	4.0<br>(2.6 to 5.6)	4.6<br>(3.1 to 6.5)
Hodgkin lymphoma	Terminal phase of Hodgkin disease	2.2<br>(2.0 to 2.6)	2.2<br>(2.0 to 2.6)	--	--	1.2<br>(0.8 to 1.6)	1.2<br>(0.8 to 1.6)
Non-Hodgkin's lymphoma	Metastatic phase of non-Hodgkin lymphoma	75.2<br>(71.7 to 78.6)	102.6<br>(95.1 to 106.6)	--	--	32.4<br>(22.2 to 42.9)	44.0<br>(29.8 to 58.6)
Non-Hodgkin's lymphoma	Controlled phase of non-Hodgkin lymphoma	951.1<br>(895.6 to 994.8)	1,447.4<br>(1,339.0 to 1,515.7)	--	--	43.6<br>(28.1 to 63.9)	66.1<br>(41.8 to 95.8)
Non-Hodgkin's lymphoma	Diagnosis and primary therapy phase of non-Hodgkin lymphoma	68.0<br>(64.2 to 71.0)	102.3<br>(94.7 to 107.0)	319.1<br>(304.4 to 333.8)	461.2<br>(427.6 to 482.0)	18.5<br>(12.6 to 25.4)	27.7<br>(18.8 to 38.2)
Non-Hodgkin's lymphoma	Terminal phase of non-Hodgkin lymphoma	12.6<br>(12.1 to 13.2)	17.1<br>(15.9 to 17.7)	--	--	6.8<br>(4.7 to 8.8)	9.2<br>(6.4 to 11.8)
Multiple myeloma	Terminal phase of multiple myeloma	6.1<br>(5.5 to 6.9)	8.1<br>(7.3 to 9.2)	--	--	3.3<br>(2.3 to 4.3)	4.4<br>(3.0 to 5.7)
Multiple myeloma	Diagnosis and primary therapy phase of multiple myeloma	20.0<br>(16.7 to 21.6)	30.4<br>(25.7 to 33.8)	100.3<br>(88.2 to 111.4)	138.5<br>(121.0 to 155.5)	5.6<br>(3.6 to 7.8)	8.4<br>(5.5 to 11.8)
Multiple myeloma	Controlled phase of multiple myeloma	134.9<br>(112.7 to 144.9)	205.7<br>(173.7 to 228.6)	--	--	6.2<br>(3.9 to 9.0)	9.4<br>(5.9 to 13.8)
Multiple myeloma	Metastatic phase of multiple myeloma	80.8<br>(70.4 to 89.4)	113.8<br>(98.9 to 128.2)	--	--	33.7<br>(22.8 to 44.4)	47.4<br>(31.9 to 62.8)
Acute lymphoid leukaemia	Controlled phase of acute lymphoid leukaemia	162.7<br>(153.7 to 188.4)	215.4<br>(188.5 to 229.6)	--	--	7.9<br>(5.0 to 11.8)	10.5<br>(6.6 to 15.6)
Acute lymphoid leukaemia	Diagnosis and primary therapy phase of acute lymphoid leukaemia	44.6<br>(42.3 to 51.2)	58.8<br>(51.7 to 62.5)	58.1<br>(55.3 to 65.9)	76.0<br>(66.5 to 80.3)	12.8<br>(8.6 to 18.2)	16.9<br>(11.2 to 23.7)
Acute lymphoid leukaemia	Terminal phase of acute lymphoid leukaemia	2.0<br>(1.8 to 2.2)	2.6<br>(2.2 to 2.7)	--	--	1.1<br>(0.7 to 1.4)	1.4<br>(0.9 to 1.8)
Acute lymphoid leukaemia	Metastatic phase of acute lymphoid leukaemia	11.9<br>(11.0 to 13.2)	15.2<br>(13.3 to 16.0)	--	--	5.4<br>(3.6 to 7.2)	6.9<br>(4.6 to 9.3)
Chronic lymphoid leukaemia	Controlled phase of chronic lymphoid leukaemia	251.0<br>(238.7 to 275.2)	348.8<br>(325.3 to 376.1)	--	--	11.5<br>(7.4 to 16.9)	16.0<br>(10.4 to 23.4)
Chronic lymphoid leukaemia	Terminal phase of chronic lymphoid leukaemia	2.2<br>(2.1 to 2.4)	3.0<br>(2.8 to 3.2)	--	--	1.2<br>(0.8 to 1.5)	1.6<br>(1.1 to 2.1)
Chronic lymphoid leukaemia	Diagnosis and primary therapy phase of chronic lymphoid leukaemia	26.8<br>(25.5 to 29.4)	37.2<br>(34.7 to 40.2)	76.3<br>(72.5 to 83.1)	105.3<br>(98.3 to 113.3)	7.5<br>(5.0 to 10.3)	10.3<br>(6.9 to 14.2)
Chronic lymphoid leukaemia	Metastatic phase of chronic lymphoid leukaemia	51.8<br>(49.0 to 56.6)	70.8<br>(66.1 to 76.4)	--	--	21.4<br>(14.8 to 28.1)	29.2<br>(20.0 to 38.5)
Acute myeloid leukaemia	Terminal phase of acute myeloid leukaemia	5.4<br>(4.9 to 5.7)	7.2<br>(6.4 to 7.6)	--	--	2.9<br>(2.0 to 3.8)	3.9<br>(2.7 to 5.0)
Acute myeloid leukaemia	Diagnosis and primary therapy phase of acute myeloid leukaemia	14.3<br>(13.3 to 15.3)	19.0<br>(16.8 to 20.1)	77.3<br>(71.1 to 82.1)	103.0<br>(91.1 to 107.6)	4.1<br>(2.7 to 5.8)	5.4<br>(3.6 to 7.7)
Acute myeloid leukaemia	Metastatic phase of acute myeloid leukaemia	24.9<br>(22.7 to 26.3)	33.3<br>(29.6 to 34.8)	--	--	11.1<br>(7.5 to 15.0)	14.8<br>(9.9 to 20.1)
Acute myeloid leukaemia	Controlled phase of acute myeloid leukaemia	76.0<br>(70.6 to 82.7)	100.0<br>(87.8 to 107.7)	--	--	3.7<br>(2.3 to 5.4)	4.8<br>(3.0 to 7.2)
Chronic myeloid leukaemia	Controlled phase of chronic myeloid leukaemia	65.3<br>(60.9 to 69.1)	70.3<br>(64.3 to 75.2)	--	--	3.1<br>(2.0 to 4.7)	3.4<br>(2.1 to 5.1)
Chronic myeloid leukaemia	Metastatic phase of chronic myeloid leukaemia	8.2<br>(7.4 to 8.8)	8.6<br>(7.7 to 9.2)	--	--	3.7<br>(2.5 to 5.0)	3.9<br>(2.6 to 5.2)
Chronic myeloid leukaemia	Terminal phase of chronic myeloid leukaemia	1.8<br>(1.6 to 1.9)	1.9<br>(1.7 to 2.0)	--	--	1.0<br>(0.7 to 1.2)	1.0<br>(0.7 to 1.3)
Chronic myeloid leukaemia	Diagnosis and primary therapy phase of chronic myeloid leukaemia	10.8<br>(10.0 to 11.4)	11.6<br>(10.5 to 12.3)	30.4<br>(27.8 to 32.3)	32.4<br>(29.3 to 34.3)	3.1<br>(2.0 to 4.4)	3.3<br>(2.2 to 4.6)
Other leukaemia	Metastatic phase of other leukaemia	76.0<br>(65.1 to 79.4)	90.5<br>(77.9 to 96.6)	--	--	32.9<br>(22.0 to 43.9)	38.6<br>(26.1 to 51.9)
Other leukaemia	Diagnosis and primary therapy phase of other leukaemia	47.6<br>(40.1 to 50.3)	55.2<br>(46.2 to 59.5)	128.4<br>(109.0 to 135.2)	150.1<br>(127.1 to 161.3)	13.6<br>(9.0 to 19.3)	15.7<br>(10.1 to 22.3)
Other leukaemia	Terminal phase of other leukaemia	3.2<br>(2.7 to 3.3)	3.8<br>(3.3 to 4.1)	--	--	1.7<br>(1.2 to 2.2)	2.1<br>(1.4 to 2.7)
Other leukaemia	Controlled phase of other leukaemia	450.3<br>(378.8 to 476.5)	522.0<br>(436.2 to 562.6)	--	--	21.2<br>(13.4 to 31.1)	24.4<br>(15.4 to 36.4)
Other neoplasms	Terminal phase of other neoplasms	20.7<br>(19.8 to 21.1)	29.1<br>(26.7 to 29.9)	--	--	11.1<br>(7.8 to 14.2)	15.6<br>(10.8 to 20.0)
Other neoplasms	Controlled phase of other neoplasms	1,447.9<br>(1,341.8 to 1,475.3)	2,343.0<br>(2,115.0 to 2,423.1)	--	--	66.7<br>(42.7 to 96.5)	107.6<br>(69.1 to 157.6)
Other neoplasms	Metastatic phase of other neoplasms	121.4<br>(115.8 to 123.6)	171.8<br>(157.1 to 176.5)	--	--	52.4<br>(36.1 to 69.4)	73.5<br>(50.4 to 97.1)
Other neoplasms	Diagnosis and primary therapy phase of other neoplasms	55.2<br>(51.6 to 56.2)	88.0<br>(79.5 to 90.9)	495.0<br>(468.8 to 503.0)	750.3<br>(681.9 to 772.5)	15.6<br>(10.4 to 21.7)	24.7<br>(16.5 to 34.2)
Rheumatic heart disease	Asymptomatic and mild heart failure due to rheumatic heart disease	700.7<br>(599.6 to 816.1)	924.6<br>(797.2 to 1,074.7)	< 0.05<br>(0.0 to < 0.05)	< 0.05<br>(0.0 to < 0.05)	27.0<br>(17.0 to 40.2)	35.6<br>(22.4 to 52.8)
Rheumatic heart disease	Severe heart failure due to rheumatic heart disease	410.3<br>(344.3 to 485.5)	541.5<br>(454.9 to 643.4)	< 0.05<br>(0.0 to < 0.05)	< 0.05<br>(0.0 to < 0.05)	66.6<br>(43.7 to 94.1)	87.8<br>(57.3 to 124.5)

Rheumatic heart disease	Rheumatic heart disease, without heart failure	24,162.5 (21,982.4 to 26,548.7)	28,007.9 (25,119.7 to 31,316.5)	1,100.2 (1,023.5 to 1,186.3)	1,195.9 (1,095.4 to 1,307.5)	1,136.4 (717.9 to 1,680.3)	1,318.4 (826.0 to 1,961.6)
Rheumatic heart disease	Moderate heart failure due to rheumatic heart disease	153.8 (109.0 to 206.2)	203.0 (144.0 to 273.1)	< 0.05 (0.0 to < 0.05)	< 0.05 (0.0 to < 0.05)	10.4 (6.2 to 16.2)	13.7 (8.2 to 21.3)
Ischaemic heart disease	Asymptomatic angina due to ischaemic heart disease	29,880.7 (26,895.3 to 33,029.8)	38,656.5 (34,737.0 to 42,727.4)	2,732.0 (2,460.5 to 3,012.4)	3,439.6 (3,100.0 to 3,798.8)	< 0.05 (0.0 to < 0.05)	< 0.05 (0.0 to < 0.05)
Ischaemic heart disease	Acute myocardial infarction first 2 days	46.7 (42.2 to 51.5)	51.3 (45.6 to 57.5)	11,773.3 (10,988.1 to 12,650.4)	12,967.2 (11,920.7 to 14,225.9)	19.4 (13.0 to 26.4)	21.5 (14.2 to 29.2)
Ischaemic heart disease	Severe heart failure due to ischaemic heart disease	6,524.5 (5,511.8 to 7,699.7)	8,497.2 (7,151.1 to 10,085.3)	--	--	1,024.6 (689.0 to 1,427.1)	1,333.2 (900.8 to 1,852.9)
Ischaemic heart disease	Acute myocardial infarction 3 to 28 days	607.3 (562.4 to 658.0)	667.2 (608.1 to 734.8)	< 0.05 (0.0 to < 0.05)	< 0.05 (0.0 to < 0.05)	40.9 (27.8 to 57.7)	45.0 (30.7 to 63.6)
Ischaemic heart disease	Severe angina due to ischaemic heart disease	18,734.6 (16,096.0 to 21,462.5)	24,236.9 (20,794.0 to 27,788.7)	1,704.8 (1,472.4 to 1,948.5)	2,146.4 (1,850.6 to 2,461.0)	2,815.3 (1,819.3 to 4,055.0)	3,639.0 (2,355.9 to 5,247.1)
Ischaemic heart disease	Mild angina due to ischaemic heart disease	12,355.7 (9,588.9 to 15,500.7)	15,984.6 (12,395.5 to 20,059.0)	1,122.4 (866.2 to 1,392.8)	1,413.2 (1,085.5 to 1,745.0)	382.6 (218.9 to 603.0)	494.6 (284.3 to 776.8)
Ischaemic heart disease	Moderate heart failure due to ischaemic heart disease	2,446.6 (1,736.0 to 3,260.1)	3,186.3 (2,254.7 to 4,239.4)	--	--	158.1 (93.9 to 246.6)	205.7 (121.8 to 319.0)
Ischaemic heart disease	Asymptomatic and mild heart failure due to ischaemic heart disease	11,140.4 (9,624.1 to 12,836.1)	14,508.7 (12,455.5 to 16,786.2)	--	--	420.4 (263.7 to 617.4)	547.2 (347.4 to 803.1)
Ischaemic heart disease	Asymptomatic ischaemic heart disease following myocardial infarction	34,956.8 (31,677.6 to 38,056.7)	39,114.6 (33,792.3 to 44,501.4)	--	--	< 0.05 (0.0 to < 0.05)	< 0.05 (0.0 to < 0.05)
Ischaemic heart disease	Moderate angina due to ischaemic heart disease	6,670.8 (5,008.4 to 8,551.8)	8,629.9 (6,486.6 to 11,069.6)	607.1 (456.0 to 771.7)	764.4 (572.6 to 971.8)	487.1 (303.7 to 716.2)	629.7 (393.5 to 925.3)
Ischaemic stroke	Asymptomatic chronic ischaemic stroke	9,404.0 (8,738.6 to 10,092.5)	12,471.1 (11,189.8 to 13,771.5)	< 0.05 (0.0 to < 0.05)	< 0.05 (0.0 to < 0.05)	< 0.05 (0.0 to < 0.05)	< 0.05 (0.0 to < 0.05)
Ischaemic stroke	Chronic ischaemic stroke severity level 2	7,829.4 (7,244.2 to 8,426.7)	10,354.6 (9,316.3 to 11,459.6)	< 0.05 (0.0 to < 0.05)	< 0.05 (0.0 to < 0.05)	499.6 (337.1 to 690.9)	661.2 (441.8 to 911.6)
Ischaemic stroke	Chronic ischaemic stroke severity level 4	4,834.7 (4,474.6 to 5,222.8)	6,519.6 (5,869.2 to 7,235.9)	< 0.05 (0.0 to < 0.05)	< 0.05 (0.0 to < 0.05)	2,275.4 (1,566.5 to 2,894.4)	3,069.6 (2,082.3 to 3,955.8)
Ischaemic stroke	Acute ischaemic stroke severity level 5	84.0 (76.4 to 92.0)	115.8 (103.2 to 128.9)	1,140.8 (1,035.9 to 1,246.0)	1,561.1 (1,391.3 to 1,724.7)	45.9 (32.0 to 58.4)	63.2 (44.1 to 81.6)
Ischaemic stroke	Acute ischaemic stroke severity level 2	75.9 (69.0 to 83.5)	105.4 (93.4 to 118.2)	1,014.1 (923.1 to 1,115.6)	1,398.6 (1,243.6 to 1,569.1)	5.2 (3.4 to 7.5)	7.2 (4.7 to 10.4)
Ischaemic stroke	Acute ischaemic stroke severity level 1	177.4 (162.5 to 193.3)	246.3 (220.8 to 273.3)	2,399.5 (2,211.5 to 2,602.1)	3,306.4 (2,969.1 to 3,657.2)	3.2 (1.7 to 5.4)	4.4 (2.4 to 7.6)
Ischaemic stroke	Acute ischaemic stroke severity level 4	84.7 (76.9 to 93.2)	117.6 (104.3 to 131.4)	1,140.4 (1,036.5 to 1,260.9)	1,572.0 (1,395.2 to 1,749.4)	44.2 (30.5 to 57.4)	61.3 (41.8 to 80.4)
Ischaemic stroke	Acute ischaemic stroke severity level 3	92.5 (84.5 to 101.2)	127.4 (113.5 to 142.2)	1,256.2 (1,143.4 to 1,376.5)	1,718.3 (1,538.3 to 1,903.9)	27.2 (17.9 to 38.0)	37.5 (25.0 to 53.0)
Ischaemic stroke	Chronic ischaemic stroke severity level 5	5,744.2 (5,310.0 to 6,188.5)	7,778.3 (6,999.5 to 8,622.7)	< 0.05 (0.0 to < 0.05)	< 0.05 (0.0 to < 0.05)	2,875.8 (2,043.5 to 3,566.8)	3,897.1 (2,733.4 to 4,935.6)
Ischaemic stroke	Chronic ischaemic stroke severity level 3	9,883.1 (9,130.9 to 10,651.2)	13,419.1 (12,061.2 to 14,882.0)	< 0.05 (0.0 to < 0.05)	< 0.05 (0.0 to < 0.05)	2,729.8 (1,827.0 to 3,691.8)	3,709.3 (2,463.8 to 5,069.1)
Ischaemic stroke	Chronic ischaemic stroke severity level 1	12,438.0 (11,585.8 to 13,343.9)	16,340.0 (14,656.7 to 18,041.3)	< 0.05 (0.0 to < 0.05)	< 0.05 (0.0 to < 0.05)	221.7 (120.4 to 377.1)	291.5 (154.5 to 494.2)
Hemorrhagic stroke	Chronic hemorrhagic stroke severity level 3	2,333.1 (2,159.9 to 2,531.9)	2,973.0 (2,679.9 to 3,301.2)	< 0.05 (0.0 to < 0.05)	< 0.05 (0.0 to < 0.05)	653.0 (438.9 to 897.0)	830.0 (554.5 to 1,133.7)
Hemorrhagic stroke	Acute hemorrhagic stroke severity level 5	32.9 (30.3 to 35.9)	40.8 (36.5 to 45.2)	560.6 (519.5 to 605.0)	672.9 (608.2 to 742.8)	19.1 (13.2 to 24.7)	23.8 (16.3 to 31.1)
Hemorrhagic stroke	Acute hemorrhagic stroke severity level 2	30.5 (27.9 to 33.3)	37.9 (33.9 to 42.3)	506.9 (468.3 to 550.1)	610.8 (547.3 to 678.5)	2.1 (1.4 to 3.1)	2.7 (1.7 to 3.8)
Hemorrhagic stroke	Chronic hemorrhagic stroke severity level 1	3,056.5 (2,852.1 to 3,305.9)	3,806.1 (3,442.3 to 4,216.8)	< 0.05 (0.0 to < 0.05)	< 0.05 (0.0 to < 0.05)	55.0 (29.3 to 92.3)	68.4 (35.9 to 116.3)
Hemorrhagic stroke	Chronic hemorrhagic stroke severity level 5	1,327.7 (1,233.1 to 1,439.6)	1,687.5 (1,522.4 to 1,874.5)	< 0.05 (0.0 to < 0.05)	< 0.05 (0.0 to < 0.05)	677.4 (484.2 to 843.2)	858.5 (610.1 to 1,084.1)
Hemorrhagic stroke	Asymptomatic chronic hemorrhagic stroke	2,307.8 (2,152.1 to 2,499.8)	2,878.5 (2,596.4 to 3,194.6)	< 0.05 (0.0 to < 0.05)	< 0.05 (0.0 to < 0.05)	< 0.05 (0.0 to < 0.05)	< 0.05 (0.0 to < 0.05)
Hemorrhagic stroke	Chronic hemorrhagic stroke severity level 4	1,120.2 (1,039.8 to 1,216.4)	1,419.6 (1,278.3 to 1,574.6)	< 0.05 (0.0 to < 0.05)	< 0.05 (0.0 to < 0.05)	538.1 (372.7 to 684.1)	679.7 (464.4 to 868.0)
Hemorrhagic stroke	Chronic hemorrhagic stroke severity level 2	1,819.7 (1,687.3 to 1,974.2)	2,293.9 (2,072.0 to 2,541.3)	< 0.05 (0.0 to < 0.05)	< 0.05 (0.0 to < 0.05)	117.4 (78.7 to 163.5)	147.7 (99.5 to 202.0)
Hemorrhagic stroke	Acute hemorrhagic stroke severity level 4	36.1 (33.0 to 39.3)	44.8 (40.2 to 49.8)	615.3 (570.0 to 662.6)	739.2 (667.5 to 811.5)	19.6 (13.4 to 25.8)	24.5 (16.8 to 32.1)
Hemorrhagic stroke	Acute hemorrhagic stroke severity level 3	33.6 (30.8 to 36.7)	41.9 (37.6 to 46.8)	564.3 (520.3 to 609.9)	682.1 (617.1 to 762.2)	10.6 (6.9 to 14.9)	13.2 (8.7 to 18.6)
Hemorrhagic stroke	Acute hemorrhagic stroke severity level 1	68.8 (63.5 to 74.0)	86.3 (78.0 to 95.1)	1,167.7 (1,089.4 to 1,251.8)	1,415.3 (1,277.2 to 1,557.5)	1.3 (0.7 to 2.2)	1.6 (0.9 to 2.7)
Hypertensive heart disease	Asymptomatic and mild heart failure due to hypertensive heart disease	7,228.0 (6,111.8 to 8,589.8)	9,522.7 (8,036.9 to 11,292.0)	--	--	274.8 (173.4 to 411.6)	361.6 (226.9 to 541.8)
Hypertensive heart disease	Moderate heart failure due to hypertensive heart disease	1,587.3 (1,133.5 to 2,147.3)	2,091.2 (1,497.2 to 2,834.8)	--	--	103.4 (61.2 to 161.8)	136.1 (80.9 to 212.8)
Hypertensive heart disease	Severe heart failure due to hypertensive heart disease	4,232.4 (3,518.3 to 5,022.7)	5,576.1 (4,622.1 to 6,638.6)	--	--	670.4 (456.6 to 941.6)	882.4 (596.3 to 1,240.9)
Myocarditis	Severe heart failure due to myocarditis	345.8 (287.1 to 415.6)	386.0 (319.9 to 464.3)	--	--	60.4 (39.4 to 89.2)	67.4 (43.6 to 99.2)
Myocarditis	Moderate heart failure due to myocarditis	129.6 (90.9 to 174.0)	144.7 (101.4 to 194.2)	--	--	9.2 (5.2 to 15.1)	10.3 (5.8 to 16.7)
Myocarditis	Asymptomatic and mild heart failure due to myocarditis	590.3 (495.1 to 693.3)	658.9 (554.2 to 771.3)	--	--	23.8 (14.4 to 36.3)	26.6 (16.2 to 40.6)
Myocarditis	Acute myocarditis	498.8 (447.9 to 557.8)	596.1 (533.4 to 670.5)	2,084.7 (1,867.4 to 2,338.0)	2,482.7 (2,216.6 to 2,784.2)	24.3 (15.4 to 35.6)	28.9 (18.3 to 42.7)
Alcoholic cardiomyopathy	Moderate heart failure due to alcoholic cardiomyopathy	117.6 (82.0 to 157.1)	141.7 (98.3 to 189.3)	--	--	8.0 (4.7 to 12.7)	9.6 (5.6 to 15.3)
Alcoholic cardiomyopathy	Severe heart failure due to alcoholic cardiomyopathy	313.7 (257.9 to 380.7)	378.1 (309.8 to 461.9)	--	--	51.5 (32.9 to 75.8)	62.2 (40.4 to 90.7)

Alcoholic cardiomyopathy	Asymptomatic and mild heart failure due to alcoholic cardiomyopathy	535.4 (454.5 to 635.0)	645.4 (540.8 to 768.2)	--	--	20.8 (12.9 to 31.0)	25.1 (15.5 to 37.5)
Other cardiomyopathy	Moderate heart failure due to other cardiomyopathy	402.7 (283.2 to 533.0)	487.2 (342.9 to 646.5)	--	--	27.1 (15.8 to 42.6)	32.7 (19.0 to 51.5)
Other cardiomyopathy	Asymptomatic and mild heart failure due to other cardiomyopathy	1,833.9 (1,592.7 to 2,120.0)	2,218.5 (1,926.4 to 2,566.3)	--	--	70.9 (44.2 to 104.6)	85.5 (53.4 to 126.6)
Other cardiomyopathy	Severe heart failure due to other cardiomyopathy	1,074.3 (901.2 to 1,280.4)	1,299.6 (1,084.8 to 1,549.7)	--	--	175.0 (114.9 to 252.7)	210.9 (138.2 to 304.1)
Atrial fibrillation and flutter	Symptomatic atrial fibrillation and flutter	14,025.3 (12,498.8 to 15,800.2)	18,519.3 (16,465.7 to 20,927.3)	1,173.1 (1,027.6 to 1,342.4)	1,536.1 (1,345.0 to 1,758.4)	2,739.0 (1,859.9 to 3,800.8)	3,614.4 (2,439.1 to 5,023.8)
Atrial fibrillation and flutter	Asymptomatic atrial fibrillation and flutter	21,047.4 (18,821.1 to 23,708.7)	27,791.3 (24,849.1 to 31,270.4)	1,760.3 (1,543.3 to 2,012.1)	2,305.0 (2,022.6 to 2,638.1)	< 0.05 (0.0 to < 0.05)	< 0.05 (0.0 to < 0.05)
Peripheral vascular disease	Asymptomatic peripheral vascular disease	60,685.4 (51,889.2 to 70,723.2)	80,346.9 (68,890.6 to 93,584.2)	8,579.6 (7,452.6 to 9,901.5)	11,020.3 (9,557.3 to 12,777.9)	< 0.05 (0.0 to < 0.05)	< 0.05 (0.0 to < 0.05)
Peripheral vascular disease	Symptomatic claudication due to peripheral vascular disease	31,716.5 (25,798.2 to 38,967.5)	39,797.7 (32,281.0 to 48,503.0)	--	--	414.1 (194.7 to 751.7)	519.6 (244.4 to 940.9)
Endocarditis	Moderate endocarditis	63.2 (57.3 to 69.4)	78.9 (71.6 to 86.5)	877.0 (800.5 to 952.1)	1,091.4 (993.8 to 1,191.7)	3.2 (2.0 to 4.7)	4.0 (2.5 to 5.9)
Endocarditis	Asymptomatic and mild heart failure due to endocarditis	187.1 (158.9 to 219.6)	230.0 (194.9 to 269.3)	--	--	7.3 (4.5 to 11.0)	9.0 (5.5 to 13.6)
Endocarditis	Severe heart failure due to endocarditis	109.6 (91.2 to 131.4)	134.7 (112.1 to 162.0)	--	--	18.2 (11.9 to 26.3)	22.4 (14.6 to 32.5)
Endocarditis	Severe endocarditis	4.8 (4.3 to 5.3)	5.8 (5.3 to 6.5)	66.2 (60.3 to 72.6)	81.0 (73.5 to 89.1)	0.6 (0.4 to 0.9)	0.8 (0.5 to 1.1)
Endocarditis	Moderate heart failure due to endocarditis	41.1 (29.1 to 55.3)	50.5 (36.0 to 68.1)	--	--	2.8 (1.6 to 4.6)	3.5 (2.0 to 5.7)
Other cardiovascular and circulatory diseases	Asymptomatic and mild heart failure due to other cardiovascular diseases	877.7 (735.0 to 1,041.9)	1,103.4 (919.7 to 1,315.4)	--	--	33.1 (20.9 to 50.4)	41.6 (26.2 to 63.0)
Other cardiovascular and circulatory diseases	Moderate heart failure due to other cardiovascular diseases	192.8 (137.5 to 260.9)	242.3 (170.0 to 325.2)	--	--	12.6 (7.4 to 20.0)	15.8 (9.3 to 25.4)
Other cardiovascular and circulatory diseases	Asymptomatic other cardiovascular diseases	27,512.9 (23,251.6 to 31,799.3)	33,163.1 (27,992.1 to 38,275.5)	--	--	< 0.05 (0.0 to < 0.05)	< 0.05 (0.0 to < 0.05)
Other cardiovascular and circulatory diseases	Mild other cardiovascular diseases	21,262.3 (17,093.6 to 25,893.9)	25,629.5 (20,549.0 to 31,369.3)	--	--	828.3 (501.5 to 1,255.1)	997.5 (606.1 to 1,514.2)
Other cardiovascular and circulatory diseases	Severe heart failure due to other cardiovascular diseases	514.0 (419.2 to 629.6)	646.2 (526.4 to 791.4)	--	--	81.1 (53.3 to 116.1)	101.8 (66.4 to 145.4)
Other cardiovascular and circulatory diseases	Moderate other cardiovascular diseases	7,761.3 (5,314.7 to 10,313.1)	9,355.4 (6,371.3 to 12,368.3)	--	--	517.1 (298.9 to 790.8)	622.8 (360.4 to 949.8)
Other cardiovascular and circulatory diseases	Severe other cardiovascular diseases	14,088.9 (11,365.3 to 17,180.2)	16,982.1 (13,658.5 to 20,704.5)	--	--	2,297.2 (1,480.5 to 3,338.0)	2,766.1 (1,803.3 to 4,014.2)
Chronic obstructive pulmonary disease	Moderate heart failure due to severe chronic obstructive pulmonary disease	791.6 (549.6 to 1,109.7)	1,044.5 (729.9 to 1,467.9)	< 0.05 (0.0 to < 0.05)	< 0.05 (0.0 to < 0.05)	296.5 (202.4 to 407.0)	390.5 (268.3 to 535.2)
Chronic obstructive pulmonary disease	Mild chronic obstructive pulmonary disease	58,770.3 (49,861.7 to 67,585.3)	73,901.6 (62,014.4 to 84,844.7)	4,679.3 (3,982.7 to 5,369.2)	5,726.3 (4,813.0 to 6,606.4)	1,068.5 (576.2 to 1,811.5)	1,341.9 (728.8 to 2,265.9)
Chronic obstructive pulmonary disease	Moderate chronic obstructive pulmonary disease	14,432.0 (11,354.3 to 17,169.8)	18,146.1 (14,294.0 to 21,622.6)	1,149.0 (904.4 to 1,368.0)	1,406.0 (1,100.1 to 1,675.3)	2,929.2 (1,706.8 to 4,370.8)	3,676.7 (2,135.9 to 5,480.0)
Chronic obstructive pulmonary disease	Asymptomatic and mild heart failure due to severe chronic obstructive pulmonary disease	3,603.2 (2,950.9 to 4,396.5)	4,754.4 (3,911.4 to 5,771.3)	< 0.05 (0.0 to < 0.05)	< 0.05 (0.0 to < 0.05)	1,296.3 (990.7 to 1,585.2)	1,707.6 (1,310.3 to 2,089.9)
Chronic obstructive pulmonary disease	Asymptomatic chronic obstructive pulmonary disease	101,255.8 (91,231.7 to 111,354.0)	128,131.5 (114,800.1 to 141,671.8)	7,485.5 (6,763.0 to 8,218.1)	9,188.6 (8,279.6 to 10,175.6)	< 0.05 (0.0 to < 0.05)	< 0.05 (0.0 to < 0.05)
Chronic obstructive pulmonary disease	Severe heart failure due to severe chronic obstructive pulmonary disease	2,110.2 (1,690.7 to 2,585.3)	2,784.3 (2,234.4 to 3,418.3)	< 0.05 (0.0 to < 0.05)	< 0.05 (0.0 to < 0.05)	894.1 (681.6 to 1,101.9)	1,177.4 (898.4 to 1,454.6)
Chronic obstructive pulmonary disease	Severe chronic obstructive pulmonary disease without heart failure	17,602.9 (10,551.1 to 26,250.1)	22,868.2 (13,432.8 to 34,504.8)	1,895.2 (1,267.6 to 2,605.5)	2,400.4 (1,592.1 to 3,321.2)	6,164.4 (4,819.3 to 7,313.3)	7,994.0 (6,232.8 to 9,496.8)
Silicosis	Moderate silicosis	42.6 (28.1 to 58.5)	56.4 (37.3 to 77.2)	4.6 (3.2 to 6.4)	6.0 (4.2 to 8.3)	8.9 (5.0 to 13.9)	11.7 (6.5 to 18.3)
Silicosis	Mild silicosis	108.8 (90.5 to 126.7)	144.0 (119.1 to 168.0)	11.8 (9.9 to 13.8)	15.2 (12.8 to 17.8)	2.0 (1.1 to 3.3)	2.6 (1.4 to 4.4)
Silicosis	Severe heart failure due to severe silicosis	2.2 (1.8 to 2.7)	2.7 (2.2 to 3.2)	< 0.05 (0.0 to < 0.05)	< 0.05 (0.0 to < 0.05)	1.1 (0.8 to 1.6)	1.4 (0.9 to 1.9)
Silicosis	Severe silicosis without heart failure	86.8 (68.0 to 105.9)	115.8 (90.6 to 141.4)	10.2 (8.1 to 12.3)	13.1 (10.5 to 16.0)	31.9 (20.4 to 46.8)	42.4 (26.9 to 62.5)
Silicosis	Asymptomatic silicosis	72.8 (61.9 to 84.9)	96.4 (81.5 to 112.3)	7.9 (6.8 to 9.2)	10.2 (8.7 to 11.9)	< 0.05 (0.0 to < 0.05)	< 0.05 (0.0 to < 0.05)
Silicosis	Asymptomatic and mild heart failure due to severe silicosis	3.8 (3.2 to 4.5)	4.6 (3.9 to 5.4)	< 0.05 (0.0 to < 0.05)	< 0.05 (0.0 to < 0.05)	1.6 (1.1 to 2.2)	1.9 (1.3 to 2.7)
Silicosis	Moderate heart failure due to severe silicosis	0.8 (0.6 to 1.1)	1.0 (0.7 to 1.4)	< 0.05 (0.0 to < 0.05)	< 0.05 (0.0 to < 0.05)	0.4 (0.2 to 0.6)	0.4 (0.3 to 0.7)
Asbestosis	Moderate heart failure due to severe asbestosis	0.4 (0.3 to 0.5)	0.5 (0.3 to 0.6)	< 0.05 (0.0 to < 0.05)	< 0.05 (0.0 to < 0.05)	0.2 (0.1 to 0.2)	0.2 (0.1 to 0.3)
Asbestosis	Severe asbestosis without heart failure	32.3 (25.3 to 40.1)	41.2 (32.2 to 51.0)	2.7 (2.1 to 3.3)	3.5 (2.7 to 4.3)	12.4 (8.0 to 18.2)	15.8 (10.1 to 23.1)
Asbestosis	Mild asbestosis	41.1 (33.8 to 48.7)	52.2 (43.0 to 61.9)	3.1 (2.6 to 3.6)	4.0 (3.4 to 4.8)	0.8 (0.4 to 1.3)	1.0 (0.5 to 1.7)
Asbestosis	Moderate asbestosis	16.1 (11.0 to 21.9)	20.4 (14.0 to 28.0)	1.2 (0.8 to 1.7)	1.6 (1.1 to 2.2)	3.5 (2.0 to 5.4)	4.4 (2.5 to 6.8)
Asbestosis	Asymptomatic asbestosis	27.5 (23.2 to 32.4)	34.9 (29.4 to 41.2)	2.1 (1.7 to 2.4)	2.7 (2.3 to 3.2)	< 0.05 (0.0 to < 0.05)	< 0.05 (0.0 to < 0.05)
Asbestosis	Severe heart failure due to severe asbestosis	1.0 (0.8 to 1.2)	1.2 (1.0 to 1.4)	< 0.05 (0.0 to < 0.05)	< 0.05 (0.0 to < 0.05)	0.5 (0.3 to 0.7)	0.6 (0.4 to 0.8)
Asbestosis	Asymptomatic and mild heart failure due to severe asbestosis	1.7 (1.5 to 2.0)	2.1 (1.8 to 2.4)	< 0.05 (0.0 to < 0.05)	< 0.05 (0.0 to < 0.05)	0.7 (0.5 to 1.0)	0.9 (0.6 to 1.2)
Coal workers pneumoconiosis	Severe heart failure due to severe coal workers pneumoconiosis	1.1 (0.9 to 1.3)	1.3 (1.1 to 1.6)	< 0.05 (0.0 to < 0.05)	< 0.05 (0.0 to < 0.05)	0.5 (0.4 to 0.7)	0.7 (0.5 to 0.9)
Coal workers pneumoconiosis	Severe coal workers pneumoconiosis without heart failure	60.9 (46.9 to 76.5)	82.6 (63.4 to 104.5)	4.9 (3.8 to 6.1)	6.3 (4.8 to 7.9)	22.6 (14.4 to 33.4)	30.5 (19.6 to 44.8)

Coal workers pneumoconiosis	Asymptomatic coal workers pneumoconiosis	49.9 (41.8 to 59.7)	67.4 (55.9 to 80.9)	3.8 (3.2 to 4.5)	4.9 (4.0 to 5.9)	< 0.05 (0.0 to < 0.05)	< 0.05 (0.0 to < 0.05)
Coal workers pneumoconiosis	Moderate coal workers pneumoconiosis	29.2 (19.6 to 39.5)	39.4 (26.4 to 53.9)	2.2 (1.5 to 3.1)	2.9 (1.9 to 4.0)	6.1 (3.4 to 9.4)	8.3 (4.6 to 12.7)
Coal workers pneumoconiosis	Mild coal workers pneumoconiosis	74.6 (60.8 to 90.8)	100.7 (82.0 to 123.5)	5.7 (4.6 to 6.8)	7.3 (5.9 to 8.8)	1.4 (0.8 to 2.3)	1.9 (1.0 to 3.2)
Coal workers pneumoconiosis	Asymptomatic and mild heart failure due to severe coal workers pneumoconiosis	1.9 (1.6 to 2.2)	2.3 (1.9 to 2.7)	< 0.05 (0.0 to < 0.05)	< 0.05 (0.0 to < 0.05)	0.8 (0.5 to 1.0)	0.9 (0.7 to 1.3)
Coal workers pneumoconiosis	Moderate heart failure due to severe coal workers pneumoconiosis	0.4 (0.3 to 0.6)	0.5 (0.4 to 0.7)	< 0.05 (0.0 to < 0.05)	< 0.05 (0.0 to < 0.05)	0.2 (0.1 to 0.3)	0.2 (0.1 to 0.3)
Other pneumoconiosis	Mild other pneumoconiosis	62.8 (51.4 to 75.2)	83.3 (67.9 to 100.5)	5.6 (4.6 to 6.7)	7.2 (5.8 to 8.7)	1.2 (0.6 to 2.0)	1.6 (0.8 to 2.7)
Other pneumoconiosis	Asymptomatic other pneumoconiosis	42.0 (34.9 to 50.0)	55.8 (45.8 to 66.5)	3.8 (3.1 to 4.5)	4.8 (4.0 to 5.9)	< 0.05 (0.0 to < 0.05)	< 0.05 (0.0 to < 0.05)
Other pneumoconiosis	Severe heart failure due to severe other pneumoconiosis	0.7 (0.5 to 0.8)	0.9 (0.7 to 1.0)	< 0.05 (0.0 to < 0.05)	< 0.05 (0.0 to < 0.05)	0.3 (0.2 to 0.5)	0.4 (0.3 to 0.6)
Other pneumoconiosis	Moderate heart failure due to severe other pneumoconiosis	0.2 (0.2 to 0.3)	0.3 (0.2 to 0.4)	< 0.05 (0.0 to < 0.05)	< 0.05 (0.0 to < 0.05)	0.1 (0.1 to 0.2)	0.1 (0.1 to 0.2)
Other pneumoconiosis	Severe other pneumoconiosis without heart failure	52.0 (40.6 to 64.9)	69.1 (54.0 to 86.9)	4.8 (3.8 to 6.0)	6.2 (4.8 to 7.8)	19.9 (12.8 to 29.1)	26.4 (16.9 to 38.5)
Other pneumoconiosis	Moderate other pneumoconiosis	24.6 (16.4 to 33.8)	32.6 (21.7 to 44.8)	2.2 (1.5 to 3.0)	2.8 (1.9 to 3.9)	5.4 (3.1 to 8.3)	7.2 (4.1 to 11.2)
Other pneumoconiosis	Asymptomatic and mild heart failure due to severe other pneumoconiosis	1.1 (1.0 to 1.3)	1.5 (1.2 to 1.7)	< 0.05 (0.0 to < 0.05)	< 0.05 (0.0 to < 0.05)	0.5 (0.3 to 0.7)	0.6 (0.4 to 0.9)
Asthma	Controlled asthma	57,567.6 (41,176.5 to 76,156.7)	67,619.0 (48,320.9 to 89,379.7)	12,611.5 (9,060.2 to 16,451.9)	14,968.7 (10,766.5 to 19,557.2)	812.9 (394.4 to 1,481.1)	953.2 (462.7 to 1,739.9)
Asthma	Asymptomatic asthma	104,618.8 (92,222.9 to 117,191.8)	122,882.9 (108,108.2 to 137,972.4)	23,091.0 (20,348.6 to 26,003.4)	27,405.1 (24,070.8 to 30,840.0)	< 0.05 (0.0 to < 0.05)	< 0.05 (0.0 to < 0.05)
Asthma	Uncontrolled asthma	67,117.0 (53,853.9 to 81,400.9)	78,835.5 (63,445.9 to 96,002.5)	14,786.2 (11,670.3 to 18,147.0)	17,548.6 (13,818.5 to 21,501.6)	8,426.6 (5,261.7 to 12,664.8)	9,874.5 (6,136.7 to 14,826.0)
Asthma	Partially controlled asthma	59,684.0 (45,853.4 to 73,613.2)	70,102.5 (53,689.6 to 86,822.5)	13,122.1 (10,176.9 to 16,385.0)	15,573.7 (12,139.7 to 19,467.2)	2,041.5 (1,163.4 to 3,248.6)	2,393.5 (1,354.1 to 3,834.8)
Interstitial lung disease and pulmonary sarcoidosis	Severe heart failure due to severe interstitial lung disease and pulmonary sarcoidosis	54.4 (40.3 to 69.6)	71.3 (52.7 to 91.1)	< 0.05 (0.0 to < 0.05)	< 0.05 (0.0 to < 0.05)	25.5 (16.8 to 36.5)	33.2 (21.8 to 47.7)
Interstitial lung disease and pulmonary sarcoidosis	Severe interstitial lung disease and pulmonary sarcoidosis without heart failure	349.2 (200.7 to 518.4)	450.4 (258.7 to 671.0)	47.9 (31.0 to 68.1)	64.5 (41.8 to 91.8)	129.9 (66.2 to 213.1)	166.2 (84.7 to 277.6)
Interstitial lung disease and pulmonary sarcoidosis	Asymptomatic interstitial lung disease and pulmonary sarcoidosis	939.3 (820.7 to 1,072.1)	1,217.5 (1,057.6 to 1,394.9)	86.2 (74.8 to 98.5)	116.0 (100.4 to 132.7)	< 0.05 (0.0 to < 0.05)	< 0.05 (0.0 to < 0.05)
Interstitial lung disease and pulmonary sarcoidosis	Asymptomatic and mild heart failure due to severe interstitial lung disease and pulmonary sarcoidosis	92.8 (68.8 to 116.6)	121.7 (90.3 to 152.7)	< 0.05 (0.0 to < 0.05)	< 0.05 (0.0 to < 0.05)	35.8 (23.9 to 51.4)	46.7 (31.1 to 66.8)
Interstitial lung disease and pulmonary sarcoidosis	Moderate heart failure due to severe interstitial lung disease and pulmonary sarcoidosis	20.4 (13.5 to 29.0)	26.7 (17.6 to 38.1)	< 0.05 (0.0 to < 0.05)	< 0.05 (0.0 to < 0.05)	9.0 (5.2 to 14.1)	11.7 (6.8 to 18.4)
Interstitial lung disease and pulmonary sarcoidosis	Moderate interstitial lung disease and pulmonary sarcoidosis	502.9 (396.0 to 617.7)	651.8 (514.3 to 803.4)	45.9 (35.8 to 56.4)	61.9 (48.1 to 76.2)	102.6 (65.4 to 145.6)	132.3 (84.2 to 187.6)
Interstitial lung disease and pulmonary sarcoidosis	Mild interstitial lung disease and pulmonary sarcoidosis	1,109.5 (956.3 to 1,273.5)	1,438.1 (1,232.5 to 1,653.0)	101.7 (86.9 to 116.7)	137.0 (116.8 to 157.4)	20.2 (10.9 to 34.0)	26.1 (14.1 to 43.4)
Other chronic respiratory diseases	Other chronic respiratory diseases	--	--	--	--	578.9 (504.5 to 639.0)	866.4 (752.1 to 954.8)
Cirrhosis and other chronic liver diseases due to hepatitis B	Cirrhosis and other chronic liver diseases due to hepatitis B, decompensated	1,898.4 (1,760.8 to 2,061.3)	2,407.0 (2,223.8 to 2,616.9)	377.3 (350.1 to 411.0)	456.6 (421.3 to 499.8)	311.3 (216.8 to 434.3)	394.2 (274.1 to 544.6)
Cirrhosis and other chronic liver diseases due to hepatitis B	Cirrhosis and other chronic liver diseases due to hepatitis B, compensated	7,723.8 (7,108.7 to 8,367.3)	10,075.3 (9,276.4 to 10,933.2)	--	--	< 0.05 (0.0 to < 0.05)	< 0.05 (0.0 to < 0.05)
Cirrhosis and other chronic liver diseases due to hepatitis C	Cirrhosis and other chronic liver diseases due to hepatitis C, decompensated	1,515.9 (1,391.6 to 1,657.9)	1,918.8 (1,751.7 to 2,108.7)	316.6 (289.2 to 347.3)	386.9 (353.0 to 425.5)	248.1 (170.6 to 342.6)	313.6 (215.3 to 432.7)
Cirrhosis and other chronic liver diseases due to hepatitis C	Cirrhosis and other chronic liver diseases due to hepatitis C, compensated	6,838.0 (6,209.8 to 7,544.2)	8,983.9 (8,154.5 to 9,924.8)	--	--	< 0.05 (0.0 to < 0.05)	< 0.05 (0.0 to < 0.05)
Cirrhosis and other chronic liver diseases due to alcohol use	Cirrhosis and other chronic liver diseases due to alcohol, compensated	7,266.0 (6,661.0 to 7,906.1)	9,460.4 (8,651.1 to 10,332.3)	--	--	< 0.05 (0.0 to < 0.05)	< 0.05 (0.0 to < 0.05)
Cirrhosis and other chronic liver diseases due to alcohol use	Cirrhosis and other chronic liver diseases due to alcohol, decompensated	1,533.2 (1,415.1 to 1,664.7)	1,931.2 (1,783.7 to 2,109.7)	333.7 (310.1 to 360.6)	403.7 (373.2 to 439.0)	249.4 (172.5 to 342.0)	313.7 (219.5 to 431.0)
Cirrhosis and other chronic liver diseases due to other causes	Cirrhosis and other chronic liver diseases due to other cause, compensated	6,138.9 (5,522.6 to 6,844.0)	7,724.4 (6,904.6 to 8,614.2)	--	--	< 0.05 (0.0 to < 0.05)	< 0.05 (0.0 to < 0.05)
Cirrhosis and other chronic liver diseases due to other causes	Cirrhosis and other chronic liver diseases due to other cause, decompensated	2,915.7 (2,735.5 to 3,109.5)	3,282.2 (3,067.0 to 3,513.5)	341.9 (318.4 to 366.0)	390.6 (362.6 to 420.7)	491.8 (335.2 to 693.5)	552.0 (375.8 to 771.8)
Peptic ulcer disease	Severe anaemia due to peptic ulcer disease	362.8 (328.4 to 403.2)	542.2 (485.5 to 612.8)	--	--	49.5 (33.1 to 68.9)	73.9 (50.2 to 103.1)
Peptic ulcer disease	Moderate anaemia due to peptic ulcer disease	7,984.1 (7,233.8 to 8,874.8)	11,044.5 (9,867.2 to 12,429.6)	--	--	388.1 (250.5 to 568.5)	536.3 (349.6 to 790.1)
Peptic ulcer disease	Mild symptomatic peptic ulcer disease with severe anaemia	2.9 (2.2 to 3.8)	3.8 (2.9 to 4.8)	--	--	0.5 (0.3 to 0.7)	0.6 (0.4 to 0.9)
Peptic ulcer disease	Asymptomatic peptic ulcer disease with mild anaemia	9,890.5 (9,029.0 to 10,874.0)	13,870.8 (12,541.5 to 15,431.6)	--	--	35.2 (12.6 to 77.2)	49.3 (17.4 to 109.2)
Peptic ulcer disease	Mild anaemia due to peptic ulcer disease	10,074.5 (9,202.8 to 11,079.8)	14,089.1 (12,733.5 to 15,684.4)	--	--	35.9 (12.7 to 78.3)	50.1 (17.8 to 111.0)
Peptic ulcer disease	Mild symptomatic peptic ulcer disease with no anaemia	85.0 (60.5 to 111.1)	93.8 (68.1 to 122.6)	3,897.9 (3,225.8 to 4,665.7)	4,422.4 (3,658.3 to 5,291.2)	0.9 (0.4 to 1.8)	1.0 (0.5 to 2.0)
Peptic ulcer disease	Moderate symptomatic peptic ulcer disease with moderate anaemia	86.1 (69.4 to 106.2)	106.0 (85.5 to 130.5)	--	--	13.7 (8.9 to 19.9)	16.7 (10.9 to 24.2)
Peptic ulcer disease	Asymptomatic peptic ulcer disease with moderate anaemia	7,826.9 (7,095.4 to 8,695.0)	10,859.7 (9,683.6 to 12,224.8)	--	--	380.4 (245.2 to 557.8)	527.1 (343.3 to 774.9)
Peptic ulcer disease	Asymptomatic peptic ulcer disease with severe anaemia	355.6 (321.8 to 394.9)	533.3 (477.3 to 602.6)	--	--	48.4 (32.5 to 69.1)	72.6 (49.7 to 103.0)
Peptic ulcer disease	Mild symptomatic peptic ulcer disease with moderate anaemia	64.0 (49.0 to 80.7)	78.8 (60.3 to 99.7)	--	--	4.0 (2.3 to 6.2)	4.9 (2.8 to 7.6)
Peptic ulcer disease	Asymptomatic peptic ulcer disease with no anaemia	18,389.0 (15,132.1 to 21,854.7)	22,763.3 (18,055.0 to 27,551.2)	< 0.05 (0.0 to < 0.05)	< 0.05 (0.0 to < 0.05)	< 0.05 (0.0 to < 0.05)	< 0.05 (0.0 to < 0.05)

Peptic ulcer disease	Moderate symptomatic peptier ulcer disease with no anaemia	114.3 (86.7 to 144.6)	126.2 (95.4 to 161.9)	5,206.2 (4,370.3 to 6,042.4)	5,906.7 (4,967.9 to 6,885.1)	13.0 (7.8 to 20.4)	14.4 (8.5 to 22.0)
Peptic ulcer disease	Moderate symptomatic peptic ulcer disease with mild anaemia	100.6 (81.3 to 123.8)	125.2 (101.4 to 154.6)	--	--	11.8 (7.5 to 17.2)	14.6 (9.3 to 21.3)
Peptic ulcer disease	Mild symptomatic peptic ulcer disease with mild anaemia	74.8 (57.4 to 94.7)	93.1 (71.2 to 117.1)	--	--	1.1 (0.5 to 2.2)	1.4 (0.6 to 2.7)
Peptic ulcer disease	Moderate symptomatic peptic ulcer disease with severe anaemia	3.9 (3.1 to 4.9)	5.1 (4.0 to 6.3)	--	--	0.6 (0.4 to 0.9)	0.8 (0.5 to 1.2)
Gastritis and duodenitis	Mild symptomatic gastritis and duodenitis with moderate anaemia	109.8 (86.0 to 142.1)	152.7 (118.7 to 197.9)	--	--	6.7 (4.1 to 10.7)	9.3 (5.6 to 14.9)
Gastritis and duodenitis	Mild anaemia due to gastritis and duodenitis	16,661.8 (15,045.5 to 18,353.4)	23,845.8 (21,417.2 to 26,185.6)	--	--	59.1 (20.9 to 132.6)	84.4 (30.0 to 188.4)
Gastritis and duodenitis	Asymptomatic gastritis and duodenitis with severe anaemia	353.2 (319.5 to 392.8)	527.5 (472.5 to 596.4)	--	--	48.2 (32.5 to 68.1)	71.9 (48.9 to 102.4)
Gastritis and duodenitis	Moderate symptomatic gastritis and duodenitis with severe anaemia	4.5 (3.4 to 5.7)	6.9 (5.2 to 8.8)	--	--	1.1 (0.7 to 1.6)	1.7 (1.1 to 2.5)
Gastritis and duodenitis	Mild symptomatic gastritis and duodenitis with severe anaemia	5.0 (3.9 to 6.4)	7.7 (6.1 to 10.0)	--	--	0.8 (0.5 to 1.2)	1.2 (0.8 to 1.8)
Gastritis and duodenitis	Severe anaemia due to gastritis and duodenitis	560.6 (499.4 to 621.6)	824.4 (731.2 to 919.8)	--	--	76.6 (52.5 to 106.4)	112.2 (77.1 to 156.3)
Gastritis and duodenitis	Moderate symptomatic gastritis and duodenitis with mild anaemia	116.9 (88.5 to 148.4)	165.2 (125.5 to 211.0)	--	--	13.6 (8.3 to 20.4)	19.1 (11.7 to 28.1)
Gastritis and duodenitis	Asymptomatic gastritis and duodenitis with moderate anaemia	7,775.4 (7,049.8 to 8,634.7)	10,755.0 (9,609.6 to 12,103.7)	--	--	378.0 (244.2 to 555.4)	522.1 (339.1 to 767.3)
Gastritis and duodenitis	Asymptomatic gastritis and duodenitis with mild anaemia	9,826.5 (8,973.3 to 10,809.7)	13,739.5 (12,432.7 to 15,286.9)	--	--	35.0 (12.5 to 76.6)	48.8 (17.3 to 108.6)
Gastritis and duodenitis	Asymptomatic gastritis and duodenitis with no anaemia	44,963.2 (39,216.8 to 51,367.7)	57,332.4 (50,043.3 to 65,129.6)	< 0.05 (0.0 to < 0.05)	< 0.05 (0.0 to < 0.05)	< 0.05 (0.0 to < 0.05)	< 0.05 (0.0 to < 0.05)
Gastritis and duodenitis	Mild symptomatic gastritis and duodenitis with no anaemia	198.0 (151.7 to 252.7)	256.5 (200.0 to 323.9)	32,196.9 (26,808.8 to 38,014.1)	40,906.1 (34,056.4 to 48,509.4)	2.1 (1.0 to 4.1)	2.7 (1.3 to 5.3)
Gastritis and duodenitis	Moderate anaemia due to gastritis and duodenitis	11,271.2 (10,093.9 to 12,456.6)	15,454.4 (13,877.3 to 17,118.1)	--	--	548.2 (351.5 to 786.1)	749.5 (479.8 to 1,083.4)
Gastritis and duodenitis	Moderate symptomatic gastritis and duodenitis with no anaemia	177.5 (129.0 to 229.2)	229.9 (173.5 to 294.2)	28,517.5 (23,111.0 to 34,071.9)	36,233.8 (29,532.6 to 43,264.3)	19.6 (12.0 to 29.4)	25.1 (15.8 to 36.9)
Gastritis and duodenitis	Moderate symptomatic gastritis and duodenitis with moderate anaemia	98.4 (74.1 to 126.6)	136.8 (103.2 to 175.5)	--	--	15.6 (9.5 to 23.2)	21.5 (13.1 to 31.8)
Gastritis and duodenitis	Mild symptomatic gastritis and duodenitis with mild anaemia	130.5 (103.4 to 166.1)	184.4 (145.7 to 235.6)	--	--	1.9 (0.8 to 3.8)	2.7 (1.2 to 5.5)
Appendicitis	Appendicitis	779.8 (722.3 to 839.5)	857.1 (795.6 to 921.6)	20,541.1 (19,030.3 to 22,107.5)	22,580.2 (20,992.8 to 24,257.6)	239.9 (158.8 to 328.0)	263.4 (176.3 to 360.2)
Paralytic ileus and intestinal obstruction	Paralytic ileus and intestinal obstruction	135.0 (123.6 to 147.7)	169.7 (155.2 to 185.7)	3,689.3 (3,385.4 to 4,021.5)	4,651.4 (4,265.0 to 5,079.5)	43.1 (28.8 to 59.2)	54.0 (36.4 to 74.5)
Inguinal, femoral, and abdominal hernia	Inguinal, femoral, and abdominal hernia, asymptomatic	24,457.8 (20,319.1 to 28,958.3)	28,863.7 (24,230.3 to 33,871.7)	--	--	< 0.05 (0.0 to < 0.05)	< 0.05 (0.0 to < 0.05)
Inguinal, femoral, and abdominal hernia	Severe symptomatic Inguinal, femoral and abdominal hernia	4,195.9 (3,307.6 to 5,241.4)	4,850.8 (3,831.2 to 6,051.8)	5,577.4 (4,328.9 to 7,018.1)	7,898.5 (6,124.9 to 9,926.4)	1,243.9 (791.0 to 1,784.5)	1,433.6 (913.5 to 2,054.5)
Inguinal, femoral, and abdominal hernia	Moderate symptomatic Inguinal, femoral and abdominal hernia	4,782.4 (3,586.1 to 6,038.2)	5,529.3 (4,129.2 to 6,975.8)	6,302.6 (4,748.0 to 8,030.6)	8,925.1 (6,693.6 to 11,434.3)	512.5 (310.5 to 767.5)	591.0 (356.2 to 891.5)
Inguinal, femoral, and abdominal hernia	Mild symptomatic Inguinal, femoral and abdominal hernia	10,374.7 (8,800.8 to 12,010.1)	11,995.3 (10,145.7 to 13,960.7)	13,710.5 (11,563.6 to 16,088.0)	19,416.9 (16,379.3 to 22,937.5)	109.9 (53.4 to 204.4)	126.9 (61.1 to 235.5)
Inflammatory bowel disease	Asymptomatic Crohn's disease	491.4 (454.7 to 530.3)	555.5 (512.4 to 602.3)	373.2 (339.0 to 413.1)	385.6 (351.3 to 425.0)	< 0.05 (0.0 to < 0.05)	< 0.05 (0.0 to < 0.05)
Inflammatory bowel disease	Asymptomatic Ulcerative colitis	1,020.8 (937.0 to 1,109.9)	1,379.2 (1,247.8 to 1,524.5)	658.0 (593.3 to 726.3)	740.8 (664.6 to 822.1)	< 0.05 (0.0 to < 0.05)	< 0.05 (0.0 to < 0.05)
Inflammatory bowel disease	Ulcerative colitis	2,040.7 (1,884.0 to 2,215.7)	2,757.3 (2,509.2 to 3,045.8)	1,314.1 (1,184.9 to 1,439.8)	1,479.5 (1,325.5 to 1,630.8)	431.4 (296.3 to 587.0)	581.7 (396.6 to 793.8)
Inflammatory bowel disease	Crohn's disease	1,163.2 (1,083.5 to 1,251.1)	1,315.1 (1,219.8 to 1,420.5)	882.5 (805.8 to 969.5)	911.9 (831.7 to 1,000.0)	249.2 (170.6 to 337.5)	281.0 (192.5 to 381.3)
Vascular intestinal disorders	Vascular intestinal disorders	82.3 (74.3 to 90.7)	103.7 (92.8 to 114.9)	470.7 (429.1 to 518.2)	582.9 (527.4 to 644.5)	25.1 (16.8 to 34.4)	31.6 (20.9 to 43.3)
Gallbladder and biliary diseases	Mild symptomatic episodes gallbladder and biliary diseases	1,273.8 (1,069.1 to 1,472.7)	1,652.6 (1,381.1 to 1,919.4)	23,605.4 (19,858.4 to 27,614.4)	30,368.1 (25,648.9 to 35,416.6)	13.4 (6.4 to 25.0)	17.4 (8.3 to 32.2)
Gallbladder and biliary diseases	Gallbladder and biliary diseases, asymptomatic	11,604.5 (10,308.6 to 13,029.8)	14,452.7 (12,781.5 to 16,258.6)	--	--	< 0.05 (0.0 to < 0.05)	< 0.05 (0.0 to < 0.05)
Gallbladder and biliary diseases	Severe symptomatic episodes gallbladder and biliary diseases	357.9 (268.1 to 456.6)	464.4 (347.2 to 594.7)	6,615.1 (4,914.6 to 8,665.2)	8,510.7 (6,315.8 to 11,215.2)	108.0 (67.9 to 164.4)	139.3 (86.8 to 214.0)
Gallbladder and biliary diseases	Moderate symptomatic episodes gallbladder and biliary diseases	538.6 (383.5 to 707.8)	698.8 (494.7 to 918.3)	9,817.2 (7,253.0 to 12,931.6)	12,629.9 (9,299.4 to 16,577.1)	57.6 (34.1 to 85.6)	74.6 (44.4 to 110.8)
Pancreatitis	Severe pancreatitis episodes	132.7 (108.1 to 162.1)	173.1 (139.8 to 212.5)	1,194.1 (962.5 to 1,448.9)	1,551.4 (1,245.3 to 1,890.7)	41.4 (27.2 to 60.1)	53.5 (34.5 to 78.0)
Pancreatitis	Moderate pancreatitis episodes	113.6 (88.4 to 142.9)	148.2 (115.0 to 187.2)	1,014.0 (811.6 to 1,244.1)	1,317.4 (1,053.7 to 1,623.1)	12.7 (7.9 to 18.8)	16.5 (10.3 to 24.4)
Pancreatitis	Mild pancreatitis episodes	201.5 (165.6 to 238.6)	262.9 (215.9 to 313.9)	1,802.2 (1,498.7 to 2,114.4)	2,341.6 (1,930.2 to 2,777.4)	2.2 (1.0 to 4.1)	2.8 (1.3 to 5.3)
Other digestive diseases	Other digestive diseases	--	--	--	--	440.8 (313.0 to 590.3)	583.6 (413.1 to 784.4)
Alzheimer's disease and other dementias	Severe Alzheimer disease and other dementias	2,995.4 (2,198.9 to 4,039.9)	4,160.9 (3,046.5 to 5,679.2)	534.6 (372.7 to 727.4)	744.2 (513.9 to 1,019.5)	1,099.9 (695.0 to 1,585.1)	1,525.8 (965.6 to 2,219.5)
Alzheimer's disease and other dementias	Moderate Alzheimer disease and other dementias	6,997.8 (5,706.5 to 8,389.4)	9,728.2 (7,918.7 to 11,740.0)	1,244.1 (1,017.9 to 1,523.7)	1,731.7 (1,410.6 to 2,127.5)	2,191.6 (1,456.5 to 2,962.7)	3,043.9 (2,013.1 to 4,138.3)
Alzheimer's disease and other dementias	Mild Alzheimer disease and other dementias	21,896.3 (18,714.7 to 25,405.1)	29,946.6 (25,545.5 to 34,963.7)	3,870.6 (3,291.4 to 4,567.5)	5,303.4 (4,497.2 to 6,264.3)	1,350.4 (894.2 to 1,903.3)	1,845.7 (1,221.0 to 2,606.1)
Parkinson's disease	Severe Parkinson disease	154.1 (78.2 to 256.8)	210.1 (107.5 to 351.0)	17.6 (9.0 to 30.3)	23.8 (12.1 to 40.9)	76.8 (36.4 to 135.5)	104.7 (49.6 to 183.2)
Parkinson's disease	Mild Parkinson disease	2,477.9 (2,038.7 to 3,033.3)	3,378.2 (2,762.4 to 4,156.9)	287.0 (230.8 to 353.8)	387.0 (311.2 to 479.0)	22.8 (10.5 to 45.4)	31.0 (14.4 to 61.1)

Parkinson's disease	Moderate Parkinson disease	1,815.1 (1,453.0 to 2,226.9)	2,474.6 (1,979.8 to 3,059.4)	211.4 (168.8 to 262.9)	285.0 (227.0 to 356.6)	418.8 (266.3 to 590.3)	570.7 (363.3 to 805.5)
Epilepsy	Severe epilepsy	9,399.7 (7,231.8 to 11,653.2)	9,935.8 (7,585.3 to 12,520.9)	1,140.3 (870.1 to 1,446.0)	1,174.2 (895.8 to 1,520.2)	4,797.8 (3,132.7 to 6,758.8)	5,057.1 (3,297.6 to 7,203.0)
Epilepsy	Seizure-free, treated epilepsy	3,352.4 (2,563.4 to 4,330.2)	4,794.7 (3,679.6 to 6,037.1)	361.1 (272.9 to 465.5)	509.2 (387.2 to 645.0)	157.6 (92.2 to 247.5)	224.9 (133.4 to 351.1)
Epilepsy	Less severe epilepsy	8,057.7 (6,310.1 to 10,182.3)	9,231.9 (7,225.8 to 11,400.4)	970.9 (754.5 to 1,231.8)	1,077.9 (846.2 to 1,356.4)	1,983.0 (1,234.5 to 2,936.0)	2,264.8 (1,427.9 to 3,302.4)
Multiple sclerosis	Asymptomatic multiple sclerosis	405.6 (354.1 to 456.6)	506.4 (440.1 to 575.2)	13.5 (11.8 to 15.2)	15.7 (13.7 to 17.8)	< 0.05 (0.0 to < 0.05)	< 0.05 (0.0 to < 0.05)
Multiple sclerosis	Severe multiple sclerosis	224.0 (176.7 to 279.7)	279.7 (221.1 to 350.3)	7.4 (5.8 to 9.1)	8.6 (6.7 to 10.7)	149.3 (103.9 to 200.7)	185.9 (129.7 to 247.9)
Multiple sclerosis	Moderate multiple sclerosis	488.3 (417.9 to 565.9)	609.7 (518.2 to 709.7)	16.1 (13.6 to 18.9)	18.9 (15.8 to 22.2)	207.6 (134.6 to 280.9)	258.2 (166.3 to 349.6)
Multiple sclerosis	Mild multiple sclerosis	661.2 (583.3 to 744.3)	825.5 (722.4 to 935.3)	21.9 (19.4 to 24.6)	25.6 (22.4 to 28.9)	112.4 (74.8 to 156.7)	140.0 (92.6 to 196.0)
Motor neuron disease	Moderate motor impairment, severe respiratory problems, and speech problems due to motor neuron disease	0.8 (0.6 to 0.9)	1.0 (0.8 to 1.2)	0.1 (0.1 to 0.2)	0.2 (0.1 to 0.2)	0.4 (0.3 to 0.5)	0.4 (0.3 to 0.6)
Motor neuron disease	Severe respiratory problems due to motor neuron disease	< 0.05 (< 0.05 to < 0.05)	< 0.05 (< 0.05 to < 0.05)	< 0.05 (< 0.05 to < 0.05)	< 0.05 (< 0.05 to < 0.05)	< 0.05 (< 0.05 to < 0.05)	< 0.05 (< 0.05 to < 0.05)
Motor neuron disease	Speech problems due to motor neuron disease	6.7 (5.9 to 7.6)	8.2 (7.2 to 9.2)	1.1 (1.0 to 1.2)	1.4 (1.3 to 1.6)	0.3 (0.2 to 0.5)	0.4 (0.3 to 0.6)
Motor neuron disease	Severe motor impairment and mild respiratory problems due to motor neuron disease	18.9 (17.1 to 21.1)	22.9 (20.7 to 25.6)	3.1 (2.9 to 3.4)	4.0 (3.6 to 4.4)	7.8 (5.3 to 10.6)	9.5 (6.4 to 12.9)
Motor neuron disease	Mild motor impairment, moderate respiratory problems, and speech problems due to motor neuron disease	0.7 (0.6 to 0.9)	0.9 (0.7 to 1.1)	0.1 (0.1 to 0.1)	0.2 (0.1 to 0.2)	0.2 (0.1 to 0.3)	0.2 (0.2 to 0.3)
Motor neuron disease	Moderate motor impairment and mild respiratory problems due to motor neuron disease	18.5 (16.6 to 20.6)	22.4 (20.2 to 25.0)	3.1 (2.8 to 3.3)	3.9 (3.5 to 4.3)	0.5 (0.3 to 1.0)	0.7 (0.3 to 1.2)
Motor neuron disease	Moderate motor impairment, moderate respiratory problems, and speech problems due to motor neuron disease	2.0 (1.7 to 2.3)	2.4 (2.0 to 2.7)	0.3 (0.3 to 0.4)	0.4 (0.4 to 0.5)	0.6 (0.4 to 0.8)	0.7 (0.5 to 1.0)
Motor neuron disease	Moderate motor impairment and speech problems due to motor neuron disease	8.3 (7.4 to 9.4)	10.1 (9.0 to 11.4)	1.4 (1.3 to 1.5)	1.8 (1.6 to 1.9)	0.9 (0.6 to 1.3)	1.1 (0.7 to 1.6)
Motor neuron disease	Severe motor impairment, moderate respiratory problems, and speech problems due to motor neuron disease	6.1 (5.5 to 6.9)	7.4 (6.6 to 8.4)	1.0 (0.9 to 1.1)	1.3 (1.2 to 1.4)	3.4 (2.5 to 4.5)	4.1 (3.0 to 5.4)
Motor neuron disease	Severe motor impairment and speech problems due to motor neuron disease	13.0 (11.7 to 14.6)	15.8 (14.1 to 17.8)	2.2 (2.0 to 2.4)	2.7 (2.5 to 3.0)	5.6 (3.9 to 7.6)	6.8 (4.7 to 9.2)
Motor neuron disease	Severe motor impairment, mild respiratory problems, and speech problems due to motor neuron disease	14.4 (12.9 to 15.9)	17.4 (15.7 to 19.4)	2.4 (2.2 to 2.6)	3.0 (2.7 to 3.3)	6.4 (4.4 to 8.5)	7.7 (5.4 to 10.3)
Motor neuron disease	Mild motor impairment and severe respiratory problems due to motor neuron disease	0.2 (0.1 to 0.3)	0.2 (0.1 to 0.3)	< 0.05 (< 0.05 to < 0.05)	< 0.05 (< 0.05 to 0.1)	0.1 (< 0.05 to 0.1)	0.1 (0.1 to 0.2)
Motor neuron disease	Moderate motor impairment due to motor neuron disease	55.1 (49.8 to 61.4)	66.8 (60.3 to 74.4)	9.1 (8.4 to 10.0)	11.6 (10.6 to 12.8)	3.3 (2.2 to 5.0)	4.0 (2.6 to 6.0)
Motor neuron disease	Mild respiratory problems due to motor neuron disease	1.8 (1.5 to 2.0)	2.1 (1.8 to 2.5)	0.3 (0.3 to 0.3)	0.4 (0.3 to 0.4)	< 0.05 (< 0.05 to 0.1)	< 0.05 (< 0.05 to 0.1)
Motor neuron disease	Mild motor impairment and mild respiratory problems due to motor neuron disease	6.1 (5.4 to 6.9)	7.4 (6.6 to 8.4)	1.0 (0.9 to 1.1)	1.3 (1.2 to 1.5)	0.5 (0.3 to 0.7)	0.6 (0.4 to 0.9)
Motor neuron disease	Mild motor impairment and speech problems due to motor neuron disease	5.6 (5.0 to 6.4)	6.8 (6.0 to 7.7)	0.9 (0.8 to 1.0)	1.2 (1.1 to 1.3)	0.3 (0.2 to 0.5)	0.4 (0.3 to 0.7)
Motor neuron disease	Severe motor impairment, severe respiratory problems, and speech problems due to motor neuron disease	3.7 (3.3 to 4.3)	4.5 (4.0 to 5.2)	0.6 (0.5 to 0.7)	0.8 (0.7 to 0.9)	2.5 (1.8 to 3.2)	3.0 (2.2 to 3.8)
Motor neuron disease	Moderate motor impairment and moderate respiratory problems due to motor neuron disease	3.6 (3.1 to 4.1)	4.3 (3.8 to 4.9)	0.6 (0.5 to 0.7)	0.7 (0.7 to 0.8)	0.8 (0.6 to 1.2)	1.0 (0.7 to 1.4)
Motor neuron disease	Moderate motor impairment, mild respiratory problems, and speech problems due to motor neuron disease	9.2 (8.2 to 10.3)	11.2 (9.9 to 12.5)	1.5 (1.4 to 1.7)	1.9 (1.8 to 2.2)	1.2 (0.7 to 1.7)	1.4 (0.9 to 2.1)
Motor neuron disease	Severe motor impairment and moderate respiratory problems due to motor neuron disease	5.5 (4.9 to 6.2)	6.7 (5.9 to 7.5)	0.9 (0.8 to 1.0)	1.2 (1.0 to 1.3)	2.9 (2.0 to 3.9)	3.6 (2.5 to 4.7)
Motor neuron disease	Moderate respiratory problems and speech problems due to motor neuron disease	0.4 (0.3 to 0.5)	0.5 (0.4 to 0.6)	0.1 (< 0.05 to 0.1)	0.1 (0.1 to 0.1)	0.1 (0.1 to 0.2)	0.1 (0.1 to 0.2)
Motor neuron disease	Moderate respiratory problems due to motor neuron disease	0.1 (0.1 to 0.2)	0.1 (0.1 to 0.2)	< 0.05 (< 0.05 to < 0.05)	< 0.05 (< 0.05 to < 0.05)	< 0.05 (< 0.05 to < 0.05)	< 0.05 (< 0.05 to 0.1)
Motor neuron disease	Mild motor impairment, mild respiratory problems, and speech problems due to motor neuron disease	4.9 (4.3 to 5.5)	5.9 (5.2 to 6.7)	0.8 (0.7 to 0.9)	1.0 (0.9 to 1.1)	0.4 (0.2 to 0.6)	0.5 (0.3 to 0.7)
Motor neuron disease	Diagnosis of motor neuron disease	10.2 (9.2 to 11.5)	12.4 (11.1 to 13.9)	1.7 (1.5 to 1.9)	2.2 (1.9 to 2.4)	0.1 (0.1 to 0.2)	0.2 (0.1 to 0.3)
Motor neuron disease	Severe motor impairment due to motor neuron disease	41.1 (37.3 to 45.9)	49.9 (45.2 to 55.6)	6.8 (6.2 to 7.4)	8.7 (7.9 to 9.5)	16.5 (11.0 to 22.6)	19.9 (13.3 to 27.3)
Motor neuron disease	Mild motor impairment due to motor neuron disease	28.4 (25.8 to 31.8)	34.5 (31.3 to 38.3)	4.7 (4.3 to 5.1)	6.0 (5.5 to 6.6)	0.3 (0.1 to 0.5)	0.3 (0.2 to 0.7)
Motor neuron disease	Mild respiratory problems and speech problems due to motor neuron disease	3.2 (2.8 to 3.6)	3.8 (3.4 to 4.4)	0.5 (0.5 to 0.6)	0.7 (0.6 to 0.8)	0.2 (0.1 to 0.3)	0.3 (0.2 to 0.4)
Motor neuron disease	Mild motor impairment, severe respiratory problems and speech problems due to motor neuron disease	0.2 (0.2 to 0.3)	0.3 (0.2 to 0.4)	< 0.05 (< 0.05 to 0.1)	< 0.05 (< 0.05 to 0.1)	0.1 (0.1 to 0.2)	0.1 (0.1 to 0.2)
Motor neuron disease	Severe respiratory problems and speech problems due to motor neuron disease	0.1 (< 0.05 to 0.1)	0.1 (< 0.05 to 0.1)	< 0.05 (< 0.05 to < 0.05)	< 0.05 (< 0.05 to < 0.05)	< 0.05 (< 0.05 to 0.1)	< 0.05 (< 0.05 to 0.1)
Motor neuron disease	Severe motor impairment and severe respiratory problems due to motor neuron disease	2.9 (2.5 to 3.3)	3.5 (3.1 to 4.0)	0.5 (0.4 to 0.5)	0.6 (0.5 to 0.7)	1.9 (1.3 to 2.4)	2.3 (1.6 to 2.9)
Motor neuron disease	Moderate motor impairment and severe respiratory problems due to motor neuron disease	1.1 (0.9 to 1.3)	1.4 (1.1 to 1.6)	0.2 (0.2 to 0.2)	0.2 (0.2 to 0.3)	0.5 (0.3 to 0.7)	0.6 (0.4 to 0.8)
Migraine	Asymptomatic migraine	786,465.0 (745,040.4 to 828,780.8)	887,754.7 (840,969.9 to 934,893.9)	73,480.3 (69,030.6 to 78,103.7)	80,165.6 (75,409.0 to 85,215.1)	< 0.05 (0.0 to < 0.05)	< 0.05 (0.0 to < 0.05)
Migraine	Symptomatic medication overuse due to migraine	47,774.4 (41,695.4 to 53,963.7)	56,915.4 (49,703.1 to 64,176.9)	14,712.7 (12,999.3 to 16,514.9)	17,243.0 (15,276.9 to 19,331.0)	9,883.1 (6,600.2 to 13,768.4)	11,755.2 (7,858.5 to 16,344.4)
Migraine	Symptomatic migraine	72,681.5 (53,238.2 to 95,422.9)	82,042.7 (60,082.2 to 107,639.4)	6,820.8 (4,906.9 to 8,841.5)	7,441.3 (5,367.1 to 9,648.3)	29,602.2 (18,156.8 to 43,262.7)	33,366.7 (20,434.2 to 48,835.7)
Migraine	Asymptomatic medication overuse headache due to migraine	15,158.1 (12,367.0 to 18,250.9)	18,058.6 (14,759.9 to 21,767.8)	4,664.4 (3,797.3 to 5,556.2)	5,466.6 (4,445.0 to 6,504.4)	< 0.05 (0.0 to < 0.05)	< 0.05 (0.0 to < 0.05)

Tension-type headache	Asymptomatic tension-type headache	1,615,192.2<br>(1,447,943.4 to 1,802,664.5)	1,783,528.3<br>(1,599,157.7 to 1,984,194.6)	934,851.9<br>(783,440.0 to 1,097,320.0)	1,009,705.1<br>(849,626.5 to 1,182,936.3)	< 0.05<br>(0.0 to < 0.05)	< 0.05<br>(0.0 to < 0.05)
Tension-type headache	Symptomatic tension-type headache	71,883.7<br>(42,262.5 to 109,583.2)	79,379.3<br>(47,020.0 to 121,269.9)	40,687.7<br>(22,219.2 to 65,220.6)	43,945.5<br>(24,058.0 to 70,380.4)	2,579.8<br>(1,210.7 to 4,787.2)	2,846.4<br>(1,340.2 to 5,273.2)
Tension-type headache	Symptomatic medication overuse due to tension-type headache	17,678.1<br>(12,833.0 to 23,035.3)	21,061.1<br>(15,311.1 to 27,506.5)	5,402.9<br>(3,877.4 to 6,999.2)	6,332.3<br>(4,543.6 to 8,207.8)	3,656.4<br>(2,194.1 to 5,589.8)	4,348.7<br>(2,616.2 to 6,624.7)
Tension-type headache	Asymptomatic medication overuse headache due to tension-type headache	5,625.5<br>(3,851.3 to 7,699.9)	6,701.7<br>(4,581.0 to 9,144.4)	1,719.7<br>(1,165.0 to 2,412.7)	2,015.4<br>(1,365.5 to 2,824.1)	< 0.05<br>(0.0 to < 0.05)	< 0.05<br>(0.0 to < 0.05)
Other neurological disorders	Other neurological disorders	--	--	--	--	1,367.0<br>(925.7 to 1,862.2)	1,778.6<br>(1,208.1 to 2,423.8)
Other neurological disorders	Guillain-Barre syndrome due to other neurological disorders	20.6<br>(13.5 to 28.4)	24.0<br>(15.6 to 33.2)	41.5<br>(27.2 to 57.0)	48.5<br>(31.8 to 66.5)	6.1<br>(3.2 to 10.0)	7.1<br>(3.8 to 11.5)
Schizophrenia	Schizophrenia residual state	6,563.6<br>(2,719.0 to 10,808.4)	7,676.4<br>(3,197.7 to 12,676.8)	387.4<br>(158.5 to 647.2)	418.6<br>(170.7 to 700.6)	3,520.9<br>(1,383.7 to 6,149.2)	4,109.9<br>(1,624.2 to 7,209.6)
Schizophrenia	Schizophrenia acute state	11,293.2<br>(6,962.7 to 15,675.0)	13,206.6<br>(8,205.2 to 18,302.8)	666.7<br>(407.4 to 907.0)	720.3<br>(438.5 to 980.4)	7,971.7<br>(4,604.6 to 11,500.7)	9,304.4<br>(5,400.0 to 13,360.2)
Alcohol use disorders	Very mild alcohol dependence	42,786.8<br>(38,125.1 to 47,444.6)	46,952.7<br>(41,570.6 to 52,590.6)	21,426.9<br>(18,744.5 to 24,337.0)	23,586.4<br>(20,573.2 to 26,850.3)	4,974.8<br>(3,323.4 to 7,049.8)	5,455.9<br>(3,628.2 to 7,764.5)
Alcohol use disorders	Moderate alcohol dependence	3,084.8<br>(2,142.4 to 4,201.7)	3,385.2<br>(2,353.0 to 4,606.3)	1,553.3<br>(1,082.0 to 2,111.2)	1,709.9<br>(1,184.8 to 2,316.6)	1,066.9<br>(646.4 to 1,623.6)	1,169.5<br>(710.5 to 1,787.9)
Alcohol use disorders	Mild fetal alcohol syndrome	98.6<br>(73.4 to 122.9)	109.2<br>(81.5 to 136.4)	--	--	1.6<br>(0.7 to 3.0)	1.8<br>(0.8 to 3.4)
Alcohol use disorders	Asymptomatic fetal alcohol syndrome	43.3<br>(31.3 to 57.8)	48.0<br>(34.6 to 63.9)	< 0.05<br>(0.0 to < 0.05)	< 0.05<br>(0.0 to < 0.05)	< 0.05<br>(0.0 to < 0.05)	< 0.05<br>(0.0 to < 0.05)
Alcohol use disorders	Mild alcohol dependence	3,630.5<br>(2,038.7 to 5,771.7)	3,983.7<br>(2,240.7 to 6,350.6)	1,846.2<br>(1,025.6 to 2,865.5)	2,031.8<br>(1,138.3 to 3,169.7)	799.0<br>(387.0 to 1,413.3)	876.6<br>(432.8 to 1,547.6)
Alcohol use disorders	Severe alcohol dependence	4,368.4<br>(2,713.7 to 6,280.9)	4,794.4<br>(2,992.8 to 6,901.2)	2,228.4<br>(1,405.2 to 3,231.3)	2,453.2<br>(1,543.4 to 3,570.6)	2,285.5<br>(1,264.6 to 3,462.5)	2,508.0<br>(1,373.1 to 3,789.1)
Alcohol use disorders	Asymptomatic alcohol dependence	37,247.2<br>(33,064.1 to 41,958.9)	40,872.9<br>(35,924.0 to 46,103.2)	18,759.9<br>(16,310.5 to 21,212.7)	20,650.9<br>(17,846.2 to 23,467.7)	< 0.05<br>(0.0 to < 0.05)	< 0.05<br>(0.0 to < 0.05)
Alcohol use disorders	Severe fetal alcohol syndrome	41.1<br>(28.0 to 55.7)	44.2<br>(29.6 to 60.6)	--	--	7.4<br>(4.1 to 11.7)	7.9<br>(4.4 to 12.6)
Alcohol use disorders	Moderate fetal alcohol syndrome	178.9<br>(145.9 to 211.3)	199.0<br>(162.0 to 234.5)	--	--	9.9<br>(6.1 to 15.1)	11.0<br>(6.9 to 16.8)
Opioid use disorders	Mild opioid dependence	8,260.2<br>(5,388.1 to 11,347.2)	9,757.3<br>(6,352.0 to 13,414.7)	905.4<br>(550.7 to 1,270.7)	1,014.8<br>(620.5 to 1,431.7)	2,570.7<br>(1,425.9 to 3,970.3)	3,032.0<br>(1,694.4 to 4,657.2)
Opioid use disorders	Severe opioid dependence	10,769.1<br>(7,754.1 to 13,872.7)	12,721.0<br>(9,238.6 to 16,412.6)	1,188.1<br>(847.5 to 1,547.9)	1,331.7<br>(950.6 to 1,740.1)	6,865.3<br>(4,401.2 to 9,539.2)	8,099.9<br>(5,193.9 to 11,253.9)
Opioid use disorders	Asymptomatic opioid dependence	3,687.7<br>(2,695.8 to 5,037.9)	4,356.1<br>(3,171.4 to 5,961.4)	400.4<br>(289.1 to 548.5)	448.8<br>(324.2 to 610.5)	< 0.05<br>(0.0 to < 0.05)	< 0.05<br>(0.0 to < 0.05)
Cocaine use disorders	Asymptomatic cocaine dependence	2,623.5<br>(2,121.4 to 3,160.9)	2,938.2<br>(2,373.3 to 3,548.7)	168.7<br>(134.3 to 205.3)	174.2<br>(138.6 to 210.9)	< 0.05<br>(0.0 to < 0.05)	< 0.05<br>(0.0 to < 0.05)
Cocaine use disorders	Mild cocaine dependence	1,317.1<br>(972.2 to 1,717.3)	1,475.2<br>(1,089.1 to 1,930.6)	85.0<br>(62.3 to 111.4)	87.8<br>(64.4 to 115.4)	145.4<br>(84.1 to 226.8)	162.7<br>(94.6 to 254.0)
Cocaine use disorders	Severe cocaine dependence	1,274.1<br>(900.9 to 1,690.8)	1,426.9<br>(1,009.5 to 1,900.8)	82.2<br>(59.7 to 111.6)	84.9<br>(61.9 to 114.8)	567.6<br>(335.5 to 836.0)	634.5<br>(377.5 to 936.4)
Amphetamine use disorders	Severe amphetamine dependence	1,223.2<br>(797.2 to 1,785.1)	1,267.5<br>(811.7 to 1,855.8)	118.3<br>(75.0 to 178.4)	111.6<br>(71.5 to 166.0)	562.3<br>(316.4 to 904.2)	583.1<br>(329.6 to 938.6)
Amphetamine use disorders	Asymptomatic amphetamine dependence	2,624.8<br>(1,811.0 to 3,608.9)	2,720.2<br>(1,856.2 to 3,756.9)	257.6<br>(183.2 to 351.3)	243.1<br>(173.1 to 329.8)	< 0.05<br>(0.0 to < 0.05)	< 0.05<br>(0.0 to < 0.05)
Amphetamine use disorders	Mild amphetamine dependence	933.6<br>(588.4 to 1,446.3)	967.4<br>(606.8 to 1,502.2)	90.3<br>(57.7 to 130.2)	85.2<br>(54.5 to 123.3)	71.8<br>(36.5 to 123.3)	74.4<br>(37.7 to 128.1)
Cannabis use disorders	Severe cannabis dependence	1,300.4<br>(798.9 to 1,924.2)	1,347.9<br>(830.7 to 2,009.2)	194.5<br>(122.6 to 282.4)	192.2<br>(120.5 to 278.3)	329.2<br>(179.1 to 535.5)	341.5<br>(184.3 to 550.1)
Cannabis use disorders	Mild cannabis dependence	7,689.2<br>(6,303.0 to 9,207.2)	7,969.7<br>(6,562.6 to 9,543.4)	1,159.7<br>(931.1 to 1,439.6)	1,146.2<br>(934.3 to 1,423.5)	294.7<br>(168.7 to 468.7)	305.4<br>(174.8 to 485.2)
Cannabis use disorders	Asymptomatic cannabis dependence	12,327.1<br>(10,298.4 to 14,673.6)	12,776.9<br>(10,689.4 to 15,191.7)	1,866.5<br>(1,536.7 to 2,232.1)	1,844.6<br>(1,532.4 to 2,196.7)	< 0.05<br>(0.0 to < 0.05)	< 0.05<br>(0.0 to < 0.05)
Other drug use disorders	Other drug use disorders	3,365.5<br>(3,029.6 to 3,705.6)	3,944.0<br>(3,536.6 to 4,361.6)	607.9<br>(546.2 to 674.4)	694.9<br>(624.4 to 769.6)	367.5<br>(237.8 to 518.1)	429.9<br>(277.0 to 609.6)
Major depressive disorder	Mild major depressive disorder	89,755.5<br>(76,511.2 to 103,967.5)	99,992.2<br>(85,488.7 to 115,717.5)	137,965.6<br>(119,433.9 to 160,873.1)	153,275.8<br>(132,133.0 to 178,557.7)	12,185.8<br>(8,055.3 to 17,459.6)	13,553.4<br>(8,971.0 to 19,504.8)
Major depressive disorder	Severe major depressive disorder	15,391.6<br>(6,083.9 to 27,676.5)	17,148.8<br>(6,757.7 to 31,130.7)	23,396.5<br>(8,140.8 to 42,888.7)	25,995.1<br>(9,030.7 to 47,828.1)	9,101.9<br>(3,674.0 to 16,835.7)	10,118.3<br>(4,063.2 to 18,783.7)
Major depressive disorder	Moderate major depressive disorder	26,018.3<br>(19,353.0 to 33,314.4)	28,987.6<br>(21,489.5 to 37,214.7)	40,303.0<br>(29,835.5 to 52,153.2)	44,776.3<br>(33,152.4 to 58,156.1)	9,382.6<br>(5,871.7 to 13,355.2)	10,433.0<br>(6,511.1 to 14,918.4)
Major depressive disorder	Major depressive disorder, currently without symptoms	19,485.2<br>(14,651.4 to 25,103.4)	21,707.8<br>(16,284.4 to 28,014.0)	30,352.1<br>(22,420.3 to 39,256.1)	33,721.0<br>(24,898.9 to 43,513.6)	< 0.05<br>(0.0 to < 0.05)	< 0.05<br>(0.0 to < 0.05)
Dysthymia	Symptomatic dysthymia	61,889.0<br>(53,478.0 to 71,388.7)	74,732.6<br>(64,676.2 to 86,618.5)	10,176.4<br>(8,866.3 to 11,773.8)	11,996.0<br>(10,420.7 to 13,859.7)	8,382.0<br>(5,676.3 to 12,123.1)	10,103.8<br>(6,860.6 to 14,611.5)
Dysthymia	Dysthymia, currently without symptoms	25,545.9<br>(20,488.3 to 31,587.4)	30,847.7<br>(24,649.6 to 38,256.9)	4,190.1<br>(3,328.0 to 5,111.6)	4,939.4<br>(3,937.4 to 6,027.2)	< 0.05<br>(0.0 to < 0.05)	< 0.05<br>(0.0 to < 0.05)
Bipolar disorder	Bipolar disorder residual state	20,535.5<br>(14,177.6 to 27,046.9)	23,617.7<br>(16,317.5 to 30,991.7)	1,847.9<br>(1,263.0 to 2,436.5)	2,042.6<br>(1,396.5 to 2,691.4)	630.2<br>(346.9 to 1,035.0)	724.1<br>(401.6 to 1,185.5)
Bipolar disorder	Bipolar disorder manic state	8,355.9<br>(4,686.3 to 13,373.0)	9,610.7<br>(5,365.3 to 15,373.2)	753.0<br>(420.0 to 1,200.7)	832.3<br>(465.5 to 1,323.5)	3,769.6<br>(1,926.9 to 6,421.3)	4,329.5<br>(2,220.3 to 7,379.7)
Bipolar disorder	Bipolar disorder depressive state	9,285.4<br>(4,667.5 to 15,358.6)	10,679.3<br>(5,362.5 to 17,693.8)	815.8<br>(409.8 to 1,293.0)	901.8<br>(450.9 to 1,425.2)	3,395.3<br>(1,541.5 to 6,138.7)	3,900.4<br>(1,762.4 to 7,047.4)
Anxiety disorders	Moderate anxiety disorders	46,436.7<br>(38,744.1 to 54,784.2)	52,609.6<br>(43,933.6 to 62,198.5)	7,268.8<br>(5,978.8 to 8,606.7)	8,111.1<br>(6,676.4 to 9,622.0)	5,846.1<br>(3,765.0 to 8,341.7)	6,611.7<br>(4,251.2 to 9,437.3)
Anxiety disorders	Severe anxiety disorders	30,813.1<br>(22,693.9 to 39,625.5)	34,910.4<br>(25,710.7 to 44,894.8)	4,820.0<br>(3,575.8 to 6,135.9)	5,378.6<br>(3,983.6 to 6,846.8)	14,805.1<br>(9,241.1 to 21,303.1)	16,735.7<br>(10,437.8 to 24,040.0)
Anxiety disorders	Anxiety disorders, currently without symptoms	69,999.4<br>(63,063.2 to 77,350.1)	79,305.9<br>(71,309.4 to 87,486.0)	10,992.7<br>(9,912.3 to 12,241.5)	12,266.7<br>(11,081.0 to 13,665.7)	< 0.05<br>(0.0 to < 0.05)	< 0.05<br>(0.0 to < 0.05)
Anxiety disorders	Mild anxiety disorders	95,140.8<br>(83,424.0 to 107,047.7)	107,789.4<br>(94,710.7 to 120,866.2)	14,921.7<br>(13,145.1 to 16,786.5)	16,651.0<br>(14,597.4 to 18,759.5)	2,713.3<br>(1,631.5 to 4,220.9)	3,070.0<br>(1,826.1 to 4,779.3)

Anorexia nervosa	Anorexia nervosa	2,322.8 (1,724.9 to 3,049.9)	2,606.1 (1,931.7 to 3,450.1)	808.6 (594.6 to 1,074.4)	897.1 (665.0 to 1,182.9)	495.1 (303.1 to 745.4)	555.5 (337.9 to 829.5)
Bulimia nervosa	Bulimia nervosa	6,341.2 (4,818.2 to 8,340.8)	7,558.8 (5,764.9 to 9,931.0)	4,112.3 (2,851.2 to 5,750.5)	4,542.7 (3,168.7 to 6,256.5)	1,335.4 (815.8 to 1,994.0)	1,592.2 (971.6 to 2,391.5)
Autism	Autism with moderate intellectual disability	3,301.5 (1,950.7 to 4,844.3)	3,679.9 (2,178.2 to 5,401.5)	--	--	1,015.0 (539.3 to 1,612.1)	1,128.8 (599.8 to 1,787.0)
Autism	Autism without intellectual disability	2,767.4 (1,615.4 to 4,241.5)	3,084.6 (1,793.6 to 4,728.9)	48.0 (24.9 to 81.1)	50.9 (26.4 to 85.8)	554.4 (264.1 to 951.2)	616.6 (294.3 to 1,055.4)
Autism	Autism with mild intellectual disability	6,437.6 (4,152.3 to 8,650.3)	7,175.3 (4,617.2 to 9,654.7)	--	--	1,538.3 (854.0 to 2,331.9)	1,711.5 (957.8 to 2,591.4)
Autism	Autism with severe intellectual disability	987.1 (0.1 to 4,875.4)	1,100.5 (0.1 to 5,418.9)	--	--	404.6 (< 0.05 to 1,889.1)	450.7 (< 0.05 to 2,117.9)
Autism	Autism with profound intellectual disability	177.0 (< 0.05 to 2,014.4)	197.3 (< 0.05 to 2,264.9)	--	--	94.5 (< 0.05 to 1,071.1)	105.4 (< 0.05 to 1,192.5)
Autism	Autism with borderline intellectual disability	2,749.5 (1,521.6 to 4,145.4)	3,064.5 (1,700.7 to 4,632.5)	--	--	571.4 (272.7 to 958.9)	636.0 (307.2 to 1,068.2)
Asperger syndrome and other autistic spectrum disorders	Asperger syndrome and other autistic spectrum disorders	39,237.1 (32,582.9 to 46,974.0)	43,797.9 (36,424.7 to 52,402.6)	381.0 (246.4 to 543.7)	399.4 (257.6 to 572.3)	3,926.4 (2,574.5 to 5,735.4)	4,376.7 (2,864.1 to 6,392.9)
Attention-deficit/hyperactivity disorder	Attention-deficit/hyperactivity disorder, currently without symptoms	42,606.8 (37,701.6 to 47,656.2)	45,221.9 (40,136.3 to 50,646.4)	1,900.3 (1,654.3 to 2,226.8)	2,072.1 (1,808.1 to 2,415.8)	< 0.05 (0.0 to < 0.05)	< 0.05 (0.0 to < 0.05)
Attention-deficit/hyperactivity disorder	Symptomatic attention-deficit/hyperactivity disorder	16,395.9 (13,045.2 to 20,043.5)	17,402.3 (13,847.1 to 21,281.0)	734.0 (573.9 to 905.0)	800.4 (625.8 to 986.6)	711.9 (426.0 to 1,134.5)	755.2 (452.2 to 1,196.6)
Conduct disorder	Conduct disorder, currently without symptoms	22,898.3 (17,449.6 to 28,738.9)	23,383.8 (17,818.0 to 29,452.5)	7,770.6 (5,660.8 to 10,115.9)	8,091.4 (5,906.7 to 10,552.3)	< 0.05 (0.0 to < 0.05)	< 0.05 (0.0 to < 0.05)
Conduct disorder	Symptomatic conduct disorder	25,089.2 (19,612.2 to 31,427.0)	25,621.2 (19,987.0 to 32,004.9)	8,446.4 (6,315.7 to 10,897.9)	8,795.2 (6,588.2 to 11,350.7)	5,820.5 (3,615.8 to 8,803.8)	5,947.3 (3,701.9 to 8,998.5)
Idiopathic developmental intellectual disability	Severe idiopathic developmental intellectual disability	724.0 (317.8 to 1,344.1)	664.2 (331.0 to 1,232.7)	--	--	110.3 (43.9 to 214.3)	101.6 (44.4 to 198.8)
Idiopathic developmental intellectual disability	Mild idiopathic developmental intellectual disability	67,421.9 (24,202.5 to 110,002.3)	71,212.7 (25,590.2 to 116,100.0)	--	--	2,788.1 (836.9 to 5,261.6)	2,942.8 (880.7 to 5,536.1)
Idiopathic developmental intellectual disability	Profound idiopathic developmental intellectual disability	3,160.5 (979.2 to 5,857.6)	3,347.1 (1,019.5 to 6,186.3)	--	--	599.3 (161.4 to 1,238.3)	633.6 (168.4 to 1,312.5)
Idiopathic developmental intellectual disability	Borderline idiopathic developmental intellectual disability	32,193.1 (23,557.6 to 40,492.5)	33,477.2 (24,405.0 to 42,145.0)	--	--	339.5 (154.5 to 635.3)	352.7 (159.5 to 653.3)
Idiopathic developmental intellectual disability	Moderate idiopathic developmental intellectual disability	6,964.3 (3,150.4 to 11,564.1)	6,096.0 (2,329.7 to 10,914.2)	--	--	663.2 (270.4 to 1,230.8)	580.2 (193.9 to 1,137.1)
Other mental and substance use disorders	Other mental disorders, currently without symptoms	16,282.1 (12,437.5 to 20,571.1)	19,213.1 (14,674.2 to 24,268.0)	587.0 (449.5 to 743.9)	620.1 (473.6 to 784.9)	< 0.05 (0.0 to < 0.05)	< 0.05 (0.0 to < 0.05)
Other mental and substance use disorders	Mild other mental disorders	74,542.1 (63,917.2 to 86,383.7)	87,959.3 (75,498.6 to 101,889.2)	2,702.9 (2,327.0 to 3,110.5)	2,855.4 (2,469.1 to 3,280.4)	2,120.1 (1,237.4 to 3,311.6)	2,498.8 (1,459.5 to 3,897.6)
Other mental and substance use disorders	Severe other mental disorders	9,238.2 (5,591.0 to 13,786.3)	10,901.1 (6,608.7 to 16,272.1)	336.4 (201.0 to 500.9)	355.4 (212.7 to 530.0)	4,399.7 (2,439.4 to 7,353.6)	5,180.7 (2,891.6 to 8,633.2)
Other mental and substance use disorders	Moderate other mental disorders	18,162.3 (13,976.0 to 22,938.1)	21,431.4 (16,480.2 to 27,035.8)	655.5 (509.8 to 821.2)	692.5 (538.5 to 862.1)	2,272.6 (1,463.8 to 3,302.6)	2,677.6 (1,729.3 to 3,873.8)
Diabetes mellitus	Diabetic neuropathy and amputation without treatment	3,456.4 (3,022.7 to 3,939.1)	4,259.3 (3,693.7 to 4,869.8)	< 0.05 (0.0 to < 0.05)	< 0.05 (0.0 to < 0.05)	866.4 (606.5 to 1,168.9)	1,066.6 (753.2 to 1,436.6)
Diabetes mellitus	Moderate vision impairment due to diabetes mellitus	1,430.1 (1,153.6 to 1,751.1)	1,852.1 (1,474.9 to 2,297.9)	--	--	41.3 (25.0 to 67.0)	53.4 (32.3 to 86.9)
Diabetes mellitus	Diabetic neuropathy	83,054.0 (67,570.0 to 100,466.6)	105,576.3 (85,515.4 to 127,846.9)	< 0.05 (0.0 to < 0.05)	< 0.05 (0.0 to < 0.05)	10,143.2 (6,498.9 to 14,756.1)	12,880.5 (8,300.7 to 18,842.7)
Diabetes mellitus	Diabetic neuropathy and amputation with treatment	2,001.6 (1,652.5 to 2,398.5)	2,533.6 (2,089.4 to 3,035.6)	< 0.05 (0.0 to < 0.05)	< 0.05 (0.0 to < 0.05)	300.8 (203.3 to 416.3)	380.1 (255.7 to 529.0)
Diabetes mellitus	Diabetic foot due to neuropathy	14,670.6 (11,894.3 to 17,989.3)	18,582.6 (14,983.9 to 22,879.5)	< 0.05 (0.0 to < 0.05)	< 0.05 (0.0 to < 0.05)	1,984.9 (1,316.1 to 2,827.6)	2,510.3 (1,655.3 to 3,562.8)
Diabetes mellitus	Blindness due to diabetes mellitus	329.8 (254.0 to 415.3)	494.2 (378.6 to 626.9)	--	--	56.5 (35.5 to 82.4)	84.2 (52.3 to 122.3)
Diabetes mellitus	Severe vision impairment due to diabetes mellitus	290.8 (223.5 to 376.5)	384.1 (294.3 to 493.6)	--	--	48.8 (30.7 to 71.3)	64.4 (40.4 to 95.2)
Diabetes mellitus	Uncomplicated diabetes mellitus	209,093.1 (186,354.5 to 231,010.2)	249,770.8 (220,977.0 to 277,814.1)	17,571.8 (16,272.3 to 18,967.2)	20,827.8 (19,160.5 to 22,734.0)	9,680.1 (6,139.6 to 14,322.5)	11,544.1 (7,349.6 to 16,900.6)
Acute glomerulonephritis	Acute glomerulonephritis	99.2 (86.5 to 113.3)	103.5 (90.1 to 117.3)	1,454.9 (1,281.4 to 1,661.1)	1,530.2 (1,356.6 to 1,728.8)	5.0 (3.1 to 7.5)	5.2 (3.2 to 7.8)
Chronic kidney disease due to diabetes mellitus	Stage IV chronic kidney disease untreated without anaemia due to diabetes mellitus	1,586.4 (1,250.7 to 1,972.9)	1,754.8 (1,382.5 to 2,214.5)	< 0.05 (0.0 to < 0.05)	< 0.05 (0.0 to < 0.05)	150.9 (97.6 to 221.6)	166.7 (107.5 to 244.3)
Chronic kidney disease due to diabetes mellitus	Stage IV chronic kidney disease untreated and mild anaemia due to diabetes mellitus	311.8 (252.5 to 378.2)	399.9 (320.6 to 485.8)	< 0.05 (0.0 to < 0.05)	< 0.05 (0.0 to < 0.05)	30.3 (19.9 to 43.7)	38.9 (25.8 to 55.7)
Chronic kidney disease due to diabetes mellitus	End-stage renal disease after transplant due to diabetes mellitus	292.0 (261.0 to 323.3)	365.6 (324.0 to 410.6)	--	--	6.9 (3.9 to 11.1)	8.6 (4.8 to 14.0)
Chronic kidney disease due to diabetes mellitus	Stage V chronic kidney disease untreated and moderate anaemia due to diabetes mellitus	1,303.2 (1,103.4 to 1,544.9)	1,642.7 (1,379.8 to 1,950.9)	< 0.05 (0.0 to < 0.05)	< 0.05 (0.0 to < 0.05)	673.7 (476.6 to 880.6)	848.0 (600.3 to 1,113.1)
Chronic kidney disease due to diabetes mellitus	Stage V chronic kidney disease untreated without anaemia due to diabetes mellitus	447.5 (340.2 to 578.6)	509.5 (388.2 to 666.4)	< 0.05 (0.0 to < 0.05)	< 0.05 (0.0 to < 0.05)	227.7 (145.7 to 320.5)	258.6 (165.3 to 368.0)
Chronic kidney disease due to diabetes mellitus	Stage IV chronic kidney disease untreated and moderate anaemia due to diabetes mellitus	2,171.8 (1,780.6 to 2,633.9)	2,777.2 (2,256.3 to 3,352.7)	< 0.05 (0.0 to < 0.05)	< 0.05 (0.0 to < 0.05)	292.6 (196.9 to 412.2)	374.0 (251.8 to 532.7)
Chronic kidney disease due to diabetes mellitus	Stage III chronic kidney disease and mild anaemia due to diabetes mellitus	17,483.4 (15,483.2 to 19,704.7)	26,779.4 (23,508.0 to 30,581.3)	< 0.05 (0.0 to < 0.05)	< 0.05 (0.0 to < 0.05)	60.6 (21.6 to 133.5)	92.8 (32.6 to 204.1)
Chronic kidney disease due to diabetes mellitus	Stage III chronic kidney disease and severe anaemia due to diabetes mellitus	422.0 (368.2 to 481.7)	678.1 (590.1 to 774.9)	< 0.05 (0.0 to < 0.05)	< 0.05 (0.0 to < 0.05)	56.4 (38.4 to 78.2)	90.6 (61.3 to 126.1)
Chronic kidney disease due to diabetes mellitus	Stage III chronic kidney disease and moderate anaemia due to diabetes mellitus	11,104.8 (9,677.9 to 12,697.3)	15,333.3 (13,282.8 to 17,585.2)	< 0.05 (0.0 to < 0.05)	< 0.05 (0.0 to < 0.05)	528.5 (344.0 to 768.8)	729.7 (470.9 to 1,058.3)
Chronic kidney disease due to diabetes mellitus	Stage V chronic kidney disease untreated and mild anaemia due to diabetes mellitus	48.3 (40.3 to 56.9)	67.5 (56.7 to 79.6)	< 0.05 (0.0 to < 0.05)	< 0.05 (0.0 to < 0.05)	25.7 (17.6 to 34.6)	36.0 (24.2 to 48.4)
Chronic kidney disease due to diabetes mellitus	Stage V chronic kidney disease untreated and severe anaemia due to diabetes mellitus	226.9 (189.3 to 270.5)	400.5 (334.8 to 478.9)	< 0.05 (0.0 to < 0.05)	< 0.05 (0.0 to < 0.05)	125.9 (90.0 to 164.0)	223.2 (158.9 to 291.4)
Chronic kidney disease due to diabetes mellitus	End-stage renal disease on dialysis due to diabetes mellitus	1,114.0 (999.3 to 1,229.5)	1,535.1 (1,367.6 to 1,710.9)	--	--	555.8 (388.5 to 710.6)	764.6 (536.3 to 982.7)

Chronic kidney disease due to diabetes mellitus	Stage IV chronic kidney disease untreated and severe anaemia due to diabetes mellitus	167.6 (137.9 to 204.1)	299.0 (242.0 to 367.4)	< 0.05 (0.0 to < 0.05)	< 0.05 (0.0 to < 0.05)	35.8 (24.4 to 50.0)	63.8 (42.9 to 89.3)
Chronic kidney disease due to diabetes mellitus	Stage III chronic kidney disease without anaemia due to diabetes mellitus	49,147.8 (43,058.6 to 55,630.6)	62,813.4 (54,835.4 to 71,956.3)	6,635.3 (5,795.1 to 7,610.6)	8,978.8 (7,741.6 to 10,376.8)	< 0.05 (0.0 to < 0.05)	< 0.05 (0.0 to < 0.05)
Chronic kidney disease due to hypertension	Stage V chronic kidney disease untreated and severe anaemia due to hypertension	122.7 (102.0 to 144.2)	205.0 (169.1 to 243.3)	< 0.05 (0.0 to < 0.05)	< 0.05 (0.0 to < 0.05)	68.8 (48.4 to 90.7)	114.7 (80.2 to 152.4)
Chronic kidney disease due to hypertension	Stage IV chronic kidney disease untreated without anaemia due to hypertension	722.5 (566.0 to 894.6)	823.9 (648.1 to 1,032.7)	< 0.05 (0.0 to < 0.05)	< 0.05 (0.0 to < 0.05)	68.1 (43.0 to 98.5)	77.5 (48.2 to 112.1)
Chronic kidney disease due to hypertension	Stage IV chronic kidney disease untreated and severe anaemia due to hypertension	102.4 (84.3 to 123.4)	169.7 (137.3 to 204.2)	< 0.05 (0.0 to < 0.05)	< 0.05 (0.0 to < 0.05)	22.2 (14.7 to 30.8)	36.3 (24.1 to 50.7)
Chronic kidney disease due to hypertension	Stage IV chronic kidney disease untreated and moderate anaemia due to hypertension	1,080.8 (865.2 to 1,324.4)	1,410.9 (1,117.3 to 1,747.5)	< 0.05 (0.0 to < 0.05)	< 0.05 (0.0 to < 0.05)	143.0 (93.4 to 200.3)	186.6 (120.8 to 261.8)
Chronic kidney disease due to hypertension	Stage V chronic kidney disease untreated without anaemia due to hypertension	166.7 (127.0 to 211.6)	198.2 (151.0 to 254.6)	< 0.05 (0.0 to < 0.05)	< 0.05 (0.0 to < 0.05)	86.9 (55.8 to 124.7)	103.0 (65.4 to 148.6)
Chronic kidney disease due to hypertension	Stage III chronic kidney disease and severe anaemia due to hypertension	241.5 (205.2 to 279.0)	347.3 (293.8 to 401.6)	< 0.05 (0.0 to < 0.05)	< 0.05 (0.0 to < 0.05)	31.6 (20.7 to 43.7)	45.5 (29.5 to 62.8)
Chronic kidney disease due to hypertension	Stage III chronic kidney disease and moderate anaemia due to hypertension	5,959.1 (5,141.1 to 6,843.5)	8,436.3 (7,237.6 to 9,707.0)	< 0.05 (0.0 to < 0.05)	< 0.05 (0.0 to < 0.05)	279.9 (175.4 to 402.5)	396.5 (245.9 to 570.4)
Chronic kidney disease due to hypertension	Stage V chronic kidney disease untreated and moderate anaemia due to hypertension	488.7 (404.3 to 577.1)	630.8 (517.2 to 748.6)	< 0.05 (0.0 to < 0.05)	< 0.05 (0.0 to < 0.05)	249.9 (174.1 to 331.0)	321.6 (220.1 to 429.1)
Chronic kidney disease due to hypertension	End-stage renal disease on dialysis due to hypertension	411.6 (354.3 to 473.4)	594.2 (505.4 to 685.7)	--	--	204.1 (139.9 to 267.3)	294.2 (201.3 to 388.0)
Chronic kidney disease due to hypertension	End-stage renal disease after transplant due to hypertension	71.6 (61.4 to 84.0)	91.0 (77.1 to 108.1)	--	--	1.7 (1.0 to 2.8)	2.2 (1.2 to 3.6)
Chronic kidney disease due to hypertension	Stage IV chronic kidney disease untreated and mild anaemia due to hypertension	192.1 (154.8 to 235.5)	251.3 (200.6 to 310.2)	< 0.05 (0.0 to < 0.05)	< 0.05 (0.0 to < 0.05)	18.6 (12.0 to 26.3)	24.3 (15.6 to 34.5)
Chronic kidney disease due to hypertension	Stage V chronic kidney disease untreated and mild anaemia due to hypertension	48.9 (40.6 to 58.3)	63.4 (52.3 to 75.9)	< 0.05 (0.0 to < 0.05)	< 0.05 (0.0 to < 0.05)	26.5 (17.3 to 36.1)	34.4 (22.4 to 47.1)
Chronic kidney disease due to hypertension	Stage III chronic kidney disease and mild anaemia due to hypertension	8,175.8 (7,026.0 to 9,319.7)	12,518.4 (10,649.9 to 14,420.0)	< 0.05 (0.0 to < 0.05)	< 0.05 (0.0 to < 0.05)	27.9 (10.2 to 62.8)	42.8 (15.6 to 95.8)
Chronic kidney disease due to hypertension	Stage III chronic kidney disease without anaemia due to hypertension	21,502.6 (18,661.0 to 24,416.4)	24,356.5 (20,807.7 to 28,120.2)	3,087.3 (2,705.4 to 3,478.0)	3,626.2 (3,131.3 to 4,163.4)	< 0.05 (0.0 to < 0.05)	< 0.05 (0.0 to < 0.05)
Chronic kidney disease due to glomerulonephritis	Stage V chronic kidney disease untreated and moderate anaemia due to glomerulonephritis	550.7 (456.0 to 659.3)	645.1 (529.6 to 782.6)	< 0.05 (0.0 to < 0.05)	< 0.05 (0.0 to < 0.05)	296.3 (204.5 to 393.2)	347.3 (238.5 to 463.9)
Chronic kidney disease due to glomerulonephritis	Stage III chronic kidney disease and moderate anaemia due to glomerulonephritis	5,383.1 (4,674.8 to 6,162.9)	6,783.3 (5,805.8 to 7,848.0)	< 0.05 (0.0 to < 0.05)	< 0.05 (0.0 to < 0.05)	258.2 (167.5 to 379.6)	325.6 (211.4 to 477.0)
Chronic kidney disease due to glomerulonephritis	Stage IV chronic kidney disease untreated and mild anaemia due to glomerulonephritis	207.7 (170.1 to 251.8)	243.3 (199.4 to 293.1)	< 0.05 (0.0 to < 0.05)	< 0.05 (0.0 to < 0.05)	21.1 (13.9 to 30.1)	24.9 (16.4 to 35.8)
Chronic kidney disease due to glomerulonephritis	Stage IV chronic kidney disease untreated without anaemia due to glomerulonephritis	730.0 (582.9 to 907.1)	739.7 (591.6 to 917.9)	< 0.05 (0.0 to < 0.05)	< 0.05 (0.0 to < 0.05)	71.3 (45.7 to 104.4)	72.3 (46.1 to 105.5)
Chronic kidney disease due to glomerulonephritis	Stage III chronic kidney disease without anaemia due to glomerulonephritis	22,368.7 (19,199.7 to 25,632.3)	29,126.6 (24,998.5 to 33,505.6)	2,833.6 (2,409.5 to 3,284.5)	3,893.1 (3,261.3 to 4,529.1)	< 0.05 (0.0 to < 0.05)	< 0.05 (0.0 to < 0.05)
Chronic kidney disease due to glomerulonephritis	Stage IV chronic kidney disease untreated and moderate anaemia due to glomerulonephritis	883.8 (728.3 to 1,060.8)	998.3 (811.8 to 1,205.5)	< 0.05 (0.0 to < 0.05)	< 0.05 (0.0 to < 0.05)	121.3 (82.3 to 168.6)	137.1 (92.2 to 193.9)
Chronic kidney disease due to glomerulonephritis	Stage V chronic kidney disease untreated and mild anaemia due to glomerulonephritis	78.7 (63.5 to 95.6)	97.8 (79.0 to 119.1)	< 0.05 (0.0 to < 0.05)	< 0.05 (0.0 to < 0.05)	44.0 (29.3 to 60.5)	54.6 (36.1 to 75.4)
Chronic kidney disease due to glomerulonephritis	End-stage renal disease after transplant due to glomerulonephritis	127.2 (110.5 to 145.3)	147.1 (126.4 to 170.2)	--	--	3.1 (1.7 to 5.0)	3.5 (1.9 to 5.9)
Chronic kidney disease due to glomerulonephritis	Stage V chronic kidney disease untreated and severe anaemia due to glomerulonephritis	132.8 (110.7 to 158.3)	190.2 (158.1 to 230.4)	< 0.05 (0.0 to < 0.05)	< 0.05 (0.0 to < 0.05)	79.2 (55.7 to 103.3)	113.6 (79.7 to 149.4)
Chronic kidney disease due to glomerulonephritis	Stage IV chronic kidney disease untreated and severe anaemia due to glomerulonephritis	109.9 (88.6 to 134.9)	157.8 (128.2 to 195.1)	< 0.05 (0.0 to < 0.05)	< 0.05 (0.0 to < 0.05)	25.2 (16.8 to 35.7)	36.3 (24.3 to 52.4)
Chronic kidney disease due to glomerulonephritis	Stage V chronic kidney disease untreated without anaemia due to glomerulonephritis	232.3 (178.1 to 293.7)	237.7 (181.9 to 300.5)	< 0.05 (0.0 to < 0.05)	< 0.05 (0.0 to < 0.05)	125.7 (80.1 to 179.5)	129.6 (83.6 to 184.8)
Chronic kidney disease due to glomerulonephritis	Stage III chronic kidney disease and severe anaemia due to glomerulonephritis	176.4 (151.3 to 202.1)	239.8 (205.0 to 276.8)	< 0.05 (0.0 to < 0.05)	< 0.05 (0.0 to < 0.05)	23.9 (16.2 to 33.5)	32.6 (21.9 to 46.0)
Chronic kidney disease due to glomerulonephritis	Stage III chronic kidney disease and mild anaemia due to glomerulonephritis	7,602.8 (6,674.1 to 8,609.9)	9,685.3 (8,396.4 to 11,109.2)	< 0.05 (0.0 to < 0.05)	< 0.05 (0.0 to < 0.05)	26.6 (9.3 to 61.1)	33.9 (12.0 to 77.0)
Chronic kidney disease due to glomerulonephritis	End-stage renal disease on dialysis due to glomerulonephritis	250.8 (217.3 to 286.7)	322.5 (274.7 to 376.4)	--	--	130.7 (91.2 to 170.7)	167.8 (117.0 to 222.4)
Chronic kidney disease due to other causes	Stage III chronic kidney disease and mild anaemia due to other causes	9,575.1 (8,351.1 to 10,800.0)	13,762.3 (11,899.7 to 15,676.4)	< 0.05 (0.0 to < 0.05)	< 0.05 (0.0 to < 0.05)	33.3 (11.9 to 72.8)	47.8 (16.9 to 106.1)
Chronic kidney disease due to other causes	Stage V chronic kidney disease untreated and severe anaemia due to other causes	153.9 (128.6 to 183.3)	235.6 (196.0 to 281.6)	< 0.05 (0.0 to < 0.05)	< 0.05 (0.0 to < 0.05)	89.7 (63.4 to 117.6)	136.7 (95.8 to 180.3)
Chronic kidney disease due to other causes	Stage IV chronic kidney disease untreated and mild anaemia due to other causes	262.6 (216.2 to 314.2)	339.7 (279.1 to 410.6)	< 0.05 (0.0 to < 0.05)	< 0.05 (0.0 to < 0.05)	26.2 (17.0 to 37.7)	33.8 (22.2 to 47.8)
Chronic kidney disease due to other causes	Stage III chronic kidney disease and severe anaemia due to other causes	274.3 (233.0 to 318.9)	362.1 (307.3 to 421.8)	< 0.05 (0.0 to < 0.05)	< 0.05 (0.0 to < 0.05)	36.6 (24.4 to 51.9)	48.2 (32.2 to 68.2)
Chronic kidney disease due to other causes	End-stage renal disease on dialysis due to other causes	374.6 (325.8 to 429.9)	508.8 (439.3 to 593.5)	--	--	190.5 (132.6 to 249.1)	257.4 (178.6 to 337.9)
Chronic kidney disease due to other causes	Stage V chronic kidney disease untreated and moderate anaemia due to other causes	659.2 (545.4 to 786.0)	815.7 (670.5 to 973.1)	< 0.05 (0.0 to < 0.05)	< 0.05 (0.0 to < 0.05)	348.5 (247.2 to 460.3)	430.5 (302.1 to 575.2)
Chronic kidney disease due to other causes	Stage V chronic kidney disease untreated and mild anaemia due to other causes	87.1 (71.4 to 105.5)	112.3 (92.5 to 135.2)	< 0.05 (0.0 to < 0.05)	< 0.05 (0.0 to < 0.05)	47.6 (32.1 to 66.0)	61.4 (41.6 to 85.6)
Chronic kidney disease due to other causes	Stage III chronic kidney disease without anaemia due to other causes	26,872.9 (23,201.7 to 30,475.7)	33,333.0 (28,646.7 to 38,080.8)	3,756.3 (3,259.8 to 4,245.3)	4,831.0 (4,135.1 to 5,525.8)	< 0.05 (0.0 to < 0.05)	< 0.05 (0.0 to < 0.05)
Chronic kidney disease due to other causes	Stage IV chronic kidney disease untreated and moderate anaemia due to other causes	1,138.9 (941.6 to 1,365.0)	1,405.7 (1,155.6 to 1,694.1)	< 0.05 (0.0 to < 0.05)	< 0.05 (0.0 to < 0.05)	154.4 (102.9 to 217.5)	190.2 (125.8 to 269.3)
Chronic kidney disease due to other causes	Stage III chronic kidney disease and moderate anaemia due to other causes	6,125.8 (5,234.7 to 7,018.6)	8,271.5 (7,074.5 to 9,500.9)	< 0.05 (0.0 to < 0.05)	< 0.05 (0.0 to < 0.05)	290.7 (187.7 to 422.2)	392.4 (254.2 to 569.7)
Chronic kidney disease due to other causes	Stage V chronic kidney disease untreated without anaemia due to other causes	294.8 (228.3 to 377.9)	326.8 (255.2 to 419.6)	< 0.05 (0.0 to < 0.05)	< 0.05 (0.0 to < 0.05)	156.9 (103.9 to 222.8)	174.3 (114.9 to 250.9)
Chronic kidney disease due to other causes	Stage IV chronic kidney disease untreated without anaemia due to other causes	952.5 (765.5 to 1,174.5)	1,038.5 (841.5 to 1,288.8)	< 0.05 (0.0 to < 0.05)	< 0.05 (0.0 to < 0.05)	92.2 (58.0 to 136.1)	100.3 (63.1 to 147.4)
Chronic kidney disease due to other causes	End-stage renal disease after transplant due to other causes	131.0 (115.5 to 147.9)	167.8 (146.5 to 191.7)	--	--	3.1 (1.7 to 5.1)	4.0 (2.2 to 6.5)

Chronic kidney disease due to other causes	Stage IV chronic kidney disease untreated and severe anaemia due to other causes	125.8 (101.7 to 153.4)	182.4 (148.9 to 220.5)	< 0.05 (0.0 to < 0.05)	< 0.05 (0.0 to < 0.05)	28.4 (18.6 to 41.0)	40.8 (27.1 to 58.4)
Interstitial nephritis and urinary tract infections	Mild interstitial nephritis and urinary tract infections	2,176.1 (1,641.6 to 2,773.4)	2,486.7 (1,875.1 to 3,164.1)	114,528.9 (88,192.7 to 144,584.1)	130,877.7 (101,594.9 to 164,983.0)	12.3 (4.7 to 26.5)	14.0 (5.3 to 29.9)
Interstitial nephritis and urinary tract infections	Moderate interstitial nephritis and urinary tract infections	3,873.7 (3,256.5 to 4,546.8)	4,426.5 (3,724.7 to 5,197.1)	201,216.8 (169,412.5 to 234,129.3)	229,937.8 (194,786.1 to 268,004.5)	187.8 (116.1 to 281.5)	214.3 (132.3 to 324.1)
Urolithiasis	Moderate urolithiasis episodes	506.2 (357.8 to 686.1)	618.1 (437.3 to 834.1)	13,323.1 (9,509.8 to 17,372.5)	16,251.9 (11,644.2 to 21,237.4)	54.7 (32.9 to 85.7)	66.6 (39.9 to 104.2)
Urolithiasis	Severe urolithiasis episodes	326.8 (246.3 to 422.3)	399.0 (301.1 to 515.4)	8,707.3 (6,620.4 to 11,178.5)	10,621.5 (8,071.4 to 13,735.8)	100.3 (62.0 to 150.1)	121.7 (75.1 to 180.6)
Urolithiasis	Mild urolithiasis episodes	1,491.7 (1,276.4 to 1,722.4)	1,821.6 (1,564.2 to 2,090.1)	39,631.9 (34,546.6 to 45,687.8)	48,344.5 (42,082.5 to 55,799.9)	15.7 (7.5 to 29.4)	19.2 (9.1 to 35.8)
Benign prostatic hyperplasia	Symptomatic benign prostatic hyperplasia	42,435.2 (37,589.6 to 47,330.5)	54,713.5 (48,498.8 to 61,142.1)	5,480.7 (4,733.9 to 6,275.1)	6,971.0 (6,039.3 to 8,020.0)	2,625.2 (1,692.9 to 3,763.4)	3,383.9 (2,178.0 to 4,835.1)
Benign prostatic hyperplasia	Asymptomatic benign prostatic hyperplasia	37,958.9 (33,620.2 to 42,209.5)	48,941.9 (43,484.2 to 54,560.4)	4,903.8 (4,222.2 to 5,636.8)	6,237.2 (5,358.4 to 7,186.2)	< 0.05 (0.0 to < 0.05)	< 0.05 (0.0 to < 0.05)
Male infertility	Primary male infertility	7,916.9 (5,868.2 to 10,403.0)	9,665.8 (6,995.6 to 13,092.2)	--	--	59.0 (24.6 to 117.5)	72.1 (29.5 to 148.0)
Male infertility	Secondary male infertility	15,686.2 (11,935.4 to 20,653.3)	18,378.5 (13,754.6 to 24,927.7)	--	--	79.3 (29.6 to 170.0)	92.9 (34.5 to 200.9)
Other urinary diseases	Other urinary diseases	--	--	--	--	--	--
Uterine fibroids	Mild abdominal pain due to uterine fibroids, with moderate anaemia	9,363.7 (8,638.9 to 10,128.6)	10,949.1 (10,102.7 to 11,843.6)	< 0.05 (0.0 to < 0.05)	< 0.05 (0.0 to < 0.05)	548.8 (354.5 to 798.8)	641.9 (414.4 to 940.3)
Uterine fibroids	Mild abdominal pain due to uterine fibroids, with severe anaemia	756.4 (682.0 to 834.7)	896.5 (805.9 to 993.3)	< 0.05 (0.0 to < 0.05)	< 0.05 (0.0 to < 0.05)	110.7 (77.7 to 151.1)	131.4 (93.1 to 179.5)
Uterine fibroids	Mild abdominal pain due to uterine fibroids, with mild anaemia	12,706.5 (12,033.6 to 13,417.5)	14,670.4 (13,902.6 to 15,516.6)	< 0.05 (0.0 to < 0.05)	< 0.05 (0.0 to < 0.05)	179.6 (82.4 to 347.6)	207.4 (95.6 to 402.8)
Uterine fibroids	Asymptomatic uterine fibroids	22,642.8 (20,194.6 to 25,159.5)	27,293.3 (24,341.9 to 30,402.0)	2,540.8 (2,210.8 to 2,895.8)	2,819.5 (2,463.4 to 3,214.2)	< 0.05 (0.0 to < 0.05)	< 0.05 (0.0 to < 0.05)
Uterine fibroids	Mild abdominal pain due to uterine fibroids, without anaemia	29,784.5 (25,513.5 to 34,625.2)	37,202.8 (31,933.6 to 43,162.4)	5,925.1 (5,216.7 to 6,723.3)	6,575.0 (5,778.2 to 7,441.6)	315.6 (151.6 to 597.0)	393.9 (189.0 to 746.0)
Polycystic ovarian syndrome	Hirsutism and primary infertility due to polycystic ovarian syndrome	418.0 (266.8 to 608.8)	476.9 (298.5 to 697.6)	--	--	7.5 (3.1 to 15.0)	8.6 (3.5 to 17.5)
Polycystic ovarian syndrome	Primary infertility due to polycystic ovarian syndrome	101.7 (8.9 to 283.2)	116.0 (10.2 to 321.8)	--	--	0.8 (0.1 to 2.4)	0.9 (0.1 to 2.7)
Polycystic ovarian syndrome	Asymptomatic polycystic ovarian syndrome	1,612.7 (742.3 to 2,788.0)	1,711.5 (784.7 to 2,949.7)	6,474.0 (4,717.1 to 8,508.5)	7,651.0 (5,517.8 to 10,179.4)	< 0.05 (0.0 to < 0.05)	< 0.05 (0.0 to < 0.05)
Polycystic ovarian syndrome	Hirsutism and secondary infertility due to polycystic ovarian syndrome	978.3 (631.9 to 1,388.0)	1,126.8 (723.2 to 1,596.7)	--	--	4.9 (1.8 to 10.5)	5.6 (2.1 to 11.8)
Polycystic ovarian syndrome	Secondary infertility due to polycystic ovarian syndrome	240.4 (20.5 to 698.8)	276.8 (23.4 to 803.3)	--	--	3.7 (0.3 to 11.9)	4.3 (0.3 to 13.6)
Polycystic ovarian syndrome	Hirsutism due to polycystic ovarian syndrome	6,534.4 (5,090.6 to 8,073.0)	6,933.8 (5,380.2 to 8,594.9)	--	--	70.1 (31.6 to 131.0)	74.4 (33.7 to 137.7)
Female infertility	Idiopathic primary female infertility	6,551.5 (4,325.7 to 9,659.2)	8,811.4 (5,922.9 to 12,837.0)	--	--	48.4 (18.9 to 100.7)	65.4 (25.8 to 138.9)
Female infertility	Idiopathic secondary female infertility	16,200.9 (8,390.7 to 29,282.5)	22,665.4 (11,753.8 to 39,812.4)	--	--	81.5 (24.7 to 202.2)	114.4 (36.7 to 279.3)
Endometriosis	Mild abdominal pain due to endometriosis	178.4 (119.9 to 256.8)	192.8 (129.4 to 279.2)	--	--	1.9 (0.8 to 3.9)	2.1 (0.9 to 4.2)
Endometriosis	Moderate abdominal pain and primary infertility due to endometriosis	24.7 (14.8 to 38.0)	25.3 (15.4 to 38.6)	--	--	3.0 (1.6 to 5.2)	3.1 (1.6 to 5.3)
Endometriosis	Primary infertility due to endometriosis	14.4 (8.8 to 21.4)	14.8 (9.0 to 21.9)	--	--	0.1 (< 0.05 to 0.2)	0.1 (< 0.05 to 0.2)
Endometriosis	Moderate abdominal pain and secondary infertility due to endometriosis	55.4 (36.4 to 82.0)	59.5 (39.0 to 88.8)	--	--	6.5 (3.5 to 10.6)	7.0 (3.8 to 11.4)
Endometriosis	Severe abdominal pain and secondary infertility due to endometriosis	12.3 (7.7 to 18.0)	13.2 (8.2 to 19.5)	--	--	4.0 (2.2 to 6.5)	4.3 (2.3 to 6.9)
Endometriosis	Severe endometriosis	367.1 (249.3 to 517.9)	396.8 (270.4 to 560.3)	--	--	112.0 (63.1 to 178.3)	120.9 (68.3 to 191.8)
Endometriosis	Secondary infertility due to endometriosis	32.3 (21.0 to 46.6)	34.7 (22.6 to 50.1)	--	--	0.2 (0.1 to 0.4)	0.2 (0.1 to 0.4)
Endometriosis	Moderate abdominal pain due to endometriosis	1,636.2 (1,297.3 to 2,007.5)	1,768.5 (1,405.3 to 2,161.8)	--	--	178.1 (112.8 to 261.5)	192.2 (121.8 to 281.2)
Endometriosis	Mild abdominal pain and primary infertility due to endometriosis	2.7 (1.6 to 4.3)	2.8 (1.6 to 4.4)	--	--	< 0.05 (< 0.05 to 0.1)	0.1 (< 0.05 to 0.1)
Endometriosis	Asymptomatic endometriosis	956.4 (688.2 to 1,255.7)	1,033.8 (743.5 to 1,356.0)	9,891.7 (8,423.2 to 11,767.6)	9,261.8 (7,894.1 to 10,959.7)	< 0.05 (0.0 to < 0.05)	< 0.05 (0.0 to < 0.05)
Endometriosis	Severe abdominal pain and primary infertility due to endometriosis	5.5 (3.3 to 8.3)	5.6 (3.4 to 8.5)	--	--	1.8 (1.0 to 3.0)	1.9 (1.0 to 3.0)
Endometriosis	Mild abdominal pain and secondary infertility due to endometriosis	6.0 (3.8 to 9.0)	6.5 (4.1 to 9.7)	--	--	0.1 (< 0.05 to 0.2)	0.1 (< 0.05 to 0.2)
Genital prolapse	Asymptomatic genital prolapse	176,567.3 (151,494.2 to 202,175.0)	202,619.3 (174,362.3 to 232,530.3)	12,607.9 (10,603.2 to 14,728.3)	13,441.1 (11,326.1 to 15,698.4)	< 0.05 (0.0 to < 0.05)	< 0.05 (0.0 to < 0.05)
Genital prolapse	Stress incontinence due to genital prolapse	24,368.2 (14,490.4 to 35,836.6)	27,964.7 (16,659.4 to 40,875.2)	1,718.6 (990.3 to 2,582.1)	1,832.2 (1,056.3 to 2,748.4)	458.2 (208.9 to 837.7)	526.0 (240.0 to 960.4)
Genital prolapse	Abdominal pain due to genital prolapse	15,153.1 (3,566.3 to 34,973.7)	17,388.9 (4,073.3 to 40,138.8)	1,100.4 (243.4 to 2,471.4)	1,173.3 (262.1 to 2,643.4)	157.8 (30.7 to 410.7)	181.1 (35.3 to 470.7)
Genital prolapse	Abdominal pain and stress incontinence due to genital prolapse	2,130.8 (427.0 to 5,183.5)	2,445.2 (491.6 to 5,927.7)	151.7 (30.4 to 358.8)	161.8 (32.3 to 383.1)	62.0 (11.8 to 176.9)	71.1 (13.4 to 201.4)
Premenstrual syndrome	Asymptomatic premenstrual syndrome	369,732.5 (351,072.0 to 387,671.2)	404,045.6 (381,561.7 to 425,457.5)	135,485.8 (127,327.8 to 143,270.1)	144,343.5 (135,417.6 to 152,916.6)	< 0.05 (0.0 to < 0.05)	< 0.05 (0.0 to < 0.05)
Premenstrual syndrome	Abdominal pain and depression due to premenstrual syndrome	16,670.8 (12,342.3 to 21,445.1)	18,218.5 (13,514.2 to 23,414.0)	6,121.3 (4,477.1 to 7,896.1)	6,521.3 (4,780.3 to 8,401.6)	2,436.1 (1,475.1 to 3,549.7)	2,661.0 (1,599.2 to 3,886.7)
Premenstrual syndrome	Depression due to premenstrual syndrome	5,650.0 (1,772.2 to 11,464.4)	6,173.8 (1,931.4 to 12,482.8)	2,038.6 (643.5 to 4,192.1)	2,171.9 (687.0 to 4,470.6)	781.2 (220.6 to 1,784.8)	853.2 (242.9 to 1,940.7)

Premenstrual syndrome	Abdominal pain due to premenstrual syndrome	23,660.8<br>(17,232.7 to 31,118.2)	25,857.6<br>(18,748.8 to 34,053.2)	8,821.6<br>(6,269.6 to 11,754.2)	9,398.2<br>(6,702.6 to 12,657.6)	253.4<br>(114.6 to 494.4)	276.9<br>(124.8 to 540.1)
Other gynecological diseases	Mild anaemia due to other gynecological diseases	9,376.3<br>(9,119.0 to 9,641.0)	10,442.6<br>(10,132.4 to 10,767.3)	--	--	33.8<br>(12.1 to 73.4)	37.7<br>(13.4 to 81.8)
Other gynecological diseases	Severe other gynecological disorders	4,846.0<br>(3,454.8 to 6,499.6)	5,383.0<br>(3,828.3 to 7,227.2)	5,438.5<br>(3,807.6 to 7,524.2)	5,990.9<br>(4,210.4 to 8,239.3)	1,458.9<br>(878.2 to 2,275.2)	1,620.3<br>(974.4 to 2,506.0)
Other gynecological diseases	Mild other gynecological disorders	32,187.8<br>(26,414.6 to 38,487.3)	35,754.2<br>(29,840.8 to 42,023.0)	36,222.6<br>(29,595.3 to 43,299.5)	39,903.0<br>(32,950.7 to 47,259.5)	344.8<br>(161.6 to 666.9)	382.9<br>(180.2 to 733.7)
Other gynecological diseases	Moderate anaemia due to other gynecological diseases	7,290.3<br>(7,012.3 to 7,604.0)	8,193.1<br>(7,842.5 to 8,557.9)	--	--	360.3<br>(238.9 to 521.3)	405.1<br>(269.5 to 584.4)
Other gynecological diseases	Severe anaemia due to other gynecological diseases	595.0<br>(562.6 to 633.1)	648.7<br>(609.2 to 692.4)	--	--	83.2<br>(57.4 to 114.8)	90.8<br>(62.8 to 125.5)
Other gynecological diseases	Moderate other gynecological disorders	9,215.7<br>(6,036.8 to 13,023.6)	10,237.3<br>(6,696.7 to 14,334.8)	10,496.5<br>(6,741.6 to 15,315.5)	11,562.8<br>(7,495.9 to 16,706.4)	998.4<br>(561.7 to 1,588.0)	1,108.6<br>(626.0 to 1,752.2)
Other gynecological diseases	Asymptomatic other gynecological disorders	30,637.2<br>(25,787.0 to 36,244.3)	34,033.1<br>(28,977.9 to 39,781.8)	34,511.5<br>(28,541.6 to 40,945.6)	38,018.6<br>(31,808.8 to 44,402.2)	< 0.05<br>(0.0 to < 0.05)	< 0.05<br>(0.0 to < 0.05)
Thalassemias	Beta-thalassemia major, with moderate anaemia	121.7<br>(104.4 to 133.6)	119.5<br>(100.1 to 132.7)	< 0.05<br>(0.0 to < 0.05)	< 0.05<br>(0.0 to < 0.05)	7.6<br>(4.8 to 11.7)	7.5<br>(4.7 to 11.4)
Thalassemias	Asymptomatic and mild heart failure due to thalassemias	2.4<br>(1.9 to 3.0)	2.3<br>(1.8 to 2.9)	--	--	0.1<br>(0.1 to 0.2)	0.1<br>(0.1 to 0.2)
Thalassemias	Hemoglobin E/Beta-thalassemia, without anaemia	4.8<br>(1.5 to 10.8)	5.7<br>(2.0 to 12.1)	< 0.05<br>(0.0 to < 0.05)	< 0.05<br>(0.0 to < 0.05)	0.1<br>(< 0.05 to 0.2)	0.1<br>(< 0.05 to 0.2)
Thalassemias	Beta-thalassemia major, with mild anaemia	25.0<br>(22.3 to 28.0)	20.7<br>(18.2 to 23.9)	< 0.05<br>(0.0 to < 0.05)	< 0.05<br>(0.0 to < 0.05)	0.4<br>(0.2 to 0.8)	0.3<br>(0.2 to 0.6)
Thalassemias	Hemoglobin E/beta-thalassemia, with mild anaemia	9.4<br>(8.0 to 10.7)	11.3<br>(9.7 to 13.0)	< 0.05<br>(0.0 to < 0.05)	< 0.05<br>(0.0 to < 0.05)	0.1<br>(0.1 to 0.3)	0.2<br>(0.1 to 0.4)
Thalassemias	Severe heart failure due to thalassemias	1.4<br>(1.1 to 1.8)	1.4<br>(1.0 to 1.7)	--	--	0.3<br>(0.2 to 0.4)	0.2<br>(0.2 to 0.4)
Thalassemias	Hemoglobin E/beta-thalassemia, with severe anaemia	6.9<br>(6.1 to 7.9)	6.2<br>(5.3 to 7.0)	< 0.05<br>(0.0 to < 0.05)	< 0.05<br>(0.0 to < 0.05)	1.1<br>(0.7 to 1.6)	1.0<br>(0.7 to 1.4)
Thalassemias	Moderate heart failure due to thalassemias	0.5<br>(0.3 to 0.7)	0.5<br>(0.3 to 0.7)	--	--	< 0.05<br>(< 0.05 to 0.1)	< 0.05<br>(< 0.05 to 0.1)
Thalassemias	Hemoglobin H disease, with severe anaemia	15.3<br>(12.0 to 19.2)	14.4<br>(10.9 to 18.5)	< 0.05<br>(0.0 to < 0.05)	< 0.05<br>(0.0 to < 0.05)	2.4<br>(1.6 to 3.5)	2.3<br>(1.5 to 3.4)
Thalassemias	Hemoglobin H disease, severe infection with severe anaemia	0.4<br>(0.1 to 0.8)	0.4<br>(0.1 to 0.8)	< 0.05<br>(0.0 to < 0.05)	< 0.05<br>(0.0 to < 0.05)	0.1<br>(< 0.05 to 0.2)	0.1<br>(< 0.05 to 0.2)
Thalassemias	Hemoglobin H disease, with mild anaemia	21.0<br>(17.0 to 26.2)	22.6<br>(18.2 to 28.1)	< 0.05<br>(0.0 to < 0.05)	< 0.05<br>(0.0 to < 0.05)	0.3<br>(0.2 to 0.6)	0.4<br>(0.2 to 0.7)
Thalassemias	Beta-thalassemia major, without anaemia	22.1<br>(8.0 to 45.6)	22.9<br>(7.7 to 46.6)	< 0.05<br>(0.0 to < 0.05)	< 0.05<br>(0.0 to < 0.05)	0.3<br>(0.1 to 0.7)	0.3<br>(0.1 to 0.7)
Thalassemias	Hemoglobin E/beta-thalassemia, with moderate anaemia	43.2<br>(36.6 to 49.5)	45.2<br>(37.6 to 52.1)	< 0.05<br>(0.0 to < 0.05)	< 0.05<br>(0.0 to < 0.05)	2.7<br>(1.6 to 4.2)	2.8<br>(1.7 to 4.4)
Thalassemias	Hemoglobin H disease, with moderate anaemia	64.4<br>(51.0 to 81.1)	54.9<br>(42.3 to 70.0)	< 0.05<br>(0.0 to < 0.05)	< 0.05<br>(0.0 to < 0.05)	4.0<br>(2.4 to 6.4)	3.4<br>(2.1 to 5.5)
Thalassemias	Beta-thalassemia major, severe infection with severe anaemia	1.1<br>(0.4 to 2.2)	1.0<br>(0.3 to 2.0)	< 0.05<br>(0.0 to < 0.05)	< 0.05<br>(0.0 to < 0.05)	0.3<br>(0.1 to 0.6)	0.3<br>(0.1 to 0.6)
Thalassemias	Hemoglobin H disease, without anaemia	16.6<br>(6.6 to 32.2)	15.3<br>(6.3 to 29.8)	< 0.05<br>(0.0 to < 0.05)	< 0.05<br>(0.0 to < 0.05)	0.2<br>(0.1 to 0.5)	0.2<br>(0.1 to 0.4)
Thalassemias	Hemoglobin E/beta-thalassemia, severe infection with severe anaemia	0.2<br>(0.1 to 0.3)	0.2<br>(0.1 to 0.3)	< 0.05<br>(0.0 to < 0.05)	< 0.05<br>(0.0 to < 0.05)	< 0.05<br>(< 0.05 to 0.1)	< 0.05<br>(< 0.05 to 0.1)
Thalassemias	Beta-thalassemia major, with severe anaemia	22.6<br>(18.2 to 27.3)	21.8<br>(17.5 to 26.6)	< 0.05<br>(0.0 to < 0.05)	< 0.05<br>(0.0 to < 0.05)	3.6<br>(2.3 to 5.1)	3.5<br>(2.2 to 4.9)
Thalassaemias trait	Mild anaemia due to B-thalassemia trait	66,811.7<br>(63,653.3 to 70,066.9)	80,501.5<br>(76,005.6 to 85,233.4)	--	--	241.7<br>(86.5 to 521.8)	290.8<br>(103.7 to 623.2)
Thalassaemias trait	Moderate anaemia due to B-thalassemia trait	45,198.7<br>(42,309.3 to 48,432.7)	49,729.9<br>(46,134.5 to 53,897.3)	--	--	2,246.0<br>(1,483.2 to 3,231.5)	2,462.9<br>(1,625.1 to 3,556.3)
Thalassaemias trait	Moderate anaemia due to hemoglobin E trait	2,401.9<br>(2,239.4 to 2,593.4)	2,653.7<br>(2,444.5 to 2,888.8)	--	--	120.3<br>(78.4 to 174.6)	132.7<br>(85.4 to 193.0)
Thalassaemias trait	Severe anaemia due to hemoglobin E trait	114.0<br>(100.5 to 131.5)	166.8<br>(148.0 to 193.9)	--	--	16.7<br>(11.0 to 23.6)	24.4<br>(16.0 to 34.2)
Thalassaemias trait	Asymptomatic B-thalassemia trait	95,028.5<br>(88,325.9 to 101,722.9)	99,467.0<br>(92,444.3 to 106,890.4)	< 0.05<br>(0.0 to < 0.05)	< 0.05<br>(0.0 to < 0.05)	< 0.05<br>(0.0 to < 0.05)	< 0.05<br>(0.0 to < 0.05)
Thalassaemias trait	Severe anaemia due to B-thalassemia trait	2,207.3<br>(2,018.3 to 2,481.5)	2,444.8<br>(2,222.5 to 2,770.0)	--	--	311.6<br>(210.1 to 431.2)	343.5<br>(231.9 to 469.8)
Thalassaemias trait	Asymptomatic hemoglobin E trait	40,479.9<br>(38,474.7 to 42,660.7)	44,889.6<br>(42,637.6 to 47,266.9)	< 0.05<br>(0.0 to < 0.05)	< 0.05<br>(0.0 to < 0.05)	< 0.05<br>(0.0 to < 0.05)	< 0.05<br>(0.0 to < 0.05)
Thalassaemias trait	Mild anaemia due to hemoglobin E trait	5,755.4<br>(5,446.6 to 6,089.6)	7,253.5<br>(6,796.2 to 7,746.4)	--	--	20.8<br>(7.4 to 45.3)	26.2<br>(9.4 to 57.3)
Sickle cell disorders	Homozygous sickle cell and severe sickle cell/beta-thalassemia, with vaso-occlusive crisis and stroke, without anaemia	4.9<br>(2.8 to 8.0)	7.2<br>(4.2 to 11.2)	< 0.05<br>(0.0 to < 0.05)	< 0.05<br>(0.0 to < 0.05)	2.6<br>(1.3 to 4.4)	3.9<br>(2.0 to 6.4)
Sickle cell disorders	Mild sickle cell/beta-thalassemia, with stroke and severe anaemia	0.2<br>(0.2 to 0.3)	0.3<br>(0.2 to 0.3)	< 0.05<br>(0.0 to < 0.05)	< 0.05<br>(0.0 to < 0.05)	0.1<br>(0.1 to 0.1)	0.1<br>(0.1 to 0.2)
Sickle cell disorders	Mild sickle cell/beta-thalassemia, with mild anaemia	1.2<br>(1.1 to 1.4)	2.0<br>(1.7 to 2.3)	< 0.05<br>(0.0 to < 0.05)	< 0.05<br>(0.0 to < 0.05)	< 0.05<br>(< 0.05 to < 0.05)	< 0.05<br>(< 0.05 to 0.1)
Sickle cell disorders	Mild sickle cell/beta-thalassemia, with vaso-occlusive crisis and severe anaemia	0.2<br>(0.2 to 0.3)	0.3<br>(0.2 to 0.4)	< 0.05<br>(0.0 to < 0.05)	< 0.05<br>(0.0 to < 0.05)	0.1<br>(0.1 to 0.1)	0.1<br>(0.1 to 0.2)
Sickle cell disorders	Hemoglobin SC disease, with severe anaemia	17.0<br>(13.5 to 21.1)	20.5<br>(14.7 to 29.3)	< 0.05<br>(0.0 to < 0.05)	< 0.05<br>(0.0 to < 0.05)	2.6<br>(1.6 to 3.9)	3.1<br>(1.9 to 5.1)
Sickle cell disorders	Mild sickle cell/beta-thalassemia, with moderate anaemia	8.6<br>(7.6 to 9.7)	10.7<br>(9.4 to 12.0)	< 0.05<br>(0.0 to < 0.05)	< 0.05<br>(0.0 to < 0.05)	0.5<br>(0.3 to 0.8)	0.7<br>(0.4 to 1.0)
Sickle cell disorders	Hemoglobin SC disease, without anaemia	< 0.05<br>(0.0 to < 0.05)	< 0.05<br>(0.0 to < 0.05)	< 0.05<br>(0.0 to < 0.05)	< 0.05<br>(0.0 to < 0.05)	< 0.05<br>(0.0 to < 0.05)	< 0.05<br>(0.0 to < 0.05)
Sickle cell disorders	Homozygous sickle cell and severe sickle cell/beta-thalassemia, with stroke, without anaemia	49.2<br>(30.0 to 73.4)	69.2<br>(45.1 to 99.3)	< 0.05<br>(0.0 to < 0.05)	< 0.05<br>(0.0 to < 0.05)	15.5<br>(8.6 to 25.3)	21.7<br>(12.4 to 34.3)
Sickle cell disorders	Hemoglobin SC disease, with stroke and severe anaemia	0.5<br>(0.4 to 0.7)	1.8<br>(1.4 to 2.3)	< 0.05<br>(0.0 to < 0.05)	< 0.05<br>(0.0 to < 0.05)	0.2<br>(0.1 to 0.3)	0.8<br>(0.5 to 1.1)

Sickle cell disorders	Mild sickle cell/beta-thalassemia, without anaemia	< 0.05 (0.0 to < 0.05)	< 0.05 (0.0 to < 0.05)	< 0.05 (0.0 to < 0.05)	< 0.05 (0.0 to < 0.05)	< 0.05 (0.0 to < 0.05)	< 0.05 (0.0 to < 0.05)
Sickle cell disorders	Homozygous sickle cell and severe sickle cell/beta-thalassemia, with vaso-occlusive crisis and severe anaemia	50.5 (41.6 to 59.0)	59.1 (49.3 to 69.2)	< 0.05 (0.0 to < 0.05)	< 0.05 (0.0 to < 0.05)	20.9 (14.0 to 29.0)	24.6 (16.4 to 34.2)
Sickle cell disorders	Mild sickle cell/beta-thalassemia, with vaso-occlusive crisis, stroke, and severe anaemia	< 0.05 (< 0.05 to < 0.05)	< 0.05 (< 0.05 to < 0.05)	< 0.05 (0.0 to < 0.05)	< 0.05 (0.0 to < 0.05)	< 0.05 (< 0.05 to < 0.05)	< 0.05 (< 0.05 to < 0.05)
Sickle cell disorders	Mild sickle cell/beta-thalassemia, with vaso-occlusive crisis, without anaemia	0.1 (0.1 to 0.1)	0.1 (0.1 to 0.1)	< 0.05 (0.0 to < 0.05)	< 0.05 (0.0 to < 0.05)	< 0.05 (< 0.05 to 0.1)	< 0.05 (< 0.05 to 0.1)
Sickle cell disorders	Mild sickle cell/beta-thalassemia, with severe anaemia	4.6 (3.7 to 5.6)	5.2 (4.0 to 6.6)	< 0.05 (0.0 to < 0.05)	< 0.05 (0.0 to < 0.05)	0.7 (0.5 to 1.1)	0.8 (0.5 to 1.2)
Sickle cell disorders	Homozygous sickle cell and severe sickle cell/beta-thalassemia, with vaso-occlusive crisis, without anaemia	92.9 (56.3 to 142.9)	124.1 (79.3 to 185.4)	< 0.05 (0.0 to < 0.05)	< 0.05 (0.0 to < 0.05)	30.0 (15.8 to 49.9)	40.0 (21.3 to 66.1)
Sickle cell disorders	Homozygous sickle cell and severe sickle cell/beta-thalassemia, without anaemia	< 0.05 (0.0 to < 0.05)	< 0.05 (0.0 to < 0.05)	< 0.05 (0.0 to < 0.05)	< 0.05 (0.0 to < 0.05)	< 0.05 (0.0 to < 0.05)	< 0.05 (0.0 to < 0.05)
Sickle cell disorders	Hemoglobin SC disease, with vaso-occlusive crisis, stroke, and severe anaemia	0.1 (< 0.05 to 0.1)	0.3 (0.2 to 0.4)	< 0.05 (0.0 to < 0.05)	< 0.05 (0.0 to < 0.05)	< 0.05 (< 0.05 to 0.1)	0.2 (0.1 to 0.2)
Sickle cell disorders	Mild sickle cell/beta-thalassemia, with stroke, without anaemia	0.1 (0.1 to 0.2)	0.1 (0.1 to 0.2)	< 0.05 (0.0 to < 0.05)	< 0.05 (0.0 to < 0.05)	< 0.05 (< 0.05 to 0.1)	< 0.05 (< 0.05 to 0.1)
Sickle cell disorders	Homozygous sickle cell and severe sickle cell/beta-thalassemia, with stroke and severe anaemia	19.3 (14.6 to 24.1)	24.3 (18.2 to 30.2)	< 0.05 (0.0 to < 0.05)	< 0.05 (0.0 to < 0.05)	8.0 (5.0 to 11.5)	10.1 (6.2 to 14.5)
Sickle cell disorders	Mild sickle cell/beta-thalassemia, with vaso-occlusive crisis and stroke, without anaemia	< 0.05 (< 0.05 to < 0.05)	< 0.05 (< 0.05 to < 0.05)	< 0.05 (0.0 to < 0.05)	< 0.05 (0.0 to < 0.05)	< 0.05 (< 0.05 to < 0.05)	< 0.05 (< 0.05 to < 0.05)
Sickle cell disorders	Homozygous sickle cell and severe sickle cell/beta-thalassemia, with moderate anaemia	2,219.8 (2,040.2 to 2,565.4)	2,512.3 (2,301.3 to 2,866.4)	< 0.05 (0.0 to < 0.05)	< 0.05 (0.0 to < 0.05)	136.5 (88.0 to 203.2)	154.7 (98.6 to 233.2)
Sickle cell disorders	Hemoglobin SC disease, with vaso-occlusive crisis, without anaemia	8.9 (5.5 to 12.6)	10.0 (6.6 to 14.7)	< 0.05 (0.0 to < 0.05)	< 0.05 (0.0 to < 0.05)	2.9 (1.5 to 5.0)	3.3 (1.7 to 5.6)
Sickle cell disorders	Homozygous sickle cell and severe sickle cell/beta-thalassemia, with severe anaemia	109.9 (92.8 to 130.3)	135.3 (108.1 to 173.3)	< 0.05 (0.0 to < 0.05)	< 0.05 (0.0 to < 0.05)	16.9 (11.3 to 23.7)	20.8 (13.5 to 30.0)
Sickle cell disorders	Hemoglobin SC disease, with moderate anaemia	209.1 (188.5 to 234.6)	266.1 (239.1 to 298.1)	< 0.05 (0.0 to < 0.05)	< 0.05 (0.0 to < 0.05)	12.9 (8.1 to 19.4)	16.4 (10.3 to 24.9)
Sickle cell disorders	Hemoglobin SC disease, with vaso-occlusive crisis and stroke, without anaemia	0.5 (0.3 to 0.7)	0.5 (0.3 to 0.8)	< 0.05 (0.0 to < 0.05)	< 0.05 (0.0 to < 0.05)	0.3 (0.1 to 0.4)	0.3 (0.1 to 0.5)
Sickle cell disorders	Homozygous sickle cell and severe sickle cell/beta-thalassemia, with mild anaemia	346.8 (298.9 to 478.3)	517.6 (447.9 to 690.3)	< 0.05 (0.0 to < 0.05)	< 0.05 (0.0 to < 0.05)	5.5 (2.6 to 10.8)	8.2 (3.9 to 16.3)
Sickle cell disorders	Homozygous sickle cell and severe sickle cell/beta-thalassemia, with vaso-occlusive crisis, stroke, and severe anaemia	2.8 (1.7 to 4.2)	3.7 (2.2 to 5.4)	< 0.05 (0.0 to < 0.05)	< 0.05 (0.0 to < 0.05)	1.7 (1.0 to 2.7)	2.2 (1.2 to 3.5)
Sickle cell disorders	Hemoglobin SC disease, with vaso-occlusive crisis and severe anaemia	1.5 (1.1 to 1.9)	4.7 (3.7 to 5.6)	< 0.05 (0.0 to < 0.05)	< 0.05 (0.0 to < 0.05)	0.6 (0.4 to 0.9)	2.0 (1.3 to 2.8)
Sickle cell disorders	Hemoglobin SC disease, with mild anaemia	83.4 (74.8 to 93.4)	107.7 (95.5 to 122.1)	< 0.05 (0.0 to < 0.05)	< 0.05 (0.0 to < 0.05)	1.3 (0.6 to 2.6)	1.7 (0.8 to 3.3)
Sickle cell disorders	Hemoglobin SC disease, with stroke, without anaemia	4.4 (2.9 to 6.0)	4.7 (3.1 to 6.7)	< 0.05 (0.0 to < 0.05)	< 0.05 (0.0 to < 0.05)	1.4 (0.8 to 2.3)	1.5 (0.8 to 2.5)
Sickle cell trait	Moderate anaemia due to sickle cell trait	21,000.7 (19,192.3 to 23,385.2)	24,311.7 (22,291.2 to 27,003.5)	--	--	1,044.9 (668.6 to 1,553.9)	1,209.2 (776.2 to 1,795.1)
Sickle cell trait	Asymptomatic sickle cell trait	342,924.5 (312,705.3 to 384,216.7)	400,567.6 (363,636.4 to 450,366.3)	< 0.05 (0.0 to < 0.05)	< 0.05 (0.0 to < 0.05)	< 0.05 (0.0 to < 0.05)	< 0.05 (0.0 to < 0.05)
Sickle cell trait	Severe anaemia due to sickle cell trait	1,344.5 (1,235.5 to 1,476.1)	1,578.1 (1,421.7 to 1,771.2)	--	--	188.7 (127.6 to 265.0)	221.0 (149.8 to 307.3)
Sickle cell trait	Mild anaemia due to sickle cell trait	27,156.6 (24,598.3 to 30,525.9)	34,666.4 (31,192.3 to 39,308.8)	--	--	97.8 (34.0 to 221.1)	124.8 (44.1 to 284.2)
G6PD deficiency	Asymptomatic and mild heart failure due to G6PD deficiency	5.1 (4.2 to 6.3)	6.9 (5.6 to 8.5)	< 0.05 (0.0 to < 0.05)	< 0.05 (0.0 to < 0.05)	0.2 (0.1 to 0.3)	0.3 (0.2 to 0.4)
G6PD deficiency	Moderate heart failure due to G6PD deficiency	1.1 (0.8 to 1.5)	1.5 (1.0 to 2.1)	< 0.05 (0.0 to < 0.05)	< 0.05 (0.0 to < 0.05)	0.1 (< 0.05 to 0.1)	0.1 (0.1 to 0.2)
G6PD deficiency	Severe heart failure due to G6PD deficiency	3.0 (2.4 to 3.7)	4.0 (3.2 to 5.0)	< 0.05 (0.0 to < 0.05)	< 0.05 (0.0 to < 0.05)	0.5 (0.3 to 0.8)	0.7 (0.5 to 1.1)
G6PD deficiency	Asymptomatic G6PD deficiency	292,537.3 (281,285.6 to 305,131.1)	330,814.8 (318,421.4 to 345,602.6)	< 0.05 (0.0 to < 0.05)	< 0.05 (0.0 to < 0.05)	< 0.05 (0.0 to < 0.05)	< 0.05 (0.0 to < 0.05)
G6PD deficiency	Mild anaemia due to G6PD deficiency	323.3 (307.0 to 339.8)	437.3 (412.8 to 463.0)	< 0.05 (0.0 to < 0.05)	< 0.05 (0.0 to < 0.05)	1.2 (0.4 to 2.6)	1.6 (0.6 to 3.5)
G6PD deficiency	Moderate anaemia due to G6PD deficiency	125.5 (117.3 to 134.2)	139.8 (130.3 to 150.4)	< 0.05 (0.0 to < 0.05)	< 0.05 (0.0 to < 0.05)	6.4 (4.2 to 9.4)	7.2 (4.6 to 10.6)
G6PD deficiency	Severe anaemia due to G6PD deficiency	96.2 (87.6 to 107.2)	108.7 (98.5 to 122.5)	< 0.05 (0.0 to < 0.05)	< 0.05 (0.0 to < 0.05)	14.3 (9.3 to 20.3)	16.1 (10.6 to 22.7)
G6PD trait	Moderate anaemia due to hemizygous G6PD deficiency	2.2 (2.1 to 2.3)	2.6 (2.5 to 2.8)	--	--	0.1 (0.1 to 0.2)	0.1 (0.1 to 0.2)
G6PD trait	Mild anaemia due to hemizygous G6PD deficiency	2.2 (2.2 to 2.3)	2.6 (2.5 to 2.7)	--	--	< 0.05 (< 0.05 to < 0.05)	< 0.05 (< 0.05 to < 0.05)
G6PD trait	Asymptomatic hemizygous G6PD deficiency	769,743.0 (757,113.9 to 784,069.9)	865,919.1 (851,710.2 to 882,239.1)	< 0.05 (0.0 to < 0.05)	< 0.05 (0.0 to < 0.05)	< 0.05 (0.0 to < 0.05)	< 0.05 (0.0 to < 0.05)
G6PD trait	Severe anaemia due to hemizygous G6PD deficiency	2.4 (2.2 to 2.6)	2.8 (2.6 to 3.1)	--	--	0.4 (0.2 to 0.5)	0.4 (0.3 to 0.6)
Other hemoglobinopathies and hemolytic anaemias	Asymptomatic and mild heart failure due to other hemoglobinopathies and hemolytic anaemias	77.7 (65.1 to 92.3)	99.3 (83.2 to 118.7)	--	--	3.1 (1.9 to 4.7)	3.9 (2.4 to 5.9)
Other hemoglobinopathies and hemolytic anaemias	Moderate heart failure due to other hemoglobinopathies and hemolytic anaemias	17.1 (12.1 to 23.3)	21.8 (15.4 to 29.5)	--	--	1.2 (0.7 to 2.0)	1.5 (0.9 to 2.5)
Other hemoglobinopathies and hemolytic anaemias	Moderate anaemia due to other hemoglobinopathies and hemolytic anaemias	17,244.0 (16,509.2 to 18,080.1)	17,540.9 (16,750.1 to 18,356.1)	--	--	855.4 (562.8 to 1,239.2)	867.6 (568.2 to 1,253.5)
Other hemoglobinopathies and hemolytic anaemias	Mild anaemia due to other hemoglobinopathies and hemolytic anaemias	36,277.2 (35,198.4 to 37,364.8)	41,750.7 (40,469.0 to 43,071.9)	--	--	130.1 (46.0 to 282.4)	149.6 (53.1 to 324.8)
Other hemoglobinopathies and hemolytic anaemias	Severe heart failure due to other hemoglobinopathies and hemolytic anaemias	45.5 (37.7 to 55.2)	58.2 (48.1 to 70.6)	--	--	7.7 (5.0 to 11.2)	9.8 (6.3 to 14.5)
Other hemoglobinopathies and hemolytic anaemias	Other hemoglobinopathies and hemolytic anaemias	--	--	--	--	41.6 (29.5 to 57.4)	36.9 (26.1 to 50.8)
Other hemoglobinopathies and hemolytic anaemias	Severe anaemia due to other hemoglobinopathies and hemolytic anaemias	1,364.4 (1,275.9 to 1,469.1)	2,153.9 (2,001.0 to 2,347.8)	--	--	188.9 (129.3 to 259.8)	301.0 (207.9 to 417.2)

Endocrine, metabolic, blood, and immune disorders	Severe anaemia due to endocrine, metabolic, blood, and immune disorders	1,454.4 (1,354.6 to 1,579.6)	1,654.3 (1,520.7 to 1,832.1)	--	--	201.7 (137.7 to 278.9)	228.9 (154.9 to 313.0)
Endocrine, metabolic, blood, and immune disorders	Severe endocrine, metabolic, blood, and immune disorders	8,879.6 (7,071.8 to 10,938.1)	11,089.9 (8,759.3 to 13,649.1)	1,996.7 (1,600.5 to 2,464.8)	2,493.6 (2,002.1 to 3,080.6)	1,285.0 (816.3 to 1,877.2)	1,600.5 (1,010.5 to 2,331.4)
Endocrine, metabolic, blood, and immune disorders	Asymptomatic and mild heart failure due to endocrine, metabolic, blood, and immune disorders	126.2 (106.8 to 148.1)	156.3 (131.6 to 184.0)	--	--	4.8 (3.1 to 7.3)	6.0 (3.8 to 9.0)
Endocrine, metabolic, blood, and immune disorders	Asymptomatic endocrine, metabolic, blood, and immune disorders	25,627.1 (22,708.7 to 28,734.0)	32,006.4 (28,261.9 to 36,032.6)	5,781.0 (5,080.0 to 6,494.7)	7,219.8 (6,348.0 to 8,142.7)	< 0.05 (0.0 to < 0.05)	< 0.05 (0.0 to < 0.05)
Endocrine, metabolic, blood, and immune disorders	Moderate heart failure due to endocrine, metabolic, blood, and immune disorders	27.7 (19.8 to 37.1)	34.3 (24.3 to 46.0)	--	--	1.9 (1.1 to 3.0)	2.3 (1.4 to 3.8)
Endocrine, metabolic, blood, and immune disorders	Mild anaemia due to endocrine, metabolic, blood, and immune disorders	26,362.1 (25,694.0 to 27,022.9)	29,609.3 (28,810.7 to 30,453.3)	--	--	94.7 (33.4 to 206.6)	106.2 (37.4 to 230.6)
Endocrine, metabolic, blood, and immune disorders	Moderate anaemia due to endocrine, metabolic, blood, and immune disorders	16,873.0 (16,122.1 to 17,697.1)	18,022.0 (17,201.9 to 18,974.6)	--	--	841.4 (550.0 to 1,222.6)	898.0 (585.3 to 1,295.6)
Endocrine, metabolic, blood, and immune disorders	Moderate endocrine, metabolic, blood, and immune disorders	3,852.3 (2,476.6 to 5,538.2)	4,810.6 (3,084.6 to 6,944.3)	862.9 (550.3 to 1,251.2)	1,077.6 (690.6 to 1,566.7)	510.2 (282.7 to 792.6)	635.4 (352.5 to 985.1)
Endocrine, metabolic, blood, and immune disorders	Severe heart failure due to endocrine, metabolic, blood, and immune disorders	73.9 (61.1 to 89.6)	91.5 (75.3 to 110.8)	--	--	12.0 (7.8 to 17.3)	14.8 (9.6 to 21.4)
Endocrine, metabolic, blood, and immune disorders	Mild endocrine, metabolic, blood, and immune disorders	24,035.5 (20,881.2 to 27,408.3)	30,018.2 (25,939.3 to 34,366.2)	5,427.4 (4,702.7 to 6,188.6)	6,778.2 (5,867.4 to 7,749.4)	423.1 (224.6 to 716.5)	527.4 (281.3 to 889.0)
Rheumatoid arthritis	Moderate rheumatoid arthritis	6,492.2 (5,541.3 to 7,628.2)	8,385.4 (7,110.0 to 9,858.7)	369.5 (319.3 to 426.8)	462.5 (397.4 to 533.8)	1,844.9 (1,230.9 to 2,538.5)	2,378.0 (1,576.6 to 3,276.2)
Rheumatoid arthritis	Severe rheumatoid arthritis	2,338.7 (1,577.5 to 3,212.3)	3,020.8 (2,031.2 to 4,145.3)	132.0 (90.1 to 182.4)	165.2 (112.4 to 228.8)	1,198.7 (716.7 to 1,806.9)	1,542.9 (924.7 to 2,322.5)
Rheumatoid arthritis	Mild rheumatoid arthritis	7,688.4 (6,461.0 to 9,031.4)	9,930.5 (8,350.4 to 11,718.3)	436.4 (371.0 to 507.7)	546.3 (464.1 to 638.5)	828.6 (556.9 to 1,178.2)	1,068.4 (720.9 to 1,514.3)
Osteoarthritis	Moderate osteoarthritis of the knee	65,728.8 (58,868.9 to 72,432.6)	86,053.9 (77,434.3 to 94,783.1)	3,308.1 (2,972.3 to 3,674.2)	4,174.0 (3,753.5 to 4,630.1)	4,769.2 (3,281.6 to 6,501.6)	6,240.1 (4,294.0 to 8,505.1)
Osteoarthritis	Severe osteoarthritis of the knee	24,953.1 (21,891.1 to 28,433.2)	33,377.7 (29,270.2 to 38,119.8)	1,285.6 (1,113.4 to 1,455.4)	1,655.4 (1,428.3 to 1,871.8)	3,727.9 (2,540.2 to 5,169.6)	4,983.9 (3,376.1 to 6,891.4)
Osteoarthritis	Moderate osteoarthritis of the hip	9,553.6 (8,329.1 to 10,887.4)	12,644.5 (11,092.8 to 14,333.5)	523.0 (459.2 to 592.7)	681.5 (600.4 to 769.6)	686.0 (459.8 to 940.4)	908.0 (609.6 to 1,236.6)
Osteoarthritis	Severe osteoarthritis of the hip	3,175.1 (2,764.5 to 3,635.1)	4,351.1 (3,789.2 to 4,985.1)	183.5 (158.1 to 211.7)	247.8 (213.6 to 286.5)	468.6 (315.7 to 646.7)	642.5 (429.4 to 886.4)
Osteoarthritis	Mild osteoarthritis of the hip	17,928.8 (16,166.6 to 19,666.4)	23,131.0 (20,935.9 to 25,367.6)	945.5 (850.6 to 1,047.5)	1,195.2 (1,073.9 to 1,321.3)	378.3 (212.8 to 602.5)	487.9 (273.7 to 779.2)
Osteoarthritis	Mild osteoarthritis of the knee	110,646.1 (102,312.2 to 119,604.7)	142,009.0 (131,394.2 to 153,339.3)	5,454.2 (5,047.8 to 5,905.0)	6,741.8 (6,230.4 to 7,288.2)	2,355.2 (1,362.7 to 3,878.7)	3,020.4 (1,749.9 to 4,952.8)
Low back pain	Most severe low back pain with leg pain	32,554.0 (28,173.1 to 37,423.7)	38,978.0 (33,376.6 to 45,022.8)	15,862.5 (13,389.9 to 18,777.0)	18,969.0 (15,945.0 to 22,638.9)	11,255.1 (7,605.9 to 15,235.5)	13,449.2 (9,096.7 to 18,146.5)
Low back pain	Moderate low back pain without leg pain	101,868.2 (90,696.6 to 114,939.5)	119,407.8 (105,462.8 to 135,857.4)	50,153.1 (43,805.5 to 56,970.8)	58,688.9 (50,631.9 to 66,859.8)	5,203.5 (3,325.3 to 7,499.1)	6,088.4 (3,878.6 to 8,831.6)
Low back pain	Moderate low back pain with leg pain	50,549.3 (44,861.6 to 56,825.8)	60,521.9 (53,123.3 to 68,611.2)	24,612.2 (21,402.6 to 28,342.4)	29,436.2 (25,307.4 to 34,118.1)	2,571.5 (1,629.1 to 3,739.3)	3,074.5 (1,916.4 to 4,457.4)
Low back pain	Mild low back pain without leg pain	120,194.5 (106,852.9 to 135,168.1)	140,905.7 (124,083.9 to 159,983.1)	59,043.7 (51,436.3 to 67,394.7)	69,094.6 (59,851.2 to 79,426.5)	2,295.4 (1,217.2 to 3,785.5)	2,686.4 (1,430.9 to 4,417.0)
Low back pain	Mild low back pain with leg pain	37,821.5 (33,023.0 to 43,222.5)	45,287.4 (39,064.5 to 52,102.7)	18,491.4 (15,764.5 to 21,438.9)	22,118.6 (18,762.7 to 25,853.7)	719.8 (385.6 to 1,216.1)	860.9 (459.8 to 1,454.2)
Low back pain	Severe low back pain without leg pain	30,347.8 (26,832.4 to 34,134.5)	35,577.1 (31,076.1 to 40,284.5)	14,938.7 (13,103.9 to 16,870.7)	17,483.0 (15,203.0 to 19,893.0)	7,545.1 (5,127.9 to 10,316.2)	8,822.2 (5,951.8 to 12,042.5)
Low back pain	Most severe low back pain without leg pain	40,334.0 (34,679.7 to 46,151.4)	47,289.2 (40,431.8 to 54,336.7)	19,877.2 (16,868.4 to 23,119.7)	23,263.7 (19,658.0 to 27,251.3)	13,602.1 (9,275.8 to 18,378.7)	15,901.2 (10,742.4 to 21,672.4)
Low back pain	Severe low back pain with leg pain	19,274.9 (17,080.4 to 21,859.9)	23,080.9 (20,359.8 to 26,452.5)	9,383.4 (8,120.6 to 10,792.9)	11,222.8 (9,664.3 to 13,102.6)	5,661.0 (3,872.0 to 7,827.7)	6,765.4 (4,611.1 to 9,414.1)
Neck pain	Moderate neck pain	28,517.5 (18,134.9 to 41,342.1)	34,813.7 (21,918.9 to 50,358.4)	6,432.1 (3,951.9 to 9,627.1)	7,702.3 (4,716.6 to 11,509.8)	3,023.7 (1,598.3 to 4,814.2)	3,685.0 (1,940.0 to 5,865.8)
Neck pain	Severe neck pain	14,129.6 (10,666.7 to 18,072.4)	17,247.9 (12,984.0 to 22,145.1)	3,093.7 (2,336.8 to 3,942.4)	3,704.5 (2,798.3 to 4,742.2)	2,967.2 (1,807.8 to 4,385.1)	3,615.0 (2,207.3 to 5,344.9)
Neck pain	Most severe neck pain	35,293.1 (24,559.6 to 48,448.3)	43,085.7 (29,982.7 to 59,462.8)	7,742.3 (5,385.8 to 10,416.2)	9,271.0 (6,508.0 to 12,478.2)	9,764.1 (5,687.3 to 15,178.0)	11,895.3 (6,913.3 to 18,343.1)
Neck pain	Mild neck pain	160,051.9 (135,628.5 to 186,789.8)	195,376.7 (165,191.1 to 228,857.9)	35,473.9 (30,164.4 to 42,484.7)	42,479.4 (35,932.1 to 50,922.3)	7,990.9 (5,129.2 to 11,807.3)	9,741.0 (6,227.2 to 14,437.8)
Gout	Polyarticular gout	368.4 (329.6 to 411.2)	466.3 (417.2 to 520.9)	70.1 (61.6 to 79.0)	88.3 (77.6 to 99.2)	192.9 (132.8 to 250.9)	243.1 (168.2 to 314.0)
Gout	Asymptomatic gout	23,998.4 (21,558.0 to 26,830.5)	30,368.6 (27,313.4 to 33,982.1)	4,563.8 (4,027.9 to 5,139.2)	5,751.3 (5,055.8 to 6,475.5)	< 0.05 (0.0 to < 0.05)	< 0.05 (0.0 to < 0.05)
Gout	Symptomatic episodes of gout	2,483.4 (2,089.4 to 2,932.1)	3,142.5 (2,643.5 to 3,705.9)	472.2 (390.4 to 562.7)	595.1 (493.3 to 711.8)	656.0 (434.4 to 912.5)	828.1 (549.4 to 1,153.4)
Other musculoskeletal disorders	Other musculoskeletal disorders severity level 4	16,852.6 (12,174.4 to 22,527.5)	19,312.1 (13,815.1 to 25,900.0)	15,423.7 (11,528.9 to 20,293.2)	17,994.1 (13,428.7 to 23,813.0)	2,580.8 (1,575.0 to 3,888.8)	2,953.1 (1,783.6 to 4,522.6)
Other musculoskeletal disorders	Other musculoskeletal disorders severity level 6	19,343.8 (10,642.1 to 29,842.5)	22,167.3 (12,158.7 to 34,151.2)	18,003.9 (9,906.0 to 29,003.1)	21,007.8 (11,550.1 to 34,260.7)	10,154.8 (5,274.5 to 17,130.0)	11,612.8 (5,985.0 to 19,626.9)
Other musculoskeletal disorders	Other musculoskeletal disorders severity level 2	59,822.7 (45,226.7 to 75,942.8)	68,550.5 (51,813.7 to 87,552.0)	55,260.2 (40,315.7 to 71,118.8)	64,471.6 (46,959.2 to 83,381.6)	1,784.7 (979.5 to 2,809.8)	2,014.3 (1,094.5 to 3,220.0)
Other musculoskeletal disorders	Other musculoskeletal disorders severity level 3	30,346.9 (19,087.9 to 43,533.7)	34,781.8 (21,706.4 to 50,092.0)	27,862.8 (17,382.1 to 39,428.5)	32,505.3 (20,394.1 to 46,406.4)	3,323.7 (1,839.9 to 5,422.6)	3,803.5 (2,112.7 to 6,230.3)
Other musculoskeletal disorders	Other musculoskeletal disorders severity level 5	21,249.3 (16,100.6 to 27,439.4)	24,351.4 (18,334.1 to 31,731.8)	19,451.4 (14,728.6 to 25,133.4)	22,694.5 (17,087.5 to 29,744.2)	6,173.4 (3,869.9 to 9,063.6)	7,061.5 (4,399.0 to 10,454.7)
Other musculoskeletal disorders	Asymptomatic other musculoskeletal disorders	82,384.8 (68,278.2 to 98,370.0)	94,409.3 (76,857.4 to 113,407.7)	75,644.9 (62,289.0 to 91,141.0)	88,257.4 (72,154.9 to 106,413.0)	< 0.05 (0.0 to < 0.05)	< 0.05 (0.0 to < 0.05)
Other musculoskeletal disorders	Other musculoskeletal disorders severity level 1	64,828.6 (44,593.6 to 87,418.7)	74,287.0 (50,825.8 to 100,236.4)	59,428.2 (40,845.4 to 78,388.1)	69,336.2 (47,818.2 to 92,511.3)	1,410.6 (722.9 to 2,552.4)	1,614.4 (834.4 to 2,916.4)
Neural tube defects	Moderate motor impairment and incontinence due to encephalocele	1.2 (< 0.05 to 5.9)	1.3 (< 0.05 to 6.3)	--	--	0.2 (< 0.05 to 1.2)	0.2 (< 0.05 to 1.3)
Neural tube defects	Moderate motor impairment and severe intellectual disability due to encephalocele	3.3 (0.5 to 8.4)	3.5 (0.5 to 9.0)	--	--	0.7 (0.1 to 1.9)	0.7 (0.1 to 2.0)

Neural tube defects	Severe intellectual disability and incontinence due to encephalocele	3.4 (0.6 to 8.7)	3.7 (0.7 to 9.2)	--	--	1.0 (0.2 to 2.5)	1.0 (0.2 to 2.7)
Neural tube defects	Severe motor impairment and borderline intellectual disability due to spina bifida	8.4 (< 0.05 to 93.0)	9.6 (< 0.05 to 106.6)	--	--	3.4 (< 0.05 to 38.1)	3.9 (< 0.05 to 43.9)
Neural tube defects	Severe motor impairment and severe intellectual disability due to spina bifida	22.7 (< 0.05 to 145.5)	26.1 (< 0.05 to 166.5)	--	--	11.2 (< 0.05 to 72.1)	12.9 (< 0.05 to 82.2)
Neural tube defects	Moderate motor impairment and profound intellectual disability due to encephalocele	2.8 (0.3 to 7.9)	3.0 (0.3 to 8.3)	--	--	1.3 (0.1 to 3.9)	1.4 (0.2 to 4.3)
Neural tube defects	Mild intellectual disability due to encephalocele	24.7 (16.3 to 35.7)	26.4 (17.4 to 38.0)	--	--	1.1 (0.5 to 1.8)	1.1 (0.6 to 1.9)
Neural tube defects	Mild motor impairment and severe intellectual disability due to spina bifida	15.6 (< 0.05 to 122.8)	18.0 (< 0.05 to 139.8)	--	--	2.6 (< 0.05 to 19.9)	3.0 (< 0.05 to 22.9)
Neural tube defects	Severe motor impairment and profound intellectual disability due to spina bifida	16.8 (< 0.05 to 138.4)	19.3 (< 0.05 to 160.2)	--	--	8.7 (< 0.05 to 72.2)	10.0 (< 0.05 to 82.7)
Neural tube defects	Mild motor impairment, profound intellectual disability and incontinence due to spina bifida	2.9 (< 0.05 to 3.0)	3.3 (< 0.05 to 3.3)	--	--	1.0 (< 0.05 to 1.0)	1.1 (< 0.05 to 1.1)
Neural tube defects	Moderate motor impairment, severe intellectual disability and incontinence due to spina bifida	90.3 (15.0 to 213.6)	103.8 (17.2 to 244.1)	--	--	28.9 (4.9 to 72.3)	33.2 (5.7 to 83.6)
Neural tube defects	Severe motor impairment and incontinence due to spina bifida	1,393.5 (1,113.0 to 1,708.4)	1,602.0 (1,276.9 to 1,962.4)	--	--	627.5 (429.9 to 872.2)	720.0 (491.6 to 991.5)
Neural tube defects	Severe motor impairment and moderate intellectual disability due to encephalocele	8.2 (3.5 to 14.3)	8.8 (3.7 to 15.3)	--	--	3.8 (1.5 to 6.8)	4.0 (1.6 to 7.2)
Neural tube defects	Severe motor impairment, severe intellectual disability and incontinence due to encephalocele	1.1 (< 0.05 to 5.8)	1.2 (< 0.05 to 6.1)	--	--	0.6 (< 0.05 to 3.2)	0.7 (< 0.05 to 3.4)
Neural tube defects	Moderate motor impairment, profound intellectual disability and incontinence due to encephalocele	0.4 (< 0.05 to 4.0)	0.4 (< 0.05 to 4.2)	--	--	0.1 (< 0.05 to 1.4)	0.1 (< 0.05 to 1.4)
Neural tube defects	Asymptomatic encephalocele following treatment	55.8 (41.0 to 73.0)	59.5 (43.9 to 77.6)	--	--	< 0.05 (0.0 to < 0.05)	< 0.05 (0.0 to < 0.05)
Neural tube defects	Moderate motor impairment and mild intellectual disability due to spina bifida	2.4 (< 0.05 to 7.9)	2.7 (< 0.05 to 9.0)	--	--	0.2 (< 0.05 to 0.8)	0.3 (< 0.05 to 1.0)
Neural tube defects	Moderate motor impairment and mild intellectual disability due to encephalocele	3.5 (0.8 to 7.9)	3.7 (0.8 to 8.5)	--	--	0.3 (0.1 to 0.9)	0.4 (0.1 to 0.9)
Neural tube defects	Severe motor impairment and mild intellectual disability due to spina bifida	23.1 (< 0.05 to 146.4)	26.6 (< 0.05 to 170.0)	--	--	9.8 (< 0.05 to 63.0)	11.3 (< 0.05 to 72.0)
Neural tube defects	Mild motor impairment and borderline intellectual disability due to encephalocele	0.3 (< 0.05 to 3.0)	0.3 (< 0.05 to 3.1)	--	--	< 0.05 (< 0.05 to 0.1)	< 0.05 (< 0.05 to 0.1)
Neural tube defects	Mild motor impairment, severe intellectual disability and incontinence due to encephalocele	0.1 (< 0.05 to 0.1)	0.1 (< 0.05 to 0.1)	--	--	< 0.05 (0.0 to < 0.05)	< 0.05 (< 0.05 to < 0.05)
Neural tube defects	Severe intellectual disability due to encephalocele	23.2 (14.7 to 34.7)	24.8 (15.6 to 36.8)	--	--	3.7 (2.0 to 6.2)	4.0 (2.2 to 6.6)
Neural tube defects	Severe motor impairment and profound intellectual disability due to encephalocele	5.9 (2.0 to 12.1)	6.3 (2.2 to 13.0)	--	--	2.8 (0.9 to 6.0)	3.0 (1.0 to 6.4)
Neural tube defects	Profound intellectual disability and incontinence due to encephalocele	2.6 (0.3 to 7.5)	2.8 (0.3 to 8.0)	--	--	0.8 (0.1 to 2.5)	0.9 (0.1 to 2.7)
Neural tube defects	Moderate motor impairment due to encephalocele	8.1 (3.4 to 15.2)	8.6 (3.6 to 16.2)	--	--	0.5 (0.2 to 1.0)	0.5 (0.2 to 1.1)
Neural tube defects	Moderate motor impairment and moderate intellectual disability due to encephalocele	3.9 (0.9 to 8.8)	4.2 (0.9 to 9.4)	--	--	0.8 (0.2 to 1.9)	0.8 (0.2 to 2.0)
Neural tube defects	Mild motor impairment, moderate intellectual disability and incontinence due to encephalocele	0.1 (< 0.05 to 0.7)	0.1 (< 0.05 to 0.7)	--	--	< 0.05 (< 0.05 to 0.2)	< 0.05 (< 0.05 to 0.2)
Neural tube defects	Mild intellectual disability and incontinence due to encephalocele	3.5 (0.7 to 8.5)	3.7 (0.7 to 9.1)	--	--	0.6 (0.1 to 1.6)	0.7 (0.1 to 1.7)
Neural tube defects	Severe motor impairment and severe intellectual disability due to encephalocele	7.3 (2.8 to 14.0)	7.8 (3.0 to 14.9)	--	--	3.6 (1.3 to 7.3)	3.9 (1.4 to 7.7)
Neural tube defects	Mild motor impairment, profound intellectual disability and incontinence due to encephalocele	0.1 (0.0 to 0.0)	0.1 (0.0 to 0.0)	--	--	< 0.05 (0.0 to < 0.05)	< 0.05 (0.0 to < 0.05)
Neural tube defects	Severe motor and cognitive impairment due to anencephaly	0.2 (0.2 to 0.3)	0.2 (0.2 to 0.3)	--	--	0.1 (0.1 to 0.2)	0.1 (0.1 to 0.2)
Neural tube defects	Incontinence due to encephalocele	8.0 (3.4 to 14.5)	8.5 (3.7 to 15.5)	--	--	1.1 (0.4 to 2.2)	1.2 (0.5 to 2.3)
Neural tube defects	Mild motor impairment and borderline intellectual disability due to spina bifida	5.5 (< 0.05 to 63.8)	6.4 (< 0.05 to 72.7)	--	--	0.1 (< 0.05 to 1.4)	0.1 (< 0.05 to 1.6)
Neural tube defects	Severe motor impairment and incontinence due to encephalocele	2.4 (0.2 to 7.1)	2.6 (0.2 to 7.6)	--	--	1.2 (0.1 to 3.7)	1.2 (0.1 to 3.9)
Neural tube defects	Moderate motor impairment, mild intellectual disability and incontinence due to spina bifida	96.4 (21.8 to 233.5)	110.8 (25.0 to 268.5)	--	--	26.2 (5.2 to 66.8)	30.1 (5.9 to 76.6)
Neural tube defects	Mild motor impairment, moderate intellectual disability and incontinence due to spina bifida	3.5 (< 0.05 to 24.8)	4.0 (< 0.05 to 28.4)	--	--	0.8 (< 0.05 to 5.6)	0.9 (< 0.05 to 6.5)
Neural tube defects	Severe motor impairment and mild intellectual disability due to encephalocele	7.6 (3.1 to 14.0)	8.1 (3.4 to 14.8)	--	--	3.2 (1.2 to 6.3)	3.5 (1.3 to 6.7)
Neural tube defects	Mild motor impairment, borderline intellectual disability and incontinence due to spina bifida	1.8 (0.0 to 0.0)	2.1 (0.0 to 0.0)	--	--	0.3 (0.0 to 0.0)	0.4 (0.0 to 0.0)
Neural tube defects	Borderline intellectual disability due to encephalocele	9.3 (4.4 to 16.7)	9.9 (4.7 to 17.8)	--	--	0.1 (< 0.05 to 0.2)	0.1 (< 0.05 to 0.3)
Neural tube defects	Severe motor impairment, severe intellectual disability and incontinence due to spina bifida	77.3 (10.5 to 204.3)	88.9 (12.1 to 236.0)	--	--	44.0 (5.6 to 117.9)	50.6 (6.5 to 135.3)
Neural tube defects	Severe motor impairment and borderline intellectual disability due to encephalocele	2.8 (0.4 to 7.4)	3.0 (0.4 to 7.9)	--	--	1.2 (0.2 to 3.3)	1.2 (0.2 to 3.5)
Neural tube defects	Severe motor impairment, moderate intellectual disability and incontinence due to spina bifida	89.2 (15.0 to 229.2)	102.5 (17.3 to 266.6)	--	--	47.6 (7.3 to 122.2)	54.7 (8.3 to 141.1)
Neural tube defects	Moderate motor impairment and borderline intellectual disability due to encephalocele	1.3 (< 0.05 to 5.7)	1.4 (< 0.05 to 6.0)	--	--	0.1 (< 0.05 to 0.4)	0.1 (< 0.05 to 0.4)
Neural tube defects	Mild motor impairment and incontinence due to spina bifida	48.3 (2.1 to 174.4)	55.6 (2.5 to 200.3)	--	--	7.2 (0.3 to 28.0)	8.2 (0.4 to 31.8)
Neural tube defects	Mild motor impairment, mild intellectual disability and incontinence due to spina bifida	4.8 (< 0.05 to 6.8)	5.5 (< 0.05 to 7.7)	--	--	0.8 (< 0.05 to 1.1)	1.0 (< 0.05 to 1.3)

Neural tube defects	Mild motor impairment, mild intellectual disability and incontinence due to encephalocele	0.1 (< 0.05 to 0.3)	0.1 (< 0.05 to 0.3)	--	--	< 0.05 (< 0.05 to 0.1)	< 0.05 (< 0.05 to 0.1)
Neural tube defects	Borderline intellectual disability and incontinence due to encephalocele	1.3 (< 0.05 to 6.1)	1.4 (< 0.05 to 6.5)	--	--	0.2 (< 0.05 to 0.9)	0.2 (< 0.05 to 1.0)
Neural tube defects	Profound intellectual disability due to encephalocele	18.9 (11.3 to 28.2)	20.2 (12.1 to 30.0)	--	--	3.8 (1.9 to 6.4)	4.0 (2.1 to 6.8)
Neural tube defects	Mild motor impairment due to spina bifida	294.4 (149.7 to 458.5)	338.5 (172.0 to 527.0)	--	--	3.0 (1.1 to 6.6)	3.4 (1.2 to 7.6)
Neural tube defects	Moderate motor impairment, profound intellectual disability and incontinence due to spina bifida	77.6 (7.6 to 213.0)	89.2 (8.7 to 241.1)	--	--	27.3 (2.6 to 73.2)	31.4 (3.0 to 84.4)
Neural tube defects	Moderate motor impairment and borderline intellectual disability due to spina bifida	2.1 (0.0 to 0.0)	2.5 (0.0 to 0.0)	--	--	0.2 (0.0 to 0.0)	0.2 (0.0 to 0.0)
Neural tube defects	Mild motor impairment and mild intellectual disability due to encephalocele	0.7 (< 0.05 to 5.1)	0.7 (< 0.05 to 5.5)	--	--	< 0.05 (< 0.05 to 0.2)	< 0.05 (< 0.05 to 0.2)
Neural tube defects	Severe motor impairment due to spina bifida	405.3 (237.2 to 611.7)	465.9 (271.5 to 703.5)	--	--	158.3 (81.4 to 258.5)	181.8 (93.9 to 298.3)
Neural tube defects	Severe motor impairment, borderline intellectual disability and incontinence due to encephalocele	0.4 (< 0.05 to 4.1)	0.4 (< 0.05 to 4.5)	--	--	0.2 (< 0.05 to 2.0)	0.2 (< 0.05 to 2.1)
Neural tube defects	Moderate intellectual disability due to encephalocele	26.9 (17.8 to 38.2)	28.7 (19.0 to 40.9)	--	--	2.7 (1.5 to 4.3)	2.9 (1.6 to 4.6)
Neural tube defects	Severe motor impairment and moderate intellectual disability due to spina bifida	25.8 (< 0.05 to 149.9)	29.6 (< 0.05 to 171.5)	--	--	11.8 (< 0.05 to 67.6)	13.6 (< 0.05 to 77.9)
Neural tube defects	Moderate motor impairment due to spina bifida	47.9 (1.3 to 166.1)	55.1 (1.5 to 189.6)	--	--	2.9 (0.1 to 9.8)	3.3 (0.1 to 11.2)
Neural tube defects	Mild motor impairment and incontinence due to encephalocele	0.2 (< 0.05 to 2.2)	0.2 (< 0.05 to 2.3)	--	--	< 0.05 (< 0.05 to 0.4)	< 0.05 (< 0.05 to 0.4)
Neural tube defects	Mild motor impairment and moderate intellectual disability due to encephalocele	0.8 (< 0.05 to 5.5)	0.8 (< 0.05 to 5.9)	--	--	0.1 (< 0.05 to 0.6)	0.1 (< 0.05 to 0.6)
Neural tube defects	profound motor impairment, profound intellectual disability and incontinence due to spina bifida	60.8 (4.2 to 186.0)	69.9 (4.9 to 211.7)	--	--	35.6 (2.4 to 113.2)	40.9 (2.8 to 131.3)
Neural tube defects	Mild motor impairment and profound intellectual disability due to encephalocele	0.5 (< 0.05 to 4.0)	0.6 (< 0.05 to 4.2)	--	--	0.3 (< 0.05 to 2.0)	0.3 (< 0.05 to 2.1)
Neural tube defects	Moderate motor impairment and profound intellectual disability due to spina bifida	2.5 (< 0.05 to 0.7)	2.9 (< 0.05 to 0.8)	--	--	0.6 (< 0.05 to 0.2)	0.7 (< 0.05 to 0.2)
Neural tube defects	Moderate motor impairment, mild intellectual disability and incontinence due to encephalocele	0.5 (< 0.05 to 3.8)	0.5 (< 0.05 to 4.1)	--	--	0.1 (< 0.05 to 1.0)	0.1 (< 0.05 to 1.1)
Neural tube defects	Moderate motor impairment, borderline intellectual disability and incontinence due to encephalocele	0.2 (< 0.05 to 2.0)	0.2 (< 0.05 to 2.2)	--	--	< 0.05 (< 0.05 to 0.4)	< 0.05 (< 0.05 to 0.4)
Neural tube defects	Severe motor impairment, moderate intellectual disability and incontinence due to encephalocele	1.1 (< 0.05 to 5.4)	1.2 (< 0.05 to 5.8)	--	--	0.6 (< 0.05 to 2.8)	0.6 (< 0.05 to 2.9)
Neural tube defects	Severe motor impairment, mild intellectual disability and incontinence due to encephalocele	1.1 (< 0.05 to 5.0)	1.2 (< 0.05 to 5.4)	--	--	0.5 (< 0.05 to 2.5)	0.6 (< 0.05 to 2.7)
Neural tube defects	Mild motor impairment, severe intellectual disability and incontinence due to spina bifida	3.6 (< 0.05 to 17.9)	4.1 (< 0.05 to 20.7)	--	--	0.9 (< 0.05 to 5.7)	1.1 (< 0.05 to 6.5)
Neural tube defects	Severe motor impairment, profound intellectual disability and incontinence due to encephalocele	0.9 (< 0.05 to 5.5)	1.0 (< 0.05 to 5.8)	--	--	0.5 (< 0.05 to 3.2)	0.6 (< 0.05 to 3.4)
Neural tube defects	Mild motor impairment due to encephalocele	1.5 (< 0.05 to 5.8)	1.6 (< 0.05 to 6.2)	--	--	< 0.05 (< 0.05 to 0.1)	< 0.05 (< 0.05 to 0.1)
Neural tube defects	Mild motor impairment, borderline intellectual disability and incontinence due to encephalocele	< 0.05 (0.0 to < 0.05)	< 0.05 (0.0 to < 0.05)	--	--	< 0.05 (0.0 to < 0.05)	< 0.05 (0.0 to < 0.05)
Neural tube defects	Severe motor impairment due to encephalocele	17.3 (10.1 to 26.9)	18.4 (10.8 to 28.3)	--	--	6.9 (3.4 to 12.0)	7.4 (3.7 to 12.6)
Neural tube defects	Moderate motor impairment, moderate intellectual disability and incontinence due to spina bifida	103.7 (22.6 to 236.6)	119.2 (26.0 to 270.1)	--	--	28.2 (6.0 to 69.1)	32.5 (6.9 to 78.9)
Neural tube defects	Mild motor impairment and severe intellectual disability due to encephalocele	0.6 (< 0.05 to 4.5)	0.6 (< 0.05 to 4.8)	--	--	0.1 (< 0.05 to 0.7)	0.1 (< 0.05 to 0.8)
Neural tube defects	Severe motor impairment, mild intellectual disability and incontinence due to spina bifida	82.5 (13.3 to 211.1)	94.9 (15.3 to 242.8)	--	--	41.8 (5.9 to 112.5)	48.1 (6.8 to 128.1)
Neural tube defects	Severe motor impairment, borderline intellectual disability and incontinence due to spina bifida	29.8 (0.1 to 147.1)	34.2 (0.1 to 169.1)	--	--	14.6 (< 0.05 to 80.1)	16.8 (< 0.05 to 91.5)
Neural tube defects	Moderate motor impairment, borderline intellectual disability and incontinence due to spina bifida	36.6 (0.2 to 158.4)	42.1 (0.2 to 182.7)	--	--	7.3 (< 0.05 to 30.5)	8.4 (0.1 to 35.2)
Neural tube defects	Moderate motor impairment and severe intellectual disability due to spina bifida	2.7 (< 0.05 to 2.0)	3.1 (< 0.05 to 2.3)	--	--	0.6 (< 0.05 to 0.4)	0.7 (< 0.05 to 0.5)
Neural tube defects	Mild motor impairment and moderate intellectual disability due to spina bifida	18.2 (< 0.05 to 119.4)	21.0 (< 0.05 to 137.6)	--	--	1.9 (< 0.05 to 12.7)	2.2 (< 0.05 to 14.5)
Neural tube defects	Mild motor impairment and profound intellectual disability due to spina bifida	13.4 (< 0.05 to 122.2)	15.5 (< 0.05 to 141.8)	--	--	2.8 (< 0.05 to 23.2)	3.2 (< 0.05 to 26.4)
Neural tube defects	Mild motor impairment and mild intellectual disability due to spina bifida	17.4 (< 0.05 to 125.3)	20.1 (< 0.05 to 146.6)	--	--	0.6 (< 0.05 to 3.9)	0.6 (< 0.05 to 4.5)
Neural tube defects	Moderate motor impairment and moderate intellectual disability due to spina bifida	2.2 (< 0.05 to 7.7)	2.5 (< 0.05 to 8.8)	--	--	0.4 (< 0.05 to 1.6)	0.5 (< 0.05 to 1.8)
Neural tube defects	Moderate motor impairment and incontinence due to spina bifida	1,650.2 (1,341.3 to 1,971.4)	1,897.2 (1,533.3 to 2,273.9)	--	--	298.2 (196.8 to 416.2)	342.3 (227.2 to 476.7)
Neural tube defects	Moderate intellectual disability and incontinence due to encephalocele	3.8 (0.7 to 9.5)	4.1 (0.8 to 10.1)	--	--	0.9 (0.2 to 2.1)	0.9 (0.2 to 2.2)
Neural tube defects	Moderate motor impairment, moderate intellectual disability and incontinence due to encephalocele	0.6 (< 0.05 to 5.5)	0.7 (< 0.05 to 5.9)	--	--	0.2 (< 0.05 to 1.4)	0.2 (< 0.05 to 1.5)
Neural tube defects	Moderate motor impairment, severe intellectual disability and incontinence due to encephalocele	0.5 (< 0.05 to 5.0)	0.6 (< 0.05 to 5.3)	--	--	0.2 (< 0.05 to 1.7)	0.2 (< 0.05 to 1.8)
Congenital heart anomalies	Congenital heart disease and profound intellectual disability without heart failure due to severe congenital heart anomalies excluding single ventricle heart defects	10.9 (7.2 to 14.9)	11.3 (7.4 to 15.4)	--	--	2.2 (1.2 to 3.4)	2.3 (1.1 to 3.5)
Congenital heart anomalies	Congenital heart disease, mild intellectual disability and severe heart failure due to single ventricle and single ventricle pathway heart defects	0.1 (0.1 to 0.2)	0.1 (0.1 to 0.2)	--	--	< 0.05 (< 0.05 to < 0.05)	< 0.05 (< 0.05 to < 0.05)
Congenital heart anomalies	Congenital heart disease, severe intellectual disability and mild heart failure due to critical malformations of great vessels, congenital valvular heart disease and patent	< 0.05 (< 0.05 to < 0.05)	< 0.05 (< 0.05 to < 0.05)	--	--	< 0.05 (< 0.05 to < 0.05)	< 0.05 (< 0.05 to < 0.05)

Congenital heart anomalies	Congenital heart disease, mild intellectual disability and moderate heart failure due to severe congenital heart anomalies excluding single ventricle heart defects	0.2 (0.1 to 0.3)	0.2 (0.1 to 0.3)	--	--	< 0.05 (< 0.05 to < 0.05)	< 0.05 (< 0.05 to < 0.05)
Congenital heart anomalies	Congenital heart disease, severe intellectual disability and mild heart failure due to single ventricle and single ventricle pathway heart defects	< 0.05 (< 0.05 to < 0.05)	< 0.05 (< 0.05 to < 0.05)	--	--	< 0.05 (< 0.05 to < 0.05)	< 0.05 (< 0.05 to < 0.05)
Congenital heart anomalies	Congenital heart disease, severe intellectual disability and mild heart failure due to ventricular septal defect and atrial septal defect	< 0.05 (< 0.05 to < 0.05)	< 0.05 (< 0.05 to < 0.05)	--	--	< 0.05 (< 0.05 to < 0.05)	< 0.05 (< 0.05 to < 0.05)
Congenital heart anomalies	Congenital heart disease, boderline intellectual disability and mild heart failure due to ventricular septal defect and atrial septal defect	0.6 (0.3 to 1.0)	0.6 (0.3 to 0.9)	--	--	< 0.05 (< 0.05 to 0.1)	< 0.05 (< 0.05 to 0.1)
Congenital heart anomalies	Congenital heart disease, profound intellectual disability and severe heart failure due to critical malformations of great vessels, congenital valvular heart disease and patent	0.1 (< 0.05 to 0.1)	0.1 (< 0.05 to 0.1)	--	--	< 0.05 (< 0.05 to < 0.05)	< 0.05 (< 0.05 to < 0.05)
Congenital heart anomalies	Congenital heart disease, moderate intellectual disability and severe heart failure due to ventricular septal defect and atrial septal defect	0.1 (< 0.05 to 0.2)	0.1 (< 0.05 to 0.1)	--	--	< 0.05 (< 0.05 to < 0.05)	< 0.05 (< 0.05 to < 0.05)
Congenital heart anomalies	Congenital heart disease, moderate intellectual disability and mild heart failure due to ventricular septal defect and atrial septal defect	< 0.05 (< 0.05 to 0.1)	< 0.05 (< 0.05 to 0.1)	--	--	< 0.05 (< 0.05 to < 0.05)	< 0.05 (< 0.05 to < 0.05)
Congenital heart anomalies	Asymptomatic ventricular septal defect and atrial septal defect	2,231.6 (1,941.2 to 2,555.2)	2,244.8 (1,925.4 to 2,589.5)	--	--	< 0.05 (0.0 to < 0.05)	< 0.05 (0.0 to < 0.05)
Congenital heart anomalies	Congenital heart disease, profound intellectual disability and moderate heart failure due to ventricular septal defect and atrial septal defect	< 0.05 (< 0.05 to < 0.05)	< 0.05 (< 0.05 to < 0.05)	--	--	< 0.05 (< 0.05 to < 0.05)	< 0.05 (< 0.05 to < 0.05)
Congenital heart anomalies	Congenital heart disease, profound intellectual disability and mild heart failure due to critical malformations of great vessels, congenital valvular heart disease and patent	< 0.05 (< 0.05 to < 0.05)	< 0.05 (< 0.05 to 0.1)	--	--	< 0.05 (< 0.05 to < 0.05)	< 0.05 (< 0.05 to < 0.05)
Congenital heart anomalies	Congenital heart disease, profound intellectual disability and mild heart failure due to severe congenital heart anomalies excluding single ventricle heart defects	< 0.05 (< 0.05 to 0.1)	< 0.05 (< 0.05 to 0.1)	--	--	< 0.05 (< 0.05 to < 0.05)	< 0.05 (< 0.05 to < 0.05)
Congenital heart anomalies	Congenital heart disease, profound intellectual disability and mild heart failure due to single ventricle and single ventricle pathway heart defects	< 0.05 (< 0.05 to < 0.05)	< 0.05 (< 0.05 to < 0.05)	--	--	< 0.05 (< 0.05 to < 0.05)	< 0.05 (< 0.05 to < 0.05)
Congenital heart anomalies	Congenital heart disease and moderate intellectual disability without heart failure due to single ventricle and single ventricle pathway heart defects	3.4 (2.1 to 5.0)	2.9 (1.8 to 4.4)	--	--	0.3 (0.2 to 0.6)	0.3 (0.2 to 0.5)
Congenital heart anomalies	Congenital heart disease and severe intellectual disability without heart failure due to severe congenital heart anomalies excluding single ventricle heart defects	6.7 (2.3 to 10.7)	6.5 (1.9 to 10.8)	--	--	1.1 (0.3 to 1.8)	1.0 (0.3 to 1.9)
Congenital heart anomalies	Congenital heart disease and mild intellectual disability without heart failure due to other congenital cardiovascular anomalies	74.7 (62.5 to 86.4)	79.8 (66.6 to 92.7)	--	--	3.2 (1.9 to 4.8)	3.4 (2.0 to 5.2)
Congenital heart anomalies	Congenital heart disease and profound intellectual disability without heart failure due to single ventricle and single ventricle pathway heart defects	2.0 (1.2 to 2.9)	1.8 (1.1 to 2.6)	--	--	0.4 (0.2 to 0.7)	0.4 (0.2 to 0.6)
Congenital heart anomalies	Congenital heart disease, mild intellectual disability and mild heart failure due to severe congenital heart anomalies excluding single ventricle heart defects	0.3 (0.2 to 0.4)	0.3 (0.2 to 0.4)	--	--	< 0.05 (< 0.05 to < 0.05)	< 0.05 (< 0.05 to < 0.05)
Congenital heart anomalies	Congenital heart disease, moderate intellectual disability and moderate heart failure due to single ventricle and single ventricle pathway heart defects	< 0.05 (< 0.05 to < 0.05)	< 0.05 (< 0.05 to < 0.05)	--	--	< 0.05 (< 0.05 to < 0.05)	< 0.05 (< 0.05 to < 0.05)
Congenital heart anomalies	Congenital heart disease, borderline intellectual disability and moderate heart failure due to critical malformations of great vessels, congenital valvular heart disease and	0.4 (0.3 to 0.7)	0.5 (0.3 to 0.7)	--	--	< 0.05 (< 0.05 to 0.1)	< 0.05 (< 0.05 to 0.1)
Congenital heart anomalies	Congenital heart disease, mild intellectual disability and severe heart failure due to critical malformations of great vessels, congenital valvular heart disease and patent	0.4 (0.3 to 0.5)	0.4 (0.3 to 0.6)	--	--	0.1 (0.1 to 0.1)	0.1 (0.1 to 0.1)
Congenital heart anomalies	Congenital heart disease, mild intellectual disability and moderate heart failure due to single ventricle and single ventricle pathway heart defects	< 0.05 (< 0.05 to 0.1)	< 0.05 (< 0.05 to 0.1)	--	--	< 0.05 (< 0.05 to < 0.05)	< 0.05 (< 0.05 to < 0.05)
Congenital heart anomalies	Congenital heart disease, boderline intellectual disability and mild heart failure due to severe congenital heart anomalies excluding single ventricle heart defects	0.6 (0.3 to 1.0)	0.7 (0.4 to 1.0)	--	--	< 0.05 (< 0.05 to 0.1)	< 0.05 (< 0.05 to 0.1)
Congenital heart anomalies	Congenital heart disease, severe intellectual disability and moderate heart failure due to ventricular septal defect and atrial septal defect	< 0.05 (< 0.05 to < 0.05)	< 0.05 (< 0.05 to < 0.05)	--	--	< 0.05 (< 0.05 to < 0.05)	< 0.05 (< 0.05 to < 0.05)
Congenital heart anomalies	Congenital heart disease, profound intellectual disability and moderate heart failure due to severe congenital heart anomalies excluding single ventricle heart defects	< 0.05 (< 0.05 to 0.1)	< 0.05 (< 0.05 to 0.1)	--	--	< 0.05 (< 0.05 to < 0.05)	< 0.05 (< 0.05 to < 0.05)
Congenital heart anomalies	Congenital heart disease, borderline intellectual disability and severe heart failure due to severe congenital heart anomalies excluding single ventricle heart defects	1.4 (0.9 to 1.9)	1.5 (1.0 to 2.1)	--	--	0.3 (0.1 to 0.4)	0.3 (0.2 to 0.4)
Congenital heart anomalies	Congenital heart disease, severe intellectual disability and mild heart failure due to severe congenital heart anomalies excluding single ventricle heart defects	< 0.05 (< 0.05 to 0.1)	< 0.05 (< 0.05 to 0.1)	--	--	< 0.05 (< 0.05 to < 0.05)	< 0.05 (< 0.05 to < 0.05)
Congenital heart anomalies	Congenital heart disease, severe intellectual disability and moderate heart failure due to severe congenital heart anomalies excluding single ventricle heart defects	< 0.05 (< 0.05 to < 0.05)	< 0.05 (< 0.05 to < 0.05)	--	--	< 0.05 (< 0.05 to < 0.05)	< 0.05 (< 0.05 to < 0.05)
Congenital heart anomalies	Congenital heart disease and moderate heart failure without intellectual disability due to critical malformations of great vessels, congenital valvular heart disease and patent	7.1 (4.8 to 9.8)	7.6 (5.2 to 10.5)	--	--	0.5 (0.3 to 0.8)	0.5 (0.3 to 0.9)
Congenital heart anomalies	Congenital heart disease and borderline intellectual disability without heart failure due to other congenital cardiovascular anomalies	157.6 (125.7 to 185.0)	160.6 (126.9 to 190.3)	--	--	1.7 (0.8 to 3.2)	1.7 (0.8 to 3.3)
Congenital heart anomalies	Congenital heart disease, borderline intellectual disability and moderate heart failure due to severe congenital heart anomalies excluding single ventricle heart defects	0.5 (0.3 to 0.8)	0.5 (0.3 to 0.8)	--	--	< 0.05 (< 0.05 to 0.1)	< 0.05 (< 0.05 to 0.1)
Congenital heart anomalies	Congenital heart disease and moderate heart failure without intellectual disability due to ventricular septal defect and atrial septal defect	3.2 (2.1 to 4.6)	3.0 (1.9 to 4.5)	--	--	0.2 (0.1 to 0.4)	0.2 (0.1 to 0.4)
Congenital heart anomalies	Congenital heart disease and mild heart failure without intellectual disability due to single ventricle and single ventricle pathway heart defects	2.6 (1.7 to 3.7)	2.7 (1.7 to 3.8)	--	--	0.1 (0.1 to 0.2)	0.1 (0.1 to 0.2)
Congenital heart anomalies	Congenital heart disease, mild intellectual disability and moderate heart failure due to critical malformations of great vessels, congenital valvular heart disease and patent	0.1 (0.1 to 0.2)	0.2 (0.1 to 0.2)	--	--	< 0.05 (< 0.05 to < 0.05)	< 0.05 (< 0.05 to < 0.05)
Congenital heart anomalies	Congenital heart disease, profound intellectual disability and moderate heart failure due to critical malformations of great vessels, congenital valvular heart disease and	< 0.05 (< 0.05 to < 0.05)	< 0.05 (< 0.05 to < 0.05)	--	--	< 0.05 (< 0.05 to < 0.05)	< 0.05 (< 0.05 to < 0.05)
Congenital heart anomalies	Congenital heart disease, moderate intellectual disability and mild heart failure due to critical malformations of great vessels, congenital valvular heart disease and patent	0.1 (< 0.05 to 0.1)	0.1 (< 0.05 to 0.1)	--	--	< 0.05 (< 0.05 to < 0.05)	< 0.05 (< 0.05 to < 0.05)
Congenital heart anomalies	Congenital heart disease, moderate intellectual disability and severe heart failure due to critical malformations of great vessels, congenital valvular heart disease and patent	0.1 (0.1 to 0.2)	0.1 (0.1 to 0.2)	--	--	< 0.05 (< 0.05 to 0.1)	< 0.05 (< 0.05 to 0.1)
Congenital heart anomalies	Congenital heart disease, profound intellectual disability and severe heart failure due to severe congenital heart anomalies excluding single ventricle heart defects	0.1 (0.1 to 0.1)	0.1 (0.1 to 0.2)	--	--	< 0.05 (< 0.05 to 0.1)	< 0.05 (< 0.05 to 0.1)
Congenital heart anomalies	Congenital heart disease without heart failure or intellectual disability due to ventricular septal defect and atrial septal defect	2,397.8 (2,089.1 to 2,747.0)	2,419.8 (2,097.0 to 2,771.6)	--	--	142.6 (55.5 to 251.7)	143.9 (56.7 to 249.3)
Congenital heart anomalies	Congenital heart disease, borderline intellectual disability and moderate heart failure due to ventricular septal defect and atrial septal defect	0.5 (0.3 to 0.8)	0.5 (0.3 to 0.7)	--	--	< 0.05 (< 0.05 to 0.1)	< 0.05 (< 0.05 to 0.1)
Congenital heart anomalies	Congenital heart disease, borderline intellectual disability and severe heart failure due to ventricular septal defect and atrial septal defect	1.3 (0.9 to 1.9)	1.3 (0.8 to 1.9)	--	--	0.3 (0.1 to 0.4)	0.2 (0.1 to 0.4)
Congenital heart anomalies	Congenital heart disease without intellectual disability or heart failure due to single ventricle and single ventricle pathway heart defects	349.2 (281.5 to 420.9)	367.3 (294.0 to 446.9)	--	--	21.0 (8.1 to 38.0)	22.1 (8.4 to 39.8)
Congenital heart anomalies	Congenital heart disease and borderline intellectual disability without heart failure due to ventricular septal defect and atrial septal defect	215.1 (161.9 to 261.4)	214.9 (159.8 to 262.8)	--	--	2.3 (1.0 to 4.5)	2.3 (1.0 to 4.4)
Congenital heart anomalies	Congenital heart disease and moderate intellectual disability without heart failure due to other congenital cardiovascular anomalies	23.3 (16.6 to 31.0)	23.4 (16.3 to 31.7)	--	--	2.3 (1.4 to 3.8)	2.3 (1.4 to 3.8)

Congenital heart anomalies	Congenital heart disease and borderline intellectual disability without heart failure due to severe congenital heart anomalies excluding single ventricle heart defects	146.1 (110.7 to 181.0)	150.8 (113.5 to 188.1)	--	--	1.6 (0.7 to 2.9)	1.6 (0.7 to 3.0)
Congenital heart anomalies	Congenital heart disease, profound intellectual disability and severe heart failure due to single ventricle and single ventricle pathway heart defects	< 0.05 (< 0.05 to < 0.05)	< 0.05 (< 0.05 to < 0.05)	--	--	< 0.05 (< 0.05 to < 0.05)	< 0.05 (< 0.05 to < 0.05)
Congenital heart anomalies	Congenital heart disease, mild intellectual disability and mild heart failure due to critical malformations of great vessels, congenital valvular heart disease and patent ductus	0.2 (0.1 to 0.3)	0.2 (0.1 to 0.3)	--	--	< 0.05 (< 0.05 to < 0.05)	< 0.05 (< 0.05 to < 0.05)
Congenital heart anomalies	Congenital heart disease without intellectual disability or heart failure due to other congenital cardiovascular anomalies	3,994.7 (3,611.6 to 4,394.9)	4,467.3 (4,003.3 to 4,962.8)	--	--	233.6 (93.2 to 409.2)	260.5 (104.5 to 455.3)
Congenital heart anomalies	Congenital heart disease, mild intellectual disability and moderate heart failure due to ventricular septal defect and atrial septal defect	0.1 (0.1 to 0.2)	0.1 (0.1 to 0.2)	--	--	< 0.05 (< 0.05 to < 0.05)	< 0.05 (< 0.05 to < 0.05)
Congenital heart anomalies	Congenital heart disease, moderate intellectual disability and mild heart failure due to severe congenital heart anomalies excluding single ventricle heart defects	0.1 (0.1 to 0.1)	0.1 (0.1 to 0.2)	--	--	< 0.05 (< 0.05 to < 0.05)	< 0.05 (< 0.05 to < 0.05)
Congenital heart anomalies	Congenital heart disease, mild intellectual disability and severe heart failure due to ventricular septal defect and atrial septal defect	0.3 (0.2 to 0.5)	0.3 (0.2 to 0.4)	--	--	0.1 (< 0.05 to 0.1)	0.1 (< 0.05 to 0.1)
Congenital heart anomalies	Congenital heart disease, moderate intellectual disability and severe heart failure due to severe congenital heart anomalies excluding single ventricle heart defects	0.2 (0.1 to 0.3)	0.2 (0.1 to 0.3)	--	--	0.1 (< 0.05 to 0.1)	0.1 (< 0.05 to 0.1)
Congenital heart anomalies	Congenital heart disease and profound intellectual disability without heart failure due to critical malformations of great vessels, congenital valvular heart disease and patent	6.8 (4.4 to 9.4)	6.9 (4.1 to 9.5)	--	--	1.4 (0.7 to 2.2)	1.4 (0.7 to 2.2)
Congenital heart anomalies	Congenital heart disease, profound intellectual disability and mild heart failure due to ventricular septal defect and atrial septal defect	< 0.05 (< 0.05 to 0.1)	< 0.05 (< 0.05 to 0.1)	--	--	< 0.05 (< 0.05 to < 0.05)	< 0.05 (< 0.05 to < 0.05)
Congenital heart anomalies	Congenital heart disease, profound intellectual disability and moderate heart failure due to single ventricle and single ventricle pathway heart defects	< 0.05 (< 0.05 to < 0.05)	< 0.05 (< 0.05 to < 0.05)	--	--	< 0.05 (< 0.05 to < 0.05)	< 0.05 (< 0.05 to < 0.05)
Congenital heart anomalies	Congenital heart disease and mild heart failure without intellectual disability due to severe congenital heart anomalies excluding single ventricle heart defects	11.1 (6.9 to 15.5)	11.7 (7.4 to 16.5)	--	--	0.5 (0.2 to 0.8)	0.5 (0.3 to 0.8)
Congenital heart anomalies	Congenital heart disease and severe intellectual disability without heart failure due to single ventricle and single ventricle pathway heart defects	1.0 (0.4 to 1.6)	0.8 (0.3 to 1.4)	--	--	0.2 (0.1 to 0.3)	0.1 (< 0.05 to 0.2)
Congenital heart anomalies	Congenital heart disease, moderate intellectual disability and moderate heart failure due to critical malformations of great vessels, congenital valvular heart disease and	< 0.05 (< 0.05 to 0.1)	< 0.05 (< 0.05 to 0.1)	--	--	< 0.05 (< 0.05 to < 0.05)	< 0.05 (< 0.05 to < 0.05)
Congenital heart anomalies	Congenital heart disease, mild intellectual disability and severe heart failure due to severe congenital heart anomalies excluding single ventricle heart defects	0.6 (0.4 to 0.8)	0.6 (0.4 to 0.8)	--	--	0.1 (0.1 to 0.2)	0.1 (0.1 to 0.2)
Congenital heart anomalies	Congenital heart disease, profound intellectual disability and severe heart failure due to ventricular septal defect and atrial septal defect	0.1 (< 0.05 to 0.1)	0.1 (< 0.05 to 0.1)	--	--	< 0.05 (< 0.05 to < 0.05)	< 0.05 (< 0.05 to < 0.05)
Congenital heart anomalies	Congenital heart disease and borderline intellectual disability without heart failure due to single ventricle and single ventricle pathway heart defects	30.4 (22.1 to 38.7)	29.1 (21.1 to 37.0)	--	--	0.3 (0.1 to 0.6)	0.3 (0.1 to 0.6)
Congenital heart anomalies	Congenital heart disease, mild intellectual disability and mild heart failure due to single ventricle and single ventricle pathway heart defects	0.1 (< 0.05 to 0.1)	0.1 (< 0.05 to 0.1)	--	--	< 0.05 (< 0.05 to < 0.05)	< 0.05 (< 0.05 to < 0.05)
Congenital heart anomalies	Congenital heart disease, severe intellectual disability and severe heart failure due to ventricular septal defect and atrial septal defect	< 0.05 (< 0.05 to 0.1)	< 0.05 (< 0.05 to 0.1)	--	--	< 0.05 (< 0.05 to < 0.05)	< 0.05 (< 0.05 to < 0.05)
Congenital heart anomalies	Congenital heart disease, mild intellectual disability and mild heart failure due to ventricular septal defect and atrial septal defect	0.1 (0.1 to 0.2)	0.1 (0.1 to 0.2)	--	--	< 0.05 (< 0.05 to < 0.05)	< 0.05 (< 0.05 to < 0.05)
Congenital heart anomalies	Congenital heart disease and moderate heart failure without intellectual disability due to single ventricle and single ventricle pathway heart defects	2.2 (1.5 to 3.0)	2.2 (1.5 to 3.0)	--	--	0.2 (0.1 to 0.3)	0.2 (0.1 to 0.3)
Congenital heart anomalies	Congenital heart disease and severe heart failure without intellectual disability due to severe congenital heart anomalies excluding single ventricle heart defects	24.5 (19.5 to 30.4)	26.0 (20.6 to 32.3)	--	--	4.4 (2.8 to 6.5)	4.7 (3.0 to 6.8)
Congenital heart anomalies	Congenital heart disease and moderate heart failure without intellectual disability due to severe congenital heart anomalies excluding single ventricle heart defects	9.0 (6.3 to 12.2)	9.6 (6.7 to 12.9)	--	--	0.6 (0.4 to 1.0)	0.7 (0.4 to 1.1)
Congenital heart anomalies	Congenital heart disease, moderate intellectual disability and moderate heart failure due to ventricular septal defect and atrial septal defect	< 0.05 (< 0.05 to 0.1)	< 0.05 (< 0.05 to 0.1)	--	--	< 0.05 (< 0.05 to < 0.05)	< 0.05 (< 0.05 to < 0.05)
Congenital heart anomalies	Congenital heart disease and severe heart failure without intellectual disability due to single ventricle and single ventricle pathway heart defects	5.8 (4.5 to 7.5)	5.9 (4.5 to 7.6)	--	--	1.0 (0.6 to 1.6)	1.1 (0.6 to 1.6)
Congenital heart anomalies	Congenital heart disease and moderate intellectual disability without heart failure due to critical malformations of great vessels, congenital valvular heart disease and patent	12.2 (8.1 to 17.1)	11.7 (7.5 to 16.7)	--	--	1.2 (0.7 to 1.9)	1.2 (0.6 to 1.9)
Congenital heart anomalies	Congenital heart disease, moderate intellectual disability and moderate heart failure due to severe congenital heart anomalies excluding single ventricle heart defects	0.1 (< 0.05 to 0.1)	0.1 (< 0.05 to 0.1)	--	--	< 0.05 (< 0.05 to < 0.05)	< 0.05 (< 0.05 to < 0.05)
Congenital heart anomalies	Congenital heart disease and mild intellectual disability without heart failure due to severe congenital heart anomalies excluding single ventricle heart defects	59.6 (47.5 to 72.1)	63.8 (50.9 to 77.0)	--	--	2.5 (1.4 to 3.9)	2.7 (1.5 to 4.3)
Congenital heart anomalies	Congenital heart disease, severe intellectual disability and severe heart failure due to critical malformations of great vessels, congenital valvular heart disease and patent	< 0.05 (< 0.05 to 0.1)	< 0.05 (< 0.05 to 0.1)	--	--	< 0.05 (< 0.05 to < 0.05)	< 0.05 (< 0.05 to < 0.05)
Congenital heart anomalies	Congenital heart disease, borderline intellectual disability and severe heart failure due to critical malformations of great vessels, congenital valvular heart disease and patent	1.2 (0.8 to 1.7)	1.3 (0.9 to 1.9)	--	--	0.2 (0.1 to 0.3)	0.2 (0.1 to 0.4)
Congenital heart anomalies	Congenital heart disease and profound intellectual disability without heart failure due to other congenital cardiovascular anomalies	11.7 (7.4 to 15.4)	12.1 (7.6 to 16.0)	--	--	2.3 (1.2 to 3.6)	2.4 (1.2 to 3.7)
Congenital heart anomalies	Congenital heart disease, borderline intellectual disability and moderate heart failure due to single ventricle and single ventricle pathway heart defects	0.1 (0.1 to 0.2)	0.1 (0.1 to 0.2)	--	--	< 0.05 (< 0.05 to < 0.05)	< 0.05 (< 0.05 to < 0.05)
Congenital heart anomalies	Congenital heart disease without intellectual disability or heart failure due to severe congenital heart anomalies excluding single ventricle heart defects	2,547.8 (2,209.9 to 2,926.6)	2,892.7 (2,494.3 to 3,333.4)	--	--	149.2 (58.5 to 262.6)	169.1 (66.9 to 297.1)
Congenital heart anomalies	Congenital heart disease, severe intellectual disability and moderate heart failure due to single ventricle and single ventricle pathway heart defects	< 0.05 (< 0.05 to < 0.05)	< 0.05 (< 0.05 to < 0.05)	--	--	< 0.05 (< 0.05 to < 0.05)	< 0.05 (< 0.05 to < 0.05)
Congenital heart anomalies	Congenital heart disease, boderline intellectual disability and mild heart failure due to single ventricle and single ventricle pathway heart defects	0.1 (0.1 to 0.2)	0.1 (0.1 to 0.2)	--	--	< 0.05 (< 0.05 to < 0.05)	< 0.05 (< 0.05 to < 0.05)
Congenital heart anomalies	Congenital heart disease and severe heart failure without intellectual disability due to critical malformations of great vessels, congenital valvular heart disease and patent	19.3 (15.1 to 24.3)	20.6 (16.0 to 25.9)	--	--	3.5 (2.2 to 5.1)	3.7 (2.3 to 5.5)
Congenital heart anomalies	Congenital heart disease and mild intellectual disability without heart failure due to single ventricle and single ventricle pathway heart defects	10.5 (7.9 to 13.5)	10.2 (7.6 to 13.0)	--	--	0.4 (0.3 to 0.7)	0.4 (0.2 to 0.7)
Congenital heart anomalies	Congenital heart disease and mild heart failure without intellectual disability due to critical malformations of great vessels, congenital valvular heart disease and patent	8.7 (5.6 to 12.4)	9.3 (6.0 to 13.2)	--	--	0.4 (0.2 to 0.6)	0.4 (0.2 to 0.7)
Congenital heart anomalies	Congenital heart disease and severe intellectual disability without heart failure due to ventricular septal defect and atrial septal defect	5.4 (1.8 to 8.8)	4.7 (1.4 to 8.0)	--	--	0.9 (0.3 to 1.5)	0.7 (0.2 to 1.4)
Congenital heart anomalies	Congenital heart disease, boderline intellectual disability and mild heart failure due to critical malformations of great vessels, congenital valvular heart disease and patent	0.5 (0.3 to 0.8)	0.6 (0.3 to 0.9)	--	--	< 0.05 (< 0.05 to 0.1)	< 0.05 (< 0.05 to 0.1)
Congenital heart anomalies	Congenital heart disease, borderline intellectual disability and severe heart failure due to single ventricle and single ventricle pathway heart defects	0.3 (0.2 to 0.4)	0.3 (0.2 to 0.5)	--	--	0.1 (< 0.05 to 0.1)	0.1 (< 0.05 to 0.1)
Congenital heart anomalies	Congenital heart disease, moderate intellectual disability and severe heart failure due to single ventricle and single ventricle pathway heart defects	< 0.05 (< 0.05 to 0.1)	< 0.05 (< 0.05 to 0.1)	--	--	< 0.05 (< 0.05 to < 0.05)	< 0.05 (< 0.05 to < 0.05)
Congenital heart anomalies	Congenital heart disease, severe intellectual disability and severe heart failure due to severe congenital heart anomalies excluding single ventricle heart defects	0.1 (< 0.05 to 0.1)	0.1 (< 0.05 to 0.1)	--	--	< 0.05 (< 0.05 to < 0.05)	< 0.05 (< 0.05 to < 0.05)

Congenital heart anomalies	Congenital heart disease, severe intellectual disability and moderate heart failure due to critical malformations of great vessels, congenital valvular heart disease and patent	< 0.05 (< 0.05 to < 0.05)	< 0.05 (< 0.05 to < 0.05)	--	--	< 0.05 (< 0.05 to < 0.05)	< 0.05 (< 0.05 to < 0.05)
Congenital heart anomalies	Congenital heart disease and moderate intellectual disability without heart failure due to severe congenital heart anomalies excluding single ventricle heart defects	20.8 (14.1 to 28.5)	20.6 (13.3 to 29.2)	--	--	2.1 (1.2 to 3.3)	2.1 (1.1 to 3.3)
Congenital heart anomalies	Congenital heart disease, moderate intellectual disability and mild heart failure due to single ventricle and single ventricle pathway heart defects	< 0.05 (< 0.05 to < 0.05)	< 0.05 (< 0.05 to < 0.05)	--	--	< 0.05 (< 0.05 to < 0.05)	< 0.05 (< 0.05 to < 0.05)
Congenital heart anomalies	Congenital heart disease and mild intellectual disability without heart failure due to ventricular septal defect and atrial septal defect	59.9 (46.4 to 74.7)	58.4 (44.6 to 73.5)	--	--	2.5 (1.5 to 3.9)	2.5 (1.4 to 3.9)
Congenital heart anomalies	Congenital heart disease and mild heart failure without intellectual disability due to ventricular septal defect and atrial septal defect	3.9 (2.4 to 5.9)	3.7 (2.3 to 5.7)	--	--	0.2 (0.1 to 0.3)	0.2 (0.1 to 0.3)
Congenital heart anomalies	Congenital heart disease and mild intellectual disability without heart failure due to critical malformations of great vessels, congenital valvular heart disease and patent	37.1 (29.5 to 46.2)	38.8 (30.5 to 48.4)	--	--	1.6 (0.9 to 2.5)	1.6 (1.0 to 2.6)
Congenital heart anomalies	Congenital heart disease and severe intellectual disability without heart failure due to critical malformations of great vessels, congenital valvular heart disease and patent	3.8 (1.4 to 6.0)	3.6 (1.2 to 6.0)	--	--	0.6 (0.2 to 1.1)	0.6 (0.2 to 1.1)
Congenital heart anomalies	Congenital heart disease and profound intellectual disability without heart failure due to ventricular septal defect and atrial septal defect	10.9 (5.7 to 15.9)	10.4 (5.2 to 15.1)	--	--	2.2 (1.0 to 3.7)	2.1 (1.0 to 3.6)
Congenital heart anomalies	Congenital heart disease and moderate intellectual disability without heart failure due to ventricular septal defect and atrial septal defect	18.0 (11.1 to 26.8)	16.0 (9.5 to 24.2)	--	--	1.8 (0.9 to 3.1)	1.6 (0.8 to 2.8)
Congenital heart anomalies	Congenital heart disease and severe intellectual disability without heart failure due to other congenital cardiovascular anomalies	8.1 (2.8 to 12.4)	7.9 (2.4 to 12.5)	--	--	1.3 (0.4 to 2.2)	1.3 (0.4 to 2.2)
Congenital heart anomalies	Congenital heart disease, severe intellectual disability and severe heart failure due to single ventricle and single ventricle pathway heart defects	< 0.05 (< 0.05 to < 0.05)	< 0.05 (< 0.05 to < 0.05)	--	--	< 0.05 (< 0.05 to < 0.05)	< 0.05 (< 0.05 to < 0.05)
Congenital heart anomalies	Congenital heart disease without heart failure or intellectual disability due to critical malformations of great vessels, congenital valvular heart disease and patent ductus	1,640.1 (1,407.7 to 1,914.8)	1,807.2 (1,539.3 to 2,133.0)	--	--	96.6 (37.5 to 169.3)	106.1 (40.6 to 187.1)
Congenital heart anomalies	Congenital heart disease and borderline intellectual disability without heart failure due to critical malformations of great vessels, congenital valvular heart disease and patent	106.1 (79.2 to 136.6)	107.7 (79.5 to 139.8)	--	--	1.1 (0.5 to 2.2)	1.1 (0.5 to 2.3)
Congenital heart anomalies	Congenital heart disease and severe heart without intellectual disability due to ventricular septal defect and atrial septal defect	8.6 (6.3 to 11.8)	8.3 (6.0 to 11.4)	--	--	1.5 (0.9 to 2.3)	1.5 (0.9 to 2.3)
Orofacial clefts	Disfigurement level 2 and speech problems due to orofacial clefts	233.4 (209.7 to 257.8)	241.5 (215.2 to 268.2)	--	--	26.7 (17.6 to 38.7)	27.6 (18.2 to 39.5)
Orofacial clefts	Asymptomatic orofacial clefts	2,538.0 (2,287.1 to 2,804.3)	2,866.2 (2,574.9 to 3,165.3)	--	--	< 0.05 (0.0 to < 0.05)	< 0.05 (0.0 to < 0.05)
Orofacial clefts	Disfigurement level 2 due to orofacial clefts	233.5 (210.2 to 258.7)	241.4 (216.0 to 267.5)	--	--	15.6 (10.3 to 22.3)	16.2 (10.6 to 23.2)
Orofacial clefts	Disfigurement level 1 due to orofacial clefts	233.5 (208.9 to 258.2)	241.3 (216.4 to 267.8)	--	--	2.6 (1.2 to 4.8)	2.7 (1.2 to 5.0)
Down's syndrome	Severe intellectual disability, moderate dementia, and congenital heart disease due to Down syndrome	0.5 (0.1 to 0.7)	0.6 (0.1 to 0.8)	--	--	0.2 (0.1 to 0.3)	0.3 (0.1 to 0.4)
Down's syndrome	Moderate intellectual disability with congenital heart disease due to Down syndrome	197.9 (170.9 to 229.4)	210.5 (181.0 to 245.8)	--	--	19.6 (12.7 to 28.6)	20.9 (13.5 to 30.6)
Down's syndrome	Borderline intellectual disability, mild dementia, and congenital heart disease due to Down syndrome	3.2 (2.8 to 3.8)	4.3 (3.6 to 5.0)	--	--	0.3 (0.2 to 0.4)	0.3 (0.2 to 0.5)
Down's syndrome	Congenital heart disease and severe dementia due to Down syndrome	0.1 (0.1 to 0.1)	0.1 (0.1 to 0.1)	--	--	< 0.05 (< 0.05 to 0.1)	0.1 (< 0.05 to 0.1)
Down's syndrome	Profound intellectual disability with congenital heart disease due to Down syndrome	67.6 (57.8 to 78.6)	71.8 (61.3 to 84.2)	--	--	13.5 (8.7 to 19.4)	14.4 (9.2 to 20.6)
Down's syndrome	Borderline intellectual disability due to Down syndrome	127.2 (108.3 to 149.3)	135.2 (114.4 to 160.0)	--	--	1.4 (0.7 to 2.6)	1.5 (0.7 to 2.8)
Down's syndrome	Severe intellectual disability, severe dementia, and congenital heart disease due to Down syndrome	0.1 (< 0.05 to 0.2)	0.2 (< 0.05 to 0.3)	--	--	0.1 (< 0.05 to 0.1)	0.1 (< 0.05 to 0.1)
Down's syndrome	Mild intellectual disability, severe dementia, and congenital heart disease due to Down syndrome	0.5 (0.4 to 0.6)	0.7 (0.5 to 0.8)	--	--	0.2 (0.2 to 0.3)	0.3 (0.2 to 0.4)
Down's syndrome	Moderate intellectual disability, severe dementia, and congenital heart disease due to Down syndrome	0.4 (0.3 to 0.5)	0.5 (0.3 to 0.6)	--	--	0.2 (0.1 to 0.3)	0.2 (0.1 to 0.3)
Down's syndrome	Profound intellectual disability and moderate dementia due to Down syndrome	0.7 (0.4 to 0.8)	0.9 (0.6 to 1.1)	--	--	0.3 (0.2 to 0.5)	0.4 (0.3 to 0.6)
Down's syndrome	Severe intellectual disability and moderate dementia due to Down syndrome	0.6 (0.2 to 0.9)	0.8 (0.2 to 1.2)	--	--	0.3 (0.1 to 0.5)	0.4 (0.1 to 0.6)
Down's syndrome	Mild intellectual disability and moderate dementia due to Down syndrome	2.4 (2.0 to 2.8)	3.1 (2.6 to 3.7)	--	--	1.0 (0.6 to 1.3)	1.3 (0.8 to 1.8)
Down's syndrome	Moderate intellectual disability, mild dementia, and congenital heart disease due to Down syndrome	5.7 (4.0 to 7.2)	7.1 (4.8 to 9.3)	--	--	0.9 (0.6 to 1.3)	1.1 (0.7 to 1.7)
Down's syndrome	Borderline intellectual disability and mild dementia due to Down syndrome	4.5 (3.8 to 5.2)	5.9 (5.0 to 6.9)	--	--	0.4 (0.2 to 0.5)	0.5 (0.3 to 0.7)
Down's syndrome	Moderate intellectual disability and moderate dementia due to Down syndrome	1.7 (1.2 to 2.2)	2.1 (1.4 to 2.8)	--	--	0.8 (0.5 to 1.1)	0.9 (0.6 to 1.3)
Down's syndrome	Mild intellectual disability with congenital heart disease due to Down syndrome	243.6 (208.0 to 283.1)	259.0 (219.1 to 302.8)	--	--	10.3 (6.2 to 15.6)	10.9 (6.6 to 16.8)
Down's syndrome	Profound intellectual disability and mild dementia due to Down syndrome	3.1 (2.0 to 3.7)	4.1 (2.6 to 5.0)	--	--	0.8 (0.5 to 1.1)	1.1 (0.6 to 1.5)
Down's syndrome	Congenital heart disease and mild dementia due to Down syndrome	1.4 (1.2 to 1.6)	1.8 (1.5 to 2.1)	--	--	0.1 (0.1 to 0.1)	0.1 (0.1 to 0.2)
Down's syndrome	Borderline intellectual disability, severe dementia, and congenital heart disease due to Down syndrome	0.2 (0.2 to 0.2)	0.3 (0.2 to 0.3)	--	--	0.1 (0.1 to 0.1)	0.1 (0.1 to 0.2)
Down's syndrome	Mild intellectual disability, moderate dementia, and congenital heart disease due to Down syndrome	1.7 (1.4 to 2.0)	2.3 (1.9 to 2.7)	--	--	0.7 (0.5 to 1.0)	0.9 (0.6 to 1.3)
Down's syndrome	Severe intellectual disability and mild dementia due to Down syndrome	2.9 (0.7 to 4.4)	3.5 (0.7 to 5.4)	--	--	0.6 (0.1 to 1.0)	0.8 (0.2 to 1.3)
Down's syndrome	Moderate dementia due to Down syndrome	0.4 (0.3 to 0.5)	0.5 (0.5 to 0.6)	--	--	0.2 (0.1 to 0.2)	0.2 (0.1 to 0.3)
Down's syndrome	Severe intellectual disability and severe dementia due to Down syndrome	0.2 (< 0.05 to 0.3)	0.2 (< 0.05 to 0.3)	--	--	0.1 (< 0.05 to 0.2)	0.1 (< 0.05 to 0.2)
Down's syndrome	Severe intellectual disability, mild dementia, and congenital heart disease due to Down syndrome	2.1 (0.5 to 3.2)	2.5 (0.5 to 3.9)	--	--	0.5 (0.1 to 0.7)	0.6 (0.1 to 0.9)
Down's syndrome	Mild intellectual disability and severe dementia due to Down syndrome	0.7 (0.6 to 0.8)	0.9 (0.8 to 1.1)	--	--	0.3 (0.2 to 0.5)	0.4 (0.3 to 0.6)

Down's syndrome	Borderline intellectual disability and moderate dementia due to Down syndrome	1.0 (0.8 to 1.1)	1.3 (1.1 to 1.5)	--	--	0.4 (0.3 to 0.5)	0.5 (0.3 to 0.7)
Down's syndrome	Borderline intellectual disability with congenital heart disease due to Down syndrome	92.2 (78.5 to 106.9)	98.1 (82.6 to 114.6)	--	--	1.0 (0.5 to 1.9)	1.1 (0.5 to 2.1)
Down's syndrome	Borderline intellectual disability and severe dementia due to Down syndrome	0.3 (0.2 to 0.3)	0.4 (0.3 to 0.4)	--	--	0.1 (0.1 to 0.2)	0.2 (0.1 to 0.2)
Down's syndrome	Mild intellectual disability and mild dementia due to Down syndrome	10.9 (9.0 to 12.8)	14.4 (11.7 to 17.1)	--	--	1.2 (0.8 to 1.7)	1.6 (1.0 to 2.3)
Down's syndrome	Severe intellectual disability with congenital heart disease due to Down syndrome	128.1 (109.1 to 149.7)	136.2 (115.2 to 160.2)	--	--	20.4 (13.2 to 29.8)	21.8 (14.0 to 32.4)
Down's syndrome	Mild dementia due to Down syndrome	1.9 (1.6 to 2.2)	2.5 (2.1 to 2.9)	--	--	0.1 (0.1 to 0.2)	0.2 (0.1 to 0.2)
Down's syndrome	Congenital heart disease and moderate dementia due to Down syndrome	0.3 (0.3 to 0.3)	0.4 (0.3 to 0.5)	--	--	0.1 (0.1 to 0.2)	0.1 (0.1 to 0.2)
Down's syndrome	Mild intellectual disability due to Down syndrome	335.3 (289.5 to 388.8)	356.5 (304.5 to 415.2)	--	--	14.1 (8.4 to 21.3)	15.0 (9.0 to 22.9)
Down's syndrome	Borderline intellectual disability, moderate dementia, and congenital heart disease due to Down syndrome	0.7 (0.6 to 0.8)	0.9 (0.8 to 1.1)	--	--	0.3 (0.2 to 0.4)	0.4 (0.2 to 0.5)
Down's syndrome	Moderate intellectual disability, moderate dementia, and congenital heart disease due to Down syndrome	1.2 (0.9 to 1.6)	1.6 (1.0 to 2.0)	--	--	0.5 (0.3 to 0.8)	0.7 (0.4 to 1.0)
Down's syndrome	Asymptomatic Down syndrome	53.0 (45.4 to 61.4)	56.4 (47.9 to 66.0)	--	--	< 0.05 (0.0 to < 0.05)	< 0.05 (0.0 to < 0.05)
Down's syndrome	Severe intellectual disability due to Down syndrome	176.5 (149.0 to 206.1)	187.7 (158.0 to 220.2)	--	--	28.0 (18.3 to 40.4)	29.8 (19.3 to 43.6)
Down's syndrome	Profound intellectual disability, severe dementia, and congenital heart disease due to Down syndrome	0.1 (0.1 to 0.2)	0.2 (0.1 to 0.2)	--	--	0.1 (< 0.05 to 0.1)	0.1 (0.1 to 0.1)
Down's syndrome	Profound intellectual disability, mild dementia, and congenital heart disease due to Down syndrome	2.3 (1.5 to 2.7)	3.0 (2.0 to 3.6)	--	--	0.6 (0.3 to 0.8)	0.8 (0.4 to 1.1)
Down's syndrome	Severe dementia due to Down syndrome	0.1 (0.1 to 0.1)	0.2 (0.1 to 0.2)	--	--	0.1 (< 0.05 to 0.1)	0.1 (< 0.05 to 0.1)
Down's syndrome	Profound intellectual disability, moderate dementia, and congenital heart disease due to Down syndrome	0.5 (0.3 to 0.6)	0.7 (0.4 to 0.8)	--	--	0.2 (0.1 to 0.3)	0.3 (0.2 to 0.4)
Down's syndrome	Moderate intellectual disability and mild dementia due to Down syndrome	7.9 (5.6 to 9.9)	9.8 (6.6 to 12.6)	--	--	1.3 (0.8 to 1.9)	1.6 (0.9 to 2.4)
Down's syndrome	Isolated congenital heart disease due to Down syndrome	38.4 (32.7 to 44.9)	40.8 (34.6 to 47.9)	--	--	2.3 (0.9 to 4.1)	2.5 (1.0 to 4.4)
Down's syndrome	Moderate intellectual disability due to Down syndrome	273.5 (234.2 to 316.5)	290.8 (247.0 to 339.0)	--	--	27.0 (17.5 to 39.0)	28.8 (18.5 to 41.6)
Down's syndrome	Mild intellectual disability, mild dementia, and congenital heart disease due to Down syndrome	7.9 (6.6 to 9.3)	10.4 (8.5 to 12.3)	--	--	0.9 (0.6 to 1.2)	1.1 (0.7 to 1.6)
Down's syndrome	Profound intellectual disability due to Down syndrome	93.2 (79.8 to 109.8)	99.1 (84.3 to 118.2)	--	--	18.7 (11.9 to 27.1)	19.8 (12.5 to 29.0)
Down's syndrome	Moderate intellectual disability and severe dementia due to Down syndrome	0.5 (0.4 to 0.6)	0.6 (0.4 to 0.8)	--	--	0.3 (0.2 to 0.4)	0.3 (0.2 to 0.5)
Down's syndrome	Profound intellectual disability and severe dementia due to Down syndrome	0.2 (0.1 to 0.2)	0.3 (0.2 to 0.3)	--	--	0.1 (0.1 to 0.2)	0.1 (0.1 to 0.2)
Turner syndrome	Congenital heart disease due to Turner syndrome	268.7 (223.2 to 317.6)	282.5 (236.3 to 331.9)	--	--	16.0 (6.3 to 28.0)	16.8 (6.7 to 29.5)
Turner syndrome	Asymptomatic Turner syndrome	913.9 (799.0 to 1,041.4)	960.6 (836.0 to 1,093.8)	--	--	< 0.05 (0.0 to < 0.05)	< 0.05 (0.0 to < 0.05)
Turner syndrome	Primary infertility due to Turner syndrome	1,058.1 (923.2 to 1,208.4)	1,139.8 (987.8 to 1,307.9)	--	--	7.8 (3.3 to 15.3)	8.4 (3.7 to 16.6)
Turner syndrome	Congenital heart disease with infertility due to Turner syndrome	312.2 (262.8 to 373.0)	336.3 (280.9 to 404.8)	--	--	20.5 (9.0 to 35.0)	22.0 (9.7 to 37.3)
Klinefelter syndrome	Primary infertility due to Klinefelter syndrome	874.2 (740.6 to 1,030.8)	967.6 (817.4 to 1,144.8)	--	--	6.5 (2.8 to 13.2)	7.2 (3.1 to 14.6)
Klinefelter syndrome	Mild intellectual disability due to Klinefelter syndrome	28.0 (15.2 to 44.9)	29.3 (16.0 to 46.8)	--	--	1.2 (0.5 to 2.1)	1.2 (0.6 to 2.2)
Klinefelter syndrome	Asymptomatic Klinefelter syndrome	1,390.4 (1,166.4 to 1,634.7)	1,455.2 (1,220.3 to 1,719.1)	--	--	< 0.05 (0.0 to < 0.05)	< 0.05 (0.0 to < 0.05)
Klinefelter syndrome	Mild intellectual disability with infertility due to Klinefelter syndrome	17.6 (9.6 to 28.6)	19.5 (10.6 to 31.7)	--	--	0.9 (0.4 to 1.6)	1.0 (0.4 to 1.8)
Klinefelter syndrome	Borderline intellectual disability due to Klinefelter syndrome	327.6 (206.9 to 457.3)	342.9 (216.7 to 476.9)	--	--	3.5 (1.5 to 7.0)	3.6 (1.5 to 7.4)
Klinefelter syndrome	Borderline intellectual disability with infertility due to Klinefelter syndrome	206.1 (121.8 to 290.2)	228.1 (135.3 to 321.1)	--	--	3.7 (1.6 to 7.5)	4.1 (1.7 to 8.3)
Other chromosomal abnormalities	Moderate intellectual disability and moderate dementia due to other chromosomal abnormalities	< 0.05 (< 0.05 to < 0.05)	< 0.05 (< 0.05 to < 0.05)	--	--	< 0.05 (< 0.05 to < 0.05)	< 0.05 (< 0.05 to < 0.05)
Other chromosomal abnormalities	Borderline intellectual disability due to other chromosomal abnormalities	270.0 (223.5 to 322.0)	279.6 (229.9 to 333.4)	--	--	2.9 (1.4 to 5.6)	3.0 (1.4 to 5.9)
Other chromosomal abnormalities	Mild intellectual disability with congenital heart disease due to other chromosomal abnormalities	517.2 (433.0 to 620.2)	535.5 (444.2 to 644.5)	--	--	21.7 (13.0 to 33.6)	22.4 (13.6 to 34.5)
Other chromosomal abnormalities	Profound intellectual disability and severe dementia due to other chromosomal abnormalities	< 0.05 (< 0.05 to < 0.05)	< 0.05 (< 0.05 to < 0.05)	--	--	< 0.05 (< 0.05 to < 0.05)	< 0.05 (< 0.05 to < 0.05)
Other chromosomal abnormalities	Severe motor and cognitive impairment due to Edward Syndrome or Patau Syndrome	216.6 (162.0 to 289.0)	228.9 (170.4 to 305.5)	--	--	113.5 (69.5 to 167.1)	120.1 (74.4 to 174.1)
Other chromosomal abnormalities	Profound intellectual disability, moderate dementia, and congenital heart disease due to other chromosomal abnormalities	< 0.05 (< 0.05 to < 0.05)	< 0.05 (< 0.05 to < 0.05)	--	--	< 0.05 (< 0.05 to < 0.05)	< 0.05 (< 0.05 to < 0.05)
Other chromosomal abnormalities	Borderline intellectual disability and moderate dementia due to other chromosomal abnormalities	< 0.05 (< 0.05 to < 0.05)	< 0.05 (< 0.05 to < 0.05)	--	--	< 0.05 (< 0.05 to < 0.05)	< 0.05 (< 0.05 to < 0.05)
Other chromosomal abnormalities	Mild intellectual disability, mild dementia, and congenital heart disease due to other chromosomal abnormalities	< 0.05 (< 0.05 to < 0.05)	< 0.05 (< 0.05 to < 0.05)	--	--	< 0.05 (< 0.05 to < 0.05)	< 0.05 (< 0.05 to < 0.05)
Other chromosomal abnormalities	Borderline intellectual disability with congenital heart disease due to other chromosomal abnormalities	195.9 (161.0 to 234.5)	202.8 (166.6 to 241.9)	--	--	2.1 (1.0 to 4.2)	2.2 (1.0 to 4.3)
Other chromosomal abnormalities	Moderate intellectual disability and severe dementia due to other chromosomal abnormalities	< 0.05 (< 0.05 to < 0.05)	< 0.05 (< 0.05 to < 0.05)	--	--	< 0.05 (< 0.05 to < 0.05)	< 0.05 (< 0.05 to < 0.05)

Other chromosomal abnormalities	Borderline intellectual disability and severe dementia due to other chromosomal abnormalities	< 0.05 (< 0.05 to < 0.05)	< 0.05 (< 0.05 to < 0.05)	--	--	< 0.05 (< 0.05 to < 0.05)	< 0.05 (< 0.05 to < 0.05)
Other chromosomal abnormalities	Moderate intellectual disability, moderate dementia, and congenital heart disease due to other chromosomal abnormalities	< 0.05 (< 0.05 to < 0.05)	< 0.05 (< 0.05 to < 0.05)	--	--	< 0.05 (< 0.05 to < 0.05)	< 0.05 (< 0.05 to < 0.05)
Other chromosomal abnormalities	Mild intellectual disability, severe dementia, and congenital heart disease due to other chromosomal abnormalities	< 0.05 (< 0.05 to < 0.05)	< 0.05 (< 0.05 to < 0.05)	--	--	< 0.05 (< 0.05 to < 0.05)	< 0.05 (< 0.05 to < 0.05)
Other chromosomal abnormalities	Severe intellectual disability, severe dementia, and congenital heart disease due to other chromosomal abnormalities	< 0.05 (< 0.05 to < 0.05)	< 0.05 (< 0.05 to < 0.05)	--	--	< 0.05 (< 0.05 to < 0.05)	< 0.05 (< 0.05 to < 0.05)
Other chromosomal abnormalities	Profound intellectual disability and moderate dementia due to other chromosomal abnormalities	< 0.05 (< 0.05 to < 0.05)	< 0.05 (< 0.05 to < 0.05)	--	--	< 0.05 (< 0.05 to < 0.05)	< 0.05 (< 0.05 to < 0.05)
Other chromosomal abnormalities	Asymptomatic other chromosomal abnormalities	112.5 (94.3 to 134.9)	116.5 (97.4 to 139.3)	--	--	< 0.05 (0.0 to < 0.05)	< 0.05 (0.0 to < 0.05)
Other chromosomal abnormalities	Severe intellectual disability, mild dementia, and congenital heart disease due to other chromosomal abnormalities	< 0.05 (< 0.05 to < 0.05)	< 0.05 (< 0.05 to < 0.05)	--	--	< 0.05 (< 0.05 to < 0.05)	< 0.05 (< 0.05 to < 0.05)
Other chromosomal abnormalities	Severe intellectual disability and mild dementia due to other chromosomal abnormalities	< 0.05 (< 0.05 to < 0.05)	< 0.05 (< 0.05 to < 0.05)	--	--	< 0.05 (< 0.05 to < 0.05)	< 0.05 (< 0.05 to < 0.05)
Other chromosomal abnormalities	Moderate intellectual disability and mild dementia due to other chromosomal abnormalities	< 0.05 (< 0.05 to < 0.05)	< 0.05 (< 0.05 to < 0.05)	--	--	< 0.05 (< 0.05 to < 0.05)	< 0.05 (< 0.05 to < 0.05)
Other chromosomal abnormalities	Mild intellectual disability and mild dementia due to other chromosomal abnormalities	< 0.05 (< 0.05 to < 0.05)	< 0.05 (< 0.05 to < 0.05)	--	--	< 0.05 (< 0.05 to < 0.05)	< 0.05 (< 0.05 to < 0.05)
Other chromosomal abnormalities	Severe intellectual disability due to other chromosomal abnormalities	374.8 (311.4 to 445.0)	388.1 (319.9 to 462.0)	--	--	58.5 (37.2 to 84.3)	60.6 (38.5 to 88.2)
Other chromosomal abnormalities	Mild intellectual disability due to other chromosomal abnormalities	711.7 (593.3 to 847.1)	737.0 (614.6 to 875.5)	--	--	29.8 (17.5 to 46.4)	30.9 (18.2 to 48.1)
Other chromosomal abnormalities	Borderline intellectual disability, severe dementia, and congenital heart disease due to other chromosomal abnormalities	< 0.05 (< 0.05 to < 0.05)	< 0.05 (< 0.05 to < 0.05)	--	--	< 0.05 (< 0.05 to < 0.05)	< 0.05 (< 0.05 to < 0.05)
Other chromosomal abnormalities	Severe intellectual disability with congenital heart disease due to other chromosomal abnormalities	271.9 (224.8 to 327.8)	281.6 (232.8 to 339.3)	--	--	42.5 (26.8 to 61.4)	44.0 (28.0 to 65.0)
Other chromosomal abnormalities	Moderate intellectual disability, mild dementia, and congenital heart disease due to other chromosomal abnormalities	< 0.05 (< 0.05 to < 0.05)	< 0.05 (< 0.05 to < 0.05)	--	--	< 0.05 (< 0.05 to < 0.05)	< 0.05 (< 0.05 to < 0.05)
Other chromosomal abnormalities	Borderline intellectual disability, moderate dementia, and congenital heart disease due to other chromosomal abnormalities	< 0.05 (< 0.05 to < 0.05)	< 0.05 (< 0.05 to < 0.05)	--	--	< 0.05 (< 0.05 to < 0.05)	< 0.05 (< 0.05 to < 0.05)
Other chromosomal abnormalities	Profound intellectual disability due to other chromosomal abnormalities	197.8 (164.7 to 237.9)	204.8 (169.7 to 245.6)	--	--	38.7 (24.9 to 55.7)	40.1 (25.5 to 58.3)
Other chromosomal abnormalities	Moderate intellectual disability, severe dementia, and congenital heart disease due to other chromosomal abnormalities	< 0.05 (< 0.05 to < 0.05)	< 0.05 (< 0.05 to < 0.05)	--	--	< 0.05 (< 0.05 to < 0.05)	< 0.05 (< 0.05 to < 0.05)
Other chromosomal abnormalities	Isolated congenital heart disease due to other chromosomal abnormalities	81.5 (67.9 to 97.6)	84.4 (70.2 to 101.5)	--	--	4.9 (1.9 to 8.8)	5.1 (2.0 to 9.2)
Other chromosomal abnormalities	Congenital heart disease and severe dementia due to other chromosomal abnormalities	< 0.05 (< 0.05 to < 0.05)	< 0.05 (< 0.05 to < 0.05)	--	--	< 0.05 (< 0.05 to < 0.05)	< 0.05 (< 0.05 to < 0.05)
Other chromosomal abnormalities	Moderate intellectual disability due to other chromosomal abnormalities	580.6 (482.9 to 696.6)	601.3 (500.7 to 723.7)	--	--	56.8 (36.0 to 85.2)	58.8 (37.4 to 87.9)
Other chromosomal abnormalities	Severe motor and cognitive impairment with congenital heart disease due to Edward Syndrome or Patau Syndrome	62.7 (42.5 to 89.8)	66.3 (44.8 to 94.6)	--	--	33.4 (19.2 to 51.3)	35.3 (20.3 to 53.9)
Other chromosomal abnormalities	Moderate dementia due to other chromosomal abnormalities	< 0.05 (< 0.05 to < 0.05)	< 0.05 (< 0.05 to < 0.05)	--	--	< 0.05 (< 0.05 to < 0.05)	< 0.05 (< 0.05 to < 0.05)
Other chromosomal abnormalities	Mild intellectual disability and severe dementia due to other chromosomal abnormalities	< 0.05 (< 0.05 to < 0.05)	< 0.05 (< 0.05 to < 0.05)	--	--	< 0.05 (< 0.05 to < 0.05)	< 0.05 (< 0.05 to < 0.05)
Other chromosomal abnormalities	Profound intellectual disability, severe dementia, and congenital heart disease due to other chromosomal abnormalities	< 0.05 (< 0.05 to < 0.05)	< 0.05 (< 0.05 to < 0.05)	--	--	< 0.05 (< 0.05 to < 0.05)	< 0.05 (< 0.05 to < 0.05)
Other chromosomal abnormalities	Severe intellectual disability and severe dementia due to other chromosomal abnormalities	< 0.05 (< 0.05 to < 0.05)	< 0.05 (< 0.05 to < 0.05)	--	--	< 0.05 (< 0.05 to < 0.05)	< 0.05 (< 0.05 to < 0.05)
Other chromosomal abnormalities	Profound intellectual disability with congenital heart disease due to other chromosomal abnormalities	143.5 (119.5 to 173.1)	148.5 (122.5 to 179.8)	--	--	28.1 (18.4 to 40.7)	29.2 (18.7 to 42.7)
Other chromosomal abnormalities	Severe intellectual disability, moderate dementia, and congenital heart disease due to other chromosomal abnormalities	< 0.05 (< 0.05 to < 0.05)	< 0.05 (< 0.05 to < 0.05)	--	--	< 0.05 (< 0.05 to < 0.05)	< 0.05 (< 0.05 to < 0.05)
Other chromosomal abnormalities	Mild intellectual disability, moderate dementia, and congenital heart disease due to other chromosomal abnormalities	< 0.05 (< 0.05 to < 0.05)	< 0.05 (< 0.05 to < 0.05)	--	--	< 0.05 (< 0.05 to < 0.05)	< 0.05 (< 0.05 to < 0.05)
Other chromosomal abnormalities	Congenital heart disease and moderate dementia due to other chromosomal abnormalities	< 0.05 (< 0.05 to < 0.05)	< 0.05 (< 0.05 to < 0.05)	--	--	< 0.05 (< 0.05 to < 0.05)	< 0.05 (< 0.05 to < 0.05)
Other chromosomal abnormalities	Borderline intellectual disability, mild dementia, and congenital heart disease due to other chromosomal abnormalities	< 0.05 (< 0.05 to < 0.05)	< 0.05 (< 0.05 to < 0.05)	--	--	< 0.05 (< 0.05 to < 0.05)	< 0.05 (< 0.05 to < 0.05)
Other chromosomal abnormalities	Borderline intellectual disability and mild dementia due to other chromosomal abnormalities	< 0.05 (< 0.05 to < 0.05)	< 0.05 (< 0.05 to < 0.05)	--	--	< 0.05 (< 0.05 to < 0.05)	< 0.05 (< 0.05 to < 0.05)
Other chromosomal abnormalities	Moderate intellectual disability with congenital heart disease due to other chromosomal abnormalities	420.1 (352.7 to 501.1)	435.0 (365.2 to 522.8)	--	--	41.1 (26.0 to 61.9)	42.6 (27.0 to 63.9)
Other chromosomal abnormalities	Severe intellectual disability and moderate dementia due to other chromosomal abnormalities	< 0.05 (< 0.05 to < 0.05)	< 0.05 (< 0.05 to < 0.05)	--	--	< 0.05 (< 0.05 to < 0.05)	< 0.05 (< 0.05 to < 0.05)
Other chromosomal abnormalities	Severe dementia due to other chromosomal abnormalities	< 0.05 (< 0.05 to < 0.05)	< 0.05 (< 0.05 to < 0.05)	--	--	< 0.05 (< 0.05 to < 0.05)	< 0.05 (< 0.05 to < 0.05)
Other chromosomal abnormalities	Mild dementia due to other chromosomal abnormalities	< 0.05 (< 0.05 to < 0.05)	< 0.05 (< 0.05 to < 0.05)	--	--	< 0.05 (< 0.05 to < 0.05)	< 0.05 (< 0.05 to < 0.05)
Other chromosomal abnormalities	Congenital heart disease and mild dementia due to other chromosomal abnormalities	< 0.05 (< 0.05 to < 0.05)	< 0.05 (< 0.05 to < 0.05)	--	--	< 0.05 (< 0.05 to < 0.05)	< 0.05 (< 0.05 to < 0.05)
Other chromosomal abnormalities	Profound intellectual disability, mild dementia, and congenital heart disease due to other chromosomal abnormalities	< 0.05 (< 0.05 to < 0.05)	< 0.05 (< 0.05 to < 0.05)	--	--	< 0.05 (< 0.05 to < 0.05)	< 0.05 (< 0.05 to < 0.05)
Other chromosomal abnormalities	Profound intellectual disability and mild dementia due to other chromosomal abnormalities	< 0.05 (< 0.05 to < 0.05)	< 0.05 (< 0.05 to < 0.05)	--	--	< 0.05 (< 0.05 to < 0.05)	< 0.05 (< 0.05 to < 0.05)
Other chromosomal abnormalities	Mild intellectual disability and moderate dementia due to other chromosomal abnormalities	< 0.05 (< 0.05 to < 0.05)	< 0.05 (< 0.05 to < 0.05)	--	--	< 0.05 (< 0.05 to < 0.05)	< 0.05 (< 0.05 to < 0.05)
Congenital musculoskeletal and limb anomalies	Disfigurement level 2 and mild motor impairment due to other congenital musculoskeletal anomalies	292.1 (197.4 to 411.4)	331.9 (222.8 to 470.0)	--	--	21.8 (12.1 to 37.0)	24.8 (13.7 to 41.7)
Congenital musculoskeletal and limb anomalies	Disfigurement level 2 and mild motor impairment due to congenital limb deficiency	303.6 (197.2 to 427.6)	344.2 (223.5 to 487.6)	--	--	23.1 (12.4 to 38.2)	26.1 (14.1 to 42.7)
Congenital musculoskeletal and limb anomalies	Disfigurement level 2 with pain and mild motor impairment due to other congenital musculoskeletal anomalies	517.0 (388.9 to 662.7)	587.4 (440.0 to 756.0)	--	--	97.0 (59.5 to 148.8)	109.8 (66.6 to 166.9)

Congenital musculoskeletal and limb anomalies	Disfigurement level 2 due to other congenital musculoskeletal anomalies	913.3 (746.4 to 1,088.3)	1,037.4 (837.6 to 1,251.9)	--	--	58.7 (38.2 to 86.5)	66.5 (42.7 to 99.4)
Congenital musculoskeletal and limb anomalies	Disfigurement level 2 with pain and moderate motor impairment due to congenital limb deficiency	689.0 (534.7 to 868.3)	781.1 (606.6 to 989.0)	--	--	157.3 (99.9 to 233.3)	178.0 (112.4 to 267.4)
Congenital musculoskeletal and limb anomalies	Disfigurement level 2 with pain due to other congenital musculoskeletal anomalies	1,624.4 (1,412.2 to 1,847.7)	1,845.1 (1,582.2 to 2,119.7)	--	--	286.2 (190.1 to 404.1)	324.0 (214.5 to 455.3)
Congenital musculoskeletal and limb anomalies	Disfigurement level 1 due to polydactyly and syndactyly	671.3 (574.4 to 774.7)	667.2 (565.3 to 782.1)	--	--	7.3 (3.3 to 13.8)	7.3 (3.2 to 13.7)
Congenital musculoskeletal and limb anomalies	Disfigurement level 2 with pain and moderate motor impairment due to other congenital musculoskeletal anomalies	663.4 (520.8 to 813.9)	753.6 (587.0 to 927.4)	--	--	149.0 (96.4 to 217.4)	168.7 (109.1 to 245.5)
Congenital musculoskeletal and limb anomalies	Disfigurement level 2 due to congenital limb deficiency	948.6 (752.4 to 1,151.1)	1,075.3 (856.7 to 1,315.4)	--	--	61.5 (39.1 to 91.5)	69.6 (44.2 to 102.9)
Congenital musculoskeletal and limb anomalies	Disfigurement level 2 and moderate motor impairment due to other congenital musculoskeletal anomalies	375.1 (263.9 to 495.0)	426.1 (299.7 to 563.3)	--	--	44.9 (26.2 to 70.7)	50.8 (29.6 to 79.7)
Congenital musculoskeletal and limb anomalies	Disfigurement level 2 with pain and mild motor impairment due to congenital limb deficiency	537.0 (402.7 to 692.9)	608.8 (452.4 to 789.4)	--	--	102.5 (61.4 to 158.2)	116.3 (68.3 to 180.9)
Congenital musculoskeletal and limb anomalies	Disfigurement level 2 and moderate motor impairment due to congenital limb deficiency	389.5 (277.6 to 518.8)	441.5 (315.1 to 591.9)	--	--	47.4 (26.7 to 74.8)	53.8 (30.6 to 85.6)
Congenital musculoskeletal and limb anomalies	Disfigurement level 2 with pain due to congenital limb deficiency	1,687.3 (1,425.7 to 1,964.8)	1,912.7 (1,606.5 to 2,244.0)	--	--	300.8 (193.8 to 426.8)	340.7 (220.3 to 486.6)
Urogenital congenital anomalies	Atypical genitalia, recurrent urinary tract infections or other abdominal issues and impotence due to congenital genital anomalies	232.6 (129.4 to 371.3)	247.7 (137.5 to 395.3)	--	--	9.0 (3.6 to 17.9)	9.6 (3.9 to 19.0)
Urogenital congenital anomalies	Atypical genitalia and impotence due to congenital anomalies of the urinary tract	148.7 (76.4 to 233.1)	146.9 (75.9 to 230.1)	--	--	4.2 (1.7 to 8.9)	4.1 (1.7 to 8.8)
Urogenital congenital anomalies	Atypical genitalia due to congenital anomalies of the urinary tract	514.9 (378.2 to 680.7)	509.0 (371.6 to 676.2)	--	--	5.6 (2.4 to 11.1)	5.6 (2.4 to 11.2)
Urogenital congenital anomalies	Incontinence due to congenital anomalies of the urinary tract	155.6 (86.2 to 251.9)	153.8 (84.8 to 249.6)	--	--	21.5 (10.0 to 38.5)	21.3 (10.1 to 38.3)
Urogenital congenital anomalies	Impotence due to congenital anomalies of the urinary tract	136.5 (70.9 to 221.0)	135.0 (70.1 to 219.4)	--	--	2.4 (0.9 to 4.8)	2.3 (0.9 to 4.7)
Urogenital congenital anomalies	Atypical genitalia and primary infertility due to congenital genital anomalies	136.4 (50.8 to 253.0)	145.2 (54.4 to 269.3)	--	--	2.5 (0.8 to 6.2)	2.7 (0.8 to 6.6)
Urogenital congenital anomalies	Atypical genital and recurrent urinary tract infections and other abdominal issues due to congenital genital anomalies	777.9 (568.7 to 1,019.4)	828.3 (605.4 to 1,088.9)	--	--	16.8 (7.4 to 33.5)	17.9 (7.7 to 35.2)
Urogenital congenital anomalies	Atypical genitalia, infertility and impotence due to congenital genital anomalies	40.8 (1.6 to 158.5)	43.5 (1.7 to 168.5)	--	--	1.2 (< 0.05 to 4.9)	1.3 (< 0.05 to 5.1)
Urogenital congenital anomalies	Atypical genitalia, incontinence and impotence due to congenital anomalies of the urinary tract	73.5 (29.1 to 143.8)	72.7 (28.7 to 142.1)	--	--	11.5 (4.3 to 24.0)	11.4 (4.3 to 24.1)
Urogenital congenital anomalies	Recurrent urinary tract infections or other abdominal issues due to congenital genital anomalies	210.6 (99.6 to 356.8)	224.2 (105.0 to 380.8)	--	--	2.3 (0.9 to 5.0)	2.5 (0.9 to 5.4)
Urogenital congenital anomalies	Asymptomatic congenital genital anomalies	731.9 (537.5 to 945.9)	779.4 (573.2 to 1,010.4)	--	--	< 0.05 (0.0 to < 0.05)	< 0.05 (0.0 to < 0.05)
Urogenital congenital anomalies	Atypical genitalia, incontinence, impotence, and recurrent urinary tract infections or other abdominal issues and impotence due to congenital anomalies of the urinary tract	43.1 (9.3 to 103.7)	42.6 (9.2 to 101.6)	--	--	7.4 (1.5 to 19.2)	7.3 (1.5 to 19.0)
Urogenital congenital anomalies	Atypical genitalia and incontinence due to congenital anomalies of the urinary tract	251.5 (158.7 to 361.9)	248.6 (155.9 to 358.9)	--	--	5.4 (2.3 to 10.8)	5.4 (2.3 to 10.5)
Urogenital congenital anomalies	Incontinence and impotence due to congenital anomalies of the urinary tract	39.6 (6.9 to 95.0)	39.1 (6.9 to 93.8)	--	--	6.1 (1.0 to 14.9)	6.0 (1.0 to 14.9)
Urogenital congenital anomalies	Infertility and impotence due to congenital genital anomalies	21.6 (< 0.05 to 111.6)	23.0 (< 0.05 to 119.1)	--	--	0.5 (< 0.05 to 3.0)	0.6 (< 0.05 to 3.2)
Urogenital congenital anomalies	Atypical genitalia, infertility, impotence, and recurrent urinary tract infections or other abdominal issues and impotence due to congenital genital anomalies	21.1 (< 0.05 to 117.7)	22.5 (< 0.05 to 125.4)	--	--	1.0 (< 0.05 to 5.7)	1.0 (< 0.05 to 6.1)
Urogenital congenital anomalies	Impotence and recurrent urinary tract infections or other abdominal issues due to congenital genital anomalies	121.5 (39.7 to 257.8)	129.4 (42.1 to 274.1)	--	--	3.4 (0.9 to 7.9)	3.6 (1.0 to 8.4)
Urogenital congenital anomalies	Incontinence and recurrent urinary tract infections or other abdominal issues due to congenital anomalies of the urinary tract	78.1 (32.5 to 147.2)	77.2 (32.1 to 145.4)	--	--	11.5 (4.4 to 23.4)	11.4 (4.3 to 23.2)
Urogenital congenital anomalies	Atypical genitalia and impotence due to congenital genital anomalies	406.6 (261.7 to 594.4)	433.0 (278.5 to 630.7)	--	--	11.3 (5.1 to 21.7)	12.1 (5.4 to 23.1)
Urogenital congenital anomalies	Atypical genitalia, recurrent urinary tract infections or other abdominal issues and incontinence due to congenital anomalies of the urinary tract	147.1 (79.1 to 235.1)	145.4 (78.5 to 234.1)	--	--	23.1 (10.8 to 40.7)	22.8 (10.8 to 40.3)
Urogenital congenital anomalies	Infertility, impotence, and recurrent urinary tract infections or other abdominal issues and impotence due to congenital genital anomalies	10.2 (< 0.05 to 85.3)	10.9 (< 0.05 to 90.7)	--	--	0.4 (< 0.05 to 3.1)	0.4 (< 0.05 to 3.4)
Urogenital congenital anomalies	Primary infertility and recurrent urinary tract infections or other abdominal issues due to congenital genital anomalies	41.7 (1.5 to 145.1)	44.4 (1.6 to 155.1)	--	--	0.8 (< 0.05 to 2.9)	0.8 (< 0.05 to 3.1)
Urogenital congenital anomalies	Atypical genitalia, recurrent urinary tract infections or other abdominal issues and impotence due to congenital anomalies of the urinary tract	39.2 (7.0 to 97.6)	38.7 (6.9 to 96.9)	--	--	1.5 (0.2 to 4.4)	1.5 (0.2 to 4.4)
Urogenital congenital anomalies	Atypical genital and recurrent urinary tract infections and other abdominal issues due to congenital anomalies of the urinary tract	293.0 (195.1 to 416.6)	289.7 (194.0 to 413.4)	--	--	6.4 (2.8 to 12.8)	6.3 (2.7 to 12.8)
Urogenital congenital anomalies	Impotence due to congenital genital anomalies	72.4 (13.5 to 176.7)	77.1 (14.3 to 188.2)	--	--	1.3 (0.2 to 3.8)	1.3 (0.2 to 4.0)
Urogenital congenital anomalies	Atypical genitalia, recurrent urinary tract infections or other abdominal issues and infertility due to congenital genital anomalies	75.6 (12.8 to 187.9)	80.6 (13.6 to 199.8)	--	--	2.2 (0.3 to 6.2)	2.4 (0.3 to 6.6)
Urogenital congenital anomalies	Impotence and recurrent urinary tract infections or other abdominal issues due to congenital anomalies of the urinary tract	148.9 (82.6 to 239.0)	147.2 (81.3 to 237.0)	--	--	4.2 (1.7 to 8.4)	4.1 (1.6 to 8.2)
Urogenital congenital anomalies	Incontinence, impotence, and recurrent urinary tract infections or other abdominal issues and impotence due to congenital anomalies of the urinary tract	23.3 (1.0 to 79.8)	23.0 (1.0 to 78.1)	--	--	3.8 (0.2 to 13.5)	3.8 (0.1 to 13.4)
Urogenital congenital anomalies	Primary infertility due to congenital genital anomalies	418.4 (259.2 to 615.2)	445.6 (275.8 to 650.2)	--	--	3.1 (1.2 to 6.7)	3.3 (1.3 to 7.1)
Urogenital congenital anomalies	Asymptomatic congenital anomalies of the urinary tract	43.4 (8.7 to 100.5)	42.9 (8.6 to 100.0)	--	--	< 0.05 (0.0 to < 0.05)	< 0.05 (0.0 to < 0.05)
Urogenital congenital anomalies	Atypical genitalia due to congenital genital anomalies	1,359.1 (1,101.9 to 1,651.2)	1,447.3 (1,173.7 to 1,755.1)	--	--	14.7 (6.8 to 28.1)	15.7 (7.2 to 29.8)
Urogenital congenital anomalies	Recurrent urinary tract infections or other abdominal issues due to congenital anomalies of the urinary tract	79.2 (30.7 to 145.6)	78.3 (30.3 to 143.3)	--	--	0.9 (0.3 to 2.2)	0.9 (0.3 to 2.2)
Digestive congenital anomalies	Chronic abdominal pain and mild chronic respiratory problems due to congenital diaphragmatic hernia	22.9 (10.1 to 40.4)	25.3 (11.2 to 45.7)	--	--	0.7 (0.2 to 1.5)	0.8 (0.3 to 1.7)

Digestive congenital anomalies	Acid reflux, dyspahgia, and/or constipation due to other congenital malformations of the digestive tract	4,188.9 (3,730.4 to 4,687.1)	4,556.6 (4,016.4 to 5,141.4)	--	--	45.0 (21.9 to 83.9)	48.9 (23.5 to 91.3)
Digestive congenital anomalies	Chronic abdominal pain and developmental delay due to congenital diaphragmatic hernia	32.9 (18.3 to 53.9)	36.4 (20.3 to 59.9)	--	--	1.7 (0.8 to 3.3)	1.9 (0.9 to 3.7)
Digestive congenital anomalies	Dysphagia or acid reflux, chronic abdominal pain and chronic respiratory problems due to congenital atresia and/or stenosis of the digestive tract	117.8 (94.1 to 142.9)	124.7 (98.7 to 153.2)	--	--	16.6 (10.3 to 24.5)	17.6 (10.9 to 26.2)
Digestive congenital anomalies	Constipation and chronic abdominal pain due to congenital malformations of the abdominal wall	25.1 (10.9 to 44.5)	29.8 (13.0 to 52.9)	--	--	3.1 (1.3 to 6.1)	3.7 (1.5 to 7.2)
Digestive congenital anomalies	Chronic respiratory problems and abdominal pain due to congenital atresia and/or stenosis of the digestive tract	16.8 (9.4 to 25.6)	17.7 (9.9 to 27.4)	--	--	0.5 (0.2 to 1.0)	0.5 (0.2 to 1.0)
Digestive congenital anomalies	Constipation due to congenital malformations of the abdominal wall	65.7 (42.2 to 91.7)	77.9 (50.3 to 109.3)	--	--	7.5 (4.2 to 12.3)	8.9 (5.0 to 14.5)
Digestive congenital anomalies	Dysphagia or acid reflux due to congenital atresia and/or stenosis of the digestive tract	60.3 (45.7 to 77.1)	63.8 (48.3 to 81.6)	--	--	0.7 (0.3 to 1.3)	0.7 (0.3 to 1.4)
Digestive congenital anomalies	Chronic abdominal pain due to congenital malformations of the abdominal wall	60.6 (39.0 to 86.2)	71.9 (46.7 to 102.4)	--	--	0.7 (0.3 to 1.5)	0.8 (0.3 to 1.7)
Digestive congenital anomalies	Chronic respiratory problems and dysphagia or acid reflux due to congenital atresia and/or stenosis of the digestive tract	94.4 (75.1 to 117.1)	99.9 (79.4 to 126.0)	--	--	2.8 (1.4 to 5.2)	3.0 (1.5 to 5.5)
Digestive congenital anomalies	Mild chronic respiratory problems and developmental delay due to congenital diaphragmatic hernia	20.4 (8.8 to 37.9)	22.7 (9.8 to 41.9)	--	--	1.3 (0.5 to 2.7)	1.4 (0.5 to 3.0)
Digestive congenital anomalies	Chronic abdominal pain due to congenital atresia and/or stenosis of the digestive tract	13.1 (6.7 to 21.4)	13.9 (7.1 to 22.8)	--	--	1.5 (0.7 to 2.7)	1.6 (0.7 to 2.9)
Digestive congenital anomalies	Chronic abdominal pain, disfigurement and chronic respiratory problems due to congenital diaphragmatic hernia	8.0 (1.3 to 21.4)	8.8 (1.4 to 23.6)	--	--	0.4 (0.1 to 1.3)	0.5 (0.1 to 1.4)
Digestive congenital anomalies	Chronic abdominal pain, disfigurement, developmental delay and chronic respiratory problems due to congenital diaphragmatic hernia	2.2 (< 0.05 to 13.7)	2.4 (< 0.05 to 15.3)	--	--	0.2 (< 0.05 to 1.3)	0.2 (< 0.05 to 1.4)
Digestive congenital anomalies	Asymptomatic congenital diaphragmatic hernia	387.6 (331.1 to 450.1)	429.4 (365.6 to 497.9)	--	--	< 0.05 (0.0 to < 0.05)	< 0.05 (0.0 to < 0.05)
Digestive congenital anomalies	Disfigurement due to congenital diaphragmatic hernia	133.2 (101.9 to 169.3)	147.5 (112.9 to 189.2)	--	--	1.5 (0.7 to 2.8)	1.6 (0.7 to 3.1)
Digestive congenital anomalies	Mild chronic respiratory problems and breathlessness due to congenital diaphragmatic hernia	75.2 (51.6 to 102.4)	83.3 (57.4 to 113.9)	--	--	1.5 (0.7 to 2.5)	1.6 (0.8 to 2.8)
Digestive congenital anomalies	Disfigurement, chronic respiratory problems and developmental delay due to congenital diaphragmatic hernia	7.5 (1.0 to 21.7)	8.3 (1.1 to 24.2)	--	--	0.6 (0.1 to 2.0)	0.7 (0.1 to 2.2)
Digestive congenital anomalies	Asymptomatic other congenital malformations of the digestive tract	397.0 (252.3 to 566.6)	431.9 (273.9 to 617.0)	--	--	< 0.05 (0.0 to < 0.05)	< 0.05 (0.0 to < 0.05)
Digestive congenital anomalies	Disfigurement and mild chronic respiratory problems due to congenital diaphragmatic hernia	25.6 (12.0 to 44.2)	28.4 (13.1 to 48.7)	--	--	1.2 (0.4 to 2.3)	1.3 (0.5 to 2.6)
Digestive congenital anomalies	Chronic abdominal pain due to congenital diaphragmatic hernia	115.1 (85.5 to 150.0)	127.5 (95.2 to 166.4)	--	--	1.3 (0.6 to 2.4)	1.4 (0.6 to 2.6)
Digestive congenital anomalies	Chronic abdominal pain and disfigurement due to congenital diaphragmatic hernia	39.5 (22.4 to 61.2)	43.7 (24.6 to 68.0)	--	--	1.5 (0.6 to 2.8)	1.6 (0.7 to 3.1)
Digestive congenital anomalies	Chronic abdominal pain and concern about scars due to congenital malformations of the abdominal wall	46.6 (27.5 to 71.6)	55.2 (32.6 to 84.7)	--	--	5.8 (3.0 to 10.2)	6.9 (3.6 to 12.1)
Digestive congenital anomalies	Chronic respiratory problems including difficulty breaking and recurrent upper respiratory infections due to atresia and/or stenosis of the digestive tract	13.3 (6.9 to 22.1)	14.1 (7.3 to 23.8)	--	--	0.3 (0.1 to 0.5)	0.3 (0.1 to 0.5)
Digestive congenital anomalies	Chronic abdominal pain and/or nausea with acid reflux, dyspahgia, and/or constipation due to other congenital malformations of the digestive tract	2,100.0 (1,803.2 to 2,441.0)	2,284.2 (1,948.7 to 2,660.9)	--	--	248.4 (162.7 to 352.9)	269.4 (176.8 to 383.3)
Digestive congenital anomalies	Chronic abdominal pain, disfigurement and developmental delay due to congenital diaphragmatic hernia	11.2 (3.0 to 26.3)	12.4 (3.3 to 29.3)	--	--	0.9 (0.2 to 2.1)	1.0 (0.2 to 2.4)
Digestive congenital anomalies	Chronic abdominal pain, chronic respiratory problems and developmental delay due to congenital diaphragmatic hernia	6.3 (0.6 to 19.9)	7.0 (0.7 to 21.8)	--	--	0.3 (< 0.05 to 1.1)	0.4 (< 0.05 to 1.3)
Digestive congenital anomalies	Constipation, chronic abdominal pain and concern about scars due to congenital malformations of the abdominal wall	19.3 (6.9 to 36.3)	22.9 (8.3 to 43.0)	--	--	4.0 (1.4 to 7.8)	4.7 (1.7 to 9.2)
Digestive congenital anomalies	Disfigurement from scars following treatment for congenital malformations of the abdominal wall	123.4 (91.9 to 159.6)	146.3 (108.9 to 188.5)	--	--	1.4 (0.6 to 2.7)	1.6 (0.7 to 3.2)
Digestive congenital anomalies	Asymptomatic congenital malformations of the abdominal wall after treatment	158.4 (122.1 to 197.0)	187.7 (145.0 to 234.9)	--	--	< 0.05 (0.0 to < 0.05)	< 0.05 (0.0 to < 0.05)
Digestive congenital anomalies	Dysphagia or acid reflux and chronic abdominal pain due to congenital atresia and/or stenosis of the digestive tract	74.0 (56.8 to 93.0)	78.3 (59.6 to 99.0)	--	--	9.2 (5.5 to 13.3)	9.7 (5.9 to 14.2)
Digestive congenital anomalies	Chronic abdominal pain and/or nausea due to other congenital malformations of the digestive tract	202.0 (98.8 to 334.9)	219.7 (106.0 to 365.2)	--	--	23.2 (9.8 to 41.9)	25.2 (10.7 to 45.7)
Digestive congenital anomalies	Asymptomatic congenital atresia and/or stenosis of the digestive tract	5.6 (1.7 to 11.5)	5.9 (1.8 to 12.1)	--	--	< 0.05 (0.0 to < 0.05)	< 0.05 (0.0 to < 0.05)
Digestive congenital anomalies	Constipation and concern about scars due to congenital malformations of the abdominal wall	51.5 (31.4 to 76.4)	61.0 (37.4 to 90.4)	--	--	1.1 (0.5 to 2.2)	1.3 (0.6 to 2.7)
Digestive congenital anomalies	Developmental delay or mild intellectual disability due to congenital diaphragmatic hernia	109.7 (81.5 to 142.6)	121.6 (89.6 to 158.8)	--	--	4.7 (2.6 to 7.5)	5.2 (2.9 to 8.3)
Digestive congenital anomalies	Disfigurement and developmental delay due to congenital diaphragmatic hernia	38.5 (22.1 to 61.3)	42.7 (24.4 to 67.3)	--	--	2.6 (1.2 to 4.9)	2.9 (1.4 to 5.5)
Other congenital anomalies	Moderate hearing loss with ringing due to other congenital anomalies	1,325.6 (684.6 to 2,153.8)	1,500.8 (815.3 to 2,387.5)	--	--	93.7 (45.9 to 169.6)	105.8 (53.0 to 187.5)
Other congenital anomalies	Severe hearing loss due to other congenital anomalies	1,058.7 (477.7 to 1,910.4)	1,077.4 (483.7 to 1,944.6)	--	--	159.7 (63.6 to 310.4)	162.8 (65.3 to 313.9)
Other congenital anomalies	Profound hearing loss with ringing due to other congenital anomalies	3,371.2 (2,024.1 to 5,408.0)	3,482.0 (2,096.6 to 5,573.7)	--	--	875.8 (453.0 to 1,508.3)	903.4 (461.9 to 1,544.1)
Other congenital anomalies	Moderately severe hearing loss with ringing due to other congenital anomalies	2,650.8 (1,678.6 to 4,160.3)	2,714.9 (1,735.8 to 4,267.0)	--	--	420.4 (226.6 to 701.7)	429.7 (231.9 to 706.5)
Other congenital anomalies	Mild hearing loss due to other congenital anomalies	8,507.1 (3,673.6 to 15,768.0)	9,785.2 (4,165.0 to 18,086.0)	--	--	79.2 (24.8 to 188.4)	90.9 (28.9 to 215.1)
Other congenital anomalies	Moderate hearing loss due to other congenital anomalies	3,062.5 (1,563.3 to 4,961.1)	3,463.8 (1,886.3 to 5,483.0)	--	--	78.3 (35.2 to 148.8)	88.5 (41.9 to 165.8)
Other congenital anomalies	Profound hearing loss due to other congenital anomalies	1,887.0 (1,141.9 to 3,093.3)	1,960.2 (1,155.1 to 3,160.0)	--	--	362.9 (191.4 to 649.1)	377.3 (193.3 to 671.9)
Other congenital anomalies	Complete hearing loss due to other congenital anomalies	999.3 (615.3 to 1,492.7)	982.2 (606.3 to 1,514.6)	--	--	203.5 (110.1 to 343.6)	200.9 (110.1 to 341.2)

Other congenital anomalies	Mild hearing loss with ringing due to other congenital anomalies	2,251.7 (967.9 to 4,164.4)	2,589.7 (1,101.9 to 4,743.2)	--	--	46.8 (16.9 to 99.5)	53.8 (19.6 to 114.3)
Other congenital anomalies	Complete hearing loss with ringing due to other congenital anomalies	1,199.6 (754.1 to 1,788.1)	1,175.2 (732.6 to 1,747.2)	--	--	355.9 (193.5 to 587.9)	348.7 (183.5 to 582.3)
Other congenital anomalies	Severe hearing loss with ringing due to other congenital anomalies	599.0 (265.6 to 1,068.2)	610.8 (278.4 to 1,108.4)	--	--	150.0 (60.1 to 290.8)	153.4 (61.1 to 293.8)
Other congenital anomalies	Other congenital birth defects	--	--	--	--	715.6 (501.4 to 937.6)	888.7 (628.7 to 1,163.4)
Other congenital anomalies	Moderately severe hearing loss due to other congenital anomalies	5,507.5 (3,444.7 to 8,619.5)	5,641.3 (3,565.5 to 8,706.5)	--	--	486.8 (262.8 to 816.4)	497.9 (266.9 to 831.7)
Dermatitis	Mild contact dermatitis	32,501.8 (28,078.2 to 37,426.6)	37,276.1 (32,267.8 to 42,789.4)	89,871.5 (77,823.9 to 102,855.3)	103,898.3 (90,106.4 to 118,211.2)	827.7 (468.9 to 1,331.7)	947.6 (535.6 to 1,527.5)
Dermatitis	Mild atopic dermatitis	167,477.6 (150,633.9 to 183,341.7)	185,968.9 (166,835.8 to 204,263.2)	23,198.2 (20,665.6 to 25,490.6)	26,120.8 (23,203.3 to 28,775.0)	4,326.2 (2,442.2 to 6,875.2)	4,799.7 (2,726.9 to 7,603.3)
Dermatitis	Asymptomatic seborrhoeic dermatitis	11,552.2 (10,615.9 to 12,604.9)	13,244.2 (12,213.7 to 14,417.7)	80,196.8 (71,731.0 to 88,566.2)	90,249.1 (80,771.5 to 99,564.1)	< 0.05 (0.0 to < 0.05)	< 0.05 (0.0 to < 0.05)
Dermatitis	Moderate contact dermatitis	4,633.9 (3,035.1 to 6,498.2)	5,314.7 (3,476.7 to 7,473.4)	12,774.9 (8,223.4 to 18,370.3)	14,769.0 (9,509.5 to 21,271.8)	811.8 (454.5 to 1,266.7)	928.6 (513.8 to 1,448.1)
Dermatitis	Symptomatic seborrhoeic dermatitis	13,033.0 (12,008.4 to 14,143.5)	14,941.9 (13,751.9 to 16,183.1)	90,427.9 (81,999.7 to 99,913.8)	101,762.6 (92,192.5 to 112,308.5)	333.0 (189.9 to 539.9)	381.1 (216.6 to 616.2)
Dermatitis	Asymptomatic contact dermatitis	28,486.3 (24,565.1 to 32,738.5)	32,670.6 (28,400.2 to 37,519.8)	78,931.4 (68,339.9 to 90,026.1)	91,251.9 (79,332.3 to 103,381.0)	< 0.05 (0.0 to < 0.05)	< 0.05 (0.0 to < 0.05)
Dermatitis	Severe atopic dermatitis	2,805.6 (22.1 to 11,966.8)	3,115.9 (24.5 to 13,339.7)	379.2 (1.9 to 1,600.0)	427.1 (2.1 to 1,796.2)	1,511.2 (11.1 to 6,708.7)	1,676.2 (12.0 to 7,464.0)
Dermatitis	Moderate atopic dermatitis	12,451.8 (3,113.1 to 26,102.7)	13,826.7 (3,471.2 to 28,975.4)	1,684.7 (395.9 to 3,473.1)	1,897.0 (445.7 to 3,911.0)	2,233.5 (511.7 to 5,125.3)	2,477.0 (569.8 to 5,692.3)
Psoriasis	Moderate psoriasis	6,316.4 (4,575.7 to 8,341.2)	7,703.0 (5,587.1 to 10,170.8)	794.1 (584.5 to 1,016.9)	963.4 (710.3 to 1,235.1)	1,106.3 (670.2 to 1,675.8)	1,346.1 (818.4 to 2,036.1)
Psoriasis	Mild psoriasis	42,416.5 (39,698.9 to 44,914.3)	51,727.7 (48,447.7 to 54,788.5)	5,352.2 (5,037.7 to 5,686.4)	6,493.2 (6,104.4 to 6,897.4)	1,080.3 (619.1 to 1,716.6)	1,315.5 (754.1 to 2,085.5)
Psoriasis	Severe psoriasis	4,677.5 (3,350.7 to 6,148.6)	5,704.2 (4,083.8 to 7,490.1)	588.0 (425.6 to 777.1)	713.4 (516.8 to 943.2)	2,452.2 (1,573.6 to 3,534.8)	2,981.9 (1,920.8 to 4,302.7)
Cellulitis	Moderate cellulitis	886.1 (726.2 to 1,061.9)	1,013.4 (831.9 to 1,213.1)	17,893.0 (14,944.1 to 21,367.0)	20,482.2 (17,079.0 to 24,483.9)	43.2 (26.5 to 65.6)	49.2 (30.3 to 75.2)
Cellulitis	Mild cellulitis	962.3 (760.6 to 1,174.4)	1,100.5 (873.9 to 1,340.5)	19,534.1 (15,790.2 to 23,354.4)	22,360.6 (18,016.3 to 26,718.6)	5.4 (2.1 to 11.5)	6.2 (2.4 to 13.3)
Cellulitis	Severe cellulitis	791.0 (632.6 to 964.7)	904.6 (721.6 to 1,102.9)	16,152.8 (12,730.5 to 19,789.6)	18,490.4 (14,558.6 to 22,642.0)	100.8 (63.6 to 149.4)	114.8 (72.8 to 170.6)
Pyoderma	Impetigo	4,776.0 (4,483.5 to 5,075.2)	5,736.3 (5,377.9 to 6,116.8)	87,019.2 (81,504.7 to 92,337.0)	104,530.8 (97,667.1 to 110,882.8)	27.3 (10.8 to 57.4)	32.8 (13.0 to 68.9)
Pyoderma	Abscess and other bacterial skin diseases	12,323.3 (12,035.9 to 12,636.3)	15,283.9 (14,916.2 to 15,679.7)	298,892.1 (290,095.3 to 308,898.4)	369,853.7 (358,588.9 to 382,210.0)	68.2 (27.5 to 143.4)	84.5 (34.2 to 177.4)
Scabies	Scabies	142,060.1 (123,104.5 to 165,509.8)	146,784.6 (127,773.0 to 170,008.8)	442,527.8 (382,259.9 to 517,585.7)	454,670.6 (392,690.4 to 529,184.4)	3,670.6 (2,034.7 to 5,849.4)	3,787.8 (2,103.6 to 6,029.0)
Fungal skin diseases	Other fungal skin diseases	418,610.4 (373,740.9 to 463,882.3)	500,640.4 (448,978.5 to 556,178.3)	1,483,855.5 (1,316,166.1 to 1,671,368.9)	1,793,878.4 (1,588,423.6 to 2,022,376.4)	2,336.9 (930.5 to 4,930.5)	2,789.0 (1,107.3 to 5,865.2)
Fungal skin diseases	Tinea capitis	111,490.0 (89,416.9 to 139,892.7)	126,059.5 (101,477.8 to 158,585.4)	275,986.1 (217,498.7 to 344,658.2)	304,864.9 (239,365.6 to 381,762.5)	636.5 (247.9 to 1,357.1)	719.8 (281.5 to 1,537.0)
Viral skin diseases	Severe viral warts	58,088.9 (46,269.8 to 70,863.2)	63,966.8 (51,048.2 to 77,759.5)	91,142.7 (73,372.7 to 109,472.2)	100,489.5 (80,880.2 to 120,638.0)	3,737.8 (2,341.6 to 5,445.2)	4,110.4 (2,569.2 to 6,005.9)
Viral skin diseases	Mild molluscum contagiosum	23,051.5 (19,418.1 to 26,797.3)	24,386.5 (20,552.3 to 28,364.0)	22,473.6 (19,022.4 to 26,139.8)	23,746.6 (20,112.5 to 27,586.0)	132.0 (52.8 to 281.1)	139.7 (55.9 to 296.6)
Viral skin diseases	Mild viral warts	78,876.6 (66,700.6 to 92,005.1)	86,856.7 (73,418.3 to 101,217.3)	122,284.5 (103,187.6 to 141,619.2)	134,823.9 (113,782.7 to 155,943.0)	446.3 (176.9 to 927.6)	490.8 (194.8 to 1,020.1)
Viral skin diseases	Severe molluscum contagiosum	16,977.4 (13,397.2 to 20,748.3)	17,960.7 (14,173.3 to 21,930.3)	16,746.4 (13,382.0 to 20,473.8)	17,694.9 (14,166.8 to 21,607.0)	1,109.7 (689.6 to 1,637.2)	1,174.3 (726.6 to 1,732.9)
Acne vulgaris	Severe acne vulgaris	13,546.1 (9,511.9 to 18,638.0)	14,242.1 (9,992.2 to 19,608.8)	9,599.8 (6,394.1 to 13,762.1)	10,031.2 (6,693.7 to 14,393.8)	5,184.5 (3,064.7 to 7,883.6)	5,448.0 (3,211.0 to 8,295.8)
Acne vulgaris	Moderate acne vulgaris	68,131.8 (45,981.1 to 95,484.6)	71,637.6 (48,296.0 to 100,415.1)	47,471.8 (30,894.5 to 66,379.5)	49,605.8 (32,101.5 to 69,321.3)	4,434.2 (2,485.0 to 7,171.8)	4,660.9 (2,612.2 to 7,511.2)
Acne vulgaris	Mild acne vulgaris	503,035.5 (452,817.7 to 558,002.7)	528,891.1 (477,879.4 to 584,131.4)	354,266.3 (296,303.1 to 430,980.3)	370,185.2 (309,524.4 to 452,905.4)	5,448.8 (2,568.4 to 10,042.6)	5,727.1 (2,711.6 to 10,541.5)
Alopecia areata	Severe alopecia areata	5,632.2 (4,414.4 to 6,834.3)	6,359.7 (4,982.2 to 7,733.9)	9,905.3 (7,920.5 to 11,954.6)	11,160.9 (8,919.1 to 13,465.6)	361.1 (228.8 to 537.3)	407.3 (259.4 to 609.6)
Alopecia areata	Mild alopecia areata	8,020.1 (6,825.4 to 9,226.3)	9,055.8 (7,698.5 to 10,404.5)	14,276.3 (12,207.1 to 16,345.5)	16,086.0 (13,747.2 to 18,445.9)	85.9 (40.9 to 160.7)	96.9 (46.2 to 181.7)
Pruritus	Pruritus	56,391.3 (50,120.8 to 63,167.1)	66,780.0 (59,262.3 to 74,868.4)	44,914.5 (40,507.0 to 50,267.5)	53,046.2 (47,751.0 to 59,503.3)	599.8 (278.6 to 1,087.3)	709.1 (329.7 to 1,298.7)
Urticaria	Mild urticaria	47,515.2 (41,049.2 to 54,818.0)	52,042.0 (45,048.8 to 59,841.8)	84,721.2 (73,341.5 to 99,090.1)	92,551.6 (80,211.2 to 107,584.6)	1,226.5 (692.0 to 1,948.3)	1,341.9 (757.2 to 2,120.2)
Urticaria	Severe urticaria	13,712.0 (10,388.2 to 17,462.5)	15,017.7 (11,390.9 to 19,124.0)	24,579.7 (18,282.6 to 31,302.1)	26,851.5 (19,989.4 to 34,126.5)	2,457.9 (1,459.5 to 3,678.0)	2,688.1 (1,592.0 to 4,016.0)
Decubitus ulcer	Mild decubitus ulcer	800.8 (687.4 to 928.5)	1,024.5 (873.9 to 1,191.1)	2,924.9 (2,508.6 to 3,357.3)	3,751.4 (3,204.3 to 4,323.9)	19.8 (11.4 to 31.0)	25.2 (14.5 to 39.9)
Decubitus ulcer	Moderate decubitus ulcer	342.5 (262.8 to 435.8)	438.1 (334.8 to 556.2)	1,250.6 (963.4 to 1,550.8)	1,604.0 (1,234.9 to 1,991.9)	58.7 (37.0 to 88.7)	74.7 (46.7 to 111.7)
Decubitus ulcer	Severe decubitus ulcer	292.0 (212.6 to 383.4)	373.6 (270.7 to 489.8)	1,065.6 (786.0 to 1,372.4)	1,366.6 (1,003.1 to 1,764.8)	149.6 (95.2 to 214.7)	189.9 (121.9 to 275.8)
Other skin and subcutaneous diseases	Asymptomatic other skin and subcutaneous diseases	204,603.6 (198,564.7 to 210,336.8)	256,801.4 (249,310.3 to 263,819.9)	240,826.5 (232,300.8 to 249,042.5)	305,593.1 (294,679.9 to 315,834.1)	< 0.05 (0.0 to < 0.05)	< 0.05 (0.0 to < 0.05)
Other skin and subcutaneous diseases	Symptomatic other skin and subcutaneous diseases	219,891.2 (212,806.9 to 226,520.4)	275,988.8 (267,208.3 to 284,380.8)	258,751.8 (250,211.1 to 267,430.7)	328,339.2 (317,517.1 to 339,161.1)	2,324.9 (1,120.7 to 4,256.7)	2,912.8 (1,409.4 to 5,325.1)
Glaucoma	Severe vision impairment due to glaucoma	378.0 (280.7 to 502.9)	479.0 (353.7 to 641.6)	--	--	61.1 (38.3 to 91.9)	77.5 (48.6 to 117.6)
Glaucoma	Blindness due to glaucoma	1,390.8 (1,093.5 to 1,784.7)	1,929.2 (1,517.1 to 2,471.9)	--	--	228.4 (146.6 to 332.5)	316.9 (202.9 to 465.2)

Glaucoma	Moderate vision impairment due to glaucoma	1,773.5 (1,385.7 to 2,216.5)	2,353.0 (1,819.6 to 2,970.8)	--	--	50.2 (29.2 to 82.8)	66.8 (38.6 to 111.8)
Cataract	Moderate vision impairment due to cataract	43,496.3 (36,955.7 to 50,517.4)	57,963.7 (48,642.6 to 67,794.5)	--	--	1,225.9 (762.5 to 1,927.1)	1,634.9 (1,013.9 to 2,584.9)
Cataract	Severe vision impairment due to cataract	10,281.9 (8,391.3 to 12,233.4)	13,650.0 (11,067.8 to 16,261.4)	--	--	1,645.4 (1,082.6 to 2,319.7)	2,184.8 (1,441.8 to 3,088.0)
Cataract	Blindness due to cataract	9,417.7 (8,194.6 to 10,940.2)	12,014.8 (10,403.9 to 13,956.0)	--	--	1,546.9 (1,046.5 to 2,140.4)	1,969.2 (1,324.3 to 2,731.3)
Macular degeneration	Blindness due to macular degeneration	690.3 (527.8 to 868.5)	964.7 (746.1 to 1,217.6)	--	--	113.0 (72.9 to 161.3)	157.9 (101.5 to 226.8)
Macular degeneration	Severe vision impairment due to macular degeneration	615.5 (456.2 to 809.2)	778.2 (573.9 to 1,028.2)	--	--	99.3 (62.6 to 145.6)	125.5 (78.5 to 186.0)
Macular degeneration	Moderate vision impairment due to macular degeneration	2,992.7 (2,346.3 to 3,788.0)	4,404.1 (3,449.2 to 5,536.9)	--	--	84.8 (50.9 to 136.0)	125.1 (74.4 to 203.3)
Refraction and accommodation disorders	Severe vision impairment due to uncorrected refractive error	11,982.3 (10,637.6 to 13,390.5)	13,728.0 (12,112.8 to 15,513.4)	--	--	2,002.3 (1,389.0 to 2,767.2)	2,293.1 (1,585.6 to 3,186.6)
Refraction and accommodation disorders	Moderate vision impairment due to uncorrected refractive error	145,110.2 (129,222.6 to 161,547.5)	161,812.5 (143,336.4 to 180,473.5)	--	--	4,274.9 (2,623.9 to 6,710.4)	4,762.5 (2,918.2 to 7,487.9)
Refraction and accommodation disorders	Blindness due to uncorrected refractive error	6,059.4 (5,414.2 to 6,780.0)	6,581.2 (5,882.3 to 7,391.3)	--	--	1,040.2 (705.9 to 1,444.2)	1,128.8 (760.8 to 1,557.8)
Refraction and accommodation disorders	Near vision impairment	565,256.6 (545,508.3 to 585,259.1)	672,177.0 (648,254.1 to 697,017.0)	--	--	5,718.1 (2,692.7 to 10,550.5)	6,788.1 (3,196.5 to 12,474.2)
Age-related and other hearing loss	Profound hearing loss with ringing due to age-related and other hearing loss	3,731.9 (2,235.7 to 5,232.1)	4,308.9 (2,702.7 to 5,918.2)	--	--	945.0 (476.9 to 1,515.4)	1,083.4 (568.4 to 1,711.2)
Age-related and other hearing loss	Moderate hearing loss with ringing due to age-related and other hearing loss	61,044.4 (56,010.5 to 66,314.0)	75,485.2 (69,178.9 to 82,036.7)	--	--	4,154.1 (2,739.4 to 5,894.4)	5,127.8 (3,431.2 to 7,279.4)
Age-related and other hearing loss	Mild hearing loss due to age-related and other hearing loss	555,337.5 (511,966.3 to 602,990.1)	707,843.3 (655,810.4 to 765,689.4)	--	--	5,062.6 (2,294.0 to 9,842.9)	6,448.7 (2,895.1 to 12,564.8)
Age-related and other hearing loss	Severe hearing loss due to age-related and other hearing loss	8,288.1 (6,808.0 to 9,838.9)	9,501.4 (7,894.3 to 11,267.6)	--	--	1,195.4 (761.5 to 1,739.4)	1,363.8 (866.7 to 1,957.3)
Age-related and other hearing loss	Severe hearing loss with ringing due to age-related and other hearing loss	4,688.8 (3,874.5 to 5,639.2)	5,391.8 (4,473.1 to 6,448.2)	--	--	1,098.0 (707.0 to 1,578.8)	1,255.0 (796.7 to 1,787.7)
Age-related and other hearing loss	Moderate hearing loss due to age-related and other hearing loss	140,963.7 (130,645.3 to 152,356.6)	174,247.7 (161,150.1 to 188,304.3)	--	--	3,504.8 (2,067.3 to 5,489.3)	4,325.5 (2,532.0 to 6,802.3)
Age-related and other hearing loss	Complete hearing loss with ringing due to age-related and other hearing loss	4,728.3 (3,836.9 to 5,696.8)	5,320.6 (4,407.4 to 6,328.3)	--	--	1,361.2 (869.1 to 1,944.2)	1,526.2 (996.1 to 2,151.4)
Age-related and other hearing loss	Moderately severe hearing loss with ringing due to age-related and other hearing loss	25,126.6 (21,844.8 to 28,449.3)	30,918.1 (26,949.5 to 34,994.3)	--	--	3,772.6 (2,562.0 to 5,127.6)	4,623.4 (3,145.4 to 6,229.1)
Age-related and other hearing loss	Moderately severe hearing loss due to age-related and other hearing loss	52,335.7 (45,841.3 to 59,148.0)	64,251.3 (56,351.9 to 72,491.5)	--	--	4,413.0 (3,007.6 to 6,063.5)	5,399.8 (3,653.6 to 7,459.8)
Age-related and other hearing loss	Complete hearing loss due to age-related and other hearing loss	3,958.8 (3,213.0 to 4,779.5)	4,431.1 (3,586.9 to 5,329.5)	--	--	784.2 (489.5 to 1,137.6)	874.8 (535.5 to 1,259.0)
Age-related and other hearing loss	Profound hearing loss due to age-related and other hearing loss	2,094.4 (1,265.8 to 2,952.9)	2,420.2 (1,474.4 to 3,326.6)	--	--	393.4 (197.4 to 637.1)	451.6 (241.8 to 725.8)
Age-related and other hearing loss	Mild hearing loss with ringing due to age-related and other hearing loss	147,103.0 (135,115.8 to 159,424.3)	187,555.3 (173,349.3 to 202,585.5)	--	--	2,988.7 (1,631.0 to 4,958.5)	3,807.5 (2,108.5 to 6,340.8)
Other vision loss	Severe vision impairment due to other vision loss	2,674.6 (2,168.4 to 3,256.0)	3,277.4 (2,633.3 to 4,025.0)	--	--	442.9 (293.6 to 625.2)	542.2 (356.5 to 768.8)
Other vision loss	Blindness due to other vision loss	5,239.1 (4,363.4 to 6,226.2)	6,743.1 (5,612.9 to 7,985.8)	--	--	890.0 (582.4 to 1,243.7)	1,141.7 (752.8 to 1,597.0)
Other vision loss	Moderate vision impairment due to other vision loss	15,296.3 (13,019.6 to 17,835.8)	19,228.6 (16,187.2 to 22,515.7)	--	--	444.0 (271.7 to 710.0)	557.3 (341.0 to 902.0)
Other sense organ diseases	Moderate acute sense organ diseases	1,499.4 (825.7 to 2,237.3)	1,715.1 (941.9 to 2,558.2)	76,348.5 (39,713.6 to 118,867.2)	87,472.5 (45,450.7 to 136,206.1)	16.1 (6.1 to 34.2)	18.4 (7.0 to 39.1)
Other sense organ diseases	Severe chronic sense organ diseases	47,459.9 (35,147.3 to 59,684.5)	58,893.9 (43,706.2 to 74,237.8)	23,658.7 (17,680.7 to 29,690.8)	29,520.1 (22,082.8 to 37,038.3)	4,979.0 (3,006.0 to 7,619.5)	6,164.8 (3,714.3 to 9,418.4)
Other sense organ diseases	Moderate chronic sense organ diseases	25,690.2 (14,169.8 to 38,616.9)	31,879.9 (17,640.7 to 47,964.5)	12,584.5 (6,518.5 to 19,407.9)	15,701.9 (8,125.0 to 24,262.9)	270.4 (103.9 to 579.2)	335.0 (128.5 to 719.2)
Other sense organ diseases	Asymptomatic chronic sense organ diseases	87,156.3 (77,661.9 to 98,039.7)	108,153.9 (96,323.5 to 121,469.5)	43,337.5 (38,383.0 to 48,379.4)	54,074.2 (48,024.6 to 60,523.6)	< 0.05 (0.0 to < 0.05)	< 0.05 (0.0 to < 0.05)
Other sense organ diseases	Asymptomatic acute sense organ diseases	5,086.3 (4,501.1 to 5,732.1)	5,817.6 (5,158.4 to 6,546.4)	262,907.5 (233,432.3 to 294,034.5)	301,201.9 (267,506.8 to 336,233.9)	< 0.05 (0.0 to < 0.05)	< 0.05 (0.0 to < 0.05)
Other sense organ diseases	Mild acute sense organ diseases	3,704.0 (2,996.5 to 4,419.0)	4,236.6 (3,434.5 to 5,067.5)	192,055.1 (159,641.8 to 228,357.8)	220,033.7 (182,951.1 to 261,595.7)	20.8 (8.3 to 42.6)	23.8 (9.5 to 49.0)
Caries of deciduous teeth	Asymptomatic caries of deciduous teeth	441,289.6 (367,563.8 to 516,303.2)	473,458.0 (387,840.6 to 558,474.3)	1,586,636.4 (1,151,068.7 to 2,132,014.2)	1,716,300.9 (1,229,843.5 to 2,327,542.4)	< 0.05 (0.0 to < 0.05)	< 0.05 (0.0 to < 0.05)
Caries of deciduous teeth	Pain due to caries of deciduous teeth	11,836.1 (9,551.6 to 14,363.9)	12,636.6 (10,047.0 to 15,432.9)	42,646.5 (30,327.4 to 58,595.8)	45,963.6 (31,863.0 to 63,976.1)	119.1 (52.5 to 231.1)	127.2 (55.9 to 249.1)
Caries of permanent teeth	Asymptomatic caries of permanent teeth	2,067,749.5 (1,935,040.0 to 2,197,910.7)	2,262,473.1 (2,124,069.5 to 2,404,938.9)	6,060,927.5 (5,597,619.1 to 6,567,544.9)	6,745,370.5 (6,222,408.4 to 7,294,862.5)	< 0.05 (0.0 to < 0.05)	< 0.05 (0.0 to < 0.05)
Caries of permanent teeth	Pain due to caries of permanent teeth	159,317.3 (140,114.7 to 179,637.2)	173,376.6 (152,582.2 to 195,557.0)	468,392.5 (407,769.1 to 532,250.1)	518,621.5 (451,022.1 to 587,744.5)	1,571.3 (699.0 to 3,047.3)	1,707.6 (760.1 to 3,324.9)
Periodontal disease	Chronic periodontal diseases	596,506.9 (504,569.1 to 695,735.9)	750,847.0 (633,974.8 to 874,076.6)	72,364.2 (59,874.3 to 86,116.6)	89,840.5 (74,127.9 to 106,715.8)	3,893.0 (1,550.6 to 8,078.4)	4,898.0 (1,946.8 to 10,208.7)
Edentulism and severe tooth loss	Asymptomatic edentulism and severe tooth loss	132,022.1 (115,327.7 to 148,124.3)	168,096.5 (146,241.4 to 188,915.8)	10,270.0 (8,734.0 to 11,860.7)	13,017.4 (11,015.6 to 15,153.5)	< 0.05 (0.0 to < 0.05)	< 0.05 (0.0 to < 0.05)
Edentulism and severe tooth loss	Difficulty eating due to edentulism and severe tooth loss	105,436.4 (92,522.0 to 117,971.4)	134,246.0 (117,063.8 to 150,700.6)	8,202.0 (6,989.1 to 9,459.9)	10,396.2 (8,815.9 to 12,068.3)	6,553.4 (4,335.5 to 9,234.5)	8,338.4 (5,467.4 to 11,760.4)
Other oral disorders	Severe other oral disorders	64,490.9 (55,681.0 to 73,794.5)	74,395.9 (64,254.3 to 84,888.2)	--	--	3,123.1 (1,950.9 to 4,604.7)	3,597.6 (2,246.6 to 5,311.2)
Other oral disorders	Mild other oral disorders	52,065.0 (43,002.0 to 60,731.0)	60,061.4 (49,574.3 to 70,169.2)	--	--	292.9 (115.7 to 608.7)	337.4 (133.1 to 702.7)

SR Table 3: Global prevalence and years lived with disability (YLDs) for 2016, percentage change of YLD counts and percentage change of age-standardised YLD rates between 2006 and 2016 of anaemia by cause, all ages and both sexes combined				
Cause	Prevalence in thousands (95% uncertainty interval)	YLDs in thousands (95% uncertainty interval)		
	2016	2016	Percentage change in counts between 2006 and 2016	Percentage change in age-standardised rates between 2006 and 2016
Iron-deficiency anaemia	1,244,775.1 (1,205,789.8 to 1,284,420.6)	34,726.9 (22,959.4 to 49,578.8)	7.5% (6.2% to 8.9%)	-1.8% (-3.0% to -0.5%)
HIV/AIDS resulting in other diseases	35,133.7 (33,015.6 to 37,885.9)	3,569.9 (2,444.5 to 5,013.0)	-8.3% (-25.6% to 12.2%)	-20.0% (-35.3% to -1.1%)
Thalassaemias trait	142,750.2 (134,359.7 to 151,953.9)	3,280.4 (2,156.8 to 4,823.8)	10.9% (8.0% to 13.9%)	-0.6% (-3.1% to 2.0%)
Chronic kidney disease due to diabetes mellitus	48,377.6 (42,538.3 to 54,909.6)	2,497.0 (1,821.0 to 3,268.5)	36.5% (33.2% to 39.7%)	7.5% (5.3% to 10.0%)
Gastritis and duodenitis	66,272.2 (60,912.2 to 71,950.5)	1,669.5 (1,092.3 to 2,415.1)	38.6% (34.5% to 42.9%)	14.0% (10.8% to 17.2%)
Sickle cell trait	60,556.2 (54,907.8 to 67,904.9)	1,555.0 (1,014.1 to 2,282.9)	16.8% (14.8% to 18.8%)	7.3% (5.4% to 9.1%)
Other neglected tropical diseases	49,492.8 (47,573.7 to 51,780.9)	1,420.7 (926.9 to 2,062.2)	5.2% (2.8% to 7.4%)	-1.4% (-3.7% to 0.8%)
Chronic kidney disease due to other causes	25,487.2 (21,939.7 to 29,026.5)	1,381.6 (1,002.2 to 1,821.5)	30.9% (27.8% to 34.3%)	6.2% (3.8% to 8.9%)
Peptic ulcer disease	51,460.3 (46,176.1 to 57,534.0)	1,346.1 (877.8 to 2,002.4)	39.0% (34.4% to 43.7%)	14.2% (10.7% to 17.9%)
Other hemoglobinopathies and hemolytic anaemias	61,445.5 (59,501.4 to 63,452.6)	1,318.2 (871.7 to 1,904.3)	12.2% (9.9% to 15.2%)	0.8% (-1.2% to 3.4%)
Endocrine, metabolic, blood, and immune disorders	49,285.6 (47,765.4 to 50,931.5)	1,233.2 (814.9 to 1,770.7)	8.4% (6.7% to 10.1%)	-1.3% (-2.7% to 0.3%)
Chronic kidney disease due to hypertension	24,033.2 (20,625.7 to 27,469.3)	1,202.7 (864.0 to 1,569.8)	38.5% (34.8% to 42.4%)	6.1% (3.3% to 9.0%)
Other infectious diseases	44,421.5 (42,781.1 to 46,292.5)	1,169.0 (764.8 to 1,692.0)	11.8% (9.6% to 14.2%)	4.3% (2.2% to 6.7%)
Chronic kidney disease due to glomerulonephritis	19,041.0 (16,476.9 to 21,825.7)	1,106.0 (811.9 to 1,459.4)	23.5% (20.1% to 27.0%)	1.9% (-1.1% to 4.8%)
Malaria	26,691.4 (23,190.2 to 30,341.1)	1,057.1 (675.8 to 1,573.8)	-23.0% (-28.8% to -17.4%)	-28.2% (-33.8% to -23.0%)
Uterine fibroids	26,516.0 (24,820.5 to 28,336.6)	980.7 (627.4 to 1,484.5)	16.9% (15.5% to 18.3%)	-0.0% (-1.2% to 1.2%)
Hookworm disease	27,370.5 (25,583.5 to 29,444.7)	578.1 (374.4 to 847.6)	1.5% (-6.9% to 11.2%)	-7.3% (-15.0% to 1.4%)
Other gynecological diseases	19,284.4 (18,624.1 to 20,000.7)	533.6 (359.7 to 769.5)	11.8% (9.7% to 14.1%)	2.0% (0.3% to 4.0%)
Drug-susceptible HIV/AIDS - Tuberculosis	1,064.5 (752.5 to 1,449.9)	409.8 (246.5 to 625.1)	-20.9% (-24.7% to -17.4%)	-30.0% (-33.4% to -26.9%)
Schistosomiasis	13,367.3 (12,502.4 to 14,318.9)	283.3 (189.8 to 408.1)	-24.4% (-27.0% to -22.2%)	-33.2% (-35.4% to -31.1%)
Sickle cell disorders	3,671.7 (3,373.2 to 4,220.8)	246.6 (168.4 to 357.2)	18.2% (13.5% to 22.9%)	12.1% (7.7% to 16.6%)
G6PD deficiency	685.8 (645.0 to 730.7)	24.9 (16.7 to 35.2)	13.8% (10.7% to 17.0%)	3.1% (0.3% to 6.0%)
Maternal haemorrhage	1,189.7 (962.7 to 1,469.9)	24.7 (15.2 to 37.4)	4.8% (-1.5% to 12.5%)	-4.1% (-9.8% to 2.7%)
Thalassemias	318.2 (272.9 to 355.5)	21.8 (14.0 to 32.2)	-4.5% (-8.9% to 0.0%)	-8.7% (-12.9% to -4.4%)
Multidrug-resistant HIV/AIDS - Tuberculosis without extensive drug resistance	27.5 (18.2 to 40.7)	11.2 (6.5 to 18.5)	-27.9% (-39.8% to -15.2%)	-36.3% (-46.8% to -25.2%)
G6PD trait	8.0 (7.6 to 8.6)	0.6 (0.4 to 0.8)	17.6% (14.9% to 20.7%)	4.9% (2.6% to 7.5%)
Extensively drug-resistant HIV/AIDS - Tuberculosis	0.3 (0.2 to 0.5)	0.1 (0.1 to 0.2)	244.6% (187.5% to 310.0%)	205.0% (155.4% to 263.0%)
**All causes**	**2,042,727.5 (1,979,002.8 to 2,110,036.9)**	**61,648.6 (41,483.4 to 86,424.1)**	**9.2% (6.4% to 11.2%)**	**-2.2% (-4.8% to -0.3%)**

SR Table 4: Global prevalence and years lived with disability (YLDs) for 2016, percentage change of YLD counts and percentage change of age-standardised YLD rates between 2006 and 2016 of developmental intellectual disability by cause, all ages and both sexes combined				
**Cause**	**Prevalence in thousands (95% uncertainty interval)**	**YLDs in thousands (95% uncertainty interval)**		
	**2016**	**2016**	**Percentage change in counts between 2006 and 2016**	**Percentage change in age-standardised rates between 2006 and 2016**
Idiopathic developmental intellectual disability	114,797.1 (69,505.6 to 161,352.7)	4,611.0 (2,201.5 to 7,932.1)	2.5% (-0.9% to 4.6%)	-6.9% (-10.0% to -4.9%)
Autism	15,217.5 (12,821.1 to 18,264.6)	4,032.4 (2,587.1 to 5,998.1)	11.3% (10.2% to 12.4%)	-0.1% (-1.0% to 0.9%)
Neonatal preterm birth complications	17,607.7 (12,984.2 to 22,527.1)	3,217.2 (2,051.1 to 4,616.1)	13.0% (-2.1% to 29.6%)	3.0% (-10.7% to 18.2%)
Neonatal sepsis and other neonatal infections	6,454.3 (2,523.0 to 14,864.5)	2,640.6 (881.0 to 6,761.8)	30.0% (22.9% to 40.0%)	19.7% (13.3% to 28.6%)
Iodine deficiency	2,513.9 (1,764.9 to 3,103.2)	808.0 (483.8 to 1,156.5)	-1.7% (-6.0% to 2.2%)	-9.5% (-13.6% to -5.8%)
Congenital heart anomalies	13,088.9 (11,645.3 to 14,563.9)	746.0 (322.1 to 1,278.0)	8.6% (6.4% to 10.5%)	-0.9% (-2.9% to 0.8%)
Encephalitis	6,440.5 (3,497.3 to 10,677.3)	652.9 (414.6 to 906.0)	1.5% (-5.8% to 7.4%)	-11.6% (-17.9% to -6.6%)
Other chromosomal abnormalities	4,417.8 (3,852.5 to 5,098.8)	649.9 (454.9 to 872.5)	2.4% (0.1% to 4.6%)	-1.0% (-3.4% to 1.2%)
Neonatal encephalopathy due to birth asphyxia and trauma	4,355.4 (2,627.9 to 6,786.6)	628.1 (235.4 to 1,430.5)	28.1% (15.3% to 43.9%)	14.9% (3.8% to 27.7%)
Neural tube defects	1,589.0 (1,193.3 to 2,098.1)	554.1 (349.4 to 823.8)	14.1% (11.6% to 16.7%)	2.8% (0.6% to 5.2%)
Malaria	700.7 (554.5 to 841.1)	320.1 (212.1 to 428.4)	17.6% (9.4% to 25.0%)	9.0% (1.3% to 15.8%)
Hemolytic disease and other neonatal jaundice	601.7 (432.7 to 740.5)	269.1 (164.8 to 378.1)	14.8% (7.4% to 20.3%)	3.8% (-3.1% to 8.9%)
Pneumococcal meningitis	2,269.1 (1,785.4 to 2,847.5)	246.1 (156.8 to 352.6)	6.9% (0.0% to 13.1%)	-3.8% (-10.1% to 1.9%)
Down's syndrome	1,928.3 (1,687.3 to 2,198.1)	181.5 (121.7 to 254.6)	8.3% (5.9% to 10.7%)	-1.1% (-3.4% to 1.1%)
Other meningitis	1,607.7 (984.2 to 2,263.0)	152.5 (92.6 to 222.0)	25.5% (16.9% to 33.5%)	12.3% (4.6% to 19.4%)
H influenzae type B meningitis	811.2 (577.3 to 1,088.4)	100.4 (62.0 to 144.4)	-14.2% (-20.8% to -8.0%)	-22.0% (-28.3% to -16.5%)
Meningococcal infection	573.9 (344.5 to 854.1)	62.1 (38.1 to 93.5)	14.3% (7.2% to 27.4%)	3.1% (-3.6% to 17.3%)
Alcohol use disorders	352.4 (322.5 to 377.5)	20.8 (13.5 to 30.1)	9.8% (6.1% to 12.3%)	-3.5% (-6.8% to -1.2%)
Digestive congenital anomalies	203.8 (160.1 to 254.2)	10.4 (5.9 to 16.6)	10.8% (8.3% to 13.2%)	-4.5% (-6.7% to -2.3%)
Klinefelter syndrome	619.8 (451.1 to 793.9)	10.0 (5.0 to 18.0)	7.7% (4.0% to 11.5%)	0.4% (-3.0% to 3.9%)
Zika virus disease	16.6 (11.1 to 33.5)	9.8 (5.8 to 17.7)	--	--
Tetanus	75.5 (47.8 to 102.7)	2.3 (1.2 to 4.0)	15.7% (3.7% to 30.1%)	4.7% (-5.8% to 17.4%)
**All causes**	**196,242.7 (150,264.0 to 242,144.5)**	**19,925.3 (14,584.2 to 26,433.7)**	**10.7% (8.3% to 14.2%)**	**0.6% (-1.6% to 3.9%)**

SR Table 5: Global prevalence and years lived with disability (YLDs) for 2016, percentage change of YLD counts and percentage change of age-standardised YLD rates between 2006 and 2016 of epilepsy by cause, all ages and both sexes combined				
**Cause**	**Prevalence in thousands (95% uncertainty interval)**	**YLDs in thousands (95% uncertainty interval)**		
	**2016**	**2016**	**Percentage change in counts between 2006 and 2016**	**Percentage change in age-standardised rates between 2006 and 2016**
Epilepsy	23,962.4 (20,401.8 to 27,737.0)	7,546.8 (5,111.2 to 10,458.3)	8.8% (-2.1% to 22.0%)	-2.6% (-12.3% to 9.2%)
Neonatal preterm birth complications	8,646.3 (6,173.2 to 11,386.0)	3,297.6 (1,989.0 to 4,850.0)	13.2% (-4.3% to 32.1%)	3.8% (-12.2% to 21.1%)
Neonatal sepsis and other neonatal infections	5,582.1 (1,957.3 to 13,536.3)	2,522.1 (814.8 to 6,470.3)	29.9% (22.8% to 40.2%)	19.8% (13.3% to 29.0%)
Neonatal encephalopathy due to birth asphyxia and trauma	1,705.0 (562.4 to 3,937.0)	646.5 (199.3 to 1,600.2)	31.6% (18.5% to 54.4%)	18.5% (7.4% to 37.2%)
Cysticercosis	2,675.5 (2,231.9 to 3,148.7)	420.9 (274.1 to 580.8)	-4.6% (-11.0% to 1.4%)	-19.9% (-25.3% to -15.1%)
Malaria	868.0 (726.3 to 1,015.3)	361.6 (239.3 to 485.0)	17.1% (10.1% to 23.4%)	9.6% (3.0% to 15.5%)
Hemolytic disease and other neonatal jaundice	792.1 (635.6 to 936.9)	314.3 (203.8 to 437.3)	14.7% (8.1% to 20.0%)	4.2% (-1.8% to 9.0%)
Encephalitis	871.7 (775.2 to 981.5)	140.0 (95.2 to 187.3)	1.9% (-4.2% to 7.6%)	-9.9% (-14.7% to -5.2%)
Pneumococcal meningitis	262.4 (224.4 to 305.8)	46.7 (31.5 to 65.4)	3.4% (-3.3% to 10.1%)	-4.9% (-10.9% to 1.4%)
Food-borne trematodiases	249.1 (75.4 to 504.4)	37.3 (10.5 to 81.7)	-13.8% (-25.7% to -1.7%)	-23.3% (-33.9% to -12.6%)
Other meningitis	131.3 (111.5 to 152.5)	23.5 (15.6 to 33.1)	17.1% (10.3% to 23.6%)	6.9% (0.6% to 12.7%)
H influenzae type B meningitis	122.8 (102.3 to 148.5)	22.6 (14.9 to 32.4)	-0.7% (-9.8% to 8.5%)	-6.7% (-15.2% to 1.7%)
Meningococcal infection	46.6 (38.3 to 57.3)	8.6 (5.5 to 12.3)	14.1% (8.8% to 18.6%)	5.7% (0.8% to 10.0%)
Zika virus disease	2.4 (1.6 to 4.5)	1.6 (1.0 to 2.8)	--	--
Cystic echinococcosis	8.1 (2.0 to 18.3)	1.6 (0.4 to 3.7)	-41.3% (-62.4% to -3.1%)	-48.3% (-66.9% to -16.1%)
Tetanus	<0.05 (<0.05 to <0.05)	<0.05 (<0.05 to <0.05)	-21.1% (-30.6% to -11.5%)	-21.7% (-31.2% to -12.3%)
**All causes**	**45,925.8 (39,941.5 to 54,564.0)**	**15,391.7 (11,593.7 to 20,379.3)**	**13.3% (5.9% to 20.9%)**	**2.3% (-4.7% to 9.2%)**

SR Table 6: Global prevalence and years lived with disability (YLDs) for 2016, percentage change of YLD counts and percentage change of age-standardised YLD rates between 2006 and 2016 of Guillain-Barré syndrome by cause, all ages and both sexes combined				
Cause	Prevalence in thousands (95% uncertainty interval)	YLDs in thousands (95% uncertainty interval)		
	2016	2016	Percentage change in counts between 2006 and 2016	Percentage change in age-standardised rates between 2006 and 2016
Other neurological disorders	24.0 (15.6 to 33.2)	7.1 (3.8 to 11.5)	16.8% (14.1% to 19.6%)	0.5% (-0.4% to 1.2%)
Upper respiratory infections	20.6 (14.9 to 27.7)	6.1 (3.6 to 9.5)	16.6% (14.1% to 19.4%)	0.5% (-0.4% to 1.2%)
Lower respiratory infections	7.6 (4.1 to 11.7)	2.3 (1.1 to 3.9)	16.6% (14.1% to 19.4%)	0.5% (-0.4% to 1.2%)
Diarrhoeal diseases	7.0 (4.7 to 9.7)	2.1 (1.2 to 3.2)	16.6% (14.0% to 19.4%)	0.5% (-0.4% to 1.2%)
Other infectious diseases	4.5 (3.0 to 6.6)	1.3 (0.8 to 2.2)	16.6% (14.0% to 19.4%)	0.5% (-0.4% to 1.2%)
Zika virus disease	0.9 (0.7 to 1.3)	0.3 (0.2 to 0.5)	--	--
**All causes**	**64.7 (51.6 to 81.6)**	**19.2 (11.8 to 28.4)**	**18.4% (15.6% to 21.4%)**	**1.9% (1.2% to 2.7%)**

SR Table 7: Global prevalence and years lived with disability (YLDs) for 2016, percentage change of YLD counts and percentage change of age-standardised YLD rates between 2006 and 2016 of hearing loss by cause, all ages and both sexes combined				
**Cause**	**Prevalence in thousands (95% uncertainty interval)**	**YLDs in thousands (95% uncertainty interval)**		
	**2016**	**2016**	**Percentage change in counts between 2006 and 2016**	**Percentage change in age-standardised rates between 2006 and 2016**
Age-related and other hearing loss	1,271,674.9 (1,210,237.4 to 1,337,731.0)	36,287.5 (25,341.8 to 50,893.6)	22.3% (20.4% to 24.0%)	-1.7% (-2.9% to -0.6%)
Other congenital anomalies	34,983.5 (24,583.5 to 49,401.8)	3,413.0 (2,101.2 to 5,262.8)	3.0% (0.5% to 5.9%)	-7.3% (-9.6% to -4.7%)
Otitis media	81,517.9 (72,281.5 to 91,965.5)	2,815.5 (1,774.2 to 4,133.9)	1.3% (-1.3% to 4.1%)	-6.6% (-9.0% to -4.2%)
Pneumococcal meningitis	983.3 (504.4 to 1,332.8)	47.5 (8.2 to 76.7)	-17.0% (-22.0% to -10.6%)	-25.7% (-30.1% to -20.6%)
Other meningitis	760.6 (392.6 to 1,004.4)	37.3 (6.4 to 59.3)	80.7% (69.1% to 93.3%)	60.3% (49.7% to 71.2%)
Meningococcal infection	289.4 (140.7 to 403.4)	14.7 (2.5 to 24.1)	9.3% (2.5% to 17.3%)	-1.6% (-7.7% to 5.5%)
H influenzae type B meningitis	272.4 (134.5 to 400.0)	14.3 (2.3 to 24.9)	-46.1% (-51.9% to -39.7%)	-51.1% (-56.7% to -45.3%)
**All causes**	**1,390,482.0 (1,329,902.3 to 1,455,762.3)**	**42,629.8 (30,019.2 to 59,264.5)**	**18.8% (17.0% to 20.4%)**	**-2.5% (-3.6% to -1.5%)**

SR Table 8: Global prevalence and years lived with disability (YLDs) for 2016, percentage change of YLD counts and percentage change of age-standardised YLD rates between 2006 and 2016 of heart failure by cause, all ages and both sexes combined				
**Cause**	**Prevalence in thousands (95% uncertainty interval)**	**YLDs in thousands (95% uncertainty interval)**		
	**2016**	**2016**	**Percentage change in counts between 2006 and 2016**	**Percentage change in age-standardised rates between 2006 and 2016**
Chronic obstructive pulmonary disease	8,583.2 (7,135.0 to 10,259.9)	3,275.5 (2,572.0 to 3,907.8)	31.7% (29.3% to 33.9%)	0.2% (-1.6% to 2.0%)
Ischaemic heart disease	26,192.3 (23,036.1 to 29,655.3)	2,086.1 (1,446.1 to 2,826.8)	30.1% (28.1% to 32.2%)	-1.6% (-3.0% to -0.1%)
Hypertensive heart disease	17,190.0 (14,948.1 to 19,774.1)	1,380.1 (958.6 to 1,894.5)	31.6% (29.7% to 33.5%)	0.4% (-0.8% to 1.6%)
Other cardiomyopathy	4,005.3 (3,533.6 to 4,524.6)	329.1 (226.3 to 462.2)	20.6% (18.3% to 22.9%)	-4.5% (-6.1% to -2.9%)
Other cardiovascular and circulatory diseases	1,992.0 (1,706.5 to 2,341.3)	159.2 (106.4 to 225.9)	25.6% (22.5% to 28.8%)	-5.9% (-7.9% to -4.0%)
Rheumatic heart disease	1,669.0 (1,475.3 to 1,881.0)	137.1 (93.1 to 188.3)	31.8% (28.5% to 35.1%)	3.2% (0.6% to 5.6%)
Myocarditis	1,189.5 (1,033.8 to 1,364.8)	104.3 (69.1 to 148.5)	11.6% (9.2% to 13.8%)	-1.8% (-3.6% to -0.0%)
Alcoholic cardiomyopathy	1,165.3 (994.2 to 1,358.9)	96.9 (65.5 to 138.0)	20.6% (15.6% to 25.7%)	-5.4% (-9.0% to -1.7%)
Interstitial lung disease and pulmonary sarcoidosis	219.7 (165.8 to 271.0)	91.6 (61.2 to 129.3)	30.4% (27.7% to 33.2%)	-0.8% (-2.8% to 1.3%)
Endocarditis	415.3 (361.6 to 479.7)	34.8 (23.6 to 49.1)	22.8% (20.1% to 25.8%)	-4.6% (-6.7% to -2.5%)
Endocrine, metabolic, blood, and immune disorders	282.2 (244.8 to 327.6)	23.2 (15.6 to 32.8)	23.8% (20.3% to 27.6%)	-6.6% (-9.2% to -4.0%)
Congenital heart anomalies	153.1 (131.3 to 176.3)	21.5 (13.8 to 30.0)	2.7% (1.4% to 4.1%)	-3.8% (-5.0% to -2.6%)
Chagas disease	328.5 (214.7 to 474.0)	16.2 (8.8 to 26.8)	12.8% (8.8% to 16.7%)	-10.1% (-13.3% to -7.2%)
Other hemoglobinopathies and hemolytic anaemias	179.3 (153.9 to 209.9)	15.2 (10.2 to 21.8)	27.7% (24.0% to 31.3%)	-3.2% (-5.9% to -0.6%)
Silicosis	8.3 (7.1 to 9.5)	3.7 (2.6 to 5.1)	19.9% (15.9% to 24.1%)	-8.2% (-11.3% to -5.1%)
Coal workers pneumoconiosis	4.1 (3.6 to 4.8)	1.8 (1.3 to 2.5)	22.8% (17.5% to 28.2%)	-5.5% (-9.7% to -1.2%)
Asbestosis	3.8 (3.4 to 4.3)	1.7 (1.2 to 2.3)	24.1% (21.9% to 26.2%)	-7.2% (-8.8% to -5.6%)
Zika virus disease	2.8 (1.9 to 5.4)	1.7 (1.0 to 2.8)	--	--
Other pneumoconiosis	2.6 (2.3 to 3.1)	1.2 (0.8 to 1.7)	29.1% (26.7% to 31.3%)	-1.9% (-3.3% to -0.4%)
G6PD deficiency	12.4 (10.2 to 14.8)	1.1 (0.7 to 1.6)	34.3% (31.9% to 36.6%)	3.4% (1.6% to 5.2%)
Iron-deficiency anaemia	5.7 (4.9 to 6.7)	0.5 (0.3 to 0.7)	32.9% (28.7% to 37.4%)	3.3% (-0.1% to 6.9%)
Thalassemias	4.2 (3.3 to 5.2)	0.4 (0.2 to 0.6)	-2.5% (-5.4% to 0.2%)	-4.6% (-7.1% to -2.3%)
Iodine deficiency	0.2 (0.1 to 0.2)	0.1 (0.1 to 0.1)	35.4% (31.6% to 38.8%)	7.3% (4.2% to 10.0%)
**All causes**	**63,608.8 (56,943.7 to 71,388.3)**	**7,783.1 (5,857.3 to 9,788.5)**	**30.0% (28.6% to 31.3%)**	**-0.7% (-1.6% to 0.2%)**

SR Table 9: Global prevalence and years lived with disability (YLDs) for 2016, percentage change of YLD counts and percentage change of age-standardised YLD rates between 2006 and 2016 of infertility by cause, all ages and both sexes combined				
**Cause**	**Prevalence in thousands (95% uncertainty interval)**	**YLDs in thousands (95% uncertainty interval)**		
	**2016**	**2016**	**Percentage change in counts between 2006 and 2016**	**Percentage change in age-standardised rates between 2006 and 2016**
Other sexually transmitted diseases	33,717.4 (29,034.7 to 37,480.0)	184.8 (73.8 to 372.6)	11.1% (8.5% to 13.6%)	-0.5% (-2.6% to 1.6%)
Female infertility	31,476.8 (19,471.6 to 49,303.7)	179.8 (66.2 to 399.0)	38.5% (29.4% to 49.5%)	25.3% (17.7% to 34.8%)
Male infertility	28,044.3 (22,702.7 to 35,076.5)	165.0 (66.1 to 327.1)	19.3% (15.7% to 22.6%)	7.9% (4.9% to 10.6%)
Chlamydial infection	12,871.5 (11,105.2 to 14,383.6)	71.6 (29.0 to 142.5)	16.2% (13.3% to 18.8%)	4.4% (1.9% to 6.7%)
Turner syndrome	1,476.1 (1,286.3 to 1,680.4)	30.5 (15.6 to 49.2)	7.7% (3.5% to 12.1%)	-0.2% (-4.1% to 3.8%)
Gonococcal infection	4,722.3 (4,127.3 to 5,306.4)	26.1 (10.5 to 52.1)	15.2% (11.9% to 18.0%)	3.2% (0.3% to 5.8%)
Polycystic ovarian syndrome	1,996.4 (1,368.6 to 2,801.9)	19.4 (8.1 to 39.5)	14.6% (10.8% to 18.4%)	4.1% (0.6% to 7.6%)
Endometriosis	162.4 (120.6 to 218.9)	16.7 (10.1 to 26.1)	5.9% (2.0% to 10.0%)	-3.6% (-7.3% to 0.2%)
Klinefelter syndrome	1,215.2 (1,055.8 to 1,400.7)	12.3 (5.7 to 23.5)	10.8% (7.7% to 14.0%)	-0.7% (-3.5% to 2.2%)
Urogenital congenital anomalies	712.6 (474.6 to 968.2)	9.1 (3.6 to 19.3)	6.5% (4.9% to 8.0%)	-2.2% (-3.6% to -0.9%)
Maternal sepsis and other pregnancy related infections	812.2 (717.8 to 910.7)	4.1 (1.6 to 8.8)	14.1% (10.4% to 17.8%)	-0.8% (-3.9% to 2.4%)
**All causes**	**117,207.3 (94,951.8 to 145,047.5)**	**719.3 (309.5 to 1,399.9)**	**19.3% (16.0% to 22.3%)**	**7.6% (4.9% to 10.0%)**

**SR Table 10: Global prevalence and years lived with disability (YLDs) for 2016, percentage change of YLD counts and percentage change of age-standardised YLD rates between 2006 and 2016 of pelvic inflammatory disease by cause, all ages and both sexes combined**				
**Cause**	**Prevalence in thousands (95% uncertainty interval)**	**YLDs in thousands (95% uncertainty interval)**		
	**2016**	**2016**	**Percentage change in counts between 2006 and 2016**	**Percentage change in age-standardised rates between 2006 and 2016**
Other sexually transmitted diseases	2,871.1 (2,463.0 to 3,323.8)	372.8 (254.0 to 517.4)	7.0% (4.3% to 9.8%)	-6.5% (-8.7% to -4.3%)
Chlamydial infection	918.2 (787.6 to 1,071.9)	120.9 (81.2 to 171.1)	9.8% (6.7% to 13.1%)	-3.2% (-6.0% to -0.2%)
Gonococcal infection	310.1 (259.0 to 370.2)	41.4 (27.2 to 58.6)	9.0% (4.9% to 13.1%)	-3.1% (-6.7% to 0.4%)
**All causes**	**4,099.4 (3,532.9 to 4,740.1)**	**535.1 (360.2 to 742.6)**	**7.8% (5.5% to 10.1%)**	**-5.5% (-7.5% to -3.6%)**

SR Table 11: Global prevalence and years lived with disability (YLDs) for 2016, percentage change of YLD counts and percentage change of age-standardised YLD rates between 2006 and 2016 of vision loss by cause, all ages and both sexes combined				
**Cause**	**Prevalence in thousands (95% uncertainty interval)**	**YLDs in thousands (95% uncertainty interval)**		
	**2016**	**2016**	**Percentage change in counts between 2006 and 2016**	**Percentage change in age-standardised rates between 2006 and 2016**
Refraction and accommodation disorders	854,298.7 (822,109.1 to 885,544.8)	14,972.5 (9,340.6 to 23,361.5)	14.9% (13.7% to 15.9%)	-4.9% (-5.8% to -4.3%)
Near vision impairment	672,177.0 (648,254.1 to 697,017.0)	6,788.1 (3,196.5 to 12,474.2)	18.7% (18.0% to 19.4%)	-3.3% (-3.8% to -2.8%)
Cataract	83,628.5 (73,800.3 to 93,904.7)	5,789.0 (4,134.6 to 7,915.3)	31.0% (29.4% to 32.6%)	0.0% (-1.2% to 1.1%)
Other vision loss	29,249.1 (25,873.3 to 32,927.4)	2,241.2 (1,578.3 to 3,016.4)	26.1% (23.9% to 28.1%)	0.7% (-0.8% to 2.0%)
Neonatal sepsis and other neonatal infections	4,000.8 (1,411.3 to 10,076.4)	2,015.3 (670.9 to 5,234.2)	30.3% (22.7% to 40.5%)	20.4% (13.7% to 29.8%)
Neonatal preterm birth complications	10,182.7 (8,150.1 to 12,370.1)	1,620.0 (1,167.7 to 2,175.2)	17.2% (7.9% to 27.2%)	5.7% (-2.8% to 14.8%)
Glaucoma	4,761.2 (3,973.2 to 5,573.1)	461.1 (311.4 to 642.1)	35.7% (33.7% to 38.0%)	2.1% (0.7% to 3.6%)
Macular degeneration	6,146.9 (5,110.6 to 7,401.3)	408.6 (277.9 to 555.6)	37.5% (34.7% to 40.4%)	2.7% (0.7% to 4.7%)
Vitamin A deficiency	5,252.8 (4,013.2 to 6,842.2)	252.4 (158.8 to 388.3)	12.0% (8.7% to 15.5%)	2.6% (-0.3% to 5.6%)
Trachoma	3,337.6 (2,439.3 to 4,491.7)	245.2 (162.4 to 353.6)	-0.5% (-6.5% to 5.2%)	-23.8% (-28.5% to -19.3%)
Diabetes mellitus	2,730.4 (2,305.9 to 3,251.4)	202.0 (136.4 to 278.8)	37.8% (34.8% to 41.1%)	7.6% (5.5% to 10.0%)
Neonatal encephalopathy due to birth asphyxia and trauma	378.6 (127.1 to 864.6)	168.5 (52.4 to 393.7)	33.9% (20.2% to 56.8%)	20.0% (8.9% to 38.3%)
Encephalitis	2,636.0 (1,310.4 to 4,672.3)	91.1 (55.3 to 147.1)	7.1% (3.5% to 11.4%)	-10.0% (-12.8% to -6.9%)
Malaria	186.9 (151.8 to 223.1)	90.1 (64.1 to 116.5)	17.5% (10.4% to 23.1%)	9.8% (3.1% to 15.0%)
Hemolytic disease and other neonatal jaundice	187.0 (148.1 to 226.9)	84.1 (58.6 to 109.7)	17.7% (9.7% to 24.8%)	6.6% (-0.6% to 12.9%)
Onchocerciasis	781.6 (580.8 to 1,188.1)	55.2 (36.9 to 77.1)	-6.9% (-15.0% to 6.4%)	-25.5% (-32.1% to -15.0%)
Other meningitis	644.6 (354.9 to 989.1)	20.1 (11.7 to 33.0)	87.9% (75.7% to 101.6%)	61.6% (51.6% to 73.0%)
Pneumococcal meningitis	576.8 (365.3 to 870.0)	14.4 (8.1 to 23.4)	-12.3% (-18.0% to -6.5%)	-23.4% (-28.3% to -18.4%)
Meningococcal infection	306.8 (185.6 to 461.0)	13.7 (8.3 to 20.8)	16.2% (8.5% to 25.0%)	1.6% (-5.2% to 9.3%)
H influenzae type B meningitis	184.8 (80.8 to 327.0)	3.2 (1.3 to 6.5)	-44.8% (-51.1% to -38.1%)	-50.6% (-56.3% to -44.6%)
Tetanus	<0.05 (<0.05 to <0.05)	<0.05 (<0.05 to <0.05)	-25.2% (-34.6% to -15.9%)	-25.8% (-35.1% to -16.6%)
**All causes**	**1,009,471.7 (969,235.1 to 1,047,829.0)**	**28,747.6 (20,341.5 to 40,240.5)**	**20.4% (19.1% to 22.0%)**	**-1.3% (-2.4% to 0.8%)**

SR Table 12: Global prevalence, incidence, and YLDs for 2016, percentage change of YLD counts and percentage change of YLD rates between 2006 and 2016 by cause, age 0-6 days.					
Cause	Prevalence in thousands (95% UI)	Incidence in thousands (95% UI)	YLDs in thousands (95% UI)		
	2016 counts	2016 counts	2016 counts	Percentage change in counts between 2006 and 2016	Percentage change in rates between 2006 and 2016
**All causes**	**1,486.7 (1,471.7 to 1,500.1)**	**18,463.5† (16,226.6 to 20,838.1)**	**77.8 (55.1 to 104.0)**	**-7.4 (-10.5 to -4.0)**	**-6.9 (-10.1 to -3.6)**
**Communicable, maternal, neonatal, and nutritional disorders**	**1,063.8 (1,046.4 to 1,080.0)**	**16,941.6† (14,720.1 to 19,294.0)**	**63.1 (44.1 to 85.0)**	**-8.5 (-12.2 to -4.4)**	**-8.0 (-11.7 to -3.9)**
**HIV/AIDS and tuberculosis**	**280.0 (254.5 to 311.8)**	**1.2† (0.8 to 1.7)**	**< 0.05 (< 0.05 to < 0.05)**	**-14.8 (-19.3 to -9.1)**	**-14.4 (-18.9 to -8.7)**
Tuberculosis	267.4 (230.9 to 307.7)	1.2† (0.8 to 1.7)	< 0.05 (< 0.05 to < 0.05)	-14.8 (-19.3 to -9.1)	-14.4 (-18.9 to -8.7)
Drug-susceptible tuberculosis	< 0.05 (< 0.05 to < 0.05)	1.2 (0.8 to 1.7)	< 0.05 (< 0.05 to < 0.05)	-14.9 (-19.4 to -9.2)	-14.5 (-19.0 to -8.7)
Multidrug-resistant tuberculosis without extensive drug resistance	< 0.05 (< 0.05 to < 0.05)	< 0.05 (< 0.05 to < 0.05)	< 0.05 (< 0.05 to < 0.05)	-15.1 (-32.4 to 7.7)	-14.6 (-32.0 to 8.3)
Extensively drug-resistant tuberculosis	< 0.05 (< 0.05 to < 0.05)	< 0.05 (< 0.05 to < 0.05)	< 0.05 (< 0.05 to < 0.05)	206.2 (119.8 to 356.4)	207.8 (120.9 to 358.8)
Latent tuberculosis infection	267.4 (230.9 to 307.7)	--	--	--	--
HIV/AIDS	--	--	--	--	--
Drug-susceptible HIV/AIDS - Tuberculosis	--	--	--	--	--
Multidrug-resistant HIV/AIDS - Tuberculosis without extensive drug resistance	--	--	--	--	--
Extensively drug-resistant HIV/AIDS - Tuberculosis	--	--	--	--	--
HIV/AIDS resulting in other diseases	--	--	--	--	--
**Diarrhoea, lower respiratory infections, and other common infectious diseases**	**146.1 (140.7 to 151.3)**	**16,542.7† (14,316.2 to 18,893.1)**	**9.1 (6.1 to 12.8)**	**-10.9 (-13.8 to -8.0)**	**-10.5 (-13.4 to -7.5)**
Diarrhoeal diseases	38.8 (33.8 to 44.3)	6,773.5 (5,903.9 to 7,739.6)	4.5 (3.0 to 6.4)	-13.1 (-16.9 to -9.8)	-12.7 (-16.5 to -9.3)
Intestinal infectious diseases	--	--	--	--	--
Typhoid fever	--	--	--	--	--
Paratyphoid fever	--	--	--	--	--
Other intestinal infectious diseases	--	--	--	--	--
Lower respiratory infections	49.8 (40.3 to 61.6)	254.6 (196.3 to 323.7)	3.1 (1.9 to 4.7)	-5.0 (-10.8 to 0.4)	-4.5 (-10.4 to 0.9)
Upper respiratory infections	46.6 (36.5 to 58.5)	8,536.9 (6,638.6 to 10,817.2)	1.2 (0.7 to 1.9)	1.4 (-0.8 to 3.4)	1.9 (-0.2 to 4.0)
Otitis media	3.6 (2.8 to 4.7)	375.1 (270.7 to 512.3)	0.1 (< 0.05 to 0.1)	-2.9 (-6.4 to 1.0)	-2.4 (-5.9 to 1.5)
Meningitis	0.4 (0.3 to 0.5)	25.7 (20.6 to 31.6)	0.1 (< 0.05 to 0.1)	-2.5 (-12.4 to 7.4)	-1.9 (-12.0 to 7.9)
Pneumococcal meningitis	< 0.05 (< 0.05 to 0.1)	4.1 (2.6 to 5.8)	< 0.05 (< 0.05 to < 0.05)	-27.4 (-33.3 to -21.0)	-27.0 (-32.9 to -20.6)
H influenzae type B meningitis	< 0.05 (< 0.05 to 0.1)	4.8 (2.9 to 7.1)	< 0.05 (< 0.05 to < 0.05)	-44.0 (-52.4 to -34.3)	-43.7 (-52.2 to -33.9)
Meningococcal infection	< 0.05 (< 0.05 to 0.1)	5.7 (3.7 to 8.2)	< 0.05 (< 0.05 to < 0.05)	4.5 (-8.1 to 18.4)	5.1 (-7.6 to 19.1)
Other meningitis	0.3 (0.2 to 0.4)	11.1 (8.1 to 14.4)	< 0.05 (< 0.05 to 0.1)	14.0 (-1.9 to 30.9)	14.6 (-1.4 to 31.6)
Encephalitis	0.1 (< 0.05 to 0.1)	5.8 (4.7 to 6.9)	< 0.05 (< 0.05 to < 0.05)	-9.8 (-14.3 to -6.2)	-9.4 (-13.8 to -5.7)
Diphtheria	--	--	--	--	--
Whooping cough	--	--	--	--	--
Tetanus	0.9 (0.5 to 1.3)	15.8 (7.9 to 23.0)	0.1 (0.1 to 0.2)	-70.7 (-79.9 to -58.7)	-70.5 (-79.8 to -58.5)
Measles	--	--	--	--	--
Varicella and herpes zoster	10.7 (7.3 to 15.0)	555.2 (382.4 to 778.9)	0.1 (< 0.05 to 0.1)	-3.9 (-6.4 to -1.4)	-3.4 (-5.9 to -0.9)
**Neglected tropical diseases and malaria**	**4.7 (4.2 to 5.1)**	**4.6† (3.4 to 7.0)**	**< 0.05 (< 0.05 to < 0.05)**	**--**	**--**
Malaria	--	--	--	--	--
Chagas disease	--	--	--	--	--
Leishmaniasis	--	--	--	--	--
Visceral leishmaniasis	--	--	--	--	--
Cutaneous and mucocutaneous leishmaniasis	--	--	--	--	--
African trypanosomiasis	--	--	--	--	--
Schistosomiasis	--	--	--	--	--
Cysticercosis	--	--	--	--	--
Cystic echinococcosis	--	--	--	--	--
Lymphatic filariasis	4.3 (3.7 to 4.9)	0.7 (0.5 to 1.2)	--	--	--
Onchocerciasis	--	--	--	--	--
Trachoma	--	--	--	--	--
Dengue	--	--	--	--	--
Yellow fever	--	--	--	--	--
Rabies	--	--	--	--	--
Intestinal nematode infections	--	--	--	--	--
Ascariasis	--	--	--	--	--

Cause	Prevalence in thousands (95% UI)	Incidence in thousands (95% UI)	YLDs in thousands (95% UI)		
	2016 counts	2016 counts	2016 counts	Percentage change in counts between 2006 and 2016	Percentage change in rates between 2006 and 2016
Trichuriasis	--	--	--	--	--
Hookworm disease	--	--	--	--	--
Food-borne trematodiases	--	--	--	--	--
Leprosy	--	--	--	--	--
Ebola virus disease	--	--	--	--	--
Zika virus disease	0.1 (< 0.05 to 0.1)	3.9 (2.7 to 6.3)	< 0.05 (< 0.05 to < 0.05)	--	--
Guinea worm disease	--	--	--	--	--
Other neglected tropical diseases	--	--	--	--	--
**Maternal disorders**	**--**	**--**	**--**	**--**	**--**
Maternal haemorrhage	--	--	--	--	--
Maternal sepsis and other pregnancy related infections	--	--	--	--	--
Maternal hypertensive disorders	--	--	--	--	--
Maternal obstructed labour and uterine rupture	--	--	--	--	--
Maternal abortion, miscarriage, and ectopic pregnancy	--	--	--	--	--
Other maternal disorders	--	--	--	--	--
**Neonatal disorders**	**251.3 (236.5 to 263.8)**	**--**	**25.0 (17.0 to 35.2)**	**-6.5 (-13.6 to 2.7)**	**-6.0 (-13.2 to 3.3)**
Neonatal preterm birth complications	186.2 (158.9 to 214.9)	--	8.5 (5.1 to 13.1)	-6.7 (-24.9 to 16.3)	-6.2 (-24.5 to 16.9)
Neonatal encephalopathy due to birth asphyxia and trauma	27.5 (20.6 to 36.0)	--	3.3 (1.9 to 5.4)	-4.1 (-7.1 to -1.0)	-3.6 (-6.6 to -0.5)
Neonatal sepsis and other neonatal infections	43.0 (25.6 to 68.7)	--	8.6 (4.8 to 15.2)	-4.5 (-6.6 to -2.4)	-4.0 (-6.1 to -1.9)
Hemolytic disease and other neonatal jaundice	1.9 (1.6 to 2.3)	--	0.4 (0.3 to 0.6)	-0.0 (-7.5 to 7.3)	0.5 (-7.0 to 7.9)
Other neonatal disorders	--	--	4.1 (2.8 to 5.8)	-12.5 (-19.1 to -3.9)	-12.1 (-18.7 to -3.4)
**Nutritional deficiencies**	**561.2 (541.2 to 575.4)**	**377.2† (365.1 to 390.6)**	**28.2 (18.9 to 39.1)**	**-9.8 (-15.0 to -4.5)**	**-9.3 (-14.6 to -4.0)**
Protein-energy malnutrition	322.7 (312.4 to 334.6)	377.2 (365.1 to 390.6)	17.2 (11.2 to 24.3)	-12.7 (-20.1 to -4.8)	-12.2 (-19.7 to -4.3)
Iodine deficiency	--	--	--	--	--
Vitamin A deficiency	--	--	--	--	--
Iron-deficiency anaemia	305.6 (245.7 to 358.0)	--	10.9 (7.0 to 16.2)	-5.0 (-7.6 to -2.3)	-4.5 (-7.1 to -1.8)
Other nutritional deficiencies	--	--	--	--	--
**Other communicable, maternal, neonatal, and nutritional diseases**	**104.7 (78.4 to 139.5)**	**15.9 (7.8 to 25.8)**	**0.9 (0.6 to 1.2)**	**6.5 (3.3 to 9.6)**	**7.0 (3.8 to 10.2)**
Sexually transmitted diseases excluding HIV	--	--	--	--	--
Syphilis	--	--	--	--	--
Chlamydial infection	--	--	--	--	--
Gonococcal infection	--	--	--	--	--
Trichomoniasis	--	--	--	--	--
Genital herpes	--	--	--	--	--
Other sexually transmitted diseases	--	--	--	--	--
Hepatitis	79.0 (40.1 to 136.6)	15.9 (7.8 to 25.8)	< 0.05 (< 0.05 to < 0.05)	-9.2 (-41.6 to 47.9)	-8.7 (-41.3 to 48.7)
Acute hepatitis A	--	--	--	--	--
Hepatitis B	79.0 (40.1 to 136.6)	15.9 (7.8 to 25.8)	< 0.05 (< 0.05 to < 0.05)	-9.2 (-41.6 to 47.9)	-8.7 (-41.3 to 48.7)
Hepatitis C	--	--	--	--	--
Acute hepatitis E	--	--	--	--	--
Other infectious diseases	19.2 (15.5 to 22.6)	< 0.05 (< 0.05 to < 0.05)	0.8 (0.6 to 1.2)	6.5 (3.3 to 9.7)	7.1 (3.8 to 10.2)
**Non-communicable diseases**	**793.2 (785.1 to 801.9)**	**1,513.0† (1,389.8 to 1,653.5)**	**14.6 (10.6 to 19.2)**	**-2.5 (-4.1 to -0.9)**	**-2.0 (-3.6 to -0.4)**
**Neoplasms**	**--**	**--**	**--**	**--**	**--**
Lip and oral cavity cancer	--	--	--	--	--
Nasopharynx cancer	--	--	--	--	--
Other pharynx cancer	--	--	--	--	--
Oesophageal cancer	--	--	--	--	--
Stomach cancer	--	--	--	--	--
Colon and rectum cancer	--	--	--	--	--
Liver cancer	--	--	--	--	--
Liver cancer due to hepatitis B	--	--	--	--	--

Cause	Prevalence in thousands (95% UI)	Incidence in thousands (95% UI)	YLDs in thousands (95% UI)		
	2016 counts	2016 counts	2016 counts	Percentage change in counts between 2006 and 2016	Percentage change in rates between 2006 and 2016
Liver cancer due to hepatitis C	--	--	--	--	--
Liver cancer due to alcohol use	--	--	--	--	--
Liver cancer due to other causes	--	--	--	--	--
Gallbladder and biliary tract cancer	--	--	--	--	--
Pancreatic cancer	--	--	--	--	--
Larynx cancer	--	--	--	--	--
Tracheal, bronchus, and lung cancer	--	--	--	--	--
Malignant skin melanoma	--	--	--	--	--
Non-melanoma skin cancer	--	--	--	--	--
Non-melanoma skin cancer (squamous-cell carcinoma)	--	--	--	--	--
Non-melanoma skin cancer (basal-cell carcinoma)	--	--	--	--	--
Breast cancer	--	--	--	--	--
Cervical cancer	--	--	--	--	--
Uterine cancer	--	--	--	--	--
Ovarian cancer	--	--	--	--	--
Prostate cancer	--	--	--	--	--
Testicular cancer	--	--	--	--	--
Kidney cancer	--	--	--	--	--
Bladder cancer	--	--	--	--	--
Brain and nervous system cancer	--	--	--	--	--
Thyroid cancer	--	--	--	--	--
Mesothelioma	--	--	--	--	--
Hodgkin lymphoma	--	--	--	--	--
Non-Hodgkin's lymphoma	--	--	--	--	--
Multiple myeloma	--	--	--	--	--
Leukaemia	--	--	--	--	--
Acute lymphoid leukaemia	--	--	--	--	--
Chronic lymphoid leukaemia	--	--	--	--	--
Acute myeloid leukaemia	--	--	--	--	--
Chronic myeloid leukaemia	--	--	--	--	--
Other leukaemia	--	--	--	--	--
Other neoplasms	--	--	--	--	--
**Cardiovascular diseases**	**< 0.05 (< 0.05 to < 0.05)**	**0.6† (0.4 to 0.7)**	**< 0.05 (< 0.05 to < 0.05)**	**-7.7 (-11.6 to -3.5)**	**-7.2 (-11.1 to -3.0)**
Rheumatic heart disease	--	--	--	--	--
Ischaemic heart disease	--	--	--	--	--
Cerebrovascular disease	--	--	--	--	--
Ischaemic stroke	--	--	--	--	--
Hemorrhagic stroke	--	--	--	--	--
Hypertensive heart disease	--	--	--	--	--
Cardiomyopathy and myocarditis	--	--	--	--	--
Myocarditis	--	--	--	--	--
Alcoholic cardiomyopathy	--	--	--	--	--
Other cardiomyopathy	--	--	--	--	--
Atrial fibrillation and flutter	--	--	--	--	--
Peripheral vascular disease	--	--	--	--	--
Endocarditis	< 0.05 (< 0.05 to < 0.05)	0.6 (0.4 to 0.7)	< 0.05 (< 0.05 to < 0.05)	-7.7 (-11.6 to -3.5)	-7.2 (-11.1 to -3.0)
Other cardiovascular and circulatory diseases	--	--	--	--	--
**Chronic respiratory diseases**	**--**	**--**	**--**	**--**	**--**
Chronic obstructive pulmonary disease	--	--	--	--	--
Pneumoconiosis	--	--	--	--	--
Silicosis	--	--	--	--	--

Cause	Prevalence in thousands (95% UI)	Incidence in thousands (95% UI)	YLDs in thousands (95% UI)		
	2016 counts	2016 counts	2016 counts	Percentage change in counts between 2006 and 2016	Percentage change in rates between 2006 and 2016
Asbestosis	--	--	--	--	--
Coal workers pneumoconiosis	--	--	--	--	--
Other pneumoconiosis	--	--	--	--	--
Asthma	--	--	--	--	--
Interstitial lung disease and pulmonary sarcoidosis	--	--	--	--	--
Other chronic respiratory diseases	--	--	--	--	--
**Cirrhosis and other chronic liver diseases**	**< 0.05 (< 0.05 to < 0.05)**	**1.7 (1.5 to 1.9)**	**< 0.05 (< 0.05 to < 0.05)**	**1.6 (0.1 to 3.3)**	**2.1 (0.6 to 3.9)**
Cirrhosis and other chronic liver diseases due to hepatitis B	< 0.05 (< 0.05 to < 0.05)	< 0.05 (< 0.05 to < 0.05)	< 0.05 (< 0.05 to < 0.05)	6.8 (2.2 to 11.1)	7.4 (2.7 to 11.7)
Cirrhosis and other chronic liver diseases due to hepatitis C	< 0.05 (< 0.05 to < 0.05)	< 0.05 (< 0.05 to < 0.05)	< 0.05 (< 0.05 to < 0.05)	4.1 (0.0 to 8.3)	4.7 (0.5 to 8.9)
Cirrhosis and other chronic liver diseases due to alcohol use	--	--	--	--	--
Cirrhosis and other chronic liver diseases due to other causes	< 0.05 (< 0.05 to < 0.05)	1.7 (1.5 to 1.9)	< 0.05 (< 0.05 to < 0.05)	1.6 (0.1 to 3.3)	2.1 (0.6 to 3.9)
**Digestive diseases**	**0.2 (0.2 to 0.3)**	**1.4† (1.0 to 1.8)**	**0.1 (< 0.05 to 0.1)**	**-8.4 (-21.9 to 4.7)**	**-7.9 (-21.5 to 5.2)**
Peptic ulcer disease	--	--	--	--	--
Gastritis and duodenitis	--	--	--	--	--
Appendicitis	--	--	--	--	--
Paralytic ileus and intestinal obstruction	0.2 (0.1 to 0.3)	1.4 (1.0 to 1.8)	0.1 (< 0.05 to 0.1)	-8.4 (-21.9 to 4.7)	-7.9 (-21.5 to 5.2)
Inguinal, femoral, and abdominal hernia	--	--	--	--	--
Inflammatory bowel disease	--	--	--	--	--
Vascular intestinal disorders	--	--	--	--	--
Gallbladder and biliary diseases	--	--	--	--	--
Pancreatitis	--	--	--	--	--
Other digestive diseases	--	--	--	--	--
**Neurological disorders**	**0.1 (< 0.05 to 0.1)**	**5.7 (4.2 to 7.4)**	**< 0.05 (< 0.05 to < 0.05)**	**-7.5 (-19.2 to 5.9)**	**-7.1 (-18.8 to 6.4)**
Alzheimer's disease and other dementias	--	--	--	--	--
Parkinson's disease	--	--	--	--	--
Epilepsy	0.1 (< 0.05 to 0.1)	5.6 (4.1 to 7.3)	< 0.05 (< 0.05 to < 0.05)	-7.5 (-19.3 to 6.0)	-7.1 (-18.8 to 6.5)
Multiple sclerosis	--	--	--	--	--
Motor neuron disease	< 0.05 (< 0.05 to < 0.05)	0.1 (0.1 to 0.1)	< 0.05 (< 0.05 to < 0.05)	-7.4 (-9.6 to -4.8)	-6.9 (-9.1 to -4.3)
Migraine	--	--	--	--	--
Tension-type headache	--	--	--	--	--
Other neurological disorders	--	--	--	--	--
**Mental and substance use disorders**	**18.9 (16.4 to 21.0)**	**1.0† (0.6 to 1.5)**	**2.1 (1.3 to 3.1)**	**-2.0 (-4.3 to 0.3)**	**-1.5 (-3.8 to 0.8)**
Schizophrenia	--	--	--	--	--
Alcohol use disorders	0.1 (0.1 to 0.1)	--	< 0.05 (< 0.05 to < 0.05)	0.3 (-6.3 to 6.0)	0.8 (-5.8 to 6.5)
Drug use disorders	--	--	--	--	--
Opioid use disorders	--	--	--	--	--
Cocaine use disorders	--	--	--	--	--
Amphetamine use disorders	--	--	--	--	--
Cannabis use disorders	--	--	--	--	--
Other drug use disorders	--	--	--	--	--
Depressive disorders	--	--	--	--	--
Major depressive disorder	--	--	--	--	--
Dysthymia	--	--	--	--	--
Bipolar disorder	--	--	--	--	--
Anxiety disorders	--	--	--	--	--
Eating disorders	--	--	--	--	--
Anorexia nervosa	--	--	--	--	--
Bulimia nervosa	--	--	--	--	--
Autistic spectrum disorders	14.2 (12.6 to 16.4)	1.0 (0.6 to 1.5)	1.8 (1.1 to 2.8)	-0.5 (-2.6 to 1.6)	0.0 (-2.0 to 2.1)
Autism	2.5 (1.6 to 3.6)	0.2 (0.1 to 0.3)	0.7 (0.3 to 1.1)	-0.4 (-4.7 to 3.9)	0.1 (-4.2 to 4.4)
Asperger syndrome and other autistic spectrum disorders	11.4 (7.8 to 15.7)	0.8 (0.5 to 1.4)	1.2 (0.7 to 1.9)	-0.6 (-2.8 to 1.9)	-0.0 (-2.3 to 2.4)

Cause	Prevalence in thousands (95% UI)	Incidence in thousands (95% UI)	YLDs in thousands (95% UI)		
	2016 counts	2016 counts	2016 counts	Percentage change in counts between 2006 and 2016	Percentage change in rates between 2006 and 2016
Attention-deficit/hyperactivity disorder	--	--	--	--	--
Conduct disorder	--	--	--	--	--
Idiopathic developmental intellectual disability	5.2 (1.6 to 10.2)	--	0.2 (0.1 to 0.5)	-13.1 (-18.2 to -7.0)	-12.7 (-17.7 to -6.5)
Other mental and substance use disorders	--	--	--	--	--
**Diabetes, urogenital, blood, and endocrine diseases**	**676.5 (667.9 to 685.8)**	**116.7† (96.0 to 138.3)**	**2.2 (1.4 to 3.3)**	**-9.6 (-12.9 to -6.4)**	**-9.2 (-12.4 to -5.9)**
Diabetes mellitus	--	--	--	--	--
Acute glomerulonephritis	< 0.05 (< 0.05 to < 0.05)	0.1 (0.1 to 0.2)	< 0.05 (< 0.05 to < 0.05)	-6.4 (-8.1 to -4.7)	-5.9 (-7.7 to -4.2)
Chronic kidney disease	< 0.05 (< 0.05 to < 0.05)	0.3 (0.2 to 0.4)	< 0.05 (< 0.05 to < 0.05)	-6.1 (-11.5 to -0.5)	-5.6 (-11.0 to 0.0)
Chronic kidney disease due to diabetes mellitus	< 0.05 (< 0.05 to < 0.05)	< 0.05 (< 0.05 to < 0.05)	< 0.05 (< 0.05 to < 0.05)	-6.0 (-12.5 to 0.8)	-5.5 (-12.0 to 1.3)
Chronic kidney disease due to hypertension	< 0.05 (< 0.05 to < 0.05)	0.1 (0.1 to 0.2)	< 0.05 (< 0.05 to < 0.05)	-8.5 (-14.7 to -2.6)	-8.0 (-14.2 to -2.1)
Chronic kidney disease due to glomerulonephritis	< 0.05 (< 0.05 to < 0.05)	< 0.05 (< 0.05 to < 0.05)	< 0.05 (< 0.05 to < 0.05)	-5.3 (-10.8 to 0.4)	-4.8 (-10.3 to 0.9)
Chronic kidney disease due to other causes	< 0.05 (< 0.05 to < 0.05)	0.1 (0.1 to 0.2)	< 0.05 (< 0.05 to < 0.05)	-6.7 (-11.9 to -1.2)	-6.2 (-11.5 to -0.7)
Urinary diseases and male infertility	0.8 (0.7 to 0.8)	106.4† (85.8 to 128.1)	< 0.05 (< 0.05 to 0.1)	-18.6 (-26.9 to -10.2)	-18.1 (-26.6 to -9.7)
Interstitial nephritis and urinary tract infections	0.7 (0.5 to 0.8)	106.4 (85.8 to 128.1)	< 0.05 (< 0.05 to < 0.05)	-4.4 (-14.2 to 5.5)	-3.9 (-13.8 to 6.1)
Urolithiasis	--	--	--	--	--
Benign prostatic hyperplasia	--	--	--	--	--
Male infertility	--	--	--	--	--
Other urinary diseases	--	--	< 0.05 (< 0.05 to < 0.05)	-35.4 (-42.0 to -28.7)	-35.0 (-41.7 to -28.3)
Gynecological diseases	--	--	--	--	--
Uterine fibroids	--	--	--	--	--
Polycystic ovarian syndrome	--	--	--	--	--
Female infertility	--	--	--	--	--
Endometriosis	--	--	--	--	--
Genital prolapse	--	--	--	--	--
Premenstrual syndrome	--	--	--	--	--
Other gynecological diseases	--	--	--	--	--
Hemoglobinopathies and hemolytic anaemias	666.6 (657.4 to 676.0)	--	1.7 (1.1 to 2.5)	-10.7 (-14.2 to -7.1)	-10.2 (-13.8 to -6.6)
Thalassemias	1.8 (1.6 to 1.9)	--	0.1 (< 0.05 to 0.1)	-16.3 (-22.0 to -11.0)	-15.9 (-21.6 to -10.5)
Thalassaemias trait	77.2 (73.7 to 81.0)	--	0.5 (0.3 to 0.7)	-19.1 (-25.8 to -12.6)	-18.6 (-25.4 to -12.1)
Sickle cell disorders	5.4 (4.5 to 7.0)	--	0.3 (0.2 to 0.4)	-0.4 (-8.2 to 7.2)	0.1 (-7.7 to 7.8)
Sickle cell trait	207.9 (190.3 to 231.0)	--	0.5 (0.3 to 0.7)	-5.8 (-10.9 to -0.8)	-5.3 (-10.4 to -0.3)
G6PD deficiency	135.4 (129.8 to 142.1)	--	< 0.05 (< 0.05 to < 0.05)	-8.2 (-11.6 to -4.8)	-7.8 (-11.2 to -4.3)
G6PD trait	311.3 (306.0 to 317.4)	--	< 0.05 (< 0.05 to < 0.05)	-15.0 (-19.2 to -10.6)	-14.6 (-18.8 to -10.1)
Other hemoglobinopathies and hemolytic anaemias	11.1 (9.0 to 13.0)	--	0.4 (0.2 to 0.6)	-10.1 (-14.0 to -6.5)	-9.7 (-13.6 to -6.0)
Endocrine, metabolic, blood, and immune disorders	14.2 (11.4 to 16.6)	9.9 (8.1 to 11.8)	0.5 (0.3 to 0.8)	-5.1 (-8.9 to -1.6)	-4.6 (-8.4 to -1.1)
**Musculoskeletal disorders**	**--**	**--**	**--**	**--**	**--**
Rheumatoid arthritis	--	--	--	--	--
Osteoarthritis	--	--	--	--	--
Low back and neck pain	--	--	--	--	--
Low back pain	--	--	--	--	--
Neck pain	--	--	--	--	--
Gout	--	--	--	--	--
Other musculoskeletal disorders	--	--	--	--	--
**Other non-communicable diseases**	**146.9 (142.9 to 151.0)**	**1,386.1† (1,267.0 to 1,533.0)**	**10.2 (7.4 to 13.6)**	**-0.9 (-2.8 to 1.1)**	**-0.4 (-2.3 to 1.7)**
Congenital anomalies	69.0 (65.6 to 71.7)	--	8.3 (6.0 to 11.0)	0.6 (-1.6 to 2.8)	1.1 (-1.1 to 3.3)
Neural tube defects	3.4 (3.1 to 3.8)	--	1.1 (0.7 to 1.4)	3.2 (-1.3 to 8.0)	3.8 (-0.8 to 8.6)
Congenital heart anomalies	33.2 (26.7 to 40.3)	--	1.4 (0.7 to 2.6)	-1.9 (-5.0 to 0.8)	-1.4 (-4.5 to 1.3)
Orofacial clefts	2.3 (1.9 to 2.9)	--	< 0.05 (< 0.05 to 0.1)	-9.2 (-14.6 to -4.4)	-8.8 (-14.1 to -3.9)
Down's syndrome	1.5 (1.2 to 1.7)	--	0.1 (0.1 to 0.2)	-1.0 (-4.6 to 3.0)	-0.5 (-4.1 to 3.6)
Turner syndrome	1.6 (1.4 to 1.9)	--	< 0.05 (< 0.05 to < 0.05)	-5.7 (-17.1 to 7.2)	-5.2 (-16.7 to 7.7)
Klinefelter syndrome	2.1 (1.8 to 2.4)	--	< 0.05 (< 0.05 to < 0.05)	1.3 (-6.7 to 9.8)	1.8 (-6.2 to 10.4)

Cause	Prevalence in thousands (95% UI)	Incidence in thousands (95% UI)	YLDs in thousands (95% UI)		
	2016 counts	2016 counts	2016 counts	Percentage change in counts between 2006 and 2016	Percentage change in rates between 2006 and 2016
Other chromosomal abnormalities	6.3 (5.3 to 7.3)	--	1.2 (0.8 to 1.7)	-4.6 (-8.7 to -0.3)	-4.1 (-8.2 to 0.2)
Congenital musculoskeletal and limb anomalies	3.4 (3.1 to 3.7)	--	0.5 (0.3 to 0.6)	-0.0 (-3.2 to 3.4)	0.5 (-2.7 to 4.0)
Urogenital congenital anomalies	7.6 (6.7 to 8.7)	--	0.2 (0.1 to 0.3)	-4.6 (-7.0 to -2.2)	-4.1 (-6.5 to -1.7)
Digestive congenital anomalies	3.5 (3.2 to 3.9)	--	0.2 (0.1 to 0.2)	-2.8 (-7.3 to 2.0)	-2.3 (-6.9 to 2.5)
Other congenital anomalies	12.7 (8.9 to 18.0)	--	3.5 (2.4 to 4.8)	3.7 (-0.3 to 7.4)	4.2 (0.2 to 8.0)
Skin and subcutaneous diseases	12.2 (11.8 to 12.6)	1,386.1 (1,267.0 to 1,533.0)	0.2 (0.1 to 0.3)	-0.9 (-5.0 to 3.3)	-0.4 (-4.5 to 3.8)
Dermatitis	--	--	--	--	--
Psoriasis	--	--	--	--	--
Cellulitis	0.1 (0.1 to 0.1)	11.8 (10.0 to 13.6)	< 0.05 (< 0.05 to < 0.05)	2.1 (1.1 to 3.0)	2.6 (1.7 to 3.6)
Pyoderma	2.2 (2.1 to 2.4)	278.8 (264.1 to 294.6)	< 0.05 (< 0.05 to < 0.05)	10.9 (4.9 to 17.3)	11.5 (5.5 to 17.9)
Scabies	1.9 (1.4 to 2.5)	200.2 (146.4 to 264.6)	< 0.05 (< 0.05 to 0.1)	-7.2 (-12.0 to -2.1)	-6.7 (-11.6 to -1.6)
Fungal skin diseases	4.3 (3.4 to 5.3)	459.7 (367.9 to 570.1)	< 0.05 (< 0.05 to 0.1)	2.9 (-0.7 to 6.8)	3.5 (-0.1 to 7.3)
Viral skin diseases	--	--	--	--	--
Acne vulgaris	--	--	--	--	--
Alopecia areata	--	--	--	--	--
Pruritus	0.2 (0.1 to 0.2)	18.0 (13.4 to 23.4)	< 0.05 (< 0.05 to < 0.05)	0.4 (-0.8 to 1.7)	1.0 (-0.3 to 2.2)
Urticaria	1.8 (1.3 to 2.2)	185.8 (141.9 to 235.8)	0.1 (0.1 to 0.2)	-0.7 (-8.2 to 7.4)	-0.2 (-7.7 to 8.0)
Decubitus ulcer	--	--	--	--	--
Other skin and subcutaneous diseases	2.2 (2.0 to 2.4)	231.8 (215.1 to 250.1)	< 0.05 (< 0.05 to < 0.05)	4.0 (-2.6 to 10.6)	4.5 (-2.1 to 11.2)
Sense organ diseases	68.7 (65.8 to 70.8)	--	1.7 (1.1 to 2.6)	-7.4 (-9.8 to -4.7)	-6.9 (-9.4 to -4.2)
Glaucoma	--	--	--	--	--
Cataract	--	--	--	--	--
Macular degeneration	--	--	--	--	--
Refraction and accommodation disorders	54.4 (49.1 to 59.7)	--	0.6 (0.3 to 1.1)	-6.4 (-8.0 to -4.8)	-5.9 (-7.6 to -4.2)
Age-related and other hearing loss	5.6 (3.9 to 7.9)	--	0.6 (0.4 to 1.0)	-7.3 (-12.6 to -1.7)	-6.9 (-12.2 to -1.2)
Other vision loss	9.5 (5.4 to 15.5)	--	0.5 (0.3 to 0.9)	-8.5 (-14.0 to -4.1)	-8.0 (-13.5 to -3.6)
Other sense organ diseases	--	--	--	--	--
Oral disorders	--	--	--	--	--
Caries of deciduous teeth	--	--	--	--	--
Caries of permanent teeth	--	--	--	--	--
Periodontal disease	--	--	--	--	--
Edentulism and severe tooth loss	--	--	--	--	--
Other oral disorders	--	--	--	--	--
**Injuries**	**1.0 (0.9 to 1.2)**	**9.0† (8.2 to 9.8)**	**0.1 (0.1 to 0.1)**	**-6.8 (-8.6 to -5.0)**	**-6.3 (-8.1 to -4.5)**
**Transport injuries**	**< 0.05 (< 0.05 to 0.1)**	**0.3 (0.2 to 0.4)**	**< 0.05 (< 0.05 to < 0.05)**	**-4.4 (-7.5 to -1.3)**	**-3.9 (-7.0 to -0.8)**
Road injuries	< 0.05 (< 0.05 to < 0.05)	< 0.05 (< 0.05 to < 0.05)	< 0.05 (< 0.05 to < 0.05)	11.1 (4.3 to 16.7)	11.6 (4.9 to 17.3)
Pedestrian road injuries	< 0.05 (< 0.05 to < 0.05)	< 0.05 (< 0.05 to < 0.05)	< 0.05 (< 0.05 to < 0.05)	8.7 (1.0 to 16.0)	9.2 (1.5 to 16.6)
Cyclist road injuries	--	--	--	--	--
Motorcyclist road injuries	< 0.05 (< 0.05 to < 0.05)	< 0.05 (< 0.05 to < 0.05)	< 0.05 (< 0.05 to < 0.05)	12.4 (4.7 to 18.9)	13.0 (5.3 to 19.5)
Motor vehicle road injuries	< 0.05 (< 0.05 to < 0.05)	< 0.05 (< 0.05 to < 0.05)	< 0.05 (< 0.05 to < 0.05)	8.6 (1.3 to 15.3)	9.2 (1.8 to 16.0)
Other road injuries	< 0.05 (< 0.05 to < 0.05)	< 0.05 (< 0.05 to < 0.05)	< 0.05 (< 0.05 to < 0.05)	27.3 (18.7 to 35.4)	28.0 (19.4 to 36.1)
Other transport injuries	< 0.05 (< 0.05 to 0.1)	0.2 (0.2 to 0.3)	< 0.05 (< 0.05 to < 0.05)	-5.6 (-8.9 to -2.6)	-5.1 (-8.4 to -2.1)
**Unintentional injuries**	**1.0 (0.8 to 1.1)**	**8.6 (7.8 to 9.4)**	**0.1 (< 0.05 to 0.1)**	**-7.0 (-8.9 to -5.0)**	**-6.5 (-8.4 to -4.5)**
Falls	0.2 (0.2 to 0.3)	1.6 (1.2 to 2.0)	< 0.05 (< 0.05 to < 0.05)	6.3 (2.5 to 9.5)	6.9 (3.0 to 10.1)
Drowning	< 0.05 (< 0.05 to < 0.05)	< 0.05 (< 0.05 to < 0.05)	< 0.05 (< 0.05 to < 0.05)	-12.5 (-15.6 to -9.6)	-12.1 (-15.2 to -9.2)
Fire, heat, and hot substances	< 0.05 (< 0.05 to < 0.05)	0.1 (0.1 to 0.2)	< 0.05 (< 0.05 to < 0.05)	-5.4 (-10.6 to -0.1)	-4.9 (-10.1 to 0.4)
Poisonings	< 0.05 (< 0.05 to < 0.05)	0.1 (< 0.05 to 0.1)	< 0.05 (< 0.05 to < 0.05)	5.2 (1.0 to 8.2)	5.8 (1.5 to 8.7)
Exposure to mechanical forces	0.1 (0.1 to 0.1)	0.9 (0.7 to 1.1)	< 0.05 (< 0.05 to < 0.05)	-11.2 (-15.9 to -7.1)	-10.8 (-15.5 to -6.6)
Unintentional firearm injuries	< 0.05 (< 0.05 to < 0.05)	< 0.05 (< 0.05 to < 0.05)	< 0.05 (< 0.05 to < 0.05)	-3.9 (-10.9 to 3.0)	-3.4 (-10.4 to 3.5)
Unintentional suffocation	< 0.05 (< 0.05 to < 0.05)	0.2 (0.1 to 0.2)	< 0.05 (< 0.05 to < 0.05)	-19.2 (-25.1 to -14.1)	-18.8 (-24.7 to -13.6)
Other exposure to mechanical forces	0.1 (0.1 to 0.1)	0.7 (0.5 to 0.9)	< 0.05 (< 0.05 to < 0.05)	-7.3 (-12.4 to -2.8)	-6.8 (-11.9 to -2.3)

Cause	Prevalence in thousands (95% UI)	Incidence in thousands (95% UI)	YLDs in thousands (95% UI)		
	2016 counts	2016 counts	2016 counts	Percentage change in counts between 2006 and 2016	Percentage change in rates between 2006 and 2016
Adverse effects of medical treatment	0.1 (0.1 to 0.2)	1.8 (1.5 to 2.1)	< 0.05 (< 0.05 to < 0.05)	-13.2 (-17.5 to -9.4)	-12.8 (-17.1 to -8.9)
Animal contact	0.1 (< 0.05 to 0.1)	0.8 (0.6 to 1.0)	< 0.05 (< 0.05 to < 0.05)	-13.9 (-18.6 to -9.7)	-13.4 (-18.2 to -9.2)
Venomous animal contact	< 0.05 (< 0.05 to < 0.05)	0.4 (0.3 to 0.5)	< 0.05 (< 0.05 to < 0.05)	-17.1 (-22.1 to -12.7)	-16.7 (-21.7 to -12.2)
Non-venomous animal contact	< 0.05 (< 0.05 to 0.1)	0.4 (0.3 to 0.6)	< 0.05 (< 0.05 to < 0.05)	-7.2 (-12.8 to -2.6)	-6.7 (-12.3 to -2.1)
Foreign body	0.1 (0.1 to 0.1)	1.3 (1.1 to 1.6)	< 0.05 (< 0.05 to < 0.05)	-8.2 (-10.8 to -5.7)	-7.8 (-10.3 to -5.2)
Pulmonary aspiration and foreign body in airway	0.1 (0.1 to 0.1)	0.9 (0.8 to 1.2)	< 0.05 (< 0.05 to < 0.05)	-10.2 (-13.3 to -7.0)	-9.7 (-12.8 to -6.5)
Foreign body in eyes	< 0.05 (< 0.05 to < 0.05)	0.1 (0.1 to 0.2)	< 0.05 (< 0.05 to < 0.05)	3.7 (0.4 to 8.4)	4.3 (1.0 to 8.9)
Foreign body in other body part	< 0.05 (< 0.05 to < 0.05)	0.3 (0.2 to 0.4)	< 0.05 (< 0.05 to < 0.05)	-4.3 (-8.3 to -0.6)	-3.8 (-7.8 to -0.1)
Environmental heat and cold exposure	0.1 (< 0.05 to 0.1)	0.4 (0.3 to 0.6)	< 0.05 (< 0.05 to < 0.05)	-9.9 (-14.3 to -6.2)	-9.4 (-13.9 to -5.7)
Other unintentional injuries	0.2 (0.2 to 0.3)	1.6 (1.3 to 2.1)	< 0.05 (< 0.05 to < 0.05)	-2.5 (-7.1 to 2.1)	-2.0 (-6.6 to 2.6)
**Self-harm and interpersonal violence**	**< 0.05 (< 0.05 to < 0.05)**	**0.1† (0.1 to 0.1)**	**< 0.05 (< 0.05 to < 0.05)**	**-6.7 (-12.2 to -1.2)**	**-6.2 (-11.8 to -0.7)**
Self-harm	--	--	--	--	--
Self-harm by firearm	--	--	--	--	--
Self-harm by other specified means	--	--	--	--	--
Interpersonal violence	< 0.05 (< 0.05 to < 0.05)	0.1† (0.1 to 0.1)	< 0.05 (< 0.05 to < 0.05)	-6.7 (-12.2 to -1.2)	-6.2 (-11.8 to -0.7)
Assault by firearm	< 0.05 (< 0.05 to < 0.05)	< 0.05 (< 0.05 to < 0.05)	< 0.05 (< 0.05 to < 0.05)	-2.8 (-10.1 to 4.3)	-2.3 (-9.6 to 4.8)
Assault by sharp object	< 0.05 (< 0.05 to < 0.05)	< 0.05 (< 0.05 to < 0.05)	< 0.05 (< 0.05 to < 0.05)	-1.7 (-8.6 to 5.8)	-1.2 (-8.1 to 6.4)
Sexual violence	--	--	--	--	--
Assault by other means	< 0.05 (< 0.05 to < 0.05)	0.1 (0.1 to 0.1)	< 0.05 (< 0.05 to < 0.05)	-8.8 (-14.4 to -3.3)	-8.4 (-13.9 to -2.8)
**Forces of nature, conflict and terrorism, and executions and police conflict**	**--**	**--**	**--**	**--**	**--**
Forces of nature, conflict and terrorism, and state actor violence	--	--	--	--	--
Conflict and terrorism	--	--	--	--	--
Executions and police conflict	--	--	--	--	--

We did not calculate incidence for certain NTDs and causes with only birth prevalence. Blank cells mean that no estimate is available.

† Incidence for aggregate causes represents the sum of the incidence of the causes below the aggregate.

‡ Incidence estimates for stroke represent first-ever stroke only.

^ Incidence of HIV/AIDS represents new infections of HIV only and does not include new infections of Tuberculosis in HIV positive cases.

SR Table 12: Global prevalence, incidence, and YLDs for 2016, percentage change of YLD counts and percentage change of YLD rates between 2006 and 2016 by cause, age 7-27 days.					
Cause	Prevalence in thousands (95% UI)	Incidence in thousands (95% UI)	YLDs in thousands (95% UI)		
	2016 counts	2016 counts	2016 counts	Percentage change in counts between 2006 and 2016	Percentage change in rates between 2006 and 2016
**All causes**	**4,682.5 (4,650.0 to 4,726.4)**	**55,267.8† (48,706.9 to 62,004.3)**	**249.8 (179.2 to 333.1)**	**-4.8 (-7.5 to -1.8)**	**-4.6 (-7.4 to -1.6)**
**Communicable, maternal, neonatal, and nutritional disorders**	**3,481.8 (3,429.7 to 3,539.6)**	**50,704.3† (44,094.1 to 57,488.2)**	**204.7 (143.9 to 275.1)**	**-5.3 (-8.6 to -1.7)**	**-5.1 (-8.4 to -1.5)**
**HIV/AIDS and tuberculosis**	**831.4 (755.1 to 923.2)**	**3.7† (2.5 to 5.1)**	**< 0.05 (< 0.05 to < 0.05)**	**-3.9 (-13.3 to 9.4)**	**-3.8 (-13.1 to 9.6)**
Tuberculosis	794.4 (686.1 to 912.2)	3.7† (2.5 to 5.1)	< 0.05 (< 0.05 to < 0.05)	-3.9 (-13.3 to 9.4)	-3.8 (-13.1 to 9.6)
Drug-susceptible tuberculosis	0.1 (< 0.05 to 0.1)	3.6 (2.5 to 4.9)	< 0.05 (< 0.05 to < 0.05)	-3.8 (-13.2 to 9.7)	-3.6 (-13.1 to 9.8)
Multidrug-resistant tuberculosis without extensive drug resistance	< 0.05 (< 0.05 to < 0.05)	0.1 (0.1 to 0.1)	< 0.05 (< 0.05 to < 0.05)	-11.9 (-26.9 to 6.2)	-11.8 (-26.8 to 6.4)
Extensively drug-resistant tuberculosis	< 0.05 (< 0.05 to < 0.05)	< 0.05 (< 0.05 to < 0.05)	< 0.05 (< 0.05 to < 0.05)	157.7 (109.0 to 216.0)	158.1 (109.4 to 216.6)
Latent tuberculosis infection	794.3 (686.0 to 912.1)	--	--	--	--
HIV/AIDS	--	--	--	--	--
Drug-susceptible HIV/AIDS - Tuberculosis	--	--	--	--	--
Multidrug-resistant HIV/AIDS - Tuberculosis without extensive drug resistance	--	--	--	--	--
Extensively drug-resistant HIV/AIDS - Tuberculosis	--	--	--	--	--
HIV/AIDS resulting in other diseases	--	--	--	--	--
**Diarrhoea, lower respiratory infections, and other common infectious diseases**	**644.9 (617.6 to 669.9)**	**48,555.6† (42,077.8 to 55,362.6)**	**41.7 (27.5 to 57.1)**	**-9.4 (-12.1 to -7.0)**	**-9.2 (-11.9 to -6.8)**
Diarrhoeal diseases	250.6 (219.2 to 285.4)	19,416.3 (17,088.0 to 22,034.8)	29.2 (19.3 to 40.9)	-12.4 (-15.9 to -9.3)	-12.2 (-15.8 to -9.1)
Intestinal infectious diseases	--	--	--	--	--
Typhoid fever	--	--	--	--	--
Paratyphoid fever	--	--	--	--	--
Other intestinal infectious diseases	--	--	--	--	--
Lower respiratory infections	54.6 (47.0 to 64.4)	771.8 (604.1 to 971.1)	3.4 (2.2 to 5.0)	-2.7 (-7.8 to 2.3)	-2.5 (-7.7 to 2.4)
Upper respiratory infections	294.5 (233.3 to 366.9)	25,438.1 (19,987.3 to 32,132.5)	7.5 (4.4 to 11.9)	1.7 (-0.4 to 3.5)	1.9 (-0.2 to 3.6)
Otitis media	39.6 (30.8 to 50.6)	1,208.4 (918.9 to 1,605.3)	0.8 (0.4 to 1.2)	-2.8 (-5.6 to -0.2)	-2.7 (-5.4 to -0.0)
Meningitis	3.9 (3.2 to 4.7)	73.2 (58.6 to 90.1)	0.5 (0.3 to 0.7)	-1.9 (-9.8 to 6.0)	-1.7 (-9.7 to 6.2)
Pneumococcal meningitis	0.4 (0.3 to 0.6)	11.8 (7.6 to 16.4)	0.1 (< 0.05 to 0.1)	-25.6 (-36.9 to -11.6)	-25.5 (-36.8 to -11.4)
H influenzae type B meningitis	0.4 (0.3 to 0.6)	13.8 (8.3 to 20.3)	0.1 (< 0.05 to 0.1)	-42.5 (-52.0 to -32.3)	-42.4 (-51.9 to -32.2)
Meningococcal infection	0.5 (0.3 to 0.6)	16.1 (10.7 to 23.0)	0.1 (< 0.05 to 0.1)	5.4 (-16.6 to 33.0)	5.5 (-16.4 to 33.2)
Other meningitis	2.6 (2.1 to 3.2)	31.6 (23.1 to 40.5)	0.3 (0.2 to 0.5)	15.3 (3.5 to 28.8)	15.5 (3.7 to 29.0)
Encephalitis	0.5 (0.4 to 0.6)	16.8 (13.8 to 20.0)	0.1 (< 0.05 to 0.1)	-8.8 (-18.6 to 2.7)	-8.6 (-18.5 to 2.9)
Diphtheria	--	--	--	--	--
Whooping cough	--	--	--	--	--
Tetanus	0.8 (0.5 to 1.1)	11.5 (6.5 to 17.4)	0.1 (< 0.05 to 0.2)	-68.0 (-78.6 to -51.6)	-67.9 (-78.6 to -51.5)
Measles	--	--	--	--	--
Varicella and herpes zoster	31.1 (21.8 to 43.0)	1,619.4 (1,139.4 to 2,239.2)	0.2 (0.1 to 0.4)	-3.5 (-6.3 to -1.0)	-3.4 (-6.1 to -0.8)
**Neglected tropical diseases and malaria**	**111.5 (95.7 to 129.2)**	**875.6† (586.2 to 1,216.7)**	**1.6 (1.0 to 2.6)**	**-30.9 (-41.5 to -20.3)**	**-30.8 (-41.4 to -20.1)**
Malaria	94.4 (70.5 to 121.9)	868.8 (579.1 to 1,209.7)	1.5 (0.9 to 2.5)	-34.6 (-45.1 to -25.2)	-34.5 (-45.1 to -25.1)
Chagas disease	--	--	--	--	--
Leishmaniasis	--	--	--	--	--
Visceral leishmaniasis	--	--	--	--	--
Cutaneous and mucocutaneous leishmaniasis	--	--	--	--	--
African trypanosomiasis	--	--	--	--	--
Schistosomiasis	--	--	--	--	--
Cysticercosis	--	--	--	--	--
Cystic echinococcosis	--	--	--	--	--
Lymphatic filariasis	12.7 (11.1 to 14.4)	2.2 (1.3 to 3.5)	--	--	--
Onchocerciasis	--	--	--	--	--
Trachoma	--	--	--	--	--
Dengue	--	--	--	--	--
Yellow fever	< 0.05 (< 0.05 to < 0.05)	0.1 (< 0.05 to 0.2)	< 0.05 (< 0.05 to < 0.05)	-18.9 (-48.7 to 24.9)	-18.7 (-48.6 to 25.1)
Rabies	--	--	--	--	--
Intestinal nematode infections	--	--	--	--	--
Ascariasis	--	--	--	--	--

Cause	Prevalence in thousands (95% UI)	Incidence in thousands (95% UI)	YLDs in thousands (95% UI)		
	2016 counts	2016 counts	2016 counts	Percentage change in counts between 2006 and 2016	Percentage change in rates between 2006 and 2016
Trichuriasis	--	--	--	--	--
Hookworm disease	--	--	--	--	--
Food-borne trematodiases	--	--	--	--	--
Leprosy	--	--	--	--	--
Ebola virus disease	--	--	--	--	--
Zika virus disease	0.2 (0.1 to 0.4)	4.5 (2.5 to 8.7)	0.1 (< 0.05 to 0.1)	--	--
Guinea worm disease	--	--	--	--	--
Other neglected tropical diseases	--	--	< 0.05 (< 0.05 to < 0.05)	-11.3 (-44.0 to 36.5)	-11.2 (-43.9 to 36.7)
**Maternal disorders**	**--**	**--**	**--**	**--**	**--**
Maternal haemorrhage	--	--	--	--	--
Maternal sepsis and other pregnancy related infections	--	--	--	--	--
Maternal hypertensive disorders	--	--	--	--	--
Maternal obstructed labour and uterine rupture	--	--	--	--	--
Maternal abortion, miscarriage, and ectopic pregnancy	--	--	--	--	--
Other maternal disorders	--	--	--	--	--
**Neonatal disorders**	**667.3 (626.0 to 699.3)**	**--**	**58.2 (39.0 to 83.5)**	**-5.8 (-13.7 to 4.1)**	**-5.6 (-13.5 to 4.3)**
Neonatal preterm birth complications	532.5 (454.8 to 618.3)	--	20.6 (12.5 to 31.6)	-6.0 (-23.9 to 16.2)	-5.8 (-23.7 to 16.5)
Neonatal encephalopathy due to birth asphyxia and trauma	76.0 (57.3 to 99.4)	--	8.6 (5.1 to 13.7)	-2.5 (-5.7 to 0.9)	-2.3 (-5.5 to 1.1)
Neonatal sepsis and other neonatal infections	66.8 (40.3 to 107.3)	--	17.2 (8.9 to 34.2)	-3.3 (-5.8 to -0.4)	-3.1 (-5.6 to -0.2)
Hemolytic disease and other neonatal jaundice	5.5 (4.7 to 6.5)	--	1.2 (0.8 to 1.7)	0.9 (-6.1 to 8.1)	1.1 (-5.9 to 8.3)
Other neonatal disorders	--	--	10.5 (7.0 to 15.1)	-12.2 (-19.5 to -3.0)	-12.0 (-19.4 to -2.8)
**Nutritional deficiencies**	**1,936.4 (1,897.4 to 1,972.4)**	**1,222.2† (1,189.8 to 1,255.1)**	**100.1 (68.0 to 139.4)**	**-2.9 (-7.5 to 1.4)**	**-2.7 (-7.3 to 1.6)**
Protein-energy malnutrition	1,020.5 (991.5 to 1,050.3)	1,222.2 (1,189.8 to 1,255.1)	56.8 (37.0 to 81.1)	-3.2 (-10.7 to 4.6)	-3.0 (-10.5 to 4.8)
Iodine deficiency	--	--	--	--	--
Vitamin A deficiency	--	--	--	--	--
Iron-deficiency anaemia	1,204.2 (1,055.1 to 1,334.1)	--	43.3 (28.1 to 62.3)	-2.5 (-5.2 to -0.0)	-2.3 (-5.0 to 0.1)
Other nutritional deficiencies	--	--	--	--	--
**Other communicable, maternal, neonatal, and nutritional diseases**	**325.1 (255.0 to 421.0)**	**47.1 (23.8 to 76.5)**	**3.1 (2.0 to 4.4)**	**8.3 (4.9 to 11.9)**	**8.5 (5.1 to 12.1)**
Sexually transmitted diseases excluding HIV	--	--	--	--	--
Syphilis	--	--	--	--	--
Chlamydial infection	--	--	--	--	--
Gonococcal infection	--	--	--	--	--
Trichomoniasis	--	--	--	--	--
Genital herpes	--	--	--	--	--
Other sexually transmitted diseases	--	--	--	--	--
Hepatitis	234.6 (119.5 to 404.8)	47.1 (23.8 to 76.5)	< 0.05 (< 0.05 to < 0.05)	-8.5 (-14.8 to -2.2)	-8.3 (-14.6 to -2.0)
Acute hepatitis A	--	--	--	--	--
Hepatitis B	234.6 (119.5 to 404.8)	47.1 (23.8 to 76.5)	< 0.05 (< 0.05 to < 0.05)	-8.5 (-14.8 to -2.2)	-8.3 (-14.6 to -2.0)
Hepatitis C	--	--	--	--	--
Acute hepatitis E	--	--	--	--	--
Other infectious diseases	75.9 (66.5 to 84.0)	< 0.05 (< 0.05 to < 0.05)	3.0 (2.0 to 4.3)	8.5 (5.0 to 12.1)	8.7 (5.2 to 12.3)
**Non-communicable diseases**	**2,449.9 (2,427.2 to 2,475.6)**	**4,484.5† (4,132.0 to 4,896.1)**	**44.4 (31.9 to 59.0)**	**-2.6 (-3.8 to -1.4)**	**-2.4 (-3.7 to -1.2)**
**Neoplasms**	**--**	**--**	**--**	**--**	**--**
Lip and oral cavity cancer	--	--	--	--	--
Nasopharynx cancer	--	--	--	--	--
Other pharynx cancer	--	--	--	--	--
Oesophageal cancer	--	--	--	--	--
Stomach cancer	--	--	--	--	--
Colon and rectum cancer	--	--	--	--	--
Liver cancer	--	--	--	--	--
Liver cancer due to hepatitis B	--	--	--	--	--

Cause	Prevalence in thousands (95% UI)	Incidence in thousands (95% UI)	YLDs in thousands (95% UI)		
	2016 counts	2016 counts	2016 counts	Percentage change in counts between 2006 and 2016	Percentage change in rates between 2006 and 2016
Liver cancer due to hepatitis C	--	--	--	--	--
Liver cancer due to alcohol use	--	--	--	--	--
Liver cancer due to other causes	--	--	--	--	--
Gallbladder and biliary tract cancer	--	--	--	--	--
Pancreatic cancer	--	--	--	--	--
Larynx cancer	--	--	--	--	--
Tracheal, bronchus, and lung cancer	--	--	--	--	--
Malignant skin melanoma	--	--	--	--	--
Non-melanoma skin cancer	--	--	--	--	--
Non-melanoma skin cancer (squamous-cell carcinoma)	--	--	--	--	--
Non-melanoma skin cancer (basal-cell carcinoma)	--	--	--	--	--
Breast cancer	--	--	--	--	--
Cervical cancer	--	--	--	--	--
Uterine cancer	--	--	--	--	--
Ovarian cancer	--	--	--	--	--
Prostate cancer	--	--	--	--	--
Testicular cancer	--	--	--	--	--
Kidney cancer	--	--	--	--	--
Bladder cancer	--	--	--	--	--
Brain and nervous system cancer	--	--	--	--	--
Thyroid cancer	--	--	--	--	--
Mesothelioma	--	--	--	--	--
Hodgkin lymphoma	--	--	--	--	--
Non-Hodgkin's lymphoma	--	--	--	--	--
Multiple myeloma	--	--	--	--	--
Leukaemia	--	--	--	--	--
Acute lymphoid leukaemia	--	--	--	--	--
Chronic lymphoid leukaemia	--	--	--	--	--
Acute myeloid leukaemia	--	--	--	--	--
Chronic myeloid leukaemia	--	--	--	--	--
Other leukaemia	--	--	--	--	--
Other neoplasms	--	--	--	--	--
**Cardiovascular diseases**	**< 0.05 (< 0.05 to 0.1)**	**1.6† (1.3 to 2.0)**	**< 0.05 (< 0.05 to < 0.05)**	**-7.2 (-11.0 to -3.2)**	**-7.0 (-10.9 to -3.0)**
Rheumatic heart disease	--	--	--	--	--
Ischaemic heart disease	--	--	--	--	--
Cerebrovascular disease	--	--	--	--	--
Ischaemic stroke	--	--	--	--	--
Hemorrhagic stroke	--	--	--	--	--
Hypertensive heart disease	--	--	--	--	--
Cardiomyopathy and myocarditis	--	--	--	--	--
Myocarditis	--	--	--	--	--
Alcoholic cardiomyopathy	--	--	--	--	--
Other cardiomyopathy	--	--	--	--	--
Atrial fibrillation and flutter	--	--	--	--	--
Peripheral vascular disease	--	--	--	--	--
Endocarditis	< 0.05 (< 0.05 to 0.1)	1.6 (1.3 to 2.0)	< 0.05 (< 0.05 to < 0.05)	-7.2 (-11.0 to -3.2)	-7.0 (-10.9 to -3.0)
Other cardiovascular and circulatory diseases	--	--	--	--	--
**Chronic respiratory diseases**	**--**	**--**	**--**	**--**	**--**
Chronic obstructive pulmonary disease	--	--	--	--	--
Pneumoconiosis	--	--	--	--	--
Silicosis	--	--	--	--	--

Cause	Prevalence in thousands (95% UI)	Incidence in thousands (95% UI)	YLDs in thousands (95% UI)		
	2016 counts	2016 counts	2016 counts	Percentage change in counts between 2006 and 2016	Percentage change in rates between 2006 and 2016
Asbestosis	--	--	--	--	--
Coal workers pneumoconiosis	--	--	--	--	--
Other pneumoconiosis	--	--	--	--	--
Asthma	--	--	--	--	--
Interstitial lung disease and pulmonary sarcoidosis	--	--	--	--	--
Other chronic respiratory diseases	--	--	--	--	--
**Cirrhosis and other chronic liver diseases**	**0.2 (0.2 to 0.3)**	**4.9 (4.3 to 5.5)**	**< 0.05 (< 0.05 to 0.1)**	**1.9 (0.5 to 3.6)**	**2.1 (0.7 to 3.8)**
Cirrhosis and other chronic liver diseases due to hepatitis B	< 0.05 (< 0.05 to < 0.05)	< 0.05 (< 0.05 to < 0.05)	< 0.05 (< 0.05 to < 0.05)	7.2 (2.6 to 11.6)	7.4 (2.8 to 11.8)
Cirrhosis and other chronic liver diseases due to hepatitis C	< 0.05 (< 0.05 to < 0.05)	< 0.05 (< 0.05 to < 0.05)	< 0.05 (< 0.05 to < 0.05)	4.5 (0.4 to 8.6)	4.7 (0.6 to 8.8)
Cirrhosis and other chronic liver diseases due to alcohol use	--	--	--	--	--
Cirrhosis and other chronic liver diseases due to other causes	0.2 (0.2 to 0.3)	4.9 (4.3 to 5.5)	< 0.05 (< 0.05 to 0.1)	1.9 (0.5 to 3.6)	2.1 (0.7 to 3.8)
**Digestive diseases**	**0.3 (0.2 to 0.4)**	**4.0† (3.0 to 5.2)**	**0.1 (0.1 to 0.1)**	**-7.0 (-12.9 to -1.3)**	**-6.8 (-12.7 to -1.1)**
Peptic ulcer disease	--	--	--	--	--
Gastritis and duodenitis	--	--	--	--	--
Appendicitis	--	--	--	--	--
Paralytic ileus and intestinal obstruction	0.3 (0.2 to 0.4)	4.0 (3.0 to 5.2)	0.1 (0.1 to 0.1)	-7.0 (-12.9 to -1.3)	-6.8 (-12.7 to -1.1)
Inguinal, femoral, and abdominal hernia	--	--	--	--	--
Inflammatory bowel disease	--	--	--	--	--
Vascular intestinal disorders	--	--	--	--	--
Gallbladder and biliary diseases	--	--	--	--	--
Pancreatitis	--	--	--	--	--
Other digestive diseases	--	--	--	--	--
**Neurological disorders**	**0.8 (0.6 to 1.0)**	**16.6 (12.6 to 21.5)**	**0.3 (0.2 to 0.4)**	**-6.8 (-18.4 to 6.8)**	**-6.6 (-18.3 to 7.0)**
Alzheimer's disease and other dementias	--	--	--	--	--
Parkinson's disease	--	--	--	--	--
Epilepsy	0.8 (0.6 to 1.0)	16.4 (12.4 to 21.2)	0.3 (0.2 to 0.4)	-6.8 (-18.5 to 6.9)	-6.6 (-18.4 to 7.1)
Multiple sclerosis	--	--	--	--	--
Motor neuron disease	< 0.05 (< 0.05 to < 0.05)	0.2 (0.2 to 0.3)	< 0.05 (< 0.05 to < 0.05)	-6.5 (-8.6 to -3.9)	-6.3 (-8.5 to -3.7)
Migraine	--	--	--	--	--
Tension-type headache	--	--	--	--	--
Other neurological disorders	--	--	--	--	--
**Mental and substance use disorders**	**56.9 (49.1 to 63.0)**	**2.9† (1.8 to 4.5)**	**6.2 (3.9 to 9.3)**	**-1.8 (-4.1 to 0.6)**	**-1.6 (-4.0 to 0.8)**
Schizophrenia	--	--	--	--	--
Alcohol use disorders	0.3 (0.2 to 0.3)	--	< 0.05 (< 0.05 to < 0.05)	0.8 (-5.6 to 6.5)	1.0 (-5.4 to 6.7)
Drug use disorders	--	--	--	--	--
Opioid use disorders	--	--	--	--	--
Cocaine use disorders	--	--	--	--	--
Amphetamine use disorders	--	--	--	--	--
Cannabis use disorders	--	--	--	--	--
Other drug use disorders	--	--	--	--	--
Depressive disorders	--	--	--	--	--
Major depressive disorder	--	--	--	--	--
Dysthymia	--	--	--	--	--
Bipolar disorder	--	--	--	--	--
Anxiety disorders	--	--	--	--	--
Eating disorders	--	--	--	--	--
Anorexia nervosa	--	--	--	--	--
Bulimia nervosa	--	--	--	--	--
Autistic spectrum disorders	42.4 (37.5 to 49.0)	2.9 (1.8 to 4.5)	5.5 (3.4 to 8.2)	-0.2 (-2.3 to 1.9)	-0.1 (-2.1 to 2.1)
Autism	7.5 (4.7 to 10.8)	0.5 (0.2 to 0.8)	2.0 (1.0 to 3.3)	-0.2 (-4.6 to 4.3)	-0.0 (-4.4 to 4.5)
Asperger syndrome and other autistic spectrum disorders	34.0 (23.3 to 46.6)	2.5 (1.4 to 4.0)	3.5 (2.0 to 5.5)	-0.3 (-2.5 to 2.1)	-0.1 (-2.4 to 2.2)

Cause	Prevalence in thousands (95% UI)	Incidence in thousands (95% UI)	YLDs in thousands (95% UI)		
	2016 counts	2016 counts	2016 counts	Percentage change in counts between 2006 and 2016	Percentage change in rates between 2006 and 2016
Attention-deficit/hyperactivity disorder	--	--	--	--	--
Conduct disorder	--	--	--	--	--
Idiopathic developmental intellectual disability	15.9 (5.1 to 31.0)	--	0.7 (0.2 to 1.5)	-13.0 (-18.0 to -7.0)	-12.8 (-17.9 to -6.8)
Other mental and substance use disorders	--	--	--	--	--
**Diabetes, urogenital, blood, and endocrine diseases**	**2,029.0 (2,003.2 to 2,057.8)**	**344.6† (283.7 to 408.1)**	**9.1 (5.9 to 13.2)**	**-3.4 (-6.4 to -0.6)**	**-3.2 (-6.2 to -0.5)**
Diabetes mellitus	--	--	--	--	--
Acute glomerulonephritis	< 0.05 (< 0.05 to < 0.05)	0.4 (0.3 to 0.6)	< 0.05 (< 0.05 to < 0.05)	-6.2 (-8.0 to -4.5)	-6.0 (-7.8 to -4.3)
Chronic kidney disease	< 0.05 (< 0.05 to < 0.05)	0.8 (0.6 to 1.1)	< 0.05 (< 0.05 to < 0.05)	-5.5 (-10.8 to 0.1)	-5.3 (-10.6 to 0.3)
Chronic kidney disease due to diabetes mellitus	< 0.05 (< 0.05 to < 0.05)	< 0.05 (< 0.05 to < 0.05)	< 0.05 (< 0.05 to < 0.05)	-5.3 (-11.9 to 1.6)	-5.2 (-11.7 to 1.8)
Chronic kidney disease due to hypertension	< 0.05 (< 0.05 to < 0.05)	0.3 (0.2 to 0.5)	< 0.05 (< 0.05 to < 0.05)	-7.9 (-14.2 to -1.9)	-7.8 (-14.0 to -1.7)
Chronic kidney disease due to glomerulonephritis	< 0.05 (< 0.05 to < 0.05)	< 0.05 (< 0.05 to < 0.05)	< 0.05 (< 0.05 to < 0.05)	-4.6 (-10.1 to 1.1)	-4.5 (-9.9 to 1.3)
Chronic kidney disease due to other causes	< 0.05 (< 0.05 to < 0.05)	0.5 (0.3 to 0.6)	< 0.05 (< 0.05 to < 0.05)	-6.0 (-11.2 to -0.6)	-5.9 (-11.1 to -0.4)
Urinary diseases and male infertility	5.5 (5.0 to 6.3)	314.7† (254.3 to 378.4)	0.2 (0.1 to 0.4)	-17.6 (-23.9 to -11.4)	-17.4 (-23.8 to -11.3)
Interstitial nephritis and urinary tract infections	5.0 (4.0 to 6.0)	314.7 (254.3 to 378.4)	0.2 (0.1 to 0.3)	-4.0 (-11.4 to 3.1)	-3.8 (-11.2 to 3.3)
Urolithiasis	--	--	--	--	--
Benign prostatic hyperplasia	--	--	--	--	--
Male infertility	--	--	--	--	--
Other urinary diseases	--	--	0.1 (0.1 to 0.1)	-35.5 (-40.5 to -30.7)	-35.4 (-40.4 to -30.6)
Gynecological diseases	--	--	--	--	--
Uterine fibroids	--	--	--	--	--
Polycystic ovarian syndrome	--	--	--	--	--
Female infertility	--	--	--	--	--
Endometriosis	--	--	--	--	--
Genital prolapse	--	--	--	--	--
Premenstrual syndrome	--	--	--	--	--
Other gynecological diseases	--	--	--	--	--
Hemoglobinopathies and hemolytic anaemias	1,987.1 (1,960.7 to 2,014.5)	--	6.8 (4.4 to 10.0)	-3.0 (-6.6 to 0.1)	-2.8 (-6.4 to 0.3)
Thalassemias	5.2 (4.8 to 5.6)	--	0.2 (0.2 to 0.4)	-11.9 (-16.6 to -7.0)	-11.8 (-16.5 to -6.8)
Thalassaemias trait	230.1 (219.6 to 241.5)	--	2.3 (1.4 to 3.3)	-5.8 (-11.1 to -0.4)	-5.6 (-11.0 to -0.2)
Sickle cell disorders	18.2 (15.9 to 23.1)	--	1.0 (0.6 to 1.5)	2.8 (-3.8 to 9.5)	3.0 (-3.6 to 9.7)
Sickle cell trait	616.0 (563.9 to 684.4)	--	1.8 (1.2 to 2.7)	2.5 (-2.1 to 7.2)	2.6 (-1.9 to 7.4)
G6PD deficiency	401.4 (384.8 to 421.3)	--	< 0.05 (< 0.05 to < 0.05)	-6.9 (-10.5 to -3.9)	-6.7 (-10.3 to -3.7)
G6PD trait	925.7 (910.0 to 943.7)	--	< 0.05 (< 0.05 to < 0.05)	1.2 (-2.7 to 5.4)	1.4 (-2.5 to 5.6)
Other hemoglobinopathies and hemolytic anaemias	43.8 (38.5 to 48.4)	--	1.5 (1.0 to 2.1)	-6.6 (-10.3 to -2.8)	-6.4 (-10.1 to -2.7)
Endocrine, metabolic, blood, and immune disorders	56.7 (49.9 to 62.7)	28.7 (23.7 to 34.2)	2.0 (1.3 to 2.9)	-2.5 (-6.3 to 0.9)	-2.3 (-6.1 to 1.1)
**Musculoskeletal disorders**	**--**	**--**	**--**	**--**	**--**
Rheumatoid arthritis	--	--	--	--	--
Osteoarthritis	--	--	--	--	--
Low back and neck pain	--	--	--	--	--
Low back pain	--	--	--	--	--
Neck pain	--	--	--	--	--
Gout	--	--	--	--	--
Other musculoskeletal disorders	--	--	--	--	--
**Other non-communicable diseases**	**543.0 (534.2 to 553.0)**	**4,109.8† (3,756.3 to 4,531.2)**	**28.7 (20.8 to 38.1)**	**-2.5 (-4.0 to -0.9)**	**-2.3 (-3.9 to -0.7)**
Congenital anomalies	199.5 (190.2 to 207.2)	--	21.0 (15.3 to 27.9)	-1.7 (-3.7 to 0.1)	-1.5 (-3.5 to 0.3)
Neural tube defects	9.3 (8.4 to 10.4)	--	2.8 (2.0 to 3.7)	4.2 (-0.5 to 9.2)	4.4 (-0.3 to 9.4)
Congenital heart anomalies	94.4 (76.0 to 114.5)	--	4.1 (1.8 to 7.2)	-1.9 (-4.8 to 1.0)	-1.7 (-4.6 to 1.2)
Orofacial clefts	6.9 (5.6 to 8.6)	--	0.1 (0.1 to 0.1)	-9.0 (-13.9 to -3.9)	-8.8 (-13.7 to -3.7)
Down's syndrome	4.3 (3.6 to 5.1)	--	0.4 (0.2 to 0.5)	-0.7 (-4.7 to 3.2)	-0.5 (-4.5 to 3.4)
Turner syndrome	4.8 (4.1 to 5.5)	--	0.1 (< 0.05 to 0.1)	-5.8 (-17.3 to 7.7)	-5.6 (-17.2 to 7.9)
Klinefelter syndrome	6.2 (5.4 to 7.2)	--	< 0.05 (< 0.05 to < 0.05)	2.0 (-7.0 to 12.0)	2.2 (-6.8 to 12.2)

Cause	Prevalence in thousands (95% UI)	Incidence in thousands (95% UI)	YLDs in thousands (95% UI)		
	2016 counts	2016 counts	2016 counts	Percentage change in counts between 2006 and 2016	Percentage change in rates between 2006 and 2016
Other chromosomal abnormalities	18.4<br>(15.7 to 21.4)	--	3.7<br>(2.5 to 5.2)	-4.2<br>(-8.4 to 0.1)	-4.1<br>(-8.3 to 0.2)
Congenital musculoskeletal and limb anomalies	10.1<br>(9.1 to 10.9)	--	1.3<br>(0.9 to 1.8)	0.5<br>(-2.9 to 4.0)	0.7<br>(-2.7 to 4.1)
Urogenital congenital anomalies	22.5<br>(19.8 to 25.7)	--	0.6<br>(0.3 to 0.9)	-4.3<br>(-6.8 to -2.0)	-4.2<br>(-6.6 to -1.8)
Digestive congenital anomalies	10.0<br>(9.0 to 11.2)	--	0.5<br>(0.3 to 0.7)	-2.2<br>(-6.7 to 2.5)	-2.0<br>(-6.5 to 2.7)
Other congenital anomalies	37.6<br>(26.4 to 53.4)	--	7.6<br>(5.2 to 10.6)	-2.3<br>(-5.9 to 0.8)	-2.2<br>(-5.7 to 1.0)
Skin and subcutaneous diseases	158.5<br>(154.2 to 163.2)	4,109.8<br>(3,756.3 to 4,531.2)	2.8<br>(1.7 to 4.4)	-0.7<br>(-2.8 to 1.5)	-0.5<br>(-2.6 to 1.7)
Dermatitis	--	--	--	--	--
Psoriasis	--	--	--	--	--
Cellulitis	1.0<br>(0.9 to 1.2)	35.4<br>(30.4 to 40.8)	0.1<br>(< 0.05 to 0.1)	2.5<br>(0.4 to 4.9)	2.7<br>(0.6 to 5.1)
Pyoderma	22.7<br>(21.5 to 23.9)	820.1<br>(779.2 to 864.9)	0.1<br>(0.1 to 0.3)	11.4<br>(7.9 to 15.1)	11.6<br>(8.1 to 15.3)
Scabies	24.5<br>(18.2 to 32.0)	595.4<br>(441.1 to 779.6)	0.6<br>(0.3 to 1.1)	-6.9<br>(-9.6 to -4.1)	-6.7<br>(-9.4 to -3.9)
Fungal skin diseases	56.8<br>(46.3 to 69.9)	1,380.1<br>(1,119.5 to 1,705.6)	0.3<br>(0.1 to 0.7)	3.3<br>(1.3 to 5.3)	3.5<br>(1.5 to 5.5)
Viral skin diseases	--	--	--	--	--
Acne vulgaris	--	--	--	--	--
Alopecia areata	--	--	--	--	--
Pruritus	2.4<br>(1.8 to 3.1)	53.2<br>(40.1 to 69.2)	< 0.05<br>(< 0.05 to 0.1)	1.0<br>(-7.4 to 9.4)	1.1<br>(-7.2 to 9.6)
Urticaria	23.8<br>(18.3 to 30.2)	548.6<br>(421.9 to 696.1)	1.5<br>(0.9 to 2.2)	-0.4<br>(-4.1 to 3.3)	-0.2<br>(-3.9 to 3.5)
Decubitus ulcer	--	--	--	--	--
Other skin and subcutaneous diseases	30.4<br>(28.3 to 32.7)	677.0<br>(630.4 to 729.2)	0.2<br>(0.1 to 0.3)	4.4<br>(1.4 to 7.7)	4.5<br>(1.6 to 7.8)
Sense organ diseases	200.8<br>(192.6 to 207.3)	--	4.9<br>(3.1 to 7.4)	-6.7<br>(-9.1 to -4.3)	-6.5<br>(-8.9 to -4.1)
Glaucoma	--	--	--	--	--
Cataract	--	--	--	--	--
Macular degeneration	--	--	--	--	--
Refraction and accommodation disorders	170.6<br>(153.5 to 187.4)	--	2.1<br>(1.1 to 3.9)	-6.7<br>(-8.5 to -4.9)	-6.5<br>(-8.3 to -4.8)
Age-related and other hearing loss	11.3<br>(8.1 to 15.7)	--	1.5<br>(0.9 to 2.2)	-6.0<br>(-11.9 to -0.4)	-5.8<br>(-11.7 to -0.2)
Other vision loss	21.3<br>(12.8 to 33.4)	--	1.3<br>(0.7 to 2.2)	-7.4<br>(-12.8 to -2.5)	-7.3<br>(-12.6 to -2.3)
Other sense organ diseases	--	--	--	--	--
Oral disorders	--	--	--	--	--
Caries of deciduous teeth	--	--	--	--	--
Caries of permanent teeth	--	--	--	--	--
Periodontal disease	--	--	--	--	--
Edentulism and severe tooth loss	--	--	--	--	--
Other oral disorders	--	--	--	--	--
**Injuries**	**10.6<br>(9.2 to 12.0)**	**79.0†<br>(72.4 to 86.5)**	**0.7<br>(0.5 to 1.0)**	**-6.2<br>(-8.0 to -4.5)**	**-6.0<br>(-7.8 to -4.3)**
**Transport injuries**	**0.5<br>(0.4 to 0.7)**	**2.4<br>(1.8 to 3.1)**	**< 0.05<br>(< 0.05 to 0.1)**	**-4.0<br>(-6.8 to -1.3)**	**-3.8<br>(-6.6 to -1.1)**
Road injuries	< 0.05<br>(< 0.05 to 0.1)	0.2<br>(0.2 to 0.3)	< 0.05<br>(< 0.05 to < 0.05)	12.1<br>(6.1 to 17.3)	12.2<br>(6.3 to 17.5)
Pedestrian road injuries	< 0.05<br>(< 0.05 to < 0.05)	< 0.05<br>(< 0.05 to 0.1)	< 0.05<br>(< 0.05 to < 0.05)	9.5<br>(2.8 to 15.8)	9.7<br>(3.0 to 16.0)
Cyclist road injuries	--	--	--	--	--
Motorcyclist road injuries	< 0.05<br>(< 0.05 to < 0.05)	0.1<br>(< 0.05 to 0.1)	< 0.05<br>(< 0.05 to < 0.05)	13.3<br>(6.2 to 19.4)	13.5<br>(6.4 to 19.6)
Motor vehicle road injuries	< 0.05<br>(< 0.05 to < 0.05)	0.1<br>(0.1 to 0.2)	< 0.05<br>(< 0.05 to < 0.05)	9.6<br>(2.8 to 15.9)	9.8<br>(2.9 to 16.1)
Other road injuries	< 0.05<br>(< 0.05 to < 0.05)	< 0.05<br>(< 0.05 to < 0.05)	< 0.05<br>(< 0.05 to < 0.05)	28.7<br>(20.5 to 36.1)	28.9<br>(20.7 to 36.3)
Other transport injuries	0.5<br>(0.3 to 0.7)	2.1<br>(1.6 to 2.9)	< 0.05<br>(< 0.05 to 0.1)	-5.2<br>(-8.1 to -2.5)	-5.0<br>(-8.0 to -2.4)
**Unintentional injuries**	**9.9<br>(8.6 to 11.3)**	**75.7<br>(69.1 to 83.0)**	**0.7<br>(0.5 to 0.9)**	**-6.4<br>(-8.3 to -4.5)**	**-6.2<br>(-8.1 to -4.3)**
Falls	2.3<br>(1.7 to 3.1)	13.8<br>(10.4 to 17.6)	0.1<br>(0.1 to 0.2)	6.9<br>(3.4 to 9.8)	7.0<br>(3.6 to 10.0)
Drowning	< 0.05<br>(< 0.05 to < 0.05)	0.1<br>(0.1 to 0.1)	< 0.05<br>(< 0.05 to < 0.05)	-12.6<br>(-15.1 to -9.9)	-12.5<br>(-15.0 to -9.7)
Fire, heat, and hot substances	0.2<br>(0.1 to 0.3)	1.2<br>(0.9 to 1.5)	< 0.05<br>(< 0.05 to < 0.05)	-4.9<br>(-9.6 to -0.3)	-4.8<br>(-9.4 to -0.1)
Poisonings	< 0.05<br>(< 0.05 to < 0.05)	0.6<br>(0.4 to 0.8)	< 0.05<br>(< 0.05 to < 0.05)	5.8<br>(1.9 to 8.2)	6.0<br>(2.0 to 8.4)
Exposure to mechanical forces	1.3<br>(1.0 to 1.6)	7.8<br>(6.1 to 9.8)	0.1<br>(< 0.05 to 0.1)	-11.2<br>(-15.5 to -7.5)	-11.1<br>(-15.4 to -7.3)
Unintentional firearm injuries	< 0.05<br>(< 0.05 to < 0.05)	0.1<br>(< 0.05 to 0.1)	< 0.05<br>(< 0.05 to < 0.05)	-3.8<br>(-10.0 to 2.4)	-3.6<br>(-9.8 to 2.6)
Unintentional suffocation	0.3<br>(0.2 to 0.4)	1.5<br>(1.1 to 2.0)	< 0.05<br>(< 0.05 to < 0.05)	-19.3<br>(-24.8 to -14.6)	-19.2<br>(-24.6 to -14.4)
Other exposure to mechanical forces	1.0<br>(0.7 to 1.3)	6.2<br>(4.8 to 7.9)	< 0.05<br>(< 0.05 to 0.1)	-7.2<br>(-11.7 to -3.2)	-7.1<br>(-11.6 to -3.0)

Cause	Prevalence in thousands (95% UI)	Incidence in thousands (95% UI)	YLDs in thousands (95% UI)		
	2016 counts	2016 counts	2016 counts	Percentage change in counts between 2006 and 2016	Percentage change in rates between 2006 and 2016
Adverse effects of medical treatment	1.2<br>(0.9 to 1.5)	15.6<br>(12.9 to 18.7)	0.2<br>(0.1 to 0.2)	-12.6<br>(-16.9 to -8.7)	-12.4<br>(-16.7 to -8.5)
Animal contact	0.6<br>(0.4 to 0.8)	6.7<br>(5.2 to 8.4)	< 0.05<br>(< 0.05 to < 0.05)	-13.3<br>(-17.7 to -9.3)	-13.1<br>(-17.5 to -9.2)
Venomous animal contact	0.2<br>(0.1 to 0.3)	3.1<br>(2.3 to 4.0)	< 0.05<br>(< 0.05 to < 0.05)	-16.2<br>(-21.0 to -12.1)	-16.0<br>(-20.8 to -11.9)
Non-venomous animal contact	0.4<br>(0.3 to 0.5)	3.6<br>(2.8 to 4.8)	< 0.05<br>(< 0.05 to < 0.05)	-6.7<br>(-12.0 to -2.2)	-6.5<br>(-11.8 to -2.0)
Foreign body	1.3<br>(1.0 to 1.7)	11.8<br>(10.0 to 13.8)	0.1<br>(0.1 to 0.1)	-8.4<br>(-10.7 to -5.9)	-8.2<br>(-10.6 to -5.8)
Pulmonary aspiration and foreign body in airway	0.9<br>(0.6 to 1.3)	8.2<br>(6.7 to 10.1)	0.1<br>(< 0.05 to 0.1)	-10.2<br>(-13.0 to -7.3)	-10.0<br>(-12.8 to -7.2)
Foreign body in eyes	0.1<br>(< 0.05 to 0.2)	0.9<br>(0.5 to 1.5)	< 0.05<br>(< 0.05 to < 0.05)	4.5<br>(1.2 to 9.1)	4.7<br>(1.4 to 9.3)
Foreign body in other body part	0.3<br>(0.2 to 0.4)	2.7<br>(2.0 to 3.4)	< 0.05<br>(< 0.05 to < 0.05)	-4.3<br>(-8.1 to -0.9)	-4.2<br>(-8.0 to -0.7)
Environmental heat and cold exposure	0.8<br>(0.5 to 1.1)	3.7<br>(2.8 to 4.9)	0.1<br>(< 0.05 to 0.1)	-9.5<br>(-13.5 to -6.2)	-9.4<br>(-13.3 to -6.0)
Other unintentional injuries	2.2<br>(1.6 to 3.0)	14.5<br>(11.2 to 18.8)	0.1<br>(0.1 to 0.2)	-1.9<br>(-6.0 to 2.4)	-1.8<br>(-5.9 to 2.6)
**Self-harm and interpersonal violence**	**0.1<br>(0.1 to 0.2)**	**1.0†<br>(0.8 to 1.3)**	**< 0.05<br>(< 0.05 to < 0.05)**	**-6.3<br>(-11.5 to -1.2)**	**-6.1<br>(-11.3 to -1.0)**
Self-harm	--	--	--	--	--
Self-harm by firearm	--	--	--	--	--
Self-harm by other specified means	--	--	--	--	--
Interpersonal violence	0.1<br>(0.1 to 0.2)	1.0†<br>(0.8 to 1.3)	< 0.05<br>(< 0.05 to < 0.05)	-6.3<br>(-11.5 to -1.2)	-6.1<br>(-11.3 to -1.0)
Assault by firearm	< 0.05<br>(< 0.05 to < 0.05)	< 0.05<br>(< 0.05 to 0.1)	< 0.05<br>(< 0.05 to < 0.05)	-2.2<br>(-9.1 to 4.4)	-2.0<br>(-9.0 to 4.6)
Assault by sharp object	< 0.05<br>(< 0.05 to < 0.05)	0.2<br>(0.1 to 0.3)	< 0.05<br>(< 0.05 to < 0.05)	-1.1<br>(-7.6 to 6.0)	-0.9<br>(-7.5 to 6.2)
Sexual violence	--	--	--	--	--
Assault by other means	0.1<br>(0.1 to 0.1)	0.7<br>(0.6 to 1.0)	< 0.05<br>(< 0.05 to < 0.05)	-8.4<br>(-13.8 to -3.4)	-8.3<br>(-13.7 to -3.3)
**Forces of nature, conflict and terrorism, and executions and police conflict**	**--**	**--**	**--**	**--**	**--**
Forces of nature, conflict and terrorism, and state actor violence	--	--	--	--	--
Conflict and terrorism	--	--	--	--	--
Executions and police conflict	--	--	--	--	--

We did not calculate incidence for certain NTDs and causes with only birth prevalence. Blank cells mean that no estimate is available.
† Incidence for aggregate causes represents the sum of the incidence of the causes below the aggregate.
‡ Incidence estimates for stroke represent first-ever stroke only.
^ Incidence of HIV/AIDS represents new infections of HIV only and does not include new infections of Tuberculosis in HIV positive cases.

SR Table 12: Global prevalence, incidence, and YLDs for 2016, percentage change of YLD counts and percentage change of YLD rates between 2006 and 2016 by cause, age 28-364 days.					
**Cause**	**Prevalence in thousands (95% UI)**	**Incidence in thousands (95% UI)**	**YLDs in thousands (95% UI)**		
	**2016 counts**	**2016 counts**	**2016 counts**	**Percentage change in counts between 2006 and 2016**	**Percentage change in rates between 2006 and 2016**
**All causes**	**92,760.0 (91,394.5 to 95,294.4)**	**1,012,306.0† (847,811.2 to 1,206,168.4)**	**5,169.0 (3,672.0 to 7,071.8)**	**-3.5 (-5.2 to -1.5)**	**-4.2 (-5.9 to -2.3)**
**Communicable, maternal, neonatal, and nutritional disorders**	**78,794.9 (76,901.4 to 81,851.2)**	**923,246.7† (759,626.1 to 1,114,964.5)**	**3,884.5 (2,736.9 to 5,310.2)**	**-4.4 (-6.6 to -1.8)**	**-5.2 (-7.4 to -2.6)**
**HIV/AIDS and tuberculosis**	**13,256.5 (12,166.7 to 14,563.8)**	**196.6† (178.3 to 216.2)**	**23.0 (16.1 to 30.5)**	**-25.3 (-30.7 to -19.8)**	**-25.9 (-31.2 to -20.5)**
Tuberculosis	12,656.5 (11,122.1 to 14,428.7)	61.4† (45.9 to 78.6)	5.2 (3.0 to 8.2)	-21.5 (-31.3 to -7.4)	-22.1 (-31.9 to -8.2)
Drug-susceptible tuberculosis	15.7 (10.9 to 21.3)	59.8 (44.7 to 76.5)	5.1 (2.9 to 8.0)	-21.4 (-31.5 to -7.0)	-22.1 (-32.1 to -7.7)
Multidrug-resistant tuberculosis without extensive drug resistance	0.4 (0.3 to 0.6)	1.6 (1.2 to 2.1)	0.1 (0.1 to 0.2)	-25.1 (-37.5 to -9.8)	-25.7 (-38.0 to -10.5)
Extensively drug-resistant tuberculosis	< 0.05 (< 0.05 to < 0.05)	0.1 (< 0.05 to 0.1)	< 0.05 (< 0.05 to < 0.05)	124.9 (92.3 to 161.5)	123.0 (90.7 to 159.4)
Latent tuberculosis infection	12,640.3 (11,105.7 to 14,414.3)	--	--	--	--
HIV/AIDS	154.1 (144.7 to 163.7)	135.2^ (125.5 to 145.5)	17.8 (12.7 to 23.2)	-26.3 (-32.7 to -19.8)	-26.9 (-33.3 to -20.4)
Drug-susceptible HIV/AIDS - Tuberculosis	4.0 (2.8 to 5.6)	6.0 (4.1 to 8.3)	1.7 (1.0 to 2.6)	-55.1 (-60.1 to -50.3)	-55.5 (-60.4 to -50.7)
Multidrug-resistant HIV/AIDS - Tuberculosis without extensive drug resistance	0.1 (0.1 to 0.2)	0.2 (0.1 to 0.2)	< 0.05 (< 0.05 to 0.1)	-53.8 (-64.3 to -42.1)	-54.2 (-64.5 to -42.6)
Extensively drug-resistant HIV/AIDS - Tuberculosis	< 0.05 (< 0.05 to < 0.05)	< 0.05 (< 0.05 to < 0.05)	< 0.05 (< 0.05 to < 0.05)	87.9 (53.9 to 129.1)	86.3 (52.6 to 127.2)
HIV/AIDS resulting in other diseases	149.9 (140.3 to 159.9)	135.2 (125.5 to 145.5)	16.1 (11.5 to 21.1)	-20.9 (-28.5 to -13.1)	-21.6 (-29.1 to -13.8)
**Diarrhoea, lower respiratory infections, and other common infectious diseases**	**12,469.1 (12,050.5 to 12,855.9)**	**755,863.2† (655,131.7 to 867,814.9)**	**745.1 (503.2 to 1,013.1)**	**-8.5 (-10.9 to -6.6)**	**-9.2 (-11.6 to -7.4)**
Diarrhoeal diseases	4,108.9 (3,578.2 to 4,659.0)	265,772.2 (216,916.4 to 314,371.2)	474.8 (320.6 to 660.0)	-9.4 (-12.4 to -6.9)	-10.2 (-13.1 to -7.6)
Intestinal infectious diseases	0.7 (0.5 to 0.9)	13.3† (10.4 to 17.0)	0.6 (0.4 to 0.9)	-42.5 (-56.1 to -24.2)	-42.9 (-56.4 to -24.9)
Typhoid fever	0.7 (0.5 to 0.9)	10.3 (8.1 to 13.5)	0.1 (0.1 to 0.1)	-16.9 (-37.9 to 11.8)	-17.6 (-38.4 to 10.9)
Paratyphoid fever	0.1 (0.1 to 0.2)	2.9 (2.2 to 3.9)	< 0.05 (< 0.05 to < 0.05)	-14.5 (-35.6 to 12.3)	-15.2 (-36.1 to 11.4)
Other intestinal infectious diseases	--	--	0.5 (0.3 to 0.7)	-46.3 (-59.0 to -29.3)	-46.8 (-59.3 to -29.9)
Lower respiratory infections	346.0 (300.8 to 398.4)	12,795.4 (10,190.7 to 15,702.7)	21.4 (13.8 to 30.5)	-5.0 (-9.1 to -0.9)	-5.7 (-9.8 to -1.7)
Upper respiratory infections	5,294.0 (4,291.1 to 6,525.0)	412,143.6 (325,900.4 to 516,654.0)	134.2 (77.1 to 213.7)	2.8 (1.0 to 4.4)	2.0 (0.1 to 3.5)
Otitis media	2,178.3 (1,723.3 to 2,746.1)	37,005.3 (25,448.5 to 52,515.9)	57.6 (35.6 to 87.0)	-2.3 (-5.4 to 0.8)	-3.1 (-6.2 to -0.0)
Meningitis	108.3 (93.7 to 127.1)	673.3 (542.9 to 841.8)	12.4 (8.5 to 17.1)	-0.7 (-5.6 to 4.9)	-1.5 (-6.4 to 4.0)
Pneumococcal meningitis	25.1 (20.3 to 30.7)	113.1 (74.0 to 159.5)	2.8 (1.8 to 3.9)	-14.9 (-21.5 to -8.1)	-15.6 (-22.2 to -8.9)
H influenzae type B meningitis	15.8 (12.2 to 20.5)	126.1 (80.0 to 184.5)	1.9 (1.2 to 2.6)	-33.4 (-40.4 to -26.8)	-33.9 (-40.9 to -27.4)
Meningococcal infection	13.9 (10.2 to 18.7)	146.9 (100.5 to 210.1)	1.5 (1.0 to 2.2)	8.6 (-4.3 to 23.8)	7.7 (-5.1 to 22.8)
Other meningitis	53.4 (43.8 to 65.7)	287.3 (212.3 to 372.0)	6.2 (4.1 to 9.1)	23.9 (13.7 to 34.3)	22.9 (12.8 to 33.2)
Encephalitis	19.2 (15.5 to 24.0)	204.6 (165.6 to 246.2)	2.3 (1.6 to 3.3)	-6.7 (-12.9 to -0.7)	-7.5 (-13.6 to -1.5)
Diphtheria	0.1 (< 0.05 to 0.1)	0.8 (0.5 to 1.2)	< 0.05 (< 0.05 to < 0.05)	-62.5 (-77.0 to -35.4)	-62.8 (-77.2 to -35.9)
Whooping cough	694.6 (541.7 to 872.3)	5,070.9 (3,954.7 to 6,367.6)	34.5 (20.5 to 54.2)	-24.3 (-26.6 to -21.9)	-25.0 (-27.2 to -22.5)
Tetanus	3.2 (3.1 to 3.4)	4.8 (2.5 to 7.5)	0.1 (0.1 to 0.2)	-42.4 (-56.6 to -25.7)	-42.9 (-57.0 to -26.3)
Measles	55.7 (20.2 to 120.7)	2,032.8 (739.0 to 4,403.9)	5.0 (1.6 to 11.5)	-64.2 (-69.0 to -59.0)	-64.5 (-69.2 to -59.4)
Varicella and herpes zoster	387.4 (286.2 to 510.6)	20,146.3 (14,881.8 to 26,588.5)	2.3 (0.9 to 4.9)	-2.0 (-4.7 to 0.4)	-2.8 (-5.5 to -0.4)
**Neglected tropical diseases and malaria**	**22,919.4 (22,206.5 to 23,649.2)**	**19,559.0† (13,547.4 to 26,650.9)**	**238.2 (160.1 to 347.6)**	**-7.4 (-11.3 to -3.1)**	**-8.2 (-12.0 to -3.9)**
Malaria	2,188.1 (1,638.5 to 2,980.8)	17,389.2 (11,496.5 to 24,441.6)	46.0 (28.0 to 70.5)	-28.4 (-38.9 to -18.7)	-29.0 (-39.4 to -19.4)
Chagas disease	7.5 (5.9 to 9.5)	3.8 (2.5 to 5.3)	< 0.05 (< 0.05 to < 0.05)	-26.1 (-32.5 to -21.0)	-26.8 (-33.1 to -21.7)
Leishmaniasis	5.5 (4.4 to 6.8)	15.0 (11.3 to 19.8)	0.3 (0.2 to 0.5)	-45.1 (-60.0 to -24.0)	-45.6 (-60.4 to -24.6)
Visceral leishmaniasis	3.1 (2.2 to 4.3)	12.2 (8.7 to 17.1)	0.2 (0.1 to 0.3)	-54.1 (-69.0 to -31.8)	-54.5 (-69.3 to -32.4)
Cutaneous and mucocutaneous leishmaniasis	1.6 (1.1 to 2.2)	2.8 (2.0 to 3.9)	0.1 (0.1 to 0.2)	-8.6 (-36.7 to 36.3)	-9.3 (-37.2 to 35.1)
African trypanosomiasis	--	--	--	--	--
Schistosomiasis	0.3 (0.2 to 0.3)	605.7 (605.6 to 605.7)	< 0.05 (< 0.05 to < 0.05)	8.8 (3.0 to 14.9)	7.9 (2.1 to 13.9)
Cysticercosis	--	--	--	--	--
Cystic echinococcosis	--	--	--	--	--
Lymphatic filariasis	215.8 (191.1 to 242.6)	42.0 (26.4 to 64.5)	--	--	--
Onchocerciasis	--	--	--	--	--
Trachoma	--	--	--	--	--
Dengue	85.6 (46.6 to 145.0)	1,430.4 (870.3 to 2,170.0)	14.3 (6.3 to 26.6)	43.7 (16.1 to 225.4)	42.6 (15.1 to 222.7)
Yellow fever	< 0.05 (< 0.05 to 0.1)	1.1 (0.3 to 2.9)	< 0.05 (< 0.05 to < 0.05)	-19.0 (-47.3 to 26.8)	-19.6 (-47.7 to 25.7)
Rabies	< 0.05 (< 0.05 to < 0.05)	0.2 (0.1 to 0.3)	< 0.05 (< 0.05 to < 0.05)	-65.8 (-75.9 to -50.0)	-66.1 (-76.1 to -50.4)
Intestinal nematode infections	18,288.1 (17,476.1 to 19,094.8)	--	43.5 (26.3 to 68.1)	-19.0 (-24.7 to -12.2)	-19.6 (-25.3 to -13.0)
Ascariasis	9,071.7 (8,281.6 to 9,932.5)	--	15.0 (8.6 to 24.6)	-28.2 (-37.0 to -18.1)	-28.8 (-37.5 to -18.7)

Cause	Prevalence in thousands (95% UI)	Incidence in thousands (95% UI)	YLDs in thousands (95% UI)		
	2016 counts	2016 counts	2016 counts	Percentage change in counts between 2006 and 2016	Percentage change in rates between 2006 and 2016
Trichuriasis	5,591.2 (5,163.6 to 6,051.8)	--	3.7 (2.1 to 6.3)	-27.4 (-39.1 to -14.9)	-27.9 (-39.6 to -15.6)
Hookworm disease	4,881.2 (4,486.4 to 5,339.6)	--	24.7 (15.3 to 37.5)	-10.4 (-18.9 to -0.7)	-11.1 (-19.5 to -1.5)
Food-borne trematodiases	--	--	--	--	--
Leprosy	--	--	--	--	--
Ebola virus disease	< 0.05 (< 0.05 to 0.3)	--	< 0.05 (< 0.05 to 0.1)	--	--
Zika virus disease	3.4 (2.4 to 5.5)	71.6 (41.4 to 125.4)	1.3 (0.8 to 2.3)	--	--
Guinea worm disease	--	--	--	--	--
Other neglected tropical diseases	3,755.9 (3,414.9 to 4,134.5)	--	132.7 (86.1 to 194.5)	3.1 (0.1 to 5.9)	2.2 (-0.7 to 5.1)
**Maternal disorders**	**--**	**--**	**--**	**--**	**--**
Maternal haemorrhage	--	--	--	--	--
Maternal sepsis and other pregnancy related infections	--	--	--	--	--
Maternal hypertensive disorders	--	--	--	--	--
Maternal obstructed labour and uterine rupture	--	--	--	--	--
Maternal abortion, miscarriage, and ectopic pregnancy	--	--	--	--	--
Other maternal disorders	--	--	--	--	--
**Neonatal disorders**	**9,285.0 (8,679.0 to 9,831.2)**	**--**	**597.0 (403.7 to 892.4)**	**1.0 (-8.1 to 11.5)**	**0.2 (-8.8 to 10.6)**
Neonatal preterm birth complications	7,966.0 (6,806.4 to 9,233.6)	--	225.0 (143.6 to 335.8)	-5.1 (-22.3 to 15.7)	-5.9 (-23.0 to 14.8)
Neonatal encephalopathy due to birth asphyxia and trauma	888.1 (628.0 to 1,210.5)	--	70.4 (34.3 to 134.0)	7.3 (-0.6 to 18.1)	6.4 (-1.4 to 17.1)
Neonatal sepsis and other neonatal infections	443.1 (252.9 to 832.9)	--	155.6 (67.6 to 356.0)	5.6 (0.4 to 11.9)	4.7 (-0.5 to 11.0)
Hemolytic disease and other neonatal jaundice	69.8 (60.0 to 80.6)	--	16.8 (11.8 to 22.1)	4.1 (-2.8 to 12.0)	3.2 (-3.6 to 11.0)
Other neonatal disorders	--	--	129.2 (87.4 to 193.2)	3.5 (-5.8 to 14.2)	2.6 (-6.6 to 13.3)
**Nutritional deficiencies**	**47,366.9 (45,211.2 to 49,554.5)**	**14,881.2† (14,548.5 to 15,245.8)**	**2,170.1 (1,455.0 to 3,098.9)**	**-3.8 (-6.7 to -1.0)**	**-4.6 (-7.4 to -1.8)**
Protein-energy malnutrition	14,050.9 (13,723.0 to 14,402.1)	14,881.2 (14,548.5 to 15,245.8)	693.5 (444.1 to 977.7)	-10.8 (-16.1 to -5.4)	-11.6 (-16.8 to -6.2)
Iodine deficiency	--	--	--	--	--
Vitamin A deficiency	--	--	--	--	--
Iron-deficiency anaemia	42,772.2 (38,533.5 to 47,596.6)	--	1,455.7 (928.2 to 2,125.9)	0.1 (-2.4 to 2.6)	-0.7 (-3.2 to 1.7)
Other nutritional deficiencies	--	--	21.0 (13.4 to 29.6)	-18.2 (-23.1 to -13.3)	-18.9 (-23.7 to -14.0)
**Other communicable, maternal, neonatal, and nutritional diseases**	**20,353.1 (12,095.1 to 29,554.6)**	**132,746.7 (21,826.7 to 301,248.0)**	**111.1 (72.2 to 160.5)**	**-2.6 (-10.6 to 5.3)**	**-3.4 (-11.3 to 4.4)**
Sexually transmitted diseases excluding HIV	--	--	--	--	--
Syphilis	--	--	--	--	--
Chlamydial infection	--	--	--	--	--
Gonococcal infection	--	--	--	--	--
Trichomoniasis	--	--	--	--	--
Genital herpes	--	--	--	--	--
Other sexually transmitted diseases	--	--	--	--	--
Hepatitis	14,853.2 (5,592.2 to 28,206.0)	132,746.5 (21,826.6 to 301,248.0)	15.1 (7.9 to 26.7)	-48.9 (-59.5 to -35.0)	-49.4 (-59.8 to -35.6)
Acute hepatitis A	8,895.5 (455.1 to 21,920.7)	115,641.6 (5,916.0 to 284,969.5)	1.5 (0.1 to 4.3)	-53.2 (-97.7 to 641.3)	-53.6 (-97.7 to 635.2)
Hepatitis B	5,600.3 (3,645.0 to 8,216.7)	16,015.3 (9,077.1 to 25,289.5)	12.6 (6.2 to 22.8)	-50.6 (-56.6 to -43.6)	-51.0 (-57.0 to -44.1)
Hepatitis C	357.3 (304.1 to 417.2)	1,089.6 (921.0 to 1,280.0)	1.0 (0.5 to 2.0)	18.2 (5.3 to 33.5)	17.3 (4.5 to 32.4)
Acute hepatitis E	--	--	--	--	--
Other infectious diseases	2,694.5 (2,427.9 to 2,997.6)	0.1 (0.1 to 0.2)	96.0 (61.3 to 140.1)	13.7 (10.4 to 17.2)	12.7 (9.5 to 16.2)
**Non-communicable diseases**	**47,439.0 (47,060.6 to 47,892.6)**	**68,997.6† (62,014.6 to 76,482.2)**	**1,115.6 (794.0 to 1,510.1)**	**0.1 (-1.2 to 1.7)**	**-0.7 (-2.0 to 0.8)**
**Neoplasms**	**24.5 (21.7 to 27.0)**	**12.7 (9.8 to 14.7)**	**5.6 (3.5 to 8.0)**	**41.2 (9.4 to 66.1)**	**40.0 (8.5 to 64.7)**
Lip and oral cavity cancer	--	--	--	--	--
Nasopharynx cancer	--	--	--	--	--
Other pharynx cancer	--	--	--	--	--
Oesophageal cancer	--	--	--	--	--
Stomach cancer	--	--	--	--	--
Colon and rectum cancer	--	--	--	--	--
Liver cancer	--	--	--	--	--
Liver cancer due to hepatitis B	--	--	--	--	--

Cause	Prevalence in thousands (95% UI)	Incidence in thousands (95% UI)	YLDs in thousands (95% UI)		
	2016 counts	2016 counts	2016 counts	Percentage change in counts between 2006 and 2016	Percentage change in rates between 2006 and 2016
Liver cancer due to hepatitis C	--	--	--	--	--
Liver cancer due to alcohol use	--	--	--	--	--
Liver cancer due to other causes	--	--	--	--	--
Gallbladder and biliary tract cancer	--	--	--	--	--
Pancreatic cancer	--	--	--	--	--
Larynx cancer	--	--	--	--	--
Tracheal, bronchus, and lung cancer	--	--	--	--	--
Malignant skin melanoma	--	--	--	--	--
Non-melanoma skin cancer	--	--	--	--	--
Non-melanoma skin cancer (squamous-cell carcinoma)	--	--	--	--	--
Non-melanoma skin cancer (basal-cell carcinoma)	--	--	--	--	--
Breast cancer	--	--	--	--	--
Cervical cancer	--	--	--	--	--
Uterine cancer	--	--	--	--	--
Ovarian cancer	--	--	--	--	--
Prostate cancer	--	--	--	--	--
Testicular cancer	--	--	--	--	--
Kidney cancer	--	--	--	--	--
Bladder cancer	--	--	--	--	--
Brain and nervous system cancer	--	--	--	--	--
Thyroid cancer	--	--	--	--	--
Mesothelioma	--	--	--	--	--
Hodgkin lymphoma	0.4<br>(0.3 to 0.5)	0.2<br>(0.1 to 0.2)	0.1<br>(0.1 to 0.1)	7.1<br>(-9.3 to 27.5)	6.2<br>(-10.0 to 26.4)
Non-Hodgkin's lymphoma	--	--	--	--	--
Multiple myeloma	--	--	--	--	--
Leukaemia	15.6<br>(9.9 to 20.1)	6.8<br>(4.4 to 8.7)	3.6<br>(2.0 to 5.5)	47.3<br>(1.9 to 83.3)	46.1<br>(1.0 to 81.8)
Acute lymphoid leukaemia	6.6<br>(4.9 to 8.1)	3.0<br>(2.2 to 3.6)	1.3<br>(0.8 to 1.9)	71.0<br>(24.4 to 104.9)	69.6<br>(23.4 to 103.2)
Chronic lymphoid leukaemia	--	--	--	--	--
Acute myeloid leukaemia	1.1<br>(0.8 to 1.5)	1.1<br>(0.8 to 1.4)	0.3<br>(0.2 to 0.5)	38.0<br>(2.0 to 68.2)	36.9<br>(1.2 to 66.8)
Chronic myeloid leukaemia	--	--	--	--	--
Other leukaemia	7.9<br>(4.2 to 10.7)	2.8<br>(1.4 to 3.8)	2.0<br>(0.9 to 3.1)	36.2<br>(-13.1 to 83.0)	35.1<br>(-13.8 to 81.4)
Other neoplasms	12.7<br>(11.4 to 13.8)	5.7<br>(5.1 to 6.1)	1.9<br>(1.3 to 2.5)	32.4<br>(7.4 to 47.5)	31.3<br>(6.5 to 46.3)
**Cardiovascular diseases**	**1.6<br>(1.3 to 1.9)**	**24.8†<br>(20.1 to 30.9)**	**0.1<br>(0.1 to 0.1)**	**-4.0<br>(-6.8 to -1.2)**	**-4.8<br>(-7.6 to -2.0)**
Rheumatic heart disease	--	--	--	--	--
Ischaemic heart disease	--	--	--	--	--
Cerebrovascular disease	--	--	--	--	--
Ischaemic stroke	--	--	--	--	--
Hemorrhagic stroke	--	--	--	--	--
Hypertensive heart disease	--	--	--	--	--
Cardiomyopathy and myocarditis	--	--	--	--	--
Myocarditis	--	--	--	--	--
Alcoholic cardiomyopathy	--	--	--	--	--
Other cardiomyopathy	--	--	--	--	--
Atrial fibrillation and flutter	--	--	--	--	--
Peripheral vascular disease	--	--	--	--	--
Endocarditis	1.6<br>(1.3 to 1.9)	24.8<br>(20.1 to 30.9)	0.1<br>(0.1 to 0.1)	-4.0<br>(-6.8 to -1.2)	-4.8<br>(-7.6 to -2.0)
Other cardiovascular and circulatory diseases	--	--	--	--	--
**Chronic respiratory diseases**	**--**	**--**	**--**	**--**	**--**
Chronic obstructive pulmonary disease	--	--	--	--	--
Pneumoconiosis	--	--	--	--	--
Silicosis	--	--	--	--	--

Cause	Prevalence in thousands (95% UI)	Incidence in thousands (95% UI)	YLDs in thousands (95% UI)		
	2016 counts	2016 counts	2016 counts	Percentage change in counts between 2006 and 2016	Percentage change in rates between 2006 and 2016
Asbestosis	--	--	--	--	--
Coal workers pneumoconiosis	--	--	--	--	--
Other pneumoconiosis	--	--	--	--	--
Asthma	--	--	--	--	--
Interstitial lung disease and pulmonary sarcoidosis	--	--	--	--	--
Other chronic respiratory diseases	--	--	--	--	--
**Cirrhosis and other chronic liver diseases**	**33.0 (30.2 to 36.1)**	**50.6 (46.2 to 55.2)**	**5.5 (3.6 to 7.9)**	**2.8 (-6.5 to 13.2)**	**2.0 (-7.2 to 12.3)**
Cirrhosis and other chronic liver diseases due to hepatitis B	< 0.05 (< 0.05 to 0.1)	0.1 (< 0.05 to 0.1)	< 0.05 (< 0.05 to < 0.05)	8.1 (3.8 to 12.2)	7.2 (2.9 to 11.3)
Cirrhosis and other chronic liver diseases due to hepatitis C	< 0.05 (< 0.05 to < 0.05)	0.1 (< 0.05 to 0.1)	< 0.05 (< 0.05 to < 0.05)	5.4 (1.4 to 9.2)	4.6 (0.6 to 8.3)
Cirrhosis and other chronic liver diseases due to alcohol use	--	--	--	--	--
Cirrhosis and other chronic liver diseases due to other causes	33.0 (30.1 to 36.1)	50.5 (46.1 to 55.1)	5.5 (3.6 to 7.9)	2.8 (-6.5 to 13.2)	2.0 (-7.2 to 12.3)
**Digestive diseases**	**2.4 (1.9 to 3.0)**	**59.0† (45.0 to 76.7)**	**0.8 (0.5 to 1.1)**	**-3.2 (-5.8 to -0.5)**	**-4.0 (-6.5 to -1.3)**
Peptic ulcer disease	--	--	--	--	--
Gastritis and duodenitis	--	--	--	--	--
Appendicitis	--	--	--	--	--
Paralytic ileus and intestinal obstruction	2.4 (1.9 to 3.0)	59.0 (45.0 to 76.7)	0.8 (0.5 to 1.1)	-3.2 (-5.8 to -0.5)	-4.0 (-6.5 to -1.3)
Inguinal, femoral, and abdominal hernia	--	--	--	--	--
Inflammatory bowel disease	--	--	--	--	--
Vascular intestinal disorders	--	--	--	--	--
Gallbladder and biliary diseases	--	--	--	--	--
Pancreatitis	--	--	--	--	--
Other digestive diseases	--	--	--	--	--
**Neurological disorders**	**144.0 (115.6 to 167.7)**	**225.8 (172.5 to 288.6)**	**64.8 (41.7 to 92.6)**	**7.9 (-6.0 to 24.9)**	**7.1 (-6.7 to 23.9)**
Alzheimer's disease and other dementias	--	--	--	--	--
Parkinson's disease	--	--	--	--	--
Epilepsy	118.8 (91.8 to 150.9)	223.6 (170.3 to 286.3)	41.4 (26.7 to 59.2)	-2.5 (-15.1 to 12.9)	-3.3 (-15.8 to 12.0)
Multiple sclerosis	--	--	--	--	--
Motor neuron disease	1.0 (0.8 to 1.2)	2.2 (1.9 to 2.8)	0.2 (0.1 to 0.3)	-2.4 (-4.4 to -0.2)	-3.2 (-5.1 to -1.0)
Migraine	--	--	--	--	--
Tension-type headache	--	--	--	--	--
Other neurological disorders	--	--	23.2 (14.9 to 33.1)	33.7 (16.4 to 54.9)	32.6 (15.5 to 53.6)
**Mental and substance use disorders**	**1,022.6 (880.7 to 1,132.1)**	**47.6† (31.3 to 70.2)**	**108.4 (69.3 to 161.8)**	**-1.0 (-3.1 to 1.4)**	**-1.8 (-4.0 to 0.6)**
Schizophrenia	--	--	--	--	--
Alcohol use disorders	4.6 (3.8 to 5.3)	--	0.2 (0.1 to 0.2)	2.6 (-3.2 to 7.9)	1.8 (-4.0 to 7.1)
Drug use disorders	--	--	--	--	--
Opioid use disorders	--	--	--	--	--
Cocaine use disorders	--	--	--	--	--
Amphetamine use disorders	--	--	--	--	--
Cannabis use disorders	--	--	--	--	--
Other drug use disorders	--	--	--	--	--
Depressive disorders	--	--	--	--	--
Major depressive disorder	--	--	--	--	--
Dysthymia	--	--	--	--	--
Bipolar disorder	--	--	--	--	--
Anxiety disorders	--	--	--	--	--
Eating disorders	--	--	--	--	--
Anorexia nervosa	--	--	--	--	--
Bulimia nervosa	--	--	--	--	--
Autistic spectrum disorders	709.7 (642.0 to 805.5)	47.6 (31.3 to 70.2)	94.0 (59.2 to 137.2)	0.8 (-1.1 to 2.8)	0.0 (-1.9 to 2.0)
Autism	139.9 (94.8 to 191.8)	7.2 (3.3 to 12.4)	36.9 (20.7 to 58.8)	0.9 (-3.1 to 4.7)	0.1 (-3.9 to 3.9)
Asperger syndrome and other autistic spectrum disorders	558.7 (392.0 to 758.8)	40.5 (25.1 to 62.8)	57.1 (33.3 to 90.0)	0.8 (-1.4 to 2.9)	-0.1 (-2.2 to 2.1)

Cause	Prevalence in thousands (95% UI)	Incidence in thousands (95% UI)	YLDs in thousands (95% UI)		
	2016 counts	2016 counts	2016 counts	Percentage change in counts between 2006 and 2016	Percentage change in rates between 2006 and 2016
Attention-deficit/hyperactivity disorder	--	--	--	--	--
Conduct disorder	--	--	--	--	--
Idiopathic developmental intellectual disability	335.3 (115.6 to 615.5)	--	14.2 (4.1 to 30.5)	-11.3 (-16.2 to -7.1)	-12.1 (-16.9 to -7.9)
Other mental and substance use disorders	--	--	--	--	--
**Diabetes, urogenital, blood, and endocrine diseases**	**33,654.0 (33,262.1 to 34,074.8)**	**5,095.8† (4,268.6 to 6,015.2)**	**309.2 (199.4 to 450.4)**	**-0.0 (-2.8 to 3.1)**	**-0.8 (-3.6 to 2.3)**
Diabetes mellitus	--	--	--	--	--
Acute glomerulonephritis	0.4 (0.3 to 0.6)	7.2 (5.0 to 9.8)	< 0.05 (< 0.05 to < 0.05)	-5.5 (-8.0 to -3.5)	-6.3 (-8.7 to -4.3)
Chronic kidney disease	4.1 (3.1 to 5.4)	11.0 (8.2 to 14.7)	0.8 (0.5 to 1.3)	-1.6 (-7.2 to 3.6)	-2.4 (-7.9 to 2.8)
Chronic kidney disease due to diabetes mellitus	0.1 (0.1 to 0.2)	0.3 (0.2 to 0.5)	< 0.05 (< 0.05 to < 0.05)	-1.1 (-8.2 to 6.1)	-1.9 (-8.9 to 5.2)
Chronic kidney disease due to hypertension	0.5 (0.3 to 0.7)	4.5 (3.1 to 6.2)	< 0.05 (< 0.05 to < 0.05)	-4.6 (-10.7 to 1.4)	-5.4 (-11.4 to 0.6)
Chronic kidney disease due to glomerulonephritis	1.2 (0.8 to 1.7)	0.1 (< 0.05 to 0.1)	0.3 (0.2 to 0.5)	-0.8 (-6.5 to 4.6)	-1.6 (-7.3 to 3.8)
Chronic kidney disease due to other causes	2.3 (1.6 to 3.1)	6.1 (4.3 to 8.5)	0.5 (0.2 to 0.7)	-2.2 (-7.6 to 2.9)	-3.0 (-8.3 to 2.0)
Urinary diseases and male infertility	102.1 (91.7 to 114.4)	4,777.8† (3,945.9 to 5,706.7)	4.0 (2.3 to 6.3)	-14.8 (-20.7 to -8.3)	-15.5 (-21.4 to -9.1)
Interstitial nephritis and urinary tract infections	91.9 (75.0 to 110.5)	4,777.8 (3,945.9 to 5,706.7)	3.1 (1.8 to 4.9)	-2.7 (-9.5 to 4.7)	-3.5 (-10.2 to 3.9)
Urolithiasis	--	--	--	--	--
Benign prostatic hyperplasia	--	--	--	--	--
Male infertility	--	--	--	--	--
Other urinary diseases	--	--	0.9 (0.5 to 1.4)	-39.9 (-44.0 to -35.2)	-40.3 (-44.5 to -35.8)
Gynecological diseases	--	--	--	--	--
Uterine fibroids	--	--	--	--	--
Polycystic ovarian syndrome	--	--	--	--	--
Female infertility	--	--	--	--	--
Endometriosis	--	--	--	--	--
Genital prolapse	--	--	--	--	--
Premenstrual syndrome	--	--	--	--	--
Other gynecological diseases	--	--	--	--	--
Hemoglobinopathies and hemolytic anaemias	32,129.2 (31,725.5 to 32,564.9)	--	232.5 (148.9 to 340.4)	0.1 (-3.0 to 3.7)	-0.7 (-3.8 to 2.9)
Thalassemias	71.8 (66.5 to 78.0)	--	4.9 (3.2 to 7.1)	-8.9 (-13.8 to -4.1)	-9.6 (-14.5 to -4.9)
Thalassaemias trait	3,766.3 (3,561.9 to 4,042.7)	--	91.0 (56.0 to 136.4)	-2.4 (-7.7 to 4.3)	-3.2 (-8.4 to 3.5)
Sickle cell disorders	323.8 (279.6 to 428.5)	--	21.9 (14.0 to 32.7)	8.2 (1.9 to 14.4)	7.3 (1.1 to 13.5)
Sickle cell trait	9,739.3 (8,908.5 to 10,833.5)	--	64.1 (41.3 to 95.8)	7.0 (3.2 to 10.6)	6.1 (2.4 to 9.7)
G6PD deficiency	6,359.2 (6,096.8 to 6,673.4)	--	1.0 (0.6 to 1.4)	-5.9 (-14.7 to 2.2)	-6.7 (-15.4 to 1.4)
G6PD trait	14,714.1 (14,464.4 to 14,999.9)	--	< 0.05 (< 0.05 to < 0.05)	3.0 (-1.4 to 7.8)	2.2 (-2.2 to 6.9)
Other hemoglobinopathies and hemolytic anaemias	1,558.2 (1,410.0 to 1,728.8)	--	49.7 (31.8 to 72.8)	-5.3 (-8.6 to -2.2)	-6.1 (-9.3 to -3.0)
Endocrine, metabolic, blood, and immune disorders	2,114.2 (1,920.3 to 2,337.0)	299.9 (251.8 to 357.7)	71.8 (46.5 to 104.8)	0.5 (-2.3 to 3.3)	-0.3 (-3.1 to 2.4)
**Musculoskeletal disorders**	**--**	**--**	**--**	**--**	**--**
Rheumatoid arthritis	--	--	--	--	--
Osteoarthritis	--	--	--	--	--
Low back and neck pain	--	--	--	--	--
Low back pain	--	--	--	--	--
Neck pain	--	--	--	--	--
Gout	--	--	--	--	--
Other musculoskeletal disorders	--	--	--	--	--
**Other non-communicable diseases**	**18,698.7 (18,303.2 to 19,110.6)**	**63,481.2† (56,559.2 to 70,841.2)**	**621.3 (436.7 to 841.6)**	**-0.6 (-1.7 to 0.4)**	**-1.4 (-2.5 to -0.4)**
Congenital anomalies	2,714.6 (2,618.0 to 2,824.6)	--	286.2 (208.0 to 378.2)	-0.4 (-2.4 to 1.6)	-1.2 (-3.1 to 0.8)
Neural tube defects	128.7 (116.2 to 142.5)	--	37.8 (26.4 to 51.1)	5.3 (0.2 to 10.3)	4.4 (-0.6 to 9.4)
Congenital heart anomalies	1,129.7 (921.9 to 1,373.8)	--	47.1 (21.4 to 79.8)	-1.6 (-4.4 to 1.4)	-2.4 (-5.2 to 0.6)
Orofacial clefts	105.5 (85.4 to 129.1)	--	1.5 (0.9 to 2.2)	-7.6 (-12.6 to -2.8)	-8.3 (-13.3 to -3.6)
Down's syndrome	57.1 (48.6 to 67.1)	--	4.9 (3.3 to 7.0)	1.4 (-2.1 to 5.0)	0.6 (-2.9 to 4.2)
Turner syndrome	74.0 (63.7 to 84.9)	--	1.0 (0.4 to 1.8)	-4.1 (-15.3 to 9.0)	-4.9 (-16.0 to 8.1)
Klinefelter syndrome	97.2 (83.6 to 112.6)	--	0.3 (0.1 to 0.5)	2.5 (-7.0 to 11.7)	1.6 (-7.8 to 10.8)

Cause	Prevalence in thousands (95% UI)	Incidence in thousands (95% UI)	YLDs in thousands (95% UI)		
	2016 counts	2016 counts	2016 counts	Percentage change in counts between 2006 and 2016	Percentage change in rates between 2006 and 2016
Other chromosomal abnormalities	268.5 (231.7 to 310.7)	--	52.8 (36.0 to 73.1)	-1.9 (-6.0 to 2.5)	-2.7 (-6.8 to 1.7)
Congenital musculoskeletal and limb anomalies	155.1 (141.4 to 168.6)	--	20.3 (13.8 to 28.0)	1.4 (-2.0 to 4.5)	0.6 (-2.8 to 3.6)
Urogenital congenital anomalies	342.3 (301.0 to 389.7)	--	9.1 (5.3 to 14.5)	-3.3 (-5.7 to -1.0)	-4.1 (-6.5 to -1.8)
Digestive congenital anomalies	144.8 (129.8 to 160.7)	--	7.1 (4.6 to 10.1)	-1.1 (-6.2 to 4.1)	-1.9 (-6.9 to 3.3)
Other congenital anomalies	598.1 (420.9 to 849.7)	--	104.3 (71.4 to 146.1)	-0.9 (-4.8 to 3.0)	-1.7 (-5.6 to 2.2)
Skin and subcutaneous diseases	13,547.5 (13,110.4 to 13,953.1)	63,481.2 (56,559.2 to 70,841.2)	265.4 (163.8 to 400.7)	0.2 (-0.8 to 1.2)	-0.6 (-1.7 to 0.4)
Dermatitis	--	--	--	--	--
Psoriasis	--	--	--	--	--
Cellulitis	34.3 (28.9 to 39.9)	639.6 (543.1 to 756.3)	2.0 (1.3 to 3.0)	4.0 (-1.2 to 9.1)	3.1 (-2.0 to 8.2)
Pyoderma	573.1 (541.0 to 606.0)	11,564.4 (10,846.6 to 12,346.0)	3.3 (1.3 to 6.9)	12.7 (10.0 to 15.2)	11.8 (9.1 to 14.2)
Scabies	2,111.6 (1,566.1 to 2,733.3)	9,522.9 (6,998.1 to 12,493.6)	55.2 (28.4 to 93.5)	-5.7 (-7.2 to -4.2)	-6.5 (-7.9 to -5.0)
Fungal skin diseases	5,418.8 (4,289.8 to 6,799.1)	24,872.2 (19,107.9 to 31,567.1)	31.1 (12.3 to 67.2)	4.9 (3.9 to 5.8)	4.0 (3.0 to 4.9)
Viral skin diseases	--	--	--	--	--
Acne vulgaris	--	--	--	--	--
Alopecia areata	--	--	--	--	--
Pruritus	333.7 (255.5 to 429.5)	802.7 (612.4 to 1,042.3)	3.6 (1.7 to 7.1)	1.8 (-0.8 to 4.6)	0.9 (-1.6 to 3.7)
Urticaria	2,460.7 (1,946.1 to 3,065.3)	7,891.8 (6,115.2 to 9,806.8)	151.1 (92.5 to 222.4)	0.7 (-0.7 to 2.0)	-0.2 (-1.5 to 1.2)
Decubitus ulcer	--	--	--	--	--
Other skin and subcutaneous diseases	3,364.7 (3,162.7 to 3,574.6)	8,187.7 (7,621.6 to 8,724.2)	19.0 (8.9 to 34.9)	5.5 (4.3 to 6.9)	4.6 (3.4 to 6.1)
Sense organ diseases	3,251.4 (3,097.3 to 3,399.6)	--	69.7 (43.6 to 106.9)	-4.6 (-7.2 to -1.9)	-5.4 (-8.0 to -2.6)
Glaucoma	--	--	--	--	--
Cataract	--	--	--	--	--
Macular degeneration	--	--	--	--	--
Refraction and accommodation disorders	3,008.3 (2,728.1 to 3,299.8)	--	43.6 (24.5 to 76.0)	-5.8 (-7.5 to -4.2)	-6.6 (-8.3 to -5.0)
Age-related and other hearing loss	146.0 (82.3 to 210.3)	--	21.0 (10.2 to 34.3)	-1.9 (-10.0 to 6.4)	-2.7 (-10.7 to 5.5)
Other vision loss	92.7 (62.5 to 131.3)	--	5.0 (2.9 to 8.2)	-5.0 (-11.6 to 1.5)	-5.8 (-12.4 to 0.6)
Other sense organ diseases	--	--	--	--	--
Oral disorders	--	--	--	--	--
Caries of deciduous teeth	--	--	--	--	--
Caries of permanent teeth	--	--	--	--	--
Periodontal disease	--	--	--	--	--
Edentulism and severe tooth loss	--	--	--	--	--
Other oral disorders	--	--	--	--	--
**Injuries**	**2,359.8 (2,115.7 to 2,636.0)**	**20,061.7† (18,382.1 to 21,966.6)**	**169.0 (116.3 to 233.6)**	**-4.0 (-5.8 to -2.2)**	**-4.8 (-6.5 to -3.0)**
**Transport injuries**	**108.5 (84.6 to 138.4)**	**599.2 (464.9 to 787.5)**	**9.9 (6.4 to 14.8)**	**-1.5 (-4.6 to 1.6)**	**-2.3 (-5.4 to 0.8)**
Road injuries	9.8 (7.3 to 12.9)	60.6 (41.4 to 85.3)	0.8 (0.5 to 1.3)	14.2 (7.8 to 19.7)	13.2 (6.9 to 18.7)
Pedestrian road injuries	1.5 (1.0 to 2.1)	8.4 (4.9 to 12.9)	0.1 (0.1 to 0.2)	11.4 (4.4 to 18.5)	10.5 (3.5 to 17.5)
Cyclist road injuries	--	--	--	--	--
Motorcyclist road injuries	3.0 (2.0 to 4.1)	15.7 (9.6 to 23.5)	0.2 (0.1 to 0.4)	15.4 (8.4 to 21.8)	14.4 (7.5 to 20.8)
Motor vehicle road injuries	4.2 (2.9 to 5.9)	28.8 (18.5 to 42.1)	0.4 (0.2 to 0.6)	11.8 (4.6 to 18.5)	10.8 (3.7 to 17.5)
Other road injuries	1.1 (0.7 to 1.7)	7.6 (4.3 to 12.5)	0.1 (< 0.05 to 0.1)	31.2 (22.9 to 39.6)	30.2 (21.9 to 38.4)
Other transport injuries	98.6 (75.3 to 128.9)	538.6 (407.2 to 732.4)	9.1 (5.8 to 13.6)	-2.7 (-6.2 to 0.6)	-3.5 (-6.9 to -0.3)
**Unintentional injuries**	**2,219.7 (1,981.9 to 2,500.2)**	**19,211.8 (17,529.8 to 21,074.5)**	**156.6 (107.1 to 216.9)**	**-4.2 (-6.1 to -2.3)**	**-5.0 (-6.8 to -3.1)**
Falls	522.5 (402.5 to 660.0)	3,501.9 (2,649.5 to 4,466.1)	27.1 (17.0 to 40.4)	9.3 (5.5 to 12.5)	8.4 (4.7 to 11.6)
Drowning	3.7 (2.7 to 5.3)	24.1 (19.6 to 29.9)	0.3 (0.2 to 0.5)	-11.3 (-13.9 to -8.3)	-12.1 (-14.6 to -9.1)
Fire, heat, and hot substances	46.3 (35.8 to 59.0)	292.6 (225.4 to 374.4)	4.7 (2.9 to 7.1)	-2.3 (-7.2 to 2.6)	-3.1 (-7.9 to 1.7)
Poisonings	4.5 (3.3 to 6.3)	152.1 (111.4 to 198.1)	0.6 (0.3 to 0.9)	6.5 (-0.2 to 13.4)	5.6 (-1.0 to 12.5)
Exposure to mechanical forces	271.0 (211.5 to 338.2)	1,978.6 (1,535.7 to 2,492.2)	11.3 (7.5 to 17.1)	-8.7 (-13.3 to -4.5)	-9.4 (-14.0 to -5.3)
Unintentional firearm injuries	3.3 (2.3 to 4.9)	19.7 (12.2 to 29.8)	0.3 (0.1 to 0.5)	-1.5 (-8.6 to 5.4)	-2.3 (-9.3 to 4.5)
Unintentional suffocation	59.8 (43.7 to 77.9)	392.7 (286.0 to 518.0)	3.5 (2.2 to 5.5)	-17.0 (-22.6 to -11.8)	-17.7 (-23.2 to -12.6)
Other exposure to mechanical forces	207.9 (159.8 to 269.0)	1,566.3 (1,200.8 to 1,993.3)	7.5 (4.9 to 11.3)	-4.5 (-9.7 to -0.1)	-5.3 (-10.4 to -0.9)

Cause	Prevalence in thousands (95% UI)	Incidence in thousands (95% UI)	YLDs in thousands (95% UI)		
	2016 counts	2016 counts	2016 counts	Percentage change in counts between 2006 and 2016	Percentage change in rates between 2006 and 2016
Adverse effects of medical treatment	302.1 (230.5 to 384.7)	3,958.2 (3,279.2 to 4,755.5)	40.3 (24.8 to 62.5)	-10.8 (-15.2 to -6.8)	-11.6 (-15.8 to -7.6)
Animal contact	139.0 (104.1 to 188.8)	1,705.4 (1,314.6 to 2,129.9)	7.4 (4.6 to 11.0)	-11.1 (-15.7 to -6.6)	-11.8 (-16.4 to -7.4)
Venomous animal contact	47.4 (35.1 to 65.2)	777.9 (581.6 to 1,019.7)	4.8 (3.0 to 7.0)	-14.1 (-19.2 to -9.4)	-14.8 (-19.8 to -10.1)
Non-venomous animal contact	91.7 (63.5 to 129.0)	927.5 (709.0 to 1,223.2)	2.6 (1.5 to 4.1)	-4.9 (-10.3 to -0.2)	-5.7 (-11.1 to -1.0)
Foreign body	272.1 (214.5 to 333.1)	2,983.8 (2,537.3 to 3,496.4)	22.7 (15.3 to 31.9)	-5.6 (-8.2 to -3.0)	-6.4 (-8.9 to -3.8)
Pulmonary aspiration and foreign body in airway	177.3 (133.4 to 223.6)	2,079.4 (1,693.5 to 2,560.8)	16.9 (11.0 to 23.9)	-7.7 (-10.8 to -4.4)	-8.4 (-11.5 to -5.1)
Foreign body in eyes	30.8 (9.5 to 62.9)	227.9 (129.5 to 389.2)	1.7 (0.5 to 3.7)	6.2 (2.8 to 10.9)	5.3 (2.0 to 10.0)
Foreign body in other body part	64.0 (47.8 to 83.0)	676.5 (508.3 to 871.6)	4.1 (2.6 to 6.1)	-1.3 (-5.3 to 2.4)	-2.1 (-6.0 to 1.5)
Environmental heat and cold exposure	160.8 (120.7 to 218.9)	949.6 (709.1 to 1,242.9)	11.6 (7.4 to 17.2)	-6.9 (-11.3 to -3.3)	-7.7 (-12.0 to -4.1)
Other unintentional injuries	497.5 (388.8 to 641.2)	3,665.6 (2,837.5 to 4,760.6)	30.6 (19.7 to 45.2)	0.2 (-4.4 to 4.8)	-0.6 (-5.2 to 3.9)
**Self-harm and interpersonal violence**	**31.7 (24.1 to 40.9)**	**250.7† (189.7 to 329.4)**	**2.5 (1.6 to 3.7)**	**-4.2 (-9.7 to 1.3)**	**-5.0 (-10.5 to 0.5)**
Self-harm	--	--	--	--	--
Self-harm by firearm	--	--	--	--	--
Self-harm by other specified means	--	--	--	--	--
Interpersonal violence	31.7 (24.1 to 40.9)	250.7† (189.7 to 329.4)	2.5 (1.6 to 3.7)	-4.2 (-9.7 to 1.3)	-5.0 (-10.5 to 0.5)
Assault by firearm	1.5 (1.0 to 2.2)	9.7 (5.6 to 15.3)	0.2 (0.1 to 0.3)	-0.5 (-7.9 to 6.6)	-1.4 (-8.7 to 5.8)
Assault by sharp object	7.4 (5.2 to 10.1)	55.0 (35.5 to 80.5)	0.6 (0.4 to 1.0)	0.8 (-6.1 to 8.4)	-0.0 (-6.9 to 7.5)
Sexual violence	--	--	--	--	--
Assault by other means	22.7 (17.0 to 30.3)	186.1 (138.8 to 241.3)	1.7 (1.0 to 2.5)	-6.4 (-11.9 to -1.0)	-7.1 (-12.6 to -1.8)
**Forces of nature, conflict and terrorism, and executions and police conflict**	**--**	**--**	**--**	**--**	**--**
Forces of nature, conflict and terrorism, and state actor violence	--	--	--	--	--
Conflict and terrorism	--	--	--	--	--
Executions and police conflict	--	--	--	--	--

We did not calculate incidence for certain NTDs and causes with only birth prevalence. Blank cells mean that no estimate is available.
† Incidence for aggregate causes represents the sum of the incidence of the causes below the aggregate.
‡ Incidence estimates for stroke represent first-ever stroke only.
^ Incidence of HIV/AIDS represents new infections of HIV only and does not include new infections of Tuberculosis in HIV positive cases.

SR Table 12: Global prevalence, incidence, and YLDs for 2016, percentage change of YLD counts and percentage change of YLD rates between 2006 and 2016 by cause, age 1-4 years.					
Cause	Prevalence in thousands (95% UI)	Incidence in thousands (95% UI)	YLDs in thousands (95% UI)		
	2016 counts	2016 counts	2016 counts	Percentage change in counts between 2006 and 2016	Percentage change in rates between 2006 and 2016
**All causes**	**452,157.7 (449,874.8 to 454,742.4)**	**4,099,632.5† (3,566,556.6 to 4,692,859.3)**	**23,797.6 (16,715.9 to 32,421.5)**	**4.7 (2.9 to 6.7)**	**-0.9 (-2.6 to 1.0)**
**Communicable, maternal, neonatal, and nutritional disorders**	**324,063.5 (320,029.4 to 327,725.9)**	**3,033,738.2† (2,583,621.0 to 3,559,977.8)**	**13,543.3 (9,403.3 to 18,447.0)**	**3.1 (0.4 to 6.1)**	**-2.4 (-4.9 to 0.5)**
**HIV/AIDS and tuberculosis**	**58,197.5 (51,158.3 to 66,353.8)**	**219.3† (152.6 to 307.2)**	**110.0 (76.4 to 150.2)**	**-26.4 (-30.9 to -21.1)**	**-30.3 (-34.6 to -25.3)**
Tuberculosis	54,835.5 (45,312.1 to 65,686.4)	219.3† (152.6 to 307.2)	39.0 (21.5 to 63.3)	-31.4 (-38.0 to -23.4)	-35.0 (-41.3 to -27.5)
Drug-susceptible tuberculosis	117.7 (77.8 to 170.1)	213.3 (148.4 to 299.7)	37.9 (20.8 to 61.6)	-31.4 (-38.4 to -23.0)	-35.1 (-41.6 to -27.1)
Multidrug-resistant tuberculosis without extensive drug resistance	3.2 (2.2 to 4.8)	5.8 (3.9 to 8.2)	1.1 (0.6 to 1.8)	-31.6 (-42.2 to -18.6)	-35.2 (-45.2 to -22.9)
Extensively drug-resistant tuberculosis	0.1 (0.1 to 0.2)	0.2 (0.1 to 0.3)	< 0.05 (< 0.05 to 0.1)	121.8 (91.9 to 153.7)	110.1 (81.8 to 140.3)
Latent tuberculosis infection	54,714.5 (45,236.8 to 65,571.1)	--	--	--	--
HIV/AIDS	630.3 (593.7 to 669.2)	--	71.0 (51.0 to 92.9)	-23.4 (-29.5 to -16.8)	-27.4 (-33.2 to -21.2)
Drug-susceptible HIV/AIDS - Tuberculosis	17.0 (11.7 to 23.4)	24.7 (17.1 to 34.2)	6.9 (4.0 to 11.0)	-52.8 (-57.7 to -47.6)	-55.3 (-60.0 to -50.4)
Multidrug-resistant HIV/AIDS - Tuberculosis without extensive drug resistance	0.5 (0.3 to 0.7)	0.7 (0.4 to 1.0)	0.2 (0.1 to 0.3)	-51.6 (-62.3 to -39.4)	-54.1 (-64.3 to -42.6)
Extensively drug-resistant HIV/AIDS - Tuberculosis	< 0.05 (< 0.05 to < 0.05)	< 0.05 (< 0.05 to < 0.05)	< 0.05 (< 0.05 to < 0.05)	103.3 (66.3 to 149.0)	92.6 (57.6 to 135.9)
HIV/AIDS resulting in other diseases	612.9 (573.7 to 652.8)	--	63.9 (45.5 to 83.8)	-17.7 (-25.2 to -9.7)	-22.0 (-29.2 to -14.4)
**Diarrhoea, lower respiratory infections, and other common infectious diseases**	**53,556.1 (51,373.7 to 55,884.1)**	**2,792,326.5† (2,343,495.3 to 3,317,938.9)**	**2,889.0 (1,971.4 to 3,969.1)**	**-1.1 (-3.0 to 0.7)**	**-6.3 (-8.1 to -4.6)**
Diarrhoeal diseases	12,929.2 (11,041.7 to 15,232.1)	813,444.9 (685,933.6 to 958,579.5)	1,491.4 (1,000.1 to 2,084.0)	-2.8 (-5.6 to -0.1)	-7.9 (-10.6 to -5.4)
Intestinal infectious diseases	99.2 (74.4 to 137.2)	1,798.6† (1,209.5 to 2,617.5)	15.0 (8.6 to 24.2)	-26.5 (-41.2 to -8.1)	-30.4 (-44.3 to -12.9)
Typhoid fever	89.7 (57.6 to 135.3)	1,421.2 (940.5 to 2,078.9)	13.6 (7.7 to 22.0)	-25.7 (-41.4 to -5.4)	-29.6 (-44.5 to -10.3)
Paratyphoid fever	16.6 (10.6 to 25.3)	377.4 (242.5 to 554.4)	1.0 (0.6 to 1.7)	-22.7 (-39.2 to -1.6)	-26.8 (-42.4 to -6.8)
Other intestinal infectious diseases	--	--	0.4 (0.2 to 0.6)	-52.1 (-61.6 to -40.1)	-54.6 (-63.7 to -43.2)
Lower respiratory infections	1,294.9 (1,055.2 to 1,568.5)	54,236.6 (43,084.4 to 67,283.2)	80.2 (50.6 to 121.0)	-7.1 (-11.9 to -2.4)	-12.0 (-16.5 to -7.5)
Upper respiratory infections	22,324.5 (17,279.4 to 28,255.4)	1,694,630.9 (1,282,770.6 to 2,188,180.1)	565.3 (320.1 to 935.5)	7.3 (5.1 to 9.1)	1.6 (-0.5 to 3.4)
Otitis media	17,698.8 (14,151.6 to 22,212.4)	173,838.8 (116,385.0 to 253,242.7)	551.4 (341.3 to 837.9)	2.7 (-0.0 to 5.4)	-2.7 (-5.3 to -0.2)
Meningitis	820.7 (711.3 to 962.3)	753.0 (565.1 to 1,032.8)	95.3 (67.3 to 127.4)	4.0 (-0.1 to 8.2)	-1.5 (-5.4 to 2.5)
Pneumococcal meningitis	276.4 (230.5 to 335.3)	147.9 (92.2 to 226.6)	32.9 (22.2 to 44.9)	-2.2 (-9.5 to 6.1)	-7.4 (-14.3 to 0.5)
H influenzae type B meningitis	138.6 (110.7 to 173.6)	134.6 (80.7 to 209.6)	18.0 (12.5 to 24.1)	-21.1 (-27.5 to -14.3)	-25.3 (-31.4 to -18.9)
Meningococcal infection	100.2 (72.9 to 133.7)	155.7 (97.4 to 233.7)	10.7 (7.4 to 14.7)	12.6 (8.0 to 17.2)	6.7 (2.3 to 11.0)
Other meningitis	305.4 (237.8 to 389.5)	314.8 (215.2 to 451.3)	33.7 (23.1 to 47.0)	31.2 (22.7 to 39.9)	24.2 (16.2 to 32.5)
Encephalitis	252.5 (190.6 to 341.2)	727.4 (552.8 to 926.0)	30.5 (21.0 to 40.9)	-3.2 (-8.3 to 2.0)	-8.3 (-13.2 to -3.4)
Diphtheria	0.1 (0.1 to 0.2)	1.4 (0.8 to 2.3)	< 0.05 (< 0.05 to < 0.05)	-65.8 (-83.1 to -29.7)	-67.6 (-84.0 to -33.4)
Whooping cough	781.7 (609.7 to 982.0)	5,706.3 (4,450.6 to 7,168.8)	38.7 (22.9 to 61.4)	-20.9 (-24.6 to -17.1)	-25.1 (-28.6 to -21.5)
Tetanus	12.8 (12.6 to 13.0)	3.4 (1.6 to 6.3)	0.3 (0.1 to 0.5)	-12.0 (-36.8 to 19.3)	-16.7 (-40.1 to 13.0)
Measles	152.2 (55.2 to 330.1)	5,555.1 (2,016.7 to 12,048.5)	13.7 (4.5 to 31.7)	-62.1 (-67.2 to -56.5)	-64.1 (-68.9 to -58.8)
Varicella and herpes zoster	839.6 (648.2 to 1,031.2)	41,630.2 (31,721.9 to 51,673.9)	7.2 (3.7 to 13.6)	4.3 (0.8 to 7.5)	-1.2 (-4.6 to 1.9)
**Neglected tropical diseases and malaria**	**104,290.6 (100,575.6 to 107,788.6)**	**74,749.9† (52,278.1 to 100,568.8)**	**955.9 (630.6 to 1,413.0)**	**-7.0 (-11.7 to -2.6)**	**-12.0 (-16.3 to -7.8)**
Malaria	19,410.3 (13,686.3 to 25,193.2)	64,064.0 (42,138.4 to 89,747.0)	276.3 (179.2 to 414.0)	-19.9 (-30.9 to -10.8)	-24.1 (-34.5 to -15.5)
Chagas disease	73.7 (56.9 to 90.9)	16.4 (11.5 to 21.9)	< 0.05 (< 0.05 to < 0.05)	-23.2 (-28.3 to -19.2)	-27.3 (-32.1 to -23.5)
Leishmaniasis	84.4 (71.6 to 98.6)	69.9 (52.8 to 89.2)	4.9 (3.2 to 7.4)	7.1 (-13.7 to 32.6)	1.4 (-18.3 to 25.6)
Visceral leishmaniasis	3.9 (2.7 to 5.6)	15.8 (11.0 to 22.5)	0.3 (0.1 to 0.5)	-61.9 (-75.9 to -39.7)	-63.9 (-77.1 to -42.9)
Cutaneous and mucocutaneous leishmaniasis	70.9 (56.1 to 88.2)	54.1 (38.4 to 73.4)	4.7 (3.0 to 7.0)	19.9 (-4.0 to 49.4)	13.6 (-9.1 to 41.5)
African trypanosomiasis	0.3 (< 0.05 to 1.0)	0.2 (< 0.05 to 0.7)	0.1 (< 0.05 to 0.3)	-75.0 (-96.6 to 30.4)	-76.3 (-96.8 to 23.5)
Schistosomiasis	279.8 (231.4 to 330.8)	4,511.8 (4,417.8 to 4,629.2)	2.0 (1.0 to 3.8)	-12.1 (-18.9 to -5.4)	-16.7 (-23.2 to -10.4)
Cysticercosis	--	--	--	--	--
Cystic echinococcosis	49.4 (24.4 to 106.0)	10.3 (4.4 to 27.8)	4.7 (2.0 to 11.9)	-39.8 (-79.8 to 80.5)	-42.9 (-80.9 to 71.0)
Lymphatic filariasis	1,069.5 (948.8 to 1,204.9)	310.9 (178.1 to 477.9)	--	--	--
Onchocerciasis	--	--	--	--	--
Trachoma	--	--	--	--	--
Dengue	311.2 (169.5 to 527.2)	5,201.9 (3,164.0 to 7,892.3)	51.8 (23.0 to 97.7)	51.3 (21.7 to 245.8)	43.3 (15.3 to 227.5)
Yellow fever	0.3 (0.1 to 0.8)	10.6 (3.0 to 28.0)	< 0.05 (< 0.05 to < 0.05)	-16.8 (-48.4 to 27.2)	-21.2 (-51.2 to 20.4)
Rabies	0.1 (< 0.05 to 0.1)	1.5 (0.7 to 2.7)	< 0.05 (< 0.05 to < 0.05)	-43.9 (-64.7 to -6.5)	-46.9 (-66.5 to -11.5)
Intestinal nematode infections	78,323.5 (74,543.7 to 81,997.9)	--	168.9 (101.6 to 270.6)	-15.4 (-21.8 to -8.4)	-19.9 (-25.9 to -13.3)
Ascariasis	39,190.1 (35,663.1 to 43,027.3)	--	62.6 (35.8 to 104.4)	-24.1 (-33.7 to -13.0)	-28.1 (-37.2 to -17.6)

Cause	Prevalence in thousands (95% UI)	Incidence in thousands (95% UI)	YLDs in thousands (95% UI)		
	2016 counts	2016 counts	2016 counts	Percentage change in counts between 2006 and 2016	Percentage change in rates between 2006 and 2016
Trichuriasis	23,744.6<br>(21,903.4 to 25,696.2)	--	15.4<br>(8.7 to 26.0)	-25.1<br>(-37.5 to -11.7)	-29.0<br>(-40.8 to -16.3)
Hookworm disease	20,717.9<br>(18,923.1 to 22,788.2)	--	90.8<br>(56.0 to 143.8)	-5.9<br>(-14.4 to 4.4)	-10.9<br>(-18.9 to -1.1)
Food-borne trematodiases	658.2<br>(580.8 to 749.1)	210.3<br>(183.0 to 245.8)	5.5<br>(3.0 to 8.5)	-5.1<br>(-18.4 to 9.8)	-10.1<br>(-22.7 to 4.0)
Leprosy	0.3<br>(0.2 to 0.4)	0.1<br>(0.1 to 0.2)	< 0.05<br>(< 0.05 to < 0.05)	-24.2<br>(-25.7 to -22.5)	-28.2<br>(-29.7 to -26.6)
Ebola virus disease	0.1<br>(< 0.05 to 0.4)	--	< 0.05<br>(< 0.05 to 0.1)	--	--
Zika virus disease	6.1<br>(3.7 to 10.4)	341.9<br>(200.1 to 604.6)	0.4<br>(0.2 to 0.6)	--	--
Guinea worm disease	< 0.05<br>(< 0.05 to < 0.05)	< 0.05<br>(< 0.05 to < 0.05)	< 0.05<br>(< 0.05 to < 0.05)	-99.6<br>(-99.7 to -99.4)	-99.6<br>(-99.7 to -99.4)
Other neglected tropical diseases	13,518.1<br>(12,450.5 to 14,909.0)	--	441.2<br>(276.8 to 659.7)	3.0<br>(-1.5 to 7.5)	-2.5<br>(-6.7 to 1.8)
**Maternal disorders**	**--**	**--**	**--**	**--**	**--**
Maternal haemorrhage	--	--	--	--	--
Maternal sepsis and other pregnancy related infections	--	--	--	--	--
Maternal hypertensive disorders	--	--	--	--	--
Maternal obstructed labour and uterine rupture	--	--	--	--	--
Maternal abortion, miscarriage, and ectopic pregnancy	--	--	--	--	--
Other maternal disorders	--	--	--	--	--
**Neonatal disorders**	**6,784.7<br>(6,150.4 to 7,366.2)**	**--**	**1,692.3<br>(1,148.9 to 2,540.9)**	**11.6<br>(-0.1 to 24.5)**	**5.7<br>(-5.4 to 17.9)**
Neonatal preterm birth complications	5,099.5<br>(4,154.2 to 6,261.9)	--	755.8<br>(502.5 to 1,096.9)	3.9<br>(-13.4 to 25.2)	-1.6<br>(-17.9 to 18.6)
Neonatal encephalopathy due to birth asphyxia and trauma	1,113.2<br>(635.1 to 1,953.4)	--	146.8<br>(41.0 to 368.8)	31.7<br>(16.5 to 61.3)	24.8<br>(10.4 to 52.8)
Neonatal sepsis and other neonatal infections	792.3<br>(324.8 to 1,826.3)	--	363.9<br>(128.1 to 881.6)	25.9<br>(16.9 to 39.4)	19.3<br>(10.7 to 32.1)
Hemolytic disease and other neonatal jaundice	215.6<br>(190.3 to 241.8)	--	58.1<br>(43.4 to 74.7)	10.6<br>(4.0 to 18.5)	4.8<br>(-1.5 to 12.3)
Other neonatal disorders	--	--	367.7<br>(249.6 to 552.1)	9.3<br>(-2.2 to 21.9)	3.5<br>(-7.4 to 15.5)
**Nutritional deficiencies**	**217,236.7<br>(211,132.0 to 222,265.3)**	**132,588.6†<br>(106,691.8 to 161,107.7)**	**7,560.4<br>(5,018.6 to 10,658.3)**	**4.9<br>(1.4 to 8.7)**	**-0.7<br>(-4.0 to 3.0)**
Protein-energy malnutrition	47,741.0<br>(46,817.4 to 48,730.3)	27,109.0<br>(26,610.8 to 27,673.2)	2,437.4<br>(1,577.2 to 3,420.4)	15.9<br>(9.0 to 23.3)	9.8<br>(3.3 to 16.8)
Iodine deficiency	568.3<br>(496.1 to 654.1)	336.6<br>(293.6 to 385.2)	14.2<br>(9.0 to 20.9)	-23.7<br>(-29.4 to -17.8)	-27.7<br>(-33.1 to -22.2)
Vitamin A deficiency	84,267.0<br>(66,349.2 to 103,250.0)	105,142.9<br>(79,406.0 to 133,429.4)	59.6<br>(33.9 to 97.2)	4.6<br>(-0.6 to 9.9)	-0.9<br>(-5.8 to 4.1)
Iron-deficiency anaemia	154,787.1<br>(141,563.8 to 172,446.3)	--	4,961.5<br>(3,095.6 to 7,427.5)	0.1<br>(-4.3 to 4.7)	-5.2<br>(-9.3 to -0.8)
Other nutritional deficiencies	--	--	87.7<br>(56.7 to 123.0)	17.7<br>(10.7 to 25.2)	11.5<br>(4.9 to 18.6)
**Other communicable, maternal, neonatal, and nutritional diseases**	**35,869.2<br>(31,821.6 to 40,542.0)**	**33,854.0<br>(12,112.2 to 68,462.9)**	**335.7<br>(210.2 to 506.7)**	**7.2<br>(-3.5 to 16.7)**	**1.5<br>(-8.6 to 10.6)**
Sexually transmitted diseases excluding HIV	--	--	--	--	--
Syphilis	--	--	--	--	--
Chlamydial infection	--	--	--	--	--
Gonococcal infection	--	--	--	--	--
Trichomoniasis	--	--	--	--	--
Genital herpes	--	--	--	--	--
Other sexually transmitted diseases	--	--	--	--	--
Hepatitis	26,303.9<br>(18,637.4 to 36,264.8)	33,853.4<br>(12,111.4 to 68,462.1)	14.6<br>(5.8 to 30.3)	-49.5<br>(-82.0 to 53.4)	-52.2<br>(-83.0 to 45.3)
Acute hepatitis A	1,681.0<br>(87.7 to 4,376.5)	21,853.4<br>(1,140.0 to 56,894.3)	7.1<br>(0.3 to 21.0)	-54.6<br>(-98.2 to 974.2)	-57.0<br>(-98.3 to 917.5)
Hepatitis B	19,222.5<br>(11,930.4 to 28,941.7)	7,197.3<br>(4,146.9 to 11,675.8)	4.7<br>(2.2 to 8.8)	-55.4<br>(-64.8 to -42.8)	-57.7<br>(-66.6 to -45.8)
Hepatitis C	5,246.2<br>(4,434.4 to 6,199.7)	2,799.4<br>(2,383.7 to 3,295.9)	2.6<br>(1.2 to 5.0)	20.7<br>(5.0 to 39.3)	14.3<br>(-0.5 to 31.9)
Acute hepatitis E	154.1<br>(125.9 to 181.8)	2,003.4<br>(1,636.1 to 2,363.2)	0.3<br>(0.2 to 0.5)	-60.1<br>(-75.5 to -32.9)	-62.2<br>(-76.8 to -36.5)
Other infectious diseases	9,746.1<br>(8,917.5 to 10,849.7)	0.6<br>(0.3 to 1.0)	321.0<br>(200.0 to 481.8)	13.0<br>(8.1 to 18.7)	7.0<br>(2.4 to 12.4)
**Non-communicable diseases**	**372,186.9<br>(366,430.7 to 379,566.8)**	**1,013,306.7†<br>(748,363.8 to 1,328,734.6)**	**9,513.8<br>(6,609.1 to 13,014.4)**	**6.6<br>(5.2 to 8.1)**	**0.9<br>(-0.3 to 2.4)**
**Neoplasms**	**320.7<br>(305.2 to 337.6)**	**74.8<br>(65.3 to 80.7)**	**28.4<br>(18.8 to 40.0)**	**24.2<br>(2.6 to 41.9)**	**17.6<br>(-2.8 to 34.4)**
Lip and oral cavity cancer	--	--	--	--	--
Nasopharynx cancer	--	--	--	--	--
Other pharynx cancer	--	--	--	--	--
Oesophageal cancer	--	--	--	--	--
Stomach cancer	--	--	--	--	--
Colon and rectum cancer	--	--	--	--	--
Liver cancer	--	--	--	--	--
Liver cancer due to hepatitis B	--	--	--	--	--

Cause	Prevalence in thousands (95% UI)	Incidence in thousands (95% UI)	YLDs in thousands (95% UI)		
	2016 counts	2016 counts	2016 counts	Percentage change in counts between 2006 and 2016	Percentage change in rates between 2006 and 2016
Liver cancer due to hepatitis C	--	--	--	--	--
Liver cancer due to alcohol use	--	--	--	--	--
Liver cancer due to other causes	--	--	--	--	--
Gallbladder and biliary tract cancer	--	--	--	--	--
Pancreatic cancer	--	--	--	--	--
Larynx cancer	--	--	--	--	--
Tracheal, bronchus, and lung cancer	--	--	--	--	--
Malignant skin melanoma	--	--	--	--	--
Non-melanoma skin cancer	--	--	--	--	--
Non-melanoma skin cancer (squamous-cell carcinoma)	--	--	--	--	--
Non-melanoma skin cancer (basal-cell carcinoma)	--	--	--	--	--
Breast cancer	--	--	--	--	--
Cervical cancer	--	--	--	--	--
Uterine cancer	--	--	--	--	--
Ovarian cancer	--	--	--	--	--
Prostate cancer	--	--	--	--	--
Testicular cancer	--	--	--	--	--
Kidney cancer	46.4 (40.0 to 51.6)	8.4 (7.3 to 9.4)	3.1 (2.0 to 4.5)	7.2 (-15.5 to 29.3)	1.6 (-19.9 to 22.4)
Bladder cancer	--	--	--	--	--
Brain and nervous system cancer	55.8 (47.7 to 60.9)	10.1 (8.7 to 11.1)	3.7 (2.5 to 5.2)	20.9 (0.7 to 38.0)	14.5 (-4.6 to 30.7)
Thyroid cancer	--	--	--	--	--
Mesothelioma	--	--	--	--	--
Hodgkin lymphoma	4.8 (3.8 to 5.9)	0.9 (0.7 to 1.1)	0.3 (0.2 to 0.5)	-11.3 (-26.7 to 15.5)	-16.0 (-30.5 to 9.4)
Non-Hodgkin's lymphoma	31.8 (25.2 to 35.7)	5.8 (4.6 to 6.5)	2.0 (1.2 to 2.8)	12.9 (-17.2 to 34.5)	7.0 (-21.6 to 27.4)
Multiple myeloma	--	--	--	--	--
Leukaemia	158.1 (126.2 to 180.2)	28.7 (22.9 to 32.8)	12.9 (8.7 to 18.6)	30.1 (-1.1 to 58.6)	23.2 (-6.3 to 50.2)
Acute lymphoid leukaemia	81.6 (68.9 to 91.7)	14.8 (12.5 to 16.7)	7.5 (5.0 to 10.6)	45.1 (7.4 to 74.5)	37.4 (1.7 to 65.2)
Chronic lymphoid leukaemia	--	--	--	--	--
Acute myeloid leukaemia	28.4 (22.7 to 33.0)	5.2 (4.1 to 6.0)	2.0 (1.3 to 2.9)	20.8 (-8.9 to 47.4)	14.4 (-13.8 to 39.7)
Chronic myeloid leukaemia	--	--	--	--	--
Other leukaemia	48.1 (32.9 to 58.5)	8.7 (6.0 to 10.6)	3.4 (2.0 to 5.2)	10.0 (-21.7 to 53.2)	4.2 (-25.8 to 45.1)
Other neoplasms	114.4 (102.6 to 122.8)	20.8 (18.7 to 22.3)	6.4 (4.1 to 9.3)	30.8 (0.4 to 50.0)	23.9 (-4.9 to 42.1)
**Cardiovascular diseases**	**865.5 (725.5 to 1,081.3)**	**540.5† (500.2 to 586.2)**	**71.4 (46.8 to 101.8)**	**3.0 (-1.8 to 7.3)**	**-2.5 (-7.0 to 1.6)**
Rheumatic heart disease	221.4 (194.2 to 249.2)	126.4 (110.8 to 142.5)	10.6 (6.5 to 15.8)	12.8 (4.1 to 22.8)	6.8 (-1.4 to 16.3)
Ischaemic heart disease	136.2 (121.0 to 153.6)	162.3 (140.1 to 186.1)	8.3 (5.4 to 12.2)	6.4 (3.1 to 9.6)	0.8 (-2.3 to 3.8)
Cerebrovascular disease	64.9 (51.5 to 81.0)	59.7‡ (42.9 to 83.5)	14.1 (9.5 to 19.3)	-21.8 (-29.2 to -14.1)	-25.9 (-32.9 to -18.7)
Ischaemic stroke	28.1 (19.9 to 37.2)	22.0‡ (9.7 to 43.0)	4.5 (2.8 to 6.7)	-0.9 (-7.3 to 4.3)	-6.1 (-12.2 to -1.2)
Hemorrhagic stroke	64.5 (50.8 to 81.0)	37.7‡ (27.6 to 52.8)	9.6 (6.3 to 13.4)	-28.8 (-38.4 to -19.0)	-32.5 (-41.7 to -23.2)
Hypertensive heart disease	0.1 (< 0.05 to 0.1)	--	< 0.05 (< 0.05 to < 0.05)	24.2 (18.6 to 30.4)	17.7 (12.3 to 23.6)
Cardiomyopathy and myocarditis	56.8 (44.2 to 72.8)	105.7† (85.8 to 130.5)	6.5 (4.1 to 9.8)	7.2 (5.1 to 9.3)	1.5 (-0.5 to 3.5)
Myocarditis	38.2 (31.7 to 46.2)	105.7 (85.8 to 130.5)	2.4 (1.5 to 3.6)	5.8 (2.1 to 9.5)	0.2 (-3.3 to 3.8)
Alcoholic cardiomyopathy	--	--	--	--	--
Other cardiomyopathy	45.2 (31.5 to 62.4)	--	4.0 (2.4 to 6.3)	8.1 (5.8 to 10.2)	2.3 (0.2 to 4.4)
Atrial fibrillation and flutter	--	--	--	--	--
Peripheral vascular disease	--	--	--	--	--
Endocarditis	11.6 (9.6 to 14.1)	86.4 (71.0 to 105.8)	0.9 (0.6 to 1.3)	5.9 (3.7 to 8.1)	0.3 (-1.8 to 2.4)
Other cardiovascular and circulatory diseases	559.7 (335.8 to 909.8)	--	31.0 (16.0 to 54.9)	14.0 (6.9 to 21.0)	8.0 (1.2 to 14.6)
**Chronic respiratory diseases**	**17,963.4 (16,457.8 to 20,025.4)**	**12,975.6† (10,797.0 to 15,437.3)**	**750.3 (491.0 to 1,100.9)**	**11.1 (8.4 to 13.8)**	**5.2 (2.6 to 7.8)**
Chronic obstructive pulmonary disease	312.6 (266.8 to 358.4)	116.3 (101.2 to 132.0)	27.6 (21.9 to 33.2)	-2.0 (-10.9 to 7.5)	-7.2 (-15.6 to 1.9)
Pneumoconiosis	--	--	--	--	--
Silicosis	--	--	--	--	--

Cause	Prevalence in thousands (95% UI)	Incidence in thousands (95% UI)	YLDs in thousands (95% UI)		
	2016 counts	2016 counts	2016 counts	Percentage change in counts between 2006 and 2016	Percentage change in rates between 2006 and 2016
Asbestosis	--	--	--	--	--
Coal workers pneumoconiosis	--	--	--	--	--
Other pneumoconiosis	--	--	--	--	--
Asthma	17,164.3 (14,736.3 to 19,937.2)	12,852.2 (10,680.0 to 15,325.1)	690.7 (436.6 to 1,039.5)	10.8 (7.9 to 13.6)	5.0 (2.2 to 7.6)
Interstitial lung disease and pulmonary sarcoidosis	30.7 (24.4 to 38.1)	7.0 (5.3 to 9.2)	3.5 (2.2 to 5.2)	1.0 (-0.2 to 2.1)	-4.3 (-5.4 to -3.3)
Other chronic respiratory diseases	--	--	28.6 (22.6 to 34.2)	38.1 (26.8 to 50.0)	30.8 (20.1 to 42.1)
**Cirrhosis and other chronic liver diseases**	**377.0 (350.8 to 406.0)**	**58.3 (49.8 to 67.3)**	**59.6 (40.4 to 83.9)**	**7.6 (1.4 to 14.0)**	**1.9 (-4.0 to 8.0)**
Cirrhosis and other chronic liver diseases due to hepatitis B	2.9 (2.1 to 3.7)	0.5 (0.4 to 0.7)	0.5 (0.3 to 0.8)	12.9 (8.8 to 17.2)	7.0 (3.0 to 11.0)
Cirrhosis and other chronic liver diseases due to hepatitis C	2.4 (1.7 to 3.2)	0.4 (0.3 to 0.5)	0.4 (0.2 to 0.6)	10.8 (6.6 to 14.7)	5.0 (0.9 to 8.6)
Cirrhosis and other chronic liver diseases due to alcohol use	--	--	--	--	--
Cirrhosis and other chronic liver diseases due to other causes	371.8 (346.1 to 400.7)	57.5 (49.0 to 66.2)	58.8 (39.9 to 82.7)	7.5 (1.2 to 14.0)	1.8 (-4.1 to 8.0)
**Digestive diseases**	**5,600.0 (4,882.2 to 6,316.6)**	**2,912.7† (2,467.1 to 3,383.5)**	**244.3 (160.0 to 347.8)**	**1.6 (-2.2 to 5.6)**	**-3.8 (-7.4 to 0.0)**
Peptic ulcer disease	--	--	--	--	--
Gastritis and duodenitis	2,435.2 (1,808.1 to 3,224.7)	797.6 (583.0 to 1,062.6)	24.5 (12.8 to 42.2)	17.6 (7.7 to 30.8)	11.4 (2.0 to 23.9)
Appendicitis	17.8 (14.7 to 21.4)	548.9 (452.5 to 661.7)	5.7 (3.5 to 8.2)	3.6 (-7.3 to 16.0)	-1.9 (-12.2 to 9.8)
Paralytic ileus and intestinal obstruction	7.6 (5.5 to 10.3)	205.0 (143.1 to 281.5)	2.5 (1.4 to 3.7)	5.0 (2.8 to 7.1)	-0.5 (-2.7 to 1.5)
Inguinal, femoral, and abdominal hernia	3,042.8 (1,857.9 to 4,577.3)	1,147.7 (813.0 to 1,535.5)	166.2 (103.6 to 245.0)	0.3 (-4.1 to 4.4)	-5.0 (-9.2 to -1.2)
Inflammatory bowel disease	5.1 (4.0 to 6.5)	20.6 (16.7 to 24.9)	0.8 (0.5 to 1.2)	-1.3 (-3.7 to 0.6)	-6.5 (-8.8 to -4.7)
Vascular intestinal disorders	5.4 (3.5 to 7.7)	21.2 (14.5 to 29.2)	1.8 (1.0 to 2.9)	9.7 (7.9 to 11.5)	3.9 (2.2 to 5.6)
Gallbladder and biliary diseases	92.7 (66.6 to 125.8)	143.4 (86.7 to 218.4)	0.8 (0.4 to 1.3)	-1.5 (-5.0 to 1.1)	-6.7 (-10.1 to -4.2)
Pancreatitis	3.1 (2.1 to 4.8)	28.4 (18.1 to 43.5)	0.4 (0.2 to 0.7)	-3.9 (-10.7 to 1.2)	-8.9 (-15.5 to -4.2)
Other digestive diseases	--	--	41.8 (27.4 to 59.5)	-1.6 (-5.4 to 2.2)	-6.8 (-10.3 to -3.2)
**Neurological disorders**	**1,912.7 (1,469.4 to 2,274.4)**	**531.6 (370.1 to 717.4)**	**729.5 (462.5 to 1,097.4)**	**14.6 (0.8 to 31.0)**	**8.5 (-4.6 to 24.1)**
Alzheimer's disease and other dementias	--	--	--	--	--
Parkinson's disease	--	--	--	--	--
Epilepsy	1,568.9 (1,180.5 to 2,073.8)	525.6 (364.9 to 712.3)	545.0 (344.9 to 820.0)	8.2 (-4.9 to 23.8)	2.5 (-9.9 to 17.2)
Multiple sclerosis	--	--	--	--	--
Motor neuron disease	8.1 (6.4 to 10.2)	3.0 (2.2 to 4.0)	1.7 (1.1 to 2.5)	4.6 (3.1 to 6.2)	-1.0 (-2.3 to 0.6)
Migraine	--	--	--	--	--
Tension-type headache	--	--	--	--	--
Other neurological disorders	1.4 (0.7 to 2.3)	3.0 (1.5 to 5.2)	182.8 (115.8 to 275.1)	39.0 (22.2 to 58.9)	31.6 (15.8 to 50.5)
**Mental and substance use disorders**	**8,499.5 (7,530.3 to 9,265.5)**	**1,748.7† (1,578.2 to 1,941.2)**	**754.6 (501.6 to 1,074.2)**	**4.2 (2.4 to 5.9)**	**-1.4 (-3.0 to 0.3)**
Schizophrenia	--	--	--	--	--
Alcohol use disorders	23.3 (20.8 to 25.4)	--	1.0 (0.6 to 1.4)	7.0 (2.5 to 11.0)	1.4 (-2.9 to 5.1)
Drug use disorders	--	--	--	--	--
Opioid use disorders	--	--	--	--	--
Cocaine use disorders	--	--	--	--	--
Amphetamine use disorders	--	--	--	--	--
Cannabis use disorders	--	--	--	--	--
Other drug use disorders	--	--	--	--	--
Depressive disorders	5.7 (2.6 to 11.7)	20.0 (9.1 to 38.0)	1.5 (0.7 to 3.2)	8.7 (7.1 to 10.0)	2.9 (1.5 to 4.2)
Major depressive disorder	5.5 (1.9 to 11.7)	15.9 (5.3 to 33.7)	1.2 (0.4 to 2.7)	9.3 (7.6 to 10.8)	3.5 (1.9 to 4.9)
Dysthymia	2.7 (0.9 to 5.8)	4.1 (1.4 to 8.7)	0.3 (0.1 to 0.6)	6.1 (3.2 to 9.3)	0.5 (-2.3 to 3.5)
Bipolar disorder	--	--	--	--	--
Anxiety disorders	624.5 (534.4 to 722.5)	661.1 (566.1 to 765.6)	62.9 (42.8 to 88.4)	6.2 (-2.0 to 14.4)	0.5 (-7.2 to 8.4)
Eating disorders	--	--	--	--	--
Anorexia nervosa	--	--	--	--	--
Bulimia nervosa	--	--	--	--	--
Autistic spectrum disorders	3,837.2 (3,568.4 to 4,131.4)	210.3 (130.6 to 334.0)	543.0 (348.4 to 784.7)	5.6 (3.8 to 7.5)	0.1 (-1.7 to 1.8)
Autism	962.8 (761.1 to 1,178.1)	24.2 (11.6 to 41.6)	252.5 (154.8 to 382.9)	5.6 (2.7 to 8.9)	0.1 (-2.7 to 3.1)
Asperger syndrome and other autistic spectrum disorders	2,846.8 (2,206.6 to 3,653.1)	186.1 (107.2 to 310.1)	290.4 (180.6 to 437.6)	5.6 (3.4 to 7.8)	0.1 (-2.0 to 2.1)

Cause	Prevalence in thousands (95% UI)	Incidence in thousands (95% UI)	YLDs in thousands (95% UI)		
	2016 counts	2016 counts	2016 counts	Percentage change in counts between 2006 and 2016	Percentage change in rates between 2006 and 2016
Attention-deficit/hyperactivity disorder	890.2 (794.1 to 1,022.2)	857.2 (764.4 to 984.6)	10.8 (6.5 to 17.1)	7.7 (1.3 to 14.6)	2.0 (-4.0 to 8.5)
Conduct disorder	--	--	--	--	--
Idiopathic developmental intellectual disability	3,277.7 (1,484.6 to 5,212.9)	--	135.4 (50.9 to 255.7)	-2.5 (-6.9 to 0.5)	-7.7 (-11.8 to -4.8)
Other mental and substance use disorders	--	--	--	--	--
**Diabetes, urogenital, blood, and endocrine diseases**	**143,754.8 (141,893.4 to 145,596.6)**	**16,576.8† (13,266.0 to 20,291.1)**	**1,072.3 (672.4 to 1,613.3)**	**5.5 (0.5 to 11.5)**	**-0.1 (-4.8 to 5.6)**
Diabetes mellitus	30.4 (17.4 to 51.3)	22.4 (12.8 to 37.7)	1.5 (0.7 to 2.8)	-1.0 (-12.3 to 8.3)	-6.2 (-16.9 to 2.6)
Acute glomerulonephritis	3.0 (1.9 to 4.3)	46.6 (29.2 to 68.8)	0.1 (0.1 to 0.3)	-1.0 (-3.3 to 1.1)	-6.2 (-8.4 to -4.2)
Chronic kidney disease	60.9 (42.0 to 84.5)	42.6 (27.8 to 60.2)	7.5 (4.1 to 12.2)	11.2 (5.2 to 17.2)	5.4 (-0.4 to 11.0)
Chronic kidney disease due to diabetes mellitus	2.0 (1.1 to 3.1)	1.3 (0.7 to 2.1)	0.3 (0.1 to 0.5)	12.3 (4.4 to 19.7)	6.3 (-1.1 to 13.4)
Chronic kidney disease due to hypertension	9.0 (4.9 to 14.7)	17.2 (10.3 to 25.3)	0.1 (< 0.05 to 0.1)	8.4 (1.5 to 15.1)	2.6 (-3.9 to 9.0)
Chronic kidney disease due to glomerulonephritis	16.0 (9.5 to 24.4)	0.4 (0.2 to 0.7)	3.0 (1.6 to 5.2)	12.0 (5.8 to 17.9)	6.0 (0.2 to 11.7)
Chronic kidney disease due to other causes	33.9 (23.0 to 48.8)	23.6 (15.2 to 34.3)	4.1 (2.2 to 6.8)	10.7 (4.8 to 16.8)	4.9 (-0.8 to 10.6)
Urinary diseases and male infertility	358.9 (326.2 to 402.0)	15,997.3† (12,693.2 to 19,680.9)	18.2 (11.3 to 28.5)	-8.1 (-14.2 to -2.0)	-12.9 (-18.7 to -7.2)
Interstitial nephritis and urinary tract infections	319.7 (259.6 to 384.6)	15,667.3 (12,370.4 to 19,347.0)	10.8 (6.5 to 17.4)	3.9 (-3.7 to 11.8)	-1.6 (-8.8 to 5.9)
Urolithiasis	12.5 (8.8 to 16.9)	330.0 (228.8 to 457.9)	1.0 (0.6 to 1.5)	3.4 (2.4 to 4.4)	-2.1 (-3.0 to -1.1)
Benign prostatic hyperplasia	--	--	--	--	--
Male infertility	--	--	--	--	--
Other urinary diseases	--	--	6.4 (4.0 to 10.0)	-24.2 (-29.2 to -19.1)	-28.2 (-32.9 to -23.4)
Gynecological diseases	--	--	--	--	--
Uterine fibroids	--	--	--	--	--
Polycystic ovarian syndrome	--	--	--	--	--
Female infertility	--	--	--	--	--
Endometriosis	--	--	--	--	--
Genital prolapse	--	--	--	--	--
Premenstrual syndrome	--	--	--	--	--
Other gynecological diseases	--	--	--	--	--
Hemoglobinopathies and hemolytic anaemias	137,642.8 (135,808.3 to 139,548.9)	--	766.2 (473.9 to 1,191.7)	6.8 (1.1 to 13.7)	1.2 (-4.2 to 7.7)
Thalassemias	172.3 (160.0 to 186.3)	--	10.0 (6.0 to 15.1)	-3.0 (-10.3 to 4.0)	-8.1 (-15.0 to -1.5)
Thalassaemias trait	16,974.0 (16,021.6 to 18,665.9)	--	301.6 (171.5 to 495.7)	8.2 (-0.0 to 19.7)	2.5 (-5.3 to 13.3)
Sickle cell disorders	1,013.5 (895.9 to 1,269.2)	--	63.3 (40.8 to 94.8)	13.9 (6.0 to 22.5)	7.9 (0.4 to 16.0)
Sickle cell trait	41,377.0 (37,755.4 to 46,111.2)	--	216.0 (134.9 to 321.3)	12.6 (6.7 to 19.2)	6.7 (1.1 to 12.9)
G6PD deficiency	27,187.3 (26,084.2 to 28,511.2)	--	3.6 (2.1 to 5.8)	4.4 (-9.7 to 16.7)	-1.1 (-14.4 to 10.6)
G6PD trait	63,499.9 (62,440.1 to 64,714.3)	--	< 0.05 (< 0.05 to 0.1)	12.0 (1.4 to 21.9)	6.1 (-4.0 to 15.5)
Other hemoglobinopathies and hemolytic anaemias	5,634.4 (5,174.4 to 6,243.6)	--	171.7 (107.3 to 254.3)	-3.2 (-8.8 to 3.7)	-8.3 (-13.6 to -1.8)
Endocrine, metabolic, blood, and immune disorders	8,519.0 (7,831.7 to 9,349.9)	467.9 (354.8 to 616.6)	278.8 (178.7 to 406.5)	2.8 (-1.6 to 7.1)	-2.6 (-6.8 to 1.5)
**Musculoskeletal disorders**	**--**	**--**	**--**	**--**	**--**
Rheumatoid arthritis	--	--	--	--	--
Osteoarthritis	--	--	--	--	--
Low back and neck pain	--	--	--	--	--
Low back pain	--	--	--	--	--
Neck pain	--	--	--	--	--
Gout	--	--	--	--	--
Other musculoskeletal disorders	--	--	--	--	--
**Other non-communicable diseases**	**308,179.6 (298,984.2 to 320,347.2)**	**977,887.8† (715,093.0 to 1,291,771.9)**	**5,803.3 (3,835.6 to 8,235.9)**	**5.8 (5.0 to 6.7)**	**0.2 (-0.5 to 1.0)**
Congenital anomalies	8,991.6 (8,624.1 to 9,376.1)	--	1,017.0 (744.2 to 1,336.2)	4.6 (2.6 to 6.9)	-0.9 (-2.8 to 1.3)
Neural tube defects	495.7 (447.6 to 549.9)	--	146.4 (102.7 to 196.3)	10.1 (5.1 to 15.2)	4.3 (-0.4 to 9.1)
Congenital heart anomalies	2,828.7 (2,360.1 to 3,316.3)	--	115.5 (56.2 to 188.0)	2.7 (-0.5 to 5.5)	-2.8 (-5.8 to -0.0)
Orofacial clefts	305.1 (265.4 to 352.5)	--	4.3 (2.8 to 6.2)	-3.1 (-6.3 to -0.1)	-8.2 (-11.2 to -5.4)
Down's syndrome	192.8 (168.0 to 221.5)	--	16.6 (11.1 to 23.4)	7.2 (4.0 to 10.3)	1.6 (-1.5 to 4.5)
Turner syndrome	292.6 (254.0 to 334.6)	--	4.0 (1.6 to 7.1)	2.2 (-10.0 to 15.7)	-3.2 (-14.7 to 9.6)
Klinefelter syndrome	391.3 (336.8 to 451.6)	--	1.1 (0.5 to 2.0)	6.8 (-2.4 to 16.4)	1.2 (-7.5 to 10.2)

Cause	Prevalence in thousands (95% UI)	Incidence in thousands (95% UI)	YLDs in thousands (95% UI)		
	2016 counts	2016 counts	2016 counts	Percentage change in counts between 2006 and 2016	Percentage change in rates between 2006 and 2016
Other chromosomal abnormalities	956.0 (836.5 to 1,099.9)	--	154.7 (107.6 to 211.3)	8.5 (4.1 to 13.1)	2.8 (-1.4 to 7.1)
Congenital musculoskeletal and limb anomalies	661.7 (596.5 to 733.1)	--	78.5 (52.6 to 109.4)	6.5 (2.7 to 10.4)	0.9 (-2.7 to 4.6)
Urogenital congenital anomalies	1,263.8 (1,117.5 to 1,425.7)	--	36.2 (21.5 to 56.9)	1.5 (-0.9 to 4.1)	-3.9 (-6.2 to -1.4)
Digestive congenital anomalies	593.3 (530.9 to 656.1)	--	28.5 (18.4 to 41.1)	3.2 (-2.2 to 8.6)	-2.2 (-7.4 to 2.9)
Other congenital anomalies	2,606.1 (1,834.1 to 3,702.7)	--	431.4 (294.9 to 607.8)	2.2 (-1.4 to 5.8)	-3.2 (-6.6 to 0.2)
Skin and subcutaneous diseases	136,264.8 (132,751.8 to 139,604.7)	310,162.1 (270,412.8 to 358,102.2)	3,948.7 (2,467.0 to 5,921.0)	6.9 (6.0 to 7.8)	1.2 (0.4 to 2.1)
Dermatitis	38,985.4 (35,260.0 to 43,058.4)	13,637.8 (11,979.9 to 15,345.6)	1,732.6 (967.4 to 2,923.5)	8.5 (7.0 to 10.0)	2.8 (1.3 to 4.2)
Psoriasis	1,095.2 (1,002.9 to 1,192.8)	421.5 (374.3 to 466.1)	99.9 (69.1 to 134.6)	10.1 (4.2 to 16.7)	4.3 (-1.3 to 10.6)
Cellulitis	179.6 (148.4 to 216.8)	3,197.6 (2,663.3 to 3,810.3)	10.5 (6.5 to 15.9)	7.9 (1.5 to 14.6)	2.2 (-3.9 to 8.6)
Pyoderma	2,145.8 (1,968.1 to 2,336.4)	40,591.4 (37,308.4 to 44,042.3)	12.3 (5.0 to 25.7)	18.4 (15.2 to 21.5)	12.2 (9.1 to 15.1)
Scabies	12,947.7 (9,554.0 to 17,138.2)	40,061.0 (29,084.0 to 54,654.4)	338.2 (179.3 to 569.8)	-1.1 (-2.5 to 0.4)	-6.3 (-7.6 to -4.9)
Fungal skin diseases	48,890.2 (38,241.2 to 61,919.2)	148,843.4 (113,439.6 to 193,438.0)	280.2 (112.1 to 593.6)	10.8 (10.0 to 11.7)	5.0 (4.2 to 5.8)
Viral skin diseases	15,536.4 (14,374.7 to 16,879.9)	19,451.7 (17,657.2 to 21,342.7)	483.8 (296.3 to 731.1)	5.8 (4.6 to 6.9)	0.2 (-0.9 to 1.2)
Acne vulgaris	--	--	--	--	--
Alopecia areata	312.1 (276.4 to 348.1)	545.7 (476.0 to 614.3)	10.5 (6.5 to 15.9)	5.5 (-1.5 to 13.0)	-0.1 (-6.7 to 7.1)
Pruritus	3,270.0 (2,417.8 to 4,285.3)	2,841.4 (2,027.8 to 3,871.1)	35.6 (16.0 to 66.6)	6.3 (4.2 to 8.2)	0.7 (-1.3 to 2.5)
Urticaria	13,540.2 (10,197.2 to 17,576.1)	22,587.6 (16,545.9 to 29,987.1)	830.0 (488.2 to 1,288.6)	5.3 (4.0 to 6.7)	-0.2 (-1.5 to 1.0)
Decubitus ulcer	39.0 (27.4 to 52.3)	129.8 (90.1 to 173.8)	6.9 (4.2 to 10.4)	4.4 (3.3 to 5.4)	-1.1 (-2.1 to -0.1)
Other skin and subcutaneous diseases	19,168.0 (17,509.2 to 20,833.2)	17,853.2 (16,105.7 to 19,691.6)	108.2 (51.2 to 199.6)	10.8 (9.6 to 12.1)	5.0 (3.8 to 6.2)
Sense organ diseases	26,076.1 (25,314.1 to 26,774.9)	79,979.8† (71,238.8 to 89,088.7)	782.1 (507.5 to 1,143.0)	2.1 (0.2 to 4.2)	-3.3 (-5.1 to -1.2)
Glaucoma	--	--	--	--	--
Cataract	--	--	--	--	--
Macular degeneration	--	--	--	--	--
Refraction and accommodation disorders	18,253.6 (16,391.3 to 20,497.9)	--	370.2 (218.5 to 603.3)	0.6 (-1.0 to 2.0)	-4.7 (-6.2 to -3.4)
Age-related and other hearing loss	3,141.4 (2,045.8 to 4,306.5)	--	266.6 (146.1 to 411.2)	2.1 (-3.1 to 7.5)	-3.3 (-8.2 to 1.8)
Other vision loss	368.3 (265.4 to 502.9)	--	17.9 (10.9 to 27.2)	3.4 (-1.5 to 8.7)	-2.1 (-6.7 to 3.0)
Other sense organ diseases	5,073.9 (4,604.9 to 5,603.0)	79,979.8 (71,238.8 to 89,088.7)	127.4 (78.1 to 193.2)	6.8 (3.7 to 9.6)	1.1 (-1.8 to 3.8)
Oral disorders	229,608.9 (204,189.2 to 269,076.5)	587,745.9† (319,424.7 to 893,620.5)	55.5 (22.1 to 110.2)	6.0 (2.9 to 8.2)	0.4 (-2.5 to 2.5)
Caries of deciduous teeth	213,863.0 (161,903.0 to 268,105.0)	587,745.9 (319,424.7 to 893,620.5)	55.5 (22.1 to 110.2)	6.0 (2.9 to 8.2)	0.4 (-2.5 to 2.5)
Caries of permanent teeth	--	--	--	--	--
Periodontal disease	--	--	--	--	--
Edentulism and severe tooth loss	--	--	--	--	--
Other oral disorders	--	--	--	--	--
**Injuries**	**16,155.6 (14,336.9 to 18,043.0)**	**52,587.6† (46,469.8 to 59,812.3)**	**740.5 (490.4 to 1,076.6)**	**9.8 (8.2 to 11.5)**	**4.0 (2.5 to 5.6)**
**Transport injuries**	**933.4 (747.4 to 1,180.5)**	**2,366.8 (1,771.7 to 3,138.4)**	**64.7 (40.6 to 98.4)**	**4.5 (2.1 to 6.8)**	**-1.0 (-3.3 to 1.2)**
Road injuries	212.6 (175.2 to 255.7)	875.3 (586.6 to 1,223.3)	14.9 (9.1 to 23.2)	3.4 (-2.2 to 8.4)	-2.0 (-7.4 to 2.7)
Pedestrian road injuries	38.5 (28.4 to 52.1)	140.2 (84.3 to 219.7)	2.7 (1.6 to 4.4)	-4.2 (-10.4 to 1.3)	-9.3 (-15.2 to -4.0)
Cyclist road injuries	43.2 (30.3 to 58.2)	214.1 (116.5 to 338.8)	2.7 (1.5 to 4.5)	4.5 (-2.1 to 10.2)	-1.0 (-7.2 to 4.4)
Motorcyclist road injuries	46.3 (34.5 to 60.9)	138.3 (78.8 to 220.4)	3.1 (1.8 to 4.9)	10.4 (5.2 to 14.8)	4.6 (-0.4 to 8.8)
Motor vehicle road injuries	61.0 (46.1 to 80.8)	272.6 (166.9 to 417.9)	5.0 (3.0 to 8.0)	1.3 (-4.9 to 6.7)	-4.1 (-9.9 to 1.1)
Other road injuries	23.5 (16.0 to 33.9)	110.0 (56.8 to 189.4)	1.4 (0.7 to 2.4)	11.5 (4.1 to 17.7)	5.6 (-1.4 to 11.5)
Other transport injuries	720.8 (540.1 to 966.0)	1,491.5 (984.4 to 2,183.2)	49.8 (30.5 to 76.2)	4.9 (2.4 to 7.4)	-0.6 (-3.0 to 1.7)
**Unintentional injuries**	**14,184.1 (12,474.2 to 16,039.8)**	**49,328.2 (43,242.1 to 56,582.0)**	**641.4 (425.7 to 934.9)**	**9.8 (8.3 to 11.4)**	**4.0 (2.6 to 5.5)**
Falls	3,946.9 (2,997.0 to 5,244.8)	13,528.6 (9,713.5 to 19,386.0)	168.2 (106.7 to 251.3)	16.0 (13.3 to 18.7)	9.9 (7.4 to 12.4)
Drowning	43.9 (32.3 to 61.5)	97.2 (78.0 to 118.8)	2.9 (1.7 to 4.6)	-8.5 (-10.7 to -6.2)	-13.3 (-15.4 to -11.2)
Fire, heat, and hot substances	864.9 (598.0 to 1,177.0)	1,351.6 (913.0 to 1,862.5)	35.3 (21.6 to 52.7)	7.5 (3.2 to 11.4)	1.8 (-2.2 to 5.5)
Poisonings	54.9 (40.8 to 75.0)	609.9 (408.4 to 872.1)	6.6 (3.9 to 10.4)	13.5 (8.2 to 18.9)	7.5 (2.5 to 12.6)
Exposure to mechanical forces	1,689.4 (1,281.8 to 2,158.9)	5,008.7 (3,466.8 to 6,947.0)	50.5 (30.5 to 78.3)	8.8 (5.5 to 12.3)	3.1 (-0.1 to 6.4)
Unintentional firearm injuries	30.7 (22.2 to 40.1)	79.8 (44.1 to 135.2)	1.8 (1.0 to 2.9)	10.1 (5.5 to 15.1)	4.3 (-0.0 to 9.0)
Unintentional suffocation	332.0 (224.6 to 456.1)	553.4 (319.7 to 881.7)	12.4 (7.2 to 20.0)	4.4 (0.7 to 8.5)	-1.2 (-4.6 to 2.8)
Other exposure to mechanical forces	1,326.6 (979.5 to 1,760.4)	4,375.5 (3,009.4 to 6,026.9)	36.3 (22.0 to 57.1)	10.4 (6.6 to 13.9)	4.5 (1.0 to 7.9)

Cause	Prevalence in thousands (95% UI)	Incidence in thousands (95% UI)	YLDs in thousands (95% UI)		
	2016 counts	2016 counts	2016 counts	Percentage change in counts between 2006 and 2016	Percentage change in rates between 2006 and 2016
Adverse effects of medical treatment	462.7<br>(317.3 to 651.5)	6,050.9<br>(4,384.9 to 8,056.9)	61.7<br>(33.6 to 100.4)	8.4<br>(4.0 to 12.9)	2.7<br>(-1.5 to 7.0)
Animal contact	795.3<br>(594.1 to 1,081.9)	5,362.0<br>(3,830.3 to 7,192.1)	40.7<br>(25.7 to 59.4)	1.1<br>(-2.7 to 5.0)	-4.2<br>(-7.8 to -0.6)
Venomous animal contact	324.7<br>(231.1 to 448.9)	2,335.1<br>(1,599.1 to 3,294.7)	29.6<br>(18.3 to 43.1)	0.1<br>(-4.7 to 5.1)	-5.2<br>(-9.7 to -0.5)
Non-venomous animal contact	470.6<br>(321.5 to 699.8)	3,026.9<br>(2,065.3 to 4,202.0)	11.1<br>(6.2 to 17.7)	3.8<br>(-0.1 to 7.2)	-1.7<br>(-5.4 to 1.5)
Foreign body	1,827.5<br>(1,399.4 to 2,287.1)	5,839.3<br>(4,585.4 to 7,392.3)	93.8<br>(59.5 to 142.2)	6.1<br>(3.8 to 8.4)	0.5<br>(-1.7 to 2.6)
Pulmonary aspiration and foreign body in airway	1,109.0<br>(764.4 to 1,562.9)	2,837.2<br>(1,988.0 to 4,029.1)	62.3<br>(38.9 to 94.8)	4.0<br>(1.5 to 6.6)	-1.4<br>(-3.9 to 1.0)
Foreign body in eyes	145.5<br>(45.1 to 311.2)	1,050.5<br>(577.1 to 1,822.7)	8.1<br>(2.5 to 17.9)	10.7<br>(7.3 to 15.5)	4.9<br>(1.7 to 9.3)
Foreign body in other body part	573.1<br>(400.3 to 783.6)	1,951.7<br>(1,369.1 to 2,767.2)	23.4<br>(14.3 to 35.2)	10.3<br>(7.5 to 13.1)	4.5<br>(1.8 to 7.1)
Environmental heat and cold exposure	1,277.4<br>(907.2 to 1,922.5)	2,846.0<br>(1,955.1 to 3,916.3)	61.6<br>(37.5 to 95.1)	5.4<br>(3.1 to 7.4)	-0.2<br>(-2.3 to 1.7)
Other unintentional injuries	3,221.2<br>(2,300.8 to 4,642.0)	8,634.0<br>(5,954.1 to 12,422.1)	120.1<br>(74.5 to 184.5)	12.3<br>(9.6 to 15.0)	6.3<br>(3.8 to 8.9)
**Self-harm and interpersonal violence**	**1,128.7<br>(901.9 to 1,384.4)**	**855.8†<br>(562.7 to 1,210.6)**	**21.4<br>(13.6 to 31.7)**	**4.5<br>(2.2 to 6.9)**	**-1.1<br>(-3.2 to 1.2)**
Self-harm	--	--	--	--	--
Self-harm by firearm	--	--	--	--	--
Self-harm by other specified means	--	--	--	--	--
Interpersonal violence	1,128.7<br>(901.9 to 1,384.4)	855.8†<br>(562.7 to 1,210.6)	21.4<br>(13.6 to 31.7)	4.5<br>(2.2 to 6.9)	-1.1<br>(-3.2 to 1.2)
Assault by firearm	10.9<br>(8.1 to 14.6)	28.6<br>(14.6 to 50.5)	0.8<br>(0.4 to 1.3)	10.3<br>(6.4 to 14.3)	4.5<br>(0.8 to 8.3)
Assault by sharp object	70.4<br>(48.8 to 97.9)	229.2<br>(125.5 to 377.1)	3.6<br>(2.0 to 6.0)	11.4<br>(7.5 to 15.6)	5.5<br>(1.9 to 9.5)
Sexual violence	899.9<br>(677.8 to 1,146.5)	--	9.1<br>(5.5 to 13.7)	-1.5<br>(-2.7 to -0.3)	-6.7<br>(-7.9 to -5.6)
Assault by other means	147.5<br>(108.2 to 202.5)	598.1<br>(389.1 to 872.8)	8.0<br>(4.6 to 12.7)	8.4<br>(5.0 to 11.8)	2.6<br>(-0.5 to 5.9)
**Forces of nature, conflict and terrorism, and executions and police conflict**	**809.4<br>(463.9 to 1,322.5)**	**36.9†<br>(23.9 to 53.9)**	**12.9<br>(5.6 to 23.8)**	**73.5<br>(34.1 to 147.5)**	**64.3<br>(27.0 to 134.5)**
Forces of nature, conflict and terrorism, and state actor violence	7.3<br>(4.1 to 11.8)	15.8<br>(8.7 to 25.0)	0.4<br>(0.2 to 0.8)	-84.7<br>(-88.4 to -79.9)	-85.5<br>(-89.0 to -80.9)
Conflict and terrorism	795.5<br>(452.3 to 1,300.7)	--	12.5<br>(5.3 to 22.8)	173.5<br>(97.6 to 383.1)	159.0<br>(87.2 to 357.6)
Executions and police conflict	6.6<br>(3.5 to 12.5)	21.1<br>(11.5 to 36.4)	0.3<br>(0.1 to 0.7)	-8.1<br>(-32.7 to 30.1)	-13.0<br>(-36.3 to 23.2)

We did not calculate incidence for certain NTDs and causes with only birth prevalence. Blank cells mean that no estimate is available.

† Incidence for aggregate causes represents the sum of the incidence of the causes below the aggregate.

‡ Incidence estimates for stroke represent first-ever stroke only.

^ Incidence of HIV/AIDS represents new infections of HIV only and does not include new infections of Tuberculosis in HIV positive cases.

SR Table 12: Global prevalence, incidence, and YLDs for 2016, percentage change of YLD counts and percentage change of YLD rates between 2006 and 2016 by cause, age 5-9 years.					
Cause	Prevalence in thousands (95% UI)	Incidence in thousands (95% UI)	YLDs in thousands (95% UI)		
	2016 counts	2016 counts	2016 counts	Percentage change in counts between 2006 and 2016	Percentage change in rates between 2006 and 2016
**All causes**	**589,935.9<br>(586,136.3 to 593,904.1)**	**4,375,271.5†<br>(3,627,247.2 to 5,231,851.3)**	**30,681.7<br>(21,602.1 to 42,231.1)**	**6.2<br>(5.0 to 7.4)**	**-1.4<br>(-2.5 to -0.2)**
**Communicable, maternal, neonatal, and nutritional disorders**	**392,128.0<br>(387,390.8 to 396,274.1)**	**2,502,574.3†<br>(1,940,494.0 to 3,163,102.7)**	**12,068.4<br>(8,301.2 to 17,086.0)**	**2.6<br>(0.4 to 5.2)**	**-4.7<br>(-6.7 to -2.3)**
**HIV/AIDS and tuberculosis**	**88,994.0<br>(79,927.3 to 101,118.7)**	**176.2†<br>(116.7 to 255.0)**	**161.8<br>(116.4 to 213.6)**	**-6.2<br>(-14.2 to 3.3)**	**-12.9<br>(-20.3 to -4.1)**
Tuberculosis	83,418.2<br>(68,909.3 to 100,087.2)	176.2†<br>(116.7 to 255.0)	34.6<br>(19.0 to 56.3)	-13.8<br>(-23.2 to -2.2)	-19.9<br>(-28.7 to -9.2)
Drug-susceptible tuberculosis	103.8<br>(65.9 to 152.7)	170.9<br>(113.2 to 247.4)	33.4<br>(18.3 to 54.4)	-13.8<br>(-23.6 to -2.1)	-19.9<br>(-29.1 to -9.0)
Multidrug-resistant tuberculosis without extensive drug resistance	3.3<br>(2.1 to 4.7)	5.0<br>(3.4 to 7.3)	1.1<br>(0.6 to 1.8)	-15.2<br>(-24.8 to -3.8)	-21.3<br>(-30.2 to -10.7)
Extensively drug-resistant tuberculosis	0.2<br>(0.1 to 0.2)	0.2<br>(0.2 to 0.3)	0.1<br>(< 0.05 to 0.1)	170.9<br>(133.1 to 215.1)	151.6<br>(116.5 to 192.6)
Latent tuberculosis infection	83,311.0<br>(68,800.3 to 99,965.1)	--	--	--	--
HIV/AIDS	914.2<br>(873.1 to 958.6)	--	127.2<br>(92.5 to 165.9)	-3.9<br>(-13.8 to 7.6)	-10.7<br>(-19.9 to -0.1)
Drug-susceptible HIV/AIDS - Tuberculosis	24.4<br>(16.8 to 33.9)	36.6<br>(25.3 to 50.8)	9.9<br>(5.7 to 15.9)	-26.1<br>(-35.6 to -16.3)	-31.4<br>(-40.2 to -22.3)
Multidrug-resistant HIV/AIDS - Tuberculosis without extensive drug resistance	0.6<br>(0.4 to 1.0)	1.0<br>(0.6 to 1.5)	0.3<br>(0.1 to 0.4)	-24.9<br>(-41.2 to -7.1)	-30.3<br>(-45.4 to -13.7)
Extensively drug-resistant HIV/AIDS - Tuberculosis	< 0.05<br>(< 0.05 to < 0.05)	< 0.05<br>(< 0.05 to < 0.05)	< 0.05<br>(< 0.05 to < 0.05)	230.8<br>(173.3 to 301.2)	207.2<br>(153.8 to 272.7)
HIV/AIDS resulting in other diseases	889.2<br>(847.5 to 934.9)	--	117.0<br>(84.7 to 151.1)	-1.3<br>(-12.4 to 12.3)	-8.3<br>(-18.7 to 4.3)
**Diarrhoea, lower respiratory infections, and other common infectious diseases**	**49,956.9<br>(46,465.0 to 52,748.8)**	**2,335,124.8†<br>(1,779,261.9 to 2,990,245.8)**	**2,136.0<br>(1,424.3 to 3,079.5)**	**4.8<br>(3.2 to 6.3)**	**-2.6<br>(-4.1 to -1.3)**
Diarrhoeal diseases	4,753.6<br>(3,914.5 to 5,687.2)	312,303.3<br>(255,510.0 to 373,349.5)	548.5<br>(363.8 to 781.1)	5.0<br>(2.5 to 7.3)	-2.5<br>(-4.8 to -0.4)
Intestinal infectious diseases	222.9<br>(182.9 to 280.3)	3,744.2†<br>(2,754.8 to 4,988.4)	31.3<br>(18.3 to 48.8)	-24.5<br>(-38.9 to -5.4)	-29.9<br>(-43.3 to -12.1)
Typhoid fever	193.6<br>(137.8 to 264.9)	3,050.9<br>(2,249.1 to 4,126.1)	29.3<br>(17.1 to 45.7)	-24.8<br>(-39.9 to -4.3)	-30.1<br>(-44.1 to -11.2)
Paratyphoid fever	30.4<br>(21.1 to 42.9)	693.4<br>(488.6 to 969.2)	1.9<br>(1.1 to 3.2)	-20.1<br>(-37.2 to 1.2)	-25.8<br>(-41.7 to -6.0)
Other intestinal infectious diseases	--	--	0.1<br>(< 0.05 to 0.1)	-37.6<br>(-49.5 to -21.8)	-42.1<br>(-53.1 to -27.4)
Lower respiratory infections	870.4<br>(688.4 to 1,101.2)	34,679.5<br>(27,339.1 to 44,478.8)	53.9<br>(33.0 to 83.6)	14.1<br>(7.4 to 21.1)	6.0<br>(-0.3 to 12.4)
Upper respiratory infections	24,705.4<br>(17,683.5 to 32,769.3)	1,863,359.9<br>(1,302,060.1 to 2,504,876.1)	625.1<br>(334.1 to 1,077.6)	8.2<br>(6.0 to 10.1)	0.5<br>(-1.6 to 2.2)
Otitis media	19,790.0<br>(16,155.4 to 24,080.6)	96,968.5<br>(63,394.0 to 145,302.2)	626.8<br>(374.8 to 979.0)	4.4<br>(1.5 to 7.1)	-3.0<br>(-5.7 to -0.5)
Meningitis	1,296.7<br>(1,108.8 to 1,522.6)	274.1<br>(193.8 to 378.7)	149.1<br>(105.2 to 197.9)	4.8<br>(0.4 to 9.1)	-2.6<br>(-6.8 to 1.3)
Pneumococcal meningitis	509.1<br>(422.3 to 623.4)	67.3<br>(40.8 to 101.5)	60.2<br>(41.5 to 82.7)	2.2<br>(-5.9 to 10.4)	-5.1<br>(-12.6 to 2.5)
H influenzae type B meningitis	223.9<br>(177.9 to 282.6)	38.9<br>(21.4 to 65.9)	29.6<br>(20.0 to 40.3)	-19.0<br>(-25.1 to -13.2)	-24.8<br>(-30.5 to -19.4)
Meningococcal infection	159.4<br>(113.6 to 216.2)	52.5<br>(30.4 to 81.6)	16.9<br>(11.7 to 23.0)	13.5<br>(9.9 to 17.1)	5.4<br>(2.1 to 8.7)
Other meningitis	404.3<br>(302.4 to 521.8)	115.4<br>(78.5 to 168.4)	42.3<br>(28.9 to 58.0)	33.1<br>(25.7 to 41.0)	23.6<br>(16.8 to 31.0)
Encephalitis	665.1<br>(482.6 to 918.9)	891.9<br>(703.9 to 1,119.7)	83.1<br>(57.9 to 110.8)	-1.1<br>(-6.8 to 4.9)	-8.1<br>(-13.4 to -2.6)
Diphtheria	< 0.05<br>(< 0.05 to < 0.05)	0.3<br>(0.2 to 0.6)	< 0.05<br>(< 0.05 to < 0.05)	-61.7<br>(-76.6 to -32.7)	-64.4<br>(-78.3 to -37.5)
Whooping cough	79.3<br>(61.9 to 99.7)	579.2<br>(451.6 to 727.5)	3.9<br>(2.3 to 6.2)	-22.2<br>(-31.6 to -12.0)	-27.8<br>(-36.4 to -18.2)
Tetanus	17.1<br>(16.9 to 17.4)	2.9<br>(1.2 to 5.3)	0.4<br>(0.2 to 0.6)	-4.3<br>(-30.1 to 30.9)	-11.1<br>(-35.1 to 21.6)
Measles	25.5<br>(9.2 to 55.2)	931.2<br>(337.1 to 2,014.7)	2.4<br>(0.8 to 5.4)	-61.0<br>(-66.6 to -53.9)	-63.7<br>(-69.0 to -57.2)
Varicella and herpes zoster	560.2<br>(398.0 to 742.9)	21,389.9<br>(13,644.8 to 30,369.8)	11.7<br>(6.6 to 18.8)	6.9<br>(-0.5 to 14.9)	-0.7<br>(-7.5 to 6.7)
**Neglected tropical diseases and malaria**	**169,890.6<br>(164,618.8 to 174,765.7)**	**61,247.3†<br>(47,858.0 to 82,142.9)**	**1,562.1<br>(1,051.0 to 2,284.8)**	**-10.4<br>(-14.3 to -6.2)**	**-16.8<br>(-20.4 to -12.8)**
Malaria	28,222.0<br>(20,819.3 to 35,386.2)	39,277.5<br>(26,543.4 to 60,065.6)	391.2<br>(262.1 to 578.5)	-14.7<br>(-22.0 to -7.3)	-20.8<br>(-27.5 to -13.9)
Chagas disease	177.1<br>(135.2 to 223.2)	19.2<br>(13.6 to 25.9)	0.1<br>(0.1 to 0.2)	-25.3<br>(-35.1 to -14.7)	-30.6<br>(-39.7 to -20.8)
Leishmaniasis	273.1<br>(242.4 to 308.3)	115.4<br>(80.2 to 159.3)	16.6<br>(10.5 to 24.1)	18.1<br>(-1.8 to 43.0)	9.7<br>(-8.8 to 32.8)
Visceral leishmaniasis	1.9<br>(1.3 to 2.7)	7.7<br>(5.2 to 10.8)	0.1<br>(0.1 to 0.2)	-65.8<br>(-77.0 to -47.5)	-68.2<br>(-78.7 to -51.2)
Cutaneous and mucocutaneous leishmaniasis	250.3<br>(210.7 to 297.7)	107.7<br>(71.8 to 151.7)	16.4<br>(10.4 to 23.9)	20.5<br>(-0.2 to 46.6)	12.0<br>(-7.3 to 36.1)
African trypanosomiasis	0.7<br>(0.1 to 2.4)	0.5<br>(0.1 to 1.7)	0.2<br>(< 0.05 to 0.7)	-75.2<br>(-95.8 to 31.3)	-77.0<br>(-96.1 to 22.0)
Schistosomiasis	3,785.1<br>(3,290.4 to 4,293.7)	8,205.1<br>(7,198.6 to 9,440.5)	26.9<br>(12.2 to 53.5)	-26.0<br>(-29.5 to -23.1)	-31.3<br>(-34.6 to -28.5)
Cysticercosis	0.4<br>(0.1 to 0.8)	--	0.1<br>(< 0.05 to 0.1)	-2.4<br>(-17.6 to 12.8)	-9.3<br>(-23.5 to 4.7)
Cystic echinococcosis	68.1<br>(32.3 to 159.2)	14.8<br>(6.1 to 36.7)	6.5<br>(2.8 to 15.5)	-41.9<br>(-80.9 to 57.3)	-46.1<br>(-82.2 to 46.1)
Lymphatic filariasis	2,200.8<br>(1,880.3 to 2,625.0)	624.1<br>(352.4 to 996.1)	56.8<br>(24.9 to 110.8)	-42.4<br>(-54.0 to -32.3)	-46.5<br>(-57.3 to -37.1)
Onchocerciasis	1,043.2<br>(567.8 to 1,598.0)	493.5<br>(305.5 to 830.5)	72.7<br>(25.9 to 138.0)	-31.4<br>(-52.4 to -14.9)	-36.2<br>(-55.8 to -20.9)
Trachoma	--	--	--	--	--
Dengue	701.4<br>(381.1 to 1,189.5)	11,724.6<br>(7,116.6 to 17,813.3)	116.9<br>(51.2 to 219.0)	58.1<br>(26.7 to 266.0)	46.9<br>(17.6 to 239.9)
Yellow fever	0.6<br>(0.1 to 1.5)	20.7<br>(5.6 to 57.5)	< 0.05<br>(< 0.05 to 0.1)	-18.9<br>(-48.3 to 22.7)	-24.6<br>(-52.0 to 13.9)
Rabies	0.1<br>(< 0.05 to 0.1)	2.1<br>(0.8 to 3.9)	< 0.05<br>(< 0.05 to < 0.05)	-49.8<br>(-69.7 to -10.8)	-53.4<br>(-71.9 to -17.1)
Intestinal nematode infections	137,644.5<br>(131,797.5 to 142,951.4)	--	322.3<br>(186.0 to 523.6)	-20.4<br>(-26.0 to -15.2)	-26.1<br>(-31.3 to -21.3)
Ascariasis	73,279.6<br>(66,489.1 to 81,606.1)	--	118.5<br>(64.2 to 199.3)	-27.4<br>(-36.1 to -17.6)	-32.5<br>(-40.7 to -23.4)

Cause	Prevalence in thousands (95% UI)	Incidence in thousands (95% UI)	YLDs in thousands (95% UI)		
	2016 counts	2016 counts	2016 counts	Percentage change in counts between 2006 and 2016	Percentage change in rates between 2006 and 2016
Trichuriasis	43,745.8 (40,537.0 to 46,905.2)	--	40.3 (22.1 to 70.0)	-26.4 (-35.2 to -16.4)	-31.7 (-39.8 to -22.4)
Hookworm disease	37,109.4 (34,168.8 to 40,690.5)	--	163.5 (99.1 to 252.1)	-12.6 (-20.3 to -4.6)	-18.8 (-26.0 to -11.4)
Food-borne trematodiases	1,736.1 (1,530.3 to 1,999.7)	280.8 (239.2 to 335.0)	18.7 (9.5 to 32.5)	-18.9 (-27.8 to -6.2)	-24.7 (-33.0 to -12.9)
Leprosy	0.9 (0.8 to 1.0)	0.5 (0.4 to 0.6)	< 0.05 (< 0.05 to 0.1)	-32.9 (-34.3 to -31.4)	-37.7 (-39.0 to -36.3)
Ebola virus disease	< 0.05 (< 0.05 to 0.2)	--	< 0.05 (0.0 to < 0.05)	--	--
Zika virus disease	7.8 (4.4 to 14.0)	468.6 (265.9 to 847.7)	0.1 (0.1 to 0.3)	--	--
Guinea worm disease	< 0.05 (< 0.05 to < 0.05)	< 0.05 (< 0.05 to < 0.05)	< 0.05 (< 0.05 to < 0.05)	-99.5 (-99.7 to -99.3)	-99.5 (-99.7 to -99.4)
Other neglected tropical diseases	13,238.1 (12,322.8 to 14,154.6)	--	532.9 (346.3 to 787.9)	3.5 (0.4 to 6.8)	-3.9 (-6.8 to -0.8)
**Maternal disorders**	**--**	**--**	**--**	**--**	**--**
Maternal haemorrhage	--	--	--	--	--
Maternal sepsis and other pregnancy related infections	--	--	--	--	--
Maternal hypertensive disorders	--	--	--	--	--
Maternal obstructed labour and uterine rupture	--	--	--	--	--
Maternal abortion, miscarriage, and ectopic pregnancy	--	--	--	--	--
Other maternal disorders	--	--	--	--	--
**Neonatal disorders**	**7,696.7 (7,016.2 to 8,432.1)**	**--**	**1,506.4 (1,030.1 to 2,205.1)**	**19.4 (6.0 to 33.8)**	**10.9 (-1.6 to 24.2)**
Neonatal preterm birth complications	6,068.8 (4,988.5 to 7,433.5)	--	950.0 (650.4 to 1,364.6)	13.4 (-4.9 to 34.5)	5.3 (-11.7 to 24.9)
Neonatal encephalopathy due to birth asphyxia and trauma	1,238.0 (723.4 to 2,148.6)	--	162.5 (45.5 to 439.7)	36.7 (21.7 to 61.8)	26.9 (13.1 to 50.3)
Neonatal sepsis and other neonatal infections	756.3 (253.1 to 1,833.8)	--	322.0 (93.4 to 826.9)	33.1 (22.2 to 51.4)	23.6 (13.5 to 40.6)
Hemolytic disease and other neonatal jaundice	237.6 (210.3 to 265.4)	--	71.9 (54.8 to 91.4)	14.2 (7.4 to 21.3)	6.0 (-0.2 to 12.7)
Other neonatal disorders	--	--	--	--	--
**Nutritional deficiencies**	**217,839.4 (209,759.0 to 223,459.5)**	**97,729.2† (76,185.7 to 126,674.8)**	**6,298.6 (4,084.2 to 9,348.8)**	**2.1 (-0.3 to 5.0)**	**-5.2 (-7.4 to -2.5)**
Protein-energy malnutrition	--	--	--	--	--
Iodine deficiency	3,968.8 (3,468.4 to 4,550.6)	1,040.6 (910.1 to 1,186.6)	107.4 (69.3 to 155.7)	-23.4 (-28.1 to -18.7)	-28.9 (-33.2 to -24.4)
Vitamin A deficiency	106,005.9 (84,529.4 to 133,546.9)	96,688.6 (75,131.6 to 125,556.5)	43.3 (24.2 to 74.6)	8.3 (1.8 to 15.1)	0.6 (-5.5 to 6.9)
Iron-deficiency anaemia	153,365.3 (142,685.5 to 164,251.0)	--	6,147.9 (3,986.2 to 9,111.1)	2.6 (0.3 to 5.6)	-4.7 (-6.9 to -1.9)
Other nutritional deficiencies	--	--	--	--	--
**Other communicable, maternal, neonatal, and nutritional diseases**	**47,817.5 (42,440.2 to 52,938.3)**	**8,296.8 (4,817.6 to 12,658.1)**	**403.5 (260.9 to 594.3)**	**6.7 (2.5 to 11.1)**	**-0.9 (-4.8 to 3.2)**
Sexually transmitted diseases excluding HIV	54.0 (37.2 to 87.3)	74.1 (42.8 to 141.1)	< 0.05 (< 0.05 to < 0.05)	5.1 (-6.1 to 13.1)	-2.4 (-12.8 to 5.0)
Syphilis	46.4 (26.2 to 84.7)	74.1 (42.8 to 141.1)	< 0.05 (< 0.05 to < 0.05)	5.1 (-6.1 to 13.1)	-2.4 (-12.8 to 5.0)
Chlamydial infection	--	--	--	--	--
Gonococcal infection	--	--	--	--	--
Trichomoniasis	--	--	--	--	--
Genital herpes	--	--	--	--	--
Other sexually transmitted diseases	--	--	--	--	--
Hepatitis	38,110.6 (29,114.1 to 48,416.6)	8,221.9 (4,742.5 to 12,589.9)	7.6 (3.0 to 15.1)	-44.6 (-79.7 to 50.3)	-48.5 (-81.1 to 39.6)
Acute hepatitis A	233.7 (22.6 to 528.5)	3,038.1 (293.7 to 6,870.1)	4.4 (0.4 to 10.8)	-43.4 (-95.4 to 650.6)	-47.5 (-95.8 to 597.1)
Hepatitis B	28,243.4 (19,371.2 to 38,396.5)	3,538.5 (1,990.8 to 5,641.8)	1.7 (0.8 to 3.0)	-44.5 (-58.0 to -25.2)	-48.4 (-61.0 to -30.5)
Hepatitis C	9,523.2 (8,145.7 to 11,025.9)	210.8 (142.6 to 303.9)	0.2 (0.1 to 0.4)	-36.5 (-39.1 to -34.8)	-41.0 (-43.5 to -39.5)
Acute hepatitis E	110.4 (89.8 to 131.5)	1,434.5 (1,167.3 to 1,709.3)	1.3 (0.7 to 2.2)	-49.2 (-66.0 to -22.4)	-52.8 (-68.4 to -27.9)
Other infectious diseases	9,659.1 (8,987.0 to 10,343.9)	0.8 (0.4 to 1.3)	395.9 (256.1 to 581.5)	8.7 (5.6 to 12.2)	0.9 (-1.9 to 4.2)
**Non-communicable diseases**	**517,935.1 (508,172.2 to 527,920.7)**	**1,825,947.0† (1,392,250.4 to 2,329,278.3)**	**17,286.6 (12,210.7 to 23,494.2)**	**8.5 (7.5 to 9.7)**	**0.8 (-0.2 to 1.8)**
**Neoplasms**	**108.7 (97.5 to 116.6)**	**57.5 (50.2 to 61.7)**	**19.9 (14.0 to 27.2)**	**15.0 (3.2 to 24.8)**	**6.8 (-4.2 to 15.9)**
Lip and oral cavity cancer	--	--	--	--	--
Nasopharynx cancer	2.0 (1.8 to 2.2)	0.5 (0.5 to 0.6)	0.2 (0.1 to 0.3)	-6.0 (-18.4 to 10.7)	-12.7 (-24.3 to 2.8)
Other pharynx cancer	--	--	--	--	--
Oesophageal cancer	--	--	--	--	--
Stomach cancer	--	--	--	--	--
Colon and rectum cancer	--	--	--	--	--
Liver cancer	0.7 (0.6 to 0.8)	0.8 (0.7 to 0.8)	0.2 (0.1 to 0.2)	12.8 (-7.1 to 29.0)	4.8 (-13.7 to 19.8)
Liver cancer due to hepatitis B	0.3 (0.2 to 0.4)	0.2 (0.1 to 0.3)	< 0.05 (< 0.05 to 0.1)	16.1 (-2.4 to 36.4)	7.8 (-9.3 to 26.7)

Cause	Prevalence in thousands (95% UI)	Incidence in thousands (95% UI)	YLDs in thousands (95% UI)		
	2016 counts	2016 counts	2016 counts	Percentage change in counts between 2006 and 2016	Percentage change in rates between 2006 and 2016
Liver cancer due to hepatitis C	< 0.05 (< 0.05 to < 0.05)	< 0.05 (< 0.05 to < 0.05)	< 0.05 (< 0.05 to < 0.05)	9.1 (-7.9 to 25.8)	1.4 (-14.5 to 16.8)
Liver cancer due to alcohol use	--	--	--	--	--
Liver cancer due to other causes	0.6 (0.5 to 0.7)	0.5 (0.5 to 0.6)	0.1 (0.1 to 0.2)	11.8 (-8.9 to 27.3)	3.8 (-15.4 to 18.2)
Gallbladder and biliary tract cancer	--	--	--	--	--
Pancreatic cancer	--	--	--	--	--
Larynx cancer	--	--	--	--	--
Tracheal, bronchus, and lung cancer	--	--	--	--	--
Malignant skin melanoma	--	--	--	--	--
Non-melanoma skin cancer	--	--	--	--	--
Non-melanoma skin cancer (squamous-cell carcinoma)	--	--	--	--	--
Non-melanoma skin cancer (basal-cell carcinoma)	--	--	--	--	--
Breast cancer	--	--	--	--	--
Cervical cancer	--	--	--	--	--
Uterine cancer	--	--	--	--	--
Ovarian cancer	--	--	--	--	--
Prostate cancer	--	--	--	--	--
Testicular cancer	--	--	--	--	--
Kidney cancer	16.8 (14.8 to 18.4)	4.0 (3.6 to 4.4)	1.4 (0.9 to 1.9)	14.9 (2.2 to 25.6)	6.7 (-5.1 to 16.6)
Bladder cancer	--	--	--	--	--
Brain and nervous system cancer	29.3 (24.5 to 32.3)	12.1 (10.4 to 13.2)	3.8 (2.6 to 5.2)	24.8 (15.2 to 34.6)	15.9 (7.0 to 25.0)
Thyroid cancer	--	--	--	--	--
Mesothelioma	--	--	--	--	--
Hodgkin lymphoma	7.1 (5.1 to 9.1)	1.9 (1.4 to 2.5)	0.7 (0.4 to 1.0)	-17.0 (-31.4 to 4.8)	-22.9 (-36.3 to -2.6)
Non-Hodgkin's lymphoma	24.5 (19.5 to 27.9)	7.3 (5.9 to 8.3)	2.3 (1.5 to 3.1)	18.8 (1.1 to 34.7)	10.3 (-6.1 to 25.1)
Multiple myeloma	--	--	--	--	--
Leukaemia	70.8 (57.8 to 79.4)	18.4 (15.3 to 20.3)	7.7 (5.2 to 10.7)	9.2 (-7.8 to 25.4)	1.4 (-14.4 to 16.5)
Acute lymphoid leukaemia	35.8 (30.9 to 39.0)	9.2 (8.0 to 10.0)	4.2 (2.9 to 5.8)	17.8 (-3.9 to 33.5)	9.4 (-10.7 to 24.0)
Chronic lymphoid leukaemia	--	--	--	--	--
Acute myeloid leukaemia	4.6 (3.7 to 5.2)	3.2 (2.6 to 3.6)	0.9 (0.6 to 1.2)	11.2 (-5.6 to 25.5)	3.3 (-12.3 to 16.6)
Chronic myeloid leukaemia	--	--	--	--	--
Other leukaemia	30.4 (22.5 to 36.1)	5.9 (4.4 to 7.0)	2.6 (1.6 to 3.7)	-3.1 (-19.3 to 19.0)	-10.0 (-25.1 to 10.5)
Other neoplasms	46.8 (40.6 to 50.4)	12.5 (11.0 to 13.4)	3.7 (2.5 to 5.1)	26.7 (9.9 to 38.4)	17.7 (2.0 to 28.5)
**Cardiovascular diseases**	**2,976.9 (2,620.8 to 3,359.9)**	**659.8† (601.8 to 727.3)**	**197.0 (130.2 to 282.7)**	**11.9 (8.9 to 14.9)**	**3.9 (1.1 to 6.7)**
Rheumatic heart disease	1,229.7 (1,079.8 to 1,386.0)	237.5 (209.6 to 267.2)	59.0 (36.5 to 86.9)	11.8 (6.4 to 17.4)	3.9 (-1.2 to 9.0)
Ischaemic heart disease	278.0 (252.2 to 307.1)	201.3 (174.5 to 234.5)	16.6 (10.9 to 24.5)	9.1 (1.2 to 17.9)	1.3 (-6.0 to 9.5)
Cerebrovascular disease	106.8 (82.3 to 137.7)	51.7‡ (29.3 to 79.9)	27.9 (18.1 to 40.0)	2.3 (-4.0 to 7.3)	-5.0 (-10.8 to -0.4)
Ischaemic stroke	98.0 (66.3 to 135.8)	32.0‡ (13.1 to 57.5)	15.3 (9.1 to 23.6)	7.5 (0.6 to 12.8)	-0.1 (-6.6 to 4.7)
Hemorrhagic stroke	83.5 (60.1 to 112.1)	19.7‡ (8.2 to 36.0)	12.6 (8.0 to 18.6)	-3.5 (-13.4 to 5.2)	-10.3 (-19.6 to -2.3)
Hypertensive heart disease	0.1 (< 0.05 to 0.1)	--	< 0.05 (< 0.05 to < 0.05)	24.2 (18.4 to 30.8)	15.3 (9.9 to 21.5)
Cardiomyopathy and myocarditis	94.9 (71.5 to 125.6)	109.1† (81.0 to 143.7)	10.0 (5.9 to 16.1)	8.8 (7.0 to 10.6)	1.1 (-0.6 to 2.7)
Myocarditis	92.7 (68.9 to 122.8)	109.1 (81.0 to 143.7)	7.3 (4.3 to 11.6)	8.8 (6.6 to 11.0)	1.0 (-1.0 to 3.1)
Alcoholic cardiomyopathy	--	--	--	--	--
Other cardiomyopathy	30.3 (20.1 to 43.8)	--	2.7 (1.6 to 4.5)	9.0 (7.4 to 10.4)	1.2 (-0.2 to 2.6)
Atrial fibrillation and flutter	--	--	--	--	--
Peripheral vascular disease	--	--	--	--	--
Endocarditis	4.6 (3.5 to 5.7)	60.1 (44.4 to 74.9)	0.2 (0.1 to 0.4)	2.3 (-1.1 to 5.3)	-5.0 (-8.2 to -2.2)
Other cardiovascular and circulatory diseases	1,510.8 (920.3 to 2,297.6)	--	83.2 (44.4 to 137.8)	16.6 (11.3 to 21.8)	8.3 (3.4 to 13.1)
**Chronic respiratory diseases**	**49,863.2 (45,965.3 to 55,587.5)**	**9,406.2† (7,763.6 to 11,587.5)**	**2,150.8 (1,433.0 to 3,094.4)**	**15.7 (13.4 to 18.1)**	**7.5 (5.3 to 9.6)**
Chronic obstructive pulmonary disease	1,016.9 (909.4 to 1,133.4)	134.9 (116.6 to 154.8)	89.9 (75.4 to 104.0)	2.5 (-4.7 to 10.6)	-4.8 (-11.5 to 2.7)
Pneumoconiosis	--	--	--	--	--
Silicosis	--	--	--	--	--

Cause	Prevalence in thousands (95% UI)	Incidence in thousands (95% UI)	YLDs in thousands (95% UI)		
	2016 counts	2016 counts	2016 counts	Percentage change in counts between 2006 and 2016	Percentage change in rates between 2006 and 2016
Asbestosis	--	--	--	--	--
Coal workers pneumoconiosis	--	--	--	--	--
Other pneumoconiosis	--	--	--	--	--
Asthma	47,965.0<br>(41,179.9 to 55,484.7)	9,268.2<br>(7,617.1 to 11,463.8)	1,928.3<br>(1,217.2 to 2,869.2)	15.1<br>(12.7 to 17.6)	6.9<br>(4.6 to 9.2)
Interstitial lung disease and pulmonary sarcoidosis	57.8<br>(46.1 to 71.5)	3.1<br>(1.9 to 4.5)	6.5<br>(4.0 to 9.7)	4.7<br>(3.5 to 5.9)	-2.8<br>(-3.9 to -1.7)
Other chronic respiratory diseases	--	--	126.1<br>(106.3 to 145.5)	40.6<br>(31.3 to 51.0)	30.6<br>(22.0 to 40.3)
**Cirrhosis and other chronic liver diseases**	**600.4<br>(555.8 to 650.3)**	**7.1<br>(5.0 to 9.8)**	**87.5<br>(59.5 to 124.4)**	**8.6<br>(2.8 to 14.3)**	**0.8<br>(-4.5 to 6.2)**
Cirrhosis and other chronic liver diseases due to hepatitis B	12.0<br>(9.1 to 15.3)	0.2<br>(0.1 to 0.2)	2.0<br>(1.2 to 2.9)	12.9<br>(7.9 to 17.9)	4.9<br>(0.2 to 9.5)
Cirrhosis and other chronic liver diseases due to hepatitis C	10.0<br>(7.4 to 13.3)	0.1<br>(0.1 to 0.2)	1.6<br>(0.9 to 2.4)	10.7<br>(6.9 to 14.1)	2.8<br>(-0.7 to 6.0)
Cirrhosis and other chronic liver diseases due to alcohol use	--	--	--	--	--
Cirrhosis and other chronic liver diseases due to other causes	578.4<br>(534.6 to 627.0)	6.8<br>(4.8 to 9.4)	84.0<br>(56.8 to 119.6)	8.4<br>(2.3 to 14.3)	0.7<br>(-5.0 to 6.2)
**Digestive diseases**	**7,242.6<br>(6,349.0 to 8,127.0)**	**6,063.2†<br>(5,201.7 to 6,978.3)**	**285.2<br>(179.6 to 414.3)**	**3.3<br>(-0.5 to 7.3)**	**-4.0<br>(-7.6 to -0.3)**
Peptic ulcer disease	264.8<br>(192.8 to 348.2)	209.9<br>(136.4 to 291.0)	8.2<br>(4.4 to 13.4)	-11.9<br>(-19.1 to -4.1)	-18.2<br>(-24.9 to -10.9)
Gastritis and duodenitis	3,162.6<br>(2,262.3 to 4,262.9)	2,221.9<br>(1,670.5 to 2,885.1)	40.0<br>(23.4 to 64.0)	1.6<br>(-5.6 to 9.6)	-5.7<br>(-12.3 to 1.8)
Appendicitis	68.6<br>(56.1 to 83.0)	1,936.0<br>(1,536.3 to 2,367.9)	21.6<br>(13.8 to 30.8)	5.2<br>(-6.8 to 17.8)	-2.2<br>(-13.4 to 9.4)
Paralytic ileus and intestinal obstruction	8.3<br>(5.4 to 12.2)	216.0<br>(138.6 to 315.0)	2.7<br>(1.5 to 4.4)	10.2<br>(7.5 to 12.2)	2.3<br>(-0.1 to 4.2)
Inguinal, femoral, and abdominal hernia	3,817.3<br>(2,336.0 to 5,566.5)	1,092.2<br>(737.7 to 1,494.8)	169.7<br>(97.8 to 264.6)	2.5<br>(-2.3 to 7.6)	-4.8<br>(-9.3 to -0.0)
Inflammatory bowel disease	28.5<br>(23.4 to 34.0)	91.6<br>(76.3 to 111.2)	4.5<br>(2.9 to 6.7)	0.3<br>(-5.5 to 6.0)	-6.8<br>(-12.2 to -1.5)
Vascular intestinal disorders	2.8<br>(1.6 to 4.5)	14.9<br>(9.4 to 22.4)	0.9<br>(0.4 to 1.7)	9.7<br>(8.2 to 11.2)	1.9<br>(0.5 to 3.3)
Gallbladder and biliary diseases	151.0<br>(103.8 to 213.6)	222.8<br>(132.6 to 332.6)	1.1<br>(0.6 to 1.9)	0.7<br>(-2.7 to 3.6)	-6.5<br>(-9.6 to -3.8)
Pancreatitis	6.6<br>(4.6 to 9.2)	57.8<br>(39.9 to 79.6)	0.9<br>(0.5 to 1.3)	3.1<br>(-4.2 to 8.1)	-4.2<br>(-11.1 to 0.4)
Other digestive diseases	--	--	35.6<br>(22.4 to 51.6)	13.2<br>(9.0 to 17.5)	5.1<br>(1.2 to 9.2)
**Neurological disorders**	**59,371.6<br>(52,973.2 to 66,350.3)**	**49,338.8<br>(36,288.7 to 66,500.3)**	**1,661.1<br>(1,090.3 to 2,360.4)**	**11.9<br>(3.2 to 22.1)**	**3.9<br>(-4.1 to 13.4)**
Alzheimer's disease and other dementias	--	--	--	--	--
Parkinson's disease	--	--	--	--	--
Epilepsy	2,406.8<br>(1,798.7 to 3,146.4)	341.3<br>(229.7 to 480.9)	827.9<br>(519.9 to 1,251.8)	10.7<br>(-2.8 to 26.6)	2.8<br>(-9.7 to 17.6)
Multiple sclerosis	3.6<br>(2.6 to 4.8)	1.0<br>(0.7 to 1.4)	1.0<br>(0.6 to 1.5)	7.7<br>(5.4 to 9.5)	0.0<br>(-2.1 to 1.7)
Motor neuron disease	12.7<br>(10.0 to 15.8)	0.6<br>(0.4 to 0.9)	2.7<br>(1.8 to 3.9)	8.2<br>(7.0 to 9.6)	0.5<br>(-0.6 to 1.8)
Migraine	14,539.3<br>(13,058.8 to 15,923.5)	9,359.2<br>(8,373.5 to 10,291.4)	539.6<br>(323.5 to 804.9)	5.9<br>(2.5 to 9.2)	-1.7<br>(-4.8 to 1.5)
Tension-type headache	42,756.6<br>(29,721.8 to 59,128.1)	39,632.7<br>(26,653.4 to 57,031.4)	74.4<br>(36.8 to 135.7)	3.5<br>(1.0 to 6.2)	-3.9<br>(-6.2 to -1.3)
Other neurological disorders	2.0<br>(0.9 to 3.9)	4.0<br>(1.9 to 7.0)	215.5<br>(135.7 to 325.2)	41.7<br>(24.5 to 61.9)	31.6<br>(15.7 to 50.4)
**Mental and substance use disorders**	**43,639.7<br>(41,908.6 to 45,509.6)**	**14,192.9†<br>(12,027.0 to 16,543.4)**	**3,598.6<br>(2,453.3 to 4,932.7)**	**7.7<br>(6.4 to 9.0)**	**0.0<br>(-1.1 to 1.2)**
Schizophrenia	--	--	--	--	--
Alcohol use disorders	32.3<br>(29.9 to 34.9)	--	1.6<br>(1.0 to 2.3)	10.3<br>(6.5 to 12.7)	2.4<br>(-1.1 to 4.7)
Drug use disorders	--	--	--	--	--
Opioid use disorders	--	--	--	--	--
Cocaine use disorders	--	--	--	--	--
Amphetamine use disorders	--	--	--	--	--
Cannabis use disorders	--	--	--	--	--
Other drug use disorders	--	--	--	--	--
Depressive disorders	925.4<br>(766.2 to 1,157.4)	1,637.6<br>(1,026.1 to 2,489.1)	177.1<br>(99.7 to 289.2)	15.2<br>(10.3 to 21.1)	7.0<br>(2.5 to 12.4)
Major depressive disorder	704.2<br>(418.0 to 1,123.4)	1,467.7<br>(845.1 to 2,311.9)	151.8<br>(78.6 to 261.3)	16.4<br>(10.6 to 22.9)	8.1<br>(2.7 to 14.1)
Dysthymia	251.7<br>(163.0 to 363.2)	169.9<br>(105.2 to 257.2)	25.3<br>(14.1 to 41.4)	8.5<br>(-0.6 to 18.8)	0.7<br>(-7.7 to 10.3)
Bipolar disorder	--	--	--	--	--
Anxiety disorders	9,252.2<br>(7,965.9 to 10,726.2)	4,101.7<br>(3,522.3 to 4,741.8)	930.5<br>(635.6 to 1,283.1)	8.1<br>(5.2 to 10.9)	0.4<br>(-2.3 to 3.0)
Eating disorders	67.2<br>(47.8 to 101.6)	76.4<br>(46.8 to 118.9)	13.5<br>(6.6 to 24.8)	15.5<br>(5.1 to 26.9)	7.3<br>(-2.4 to 17.9)
Anorexia nervosa	56.6<br>(29.4 to 98.8)	49.9<br>(25.0 to 86.4)	12.5<br>(5.7 to 23.7)	15.3<br>(4.2 to 27.8)	7.0<br>(-3.3 to 18.7)
Bulimia nervosa	4.5<br>(2.1 to 8.4)	26.5<br>(12.9 to 47.6)	1.0<br>(0.4 to 2.1)	18.6<br>(16.6 to 20.2)	10.1<br>(8.3 to 11.6)
Autistic spectrum disorders	6,088.7<br>(5,772.6 to 6,327.9)	151.2<br>(82.7 to 242.9)	890.8<br>(591.7 to 1,259.5)	7.7<br>(6.1 to 9.4)	0.1<br>(-1.4 to 1.6)
Autism	1,715.3<br>(1,449.9 to 2,017.6)	15.7<br>(7.2 to 29.0)	448.7<br>(285.5 to 663.2)	7.6<br>(4.9 to 10.4)	-0.1<br>(-2.5 to 2.6)
Asperger syndrome and other autistic spectrum disorders	4,335.3<br>(3,524.9 to 5,256.4)	135.5<br>(66.9 to 227.9)	442.1<br>(287.9 to 652.1)	7.9<br>(6.2 to 9.7)	0.2<br>(-1.4 to 1.9)

Cause	Prevalence in thousands (95% UI)	Incidence in thousands (95% UI)	YLDs in thousands (95% UI)		
	2016 counts	2016 counts	2016 counts	Percentage change in counts between 2006 and 2016	Percentage change in rates between 2006 and 2016
Attention-deficit/hyperactivity disorder	9,558.7 (8,533.6 to 10,936.6)	1,860.4 (1,660.4 to 2,132.2)	116.4 (69.7 to 184.4)	10.5 (8.0 to 13.0)	2.6 (0.3 to 4.9)
Conduct disorder	8,522.9 (6,256.9 to 11,296.5)	6,365.6 (4,493.2 to 8,520.7)	1,043.3 (598.0 to 1,664.1)	9.2 (7.0 to 11.6)	1.4 (-0.6 to 3.7)
Idiopathic developmental intellectual disability	10,735.9 (6,519.2 to 15,306.6)	--	425.6 (196.6 to 737.0)	-0.2 (-4.4 to 2.7)	-7.3 (-11.2 to -4.7)
Other mental and substance use disorders	--	--	--	--	--
**Diabetes, urogenital, blood, and endocrine diseases**	**178,779.3 (176,721.1 to 181,185.3)**	**15,170.8† (11,205.4 to 20,161.3)**	**1,380.2 (897.9 to 2,023.2)**	**3.4 (0.6 to 6.3)**	**-4.0 (-6.5 to -1.3)**
Diabetes mellitus	456.8 (316.0 to 622.2)	179.2 (114.3 to 251.4)	22.0 (11.8 to 36.5)	8.1 (2.3 to 13.7)	0.4 (-5.0 to 5.6)
Acute glomerulonephritis	6.3 (4.2 to 8.9)	96.5 (65.8 to 134.6)	0.3 (0.2 to 0.5)	3.0 (0.7 to 5.9)	-4.4 (-6.5 to -1.6)
Chronic kidney disease	345.6 (244.7 to 471.0)	108.3 (73.0 to 155.5)	25.3 (14.5 to 40.1)	8.3 (3.7 to 12.6)	0.6 (-3.6 to 4.5)
Chronic kidney disease due to diabetes mellitus	12.8 (6.8 to 21.4)	3.9 (2.0 to 6.7)	1.0 (0.5 to 1.8)	6.0 (-0.1 to 12.5)	-1.6 (-7.2 to 4.5)
Chronic kidney disease due to hypertension	72.4 (44.4 to 108.8)	43.6 (25.5 to 64.9)	0.4 (0.2 to 0.7)	4.5 (-1.1 to 9.6)	-3.0 (-8.2 to 1.8)
Chronic kidney disease due to glomerulonephritis	71.1 (43.2 to 105.5)	1.7 (0.9 to 2.7)	10.3 (5.4 to 17.1)	8.9 (4.5 to 13.3)	1.2 (-3.0 to 5.2)
Chronic kidney disease due to other causes	189.3 (124.0 to 276.9)	59.2 (38.1 to 89.8)	13.6 (7.7 to 22.6)	8.2 (3.3 to 12.5)	0.5 (-4.0 to 4.5)
Urinary diseases and male infertility	314.3 (270.5 to 383.3)	14,491.1† (10,507.7 to 19,483.3)	15.0 (9.1 to 24.3)	-4.0 (-10.0 to 3.1)	-10.8 (-16.4 to -4.2)
Interstitial nephritis and urinary tract infections	270.8 (192.7 to 367.2)	13,968.7 (9,980.2 to 18,944.0)	9.2 (5.2 to 15.6)	6.6 (-1.1 to 15.7)	-1.0 (-8.1 to 7.4)
Urolithiasis	19.5 (13.4 to 27.2)	522.3 (360.5 to 748.9)	1.5 (0.9 to 2.4)	2.8 (1.7 to 3.9)	-4.5 (-5.6 to -3.5)
Benign prostatic hyperplasia	--	--	--	--	--
Male infertility	--	--	--	--	--
Other urinary diseases	--	--	4.3 (2.6 to 7.0)	-22.1 (-27.0 to -16.4)	-27.7 (-32.2 to -22.3)
Gynecological diseases	--	--	--	--	--
Uterine fibroids	--	--	--	--	--
Polycystic ovarian syndrome	--	--	--	--	--
Female infertility	--	--	--	--	--
Endometriosis	--	--	--	--	--
Genital prolapse	--	--	--	--	--
Premenstrual syndrome	--	--	--	--	--
Other gynecological diseases	--	--	--	--	--
Hemoglobinopathies and hemolytic anaemias	172,031.6 (169,839.1 to 174,424.6)	--	967.6 (630.8 to 1,445.0)	2.7 (-0.7 to 6.2)	-4.6 (-7.8 to -1.4)
Thalassemias	69.8 (64.7 to 75.7)	--	4.4 (2.9 to 6.5)	-2.4 (-9.8 to 5.3)	-9.3 (-16.2 to -2.2)
Thalassaemias trait	22,678.8 (21,819.5 to 23,683.5)	--	385.3 (242.8 to 579.2)	-0.8 (-6.5 to 5.0)	-7.9 (-13.2 to -2.5)
Sickle cell disorders	928.2 (840.6 to 1,070.2)	--	77.5 (52.3 to 110.8)	17.7 (9.0 to 26.7)	9.3 (1.2 to 17.7)
Sickle cell trait	51,379.8 (46,907.9 to 57,162.0)	--	273.8 (176.2 to 407.4)	4.2 (0.5 to 7.9)	-3.2 (-6.7 to 0.2)
G6PD deficiency	33,857.8 (32,511.5 to 35,468.9)	--	3.6 (2.4 to 5.3)	-7.9 (-14.9 to -0.7)	-14.5 (-21.0 to -7.8)
G6PD trait	79,732.6 (78,413.0 to 81,221.3)	--	< 0.05 (< 0.05 to 0.1)	8.9 (3.9 to 14.5)	1.2 (-3.5 to 6.3)
Other hemoglobinopathies and hemolytic anaemias	5,575.0 (5,195.5 to 5,961.8)	--	222.9 (145.2 to 326.5)	3.0 (-0.7 to 7.6)	-4.3 (-7.8 to -0.1)
Endocrine, metabolic, blood, and immune disorders	8,894.2 (8,246.8 to 9,620.1)	295.6 (191.6 to 433.7)	350.0 (226.3 to 502.3)	4.9 (2.1 to 7.8)	-2.6 (-5.1 to 0.1)
**Musculoskeletal disorders**	**2,257.2 (1,774.6 to 2,950.8)**	**2,028.0 (1,321.4 to 2,943.9)**	**251.5 (145.6 to 400.1)**	**4.0 (-0.2 to 7.7)**	**-3.4 (-7.4 to 0.0)**
Rheumatoid arthritis	32.0 (21.4 to 44.4)	19.2 (12.8 to 26.6)	8.4 (4.9 to 12.8)	8.0 (4.7 to 10.7)	0.3 (-2.8 to 2.9)
Osteoarthritis	--	--	--	--	--
Low back and neck pain	2,255.2 (1,772.3 to 2,950.0)	2,008.7 (1,303.6 to 2,926.8)	243.2 (139.4 to 391.1)	3.9 (-0.6 to 7.7)	-3.5 (-7.6 to 0.0)
Low back pain	1,940.2 (1,191.9 to 2,927.4)	1,779.1 (1,099.6 to 2,682.4)	210.5 (113.7 to 352.1)	3.4 (-1.6 to 7.8)	-3.9 (-8.7 to 0.1)
Neck pain	305.2 (191.0 to 443.9)	229.6 (142.1 to 331.0)	32.7 (16.7 to 53.7)	6.7 (2.2 to 11.0)	-0.9 (-5.0 to 3.1)
Gout	--	--	--	--	--
Other musculoskeletal disorders	--	--	--	--	--
**Other non-communicable diseases**	**420,125.7 (402,355.7 to 439,008.1)**	**1,729,022.9† (1,298,351.5 to 2,233,415.6)**	**7,654.8 (5,104.6 to 10,892.2)**	**7.6 (6.7 to 8.3)**	**-0.1 (-0.9 to 0.6)**
Congenital anomalies	9,600.5 (9,152.5 to 10,028.3)	--	1,075.1 (773.6 to 1,431.5)	5.6 (3.2 to 8.0)	-1.9 (-4.2 to 0.3)
Neural tube defects	589.1 (531.5 to 652.4)	--	175.7 (121.9 to 238.1)	10.5 (5.4 to 15.8)	2.6 (-2.1 to 7.5)
Congenital heart anomalies	2,147.3 (1,865.5 to 2,474.6)	--	101.9 (41.5 to 174.7)	5.9 (2.5 to 9.3)	-1.7 (-4.8 to 1.5)
Orofacial clefts	305.9 (271.1 to 341.0)	--	4.2 (2.8 to 6.1)	-1.8 (-4.9 to 1.4)	-8.8 (-11.7 to -5.8)
Down's syndrome	223.9 (195.2 to 259.1)	--	19.2 (12.8 to 27.3)	7.2 (4.0 to 10.3)	-0.5 (-3.4 to 2.4)
Turner syndrome	346.7 (302.9 to 396.4)	--	4.7 (1.9 to 8.5)	3.8 (-8.9 to 18.4)	-3.6 (-15.4 to 10.0)
Klinefelter syndrome	456.7 (393.5 to 526.6)	--	1.2 (0.6 to 2.3)	8.2 (-1.2 to 18.2)	0.5 (-8.2 to 9.8)

Cause	Prevalence in thousands (95% UI)	Incidence in thousands (95% UI)	YLDs in thousands (95% UI)		
	2016 counts	2016 counts	2016 counts	Percentage change in counts between 2006 and 2016	Percentage change in rates between 2006 and 2016
Other chromosomal abnormalities	995.7 (850.5 to 1,158.6)	--	105.8 (71.8 to 144.0)	13.1 (9.7 to 16.5)	5.0 (1.8 to 8.2)
Congenital musculoskeletal and limb anomalies	940.6 (854.5 to 1,034.5)	--	129.0 (86.9 to 179.3)	9.1 (5.0 to 13.1)	1.3 (-2.4 to 5.0)
Urogenital congenital anomalies	1,258.4 (1,119.8 to 1,422.9)	--	38.6 (23.1 to 59.9)	5.3 (2.9 to 7.6)	-2.2 (-4.5 to -0.1)
Digestive congenital anomalies	755.8 (676.5 to 837.0)	--	35.2 (22.9 to 49.9)	6.1 (0.9 to 11.8)	-1.4 (-6.3 to 3.8)
Other congenital anomalies	3,318.3 (2,345.3 to 4,695.9)	--	459.5 (304.0 to 668.3)	1.3 (-2.7 to 5.6)	-5.9 (-9.6 to -1.9)
Skin and subcutaneous diseases	174,715.9 (167,354.6 to 181,192.8)	369,758.8 (318,604.8 to 429,378.5)	4,799.2 (3,023.9 to 7,064.4)	9.2 (8.4 to 10.1)	1.4 (0.7 to 2.3)
Dermatitis	41,988.6 (37,439.5 to 46,865.3)	12,081.5 (9,954.4 to 14,307.1)	1,821.8 (1,025.4 to 3,080.4)	10.8 (9.1 to 12.4)	2.9 (1.3 to 4.4)
Psoriasis	2,789.3 (2,497.7 to 3,057.8)	504.2 (436.8 to 578.0)	254.2 (179.2 to 344.6)	13.8 (9.1 to 18.7)	5.7 (1.3 to 10.2)
Cellulitis	214.9 (170.7 to 265.0)	3,945.5 (3,179.9 to 4,760.4)	12.6 (7.7 to 19.1)	7.7 (2.7 to 13.5)	0.0 (-4.6 to 5.4)
Pyoderma	2,039.8 (1,826.2 to 2,251.5)	38,081.7 (34,496.4 to 41,445.6)	11.7 (4.6 to 25.4)	19.6 (16.1 to 22.9)	11.1 (7.9 to 14.2)
Scabies	15,919.3 (10,806.4 to 22,347.9)	49,410.0 (33,420.3 to 70,115.8)	415.6 (213.0 to 729.6)	2.0 (0.5 to 3.7)	-5.3 (-6.7 to -3.7)
Fungal skin diseases	72,070.2 (53,712.6 to 95,660.3)	185,156.5 (139,832.2 to 241,961.1)	413.4 (156.9 to 877.6)	12.7 (11.7 to 13.5)	4.6 (3.7 to 5.4)
Viral skin diseases	31,469.0 (28,758.4 to 34,434.4)	36,431.5 (32,907.5 to 40,391.9)	979.8 (595.3 to 1,469.0)	7.6 (6.7 to 8.5)	-0.0 (-0.9 to 0.8)
Acne vulgaris	7,567.4 (6,280.2 to 8,987.0)	10,620.9 (8,601.1 to 12,703.9)	197.8 (128.4 to 304.0)	11.1 (7.7 to 14.7)	3.2 (-0.0 to 6.6)
Alopecia areata	402.7 (356.0 to 456.9)	711.0 (621.5 to 806.0)	13.5 (8.3 to 20.8)	7.7 (1.4 to 14.7)	0.0 (-5.9 to 6.5)
Pruritus	3,624.0 (2,408.7 to 5,396.6)	2,639.3 (1,715.0 to 3,993.3)	39.5 (15.7 to 80.0)	8.3 (6.0 to 10.4)	0.6 (-1.5 to 2.6)
Urticaria	8,914.6 (6,234.9 to 12,614.1)	14,563.3 (10,302.5 to 20,463.9)	546.2 (301.7 to 871.8)	7.2 (5.3 to 8.9)	-0.5 (-2.2 to 1.2)
Decubitus ulcer	33.9 (22.6 to 47.0)	116.7 (79.0 to 162.8)	6.0 (3.5 to 9.5)	6.0 (4.8 to 7.0)	-1.5 (-2.6 to -0.7)
Other skin and subcutaneous diseases	15,417.4 (13,974.0 to 17,016.2)	15,496.7 (13,946.6 to 17,099.9)	87.2 (41.1 to 163.1)	13.3 (11.8 to 14.9)	5.2 (3.8 to 6.7)
Sense organ diseases	43,986.5 (41,928.2 to 46,056.3)	72,349.7† (64,169.6 to 80,991.9)	1,450.7 (972.5 to 2,105.4)	4.0 (2.0 to 6.0)	-3.4 (-5.3 to -1.5)
Glaucoma	--	--	--	--	--
Cataract	--	--	--	--	--
Macular degeneration	--	--	--	--	--
Refraction and accommodation disorders	27,796.0 (24,550.7 to 31,481.0)	--	622.6 (373.6 to 990.0)	3.5 (1.9 to 4.9)	-3.9 (-5.3 to -2.6)
Age-related and other hearing loss	12,024.2 (7,992.8 to 16,633.2)	--	663.1 (415.8 to 981.5)	3.4 (-0.6 to 7.4)	-4.0 (-7.6 to -0.3)
Other vision loss	467.4 (316.6 to 653.7)	--	21.5 (12.4 to 32.6)	8.1 (2.8 to 13.8)	0.4 (-4.5 to 5.7)
Other sense organ diseases	5,412.9 (4,774.8 to 6,083.9)	72,349.7 (64,169.6 to 80,991.9)	143.5 (86.9 to 220.0)	9.0 (5.8 to 12.3)	1.3 (-1.7 to 4.3)
Oral disorders	316,401.4 (286,327.5 to 352,581.8)	1,286,914.5† (873,624.0 to 1,798,610.1)	329.8 (187.2 to 539.6)	6.6 (5.1 to 7.9)	-1.0 (-2.4 to 0.2)
Caries of deciduous teeth	261,410.8 (191,631.7 to 339,769.4)	1,121,157.4 (713,500.7 to 1,612,830.8)	68.9 (28.7 to 139.2)	8.2 (4.8 to 10.6)	0.5 (-2.7 to 2.7)
Caries of permanent teeth	77,744.2 (55,549.4 to 104,471.1)	165,757.1 (96,478.4 to 260,406.4)	54.5 (22.6 to 112.5)	0.8 (-1.9 to 3.2)	-6.4 (-8.9 to -4.1)
Periodontal disease	--	--	--	--	--
Edentulism and severe tooth loss	--	--	--	--	--
Other oral disorders	6,873.6 (5,878.9 to 8,012.7)	--	206.3 (125.3 to 323.6)	7.7 (5.9 to 9.4)	0.0 (-1.6 to 1.6)
**Injuries**	**32,842.1 (29,336.2 to 36,963.0)**	**46,750.3† (39,950.9 to 54,884.2)**	**1,326.7 (859.0 to 1,978.6)**	**10.4 (8.7 to 12.1)**	**2.6 (1.0 to 4.1)**
**Transport injuries**	**2,440.0 (2,027.9 to 3,013.3)**	**3,711.8 (2,818.5 to 4,799.0)**	**151.0 (95.4 to 230.7)**	**5.8 (3.5 to 7.9)**	**-1.8 (-3.9 to 0.3)**
Road injuries	1,011.4 (868.1 to 1,150.1)	2,709.7 (1,858.0 to 3,619.4)	64.7 (39.8 to 98.9)	3.4 (-0.3 to 6.9)	-3.9 (-7.4 to -0.8)
Pedestrian road injuries	227.6 (171.6 to 292.7)	530.5 (316.7 to 817.2)	14.8 (8.7 to 22.9)	-5.2 (-9.5 to -0.6)	-11.9 (-16.0 to -7.7)
Cyclist road injuries	256.5 (190.3 to 321.4)	901.4 (532.6 to 1,353.0)	15.5 (9.0 to 24.1)	4.4 (-0.4 to 9.0)	-3.1 (-7.5 to 1.2)
Motorcyclist road injuries	181.8 (139.6 to 235.3)	297.1 (160.2 to 501.1)	10.8 (6.4 to 17.5)	12.5 (9.0 to 15.8)	4.5 (1.2 to 7.6)
Motor vehicle road injuries	230.9 (178.2 to 294.8)	626.9 (386.2 to 950.2)	17.0 (10.1 to 25.4)	1.7 (-2.6 to 5.9)	-5.6 (-9.6 to -1.6)
Other road injuries	114.6 (79.7 to 163.7)	353.8 (186.3 to 601.8)	6.5 (3.8 to 10.8)	14.4 (9.7 to 18.8)	6.3 (1.9 to 10.4)
Other transport injuries	1,428.5 (1,047.5 to 1,954.1)	1,002.1 (624.3 to 1,535.0)	86.3 (54.0 to 135.2)	7.6 (4.9 to 10.0)	-0.1 (-2.6 to 2.1)
**Unintentional injuries**	**28,410.9 (25,026.2 to 32,451.9)**	**42,054.8 (35,680.1 to 50,087.3)**	**1,054.0 (675.8 to 1,564.1)**	**11.4 (9.6 to 13.0)**	**3.5 (1.8 to 5.0)**
Falls	9,187.2 (7,178.4 to 11,834.1)	16,745.3 (11,427.6 to 23,792.7)	359.2 (234.3 to 522.7)	14.6 (12.3 to 16.8)	6.5 (4.3 to 8.4)
Drowning	111.4 (81.8 to 144.2)	93.4 (71.6 to 120.7)	6.7 (4.1 to 10.3)	-7.8 (-10.4 to -5.4)	-14.4 (-16.8 to -12.2)
Fire, heat, and hot substances	2,369.5 (1,737.8 to 3,254.0)	1,544.6 (1,017.6 to 2,228.6)	79.5 (50.3 to 118.4)	9.5 (5.8 to 13.0)	1.7 (-1.7 to 5.0)
Poisonings	131.2 (96.6 to 169.5)	529.8 (312.8 to 801.6)	15.6 (9.5 to 24.2)	20.0 (15.2 to 24.7)	11.5 (7.0 to 15.8)
Exposure to mechanical forces	3,535.2 (2,778.2 to 4,645.5)	4,826.6 (3,029.4 to 7,053.7)	91.2 (55.1 to 144.5)	11.8 (9.4 to 14.4)	3.8 (1.6 to 6.3)
Unintentional firearm injuries	86.1 (61.3 to 123.4)	101.0 (43.0 to 199.1)	3.9 (2.2 to 6.7)	13.9 (10.6 to 17.5)	5.8 (2.8 to 9.2)
Unintentional suffocation	530.0 (356.6 to 729.9)	174.6 (80.6 to 342.4)	16.6 (9.3 to 27.6)	6.3 (4.2 to 9.0)	-1.2 (-3.2 to 1.2)
Other exposure to mechanical forces	2,919.1 (2,230.2 to 4,025.1)	4,551.0 (2,859.7 to 6,654.4)	70.7 (43.5 to 109.7)	13.1 (10.4 to 15.7)	5.0 (2.5 to 7.4)

Cause	Prevalence in thousands (95% UI)	Incidence in thousands (95% UI)	YLDs in thousands (95% UI)		
	2016 counts	2016 counts	2016 counts	Percentage change in counts between 2006 and 2016	Percentage change in rates between 2006 and 2016
Adverse effects of medical treatment	149.9<br>(93.6 to 240.2)	1,960.7<br>(1,269.3 to 3,003.7)	20.0<br>(10.7 to 34.2)	17.0<br>(13.0 to 20.6)	8.7<br>(5.0 to 12.0)
Animal contact	1,576.3<br>(1,257.8 to 1,999.0)	5,993.7<br>(4,229.9 to 8,452.5)	79.1<br>(50.6 to 116.7)	1.8<br>(-1.7 to 5.2)	-5.5<br>(-8.7 to -2.3)
Venomous animal contact	750.3<br>(539.8 to 1,028.5)	2,749.3<br>(1,814.7 to 4,021.1)	62.8<br>(39.6 to 93.0)	1.9<br>(-2.2 to 6.0)	-5.4<br>(-9.2 to -1.6)
Non-venomous animal contact	826.0<br>(596.3 to 1,209.8)	3,244.3<br>(2,223.4 to 4,698.6)	16.3<br>(9.5 to 26.0)	1.3<br>(-2.2 to 4.5)	-5.9<br>(-9.2 to -3.0)
Foreign body	3,092.2<br>(2,356.4 to 3,860.9)	3,097.1<br>(2,210.9 to 4,183.1)	131.3<br>(81.0 to 207.7)	8.0<br>(5.4 to 10.5)	0.3<br>(-2.1 to 2.6)
Pulmonary aspiration and foreign body in airway	1,742.5<br>(1,182.3 to 2,600.9)	689.2<br>(399.1 to 1,093.0)	82.0<br>(49.1 to 129.4)	5.4<br>(2.7 to 7.9)	-2.1<br>(-4.6 to 0.2)
Foreign body in eyes	179.0<br>(58.9 to 368.0)	1,238.2<br>(624.4 to 2,156.6)	10.0<br>(3.1 to 22.5)	12.4<br>(9.0 to 16.5)	4.4<br>(1.2 to 8.2)
Foreign body in other body part	1,170.8<br>(816.8 to 1,619.9)	1,169.6<br>(744.6 to 1,736.2)	39.3<br>(24.0 to 61.9)	12.8<br>(9.9 to 15.7)	4.8<br>(2.1 to 7.4)
Environmental heat and cold exposure	2,536.7<br>(1,874.1 to 3,725.3)	2,224.1<br>(1,327.5 to 3,477.3)	105.8<br>(62.6 to 165.2)	8.6<br>(6.4 to 10.8)	0.9<br>(-1.2 to 2.9)
Other unintentional injuries	5,721.2<br>(4,015.8 to 8,352.5)	5,039.7<br>(3,135.6 to 7,632.0)	165.6<br>(101.9 to 260.4)	14.6<br>(12.7 to 16.3)	6.4<br>(4.7 to 8.0)
**Self-harm and interpersonal violence**	**7,479.2**<br>**(5,856.9 to 9,322.7)**	**912.5†**<br>**(511.6 to 1,468.3)**	**90.7**<br>**(57.4 to 129.3)**	**2.5**<br>**(1.1 to 4.2)**	**-4.8**<br>**(-6.1 to -3.2)**
Self-harm	--	--	--	--	--
Self-harm by firearm	--	--	--	--	--
Self-harm by other specified means	--	--	--	--	--
Interpersonal violence	7,479.2<br>(5,856.9 to 9,322.7)	912.5†<br>(511.6 to 1,468.3)	90.7<br>(57.4 to 129.3)	2.5<br>(1.1 to 4.2)	-4.8<br>(-6.1 to -3.2)
Assault by firearm	23.0<br>(17.7 to 31.4)	25.2<br>(10.2 to 53.2)	1.2<br>(0.6 to 2.0)	12.0<br>(8.7 to 15.6)	4.0<br>(1.0 to 7.4)
Assault by sharp object	164.2<br>(114.1 to 229.2)	265.1<br>(122.9 to 492.4)	6.4<br>(3.6 to 10.5)	12.6<br>(8.7 to 16.0)	4.5<br>(1.0 to 7.7)
Sexual violence	6,973.3<br>(5,363.2 to 8,760.1)	--	68.6<br>(42.3 to 101.7)	-0.1<br>(-1.2 to 1.0)	-7.2<br>(-8.3 to -6.2)
Assault by other means	318.7<br>(236.7 to 433.0)	622.2<br>(342.8 to 1,002.8)	14.5<br>(8.5 to 23.1)	11.2<br>(8.3 to 14.1)	3.2<br>(0.6 to 6.0)
**Forces of nature, conflict and terrorism, and executions and police conflict**	**1,485.4**<br>**(845.8 to 2,474.1)**	**71.2†**<br>**(47.9 to 101.4)**	**31.0**<br>**(12.6 to 60.1)**	**29.6**<br>**(4.1 to 85.9)**	**20.4**<br>**(-3.3 to 72.6)**
Forces of nature, conflict and terrorism, and state actor violence	98.5<br>(45.1 to 182.4)	42.2<br>(24.9 to 64.8)	3.7<br>(1.2 to 7.8)	-39.9<br>(-55.2 to -26.5)	-44.2<br>(-58.4 to -31.7)
Conflict and terrorism	1,368.1<br>(765.7 to 2,336.8)	--	27.2<br>(11.1 to 52.1)	53.2<br>(17.1 to 149.3)	42.2<br>(8.7 to 131.5)
Executions and police conflict	18.8<br>(10.2 to 33.3)	29.1<br>(14.8 to 48.4)	0.7<br>(0.3 to 1.8)	-8.7<br>(-18.4 to 4.8)	-15.2<br>(-24.2 to -2.7)

We did not calculate incidence for certain NTDs and causes with only birth prevalence. Blank cells mean that no estimate is available.

† Incidence for aggregate causes represents the sum of the incidence of the causes below the aggregate.

‡ Incidence estimates for stroke represent first-ever stroke only.

^ Incidence of HIV/AIDS represents new infections of HIV only and does not include new infections of Tuberculosis in HIV positive cases.

SR Table 12: Global prevalence, incidence, and YLDs for 2016, percentage change of YLD counts and percentage change of YLD rates between 2006 and 2016 by cause, age 10-14 years.					
Cause	Prevalence in thousands (95% UI)	Incidence in thousands (95% UI)	YLDs in thousands (95% UI)		
	2016 counts	2016 counts	2016 counts	Percentage change in counts between 2006 and 2016	Percentage change in rates between 2006 and 2016
**All causes**	**571,634.4 (569,411.0 to 573,455.0)**	**3,377,584.2† (2,851,917.1 to 3,998,623.9)**	**37,275.2 (26,987.5 to 49,652.8)**	**1.2 (0.4 to 2.1)**	**0.4 (-0.4 to 1.3)**
**Communicable, maternal, neonatal, and nutritional disorders**	**354,223.6 (349,570.2 to 359,243.1)**	**2,126,713.5† (1,612,700.7 to 2,688,154.5)**	**8,566.0 (6,010.7 to 11,929.3)**	**-1.5 (-4.0 to 1.8)**	**-2.3 (-4.8 to 0.9)**
**HIV/AIDS and tuberculosis**	**112,544.8 (99,305.8 to 129,181.0)**	**239.0† (155.0 to 363.9)**	**168.6 (117.6 to 225.3)**	**16.8 (5.7 to 30.2)**	**15.8 (4.9 to 29.1)**
Tuberculosis	104,913.1 (84,576.5 to 127,963.7)	239.0† (155.0 to 363.9)	45.3 (24.2 to 76.6)	-15.6 (-22.7 to -7.7)	-16.3 (-23.4 to -8.5)
Drug-susceptible tuberculosis	137.2 (87.1 to 202.6)	232.0 (150.3 to 353.1)	43.8 (23.4 to 74.0)	-15.5 (-22.8 to -7.5)	-16.2 (-23.4 to -8.3)
Multidrug-resistant tuberculosis without extensive drug resistance	4.2 (2.7 to 6.1)	6.7 (4.4 to 10.5)	1.4 (0.8 to 2.3)	-22.2 (-31.6 to -11.5)	-22.8 (-32.1 to -12.2)
Extensively drug-resistant tuberculosis	0.2 (0.1 to 0.3)	0.3 (0.2 to 0.4)	0.1 (< 0.05 to 0.1)	116.8 (82.3 to 155.1)	115.0 (80.8 to 153.0)
Latent tuberculosis infection	104,771.5 (84,418.6 to 127,826.1)	--	--	--	--
HIV/AIDS	651.2 (619.7 to 684.6)	--	123.3 (89.6 to 160.3)	35.9 (18.4 to 56.3)	34.8 (17.5 to 55.0)
Drug-susceptible HIV/AIDS - Tuberculosis	15.9 (10.8 to 22.4)	24.7 (16.9 to 34.7)	6.4 (3.6 to 10.3)	9.7 (-4.8 to 24.4)	8.8 (-5.5 to 23.4)
Multidrug-resistant HIV/AIDS - Tuberculosis without extensive drug resistance	0.4 (0.3 to 0.7)	0.7 (0.4 to 1.0)	0.2 (0.1 to 0.3)	21.7 (-7.0 to 53.4)	20.7 (-7.8 to 52.1)
Extensively drug-resistant HIV/AIDS - Tuberculosis	< 0.05 (< 0.05 to < 0.05)	< 0.05 (< 0.05 to < 0.05)	< 0.05 (< 0.05 to < 0.05)	423.5 (332.1 to 529.9)	419.2 (328.5 to 524.7)
HIV/AIDS resulting in other diseases	634.8 (601.4 to 668.5)	--	116.7 (84.7 to 151.2)	37.8 (18.8 to 60.1)	36.6 (17.8 to 58.8)
**Diarrhoea, lower respiratory infections, and other common infectious diseases**	**40,051.6 (37,880.5 to 42,601.9)**	**2,001,147.3† (1,497,551.7 to 2,561,030.8)**	**1,802.5 (1,224.4 to 2,571.2)**	**-1.1 (-2.3 to 0.2)**	**-1.9 (-3.1 to -0.7)**
Diarrhoeal diseases	4,518.2 (4,016.6 to 5,080.7)	306,160.2 (272,371.0 to 343,139.9)	519.0 (358.0 to 719.6)	1.6 (-0.4 to 3.6)	0.8 (-1.2 to 2.8)
Intestinal infectious diseases	141.3 (111.6 to 183.0)	2,514.7† (1,914.9 to 3,316.9)	20.9 (12.2 to 33.6)	-20.1 (-35.4 to -1.6)	-20.8 (-35.9 to -2.5)
Typhoid fever	128.7 (94.4 to 177.9)	2,028.5 (1,530.9 to 2,694.0)	19.6 (11.4 to 31.5)	-20.4 (-36.2 to -0.8)	-21.0 (-36.8 to -1.6)
Paratyphoid fever	21.4 (15.0 to 30.8)	486.1 (349.2 to 688.9)	1.4 (0.8 to 2.3)	-15.9 (-33.7 to 7.3)	-16.6 (-34.3 to 6.4)
Other intestinal infectious diseases	--	--	< 0.05 (< 0.05 to < 0.05)	-34.5 (-47.1 to -19.4)	-35.1 (-47.5 to -20.1)
Lower respiratory infections	459.9 (377.4 to 552.1)	18,873.4 (15,258.4 to 22,989.0)	28.5 (17.7 to 42.1)	17.7 (11.2 to 24.9)	16.7 (10.3 to 23.9)
Upper respiratory infections	21,569.7 (15,289.4 to 28,855.8)	1,632,387.2 (1,134,542.6 to 2,188,066.4)	543.0 (306.7 to 909.3)	-0.7 (-2.2 to 0.8)	-1.5 (-3.0 to -0.1)
Otitis media	12,723.9 (10,332.1 to 15,407.6)	31,203.2 (21,560.9 to 46,859.6)	407.4 (239.8 to 644.9)	-3.2 (-7.0 to -0.0)	-4.0 (-7.8 to -0.9)
Meningitis	1,356.0 (1,166.0 to 1,580.3)	166.0 (114.6 to 231.6)	153.6 (108.8 to 201.3)	3.3 (-0.2 to 6.9)	2.5 (-1.1 to 6.0)
Pneumococcal meningitis	560.5 (464.9 to 683.2)	46.0 (28.4 to 73.5)	64.9 (45.9 to 88.5)	1.1 (-5.7 to 9.3)	0.2 (-6.5 to 8.4)
H influenzae type B meningitis	222.5 (173.7 to 282.2)	17.6 (9.2 to 29.7)	29.2 (19.9 to 39.1)	-19.7 (-25.1 to -14.7)	-20.3 (-25.7 to -15.4)
Meningococcal infection	160.2 (113.5 to 218.2)	31.4 (18.0 to 49.8)	17.0 (11.7 to 23.1)	11.2 (8.3 to 14.1)	10.3 (7.4 to 13.2)
Other meningitis	412.9 (308.8 to 532.2)	71.1 (43.9 to 105.5)	42.5 (29.3 to 57.5)	29.4 (22.9 to 35.9)	28.3 (21.9 to 34.8)
Encephalitis	899.0 (635.5 to 1,258.8)	656.2 (478.1 to 876.1)	114.6 (81.5 to 152.6)	-10.8 (-15.9 to -5.8)	-11.6 (-16.6 to -6.6)
Diphtheria	< 0.05 (< 0.05 to < 0.05)	0.2 (0.1 to 0.3)	< 0.05 (< 0.05 to < 0.05)	-59.0 (-71.6 to -41.5)	-59.4 (-71.8 to -42.0)
Whooping cough	14.7 (11.4 to 18.4)	107.1 (83.5 to 134.6)	0.7 (0.4 to 1.2)	-23.0 (-24.9 to -21.1)	-23.6 (-25.5 to -21.7)
Tetanus	16.7 (16.4 to 17.0)	3.6 (1.6 to 6.8)	0.4 (0.2 to 0.6)	-0.7 (-23.7 to 26.7)	-1.5 (-24.4 to 25.7)
Measles	6.7 (2.4 to 14.4)	242.7 (87.7 to 524.4)	0.6 (0.2 to 1.4)	-60.8 (-65.0 to -56.0)	-61.1 (-65.3 to -56.4)
Varicella and herpes zoster	377.4 (304.3 to 466.9)	8,832.8 (6,464.1 to 12,296.1)	13.8 (7.9 to 22.4)	0.4 (-6.1 to 7.1)	-0.4 (-6.8 to 6.2)
**Neglected tropical diseases and malaria**	**165,772.2 (161,182.2 to 169,989.5)**	**46,434.4† (37,369.3 to 59,670.6)**	**1,084.3 (732.5 to 1,540.1)**	**-16.5 (-21.2 to -10.7)**	**-17.2 (-21.8 to -11.5)**
Malaria	18,540.0 (14,526.6 to 24,896.2)	23,335.6 (16,113.1 to 35,811.7)	243.3 (179.9 to 325.9)	-8.7 (-16.0 to -0.1)	-9.4 (-16.7 to -1.0)
Chagas disease	285.6 (216.8 to 365.4)	19.3 (13.1 to 26.2)	0.7 (0.4 to 1.1)	-18.8 (-32.8 to -2.0)	-19.5 (-33.3 to -2.8)
Leishmaniasis	391.9 (351.9 to 437.5)	99.5 (67.9 to 141.4)	24.0 (15.6 to 35.1)	27.9 (8.5 to 50.7)	26.9 (7.6 to 49.5)
Visceral leishmaniasis	1.1 (0.8 to 1.6)	4.5 (3.0 to 6.3)	0.1 (< 0.05 to 0.1)	-64.7 (-77.0 to -44.2)	-65.0 (-77.2 to -44.7)
Cutaneous and mucocutaneous leishmaniasis	366.5 (317.1 to 427.0)	95.0 (62.9 to 136.9)	23.9 (15.5 to 35.1)	29.1 (9.3 to 52.3)	28.0 (8.4 to 51.1)
African trypanosomiasis	0.7 (0.2 to 2.3)	0.5 (0.1 to 1.7)	0.2 (< 0.05 to 0.6)	-75.0 (-95.4 to 26.0)	-75.2 (-95.4 to 25.0)
Schistosomiasis	13,573.9 (12,279.4 to 14,857.8)	8,938.0 (7,364.8 to 11,177.6)	97.3 (45.2 to 191.9)	-28.9 (-31.7 to -26.7)	-29.4 (-32.2 to -27.3)
Cysticercosis	13.2 (6.4 to 22.2)	--	2.4 (1.1 to 4.3)	-9.7 (-24.8 to 5.0)	-10.4 (-25.4 to 4.2)
Cystic echinococcosis	74.3 (36.1 to 163.0)	16.2 (6.9 to 44.6)	7.1 (3.1 to 15.7)	-40.5 (-78.3 to 62.4)	-41.0 (-78.5 to 61.1)
Lymphatic filariasis	2,610.4 (2,248.8 to 3,110.1)	767.3 (441.0 to 1,236.9)	75.5 (35.7 to 140.8)	-38.8 (-51.2 to -29.0)	-39.3 (-51.6 to -29.6)
Onchocerciasis	1,424.7 (880.7 to 2,265.9)	517.1 (242.6 to 1,027.2)	91.4 (38.0 to 170.2)	-32.3 (-50.5 to -17.4)	-32.8 (-50.9 to -18.1)
Trachoma	--	--	--	--	--
Dengue	709.0 (385.5 to 1,202.1)	11,851.3 (7,197.5 to 17,998.3)	117.7 (51.3 to 221.3)	61.9 (32.0 to 269.6)	60.6 (30.9 to 266.6)
Yellow fever	0.5 (0.1 to 1.2)	17.1 (4.7 to 45.0)	< 0.05 (< 0.05 to < 0.05)	-19.0 (-49.4 to 29.5)	-19.7 (-49.9 to 28.4)
Rabies	< 0.05 (< 0.05 to 0.1)	1.1 (0.5 to 1.8)	< 0.05 (< 0.05 to < 0.05)	-49.2 (-65.5 to -25.3)	-49.6 (-65.8 to -25.9)
Intestinal nematode infections	140,427.0 (135,334.1 to 145,076.5)	--	288.4 (169.2 to 463.0)	-22.1 (-27.5 to -16.1)	-22.8 (-28.1 to -16.8)
Ascariasis	74,623.4 (67,920.7 to 82,262.5)	--	93.9 (51.6 to 158.5)	-32.7 (-42.0 to -23.2)	-33.3 (-42.5 to -23.8)

Cause	Prevalence in thousands (95% UI)	Incidence in thousands (95% UI)	YLDs in thousands (95% UI)		
	2016 counts	2016 counts	2016 counts	Percentage change in counts between 2006 and 2016	Percentage change in rates between 2006 and 2016
Trichuriasis	43,497.4 (40,124.9 to 46,960.8)	--	36.0 (19.6 to 60.6)	-26.8 (-35.6 to -16.6)	-27.4 (-36.1 to -17.3)
Hookworm disease	41,728.0 (38,496.8 to 45,257.4)	--	158.5 (96.2 to 247.0)	-12.7 (-20.5 to -4.0)	-13.4 (-21.1 to -4.8)
Food-borne trematodiases	2,810.5 (2,565.2 to 3,119.8)	330.2 (296.9 to 367.4)	46.1 (24.1 to 82.8)	-35.2 (-42.2 to -23.7)	-35.8 (-42.7 to -24.3)
Leprosy	2.7 (2.4 to 3.0)	1.6 (1.4 to 1.8)	0.1 (0.1 to 0.2)	-27.7 (-28.9 to -26.4)	-28.3 (-29.5 to -27.0)
Ebola virus disease	< 0.05 (< 0.05 to 0.2)	< 0.05 (< 0.05 to < 0.05)	< 0.05 (< 0.05 to < 0.05)	--	--
Zika virus disease	8.9 (5.4 to 15.7)	539.5 (326.8 to 943.1)	0.2 (0.1 to 0.3)	--	--
Guinea worm disease	< 0.05 (< 0.05 to < 0.05)	< 0.05 (< 0.05 to < 0.05)	< 0.05 (< 0.05 to < 0.05)	-99.5 (-99.7 to -99.3)	-99.5 (-99.7 to -99.3)
Other neglected tropical diseases	2,352.0 (2,073.0 to 2,649.2)	--	89.9 (57.0 to 130.9)	2.5 (-2.5 to 8.0)	1.7 (-3.3 to 7.1)
**Maternal disorders**	**5.6 (3.9 to 7.5)**	**114.6† (93.4 to 139.5)**	**1.4 (0.9 to 2.2)**	**-15.0 (-25.3 to -3.2)**	**-15.7 (-25.9 to -4.0)**
Maternal haemorrhage	1.0 (0.7 to 1.5)	9.1 (6.0 to 13.9)	0.1 (0.1 to 0.1)	-16.7 (-28.9 to -2.3)	-17.3 (-29.5 to -3.1)
Maternal sepsis and other pregnancy related infections	2.5 (1.6 to 3.9)	19.9 (13.2 to 27.9)	0.1 (< 0.05 to 0.2)	-10.6 (-53.6 to 80.7)	-11.4 (-54.0 to 79.2)
Maternal hypertensive disorders	5.0 (2.9 to 7.2)	24.2 (18.5 to 29.9)	0.2 (0.1 to 0.4)	-16.9 (-47.4 to 32.3)	-17.6 (-47.9 to 31.2)
Maternal obstructed labour and uterine rupture	2.0 (1.1 to 3.4)	9.1 (6.4 to 12.3)	0.7 (0.3 to 1.3)	-10.8 (-17.5 to -3.7)	-11.5 (-18.2 to -4.5)
Maternal abortion, miscarriage, and ectopic pregnancy	0.4 (0.2 to 0.7)	52.4 (35.0 to 75.6)	< 0.05 (< 0.05 to 0.1)	-23.0 (-52.1 to 20.4)	-23.7 (-52.5 to 19.4)
Other maternal disorders	--	--	0.2 (0.1 to 0.4)	-22.9 (-32.2 to -12.2)	-23.5 (-32.8 to -12.9)
**Neonatal disorders**	**7,036.2 (6,404.0 to 7,713.6)**	**--**	**1,384.3 (947.4 to 2,002.6)**	**14.0 (0.8 to 28.7)**	**13.1 (-0.1 to 27.7)**
Neonatal preterm birth complications	5,651.2 (4,663.0 to 6,904.7)	--	897.3 (607.8 to 1,294.3)	9.5 (-8.7 to 30.8)	8.6 (-9.5 to 29.7)
Neonatal encephalopathy due to birth asphyxia and trauma	1,115.9 (657.8 to 1,917.3)	--	148.5 (42.6 to 398.0)	26.7 (15.2 to 43.2)	25.6 (14.3 to 42.0)
Neonatal sepsis and other neonatal infections	657.9 (208.6 to 1,602.7)	--	270.2 (73.3 to 711.8)	25.7 (16.7 to 40.9)	24.7 (15.7 to 39.8)
Hemolytic disease and other neonatal jaundice	216.1 (191.1 to 241.5)	--	68.2 (52.1 to 85.9)	9.4 (3.8 to 15.5)	8.5 (3.0 to 14.6)
Other neonatal disorders	--	--	--	--	--
**Nutritional deficiencies**	**141,028.8 (134,938.4 to 144,750.3)**	**60,388.8† (48,831.4 to 76,778.2)**	**3,991.2 (2,554.2 to 5,800.2)**	**-2.1 (-4.9 to 0.6)**	**-3.0 (-5.7 to -0.2)**
Protein-energy malnutrition	--	--	--	--	--
Iodine deficiency	8,960.5 (7,851.7 to 10,244.4)	969.8 (852.4 to 1,100.2)	245.2 (161.6 to 350.8)	-20.6 (-23.6 to -17.3)	-21.3 (-24.3 to -18.0)
Vitamin A deficiency	54,464.5 (46,094.1 to 66,408.1)	59,419.0 (47,752.5 to 75,717.9)	25.4 (14.2 to 41.6)	5.6 (-1.4 to 12.8)	4.8 (-2.2 to 11.9)
Iron-deficiency anaemia	97,982.7 (89,090.4 to 108,171.7)	--	3,720.6 (2,386.0 to 5,439.3)	-0.7 (-3.7 to 2.4)	-1.5 (-4.5 to 1.5)
Other nutritional deficiencies	--	--	--	--	--
**Other communicable, maternal, neonatal, and nutritional diseases**	**49,394.8 (44,438.4 to 53,993.3)**	**18,389.5 (14,361.9 to 23,330.4)**	**133.5 (84.9 to 197.8)**	**-1.5 (-11.2 to 8.3)**	**-2.3 (-12.0 to 7.4)**
Sexually transmitted diseases excluding HIV	6,247.9 (5,791.1 to 6,666.5)	9,425.4 (7,755.8 to 11,643.8)	13.2 (8.0 to 21.3)	2.5 (-4.1 to 9.3)	1.6 (-4.9 to 8.4)
Syphilis	816.7 (471.6 to 1,228.6)	1,070.3 (590.8 to 1,673.0)	0.2 (< 0.05 to 0.5)	4.5 (-16.5 to 25.6)	3.6 (-17.2 to 24.6)
Chlamydial infection	303.0 (184.0 to 458.6)	574.2 (406.6 to 757.4)	3.0 (1.9 to 4.6)	1.4 (-1.3 to 4.4)	0.6 (-2.1 to 3.5)
Gonococcal infection	331.5 (148.4 to 709.5)	1,459.2 (667.8 to 2,916.5)	2.6 (1.2 to 5.2)	3.0 (-21.6 to 19.4)	2.1 (-22.2 to 18.4)
Trichomoniasis	965.5 (586.6 to 1,472.6)	1,268.8 (767.6 to 1,926.6)	1.2 (0.4 to 2.8)	7.8 (0.2 to 15.7)	6.9 (-0.7 to 14.8)
Genital herpes	3,956.3 (2,979.5 to 5,062.9)	4,670.0 (3,506.4 to 5,987.8)	2.1 (0.8 to 4.5)	4.3 (-4.6 to 14.0)	3.4 (-5.4 to 13.0)
Other sexually transmitted diseases	20.7 (15.0 to 28.1)	382.8 (285.2 to 511.2)	4.0 (2.5 to 6.0)	0.4 (-9.3 to 11.0)	-0.5 (-10.0 to 10.1)
Hepatitis	40,874.8 (31,913.2 to 50,424.3)	8,963.5 (5,465.1 to 13,183.5)	12.0 (5.3 to 22.0)	-35.7 (-72.1 to 42.8)	-36.3 (-72.4 to 41.6)
Acute hepatitis A	168.8 (15.3 to 406.8)	2,195.1 (198.4 to 5,288.0)	5.6 (0.5 to 14.1)	-35.1 (-95.0 to 645.1)	-35.6 (-95.0 to 639.0)
Hepatitis B	30,826.4 (21,779.7 to 40,137.3)	4,601.3 (2,419.6 to 7,698.7)	2.8 (1.2 to 5.2)	-32.6 (-53.8 to -1.0)	-33.2 (-54.2 to -1.8)
Hepatitis C	9,721.0 (8,363.9 to 11,101.4)	105.1 (73.3 to 145.7)	0.1 (< 0.05 to 0.2)	-29.6 (-30.2 to -29.0)	-30.2 (-30.8 to -29.6)
Acute hepatitis E	158.6 (136.1 to 181.1)	2,062.0 (1,769.4 to 2,354.6)	3.5 (2.1 to 5.4)	-39.1 (-55.0 to -17.0)	-39.6 (-55.3 to -17.7)
Other infectious diseases	2,620.0 (2,314.5 to 2,955.3)	0.7 (0.3 to 1.2)	108.4 (69.5 to 158.9)	4.2 (-0.2 to 8.8)	3.3 (-1.1 to 7.9)
**Non-communicable diseases**	**525,035.3 (520,270.4 to 529,562.6)**	**1,209,348.6† (1,016,405.6 to 1,411,635.7)**	**26,931.1 (19,310.5 to 35,741.0)**	**2.0 (1.3 to 2.7)**	**1.2 (0.5 to 1.8)**
**Neoplasms**	**88.3 (80.7 to 93.9)**	**53.3 (48.6 to 56.1)**	**19.0 (13.4 to 25.6)**	**6.9 (0.8 to 12.8)**	**6.0 (0.0 to 11.8)**
Lip and oral cavity cancer	--	--	--	--	--
Nasopharynx cancer	3.6 (3.2 to 4.1)	1.0 (0.9 to 1.2)	0.4 (0.3 to 0.6)	-12.4 (-22.7 to 1.1)	-13.1 (-23.3 to 0.3)
Other pharynx cancer	--	--	--	--	--
Oesophageal cancer	--	--	--	--	--
Stomach cancer	--	--	--	--	--
Colon and rectum cancer	--	--	--	--	--
Liver cancer	0.7 (0.6 to 0.8)	0.9 (0.8 to 1.1)	0.2 (0.1 to 0.3)	0.8 (-12.2 to 14.3)	-0.1 (-12.9 to 13.4)
Liver cancer due to hepatitis B	0.4 (0.3 to 0.6)	0.4 (0.3 to 0.5)	0.1 (0.1 to 0.1)	-1.6 (-15.5 to 15.5)	-2.4 (-16.1 to 14.6)

Cause	Prevalence in thousands (95% UI)	Incidence in thousands (95% UI)	YLDs in thousands (95% UI)		
	2016 counts	2016 counts	2016 counts	Percentage change in counts between 2006 and 2016	Percentage change in rates between 2006 and 2016
Liver cancer due to hepatitis C	< 0.05 (< 0.05 to < 0.05)	< 0.05 (< 0.05 to < 0.05)	< 0.05 (< 0.05 to < 0.05)	3.0 (-9.2 to 17.4)	2.1 (-9.9 to 16.4)
Liver cancer due to alcohol use	--	--	--	--	--
Liver cancer due to other causes	0.6 (0.5 to 0.8)	0.6 (0.5 to 0.7)	0.1 (0.1 to 0.2)	2.3 (-10.7 to 15.3)	1.4 (-11.4 to 14.3)
Gallbladder and biliary tract cancer	--	--	--	--	--
Pancreatic cancer	--	--	--	--	--
Larynx cancer	--	--	--	--	--
Tracheal, bronchus, and lung cancer	--	--	--	--	--
Malignant skin melanoma	--	--	--	--	--
Non-melanoma skin cancer	--	--	--	--	--
Non-melanoma skin cancer (squamous-cell carcinoma)	--	--	--	--	--
Non-melanoma skin cancer (basal-cell carcinoma)	--	--	--	--	--
Breast cancer	--	--	--	--	--
Cervical cancer	--	--	--	--	--
Uterine cancer	--	--	--	--	--
Ovarian cancer	--	--	--	--	--
Prostate cancer	--	--	--	--	--
Testicular cancer	--	--	--	--	--
Kidney cancer	6.5 (5.9 to 7.0)	1.7 (1.6 to 1.8)	0.6 (0.4 to 0.8)	10.8 (4.1 to 17.2)	9.9 (3.3 to 16.2)
Bladder cancer	--	--	--	--	--
Brain and nervous system cancer	22.8 (19.5 to 24.9)	10.3 (9.0 to 11.1)	3.3 (2.3 to 4.4)	16.9 (10.4 to 24.1)	16.0 (9.5 to 23.1)
Thyroid cancer	5.7 (4.9 to 6.2)	1.1 (0.9 to 1.2)	0.4 (0.2 to 0.5)	29.6 (19.7 to 40.7)	28.5 (18.7 to 39.5)
Mesothelioma	--	--	--	--	--
Hodgkin lymphoma	7.6 (5.9 to 8.8)	2.2 (1.7 to 2.6)	0.9 (0.6 to 1.2)	-9.3 (-16.8 to -0.5)	-10.1 (-17.4 to -1.3)
Non-Hodgkin's lymphoma	17.4 (14.4 to 19.3)	5.7 (4.8 to 6.3)	1.8 (1.2 to 2.5)	17.2 (6.0 to 28.1)	16.2 (5.2 to 27.1)
Multiple myeloma	--	--	--	--	--
Leukaemia	46.5 (40.8 to 50.6)	13.5 (12.0 to 14.6)	6.2 (4.3 to 8.6)	-9.8 (-17.0 to -1.1)	-10.6 (-17.7 to -1.9)
Acute lymphoid leukaemia	20.7 (18.5 to 22.7)	6.0 (5.3 to 6.6)	2.8 (2.0 to 3.8)	3.6 (-8.9 to 13.8)	2.8 (-9.7 to 12.8)
Chronic lymphoid leukaemia	--	--	--	--	--
Acute myeloid leukaemia	3.6 (3.1 to 4.0)	2.7 (2.3 to 3.0)	0.8 (0.5 to 1.0)	-0.4 (-8.3 to 8.1)	-1.2 (-9.1 to 7.2)
Chronic myeloid leukaemia	--	--	--	--	--
Other leukaemia	22.2 (18.2 to 25.1)	4.9 (4.0 to 5.5)	2.7 (1.8 to 3.7)	-22.2 (-30.8 to -11.8)	-22.9 (-31.4 to -12.5)
Other neoplasms	57.9 (50.3 to 62.2)	16.9 (14.8 to 18.1)	5.1 (3.6 to 7.0)	29.0 (17.5 to 39.9)	28.0 (16.5 to 38.8)
**Cardiovascular diseases**	**4,087.1 (3,734.9 to 4,352.2)**	**558.4† (500.8 to 619.0)**	**267.6 (178.8 to 385.9)**	**4.7 (1.6 to 7.5)**	**3.8 (0.8 to 6.6)**
Rheumatic heart disease	2,153.1 (1,903.9 to 2,420.8)	193.5 (172.6 to 216.3)	103.1 (64.5 to 152.6)	10.3 (5.4 to 15.0)	9.4 (4.6 to 14.0)
Ischaemic heart disease	397.4 (357.9 to 437.8)	191.4 (163.5 to 224.2)	23.4 (15.3 to 34.3)	2.8 (-4.8 to 11.1)	1.9 (-5.6 to 10.2)
Cerebrovascular disease	207.1 (159.8 to 271.8)	48.0‡ (26.7 to 75.3)	45.9 (30.4 to 64.6)	-7.7 (-13.3 to -3.0)	-8.5 (-14.0 to -3.8)
Ischaemic stroke	201.9 (146.1 to 270.0)	30.4‡ (13.6 to 54.7)	31.1 (19.5 to 46.1)	-7.7 (-15.5 to -1.3)	-8.4 (-16.2 to -2.1)
Hemorrhagic stroke	97.5 (71.7 to 127.3)	17.6‡ (7.5 to 33.1)	14.8 (9.6 to 21.7)	-7.9 (-15.1 to -1.3)	-8.6 (-15.8 to -2.1)
Hypertensive heart disease	0.1 (< 0.05 to 0.1)	--	< 0.05 (< 0.05 to < 0.05)	22.9 (17.0 to 29.8)	21.9 (16.0 to 28.8)
Cardiomyopathy and myocarditis	107.6 (83.0 to 140.2)	93.9† (67.4 to 125.7)	12.3 (7.4 to 19.4)	-1.0 (-3.5 to 1.2)	-1.9 (-4.3 to 0.4)
Myocarditis	102.1 (77.3 to 133.4)	93.9 (67.4 to 125.7)	8.3 (5.0 to 13.1)	-1.8 (-5.1 to 1.2)	-2.6 (-5.9 to 0.4)
Alcoholic cardiomyopathy	--	--	--	--	--
Other cardiomyopathy	44.5 (31.1 to 60.3)	--	4.0 (2.3 to 6.4)	0.6 (-1.3 to 2.4)	-0.2 (-2.1 to 1.6)
Atrial fibrillation and flutter	--	--	--	--	--
Peripheral vascular disease	--	--	--	--	--
Endocarditis	9.7 (7.0 to 12.7)	31.6 (17.6 to 46.4)	0.8 (0.5 to 1.2)	7.1 (5.1 to 9.2)	6.2 (4.2 to 8.3)
Other cardiovascular and circulatory diseases	1,500.5 (936.0 to 2,356.9)	--	82.2 (43.7 to 138.6)	7.3 (2.3 to 12.6)	6.4 (1.4 to 11.7)
**Chronic respiratory diseases**	**37,716.0 (34,581.7 to 41,361.9)**	**5,256.5† (4,110.8 to 6,546.7)**	**1,749.0 (1,208.0 to 2,411.6)**	**8.4 (6.3 to 10.7)**	**7.5 (5.5 to 9.8)**
Chronic obstructive pulmonary disease	1,592.4 (1,440.2 to 1,754.9)	123.5 (104.2 to 143.9)	137.5 (117.0 to 157.3)	1.6 (-4.5 to 7.4)	0.7 (-5.3 to 6.5)
Pneumoconiosis	0.2 (0.1 to 0.2)	0.1 (0.1 to 0.1)	< 0.05 (< 0.05 to < 0.05)	-1.8 (-3.9 to 0.3)	-2.6 (-4.7 to -0.6)
Silicosis	--	--	--	--	--

Cause	Prevalence in thousands (95% UI)	Incidence in thousands (95% UI)	YLDs in thousands (95% UI)		
	2016 counts	2016 counts	2016 counts	Percentage change in counts between 2006 and 2016	Percentage change in rates between 2006 and 2016
Asbestosis	0.1<br>(< 0.05 to 0.1)	< 0.05<br>(< 0.05 to 0.1)	< 0.05<br>(< 0.05 to < 0.05)	1.2<br>(-2.7 to 4.6)	0.3<br>(-3.5 to 3.8)
Coal workers pneumoconiosis	< 0.05<br>(< 0.05 to < 0.05)	< 0.05<br>(< 0.05 to < 0.05)	< 0.05<br>(< 0.05 to < 0.05)	-7.2<br>(-9.9 to -4.3)	-7.9<br>(-10.7 to -5.1)
Other pneumoconiosis	0.1<br>(0.1 to 0.1)	0.1<br>(< 0.05 to 0.1)	< 0.05<br>(< 0.05 to < 0.05)	-3.0<br>(-5.7 to -0.4)	-3.8<br>(-6.5 to -1.2)
Asthma	35,680.9<br>(30,944.3 to 41,009.1)	5,131.1<br>(3,990.3 to 6,427.5)	1,426.7<br>(919.0 to 2,081.7)	6.2<br>(3.9 to 8.5)	5.3<br>(3.0 to 7.6)
Interstitial lung disease and pulmonary sarcoidosis	54.9<br>(44.4 to 66.3)	1.8<br>(1.1 to 2.7)	6.2<br>(3.8 to 9.3)	-6.1<br>(-8.3 to -4.3)	-6.9<br>(-9.1 to -5.1)
Other chronic respiratory diseases	--	--	178.6<br>(151.7 to 204.3)	39.2<br>(31.3 to 46.8)	38.0<br>(30.2 to 45.6)
**Cirrhosis and other chronic liver diseases**	**649.2<br>(591.9 to 721.6)**	**3.8<br>(2.5 to 5.3)**	**81.3<br>(55.6 to 115.4)**	**4.1<br>(-1.2 to 9.4)**	**3.2<br>(-2.0 to 8.5)**
Cirrhosis and other chronic liver diseases due to hepatitis B	23.1<br>(17.3 to 29.9)	0.1<br>(0.1 to 0.2)	3.4<br>(2.0 to 5.2)	9.1<br>(-6.9 to 28.4)	8.2<br>(-7.7 to 27.3)
Cirrhosis and other chronic liver diseases due to hepatitis C	20.1<br>(14.9 to 26.8)	0.1<br>(0.1 to 0.2)	2.7<br>(1.5 to 4.2)	8.3<br>(-7.7 to 27.1)	7.4<br>(-8.4 to 26.0)
Cirrhosis and other chronic liver diseases due to alcohol use	--	--	--	--	--
Cirrhosis and other chronic liver diseases due to other causes	606.0<br>(550.5 to 674.6)	3.5<br>(2.3 to 4.9)	75.2<br>(51.3 to 107.0)	3.7<br>(-1.9 to 9.4)	2.9<br>(-2.7 to 8.5)
**Digestive diseases**	**5,953.9<br>(5,480.9 to 6,426.6)**	**7,383.7†<br>(6,408.4 to 8,406.5)**	**194.5<br>(133.5 to 274.5)**	**-6.6<br>(-10.0 to -2.9)**	**-7.4<br>(-10.8 to -3.7)**
Peptic ulcer disease	1,069.8<br>(839.9 to 1,355.5)	454.4<br>(323.2 to 615.8)	20.3<br>(11.9 to 33.2)	-7.4<br>(-14.0 to -0.8)	-8.2<br>(-14.7 to -1.6)
Gastritis and duodenitis	2,723.6<br>(1,999.6 to 3,765.9)	2,453.9<br>(1,757.0 to 3,292.7)	33.4<br>(20.2 to 52.1)	-12.0<br>(-17.7 to -5.3)	-12.7<br>(-18.3 to -6.1)
Appendicitis	116.0<br>(96.8 to 137.9)	3,127.1<br>(2,590.7 to 3,753.8)	36.1<br>(22.7 to 51.6)	-0.1<br>(-9.3 to 9.5)	-1.0<br>(-10.0 to 8.6)
Paralytic ileus and intestinal obstruction	7.6<br>(5.0 to 10.6)	196.7<br>(128.7 to 272.1)	2.5<br>(1.5 to 3.9)	6.3<br>(4.0 to 8.3)	5.5<br>(3.1 to 7.4)
Inguinal, femoral, and abdominal hernia	2,016.4<br>(1,200.4 to 3,158.8)	554.1<br>(380.4 to 769.5)	71.1<br>(41.2 to 113.3)	-9.0<br>(-14.6 to -3.3)	-9.7<br>(-15.3 to -4.1)
Inflammatory bowel disease	69.8<br>(59.1 to 83.8)	174.8<br>(143.8 to 212.2)	11.0<br>(7.0 to 15.8)	-3.3<br>(-8.1 to 2.0)	-4.1<br>(-8.9 to 1.2)
Vascular intestinal disorders	1.7<br>(0.8 to 2.8)	10.7<br>(6.4 to 16.8)	0.5<br>(0.2 to 1.0)	2.1<br>(0.1 to 3.7)	1.2<br>(-0.8 to 2.9)
Gallbladder and biliary diseases	283.9<br>(203.6 to 393.8)	342.7<br>(224.9 to 510.0)	1.6<br>(0.9 to 2.8)	-2.1<br>(-3.9 to -0.4)	-2.9<br>(-4.7 to -1.2)
Pancreatitis	8.0<br>(5.5 to 10.9)	69.3<br>(48.1 to 95.4)	1.0<br>(0.6 to 1.7)	-9.8<br>(-17.0 to -4.2)	-10.5<br>(-17.7 to -5.0)
Other digestive diseases	--	--	16.9<br>(11.6 to 23.9)	-0.9<br>(-4.5 to 3.0)	-1.7<br>(-5.3 to 2.2)
**Neurological disorders**	**182,073.9<br>(171,065.0 to 195,237.8)**	**93,818.9<br>(65,602.7 to 127,379.4)**	**3,794.2<br>(2,549.7 to 5,257.4)**	**3.0<br>(-0.6 to 7.2)**	**2.1<br>(-1.4 to 6.3)**
Alzheimer's disease and other dementias	--	--	--	--	--
Parkinson's disease	--	--	--	--	--
Epilepsy	2,271.1<br>(1,756.6 to 2,843.9)	264.4<br>(173.9 to 373.4)	768.5<br>(487.2 to 1,114.2)	0.6<br>(-11.3 to 14.5)	-0.2<br>(-12.0 to 13.6)
Multiple sclerosis	10.9<br>(8.0 to 14.0)	2.1<br>(1.5 to 3.0)	3.1<br>(1.9 to 4.4)	2.1<br>(0.6 to 3.6)	1.3<br>(-0.2 to 2.8)
Motor neuron disease	13.0<br>(10.4 to 16.2)	0.2<br>(0.1 to 0.4)	2.8<br>(1.9 to 3.9)	-1.0<br>(-1.9 to 0.2)	-1.8<br>(-2.8 to -0.6)
Migraine	63,260.8<br>(58,214.2 to 68,225.5)	14,055.0<br>(12,487.9 to 15,685.3)	2,468.2<br>(1,532.8 to 3,607.8)	1.6<br>(0.3 to 3.1)	0.8<br>(-0.6 to 2.2)
Tension-type headache	131,709.2<br>(99,562.3 to 166,599.7)	79,493.8<br>(51,243.3 to 113,394.4)	301.9<br>(179.3 to 496.2)	0.9<br>(-0.9 to 3.2)	0.1<br>(-1.7 to 2.4)
Other neurological disorders	1.8<br>(0.8 to 3.5)	3.4<br>(1.6 to 6.0)	249.7<br>(158.5 to 361.4)	33.0<br>(17.3 to 51.3)	31.9<br>(16.4 to 50.0)
**Mental and substance use disorders**	**81,261.7<br>(78,765.3 to 83,517.1)**	**28,256.5†<br>(24,011.7 to 33,074.7)**	**8,402.1<br>(5,798.2 to 11,366.1)**	**2.1<br>(1.2 to 3.1)**	**1.3<br>(0.4 to 2.2)**
Schizophrenia	27.9<br>(15.9 to 44.8)	16.9<br>(9.6 to 27.1)	19.8<br>(10.2 to 33.6)	-0.5<br>(-1.6 to 0.9)	-1.3<br>(-2.4 to 0.1)
Alcohol use disorders	464.7<br>(285.8 to 723.0)	433.6<br>(257.4 to 698.4)	47.2<br>(25.1 to 81.3)	0.8<br>(-3.5 to 4.9)	-0.1<br>(-4.3 to 4.0)
Drug use disorders	397.0<br>(310.2 to 552.2)	470.4<br>(295.7 to 697.4)	15.6<br>(9.2 to 24.4)	-9.1<br>(-15.3 to -4.5)	-9.9<br>(-16.0 to -5.3)
Opioid use disorders	--	--	--	--	--
Cocaine use disorders	--	--	--	--	--
Amphetamine use disorders	21.7<br>(13.6 to 32.0)	43.4<br>(27.1 to 64.2)	3.0<br>(1.6 to 5.2)	-8.5<br>(-16.0 to -2.8)	-9.3<br>(-16.7 to -3.6)
Cannabis use disorders	352.3<br>(206.5 to 545.1)	426.4<br>(254.8 to 653.2)	10.6<br>(5.2 to 18.4)	-9.5<br>(-17.4 to -3.4)	-10.2<br>(-18.1 to -4.1)
Other drug use disorders	0.3<br>(0.1 to 0.6)	0.6<br>(0.2 to 1.3)	2.0<br>(1.1 to 3.4)	-8.4<br>(-15.8 to -1.5)	-9.2<br>(-16.4 to -2.3)
Depressive disorders	7,952.0<br>(7,197.3 to 8,798.0)	11,734.7<br>(8,589.9 to 15,707.7)	1,454.2<br>(931.1 to 2,168.8)	4.5<br>(2.6 to 6.5)	3.6<br>(1.7 to 5.7)
Major depressive disorder	6,006.1<br>(4,381.4 to 8,076.5)	10,995.8<br>(7,800.9 to 15,015.6)	1,280.2<br>(788.4 to 1,970.7)	4.7<br>(2.5 to 7.0)	3.8<br>(1.6 to 6.2)
Dysthymia	1,746.2<br>(1,264.4 to 2,406.5)	738.9<br>(512.3 to 1,030.9)	174.0<br>(105.1 to 276.9)	3.0<br>(-0.4 to 6.2)	2.1<br>(-1.2 to 5.4)
Bipolar disorder	1,521.5<br>(1,121.7 to 1,984.5)	835.5<br>(615.8 to 1,092.2)	323.0<br>(192.9 to 510.1)	3.8<br>(-0.1 to 7.2)	2.9<br>(-0.9 to 6.3)
Anxiety disorders	21,635.6<br>(18,709.0 to 25,192.7)	5,089.2<br>(4,495.3 to 5,748.0)	2,161.7<br>(1,482.1 to 2,985.6)	1.1<br>(-1.1 to 3.2)	0.3<br>(-1.9 to 2.3)
Eating disorders	664.3<br>(579.4 to 778.7)	1,052.1<br>(645.2 to 1,637.2)	137.8<br>(82.1 to 223.5)	9.9<br>(5.7 to 14.0)	9.0<br>(4.8 to 13.1)
Anorexia nervosa	290.8<br>(200.4 to 413.7)	180.3<br>(125.3 to 251.2)	63.0<br>(36.0 to 98.7)	8.3<br>(1.9 to 14.8)	7.4<br>(1.1 to 13.8)
Bulimia nervosa	348.2<br>(185.9 to 591.7)	871.8<br>(485.7 to 1,431.5)	74.8<br>(36.0 to 138.1)	11.4<br>(6.0 to 17.6)	10.4<br>(5.1 to 16.6)
Autistic spectrum disorders	6,168.9<br>(5,889.2 to 6,412.8)	37.2<br>(17.0 to 80.0)	909.7<br>(614.1 to 1,282.0)	1.4<br>(-0.1 to 3.0)	0.6<br>(-0.9 to 2.2)
Autism	1,793.9<br>(1,544.6 to 2,087.0)	3.1<br>(1.0 to 7.3)	466.6<br>(299.5 to 677.9)	1.5<br>(-1.0 to 4.1)	0.6<br>(-1.8 to 3.2)
Asperger syndrome and other autistic spectrum disorders	4,358.5<br>(3,629.1 to 5,229.2)	34.0<br>(13.9 to 74.4)	443.2<br>(290.8 to 645.8)	1.3<br>(-0.4 to 2.9)	0.5<br>(-1.2 to 2.1)

Cause	Prevalence in thousands (95% UI)	Incidence in thousands (95% UI)	YLDs in thousands (95% UI)		
	2016 counts	2016 counts	2016 counts	Percentage change in counts between 2006 and 2016	Percentage change in rates between 2006 and 2016
Attention-deficit/hyperactivity disorder	12,017.4 (10,676.7 to 13,643.7)	155.0 (138.3 to 177.5)	145.9 (88.1 to 232.3)	-0.2 (-2.1 to 1.6)	-1.0 (-2.9 to 0.8)
Conduct disorder	21,122.2 (16,392.3 to 26,578.2)	8,006.2 (5,621.7 to 10,185.9)	2,571.7 (1,507.2 to 4,052.9)	3.4 (1.8 to 4.9)	2.5 (0.9 to 4.0)
Idiopathic developmental intellectual disability	14,318.2 (8,889.3 to 19,655.1)	--	559.6 (260.8 to 960.6)	-4.9 (-8.4 to -2.5)	-5.7 (-9.2 to -3.3)
Other mental and substance use disorders	716.6 (590.5 to 855.2)	426.0 (350.8 to 508.8)	56.0 (37.3 to 81.5)	1.1 (-4.7 to 7.4)	0.3 (-5.5 to 6.5)
**Diabetes, urogenital, blood, and endocrine diseases**	**168,627.0 (166,560.1 to 171,125.9)**	**20,768.2† (16,317.8 to 26,175.2)**	**979.6 (643.1 to 1,405.8)**	**0.1 (-3.1 to 3.2)**	**-0.7 (-3.9 to 2.3)**
Diabetes mellitus	1,955.9 (1,425.6 to 2,569.1)	457.1 (326.3 to 624.5)	99.2 (56.6 to 158.6)	8.0 (4.4 to 11.1)	7.1 (3.5 to 10.2)
Acute glomerulonephritis	7.2 (4.9 to 10.2)	104.8 (71.6 to 147.5)	0.4 (0.2 to 0.6)	-4.8 (-7.6 to -1.3)	-5.6 (-8.4 to -2.1)
Chronic kidney disease	1,137.5 (856.7 to 1,457.6)	249.5 (173.7 to 346.6)	56.8 (34.2 to 87.4)	0.9 (-3.8 to 5.5)	0.1 (-4.6 to 4.6)
Chronic kidney disease due to diabetes mellitus	50.6 (28.9 to 82.9)	10.4 (5.6 to 18.2)	2.7 (1.4 to 4.8)	-1.7 (-7.1 to 4.2)	-2.5 (-7.8 to 3.3)
Chronic kidney disease due to hypertension	316.1 (206.2 to 444.6)	105.4 (64.9 to 159.2)	1.5 (0.8 to 2.7)	-2.4 (-7.4 to 2.8)	-3.1 (-8.2 to 1.9)
Chronic kidney disease due to glomerulonephritis	193.6 (127.8 to 269.4)	6.2 (3.6 to 10.4)	24.0 (13.1 to 38.8)	0.7 (-3.7 to 5.3)	-0.1 (-4.5 to 4.4)
Chronic kidney disease due to other causes	577.3 (397.1 to 809.3)	127.5 (82.8 to 185.5)	28.6 (15.9 to 45.3)	1.5 (-3.5 to 6.7)	0.7 (-4.3 to 5.8)
Urinary diseases and male infertility	389.1 (344.3 to 466.7)	19,746.4† (15,249.6 to 25,190.9)	19.0 (11.4 to 30.2)	-7.0 (-12.2 to -1.4)	-7.8 (-12.9 to -2.2)
Interstitial nephritis and urinary tract infections	344.7 (258.4 to 445.3)	19,011.9 (14,515.0 to 24,329.7)	11.6 (6.7 to 19.0)	0.7 (-6.0 to 7.7)	-0.2 (-6.8 to 6.8)
Urolithiasis	27.1 (18.6 to 37.8)	734.6 (507.9 to 1,044.6)	2.1 (1.2 to 3.4)	-3.2 (-4.3 to -2.0)	-4.0 (-5.1 to -2.8)
Benign prostatic hyperplasia	--	--	--	--	--
Male infertility	--	--	--	--	--
Other urinary diseases	--	--	5.3 (3.2 to 8.4)	-21.4 (-25.7 to -16.6)	-22.0 (-26.4 to -17.3)
Gynecological diseases	--	--	--	--	--
Uterine fibroids	--	--	--	--	--
Polycystic ovarian syndrome	--	--	--	--	--
Female infertility	--	--	--	--	--
Endometriosis	--	--	--	--	--
Genital prolapse	--	--	--	--	--
Premenstrual syndrome	--	--	--	--	--
Other gynecological diseases	--	--	--	--	--
Hemoglobinopathies and hemolytic anaemias	163,124.2 (160,753.0 to 165,566.5)	--	625.8 (407.6 to 905.8)	-0.8 (-4.9 to 3.3)	-1.6 (-5.7 to 2.5)
Thalassemias	20.8 (19.3 to 22.7)	--	1.1 (0.7 to 1.6)	-8.9 (-16.1 to -2.2)	-9.7 (-16.8 to -3.0)
Thalassaemias trait	22,274.6 (21,459.3 to 23,157.9)	--	224.2 (144.9 to 324.8)	-10.8 (-16.4 to -4.8)	-11.5 (-17.1 to -5.5)
Sickle cell disorders	577.7 (527.9 to 645.6)	--	44.4 (29.5 to 63.2)	19.8 (10.5 to 31.5)	18.8 (9.6 to 30.4)
Sickle cell trait	48,141.9 (43,658.2 to 53,992.0)	--	166.4 (107.7 to 249.3)	7.2 (3.3 to 11.4)	6.4 (2.5 to 10.5)
G6PD deficiency	31,773.5 (30,522.2 to 33,267.5)	--	2.1 (1.3 to 3.0)	1.2 (-4.6 to 8.5)	0.4 (-5.4 to 7.6)
G6PD trait	75,574.6 (74,340.7 to 76,956.1)	--	< 0.05 (< 0.05 to < 0.05)	-2.1 (-6.4 to 1.8)	-3.0 (-7.1 to 1.0)
Other hemoglobinopathies and hemolytic anaemias	4,838.2 (4,259.6 to 5,465.8)	--	187.6 (121.1 to 273.4)	2.0 (-4.3 to 9.0)	1.1 (-5.1 to 8.1)
Endocrine, metabolic, blood, and immune disorders	4,734.9 (4,206.9 to 5,304.3)	210.3 (131.9 to 308.8)	178.3 (114.0 to 259.2)	-0.3 (-3.6 to 3.2)	-1.1 (-4.4 to 2.3)
**Musculoskeletal disorders**	**13,604.3 (12,343.5 to 14,805.0)**	**7,948.8 (5,950.2 to 10,306.6)**	**1,443.9 (932.9 to 2,059.7)**	**-4.6 (-7.2 to -2.6)**	**-5.4 (-7.9 to -3.4)**
Rheumatoid arthritis	180.0 (131.8 to 238.8)	39.6 (31.2 to 49.4)	46.2 (28.5 to 69.1)	1.7 (-3.9 to 7.0)	0.8 (-4.7 to 6.1)
Osteoarthritis	--	--	--	--	--
Low back and neck pain	11,362.4 (9,955.1 to 13,136.6)	6,349.1 (4,526.4 to 8,568.1)	1,183.3 (728.6 to 1,754.7)	-2.8 (-5.6 to -0.6)	-3.6 (-6.4 to -1.4)
Low back pain	9,436.3 (6,589.1 to 12,629.1)	5,710.7 (3,869.5 to 7,900.8)	1,020.3 (606.9 to 1,540.0)	-2.9 (-6.2 to -0.4)	-3.7 (-7.0 to -1.2)
Neck pain	1,561.2 (1,138.8 to 2,120.2)	638.4 (424.2 to 907.7)	163.1 (97.2 to 251.2)	-2.1 (-5.8 to 1.6)	-2.9 (-6.5 to 0.8)
Gout	--	--	--	--	--
Other musculoskeletal disorders	2,387.5 (1,425.7 to 3,685.5)	1,560.1 (1,040.0 to 2,389.1)	214.4 (113.2 to 354.7)	-14.6 (-22.4 to -8.7)	-15.3 (-23.0 to -9.5)
**Other non-communicable diseases**	**402,907.7 (392,186.5 to 415,534.0)**	**1,045,300.5† (853,140.3 to 1,244,088.8)**	**10,000.1 (6,765.9 to 14,385.1)**	**1.8 (1.0 to 2.4)**	**0.9 (0.2 to 1.6)**
Congenital anomalies	8,189.1 (7,723.5 to 8,599.5)	--	925.1 (654.6 to 1,240.3)	-0.6 (-2.8 to 1.8)	-1.4 (-3.6 to 0.9)
Neural tube defects	542.3 (488.6 to 601.2)	--	162.7 (113.7 to 218.8)	5.8 (1.1 to 11.0)	4.9 (0.3 to 10.1)
Congenital heart anomalies	1,418.4 (1,240.2 to 1,606.9)	--	69.4 (29.0 to 119.0)	-2.9 (-6.0 to 0.3)	-3.7 (-6.8 to -0.6)
Orofacial clefts	276.1 (245.2 to 308.2)	--	3.8 (2.5 to 5.5)	-7.1 (-9.9 to -4.2)	-7.9 (-10.6 to -5.0)
Down's syndrome	210.6 (183.8 to 243.1)	--	18.1 (12.2 to 25.5)	-0.6 (-3.8 to 2.4)	-1.4 (-4.6 to 1.5)
Turner syndrome	316.1 (276.4 to 361.4)	--	4.3 (1.7 to 7.4)	-1.4 (-12.7 to 11.3)	-2.2 (-13.4 to 10.4)
Klinefelter syndrome	413.2 (355.2 to 475.0)	--	1.1 (0.5 to 2.1)	2.9 (-5.2 to 12.8)	2.1 (-6.0 to 11.8)

Cause	Prevalence in thousands (95% UI)	Incidence in thousands (95% UI)	YLDs in thousands (95% UI)		
	2016 counts	2016 counts	2016 counts	Percentage change in counts between 2006 and 2016	Percentage change in rates between 2006 and 2016
Other chromosomal abnormalities	895.8<br>(761.3 to 1,048.0)	--	77.5<br>(51.3 to 109.3)	1.8<br>(-1.8 to 5.2)	1.0<br>(-2.6 to 4.3)
Congenital musculoskeletal and limb anomalies	889.4<br>(813.9 to 968.9)	--	127.2<br>(86.1 to 176.7)	2.4<br>(-0.9 to 5.8)	1.6<br>(-1.8 to 4.9)
Urogenital congenital anomalies	944.4<br>(844.4 to 1,062.2)	--	29.9<br>(18.0 to 46.3)	-2.9<br>(-5.2 to -0.6)	-3.7<br>(-6.0 to -1.4)
Digestive congenital anomalies	717.2<br>(642.6 to 793.5)	--	32.7<br>(21.5 to 46.7)	-5.8<br>(-10.5 to -0.8)	-6.5<br>(-11.2 to -1.7)
Other congenital anomalies	3,166.6<br>(2,243.0 to 4,461.5)	--	398.4<br>(258.6 to 592.5)	-3.2<br>(-7.3 to 1.0)	-4.0<br>(-8.1 to 0.2)
Skin and subcutaneous diseases	240,107.4<br>(234,956.2 to 245,651.5)	457,925.9<br>(396,896.8 to 539,475.4)	7,104.2<br>(4,682.2 to 10,421.1)	3.0<br>(2.2 to 3.6)	2.1<br>(1.3 to 2.8)
Dermatitis	31,501.0<br>(27,912.1 to 35,191.9)	20,270.6<br>(16,464.3 to 24,991.6)	1,267.1<br>(721.7 to 2,096.6)	3.7<br>(1.6 to 5.6)	2.8<br>(0.8 to 4.8)
Psoriasis	3,406.4<br>(3,089.8 to 3,759.5)	465.6<br>(398.7 to 544.3)	308.2<br>(213.7 to 413.1)	6.8<br>(3.2 to 10.3)	5.9<br>(2.3 to 9.4)
Cellulitis	213.4<br>(169.3 to 265.6)	4,003.7<br>(3,287.0 to 4,907.9)	12.4<br>(7.8 to 18.8)	2.2<br>(-3.3 to 8.0)	1.3<br>(-4.1 to 7.1)
Pyoderma	1,256.2<br>(1,107.2 to 1,414.3)	23,577.0<br>(21,065.3 to 26,280.6)	7.2<br>(2.8 to 14.3)	16.0<br>(11.8 to 20.4)	15.0<br>(10.9 to 19.4)
Scabies	15,766.5<br>(10,862.0 to 21,673.0)	49,181.3<br>(33,346.0 to 69,123.8)	409.9<br>(214.0 to 708.7)	-4.6<br>(-6.3 to -3.0)	-5.4<br>(-7.1 to -3.8)
Fungal skin diseases	49,677.8<br>(36,456.7 to 64,469.9)	129,520.4<br>(97,045.6 to 165,904.6)	285.2<br>(109.2 to 618.9)	7.1<br>(6.0 to 8.2)	6.2<br>(5.1 to 7.2)
Viral skin diseases	26,845.1<br>(23,603.8 to 30,623.0)	37,190.0<br>(33,067.4 to 41,607.9)	833.4<br>(508.2 to 1,263.8)	0.5<br>(-0.4 to 1.4)	-0.3<br>(-1.2 to 0.6)
Acne vulgaris	133,810.6<br>(114,133.4 to 156,989.5)	160,645.8<br>(111,054.9 to 233,032.5)	3,483.4<br>(2,269.9 to 5,141.7)	3.8<br>(2.6 to 4.9)	3.0<br>(1.8 to 4.0)
Alopecia areata	493.2<br>(439.5 to 551.5)	916.8<br>(794.7 to 1,039.6)	16.5<br>(10.3 to 24.8)	0.5<br>(-5.2 to 6.7)	-0.3<br>(-5.9 to 5.9)
Pruritus	2,992.8<br>(1,999.0 to 4,234.8)	2,255.3<br>(1,530.2 to 3,083.7)	32.6<br>(13.9 to 67.4)	1.3<br>(-0.8 to 3.6)	0.5<br>(-1.6 to 2.8)
Urticaria	5,570.4<br>(3,838.9 to 7,539.7)	9,516.7<br>(6,576.1 to 12,779.6)	339.7<br>(191.8 to 541.2)	0.2<br>(-1.9 to 2.2)	-0.6<br>(-2.7 to 1.4)
Decubitus ulcer	30.7<br>(20.4 to 44.7)	107.2<br>(69.2 to 153.1)	5.4<br>(3.1 to 9.0)	2.4<br>(1.4 to 3.2)	1.6<br>(0.6 to 2.4)
Other skin and subcutaneous diseases	18,304.5<br>(16,652.3 to 20,073.6)	20,275.6<br>(18,332.9 to 22,410.3)	103.2<br>(48.5 to 191.0)	5.7<br>(4.5 to 7.0)	4.8<br>(3.6 to 6.1)
Sense organ diseases	47,783.3<br>(46,026.1 to 49,742.3)	50,314.5†<br>(44,607.2 to 57,272.7)	1,621.8<br>(1,080.8 to 2,302.5)	-1.1<br>(-3.2 to 0.7)	-1.9<br>(-4.0 to -0.1)
Glaucoma	--	--	--	--	--
Cataract	--	--	--	--	--
Macular degeneration	--	--	--	--	--
Refraction and accommodation disorders	27,645.7<br>(24,412.8 to 31,110.6)	--	608.8<br>(371.9 to 960.1)	-1.8<br>(-3.1 to -0.3)	-2.6<br>(-3.9 to -1.2)
Age-related and other hearing loss	16,310.2<br>(12,260.5 to 21,034.3)	--	838.5<br>(543.3 to 1,198.9)	-1.2<br>(-4.9 to 2.3)	-2.0<br>(-5.7 to 1.5)
Other vision loss	450.4<br>(313.0 to 621.1)	--	22.0<br>(13.0 to 34.2)	-0.1<br>(-6.4 to 6.6)	-0.9<br>(-7.2 to 5.8)
Other sense organ diseases	5,283.3<br>(4,662.8 to 5,969.7)	50,314.5<br>(44,607.2 to 57,272.7)	152.4<br>(92.9 to 233.4)	1.5<br>(-1.1 to 4.0)	0.6<br>(-1.9 to 3.2)
Oral disorders	246,054.3<br>(214,832.4 to 281,821.9)	537,060.2†<br>(361,955.4 to 728,053.3)	349.1<br>(193.4 to 593.0)	-2.0<br>(-3.3 to -0.5)	-2.8<br>(-4.1 to -1.4)
Caries of deciduous teeth	10,820.8<br>(1,094.7 to 23,506.6)	53,361.2<br>(3,325.2 to 193,327.0)	2.8<br>(0.3 to 8.1)	-7.5<br>(-52.7 to 1.6)	-8.3<br>(-53.1 to 0.7)
Caries of permanent teeth	226,313.7<br>(172,956.8 to 280,080.8)	483,699.0<br>(333,330.0 to 628,973.5)	155.1<br>(67.5 to 308.9)	-5.1<br>(-7.4 to -3.2)	-5.9<br>(-8.2 to -4.0)
Periodontal disease	--	--	--	--	--
Edentulism and severe tooth loss	--	--	--	--	--
Other oral disorders	6,380.9<br>(5,439.9 to 7,439.9)	--	191.2<br>(117.3 to 297.6)	0.8<br>(-0.9 to 2.4)	-0.0<br>(-1.7 to 1.6)
**Injuries**	**44,366.2<br>(39,664.3 to 49,263.6)**	**41,522.1†<br>(35,136.4 to 49,039.6)**	**1,778.1<br>(1,136.5 to 2,613.1)**	**3.6<br>(2.1 to 4.8)**	**2.7<br>(1.3 to 3.9)**
**Transport injuries**	**4,073.4<br>(3,520.7 to 4,695.5)**	**4,784.5<br>(3,605.0 to 6,232.1)**	**242.7<br>(151.8 to 367.1)**	**-1.0<br>(-2.9 to 0.9)**	**-1.8<br>(-3.7 to 0.1)**
Road injuries	2,253.8<br>(1,975.9 to 2,546.9)	4,049.9<br>(2,996.6 to 5,434.1)	137.0<br>(84.5 to 207.8)	-1.4<br>(-4.0 to 1.5)	-2.2<br>(-4.8 to 0.7)
Pedestrian road injuries	549.5<br>(409.6 to 714.3)	771.0<br>(485.8 to 1,205.5)	33.7<br>(20.3 to 53.2)	-8.0<br>(-10.8 to -4.8)	-8.7<br>(-11.5 to -5.5)
Cyclist road injuries	610.1<br>(446.9 to 816.2)	1,372.2<br>(860.8 to 2,031.2)	34.9<br>(21.1 to 52.5)	0.0<br>(-3.2 to 3.8)	-0.8<br>(-4.0 to 2.9)
Motorcyclist road injuries	389.7<br>(306.5 to 493.0)	478.0<br>(273.0 to 757.9)	22.7<br>(13.6 to 35.0)	4.9<br>(2.3 to 7.7)	4.0<br>(1.5 to 6.8)
Motor vehicle road injuries	467.2<br>(369.7 to 591.1)	937.0<br>(581.8 to 1,413.0)	32.7<br>(20.3 to 48.9)	-3.5<br>(-7.0 to 0.5)	-4.3<br>(-7.8 to -0.4)
Other road injuries	237.4<br>(180.9 to 319.7)	491.7<br>(262.1 to 812.8)	13.0<br>(7.6 to 19.8)	9.3<br>(6.2 to 13.2)	8.4<br>(5.4 to 12.3)
Other transport injuries	1,819.6<br>(1,377.6 to 2,356.6)	734.6<br>(453.4 to 1,122.9)	105.7<br>(66.1 to 160.3)	-0.5<br>(-2.7 to 1.4)	-1.3<br>(-3.5 to 0.5)
**Unintentional injuries**	**37,291.7<br>(32,731.5 to 41,996.9)**	**34,964.8<br>(28,881.6 to 42,319.3)**	**1,317.8<br>(852.1 to 1,933.5)**	**5.2<br>(3.8 to 6.3)**	**4.3<br>(3.0 to 5.5)**
Falls	13,343.8<br>(10,614.8 to 16,386.2)	14,754.4<br>(9,620.1 to 20,848.0)	506.1<br>(335.4 to 737.2)	7.4<br>(5.9 to 8.8)	6.5<br>(5.0 to 8.0)
Drowning	154.2<br>(117.6 to 197.8)	69.3<br>(53.6 to 89.7)	8.9<br>(5.6 to 13.5)	-14.7<br>(-16.6 to -12.9)	-15.4<br>(-17.3 to -13.6)
Fire, heat, and hot substances	3,416.5<br>(2,570.2 to 4,570.8)	1,125.1<br>(677.6 to 1,765.7)	106.4<br>(68.9 to 159.5)	5.0<br>(2.0 to 7.5)	4.2<br>(1.2 to 6.6)
Poisonings	167.5<br>(126.3 to 214.6)	386.1<br>(223.3 to 597.7)	19.2<br>(11.8 to 29.2)	14.0<br>(10.4 to 17.4)	13.0<br>(9.5 to 16.4)
Exposure to mechanical forces	4,918.1<br>(3,795.8 to 6,204.7)	4,808.1<br>(3,044.1 to 7,456.3)	121.1<br>(73.0 to 190.9)	4.5<br>(2.7 to 6.1)	3.6<br>(1.9 to 5.2)
Unintentional firearm injuries	141.9<br>(103.6 to 193.5)	118.8<br>(53.2 to 231.8)	6.1<br>(3.6 to 10.0)	6.5<br>(4.1 to 9.0)	5.6<br>(3.3 to 8.1)
Unintentional suffocation	555.0<br>(382.0 to 764.1)	75.0<br>(34.6 to 143.5)	17.0<br>(9.6 to 28.4)	-5.1<br>(-6.9 to -3.4)	-5.9<br>(-7.6 to -4.2)
Other exposure to mechanical forces	4,221.2<br>(3,149.3 to 5,503.7)	4,614.2<br>(2,915.4 to 7,110.7)	98.0<br>(59.6 to 152.0)	6.2<br>(4.3 to 7.9)	5.3<br>(3.4 to 7.0)

Cause	Prevalence in thousands (95% UI)	Incidence in thousands (95% UI)	YLDs in thousands (95% UI)		
	2016 counts	2016 counts	2016 counts	Percentage change in counts between 2006 and 2016	Percentage change in rates between 2006 and 2016
Adverse effects of medical treatment	78.5 (48.2 to 120.8)	1,026.7 (660.6 to 1,555.7)	10.5 (5.3 to 18.2)	6.2 (1.3 to 10.2)	5.3 (0.5 to 9.3)
Animal contact	2,190.3 (1,782.2 to 2,714.8)	5,845.8 (4,047.2 to 8,304.6)	104.8 (68.1 to 150.8)	-0.1 (-3.1 to 2.7)	-1.0 (-3.9 to 1.8)
Venomous animal contact	1,100.9 (819.6 to 1,500.0)	2,878.0 (1,885.4 to 4,236.9)	85.3 (55.8 to 123.5)	0.5 (-3.0 to 3.9)	-0.3 (-3.8 to 3.0)
Non-venomous animal contact	1,089.4 (817.6 to 1,512.2)	2,967.8 (1,974.3 to 4,339.9)	19.5 (11.7 to 31.2)	-3.1 (-5.9 to -0.8)	-3.9 (-6.7 to -1.6)
Foreign body	3,257.9 (2,494.9 to 4,051.5)	1,935.0 (1,350.8 to 2,733.6)	132.2 (79.8 to 208.5)	3.6 (1.4 to 5.5)	2.8 (0.6 to 4.6)
Pulmonary aspiration and foreign body in airway	1,750.3 (1,197.9 to 2,552.5)	270.6 (154.8 to 456.3)	80.2 (47.3 to 124.8)	0.3 (-1.7 to 2.1)	-0.5 (-2.5 to 1.3)
Foreign body in eyes	161.7 (63.0 to 309.2)	1,063.2 (573.3 to 1,749.4)	9.1 (3.4 to 18.5)	10.9 (7.3 to 16.1)	10.0 (6.4 to 15.1)
Foreign body in other body part	1,345.9 (995.6 to 1,866.8)	601.2 (345.1 to 933.5)	42.9 (26.0 to 67.9)	8.8 (6.3 to 11.0)	7.9 (5.4 to 10.1)
Environmental heat and cold exposure	3,228.8 (2,462.8 to 4,271.6)	1,543.1 (915.8 to 2,362.5)	127.2 (78.4 to 196.6)	2.1 (0.2 to 3.7)	1.3 (-0.6 to 2.8)
Other unintentional injuries	6,536.1 (4,657.4 to 9,364.7)	3,471.3 (2,081.2 to 5,257.8)	181.5 (111.6 to 278.7)	6.6 (4.8 to 8.2)	5.7 (4.0 to 7.3)
**Self-harm and interpersonal violence**	**14,001.9 (10,965.8 to 17,276.7)**	**1,670.2† (1,122.3 to 2,301.2)**	**170.0 (108.1 to 239.1)**	**-2.5 (-3.7 to -1.2)**	**-3.3 (-4.5 to -2.0)**
Self-harm	131.9 (101.4 to 173.6)	315.5 (209.3 to 459.4)	9.5 (5.6 to 15.0)	3.4 (1.3 to 5.3)	2.5 (0.5 to 4.4)
Self-harm by firearm	13.0 (9.9 to 17.4)	16.8 (6.8 to 32.5)	0.6 (0.3 to 0.9)	-0.4 (-3.4 to 2.9)	-1.2 (-4.2 to 2.0)
Self-harm by other specified means	118.9 (89.0 to 161.0)	298.7 (196.8 to 435.6)	9.0 (5.2 to 14.1)	3.6 (1.5 to 5.6)	2.8 (0.7 to 4.8)
Interpersonal violence	13,870.1 (10,837.3 to 17,159.2)	1,354.7† (829.0 to 1,986.7)	160.5 (102.3 to 226.2)	-2.8 (-4.0 to -1.5)	-3.6 (-4.8 to -2.3)
Assault by firearm	36.7 (29.1 to 49.4)	32.9 (13.7 to 63.6)	1.8 (1.0 to 2.9)	4.8 (2.0 to 7.6)	3.9 (1.2 to 6.8)
Assault by sharp object	272.8 (195.8 to 367.2)	351.9 (172.8 to 620.2)	10.0 (6.0 to 15.5)	4.5 (1.2 to 7.8)	3.7 (0.4 to 6.9)
Sexual violence	13,023.4 (10,012.2 to 16,223.2)	--	124.4 (76.5 to 182.5)	-4.7 (-5.7 to -3.7)	-5.5 (-6.5 to -4.5)
Assault by other means	537.2 (404.8 to 706.5)	969.9 (558.7 to 1,469.4)	24.2 (14.5 to 37.5)	4.4 (1.7 to 6.6)	3.5 (0.9 to 5.7)
**Forces of nature, conflict and terrorism, and executions and police conflict**	**2,022.5 (1,190.2 to 3,156.9)**	**102.5† (70.3 to 139.9)**	**47.6 (17.9 to 92.0)**	**6.9 (-8.3 to 33.2)**	**6.0 (-9.1 to 32.1)**
Forces of nature, conflict and terrorism, and state actor violence	303.1 (158.6 to 491.1)	69.6 (40.7 to 104.5)	11.7 (3.9 to 23.2)	-14.6 (-32.0 to 8.7)	-15.3 (-32.6 to 7.8)
Conflict and terrorism	1,687.2 (937.3 to 2,781.9)	--	35.7 (13.5 to 68.5)	14.0 (-6.6 to 53.6)	13.0 (-7.4 to 52.4)
Executions and police conflict	32.2 (17.9 to 55.3)	33.0 (17.7 to 52.2)	1.1 (0.4 to 2.8)	-18.6 (-25.1 to -6.9)	-19.3 (-25.8 to -7.6)

We did not calculate incidence for certain NTDs and causes with only birth prevalence. Blank cells mean that no estimate is available.
† Incidence for aggregate causes represents the sum of the incidence of the causes below the aggregate.
‡ Incidence estimates for stroke represent first-ever stroke only.
^ Incidence of HIV/AIDS represents new infections of HIV only and does not include new infections of Tuberculosis in HIV positive cases.

SR Table 12: Global prevalence, incidence, and YLDs for 2016, percentage change of YLD counts and percentage change of YLD rates between 2006 and 2016 by cause, age 15-19 years.

Cause	Prevalence in thousands (95% UI)	Incidence in thousands (95% UI)	YLDs in thousands (95% UI)		
	2016 counts	2016 counts	2016 counts	Percentage change in counts between 2006 and 2016	Percentage change in rates between 2006 and 2016
**All causes**	**571,836.5 (570,897.1 to 573,090.5)**	**3,463,494.0† (2,941,436.9 to 4,050,623.3)**	**46,098.8 (34,011.1 to 60,449.0)**	**-4.6 (-5.2 to -4.0)**	**0.9 (0.3 to 1.6)**
**Communicable, maternal, neonatal, and nutritional disorders**	**382,272.8 (377,923.3 to 387,163.8)**	**2,023,173.4† (1,564,744.1 to 2,538,304.8)**	**7,324.1 (5,273.5 to 10,028.5)**	**-4.8 (-7.4 to -1.7)**	**0.7 (-1.9 to 4.0)**
**HIV/AIDS and tuberculosis**	**142,735.2 (123,964.0 to 167,574.0)**	**1,018.6† (779.4 to 1,298.4)**	**248.8 (155.8 to 367.6)**	**1.8 (-6.9 to 15.9)**	**7.8 (-1.5 to 22.7)**
Tuberculosis	133,084.9 (107,469.8 to 166,245.3)	855.1† (620.4 to 1,134.1)	153.6 (87.6 to 240.4)	-10.2 (-14.7 to -4.8)	-4.9 (-9.7 to 0.7)
Drug-susceptible tuberculosis	468.2 (322.4 to 654.8)	828.6 (600.4 to 1,099.7)	148.3 (84.7 to 232.4)	-9.9 (-14.6 to -4.4)	-4.6 (-9.6 to 1.2)
Multidrug-resistant tuberculosis without extensive drug resistance	15.1 (10.7 to 20.5)	25.4 (18.5 to 33.7)	5.0 (2.9 to 7.7)	-19.4 (-27.1 to -10.5)	-14.6 (-22.8 to -5.2)
Extensively drug-resistant tuberculosis	0.8 (0.6 to 1.0)	1.1 (0.8 to 1.5)	0.2 (0.1 to 0.4)	102.2 (76.6 to 130.6)	114.0 (86.9 to 144.1)
Latent tuberculosis infection	132,600.9 (107,081.0 to 165,707.7)	--	--	--	--
HIV/AIDS	774.8 (701.9 to 865.5)	163.5^ (144.2 to 189.6)	95.2 (60.9 to 148.7)	29.8 (5.5 to 65.5)	37.4 (11.7 to 75.2)
Drug-susceptible HIV/AIDS - Tuberculosis	32.1 (22.8 to 43.6)	40.6 (28.7 to 56.1)	12.8 (7.6 to 19.9)	-18.9 (-25.8 to -11.1)	-14.2 (-21.4 to -5.9)
Multidrug-resistant HIV/AIDS - Tuberculosis without extensive drug resistance	0.6 (0.4 to 0.9)	0.9 (0.6 to 1.4)	0.3 (0.1 to 0.4)	-44.6 (-54.4 to -33.1)	-41.4 (-51.7 to -29.2)
Extensively drug-resistant HIV/AIDS - Tuberculosis	< 0.05 (< 0.05 to < 0.05)	< 0.05 (< 0.05 to < 0.05)	< 0.05 (< 0.05 to < 0.05)	41.7 (9.5 to 89.9)	50.0 (15.9 to 101.0)
HIV/AIDS resulting in other diseases	742.0 (670.2 to 831.6)	163.5 (144.2 to 189.6)	82.1 (51.9 to 134.0)	43.9 (12.9 to 89.5)	52.3 (19.5 to 100.5)
**Diarrhoea, lower respiratory infections, and other common infectious diseases**	**36,198.7 (33,729.6 to 38,120.7)**	**1,851,628.0† (1,386,335.5 to 2,365,263.1)**	**1,633.5 (1,122.0 to 2,294.0)**	**-6.4 (-7.5 to -5.2)**	**-0.9 (-2.1 to 0.4)**
Diarrhoeal diseases	4,369.6 (3,870.5 to 4,918.8)	295,961.5 (261,761.7 to 335,008.2)	498.3 (336.2 to 698.9)	-1.4 (-3.7 to 0.9)	4.3 (2.0 to 6.8)
Intestinal infectious diseases	89.7 (67.5 to 120.2)	1,687.4† (1,222.4 to 2,303.7)	13.7 (7.9 to 21.9)	-15.8 (-33.2 to 6.9)	-10.9 (-29.3 to 13.1)
Typhoid fever	83.3 (58.4 to 116.4)	1,315.8 (940.3 to 1,793.2)	12.7 (7.2 to 20.4)	-16.0 (-35.0 to 8.2)	-11.1 (-31.2 to 14.5)
Paratyphoid fever	16.4 (11.2 to 23.0)	371.6 (256.5 to 527.7)	1.0 (0.6 to 1.7)	-13.4 (-29.6 to 7.8)	-8.3 (-25.5 to 14.1)
Other intestinal infectious diseases	--	--	< 0.05 (< 0.05 to < 0.05)	-34.1 (-47.7 to -16.3)	-30.3 (-44.7 to -11.4)
Lower respiratory infections	326.5 (286.1 to 372.8)	13,410.9 (11,537.5 to 15,748.7)	20.2 (13.0 to 29.2)	10.8 (4.8 to 17.2)	17.2 (10.9 to 24.0)
Upper respiratory infections	20,053.7 (14,209.8 to 26,316.0)	1,512,361.0 (1,062,862.5 to 2,032,780.9)	502.4 (278.7 to 827.6)	-7.8 (-9.5 to -6.5)	-2.4 (-4.2 to -1.1)
Otitis media	10,081.1 (8,205.0 to 12,167.9)	22,214.0 (17,440.8 to 28,846.8)	304.8 (176.9 to 494.2)	-9.5 (-13.2 to -6.3)	-4.2 (-8.1 to -0.8)
Meningitis	1,361.7 (1,175.4 to 1,578.8)	130.2 (95.8 to 169.7)	150.0 (105.3 to 198.8)	0.4 (-3.1 to 4.0)	6.3 (2.6 to 10.0)
Pneumococcal meningitis	581.1 (481.6 to 703.1)	37.5 (23.3 to 56.6)	64.9 (45.0 to 88.2)	-1.9 (-9.0 to 5.2)	3.9 (-3.7 to 11.3)
H influenzae type B meningitis	211.4 (164.2 to 268.4)	11.8 (6.4 to 19.6)	27.2 (18.2 to 36.3)	-21.1 (-26.6 to -16.5)	-16.5 (-22.3 to -11.6)
Meningococcal infection	156.1 (110.9 to 209.1)	24.5 (15.0 to 37.5)	16.3 (11.1 to 22.4)	6.6 (3.9 to 9.5)	12.9 (9.9 to 15.8)
Other meningitis	413.2 (309.7 to 531.5)	56.4 (38.9 to 78.0)	41.5 (28.5 to 57.1)	24.3 (18.4 to 30.9)	31.6 (25.3 to 38.5)
Encephalitis	1,031.5 (725.5 to 1,469.7)	414.8 (320.8 to 526.0)	130.0 (92.0 to 171.5)	-17.7 (-22.3 to -13.0)	-12.9 (-17.8 to -7.9)
Diphtheria	< 0.05 (< 0.05 to < 0.05)	0.1 (0.1 to 0.2)	< 0.05 (< 0.05 to < 0.05)	-56.3 (-68.2 to -40.8)	-53.7 (-66.3 to -37.3)
Whooping cough	5.4 (4.2 to 6.8)	39.5 (30.8 to 49.6)	0.3 (0.2 to 0.4)	-23.5 (-25.0 to -21.9)	-19.0 (-20.6 to -17.4)
Tetanus	15.9 (15.6 to 16.1)	5.0 (2.0 to 9.2)	0.4 (0.2 to 0.6)	-0.6 (-27.8 to 35.9)	5.2 (-23.5 to 43.8)
Measles	2.3 (0.8 to 5.0)	83.8 (30.3 to 181.0)	0.2 (0.1 to 0.5)	-60.5 (-64.8 to -55.6)	-58.2 (-62.7 to -53.1)
Varicella and herpes zoster	306.0 (245.3 to 371.4)	5,319.8 (3,349.1 to 8,100.2)	13.2 (7.8 to 20.8)	-6.8 (-12.5 to 0.0)	-1.3 (-7.4 to 5.9)
**Neglected tropical diseases and malaria**	**173,349.0 (168,202.1 to 177,881.2)**	**36,263.4† (30,137.7 to 43,284.9)**	**1,097.1 (748.8 to 1,581.4)**	**-20.7 (-25.4 to -15.1)**	**-16.1 (-21.0 to -10.1)**
Malaria	13,649.6 (11,447.1 to 16,553.8)	14,664.1 (10,965.0 to 18,779.8)	178.0 (135.3 to 227.3)	-5.5 (-11.6 to 1.9)	0.1 (-6.4 to 7.8)
Chagas disease	385.4 (299.4 to 483.6)	18.5 (12.8 to 26.2)	1.6 (0.9 to 2.4)	-16.3 (-26.8 to -4.4)	-11.4 (-22.5 to 1.2)
Leishmaniasis	439.6 (399.4 to 482.9)	75.5 (53.0 to 103.9)	26.9 (17.7 to 38.8)	30.1 (14.7 to 49.2)	37.7 (21.4 to 57.9)
Visceral leishmaniasis	1.3 (0.9 to 1.9)	5.2 (3.5 to 7.4)	0.1 (0.1 to 0.1)	-62.2 (-75.5 to -41.2)	-60.0 (-74.1 to -37.7)
Cutaneous and mucocutaneous leishmaniasis	413.7 (364.5 to 469.7)	70.2 (47.8 to 99.6)	26.8 (17.6 to 38.6)	31.2 (15.5 to 50.4)	38.9 (22.2 to 59.2)
African trypanosomiasis	0.8 (0.2 to 2.6)	0.5 (0.1 to 1.6)	0.2 (< 0.05 to 0.8)	-72.5 (-94.9 to 72.7)	-70.9 (-94.6 to 82.8)
Schistosomiasis	22,033.4 (20,059.7 to 24,212.1)	8,563.9 (7,007.9 to 10,488.4)	173.8 (84.4 to 330.7)	-26.1 (-28.3 to -23.9)	-21.8 (-24.1 to -19.4)
Cysticercosis	82.2 (51.6 to 122.3)	--	14.2 (7.7 to 24.0)	-17.2 (-32.0 to -4.7)	-12.3 (-28.0 to 0.9)
Cystic echinococcosis	79.8 (37.9 to 181.8)	17.4 (7.2 to 48.3)	7.5 (3.1 to 17.4)	-41.1 (-79.1 to 70.5)	-37.7 (-77.9 to 80.5)
Lymphatic filariasis	2,662.6 (2,295.4 to 3,155.3)	814.9 (485.9 to 1,282.1)	83.4 (40.0 to 151.5)	-38.6 (-52.9 to -28.1)	-35.0 (-50.1 to -23.8)
Onchocerciasis	1,629.3 (1,012.6 to 2,728.1)	492.9 (215.6 to 992.5)	98.2 (42.0 to 176.8)	-32.8 (-49.6 to -17.9)	-28.9 (-46.7 to -13.1)
Trachoma	5.5 (1.5 to 13.2)	--	0.3 (0.1 to 0.6)	-40.9 (-62.9 to -12.5)	-37.4 (-60.7 to -7.4)
Dengue	633.7 (344.7 to 1,074.4)	10,592.8 (6,434.5 to 16,084.0)	104.5 (45.8 to 197.3)	61.9 (31.9 to 272.0)	71.4 (39.7 to 293.7)
Yellow fever	0.4 (0.1 to 1.1)	13.9 (3.7 to 38.1)	< 0.05 (< 0.05 to < 0.05)	-17.5 (-47.8 to 29.5)	-12.7 (-44.7 to 37.1)
Rabies	< 0.05 (< 0.05 to < 0.05)	0.9 (0.4 to 1.3)	< 0.05 (< 0.05 to < 0.05)	-49.9 (-64.5 to -29.7)	-46.9 (-62.4 to -25.6)
Intestinal nematode infections	145,668.5 (139,684.3 to 150,264.5)	--	294.0 (167.5 to 481.4)	-23.0 (-28.5 to -16.4)	-18.5 (-24.4 to -11.5)
Ascariasis	76,579.4 (69,687.6 to 84,692.8)	--	84.9 (45.6 to 144.7)	-36.0 (-44.0 to -26.6)	-32.3 (-40.8 to -22.3)

Cause	Prevalence in thousands (95% UI)	Incidence in thousands (95% UI)	YLDs in thousands (95% UI)		
	2016 counts	2016 counts	2016 counts	Percentage change in counts between 2006 and 2016	Percentage change in rates between 2006 and 2016
Trichuriasis	41,775.8 (38,713.2 to 45,450.6)	--	33.8 (18.4 to 58.4)	-26.6 (-36.7 to -15.6)	-22.3 (-33.0 to -10.7)
Hookworm disease	47,478.2 (43,859.3 to 51,849.7)	--	175.4 (102.4 to 272.8)	-13.8 (-22.3 to -3.8)	-8.8 (-17.7 to 1.8)
Food-borne trematodiases	3,719.4 (3,439.5 to 4,039.7)	377.6 (330.9 to 427.3)	69.3 (31.2 to 136.1)	-49.2 (-52.7 to -42.6)	-46.2 (-49.9 to -39.2)
Leprosy	6.3 (5.8 to 6.9)	3.0 (2.8 to 3.4)	0.4 (0.2 to 0.5)	-26.0 (-30.4 to -21.1)	-21.6 (-26.3 to -16.5)
Ebola virus disease	< 0.05 (< 0.05 to 0.3)	--	< 0.05 (< 0.05 to 0.1)	--	--
Zika virus disease	10.4 (6.1 to 19.3)	627.4 (366.8 to 1,167.0)	0.2 (0.1 to 0.4)	--	--
Guinea worm disease	< 0.05 (< 0.05 to < 0.05)	< 0.05 (< 0.05 to < 0.05)	< 0.05 (< 0.05 to < 0.05)	-99.5 (-99.7 to -99.3)	-99.5 (-99.7 to -99.2)
Other neglected tropical diseases	2,582.7 (2,305.9 to 2,888.4)	--	44.5 (28.1 to 67.7)	9.7 (3.1 to 17.0)	16.1 (9.2 to 23.8)
**Maternal disorders**	**756.8 (671.7 to 836.8)**	**10,617.2† (8,765.0 to 12,653.1)**	**69.1 (44.7 to 99.4)**	**-18.0 (-33.2 to 1.8)**	**-13.2 (-29.4 to 7.8)**
Maternal haemorrhage	99.3 (73.0 to 131.9)	832.1 (563.9 to 1,205.6)	5.4 (3.4 to 8.1)	-18.4 (-36.7 to 3.6)	-13.6 (-33.0 to 9.6)
Maternal sepsis and other pregnancy related infections	133.6 (74.2 to 213.8)	1,479.9 (1,064.0 to 1,985.1)	6.0 (2.9 to 11.2)	-11.6 (-55.6 to 82.4)	-6.4 (-53.0 to 93.1)
Maternal hypertensive disorders	467.8 (287.1 to 678.7)	2,240.3 (1,757.3 to 2,727.6)	22.9 (11.9 to 38.0)	-15.5 (-46.5 to 34.9)	-10.6 (-43.4 to 42.8)
Maternal obstructed labour and uterine rupture	55.7 (40.8 to 73.3)	673.6 (463.0 to 913.6)	18.4 (11.4 to 28.7)	-17.9 (-30.8 to -4.7)	-13.1 (-26.8 to 0.9)
Maternal abortion, miscarriage, and ectopic pregnancy	44.2 (26.8 to 66.4)	5,391.2 (3,747.3 to 7,375.3)	4.9 (2.5 to 8.3)	-23.1 (-52.1 to 20.9)	-18.6 (-49.3 to 27.9)
Other maternal disorders	--	--	11.5 (7.4 to 16.5)	-23.3 (-37.6 to -4.8)	-18.8 (-33.9 to 0.8)
**Neonatal disorders**	**6,528.1 (5,925.3 to 7,139.4)**	**--**	**1,257.0 (870.3 to 1,814.2)**	**8.5 (-3.5 to 22.2)**	**14.9 (2.1 to 29.3)**
Neonatal preterm birth complications	5,300.3 (4,407.9 to 6,445.0)	--	819.2 (560.4 to 1,175.3)	5.4 (-11.7 to 25.6)	11.6 (-6.6 to 33.0)
Neonatal encephalopathy due to birth asphyxia and trauma	1,022.9 (608.9 to 1,732.0)	--	135.9 (41.3 to 353.8)	14.5 (5.1 to 25.5)	21.2 (11.2 to 32.9)
Neonatal sepsis and other neonatal infections	591.9 (198.8 to 1,429.8)	--	239.2 (66.6 to 627.4)	18.2 (10.2 to 30.2)	25.1 (16.6 to 37.9)
Hemolytic disease and other neonatal jaundice	197.7 (175.4 to 221.1)	--	62.7 (48.3 to 79.2)	4.5 (-0.9 to 10.1)	10.6 (4.9 to 16.5)
Other neonatal disorders	--	--	--	--	--
**Nutritional deficiencies**	**144,447.4 (139,904.3 to 148,384.4)**	**51,958.2† (42,379.1 to 65,675.5)**	**2,805.2 (1,830.6 to 4,124.2)**	**-1.6 (-4.5 to 1.7)**	**4.2 (1.1 to 7.6)**
Protein-energy malnutrition	--	--	--	--	--
Iodine deficiency	11,448.9 (10,054.0 to 13,054.8)	334.1 (297.0 to 375.3)	309.2 (204.8 to 442.0)	-22.3 (-25.4 to -19.4)	-17.7 (-21.0 to -14.7)
Vitamin A deficiency	45,540.4 (38,603.9 to 55,159.5)	51,624.1 (42,033.3 to 65,353.2)	18.5 (11.0 to 29.7)	1.9 (-4.7 to 9.0)	7.9 (0.9 to 15.4)
Iron-deficiency anaemia	107,467.2 (97,780.7 to 118,249.2)	--	2,477.5 (1,604.3 to 3,642.2)	1.8 (-1.5 to 5.5)	7.7 (4.3 to 11.6)
Other nutritional deficiencies	--	--	--	--	--
**Other communicable, maternal, neonatal, and nutritional diseases**	**87,851.9 (83,763.8 to 92,209.5)**	**71,688.2 (60,331.8 to 86,324.7)**	**210.7 (131.8 to 322.8)**	**-6.6 (-17.9 to 4.7)**	**-1.1 (-13.2 to 10.8)**
Sexually transmitted diseases excluding HIV	45,677.2 (43,137.8 to 48,127.8)	52,942.8 (43,096.8 to 64,789.0)	118.3 (70.3 to 190.1)	-3.9 (-9.4 to 1.3)	1.7 (-4.1 to 7.2)
Syphilis	3,776.4 (2,578.0 to 5,640.5)	3,510.8 (2,248.9 to 5,723.9)	1.2 (0.5 to 2.7)	2.9 (-6.2 to 11.7)	8.9 (-0.7 to 18.2)
Chlamydial infection	5,796.6 (3,933.7 to 8,212.8)	7,042.8 (4,743.4 to 10,192.5)	29.1 (17.2 to 47.9)	-8.0 (-13.1 to -1.5)	-2.6 (-8.1 to 4.2)
Gonococcal infection	3,786.3 (2,236.7 to 5,870.0)	15,501.4 (8,509.7 to 25,298.8)	25.8 (12.8 to 48.8)	0.4 (-16.1 to 11.7)	6.2 (-11.2 to 18.2)
Trichomoniasis	10,011.2 (7,033.0 to 13,707.2)	12,345.0 (8,155.5 to 17,572.4)	12.8 (4.6 to 28.4)	3.0 (0.2 to 5.9)	9.0 (6.1 to 12.1)
Genital herpes	24,446.6 (18,544.3 to 31,008.6)	13,088.8 (9,812.0 to 16,773.8)	9.3 (3.3 to 19.5)	-1.4 (-5.7 to 3.0)	4.4 (-0.2 to 9.0)
Other sexually transmitted diseases	475.7 (364.9 to 581.2)	1,454.0 (1,074.7 to 1,943.8)	40.2 (25.4 to 61.0)	-6.1 (-12.3 to -0.2)	-0.6 (-7.2 to 5.6)
Hepatitis	44,167.8 (35,577.2 to 53,872.1)	18,744.8 (12,276.9 to 26,887.4)	27.0 (13.2 to 50.1)	-30.0 (-67.3 to 46.2)	-25.9 (-65.4 to 54.8)
Acute hepatitis A	270.1 (21.6 to 655.3)	3,511.3 (280.5 to 8,519.2)	10.8 (0.7 to 30.0)	-29.6 (-95.2 to 705.2)	-25.4 (-94.9 to 752.4)
Hepatitis B	33,822.5 (25,534.7 to 43,503.0)	11,591.9 (7,200.8 to 18,798.5)	8.6 (4.5 to 15.9)	-25.7 (-51.6 to 19.4)	-21.4 (-48.7 to 26.4)
Hepatitis C	9,809.5 (8,501.5 to 11,075.3)	188.8 (133.2 to 251.3)	0.2 (0.1 to 0.4)	-24.3 (-24.9 to -23.7)	-19.9 (-20.5 to -19.3)
Acute hepatitis E	265.6 (209.0 to 321.1)	3,452.8 (2,717.5 to 4,174.3)	7.4 (4.4 to 11.5)	-35.0 (-48.7 to -18.9)	-31.2 (-45.7 to -14.1)
Other infectious diseases	2,923.3 (2,605.3 to 3,271.1)	0.5 (0.3 to 0.9)	65.4 (41.6 to 100.0)	2.3 (-2.0 to 7.5)	8.3 (3.7 to 13.8)
**Non-communicable diseases**	**541,744.3 (538,796.9 to 544,490.1)**	**1,399,489.0† (1,231,289.9 to 1,594,710.0)**	**36,515.7 (26,686.4 to 47,766.1)**	**-4.7 (-5.2 to -4.2)**	**0.9 (0.4 to 1.4)**
**Neoplasms**	**87.9 (81.6 to 92.3)**	**71.4 (66.2 to 76.8)**	**24.6 (17.7 to 32.9)**	**-5.6 (-9.1 to -1.1)**	**-0.0 (-3.8 to 4.7)**
Lip and oral cavity cancer	5.1 (4.7 to 5.5)	1.4 (1.3 to 1.5)	0.5 (0.4 to 0.7)	0.7 (-7.9 to 9.7)	6.6 (-2.6 to 16.1)
Nasopharynx cancer	4.8 (4.5 to 5.3)	1.4 (1.3 to 1.5)	0.6 (0.4 to 0.8)	-19.5 (-27.3 to -10.0)	-14.8 (-23.0 to -4.7)
Other pharynx cancer	1.0 (0.9 to 1.2)	0.4 (0.4 to 0.5)	0.1 (0.1 to 0.2)	-4.0 (-17.3 to 10.1)	1.6 (-12.4 to 16.6)
Oesophageal cancer	0.4 (0.4 to 0.4)	0.3 (0.3 to 0.4)	0.1 (0.1 to 0.1)	-26.2 (-32.2 to -18.7)	-21.9 (-28.2 to -14.0)
Stomach cancer	1.8 (1.7 to 2.0)	1.1 (1.0 to 1.2)	0.3 (0.2 to 0.4)	-27.4 (-32.0 to -20.4)	-23.1 (-28.0 to -15.8)
Colon and rectum cancer	5.0 (4.6 to 5.4)	1.5 (1.4 to 1.6)	0.5 (0.4 to 0.7)	-8.5 (-16.6 to 0.9)	-3.2 (-11.7 to 6.8)
Liver cancer	1.2 (1.0 to 1.3)	1.6 (1.4 to 1.8)	0.4 (0.2 to 0.5)	-11.0 (-20.7 to 1.2)	-5.8 (-16.0 to 7.1)
Liver cancer due to hepatitis B	0.9 (0.7 to 1.1)	0.7 (0.6 to 0.9)	0.2 (0.1 to 0.2)	-14.9 (-26.1 to -0.9)	-10.0 (-21.8 to 4.9)

Cause	Prevalence in thousands (95% UI)	Incidence in thousands (95% UI)	YLDs in thousands (95% UI)		
	2016 counts	2016 counts	2016 counts	Percentage change in counts between 2006 and 2016	Percentage change in rates between 2006 and 2016
Liver cancer due to hepatitis C	0.1 (< 0.05 to 0.1)	0.1 (< 0.05 to 0.1)	< 0.05 (< 0.05 to < 0.05)	-4.6 (-17.4 to 11.7)	1.0 (-12.6 to 18.2)
Liver cancer due to alcohol use	< 0.05 (< 0.05 to < 0.05)	< 0.05 (< 0.05 to < 0.05)	< 0.05 (< 0.05 to < 0.05)	-2.3 (-16.0 to 12.6)	3.4 (-11.1 to 19.2)
Liver cancer due to other causes	0.9 (0.7 to 1.2)	0.8 (0.6 to 1.0)	0.2 (0.1 to 0.2)	-7.5 (-17.0 to 4.4)	-2.1 (-12.1 to 10.5)
Gallbladder and biliary tract cancer	0.1 (0.1 to 0.1)	0.1 (0.1 to 0.1)	< 0.05 (< 0.05 to < 0.05)	-11.7 (-17.3 to -7.0)	-6.6 (-12.5 to -1.5)
Pancreatic cancer	0.1 (0.1 to 0.1)	0.2 (0.2 to 0.2)	< 0.05 (< 0.05 to 0.1)	-9.7 (-14.0 to -5.3)	-4.4 (-8.9 to 0.2)
Larynx cancer	0.8 (0.8 to 0.8)	0.2 (0.2 to 0.2)	0.1 (0.1 to 0.1)	-11.9 (-15.6 to -7.3)	-6.7 (-10.7 to -1.9)
Tracheal, bronchus, and lung cancer	1.2 (1.2 to 1.3)	1.0 (1.0 to 1.1)	0.3 (0.2 to 0.3)	-15.3 (-21.3 to -8.6)	-10.4 (-16.7 to -3.2)
Malignant skin melanoma	5.9 (5.0 to 6.7)	1.2 (1.1 to 1.4)	0.4 (0.3 to 0.6)	3.7 (-4.6 to 12.7)	9.8 (1.0 to 19.2)
Non-melanoma skin cancer	0.5 (0.1 to 1.3)	2.6 (0.8 to 7.8)	< 0.05 (< 0.05 to 0.1)	29.2 (10.6 to 41.6)	36.7 (17.0 to 49.9)
Non-melanoma skin cancer (squamous-cell carcinoma)	0.5 (0.1 to 1.3)	1.1 (0.2 to 2.5)	< 0.05 (< 0.05 to 0.1)	31.6 (13.5 to 44.4)	39.2 (20.1 to 52.9)
Non-melanoma skin cancer (basal-cell carcinoma)	0.1 (< 0.05 to 0.7)	1.5 (0.2 to 6.9)	< 0.05 (< 0.05 to < 0.05)	2.0 (-20.5 to 9.8)	8.0 (-15.9 to 16.2)
Breast cancer	4.7 (3.9 to 5.5)	1.0 (0.8 to 1.2)	0.4 (0.3 to 0.6)	5.2 (-10.3 to 23.6)	11.3 (-5.1 to 30.8)
Cervical cancer	3.1 (2.1 to 3.7)	0.8 (0.6 to 1.0)	0.3 (0.2 to 0.4)	-24.3 (-30.9 to -10.4)	-19.9 (-26.9 to -5.1)
Uterine cancer	1.5 (1.4 to 1.7)	0.3 (0.3 to 0.4)	0.1 (0.1 to 0.2)	-9.6 (-17.9 to 2.3)	-4.3 (-13.1 to 8.3)
Ovarian cancer	8.2 (7.2 to 9.0)	2.6 (2.4 to 2.9)	1.3 (0.9 to 1.8)	1.8 (-10.2 to 11.7)	7.7 (-5.0 to 18.2)
Prostate cancer	2.0 (1.7 to 2.2)	0.4 (0.4 to 0.5)	0.2 (0.1 to 0.2)	13.8 (4.3 to 20.3)	20.5 (10.4 to 27.3)
Testicular cancer	13.1 (12.2 to 14.4)	2.6 (2.4 to 2.8)	0.9 (0.6 to 1.3)	14.1 (1.8 to 28.9)	20.8 (7.8 to 36.4)
Kidney cancer	4.7 (4.2 to 4.9)	1.2 (1.2 to 1.3)	0.4 (0.3 to 0.6)	-0.8 (-7.0 to 5.2)	5.0 (-1.6 to 11.4)
Bladder cancer	1.9 (1.8 to 2.0)	0.5 (0.4 to 0.5)	0.2 (0.1 to 0.2)	2.0 (-4.4 to 8.9)	8.0 (1.2 to 15.3)
Brain and nervous system cancer	17.1 (14.8 to 18.6)	7.6 (6.7 to 8.3)	2.5 (1.7 to 3.3)	5.3 (-2.5 to 12.6)	11.4 (3.2 to 19.1)
Thyroid cancer	8.8 (8.0 to 10.1)	1.7 (1.5 to 1.9)	0.6 (0.4 to 0.8)	14.1 (2.7 to 30.8)	20.8 (8.7 to 38.5)
Mesothelioma	0.2 (0.2 to 0.2)	0.1 (0.1 to 0.1)	< 0.05 (< 0.05 to 0.1)	-10.8 (-16.3 to -5.2)	-5.5 (-11.5 to 0.3)
Hodgkin lymphoma	14.4 (12.2 to 16.2)	3.7 (3.1 to 4.2)	1.5 (1.0 to 2.0)	-9.9 (-16.2 to -2.4)	-4.6 (-11.3 to 3.4)
Non-Hodgkin's lymphoma	17.5 (15.2 to 18.7)	5.5 (4.8 to 5.8)	1.8 (1.2 to 2.4)	7.7 (0.9 to 13.5)	14.0 (6.8 to 20.1)
Multiple myeloma	0.8 (0.5 to 0.9)	0.3 (0.3 to 0.4)	0.2 (0.1 to 0.2)	4.2 (-6.4 to 15.4)	10.2 (-0.9 to 22.2)
Leukaemia	40.8 (36.1 to 44.4)	12.1 (10.9 to 13.2)	5.5 (3.8 to 7.4)	-24.0 (-29.5 to -17.7)	-19.6 (-25.4 to -12.9)
Acute lymphoid leukaemia	14.4 (12.8 to 15.7)	4.2 (3.7 to 4.5)	1.9 (1.3 to 2.6)	-4.9 (-14.8 to 2.6)	0.7 (-9.8 to 8.6)
Chronic lymphoid leukaemia	2.5 (2.3 to 2.9)	0.6 (0.5 to 0.6)	0.3 (0.2 to 0.4)	-24.3 (-34.7 to -15.0)	-19.9 (-30.9 to -10.0)
Acute myeloid leukaemia	3.6 (3.2 to 4.1)	2.8 (2.4 to 3.1)	0.8 (0.5 to 1.0)	-5.5 (-11.6 to 1.3)	0.0 (-6.5 to 7.2)
Chronic myeloid leukaemia	1.7 (1.4 to 1.9)	0.6 (0.5 to 0.7)	0.2 (0.1 to 0.3)	-23.6 (-32.7 to -11.1)	-19.2 (-28.8 to -5.9)
Other leukaemia	18.6 (15.6 to 20.4)	4.1 (3.4 to 4.5)	2.3 (1.6 to 3.1)	-38.6 (-45.3 to -30.2)	-35.0 (-42.1 to -26.1)
Other neoplasms	58.5 (51.9 to 61.8)	17.7 (15.8 to 18.7)	5.4 (3.9 to 7.2)	10.0 (1.6 to 17.9)	16.4 (7.5 to 24.8)
**Cardiovascular diseases**	**8,566.9 (7,911.9 to 9,254.3)**	**521.4† (467.5 to 578.7)**	**526.8 (351.4 to 760.9)**	**-0.2 (-2.9 to 2.5)**	**5.7 (2.8 to 8.5)**
Rheumatic heart disease	2,762.3 (2,456.9 to 3,102.0)	152.7 (137.0 to 169.8)	131.5 (82.0 to 194.5)	7.4 (2.7 to 11.8)	13.7 (8.7 to 18.4)
Ischaemic heart disease	628.0 (556.2 to 705.1)	187.2 (157.3 to 219.1)	36.6 (23.8 to 54.3)	-2.6 (-8.2 to 3.9)	3.1 (-2.9 to 10.0)
Cerebrovascular disease	344.4 (287.2 to 430.4)	45.9‡ (26.4 to 72.3)	70.0 (46.7 to 98.7)	-19.1 (-23.8 to -14.5)	-14.3 (-19.3 to -9.5)
Ischaemic stroke	325.7 (250.0 to 424.6)	29.3‡ (13.8 to 49.5)	49.9 (32.4 to 71.5)	-19.1 (-25.9 to -13.0)	-14.4 (-21.5 to -7.9)
Hemorrhagic stroke	130.8 (101.1 to 165.4)	16.5‡ (7.1 to 31.7)	20.1 (13.3 to 28.7)	-18.9 (-24.6 to -13.5)	-14.2 (-20.2 to -8.4)
Hypertensive heart disease	0.1 (< 0.05 to 0.1)	--	< 0.05 (< 0.05 to < 0.05)	18.4 (12.5 to 25.4)	25.3 (19.1 to 32.7)
Cardiomyopathy and myocarditis	153.7 (127.9 to 186.9)	104.9† (75.6 to 144.0)	18.8 (12.0 to 28.9)	-8.0 (-10.9 to -5.2)	-2.6 (-5.7 to 0.4)
Myocarditis	129.9 (103.8 to 161.3)	104.9 (75.6 to 144.0)	10.6 (6.8 to 16.3)	-9.6 (-13.8 to -5.3)	-4.3 (-8.8 to 0.2)
Alcoholic cardiomyopathy	< 0.05 (< 0.05 to < 0.05)	--	< 0.05 (< 0.05 to < 0.05)	3.2 (-8.6 to 15.8)	9.3 (-3.2 to 22.5)
Other cardiomyopathy	92.1 (70.5 to 118.0)	--	8.2 (5.1 to 12.8)	-5.9 (-9.7 to -2.0)	-0.4 (-4.4 to 3.7)
Atrial fibrillation and flutter	--	--	--	--	--
Peripheral vascular disease	--	--	--	--	--
Endocarditis	7.8 (6.0 to 9.9)	30.7 (19.2 to 44.5)	0.6 (0.4 to 0.9)	6.0 (4.1 to 8.0)	12.2 (10.2 to 14.3)
Other cardiovascular and circulatory diseases	4,966.4 (3,460.4 to 6,813.4)	--	269.2 (159.9 to 428.5)	3.5 (-0.4 to 7.2)	9.5 (5.4 to 13.5)
**Chronic respiratory diseases**	**29,198.6 (27,376.2 to 31,582.4)**	**4,117.5† (3,460.2 to 4,871.3)**	**1,292.6 (900.8 to 1,808.4)**	**-1.2 (-3.1 to 0.7)**	**4.6 (2.6 to 6.6)**
Chronic obstructive pulmonary disease	2,134.4 (1,945.2 to 2,342.5)	121.8 (98.7 to 148.0)	177.6 (150.7 to 202.5)	-1.2 (-6.2 to 3.8)	4.5 (-0.7 to 9.9)
Pneumoconiosis	1.3 (1.0 to 1.7)	0.4 (0.3 to 0.5)	0.2 (0.1 to 0.3)	-5.2 (-7.5 to -3.0)	0.3 (-2.1 to 2.7)
Silicosis	0.1 (0.1 to 0.1)	0.1 (< 0.05 to 0.1)	< 0.05 (< 0.05 to < 0.05)	-12.3 (-15.4 to -9.5)	-7.1 (-10.4 to -4.2)

Cause	Prevalence in thousands (95% UI)	Incidence in thousands (95% UI)	YLDs in thousands (95% UI)		
	2016 counts	2016 counts	2016 counts	Percentage change in counts between 2006 and 2016	Percentage change in rates between 2006 and 2016
Asbestosis	0.5 (0.3 to 0.8)	0.1 (0.1 to 0.2)	0.1 (< 0.05 to 0.1)	-0.5 (-4.4 to 3.7)	5.3 (1.2 to 9.7)
Coal workers pneumoconiosis	0.1 (0.1 to 0.2)	< 0.05 (< 0.05 to < 0.05)	< 0.05 (< 0.05 to < 0.05)	-11.6 (-14.5 to -8.7)	-6.4 (-9.4 to -3.3)
Other pneumoconiosis	0.6 (0.4 to 0.8)	0.1 (0.1 to 0.2)	0.1 (< 0.05 to 0.1)	-6.4 (-9.6 to -2.9)	-0.9 (-4.3 to 2.8)
Asthma	26,918.6 (23,273.6 to 31,067.8)	3,993.5 (3,327.8 to 4,744.7)	1,068.7 (684.0 to 1,574.7)	-2.1 (-4.2 to 0.1)	3.7 (1.4 to 5.9)
Interstitial lung disease and pulmonary sarcoidosis	51.4 (42.1 to 60.6)	1.8 (1.1 to 2.6)	5.8 (3.6 to 8.6)	-11.2 (-14.4 to -8.4)	-6.0 (-9.4 to -3.1)
Other chronic respiratory diseases	--	--	40.3 (34.1 to 45.8)	31.2 (24.8 to 37.7)	38.9 (32.1 to 45.8)
**Cirrhosis and other chronic liver diseases**	**841.2 (757.2 to 936.0)**	**7.4 (5.3 to 10.0)**	**76.5 (52.6 to 107.5)**	**-0.4 (-5.6 to 4.5)**	**5.4 (-0.1 to 10.6)**
Cirrhosis and other chronic liver diseases due to hepatitis B	47.4 (33.5 to 64.4)	0.5 (0.3 to 0.7)	4.9 (2.8 to 7.9)	3.8 (-11.2 to 21.3)	9.8 (-6.0 to 28.4)
Cirrhosis and other chronic liver diseases due to hepatitis C	42.4 (28.9 to 58.2)	0.4 (0.2 to 0.6)	4.1 (2.3 to 6.5)	4.4 (-9.2 to 20.3)	10.5 (-3.9 to 27.4)
Cirrhosis and other chronic liver diseases due to alcohol use	11.8 (6.8 to 17.9)	0.1 (0.1 to 0.2)	1.1 (0.6 to 2.0)	3.9 (-0.6 to 8.8)	10.0 (5.2 to 15.2)
Cirrhosis and other chronic liver diseases due to other causes	739.7 (662.5 to 833.6)	6.4 (4.6 to 8.6)	66.4 (45.4 to 94.4)	-1.1 (-6.3 to 4.3)	4.7 (-0.8 to 10.4)
**Digestive diseases**	**6,696.9 (6,298.1 to 7,058.9)**	**7,708.7† (6,696.8 to 8,673.4)**	**176.9 (123.6 to 246.7)**	**-9.1 (-12.4 to -5.6)**	**-3.8 (-7.3 to -0.1)**
Peptic ulcer disease	1,802.3 (1,426.8 to 2,311.9)	549.8 (375.8 to 788.8)	23.0 (13.2 to 38.0)	-8.9 (-15.8 to -1.7)	-3.6 (-10.8 to 4.1)
Gastritis and duodenitis	2,737.7 (2,034.4 to 3,606.0)	2,300.7 (1,607.0 to 3,118.9)	27.2 (16.2 to 42.2)	-17.2 (-23.8 to -10.3)	-12.3 (-19.4 to -5.1)
Appendicitis	126.0 (104.8 to 149.5)	3,249.3 (2,676.2 to 3,846.1)	38.9 (24.8 to 56.0)	-4.0 (-12.5 to 4.1)	1.6 (-7.4 to 10.2)
Paralytic ileus and intestinal obstruction	6.8 (4.6 to 9.5)	176.8 (119.5 to 245.8)	2.2 (1.2 to 3.5)	1.6 (-0.6 to 3.4)	7.5 (5.3 to 9.4)
Inguinal, femoral, and abdominal hernia	1,674.0 (1,024.8 to 2,478.1)	496.2 (322.8 to 696.0)	47.3 (26.9 to 78.2)	-12.0 (-18.2 to -5.2)	-6.9 (-13.4 to 0.4)
Inflammatory bowel disease	132.2 (113.1 to 153.4)	254.1 (214.3 to 304.0)	20.4 (13.5 to 29.1)	-9.8 (-14.2 to -5.2)	-4.5 (-9.2 to 0.3)
Vascular intestinal disorders	1.5 (0.8 to 2.7)	10.5 (6.4 to 16.1)	0.5 (0.2 to 0.9)	-3.6 (-5.7 to -1.6)	2.0 (-0.2 to 4.1)
Gallbladder and biliary diseases	488.0 (360.0 to 636.8)	583.3 (405.2 to 829.2)	2.8 (1.5 to 4.5)	-8.2 (-10.2 to -6.4)	-2.9 (-4.9 to -1.0)
Pancreatitis	10.0 (6.9 to 13.2)	88.0 (61.1 to 117.5)	1.3 (0.7 to 2.0)	-17.4 (-24.3 to -12.6)	-12.6 (-19.9 to -7.5)
Other digestive diseases	--	--	13.4 (9.4 to 18.7)	7.1 (3.3 to 11.3)	13.3 (9.3 to 17.8)
**Neurological disorders**	**229,667.2 (212,250.0 to 246,167.2)**	**114,161.6 (76,011.7 to 176,325.0)**	**5,116.1 (3,426.2 to 7,077.6)**	**-3.4 (-5.5 to -1.0)**	**2.2 (0.1 to 4.8)**
Alzheimer's disease and other dementias	--	--	--	--	--
Parkinson's disease	--	--	--	--	--
Epilepsy	2,136.2 (1,699.5 to 2,646.5)	219.3 (158.5 to 293.3)	707.9 (450.5 to 1,026.7)	-7.1 (-17.2 to 5.9)	-1.7 (-12.3 to 12.1)
Multiple sclerosis	28.0 (21.6 to 35.2)	5.4 (3.6 to 7.4)	8.0 (5.2 to 11.3)	-2.0 (-4.5 to 0.2)	3.8 (1.1 to 6.0)
Motor neuron disease	13.5 (10.8 to 16.6)	0.2 (0.1 to 0.4)	2.9 (1.9 to 4.0)	-7.5 (-8.4 to -6.3)	-2.0 (-3.0 to -0.8)
Migraine	85,153.6 (77,677.9 to 92,901.8)	10,283.4 (8,743.0 to 11,837.3)	3,645.2 (2,302.5 to 5,178.2)	-3.2 (-4.2 to -2.1)	2.5 (1.4 to 3.6)
Tension-type headache	169,459.7 (122,071.6 to 224,789.0)	103,650.5 (65,729.9 to 166,183.9)	561.2 (340.2 to 859.7)	-3.6 (-5.2 to -1.7)	2.0 (0.3 to 4.0)
Other neurological disorders	1.4 (0.8 to 2.5)	2.9 (1.5 to 5.0)	191.0 (121.6 to 276.2)	8.1 (-3.4 to 23.0)	14.4 (2.2 to 30.2)
**Mental and substance use disorders**	**101,583.2 (99,282.1 to 104,163.5)**	**36,558.2† (31,538.9 to 42,282.4)**	**12,326.1 (8,781.9 to 16,389.7)**	**-4.9 (-5.7 to -4.2)**	**0.7 (-0.1 to 1.4)**
Schizophrenia	281.6 (199.4 to 383.8)	124.7 (82.9 to 171.5)	188.2 (119.5 to 276.2)	-12.6 (-17.8 to -7.4)	-7.4 (-12.9 to -2.0)
Alcohol use disorders	4,695.9 (3,493.7 to 6,163.1)	3,340.0 (2,362.4 to 4,422.5)	479.6 (296.9 to 719.1)	-6.1 (-9.0 to -3.4)	-0.6 (-3.6 to 2.2)
Drug use disorders	6,624.7 (6,128.0 to 7,254.0)	2,118.0 (1,813.1 to 2,480.9)	813.6 (574.9 to 1,111.1)	-9.4 (-11.9 to -6.7)	-4.1 (-6.8 to -1.2)
Opioid use disorders	718.6 (574.0 to 936.5)	417.9 (330.7 to 542.4)	306.5 (198.6 to 425.8)	-6.5 (-11.7 to -1.8)	-1.0 (-6.5 to 3.9)
Cocaine use disorders	703.0 (577.3 to 840.6)	213.2 (177.8 to 255.4)	98.2 (59.8 to 147.6)	-2.3 (-6.0 to 1.9)	3.5 (-0.5 to 7.8)
Amphetamine use disorders	948.6 (610.5 to 1,381.9)	238.8 (157.8 to 342.7)	126.4 (66.9 to 212.4)	-14.9 (-20.1 to -10.1)	-9.9 (-15.4 to -4.9)
Cannabis use disorders	4,338.7 (3,104.2 to 5,931.2)	1,193.7 (918.1 to 1,511.0)	127.6 (74.1 to 201.6)	-14.3 (-18.4 to -10.7)	-9.3 (-13.7 to -5.5)
Other drug use disorders	82.9 (64.0 to 103.5)	54.4 (41.8 to 69.0)	155.0 (97.0 to 232.0)	-9.9 (-13.8 to -5.8)	-4.7 (-8.8 to -0.3)
Depressive disorders	17,139.2 (15,782.0 to 18,343.9)	20,846.5 (16,138.9 to 26,004.5)	3,110.9 (2,022.9 to 4,420.4)	-5.2 (-6.9 to -3.6)	0.4 (-1.4 to 2.0)
Major depressive disorder	12,652.4 (9,696.1 to 15,965.7)	19,664.0 (14,922.8 to 24,787.3)	2,667.5 (1,722.0 to 3,857.5)	-5.7 (-7.6 to -3.9)	-0.1 (-2.2 to 1.7)
Dysthymia	4,487.8 (3,328.2 to 5,993.3)	1,182.5 (876.6 to 1,584.4)	443.4 (269.2 to 676.1)	-2.0 (-4.8 to 0.5)	3.8 (0.8 to 6.4)
Bipolar disorder	4,905.2 (3,716.9 to 6,295.8)	841.6 (673.8 to 1,045.6)	1,029.7 (621.1 to 1,595.4)	-0.8 (-2.7 to 0.9)	5.0 (3.0 to 6.8)
Anxiety disorders	25,134.3 (22,200.7 to 28,547.4)	3,777.8 (3,239.0 to 4,431.7)	2,485.4 (1,707.4 to 3,428.3)	-4.9 (-6.8 to -3.1)	0.7 (-1.3 to 2.5)
Eating disorders	1,724.2 (1,484.1 to 2,090.4)	1,759.8 (1,089.0 to 2,753.5)	354.4 (205.8 to 556.6)	4.2 (1.6 to 6.6)	10.3 (7.6 to 12.8)
Anorexia nervosa	586.8 (383.9 to 836.8)	233.9 (142.6 to 345.0)	125.5 (70.8 to 203.0)	2.3 (-1.3 to 5.9)	8.3 (4.5 to 12.1)
Bulimia nervosa	1,077.0 (600.8 to 1,755.6)	1,525.9 (859.7 to 2,518.8)	228.9 (116.8 to 411.4)	5.4 (2.2 to 8.6)	11.5 (8.1 to 14.9)
Autistic spectrum disorders	5,826.0 (5,534.3 to 6,089.6)	--	856.1 (579.0 to 1,204.1)	-4.8 (-6.2 to -3.4)	0.8 (-0.7 to 2.3)
Autism	1,705.1 (1,469.7 to 1,990.5)	--	439.9 (283.6 to 640.2)	-4.8 (-7.1 to -2.5)	0.8 (-1.7 to 3.2)
Asperger syndrome and other autistic spectrum disorders	4,114.4 (3,448.5 to 4,939.2)	--	416.1 (271.3 to 605.5)	-4.8 (-6.4 to -3.3)	0.8 (-0.9 to 2.4)

Cause	Prevalence in thousands (95% UI)	Incidence in thousands (95% UI)	YLDs in thousands (95% UI)		
	2016 counts	2016 counts	2016 counts	Percentage change in counts between 2006 and 2016	Percentage change in rates between 2006 and 2016
Attention-deficit/hyperactivity disorder	9,407.2 (8,409.6 to 10,549.0)	--	113.7 (68.0 to 182.1)	-9.6 (-11.5 to -7.7)	-4.3 (-6.3 to -2.3)
Conduct disorder	16,548.8 (13,335.5 to 20,114.2)	2,514.8 (1,582.0 to 3,370.0)	1,994.0 (1,228.2 to 3,041.9)	-3.1 (-4.8 to -1.3)	2.6 (0.7 to 4.5)
Idiopathic developmental intellectual disability	13,913.4 (8,840.6 to 19,167.8)	--	539.9 (259.0 to 930.0)	-10.1 (-13.5 to -8.0)	-4.9 (-8.4 to -2.6)
Other mental and substance use disorders	4,701.0 (3,882.4 to 5,604.8)	1,234.9 (1,016.9 to 1,473.9)	360.6 (240.7 to 524.6)	-5.1 (-7.8 to -2.4)	0.4 (-2.5 to 3.3)
**Diabetes, urogenital, blood, and endocrine diseases**	**200,013.1 (197,827.5 to 202,355.4)**	**72,510.8† (63,757.1 to 82,642.3)**	**1,596.0 (1,087.1 to 2,292.7)**	**-3.1 (-5.1 to -1.0)**	**2.6 (0.5 to 4.8)**
Diabetes mellitus	4,896.7 (3,768.1 to 6,195.6)	758.1 (550.2 to 1,009.9)	270.2 (165.3 to 414.9)	-0.2 (-2.9 to 2.4)	5.6 (2.8 to 8.4)
Acute glomerulonephritis	5.9 (3.8 to 8.6)	84.5 (53.9 to 121.5)	0.3 (0.1 to 0.5)	-17.1 (-19.8 to -14.8)	-12.2 (-15.1 to -9.8)
Chronic kidney disease	2,648.9 (2,065.1 to 3,339.2)	390.4 (270.5 to 527.1)	119.9 (75.2 to 184.0)	-0.5 (-5.2 to 4.2)	5.4 (0.3 to 10.2)
Chronic kidney disease due to diabetes mellitus	171.2 (95.9 to 275.4)	23.4 (12.2 to 38.9)	10.2 (5.0 to 18.4)	-2.6 (-8.5 to 4.6)	3.1 (-3.1 to 10.7)
Chronic kidney disease due to hypertension	672.5 (458.2 to 948.7)	172.4 (108.0 to 258.1)	5.9 (3.1 to 9.9)	-1.8 (-7.7 to 3.7)	3.9 (-2.3 to 9.8)
Chronic kidney disease due to glomerulonephritis	605.5 (411.4 to 835.2)	15.1 (8.8 to 23.7)	52.7 (31.4 to 85.1)	-1.3 (-6.4 to 3.5)	4.5 (-0.9 to 9.6)
Chronic kidney disease due to other causes	1,199.7 (810.0 to 1,682.4)	179.6 (113.6 to 259.9)	51.1 (28.7 to 81.7)	1.1 (-4.3 to 5.9)	7.0 (1.3 to 12.1)
Urinary diseases and male infertility	1,672.2 (1,384.7 to 2,046.0)	31,239.4† (23,190.8 to 40,422.3)	36.8 (21.4 to 59.7)	-6.5 (-10.8 to -1.7)	-1.1 (-5.6 to 4.0)
Interstitial nephritis and urinary tract infections	551.4 (403.4 to 717.9)	29,816.5 (21,592.0 to 39,199.1)	18.5 (10.3 to 30.2)	-5.1 (-9.7 to -0.0)	0.5 (-4.4 to 5.8)
Urolithiasis	50.7 (36.0 to 67.3)	1,422.8 (1,018.4 to 1,922.8)	3.9 (2.4 to 6.2)	-7.6 (-8.8 to -6.3)	-2.2 (-3.5 to -0.8)
Benign prostatic hyperplasia	--	--	--	--	--
Male infertility	1,041.5 (628.3 to 1,600.0)	--	7.3 (2.7 to 16.2)	12.2 (1.5 to 22.2)	18.7 (7.5 to 29.3)
Other urinary diseases	--	--	7.0 (4.2 to 11.0)	-22.6 (-25.7 to -19.2)	-18.1 (-21.4 to -14.5)
Gynecological diseases	56,083.3 (54,041.5 to 58,334.3)	39,829.4† (35,935.5 to 44,201.4)	611.8 (402.7 to 884.5)	-5.7 (-7.8 to -3.4)	-0.2 (-2.4 to 2.2)
Uterine fibroids	288.5 (220.6 to 369.9)	71.0 (44.6 to 102.5)	7.0 (4.0 to 11.5)	-5.5 (-12.5 to 2.1)	0.0 (-7.4 to 8.0)
Polycystic ovarian syndrome	446.7 (323.6 to 628.3)	797.9 (571.5 to 1,110.6)	4.1 (1.8 to 8.3)	-6.3 (-10.2 to -2.4)	-0.8 (-5.0 to 3.3)
Female infertility	1,021.7 (505.2 to 1,775.6)	--	6.8 (2.2 to 15.4)	24.2 (-29.0 to 74.9)	31.4 (-24.8 to 85.1)
Endometriosis	242.9 (190.3 to 301.8)	2,844.1 (2,139.8 to 3,760.8)	23.2 (14.8 to 33.9)	-18.6 (-24.4 to -11.9)	-13.8 (-20.0 to -6.7)
Genital prolapse	2,070.5 (1,448.5 to 2,801.8)	1,080.9 (804.4 to 1,399.2)	6.7 (2.9 to 13.3)	-12.2 (-17.0 to -7.5)	-7.0 (-12.2 to -2.0)
Premenstrual syndrome	49,808.1 (44,585.0 to 55,774.1)	32,692.1 (28,909.3 to 37,095.5)	421.5 (260.9 to 649.7)	-7.5 (-10.0 to -5.2)	-2.0 (-4.7 to 0.3)
Other gynecological diseases	4,380.3 (3,840.1 to 4,954.9)	2,343.4 (1,555.9 to 3,219.9)	142.4 (94.7 to 201.0)	1.7 (-2.8 to 6.6)	7.7 (2.9 to 12.8)
Hemoglobinopathies and hemolytic anaemias	154,702.5 (152,604.1 to 157,066.6)	--	440.7 (286.0 to 652.1)	-1.6 (-6.4 to 3.6)	4.1 (-0.9 to 9.7)
Thalassemias	8.9 (8.3 to 9.6)	--	0.7 (0.5 to 1.0)	-11.9 (-18.9 to -5.6)	-6.7 (-14.2 to -0.1)
Thalassaemias trait	21,399.7 (20,565.2 to 22,322.6)	--	178.5 (111.2 to 275.3)	-19.2 (-26.3 to -12.1)	-14.4 (-22.0 to -7.0)
Sickle cell disorders	425.7 (392.6 to 466.9)	--	38.1 (26.1 to 52.0)	21.2 (11.9 to 31.2)	28.3 (18.4 to 38.9)
Sickle cell trait	44,385.2 (40,102.1 to 50,048.8)	--	119.2 (76.5 to 176.9)	11.4 (6.9 to 16.2)	18.0 (13.2 to 23.0)
G6PD deficiency	29,718.8 (28,561.4 to 31,106.5)	--	1.9 (1.2 to 2.8)	7.2 (2.5 to 12.1)	13.4 (8.5 to 18.6)
G6PD trait	72,093.3 (70,911.4 to 73,419.2)	--	< 0.05 (< 0.05 to 0.1)	-8.5 (-14.0 to -2.4)	-3.1 (-9.0 to 3.3)
Other hemoglobinopathies and hemolytic anaemias	5,419.4 (4,807.1 to 6,086.8)	--	102.3 (66.5 to 151.1)	18.6 (10.6 to 28.2)	25.5 (17.1 to 35.8)
Endocrine, metabolic, blood, and immune disorders	4,860.7 (4,344.6 to 5,434.7)	208.9 (135.1 to 310.2)	116.3 (76.0 to 165.9)	-1.8 (-5.4 to 2.1)	4.0 (0.1 to 8.1)
**Musculoskeletal disorders**	**36,872.4 (34,129.1 to 39,374.9)**	**20,602.5 (15,646.8 to 26,164.0)**	**3,830.7 (2,565.0 to 5,288.7)**	**-8.9 (-11.2 to -7.0)**	**-3.6 (-6.0 to -1.5)**
Rheumatoid arthritis	358.9 (285.9 to 442.2)	43.8 (30.5 to 61.0)	90.0 (58.5 to 128.2)	-3.4 (-7.9 to 1.6)	2.2 (-2.5 to 7.6)
Osteoarthritis	--	--	--	--	--
Low back and neck pain	22,365.9 (20,236.1 to 25,180.2)	10,477.3† (7,424.6 to 13,575.0)	2,389.5 (1,522.8 to 3,456.9)	-7.7 (-10.1 to -5.4)	-2.2 (-4.8 to 0.1)
Low back pain	18,360.6 (13,116.7 to 24,119.0)	9,115.5 (6,170.3 to 12,345.5)	2,011.3 (1,229.9 to 2,965.5)	-7.1 (-10.0 to -4.6)	-1.6 (-4.8 to 1.0)
Neck pain	3,651.4 (2,531.9 to 4,973.7)	1,361.8 (852.4 to 1,955.4)	378.2 (231.1 to 589.4)	-10.6 (-13.3 to -7.9)	-5.4 (-8.2 to -2.5)
Gout	40.7 (24.3 to 63.0)	28.4 (16.9 to 44.4)	1.4 (0.7 to 2.4)	-5.8 (-7.1 to -4.6)	-0.3 (-1.7 to 1.0)
Other musculoskeletal disorders	15,216.8 (10,493.7 to 21,054.5)	10,052.9 (6,336.7 to 14,521.7)	1,349.8 (797.0 to 2,035.5)	-11.4 (-16.2 to -7.8)	-6.2 (-11.3 to -2.4)
**Other non-communicable diseases**	**432,269.7 (424,918.1 to 440,008.3)**	**1,143,229.7† (984,257.0 to 1,338,697.3)**	**11,549.3 (7,864.3 to 16,392.5)**	**-4.3 (-4.9 to -3.6)**	**1.3 (0.7 to 2.0)**
Congenital anomalies	7,369.1 (6,972.1 to 7,715.5)	--	854.0 (603.2 to 1,138.7)	-6.0 (-8.1 to -3.8)	-0.5 (-2.7 to 1.9)
Neural tube defects	509.0 (459.2 to 564.5)	--	152.7 (107.5 to 205.9)	1.3 (-3.2 to 5.9)	7.2 (2.4 to 12.1)
Congenital heart anomalies	1,065.6 (944.5 to 1,199.8)	--	53.7 (23.0 to 92.5)	-12.3 (-15.4 to -9.1)	-7.1 (-10.5 to -3.8)
Orofacial clefts	263.1 (234.2 to 293.0)	--	3.6 (2.3 to 5.2)	-12.8 (-15.5 to -10.2)	-7.7 (-10.6 to -4.9)
Down's syndrome	203.1 (177.3 to 234.4)	--	17.4 (11.8 to 24.6)	-7.3 (-10.2 to -4.2)	-1.8 (-4.9 to 1.4)
Turner syndrome	294.7 (257.6 to 336.1)	--	6.1 (3.1 to 9.9)	-6.3 (-14.6 to 2.1)	-0.8 (-9.6 to 8.1)
Klinefelter syndrome	378.4 (326.0 to 433.7)	--	1.0 (0.5 to 1.9)	-2.4 (-10.8 to 6.8)	3.4 (-5.6 to 13.1)

Cause	Prevalence in thousands (95% UI)	Incidence in thousands (95% UI)	YLDs in thousands (95% UI)		
	2016 counts	2016 counts	2016 counts	Percentage change in counts between 2006 and 2016	Percentage change in rates between 2006 and 2016
Other chromosomal abnormalities	870.9<br>(740.1 to 1,018.1)	--	73.0<br>(48.8 to 104.5)	-6.2<br>(-9.4 to -2.6)	-0.7<br>(-4.1 to 3.1)
Congenital musculoskeletal and limb anomalies	820.8<br>(756.5 to 891.9)	--	118.9<br>(81.3 to 164.2)	-5.4<br>(-8.5 to -2.2)	0.1<br>(-3.2 to 3.6)
Urogenital congenital anomalies	735.6<br>(653.3 to 823.0)	--	23.2<br>(14.0 to 36.0)	-11.8<br>(-13.9 to -9.8)	-6.6<br>(-8.9 to -4.5)
Digestive congenital anomalies	703.9<br>(631.7 to 778.6)	--	31.7<br>(20.7 to 45.1)	-15.7<br>(-19.8 to -11.1)	-10.8<br>(-15.1 to -5.9)
Other congenital anomalies	3,026.2<br>(2,138.7 to 4,264.3)	--	372.7<br>(243.3 to 549.0)	-6.5<br>(-10.5 to -2.2)	-1.0<br>(-5.2 to 3.5)
Skin and subcutaneous diseases	281,874.6<br>(272,835.6 to 292,013.2)	422,609.6<br>(373,425.0 to 482,853.6)	8,477.2<br>(5,723.1 to 12,252.9)	-3.6<br>(-4.3 to -2.8)	2.1<br>(1.3 to 2.9)
Dermatitis	24,814.1<br>(21,608.1 to 28,453.9)	29,453.5<br>(23,704.8 to 35,465.7)	933.4<br>(551.8 to 1,506.7)	-1.4<br>(-3.3 to 0.5)	4.4<br>(2.4 to 6.4)
Psoriasis	3,689.3<br>(3,346.8 to 4,055.5)	493.5<br>(423.5 to 567.5)	330.4<br>(228.6 to 441.2)	0.2<br>(-2.8 to 3.4)	6.1<br>(2.9 to 9.4)
Cellulitis	240.4<br>(192.5 to 293.6)	4,463.2<br>(3,713.7 to 5,327.1)	13.8<br>(8.6 to 21.1)	-2.8<br>(-8.7 to 4.1)	2.9<br>(-3.3 to 10.2)
Pyoderma	868.4<br>(789.0 to 949.3)	17,290.9<br>(15,753.6 to 18,942.1)	5.0<br>(2.0 to 10.7)	6.6<br>(2.6 to 10.4)	12.8<br>(8.7 to 16.8)
Scabies	16,728.3<br>(11,608.1 to 23,596.0)	52,626.5<br>(36,274.7 to 74,697.9)	434.4<br>(219.6 to 761.6)	-12.7<br>(-14.0 to -11.3)	-7.6<br>(-8.9 to -6.2)
Fungal skin diseases	37,857.2<br>(27,961.4 to 50,763.3)	108,572.2<br>(79,712.9 to 144,751.4)	215.9<br>(83.4 to 481.4)	-0.8<br>(-2.0 to 0.4)	5.0<br>(3.8 to 6.2)
Viral skin diseases	21,509.3<br>(18,492.7 to 25,194.3)	31,877.8<br>(27,561.0 to 36,439.5)	663.0<br>(408.8 to 977.5)	-5.5<br>(-6.7 to -4.4)	-0.0<br>(-1.3 to 1.2)
Acne vulgaris	209,587.1<br>(175,308.5 to 246,418.1)	141,922.0<br>(110,224.5 to 184,521.2)	5,414.6<br>(3,581.7 to 7,931.2)	-3.1<br>(-4.1 to -2.1)	2.6<br>(1.5 to 3.6)
Alopecia areata	876.9<br>(779.2 to 981.7)	1,690.5<br>(1,492.2 to 1,893.7)	29.1<br>(18.1 to 44.9)	-5.7<br>(-9.6 to -1.4)	-0.2<br>(-4.3 to 4.4)
Pruritus	3,181.5<br>(2,143.0 to 4,529.0)	2,475.5<br>(1,670.5 to 3,374.1)	34.5<br>(14.8 to 65.3)	-5.0<br>(-6.9 to -3.2)	0.5<br>(-1.4 to 2.4)
Urticaria	4,488.8<br>(3,094.1 to 6,269.2)	7,779.0<br>(5,327.8 to 10,755.3)	271.8<br>(151.0 to 443.3)	-6.6<br>(-8.9 to -4.4)	-1.1<br>(-3.6 to 1.2)
Decubitus ulcer	30.2<br>(20.7 to 41.8)	106.1<br>(73.6 to 146.9)	5.3<br>(3.1 to 8.3)	-3.5<br>(-4.4 to -2.6)	2.2<br>(1.2 to 3.1)
Other skin and subcutaneous diseases	22,446.0<br>(20,099.0 to 25,074.5)	23,858.9<br>(21,531.5 to 26,586.2)	125.9<br>(59.8 to 228.6)	-0.8<br>(-1.9 to 0.3)	5.0<br>(3.9 to 6.1)
Sense organ diseases	51,850.3<br>(50,049.5 to 53,638.6)	39,986.3†<br>(35,587.5 to 44,735.2)	1,746.7<br>(1,178.0 to 2,473.9)	-6.4<br>(-8.4 to -4.6)	-0.9<br>(-3.0 to 0.9)
Glaucoma	--	--	--	--	--
Cataract	--	--	--	--	--
Macular degeneration	--	--	--	--	--
Refraction and accommodation disorders	27,886.2<br>(24,961.7 to 30,919.3)	--	595.7<br>(369.7 to 930.6)	-6.9<br>(-8.2 to -5.7)	-1.5<br>(-2.8 to -0.1)
Age-related and other hearing loss	19,607.8<br>(15,285.1 to 24,465.1)	--	939.0<br>(621.8 to 1,342.5)	-6.2<br>(-9.9 to -3.1)	-0.8<br>(-4.7 to 2.6)
Other vision loss	513.5<br>(360.7 to 719.1)	--	32.1<br>(18.4 to 49.9)	-8.0<br>(-14.6 to -1.2)	-2.6<br>(-9.6 to 4.6)
Other sense organ diseases	5,932.6<br>(5,237.6 to 6,668.3)	39,986.3<br>(35,587.5 to 44,735.2)	180.0<br>(110.2 to 277.4)	-4.9<br>(-7.2 to -2.6)	0.7<br>(-1.7 to 3.1)
Oral disorders	264,786.4<br>(236,765.2 to 297,720.2)	680,633.8†<br>(526,602.8 to 872,978.9)	471.4<br>(262.1 to 772.8)	-5.7<br>(-6.7 to -4.7)	-0.2<br>(-1.2 to 0.9)
Caries of deciduous teeth	--	--	--	--	--
Caries of permanent teeth	248,543.6<br>(198,444.3 to 296,907.5)	679,138.1<br>(524,976.2 to 871,518.7)	168.3<br>(71.0 to 324.8)	-7.4<br>(-8.9 to -6.1)	-2.0<br>(-3.6 to -0.6)
Periodontal disease	4,706.4<br>(2,564.3 to 7,529.1)	1,495.7<br>(841.1 to 2,359.6)	31.6<br>(10.5 to 73.9)	4.0<br>(1.6 to 6.4)	10.1<br>(7.6 to 12.6)
Edentulism and severe tooth loss	--	--	--	--	--
Other oral disorders	9,110.9<br>(8,002.8 to 10,381.9)	--	271.5<br>(163.1 to 416.7)	-5.6<br>(-6.9 to -4.3)	-0.1<br>(-1.5 to 1.3)
**Injuries**	**56,862.8<br>(51,712.1 to 62,660.9)**	**40,831.5†<br>(35,080.5 to 47,427.7)**	**2,259.1<br>(1,476.5 to 3,288.6)**	**-3.4<br>(-4.6 to -2.3)**	**2.2<br>(1.0 to 3.4)**
**Transport injuries**	**6,376.8<br>(5,448.0 to 7,247.4)**	**6,587.3<br>(5,031.8 to 8,171.9)**	**370.0<br>(237.6 to 550.4)**	**-5.5<br>(-7.3 to -3.8)**	**0.1<br>(-1.9 to 1.8)**
Road injuries	4,226.8<br>(3,643.8 to 4,805.9)	5,924.8<br>(4,403.7 to 7,488.4)	248.8<br>(158.7 to 367.9)	-4.5<br>(-6.7 to -2.4)	1.1<br>(-1.2 to 3.3)
Pedestrian road injuries	995.6<br>(757.2 to 1,278.3)	973.9<br>(620.8 to 1,433.6)	59.1<br>(37.2 to 89.3)	-8.4<br>(-10.6 to -5.9)	-3.0<br>(-5.4 to -0.4)
Cyclist road injuries	1,079.5<br>(804.8 to 1,420.9)	1,553.1<br>(961.7 to 2,296.0)	57.5<br>(35.6 to 86.5)	-2.0<br>(-4.5 to 1.0)	3.7<br>(1.1 to 6.9)
Motorcyclist road injuries	822.3<br>(655.0 to 1,021.0)	1,074.8<br>(690.6 to 1,565.0)	48.9<br>(30.8 to 72.3)	-1.1<br>(-3.6 to 1.6)	4.7<br>(2.0 to 7.6)
Motor vehicle road injuries	929.0<br>(735.1 to 1,191.7)	1,708.3<br>(1,171.4 to 2,378.1)	62.2<br>(39.5 to 90.3)	-8.5<br>(-11.6 to -5.5)	-3.1<br>(-6.5 to -0.0)
Other road injuries	400.3<br>(306.2 to 530.8)	614.7<br>(335.2 to 975.7)	21.0<br>(12.7 to 31.3)	5.5<br>(2.9 to 8.6)	11.6<br>(8.9 to 15.0)
Other transport injuries	2,149.9<br>(1,667.3 to 2,741.7)	662.5<br>(421.2 to 942.7)	121.2<br>(78.5 to 180.1)	-7.3<br>(-9.2 to -5.5)	-1.9<br>(-3.9 to 0.1)
**Unintentional injuries**	**46,199.0<br>(41,119.1 to 51,685.3)**	**30,980.2<br>(25,768.0 to 37,158.1)**	**1,566.1<br>(1,019.5 to 2,276.4)**	**-1.2<br>(-2.4 to -0.2)**	**4.6<br>(3.3 to 5.7)**
Falls	16,841.5<br>(13,547.5 to 20,469.1)	11,793.7<br>(8,014.2 to 16,975.7)	624.3<br>(417.5 to 900.6)	0.0<br>(-1.3 to 1.2)	5.9<br>(4.5 to 7.1)
Drowning	191.3<br>(151.5 to 235.9)	55.6<br>(42.6 to 71.9)	10.7<br>(6.8 to 16.0)	-20.8<br>(-22.4 to -19.2)	-16.1<br>(-17.8 to -14.5)
Fire, heat, and hot substances	4,636.3<br>(3,566.7 to 5,919.9)	917.8<br>(608.6 to 1,338.3)	133.7<br>(87.9 to 194.6)	-0.7<br>(-3.0 to 1.3)	5.1<br>(2.6 to 7.2)
Poisonings	193.2<br>(150.0 to 244.6)	350.4<br>(213.9 to 556.1)	21.2<br>(13.1 to 32.1)	7.4<br>(4.2 to 10.4)	13.6<br>(10.3 to 16.9)
Exposure to mechanical forces	6,754.5<br>(5,176.2 to 9,749.6)	5,259.5<br>(3,573.1 to 7,417.2)	160.8<br>(98.8 to 256.0)	-2.6<br>(-4.1 to -1.2)	3.1<br>(1.6 to 4.6)
Unintentional firearm injuries	213.6<br>(160.4 to 293.7)	147.8<br>(75.4 to 275.2)	8.8<br>(5.4 to 13.5)	-1.1<br>(-3.2 to 1.2)	4.7<br>(2.5 to 7.1)
Unintentional suffocation	567.8<br>(398.0 to 770.6)	50.4<br>(23.0 to 96.2)	17.2<br>(9.7 to 28.1)	-15.4<br>(-16.8 to -13.9)	-10.4<br>(-11.9 to -8.8)
Other exposure to mechanical forces	5,973.0<br>(4,463.4 to 8,972.8)	5,061.4<br>(3,418.7 to 7,104.3)	134.9<br>(83.3 to 214.1)	-0.8<br>(-2.4 to 0.8)	5.0<br>(3.3 to 6.7)

Cause	Prevalence in thousands (95% UI)	Incidence in thousands (95% UI)	YLDs in thousands (95% UI)		
	2016 counts	2016 counts	2016 counts	Percentage change in counts between 2006 and 2016	Percentage change in rates between 2006 and 2016
Adverse effects of medical treatment	67.3<br>(40.2 to 99.8)	880.2<br>(565.3 to 1,262.7)	9.0<br>(4.6 to 15.2)	-3.1<br>(-9.0 to 1.1)	2.6<br>(-3.7 to 7.0)
Animal contact	2,706.7<br>(2,238.8 to 3,380.9)	5,606.5<br>(3,763.1 to 8,009.7)	120.9<br>(79.8 to 171.3)	-1.5<br>(-4.4 to 1.4)	4.2<br>(1.2 to 7.4)
Venomous animal contact	1,381.4<br>(1,058.4 to 1,755.3)	2,845.4<br>(1,821.8 to 4,216.4)	98.4<br>(64.4 to 139.9)	-0.4<br>(-4.0 to 3.0)	5.4<br>(1.7 to 9.0)
Non-venomous animal contact	1,325.3<br>(961.3 to 1,839.4)	2,761.1<br>(1,763.5 to 4,070.7)	22.5<br>(13.3 to 35.9)	-6.0<br>(-8.6 to -3.7)	-0.6<br>(-3.3 to 1.9)
Foreign body	3,388.7<br>(2,635.2 to 4,154.2)	1,577.9<br>(1,030.3 to 2,392.2)	133.1<br>(81.9 to 206.1)	-1.2<br>(-3.0 to 0.6)	4.6<br>(2.6 to 6.5)
Pulmonary aspiration and foreign body in airway	1,751.9<br>(1,222.8 to 2,497.6)	195.7<br>(111.5 to 328.8)	78.6<br>(46.7 to 124.8)	-5.3<br>(-6.9 to -3.9)	0.3<br>(-1.4 to 1.7)
Foreign body in eyes	154.8<br>(65.8 to 296.0)	958.3<br>(469.3 to 1,764.3)	8.8<br>(3.4 to 18.5)	8.6<br>(4.4 to 13.2)	14.9<br>(10.5 to 19.8)
Foreign body in other body part	1,482.0<br>(1,114.2 to 2,026.9)	423.9<br>(255.1 to 662.7)	45.7<br>(28.2 to 71.0)	4.8<br>(2.6 to 6.9)	10.9<br>(8.6 to 13.2)
Environmental heat and cold exposure	3,754.8<br>(2,920.5 to 4,795.2)	1,214.4<br>(704.6 to 1,832.5)	143.8<br>(90.1 to 216.4)	-4.5<br>(-6.0 to -3.0)	1.1<br>(-0.5 to 2.7)
Other unintentional injuries	7,664.7<br>(5,580.7 to 10,475.0)	3,324.4<br>(2,174.5 to 4,921.6)	208.5<br>(132.0 to 315.3)	-1.1<br>(-2.5 to 0.1)	4.7<br>(3.2 to 6.0)
**Self-harm and interpersonal violence**	**20,799.7**<br>**(15,947.6 to 26,779.4)**	**3,182.3†**<br>**(2,413.7 to 4,170.3)**	**260.2**<br>**(169.6 to 382.6)**	**-8.8**<br>**(-9.9 to -7.8)**	**-3.5**<br>**(-4.6 to -2.4)**
Self-harm	265.4<br>(203.7 to 344.9)	615.8<br>(455.0 to 807.2)	17.9<br>(11.0 to 27.2)	-5.9<br>(-7.8 to -4.1)	-0.4<br>(-2.4 to 1.5)
Self-harm by firearm	20.7<br>(16.0 to 26.2)	14.0<br>(4.6 to 29.4)	0.8<br>(0.5 to 1.3)	-8.1<br>(-10.8 to -5.3)	-2.7<br>(-5.6 to 0.3)
Self-harm by other specified means	244.7<br>(184.9 to 322.7)	601.8<br>(444.7 to 791.0)	17.1<br>(10.5 to 25.9)	-5.8<br>(-7.7 to -4.0)	-0.3<br>(-2.3 to 1.7)
Interpersonal violence	20,534.2<br>(15,680.2 to 26,519.6)	2,566.5†<br>(1,796.8 to 3,538.8)	242.3<br>(157.7 to 356.7)	-9.0<br>(-10.1 to -8.0)	-3.7<br>(-4.9 to -2.6)
Assault by firearm	69.4<br>(55.2 to 91.3)	90.8<br>(54.5 to 143.5)	3.5<br>(2.2 to 5.4)	-3.1<br>(-6.7 to 0.8)	2.6<br>(-1.2 to 6.7)
Assault by sharp object	466.3<br>(358.8 to 628.5)	625.1<br>(376.8 to 998.5)	17.0<br>(10.7 to 25.2)	-6.7<br>(-10.0 to -3.4)	-1.2<br>(-4.7 to 2.3)
Sexual violence	19,005.9<br>(14,200.2 to 24,991.7)	--	177.3<br>(109.7 to 275.4)	-9.8<br>(-10.9 to -8.8)	-4.5<br>(-5.7 to -3.4)
Assault by other means	992.6<br>(789.1 to 1,259.4)	1,850.6<br>(1,272.6 to 2,571.4)	44.4<br>(28.2 to 65.6)	-7.3<br>(-10.0 to -4.9)	-1.9<br>(-4.7 to 0.7)
**Forces of nature, conflict and terrorism, and executions and police conflict**	**2,493.2**<br>**(1,542.2 to 3,744.2)**	**81.6†**<br>**(57.2 to 111.8)**	**62.8**<br>**(21.9 to 126.5)**	**-18.4**<br>**(-27.5 to -7.2)**	**-13.7**<br>**(-23.3 to -1.8)**
Forces of nature, conflict and terrorism, and state actor violence	475.6<br>(254.2 to 768.2)	37.8<br>(21.8 to 56.7)	17.6<br>(5.4 to 35.2)	-2.7<br>(-22.5 to 24.9)	3.0<br>(-17.9 to 32.1)
Conflict and terrorism	1,967.8<br>(1,163.6 to 3,134.1)	--	44.9<br>(16.4 to 89.9)	-24.6<br>(-35.1 to -11.1)	-20.2<br>(-31.3 to -5.9)
Executions and police conflict	49.8<br>(29.2 to 82.4)	43.8<br>(25.1 to 67.8)	1.5<br>(0.5 to 4.1)	-37.0<br>(-41.2 to -33.2)	-33.3<br>(-37.8 to -29.3)

We did not calculate incidence for certain NTDs and causes with only birth prevalence. Blank cells mean that no estimate is available.
† Incidence for aggregate causes represents the sum of the incidence of the causes below the aggregate.
‡ Incidence estimates for stroke represent first-ever stroke only.
^ Incidence of HIV/AIDS represents new infections of HIV only and does not include new infections of Tuberculosis in HIV positive cases.

SR Table 12: Global prevalence, incidence, and YLDs for 2016, percentage change of YLD counts and percentage change of YLD rates between 2006 and 2016 by cause, age 20-24 years.					
Cause	Prevalence in thousands (95% UI)	Incidence in thousands (95% UI)	YLDs in thousands (95% UI)		
	2016 counts	2016 counts	2016 counts	Percentage change in counts between 2006 and 2016	Percentage change in rates between 2006 and 2016
**All causes**	**581,199.6<br>(580,166.2 to 582,361.1)**	**3,436,189.4†<br>(2,930,909.1 to 4,064,037.9)**	**50,427.3<br>(37,154.2 to 65,877.4)**	**3.2<br>(2.6 to 3.9)**	**-1.5<br>(-2.1 to -0.9)**
**Communicable, maternal, neonatal, and nutritional disorders**	**417,286.9<br>(412,633.4 to 422,033.3)**	**2,025,069.7†<br>(1,555,485.0 to 2,652,644.2)**	**7,863.1<br>(5,622.6 to 10,743.9)**	**0.6<br>(-2.1 to 3.8)**	**-4.1<br>(-6.6 to -1.0)**
**HIV/AIDS and tuberculosis**	**176,233.7<br>(155,260.8 to 200,050.5)**	**1,547.4†<br>(1,206.8 to 1,937.4)**	**521.3<br>(351.5 to 725.6)**	**-6.1<br>(-11.1 to 0.8)**	**-10.4<br>(-15.2 to -3.9)**
Tuberculosis	165,397.0<br>(133,409.2 to 198,938.9)	1,158.5†<br>(819.5 to 1,547.1)	262.1<br>(151.7 to 402.6)	4.4<br>(-1.3 to 12.0)	-0.4<br>(-5.9 to 6.8)
Drug-susceptible tuberculosis	798.3<br>(550.2 to 1,103.6)	1,121.0<br>(791.5 to 1,497.2)	252.8<br>(146.2 to 388.1)	4.6<br>(-1.2 to 12.3)	-0.2<br>(-5.8 to 7.1)
Multidrug-resistant tuberculosis without extensive drug resistance	27.1<br>(19.6 to 36.8)	35.8<br>(26.1 to 47.3)	8.8<br>(5.2 to 13.7)	-4.6<br>(-17.2 to 12.9)	-9.0<br>(-21.0 to 7.7)
Extensively drug-resistant tuberculosis	1.6<br>(1.2 to 2.1)	1.8<br>(1.3 to 2.4)	0.5<br>(0.3 to 0.8)	154.5<br>(119.9 to 190.8)	142.8<br>(109.7 to 177.4)
Latent tuberculosis infection	164,570.0<br>(132,586.3 to 197,749.6)	--	--	--	--
HIV/AIDS	2,567.9<br>(2,366.8 to 2,802.6)	388.8^<br>(350.0 to 441.7)	259.1<br>(186.2 to 345.6)	-14.7<br>(-22.2 to -3.2)	-18.6<br>(-25.7 to -7.7)
Drug-susceptible HIV/AIDS - Tuberculosis	101.8<br>(72.1 to 139.2)	128.5<br>(90.9 to 177.0)	40.1<br>(23.7 to 62.4)	-37.1<br>(-41.4 to -32.5)	-40.0<br>(-44.1 to -35.6)
Multidrug-resistant HIV/AIDS - Tuberculosis without extensive drug resistance	2.2<br>(1.5 to 3.4)	3.1<br>(2.0 to 4.6)	0.9<br>(0.5 to 1.6)	-51.1<br>(-60.1 to -40.8)	-53.4<br>(-61.9 to -43.5)
Extensively drug-resistant HIV/AIDS - Tuberculosis	0.1<br>(0.1 to 0.1)	0.1<br>(0.1 to 0.1)	< 0.05<br>(< 0.05 to 0.1)	44.6<br>(15.9 to 83.1)	38.0<br>(10.6 to 74.6)
HIV/AIDS resulting in other diseases	2,463.8<br>(2,264.5 to 2,703.3)	388.8<br>(350.0 to 441.7)	218.1<br>(154.4 to 291.0)	-8.4<br>(-18.2 to 7.1)	-12.6<br>(-22.0 to 2.1)
**Diarrhoea, lower respiratory infections, and other common infectious diseases**	**33,613.7<br>(30,929.8 to 36,089.8)**	**1,793,505.8†<br>(1,325,484.4 to 2,414,177.7)**	**1,532.3<br>(1,032.7 to 2,156.9)**	**1.5<br>(0.1 to 2.8)**	**-3.2<br>(-4.5 to -2.0)**
Diarrhoeal diseases	4,168.4<br>(3,633.1 to 4,760.2)	281,438.0<br>(244,472.6 to 321,376.9)	474.6<br>(319.9 to 670.6)	5.0<br>(2.5 to 7.3)	0.1<br>(-2.2 to 2.4)
Intestinal infectious diseases	54.1<br>(39.2 to 72.2)	1,109.0†<br>(809.4 to 1,449.4)	8.8<br>(5.2 to 13.8)	-8.3<br>(-28.1 to 14.2)	-12.5<br>(-31.4 to 8.9)
Typhoid fever	52.3<br>(36.0 to 71.1)	824.5<br>(594.5 to 1,076.3)	8.0<br>(4.7 to 12.7)	-8.3<br>(-29.6 to 16.9)	-12.5<br>(-32.9 to 11.5)
Paratyphoid fever	12.5<br>(8.7 to 17.4)	284.5<br>(204.4 to 379.3)	0.8<br>(0.4 to 1.3)	-7.2<br>(-25.6 to 18.2)	-11.5<br>(-29.1 to 12.8)
Other intestinal infectious diseases	--	--	< 0.05<br>(< 0.05 to < 0.05)	-36.2<br>(-50.0 to -20.6)	-39.2<br>(-52.3 to -24.3)
Lower respiratory infections	257.9<br>(217.6 to 305.8)	10,991.0<br>(8,872.6 to 13,326.4)	16.0<br>(10.0 to 23.5)	19.0<br>(11.6 to 26.1)	13.5<br>(6.4 to 20.3)
Upper respiratory infections	19,587.5<br>(13,293.9 to 27,449.8)	1,477,047.4<br>(1,002,667.1 to 2,090,335.7)	491.9<br>(252.6 to 855.5)	2.8<br>(1.4 to 4.2)	-2.0<br>(-3.3 to -0.6)
Otitis media	8,362.1<br>(6,141.6 to 11,216.3)	17,026.6<br>(13,335.4 to 21,668.1)	233.8<br>(131.9 to 379.4)	-5.5<br>(-9.5 to -1.3)	-9.9<br>(-13.7 to -5.9)
Meningitis	1,363.7<br>(1,177.9 to 1,580.7)	107.3<br>(76.8 to 148.4)	147.2<br>(102.6 to 195.0)	3.9<br>(0.3 to 7.5)	-0.9<br>(-4.4 to 2.6)
Pneumococcal meningitis	594.2<br>(490.7 to 714.4)	31.2<br>(19.3 to 48.0)	64.7<br>(44.9 to 86.4)	0.5<br>(-6.4 to 7.8)	-4.1<br>(-10.7 to 2.8)
H influenzae type B meningitis	200.4<br>(156.5 to 255.8)	8.6<br>(4.5 to 15.0)	25.5<br>(17.0 to 34.5)	-18.4<br>(-23.6 to -13.4)	-22.1<br>(-27.1 to -17.4)
Meningococcal infection	154.3<br>(110.3 to 206.1)	19.6<br>(11.6 to 30.5)	15.9<br>(10.7 to 21.5)	8.7<br>(5.7 to 11.7)	3.7<br>(0.8 to 6.5)
Other meningitis	414.8<br>(313.8 to 536.1)	47.9<br>(31.4 to 69.2)	41.1<br>(27.9 to 56.9)	31.0<br>(24.6 to 37.5)	24.9<br>(18.8 to 31.2)
Encephalitis	1,159.1<br>(802.5 to 1,671.4)	308.3<br>(224.2 to 409.9)	145.5<br>(102.8 to 191.4)	-5.1<br>(-10.1 to 0.1)	-9.4<br>(-14.2 to -4.5)
Diphtheria	< 0.05<br>(< 0.05 to < 0.05)	0.1<br>(0.1 to 0.2)	< 0.05<br>(< 0.05 to < 0.05)	-52.6<br>(-65.2 to -35.6)	-54.8<br>(-66.8 to -38.6)
Whooping cough	4.1<br>(3.2 to 5.2)	30.0<br>(23.4 to 37.7)	0.2<br>(0.1 to 0.3)	-21.0<br>(-22.8 to -19.3)	-24.7<br>(-26.3 to -23.0)
Tetanus	13.8<br>(13.5 to 14.0)	3.5<br>(1.6 to 6.0)	0.3<br>(0.2 to 0.5)	-0.1<br>(-23.9 to 32.1)	-4.7<br>(-27.4 to 26.0)
Measles	1.2<br>(0.4 to 2.6)	43.3<br>(15.6 to 93.2)	0.1<br>(< 0.05 to 0.2)	-59.6<br>(-64.0 to -54.5)	-61.4<br>(-65.7 to -56.6)
Varicella and herpes zoster	315.4<br>(243.6 to 394.8)	5,401.3<br>(3,511.8 to 7,686.4)	13.8<br>(7.7 to 22.5)	4.6<br>(-1.9 to 11.8)	-0.2<br>(-6.4 to 6.7)
**Neglected tropical diseases and malaria**	**167,566.7<br>(162,192.8 to 172,097.3)**	**31,712.7†<br>(26,475.6 to 37,887.1)**	**1,144.7<br>(779.7 to 1,626.2)**	**-17.1<br>(-21.4 to -12.4)**	**-20.9<br>(-25.1 to -16.5)**
Malaria	10,423.1<br>(8,753.0 to 12,479.4)	12,029.8<br>(9,003.4 to 15,393.4)	143.2<br>(109.5 to 181.1)	-6.6<br>(-13.1 to 0.6)	-10.9<br>(-17.1 to -4.1)
Chagas disease	469.2<br>(368.9 to 583.4)	16.9<br>(11.5 to 23.2)	2.4<br>(1.4 to 3.6)	-15.7<br>(-24.7 to -5.6)	-19.6<br>(-28.2 to -9.9)
Leishmaniasis	435.4<br>(393.9 to 479.7)	58.9<br>(42.4 to 80.7)	26.4<br>(17.5 to 37.8)	25.5<br>(7.8 to 45.9)	19.7<br>(2.8 to 39.1)
Visceral leishmaniasis	1.6<br>(1.1 to 2.3)	6.5<br>(4.5 to 9.1)	0.1<br>(0.1 to 0.2)	-57.1<br>(-71.4 to -34.6)	-59.1<br>(-72.7 to -37.7)
Cutaneous and mucocutaneous leishmaniasis	406.9<br>(356.8 to 465.6)	52.4<br>(35.6 to 73.8)	26.3<br>(17.4 to 37.7)	26.6<br>(8.5 to 47.7)	20.8<br>(3.5 to 40.9)
African trypanosomiasis	0.8<br>(0.2 to 2.5)	0.5<br>(0.1 to 1.8)	0.2<br>(< 0.05 to 0.7)	-71.0<br>(-95.1 to 57.3)	-72.4<br>(-95.3 to 50.0)
Schistosomiasis	23,533.0<br>(21,221.9 to 25,844.9)	7,697.7<br>(6,365.9 to 9,609.5)	195.1<br>(95.9 to 364.3)	-25.9<br>(-27.9 to -23.4)	-29.4<br>(-31.2 to -27.0)
Cysticercosis	180.3<br>(117.7 to 253.5)	--	30.5<br>(16.9 to 47.9)	-14.8<br>(-26.2 to -5.0)	-18.8<br>(-29.6 to -9.4)
Cystic echinococcosis	83.7<br>(41.7 to 194.1)	18.1<br>(8.0 to 50.2)	7.9<br>(3.4 to 18.5)	-40.3<br>(-77.5 to 73.1)	-43.1<br>(-78.5 to 65.1)
Lymphatic filariasis	2,573.4<br>(2,190.6 to 3,120.7)	792.1<br>(437.8 to 1,253.9)	89.5<br>(43.9 to 160.8)	-41.0<br>(-54.6 to -29.9)	-43.8<br>(-56.7 to -33.1)
Onchocerciasis	1,817.4<br>(1,090.6 to 3,192.4)	447.8<br>(186.1 to 893.8)	101.7<br>(43.8 to 184.9)	-28.1<br>(-46.2 to -11.5)	-31.4<br>(-48.7 to -15.5)
Trachoma	10.4<br>(3.6 to 24.3)	--	0.9<br>(0.4 to 1.6)	-36.7<br>(-54.7 to -15.4)	-39.6<br>(-56.8 to -19.3)
Dengue	563.8<br>(306.9 to 955.5)	9,424.3<br>(5,729.2 to 14,301.3)	93.0<br>(40.7 to 173.6)	66.4<br>(34.2 to 277.9)	58.7<br>(28.0 to 260.5)
Yellow fever	0.3<br>(0.1 to 0.8)	11.0<br>(3.1 to 30.8)	< 0.05<br>(< 0.05 to < 0.05)	-18.4<br>(-48.1 to 30.4)	-22.2<br>(-50.5 to 24.4)
Rabies	< 0.05<br>(< 0.05 to < 0.05)	0.9<br>(0.5 to 1.3)	< 0.05<br>(< 0.05 to < 0.05)	-46.3<br>(-60.9 to -26.3)	-48.8<br>(-62.7 to -29.7)
Intestinal nematode infections	140,013.2<br>(133,974.3 to 145,129.0)	--	284.1<br>(164.7 to 462.1)	-18.5<br>(-24.4 to -12.3)	-22.2<br>(-27.9 to -16.3)
Ascariasis	74,070.5<br>(67,120.9 to 82,418.4)	--	81.9<br>(44.2 to 140.1)	-31.7<br>(-40.3 to -21.4)	-34.8<br>(-43.0 to -25.0)

Cause	Prevalence in thousands (95% UI)	Incidence in thousands (95% UI)	YLDs in thousands (95% UI)		
	2016 counts	2016 counts	2016 counts	Percentage change in counts between 2006 and 2016	Percentage change in rates between 2006 and 2016
Trichuriasis	40,041.9 (37,161.7 to 43,455.8)	--	32.4 (17.7 to 56.0)	-23.0 (-33.8 to -11.6)	-26.6 (-36.8 to -15.7)
Hookworm disease	44,314.3 (41,088.6 to 47,836.2)	--	169.8 (101.1 to 267.3)	-9.0 (-17.9 to 0.7)	-13.2 (-21.7 to -3.9)
Food-borne trematodiases	5,465.5 (5,068.2 to 5,928.2)	542.7 (461.9 to 620.8)	133.7 (64.6 to 248.7)	-16.6 (-21.3 to -9.3)	-20.4 (-24.9 to -13.5)
Leprosy	12.4 (11.5 to 13.4)	4.8 (4.4 to 5.3)	0.7 (0.5 to 1.1)	-27.6 (-33.9 to -19.9)	-30.9 (-37.0 to -23.6)
Ebola virus disease	< 0.05 (< 0.05 to 0.3)	< 0.05 (< 0.05 to < 0.05)	< 0.05 (< 0.05 to 0.1)	--	--
Zika virus disease	11.1 (6.5 to 19.9)	667.1 (391.5 to 1,205.2)	0.2 (0.1 to 0.4)	--	--
Guinea worm disease	< 0.05 (< 0.05 to < 0.05)	< 0.05 (< 0.05 to < 0.05)	< 0.05 (< 0.05 to < 0.05)	-99.5 (-99.7 to -99.3)	-99.5 (-99.7 to -99.3)
Other neglected tropical diseases	2,058.0 (1,775.9 to 2,358.3)	--	35.2 (22.2 to 53.7)	17.6 (11.0 to 25.2)	12.2 (5.9 to 19.5)
**Maternal disorders**	**2,163.6 (1,972.2 to 2,349.8)**	**33,126.7† (26,638.3 to 40,106.1)**	**199.3 (132.7 to 285.1)**	**-12.5 (-27.1 to 6.8)**	**-16.5 (-30.4 to 1.9)**
Maternal haemorrhage	334.4 (243.9 to 458.2)	2,500.4 (1,788.2 to 3,493.2)	16.2 (10.2 to 24.0)	-8.0 (-28.7 to 17.9)	-12.3 (-32.0 to 12.4)
Maternal sepsis and other pregnancy related infections	317.9 (184.3 to 499.0)	3,206.1 (2,226.8 to 4,627.2)	13.2 (6.1 to 24.6)	-6.0 (-52.6 to 90.2)	-10.4 (-54.8 to 81.5)
Maternal hypertensive disorders	1,240.3 (764.2 to 1,818.2)	5,855.1 (4,588.7 to 7,246.3)	60.4 (30.9 to 100.2)	-6.1 (-40.8 to 51.0)	-10.5 (-43.6 to 44.0)
Maternal obstructed labour and uterine rupture	182.6 (136.7 to 242.1)	2,072.5 (1,486.6 to 2,805.7)	59.5 (36.6 to 90.7)	-18.2 (-28.0 to -7.8)	-22.0 (-31.3 to -12.1)
Maternal abortion, miscarriage, and ectopic pregnancy	159.8 (97.0 to 243.9)	19,492.7 (13,417.5 to 26,282.3)	17.5 (9.4 to 30.2)	-13.8 (-46.6 to 36.8)	-17.8 (-49.0 to 30.4)
Other maternal disorders	--	--	32.5 (21.6 to 46.4)	-16.0 (-30.0 to 2.6)	-19.9 (-33.2 to -2.2)
**Neonatal disorders**	**6,267.6 (5,723.4 to 6,853.7)**	**--**	**1,212.9 (852.1 to 1,722.0)**	**17.9 (5.3 to 33.2)**	**12.5 (0.4 to 27.0)**
Neonatal preterm birth complications	5,168.3 (4,328.0 to 6,195.9)	--	793.9 (544.0 to 1,134.6)	15.6 (-2.9 to 38.7)	10.2 (-7.3 to 32.3)
Neonatal encephalopathy due to birth asphyxia and trauma	980.0 (593.2 to 1,644.0)	--	138.0 (46.7 to 337.8)	23.0 (14.2 to 36.8)	17.3 (8.9 to 30.4)
Neonatal sepsis and other neonatal infections	555.9 (195.8 to 1,322.9)	--	219.8 (65.1 to 574.6)	25.4 (15.8 to 39.3)	19.6 (10.5 to 32.9)
Hemolytic disease and other neonatal jaundice	187.7 (166.4 to 210.1)	--	61.2 (47.0 to 76.8)	13.5 (7.9 to 19.4)	8.2 (2.9 to 13.9)
Other neonatal disorders	--	--	--	--	--
**Nutritional deficiencies**	**138,225.2 (132,544.9 to 143,645.4)**	**44,306.9† (36,308.6 to 55,760.2)**	**2,852.9 (1,879.5 to 4,166.1)**	**5.0 (1.1 to 9.1)**	**0.2 (-3.6 to 4.1)**
Protein-energy malnutrition	--	--	--	--	--
Iodine deficiency	11,817.9 (10,392.6 to 13,428.2)	54.2 (44.2 to 65.6)	322.4 (215.0 to 455.1)	-15.5 (-18.3 to -12.9)	-19.4 (-22.1 to -16.9)
Vitamin A deficiency	39,536.0 (33,544.3 to 47,544.4)	44,252.7 (36,262.4 to 55,708.0)	15.6 (9.3 to 25.0)	8.5 (1.5 to 16.1)	3.5 (-3.1 to 10.8)
Iron-deficiency anaemia	103,050.1 (90,946.1 to 115,759.2)	--	2,515.0 (1,636.2 to 3,738.0)	8.4 (3.9 to 13.2)	3.4 (-0.8 to 7.9)
Other nutritional deficiencies	--	--	--	--	--
**Other communicable, maternal, neonatal, and nutritional diseases**	**149,861.4 (145,377.7 to 154,678.6)**	**120,870.3 (101,903.2 to 143,989.8)**	**389.4 (241.5 to 622.0)**	**2.2 (-5.2 to 10.4)**	**-2.5 (-9.6 to 5.3)**
Sexually transmitted diseases excluding HIV	108,456.6 (103,593.2 to 112,369.8)	95,824.8 (80,636.9 to 113,647.8)	299.5 (181.0 to 488.0)	5.2 (1.8 to 8.8)	0.4 (-2.9 to 3.8)
Syphilis	8,243.6 (5,415.2 to 12,991.3)	5,569.1 (3,411.1 to 8,684.0)	4.0 (2.3 to 7.2)	7.9 (2.4 to 12.4)	2.9 (-2.3 to 7.2)
Chlamydial infection	20,318.3 (14,881.1 to 27,136.2)	18,509.9 (13,202.3 to 25,258.6)	92.5 (54.3 to 150.6)	3.6 (0.0 to 7.2)	-1.2 (-4.6 to 2.3)
Gonococcal infection	8,396.7 (5,691.3 to 12,246.4)	29,097.2 (18,484.4 to 44,307.5)	66.2 (35.7 to 117.1)	7.8 (-2.5 to 15.8)	2.8 (-7.0 to 10.4)
Trichomoniasis	25,520.1 (18,860.4 to 33,541.1)	24,139.2 (17,282.7 to 31,733.5)	31.4 (11.9 to 66.9)	8.0 (6.0 to 10.2)	3.1 (1.1 to 5.1)
Genital herpes	56,684.3 (44,153.1 to 70,660.1)	15,181.8 (12,469.0 to 18,376.0)	17.7 (6.3 to 38.5)	5.4 (2.1 to 8.9)	0.5 (-2.6 to 3.9)
Other sexually transmitted diseases	2,034.0 (1,840.6 to 2,225.5)	3,327.7 (2,665.9 to 4,088.0)	87.8 (56.9 to 133.5)	3.9 (0.0 to 8.0)	-0.9 (-4.6 to 3.0)
Hepatitis	50,086.3 (41,998.9 to 60,041.5)	25,045.0 (15,492.9 to 38,432.2)	34.9 (16.4 to 61.0)	-22.9 (-59.2 to 46.4)	-26.4 (-61.0 to 39.6)
Acute hepatitis A	270.5 (30.9 to 673.3)	3,516.1 (401.9 to 8,752.7)	11.3 (1.1 to 29.0)	-25.4 (-93.3 to 816.6)	-28.9 (-93.6 to 774.3)
Hepatitis B	38,939.9 (30,921.9 to 48,784.2)	18,054.7 (10,025.4 to 31,116.2)	15.8 (7.2 to 30.7)	-18.2 (-52.7 to 39.6)	-22.0 (-54.9 to 33.2)
Hepatitis C	10,628.7 (9,284.9 to 11,959.2)	259.4 (185.7 to 345.3)	0.2 (0.1 to 0.5)	-16.8 (-17.4 to -16.2)	-20.7 (-21.2 to -20.1)
Acute hepatitis E	247.3 (162.4 to 329.8)	3,214.7 (2,111.2 to 4,287.8)	7.5 (4.1 to 12.5)	-28.1 (-44.0 to -9.5)	-31.4 (-46.5 to -13.7)
Other infectious diseases	2,378.4 (2,050.1 to 2,742.5)	0.5 (0.3 to 0.9)	55.1 (34.0 to 85.0)	7.9 (3.5 to 12.7)	2.9 (-1.3 to 7.5)
**Non-communicable diseases**	**543,552.2 (540,289.4 to 547,300.3)**	**1,367,273.4† (1,169,435.2 to 1,566,371.5)**	**39,698.7 (29,043.3 to 51,574.2)**	**3.8 (3.2 to 4.3)**	**-1.0 (-1.6 to -0.5)**
**Neoplasms**	**152.5 (144.8 to 159.3)**	**117.7 (109.9 to 128.2)**	**39.5 (28.5 to 52.4)**	**9.1 (5.7 to 13.6)**	**4.1 (0.8 to 8.3)**
Lip and oral cavity cancer	11.9 (11.1 to 12.7)	3.2 (3.0 to 3.4)	1.2 (0.9 to 1.7)	9.1 (0.5 to 18.1)	4.1 (-4.1 to 12.7)
Nasopharynx cancer	7.6 (7.0 to 8.3)	2.2 (2.0 to 2.4)	0.9 (0.6 to 1.2)	-5.9 (-15.4 to 5.3)	-10.2 (-19.3 to 0.5)
Other pharynx cancer	1.9 (1.7 to 2.1)	0.8 (0.7 to 0.8)	0.3 (0.2 to 0.4)	4.9 (-8.7 to 16.9)	0.1 (-12.9 to 11.5)
Oesophageal cancer	0.9 (0.8 to 1.0)	0.8 (0.7 to 0.8)	0.2 (0.1 to 0.3)	-12.1 (-17.4 to -6.1)	-16.1 (-21.3 to -10.4)
Stomach cancer	5.3 (5.0 to 5.7)	3.0 (2.9 to 3.2)	0.8 (0.6 to 1.1)	-11.7 (-16.6 to -6.0)	-15.7 (-20.4 to -10.4)
Colon and rectum cancer	14.5 (13.6 to 15.3)	4.2 (4.0 to 4.5)	1.6 (1.1 to 2.1)	6.6 (-1.8 to 14.2)	1.7 (-6.3 to 8.9)
Liver cancer	3.2 (2.7 to 3.8)	4.2 (3.7 to 4.7)	1.0 (0.7 to 1.3)	11.8 (-2.0 to 31.0)	6.7 (-6.5 to 24.9)
Liver cancer due to hepatitis B	2.9 (2.3 to 3.6)	2.4 (1.9 to 2.8)	0.5 (0.4 to 0.7)	10.9 (-3.8 to 30.8)	5.8 (-8.2 to 24.7)

Cause	Prevalence in thousands (95% UI)	Incidence in thousands (95% UI)	YLDs in thousands (95% UI)		
	2016 counts	2016 counts	2016 counts	Percentage change in counts between 2006 and 2016	Percentage change in rates between 2006 and 2016
Liver cancer due to hepatitis C	0.2 (0.2 to 0.3)	0.2 (0.1 to 0.2)	< 0.05 (< 0.05 to 0.1)	13.4 (-1.0 to 36.3)	8.1 (-5.6 to 30.0)
Liver cancer due to alcohol use	0.1 (0.1 to 0.1)	0.1 (< 0.05 to 0.1)	< 0.05 (< 0.05 to < 0.05)	15.5 (-1.4 to 34.9)	10.1 (-6.0 to 28.7)
Liver cancer due to other causes	1.9 (1.5 to 2.5)	1.6 (1.2 to 1.9)	0.4 (0.2 to 0.5)	12.9 (-0.4 to 32.4)	7.7 (-5.0 to 26.3)
Gallbladder and biliary tract cancer	0.2 (0.2 to 0.2)	0.2 (0.2 to 0.2)	0.1 (< 0.05 to 0.1)	-5.6 (-13.8 to 2.6)	-10.0 (-17.7 to -2.1)
Pancreatic cancer	0.4 (0.3 to 0.4)	0.5 (0.4 to 0.5)	0.1 (0.1 to 0.1)	2.0 (-3.0 to 7.1)	-2.7 (-7.5 to 2.2)
Larynx cancer	1.2 (1.2 to 1.3)	0.4 (0.3 to 0.4)	0.2 (0.1 to 0.2)	-3.1 (-7.6 to 2.1)	-7.5 (-11.8 to -2.6)
Tracheal, bronchus, and lung cancer	3.0 (2.8 to 3.2)	2.4 (2.2 to 2.5)	0.6 (0.4 to 0.8)	0.8 (-6.8 to 8.4)	-3.8 (-11.1 to 3.4)
Malignant skin melanoma	19.0 (16.8 to 23.7)	3.9 (3.5 to 4.8)	1.3 (0.9 to 1.8)	17.7 (6.4 to 30.7)	12.2 (1.5 to 24.6)
Non-melanoma skin cancer	0.9 (0.3 to 2.0)	7.4 (2.7 to 17.2)	< 0.05 (< 0.05 to 0.1)	39.1 (17.7 to 55.2)	32.6 (12.3 to 48.0)
Non-melanoma skin cancer (squamous-cell carcinoma)	0.6 (0.1 to 1.5)	1.1 (0.3 to 2.8)	< 0.05 (< 0.05 to 0.1)	48.1 (27.3 to 62.3)	41.3 (21.5 to 54.8)
Non-melanoma skin cancer (basal-cell carcinoma)	0.6 (0.2 to 1.7)	6.3 (1.8 to 15.9)	< 0.05 (< 0.05 to < 0.05)	9.2 (-3.7 to 17.4)	4.2 (-8.1 to 12.0)
Breast cancer	19.4 (16.4 to 22.3)	4.4 (3.8 to 5.0)	1.8 (1.2 to 2.5)	16.6 (-0.8 to 38.4)	11.2 (-5.4 to 32.0)
Cervical cancer	16.0 (11.1 to 18.7)	4.2 (3.0 to 4.8)	1.6 (1.0 to 2.2)	-13.0 (-21.7 to 2.3)	-17.0 (-25.3 to -2.4)
Uterine cancer	4.4 (4.0 to 5.0)	0.9 (0.8 to 1.1)	0.4 (0.2 to 0.5)	5.4 (-5.0 to 22.5)	0.6 (-9.4 to 16.9)
Ovarian cancer	15.2 (13.6 to 16.4)	4.9 (4.4 to 5.3)	2.4 (1.7 to 3.2)	14.5 (0.0 to 25.0)	9.2 (-4.6 to 19.3)
Prostate cancer	3.1 (2.5 to 3.4)	0.7 (0.5 to 0.7)	0.3 (0.2 to 0.4)	31.9 (20.7 to 39.3)	25.8 (15.2 to 32.9)
Testicular cancer	46.2 (43.5 to 49.3)	9.0 (8.5 to 9.6)	3.2 (2.2 to 4.4)	28.6 (17.2 to 41.2)	22.7 (11.8 to 34.7)
Kidney cancer	8.3 (7.6 to 8.6)	2.2 (2.0 to 2.3)	0.8 (0.5 to 1.0)	16.0 (10.6 to 22.0)	10.7 (5.5 to 16.3)
Bladder cancer	3.7 (3.4 to 4.0)	0.9 (0.8 to 1.0)	0.4 (0.2 to 0.5)	16.6 (7.4 to 25.3)	11.2 (2.4 to 19.5)
Brain and nervous system cancer	25.2 (21.6 to 27.8)	11.0 (9.6 to 12.2)	3.6 (2.5 to 4.7)	40.1 (29.6 to 51.4)	33.7 (23.6 to 44.5)
Thyroid cancer	21.1 (19.3 to 24.0)	4.0 (3.7 to 4.6)	1.3 (0.9 to 1.9)	33.2 (17.8 to 55.1)	27.1 (12.3 to 47.9)
Mesothelioma	0.3 (0.3 to 0.4)	0.2 (0.2 to 0.2)	0.1 (< 0.05 to 0.1)	-3.7 (-10.4 to 4.1)	-8.1 (-14.5 to -0.7)
Hodgkin lymphoma	30.3 (27.3 to 35.6)	7.2 (6.5 to 8.5)	2.8 (2.0 to 3.8)	0.7 (-7.1 to 8.7)	-4.0 (-11.4 to 3.7)
Non-Hodgkin's lymphoma	20.0 (17.8 to 21.3)	6.3 (5.6 to 6.7)	2.0 (1.4 to 2.7)	12.6 (5.9 to 18.3)	7.4 (1.0 to 12.8)
Multiple myeloma	1.3 (0.9 to 1.5)	0.6 (0.5 to 0.6)	0.3 (0.2 to 0.4)	33.3 (18.2 to 46.8)	27.1 (12.8 to 40.1)
Leukaemia	41.0 (35.5 to 44.0)	12.1 (10.5 to 12.9)	5.5 (3.8 to 7.4)	-12.8 (-18.2 to -6.8)	-16.8 (-22.0 to -11.1)
Acute lymphoid leukaemia	11.2 (9.8 to 12.2)	3.2 (2.8 to 3.5)	1.5 (1.0 to 2.0)	3.7 (-6.0 to 11.2)	-1.1 (-10.3 to 6.0)
Chronic lymphoid leukaemia	5.5 (4.8 to 6.3)	1.2 (1.0 to 1.4)	0.7 (0.5 to 0.9)	8.8 (-4.8 to 21.2)	3.8 (-9.2 to 15.6)
Acute myeloid leukaemia	3.7 (3.2 to 4.1)	2.8 (2.4 to 3.1)	0.8 (0.5 to 1.1)	2.0 (-3.5 to 8.0)	-2.8 (-8.0 to 3.1)
Chronic myeloid leukaemia	2.3 (2.0 to 2.6)	0.8 (0.7 to 0.9)	0.3 (0.2 to 0.4)	-18.8 (-27.0 to -9.1)	-22.6 (-30.3 to -13.3)
Other leukaemia	18.3 (14.6 to 19.8)	4.0 (3.3 to 4.4)	2.2 (1.5 to 3.0)	-27.7 (-34.4 to -19.6)	-31.1 (-37.4 to -23.3)
Other neoplasms	52.8 (46.6 to 55.9)	16.0 (14.2 to 16.9)	4.9 (3.5 to 6.6)	20.7 (13.6 to 28.0)	15.1 (8.3 to 22.1)
**Cardiovascular diseases**	**8,571.6 (8,119.6 to 9,025.2)**	**544.3† (492.1 to 600.5)**	**561.6 (384.4 to 787.8)**	**6.0 (3.7 to 8.1)**	**1.1 (-1.1 to 3.1)**
Rheumatic heart disease	3,138.0 (2,797.7 to 3,512.4)	117.9 (106.7 to 131.0)	149.3 (93.6 to 222.1)	10.8 (6.3 to 14.9)	5.7 (1.4 to 9.6)
Ischaemic heart disease	1,127.8 (993.3 to 1,278.2)	220.4 (192.2 to 251.6)	65.4 (42.7 to 96.0)	6.9 (2.2 to 12.0)	2.0 (-2.5 to 6.9)
Cerebrovascular disease	558.6 (497.0 to 656.1)	53.5‡ (33.2 to 81.1)	108.8 (73.7 to 146.3)	-2.7 (-7.9 to 2.8)	-7.2 (-12.2 to -2.0)
Ischaemic stroke	503.7 (402.0 to 634.9)	34.3‡ (17.5 to 58.0)	78.5 (52.6 to 110.3)	-1.9 (-9.3 to 4.9)	-6.4 (-13.5 to 0.1)
Hemorrhagic stroke	193.1 (148.7 to 239.8)	19.1‡ (9.6 to 33.4)	30.3 (19.9 to 43.4)	-4.7 (-12.3 to 2.5)	-9.1 (-16.3 to -2.2)
Hypertensive heart disease	0.2 (0.1 to 0.4)	--	< 0.05 (< 0.05 to < 0.05)	17.8 (12.6 to 22.8)	12.3 (7.4 to 17.2)
Cardiomyopathy and myocarditis	251.0 (220.5 to 291.4)	116.5† (86.6 to 157.3)	28.9 (18.0 to 43.5)	4.5 (-0.4 to 9.1)	-0.3 (-5.0 to 4.1)
Myocarditis	150.6 (121.2 to 185.7)	116.5 (86.6 to 157.3)	12.3 (7.7 to 18.7)	4.7 (-1.0 to 10.9)	-0.1 (-5.6 to 5.8)
Alcoholic cardiomyopathy	< 0.05 (< 0.05 to < 0.05)	--	< 0.05 (< 0.05 to < 0.05)	8.3 (-1.3 to 18.3)	3.3 (-5.8 to 12.8)
Other cardiomyopathy	190.5 (147.9 to 241.8)	--	16.7 (10.3 to 25.0)	4.5 (-1.8 to 11.5)	-0.4 (-6.4 to 6.3)
Atrial fibrillation and flutter	--	--	--	--	--
Peripheral vascular disease	--	--	--	--	--
Endocarditis	17.7 (13.2 to 22.7)	36.1 (24.9 to 49.8)	1.5 (0.9 to 2.3)	9.4 (7.6 to 11.3)	4.4 (2.6 to 6.1)
Other cardiovascular and circulatory diseases	3,826.0 (2,675.1 to 5,222.2)	--	207.7 (120.6 to 324.4)	7.5 (3.7 to 10.8)	2.5 (-1.1 to 5.7)
**Chronic respiratory diseases**	**22,841.7 (21,617.5 to 24,321.8)**	**3,921.7† (3,216.2 to 4,684.2)**	**1,076.4 (774.8 to 1,449.7)**	**8.1 (6.3 to 9.9)**	**3.1 (1.4 to 4.9)**
Chronic obstructive pulmonary disease	2,907.1 (2,654.7 to 3,191.9)	203.6 (177.8 to 233.1)	228.2 (195.0 to 258.6)	7.3 (3.0 to 12.3)	2.4 (-1.8 to 7.1)
Pneumoconiosis	3.9 (3.1 to 4.7)	0.6 (0.5 to 0.7)	0.6 (0.4 to 0.9)	3.4 (1.9 to 5.0)	-1.4 (-2.8 to 0.1)
Silicosis	0.7 (0.5 to 0.9)	0.2 (0.1 to 0.2)	0.1 (0.1 to 0.2)	0.5 (-1.4 to 2.4)	-4.1 (-5.9 to -2.4)

Cause	Prevalence in thousands (95% UI)	Incidence in thousands (95% UI)	YLDs in thousands (95% UI)		
	2016 counts	2016 counts	2016 counts	Percentage change in counts between 2006 and 2016	Percentage change in rates between 2006 and 2016
Asbestosis	1.3 (0.8 to 1.9)	0.2 (0.1 to 0.2)	0.2 (0.1 to 0.3)	6.0 (3.0 to 9.5)	1.2 (-1.8 to 4.5)
Coal workers pneumoconiosis	0.4 (0.3 to 0.5)	0.1 (< 0.05 to 0.1)	0.1 (< 0.05 to 0.1)	1.4 (-1.9 to 4.3)	-3.3 (-6.5 to -0.5)
Other pneumoconiosis	1.6 (1.1 to 2.2)	0.2 (0.2 to 0.3)	0.2 (0.1 to 0.4)	3.1 (0.7 to 5.5)	-1.7 (-4.0 to 0.6)
Asthma	19,941.2 (17,042.6 to 22,942.7)	3,714.9 (3,013.6 to 4,477.3)	790.3 (505.6 to 1,155.6)	6.6 (4.6 to 8.5)	1.7 (-0.2 to 3.5)
Interstitial lung disease and pulmonary sarcoidosis	57.5 (47.5 to 67.5)	2.5 (1.6 to 3.7)	6.5 (4.1 to 9.7)	-0.8 (-4.8 to 2.9)	-5.4 (-9.2 to -1.8)
Other chronic respiratory diseases	--	--	50.9 (43.3 to 57.5)	44.1 (38.4 to 50.7)	37.5 (32.0 to 43.8)
**Cirrhosis and other chronic liver diseases**	**1,289.7 (1,164.3 to 1,423.6)**	**15.8 (11.7 to 20.1)**	**73.9 (50.2 to 104.7)**	**7.4 (2.4 to 12.7)**	**2.4 (-2.4 to 7.5)**
Cirrhosis and other chronic liver diseases due to hepatitis B	235.4 (173.5 to 304.4)	3.2 (2.2 to 4.6)	14.5 (9.0 to 22.0)	9.6 (-2.5 to 22.0)	4.5 (-7.0 to 16.4)
Cirrhosis and other chronic liver diseases due to hepatitis C	181.6 (134.4 to 234.9)	2.3 (1.6 to 3.3)	10.7 (6.4 to 16.9)	9.5 (-4.0 to 24.3)	4.4 (-8.4 to 18.6)
Cirrhosis and other chronic liver diseases due to alcohol use	90.1 (66.0 to 119.3)	1.2 (0.8 to 1.7)	5.2 (3.1 to 8.1)	7.6 (-5.1 to 22.9)	2.7 (-9.5 to 17.2)
Cirrhosis and other chronic liver diseases due to other causes	782.5 (662.4 to 911.9)	9.0 (6.6 to 11.8)	43.5 (28.8 to 62.1)	6.2 (-0.6 to 13.0)	1.3 (-5.2 to 7.7)
**Digestive diseases**	**8,449.0 (7,918.0 to 9,010.3)**	**8,200.2† (7,195.2 to 9,391.1)**	**225.2 (151.2 to 311.4)**	**5.0 (1.3 to 8.9)**	**0.1 (-3.4 to 3.9)**
Peptic ulcer disease	2,544.1 (2,002.8 to 3,163.2)	706.7 (500.5 to 993.8)	38.2 (22.0 to 63.1)	11.6 (3.6 to 20.5)	6.4 (-1.2 to 15.0)
Gastritis and duodenitis	2,980.3 (2,210.3 to 3,960.9)	2,470.3 (1,742.9 to 3,330.0)	36.9 (22.3 to 59.0)	10.8 (2.8 to 19.6)	5.7 (-2.0 to 14.1)
Appendicitis	102.8 (81.5 to 124.5)	2,620.4 (2,095.9 to 3,163.0)	31.8 (20.1 to 45.8)	3.0 (-6.4 to 13.6)	-1.8 (-10.7 to 8.3)
Paralytic ileus and intestinal obstruction	6.6 (4.5 to 9.1)	175.0 (120.5 to 239.2)	2.1 (1.2 to 3.3)	9.8 (7.4 to 11.8)	4.7 (2.4 to 6.7)
Inguinal, femoral, and abdominal hernia	2,201.7 (1,374.6 to 3,218.3)	676.5 (463.1 to 941.0)	64.4 (37.2 to 102.6)	-1.8 (-7.8 to 4.5)	-6.4 (-12.0 to -0.3)
Inflammatory bowel disease	207.6 (180.8 to 238.3)	323.1 (265.9 to 391.8)	31.7 (21.1 to 45.7)	6.4 (1.9 to 11.6)	1.5 (-2.9 to 6.4)
Vascular intestinal disorders	1.8 (1.0 to 3.0)	12.5 (8.1 to 18.0)	0.6 (0.3 to 1.0)	7.9 (5.9 to 9.5)	2.9 (1.0 to 4.5)
Gallbladder and biliary diseases	682.2 (476.3 to 956.1)	1,081.7 (766.2 to 1,469.1)	5.2 (3.1 to 8.1)	2.9 (0.5 to 5.4)	-1.9 (-4.1 to 0.5)
Pancreatitis	15.1 (11.0 to 20.5)	134.1 (96.2 to 186.7)	2.0 (1.2 to 3.1)	2.0 (-3.3 to 6.8)	-2.7 (-7.7 to 1.9)
Other digestive diseases	--	--	12.3 (8.3 to 17.1)	9.6 (5.8 to 13.7)	4.5 (0.9 to 8.4)
**Neurological disorders**	**268,288.3 (252,623.4 to 290,540.3)**	**143,334.2 (96,059.4 to 207,256.9)**	**5,757.1 (3,847.1 to 7,947.3)**	**4.1 (2.3 to 6.1)**	**-0.7 (-2.4 to 1.2)**
Alzheimer's disease and other dementias	--	--	--	--	--
Parkinson's disease	1.4 (0.6 to 2.5)	0.8 (0.4 to 1.5)	0.2 (0.1 to 0.4)	10.3 (2.7 to 19.6)	5.2 (-2.0 to 14.1)
Epilepsy	2,050.8 (1,598.0 to 2,549.5)	180.8 (125.0 to 248.6)	668.9 (431.0 to 943.5)	-0.1 (-11.0 to 12.8)	-4.7 (-15.2 to 7.6)
Multiple sclerosis	71.2 (57.2 to 89.8)	11.1 (8.1 to 14.2)	19.9 (13.3 to 28.4)	8.4 (2.2 to 14.2)	3.4 (-2.5 to 8.9)
Motor neuron disease	15.1 (12.2 to 18.5)	0.3 (0.2 to 0.6)	3.2 (2.2 to 4.5)	6.2 (5.2 to 7.4)	1.3 (0.3 to 2.5)
Migraine	99,718.9 (91,617.8 to 107,875.4)	11,701.7 (9,930.0 to 13,702.8)	4,256.3 (2,687.6 to 6,127.9)	4.4 (3.4 to 5.5)	-0.4 (-1.4 to 0.6)
Tension-type headache	208,790.0 (157,312.5 to 270,309.5)	131,436.7 (84,742.4 to 195,771.3)	672.6 (401.6 to 1,043.2)	3.9 (2.3 to 5.6)	-0.9 (-2.4 to 0.7)
Other neurological disorders	1.3 (0.7 to 2.4)	2.7 (1.4 to 4.6)	135.9 (88.1 to 190.1)	20.6 (7.9 to 35.6)	15.1 (3.0 to 29.4)
**Mental and substance use disorders**	**108,209.2 (105,929.5 to 110,180.7)**	**37,353.5† (32,086.6 to 44,045.0)**	**14,782.9 (10,608.9 to 19,378.6)**	**3.2 (2.4 to 4.0)**	**-1.6 (-2.4 to -0.8)**
Schizophrenia	1,308.4 (956.7 to 1,679.4)	261.4 (192.1 to 343.0)	874.2 (561.6 to 1,204.1)	2.3 (-0.8 to 5.5)	-2.4 (-5.4 to 0.6)
Alcohol use disorders	12,482.0 (9,161.7 to 16,398.6)	6,484.1 (4,972.4 to 8,088.7)	1,275.9 (793.6 to 1,906.0)	1.7 (-1.4 to 4.6)	-3.0 (-6.0 to -0.3)
Drug use disorders	11,826.5 (11,320.5 to 12,441.6)	1,590.8 (1,314.6 to 1,918.5)	2,358.9 (1,643.2 to 3,142.5)	4.0 (1.5 to 6.6)	-0.8 (-3.1 to 1.7)
Opioid use disorders	3,850.5 (3,094.0 to 4,862.4)	723.7 (592.8 to 894.3)	1,638.2 (1,086.4 to 2,273.2)	4.5 (1.0 to 7.9)	-0.3 (-3.6 to 2.9)
Cocaine use disorders	901.2 (782.0 to 1,038.1)	36.4 (22.5 to 53.6)	125.3 (79.2 to 184.3)	5.8 (1.8 to 9.8)	0.9 (-2.9 to 4.7)
Amphetamine use disorders	1,398.0 (949.0 to 1,942.9)	35.8 (15.1 to 64.5)	186.0 (103.6 to 306.8)	0.8 (-4.6 to 5.7)	-3.9 (-9.0 to 0.8)
Cannabis use disorders	5,612.2 (4,391.6 to 7,041.4)	687.4 (448.8 to 968.9)	164.8 (99.7 to 247.3)	3.8 (0.5 to 7.4)	-1.0 (-4.2 to 2.5)
Other drug use disorders	382.4 (304.1 to 471.8)	107.5 (89.2 to 128.9)	244.5 (156.1 to 355.1)	2.6 (-0.9 to 6.4)	-2.1 (-5.5 to 1.5)
Depressive disorders	20,739.1 (19,266.1 to 22,006.8)	22,863.5 (17,864.7 to 29,029.6)	3,693.0 (2,408.4 to 5,371.2)	0.4 (-1.5 to 2.3)	-4.2 (-6.1 to -2.5)
Major depressive disorder	14,392.5 (10,947.9 to 18,645.9)	21,505.7 (16,472.9 to 27,813.0)	3,026.8 (1,924.7 to 4,514.8)	-1.0 (-3.4 to 1.2)	-5.5 (-7.9 to -3.5)
Dysthymia	6,744.6 (5,204.2 to 8,639.5)	1,357.8 (951.8 to 1,811.2)	666.2 (418.7 to 1,018.7)	7.3 (4.5 to 10.1)	2.3 (-0.3 to 5.0)
Bipolar disorder	5,150.1 (4,112.4 to 6,330.9)	283.4 (159.0 to 441.3)	1,078.4 (643.6 to 1,634.2)	5.5 (3.5 to 7.5)	0.6 (-1.2 to 2.5)
Anxiety disorders	24,699.5 (21,543.9 to 28,395.4)	3,392.3 (2,768.8 to 4,109.1)	2,434.7 (1,652.7 to 3,397.7)	4.3 (2.4 to 6.1)	-0.5 (-2.4 to 1.2)
Eating disorders	1,941.7 (1,619.7 to 2,362.8)	1,134.3 (628.0 to 1,799.5)	403.1 (231.8 to 634.5)	15.8 (13.1 to 18.6)	10.5 (7.9 to 13.2)
Anorexia nervosa	601.0 (400.9 to 868.8)	177.8 (119.9 to 248.5)	128.2 (74.2 to 204.7)	14.1 (9.6 to 18.6)	8.8 (4.5 to 13.1)
Bulimia nervosa	1,297.3 (755.1 to 2,105.2)	956.5 (459.5 to 1,634.4)	275.0 (139.8 to 469.5)	16.7 (13.1 to 20.7)	11.3 (7.9 to 15.1)
Autistic spectrum disorders	5,694.4 (5,411.8 to 5,930.2)	--	838.1 (566.8 to 1,179.9)	5.4 (3.9 to 7.0)	0.6 (-0.9 to 2.1)
Autism	1,675.9 (1,440.2 to 1,946.6)	--	431.8 (279.7 to 623.4)	5.2 (2.7 to 7.7)	0.3 (-2.0 to 2.8)
Asperger syndrome and other autistic spectrum disorders	4,017.6 (3,361.4 to 4,829.3)	--	406.2 (266.2 to 592.9)	5.7 (3.9 to 7.5)	0.8 (-0.9 to 2.6)

Cause	Prevalence in thousands (95% UI)	Incidence in thousands (95% UI)	YLDs in thousands (95% UI)		
	2016 counts	2016 counts	2016 counts	Percentage change in counts between 2006 and 2016	Percentage change in rates between 2006 and 2016
Attention-deficit/hyperactivity disorder	7,473.8 (6,692.4 to 8,385.8)	--	90.3 (53.2 to 143.0)	4.3 (2.0 to 6.6)	-0.5 (-2.7 to 1.7)
Conduct disorder	2,811.1 (2,279.3 to 3,400.5)	--	338.3 (204.7 to 515.0)	5.9 (2.6 to 9.0)	1.0 (-2.2 to 3.9)
Idiopathic developmental intellectual disability	13,182.9 (8,414.5 to 18,141.6)	--	519.0 (251.3 to 888.7)	-2.2 (-5.7 to 0.1)	-6.7 (-10.1 to -4.5)
Other mental and substance use disorders	11,474.5 (9,507.1 to 13,639.3)	1,343.7 (1,141.4 to 1,584.7)	878.9 (583.3 to 1,270.7)	5.2 (3.3 to 7.1)	0.4 (-1.5 to 2.2)
**Diabetes, urogenital, blood, and endocrine diseases**	**217,564.1 (215,245.8 to 219,867.5)**	**70,152.2† (61,263.2 to 80,630.2)**	**2,396.3 (1,635.2 to 3,394.3)**	**6.7 (4.7 to 8.8)**	**1.7 (-0.1 to 3.8)**
Diabetes mellitus	9,472.9 (7,623.7 to 11,694.0)	966.2 (679.0 to 1,279.1)	564.5 (360.5 to 858.6)	7.0 (4.0 to 10.0)	2.1 (-0.8 to 4.9)
Acute glomerulonephritis	5.5 (3.4 to 8.1)	80.4 (51.3 to 115.5)	0.3 (0.1 to 0.5)	-6.5 (-8.8 to -4.5)	-10.9 (-13.0 to -8.9)
Chronic kidney disease	4,631.8 (3,742.2 to 5,691.9)	452.2 (305.7 to 608.2)	229.7 (147.3 to 334.5)	9.6 (4.4 to 14.7)	4.5 (-0.4 to 9.4)
Chronic kidney disease due to diabetes mellitus	508.1 (305.7 to 758.9)	46.4 (25.5 to 75.5)	30.4 (16.3 to 50.7)	16.0 (9.5 to 24.3)	10.6 (4.4 to 18.6)
Chronic kidney disease due to hypertension	1,218.0 (854.3 to 1,643.2)	199.6 (120.1 to 299.0)	15.1 (8.4 to 24.9)	7.7 (2.0 to 13.0)	2.8 (-2.7 to 7.8)
Chronic kidney disease due to glomerulonephritis	1,083.2 (770.5 to 1,451.0)	24.9 (14.5 to 38.3)	100.2 (59.4 to 152.3)	7.1 (1.3 to 13.1)	2.2 (-3.4 to 7.9)
Chronic kidney disease due to other causes	1,822.5 (1,210.2 to 2,499.6)	181.3 (108.9 to 268.9)	84.0 (47.3 to 132.3)	10.8 (4.5 to 17.5)	5.7 (-0.3 to 12.1)
Urinary diseases and male infertility	5,534.2 (4,445.1 to 6,885.9)	39,904.3† (32,170.8 to 49,780.1)	71.5 (40.8 to 119.9)	5.3 (-0.2 to 11.2)	0.5 (-4.8 to 6.1)
Interstitial nephritis and urinary tract infections	706.3 (545.2 to 907.6)	37,096.3 (29,265.2 to 46,941.8)	23.7 (13.4 to 37.8)	1.9 (-3.0 to 6.5)	-2.8 (-7.4 to 1.6)
Urolithiasis	102.2 (74.7 to 136.7)	2,808.1 (2,027.3 to 3,830.3)	7.9 (4.9 to 12.4)	2.8 (1.3 to 4.4)	-2.0 (-3.4 to -0.4)
Benign prostatic hyperplasia	--	--	--	--	--
Male infertility	4,431.7 (2,994.6 to 6,293.7)	--	29.4 (10.9 to 62.3)	21.2 (13.6 to 28.3)	15.7 (8.3 to 22.4)
Other urinary diseases	--	--	10.5 (6.4 to 16.1)	-17.4 (-20.2 to -14.6)	-21.2 (-23.9 to -18.5)
Gynecological diseases	77,995.9 (76,135.5 to 79,759.7)	28,438.5† (25,084.1 to 31,946.0)	979.8 (653.1 to 1,421.0)	5.2 (2.7 to 7.8)	0.3 (-2.0 to 2.8)
Uterine fibroids	820.4 (635.6 to 1,030.3)	234.1 (149.1 to 329.4)	20.8 (12.3 to 33.7)	5.6 (0.6 to 10.8)	0.7 (-4.0 to 5.7)
Polycystic ovarian syndrome	1,179.2 (892.3 to 1,563.4)	409.5 (214.2 to 655.8)	10.9 (4.8 to 21.6)	4.4 (1.3 to 7.5)	-0.4 (-3.4 to 2.5)
Female infertility	6,529.8 (3,772.0 to 9,869.6)	--	41.0 (14.8 to 93.6)	30.1 (19.8 to 41.9)	24.1 (14.3 to 35.4)
Endometriosis	537.8 (448.8 to 633.6)	1,056.3 (670.6 to 1,559.4)	50.8 (33.0 to 72.7)	-1.4 (-6.8 to 4.4)	-6.0 (-11.1 to -0.5)
Genital prolapse	5,956.9 (4,313.2 to 7,950.3)	980.2 (550.4 to 1,477.6)	19.2 (8.4 to 36.6)	-3.5 (-7.1 to 0.5)	-7.9 (-11.4 to -4.2)
Premenstrual syndrome	60,311.2 (53,440.8 to 67,084.4)	18,447.2 (15,425.3 to 21,579.3)	509.6 (313.8 to 791.8)	1.8 (-1.3 to 4.5)	-2.9 (-5.8 to -0.3)
Other gynecological diseases	9,441.4 (7,960.2 to 10,913.4)	7,311.1 (5,790.7 to 9,021.4)	327.5 (213.4 to 466.0)	10.0 (5.4 to 16.0)	4.9 (0.5 to 10.6)
Hemoglobinopathies and hemolytic anaemias	150,344.5 (148,306.6 to 152,461.4)	--	436.4 (283.6 to 647.6)	8.5 (2.3 to 15.3)	3.5 (-2.4 to 10.0)
Thalassemias	4.8 (4.5 to 5.1)	--	0.4 (0.2 to 0.5)	-1.4 (-8.5 to 7.2)	-6.0 (-12.7 to 2.3)
Thalassaemias trait	22,367.4 (21,488.0 to 23,312.4)	--	214.9 (136.2 to 325.1)	-2.3 (-10.9 to 7.5)	-6.8 (-15.1 to 2.5)
Sickle cell disorders	244.0 (224.0 to 265.6)	--	23.7 (16.6 to 32.2)	29.0 (18.6 to 40.2)	23.1 (13.2 to 33.8)
Sickle cell trait	40,777.2 (36,840.2 to 45,990.1)	--	116.9 (73.9 to 172.0)	17.6 (12.3 to 23.1)	12.1 (7.1 to 17.4)
G6PD deficiency	28,596.8 (27,517.5 to 29,894.1)	--	1.7 (1.1 to 2.4)	15.2 (10.0 to 20.6)	9.9 (4.9 to 15.0)
G6PD trait	71,350.4 (70,181.1 to 72,679.8)	--	< 0.05 (< 0.05 to 0.1)	10.4 (4.7 to 17.5)	5.3 (-0.1 to 12.1)
Other hemoglobinopathies and hemolytic anaemias	4,272.1 (3,661.3 to 4,952.7)	--	78.8 (50.6 to 117.7)	25.6 (16.8 to 35.9)	19.8 (11.4 to 29.6)
Endocrine, metabolic, blood, and immune disorders	4,580.3 (4,015.8 to 5,208.5)	310.5 (205.8 to 449.9)	114.2 (74.1 to 167.0)	5.9 (2.1 to 9.5)	1.0 (-2.6 to 4.5)
**Musculoskeletal disorders**	**58,167.5 (54,955.8 to 61,301.8)**	**33,242.1 (26,690.8 to 40,310.1)**	**6,228.9 (4,222.4 to 8,512.7)**	**1.8 (-0.3 to 3.8)**	**-2.9 (-4.9 to -1.0)**
Rheumatoid arthritis	567.1 (446.1 to 713.1)	51.6 (36.5 to 69.9)	141.0 (90.2 to 198.7)	7.2 (2.8 to 12.2)	2.3 (-1.9 to 7.1)
Osteoarthritis	--	--	--	--	--
Low back and neck pain	33,173.6 (30,485.5 to 37,610.5)	14,922.1 (10,748.9 to 20,302.9)	3,731.8 (2,447.0 to 5,422.8)	3.1 (0.7 to 5.6)	-1.7 (-3.9 to 0.7)
Low back pain	25,933.6 (19,051.4 to 35,401.6)	12,474.0 (8,557.8 to 17,831.3)	2,935.3 (1,832.0 to 4,437.6)	2.9 (-0.1 to 6.1)	-1.8 (-4.7 to 1.2)
Neck pain	7,696.0 (5,410.7 to 10,365.6)	2,448.1 (1,516.0 to 3,567.4)	796.6 (481.6 to 1,235.6)	3.6 (0.9 to 6.3)	-1.2 (-3.8 to 1.4)
Gout	272.8 (199.8 to 369.6)	107.4 (83.2 to 134.5)	9.6 (5.7 to 14.8)	5.2 (1.7 to 8.7)	0.3 (-3.0 to 3.7)
Other musculoskeletal disorders	26,497.5 (19,014.1 to 34,394.9)	18,160.9 (13,149.5 to 24,027.3)	2,346.5 (1,430.0 to 3,493.6)	-0.6 (-4.6 to 2.9)	-5.1 (-9.0 to -1.9)
**Other non-communicable diseases**	**384,191.8 (375,702.3 to 391,831.3)**	**1,070,392.0† (877,478.1 to 1,264,498.1)**	**8,557.1 (5,794.9 to 12,369.3)**	**4.5 (3.8 to 5.1)**	**-0.3 (-1.0 to 0.3)**
Congenital anomalies	6,553.5 (6,147.2 to 6,945.0)	--	771.3 (543.1 to 1,028.0)	2.2 (-0.3 to 4.6)	-2.5 (-4.9 to -0.2)
Neural tube defects	490.4 (442.7 to 543.6)	--	147.8 (103.7 to 200.5)	7.6 (2.8 to 12.7)	2.6 (-1.9 to 7.5)
Congenital heart anomalies	949.9 (846.3 to 1,061.2)	--	49.3 (21.0 to 84.7)	2.6 (-0.9 to 6.1)	-2.2 (-5.5 to 1.2)
Orofacial clefts	269.6 (240.0 to 299.8)	--	3.6 (2.3 to 5.2)	-3.6 (-6.5 to -0.8)	-8.1 (-10.8 to -5.3)
Down's syndrome	205.9 (178.9 to 236.8)	--	17.7 (11.9 to 24.8)	0.6 (-3.3 to 4.2)	-4.1 (-7.7 to -0.6)
Turner syndrome	284.3 (248.6 to 323.4)	--	5.9 (3.0 to 9.6)	2.2 (-7.1 to 11.9)	-2.5 (-11.4 to 6.7)
Klinefelter syndrome	353.6 (305.8 to 407.4)	--	3.6 (1.7 to 6.9)	5.2 (-0.4 to 10.7)	0.3 (-5.0 to 5.6)

Cause	Prevalence in thousands (95% UI)	Incidence in thousands (95% UI)	YLDs in thousands (95% UI)		
	2016 counts	2016 counts	2016 counts	Percentage change in counts between 2006 and 2016	Percentage change in rates between 2006 and 2016
Other chromosomal abnormalities	297.7 (256.8 to 347.2)	--	25.2 (16.7 to 34.9)	2.0 (-2.2 to 6.2)	-2.7 (-6.7 to 1.3)
Congenital musculoskeletal and limb anomalies	840.8 (774.5 to 914.1)	--	122.1 (83.5 to 170.0)	4.2 (0.9 to 7.5)	-0.7 (-3.8 to 2.5)
Urogenital congenital anomalies	576.3 (512.7 to 644.0)	--	16.8 (10.2 to 25.9)	-2.0 (-4.0 to -0.2)	-6.6 (-8.4 to -4.8)
Digestive congenital anomalies	758.4 (682.2 to 839.1)	--	34.0 (22.2 to 49.0)	0.9 (-4.1 to 6.0)	-3.8 (-8.5 to 1.1)
Other congenital anomalies	2,979.1 (2,098.1 to 4,206.4)	--	345.5 (223.4 to 514.3)	-0.2 (-4.4 to 4.2)	-4.8 (-8.8 to -0.7)
Skin and subcutaneous diseases	188,287.8 (184,433.8 to 192,020.4)	318,512.4 (288,699.7 to 353,305.0)	5,134.2 (3,379.8 to 7,492.1)	5.7 (5.0 to 6.4)	0.8 (0.2 to 1.5)
Dermatitis	20,932.5 (18,108.1 to 24,164.5)	38,582.8 (31,014.7 to 47,661.1)	741.6 (438.5 to 1,190.0)	6.1 (4.2 to 7.9)	1.2 (-0.6 to 3.0)
Psoriasis	4,277.1 (3,890.4 to 4,692.8)	607.0 (531.1 to 689.2)	382.0 (267.9 to 509.7)	11.0 (7.9 to 14.2)	5.9 (2.9 to 8.9)
Cellulitis	239.1 (193.9 to 295.0)	4,371.4 (3,628.6 to 5,201.6)	13.8 (8.4 to 20.7)	6.1 (-0.3 to 12.7)	1.2 (-4.9 to 7.5)
Pyoderma	751.6 (686.9 to 819.7)	15,776.8 (14,338.8 to 17,467.6)	4.3 (1.7 to 9.2)	9.6 (5.8 to 13.4)	4.5 (0.9 to 8.2)
Scabies	17,155.2 (12,092.0 to 23,490.4)	52,491.1 (36,106.9 to 72,417.2)	446.2 (223.5 to 793.6)	-1.5 (-2.8 to -0.1)	-6.0 (-7.3 to -4.7)
Fungal skin diseases	33,417.1 (26,869.4 to 41,168.8)	104,813.9 (83,978.1 to 131,518.4)	190.5 (73.5 to 395.5)	7.0 (6.3 to 7.7)	2.1 (1.4 to 2.7)
Viral skin diseases	17,214.5 (14,662.6 to 20,104.7)	25,755.1 (22,032.1 to 29,998.3)	530.2 (325.7 to 793.2)	4.7 (3.5 to 5.9)	-0.1 (-1.2 to 1.0)
Acne vulgaris	90,264.8 (77,966.5 to 103,089.9)	35,895.5 (29,359.2 to 43,208.2)	2,329.2 (1,518.3 to 3,427.1)	6.2 (5.1 to 7.2)	1.3 (0.3 to 2.3)
Alopecia areata	1,557.3 (1,399.1 to 1,721.7)	2,892.3 (2,576.5 to 3,237.2)	51.7 (32.2 to 78.3)	4.7 (1.5 to 7.7)	-0.1 (-3.2 to 2.7)
Pruritus	3,841.3 (2,747.9 to 5,245.6)	3,070.2 (2,140.8 to 4,188.1)	41.5 (19.3 to 78.5)	6.0 (4.1 to 7.8)	1.1 (-0.7 to 2.8)
Urticaria	4,255.4 (2,860.0 to 5,942.8)	7,513.1 (5,085.1 to 10,259.5)	257.8 (142.6 to 416.7)	4.3 (2.1 to 6.6)	-0.5 (-2.6 to 1.7)
Decubitus ulcer	31.0 (21.5 to 42.4)	108.9 (76.5 to 150.0)	5.5 (3.3 to 8.6)	3.2 (2.1 to 4.3)	-1.6 (-2.6 to -0.6)
Other skin and subcutaneous diseases	24,960.4 (22,450.5 to 27,759.7)	26,634.3 (23,968.4 to 29,491.8)	140.1 (64.9 to 258.1)	10.5 (9.4 to 11.6)	5.4 (4.4 to 6.5)
Sense organ diseases	60,206.1 (58,021.8 to 62,376.6)	37,154.5† (33,144.8 to 41,447.7)	1,975.3 (1,335.5 to 2,797.2)	2.5 (0.4 to 4.5)	-2.2 (-4.3 to -0.3)
Glaucoma	--	--	--	--	--
Cataract	33.5 (20.6 to 49.3)	--	3.6 (1.8 to 6.5)	-2.6 (-13.4 to 5.5)	-7.0 (-17.4 to 0.6)
Macular degeneration	--	--	--	--	--
Refraction and accommodation disorders	29,592.7 (26,527.3 to 33,263.9)	--	617.9 (381.7 to 961.3)	0.9 (-0.3 to 2.1)	-3.7 (-4.9 to -2.6)
Age-related and other hearing loss	25,409.0 (18,796.4 to 32,528.2)	--	1,073.2 (715.0 to 1,553.5)	2.8 (-1.0 to 6.5)	-1.9 (-5.6 to 1.5)
Other vision loss	674.5 (480.7 to 917.2)	--	51.5 (29.7 to 81.7)	1.2 (-6.2 to 8.0)	-3.5 (-10.5 to 3.0)
Other sense organ diseases	7,300.7 (6,431.6 to 8,166.4)	37,154.5 (33,144.8 to 41,447.7)	229.1 (139.0 to 351.1)	5.9 (3.5 to 8.2)	1.0 (-1.2 to 3.2)
Oral disorders	254,443.7 (235,016.4 to 282,231.7)	714,725.1† (527,015.1 to 910,255.8)	676.2 (387.9 to 1,061.9)	4.0 (3.1 to 5.0)	-0.8 (-1.7 to 0.1)
Caries of deciduous teeth	--	--	--	--	--
Caries of permanent teeth	235,525.5 (193,355.7 to 280,441.9)	710,629.8 (521,891.8 to 906,349.3)	161.8 (70.6 to 309.2)	0.3 (-1.0 to 1.5)	-4.3 (-5.5 to -3.1)
Periodontal disease	14,596.9 (9,245.3 to 21,002.3)	3,507.4 (1,925.4 to 5,665.7)	97.5 (35.3 to 214.5)	8.7 (7.0 to 10.3)	3.7 (2.1 to 5.2)
Edentulism and severe tooth loss	2,641.2 (1,876.7 to 3,507.7)	587.9 (430.6 to 797.2)	76.5 (44.2 to 117.5)	3.4 (-0.1 to 6.7)	-1.4 (-4.7 to 1.8)
Other oral disorders	11,429.6 (10,270.1 to 12,701.5)	--	340.4 (205.4 to 523.0)	4.7 (3.5 to 5.9)	-0.1 (-1.2 to 1.0)
**Injuries**	**72,665.8 (66,355.9 to 79,418.9)**	**43,846.3† (38,325.4 to 49,732.2)**	**2,865.5 (1,890.4 to 4,176.5)**	**3.2 (0.7 to 5.2)**	**-1.6 (-3.9 to 0.4)**
**Transport injuries**	**9,912.2 (8,498.9 to 11,012.1)**	**7,860.4 (6,064.0 to 9,773.5)**	**562.4 (363.6 to 825.6)**	**6.0 (4.3 to 7.5)**	**1.1 (-0.5 to 2.6)**
Road injuries	7,246.3 (6,181.4 to 8,152.2)	7,216.1 (5,516.4 to 9,174.2)	416.3 (268.9 to 614.1)	6.8 (5.0 to 8.5)	1.9 (0.1 to 3.5)
Pedestrian road injuries	1,613.0 (1,275.9 to 2,007.7)	1,092.2 (696.9 to 1,577.3)	93.0 (58.8 to 138.6)	4.2 (2.3 to 6.2)	-0.6 (-2.4 to 1.3)
Cyclist road injuries	1,593.3 (1,191.7 to 2,037.0)	1,478.9 (923.7 to 2,182.9)	83.5 (52.8 to 126.5)	9.8 (7.9 to 12.0)	4.7 (2.9 to 6.8)
Motorcyclist road injuries	1,696.5 (1,328.2 to 2,112.6)	1,583.8 (1,085.6 to 2,259.6)	97.9 (62.8 to 144.8)	9.0 (6.8 to 11.1)	4.0 (1.9 to 6.0)
Motor vehicle road injuries	1,714.9 (1,382.7 to 2,114.4)	2,389.3 (1,689.3 to 3,165.8)	110.0 (71.5 to 158.8)	1.3 (-1.1 to 3.9)	-3.3 (-5.6 to -0.9)
Other road injuries	628.5 (464.4 to 815.5)	671.8 (361.2 to 1,068.9)	32.0 (19.9 to 48.7)	22.4 (20.0 to 24.8)	16.8 (14.5 to 19.0)
Other transport injuries	2,665.9 (2,129.1 to 3,389.5)	644.3 (398.3 to 946.8)	146.1 (96.6 to 212.3)	3.6 (1.6 to 5.5)	-1.2 (-3.1 to 0.6)
**Unintentional injuries**	**56,531.4 (50,630.1 to 63,169.4)**	**31,572.7 (26,654.2 to 36,763.0)**	**1,875.2 (1,225.2 to 2,694.2)**	**6.6 (4.9 to 8.0)**	**1.7 (0.1 to 3.0)**
Falls	20,864.3 (16,812.1 to 25,065.9)	11,045.0 (7,581.2 to 15,221.8)	768.9 (511.6 to 1,092.4)	7.0 (5.4 to 8.4)	2.1 (0.6 to 3.4)
Drowning	243.4 (194.2 to 295.3)	50.6 (39.4 to 66.0)	13.1 (8.4 to 19.2)	-9.2 (-11.5 to -7.2)	-13.4 (-15.6 to -11.5)
Fire, heat, and hot substances	5,546.0 (4,337.9 to 7,157.7)	848.1 (564.6 to 1,246.2)	157.3 (105.3 to 226.9)	6.8 (3.3 to 9.7)	1.8 (-1.5 to 4.7)
Poisonings	228.8 (183.1 to 282.5)	373.4 (208.3 to 609.5)	24.0 (15.3 to 35.7)	16.6 (13.1 to 19.7)	11.2 (7.9 to 14.2)
Exposure to mechanical forces	9,326.9 (7,538.4 to 12,899.1)	6,282.7 (4,327.1 to 8,646.4)	217.7 (134.7 to 338.0)	7.5 (5.7 to 9.2)	2.6 (0.8 to 4.2)
Unintentional firearm injuries	337.0 (247.2 to 449.1)	175.7 (88.2 to 307.9)	13.1 (7.9 to 20.8)	8.3 (6.1 to 10.5)	3.3 (1.2 to 5.4)
Unintentional suffocation	640.6 (461.8 to 861.8)	52.0 (23.6 to 101.4)	19.1 (11.0 to 30.9)	-0.6 (-2.4 to 1.3)	-5.2 (-6.9 to -3.4)
Other exposure to mechanical forces	8,349.3 (6,530.1 to 11,967.9)	6,055.0 (4,158.7 to 8,309.6)	185.5 (116.0 to 287.2)	8.4 (6.4 to 10.2)	3.4 (1.5 to 5.1)

Cause	Prevalence in thousands (95% UI)	Incidence in thousands (95% UI)	YLDs in thousands (95% UI)		
	2016 counts	2016 counts	2016 counts	Percentage change in counts between 2006 and 2016	Percentage change in rates between 2006 and 2016
Adverse effects of medical treatment	86.2 (55.1 to 127.4)	1,127.1 (761.2 to 1,576.1)	11.5 (6.2 to 18.9)	3.9 (-2.0 to 8.2)	-0.9 (-6.5 to 3.2)
Animal contact	3,222.4 (2,709.4 to 3,866.0)	5,513.9 (3,623.8 to 7,902.6)	133.4 (88.6 to 188.7)	3.3 (0.8 to 5.8)	-1.4 (-3.8 to 0.9)
Venomous animal contact	1,624.5 (1,236.2 to 2,051.4)	2,825.4 (1,819.3 to 4,174.3)	107.1 (71.4 to 151.3)	4.4 (1.4 to 7.3)	-0.4 (-3.3 to 2.4)
Non-venomous animal contact	1,597.9 (1,201.2 to 2,137.6)	2,688.5 (1,705.6 to 3,979.0)	26.4 (15.8 to 41.4)	-0.8 (-3.4 to 1.4)	-5.3 (-7.9 to -3.3)
Foreign body	3,621.3 (2,868.7 to 4,412.6)	1,730.0 (1,083.8 to 2,584.9)	139.5 (87.1 to 213.1)	6.3 (4.0 to 8.6)	1.4 (-0.8 to 3.6)
Pulmonary aspiration and foreign body in airway	1,819.5 (1,289.7 to 2,556.2)	203.4 (116.7 to 325.5)	79.9 (47.6 to 124.7)	2.6 (0.6 to 4.4)	-2.1 (-4.0 to -0.4)
Foreign body in eyes	183.5 (81.1 to 349.3)	1,113.0 (523.6 to 1,936.0)	10.5 (4.1 to 21.7)	14.5 (10.6 to 18.8)	9.2 (5.5 to 13.4)
Foreign body in other body part	1,618.2 (1,227.4 to 2,167.1)	413.7 (240.7 to 618.8)	49.1 (30.7 to 75.4)	11.2 (8.2 to 13.9)	6.0 (3.2 to 8.7)
Environmental heat and cold exposure	4,438.5 (3,533.2 to 5,532.1)	1,066.6 (636.7 to 1,621.6)	166.1 (105.2 to 245.4)	6.2 (4.0 to 8.0)	1.2 (-0.8 to 3.0)
Other unintentional injuries	8,953.6 (6,791.7 to 11,619.4)	3,535.4 (2,382.6 to 5,091.3)	243.7 (155.7 to 362.5)	6.9 (4.7 to 8.3)	1.9 (-0.1 to 3.3)
**Self-harm and interpersonal violence**	**26,039.0 (19,653.9 to 34,915.5)**	**4,293.9† (3,300.6 to 5,546.9)**	**345.3 (227.8 to 505.5)**	**-0.8 (-2.0 to 0.2)**	**-5.4 (-6.5 to -4.4)**
Self-harm	473.2 (374.2 to 612.9)	771.9 (577.4 to 1,033.7)	29.3 (18.6 to 44.0)	0.7 (-1.5 to 2.9)	-4.0 (-6.0 to -1.8)
Self-harm by firearm	25.9 (20.7 to 31.8)	8.3 (2.3 to 21.9)	1.0 (0.6 to 1.6)	-3.4 (-6.2 to -0.8)	-7.9 (-10.5 to -5.4)
Self-harm by other specified means	447.3 (346.8 to 583.8)	763.7 (570.5 to 1,027.7)	28.4 (18.0 to 42.5)	0.8 (-1.3 to 3.1)	-3.8 (-5.9 to -1.7)
Interpersonal violence	25,565.8 (19,242.2 to 34,458.9)	3,522.0† (2,572.7 to 4,751.8)	316.0 (208.3 to 465.5)	-1.0 (-2.2 to 0.1)	-5.5 (-6.7 to -4.5)
Assault by firearm	135.5 (97.9 to 185.6)	116.6 (72.4 to 175.1)	6.1 (3.7 to 9.7)	1.8 (-0.8 to 4.5)	-2.9 (-5.4 to -0.3)
Assault by sharp object	744.1 (568.7 to 976.3)	839.9 (532.4 to 1,223.5)	26.0 (16.6 to 39.4)	-0.2 (-3.2 to 2.5)	-4.8 (-7.7 to -2.2)
Sexual violence	22,939.6 (16,523.1 to 31,844.7)	--	209.1 (123.9 to 331.1)	-1.8 (-3.3 to -0.6)	-6.4 (-7.8 to -5.2)
Assault by other means	1,746.6 (1,379.5 to 2,274.5)	2,565.4 (1,798.6 to 3,538.4)	74.8 (48.2 to 112.2)	1.0 (-1.0 to 3.1)	-3.7 (-5.6 to -1.7)
**Forces of nature, conflict and terrorism, and executions and police conflict**	**3,122.8 (1,989.4 to 4,558.4)**	**119.3† (78.3 to 169.4)**	**82.5 (29.6 to 169.2)**	**-40.4 (-54.0 to -30.9)**	**-43.1 (-56.1 to -34.0)**
Forces of nature, conflict and terrorism, and state actor violence	599.3 (319.1 to 972.7)	28.3 (16.5 to 42.5)	20.9 (6.2 to 46.9)	2.7 (-5.6 to 12.9)	-2.0 (-10.0 to 7.7)
Conflict and terrorism	2,432.5 (1,479.6 to 3,741.2)	--	61.5 (23.2 to 123.5)	-48.2 (-61.2 to -38.2)	-50.6 (-63.0 to -41.1)
Executions and police conflict	90.9 (54.9 to 141.8)	91.0 (53.1 to 139.3)	2.8 (1.1 to 7.0)	-43.3 (-48.3 to -36.2)	-45.9 (-50.7 to -39.1)

We did not calculate incidence for certain NTDs and causes with only birth prevalence. Blank cells mean that no estimate is available.

† Incidence for aggregate causes represents the sum of the incidence of the causes below the aggregate.

‡ Incidence estimates for stroke represent first-ever stroke only.

^ Incidence of HIV/AIDS represents new infections of HIV only and does not include new infections of Tuberculosis in HIV positive cases.

SR Table 12: Global prevalence, incidence, and YLDs for 2016, percentage change of YLD counts and percentage change of YLD rates between 2006 and 2016 by cause, age 25-29 years.

Cause	Prevalence in thousands (95% UI)	Incidence in thousands (95% UI)	YLDs in thousands (95% UI)		
	2016 counts	2016 counts	2016 counts	Percentage change in counts between 2006 and 2016	Percentage change in rates between 2006 and 2016
**All causes**	**596,297.5 (595,217.1 to 597,277.2)**	**3,442,263.6† (2,965,019.5 to 4,019,040.6)**	**55,802.9 (40,811.7 to 72,594.9)**	**15.4 (14.7 to 16.0)**	**-2.8 (-3.4 to -2.3)**
**Communicable, maternal, neonatal, and nutritional disorders**	**433,103.4 (427,674.6 to 438,492.4)**	**1,982,526.0† (1,540,285.5 to 2,528,888.2)**	**7,658.9 (5,529.9 to 10,394.6)**	**7.9 (4.9 to 11.3)**	**-9.1 (-11.6 to -6.2)**
**HIV/AIDS and tuberculosis**	**189,273.8 (168,190.5 to 216,258.6)**	**1,323.5† (1,034.7 to 1,718.7)**	**701.1 (483.2 to 947.0)**	**-11.2 (-20.6 to -3.7)**	**-25.2 (-33.1 to -18.9)**
Tuberculosis	174,888.6 (140,632.8 to 215,186.1)	963.7† (672.2 to 1,350.3)	213.4 (121.2 to 332.9)	14.9 (6.6 to 28.3)	-3.2 (-10.2 to 8.1)
Drug-susceptible tuberculosis	652.3 (431.0 to 940.6)	929.9 (648.9 to 1,305.3)	205.5 (116.3 to 320.0)	15.0 (6.4 to 28.5)	-3.1 (-10.4 to 8.3)
Multidrug-resistant tuberculosis without extensive drug resistance	22.7 (15.7 to 31.9)	31.9 (22.9 to 43.5)	7.5 (4.3 to 11.7)	8.6 (-6.2 to 26.7)	-8.5 (-21.0 to 6.7)
Extensively drug-resistant tuberculosis	1.5 (1.0 to 2.0)	2.0 (1.4 to 2.7)	0.5 (0.3 to 0.8)	205.7 (163.1 to 257.3)	157.5 (121.6 to 201.0)
Latent tuberculosis infection	174,212.1 (139,944.8 to 214,513.1)	--	--	--	--
HIV/AIDS	4,584.3 (4,326.3 to 4,847.2)	359.8^ (322.1 to 405.2)	487.7 (344.7 to 652.7)	-19.3 (-29.1 to -10.5)	-32.0 (-40.3 to -24.6)
Drug-susceptible HIV/AIDS - Tuberculosis	158.1 (113.2 to 214.7)	207.8 (147.4 to 283.9)	61.6 (36.6 to 95.3)	-35.4 (-39.6 to -30.8)	-45.6 (-49.1 to -41.7)
Multidrug-resistant HIV/AIDS - Tuberculosis without extensive drug resistance	3.9 (2.6 to 5.9)	5.3 (3.5 to 8.0)	1.6 (0.9 to 2.7)	-42.1 (-53.1 to -29.1)	-51.2 (-60.5 to -40.3)
Extensively drug-resistant HIV/AIDS - Tuberculosis	0.2 (0.1 to 0.3)	0.2 (0.1 to 0.3)	0.1 (< 0.05 to 0.1)	87.1 (53.0 to 127.2)	57.6 (28.9 to 91.4)
HIV/AIDS resulting in other diseases	4,422.1 (4,155.6 to 4,691.6)	359.8 (322.1 to 405.2)	424.4 (300.3 to 569.8)	-16.1 (-28.3 to -4.5)	-29.4 (-39.6 to -19.6)
**Diarrhoea, lower respiratory infections, and other common infectious diseases**	**31,299.5 (29,073.4 to 33,449.6)**	**1,760,326.8† (1,314,866.7 to 2,312,866.2)**	**1,433.8 (980.6 to 2,056.6)**	**13.5 (12.0 to 15.0)**	**-4.4 (-5.7 to -3.1)**
Diarrhoeal diseases	3,889.9 (3,495.2 to 4,290.1)	262,511.8 (236,879.1 to 289,091.0)	441.4 (297.8 to 614.3)	14.0 (11.5 to 16.4)	-4.0 (-6.0 to -1.9)
Intestinal infectious diseases	37.6 (29.2 to 48.5)	835.2† (690.3 to 1,037.0)	6.4 (4.0 to 9.5)	-4.2 (-22.4 to 17.8)	-19.3 (-34.6 to -0.8)
Typhoid fever	36.9 (28.2 to 48.0)	582.4 (473.2 to 725.9)	5.7 (3.5 to 8.5)	-4.0 (-24.5 to 21.2)	-19.2 (-36.4 to 2.1)
Paratyphoid fever	11.1 (8.6 to 13.9)	252.8 (204.8 to 315.8)	0.7 (0.4 to 1.0)	-4.8 (-22.2 to 15.6)	-19.8 (-34.4 to -2.7)
Other intestinal infectious diseases	--	--	< 0.05 (< 0.05 to < 0.05)	-29.3 (-42.7 to -13.1)	-40.5 (-51.8 to -26.8)
Lower respiratory infections	261.9 (231.4 to 294.4)	11,267.8 (9,790.8 to 12,828.2)	16.2 (10.5 to 23.3)	32.3 (25.1 to 40.3)	11.4 (5.4 to 18.2)
Upper respiratory infections	19,443.1 (13,830.2 to 26,775.7)	1,464,922.8 (1,018,666.2 to 2,021,029.1)	487.5 (254.7 to 823.4)	16.7 (15.3 to 18.2)	-1.7 (-2.9 to -0.4)
Otitis media	5,960.0 (4,490.8 to 7,969.9)	14,744.1 (12,185.3 to 17,745.1)	163.8 (93.5 to 258.1)	5.2 (0.7 to 9.8)	-11.4 (-15.2 to -7.5)
Meningitis	1,320.0 (1,134.3 to 1,525.4)	96.5 (69.0 to 129.0)	142.0 (99.3 to 185.7)	12.9 (9.3 to 16.7)	-4.9 (-7.9 to -1.7)
Pneumococcal meningitis	584.8 (485.6 to 698.2)	27.5 (17.0 to 41.8)	62.9 (43.1 to 83.6)	8.7 (1.8 to 15.6)	-8.4 (-14.3 to -2.6)
H influenzae type B meningitis	185.5 (145.4 to 235.1)	7.0 (3.7 to 11.8)	23.8 (15.8 to 32.2)	-10.5 (-16.4 to -5.6)	-24.6 (-29.5 to -20.5)
Meningococcal infection	146.9 (106.4 to 195.5)	16.6 (9.4 to 25.8)	15.3 (10.4 to 20.8)	17.7 (13.8 to 21.6)	-0.8 (-4.1 to 2.4)
Other meningitis	402.9 (304.5 to 514.0)	45.4 (30.8 to 63.0)	40.0 (26.9 to 54.8)	41.5 (35.6 to 48.4)	19.2 (14.2 to 25.0)
Encephalitis	1,280.9 (878.7 to 1,842.3)	322.5 (239.5 to 423.4)	160.5 (114.0 to 212.7)	11.5 (4.9 to 17.5)	-6.1 (-11.7 to -1.0)
Diphtheria	< 0.05 (< 0.05 to < 0.05)	0.1 (0.1 to 0.2)	< 0.05 (< 0.05 to < 0.05)	-47.2 (-60.4 to -29.1)	-55.5 (-66.7 to -40.3)
Whooping cough	2.7 (2.1 to 3.5)	20.0 (15.6 to 25.2)	0.1 (0.1 to 0.2)	-16.2 (-18.2 to -14.2)	-29.4 (-31.1 to -27.7)
Tetanus	12.6 (12.4 to 12.8)	3.1 (1.7 to 5.0)	0.3 (0.1 to 0.4)	7.4 (-17.4 to 38.9)	-9.5 (-30.4 to 17.0)
Measles	0.6 (0.2 to 1.4)	23.3 (8.4 to 50.1)	0.1 (< 0.05 to 0.1)	-56.7 (-61.5 to -51.2)	-63.5 (-67.6 to -58.9)
Varicella and herpes zoster	346.2 (277.0 to 428.6)	5,579.4 (3,758.8 to 7,608.1)	15.5 (8.6 to 25.4)	19.8 (12.3 to 27.3)	0.9 (-5.4 to 7.2)
**Neglected tropical diseases and malaria**	**164,138.2 (159,117.0 to 168,667.7)**	**26,644.2† (22,266.1 to 31,999.2)**	**1,169.7 (792.5 to 1,658.6)**	**-8.2 (-14.4 to -1.4)**	**-22.7 (-27.9 to -17.0)**
Malaria	8,489.4 (7,273.2 to 9,983.4)	8,462.6 (6,344.5 to 10,808.3)	112.1 (85.0 to 141.7)	-3.3 (-9.9 to 3.6)	-18.5 (-24.1 to -12.7)
Chagas disease	556.0 (451.3 to 684.5)	15.9 (10.5 to 22.6)	3.2 (2.0 to 4.8)	-10.6 (-18.7 to -1.7)	-24.7 (-31.6 to -17.2)
Leishmaniasis	421.9 (375.5 to 477.6)	50.1 (36.2 to 67.9)	25.1 (16.4 to 36.4)	26.9 (8.6 to 50.3)	6.9 (-8.5 to 26.6)
Visceral leishmaniasis	1.4 (0.9 to 1.9)	5.5 (3.7 to 7.6)	0.1 (0.1 to 0.2)	-55.7 (-70.5 to -33.3)	-62.7 (-75.2 to -43.8)
Cutaneous and mucocutaneous leishmaniasis	389.0 (334.3 to 453.9)	44.7 (31.2 to 62.5)	25.0 (16.3 to 36.3)	27.9 (9.2 to 51.9)	7.7 (-8.1 to 27.9)
African trypanosomiasis	0.7 (0.1 to 2.2)	0.5 (0.1 to 1.5)	0.2 (< 0.05 to 0.6)	-71.9 (-94.9 to 69.6)	-76.3 (-95.7 to 42.9)
Schistosomiasis	23,066.1 (20,996.5 to 25,128.5)	6,712.6 (5,682.6 to 8,117.7)	192.3 (97.3 to 354.4)	-22.6 (-25.1 to -19.6)	-34.8 (-36.9 to -32.3)
Cysticercosis	239.6 (155.6 to 339.4)	--	39.9 (22.3 to 62.5)	-8.4 (-19.9 to 1.4)	-22.8 (-32.5 to -14.6)
Cystic echinococcosis	86.9 (44.4 to 192.3)	18.7 (8.0 to 49.0)	8.2 (3.5 to 18.8)	-37.4 (-76.2 to 64.8)	-47.2 (-80.0 to 38.8)
Lymphatic filariasis	2,538.9 (2,125.2 to 3,120.6)	750.4 (433.7 to 1,165.0)	101.4 (49.2 to 185.3)	-42.2 (-56.5 to -31.2)	-51.4 (-63.3 to -42.0)
Onchocerciasis	1,785.9 (1,046.9 to 3,132.8)	393.4 (169.4 to 774.2)	100.2 (43.5 to 180.0)	-20.3 (-40.6 to -0.7)	-32.8 (-50.0 to -16.3)
Trachoma	11.3 (5.2 to 22.1)	--	1.2 (0.6 to 2.1)	-26.7 (-45.8 to -5.8)	-38.2 (-54.4 to -20.6)
Dengue	515.1 (280.7 to 872.3)	8,610.4 (5,239.3 to 13,057.8)	84.6 (37.2 to 158.3)	78.0 (43.9 to 302.2)	50.0 (21.2 to 238.8)
Yellow fever	0.2 (0.1 to 0.6)	8.8 (2.4 to 23.5)	< 0.05 (< 0.05 to < 0.05)	-18.6 (-46.8 to 26.8)	-31.4 (-55.1 to 6.8)
Rabies	< 0.05 (< 0.05 to < 0.05)	0.4 (0.2 to 0.5)	< 0.05 (< 0.05 to < 0.05)	-47.8 (-57.4 to -34.8)	-56.0 (-64.1 to -45.1)
Intestinal nematode infections	135,889.4 (129,780.3 to 141,060.3)	--	264.5 (152.8 to 434.7)	-11.9 (-18.6 to -5.0)	-25.8 (-31.4 to -20.0)
Ascariasis	72,190.2 (65,074.7 to 80,152.0)	--	78.1 (41.5 to 133.0)	-26.4 (-35.8 to -15.4)	-38.0 (-45.9 to -28.7)

Cause	Prevalence in thousands (95% UI)	Incidence in thousands (95% UI)	YLDs in thousands (95% UI)		
	2016 counts	2016 counts	2016 counts	Percentage change in counts between 2006 and 2016	Percentage change in rates between 2006 and 2016
Trichuriasis	38,497.6 (35,492.8 to 42,103.0)	--	30.1 (16.4 to 52.0)	-19.5 (-31.0 to -7.5)	-32.2 (-41.9 to -22.1)
Hookworm disease	41,879.1 (38,661.3 to 45,524.9)	--	156.3 (92.6 to 248.7)	-0.2 (-10.3 to 9.9)	-16.0 (-24.4 to -7.4)
Food-borne trematodiases	8,494.3 (7,979.9 to 9,078.3)	904.9 (678.5 to 1,080.9)	208.2 (99.3 to 390.1)	34.2 (26.2 to 45.3)	13.0 (6.3 to 22.4)
Leprosy	18.9 (17.6 to 20.4)	4.4 (4.1 to 4.8)	1.2 (0.8 to 1.7)	-25.1 (-34.0 to -14.4)	-36.9 (-44.4 to -27.9)
Ebola virus disease	0.1 (< 0.05 to 0.5)	--	< 0.05 (< 0.05 to 0.1)	--	--
Zika virus disease	11.8 (6.9 to 19.9)	711.1 (414.9 to 1,207.6)	0.2 (0.1 to 0.4)	--	--
Guinea worm disease	< 0.05 (< 0.05 to < 0.05)	< 0.05 (< 0.05 to < 0.05)	< 0.05 (< 0.05 to < 0.05)	-99.5 (-99.7 to -99.3)	-99.6 (-99.7 to -99.4)
Other neglected tropical diseases	1,717.0 (1,478.0 to 1,997.8)	--	27.3 (17.2 to 42.0)	21.4 (14.6 to 29.2)	2.2 (-3.5 to 8.9)
**Maternal disorders**	**2,260.3 (2,062.8 to 2,451.5)**	**34,459.2† (28,176.6 to 43,379.6)**	**214.5 (143.5 to 296.7)**	**0.9 (-14.3 to 19.9)**	**-15.0 (-27.8 to 1.0)**
Maternal haemorrhage	405.0 (304.2 to 538.8)	2,988.8 (2,224.7 to 4,007.8)	17.8 (11.0 to 26.8)	13.5 (-12.9 to 49.0)	-4.4 (-26.7 to 25.5)
Maternal sepsis and other pregnancy related infections	327.7 (214.2 to 477.9)	2,792.6 (2,037.5 to 3,816.3)	11.7 (5.4 to 22.5)	10.3 (-44.0 to 118.7)	-7.1 (-52.8 to 84.2)
Maternal hypertensive disorders	1,233.7 (765.9 to 1,762.1)	5,746.2 (4,611.2 to 6,924.4)	60.0 (31.3 to 100.5)	19.7 (-24.7 to 90.1)	0.9 (-36.5 to 60.1)
Maternal obstructed labour and uterine rupture	227.4 (179.7 to 282.0)	2,239.2 (1,648.8 to 3,032.9)	74.0 (47.1 to 107.5)	-13.1 (-21.4 to -4.2)	-26.8 (-33.8 to -19.3)
Maternal abortion, miscarriage, and ectopic pregnancy	169.8 (101.3 to 265.8)	20,692.3 (14,869.5 to 29,078.7)	18.6 (9.7 to 32.3)	5.0 (-34.9 to 65.5)	-11.5 (-45.1 to 39.4)
Other maternal disorders	--	--	32.4 (21.7 to 44.8)	-2.7 (-17.4 to 15.6)	-18.1 (-30.4 to -2.6)
**Neonatal disorders**	**6,156.6 (5,643.0 to 6,741.8)**	**--**	**1,190.3 (839.9 to 1,678.5)**	**32.3 (18.2 to 49.4)**	**11.5 (-0.4 to 25.8)**
Neonatal preterm birth complications	5,117.1 (4,286.6 to 6,130.0)	--	781.6 (543.6 to 1,110.7)	29.7 (8.5 to 55.2)	9.3 (-8.6 to 30.7)
Neonatal encephalopathy due to birth asphyxia and trauma	962.7 (595.3 to 1,559.7)	--	140.8 (51.9 to 327.9)	39.8 (28.8 to 60.5)	17.8 (8.5 to 35.2)
Neonatal sepsis and other neonatal infections	531.7 (197.8 to 1,275.2)	--	208.1 (62.3 to 540.3)	39.2 (28.4 to 59.4)	17.3 (8.1 to 34.3)
Hemolytic disease and other neonatal jaundice	183.0 (162.3 to 204.1)	--	59.8 (45.6 to 75.8)	27.8 (21.6 to 34.5)	7.7 (2.4 to 13.2)
Other neonatal disorders	--	--	--	--	--
**Nutritional deficiencies**	**121,322.7 (116,682.3 to 125,793.9)**	**38,753.5† (32,028.1 to 48,188.0)**	**2,422.6 (1,588.0 to 3,545.4)**	**10.3 (6.6 to 14.0)**	**-7.1 (-10.2 to -4.0)**
Protein-energy malnutrition	--	--	--	--	--
Iodine deficiency	11,871.9 (10,485.8 to 13,413.7)	55.4 (45.9 to 66.2)	321.9 (215.9 to 454.1)	-4.9 (-7.8 to -2.1)	-19.9 (-22.3 to -17.5)
Vitamin A deficiency	34,866.7 (29,703.9 to 41,691.3)	38,698.1 (31,979.9 to 48,134.6)	14.8 (8.9 to 23.3)	20.2 (12.3 to 28.3)	1.3 (-5.4 to 8.1)
Iron-deficiency anaemia	88,606.2 (78,161.7 to 101,225.6)	--	2,085.9 (1,344.4 to 3,083.1)	13.0 (8.9 to 17.2)	-4.8 (-8.2 to -1.3)
Other nutritional deficiencies	--	--	--	--	--
**Other communicable, maternal, neonatal, and nutritional diseases**	**188,076.3 (182,772.7 to 193,464.2)**	**121,018.8 (101,607.4 to 144,504.9)**	**510.2 (320.8 to 811.4)**	**16.9 (11.4 to 21.8)**	**-1.5 (-6.2 to 2.6)**
Sexually transmitted diseases excluding HIV	145,837.1 (140,132.1 to 152,204.0)	105,013.6 (86,951.9 to 126,582.0)	437.9 (267.7 to 707.6)	20.0 (16.9 to 23.2)	1.1 (-1.6 to 3.8)
Syphilis	9,567.3 (6,209.5 to 14,297.6)	6,105.3 (3,844.4 to 9,870.9)	7.6 (5.0 to 11.4)	20.8 (15.2 to 25.8)	1.8 (-3.0 to 6.0)
Chlamydial infection	28,298.7 (19,952.4 to 38,841.4)	21,046.4 (14,941.0 to 29,457.2)	134.5 (74.9 to 226.1)	20.6 (17.0 to 24.9)	1.6 (-1.5 to 5.2)
Gonococcal infection	10,279.3 (6,575.0 to 15,366.2)	35,375.1 (22,289.0 to 53,547.6)	100.1 (53.6 to 171.8)	22.5 (13.6 to 29.5)	3.1 (-4.3 to 9.1)
Trichomoniasis	28,885.3 (20,367.8 to 38,891.1)	24,644.0 (16,773.9 to 34,299.3)	33.9 (13.1 to 73.1)	18.5 (16.3 to 20.6)	-0.2 (-2.0 to 1.6)
Genital herpes	83,443.8 (69,739.4 to 99,385.5)	11,564.7 (8,890.4 to 15,112.4)	23.4 (7.8 to 52.6)	17.5 (14.7 to 20.6)	-1.1 (-3.4 to 1.6)
Other sexually transmitted diseases	4,625.8 (4,142.5 to 5,194.4)	6,278.1 (4,656.2 to 8,456.4)	138.3 (90.0 to 211.0)	18.3 (14.5 to 21.9)	-0.3 (-3.6 to 2.7)
Hepatitis	55,363.2 (46,621.6 to 65,980.6)	16,004.6 (10,125.7 to 25,156.9)	26.0 (13.5 to 44.9)	-16.1 (-53.1 to 46.4)	-29.4 (-60.5 to 23.3)
Acute hepatitis A	145.4 (13.4 to 359.6)	1,890.1 (174.3 to 4,674.8)	6.2 (0.5 to 16.1)	-19.4 (-92.8 to 677.8)	-32.1 (-94.0 to 555.2)
Hepatitis B	43,327.7 (34,585.3 to 54,040.5)	12,064.4 (6,802.9 to 20,740.8)	15.2 (7.0 to 30.2)	-12.4 (-53.6 to 73.5)	-26.2 (-60.9 to 46.1)
Hepatitis C	11,747.6 (10,373.4 to 13,193.5)	197.1 (134.7 to 274.9)	0.2 (0.1 to 0.4)	-11.3 (-11.9 to -10.6)	-25.2 (-25.8 to -24.7)
Acute hepatitis E	142.5 (110.1 to 179.7)	1,853.0 (1,431.9 to 2,336.4)	4.4 (2.6 to 6.9)	-23.3 (-39.7 to -4.6)	-35.4 (-49.2 to -19.7)
Other infectious diseases	1,996.5 (1,716.3 to 2,336.3)	0.5 (0.3 to 0.9)	46.3 (28.6 to 72.1)	14.6 (10.1 to 19.1)	-3.5 (-7.3 to 0.4)
**Non-communicable diseases**	**559,300.1 (555,488.3 to 562,516.6)**	**1,414,856.4† (1,252,112.2 to 1,608,214.9)**	**44,650.6 (32,813.5 to 58,147.0)**	**16.7 (16.1 to 17.2)**	**-1.7 (-2.2 to -1.2)**
**Neoplasms**	**378.7 (364.2 to 392.7)**	**215.5 (202.1 to 239.5)**	**71.6 (51.8 to 95.0)**	**26.9 (22.8 to 32.2)**	**6.9 (3.4 to 11.4)**
Lip and oral cavity cancer	24.1 (22.4 to 26.0)	6.5 (6.0 to 7.0)	2.5 (1.8 to 3.4)	20.8 (8.2 to 34.3)	1.8 (-8.8 to 13.1)
Nasopharynx cancer	13.1 (11.8 to 14.6)	3.7 (3.4 to 4.1)	1.6 (1.1 to 2.1)	15.9 (1.5 to 32.4)	-2.4 (-14.5 to 11.5)
Other pharynx cancer	3.4 (3.0 to 3.7)	1.3 (1.2 to 1.5)	0.5 (0.3 to 0.6)	14.4 (0.7 to 26.2)	-3.6 (-15.2 to 6.4)
Oesophageal cancer	1.7 (1.6 to 1.8)	1.4 (1.4 to 1.5)	0.4 (0.3 to 0.5)	2.2 (-3.8 to 8.9)	-13.9 (-18.9 to -8.3)
Stomach cancer	13.4 (12.8 to 14.0)	7.4 (7.1 to 7.8)	2.1 (1.5 to 2.7)	8.1 (2.9 to 13.7)	-8.9 (-13.3 to -4.2)
Colon and rectum cancer	33.8 (31.9 to 35.9)	9.5 (9.0 to 10.1)	3.6 (2.6 to 4.8)	28.8 (18.6 to 37.7)	8.5 (-0.1 to 16.0)
Liver cancer	8.8 (7.4 to 10.2)	10.4 (9.5 to 11.5)	2.4 (1.7 to 3.3)	31.3 (17.4 to 47.0)	10.6 (-1.1 to 23.9)
Liver cancer due to hepatitis B	8.4 (6.7 to 10.1)	6.5 (5.2 to 7.7)	1.5 (1.0 to 2.1)	31.1 (16.2 to 47.9)	10.4 (-2.1 to 24.6)

Cause	Prevalence in thousands (95% UI)	Incidence in thousands (95% UI)	YLDs in thousands (95% UI)		
	2016 counts	2016 counts	2016 counts	Percentage change in counts between 2006 and 2016	Percentage change in rates between 2006 and 2016
Liver cancer due to hepatitis C	0.6 (0.4 to 0.8)	0.5 (0.3 to 0.7)	0.1 (0.1 to 0.2)	28.5 (14.0 to 46.4)	8.2 (-4.0 to 23.3)
Liver cancer due to alcohol use	0.3 (0.2 to 0.5)	0.3 (0.1 to 0.4)	0.1 (< 0.05 to 0.1)	22.2 (2.2 to 41.6)	2.9 (-13.9 to 19.2)
Liver cancer due to other causes	4.2 (3.2 to 5.6)	3.2 (2.4 to 4.2)	0.7 (0.5 to 1.1)	33.0 (19.4 to 50.1)	12.0 (0.6 to 26.4)
Gallbladder and biliary tract cancer	0.5 (0.4 to 0.5)	0.6 (0.5 to 0.6)	0.1 (0.1 to 0.2)	8.1 (-2.1 to 19.3)	-9.0 (-17.6 to 0.5)
Pancreatic cancer	0.9 (0.9 to 0.9)	1.1 (1.1 to 1.2)	0.2 (0.2 to 0.3)	22.4 (16.8 to 28.1)	3.1 (-1.6 to 7.9)
Larynx cancer	2.2 (2.1 to 2.3)	0.7 (0.6 to 0.7)	0.3 (0.2 to 0.4)	15.1 (9.0 to 22.4)	-3.0 (-8.2 to 3.1)
Tracheal, bronchus, and lung cancer	7.6 (7.1 to 8.1)	5.8 (5.4 to 6.2)	1.5 (1.1 to 2.0)	20.1 (12.2 to 28.8)	1.2 (-5.5 to 8.5)
Malignant skin melanoma	45.8 (40.1 to 56.1)	9.3 (8.2 to 11.3)	3.0 (2.0 to 4.3)	32.9 (21.7 to 44.4)	12.0 (2.5 to 21.6)
Non-melanoma skin cancer	1.7 (0.6 to 4.1)	14.0 (4.5 to 36.8)	0.1 (< 0.05 to 0.1)	44.3 (24.6 to 63.1)	21.6 (4.9 to 37.4)
Non-melanoma skin cancer (squamous-cell carcinoma)	1.0 (0.3 to 2.5)	1.4 (0.4 to 3.8)	< 0.05 (< 0.05 to 0.1)	55.8 (34.7 to 71.0)	31.2 (13.4 to 44.1)
Non-melanoma skin cancer (basal-cell carcinoma)	1.4 (0.4 to 3.9)	12.6 (3.4 to 34.9)	< 0.05 (< 0.05 to < 0.05)	16.8 (5.4 to 24.4)	-1.6 (-11.2 to 4.8)
Breast cancer	94.2 (79.0 to 104.4)	19.8 (16.7 to 21.9)	8.3 (5.8 to 11.4)	33.4 (17.3 to 51.0)	12.3 (-1.2 to 27.2)
Cervical cancer	66.8 (50.1 to 73.8)	17.4 (13.3 to 18.9)	6.5 (4.4 to 8.8)	2.9 (-7.2 to 16.4)	-13.3 (-21.8 to -1.9)
Uterine cancer	13.1 (11.9 to 14.6)	2.7 (2.5 to 3.1)	1.0 (0.7 to 1.4)	39.0 (18.7 to 61.0)	17.1 (0.0 to 35.7)
Ovarian cancer	18.7 (16.9 to 20.0)	6.0 (5.5 to 6.4)	3.0 (2.1 to 4.0)	24.8 (12.9 to 34.6)	5.1 (-4.9 to 13.4)
Prostate cancer	4.8 (3.7 to 5.2)	1.1 (0.9 to 1.2)	0.4 (0.3 to 0.6)	54.9 (38.4 to 66.3)	30.5 (16.6 to 40.1)
Testicular cancer	70.5 (66.3 to 75.0)	13.7 (12.9 to 14.6)	4.8 (3.3 to 6.7)	37.3 (25.5 to 49.6)	15.7 (5.7 to 26.0)
Kidney cancer	15.3 (14.1 to 16.0)	4.0 (3.8 to 4.2)	1.4 (1.0 to 1.9)	37.3 (31.2 to 43.3)	15.7 (10.5 to 20.7)
Bladder cancer	7.3 (6.7 to 7.9)	1.8 (1.6 to 1.9)	0.7 (0.5 to 1.0)	35.5 (22.1 to 49.8)	14.1 (2.8 to 26.2)
Brain and nervous system cancer	29.5 (25.8 to 32.6)	13.0 (11.6 to 14.4)	4.2 (2.9 to 5.6)	49.1 (37.4 to 63.5)	25.6 (15.8 to 37.7)
Thyroid cancer	45.8 (42.5 to 50.9)	8.7 (8.1 to 9.6)	2.9 (1.9 to 4.1)	61.5 (43.2 to 83.8)	36.0 (20.6 to 54.8)
Mesothelioma	0.7 (0.6 to 0.7)	0.4 (0.4 to 0.5)	0.1 (0.1 to 0.2)	13.0 (5.2 to 22.2)	-4.8 (-11.3 to 2.9)
Hodgkin lymphoma	30.3 (27.6 to 35.5)	7.3 (6.6 to 8.5)	2.9 (2.0 to 3.9)	14.1 (5.7 to 23.1)	-3.9 (-10.9 to 3.7)
Non-Hodgkin's lymphoma	26.4 (23.6 to 27.9)	8.2 (7.3 to 8.6)	2.7 (1.9 to 3.6)	26.7 (18.9 to 33.8)	6.8 (0.1 to 12.7)
Multiple myeloma	2.0 (1.4 to 2.3)	0.9 (0.7 to 0.9)	0.5 (0.3 to 0.6)	69.4 (45.8 to 86.2)	42.7 (22.8 to 56.9)
Leukaemia	49.2 (42.0 to 52.8)	14.6 (12.6 to 15.5)	6.6 (4.6 to 8.8)	9.8 (3.5 to 16.8)	-7.5 (-12.8 to -1.6)
Acute lymphoid leukaemia	9.6 (8.2 to 10.8)	2.8 (2.4 to 3.1)	1.3 (0.9 to 1.8)	21.8 (7.8 to 31.0)	2.6 (-9.2 to 10.3)
Chronic lymphoid leukaemia	7.6 (6.6 to 8.5)	1.7 (1.4 to 1.9)	0.9 (0.7 to 1.3)	36.6 (21.0 to 51.9)	15.0 (1.9 to 27.9)
Acute myeloid leukaemia	4.6 (4.0 to 5.1)	3.5 (3.0 to 3.9)	1.0 (0.7 to 1.3)	18.6 (11.7 to 24.6)	-0.1 (-5.9 to 5.0)
Chronic myeloid leukaemia	4.7 (4.0 to 5.2)	1.7 (1.4 to 1.8)	0.6 (0.4 to 0.8)	-2.4 (-11.3 to 8.4)	-17.8 (-25.2 to -8.7)
Other leukaemia	22.7 (17.7 to 24.6)	5.0 (3.9 to 5.4)	2.8 (1.9 to 3.8)	-1.0 (-9.8 to 7.8)	-16.6 (-24.0 to -9.2)
Other neoplasms	82.6 (73.8 to 88.5)	24.1 (21.5 to 25.7)	7.3 (5.2 to 9.9)	50.3 (39.6 to 61.5)	26.6 (17.6 to 36.0)
**Cardiovascular diseases**	**10,398.9 (9,958.3 to 10,863.9)**	**610.4† (545.7 to 669.5)**	**703.8 (483.2 to 988.6)**	**19.2 (17.0 to 21.5)**	**0.4 (-1.4 to 2.4)**
Rheumatic heart disease	3,355.5 (3,013.1 to 3,742.8)	89.1 (80.2 to 100.2)	159.3 (99.4 to 235.1)	18.8 (14.5 to 23.0)	0.1 (-3.6 to 3.6)
Ischaemic heart disease	1,852.1 (1,685.0 to 2,048.8)	287.5 (251.6 to 324.9)	107.0 (70.5 to 156.2)	20.2 (16.0 to 24.5)	1.2 (-2.3 to 4.9)
Cerebrovascular disease	874.6 (806.5 to 975.9)	68.2‡ (40.4 to 103.4)	161.3 (111.2 to 216.3)	16.5 (10.1 to 23.0)	-1.9 (-7.3 to 3.6)
Ischaemic stroke	749.3 (604.5 to 912.6)	43.6‡ (20.3 to 77.0)	118.4 (80.5 to 161.7)	17.7 (9.1 to 26.3)	-0.8 (-8.1 to 6.4)
Hemorrhagic stroke	267.4 (213.1 to 327.1)	24.6‡ (12.7 to 40.7)	43.0 (28.7 to 60.7)	13.2 (4.7 to 22.1)	-4.7 (-11.8 to 2.9)
Hypertensive heart disease	0.8 (0.4 to 1.3)	--	0.1 (< 0.05 to 0.1)	20.5 (12.6 to 27.5)	1.5 (-5.1 to 7.4)
Cardiomyopathy and myocarditis	402.8 (357.7 to 458.9)	118.5† (85.1 to 157.7)	42.4 (25.5 to 66.0)	20.5 (15.1 to 26.0)	1.5 (-3.0 to 6.2)
Myocarditis	224.7 (172.3 to 285.0)	118.5 (85.1 to 157.7)	18.6 (11.2 to 28.7)	23.8 (15.8 to 32.1)	4.3 (-2.5 to 11.3)
Alcoholic cardiomyopathy	< 0.05 (< 0.05 to < 0.05)	--	< 0.05 (< 0.05 to < 0.05)	3.0 (-6.5 to 13.5)	-13.3 (-21.2 to -4.4)
Other cardiomyopathy	273.6 (201.1 to 357.3)	--	23.8 (14.0 to 36.8)	18.0 (10.7 to 25.8)	-0.6 (-6.8 to 6.0)
Atrial fibrillation and flutter	--	--	--	--	--
Peripheral vascular disease	--	--	--	--	--
Endocarditis	34.5 (24.0 to 46.2)	47.1 (32.0 to 67.9)	3.0 (1.7 to 4.8)	17.4 (15.0 to 19.7)	-1.1 (-3.2 to 0.8)
Other cardiovascular and circulatory diseases	4,261.8 (2,826.8 to 5,937.4)	--	230.7 (127.8 to 366.1)	20.8 (17.1 to 24.6)	1.8 (-1.3 to 4.9)
**Chronic respiratory diseases**	**23,138.3 (22,132.6 to 23,913.7)**	**4,750.4† (4,034.2 to 5,552.9)**	**1,120.6 (834.1 to 1,462.3)**	**20.5 (18.4 to 22.5)**	**1.5 (-0.3 to 3.2)**
Chronic obstructive pulmonary disease	4,357.8 (4,047.5 to 4,733.4)	361.1 (302.4 to 425.1)	320.8 (273.3 to 361.9)	19.7 (15.0 to 24.1)	0.8 (-3.2 to 4.6)
Pneumoconiosis	7.5 (6.3 to 8.6)	0.8 (0.6 to 1.1)	1.2 (0.8 to 1.7)	14.4 (12.9 to 15.9)	-3.6 (-4.9 to -2.3)
Silicosis	1.8 (1.4 to 2.4)	0.3 (0.2 to 0.4)	0.3 (0.2 to 0.4)	17.4 (15.5 to 19.1)	-1.1 (-2.7 to 0.3)

Cause	Prevalence in thousands (95% UI)	Incidence in thousands (95% UI)	YLDs in thousands (95% UI)		
	2016 counts	2016 counts	2016 counts	Percentage change in counts between 2006 and 2016	Percentage change in rates between 2006 and 2016
Asbestosis	2.1 (1.5 to 2.9)	0.2 (0.1 to 0.3)	0.3 (0.2 to 0.5)	15.3 (12.5 to 17.8)	-2.9 (-5.3 to -0.7)
Coal workers pneumoconiosis	0.7 (0.5 to 0.9)	0.1 (< 0.05 to 0.1)	0.1 (0.1 to 0.2)	10.9 (3.3 to 17.9)	-6.6 (-13.0 to -0.7)
Other pneumoconiosis	2.8 (2.1 to 3.6)	0.3 (0.1 to 0.5)	0.4 (0.3 to 0.7)	12.8 (9.6 to 15.7)	-5.0 (-7.7 to -2.5)
Asthma	18,876.1 (16,803.7 to 21,362.9)	4,384.6 (3,687.4 to 5,201.2)	746.5 (485.1 to 1,076.3)	19.5 (17.4 to 21.6)	0.6 (-1.1 to 2.4)
Interstitial lung disease and pulmonary sarcoidosis	71.0 (59.0 to 83.4)	3.9 (2.6 to 5.7)	8.0 (5.0 to 11.9)	15.8 (12.0 to 19.5)	-2.5 (-5.7 to 0.7)
Other chronic respiratory diseases	--	--	44.2 (37.6 to 49.9)	52.3 (46.5 to 57.8)	28.3 (23.4 to 32.9)
**Cirrhosis and other chronic liver diseases**	**2,051.6 (1,844.8 to 2,277.4)**	**32.2 (25.6 to 40.1)**	**74.2 (50.5 to 104.2)**	**21.2 (15.4 to 26.9)**	**2.1 (-2.8 to 6.9)**
Cirrhosis and other chronic liver diseases due to hepatitis B	565.1 (430.5 to 708.6)	10.0 (7.0 to 13.9)	21.3 (13.6 to 32.0)	22.3 (11.8 to 34.5)	3.1 (-5.9 to 13.3)
Cirrhosis and other chronic liver diseases due to hepatitis C	425.3 (324.1 to 548.3)	6.9 (4.9 to 9.5)	15.5 (9.6 to 24.0)	20.7 (8.3 to 34.0)	1.7 (-8.8 to 12.9)
Cirrhosis and other chronic liver diseases due to alcohol use	231.1 (170.1 to 304.5)	3.7 (2.5 to 5.4)	8.2 (4.9 to 12.5)	18.1 (4.8 to 32.2)	-0.5 (-11.7 to 11.4)
Cirrhosis and other chronic liver diseases due to other causes	830.1 (658.1 to 1,019.0)	11.6 (8.4 to 15.2)	29.2 (19.0 to 42.8)	21.5 (12.8 to 31.1)	2.4 (-5.0 to 10.4)
**Digestive diseases**	**10,523.2 (9,921.6 to 11,190.6)**	**9,512.2† (8,297.8 to 10,874.2)**	**285.8 (194.1 to 397.7)**	**19.2 (15.0 to 23.8)**	**0.4 (-3.2 to 4.3)**
Peptic ulcer disease	3,493.6 (2,770.8 to 4,350.0)	873.9 (633.5 to 1,178.5)	52.1 (30.5 to 83.9)	29.8 (21.0 to 38.1)	9.4 (1.9 to 16.3)
Gastritis and duodenitis	3,679.7 (2,720.7 to 4,826.6)	3,051.5 (2,188.9 to 4,107.5)	47.0 (27.9 to 73.2)	32.6 (24.2 to 40.9)	11.7 (4.6 to 18.7)
Appendicitis	80.5 (64.2 to 99.9)	2,056.4 (1,653.9 to 2,527.8)	24.9 (15.5 to 37.3)	13.6 (1.5 to 25.7)	-4.3 (-14.5 to 5.9)
Paralytic ileus and intestinal obstruction	7.1 (5.0 to 9.8)	189.4 (134.2 to 262.2)	2.3 (1.3 to 3.5)	20.8 (18.0 to 23.2)	1.7 (-0.6 to 3.8)
Inguinal, femoral, and abdominal hernia	2,461.4 (1,722.0 to 3,366.5)	952.5 (651.8 to 1,348.5)	87.6 (51.5 to 135.7)	9.9 (4.3 to 15.8)	-7.4 (-12.1 to -2.5)
Inflammatory bowel disease	280.5 (240.1 to 325.2)	371.4 (308.6 to 452.5)	42.4 (27.8 to 59.4)	18.1 (12.5 to 24.5)	-0.5 (-5.2 to 4.9)
Vascular intestinal disorders	2.3 (1.3 to 3.6)	15.5 (9.8 to 22.5)	0.7 (0.4 to 1.3)	20.2 (17.7 to 22.2)	1.2 (-0.9 to 2.9)
Gallbladder and biliary diseases	899.9 (623.5 to 1,225.0)	1,790.1 (1,289.5 to 2,441.3)	8.9 (5.2 to 14.3)	15.0 (10.6 to 19.6)	-3.2 (-6.8 to 0.8)
Pancreatitis	23.4 (17.2 to 30.5)	211.4 (153.6 to 275.6)	3.0 (1.9 to 4.6)	20.1 (14.0 to 25.4)	1.2 (-4.0 to 5.6)
Other digestive diseases	--	--	16.9 (11.5 to 23.5)	20.4 (16.2 to 25.1)	1.4 (-2.1 to 5.4)
**Neurological disorders**	**290,050.9 (268,106.6 to 313,303.8)**	**149,098.9 (98,023.7 to 217,452.8)**	**6,331.4 (4,167.8 to 8,754.8)**	**16.1 (14.4 to 17.8)**	**-2.2 (-3.6 to -0.8)**
Alzheimer's disease and other dementias	--	--	--	--	--
Parkinson's disease	9.5 (4.4 to 17.3)	2.4 (1.1 to 4.5)	1.3 (0.5 to 2.5)	22.4 (12.9 to 31.6)	3.1 (-4.9 to 10.8)
Epilepsy	1,910.3 (1,524.2 to 2,328.8)	141.9 (97.3 to 192.9)	613.8 (400.2 to 861.9)	10.7 (-0.7 to 24.5)	-6.7 (-16.3 to 4.9)
Multiple sclerosis	141.3 (117.6 to 166.5)	15.2 (11.1 to 19.2)	38.8 (26.1 to 52.5)	20.0 (12.7 to 27.7)	1.1 (-5.0 to 7.6)
Motor neuron disease	17.1 (14.1 to 20.7)	0.6 (0.3 to 0.9)	3.6 (2.4 to 5.1)	20.0 (18.7 to 21.3)	1.1 (0.0 to 2.2)
Migraine	117,510.8 (108,194.4 to 127,095.3)	12,891.3 (10,923.6 to 14,861.6)	4,876.7 (3,069.8 to 6,954.1)	16.4 (15.3 to 17.5)	-2.0 (-2.9 to -1.1)
Tension-type headache	224,855.1 (166,445.5 to 291,409.9)	136,044.6 (84,517.7 to 204,119.9)	700.8 (416.2 to 1,076.6)	16.7 (14.9 to 18.9)	-1.7 (-3.2 to 0.1)
Other neurological disorders	1.4 (0.7 to 2.4)	2.9 (1.5 to 4.8)	96.4 (64.2 to 134.3)	35.0 (22.1 to 50.9)	13.7 (2.9 to 27.1)
**Mental and substance use disorders**	**112,821.4 (110,736.8 to 115,185.7)**	**37,773.5† (32,925.3 to 43,508.9)**	**16,508.7 (12,056.5 to 21,443.3)**	**16.6 (15.7 to 17.4)**	**-1.8 (-2.6 to -1.1)**
Schizophrenia	2,414.2 (1,878.5 to 3,003.6)	224.5 (158.2 to 300.7)	1,604.9 (1,082.4 to 2,133.5)	22.0 (19.2 to 24.7)	2.8 (0.4 to 5.0)
Alcohol use disorders	15,170.1 (12,244.1 to 18,521.6)	8,148.9 (6,401.4 to 10,192.1)	1,544.5 (1,033.7 to 2,237.1)	13.5 (9.9 to 16.8)	-4.4 (-7.4 to -1.6)
Drug use disorders	11,929.6 (11,338.5 to 12,524.5)	912.2 (750.9 to 1,087.4)	3,008.9 (2,107.0 to 4,035.0)	19.2 (16.4 to 21.8)	0.5 (-2.0 to 2.6)
Opioid use disorders	5,658.0 (4,709.3 to 6,890.4)	477.4 (376.3 to 607.6)	2,397.8 (1,622.5 to 3,257.1)	19.7 (16.1 to 22.7)	0.9 (-2.2 to 3.4)
Cocaine use disorders	802.9 (705.7 to 920.5)	23.9 (16.9 to 31.5)	111.3 (69.5 to 159.9)	15.1 (10.3 to 20.0)	-3.1 (-7.1 to 1.1)
Amphetamine use disorders	1,112.3 (802.3 to 1,477.3)	24.2 (12.2 to 41.0)	147.5 (85.5 to 235.2)	18.4 (11.3 to 25.0)	-0.3 (-6.2 to 5.3)
Cannabis use disorders	4,071.2 (3,067.3 to 5,269.0)	278.3 (169.5 to 411.5)	119.4 (69.9 to 185.7)	17.6 (13.3 to 22.3)	-0.9 (-4.6 to 3.0)
Other drug use disorders	586.9 (503.4 to 688.3)	108.5 (84.0 to 132.6)	233.0 (155.5 to 329.5)	17.7 (13.8 to 21.8)	-0.9 (-4.2 to 2.6)
Depressive disorders	22,360.7 (21,042.3 to 23,354.7)	22,988.2 (18,447.3 to 28,324.4)	3,847.8 (2,603.9 to 5,428.1)	12.6 (10.7 to 14.3)	-5.1 (-6.8 to -3.7)
Major depressive disorder	14,418.0 (11,812.8 to 17,698.4)	21,492.6 (16,949.7 to 26,766.3)	3,021.9 (2,002.1 to 4,372.4)	10.8 (8.4 to 12.8)	-6.7 (-8.7 to -5.0)
Dysthymia	8,380.5 (6,506.0 to 10,800.0)	1,495.6 (1,209.5 to 1,824.9)	825.9 (520.0 to 1,281.5)	19.9 (16.9 to 22.9)	1.0 (-1.6 to 3.6)
Bipolar disorder	4,842.1 (3,974.0 to 5,941.2)	250.2 (155.7 to 360.9)	1,009.1 (612.2 to 1,506.5)	16.1 (13.8 to 18.2)	-2.2 (-4.1 to -0.4)
Anxiety disorders	25,082.4 (21,805.2 to 28,860.8)	3,752.0 (3,311.3 to 4,258.1)	2,463.0 (1,669.9 to 3,448.3)	16.2 (14.1 to 18.4)	-2.1 (-3.9 to -0.3)
Eating disorders	1,981.6 (1,714.6 to 2,399.0)	798.5 (565.1 to 1,067.1)	409.7 (244.6 to 640.1)	27.7 (24.8 to 30.5)	7.5 (5.1 to 9.9)
Anorexia nervosa	471.3 (339.4 to 654.4)	115.1 (87.9 to 146.2)	100.1 (59.5 to 153.4)	23.7 (18.2 to 28.9)	4.2 (-0.4 to 8.5)
Bulimia nervosa	1,465.5 (980.5 to 2,208.4)	683.3 (444.2 to 943.2)	309.6 (173.1 to 502.9)	29.0 (25.8 to 32.2)	8.7 (6.0 to 11.4)
Autistic spectrum disorders	5,585.3 (5,350.6 to 5,781.8)	--	820.7 (560.4 to 1,153.5)	19.1 (17.4 to 20.8)	0.4 (-1.1 to 1.8)
Autism	1,649.9 (1,417.6 to 1,918.0)	--	423.9 (275.0 to 618.0)	18.8 (16.0 to 21.6)	0.0 (-2.3 to 2.4)
Asperger syndrome and other autistic spectrum disorders	3,933.8 (3,287.7 to 4,715.3)	--	396.8 (260.1 to 578.9)	19.5 (17.7 to 21.4)	0.7 (-0.8 to 2.3)

Cause	Prevalence in thousands (95% UI)	Incidence in thousands (95% UI)	YLDs in thousands (95% UI)		
	2016 counts	2016 counts	2016 counts	Percentage change in counts between 2006 and 2016	Percentage change in rates between 2006 and 2016
Attention-deficit/hyperactivity disorder	6,290.8 (5,632.9 to 7,139.9)	--	75.9 (45.0 to 118.0)	22.5 (19.8 to 25.4)	3.2 (0.9 to 5.7)
Conduct disorder	--	--	--	--	--
Idiopathic developmental intellectual disability	12,277.1 (7,785.9 to 16,947.2)	--	487.6 (238.0 to 827.8)	9.8 (5.7 to 12.6)	-7.5 (-11.0 to -5.2)
Other mental and substance use disorders	16,193.6 (13,816.9 to 19,036.2)	699.1 (585.9 to 838.3)	1,236.7 (828.7 to 1,788.6)	18.8 (17.2 to 20.7)	0.1 (-1.3 to 1.6)
**Diabetes, urogenital, blood, and endocrine diseases**	**228,756.9 (226,354.6 to 231,270.6)**	**85,373.8† (74,606.7 to 98,018.7)**	**3,080.7 (2,121.7 to 4,329.0)**	**20.0 (18.0 to 22.1)**	**1.1 (-0.6 to 2.9)**
Diabetes mellitus	14,905.7 (12,394.2 to 18,143.7)	1,098.2 (751.7 to 1,501.5)	956.8 (614.7 to 1,396.4)	20.4 (16.7 to 23.7)	1.4 (-1.7 to 4.2)
Acute glomerulonephritis	6.1 (3.8 to 9.0)	89.7 (58.6 to 130.4)	0.3 (0.2 to 0.5)	9.6 (7.1 to 11.7)	-7.7 (-9.8 to -5.9)
Chronic kidney disease	6,763.8 (5,543.0 to 8,090.9)	511.7 (335.0 to 728.3)	326.3 (219.8 to 453.7)	22.1 (16.7 to 27.8)	2.8 (-1.7 to 7.6)
Chronic kidney disease due to diabetes mellitus	1,239.5 (818.4 to 1,750.1)	89.6 (50.6 to 141.9)	67.6 (39.7 to 105.5)	30.8 (22.9 to 40.3)	10.2 (3.6 to 18.2)
Chronic kidney disease due to hypertension	1,716.9 (1,223.1 to 2,318.9)	204.5 (118.3 to 310.8)	25.7 (14.8 to 41.4)	19.6 (13.4 to 26.3)	0.7 (-4.5 to 6.4)
Chronic kidney disease due to glomerulonephritis	1,450.4 (1,042.4 to 1,937.7)	36.4 (20.6 to 58.4)	126.5 (75.2 to 201.4)	19.8 (12.8 to 26.4)	0.9 (-5.0 to 6.5)
Chronic kidney disease due to other causes	2,357.1 (1,616.1 to 3,280.4)	181.2 (109.7 to 281.2)	106.5 (61.6 to 165.8)	20.3 (13.3 to 28.0)	1.3 (-4.5 to 7.8)
Urinary diseases and male infertility	6,650.6 (5,440.8 to 8,315.2)	44,860.4† (35,019.9 to 56,920.6)	86.3 (50.5 to 144.9)	15.8 (10.2 to 21.6)	-2.5 (-7.2 to 2.4)
Interstitial nephritis and urinary tract infections	775.3 (600.0 to 992.4)	40,613.5 (31,028.9 to 52,329.0)	25.9 (15.3 to 41.0)	14.6 (9.6 to 19.7)	-3.5 (-7.6 to 0.8)
Urolithiasis	158.7 (111.8 to 212.0)	4,246.9 (2,943.4 to 5,688.6)	12.3 (7.3 to 19.1)	15.0 (12.1 to 18.0)	-3.1 (-5.6 to -0.6)
Benign prostatic hyperplasia	--	--	--	--	--
Male infertility	5,256.9 (3,526.7 to 7,573.8)	--	31.9 (11.8 to 71.7)	32.9 (25.5 to 39.0)	12.0 (5.8 to 17.1)
Other urinary diseases	--	--	16.2 (10.3 to 24.1)	-6.0 (-8.8 to -3.0)	-20.8 (-23.2 to -18.3)
Gynecological diseases	87,234.0 (84,985.1 to 88,711.5)	38,320.9† (34,238.0 to 42,503.8)	1,200.9 (801.5 to 1,739.4)	17.4 (14.8 to 19.7)	-1.1 (-3.3 to 0.9)
Uterine fibroids	2,234.4 (1,749.5 to 2,790.8)	571.4 (409.0 to 774.7)	53.4 (31.6 to 87.7)	18.0 (14.2 to 21.9)	-0.6 (-3.8 to 2.7)
Polycystic ovarian syndrome	1,554.1 (1,196.5 to 2,058.7)	333.4 (164.8 to 567.2)	14.0 (6.3 to 28.1)	17.7 (14.3 to 21.3)	-0.9 (-3.7 to 2.2)
Female infertility	7,382.6 (3,316.0 to 13,874.7)	--	42.7 (12.2 to 113.4)	55.0 (42.0 to 80.1)	30.5 (19.6 to 51.7)
Endometriosis	563.9 (474.9 to 658.3)	746.1 (537.0 to 985.9)	53.1 (34.7 to 75.6)	20.3 (13.8 to 27.0)	1.3 (-4.2 to 7.0)
Genital prolapse	7,427.6 (5,626.9 to 9,634.9)	703.4 (431.0 to 1,111.7)	23.9 (10.9 to 45.9)	6.4 (3.2 to 9.8)	-10.4 (-13.0 to -7.5)
Premenstrual syndrome	63,442.3 (57,297.6 to 69,918.4)	23,761.2 (21,107.0 to 26,651.8)	535.0 (328.4 to 834.3)	15.4 (11.4 to 18.9)	-2.8 (-6.1 to 0.2)
Other gynecological diseases	13,604.0 (11,125.9 to 16,496.7)	12,205.5 (9,155.4 to 15,844.5)	478.8 (312.8 to 697.1)	17.4 (13.7 to 20.8)	-1.2 (-4.2 to 1.8)
Hemoglobinopathies and hemolytic anaemias	148,568.2 (146,729.1 to 150,617.3)	--	386.7 (247.2 to 577.9)	28.2 (20.9 to 35.4)	8.0 (1.9 to 14.1)
Thalassemias	3.4 (3.3 to 3.6)	--	0.3 (0.2 to 0.4)	20.0 (10.4 to 30.9)	1.1 (-7.0 to 10.3)
Thalassaemias trait	24,430.2 (23,568.4 to 25,337.4)	--	214.4 (136.5 to 323.7)	30.5 (19.2 to 41.5)	9.9 (0.5 to 19.2)
Sickle cell disorders	145.3 (131.7 to 159.3)	--	16.7 (11.5 to 22.7)	41.6 (32.8 to 50.6)	19.3 (11.9 to 26.9)
Sickle cell trait	37,916.4 (34,301.6 to 42,739.0)	--	96.4 (61.8 to 145.2)	23.6 (18.4 to 28.9)	4.1 (-0.2 to 8.6)
G6PD deficiency	27,788.5 (26,764.6 to 28,993.6)	--	1.5 (0.9 to 2.2)	35.8 (29.9 to 42.2)	14.4 (9.4 to 19.8)
G6PD trait	71,279.1 (70,115.9 to 72,607.4)	--	< 0.05 (< 0.05 to 0.1)	26.9 (20.6 to 33.0)	6.9 (1.6 to 12.0)
Other hemoglobinopathies and hemolytic anaemias	3,571.7 (3,038.4 to 4,211.5)	--	57.3 (36.1 to 86.7)	24.6 (17.8 to 32.6)	5.0 (-0.8 to 11.7)
Endocrine, metabolic, blood, and immune disorders	4,716.9 (4,073.9 to 5,401.5)	492.9 (333.4 to 673.8)	123.5 (79.4 to 181.5)	16.6 (13.0 to 20.1)	-1.8 (-4.8 to 1.2)
**Musculoskeletal disorders**	**75,513.1 (69,738.9 to 79,734.9)**	**44,892.5 (34,073.3 to 57,772.9)**	**8,316.8 (5,558.3 to 11,542.0)**	**14.7 (12.4 to 16.8)**	**-3.4 (-5.3 to -1.6)**
Rheumatoid arthritis	808.4 (642.2 to 990.6)	61.7 (40.8 to 88.4)	199.7 (129.0 to 281.3)	21.3 (16.1 to 26.1)	2.2 (-2.2 to 6.2)
Osteoarthritis	--	--	--	--	--
Low back and neck pain	45,800.9 (41,475.5 to 51,235.7)	20,088.1 (14,789.9 to 26,572.9)	5,282.7 (3,441.1 to 7,706.4)	14.3 (11.4 to 17.1)	-3.8 (-6.2 to -1.3)
Low back pain	34,186.5 (24,667.6 to 47,103.7)	16,259.5 (11,279.6 to 22,553.9)	3,933.6 (2,410.4 to 6,011.9)	12.5 (8.4 to 16.1)	-5.3 (-8.7 to -2.2)
Neck pain	13,056.1 (9,428.5 to 18,047.5)	3,828.6 (2,409.2 to 5,602.7)	1,349.1 (829.2 to 2,095.4)	19.8 (16.6 to 22.8)	0.9 (-1.7 to 3.5)
Gout	751.1 (573.9 to 968.9)	209.9 (146.1 to 285.3)	25.8 (16.2 to 37.9)	18.4 (9.2 to 27.5)	-0.3 (-8.0 to 7.4)
Other musculoskeletal disorders	31,797.2 (21,993.2 to 41,840.1)	24,532.8 (15,043.6 to 35,936.1)	2,808.6 (1,742.0 to 4,286.4)	14.9 (10.4 to 18.9)	-3.2 (-7.0 to 0.1)
**Other non-communicable diseases**	**386,243.4 (377,307.2 to 394,631.7)**	**1,082,597.0† (929,451.6 to 1,267,593.0)**	**8,156.9 (5,511.5 to 11,661.2)**	**17.2 (16.4 to 18.0)**	**-1.2 (-1.9 to -0.6)**
Congenital anomalies	6,220.0 (5,790.8 to 6,598.3)	--	718.0 (505.1 to 963.6)	14.6 (11.6 to 17.6)	-3.5 (-6.0 to -0.9)
Neural tube defects	475.8 (429.8 to 527.1)	--	143.5 (101.7 to 191.8)	18.7 (13.8 to 24.0)	0.0 (-4.2 to 4.5)
Congenital heart anomalies	902.0 (803.6 to 1,004.0)	--	47.9 (20.2 to 82.5)	22.4 (18.3 to 26.9)	3.1 (-0.3 to 6.9)
Orofacial clefts	280.2 (249.3 to 311.0)	--	3.6 (2.3 to 5.3)	9.1 (5.7 to 12.2)	-8.1 (-11.0 to -5.5)
Down's syndrome	207.3 (180.3 to 237.7)	--	17.8 (12.0 to 24.8)	13.7 (9.2 to 18.2)	-4.2 (-8.0 to -0.4)
Turner syndrome	267.3 (232.3 to 303.5)	--	5.5 (2.8 to 8.9)	16.3 (5.5 to 26.7)	-2.0 (-11.1 to 6.7)
Klinefelter syndrome	309.1 (267.6 to 355.7)	--	3.1 (1.4 to 6.0)	16.1 (9.8 to 22.9)	-2.2 (-7.5 to 3.6)

Cause	Prevalence in thousands (95% UI)	Incidence in thousands (95% UI)	YLDs in thousands (95% UI)		
	2016 counts	2016 counts	2016 counts	Percentage change in counts between 2006 and 2016	Percentage change in rates between 2006 and 2016
Other chromosomal abnormalities	0.8 (0.7 to 0.9)	--	0.2 (0.1 to 0.2)	51.9 (0.5 to 121.6)	28.0 (-15.3 to 86.7)
Congenital musculoskeletal and limb anomalies	862.8 (795.8 to 935.7)	--	125.7 (85.8 to 173.0)	18.4 (14.7 to 22.2)	-0.3 (-3.4 to 2.9)
Urogenital congenital anomalies	418.7 (375.9 to 462.4)	--	9.7 (5.5 to 15.6)	12.6 (9.5 to 15.6)	-5.1 (-7.8 to -2.6)
Digestive congenital anomalies	823.7 (742.3 to 910.1)	--	36.7 (23.8 to 52.7)	21.4 (15.8 to 27.4)	2.3 (-2.4 to 7.4)
Other congenital anomalies	2,959.8 (2,076.7 to 4,181.7)	--	324.2 (209.3 to 487.6)	9.9 (5.3 to 15.0)	-7.4 (-11.3 to -3.2)
Skin and subcutaneous diseases	162,061.7 (157,992.0 to 165,405.0)	316,287.5 (281,112.9 to 352,137.3)	4,137.1 (2,755.4 to 5,958.3)	19.2 (18.4 to 20.1)	0.4 (-0.3 to 1.1)
Dermatitis	19,838.3 (17,168.4 to 22,991.9)	45,895.0 (37,552.7 to 55,177.2)	666.1 (402.1 to 1,039.9)	17.0 (15.2 to 18.7)	-1.4 (-2.9 to 0.0)
Psoriasis	5,106.9 (4,681.0 to 5,577.4)	702.2 (615.5 to 793.5)	454.7 (316.3 to 605.3)	24.3 (21.1 to 27.6)	4.7 (2.0 to 7.5)
Cellulitis	217.0 (172.2 to 265.4)	4,083.7 (3,419.4 to 4,891.5)	12.5 (7.7 to 18.4)	15.4 (8.6 to 23.1)	-2.8 (-8.5 to 3.7)
Pyoderma	732.9 (662.5 to 806.7)	16,200.8 (14,576.4 to 17,988.7)	4.2 (1.6 to 8.9)	18.2 (13.8 to 22.2)	-0.5 (-4.1 to 2.9)
Scabies	14,317.2 (9,483.1 to 20,692.5)	43,020.6 (28,851.9 to 61,310.2)	372.0 (179.0 to 649.6)	13.8 (12.0 to 15.7)	-4.1 (-5.7 to -2.6)
Fungal skin diseases	34,535.0 (26,671.3 to 43,077.1)	111,801.9 (83,437.2 to 143,791.0)	196.5 (76.2 to 406.2)	19.5 (18.8 to 20.2)	0.7 (0.1 to 1.2)
Viral skin diseases	14,244.5 (12,179.6 to 16,783.4)	22,252.8 (19,156.5 to 25,631.2)	438.2 (268.6 to 663.8)	18.1 (16.8 to 19.5)	-0.5 (-1.6 to 0.7)
Acne vulgaris	56,019.6 (48,916.7 to 63,523.8)	26,553.0 (22,496.0 to 30,733.5)	1,441.4 (943.7 to 2,144.3)	20.0 (18.4 to 21.6)	1.1 (-0.2 to 2.4)
Alopecia areata	2,145.2 (1,912.5 to 2,384.8)	3,880.0 (3,505.6 to 4,265.4)	71.0 (44.8 to 109.0)	17.2 (14.2 to 20.3)	-1.3 (-3.8 to 1.3)
Pruritus	4,761.9 (3,372.0 to 6,541.0)	3,877.3 (2,697.3 to 5,342.1)	51.3 (22.9 to 97.8)	20.5 (18.5 to 22.3)	1.5 (-0.1 to 3.0)
Urticaria	4,407.9 (3,053.8 to 6,043.6)	7,797.9 (5,443.4 to 10,748.0)	265.3 (154.2 to 403.1)	18.8 (16.2 to 21.3)	0.1 (-2.1 to 2.2)
Decubitus ulcer	33.8 (23.4 to 48.0)	120.5 (84.2 to 172.4)	6.0 (3.5 to 9.4)	13.9 (12.6 to 15.1)	-4.1 (-5.2 to -3.0)
Other skin and subcutaneous diseases	28,194.1 (25,508.5 to 31,142.3)	30,101.7 (27,050.5 to 33,352.7)	158.0 (75.8 to 290.0)	25.0 (23.8 to 26.2)	5.3 (4.3 to 6.3)
Sense organ diseases	76,628.9 (73,711.2 to 78,716.5)	39,916.1† (35,722.3 to 43,984.9)	2,369.1 (1,576.8 to 3,400.8)	15.0 (12.7 to 16.9)	-3.1 (-5.0 to -1.5)
Glaucoma	--	--	--	--	--
Cataract	205.5 (135.7 to 291.7)	--	22.9 (12.0 to 38.2)	10.6 (0.1 to 19.4)	-6.9 (-15.7 to 0.6)
Macular degeneration	--	--	--	--	--
Refraction and accommodation disorders	32,619.4 (29,605.3 to 36,081.6)	--	664.8 (408.4 to 1,047.7)	12.9 (11.5 to 14.3)	-4.9 (-6.1 to -3.7)
Age-related and other hearing loss	37,276.9 (29,915.8 to 46,229.8)	--	1,291.9 (846.6 to 1,868.0)	14.9 (11.0 to 18.6)	-3.2 (-6.5 to -0.1)
Other vision loss	869.0 (631.4 to 1,166.5)	--	71.5 (43.7 to 110.9)	15.2 (8.3 to 21.8)	-3.0 (-8.7 to 2.6)
Other sense organ diseases	9,955.1 (8,808.9 to 11,283.8)	39,916.1 (35,722.3 to 43,984.9)	318.0 (197.9 to 494.0)	20.4 (18.1 to 22.7)	1.4 (-0.5 to 3.4)
Oral disorders	262,280.6 (240,221.5 to 286,074.0)	726,393.4† (573,726.7 to 914,305.7)	932.7 (529.4 to 1,512.2)	16.5 (15.6 to 17.3)	-1.9 (-2.6 to -1.2)
Caries of deciduous teeth	--	--	--	--	--
Caries of permanent teeth	230,791.4 (187,366.2 to 276,376.1)	719,475.4 (568,269.7 to 908,168.9)	161.7 (70.1 to 317.3)	11.7 (10.0 to 13.3)	-5.9 (-7.4 to -4.5)
Periodontal disease	32,446.5 (21,369.4 to 46,782.6)	6,270.2 (3,685.1 to 9,952.0)	216.9 (79.3 to 471.2)	18.4 (17.2 to 19.5)	-0.2 (-1.3 to 0.7)
Edentulism and severe tooth loss	5,741.6 (4,361.9 to 7,550.9)	647.9 (425.9 to 942.8)	166.1 (100.9 to 254.3)	13.6 (10.6 to 16.3)	-4.3 (-6.8 to -2.1)
Other oral disorders	13,045.6 (11,616.5 to 14,597.8)	--	388.0 (237.5 to 593.1)	18.8 (17.5 to 20.0)	0.1 (-1.0 to 1.1)
**Injuries**	**91,624.1 (84,973.1 to 99,701.7)**	**44,881.3† (39,290.4 to 51,319.6)**	**3,493.4 (2,307.8 to 5,074.6)**	**16.5 (13.1 to 19.0)**	**-1.9 (-4.7 to 0.2)**
**Transport injuries**	**13,907.4 (12,324.8 to 15,197.0)**	**7,581.5 (5,877.6 to 9,765.9)**	**761.5 (497.9 to 1,113.8)**	**21.3 (19.4 to 23.1)**	**2.2 (0.6 to 3.7)**
Road injuries	10,728.9 (9,507.1 to 11,884.1)	6,948.7 (5,244.9 to 9,129.2)	591.6 (379.1 to 871.9)	22.4 (20.5 to 24.3)	3.1 (1.5 to 4.7)
Pedestrian road injuries	2,301.9 (1,849.0 to 2,835.4)	1,069.3 (684.2 to 1,580.0)	128.2 (81.9 to 189.3)	21.8 (19.5 to 24.0)	2.6 (0.6 to 4.5)
Cyclist road injuries	2,200.9 (1,686.2 to 2,838.0)	1,363.8 (837.2 to 2,099.8)	110.9 (70.9 to 167.2)	26.0 (24.1 to 28.1)	6.1 (4.5 to 7.9)
Motorcyclist road injuries	2,713.7 (2,029.1 to 3,567.2)	1,516.7 (1,023.4 to 2,139.2)	148.8 (96.0 to 217.8)	23.0 (20.9 to 25.0)	3.6 (1.9 to 5.3)
Motor vehicle road injuries	2,638.2 (2,095.6 to 3,347.5)	2,330.7 (1,644.9 to 3,207.6)	160.3 (103.9 to 233.2)	15.7 (13.6 to 18.0)	-2.6 (-4.4 to -0.6)
Other road injuries	874.1 (678.9 to 1,082.6)	668.2 (375.4 to 1,040.1)	43.3 (27.4 to 65.9)	43.5 (40.6 to 46.5)	20.9 (18.4 to 23.4)
Other transport injuries	3,178.6 (2,563.0 to 3,962.4)	632.9 (417.9 to 924.7)	169.9 (112.5 to 246.8)	17.6 (15.0 to 20.0)	-1.0 (-3.1 to 1.1)
**Unintentional injuries**	**69,429.5 (63,250.7 to 76,908.6)**	**33,013.4 (27,875.1 to 38,930.4)**	**2,255.0 (1,482.7 to 3,202.8)**	**20.2 (18.0 to 21.9)**	**1.2 (-0.6 to 2.7)**
Falls	25,989.8 (21,434.8 to 30,947.1)	11,320.2 (7,875.6 to 15,376.9)	958.4 (645.8 to 1,342.2)	20.8 (18.8 to 22.4)	1.8 (0.1 to 3.1)
Drowning	301.7 (250.8 to 362.1)	48.4 (36.8 to 63.4)	15.7 (10.1 to 23.0)	5.5 (2.5 to 8.2)	-11.2 (-13.7 to -8.9)
Fire, heat, and hot substances	6,898.5 (5,632.9 to 8,589.4)	808.9 (516.6 to 1,159.8)	188.5 (125.2 to 272.8)	20.5 (16.6 to 23.6)	1.5 (-1.7 to 4.1)
Poisonings	261.7 (209.9 to 320.1)	390.9 (231.7 to 609.6)	25.9 (16.5 to 38.4)	29.8 (26.2 to 33.4)	9.3 (6.3 to 12.4)
Exposure to mechanical forces	12,499.0 (10,210.6 to 16,403.1)	6,849.7 (4,800.9 to 9,562.8)	287.7 (181.6 to 438.8)	23.2 (21.0 to 25.2)	3.8 (1.9 to 5.5)
Unintentional firearm injuries	464.0 (359.9 to 620.1)	179.2 (92.5 to 317.8)	17.4 (10.8 to 27.1)	22.4 (19.9 to 24.9)	3.1 (1.0 to 5.2)
Unintentional suffocation	731.9 (539.0 to 968.9)	62.0 (29.8 to 118.7)	21.7 (12.9 to 34.5)	19.0 (16.4 to 21.4)	0.2 (-2.0 to 2.2)
Other exposure to mechanical forces	11,303.1 (9,104.0 to 15,292.9)	6,608.5 (4,636.2 to 9,237.1)	248.6 (158.3 to 376.1)	23.7 (21.5 to 25.7)	4.2 (2.3 to 5.9)

Cause	Prevalence in thousands (95% UI)	Incidence in thousands (95% UI)	YLDs in thousands (95% UI)		
	2016 counts	2016 counts	2016 counts	Percentage change in counts between 2006 and 2016	Percentage change in rates between 2006 and 2016
Adverse effects of medical treatment	111.7<br>(76.1 to 154.9)	1,460.9<br>(1,048.3 to 1,985.1)	14.9<br>(8.4 to 24.0)	16.5<br>(11.6 to 20.9)	-1.9<br>(-6.0 to 1.9)
Animal contact	3,811.4<br>(3,227.3 to 4,564.4)	5,491.4<br>(3,640.6 to 7,733.2)	142.6<br>(94.6 to 203.0)	12.3<br>(9.7 to 14.8)	-5.4<br>(-7.6 to -3.2)
Venomous animal contact	1,896.7<br>(1,468.2 to 2,413.1)	2,806.5<br>(1,756.3 to 4,150.1)	111.8<br>(74.9 to 157.0)	13.7<br>(10.7 to 16.6)	-4.2<br>(-6.8 to -1.8)
Non-venomous animal contact	1,914.8<br>(1,470.9 to 2,417.5)	2,685.0<br>(1,666.0 to 3,974.2)	30.8<br>(18.6 to 47.7)	7.8<br>(4.7 to 10.3)	-9.2<br>(-11.8 to -7.1)
Foreign body	3,946.1<br>(3,171.4 to 4,756.7)	2,051.0<br>(1,295.0 to 3,113.8)	149.0<br>(92.6 to 225.2)	18.6<br>(15.6 to 21.5)	-0.1<br>(-2.6 to 2.3)
Pulmonary aspiration and foreign body in airway	1,928.4<br>(1,378.1 to 2,664.3)	247.3<br>(143.0 to 390.7)	82.7<br>(50.1 to 127.5)	15.6<br>(13.0 to 18.0)	-2.6<br>(-4.9 to -0.6)
Foreign body in eyes	226.0<br>(101.1 to 413.5)	1,350.7<br>(634.9 to 2,354.6)	13.0<br>(5.3 to 25.5)	26.0<br>(22.3 to 30.1)	6.1<br>(3.0 to 9.6)
Foreign body in other body part	1,791.6<br>(1,383.8 to 2,383.0)	453.0<br>(261.9 to 711.2)	53.3<br>(33.5 to 81.6)	21.8<br>(18.0 to 25.0)	2.6<br>(-0.6 to 5.3)
Environmental heat and cold exposure	5,197.4<br>(4,235.5 to 6,375.8)	1,022.0<br>(650.1 to 1,554.0)	190.1<br>(122.0 to 278.2)	21.9<br>(18.9 to 24.5)	2.7<br>(0.1 to 4.9)
Other unintentional injuries	10,412.1<br>(8,268.0 to 13,097.2)	3,570.1<br>(2,317.5 to 5,010.8)	282.3<br>(183.6 to 417.3)	19.2<br>(17.0 to 20.9)	0.4<br>(-1.5 to 1.9)
**Self-harm and interpersonal violence**	**25,843.0**<br>**(19,252.1 to 33,893.2)**	**4,140.4†**<br>**(3,145.8 to 5,454.3)**	**371.5**<br>**(248.2 to 527.1)**	**11.6**<br>**(10.2 to 12.8)**	**-6.0**<br>**(-7.2 to -5.0)**
Self-harm	655.5<br>(530.8 to 809.7)	684.7<br>(494.1 to 902.8)	37.2<br>(23.8 to 55.0)	9.5<br>(7.3 to 11.8)	-7.8<br>(-9.6 to -5.8)
Self-harm by firearm	29.8<br>(24.4 to 36.0)	7.2<br>(1.8 to 18.7)	1.1<br>(0.7 to 1.7)	4.8<br>(1.9 to 7.6)	-11.7<br>(-14.2 to -9.4)
Self-harm by other specified means	625.6<br>(501.9 to 781.3)	677.6<br>(490.2 to 890.4)	36.1<br>(23.1 to 53.4)	9.7<br>(7.4 to 12.0)	-7.6<br>(-9.5 to -5.7)
Interpersonal violence	25,187.5<br>(18,639.9 to 33,127.5)	3,455.7†<br>(2,478.9 to 4,687.1)	334.3<br>(220.3 to 472.6)	11.8<br>(10.4 to 13.1)	-5.8<br>(-7.0 to -4.8)
Assault by firearm	189.8<br>(135.8 to 245.1)	93.0<br>(54.4 to 150.5)	7.9<br>(4.8 to 12.2)	9.5<br>(7.1 to 12.0)	-7.7<br>(-9.8 to -5.6)
Assault by sharp object	1,053.5<br>(790.5 to 1,337.0)	801.4<br>(511.0 to 1,216.1)	33.5<br>(21.1 to 49.8)	11.0<br>(8.1 to 13.9)	-6.5<br>(-9.0 to -4.0)
Sexual violence	21,435.5<br>(14,849.5 to 29,482.7)	--	188.6<br>(111.0 to 302.8)	10.2<br>(8.0 to 11.7)	-7.2<br>(-9.0 to -5.9)
Assault by other means	2,508.7<br>(2,006.7 to 3,223.2)	2,561.3<br>(1,780.1 to 3,524.1)	104.2<br>(68.1 to 154.5)	15.4<br>(13.2 to 17.4)	-2.8<br>(-4.6 to -1.1)
**Forces of nature, conflict and terrorism, and executions and police conflict**	**3,879.8**<br>**(2,475.9 to 5,605.9)**	**145.9†**<br>**(88.3 to 211.5)**	**105.4**<br>**(38.3 to 218.4)**	**-35.3**<br>**(-51.0 to -23.3)**	**-45.5**<br>**(-58.7 to -35.4)**
Forces of nature, conflict and terrorism, and state actor violence	674.4<br>(374.0 to 1,092.8)	28.7<br>(16.4 to 44.0)	23.4<br>(6.6 to 54.7)	-2.4<br>(-13.7 to 3.0)	-17.8<br>(-27.3 to -13.2)
Conflict and terrorism	3,037.3<br>(1,838.5 to 4,631.8)	--	82.1<br>(31.7 to 164.8)	-40.8<br>(-56.2 to -27.7)	-50.1<br>(-63.1 to -39.1)
Executions and police conflict	168.1<br>(99.3 to 260.0)	117.2<br>(60.5 to 181.7)	4.6<br>(1.7 to 11.6)	-40.5<br>(-44.5 to -24.4)	-49.9<br>(-53.2 to -36.3)

We did not calculate incidence for certain NTDs and causes with only birth prevalence. Blank cells mean that no estimate is available.

† Incidence for aggregate causes represents the sum of the incidence of the causes below the aggregate.

‡ Incidence estimates for stroke represent first-ever stroke only.

^ Incidence of HIV/AIDS represents new infections of HIV only and does not include new infections of Tuberculosis in HIV positive cases.

SR Table 12: Global prevalence, incidence, and YLDs for 2016, percentage change of YLD counts and percentage change of YLD rates between 2006 and 2016 by cause, age 30-34 years.

Cause	Prevalence in thousands (95% UI)	Incidence in thousands (95% UI)	YLDs in thousands (95% UI)		
	2016 counts	2016 counts	2016 counts	Percentage change in counts between 2006 and 2016	Percentage change in rates between 2006 and 2016
**All causes**	**550,412.2<br>(549,460.1 to 551,191.6)**	**3,235,579.9†<br>(2,823,430.4 to 3,768,527.7)**	**57,197.8<br>(42,248.5 to 74,516.2)**	**12.3<br>(11.5 to 13.0)**	**-0.5<br>(-1.2 to 0.1)**
**Communicable, maternal, neonatal, and nutritional disorders**	**405,140.1<br>(399,842.3 to 409,638.2)**	**1,796,816.6†<br>(1,424,611.2 to 2,283,829.1)**	**7,302.4<br>(5,227.4 to 10,025.4)**	**7.5<br>(2.8 to 11.0)**	**-4.8<br>(-9.0 to -1.7)**
**HIV/AIDS and tuberculosis**	**186,844.1<br>(166,320.9 to 210,014.7)**	**1,193.5†<br>(914.4 to 1,554.4)**	**849.7<br>(583.2 to 1,169.8)**	**-10.4<br>(-26.4 to 2.7)**	**-20.7<br>(-34.8 to -9.1)**
Tuberculosis	172,633.6<br>(143,096.7 to 207,606.1)	902.9†<br>(628.8 to 1,263.5)	240.7<br>(140.0 to 373.1)	6.8<br>(1.1 to 14.4)	-5.4<br>(-10.5 to 1.3)
Drug-susceptible tuberculosis	741.0<br>(510.9 to 1,028.8)	870.2<br>(604.6 to 1,220.5)	231.8<br>(134.3 to 358.7)	6.9<br>(1.1 to 15.0)	-5.3<br>(-10.5 to 1.8)
Multidrug-resistant tuberculosis without extensive drug resistance	25.8<br>(18.6 to 34.5)	30.7<br>(22.3 to 41.6)	8.4<br>(5.0 to 13.1)	-0.5<br>(-15.3 to 17.9)	-11.9<br>(-25.0 to 4.4)
Extensively drug-resistant tuberculosis	1.7<br>(1.2 to 2.2)	2.0<br>(1.5 to 2.7)	0.6<br>(0.3 to 0.8)	173.0<br>(137.1 to 212.9)	141.8<br>(110.0 to 177.1)
Latent tuberculosis infection	171,865.2<br>(142,362.4 to 206,921.1)	--	--	--	--
HIV/AIDS	5,579.1<br>(5,218.7 to 5,964.2)	290.6^<br>(259.2 to 332.3)	609.0<br>(420.4 to 846.5)	-15.8<br>(-33.1 to 0.3)	-25.4<br>(-40.7 to -11.2)
Drug-susceptible HIV/AIDS - Tuberculosis	184.0<br>(131.2 to 252.2)	235.0<br>(169.7 to 314.9)	71.1<br>(43.4 to 109.3)	-25.8<br>(-30.5 to -21.5)	-34.3<br>(-38.5 to -30.5)
Multidrug-resistant HIV/AIDS - Tuberculosis without extensive drug resistance	4.6<br>(3.1 to 6.8)	6.2<br>(4.0 to 8.9)	1.9<br>(1.1 to 3.1)	-33.7<br>(-45.9 to -20.4)	-41.2<br>(-52.1 to -29.5)
Extensively drug-resistant HIV/AIDS - Tuberculosis	0.2<br>(0.1 to 0.3)	0.2<br>(0.1 to 0.4)	0.1<br>(< 0.05 to 0.1)	124.2<br>(84.4 to 170.3)	98.6<br>(63.3 to 139.4)
HIV/AIDS resulting in other diseases	5,390.3<br>(5,040.2 to 5,780.0)	290.6<br>(259.2 to 332.3)	536.0<br>(364.6 to 753.2)	-14.2<br>(-34.0 to 6.0)	-24.0<br>(-41.6 to -6.1)
**Diarrhoea, lower respiratory infections, and other common infectious diseases**	**27,039.2<br>(25,104.6 to 28,698.2)**	**1,616,679.2†<br>(1,247,188.0 to 2,091,416.5)**	**1,261.2<br>(870.0 to 1,763.1)**	**11.0<br>(9.6 to 12.4)**	**-1.7<br>(-3.0 to -0.5)**
Diarrhoeal diseases	3,438.3<br>(3,102.8 to 3,776.9)	231,972.4<br>(208,497.3 to 255,265.5)	388.1<br>(265.4 to 533.9)	14.9<br>(12.5 to 17.3)	1.7<br>(-0.4 to 3.9)
Intestinal infectious diseases	28.7<br>(21.9 to 37.6)	669.7†<br>(542.2 to 845.1)	5.0<br>(3.1 to 7.7)	-2.7<br>(-20.8 to 19.8)	-13.8<br>(-29.9 to 6.1)
Typhoid fever	28.2<br>(21.4 to 37.5)	445.5<br>(353.9 to 566.9)	4.4<br>(2.6 to 6.7)	-2.4<br>(-23.0 to 23.2)	-13.6<br>(-31.8 to 9.1)
Paratyphoid fever	9.8<br>(7.6 to 12.8)	224.2<br>(176.8 to 283.8)	0.6<br>(0.4 to 1.0)	-3.6<br>(-21.1 to 18.8)	-14.7<br>(-30.1 to 5.2)
Other intestinal infectious diseases	--	--	< 0.05<br>(< 0.05 to < 0.05)	-21.1<br>(-35.8 to -2.8)	-30.1<br>(-43.1 to -13.9)
Lower respiratory infections	249.9<br>(226.4 to 276.6)	10,777.2<br>(9,593.6 to 12,221.0)	15.4<br>(10.2 to 22.1)	33.1<br>(26.0 to 40.1)	17.9<br>(11.6 to 24.1)
Upper respiratory infections	17,847.4<br>(13,012.4 to 23,663.3)	1,355,522.0<br>(981,732.8 to 1,823,514.4)	445.1<br>(252.1 to 749.3)	11.2<br>(9.8 to 12.5)	-1.6<br>(-2.8 to -0.4)
Otitis media	4,035.1<br>(2,912.5 to 5,490.0)	12,057.3<br>(9,964.8 to 14,658.2)	108.4<br>(61.2 to 180.0)	1.7<br>(-3.1 to 6.8)	-9.9<br>(-14.2 to -5.4)
Meningitis	1,228.5<br>(1,057.9 to 1,420.5)	81.8<br>(58.1 to 110.2)	129.8<br>(90.9 to 170.2)	15.6<br>(11.9 to 19.4)	2.4<br>(-0.9 to 5.7)
Pneumococcal meningitis	553.4<br>(464.6 to 660.0)	22.5<br>(13.6 to 34.4)	58.1<br>(39.8 to 78.1)	12.9<br>(6.0 to 20.2)	-0.0<br>(-6.2 to 6.4)
H influenzae type B meningitis	166.7<br>(131.1 to 211.9)	5.6<br>(3.1 to 9.6)	21.2<br>(14.3 to 29.1)	-7.2<br>(-12.8 to -2.1)	-17.8<br>(-22.7 to -13.3)
Meningococcal infection	135.0<br>(97.7 to 179.6)	13.8<br>(7.6 to 22.8)	14.0<br>(9.4 to 19.2)	21.6<br>(17.8 to 25.5)	7.7<br>(4.4 to 11.1)
Other meningitis	373.5<br>(279.6 to 475.1)	39.9<br>(27.2 to 55.9)	36.4<br>(24.4 to 50.1)	38.0<br>(31.6 to 44.9)	22.2<br>(16.6 to 28.3)
Encephalitis	1,237.2<br>(838.7 to 1,784.7)	309.9<br>(230.6 to 406.6)	152.5<br>(108.6 to 200.8)	3.3<br>(-2.4 to 9.0)	-8.5<br>(-13.6 to -3.5)
Diphtheria	< 0.05<br>(< 0.05 to < 0.05)	0.1<br>(0.1 to 0.2)	< 0.05<br>(< 0.05 to < 0.05)	-45.0<br>(-59.0 to -28.0)	-51.3<br>(-63.7 to -36.2)
Whooping cough	2.2<br>(1.7 to 2.7)	15.7<br>(12.3 to 19.8)	0.1<br>(0.1 to 0.2)	-14.5<br>(-16.3 to -12.7)	-24.3<br>(-25.9 to -22.7)
Tetanus	11.8<br>(11.6 to 12.0)	3.2<br>(1.8 to 4.6)	0.3<br>(0.1 to 0.4)	8.8<br>(-16.2 to 38.9)	-3.7<br>(-25.8 to 23.0)
Measles	0.4<br>(0.1 to 0.9)	15.1<br>(5.5 to 32.5)	< 0.05<br>(< 0.05 to 0.1)	-54.9<br>(-59.9 to -49.2)	-60.1<br>(-64.5 to -55.1)
Varicella and herpes zoster	357.7<br>(303.5 to 424.5)	5,254.7<br>(3,862.3 to 6,807.9)	16.5<br>(9.7 to 26.0)	11.5<br>(5.2 to 17.7)	-1.3<br>(-6.8 to 4.2)
**Neglected tropical diseases and malaria**	**147,870.0<br>(143,432.1 to 151,544.9)**	**22,802.9†<br>(19,093.7 to 27,331.0)**	**1,104.4<br>(745.4 to 1,574.7)**	**-12.2<br>(-17.9 to -6.6)**	**-22.2<br>(-27.3 to -17.3)**
Malaria	6,881.6<br>(5,951.1 to 7,869.6)	6,721.5<br>(5,034.9 to 8,587.4)	94.2<br>(71.0 to 120.2)	-0.3<br>(-6.6 to 7.3)	-11.7<br>(-17.3 to -5.0)
Chagas disease	629.3<br>(521.1 to 765.9)	14.8<br>(9.8 to 21.2)	4.0<br>(2.5 to 6.0)	-1.4<br>(-8.9 to 6.9)	-12.6<br>(-19.3 to -5.3)
Leishmaniasis	412.1<br>(354.3 to 479.4)	45.5<br>(33.5 to 61.0)	23.9<br>(15.3 to 35.0)	34.4<br>(14.8 to 59.8)	19.0<br>(1.6 to 41.6)
Visceral leishmaniasis	1.2<br>(0.8 to 1.7)	4.9<br>(3.4 to 6.8)	0.1<br>(< 0.05 to 0.1)	-56.8<br>(-71.0 to -36.0)	-61.7<br>(-74.4 to -43.3)
Cutaneous and mucocutaneous leishmaniasis	373.7<br>(309.9 to 455.5)	40.6<br>(28.6 to 55.8)	23.9<br>(15.2 to 34.9)	35.4<br>(15.3 to 62.1)	19.9<br>(2.1 to 43.6)
African trypanosomiasis	0.6<br>(0.1 to 2.0)	0.4<br>(0.1 to 1.3)	0.2<br>(< 0.05 to 0.6)	-71.0<br>(-94.5 to 54.8)	-74.3<br>(-95.2 to 37.1)
Schistosomiasis	21,521.4<br>(19,665.1 to 23,300.1)	5,733.7<br>(4,952.5 to 6,868.1)	180.8<br>(91.4 to 331.6)	-19.6<br>(-22.2 to -16.3)	-28.8<br>(-31.1 to -25.9)
Cysticercosis	245.5<br>(162.2 to 349.0)	--	40.4<br>(22.5 to 62.9)	-13.9<br>(-24.8 to -4.3)	-23.8<br>(-33.4 to -15.3)
Cystic echinococcosis	86.6<br>(46.1 to 190.8)	18.6<br>(8.4 to 46.2)	8.1<br>(3.6 to 18.1)	-36.0<br>(-76.3 to 57.7)	-43.3<br>(-79.0 to 39.6)
Lymphatic filariasis	2,594.2<br>(2,121.8 to 3,320.3)	696.6<br>(406.1 to 1,113.6)	119.6<br>(57.4 to 216.2)	-40.2<br>(-54.5 to -28.9)	-47.1<br>(-59.7 to -37.0)
Onchocerciasis	1,505.2<br>(916.7 to 2,592.9)	333.2<br>(139.7 to 668.4)	91.1<br>(40.2 to 164.4)	-16.2<br>(-38.1 to 4.1)	-25.8<br>(-45.2 to -7.8)
Trachoma	13.0<br>(6.8 to 24.0)	--	1.6<br>(0.9 to 2.7)	-19.8<br>(-38.0 to 2.2)	-28.9<br>(-45.1 to -9.5)
Dengue	447.4<br>(243.8 to 757.8)	7,479.0<br>(4,550.3 to 11,342.7)	73.0<br>(31.8 to 135.7)	86.5<br>(50.1 to 335.8)	65.2<br>(33.0 to 285.9)
Yellow fever	0.2<br>(0.1 to 0.6)	7.1<br>(2.0 to 20.5)	< 0.05<br>(< 0.05 to < 0.05)	-16.0<br>(-45.8 to 27.5)	-25.6<br>(-52.0 to 12.9)
Rabies	< 0.05<br>(< 0.05 to < 0.05)	0.5<br>(0.3 to 0.7)	< 0.05<br>(< 0.05 to < 0.05)	-50.9<br>(-59.3 to -39.5)	-56.5<br>(-64.0 to -46.5)
Intestinal nematode infections	122,189.6<br>(116,899.4 to 126,232.9)	--	243.1<br>(139.8 to 393.7)	-12.5<br>(-18.9 to -4.8)	-22.5<br>(-28.2 to -15.7)
Ascariasis	65,236.1<br>(58,789.4 to 72,713.3)	--	70.9<br>(38.1 to 121.0)	-25.4<br>(-35.0 to -15.0)	-33.9<br>(-42.5 to -24.7)

Cause	Prevalence in thousands (95% UI)	Incidence in thousands (95% UI)	YLDs in thousands (95% UI)		
	2016 counts	2016 counts	2016 counts	Percentage change in counts between 2006 and 2016	Percentage change in rates between 2006 and 2016
Trichuriasis	34,381.6 (31,667.2 to 37,486.6)	--	26.8 (14.5 to 45.7)	-18.1 (-29.6 to -5.3)	-27.5 (-37.7 to -16.1)
Hookworm disease	37,570.5 (34,660.8 to 40,862.5)	--	145.4 (85.3 to 228.3)	-3.1 (-12.4 to 7.5)	-14.2 (-22.4 to -4.8)
Food-borne trematodiases	7,295.4 (6,834.2 to 7,877.2)	1,001.5 (696.2 to 1,263.0)	196.8 (99.4 to 356.1)	-4.7 (-10.2 to 3.4)	-15.6 (-20.4 to -8.4)
Leprosy	26.5 (24.7 to 28.7)	4.1 (3.8 to 4.4)	1.7 (1.1 to 2.4)	-18.1 (-28.0 to -7.7)	-27.5 (-36.2 to -18.2)
Ebola virus disease	< 0.05 (< 0.05 to 0.3)	--	< 0.05 (< 0.05 to 0.1)	--	--
Zika virus disease	12.4 (7.0 to 22.4)	746.4 (423.4 to 1,356.8)	0.2 (0.1 to 0.4)	--	--
Guinea worm disease	< 0.05 (< 0.05 to < 0.05)	< 0.05 (< 0.05 to < 0.05)	< 0.05 (< 0.05 to < 0.05)	-99.5 (-99.7 to -99.4)	-99.6 (-99.7 to -99.4)
Other neglected tropical diseases	1,728.6 (1,491.7 to 2,004.3)	--	25.8 (15.8 to 40.7)	27.2 (20.2 to 34.6)	12.7 (6.5 to 19.2)
**Maternal disorders**	**1,715.2 (1,592.5 to 1,823.0)**	**22,775.5† (18,735.2 to 27,453.8)**	**166.9 (114.2 to 230.3)**	**2.0 (-11.5 to 19.4)**	**-9.7 (-21.6 to 5.8)**
Maternal haemorrhage	342.7 (252.5 to 471.1)	1,989.3 (1,476.4 to 2,633.6)	12.2 (7.4 to 18.3)	14.6 (-12.2 to 48.4)	1.5 (-22.3 to 31.5)
Maternal sepsis and other pregnancy related infections	263.7 (195.0 to 360.3)	1,670.9 (1,178.7 to 2,314.1)	7.4 (3.4 to 13.7)	16.7 (-38.0 to 129.5)	3.4 (-45.1 to 103.2)
Maternal hypertensive disorders	855.2 (562.1 to 1,210.3)	3,941.3 (3,252.8 to 4,675.5)	41.3 (22.3 to 66.6)	26.4 (-20.2 to 102.3)	12.0 (-29.4 to 79.1)
Maternal obstructed labour and uterine rupture	215.3 (171.2 to 265.9)	1,375.4 (978.3 to 1,942.9)	69.6 (43.1 to 101.2)	-10.1 (-18.0 to -1.2)	-20.3 (-27.4 to -12.5)
Maternal abortion, miscarriage, and ectopic pregnancy	113.3 (69.5 to 169.9)	13,798.7 (10,025.1 to 18,365.7)	12.4 (6.4 to 20.4)	3.1 (-34.4 to 66.6)	-8.7 (-41.9 to 47.6)
Other maternal disorders	--	--	24.0 (16.4 to 33.1)	-2.4 (-15.3 to 14.3)	-13.6 (-25.0 to 1.2)
**Neonatal disorders**	**5,547.1 (5,081.5 to 6,059.3)**	**--**	**1,043.5 (734.1 to 1,479.0)**	**29.8 (15.4 to 46.4)**	**14.9 (2.2 to 29.7)**
Neonatal preterm birth complications	4,618.8 (3,883.7 to 5,517.3)	--	681.6 (472.4 to 961.5)	26.7 (5.8 to 52.1)	12.2 (-6.3 to 34.8)
Neonatal encephalopathy due to birth asphyxia and trauma	867.1 (544.1 to 1,380.9)	--	125.4 (47.9 to 284.7)	35.2 (26.1 to 53.1)	19.7 (11.7 to 35.6)
Neonatal sepsis and other neonatal infections	471.5 (179.4 to 1,132.9)	--	183.6 (58.1 to 478.8)	39.8 (30.3 to 54.9)	23.8 (15.4 to 37.2)
Hemolytic disease and other neonatal jaundice	163.0 (144.5 to 182.0)	--	52.9 (40.6 to 66.3)	26.4 (20.3 to 32.5)	11.9 (6.5 to 17.4)
Other neonatal disorders	--	--	--	--	--
**Nutritional deficiencies**	**114,879.2 (110,198.8 to 119,576.2)**	**33,670.0† (28,012.8 to 41,331.0)**	**2,341.8 (1,511.3 to 3,429.4)**	**17.2 (13.5 to 20.7)**	**3.8 (0.5 to 6.9)**
Protein-energy malnutrition	--	--	--	--	--
Iodine deficiency	10,997.9 (9,732.5 to 12,396.0)	46.8 (39.0 to 56.1)	294.8 (198.2 to 412.6)	-5.2 (-8.1 to -2.6)	-16.0 (-18.6 to -13.8)
Vitamin A deficiency	30,454.5 (26,193.1 to 36,123.8)	33,623.3 (27,970.4 to 41,285.6)	13.3 (8.1 to 20.2)	20.5 (12.7 to 29.2)	6.8 (-0.2 to 14.4)
Iron-deficiency anaemia	86,355.9 (75,807.6 to 98,633.8)	--	2,033.8 (1,294.4 to 3,003.7)	21.3 (17.1 to 25.5)	7.5 (3.7 to 11.1)
Other nutritional deficiencies	--	--	--	--	--
**Other communicable, maternal, neonatal, and nutritional diseases**	**183,180.0 (179,671.2 to 186,804.9)**	**99,695.6 (82,372.8 to 122,769.3)**	**513.7 (323.2 to 796.8)**	**14.4 (9.8 to 19.6)**	**1.3 (-2.8 to 5.9)**
Sexually transmitted diseases excluding HIV	144,550.8 (140,205.2 to 148,568.5)	89,641.7 (72,148.9 to 112,174.5)	447.7 (278.7 to 696.4)	16.1 (12.3 to 20.7)	2.9 (-0.5 to 6.9)
Syphilis	8,776.3 (5,681.6 to 14,259.9)	5,744.6 (3,482.1 to 9,575.0)	11.0 (7.4 to 15.5)	19.3 (15.2 to 23.2)	5.6 (2.0 to 9.2)
Chlamydial infection	18,678.8 (13,447.4 to 26,119.2)	11,928.3 (8,312.8 to 16,974.5)	97.5 (56.3 to 158.1)	8.7 (4.7 to 12.4)	-3.8 (-7.3 to -0.4)
Gonococcal infection	11,368.5 (7,557.6 to 16,909.0)	37,865.4 (23,771.8 to 59,118.6)	115.0 (64.0 to 195.0)	29.8 (21.8 to 36.5)	15.0 (7.9 to 20.9)
Trichomoniasis	23,390.0 (15,477.0 to 32,229.1)	19,332.2 (12,332.9 to 27,134.7)	26.9 (10.1 to 58.4)	15.1 (13.1 to 17.0)	1.9 (0.1 to 3.6)
Genital herpes	93,976.5 (81,230.2 to 109,028.3)	7,247.8 (4,527.9 to 10,933.7)	24.9 (8.0 to 56.9)	14.7 (12.4 to 17.0)	1.6 (-0.5 to 3.6)
Other sexually transmitted diseases	7,200.9 (6,230.2 to 8,209.3)	7,523.4 (5,885.0 to 9,560.9)	172.5 (111.2 to 259.8)	12.8 (9.2 to 17.1)	-0.1 (-3.3 to 3.7)
Hepatitis	51,585.8 (43,842.1 to 61,566.2)	10,053.3 (6,700.7 to 14,911.8)	21.2 (10.9 to 37.4)	-13.8 (-52.2 to 46.0)	-23.6 (-57.6 to 29.3)
Acute hepatitis A	88.7 (10.2 to 211.1)	1,152.7 (133.2 to 2,744.4)	3.7 (0.4 to 9.2)	-17.0 (-91.7 to 576.1)	-26.5 (-92.6 to 498.8)
Hepatitis B	40,206.1 (32,465.0 to 49,745.2)	7,681.8 (4,669.6 to 12,316.6)	14.8 (6.5 to 29.6)	-11.8 (-57.3 to 72.5)	-21.9 (-62.2 to 52.8)
Hepatitis C	11,210.0 (9,948.1 to 12,539.6)	167.0 (122.0 to 219.7)	0.1 (0.1 to 0.3)	-9.8 (-10.5 to -9.2)	-20.2 (-20.8 to -19.6)
Acute hepatitis E	80.9 (66.3 to 99.0)	1,051.9 (861.7 to 1,287.3)	2.5 (1.5 to 3.9)	-20.0 (-36.6 to -0.1)	-29.1 (-43.9 to -11.5)
Other infectious diseases	2,045.0 (1,754.9 to 2,383.8)	0.6 (0.3 to 0.9)	44.9 (27.6 to 70.6)	14.9 (10.7 to 19.5)	1.8 (-2.0 to 5.8)
**Non-communicable diseases**	**523,198.5 (520,807.0 to 525,736.9)**	**1,397,318.4† (1,220,934.2 to 1,573,243.6)**	**46,052.8 (33,928.4 to 60,148.9)**	**12.8 (12.3 to 13.4)**	**-0.1 (-0.5 to 0.4)**
**Neoplasms**	**654.2 (635.3 to 672.6)**	**315.2 (300.4 to 336.5)**	**104.9 (76.2 to 138.5)**	**16.2 (12.6 to 21.6)**	**2.9 (-0.3 to 7.7)**
Lip and oral cavity cancer	39.3 (36.8 to 42.2)	10.6 (9.9 to 11.4)	4.1 (2.9 to 5.5)	19.9 (8.6 to 32.4)	6.2 (-3.9 to 17.2)
Nasopharynx cancer	19.6 (17.4 to 22.2)	5.5 (4.9 to 6.3)	2.3 (1.6 to 3.2)	-7.1 (-19.3 to 7.3)	-17.7 (-28.5 to -5.0)
Other pharynx cancer	5.8 (5.2 to 6.4)	2.3 (2.0 to 2.5)	0.8 (0.6 to 1.1)	20.6 (6.3 to 31.4)	6.8 (-5.9 to 16.4)
Oesophageal cancer	3.1 (2.9 to 3.3)	2.6 (2.5 to 2.7)	0.7 (0.5 to 0.9)	-5.7 (-11.5 to 0.7)	-16.5 (-21.6 to -10.8)
Stomach cancer	21.6 (20.7 to 22.5)	12.1 (11.7 to 12.6)	3.4 (2.4 to 4.5)	-10.0 (-13.9 to -5.7)	-20.2 (-23.7 to -16.5)
Colon and rectum cancer	55.5 (52.7 to 58.6)	15.6 (14.8 to 16.5)	5.9 (4.2 to 7.9)	14.7 (5.9 to 24.7)	1.6 (-6.2 to 10.5)
Liver cancer	16.3 (13.4 to 20.1)	17.7 (15.8 to 19.9)	4.1 (2.9 to 5.6)	5.4 (-5.7 to 20.0)	-6.7 (-16.5 to 6.3)
Liver cancer due to hepatitis B	14.6 (11.9 to 17.8)	11.0 (9.1 to 12.9)	2.6 (1.7 to 3.5)	4.3 (-6.9 to 20.4)	-7.6 (-17.5 to 6.7)

Cause	Prevalence in thousands (95% UI)	Incidence in thousands (95% UI)	YLDs in thousands (95% UI)		
	2016 counts	2016 counts	2016 counts	Percentage change in counts between 2006 and 2016	Percentage change in rates between 2006 and 2016
Liver cancer due to hepatitis C	1.2 (0.9 to 1.6)	1.0 (0.7 to 1.3)	0.2 (0.1 to 0.3)	16.5 (3.4 to 36.0)	3.2 (-8.4 to 20.5)
Liver cancer due to alcohol use	1.0 (0.7 to 1.3)	0.8 (0.6 to 1.1)	0.2 (0.1 to 0.3)	17.1 (2.5 to 33.0)	3.7 (-9.2 to 17.8)
Liver cancer due to other causes	6.7 (5.0 to 9.0)	4.9 (3.7 to 6.4)	1.2 (0.7 to 1.7)	4.1 (-9.7 to 23.2)	-7.8 (-20.0 to 9.1)
Gallbladder and biliary tract cancer	1.2 (1.0 to 1.2)	1.4 (1.2 to 1.5)	0.3 (0.2 to 0.4)	5.1 (-3.8 to 14.5)	-6.9 (-14.8 to 1.4)
Pancreatic cancer	1.9 (1.8 to 1.9)	2.4 (2.3 to 2.4)	0.5 (0.4 to 0.7)	14.7 (9.8 to 19.5)	1.6 (-2.7 to 5.9)
Larynx cancer	4.1 (3.9 to 4.4)	1.2 (1.2 to 1.3)	0.5 (0.4 to 0.7)	6.8 (-0.3 to 14.9)	-5.4 (-11.7 to 1.7)
Tracheal, bronchus, and lung cancer	13.9 (12.8 to 14.9)	10.6 (9.8 to 11.2)	2.8 (2.0 to 3.6)	3.1 (-3.8 to 10.1)	-8.7 (-14.8 to -2.5)
Malignant skin melanoma	56.1 (50.0 to 68.9)	11.3 (10.2 to 13.9)	3.7 (2.5 to 5.3)	28.0 (17.5 to 39.8)	13.4 (4.1 to 23.8)
Non-melanoma skin cancer	2.5 (1.1 to 5.2)	18.9 (9.3 to 39.7)	0.1 (< 0.05 to 0.2)	29.6 (13.1 to 46.9)	14.8 (0.1 to 30.1)
Non-melanoma skin cancer (squamous-cell carcinoma)	1.7 (0.6 to 3.9)	2.2 (0.9 to 4.7)	0.1 (< 0.05 to 0.2)	34.6 (14.3 to 53.1)	19.2 (1.3 to 35.6)
Non-melanoma skin cancer (basal-cell carcinoma)	1.9 (0.7 to 4.5)	16.7 (7.4 to 37.5)	< 0.05 (< 0.05 to 0.1)	12.5 (2.8 to 21.0)	-0.4 (-9.0 to 7.1)
Breast cancer	200.4 (182.2 to 216.0)	41.9 (38.4 to 45.1)	17.6 (12.5 to 23.9)	19.9 (8.0 to 33.6)	6.2 (-4.3 to 18.3)
Cervical cancer	139.6 (107.2 to 152.7)	36.3 (28.4 to 39.2)	13.6 (9.1 to 18.3)	0.5 (-8.4 to 12.5)	-11.0 (-18.9 to -0.4)
Uterine cancer	22.1 (20.4 to 24.4)	4.6 (4.3 to 5.1)	1.7 (1.2 to 2.4)	18.4 (3.9 to 37.6)	4.9 (-8.0 to 21.9)
Ovarian cancer	23.6 (21.9 to 24.9)	7.6 (7.2 to 8.0)	3.8 (2.6 to 5.0)	21.8 (10.6 to 30.2)	7.9 (-2.1 to 15.3)
Prostate cancer	5.6 (4.5 to 6.2)	1.4 (1.2 to 1.5)	0.6 (0.4 to 0.8)	41.3 (28.3 to 50.0)	25.2 (13.6 to 32.8)
Testicular cancer	60.6 (57.1 to 64.5)	11.8 (11.1 to 12.6)	4.1 (2.9 to 5.7)	32.2 (20.5 to 45.8)	17.1 (6.8 to 29.1)
Kidney cancer	19.4 (18.1 to 20.2)	5.1 (4.9 to 5.3)	1.8 (1.3 to 2.4)	22.3 (17.4 to 27.8)	8.3 (4.0 to 13.2)
Bladder cancer	14.0 (12.9 to 15.1)	3.4 (3.2 to 3.7)	1.4 (1.0 to 1.8)	26.3 (15.2 to 37.9)	11.9 (2.0 to 22.1)
Brain and nervous system cancer	38.2 (34.2 to 42.1)	16.8 (15.2 to 18.6)	5.4 (3.9 to 7.1)	39.1 (28.7 to 51.4)	23.2 (14.0 to 34.1)
Thyroid cancer	68.2 (63.2 to 74.9)	12.9 (12.0 to 14.2)	4.2 (2.9 to 6.0)	50.4 (35.4 to 69.2)	33.2 (19.9 to 49.9)
Mesothelioma	0.7 (0.6 to 0.8)	0.5 (0.4 to 0.5)	0.1 (0.1 to 0.2)	9.0 (2.1 to 16.2)	-3.5 (-9.6 to 2.9)
Hodgkin lymphoma	28.2 (25.5 to 32.8)	6.8 (6.1 to 7.9)	2.6 (1.9 to 3.6)	17.7 (7.6 to 28.2)	4.2 (-4.7 to 13.5)
Non-Hodgkin's lymphoma	37.9 (34.0 to 40.6)	11.3 (10.1 to 12.0)	3.7 (2.6 to 4.9)	32.7 (25.4 to 39.7)	17.5 (11.0 to 23.7)
Multiple myeloma	2.6 (2.1 to 2.9)	1.2 (1.0 to 1.3)	0.6 (0.4 to 0.8)	37.7 (27.8 to 46.8)	21.9 (13.2 to 30.0)
Leukaemia	43.0 (36.3 to 46.0)	12.9 (11.1 to 13.8)	5.7 (4.0 to 7.7)	-5.3 (-10.7 to 0.9)	-16.1 (-20.9 to -10.7)
Acute lymphoid leukaemia	8.3 (7.0 to 9.1)	2.4 (2.0 to 2.7)	1.1 (0.8 to 1.5)	15.0 (-0.4 to 23.0)	1.8 (-11.7 to 8.9)
Chronic lymphoid leukaemia	6.6 (5.6 to 7.3)	1.4 (1.2 to 1.6)	0.8 (0.6 to 1.1)	11.2 (-3.0 to 24.0)	-1.5 (-14.1 to 9.8)
Acute myeloid leukaemia	4.2 (3.7 to 4.7)	3.2 (2.8 to 3.5)	0.9 (0.6 to 1.2)	14.3 (8.1 to 20.2)	1.2 (-4.2 to 6.4)
Chronic myeloid leukaemia	4.7 (4.0 to 5.2)	1.7 (1.4 to 1.8)	0.6 (0.4 to 0.8)	-3.9 (-12.0 to 5.4)	-14.9 (-22.0 to -6.7)
Other leukaemia	19.2 (15.3 to 20.8)	4.2 (3.4 to 4.6)	2.4 (1.6 to 3.2)	-21.2 (-28.2 to -13.3)	-30.2 (-36.4 to -23.2)
Other neoplasms	97.5 (86.9 to 104.2)	28.5 (25.6 to 30.3)	8.7 (6.1 to 11.6)	42.9 (33.1 to 52.7)	26.5 (17.9 to 35.2)
**Cardiovascular diseases**	**10,494.9 (10,119.9 to 10,871.4)**	**735.9† (667.7 to 804.9)**	**758.7 (530.6 to 1,041.2)**	**12.9 (10.4 to 15.3)**	**0.0 (-2.2 to 2.1)**
Rheumatic heart disease	3,306.3 (2,973.2 to 3,677.7)	62.6 (54.1 to 74.2)	157.6 (99.6 to 231.4)	21.8 (17.2 to 26.2)	7.8 (3.8 to 11.8)
Ischaemic heart disease	2,770.6 (2,541.7 to 3,017.0)	353.6 (306.3 to 401.5)	165.1 (110.3 to 233.1)	18.3 (15.2 to 21.4)	4.8 (2.0 to 7.5)
Cerebrovascular disease	1,173.6 (1,106.8 to 1,273.0)	122.5‡ (90.4 to 158.7)	200.5 (139.6 to 272.1)	2.6 (-2.9 to 8.3)	-9.2 (-14.0 to -4.0)
Ischaemic stroke	964.4 (791.6 to 1,169.8)	78.3‡ (50.4 to 110.3)	148.8 (100.4 to 206.1)	2.4 (-4.8 to 9.7)	-9.3 (-15.6 to -2.8)
Hemorrhagic stroke	323.2 (262.5 to 398.7)	44.1‡ (30.0 to 60.0)	51.7 (34.2 to 72.0)	3.2 (-5.3 to 12.3)	-8.6 (-16.1 to -0.6)
Hypertensive heart disease	167.6 (119.2 to 221.1)	--	14.6 (8.7 to 22.8)	12.9 (4.3 to 21.7)	0.0 (-7.6 to 7.8)
Cardiomyopathy and myocarditis	268.7 (234.9 to 308.1)	117.4† (87.6 to 153.5)	30.9 (18.9 to 46.5)	10.7 (6.0 to 15.6)	-1.9 (-6.1 to 2.4)
Myocarditis	127.4 (100.4 to 155.2)	117.4 (87.6 to 153.5)	10.2 (6.2 to 15.3)	7.4 (1.1 to 13.9)	-4.9 (-10.5 to 0.9)
Alcoholic cardiomyopathy	37.6 (28.4 to 47.1)	--	3.3 (2.0 to 5.0)	7.6 (-6.9 to 22.2)	-4.7 (-17.6 to 8.2)
Other cardiomyopathy	201.1 (152.5 to 253.1)	--	17.5 (10.7 to 26.3)	13.4 (6.9 to 20.9)	0.5 (-5.4 to 7.0)
Atrial fibrillation and flutter	491.8 (382.6 to 602.6)	27.3 (20.9 to 34.9)	41.7 (25.8 to 60.9)	12.6 (6.0 to 20.3)	-0.3 (-6.2 to 6.5)
Peripheral vascular disease	--	--	--	--	--
Endocarditis	11.9 (8.7 to 16.0)	52.5 (37.9 to 72.6)	0.9 (0.6 to 1.4)	15.0 (13.1 to 16.9)	1.9 (0.1 to 3.5)
Other cardiovascular and circulatory diseases	2,735.0 (1,740.4 to 4,066.1)	--	147.3 (78.6 to 242.0)	14.4 (10.3 to 18.5)	1.4 (-2.3 to 4.9)
**Chronic respiratory diseases**	**24,585.3 (23,468.5 to 25,397.0)**	**4,696.8† (4,052.6 to 5,350.9)**	**1,231.6 (933.8 to 1,593.6)**	**18.4 (16.4 to 20.6)**	**4.8 (3.0 to 6.8)**
Chronic obstructive pulmonary disease	5,972.5 (5,523.9 to 6,466.9)	482.5 (405.2 to 573.1)	429.3 (363.8 to 487.6)	17.2 (13.3 to 21.2)	3.8 (0.4 to 7.3)
Pneumoconiosis	11.3 (9.7 to 13.1)	1.2 (1.0 to 1.5)	1.8 (1.2 to 2.5)	10.4 (8.9 to 12.0)	-2.2 (-3.6 to -0.8)
Silicosis	3.2 (2.4 to 4.1)	0.4 (0.3 to 0.6)	0.5 (0.3 to 0.8)	5.5 (3.4 to 7.7)	-6.6 (-8.4 to -4.7)

Cause	Prevalence in thousands (95% UI)	Incidence in thousands (95% UI)	YLDs in thousands (95% UI)		
	2016 counts	2016 counts	2016 counts	Percentage change in counts between 2006 and 2016	Percentage change in rates between 2006 and 2016
Asbestosis	2.9<br>(2.1 to 3.8)	0.2<br>(0.1 to 0.3)	0.5<br>(0.3 to 0.7)	16.9<br>(14.0 to 19.6)	3.6<br>(0.9 to 5.9)
Coal workers pneumoconiosis	1.1<br>(0.8 to 1.4)	0.1<br>(0.1 to 0.2)	0.2<br>(0.1 to 0.2)	1.6<br>(-2.7 to 6.5)	-10.0<br>(-13.8 to -5.7)
Other pneumoconiosis	4.1<br>(3.1 to 5.5)	0.4<br>(0.3 to 0.6)	0.7<br>(0.4 to 1.0)	12.6<br>(9.6 to 15.9)	-0.3<br>(-3.0 to 2.6)
Asthma	18,705.9<br>(16,282.1 to 21,496.8)	4,207.4<br>(3,588.2 to 4,876.0)	736.1<br>(470.9 to 1,079.8)	16.9<br>(14.6 to 19.1)	3.6<br>(1.5 to 5.5)
Interstitial lung disease and pulmonary sarcoidosis	83.4<br>(70.3 to 97.9)	5.7<br>(3.8 to 8.1)	9.3<br>(5.9 to 14.0)	14.7<br>(5.6 to 25.2)	1.6<br>(-6.5 to 10.9)
Other chronic respiratory diseases	--	--	55.1<br>(46.5 to 62.5)	58.9<br>(53.7 to 64.2)	40.7<br>(36.1 to 45.4)
**Cirrhosis and other chronic liver diseases**	**2,779.0<br>(2,536.1 to 3,074.6)**	**58.1<br>(46.4 to 69.7)**	**74.7<br>(51.1 to 103.7)**	**18.9<br>(13.1 to 24.2)**	**5.2<br>(0.2 to 10.0)**
Cirrhosis and other chronic liver diseases due to hepatitis B	841.4<br>(706.7 to 991.1)	20.0<br>(15.6 to 25.2)	24.4<br>(16.1 to 35.3)	17.8<br>(8.2 to 28.8)	4.3<br>(-4.2 to 14.1)
Cirrhosis and other chronic liver diseases due to hepatitis C	692.3<br>(569.8 to 827.1)	14.3<br>(10.8 to 18.3)	18.3<br>(11.8 to 26.3)	22.0<br>(11.4 to 33.1)	8.1<br>(-1.4 to 17.9)
Cirrhosis and other chronic liver diseases due to alcohol use	542.6<br>(451.1 to 639.5)	11.3<br>(8.6 to 14.2)	13.9<br>(9.2 to 20.3)	18.9<br>(8.2 to 30.1)	5.3<br>(-4.2 to 15.2)
Cirrhosis and other chronic liver diseases due to other causes	702.7<br>(552.7 to 863.7)	12.6<br>(9.2 to 16.4)	18.0<br>(11.2 to 26.1)	17.2<br>(6.6 to 28.1)	3.8<br>(-5.6 to 13.4)
**Digestive diseases**	**13,626.4<br>(12,918.1 to 14,379.0)**	**10,587.4†<br>(9,130.5 to 12,144.1)**	**395.6<br>(269.1 to 562.4)**	**9.2<br>(4.6 to 14.6)**	**-3.3<br>(-7.4 to 1.5)**
Peptic ulcer disease	4,789.6<br>(3,791.2 to 6,039.2)	845.4<br>(608.3 to 1,136.6)	91.7<br>(53.0 to 149.6)	8.6<br>(-1.2 to 18.6)	-3.9<br>(-12.5 to 5.0)
Gastritis and duodenitis	5,127.2<br>(3,845.7 to 6,692.6)	3,734.9<br>(2,749.5 to 5,035.3)	83.6<br>(47.2 to 133.2)	2.9<br>(-7.3 to 13.1)	-8.9<br>(-17.9 to 0.2)
Appendicitis	59.8<br>(47.1 to 73.0)	1,520.5<br>(1,200.8 to 1,838.7)	18.4<br>(11.5 to 26.9)	15.5<br>(3.2 to 27.6)	2.2<br>(-8.6 to 13.0)
Paralytic ileus and intestinal obstruction	7.5<br>(5.5 to 10.0)	203.1<br>(146.3 to 272.4)	2.5<br>(1.4 to 3.7)	19.3<br>(16.8 to 21.8)	5.7<br>(3.4 to 7.9)
Inguinal, femoral, and abdominal hernia	2,760.6<br>(1,909.9 to 3,824.9)	1,178.6<br>(794.2 to 1,684.3)	106.4<br>(60.4 to 166.5)	11.5<br>(5.8 to 17.1)	-1.3<br>(-6.4 to 3.7)
Inflammatory bowel disease	325.0<br>(280.5 to 376.0)	353.4<br>(288.3 to 434.5)	48.7<br>(31.9 to 69.3)	12.6<br>(7.5 to 17.7)	-0.3<br>(-4.8 to 4.2)
Vascular intestinal disorders	2.7<br>(1.6 to 4.4)	16.6<br>(10.7 to 23.8)	0.9<br>(0.4 to 1.6)	16.3<br>(13.8 to 18.2)	3.0<br>(0.8 to 4.7)
Gallbladder and biliary diseases	1,045.9<br>(762.4 to 1,381.2)	2,476.9<br>(1,809.5 to 3,353.8)	12.3<br>(7.0 to 19.6)	11.8<br>(5.5 to 17.9)	-1.0<br>(-6.6 to 4.4)
Pancreatitis	29.1<br>(21.5 to 38.9)	258.1<br>(188.8 to 346.7)	3.8<br>(2.3 to 5.7)	14.4<br>(7.9 to 20.2)	1.3<br>(-4.5 to 6.5)
Other digestive diseases	--	--	27.4<br>(18.6 to 38.9)	11.0<br>(6.3 to 16.5)	-1.7<br>(-5.8 to 3.2)
**Neurological disorders**	**274,616.8<br>(258,574.1 to 297,508.4)**	**136,321.3<br>(89,288.5 to 196,387.1)**	**6,545.0<br>(4,361.0 to 8,988.8)**	**14.4<br>(12.9 to 15.9)**	**1.3<br>(0.0 to 2.7)**
Alzheimer's disease and other dementias	--	--	--	--	--
Parkinson's disease	24.1<br>(13.4 to 40.9)	4.0<br>(2.4 to 6.4)	3.2<br>(1.6 to 6.0)	19.3<br>(12.3 to 26.4)	5.7<br>(-0.6 to 11.9)
Epilepsy	1,605.1<br>(1,303.5 to 1,915.5)	114.4<br>(72.1 to 161.6)	509.3<br>(334.1 to 716.5)	8.0<br>(-3.6 to 21.1)	-4.4<br>(-14.6 to 7.3)
Multiple sclerosis	207.1<br>(177.5 to 236.4)	12.9<br>(9.4 to 16.6)	56.1<br>(38.3 to 76.0)	22.6<br>(16.0 to 29.5)	8.6<br>(2.7 to 14.7)
Motor neuron disease	17.7<br>(14.6 to 21.2)	0.9<br>(0.6 to 1.4)	3.8<br>(2.5 to 5.2)	11.7<br>(10.8 to 12.7)	-1.1<br>(-1.9 to -0.2)
Migraine	120,909.4<br>(111,727.5 to 129,951.1)	11,259.6<br>(9,445.0 to 13,178.8)	5,137.9<br>(3,275.7 to 7,224.9)	14.7<br>(13.7 to 15.7)	1.5<br>(0.7 to 2.4)
Tension-type headache	208,325.1<br>(156,814.3 to 277,938.4)	124,926.7<br>(77,366.8 to 185,518.3)	755.4<br>(462.4 to 1,142.7)	15.2<br>(13.4 to 17.4)	2.0<br>(0.5 to 4.0)
Other neurological disorders	1.4<br>(0.7 to 2.6)	2.9<br>(1.5 to 4.8)	79.3<br>(53.0 to 109.9)	31.8<br>(19.2 to 46.4)	16.7<br>(5.6 to 29.6)
**Mental and substance use disorders**	**104,369.1<br>(102,454.4 to 106,410.6)**	**35,906.6†<br>(31,172.8 to 41,906.1)**	**15,466.8<br>(11,344.9 to 20,140.3)**	**12.7<br>(11.9 to 13.6)**	**-0.2<br>(-0.9 to 0.6)**
Schizophrenia	2,663.5<br>(2,194.6 to 3,155.9)	152.6<br>(95.5 to 224.8)	1,753.7<br>(1,247.2 to 2,266.4)	8.0<br>(5.9 to 10.1)	-4.3<br>(-6.2 to -2.5)
Alcohol use disorders	14,126.7<br>(11,356.4 to 17,244.7)	7,751.7<br>(6,098.7 to 9,561.7)	1,429.5<br>(948.9 to 2,086.4)	10.8<br>(6.9 to 14.3)	-1.9<br>(-5.3 to 1.2)
Drug use disorders	8,911.0<br>(8,544.8 to 9,179.0)	579.3<br>(454.4 to 725.1)	2,422.9<br>(1,688.6 to 3,246.3)	17.9<br>(15.0 to 20.6)	4.4<br>(1.9 to 6.8)
Opioid use disorders	4,756.4<br>(3,814.5 to 5,712.0)	281.9<br>(186.8 to 401.4)	1,999.4<br>(1,364.3 to 2,731.9)	19.1<br>(15.9 to 22.3)	5.5<br>(2.6 to 8.3)
Cocaine use disorders	678.9<br>(593.1 to 773.0)	14.8<br>(9.2 to 21.6)	93.7<br>(57.6 to 134.9)	14.8<br>(9.7 to 20.3)	1.7<br>(-2.9 to 6.5)
Amphetamine use disorders	610.7<br>(430.3 to 845.4)	16.2<br>(6.8 to 30.4)	80.3<br>(44.9 to 127.1)	11.4<br>(3.7 to 18.7)	-1.4<br>(-8.2 to 5.1)
Cannabis use disorders	2,458.7<br>(1,923.1 to 3,118.2)	173.4<br>(111.5 to 255.7)	71.8<br>(42.2 to 108.8)	7.9<br>(3.3 to 12.5)	-4.4<br>(-8.5 to -0.4)
Other drug use disorders	587.6<br>(482.3 to 702.7)	93.0<br>(70.7 to 114.7)	177.7<br>(119.2 to 243.5)	13.4<br>(9.7 to 17.4)	0.4<br>(-2.8 to 3.9)
Depressive disorders	22,352.8<br>(21,031.3 to 23,602.2)	22,720.1<br>(18,267.5 to 28,211.6)	3,765.9<br>(2,463.6 to 5,380.8)	10.4<br>(8.7 to 12.0)	-2.2<br>(-3.7 to -0.8)
Major depressive disorder	13,937.5<br>(10,938.9 to 17,467.4)	21,242.0<br>(16,737.7 to 26,768.4)	2,901.2<br>(1,863.9 to 4,294.8)	9.4<br>(7.0 to 11.3)	-3.1<br>(-5.3 to -1.4)
Dysthymia	8,823.5<br>(7,195.5 to 10,882.9)	1,478.1<br>(1,190.8 to 1,796.4)	864.8<br>(555.6 to 1,304.5)	13.9<br>(11.2 to 16.5)	0.9<br>(-1.5 to 3.1)
Bipolar disorder	4,444.7<br>(3,675.1 to 5,427.2)	231.1<br>(145.4 to 342.0)	919.3<br>(552.7 to 1,377.2)	16.1<br>(13.9 to 18.2)	2.9<br>(0.9 to 4.7)
Anxiety disorders	24,429.7<br>(21,750.9 to 27,508.1)	3,764.3<br>(3,376.9 to 4,210.4)	2,380.9<br>(1,638.4 to 3,281.3)	14.6<br>(12.6 to 16.4)	1.5<br>(-0.3 to 3.1)
Eating disorders	1,867.7<br>(1,608.7 to 2,237.5)	433.2<br>(281.9 to 636.4)	378.2<br>(221.2 to 579.4)	23.5<br>(20.6 to 26.4)	9.4<br>(6.8 to 12.0)
Anorexia nervosa	293.1<br>(205.7 to 425.2)	65.1<br>(43.4 to 91.0)	61.9<br>(36.4 to 99.2)	16.9<br>(10.0 to 23.2)	3.5<br>(-2.6 to 9.1)
Bulimia nervosa	1,506.5<br>(1,026.5 to 2,123.4)	368.1<br>(213.5 to 573.3)	316.4<br>(178.8 to 502.5)	24.9<br>(21.5 to 28.1)	10.6<br>(7.6 to 13.5)
Autistic spectrum disorders	4,919.5<br>(4,684.0 to 5,130.5)	--	718.9<br>(490.3 to 1,009.4)	13.1<br>(11.5 to 14.8)	0.1<br>(-1.3 to 1.6)
Autism	1,454.2<br>(1,251.2 to 1,691.3)	--	371.5<br>(239.6 to 538.6)	12.7<br>(10.0 to 15.5)	-0.2<br>(-2.5 to 2.3)
Asperger syndrome and other autistic spectrum disorders	3,460.8<br>(2,884.9 to 4,153.8)	--	347.4<br>(228.4 to 503.2)	13.5<br>(11.9 to 15.3)	0.6<br>(-0.9 to 2.1)

Cause	Prevalence in thousands (95% UI)	Incidence in thousands (95% UI)	YLDs in thousands (95% UI)		
	2016 counts	2016 counts	2016 counts	Percentage change in counts between 2006 and 2016	Percentage change in rates between 2006 and 2016
Attention-deficit/hyperactivity disorder	4,715.6 (4,212.6 to 5,343.1)	--	56.7 (33.7 to 89.2)	11.1 (8.3 to 13.9)	-1.6 (-4.1 to 0.9)
Conduct disorder	--	--	--	--	--
Idiopathic developmental intellectual disability	10,216.1 (6,323.2 to 14,187.5)	--	405.7 (196.1 to 689.1)	4.9 (0.7 to 7.7)	-7.1 (-10.8 to -4.6)
Other mental and substance use disorders	16,281.9 (14,193.9 to 18,875.9)	274.2 (178.1 to 410.8)	1,235.1 (841.4 to 1,787.7)	13.0 (11.4 to 14.7)	0.1 (-1.4 to 1.6)
**Diabetes, urogenital, blood, and endocrine diseases**	**223,291.7 (220,879.0 to 225,901.1)**	**85,754.5† (75,551.4 to 97,612.0)**	**3,688.5 (2,550.5 to 5,137.9)**	**11.3 (9.3 to 13.3)**	**-1.5 (-3.2 to 0.4)**
Diabetes mellitus	18,813.8 (15,923.2 to 22,110.4)	1,421.6 (1,035.5 to 1,865.4)	1,296.7 (841.2 to 1,869.2)	10.2 (6.8 to 13.2)	-2.4 (-5.4 to 0.3)
Acute glomerulonephritis	5.9 (3.9 to 8.5)	87.2 (57.7 to 124.2)	0.3 (0.2 to 0.5)	-2.4 (-5.0 to 0.1)	-13.5 (-15.8 to -11.4)
Chronic kidney disease	8,635.7 (7,172.4 to 10,261.8)	590.4 (376.1 to 832.4)	427.2 (298.1 to 595.1)	21.1 (16.3 to 26.0)	7.2 (3.0 to 11.6)
Chronic kidney disease due to diabetes mellitus	2,266.4 (1,579.5 to 3,204.8)	150.3 (83.4 to 237.9)	122.7 (74.4 to 189.1)	20.2 (13.4 to 26.9)	6.5 (0.4 to 12.4)
Chronic kidney disease due to hypertension	1,764.4 (1,269.3 to 2,392.1)	194.6 (111.1 to 308.9)	39.3 (23.8 to 61.2)	17.9 (11.4 to 25.3)	4.4 (-1.3 to 11.0)
Chronic kidney disease due to glomerulonephritis	1,778.5 (1,277.5 to 2,342.7)	49.9 (28.7 to 78.4)	134.2 (80.2 to 200.0)	21.1 (14.8 to 27.6)	7.3 (1.6 to 13.0)
Chronic kidney disease due to other causes	2,826.4 (1,986.9 to 3,926.6)	195.6 (116.7 to 305.0)	131.1 (80.5 to 202.3)	22.9 (16.3 to 30.1)	8.9 (3.0 to 15.2)
Urinary diseases and male infertility	7,170.4 (5,927.5 to 9,003.3)	41,788.5† (32,388.9 to 53,021.0)	89.3 (52.5 to 147.4)	12.3 (8.4 to 16.3)	-0.6 (-4.0 to 3.0)
Interstitial nephritis and urinary tract infections	712.2 (525.3 to 941.6)	36,596.2 (27,320.4 to 47,627.3)	23.7 (13.7 to 37.9)	14.2 (9.5 to 19.0)	1.1 (-3.0 to 5.4)
Urolithiasis	194.0 (138.4 to 259.5)	5,192.4 (3,708.4 to 6,916.6)	14.9 (9.0 to 22.3)	11.6 (7.1 to 16.6)	-1.2 (-5.2 to 3.2)
Benign prostatic hyperplasia	--	--	--	--	--
Male infertility	5,747.5 (3,794.3 to 8,283.2)	--	33.0 (12.5 to 72.6)	21.7 (15.8 to 26.8)	7.8 (2.6 to 12.3)
Other urinary diseases	--	--	17.7 (11.2 to 26.0)	-3.4 (-6.1 to -0.5)	-14.4 (-16.8 to -11.9)
Gynecological diseases	90,820.4 (87,980.9 to 92,695.0)	41,227.6† (37,117.6 to 45,651.0)	1,313.7 (876.1 to 1,889.4)	9.4 (6.6 to 11.9)	-3.1 (-5.6 to -0.9)
Uterine fibroids	4,921.9 (3,976.1 to 5,968.5)	1,219.4 (852.2 to 1,610.1)	112.3 (64.8 to 179.0)	21.3 (18.3 to 24.9)	7.4 (4.8 to 10.6)
Polycystic ovarian syndrome	1,902.2 (1,499.5 to 2,388.2)	1,324.4 (900.3 to 1,825.2)	16.8 (7.7 to 31.7)	13.2 (10.5 to 16.1)	0.3 (-2.2 to 2.8)
Female infertility	7,124.9 (2,363.7 to 14,286.5)	--	39.3 (9.1 to 106.5)	46.8 (31.0 to 95.0)	30.0 (16.0 to 72.7)
Endometriosis	413.8 (347.4 to 498.2)	980.0 (717.4 to 1,267.4)	38.8 (25.4 to 55.1)	7.5 (0.6 to 14.5)	-4.8 (-11.0 to 1.4)
Genital prolapse	7,987.7 (6,209.3 to 10,253.9)	1,003.4 (684.7 to 1,424.0)	25.6 (11.6 to 49.8)	10.2 (7.3 to 13.3)	-2.4 (-5.0 to 0.3)
Premenstrual syndrome	64,622.7 (58,249.9 to 70,866.9)	22,461.5 (19,966.7 to 25,146.8)	541.5 (330.9 to 823.6)	9.8 (5.8 to 13.3)	-2.8 (-6.3 to 0.3)
Other gynecological diseases	15,343.6 (12,558.4 to 18,484.9)	14,239.0 (11,234.8 to 17,774.4)	539.5 (353.0 to 786.9)	4.9 (1.2 to 8.1)	-7.2 (-10.4 to -4.3)
Hemoglobinopathies and hemolytic anaemias	136,537.1 (134,851.9 to 138,467.4)	--	415.7 (259.4 to 625.5)	9.4 (0.9 to 17.6)	-3.1 (-10.7 to 4.2)
Thalassemias	2.3 (2.2 to 2.4)	--	0.2 (0.1 to 0.3)	0.3 (-17.2 to 18.0)	-11.2 (-26.6 to 4.5)
Thalassaemias trait	22,485.9 (21,611.8 to 23,503.6)	--	247.7 (152.3 to 380.9)	-1.8 (-13.0 to 8.6)	-13.1 (-22.9 to -3.9)
Sickle cell disorders	89.5 (81.4 to 98.2)	--	11.7 (8.3 to 15.8)	53.6 (42.4 to 65.4)	36.0 (26.1 to 46.5)
Sickle cell trait	34,566.3 (31,271.1 to 38,994.2)	--	100.7 (63.5 to 150.3)	29.1 (23.9 to 34.9)	14.3 (9.7 to 19.4)
G6PD deficiency	25,288.7 (24,363.3 to 26,382.8)	--	1.6 (1.0 to 2.4)	29.9 (23.2 to 36.6)	15.0 (9.1 to 20.9)
G6PD trait	65,677.8 (64,596.0 to 66,897.2)	--	0.1 (< 0.05 to 0.1)	12.5 (4.1 to 22.5)	-0.3 (-7.8 to 8.5)
Other hemoglobinopathies and hemolytic anaemias	3,697.7 (3,154.9 to 4,336.3)	--	53.7 (33.5 to 84.0)	32.9 (26.3 to 39.9)	17.7 (11.8 to 23.9)
Endocrine, metabolic, blood, and immune disorders	5,386.4 (4,577.6 to 6,290.9)	639.2 (426.6 to 910.9)	145.7 (92.6 to 215.2)	16.9 (13.6 to 20.6)	3.5 (0.6 to 6.8)
**Musculoskeletal disorders**	**91,735.0 (88,054.9 to 96,775.4)**	**51,734.5 (39,842.6 to 63,950.5)**	**9,995.9 (6,815.2 to 13,855.9)**	**11.8 (10.0 to 13.5)**	**-1.0 (-2.6 to 0.6)**
Rheumatoid arthritis	1,005.0 (802.9 to 1,233.9)	75.0 (54.8 to 100.5)	246.1 (159.1 to 348.0)	18.2 (13.8 to 22.2)	4.7 (0.8 to 8.3)
Osteoarthritis	7,314.8 (6,688.6 to 7,983.5)	706.6 (607.0 to 824.3)	441.3 (306.4 to 607.3)	15.8 (14.0 to 17.6)	2.5 (1.0 to 4.2)
Low back and neck pain	55,187.1 (50,988.6 to 59,323.4)	23,352.1 (17,396.1 to 30,169.8)	6,405.2 (4,172.9 to 8,876.5)	11.1 (8.7 to 13.1)	-1.7 (-3.8 to 0.2)
Low back pain	38,618.2 (28,335.5 to 50,977.8)	17,859.5 (12,572.7 to 24,461.4)	4,474.2 (2,735.0 to 6,588.9)	10.5 (7.3 to 13.3)	-2.1 (-5.0 to 0.3)
Neck pain	18,812.3 (13,279.3 to 25,356.1)	5,492.5 (3,598.4 to 8,178.1)	1,931.0 (1,142.0 to 2,982.4)	12.3 (9.6 to 14.9)	-0.6 (-3.0 to 1.7)
Gout	1,291.5 (971.9 to 1,684.9)	293.0 (229.2 to 371.7)	43.8 (27.0 to 65.0)	10.7 (4.6 to 17.2)	-2.0 (-7.4 to 3.8)
Other musculoskeletal disorders	32,580.8 (22,030.9 to 44,938.1)	27,307.8 (17,374.0 to 38,924.7)	2,859.5 (1,678.5 to 4,629.1)	12.4 (8.0 to 16.5)	-0.4 (-4.4 to 3.2)
**Other non-communicable diseases**	**377,910.9 (369,368.3 to 385,857.7)**	**1,071,208.2† (904,681.8 to 1,233,790.4)**	**7,791.0 (5,227.9 to 11,169.6)**	**13.2 (12.3 to 14.0)**	**0.2 (-0.6 to 0.9)**
Congenital anomalies	5,479.7 (5,106.6 to 5,812.2)	--	648.4 (457.7 to 865.2)	12.2 (9.6 to 15.1)	-0.7 (-3.0 to 1.9)
Neural tube defects	432.1 (390.5 to 478.7)	--	130.2 (91.5 to 173.1)	19.8 (14.8 to 25.4)	6.1 (1.7 to 11.0)
Congenital heart anomalies	748.7 (665.2 to 832.3)	--	40.4 (16.8 to 69.7)	11.7 (7.9 to 15.6)	-1.1 (-4.5 to 2.4)
Orofacial clefts	257.9 (229.8 to 286.7)	--	3.3 (2.1 to 4.8)	4.0 (0.6 to 7.1)	-7.9 (-10.9 to -5.1)
Down's syndrome	186.9 (162.7 to 214.1)	--	16.0 (10.8 to 22.6)	14.1 (9.7 to 18.8)	1.0 (-2.9 to 5.2)
Turner syndrome	220.2 (190.9 to 250.9)	--	4.5 (2.3 to 7.2)	14.4 (3.4 to 26.4)	1.3 (-8.4 to 12.0)
Klinefelter syndrome	230.8 (198.1 to 269.2)	--	2.3 (1.1 to 4.5)	13.6 (6.6 to 20.3)	0.6 (-5.6 to 6.5)

Cause	Prevalence in thousands (95% UI)	Incidence in thousands (95% UI)	YLDs in thousands (95% UI)		
	2016 counts	2016 counts	2016 counts	Percentage change in counts between 2006 and 2016	Percentage change in rates between 2006 and 2016
Other chromosomal abnormalities	0.1 (0.1 to 0.1)	--	0.1 (< 0.05 to 0.1)	15.3 (-41.5 to 121.8)	2.1 (-48.2 to 96.4)
Congenital musculoskeletal and limb anomalies	798.8 (736.3 to 867.0)	--	116.8 (80.1 to 160.6)	16.7 (13.0 to 20.4)	3.3 (0.1 to 6.6)
Urogenital congenital anomalies	285.9 (258.1 to 314.8)	--	5.0 (2.6 to 8.6)	9.8 (6.3 to 12.9)	-2.8 (-5.9 to 0.0)
Digestive congenital anomalies	739.3 (665.5 to 818.0)	--	32.5 (21.2 to 46.5)	7.0 (1.7 to 12.1)	-5.2 (-10.0 to -0.7)
Other congenital anomalies	2,675.1 (1,880.3 to 3,785.8)	--	297.3 (191.7 to 442.2)	8.1 (3.9 to 13.0)	-4.2 (-8.0 to 0.1)
Skin and subcutaneous diseases	139,198.8 (135,713.6 to 142,256.9)	289,418.1 (259,478.5 to 321,830.0)	3,377.1 (2,265.6 to 4,789.1)	13.7 (12.9 to 14.5)	0.7 (-0.0 to 1.4)
Dermatitis	18,019.7 (15,338.9 to 20,808.7)	44,440.9 (35,986.3 to 53,240.3)	590.1 (346.3 to 919.6)	12.6 (10.8 to 14.4)	-0.3 (-1.9 to 1.3)
Psoriasis	5,274.6 (4,812.8 to 5,733.8)	661.7 (576.6 to 749.6)	466.7 (328.0 to 618.6)	19.6 (16.5 to 22.6)	5.9 (3.2 to 8.5)
Cellulitis	199.6 (162.4 to 242.7)	3,794.8 (3,133.8 to 4,494.7)	11.4 (7.3 to 17.1)	15.5 (8.0 to 23.0)	2.2 (-4.3 to 9.0)
Pyoderma	830.0 (762.6 to 904.4)	18,591.5 (16,789.2 to 20,731.4)	4.7 (1.9 to 9.8)	17.8 (13.9 to 21.7)	4.3 (0.8 to 7.8)
Scabies	9,940.3 (6,802.6 to 13,946.9)	29,749.8 (20,122.9 to 41,716.7)	257.5 (124.4 to 451.4)	3.1 (1.6 to 4.6)	-8.7 (-10.1 to -7.4)
Fungal skin diseases	33,113.3 (25,167.7 to 42,481.7)	109,057.9 (81,115.1 to 139,231.3)	187.8 (74.2 to 394.1)	15.1 (14.3 to 15.9)	1.9 (1.2 to 2.6)
Viral skin diseases	11,649.4 (9,732.6 to 13,807.2)	18,399.7 (15,624.9 to 21,704.8)	356.8 (219.2 to 538.1)	13.2 (11.8 to 14.7)	0.2 (-1.0 to 1.6)
Acne vulgaris	38,115.6 (33,137.5 to 44,287.8)	18,447.1 (15,660.1 to 21,349.5)	974.5 (641.9 to 1,435.9)	14.2 (12.6 to 15.8)	1.1 (-0.3 to 2.6)
Alopecia areata	2,094.8 (1,866.1 to 2,324.6)	3,623.2 (3,291.6 to 4,013.0)	69.0 (43.2 to 104.2)	11.6 (8.6 to 14.5)	-1.2 (-3.8 to 1.4)
Pruritus	4,962.8 (3,471.1 to 6,825.6)	3,938.8 (2,712.6 to 5,442.9)	53.3 (22.8 to 103.7)	14.3 (12.5 to 16.1)	1.2 (-0.4 to 2.8)
Urticaria	3,958.8 (2,781.3 to 5,513.6)	6,911.8 (4,879.8 to 9,573.7)	236.8 (136.8 to 368.8)	13.2 (10.8 to 15.7)	0.2 (-1.8 to 2.5)
Decubitus ulcer	38.5 (25.9 to 54.7)	138.9 (90.5 to 197.5)	6.8 (3.9 to 10.5)	14.2 (12.8 to 15.6)	1.2 (-0.1 to 2.4)
Other skin and subcutaneous diseases	28,956.0 (26,154.1 to 31,955.3)	31,661.8 (28,360.9 to 34,697.2)	161.7 (77.0 to 297.5)	18.7 (17.6 to 19.8)	5.2 (4.1 to 6.1)
Sense organ diseases	88,815.0 (85,495.0 to 91,942.9)	38,162.6† (34,322.3 to 42,293.3)	2,624.7 (1,729.2 to 3,739.5)	12.0 (9.8 to 14.0)	-0.8 (-2.7 to 0.9)
Glaucoma	--	--	--	--	--
Cataract	511.1 (365.8 to 680.1)	--	54.2 (31.2 to 85.3)	11.9 (4.1 to 18.9)	-0.9 (-7.8 to 5.4)
Macular degeneration	--	--	--	--	--
Refraction and accommodation disorders	35,052.5 (31,632.2 to 38,923.7)	--	690.5 (423.1 to 1,079.2)	11.7 (10.4 to 13.1)	-1.1 (-2.2 to 0.2)
Age-related and other hearing loss	47,989.4 (38,545.3 to 60,363.6)	--	1,444.2 (936.2 to 2,104.5)	11.5 (7.7 to 15.1)	-1.2 (-4.6 to 1.9)
Other vision loss	1,038.2 (789.0 to 1,352.2)	--	88.9 (56.5 to 132.6)	13.7 (7.6 to 19.4)	0.7 (-4.7 to 5.8)
Other sense organ diseases	10,819.9 (9,507.9 to 12,201.4)	38,162.6 (34,322.3 to 42,293.3)	346.9 (214.1 to 535.6)	14.0 (12.1 to 16.1)	1.0 (-0.7 to 2.8)
Oral disorders	271,807.3 (254,197.1 to 289,969.9)	743,627.6† (582,412.6 to 906,165.2)	1,140.8 (642.7 to 1,883.6)	15.0 (13.8 to 16.1)	1.8 (0.8 to 2.8)
Caries of deciduous teeth	--	--	--	--	--
Caries of permanent teeth	231,036.3 (190,179.0 to 275,346.6)	734,504.5 (574,892.3 to 897,418.9)	163.5 (69.6 to 322.0)	9.1 (7.9 to 10.2)	-3.4 (-4.5 to -2.4)
Periodontal disease	54,060.8 (38,835.6 to 75,864.5)	8,526.5 (4,502.1 to 12,772.6)	360.9 (131.5 to 799.4)	20.5 (19.4 to 21.5)	6.7 (5.8 to 7.6)
Edentulism and severe tooth loss	8,214.3 (6,286.5 to 10,558.0)	596.5 (364.8 to 923.8)	236.5 (144.0 to 358.9)	14.4 (12.4 to 16.3)	1.4 (-0.5 to 3.0)
Other oral disorders	12,833.0 (11,571.2 to 14,230.7)	--	380.0 (234.6 to 573.6)	13.1 (11.8 to 14.3)	0.1 (-1.0 to 1.2)
**Injuries**	**104,783.5 (98,042.9 to 112,995.8)**	**41,444.8† (36,279.4 to 46,742.6)**	**3,842.7 (2,537.6 to 5,493.8)**	**15.6 (13.9 to 17.0)**	**2.4 (0.9 to 3.6)**
**Transport injuries**	**16,402.4 (14,824.0 to 18,011.5)**	**6,669.0 (5,156.9 to 8,458.1)**	**878.1 (578.6 to 1,275.5)**	**16.2 (14.7 to 17.4)**	**2.9 (1.6 to 4.0)**
Road injuries	13,036.3 (11,672.7 to 14,501.4)	6,114.9 (4,605.8 to 7,866.3)	701.3 (458.3 to 1,021.0)	17.8 (16.4 to 19.0)	4.3 (3.0 to 5.4)
Pedestrian road injuries	2,742.5 (2,289.1 to 3,238.5)	938.5 (564.3 to 1,388.6)	149.6 (98.2 to 217.4)	16.9 (15.3 to 18.4)	3.6 (2.1 to 4.9)
Cyclist road injuries	2,520.8 (1,990.8 to 3,167.3)	1,157.1 (707.4 to 1,726.2)	124.4 (81.0 to 184.4)	19.2 (17.8 to 20.7)	5.6 (4.3 to 6.9)
Motorcyclist road injuries	3,454.4 (2,716.5 to 4,473.2)	1,321.0 (854.1 to 1,856.7)	183.7 (119.2 to 267.6)	17.4 (15.8 to 18.9)	4.0 (2.5 to 5.3)
Motor vehicle road injuries	3,334.7 (2,726.6 to 4,084.1)	2,101.2 (1,464.4 to 2,893.0)	195.1 (127.7 to 281.4)	14.5 (12.7 to 16.2)	1.4 (-0.2 to 2.9)
Other road injuries	984.0 (779.3 to 1,217.0)	597.2 (337.9 to 992.9)	48.5 (31.2 to 72.6)	34.1 (32.1 to 36.1)	18.7 (17.0 to 20.5)
Other transport injuries	3,366.0 (2,747.5 to 4,081.2)	554.0 (369.4 to 798.6)	176.8 (118.1 to 254.7)	10.1 (8.2 to 11.8)	-2.5 (-4.2 to -1.0)
**Unintentional injuries**	**77,037.1 (71,018.5 to 84,655.8)**	**31,227.7 (26,494.5 to 36,234.6)**	**2,439.8 (1,622.7 to 3,482.8)**	**17.7 (16.3 to 18.7)**	**4.2 (3.0 to 5.2)**
Falls	28,335.0 (23,852.9 to 32,971.4)	10,960.4 (8,103.6 to 14,413.4)	1,049.3 (716.4 to 1,473.0)	19.1 (17.9 to 20.2)	5.5 (4.4 to 6.5)
Drowning	318.2 (270.7 to 377.8)	40.9 (30.9 to 55.0)	16.3 (10.6 to 23.6)	-1.5 (-3.6 to 0.3)	-12.8 (-14.6 to -11.1)
Fire, heat, and hot substances	8,662.0 (7,100.8 to 10,659.1)	734.4 (509.9 to 1,039.4)	216.3 (141.4 to 322.1)	17.9 (15.7 to 19.7)	4.4 (2.4 to 6.0)
Poisonings	272.5 (225.4 to 326.2)	343.7 (202.5 to 526.5)	24.9 (15.9 to 36.8)	26.3 (23.0 to 29.6)	11.9 (9.0 to 14.8)
Exposure to mechanical forces	14,186.5 (11,668.1 to 18,167.5)	6,011.4 (4,067.9 to 8,301.2)	322.7 (204.2 to 485.7)	18.7 (17.2 to 20.1)	5.1 (3.8 to 6.4)
Unintentional firearm injuries	554.3 (441.0 to 685.8)	146.9 (73.5 to 250.2)	19.8 (12.2 to 29.7)	18.8 (17.1 to 20.4)	5.2 (3.7 to 6.7)
Unintentional suffocation	694.6 (517.0 to 914.1)	59.7 (28.0 to 110.1)	20.8 (12.3 to 32.5)	6.8 (5.2 to 8.4)	-5.4 (-6.8 to -4.0)
Other exposure to mechanical forces	12,937.5 (10,564.2 to 16,787.7)	5,804.9 (3,939.5 to 8,010.4)	282.1 (179.1 to 421.7)	19.7 (18.2 to 21.1)	6.0 (4.7 to 7.2)

Cause	Prevalence in thousands (95% UI)	Incidence in thousands (95% UI)	YLDs in thousands (95% UI)		
	2016 counts	2016 counts	2016 counts	Percentage change in counts between 2006 and 2016	Percentage change in rates between 2006 and 2016
Adverse effects of medical treatment	116.2 (79.7 to 166.1)	1,519.0 (1,065.5 to 2,100.7)	15.5 (8.7 to 25.9)	9.8 (5.2 to 14.1)	-2.8 (-6.9 to 1.1)
Animal contact	4,140.7 (3,527.1 to 4,964.9)	5,044.3 (3,132.9 to 7,280.6)	141.0 (94.9 to 197.6)	13.8 (11.1 to 16.1)	0.8 (-1.6 to 2.9)
Venomous animal contact	2,041.8 (1,627.1 to 2,600.5)	2,500.7 (1,510.8 to 3,696.7)	107.2 (72.1 to 151.5)	15.9 (12.5 to 19.0)	2.6 (-0.3 to 5.4)
Non-venomous animal contact	2,098.9 (1,632.1 to 2,684.0)	2,543.6 (1,517.7 to 3,795.2)	33.7 (20.9 to 51.5)	7.7 (4.9 to 9.7)	-4.6 (-7.1 to -2.8)
Foreign body	3,944.9 (3,224.7 to 4,701.5)	2,115.6 (1,436.2 to 3,109.2)	147.2 (92.4 to 221.7)	17.3 (15.3 to 19.3)	3.9 (2.1 to 5.7)
Pulmonary aspiration and foreign body in airway	1,857.2 (1,352.7 to 2,537.9)	273.8 (171.6 to 400.5)	78.6 (47.8 to 119.0)	12.7 (10.9 to 14.5)	-0.2 (-1.8 to 1.4)
Foreign body in eyes	247.8 (120.3 to 426.4)	1,414.2 (764.1 to 2,345.3)	14.3 (6.2 to 27.4)	26.4 (22.8 to 29.7)	12.0 (8.8 to 14.9)
Foreign body in other body part	1,839.8 (1,459.7 to 2,399.4)	427.6 (263.0 to 655.9)	54.2 (34.0 to 81.6)	22.2 (19.9 to 24.2)	8.3 (6.2 to 10.0)
Environmental heat and cold exposure	5,395.3 (4,433.2 to 6,518.4)	978.5 (602.3 to 1,465.1)	195.4 (127.0 to 284.2)	16.0 (14.0 to 17.8)	2.7 (1.0 to 4.3)
Other unintentional injuries	11,666.1 (9,398.5 to 14,272.1)	3,479.3 (2,381.8 to 4,956.8)	311.2 (202.7 to 457.8)	15.5 (13.9 to 17.0)	2.3 (0.8 to 3.6)
**Self-harm and interpersonal violence**	**23,945.4 (17,958.0 to 31,047.3)**	**3,422.9† (2,527.3 to 4,508.2)**	**366.3 (240.9 to 527.5)**	**7.6 (6.5 to 8.6)**	**-4.7 (-5.7 to -3.8)**
Self-harm	802.4 (654.5 to 984.0)	602.9 (456.5 to 810.8)	41.4 (26.2 to 61.1)	7.8 (6.1 to 9.5)	-4.5 (-6.0 to -3.1)
Self-harm by firearm	31.3 (26.1 to 37.1)	7.4 (1.8 to 20.5)	1.1 (0.7 to 1.7)	4.1 (1.5 to 6.4)	-7.8 (-10.1 to -5.7)
Self-harm by other specified means	771.1 (622.9 to 955.4)	595.5 (451.2 to 799.7)	40.2 (25.5 to 59.4)	7.9 (6.2 to 9.6)	-4.4 (-5.9 to -3.0)
Interpersonal violence	23,143.0 (17,105.0 to 30,257.3)	2,820.0† (1,943.1 to 3,858.6)	324.9 (211.5 to 469.4)	7.5 (6.4 to 8.6)	-4.8 (-5.8 to -3.8)
Assault by firearm	225.6 (168.2 to 295.9)	74.0 (42.8 to 121.9)	8.9 (5.5 to 13.8)	10.8 (9.1 to 12.6)	-1.9 (-3.4 to -0.3)
Assault by sharp object	1,250.0 (950.7 to 1,569.8)	630.4 (368.4 to 1,007.2)	37.1 (23.6 to 56.4)	4.9 (2.7 to 7.0)	-7.1 (-9.1 to -5.2)
Sexual violence	18,549.3 (12,301.5 to 25,674.1)	--	157.9 (90.3 to 245.1)	7.6 (5.6 to 9.1)	-4.7 (-6.5 to -3.4)
Assault by other means	3,118.0 (2,481.1 to 4,009.8)	2,115.6 (1,464.5 to 2,929.1)	121.0 (78.2 to 179.4)	8.1 (6.6 to 9.4)	-4.3 (-5.6 to -3.1)
**Forces of nature, conflict and terrorism, and executions and police conflict**	**5,948.0 (3,720.2 to 8,882.7)**	**125.4† (70.5 to 182.9)**	**158.5 (60.6 to 322.0)**	**3.6 (-7.8 to 14.5)**	**-8.3 (-18.4 to 1.4)**
Forces of nature, conflict and terrorism, and state actor violence	736.1 (403.1 to 1,170.4)	25.8 (15.0 to 40.2)	25.0 (6.8 to 59.0)	13.7 (-3.6 to 21.0)	0.7 (-14.6 to 7.2)
Conflict and terrorism	4,957.0 (2,876.0 to 7,729.5)	--	134.6 (53.5 to 261.4)	8.2 (-6.5 to 22.7)	-4.2 (-17.2 to 8.7)
Executions and police conflict	254.8 (140.8 to 395.2)	99.5 (45.6 to 155.9)	6.4 (2.1 to 16.6)	-56.3 (-59.4 to -39.0)	-61.3 (-64.1 to -46.0)

We did not calculate incidence for certain NTDs and causes with only birth prevalence. Blank cells mean that no estimate is available.

† Incidence for aggregate causes represents the sum of the incidence of the causes below the aggregate.

‡ Incidence estimates for stroke represent first-ever stroke only.

^ Incidence of HIV/AIDS represents new infections of HIV only and does not include new infections of Tuberculosis in HIV positive cases.

SR Table 12: Global prevalence, incidence, and YLDs for 2016, percentage change of YLD counts and percentage change of YLD rates between 2006 and 2016 by cause, age 35-39 years.					
Cause	Prevalence in thousands (95% UI)	Incidence in thousands (95% UI)	YLDs in thousands (95% UI)		
	2016 counts	2016 counts	2016 counts	Percentage change in counts between 2006 and 2016	Percentage change in rates between 2006 and 2016
**All causes**	**495,336.1 (494,934.0 to 495,829.7)**	**2,861,344.7† (2,449,840.7 to 3,373,499.4)**	**56,138.3 (41,533.9 to 72,995.4)**	**6.1 (5.3 to 6.8)**	**0.3 (-0.4 to 0.9)**
**Communicable, maternal, neonatal, and nutritional disorders**	**365,996.1 (360,626.3 to 370,544.3)**	**1,565,506.4† (1,204,100.9 to 2,047,537.9)**	**6,427.6 (4,615.5 to 8,602.6)**	**1.5 (-3.2 to 5.8)**	**-4.0 (-8.5 to -0.0)**
**HIV/AIDS and tuberculosis**	**179,606.1 (154,867.9 to 207,011.8)**	**860.3† (643.3 to 1,176.2)**	**835.3 (571.7 to 1,176.5)**	**-13.1 (-28.1 to 3.9)**	**-17.8 (-32.0 to -1.8)**
Tuberculosis	166,938.9 (134,446.4 to 205,398.8)	669.7† (446.7 to 985.9)	209.4 (121.1 to 328.0)	-2.9 (-7.1 to 2.6)	-8.2 (-12.2 to -3.0)
Drug-susceptible tuberculosis	649.7 (430.5 to 945.8)	644.7 (428.9 to 951.8)	201.6 (116.2 to 316.8)	-2.7 (-7.0 to 2.7)	-8.1 (-12.0 to -2.9)
Multidrug-resistant tuberculosis without extensive drug resistance	22.8 (16.0 to 31.7)	23.4 (16.4 to 32.7)	7.3 (4.2 to 11.2)	-10.5 (-24.6 to 7.2)	-15.4 (-28.7 to 1.3)
Extensively drug-resistant tuberculosis	1.4 (1.0 to 1.9)	1.6 (1.1 to 2.3)	0.5 (0.3 to 0.7)	144.2 (112.2 to 180.1)	130.8 (100.6 to 164.8)
Latent tuberculosis infection	166,265.0 (133,736.8 to 204,462.9)	--	--	--	--
HIV/AIDS	5,535.5 (5,200.4 to 5,885.5)	190.5^ (162.9 to 228.4)	625.9 (422.7 to 897.6)	-16.0 (-33.2 to 7.1)	-20.6 (-36.8 to 1.3)
Drug-susceptible HIV/AIDS - Tuberculosis	164.1 (116.1 to 227.6)	203.3 (147.4 to 280.0)	62.9 (38.7 to 96.2)	-17.2 (-22.2 to -12.0)	-21.7 (-26.5 to -16.8)
Multidrug-resistant HIV/AIDS - Tuberculosis without extensive drug resistance	4.1 (2.7 to 6.1)	5.3 (3.5 to 7.7)	1.7 (1.0 to 2.7)	-26.9 (-39.1 to -13.6)	-30.9 (-42.4 to -18.3)
Extensively drug-resistant HIV/AIDS - Tuberculosis	0.2 (0.1 to 0.3)	0.2 (0.1 to 0.4)	0.1 (< 0.05 to 0.1)	145.7 (104.5 to 198.2)	132.3 (93.3 to 181.8)
HIV/AIDS resulting in other diseases	5,367.1 (5,034.9 to 5,716.1)	190.5 (162.9 to 228.4)	561.2 (373.6 to 816.0)	-15.9 (-34.3 to 11.9)	-20.5 (-37.9 to 5.8)
**Diarrhoea, lower respiratory infections, and other common infectious diseases**	**24,159.0 (22,226.7 to 26,263.9)**	**1,427,067.3† (1,060,738.4 to 1,906,208.0)**	**1,104.1 (761.6 to 1,532.5)**	**5.3 (4.0 to 6.7)**	**-0.5 (-1.7 to 0.8)**
Diarrhoeal diseases	2,898.3 (2,520.7 to 3,314.2)	195,506.3 (168,158.0 to 224,118.1)	325.0 (218.5 to 451.6)	11.3 (8.7 to 14.2)	5.2 (2.7 to 7.9)
Intestinal infectious diseases	23.4 (18.0 to 30.2)	561.9† (447.7 to 698.9)	4.1 (2.5 to 6.2)	-3.7 (-20.9 to 16.7)	-9.0 (-25.2 to 10.3)
Typhoid fever	22.9 (17.3 to 29.9)	362.9 (284.9 to 456.6)	3.5 (2.1 to 5.3)	-3.3 (-22.6 to 21.5)	-8.6 (-26.8 to 14.8)
Paratyphoid fever	8.7 (6.8 to 10.7)	199.0 (157.8 to 245.4)	0.6 (0.3 to 0.8)	-4.6 (-20.2 to 16.1)	-9.8 (-24.5 to 9.8)
Other intestinal infectious diseases	--	--	< 0.05 (< 0.05 to 0.1)	-24.0 (-37.6 to -7.9)	-28.2 (-41.0 to -13.0)
Lower respiratory infections	229.0 (201.0 to 261.5)	9,901.3 (8,449.2 to 11,778.2)	14.1 (9.3 to 20.2)	27.5 (20.8 to 34.8)	20.6 (14.2 to 27.4)
Upper respiratory infections	16,035.8 (11,250.8 to 22,080.4)	1,206,306.9 (847,395.8 to 1,691,813.1)	398.1 (222.3 to 670.7)	3.7 (2.4 to 5.1)	-1.9 (-3.2 to -0.7)
Otitis media	3,459.4 (2,679.1 to 4,440.1)	9,812.4 (8,272.2 to 11,927.2)	92.9 (54.4 to 147.4)	-3.5 (-7.9 to 1.0)	-8.8 (-12.9 to -4.6)
Meningitis	1,077.2 (926.4 to 1,249.9)	63.8 (44.6 to 85.4)	111.5 (77.8 to 147.7)	13.2 (10.0 to 16.8)	7.0 (3.9 to 10.4)
Pneumococcal meningitis	490.1 (411.6 to 584.4)	16.5 (10.0 to 24.4)	50.3 (34.5 to 68.2)	11.8 (5.0 to 18.4)	5.7 (-0.8 to 11.9)
H influenzae type B meningitis	141.5 (111.0 to 180.8)	4.2 (2.4 to 7.2)	17.9 (12.0 to 24.6)	-7.8 (-13.1 to -3.6)	-12.9 (-17.8 to -8.9)
Meningococcal infection	117.6 (85.5 to 155.3)	10.8 (6.4 to 17.2)	12.1 (8.0 to 16.6)	20.0 (16.4 to 23.4)	13.4 (10.1 to 16.6)
Other meningitis	328.0 (244.7 to 414.9)	32.1 (21.6 to 44.6)	31.3 (21.3 to 42.6)	30.0 (24.2 to 36.4)	22.9 (17.4 to 29.0)
Encephalitis	1,171.8 (793.1 to 1,692.0)	285.5 (227.0 to 346.2)	141.2 (99.6 to 185.7)	-3.4 (-8.3 to 1.6)	-8.7 (-13.3 to -4.0)
Diphtheria	< 0.05 (< 0.05 to < 0.05)	0.2 (0.1 to 0.3)	< 0.05 (< 0.05 to < 0.05)	-44.9 (-57.5 to -28.0)	-47.9 (-59.8 to -32.0)
Whooping cough	1.6 (1.2 to 2.0)	11.7 (9.1 to 14.7)	0.1 (< 0.05 to 0.1)	-15.8 (-17.3 to -14.2)	-20.4 (-21.9 to -18.9)
Tetanus	10.5 (10.3 to 10.7)	3.6 (1.9 to 5.5)	0.2 (0.1 to 0.4)	6.0 (-18.1 to 36.7)	0.2 (-22.6 to 29.2)
Measles	0.3 (0.1 to 0.6)	9.4 (3.4 to 20.2)	< 0.05 (< 0.05 to 0.1)	-55.1 (-60.0 to -49.5)	-57.6 (-62.2 to -52.3)
Varicella and herpes zoster	353.6 (293.4 to 423.2)	4,604.3 (3,441.4 to 6,148.9)	16.9 (10.0 to 26.9)	3.0 (-2.9 to 8.9)	-2.7 (-8.2 to 3.0)
**Neglected tropical diseases and malaria**	**130,191.6 (126,256.6 to 133,598.4)**	**20,060.6† (16,733.9 to 24,004.5)**	**975.4 (665.8 to 1,389.8)**	**-17.2 (-23.0 to -11.4)**	**-21.7 (-27.2 to -16.2)**
Malaria	5,435.2 (4,711.3 to 6,216.7)	5,888.1 (4,395.0 to 7,543.8)	72.3 (55.0 to 92.2)	-0.1 (-6.8 to 7.9)	-5.6 (-11.9 to 2.0)
Chagas disease	667.2 (556.9 to 809.6)	12.8 (8.4 to 18.1)	4.6 (2.8 to 6.8)	2.4 (-5.5 to 10.1)	-3.2 (-10.7 to 4.0)
Leishmaniasis	390.0 (322.2 to 466.9)	41.8 (30.8 to 54.6)	22.1 (13.8 to 33.9)	33.6 (18.5 to 57.0)	26.3 (12.0 to 48.5)
Visceral leishmaniasis	1.5 (1.0 to 2.0)	5.8 (4.0 to 8.2)	0.1 (0.1 to 0.2)	-57.6 (-71.4 to -37.9)	-59.9 (-73.0 to -41.3)
Cutaneous and mucocutaneous leishmaniasis	346.9 (276.5 to 440.6)	36.0 (25.5 to 49.3)	22.0 (13.8 to 33.8)	35.0 (19.2 to 58.9)	27.6 (12.6 to 50.2)
African trypanosomiasis	0.5 (0.1 to 2.0)	0.4 (0.1 to 1.3)	0.1 (< 0.05 to 0.5)	-71.8 (-94.6 to 61.6)	-73.3 (-94.9 to 52.8)
Schistosomiasis	18,595.2 (17,108.1 to 19,939.8)	4,733.9 (4,192.4 to 5,491.7)	152.5 (77.9 to 284.3)	-20.3 (-22.9 to -17.1)	-24.7 (-27.1 to -21.6)
Cysticercosis	238.1 (157.2 to 332.8)	--	38.6 (22.6 to 59.2)	-19.2 (-29.1 to -10.2)	-23.6 (-33.0 to -15.1)
Cystic echinococcosis	82.6 (43.8 to 173.4)	17.4 (7.9 to 43.9)	7.7 (3.5 to 17.4)	-36.2 (-75.1 to 62.1)	-39.7 (-76.5 to 53.3)
Lymphatic filariasis	2,528.9 (2,023.8 to 3,276.3)	610.4 (343.8 to 960.4)	129.9 (63.4 to 233.4)	-39.3 (-54.2 to -27.8)	-42.6 (-56.8 to -31.7)
Onchocerciasis	1,176.7 (731.5 to 2,020.9)	273.0 (112.9 to 546.8)	76.5 (34.0 to 138.3)	-19.2 (-40.6 to 0.9)	-23.6 (-43.8 to -4.7)
Trachoma	19.1 (10.7 to 32.6)	--	2.6 (1.4 to 4.4)	-13.7 (-29.5 to 3.4)	-18.5 (-33.3 to -2.3)
Dengue	394.4 (214.9 to 667.9)	6,591.9 (4,010.3 to 9,997.8)	63.9 (28.2 to 119.6)	84.4 (48.4 to 321.2)	74.3 (40.2 to 298.1)
Yellow fever	0.1 (< 0.05 to 0.4)	5.6 (1.6 to 15.7)	< 0.05 (< 0.05 to < 0.05)	-14.6 (-45.3 to 32.8)	-19.2 (-48.3 to 25.6)
Rabies	< 0.05 (< 0.05 to < 0.05)	0.6 (0.3 to 0.9)	< 0.05 (< 0.05 to < 0.05)	-47.5 (-58.0 to -30.8)	-50.4 (-60.3 to -34.6)
Intestinal nematode infections	107,799.4 (102,933.6 to 111,570.9)	--	209.6 (120.0 to 343.5)	-14.7 (-21.6 to -7.9)	-19.4 (-25.9 to -13.0)
Ascariasis	57,765.5 (51,964.8 to 64,482.9)	--	62.2 (33.8 to 105.4)	-28.0 (-37.3 to -17.8)	-32.0 (-40.7 to -22.3)

Cause	Prevalence in thousands (95% UI)	Incidence in thousands (95% UI)	YLDs in thousands (95% UI)		
	2016 counts	2016 counts	2016 counts	Percentage change in counts between 2006 and 2016	Percentage change in rates between 2006 and 2016
Trichuriasis	30,310.5 (27,910.6 to 33,193.2)	--	23.5 (12.7 to 40.6)	-18.9 (-30.5 to -5.8)	-23.3 (-34.3 to -10.9)
Hookworm disease	32,815.6 (30,373.3 to 35,876.2)	--	123.9 (72.5 to 196.4)	-5.0 (-14.5 to 6.1)	-10.2 (-19.2 to 0.3)
Food-borne trematodiases	6,388.2 (5,968.6 to 6,885.4)	1,187.1 (776.7 to 1,528.1)	169.9 (87.4 to 308.2)	-22.5 (-26.7 to -16.4)	-26.7 (-30.7 to -20.9)
Leprosy	33.4 (31.2 to 36.0)	3.6 (3.2 to 3.9)	2.1 (1.4 to 3.0)	-10.5 (-19.9 to 0.3)	-15.4 (-24.3 to -5.2)
Ebola virus disease	0.1 (< 0.05 to 0.4)	< 0.05 (< 0.05 to < 0.05)	< 0.05 (< 0.05 to 0.1)	--	--
Zika virus disease	11.5 (6.7 to 20.4)	694.0 (403.8 to 1,231.7)	0.2 (0.1 to 0.4)	--	--
Guinea worm disease	< 0.05 (< 0.05 to < 0.05)	< 0.05 (< 0.05 to < 0.05)	< 0.05 (< 0.05 to < 0.05)	-99.6 (-99.7 to -99.4)	-99.6 (-99.7 to -99.4)
Other neglected tropical diseases	1,519.3 (1,324.9 to 1,722.4)	--	22.8 (13.9 to 36.0)	22.2 (14.8 to 29.9)	15.5 (8.5 to 22.8)
**Maternal disorders**	**996.7 (927.7 to 1,069.2)**	**12,092.9† (9,778.6 to 14,555.1)**	**105.8 (72.7 to 145.4)**	**-1.0 (-12.8 to 13.2)**	**-6.4 (-17.6 to 7.0)**
Maternal haemorrhage	158.3 (112.2 to 219.2)	921.2 (664.5 to 1,291.8)	5.4 (3.3 to 8.3)	15.6 (-13.2 to 51.7)	9.2 (-17.9 to 43.4)
Maternal sepsis and other pregnancy related infections	201.5 (164.8 to 250.6)	797.8 (541.5 to 1,129.5)	3.9 (1.9 to 7.0)	14.6 (-37.7 to 106.0)	8.3 (-41.1 to 94.7)
Maternal hypertensive disorders	464.2 (302.3 to 651.0)	2,131.8 (1,705.0 to 2,563.0)	22.4 (12.0 to 37.8)	24.9 (-20.4 to 97.9)	18.0 (-24.7 to 87.1)
Maternal obstructed labour and uterine rupture	161.4 (128.8 to 200.3)	627.0 (446.5 to 871.3)	51.9 (31.6 to 75.4)	-10.3 (-18.3 to -1.8)	-15.2 (-22.8 to -7.2)
Maternal abortion, miscarriage, and ectopic pregnancy	62.5 (37.7 to 92.0)	7,615.1 (5,400.1 to 9,936.4)	6.8 (3.6 to 11.2)	2.7 (-36.4 to 64.7)	-2.9 (-39.9 to 55.7)
Other maternal disorders	--	--	15.5 (10.6 to 21.2)	-5.8 (-17.1 to 7.7)	-10.9 (-21.6 to 1.8)
**Neonatal disorders**	**4,802.2 (4,387.3 to 5,245.7)**	**--**	**878.4 (619.3 to 1,237.7)**	**22.6 (9.5 to 37.9)**	**15.9 (3.5 to 30.3)**
Neonatal preterm birth complications	4,015.6 (3,390.7 to 4,789.3)	--	570.8 (395.6 to 805.9)	19.3 (-0.1 to 43.0)	12.8 (-5.6 to 35.2)
Neonatal encephalopathy due to birth asphyxia and trauma	755.5 (478.7 to 1,180.9)	--	108.3 (42.9 to 232.3)	26.8 (18.6 to 42.5)	19.9 (12.1 to 34.7)
Neonatal sepsis and other neonatal infections	398.1 (155.6 to 944.0)	--	154.2 (50.1 to 396.7)	33.7 (24.9 to 47.2)	26.4 (18.1 to 39.2)
Hemolytic disease and other neonatal jaundice	139.6 (124.1 to 155.9)	--	45.0 (34.6 to 57.2)	21.0 (15.5 to 27.0)	14.4 (9.1 to 20.1)
Other neonatal disorders	--	--	--	--	--
**Nutritional deficiencies**	**100,567.3 (94,901.9 to 104,782.3)**	**28,003.7† (23,656.9 to 33,931.8)**	**2,068.1 (1,358.4 to 3,010.1)**	**9.8 (6.2 to 13.7)**	**3.8 (0.4 to 7.5)**
Protein-energy malnutrition	--	--	--	--	--
Iodine deficiency	9,903.0 (8,771.0 to 11,104.6)	39.7 (33.2 to 47.4)	262.6 (178.2 to 368.0)	-7.1 (-9.9 to -4.6)	-12.2 (-14.8 to -9.8)
Vitamin A deficiency	25,490.5 (22,094.5 to 29,967.6)	27,964.0 (23,612.5 to 33,892.9)	11.8 (7.2 to 17.7)	17.0 (8.8 to 26.2)	10.6 (2.9 to 19.3)
Iron-deficiency anaemia	75,861.5 (67,087.6 to 85,343.7)	--	1,793.7 (1,163.6 to 2,628.5)	12.8 (8.7 to 17.2)	6.6 (2.7 to 10.8)
Other nutritional deficiencies	--	--	--	--	--
**Other communicable, maternal, neonatal, and nutritional diseases**	**168,477.7 (163,498.9 to 173,429.6)**	**77,421.7 (62,453.1 to 96,791.1)**	**443.0 (276.1 to 687.2)**	**8.9 (3.7 to 14.2)**	**2.9 (-1.9 to 8.0)**
Sexually transmitted diseases excluding HIV	133,337.7 (128,013.8 to 137,898.7)	69,992.0 (55,172.7 to 88,600.3)	386.7 (239.0 to 602.8)	10.1 (5.7 to 14.8)	4.0 (-0.1 to 8.6)
Syphilis	7,517.8 (4,994.4 to 11,143.1)	5,203.7 (3,333.9 to 8,263.0)	13.6 (9.3 to 18.7)	14.1 (10.6 to 17.6)	7.9 (4.5 to 11.2)
Chlamydial infection	10,352.8 (7,718.9 to 13,706.1)	6,265.0 (4,556.4 to 8,324.5)	65.1 (39.1 to 104.5)	3.1 (-1.6 to 7.3)	-2.6 (-7.0 to 1.4)
Gonococcal infection	9,968.7 (6,375.4 to 15,020.2)	31,648.5 (18,607.1 to 49,765.8)	100.4 (55.2 to 170.0)	27.5 (19.0 to 34.2)	20.6 (12.5 to 26.8)
Trichomoniasis	19,141.0 (13,725.1 to 26,626.4)	16,202.8 (11,287.0 to 22,607.0)	21.7 (7.9 to 47.4)	9.0 (7.1 to 10.9)	3.0 (1.3 to 4.8)
Genital herpes	92,979.6 (80,740.1 to 107,746.1)	3,354.1 (2,336.4 to 4,538.7)	23.5 (7.2 to 54.0)	7.8 (5.8 to 9.9)	1.9 (-0.0 to 3.9)
Other sexually transmitted diseases	9,050.1 (7,022.8 to 10,519.3)	7,318.0 (5,567.3 to 9,355.6)	162.3 (103.9 to 243.1)	4.2 (-0.2 to 8.9)	-1.5 (-5.7 to 2.9)
Hepatitis	47,488.5 (40,612.1 to 56,055.6)	7,429.2 (4,916.8 to 11,407.9)	17.3 (8.7 to 31.2)	-14.4 (-47.8 to 43.2)	-19.1 (-50.7 to 35.3)
Acute hepatitis A	63.7 (9.5 to 149.9)	827.6 (124.1 to 1,948.6)	2.7 (0.4 to 6.8)	-19.5 (-88.8 to 416.6)	-23.9 (-89.5 to 388.3)
Hepatitis B	36,651.3 (30,015.2 to 45,134.5)	5,820.2 (3,519.3 to 9,550.6)	13.0 (6.0 to 25.6)	-13.3 (-51.5 to 58.7)	-18.1 (-54.2 to 50.1)
Hepatitis C	10,726.4 (9,505.1 to 11,985.8)	168.3 (119.2 to 221.8)	0.2 (0.1 to 0.3)	-11.5 (-12.2 to -10.8)	-16.3 (-17.0 to -15.7)
Acute hepatitis E	47.2 (33.0 to 64.9)	613.1 (428.7 to 843.6)	1.5 (0.8 to 2.4)	-14.1 (-33.5 to 9.4)	-18.8 (-37.1 to 3.4)
Other infectious diseases	1,802.2 (1,564.0 to 2,058.9)	0.5 (0.3 to 0.9)	39.0 (23.7 to 59.5)	10.6 (5.9 to 15.8)	4.6 (0.1 to 9.5)
**Non-communicable diseases**	**475,936.6 (474,451.9 to 477,535.4)**	**1,258,375.7† (1,113,606.6 to 1,396,540.6)**	**45,731.3 (34,024.8 to 58,964.8)**	**6.4 (5.8 to 6.9)**	**0.5 (0.0 to 1.1)**
**Neoplasms**	**1,024.5 (999.6 to 1,048.3)**	**428.7 (412.2 to 453.1)**	**145.2 (105.0 to 192.3)**	**4.9 (1.1 to 10.2)**	**-0.9 (-4.4 to 4.2)**
Lip and oral cavity cancer	59.5 (56.0 to 63.6)	16.1 (15.2 to 17.2)	6.3 (4.5 to 8.2)	17.6 (7.9 to 28.3)	11.1 (2.0 to 21.3)
Nasopharynx cancer	25.9 (23.3 to 29.0)	7.4 (6.6 to 8.2)	3.1 (2.1 to 4.2)	-22.5 (-33.4 to -10.4)	-26.7 (-37.1 to -15.3)
Other pharynx cancer	10.2 (9.2 to 10.9)	3.9 (3.5 to 4.2)	1.4 (1.0 to 1.8)	19.9 (6.0 to 30.1)	13.3 (0.2 to 23.0)
Oesophageal cancer	5.5 (5.2 to 5.9)	4.7 (4.4 to 4.9)	1.2 (0.9 to 1.7)	-14.5 (-19.5 to -8.0)	-19.1 (-23.9 to -13.0)
Stomach cancer	29.8 (28.7 to 31.0)	16.8 (16.2 to 17.4)	4.7 (3.3 to 6.2)	-23.7 (-27.2 to -19.7)	-27.9 (-31.2 to -24.1)
Colon and rectum cancer	93.2 (88.5 to 98.1)	25.8 (24.5 to 27.1)	9.7 (7.0 to 12.8)	7.4 (-0.7 to 15.8)	1.5 (-6.1 to 9.5)
Liver cancer	22.2 (18.8 to 25.6)	24.6 (22.4 to 26.9)	5.7 (4.0 to 7.6)	-14.9 (-23.3 to -5.8)	-19.6 (-27.5 to -11.0)
Liver cancer due to hepatitis B	19.4 (15.6 to 23.1)	14.7 (11.9 to 17.2)	3.4 (2.3 to 4.6)	-16.9 (-25.4 to -7.5)	-21.5 (-29.5 to -12.6)

Cause	Prevalence in thousands (95% UI)	Incidence in thousands (95% UI)	YLDs in thousands (95% UI)		
	2016 counts	2016 counts	2016 counts	Percentage change in counts between 2006 and 2016	Percentage change in rates between 2006 and 2016
Liver cancer due to hepatitis C	2.0 (1.4 to 2.6)	1.6 (1.2 to 2.2)	0.4 (0.2 to 0.6)	-0.6 (-11.9 to 13.4)	-6.0 (-16.7 to 7.2)
Liver cancer due to alcohol use	2.2 (1.4 to 3.1)	1.8 (1.2 to 2.6)	0.4 (0.2 to 0.7)	3.8 (-6.2 to 14.5)	-1.9 (-11.4 to 8.2)
Liver cancer due to other causes	8.8 (6.9 to 11.4)	6.5 (5.1 to 8.3)	1.5 (1.0 to 2.2)	-17.4 (-27.5 to -6.0)	-21.9 (-31.5 to -11.2)
Gallbladder and biliary tract cancer	2.1 (1.8 to 2.3)	2.6 (2.3 to 2.8)	0.6 (0.4 to 0.8)	0.4 (-7.6 to 9.0)	-5.1 (-12.7 to 3.0)
Pancreatic cancer	3.2 (3.1 to 3.3)	4.0 (3.9 to 4.1)	0.9 (0.6 to 1.1)	2.1 (-1.4 to 5.3)	-3.5 (-6.8 to -0.4)
Larynx cancer	7.4 (7.0 to 7.8)	2.2 (2.1 to 2.3)	0.9 (0.7 to 1.2)	-2.0 (-7.1 to 3.6)	-7.4 (-12.2 to -2.1)
Tracheal, bronchus, and lung cancer	20.7 (19.6 to 21.8)	15.7 (15.0 to 16.4)	4.1 (2.9 to 5.4)	-18.7 (-23.0 to -13.1)	-23.1 (-27.2 to -17.9)
Malignant skin melanoma	63.4 (55.6 to 77.2)	12.8 (11.3 to 15.6)	4.2 (2.8 to 5.9)	13.1 (3.7 to 22.7)	6.9 (-2.0 to 16.0)
Non-melanoma skin cancer	3.7 (1.9 to 7.1)	22.0 (11.1 to 43.1)	0.2 (0.1 to 0.4)	12.1 (0.4 to 26.2)	6.0 (-5.1 to 19.3)
Non-melanoma skin cancer (squamous-cell carcinoma)	3.1 (1.2 to 7.0)	3.4 (1.4 to 6.9)	0.1 (0.1 to 0.3)	13.8 (-1.0 to 28.7)	7.5 (-6.4 to 21.6)
Non-melanoma skin cancer (basal-cell carcinoma)	2.1 (0.9 to 4.6)	18.6 (8.1 to 39.5)	< 0.05 (< 0.05 to 0.1)	2.7 (-4.5 to 10.1)	-2.9 (-9.7 to 4.0)
Breast cancer	370.6 (345.2 to 398.9)	77.4 (72.2 to 83.4)	32.8 (23.1 to 44.5)	9.1 (-1.0 to 21.4)	3.1 (-6.4 to 14.8)
Cervical cancer	197.1 (151.7 to 213.9)	51.5 (40.2 to 55.5)	19.0 (12.9 to 25.4)	-3.4 (-10.7 to 6.9)	-8.7 (-15.6 to 1.0)
Uterine cancer	42.9 (40.0 to 47.0)	9.0 (8.4 to 9.8)	3.3 (2.3 to 4.6)	7.2 (-5.5 to 23.3)	1.3 (-10.6 to 16.6)
Ovarian cancer	32.8 (30.4 to 34.4)	10.6 (10.0 to 11.1)	5.2 (3.7 to 6.9)	15.2 (6.1 to 22.8)	8.9 (0.3 to 16.1)
Prostate cancer	8.1 (6.6 to 8.8)	2.1 (1.7 to 2.2)	0.8 (0.5 to 1.1)	31.6 (20.0 to 39.1)	24.4 (13.5 to 31.5)
Testicular cancer	42.3 (39.7 to 45.6)	8.3 (7.8 to 8.9)	2.9 (2.0 to 4.0)	17.1 (6.4 to 29.8)	10.7 (0.6 to 22.7)
Kidney cancer	29.2 (27.8 to 30.4)	7.8 (7.5 to 8.0)	2.7 (1.9 to 3.6)	10.4 (4.4 to 16.8)	4.4 (-1.3 to 10.4)
Bladder cancer	22.9 (21.3 to 24.5)	5.6 (5.2 to 6.0)	2.2 (1.6 to 3.0)	13.5 (4.5 to 22.7)	7.3 (-1.3 to 16.0)
Brain and nervous system cancer	37.9 (33.5 to 41.8)	16.8 (15.1 to 18.6)	5.4 (3.8 to 7.2)	20.5 (12.5 to 29.5)	14.0 (6.3 to 22.4)
Thyroid cancer	98.1 (92.0 to 107.5)	18.6 (17.4 to 20.3)	6.0 (4.1 to 8.5)	34.6 (21.4 to 51.4)	27.2 (14.7 to 43.1)
Mesothelioma	1.1 (1.0 to 1.1)	0.7 (0.7 to 0.8)	0.2 (0.2 to 0.3)	5.9 (-0.1 to 12.4)	0.1 (-5.5 to 6.3)
Hodgkin lymphoma	23.2 (21.1 to 26.7)	5.7 (5.1 to 6.6)	2.2 (1.6 to 3.0)	7.5 (-0.2 to 16.7)	1.7 (-5.6 to 10.3)
Non-Hodgkin's lymphoma	46.8 (42.3 to 51.9)	13.6 (12.3 to 15.0)	4.5 (3.1 to 6.0)	24.5 (16.6 to 31.3)	17.7 (10.2 to 24.1)
Multiple myeloma	4.0 (3.4 to 4.4)	1.8 (1.6 to 2.0)	0.9 (0.6 to 1.2)	22.3 (15.4 to 29.0)	15.6 (9.1 to 21.9)
Leukaemia	40.6 (35.5 to 44.1)	12.4 (11.0 to 13.5)	5.5 (3.8 to 7.3)	-14.8 (-20.8 to -8.0)	-19.5 (-25.1 to -13.0)
Acute lymphoid leukaemia	6.9 (6.1 to 7.8)	2.0 (1.8 to 2.3)	0.9 (0.7 to 1.2)	4.2 (-7.0 to 11.8)	-1.4 (-12.1 to 5.7)
Chronic lymphoid leukaemia	7.5 (6.5 to 8.6)	1.7 (1.4 to 1.9)	0.9 (0.6 to 1.2)	3.4 (-9.8 to 14.8)	-2.3 (-14.7 to 8.5)
Acute myeloid leukaemia	4.4 (3.9 to 4.8)	3.3 (2.9 to 3.6)	0.9 (0.6 to 1.2)	10.6 (4.8 to 17.2)	4.5 (-1.0 to 10.8)
Chronic myeloid leukaemia	4.5 (3.9 to 5.0)	1.6 (1.4 to 1.8)	0.6 (0.4 to 0.8)	-9.3 (-15.4 to -2.2)	-14.3 (-20.0 to -7.5)
Other leukaemia	17.3 (14.5 to 19.0)	3.8 (3.2 to 4.2)	2.1 (1.5 to 2.8)	-33.0 (-40.4 to -23.9)	-36.6 (-43.7 to -28.1)
Other neoplasms	96.4 (86.8 to 102.9)	28.3 (25.6 to 30.1)	8.6 (6.1 to 11.5)	31.3 (23.0 to 41.0)	24.1 (16.3 to 33.3)
**Cardiovascular diseases**	**12,415.3 (11,966.0 to 12,674.9)**	**903.5† (800.6 to 1,019.4)**	**910.4 (634.7 to 1,219.3)**	**5.2 (2.8 to 7.4)**	**-0.5 (-2.8 to 1.6)**
Rheumatic heart disease	3,012.2 (2,727.3 to 3,339.3)	39.9 (31.1 to 52.0)	144.3 (91.3 to 210.8)	17.2 (13.0 to 21.2)	10.8 (6.8 to 14.6)
Ischaemic heart disease	3,931.2 (3,595.8 to 4,296.9)	420.1 (350.1 to 494.3)	221.5 (147.0 to 313.2)	12.4 (9.9 to 15.1)	6.2 (3.9 to 8.8)
Cerebrovascular disease	1,703.3 (1,624.7 to 1,801.7)	207.7‡ (147.5 to 275.3)	274.6 (191.9 to 359.5)	-5.4 (-11.1 to 0.0)	-10.5 (-16.0 to -5.5)
Ischaemic stroke	1,358.4 (1,149.2 to 1,620.2)	132.9‡ (82.8 to 196.9)	207.5 (142.7 to 277.7)	-5.9 (-13.0 to 0.9)	-11.1 (-17.7 to -4.6)
Hemorrhagic stroke	425.8 (353.0 to 512.0)	74.8‡ (48.9 to 104.8)	67.1 (45.3 to 91.2)	-3.5 (-12.3 to 5.9)	-8.8 (-17.1 to 0.1)
Hypertensive heart disease	412.0 (305.8 to 547.1)	--	35.2 (21.5 to 53.6)	4.4 (-1.2 to 10.2)	-1.3 (-6.6 to 4.2)
Cardiomyopathy and myocarditis	188.8 (163.1 to 225.6)	124.5† (91.8 to 165.0)	22.0 (13.1 to 33.1)	3.7 (-0.9 to 8.3)	-2.0 (-6.3 to 2.3)
Myocarditis	99.4 (78.2 to 124.2)	124.5 (91.8 to 165.0)	7.6 (4.6 to 11.3)	-0.1 (-5.8 to 6.2)	-5.6 (-11.0 to 0.4)
Alcoholic cardiomyopathy	47.8 (34.8 to 64.0)	--	4.2 (2.4 to 6.6)	4.2 (-8.4 to 19.3)	-1.5 (-13.5 to 12.8)
Other cardiomyopathy	117.3 (86.0 to 155.4)	--	10.2 (6.0 to 15.5)	6.5 (0.6 to 12.8)	0.7 (-4.9 to 6.6)
Atrial fibrillation and flutter	649.5 (534.1 to 771.8)	58.4 (45.4 to 75.6)	54.7 (34.7 to 78.5)	5.7 (-0.1 to 11.5)	-0.1 (-5.5 to 5.4)
Peripheral vascular disease	--	--	--	--	--
Endocarditis	6.4 (4.9 to 8.3)	53.0 (36.3 to 74.0)	0.4 (0.3 to 0.7)	10.9 (9.2 to 12.7)	4.9 (3.2 to 6.5)
Other cardiovascular and circulatory diseases	2,945.7 (2,008.1 to 4,098.6)	--	157.6 (92.0 to 249.9)	6.8 (3.0 to 10.4)	0.9 (-2.6 to 4.4)
**Chronic respiratory diseases**	**25,612.4 (24,789.1 to 26,149.6)**	**4,310.4† (3,740.7 to 4,968.4)**	**1,340.8 (1,033.6 to 1,702.1)**	**12.7 (10.6 to 14.8)**	**6.5 (4.6 to 8.5)**
Chronic obstructive pulmonary disease	7,976.4 (7,350.7 to 8,620.9)	706.2 (580.1 to 842.3)	567.2 (479.8 to 650.0)	11.4 (7.6 to 15.3)	5.3 (1.8 to 9.0)
Pneumoconiosis	16.8 (14.8 to 19.1)	2.0 (1.5 to 2.5)	2.6 (1.8 to 3.7)	-1.2 (-2.9 to 0.6)	-6.6 (-8.2 to -4.9)
Silicosis	5.0 (3.9 to 6.4)	0.7 (0.5 to 1.0)	0.8 (0.5 to 1.2)	-9.2 (-11.4 to -6.9)	-14.1 (-16.2 to -12.0)

Cause	Prevalence in thousands (95% UI)	Incidence in thousands (95% UI)	YLDs in thousands (95% UI)		
	2016 counts	2016 counts	2016 counts	Percentage change in counts between 2006 and 2016	Percentage change in rates between 2006 and 2016
Asbestosis	3.8 (2.9 to 4.8)	0.3 (0.2 to 0.5)	0.6 (0.4 to 0.9)	11.1 (8.5 to 13.1)	5.0 (2.6 to 7.0)
Coal workers pneumoconiosis	2.0 (1.5 to 2.4)	0.3 (0.2 to 0.4)	0.3 (0.2 to 0.5)	-12.0 (-14.5 to -9.4)	-16.8 (-19.2 to -14.3)
Other pneumoconiosis	6.1 (4.7 to 7.9)	0.7 (0.4 to 1.0)	1.0 (0.6 to 1.4)	3.4 (0.3 to 6.2)	-2.3 (-5.2 to 0.4)
Asthma	17,968.7 (15,947.4 to 20,219.7)	3,594.2 (3,013.9 to 4,270.9)	703.2 (456.4 to 1,009.0)	11.5 (9.5 to 13.3)	5.4 (3.5 to 7.1)
Interstitial lung disease and pulmonary sarcoidosis	100.3 (83.6 to 119.8)	8.0 (5.2 to 11.5)	11.2 (7.2 to 16.9)	6.7 (-0.2 to 14.5)	0.9 (-5.7 to 8.2)
Other chronic respiratory diseases	--	--	56.5 (47.7 to 65.0)	54.7 (49.6 to 60.1)	46.2 (41.4 to 51.3)
**Cirrhosis and other chronic liver diseases**	**3,438.8 (3,145.0 to 3,739.0)**	**88.7 (72.8 to 105.3)**	**81.4 (55.3 to 113.6)**	**10.8 (6.1 to 15.5)**	**4.7 (0.3 to 9.2)**
Cirrhosis and other chronic liver diseases due to hepatitis B	1,068.8 (897.7 to 1,241.6)	31.2 (24.6 to 39.4)	27.9 (18.8 to 40.4)	8.3 (-0.2 to 17.1)	2.3 (-5.7 to 10.7)
Cirrhosis and other chronic liver diseases due to hepatitis C	919.0 (738.3 to 1,114.6)	22.8 (17.5 to 28.8)	20.9 (13.3 to 30.6)	15.8 (7.3 to 25.1)	9.5 (1.5 to 18.2)
Cirrhosis and other chronic liver diseases due to alcohol use	886.7 (726.4 to 1,059.2)	22.2 (17.2 to 28.4)	19.9 (13.1 to 29.1)	12.3 (3.9 to 21.7)	6.2 (-1.8 to 15.1)
Cirrhosis and other chronic liver diseases due to other causes	564.3 (418.2 to 739.2)	12.5 (8.8 to 16.9)	12.6 (7.7 to 19.3)	6.2 (-4.1 to 18.3)	0.4 (-9.4 to 11.8)
**Digestive diseases**	**16,380.7 (15,412.5 to 17,217.5)**	**11,862.5† (10,118.1 to 13,760.1)**	**478.2 (315.9 to 675.9)**	**10.3 (4.6 to 16.5)**	**4.2 (-1.1 to 10.1)**
Peptic ulcer disease	5,539.4 (4,423.5 to 6,852.9)	777.8 (566.6 to 1,036.6)	117.8 (65.9 to 190.8)	17.3 (6.0 to 28.3)	10.9 (0.2 to 21.3)
Gastritis and duodenitis	6,368.5 (4,786.7 to 8,209.3)	4,253.0 (2,911.0 to 5,729.6)	112.8 (67.7 to 176.5)	11.5 (0.4 to 22.2)	5.4 (-5.0 to 15.5)
Appendicitis	47.9 (38.3 to 57.9)	1,259.4 (1,021.2 to 1,540.5)	14.8 (9.4 to 21.7)	10.4 (0.0 to 21.7)	4.3 (-5.5 to 15.0)
Paralytic ileus and intestinal obstruction	8.2 (6.0 to 11.0)	224.0 (160.8 to 298.0)	2.7 (1.6 to 4.2)	11.2 (9.2 to 13.2)	5.1 (3.2 to 7.0)
Inguinal, femoral, and abdominal hernia	3,302.6 (2,299.7 to 4,541.2)	1,465.3 (1,011.3 to 2,039.7)	123.5 (73.1 to 196.0)	9.1 (4.3 to 13.9)	3.2 (-1.4 to 7.7)
Inflammatory bowel disease	366.1 (313.2 to 427.6)	316.4 (250.0 to 387.6)	54.4 (35.2 to 77.0)	-2.3 (-6.6 to 2.1)	-7.7 (-11.7 to -3.5)
Vascular intestinal disorders	2.9 (1.8 to 4.8)	17.0 (11.6 to 23.9)	1.0 (0.5 to 1.7)	10.1 (7.3 to 12.2)	4.0 (1.4 to 6.0)
Gallbladder and biliary diseases	1,189.5 (867.6 to 1,529.0)	3,249.9 (2,348.8 to 4,350.2)	16.0 (9.8 to 24.8)	5.2 (-1.7 to 11.6)	-0.6 (-7.0 to 5.5)
Pancreatitis	33.0 (25.1 to 42.7)	299.9 (226.5 to 388.4)	4.3 (2.7 to 6.6)	2.6 (-3.8 to 8.7)	-3.0 (-9.1 to 2.8)
Other digestive diseases	--	--	31.0 (20.5 to 43.9)	14.2 (8.4 to 20.7)	8.0 (2.5 to 14.1)
**Neurological disorders**	**254,770.1 (243,637.6 to 270,107.2)**	**121,260.4 (78,173.6 to 170,865.7)**	**6,353.7 (4,186.5 to 8,769.1)**	**8.8 (7.5 to 10.2)**	**2.9 (1.7 to 4.2)**
Alzheimer's disease and other dementias	--	--	--	--	--
Parkinson's disease	43.2 (26.3 to 69.4)	5.2 (2.2 to 9.6)	5.8 (3.0 to 9.9)	12.9 (7.9 to 19.2)	6.8 (2.0 to 12.7)
Epilepsy	1,360.5 (1,116.4 to 1,631.7)	103.6 (71.2 to 144.3)	425.9 (281.9 to 596.1)	1.8 (-8.8 to 13.8)	-3.8 (-13.8 to 7.5)
Multiple sclerosis	233.3 (205.3 to 263.8)	7.3 (5.2 to 10.4)	62.5 (43.4 to 83.2)	13.5 (7.0 to 20.4)	7.3 (1.2 to 13.8)
Motor neuron disease	17.7 (14.6 to 21.4)	1.3 (0.8 to 2.1)	3.8 (2.6 to 5.3)	2.5 (1.7 to 3.3)	-3.1 (-3.8 to -2.3)
Migraine	112,363.8 (104,010.9 to 121,695.2)	9,676.8 (8,313.6 to 11,261.7)	4,976.7 (3,118.0 to 6,928.8)	9.2 (8.1 to 10.2)	3.2 (2.2 to 4.2)
Tension-type headache	191,698.7 (139,588.4 to 244,476.4)	111,463.3 (68,568.2 to 160,565.2)	807.0 (494.2 to 1,251.5)	9.7 (7.9 to 11.9)	3.7 (2.0 to 5.8)
Other neurological disorders	1.4 (0.7 to 2.5)	2.9 (1.5 to 4.7)	72.0 (49.1 to 98.6)	18.1 (7.7 to 30.3)	11.7 (1.8 to 23.1)
**Mental and substance use disorders**	**93,134.9 (91,597.1 to 94,356.9)**	**33,708.5† (29,097.9 to 38,192.0)**	**13,724.5 (10,092.0 to 17,677.8)**	**5.4 (4.6 to 6.2)**	**-0.4 (-1.1 to 0.4)**
Schizophrenia	2,580.5 (2,176.1 to 3,024.0)	103.6 (79.2 to 131.0)	1,684.5 (1,208.6 to 2,133.1)	-0.8 (-2.8 to 1.1)	-6.3 (-8.1 to -4.5)
Alcohol use disorders	11,781.4 (9,797.0 to 13,986.0)	6,088.6 (4,751.7 to 7,541.7)	1,184.9 (785.2 to 1,667.4)	3.3 (-0.0 to 6.4)	-2.4 (-5.5 to 0.6)
Drug use disorders	6,033.0 (5,767.5 to 6,255.2)	409.1 (329.4 to 496.4)	1,622.3 (1,128.9 to 2,164.5)	10.7 (8.0 to 13.6)	4.7 (2.1 to 7.4)
Opioid use disorders	3,181.5 (2,581.2 to 3,815.0)	184.3 (128.5 to 254.5)	1,326.5 (900.3 to 1,798.3)	12.2 (8.8 to 15.5)	6.1 (2.9 to 9.2)
Cocaine use disorders	551.4 (483.3 to 627.9)	12.0 (7.5 to 17.6)	75.7 (47.1 to 108.8)	6.4 (1.7 to 11.3)	0.6 (-3.9 to 5.2)
Amphetamine use disorders	314.4 (221.3 to 436.7)	15.6 (7.4 to 26.9)	41.3 (23.4 to 66.6)	4.3 (-4.1 to 12.9)	-1.4 (-9.3 to 6.8)
Cannabis use disorders	1,594.2 (1,213.6 to 2,030.7)	122.3 (77.2 to 172.6)	46.4 (27.5 to 70.2)	-0.9 (-5.7 to 3.8)	-6.3 (-10.8 to -1.9)
Other drug use disorders	515.8 (431.3 to 608.6)	74.9 (58.8 to 92.9)	132.5 (89.6 to 181.5)	5.8 (2.3 to 9.2)	-0.0 (-3.3 to 3.2)
Depressive disorders	22,373.2 (21,476.5 to 23,293.7)	22,942.2 (18,723.2 to 27,286.2)	3,764.5 (2,521.9 to 5,242.4)	3.9 (2.6 to 5.3)	-1.8 (-3.1 to -0.5)
Major depressive disorder	14,067.0 (11,716.7 to 16,672.6)	21,524.0 (17,272.9 to 25,960.4)	2,906.6 (1,875.2 to 4,174.1)	3.8 (2.1 to 5.5)	-1.9 (-3.5 to -0.3)
Dysthymia	8,799.3 (7,125.6 to 10,826.5)	1,418.2 (1,042.6 to 1,814.3)	857.9 (557.3 to 1,290.9)	4.4 (1.6 to 7.1)	-1.4 (-4.0 to 1.2)
Bipolar disorder	4,033.0 (3,339.7 to 4,856.2)	231.3 (141.2 to 335.8)	829.0 (498.8 to 1,246.2)	11.2 (9.1 to 13.4)	5.2 (3.2 to 7.2)
Anxiety disorders	23,308.4 (20,997.8 to 25,893.2)	3,641.0 (3,153.7 to 4,203.8)	2,256.3 (1,571.3 to 3,084.8)	8.1 (6.0 to 9.9)	2.1 (0.2 to 3.9)
Eating disorders	1,325.4 (1,113.4 to 1,625.0)	152.9 (105.9 to 214.4)	264.2 (157.2 to 406.8)	15.9 (12.7 to 19.0)	9.6 (6.5 to 12.5)
Anorexia nervosa	169.4 (119.0 to 245.3)	42.2 (28.2 to 59.0)	35.6 (20.8 to 56.3)	7.9 (1.0 to 15.0)	2.0 (-4.5 to 8.7)
Bulimia nervosa	1,095.6 (745.8 to 1,552.5)	110.7 (64.3 to 172.2)	228.6 (128.5 to 359.0)	17.3 (13.6 to 20.8)	10.8 (7.4 to 14.2)
Autistic spectrum disorders	4,239.7 (4,052.9 to 4,432.9)	--	616.9 (421.6 to 868.9)	5.9 (4.2 to 7.5)	0.1 (-1.5 to 1.6)
Autism	1,258.5 (1,086.0 to 1,461.6)	--	319.4 (207.5 to 460.1)	5.2 (2.7 to 7.8)	-0.6 (-3.0 to 1.9)
Asperger syndrome and other autistic spectrum disorders	2,978.5 (2,480.2 to 3,567.6)	--	297.5 (194.7 to 431.8)	6.6 (5.0 to 8.5)	0.8 (-0.7 to 2.5)

Cause	Prevalence in thousands (95% UI)	Incidence in thousands (95% UI)	YLDs in thousands (95% UI)		
	2016 counts	2016 counts	2016 counts	Percentage change in counts between 2006 and 2016	Percentage change in rates between 2006 and 2016
Attention-deficit/hyperactivity disorder	3,548.1<br>(3,143.3 to 4,007.9)	--	42.5<br>(25.4 to 66.6)	1.6<br>(-1.1 to 4.5)	-3.9<br>(-6.5 to -1.2)
Conduct disorder	--	--	--	--	--
Idiopathic developmental intellectual disability	8,287.4<br>(5,049.1 to 11,564.2)	--	330.7<br>(159.3 to 564.9)	-1.2<br>(-5.4 to 1.5)	-6.6<br>(-10.6 to -4.0)
Other mental and substance use disorders	14,970.7<br>(13,132.8 to 17,157.0)	139.8<br>(95.4 to 197.0)	1,128.8<br>(763.6 to 1,627.8)	5.8<br>(4.2 to 7.3)	-0.0<br>(-1.5 to 1.4)
**Diabetes, urogenital, blood, and endocrine diseases**	**209,667.8<br>(207,188.9 to 211,818.0)**	**78,670.9†<br>(70,207.9 to 87,652.5)**	**4,180.3<br>(2,882.9 to 5,796.8)**	**5.0<br>(3.2 to 6.8)**	**-0.8<br>(-2.4 to 1.0)**
Diabetes mellitus	23,744.0<br>(20,403.9 to 27,492.3)	1,863.9<br>(1,363.9 to 2,493.3)	1,711.5<br>(1,146.0 to 2,433.2)	3.3<br>(0.2 to 6.2)	-2.4<br>(-5.2 to 0.4)
Acute glomerulonephritis	6.0<br>(3.8 to 8.9)	89.6<br>(57.2 to 133.6)	0.3<br>(0.2 to 0.5)	-13.6<br>(-16.4 to -11.3)	-18.4<br>(-20.9 to -16.1)
Chronic kidney disease	10,405.5<br>(8,789.6 to 12,285.7)	709.4<br>(469.8 to 968.6)	495.9<br>(342.9 to 695.4)	18.6<br>(14.0 to 23.2)	12.1<br>(7.8 to 16.5)
Chronic kidney disease due to diabetes mellitus	3,531.2<br>(2,484.8 to 4,642.0)	234.7<br>(145.7 to 346.7)	183.5<br>(116.4 to 273.5)	16.0<br>(10.2 to 22.1)	9.7<br>(4.2 to 15.4)
Chronic kidney disease due to hypertension	1,881.2<br>(1,327.3 to 2,468.4)	191.3<br>(113.8 to 297.9)	52.5<br>(32.6 to 77.8)	15.1<br>(8.5 to 21.7)	8.8<br>(2.5 to 15.1)
Chronic kidney disease due to glomerulonephritis	1,912.5<br>(1,395.0 to 2,503.6)	71.0<br>(41.9 to 106.9)	123.7<br>(72.4 to 182.9)	20.1<br>(13.8 to 26.2)	13.5<br>(7.6 to 19.3)
Chronic kidney disease due to other causes	3,080.5<br>(2,188.3 to 4,113.4)	212.4<br>(126.9 to 327.4)	136.2<br>(82.5 to 204.2)	22.2<br>(16.2 to 29.2)	15.6<br>(9.8 to 22.1)
Urinary diseases and male infertility	6,926.6<br>(5,356.2 to 9,301.6)	34,182.9†<br>(26,339.2 to 42,148.1)	80.2<br>(48.9 to 126.1)	5.3<br>(2.0 to 8.6)	-0.4<br>(-3.6 to 2.6)
Interstitial nephritis and urinary tract infections	552.2<br>(420.3 to 699.1)	28,238.8<br>(21,200.9 to 36,177.3)	18.3<br>(10.8 to 28.1)	9.7<br>(4.4 to 15.1)	3.7<br>(-1.3 to 8.8)
Urolithiasis	223.2<br>(163.4 to 302.2)	5,944.1<br>(4,341.3 to 8,107.7)	16.9<br>(10.5 to 26.1)	3.3<br>(-1.3 to 8.5)	-2.3<br>(-6.7 to 2.6)
Benign prostatic hyperplasia	--	--	--	--	--
Male infertility	5,534.0<br>(3,396.4 to 8,685.7)	--	30.6<br>(11.3 to 64.4)	8.8<br>(3.6 to 13.3)	2.9<br>(-2.1 to 7.1)
Other urinary diseases	--	--	14.5<br>(9.3 to 20.9)	-3.9<br>(-7.1 to -0.6)	-9.2<br>(-12.2 to -6.1)
Gynecological diseases	87,749.8<br>(84,992.7 to 89,676.0)	41,060.7†<br>(36,283.5 to 45,976.2)	1,350.4<br>(910.6 to 1,949.0)	1.2<br>(-1.3 to 3.7)	-4.4<br>(-6.7 to -2.0)
Uterine fibroids	9,930.9<br>(8,038.1 to 11,906.8)	2,363.6<br>(1,713.6 to 3,094.8)	197.8<br>(115.8 to 317.5)	9.2<br>(6.5 to 12.0)	3.2<br>(0.7 to 5.9)
Polycystic ovarian syndrome	2,964.8<br>(2,356.8 to 3,655.0)	3,015.2<br>(2,009.3 to 4,279.5)	25.7<br>(11.8 to 47.5)	3.8<br>(1.8 to 5.8)	-1.9<br>(-3.8 to 0.1)
Female infertility	5,312.6<br>(1,115.7 to 14,738.0)	--	27.9<br>(4.6 to 82.3)	25.8<br>(8.0 to 60.4)	18.9<br>(2.0 to 51.6)
Endometriosis	417.4<br>(354.4 to 488.6)	1,720.8<br>(1,252.0 to 2,271.9)	38.9<br>(25.5 to 55.7)	-8.7<br>(-14.3 to -2.8)	-13.7<br>(-19.0 to -8.1)
Genital prolapse	10,361.6<br>(8,273.6 to 12,685.1)	1,672.0<br>(1,088.9 to 2,357.8)	33.1<br>(16.0 to 62.6)	7.4<br>(4.9 to 10.2)	1.5<br>(-0.8 to 4.2)
Premenstrual syndrome	56,713.7<br>(52,669.9 to 61,462.5)	17,051.1<br>(14,884.4 to 19,667.7)	472.3<br>(295.3 to 719.5)	3.7<br>(1.5 to 6.0)	-2.0<br>(-4.0 to 0.1)
Other gynecological diseases	15,782.5<br>(12,743.8 to 18,489.8)	15,238.0<br>(11,163.2 to 18,915.1)	554.7<br>(354.1 to 810.5)	-4.0<br>(-7.5 to 0.7)	-9.2<br>(-12.6 to -4.9)
Hemoglobinopathies and hemolytic anaemias	121,079.8<br>(119,729.4 to 122,786.6)	--	379.6<br>(240.1 to 575.3)	9.4<br>(1.4 to 17.6)	3.4<br>(-4.2 to 11.1)
Thalassemias	1.8<br>(1.7 to 1.8)	--	0.1<br>(0.1 to 0.2)	2.4<br>(-13.5 to 25.7)	-3.2<br>(-18.2 to 18.9)
Thalassaemias trait	20,469.5<br>(19,697.7 to 21,402.1)	--	236.1<br>(146.1 to 354.7)	0.4<br>(-9.7 to 10.9)	-5.1<br>(-14.6 to 4.9)
Sickle cell disorders	52.6<br>(47.5 to 58.0)	--	8.1<br>(5.7 to 10.8)	50.1<br>(37.6 to 63.4)	41.9<br>(30.1 to 54.4)
Sickle cell trait	30,067.7<br>(27,174.8 to 33,967.2)	--	88.5<br>(56.0 to 134.2)	30.1<br>(24.8 to 35.4)	23.0<br>(18.0 to 28.0)
G6PD deficiency	22,127.8<br>(21,316.1 to 23,073.4)	--	1.4<br>(0.9 to 2.0)	37.6<br>(30.5 to 44.7)	30.1<br>(23.4 to 36.8)
G6PD trait	58,451.0<br>(57,496.7 to 59,537.0)	--	< 0.05<br>(< 0.05 to 0.1)	6.1<br>(-0.0 to 13.0)	0.2<br>(-5.5 to 6.9)
Other hemoglobinopathies and hemolytic anaemias	3,265.0<br>(2,815.8 to 3,745.1)	--	45.3<br>(27.7 to 71.0)	21.3<br>(14.9 to 28.2)	14.7<br>(8.6 to 21.2)
Endocrine, metabolic, blood, and immune disorders	5,690.0<br>(4,775.1 to 6,862.3)	764.4<br>(520.7 to 1,098.3)	162.4<br>(103.9 to 246.5)	8.9<br>(6.1 to 11.8)	3.0<br>(0.3 to 5.7)
**Musculoskeletal disorders**	**101,597.5<br>(95,832.1 to 107,736.0)**	**55,958.2<br>(44,075.1 to 70,056.9)**	**11,068.3<br>(7,513.2 to 15,304.9)**	**5.4<br>(3.5 to 7.1)**	**-0.4<br>(-2.1 to 1.2)**
Rheumatoid arthritis	1,222.3<br>(998.4 to 1,469.5)	92.1<br>(62.0 to 128.6)	296.9<br>(198.2 to 409.9)	11.2<br>(7.2 to 14.7)	5.1<br>(1.4 to 8.4)
Osteoarthritis	11,178.4<br>(10,264.0 to 12,131.5)	1,238.9<br>(1,027.3 to 1,473.5)	667.1<br>(465.4 to 929.2)	7.9<br>(6.3 to 9.6)	2.0<br>(0.5 to 3.6)
Low back and neck pain	63,192.8<br>(57,333.6 to 68,509.5)	26,808.7<br>(19,782.6 to 35,019.9)	7,322.4<br>(4,832.5 to 10,499.7)	5.2<br>(2.8 to 7.4)	-0.6<br>(-2.9 to 1.5)
Low back pain	41,033.1<br>(30,524.6 to 56,180.0)	19,880.3<br>(13,729.4 to 28,042.3)	4,755.1<br>(2,974.9 to 7,209.6)	6.2<br>(2.8 to 9.0)	0.4<br>(-2.9 to 3.1)
Neck pain	25,149.5<br>(17,771.4 to 34,650.0)	6,928.5<br>(4,178.8 to 10,032.3)	2,567.3<br>(1,546.8 to 3,913.5)	3.3<br>(0.6 to 5.9)	-2.4<br>(-4.9 to 0.1)
Gout	1,828.4<br>(1,478.2 to 2,226.6)	362.7<br>(262.4 to 484.9)	61.1<br>(39.1 to 86.7)	2.9<br>(-2.2 to 8.5)	-2.7<br>(-7.5 to 2.5)
Other musculoskeletal disorders	31,185.9<br>(21,631.7 to 43,106.1)	27,455.7<br>(17,775.6 to 38,913.9)	2,720.8<br>(1,624.2 to 4,256.5)	4.7<br>(0.5 to 8.6)	-1.0<br>(-5.0 to 2.6)
**Other non-communicable diseases**	**355,645.3<br>(348,808.3 to 362,330.9)**	**951,183.8†<br>(813,753.1 to 1,081,929.2)**	**7,448.6<br>(4,950.1 to 10,721.8)**	**7.2<br>(6.3 to 7.9)**	**1.3<br>(0.5 to 2.0)**
Congenital anomalies	4,694.4<br>(4,354.7 to 4,993.2)	--	559.7<br>(394.7 to 749.5)	5.4<br>(2.7 to 8.0)	-0.4<br>(-2.9 to 2.1)
Neural tube defects	379.3<br>(343.2 to 420.2)	--	114.0<br>(80.5 to 151.9)	15.4<br>(10.7 to 20.7)	9.1<br>(4.6 to 14.1)
Congenital heart anomalies	627.6<br>(558.5 to 698.0)	--	34.4<br>(14.4 to 60.1)	2.3<br>(-1.7 to 6.0)	-3.3<br>(-7.1 to 0.2)
Orofacial clefts	233.7<br>(208.2 to 259.2)	--	3.0<br>(1.9 to 4.3)	-2.6<br>(-5.3 to 0.3)	-7.9<br>(-10.5 to -5.2)
Down's syndrome	158.9<br>(138.9 to 182.6)	--	13.6<br>(9.2 to 19.3)	10.2<br>(6.2 to 14.6)	4.2<br>(0.4 to 8.3)
Turner syndrome	169.5<br>(147.1 to 193.8)	--	3.5<br>(1.8 to 5.5)	9.4<br>(-0.6 to 21.2)	3.4<br>(-6.1 to 14.6)
Klinefelter syndrome	154.6<br>(133.1 to 181.3)	--	1.6<br>(0.7 to 3.0)	6.9<br>(0.4 to 13.9)	1.1<br>(-5.1 to 7.7)

Cause	Prevalence in thousands (95% UI)	Incidence in thousands (95% UI)	YLDs in thousands (95% UI)		
	2016 counts	2016 counts	2016 counts	Percentage change in counts between 2006 and 2016	Percentage change in rates between 2006 and 2016
Other chromosomal abnormalities	0.1<br>(0.1 to 0.1)	--	< 0.05<br>(< 0.05 to 0.1)	-13.8<br>(-63.5 to 98.9)	-18.5<br>(-65.5 to 88.0)
Congenital musculoskeletal and limb anomalies	713.8<br>(657.9 to 774.7)	--	104.6<br>(71.0 to 143.7)	8.5<br>(4.7 to 12.3)	2.5<br>(-1.0 to 6.2)
Urogenital congenital anomalies	223.2<br>(200.5 to 245.6)	--	3.5<br>(1.7 to 6.2)	4.0<br>(1.1 to 6.9)	-1.7<br>(-4.4 to 1.1)
Digestive congenital anomalies	660.0<br>(594.0 to 729.5)	--	28.5<br>(18.7 to 40.8)	-4.2<br>(-8.7 to 0.8)	-9.4<br>(-13.7 to -4.8)
Other congenital anomalies	2,361.9<br>(1,661.7 to 3,336.1)	--	253.1<br>(163.3 to 380.4)	1.6<br>(-2.6 to 6.4)	-3.9<br>(-7.9 to 0.6)
Skin and subcutaneous diseases	120,457.6<br>(118,152.1 to 123,193.8)	263,949.6<br>(241,491.5 to 288,994.5)	2,793.4<br>(1,865.5 to 3,999.5)	6.6<br>(5.8 to 7.5)	0.8<br>(0.0 to 1.6)
Dermatitis	15,904.2<br>(13,692.6 to 18,445.3)	40,877.0<br>(33,274.6 to 48,821.8)	514.0<br>(314.0 to 795.0)	5.4<br>(3.7 to 7.0)	-0.4<br>(-2.0 to 1.2)
Psoriasis	5,046.9<br>(4,570.3 to 5,522.1)	583.8<br>(503.5 to 674.1)	443.6<br>(315.0 to 588.2)	11.4<br>(8.6 to 14.2)	5.3<br>(2.7 to 7.9)
Cellulitis	164.6<br>(133.4 to 199.7)	3,276.8<br>(2,742.4 to 3,857.4)	9.4<br>(6.0 to 14.0)	8.9<br>(2.9 to 15.2)	3.0<br>(-2.8 to 8.8)
Pyoderma	915.6<br>(834.3 to 1,002.8)	20,679.0<br>(18,434.3 to 23,014.3)	5.2<br>(2.1 to 10.8)	11.1<br>(7.8 to 14.5)	5.0<br>(1.9 to 8.3)
Scabies	7,459.0<br>(5,058.0 to 10,964.3)	22,571.6<br>(15,438.3 to 32,623.0)	192.5<br>(93.9 to 350.0)	-5.3<br>(-6.8 to -3.8)	-10.4<br>(-11.9 to -9.0)
Fungal skin diseases	31,045.9<br>(25,465.8 to 37,295.7)	103,740.3<br>(84,830.2 to 124,287.0)	175.6<br>(70.4 to 358.5)	8.9<br>(8.1 to 9.8)	3.0<br>(2.1 to 3.8)
Viral skin diseases	9,582.7<br>(8,165.2 to 11,260.2)	15,054.1<br>(12,844.4 to 17,508.5)	292.1<br>(178.7 to 438.4)	6.0<br>(4.5 to 7.5)	0.2<br>(-1.2 to 1.6)
Acne vulgaris	26,860.2<br>(23,524.5 to 30,462.7)	12,407.9<br>(10,561.5 to 14,158.6)	683.9<br>(443.7 to 989.4)	7.2<br>(5.5 to 8.9)	1.4<br>(-0.3 to 2.9)
Alopecia areata	1,668.7<br>(1,492.2 to 1,871.3)	2,891.3<br>(2,625.0 to 3,200.4)	54.8<br>(35.0 to 83.4)	4.2<br>(1.7 to 7.0)	-1.5<br>(-3.9 to 1.1)
Pruritus	4,665.9<br>(3,303.9 to 6,499.4)	3,646.3<br>(2,517.2 to 4,956.5)	50.2<br>(21.8 to 99.4)	7.0<br>(5.2 to 8.7)	1.2<br>(-0.5 to 2.7)
Urticaria	3,343.5<br>(2,358.6 to 4,645.5)	5,862.6<br>(4,165.9 to 8,145.8)	199.1<br>(115.5 to 305.9)	6.1<br>(3.6 to 8.4)	0.3<br>(-2.1 to 2.5)
Decubitus ulcer	44.5<br>(29.7 to 62.1)	160.4<br>(107.0 to 219.2)	7.9<br>(4.5 to 12.3)	7.9<br>(6.5 to 9.1)	2.0<br>(0.7 to 3.1)
Other skin and subcutaneous diseases	29,665.2<br>(26,777.2 to 32,505.6)	32,198.6<br>(28,778.6 to 35,755.7)	165.2<br>(78.1 to 308.4)	10.9<br>(9.8 to 11.9)	4.9<br>(3.8 to 5.8)
Sense organ diseases	102,607.2<br>(98,714.2 to 105,810.6)	36,546.0†<br>(32,830.5 to 40,431.5)	2,825.6<br>(1,884.0 to 4,020.9)	6.7<br>(5.1 to 8.2)	0.9<br>(-0.6 to 2.3)
Glaucoma	--	--	--	--	--
Cataract	1,054.1<br>(788.6 to 1,407.6)	--	99.4<br>(61.2 to 149.7)	9.3<br>(4.0 to 14.1)	3.3<br>(-1.7 to 7.9)
Macular degeneration	--	--	--	--	--
Refraction and accommodation disorders	41,163.6<br>(37,159.2 to 45,739.3)	--	769.7<br>(476.1 to 1,210.0)	7.6<br>(6.3 to 8.8)	1.7<br>(0.5 to 2.9)
Age-related and other hearing loss	59,845.0<br>(49,074.9 to 72,381.6)	--	1,527.6<br>(1,012.6 to 2,258.4)	6.0<br>(2.9 to 8.7)	0.2<br>(-2.7 to 2.7)
Other vision loss	1,257.5<br>(944.5 to 1,623.4)	--	109.6<br>(68.0 to 166.1)	8.9<br>(4.5 to 13.5)	3.0<br>(-1.2 to 7.2)
Other sense organ diseases	10,036.9<br>(8,808.9 to 11,481.2)	36,546.0<br>(32,830.5 to 40,431.5)	319.3<br>(194.0 to 485.1)	6.7<br>(4.9 to 8.7)	0.9<br>(-0.9 to 2.8)
Oral disorders	258,916.7<br>(245,070.6 to 270,628.0)	650,688.2†<br>(519,601.7 to 777,914.3)	1,269.8<br>(709.8 to 2,126.3)	10.1<br>(8.8 to 11.3)	4.1<br>(2.9 to 5.2)
Caries of deciduous teeth	--	--	--	--	--
Caries of permanent teeth	208,263.9<br>(172,393.5 to 244,975.8)	640,668.5<br>(507,360.5 to 767,326.8)	147.6<br>(65.0 to 287.6)	4.0<br>(2.9 to 5.1)	-1.7<br>(-2.7 to -0.6)
Periodontal disease	71,789.4<br>(51,848.9 to 95,143.7)	9,352.2<br>(5,094.4 to 13,394.1)	477.3<br>(180.3 to 1,038.0)	15.6<br>(14.3 to 16.6)	9.2<br>(8.1 to 10.2)
Edentulism and severe tooth loss	10,215.8<br>(7,987.9 to 12,941.7)	667.5<br>(386.3 to 1,027.4)	293.0<br>(181.7 to 434.8)	10.3<br>(8.7 to 11.9)	4.3<br>(2.7 to 5.8)
Other oral disorders	11,942.5<br>(10,652.6 to 13,269.5)	--	352.0<br>(218.7 to 533.0)	5.8<br>(4.6 to 7.0)	0.0<br>(-1.1 to 1.1)
**Injuries**	**109,550.1<br>(102,920.9 to 117,674.1)**	**37,462.6†<br>(33,124.6 to 42,457.2)**	**3,979.4<br>(2,646.1 to 5,657.6)**	**10.6<br>(9.1 to 12.1)**	**4.5<br>(3.1 to 6.0)**
**Transport injuries**	**17,893.0<br>(16,328.1 to 19,615.8)**	**5,991.0<br>(4,590.9 to 7,715.7)**	**950.6<br>(632.6 to 1,375.2)**	**9.3<br>(8.0 to 10.7)**	**3.4<br>(2.1 to 4.6)**
Road injuries	14,486.2<br>(13,157.9 to 16,004.8)	5,473.8<br>(4,061.9 to 7,262.5)	774.0<br>(514.7 to 1,120.1)	11.1<br>(9.8 to 12.4)	5.0<br>(3.8 to 6.2)
Pedestrian road injuries	2,999.4<br>(2,505.5 to 3,503.1)	871.5<br>(544.1 to 1,294.6)	162.2<br>(107.8 to 232.7)	10.0<br>(8.6 to 11.5)	4.0<br>(2.6 to 5.4)
Cyclist road injuries	2,744.6<br>(2,220.3 to 3,417.3)	1,053.4<br>(637.0 to 1,557.0)	134.7<br>(87.8 to 197.8)	11.8<br>(10.4 to 13.2)	5.7<br>(4.3 to 7.0)
Motorcyclist road injuries	3,872.1<br>(3,178.4 to 5,001.2)	1,121.6<br>(751.5 to 1,633.8)	205.2<br>(136.8 to 297.8)	10.2<br>(8.9 to 11.6)	4.2<br>(2.9 to 5.5)
Motor vehicle road injuries	3,781.9<br>(3,050.2 to 4,542.6)	1,900.9<br>(1,348.2 to 2,630.7)	218.9<br>(145.4 to 315.6)	9.1<br>(7.4 to 10.8)	3.1<br>(1.5 to 4.7)
Other road injuries	1,088.1<br>(874.1 to 1,321.8)	526.4<br>(273.9 to 894.5)	53.1<br>(34.3 to 78.7)	26.3<br>(24.6 to 28.0)	19.4<br>(17.7 to 21.0)
Other transport injuries	3,406.8<br>(2,777.5 to 4,111.4)	517.2<br>(335.0 to 766.8)	176.6<br>(118.7 to 253.5)	2.3<br>(0.7 to 3.9)	-3.3<br>(-4.8 to -1.8)
**Unintentional injuries**	**79,192.1<br>(73,042.0 to 86,432.7)**	**28,525.5<br>(24,380.2 to 33,268.8)**	**2,509.2<br>(1,671.4 to 3,556.9)**	**12.1<br>(11.0 to 13.2)**	**6.0<br>(4.9 to 7.0)**
Falls	29,721.4<br>(25,348.1 to 34,135.6)	10,243.3<br>(7,257.1 to 14,106.8)	1,117.5<br>(771.6 to 1,554.7)	14.3<br>(13.2 to 15.5)	8.1<br>(7.0 to 9.2)
Drowning	321.3<br>(273.8 to 378.0)	34.6<br>(26.4 to 45.0)	16.3<br>(10.8 to 23.5)	-9.1<br>(-10.9 to -7.5)	-14.1<br>(-15.8 to -12.5)
Fire, heat, and hot substances	8,693.4<br>(7,202.4 to 10,634.4)	640.5<br>(411.0 to 911.6)	213.8<br>(139.5 to 320.4)	11.4<br>(9.5 to 13.1)	5.3<br>(3.5 to 6.9)
Poisonings	268.9<br>(223.7 to 319.4)	296.3<br>(177.7 to 447.6)	23.2<br>(15.0 to 33.8)	19.2<br>(15.8 to 23.1)	12.7<br>(9.5 to 16.4)
Exposure to mechanical forces	14,889.0<br>(12,315.1 to 19,029.8)	5,044.5<br>(3,459.3 to 7,022.2)	338.2<br>(214.1 to 503.8)	11.6<br>(10.3 to 12.8)	5.5<br>(4.3 to 6.6)
Unintentional firearm injuries	589.5<br>(460.1 to 752.6)	110.5<br>(55.9 to 189.3)	20.4<br>(12.6 to 30.9)	11.5<br>(10.0 to 13.1)	5.4<br>(4.0 to 6.9)
Unintentional suffocation	650.8<br>(496.4 to 838.3)	55.6<br>(25.4 to 102.7)	19.6<br>(12.0 to 30.4)	-2.9<br>(-4.3 to -1.3)	-8.2<br>(-9.5 to -6.7)
Other exposure to mechanical forces	13,648.8<br>(11,132.9 to 17,753.5)	4,878.4<br>(3,354.0 to 6,764.2)	298.2<br>(189.4 to 442.6)	12.8<br>(11.4 to 13.9)	6.6<br>(5.3 to 7.7)

Cause	Prevalence in thousands (95% UI)	Incidence in thousands (95% UI)	YLDs in thousands (95% UI)		
	2016 counts	2016 counts	2016 counts	Percentage change in counts between 2006 and 2016	Percentage change in rates between 2006 and 2016
Adverse effects of medical treatment	113.1<br>(74.7 to 160.8)	1,478.2<br>(1,008.2 to 2,011.0)	15.1<br>(8.5 to 24.1)	1.0<br>(-4.2 to 5.0)	-4.5<br>(-9.5 to -0.8)
Animal contact	4,224.6<br>(3,635.4 to 5,028.3)	4,569.5<br>(3,051.4 to 6,564.4)	133.1<br>(90.0 to 184.8)	10.9<br>(8.5 to 13.3)	4.9<br>(2.6 to 7.1)
Venomous animal contact	2,022.9<br>(1,644.3 to 2,466.9)	2,196.1<br>(1,396.4 to 3,271.3)	97.7<br>(65.7 to 135.2)	13.6<br>(10.3 to 16.8)	7.4<br>(4.3 to 10.4)
Non-venomous animal contact	2,201.7<br>(1,741.0 to 2,774.8)	2,373.4<br>(1,495.8 to 3,493.9)	35.4<br>(22.3 to 53.2)	4.2<br>(2.0 to 6.1)	-1.5<br>(-3.6 to 0.3)
Foreign body	3,817.0<br>(3,123.0 to 4,492.3)	1,976.0<br>(1,345.4 to 2,929.6)	140.6<br>(89.2 to 212.0)	12.6<br>(10.7 to 14.5)	6.4<br>(4.6 to 8.3)
Pulmonary aspiration and foreign body in airway	1,763.2<br>(1,320.0 to 2,365.3)	290.5<br>(188.8 to 417.6)	73.6<br>(45.9 to 110.9)	7.5<br>(5.6 to 9.3)	1.6<br>(-0.1 to 3.3)
Foreign body in eyes	244.1<br>(121.7 to 437.1)	1,311.7<br>(721.0 to 2,235.0)	14.3<br>(6.7 to 27.0)	22.3<br>(18.6 to 25.7)	15.6<br>(12.1 to 18.8)
Foreign body in other body part	1,809.7<br>(1,438.0 to 2,300.1)	373.8<br>(220.6 to 579.8)	52.7<br>(33.7 to 78.8)	17.7<br>(15.8 to 19.5)	11.3<br>(9.4 to 13.0)
Environmental heat and cold exposure	5,333.7<br>(4,411.9 to 6,363.2)	958.6<br>(594.5 to 1,458.5)	193.8<br>(128.8 to 279.6)	9.2<br>(7.5 to 10.8)	3.2<br>(1.6 to 4.8)
Other unintentional injuries	11,809.7<br>(9,635.4 to 14,237.8)	3,284.2<br>(2,108.9 to 4,826.8)	317.6<br>(206.0 to 463.6)	9.2<br>(7.7 to 10.8)	3.2<br>(1.8 to 4.7)
**Self-harm and interpersonal violence**	**20,698.1<br>(15,988.3 to 26,339.3)**	**2,849.9†<br>(2,102.1 to 3,779.4)**	**341.2<br>(221.3 to 489.6)**	**0.6<br>(-0.5 to 1.5)**	**-4.9<br>(-5.9 to -4.1)**
Self-harm	889.6<br>(730.7 to 1,063.0)	558.3<br>(406.8 to 746.9)	43.2<br>(27.7 to 63.1)	2.0<br>(0.4 to 3.5)	-3.6<br>(-5.1 to -2.1)
Self-harm by firearm	31.5<br>(26.7 to 36.8)	6.8<br>(1.8 to 19.9)	1.2<br>(0.7 to 1.7)	-0.4<br>(-2.7 to 1.7)	-5.9<br>(-8.0 to -3.9)
Self-harm by other specified means	858.1<br>(697.1 to 1,028.6)	551.5<br>(401.1 to 739.2)	42.1<br>(27.0 to 61.3)	2.0<br>(0.5 to 3.6)	-3.5<br>(-5.0 to -2.1)
Interpersonal violence	19,808.5<br>(15,065.7 to 25,409.2)	2,291.6†<br>(1,591.0 to 3,214.2)	298.0<br>(192.6 to 431.3)	0.4<br>(-0.8 to 1.4)	-5.1<br>(-6.2 to -4.1)
Assault by firearm	245.9<br>(191.5 to 312.3)	58.0<br>(33.1 to 94.5)	9.5<br>(5.9 to 14.3)	4.2<br>(2.8 to 5.8)	-1.5<br>(-2.8 to 0.0)
Assault by sharp object	1,315.4<br>(1,022.6 to 1,611.9)	490.6<br>(283.9 to 757.1)	37.7<br>(24.2 to 56.8)	-2.6<br>(-4.5 to -0.8)	-8.0<br>(-9.7 to -6.2)
Sexual violence	14,947.8<br>(10,072.0 to 20,720.7)	--	123.0<br>(68.5 to 202.6)	2.1<br>(0.6 to 3.5)	-3.5<br>(-4.9 to -2.2)
Assault by other means	3,299.4<br>(2,661.4 to 4,241.5)	1,743.0<br>(1,193.3 to 2,477.2)	127.8<br>(83.6 to 188.7)	-0.7<br>(-2.0 to 0.6)	-6.1<br>(-7.4 to -4.9)
**Forces of nature, conflict and terrorism, and executions and police conflict**	**6,714.7<br>(4,136.0 to 10,038.2)**	**96.2†<br>(53.0 to 138.3)**	**178.4<br>(67.6 to 356.6)**	**17.1<br>(5.0 to 33.1)**	**10.7<br>(-0.7 to 25.8)**
Forces of nature, conflict and terrorism, and state actor violence	925.1<br>(504.3 to 1,446.6)	22.4<br>(13.2 to 34.8)	30.0<br>(8.2 to 71.2)	64.7<br>(50.4 to 72.9)	55.6<br>(42.2 to 63.4)
Conflict and terrorism	5,429.6<br>(3,194.0 to 8,697.1)	--	148.7<br>(60.5 to 287.2)	25.9<br>(9.0 to 44.5)	19.0<br>(3.0 to 36.6)
Executions and police conflict	360.1<br>(172.3 to 557.1)	73.9<br>(32.0 to 116.0)	8.5<br>(2.4 to 22.3)	-63.2<br>(-66.0 to -50.3)	-65.2<br>(-67.9 to -53.0)

We did not calculate incidence for certain NTDs and causes with only birth prevalence. Blank cells mean that no estimate is available.

† Incidence for aggregate causes represents the sum of the incidence of the causes below the aggregate.

‡ Incidence estimates for stroke represent first-ever stroke only.

^ Incidence of HIV/AIDS represents new infections of HIV only and does not include new infections of Tuberculosis in HIV positive cases.

SR Table 12: Global prevalence, incidence, and YLDs for 2016, percentage change of YLD counts and percentage change of YLD rates between 2006 and 2016 by cause, age 40-44 years.					
Cause	Prevalence in thousands (95% UI)	Incidence in thousands (95% UI)	YLDs in thousands (95% UI)		
	2016 counts	2016 counts	2016 counts	Percentage change in counts between 2006 and 2016	Percentage change in rates between 2006 and 2016
**All causes**	**479,809.5 (479,313.8 to 480,260.1)**	**2,652,150.2† (2,312,235.6 to 3,082,333.4)**	**59,216.8 (43,971.0 to 77,096.8)**	**12.5 (11.5 to 13.2)**	**-1.2 (-2.0 to -0.6)**
**Communicable, maternal, neonatal, and nutritional disorders**	**352,093.3 (347,836.8 to 356,708.0)**	**1,405,018.1† (1,094,907.7 to 1,833,654.2)**	**5,879.6 (4,257.5 to 7,872.6)**	**5.6 (0.2 to 9.8)**	**-7.2 (-12.0 to -3.5)**
**HIV/AIDS and tuberculosis**	**176,078.6 (155,936.7 to 199,781.5)**	**740.4† (542.6 to 989.1)**	**747.7 (497.3 to 1,064.6)**	**-7.8 (-25.5 to 13.5)**	**-19.0 (-34.6 to -0.4)**
Tuberculosis	164,875.9 (135,266.7 to 197,557.6)	621.5† (418.4 to 871.1)	221.5 (130.7 to 339.9)	2.6 (-1.6 to 7.9)	-9.9 (-13.6 to -5.2)
Drug-susceptible tuberculosis	692.9 (465.1 to 997.4)	597.8 (401.7 to 839.8)	213.1 (125.3 to 328.0)	2.7 (-1.6 to 8.0)	-9.8 (-13.6 to -5.1)
Multidrug-resistant tuberculosis without extensive drug resistance	24.6 (17.0 to 34.1)	22.1 (15.7 to 30.4)	7.9 (4.6 to 12.1)	-3.0 (-17.8 to 14.0)	-14.8 (-27.8 to 0.1)
Extensively drug-resistant tuberculosis	1.5 (1.0 to 2.0)	1.6 (1.1 to 2.2)	0.5 (0.3 to 0.8)	167.0 (132.5 to 205.0)	134.5 (104.2 to 167.8)
Latent tuberculosis infection	164,157.0 (134,581.8 to 196,983.8)	--	--	--	--
HIV/AIDS	4,707.0 (4,203.2 to 5,260.6)	119.0^ (98.9 to 149.5)	526.2 (341.3 to 782.0)	-11.6 (-32.0 to 18.1)	-22.3 (-40.3 to 3.7)
Drug-susceptible HIV/AIDS - Tuberculosis	128.5 (90.8 to 180.8)	157.4 (113.4 to 214.2)	49.1 (28.9 to 76.1)	-7.9 (-13.8 to -1.6)	-19.1 (-24.3 to -13.6)
Multidrug-resistant HIV/AIDS - Tuberculosis without extensive drug resistance	3.4 (2.2 to 5.1)	4.2 (2.7 to 6.1)	1.4 (0.8 to 2.3)	-16.3 (-31.2 to 0.4)	-26.5 (-39.6 to -11.9)
Extensively drug-resistant HIV/AIDS - Tuberculosis	0.2 (0.1 to 0.2)	0.2 (0.1 to 0.3)	0.1 (< 0.05 to 0.1)	191.2 (143.7 to 252.2)	155.7 (114.0 to 209.3)
HIV/AIDS resulting in other diseases	4,575.0 (4,079.6 to 5,121.2)	119.0 (98.9 to 149.5)	475.7 (302.1 to 722.2)	-11.9 (-33.4 to 22.8)	-22.6 (-41.5 to 7.8)
**Diarrhoea, lower respiratory infections, and other common infectious diseases**	**22,809.7 (20,672.0 to 24,772.2)**	**1,300,167.4† (987,439.0 to 1,728,026.4)**	**1,040.5 (719.0 to 1,448.3)**	**11.0 (9.6 to 12.3)**	**-2.5 (-3.7 to -1.4)**
Diarrhoeal diseases	2,736.8 (2,370.5 to 3,122.6)	188,557.1 (164,230.8 to 213,536.3)	304.8 (206.3 to 422.6)	12.8 (10.3 to 15.5)	-0.9 (-3.1 to 1.4)
Intestinal infectious diseases	20.3 (15.8 to 25.5)	502.5† (394.8 to 612.5)	3.6 (2.2 to 5.4)	-2.9 (-19.6 to 17.4)	-14.7 (-29.4 to 3.1)
Typhoid fever	19.9 (15.1 to 25.2)	314.7 (246.7 to 387.3)	3.1 (1.9 to 4.7)	-2.5 (-21.6 to 23.3)	-14.4 (-31.2 to 8.3)
Paratyphoid fever	8.2 (6.5 to 10.2)	187.8 (147.5 to 228.7)	0.5 (0.3 to 0.8)	-4.1 (-19.8 to 13.7)	-15.7 (-29.6 to -0.1)
Other intestinal infectious diseases	--	--	< 0.05 (< 0.05 to 0.1)	-18.3 (-32.3 to -1.2)	-28.2 (-40.5 to -13.2)
Lower respiratory infections	252.6 (224.5 to 286.3)	11,213.9 (9,806.6 to 12,892.3)	15.4 (10.2 to 22.0)	30.3 (23.8 to 37.1)	14.5 (8.8 to 20.4)
Upper respiratory infections	14,519.1 (10,386.2 to 20,257.0)	1,086,944.0 (777,175.2 to 1,512,037.3)	358.0 (198.1 to 593.4)	11.3 (9.6 to 12.7)	-2.3 (-3.7 to -1.0)
Otitis media	3,712.4 (2,637.8 to 5,044.0)	8,367.5 (6,915.7 to 10,028.7)	100.8 (57.8 to 169.2)	3.1 (-1.6 to 8.0)	-9.4 (-13.6 to -5.1)
Meningitis	945.5 (810.6 to 1,091.9)	55.4 (39.4 to 75.2)	96.6 (67.2 to 128.5)	15.0 (11.5 to 18.5)	1.0 (-2.1 to 4.0)
Pneumococcal meningitis	431.3 (359.9 to 513.2)	13.6 (8.4 to 20.5)	43.5 (29.6 to 58.1)	12.9 (6.3 to 19.7)	-0.9 (-6.6 to 5.1)
H influenzae type B meningitis	120.0 (94.0 to 153.3)	3.7 (2.1 to 6.4)	15.1 (10.0 to 21.0)	-6.1 (-11.8 to -1.7)	-17.5 (-22.5 to -13.7)
Meningococcal infection	101.7 (74.1 to 133.8)	9.4 (5.5 to 14.3)	10.4 (7.1 to 14.3)	19.7 (16.9 to 22.4)	5.1 (2.7 to 7.5)
Other meningitis	292.5 (219.9 to 369.7)	28.7 (19.6 to 40.1)	27.7 (18.8 to 37.5)	33.3 (27.8 to 39.2)	17.0 (12.3 to 22.2)
Encephalitis	1,214.8 (816.3 to 1,769.5)	298.0 (243.2 to 359.1)	142.3 (100.3 to 187.0)	8.4 (3.0 to 13.9)	-4.7 (-9.5 to 0.0)
Diphtheria	< 0.05 (< 0.05 to < 0.05)	0.2 (0.1 to 0.3)	< 0.05 (< 0.05 to < 0.05)	-40.3 (-54.7 to -22.7)	-47.6 (-60.2 to -32.1)
Whooping cough	1.7 (1.4 to 2.2)	12.7 (9.9 to 16.0)	0.1 (0.1 to 0.1)	-15.7 (-17.4 to -14.0)	-25.9 (-27.4 to -24.5)
Tetanus	9.3 (9.1 to 9.5)	4.4 (2.3 to 6.5)	0.2 (0.1 to 0.4)	0.8 (-20.9 to 30.5)	-11.5 (-30.5 to 14.7)
Measles	0.2 (0.1 to 0.4)	6.6 (2.4 to 14.3)	< 0.05 (< 0.05 to < 0.05)	-54.6 (-59.5 to -48.9)	-60.1 (-64.4 to -55.1)
Varicella and herpes zoster	377.0 (292.7 to 469.3)	4,205.0 (3,100.5 to 5,491.8)	18.6 (10.5 to 29.9)	13.3 (6.4 to 20.2)	-0.5 (-6.5 to 5.6)
**Neglected tropical diseases and malaria**	**119,885.2 (115,814.9 to 123,392.9)**	**17,007.0† (14,204.5 to 20,278.7)**	**911.4 (617.8 to 1,274.1)**	**-11.7 (-18.5 to -5.5)**	**-22.4 (-28.4 to -17.0)**
Malaria	4,191.2 (3,625.0 to 4,801.5)	4,902.6 (3,647.0 to 6,298.2)	57.2 (42.9 to 73.5)	-1.5 (-7.9 to 6.6)	-13.5 (-19.1 to -6.4)
Chagas disease	653.3 (548.4 to 792.0)	10.3 (6.7 to 14.6)	4.9 (2.9 to 7.2)	-0.7 (-7.9 to 6.7)	-12.8 (-19.1 to -6.3)
Leishmaniasis	351.3 (277.5 to 431.9)	36.6 (26.8 to 48.3)	19.5 (11.9 to 30.2)	26.2 (15.5 to 43.5)	10.8 (1.5 to 26.0)
Visceral leishmaniasis	1.5 (1.0 to 2.1)	5.9 (4.1 to 8.2)	0.1 (0.1 to 0.2)	-55.5 (-69.8 to -34.5)	-61.0 (-73.4 to -42.5)
Cutaneous and mucocutaneous leishmaniasis	307.8 (237.2 to 406.2)	30.7 (21.3 to 41.5)	19.4 (11.9 to 30.1)	27.4 (16.4 to 45.7)	11.9 (2.2 to 28.0)
African trypanosomiasis	0.5 (0.1 to 1.7)	0.3 (0.1 to 1.0)	0.1 (< 0.05 to 0.4)	-70.5 (-95.3 to 111.1)	-74.1 (-95.9 to 85.4)
Schistosomiasis	15,449.9 (14,228.0 to 16,652.5)	3,821.3 (3,405.7 to 4,362.5)	125.8 (64.6 to 231.0)	-22.6 (-25.3 to -18.9)	-32.0 (-34.4 to -28.8)
Cysticercosis	248.7 (170.5 to 350.7)	--	39.6 (23.3 to 61.7)	-11.4 (-21.8 to -3.0)	-22.2 (-31.3 to -14.8)
Cystic echinococcosis	75.8 (40.1 to 159.7)	15.8 (7.2 to 37.8)	7.0 (3.2 to 15.2)	-37.7 (-75.3 to 59.9)	-45.3 (-78.3 to 40.5)
Lymphatic filariasis	2,360.3 (1,872.8 to 3,134.2)	514.0 (286.2 to 812.8)	129.8 (65.0 to 238.4)	-38.3 (-53.2 to -26.5)	-45.8 (-58.9 to -35.5)
Onchocerciasis	935.1 (593.1 to 1,599.6)	221.2 (90.6 to 449.2)	64.4 (29.5 to 118.3)	-21.8 (-41.6 to -2.8)	-31.3 (-48.7 to -14.6)
Trachoma	38.6 (22.9 to 68.5)	--	5.4 (2.9 to 8.9)	-7.4 (-21.4 to 9.2)	-18.7 (-31.0 to -4.1)
Dengue	331.8 (180.7 to 562.0)	5,545.9 (3,373.2 to 8,412.5)	53.3 (24.0 to 100.5)	85.5 (49.7 to 326.2)	62.9 (31.5 to 274.3)
Yellow fever	0.1 (< 0.05 to 0.3)	4.4 (1.3 to 11.9)	< 0.05 (< 0.05 to < 0.05)	-15.6 (-44.6 to 29.7)	-25.9 (-51.3 to 13.9)
Rabies	< 0.05 (< 0.05 to < 0.05)	0.7 (0.4 to 0.9)	< 0.05 (< 0.05 to < 0.05)	-50.9 (-58.9 to -40.5)	-56.9 (-63.9 to -47.7)
Intestinal nematode infections	99,453.1 (94,283.5 to 103,191.7)	--	187.9 (109.6 to 309.9)	-11.0 (-17.9 to -3.5)	-21.8 (-27.9 to -15.2)
Ascariasis	53,203.0 (47,633.7 to 59,982.6)	--	55.1 (30.2 to 93.1)	-26.7 (-36.2 to -15.7)	-35.6 (-44.0 to -26.0)

Cause	Prevalence in thousands (95% UI)	Incidence in thousands (95% UI)	YLDs in thousands (95% UI)		
	2016 counts	2016 counts	2016 counts	Percentage change in counts between 2006 and 2016	Percentage change in rates between 2006 and 2016
Trichuriasis	27,732.0<br>(25,562.5 to 30,390.1)	--	20.6<br>(11.2 to 34.8)	-18.1<br>(-29.6 to -4.4)	-28.1<br>(-38.1 to -16.0)
Hookworm disease	29,827.4<br>(27,422.4 to 32,625.6)	--	112.3<br>(67.6 to 178.5)	1.4<br>(-9.3 to 12.9)	-11.0<br>(-20.4 to -0.8)
Food-borne trematodiases	7,383.7<br>(6,736.1 to 8,108.3)	1,304.3<br>(708.6 to 1,788.5)	192.5<br>(94.2 to 356.1)	8.7<br>(3.2 to 15.8)	-4.6<br>(-9.4 to 1.7)
Leprosy	41.1<br>(38.5 to 44.0)	4.9<br>(4.5 to 5.4)	2.6<br>(1.7 to 3.6)	-4.6<br>(-14.0 to 5.5)	-16.2<br>(-24.4 to -7.4)
Ebola virus disease	0.1<br>(< 0.05 to 0.3)	< 0.05<br>(< 0.05 to < 0.05)	< 0.05<br>(< 0.05 to 0.1)	--	--
Zika virus disease	10.3<br>(6.0 to 20.3)	624.7<br>(362.1 to 1,227.2)	0.2<br>(0.1 to 0.4)	--	--
Guinea worm disease	< 0.05<br>(< 0.05 to < 0.05)	< 0.05<br>(< 0.05 to < 0.05)	< 0.05<br>(< 0.05 to < 0.05)	-99.6<br>(-99.7 to -99.4)	-99.6<br>(-99.8 to -99.5)
Other neglected tropical diseases	1,510.3<br>(1,325.8 to 1,715.2)	--	21.2<br>(13.5 to 33.0)	27.4<br>(20.4 to 35.3)	11.8<br>(5.8 to 18.9)
**Maternal disorders**	**444.2<br>(420.8 to 469.2)**	**4,089.4†<br>(3,380.2 to 4,826.5)**	**53.7<br>(37.1 to 72.8)**	**-9.0<br>(-18.2 to 0.5)**	**-20.0<br>(-28.1 to -11.7)**
Maternal haemorrhage	51.8<br>(38.0 to 68.3)	304.0<br>(215.5 to 419.7)	1.9<br>(1.2 to 2.9)	9.4<br>(-15.4 to 40.3)	-3.9<br>(-25.7 to 23.2)
Maternal sepsis and other pregnancy related infections	160.2<br>(140.3 to 182.8)	304.1<br>(218.5 to 408.8)	1.9<br>(0.9 to 3.5)	4.4<br>(-34.8 to 67.1)	-8.3<br>(-42.7 to 46.7)
Maternal hypertensive disorders	148.0<br>(92.7 to 211.1)	698.1<br>(572.3 to 826.9)	7.1<br>(3.9 to 11.9)	13.9<br>(-27.8 to 81.1)	0.1<br>(-36.6 to 59.0)
Maternal obstructed labour and uterine rupture	102.0<br>(85.1 to 122.1)	193.8<br>(134.6 to 266.9)	32.5<br>(21.0 to 46.1)	-13.5<br>(-21.5 to -4.4)	-24.0<br>(-31.1 to -16.0)
Maternal abortion, miscarriage, and ectopic pregnancy	21.3<br>(13.0 to 30.7)	2,589.3<br>(1,921.2 to 3,313.7)	2.4<br>(1.3 to 3.9)	-0.7<br>(-38.2 to 58.8)	-12.8<br>(-45.7 to 39.5)
Other maternal disorders	--	--	7.9<br>(5.4 to 10.7)	-14.4<br>(-23.1 to -5.5)	-24.9<br>(-32.4 to -17.0)
**Neonatal disorders**	**4,342.7<br>(3,976.2 to 4,749.3)**	**--**	**769.2<br>(545.3 to 1,070.4)**	**29.0<br>(15.5 to 45.2)**	**13.3<br>(1.5 to 27.5)**
Neonatal preterm birth complications	3,659.4<br>(3,089.5 to 4,329.6)	--	498.2<br>(344.5 to 705.2)	27.4<br>(6.6 to 52.8)	11.9<br>(-6.3 to 34.2)
Neonatal encephalopathy due to birth asphyxia and trauma	682.6<br>(437.0 to 1,040.3)	--	98.5<br>(41.6 to 207.3)	30.5<br>(22.1 to 45.6)	14.6<br>(7.2 to 27.9)
Neonatal sepsis and other neonatal infections	343.9<br>(136.3 to 809.3)	--	132.4<br>(44.6 to 342.9)	34.4<br>(25.4 to 48.8)	18.0<br>(10.1 to 30.7)
Hemolytic disease and other neonatal jaundice	124.3<br>(110.5 to 138.3)	--	40.1<br>(30.9 to 50.6)	28.4<br>(22.6 to 34.6)	12.8<br>(7.7 to 18.3)
Other neonatal disorders	--	--	--	--	--
**Nutritional deficiencies**	**94,255.5<br>(90,491.6 to 97,851.0)**	**23,215.5†<br>(19,713.8 to 27,837.5)**	**1,966.3<br>(1,323.2 to 2,898.2)**	**10.4<br>(6.8 to 14.1)**	**-3.1<br>(-6.2 to 0.2)**
Protein-energy malnutrition	--	--	--	--	--
Iodine deficiency	9,336.6<br>(8,353.6 to 10,436.2)	38.4<br>(32.5 to 45.4)	244.9<br>(166.8 to 342.4)	-2.2<br>(-4.6 to 0.4)	-14.1<br>(-16.2 to -11.8)
Vitamin A deficiency	21,319.9<br>(18,630.0 to 24,833.0)	23,177.2<br>(19,678.7 to 27,799.5)	10.5<br>(6.4 to 15.8)	22.7<br>(14.4 to 31.3)	7.7<br>(0.5 to 15.4)
Iron-deficiency anaemia	73,083.7<br>(64,780.0 to 82,314.5)	--	1,710.9<br>(1,131.1 to 2,556.0)	12.4<br>(8.4 to 16.6)	-1.3<br>(-4.8 to 2.5)
Other nutritional deficiencies	--	--	--	--	--
**Other communicable, maternal, neonatal, and nutritional diseases**	**164,308.0<br>(159,935.8 to 169,966.2)**	**59,798.3<br>(47,927.0 to 74,411.9)**	**378.0<br>(233.7 to 595.5)**	**15.1<br>(10.1 to 19.8)**	**1.1<br>(-3.3 to 5.2)**
Sexually transmitted diseases excluding HIV	127,436.4<br>(122,957.3 to 133,151.0)	52,710.3<br>(41,557.6 to 66,455.4)	326.4<br>(200.3 to 527.8)	16.4<br>(12.8 to 20.3)	2.3<br>(-1.0 to 5.7)
Syphilis	6,362.1<br>(4,167.4 to 9,503.7)	4,556.3<br>(2,769.6 to 7,157.8)	16.4<br>(11.3 to 21.9)	19.0<br>(15.8 to 22.6)	4.5<br>(1.7 to 7.7)
Chlamydial infection	6,865.8<br>(5,346.7 to 8,604.5)	3,906.6<br>(2,988.9 to 5,001.8)	51.9<br>(31.5 to 82.5)	13.6<br>(8.9 to 18.0)	-0.2<br>(-4.3 to 3.6)
Gonococcal infection	7,345.5<br>(4,790.1 to 11,047.9)	21,299.8<br>(12,256.1 to 33,347.2)	73.6<br>(39.3 to 127.2)	27.4<br>(18.9 to 34.1)	11.9<br>(4.4 to 17.8)
Trichomoniasis	18,021.0<br>(12,524.4 to 24,563.4)	15,005.5<br>(10,330.0 to 20,811.8)	20.0<br>(7.5 to 41.8)	15.4<br>(13.6 to 17.2)	1.4<br>(-0.2 to 2.9)
Genital herpes	91,845.6<br>(80,287.6 to 106,587.2)	1,353.4<br>(756.7 to 2,106.1)	22.7<br>(6.7 to 51.8)	13.2<br>(11.0 to 15.4)	-0.6<br>(-2.5 to 1.4)
Other sexually transmitted diseases	10,978.9<br>(7,943.9 to 13,084.4)	6,588.7<br>(4,898.7 to 8,574.1)	141.8<br>(86.6 to 222.6)	12.8<br>(8.4 to 17.2)	-0.9<br>(-4.8 to 2.9)
Hepatitis	49,594.2<br>(42,345.8 to 57,919.3)	7,087.4<br>(4,480.6 to 10,906.2)	16.8<br>(8.6 to 29.8)	-11.5<br>(-47.6 to 51.5)	-22.2<br>(-54.0 to 33.1)
Acute hepatitis A	61.1<br>(10.3 to 135.3)	795.0<br>(134.5 to 1,759.5)	2.6<br>(0.4 to 6.3)	-21.7<br>(-90.3 to 274.2)	-31.2<br>(-91.5 to 228.6)
Hepatitis B	37,939.6<br>(30,974.0 to 46,254.8)	5,584.2<br>(3,239.7 to 9,179.1)	12.8<br>(6.0 to 24.3)	-9.5<br>(-50.5 to 63.6)	-20.5<br>(-56.5 to 43.7)
Hepatitis C	11,555.2<br>(10,270.8 to 12,896.2)	210.7<br>(157.2 to 272.5)	0.2<br>(0.1 to 0.4)	-9.7<br>(-10.4 to -9.1)	-20.7<br>(-21.3 to -20.2)
Acute hepatitis E	38.3<br>(23.5 to 57.1)	497.6<br>(305.9 to 742.2)	1.2<br>(0.6 to 2.0)	-8.0<br>(-27.3 to 16.9)	-19.2<br>(-36.2 to 2.7)
Other infectious diseases	1,804.2<br>(1,575.0 to 2,065.3)	0.6<br>(0.3 to 0.9)	34.9<br>(21.7 to 54.3)	19.2<br>(14.3 to 24.5)	4.7<br>(0.4 to 9.4)
**Non-communicable diseases**	**464,218.0<br>(463,182.1 to 465,385.3)**	**1,210,583.5†<br>(1,076,431.4 to 1,346,384.6)**	**49,060.2<br>(36,380.1 to 63,821.7)**	**13.1<br>(12.4 to 13.7)**	**-0.7<br>(-1.3 to -0.1)**
**Neoplasms**	**1,750.8<br>(1,712.7 to 1,787.9)**	**685.5<br>(660.0 to 722.8)**	**228.0<br>(165.5 to 300.2)**	**13.1<br>(8.9 to 18.4)**	**-0.7<br>(-4.3 to 4.0)**
Lip and oral cavity cancer	73.1<br>(68.8 to 77.8)	19.8<br>(18.7 to 21.2)	7.7<br>(5.5 to 10.2)	17.1<br>(8.1 to 27.1)	2.9<br>(-5.1 to 11.7)
Nasopharynx cancer	35.2<br>(31.4 to 39.8)	9.9<br>(8.9 to 11.2)	4.1<br>(2.9 to 5.5)	-7.8<br>(-21.6 to 8.5)	-19.0<br>(-31.1 to -4.7)
Other pharynx cancer	20.8<br>(18.9 to 22.1)	7.6<br>(6.9 to 8.2)	2.7<br>(1.9 to 3.5)	18.3<br>(5.5 to 28.9)	3.9<br>(-7.4 to 13.3)
Oesophageal cancer	12.2<br>(11.5 to 13.0)	10.1<br>(9.6 to 10.7)	2.7<br>(1.9 to 3.6)	-12.4<br>(-18.0 to -4.9)	-23.0<br>(-27.9 to -16.4)
Stomach cancer	57.9<br>(55.3 to 60.9)	31.8<br>(30.4 to 33.3)	8.9<br>(6.4 to 11.7)	-9.5<br>(-14.6 to -4.4)	-20.6<br>(-25.0 to -16.1)
Colon and rectum cancer	167.7<br>(159.3 to 177.0)	45.6<br>(43.3 to 48.1)	17.0<br>(12.2 to 22.7)	19.0<br>(10.8 to 28.4)	4.5<br>(-2.7 to 12.8)
Liver cancer	48.7<br>(41.6 to 55.7)	50.6<br>(45.8 to 55.7)	11.8<br>(8.1 to 16.1)	21.1<br>(8.0 to 35.9)	6.3<br>(-5.2 to 19.4)
Liver cancer due to hepatitis B	40.6<br>(32.4 to 48.2)	29.4<br>(23.4 to 34.5)	6.9<br>(4.5 to 9.6)	20.1<br>(5.7 to 37.2)	5.5<br>(-7.2 to 20.5)

Cause	Prevalence in thousands (95% UI)	Incidence in thousands (95% UI)	YLDs in thousands (95% UI)		
	2016 counts	2016 counts	2016 counts	Percentage change in counts between 2006 and 2016	Percentage change in rates between 2006 and 2016
Liver cancer due to hepatitis C	4.7 (3.6 to 6.0)	3.7 (2.9 to 4.7)	0.8 (0.6 to 1.2)	22.2 (10.1 to 34.9)	7.4 (-3.3 to 18.5)
Liver cancer due to alcohol use	5.5 (3.8 to 7.5)	4.4 (3.1 to 6.1)	1.0 (0.6 to 1.6)	27.6 (14.9 to 40.9)	12.1 (0.9 to 23.7)
Liver cancer due to other causes	18.4 (14.3 to 24.0)	13.1 (10.4 to 16.9)	3.1 (2.0 to 4.4)	20.8 (4.5 to 37.9)	6.1 (-8.2 to 21.1)
Gallbladder and biliary tract cancer	4.0 (3.4 to 4.3)	4.8 (4.2 to 5.1)	1.1 (0.8 to 1.5)	6.4 (-1.4 to 14.1)	-6.5 (-13.4 to 0.2)
Pancreatic cancer	6.3 (6.1 to 6.5)	7.8 (7.7 to 8.0)	1.7 (1.2 to 2.2)	8.2 (4.3 to 11.6)	-5.0 (-8.4 to -2.0)
Larynx cancer	16.8 (16.1 to 17.6)	5.0 (4.8 to 5.2)	2.1 (1.5 to 2.8)	-3.6 (-8.5 to 2.6)	-15.3 (-19.7 to -9.9)
Tracheal, bronchus, and lung cancer	45.3 (43.1 to 47.7)	33.8 (32.3 to 35.4)	8.9 (6.2 to 11.6)	-11.3 (-16.3 to -5.1)	-22.1 (-26.5 to -16.6)
Malignant skin melanoma	86.0 (74.7 to 103.6)	17.4 (15.2 to 21.0)	5.7 (3.9 to 7.9)	14.0 (5.4 to 22.5)	0.1 (-7.4 to 7.6)
Non-melanoma skin cancer	7.1 (4.0 to 13.2)	38.1 (20.4 to 74.7)	0.4 (0.2 to 0.7)	1.9 (-10.5 to 15.5)	-10.5 (-21.4 to 1.5)
Non-melanoma skin cancer (squamous-cell carcinoma)	6.5 (3.0 to 12.7)	6.8 (3.6 to 11.9)	0.3 (0.1 to 0.7)	1.4 (-13.6 to 16.4)	-10.9 (-24.1 to 2.2)
Non-melanoma skin cancer (basal-cell carcinoma)	3.5 (1.5 to 7.5)	31.3 (14.3 to 66.9)	< 0.05 (< 0.05 to 0.1)	5.9 (0.6 to 11.2)	-7.0 (-11.7 to -2.3)
Breast cancer	686.3 (637.6 to 741.1)	142.3 (132.8 to 153.5)	59.0 (42.2 to 79.3)	15.2 (5.2 to 26.6)	1.2 (-7.6 to 11.2)
Cervical cancer	249.8 (193.3 to 267.8)	65.3 (51.3 to 69.8)	24.0 (16.2 to 32.2)	2.9 (-5.2 to 12.5)	-9.6 (-16.8 to -1.2)
Uterine cancer	81.2 (75.1 to 88.8)	17.0 (15.8 to 18.6)	6.3 (4.4 to 8.7)	18.1 (5.8 to 34.8)	3.7 (-7.1 to 18.4)
Ovarian cancer	55.4 (51.5 to 58.0)	17.9 (16.8 to 18.7)	8.7 (6.1 to 11.4)	18.3 (6.8 to 30.2)	3.9 (-6.2 to 14.4)
Prostate cancer	20.0 (17.0 to 22.0)	5.8 (5.1 to 6.3)	1.9 (1.3 to 2.6)	25.1 (13.6 to 34.8)	9.8 (-0.2 to 18.4)
Testicular cancer	29.9 (27.8 to 32.7)	5.8 (5.4 to 6.4)	2.0 (1.4 to 2.9)	13.7 (1.0 to 28.9)	-0.1 (-11.3 to 13.2)
Kidney cancer	50.4 (47.7 to 52.6)	13.3 (12.8 to 13.9)	4.6 (3.3 to 6.1)	10.8 (4.1 to 17.6)	-2.7 (-8.6 to 3.3)
Bladder cancer	33.5 (31.5 to 35.4)	8.2 (7.7 to 8.7)	3.3 (2.4 to 4.4)	13.4 (5.3 to 21.4)	-0.4 (-7.5 to 6.6)
Brain and nervous system cancer	44.9 (40.1 to 50.4)	19.9 (18.0 to 22.4)	6.4 (4.5 to 8.7)	28.9 (20.5 to 39.2)	13.3 (5.8 to 22.3)
Thyroid cancer	120.6 (112.3 to 131.8)	22.8 (21.3 to 24.9)	7.4 (5.1 to 10.5)	42.6 (29.4 to 59.0)	25.2 (13.7 to 39.7)
Mesothelioma	1.7 (1.5 to 1.8)	1.1 (1.0 to 1.2)	0.4 (0.2 to 0.5)	10.3 (3.9 to 16.6)	-3.1 (-8.7 to 2.4)
Hodgkin lymphoma	19.2 (17.1 to 21.7)	4.8 (4.2 to 5.5)	1.9 (1.3 to 2.5)	3.2 (-4.5 to 12.0)	-9.3 (-16.1 to -1.6)
Non-Hodgkin's lymphoma	67.0 (61.4 to 73.4)	19.2 (17.6 to 20.8)	6.3 (4.5 to 8.5)	31.1 (22.7 to 38.9)	15.1 (7.7 to 21.9)
Multiple myeloma	8.0 (6.8 to 8.7)	3.5 (3.1 to 3.9)	1.8 (1.2 to 2.4)	24.8 (18.3 to 32.1)	9.6 (3.9 to 16.1)
Leukaemia	53.5 (47.6 to 57.3)	15.7 (14.1 to 16.7)	7.1 (5.0 to 9.6)	5.1 (-1.7 to 12.9)	-7.7 (-13.6 to -0.9)
Acute lymphoid leukaemia	8.1 (7.1 to 9.0)	2.4 (2.1 to 2.6)	1.1 (0.8 to 1.5)	22.5 (10.5 to 30.7)	7.6 (-3.0 to 14.8)
Chronic lymphoid leukaemia	11.5 (10.1 to 12.7)	2.5 (2.2 to 2.8)	1.4 (1.0 to 1.9)	24.5 (11.3 to 36.8)	9.3 (-2.2 to 20.1)
Acute myeloid leukaemia	5.0 (4.5 to 5.6)	3.8 (3.3 to 4.2)	1.1 (0.7 to 1.4)	18.9 (12.8 to 25.3)	4.5 (-0.9 to 10.0)
Chronic myeloid leukaemia	5.3 (4.6 to 5.9)	1.9 (1.6 to 2.0)	0.7 (0.5 to 0.9)	-4.4 (-10.8 to 2.7)	-16.1 (-21.7 to -9.8)
Other leukaemia	23.6 (20.1 to 26.0)	5.2 (4.5 to 5.7)	2.9 (2.0 to 4.0)	-8.4 (-17.8 to 2.6)	-19.6 (-27.8 to -9.8)
Other neoplasms	140.5 (127.5 to 150.1)	40.4 (36.8 to 43.0)	12.2 (8.7 to 16.3)	46.8 (36.7 to 57.8)	28.9 (20.1 to 38.6)
**Cardiovascular diseases**	**20,421.4 (19,680.9 to 20,930.7)**	**2,021.4† (1,849.5 to 2,219.6)**	**1,288.7 (898.6 to 1,731.0)**	**14.0 (11.5 to 16.6)**	**0.2 (-2.0 to 2.4)**
Rheumatic heart disease	2,678.0 (2,432.0 to 2,962.2)	25.2 (18.7 to 34.1)	129.2 (81.2 to 188.0)	17.5 (13.2 to 21.3)	3.2 (-0.5 to 6.5)
Ischaemic heart disease	5,681.8 (5,182.7 to 6,172.8)	682.2 (604.9 to 775.4)	307.8 (203.6 to 432.0)	18.4 (16.2 to 20.5)	4.0 (2.1 to 5.8)
Cerebrovascular disease	2,795.2 (2,650.5 to 2,950.6)	453.2‡ (378.9 to 528.2)	447.3 (312.2 to 588.8)	11.7 (5.2 to 17.9)	-1.9 (-7.6 to 3.6)
Ischaemic stroke	2,224.0 (1,876.7 to 2,652.5)	295.4‡ (229.2 to 369.6)	344.4 (237.0 to 457.1)	11.6 (3.7 to 19.3)	-2.0 (-8.9 to 4.8)
Hemorrhagic stroke	651.7 (539.5 to 782.7)	157.8‡ (128.3 to 189.1)	102.9 (70.4 to 139.8)	12.0 (1.7 to 22.9)	-1.6 (-10.7 to 8.0)
Hypertensive heart disease	646.6 (499.1 to 833.7)	--	54.8 (33.7 to 81.4)	15.6 (10.6 to 21.0)	1.5 (-2.8 to 6.2)
Cardiomyopathy and myocarditis	199.5 (173.0 to 234.6)	143.2† (106.1 to 186.4)	22.8 (13.7 to 34.0)	10.9 (5.6 to 16.3)	-2.6 (-7.3 to 2.2)
Myocarditis	101.0 (79.0 to 127.9)	143.2 (106.1 to 186.4)	7.6 (4.7 to 11.5)	15.2 (9.8 to 20.5)	1.2 (-3.6 to 5.8)
Alcoholic cardiomyopathy	55.8 (41.6 to 74.1)	--	4.9 (2.8 to 7.5)	2.0 (-12.3 to 19.9)	-10.4 (-23.0 to 5.3)
Other cardiomyopathy	119.3 (86.4 to 160.9)	--	10.3 (6.0 to 15.6)	12.4 (6.0 to 18.7)	-1.3 (-6.9 to 4.2)
Atrial fibrillation and flutter	994.6 (825.6 to 1,190.0)	89.8 (68.4 to 116.8)	83.3 (53.7 to 119.5)	13.1 (8.2 to 17.8)	-0.7 (-4.9 to 3.5)
Peripheral vascular disease	3,520.0 (2,576.5 to 4,698.4)	570.7 (444.7 to 724.5)	0.1 (< 0.05 to 0.2)	3.8 (0.6 to 6.3)	-8.8 (-11.6 to -6.6)
Endocarditis	11.7 (9.0 to 14.9)	57.1 (41.7 to 76.8)	0.9 (0.6 to 1.4)	14.8 (11.1 to 18.3)	0.8 (-2.4 to 3.9)
Other cardiovascular and circulatory diseases	4,564.7 (3,097.8 to 6,451.8)	--	242.4 (140.4 to 376.7)	11.8 (8.6 to 14.7)	-1.8 (-4.7 to 0.8)
**Chronic respiratory diseases**	**29,540.3 (28,884.7 to 30,243.5)**	**4,693.4† (4,082.7 to 5,339.1)**	**1,626.9 (1,283.5 to 2,030.2)**	**18.0 (15.8 to 20.3)**	**3.7 (1.8 to 5.6)**
Chronic obstructive pulmonary disease	12,012.2 (11,140.6 to 12,986.9)	1,239.4 (1,066.8 to 1,440.7)	835.4 (707.8 to 961.9)	16.5 (13.0 to 20.1)	2.3 (-0.7 to 5.5)
Pneumoconiosis	28.7 (25.2 to 32.7)	4.1 (3.5 to 4.8)	4.5 (3.0 to 6.4)	9.6 (8.3 to 11.0)	-3.7 (-4.9 to -2.5)
Silicosis	9.1 (7.0 to 11.4)	1.4 (1.0 to 2.0)	1.4 (0.9 to 2.1)	6.5 (4.5 to 8.6)	-6.4 (-8.2 to -4.6)

Cause	Prevalence in thousands (95% UI)	Incidence in thousands (95% UI)	YLDs in thousands (95% UI)		
	2016 counts	2016 counts	2016 counts	Percentage change in counts between 2006 and 2016	Percentage change in rates between 2006 and 2016
Asbestosis	5.0<br>(3.9 to 6.3)	0.4<br>(0.3 to 0.6)	0.8<br>(0.5 to 1.2)	11.2<br>(9.3 to 12.7)	-2.3<br>(-4.0 to -1.0)
Coal workers pneumoconiosis	5.1<br>(4.1 to 6.1)	1.1<br>(0.9 to 1.4)	0.8<br>(0.5 to 1.2)	8.0<br>(5.8 to 10.1)	-5.1<br>(-7.0 to -3.3)
Other pneumoconiosis	9.6<br>(7.3 to 12.5)	1.1<br>(0.8 to 1.5)	1.5<br>(1.0 to 2.3)	12.8<br>(9.8 to 15.9)	-0.9<br>(-3.5 to 1.8)
Asthma	18,247.1<br>(15,965.2 to 20,803.8)	3,437.3<br>(2,861.3 to 4,054.1)	709.8<br>(458.7 to 1,035.6)	17.4<br>(15.0 to 19.8)	3.1<br>(1.0 to 5.2)
Interstitial lung disease and pulmonary sarcoidosis	137.1<br>(113.2 to 164.7)	12.6<br>(8.5 to 18.0)	15.4<br>(9.8 to 23.2)	10.1<br>(3.5 to 16.2)	-3.3<br>(-9.1 to 2.0)
Other chronic respiratory diseases	--	--	61.8<br>(52.1 to 71.2)	59.5<br>(54.8 to 64.4)	40.1<br>(36.0 to 44.4)
**Cirrhosis and other chronic liver diseases**	**4,570.8<br>(4,214.5 to 4,965.1)**	**125.8<br>(106.6 to 145.8)**	**102.1<br>(69.5 to 144.9)**	**15.0<br>(10.1 to 19.9)**	**1.0<br>(-3.3 to 5.3)**
Cirrhosis and other chronic liver diseases due to hepatitis B	1,435.8<br>(1,249.4 to 1,636.4)	43.0<br>(34.5 to 52.0)	34.8<br>(23.0 to 50.7)	16.5<br>(8.9 to 25.1)	2.3<br>(-4.4 to 9.9)
Cirrhosis and other chronic liver diseases due to hepatitis C	1,212.5<br>(1,019.1 to 1,427.2)	32.7<br>(25.5 to 40.5)	26.2<br>(17.1 to 37.9)	16.7<br>(8.5 to 24.8)	2.5<br>(-4.7 to 9.6)
Cirrhosis and other chronic liver diseases due to alcohol use	1,262.2<br>(1,086.3 to 1,467.7)	34.1<br>(27.4 to 41.6)	27.1<br>(17.9 to 39.7)	13.0<br>(5.5 to 21.4)	-0.8<br>(-7.3 to 6.7)
Cirrhosis and other chronic liver diseases due to other causes	660.3<br>(496.0 to 862.5)	15.9<br>(11.4 to 21.2)	14.0<br>(8.2 to 21.9)	12.1<br>(1.5 to 24.1)	-1.5<br>(-10.8 to 9.0)
**Digestive diseases**	**21,843.4<br>(20,453.6 to 23,020.5)**	**14,926.9†<br>(12,701.8 to 17,265.4)**	**658.2<br>(436.0 to 930.4)**	**29.1<br>(21.4 to 37.5)**	**13.4<br>(6.7 to 20.7)**
Peptic ulcer disease	7,182.5<br>(5,789.4 to 8,886.3)	767.2<br>(560.9 to 1,011.6)	172.8<br>(99.1 to 278.2)	45.2<br>(32.6 to 57.6)	27.6<br>(16.5 to 38.5)
Gastritis and duodenitis	9,196.4<br>(7,071.1 to 11,687.1)	5,519.8<br>(3,963.1 to 7,258.7)	184.6<br>(111.2 to 292.5)	44.6<br>(30.9 to 59.7)	27.0<br>(15.0 to 40.2)
Appendicitis	45.3<br>(37.0 to 55.3)	1,172.5<br>(958.9 to 1,422.4)	14.0<br>(8.8 to 20.2)	15.3<br>(4.9 to 26.6)	1.2<br>(-7.8 to 11.2)
Paralytic ileus and intestinal obstruction	9.9<br>(7.2 to 13.0)	268.2<br>(197.1 to 350.8)	3.2<br>(2.0 to 4.9)	17.1<br>(14.4 to 19.6)	2.8<br>(0.5 to 5.0)
Inguinal, femoral, and abdominal hernia	3,961.9<br>(2,818.8 to 5,339.2)	2,130.2<br>(1,493.6 to 2,948.3)	143.2<br>(83.4 to 228.0)	11.5<br>(6.6 to 16.2)	-2.1<br>(-6.3 to 2.1)
Inflammatory bowel disease	488.7<br>(411.5 to 573.7)	308.6<br>(247.5 to 377.4)	71.8<br>(47.1 to 101.2)	9.9<br>(4.4 to 16.3)	-3.4<br>(-8.3 to 2.1)
Vascular intestinal disorders	3.6<br>(2.2 to 5.6)	20.9<br>(14.0 to 29.3)	1.2<br>(0.6 to 2.0)	14.8<br>(11.2 to 17.8)	0.8<br>(-2.3 to 3.5)
Gallbladder and biliary diseases	1,578.0<br>(1,145.3 to 2,097.6)	4,334.7<br>(3,171.2 to 5,811.7)	20.9<br>(12.3 to 33.1)	15.7<br>(7.3 to 24.1)	1.6<br>(-5.8 to 9.0)
Pancreatitis	44.9<br>(33.3 to 57.8)	405.0<br>(300.0 to 519.8)	5.8<br>(3.5 to 8.7)	13.4<br>(4.5 to 20.9)	-0.4<br>(-8.2 to 6.2)
Other digestive diseases	--	--	40.8<br>(27.1 to 57.7)	35.0<br>(26.9 to 43.7)	18.6<br>(11.5 to 26.2)
**Neurological disorders**	**235,079.4<br>(224,656.6 to 248,589.9)**	**98,514.9<br>(62,797.3 to 142,431.2)**	**6,038.7<br>(4,022.4 to 8,313.0)**	**13.8<br>(12.5 to 15.1)**	**-0.1<br>(-1.2 to 1.1)**
Alzheimer's disease and other dementias	46.1<br>(26.4 to 77.7)	27.2<br>(15.6 to 45.8)	6.3<br>(3.2 to 11.3)	6.0<br>(1.1 to 9.6)	-6.9<br>(-11.2 to -3.7)
Parkinson's disease	76.5<br>(47.3 to 118.3)	10.7<br>(6.3 to 16.1)	10.2<br>(5.6 to 17.1)	21.6<br>(15.0 to 28.5)	6.8<br>(1.0 to 12.9)
Epilepsy	1,278.1<br>(1,058.2 to 1,545.3)	100.2<br>(70.3 to 139.1)	393.2<br>(266.4 to 552.7)	7.4<br>(-3.7 to 19.9)	-5.7<br>(-15.5 to 5.4)
Multiple sclerosis	247.4<br>(223.3 to 276.5)	4.4<br>(3.1 to 6.0)	65.9<br>(45.4 to 86.8)	12.4<br>(6.2 to 18.3)	-1.3<br>(-6.7 to 3.9)
Motor neuron disease	19.3<br>(15.6 to 24.5)	2.2<br>(1.4 to 3.3)	4.1<br>(2.7 to 5.8)	11.7<br>(10.4 to 12.8)	-1.9<br>(-3.0 to -0.9)
Migraine	105,267.9<br>(97,313.9 to 113,388.9)	8,960.1<br>(7,789.9 to 10,308.1)	4,693.4<br>(3,014.7 to 6,573.9)	14.0<br>(13.0 to 15.0)	0.1<br>(-0.7 to 1.0)
Tension-type headache	172,730.1<br>(126,598.8 to 220,525.4)	89,407.1<br>(53,889.3 to 133,276.1)	772.7<br>(484.1 to 1,175.2)	14.9<br>(13.1 to 16.8)	0.9<br>(-0.6 to 2.6)
Other neurological disorders	1.5<br>(0.7 to 2.8)	3.0<br>(1.6 to 4.7)	92.9<br>(64.2 to 126.5)	22.9<br>(12.4 to 34.9)	8.0<br>(-1.2 to 18.5)
**Mental and substance use disorders**	**88,609.9<br>(86,685.0 to 90,054.7)**	**32,996.7†<br>(28,117.7 to 37,666.2)**	**12,855.9<br>(9,447.6 to 16,740.1)**	**11.1<br>(10.2 to 12.1)**	**-2.4<br>(-3.2 to -1.6)**
Schizophrenia	2,602.8<br>(2,251.9 to 2,975.4)	78.6<br>(54.7 to 108.8)	1,682.9<br>(1,231.5 to 2,111.0)	12.5<br>(10.5 to 14.6)	-1.2<br>(-2.9 to 0.6)
Alcohol use disorders	10,364.9<br>(8,173.0 to 12,978.6)	5,109.2<br>(3,941.9 to 6,398.9)	1,036.2<br>(663.0 to 1,487.2)	5.8<br>(0.7 to 10.8)	-7.1<br>(-11.6 to -2.7)
Drug use disorders	4,244.3<br>(4,061.4 to 4,430.1)	356.6<br>(286.0 to 438.2)	1,083.4<br>(761.7 to 1,424.2)	14.8<br>(11.4 to 18.1)	0.8<br>(-2.1 to 3.7)
Opioid use disorders	2,061.3<br>(1,686.9 to 2,512.7)	144.4<br>(92.2 to 208.4)	851.2<br>(580.6 to 1,144.9)	16.5<br>(12.5 to 20.4)	2.3<br>(-1.2 to 5.8)
Cocaine use disorders	475.4<br>(417.8 to 543.8)	10.9<br>(6.4 to 16.5)	64.9<br>(39.8 to 94.6)	6.7<br>(1.2 to 11.9)	-6.3<br>(-11.1 to -1.7)
Amphetamine use disorders	187.0<br>(128.1 to 265.1)	15.5<br>(8.0 to 25.9)	24.6<br>(13.7 to 41.0)	9.9<br>(-0.0 to 19.4)	-3.5<br>(-12.2 to 4.9)
Cannabis use disorders	1,163.3<br>(901.8 to 1,482.6)	121.8<br>(83.5 to 174.0)	33.8<br>(20.5 to 51.4)	7.4<br>(1.3 to 13.5)	-5.7<br>(-11.1 to -0.3)
Other drug use disorders	466.8<br>(391.9 to 558.6)	64.1<br>(49.1 to 82.2)	109.0<br>(72.2 to 148.3)	10.5<br>(6.6 to 14.5)	-2.9<br>(-6.4 to 0.6)
Depressive disorders	23,614.1<br>(22,018.3 to 24,821.1)	23,691.0<br>(19,181.9 to 28,351.8)	3,912.2<br>(2,615.9 to 5,539.2)	8.6<br>(6.9 to 10.3)	-4.6<br>(-6.1 to -3.1)
Major depressive disorder	14,570.7<br>(11,690.8 to 17,630.8)	22,188.5<br>(17,548.4 to 26,900.8)	2,987.5<br>(1,939.9 to 4,363.7)	7.6<br>(5.4 to 9.4)	-5.5<br>(-7.5 to -3.9)
Dysthymia	9,540.8<br>(7,379.8 to 11,992.6)	1,502.4<br>(1,132.0 to 1,909.6)	924.6<br>(584.1 to 1,399.1)	12.1<br>(8.7 to 15.5)	-1.5<br>(-4.6 to 1.5)
Bipolar disorder	3,853.6<br>(3,120.2 to 4,653.5)	227.9<br>(144.9 to 318.3)	785.3<br>(476.5 to 1,176.0)	14.2<br>(12.1 to 16.3)	0.3<br>(-1.6 to 2.1)
Anxiety disorders	23,344.5<br>(20,805.6 to 26,276.1)	3,430.2<br>(2,942.5 to 3,949.5)	2,242.7<br>(1,544.3 to 3,058.4)	12.6<br>(10.4 to 15.1)	-1.1<br>(-3.1 to 1.1)
Eating disorders	700.3<br>(566.5 to 858.3)	24.8<br>(16.6 to 34.7)	136.8<br>(80.7 to 208.1)	21.7<br>(17.6 to 25.8)	6.9<br>(3.3 to 10.5)
Anorexia nervosa	95.8<br>(67.5 to 133.2)	24.8<br>(16.6 to 34.7)	20.1<br>(11.8 to 31.0)	15.5<br>(6.8 to 25.8)	1.4<br>(-6.2 to 10.5)
Bulimia nervosa	564.4<br>(365.8 to 817.4)	--	116.8<br>(65.0 to 184.6)	22.9<br>(18.4 to 27.6)	7.9<br>(4.0 to 12.1)
Autistic spectrum disorders	3,942.3<br>(3,774.7 to 4,102.0)	--	572.4<br>(388.6 to 809.4)	13.8<br>(12.1 to 15.7)	-0.0<br>(-1.6 to 1.6)
Autism	1,181.3<br>(1,017.2 to 1,378.3)	--	298.1<br>(190.9 to 431.1)	12.8<br>(10.0 to 15.8)	-0.9<br>(-3.4 to 1.7)
Asperger syndrome and other autistic spectrum disorders	2,760.9<br>(2,311.8 to 3,300.9)	--	274.3<br>(178.7 to 398.6)	14.9<br>(13.0 to 16.8)	0.9<br>(-0.7 to 2.6)

Cause	Prevalence in thousands (95% UI)	Incidence in thousands (95% UI)	YLDs in thousands (95% UI)		
	2016 counts	2016 counts	2016 counts	Percentage change in counts between 2006 and 2016	Percentage change in rates between 2006 and 2016
Attention-deficit/hyperactivity disorder	2,969.0<br>(2,639.0 to 3,347.8)	--	35.4<br>(21.3 to 55.6)	13.6<br>(10.0 to 17.1)	-0.3<br>(-3.4 to 2.9)
Conduct disorder	--	--	--	--	--
Idiopathic developmental intellectual disability	7,191.1<br>(4,325.7 to 10,078.4)	--	290.0<br>(140.7 to 496.2)	6.2<br>(1.8 to 9.1)	-6.7<br>(-10.6 to -4.2)
Other mental and substance use disorders	14,402.4<br>(12,684.0 to 16,454.3)	78.4<br>(45.0 to 122.4)	1,078.5<br>(735.8 to 1,548.3)	13.7<br>(12.2 to 15.2)	-0.1<br>(-1.4 to 1.2)
**Diabetes, urogenital, blood, and endocrine diseases**	**217,459.7<br>(215,054.3 to 219,216.0)**	**90,565.4†<br>(82,675.3 to 99,884.9)**	**5,207.3<br>(3,620.8 to 7,260.8)**	**14.4<br>(12.2 to 16.4)**	**0.5<br>(-1.4 to 2.3)**
Diabetes mellitus	31,633.7<br>(27,216.6 to 36,549.3)	2,267.5<br>(1,783.4 to 2,811.6)	2,334.1<br>(1,554.2 to 3,345.7)	11.3<br>(7.5 to 14.8)	-2.3<br>(-5.6 to 0.8)
Acute glomerulonephritis	7.0<br>(4.7 to 10.0)	103.3<br>(68.9 to 147.8)	0.4<br>(0.2 to 0.6)	-3.3<br>(-5.8 to -1.0)	-15.1<br>(-17.3 to -13.1)
Chronic kidney disease	12,899.0<br>(11,052.7 to 15,068.6)	905.0<br>(624.9 to 1,235.7)	578.4<br>(413.4 to 776.2)	23.6<br>(19.4 to 28.6)	8.6<br>(4.9 to 12.9)
Chronic kidney disease due to diabetes mellitus	5,320.5<br>(3,894.2 to 6,901.6)	367.2<br>(228.3 to 525.5)	259.7<br>(169.9 to 368.0)	24.6<br>(19.0 to 30.6)	9.4<br>(4.5 to 14.7)
Chronic kidney disease due to hypertension	2,120.7<br>(1,544.5 to 2,722.7)	196.1<br>(112.0 to 301.2)	72.3<br>(45.1 to 108.0)	24.1<br>(17.7 to 31.6)	9.0<br>(3.4 to 15.6)
Chronic kidney disease due to glomerulonephritis	2,167.9<br>(1,590.5 to 2,777.4)	108.1<br>(67.3 to 161.2)	113.0<br>(64.7 to 174.6)	21.2<br>(15.5 to 27.1)	6.5<br>(1.4 to 11.7)
Chronic kidney disease due to other causes	3,289.9<br>(2,241.5 to 4,552.5)	233.5<br>(135.7 to 352.0)	133.4<br>(80.2 to 200.9)	23.6<br>(18.0 to 29.5)	8.5<br>(3.6 to 13.7)
Urinary diseases and male infertility	8,110.0<br>(7,209.9 to 9,300.6)	32,330.8†<br>(25,786.0 to 39,975.6)	142.8<br>(90.1 to 209.0)	11.7<br>(8.2 to 14.9)	-1.9<br>(-5.0 to 0.9)
Interstitial nephritis and urinary tract infections	471.1<br>(339.5 to 615.1)	24,423.9<br>(18,120.1 to 31,497.7)	15.5<br>(8.8 to 24.9)	15.3<br>(9.7 to 21.0)	1.2<br>(-3.7 to 6.3)
Urolithiasis	264.0<br>(192.9 to 351.6)	7,009.1<br>(5,113.7 to 9,208.9)	19.8<br>(12.1 to 30.2)	11.2<br>(4.1 to 19.0)	-2.3<br>(-8.6 to 4.5)
Benign prostatic hyperplasia	1,881.2<br>(1,432.4 to 2,405.6)	897.9<br>(716.3 to 1,111.0)	64.0<br>(39.2 to 97.2)	11.7<br>(5.8 to 16.8)	-1.9<br>(-7.1 to 2.6)
Male infertility	5,486.8<br>(3,676.0 to 7,917.3)	--	30.1<br>(11.1 to 65.4)	15.4<br>(9.2 to 20.8)	1.3<br>(-4.1 to 6.1)
Other urinary diseases	--	--	13.4<br>(8.8 to 19.2)	1.2<br>(-2.9 to 5.6)	-11.1<br>(-14.7 to -7.3)
Gynecological diseases	98,646.3<br>(96,423.7 to 100,858.4)	53,952.8†<br>(49,372.9 to 58,581.4)	1,543.6<br>(1,062.0 to 2,188.5)	12.6<br>(10.4 to 15.6)	-1.1<br>(-3.0 to 1.5)
Uterine fibroids	17,919.1<br>(14,688.9 to 21,363.6)	2,901.7<br>(2,226.9 to 3,629.1)	284.0<br>(168.2 to 468.6)	12.7<br>(10.7 to 14.6)	-1.1<br>(-2.8 to 0.7)
Polycystic ovarian syndrome	2,503.2<br>(1,918.8 to 3,193.2)	1,770.7<br>(1,182.9 to 2,513.3)	21.5<br>(9.7 to 40.1)	7.7<br>(5.0 to 10.3)	-5.4<br>(-7.7 to -3.1)
Female infertility	3,887.0<br>(1,557.8 to 10,320.0)	--	20.9<br>(5.3 to 62.1)	35.5<br>(15.8 to 61.6)	19.1<br>(1.7 to 42.0)
Endometriosis	576.2<br>(482.8 to 691.5)	1,534.4<br>(1,172.9 to 1,987.0)	53.3<br>(34.9 to 77.1)	8.4<br>(3.0 to 14.5)	-4.8<br>(-9.6 to 0.6)
Genital prolapse	15,207.4<br>(12,050.9 to 18,817.1)	2,773.6<br>(2,244.8 to 3,391.2)	48.2<br>(23.5 to 89.0)	6.5<br>(4.2 to 8.6)	-6.5<br>(-8.4 to -4.7)
Premenstrual syndrome	61,116.0<br>(56,404.5 to 66,257.3)	28,043.7<br>(25,809.8 to 30,268.7)	505.4<br>(312.3 to 781.5)	12.4<br>(10.3 to 14.3)	-1.3<br>(-3.1 to 0.4)
Other gynecological diseases	17,410.7<br>(13,951.5 to 20,557.7)	16,928.9<br>(13,101.5 to 20,883.1)	610.3<br>(395.8 to 886.6)	13.3<br>(8.6 to 20.5)	-0.5<br>(-4.7 to 5.9)
Hemoglobinopathies and hemolytic anaemias	113,235.9<br>(111,899.4 to 114,710.2)	--	410.0<br>(264.4 to 615.1)	30.7<br>(21.3 to 40.7)	14.8<br>(6.5 to 23.6)
Thalassemias	1.4<br>(1.3 to 1.4)	--	0.1<br>(0.1 to 0.2)	27.1<br>(-5.1 to 65.3)	11.6<br>(-16.6 to 45.2)
Thalassaemias trait	21,098.4<br>(20,265.2 to 21,989.5)	--	279.4<br>(177.7 to 420.2)	29.9<br>(17.7 to 42.8)	14.1<br>(3.4 to 25.4)
Sickle cell disorders	31.0<br>(27.6 to 34.4)	--	5.3<br>(3.7 to 7.1)	56.2<br>(45.1 to 68.3)	37.2<br>(27.5 to 47.9)
Sickle cell trait	25,979.6<br>(23,552.1 to 29,250.7)	--	81.9<br>(52.4 to 123.7)	34.3<br>(28.7 to 39.9)	17.9<br>(13.0 to 22.9)
G6PD deficiency	20,242.0<br>(19,510.8 to 21,081.8)	--	1.4<br>(0.9 to 2.0)	43.4<br>(36.8 to 50.0)	25.9<br>(20.2 to 31.7)
G6PD trait	54,696.6<br>(53,829.2 to 55,707.6)	--	0.1<br>(< 0.05 to 0.1)	39.3<br>(30.2 to 49.9)	22.4<br>(14.4 to 31.7)
Other hemoglobinopathies and hemolytic anaemias	3,300.3<br>(2,870.1 to 3,788.8)	--	41.9<br>(26.6 to 65.5)	25.9<br>(19.5 to 33.1)	10.6<br>(5.0 to 16.9)
Endocrine, metabolic, blood, and immune disorders	6,681.8<br>(5,583.9 to 8,067.2)	1,006.0<br>(712.1 to 1,349.7)	198.1<br>(124.8 to 298.0)	13.6<br>(10.8 to 16.6)	-0.2<br>(-2.7 to 2.4)
**Musculoskeletal disorders**	**119,510.2<br>(112,739.4 to 126,336.4)**	**63,621.4<br>(50,765.5 to 78,027.5)**	**13,199.9<br>(8,952.3 to 18,119.0)**	**12.6<br>(10.7 to 14.4)**	**-1.1<br>(-2.8 to 0.5)**
Rheumatoid arthritis	1,579.4<br>(1,297.2 to 1,897.6)	117.0<br>(88.0 to 151.0)	380.4<br>(254.4 to 518.6)	17.4<br>(14.0 to 20.9)	3.1<br>(0.1 to 6.2)
Osteoarthritis	18,189.0<br>(16,464.0 to 20,150.6)	1,801.7<br>(1,586.2 to 2,035.9)	1,073.0<br>(741.7 to 1,474.1)	17.1<br>(15.8 to 18.5)	2.9<br>(1.8 to 4.1)
Low back and neck pain	75,746.3<br>(69,984.2 to 81,350.1)	31,517.1<br>(23,743.9 to 41,848.3)	8,890.4<br>(5,911.2 to 12,661.0)	12.5<br>(10.0 to 15.0)	-1.2<br>(-3.4 to 1.0)
Low back pain	47,376.7<br>(35,209.6 to 62,669.4)	22,871.0<br>(15,810.9 to 32,846.6)	5,499.4<br>(3,462.5 to 8,236.7)	12.5<br>(8.7 to 15.9)	-1.2<br>(-4.5 to 1.8)
Neck pain	33,416.3<br>(23,673.4 to 44,992.0)	8,646.1<br>(5,593.0 to 12,091.9)	3,391.0<br>(2,049.2 to 5,342.1)	12.7<br>(9.3 to 15.5)	-1.1<br>(-4.0 to 1.5)
Gout	2,532.7<br>(1,976.0 to 3,198.0)	470.9<br>(370.0 to 591.8)	83.9<br>(52.4 to 122.0)	12.2<br>(6.7 to 17.8)	-1.4<br>(-6.2 to 3.4)
Other musculoskeletal disorders	32,021.0<br>(21,634.8 to 45,231.7)	29,714.7<br>(20,540.3 to 40,579.7)	2,772.2<br>(1,637.5 to 4,433.2)	10.3<br>(4.7 to 14.7)	-3.1<br>(-8.0 to 0.8)
**Other non-communicable diseases**	**355,481.5<br>(350,836.6 to 362,150.5)**	**902,432.2†<br>(778,108.0 to 1,033,955.0)**	**7,854.5<br>(5,182.4 to 11,495.1)**	**13.4<br>(12.5 to 14.1)**	**-0.4<br>(-1.2 to 0.3)**
Congenital anomalies	4,366.1<br>(4,042.5 to 4,658.1)	--	516.5<br>(364.7 to 693.5)	10.8<br>(8.2 to 13.7)	-2.7<br>(-5.0 to -0.2)
Neural tube defects	341.4<br>(309.5 to 377.9)	--	102.2<br>(73.2 to 136.1)	17.2<br>(12.5 to 22.2)	3.0<br>(-1.2 to 7.3)
Congenital heart anomalies	606.6<br>(541.9 to 675.4)	--	33.4<br>(14.2 to 57.8)	14.9<br>(10.4 to 19.5)	1.0<br>(-3.1 to 5.0)
Orofacial clefts	232.1<br>(207.0 to 257.0)	--	2.9<br>(1.9 to 4.2)	6.5<br>(3.5 to 9.6)	-6.4<br>(-9.1 to -3.8)
Down's syndrome	136.0<br>(118.6 to 155.7)	--	11.6<br>(7.9 to 16.5)	13.0<br>(8.0 to 18.2)	-0.8<br>(-5.1 to 3.8)
Turner syndrome	135.6<br>(118.0 to 154.7)	--	2.8<br>(1.4 to 4.5)	16.5<br>(4.4 to 29.4)	2.3<br>(-8.3 to 13.7)
Klinefelter syndrome	102.2<br>(86.3 to 119.8)	--	1.0<br>(0.5 to 2.0)	10.5<br>(3.5 to 17.9)	-3.0<br>(-9.1 to 3.6)

Cause	Prevalence in thousands (95% UI)	Incidence in thousands (95% UI)	YLDs in thousands (95% UI)		
	2016 counts	2016 counts	2016 counts	Percentage change in counts between 2006 and 2016	Percentage change in rates between 2006 and 2016
Other chromosomal abnormalities	0.1 (0.1 to 0.1)	--	< 0.05 (< 0.05 to < 0.05)	18.6 (-53.6 to 191.7)	4.1 (-59.2 to 156.2)
Congenital musculoskeletal and limb anomalies	682.7 (629.3 to 740.8)	--	99.9 (68.3 to 137.4)	10.7 (6.9 to 14.6)	-2.8 (-6.1 to 0.7)
Urogenital congenital anomalies	204.3 (183.8 to 225.3)	--	3.1 (1.5 to 5.7)	13.1 (9.6 to 16.8)	-0.7 (-3.7 to 2.6)
Digestive congenital anomalies	673.7 (607.3 to 745.5)	--	28.8 (19.0 to 40.7)	9.3 (4.0 to 14.8)	-4.0 (-8.7 to 0.9)
Other congenital anomalies	2,217.6 (1,556.5 to 3,116.9)	--	230.6 (150.5 to 348.1)	7.7 (3.3 to 12.9)	-5.4 (-9.3 to -0.9)
Skin and subcutaneous diseases	114,929.4 (111,881.8 to 117,281.2)	266,161.6 (242,762.7 to 295,470.6)	2,512.6 (1,689.2 to 3,687.7)	14.2 (13.4 to 15.0)	0.3 (-0.4 to 1.0)
Dermatitis	15,331.5 (13,022.5 to 18,285.8)	40,509.6 (33,022.9 to 48,953.5)	486.8 (293.6 to 752.1)	11.9 (10.1 to 13.7)	-1.7 (-3.3 to -0.2)
Psoriasis	5,092.5 (4,670.0 to 5,563.8)	567.9 (490.2 to 665.2)	444.4 (313.7 to 585.7)	17.5 (14.9 to 20.2)	3.2 (0.9 to 5.6)
Cellulitis	156.6 (127.1 to 192.9)	3,258.9 (2,725.5 to 3,869.9)	9.0 (5.6 to 13.2)	9.7 (3.5 to 16.4)	-3.6 (-9.1 to 2.2)
Pyoderma	1,051.0 (957.6 to 1,143.3)	24,653.9 (22,215.2 to 27,242.7)	5.9 (2.4 to 12.2)	16.0 (12.7 to 19.1)	1.9 (-1.0 to 4.6)
Scabies	7,196.6 (5,066.9 to 9,891.2)	22,012.7 (15,315.9 to 30,537.7)	184.6 (94.2 to 325.4)	8.5 (6.5 to 10.1)	-4.7 (-6.5 to -3.3)
Fungal skin diseases	30,961.7 (25,259.0 to 37,861.9)	104,459.8 (84,604.2 to 131,006.6)	174.6 (67.8 to 361.1)	16.3 (15.7 to 17.0)	2.2 (1.6 to 2.7)
Viral skin diseases	8,510.6 (7,193.4 to 10,126.8)	12,919.8 (10,908.7 to 15,249.4)	257.9 (158.6 to 385.4)	13.4 (12.0 to 15.0)	-0.4 (-1.6 to 1.0)
Acne vulgaris	18,630.8 (16,211.9 to 21,326.6)	8,363.5 (7,071.3 to 9,727.9)	470.7 (314.9 to 689.6)	14.5 (12.6 to 16.7)	0.6 (-1.1 to 2.5)
Alopecia areata	1,393.2 (1,242.2 to 1,549.6)	2,354.5 (2,115.0 to 2,594.3)	45.5 (29.0 to 68.7)	11.8 (8.6 to 15.1)	-1.8 (-4.6 to 1.1)
Pruritus	4,932.3 (3,347.3 to 7,100.0)	3,821.9 (2,587.4 to 5,360.5)	52.9 (22.4 to 106.9)	15.7 (13.9 to 17.4)	1.7 (0.0 to 3.1)
Urticaria	3,147.2 (2,183.9 to 4,400.2)	5,536.0 (3,845.6 to 7,775.0)	186.2 (107.6 to 288.4)	13.8 (11.1 to 16.3)	-0.1 (-2.4 to 2.1)
Decubitus ulcer	56.0 (39.6 to 75.1)	202.5 (145.8 to 270.5)	9.9 (5.9 to 15.3)	11.7 (10.5 to 12.8)	-1.9 (-3.0 to -0.9)
Other skin and subcutaneous diseases	33,175.5 (29,885.5 to 36,603.9)	37,500.7 (33,850.8 to 41,713.0)	184.2 (84.4 to 351.4)	18.8 (17.7 to 19.8)	4.4 (3.4 to 5.2)
Sense organ diseases	129,319.6 (124,116.0 to 133,669.3)	37,197.1† (33,540.1 to 40,826.3)	3,392.6 (2,231.0 to 4,928.3)	12.7 (11.0 to 14.2)	-1.0 (-2.5 to 0.3)
Glaucoma	--	--	--	--	--
Cataract	1,798.2 (1,333.3 to 2,385.5)	--	155.0 (97.3 to 227.6)	14.6 (10.9 to 18.1)	0.7 (-2.6 to 3.7)
Macular degeneration	--	--	--	--	--
Refraction and accommodation disorders	54,652.6 (49,001.9 to 60,756.7)	--	954.0 (590.1 to 1,536.7)	10.7 (9.3 to 11.9)	-2.8 (-4.0 to -1.7)
Age-related and other hearing loss	76,577.7 (61,565.5 to 93,710.6)	--	1,738.1 (1,133.7 to 2,609.1)	12.7 (9.6 to 15.3)	-1.0 (-3.8 to 1.3)
Other vision loss	1,643.8 (1,255.7 to 2,105.4)	--	143.3 (92.0 to 206.6)	17.1 (13.1 to 21.1)	2.9 (-0.7 to 6.4)
Other sense organ diseases	12,565.3 (10,964.2 to 14,265.9)	37,197.1 (33,540.1 to 40,826.3)	402.2 (244.3 to 605.3)	15.0 (13.3 to 16.8)	1.0 (-0.5 to 2.6)
Oral disorders	252,633.5 (243,174.5 to 262,305.4)	599,073.6† (477,218.2 to 722,268.6)	1,432.9 (791.4 to 2,372.7)	14.8 (13.8 to 15.6)	0.8 (-0.1 to 1.5)
Caries of deciduous teeth	--	--	--	--	--
Caries of permanent teeth	189,090.5 (158,503.9 to 223,818.3)	588,391.7 (467,178.1 to 710,670.0)	133.4 (57.2 to 255.6)	9.8 (8.4 to 11.0)	-3.6 (-4.8 to -2.5)
Periodontal disease	86,733.0 (62,484.6 to 109,667.6)	9,703.0 (5,554.4 to 13,986.6)	574.0 (218.7 to 1,229.8)	17.4 (16.4 to 18.4)	3.1 (2.2 to 4.0)
Edentulism and severe tooth loss	13,313.2 (10,561.4 to 16,389.5)	978.9 (603.8 to 1,447.1)	380.1 (238.6 to 565.8)	13.4 (11.9 to 14.8)	-0.4 (-1.8 to 0.9)
Other oral disorders	11,780.0 (10,517.8 to 13,008.0)	--	345.4 (215.1 to 519.5)	14.0 (12.7 to 15.2)	0.1 (-1.0 to 1.2)
**Injuries**	**118,148.7 (111,589.9 to 125,891.8)**	**36,548.7† (32,049.0 to 41,267.9)**	**4,277.0 (2,871.2 to 6,096.7)**	**15.3 (13.9 to 16.5)**	**1.3 (0.1 to 2.3)**
**Transport injuries**	**20,925.1 (19,264.2 to 22,726.9)**	**5,916.6 (4,521.9 to 7,516.9)**	**1,100.9 (740.1 to 1,574.9)**	**18.1 (16.9 to 19.3)**	**3.8 (2.7 to 4.8)**
Road injuries	17,144.6 (15,665.5 to 18,677.5)	5,405.2 (4,067.7 to 6,995.5)	908.7 (609.1 to 1,301.4)	19.6 (18.3 to 20.8)	5.0 (3.9 to 6.1)
Pedestrian road injuries	3,593.6 (2,979.9 to 4,205.4)	929.1 (588.8 to 1,362.8)	194.2 (130.3 to 278.9)	20.6 (19.1 to 22.2)	5.9 (4.6 to 7.3)
Cyclist road injuries	3,269.1 (2,698.6 to 3,936.9)	1,169.8 (733.7 to 1,736.4)	160.1 (106.1 to 232.2)	22.7 (21.4 to 24.2)	7.8 (6.6 to 9.1)
Motorcyclist road injuries	4,513.9 (3,815.0 to 5,688.5)	965.0 (619.8 to 1,400.6)	236.0 (157.5 to 334.9)	19.1 (17.7 to 20.4)	4.6 (3.4 to 5.7)
Motor vehicle road injuries	4,473.7 (3,736.3 to 5,259.0)	1,833.1 (1,262.7 to 2,575.0)	255.2 (172.8 to 360.6)	13.2 (11.8 to 14.7)	-0.5 (-1.8 to 0.7)
Other road injuries	1,294.2 (1,046.5 to 1,608.6)	508.2 (270.6 to 852.7)	63.1 (41.1 to 93.6)	40.7 (38.9 to 42.5)	23.6 (22.0 to 25.2)
Other transport injuries	3,780.6 (3,079.8 to 4,520.3)	511.4 (351.4 to 735.4)	192.2 (129.4 to 273.4)	11.7 (9.9 to 13.4)	-1.9 (-3.5 to -0.4)
**Unintentional injuries**	**84,948.1 (78,403.5 to 91,883.5)**	**27,967.1 (24,194.8 to 32,549.7)**	**2,687.0 (1,821.8 to 3,799.0)**	**15.9 (14.7 to 17.0)**	**1.8 (0.8 to 2.7)**
Falls	32,205.7 (27,679.1 to 36,777.7)	10,444.5 (7,757.7 to 14,187.0)	1,224.6 (849.0 to 1,698.1)	16.7 (15.5 to 17.9)	2.5 (1.4 to 3.6)
Drowning	352.8 (303.4 to 411.0)	33.0 (25.0 to 42.8)	17.6 (11.6 to 25.2)	-0.7 (-2.9 to 1.4)	-12.8 (-14.7 to -11.0)
Fire, heat, and hot substances	8,763.4 (7,363.4 to 10,589.6)	576.9 (366.5 to 832.2)	213.6 (139.9 to 320.1)	14.8 (12.9 to 16.4)	0.8 (-0.8 to 2.2)
Poisonings	281.8 (238.8 to 328.1)	274.9 (180.4 to 414.6)	22.9 (14.8 to 33.0)	23.2 (20.1 to 26.6)	8.2 (5.5 to 11.2)
Exposure to mechanical forces	16,940.9 (14,272.3 to 21,191.2)	4,702.4 (3,252.7 to 6,642.2)	380.2 (246.3 to 560.8)	17.3 (16.1 to 18.3)	3.0 (1.9 to 3.9)
Unintentional firearm injuries	643.7 (510.8 to 821.2)	90.3 (45.5 to 165.8)	21.5 (13.6 to 32.6)	16.3 (15.0 to 17.7)	2.1 (1.0 to 3.4)
Unintentional suffocation	699.1 (534.6 to 903.1)	54.8 (24.9 to 101.5)	20.8 (12.9 to 31.7)	8.3 (6.7 to 9.8)	-4.9 (-6.3 to -3.5)
Other exposure to mechanical forces	15,598.1 (12,948.4 to 19,830.1)	4,557.4 (3,159.2 to 6,418.8)	337.9 (219.5 to 496.9)	18.0 (16.7 to 19.1)	3.6 (2.5 to 4.6)

Cause	Prevalence in thousands (95% UI)	Incidence in thousands (95% UI)	YLDs in thousands (95% UI)		
	2016 counts	2016 counts	2016 counts	Percentage change in counts between 2006 and 2016	Percentage change in rates between 2006 and 2016
Adverse effects of medical treatment	123.1 (82.8 to 184.6)	1,609.2 (1,115.9 to 2,295.7)	16.4 (9.6 to 27.5)	8.2 (3.2 to 12.8)	-5.0 (-9.4 to -1.0)
Animal contact	4,311.2 (3,733.1 to 5,039.4)	4,255.2 (2,920.8 to 5,930.8)	126.9 (85.9 to 178.3)	10.5 (8.4 to 12.4)	-3.0 (-4.8 to -1.2)
Venomous animal contact	2,025.1 (1,696.2 to 2,414.0)	2,047.3 (1,340.0 to 3,032.9)	90.5 (62.0 to 125.8)	12.6 (9.9 to 15.2)	-1.1 (-3.5 to 1.2)
Non-venomous animal contact	2,286.1 (1,825.9 to 2,950.0)	2,207.9 (1,414.3 to 3,218.9)	36.4 (23.1 to 55.7)	5.6 (3.4 to 7.5)	-7.2 (-9.2 to -5.6)
Foreign body	3,850.5 (3,200.4 to 4,498.3)	1,823.8 (1,283.9 to 2,671.4)	139.2 (89.8 to 207.5)	16.6 (14.6 to 18.5)	2.4 (0.6 to 4.1)
Pulmonary aspiration and foreign body in airway	1,787.8 (1,356.2 to 2,340.3)	311.7 (204.7 to 463.9)	72.8 (46.4 to 108.9)	13.1 (11.1 to 15.0)	-0.7 (-2.4 to 1.0)
Foreign body in eyes	236.5 (133.8 to 393.6)	1,154.4 (640.1 to 1,963.4)	14.0 (7.4 to 25.5)	25.6 (21.8 to 30.7)	10.3 (7.0 to 14.8)
Foreign body in other body part	1,826.3 (1,479.6 to 2,320.9)	357.7 (213.6 to 545.3)	52.4 (33.9 to 78.3)	19.5 (17.5 to 21.1)	4.9 (3.2 to 6.4)
Environmental heat and cold exposure	5,704.5 (4,699.3 to 6,720.9)	985.4 (612.8 to 1,422.7)	206.8 (138.0 to 297.1)	16.7 (14.7 to 18.6)	2.5 (0.7 to 4.2)
Other unintentional injuries	12,414.2 (9,915.0 to 15,209.1)	3,261.8 (2,122.5 to 4,848.3)	338.8 (220.7 to 495.8)	14.7 (13.3 to 16.0)	0.7 (-0.5 to 1.9)
**Self-harm and interpersonal violence**	**18,305.0 (14,348.4 to 23,227.0)**	**2,578.7† (1,960.8 to 3,355.8)**	**332.4 (218.5 to 474.8)**	**5.4 (4.3 to 6.4)**	**-7.4 (-8.4 to -6.6)**
Self-harm	997.5 (811.7 to 1,202.5)	554.6 (416.0 to 718.8)	46.3 (30.1 to 67.6)	4.3 (2.7 to 5.9)	-8.4 (-9.8 to -7.0)
Self-harm by firearm	32.2 (27.2 to 37.6)	5.5 (1.6 to 13.7)	1.2 (0.7 to 1.7)	0.0 (-2.2 to 1.9)	-12.1 (-14.1 to -10.5)
Self-harm by other specified means	965.3 (779.4 to 1,170.2)	549.1 (412.3 to 711.9)	45.1 (29.3 to 66.0)	4.4 (2.8 to 6.0)	-8.3 (-9.7 to -6.9)
Interpersonal violence	17,307.5 (13,436.6 to 22,116.3)	2,024.1† (1,426.4 to 2,781.5)	286.1 (189.5 to 409.0)	5.6 (4.4 to 6.6)	-7.3 (-8.3 to -6.4)
Assault by firearm	265.7 (211.8 to 323.9)	45.4 (24.8 to 77.8)	9.9 (6.2 to 15.0)	4.2 (2.9 to 5.8)	-8.4 (-9.6 to -7.1)
Assault by sharp object	1,450.2 (1,154.4 to 1,739.5)	399.4 (228.9 to 637.2)	40.2 (25.9 to 59.5)	2.1 (0.2 to 3.8)	-10.3 (-12.0 to -8.8)
Sexual violence	11,861.7 (8,135.1 to 16,697.9)	--	93.5 (52.0 to 155.1)	8.2 (6.8 to 9.6)	-5.0 (-6.2 to -3.8)
Assault by other means	3,729.8 (3,030.9 to 4,629.8)	1,579.2 (1,091.8 to 2,180.5)	142.4 (94.6 to 208.7)	5.0 (3.6 to 6.1)	-7.8 (-9.0 to -6.8)
**Forces of nature, conflict and terrorism, and executions and police conflict**	**5,832.1 (3,672.3 to 8,452.2)**	**86.3† (50.5 to 124.2)**	**156.8 (56.2 to 320.9)**	**9.2 (-1.6 to 19.1)**	**-4.1 (-13.5 to 4.6)**
Forces of nature, conflict and terrorism, and state actor violence	838.3 (466.1 to 1,298.8)	23.2 (12.6 to 36.1)	27.2 (7.5 to 64.6)	73.0 (60.6 to 81.3)	52.0 (41.1 to 59.2)
Conflict and terrorism	4,375.3 (2,542.9 to 6,798.1)	--	123.8 (47.8 to 242.2)	12.5 (-2.0 to 24.9)	-1.2 (-13.9 to 9.7)
Executions and police conflict	618.5 (213.9 to 972.9)	63.1 (28.3 to 101.6)	13.7 (3.1 to 35.4)	-43.6 (-47.1 to -29.9)	-50.5 (-53.6 to -38.4)

We did not calculate incidence for certain NTDs and causes with only birth prevalence. Blank cells mean that no estimate is available.

† Incidence for aggregate causes represents the sum of the incidence of the causes below the aggregate.

‡ Incidence estimates for stroke represent first-ever stroke only.

^ Incidence of HIV/AIDS represents new infections of HIV only and does not include new infections of Tuberculosis in HIV positive cases.

SR Table 12: Global prevalence, incidence, and YLDs for 2016, percentage change of YLD counts and percentage change of YLD rates between 2006 and 2016 by cause, age 45-49 years.					
Cause	Prevalence in thousands (95% UI)	Incidence in thousands (95% UI)	YLDs in thousands (95% UI)		
	2016 counts	2016 counts	2016 counts	Percentage change in counts between 2006 and 2016	Percentage change in rates between 2006 and 2016
**All causes**	**455,607.8<br>(455,279.4 to 455,987.2)**	**2,420,797.2†<br>(2,110,052.5 to 2,778,929.4)**	**61,321.9<br>(45,588.5 to 79,275.2)**	**22.0<br>(21.2 to 22.7)**	**-2.2<br>(-2.9 to -1.6)**
**Communicable, maternal, neonatal, and nutritional disorders**	**326,087.3<br>(320,860.1 to 329,942.9)**	**1,247,676.0†<br>(966,469.7 to 1,588,208.9)**	**5,052.9<br>(3,652.7 to 6,816.7)**	**12.6<br>(7.6 to 16.9)**	**-9.7<br>(-13.7 to -6.3)**
**HIV/AIDS and tuberculosis**	**167,134.2<br>(146,824.5 to 191,601.4)**	**684.6†<br>(492.6 to 919.3)**	**635.1<br>(422.2 to 917.1)**	**-3.2<br>(-19.5 to 16.8)**	**-22.4<br>(-35.5 to -6.3)**
Tuberculosis	156,416.0<br>(127,438.2 to 190,581.2)	621.4†<br>(428.9 to 860.9)	230.3<br>(137.0 to 358.0)	10.9<br>(6.3 to 16.4)	-11.1<br>(-14.8 to -6.7)
Drug-susceptible tuberculosis	727.5<br>(506.3 to 1,009.4)	598.4<br>(412.4 to 831.5)	221.8<br>(131.9 to 345.4)	11.0<br>(6.4 to 16.6)	-11.0<br>(-14.7 to -6.5)
Multidrug-resistant tuberculosis without extensive drug resistance	25.4<br>(18.5 to 34.1)	21.5<br>(15.4 to 28.5)	8.0<br>(4.7 to 12.2)	3.6<br>(-11.6 to 21.7)	-16.9<br>(-29.1 to -2.4)
Extensively drug-resistant tuberculosis	1.6<br>(1.2 to 2.1)	1.5<br>(1.1 to 2.0)	0.5<br>(0.3 to 0.8)	186.9<br>(148.5 to 229.5)	130.1<br>(99.2 to 164.2)
Latent tuberculosis infection	155,661.5<br>(126,666.5 to 189,834.9)	--	--	--	--
HIV/AIDS	3,612.5<br>(3,157.6 to 4,169.9)	63.2^<br>(50.4 to 81.0)	404.8<br>(258.0 to 601.6)	-9.7<br>(-28.9 to 20.9)	-27.6<br>(-43.0 to -3.1)
Drug-susceptible HIV/AIDS - Tuberculosis	91.2<br>(63.9 to 125.8)	112.6<br>(81.2 to 153.0)	34.6<br>(20.2 to 53.3)	1.6<br>(-6.2 to 9.5)	-18.6<br>(-24.8 to -12.2)
Multidrug-resistant HIV/AIDS - Tuberculosis without extensive drug resistance	2.5<br>(1.7 to 3.7)	3.0<br>(2.0 to 4.4)	1.0<br>(0.6 to 1.7)	-6.5<br>(-22.2 to 11.3)	-25.0<br>(-37.6 to -10.8)
Extensively drug-resistant HIV/AIDS - Tuberculosis	0.1<br>(0.1 to 0.2)	0.1<br>(0.1 to 0.2)	< 0.05<br>(< 0.05 to 0.1)	236.5<br>(184.4 to 304.8)	169.9<br>(128.1 to 224.6)
HIV/AIDS resulting in other diseases	3,518.7<br>(3,069.9 to 4,064.7)	63.2<br>(50.4 to 81.0)	369.1<br>(227.1 to 559.3)	-10.7<br>(-30.4 to 23.4)	-28.4<br>(-44.2 to -1.0)
**Diarrhoea, lower respiratory infections, and other common infectious diseases**	**21,711.4<br>(20,377.3 to 22,675.9)**	**1,171,056.9†<br>(889,613.2 to 1,509,088.7)**	**1,017.0<br>(706.5 to 1,405.2)**	**19.5<br>(18.0 to 21.2)**	**-4.2<br>(-5.4 to -2.8)**
Diarrhoeal diseases	2,895.7<br>(2,625.0 to 3,171.1)	199,414.4<br>(180,606.6 to 217,825.4)	320.0<br>(219.8 to 439.4)	17.6<br>(15.3 to 20.0)	-5.7<br>(-7.5 to -3.8)
Intestinal infectious diseases	18.5<br>(14.8 to 22.9)	473.0†<br>(386.1 to 566.4)	3.4<br>(2.1 to 4.9)	0.4<br>(-16.7 to 20.6)	-19.5<br>(-33.2 to -3.3)
Typhoid fever	18.2<br>(14.2 to 22.6)	288.0<br>(232.7 to 348.5)	2.8<br>(1.8 to 4.2)	1.0<br>(-19.2 to 25.3)	-19.0<br>(-35.2 to 0.5)
Paratyphoid fever	8.1<br>(6.4 to 10.0)	185.0<br>(150.8 to 225.9)	0.5<br>(0.3 to 0.8)	-1.3<br>(-17.6 to 17.2)	-20.9<br>(-34.0 to -6.0)
Other intestinal infectious diseases	--	--	< 0.05<br>(< 0.05 to 0.1)	-16.9<br>(-31.1 to -0.2)	-33.4<br>(-44.7 to -20.0)
Lower respiratory infections	312.3<br>(288.3 to 341.3)	13,741.0<br>(12,401.0 to 15,304.5)	18.9<br>(12.7 to 26.8)	36.2<br>(29.7 to 43.0)	9.2<br>(4.0 to 14.6)
Upper respiratory infections	12,675.7<br>(8,926.7 to 17,064.7)	945,613.2<br>(663,130.8 to 1,285,943.3)	311.8<br>(165.6 to 510.6)	21.3<br>(19.4 to 23.1)	-2.8<br>(-4.2 to -1.3)
Otitis media	4,338.7<br>(3,312.5 to 5,532.1)	7,114.3<br>(5,857.2 to 8,506.7)	119.4<br>(71.0 to 189.0)	13.1<br>(8.2 to 18.1)	-9.3<br>(-13.2 to -5.3)
Meningitis	830.8<br>(708.1 to 957.7)	53.2<br>(37.4 to 72.1)	82.7<br>(57.8 to 110.1)	20.2<br>(16.4 to 24.0)	-3.6<br>(-6.7 to -0.5)
Pneumococcal meningitis	378.9<br>(315.7 to 454.2)	12.6<br>(7.5 to 19.3)	37.1<br>(25.6 to 50.6)	17.3<br>(10.6 to 24.4)	-6.0<br>(-11.3 to -0.2)
H influenzae type B meningitis	101.2<br>(78.5 to 129.5)	3.6<br>(2.0 to 5.8)	12.4<br>(8.2 to 17.3)	-2.2<br>(-8.6 to 2.9)	-21.6<br>(-26.7 to -17.5)
Meningococcal infection	88.3<br>(65.0 to 115.7)	9.0<br>(5.1 to 14.3)	8.9<br>(6.1 to 12.3)	21.8<br>(18.9 to 24.4)	-2.3<br>(-4.6 to -0.2)
Other meningitis	262.4<br>(198.5 to 330.6)	28.1<br>(19.6 to 40.2)	24.3<br>(16.6 to 33.0)	41.2<br>(35.8 to 47.3)	13.3<br>(8.9 to 18.2)
Encephalitis	1,219.0<br>(813.6 to 1,778.7)	318.3<br>(262.6 to 376.2)	138.3<br>(96.4 to 180.7)	22.7<br>(17.2 to 28.4)	-1.6<br>(-6.0 to 3.0)
Diphtheria	< 0.05<br>(< 0.05 to < 0.05)	0.2<br>(0.2 to 0.3)	< 0.05<br>(< 0.05 to < 0.05)	-34.8<br>(-47.8 to -15.1)	-47.7<br>(-58.1 to -31.9)
Whooping cough	1.5<br>(1.2 to 1.9)	11.2<br>(8.7 to 14.0)	0.1<br>(< 0.05 to 0.1)	-14.0<br>(-15.9 to -12.1)	-31.1<br>(-32.6 to -29.5)
Tetanus	8.1<br>(7.9 to 8.3)	4.6<br>(2.2 to 6.8)	0.2<br>(0.1 to 0.3)	3.1<br>(-19.0 to 28.6)	-17.4<br>(-35.0 to 3.1)
Measles	0.1<br>(< 0.05 to 0.3)	4.8<br>(1.7 to 10.3)	< 0.05<br>(< 0.05 to < 0.05)	-53.3<br>(-58.7 to -47.3)	-62.6<br>(-66.9 to -57.8)
Varicella and herpes zoster	438.9<br>(349.0 to 541.4)	4,308.9<br>(3,427.0 to 5,243.1)	22.3<br>(12.9 to 35.9)	29.0<br>(22.1 to 36.0)	3.5<br>(-2.1 to 9.1)
**Neglected tropical diseases and malaria**	**109,501.6<br>(105,676.8 to 112,676.4)**	**13,934.1†<br>(11,587.3 to 16,639.2)**	**818.2<br>(548.8 to 1,150.8)**	**-3.6<br>(-12.5 to 6.4)**	**-22.7<br>(-29.9 to -14.7)**
Malaria	3,213.8<br>(2,797.8 to 3,633.1)	3,444.0<br>(2,544.3 to 4,442.1)	40.1<br>(29.9 to 51.3)	-6.0<br>(-12.4 to 1.7)	-24.6<br>(-29.8 to -18.5)
Chagas disease	636.1<br>(533.6 to 761.0)	8.5<br>(5.7 to 12.0)	5.2<br>(3.3 to 7.6)	4.3<br>(-3.2 to 11.4)	-16.4<br>(-22.4 to -10.6)
Leishmaniasis	322.8<br>(248.6 to 399.8)	33.4<br>(24.9 to 44.1)	17.5<br>(10.7 to 27.7)	22.1<br>(13.1 to 36.7)	-2.1<br>(-9.3 to 9.7)
Visceral leishmaniasis	1.4<br>(0.9 to 1.9)	5.5<br>(3.8 to 7.8)	0.1<br>(0.1 to 0.1)	-55.2<br>(-70.6 to -28.1)	-64.1<br>(-76.4 to -42.3)
Cutaneous and mucocutaneous leishmaniasis	279.1<br>(208.8 to 375.1)	27.9<br>(19.8 to 38.2)	17.4<br>(10.6 to 27.6)	23.3<br>(14.0 to 38.8)	-1.2<br>(-8.6 to 11.3)
African trypanosomiasis	0.4<br>(0.1 to 1.3)	0.3<br>(0.1 to 1.0)	0.1<br>(< 0.05 to 0.4)	-71.7<br>(-96.2 to 81.6)	-77.3<br>(-97.0 to 45.6)
Schistosomiasis	12,343.9<br>(11,581.6 to 13,167.0)	3,084.5<br>(2,820.0 to 3,422.6)	95.4<br>(47.9 to 177.8)	-26.4<br>(-28.5 to -23.7)	-41.0<br>(-42.6 to -38.8)
Cysticercosis	261.7<br>(175.7 to 374.6)	--	41.0<br>(24.7 to 65.0)	-0.6<br>(-9.5 to 7.6)	-20.3<br>(-27.5 to -13.7)
Cystic echinococcosis	67.7<br>(34.9 to 134.4)	13.8<br>(6.6 to 32.0)	6.2<br>(2.7 to 13.3)	-38.6<br>(-76.8 to 54.3)	-50.7<br>(-81.4 to 23.8)
Lymphatic filariasis	2,094.3<br>(1,650.9 to 2,763.8)	423.7<br>(245.3 to 665.7)	118.4<br>(60.1 to 211.3)	-35.8<br>(-50.9 to -23.3)	-48.5<br>(-60.6 to -38.5)
Onchocerciasis	784.3<br>(510.0 to 1,325.0)	177.6<br>(73.3 to 349.8)	57.2<br>(27.1 to 103.7)	-20.3<br>(-39.8 to -1.2)	-36.1<br>(-51.8 to -20.7)
Trachoma	61.8<br>(34.1 to 117.2)	--	7.8<br>(4.4 to 12.8)	-3.6<br>(-17.4 to 11.8)	-22.7<br>(-33.7 to -10.4)
Dengue	286.4<br>(156.2 to 485.0)	4,788.2<br>(2,914.3 to 7,259.8)	45.7<br>(20.0 to 85.6)	94.6<br>(57.2 to 345.7)	56.1<br>(26.0 to 257.4)
Yellow fever	0.1<br>(< 0.05 to 0.2)	3.3<br>(0.9 to 8.7)	< 0.05<br>(< 0.05 to < 0.05)	-20.1<br>(-48.0 to 20.9)	-35.9<br>(-58.3 to -3.0)
Rabies	< 0.05<br>(< 0.05 to 0.1)	1.0<br>(0.5 to 1.4)	< 0.05<br>(< 0.05 to < 0.05)	-40.6<br>(-51.8 to -25.9)	-52.4<br>(-61.4 to -40.6)
Intestinal nematode infections	91,399.9<br>(86,741.5 to 94,893.1)	--	160.1<br>(91.1 to 262.2)	-5.8<br>(-13.4 to 3.0)	-24.4<br>(-30.6 to -17.4)
Ascariasis	48,880.6<br>(43,618.7 to 55,473.5)	--	49.3<br>(26.5 to 82.5)	-20.8<br>(-31.4 to -8.6)	-36.5<br>(-45.0 to -26.7)

Cause	Prevalence in thousands (95% UI)	Incidence in thousands (95% UI)	YLDs in thousands (95% UI)		
	2016 counts	2016 counts	2016 counts	Percentage change in counts between 2006 and 2016	Percentage change in rates between 2006 and 2016
Trichuriasis	25,471.9 (23,254.8 to 27,986.5)	--	18.3 (10.1 to 31.1)	-12.1 (-25.2 to 2.5)	-29.5 (-40.0 to -17.8)
Hookworm disease	26,966.2 (24,816.4 to 29,237.8)	--	92.4 (54.3 to 148.9)	6.5 (-5.2 to 19.8)	-14.6 (-24.0 to -3.9)
Food-borne trematodiases	7,893.1 (7,247.7 to 8,610.2)	1,399.4 (725.8 to 1,932.0)	204.2 (99.2 to 371.2)	54.6 (47.6 to 62.1)	24.0 (18.4 to 30.0)
Leprosy	48.3 (45.6 to 51.2)	5.0 (4.7 to 5.4)	3.0 (2.0 to 4.3)	-0.1 (-9.0 to 9.6)	-19.9 (-27.0 to -12.1)
Ebola virus disease	< 0.05 (< 0.05 to 0.2)	--	< 0.05 (< 0.05 to 0.1)	--	--
Zika virus disease	9.1 (5.2 to 17.1)	551.4 (315.8 to 1,033.1)	0.2 (0.1 to 0.3)	--	--
Guinea worm disease	< 0.05 (< 0.05 to < 0.05)	< 0.05 (< 0.05 to < 0.05)	< 0.05 (< 0.05 to < 0.05)	-99.6 (-99.7 to -99.3)	-99.7 (-99.8 to -99.5)
Other neglected tropical diseases	1,313.4 (1,146.2 to 1,490.0)	--	16.2 (9.5 to 25.5)	30.5 (22.5 to 38.7)	4.6 (-1.8 to 11.2)
**Maternal disorders**	**229.4 (218.7 to 240.3)**	**977.7† (805.6 to 1,157.2)**	**27.4 (18.8 to 37.9)**	**-14.7 (-22.3 to -6.2)**	**-31.6 (-37.7 to -24.8)**
Maternal haemorrhage	10.0 (6.8 to 14.2)	76.4 (47.3 to 113.3)	0.7 (0.4 to 1.1)	0.1 (-16.3 to 20.1)	-19.7 (-32.9 to -3.7)
Maternal sepsis and other pregnancy related infections	135.6 (120.2 to 151.7)	99.5 (62.8 to 147.7)	1.0 (0.5 to 1.9)	3.2 (-25.9 to 40.5)	-17.2 (-40.5 to 12.7)
Maternal hypertensive disorders	33.4 (21.4 to 47.7)	167.1 (137.3 to 195.8)	1.6 (0.9 to 2.6)	3.1 (-35.1 to 65.5)	-17.4 (-47.9 to 32.7)
Maternal obstructed labour and uterine rupture	60.6 (50.9 to 72.1)	45.8 (29.7 to 69.4)	19.2 (12.3 to 27.5)	-16.3 (-24.6 to -7.3)	-32.9 (-39.6 to -25.7)
Maternal abortion, miscarriage, and ectopic pregnancy	4.8 (3.1 to 7.4)	588.9 (431.5 to 757.0)	0.5 (0.3 to 0.9)	-10.1 (-43.6 to 43.3)	-27.9 (-54.8 to 15.0)
Other maternal disorders	--	--	4.3 (3.0 to 6.0)	-19.1 (-26.3 to -11.0)	-35.1 (-40.9 to -28.6)
**Neonatal disorders**	**3,875.7 (3,551.2 to 4,245.0)**	**--**	**662.6 (467.7 to 921.3)**	**39.6 (25.0 to 57.6)**	**11.9 (0.2 to 26.4)**
Neonatal preterm birth complications	3,291.1 (2,777.0 to 3,877.2)	--	427.4 (296.4 to 605.1)	39.7 (16.1 to 67.3)	12.0 (-6.9 to 34.1)
Neonatal encephalopathy due to birth asphyxia and trauma	610.9 (399.4 to 914.4)	--	88.2 (38.1 to 178.9)	38.2 (28.9 to 55.1)	10.9 (3.4 to 24.3)
Neonatal sepsis and other neonatal infections	293.7 (120.2 to 687.4)	--	112.3 (38.4 to 291.8)	40.4 (30.8 to 56.6)	12.6 (4.9 to 25.6)
Hemolytic disease and other neonatal jaundice	107.4 (95.4 to 119.4)	--	34.8 (26.9 to 43.8)	38.8 (32.1 to 45.5)	11.3 (5.9 to 16.7)
Other neonatal disorders	--	--	--	--	--
**Nutritional deficiencies**	**81,324.2 (77,594.7 to 84,183.6)**	**19,064.3† (16,225.1 to 22,519.4)**	**1,655.6 (1,072.8 to 2,423.0)**	**16.1 (12.5 to 19.4)**	**-6.9 (-9.8 to -4.2)**
Protein-energy malnutrition	--	--	--	--	--
Iodine deficiency	8,717.7 (7,812.5 to 9,722.0)	35.1 (30.0 to 41.4)	226.5 (153.1 to 314.9)	6.0 (3.5 to 8.7)	-15.0 (-17.0 to -12.8)
Vitamin A deficiency	17,657.0 (15,527.5 to 20,350.2)	19,029.2 (16,189.0 to 22,483.9)	9.5 (5.9 to 14.4)	30.5 (21.6 to 40.6)	4.7 (-2.5 to 12.7)
Iron-deficiency anaemia	62,779.1 (54,938.5 to 70,914.4)	--	1,419.5 (900.3 to 2,101.1)	17.8 (13.7 to 21.6)	-5.6 (-8.9 to -2.5)
Other nutritional deficiencies	--	--	--	--	--
**Other communicable, maternal, neonatal, and nutritional diseases**	**145,708.7 (140,799.7 to 150,125.1)**	**41,958.4 (34,103.6 to 51,745.8)**	**229.1 (149.3 to 345.8)**	**23.5 (16.9 to 29.7)**	**-1.0 (-6.3 to 4.0)**
Sexually transmitted diseases excluding HIV	107,933.4 (103,263.2 to 112,511.2)	35,356.5 (28,189.1 to 44,304.1)	184.6 (117.3 to 284.5)	26.7 (22.6 to 30.4)	1.6 (-1.7 to 4.6)
Syphilis	5,037.6 (3,315.3 to 7,753.1)	3,652.5 (2,258.6 to 6,341.6)	18.7 (13.1 to 25.1)	27.1 (22.8 to 31.7)	1.9 (-1.6 to 5.6)
Chlamydial infection	2,031.5 (1,474.9 to 2,795.6)	2,385.6 (1,879.2 to 2,950.3)	23.8 (15.3 to 34.2)	23.1 (16.5 to 30.1)	-1.3 (-6.6 to 4.3)
Gonococcal infection	3,593.7 (2,056.5 to 5,986.2)	11,841.0 (6,893.7 to 19,639.1)	39.7 (20.6 to 72.3)	35.0 (25.3 to 42.2)	8.3 (0.5 to 14.0)
Trichomoniasis	15,409.0 (10,818.7 to 21,897.4)	12,512.4 (8,788.0 to 17,577.5)	16.9 (6.4 to 36.8)	24.8 (22.7 to 26.7)	0.1 (-1.6 to 1.6)
Genital herpes	87,703.3 (76,683.3 to 101,391.2)	544.6 (350.9 to 798.1)	21.4 (6.2 to 48.8)	22.0 (19.3 to 24.5)	-2.2 (-4.3 to -0.2)
Other sexually transmitted diseases	1,774.4 (1,157.1 to 2,553.1)	4,420.5 (3,439.1 to 5,515.8)	64.1 (42.4 to 92.9)	25.3 (19.0 to 31.4)	0.5 (-4.6 to 5.3)
Hepatitis	49,355.1 (42,321.7 to 57,496.2)	6,601.3 (4,373.1 to 9,884.9)	15.7 (8.3 to 26.6)	-4.4 (-42.8 to 62.1)	-23.4 (-54.1 to 30.0)
Acute hepatitis A	60.4 (12.0 to 130.8)	785.8 (156.5 to 1,699.9)	2.5 (0.5 to 6.1)	-15.5 (-86.4 to 284.5)	-32.2 (-89.1 to 208.3)
Hepatitis B	37,023.2 (29,942.6 to 45,093.7)	5,112.0 (2,982.6 to 8,227.8)	11.8 (5.8 to 21.8)	-1.9 (-46.9 to 82.9)	-21.3 (-57.5 to 46.6)
Hepatitis C	12,237.0 (10,941.8 to 13,532.3)	254.7 (183.9 to 338.0)	0.2 (0.1 to 0.5)	-3.3 (-5.9 to -1.0)	-22.5 (-24.6 to -20.6)
Acute hepatitis E	34.5 (23.7 to 48.4)	448.8 (307.5 to 629.7)	1.1 (0.6 to 1.7)	-2.4 (-23.6 to 23.9)	-21.7 (-38.8 to -0.7)
Other infectious diseases	1,566.0 (1,358.1 to 1,781.1)	0.6 (0.3 to 1.0)	28.8 (17.1 to 45.0)	23.2 (18.4 to 28.0)	-1.2 (-5.1 to 2.6)
**Non-communicable diseases**	**443,808.7 (442,681.6 to 444,888.2)**	**1,137,486.8† (1,006,167.0 to 1,275,026.6)**	**51,689.8 (38,085.9 to 66,780.6)**	**22.9 (22.1 to 23.6)**	**-1.5 (-2.1 to -0.9)**
**Neoplasms**	**2,667.1 (2,613.5 to 2,717.0)**	**1,034.9 (990.9 to 1,107.8)**	**337.3 (247.1 to 442.6)**	**24.8 (21.0 to 29.6)**	**0.1 (-3.0 to 3.9)**
Lip and oral cavity cancer	105.2 (100.2 to 110.7)	28.5 (27.1 to 30.1)	11.0 (7.9 to 14.5)	22.8 (13.1 to 33.2)	-1.5 (-9.3 to 6.8)
Nasopharynx cancer	44.9 (40.6 to 49.6)	12.7 (11.6 to 14.0)	5.3 (3.7 to 7.1)	22.8 (7.9 to 37.8)	-1.5 (-13.5 to 10.5)
Other pharynx cancer	37.0 (34.2 to 38.9)	13.2 (12.1 to 13.9)	4.6 (3.3 to 6.1)	19.1 (7.9 to 27.9)	-4.5 (-13.4 to 2.6)
Oesophageal cancer	25.7 (24.6 to 27.2)	21.2 (20.4 to 22.2)	5.7 (3.9 to 7.5)	1.1 (-6.3 to 8.8)	-18.9 (-24.9 to -12.8)
Stomach cancer	102.4 (97.4 to 108.1)	55.3 (52.7 to 58.0)	15.5 (10.9 to 20.5)	10.7 (2.9 to 18.7)	-11.3 (-17.5 to -4.9)
Colon and rectum cancer	278.5 (266.1 to 294.2)	75.4 (72.0 to 79.5)	28.0 (20.1 to 36.9)	30.1 (22.1 to 39.8)	4.3 (-2.1 to 12.1)
Liver cancer	81.7 (70.7 to 92.4)	81.8 (74.4 to 88.8)	19.1 (13.3 to 25.6)	49.8 (33.9 to 66.6)	20.1 (7.4 to 33.6)
Liver cancer due to hepatitis B	63.5 (50.0 to 75.1)	45.1 (36.0 to 52.7)	10.5 (6.7 to 14.3)	50.5 (30.0 to 72.2)	20.7 (4.2 to 38.1)

Cause	Prevalence in thousands (95% UI)	Incidence in thousands (95% UI)	YLDs in thousands (95% UI)		
	2016 counts	2016 counts	2016 counts	Percentage change in counts between 2006 and 2016	Percentage change in rates between 2006 and 2016
Liver cancer due to hepatitis C	9.9 (7.6 to 12.5)	7.7 (5.9 to 9.7)	1.8 (1.2 to 2.6)	38.1 (26.6 to 49.6)	10.8 (1.6 to 19.9)
Liver cancer due to alcohol use	10.1 (6.9 to 14.1)	8.1 (5.5 to 11.3)	1.9 (1.1 to 2.9)	47.0 (33.4 to 64.5)	17.8 (7.0 to 31.9)
Liver cancer due to other causes	29.8 (23.3 to 38.9)	20.9 (16.5 to 26.9)	4.9 (3.2 to 7.2)	54.0 (35.4 to 76.1)	23.5 (8.6 to 41.2)
Gallbladder and biliary tract cancer	6.3 (5.5 to 6.7)	7.5 (6.6 to 8.0)	1.7 (1.2 to 2.3)	11.7 (4.6 to 18.1)	-10.5 (-16.1 to -5.3)
Pancreatic cancer	12.0 (11.7 to 12.4)	14.8 (14.5 to 15.1)	3.2 (2.2 to 4.2)	17.1 (13.3 to 20.1)	-6.1 (-9.2 to -3.7)
Larynx cancer	36.8 (35.5 to 38.5)	10.8 (10.4 to 11.3)	4.4 (3.2 to 5.9)	3.5 (-2.2 to 9.5)	-17.0 (-21.6 to -12.2)
Tracheal, bronchus, and lung cancer	103.7 (99.0 to 108.5)	76.6 (73.4 to 79.5)	19.8 (14.0 to 25.7)	11.9 (3.5 to 21.4)	-10.3 (-17.0 to -2.6)
Malignant skin melanoma	96.4 (82.2 to 114.7)	19.6 (16.7 to 23.3)	6.4 (4.4 to 9.0)	17.4 (8.4 to 26.5)	-5.8 (-13.1 to 1.5)
Non-melanoma skin cancer	14.2 (7.9 to 25.8)	63.5 (30.2 to 130.3)	0.7 (0.3 to 1.4)	-5.6 (-19.7 to 7.9)	-24.3 (-35.6 to -13.5)
Non-melanoma skin cancer (squamous-cell carcinoma)	12.5 (5.1 to 25.1)	11.7 (5.2 to 23.4)	0.6 (0.2 to 1.3)	-7.1 (-22.7 to 7.6)	-25.5 (-38.0 to -13.7)
Non-melanoma skin cancer (basal-cell carcinoma)	5.9 (2.1 to 12.8)	51.9 (20.2 to 115.9)	0.1 (< 0.05 to 0.2)	10.9 (2.9 to 18.4)	-11.1 (-17.5 to -5.0)
Breast cancer	970.8 (910.6 to 1,043.8)	200.6 (188.1 to 215.7)	83.7 (59.5 to 112.3)	23.3 (13.3 to 33.8)	-1.2 (-9.1 to 7.3)
Cervical cancer	265.4 (204.2 to 286.5)	69.6 (54.4 to 74.2)	25.4 (17.0 to 34.0)	12.6 (3.1 to 22.2)	-9.7 (-17.3 to -2.0)
Uterine cancer	156.4 (147.3 to 168.1)	32.8 (30.8 to 35.2)	12.0 (8.5 to 16.4)	32.8 (20.1 to 49.1)	6.5 (-3.7 to 19.5)
Ovarian cancer	79.5 (74.2 to 82.9)	25.7 (24.3 to 26.7)	12.2 (8.6 to 16.0)	22.3 (9.2 to 35.4)	-1.9 (-12.4 to 8.6)
Prostate cancer	71.9 (62.6 to 83.1)	18.7 (16.6 to 20.8)	6.5 (4.5 to 8.9)	26.1 (15.0 to 36.5)	1.1 (-7.8 to 9.4)
Testicular cancer	23.0 (21.3 to 25.4)	4.5 (4.2 to 4.9)	1.6 (1.1 to 2.2)	25.4 (11.1 to 43.9)	0.6 (-10.9 to 15.4)
Kidney cancer	88.2 (83.7 to 92.3)	23.4 (22.5 to 24.4)	8.0 (5.8 to 10.8)	16.4 (9.2 to 23.0)	-6.7 (-12.4 to -1.3)
Bladder cancer	58.5 (55.7 to 61.0)	14.2 (13.5 to 14.8)	5.7 (4.0 to 7.5)	19.8 (12.5 to 27.5)	-3.9 (-9.8 to 2.3)
Brain and nervous system cancer	56.3 (50.3 to 62.4)	24.8 (22.6 to 27.8)	8.0 (5.5 to 10.6)	44.8 (34.2 to 56.8)	16.1 (7.6 to 25.7)
Thyroid cancer	138.6 (129.4 to 149.9)	26.3 (24.6 to 28.4)	8.6 (5.9 to 12.0)	48.4 (35.2 to 63.7)	19.0 (8.4 to 31.3)
Mesothelioma	2.4 (2.2 to 2.6)	1.6 (1.5 to 1.7)	0.5 (0.4 to 0.7)	21.9 (13.7 to 27.9)	-2.3 (-8.8 to 2.6)
Hodgkin lymphoma	20.0 (17.9 to 22.8)	5.0 (4.5 to 5.8)	2.0 (1.4 to 2.7)	10.1 (2.0 to 19.2)	-11.7 (-18.2 to -4.4)
Non-Hodgkin's lymphoma	99.5 (89.4 to 110.3)	28.0 (25.1 to 30.6)	9.2 (6.6 to 12.5)	46.6 (35.8 to 54.8)	17.5 (8.9 to 24.1)
Multiple myeloma	14.1 (12.3 to 15.8)	5.9 (5.3 to 6.6)	3.1 (2.1 to 4.1)	34.0 (26.8 to 41.9)	7.4 (1.7 to 13.8)
Leukaemia	78.2 (69.9 to 84.6)	22.7 (20.4 to 24.3)	10.4 (7.2 to 13.9)	31.7 (24.1 to 40.8)	5.6 (-0.5 to 12.9)
Acute lymphoid leukaemia	10.9 (9.5 to 11.9)	3.2 (2.8 to 3.5)	1.5 (1.0 to 2.0)	48.0 (34.8 to 58.7)	18.7 (8.1 to 27.3)
Chronic lymphoid leukaemia	18.9 (17.1 to 21.3)	4.2 (3.8 to 4.7)	2.3 (1.6 to 3.1)	45.3 (33.2 to 59.0)	16.5 (6.8 to 27.5)
Acute myeloid leukaemia	7.1 (6.4 to 7.9)	5.4 (4.8 to 5.9)	1.5 (1.0 to 2.0)	31.6 (25.5 to 38.1)	5.5 (0.6 to 10.7)
Chronic myeloid leukaemia	7.2 (6.3 to 8.0)	2.6 (2.2 to 2.8)	0.9 (0.6 to 1.3)	6.2 (-0.6 to 13.1)	-14.8 (-20.3 to -9.3)
Other leukaemia	34.0 (28.3 to 37.7)	7.5 (6.2 to 8.3)	4.2 (2.8 to 5.7)	26.9 (14.3 to 41.3)	1.8 (-8.3 to 13.3)
Other neoplasms	176.5 (156.5 to 187.6)	50.4 (45.0 to 53.4)	15.2 (10.8 to 20.2)	60.9 (49.9 to 71.4)	29.0 (20.2 to 37.5)
**Cardiovascular diseases**	**28,505.4 (27,610.3 to 29,225.0)**	**3,029.6† (2,690.5 to 3,442.1)**	**1,794.9 (1,266.9 to 2,349.2)**	**28.1 (24.8 to 31.6)**	**2.7 (0.1 to 5.5)**
Rheumatic heart disease	2,284.3 (2,070.3 to 2,537.2)	15.4 (12.0 to 19.0)	111.6 (70.8 to 160.9)	21.3 (17.3 to 25.1)	-2.8 (-6.0 to 0.3)
Ischaemic heart disease	8,560.5 (7,914.9 to 9,209.2)	1,066.7 (881.6 to 1,271.6)	443.4 (296.2 to 623.7)	26.9 (25.1 to 28.9)	1.8 (0.3 to 3.4)
Cerebrovascular disease	4,630.6 (4,397.8 to 4,827.1)	836.6‡ (686.5 to 1,021.5)	743.3 (533.1 to 968.2)	32.9 (25.2 to 40.7)	6.6 (0.4 to 12.8)
Ischaemic stroke	3,722.0 (3,199.5 to 4,302.1)	553.6‡ (413.6 to 725.7)	581.9 (412.4 to 760.4)	34.2 (25.4 to 43.2)	7.6 (0.5 to 14.9)
Hemorrhagic stroke	1,026.2 (880.7 to 1,221.2)	283.0‡ (220.0 to 355.2)	161.5 (111.6 to 214.3)	28.4 (17.2 to 40.4)	2.9 (-6.0 to 12.6)
Hypertensive heart disease	997.0 (797.8 to 1,215.1)	--	83.9 (54.5 to 119.9)	29.6 (24.9 to 33.9)	3.9 (0.1 to 7.4)
Cardiomyopathy and myocarditis	333.4 (304.2 to 367.9)	151.9† (106.7 to 211.2)	35.2 (21.8 to 51.2)	16.9 (11.9 to 22.1)	-6.2 (-10.3 to -2.1)
Myocarditis	106.9 (86.8 to 129.5)	151.9 (106.7 to 211.2)	8.0 (5.1 to 11.5)	29.0 (22.1 to 36.4)	3.5 (-2.1 to 9.4)
Alcoholic cardiomyopathy	126.0 (99.2 to 156.3)	--	10.8 (6.6 to 15.9)	5.9 (-3.9 to 17.5)	-15.1 (-22.9 to -5.8)
Other cardiomyopathy	191.2 (147.5 to 239.8)	--	16.3 (10.0 to 24.1)	19.7 (13.1 to 26.2)	-4.0 (-9.3 to 1.2)
Atrial fibrillation and flutter	1,452.5 (1,191.5 to 1,784.8)	117.4 (87.9 to 153.0)	120.7 (76.9 to 174.5)	23.3 (19.2 to 27.4)	-1.1 (-4.4 to 2.2)
Peripheral vascular disease	6,600.3 (5,158.1 to 8,259.8)	779.8 (544.0 to 1,082.1)	0.4 (0.1 to 0.9)	12.7 (4.3 to 21.5)	-9.7 (-16.4 to -2.6)
Endocarditis	18.9 (14.8 to 23.9)	61.9 (42.4 to 81.2)	1.5 (1.0 to 2.2)	20.5 (17.1 to 24.3)	-3.4 (-6.1 to -0.3)
Other cardiovascular and circulatory diseases	4,810.1 (3,487.1 to 6,553.6)	--	254.8 (159.8 to 391.5)	23.8 (20.7 to 26.9)	-0.7 (-3.2 to 1.8)
**Chronic respiratory diseases**	**35,420.1 (34,619.5 to 36,170.7)**	**5,152.9† (4,477.7 to 5,885.4)**	**2,044.0 (1,650.5 to 2,477.2)**	**24.7 (22.1 to 27.3)**	**-0.0 (-2.1 to 2.0)**
Chronic obstructive pulmonary disease	18,181.4 (16,906.4 to 19,523.3)	1,773.0 (1,525.1 to 2,014.4)	1,245.1 (1,056.7 to 1,433.1)	23.2 (19.5 to 27.3)	-1.2 (-4.2 to 2.1)
Pneumoconiosis	51.7 (45.4 to 58.1)	7.4 (6.0 to 8.9)	8.1 (5.3 to 11.5)	29.1 (23.9 to 34.3)	3.5 (-0.6 to 7.7)
Silicosis	15.3 (12.2 to 19.1)	2.4 (1.6 to 3.3)	2.4 (1.5 to 3.6)	31.9 (21.4 to 44.0)	5.8 (-2.7 to 15.5)

Cause	Prevalence in thousands (95% UI)	Incidence in thousands (95% UI)	YLDs in thousands (95% UI)		
	2016 counts	2016 counts	2016 counts	Percentage change in counts between 2006 and 2016	Percentage change in rates between 2006 and 2016
Asbestosis	6.9 (5.5 to 8.5)	0.6 (0.4 to 0.9)	1.1 (0.7 to 1.6)	12.1 (9.3 to 14.2)	-10.1 (-12.4 to -8.4)
Coal workers pneumoconiosis	14.6 (11.6 to 18.0)	2.7 (2.0 to 3.6)	2.3 (1.4 to 3.4)	36.5 (22.6 to 52.4)	9.5 (-1.7 to 22.2)
Other pneumoconiosis	15.0 (11.3 to 18.9)	1.6 (0.8 to 2.5)	2.4 (1.5 to 3.4)	28.6 (25.2 to 31.7)	3.1 (0.4 to 5.6)
Asthma	18,464.8 (16,481.2 to 20,687.2)	3,354.1 (2,724.8 to 3,986.7)	714.0 (462.1 to 1,025.2)	25.5 (22.9 to 28.0)	0.7 (-1.4 to 2.6)
Interstitial lung disease and pulmonary sarcoidosis	191.5 (157.7 to 236.0)	18.3 (12.2 to 26.4)	21.5 (13.6 to 32.4)	19.3 (11.9 to 26.3)	-4.4 (-10.3 to 1.3)
Other chronic respiratory diseases	--	--	55.4 (46.8 to 63.8)	54.9 (50.4 to 60.0)	24.2 (20.6 to 28.3)
**Cirrhosis and other chronic liver diseases**	**5,359.6 (4,983.2 to 5,774.2)**	**160.4 (134.1 to 188.9)**	**127.7 (86.4 to 178.8)**	**24.2 (19.0 to 29.0)**	**-0.4 (-4.6 to 3.5)**
Cirrhosis and other chronic liver diseases due to hepatitis B	1,592.8 (1,366.8 to 1,837.3)	50.0 (39.3 to 61.8)	40.8 (26.7 to 58.7)	31.2 (22.9 to 39.8)	5.2 (-1.4 to 12.1)
Cirrhosis and other chronic liver diseases due to hepatitis C	1,399.7 (1,156.0 to 1,668.3)	42.5 (33.0 to 53.5)	32.8 (21.1 to 47.6)	22.7 (14.8 to 30.6)	-1.6 (-7.9 to 4.8)
Cirrhosis and other chronic liver diseases due to alcohol use	1,539.6 (1,312.6 to 1,784.1)	46.0 (36.1 to 57.2)	35.5 (23.2 to 51.2)	18.7 (11.5 to 26.7)	-4.8 (-10.6 to 1.6)
Cirrhosis and other chronic liver diseases due to other causes	827.5 (615.1 to 1,084.9)	21.8 (15.2 to 28.9)	18.6 (11.1 to 28.7)	23.4 (13.3 to 34.2)	-1.0 (-9.1 to 7.6)
**Digestive diseases**	**25,322.5 (23,757.5 to 26,443.5)**	**18,168.5† (15,483.1 to 21,198.6)**	**716.0 (474.9 to 993.1)**	**42.0 (33.6 to 51.0)**	**13.8 (7.2 to 21.1)**
Peptic ulcer disease	8,167.9 (6,606.6 to 10,038.1)	783.4 (581.5 to 1,032.0)	173.2 (98.1 to 274.4)	60.5 (47.4 to 72.9)	28.7 (18.2 to 38.6)
Gastritis and duodenitis	11,499.5 (8,568.7 to 15,812.9)	7,057.3 (5,214.9 to 9,536.9)	196.8 (114.0 to 308.9)	65.3 (50.6 to 79.7)	32.5 (20.8 to 44.1)
Appendicitis	36.8 (29.2 to 45.0)	950.2 (767.7 to 1,150.1)	11.4 (7.2 to 16.3)	27.3 (16.5 to 39.7)	2.1 (-6.6 to 12.0)
Paralytic ileus and intestinal obstruction	11.4 (8.5 to 14.7)	309.1 (229.6 to 398.8)	3.7 (2.3 to 5.5)	29.7 (26.7 to 33.1)	4.0 (1.6 to 6.7)
Inguinal, femoral, and abdominal hernia	4,114.7 (2,958.1 to 5,465.8)	2,888.7 (1,962.3 to 3,987.2)	161.2 (96.4 to 248.0)	16.1 (11.8 to 20.7)	-6.9 (-10.3 to -3.2)
Inflammatory bowel disease	606.8 (515.5 to 728.2)	301.9 (240.6 to 365.6)	88.2 (59.2 to 124.8)	28.4 (21.5 to 35.9)	3.0 (-2.6 to 8.9)
Vascular intestinal disorders	4.5 (2.7 to 6.8)	26.3 (17.7 to 37.4)	1.5 (0.8 to 2.4)	20.3 (14.3 to 24.7)	-3.6 (-8.3 to -0.1)
Gallbladder and biliary diseases	1,834.8 (1,346.2 to 2,452.8)	5,378.4 (3,983.3 to 6,988.8)	25.2 (15.6 to 39.3)	29.9 (21.4 to 39.1)	4.2 (-2.7 to 11.5)
Pancreatitis	53.2 (39.9 to 68.4)	473.3 (356.6 to 607.0)	6.8 (4.2 to 10.4)	26.0 (14.1 to 37.6)	1.1 (-8.5 to 10.3)
Other digestive diseases	--	--	47.8 (31.7 to 66.3)	50.0 (41.2 to 59.6)	20.3 (13.2 to 28.0)
**Neurological disorders**	**201,856.8 (190,257.9 to 213,689.6)**	**75,659.6 (46,762.4 to 109,777.3)**	**5,321.1 (3,620.1 to 7,352.4)**	**21.6 (20.2 to 23.2)**	**-2.5 (-3.6 to -1.2)**
Alzheimer's disease and other dementias	312.0 (211.8 to 453.6)	84.0 (53.8 to 118.8)	40.5 (23.7 to 65.6)	19.2 (12.8 to 24.9)	-4.4 (-9.5 to 0.2)
Parkinson's disease	145.3 (97.1 to 205.0)	19.8 (10.4 to 31.9)	18.5 (10.8 to 29.9)	34.5 (22.9 to 45.8)	7.8 (-1.4 to 16.9)
Epilepsy	1,237.3 (1,021.2 to 1,511.5)	97.6 (67.0 to 132.2)	373.5 (257.4 to 521.5)	16.7 (4.8 to 30.5)	-6.4 (-16.0 to 4.6)
Multiple sclerosis	249.2 (227.9 to 275.5)	2.7 (2.0 to 3.6)	65.9 (46.1 to 86.2)	17.8 (11.6 to 24.0)	-5.5 (-10.5 to -0.5)
Motor neuron disease	21.6 (16.8 to 27.8)	3.5 (2.3 to 4.9)	4.6 (3.1 to 6.8)	23.1 (20.7 to 25.5)	-1.3 (-3.2 to 0.7)
Migraine	94,052.8 (87,250.3 to 100,531.5)	7,336.1 (6,086.6 to 8,602.0)	4,074.5 (2,629.3 to 5,714.4)	21.6 (20.6 to 22.7)	-2.5 (-3.3 to -1.6)
Tension-type headache	141,739.5 (105,146.3 to 186,954.2)	68,113.1 (39,919.0 to 102,537.4)	629.3 (389.9 to 955.5)	22.7 (20.9 to 24.8)	-1.6 (-3.1 to 0.0)
Other neurological disorders	1.5 (0.7 to 2.6)	3.0 (1.6 to 5.0)	114.3 (80.2 to 154.3)	33.9 (23.4 to 45.3)	7.4 (-1.1 to 16.5)
**Mental and substance use disorders**	**82,944.6 (81,387.9 to 84,381.6)**	**31,576.5† (28,163.3 to 35,167.1)**	**11,888.7 (8,696.1 to 15,401.3)**	**20.3 (19.4 to 21.3)**	**-3.5 (-4.2 to -2.7)**
Schizophrenia	2,452.6 (2,140.9 to 2,779.4)	63.5 (47.5 to 82.9)	1,569.5 (1,179.1 to 1,964.1)	27.8 (25.6 to 29.9)	2.4 (0.7 to 4.2)
Alcohol use disorders	8,925.2 (7,449.0 to 10,630.3)	4,180.7 (3,310.4 to 5,156.8)	885.6 (581.8 to 1,228.0)	10.3 (5.2 to 14.6)	-11.6 (-15.7 to -8.1)
Drug use disorders	3,182.7 (3,041.1 to 3,273.5)	304.0 (248.9 to 373.3)	776.9 (545.2 to 1,007.8)	21.8 (18.3 to 25.6)	-2.4 (-5.1 to 0.7)
Opioid use disorders	1,444.5 (1,199.5 to 1,743.8)	136.2 (93.0 to 192.2)	590.6 (401.8 to 783.6)	24.0 (19.5 to 28.7)	-0.5 (-4.2 to 3.2)
Cocaine use disorders	404.0 (354.1 to 461.0)	10.0 (6.2 to 14.8)	54.7 (33.9 to 78.2)	10.5 (5.3 to 16.2)	-11.4 (-15.6 to -6.8)
Amphetamine use disorders	121.5 (86.1 to 169.0)	13.8 (8.1 to 20.7)	16.1 (8.8 to 27.1)	14.5 (3.5 to 25.4)	-8.2 (-17.0 to 0.6)
Cannabis use disorders	922.4 (727.0 to 1,157.0)	92.4 (61.8 to 131.0)	26.6 (16.3 to 39.7)	18.5 (11.9 to 25.3)	-5.0 (-10.3 to 0.5)
Other drug use disorders	395.1 (331.6 to 466.9)	51.6 (40.1 to 64.1)	88.8 (59.0 to 121.0)	17.3 (12.9 to 21.6)	-6.0 (-9.5 to -2.5)
Depressive disorders	24,376.6 (23,160.5 to 25,500.2)	23,833.0 (20,550.1 to 27,293.1)	3,962.3 (2,694.5 to 5,470.0)	17.6 (16.0 to 19.3)	-5.7 (-7.0 to -4.3)
Major depressive disorder	14,612.9 (12,553.1 to 16,853.5)	22,261.8 (19,040.5 to 25,799.8)	2,973.3 (1,991.2 to 4,224.4)	15.2 (13.4 to 16.9)	-7.6 (-9.1 to -6.3)
Dysthymia	10,268.9 (8,139.7 to 12,738.0)	1,571.2 (1,298.4 to 1,899.8)	989.0 (625.8 to 1,466.6)	25.5 (21.9 to 28.9)	0.6 (-2.3 to 3.4)
Bipolar disorder	3,584.8 (2,928.0 to 4,284.3)	211.8 (141.8 to 298.8)	724.7 (443.3 to 1,087.5)	21.0 (18.9 to 23.1)	-3.0 (-4.7 to -1.3)
Anxiety disorders	22,242.9 (19,894.4 to 24,947.3)	2,912.2 (2,584.6 to 3,263.0)	2,120.6 (1,466.5 to 2,876.8)	21.6 (19.1 to 24.2)	-2.5 (-4.5 to -0.4)
Eating disorders	250.2 (205.7 to 319.5)	7.9 (5.3 to 11.1)	49.9 (30.1 to 78.2)	31.4 (24.3 to 38.4)	5.4 (-0.4 to 11.0)
Anorexia nervosa	41.2 (27.7 to 59.4)	7.9 (5.3 to 11.1)	8.8 (5.0 to 13.9)	27.0 (15.6 to 39.4)	1.8 (-7.3 to 11.8)
Bulimia nervosa	199.9 (126.0 to 298.6)	--	41.2 (22.7 to 66.6)	32.4 (24.4 to 40.9)	6.1 (-0.3 to 13.0)
Autistic spectrum disorders	3,588.8 (3,424.8 to 3,726.3)	--	519.8 (356.3 to 731.9)	24.5 (22.4 to 26.6)	-0.2 (-1.8 to 1.5)
Autism	1,089.7 (937.9 to 1,271.2)	--	272.8 (174.8 to 394.4)	23.6 (20.5 to 27.0)	-0.9 (-3.4 to 1.8)
Asperger syndrome and other autistic spectrum disorders	2,501.8 (2,100.2 to 2,987.9)	--	247.0 (161.3 to 358.8)	25.5 (23.4 to 27.5)	0.6 (-1.1 to 2.2)

Cause	Prevalence in thousands (95% UI)	Incidence in thousands (95% UI)	YLDs in thousands (95% UI)		
	2016 counts	2016 counts	2016 counts	Percentage change in counts between 2006 and 2016	Percentage change in rates between 2006 and 2016
Attention-deficit/hyperactivity disorder	2,366.2 (2,089.7 to 2,666.1)	--	28.0 (17.0 to 44.3)	28.3 (24.3 to 32.6)	2.9 (-0.3 to 6.4)
Conduct disorder	--	--	--	--	--
Idiopathic developmental intellectual disability	6,118.8 (3,627.6 to 8,621.6)	--	249.7 (121.2 to 424.1)	15.7 (11.6 to 18.6)	-7.2 (-10.5 to -4.9)
Other mental and substance use disorders	13,474.8 (11,873.5 to 15,419.8)	63.3 (38.8 to 90.6)	1,001.7 (684.9 to 1,430.2)	24.4 (22.8 to 26.1)	-0.2 (-1.5 to 1.1)
**Diabetes, urogenital, blood, and endocrine diseases**	**221,586.2 (220,090.7 to 222,964.0)**	**78,376.7† (71,297.3 to 85,776.9)**	**6,074.3 (4,234.3 to 8,397.4)**	**23.9 (21.5 to 26.1)**	**-0.7 (-2.5 to 1.2)**
Diabetes mellitus	39,423.8 (34,507.8 to 44,831.1)	2,567.7 (1,921.7 to 3,379.2)	2,969.2 (1,993.2 to 4,281.4)	21.5 (17.4 to 25.5)	-2.6 (-5.8 to 0.6)
Acute glomerulonephritis	7.5 (5.0 to 10.8)	109.8 (71.7 to 158.0)	0.4 (0.2 to 0.6)	11.0 (8.0 to 13.3)	-11.0 (-13.4 to -9.2)
Chronic kidney disease	16,046.4 (13,770.4 to 18,685.2)	1,175.0 (790.6 to 1,648.8)	666.6 (470.5 to 878.0)	28.6 (24.1 to 33.5)	3.1 (-0.5 to 7.1)
Chronic kidney disease due to diabetes mellitus	7,488.9 (5,694.8 to 9,546.4)	547.0 (345.9 to 786.5)	332.7 (226.5 to 458.5)	30.8 (25.1 to 36.9)	4.8 (0.3 to 9.8)
Chronic kidney disease due to hypertension	2,458.6 (1,810.7 to 3,180.3)	205.6 (117.5 to 329.3)	95.3 (61.0 to 138.4)	35.5 (28.1 to 44.7)	8.7 (2.8 to 16.1)
Chronic kidney disease due to glomerulonephritis	2,538.7 (1,901.1 to 3,236.6)	160.0 (100.0 to 238.0)	104.5 (60.2 to 164.8)	19.2 (12.4 to 25.7)	-4.4 (-9.9 to 0.8)
Chronic kidney disease due to other causes	3,560.2 (2,298.3 to 5,097.2)	262.6 (149.4 to 417.7)	134.1 (72.9 to 207.2)	26.5 (20.7 to 32.5)	1.5 (-3.2 to 6.3)
Urinary diseases and male infertility	8,628.5 (7,920.0 to 9,343.8)	31,639.2† (25,900.1 to 38,243.2)	296.9 (191.6 to 428.4)	20.0 (17.4 to 22.7)	-3.8 (-5.8 to -1.6)
Interstitial nephritis and urinary tract infections	434.4 (336.2 to 545.3)	22,576.9 (17,164.7 to 28,660.5)	14.2 (8.3 to 22.5)	24.1 (17.8 to 30.2)	-0.5 (-5.5 to 4.5)
Urolithiasis	292.6 (217.3 to 388.1)	7,748.0 (5,740.2 to 10,116.5)	21.7 (13.2 to 34.1)	21.8 (13.1 to 32.1)	-2.3 (-9.3 to 5.9)
Benign prostatic hyperplasia	7,246.5 (5,945.7 to 8,596.6)	1,314.4 (1,023.4 to 1,709.5)	244.9 (154.7 to 360.7)	20.2 (17.4 to 23.2)	-3.6 (-5.9 to -1.2)
Male infertility	545.9 (296.2 to 885.8)	--	2.8 (0.9 to 6.5)	23.0 (9.6 to 34.1)	-1.4 (-12.1 to 7.5)
Other urinary diseases	--	--	13.3 (8.7 to 19.0)	8.6 (3.4 to 14.2)	-12.9 (-17.1 to -8.4)
Gynecological diseases	108,340.4 (107,090.6 to 109,831.7)	41,563.1† (37,309.2 to 45,487.1)	1,565.9 (1,082.4 to 2,218.9)	24.2 (22.2 to 26.4)	-0.4 (-2.0 to 1.4)
Uterine fibroids	20,595.4 (17,632.4 to 23,936.5)	1,574.4 (1,096.5 to 2,147.4)	288.9 (169.3 to 468.8)	22.3 (19.8 to 24.4)	-1.9 (-3.9 to -0.2)
Polycystic ovarian syndrome	90.8 (67.3 to 120.0)	--	0.8 (0.3 to 1.5)	10.0 (0.6 to 19.0)	-11.8 (-19.3 to -4.6)
Female infertility	218.3 (127.6 to 369.0)	--	1.3 (0.5 to 2.8)	29.9 (16.5 to 42.4)	4.1 (-6.6 to 14.2)
Endometriosis	540.8 (459.5 to 625.1)	371.4 (220.2 to 582.6)	49.7 (32.4 to 71.6)	35.9 (28.6 to 43.6)	9.0 (3.2 to 15.1)
Genital prolapse	25,050.9 (21,555.3 to 28,790.2)	4,160.5 (3,400.3 to 5,102.1)	79.1 (38.5 to 144.5)	10.3 (8.7 to 11.8)	-11.6 (-12.9 to -10.3)
Premenstrual syndrome	71,237.2 (67,156.2 to 75,433.6)	19,978.1 (17,842.6 to 21,934.0)	585.2 (365.7 to 896.3)	23.5 (21.5 to 25.5)	-1.0 (-2.6 to 0.6)
Other gynecological diseases	16,171.6 (13,457.4 to 18,709.9)	15,478.8 (11,988.2 to 19,175.7)	560.9 (369.0 to 806.0)	27.4 (22.8 to 33.2)	2.1 (-1.6 to 6.8)
Hemoglobinopathies and hemolytic anaemias	104,233.5 (103,078.8 to 105,520.7)	--	335.6 (207.1 to 518.0)	40.7 (31.2 to 49.6)	12.8 (5.2 to 20.0)
Thalassemias	1.0 (0.9 to 1.0)	--	0.1 (0.1 to 0.2)	28.1 (-40.1 to 178.4)	2.8 (-52.0 to 123.2)
Thalassaemias trait	20,324.8 (19,589.6 to 21,103.4)	--	234.8 (143.9 to 363.6)	44.0 (31.8 to 56.1)	15.5 (5.7 to 25.2)
Sickle cell disorders	16.7 (14.9 to 18.4)	--	2.8 (2.0 to 3.8)	54.1 (42.6 to 66.3)	23.6 (14.4 to 33.4)
Sickle cell trait	22,436.0 (20,296.8 to 25,303.9)	--	64.5 (40.0 to 95.9)	32.8 (27.8 to 38.3)	6.5 (2.5 to 10.9)
G6PD deficiency	18,355.9 (17,698.9 to 19,108.7)	--	1.2 (0.8 to 1.8)	42.3 (36.6 to 48.8)	14.1 (9.5 to 19.3)
G6PD trait	50,996.0 (50,178.3 to 51,951.9)	--	< 0.05 (< 0.05 to 0.1)	31.6 (20.0 to 44.0)	5.5 (-3.7 to 15.4)
Other hemoglobinopathies and hemolytic anaemias	2,881.4 (2,495.2 to 3,280.2)	--	32.1 (19.4 to 49.8)	32.6 (25.5 to 40.8)	6.4 (0.6 to 12.9)
Endocrine, metabolic, blood, and immune disorders	7,715.8 (6,281.1 to 9,478.7)	1,321.8 (941.3 to 1,802.2)	239.6 (148.8 to 351.4)	22.5 (19.2 to 25.4)	-1.8 (-4.4 to 0.6)
**Musculoskeletal disorders**	**132,595.2 (126,981.8 to 137,155.9)**	**67,772.1 (53,446.5 to 84,397.0)**	**14,775.4 (10,321.5 to 20,117.5)**	**23.5 (21.5 to 25.4)**	**-1.0 (-2.6 to 0.6)**
Rheumatoid arthritis	1,993.7 (1,698.0 to 2,329.1)	142.4 (104.6 to 187.7)	476.6 (322.9 to 656.5)	27.0 (23.3 to 30.6)	1.9 (-1.2 to 4.7)
Osteoarthritis	27,001.9 (24,719.5 to 29,324.8)	2,302.4 (1,945.6 to 2,668.4)	1,569.9 (1,094.9 to 2,170.5)	29.0 (27.6 to 30.5)	3.5 (2.3 to 4.6)
Low back and neck pain	82,870.8 (76,545.2 to 89,245.7)	32,790.8 (24,248.8 to 43,353.5)	9,787.1 (6,529.4 to 13,881.4)	23.3 (20.4 to 25.9)	-1.1 (-3.5 to 1.0)
Low back pain	49,988.1 (37,469.0 to 66,821.8)	23,938.4 (16,054.8 to 34,291.6)	5,755.7 (3,677.7 to 8,544.8)	21.0 (16.9 to 24.5)	-3.0 (-6.2 to -0.1)
Neck pain	39,933.8 (28,234.9 to 52,711.9)	8,852.4 (5,980.7 to 12,592.2)	4,031.4 (2,390.7 to 6,125.7)	26.8 (22.7 to 30.5)	1.6 (-1.6 to 4.7)
Gout	3,158.3 (2,579.7 to 3,834.2)	575.5 (397.2 to 777.1)	103.1 (66.7 to 149.6)	24.3 (18.6 to 30.2)	-0.3 (-4.9 to 4.4)
Other musculoskeletal disorders	33,071.1 (23,080.1 to 44,721.0)	31,961.0 (20,907.4 to 46,166.7)	2,838.6 (1,709.0 to 4,376.3)	20.6 (14.7 to 24.9)	-3.3 (-8.0 to 0.1)
**Other non-communicable diseases**	**352,630.0 (348,375.5 to 357,765.4)**	**856,555.7† (722,512.1 to 987,611.5)**	**8,610.4 (5,745.6 to 12,698.3)**	**22.5 (21.6 to 23.4)**	**-1.8 (-2.5 to -1.1)**
Congenital anomalies	4,053.3 (3,775.6 to 4,338.7)	--	519.6 (372.1 to 692.7)	22.2 (19.2 to 25.6)	-2.0 (-4.4 to 0.7)
Neural tube defects	307.9 (279.8 to 340.2)	--	91.9 (65.2 to 122.4)	22.9 (17.6 to 28.5)	-1.5 (-5.7 to 3.0)
Congenital heart anomalies	582.9 (521.8 to 651.0)	--	32.2 (13.4 to 55.4)	31.4 (26.1 to 36.9)	5.4 (1.1 to 9.8)
Orofacial clefts	223.5 (199.5 to 247.2)	--	2.7 (1.8 to 3.9)	18.0 (14.9 to 21.1)	-5.4 (-7.8 to -2.9)
Down's syndrome	102.7 (88.7 to 118.0)	--	9.6 (6.5 to 13.4)	16.9 (11.5 to 21.8)	-6.3 (-10.6 to -2.3)
Turner syndrome	104.5 (91.1 to 120.0)	--	2.2 (1.1 to 3.5)	25.1 (12.1 to 39.2)	0.3 (-10.1 to 11.6)
Klinefelter syndrome	64.9 (54.3 to 76.5)	--	0.7 (0.3 to 1.2)	19.0 (10.5 to 27.7)	-4.5 (-11.4 to 2.4)

Cause	Prevalence in thousands (95% UI)	Incidence in thousands (95% UI)	YLDs in thousands (95% UI)		
	2016 counts	2016 counts	2016 counts	Percentage change in counts between 2006 and 2016	Percentage change in rates between 2006 and 2016
Other chromosomal abnormalities	0.1 (0.1 to 0.1)	--	< 0.05 (< 0.05 to < 0.05)	105.2 (-23.2 to 407.0)	64.5 (-38.4 to 306.6)
Congenital musculoskeletal and limb anomalies	654.2 (604.1 to 708.1)	--	95.5 (65.4 to 130.8)	16.8 (12.9 to 20.8)	-6.3 (-9.5 to -3.1)
Urogenital congenital anomalies	188.9 (170.6 to 208.5)	--	2.9 (1.4 to 5.2)	25.5 (21.7 to 29.6)	0.6 (-2.4 to 3.9)
Digestive congenital anomalies	668.5 (603.5 to 739.2)	--	28.4 (18.9 to 40.7)	26.7 (19.9 to 33.7)	1.6 (-3.8 to 7.2)
Other congenital anomalies	2,062.4 (1,445.3 to 2,888.1)	--	253.5 (170.6 to 365.0)	22.8 (17.5 to 28.8)	-1.6 (-5.8 to 3.2)
Skin and subcutaneous diseases	111,548.2 (108,614.6 to 114,312.4)	266,014.1 (237,025.0 to 302,240.8)	2,299.3 (1,551.6 to 3,355.2)	25.0 (24.0 to 25.9)	0.3 (-0.6 to 0.9)
Dermatitis	14,674.6 (12,471.9 to 17,141.0)	37,881.4 (31,074.0 to 46,200.5)	463.3 (273.1 to 717.2)	21.2 (19.5 to 22.9)	-2.8 (-4.2 to -1.4)
Psoriasis	5,099.2 (4,660.6 to 5,579.0)	582.1 (499.8 to 662.8)	441.9 (308.1 to 591.2)	27.5 (24.6 to 30.2)	2.2 (-0.1 to 4.4)
Cellulitis	164.0 (133.7 to 198.4)	3,381.0 (2,847.9 to 4,011.9)	9.3 (5.9 to 14.2)	13.6 (6.7 to 20.6)	-8.9 (-14.4 to -3.3)
Pyoderma	1,229.1 (1,130.8 to 1,339.5)	28,919.7 (26,027.2 to 32,096.2)	6.9 (2.7 to 14.6)	25.8 (22.6 to 28.8)	0.8 (-1.7 to 3.3)
Scabies	6,862.7 (4,752.3 to 9,677.4)	20,901.9 (14,332.8 to 29,858.2)	175.4 (84.8 to 315.3)	25.6 (23.4 to 27.8)	0.7 (-1.0 to 2.4)
Fungal skin diseases	30,098.4 (23,007.6 to 39,846.0)	102,304.9 (75,941.1 to 138,282.9)	169.0 (62.8 to 364.3)	26.4 (25.5 to 27.2)	1.4 (0.7 to 2.0)
Viral skin diseases	7,419.2 (6,263.5 to 8,786.8)	11,346.7 (9,606.6 to 13,382.9)	223.7 (137.2 to 341.0)	24.2 (22.5 to 25.8)	-0.4 (-1.8 to 0.9)
Acne vulgaris	12,704.1 (11,252.4 to 14,365.2)	5,435.0 (4,688.9 to 6,328.3)	319.2 (212.5 to 474.4)	24.4 (21.9 to 26.9)	-0.2 (-2.3 to 1.7)
Alopecia areata	1,106.9 (986.2 to 1,249.8)	1,934.3 (1,746.6 to 2,157.0)	36.0 (22.8 to 54.9)	21.6 (18.2 to 25.2)	-2.5 (-5.2 to 0.4)
Pruritus	5,027.8 (3,382.2 to 7,143.1)	3,882.4 (2,525.3 to 5,605.8)	53.6 (23.1 to 111.7)	27.1 (25.1 to 29.1)	1.9 (0.3 to 3.5)
Urticaria	2,960.5 (2,095.4 to 3,979.6)	5,221.9 (3,707.8 to 7,020.9)	174.3 (99.4 to 273.3)	24.5 (21.5 to 27.2)	-0.1 (-2.6 to 2.0)
Decubitus ulcer	71.3 (51.5 to 95.3)	258.3 (183.4 to 352.8)	12.6 (7.3 to 19.5)	19.0 (16.3 to 21.4)	-4.6 (-6.7 to -2.7)
Other skin and subcutaneous diseases	38,753.9 (34,940.2 to 42,693.9)	43,964.5 (39,420.2 to 48,712.1)	214.0 (100.5 to 388.9)	30.2 (29.2 to 31.2)	4.4 (3.6 to 5.2)
Sense organ diseases	157,821.6 (151,480.7 to 162,474.1)	38,146.1† (34,425.0 to 42,237.9)	4,200.3 (2,822.9 to 6,177.6)	21.2 (19.5 to 22.8)	-2.8 (-4.2 to -1.5)
Glaucoma	24.1 (15.9 to 34.4)	--	2.8 (1.5 to 4.6)	26.6 (19.6 to 33.2)	1.5 (-4.1 to 6.9)
Cataract	2,756.6 (2,051.5 to 3,583.6)	--	218.7 (143.1 to 321.9)	22.2 (19.3 to 25.2)	-2.0 (-4.4 to 0.4)
Macular degeneration	22.6 (13.8 to 32.8)	--	2.6 (1.4 to 4.4)	29.6 (26.8 to 32.2)	3.9 (1.6 to 6.0)
Refraction and accommodation disorders	69,627.4 (62,891.5 to 76,737.0)	--	1,146.5 (689.4 to 1,856.6)	16.2 (14.6 to 17.6)	-6.8 (-8.1 to -5.7)
Age-related and other hearing loss	97,763.0 (81,418.0 to 115,473.4)	--	2,189.5 (1,425.0 to 3,291.7)	22.4 (18.9 to 25.3)	-1.9 (-4.7 to 0.5)
Other vision loss	2,109.4 (1,633.3 to 2,670.7)	--	177.9 (116.6 to 266.5)	27.2 (23.6 to 30.7)	2.0 (-0.9 to 4.8)
Other sense organ diseases	14,421.7 (12,687.5 to 16,448.5)	38,146.1 (34,425.0 to 42,237.9)	462.5 (283.8 to 719.9)	26.2 (24.3 to 28.0)	1.2 (-0.3 to 2.7)
Oral disorders	244,012.3 (234,962.3 to 253,352.0)	552,395.5† (423,638.1 to 677,574.4)	1,591.2 (888.5 to 2,640.7)	22.4 (21.5 to 23.2)	-1.8 (-2.6 to -1.2)
Caries of deciduous teeth	--	--	--	--	--
Caries of permanent teeth	171,650.5 (140,549.5 to 204,938.7)	541,019.7 (412,232.2 to 667,545.3)	121.0 (50.1 to 239.7)	17.7 (16.0 to 19.4)	-5.6 (-7.0 to -4.2)
Periodontal disease	95,957.5 (73,958.7 to 119,283.0)	9,799.3 (5,469.9 to 14,241.4)	632.5 (239.1 to 1,340.4)	24.9 (24.0 to 25.8)	0.2 (-0.6 to 0.9)
Edentulism and severe tooth loss	17,966.9 (14,240.9 to 21,692.4)	1,576.5 (1,025.0 to 2,304.5)	510.5 (317.8 to 757.1)	19.1 (17.9 to 20.4)	-4.5 (-5.5 to -3.4)
Other oral disorders	11,218.0 (10,003.7 to 12,395.0)	--	327.2 (202.6 to 491.6)	24.9 (23.6 to 26.3)	0.2 (-0.9 to 1.3)
**Injuries**	**126,271.1 (118,828.6 to 134,333.1)**	**35,634.5† (31,457.1 to 40,420.7)**	**4,579.2 (3,073.8 to 6,536.9)**	**23.2 (21.8 to 24.5)**	**-1.2 (-2.3 to -0.1)**
**Transport injuries**	**23,527.3 (21,684.8 to 25,340.5)**	**5,606.1 (4,256.4 to 7,261.8)**	**1,223.6 (827.6 to 1,747.6)**	**29.6 (28.2 to 31.0)**	**3.9 (2.8 to 5.0)**
Road injuries	19,490.0 (17,945.1 to 21,016.8)	5,135.6 (3,795.2 to 6,748.2)	1,022.9 (693.1 to 1,460.2)	30.8 (29.4 to 32.1)	4.9 (3.8 to 5.9)
Pedestrian road injuries	4,236.8 (3,564.1 to 4,954.4)	918.2 (570.6 to 1,367.8)	226.9 (152.1 to 321.5)	33.8 (32.1 to 35.5)	7.3 (5.9 to 8.6)
Cyclist road injuries	3,719.3 (3,093.4 to 4,447.1)	1,191.4 (774.7 to 1,777.7)	182.8 (121.9 to 263.4)	36.6 (35.2 to 38.0)	9.5 (8.4 to 10.7)
Motorcyclist road injuries	5,021.7 (4,237.4 to 6,262.0)	822.1 (520.7 to 1,248.6)	259.0 (173.6 to 373.7)	32.7 (31.2 to 34.1)	6.4 (5.2 to 7.6)
Motor vehicle road injuries	5,065.4 (4,221.4 to 5,895.0)	1,696.6 (1,147.6 to 2,368.7)	284.0 (192.4 to 400.7)	18.5 (16.9 to 20.0)	-5.0 (-6.2 to -3.7)
Other road injuries	1,446.8 (1,169.6 to 1,761.0)	507.3 (251.5 to 817.6)	70.2 (46.6 to 104.1)	59.2 (57.2 to 61.1)	27.7 (26.1 to 29.2)
Other transport injuries	4,037.3 (3,315.1 to 4,874.2)	470.5 (318.5 to 649.2)	200.7 (136.3 to 284.2)	24.1 (21.9 to 26.1)	-0.5 (-2.2 to 1.1)
**Unintentional injuries**	**90,192.9 (83,075.9 to 97,402.9)**	**27,668.0 (23,822.6 to 32,134.8)**	**2,874.1 (1,962.1 to 4,055.2)**	**21.6 (20.3 to 22.8)**	**-2.5 (-3.5 to -1.5)**
Falls	35,144.5 (30,596.4 to 40,046.1)	11,388.5 (8,420.3 to 15,030.6)	1,362.9 (935.2 to 1,887.3)	20.7 (19.4 to 21.9)	-3.2 (-4.3 to -2.2)
Drowning	385.2 (332.8 to 445.2)	32.5 (24.8 to 41.8)	18.9 (12.5 to 26.8)	11.9 (9.4 to 14.3)	-10.2 (-12.3 to -8.3)
Fire, heat, and hot substances	8,787.5 (7,510.5 to 10,506.1)	500.9 (328.6 to 723.1)	210.5 (138.4 to 316.3)	20.4 (18.3 to 22.2)	-3.4 (-5.1 to -2.0)
Poisonings	286.8 (241.9 to 329.5)	237.0 (151.1 to 365.9)	22.0 (14.2 to 31.7)	29.6 (26.2 to 32.9)	3.9 (1.2 to 6.6)
Exposure to mechanical forces	18,185.7 (15,199.2 to 22,227.2)	4,294.6 (2,909.1 to 5,960.7)	407.6 (265.1 to 594.3)	25.7 (24.5 to 26.8)	0.8 (-0.2 to 1.7)
Unintentional firearm injuries	685.2 (553.0 to 852.4)	75.7 (37.1 to 136.6)	22.2 (14.3 to 33.2)	27.3 (26.0 to 28.8)	2.1 (1.1 to 3.2)
Unintentional suffocation	727.6 (563.3 to 933.1)	48.0 (22.6 to 89.8)	21.4 (13.4 to 32.3)	22.6 (20.9 to 24.2)	-1.7 (-3.0 to -0.4)
Other exposure to mechanical forces	16,772.9 (13,841.2 to 20,691.0)	4,171.0 (2,846.0 to 5,756.8)	363.9 (236.4 to 528.5)	25.8 (24.5 to 26.9)	0.9 (-0.1 to 1.8)

Cause	Prevalence in thousands (95% UI)	Incidence in thousands (95% UI)	YLDs in thousands (95% UI)		
	2016 counts	2016 counts	2016 counts	Percentage change in counts between 2006 and 2016	Percentage change in rates between 2006 and 2016
Adverse effects of medical treatment	137.9 (88.9 to 203.4)	1,801.8 (1,234.0 to 2,583.5)	18.3 (10.4 to 30.0)	17.6 (13.6 to 22.1)	-5.7 (-8.9 to -2.0)
Animal contact	4,451.6 (3,877.6 to 5,138.7)	3,605.5 (2,531.1 to 4,930.2)	121.7 (81.8 to 170.6)	13.1 (11.0 to 15.2)	-9.3 (-11.0 to -7.6)
Venomous animal contact	2,054.8 (1,688.6 to 2,440.2)	1,731.3 (1,139.0 to 2,415.8)	84.1 (57.3 to 116.5)	15.0 (12.2 to 17.7)	-7.8 (-10.0 to -5.6)
Non-venomous animal contact	2,396.8 (1,970.5 to 2,988.6)	1,874.2 (1,240.0 to 2,692.5)	37.6 (24.1 to 58.2)	9.1 (6.8 to 11.0)	-12.5 (-14.4 to -11.0)
Foreign body	3,861.0 (3,215.0 to 4,466.2)	1,626.8 (1,125.5 to 2,380.2)	137.0 (87.4 to 203.3)	22.1 (20.1 to 24.2)	-2.1 (-3.7 to -0.4)
Pulmonary aspiration and foreign body in airway	1,790.5 (1,368.7 to 2,306.4)	303.5 (194.7 to 441.8)	71.4 (45.8 to 105.5)	19.4 (17.3 to 21.4)	-4.3 (-6.0 to -2.7)
Foreign body in eyes	222.7 (131.6 to 367.1)	965.5 (521.3 to 1,750.2)	13.3 (7.2 to 24.0)	31.5 (27.1 to 37.3)	5.4 (1.9 to 10.1)
Foreign body in other body part	1,847.8 (1,503.2 to 2,334.3)	357.9 (226.6 to 528.6)	52.3 (33.9 to 77.2)	23.8 (21.8 to 25.4)	-0.8 (-2.3 to 0.6)
Environmental heat and cold exposure	6,066.2 (5,051.9 to 7,077.9)	977.7 (622.1 to 1,450.6)	219.8 (146.7 to 311.6)	24.5 (22.2 to 26.8)	-0.1 (-2.0 to 1.7)
Other unintentional injuries	12,886.4 (10,394.5 to 15,810.9)	3,202.7 (2,140.3 to 4,625.8)	355.4 (234.6 to 522.5)	22.9 (21.5 to 24.2)	-1.4 (-2.6 to -0.4)
**Self-harm and interpersonal violence**	**17,258.4 (13,637.3 to 21,608.5)**	**2,288.5† (1,710.5 to 2,919.4)**	**332.0 (222.5 to 471.5)**	**13.3 (12.2 to 14.4)**	**-9.1 (-10.1 to -8.3)**
Self-harm	1,083.2 (894.6 to 1,302.2)	531.6 (403.5 to 694.8)	48.1 (31.1 to 69.9)	7.3 (5.8 to 8.7)	-14.0 (-15.2 to -12.8)
Self-harm by firearm	32.6 (27.5 to 37.9)	5.2 (1.4 to 14.5)	1.1 (0.7 to 1.7)	2.4 (0.1 to 4.5)	-17.9 (-19.8 to -16.2)
Self-harm by other specified means	1,050.6 (861.4 to 1,269.5)	526.5 (400.5 to 690.5)	47.0 (30.3 to 68.3)	7.4 (5.9 to 8.8)	-13.9 (-15.1 to -12.7)
Interpersonal violence	16,175.2 (12,609.9 to 20,547.6)	1,756.9† (1,211.5 to 2,360.6)	283.8 (190.4 to 404.1)	14.4 (13.3 to 15.4)	-8.2 (-9.2 to -7.4)
Assault by firearm	282.9 (226.9 to 340.8)	34.8 (18.1 to 58.9)	10.2 (6.6 to 15.2)	11.0 (9.7 to 12.7)	-11.0 (-12.1 to -9.7)
Assault by sharp object	1,540.9 (1,239.9 to 1,847.2)	310.5 (178.6 to 500.3)	41.3 (26.9 to 60.1)	11.2 (9.4 to 12.9)	-10.9 (-12.3 to -9.5)
Sexual violence	10,264.3 (6,861.2 to 14,649.8)	--	77.5 (42.1 to 124.3)	18.3 (17.0 to 19.4)	-5.2 (-6.2 to -4.2)
Assault by other means	4,087.2 (3,345.4 to 5,015.4)	1,411.5 (969.5 to 1,903.2)	154.7 (103.9 to 224.7)	13.7 (12.5 to 14.8)	-8.9 (-9.8 to -7.9)
**Forces of nature, conflict and terrorism, and executions and police conflict**	**5,556.8 (3,555.7 to 7,953.5)**	**71.8† (42.9 to 99.8)**	**149.5 (51.5 to 314.1)**	**28.1 (16.4 to 37.8)**	**2.8 (-6.7 to 10.5)**
Forces of nature, conflict and terrorism, and state actor violence	671.4 (373.2 to 1,043.6)	22.1 (11.6 to 34.8)	22.7 (6.1 to 55.1)	67.4 (51.8 to 74.5)	34.3 (21.7 to 39.9)
Conflict and terrorism	3,933.4 (2,304.5 to 6,082.1)	--	113.0 (44.2 to 221.0)	28.8 (13.8 to 41.6)	3.3 (-8.7 to 13.6)
Executions and police conflict	952.1 (255.3 to 1,488.9)	49.7 (24.8 to 74.2)	20.4 (3.8 to 53.3)	0.6 (-4.1 to 9.2)	-19.4 (-23.1 to -12.4)

We did not calculate incidence for certain NTDs and causes with only birth prevalence. Blank cells mean that no estimate is available.

† Incidence for aggregate causes represents the sum of the incidence of the causes below the aggregate.

‡ Incidence estimates for stroke represent first-ever stroke only.

^ Incidence of HIV/AIDS represents new infections of HIV only and does not include new infections of Tuberculosis in HIV positive cases.

SR Table 12: Global prevalence, incidence, and YLDs for 2016, percentage change of YLD counts and percentage change of YLD rates between 2006 and 2016 by cause, age 50-54 years.					
Cause	Prevalence in thousands (95% UI)	Incidence in thousands (95% UI)	YLDs in thousands (95% UI)		
	2016 counts	2016 counts	2016 counts	Percentage change in counts between 2006 and 2016	Percentage change in rates between 2006 and 2016
**All causes**	**407,104.3 (406,839.4 to 407,421.2)**	**2,156,823.5† (1,855,168.0 to 2,497,606.5)**	**59,915.4 (44,199.3 to 76,981.2)**	**23.1 (22.3 to 23.7)**	**-1.2 (-1.8 to -0.7)**
**Communicable, maternal, neonatal, and nutritional disorders**	**282,864.3 (279,484.5 to 286,443.8)**	**1,098,692.8† (825,715.9 to 1,416,292.2)**	**3,950.8 (2,888.0 to 5,284.1)**	**12.1 (7.8 to 16.1)**	**-10.0 (-13.5 to -6.8)**
**HIV/AIDS and tuberculosis**	**148,388.9 (130,633.1 to 171,974.9)**	**617.2† (460.9 to 827.0)**	**495.2 (326.6 to 705.0)**	**5.0 (-13.8 to 27.2)**	**-15.7 (-30.8 to 2.1)**
Tuberculosis	139,034.5 (110,912.6 to 171,289.4)	568.1† (412.0 to 780.5)	227.4 (135.2 to 361.9)	7.5 (3.2 to 12.4)	-13.7 (-17.2 to -9.8)
Drug-susceptible tuberculosis	726.0 (500.1 to 1,051.8)	546.8 (395.6 to 752.4)	219.0 (129.7 to 348.4)	7.7 (3.4 to 12.7)	-13.5 (-17.0 to -9.5)
Multidrug-resistant tuberculosis without extensive drug resistance	25.3 (17.8 to 35.4)	19.8 (14.9 to 26.4)	7.9 (4.8 to 12.4)	-1.1 (-15.3 to 17.6)	-20.6 (-32.0 to -5.6)
Extensively drug-resistant tuberculosis	1.6 (1.1 to 2.2)	1.5 (1.1 to 1.9)	0.5 (0.3 to 0.8)	178.6 (142.1 to 219.2)	123.7 (94.4 to 156.3)
Latent tuberculosis infection	138,281.7 (110,206.7 to 170,372.1)	--	--	--	--
HIV/AIDS	2,533.1 (2,183.4 to 2,973.0)	49.1^ (39.0 to 61.5)	267.8 (167.6 to 405.2)	2.9 (-24.7 to 49.9)	-17.4 (-39.5 to 20.4)
Drug-susceptible HIV/AIDS - Tuberculosis	56.6 (39.6 to 79.0)	71.6 (51.5 to 97.0)	21.4 (12.6 to 33.2)	7.5 (-1.3 to 16.5)	-13.7 (-20.7 to -6.4)
Multidrug-resistant HIV/AIDS - Tuberculosis without extensive drug resistance	1.6 (1.1 to 2.4)	2.0 (1.3 to 2.9)	0.7 (0.4 to 1.1)	-0.2 (-16.9 to 16.9)	-19.9 (-33.2 to -6.1)
Extensively drug-resistant HIV/AIDS - Tuberculosis	0.1 (< 0.05 to 0.1)	0.1 (< 0.05 to 0.1)	< 0.05 (< 0.05 to 0.1)	250.8 (200.0 to 314.6)	181.7 (140.9 to 232.9)
HIV/AIDS resulting in other diseases	2,474.8 (2,122.2 to 2,911.1)	49.1 (39.0 to 61.5)	245.7 (151.1 to 376.7)	2.5 (-26.4 to 57.1)	-17.7 (-40.9 to 26.2)
**Diarrhoea, lower respiratory infections, and other common infectious diseases**	**20,054.5 (18,691.3 to 21,070.7)**	**1,042,243.6† (769,238.8 to 1,361,255.9)**	**947.6 (658.6 to 1,318.4)**	**20.3 (18.7 to 21.9)**	**-3.4 (-4.7 to -2.1)**
Diarrhoeal diseases	2,915.5 (2,617.4 to 3,234.4)	200,371.9 (179,023.2 to 222,792.1)	319.4 (220.1 to 436.9)	19.2 (16.9 to 21.8)	-4.3 (-6.2 to -2.2)
Intestinal infectious diseases	15.9 (12.6 to 19.7)	396.0† (321.0 to 478.4)	2.8 (1.8 to 4.2)	1.6 (-15.7 to 21.1)	-18.4 (-32.3 to -2.8)
Typhoid fever	15.5 (12.0 to 19.5)	245.6 (196.6 to 297.8)	2.4 (1.5 to 3.6)	2.0 (-17.8 to 25.1)	-18.1 (-34.0 to 0.4)
Paratyphoid fever	6.6 (5.2 to 8.2)	150.4 (119.9 to 183.6)	0.4 (0.3 to 0.6)	0.1 (-15.0 to 19.2)	-19.7 (-31.8 to -4.3)
Other intestinal infectious diseases	--	--	< 0.05 (< 0.05 to < 0.05)	-18.4 (-32.3 to -2.7)	-34.5 (-45.6 to -21.9)
Lower respiratory infections	356.9 (326.5 to 392.6)	15,560.1 (13,869.1 to 17,543.2)	21.4 (14.1 to 30.2)	41.4 (33.7 to 48.8)	13.5 (7.4 to 19.5)
Upper respiratory infections	10,849.5 (7,300.7 to 14,801.1)	815,350.2 (542,287.8 to 1,132,202.1)	266.2 (137.1 to 447.1)	22.9 (21.2 to 24.9)	-1.3 (-2.7 to 0.3)
Otitis media	4,438.2 (3,153.8 to 5,916.8)	5,858.9 (4,835.5 to 6,962.4)	122.4 (70.9 to 191.2)	12.8 (7.7 to 18.0)	-9.5 (-13.5 to -5.2)
Meningitis	718.4 (608.2 to 830.2)	51.8 (38.2 to 68.3)	68.1 (47.9 to 90.3)	23.5 (19.9 to 27.3)	-0.8 (-3.8 to 2.2)
Pneumococcal meningitis	327.6 (271.3 to 395.2)	12.0 (7.5 to 18.1)	30.6 (21.2 to 42.2)	21.5 (15.3 to 28.4)	-2.5 (-7.4 to 3.1)
H influenzae type B meningitis	83.4 (64.1 to 106.8)	3.5 (1.9 to 5.7)	9.7 (6.4 to 13.5)	0.7 (-6.5 to 6.6)	-19.2 (-24.9 to -14.4)
Meningococcal infection	77.5 (56.7 to 101.3)	9.0 (5.2 to 13.4)	7.5 (5.1 to 10.4)	25.5 (22.6 to 28.5)	0.8 (-1.6 to 3.1)
Other meningitis	230.0 (172.3 to 291.3)	27.4 (19.5 to 37.0)	20.3 (13.7 to 27.7)	41.5 (36.6 to 46.7)	13.6 (9.7 to 17.8)
Encephalitis	1,118.4 (737.0 to 1,639.0)	327.4 (279.8 to 381.4)	120.9 (84.3 to 159.3)	19.9 (14.0 to 26.3)	-3.7 (-8.4 to 1.4)
Diphtheria	< 0.05 (< 0.05 to < 0.05)	0.2 (0.2 to 0.3)	< 0.05 (< 0.05 to < 0.05)	-31.4 (-46.0 to -12.3)	-44.9 (-56.7 to -29.6)
Whooping cough	1.4 (1.1 to 1.8)	10.4 (8.1 to 13.1)	0.1 (< 0.05 to 0.1)	-10.1 (-12.0 to -8.2)	-27.8 (-29.3 to -26.3)
Tetanus	7.0 (6.8 to 7.1)	4.6 (2.3 to 7.0)	0.2 (0.1 to 0.3)	-1.4 (-22.8 to 24.1)	-20.8 (-38.1 to -0.4)
Measles	0.1 (< 0.05 to 0.2)	3.7 (1.3 to 7.9)	< 0.05 (< 0.05 to < 0.05)	-53.0 (-58.4 to -47.0)	-62.2 (-66.6 to -57.4)
Varicella and herpes zoster	505.4 (420.9 to 602.0)	4,308.2 (3,506.8 to 5,233.4)	26.2 (15.8 to 41.5)	28.5 (22.6 to 34.3)	3.2 (-1.6 to 7.8)
**Neglected tropical diseases and malaria**	**92,793.8 (89,392.4 to 95,629.5)**	**13,428.7† (10,951.1 to 16,100.9)**	**660.4 (446.5 to 930.3)**	**-5.4 (-13.2 to 2.1)**	**-24.1 (-30.3 to -18.0)**
Malaria	2,509.1 (2,204.5 to 2,813.7)	3,182.1 (2,344.8 to 4,109.8)	28.6 (21.4 to 36.9)	-4.8 (-11.8 to 3.2)	-23.6 (-29.2 to -17.1)
Chagas disease	612.2 (516.7 to 725.2)	7.0 (4.7 to 10.2)	5.6 (3.6 to 8.2)	12.4 (5.2 to 20.1)	-9.7 (-15.6 to -3.6)
Leishmaniasis	299.0 (227.8 to 369.9)	32.1 (24.4 to 42.0)	16.0 (9.7 to 25.2)	22.9 (14.5 to 35.7)	-1.3 (-8.0 to 9.0)
Visceral leishmaniasis	1.4 (1.0 to 1.9)	5.5 (3.9 to 7.7)	0.1 (0.1 to 0.2)	-56.0 (-71.1 to -32.9)	-64.6 (-76.8 to -46.1)
Cutaneous and mucocutaneous leishmaniasis	256.8 (188.4 to 345.8)	26.6 (19.0 to 36.0)	15.9 (9.6 to 25.1)	24.3 (15.6 to 37.9)	-0.2 (-7.2 to 10.7)
African trypanosomiasis	0.3 (0.1 to 1.1)	0.2 (< 0.05 to 0.7)	0.1 (< 0.05 to 0.3)	-71.7 (-95.6 to 64.2)	-77.3 (-96.5 to 31.8)
Schistosomiasis	9,947.7 (9,321.4 to 10,620.2)	2,504.4 (2,309.7 to 2,764.0)	73.7 (36.6 to 139.5)	-25.5 (-28.2 to -22.0)	-40.2 (-42.3 to -37.4)
Cysticercosis	258.6 (176.5 to 355.3)	--	39.8 (24.0 to 61.6)	-3.0 (-11.5 to 4.5)	-22.1 (-28.9 to -16.1)
Cystic echinococcosis	59.4 (31.1 to 113.6)	11.9 (5.5 to 28.4)	5.4 (2.5 to 11.0)	-38.1 (-75.6 to 46.3)	-50.3 (-80.4 to 17.5)
Lymphatic filariasis	1,783.2 (1,405.1 to 2,328.7)	348.8 (195.3 to 559.1)	99.7 (50.3 to 175.2)	-32.4 (-48.2 to -19.2)	-45.7 (-58.4 to -35.1)
Onchocerciasis	643.6 (429.3 to 1,037.8)	140.7 (56.4 to 279.7)	50.1 (24.4 to 89.9)	-20.0 (-38.6 to -0.1)	-35.8 (-50.7 to -19.8)
Trachoma	111.0 (47.6 to 227.0)	--	10.9 (5.6 to 19.6)	-0.4 (-13.1 to 12.7)	-20.0 (-30.2 to -9.5)
Dengue	261.7 (142.8 to 442.8)	4,374.8 (2,664.7 to 6,629.3)	41.4 (18.5 to 77.5)	100.4 (61.9 to 355.9)	60.9 (30.0 to 266.1)
Yellow fever	0.1 (< 0.05 to 0.2)	2.6 (0.7 to 6.9)	< 0.05 (< 0.05 to < 0.05)	-19.8 (-48.6 to 24.5)	-35.6 (-58.7 to -0.1)
Rabies	< 0.05 (< 0.05 to < 0.05)	0.7 (0.4 to 1.1)	< 0.05 (< 0.05 to < 0.05)	-49.9 (-56.7 to -41.0)	-59.7 (-65.2 to -52.6)
Intestinal nematode infections	78,149.3 (74,126.1 to 81,248.3)	--	131.0 (74.7 to 217.5)	-2.7 (-10.1 to 5.5)	-21.9 (-27.8 to -15.2)
Ascariasis	41,874.5 (37,309.8 to 47,389.8)	--	42.4 (23.2 to 72.0)	-16.2 (-27.6 to -4.4)	-32.7 (-41.9 to -23.2)

Cause	Prevalence in thousands (95% UI)	Incidence in thousands (95% UI)	YLDs in thousands (95% UI)		
	2016 counts	2016 counts	2016 counts	Percentage change in counts between 2006 and 2016	Percentage change in rates between 2006 and 2016
Trichuriasis	21,693.5 (19,894.7 to 23,843.6)	--	15.7 (8.6 to 26.7)	-7.7 (-21.3 to 7.7)	-25.9 (-36.8 to -13.5)
Hookworm disease	22,921.9 (21,185.5 to 25,107.9)	--	73.0 (41.4 to 119.5)	8.8 (-3.1 to 21.9)	-12.7 (-22.2 to -2.1)
Food-borne trematodiases	6,151.7 (5,650.6 to 6,698.7)	2,335.2 (1,034.2 to 3,322.6)	142.8 (65.0 to 269.4)	27.7 (21.5 to 34.4)	2.5 (-2.5 to 7.9)
Leprosy	53.5 (50.6 to 56.4)	5.0 (4.6 to 5.4)	3.3 (2.2 to 4.6)	1.4 (-6.3 to 10.3)	-18.5 (-24.8 to -11.4)
Ebola virus disease	< 0.05 (< 0.05 to 0.2)	--	< 0.05 (0.0 to < 0.05)	--	--
Zika virus disease	8.0 (4.5 to 15.1)	483.1 (273.7 to 915.4)	0.1 (0.1 to 0.3)	--	--
Guinea worm disease	< 0.05 (< 0.05 to < 0.05)	< 0.05 (< 0.05 to < 0.05)	< 0.05 (< 0.05 to < 0.05)	-99.5 (-99.7 to -99.2)	-99.6 (-99.8 to -99.4)
Other neglected tropical diseases	1,054.7 (927.0 to 1,197.7)	--	11.8 (7.2 to 19.3)	18.8 (10.6 to 26.8)	-4.6 (-11.2 to 1.8)
**Maternal disorders**	**158.6 (149.9 to 167.2)**	**43.5† (37.1 to 50.6)**	**14.2 (9.6 to 19.6)**	**-18.4 (-27.9 to -8.1)**	**-34.5 (-42.1 to -26.2)**
Maternal haemorrhage	0.6 (0.4 to 0.7)	4.4 (3.2 to 5.9)	< 0.05 (< 0.05 to 0.1)	10.6 (-8.4 to 32.2)	-11.2 (-26.5 to 6.1)
Maternal sepsis and other pregnancy related infections	119.1 (106.2 to 133.8)	5.9 (4.4 to 7.8)	0.6 (0.2 to 1.3)	16.6 (7.8 to 25.1)	-6.4 (-13.5 to 0.5)
Maternal hypertensive disorders	1.3 (0.8 to 1.8)	7.0 (5.8 to 8.2)	0.1 (< 0.05 to 0.1)	-2.0 (-35.3 to 49.8)	-21.3 (-48.0 to 20.2)
Maternal obstructed labour and uterine rupture	34.4 (28.9 to 41.1)	2.2 (1.5 to 3.0)	10.8 (6.9 to 15.3)	-19.4 (-28.6 to -8.7)	-35.3 (-42.7 to -26.7)
Maternal abortion, miscarriage, and ectopic pregnancy	0.2 (0.1 to 0.3)	24.0 (18.3 to 30.4)	< 0.05 (< 0.05 to < 0.05)	-7.4 (-38.6 to 37.6)	-25.7 (-50.7 to 10.5)
Other maternal disorders	--	--	2.7 (1.8 to 3.7)	-20.9 (-30.1 to -10.9)	-36.5 (-43.9 to -28.4)
**Neonatal disorders**	**3,262.4 (2,985.1 to 3,568.1)**	**--**	**530.7 (376.5 to 735.1)**	**42.0 (27.3 to 60.0)**	**14.0 (2.2 to 28.4)**
Neonatal preterm birth complications	2,780.1 (2,370.5 to 3,263.8)	--	334.6 (233.4 to 471.8)	41.0 (17.2 to 68.8)	13.2 (-5.9 to 35.5)
Neonatal encephalopathy due to birth asphyxia and trauma	534.7 (357.1 to 788.9)	--	75.9 (34.1 to 152.3)	43.5 (33.3 to 64.5)	15.2 (7.0 to 32.0)
Neonatal sepsis and other neonatal infections	243.1 (103.4 to 561.2)	--	92.0 (32.6 to 236.2)	45.5 (34.5 to 63.0)	16.8 (8.0 to 30.9)
Hemolytic disease and other neonatal jaundice	86.4 (77.1 to 96.5)	--	28.2 (21.8 to 35.8)	39.1 (32.9 to 45.1)	11.7 (6.7 to 16.5)
Other neonatal disorders	--	--	--	--	--
**Nutritional deficiencies**	**62,449.6 (59,973.8 to 64,575.2)**	**15,398.2† (13,185.5 to 18,112.3)**	**1,148.9 (756.6 to 1,704.1)**	**10.0 (6.5 to 13.4)**	**-11.7 (-14.5 to -8.9)**
Protein-energy malnutrition	--	--	--	--	--
Iodine deficiency	7,713.5 (6,913.4 to 8,580.4)	28.1 (24.1 to 33.0)	199.0 (135.4 to 276.9)	5.7 (3.3 to 8.3)	-15.1 (-17.1 to -13.1)
Vitamin A deficiency	14,321.2 (12,654.1 to 16,363.5)	15,370.1 (13,155.8 to 18,082.9)	8.2 (5.2 to 12.5)	28.2 (19.0 to 38.0)	3.0 (-4.4 to 10.8)
Iron-deficiency anaemia	46,463.0 (40,610.9 to 53,182.1)	--	941.7 (596.9 to 1,439.1)	10.8 (6.7 to 14.8)	-11.0 (-14.3 to -7.9)
Other nutritional deficiencies	--	--	--	--	--
**Other communicable, maternal, neonatal, and nutritional diseases**	**125,060.4 (120,812.3 to 129,577.6)**	**26,961.6 (22,445.1 to 32,733.5)**	**150.2 (100.9 to 222.2)**	**20.0 (11.6 to 28.6)**	**-3.6 (-10.4 to 3.2)**
Sexually transmitted diseases excluding HIV	92,001.2 (87,045.5 to 96,950.4)	20,835.4 (17,141.2 to 25,510.9)	112.5 (74.8 to 168.4)	24.4 (19.9 to 29.1)	-0.2 (-3.7 to 3.7)
Syphilis	3,503.2 (2,207.8 to 5,406.2)	2,364.3 (1,458.5 to 3,833.2)	20.0 (13.6 to 27.1)	30.7 (25.6 to 36.2)	4.9 (0.8 to 9.3)
Chlamydial infection	603.8 (414.1 to 857.4)	1,218.5 (888.9 to 1,597.3)	10.9 (6.8 to 16.3)	17.5 (9.7 to 26.2)	-5.6 (-11.9 to 1.3)
Gonococcal infection	1,539.7 (940.4 to 2,439.9)	5,078.7 (3,250.2 to 7,587.2)	18.2 (9.8 to 32.0)	37.4 (25.6 to 47.8)	10.3 (0.8 to 18.7)
Trichomoniasis	11,703.6 (8,357.8 to 15,981.3)	9,252.0 (6,368.5 to 13,004.5)	12.8 (4.8 to 28.4)	25.2 (23.0 to 27.4)	0.5 (-1.2 to 2.3)
Genital herpes	79,050.6 (69,204.4 to 91,411.6)	117.9 (65.8 to 192.9)	19.1 (5.6 to 43.6)	23.6 (21.2 to 26.2)	-0.8 (-2.7 to 1.3)
Other sexually transmitted diseases	191.2 (136.1 to 262.9)	2,803.9 (2,035.5 to 3,771.2)	31.5 (20.6 to 46.9)	16.8 (9.3 to 25.2)	-6.2 (-12.3 to 0.5)
Hepatitis	43,254.3 (37,196.7 to 50,308.3)	6,125.7 (3,966.9 to 9,497.3)	14.7 (7.8 to 25.8)	-2.9 (-42.2 to 63.3)	-22.0 (-53.6 to 31.1)
Acute hepatitis A	64.1 (14.2 to 131.9)	833.6 (184.4 to 1,714.1)	2.7 (0.6 to 6.0)	-8.7 (-81.8 to 270.5)	-26.7 (-85.4 to 197.5)
Hepatitis B	31,271.5 (25,449.5 to 37,758.5)	4,594.1 (2,610.6 to 7,783.2)	10.9 (5.2 to 20.2)	-1.9 (-45.2 to 93.2)	-21.2 (-56.0 to 55.1)
Hepatitis C	11,887.8 (10,715.8 to 13,184.9)	296.1 (219.0 to 389.3)	0.3 (0.1 to 0.6)	0.3 (-3.4 to 3.9)	-19.5 (-22.4 to -16.6)
Acute hepatitis E	30.9 (21.9 to 42.7)	401.9 (285.4 to 554.7)	1.0 (0.5 to 1.6)	2.0 (-18.9 to 27.8)	-18.1 (-34.9 to 2.6)
Other infectious diseases	1,242.9 (1,094.0 to 1,423.1)	0.6 (0.3 to 1.0)	22.9 (14.1 to 36.2)	17.8 (13.0 to 22.7)	-5.4 (-9.3 to -1.5)
**Non-communicable diseases**	**399,506.9 (398,845.7 to 400,290.2)**	**1,023,906.3† (910,851.5 to 1,138,083.5)**	**51,238.9 (37,719.7 to 65,758.8)**	**23.7 (23.0 to 24.4)**	**-0.7 (-1.2 to -0.2)**
**Neoplasms**	**3,667.6 (3,608.8 to 3,730.2)**	**1,429.3 (1,378.1 to 1,503.5)**	**455.3 (333.9 to 594.4)**	**25.4 (22.0 to 29.4)**	**0.7 (-2.1 to 3.9)**
Lip and oral cavity cancer	149.1 (142.4 to 155.5)	40.5 (38.6 to 42.3)	15.5 (11.1 to 20.4)	26.8 (17.6 to 36.2)	1.8 (-5.5 to 9.4)
Nasopharynx cancer	38.1 (35.5 to 41.2)	10.9 (10.2 to 11.8)	4.5 (3.2 to 6.2)	7.7 (-5.3 to 21.8)	-13.6 (-23.9 to -2.2)
Other pharynx cancer	65.2 (61.1 to 68.4)	22.6 (21.1 to 23.7)	7.9 (5.7 to 10.3)	29.4 (18.3 to 39.5)	3.9 (-5.0 to 12.0)
Oesophageal cancer	42.9 (41.3 to 45.0)	35.4 (34.1 to 36.9)	9.4 (6.6 to 12.5)	-7.0 (-15.5 to 2.1)	-25.3 (-32.2 to -18.0)
Stomach cancer	159.6 (150.7 to 168.9)	85.6 (81.2 to 90.2)	23.6 (16.9 to 31.0)	5.9 (-2.9 to 15.1)	-15.0 (-22.0 to -7.6)
Colon and rectum cancer	449.0 (430.2 to 471.7)	120.8 (115.6 to 126.7)	43.7 (31.9 to 58.0)	30.3 (21.5 to 40.8)	4.7 (-2.4 to 13.0)
Liver cancer	113.1 (99.7 to 127.1)	109.8 (100.8 to 117.7)	25.3 (17.8 to 33.3)	36.1 (22.7 to 51.0)	9.2 (-1.5 to 21.2)
Liver cancer due to hepatitis B	78.6 (61.5 to 93.8)	55.7 (43.8 to 66.1)	12.8 (8.2 to 17.7)	34.8 (17.0 to 55.4)	8.2 (-6.0 to 24.8)

Cause	Prevalence in thousands (95% UI)	Incidence in thousands (95% UI)	YLDs in thousands (95% UI)		
	2016 counts	2016 counts	2016 counts	Percentage change in counts between 2006 and 2016	Percentage change in rates between 2006 and 2016
Liver cancer due to hepatitis C	18.5 (14.5 to 22.4)	14.1 (11.1 to 17.2)	3.3 (2.1 to 4.7)	34.9 (22.7 to 48.0)	8.3 (-1.5 to 18.9)
Liver cancer due to alcohol use	16.3 (11.4 to 22.0)	13.1 (9.2 to 17.6)	3.0 (1.8 to 4.5)	43.2 (29.4 to 62.2)	15.0 (3.9 to 30.2)
Liver cancer due to other causes	38.4 (29.9 to 49.3)	26.9 (21.3 to 34.3)	6.3 (4.1 to 9.0)	36.0 (19.2 to 56.4)	9.2 (-4.3 to 25.6)
Gallbladder and biliary tract cancer	9.5 (8.2 to 10.1)	11.4 (10.0 to 12.0)	2.6 (1.8 to 3.5)	13.4 (7.0 to 19.5)	-8.9 (-14.1 to -4.1)
Pancreatic cancer	21.4 (20.8 to 22.0)	26.0 (25.5 to 26.5)	5.6 (3.9 to 7.4)	20.6 (17.0 to 23.9)	-3.1 (-6.0 to -0.5)
Larynx cancer	67.7 (65.4 to 70.5)	19.7 (19.1 to 20.5)	8.1 (5.9 to 10.7)	10.6 (4.3 to 17.6)	-11.2 (-16.3 to -5.6)
Tracheal, bronchus, and lung cancer	190.5 (182.2 to 198.5)	139.9 (134.3 to 145.2)	35.3 (24.7 to 45.8)	13.2 (4.7 to 22.8)	-9.1 (-16.0 to -1.4)
Malignant skin melanoma	125.0 (107.2 to 145.8)	25.5 (22.0 to 29.7)	8.3 (5.7 to 11.5)	31.8 (22.5 to 41.5)	5.8 (-1.6 to 13.6)
Non-melanoma skin cancer	27.9 (18.4 to 45.9)	96.7 (55.2 to 162.8)	1.2 (0.6 to 2.5)	-7.8 (-21.5 to 6.9)	-25.9 (-37.0 to -14.2)
Non-melanoma skin cancer (squamous-cell carcinoma)	23.9 (12.0 to 45.1)	23.5 (12.8 to 41.3)	1.1 (0.5 to 2.4)	-9.4 (-24.5 to 5.6)	-27.2 (-39.4 to -15.2)
Non-melanoma skin cancer (basal-cell carcinoma)	8.4 (3.6 to 16.9)	73.2 (35.4 to 132.8)	0.1 (< 0.05 to 0.2)	18.2 (8.0 to 27.0)	-5.1 (-13.3 to 2.0)
Breast cancer	1,051.2 (989.2 to 1,114.4)	217.2 (204.3 to 230.4)	93.2 (65.6 to 125.5)	25.6 (16.9 to 35.2)	0.8 (-6.2 to 8.5)
Cervical cancer	250.7 (192.2 to 270.6)	66.0 (51.5 to 70.3)	23.9 (16.1 to 31.8)	13.2 (5.3 to 22.4)	-9.1 (-15.5 to -1.7)
Uterine cancer	265.7 (249.6 to 286.0)	55.7 (52.4 to 60.0)	20.2 (14.3 to 27.4)	36.8 (24.4 to 52.4)	9.8 (-0.1 to 22.4)
Ovarian cancer	92.9 (86.2 to 96.9)	30.2 (28.3 to 31.3)	14.1 (10.1 to 18.4)	24.7 (12.2 to 37.9)	0.1 (-9.9 to 10.7)
Prostate cancer	229.2 (201.8 to 268.8)	52.9 (47.0 to 60.6)	19.4 (13.2 to 26.7)	34.4 (23.2 to 46.1)	7.9 (-1.1 to 17.3)
Testicular cancer	16.6 (15.5 to 18.1)	3.3 (3.1 to 3.6)	1.2 (0.8 to 1.6)	38.2 (21.4 to 57.8)	11.0 (-2.5 to 26.7)
Kidney cancer	123.2 (116.9 to 129.0)	32.7 (31.2 to 34.2)	11.1 (8.0 to 14.9)	22.0 (14.2 to 30.0)	-2.0 (-8.3 to 4.4)
Bladder cancer	109.4 (105.5 to 112.7)	26.4 (25.5 to 27.3)	10.5 (7.5 to 14.0)	24.5 (17.5 to 32.0)	0.0 (-5.7 to 6.0)
Brain and nervous system cancer	64.0 (57.7 to 71.8)	28.4 (26.1 to 32.4)	9.1 (6.5 to 12.1)	35.5 (27.0 to 47.6)	8.8 (2.0 to 18.5)
Thyroid cancer	155.1 (146.3 to 164.9)	29.6 (27.9 to 31.4)	9.7 (6.7 to 13.5)	52.5 (41.1 to 64.7)	22.4 (13.3 to 32.2)
Mesothelioma	3.8 (3.5 to 4.1)	2.5 (2.3 to 2.7)	0.8 (0.6 to 1.1)	23.4 (15.1 to 29.6)	-0.9 (-7.6 to 4.1)
Hodgkin lymphoma	21.6 (19.4 to 24.4)	5.5 (4.9 to 6.3)	2.2 (1.5 to 2.9)	19.9 (10.7 to 29.2)	-3.7 (-11.1 to 3.8)
Non-Hodgkin's lymphoma	137.2 (124.6 to 152.3)	38.2 (34.7 to 41.9)	12.5 (9.0 to 16.8)	52.5 (42.3 to 60.8)	22.4 (14.2 to 29.1)
Multiple myeloma	26.3 (23.2 to 29.9)	10.7 (9.6 to 12.4)	5.7 (3.9 to 7.6)	41.5 (34.1 to 49.7)	13.6 (7.7 to 20.2)
Leukaemia	106.2 (93.4 to 113.6)	30.5 (26.9 to 32.4)	14.1 (10.0 to 19.0)	36.0 (27.7 to 45.6)	9.2 (2.5 to 16.9)
Acute lymphoid leukaemia	12.8 (11.0 to 13.8)	3.7 (3.2 to 4.0)	1.7 (1.2 to 2.3)	55.0 (41.3 to 65.9)	24.4 (13.4 to 33.2)
Chronic lymphoid leukaemia	33.2 (30.3 to 36.5)	7.4 (6.7 to 8.1)	4.1 (2.9 to 5.5)	45.1 (34.5 to 56.9)	16.5 (8.0 to 26.0)
Acute myeloid leukaemia	9.2 (8.3 to 10.1)	7.0 (6.2 to 7.6)	1.9 (1.3 to 2.6)	41.7 (34.5 to 48.9)	13.8 (8.0 to 19.6)
Chronic myeloid leukaemia	8.6 (7.5 to 9.5)	3.0 (2.6 to 3.3)	1.1 (0.8 to 1.5)	12.5 (6.1 to 19.0)	-9.7 (-14.8 to -4.5)
Other leukaemia	42.4 (34.0 to 47.0)	9.4 (7.5 to 10.4)	5.2 (3.6 to 7.1)	28.1 (14.7 to 44.5)	2.9 (-7.9 to 16.0)
Other neoplasms	189.3 (171.3 to 199.2)	54.8 (49.6 to 57.4)	16.5 (11.8 to 21.7)	55.3 (46.1 to 64.5)	24.7 (17.3 to 32.1)
**Cardiovascular diseases**	**37,851.0 (36,605.9 to 38,801.2)**	**4,126.6† (3,763.6 to 4,503.2)**	**2,379.7 (1,697.9 to 3,112.1)**	**27.8 (24.0 to 32.0)**	**2.6 (-0.4 to 6.0)**
Rheumatic heart disease	1,802.5 (1,631.6 to 2,007.0)	12.7 (10.4 to 15.0)	89.4 (58.1 to 128.9)	20.7 (16.3 to 24.6)	-3.1 (-6.6 to 0.0)
Ischaemic heart disease	12,174.8 (11,162.3 to 13,224.5)	1,463.4 (1,273.8 to 1,658.5)	579.9 (389.7 to 819.9)	28.0 (26.4 to 29.7)	2.8 (1.5 to 4.2)
Cerebrovascular disease	6,633.5 (6,310.7 to 6,919.1)	1,173.5‡ (1,018.5 to 1,342.2)	1,082.7 (767.4 to 1,411.2)	30.6 (22.4 to 39.4)	4.8 (-1.8 to 12.0)
Ischaemic stroke	5,440.1 (4,663.3 to 6,317.2)	805.5‡ (656.3 to 962.0)	864.9 (610.0 to 1,150.5)	33.0 (23.4 to 43.5)	6.8 (-0.9 to 15.2)
Hemorrhagic stroke	1,371.5 (1,144.6 to 1,652.0)	368.0‡ (307.6 to 433.7)	217.8 (147.3 to 290.3)	21.6 (9.6 to 34.4)	-2.4 (-12.0 to 7.9)
Hypertensive heart disease	1,264.3 (981.5 to 1,582.1)	--	105.8 (68.6 to 153.6)	26.2 (22.2 to 30.3)	1.3 (-1.9 to 4.6)
Cardiomyopathy and myocarditis	443.8 (400.9 to 494.5)	173.8† (127.2 to 235.0)	43.1 (27.0 to 62.7)	21.7 (16.3 to 26.9)	-2.3 (-6.6 to 1.9)
Myocarditis	98.4 (79.3 to 121.1)	173.8 (127.2 to 235.0)	7.1 (4.5 to 10.2)	21.1 (14.2 to 28.1)	-2.8 (-8.3 to 2.8)
Alcoholic cardiomyopathy	175.7 (134.4 to 225.2)	--	14.9 (9.3 to 21.9)	20.0 (9.3 to 31.3)	-3.7 (-12.2 to 5.4)
Other cardiomyopathy	250.6 (186.0 to 323.4)	--	21.1 (13.0 to 30.9)	23.2 (16.7 to 29.5)	-1.1 (-6.3 to 4.0)
Atrial fibrillation and flutter	2,048.8 (1,716.4 to 2,454.4)	218.5 (170.9 to 268.5)	168.8 (108.7 to 244.2)	25.4 (21.9 to 28.8)	0.7 (-2.1 to 3.4)
Peripheral vascular disease	9,909.7 (7,890.5 to 12,465.9)	1,017.3 (769.6 to 1,306.4)	10.6 (4.6 to 19.4)	18.0 (14.5 to 21.7)	-5.2 (-8.1 to -2.3)
Endocarditis	23.4 (18.2 to 29.7)	67.3 (50.9 to 84.8)	1.9 (1.2 to 2.9)	24.4 (21.2 to 28.0)	-0.1 (-2.7 to 2.8)
Other cardiovascular and circulatory diseases	5,643.0 (4,056.8 to 7,609.0)	--	297.6 (184.2 to 454.0)	23.2 (20.0 to 26.1)	-1.0 (-3.6 to 1.3)
**Chronic respiratory diseases**	**41,113.5 (40,124.7 to 41,897.6)**	**5,632.5† (5,022.0 to 6,243.4)**	**2,470.9 (2,031.5 to 2,906.6)**	**26.1 (23.3 to 29.2)**	**1.3 (-1.0 to 3.8)**
Chronic obstructive pulmonary disease	24,558.2 (22,889.0 to 26,195.6)	2,110.9 (1,824.2 to 2,436.8)	1,676.7 (1,426.0 to 1,902.8)	24.2 (20.3 to 28.2)	-0.3 (-3.4 to 3.0)
Pneumoconiosis	81.7 (72.0 to 92.5)	11.9 (10.3 to 13.8)	12.7 (8.3 to 18.2)	22.5 (14.1 to 31.7)	-1.6 (-8.4 to 5.7)
Silicosis	22.9 (18.2 to 28.3)	4.0 (2.8 to 5.7)	3.6 (2.2 to 5.4)	24.8 (12.8 to 38.0)	0.2 (-9.4 to 10.8)

Cause	Prevalence in thousands (95% UI)	Incidence in thousands (95% UI)	YLDs in thousands (95% UI)		
	2016 counts	2016 counts	2016 counts	Percentage change in counts between 2006 and 2016	Percentage change in rates between 2006 and 2016
Asbestosis	9.8 (7.9 to 12.0)	1.1 (0.8 to 1.4)	1.5 (1.0 to 2.2)	12.9 (10.8 to 14.8)	-9.4 (-11.0 to -7.9)
Coal workers pneumoconiosis	27.7 (21.9 to 34.4)	4.2 (3.4 to 5.0)	4.2 (2.6 to 6.2)	21.6 (-0.2 to 46.9)	-2.4 (-19.8 to 17.9)
Other pneumoconiosis	21.3 (16.0 to 27.3)	2.7 (2.0 to 3.5)	3.4 (2.0 to 5.0)	26.3 (21.5 to 30.2)	1.4 (-2.5 to 4.5)
Asthma	18,444.5 (16,063.4 to 20,903.3)	3,484.4 (2,961.2 to 4,047.2)	708.2 (459.2 to 1,032.7)	29.5 (26.5 to 32.3)	4.0 (1.6 to 6.2)
Interstitial lung disease and pulmonary sarcoidosis	256.7 (211.7 to 307.2)	25.2 (17.1 to 35.0)	28.3 (17.8 to 43.2)	22.7 (15.1 to 29.9)	-1.4 (-7.6 to 4.3)
Other chronic respiratory diseases	--	--	45.0 (38.0 to 51.0)	60.4 (55.5 to 65.5)	28.8 (24.9 to 32.9)
**Cirrhosis and other chronic liver diseases**	**5,465.3 (5,102.8 to 5,850.3)**	**178.6 (151.4 to 209.4)**	**145.2 (97.6 to 203.3)**	**24.9 (20.1 to 29.4)**	**0.3 (-3.6 to 3.9)**
Cirrhosis and other chronic liver diseases due to hepatitis B	1,517.5 (1,292.9 to 1,750.3)	51.4 (40.7 to 64.1)	43.9 (29.2 to 63.1)	26.7 (19.5 to 34.4)	1.7 (-4.0 to 7.9)
Cirrhosis and other chronic liver diseases due to hepatitis C	1,421.5 (1,173.3 to 1,676.9)	48.0 (37.5 to 59.8)	37.4 (24.2 to 53.9)	26.5 (19.4 to 34.5)	1.6 (-4.1 to 8.0)
Cirrhosis and other chronic liver diseases due to alcohol use	1,615.4 (1,390.4 to 1,869.7)	53.0 (42.1 to 65.3)	41.6 (27.7 to 60.8)	23.2 (16.1 to 30.3)	-1.1 (-6.8 to 4.6)
Cirrhosis and other chronic liver diseases due to other causes	910.9 (688.3 to 1,170.8)	26.2 (19.1 to 34.6)	22.4 (14.0 to 34.1)	22.2 (12.8 to 32.0)	-1.9 (-9.5 to 6.0)
**Digestive diseases**	**25,249.5 (23,485.0 to 26,985.5)**	**19,958.7† (17,124.7 to 23,356.2)**	**647.4 (435.5 to 895.8)**	**39.2 (31.0 to 48.0)**	**11.8 (5.2 to 18.8)**
Peptic ulcer disease	7,702.3 (6,287.5 to 9,359.6)	778.8 (587.0 to 982.6)	128.9 (70.7 to 208.4)	60.9 (45.8 to 76.9)	29.2 (17.1 to 42.1)
Gastritis and duodenitis	11,733.2 (8,823.2 to 14,998.4)	7,645.1 (5,736.4 to 10,225.3)	150.2 (88.2 to 234.8)	61.8 (46.9 to 76.8)	29.9 (17.9 to 42.0)
Appendicitis	28.9 (23.2 to 35.1)	751.8 (609.1 to 907.2)	8.9 (5.7 to 13.0)	27.3 (16.6 to 38.7)	2.2 (-6.4 to 11.3)
Paralytic ileus and intestinal obstruction	12.6 (9.5 to 16.2)	344.7 (255.3 to 438.9)	4.1 (2.5 to 6.1)	31.0 (27.8 to 34.0)	5.2 (2.6 to 7.6)
Inguinal, femoral, and abdominal hernia	4,167.1 (3,088.2 to 5,353.9)	3,596.5 (2,533.2 to 4,836.0)	174.0 (107.0 to 265.6)	18.4 (13.3 to 23.0)	-4.9 (-9.1 to -1.2)
Inflammatory bowel disease	687.3 (579.8 to 812.3)	284.0 (226.4 to 351.4)	99.0 (66.2 to 139.0)	29.9 (23.3 to 36.8)	4.3 (-1.0 to 9.8)
Vascular intestinal disorders	5.9 (3.8 to 9.0)	33.9 (23.1 to 48.6)	1.9 (1.1 to 3.2)	26.1 (20.4 to 30.7)	1.2 (-3.4 to 4.9)
Gallbladder and biliary diseases	1,881.5 (1,426.0 to 2,510.0)	6,023.7 (4,600.6 to 7,812.6)	27.3 (16.5 to 41.8)	32.2 (23.4 to 41.5)	6.1 (-0.9 to 13.6)
Pancreatitis	56.4 (42.7 to 71.9)	500.1 (380.2 to 639.9)	7.1 (4.3 to 10.7)	28.4 (14.5 to 41.6)	3.1 (-8.1 to 13.7)
Other digestive diseases	--	--	45.8 (30.8 to 63.4)	48.5 (39.7 to 57.8)	19.2 (12.2 to 26.7)
**Neurological disorders**	**169,972.0 (159,135.5 to 180,646.1)**	**61,595.8 (40,189.0 to 91,359.1)**	**4,546.7 (3,091.7 to 6,167.2)**	**24.2 (22.7 to 25.8)**	**-0.3 (-1.5 to 1.0)**
Alzheimer's disease and other dementias	782.5 (577.0 to 1,023.4)	139.4 (93.5 to 201.3)	99.3 (60.1 to 150.2)	22.4 (17.7 to 26.6)	-1.8 (-5.5 to 1.7)
Parkinson's disease	247.4 (172.8 to 350.5)	34.3 (22.7 to 48.1)	31.0 (18.4 to 47.8)	32.5 (23.9 to 42.0)	6.4 (-0.5 to 14.0)
Epilepsy	1,168.9 (953.4 to 1,426.3)	90.1 (62.3 to 121.1)	345.8 (238.0 to 485.5)	18.1 (6.1 to 31.1)	-5.2 (-14.8 to 5.2)
Multiple sclerosis	241.8 (221.9 to 264.6)	1.7 (1.0 to 2.3)	63.5 (44.8 to 82.9)	28.9 (22.2 to 36.1)	3.5 (-1.9 to 9.3)
Motor neuron disease	24.1 (18.7 to 31.1)	4.6 (3.2 to 6.5)	5.1 (3.4 to 7.4)	26.3 (23.6 to 28.6)	1.4 (-0.7 to 3.3)
Migraine	77,102.8 (71,263.1 to 82,744.4)	5,336.8 (4,533.5 to 6,292.7)	3,364.7 (2,182.2 to 4,728.5)	24.3 (23.2 to 25.5)	-0.2 (-1.1 to 0.8)
Tension-type headache	118,597.4 (84,501.6 to 157,672.9)	55,985.7 (34,619.6 to 85,843.1)	541.2 (336.4 to 811.2)	24.9 (23.1 to 27.0)	0.3 (-1.1 to 2.0)
Other neurological disorders	1.6 (0.8 to 2.8)	3.2 (1.7 to 5.0)	96.0 (67.5 to 129.0)	33.3 (24.2 to 42.9)	7.0 (-0.3 to 14.8)
**Mental and substance use disorders**	**73,359.8 (71,942.1 to 74,740.7)**	**28,817.6† (25,727.5 to 32,160.0)**	**10,462.4 (7,647.4 to 13,555.3)**	**20.9 (19.9 to 21.9)**	**-2.9 (-3.7 to -2.1)**
Schizophrenia	2,066.3 (1,809.5 to 2,352.2)	46.4 (38.0 to 56.9)	1,307.5 (986.6 to 1,619.8)	25.0 (22.7 to 27.3)	0.3 (-1.4 to 2.2)
Alcohol use disorders	7,452.2 (5,940.6 to 9,080.6)	3,277.0 (2,538.4 to 4,103.2)	731.9 (474.8 to 1,025.0)	13.7 (8.5 to 18.4)	-8.7 (-12.9 to -4.9)
Drug use disorders	2,376.4 (2,269.0 to 2,462.8)	238.7 (197.9 to 294.9)	610.8 (423.9 to 790.4)	26.2 (22.6 to 30.0)	1.3 (-1.6 to 4.4)
Opioid use disorders	1,147.3 (916.5 to 1,395.0)	141.5 (104.0 to 191.7)	464.0 (313.8 to 621.8)	27.5 (22.8 to 32.1)	2.4 (-1.4 to 6.0)
Cocaine use disorders	349.2 (304.8 to 394.7)	9.4 (5.9 to 13.4)	46.9 (28.9 to 66.3)	20.7 (14.8 to 27.0)	-3.1 (-7.8 to 2.0)
Amphetamine use disorders	88.0 (63.0 to 118.7)	11.1 (5.5 to 17.9)	11.7 (6.2 to 19.4)	20.0 (8.3 to 30.4)	-3.7 (-13.0 to 4.7)
Cannabis use disorders	593.4 (479.4 to 738.2)	39.7 (23.9 to 60.5)	17.2 (10.5 to 25.7)	20.8 (13.5 to 29.1)	-3.0 (-8.9 to 3.7)
Other drug use disorders	306.4 (254.2 to 365.8)	37.1 (27.3 to 50.0)	71.0 (47.1 to 96.8)	23.7 (19.3 to 28.4)	-0.7 (-4.2 to 3.1)
Depressive disorders	23,607.0 (22,582.9 to 24,424.0)	22,708.3 (19,678.5 to 25,925.3)	3,779.8 (2,549.6 to 5,150.3)	17.8 (16.1 to 19.4)	-5.4 (-6.8 to -4.2)
Major depressive disorder	13,894.7 (12,117.5 to 15,865.3)	21,195.6 (18,257.0 to 24,423.7)	2,803.1 (1,892.0 to 3,896.4)	15.5 (13.7 to 17.2)	-7.3 (-8.7 to -5.9)
Dysthymia	10,215.9 (8,460.8 to 12,421.1)	1,512.7 (1,207.7 to 1,850.6)	976.7 (627.5 to 1,435.3)	24.9 (21.8 to 28.5)	0.3 (-2.2 to 3.1)
Bipolar disorder	3,183.5 (2,646.0 to 3,845.1)	185.2 (122.2 to 264.6)	637.8 (392.6 to 966.2)	24.5 (22.2 to 27.0)	-0.0 (-1.9 to 2.0)
Anxiety disorders	19,643.2 (17,749.1 to 21,730.0)	2,310.5 (2,067.3 to 2,566.9)	1,857.4 (1,291.5 to 2,519.3)	23.3 (20.8 to 25.8)	-1.0 (-3.0 to 1.0)
Eating disorders	--	--	--	--	--
Anorexia nervosa	--	--	--	--	--
Bulimia nervosa	--	--	--	--	--
Autistic spectrum disorders	3,077.0 (2,950.5 to 3,186.6)	--	444.4 (304.3 to 624.3)	24.6 (22.5 to 26.6)	0.1 (-1.6 to 1.7)
Autism	948.8 (815.6 to 1,108.4)	--	235.3 (151.4 to 339.7)	24.2 (20.9 to 27.4)	-0.3 (-2.9 to 2.3)
Asperger syndrome and other autistic spectrum disorders	2,134.4 (1,787.0 to 2,535.5)	--	209.1 (136.4 to 303.0)	25.1 (22.8 to 27.3)	0.4 (-1.4 to 2.2)

Cause	Prevalence in thousands (95% UI)	Incidence in thousands (95% UI)	YLDs in thousands (95% UI)		
	2016 counts	2016 counts	2016 counts	Percentage change in counts between 2006 and 2016	Percentage change in rates between 2006 and 2016
Attention-deficit/hyperactivity disorder	1,649.4 (1,437.3 to 1,877.2)	--	19.4 (11.7 to 30.9)	24.7 (20.3 to 29.4)	0.1 (-3.4 to 3.9)
Conduct disorder	--	--	--	--	--
Idiopathic developmental intellectual disability	4,838.2 (2,802.0 to 6,867.4)	--	200.4 (97.2 to 342.3)	13.3 (8.2 to 16.2)	-9.0 (-13.1 to -6.7)
Other mental and substance use disorders	11,852.4 (10,434.7 to 13,529.2)	51.5 (31.9 to 74.9)	872.9 (595.6 to 1,246.0)	24.5 (22.7 to 26.3)	-0.0 (-1.5 to 1.4)
**Diabetes, urogenital, blood, and endocrine diseases**	**195,480.1 (194,141.8 to 196,929.2)**	**50,855.1† (44,189.6 to 57,409.6)**	**6,037.4 (4,266.2 to 8,374.3)**	**23.3 (20.9 to 25.9)**	**-1.0 (-2.9 to 1.1)**
Diabetes mellitus	44,201.7 (38,494.2 to 49,901.0)	2,570.9 (2,034.7 to 3,228.2)	3,379.6 (2,274.7 to 4,788.4)	22.3 (18.3 to 26.7)	-1.8 (-5.0 to 1.7)
Acute glomerulonephritis	7.3 (4.8 to 10.3)	106.6 (69.7 to 155.3)	0.4 (0.2 to 0.6)	5.7 (2.7 to 8.3)	-15.2 (-17.6 to -13.0)
Chronic kidney disease	20,038.2 (17,131.9 to 23,606.5)	1,651.0 (1,103.2 to 2,294.3)	714.1 (513.5 to 944.5)	31.3 (27.1 to 35.8)	5.4 (2.1 to 9.1)
Chronic kidney disease due to diabetes mellitus	9,733.9 (7,249.9 to 12,245.8)	804.7 (508.4 to 1,188.0)	364.7 (244.4 to 509.4)	31.4 (26.2 to 37.3)	5.5 (1.4 to 10.2)
Chronic kidney disease due to hypertension	3,007.4 (2,217.8 to 3,948.0)	259.4 (148.4 to 414.3)	114.7 (73.3 to 168.1)	37.1 (29.3 to 46.3)	10.0 (3.9 to 17.5)
Chronic kidney disease due to glomerulonephritis	3,148.4 (2,390.6 to 4,052.6)	245.1 (152.9 to 376.9)	99.5 (56.6 to 152.1)	25.1 (17.6 to 32.4)	0.5 (-5.6 to 6.3)
Chronic kidney disease due to other causes	4,148.5 (2,671.3 to 5,987.5)	341.8 (193.5 to 548.4)	135.2 (81.1 to 209.7)	31.1 (25.1 to 37.5)	5.3 (0.4 to 10.4)
Urinary diseases and male infertility	13,535.8 (11,958.6 to 15,377.6)	29,604.0† (24,107.6 to 35,237.4)	458.6 (295.6 to 675.6)	20.2 (17.5 to 22.8)	-3.5 (-5.7 to -1.4)
Interstitial nephritis and urinary tract infections	390.4 (292.7 to 494.6)	20,316.6 (15,316.7 to 25,491.1)	12.7 (7.2 to 20.4)	28.4 (21.7 to 34.8)	3.1 (-2.3 to 8.2)
Urolithiasis	294.8 (219.2 to 387.6)	7,774.9 (5,742.5 to 10,308.8)	21.6 (12.9 to 32.5)	21.5 (12.5 to 31.6)	-2.5 (-9.7 to 5.7)
Benign prostatic hyperplasia	12,346.2 (10,251.5 to 14,822.0)	1,512.5 (1,204.2 to 1,885.3)	413.9 (256.5 to 610.0)	20.3 (17.5 to 23.3)	-3.4 (-5.7 to -1.0)
Male infertility	--	--	--	--	--
Other urinary diseases	--	--	10.4 (6.8 to 15.1)	4.7 (-0.8 to 10.5)	-16.0 (-20.3 to -11.3)
Gynecological diseases	76,204.4 (74,708.2 to 77,341.8)	15,363.2† (12,181.2 to 18,885.3)	991.5 (673.6 to 1,401.6)	19.9 (17.4 to 22.5)	-3.7 (-5.8 to -1.7)
Uterine fibroids	15,189.9 (13,400.3 to 17,410.2)	325.5 (202.0 to 490.0)	202.9 (119.3 to 339.5)	21.1 (18.5 to 23.3)	-2.7 (-4.9 to -1.0)
Polycystic ovarian syndrome	0.7 (0.6 to 0.9)	--	< 0.05 (< 0.05 to < 0.05)	16.4 (15.1 to 17.9)	-6.5 (-7.6 to -5.4)
Female infertility	--	--	--	--	--
Endometriosis	261.7 (228.3 to 303.7)	8.7 (0.5 to 39.6)	24.0 (15.9 to 34.4)	26.1 (16.9 to 35.1)	1.2 (-6.2 to 8.4)
Genital prolapse	35,806.1 (31,467.5 to 40,568.5)	3,298.3 (2,584.5 to 4,154.4)	112.6 (54.3 to 204.0)	13.7 (12.3 to 15.2)	-8.7 (-9.9 to -7.5)
Premenstrual syndrome	27,044.3 (25,258.6 to 28,885.7)	--	220.8 (138.6 to 338.6)	23.5 (20.9 to 26.1)	-0.8 (-2.9 to 1.2)
Other gynecological diseases	12,558.0 (9,788.9 to 15,797.3)	11,730.7 (8,836.4 to 15,223.0)	431.3 (277.2 to 649.9)	19.1 (13.9 to 24.2)	-4.4 (-8.5 to -0.2)
Hemoglobinopathies and hemolytic anaemias	90,563.3 (89,636.4 to 91,637.3)	--	217.2 (133.2 to 341.5)	38.3 (28.0 to 47.0)	11.1 (2.8 to 18.0)
Thalassemias	0.5 (0.5 to 0.5)	--	0.1 (< 0.05 to 0.1)	27.7 (9.4 to 67.1)	2.6 (-12.2 to 34.2)
Thalassaemias trait	16,920.2 (16,282.1 to 17,610.6)	--	143.9 (88.0 to 228.7)	42.8 (29.3 to 54.7)	14.7 (3.8 to 24.2)
Sickle cell disorders	8.7 (7.7 to 9.6)	--	1.4 (1.0 to 1.9)	61.2 (48.8 to 73.7)	29.4 (19.4 to 39.5)
Sickle cell trait	19,429.4 (17,647.2 to 21,826.6)	--	44.8 (28.2 to 68.2)	36.6 (30.7 to 42.6)	9.7 (4.9 to 14.5)
G6PD deficiency	15,810.5 (15,241.4 to 16,467.1)	--	1.0 (0.6 to 1.4)	30.2 (22.2 to 37.2)	4.5 (-1.9 to 10.2)
G6PD trait	45,150.3 (44,413.1 to 46,017.0)	--	< 0.05 (< 0.05 to 0.1)	66.0 (46.1 to 91.7)	33.3 (17.3 to 53.9)
Other hemoglobinopathies and hemolytic anaemias	2,329.2 (2,047.4 to 2,667.5)	--	26.1 (15.8 to 41.9)	19.6 (12.4 to 26.1)	-4.0 (-9.8 to 1.3)
Endocrine, metabolic, blood, and immune disorders	8,544.0 (6,898.1 to 10,882.5)	1,559.5 (1,104.5 to 2,096.2)	276.0 (173.1 to 404.8)	23.3 (20.2 to 26.3)	-1.0 (-3.5 to 1.4)
**Musculoskeletal disorders**	**133,304.2 (126,948.2 to 139,726.9)**	**65,556.6 (51,139.3 to 82,292.3)**	**14,829.4 (10,384.8 to 19,749.7)**	**24.3 (22.4 to 26.2)**	**-0.2 (-1.7 to 1.3)**
Rheumatoid arthritis	2,337.5 (2,008.4 to 2,704.0)	144.1 (110.9 to 183.2)	554.1 (377.2 to 762.7)	29.7 (25.9 to 33.3)	4.1 (1.1 to 7.0)
Osteoarthritis	34,890.2 (31,994.3 to 37,869.2)	2,380.4 (2,062.1 to 2,710.4)	1,985.5 (1,392.7 to 2,712.8)	27.3 (26.3 to 28.4)	2.2 (1.4 to 3.1)
Low back and neck pain	80,169.1 (74,503.6 to 86,149.4)	30,303.1 (22,169.9 to 41,161.5)	9,459.1 (6,343.7 to 13,351.8)	24.2 (21.5 to 26.5)	-0.2 (-2.4 to 1.6)
Low back pain	49,570.2 (36,157.7 to 66,904.4)	23,952.6 (16,266.8 to 34,325.9)	5,679.8 (3,627.9 to 8,424.0)	24.1 (20.2 to 27.3)	-0.3 (-3.5 to 2.2)
Neck pain	37,758.1 (27,626.6 to 49,591.2)	6,350.5 (3,906.4 to 9,481.6)	3,779.3 (2,323.3 to 5,765.7)	24.4 (20.9 to 27.8)	-0.1 (-3.0 to 2.6)
Gout	3,571.9 (2,796.9 to 4,417.2)	692.4 (524.2 to 886.5)	115.2 (73.3 to 167.3)	25.2 (20.0 to 31.1)	0.5 (-3.6 to 5.3)
Other musculoskeletal disorders	31,934.7 (20,274.9 to 45,313.5)	32,036.6 (21,792.0 to 44,977.0)	2,715.4 (1,511.5 to 4,343.5)	21.6 (14.8 to 26.7)	-2.4 (-7.8 to 1.7)
**Other non-communicable diseases**	**332,441.4 (328,588.5 to 336,481.1)**	**785,755.6† (680,153.3 to 902,610.1)**	**9,264.6 (6,227.4 to 13,643.7)**	**22.9 (21.8 to 23.9)**	**-1.3 (-2.2 to -0.6)**
Congenital anomalies	3,582.5 (3,326.1 to 3,832.7)	--	461.2 (328.2 to 608.2)	24.2 (21.1 to 27.4)	-0.3 (-2.8 to 2.3)
Neural tube defects	271.9 (247.4 to 300.4)	--	80.8 (57.6 to 107.0)	25.8 (21.0 to 31.0)	1.0 (-2.8 to 5.2)
Congenital heart anomalies	520.7 (466.2 to 581.7)	--	29.0 (12.1 to 50.3)	30.1 (25.0 to 35.0)	4.5 (0.4 to 8.4)
Orofacial clefts	197.8 (176.6 to 219.1)	--	2.4 (1.6 to 3.5)	16.3 (13.0 to 19.6)	-6.6 (-9.3 to -4.0)
Down's syndrome	67.2 (57.2 to 78.4)	--	9.4 (6.4 to 12.8)	23.4 (19.4 to 27.1)	-0.9 (-4.1 to 2.1)
Turner syndrome	76.2 (65.8 to 88.2)	--	1.0 (0.4 to 1.9)	27.5 (9.8 to 47.5)	2.4 (-11.8 to 18.4)
Klinefelter syndrome	38.9 (32.1 to 46.3)	--	0.1 (0.1 to 0.2)	23.9 (11.2 to 37.1)	-0.5 (-10.7 to 10.1)

Cause	Prevalence in thousands (95% UI)	Incidence in thousands (95% UI)	YLDs in thousands (95% UI)		
	2016 counts	2016 counts	2016 counts	Percentage change in counts between 2006 and 2016	Percentage change in rates between 2006 and 2016
Other chromosomal abnormalities	< 0.05<br>(< 0.05 to < 0.05)	--	< 0.05<br>(< 0.05 to < 0.05)	26.5<br>(-53.9 to 279.7)	1.6<br>(-63.0 to 204.9)
Congenital musculoskeletal and limb anomalies	617.3<br>(570.1 to 668.8)	--	89.9<br>(62.4 to 123.0)	22.7<br>(18.2 to 27.0)	-1.5<br>(-5.1 to 2.0)
Urogenital congenital anomalies	167.4<br>(151.2 to 184.8)	--	2.6<br>(1.3 to 4.6)	28.4<br>(24.8 to 32.2)	3.1<br>(0.2 to 6.2)
Digestive congenital anomalies	596.8<br>(539.0 to 659.0)	--	25.5<br>(16.6 to 36.3)	23.1<br>(16.6 to 29.7)	-1.2<br>(-6.4 to 4.2)
Other congenital anomalies	1,795.2<br>(1,256.7 to 2,517.1)	--	220.5<br>(150.2 to 317.6)	23.7<br>(18.7 to 29.2)	-0.7<br>(-4.7 to 3.8)
Skin and subcutaneous diseases	105,472.6<br>(102,810.1 to 107,888.5)	260,721.0<br>(234,182.9 to 291,272.1)	2,067.1<br>(1,386.8 to 2,982.9)	25.9<br>(24.9 to 26.9)	1.1<br>(0.3 to 1.9)
Dermatitis	13,619.6<br>(11,458.1 to 16,143.0)	30,333.4<br>(24,650.0 to 37,258.7)	430.6<br>(257.3 to 655.0)	22.7<br>(20.6 to 24.6)	-1.5<br>(-3.2 to 0.0)
Psoriasis	5,042.0<br>(4,637.0 to 5,510.3)	611.2<br>(526.9 to 700.9)	433.1<br>(305.8 to 574.2)	29.8<br>(26.9 to 33.0)	4.2<br>(1.9 to 6.8)
Cellulitis	174.5<br>(142.9 to 216.7)	3,710.5<br>(3,078.2 to 4,463.1)	9.8<br>(6.2 to 15.1)	20.6<br>(12.4 to 28.9)	-3.1<br>(-9.8 to 3.5)
Pyoderma	1,303.3<br>(1,201.6 to 1,412.7)	30,994.8<br>(28,006.4 to 34,151.1)	7.3<br>(2.9 to 15.0)	26.0<br>(23.0 to 29.1)	1.2<br>(-1.3 to 3.6)
Scabies	5,604.4<br>(3,787.2 to 7,853.2)	17,101.1<br>(11,427.8 to 23,899.5)	142.3<br>(71.8 to 240.9)	20.3<br>(18.0 to 22.2)	-3.4<br>(-5.2 to -1.8)
Fungal skin diseases	29,861.0<br>(23,286.9 to 38,308.3)	104,466.4<br>(79,973.7 to 132,542.2)	166.9<br>(63.8 to 360.0)	26.3<br>(25.6 to 27.0)	1.4<br>(0.8 to 2.0)
Viral skin diseases	6,825.7<br>(5,779.9 to 8,063.9)	10,644.0<br>(9,044.2 to 12,321.9)	204.6<br>(127.4 to 303.3)	24.6<br>(22.9 to 26.4)	0.1<br>(-1.3 to 1.5)
Acne vulgaris	7,755.7<br>(6,769.7 to 8,856.8)	3,452.5<br>(2,975.6 to 4,010.7)	193.8<br>(127.5 to 289.0)	26.2<br>(23.2 to 29.2)	1.3<br>(-1.1 to 3.8)
Alopecia areata	929.1<br>(838.9 to 1,036.8)	1,582.6<br>(1,435.8 to 1,749.4)	30.0<br>(18.8 to 45.3)	22.7<br>(18.6 to 26.9)	-1.5<br>(-4.8 to 1.9)
Pruritus	4,742.5<br>(3,328.5 to 6,626.0)	3,831.7<br>(2,617.4 to 5,180.6)	50.4<br>(21.2 to 96.7)	26.2<br>(24.2 to 28.0)	1.3<br>(-0.3 to 2.8)
Urticaria	2,620.2<br>(1,825.1 to 3,614.1)	4,609.9<br>(3,220.0 to 6,224.8)	153.0<br>(90.0 to 236.5)	24.5<br>(21.5 to 27.5)	-0.0<br>(-2.5 to 2.4)
Decubitus ulcer	89.6<br>(65.1 to 119.2)	325.8<br>(233.2 to 434.7)	15.7<br>(9.7 to 24.6)	21.7<br>(16.1 to 27.2)	-2.3<br>(-6.8 to 2.1)
Other skin and subcutaneous diseases	41,781.8<br>(38,094.2 to 45,457.7)	49,057.1<br>(44,132.9 to 54,141.6)	229.6<br>(112.7 to 424.1)	30.8<br>(29.8 to 31.8)	5.0<br>(4.2 to 5.8)
Sense organ diseases	179,055.1<br>(174,773.8 to 182,836.4)	37,707.2†<br>(33,950.7 to 41,435.3)	5,013.1<br>(3,348.0 to 7,397.6)	21.1<br>(19.2 to 22.6)	-2.8<br>(-4.3 to -1.6)
Glaucoma	183.1<br>(125.0 to 251.4)	--	19.5<br>(11.1 to 31.1)	27.0<br>(19.0 to 35.4)	2.0<br>(-4.5 to 8.8)
Cataract	4,135.9<br>(3,111.1 to 5,395.1)	--	302.9<br>(200.0 to 440.1)	24.2<br>(21.4 to 27.2)	-0.3<br>(-2.5 to 2.1)
Macular degeneration	181.1<br>(128.0 to 253.5)	--	16.0<br>(9.3 to 25.5)	27.6<br>(18.3 to 36.8)	2.5<br>(-5.0 to 9.8)
Refraction and accommodation disorders	80,296.9<br>(73,390.2 to 87,613.1)	--	1,278.8<br>(752.8 to 2,055.3)	16.1<br>(14.1 to 17.7)	-6.8<br>(-8.4 to -5.5)
Age-related and other hearing loss	119,143.0<br>(102,229.9 to 138,158.8)	--	2,661.4<br>(1,737.8 to 3,959.2)	22.0<br>(18.5 to 24.9)	-2.0<br>(-4.8 to 0.2)
Other vision loss	2,478.9<br>(1,904.5 to 3,209.3)	--	201.8<br>(130.2 to 297.6)	25.3<br>(21.8 to 28.8)	0.6<br>(-2.2 to 3.4)
Other sense organ diseases	16,627.6<br>(14,313.6 to 19,089.1)	37,707.2<br>(33,950.7 to 41,435.3)	532.7<br>(328.5 to 819.3)	25.7<br>(24.0 to 27.4)	0.9<br>(-0.4 to 2.3)
Oral disorders	224,074.8<br>(217,535.6 to 231,425.5)	487,327.4†<br>(385,038.9 to 596,005.0)	1,723.2<br>(996.4 to 2,827.2)	24.7<br>(23.9 to 25.4)	0.2<br>(-0.5 to 0.7)
Caries of deciduous teeth	--	--	--	--	--
Caries of permanent teeth	148,280.2<br>(120,912.2 to 175,779.0)	475,439.7<br>(373,577.5 to 584,594.9)	105.5<br>(47.1 to 198.4)	19.7<br>(18.2 to 21.1)	-3.9<br>(-5.1 to -2.8)
Periodontal disease	95,906.4<br>(73,919.2 to 116,215.6)	9,561.0<br>(5,479.9 to 14,160.6)	629.0<br>(244.0 to 1,296.1)	27.2<br>(26.4 to 28.1)	2.2<br>(1.5 to 2.8)
Edentulism and severe tooth loss	24,603.0<br>(20,028.0 to 30,241.4)	2,326.7<br>(1,486.9 to 3,441.2)	694.8<br>(448.4 to 1,021.5)	23.3<br>(22.2 to 24.4)	-1.0<br>(-1.9 to -0.1)
Other oral disorders	10,130.3<br>(9,109.7 to 11,136.7)	--	293.9<br>(182.2 to 446.2)	24.7<br>(23.3 to 26.1)	0.1<br>(-1.0 to 1.2)
**Injuries**	**131,136.9<br>(122,777.1 to 139,741.8)**	**34,224.5†<br>(30,012.6 to 38,829.0)**	**4,725.7<br>(3,167.3 to 6,785.5)**	**26.7<br>(25.3 to 28.0)**	**1.7<br>(0.6 to 2.8)**
**Transport injuries**	**24,316.4<br>(22,533.8 to 26,347.8)**	**4,806.9<br>(3,749.9 to 6,099.8)**	**1,248.0<br>(838.8 to 1,780.2)**	**30.3<br>(28.9 to 31.6)**	**4.6<br>(3.5 to 5.7)**
Road injuries	20,326.4<br>(18,723.7 to 22,014.1)	4,372.2<br>(3,344.8 to 5,678.8)	1,052.7<br>(705.8 to 1,503.7)	32.0<br>(30.7 to 33.2)	6.0<br>(4.9 to 7.0)
Pedestrian road injuries	4,433.3<br>(3,825.4 to 5,171.5)	764.0<br>(494.2 to 1,087.7)	234.5<br>(158.0 to 333.6)	32.8<br>(31.2 to 34.3)	6.6<br>(5.4 to 7.8)
Cyclist road injuries	3,906.2<br>(3,215.8 to 4,599.3)	1,022.7<br>(643.0 to 1,559.3)	190.1<br>(125.9 to 274.3)	35.0<br>(33.6 to 36.4)	8.4<br>(7.3 to 9.5)
Motorcyclist road injuries	5,018.3<br>(4,258.8 to 6,075.4)	666.0<br>(421.2 to 995.6)	255.2<br>(170.7 to 365.6)	33.1<br>(31.7 to 34.4)	6.9<br>(5.8 to 7.9)
Motor vehicle road injuries	5,476.5<br>(4,559.0 to 6,400.3)	1,473.2<br>(1,017.4 to 2,059.2)	300.9<br>(202.2 to 428.8)	23.9<br>(22.4 to 25.3)	-0.5<br>(-1.7 to 0.6)
Other road injuries	1,492.1<br>(1,218.8 to 1,815.7)	446.3<br>(247.8 to 724.4)	72.1<br>(47.7 to 105.4)	58.0<br>(56.3 to 59.7)	26.9<br>(25.5 to 28.2)
Other transport injuries	3,990.0<br>(3,345.0 to 4,715.4)	434.6<br>(279.6 to 668.4)	195.3<br>(133.0 to 274.2)	22.0<br>(20.0 to 24.0)	-2.0<br>(-3.6 to -0.5)
**Unintentional injuries**	**94,558.8<br>(86,821.2 to 102,543.3)**	**27,522.3<br>(23,571.5 to 32,058.5)**	**3,038.9<br>(2,052.4 to 4,302.5)**	**25.6<br>(24.2 to 26.7)**	**0.8<br>(-0.2 to 1.7)**
Falls	38,710.5<br>(33,919.2 to 43,978.5)	12,609.4<br>(9,492.6 to 16,596.4)	1,526.4<br>(1,052.6 to 2,115.3)	26.6<br>(25.2 to 27.9)	1.7<br>(0.6 to 2.7)
Drowning	383.3<br>(332.8 to 439.9)	31.1<br>(23.4 to 42.0)	18.6<br>(12.3 to 26.7)	12.2<br>(9.7 to 14.6)	-9.9<br>(-12.0 to -8.0)
Fire, heat, and hot substances	8,916.9<br>(7,619.8 to 10,500.4)	420.0<br>(285.5 to 606.0)	205.4<br>(133.7 to 310.8)	24.1<br>(22.2 to 25.7)	-0.3<br>(-1.9 to 0.9)
Poisonings	281.3<br>(240.3 to 323.1)	193.1<br>(121.2 to 281.7)	20.7<br>(13.4 to 30.2)	33.1<br>(30.1 to 36.2)	6.9<br>(4.4 to 9.3)
Exposure to mechanical forces	18,812.5<br>(15,881.9 to 22,926.2)	3,678.0<br>(2,605.7 to 5,075.7)	417.1<br>(274.5 to 606.7)	28.8<br>(27.5 to 30.0)	3.5<br>(2.4 to 4.4)
Unintentional firearm injuries	692.3<br>(563.6 to 860.7)	57.7<br>(28.9 to 106.2)	21.6<br>(13.8 to 32.0)	29.5<br>(28.2 to 30.7)	4.0<br>(3.0 to 5.0)
Unintentional suffocation	692.5<br>(544.5 to 877.3)	40.0<br>(18.4 to 75.9)	20.4<br>(12.7 to 30.8)	22.1<br>(20.7 to 23.6)	-2.0<br>(-3.1 to -0.8)
Other exposure to mechanical forces	17,427.7<br>(14,448.8 to 21,618.6)	3,580.3<br>(2,520.6 to 4,938.7)	375.1<br>(246.8 to 545.5)	29.2<br>(27.8 to 30.4)	3.7<br>(2.6 to 4.7)

Cause	Prevalence in thousands (95% UI)	Incidence in thousands (95% UI)	YLDs in thousands (95% UI)		
	2016 counts	2016 counts	2016 counts	Percentage change in counts between 2006 and 2016	Percentage change in rates between 2006 and 2016
Adverse effects of medical treatment	146.6 (100.2 to 209.8)	1,917.7 (1,391.9 to 2,662.6)	19.5 (10.6 to 30.5)	20.1 (15.4 to 24.1)	-3.6 (-7.4 to -0.4)
Animal contact	4,426.1 (3,851.7 to 5,076.4)	3,180.3 (2,191.0 to 4,371.7)	114.8 (77.3 to 160.6)	15.7 (13.4 to 17.9)	-7.1 (-8.9 to -5.3)
Venomous animal contact	2,009.5 (1,663.8 to 2,383.5)	1,515.6 (1,018.2 to 2,184.7)	77.3 (52.5 to 106.8)	17.8 (14.9 to 20.8)	-5.4 (-7.8 to -3.0)
Non-venomous animal contact	2,416.7 (1,981.1 to 2,936.1)	1,664.8 (1,097.8 to 2,389.3)	37.6 (24.3 to 58.1)	11.6 (9.0 to 13.7)	-10.4 (-12.5 to -8.7)
Foreign body	3,802.6 (3,199.7 to 4,332.8)	1,504.0 (1,010.3 to 2,376.6)	132.8 (86.0 to 196.5)	24.0 (21.9 to 26.2)	-0.5 (-2.1 to 1.3)
Pulmonary aspiration and foreign body in airway	1,748.0 (1,351.7 to 2,213.1)	305.8 (205.0 to 436.5)	68.2 (43.8 to 100.3)	19.6 (17.6 to 21.6)	-3.9 (-5.6 to -2.4)
Foreign body in eyes	216.0 (135.0 to 342.6)	853.1 (418.1 to 1,673.9)	13.0 (7.2 to 22.5)	35.8 (31.1 to 43.7)	9.0 (5.3 to 15.4)
Foreign body in other body part	1,838.6 (1,516.8 to 2,261.7)	345.1 (198.5 to 530.6)	51.5 (33.4 to 75.2)	27.3 (25.3 to 29.2)	2.2 (0.6 to 3.7)
Environmental heat and cold exposure	6,077.0 (5,206.9 to 7,063.5)	970.0 (633.9 to 1,432.2)	221.1 (149.2 to 316.9)	23.7 (21.5 to 25.8)	-0.7 (-2.4 to 1.0)
Other unintentional injuries	13,001.8 (10,570.6 to 15,637.8)	3,018.7 (1,928.3 to 4,395.1)	362.5 (236.3 to 527.5)	24.2 (22.7 to 25.5)	-0.3 (-1.4 to 0.8)
**Self-harm and interpersonal violence**	**13,532.7 (11,085.6 to 16,387.4)**	**1,847.6† (1,418.2 to 2,379.3)**	**300.5 (199.8 to 430.0)**	**15.4 (14.5 to 16.4)**	**-7.3 (-8.1 to -6.6)**
Self-harm	1,149.0 (938.8 to 1,368.8)	472.9 (351.9 to 618.7)	49.0 (31.5 to 70.9)	10.7 (9.2 to 12.1)	-11.1 (-12.3 to -10.0)
Self-harm by firearm	32.3 (27.6 to 37.4)	5.7 (1.5 to 14.4)	1.1 (0.7 to 1.6)	4.5 (2.2 to 6.6)	-16.1 (-17.9 to -14.4)
Self-harm by other specified means	1,116.7 (907.7 to 1,336.1)	467.2 (348.8 to 611.7)	47.9 (30.7 to 69.3)	10.9 (9.4 to 12.3)	-11.0 (-12.2 to -9.9)
Interpersonal violence	12,383.7 (9,966.9 to 15,225.9)	1,374.7† (962.4 to 1,885.6)	251.6 (168.8 to 361.3)	16.4 (15.4 to 17.3)	-6.6 (-7.3 to -5.8)
Assault by firearm	292.4 (242.1 to 351.9)	27.1 (14.6 to 47.2)	10.2 (6.6 to 15.2)	18.5 (17.2 to 19.8)	-4.9 (-5.9 to -3.8)
Assault by sharp object	1,539.7 (1,274.0 to 1,821.7)	222.9 (124.9 to 356.1)	39.8 (25.7 to 58.2)	14.3 (12.6 to 15.8)	-8.2 (-9.6 to -7.0)
Sexual violence	6,324.9 (4,106.4 to 9,007.1)	--	45.4 (24.6 to 71.9)	17.4 (16.2 to 18.5)	-5.8 (-6.7 to -4.9)
Assault by other means	4,226.8 (3,524.0 to 5,053.2)	1,124.8 (782.3 to 1,549.9)	156.2 (105.0 to 226.3)	16.5 (15.3 to 17.6)	-6.5 (-7.4 to -5.6)
**Forces of nature, conflict and terrorism, and executions and police conflict**	**5,053.9 (3,284.5 to 7,204.0)**	**47.8† (30.4 to 66.7)**	**138.2 (46.9 to 292.4)**	**51.2 (41.0 to 66.6)**	**21.4 (13.2 to 33.8)**
Forces of nature, conflict and terrorism, and state actor violence	572.9 (319.2 to 881.4)	17.5 (9.2 to 27.5)	19.7 (5.1 to 47.9)	75.3 (59.9 to 83.1)	40.8 (28.4 to 47.0)
Conflict and terrorism	3,461.6 (2,038.2 to 5,306.6)	--	102.6 (39.8 to 203.7)	54.0 (40.4 to 74.0)	23.7 (12.7 to 39.8)
Executions and police conflict	1,019.4 (247.9 to 1,596.7)	30.3 (15.1 to 46.4)	21.6 (3.6 to 56.5)	25.8 (18.8 to 33.8)	1.0 (-4.6 to 7.5)

We did not calculate incidence for certain NTDs and causes with only birth prevalence. Blank cells mean that no estimate is available.

† Incidence for aggregate causes represents the sum of the incidence of the causes below the aggregate.

‡ Incidence estimates for stroke represent first-ever stroke only.

^ Incidence of HIV/AIDS represents new infections of HIV only and does not include new infections of Tuberculosis in HIV positive cases.

SR Table 12: Global prevalence, incidence, and YLDs for 2016, percentage change of YLD counts and percentage change of YLD rates between 2006 and 2016 by cause, age 55-59 years.					
Cause	Prevalence in thousands (95% UI)	Incidence in thousands (95% UI)	YLDs in thousands (95% UI)		
	2016 counts	2016 counts	2016 counts	Percentage change in counts between 2006 and 2016	Percentage change in rates between 2006 and 2016
**All causes**	**344,221.7 (344,072.4 to 344,374.1)**	**1,866,285.0† (1,624,800.3 to 2,126,246.9)**	**56,103.8 (41,788.7 to 72,698.3)**	**30.0 (29.4 to 30.7)**	**-0.4 (-0.9 to 0.1)**
**Communicable, maternal, neonatal, and nutritional disorders**	**237,129.6 (233,657.1 to 240,439.5)**	**923,622.8† (716,766.4 to 1,167,894.5)**	**3,387.5 (2,445.4 to 4,507.7)**	**23.1 (19.2 to 26.6)**	**-5.7 (-8.7 to -3.0)**
**HIV/AIDS and tuberculosis**	**125,205.4 (109,147.0 to 143,108.9)**	**578.6† (428.8 to 751.9)**	**384.9 (256.6 to 540.3)**	**14.7 (-3.1 to 33.8)**	**-12.1 (-25.8 to 2.6)**
Tuberculosis	118,084.0 (95,815.9 to 142,292.3)	541.1† (392.2 to 714.7)	213.4 (130.1 to 315.5)	13.2 (9.0 to 18.1)	-13.2 (-16.5 to -9.5)
Drug-susceptible tuberculosis	691.4 (487.8 to 942.6)	521.1 (377.7 to 689.4)	205.6 (125.3 to 303.6)	13.4 (9.2 to 18.5)	-13.1 (-16.3 to -9.2)
Multidrug-resistant tuberculosis without extensive drug resistance	24.0 (17.1 to 32.1)	18.6 (13.7 to 24.4)	7.4 (4.5 to 11.1)	4.2 (-11.7 to 22.8)	-20.2 (-32.3 to -5.9)
Extensively drug-resistant tuberculosis	1.5 (1.0 to 2.0)	1.3 (1.0 to 1.7)	0.5 (0.3 to 0.8)	197.2 (157.7 to 241.7)	127.7 (97.5 to 161.8)
Latent tuberculosis infection	117,367.1 (95,003.5 to 141,590.9)	--	--	--	--
HIV/AIDS	1,671.9 (1,403.9 to 1,998.3)	37.5^ (29.4 to 47.1)	171.5 (108.7 to 257.4)	16.4 (-15.1 to 69.4)	-10.8 (-34.9 to 29.8)
Drug-susceptible HIV/AIDS - Tuberculosis	35.6 (24.9 to 49.4)	45.4 (32.6 to 61.7)	13.4 (7.9 to 20.7)	13.9 (3.9 to 24.6)	-12.7 (-20.4 to -4.5)
Multidrug-resistant HIV/AIDS - Tuberculosis without extensive drug resistance	1.1 (0.7 to 1.6)	1.3 (0.9 to 1.9)	0.4 (0.2 to 0.7)	9.2 (-10.3 to 29.1)	-16.3 (-31.3 to -1.1)
Extensively drug-resistant HIV/AIDS - Tuberculosis	< 0.05 (< 0.05 to 0.1)	< 0.05 (< 0.05 to 0.1)	< 0.05 (< 0.05 to < 0.05)	278.9 (224.8 to 348.9)	190.3 (148.9 to 244.0)
HIV/AIDS resulting in other diseases	1,635.2 (1,366.1 to 1,964.6)	37.5 (29.4 to 47.1)	157.6 (97.4 to 239.5)	16.7 (-17.2 to 77.4)	-10.6 (-36.6 to 35.9)
**Diarrhoea, lower respiratory infections, and other common infectious diseases**	**16,580.9 (15,366.2 to 17,669.3)**	**882,225.3† (675,328.9 to 1,127,804.8)**	**807.3 (561.9 to 1,113.2)**	**27.5 (25.8 to 29.2)**	**-2.3 (-3.6 to -1.0)**
Diarrhoeal diseases	2,760.2 (2,407.2 to 3,160.8)	189,381.0 (165,328.1 to 217,294.7)	299.0 (206.8 to 409.3)	27.2 (24.4 to 30.1)	-2.5 (-4.6 to -0.3)
Intestinal infectious diseases	12.5 (9.7 to 16.1)	291.6† (236.7 to 358.6)	2.2 (1.3 to 3.2)	10.5 (-7.7 to 34.2)	-15.3 (-29.3 to 2.9)
Typhoid fever	12.2 (9.3 to 16.0)	192.4 (154.2 to 238.1)	1.9 (1.1 to 2.8)	11.1 (-9.4 to 39.2)	-14.8 (-30.6 to 6.7)
Paratyphoid fever	4.4 (3.5 to 5.4)	99.3 (80.3 to 122.7)	0.3 (0.2 to 0.4)	8.5 (-8.8 to 30.1)	-16.9 (-30.1 to -0.3)
Other intestinal infectious diseases	--	--	< 0.05 (< 0.05 to < 0.05)	-15.6 (-29.5 to 2.5)	-35.3 (-46.0 to -21.4)
Lower respiratory infections	366.5 (328.3 to 407.7)	15,870.9 (13,866.5 to 18,145.7)	21.7 (14.7 to 30.8)	50.5 (42.9 to 58.8)	15.3 (9.5 to 21.7)
Upper respiratory infections	8,942.4 (6,190.0 to 12,235.2)	667,939.1 (464,442.8 to 908,183.3)	217.4 (117.8 to 382.6)	27.8 (25.8 to 29.8)	-2.0 (-3.6 to -0.5)
Otitis media	3,331.5 (2,512.9 to 4,287.2)	4,563.3 (3,844.4 to 5,454.2)	91.0 (54.2 to 143.5)	17.9 (12.6 to 23.3)	-9.7 (-13.7 to -5.5)
Meningitis	590.3 (498.5 to 685.1)	48.8 (35.0 to 64.4)	51.5 (36.6 to 68.5)	31.9 (28.1 to 35.7)	1.1 (-1.8 to 4.0)
Pneumococcal meningitis	267.0 (219.6 to 322.6)	11.1 (6.9 to 16.6)	23.2 (15.9 to 32.1)	29.7 (23.3 to 36.0)	-0.6 (-5.5 to 4.2)
H influenzae type B meningitis	65.4 (49.4 to 84.4)	3.2 (1.8 to 5.1)	6.9 (4.7 to 9.7)	7.6 (-1.1 to 15.4)	-17.6 (-24.2 to -11.5)
Meningococcal infection	65.3 (47.4 to 85.5)	8.6 (5.2 to 13.1)	5.8 (4.0 to 8.0)	33.1 (29.4 to 37.3)	2.0 (-0.8 to 5.2)
Other meningitis	192.6 (142.4 to 244.4)	25.9 (17.8 to 35.8)	15.6 (10.5 to 21.3)	50.4 (46.0 to 56.0)	15.3 (11.9 to 19.5)
Encephalitis	978.1 (628.4 to 1,452.5)	321.9 (272.5 to 376.9)	97.3 (68.4 to 127.1)	28.9 (23.2 to 35.3)	-1.2 (-5.6 to 3.6)
Diphtheria	< 0.05 (< 0.05 to < 0.05)	0.2 (0.1 to 0.2)	< 0.05 (< 0.05 to < 0.05)	-25.4 (-43.0 to -1.8)	-42.9 (-56.3 to -24.7)
Whooping cough	1.7 (1.4 to 2.2)	12.7 (9.9 to 16.0)	0.1 (0.1 to 0.1)	-3.3 (-5.3 to -1.3)	-25.9 (-27.4 to -24.4)
Tetanus	5.9 (5.7 to 6.0)	3.9 (1.9 to 5.8)	0.1 (0.1 to 0.2)	6.7 (-17.1 to 36.5)	-18.2 (-36.4 to 4.6)
Measles	0.1 (< 0.05 to 0.2)	3.5 (1.3 to 7.6)	< 0.05 (< 0.05 to < 0.05)	-48.5 (-54.5 to -42.0)	-60.6 (-65.1 to -55.6)
Varicella and herpes zoster	515.1 (416.7 to 629.3)	3,788.3 (3,094.9 to 4,615.9)	27.1 (15.9 to 42.0)	35.4 (29.3 to 42.2)	3.7 (-0.9 to 9.0)
**Neglected tropical diseases and malaria**	**75,423.4 (72,630.9 to 77,711.1)**	**12,681.0† (10,062.8 to 15,377.5)**	**539.2 (370.6 to 756.2)**	**-1.5 (-8.8 to 5.6)**	**-24.5 (-30.1 to -19.1)**
Malaria	1,937.1 (1,673.9 to 2,203.7)	2,599.1 (1,912.9 to 3,361.0)	21.1 (15.5 to 27.5)	-14.2 (-21.0 to -5.8)	-34.2 (-39.4 to -27.8)
Chagas disease	542.3 (458.9 to 639.0)	5.4 (3.6 to 8.0)	5.7 (3.7 to 8.3)	19.7 (11.4 to 28.7)	-8.2 (-14.7 to -1.4)
Leishmaniasis	271.5 (206.1 to 334.2)	29.1 (21.7 to 38.4)	14.3 (8.6 to 22.5)	23.6 (16.6 to 35.1)	-5.3 (-10.7 to 3.6)
Visceral leishmaniasis	1.2 (0.8 to 1.6)	4.7 (3.3 to 6.5)	0.1 (< 0.05 to 0.1)	-50.7 (-67.3 to -27.8)	-62.2 (-75.0 to -44.6)
Cutaneous and mucocutaneous leishmaniasis	232.2 (169.6 to 313.3)	24.3 (17.0 to 33.7)	14.2 (8.6 to 22.4)	24.7 (17.3 to 36.8)	-4.5 (-10.1 to 4.9)
African trypanosomiasis	0.2 (< 0.05 to 0.9)	0.2 (< 0.05 to 0.6)	0.1 (< 0.05 to 0.2)	-72.5 (-95.4 to 81.6)	-79.0 (-96.5 to 39.1)
Schistosomiasis	7,987.2 (7,328.1 to 8,609.1)	2,006.8 (1,819.6 to 2,218.8)	58.5 (28.5 to 109.9)	-28.0 (-30.2 to -25.2)	-44.8 (-46.6 to -42.7)
Cysticercosis	233.5 (167.7 to 319.2)	--	35.6 (22.2 to 53.9)	7.5 (-0.3 to 14.9)	-17.7 (-23.6 to -12.0)
Cystic echinococcosis	50.0 (24.8 to 104.1)	9.9 (4.4 to 24.7)	4.5 (2.0 to 9.5)	-35.2 (-75.2 to 66.8)	-50.4 (-81.0 to 27.8)
Lymphatic filariasis	1,426.1 (1,139.8 to 1,834.5)	284.0 (156.3 to 454.5)	74.6 (37.8 to 131.6)	-27.0 (-44.2 to -12.0)	-44.1 (-57.3 to -32.6)
Onchocerciasis	541.7 (370.7 to 839.3)	110.2 (45.1 to 229.8)	43.9 (22.8 to 78.1)	-19.5 (-37.8 to 0.6)	-38.3 (-52.3 to -22.9)
Trachoma	260.9 (125.5 to 452.7)	--	20.3 (11.3 to 34.6)	5.7 (-7.4 to 17.5)	-19.0 (-29.1 to -10.0)
Dengue	222.1 (121.1 to 376.2)	3,713.4 (2,259.6 to 5,630.8)	34.6 (15.3 to 64.2)	118.5 (74.9 to 407.0)	67.4 (34.0 to 288.5)
Yellow fever	0.1 (< 0.05 to 0.1)	2.0 (0.6 to 5.2)	< 0.05 (< 0.05 to < 0.05)	-19.5 (-49.7 to 24.5)	-38.3 (-61.5 to -4.6)
Rabies	< 0.05 (< 0.05 to < 0.05)	0.7 (0.3 to 1.0)	< 0.05 (< 0.05 to < 0.05)	-42.4 (-53.7 to -28.4)	-55.9 (-64.5 to -45.1)
Intestinal nematode infections	63,680.9 (60,388.1 to 66,275.6)	--	107.3 (61.7 to 175.9)	4.9 (-3.2 to 14.3)	-19.6 (-25.8 to -12.4)
Ascariasis	34,205.8 (30,531.9 to 38,698.7)	--	34.3 (18.6 to 57.0)	-8.9 (-20.9 to 4.6)	-30.2 (-39.4 to -19.9)

Cause	Prevalence in thousands (95% UI)	Incidence in thousands (95% UI)	YLDs in thousands (95% UI)		
	2016 counts	2016 counts	2016 counts	Percentage change in counts between 2006 and 2016	Percentage change in rates between 2006 and 2016
Trichuriasis	17,561.5 (16,147.6 to 19,216.1)	--	12.7 (6.9 to 21.5)	2.3 (-12.8 to 19.8)	-21.6 (-33.2 to -8.2)
Hookworm disease	18,672.3 (17,112.3 to 20,365.4)	--	60.4 (36.0 to 97.3)	15.4 (2.7 to 29.9)	-11.5 (-21.3 to -0.4)
Food-borne trematodiases	4,417.8 (4,006.7 to 4,867.6)	3,551.2 (1,526.4 to 5,243.6)	101.4 (48.6 to 183.3)	30.0 (23.8 to 37.4)	-0.4 (-5.1 to 5.3)
Leprosy	56.6 (53.8 to 59.2)	4.6 (4.2 to 5.1)	3.5 (2.3 to 4.9)	10.1 (1.9 to 19.1)	-15.6 (-21.9 to -8.7)
Ebola virus disease	< 0.05 (< 0.05 to 0.1)	< 0.05 (< 0.05 to < 0.05)	< 0.05 (< 0.05 to < 0.05)	--	--
Zika virus disease	6.0 (3.4 to 11.3)	364.5 (202.7 to 684.1)	0.1 (0.1 to 0.2)	--	--
Guinea worm disease	< 0.05 (< 0.05 to < 0.05)	< 0.05 (< 0.05 to < 0.05)	< 0.05 (< 0.05 to < 0.05)	-99.6 (-99.7 to -99.3)	-99.7 (-99.8 to -99.5)
Other neglected tropical diseases	1,126.7 (984.8 to 1,250.8)	--	13.8 (8.4 to 21.7)	39.4 (31.5 to 47.7)	6.8 (0.8 to 13.2)
**Maternal disorders**	**--**	**--**	**--**	**--**	**--**
Maternal haemorrhage	--	--	--	--	--
Maternal sepsis and other pregnancy related infections	--	--	--	--	--
Maternal hypertensive disorders	--	--	--	--	--
Maternal obstructed labour and uterine rupture	--	--	--	--	--
Maternal abortion, miscarriage, and ectopic pregnancy	--	--	--	--	--
Other maternal disorders	--	--	--	--	--
**Neonatal disorders**	**2,571.0 (2,361.2 to 2,798.9)**	**--**	**385.7 (274.3 to 532.9)**	**50.5 (35.8 to 68.6)**	**15.3 (4.1 to 29.2)**
Neonatal preterm birth complications	2,194.3 (1,865.5 to 2,572.2)	--	236.2 (164.6 to 330.1)	49.9 (24.6 to 78.9)	14.9 (-4.5 to 37.1)
Neonatal encephalopathy due to birth asphyxia and trauma	440.3 (298.4 to 640.4)	--	60.2 (28.1 to 119.4)	49.1 (38.1 to 66.1)	14.3 (5.8 to 27.3)
Neonatal sepsis and other neonatal infections	182.7 (79.5 to 430.2)	--	68.1 (24.3 to 175.6)	54.2 (43.5 to 69.2)	18.2 (10.0 to 29.7)
Hemolytic disease and other neonatal jaundice	64.1 (56.8 to 71.8)	--	21.2 (16.3 to 26.6)	49.8 (43.1 to 57.1)	14.8 (9.7 to 20.4)
Other neonatal disorders	--	--	--	--	--
**Nutritional deficiencies**	**59,241.9 (56,686.2 to 61,428.5)**	**12,279.5† (10,534.0 to 14,292.7)**	**1,160.6 (752.8 to 1,724.3)**	**29.8 (26.2 to 33.5)**	**-0.5 (-3.3 to 2.3)**
Protein-energy malnutrition	--	--	--	--	--
Iodine deficiency	6,522.8 (5,849.6 to 7,252.6)	21.1 (18.1 to 24.7)	166.6 (114.6 to 230.7)	12.1 (9.4 to 14.9)	-14.1 (-16.2 to -12.0)
Vitamin A deficiency	11,448.1 (10,137.7 to 12,955.2)	12,258.4 (10,513.0 to 14,270.1)	7.0 (4.5 to 10.4)	36.4 (26.4 to 47.5)	4.5 (-3.1 to 13.0)
Iron-deficiency anaemia	47,157.2 (40,890.8 to 52,725.4)	--	987.1 (631.8 to 1,478.9)	33.4 (29.3 to 37.4)	2.2 (-0.9 to 5.3)
Other nutritional deficiencies	--	--	--	--	--
**Other communicable, maternal, neonatal, and nutritional diseases**	**102,232.1 (98,438.1 to 105,813.2)**	**15,858.4 (13,130.0 to 19,248.6)**	**109.7 (74.1 to 163.6)**	**28.0 (18.2 to 38.3)**	**-1.9 (-9.4 to 6.0)**
Sexually transmitted diseases excluding HIV	74,680.4 (70,025.9 to 79,905.6)	10,262.8 (8,383.5 to 12,664.1)	72.7 (48.1 to 107.1)	32.0 (28.2 to 35.8)	1.1 (-1.8 to 4.0)
Syphilis	1,627.0 (1,107.2 to 2,338.0)	860.7 (527.9 to 1,315.3)	19.7 (13.4 to 26.8)	39.2 (33.3 to 45.3)	6.7 (2.2 to 11.3)
Chlamydial infection	217.0 (150.9 to 312.3)	520.1 (420.7 to 632.7)	4.8 (3.2 to 6.8)	27.3 (16.5 to 39.2)	-2.5 (-10.7 to 6.7)
Gonococcal infection	420.6 (215.6 to 743.5)	1,292.8 (681.3 to 2,409.8)	5.1 (2.5 to 9.5)	41.1 (21.2 to 57.0)	8.2 (-7.2 to 20.3)
Trichomoniasis	7,569.1 (5,283.6 to 10,749.9)	5,630.1 (4,071.0 to 7,752.5)	8.3 (3.0 to 20.1)	31.1 (28.5 to 33.8)	0.5 (-1.5 to 2.6)
Genital herpes	67,036.2 (58,683.4 to 77,529.3)	33.1 (18.5 to 54.2)	16.0 (4.7 to 36.6)	30.2 (27.7 to 33.0)	-0.2 (-2.2 to 1.9)
Other sexually transmitted diseases	132.7 (106.3 to 165.4)	1,926.0 (1,550.2 to 2,364.0)	18.9 (12.7 to 26.5)	25.9 (18.0 to 35.1)	-3.5 (-9.6 to 3.6)
Hepatitis	35,782.3 (30,976.9 to 41,162.8)	5,595.0 (3,625.0 to 8,419.3)	13.5 (7.2 to 23.6)	6.0 (-35.2 to 87.1)	-18.8 (-50.3 to 43.4)
Acute hepatitis A	60.9 (14.0 to 127.3)	791.0 (182.2 to 1,655.5)	2.5 (0.6 to 5.8)	-0.7 (-78.9 to 352.4)	-23.9 (-83.9 to 246.7)
Hepatitis B	24,956.1 (20,466.5 to 30,154.0)	4,135.1 (2,338.1 to 6,728.4)	9.9 (4.9 to 18.3)	7.4 (-40.6 to 94.0)	-17.7 (-54.5 to 48.7)
Hepatitis C	10,739.2 (9,657.7 to 11,928.8)	328.7 (245.0 to 429.0)	0.3 (0.1 to 0.6)	6.7 (-2.1 to 16.3)	-18.2 (-25.0 to -10.9)
Acute hepatitis E	26.2 (15.0 to 43.5)	340.2 (194.4 to 565.4)	0.8 (0.4 to 1.5)	10.1 (-11.9 to 38.4)	-15.6 (-32.5 to 6.0)
Other infectious diseases	1,351.0 (1,172.7 to 1,507.5)	0.6 (0.3 to 1.0)	23.4 (14.8 to 36.1)	31.6 (26.3 to 36.8)	0.8 (-3.2 to 4.8)
**Non-communicable diseases**	**340,082.8 (339,787.0 to 340,368.0)**	**912,187.5† (821,455.6 to 1,008,685.5)**	**48,167.8 (35,793.5 to 62,385.8)**	**30.1 (29.4 to 30.8)**	**-0.3 (-0.8 to 0.2)**
**Neoplasms**	**4,514.8 (4,451.8 to 4,585.1)**	**1,763.2 (1,695.7 to 1,852.0)**	**546.1 (401.9 to 712.0)**	**28.8 (25.7 to 32.6)**	**-1.3 (-3.7 to 1.6)**
Lip and oral cavity cancer	177.1 (170.5 to 184.0)	48.4 (46.6 to 50.4)	18.2 (13.1 to 23.9)	36.1 (27.1 to 45.8)	4.3 (-2.6 to 11.7)
Nasopharynx cancer	33.5 (31.6 to 35.6)	9.7 (9.2 to 10.3)	4.0 (2.9 to 5.4)	13.0 (2.4 to 24.3)	-13.4 (-21.5 to -4.7)
Other pharynx cancer	80.9 (76.2 to 84.5)	27.9 (26.1 to 29.2)	9.6 (7.0 to 12.6)	41.3 (28.4 to 53.1)	8.3 (-1.6 to 17.3)
Oesophageal cancer	60.7 (58.3 to 63.4)	49.8 (48.0 to 51.9)	13.1 (9.2 to 17.1)	-0.6 (-10.1 to 9.4)	-23.8 (-31.1 to -16.2)
Stomach cancer	209.1 (197.4 to 222.2)	112.6 (106.9 to 118.9)	30.6 (21.9 to 39.9)	10.0 (1.6 to 19.5)	-15.7 (-22.2 to -8.4)
Colon and rectum cancer	606.0 (582.3 to 634.7)	163.5 (157.2 to 171.0)	58.3 (42.8 to 76.6)	30.9 (22.6 to 39.8)	0.3 (-6.1 to 7.2)
Liver cancer	127.2 (115.1 to 142.0)	124.4 (116.0 to 133.3)	28.4 (20.1 to 37.9)	43.2 (30.9 to 57.6)	9.8 (0.3 to 20.8)
Liver cancer due to hepatitis B	80.7 (62.8 to 96.2)	58.1 (44.9 to 68.7)	13.2 (8.3 to 18.2)	43.5 (26.1 to 64.9)	10.0 (-3.4 to 26.4)

Cause	Prevalence in thousands (95% UI)	Incidence in thousands (95% UI)	YLDs in thousands (95% UI)		
	2016 counts	2016 counts	2016 counts	Percentage change in counts between 2006 and 2016	Percentage change in rates between 2006 and 2016
Liver cancer due to hepatitis C	26.1 (21.9 to 31.5)	19.8 (16.6 to 23.9)	4.6 (3.1 to 6.4)	34.8 (24.4 to 46.5)	3.3 (-4.7 to 12.2)
Liver cancer due to alcohol use	21.0 (15.1 to 28.0)	16.9 (12.1 to 22.5)	3.9 (2.4 to 5.8)	50.3 (32.0 to 72.0)	15.2 (1.2 to 31.8)
Liver cancer due to other causes	41.4 (32.9 to 53.5)	29.6 (23.7 to 37.8)	6.8 (4.5 to 9.7)	44.9 (26.2 to 68.5)	11.0 (-3.3 to 29.2)
Gallbladder and biliary tract cancer	13.6 (11.9 to 14.5)	15.8 (14.0 to 16.8)	3.6 (2.5 to 4.9)	17.7 (11.0 to 24.3)	-9.8 (-15.0 to -4.7)
Pancreatic cancer	31.3 (30.4 to 32.2)	37.8 (37.0 to 38.5)	8.2 (5.7 to 10.7)	25.9 (22.1 to 29.6)	-3.5 (-6.4 to -0.7)
Larynx cancer	99.2 (95.9 to 102.8)	28.9 (27.9 to 29.8)	11.6 (8.4 to 15.4)	22.8 (13.9 to 32.6)	-5.9 (-12.7 to 1.6)
Tracheal, bronchus, and lung cancer	280.2 (268.9 to 290.9)	206.0 (198.6 to 213.1)	50.6 (36.2 to 64.9)	21.8 (13.8 to 30.0)	-6.7 (-12.8 to -0.4)
Malignant skin melanoma	138.1 (119.2 to 154.7)	28.3 (24.6 to 31.8)	9.1 (6.3 to 12.8)	36.2 (27.6 to 45.1)	4.4 (-2.3 to 11.2)
Non-melanoma skin cancer	51.1 (34.7 to 82.9)	127.3 (71.2 to 212.4)	2.1 (1.0 to 4.2)	-9.0 (-26.0 to 7.8)	-30.3 (-43.3 to -17.4)
Non-melanoma skin cancer (squamous-cell carcinoma)	41.7 (20.9 to 78.8)	38.6 (18.9 to 75.3)	2.0 (0.8 to 4.0)	-10.5 (-28.3 to 6.7)	-31.4 (-45.0 to -18.2)
Non-melanoma skin cancer (basal-cell carcinoma)	10.4 (4.5 to 23.3)	88.7 (41.3 to 168.7)	0.1 (< 0.05 to 0.3)	25.0 (11.9 to 35.9)	-4.2 (-14.3 to 4.1)
Breast cancer	1,024.1 (964.7 to 1,087.2)	211.8 (199.4 to 226.1)	92.3 (67.0 to 124.1)	26.9 (18.4 to 36.6)	-2.7 (-9.3 to 4.7)
Cervical cancer	221.8 (176.2 to 240.1)	58.7 (47.5 to 63.3)	21.1 (14.5 to 28.2)	16.5 (8.1 to 28.0)	-10.7 (-17.2 to -1.9)
Uterine cancer	313.9 (293.4 to 339.3)	66.0 (61.8 to 71.4)	23.7 (16.5 to 32.0)	41.8 (28.5 to 57.3)	8.6 (-1.6 to 20.6)
Ovarian cancer	94.3 (87.7 to 98.4)	30.7 (29.0 to 32.0)	14.2 (10.1 to 18.6)	27.1 (13.7 to 40.8)	-2.6 (-12.8 to 7.9)
Prostate cancer	463.7 (408.9 to 556.0)	112.7 (100.6 to 131.3)	38.9 (26.7 to 52.8)	34.3 (25.9 to 44.3)	2.9 (-3.5 to 10.6)
Testicular cancer	10.9 (10.1 to 11.9)	2.2 (2.0 to 2.4)	0.8 (0.5 to 1.1)	45.0 (27.1 to 67.7)	11.1 (-2.6 to 28.5)
Kidney cancer	146.9 (139.3 to 154.4)	39.2 (37.4 to 41.1)	13.2 (9.4 to 17.7)	27.2 (18.3 to 36.9)	-2.5 (-9.4 to 4.9)
Bladder cancer	153.8 (149.0 to 158.8)	37.2 (36.1 to 38.4)	14.7 (10.4 to 19.5)	27.2 (19.4 to 34.7)	-2.5 (-8.5 to 3.2)
Brain and nervous system cancer	66.2 (59.1 to 73.0)	29.8 (27.2 to 33.3)	9.6 (6.7 to 12.5)	34.5 (26.5 to 45.3)	3.1 (-3.1 to 11.3)
Thyroid cancer	156.3 (148.3 to 166.7)	29.9 (28.4 to 31.9)	9.9 (6.9 to 13.5)	53.7 (42.9 to 66.3)	17.8 (9.5 to 27.4)
Mesothelioma	5.4 (5.0 to 5.8)	3.6 (3.4 to 3.9)	1.2 (0.8 to 1.6)	22.3 (16.3 to 27.7)	-6.2 (-10.9 to -2.1)
Hodgkin lymphoma	19.3 (17.1 to 21.9)	5.0 (4.4 to 5.9)	2.0 (1.4 to 2.7)	20.0 (11.8 to 29.1)	-8.0 (-14.4 to -1.1)
Non-Hodgkin's lymphoma	158.4 (145.0 to 176.1)	44.0 (40.3 to 48.5)	14.3 (10.3 to 18.9)	49.4 (40.7 to 56.8)	14.5 (7.8 to 20.2)
Multiple myeloma	35.6 (31.1 to 41.7)	14.4 (13.0 to 16.8)	7.6 (5.3 to 10.1)	42.2 (34.0 to 52.2)	9.0 (2.7 to 16.6)
Leukaemia	120.9 (107.6 to 129.0)	34.1 (30.5 to 36.1)	16.1 (11.5 to 21.6)	35.4 (27.8 to 44.5)	3.8 (-2.1 to 10.7)
Acute lymphoid leukaemia	12.9 (11.0 to 14.0)	3.8 (3.2 to 4.0)	1.7 (1.2 to 2.4)	50.8 (37.2 to 61.4)	15.6 (5.1 to 23.7)
Chronic lymphoid leukaemia	44.8 (41.2 to 50.0)	10.0 (9.2 to 11.2)	5.6 (3.9 to 7.5)	40.4 (30.7 to 51.9)	7.6 (0.1 to 16.4)
Acute myeloid leukaemia	9.6 (8.4 to 10.4)	7.2 (6.4 to 7.9)	2.0 (1.4 to 2.7)	39.6 (33.0 to 46.4)	7.0 (2.0 to 12.2)
Chronic myeloid leukaemia	8.2 (7.2 to 9.0)	2.9 (2.5 to 3.2)	1.1 (0.7 to 1.4)	13.2 (6.7 to 19.9)	-13.2 (-18.2 to -8.1)
Other leukaemia	45.5 (37.2 to 50.0)	10.1 (8.3 to 11.1)	5.7 (3.9 to 7.7)	30.1 (17.1 to 44.2)	-0.3 (-10.3 to 10.5)
Other neoplasms	218.6 (197.4 to 230.5)	63.5 (57.6 to 66.7)	19.1 (13.7 to 25.4)	58.2 (49.7 to 66.5)	21.3 (14.7 to 27.6)
**Cardiovascular diseases**	**46,100.5 (44,728.6 to 47,453.7)**	**5,049.6† (4,463.1 to 5,698.9)**	**2,955.8 (2,111.6 to 3,873.3)**	**34.8 (30.2 to 38.9)**	**3.3 (-0.2 to 6.5)**
Rheumatic heart disease	1,344.8 (1,218.2 to 1,482.1)	14.7 (11.3 to 17.8)	68.6 (45.2 to 98.0)	25.8 (21.9 to 29.8)	-3.6 (-6.6 to -0.5)
Ischaemic heart disease	15,465.8 (14,318.6 to 16,625.4)	1,766.0 (1,463.0 to 2,076.8)	676.5 (454.6 to 940.4)	34.0 (32.5 to 35.7)	2.7 (1.5 to 4.0)
Cerebrovascular disease	8,532.9 (8,144.9 to 8,843.8)	1,412.1‡ (1,158.9 to 1,662.6)	1,438.2 (1,032.4 to 1,859.3)	39.3 (29.6 to 47.6)	6.8 (-0.7 to 13.1)
Ischaemic stroke	7,085.0 (6,117.1 to 8,077.7)	1,003.2‡ (768.8 to 1,263.2)	1,164.5 (822.1 to 1,513.5)	42.5 (31.1 to 52.2)	9.2 (0.4 to 16.6)
Hemorrhagic stroke	1,668.2 (1,421.1 to 1,950.4)	408.9‡ (319.0 to 523.4)	273.7 (192.3 to 357.8)	27.2 (16.2 to 39.7)	-2.5 (-10.9 to 7.0)
Hypertensive heart disease	1,399.7 (1,081.8 to 1,790.0)	--	116.0 (74.0 to 171.3)	32.3 (28.3 to 36.1)	1.4 (-1.7 to 4.3)
Cardiomyopathy and myocarditis	443.0 (394.3 to 487.4)	205.6† (146.1 to 283.6)	40.0 (25.5 to 59.3)	25.5 (19.7 to 31.1)	-3.8 (-8.3 to 0.4)
Myocarditis	104.2 (83.6 to 127.4)	205.6 (146.1 to 283.6)	7.2 (4.6 to 10.5)	22.0 (15.6 to 29.3)	-6.5 (-11.4 to -0.9)
Alcoholic cardiomyopathy	155.0 (117.7 to 199.6)	--	13.0 (8.1 to 19.7)	29.9 (16.6 to 43.7)	-0.4 (-10.6 to 10.1)
Other cardiomyopathy	237.1 (177.5 to 309.9)	--	19.8 (12.3 to 29.9)	24.0 (18.1 to 29.5)	-5.0 (-9.5 to -0.7)
Atrial fibrillation and flutter	3,070.6 (2,543.0 to 3,624.4)	353.0 (247.7 to 480.1)	250.1 (158.4 to 357.5)	31.4 (28.7 to 34.4)	0.7 (-1.4 to 3.0)
Peripheral vascular disease	13,018.5 (10,683.3 to 15,841.3)	1,227.0 (809.2 to 1,712.1)	39.0 (17.1 to 72.2)	23.2 (21.0 to 25.3)	-5.6 (-7.3 to -4.0)
Endocarditis	25.9 (19.8 to 33.4)	71.2 (52.9 to 92.5)	2.1 (1.3 to 3.3)	26.7 (21.4 to 31.8)	-2.9 (-7.0 to 1.0)
Other cardiovascular and circulatory diseases	6,224.3 (4,416.2 to 8,673.3)	--	325.2 (200.4 to 511.8)	25.9 (22.4 to 29.2)	-3.5 (-6.2 to -1.0)
**Chronic respiratory diseases**	**45,162.5 (44,294.5 to 45,880.8)**	**5,990.7† (5,230.5 to 6,855.5)**	**2,790.9 (2,336.2 to 3,251.1)**	**35.5 (32.4 to 38.7)**	**3.8 (1.5 to 6.3)**
Chronic obstructive pulmonary disease	29,491.3 (27,616.1 to 31,299.2)	2,228.4 (1,888.4 to 2,568.3)	2,010.2 (1,719.8 to 2,253.9)	35.0 (31.0 to 39.1)	3.5 (0.4 to 6.6)
Pneumoconiosis	127.7 (115.7 to 140.9)	19.7 (17.2 to 22.4)	19.1 (12.7 to 26.9)	29.1 (18.7 to 40.4)	-1.1 (-9.0 to 7.6)
Silicosis	40.9 (33.9 to 49.9)	9.1 (7.2 to 11.3)	6.0 (3.8 to 8.7)	30.5 (7.9 to 56.4)	0.0 (-17.3 to 19.9)

Cause	Prevalence in thousands (95% UI)	Incidence in thousands (95% UI)	YLDs in thousands (95% UI)		
	2016 counts	2016 counts	2016 counts	Percentage change in counts between 2006 and 2016	Percentage change in rates between 2006 and 2016
Asbestosis	14.1 (11.7 to 16.9)	1.6 (1.1 to 2.1)	2.2 (1.4 to 3.2)	20.7 (15.6 to 25.6)	-7.5 (-11.4 to -3.7)
Coal workers pneumoconiosis	42.7 (36.2 to 50.3)	5.2 (4.1 to 6.3)	6.2 (4.1 to 9.2)	27.8 (6.5 to 55.3)	-2.1 (-18.4 to 19.0)
Other pneumoconiosis	30.0 (24.1 to 36.2)	3.7 (2.6 to 5.0)	4.6 (2.9 to 6.8)	33.5 (20.6 to 46.3)	2.3 (-7.6 to 12.1)
Asthma	17,996.4 (15,974.2 to 20,114.6)	3,708.2 (3,022.9 to 4,484.7)	684.5 (446.8 to 987.7)	35.6 (32.8 to 38.5)	3.9 (1.8 to 6.1)
Interstitial lung disease and pulmonary sarcoidosis	325.4 (271.8 to 390.9)	34.4 (23.9 to 47.8)	35.1 (22.2 to 52.0)	30.9 (22.3 to 39.1)	0.3 (-6.2 to 6.6)
Other chronic respiratory diseases	--	--	42.0 (35.8 to 47.1)	74.1 (69.1 to 79.2)	33.4 (29.6 to 37.3)
**Cirrhosis and other chronic liver diseases**	**4,988.2 (4,687.4 to 5,322.8)**	**179.0 (153.6 to 205.2)**	**144.5 (100.7 to 202.4)**	**32.0 (27.6 to 36.7)**	**1.2 (-2.2 to 4.7)**
Cirrhosis and other chronic liver diseases due to hepatitis B	1,372.4 (1,182.1 to 1,585.4)	51.5 (40.7 to 63.0)	43.6 (29.3 to 62.9)	33.1 (26.2 to 40.1)	2.0 (-3.3 to 7.4)
Cirrhosis and other chronic liver diseases due to hepatitis C	1,271.9 (1,080.7 to 1,483.8)	47.2 (38.3 to 57.9)	36.3 (23.9 to 51.3)	32.0 (25.0 to 39.7)	1.2 (-4.2 to 7.1)
Cirrhosis and other chronic liver diseases due to alcohol use	1,437.8 (1,205.1 to 1,662.9)	51.3 (40.9 to 63.3)	40.2 (25.9 to 58.0)	31.2 (24.3 to 38.3)	0.6 (-4.8 to 6.0)
Cirrhosis and other chronic liver diseases due to other causes	906.2 (692.9 to 1,159.4)	29.0 (21.3 to 38.0)	24.4 (15.0 to 37.3)	31.5 (23.2 to 40.5)	0.8 (-5.5 to 7.7)
**Digestive diseases**	**25,023.2 (23,386.1 to 26,338.1)**	**20,137.4† (17,396.4 to 23,413.3)**	**657.8 (444.0 to 920.6)**	**39.0 (32.3 to 46.1)**	**6.5 (1.4 to 12.0)**
Peptic ulcer disease	7,281.1 (5,937.6 to 9,125.2)	727.3 (562.2 to 915.2)	124.9 (71.3 to 205.5)	50.0 (35.1 to 64.4)	15.0 (3.5 to 26.0)
Gastritis and duodenitis	12,062.6 (9,208.9 to 15,750.5)	7,501.3 (5,765.9 to 9,709.2)	154.0 (89.8 to 238.7)	46.5 (32.5 to 59.8)	12.3 (1.5 to 22.5)
Appendicitis	27.3 (22.3 to 32.9)	738.4 (610.5 to 890.5)	8.3 (5.4 to 12.0)	36.6 (24.6 to 50.3)	4.7 (-4.5 to 15.2)
Paralytic ileus and intestinal obstruction	13.0 (10.0 to 16.4)	356.6 (272.2 to 450.6)	4.2 (2.6 to 6.3)	39.2 (32.1 to 46.0)	6.7 (1.2 to 11.9)
Inguinal, femoral, and abdominal hernia	4,188.3 (3,158.7 to 5,468.5)	4,120.3 (2,867.0 to 5,762.9)	183.4 (111.2 to 277.6)	25.9 (20.4 to 31.2)	-3.5 (-7.7 to 0.5)
Inflammatory bowel disease	695.8 (597.2 to 818.9)	236.3 (187.6 to 288.8)	99.2 (65.8 to 139.8)	38.0 (31.7 to 44.6)	5.7 (0.9 to 10.8)
Vascular intestinal disorders	7.5 (5.2 to 10.5)	40.7 (28.5 to 55.2)	2.4 (1.4 to 3.8)	30.4 (21.6 to 37.4)	-0.1 (-6.8 to 5.3)
Gallbladder and biliary diseases	1,725.7 (1,283.6 to 2,260.7)	5,890.9 (4,405.1 to 7,679.0)	26.3 (15.9 to 40.8)	40.7 (29.7 to 51.0)	7.9 (-0.6 to 15.8)
Pancreatitis	58.7 (45.6 to 73.8)	525.6 (408.9 to 665.9)	7.3 (4.5 to 10.9)	39.6 (25.4 to 54.9)	7.0 (-3.9 to 18.7)
Other digestive diseases	--	--	47.7 (32.2 to 66.7)	48.0 (40.8 to 55.5)	13.4 (7.9 to 19.2)
**Neurological disorders**	**132,561.8 (125,239.4 to 144,607.5)**	**47,176.5 (30,506.1 to 67,366.4)**	**3,744.2 (2,572.5 to 5,053.3)**	**30.7 (29.0 to 32.3)**	**0.1 (-1.1 to 1.4)**
Alzheimer's disease and other dementias	1,399.4 (1,080.9 to 1,793.5)	220.9 (142.9 to 324.5)	174.3 (113.2 to 257.4)	30.0 (26.6 to 33.7)	-0.4 (-3.0 to 2.4)
Parkinson's disease	394.9 (284.3 to 528.5)	50.1 (28.9 to 76.5)	48.9 (29.9 to 73.3)	37.8 (30.7 to 45.2)	5.6 (0.2 to 11.3)
Epilepsy	1,055.2 (857.2 to 1,273.8)	77.8 (53.6 to 112.5)	306.9 (208.0 to 426.2)	26.6 (13.5 to 41.5)	-3.0 (-13.1 to 8.5)
Multiple sclerosis	212.1 (194.4 to 230.6)	1.2 (0.8 to 1.7)	55.1 (38.8 to 73.1)	33.5 (26.4 to 40.9)	2.3 (-3.2 to 8.0)
Motor neuron disease	25.4 (19.8 to 32.6)	5.9 (4.3 to 7.9)	5.4 (3.5 to 7.8)	27.6 (24.6 to 30.7)	-2.2 (-4.5 to 0.2)
Migraine	57,838.5 (53,437.8 to 62,337.6)	3,749.5 (3,205.0 to 4,406.2)	2,609.6 (1,699.7 to 3,585.0)	30.7 (29.5 to 31.9)	0.1 (-0.8 to 1.1)
Tension-type headache	93,009.1 (67,364.4 to 123,872.0)	43,067.8 (26,585.8 to 63,365.2)	462.4 (286.0 to 682.4)	31.7 (29.7 to 34.0)	0.9 (-0.6 to 2.7)
Other neurological disorders	1.6 (0.8 to 2.8)	3.2 (1.8 to 5.2)	81.6 (57.9 to 107.8)	35.9 (28.4 to 44.1)	4.2 (-1.6 to 10.5)
**Mental and substance use disorders**	**60,968.9 (59,860.4 to 62,008.5)**	**24,742.0† (21,505.8 to 28,607.5)**	**8,708.4 (6,354.9 to 11,296.3)**	**26.8 (25.8 to 27.9)**	**-2.8 (-3.6 to -2.0)**
Schizophrenia	1,605.0 (1,414.2 to 1,815.7)	30.6 (25.1 to 36.5)	1,001.1 (756.1 to 1,227.1)	30.5 (28.0 to 32.9)	0.0 (-1.9 to 1.9)
Alcohol use disorders	5,605.6 (4,692.8 to 6,614.9)	2,292.2 (1,819.2 to 2,834.7)	543.8 (364.9 to 752.1)	22.4 (17.5 to 27.2)	-6.2 (-9.9 to -2.5)
Drug use disorders	1,876.0 (1,803.9 to 1,935.4)	216.5 (189.3 to 248.6)	530.5 (375.1 to 688.4)	33.2 (29.3 to 37.1)	2.1 (-0.9 to 5.0)
Opioid use disorders	1,058.5 (888.8 to 1,243.5)	150.5 (124.7 to 181.0)	423.0 (294.1 to 558.4)	34.7 (29.8 to 39.5)	3.2 (-0.6 to 6.9)
Cocaine use disorders	287.0 (252.7 to 322.8)	8.8 (6.6 to 11.6)	38.1 (23.7 to 54.2)	26.2 (19.2 to 34.0)	-3.3 (-8.7 to 2.7)
Amphetamine use disorders	57.5 (40.9 to 77.7)	7.3 (4.1 to 11.0)	7.7 (4.2 to 12.4)	25.0 (13.1 to 37.6)	-4.2 (-13.3 to 5.5)
Cannabis use disorders	362.0 (289.5 to 445.8)	27.5 (21.5 to 34.9)	10.6 (6.4 to 16.2)	27.3 (21.8 to 32.8)	-2.5 (-6.7 to 1.8)
Other drug use disorders	201.1 (167.5 to 236.1)	22.4 (17.3 to 28.2)	51.1 (33.7 to 69.9)	29.4 (24.1 to 35.4)	-0.8 (-4.9 to 3.8)
Depressive disorders	21,436.1 (20,715.6 to 22,136.1)	20,317.5 (16,968.4 to 24,077.8)	3,377.1 (2,298.7 to 4,675.2)	24.0 (22.1 to 26.0)	-5.0 (-6.5 to -3.4)
Major depressive disorder	12,402.8 (10,536.6 to 14,583.9)	18,953.2 (15,586.3 to 22,710.9)	2,474.1 (1,654.8 to 3,445.2)	21.1 (18.9 to 23.3)	-7.2 (-8.9 to -5.5)
Dysthymia	9,537.3 (7,938.6 to 11,409.8)	1,364.3 (1,019.4 to 1,742.9)	903.0 (589.1 to 1,327.7)	32.6 (29.2 to 36.1)	1.6 (-1.0 to 4.3)
Bipolar disorder	2,625.7 (2,190.3 to 3,173.4)	147.5 (110.7 to 195.1)	520.1 (317.1 to 786.1)	29.5 (27.0 to 31.9)	-0.8 (-2.7 to 1.1)
Anxiety disorders	16,019.1 (14,504.1 to 17,631.6)	1,694.0 (1,442.3 to 1,960.3)	1,498.1 (1,041.7 to 2,008.6)	27.9 (25.4 to 30.2)	-2.0 (-3.9 to -0.2)
Eating disorders	--	--	--	--	--
Anorexia nervosa	--	--	--	--	--
Bulimia nervosa	--	--	--	--	--
Autistic spectrum disorders	2,493.2 (2,383.0 to 2,596.9)	--	357.2 (244.3 to 500.9)	29.9 (27.7 to 32.0)	-0.5 (-2.1 to 1.2)
Autism	775.2 (666.8 to 904.9)	--	190.2 (123.0 to 273.0)	28.4 (25.0 to 31.8)	-1.6 (-4.2 to 1.0)
Asperger syndrome and other autistic spectrum disorders	1,722.7 (1,436.5 to 2,044.4)	--	167.0 (108.5 to 238.2)	31.6 (29.3 to 33.8)	0.9 (-0.9 to 2.5)

Cause	Prevalence in thousands (95% UI)	Incidence in thousands (95% UI)	YLDs in thousands (95% UI)		
	2016 counts	2016 counts	2016 counts	Percentage change in counts between 2006 and 2016	Percentage change in rates between 2006 and 2016
Attention-deficit/hyperactivity disorder	957.9 (828.9 to 1,102.7)	--	11.2 (6.7 to 17.6)	30.8 (25.2 to 37.0)	0.2 (-4.0 to 5.0)
Conduct disorder	--	--	--	--	--
Idiopathic developmental intellectual disability	3,595.2 (2,027.2 to 5,162.7)	--	152.4 (73.9 to 259.6)	19.8 (14.3 to 23.1)	-8.2 (-12.4 to -5.7)
Other mental and substance use disorders	9,843.5 (8,669.9 to 11,231.2)	43.7 (28.4 to 65.8)	717.1 (488.1 to 1,017.7)	30.3 (28.3 to 32.4)	-0.1 (-1.7 to 1.4)
**Diabetes, urogenital, blood, and endocrine diseases**	**164,104.3 (162,410.5 to 165,729.3)**	**33,918.8† (29,539.3 to 38,982.7)**	**5,516.2 (3,923.4 to 7,492.3)**	**30.4 (27.9 to 33.2)**	**-0.0 (-1.9 to 2.1)**
Diabetes mellitus	44,904.0 (40,254.5 to 49,795.5)	2,330.2 (1,832.2 to 2,928.7)	3,447.3 (2,353.2 to 4,800.8)	30.1 (26.0 to 34.4)	-0.3 (-3.4 to 3.0)
Acute glomerulonephritis	6.8 (4.5 to 10.0)	99.4 (65.3 to 149.2)	0.3 (0.2 to 0.6)	10.4 (6.8 to 13.7)	-15.4 (-18.2 to -12.9)
Chronic kidney disease	24,859.3 (20,817.1 to 29,368.1)	2,290.0 (1,598.5 to 3,140.1)	768.9 (543.2 to 1,026.1)	35.2 (31.1 to 39.4)	3.6 (0.4 to 6.9)
Chronic kidney disease due to diabetes mellitus	12,397.3 (9,296.6 to 15,603.8)	1,146.8 (751.7 to 1,635.0)	399.8 (274.2 to 555.1)	35.2 (30.7 to 40.0)	3.6 (0.2 to 7.3)
Chronic kidney disease due to hypertension	3,731.1 (2,730.4 to 4,906.8)	321.2 (191.6 to 490.8)	137.1 (90.4 to 199.2)	41.7 (34.1 to 50.0)	8.6 (2.8 to 15.0)
Chronic kidney disease due to glomerulonephritis	3,846.3 (2,883.4 to 5,028.9)	371.0 (236.4 to 562.0)	93.0 (55.5 to 143.0)	28.5 (20.4 to 35.5)	-1.5 (-7.7 to 3.8)
Chronic kidney disease due to other causes	4,884.7 (3,299.1 to 7,037.9)	451.0 (269.6 to 707.4)	138.9 (84.0 to 221.2)	33.7 (28.0 to 40.3)	2.4 (-1.9 to 7.5)
Urinary diseases and male infertility	16,290.6 (14,815.6 to 18,181.2)	26,524.9† (22,224.8 to 31,334.6)	546.3 (349.9 to 791.3)	26.0 (23.8 to 28.5)	-3.5 (-5.1 to -1.5)
Interstitial nephritis and urinary tract infections	334.7 (268.1 to 412.7)	17,427.7 (13,810.9 to 21,679.1)	10.8 (6.4 to 16.9)	37.9 (30.5 to 44.9)	5.7 (0.0 to 11.1)
Urolithiasis	283.3 (210.7 to 374.7)	7,480.9 (5,581.9 to 10,088.9)	20.4 (12.5 to 30.4)	30.2 (20.8 to 41.1)	-0.2 (-7.5 to 8.2)
Benign prostatic hyperplasia	15,204.6 (12,887.3 to 17,757.3)	1,616.4 (1,099.7 to 2,249.7)	505.1 (318.1 to 742.6)	25.9 (23.6 to 28.6)	-3.5 (-5.3 to -1.4)
Male infertility	--	--	--	--	--
Other urinary diseases	--	--	10.0 (6.5 to 14.1)	11.4 (5.2 to 17.6)	-14.6 (-19.4 to -9.9)
Gynecological diseases	43,139.7 (41,368.5 to 45,544.6)	1,001.4† (799.0 to 1,239.6)	249.0 (142.3 to 423.4)	26.5 (24.6 to 29.0)	-3.1 (-4.5 to -1.2)
Uterine fibroids	8,935.2 (7,882.3 to 10,206.7)	65.3 (35.5 to 101.7)	133.8 (78.3 to 221.6)	34.1 (31.8 to 36.8)	2.8 (1.0 to 4.8)
Polycystic ovarian syndrome	--	--	--	--	--
Female infertility	--	--	--	--	--
Endometriosis	--	--	--	--	--
Genital prolapse	36,872.4 (32,349.1 to 41,840.9)	936.1 (734.9 to 1,176.8)	115.2 (55.1 to 207.9)	18.7 (17.2 to 20.2)	-9.1 (-10.2 to -7.9)
Premenstrual syndrome	--	--	--	--	--
Other gynecological diseases	--	--	--	--	--
Hemoglobinopathies and hemolytic anaemias	76,191.0 (75,472.1 to 76,995.3)	--	207.3 (131.1 to 317.9)	36.2 (27.6 to 43.8)	4.4 (-2.2 to 10.2)
Thalassemias	0.2 (0.2 to 0.3)	--	< 0.05 (< 0.05 to < 0.05)	-6.0 (-25.9 to 7.5)	-27.9 (-43.2 to -17.6)
Thalassaemias trait	13,917.6 (13,383.4 to 14,496.5)	--	131.4 (82.5 to 204.7)	35.4 (23.7 to 45.7)	3.7 (-5.2 to 11.6)
Sickle cell disorders	4.2 (3.7 to 4.7)	--	0.7 (0.5 to 0.9)	65.1 (59.8 to 70.6)	26.5 (22.5 to 30.7)
Sickle cell trait	16,175.9 (14,841.4 to 17,958.8)	--	40.8 (26.0 to 61.4)	39.5 (34.1 to 45.1)	6.9 (2.7 to 11.2)
G6PD deficiency	13,029.2 (12,559.5 to 13,572.3)	--	1.1 (0.7 to 1.6)	30.7 (24.8 to 36.2)	0.1 (-4.4 to 4.4)
G6PD trait	38,147.7 (37,517.5 to 38,876.7)	--	< 0.05 (< 0.05 to < 0.05)	32.7 (14.5 to 57.7)	1.7 (-12.3 to 20.8)
Other hemoglobinopathies and hemolytic anaemias	2,553.0 (2,218.5 to 2,846.8)	--	33.2 (21.0 to 50.6)	35.5 (28.2 to 42.5)	3.9 (-1.7 to 9.2)
Endocrine, metabolic, blood, and immune disorders	9,207.2 (7,493.4 to 11,334.9)	1,672.9 (1,181.1 to 2,251.5)	297.0 (189.9 to 438.4)	31.3 (27.8 to 34.7)	0.6 (-2.1 to 3.2)
**Musculoskeletal disorders**	**121,839.5 (117,155.8 to 126,369.3)**	**59,650.4 (47,300.0 to 74,549.7)**	**13,360.2 (9,503.5 to 18,042.5)**	**30.0 (28.2 to 31.7)**	**-0.4 (-1.8 to 0.9)**
Rheumatoid arthritis	2,448.9 (2,130.6 to 2,814.2)	126.0 (94.2 to 163.5)	574.1 (386.8 to 775.6)	35.5 (31.9 to 38.9)	3.9 (1.1 to 6.4)
Osteoarthritis	39,052.0 (36,119.2 to 42,114.7)	2,091.5 (1,787.2 to 2,418.9)	2,173.7 (1,525.6 to 2,961.3)	34.3 (33.1 to 35.6)	2.9 (2.0 to 3.9)
Low back and neck pain	68,616.6 (63,160.1 to 73,848.4)	26,345.5 (18,372.8 to 35,837.5)	8,084.0 (5,470.5 to 11,370.8)	29.2 (26.6 to 31.6)	-1.0 (-3.0 to 0.9)
Low back pain	45,677.6 (33,100.1 to 61,424.9)	22,248.6 (14,242.6 to 31,320.7)	5,202.6 (3,251.4 to 7,749.2)	28.6 (24.5 to 32.0)	-1.5 (-4.6 to 1.2)
Neck pain	29,077.5 (20,939.2 to 38,595.1)	4,096.9 (2,238.2 to 6,412.3)	2,881.4 (1,745.8 to 4,299.6)	30.4 (27.1 to 33.5)	-0.1 (-2.6 to 2.3)
Gout	3,929.9 (3,199.9 to 4,759.3)	790.4 (567.6 to 1,035.7)	125.3 (81.9 to 180.1)	28.8 (24.2 to 33.8)	-1.3 (-4.8 to 2.5)
Other musculoskeletal disorders	28,601.0 (20,237.5 to 38,969.6)	30,297.0 (20,641.0 to 42,527.9)	2,403.0 (1,450.9 to 3,756.9)	27.8 (22.3 to 32.9)	-2.0 (-6.2 to 1.8)
**Other non-communicable diseases**	**297,553.5 (295,260.5 to 299,258.0)**	**713,579.8† (625,604.4 to 805,364.6)**	**9,744.0 (6,556.5 to 14,292.2)**	**29.4 (28.4 to 30.4)**	**-0.8 (-1.6 to -0.1)**
Congenital anomalies	2,968.3 (2,761.5 to 3,170.1)	--	387.6 (278.8 to 516.7)	30.0 (26.6 to 33.2)	-0.4 (-3.0 to 2.1)
Neural tube defects	227.0 (206.3 to 251.1)	--	67.0 (47.9 to 89.0)	32.1 (26.3 to 38.0)	1.2 (-3.2 to 5.7)
Congenital heart anomalies	435.5 (389.2 to 486.7)	--	24.3 (10.0 to 42.4)	32.7 (27.6 to 38.1)	1.7 (-2.2 to 5.8)
Orofacial clefts	166.2 (148.3 to 184.1)	--	2.0 (1.3 to 2.9)	21.7 (18.3 to 25.0)	-6.8 (-9.4 to -4.2)
Down's syndrome	40.3 (33.9 to 46.9)	--	6.9 (4.6 to 9.4)	29.1 (24.6 to 33.3)	-1.0 (-4.5 to 2.1)
Turner syndrome	51.4 (44.2 to 59.8)	--	0.7 (0.3 to 1.2)	32.4 (11.3 to 57.3)	1.4 (-14.7 to 20.6)
Klinefelter syndrome	21.2 (17.4 to 25.4)	--	0.1 (< 0.05 to 0.1)	23.3 (19.7 to 26.9)	-5.5 (-8.3 to -2.8)

Cause	Prevalence in thousands (95% UI)	Incidence in thousands (95% UI)	YLDs in thousands (95% UI)		
	2016 counts	2016 counts	2016 counts	Percentage change in counts between 2006 and 2016	Percentage change in rates between 2006 and 2016
Other chromosomal abnormalities	< 0.05<br>(< 0.05 to < 0.05)	--	< 0.05<br>(< 0.05 to < 0.05)	-16.8<br>(-74.5 to 158.9)	-36.2<br>(-80.5 to 98.4)
Congenital musculoskeletal and limb anomalies	546.7<br>(506.4 to 591.2)	--	79.1<br>(54.8 to 108.1)	29.8<br>(25.0 to 34.8)	-0.5<br>(-4.2 to 3.3)
Urogenital congenital anomalies	140.5<br>(126.7 to 155.3)	--	2.1<br>(1.1 to 3.8)	34.6<br>(30.6 to 38.9)	3.1<br>(0.1 to 6.5)
Digestive congenital anomalies	496.7<br>(448.6 to 548.6)	--	20.9<br>(13.8 to 30.1)	28.2<br>(21.6 to 35.0)	-1.7<br>(-6.8 to 3.5)
Other congenital anomalies	1,478.4<br>(1,034.0 to 2,072.0)	--	184.5<br>(124.4 to 265.0)	29.2<br>(23.9 to 34.7)	-1.0<br>(-5.0 to 3.2)
Skin and subcutaneous diseases	99,961.8<br>(97,503.4 to 102,257.1)	251,550.2<br>(225,029.7 to 282,084.2)	1,842.1<br>(1,238.8 to 2,697.4)	31.1<br>(30.0 to 32.1)	0.5<br>(-0.4 to 1.2)
Dermatitis	11,745.4<br>(9,847.5 to 14,036.5)	20,526.4<br>(15,522.2 to 26,129.6)	374.7<br>(228.5 to 569.6)	26.8<br>(24.9 to 28.8)	-2.8<br>(-4.3 to -1.3)
Psoriasis	4,813.3<br>(4,427.2 to 5,225.2)	572.6<br>(498.2 to 649.7)	408.8<br>(293.1 to 537.4)	33.5<br>(30.6 to 36.3)	2.3<br>(0.1 to 4.5)
Cellulitis	171.9<br>(141.5 to 208.0)	3,737.3<br>(3,114.5 to 4,429.4)	9.5<br>(6.0 to 14.1)	27.5<br>(19.9 to 36.0)	-2.3<br>(-8.2 to 4.2)
Pyoderma	1,320.0<br>(1,215.6 to 1,432.1)	31,959.2<br>(28,843.4 to 35,269.3)	7.3<br>(2.9 to 15.7)	32.1<br>(29.2 to 35.0)	1.2<br>(-1.0 to 3.5)
Scabies	4,256.6<br>(2,842.5 to 6,144.6)	12,907.5<br>(8,606.0 to 18,287.4)	106.9<br>(52.2 to 180.0)	28.8<br>(26.4 to 31.4)	-1.3<br>(-3.2 to 0.7)
Fungal skin diseases	30,293.1<br>(24,257.3 to 37,510.3)	107,539.4<br>(83,499.6 to 136,846.4)	168.5<br>(62.6 to 367.6)	32.4<br>(31.7 to 33.0)	1.4<br>(1.0 to 1.9)
Viral skin diseases	6,102.7<br>(5,232.3 to 7,105.3)	9,813.8<br>(8,424.5 to 11,411.4)	181.5<br>(112.5 to 267.6)	30.2<br>(28.4 to 32.0)	-0.2<br>(-1.6 to 1.2)
Acne vulgaris	5,199.0<br>(4,597.2 to 5,874.6)	2,324.3<br>(2,030.2 to 2,661.0)	128.8<br>(86.2 to 186.7)	31.2<br>(27.5 to 34.9)	0.5<br>(-2.3 to 3.4)
Alopecia areata	763.6<br>(676.4 to 855.6)	1,379.2<br>(1,244.3 to 1,522.8)	24.5<br>(15.6 to 37.5)	26.9<br>(22.5 to 31.2)	-2.8<br>(-6.1 to 0.5)
Pruritus	4,622.2<br>(3,151.4 to 6,248.0)	3,709.2<br>(2,527.0 to 5,194.9)	48.7<br>(21.0 to 94.0)	32.5<br>(30.6 to 34.4)	1.6<br>(0.1 to 3.0)
Urticaria	2,160.1<br>(1,501.8 to 2,985.3)	3,777.5<br>(2,669.9 to 5,076.0)	125.0<br>(72.9 to 193.4)	30.6<br>(27.0 to 33.8)	0.1<br>(-2.6 to 2.5)
Decubitus ulcer	104.0<br>(74.2 to 134.6)	375.9<br>(271.3 to 491.9)	17.9<br>(10.9 to 26.6)	29.1<br>(22.0 to 35.7)	-1.1<br>(-6.5 to 4.0)
Other skin and subcutaneous diseases	43,927.1<br>(40,310.5 to 48,199.7)	52,928.1<br>(47,863.4 to 58,940.5)	240.0<br>(114.3 to 439.2)	35.7<br>(34.8 to 36.6)	4.0<br>(3.3 to 4.7)
Sense organ diseases	187,450.0<br>(184,281.1 to 189,749.7)	37,513.5†<br>(33,966.4 to 41,272.2)	5,736.4<br>(3,875.1 to 8,435.8)	28.4<br>(26.7 to 30.1)	-1.6<br>(-2.9 to -0.3)
Glaucoma	479.7<br>(372.9 to 615.1)	--	49.0<br>(30.2 to 74.0)	35.2<br>(29.6 to 41.1)	3.6<br>(-0.7 to 8.1)
Cataract	6,560.4<br>(4,970.2 to 8,396.7)	--	453.2<br>(301.0 to 656.2)	36.2<br>(32.8 to 39.5)	4.4<br>(1.8 to 6.9)
Macular degeneration	546.5<br>(409.9 to 705.9)	--	38.8<br>(24.6 to 56.9)	34.4<br>(27.8 to 40.4)	3.0<br>(-2.1 to 7.6)
Refraction and accommodation disorders	84,473.4<br>(77,821.9 to 90,640.9)	--	1,354.2<br>(807.6 to 2,173.3)	23.2<br>(21.1 to 25.0)	-5.6<br>(-7.2 to -4.2)
Age-related and other hearing loss	137,270.3<br>(122,671.9 to 150,884.8)	--	3,057.7<br>(1,999.7 to 4,571.5)	28.7<br>(25.5 to 31.5)	-1.4<br>(-3.8 to 0.8)
Other vision loss	2,819.1<br>(2,158.4 to 3,658.7)	--	222.5<br>(141.1 to 320.3)	32.9<br>(29.3 to 36.2)	1.8<br>(-0.9 to 4.4)
Other sense organ diseases	17,596.0<br>(15,288.5 to 20,252.6)	37,513.5<br>(33,966.4 to 41,272.2)	560.9<br>(349.9 to 847.9)	31.4<br>(29.8 to 33.1)	0.7<br>(-0.6 to 2.0)
Oral disorders	196,811.1<br>(191,083.7 to 202,551.8)	424,516.1†<br>(341,065.8 to 508,306.7)	1,777.9<br>(1,072.6 to 2,845.8)	30.8<br>(30.0 to 31.4)	0.2<br>(-0.4 to 0.7)
Caries of deciduous teeth	--	--	--	--	--
Caries of permanent teeth	128,021.6<br>(107,468.9 to 150,277.0)	412,806.7<br>(329,903.9 to 496,524.0)	91.5<br>(39.7 to 177.6)	25.5<br>(24.0 to 26.9)	-3.9<br>(-5.0 to -2.7)
Periodontal disease	85,623.1<br>(67,876.6 to 103,711.7)	8,566.0<br>(4,214.6 to 12,520.8)	556.8<br>(219.2 to 1,195.5)	33.1<br>(32.2 to 34.0)	2.0<br>(1.3 to 2.7)
Edentulism and severe tooth loss	31,325.9<br>(25,243.4 to 38,787.9)	3,143.3<br>(2,049.4 to 4,559.5)	878.5<br>(558.0 to 1,322.2)	30.0<br>(28.9 to 31.1)	-0.4<br>(-1.2 to 0.5)
Other oral disorders	8,718.5<br>(7,717.2 to 9,689.1)	--	251.0<br>(153.8 to 382.9)	30.3<br>(28.8 to 31.7)	-0.1<br>(-1.3 to 0.9)
**Injuries**	**125,958.6<br>(118,869.4 to 133,862.5)**	**30,474.7†<br>(26,847.9 to 34,560.9)**	**4,548.4<br>(3,066.2 to 6,491.6)**	**35.1<br>(33.7 to 36.5)**	**3.5<br>(2.5 to 4.6)**
**Transport injuries**	**23,111.0<br>(21,534.0 to 24,989.2)**	**3,678.1<br>(2,769.1 to 4,674.2)**	**1,165.6<br>(782.4 to 1,662.9)**	**38.7<br>(37.4 to 40.0)**	**6.3<br>(5.3 to 7.3)**
Road injuries	19,426.9<br>(17,850.8 to 21,058.0)	3,322.8<br>(2,431.9 to 4,314.4)	988.3<br>(663.4 to 1,411.2)	40.6<br>(39.3 to 41.7)	7.7<br>(6.7 to 8.6)
Pedestrian road injuries	4,235.2<br>(3,656.2 to 4,880.5)	586.6<br>(376.1 to 868.3)	220.6<br>(148.5 to 311.0)	42.0<br>(40.5 to 43.5)	8.8<br>(7.6 to 10.0)
Cyclist road injuries	3,771.2<br>(3,139.8 to 4,404.5)	790.9<br>(511.5 to 1,129.0)	180.8<br>(120.6 to 259.2)	42.3<br>(41.0 to 43.6)	9.1<br>(8.0 to 10.0)
Motorcyclist road injuries	4,614.2<br>(3,949.3 to 5,483.1)	470.3<br>(296.2 to 696.7)	230.9<br>(155.3 to 329.0)	42.6<br>(41.4 to 43.8)	9.3<br>(8.3 to 10.2)
Motor vehicle road injuries	5,419.9<br>(4,458.7 to 6,294.4)	1,134.4<br>(771.2 to 1,554.1)	289.8<br>(194.6 to 410.3)	32.3<br>(30.5 to 33.9)	1.4<br>(0.0 to 2.6)
Other road injuries	1,386.3<br>(1,150.0 to 1,704.2)	340.6<br>(187.2 to 558.2)	66.2<br>(43.9 to 96.2)	67.0<br>(65.1 to 68.6)	27.9<br>(26.6 to 29.2)
Other transport injuries	3,684.1<br>(3,061.7 to 4,236.2)	355.3<br>(230.1 to 521.4)	177.3<br>(120.0 to 251.2)	29.1<br>(27.3 to 31.0)	-1.0<br>(-2.5 to 0.4)
**Unintentional injuries**	**92,156.1<br>(85,726.0 to 99,502.2)**	**25,483.7<br>(21,852.6 to 29,592.5)**	**3,017.6<br>(2,058.3 to 4,240.2)**	**33.5<br>(32.4 to 34.6)**	**2.4<br>(1.5 to 3.1)**
Falls	39,851.6<br>(35,485.4 to 45,320.0)	12,534.7<br>(9,537.2 to 16,249.0)	1,612.6<br>(1,131.5 to 2,225.0)	35.3<br>(33.9 to 36.6)	3.7<br>(2.6 to 4.7)
Drowning	350.5<br>(308.1 to 399.1)	27.8<br>(20.8 to 37.0)	16.9<br>(11.3 to 24.0)	17.3<br>(14.8 to 19.6)	-10.1<br>(-12.1 to -8.3)
Fire, heat, and hot substances	8,304.0<br>(7,191.3 to 9,599.3)	322.5<br>(227.7 to 446.2)	184.0<br>(119.2 to 279.1)	30.6<br>(28.8 to 32.6)	0.1<br>(-1.3 to 1.6)
Poisonings	256.3<br>(219.5 to 294.6)	153.2<br>(94.2 to 224.8)	18.2<br>(11.9 to 26.1)	36.6<br>(32.9 to 40.4)	4.7<br>(1.9 to 7.6)
Exposure to mechanical forces	17,605.4<br>(14,963.0 to 21,345.7)	2,897.7<br>(2,129.7 to 3,892.6)	388.4<br>(255.3 to 563.2)	35.6<br>(34.4 to 36.7)	3.9<br>(3.0 to 4.7)
Unintentional firearm injuries	635.9<br>(520.5 to 773.1)	40.2<br>(20.6 to 70.9)	19.1<br>(12.4 to 27.8)	35.7<br>(34.4 to 37.0)	4.0<br>(3.0 to 5.0)
Unintentional suffocation	609.4<br>(488.1 to 765.6)	32.9<br>(16.3 to 59.6)	17.9<br>(11.4 to 26.8)	31.2<br>(29.8 to 32.7)	0.6<br>(-0.5 to 1.7)
Other exposure to mechanical forces	16,360.2<br>(13,752.9 to 20,121.8)	2,824.6<br>(2,067.8 to 3,796.3)	351.4<br>(231.3 to 509.7)	35.8<br>(34.5 to 36.9)	4.0<br>(3.1 to 4.9)

Cause	Prevalence in thousands (95% UI)	Incidence in thousands (95% UI)	YLDs in thousands (95% UI)		
	2016 counts	2016 counts	2016 counts	Percentage change in counts between 2006 and 2016	Percentage change in rates between 2006 and 2016
Adverse effects of medical treatment	149.3<br>(101.3 to 211.4)	1,952.0<br>(1,379.2 to 2,640.8)	19.9<br>(11.3 to 31.5)	22.9<br>(17.8 to 27.3)	-5.8<br>(-9.7 to -2.4)
Animal contact	4,176.3<br>(3,692.5 to 4,738.0)	2,661.6<br>(1,873.5 to 3,775.7)	103.2<br>(70.0 to 144.9)	22.9<br>(20.9 to 24.6)	-5.8<br>(-7.4 to -4.5)
Venomous animal contact	1,857.9<br>(1,577.4 to 2,205.1)	1,271.3<br>(860.8 to 1,895.4)	67.4<br>(46.1 to 92.8)	26.0<br>(23.4 to 28.4)	-3.4<br>(-5.4 to -1.6)
Non-venomous animal contact	2,318.4<br>(1,943.8 to 2,751.7)	1,390.3<br>(938.9 to 2,011.6)	35.8<br>(23.0 to 55.5)	17.5<br>(15.3 to 19.4)	-9.9<br>(-11.7 to -8.5)
Foreign body	3,531.0<br>(3,011.2 to 4,025.0)	1,353.7<br>(915.4 to 2,042.9)	121.8<br>(80.3 to 177.1)	31.2<br>(29.2 to 33.5)	0.6<br>(-1.0 to 2.3)
Pulmonary aspiration and foreign body in airway	1,599.9<br>(1,269.4 to 1,998.1)	305.9<br>(208.8 to 422.6)	61.6<br>(39.9 to 89.6)	28.2<br>(26.1 to 30.5)	-1.7<br>(-3.4 to 0.0)
Foreign body in eyes	201.1<br>(130.7 to 303.6)	744.3<br>(355.1 to 1,420.4)	12.2<br>(6.8 to 20.4)	40.5<br>(35.2 to 49.1)	7.6<br>(3.6 to 14.2)
Foreign body in other body part	1,730.0<br>(1,451.1 to 2,117.2)	303.6<br>(178.9 to 475.6)	48.0<br>(31.0 to 69.0)	32.9<br>(31.2 to 34.6)	1.9<br>(0.6 to 3.1)
Environmental heat and cold exposure	5,700.6<br>(4,957.3 to 6,542.8)	908.7<br>(587.2 to 1,293.5)	208.9<br>(140.7 to 298.3)	32.3<br>(30.2 to 34.4)	1.4<br>(-0.2 to 3.0)
Other unintentional injuries	12,231.1<br>(10,167.9 to 14,701.6)	2,672.0<br>(1,744.2 to 3,781.5)	343.6<br>(224.5 to 500.1)	31.5<br>(30.1 to 32.9)	0.8<br>(-0.3 to 1.8)
**Self-harm and interpersonal violence**	**10,440.2<br>(8,940.7 to 12,416.2)**	**1,285.1†<br>(999.6 to 1,657.8)**	**254.6<br>(169.5 to 367.7)**	**22.8<br>(21.9 to 23.7)**	**-5.9<br>(-6.6 to -5.2)**
Self-harm	1,126.4<br>(927.2 to 1,327.4)	380.6<br>(285.5 to 512.5)	46.3<br>(29.8 to 66.9)	14.8<br>(13.5 to 16.2)	-12.0<br>(-13.1 to -11.0)
Self-harm by firearm	30.8<br>(26.3 to 35.3)	4.9<br>(1.4 to 12.6)	1.0<br>(0.7 to 1.5)	10.7<br>(8.6 to 12.8)	-15.2<br>(-16.8 to -13.6)
Self-harm by other specified means	1,095.6<br>(896.2 to 1,297.9)	375.7<br>(282.9 to 506.2)	45.2<br>(29.2 to 65.4)	14.9<br>(13.5 to 16.3)	-11.9<br>(-13.0 to -10.9)
Interpersonal violence	9,313.8<br>(7,880.6 to 11,250.2)	904.5†<br>(640.5 to 1,266.8)	208.4<br>(139.8 to 299.4)	24.8<br>(23.8 to 25.7)	-4.4<br>(-5.1 to -3.7)
Assault by firearm	272.2<br>(230.6 to 321.3)	20.4<br>(10.5 to 35.5)	9.1<br>(5.9 to 13.4)	25.8<br>(24.6 to 27.2)	-3.6<br>(-4.5 to -2.5)
Assault by sharp object	1,375.5<br>(1,133.3 to 1,627.0)	143.2<br>(79.5 to 241.5)	34.2<br>(22.1 to 50.0)	23.1<br>(21.6 to 24.4)	-5.6<br>(-6.8 to -4.6)
Sexual violence	3,813.3<br>(2,485.5 to 5,648.4)	--	26.2<br>(14.6 to 43.1)	25.1<br>(23.8 to 26.3)	-4.2<br>(-5.1 to -3.2)
Assault by other means	3,852.9<br>(3,245.4 to 4,644.9)	740.9<br>(518.2 to 1,041.1)	138.8<br>(93.3 to 200.7)	25.0<br>(23.9 to 26.1)	-4.2<br>(-5.0 to -3.4)
**Forces of nature, conflict and terrorism, and executions and police conflict**	**4,064.6<br>(2,695.7 to 5,761.8)**	**27.8†<br>(18.3 to 38.9)**	**110.6<br>(36.8 to 241.5)**	**85.8<br>(71.3 to 108.1)**	**42.4<br>(31.3 to 59.5)**
Forces of nature, conflict and terrorism, and state actor violence	499.2<br>(287.2 to 762.9)	13.1<br>(7.0 to 21.1)	17.0<br>(4.3 to 41.3)	86.5<br>(72.4 to 95.2)	42.9<br>(32.1 to 49.6)
Conflict and terrorism	2,714.2<br>(1,602.3 to 4,253.3)	--	79.6<br>(30.4 to 159.5)	86.2<br>(67.7 to 117.2)	42.7<br>(28.5 to 66.5)
Executions and police conflict	851.2<br>(207.2 to 1,371.9)	14.7<br>(7.9 to 23.1)	18.0<br>(2.9 to 48.2)	76.3<br>(64.3 to 89.8)	35.1<br>(25.9 to 45.4)

We did not calculate incidence for certain NTDs and causes with only birth prevalence. Blank cells mean that no estimate is available.

† Incidence for aggregate causes represents the sum of the incidence of the causes below the aggregate.

‡ Incidence estimates for stroke represent first-ever stroke only.

^ Incidence of HIV/AIDS represents new infections of HIV only and does not include new infections of Tuberculosis in HIV positive cases.

SR Table 12: Global prevalence, incidence, and YLDs for 2016, percentage change of YLD counts and percentage change of YLD rates between 2006 and 2016 by cause, age 60-64 years.					
Cause	Prevalence in thousands (95% UI)	Incidence in thousands (95% UI)	YLDs in thousands (95% UI)		
	2016 counts	2016 counts	2016 counts	Percentage change in counts between 2006 and 2016	Percentage change in rates between 2006 and 2016
**All causes**	**299,009.8 (298,929.5 to 299,088.6)**	**1,662,103.4† (1,474,657.1 to 1,892,166.3)**	**54,084.0 (40,571.9 to 69,155.6)**	**49.4 (48.6 to 50.1)**	**-0.9 (-1.4 to -0.4)**
**Communicable, maternal, neonatal, and nutritional disorders**	**200,406.1 (197,307.1 to 203,595.7)**	**792,439.7† (630,728.6 to 993,454.7)**	**2,677.5 (1,944.1 to 3,529.2)**	**33.1 (29.6 to 36.9)**	**-11.7 (-14.1 to -9.2)**
**HIV/AIDS and tuberculosis**	**106,185.7 (93,143.0 to 119,955.4)**	**497.8† (377.8 to 637.9)**	**330.0 (212.3 to 468.9)**	**24.9 (8.5 to 41.7)**	**-17.2 (-28.0 to -6.0)**
Tuberculosis	99,777.0 (82,884.9 to 118,949.9)	466.8† (345.7 to 605.9)	223.8 (137.0 to 337.1)	29.6 (24.5 to 36.2)	-14.0 (-17.4 to -9.7)
Drug-susceptible tuberculosis	735.1 (537.2 to 968.6)	449.6 (332.1 to 584.8)	215.4 (131.7 to 324.9)	29.6 (24.4 to 36.3)	-14.1 (-17.5 to -9.6)
Multidrug-resistant tuberculosis without extensive drug resistance	26.2 (19.2 to 34.2)	16.1 (12.2 to 20.7)	7.9 (4.9 to 11.8)	24.7 (7.7 to 45.7)	-17.3 (-28.6 to -3.3)
Extensively drug-resistant tuberculosis	1.6 (1.2 to 2.1)	1.1 (0.8 to 1.4)	0.5 (0.3 to 0.8)	279.3 (229.9 to 332.1)	151.6 (118.8 to 186.6)
Latent tuberculosis infection	99,014.1 (82,016.9 to 118,031.1)	--	--	--	--
HIV/AIDS	1,059.7 (898.3 to 1,275.0)	31.0^ (24.9 to 37.7)	106.2 (68.5 to 157.0)	16.0 (-14.5 to 64.8)	-23.0 (-43.3 to 9.3)
Drug-susceptible HIV/AIDS - Tuberculosis	23.9 (16.5 to 33.6)	28.9 (20.7 to 39.3)	9.0 (5.1 to 14.1)	12.9 (1.5 to 24.4)	-25.1 (-32.7 to -17.4)
Multidrug-resistant HIV/AIDS - Tuberculosis without extensive drug resistance	0.8 (0.5 to 1.2)	0.9 (0.6 to 1.3)	0.3 (0.2 to 0.6)	21.8 (-1.5 to 45.4)	-19.2 (-34.7 to -3.6)
Extensively drug-resistant HIV/AIDS - Tuberculosis	< 0.05 (< 0.05 to 0.1)	< 0.05 (< 0.05 to 0.1)	< 0.05 (< 0.05 to < 0.05)	401.3 (329.0 to 492.1)	232.5 (184.6 to 292.7)
HIV/AIDS resulting in other diseases	1,034.9 (875.3 to 1,244.8)	31.0 (24.9 to 37.7)	96.8 (60.4 to 146.8)	16.3 (-16.4 to 73.0)	-22.9 (-44.6 to 14.8)
**Diarrhoea, lower respiratory infections, and other common infectious diseases**	**14,066.5 (13,056.0 to 15,117.2)**	**760,395.8† (599,393.8 to 963,377.0)**	**695.0 (486.9 to 948.6)**	**42.1 (40.3 to 44.1)**	**-5.7 (-6.9 to -4.5)**
Diarrhoeal diseases	2,662.9 (2,373.9 to 2,981.5)	184,473.2 (165,647.6 to 205,820.1)	285.0 (198.3 to 389.1)	35.0 (32.2 to 37.9)	-10.5 (-12.3 to -8.6)
Intestinal infectious diseases	11.6 (9.4 to 14.5)	255.8† (210.6 to 307.1)	2.0 (1.2 to 2.9)	15.0 (-4.8 to 38.9)	-23.7 (-36.9 to -7.9)
Typhoid fever	11.3 (9.0 to 14.3)	178.5 (146.2 to 215.2)	1.7 (1.1 to 2.6)	15.5 (-6.9 to 43.0)	-23.4 (-38.2 to -5.2)
Paratyphoid fever	3.4 (2.7 to 4.1)	77.2 (63.5 to 93.0)	0.2 (0.1 to 0.3)	15.1 (-2.8 to 37.8)	-23.7 (-35.5 to -8.6)
Other intestinal infectious diseases	--	--	< 0.05 (< 0.05 to < 0.05)	-12.9 (-27.9 to 5.3)	-42.2 (-52.2 to -30.2)
Lower respiratory infections	425.8 (390.1 to 463.6)	18,920.2 (17,122.6 to 20,932.3)	24.9 (16.8 to 35.1)	74.1 (67.0 to 82.1)	15.5 (10.8 to 20.8)
Upper respiratory infections	7,348.5 (5,199.3 to 10,029.1)	549,389.2 (386,232.6 to 742,572.2)	176.9 (98.1 to 304.0)	48.8 (46.5 to 51.1)	-1.3 (-2.9 to 0.2)
Otitis media	2,308.4 (1,598.8 to 3,103.7)	3,540.5 (2,924.3 to 4,209.1)	62.1 (34.7 to 102.6)	33.1 (27.1 to 39.6)	-11.7 (-15.7 to -7.4)
Meningitis	469.6 (393.7 to 549.1)	44.9 (32.8 to 57.7)	37.1 (26.3 to 49.2)	41.2 (36.4 to 46.0)	-6.4 (-9.5 to -3.1)
Pneumococcal meningitis	208.8 (169.6 to 254.7)	10.0 (6.3 to 14.7)	16.4 (11.2 to 22.7)	36.4 (29.6 to 43.8)	-9.5 (-14.1 to -4.6)
H influenzae type B meningitis	49.6 (36.8 to 64.8)	2.9 (1.6 to 4.5)	4.7 (3.2 to 6.3)	13.8 (2.6 to 25.2)	-24.5 (-32.0 to -17.0)
Meningococcal infection	53.1 (37.8 to 70.0)	8.2 (5.2 to 12.2)	4.3 (2.9 to 6.0)	43.9 (37.8 to 51.7)	-4.6 (-8.6 to 0.6)
Other meningitis	158.2 (115.7 to 203.0)	23.8 (16.7 to 31.7)	11.7 (8.0 to 16.0)	64.0 (59.0 to 69.5)	8.8 (5.4 to 12.4)
Encephalitis	875.8 (545.1 to 1,329.5)	312.9 (273.8 to 356.6)	78.7 (55.2 to 102.8)	50.8 (43.4 to 58.9)	0.0 (-4.9 to 5.4)
Diphtheria	--	--	--	--	--
Whooping cough	--	--	--	--	--
Tetanus	5.0 (4.9 to 5.1)	2.8 (1.4 to 4.1)	0.1 (0.1 to 0.2)	14.1 (-13.5 to 44.8)	-24.3 (-42.6 to -4.0)
Measles	--	--	--	--	--
Varicella and herpes zoster	533.5 (412.8 to 670.1)	3,456.4 (2,722.2 to 4,253.4)	28.3 (16.2 to 45.3)	58.1 (49.8 to 66.4)	4.9 (-0.7 to 10.4)
**Neglected tropical diseases and malaria**	**62,352.2 (60,001.1 to 64,396.4)**	**11,889.4† (9,273.1 to 14,641.9)**	**448.9 (305.5 to 620.2)**	**8.1 (-1.1 to 17.8)**	**-28.3 (-34.4 to -21.8)**
Malaria	1,446.3 (1,232.2 to 1,646.1)	2,200.7 (1,620.2 to 2,846.9)	13.8 (10.2 to 18.0)	-12.2 (-18.9 to -4.7)	-41.8 (-46.2 to -36.8)
Chagas disease	460.4 (389.2 to 539.0)	4.0 (2.6 to 5.8)	5.6 (3.6 to 8.3)	25.9 (18.1 to 33.9)	-16.5 (-21.7 to -11.2)
Leishmaniasis	235.3 (176.3 to 289.6)	26.5 (19.8 to 34.9)	12.2 (7.4 to 19.2)	29.5 (23.1 to 39.1)	-14.1 (-18.4 to -7.7)
Visceral leishmaniasis	1.3 (0.8 to 2.0)	5.4 (3.4 to 7.9)	0.1 (0.1 to 0.1)	-33.8 (-57.3 to -1.9)	-56.1 (-71.7 to -34.9)
Cutaneous and mucocutaneous leishmaniasis	200.4 (144.9 to 271.5)	21.2 (14.8 to 29.0)	12.2 (7.3 to 19.1)	30.4 (23.9 to 40.3)	-13.5 (-17.9 to -7.0)
African trypanosomiasis	0.2 (< 0.05 to 0.8)	0.1 (< 0.05 to 0.6)	0.1 (< 0.05 to 0.2)	-71.3 (-96.0 to 57.0)	-81.0 (-97.3 to 4.2)
Schistosomiasis	6,158.7 (5,678.2 to 6,656.4)	1,550.8 (1,410.3 to 1,707.4)	43.1 (21.1 to 81.7)	-26.2 (-28.7 to -23.0)	-51.1 (-52.7 to -49.0)
Cysticercosis	219.5 (160.0 to 305.6)	--	33.0 (20.2 to 50.2)	23.7 (15.2 to 32.3)	-18.0 (-23.6 to -12.2)
Cystic echinococcosis	40.4 (20.3 to 86.4)	7.9 (3.5 to 19.0)	3.6 (1.7 to 8.1)	-30.3 (-72.5 to 94.5)	-53.8 (-81.8 to 29.0)
Lymphatic filariasis	1,070.1 (875.5 to 1,352.8)	225.7 (126.6 to 371.7)	49.9 (25.4 to 86.3)	-25.9 (-44.2 to -8.3)	-50.9 (-63.0 to -39.2)
Onchocerciasis	470.5 (325.1 to 710.9)	83.8 (34.1 to 173.5)	38.8 (20.7 to 68.1)	-16.7 (-34.0 to 3.7)	-44.8 (-56.2 to -31.2)
Trachoma	456.6 (247.1 to 753.1)	--	33.0 (19.6 to 53.8)	10.3 (-0.8 to 20.7)	-26.8 (-34.2 to -20.0)
Dengue	188.9 (102.8 to 320.1)	3,157.2 (1,919.3 to 4,790.9)	29.1 (13.1 to 53.8)	125.1 (82.7 to 428.1)	49.3 (21.2 to 250.3)
Yellow fever	< 0.05 (< 0.05 to 0.1)	1.5 (0.4 to 4.0)	< 0.05 (< 0.05 to < 0.05)	-20.4 (-49.9 to 24.4)	-47.2 (-66.8 to -17.5)
Rabies	< 0.05 (< 0.05 to < 0.05)	0.5 (0.2 to 0.6)	< 0.05 (< 0.05 to < 0.05)	-40.2 (-50.8 to -28.6)	-60.3 (-67.4 to -52.7)
Intestinal nematode infections	52,700.5 (49,910.2 to 54,817.5)	--	83.6 (47.3 to 135.6)	8.4 (-1.0 to 18.2)	-28.1 (-34.3 to -21.6)
Ascariasis	28,108.9 (24,804.5 to 32,123.6)	--	26.7 (14.3 to 44.6)	-7.3 (-19.9 to 7.2)	-38.5 (-46.9 to -28.9)

Cause	Prevalence in thousands (95% UI)	Incidence in thousands (95% UI)	YLDs in thousands (95% UI)		
	2016 counts	2016 counts	2016 counts	Percentage change in counts between 2006 and 2016	Percentage change in rates between 2006 and 2016
Trichuriasis	14,450.2 (13,183.3 to 15,986.7)	--	9.8 (5.4 to 16.6)	3.9 (-11.3 to 21.0)	-31.1 (-41.2 to -19.7)
Hookworm disease	15,539.2 (14,125.8 to 17,103.8)	--	47.1 (27.2 to 74.7)	21.2 (7.2 to 37.0)	-19.6 (-28.9 to -9.1)
Food-borne trematodiases	4,019.7 (3,639.0 to 4,414.4)	4,356.3 (2,103.6 to 6,380.2)	89.9 (43.8 to 161.7)	73.6 (63.8 to 84.0)	15.2 (8.7 to 22.0)
Leprosy	57.7 (55.0 to 60.3)	4.7 (4.3 to 5.1)	3.5 (2.3 to 4.8)	16.1 (8.8 to 24.3)	-23.0 (-27.8 to -17.5)
Ebola virus disease	< 0.05 (< 0.05 to 0.1)	< 0.05 (< 0.05 to < 0.05)	< 0.05 (< 0.05 to < 0.05)	--	--
Zika virus disease	4.5 (2.4 to 8.9)	269.5 (147.0 to 539.6)	0.1 (< 0.05 to 0.2)	--	--
Guinea worm disease	< 0.05 (< 0.05 to < 0.05)	< 0.05 (< 0.05 to < 0.05)	< 0.05 (< 0.05 to < 0.05)	-99.6 (-99.8 to -99.3)	-99.7 (-99.8 to -99.5)
Other neglected tropical diseases	695.9 (637.2 to 752.9)	--	9.6 (6.1 to 14.3)	46.5 (37.3 to 55.2)	-2.9 (-8.9 to 2.9)
**Maternal disorders**	**--**	**--**	**--**	**--**	**--**
Maternal haemorrhage	--	--	--	--	--
Maternal sepsis and other pregnancy related infections	--	--	--	--	--
Maternal hypertensive disorders	--	--	--	--	--
Maternal obstructed labour and uterine rupture	--	--	--	--	--
Maternal abortion, miscarriage, and ectopic pregnancy	--	--	--	--	--
Other maternal disorders	--	--	--	--	--
**Neonatal disorders**	**2,021.1 (1,859.6 to 2,196.3)**	**--**	**270.8 (193.5 to 372.7)**	**75.1 (58.4 to 96.2)**	**16.2 (5.1 to 30.1)**
Neonatal preterm birth complications	1,734.2 (1,476.7 to 2,018.9)	--	160.7 (112.2 to 221.8)	73.2 (45.1 to 108.3)	14.9 (-3.7 to 38.2)
Neonatal encephalopathy due to birth asphyxia and trauma	356.9 (246.6 to 503.5)	--	46.1 (22.1 to 89.5)	79.9 (65.5 to 99.5)	19.3 (9.8 to 32.3)
Neonatal sepsis and other neonatal infections	131.9 (56.4 to 317.3)	--	48.4 (16.7 to 126.8)	77.2 (64.3 to 97.7)	17.5 (9.0 to 31.2)
Hemolytic disease and other neonatal jaundice	46.4 (41.0 to 52.2)	--	15.6 (12.1 to 19.7)	75.1 (68.1 to 81.7)	16.2 (11.5 to 20.5)
Other neonatal disorders	--	--	--	--	--
**Nutritional deficiencies**	**44,289.9 (42,911.1 to 45,388.4)**	**9,665.4† (8,299.0 to 11,205.4)**	**851.8 (564.6 to 1,212.6)**	**34.6 (31.6 to 37.5)**	**-10.7 (-12.7 to -8.8)**
Protein-energy malnutrition	--	--	--	--	--
Iodine deficiency	5,695.3 (5,118.7 to 6,319.0)	17.0 (14.6 to 19.8)	143.3 (98.1 to 198.8)	25.7 (22.7 to 28.7)	-16.6 (-18.6 to -14.6)
Vitamin A deficiency	9,010.9 (7,978.5 to 10,206.3)	9,648.4 (8,282.4 to 11,188.4)	5.6 (3.6 to 8.4)	46.1 (35.6 to 57.6)	-3.1 (-10.1 to 4.5)
Iron-deficiency anaemia	34,336.6 (31,135.5 to 37,565.8)	--	703.0 (457.4 to 1,009.5)	36.6 (32.8 to 39.8)	-9.4 (-11.9 to -7.2)
Other nutritional deficiencies	--	--	--	--	--
**Other communicable, maternal, neonatal, and nutritional diseases**	**86,032.8 (83,388.0 to 88,947.4)**	**9,991.3 (8,141.9 to 12,321.6)**	**81.0 (53.9 to 116.3)**	**43.9 (31.8 to 56.5)**	**-4.6 (-12.6 to 3.8)**
Sexually transmitted diseases excluding HIV	61,836.3 (57,911.0 to 67,302.2)	5,222.2 (4,611.3 to 5,884.0)	51.8 (34.8 to 75.8)	54.2 (49.7 to 59.5)	2.2 (-0.7 to 5.8)
Syphilis	404.4 (319.2 to 505.9)	108.3 (64.6 to 176.9)	19.4 (13.2 to 26.6)	58.8 (50.4 to 67.3)	5.4 (-0.3 to 11.0)
Chlamydial infection	81.3 (58.1 to 111.8)	217.8 (170.1 to 277.0)	2.1 (1.3 to 3.1)	50.8 (35.8 to 68.4)	0.0 (-9.9 to 11.7)
Gonococcal infection	73.5 (50.3 to 108.3)	247.0 (175.3 to 345.6)	1.0 (0.6 to 1.4)	52.9 (40.9 to 65.3)	1.4 (-6.5 to 9.6)
Trichomoniasis	4,598.7 (3,861.0 to 5,339.5)	3,511.1 (3,017.2 to 4,034.0)	5.0 (2.0 to 10.6)	49.9 (46.3 to 53.5)	-0.6 (-3.0 to 1.8)
Genital herpes	57,529.3 (50,326.8 to 66,551.9)	--	13.6 (4.0 to 31.2)	48.9 (46.1 to 52.5)	-1.2 (-3.1 to 1.2)
Other sexually transmitted diseases	81.2 (62.4 to 104.4)	1,137.9 (860.0 to 1,476.9)	10.7 (6.8 to 15.6)	55.7 (43.8 to 71.2)	3.3 (-4.6 to 13.6)
Hepatitis	31,874.7 (27,813.8 to 36,185.1)	4,768.5 (3,021.1 to 7,083.8)	11.5 (6.1 to 19.6)	13.8 (-29.9 to 89.9)	-24.5 (-53.5 to 26.0)
Acute hepatitis A	54.0 (12.9 to 111.5)	701.4 (168.2 to 1,450.0)	2.2 (0.5 to 4.9)	6.1 (-76.5 to 311.1)	-29.6 (-84.4 to 172.7)
Hepatitis B	21,734.5 (17,948.1 to 26,003.5)	3,504.3 (1,978.0 to 5,738.1)	8.4 (4.0 to 15.7)	16.1 (-35.8 to 109.7)	-23.0 (-57.4 to 39.1)
Hepatitis C	10,064.2 (9,003.4 to 11,175.6)	277.1 (198.7 to 370.9)	0.2 (0.1 to 0.5)	10.9 (5.6 to 15.9)	-26.4 (-30.0 to -23.1)
Acute hepatitis E	22.0 (11.5 to 38.5)	285.8 (150.1 to 500.9)	0.7 (0.3 to 1.3)	13.3 (-10.3 to 42.6)	-24.9 (-40.5 to -5.4)
Other infectious diseases	823.6 (752.2 to 893.8)	0.6 (0.3 to 1.0)	17.7 (11.3 to 26.0)	40.5 (35.4 to 45.7)	-6.8 (-10.2 to -3.4)
**Non-communicable diseases**	**296,877.7 (296,682.5 to 297,045.2)**	**842,148.1† (759,269.2 to 932,610.8)**	**47,006.2 (35,271.8 to 60,057.0)**	**49.6 (48.8 to 50.5)**	**-0.7 (-1.3 to -0.2)**
**Neoplasms**	**5,665.6 (5,587.4 to 5,754.2)**	**2,231.1 (2,154.1 to 2,337.6)**	**670.9 (491.9 to 873.5)**	**55.3 (52.0 to 59.2)**	**3.0 (0.8 to 5.6)**
Lip and oral cavity cancer	185.9 (178.3 to 193.2)	51.2 (49.1 to 53.3)	19.1 (13.8 to 24.9)	53.0 (43.0 to 63.8)	1.5 (-5.1 to 8.7)
Nasopharynx cancer	34.3 (31.9 to 36.6)	10.0 (9.4 to 10.7)	4.2 (2.9 to 5.5)	45.4 (32.2 to 60.5)	-3.5 (-12.3 to 6.4)
Other pharynx cancer	85.3 (80.0 to 89.2)	29.8 (27.8 to 31.3)	10.1 (7.2 to 13.2)	59.9 (43.7 to 74.2)	6.0 (-4.7 to 15.5)
Oesophageal cancer	87.8 (83.7 to 92.3)	71.3 (68.6 to 74.5)	18.2 (12.8 to 23.8)	31.2 (19.9 to 44.1)	-13.0 (-20.5 to -4.5)
Stomach cancer	306.0 (287.3 to 326.9)	163.8 (154.7 to 173.8)	43.4 (31.0 to 56.6)	48.1 (35.6 to 61.3)	-1.8 (-10.1 to 7.0)
Colon and rectum cancer	814.7 (781.5 to 856.5)	220.3 (211.4 to 231.3)	77.7 (57.3 to 101.7)	59.7 (50.8 to 69.0)	6.0 (0.0 to 12.1)
Liver cancer	149.5 (136.3 to 164.4)	147.3 (136.7 to 157.1)	33.3 (23.7 to 44.2)	80.0 (65.0 to 97.2)	19.4 (9.5 to 30.8)
Liver cancer due to hepatitis B	88.8 (65.4 to 107.0)	64.6 (47.2 to 77.4)	14.5 (9.1 to 20.3)	86.3 (64.4 to 112.8)	23.6 (9.0 to 41.1)

Cause	Prevalence in thousands (95% UI)	Incidence in thousands (95% UI)	YLDs in thousands (95% UI)		
	2016 counts	2016 counts	2016 counts	Percentage change in counts between 2006 and 2016	Percentage change in rates between 2006 and 2016
Liver cancer due to hepatitis C	33.6 (28.1 to 40.6)	25.4 (21.4 to 30.9)	5.8 (4.0 to 8.2)	56.0 (44.3 to 67.6)	3.4 (-4.3 to 11.2)
Liver cancer due to alcohol use	27.8 (20.6 to 36.3)	22.6 (16.7 to 29.3)	5.2 (3.3 to 7.5)	83.4 (63.5 to 106.9)	21.6 (8.4 to 37.2)
Liver cancer due to other causes	48.0 (38.7 to 62.0)	34.7 (28.2 to 44.0)	7.9 (5.3 to 11.2)	87.6 (62.7 to 117.2)	24.4 (7.9 to 44.1)
Gallbladder and biliary tract cancer	19.6 (17.1 to 21.2)	22.3 (20.0 to 24.0)	5.2 (3.5 to 6.9)	37.0 (29.1 to 45.5)	-9.1 (-14.4 to -3.5)
Pancreatic cancer	44.2 (42.6 to 45.5)	53.1 (51.6 to 54.3)	11.5 (8.0 to 15.0)	52.4 (47.6 to 57.1)	1.1 (-2.1 to 4.2)
Larynx cancer	115.8 (112.3 to 119.9)	33.9 (32.9 to 35.1)	13.6 (9.8 to 17.7)	47.9 (38.4 to 57.8)	-1.9 (-8.2 to 4.7)
Tracheal, bronchus, and lung cancer	406.4 (389.3 to 425.9)	297.6 (287.1 to 309.9)	71.5 (51.2 to 91.3)	54.3 (45.4 to 64.2)	2.4 (-3.5 to 8.9)
Malignant skin melanoma	149.2 (125.9 to 165.7)	30.8 (26.2 to 34.2)	9.9 (6.9 to 13.7)	60.4 (49.7 to 71.4)	6.4 (-0.7 to 13.7)
Non-melanoma skin cancer	84.8 (62.2 to 120.7)	171.2 (108.9 to 257.5)	3.3 (1.6 to 6.2)	8.0 (-8.1 to 26.6)	-28.3 (-39.1 to -16.1)
Non-melanoma skin cancer (squamous-cell carcinoma)	67.5 (35.5 to 115.9)	65.2 (37.1 to 112.8)	3.1 (1.5 to 6.0)	6.8 (-9.8 to 25.1)	-29.1 (-40.2 to -17.0)
Non-melanoma skin cancer (basal-cell carcinoma)	12.7 (5.5 to 28.1)	105.9 (54.6 to 181.2)	0.1 (< 0.05 to 0.3)	46.1 (28.2 to 63.3)	-3.1 (-15.0 to 8.3)
Breast cancer	1,023.0 (966.9 to 1,089.4)	211.8 (200.2 to 226.7)	92.4 (66.5 to 124.4)	50.9 (41.2 to 60.9)	0.1 (-6.3 to 6.7)
Cervical cancer	183.6 (146.1 to 201.4)	49.0 (39.3 to 53.4)	17.6 (12.2 to 23.6)	32.7 (22.3 to 46.0)	-12.0 (-18.9 to -3.1)
Uterine cancer	333.1 (310.1 to 361.5)	70.5 (65.7 to 76.5)	25.1 (17.7 to 34.1)	74.9 (59.8 to 92.9)	16.0 (6.0 to 27.9)
Ovarian cancer	92.9 (86.7 to 97.3)	30.5 (28.7 to 31.8)	14.0 (10.1 to 18.3)	46.7 (32.3 to 61.6)	-2.7 (-12.2 to 7.2)
Prostate cancer	838.5 (734.6 to 1,000.7)	209.4 (187.4 to 241.2)	72.6 (51.3 to 98.1)	52.6 (42.8 to 62.9)	1.2 (-5.3 to 8.0)
Testicular cancer	8.8 (8.2 to 9.4)	1.8 (1.7 to 1.9)	0.7 (0.5 to 0.9)	75.1 (55.4 to 96.3)	16.1 (3.1 to 30.2)
Kidney cancer	169.3 (160.8 to 177.2)	45.5 (43.8 to 47.5)	15.1 (10.8 to 19.9)	54.8 (45.2 to 64.3)	2.7 (-3.7 to 9.0)
Bladder cancer	221.9 (213.3 to 229.8)	54.1 (52.0 to 55.9)	20.8 (15.0 to 27.6)	50.7 (42.4 to 59.3)	-0.0 (-5.6 to 5.6)
Brain and nervous system cancer	75.8 (66.3 to 81.9)	34.1 (30.5 to 36.9)	10.9 (7.6 to 14.4)	67.2 (57.9 to 79.9)	10.9 (4.7 to 19.3)
Thyroid cancer	134.3 (127.2 to 143.8)	25.9 (24.5 to 27.7)	8.6 (6.0 to 11.9)	76.6 (64.8 to 90.1)	17.1 (9.3 to 26.1)
Mesothelioma	5.9 (5.4 to 6.3)	3.9 (3.6 to 4.2)	1.3 (0.9 to 1.7)	33.2 (27.1 to 40.1)	-11.6 (-15.7 to -7.1)
Hodgkin lymphoma	19.7 (17.3 to 22.2)	5.1 (4.5 to 5.9)	2.1 (1.5 to 2.8)	40.6 (29.3 to 52.5)	-6.8 (-14.3 to 1.2)
Non-Hodgkin's lymphoma	192.7 (177.2 to 208.2)	53.6 (49.4 to 58.1)	17.3 (12.5 to 22.9)	71.1 (60.2 to 80.1)	13.5 (6.3 to 19.5)
Multiple myeloma	45.9 (39.3 to 52.7)	18.5 (16.2 to 21.2)	9.6 (6.7 to 12.9)	64.8 (52.3 to 80.3)	9.3 (1.0 to 19.6)
Leukaemia	149.5 (134.1 to 158.8)	41.6 (37.5 to 44.0)	20.0 (14.1 to 26.6)	61.3 (52.5 to 70.9)	7.0 (1.1 to 13.4)
Acute lymphoid leukaemia	12.7 (10.7 to 13.7)	3.8 (3.2 to 4.1)	1.7 (1.2 to 2.4)	75.2 (58.3 to 87.6)	16.2 (5.0 to 24.4)
Chronic lymphoid leukaemia	58.5 (54.2 to 63.7)	13.2 (12.2 to 14.4)	7.3 (5.2 to 9.7)	61.2 (49.4 to 74.3)	6.9 (-0.9 to 15.6)
Acute myeloid leukaemia	11.2 (9.6 to 12.0)	8.6 (7.3 to 9.2)	2.4 (1.6 to 3.2)	59.4 (51.8 to 67.6)	5.7 (0.7 to 11.2)
Chronic myeloid leukaemia	7.7 (6.8 to 8.5)	2.8 (2.4 to 3.0)	1.0 (0.7 to 1.4)	25.9 (18.1 to 33.5)	-16.5 (-21.7 to -11.5)
Other leukaemia	59.4 (48.8 to 65.2)	13.4 (11.0 to 14.7)	7.5 (5.1 to 10.1)	65.3 (50.4 to 83.1)	9.6 (-0.2 to 21.4)
Other neoplasms	250.3 (221.9 to 264.5)	73.0 (64.8 to 76.7)	21.9 (15.6 to 28.9)	85.4 (73.1 to 96.2)	23.0 (14.8 to 30.1)
**Cardiovascular diseases**	**56,105.0 (54,528.0 to 57,430.0)**	**6,249.4† (5,711.0 to 6,877.8)**	**3,823.4 (2,770.5 to 4,960.0)**	**61.1 (55.0 to 66.7)**	**6.8 (2.8 to 10.6)**
Rheumatic heart disease	979.1 (884.6 to 1,081.5)	17.2 (14.0 to 20.0)	53.3 (35.4 to 75.0)	33.6 (29.1 to 38.2)	-11.4 (-14.4 to -8.3)
Ischaemic heart disease	18,793.0 (17,388.2 to 20,177.3)	2,143.0 (1,859.8 to 2,434.5)	796.7 (544.8 to 1,089.1)	52.3 (50.8 to 53.8)	1.0 (0.1 to 2.0)
Cerebrovascular disease	10,947.9 (10,547.5 to 11,391.0)	1,754.4‡ (1,522.3 to 1,995.5)	1,919.2 (1,352.5 to 2,489.6)	74.7 (62.3 to 85.7)	15.8 (7.6 to 23.2)
Ischaemic stroke	9,193.1 (7,862.8 to 10,476.1)	1,254.5‡ (1,035.8 to 1,480.3)	1,569.8 (1,087.7 to 2,053.7)	78.6 (63.8 to 91.0)	18.5 (8.7 to 26.7)
Hemorrhagic stroke	2,044.7 (1,673.6 to 2,462.1)	499.8‡ (411.6 to 603.0)	349.4 (241.1 to 471.9)	58.9 (42.3 to 75.0)	5.4 (-5.6 to 16.1)
Hypertensive heart disease	1,796.2 (1,394.0 to 2,276.0)	--	147.3 (95.4 to 214.4)	53.4 (49.5 to 57.2)	1.7 (-0.9 to 4.3)
Cardiomyopathy and myocarditis	503.5 (462.1 to 556.5)	238.0† (185.4 to 309.1)	42.9 (26.8 to 65.8)	46.5 (40.6 to 52.3)	-2.8 (-6.7 to 1.0)
Myocarditis	94.5 (76.0 to 114.9)	238.0 (185.4 to 309.1)	6.0 (3.9 to 8.9)	50.6 (42.2 to 59.5)	-0.1 (-5.7 to 5.8)
Alcoholic cardiomyopathy	165.8 (124.1 to 216.7)	--	13.7 (8.6 to 21.2)	59.0 (45.9 to 73.8)	5.5 (-3.2 to 15.3)
Other cardiomyopathy	280.7 (204.5 to 370.6)	--	23.2 (14.5 to 36.4)	39.0 (33.1 to 45.2)	-7.8 (-11.7 to -3.7)
Atrial fibrillation and flutter	4,675.1 (3,834.3 to 5,611.4)	547.2 (444.2 to 673.9)	376.3 (237.7 to 546.2)	52.1 (49.4 to 54.6)	0.9 (-0.9 to 2.5)
Peripheral vascular disease	16,232.2 (13,126.4 to 19,795.0)	1,463.4 (1,070.4 to 1,940.1)	72.8 (33.2 to 137.9)	40.0 (38.0 to 42.1)	-7.1 (-8.5 to -5.7)
Endocarditis	34.1 (26.7 to 43.5)	86.2 (67.5 to 105.0)	2.8 (1.8 to 4.3)	41.2 (34.0 to 48.7)	-6.4 (-11.1 to -1.4)
Other cardiovascular and circulatory diseases	7,956.0 (5,766.2 to 10,632.4)	--	412.1 (256.7 to 630.5)	44.4 (40.9 to 48.0)	-4.2 (-6.5 to -1.9)
**Chronic respiratory diseases**	**48,255.2 (47,026.8 to 49,291.5)**	**6,498.1† (5,823.8 to 7,243.5)**	**2,994.6 (2,506.0 to 3,454.1)**	**50.1 (46.5 to 53.9)**	**-0.4 (-2.8 to 2.0)**
Chronic obstructive pulmonary disease	33,641.7 (31,569.7 to 35,785.6)	2,350.9 (2,001.9 to 2,712.3)	2,223.9 (1,918.7 to 2,499.7)	48.9 (44.3 to 53.5)	-1.3 (-4.3 to 1.8)
Pneumoconiosis	210.0 (190.6 to 230.1)	23.2 (20.6 to 26.1)	30.5 (20.5 to 42.8)	59.3 (48.4 to 71.1)	5.7 (-1.6 to 13.5)
Silicosis	85.5 (72.3 to 99.0)	12.6 (10.5 to 15.0)	12.2 (8.1 to 17.5)	60.5 (40.6 to 82.5)	6.4 (-6.7 to 21.0)

Cause	Prevalence in thousands (95% UI)	Incidence in thousands (95% UI)	YLDs in thousands (95% UI)		
	2016 counts	2016 counts	2016 counts	Percentage change in counts between 2006 and 2016	Percentage change in rates between 2006 and 2016
Asbestosis	20.0 (16.2 to 24.1)	2.1 (1.7 to 2.5)	3.1 (2.0 to 4.4)	43.3 (34.5 to 53.3)	-4.9 (-10.8 to 1.7)
Coal workers pneumoconiosis	62.9 (53.7 to 73.0)	4.6 (3.6 to 5.6)	9.0 (5.9 to 12.9)	61.8 (40.4 to 86.0)	7.3 (-6.9 to 23.4)
Other pneumoconiosis	41.6 (33.3 to 50.4)	3.9 (2.9 to 5.0)	6.2 (3.9 to 9.3)	62.1 (43.6 to 83.9)	7.5 (-4.7 to 22.0)
Asthma	17,655.0 (15,279.1 to 20,139.0)	4,076.5 (3,484.4 to 4,718.4)	664.5 (432.2 to 960.9)	52.5 (48.8 to 56.2)	1.1 (-1.3 to 3.6)
Interstitial lung disease and pulmonary sarcoidosis	436.5 (362.6 to 529.2)	47.6 (33.8 to 62.9)	46.2 (28.8 to 68.0)	58.1 (49.8 to 65.7)	4.9 (-0.6 to 9.9)
Other chronic respiratory diseases	--	--	29.4 (25.2 to 33.0)	82.0 (76.8 to 87.5)	20.8 (17.2 to 24.4)
**Cirrhosis and other chronic liver diseases**	**4,542.0 (4,268.5 to 4,835.9)**	**174.6 (149.5 to 203.6)**	**139.0 (94.7 to 199.3)**	**52.4 (47.5 to 57.3)**	**1.1 (-2.2 to 4.3)**
Cirrhosis and other chronic liver diseases due to hepatitis B	1,317.2 (1,135.9 to 1,529.4)	52.7 (41.4 to 65.3)	43.7 (28.9 to 62.9)	55.2 (47.8 to 63.3)	3.0 (-1.9 to 8.3)
Cirrhosis and other chronic liver diseases due to hepatitis C	1,123.2 (937.1 to 1,339.0)	44.6 (35.3 to 55.9)	33.6 (22.1 to 49.3)	49.1 (41.6 to 56.9)	-1.1 (-6.1 to 4.1)
Cirrhosis and other chronic liver diseases due to alcohol use	1,254.2 (1,034.8 to 1,478.3)	47.6 (36.6 to 59.2)	37.2 (24.2 to 55.5)	50.4 (42.4 to 58.6)	-0.2 (-5.5 to 5.2)
Cirrhosis and other chronic liver diseases due to other causes	847.4 (630.0 to 1,112.5)	29.8 (21.3 to 39.7)	24.5 (15.3 to 37.1)	55.0 (45.5 to 64.9)	2.8 (-3.5 to 9.3)
**Digestive diseases**	**24,335.0 (23,012.8 to 25,411.8)**	**21,073.4† (18,312.7 to 24,366.1)**	**605.4 (422.9 to 836.4)**	**68.1 (62.3 to 74.9)**	**11.5 (7.6 to 16.0)**
Peptic ulcer disease	6,722.2 (5,550.9 to 8,258.2)	759.5 (585.5 to 980.2)	91.2 (55.3 to 139.6)	77.7 (63.5 to 93.4)	17.8 (8.5 to 28.2)
Gastritis and duodenitis	12,185.8 (9,278.7 to 16,233.3)	8,089.6 (6,385.1 to 10,533.8)	120.7 (74.4 to 180.6)	85.3 (71.7 to 98.9)	22.9 (13.9 to 31.9)
Appendicitis	26.9 (22.2 to 32.5)	703.5 (585.0 to 846.9)	8.1 (5.1 to 11.6)	51.7 (38.1 to 67.2)	0.6 (-8.4 to 10.9)
Paralytic ileus and intestinal obstruction	13.4 (10.2 to 16.9)	370.9 (285.5 to 469.8)	4.3 (2.7 to 6.4)	61.5 (52.5 to 70.1)	7.1 (1.2 to 12.8)
Inguinal, femoral, and abdominal hernia	4,077.1 (3,075.4 to 5,271.0)	4,462.4 (3,067.5 to 5,960.8)	193.9 (117.0 to 290.4)	50.2 (43.5 to 56.7)	-0.4 (-4.8 to 4.0)
Inflammatory bowel disease	718.3 (614.3 to 855.3)	189.3 (152.2 to 236.2)	101.2 (67.0 to 143.3)	78.0 (69.1 to 86.2)	18.0 (12.2 to 23.5)
Vascular intestinal disorders	8.8 (6.3 to 11.8)	48.8 (35.1 to 66.5)	2.8 (1.7 to 4.3)	48.3 (37.2 to 59.0)	-1.6 (-9.0 to 5.5)
Gallbladder and biliary diseases	1,656.2 (1,258.0 to 2,115.6)	5,902.4 (4,419.8 to 7,737.5)	25.4 (15.3 to 39.0)	71.0 (57.6 to 85.1)	13.4 (4.5 to 22.8)
Pancreatitis	61.8 (48.4 to 77.5)	547.1 (421.3 to 685.1)	7.6 (4.5 to 11.8)	74.5 (56.5 to 91.4)	15.8 (3.8 to 26.9)
Other digestive diseases	--	--	50.2 (35.1 to 69.4)	75.8 (69.7 to 83.0)	16.6 (12.6 to 21.4)
**Neurological disorders**	**103,525.5 (96,397.7 to 111,424.4)**	**34,325.7 (22,495.7 to 49,936.0)**	**3,065.0 (2,098.9 to 4,069.2)**	**49.5 (47.6 to 51.5)**	**-0.8 (-2.1 to 0.5)**
Alzheimer's disease and other dementias	2,544.3 (1,987.0 to 3,263.3)	422.1 (267.8 to 601.5)	312.5 (205.0 to 455.9)	54.1 (50.9 to 57.1)	2.2 (0.1 to 4.2)
Parkinson's disease	623.2 (455.5 to 836.9)	79.5 (55.0 to 107.9)	76.1 (47.9 to 113.5)	62.2 (56.0 to 68.5)	7.6 (3.5 to 11.8)
Epilepsy	989.3 (805.6 to 1,200.4)	74.1 (47.9 to 103.0)	281.7 (189.3 to 396.9)	43.0 (27.9 to 60.3)	-5.2 (-15.2 to 6.3)
Multiple sclerosis	179.5 (164.0 to 194.9)	1.0 (0.7 to 1.4)	46.2 (32.6 to 60.4)	53.9 (45.4 to 61.9)	2.1 (-3.6 to 7.4)
Motor neuron disease	28.7 (23.2 to 35.9)	7.3 (5.7 to 9.2)	6.1 (4.0 to 8.6)	47.8 (43.4 to 52.2)	-1.9 (-4.9 to 0.9)
Migraine	41,864.5 (38,858.3 to 45,121.6)	2,421.7 (2,050.8 to 2,814.5)	1,919.5 (1,272.4 to 2,665.8)	49.0 (47.5 to 50.4)	-1.2 (-2.2 to -0.2)
Tension-type headache	72,548.8 (50,919.3 to 95,866.4)	31,316.8 (19,487.3 to 47,199.0)	365.4 (228.8 to 545.3)	50.4 (47.9 to 53.1)	-0.2 (-1.9 to 1.6)
Other neurological disorders	1.6 (0.8 to 2.9)	3.2 (1.8 to 4.9)	57.4 (40.4 to 76.1)	53.4 (46.4 to 60.9)	1.8 (-2.9 to 6.7)
**Mental and substance use disorders**	**51,770.8 (50,725.2 to 52,870.0)**	**21,636.9† (18,795.2 to 24,994.3)**	**7,410.6 (5,421.4 to 9,588.4)**	**47.4 (46.1 to 48.8)**	**-2.2 (-3.1 to -1.3)**
Schizophrenia	1,267.9 (1,124.5 to 1,427.5)	19.9 (15.0 to 25.7)	778.3 (588.3 to 951.4)	53.3 (50.1 to 56.6)	1.7 (-0.4 to 3.9)
Alcohol use disorders	4,068.2 (3,233.0 to 4,951.9)	1,551.0 (1,164.5 to 1,978.9)	389.1 (255.1 to 549.7)	45.8 (39.3 to 52.3)	-3.3 (-7.6 to 1.1)
Drug use disorders	1,649.2 (1,593.4 to 1,692.9)	155.5 (129.8 to 180.4)	505.0 (362.3 to 643.5)	59.4 (55.1 to 63.5)	5.7 (2.9 to 8.4)
Opioid use disorders	1,078.8 (950.4 to 1,227.7)	110.3 (87.2 to 134.5)	425.6 (302.9 to 553.0)	61.6 (56.6 to 66.4)	7.2 (3.9 to 10.4)
Cocaine use disorders	236.0 (210.5 to 263.2)	6.1 (4.0 to 8.5)	30.9 (19.3 to 43.9)	44.8 (36.6 to 53.7)	-4.0 (-9.4 to 1.9)
Amphetamine use disorders	35.1 (24.1 to 49.2)	5.2 (2.5 to 8.8)	4.7 (2.6 to 7.8)	52.5 (39.1 to 63.7)	1.1 (-7.8 to 8.6)
Cannabis use disorders	243.3 (205.3 to 288.0)	16.1 (10.7 to 22.7)	7.1 (4.5 to 10.8)	49.8 (43.4 to 56.1)	-0.6 (-4.9 to 3.5)
Other drug use disorders	127.7 (106.7 to 150.9)	17.9 (13.0 to 23.5)	36.6 (24.5 to 50.3)	51.2 (44.2 to 58.2)	0.3 (-4.4 to 4.9)
Depressive disorders	19,884.7 (19,217.5 to 20,523.4)	18,409.2 (15,566.3 to 21,654.4)	3,075.5 (2,055.6 to 4,213.5)	44.6 (42.2 to 47.1)	-4.1 (-5.7 to -2.4)
Major depressive disorder	11,280.4 (9,524.1 to 13,306.9)	17,228.5 (14,397.0 to 20,392.0)	2,221.4 (1,476.0 to 3,088.7)	40.4 (38.0 to 42.6)	-6.9 (-8.4 to -5.4)
Dysthymia	9,116.4 (7,343.7 to 11,175.8)	1,180.7 (889.3 to 1,511.5)	854.1 (558.1 to 1,259.5)	57.1 (52.2 to 62.4)	4.2 (1.0 to 7.7)
Bipolar disorder	2,146.2 (1,809.8 to 2,550.2)	119.2 (77.4 to 165.5)	419.4 (257.5 to 630.8)	47.3 (44.1 to 50.1)	-2.3 (-4.4 to -0.4)
Anxiety disorders	13,217.5 (11,674.9 to 14,862.9)	1,342.9 (1,126.1 to 1,575.1)	1,221.1 (840.0 to 1,650.5)	45.9 (42.8 to 49.2)	-3.2 (-5.2 to -1.0)
Eating disorders	--	--	--	--	--
Anorexia nervosa	--	--	--	--	--
Bulimia nervosa	--	--	--	--	--
Autistic spectrum disorders	2,072.7 (1,975.2 to 2,162.0)	--	294.6 (202.0 to 414.7)	49.9 (47.6 to 52.4)	-0.6 (-2.1 to 1.1)
Autism	651.3 (560.0 to 759.8)	--	157.8 (101.4 to 226.5)	48.5 (44.8 to 52.3)	-1.5 (-4.0 to 1.0)
Asperger syndrome and other autistic spectrum disorders	1,426.8 (1,188.3 to 1,697.2)	--	136.8 (90.1 to 198.0)	51.6 (48.8 to 54.3)	0.6 (-1.3 to 2.4)

Cause	Prevalence in thousands (95% UI)	Incidence in thousands (95% UI)	YLDs in thousands (95% UI)		
	2016 counts	2016 counts	2016 counts	Percentage change in counts between 2006 and 2016	Percentage change in rates between 2006 and 2016
Attention-deficit/hyperactivity disorder	506.9 (434.7 to 583.3)	--	5.9 (3.6 to 9.4)	54.7 (45.6 to 64.5)	2.6 (-3.5 to 9.1)
Conduct disorder	--	--	--	--	--
Idiopathic developmental intellectual disability	2,699.5 (1,466.4 to 3,903.0)	--	118.3 (58.7 to 199.6)	36.2 (29.7 to 40.1)	-9.7 (-14.0 to -7.1)
Other mental and substance use disorders	8,388.5 (7,428.8 to 9,550.5)	39.2 (25.5 to 57.4)	603.5 (417.9 to 854.5)	50.5 (48.3 to 52.9)	-0.1 (-1.6 to 1.4)
**Diabetes, urogenital, blood, and endocrine diseases**	**152,450.1 (150,742.4 to 153,944.6)**	**31,360.9† (27,450.0 to 35,707.1)**	**5,490.0 (3,904.5 to 7,346.3)**	**47.4 (44.6 to 50.4)**	**-2.2 (-4.1 to -0.2)**
Diabetes mellitus	44,580.5 (40,450.3 to 49,064.4)	1,864.3 (1,419.4 to 2,416.2)	3,406.3 (2,319.1 to 4,680.8)	47.2 (42.9 to 51.6)	-2.4 (-5.2 to 0.6)
Acute glomerulonephritis	7.2 (4.9 to 10.4)	106.5 (71.5 to 155.6)	0.4 (0.2 to 0.6)	35.1 (30.4 to 39.6)	-10.4 (-13.5 to -7.4)
Chronic kidney disease	30,886.1 (26,124.0 to 36,416.6)	2,825.2 (1,925.2 to 3,737.2)	817.5 (600.6 to 1,088.8)	49.5 (45.5 to 53.9)	-0.9 (-3.5 to 2.1)
Chronic kidney disease due to diabetes mellitus	15,761.0 (12,352.2 to 19,701.0)	1,451.8 (972.2 to 2,008.5)	428.0 (302.3 to 576.6)	48.9 (44.0 to 54.2)	-1.2 (-4.5 to 2.3)
Chronic kidney disease due to hypertension	4,584.8 (3,409.3 to 5,914.3)	351.9 (209.5 to 530.9)	163.7 (109.4 to 233.6)	56.3 (48.6 to 65.2)	3.7 (-1.4 to 9.6)
Chronic kidney disease due to glomerulonephritis	4,907.2 (3,777.2 to 6,332.3)	504.8 (317.9 to 717.9)	87.4 (52.8 to 136.3)	45.6 (37.8 to 53.2)	-3.5 (-8.6 to 1.6)
Chronic kidney disease due to other causes	5,633.1 (3,816.3 to 8,050.0)	516.6 (305.9 to 794.1)	138.4 (83.2 to 214.0)	45.9 (39.7 to 52.4)	-3.2 (-7.3 to 1.1)
Urinary diseases and male infertility	17,832.7 (15,801.1 to 20,367.2)	24,682.3† (21,101.5 to 29,249.8)	589.6 (372.9 to 855.5)	46.3 (44.3 to 48.4)	-3.0 (-4.3 to -1.6)
Interstitial nephritis and urinary tract infections	291.2 (233.2 to 363.9)	15,194.2 (12,359.4 to 18,931.8)	9.4 (5.4 to 14.6)	61.1 (53.0 to 68.9)	6.8 (1.5 to 12.0)
Urolithiasis	283.6 (216.1 to 375.1)	7,481.9 (5,670.3 to 9,837.3)	20.1 (12.4 to 29.7)	62.2 (49.7 to 76.8)	7.6 (-0.7 to 17.3)
Benign prostatic hyperplasia	16,719.4 (13,776.7 to 19,992.6)	2,006.1 (1,520.4 to 2,626.6)	550.6 (339.8 to 808.5)	46.0 (43.8 to 48.1)	-3.2 (-4.6 to -1.8)
Male infertility	--	--	--	--	--
Other urinary diseases	--	--	9.6 (6.2 to 13.8)	27.2 (20.2 to 34.9)	-15.7 (-20.3 to -10.5)
Gynecological diseases	35,712.9 (34,024.7 to 38,152.0)	25.4† (15.0 to 38.6)	137.3 (68.5 to 245.3)	34.7 (32.5 to 37.2)	-10.7 (-12.1 to -9.0)
Uterine fibroids	5,211.1 (4,641.6 to 5,879.4)	25.4 (15.0 to 38.6)	38.1 (18.4 to 71.8)	52.8 (50.9 to 54.6)	1.4 (0.1 to 2.6)
Polycystic ovarian syndrome	--	--	--	--	--
Female infertility	--	--	--	--	--
Endometriosis	--	--	--	--	--
Genital prolapse	32,025.7 (28,100.5 to 36,340.6)	--	99.3 (47.5 to 179.8)	28.8 (27.2 to 30.5)	-14.6 (-15.6 to -13.5)
Premenstrual syndrome	--	--	--	--	--
Other gynecological diseases	--	--	--	--	--
Hemoglobinopathies and hemolytic anaemias	66,138.6 (65,616.6 to 66,716.0)	--	192.8 (123.4 to 281.1)	47.6 (41.6 to 53.2)	-2.1 (-6.1 to 1.6)
Thalassemias	0.1 (0.1 to 0.1)	--	< 0.05 (< 0.05 to < 0.05)	39.6 (19.3 to 71.4)	-7.4 (-20.9 to 13.7)
Thalassaemias trait	12,466.0 (12,015.0 to 12,951.0)	--	91.9 (57.1 to 136.1)	59.8 (49.1 to 69.9)	6.0 (-1.1 to 12.7)
Sickle cell disorders	1.9 (1.7 to 2.1)	--	0.3 (0.2 to 0.5)	74.2 (68.2 to 80.2)	15.6 (11.6 to 19.5)
Sickle cell trait	13,075.3 (12,136.4 to 14,322.6)	--	22.1 (14.0 to 32.7)	35.9 (29.4 to 42.3)	-9.8 (-14.1 to -5.6)
G6PD deficiency	10,799.6 (10,414.0 to 11,239.6)	--	0.7 (0.5 to 1.0)	49.6 (44.8 to 54.3)	-0.8 (-3.9 to 2.3)
G6PD trait	32,715.1 (32,176.0 to 33,336.3)	--	< 0.05 (< 0.05 to < 0.05)	51.0 (43.1 to 58.0)	0.1 (-5.1 to 4.8)
Other hemoglobinopathies and hemolytic anaemias	4,156.8 (3,783.6 to 4,525.7)	--	77.8 (50.8 to 111.2)	38.5 (34.3 to 42.5)	-8.1 (-10.9 to -5.5)
Endocrine, metabolic, blood, and immune disorders	10,916.1 (8,978.3 to 13,067.8)	1,857.4 (1,342.4 to 2,437.9)	346.1 (224.8 to 501.5)	52.7 (48.3 to 57.4)	1.3 (-1.6 to 4.4)
**Musculoskeletal disorders**	**113,181.8 (109,305.8 to 117,143.5)**	**56,001.4 (44,946.9 to 68,627.5)**	**12,279.1 (8,643.4 to 16,815.8)**	**49.2 (47.0 to 51.3)**	**-1.0 (-2.5 to 0.3)**
Rheumatoid arthritis	2,482.0 (2,171.6 to 2,823.7)	104.7 (77.5 to 138.8)	574.5 (386.7 to 766.6)	56.4 (52.8 to 60.0)	3.8 (1.3 to 6.1)
Osteoarthritis	42,252.3 (39,339.0 to 45,193.7)	1,733.7 (1,471.5 to 2,016.9)	2,310.6 (1,616.8 to 3,131.4)	56.7 (55.3 to 58.2)	4.0 (3.0 to 4.9)
Low back and neck pain	60,618.7 (56,491.5 to 66,094.7)	24,634.6 (17,544.3 to 32,949.4)	7,123.1 (4,749.1 to 10,163.2)	48.7 (45.2 to 51.7)	-1.4 (-3.7 to 0.7)
Low back pain	42,224.8 (31,784.0 to 56,967.8)	20,567.8 (13,707.6 to 29,105.2)	4,771.1 (3,065.1 to 7,144.5)	46.5 (41.3 to 50.6)	-2.8 (-6.3 to -0.1)
Neck pain	24,047.3 (17,846.8 to 32,684.4)	4,066.8 (2,508.7 to 5,937.9)	2,352.0 (1,475.9 to 3,565.8)	53.3 (49.6 to 57.0)	1.7 (-0.8 to 4.1)
Gout	4,316.9 (3,438.2 to 5,427.8)	826.6 (619.7 to 1,050.8)	135.6 (87.9 to 193.4)	49.2 (44.0 to 54.3)	-1.1 (-4.5 to 2.4)
Other musculoskeletal disorders	25,747.8 (17,585.1 to 35,857.7)	28,701.9 (20,946.0 to 38,903.0)	2,135.3 (1,251.4 to 3,424.8)	41.8 (33.9 to 48.1)	-5.9 (-11.2 to -1.7)
**Other non-communicable diseases**	**269,119.8 (268,072.9 to 270,325.6)**	**662,596.6† (581,488.3 to 754,082.6)**	**10,528.5 (7,085.1 to 15,078.4)**	**47.7 (46.1 to 49.0)**	**-2.0 (-3.1 to -1.2)**
Congenital anomalies	2,544.3 (2,370.1 to 2,731.0)	--	338.3 (243.4 to 447.2)	47.5 (43.6 to 51.3)	-2.2 (-4.8 to 0.4)
Neural tube defects	189.5 (172.4 to 209.3)	--	55.6 (39.5 to 73.7)	46.3 (40.4 to 52.1)	-3.0 (-6.9 to 0.9)
Congenital heart anomalies	390.6 (349.1 to 436.1)	--	21.8 (9.3 to 37.9)	59.8 (53.3 to 66.2)	6.0 (1.7 to 10.2)
Orofacial clefts	147.0 (131.2 to 162.8)	--	1.8 (1.1 to 2.5)	41.3 (37.6 to 45.0)	-6.2 (-8.7 to -3.8)
Down's syndrome	23.4 (19.4 to 27.6)	--	4.0 (2.8 to 5.5)	58.4 (52.6 to 64.0)	5.0 (1.2 to 8.8)
Turner syndrome	34.8 (29.9 to 40.4)	--	0.5 (0.2 to 0.9)	60.8 (38.2 to 88.6)	6.7 (-8.3 to 25.1)
Klinefelter syndrome	11.7 (9.6 to 14.3)	--	< 0.05 (< 0.05 to 0.1)	41.2 (38.6 to 44.2)	-6.3 (-8.1 to -4.4)

Cause	Prevalence in thousands (95% UI)	Incidence in thousands (95% UI)	YLDs in thousands (95% UI)		
	2016 counts	2016 counts	2016 counts	Percentage change in counts between 2006 and 2016	Percentage change in rates between 2006 and 2016
Other chromosomal abnormalities	< 0.05<br>(< 0.05 to < 0.05)	--	< 0.05<br>(< 0.05 to < 0.05)	37.5<br>(-66.2 to 427.2)	-8.8<br>(-77.6 to 249.7)
Congenital musculoskeletal and limb anomalies	483.6<br>(447.0 to 522.0)	--	69.5<br>(48.2 to 94.6)	53.7<br>(48.3 to 59.1)	1.9<br>(-1.6 to 5.6)
Urogenital congenital anomalies	122.2<br>(110.1 to 135.2)	--	1.9<br>(0.9 to 3.3)	60.0<br>(55.5 to 65.2)	6.1<br>(3.1 to 9.6)
Digestive congenital anomalies	442.3<br>(399.7 to 488.9)	--	18.5<br>(12.2 to 26.4)	55.3<br>(47.5 to 62.9)	3.0<br>(-2.1 to 8.0)
Other congenital anomalies	1,249.0<br>(871.8 to 1,748.2)	--	164.8<br>(113.1 to 233.7)	42.8<br>(37.2 to 48.6)	-5.3<br>(-9.0 to -1.4)
Skin and subcutaneous diseases	96,768.0<br>(93,544.9 to 99,409.4)	250,682.9<br>(215,837.0 to 291,515.1)	1,676.3<br>(1,110.9 to 2,502.8)	51.7<br>(50.6 to 52.7)	0.6<br>(-0.1 to 1.3)
Dermatitis	10,646.1<br>(9,022.1 to 12,694.1)	16,242.6<br>(12,148.9 to 21,188.6)	339.3<br>(205.8 to 516.3)	46.6<br>(44.5 to 48.7)	-2.7<br>(-4.1 to -1.4)
Psoriasis	4,531.1<br>(4,163.7 to 4,910.4)	507.9<br>(439.2 to 584.0)	380.1<br>(274.6 to 497.4)	54.2<br>(51.2 to 57.0)	2.3<br>(0.3 to 4.1)
Cellulitis	153.3<br>(125.1 to 185.8)	3,447.9<br>(2,886.7 to 4,162.4)	8.4<br>(5.3 to 12.3)	46.2<br>(37.6 to 54.6)	-3.0<br>(-8.8 to 2.6)
Pyoderma	1,390.8<br>(1,284.0 to 1,500.5)	34,639.0<br>(31,249.2 to 38,049.4)	7.7<br>(3.1 to 15.8)	56.1<br>(53.0 to 59.3)	3.6<br>(1.5 to 5.7)
Scabies	3,358.7<br>(2,374.8 to 4,713.1)	10,173.5<br>(7,361.6 to 14,188.0)	83.7<br>(43.5 to 141.4)	51.0<br>(48.1 to 53.9)	0.2<br>(-1.8 to 2.1)
Fungal skin diseases	30,344.8<br>(22,431.5 to 39,618.5)	108,678.1<br>(76,923.6 to 149,069.9)	167.7<br>(64.1 to 366.1)	51.0<br>(50.1 to 52.0)	0.2<br>(-0.4 to 0.8)
Viral skin diseases	5,552.4<br>(4,780.2 to 6,434.3)	8,946.0<br>(7,738.1 to 10,276.8)	163.8<br>(100.5 to 243.7)	50.3<br>(47.8 to 52.8)	-0.3<br>(-1.9 to 1.3)
Acne vulgaris	3,285.6<br>(3,153.2 to 3,429.0)	1,513.3<br>(1,457.2 to 1,578.3)	80.4<br>(54.9 to 115.5)	52.7<br>(48.1 to 57.5)	1.3<br>(-1.8 to 4.5)
Alopecia areata	687.5<br>(616.7 to 767.6)	1,162.4<br>(1,043.3 to 1,286.3)	21.8<br>(13.8 to 32.1)	46.4<br>(41.5 to 51.5)	-2.9<br>(-6.2 to 0.5)
Pruritus	4,451.7<br>(3,129.8 to 6,288.9)	3,418.2<br>(2,413.4 to 4,710.9)	46.6<br>(20.7 to 92.2)	53.9<br>(51.6 to 56.0)	2.1<br>(0.6 to 3.5)
Urticaria	1,805.2<br>(1,260.2 to 2,490.2)	3,160.7<br>(2,203.9 to 4,340.9)	103.5<br>(59.2 to 167.3)	51.1<br>(47.6 to 54.8)	0.2<br>(-2.1 to 2.7)
Decubitus ulcer	123.5<br>(90.8 to 162.5)	449.7<br>(328.5 to 599.4)	20.6<br>(13.1 to 30.8)	50.3<br>(42.3 to 58.2)	-0.3<br>(-5.6 to 5.0)
Other skin and subcutaneous diseases	46,676.7<br>(42,726.8 to 50,627.8)	58,343.5<br>(52,617.3 to 64,295.2)	252.8<br>(123.1 to 456.7)	56.7<br>(55.6 to 57.9)	3.9<br>(3.2 to 4.7)
Sense organ diseases	190,254.0<br>(187,818.6 to 192,142.9)	40,311.3†<br>(36,475.8 to 44,186.2)	6,655.6<br>(4,507.8 to 9,506.8)	46.2<br>(43.8 to 48.2)	-3.0<br>(-4.6 to -1.7)
Glaucoma	821.7<br>(643.8 to 1,031.0)	--	80.8<br>(51.2 to 119.5)	54.5<br>(49.8 to 59.5)	2.5<br>(-0.7 to 5.8)
Cataract	9,940.8<br>(7,725.8 to 12,484.9)	--	665.2<br>(444.0 to 948.2)	48.0<br>(44.3 to 51.9)	-1.8<br>(-4.3 to 0.7)
Macular degeneration	1,064.4<br>(798.6 to 1,380.0)	--	66.8<br>(43.6 to 97.2)	55.9<br>(48.8 to 62.8)	3.4<br>(-1.3 to 8.0)
Refraction and accommodation disorders	86,303.8<br>(79,232.9 to 92,573.4)	--	1,399.1<br>(865.7 to 2,241.6)	40.5<br>(37.6 to 42.8)	-6.8<br>(-8.8 to -5.3)
Age-related and other hearing loss	151,702.8<br>(139,765.2 to 162,110.6)	--	3,514.3<br>(2,297.2 to 5,129.6)	46.2<br>(42.1 to 49.6)	-3.0<br>(-5.7 to -0.8)
Other vision loss	3,180.3<br>(2,430.0 to 4,082.2)	--	241.9<br>(158.5 to 341.9)	53.9<br>(49.9 to 57.9)	2.1<br>(-0.6 to 4.7)
Other sense organ diseases	21,676.4<br>(18,937.4 to 24,906.3)	40,311.3<br>(36,475.8 to 44,186.2)	687.5<br>(428.9 to 1,069.5)	52.6<br>(51.0 to 54.2)	1.2<br>(0.2 to 2.3)
Oral disorders	174,759.6<br>(170,055.9 to 178,862.3)	371,602.5†<br>(297,782.0 to 447,556.8)	1,858.2<br>(1,137.7 to 2,908.3)	49.5<br>(48.6 to 50.4)	-0.8<br>(-1.4 to -0.2)
Caries of deciduous teeth	--	--	--	--	--
Caries of permanent teeth	108,999.9<br>(91,146.9 to 130,890.1)	359,993.1<br>(285,472.7 to 435,098.0)	77.3<br>(34.3 to 149.2)	45.0<br>(43.2 to 46.7)	-3.9<br>(-5.0 to -2.7)
Periodontal disease	73,004.8<br>(56,067.6 to 88,347.3)	7,509.5<br>(3,736.1 to 11,240.7)	470.2<br>(183.5 to 1,011.4)	49.5<br>(48.2 to 50.5)	-0.8<br>(-1.7 to -0.2)
Edentulism and severe tooth loss	39,459.9<br>(31,943.0 to 47,637.8)	4,099.9<br>(2,637.2 to 5,707.2)	1,097.8<br>(691.2 to 1,632.1)	49.7<br>(48.3 to 50.9)	-0.7<br>(-1.6 to 0.1)
Other oral disorders	7,461.3<br>(6,654.2 to 8,276.2)	--	212.9<br>(130.0 to 323.8)	50.7<br>(48.9 to 52.5)	-0.0<br>(-1.2 to 1.1)
**Injuries**	**121,910.0<br>(114,835.8 to 130,101.1)**	**27,515.5†<br>(24,094.4 to 31,650.5)**	**4,400.3<br>(2,974.6 to 6,184.1)**	**58.5<br>(56.7 to 60.1)**	**5.1<br>(3.9 to 6.2)**
**Transport injuries**	**22,216.8<br>(20,688.2 to 23,904.0)**	**2,870.6<br>(2,221.1 to 3,647.0)**	**1,096.3<br>(737.8 to 1,565.9)**	**63.3<br>(61.5 to 64.9)**	**8.3<br>(7.1 to 9.4)**
Road injuries	18,743.9<br>(17,254.8 to 20,272.4)	2,570.0<br>(1,930.9 to 3,386.9)	933.5<br>(628.7 to 1,332.6)	66.6<br>(65.0 to 68.0)	10.5<br>(9.5 to 11.5)
Pedestrian road injuries	4,229.6<br>(3,691.2 to 4,882.5)	514.7<br>(320.6 to 789.6)	215.9<br>(146.2 to 303.8)	70.7<br>(68.3 to 72.8)	13.2<br>(11.7 to 14.6)
Cyclist road injuries	3,720.6<br>(3,133.1 to 4,344.9)	635.3<br>(403.3 to 950.8)	175.7<br>(117.5 to 253.5)	71.6<br>(70.0 to 73.0)	13.8<br>(12.8 to 14.8)
Motorcyclist road injuries	4,310.2<br>(3,705.3 to 5,082.3)	336.2<br>(203.3 to 500.6)	210.2<br>(142.8 to 299.8)	66.3<br>(64.6 to 67.8)	10.3<br>(9.2 to 11.3)
Motor vehicle road injuries	5,129.2<br>(4,197.8 to 5,980.4)	818.7<br>(561.0 to 1,139.2)	267.9<br>(180.4 to 381.5)	56.0<br>(54.1 to 57.7)	3.5<br>(2.2 to 4.6)
Other road injuries	1,354.4<br>(1,136.8 to 1,627.3)	265.1<br>(144.0 to 431.6)	63.7<br>(42.3 to 91.2)	92.7<br>(90.0 to 95.0)	27.8<br>(26.1 to 29.4)
Other transport injuries	3,472.8<br>(2,923.2 to 3,992.6)	300.6<br>(206.5 to 419.3)	162.9<br>(111.0 to 229.1)	46.6<br>(44.0 to 49.1)	-2.8<br>(-4.5 to -1.1)
**Unintentional injuries**	**90,530.5<br>(84,408.1 to 98,195.8)**	**23,738.3<br>(20,468.5 to 27,822.3)**	**3,000.6<br>(2,055.9 to 4,175.9)**	**56.8<br>(55.2 to 58.2)**	**4.0<br>(2.9 to 4.9)**
Falls	40,982.7<br>(36,514.3 to 46,342.8)	12,206.4<br>(9,282.0 to 15,953.4)	1,682.5<br>(1,173.4 to 2,314.9)	60.7<br>(58.9 to 62.2)	6.6<br>(5.4 to 7.6)
Drowning	339.3<br>(297.2 to 381.3)	27.5<br>(20.5 to 35.3)	16.2<br>(10.9 to 23.0)	36.5<br>(33.2 to 39.8)	-9.4<br>(-11.7 to -7.3)
Fire, heat, and hot substances	7,588.7<br>(6,623.4 to 8,752.9)	251.4<br>(171.8 to 355.0)	159.8<br>(101.5 to 244.6)	52.1<br>(49.7 to 54.8)	0.9<br>(-0.7 to 2.7)
Poisonings	238.1<br>(205.5 to 273.1)	129.2<br>(75.9 to 206.8)	16.1<br>(10.6 to 23.3)	59.8<br>(55.1 to 64.2)	6.0<br>(2.9 to 8.9)
Exposure to mechanical forces	16,784.7<br>(14,476.1 to 19,982.2)	2,265.8<br>(1,655.6 to 3,013.9)	365.0<br>(240.0 to 527.8)	61.8<br>(60.3 to 63.0)	7.3<br>(6.3 to 8.1)
Unintentional firearm injuries	584.0<br>(481.4 to 701.4)	31.1<br>(15.7 to 57.3)	16.9<br>(11.0 to 24.5)	55.0<br>(53.5 to 56.3)	2.8<br>(1.8 to 3.7)
Unintentional suffocation	563.7<br>(464.5 to 699.2)	29.9<br>(14.9 to 54.4)	16.4<br>(10.4 to 24.2)	59.5<br>(57.4 to 61.5)	5.8<br>(4.4 to 7.1)
Other exposure to mechanical forces	15,637.0<br>(13,360.3 to 18,863.9)	2,204.8<br>(1,611.9 to 2,925.2)	331.8<br>(219.1 to 479.8)	62.3<br>(60.7 to 63.5)	7.7<br>(6.6 to 8.5)

Cause	Prevalence in thousands (95% UI)	Incidence in thousands (95% UI)	YLDs in thousands (95% UI)		
	2016 counts	2016 counts	2016 counts	Percentage change in counts between 2006 and 2016	Percentage change in rates between 2006 and 2016
Adverse effects of medical treatment	164.8<br>(116.4 to 225.7)	2,154.7<br>(1,591.4 to 2,827.9)	22.0<br>(13.0 to 34.3)	31.8<br>(25.9 to 37.3)	-12.6<br>(-16.5 to -8.9)
Animal contact	3,896.7<br>(3,460.6 to 4,369.8)	2,260.0<br>(1,537.3 to 3,188.0)	92.0<br>(62.9 to 128.5)	31.1<br>(28.7 to 33.5)	-13.0<br>(-14.7 to -11.5)
Venomous animal contact	1,700.1<br>(1,465.9 to 2,007.6)	1,089.3<br>(699.1 to 1,588.8)	58.4<br>(40.3 to 79.9)	32.8<br>(29.7 to 36.0)	-11.9<br>(-14.0 to -9.8)
Non-venomous animal contact	2,196.6<br>(1,882.2 to 2,586.5)	1,170.7<br>(769.9 to 1,702.5)	33.6<br>(22.0 to 51.5)	28.3<br>(25.3 to 30.7)	-14.9<br>(-16.9 to -13.3)
Foreign body	3,297.7<br>(2,858.4 to 3,721.3)	1,181.1<br>(831.6 to 1,663.6)	111.7<br>(73.5 to 161.2)	50.9<br>(48.3 to 53.4)	0.1<br>(-1.7 to 1.8)
Pulmonary aspiration and foreign body in airway	1,509.7<br>(1,229.1 to 1,835.5)	309.5<br>(219.7 to 433.3)	56.6<br>(37.1 to 82.2)	51.7<br>(48.6 to 54.8)	0.7<br>(-1.4 to 2.7)
Foreign body in eyes	185.7<br>(127.9 to 269.7)	611.6<br>(320.5 to 1,048.4)	11.3<br>(6.9 to 17.9)	55.1<br>(50.8 to 61.5)	2.8<br>(0.0 to 7.1)
Foreign body in other body part	1,602.3<br>(1,360.8 to 1,952.0)	260.0<br>(153.8 to 405.6)	43.8<br>(28.4 to 62.9)	48.9<br>(46.4 to 51.0)	-1.3<br>(-2.9 to 0.2)
Environmental heat and cold exposure	5,522.5<br>(4,859.4 to 6,328.7)	865.9<br>(560.8 to 1,240.2)	202.6<br>(136.8 to 286.0)	55.3<br>(52.2 to 58.3)	3.0<br>(0.9 to 5.0)
Other unintentional injuries	11,715.2<br>(9,698.2 to 14,099.6)	2,396.3<br>(1,549.1 to 3,498.5)	332.5<br>(219.7 to 480.4)	49.8<br>(47.9 to 51.6)	-0.6<br>(-1.9 to 0.6)
**Self-harm and interpersonal violence**	**8,748.1<br>(7,785.2 to 9,865.8)**	**887.0†<br>(686.3 to 1,135.6)**	**220.2<br>(146.1 to 312.0)**	**43.8<br>(42.4 to 45.0)**	**-4.7<br>(-5.5 to -3.8)**
Self-harm	1,096.9<br>(911.3 to 1,285.7)	302.4<br>(215.7 to 418.8)	43.2<br>(28.1 to 61.8)	33.0<br>(31.3 to 34.5)	-11.8<br>(-12.9 to -10.8)
Self-harm by firearm	28.1<br>(24.2 to 32.2)	3.7<br>(1.1 to 9.6)	0.9<br>(0.6 to 1.3)	21.0<br>(18.7 to 23.5)	-19.7<br>(-21.3 to -18.1)
Self-harm by other specified means	1,068.7<br>(884.1 to 1,255.4)	298.7<br>(211.9 to 410.6)	42.3<br>(27.5 to 60.5)	33.3<br>(31.5 to 34.7)	-11.6<br>(-12.8 to -10.6)
Interpersonal violence	7,651.2<br>(6,708.8 to 8,826.6)	584.5†<br>(413.1 to 795.0)	176.9<br>(117.8 to 250.2)	46.6<br>(45.2 to 47.9)	-2.7<br>(-3.7 to -1.9)
Assault by firearm	244.1<br>(206.0 to 288.0)	15.3<br>(7.8 to 26.9)	7.9<br>(5.1 to 11.4)	41.2<br>(39.8 to 42.6)	-6.4<br>(-7.2 to -5.4)
Assault by sharp object	1,210.3<br>(1,000.8 to 1,427.2)	89.3<br>(51.2 to 144.6)	28.9<br>(18.7 to 42.1)	42.0<br>(40.1 to 43.6)	-5.8<br>(-7.1 to -4.7)
Sexual violence	2,677.8<br>(1,928.6 to 3,652.9)	--	17.8<br>(10.1 to 28.0)	38.7<br>(36.8 to 40.5)	-8.0<br>(-9.3 to -6.8)
Assault by other means	3,519.0<br>(2,981.8 to 4,233.5)	479.9<br>(331.4 to 659.7)	122.4<br>(82.0 to 174.3)	49.4<br>(48.0 to 50.8)	-0.9<br>(-1.8 to 0.0)
**Forces of nature, conflict and terrorism, and executions and police conflict**	**3,092.5<br>(2,068.9 to 4,350.6)**	**19.7†<br>(13.0 to 27.1)**	**83.2<br>(26.1 to 189.4)**	**114.9<br>(98.1 to 141.7)**	**42.5<br>(31.4 to 60.3)**
Forces of nature, conflict and terrorism, and state actor violence	390.4<br>(226.2 to 603.8)	10.1<br>(5.6 to 16.0)	13.4<br>(3.3 to 33.7)	95.8<br>(80.3 to 105.6)	29.9<br>(19.6 to 36.4)
Conflict and terrorism	2,007.6<br>(1,200.4 to 3,147.9)	--	57.7<br>(20.8 to 119.5)	108.6<br>(87.1 to 146.5)	38.4<br>(24.1 to 63.5)
Executions and police conflict	694.5<br>(176.5 to 1,106.5)	9.5<br>(4.9 to 14.2)	14.8<br>(2.4 to 40.6)	159.0<br>(141.1 to 179.0)	71.8<br>(60.0 to 85.0)

We did not calculate incidence for certain NTDs and causes with only birth prevalence. Blank cells mean that no estimate is available.

† Incidence for aggregate causes represents the sum of the incidence of the causes below the aggregate.

‡ Incidence estimates for stroke represent first-ever stroke only.

^ Incidence of HIV/AIDS represents new infections of HIV only and does not include new infections of Tuberculosis in HIV positive cases.

SR Table 12: Global prevalence, incidence, and YLDs for 2016, percentage change of YLD counts and percentage change of YLD rates between 2006 and 2016 by cause, age 65-69 years.

Cause	Prevalence in thousands (95% UI)	Incidence in thousands (95% UI)	YLDs in thousands (95% UI)		
	2016 counts	2016 counts	2016 counts	Percentage change in counts between 2006 and 2016	Percentage change in rates between 2006 and 2016
**All causes**	**225,247.5 (225,219.7 to 225,273.7)**	**1,336,423.4† (1,197,016.9 to 1,509,562.7)**	**46,206.8 (34,854.3 to 59,193.8)**	**31.2 (30.6 to 31.9)**	**-1.0 (-1.5 to -0.5)**
**Communicable, maternal, neonatal, and nutritional disorders**	**146,704.6 (144,533.2 to 148,682.2)**	**616,961.2† (494,711.3 to 779,867.8)**	**2,058.9 (1,489.1 to 2,762.4)**	**18.7 (16.3 to 21.1)**	**-10.4 (-12.2 to -8.6)**
**HIV/AIDS and tuberculosis**	**75,219.1 (66,536.1 to 84,941.6)**	**378.0† (294.5 to 474.7)**	**246.2 (166.5 to 353.4)**	**14.0 (2.4 to 26.3)**	**-14.0 (-22.8 to -4.7)**
Tuberculosis	70,742.2 (58,452.1 to 84,384.1)	358.0† (273.9 to 454.2)	180.2 (114.5 to 266.7)	14.8 (10.2 to 20.4)	-13.3 (-16.8 to -9.1)
Drug-susceptible tuberculosis	602.7 (453.9 to 783.5)	345.3 (263.5 to 438.4)	173.4 (110.0 to 257.5)	15.0 (10.4 to 20.6)	-13.2 (-16.7 to -9.0)
Multidrug-resistant tuberculosis without extensive drug resistance	21.3 (16.0 to 27.6)	11.8 (9.3 to 14.9)	6.3 (4.0 to 9.3)	6.8 (-9.0 to 24.8)	-19.4 (-31.3 to -5.8)
Extensively drug-resistant tuberculosis	1.4 (1.0 to 1.8)	0.8 (0.6 to 1.0)	0.5 (0.3 to 0.7)	211.9 (173.8 to 257.8)	135.4 (106.6 to 170.0)
Latent tuberculosis infection	70,116.8 (57,833.8 to 83,828.9)	--	--	--	--
HIV/AIDS	660.6 (567.7 to 782.2)	20.0^ (16.3 to 24.2)	66.0 (42.4 to 96.7)	11.6 (-19.2 to 60.3)	-15.8 (-39.0 to 21.0)
Drug-susceptible HIV/AIDS - Tuberculosis	16.5 (11.3 to 23.4)	18.3 (13.0 to 25.4)	6.2 (3.6 to 9.8)	12.5 (-0.0 to 23.9)	-15.1 (-24.5 to -6.5)
Multidrug-resistant HIV/AIDS - Tuberculosis without extensive drug resistance	0.6 (0.4 to 0.9)	0.6 (0.4 to 0.8)	0.2 (0.1 to 0.4)	24.6 (0.7 to 47.6)	-5.9 (-24.0 to 11.4)
Extensively drug-resistant HIV/AIDS - Tuberculosis	< 0.05 (< 0.05 to 0.1)	< 0.05 (< 0.05 to < 0.05)	< 0.05 (< 0.05 to < 0.05)	420.9 (337.3 to 516.4)	293.2 (230.0 to 365.2)
HIV/AIDS resulting in other diseases	643.4 (549.2 to 766.2)	20.0 (16.3 to 24.2)	59.6 (37.7 to 89.8)	11.4 (-21.2 to 67.2)	-15.9 (-40.5 to 26.2)
**Diarrhoea, lower respiratory infections, and other common infectious diseases**	**10,714.9 (10,085.1 to 11,330.6)**	**593,896.0† (472,138.9 to 756,150.6)**	**537.7 (381.4 to 732.5)**	**26.1 (24.6 to 27.5)**	**-4.9 (-5.9 to -3.8)**
Diarrhoeal diseases	2,345.2 (2,163.4 to 2,561.8)	162,013.0 (149,435.4 to 176,474.1)	246.6 (171.8 to 334.4)	22.0 (19.9 to 24.1)	-7.9 (-9.5 to -6.4)
Intestinal infectious diseases	10.8 (8.6 to 13.9)	224.5† (180.7 to 278.2)	1.8 (1.1 to 2.7)	7.2 (-12.4 to 30.3)	-19.1 (-33.9 to -1.7)
Typhoid fever	10.5 (8.2 to 13.7)	165.9 (133.2 to 205.0)	1.6 (1.0 to 2.4)	7.7 (-13.5 to 33.9)	-18.7 (-34.7 to 1.1)
Paratyphoid fever	2.6 (2.0 to 3.2)	58.6 (46.6 to 73.5)	0.2 (0.1 to 0.3)	6.9 (-11.1 to 28.7)	-19.4 (-32.9 to -2.8)
Other intestinal infectious diseases	--	--	< 0.05 (< 0.05 to < 0.05)	-14.8 (-30.3 to 3.6)	-35.7 (-47.4 to -21.8)
Lower respiratory infections	464.9 (429.7 to 499.8)	20,359.3 (18,550.6 to 22,223.8)	26.7 (18.0 to 37.5)	50.1 (44.2 to 56.7)	13.3 (8.8 to 18.2)
Upper respiratory infections	5,432.0 (3,850.3 to 7,323.8)	405,890.7 (283,314.3 to 566,629.9)	129.3 (73.0 to 224.6)	28.9 (26.5 to 31.1)	-2.8 (-4.5 to -1.0)
Otitis media	1,404.2 (1,046.7 to 1,825.7)	2,435.7 (1,956.7 to 2,952.1)	37.0 (22.2 to 59.6)	18.8 (13.2 to 24.7)	-10.3 (-14.6 to -5.9)
Meningitis	338.6 (279.2 to 399.5)	38.1 (27.0 to 50.6)	22.6 (16.0 to 29.9)	24.4 (19.7 to 29.5)	-6.1 (-9.7 to -2.3)
Pneumococcal meningitis	147.2 (117.8 to 182.1)	8.2 (5.1 to 12.3)	9.8 (6.8 to 13.3)	18.8 (12.8 to 25.8)	-10.3 (-14.9 to -5.1)
H influenzae type B meningitis	33.8 (24.5 to 44.7)	2.4 (1.4 to 3.8)	2.6 (1.8 to 3.5)	-3.7 (-14.5 to 8.6)	-27.3 (-35.5 to -18.1)
Meningococcal infection	39.4 (27.5 to 52.6)	7.3 (4.4 to 11.2)	2.7 (1.8 to 3.8)	26.1 (20.1 to 36.7)	-4.8 (-9.4 to 3.1)
Other meningitis	118.2 (85.9 to 152.4)	20.1 (13.4 to 28.1)	7.4 (5.1 to 10.1)	48.0 (43.1 to 53.5)	11.7 (8.0 to 15.8)
Encephalitis	645.2 (386.9 to 1,005.6)	254.5 (222.1 to 289.7)	50.1 (35.7 to 65.4)	30.8 (24.8 to 37.8)	-1.3 (-5.8 to 4.0)
Diphtheria	--	--	--	--	--
Whooping cough	--	--	--	--	--
Tetanus	3.5 (3.4 to 3.5)	2.7 (1.5 to 4.1)	0.1 (< 0.05 to 0.1)	-4.1 (-25.3 to 24.2)	-27.7 (-43.6 to -6.3)
Measles	--	--	--	--	--
Varicella and herpes zoster	447.5 (351.8 to 568.9)	2,677.6 (2,180.8 to 3,217.3)	23.6 (13.2 to 37.3)	38.7 (31.9 to 45.6)	4.7 (-0.5 to 9.9)
**Neglected tropical diseases and malaria**	**44,585.6 (42,911.4 to 45,959.0)**	**9,368.6† (7,047.3 to 11,714.1)**	**342.9 (238.3 to 471.3)**	**-0.1 (-6.7 to 7.0)**	**-24.6 (-29.6 to -19.2)**
Malaria	1,035.1 (888.7 to 1,173.7)	1,532.2 (1,126.8 to 1,984.2)	9.8 (7.1 to 13.1)	-17.5 (-23.1 to -10.9)	-37.7 (-42.0 to -32.8)
Chagas disease	356.9 (303.3 to 418.0)	2.7 (1.7 to 4.1)	5.1 (3.3 to 7.4)	21.1 (14.0 to 28.1)	-8.6 (-14.0 to -3.3)
Leishmaniasis	194.7 (146.3 to 239.3)	23.4 (17.5 to 30.6)	10.0 (6.1 to 15.5)	36.7 (29.8 to 44.6)	3.2 (-2.0 to 9.2)
Visceral leishmaniasis	1.9 (1.1 to 2.9)	7.5 (4.4 to 11.8)	0.1 (0.1 to 0.2)	-17.5 (-49.0 to 32.9)	-37.7 (-61.5 to 0.3)
Cutaneous and mucocutaneous leishmaniasis	164.8 (118.2 to 223.7)	15.9 (11.2 to 21.7)	9.8 (6.0 to 15.3)	37.8 (30.7 to 46.4)	4.0 (-1.3 to 10.5)
African trypanosomiasis	0.2 (< 0.05 to 0.6)	0.1 (< 0.05 to 0.5)	< 0.05 (< 0.05 to 0.2)	-71.4 (-95.7 to 71.1)	-78.4 (-96.8 to 29.1)
Schistosomiasis	4,645.9 (4,350.7 to 4,961.8)	1,132.3 (1,050.8 to 1,221.9)	32.2 (15.9 to 60.7)	-28.2 (-30.6 to -25.4)	-45.9 (-47.6 to -43.7)
Cysticercosis	176.9 (126.7 to 246.0)	--	26.2 (15.7 to 40.9)	11.2 (4.0 to 18.3)	-16.1 (-21.5 to -10.7)
Cystic echinococcosis	28.7 (14.6 to 63.9)	5.6 (2.6 to 13.6)	2.5 (1.1 to 6.1)	-37.7 (-76.7 to 63.9)	-53.0 (-82.4 to 23.7)
Lymphatic filariasis	704.3 (590.0 to 863.6)	161.2 (90.8 to 251.7)	29.0 (14.9 to 50.2)	-29.8 (-47.5 to -13.6)	-47.0 (-60.4 to -34.8)
Onchocerciasis	361.4 (248.4 to 534.3)	63.8 (25.5 to 128.7)	30.2 (17.0 to 51.8)	-19.1 (-34.9 to 1.0)	-39.0 (-50.9 to -23.8)
Trachoma	609.7 (381.7 to 930.3)	--	42.3 (26.1 to 63.5)	-1.2 (-10.9 to 7.9)	-25.4 (-32.7 to -18.5)
Dengue	137.3 (74.8 to 232.4)	2,294.4 (1,396.4 to 3,478.8)	20.7 (9.4 to 38.4)	102.4 (63.7 to 368.9)	52.7 (23.5 to 253.9)
Yellow fever	< 0.05 (< 0.05 to 0.1)	1.1 (0.3 to 2.9)	< 0.05 (< 0.05 to < 0.05)	-21.2 (-49.7 to 24.1)	-40.5 (-62.0 to -6.4)
Rabies	< 0.05 (< 0.05 to < 0.05)	0.5 (0.2 to 0.7)	< 0.05 (< 0.05 to < 0.05)	-47.0 (-58.7 to -31.2)	-60.0 (-68.8 to -48.1)
Intestinal nematode infections	37,349.6 (35,431.4 to 38,894.7)	--	60.9 (35.1 to 99.5)	-0.9 (-8.9 to 8.1)	-25.2 (-31.3 to -18.4)
Ascariasis	19,949.1 (17,577.3 to 22,790.6)	--	18.6 (10.1 to 31.2)	-16.8 (-28.9 to -3.2)	-37.2 (-46.4 to -27.0)

Cause	Prevalence in thousands (95% UI)	Incidence in thousands (95% UI)	YLDs in thousands (95% UI)		
	2016 counts	2016 counts	2016 counts	Percentage change in counts between 2006 and 2016	Percentage change in rates between 2006 and 2016
Trichuriasis	10,242.8 (9,325.1 to 11,328.1)	--	6.9 (3.8 to 11.6)	-6.7 (-20.6 to 8.6)	-29.6 (-40.0 to -18.0)
Hookworm disease	11,031.6 (10,126.2 to 12,126.7)	--	35.4 (21.0 to 57.4)	11.7 (-0.2 to 26.5)	-15.7 (-24.7 to -4.5)
Food-borne trematodiases	2,831.8 (2,470.5 to 3,197.8)	3,968.9 (1,905.2 to 5,927.9)	63.0 (29.8 to 112.6)	39.5 (30.6 to 51.1)	5.3 (-1.4 to 14.0)
Leprosy	51.3 (49.1 to 53.4)	3.5 (3.2 to 3.7)	3.1 (2.1 to 4.2)	8.5 (2.2 to 15.5)	-18.1 (-22.9 to -12.8)
Ebola virus disease	< 0.05 (< 0.05 to 0.1)	--	< 0.05 (0.0 to < 0.05)	--	--
Zika virus disease	3.0 (1.7 to 5.6)	179.0 (100.4 to 341.5)	0.1 (< 0.05 to 0.1)	--	--
Guinea worm disease	< 0.05 (< 0.05 to < 0.05)	< 0.05 (< 0.05 to < 0.05)	< 0.05 (< 0.05 to < 0.05)	-99.6 (-99.8 to -99.3)	-99.7 (-99.8 to -99.5)
Other neglected tropical diseases	517.4 (487.7 to 539.2)	--	7.8 (5.0 to 11.3)	31.0 (24.6 to 37.4)	-1.1 (-6.0 to 3.7)
**Maternal disorders**	**--**	**--**	**--**	**--**	**--**
Maternal haemorrhage	--	--	--	--	--
Maternal sepsis and other pregnancy related infections	--	--	--	--	--
Maternal hypertensive disorders	--	--	--	--	--
Maternal obstructed labour and uterine rupture	--	--	--	--	--
Maternal abortion, miscarriage, and ectopic pregnancy	--	--	--	--	--
Other maternal disorders	--	--	--	--	--
**Neonatal disorders**	**1,390.9 (1,274.2 to 1,506.8)**	**--**	**151.2 (107.7 to 207.9)**	**52.5 (39.1 to 69.6)**	**15.1 (5.0 to 28.0)**
Neonatal preterm birth complications	1,200.6 (1,018.0 to 1,391.5)	--	89.9 (64.2 to 123.2)	53.5 (31.8 to 82.4)	15.9 (-0.6 to 37.6)
Neonatal encephalopathy due to birth asphyxia and trauma	230.7 (165.4 to 320.5)	--	24.3 (12.0 to 46.1)	49.4 (37.9 to 65.4)	12.8 (4.1 to 24.8)
Neonatal sepsis and other neonatal infections	78.9 (32.7 to 194.1)	--	27.8 (8.6 to 75.8)	52.5 (39.7 to 76.2)	15.1 (5.5 to 33.0)
Hemolytic disease and other neonatal jaundice	26.6 (23.0 to 30.1)	--	9.1 (6.9 to 11.5)	51.1 (45.7 to 56.8)	14.0 (9.9 to 18.3)
Other neonatal disorders	--	--	--	--	--
**Nutritional deficiencies**	**33,233.8 (32,522.1 to 33,738.1)**	**6,535.3† (5,580.1 to 7,608.3)**	**723.5 (486.5 to 1,028.1)**	**19.9 (18.2 to 21.8)**	**-9.5 (-10.8 to -8.1)**
Protein-energy malnutrition	--	--	--	--	--
Iodine deficiency	4,251.9 (3,828.7 to 4,724.6)	11.2 (9.6 to 13.1)	104.8 (71.9 to 145.0)	13.3 (10.9 to 16.1)	-14.5 (-16.3 to -12.4)
Vitamin A deficiency	6,116.1 (5,416.0 to 6,964.2)	6,524.0 (5,569.1 to 7,597.6)	3.9 (2.5 to 5.9)	31.8 (21.8 to 42.0)	-0.5 (-8.1 to 7.2)
Iron-deficiency anaemia	26,489.1 (24,604.3 to 27,879.0)	--	614.8 (408.7 to 879.4)	21.1 (19.0 to 23.2)	-8.6 (-10.2 to -7.0)
Other nutritional deficiencies	--	--	--	--	--
**Other communicable, maternal, neonatal, and nutritional diseases**	**63,543.9 (61,589.8 to 65,535.5)**	**6,783.3 (5,197.5 to 8,747.3)**	**57.5 (39.0 to 84.2)**	**23.8 (11.3 to 36.4)**	**-6.6 (-16.0 to 2.9)**
Sexually transmitted diseases excluding HIV	45,991.9 (42,920.6 to 50,423.8)	2,693.7 (2,349.3 to 3,057.0)	33.6 (22.5 to 50.3)	31.8 (27.6 to 37.2)	-0.5 (-3.7 to 3.6)
Syphilis	125.9 (107.6 to 147.9)	5.1 (3.7 to 7.0)	16.5 (11.0 to 22.7)	31.6 (24.0 to 41.3)	-0.7 (-6.4 to 6.7)
Chlamydial infection	24.2 (17.3 to 34.0)	51.2 (40.2 to 64.9)	0.6 (0.4 to 0.8)	30.1 (25.9 to 35.3)	-1.8 (-5.0 to 2.1)
Gonococcal infection	19.3 (13.4 to 27.9)	59.8 (42.6 to 83.7)	0.2 (0.2 to 0.4)	35.5 (28.2 to 42.2)	2.2 (-3.2 to 7.3)
Trichomoniasis	2,942.3 (2,489.1 to 3,404.0)	2,297.3 (1,974.5 to 2,642.5)	3.2 (1.3 to 6.8)	34.2 (30.5 to 37.8)	1.3 (-1.5 to 4.0)
Genital herpes	43,207.9 (37,798.3 to 49,982.2)	--	10.1 (3.0 to 23.2)	30.6 (28.1 to 33.5)	-1.4 (-3.4 to 0.8)
Other sexually transmitted diseases	22.5 (17.1 to 28.8)	280.3 (211.6 to 364.0)	3.0 (1.9 to 4.2)	34.8 (22.8 to 50.0)	1.8 (-7.3 to 13.2)
Hepatitis	23,252.2 (20,498.9 to 26,238.9)	4,089.1 (2,545.3 to 5,957.1)	9.9 (5.1 to 17.0)	2.8 (-39.3 to 66.4)	-22.4 (-54.2 to 25.6)
Acute hepatitis A	45.9 (10.2 to 98.5)	596.8 (133.2 to 1,280.6)	1.9 (0.4 to 4.4)	-4.0 (-80.2 to 279.0)	-27.6 (-85.0 to 186.1)
Hepatitis B	15,287.6 (12,638.7 to 18,149.9)	3,004.0 (1,671.3 to 4,706.9)	7.2 (3.3 to 13.0)	4.7 (-41.1 to 85.0)	-21.0 (-55.5 to 39.6)
Hepatitis C	7,896.8 (7,063.1 to 8,755.7)	204.0 (147.5 to 267.3)	0.2 (0.1 to 0.4)	2.1 (-3.5 to 8.4)	-22.9 (-27.1 to -18.2)
Acute hepatitis E	21.9 (14.0 to 34.0)	284.3 (181.6 to 442.1)	0.7 (0.3 to 1.2)	3.9 (-16.8 to 29.4)	-21.6 (-37.2 to -2.3)
Other infectious diseases	634.2 (593.8 to 662.5)	0.5 (0.3 to 0.8)	14.0 (9.2 to 20.6)	23.2 (19.3 to 27.7)	-7.0 (-10.0 to -3.6)
**Non-communicable diseases**	**224,407.3 (224,335.4 to 224,464.6)**	**697,246.6† (640,557.6 to 762,357.9)**	**40,494.1 (30,299.5 to 51,918.5)**	**31.6 (30.9 to 32.3)**	**-0.7 (-1.2 to -0.2)**
**Neoplasms**	**5,858.2 (5,780.7 to 5,942.8)**	**2,317.1 (2,226.9 to 2,423.5)**	**680.6 (505.3 to 877.2)**	**36.7 (33.2 to 40.8)**	**3.2 (0.6 to 6.3)**
Lip and oral cavity cancer	167.7 (161.4 to 173.9)	46.7 (45.0 to 48.5)	17.2 (12.6 to 22.6)	35.7 (27.3 to 44.9)	2.4 (-3.9 to 9.4)
Nasopharynx cancer	26.2 (24.7 to 28.0)	7.8 (7.3 to 8.3)	3.2 (2.3 to 4.3)	24.1 (12.1 to 36.8)	-6.4 (-15.4 to 3.3)
Other pharynx cancer	67.5 (63.5 to 70.6)	23.7 (22.1 to 24.9)	8.0 (5.8 to 10.4)	40.4 (27.2 to 53.2)	6.0 (-4.0 to 15.6)
Oesophageal cancer	90.8 (86.6 to 95.2)	73.4 (70.5 to 76.5)	18.3 (13.2 to 23.9)	15.5 (5.1 to 26.7)	-12.8 (-20.7 to -4.4)
Stomach cancer	321.0 (301.7 to 342.6)	172.3 (162.8 to 182.7)	44.6 (32.1 to 57.7)	23.2 (13.7 to 33.4)	-7.0 (-14.2 to 0.7)
Colon and rectum cancer	898.5 (864.5 to 952.9)	244.4 (235.1 to 258.8)	85.5 (62.8 to 109.9)	37.7 (29.8 to 46.9)	3.9 (-2.0 to 10.9)
Liver cancer	129.3 (119.7 to 139.0)	132.1 (123.9 to 140.1)	29.6 (21.1 to 38.6)	50.6 (38.6 to 63.0)	13.7 (4.6 to 23.0)
Liver cancer due to hepatitis B	69.5 (51.7 to 83.5)	52.0 (39.1 to 62.3)	11.5 (7.3 to 15.8)	53.4 (36.8 to 72.4)	15.8 (3.3 to 30.1)

Cause	Prevalence in thousands (95% UI)	Incidence in thousands (95% UI)	YLDs in thousands (95% UI)		
	2016 counts	2016 counts	2016 counts	Percentage change in counts between 2006 and 2016	Percentage change in rates between 2006 and 2016
Liver cancer due to hepatitis C	35.1 (29.9 to 40.8)	26.3 (22.4 to 30.6)	6.0 (4.1 to 8.2)	40.7 (30.7 to 51.2)	6.2 (-1.4 to 14.2)
Liver cancer due to alcohol use	29.7 (23.4 to 36.1)	24.2 (19.2 to 29.5)	5.5 (3.6 to 7.7)	49.4 (33.6 to 69.0)	12.8 (0.9 to 27.6)
Liver cancer due to other causes	40.1 (32.8 to 49.7)	29.6 (24.5 to 36.1)	6.6 (4.4 to 9.1)	56.6 (35.2 to 79.5)	18.2 (2.0 to 35.5)
Gallbladder and biliary tract cancer	21.5 (19.2 to 23.2)	24.2 (21.9 to 26.0)	5.6 (3.9 to 7.5)	20.3 (13.0 to 28.2)	-9.2 (-14.7 to -3.3)
Pancreatic cancer	50.5 (48.2 to 52.3)	59.9 (57.7 to 61.5)	12.6 (8.8 to 16.7)	35.4 (27.7 to 43.0)	2.2 (-3.6 to 7.9)
Larynx cancer	100.2 (97.1 to 103.8)	29.7 (28.8 to 30.7)	11.8 (8.6 to 15.5)	25.9 (17.3 to 35.6)	-5.0 (-11.5 to 2.3)
Tracheal, bronchus, and lung cancer	452.8 (432.0 to 473.0)	329.8 (317.5 to 343.0)	77.7 (57.0 to 98.7)	36.7 (28.6 to 44.8)	3.1 (-2.9 to 9.3)
Malignant skin melanoma	153.7 (129.0 to 169.2)	32.0 (27.1 to 35.2)	10.2 (7.1 to 14.1)	55.4 (43.3 to 67.5)	17.3 (8.2 to 26.4)
Non-melanoma skin cancer	121.2 (97.3 to 157.6)	204.8 (137.6 to 290.9)	4.4 (2.2 to 7.8)	12.4 (-0.6 to 29.2)	-15.2 (-25.0 to -2.5)
Non-melanoma skin cancer (squamous-cell carcinoma)	97.0 (54.8 to 150.2)	91.7 (53.0 to 142.2)	4.3 (2.1 to 7.7)	11.8 (-1.4 to 29.1)	-15.6 (-25.6 to -2.6)
Non-melanoma skin cancer (basal-cell carcinoma)	13.1 (6.4 to 24.5)	113.1 (62.2 to 183.5)	0.1 (< 0.05 to 0.3)	34.0 (16.0 to 49.5)	1.1 (-12.5 to 12.8)
Breast cancer	862.2 (819.4 to 920.9)	179.3 (170.2 to 191.5)	78.9 (56.8 to 105.3)	34.7 (26.6 to 43.3)	1.7 (-4.5 to 8.2)
Cervical cancer	133.6 (107.2 to 146.7)	36.0 (29.4 to 39.1)	12.9 (9.0 to 17.0)	14.4 (6.0 to 26.9)	-13.7 (-20.0 to -4.2)
Uterine cancer	264.1 (247.4 to 287.5)	56.3 (52.7 to 61.3)	19.9 (14.2 to 26.7)	45.4 (31.0 to 62.3)	9.7 (-1.1 to 22.5)
Ovarian cancer	79.7 (74.4 to 83.7)	26.4 (24.8 to 27.6)	12.0 (8.6 to 15.6)	26.2 (12.9 to 40.3)	-4.8 (-14.8 to 5.9)
Prostate cancer	1,112.4 (984.9 to 1,312.8)	279.7 (251.9 to 323.1)	99.1 (69.9 to 132.4)	41.9 (31.5 to 52.7)	7.1 (-0.7 to 15.2)
Testicular cancer	6.4 (5.9 to 6.8)	1.3 (1.2 to 1.4)	0.5 (0.4 to 0.7)	49.2 (31.7 to 69.6)	12.6 (-0.6 to 28.0)
Kidney cancer	168.2 (158.8 to 175.8)	45.6 (43.4 to 47.5)	14.9 (10.8 to 19.7)	32.7 (24.0 to 41.7)	0.2 (-6.4 to 7.0)
Bladder cancer	249.7 (240.2 to 260.3)	61.3 (58.9 to 63.8)	23.4 (17.1 to 30.9)	31.4 (23.1 to 39.8)	-0.8 (-7.1 to 5.5)
Brain and nervous system cancer	67.6 (58.5 to 72.4)	30.6 (26.8 to 32.8)	9.7 (6.8 to 12.9)	45.7 (37.6 to 54.5)	10.0 (3.9 to 16.6)
Thyroid cancer	105.9 (100.2 to 113.9)	20.6 (19.6 to 22.2)	7.0 (4.9 to 9.5)	51.0 (39.5 to 64.2)	14.0 (5.3 to 23.9)
Mesothelioma	7.2 (6.7 to 7.7)	4.9 (4.5 to 5.2)	1.6 (1.1 to 2.1)	31.1 (21.7 to 39.7)	-1.1 (-8.1 to 5.4)
Hodgkin lymphoma	16.2 (14.3 to 18.1)	4.3 (3.8 to 4.9)	1.8 (1.2 to 2.4)	19.6 (11.3 to 28.0)	-9.7 (-16.0 to -3.4)
Non-Hodgkin's lymphoma	194.8 (178.2 to 210.3)	54.1 (49.4 to 58.1)	17.2 (12.4 to 22.9)	56.2 (46.4 to 64.9)	17.9 (10.5 to 24.5)
Multiple myeloma	50.7 (42.8 to 59.6)	20.2 (17.5 to 23.7)	10.5 (7.3 to 13.9)	48.4 (35.5 to 62.3)	12.0 (2.2 to 22.5)
Leukaemia	160.4 (145.7 to 170.3)	45.7 (41.2 to 48.2)	21.7 (15.6 to 28.6)	45.0 (36.4 to 55.7)	9.4 (3.0 to 17.5)
Acute lymphoid leukaemia	12.6 (10.7 to 13.5)	3.8 (3.2 to 4.0)	1.7 (1.2 to 2.4)	61.1 (45.0 to 73.0)	21.6 (9.4 to 30.5)
Chronic lymphoid leukaemia	64.8 (59.5 to 71.0)	14.8 (13.6 to 16.2)	8.2 (5.9 to 10.8)	42.8 (29.6 to 57.6)	7.7 (-2.2 to 18.9)
Acute myeloid leukaemia	13.3 (11.3 to 14.3)	10.2 (8.6 to 10.9)	2.8 (1.9 to 3.8)	53.2 (45.0 to 60.5)	15.6 (9.4 to 21.1)
Chronic myeloid leukaemia	7.7 (6.9 to 8.3)	2.8 (2.5 to 3.0)	1.0 (0.7 to 1.4)	11.0 (3.6 to 18.1)	-16.2 (-21.8 to -10.8)
Other leukaemia	62.0 (51.8 to 68.2)	14.2 (11.9 to 15.6)	7.9 (5.6 to 10.5)	47.0 (33.0 to 62.1)	10.9 (0.4 to 22.4)
Other neoplasms	239.8 (213.6 to 253.1)	70.2 (62.7 to 73.7)	20.7 (14.8 to 27.6)	58.8 (47.7 to 68.7)	19.8 (11.5 to 27.3)
**Cardiovascular diseases**	**58,074.6 (56,877.5 to 59,390.5)**	**6,466.6† (5,741.9 to 7,265.0)**	**4,082.6 (2,978.9 to 5,318.6)**	**35.1 (30.7 to 39.2)**	**1.9 (-1.4 to 5.1)**
Rheumatic heart disease	635.8 (572.3 to 701.5)	15.5 (12.5 to 19.0)	38.0 (25.6 to 52.3)	25.4 (20.5 to 30.4)	-5.3 (-9.1 to -1.6)
Ischaemic heart disease	20,711.6 (19,289.7 to 22,131.8)	2,247.7 (1,899.7 to 2,670.8)	859.5 (597.8 to 1,165.1)	28.8 (27.5 to 30.2)	-2.8 (-3.8 to -1.8)
Cerebrovascular disease	10,895.5 (10,498.2 to 11,332.3)	1,707.3‡ (1,407.2 to 2,017.0)	1,932.4 (1,362.3 to 2,493.3)	40.7 (31.3 to 49.2)	6.2 (-0.9 to 12.6)
Ischaemic stroke	9,280.1 (8,011.1 to 10,622.6)	1,209.8‡ (936.9 to 1,507.3)	1,593.0 (1,109.4 to 2,079.7)	41.8 (30.4 to 51.9)	7.0 (-1.6 to 14.6)
Hemorrhagic stroke	1,971.6 (1,664.9 to 2,333.5)	497.5‡ (390.2 to 612.1)	339.4 (237.8 to 452.1)	35.8 (23.8 to 47.3)	2.5 (-6.6 to 11.2)
Hypertensive heart disease	2,142.6 (1,681.2 to 2,682.4)	--	173.2 (112.7 to 248.4)	31.2 (28.2 to 34.3)	-1.0 (-3.2 to 1.3)
Cardiomyopathy and myocarditis	591.1 (535.1 to 641.7)	222.6† (168.7 to 279.4)	48.2 (31.6 to 69.7)	26.5 (21.4 to 31.9)	-4.5 (-8.4 to -0.4)
Myocarditis	95.9 (79.6 to 113.7)	222.6 (168.7 to 279.4)	6.1 (4.0 to 8.8)	31.3 (24.1 to 38.0)	-0.9 (-6.3 to 4.1)
Alcoholic cardiomyopathy	155.6 (119.4 to 199.0)	--	12.7 (8.2 to 18.6)	16.8 (6.4 to 28.7)	-11.8 (-19.7 to -2.9)
Other cardiomyopathy	361.7 (275.7 to 462.7)	--	29.4 (19.1 to 43.1)	30.1 (24.6 to 35.5)	-1.8 (-6.0 to 2.2)
Atrial fibrillation and flutter	6,198.9 (5,271.2 to 7,317.3)	676.1 (492.2 to 921.0)	490.6 (319.7 to 693.4)	35.8 (34.0 to 37.6)	2.5 (1.2 to 3.8)
Peripheral vascular disease	16,869.3 (14,053.1 to 20,326.0)	1,506.2 (1,056.2 to 2,073.3)	92.9 (41.6 to 173.2)	24.2 (22.4 to 26.4)	-6.2 (-7.7 to -4.6)
Endocarditis	41.9 (32.9 to 52.1)	91.1 (69.5 to 116.5)	3.4 (2.2 to 5.3)	30.6 (21.1 to 40.4)	-1.4 (-8.6 to 6.0)
Other cardiovascular and circulatory diseases	8,646.4 (6,522.9 to 11,095.1)	--	444.5 (277.9 to 685.8)	29.5 (26.3 to 32.8)	-2.2 (-4.7 to 0.3)
**Chronic respiratory diseases**	**44,039.9 (43,221.6 to 44,773.0)**	**6,096.4† (5,452.6 to 6,805.5)**	**2,698.2 (2,271.8 to 3,090.1)**	**33.9 (30.7 to 37.0)**	**1.0 (-1.4 to 3.4)**
Chronic obstructive pulmonary disease	31,875.5 (30,008.7 to 33,653.1)	2,111.2 (1,780.9 to 2,444.6)	2,019.1 (1,760.5 to 2,257.2)	33.3 (29.2 to 37.4)	0.6 (-2.5 to 3.7)
Pneumoconiosis	210.4 (194.2 to 227.7)	13.2 (11.0 to 15.3)	30.2 (20.7 to 41.9)	35.4 (27.3 to 43.8)	2.1 (-3.9 to 8.5)
Silicosis	93.0 (82.2 to 105.4)	6.7 (4.8 to 8.6)	13.2 (8.8 to 18.7)	33.2 (19.9 to 46.1)	0.5 (-9.5 to 10.3)

Cause	Prevalence in thousands (95% UI)	Incidence in thousands (95% UI)	YLDs in thousands (95% UI)		
	2016 counts	2016 counts	2016 counts	Percentage change in counts between 2006 and 2016	Percentage change in rates between 2006 and 2016
Asbestosis	24.1 (20.8 to 27.7)	2.4 (2.1 to 2.8)	3.6 (2.4 to 5.1)	38.0 (29.8 to 47.4)	4.2 (-2.0 to 11.2)
Coal workers pneumoconiosis	54.2 (46.5 to 62.8)	1.7 (1.3 to 2.1)	7.7 (5.0 to 11.0)	38.2 (20.5 to 58.1)	4.3 (-9.0 to 19.3)
Other pneumoconiosis	39.1 (32.3 to 46.5)	2.5 (1.8 to 3.5)	5.7 (3.7 to 8.1)	35.1 (19.7 to 52.7)	1.9 (-9.7 to 15.2)
Asthma	15,594.6 (14,036.2 to 17,366.2)	3,920.0 (3,346.5 to 4,579.6)	578.3 (385.4 to 830.9)	34.3 (31.6 to 37.1)	1.3 (-0.7 to 3.5)
Interstitial lung disease and pulmonary sarcoidosis	484.3 (406.7 to 575.8)	52.0 (37.2 to 67.9)	50.5 (32.7 to 74.3)	37.4 (31.4 to 43.7)	3.7 (-0.8 to 8.4)
Other chronic respiratory diseases	--	--	20.2 (17.5 to 22.6)	68.8 (63.7 to 73.8)	27.4 (23.6 to 31.2)
**Cirrhosis and other chronic liver diseases**	**3,432.4 (3,232.2 to 3,642.7)**	**146.2 (124.0 to 168.5)**	**109.8 (74.5 to 151.8)**	**33.1 (28.4 to 37.6)**	**0.5 (-3.1 to 3.8)**
Cirrhosis and other chronic liver diseases due to hepatitis B	984.7 (849.1 to 1,137.5)	44.4 (35.1 to 54.9)	34.4 (22.8 to 48.5)	34.1 (27.7 to 40.9)	1.2 (-3.6 to 6.4)
Cirrhosis and other chronic liver diseases due to hepatitis C	877.4 (751.1 to 1,013.3)	38.4 (30.4 to 47.1)	27.2 (18.0 to 38.8)	33.7 (26.7 to 40.7)	0.9 (-4.3 to 6.2)
Cirrhosis and other chronic liver diseases due to alcohol use	958.7 (817.6 to 1,098.2)	39.0 (31.2 to 47.7)	29.4 (18.9 to 41.6)	31.1 (23.9 to 38.2)	-1.1 (-6.5 to 4.3)
Cirrhosis and other chronic liver diseases due to other causes	611.6 (458.8 to 782.5)	24.4 (17.8 to 31.9)	18.9 (11.4 to 28.0)	33.7 (25.3 to 43.1)	0.9 (-5.4 to 8.0)
**Digestive diseases**	**22,332.7 (21,182.7 to 23,291.0)**	**18,151.9† (15,992.4 to 20,448.8)**	**578.2 (407.2 to 795.5)**	**46.1 (40.6 to 51.9)**	**10.2 (6.1 to 14.6)**
Peptic ulcer disease	6,017.2 (4,964.1 to 7,215.6)	686.5 (528.7 to 844.9)	103.8 (64.6 to 158.8)	51.4 (39.9 to 63.0)	14.2 (5.6 to 23.0)
Gastritis and duodenitis	12,239.0 (9,365.2 to 15,480.9)	7,226.9 (5,824.2 to 8,892.5)	143.9 (90.0 to 212.9)	56.9 (48.1 to 65.4)	18.4 (11.8 to 24.8)
Appendicitis	21.2 (17.2 to 25.1)	571.8 (465.9 to 679.3)	6.2 (4.0 to 8.9)	33.4 (22.0 to 45.9)	0.6 (-7.9 to 10.1)
Paralytic ileus and intestinal obstruction	12.1 (9.5 to 14.8)	339.5 (267.8 to 413.0)	3.9 (2.4 to 5.6)	46.6 (39.6 to 54.4)	10.6 (5.3 to 16.5)
Inguinal, femoral, and abdominal hernia	3,459.7 (2,574.5 to 4,505.8)	3,827.7 (2,819.8 to 5,104.0)	164.5 (100.4 to 247.0)	29.6 (23.6 to 35.8)	-2.2 (-6.7 to 2.5)
Inflammatory bowel disease	578.2 (499.9 to 668.3)	123.9 (95.9 to 158.0)	80.0 (53.3 to 111.6)	54.9 (47.5 to 62.3)	16.9 (11.3 to 22.5)
Vascular intestinal disorders	10.1 (7.7 to 13.1)	55.7 (41.5 to 73.0)	3.1 (1.9 to 4.6)	38.5 (20.4 to 55.4)	4.5 (-9.1 to 17.3)
Gallbladder and biliary diseases	1,315.7 (1,016.5 to 1,689.5)	4,837.6 (3,598.3 to 6,265.0)	20.2 (12.7 to 29.5)	42.1 (30.4 to 54.2)	7.2 (-1.6 to 16.4)
Pancreatitis	54.2 (42.5 to 68.0)	482.2 (378.6 to 606.4)	6.6 (4.1 to 10.1)	54.4 (36.5 to 72.2)	16.5 (3.0 to 30.0)
Other digestive diseases	--	--	45.9 (32.3 to 63.1)	58.9 (53.0 to 65.2)	19.9 (15.4 to 24.7)
**Neurological disorders**	**69,485.6 (64,248.0 to 74,783.2)**	**22,482.2 (15,014.5 to 32,519.6)**	**2,292.6 (1,586.8 to 3,052.2)**	**31.7 (29.6 to 33.8)**	**-0.6 (-2.2 to 1.0)**
Alzheimer's disease and other dementias	3,942.4 (3,038.8 to 5,058.7)	666.1 (431.8 to 946.7)	476.4 (313.1 to 698.1)	36.1 (33.9 to 38.0)	2.7 (1.1 to 4.2)
Parkinson's disease	826.8 (617.6 to 1,069.2)	100.1 (58.3 to 152.7)	98.0 (61.8 to 144.9)	38.9 (34.1 to 43.7)	4.8 (1.2 to 8.4)
Epilepsy	846.3 (688.4 to 1,024.8)	63.7 (45.5 to 86.1)	234.0 (159.0 to 326.5)	27.3 (13.3 to 43.3)	-4.0 (-14.5 to 8.1)
Multiple sclerosis	140.2 (128.4 to 152.8)	0.8 (0.6 to 1.1)	35.5 (24.9 to 46.2)	45.8 (36.5 to 55.4)	10.1 (3.0 to 17.3)
Motor neuron disease	29.7 (24.1 to 36.3)	7.7 (6.0 to 9.7)	6.3 (4.3 to 8.7)	44.2 (40.4 to 48.2)	8.9 (6.0 to 11.9)
Migraine	25,427.6 (23,537.8 to 27,385.1)	1,440.0 (1,225.4 to 1,675.4)	1,169.3 (778.3 to 1,621.6)	29.8 (28.4 to 31.4)	-2.0 (-3.1 to -0.9)
Tension-type headache	47,272.8 (35,059.2 to 64,543.3)	20,201.1 (12,703.2 to 30,306.5)	235.2 (148.0 to 353.5)	30.4 (28.0 to 33.3)	-1.6 (-3.4 to 0.6)
Other neurological disorders	1.3 (0.7 to 2.2)	2.7 (1.5 to 4.0)	38.1 (26.6 to 50.6)	39.7 (34.9 to 44.8)	5.5 (1.8 to 9.3)
**Mental and substance use disorders**	**37,528.6 (36,950.4 to 38,126.5)**	**16,118.4† (14,359.5 to 18,123.7)**	**5,293.1 (3,881.5 to 6,858.6)**	**29.2 (28.1 to 30.3)**	**-2.5 (-3.3 to -1.6)**
Schizophrenia	801.5 (710.7 to 903.5)	10.6 (8.0 to 13.7)	481.3 (363.2 to 587.8)	36.4 (33.4 to 39.6)	2.9 (0.7 to 5.4)
Alcohol use disorders	2,418.8 (1,999.8 to 2,886.6)	847.6 (664.9 to 1,059.6)	227.4 (154.8 to 317.2)	19.4 (13.8 to 24.9)	-9.9 (-14.1 to -5.8)
Drug use disorders	1,199.4 (1,156.9 to 1,238.6)	53.4 (46.6 to 60.7)	368.7 (261.6 to 474.4)	39.3 (35.1 to 43.7)	5.1 (2.0 to 8.4)
Opioid use disorders	808.3 (708.4 to 920.1)	27.2 (21.5 to 33.2)	312.6 (221.4 to 410.7)	39.3 (34.6 to 44.5)	5.2 (1.6 to 9.0)
Cocaine use disorders	176.9 (158.0 to 198.4)	1.6 (1.0 to 2.2)	22.9 (14.2 to 32.7)	38.6 (29.8 to 47.2)	4.6 (-2.0 to 11.1)
Amphetamine use disorders	21.0 (13.7 to 30.2)	3.7 (2.2 to 5.6)	2.9 (1.6 to 5.0)	39.6 (32.1 to 46.4)	5.4 (-0.3 to 10.5)
Cannabis use disorders	158.9 (133.0 to 189.1)	4.1 (2.7 to 5.7)	4.6 (3.0 to 6.9)	36.0 (30.3 to 42.0)	2.6 (-1.7 to 7.2)
Other drug use disorders	87.2 (73.9 to 103.2)	16.9 (14.3 to 19.8)	25.8 (17.2 to 35.4)	39.7 (33.3 to 46.0)	5.5 (0.6 to 10.2)
Depressive disorders	15,171.6 (14,693.2 to 15,692.3)	14,104.7 (12,375.7 to 16,056.1)	2,318.5 (1,601.1 to 3,149.8)	26.2 (24.3 to 28.2)	-4.8 (-6.2 to -3.3)
Major depressive disorder	8,709.5 (7,657.0 to 9,957.9)	13,300.6 (11,586.5 to 15,245.3)	1,684.1 (1,134.1 to 2,324.8)	23.0 (21.1 to 24.9)	-7.1 (-8.6 to -5.7)
Dysthymia	6,876.4 (5,518.7 to 8,406.3)	804.1 (645.1 to 982.4)	634.4 (416.7 to 926.6)	35.4 (30.9 to 39.7)	2.2 (-1.2 to 5.4)
Bipolar disorder	1,508.6 (1,257.3 to 1,809.9)	84.0 (63.4 to 108.0)	289.4 (178.0 to 433.1)	31.8 (28.8 to 34.7)	-0.6 (-2.8 to 1.7)
Anxiety disorders	9,653.1 (8,458.5 to 10,913.3)	987.1 (879.3 to 1,111.3)	876.6 (607.8 to 1,183.3)	30.0 (27.2 to 32.8)	-1.9 (-4.0 to 0.2)
Eating disorders	--	--	--	--	--
Anorexia nervosa	--	--	--	--	--
Bulimia nervosa	--	--	--	--	--
Autistic spectrum disorders	1,494.1 (1,428.6 to 1,556.3)	--	210.7 (144.3 to 295.5)	33.0 (30.7 to 35.3)	0.4 (-1.3 to 2.1)
Autism	481.0 (413.5 to 561.7)	--	114.7 (73.2 to 165.3)	33.0 (29.3 to 36.8)	0.4 (-2.4 to 3.3)
Asperger syndrome and other autistic spectrum disorders	1,016.1 (849.0 to 1,212.0)	--	96.0 (63.6 to 138.0)	33.1 (30.6 to 35.6)	0.4 (-1.4 to 2.3)

Cause	Prevalence in thousands (95% UI)	Incidence in thousands (95% UI)	YLDs in thousands (95% UI)		
	2016 counts	2016 counts	2016 counts	Percentage change in counts between 2006 and 2016	Percentage change in rates between 2006 and 2016
Attention-deficit/hyperactivity disorder	195.3 (163.0 to 232.2)	--	2.2 (1.3 to 3.6)	35.9 (25.6 to 46.6)	2.6 (-5.2 to 10.7)
Conduct disorder	--	--	--	--	--
Idiopathic developmental intellectual disability	1,698.2 (873.4 to 2,505.7)	--	78.5 (39.1 to 130.5)	21.2 (16.5 to 24.9)	-8.5 (-12.1 to -5.7)
Other mental and substance use disorders	6,220.9 (5,522.1 to 7,039.0)	30.9 (19.4 to 46.2)	439.9 (304.3 to 619.1)	32.7 (30.6 to 34.8)	0.1 (-1.4 to 1.7)
**Diabetes, urogenital, blood, and endocrine diseases**	**123,104.4 (121,856.6 to 124,432.7)**	**25,872.9† (23,041.2 to 29,072.7)**	**4,827.1 (3,428.6 to 6,436.8)**	**32.0 (29.5 to 34.5)**	**-0.4 (-2.3 to 1.5)**
Diabetes mellitus	36,541.0 (33,205.4 to 40,272.3)	1,120.1 (845.0 to 1,527.4)	2,797.6 (1,907.6 to 3,889.1)	31.8 (27.9 to 36.0)	-0.5 (-3.5 to 2.6)
Acute glomerulonephritis	6.8 (4.8 to 9.5)	100.7 (68.8 to 139.5)	0.3 (0.2 to 0.6)	19.4 (8.8 to 30.6)	-9.9 (-17.9 to -1.5)
Chronic kidney disease	32,854.2 (28,204.5 to 37,715.8)	2,802.4 (1,942.3 to 3,735.7)	888.0 (644.5 to 1,172.6)	35.9 (32.2 to 39.5)	2.6 (-0.2 to 5.2)
Chronic kidney disease due to diabetes mellitus	16,304.8 (12,717.0 to 20,287.6)	1,411.7 (909.1 to 1,990.7)	447.4 (310.2 to 607.3)	37.7 (33.4 to 42.4)	4.0 (0.7 to 7.5)
Chronic kidney disease due to hypertension	5,226.7 (3,992.2 to 6,749.9)	330.2 (197.6 to 504.2)	194.2 (129.1 to 275.2)	40.4 (34.8 to 46.8)	6.0 (1.7 to 10.8)
Chronic kidney disease due to glomerulonephritis	5,365.2 (4,151.9 to 6,928.7)	553.2 (356.2 to 784.0)	94.2 (57.0 to 145.1)	24.0 (15.5 to 31.7)	-6.4 (-12.8 to -0.6)
Chronic kidney disease due to other causes	5,957.4 (4,006.0 to 8,469.5)	507.3 (310.8 to 763.8)	152.2 (93.7 to 234.5)	33.4 (27.4 to 39.8)	0.7 (-3.8 to 5.5)
Urinary diseases and male infertility	16,739.8 (15,344.1 to 18,942.5)	20,068.7† (17,247.9 to 23,225.8)	549.3 (358.7 to 793.3)	29.9 (28.4 to 31.3)	-2.0 (-3.1 to -0.9)
Interstitial nephritis and urinary tract infections	224.6 (187.0 to 272.9)	11,728.0 (9,618.3 to 14,354.7)	7.1 (4.3 to 10.8)	39.6 (31.3 to 47.8)	5.4 (-0.9 to 11.5)
Urolithiasis	235.0 (178.4 to 314.3)	6,192.5 (4,756.7 to 8,141.5)	16.4 (10.3 to 24.4)	36.6 (26.3 to 47.8)	3.1 (-4.7 to 11.6)
Benign prostatic hyperplasia	15,942.6 (13,379.6 to 18,642.3)	2,148.2 (1,448.6 to 2,865.3)	519.0 (338.6 to 756.4)	29.8 (28.4 to 31.3)	-2.0 (-3.1 to -0.9)
Male infertility	--	--	--	--	--
Other urinary diseases	--	--	6.7 (4.4 to 9.6)	12.9 (6.7 to 19.6)	-14.8 (-19.5 to -9.7)
Gynecological diseases	26,617.7 (25,188.4 to 28,744.0)	17.2† (11.5 to 24.2)	94.6 (47.1 to 167.3)	19.9 (18.3 to 21.7)	-9.5 (-10.7 to -8.2)
Uterine fibroids	2,615.2 (2,324.6 to 2,971.8)	17.2 (11.5 to 24.2)	18.9 (9.2 to 35.0)	30.9 (28.8 to 33.1)	-1.2 (-2.8 to 0.5)
Polycystic ovarian syndrome	--	--	--	--	--
Female infertility	--	--	--	--	--
Endometriosis	--	--	--	--	--
Genital prolapse	24,728.9 (21,713.5 to 28,041.3)	--	75.7 (36.3 to 137.0)	17.4 (15.8 to 19.0)	-11.4 (-12.6 to -10.2)
Premenstrual syndrome	--	--	--	--	--
Other gynecological diseases	--	--	--	--	--
Hemoglobinopathies and hemolytic anaemias	49,408.1 (48,977.1 to 49,828.5)	--	182.2 (119.8 to 264.4)	32.6 (28.7 to 36.2)	0.1 (-2.9 to 2.8)
Thalassemias	0.1 (0.1 to 0.1)	--	< 0.05 (< 0.05 to < 0.05)	22.5 (11.2 to 33.4)	-7.5 (-16.1 to 0.7)
Thalassaemias trait	9,453.3 (9,086.7 to 9,844.3)	--	98.2 (62.6 to 144.0)	43.5 (37.4 to 49.7)	8.3 (3.7 to 13.0)
Sickle cell disorders	1.0 (0.9 to 1.0)	--	0.2 (0.1 to 0.2)	70.3 (65.2 to 75.5)	28.5 (24.7 to 32.5)
Sickle cell trait	9,581.8 (8,826.3 to 10,593.9)	--	19.8 (12.8 to 28.9)	32.0 (27.5 to 36.5)	-0.4 (-3.7 to 3.0)
G6PD deficiency	7,755.1 (7,473.7 to 8,077.8)	--	0.8 (0.5 to 1.2)	32.1 (28.5 to 35.7)	-0.3 (-3.0 to 2.5)
G6PD trait	24,681.5 (24,266.5 to 25,168.0)	--	< 0.05 (< 0.05 to < 0.05)	44.4 (39.3 to 49.9)	9.0 (5.1 to 13.1)
Other hemoglobinopathies and hemolytic anaemias	3,224.7 (3,012.3 to 3,375.7)	--	63.1 (41.8 to 90.5)	18.6 (15.5 to 21.3)	-10.5 (-12.8 to -8.4)
Endocrine, metabolic, blood, and immune disorders	9,855.9 (8,282.0 to 11,675.8)	1,763.8 (1,229.5 to 2,375.0)	315.1 (209.5 to 439.8)	30.5 (26.7 to 34.4)	-1.5 (-4.4 to 1.4)
**Musculoskeletal disorders**	**92,007.2 (89,171.3 to 95,135.9)**	**46,170.5 (37,236.7 to 56,737.0)**	**9,940.8 (7,027.8 to 13,102.3)**	**31.9 (30.3 to 33.5)**	**-0.5 (-1.7 to 0.8)**
Rheumatoid arthritis	2,127.1 (1,863.3 to 2,399.8)	71.8 (52.9 to 94.2)	484.1 (334.7 to 643.5)	41.1 (38.0 to 44.2)	6.5 (4.1 to 8.8)
Osteoarthritis	37,689.4 (35,279.3 to 40,085.9)	1,160.4 (998.0 to 1,347.5)	1,979.2 (1,383.7 to 2,668.7)	35.4 (34.5 to 36.3)	2.2 (1.5 to 2.9)
Low back and neck pain	48,518.8 (45,001.6 to 52,252.7)	20,411.8 (14,409.3 to 26,783.3)	5,697.0 (3,887.5 to 7,864.0)	30.7 (28.1 to 33.2)	-1.4 (-3.3 to 0.5)
Low back pain	34,234.4 (25,591.3 to 43,698.7)	16,815.9 (10,969.6 to 23,425.8)	3,791.2 (2,414.6 to 5,551.4)	28.2 (24.7 to 31.5)	-3.2 (-5.9 to -0.8)
Neck pain	19,812.0 (14,592.8 to 26,641.7)	3,595.9 (2,339.5 to 5,306.9)	1,905.8 (1,208.5 to 2,859.3)	35.7 (32.8 to 39.2)	2.4 (0.2 to 5.1)
Gout	3,881.5 (3,159.6 to 4,743.0)	699.1 (504.0 to 938.6)	119.3 (80.4 to 167.3)	34.5 (30.1 to 38.8)	1.5 (-1.8 to 4.8)
Other musculoskeletal disorders	20,387.1 (15,011.9 to 26,915.0)	23,827.4 (17,200.5 to 31,862.8)	1,661.2 (1,051.1 to 2,487.8)	29.4 (23.2 to 35.4)	-2.3 (-7.0 to 2.2)
**Other non-communicable diseases**	**209,378.0 (208,968.5 to 209,942.0)**	**553,424.6† (500,636.9 to 618,795.7)**	**9,991.2 (6,889.3 to 14,132.4)**	**29.3 (28.0 to 30.6)**	**-2.4 (-3.4 to -1.4)**
Congenital anomalies	1,900.5 (1,768.3 to 2,032.1)	--	248.8 (180.7 to 329.2)	29.6 (26.1 to 33.0)	-2.2 (-4.8 to 0.4)
Neural tube defects	141.3 (128.7 to 156.0)	--	41.0 (29.5 to 54.2)	28.5 (22.8 to 34.2)	-3.0 (-7.4 to 1.3)
Congenital heart anomalies	303.4 (271.4 to 339.4)	--	16.9 (7.2 to 29.1)	42.4 (36.7 to 47.9)	7.5 (3.1 to 11.6)
Orofacial clefts	112.5 (100.2 to 124.5)	--	1.3 (0.9 to 1.9)	26.8 (23.5 to 30.1)	-4.3 (-6.8 to -1.8)
Down's syndrome	9.2 (7.5 to 11.0)	--	1.6 (1.1 to 2.2)	15.3 (9.8 to 20.8)	-13.0 (-17.1 to -8.8)
Turner syndrome	20.3 (17.4 to 23.6)	--	0.3 (0.1 to 0.5)	31.6 (23.4 to 41.5)	-0.7 (-6.9 to 6.8)
Klinefelter syndrome	5.9 (4.8 to 7.2)	--	< 0.05 (< 0.05 to < 0.05)	30.0 (27.2 to 33.1)	-1.9 (-4.0 to 0.5)

Cause	Prevalence in thousands (95% UI)	Incidence in thousands (95% UI)	YLDs in thousands (95% UI)		
	2016 counts	2016 counts	2016 counts	Percentage change in counts between 2006 and 2016	Percentage change in rates between 2006 and 2016
Other chromosomal abnormalities	< 0.05 (< 0.05 to < 0.05)	--	< 0.05 (< 0.05 to < 0.05)	59.0 (-63.4 to 570.1)	20.0 (-72.4 to 405.8)
Congenital musculoskeletal and limb anomalies	381.7 (352.6 to 411.7)	--	54.3 (37.8 to 73.5)	33.0 (27.7 to 38.1)	0.4 (-3.6 to 4.2)
Urogenital congenital anomalies	93.2 (84.0 to 103.2)	--	1.4 (0.7 to 2.5)	39.0 (34.3 to 44.3)	4.9 (1.3 to 8.9)
Digestive congenital anomalies	330.4 (298.5 to 365.1)	--	13.6 (9.1 to 19.3)	33.3 (26.6 to 40.5)	0.6 (-4.5 to 6.0)
Other congenital anomalies	911.8 (634.1 to 1,278.3)	--	118.5 (81.2 to 168.5)	26.7 (21.2 to 32.1)	-4.4 (-8.5 to -0.3)
Skin and subcutaneous diseases	84,014.3 (81,591.1 to 86,426.4)	236,658.8 (209,441.1 to 269,223.7)	1,369.0 (917.4 to 2,048.4)	35.2 (34.3 to 36.3)	2.1 (1.4 to 2.9)
Dermatitis	9,140.2 (7,849.2 to 10,648.4)	13,402.7 (10,124.8 to 17,131.8)	281.1 (171.2 to 427.6)	33.8 (31.9 to 35.8)	1.0 (-0.5 to 2.5)
Psoriasis	3,673.2 (3,396.0 to 3,994.1)	373.3 (320.9 to 433.6)	303.0 (219.5 to 396.5)	38.3 (35.2 to 41.3)	4.4 (2.0 to 6.7)
Cellulitis	130.3 (107.9 to 158.2)	3,092.6 (2,593.7 to 3,656.9)	7.0 (4.6 to 10.5)	35.9 (27.5 to 44.4)	2.5 (-3.8 to 9.0)
Pyoderma	1,275.3 (1,175.0 to 1,384.3)	32,540.2 (29,354.3 to 36,029.1)	6.9 (2.9 to 14.2)	31.7 (28.9 to 34.5)	-0.6 (-2.7 to 1.5)
Scabies	2,361.2 (1,648.9 to 3,249.8)	7,265.2 (5,113.1 to 10,030.6)	58.2 (29.5 to 101.2)	31.1 (28.9 to 33.5)	-1.1 (-2.7 to 0.8)
Fungal skin diseases	29,294.2 (23,014.9 to 37,571.0)	110,442.8 (85,596.2 to 142,550.9)	160.0 (62.9 to 344.9)	31.5 (30.9 to 32.2)	-0.7 (-1.2 to -0.2)
Viral skin diseases	4,238.9 (3,601.4 to 4,997.9)	6,704.1 (5,728.0 to 7,882.8)	123.4 (73.1 to 183.7)	32.5 (30.4 to 34.6)	-0.0 (-1.6 to 1.6)
Acne vulgaris	2,138.7 (2,075.2 to 2,214.0)	1,005.4 (968.3 to 1,048.6)	51.6 (35.4 to 73.9)	38.3 (33.9 to 43.3)	4.4 (1.0 to 8.1)
Alopecia areata	422.3 (375.2 to 474.8)	730.7 (659.4 to 810.9)	13.2 (8.3 to 19.6)	33.2 (27.8 to 38.6)	0.5 (-3.5 to 4.6)
Pruritus	3,641.7 (2,655.3 to 5,056.1)	2,835.6 (2,000.8 to 3,814.9)	37.5 (16.8 to 75.0)	34.6 (32.7 to 36.6)	1.6 (0.1 to 3.1)
Urticaria	1,330.2 (956.9 to 1,777.7)	2,350.6 (1,658.1 to 3,152.9)	75.2 (44.7 to 117.9)	32.5 (29.2 to 36.5)	-0.0 (-2.5 to 3.1)
Decubitus ulcer	146.4 (107.7 to 197.4)	542.9 (394.8 to 744.2)	23.3 (15.0 to 35.3)	33.2 (23.3 to 42.5)	0.5 (-6.9 to 7.5)
Other skin and subcutaneous diseases	42,760.7 (39,325.4 to 46,139.1)	55,372.7 (50,069.4 to 61,357.7)	228.7 (111.3 to 411.1)	39.1 (38.2 to 40.1)	5.0 (4.3 to 5.8)
Sense organ diseases	160,931.4 (159,890.6 to 162,101.6)	36,240.3† (33,049.8 to 39,611.1)	6,674.3 (4,578.1 to 9,328.5)	27.8 (25.9 to 29.7)	-3.6 (-5.0 to -2.1)
Glaucoma	924.3 (731.5 to 1,155.6)	--	88.2 (56.5 to 126.4)	34.2 (30.2 to 38.1)	1.3 (-1.7 to 4.2)
Cataract	12,568.3 (10,138.2 to 15,284.8)	--	835.4 (578.1 to 1,170.8)	30.3 (27.6 to 32.8)	-1.7 (-3.7 to 0.2)
Macular degeneration	1,252.2 (966.1 to 1,603.8)	--	75.3 (49.3 to 105.2)	39.5 (34.6 to 44.5)	5.3 (1.6 to 9.1)
Refraction and accommodation disorders	73,712.8 (68,471.2 to 79,251.6)	--	1,217.5 (762.5 to 1,922.6)	21.2 (18.6 to 23.2)	-8.5 (-10.5 to -7.0)
Age-related and other hearing loss	140,789.6 (134,674.2 to 146,338.0)	--	3,575.2 (2,372.0 to 5,152.1)	27.9 (24.6 to 31.4)	-3.5 (-6.0 to -0.9)
Other vision loss	3,066.8 (2,426.5 to 3,882.0)	--	227.7 (149.5 to 315.7)	33.0 (29.8 to 36.0)	0.4 (-2.0 to 2.6)
Other sense organ diseases	20,909.7 (18,425.2 to 23,763.4)	36,240.3 (33,049.8 to 39,611.1)	655.1 (409.2 to 992.5)	33.6 (32.3 to 34.9)	0.8 (-0.1 to 1.8)
Oral disorders	135,404.9 (132,814.0 to 138,313.4)	280,525.5† (232,459.1 to 335,856.8)	1,699.0 (1,080.4 to 2,547.8)	30.8 (29.8 to 31.8)	-1.3 (-2.0 to -0.5)
Caries of deciduous teeth	--	--	--	--	--
Caries of permanent teeth	83,167.0 (72,011.3 to 94,772.7)	271,003.8 (222,329.4 to 324,910.8)	59.2 (26.6 to 113.8)	25.0 (23.6 to 26.4)	-5.7 (-6.7 to -4.6)
Periodontal disease	51,960.1 (38,564.0 to 63,370.6)	5,579.7 (2,573.5 to 8,472.2)	330.4 (128.0 to 717.1)	31.3 (29.8 to 32.3)	-0.9 (-2.1 to -0.2)
Edentulism and severe tooth loss	42,144.2 (34,964.8 to 49,680.1)	3,942.0 (2,558.9 to 5,460.2)	1,158.7 (737.8 to 1,688.1)	30.8 (29.4 to 32.2)	-1.3 (-2.4 to -0.2)
Other oral disorders	5,347.9 (4,805.7 to 5,903.4)	--	150.7 (92.0 to 226.1)	32.6 (31.2 to 34.2)	0.1 (-1.0 to 1.3)
**Injuries**	**101,339.6 (95,516.7 to 106,972.9)**	**22,215.6† (19,343.6 to 25,587.2)**	**3,653.8 (2,505.0 to 5,112.1)**	**35.3 (33.8 to 36.5)**	**2.1 (1.0 to 3.1)**
**Transport injuries**	**17,823.0 (16,663.1 to 19,077.5)**	**2,002.9 (1,561.2 to 2,523.6)**	**853.2 (574.0 to 1,207.1)**	**40.7 (39.4 to 41.9)**	**6.2 (5.2 to 7.1)**
Road injuries	15,017.0 (13,951.9 to 16,202.9)	1,755.8 (1,340.0 to 2,262.4)	725.7 (487.6 to 1,028.3)	42.6 (41.5 to 43.6)	7.7 (6.8 to 8.4)
Pedestrian road injuries	3,439.7 (2,994.1 to 3,998.2)	389.2 (247.1 to 568.1)	171.6 (116.7 to 240.9)	43.5 (42.0 to 44.9)	8.3 (7.2 to 9.4)
Cyclist road injuries	3,016.1 (2,550.8 to 3,527.9)	443.6 (295.7 to 637.9)	138.5 (92.6 to 199.3)	46.9 (45.8 to 48.0)	10.9 (10.0 to 11.7)
Motorcyclist road injuries	3,311.3 (2,850.6 to 3,884.8)	208.1 (128.7 to 327.0)	156.6 (107.3 to 221.4)	46.9 (45.7 to 47.9)	10.9 (10.0 to 11.7)
Motor vehicle road injuries	4,176.2 (3,463.0 to 4,844.9)	546.4 (379.0 to 767.8)	210.2 (141.5 to 297.7)	32.5 (31.2 to 33.8)	0.0 (-1.0 to 1.0)
Other road injuries	1,073.7 (910.9 to 1,281.4)	168.5 (88.5 to 276.5)	48.8 (32.5 to 70.0)	64.0 (62.2 to 65.5)	23.8 (22.4 to 24.9)
Other transport injuries	2,806.0 (2,380.1 to 3,228.8)	247.1 (171.1 to 340.6)	127.5 (87.2 to 178.7)	30.8 (28.4 to 33.0)	-1.3 (-3.1 to 0.4)
**Unintentional injuries**	**76,918.3 (71,501.5 to 82,384.1)**	**19,625.3 (16,855.4 to 22,935.3)**	**2,581.7 (1,788.9 to 3,552.1)**	**33.6 (32.2 to 34.8)**	**0.8 (-0.2 to 1.7)**
Falls	36,667.7 (32,442.4 to 40,937.6)	10,329.9 (7,913.0 to 13,463.4)	1,524.3 (1,077.2 to 2,069.1)	36.0 (34.5 to 37.3)	2.7 (1.5 to 3.6)
Drowning	287.1 (249.7 to 321.2)	26.0 (19.4 to 33.6)	13.5 (9.0 to 19.1)	18.5 (15.9 to 20.9)	-10.6 (-12.5 to -8.8)
Fire, heat, and hot substances	5,942.5 (5,201.9 to 6,839.0)	185.6 (123.3 to 266.0)	118.0 (73.8 to 182.5)	28.3 (26.4 to 29.8)	-3.1 (-4.6 to -2.0)
Poisonings	193.2 (167.5 to 221.1)	95.7 (56.2 to 159.5)	12.7 (8.4 to 18.2)	41.3 (37.0 to 45.6)	6.6 (3.4 to 9.9)
Exposure to mechanical forces	13,639.4 (11,839.3 to 16,224.1)	1,482.0 (1,017.0 to 2,042.1)	289.5 (191.7 to 420.6)	38.1 (36.6 to 39.4)	4.2 (3.1 to 5.2)
Unintentional firearm injuries	479.4 (399.6 to 572.0)	20.7 (9.9 to 36.2)	12.8 (8.4 to 18.6)	38.3 (36.9 to 39.5)	4.4 (3.3 to 5.2)
Unintentional suffocation	435.3 (360.4 to 535.6)	23.5 (11.3 to 41.7)	12.4 (8.0 to 18.3)	32.8 (31.2 to 34.4)	0.3 (-0.9 to 1.4)
Other exposure to mechanical forces	12,724.8 (10,932.5 to 15,329.3)	1,437.9 (991.1 to 1,978.4)	264.2 (175.8 to 383.5)	38.4 (36.8 to 39.7)	4.4 (3.3 to 5.4)

Cause	Prevalence in thousands (95% UI)	Incidence in thousands (95% UI)	YLDs in thousands (95% UI)		
	2016 counts	2016 counts	2016 counts	Percentage change in counts between 2006 and 2016	Percentage change in rates between 2006 and 2016
Adverse effects of medical treatment	168.6 (119.2 to 229.9)	2,203.8 (1,657.7 to 2,837.5)	22.4 (13.4 to 35.3)	23.5 (17.8 to 28.4)	-6.8 (-11.1 to -3.1)
Animal contact	3,190.0 (2,849.3 to 3,601.8)	1,707.0 (1,202.0 to 2,321.1)	71.9 (49.8 to 100.3)	17.8 (15.6 to 19.9)	-11.1 (-12.8 to -9.5)
Venomous animal contact	1,357.2 (1,163.2 to 1,616.2)	829.5 (573.3 to 1,161.3)	44.3 (30.7 to 61.2)	17.9 (15.5 to 20.3)	-11.0 (-12.9 to -9.2)
Non-venomous animal contact	1,832.8 (1,564.4 to 2,131.4)	877.5 (587.7 to 1,224.8)	27.6 (18.3 to 41.9)	17.6 (14.5 to 19.9)	-11.3 (-13.6 to -9.5)
Foreign body	2,710.5 (2,365.0 to 3,081.8)	961.2 (700.6 to 1,351.5)	90.2 (59.4 to 130.1)	26.3 (24.3 to 28.1)	-4.7 (-6.2 to -3.3)
Pulmonary aspiration and foreign body in airway	1,216.8 (994.3 to 1,472.8)	301.1 (220.4 to 403.7)	44.9 (29.5 to 64.8)	19.1 (17.2 to 20.9)	-10.1 (-11.5 to -8.8)
Foreign body in eyes	152.8 (107.9 to 221.3)	452.1 (232.4 to 825.2)	9.3 (5.6 to 14.6)	40.1 (36.5 to 44.8)	5.8 (3.1 to 9.3)
Foreign body in other body part	1,340.9 (1,134.2 to 1,628.0)	208.0 (122.5 to 304.6)	36.0 (23.5 to 52.1)	32.9 (30.7 to 34.8)	0.3 (-1.4 to 1.7)
Environmental heat and cold exposure	4,461.8 (3,884.7 to 5,152.0)	659.8 (433.9 to 971.7)	163.8 (111.5 to 232.8)	25.8 (23.5 to 27.9)	-5.0 (-6.8 to -3.5)
Other unintentional injuries	9,657.8 (8,067.0 to 11,447.1)	1,974.4 (1,321.2 to 2,871.2)	275.2 (182.8 to 396.7)	31.4 (30.0 to 32.7)	-0.9 (-1.9 to 0.1)
**Self-harm and interpersonal violence**	**7,156.1 (6,523.4 to 7,922.9)**	**575.9† (449.9 to 738.8)**	**167.8 (112.2 to 239.3)**	**24.0 (23.0 to 25.0)**	**-6.4 (-7.2 to -5.7)**
Self-harm	951.2 (795.2 to 1,113.3)	243.0 (174.1 to 343.7)	35.9 (23.5 to 51.2)	17.5 (15.8 to 19.0)	-11.3 (-12.6 to -10.2)
Self-harm by firearm	22.9 (19.7 to 26.3)	3.2 (0.9 to 7.5)	0.7 (0.5 to 1.0)	4.9 (2.8 to 6.9)	-20.8 (-22.4 to -19.3)
Self-harm by other specified means	928.4 (772.4 to 1,090.8)	239.8 (171.3 to 339.8)	35.2 (23.0 to 50.2)	17.8 (16.1 to 19.3)	-11.1 (-12.4 to -10.0)
Interpersonal violence	6,204.9 (5,584.7 to 6,927.8)	332.9† (227.2 to 480.4)	131.8 (88.6 to 186.7)	25.9 (25.0 to 26.9)	-4.9 (-5.7 to -4.2)
Assault by firearm	201.7 (170.7 to 237.9)	10.7 (5.5 to 18.5)	6.1 (4.0 to 9.0)	35.8 (34.4 to 37.1)	2.5 (1.4 to 3.5)
Assault by sharp object	917.5 (762.1 to 1,084.4)	49.5 (25.9 to 84.5)	20.8 (13.7 to 30.3)	23.6 (21.8 to 25.1)	-6.7 (-8.1 to -5.6)
Sexual violence	2,380.2 (1,925.8 to 2,950.7)	--	15.3 (9.1 to 22.6)	25.5 (24.3 to 26.6)	-5.3 (-6.2 to -4.4)
Assault by other means	2,705.6 (2,300.5 to 3,241.4)	272.7 (185.1 to 391.5)	89.6 (60.2 to 129.3)	26.0 (24.9 to 26.9)	-4.9 (-5.8 to -4.2)
**Forces of nature, conflict and terrorism, and executions and police conflict**	**1,822.4 (1,207.9 to 2,564.0)**	**11.4† (7.5 to 16.5)**	**51.2 (15.4 to 119.4)**	**90.3 (76.8 to 113.8)**	**43.6 (33.4 to 61.4)**
Forces of nature, conflict and terrorism, and state actor violence	286.2 (162.5 to 446.1)	6.2 (3.3 to 9.9)	9.9 (2.4 to 25.4)	86.3 (73.6 to 96.2)	40.6 (31.1 to 48.1)
Conflict and terrorism	1,158.6 (698.6 to 1,795.0)	--	34.7 (11.8 to 76.0)	81.1 (63.5 to 115.5)	36.7 (23.4 to 62.7)
Executions and police conflict	377.7 (104.5 to 615.5)	5.2 (2.5 to 8.4)	8.3 (1.4 to 23.3)	129.4 (117.5 to 145.8)	73.1 (64.2 to 85.5)

We did not calculate incidence for certain NTDs and causes with only birth prevalence. Blank cells mean that no estimate is available.
† Incidence for aggregate causes represents the sum of the incidence of the causes below the aggregate.
‡ Incidence estimates for stroke represent first-ever stroke only.
^ Incidence of HIV/AIDS represents new infections of HIV only and does not include new infections of Tuberculosis in HIV positive cases.

SR Table 12: Global prevalence, incidence, and YLDs for 2016, percentage change of YLD counts and percentage change of YLD rates between 2006 and 2016 by cause, age 70-74 years.					
Cause	Prevalence in thousands (95% UI)	Incidence in thousands (95% UI)	YLDs in thousands (95% UI)		
	2016 counts	2016 counts	2016 counts	Percentage change in counts between 2006 and 2016	Percentage change in rates between 2006 and 2016
**All causes**	**157,045.4 (157,034.0 to 157,056.9)**	**988,628.5† (892,779.5 to 1,092,400.2)**	**36,344.9 (27,593.4 to 46,576.8)**	**19.0 (18.3 to 19.6)**	**-0.6 (-1.1 to -0.1)**
**Communicable, maternal, neonatal, and nutritional disorders**	**98,748.9 (97,800.0 to 100,172.5)**	**434,591.5† (351,416.5 to 538,033.9)**	**1,465.5 (1,048.5 to 1,950.0)**	**10.2 (8.1 to 12.1)**	**-7.9 (-9.6 to -6.3)**
**HIV/AIDS and tuberculosis**	**46,782.7 (42,328.2 to 52,377.6)**	**244.4† (194.2 to 304.9)**	**188.9 (128.3 to 263.0)**	**1.8 (-6.2 to 10.4)**	**-14.9 (-21.6 to -7.8)**
Tuberculosis	44,443.7 (36,883.5 to 51,933.9)	233.3† (183.9 to 294.7)	150.3 (97.3 to 216.6)	0.9 (-2.5 to 5.0)	-15.7 (-18.5 to -12.2)
Drug-susceptible tuberculosis	512.5 (400.7 to 658.1)	225.3 (177.2 to 284.7)	144.8 (93.8 to 208.6)	1.2 (-2.4 to 5.3)	-15.5 (-18.4 to -12.0)
Multidrug-resistant tuberculosis without extensive drug resistance	17.8 (13.9 to 22.5)	7.6 (6.0 to 9.5)	5.1 (3.3 to 7.5)	-9.3 (-20.9 to 5.6)	-24.2 (-33.9 to -11.8)
Extensively drug-resistant tuberculosis	1.2 (0.9 to 1.5)	0.5 (0.4 to 0.6)	0.4 (0.2 to 0.6)	165.4 (133.2 to 207.5)	121.8 (94.9 to 157.0)
Latent tuberculosis infection	43,912.2 (36,323.9 to 51,387.0)	--	--	--	--
HIV/AIDS	389.2 (337.8 to 451.2)	11.0^ (9.1 to 13.3)	38.7 (25.1 to 55.8)	5.2 (-24.0 to 52.9)	-12.0 (-36.5 to 27.8)
Drug-susceptible HIV/AIDS - Tuberculosis	11.0 (7.6 to 15.5)	11.7 (8.4 to 16.1)	4.0 (2.4 to 6.3)	3.5 (-6.0 to 13.0)	-13.5 (-21.4 to -5.6)
Multidrug-resistant HIV/AIDS - Tuberculosis without extensive drug resistance	0.4 (0.2 to 0.5)	0.3 (0.2 to 0.5)	0.1 (0.1 to 0.2)	9.6 (-10.5 to 29.4)	-8.4 (-25.2 to 8.1)
Extensively drug-resistant HIV/AIDS - Tuberculosis	< 0.05 (< 0.05 to < 0.05)	< 0.05 (< 0.05 to < 0.05)	< 0.05 (< 0.05 to < 0.05)	325.5 (244.3 to 408.2)	255.7 (187.8 to 324.8)
HIV/AIDS resulting in other diseases	377.8 (326.9 to 441.7)	11.0 (9.1 to 13.3)	34.5 (22.0 to 50.9)	5.4 (-26.4 to 62.1)	-11.9 (-38.5 to 35.5)
**Diarrhoea, lower respiratory infections, and other common infectious diseases**	**7,501.8 (7,049.2 to 7,972.3)**	**418,086.0† (333,899.6 to 520,950.7)**	**388.8 (274.4 to 526.5)**	**17.8 (16.4 to 19.4)**	**-1.6 (-2.8 to -0.2)**
Diarrhoeal diseases	1,917.9 (1,741.4 to 2,114.3)	132,135.4 (119,798.2 to 146,336.7)	198.0 (137.9 to 269.4)	16.9 (14.7 to 19.1)	-2.3 (-4.1 to -0.4)
Intestinal infectious diseases	9.2 (7.6 to 11.4)	184.6† (153.5 to 221.4)	1.5 (1.0 to 2.2)	6.4 (-13.7 to 29.5)	-11.1 (-27.9 to 8.2)
Typhoid fever	8.9 (7.1 to 11.1)	140.3 (117.0 to 169.1)	1.3 (0.9 to 2.0)	7.2 (-14.2 to 33.4)	-10.4 (-28.2 to 11.5)
Paratyphoid fever	1.9 (1.6 to 2.4)	44.3 (36.3 to 53.9)	0.1 (0.1 to 0.2)	7.2 (-11.5 to 30.1)	-10.4 (-26.1 to 8.7)
Other intestinal infectious diseases	--	--	< 0.05 (< 0.05 to < 0.05)	-22.7 (-37.3 to -5.9)	-35.4 (-47.6 to -21.4)
Lower respiratory infections	420.4 (385.1 to 460.4)	18,203.1 (16,365.3 to 20,151.7)	23.8 (16.0 to 33.3)	36.8 (31.9 to 42.1)	14.3 (10.2 to 18.8)
Upper respiratory infections	3,558.4 (2,481.3 to 5,063.2)	263,889.9 (185,547.1 to 370,478.2)	83.7 (45.9 to 139.9)	16.8 (15.0 to 18.7)	-2.4 (-3.9 to -0.8)
Otitis media	852.7 (595.3 to 1,165.1)	1,533.2 (1,213.5 to 1,863.4)	22.2 (12.4 to 37.0)	9.4 (4.3 to 15.0)	-8.6 (-12.8 to -3.9)
Meningitis	237.7 (193.0 to 282.2)	30.1 (22.7 to 38.6)	13.2 (9.1 to 17.9)	16.2 (11.1 to 28.3)	-2.9 (-7.2 to 7.2)
Pneumococcal meningitis	101.8 (79.7 to 126.5)	6.5 (4.2 to 9.6)	5.5 (3.8 to 7.5)	8.0 (2.5 to 15.6)	-9.7 (-14.4 to -3.4)
H influenzae type B meningitis	22.5 (15.7 to 30.4)	1.9 (1.1 to 3.0)	1.4 (0.9 to 1.9)	-15.9 (-25.1 to -1.4)	-29.7 (-37.4 to -17.6)
Meningococcal infection	28.6 (19.4 to 39.5)	6.0 (3.7 to 8.9)	1.8 (1.1 to 3.3)	27.0 (12.1 to 137.9)	6.2 (-6.4 to 98.8)
Other meningitis	84.8 (60.2 to 109.5)	15.8 (11.3 to 21.2)	4.5 (3.1 to 6.2)	40.7 (35.2 to 46.6)	17.6 (13.0 to 22.6)
Encephalitis	448.3 (254.4 to 718.2)	197.5 (174.6 to 223.8)	29.1 (20.7 to 38.3)	18.2 (12.4 to 24.5)	-1.2 (-6.0 to 4.0)
Diphtheria	--	--	--	--	--
Whooping cough	--	--	--	--	--
Tetanus	2.4 (2.3 to 2.5)	2.7 (1.3 to 4.2)	0.1 (< 0.05 to 0.1)	-16.6 (-36.2 to 8.8)	-30.3 (-46.7 to -9.1)
Measles	--	--	--	--	--
Varicella and herpes zoster	331.0 (274.3 to 397.2)	1,909.5 (1,596.3 to 2,247.1)	17.3 (10.3 to 26.8)	22.8 (18.0 to 28.0)	2.7 (-1.4 to 7.0)
**Neglected tropical diseases and malaria**	**30,513.6 (29,367.2 to 31,487.3)**	**7,406.4† (5,469.9 to 9,214.2)**	**251.0 (174.2 to 346.4)**	**-4.5 (-10.5 to 2.3)**	**-20.2 (-25.2 to -14.5)**
Malaria	679.2 (584.3 to 774.0)	1,265.7 (930.1 to 1,639.8)	6.4 (4.6 to 8.8)	-20.7 (-25.7 to -15.0)	-33.8 (-37.9 to -28.9)
Chagas disease	266.9 (226.5 to 311.6)	1.8 (1.1 to 2.8)	4.5 (2.9 to 6.5)	17.6 (11.1 to 24.6)	-1.7 (-7.1 to 4.1)
Leishmaniasis	140.1 (106.2 to 170.3)	17.6 (13.6 to 22.3)	7.1 (4.4 to 10.8)	32.1 (26.7 to 38.6)	10.4 (5.9 to 15.9)
Visceral leishmaniasis	1.5 (1.0 to 2.1)	6.0 (4.0 to 8.6)	0.1 (0.1 to 0.2)	-39.5 (-60.9 to -8.6)	-49.4 (-67.3 to -23.6)
Cutaneous and mucocutaneous leishmaniasis	118.9 (86.5 to 158.6)	11.6 (8.2 to 16.0)	7.0 (4.3 to 10.7)	34.3 (29.0 to 41.7)	12.2 (7.8 to 18.4)
African trypanosomiasis	0.1 (< 0.05 to 0.5)	0.1 (< 0.05 to 0.3)	< 0.05 (< 0.05 to 0.1)	-70.8 (-95.9 to 79.2)	-75.6 (-96.6 to 49.7)
Schistosomiasis	3,234.7 (3,000.3 to 3,486.3)	765.6 (704.0 to 829.6)	22.0 (11.0 to 41.4)	-31.7 (-34.3 to -28.7)	-42.9 (-45.1 to -40.4)
Cysticercosis	117.6 (84.5 to 162.4)	--	17.2 (10.2 to 25.9)	0.8 (-6.0 to 7.5)	-15.8 (-21.4 to -10.2)
Cystic echinococcosis	18.7 (9.4 to 36.9)	3.6 (1.6 to 8.4)	1.7 (0.7 to 3.4)	-39.9 (-75.9 to 42.7)	-49.7 (-79.9 to 19.3)
Lymphatic filariasis	453.0 (385.7 to 543.6)	111.2 (61.8 to 173.6)	16.3 (8.6 to 28.0)	-30.3 (-47.3 to -14.6)	-41.8 (-56.0 to -28.6)
Onchocerciasis	264.6 (181.8 to 387.2)	44.7 (18.2 to 92.2)	22.4 (12.9 to 38.7)	-16.2 (-32.4 to 4.7)	-29.9 (-43.5 to -12.5)
Trachoma	634.4 (390.2 to 981.8)	--	43.0 (26.4 to 64.9)	-7.4 (-17.6 to 1.7)	-22.6 (-31.2 to -15.0)
Dengue	98.1 (53.6 to 166.0)	1,640.4 (999.7 to 2,484.9)	14.5 (6.6 to 26.8)	93.5 (56.1 to 344.5)	61.7 (30.4 to 271.5)
Yellow fever	< 0.05 (< 0.05 to 0.1)	0.7 (0.2 to 1.9)	< 0.05 (< 0.05 to < 0.05)	-21.1 (-50.7 to 24.4)	-34.0 (-58.8 to 4.0)
Rabies	< 0.05 (< 0.05 to < 0.05)	0.3 (0.2 to 0.5)	< 0.05 (< 0.05 to < 0.05)	-49.8 (-59.9 to -37.0)	-58.1 (-66.5 to -47.4)
Intestinal nematode infections	25,362.4 (24,108.0 to 26,402.5)	--	41.3 (24.1 to 66.6)	-9.4 (-16.7 to -2.0)	-24.3 (-30.4 to -18.1)
Ascariasis	13,551.6 (12,046.6 to 15,448.4)	--	12.6 (6.9 to 21.0)	-23.8 (-33.4 to -12.7)	-36.3 (-44.3 to -27.0)

Cause	Prevalence in thousands (95% UI)	Incidence in thousands (95% UI)	YLDs in thousands (95% UI)		
	**2016 counts**	**2016 counts**	**2016 counts**	**Percentage change in counts between 2006 and 2016**	**Percentage change in rates between 2006 and 2016**
Trichuriasis	6,996.5<br>(6,400.0 to 7,718.0)	--	4.7<br>(2.6 to 7.9)	-15.3<br>(-28.5 to -0.8)	-29.2<br>(-40.2 to -17.0)
Hookworm disease	7,478.3<br>(6,861.9 to 8,199.6)	--	24.0<br>(14.2 to 37.7)	2.0<br>(-9.7 to 14.4)	-14.7<br>(-24.5 to -4.4)
Food-borne trematodiases	2,004.4<br>(1,628.6 to 2,381.0)	3,439.6<br>(1,669.4 to 4,938.5)	46.5<br>(19.3 to 85.8)	25.9<br>(13.1 to 48.9)	5.2<br>(-5.5 to 24.4)
Leprosy	43.0<br>(41.4 to 44.6)	2.6<br>(2.4 to 2.7)	2.5<br>(1.8 to 3.5)	6.4<br>(0.8 to 12.5)	-11.1<br>(-15.8 to -6.0)
Ebola virus disease	< 0.05<br>(< 0.05 to 0.1)	< 0.05<br>(< 0.05 to < 0.05)	< 0.05<br>(< 0.05 to < 0.05)	--	--
Zika virus disease	1.9<br>(1.1 to 3.4)	112.5<br>(64.3 to 205.1)	< 0.05<br>(< 0.05 to 0.1)	--	--
Guinea worm disease	< 0.05<br>(< 0.05 to < 0.05)	< 0.05<br>(< 0.05 to < 0.05)	< 0.05<br>(< 0.05 to < 0.05)	-99.6<br>(-99.7 to -99.3)	-99.6<br>(-99.8 to -99.4)
Other neglected tropical diseases	356.4<br>(342.3 to 369.3)	--	5.4<br>(3.6 to 8.1)	22.2<br>(16.1 to 28.3)	2.1<br>(-3.0 to 7.2)
**Maternal disorders**	**--**	**--**	**--**	**--**	**--**
Maternal haemorrhage	--	--	--	--	--
Maternal sepsis and other pregnancy related infections	--	--	--	--	--
Maternal hypertensive disorders	--	--	--	--	--
Maternal obstructed labour and uterine rupture	--	--	--	--	--
Maternal abortion, miscarriage, and ectopic pregnancy	--	--	--	--	--
Other maternal disorders	--	--	--	--	--
**Neonatal disorders**	**909.3<br>(825.7 to 988.3)**	**--**	**73.1<br>(52.3 to 103.1)**	**38.4<br>(27.3 to 53.0)**	**15.7<br>(6.4 to 27.9)**
Neonatal preterm birth complications	780.6<br>(664.5 to 908.3)	--	45.0<br>(32.4 to 60.7)	36.3<br>(19.1 to 58.7)	13.9<br>(-0.5 to 32.7)
Neonatal encephalopathy due to birth asphyxia and trauma	136.2<br>(100.3 to 185.3)	--	9.8<br>(4.8 to 19.0)	42.0<br>(29.8 to 56.1)	18.7<br>(8.5 to 30.4)
Neonatal sepsis and other neonatal infections	41.8<br>(16.6 to 106.9)	--	13.9<br>(3.6 to 39.8)	42.4<br>(31.7 to 64.9)	19.1<br>(10.1 to 37.9)
Hemolytic disease and other neonatal jaundice	12.7<br>(10.8 to 14.6)	--	4.4<br>(3.3 to 5.7)	40.1<br>(34.4 to 45.6)	17.1<br>(12.3 to 21.7)
Other neonatal disorders	--	--	--	--	--
**Nutritional deficiencies**	**22,930.0<br>(22,518.3 to 23,214.5)**	**4,231.4†<br>(3,590.7 to 4,953.7)**	**524.2<br>(356.7 to 733.9)**	**13.0<br>(11.5 to 14.4)**	**-5.5<br>(-6.8 to -4.4)**
Protein-energy malnutrition	--	--	--	--	--
Iodine deficiency	2,988.0<br>(2,689.8 to 3,321.1)	6.8<br>(5.7 to 8.0)	71.8<br>(49.4 to 99.2)	4.8<br>(2.5 to 7.1)	-12.4<br>(-14.3 to -10.5)
Vitamin A deficiency	3,975.7<br>(3,488.4 to 4,547.8)	4,224.6<br>(3,583.8 to 4,947.2)	2.6<br>(1.6 to 3.8)	22.8<br>(14.0 to 32.1)	2.7<br>(-4.7 to 10.4)
Iron-deficiency anaemia	18,417.2<br>(17,487.9 to 19,255.2)	--	449.8<br>(303.6 to 643.2)	14.4<br>(12.7 to 16.0)	-4.4<br>(-5.8 to -3.0)
Other nutritional deficiencies	--	--	--	--	--
**Other communicable, maternal, neonatal, and nutritional diseases**	**43,566.9<br>(42,181.2 to 45,008.4)**	**4,623.3<br>(3,506.4 to 6,091.8)**	**39.5<br>(27.3 to 56.8)**	**13.5<br>(1.2 to 27.6)**	**-5.1<br>(-15.4 to 6.7)**
Sexually transmitted diseases excluding HIV	31,897.4<br>(29,658.1 to 35,060.6)	1,362.1<br>(1,170.9 to 1,568.4)	22.3<br>(15.1 to 33.4)	20.2<br>(15.5 to 25.1)	0.5<br>(-3.5 to 4.6)
Syphilis	92.2<br>(78.0 to 111.1)	4.3<br>(3.0 to 6.0)	13.4<br>(9.0 to 18.5)	21.1<br>(13.4 to 28.7)	1.2<br>(-5.2 to 7.6)
Chlamydial infection	1.8<br>(1.1 to 2.9)	--	< 0.05<br>(< 0.05 to < 0.05)	19.9<br>(15.7 to 24.4)	0.2<br>(-3.3 to 4.0)
Gonococcal infection	0.3<br>(0.2 to 0.4)	--	< 0.05<br>(< 0.05 to < 0.05)	22.3<br>(13.1 to 29.7)	2.2<br>(-5.4 to 8.4)
Trichomoniasis	1,753.0<br>(1,482.8 to 2,028.8)	1,357.8<br>(1,166.6 to 1,563.7)	1.9<br>(0.8 to 4.0)	20.2<br>(16.5 to 23.7)	0.5<br>(-2.6 to 3.4)
Genital herpes	30,122.8<br>(26,370.8 to 34,792.7)	--	7.0<br>(2.0 to 15.9)	18.7<br>(16.4 to 21.4)	-0.8<br>(-2.7 to 1.4)
Other sexually transmitted diseases	0.2<br>(0.2 to 0.3)	--	< 0.05<br>(< 0.05 to 0.1)	16.3<br>(11.9 to 21.8)	-2.8<br>(-6.4 to 1.8)
Hepatitis	15,555.1<br>(13,748.7 to 17,437.9)	3,260.8<br>(2,186.0 to 4,696.9)	7.9<br>(4.4 to 13.2)	-4.1<br>(-40.5 to 58.7)	-19.8<br>(-50.3 to 32.7)
Acute hepatitis A	36.6<br>(8.7 to 80.8)	475.9<br>(113.3 to 1,050.0)	1.4<br>(0.3 to 3.3)	-13.1<br>(-80.5 to 241.7)	-27.4<br>(-83.7 to 185.6)
Hepatitis B	9,870.7<br>(8,102.9 to 11,663.8)	2,387.3<br>(1,394.8 to 3,809.5)	5.7<br>(2.8 to 10.2)	-1.9<br>(-44.6 to 74.1)	-18.0<br>(-53.7 to 45.6)
Hepatitis C	5,627.9<br>(5,034.6 to 6,242.2)	138.2<br>(94.2 to 196.9)	0.1<br>(0.1 to 0.2)	-3.7<br>(-9.5 to 3.0)	-19.5<br>(-24.4 to -14.0)
Acute hepatitis E	20.0<br>(11.4 to 30.7)	259.4<br>(148.3 to 398.4)	0.6<br>(0.3 to 1.1)	0.2<br>(-20.8 to 24.2)	-16.3<br>(-33.8 to 3.8)
Other infectious diseases	443.0<br>(424.4 to 460.5)	0.4<br>(0.2 to 0.6)	9.2<br>(6.1 to 13.6)	15.9<br>(12.2 to 19.9)	-3.1<br>(-6.2 to 0.2)
**Non-communicable diseases**	**156,756.2<br>(156,725.2 to 156,785.0)**	**536,502.5†<br>(489,854.2 to 589,221.6)**	**32,089.3<br>(24,328.8 to 41,048.0)**	**19.1<br>(18.4 to 19.7)**	**-0.5<br>(-1.1 to 0.1)**
**Neoplasms**	**5,043.6<br>(4,976.8 to 5,112.4)**	**2,020.4<br>(1,935.7 to 2,125.4)**	**586.3<br>(432.7 to 755.0)**	**21.6<br>(18.3 to 25.4)**	**1.6<br>(-1.1 to 4.9)**
Lip and oral cavity cancer	128.1<br>(123.4 to 133.0)	36.4<br>(35.1 to 37.8)	13.3<br>(9.8 to 17.4)	25.5<br>(18.4 to 33.4)	4.9<br>(-1.1 to 11.5)
Nasopharynx cancer	17.2<br>(16.1 to 18.4)	5.2<br>(4.9 to 5.6)	2.2<br>(1.6 to 2.9)	7.0<br>(-3.2 to 17.9)	-10.6<br>(-19.1 to -1.4)
Other pharynx cancer	45.8<br>(43.0 to 48.0)	16.6<br>(15.6 to 17.5)	5.6<br>(4.0 to 7.4)	30.6<br>(18.4 to 41.5)	9.2<br>(-1.0 to 18.3)
Oesophageal cancer	73.8<br>(70.3 to 78.2)	59.6<br>(57.2 to 62.3)	14.7<br>(10.3 to 19.0)	-2.8<br>(-10.9 to 6.5)	-18.8<br>(-25.6 to -11.0)
Stomach cancer	286.1<br>(271.5 to 301.0)	154.5<br>(147.4 to 161.8)	39.1<br>(28.3 to 50.1)	5.5<br>(-1.6 to 12.9)	-11.8<br>(-17.7 to -5.6)
Colon and rectum cancer	822.0<br>(787.5 to 878.0)	226.3<br>(216.9 to 241.0)	78.3<br>(58.0 to 101.3)	20.5<br>(13.7 to 29.2)	0.7<br>(-5.0 to 8.0)
Liver cancer	104.3<br>(97.6 to 111.9)	106.7<br>(99.5 to 112.1)	23.7<br>(16.8 to 30.9)	27.3<br>(18.1 to 36.7)	6.4<br>(-1.3 to 14.3)
Liver cancer due to hepatitis B	49.1<br>(36.0 to 59.0)	37.5<br>(27.6 to 45.4)	8.2<br>(5.1 to 11.5)	26.7<br>(13.1 to 41.6)	5.9<br>(-5.5 to 18.3)

Cause	Prevalence in thousands (95% UI)	Incidence in thousands (95% UI)	YLDs in thousands (95% UI)		
	2016 counts	2016 counts	2016 counts	Percentage change in counts between 2006 and 2016	Percentage change in rates between 2006 and 2016
Liver cancer due to hepatitis C	34.2 (29.6 to 39.6)	25.4 (21.9 to 29.5)	5.7 (4.0 to 7.7)	25.5 (15.1 to 35.4)	4.9 (-3.8 to 13.2)
Liver cancer due to alcohol use	25.5 (20.4 to 31.5)	20.8 (16.6 to 25.4)	4.7 (3.1 to 6.6)	26.9 (12.8 to 42.0)	6.0 (-5.7 to 18.7)
Liver cancer due to other causes	30.6 (25.3 to 37.4)	23.0 (19.0 to 28.0)	5.1 (3.4 to 7.0)	31.0 (16.2 to 45.7)	9.5 (-2.9 to 21.8)
Gallbladder and biliary tract cancer	21.2 (19.1 to 23.1)	23.4 (21.4 to 25.2)	5.4 (3.7 to 7.2)	8.8 (1.5 to 17.8)	-9.0 (-15.2 to -1.5)
Pancreatic cancer	49.0 (46.5 to 51.0)	57.5 (55.0 to 59.2)	11.7 (8.2 to 15.2)	22.6 (14.7 to 30.3)	2.4 (-4.2 to 8.9)
Larynx cancer	72.4 (70.2 to 74.8)	21.8 (21.2 to 22.5)	8.7 (6.2 to 11.2)	12.9 (5.7 to 19.7)	-5.6 (-11.7 to 0.1)
Tracheal, bronchus, and lung cancer	405.2 (388.8 to 422.0)	295.2 (285.0 to 305.9)	68.2 (49.8 to 86.1)	20.5 (14.4 to 26.9)	0.7 (-4.4 to 6.0)
Malignant skin melanoma	134.2 (108.4 to 147.4)	28.3 (23.0 to 31.1)	8.9 (6.2 to 12.0)	44.7 (32.0 to 55.0)	20.9 (10.3 to 29.5)
Non-melanoma skin cancer	122.0 (99.0 to 152.1)	193.2 (124.7 to 285.0)	4.4 (2.3 to 7.2)	1.8 (-9.9 to 17.1)	-14.9 (-24.7 to -2.1)
Non-melanoma skin cancer (squamous-cell carcinoma)	98.0 (56.0 to 142.8)	92.6 (53.1 to 134.6)	4.2 (2.1 to 7.1)	1.4 (-10.8 to 17.1)	-15.3 (-25.4 to -2.1)
Non-melanoma skin cancer (basal-cell carcinoma)	11.5 (5.5 to 22.7)	100.5 (52.6 to 178.4)	0.1 (< 0.05 to 0.3)	19.0 (-0.2 to 34.3)	-0.5 (-16.6 to 12.3)
Breast cancer	615.9 (586.2 to 660.9)	128.8 (122.3 to 138.9)	58.0 (42.1 to 75.8)	19.9 (13.2 to 27.6)	0.3 (-5.4 to 6.6)
Cervical cancer	79.0 (65.3 to 87.2)	21.8 (18.3 to 23.9)	7.8 (5.5 to 10.3)	1.3 (-5.7 to 11.3)	-15.3 (-21.2 to -6.9)
Uterine cancer	179.1 (168.8 to 195.0)	38.7 (36.5 to 42.0)	13.7 (10.0 to 18.2)	27.3 (15.4 to 41.9)	6.4 (-3.6 to 18.6)
Ovarian cancer	61.2 (56.5 to 64.2)	20.6 (19.2 to 21.5)	9.2 (6.7 to 11.9)	14.0 (1.5 to 24.9)	-4.8 (-15.2 to 4.4)
Prostate cancer	1,061.5 (925.3 to 1,319.7)	266.5 (235.9 to 315.2)	99.2 (69.5 to 131.8)	29.8 (20.9 to 40.7)	8.5 (1.0 to 17.6)
Testicular cancer	4.2 (4.0 to 4.5)	0.9 (0.9 to 1.0)	0.4 (0.3 to 0.5)	24.6 (14.4 to 35.5)	4.1 (-4.4 to 13.2)
Kidney cancer	140.3 (131.4 to 146.9)	38.5 (36.4 to 40.0)	12.4 (9.0 to 16.4)	20.2 (12.7 to 28.4)	0.4 (-5.8 to 7.3)
Bladder cancer	236.4 (226.4 to 246.5)	59.0 (56.6 to 61.4)	22.4 (16.4 to 29.7)	16.1 (9.3 to 23.0)	-3.0 (-8.6 to 2.8)
Brain and nervous system cancer	51.9 (43.5 to 56.1)	23.7 (20.2 to 25.4)	7.5 (5.2 to 10.0)	30.8 (22.5 to 38.7)	9.3 (2.4 to 15.9)
Thyroid cancer	70.7 (66.8 to 76.4)	14.0 (13.3 to 15.1)	4.9 (3.5 to 6.6)	29.5 (20.3 to 41.3)	8.3 (0.5 to 18.1)
Mesothelioma	7.0 (6.4 to 7.5)	4.8 (4.4 to 5.0)	1.6 (1.1 to 2.1)	22.6 (13.2 to 30.7)	2.4 (-5.4 to 9.2)
Hodgkin lymphoma	11.6 (10.5 to 12.9)	3.1 (2.8 to 3.6)	1.3 (0.9 to 1.8)	5.4 (-0.8 to 12.5)	-11.9 (-17.1 to -6.0)
Non-Hodgkin's lymphoma	180.6 (164.3 to 193.3)	50.2 (45.8 to 53.8)	15.7 (11.4 to 20.7)	40.6 (30.8 to 49.4)	17.6 (9.3 to 24.9)
Multiple myeloma	47.5 (38.7 to 54.3)	18.7 (15.8 to 21.4)	9.6 (6.5 to 12.5)	33.6 (22.6 to 46.3)	11.7 (2.4 to 22.3)
Leukaemia	144.6 (133.0 to 153.3)	42.2 (38.7 to 44.6)	19.9 (14.2 to 26.1)	26.1 (19.2 to 34.4)	5.4 (-0.4 to 12.4)
Acute lymphoid leukaemia	9.9 (8.7 to 10.7)	3.0 (2.6 to 3.2)	1.4 (1.0 to 1.9)	38.0 (26.3 to 47.4)	15.3 (5.6 to 23.2)
Chronic lymphoid leukaemia	59.4 (54.7 to 65.6)	13.8 (12.7 to 15.2)	7.6 (5.5 to 10.0)	24.7 (14.6 to 35.7)	4.2 (-4.2 to 13.4)
Acute myeloid leukaemia	12.8 (10.6 to 13.8)	9.9 (8.2 to 10.6)	2.7 (1.8 to 3.6)	38.3 (28.6 to 47.0)	15.6 (7.5 to 22.9)
Chronic myeloid leukaemia	7.0 (6.3 to 7.7)	2.6 (2.3 to 2.8)	0.9 (0.6 to 1.2)	0.7 (-6.4 to 7.6)	-15.8 (-21.8 to -10.1)
Other leukaemia	55.4 (46.8 to 60.1)	12.9 (11.0 to 14.0)	7.2 (5.1 to 9.6)	25.3 (14.5 to 37.5)	4.7 (-4.3 to 14.9)
Other neoplasms	218.6 (194.9 to 230.3)	64.5 (57.7 to 67.6)	18.7 (13.4 to 24.6)	43.1 (33.0 to 52.3)	19.6 (11.2 to 27.3)
**Cardiovascular diseases**	**52,361.3 (51,399.6 to 53,525.1)**	**6,016.3† (5,514.5 to 6,535.5)**	**3,857.1 (2,831.6 to 5,000.1)**	**18.9 (15.0 to 22.6)**	**-0.6 (-3.9 to 2.5)**
Rheumatic heart disease	383.6 (343.3 to 424.0)	14.1 (12.1 to 16.4)	24.2 (16.3 to 33.5)	21.8 (16.1 to 27.2)	1.8 (-2.9 to 6.4)
Ischaemic heart disease	19,344.8 (17,977.5 to 20,816.8)	2,170.2 (1,908.0 to 2,462.2)	805.7 (566.5 to 1,100.3)	17.5 (16.2 to 18.7)	-1.8 (-2.9 to -0.8)
Cerebrovascular disease	9,375.1 (8,929.1 to 9,863.8)	1,573.5‡ (1,379.7 to 1,779.0)	1,722.6 (1,205.7 to 2,249.1)	20.2 (11.2 to 27.9)	0.5 (-7.1 to 6.9)
Ischaemic stroke	8,099.1 (6,943.0 to 9,382.5)	1,115.6‡ (923.9 to 1,309.7)	1,431.5 (984.8 to 1,877.7)	20.7 (10.4 to 29.5)	0.9 (-7.7 to 8.2)
Hemorrhagic stroke	1,636.4 (1,329.2 to 1,970.4)	457.9‡ (385.2 to 531.0)	291.1 (205.3 to 395.2)	17.6 (4.9 to 29.4)	-1.7 (-12.3 to 8.2)
Hypertensive heart disease	2,133.7 (1,622.3 to 2,735.3)	--	169.8 (108.6 to 245.0)	18.1 (15.2 to 20.9)	-1.3 (-3.7 to 1.1)
Cardiomyopathy and myocarditis	527.1 (473.7 to 591.7)	173.1† (134.7 to 214.0)	41.6 (26.4 to 61.2)	12.6 (8.2 to 17.3)	-5.9 (-9.6 to -1.9)
Myocarditis	84.2 (67.7 to 103.2)	173.1 (134.7 to 214.0)	5.5 (3.5 to 8.0)	15.5 (8.8 to 21.7)	-3.5 (-9.1 to 1.7)
Alcoholic cardiomyopathy	100.6 (71.2 to 133.9)	--	8.1 (5.0 to 12.0)	9.2 (0.1 to 20.1)	-8.7 (-16.3 to 0.4)
Other cardiomyopathy	350.8 (247.4 to 472.1)	--	28.1 (17.8 to 41.4)	13.1 (8.2 to 18.2)	-5.5 (-9.6 to -1.2)
Atrial fibrillation and flutter	6,933.2 (5,774.4 to 8,388.5)	623.3 (495.2 to 782.9)	539.1 (361.9 to 758.1)	22.6 (21.0 to 24.0)	2.4 (1.2 to 3.7)
Peripheral vascular disease	15,445.9 (12,733.3 to 18,715.6)	1,372.2 (1,019.4 to 1,784.0)	91.2 (41.6 to 172.5)	13.6 (12.0 to 15.2)	-5.0 (-6.4 to -3.7)
Endocarditis	48.3 (37.6 to 60.3)	89.8 (74.7 to 107.2)	3.8 (2.4 to 5.9)	18.2 (9.6 to 27.3)	-1.2 (-8.4 to 6.4)
Other cardiovascular and circulatory diseases	9,022.4 (6,907.2 to 11,763.7)	--	459.0 (290.6 to 700.4)	14.9 (11.8 to 17.9)	-4.0 (-6.6 to -1.4)
**Chronic respiratory diseases**	**35,699.3 (35,069.3 to 36,300.6)**	**4,556.8† (4,052.7 to 5,115.3)**	**2,166.2 (1,845.2 to 2,470.2)**	**22.4 (19.5 to 25.2)**	**2.3 (-0.1 to 4.7)**
Chronic obstructive pulmonary disease	26,752.0 (25,232.7 to 28,228.8)	1,645.9 (1,369.6 to 1,921.9)	1,638.4 (1,439.0 to 1,819.2)	22.2 (18.5 to 25.9)	2.2 (-0.9 to 5.3)
Pneumoconiosis	156.3 (145.1 to 169.2)	6.1 (5.1 to 7.3)	22.2 (14.8 to 30.1)	16.9 (10.8 to 23.6)	-2.3 (-7.4 to 3.3)
Silicosis	66.8 (59.3 to 75.7)	2.5 (1.7 to 3.5)	9.4 (6.3 to 13.0)	13.0 (3.5 to 23.2)	-5.6 (-13.5 to 3.0)

Cause	Prevalence in thousands (95% UI)	Incidence in thousands (95% UI)	YLDs in thousands (95% UI)		
	2016 counts	2016 counts	2016 counts	Percentage change in counts between 2006 and 2016	Percentage change in rates between 2006 and 2016
Asbestosis	23.2 (20.8 to 25.8)	1.5 (1.3 to 1.8)	3.4 (2.3 to 4.6)	26.7 (19.6 to 34.2)	5.9 (0.0 to 12.2)
Coal workers pneumoconiosis	36.8 (31.8 to 42.8)	0.6 (0.4 to 0.8)	5.2 (3.5 to 7.3)	17.5 (4.6 to 33.5)	-1.8 (-12.6 to 11.6)
Other pneumoconiosis	29.5 (24.5 to 35.7)	1.5 (1.0 to 2.2)	4.2 (2.8 to 6.0)	18.1 (6.4 to 30.8)	-1.3 (-11.1 to 9.3)
Asthma	12,153.1 (10,670.1 to 13,871.5)	2,857.0 (2,435.0 to 3,337.2)	443.8 (293.3 to 634.5)	22.5 (19.7 to 25.5)	2.4 (0.1 to 4.9)
Interstitial lung disease and pulmonary sarcoidosis	472.6 (396.6 to 554.9)	47.8 (34.7 to 61.8)	48.2 (31.0 to 69.8)	21.4 (16.9 to 26.2)	1.5 (-2.3 to 5.5)
Other chronic respiratory diseases	--	--	13.7 (11.9 to 15.2)	62.4 (57.6 to 67.0)	35.7 (31.7 to 39.6)
**Cirrhosis and other chronic liver diseases**	**2,296.8 (2,163.9 to 2,439.2)**	**112.5 (95.5 to 131.2)**	**78.1 (53.1 to 107.6)**	**20.6 (16.7 to 24.4)**	**0.8 (-2.5 to 4.0)**
Cirrhosis and other chronic liver diseases due to hepatitis B	654.3 (565.7 to 755.6)	33.9 (26.7 to 42.5)	24.1 (16.0 to 34.4)	19.3 (13.8 to 24.8)	-0.3 (-4.9 to 4.3)
Cirrhosis and other chronic liver diseases due to hepatitis C	584.9 (502.6 to 675.5)	29.5 (23.4 to 36.9)	19.3 (13.0 to 27.2)	23.1 (16.9 to 30.0)	2.9 (-2.3 to 8.7)
Cirrhosis and other chronic liver diseases due to alcohol use	650.6 (542.3 to 757.5)	30.0 (23.9 to 36.8)	21.1 (13.8 to 29.6)	20.9 (14.6 to 26.9)	1.1 (-4.2 to 6.1)
Cirrhosis and other chronic liver diseases due to other causes	407.1 (308.6 to 521.5)	19.2 (13.8 to 25.3)	13.6 (8.4 to 19.9)	18.9 (12.2 to 26.1)	-0.6 (-6.2 to 5.4)
**Digestive diseases**	**16,676.3 (15,640.1 to 17,402.2)**	**13,304.2† (11,780.7 to 15,071.0)**	**421.6 (295.3 to 568.8)**	**26.6 (22.9 to 30.5)**	**5.8 (2.8 to 9.1)**
Peptic ulcer disease	4,307.5 (3,581.8 to 5,211.4)	554.4 (441.3 to 678.2)	74.2 (46.1 to 114.0)	28.4 (20.3 to 36.0)	7.4 (0.6 to 13.7)
Gastritis and duodenitis	9,523.5 (7,311.7 to 12,177.3)	5,177.9 (4,151.8 to 6,396.8)	107.1 (69.5 to 157.2)	31.3 (25.1 to 37.1)	9.7 (4.6 to 14.6)
Appendicitis	15.7 (12.9 to 18.8)	404.6 (338.9 to 476.3)	4.5 (2.9 to 6.4)	22.6 (12.0 to 34.7)	2.5 (-6.4 to 12.6)
Paralytic ileus and intestinal obstruction	10.4 (8.2 to 12.8)	297.3 (231.2 to 368.9)	3.3 (2.0 to 4.7)	31.1 (23.6 to 38.5)	9.6 (3.3 to 15.8)
Inguinal, femoral, and abdominal hernia	2,550.9 (1,883.6 to 3,397.6)	2,806.5 (2,051.8 to 3,707.3)	121.3 (75.5 to 177.8)	18.7 (11.9 to 24.7)	-0.8 (-6.4 to 4.2)
Inflammatory bowel disease	372.2 (323.4 to 424.1)	75.3 (59.4 to 93.9)	50.6 (34.2 to 70.1)	27.2 (21.8 to 33.6)	6.4 (1.8 to 11.6)
Vascular intestinal disorders	10.9 (8.6 to 13.5)	58.4 (43.8 to 75.6)	3.2 (2.0 to 4.8)	24.6 (9.1 to 42.8)	4.1 (-8.8 to 19.3)
Gallbladder and biliary diseases	940.9 (718.2 to 1,202.1)	3,510.4 (2,650.2 to 4,543.2)	14.5 (9.4 to 21.0)	25.5 (15.1 to 36.7)	4.9 (-3.8 to 14.3)
Pancreatitis	46.5 (36.2 to 58.6)	419.2 (328.0 to 529.6)	5.5 (3.5 to 8.0)	34.5 (20.3 to 48.7)	12.4 (0.5 to 24.3)
Other digestive diseases	--	--	37.4 (26.2 to 50.4)	36.6 (32.6 to 40.8)	14.2 (10.9 to 17.7)
**Neurological disorders**	**46,469.4 (44,159.6 to 48,458.5)**	**14,137.0 (9,472.6 to 20,729.3)**	**1,920.4 (1,346.4 to 2,530.8)**	**20.2 (18.5 to 21.9)**	**0.5 (-0.9 to 1.9)**
Alzheimer's disease and other dementias	5,553.9 (4,273.2 to 7,106.6)	945.1 (630.3 to 1,302.7)	780.5 (510.8 to 1,089.3)	21.8 (20.1 to 23.2)	1.8 (0.4 to 2.9)
Parkinson's disease	932.9 (686.6 to 1,233.6)	111.9 (77.2 to 151.2)	107.9 (65.0 to 158.0)	22.8 (19.3 to 26.3)	2.6 (-0.3 to 5.6)
Epilepsy	683.6 (555.2 to 821.2)	48.6 (35.4 to 63.7)	183.5 (123.7 to 255.3)	17.0 (4.0 to 32.5)	-2.2 (-13.1 to 10.8)
Multiple sclerosis	96.4 (88.2 to 105.6)	0.6 (0.4 to 0.8)	24.1 (17.3 to 31.4)	32.8 (24.7 to 41.5)	11.0 (4.2 to 18.3)
Motor neuron disease	25.1 (20.4 to 31.2)	6.6 (5.2 to 8.2)	5.3 (3.6 to 7.3)	31.8 (27.4 to 35.9)	10.2 (6.5 to 13.6)
Migraine	14,341.7 (13,220.7 to 15,549.6)	832.3 (698.6 to 984.5)	655.6 (436.1 to 909.9)	18.5 (17.1 to 20.0)	-1.0 (-2.1 to 0.3)
Tension-type headache	29,614.0 (20,706.7 to 41,431.2)	12,189.8 (7,599.2 to 18,645.0)	138.5 (87.4 to 207.1)	18.6 (16.4 to 20.8)	-0.9 (-2.7 to 1.0)
Other neurological disorders	1.0 (0.5 to 1.7)	2.1 (1.2 to 3.1)	24.9 (17.3 to 33.8)	27.9 (25.0 to 30.5)	6.9 (4.5 to 9.1)
**Mental and substance use disorders**	**24,938.3 (24,498.7 to 25,347.6)**	**11,163.0† (9,816.5 to 12,642.5)**	**3,424.2 (2,519.0 to 4,435.2)**	**16.6 (15.6 to 17.6)**	**-2.6 (-3.4 to -1.7)**
Schizophrenia	435.2 (385.4 to 491.1)	4.4 (3.3 to 5.7)	255.4 (194.2 to 311.4)	22.7 (19.4 to 26.1)	2.5 (-0.2 to 5.4)
Alcohol use disorders	1,272.4 (1,021.7 to 1,573.1)	429.5 (322.1 to 556.8)	117.7 (78.2 to 168.3)	12.5 (7.1 to 17.6)	-6.0 (-10.5 to -1.8)
Drug use disorders	727.8 (697.5 to 750.1)	16.3 (14.3 to 18.5)	216.9 (153.2 to 277.9)	23.2 (19.3 to 27.1)	3.0 (-0.3 to 6.2)
Opioid use disorders	477.4 (420.8 to 545.4)	--	180.6 (128.9 to 235.0)	22.8 (18.3 to 27.4)	2.6 (-1.1 to 6.5)
Cocaine use disorders	114.1 (101.0 to 128.8)	--	14.6 (8.9 to 20.9)	25.2 (17.2 to 32.9)	4.6 (-2.0 to 11.1)
Amphetamine use disorders	12.3 (8.5 to 17.0)	2.4 (1.5 to 3.6)	1.7 (1.0 to 2.9)	26.3 (21.6 to 30.7)	5.6 (1.6 to 9.2)
Cannabis use disorders	97.3 (81.4 to 115.2)	--	2.8 (1.8 to 4.2)	20.5 (15.6 to 25.7)	0.7 (-3.4 to 5.0)
Other drug use disorders	63.6 (55.6 to 72.9)	13.9 (12.1 to 15.9)	17.1 (11.5 to 23.2)	25.7 (19.8 to 32.2)	5.1 (0.1 to 10.5)
Depressive disorders	10,464.9 (10,063.8 to 10,811.4)	9,959.3 (8,648.4 to 11,415.6)	1,588.3 (1,092.6 to 2,177.9)	13.9 (12.3 to 15.5)	-4.8 (-6.2 to -3.5)
Major depressive disorder	6,185.3 (5,391.4 to 7,077.5)	9,454.3 (8,119.7 to 10,876.1)	1,172.6 (783.5 to 1,621.3)	11.8 (10.1 to 13.4)	-6.6 (-8.0 to -5.2)
Dysthymia	4,578.5 (3,769.0 to 5,510.3)	505.1 (414.6 to 604.1)	415.7 (274.6 to 598.3)	20.2 (16.5 to 23.8)	0.5 (-2.6 to 3.5)
Bipolar disorder	943.3 (784.4 to 1,124.8)	54.6 (43.0 to 67.9)	177.3 (109.2 to 261.4)	19.1 (16.4 to 22.0)	-0.4 (-2.7 to 1.9)
Anxiety disorders	6,553.7 (5,792.0 to 7,348.5)	672.9 (613.9 to 739.9)	583.9 (408.7 to 786.4)	17.1 (14.6 to 19.6)	-2.1 (-4.2 to -0.0)
Eating disorders	--	--	--	--	--
Anorexia nervosa	--	--	--	--	--
Bulimia nervosa	--	--	--	--	--
Autistic spectrum disorders	993.8 (952.4 to 1,031.4)	--	139.1 (95.9 to 192.2)	20.7 (18.7 to 22.9)	0.9 (-0.8 to 2.7)
Autism	327.3 (281.5 to 382.3)	--	76.7 (50.0 to 110.1)	20.7 (17.5 to 24.1)	0.9 (-1.8 to 3.7)
Asperger syndrome and other autistic spectrum disorders	670.7 (560.3 to 801.8)	--	62.4 (41.5 to 89.5)	20.8 (18.5 to 23.1)	1.0 (-1.0 to 2.9)

Cause	Prevalence in thousands (95% UI)	Incidence in thousands (95% UI)	YLDs in thousands (95% UI)		
	2016 counts	2016 counts	2016 counts	Percentage change in counts between 2006 and 2016	Percentage change in rates between 2006 and 2016
Attention-deficit/hyperactivity disorder	60.4 (47.1 to 75.5)	--	0.7 (0.4 to 1.1)	19.7 (8.6 to 32.3)	0.1 (-9.3 to 10.6)
Conduct disorder	--	--	--	--	--
Idiopathic developmental intellectual disability	978.0 (477.9 to 1,469.4)	--	48.0 (24.0 to 79.3)	9.8 (5.6 to 12.9)	-8.2 (-11.7 to -5.6)
Other mental and substance use disorders	4,278.5 (3,804.5 to 4,830.4)	26.0 (17.1 to 39.1)	297.1 (206.8 to 413.6)	20.1 (18.3 to 21.8)	0.4 (-1.1 to 1.8)
**Diabetes, urogenital, blood, and endocrine diseases**	**91,937.1 (90,995.6 to 92,720.7)**	**18,768.2† (16,589.1 to 21,115.0)**	**3,775.0 (2,686.6 to 5,057.2)**	**20.7 (18.6 to 22.8)**	**0.8 (-0.9 to 2.6)**
Diabetes mellitus	26,878.5 (24,540.2 to 29,576.6)	638.0 (442.8 to 900.3)	2,073.9 (1,429.1 to 2,853.7)	20.0 (16.5 to 23.4)	0.3 (-2.6 to 3.1)
Acute glomerulonephritis	5.7 (4.1 to 7.7)	83.2 (59.9 to 115.7)	0.3 (0.2 to 0.5)	7.4 (-4.0 to 18.9)	-10.2 (-19.7 to -0.6)
Chronic kidney disease	31,122.7 (26,970.7 to 35,290.4)	2,378.5 (1,576.8 to 3,210.1)	803.4 (593.6 to 1,064.9)	25.3 (22.3 to 28.4)	4.7 (2.2 to 7.4)
Chronic kidney disease due to diabetes mellitus	14,525.3 (11,299.1 to 17,851.3)	1,130.3 (726.8 to 1,574.9)	377.8 (265.0 to 517.7)	27.6 (23.8 to 31.5)	6.6 (3.5 to 9.9)
Chronic kidney disease due to hypertension	5,376.2 (4,189.9 to 6,694.8)	280.7 (163.6 to 439.0)	192.5 (125.8 to 270.1)	26.4 (21.5 to 31.1)	5.6 (1.5 to 9.6)
Chronic kidney disease due to glomerulonephritis	5,377.2 (4,221.5 to 6,675.5)	519.2 (319.0 to 751.0)	87.2 (54.4 to 138.7)	15.0 (7.3 to 21.7)	-3.9 (-10.3 to 1.8)
Chronic kidney disease due to other causes	5,844.1 (4,078.0 to 8,084.0)	448.3 (267.7 to 681.2)	146.0 (92.5 to 219.8)	24.8 (19.9 to 29.7)	4.3 (0.2 to 8.4)
Urinary diseases and male infertility	13,849.7 (12,664.4 to 16,210.6)	14,184.5† (12,189.8 to 16,345.4)	444.4 (285.3 to 653.8)	18.4 (17.2 to 19.6)	-1.1 (-2.1 to 0.0)
Interstitial nephritis and urinary tract infections	159.1 (128.9 to 193.8)	8,316.6 (6,847.9 to 10,042.6)	5.0 (3.0 to 7.5)	30.0 (23.0 to 36.9)	8.7 (2.8 to 14.5)
Urolithiasis	162.8 (123.6 to 208.7)	4,231.7 (3,217.5 to 5,383.0)	11.2 (7.2 to 16.5)	22.1 (13.4 to 31.8)	2.0 (-5.2 to 10.2)
Benign prostatic hyperplasia	13,201.0 (10,829.0 to 16,031.6)	1,636.2 (1,190.5 to 2,123.1)	424.2 (270.6 to 628.3)	18.3 (17.0 to 19.6)	-1.1 (-2.2 to -0.0)
Male infertility	--	--	--	--	--
Other urinary diseases	--	--	4.0 (2.7 to 5.8)	3.7 (-1.9 to 9.8)	-13.3 (-18.0 to -8.2)
Gynecological diseases	18,834.7 (17,756.3 to 20,509.5)	10.6† (7.2 to 14.5)	62.9 (31.4 to 110.5)	11.1 (9.7 to 12.6)	-7.2 (-8.3 to -5.9)
Uterine fibroids	1,246.0 (1,106.7 to 1,413.0)	10.6 (7.2 to 14.5)	8.9 (4.4 to 16.2)	17.8 (15.7 to 19.8)	-1.6 (-3.3 to 0.2)
Polycystic ovarian syndrome	--	--	--	--	--
Female infertility	--	--	--	--	--
Endometriosis	--	--	--	--	--
Genital prolapse	17,871.3 (15,681.1 to 20,256.4)	--	54.0 (25.9 to 97.1)	10.0 (8.5 to 11.6)	-8.0 (-9.3 to -6.7)
Premenstrual syndrome	--	--	--	--	--
Other gynecological diseases	--	--	--	--	--
Hemoglobinopathies and hemolytic anaemias	34,359.4 (34,050.4 to 34,687.1)	--	130.5 (87.0 to 190.4)	21.1 (18.5 to 23.7)	1.2 (-1.0 to 3.4)
Thalassemias	< 0.05 (< 0.05 to 0.1)	--	< 0.05 (< 0.05 to < 0.05)	5.5 (-1.4 to 12.9)	-11.9 (-17.6 to -5.7)
Thalassaemias trait	6,383.5 (6,123.6 to 6,667.3)	--	72.0 (46.7 to 106.8)	28.9 (24.9 to 33.1)	7.8 (4.4 to 11.3)
Sickle cell disorders	0.4 (0.3 to 0.4)	--	0.1 (< 0.05 to 0.1)	39.6 (35.5 to 43.8)	16.7 (13.2 to 20.2)
Sickle cell trait	6,598.9 (6,033.7 to 7,365.0)	--	14.6 (9.6 to 21.4)	31.6 (26.9 to 36.1)	10.0 (6.1 to 13.8)
G6PD deficiency	5,277.4 (5,080.8 to 5,501.4)	--	0.5 (0.3 to 0.7)	20.1 (15.9 to 24.4)	0.4 (-3.1 to 4.0)
G6PD trait	17,522.4 (17,214.9 to 17,881.9)	--	< 0.05 (< 0.05 to < 0.05)	27.9 (23.6 to 32.9)	6.9 (3.3 to 11.1)
Other hemoglobinopathies and hemolytic anaemias	2,247.5 (2,149.4 to 2,339.6)	--	43.3 (29.2 to 63.1)	7.4 (4.8 to 9.8)	-10.3 (-12.4 to -8.2)
Endocrine, metabolic, blood, and immune disorders	8,101.4 (6,738.0 to 9,824.5)	1,473.3 (1,025.4 to 2,021.9)	259.6 (173.9 to 365.7)	18.5 (14.4 to 22.4)	-0.9 (-4.4 to 2.3)
**Musculoskeletal disorders**	**67,996.5 (65,422.6 to 70,260.7)**	**32,352.1 (25,787.3 to 40,086.3)**	**7,211.7 (5,171.4 to 9,526.5)**	**19.3 (17.7 to 20.9)**	**-0.3 (-1.6 to 1.0)**
Rheumatoid arthritis	1,595.5 (1,408.9 to 1,785.6)	41.1 (27.8 to 57.9)	356.3 (249.4 to 464.2)	26.9 (24.2 to 29.7)	6.1 (3.8 to 8.4)
Osteoarthritis	29,691.4 (27,993.5 to 31,499.3)	662.1 (540.1 to 803.1)	1,503.9 (1,049.6 to 2,038.4)	21.2 (20.5 to 22.0)	1.3 (0.8 to 1.9)
Low back and neck pain	36,405.0 (33,471.6 to 39,783.0)	15,673.5 (11,437.8 to 20,653.0)	4,175.2 (2,856.7 to 5,863.7)	18.7 (16.7 to 20.6)	-0.8 (-2.5 to 0.8)
Low back pain	25,921.1 (19,301.7 to 34,119.4)	13,225.2 (9,110.6 to 18,227.9)	2,806.5 (1,834.7 to 4,141.1)	17.6 (14.8 to 20.1)	-1.7 (-4.0 to 0.4)
Neck pain	14,432.1 (11,023.5 to 19,255.1)	2,448.3 (1,418.5 to 3,679.1)	1,368.6 (884.5 to 2,102.4)	21.1 (18.3 to 23.6)	1.2 (-1.1 to 3.3)
Gout	3,061.2 (2,359.1 to 3,786.4)	530.8 (393.8 to 686.8)	92.1 (59.9 to 131.2)	21.2 (17.7 to 25.2)	1.3 (-1.6 to 4.7)
Other musculoskeletal disorders	13,565.8 (8,973.8 to 18,947.0)	15,444.6 (10,978.2 to 20,842.7)	1,084.2 (618.5 to 1,695.7)	16.4 (8.0 to 24.0)	-2.7 (-9.8 to 3.6)
**Other non-communicable diseases**	**149,375.2 (149,056.7 to 149,625.0)**	**434,072.1† (389,237.0 to 486,382.5)**	**8,648.8 (6,134.2 to 11,996.5)**	**17.7 (16.4 to 18.9)**	**-1.6 (-2.7 to -0.6)**
Congenital anomalies	1,328.5 (1,236.5 to 1,419.6)	--	145.2 (104.5 to 192.4)	16.8 (13.9 to 19.8)	-2.4 (-4.8 to 0.1)
Neural tube defects	99.0 (90.3 to 109.3)	--	28.4 (20.4 to 37.6)	19.0 (13.9 to 24.4)	-0.6 (-4.8 to 4.0)
Congenital heart anomalies	217.8 (195.4 to 242.8)	--	11.9 (5.0 to 20.7)	26.5 (21.7 to 31.2)	5.8 (1.7 to 9.6)
Orofacial clefts	79.0 (70.4 to 87.5)	--	0.9 (0.6 to 1.3)	14.8 (11.6 to 17.7)	-4.1 (-6.7 to -1.6)
Down's syndrome	--	--	--	--	--
Turner syndrome	10.9 (9.3 to 12.8)	--	0.1 (0.1 to 0.3)	20.1 (17.3 to 22.8)	0.4 (-1.9 to 2.7)
Klinefelter syndrome	2.7 (2.1 to 3.4)	--	< 0.05 (< 0.05 to < 0.05)	23.9 (21.0 to 27.3)	3.5 (1.2 to 6.4)

Cause	Prevalence in thousands (95% UI)	Incidence in thousands (95% UI)	YLDs in thousands (95% UI)		
	2016 counts	2016 counts	2016 counts	Percentage change in counts between 2006 and 2016	Percentage change in rates between 2006 and 2016
Other chromosomal abnormalities	< 0.05 (< 0.05 to < 0.05)	--	< 0.05 (< 0.05 to < 0.05)	11.3 (9.9 to 12.8)	-7.0 (-8.1 to -5.7)
Congenital musculoskeletal and limb anomalies	270.9 (249.9 to 292.1)	--	38.1 (26.4 to 51.5)	20.1 (15.6 to 24.9)	0.4 (-3.4 to 4.4)
Urogenital congenital anomalies	66.0 (59.5 to 73.1)	--	1.0 (0.5 to 1.8)	26.0 (21.7 to 30.3)	5.3 (1.7 to 8.9)
Digestive congenital anomalies	227.8 (206.0 to 251.7)	--	9.3 (6.2 to 13.2)	17.3 (11.4 to 23.8)	-2.0 (-6.9 to 3.5)
Other congenital anomalies	631.2 (438.2 to 885.7)	--	55.5 (35.0 to 84.2)	11.5 (6.5 to 16.9)	-6.8 (-11.0 to -2.3)
Skin and subcutaneous diseases	67,523.6 (65,049.2 to 69,480.2)	211,330.3 (182,752.1 to 246,753.7)	1,033.3 (682.8 to 1,569.2)	22.0 (21.2 to 22.9)	2.0 (1.3 to 2.7)
Dermatitis	7,031.2 (5,972.2 to 8,324.2)	9,964.7 (7,341.8 to 13,249.6)	211.0 (130.1 to 322.1)	20.5 (18.8 to 22.2)	0.7 (-0.7 to 2.1)
Psoriasis	2,592.6 (2,385.0 to 2,817.9)	239.2 (203.9 to 273.2)	209.8 (153.0 to 274.0)	25.5 (23.1 to 28.1)	4.9 (2.9 to 7.0)
Cellulitis	106.8 (89.4 to 127.3)	2,585.3 (2,206.7 to 3,057.6)	5.7 (3.7 to 8.2)	25.3 (18.1 to 33.0)	4.7 (-1.3 to 11.2)
Pyoderma	1,088.5 (992.1 to 1,188.5)	28,168.5 (25,381.2 to 31,314.4)	5.8 (2.4 to 12.0)	19.3 (17.0 to 21.7)	-0.2 (-2.2 to 1.7)
Scabies	1,697.1 (1,184.5 to 2,443.7)	5,250.1 (3,596.9 to 7,716.4)	41.4 (20.3 to 73.9)	15.1 (12.8 to 17.3)	-3.8 (-5.7 to -1.9)
Fungal skin diseases	28,127.0 (22,509.7 to 34,232.0)	109,405.8 (81,726.9 to 144,090.4)	151.4 (60.2 to 325.3)	19.4 (18.9 to 19.8)	-0.2 (-0.6 to 0.1)
Viral skin diseases	2,814.5 (2,389.1 to 3,350.9)	4,367.9 (3,737.9 to 5,092.2)	80.8 (48.9 to 119.2)	19.3 (17.6 to 21.0)	-0.3 (-1.7 to 1.1)
Acne vulgaris	1,313.9 (1,276.2 to 1,356.5)	599.2 (577.2 to 625.1)	31.2 (21.3 to 44.1)	24.6 (20.9 to 28.8)	4.2 (1.0 to 7.6)
Alopecia areata	272.6 (244.4 to 300.9)	466.5 (423.3 to 513.9)	8.4 (5.4 to 12.6)	20.0 (15.2 to 24.9)	0.3 (-3.7 to 4.4)
Pruritus	2,911.6 (2,049.9 to 4,162.1)	2,260.1 (1,571.3 to 3,109.4)	29.5 (13.3 to 56.8)	20.8 (19.1 to 22.5)	1.0 (-0.4 to 2.4)
Urticaria	899.4 (636.5 to 1,226.2)	1,563.8 (1,107.4 to 2,087.0)	49.9 (29.2 to 76.8)	19.3 (16.5 to 22.4)	-0.3 (-2.6 to 2.3)
Decubitus ulcer	181.8 (134.4 to 238.1)	683.8 (502.7 to 918.4)	27.4 (17.6 to 39.4)	22.2 (13.9 to 30.1)	2.2 (-4.8 to 8.7)
Other skin and subcutaneous diseases	34,288.9 (31,404.7 to 37,271.8)	45,775.4 (40,926.9 to 50,673.6)	181.1 (88.2 to 332.7)	25.7 (24.8 to 26.5)	5.1 (4.4 to 5.7)
Sense organ diseases	123,256.8 (122,086.7 to 124,732.0)	29,404.0† (26,687.7 to 32,057.7)	6,101.0 (4,329.4 to 8,344.1)	16.8 (15.1 to 18.4)	-2.4 (-3.8 to -1.0)
Glaucoma	799.8 (633.8 to 992.7)	--	75.9 (49.5 to 107.1)	20.8 (17.8 to 23.8)	0.9 (-1.6 to 3.5)
Cataract	13,267.6 (10,550.8 to 16,203.0)	--	885.8 (617.2 to 1,218.2)	20.2 (17.6 to 22.5)	0.5 (-1.7 to 2.4)
Macular degeneration	1,064.4 (811.5 to 1,386.9)	--	65.2 (42.8 to 90.7)	22.9 (19.1 to 26.8)	2.8 (-0.4 to 6.0)
Refraction and accommodation disorders	57,386.5 (53,243.7 to 61,783.3)	--	950.7 (594.7 to 1,506.9)	9.8 (7.5 to 11.8)	-8.2 (-10.2 to -6.5)
Age-related and other hearing loss	116,630.9 (112,964.4 to 119,989.2)	--	3,414.8 (2,376.2 to 4,765.3)	17.2 (14.2 to 20.0)	-2.1 (-4.6 to 0.3)
Other vision loss	2,580.0 (2,021.5 to 3,266.9)	--	190.8 (127.8 to 262.7)	17.4 (14.7 to 20.2)	-1.9 (-4.2 to 0.4)
Other sense organ diseases	16,789.3 (14,559.9 to 19,022.3)	29,404.0 (26,687.7 to 32,057.7)	517.8 (321.2 to 785.7)	20.7 (19.6 to 21.9)	0.9 (-0.0 to 1.9)
Oral disorders	96,739.8 (94,640.5 to 99,258.0)	193,337.8† (159,991.5 to 230,123.6)	1,369.3 (867.9 to 2,053.4)	18.8 (17.8 to 19.6)	-0.7 (-1.5 to -0.0)
Caries of deciduous teeth	--	--	--	--	--
Caries of permanent teeth	57,905.8 (48,900.2 to 68,471.1)	186,869.9 (153,518.1 to 223,751.5)	41.1 (18.8 to 78.0)	12.9 (11.4 to 14.3)	-5.7 (-6.9 to -4.5)
Periodontal disease	34,329.2 (25,522.5 to 42,276.3)	3,906.8 (2,027.1 to 5,814.0)	215.7 (84.9 to 480.5)	20.9 (19.9 to 21.7)	1.0 (0.2 to 1.7)
Edentulism and severe tooth loss	37,385.5 (31,458.2 to 44,141.6)	2,561.2 (1,587.9 to 3,710.1)	1,014.3 (646.0 to 1,466.1)	18.5 (17.3 to 19.7)	-0.9 (-2.0 to 0.0)
Other oral disorders	3,533.7 (3,153.1 to 3,925.1)	--	98.2 (60.2 to 147.6)	19.7 (18.3 to 21.1)	0.0 (-1.2 to 1.2)
**Injuries**	**77,137.2 (72,903.1 to 81,297.0)**	**17,534.5† (15,024.5 to 20,544.0)**	**2,790.1 (1,955.5 to 3,844.2)**	**23.0 (21.9 to 23.9)**	**2.8 (1.9 to 3.6)**
**Transport injuries**	**13,130.9 (12,260.3 to 13,992.7)**	**1,462.1 (1,169.0 to 1,851.0)**	**610.9 (415.6 to 866.4)**	**25.7 (24.8 to 26.6)**	**5.1 (4.3 to 5.8)**
Road injuries	11,016.7 (10,287.2 to 11,803.2)	1,244.9 (956.8 to 1,619.4)	517.1 (351.5 to 734.3)	27.0 (26.1 to 27.8)	6.2 (5.4 to 6.8)
Pedestrian road injuries	2,545.4 (2,242.7 to 2,940.7)	290.1 (193.6 to 412.4)	124.7 (85.2 to 176.2)	27.1 (26.0 to 28.2)	6.3 (5.3 to 7.1)
Cyclist road injuries	2,255.7 (1,916.0 to 2,648.3)	319.1 (212.0 to 472.3)	100.7 (67.9 to 144.6)	29.6 (28.6 to 30.5)	8.3 (7.5 to 9.1)
Motorcyclist road injuries	2,370.8 (2,043.8 to 2,787.6)	133.1 (82.8 to 204.4)	108.2 (74.7 to 153.6)	33.6 (32.8 to 34.4)	11.7 (11.0 to 12.3)
Motor vehicle road injuries	3,055.7 (2,546.9 to 3,507.1)	394.1 (275.9 to 549.4)	148.8 (100.9 to 209.3)	17.4 (16.2 to 18.4)	-1.9 (-2.9 to -1.0)
Other road injuries	789.2 (680.8 to 940.4)	108.5 (56.5 to 177.6)	34.8 (23.3 to 50.0)	46.8 (45.6 to 47.9)	22.7 (21.7 to 23.6)
Other transport injuries	2,114.2 (1,760.5 to 2,398.5)	217.2 (157.2 to 286.4)	93.8 (63.9 to 131.4)	19.2 (17.5 to 20.8)	-0.4 (-1.8 to 1.0)
**Unintentional injuries**	**59,584.8 (55,679.4 to 63,520.1)**	**15,641.1 (13,149.0 to 18,623.9)**	**2,032.6 (1,435.8 to 2,779.9)**	**22.4 (21.3 to 23.4)**	**2.3 (1.4 to 3.1)**
Falls	29,688.8 (26,227.2 to 32,712.9)	8,570.5 (6,296.2 to 11,452.3)	1,252.5 (905.5 to 1,686.8)	24.2 (22.9 to 25.5)	3.9 (2.7 to 4.9)
Drowning	233.6 (204.8 to 265.3)	25.9 (20.3 to 32.9)	10.9 (7.3 to 15.3)	9.2 (7.2 to 11.0)	-8.8 (-10.4 to -7.2)
Fire, heat, and hot substances	4,285.5 (3,760.2 to 4,922.0)	133.0 (92.0 to 190.2)	81.0 (50.6 to 126.8)	17.8 (16.6 to 18.9)	-1.5 (-2.6 to -0.6)
Poisonings	142.7 (124.1 to 162.9)	70.0 (41.1 to 114.4)	9.1 (6.0 to 13.0)	28.7 (25.5 to 31.9)	7.5 (4.9 to 10.2)
Exposure to mechanical forces	9,956.8 (8,630.6 to 11,686.6)	902.9 (616.8 to 1,234.1)	206.2 (137.2 to 299.1)	24.6 (23.6 to 25.6)	4.2 (3.3 to 5.0)
Unintentional firearm injuries	353.9 (296.7 to 419.4)	11.7 (5.8 to 20.6)	8.9 (5.8 to 13.0)	26.5 (25.5 to 27.4)	5.8 (4.9 to 6.5)
Unintentional suffocation	309.4 (258.7 to 375.4)	18.3 (8.9 to 31.6)	8.7 (5.7 to 12.8)	20.1 (18.6 to 21.4)	0.4 (-0.9 to 1.5)
Other exposure to mechanical forces	9,293.5 (7,980.8 to 10,969.1)	872.8 (601.0 to 1,199.0)	188.7 (125.6 to 273.7)	24.8 (23.7 to 25.7)	4.3 (3.4 to 5.1)

Cause	Prevalence in thousands (95% UI)	Incidence in thousands (95% UI)	YLDs in thousands (95% UI)		
	2016 counts	2016 counts	2016 counts	Percentage change in counts between 2006 and 2016	Percentage change in rates between 2006 and 2016
Adverse effects of medical treatment	152.0<br>(107.2 to 210.7)	1,987.2<br>(1,517.0 to 2,557.6)	20.3<br>(12.2 to 33.0)	13.6<br>(8.6 to 18.2)	-5.0<br>(-9.2 to -1.2)
Animal contact	2,419.8<br>(2,190.2 to 2,718.8)	1,185.8<br>(825.5 to 1,608.9)	52.8<br>(36.6 to 73.4)	12.3<br>(10.3 to 14.0)	-6.2<br>(-7.8 to -4.7)
Venomous animal contact	1,016.9<br>(868.7 to 1,206.4)	572.2<br>(385.8 to 805.2)	31.8<br>(22.1 to 43.4)	13.3<br>(10.9 to 15.5)	-5.3<br>(-7.3 to -3.5)
Non-venomous animal contact	1,402.9<br>(1,227.2 to 1,621.9)	613.6<br>(407.6 to 865.6)	21.0<br>(13.8 to 31.7)	10.7<br>(8.4 to 12.6)	-7.5<br>(-9.4 to -5.9)
Foreign body	2,067.6<br>(1,802.6 to 2,338.3)	806.5<br>(588.3 to 1,076.6)	68.3<br>(44.6 to 98.9)	16.4<br>(14.9 to 17.7)	-2.8<br>(-4.0 to -1.6)
Pulmonary aspiration and foreign body in airway	910.1<br>(737.2 to 1,098.7)	293.1<br>(216.6 to 384.5)	33.3<br>(21.6 to 48.1)	9.5<br>(8.1 to 10.8)	-8.5<br>(-9.6 to -7.4)
Foreign body in eyes	125.3<br>(91.0 to 173.4)	346.7<br>(173.8 to 600.0)	7.6<br>(4.7 to 11.9)	29.5<br>(26.2 to 33.4)	8.2<br>(5.5 to 11.5)
Foreign body in other body part	1,032.2<br>(865.1 to 1,246.3)	166.7<br>(99.2 to 250.2)	27.4<br>(17.9 to 39.7)	22.2<br>(20.6 to 23.6)	2.2<br>(0.8 to 3.3)
Environmental heat and cold exposure	3,244.7<br>(2,859.8 to 3,692.5)	459.7<br>(296.9 to 640.2)	119.4<br>(81.2 to 168.2)	15.7<br>(14.1 to 17.3)	-3.3<br>(-4.7 to -2.0)
Other unintentional injuries	7,393.3<br>(6,202.8 to 8,740.0)	1,499.7<br>(993.9 to 2,106.1)	212.1<br>(143.0 to 302.6)	21.3<br>(20.3 to 22.2)	1.4<br>(0.5 to 2.2)
**Self-harm and interpersonal violence**	**5,334.5**<br>**(4,925.3 to 5,810.0)**	**422.2†**<br>**(326.1 to 526.4)**	**116.8**<br>**(78.6 to 165.2)**	**12.1**<br>**(11.3 to 12.7)**	**-6.3**<br>**(-6.9 to -5.8)**
Self-harm	740.5<br>(626.8 to 860.7)	208.2<br>(149.8 to 285.3)	27.2<br>(17.9 to 38.3)	5.9<br>(4.7 to 6.9)	-11.5<br>(-12.5 to -10.7)
Self-harm by firearm	17.5<br>(15.0 to 20.0)	2.9<br>(0.8 to 7.1)	0.5<br>(0.4 to 0.8)	-0.0<br>(-1.9 to 1.6)	-16.5<br>(-18.0 to -15.1)
Self-harm by other specified means	723.0<br>(609.0 to 843.5)	205.3<br>(146.5 to 280.6)	26.7<br>(17.5 to 37.6)	6.0<br>(4.8 to 7.0)	-11.4<br>(-12.4 to -10.5)
Interpersonal violence	4,594.0<br>(4,196.8 to 5,075.4)	214.0†<br>(145.7 to 288.9)	89.6<br>(60.6 to 127.2)	14.1<br>(13.4 to 14.8)	-4.6<br>(-5.2 to -4.1)
Assault by firearm	148.5<br>(126.3 to 173.6)	8.7<br>(4.5 to 15.5)	4.3<br>(2.8 to 6.3)	25.1<br>(24.1 to 26.1)	4.6<br>(3.7 to 5.4)
Assault by sharp object	634.1<br>(531.7 to 748.5)	30.8<br>(16.6 to 52.2)	13.8<br>(9.0 to 20.0)	13.1<br>(11.8 to 14.2)	-5.5<br>(-6.6 to -4.6)
Sexual violence	1,936.5<br>(1,645.9 to 2,241.7)	--	12.1<br>(7.9 to 17.7)	13.6<br>(12.8 to 14.5)	-5.0<br>(-5.7 to -4.3)
Assault by other means	1,874.9<br>(1,598.9 to 2,245.1)	174.4<br>(117.7 to 242.0)	59.4<br>(39.9 to 84.9)	13.7<br>(12.9 to 14.4)	-5.0<br>(-5.6 to -4.4)
**Forces of nature, conflict and terrorism, and executions and police conflict**	**1,023.5**<br>**(675.1 to 1,453.3)**	**9.2†**<br>**(5.9 to 13.2)**	**29.9**<br>**(8.4 to 71.9)**	**65.7**<br>**(55.5 to 85.5)**	**38.5**<br>**(30.0 to 55.1)**
Forces of nature, conflict and terrorism, and state actor violence	191.9<br>(108.9 to 297.4)	5.3<br>(3.0 to 8.2)	6.7<br>(1.6 to 17.4)	71.4<br>(61.7 to 81.2)	43.3<br>(35.1 to 51.5)
Conflict and terrorism	648.4<br>(388.6 to 1,034.1)	--	20.0<br>(6.4 to 45.2)	53.8<br>(40.4 to 83.7)	28.6<br>(17.4 to 53.6)
Executions and police conflict	183.2<br>(51.7 to 299.4)	3.9<br>(1.6 to 6.8)	4.2<br>(0.7 to 12.1)	105.8<br>(94.6 to 118.5)	72.0<br>(62.6 to 82.6)

We did not calculate incidence for certain NTDs and causes with only birth prevalence. Blank cells mean that no estimate is available.

† Incidence for aggregate causes represents the sum of the incidence of the causes below the aggregate.

‡ Incidence estimates for stroke represent first-ever stroke only.

^ Incidence of HIV/AIDS represents new infections of HIV only and does not include new infections of Tuberculosis in HIV positive cases.

SR Table 12: Global prevalence, incidence, and YLDs for 2016, percentage change of YLD counts and percentage change of YLD rates between 2006 and 2016 by cause, age 75-79 years.

Cause	Prevalence in thousands (95% UI)	Incidence in thousands (95% UI)	YLDs in thousands (95% UI)		
	2016 counts	2016 counts	2016 counts	Percentage change in counts between 2006 and 2016	Percentage change in rates between 2006 and 2016
**All causes**	**116,464.5 (116,456.9 to 116,474.9)**	**771,670.2† (699,148.7 to 853,382.5)**	**29,991.6 (22,840.6 to 37,859.5)**	**23.9 (23.2 to 24.6)**	**-0.4 (-1.0 to 0.2)**
**Communicable, maternal, neonatal, and nutritional disorders**	**69,859.8 (69,140.9 to 70,756.6)**	**320,804.3† (262,197.7 to 394,238.9)**	**1,017.4 (733.7 to 1,337.5)**	**16.6 (14.5 to 19.3)**	**-6.2 (-8.0 to -4.0)**
**HIV/AIDS and tuberculosis**	**30,437.4 (28,054.9 to 33,220.5)**	**160.5† (129.7 to 195.3)**	**115.9 (77.4 to 161.9)**	**8.6 (0.6 to 16.8)**	**-12.7 (-19.1 to -6.1)**
Tuberculosis	29,076.4 (25,026.6 to 33,046.7)	154.5† (123.8 to 189.2)	94.8 (60.8 to 135.7)	8.3 (4.5 to 12.8)	-12.9 (-16.0 to -9.3)
Drug-susceptible tuberculosis	328.6 (259.2 to 408.9)	149.0 (119.2 to 182.8)	91.2 (58.5 to 130.7)	8.5 (4.6 to 13.0)	-12.8 (-15.9 to -9.2)
Multidrug-resistant tuberculosis without extensive drug resistance	11.7 (9.2 to 14.7)	5.1 (4.1 to 6.3)	3.3 (2.1 to 4.8)	-2.4 (-15.6 to 14.1)	-21.5 (-32.1 to -8.2)
Extensively drug-resistant tuberculosis	0.8 (0.7 to 1.0)	0.4 (0.3 to 0.4)	0.3 (0.2 to 0.4)	200.7 (165.2 to 242.4)	141.8 (113.3 to 175.4)
Latent tuberculosis infection	28,735.3 (24,717.4 to 32,701.0)	--	--	--	--
HIV/AIDS	213.4 (183.8 to 253.6)	6.0^ (4.7 to 7.4)	21.1 (13.7 to 30.8)	9.9 (-19.7 to 55.9)	-11.6 (-35.4 to 25.4)
Drug-susceptible HIV/AIDS - Tuberculosis	6.3 (4.3 to 8.8)	6.6 (4.7 to 9.0)	2.3 (1.3 to 3.6)	-7.8 (-15.9 to 0.8)	-25.9 (-32.4 to -18.9)
Multidrug-resistant HIV/AIDS - Tuberculosis without extensive drug resistance	0.2 (0.1 to 0.3)	0.2 (0.1 to 0.3)	0.1 (< 0.05 to 0.1)	13.2 (-12.4 to 37.0)	-9.0 (-29.6 to 10.2)
Extensively drug-resistant HIV/AIDS - Tuberculosis	< 0.05 (< 0.05 to < 0.05)	< 0.05 (< 0.05 to < 0.05)	< 0.05 (< 0.05 to < 0.05)	325.3 (202.9 to 439.7)	242.0 (143.6 to 334.0)
HIV/AIDS resulting in other diseases	206.9 (177.5 to 247.7)	6.0 (4.7 to 7.4)	18.7 (11.8 to 27.9)	12.6 (-21.0 to 70.1)	-9.5 (-36.4 to 36.8)
**Diarrhoea, lower respiratory infections, and other common infectious diseases**	**5,466.5 (5,195.1 to 5,789.2)**	**307,927.9† (249,693.2 to 380,244.3)**	**288.5 (202.7 to 387.9)**	**25.2 (23.2 to 28.6)**	**0.7 (-0.9 to 3.4)**
Diarrhoeal diseases	1,501.5 (1,326.2 to 1,696.3)	103,243.4 (90,807.8 to 117,449.5)	152.6 (106.5 to 207.6)	25.1 (22.3 to 27.8)	0.6 (-1.6 to 2.8)
Intestinal infectious diseases	7.0 (5.9 to 8.3)	138.1† (120.2 to 158.0)	1.1 (0.7 to 1.6)	16.6 (-8.0 to 47.5)	-6.2 (-26.0 to 18.6)
Typhoid fever	6.6 (5.5 to 8.0)	105.3 (91.2 to 120.9)	1.0 (0.6 to 1.4)	18.0 (-8.5 to 52.4)	-5.1 (-26.4 to 22.6)
Paratyphoid fever	1.4 (1.2 to 1.7)	32.8 (28.1 to 37.8)	0.1 (0.1 to 0.1)	20.0 (-0.3 to 46.7)	-3.5 (-19.9 to 18.0)
Other intestinal infectious diseases	--	--	< 0.05 (< 0.05 to 0.1)	-10.8 (-29.7 to 12.8)	-28.3 (-43.4 to -9.3)
Lower respiratory infections	384.2 (347.8 to 425.3)	16,487.6 (14,552.2 to 18,417.6)	21.4 (14.3 to 29.8)	45.7 (39.9 to 51.8)	17.2 (12.5 to 22.1)
Upper respiratory infections	2,497.1 (1,733.8 to 3,413.1)	185,411.3 (128,345.3 to 253,354.3)	57.9 (32.4 to 98.3)	22.0 (19.8 to 24.5)	-1.9 (-3.7 to 0.1)
Otitis media	613.8 (455.2 to 791.5)	1,000.7 (789.3 to 1,242.1)	15.8 (9.5 to 25.0)	14.8 (9.3 to 20.9)	-7.7 (-12.1 to -2.8)
Meningitis	167.5 (133.9 to 211.0)	22.7 (17.1 to 29.2)	8.5 (5.3 to 16.0)	26.2 (13.7 to 126.0)	1.5 (-8.6 to 81.7)
Pneumococcal meningitis	70.1 (53.9 to 87.7)	4.9 (3.2 to 7.2)	3.3 (2.1 to 4.5)	7.9 (2.7 to 15.0)	-13.2 (-17.4 to -7.5)
H influenzae type B meningitis	15.0 (10.3 to 20.4)	1.3 (0.8 to 2.2)	0.8 (0.5 to 1.1)	-17.8 (-27.2 to -1.3)	-33.9 (-41.5 to -20.7)
Meningococcal infection	22.3 (13.7 to 56.0)	4.5 (2.8 to 6.8)	1.6 (0.7 to 8.3)	87.2 (14.2 to 834.0)	50.6 (-8.2 to 651.1)
Other meningitis	60.1 (42.1 to 77.5)	11.9 (8.7 to 16.0)	2.8 (1.9 to 4.0)	50.2 (42.8 to 56.7)	20.8 (14.8 to 26.0)
Encephalitis	330.1 (178.6 to 540.1)	160.6 (139.7 to 181.2)	17.7 (12.5 to 23.9)	23.5 (18.6 to 28.9)	-0.7 (-4.6 to 3.6)
Diphtheria	--	--	--	--	--
Whooping cough	--	--	--	--	--
Tetanus	1.5 (1.4 to 1.6)	2.0 (0.9 to 3.0)	< 0.05 (< 0.05 to 0.1)	-13.2 (-31.7 to 11.1)	-30.2 (-45.1 to -10.7)
Measles	--	--	--	--	--
Varicella and herpes zoster	258.9 (212.9 to 310.3)	1,461.5 (1,191.8 to 1,769.4)	13.3 (7.8 to 20.6)	27.0 (22.4 to 31.9)	2.1 (-1.6 to 6.1)
**Neglected tropical diseases and malaria**	**20,653.8 (19,870.3 to 21,302.9)**	**6,710.0† (4,513.7 to 8,581.1)**	**180.2 (125.2 to 250.8)**	**2.9 (-5.4 to 13.2)**	**-17.3 (-23.9 to -9.0)**
Malaria	375.4 (318.0 to 434.8)	754.1 (553.9 to 976.8)	3.4 (2.4 to 4.8)	-23.0 (-28.6 to -17.6)	-38.1 (-42.6 to -33.7)
Chagas disease	192.0 (162.9 to 224.8)	1.2 (0.8 to 1.9)	3.8 (2.5 to 5.5)	16.2 (10.5 to 22.7)	-6.6 (-11.1 to -1.4)
Leishmaniasis	88.9 (67.8 to 106.3)	13.4 (10.1 to 17.4)	4.5 (2.8 to 6.7)	24.5 (18.6 to 31.9)	0.1 (-4.6 to 6.0)
Visceral leishmaniasis	1.3 (0.8 to 2.0)	5.3 (3.4 to 7.8)	0.1 (< 0.05 to 0.1)	-33.6 (-57.6 to 1.2)	-46.6 (-65.9 to -18.6)
Cutaneous and mucocutaneous leishmaniasis	75.2 (55.8 to 98.2)	8.1 (5.7 to 11.0)	4.4 (2.8 to 6.6)	26.7 (20.7 to 34.6)	1.9 (-3.0 to 8.2)
African trypanosomiasis	0.1 (< 0.05 to 0.3)	0.1 (< 0.05 to 0.3)	< 0.05 (< 0.05 to 0.1)	-68.8 (-95.3 to 90.5)	-74.9 (-96.2 to 53.2)
Schistosomiasis	2,010.1 (1,821.1 to 2,195.7)	461.9 (411.9 to 510.7)	13.7 (6.9 to 25.9)	-31.6 (-35.0 to -28.2)	-45.0 (-47.7 to -42.3)
Cysticercosis	76.9 (54.6 to 106.4)	--	11.0 (6.7 to 17.0)	6.7 (-0.6 to 14.0)	-14.2 (-20.0 to -8.4)
Cystic echinococcosis	11.9 (6.1 to 23.4)	2.3 (1.1 to 5.4)	1.0 (0.5 to 2.3)	-37.0 (-76.1 to 61.1)	-49.3 (-80.8 to 29.6)
Lymphatic filariasis	265.8 (229.5 to 317.8)	68.6 (39.9 to 108.6)	8.6 (4.5 to 14.9)	-29.9 (-48.0 to -13.3)	-43.7 (-58.2 to -30.3)
Onchocerciasis	157.3 (108.4 to 230.4)	26.7 (10.8 to 54.4)	13.8 (8.1 to 23.9)	-15.2 (-31.5 to 6.9)	-31.8 (-44.9 to -14.0)
Trachoma	541.6 (360.0 to 766.6)	--	36.9 (23.3 to 54.4)	-3.3 (-13.4 to 7.1)	-22.2 (-30.4 to -13.8)
Dengue	73.5 (40.2 to 124.2)	1,228.4 (749.6 to 1,859.2)	10.7 (4.8 to 19.9)	110.2 (70.2 to 380.8)	69.1 (36.9 to 286.7)
Yellow fever	< 0.05 (< 0.05 to < 0.05)	0.4 (0.1 to 1.1)	< 0.05 (< 0.05 to < 0.05)	-19.5 (-50.0 to 20.7)	-35.2 (-59.8 to -2.9)
Rabies	< 0.05 (< 0.05 to < 0.05)	0.4 (0.2 to 0.6)	< 0.05 (< 0.05 to < 0.05)	-44.7 (-58.5 to -28.6)	-55.5 (-66.6 to -42.6)
Intestinal nematode infections	17,015.8 (16,092.0 to 17,684.9)	--	27.6 (15.9 to 44.3)	-5.2 (-12.2 to 2.8)	-23.7 (-29.4 to -17.3)
Ascariasis	9,144.8 (8,095.6 to 10,424.8)	--	8.4 (4.6 to 14.0)	-20.0 (-30.6 to -8.3)	-35.6 (-44.2 to -26.3)

Cause	Prevalence in thousands (95% UI)	Incidence in thousands (95% UI)	YLDs in thousands (95% UI)		
	2016 counts	2016 counts	2016 counts	Percentage change in counts between 2006 and 2016	Percentage change in rates between 2006 and 2016
Trichuriasis	4,684.8 (4,270.4 to 5,153.9)	--	3.1 (1.8 to 5.2)	-13.7 (-26.8 to 1.5)	-30.6 (-41.1 to -18.4)
Hookworm disease	4,944.2 (4,557.6 to 5,388.6)	--	16.1 (9.5 to 25.1)	7.3 (-3.9 to 21.1)	-13.7 (-22.7 to -2.6)
Food-borne trematodiases	1,648.7 (1,232.3 to 2,058.8)	4,079.5 (1,955.3 to 5,811.6)	39.7 (14.4 to 77.3)	50.2 (28.9 to 88.5)	20.8 (3.6 to 51.6)
Leprosy	33.5 (32.3 to 34.6)	1.6 (1.5 to 1.8)	2.0 (1.4 to 2.7)	13.7 (7.9 to 19.9)	-8.6 (-13.2 to -3.6)
Ebola virus disease	< 0.05 (0.0 to < 0.05)	--	< 0.05 (0.0 to < 0.05)	--	--
Zika virus disease	1.2 (0.7 to 2.1)	71.5 (40.4 to 130.2)	< 0.05 (< 0.05 to < 0.05)	--	--
Guinea worm disease	< 0.05 (< 0.05 to < 0.05)	< 0.05 (< 0.05 to < 0.05)	< 0.05 (< 0.05 to < 0.05)	-99.5 (-99.7 to -99.2)	-99.6 (-99.8 to -99.4)
Other neglected tropical diseases	234.3 (227.3 to 242.2)	--	3.6 (2.4 to 5.3)	26.2 (19.9 to 32.8)	1.5 (-3.6 to 6.8)
**Maternal disorders**	**--**	**--**	**--**	**--**	**--**
Maternal haemorrhage	--	--	--	--	--
Maternal sepsis and other pregnancy related infections	--	--	--	--	--
Maternal hypertensive disorders	--	--	--	--	--
Maternal obstructed labour and uterine rupture	--	--	--	--	--
Maternal abortion, miscarriage, and ectopic pregnancy	--	--	--	--	--
Other maternal disorders	--	--	--	--	--
**Neonatal disorders**	**637.0 (577.5 to 686.3)**	**--**	**34.3 (24.0 to 50.3)**	**40.6 (31.7 to 50.5)**	**13.1 (5.9 to 21.0)**
Neonatal preterm birth complications	534.7 (454.6 to 624.2)	--	21.5 (15.7 to 29.0)	34.0 (23.2 to 48.0)	7.7 (-0.9 to 19.0)
Neonatal encephalopathy due to birth asphyxia and trauma	93.0 (68.5 to 125.8)	--	4.1 (1.9 to 7.9)	51.9 (32.7 to 68.0)	22.2 (6.7 to 35.1)
Neonatal sepsis and other neonatal infections	22.1 (8.6 to 52.9)	--	6.8 (1.5 to 19.7)	55.5 (44.2 to 81.7)	25.1 (16.0 to 46.1)
Hemolytic disease and other neonatal jaundice	5.6 (4.6 to 6.7)	--	2.0 (1.5 to 2.6)	50.1 (42.1 to 57.4)	20.7 (14.3 to 26.6)
Other neonatal disorders	--	--	--	--	--
**Nutritional deficiencies**	**15,455.8 (15,169.2 to 15,644.1)**	**2,519.7† (2,092.7 to 3,002.7)**	**367.9 (251.4 to 512.8)**	**18.7 (16.9 to 20.2)**	**-4.6 (-6.0 to -3.3)**
Protein-energy malnutrition	--	--	--	--	--
Iodine deficiency	2,148.6 (1,931.6 to 2,389.1)	4.4 (3.6 to 5.3)	50.6 (35.0 to 70.0)	12.1 (9.5 to 14.9)	-9.8 (-12.0 to -7.6)
Vitamin A deficiency	2,388.0 (2,065.2 to 2,782.9)	2,515.3 (2,088.6 to 2,997.7)	1.6 (1.0 to 2.3)	30.5 (20.4 to 40.5)	4.9 (-3.2 to 13.0)
Iron-deficiency anaemia	12,418.1 (11,900.0 to 12,992.4)	--	315.7 (214.3 to 443.4)	19.7 (17.7 to 21.5)	-3.7 (-5.3 to -2.3)
Other nutritional deficiencies	--	--	--	--	--
**Other communicable, maternal, neonatal, and nutritional diseases**	**31,972.3 (31,000.6 to 33,003.0)**	**3,486.3 (2,523.0 to 4,936.2)**	**30.6 (21.2 to 43.6)**	**18.5 (4.0 to 33.1)**	**-4.7 (-16.4 to 7.0)**
Sexually transmitted diseases excluding HIV	23,694.8 (21,965.3 to 26,089.6)	819.8 (705.6 to 943.5)	17.8 (12.3 to 25.6)	24.8 (19.2 to 30.9)	0.4 (-4.1 to 5.2)
Syphilis	83.1 (69.0 to 100.0)	3.6 (2.5 to 5.4)	11.5 (7.8 to 15.8)	26.1 (17.3 to 34.3)	1.4 (-5.7 to 8.0)
Chlamydial infection	0.1 (< 0.05 to 0.1)	--	< 0.05 (< 0.05 to < 0.05)	35.9 (19.7 to 57.1)	9.3 (-3.8 to 26.4)
Gonococcal infection	< 0.05 (< 0.05 to < 0.05)	--	< 0.05 (< 0.05 to < 0.05)	42.0 (-9.2 to 134.6)	14.2 (-26.9 to 88.7)
Trichomoniasis	1,066.4 (904.6 to 1,233.5)	816.2 (701.8 to 940.2)	1.2 (0.5 to 2.5)	22.4 (18.4 to 26.4)	-1.6 (-4.8 to 1.7)
Genital herpes	22,483.8 (19,680.5 to 25,944.5)	--	5.1 (1.5 to 11.6)	22.6 (20.0 to 25.4)	-1.4 (-3.5 to 0.8)
Other sexually transmitted diseases	< 0.05 (< 0.05 to < 0.05)	--	< 0.05 (< 0.05 to < 0.05)	34.7 (18.6 to 55.7)	8.3 (-4.6 to 25.2)
Hepatitis	11,147.4 (9,885.0 to 12,447.6)	2,666.1 (1,700.2 to 4,077.7)	6.4 (3.5 to 10.9)	2.6 (-37.6 to 65.8)	-17.4 (-49.8 to 33.4)
Acute hepatitis A	31.7 (8.4 to 64.6)	412.5 (109.8 to 839.5)	1.2 (0.3 to 2.7)	-8.0 (-80.8 to 189.7)	-26.0 (-84.5 to 132.9)
Hepatitis B	6,803.0 (5,614.0 to 8,020.4)	1,926.3 (1,049.7 to 3,281.5)	4.6 (2.2 to 8.8)	5.7 (-41.4 to 90.3)	-15.0 (-52.8 to 53.0)
Hepatitis C	4,294.8 (3,854.1 to 4,758.0)	95.5 (72.2 to 121.1)	0.1 (< 0.05 to 0.2)	1.6 (-4.5 to 8.7)	-18.3 (-23.2 to -12.6)
Acute hepatitis E	17.8 (7.8 to 31.1)	231.8 (101.6 to 403.7)	0.5 (0.2 to 1.0)	5.0 (-15.6 to 30.6)	-15.5 (-32.1 to 5.0)
Other infectious diseases	304.7 (294.1 to 317.3)	0.3 (0.2 to 0.5)	6.3 (4.2 to 9.1)	20.2 (16.1 to 24.7)	-3.4 (-6.6 to 0.3)
**Non-communicable diseases**	**116,343.3 (116,328.9 to 116,357.7)**	**435,309.5† (393,592.5 to 485,253.2)**	**26,580.7 (20,258.7 to 33,596.9)**	**23.8 (23.0 to 24.6)**	**-0.5 (-1.1 to 0.2)**
**Neoplasms**	**4,321.6 (4,265.5 to 4,381.4)**	**1,773.1 (1,693.0 to 1,872.6)**	**511.8 (377.9 to 650.3)**	**22.9 (20.0 to 26.2)**	**-1.2 (-3.5 to 1.5)**
Lip and oral cavity cancer	102.1 (98.9 to 105.4)	29.8 (28.9 to 30.9)	10.8 (7.9 to 14.0)	29.8 (22.4 to 37.7)	4.4 (-1.6 to 10.7)
Nasopharynx cancer	11.3 (10.7 to 12.1)	3.6 (3.4 to 3.8)	1.5 (1.1 to 2.0)	17.4 (5.8 to 28.8)	-5.6 (-14.9 to 3.6)
Other pharynx cancer	25.3 (23.8 to 26.4)	9.6 (9.0 to 10.1)	3.2 (2.4 to 4.2)	33.5 (20.6 to 45.2)	7.4 (-3.0 to 16.8)
Oesophageal cancer	61.3 (58.4 to 64.5)	50.2 (48.2 to 52.4)	12.1 (8.6 to 15.6)	6.2 (-1.9 to 15.2)	-14.6 (-21.1 to -7.4)
Stomach cancer	254.3 (244.0 to 265.3)	140.2 (135.1 to 146.0)	34.6 (25.1 to 44.2)	13.5 (7.0 to 20.6)	-8.7 (-14.0 to -3.1)
Colon and rectum cancer	779.4 (748.8 to 831.8)	221.3 (212.8 to 235.6)	75.6 (55.9 to 96.9)	23.4 (16.9 to 32.4)	-0.8 (-6.0 to 6.5)
Liver cancer	83.3 (78.1 to 88.9)	86.7 (81.4 to 91.6)	19.1 (13.5 to 24.8)	35.6 (26.5 to 45.4)	9.1 (1.7 to 16.9)
Liver cancer due to hepatitis B	36.1 (26.9 to 44.5)	28.2 (20.9 to 34.6)	6.1 (3.8 to 8.6)	38.0 (24.8 to 54.6)	11.0 (0.3 to 24.3)

Cause	Prevalence in thousands (95% UI)	Incidence in thousands (95% UI)	YLDs in thousands (95% UI)		
	2016 counts	2016 counts	2016 counts	Percentage change in counts between 2006 and 2016	Percentage change in rates between 2006 and 2016
Liver cancer due to hepatitis C	31.7<br>(27.6 to 36.0)	23.7<br>(20.7 to 27.3)	5.3<br>(3.7 to 7.0)	30.2<br>(20.4 to 40.1)	4.7<br>(-3.2 to 12.6)
Liver cancer due to alcohol use	19.9<br>(16.0 to 24.2)	16.4<br>(13.2 to 20.0)	3.7<br>(2.5 to 5.1)	36.0<br>(23.1 to 51.7)	9.4<br>(-1.0 to 22.0)
Liver cancer due to other causes	24.0<br>(20.0 to 28.8)	18.4<br>(15.5 to 22.0)	4.0<br>(2.7 to 5.5)	39.2<br>(25.1 to 54.2)	11.9<br>(0.6 to 24.0)
Gallbladder and biliary tract cancer	21.1<br>(19.2 to 22.8)	23.3<br>(21.4 to 25.0)	5.2<br>(3.6 to 7.0)	11.8<br>(3.5 to 20.8)	-10.1<br>(-16.8 to -2.9)
Pancreatic cancer	46.6<br>(44.0 to 48.6)	54.6<br>(52.2 to 56.6)	10.9<br>(7.7 to 14.1)	25.5<br>(16.4 to 33.9)	0.9<br>(-6.4 to 7.6)
Larynx cancer	53.5<br>(51.9 to 55.3)	16.6<br>(16.1 to 17.1)	6.6<br>(4.8 to 8.5)	20.3<br>(12.6 to 28.2)	-3.3<br>(-9.5 to 3.1)
Tracheal, bronchus, and lung cancer	349.9<br>(336.9 to 363.2)	258.0<br>(248.8 to 266.8)	58.4<br>(42.8 to 73.4)	23.7<br>(17.5 to 30.1)	-0.6<br>(-5.5 to 4.6)
Malignant skin melanoma	108.3<br>(86.3 to 117.8)	23.6<br>(18.9 to 25.6)	7.4<br>(5.1 to 10.0)	35.2<br>(23.7 to 46.0)	8.7<br>(-0.6 to 17.4)
Non-melanoma skin cancer	112.5<br>(90.1 to 139.4)	177.7<br>(112.4 to 272.9)	4.0<br>(2.0 to 6.6)	-6.6<br>(-18.0 to 6.9)	-24.9<br>(-34.1 to -14.1)
Non-melanoma skin cancer (squamous-cell carcinoma)	90.2<br>(52.1 to 127.8)	86.2<br>(48.9 to 124.4)	3.9<br>(1.9 to 6.5)	-7.2<br>(-19.0 to 6.8)	-25.3<br>(-34.9 to -14.1)
Non-melanoma skin cancer (basal-cell carcinoma)	10.8<br>(4.6 to 23.4)	91.5<br>(44.0 to 176.2)	0.1<br>(< 0.05 to 0.3)	17.7<br>(-2.0 to 34.4)	-5.4<br>(-21.2 to 8.1)
Breast cancer	490.0<br>(468.1 to 517.0)	104.1<br>(99.0 to 110.7)	47.7<br>(35.1 to 61.9)	15.6<br>(9.5 to 22.5)	-7.1<br>(-12.0 to -1.5)
Cervical cancer	54.8<br>(46.5 to 59.8)	15.3<br>(13.2 to 16.6)	5.5<br>(3.9 to 7.3)	4.0<br>(-2.8 to 12.8)	-16.3<br>(-21.9 to -9.3)
Uterine cancer	130.7<br>(121.0 to 142.9)	29.0<br>(26.9 to 31.7)	10.3<br>(7.4 to 13.7)	24.6<br>(11.1 to 40.7)	0.2<br>(-10.7 to 13.2)
Ovarian cancer	46.8<br>(43.5 to 49.4)	16.0<br>(15.0 to 16.8)	7.2<br>(5.2 to 9.3)	11.7<br>(-0.7 to 25.7)	-10.2<br>(-20.1 to 1.1)
Prostate cancer	872.9<br>(766.6 to 1,019.1)	221.3<br>(196.9 to 248.8)	87.7<br>(63.9 to 114.8)	26.1<br>(16.6 to 35.3)	1.4<br>(-6.2 to 8.8)
Testicular cancer	3.3<br>(3.1 to 3.5)	0.7<br>(0.7 to 0.8)	0.3<br>(0.2 to 0.4)	30.9<br>(19.1 to 44.5)	5.2<br>(-4.2 to 16.2)
Kidney cancer	120.7<br>(112.4 to 127.1)	34.2<br>(32.2 to 35.8)	10.9<br>(7.9 to 14.3)	24.2<br>(15.8 to 33.4)	-0.1<br>(-6.9 to 7.2)
Bladder cancer	234.8<br>(224.8 to 245.1)	60.4<br>(57.9 to 63.1)	22.6<br>(16.5 to 29.5)	18.3<br>(11.8 to 25.1)	-4.9<br>(-10.1 to 0.6)
Brain and nervous system cancer	41.5<br>(32.6 to 45.9)	19.3<br>(15.6 to 21.1)	6.1<br>(4.1 to 8.2)	37.4<br>(26.7 to 47.4)	10.5<br>(1.9 to 18.5)
Thyroid cancer	52.4<br>(49.5 to 56.6)	10.8<br>(10.2 to 11.6)	4.0<br>(2.9 to 5.3)	31.5<br>(22.9 to 42.5)	5.7<br>(-1.2 to 14.6)
Mesothelioma	6.7<br>(6.2 to 7.1)	4.7<br>(4.3 to 4.9)	1.5<br>(1.0 to 2.0)	26.5<br>(15.5 to 36.4)	1.7<br>(-7.1 to 9.7)
Hodgkin lymphoma	8.1<br>(7.3 to 8.7)	2.2<br>(1.9 to 2.4)	0.9<br>(0.7 to 1.2)	7.2<br>(0.8 to 14.7)	-13.8<br>(-18.9 to -7.7)
Non-Hodgkin's lymphoma	152.9<br>(136.8 to 162.1)	43.0<br>(38.8 to 45.5)	13.4<br>(9.8 to 17.6)	34.7<br>(25.6 to 43.9)	8.3<br>(1.0 to 15.7)
Multiple myeloma	45.1<br>(35.8 to 50.5)	17.8<br>(14.7 to 20.0)	9.0<br>(6.2 to 11.8)	31.0<br>(19.4 to 42.7)	5.3<br>(-4.0 to 14.8)
Leukaemia	124.5<br>(115.9 to 132.1)	37.1<br>(34.1 to 39.1)	17.6<br>(12.7 to 23.2)	23.6<br>(16.3 to 31.4)	-0.6<br>(-6.5 to 5.7)
Acute lymphoid leukaemia	6.2<br>(5.4 to 6.6)	1.9<br>(1.7 to 2.1)	0.9<br>(0.6 to 1.2)	42.3<br>(30.8 to 52.1)	14.4<br>(5.2 to 22.3)
Chronic lymphoid leukaemia	52.6<br>(48.3 to 57.2)	12.6<br>(11.5 to 13.6)	6.9<br>(4.9 to 8.9)	20.9<br>(10.0 to 33.7)	-2.8<br>(-11.5 to 7.5)
Acute myeloid leukaemia	10.8<br>(9.2 to 11.6)	8.4<br>(7.1 to 9.0)	2.3<br>(1.6 to 3.1)	33.3<br>(24.0 to 41.2)	7.2<br>(-0.3 to 13.6)
Chronic myeloid leukaemia	6.8<br>(6.1 to 7.4)	2.5<br>(2.3 to 2.8)	0.9<br>(0.6 to 1.2)	0.6<br>(-7.1 to 9.2)	-19.1<br>(-25.3 to -12.2)
Other leukaemia	48.1<br>(42.1 to 51.9)	11.6<br>(10.1 to 12.5)	6.6<br>(4.7 to 8.7)	24.9<br>(14.3 to 35.9)	0.5<br>(-8.1 to 9.3)
Other neoplasms	207.4<br>(184.3 to 220.4)	62.0<br>(55.4 to 65.6)	17.7<br>(13.1 to 22.8)	47.2<br>(34.6 to 57.4)	18.3<br>(8.3 to 26.6)
**Cardiovascular diseases**	**47,590.3<br>(46,801.6 to 48,536.7)**	**5,946.8†<br>(5,362.8 to 6,574.4)**	**3,797.8<br>(2,800.4 to 4,858.5)**	**23.9<br>(20.0 to 27.9)**	**-0.3<br>(-3.5 to 2.9)**
Rheumatic heart disease	222.6<br>(198.9 to 246.3)	14.6<br>(11.7 to 16.9)	14.8<br>(10.3 to 20.1)	26.0<br>(20.3 to 32.2)	1.4<br>(-3.3 to 6.3)
Ischaemic heart disease	18,410.3<br>(17,178.7 to 19,787.9)	2,261.6<br>(1,952.7 to 2,603.0)	759.2<br>(536.1 to 1,025.9)	24.0<br>(22.4 to 25.5)	-0.3<br>(-1.6 to 0.9)
Cerebrovascular disease	8,862.7<br>(8,412.0 to 9,387.8)	1,623.8‡<br>(1,382.7 to 1,866.5)	1,755.0<br>(1,218.2 to 2,271.3)	26.4<br>(18.2 to 34.9)	1.7<br>(-5.0 to 8.5)
Ischaemic stroke	7,837.9<br>(6,819.7 to 9,017.3)	1,171.8‡<br>(961.6 to 1,389.3)	1,481.9<br>(1,032.5 to 1,938.2)	27.2<br>(17.3 to 37.5)	2.3<br>(-5.7 to 10.6)
Hemorrhagic stroke	1,435.4<br>(1,208.6 to 1,698.8)	452.0‡<br>(379.3 to 531.2)	273.1<br>(189.4 to 365.8)	22.3<br>(12.4 to 33.2)	-1.7<br>(-9.6 to 7.2)
Hypertensive heart disease	2,120.6<br>(1,572.9 to 2,749.4)	--	166.1<br>(107.1 to 246.4)	24.3<br>(21.0 to 27.4)	-0.1<br>(-2.7 to 2.4)
Cardiomyopathy and myocarditis	504.9<br>(456.6 to 559.9)	134.1†<br>(105.3 to 163.6)	38.4<br>(24.4 to 56.8)	13.2<br>(8.0 to 18.3)	-9.0<br>(-13.2 to -4.9)
Myocarditis	68.0<br>(55.0 to 82.1)	134.1<br>(105.3 to 163.6)	4.4<br>(2.9 to 6.4)	18.4<br>(10.5 to 26.4)	-4.8<br>(-11.1 to 1.6)
Alcoholic cardiomyopathy	81.6<br>(59.7 to 106.1)	--	6.4<br>(4.0 to 9.7)	21.9<br>(8.7 to 36.5)	-2.0<br>(-12.6 to 9.8)
Other cardiomyopathy	350.2<br>(258.5 to 455.0)	--	27.5<br>(17.3 to 41.2)	10.6<br>(5.5 to 15.8)	-11.1<br>(-15.1 to -6.9)
Atrial fibrillation and flutter	7,448.3<br>(6,373.3 to 8,765.5)	552.4<br>(438.6 to 672.9)	569.8<br>(388.1 to 795.7)	24.3<br>(22.8 to 25.7)	-0.0<br>(-1.3 to 1.1)
Peripheral vascular disease	14,726.7<br>(12,284.3 to 17,334.7)	1,270.5<br>(822.5 to 1,776.8)	84.2<br>(39.2 to 158.3)	17.6<br>(15.7 to 19.4)	-5.4<br>(-7.0 to -4.0)
Endocarditis	69.0<br>(52.6 to 90.2)	89.9<br>(72.7 to 109.0)	5.4<br>(3.4 to 8.2)	15.0<br>(7.8 to 22.6)	-7.5<br>(-13.3 to -1.4)
Other cardiovascular and circulatory diseases	8,057.2<br>(5,993.4 to 10,575.6)	--	404.9<br>(259.6 to 600.3)	15.8<br>(12.7 to 18.9)	-6.9<br>(-9.4 to -4.4)
**Chronic respiratory diseases**	**28,538.4<br>(28,058.8 to 28,935.5)**	**3,176.4†<br>(2,831.7 to 3,516.0)**	**1,656.9<br>(1,417.9 to 1,881.9)**	**27.7<br>(24.8 to 30.9)**	**2.7<br>(0.3 to 5.2)**
Chronic obstructive pulmonary disease	22,259.0<br>(20,936.0 to 23,513.2)	1,337.9<br>(1,132.0 to 1,562.1)	1,269.5<br>(1,117.4 to 1,416.0)	27.8<br>(24.1 to 31.8)	2.8<br>(-0.2 to 6.0)
Pneumoconiosis	109.4<br>(101.7 to 118.1)	3.5<br>(2.9 to 4.2)	15.3<br>(10.7 to 20.9)	27.0<br>(20.1 to 34.2)	2.1<br>(-3.4 to 7.9)
Silicosis	43.7<br>(38.7 to 49.6)	1.6<br>(1.2 to 2.2)	6.0<br>(4.1 to 8.4)	26.8<br>(16.0 to 38.1)	1.9<br>(-6.7 to 11.0)

Cause	Prevalence in thousands (95% UI)	Incidence in thousands (95% UI)	YLDs in thousands (95% UI)		
	2016 counts	2016 counts	2016 counts	Percentage change in counts between 2006 and 2016	Percentage change in rates between 2006 and 2016
Asbestosis	18.8 (16.9 to 20.8)	0.6 (0.5 to 0.7)	2.7 (1.9 to 3.7)	21.8 (15.2 to 29.1)	-2.1 (-7.4 to 3.8)
Coal workers pneumoconiosis	24.9 (21.5 to 29.0)	0.4 (0.3 to 0.5)	3.5 (2.3 to 4.9)	30.1 (14.9 to 47.6)	4.6 (-7.6 to 18.7)
Other pneumoconiosis	22.0 (18.5 to 26.4)	0.9 (0.6 to 1.3)	3.1 (2.1 to 4.4)	29.0 (15.2 to 43.5)	3.7 (-7.3 to 15.4)
Asthma	8,827.0 (7,906.6 to 9,794.0)	1,794.7 (1,557.1 to 2,049.3)	317.4 (213.4 to 448.7)	26.6 (24.0 to 29.2)	1.8 (-0.3 to 3.9)
Interstitial lung disease and pulmonary sarcoidosis	458.6 (388.1 to 536.5)	40.3 (30.2 to 50.3)	45.8 (30.6 to 65.8)	28.1 (23.7 to 33.0)	3.0 (-0.6 to 6.9)
Other chronic respiratory diseases	--	--	8.8 (7.8 to 9.9)	64.1 (59.6 to 69.0)	32.0 (28.3 to 35.9)
**Cirrhosis and other chronic liver diseases**	**1,567.7 (1,473.8 to 1,667.8)**	**90.8 (76.8 to 105.6)**	**54.3 (37.4 to 74.5)**	**26.2 (21.7 to 30.8)**	**1.4 (-2.1 to 5.2)**
Cirrhosis and other chronic liver diseases due to hepatitis B	444.1 (381.2 to 505.4)	27.2 (21.7 to 33.6)	16.0 (10.6 to 22.8)	27.8 (21.4 to 34.3)	2.8 (-2.4 to 8.0)
Cirrhosis and other chronic liver diseases due to hepatitis C	370.8 (310.2 to 441.4)	22.3 (17.3 to 28.3)	12.8 (8.5 to 18.6)	25.9 (18.7 to 34.0)	1.3 (-4.5 to 7.7)
Cirrhosis and other chronic liver diseases due to alcohol use	456.7 (388.0 to 527.6)	24.5 (19.5 to 30.2)	15.3 (10.3 to 21.8)	26.2 (19.1 to 33.6)	1.5 (-4.2 to 7.4)
Cirrhosis and other chronic liver diseases due to other causes	296.1 (228.9 to 369.4)	16.7 (12.6 to 21.8)	10.2 (6.5 to 14.7)	23.7 (16.7 to 31.5)	-0.5 (-6.2 to 5.7)
**Digestive diseases**	**12,871.6 (12,238.9 to 13,403.3)**	**10,149.6† (9,106.1 to 11,290.0)**	**327.5 (226.9 to 441.3)**	**31.4 (27.5 to 35.8)**	**5.7 (2.5 to 9.2)**
Peptic ulcer disease	3,408.7 (2,853.4 to 4,058.1)	394.1 (322.9 to 470.5)	61.0 (37.5 to 93.5)	36.0 (28.0 to 44.7)	9.4 (3.0 to 16.4)
Gastritis and duodenitis	7,533.1 (5,650.9 to 9,622.2)	3,542.7 (2,982.1 to 4,161.4)	89.0 (56.0 to 128.4)	38.0 (32.0 to 44.0)	10.9 (6.2 to 15.8)
Appendicitis	12.6 (10.8 to 14.8)	349.2 (301.9 to 405.9)	3.6 (2.3 to 4.9)	27.8 (17.5 to 38.3)	2.8 (-5.5 to 11.2)
Paralytic ileus and intestinal obstruction	9.6 (7.7 to 11.7)	275.2 (221.9 to 337.8)	2.9 (1.8 to 4.3)	28.8 (19.0 to 38.3)	3.5 (-4.3 to 11.2)
Inguinal, femoral, and abdominal hernia	1,743.7 (1,266.8 to 2,376.7)	2,340.6 (1,689.8 to 3,136.5)	90.3 (57.0 to 129.4)	24.8 (18.2 to 31.7)	0.3 (-5.0 to 5.9)
Inflammatory bowel disease	244.8 (211.6 to 280.2)	51.4 (39.9 to 65.3)	32.7 (22.3 to 45.6)	23.1 (17.8 to 28.8)	-1.0 (-5.2 to 3.5)
Vascular intestinal disorders	11.4 (8.9 to 14.2)	62.6 (48.9 to 80.5)	3.2 (2.0 to 4.6)	19.8 (3.0 to 38.5)	-3.7 (-17.2 to 11.4)
Gallbladder and biliary diseases	739.2 (584.3 to 929.2)	2,785.7 (2,177.9 to 3,473.2)	11.2 (7.3 to 16.2)	31.6 (20.8 to 43.7)	5.8 (-2.9 to 15.6)
Pancreatitis	39.9 (32.4 to 49.1)	348.1 (287.3 to 418.3)	4.6 (3.0 to 6.7)	38.1 (23.5 to 54.4)	11.0 (-0.7 to 24.1)
Other digestive diseases	--	--	29.2 (20.2 to 39.3)	35.6 (31.6 to 40.2)	9.1 (5.9 to 12.8)
**Neurological disorders**	**33,249.4 (30,856.9 to 35,066.2)**	**9,879.1 (6,912.9 to 13,460.8)**	**1,875.8 (1,307.0 to 2,486.6)**	**24.7 (22.9 to 26.4)**	**0.3 (-1.2 to 1.7)**
Alzheimer's disease and other dementias	7,898.7 (6,190.2 to 9,860.6)	1,402.8 (922.4 to 2,057.3)	1,091.2 (724.8 to 1,543.1)	25.5 (23.8 to 27.1)	1.0 (-0.5 to 2.2)
Parkinson's disease	1,072.9 (811.9 to 1,366.8)	126.4 (82.9 to 185.2)	121.8 (76.0 to 174.7)	26.0 (22.3 to 29.1)	1.3 (-1.6 to 3.8)
Epilepsy	563.2 (462.1 to 678.4)	36.6 (27.0 to 47.3)	146.1 (99.8 to 200.2)	21.6 (6.4 to 40.1)	-2.2 (-14.4 to 12.6)
Multiple sclerosis	71.4 (64.7 to 78.5)	0.6 (0.5 to 0.7)	17.5 (12.5 to 22.8)	28.7 (19.8 to 38.5)	3.5 (-3.7 to 11.4)
Motor neuron disease	19.5 (15.4 to 24.4)	5.0 (3.9 to 6.2)	4.1 (2.8 to 5.7)	27.0 (22.0 to 31.7)	2.1 (-1.9 to 5.9)
Migraine	8,548.8 (7,909.5 to 9,276.3)	495.9 (403.5 to 591.5)	391.7 (261.6 to 538.3)	23.2 (21.4 to 25.0)	-1.0 (-2.4 to 0.5)
Tension-type headache	19,079.0 (12,974.4 to 25,778.4)	7,810.2 (4,903.7 to 11,296.0)	87.2 (55.3 to 129.6)	23.1 (20.5 to 26.1)	-1.0 (-3.1 to 1.4)
Other neurological disorders	0.8 (0.4 to 1.4)	1.6 (0.9 to 2.6)	16.2 (11.2 to 22.0)	27.2 (24.8 to 29.3)	2.3 (0.3 to 4.0)
**Mental and substance use disorders**	**17,789.2 (17,421.1 to 18,082.9)**	**8,412.1† (7,003.8 to 9,841.5)**	**2,380.8 (1,740.0 to 3,079.6)**	**22.4 (21.3 to 23.5)**	**-1.6 (-2.5 to -0.7)**
Schizophrenia	231.2 (202.6 to 263.8)	1.1 (0.8 to 1.4)	132.7 (100.9 to 162.6)	28.3 (23.9 to 32.4)	3.2 (-0.3 to 6.4)
Alcohol use disorders	785.0 (651.6 to 950.2)	252.9 (193.6 to 332.1)	71.4 (48.1 to 100.6)	25.2 (19.5 to 30.3)	0.7 (-3.9 to 4.8)
Drug use disorders	489.0 (469.7 to 504.7)	14.0 (11.8 to 16.3)	140.7 (99.5 to 178.9)	28.1 (24.0 to 32.6)	3.0 (-0.3 to 6.7)
Opioid use disorders	312.2 (273.7 to 358.1)	--	115.5 (80.9 to 149.1)	29.2 (24.4 to 34.5)	3.9 (0.0 to 8.1)
Cocaine use disorders	76.1 (67.0 to 86.3)	--	9.6 (6.1 to 13.8)	20.0 (11.7 to 28.6)	-3.5 (-10.2 to 3.4)
Amphetamine use disorders	8.2 (5.5 to 11.8)	1.6 (0.8 to 2.9)	1.1 (0.6 to 2.0)	25.1 (20.7 to 29.1)	0.6 (-2.9 to 3.8)
Cannabis use disorders	64.7 (53.6 to 77.0)	--	1.9 (1.2 to 2.8)	21.6 (15.8 to 27.5)	-2.2 (-6.8 to 2.5)
Other drug use disorders	54.8 (48.4 to 62.1)	12.4 (10.5 to 14.5)	12.5 (8.5 to 16.9)	26.4 (20.1 to 32.8)	1.6 (-3.4 to 6.8)
Depressive disorders	7,708.5 (7,391.6 to 7,979.8)	7,594.3 (6,223.3 to 9,030.0)	1,167.7 (797.9 to 1,610.3)	20.9 (18.9 to 22.7)	-2.8 (-4.4 to -1.3)
Major depressive disorder	4,739.5 (3,959.2 to 5,613.1)	7,256.7 (5,910.5 to 8,700.5)	881.5 (584.5 to 1,241.5)	19.4 (17.0 to 21.5)	-4.0 (-5.9 to -2.3)
Dysthymia	3,200.5 (2,638.9 to 3,876.2)	337.6 (264.5 to 420.8)	286.2 (187.1 to 411.6)	25.8 (22.5 to 29.1)	1.2 (-1.5 to 3.8)
Bipolar disorder	610.0 (510.0 to 726.8)	37.6 (27.0 to 50.5)	112.4 (69.9 to 163.7)	22.3 (18.8 to 25.6)	-1.7 (-4.5 to 1.0)
Anxiety disorders	4,750.4 (4,248.3 to 5,298.0)	487.2 (439.3 to 537.3)	415.4 (294.3 to 556.7)	21.1 (18.8 to 23.5)	-2.6 (-4.5 to -0.7)
Eating disorders	--	--	--	--	--
Anorexia nervosa	--	--	--	--	--
Bulimia nervosa	--	--	--	--	--
Autistic spectrum disorders	691.9 (661.6 to 719.8)	--	96.1 (65.7 to 132.9)	25.2 (22.8 to 27.5)	0.7 (-1.2 to 2.6)
Autism	233.3 (200.4 to 272.1)	--	53.7 (34.7 to 76.8)	24.1 (20.7 to 27.7)	-0.2 (-3.0 to 2.7)
Asperger syndrome and other autistic spectrum disorders	463.7 (386.5 to 555.1)	--	42.4 (28.4 to 60.2)	26.6 (24.0 to 29.4)	1.8 (-0.2 to 4.1)

Cause	Prevalence in thousands (95% UI)	Incidence in thousands (95% UI)	YLDs in thousands (95% UI)		
	2016 counts	2016 counts	2016 counts	Percentage change in counts between 2006 and 2016	Percentage change in rates between 2006 and 2016
Attention-deficit/hyperactivity disorder	15.5 (10.8 to 21.1)	--	0.2 (0.1 to 0.3)	25.4 (15.5 to 35.2)	0.8 (-7.1 to 8.7)
Conduct disorder	--	--	--	--	--
Idiopathic developmental intellectual disability	573.5 (265.7 to 887.4)	--	30.0 (15.0 to 48.3)	15.7 (10.7 to 19.1)	-7.0 (-11.0 to -4.3)
Other mental and substance use disorders	3,141.8 (2,787.1 to 3,539.4)	25.1 (15.1 to 41.2)	214.3 (150.7 to 296.9)	24.8 (22.7 to 26.6)	0.3 (-1.3 to 1.8)
**Diabetes, urogenital, blood, and endocrine diseases**	**71,102.0 (70,377.9 to 71,787.0)**	**14,244.0† (12,688.6 to 15,959.3)**	**3,002.8 (2,153.4 to 3,983.2)**	**24.7 (22.8 to 26.9)**	**0.3 (-1.2 to 2.0)**
Diabetes mellitus	19,894.2 (18,133.5 to 21,914.8)	381.3 (263.1 to 520.2)	1,562.4 (1,090.7 to 2,133.6)	23.4 (19.8 to 27.0)	-0.8 (-3.7 to 2.1)
Acute glomerulonephritis	4.3 (3.1 to 5.9)	61.5 (44.9 to 83.8)	0.2 (0.1 to 0.4)	16.9 (7.0 to 27.5)	-6.0 (-14.0 to 2.6)
Chronic kidney disease	29,394.5 (25,253.7 to 33,233.6)	1,870.2 (1,261.4 to 2,540.6)	728.1 (535.0 to 951.2)	28.6 (25.1 to 31.9)	3.4 (0.6 to 6.1)
Chronic kidney disease due to diabetes mellitus	12,024.5 (9,329.6 to 15,053.4)	779.4 (500.3 to 1,104.5)	302.6 (210.3 to 417.8)	31.5 (27.5 to 35.7)	5.8 (2.5 to 9.1)
Chronic kidney disease due to hypertension	5,798.6 (4,482.1 to 7,154.7)	259.4 (153.5 to 395.9)	191.2 (125.2 to 262.2)	29.8 (25.0 to 34.6)	4.4 (0.5 to 8.2)
Chronic kidney disease due to glomerulonephritis	5,599.2 (4,337.3 to 6,911.7)	447.7 (272.7 to 634.4)	91.1 (55.6 to 134.9)	17.6 (8.7 to 25.2)	-5.4 (-12.6 to 0.7)
Chronic kidney disease due to other causes	5,972.2 (4,110.1 to 8,231.9)	383.7 (224.6 to 575.8)	143.2 (89.8 to 214.8)	28.5 (22.5 to 34.2)	3.3 (-1.5 to 7.9)
Urinary diseases and male infertility	10,932.9 (9,997.5 to 12,595.8)	10,650.4† (9,326.9 to 12,211.8)	346.9 (220.9 to 519.0)	25.7 (24.4 to 26.9)	1.1 (0.0 to 2.1)
Interstitial nephritis and urinary tract infections	121.6 (102.4 to 144.2)	6,360.6 (5,318.2 to 7,638.6)	3.7 (2.3 to 5.6)	33.9 (25.7 to 41.8)	7.7 (1.1 to 14.0)
Urolithiasis	121.5 (92.6 to 156.1)	3,167.8 (2,439.1 to 4,021.4)	8.2 (5.2 to 11.9)	31.2 (20.1 to 42.2)	5.5 (-3.4 to 14.3)
Benign prostatic hyperplasia	10,486.3 (8,798.1 to 12,455.1)	1,122.0 (789.4 to 1,539.8)	332.5 (208.7 to 500.2)	25.6 (24.3 to 26.9)	1.0 (-0.1 to 2.0)
Male infertility	--	--	--	--	--
Other urinary diseases	--	--	2.5 (1.7 to 3.5)	11.9 (5.0 to 18.5)	-10.0 (-15.5 to -4.7)
Gynecological diseases	14,047.9 (13,211.4 to 15,410.9)	7.0† (4.1 to 10.4)	44.8 (22.0 to 79.0)	15.9 (14.3 to 17.6)	-6.8 (-8.1 to -5.5)
Uterine fibroids	652.4 (575.8 to 738.1)	7.0 (4.1 to 10.4)	4.6 (2.3 to 8.4)	22.5 (19.6 to 25.6)	-1.5 (-3.8 to 1.0)
Polycystic ovarian syndrome	--	--	--	--	--
Female infertility	--	--	--	--	--
Endometriosis	--	--	--	--	--
Genital prolapse	13,467.0 (11,789.7 to 15,275.9)	--	40.2 (19.4 to 72.3)	15.2 (13.5 to 16.8)	-7.4 (-8.8 to -6.0)
Premenstrual syndrome	--	--	--	--	--
Other gynecological diseases	--	--	--	--	--
Hemoglobinopathies and hemolytic anaemias	24,638.6 (24,423.3 to 24,864.4)	--	100.3 (66.9 to 145.1)	24.8 (21.9 to 27.5)	0.3 (-2.0 to 2.5)
Thalassemias	< 0.05 (< 0.05 to < 0.05)	--	< 0.05 (< 0.05 to < 0.05)	1.9 (-5.9 to 8.4)	-18.1 (-24.3 to -12.8)
Thalassaemias trait	4,522.4 (4,331.0 to 4,734.4)	--	56.9 (37.3 to 83.7)	33.3 (29.1 to 37.8)	7.2 (3.8 to 10.8)
Sickle cell disorders	0.1 (0.1 to 0.2)	--	< 0.05 (< 0.05 to < 0.05)	22.8 (19.5 to 26.5)	-1.2 (-3.9 to 1.8)
Sickle cell trait	4,400.4 (4,028.6 to 4,902.8)	--	10.7 (7.1 to 15.3)	40.0 (35.8 to 44.7)	12.6 (9.2 to 16.3)
G6PD deficiency	3,569.4 (3,433.9 to 3,722.3)	--	0.5 (0.3 to 0.7)	27.4 (23.3 to 31.3)	2.5 (-0.8 to 5.5)
G6PD trait	13,106.4 (12,867.1 to 13,383.6)	--	< 0.05 (< 0.05 to < 0.05)	18.3 (6.4 to 29.0)	-4.9 (-14.5 to 3.8)
Other hemoglobinopathies and hemolytic anaemias	1,532.4 (1,475.5 to 1,597.0)	--	32.2 (21.8 to 46.1)	8.5 (5.5 to 11.1)	-12.8 (-15.1 to -10.7)
Endocrine, metabolic, blood, and immune disorders	6,848.9 (5,682.0 to 8,440.5)	1,273.7 (914.4 to 1,704.2)	220.1 (143.6 to 314.7)	22.9 (18.0 to 27.5)	-1.2 (-5.1 to 2.5)
**Musculoskeletal disorders**	**51,543.3 (49,779.2 to 53,173.7)**	**21,817.8 (17,157.7 to 27,256.4)**	**5,342.0 (3,847.0 to 7,013.9)**	**22.5 (20.8 to 24.1)**	**-1.5 (-2.9 to -0.2)**
Rheumatoid arthritis	1,211.7 (1,072.4 to 1,355.4)	21.0 (15.1 to 27.7)	265.7 (186.3 to 346.1)	27.5 (24.4 to 30.5)	2.5 (0.0 to 4.9)
Osteoarthritis	24,072.4 (22,700.5 to 25,510.4)	344.4 (284.2 to 407.6)	1,193.3 (835.5 to 1,626.6)	26.5 (25.6 to 27.5)	1.8 (1.0 to 2.5)
Low back and neck pain	29,211.2 (26,595.1 to 31,690.3)	13,065.5 (9,454.5 to 17,167.8)	3,275.7 (2,255.0 to 4,433.0)	22.6 (19.9 to 24.8)	-1.5 (-3.6 to 0.3)
Low back pain	21,616.7 (16,072.2 to 27,820.0)	11,213.7 (7,802.0 to 15,209.0)	2,289.4 (1,493.3 to 3,243.4)	21.8 (17.9 to 24.9)	-2.0 (-5.2 to 0.4)
Neck pain	10,569.0 (7,816.0 to 14,423.5)	1,851.8 (1,121.6 to 2,704.2)	986.3 (618.3 to 1,493.9)	24.3 (21.7 to 27.0)	-0.0 (-2.1 to 2.1)
Gout	2,462.2 (1,983.9 to 2,987.9)	416.9 (300.3 to 549.9)	72.6 (48.7 to 101.0)	24.7 (20.7 to 28.7)	0.3 (-2.9 to 3.5)
Other musculoskeletal disorders	6,821.6 (4,581.8 to 9,177.9)	7,970.0 (5,216.8 to 11,196.9)	534.7 (317.2 to 808.8)	12.1 (3.4 to 20.0)	-9.9 (-16.9 to -3.5)
**Other non-communicable diseases**	**112,219.3 (112,024.9 to 112,388.2)**	**359,819.8† (317,513.4 to 411,047.7)**	**7,630.9 (5,405.3 to 10,454.9)**	**23.4 (21.8 to 25.0)**	**-0.8 (-2.1 to 0.5)**
Congenital anomalies	984.1 (921.6 to 1,045.0)	--	106.3 (77.3 to 139.4)	21.5 (18.6 to 24.6)	-2.3 (-4.7 to 0.2)
Neural tube defects	72.0 (65.6 to 79.5)	--	20.5 (14.8 to 27.0)	23.2 (17.8 to 28.9)	-0.9 (-5.3 to 3.6)
Congenital heart anomalies	167.2 (150.0 to 186.1)	--	9.1 (3.8 to 15.8)	28.8 (23.6 to 34.1)	3.6 (-0.6 to 7.8)
Orofacial clefts	58.4 (52.1 to 64.8)	--	0.7 (0.4 to 1.0)	18.3 (15.2 to 21.4)	-4.8 (-7.4 to -2.4)
Down's syndrome	--	--	--	--	--
Turner syndrome	7.1 (6.0 to 8.4)	--	0.1 (< 0.05 to 0.2)	27.4 (24.6 to 30.3)	2.5 (0.2 to 4.8)
Klinefelter syndrome	1.3 (1.0 to 1.6)	--	< 0.05 (< 0.05 to < 0.05)	29.0 (25.5 to 32.9)	3.8 (0.9 to 6.9)

Cause	Prevalence in thousands (95% UI)	Incidence in thousands (95% UI)	YLDs in thousands (95% UI)		
	2016 counts	2016 counts	2016 counts	Percentage change in counts between 2006 and 2016	Percentage change in rates between 2006 and 2016
Other chromosomal abnormalities	< 0.05<br>(< 0.05 to < 0.05)	--	< 0.05<br>(< 0.05 to < 0.05)	7.5<br>(6.0 to 9.1)	-13.6<br>(-14.8 to -12.3)
Congenital musculoskeletal and limb anomalies	216.3<br>(199.3 to 233.5)	--	29.9<br>(21.1 to 40.2)	22.6<br>(17.4 to 27.7)	-1.4<br>(-5.6 to 2.7)
Urogenital congenital anomalies	51.2<br>(46.1 to 56.7)	--	0.8<br>(0.4 to 1.4)	28.9<br>(24.4 to 34.2)	3.6<br>(0.0 to 7.9)
Digestive congenital anomalies	171.4<br>(155.1 to 189.2)	--	6.9<br>(4.6 to 9.8)	22.7<br>(16.6 to 29.6)	-1.3<br>(-6.2 to 4.2)
Other congenital anomalies	447.0<br>(309.1 to 628.3)	--	38.4<br>(24.4 to 58.3)	17.9<br>(12.5 to 24.0)	-5.2<br>(-9.5 to -0.3)
Skin and subcutaneous diseases	55,269.4<br>(53,227.3 to 57,485.7)	190,694.6<br>(156,449.7 to 236,073.3)	806.4<br>(523.4 to 1,248.3)	25.3<br>(24.4 to 26.2)	0.8<br>(0.0 to 1.5)
Dermatitis	5,566.4<br>(4,775.5 to 6,554.0)	7,791.5<br>(5,595.9 to 10,425.5)	164.3<br>(99.7 to 247.1)	21.9<br>(20.1 to 23.6)	-2.0<br>(-3.4 to -0.6)
Psoriasis	1,863.8<br>(1,705.2 to 2,021.8)	157.1<br>(134.7 to 179.8)	148.1<br>(107.0 to 191.9)	27.6<br>(24.7 to 30.4)	2.6<br>(0.3 to 4.9)
Cellulitis	95.7<br>(80.4 to 114.8)	2,315.0<br>(1,936.6 to 2,700.8)	5.0<br>(3.3 to 7.3)	22.4<br>(14.9 to 30.1)	-1.6<br>(-7.6 to 4.6)
Pyoderma	1,001.9<br>(920.2 to 1,085.9)	26,485.8<br>(23,868.8 to 28,912.3)	5.3<br>(2.2 to 11.0)	27.9<br>(25.3 to 30.3)	2.9<br>(0.8 to 4.8)
Scabies	1,368.8<br>(889.1 to 1,933.9)	4,285.3<br>(2,767.6 to 6,207.1)	32.9<br>(16.3 to 58.2)	23.3<br>(21.1 to 25.6)	-0.9<br>(-2.6 to 1.0)
Fungal skin diseases	26,215.7<br>(19,582.7 to 33,375.9)	105,116.3<br>(71,624.6 to 149,450.8)	139.1<br>(52.9 to 306.2)	25.6<br>(25.1 to 26.0)	1.0<br>(0.6 to 1.3)
Viral skin diseases	1,893.1<br>(1,587.6 to 2,280.7)	2,903.2<br>(2,463.8 to 3,437.8)	53.6<br>(33.3 to 78.6)	23.2<br>(21.3 to 25.4)	-0.9<br>(-2.5 to 0.8)
Acne vulgaris	833.3<br>(810.9 to 859.0)	365.1<br>(351.5 to 380.8)	19.5<br>(13.4 to 27.8)	26.4<br>(21.2 to 31.6)	1.6<br>(-2.5 to 5.8)
Alopecia areata	161.2<br>(142.6 to 178.9)	270.3<br>(242.7 to 299.1)	4.9<br>(3.2 to 7.3)	19.4<br>(13.9 to 25.6)	-4.0<br>(-8.4 to 1.0)
Pruritus	2,302.8<br>(1,609.0 to 3,341.7)	1,756.9<br>(1,235.8 to 2,420.2)	23.1<br>(10.2 to 45.4)	26.2<br>(24.4 to 27.9)	1.5<br>(0.0 to 2.8)
Urticaria	606.5<br>(428.8 to 844.3)	1,052.0<br>(738.3 to 1,469.2)	33.2<br>(19.5 to 52.0)	23.9<br>(20.5 to 27.5)	-0.4<br>(-3.1 to 2.5)
Decubitus ulcer	227.3<br>(173.2 to 297.1)	840.2<br>(634.8 to 1,097.8)	33.6<br>(21.9 to 48.2)	25.7<br>(18.1 to 33.2)	1.1<br>(-5.1 to 7.1)
Other skin and subcutaneous diseases	27,554.8<br>(25,117.3 to 29,783.5)	37,356.1<br>(33,361.7 to 41,328.5)	143.7<br>(69.9 to 263.5)	28.3<br>(27.5 to 29.2)	3.2<br>(2.5 to 3.9)
Sense organ diseases	97,881.8<br>(96,826.1 to 99,042.2)	25,117.1†<br>(22,831.3 to 27,484.8)	5,611.8<br>(4,019.8 to 7,581.6)	23.1<br>(21.0 to 25.3)	-1.0<br>(-2.7 to 0.8)
Glaucoma	679.5<br>(551.4 to 833.3)	--	64.4<br>(42.2 to 92.0)	27.0<br>(23.8 to 30.1)	2.1<br>(-0.5 to 4.6)
Cataract	12,702.0<br>(10,616.8 to 15,104.0)	--	847.4<br>(597.7 to 1,160.7)	26.1<br>(23.9 to 28.1)	1.4<br>(-0.4 to 3.0)
Macular degeneration	868.3<br>(689.2 to 1,114.6)	--	55.8<br>(37.0 to 78.7)	27.0<br>(23.3 to 30.1)	2.1<br>(-0.8 to 4.6)
Refraction and accommodation disorders	46,940.8<br>(43,823.6 to 50,388.4)	--	770.9<br>(485.3 to 1,202.2)	18.4<br>(16.3 to 20.2)	-4.8<br>(-6.5 to -3.4)
Age-related and other hearing loss	95,820.4<br>(93,893.6 to 97,633.7)	--	3,288.2<br>(2,328.3 to 4,450.3)	23.1<br>(19.7 to 26.9)	-1.0<br>(-3.8 to 2.0)
Other vision loss	2,294.8<br>(1,824.4 to 2,821.6)	--	168.3<br>(114.2 to 231.3)	24.4<br>(21.8 to 27.0)	0.0<br>(-2.1 to 2.1)
Other sense organ diseases	13,733.6<br>(12,043.0 to 15,530.1)	25,117.1<br>(22,831.3 to 27,484.8)	417.0<br>(264.2 to 622.0)	25.1<br>(23.8 to 26.4)	0.6<br>(-0.5 to 1.6)
Oral disorders	73,216.9<br>(71,825.6 to 74,730.3)	144,008.0†<br>(121,418.2 to 165,634.8)	1,106.4<br>(708.7 to 1,620.6)	23.4<br>(22.4 to 24.2)	-0.8<br>(-1.6 to -0.1)
Caries of deciduous teeth	--	--	--	--	--
Caries of permanent teeth	42,837.2<br>(37,435.1 to 49,095.3)	139,653.2<br>(117,826.0 to 161,173.8)	31.0<br>(14.2 to 59.2)	18.5<br>(16.9 to 20.0)	-4.7<br>(-6.0 to -3.5)
Periodontal disease	24,246.5<br>(18,387.4 to 29,938.9)	2,953.3<br>(1,613.2 to 4,248.5)	150.5<br>(58.5 to 327.1)	27.1<br>(26.2 to 28.1)	2.2<br>(1.4 to 3.0)
Edentulism and severe tooth loss	32,036.2<br>(26,826.7 to 37,682.0)	1,401.5<br>(902.8 to 2,081.9)	857.0<br>(554.6 to 1,220.5)	22.9<br>(21.5 to 24.1)	-1.2<br>(-2.3 to -0.2)
Other oral disorders	2,481.0<br>(2,213.6 to 2,731.5)	--	68.0<br>(42.5 to 102.1)	24.1<br>(22.5 to 25.8)	-0.2<br>(-1.5 to 1.1)
**Injuries**	**66,012.6<br>(62,408.2 to 69,910.7)**	**15,556.4†<br>(13,470.2 to 18,295.7)**	**2,393.5<br>(1,679.9 to 3,302.2)**	**28.3<br>(27.2 to 29.2)**	**3.2<br>(2.3 to 3.9)**
**Transport injuries**	**10,624.1<br>(9,905.8 to 11,382.4)**	**1,218.7<br>(975.1 to 1,493.5)**	**477.9<br>(327.0 to 679.8)**	**30.0<br>(29.1 to 30.9)**	**4.6<br>(3.8 to 5.2)**
Road injuries	8,955.5<br>(8,366.2 to 9,663.0)	1,018.5<br>(792.6 to 1,286.4)	405.3<br>(277.3 to 577.3)	31.2<br>(30.3 to 32.1)	5.5<br>(4.8 to 6.2)
Pedestrian road injuries	2,146.7<br>(1,891.3 to 2,511.3)	249.3<br>(172.9 to 336.2)	101.7<br>(70.0 to 144.5)	32.5<br>(31.4 to 33.6)	6.6<br>(5.7 to 7.4)
Cyclist road injuries	1,869.7<br>(1,589.5 to 2,174.8)	256.8<br>(178.2 to 364.5)	80.8<br>(54.4 to 116.2)	34.5<br>(33.6 to 35.5)	8.2<br>(7.4 to 9.0)
Motorcyclist road injuries	1,773.8<br>(1,529.0 to 2,068.0)	95.3<br>(60.8 to 141.6)	77.7<br>(53.2 to 110.9)	39.6<br>(38.8 to 40.5)	12.3<br>(11.6 to 13.0)
Motor vehicle road injuries	2,546.9<br>(2,111.1 to 2,923.9)	336.1<br>(238.1 to 450.1)	118.9<br>(81.3 to 169.2)	19.7<br>(18.4 to 20.9)	-3.8<br>(-4.8 to -2.8)
Other road injuries	618.4<br>(535.9 to 728.3)	81.0<br>(46.0 to 131.2)	26.2<br>(17.6 to 38.1)	54.4<br>(53.2 to 55.5)	24.1<br>(23.2 to 25.1)
Other transport injuries	1,668.6<br>(1,393.3 to 1,895.5)	200.1<br>(142.2 to 269.3)	72.7<br>(49.8 to 101.9)	23.6<br>(22.1 to 25.0)	-0.6<br>(-1.8 to 0.6)
**Unintentional injuries**	**52,084.8<br>(48,680.9 to 55,645.1)**	**13,949.4<br>(11,895.6 to 16,735.0)**	**1,808.1<br>(1,275.9 to 2,446.9)**	**28.1<br>(26.9 to 29.1)**	**3.0<br>(2.0 to 3.9)**
Falls	27,548.4<br>(24,336.5 to 30,912.1)	8,304.2<br>(6,518.7 to 10,910.4)	1,176.9<br>(844.1 to 1,584.0)	29.4<br>(27.7 to 30.7)	4.0<br>(2.7 to 5.1)
Drowning	212.5<br>(184.8 to 242.7)	30.2<br>(24.5 to 36.8)	9.7<br>(6.6 to 13.9)	12.2<br>(10.6 to 13.8)	-9.7<br>(-11.1 to -8.5)
Fire, heat, and hot substances	3,641.4<br>(3,201.0 to 4,172.7)	115.7<br>(85.4 to 157.9)	65.2<br>(39.3 to 103.2)	23.2<br>(22.0 to 24.6)	-0.9<br>(-1.9 to 0.2)
Poisonings	115.3<br>(100.0 to 131.7)	51.6<br>(32.7 to 75.5)	7.0<br>(4.7 to 10.0)	31.5<br>(28.1 to 34.7)	5.8<br>(3.0 to 8.3)
Exposure to mechanical forces	8,013.9<br>(6,960.2 to 9,407.8)	605.9<br>(441.7 to 801.2)	162.4<br>(107.5 to 237.1)	30.3<br>(29.4 to 31.0)	4.8<br>(4.1 to 5.3)
Unintentional firearm injuries	267.3<br>(225.4 to 314.2)	6.9<br>(3.5 to 12.6)	6.4<br>(4.2 to 9.4)	31.2<br>(30.2 to 32.2)	5.5<br>(4.7 to 6.3)
Unintentional suffocation	252.1<br>(211.1 to 301.1)	17.9<br>(10.0 to 30.0)	7.0<br>(4.6 to 10.3)	30.5<br>(29.1 to 31.8)	5.0<br>(3.8 to 6.0)
Other exposure to mechanical forces	7,494.5<br>(6,435.9 to 8,838.1)	581.0<br>(425.5 to 769.6)	149.0<br>(98.8 to 217.5)	30.2<br>(29.3 to 31.0)	4.7<br>(4.0 to 5.3)

Cause	Prevalence in thousands (95% UI)	Incidence in thousands (95% UI)	YLDs in thousands (95% UI)		
	2016 counts	2016 counts	2016 counts	Percentage change in counts between 2006 and 2016	Percentage change in rates between 2006 and 2016
Adverse effects of medical treatment	131.2 (93.0 to 179.4)	1,716.0 (1,289.7 to 2,219.8)	17.5 (10.4 to 27.4)	13.7 (7.4 to 19.5)	-8.6 (-13.6 to -3.9)
Animal contact	1,830.5 (1,655.8 to 2,058.7)	777.8 (564.6 to 1,044.8)	38.5 (26.6 to 53.2)	17.9 (16.1 to 19.5)	-5.2 (-6.7 to -3.9)
Venomous animal contact	754.0 (644.2 to 897.3)	368.3 (255.0 to 502.7)	22.6 (15.8 to 30.9)	20.8 (18.0 to 23.4)	-2.9 (-5.1 to -0.8)
Non-venomous animal contact	1,076.5 (949.2 to 1,232.0)	409.5 (282.9 to 567.7)	15.9 (10.5 to 24.0)	13.9 (12.1 to 15.3)	-8.4 (-9.9 to -7.2)
Foreign body	1,792.0 (1,576.6 to 2,010.1)	748.5 (571.5 to 982.0)	58.5 (38.8 to 85.4)	23.4 (21.9 to 24.8)	-0.8 (-2.0 to 0.3)
Pulmonary aspiration and foreign body in airway	840.2 (690.8 to 992.9)	328.6 (255.8 to 433.3)	30.1 (19.8 to 43.8)	20.9 (19.2 to 22.5)	-2.8 (-4.2 to -1.5)
Foreign body in eyes	105.5 (76.5 to 145.2)	282.6 (141.1 to 494.9)	6.4 (3.9 to 9.9)	32.6 (29.6 to 35.9)	6.7 (4.2 to 9.2)
Foreign body in other body part	846.3 (710.3 to 1,011.3)	137.3 (84.5 to 199.8)	22.1 (14.5 to 32.2)	24.3 (22.9 to 25.6)	-0.0 (-1.1 to 1.0)
Environmental heat and cold exposure	2,721.7 (2,421.2 to 3,130.7)	359.2 (250.1 to 488.2)	97.9 (66.3 to 139.5)	25.5 (23.7 to 27.2)	0.9 (-0.6 to 2.3)
Other unintentional injuries	6,077.9 (5,238.4 to 7,087.9)	1,240.4 (813.5 to 1,733.6)	174.4 (116.7 to 248.5)	27.9 (26.9 to 28.7)	2.9 (2.1 to 3.5)
**Self-harm and interpersonal violence**	**4,298.3 (3,954.2 to 4,679.0)**	**381.9† (317.0 to 455.1)**	**89.7 (60.7 to 128.1)**	**17.2 (16.6 to 17.9)**	**-5.7 (-6.2 to -5.2)**
Self-harm	635.4 (537.7 to 742.7)	222.5 (174.5 to 280.7)	22.5 (14.9 to 31.9)	8.2 (7.0 to 9.3)	-13.0 (-13.9 to -12.1)
Self-harm by firearm	13.9 (12.0 to 16.5)	2.5 (0.8 to 5.8)	0.4 (0.3 to 0.6)	4.6 (3.1 to 6.2)	-15.9 (-17.1 to -14.6)
Self-harm by other specified means	621.4 (524.4 to 729.7)	220.1 (172.1 to 276.1)	22.1 (14.7 to 31.4)	8.3 (7.1 to 9.4)	-12.9 (-13.9 to -12.0)
Interpersonal violence	3,663.0 (3,336.4 to 4,051.1)	159.3† (114.1 to 211.5)	67.2 (45.4 to 96.0)	20.6 (20.0 to 21.3)	-3.0 (-3.5 to -2.5)
Assault by firearm	110.5 (94.7 to 128.2)	7.3 (3.7 to 12.5)	3.1 (2.0 to 4.5)	23.4 (22.3 to 24.6)	-0.8 (-1.6 to 0.2)
Assault by sharp object	474.1 (395.9 to 561.5)	25.5 (13.9 to 41.3)	9.9 (6.6 to 14.4)	18.6 (17.7 to 19.5)	-4.6 (-5.4 to -3.9)
Sexual violence	1,610.8 (1,377.7 to 1,874.6)	--	9.7 (6.4 to 14.3)	20.0 (18.9 to 21.0)	-3.5 (-4.4 to -2.7)
Assault by other means	1,467.5 (1,250.0 to 1,747.8)	126.6 (90.4 to 170.1)	44.4 (29.7 to 64.3)	21.0 (20.2 to 21.9)	-2.7 (-3.3 to -2.0)
**Forces of nature, conflict and terrorism, and executions and police conflict**	**616.2 (409.6 to 880.6)**	**6.4† (4.0 to 9.4)**	**17.8 (4.8 to 43.9)**	**75.6 (65.1 to 93.9)**	**41.2 (32.8 to 55.9)**
Forces of nature, conflict and terrorism, and state actor violence	136.0 (75.8 to 207.0)	4.0 (2.2 to 6.2)	4.7 (1.0 to 12.1)	71.1 (57.7 to 82.6)	37.6 (26.8 to 46.9)
Conflict and terrorism	386.4 (227.0 to 604.1)	--	11.7 (3.5 to 27.7)	61.6 (48.2 to 91.2)	30.0 (19.2 to 53.8)
Executions and police conflict	93.9 (28.2 to 154.9)	2.5 (0.9 to 4.5)	2.2 (0.4 to 6.4)	163.4 (147.7 to 183.2)	111.8 (99.2 to 127.8)

We did not calculate incidence for certain NTDs and causes with only birth prevalence. Blank cells mean that no estimate is available.
† Incidence for aggregate causes represents the sum of the incidence of the causes below the aggregate.
‡ Incidence estimates for stroke represent first-ever stroke only.
^ Incidence of HIV/AIDS represents new infections of HIV only and does not include new infections of Tuberculosis in HIV positive cases.

SR Table 12: Global prevalence, incidence, and YLDs for 2016, percentage change of YLD counts and percentage change of YLD rates between 2006 and 2016 by cause, age 80-84 years.

Cause	Prevalence in thousands (95% UI)	Incidence in thousands (95% UI)	YLDs in thousands (95% UI)		
	2016 counts	2016 counts	2016 counts	Percentage change in counts between 2006 and 2016	Percentage change in rates between 2006 and 2016
**All causes**	**72,540.6 (72,533.4 to 72,555.1)**	**525,469.7† (480,677.4 to 574,753.3)**	**20,728.5 (15,754.3 to 26,231.2)**	**30.2 (29.4 to 31.1)**	**-0.6 (-1.2 to 0.1)**
**Communicable, maternal, neonatal, and nutritional disorders**	**41,328.1 (40,856.1 to 41,742.6)**	**205,548.1† (172,108.0 to 250,654.1)**	**623.7 (449.7 to 811.3)**	**25.8 (23.4 to 29.1)**	**-3.9 (-5.8 to -1.4)**
**HIV/AIDS and tuberculosis**	**16,591.9 (15,527.2 to 17,833.3)**	**118.4† (102.3 to 135.6)**	**66.7 (46.0 to 91.1)**	**19.7 (12.4 to 26.2)**	**-8.6 (-14.2 to -3.7)**
Tuberculosis	16,033.7 (14,379.8 to 17,737.2)	118.4† (102.3 to 135.6)	58.4 (39.2 to 80.2)	18.8 (15.4 to 22.6)	-9.3 (-11.9 to -6.4)
Drug-susceptible tuberculosis	206.3 (174.1 to 244.5)	114.2 (98.7 to 131.0)	56.1 (37.6 to 77.2)	18.9 (15.4 to 22.8)	-9.2 (-11.9 to -6.2)
Multidrug-resistant tuberculosis without extensive drug resistance	7.5 (6.2 to 8.9)	3.9 (3.3 to 4.5)	2.1 (1.4 to 2.9)	8.7 (-5.4 to 25.9)	-17.0 (-27.8 to -3.9)
Extensively drug-resistant tuberculosis	0.5 (0.4 to 0.6)	0.3 (0.2 to 0.3)	0.2 (0.1 to 0.2)	234.4 (195.1 to 279.0)	155.3 (125.3 to 189.4)
Latent tuberculosis infection	15,819.4 (14,178.6 to 17,526.4)	--	--	--	--
HIV/AIDS	81.1 (68.1 to 100.4)	--	8.3 (5.2 to 12.5)	26.2 (-13.6 to 93.7)	-3.7 (-34.0 to 47.9)
Drug-susceptible HIV/AIDS - Tuberculosis	3.1 (2.1 to 4.4)	3.3 (2.4 to 4.5)	1.1 (0.7 to 1.7)	19.2 (8.1 to 30.5)	-9.0 (-17.4 to -0.3)
Multidrug-resistant HIV/AIDS - Tuberculosis without extensive drug resistance	0.1 (0.1 to 0.1)	0.1 (0.1 to 0.1)	< 0.05 (< 0.05 to 0.1)	40.8 (2.1 to 78.5)	7.5 (-22.1 to 36.3)
Extensively drug-resistant HIV/AIDS - Tuberculosis	< 0.05 (< 0.05 to < 0.05)	< 0.05 (< 0.05 to < 0.05)	< 0.05 (< 0.05 to < 0.05)	465.3 (272.0 to 671.7)	331.7 (184.1 to 489.2)
HIV/AIDS resulting in other diseases	78.0 (64.8 to 97.0)	--	7.2 (4.4 to 11.2)	27.2 (-16.1 to 116.1)	-2.9 (-36.0 to 65.0)
**Diarrhoea, lower respiratory infections, and other common infectious diseases**	**3,527.0 (3,310.5 to 3,681.7)**	**197,350.9† (163,656.8 to 242,647.6)**	**187.2 (131.7 to 246.6)**	**33.5 (31.2 to 42.5)**	**2.0 (0.2 to 8.8)**
Diarrhoeal diseases	1,020.5 (921.7 to 1,134.9)	70,677.6 (63,815.5 to 78,372.3)	102.0 (71.4 to 137.0)	33.9 (31.2 to 36.4)	2.2 (0.2 to 4.2)
Intestinal infectious diseases	4.4 (3.7 to 5.2)	84.4† (73.3 to 97.0)	0.7 (0.4 to 1.0)	25.2 (1.0 to 56.7)	-4.4 (-22.9 to 19.6)
Typhoid fever	4.1 (3.3 to 5.0)	64.6 (56.0 to 74.5)	0.6 (0.4 to 0.8)	26.7 (0.5 to 63.2)	-3.3 (-23.3 to 24.6)
Paratyphoid fever	0.9 (0.7 to 1.0)	19.8 (17.2 to 22.9)	0.1 (< 0.05 to 0.1)	26.9 (3.7 to 54.4)	-3.1 (-20.8 to 17.9)
Other intestinal infectious diseases	--	--	< 0.05 (< 0.05 to < 0.05)	-2.2 (-21.1 to 22.4)	-25.4 (-39.8 to -6.5)
Lower respiratory infections	302.3 (279.7 to 329.4)	13,207.5 (11,978.1 to 14,485.4)	16.6 (11.2 to 23.0)	53.4 (48.0 to 58.9)	17.2 (13.0 to 21.3)
Upper respiratory infections	1,496.0 (1,057.1 to 2,103.5)	111,760.4 (78,649.1 to 157,362.3)	34.1 (19.1 to 57.2)	27.3 (24.7 to 29.9)	-2.8 (-4.8 to -0.8)
Otitis media	394.1 (277.4 to 550.0)	537.9 (410.8 to 667.8)	10.1 (5.8 to 16.9)	22.2 (16.2 to 28.8)	-6.7 (-11.3 to -1.7)
Meningitis	102.7 (77.8 to 172.0)	16.2 (12.8 to 20.4)	5.4 (2.7 to 17.1)	48.6 (18.2 to 359.4)	13.5 (-9.8 to 250.8)
Pneumococcal meningitis	42.1 (32.2 to 52.6)	3.7 (2.5 to 5.2)	1.8 (1.1 to 2.6)	11.9 (7.1 to 18.9)	-14.6 (-18.2 to -9.2)
H influenzae type B meningitis	8.5 (5.9 to 11.6)	1.0 (0.6 to 1.6)	0.4 (0.3 to 0.6)	-17.7 (-27.2 to 0.4)	-37.2 (-44.4 to -23.4)
Meningococcal infection	16.1 (7.7 to 81.9)	3.1 (1.9 to 4.5)	1.5 (0.4 to 12.5)	235.1 (17.4 to 2,510.7)	155.9 (-10.4 to 1,893.4)
Other meningitis	35.9 (24.5 to 46.4)	8.5 (6.5 to 11.1)	1.6 (1.0 to 2.2)	58.4 (50.5 to 65.9)	21.0 (14.9 to 26.7)
Encephalitis	204.5 (107.7 to 339.0)	107.4 (95.9 to 121.0)	9.6 (6.6 to 13.3)	29.4 (24.0 to 35.5)	-1.2 (-5.3 to 3.5)
Diphtheria	--	--	--	--	--
Whooping cough	--	--	--	--	--
Tetanus	0.9 (0.9 to 1.0)	1.3 (0.6 to 1.9)	< 0.05 (< 0.05 to < 0.05)	-5.9 (-27.8 to 24.2)	-28.1 (-44.9 to -5.2)
Measles	--	--	--	--	--
Varicella and herpes zoster	171.9 (131.8 to 220.8)	958.2 (735.9 to 1,234.8)	8.7 (5.0 to 13.9)	33.6 (27.6 to 40.3)	2.0 (-2.6 to 7.2)
**Neglected tropical diseases and malaria**	**12,103.6 (11,654.1 to 12,532.6)**	**4,341.6† (2,780.6 to 5,858.3)**	**106.9 (73.7 to 150.3)**	**11.7 (1.7 to 24.5)**	**-14.7 (-22.3 to -4.9)**
Malaria	153.0 (128.1 to 178.0)	349.0 (257.1 to 452.8)	1.5 (1.0 to 2.1)	-26.4 (-31.7 to -21.0)	-43.8 (-47.9 to -39.7)
Chagas disease	127.9 (108.4 to 150.4)	0.8 (0.4 to 1.1)	3.0 (1.9 to 4.4)	26.8 (19.9 to 33.1)	-3.2 (-8.4 to 1.6)
Leishmaniasis	48.1 (37.5 to 57.2)	8.3 (6.3 to 10.9)	2.4 (1.5 to 3.5)	23.8 (16.8 to 32.2)	-5.5 (-10.8 to 1.0)
Visceral leishmaniasis	0.8 (0.5 to 1.2)	3.3 (2.2 to 5.0)	0.1 (< 0.05 to 0.1)	-24.5 (-53.5 to 16.4)	-42.3 (-64.5 to -11.1)
Cutaneous and mucocutaneous leishmaniasis	40.6 (30.4 to 52.2)	4.9 (3.5 to 6.7)	2.3 (1.5 to 3.4)	25.6 (18.6 to 34.8)	-4.1 (-9.5 to 2.9)
African trypanosomiasis	< 0.05 (< 0.05 to 0.2)	< 0.05 (< 0.05 to 0.1)	< 0.05 (< 0.05 to < 0.05)	-65.6 (-96.0 to 149.8)	-73.8 (-96.9 to 90.7)
Schistosomiasis	1,025.4 (925.0 to 1,121.2)	229.9 (204.9 to 253.9)	7.1 (3.6 to 13.2)	-29.7 (-33.4 to -26.6)	-46.3 (-49.1 to -43.9)
Cysticercosis	49.0 (34.2 to 68.3)	--	6.9 (4.0 to 10.3)	14.9 (6.7 to 23.0)	-12.3 (-18.5 to -6.1)
Cystic echinococcosis	6.1 (3.1 to 12.1)	1.2 (0.5 to 3.0)	0.5 (0.2 to 1.1)	-34.2 (-73.8 to 73.2)	-49.8 (-80.0 to 32.3)
Lymphatic filariasis	133.0 (115.8 to 158.1)	35.0 (19.7 to 54.8)	3.9 (2.0 to 7.0)	-32.8 (-52.6 to -15.2)	-48.7 (-63.8 to -35.2)
Onchocerciasis	73.1 (50.0 to 109.4)	12.5 (5.3 to 24.4)	6.7 (3.9 to 11.8)	-8.5 (-25.9 to 15.1)	-30.2 (-43.4 to -12.1)
Trachoma	339.0 (217.9 to 490.6)	--	23.4 (14.9 to 35.0)	2.8 (-7.6 to 13.2)	-21.5 (-29.4 to -13.6)
Dengue	42.6 (23.4 to 71.9)	712.8 (436.2 to 1,076.6)	6.1 (2.8 to 11.1)	125.6 (83.6 to 417.7)	72.2 (40.2 to 295.3)
Yellow fever	< 0.05 (< 0.05 to < 0.05)	0.2 (< 0.05 to 0.5)	< 0.05 (< 0.05 to < 0.05)	-17.5 (-50.6 to 28.8)	-37.0 (-62.3 to -1.7)
Rabies	< 0.05 (< 0.05 to < 0.05)	0.2 (0.1 to 0.3)	< 0.05 (< 0.05 to < 0.05)	1.2 (-49.1 to 54.2)	-22.8 (-61.1 to 17.8)
Intestinal nematode infections	10,037.6 (9,507.7 to 10,468.6)	--	16.8 (9.8 to 26.9)	1.4 (-5.9 to 9.3)	-22.6 (-28.2 to -16.6)
Ascariasis	5,328.6 (4,711.3 to 6,068.9)	--	4.9 (2.7 to 8.3)	-14.7 (-25.1 to -2.4)	-34.9 (-42.8 to -25.5)

Cause	Prevalence in thousands (95% UI)	Incidence in thousands (95% UI)	YLDs in thousands (95% UI)		
	2016 counts	2016 counts	2016 counts	Percentage change in counts between 2006 and 2016	Percentage change in rates between 2006 and 2016
Trichuriasis	2,822.5 (2,575.8 to 3,101.7)	--	1.9 (1.1 to 3.2)	-7.0 (-20.5 to 8.9)	-29.0 (-39.3 to -16.9)
Hookworm disease	2,957.1 (2,708.5 to 3,237.0)	--	9.9 (5.9 to 15.3)	14.2 (3.3 to 27.9)	-12.8 (-21.1 to -2.3)
Food-borne trematodiases	1,038.1 (728.6 to 1,335.3)	2,950.7 (1,401.3 to 4,351.1)	25.2 (8.3 to 51.8)	71.3 (43.9 to 118.6)	30.8 (9.9 to 66.9)
Leprosy	21.7 (20.9 to 22.4)	0.9 (0.8 to 1.0)	1.2 (0.9 to 1.7)	24.2 (17.8 to 31.3)	-5.2 (-10.1 to 0.2)
Ebola virus disease	< 0.05 (0.0 to < 0.05)	--	< 0.05 (0.0 to < 0.05)	--	--
Zika virus disease	0.7 (0.4 to 1.2)	40.3 (23.1 to 73.4)	< 0.05 (< 0.05 to < 0.05)	--	--
Guinea worm disease	< 0.05 (< 0.05 to < 0.05)	< 0.05 (< 0.05 to < 0.05)	< 0.05 (< 0.05 to < 0.05)	-99.5 (-99.7 to -99.2)	-99.6 (-99.8 to -99.4)
Other neglected tropical diseases	131.8 (128.6 to 135.4)	--	2.2 (1.5 to 3.1)	34.7 (27.2 to 41.7)	2.8 (-2.9 to 8.2)
**Maternal disorders**	**--**	**--**	**--**	**--**	**--**
Maternal haemorrhage	--	--	--	--	--
Maternal sepsis and other pregnancy related infections	--	--	--	--	--
Maternal hypertensive disorders	--	--	--	--	--
Maternal obstructed labour and uterine rupture	--	--	--	--	--
Maternal abortion, miscarriage, and ectopic pregnancy	--	--	--	--	--
Other maternal disorders	--	--	--	--	--
**Neonatal disorders**	**119.7 (106.6 to 137.2)**	**--**	**9.5 (6.5 to 15.0)**	**51.6 (42.1 to 61.1)**	**15.8 (8.5 to 23.0)**
Neonatal preterm birth complications	109.3 (87.1 to 132.6)	--	6.3 (4.4 to 8.6)	42.4 (31.8 to 53.2)	8.7 (0.7 to 17.0)
Neonatal encephalopathy due to birth asphyxia and trauma	1.7 (0.3 to 4.9)	--	0.6 (0.1 to 1.7)	87.6 (70.0 to 136.5)	43.3 (29.8 to 80.6)
Neonatal sepsis and other neonatal infections	5.3 (0.9 to 15.7)	--	2.1 (0.4 to 6.9)	73.5 (61.2 to 110.4)	32.4 (23.1 to 60.6)
Hemolytic disease and other neonatal jaundice	1.6 (1.2 to 2.0)	--	0.6 (0.4 to 0.8)	59.5 (47.1 to 69.8)	21.8 (12.3 to 29.6)
Other neonatal disorders	--	--	--	--	--
**Nutritional deficiencies**	**8,805.8 (8,665.5 to 8,896.0)**	**1,319.1† (1,058.3 to 1,592.0)**	**233.2 (161.7 to 320.4)**	**27.8 (26.2 to 29.5)**	**-2.4 (-3.7 to -1.1)**
Protein-energy malnutrition	--	--	--	--	--
Iodine deficiency	1,316.0 (1,181.4 to 1,465.0)	2.3 (1.8 to 3.0)	30.5 (21.1 to 42.0)	18.0 (15.0 to 21.1)	-9.9 (-12.2 to -7.5)
Vitamin A deficiency	1,257.9 (1,064.6 to 1,490.5)	1,316.8 (1,056.0 to 1,589.8)	0.8 (0.5 to 1.2)	40.5 (29.1 to 52.2)	7.3 (-1.4 to 16.2)
Iron-deficiency anaemia	7,067.7 (6,821.1 to 7,338.5)	--	201.9 (138.1 to 278.9)	29.4 (27.5 to 31.2)	-1.2 (-2.6 to 0.2)
Other nutritional deficiencies	--	--	--	--	--
**Other communicable, maternal, neonatal, and nutritional diseases**	**19,395.4 (18,707.4 to 20,038.5)**	**2,418.0 (1,695.7 to 3,302.2)**	**20.2 (14.1 to 28.3)**	**31.7 (16.2 to 48.3)**	**0.6 (-11.3 to 13.2)**
Sexually transmitted diseases excluding HIV	14,531.8 (13,445.6 to 16,047.5)	401.2 (345.5 to 461.7)	11.5 (8.0 to 16.2)	30.9 (24.7 to 37.7)	-0.0 (-4.8 to 5.2)
Syphilis	58.2 (47.8 to 71.3)	2.6 (1.8 to 3.8)	7.8 (5.3 to 10.8)	33.5 (24.1 to 42.5)	1.9 (-5.3 to 8.8)
Chlamydial infection	< 0.05 (< 0.05 to < 0.05)	--	< 0.05 (< 0.05 to < 0.05)	49.5 (21.2 to 90.4)	14.1 (-7.5 to 45.4)
Gonococcal infection	< 0.05 (< 0.05 to < 0.05)	--	< 0.05 (< 0.05 to < 0.05)	68.4 (-14.4 to 283.0)	28.6 (-34.6 to 192.5)
Trichomoniasis	530.3 (451.3 to 613.9)	398.7 (343.0 to 459.3)	0.6 (0.2 to 1.3)	25.3 (20.6 to 30.0)	-4.3 (-7.9 to -0.7)
Genital herpes	13,839.6 (12,107.9 to 15,963.8)	--	3.1 (0.9 to 7.0)	26.1 (23.5 to 29.1)	-3.7 (-5.7 to -1.4)
Other sexually transmitted diseases	< 0.05 (< 0.05 to < 0.05)	--	< 0.05 (< 0.05 to < 0.05)	48.1 (20.0 to 88.5)	13.0 (-8.4 to 44.0)
Hepatitis	6,588.0 (5,842.6 to 7,361.0)	2,016.6 (1,302.5 to 2,912.0)	4.8 (2.6 to 8.2)	26.6 (-21.2 to 95.1)	-3.3 (-39.8 to 49.0)
Acute hepatitis A	23.8 (6.3 to 50.1)	309.9 (81.8 to 651.9)	0.9 (0.2 to 2.0)	13.4 (-73.4 to 269.1)	-13.4 (-79.7 to 181.8)
Hepatitis B	3,875.5 (3,188.1 to 4,589.5)	1,348.2 (714.5 to 2,223.6)	3.2 (1.5 to 5.9)	29.2 (-29.4 to 132.6)	-1.3 (-46.1 to 77.6)
Hepatitis C	2,666.1 (2,384.2 to 2,952.1)	64.4 (45.3 to 85.0)	0.1 (< 0.05 to 0.1)	26.6 (15.8 to 37.1)	-3.4 (-11.6 to 4.7)
Acute hepatitis E	22.6 (13.9 to 33.2)	294.1 (180.9 to 431.8)	0.6 (0.3 to 1.1)	35.6 (9.3 to 67.4)	3.5 (-16.5 to 27.8)
Other infectious diseases	175.6 (170.4 to 181.2)	0.2 (0.1 to 0.3)	3.9 (2.7 to 5.6)	40.9 (35.5 to 46.2)	7.5 (3.5 to 11.6)
**Non-communicable diseases**	**72,496.2 (72,487.6 to 72,512.1)**	**306,824.0† (278,950.3 to 337,204.5)**	**18,389.5 (14,035.6 to 23,260.8)**	**30.1 (29.1 to 31.1)**	**-0.7 (-1.4 to 0.1)**
**Neoplasms**	**3,622.9 (3,583.7 to 3,662.7)**	**1,173.3 (1,115.7 to 1,245.4)**	**295.3 (217.8 to 384.2)**	**31.3 (28.7 to 34.3)**	**0.3 (-1.8 to 2.5)**
Lip and oral cavity cancer	90.3 (87.6 to 93.0)	18.8 (18.3 to 19.4)	6.2 (4.5 to 8.2)	40.9 (33.1 to 48.6)	7.6 (1.6 to 13.4)
Nasopharynx cancer	10.0 (9.4 to 10.7)	2.2 (2.1 to 2.4)	0.8 (0.6 to 1.1)	29.6 (19.4 to 41.1)	-1.1 (-8.9 to 7.8)
Other pharynx cancer	18.9 (17.9 to 19.8)	5.6 (5.2 to 6.0)	1.8 (1.3 to 2.3)	46.6 (32.0 to 62.3)	12.0 (0.8 to 23.9)
Oesophageal cancer	55.9 (53.4 to 58.7)	32.9 (31.7 to 34.1)	8.0 (5.8 to 10.1)	20.8 (12.6 to 29.4)	-7.8 (-14.0 to -1.2)
Stomach cancer	253.1 (245.1 to 261.4)	93.2 (90.3 to 96.1)	23.8 (17.8 to 30.2)	26.2 (19.9 to 32.5)	-3.7 (-8.4 to 1.2)
Colon and rectum cancer	707.8 (680.8 to 748.2)	139.7 (134.5 to 147.4)	45.3 (32.5 to 60.2)	28.4 (22.3 to 36.0)	-2.0 (-6.6 to 3.8)
Liver cancer	91.9 (86.7 to 97.3)	58.2 (54.7 to 61.3)	13.2 (9.5 to 17.2)	60.4 (49.9 to 72.4)	22.5 (14.5 to 31.7)
Liver cancer due to hepatitis B	30.1 (21.4 to 38.6)	17.1 (12.1 to 21.9)	3.8 (2.3 to 5.5)	63.1 (47.1 to 83.0)	24.5 (12.3 to 39.8)

Cause	Prevalence in thousands (95% UI)	Incidence in thousands (95% UI)	YLDs in thousands (95% UI)		
	2016 counts	2016 counts	2016 counts	Percentage change in counts between 2006 and 2016	Percentage change in rates between 2006 and 2016
Liver cancer due to hepatitis C	38.8 (34.1 to 43.8)	19.1 (16.7 to 21.8)	4.4 (3.1 to 5.9)	57.0 (45.7 to 70.8)	19.9 (11.2 to 30.4)
Liver cancer due to alcohol use	18.0 (13.8 to 22.5)	10.2 (7.7 to 12.7)	2.3 (1.6 to 3.3)	58.2 (42.8 to 75.6)	20.8 (9.1 to 34.1)
Liver cancer due to other causes	21.4 (17.9 to 25.9)	11.9 (9.9 to 14.4)	2.7 (1.9 to 3.7)	64.4 (49.5 to 82.3)	25.5 (14.2 to 39.2)
Gallbladder and biliary tract cancer	24.8 (22.5 to 27.4)	19.6 (18.2 to 20.9)	4.4 (3.1 to 5.7)	23.1 (13.7 to 33.1)	-6.0 (-13.2 to 1.6)
Pancreatic cancer	50.9 (47.4 to 54.0)	44.4 (42.1 to 46.2)	8.9 (6.4 to 11.4)	39.6 (29.3 to 50.0)	6.6 (-1.3 to 14.5)
Larynx cancer	39.6 (38.5 to 41.1)	8.7 (8.4 to 9.0)	3.3 (2.4 to 4.3)	32.3 (24.7 to 40.5)	1.0 (-4.8 to 7.3)
Tracheal, bronchus, and lung cancer	351.6 (334.7 to 365.8)	176.3 (168.7 to 182.8)	40.1 (30.1 to 50.2)	44.4 (37.0 to 52.0)	10.3 (4.6 to 16.1)
Malignant skin melanoma	83.6 (65.9 to 90.1)	15.4 (12.2 to 16.6)	4.5 (3.0 to 6.3)	39.1 (25.8 to 49.4)	6.2 (-3.9 to 14.0)
Non-melanoma skin cancer	106.7 (82.7 to 129.8)	160.4 (106.9 to 230.5)	3.7 (1.9 to 6.0)	-1.6 (-14.0 to 14.6)	-24.9 (-34.3 to -12.5)
Non-melanoma skin cancer (squamous-cell carcinoma)	86.3 (49.5 to 120.4)	84.0 (50.1 to 116.4)	3.6 (1.8 to 5.9)	-2.0 (-14.5 to 14.4)	-25.2 (-34.8 to -12.6)
Non-melanoma skin cancer (basal-cell carcinoma)	8.8 (4.2 to 17.6)	76.4 (43.4 to 137.3)	0.1 (< 0.05 to 0.2)	16.3 (-2.4 to 33.3)	-11.2 (-25.5 to 1.8)
Breast cancer	341.1 (327.8 to 358.1)	60.3 (57.5 to 63.7)	24.8 (16.9 to 34.2)	14.0 (8.9 to 19.3)	-12.9 (-16.9 to -8.9)
Cervical cancer	47.7 (40.9 to 51.6)	9.5 (8.1 to 10.2)	3.1 (2.1 to 4.1)	7.5 (0.9 to 15.9)	-17.9 (-22.9 to -11.5)
Uterine cancer	79.1 (74.5 to 85.6)	14.6 (13.7 to 15.7)	4.7 (3.2 to 6.3)	21.1 (10.5 to 33.6)	-7.6 (-15.7 to 2.0)
Ovarian cancer	52.0 (48.4 to 54.8)	11.0 (10.3 to 11.6)	3.3 (2.4 to 4.4)	10.8 (2.4 to 19.9)	-15.4 (-21.9 to -8.5)
Prostate cancer	496.6 (436.0 to 565.3)	109.9 (98.0 to 122.2)	34.0 (23.5 to 46.2)	30.9 (23.2 to 38.5)	-0.1 (-5.9 to 5.7)
Testicular cancer	2.3 (2.1 to 2.5)	0.4 (0.4 to 0.5)	0.1 (0.1 to 0.2)	45.6 (27.9 to 64.8)	11.1 (-2.4 to 25.8)
Kidney cancer	78.8 (73.2 to 82.4)	16.1 (15.2 to 16.7)	5.1 (3.6 to 6.9)	27.4 (18.5 to 36.5)	-2.8 (-9.5 to 4.2)
Bladder cancer	221.7 (209.5 to 233.1)	42.3 (40.1 to 44.4)	15.7 (11.3 to 21.1)	32.3 (24.2 to 40.2)	1.0 (-5.2 to 7.0)
Brain and nervous system cancer	39.0 (27.9 to 46.0)	12.0 (8.9 to 13.8)	3.7 (2.3 to 5.1)	51.2 (36.4 to 70.2)	15.4 (4.1 to 29.9)
Thyroid cancer	27.7 (26.5 to 29.3)	5.1 (4.9 to 5.4)	1.5 (1.1 to 2.1)	32.4 (22.6 to 43.4)	1.1 (-6.4 to 9.5)
Mesothelioma	6.2 (5.7 to 6.6)	3.0 (2.8 to 3.2)	1.0 (0.7 to 1.3)	35.8 (26.1 to 44.8)	3.7 (-3.7 to 10.5)
Hodgkin lymphoma	6.0 (5.3 to 6.4)	1.2 (1.1 to 1.4)	0.4 (0.3 to 0.6)	12.1 (1.7 to 23.7)	-14.4 (-22.3 to -5.5)
Non-Hodgkin's lymphoma	150.3 (130.0 to 159.1)	30.2 (26.3 to 31.8)	9.0 (6.2 to 12.2)	38.5 (30.0 to 46.3)	5.7 (-0.7 to 11.7)
Multiple myeloma	45.6 (36.3 to 49.9)	11.2 (9.3 to 12.3)	3.9 (2.8 to 5.2)	28.0 (18.1 to 37.8)	-2.2 (-9.8 to 5.2)
Leukaemia	125.5 (118.0 to 132.9)	28.6 (26.8 to 30.1)	9.7 (7.0 to 13.1)	30.7 (24.2 to 37.7)	-0.2 (-5.2 to 5.1)
Acute lymphoid leukaemia	6.8 (6.2 to 7.4)	1.4 (1.3 to 1.6)	0.7 (0.5 to 0.9)	56.3 (43.1 to 68.5)	19.3 (9.2 to 28.7)
Chronic lymphoid leukaemia	42.9 (38.6 to 47.4)	7.9 (7.1 to 8.7)	2.8 (1.9 to 3.8)	24.4 (13.5 to 36.1)	-5.0 (-13.4 to 4.0)
Acute myeloid leukaemia	13.6 (11.7 to 14.7)	7.4 (6.3 to 7.9)	2.0 (1.4 to 2.7)	43.5 (32.8 to 53.2)	9.6 (1.4 to 16.9)
Chronic myeloid leukaemia	9.2 (8.4 to 10.2)	2.1 (1.9 to 2.3)	0.8 (0.5 to 1.0)	8.1 (-2.0 to 20.1)	-17.5 (-25.2 to -8.3)
Other leukaemia	53.0 (47.9 to 56.8)	9.8 (8.8 to 10.5)	3.5 (2.5 to 4.8)	30.9 (21.2 to 41.7)	-0.0 (-7.5 to 8.2)
Other neoplasms	204.3 (181.5 to 217.6)	42.8 (38.3 to 45.3)	11.6 (8.1 to 15.9)	53.0 (42.0 to 63.4)	16.8 (8.4 to 24.8)
**Cardiovascular diseases**	**33,876.5 (33,163.5 to 34,401.0)**	**4,754.0† (4,373.2 to 5,161.9)**	**2,834.3 (2,106.4 to 3,603.1)**	**28.0 (23.7 to 32.7)**	**-2.2 (-5.6 to 1.3)**
Rheumatic heart disease	108.2 (96.4 to 121.0)	16.4 (14.7 to 18.2)	7.5 (5.2 to 10.1)	27.2 (21.3 to 33.6)	-2.9 (-7.4 to 2.0)
Ischaemic heart disease	12,919.5 (11,941.8 to 13,995.7)	2,078.7 (1,889.2 to 2,298.6)	539.9 (383.0 to 725.7)	29.6 (27.7 to 31.6)	-1.0 (-2.5 to 0.4)
Cerebrovascular disease	6,327.4 (5,901.8 to 6,743.7)	1,231.8‡ (1,072.6 to 1,383.8)	1,307.6 (916.9 to 1,680.3)	29.0 (19.5 to 39.1)	-1.5 (-8.8 to 6.2)
Ischaemic stroke	5,611.9 (4,763.7 to 6,492.9)	871.3‡ (722.2 to 1,012.1)	1,105.7 (771.6 to 1,442.0)	29.4 (17.9 to 41.1)	-1.2 (-9.9 to 7.8)
Hemorrhagic stroke	1,014.2 (808.0 to 1,229.1)	360.4‡ (310.8 to 412.9)	201.9 (136.1 to 269.1)	26.6 (14.7 to 38.8)	-3.3 (-12.4 to 6.0)
Hypertensive heart disease	1,821.4 (1,365.0 to 2,326.1)	--	140.3 (93.8 to 207.8)	31.4 (28.2 to 34.9)	0.3 (-2.1 to 3.0)
Cardiomyopathy and myocarditis	438.4 (388.7 to 489.4)	84.0† (66.6 to 100.7)	32.3 (21.2 to 47.8)	18.6 (13.2 to 24.5)	-9.5 (-13.5 to -4.9)
Myocarditis	51.3 (41.4 to 62.7)	84.0 (66.6 to 100.7)	3.4 (2.2 to 4.9)	26.8 (18.4 to 35.4)	-3.2 (-9.6 to 3.4)
Alcoholic cardiomyopathy	40.9 (29.8 to 54.7)	--	3.2 (2.0 to 4.8)	16.0 (3.0 to 31.1)	-11.4 (-21.4 to 0.1)
Other cardiomyopathy	333.8 (245.3 to 439.4)	--	25.8 (16.7 to 38.4)	17.9 (12.2 to 23.7)	-10.0 (-14.3 to -5.6)
Atrial fibrillation and flutter	6,201.9 (5,398.5 to 7,090.6)	351.1 (265.0 to 439.8)	466.6 (321.9 to 642.4)	28.6 (26.9 to 30.2)	-1.8 (-3.1 to -0.6)
Peripheral vascular disease	11,548.4 (9,531.6 to 13,839.0)	925.1 (671.1 to 1,243.3)	63.7 (28.9 to 120.5)	22.9 (20.9 to 24.7)	-6.2 (-7.7 to -4.8)
Endocarditis	51.5 (39.4 to 66.2)	66.9 (55.4 to 79.2)	3.9 (2.5 to 5.8)	21.2 (12.8 to 28.9)	-7.5 (-13.9 to -1.6)
Other cardiovascular and circulatory diseases	5,452.8 (4,103.1 to 7,041.2)	--	272.4 (174.1 to 399.2)	20.9 (17.2 to 24.3)	-7.7 (-10.5 to -5.1)
**Chronic respiratory diseases**	**18,701.4 (18,374.1 to 19,030.0)**	**1,935.3† (1,716.5 to 2,167.1)**	**1,054.2 (915.6 to 1,185.8)**	**36.5 (32.9 to 40.0)**	**4.2 (1.5 to 6.9)**
Chronic obstructive pulmonary disease	15,241.4 (14,342.7 to 16,168.1)	908.6 (763.5 to 1,057.1)	825.0 (734.6 to 916.5)	37.0 (32.5 to 41.4)	4.6 (1.2 to 7.9)
Pneumoconiosis	60.4 (56.1 to 65.1)	2.3 (1.9 to 2.8)	8.4 (5.8 to 11.6)	41.0 (33.9 to 47.7)	7.7 (2.2 to 12.8)
Silicosis	22.6 (19.9 to 25.7)	1.3 (1.0 to 1.6)	3.1 (2.1 to 4.4)	46.0 (33.0 to 59.9)	11.4 (1.6 to 22.1)

Cause	Prevalence in thousands (95% UI)	Incidence in thousands (95% UI)	YLDs in thousands (95% UI)		
	2016 counts	2016 counts	2016 counts	Percentage change in counts between 2006 and 2016	Percentage change in rates between 2006 and 2016
Asbestosis	11.8 (10.6 to 13.0)	0.2 (0.2 to 0.3)	1.7 (1.2 to 2.3)	27.6 (20.5 to 34.8)	-2.6 (-8.0 to 2.9)
Coal workers pneumoconiosis	13.7 (11.8 to 15.9)	0.2 (0.2 to 0.3)	1.9 (1.3 to 2.6)	45.8 (29.1 to 65.0)	11.3 (-1.4 to 26.0)
Other pneumoconiosis	12.3 (10.4 to 14.9)	0.6 (0.4 to 0.9)	1.7 (1.2 to 2.5)	41.8 (28.7 to 56.3)	8.3 (-1.8 to 19.4)
Asthma	5,103.3 (4,557.2 to 5,672.4)	992.4 (839.6 to 1,159.4)	180.6 (122.5 to 252.5)	33.1 (30.1 to 35.9)	1.6 (-0.6 to 3.8)
Interstitial lung disease and pulmonary sarcoidosis	352.0 (303.7 to 406.1)	31.9 (24.8 to 39.8)	34.4 (23.2 to 49.1)	38.2 (33.7 to 42.9)	5.5 (2.1 to 9.1)
Other chronic respiratory diseases	--	--	5.8 (5.1 to 6.5)	70.1 (64.7 to 75.2)	29.9 (25.8 to 33.8)
**Cirrhosis and other chronic liver diseases**	**873.7 (813.9 to 935.8)**	**70.2 (59.9 to 80.7)**	**32.3 (22.5 to 44.9)**	**36.7 (31.8 to 42.0)**	**4.4 (0.6 to 8.4)**
Cirrhosis and other chronic liver diseases due to hepatitis B	231.2 (195.2 to 266.5)	19.9 (15.9 to 24.1)	8.6 (5.7 to 12.2)	41.3 (34.1 to 48.9)	7.9 (2.4 to 13.7)
Cirrhosis and other chronic liver diseases due to hepatitis C	201.0 (167.8 to 239.3)	17.0 (13.2 to 21.2)	7.6 (5.1 to 11.2)	36.8 (28.8 to 45.0)	4.5 (-1.7 to 10.7)
Cirrhosis and other chronic liver diseases due to alcohol use	261.2 (219.8 to 303.6)	19.2 (15.5 to 23.7)	9.5 (6.4 to 13.5)	35.2 (27.8 to 43.3)	3.3 (-2.4 to 9.4)
Cirrhosis and other chronic liver diseases due to other causes	180.2 (138.4 to 228.2)	14.1 (10.6 to 18.2)	6.6 (4.2 to 9.8)	32.9 (24.6 to 40.6)	1.5 (-4.8 to 7.4)
**Digestive diseases**	**8,051.6 (7,750.4 to 8,430.2)**	**6,402.0† (5,730.4 to 7,066.2)**	**218.7 (153.1 to 293.4)**	**37.9 (34.0 to 42.3)**	**5.3 (2.3 to 8.7)**
Peptic ulcer disease	2,193.7 (1,830.7 to 2,630.6)	235.6 (195.2 to 277.4)	44.7 (27.4 to 69.4)	41.7 (33.5 to 49.6)	8.2 (1.9 to 14.2)
Gastritis and duodenitis	4,876.5 (3,794.6 to 6,288.0)	2,205.2 (1,808.0 to 2,599.9)	67.3 (44.0 to 98.2)	45.6 (39.8 to 51.5)	11.2 (6.8 to 15.7)
Appendicitis	11.6 (10.8 to 12.7)	317.0 (294.4 to 344.2)	3.2 (2.2 to 4.2)	32.8 (24.4 to 41.6)	1.4 (-5.0 to 8.1)
Paralytic ileus and intestinal obstruction	7.2 (5.6 to 9.0)	209.7 (165.9 to 264.2)	2.1 (1.3 to 3.0)	34.5 (21.4 to 47.5)	2.7 (-7.3 to 12.6)
Inguinal, femoral, and abdominal hernia	984.3 (714.0 to 1,339.5)	1,460.7 (1,078.0 to 1,849.6)	50.3 (31.8 to 73.0)	31.6 (24.4 to 39.0)	0.5 (-5.0 to 6.1)
Inflammatory bowel disease	123.8 (105.8 to 141.3)	26.3 (21.1 to 32.5)	16.2 (10.9 to 22.2)	21.9 (16.1 to 27.2)	-7.0 (-11.3 to -2.9)
Vascular intestinal disorders	9.7 (7.8 to 11.9)	54.5 (43.3 to 68.2)	2.7 (1.7 to 3.9)	23.8 (7.3 to 41.6)	-5.5 (-18.1 to 8.2)
Gallbladder and biliary diseases	435.0 (340.5 to 556.5)	1,694.1 (1,345.2 to 2,094.8)	6.7 (4.4 to 9.6)	38.0 (26.0 to 51.4)	5.3 (-3.8 to 15.6)
Pancreatitis	22.4 (18.7 to 26.7)	198.9 (165.8 to 237.8)	2.6 (1.7 to 3.7)	43.7 (28.9 to 60.0)	9.7 (-1.6 to 22.2)
Other digestive diseases	--	--	22.9 (16.1 to 30.8)	39.3 (35.3 to 43.7)	6.4 (3.3 to 9.7)
**Neurological disorders**	**22,451.5 (21,258.5 to 23,455.2)**	**6,684.4 (4,945.9 to 8,639.6)**	**1,914.6 (1,309.3 to 2,621.6)**	**31.3 (29.3 to 33.1)**	**0.2 (-1.2 to 1.6)**
Alzheimer's disease and other dementias	8,861.2 (6,956.6 to 11,400.3)	1,550.2 (1,057.4 to 2,139.4)	1,442.9 (962.1 to 2,029.2)	32.0 (29.9 to 33.9)	0.8 (-0.8 to 2.3)
Parkinson's disease	899.5 (682.9 to 1,168.2)	88.5 (60.3 to 125.2)	100.2 (63.1 to 147.8)	31.9 (27.4 to 35.6)	0.7 (-2.7 to 3.6)
Epilepsy	385.8 (311.8 to 464.1)	21.4 (15.5 to 28.5)	96.3 (66.7 to 132.5)	27.5 (10.2 to 48.7)	-2.7 (-15.9 to 13.6)
Multiple sclerosis	46.0 (41.4 to 50.7)	0.4 (0.3 to 0.5)	11.1 (8.0 to 14.5)	28.8 (19.3 to 39.0)	-1.6 (-8.9 to 6.1)
Motor neuron disease	12.5 (9.9 to 15.3)	3.1 (2.4 to 3.8)	2.6 (1.8 to 3.7)	31.0 (25.6 to 36.0)	0.0 (-4.1 to 3.8)
Migraine	4,244.7 (3,893.3 to 4,613.5)	276.8 (230.3 to 333.4)	200.6 (134.3 to 276.2)	28.3 (26.2 to 30.6)	-2.0 (-3.6 to -0.3)
Tension-type headache	11,262.7 (8,064.9 to 15,154.6)	4,743.0 (3,077.9 to 6,594.8)	49.7 (30.9 to 75.5)	28.6 (25.7 to 31.5)	-1.8 (-4.1 to 0.4)
Other neurological disorders	0.6 (0.3 to 1.0)	1.1 (0.6 to 1.7)	11.2 (7.6 to 15.6)	34.8 (32.5 to 36.8)	2.9 (1.2 to 4.5)
**Mental and substance use disorders**	**10,618.1 (10,384.4 to 10,824.5)**	**5,063.6† (4,284.7 to 5,920.4)**	**1,367.8 (1,006.1 to 1,763.2)**	**28.7 (27.5 to 30.0)**	**-1.7 (-2.7 to -0.7)**
Schizophrenia	97.9 (83.0 to 113.3)	--	54.8 (41.0 to 67.9)	37.2 (31.1 to 43.7)	4.8 (0.1 to 9.7)
Alcohol use disorders	395.8 (312.0 to 494.0)	133.3 (101.0 to 172.5)	35.4 (23.8 to 51.0)	36.2 (29.6 to 42.0)	4.0 (-1.0 to 8.4)
Drug use disorders	282.1 (272.2 to 291.6)	11.1 (9.8 to 12.6)	76.9 (54.6 to 97.6)	33.5 (28.9 to 37.9)	1.9 (-1.6 to 5.3)
Opioid use disorders	168.7 (147.3 to 193.9)	--	61.0 (42.7 to 78.0)	34.8 (29.1 to 40.3)	3.0 (-1.5 to 7.2)
Cocaine use disorders	46.9 (41.2 to 53.0)	--	5.8 (3.6 to 8.2)	24.3 (15.2 to 33.7)	-5.1 (-12.1 to 2.1)
Amphetamine use disorders	6.0 (3.8 to 9.2)	1.7 (1.1 to 2.6)	0.8 (0.4 to 1.5)	26.8 (19.9 to 31.6)	-3.2 (-8.5 to 0.5)
Cannabis use disorders	37.7 (31.2 to 45.0)	--	1.1 (0.7 to 1.6)	27.8 (21.4 to 34.4)	-2.5 (-7.3 to 2.6)
Other drug use disorders	39.6 (34.9 to 45.6)	9.4 (8.2 to 10.7)	8.2 (5.7 to 11.2)	31.9 (25.1 to 39.0)	0.7 (-4.5 to 6.1)
Depressive disorders	4,576.2 (4,385.4 to 4,773.2)	4,613.4 (3,838.9 to 5,487.2)	688.2 (469.1 to 950.3)	27.0 (24.9 to 28.9)	-3.0 (-4.7 to -1.6)
Major depressive disorder	2,903.0 (2,401.2 to 3,457.8)	4,426.7 (3,667.1 to 5,292.3)	529.6 (350.9 to 742.2)	25.9 (23.4 to 28.3)	-3.9 (-5.8 to -2.1)
Dysthymia	1,804.2 (1,439.5 to 2,249.6)	186.7 (143.1 to 240.7)	158.7 (102.5 to 232.8)	30.8 (26.9 to 34.6)	-0.2 (-3.1 to 2.8)
Bipolar disorder	331.8 (270.9 to 402.2)	21.4 (14.6 to 29.2)	59.8 (37.4 to 88.1)	28.2 (24.5 to 32.2)	-2.1 (-4.9 to 1.0)
Anxiety disorders	2,901.7 (2,585.7 to 3,264.7)	263.2 (232.1 to 296.1)	248.6 (176.5 to 334.0)	26.6 (23.8 to 29.3)	-3.4 (-5.4 to -1.3)
Eating disorders	--	--	--	--	--
Anorexia nervosa	--	--	--	--	--
Bulimia nervosa	--	--	--	--	--
Autistic spectrum disorders	408.2 (392.2 to 422.8)	--	56.4 (39.1 to 77.7)	35.3 (32.9 to 37.9)	3.3 (1.4 to 5.3)
Autism	142.3 (122.0 to 166.5)	--	32.2 (21.1 to 46.0)	33.7 (30.1 to 37.7)	2.1 (-0.7 to 5.2)
Asperger syndrome and other autistic spectrum disorders	268.7 (223.3 to 321.7)	--	24.2 (16.2 to 34.6)	37.5 (34.7 to 40.4)	5.0 (2.8 to 7.2)

Cause	Prevalence in thousands (95% UI)	Incidence in thousands (95% UI)	YLDs in thousands (95% UI)		
	2016 counts	2016 counts	2016 counts	Percentage change in counts between 2006 and 2016	Percentage change in rates between 2006 and 2016
Attention-deficit/hyperactivity disorder	1.7 (1.0 to 2.5)	--	< 0.05 (< 0.05 to < 0.05)	38.4 (35.7 to 41.4)	5.7 (3.6 to 8.0)
Conduct disorder	--	--	--	--	--
Idiopathic developmental intellectual disability	354.8 (196.5 to 523.4)	--	16.3 (8.5 to 26.2)	24.9 (19.2 to 28.9)	-4.6 (-9.0 to -1.5)
Other mental and substance use disorders	1,960.6 (1,740.8 to 2,217.5)	21.2 (13.6 to 31.4)	131.2 (93.0 to 181.5)	32.1 (30.1 to 34.1)	0.9 (-0.7 to 2.4)
**Diabetes, urogenital, blood, and endocrine diseases**	**45,644.3 (45,204.9 to 46,174.8)**	**8,901.2† (7,947.4 to 9,980.5)**	**2,005.7 (1,444.4 to 2,654.4)**	**31.8 (29.7 to 34.0)**	**0.6 (-1.0 to 2.3)**
Diabetes mellitus	12,189.4 (11,072.7 to 13,425.0)	197.2 (130.3 to 275.4)	968.2 (679.8 to 1,315.7)	30.2 (26.5 to 34.1)	-0.6 (-3.4 to 2.4)
Acute glomerulonephritis	2.6 (1.9 to 3.3)	38.1 (28.6 to 48.3)	0.1 (0.1 to 0.2)	27.6 (17.8 to 37.5)	-2.6 (-10.0 to 5.0)
Chronic kidney disease	21,702.0 (18,691.4 to 24,369.5)	1,139.5 (797.9 to 1,630.3)	564.9 (415.7 to 741.3)	33.5 (29.2 to 37.5)	1.9 (-1.4 to 5.0)
Chronic kidney disease due to diabetes mellitus	7,630.7 (5,696.1 to 9,587.8)	406.2 (253.3 to 608.0)	200.9 (136.6 to 279.5)	38.5 (33.5 to 43.9)	5.8 (1.9 to 9.9)
Chronic kidney disease due to hypertension	4,781.0 (3,743.3 to 5,827.9)	154.2 (94.4 to 240.9)	171.6 (116.5 to 236.3)	35.5 (30.3 to 40.8)	3.5 (-0.5 to 7.5)
Chronic kidney disease due to glomerulonephritis	4,534.2 (3,572.5 to 5,535.8)	327.4 (210.4 to 501.9)	71.4 (44.6 to 106.7)	16.4 (7.1 to 24.9)	-11.2 (-18.2 to -4.7)
Chronic kidney disease due to other causes	4,756.0 (3,455.1 to 6,407.1)	251.6 (157.1 to 380.8)	121.1 (77.8 to 176.2)	34.3 (27.8 to 40.9)	2.6 (-2.4 to 7.6)
Urinary diseases and male infertility	6,746.7 (6,097.3 to 7,889.4)	6,553.7† (5,774.9 to 7,506.7)	210.3 (132.3 to 312.5)	39.3 (38.0 to 40.7)	6.4 (5.4 to 7.4)
Interstitial nephritis and urinary tract infections	79.1 (65.1 to 95.3)	4,170.5 (3,498.7 to 4,959.0)	2.4 (1.5 to 3.6)	37.9 (30.8 to 45.0)	5.3 (-0.1 to 10.7)
Urolithiasis	68.3 (52.9 to 88.3)	1,772.3 (1,355.7 to 2,266.0)	4.6 (3.0 to 6.8)	38.0 (27.1 to 50.0)	5.4 (-3.0 to 14.5)
Benign prostatic hyperplasia	6,458.8 (5,311.3 to 7,811.0)	610.9 (421.9 to 847.1)	201.9 (125.6 to 303.2)	39.5 (38.1 to 40.9)	6.5 (5.5 to 7.6)
Male infertility	--	--	--	--	--
Other urinary diseases	--	--	1.4 (1.0 to 2.0)	27.1 (20.3 to 34.4)	-2.9 (-8.2 to 2.6)
Gynecological diseases	8,828.9 (8,291.7 to 9,705.1)	4.2† (2.5 to 6.3)	27.1 (13.3 to 47.7)	17.5 (15.8 to 19.2)	-10.3 (-11.6 to -9.0)
Uterine fibroids	287.4 (252.3 to 329.1)	4.2 (2.5 to 6.3)	2.0 (1.0 to 3.6)	25.1 (21.5 to 28.9)	-4.5 (-7.2 to -1.6)
Polycystic ovarian syndrome	--	--	--	--	--
Female infertility	--	--	--	--	--
Endometriosis	--	--	--	--	--
Genital prolapse	8,517.7 (7,462.2 to 9,652.4)	--	25.1 (12.2 to 45.0)	16.9 (15.1 to 18.8)	-10.7 (-12.1 to -9.3)
Premenstrual syndrome	--	--	--	--	--
Other gynecological diseases	--	--	--	--	--
Hemoglobinopathies and hemolytic anaemias	15,125.9 (14,983.4 to 15,277.5)	--	72.9 (49.8 to 102.5)	30.6 (27.5 to 33.5)	-0.3 (-2.6 to 1.9)
Thalassemias	< 0.05 (< 0.05 to < 0.05)	--	< 0.05 (< 0.05 to < 0.05)	18.6 (11.5 to 24.5)	-9.4 (-14.9 to -4.9)
Thalassaemias trait	2,804.4 (2,682.0 to 2,939.0)	--	40.9 (27.7 to 58.5)	38.6 (34.3 to 42.9)	5.9 (2.6 to 9.1)
Sickle cell disorders	0.1 (0.1 to 0.1)	--	< 0.05 (< 0.05 to < 0.05)	17.2 (14.6 to 19.9)	-10.5 (-12.5 to -8.5)
Sickle cell trait	2,549.3 (2,321.0 to 2,864.1)	--	6.8 (4.6 to 9.9)	41.9 (37.4 to 46.8)	8.4 (4.9 to 12.1)
G6PD deficiency	2,064.1 (1,985.9 to 2,152.1)	--	0.3 (0.2 to 0.4)	31.4 (28.1 to 34.8)	0.3 (-2.2 to 2.9)
G6PD trait	8,293.7 (8,141.5 to 8,470.9)	--	< 0.05 (< 0.05 to < 0.05)	28.0 (22.4 to 33.1)	-2.3 (-6.5 to 1.7)
Other hemoglobinopathies and hemolytic anaemias	892.9 (864.5 to 923.9)	--	24.9 (17.3 to 34.7)	16.8 (13.9 to 19.7)	-10.8 (-13.1 to -8.6)
Endocrine, metabolic, blood, and immune disorders	5,025.8 (4,093.0 to 6,277.1)	968.5 (691.3 to 1,290.4)	162.2 (107.8 to 231.2)	29.8 (24.0 to 35.0)	-0.9 (-5.3 to 3.1)
**Musculoskeletal disorders**	**32,479.5 (31,559.8 to 33,436.3)**	**13,033.9 (10,042.4 to 16,664.6)**	**3,252.4 (2,333.9 to 4,238.8)**	**27.3 (25.6 to 28.9)**	**-2.8 (-4.1 to -1.6)**
Rheumatoid arthritis	762.0 (678.4 to 848.8)	11.5 (7.5 to 16.1)	163.8 (114.8 to 210.8)	31.2 (27.9 to 34.4)	0.2 (-2.3 to 2.6)
Osteoarthritis	16,045.2 (15,129.6 to 16,988.1)	156.3 (120.3 to 193.5)	759.0 (533.1 to 1,031.3)	32.2 (31.0 to 33.5)	0.9 (0.0 to 1.9)
Low back and neck pain	18,455.7 (17,278.4 to 20,011.6)	8,554.5 (6,252.4 to 11,708.4)	2,032.3 (1,402.9 to 2,768.5)	27.1 (24.7 to 29.1)	-2.9 (-4.8 to -1.4)
Low back pain	14,056.0 (10,809.9 to 18,206.9)	7,195.0 (4,927.8 to 10,191.3)	1,444.5 (938.9 to 2,048.5)	26.2 (22.9 to 28.7)	-3.6 (-6.2 to -1.7)
Neck pain	6,409.0 (4,648.3 to 8,312.4)	1,359.5 (859.4 to 1,995.0)	587.7 (371.9 to 871.4)	29.5 (26.8 to 32.0)	-1.1 (-3.2 to 0.8)
Gout	1,649.9 (1,307.4 to 2,054.4)	257.6 (185.6 to 338.6)	47.7 (31.9 to 66.3)	33.1 (29.0 to 37.5)	1.6 (-1.5 to 5.0)
Other musculoskeletal disorders	3,248.8 (2,002.2 to 4,988.1)	4,054.0 (2,426.8 to 6,179.2)	249.7 (138.5 to 406.0)	13.2 (-0.1 to 24.8)	-13.6 (-23.8 to -4.7)
**Other non-communicable diseases**	**70,444.8 (70,332.8 to 70,540.8)**	**258,806.0† (232,389.8 to 288,886.4)**	**5,414.2 (3,924.7 to 7,294.8)**	**30.7 (28.6 to 32.6)**	**-0.2 (-1.8 to 1.2)**
Congenital anomalies	604.3 (569.4 to 639.2)	--	64.5 (47.1 to 84.6)	27.0 (23.9 to 30.1)	-3.0 (-5.4 to -0.7)
Neural tube defects	43.1 (39.3 to 47.6)	--	12.1 (8.7 to 16.0)	29.4 (23.4 to 35.7)	-1.2 (-5.8 to 3.6)
Congenital heart anomalies	109.8 (98.7 to 122.1)	--	5.9 (2.4 to 10.2)	34.7 (29.3 to 40.4)	2.8 (-1.3 to 7.2)
Orofacial clefts	37.1 (33.1 to 41.2)	--	0.4 (0.3 to 0.6)	26.2 (22.4 to 29.6)	-3.6 (-6.5 to -1.0)
Down's syndrome	--	--	--	--	--
Turner syndrome	3.7 (3.1 to 4.5)	--	0.1 (< 0.05 to 0.1)	28.0 (25.4 to 31.0)	-2.2 (-4.3 to -0.0)
Klinefelter syndrome	0.5 (0.4 to 0.7)	--	< 0.05 (< 0.05 to < 0.05)	43.2 (39.0 to 47.5)	9.4 (6.1 to 12.6)

Cause	Prevalence in thousands (95% UI)	Incidence in thousands (95% UI)	YLDs in thousands (95% UI)		
	2016 counts	2016 counts	2016 counts	Percentage change in counts between 2006 and 2016	Percentage change in rates between 2006 and 2016
Other chromosomal abnormalities	< 0.05 (< 0.05 to < 0.05)	--	< 0.05 (< 0.05 to < 0.05)	41.4 (39.5 to 43.0)	7.9 (6.5 to 9.2)
Congenital musculoskeletal and limb anomalies	137.9 (127.1 to 148.6)	--	18.8 (13.2 to 25.2)	23.7 (18.5 to 29.2)	-5.5 (-9.5 to -1.4)
Urogenital congenital anomalies	32.3 (29.1 to 35.7)	--	0.5 (0.2 to 0.9)	31.7 (27.0 to 37.0)	0.6 (-3.1 to 4.6)
Digestive congenital anomalies	106.9 (96.7 to 117.8)	--	4.3 (2.9 to 6.1)	28.4 (21.8 to 35.1)	-1.9 (-7.0 to 3.1)
Other congenital anomalies	264.6 (182.6 to 371.9)	--	22.5 (14.4 to 34.4)	26.3 (20.5 to 32.6)	-3.6 (-8.0 to 1.3)
Skin and subcutaneous diseases	38,574.2 (37,487.3 to 39,502.4)	152,871.4 (130,383.1 to 180,112.2)	532.0 (343.3 to 830.3)	31.1 (29.9 to 32.0)	0.1 (-0.8 to 0.8)
Dermatitis	3,677.3 (3,149.2 to 4,269.5)	4,877.0 (3,573.7 to 6,463.2)	107.1 (66.0 to 163.1)	27.4 (25.6 to 29.1)	-2.7 (-4.1 to -1.5)
Psoriasis	1,066.9 (985.6 to 1,150.7)	78.4 (65.7 to 92.9)	83.1 (59.9 to 107.2)	32.1 (29.1 to 35.3)	0.9 (-1.4 to 3.3)
Cellulitis	74.4 (61.5 to 90.1)	1,809.8 (1,525.3 to 2,143.2)	3.8 (2.5 to 5.6)	24.1 (17.5 to 31.1)	-5.3 (-10.3 to 0.1)
Pyoderma	692.2 (631.8 to 754.7)	18,441.7 (16,697.0 to 20,213.1)	3.6 (1.5 to 7.6)	32.9 (30.3 to 35.4)	1.4 (-0.5 to 3.4)
Scabies	942.6 (633.9 to 1,341.9)	2,940.2 (1,968.4 to 4,245.0)	22.2 (11.6 to 37.6)	32.9 (30.4 to 35.2)	1.5 (-0.5 to 3.2)
Fungal skin diseases	21,454.3 (17,296.4 to 26,187.9)	95,035.7 (72,471.6 to 121,753.9)	112.2 (44.0 to 243.6)	32.7 (32.1 to 33.2)	1.3 (0.8 to 1.7)
Viral skin diseases	1,059.6 (871.5 to 1,301.7)	1,612.1 (1,332.0 to 1,956.9)	29.6 (18.5 to 45.1)	28.3 (25.9 to 30.8)	-2.0 (-3.8 to -0.2)
Acne vulgaris	436.7 (424.9 to 449.1)	180.8 (174.1 to 188.6)	10.1 (6.9 to 14.3)	31.8 (25.9 to 37.6)	0.6 (-3.9 to 5.0)
Alopecia areata	81.1 (71.7 to 91.2)	138.1 (124.3 to 152.3)	2.4 (1.5 to 3.6)	22.8 (16.1 to 29.6)	-6.2 (-11.3 to -1.0)
Pruritus	1,417.2 (1,011.1 to 1,947.5)	1,081.6 (728.1 to 1,494.0)	14.0 (6.2 to 26.8)	32.5 (30.4 to 34.6)	1.2 (-0.4 to 2.8)
Urticaria	344.4 (237.9 to 469.7)	597.4 (403.3 to 802.7)	18.6 (10.9 to 29.2)	29.8 (25.8 to 33.5)	-0.9 (-3.9 to 2.0)
Decubitus ulcer	218.5 (164.9 to 287.6)	806.3 (596.9 to 1,072.0)	31.7 (20.5 to 46.4)	28.6 (20.2 to 36.4)	-1.8 (-8.2 to 4.2)
Other skin and subcutaneous diseases	18,210.9 (16,730.1 to 19,706.2)	25,272.2 (22,589.5 to 28,084.3)	93.5 (44.7 to 171.8)	34.4 (33.6 to 35.2)	2.6 (2.0 to 3.3)
Sense organ diseases	64,060.5 (63,282.8 to 64,780.2)	17,417.4† (15,852.8 to 19,051.6)	4,126.5 (2,985.9 to 5,481.8)	30.9 (28.1 to 33.4)	-0.1 (-2.2 to 1.9)
Glaucoma	458.5 (367.8 to 564.2)	--	43.3 (29.2 to 61.8)	35.4 (32.1 to 38.5)	3.4 (0.9 to 5.7)
Cataract	9,384.7 (7,594.9 to 11,391.8)	--	637.0 (450.3 to 886.2)	33.3 (31.4 to 35.1)	1.8 (0.3 to 3.2)
Macular degeneration	597.6 (464.7 to 768.4)	--	41.9 (28.1 to 60.0)	33.7 (29.9 to 37.4)	2.1 (-0.8 to 4.9)
Refraction and accommodation disorders	31,227.6 (28,991.8 to 33,762.2)	--	505.7 (318.9 to 781.8)	27.4 (25.3 to 29.0)	-2.7 (-4.3 to -1.5)
Age-related and other hearing loss	63,451.7 (62,326.1 to 64,381.4)	--	2,491.2 (1,783.3 to 3,312.2)	30.7 (26.3 to 34.8)	-0.2 (-3.6 to 2.9)
Other vision loss	1,672.6 (1,313.1 to 2,086.0)	--	123.0 (83.9 to 166.8)	33.0 (30.4 to 35.5)	1.5 (-0.4 to 3.5)
Other sense organ diseases	9,517.6 (8,317.6 to 10,780.5)	17,417.4 (15,852.8 to 19,051.6)	284.3 (178.8 to 423.0)	31.7 (30.4 to 33.0)	0.5 (-0.5 to 1.6)
Oral disorders	45,584.6 (44,512.3 to 46,627.9)	88,517.2† (74,678.0 to 102,543.1)	691.3 (444.1 to 989.7)	29.6 (28.5 to 30.5)	-1.0 (-1.9 to -0.4)
Caries of deciduous teeth	--	--	--	--	--
Caries of permanent teeth	26,424.4 (22,734.1 to 30,575.5)	86,171.7 (71,930.7 to 99,886.0)	19.2 (8.8 to 37.8)	24.2 (22.4 to 26.0)	-5.2 (-6.5 to -3.8)
Periodontal disease	14,494.4 (10,646.3 to 17,945.9)	1,760.8 (949.5 to 2,708.2)	88.6 (33.4 to 191.7)	34.2 (33.2 to 35.4)	2.5 (1.7 to 3.3)
Edentulism and severe tooth loss	20,761.9 (17,568.8 to 24,033.9)	584.7 (364.7 to 895.6)	547.0 (361.8 to 776.3)	29.1 (27.8 to 30.2)	-1.4 (-2.4 to -0.6)
Other oral disorders	1,349.4 (1,182.1 to 1,503.7)	--	36.4 (22.9 to 54.7)	30.1 (28.1 to 32.1)	-0.7 (-2.2 to 0.9)
**Injuries**	**46,464.8 (43,981.2 to 49,203.6)**	**13,097.6† (11,526.5 to 15,007.9)**	**1,715.3 (1,197.2 to 2,325.8)**	**33.4 (32.1 to 34.4)**	**1.9 (0.8 to 2.6)**
**Transport injuries**	**7,146.5 (6,678.2 to 7,663.8)**	**874.3 (728.5 to 1,034.1)**	**312.2 (212.8 to 441.5)**	**35.7 (34.8 to 36.7)**	**3.6 (2.9 to 4.4)**
Road injuries	5,973.7 (5,569.1 to 6,472.1)	704.1 (571.2 to 857.0)	261.6 (177.5 to 371.6)	36.6 (35.6 to 37.6)	4.3 (3.5 to 5.1)
Pedestrian road injuries	1,473.5 (1,273.2 to 1,734.0)	163.3 (117.9 to 216.7)	68.2 (46.6 to 97.0)	37.8 (36.5 to 39.1)	5.2 (4.2 to 6.2)
Cyclist road injuries	1,274.2 (1,070.8 to 1,482.4)	189.0 (130.4 to 251.0)	53.4 (35.9 to 76.2)	41.9 (40.8 to 43.3)	8.4 (7.5 to 9.4)
Motorcyclist road injuries	1,122.3 (964.3 to 1,295.1)	59.1 (37.8 to 83.5)	47.2 (32.3 to 66.9)	50.1 (48.9 to 51.2)	14.6 (13.7 to 15.4)
Motor vehicle road injuries	1,702.3 (1,416.8 to 1,963.2)	239.8 (176.6 to 307.5)	76.4 (52.2 to 107.9)	21.4 (20.0 to 22.8)	-7.3 (-8.4 to -6.2)
Other road injuries	401.4 (349.9 to 470.0)	52.8 (33.4 to 80.3)	16.3 (11.0 to 23.4)	63.2 (61.8 to 64.7)	24.6 (23.5 to 25.7)
Other transport injuries	1,172.7 (989.3 to 1,353.0)	170.2 (128.1 to 215.1)	50.6 (34.6 to 70.6)	31.6 (29.9 to 33.0)	0.5 (-0.8 to 1.6)
**Unintentional injuries**	**37,255.6 (34,912.7 to 39,849.7)**	**11,919.2 (10,310.8 to 13,792.1)**	**1,337.3 (946.9 to 1,802.6)**	**33.0 (31.5 to 34.2)**	**1.6 (0.4 to 2.5)**
Falls	20,781.7 (18,606.4 to 23,294.5)	7,487.5 (5,999.9 to 9,247.4)	910.9 (651.3 to 1,236.3)	33.5 (31.6 to 35.3)	2.0 (0.4 to 3.3)
Drowning	188.4 (164.8 to 222.7)	38.6 (32.5 to 44.6)	8.6 (5.8 to 12.2)	16.9 (15.0 to 18.5)	-10.8 (-12.2 to -9.5)
Fire, heat, and hot substances	2,449.4 (2,159.7 to 2,784.4)	93.0 (71.8 to 119.7)	42.5 (25.2 to 67.3)	26.7 (25.4 to 27.9)	-3.3 (-4.2 to -2.3)
Poisonings	77.6 (67.4 to 88.3)	31.4 (20.9 to 43.8)	4.5 (3.1 to 6.4)	36.9 (32.6 to 41.3)	4.5 (1.3 to 7.9)
Exposure to mechanical forces	5,213.6 (4,528.4 to 6,121.9)	416.5 (322.8 to 529.8)	103.1 (68.5 to 149.8)	36.8 (35.8 to 37.5)	4.4 (3.7 to 5.0)
Unintentional firearm injuries	174.1 (145.7 to 206.3)	5.2 (3.1 to 8.4)	3.8 (2.5 to 5.6)	39.0 (37.8 to 40.1)	6.1 (5.2 to 7.0)
Unintentional suffocation	165.9 (137.0 to 195.4)	19.0 (12.6 to 28.4)	4.6 (3.0 to 6.6)	34.7 (33.2 to 36.1)	2.9 (1.7 to 3.9)
Other exposure to mechanical forces	4,873.6 (4,204.8 to 5,757.1)	392.4 (303.0 to 497.5)	94.8 (63.1 to 137.1)	36.8 (35.8 to 37.6)	4.4 (3.7 to 5.0)

Cause	Prevalence in thousands (95% UI)	Incidence in thousands (95% UI)	YLDs in thousands (95% UI)		
	2016 counts	2016 counts	2016 counts	Percentage change in counts between 2006 and 2016	Percentage change in rates between 2006 and 2016
Adverse effects of medical treatment	113.0 (83.0 to 149.4)	1,478.4 (1,160.1 to 1,805.4)	15.1 (9.2 to 23.8)	19.0 (12.8 to 24.6)	-9.2 (-13.8 to -4.8)
Animal contact	1,194.2 (1,083.0 to 1,331.8)	443.3 (321.7 to 576.9)	24.1 (16.7 to 33.3)	24.9 (23.2 to 26.2)	-4.7 (-5.9 to -3.6)
Venomous animal contact	471.3 (407.8 to 559.2)	207.6 (146.5 to 278.9)	13.6 (9.6 to 18.6)	28.5 (25.9 to 31.0)	-1.9 (-3.9 to 0.0)
Non-venomous animal contact	722.8 (633.3 to 817.4)	235.7 (165.2 to 327.3)	10.5 (6.9 to 15.7)	20.4 (18.8 to 21.6)	-8.0 (-9.3 to -7.2)
Foreign body	1,281.5 (1,127.5 to 1,436.2)	692.3 (566.2 to 842.0)	42.3 (28.0 to 61.2)	31.0 (29.6 to 32.3)	0.0 (-1.1 to 1.0)
Pulmonary aspiration and foreign body in airway	612.3 (496.5 to 742.4)	396.1 (316.5 to 486.1)	22.2 (14.6 to 32.0)	29.9 (28.2 to 31.4)	-0.8 (-2.1 to 0.4)
Foreign body in eyes	76.8 (57.5 to 103.9)	176.2 (90.7 to 304.0)	4.6 (2.9 to 7.0)	41.3 (38.5 to 44.8)	7.9 (5.8 to 10.6)
Foreign body in other body part	592.3 (494.7 to 720.1)	120.0 (84.3 to 162.1)	15.5 (10.3 to 22.6)	29.7 (28.2 to 31.0)	-1.0 (-2.1 to 0.0)
Environmental heat and cold exposure	1,750.8 (1,525.4 to 2,019.2)	268.9 (199.2 to 359.5)	63.6 (43.2 to 89.2)	32.2 (30.3 to 34.1)	1.0 (-0.5 to 2.4)
Other unintentional injuries	4,205.3 (3,638.0 to 4,857.0)	969.4 (660.1 to 1,389.7)	122.6 (82.7 to 174.0)	34.5 (33.5 to 35.2)	2.7 (2.0 to 3.3)
**Self-harm and interpersonal violence**	**2,848.0 (2,593.7 to 3,134.3)**	**296.2† (248.5 to 357.8)**	**56.1 (38.0 to 79.3)**	**23.2 (22.4 to 24.0)**	**-5.9 (-6.6 to -5.4)**
Self-harm	460.9 (390.6 to 549.8)	201.2 (161.7 to 246.2)	15.8 (10.5 to 22.5)	14.8 (13.2 to 16.3)	-12.3 (-13.5 to -11.2)
Self-harm by firearm	9.5 (8.0 to 11.6)	1.8 (0.6 to 4.4)	0.2 (0.2 to 0.4)	15.5 (13.8 to 17.2)	-11.8 (-13.1 to -10.5)
Self-harm by other specified means	451.4 (380.1 to 540.5)	199.5 (160.5 to 244.0)	15.5 (10.4 to 22.1)	14.8 (13.2 to 16.3)	-12.3 (-13.6 to -11.2)
Interpersonal violence	2,387.1 (2,143.1 to 2,669.8)	95.0† (66.6 to 131.4)	40.3 (27.3 to 57.3)	26.8 (26.1 to 27.5)	-3.2 (-3.7 to -2.6)
Assault by firearm	74.5 (63.3 to 85.3)	5.2 (2.6 to 9.0)	2.0 (1.3 to 2.8)	29.6 (28.2 to 31.2)	-1.0 (-2.1 to 0.1)
Assault by sharp object	292.7 (246.0 to 344.4)	19.3 (11.0 to 31.3)	5.9 (3.9 to 8.7)	26.1 (25.1 to 27.0)	-3.7 (-4.5 to -3.1)
Sexual violence	1,118.2 (931.3 to 1,341.1)	--	6.5 (4.2 to 9.7)	26.0 (24.7 to 27.3)	-3.8 (-4.8 to -2.8)
Assault by other means	901.7 (770.5 to 1,071.7)	70.4 (48.7 to 100.2)	25.9 (17.3 to 36.9)	27.0 (26.1 to 27.8)	-3.1 (-3.7 to -2.4)
**Forces of nature, conflict and terrorism, and executions and police conflict**	**333.0 (224.1 to 474.0)**	**8.0† (3.9 to 12.9)**	**9.7 (2.5 to 24.6)**	**89.8 (77.6 to 108.1)**	**44.9 (35.6 to 58.9)**
Forces of nature, conflict and terrorism, and state actor violence	91.2 (50.9 to 140.5)	2.7 (1.4 to 4.1)	3.1 (0.7 to 8.1)	96.7 (80.6 to 118.3)	50.2 (37.9 to 66.7)
Conflict and terrorism	199.4 (114.2 to 320.7)	--	6.0 (1.7 to 14.6)	71.5 (58.0 to 99.1)	31.0 (20.6 to 52.0)
Executions and police conflict	42.4 (13.4 to 69.4)	5.3 (1.5 to 10.1)	1.1 (0.2 to 3.1)	153.1 (96.2 to 188.3)	93.2 (49.8 to 120.2)

We did not calculate incidence for certain NTDs and causes with only birth prevalence. Blank cells mean that no estimate is available.

† Incidence for aggregate causes represents the sum of the incidence of the causes below the aggregate.

‡ Incidence estimates for stroke represent first-ever stroke only.

^ Incidence of HIV/AIDS represents new infections of HIV only and does not include new infections of Tuberculosis in HIV positive cases.

SR Table 12: Global prevalence, incidence, and YLDs for 2016, percentage change of YLD counts and percentage change of YLD rates between 2006 and 2016 by cause, age 85-89 years.					
Cause	Prevalence in thousands (95% UI)	Incidence in thousands (95% UI)	YLDs in thousands (95% UI)		
	2016 counts	2016 counts	2016 counts	Percentage change in counts between 2006 and 2016	Percentage change in rates between 2006 and 2016
**All causes**	**39,828.8 (39,819.9 to 39,847.6)**	**322,939.7† (295,386.1 to 355,654.2)**	**12,477.8 (9,532.3 to 15,823.0)**	**59.4 (58.3 to 60.4)**	**-0.7 (-1.4 to -0.1)**
**Communicable, maternal, neonatal, and nutritional disorders**	**21,387.8 (21,130.4 to 21,584.9)**	**118,538.8† (99,386.4 to 141,032.8)**	**320.2 (228.9 to 416.7)**	**46.1 (42.8 to 52.7)**	**-9.0 (-11.0 to -4.9)**
**HIV/AIDS and tuberculosis**	**7,816.5 (7,344.6 to 8,314.2)**	**62.0† (53.7 to 71.8)**	**30.7 (21.6 to 41.5)**	**36.6 (28.8 to 43.6)**	**-14.9 (-19.8 to -10.5)**
Tuberculosis	7,582.8 (6,940.6 to 8,282.4)	62.0† (53.7 to 71.8)	27.0 (18.5 to 36.5)	34.8 (30.9 to 39.5)	-16.0 (-18.4 to -13.1)
Drug-susceptible tuberculosis	96.7 (83.4 to 111.7)	59.9 (51.8 to 69.4)	25.9 (17.8 to 35.0)	34.8 (30.5 to 39.3)	-16.0 (-18.7 to -13.2)
Multidrug-resistant tuberculosis without extensive drug resistance	3.6 (3.0 to 4.2)	2.0 (1.8 to 2.4)	1.0 (0.7 to 1.4)	28.0 (11.6 to 49.6)	-20.3 (-30.5 to -6.8)
Extensively drug-resistant tuberculosis	0.3 (0.2 to 0.3)	0.1 (0.1 to 0.2)	0.1 (0.1 to 0.1)	320.3 (268.6 to 379.8)	161.8 (129.7 to 199.0)
Latent tuberculosis infection	7,482.3 (6,839.2 to 8,177.2)	--	--	--	--
HIV/AIDS	36.4 (30.7 to 44.8)	--	3.7 (2.4 to 5.5)	51.0 (3.0 to 129.2)	-5.9 (-35.8 to 42.8)
Drug-susceptible HIV/AIDS - Tuberculosis	1.3 (0.9 to 1.8)	1.4 (1.0 to 1.9)	0.5 (0.3 to 0.7)	29.6 (17.8 to 40.2)	-19.2 (-26.6 to -12.7)
Multidrug-resistant HIV/AIDS - Tuberculosis without extensive drug resistance	< 0.05 (< 0.05 to 0.1)	< 0.05 (< 0.05 to 0.1)	< 0.05 (< 0.05 to < 0.05)	56.3 (12.4 to 100.7)	-2.6 (-30.0 to 25.0)
Extensively drug-resistant HIV/AIDS - Tuberculosis	< 0.05 (< 0.05 to < 0.05)	< 0.05 (< 0.05 to < 0.05)	< 0.05 (< 0.05 to < 0.05)	504.7 (286.3 to 741.9)	276.8 (140.7 to 424.6)
HIV/AIDS resulting in other diseases	35.1 (29.3 to 43.6)	--	3.2 (2.0 to 5.0)	54.6 (1.1 to 155.9)	-3.6 (-37.0 to 59.5)
**Diarrhoea, lower respiratory infections, and other common infectious diseases**	**2,053.9 (1,931.0 to 2,160.0)**	**114,272.9† (95,403.0 to 136,678.9)**	**107.2 (75.2 to 146.6)**	**58.9 (55.1 to 82.0)**	**-1.0 (-3.4 to 13.4)**
Diarrhoeal diseases	580.0 (530.5 to 630.5)	40,102.7 (36,739.5 to 43,714.2)	57.2 (40.3 to 76.7)	53.5 (50.6 to 56.5)	-4.3 (-6.2 to -2.5)
Intestinal infectious diseases	1.8 (1.5 to 2.2)	35.8† (31.8 to 39.6)	0.3 (0.2 to 0.4)	29.0 (3.8 to 59.4)	-19.6 (-35.3 to -0.7)
Typhoid fever	1.7 (1.4 to 2.1)	27.5 (24.4 to 30.5)	0.2 (0.2 to 0.4)	31.3 (3.7 to 65.2)	-18.2 (-35.4 to 2.9)
Paratyphoid fever	0.4 (0.3 to 0.4)	8.3 (7.3 to 9.4)	< 0.05 (< 0.05 to < 0.05)	27.5 (5.3 to 57.4)	-20.5 (-34.4 to -1.9)
Other intestinal infectious diseases	--	--	< 0.05 (< 0.05 to < 0.05)	12.2 (-9.7 to 38.6)	-30.1 (-43.7 to -13.6)
Lower respiratory infections	219.1 (201.5 to 238.8)	9,565.2 (8,637.6 to 10,545.5)	11.9 (8.2 to 16.3)	82.8 (76.3 to 89.2)	13.9 (9.8 to 17.9)
Upper respiratory infections	852.1 (598.2 to 1,153.5)	63,727.4 (44,688.1 to 86,675.7)	19.2 (10.5 to 31.4)	60.8 (57.3 to 64.2)	0.2 (-2.0 to 2.3)
Otitis media	228.7 (172.0 to 302.1)	243.4 (184.7 to 309.8)	5.8 (3.4 to 9.1)	49.2 (41.4 to 57.3)	-7.0 (-11.9 to -2.0)
Meningitis	56.1 (38.7 to 144.3)	10.0 (8.4 to 12.0)	3.4 (1.3 to 17.5)	113.2 (33.9 to 1,025.3)	32.9 (-16.6 to 601.1)
Pneumococcal meningitis	21.5 (16.2 to 26.7)	2.4 (1.7 to 3.3)	0.9 (0.5 to 1.3)	26.4 (21.1 to 34.7)	-21.2 (-24.5 to -16.1)
H influenzae type B meningitis	4.0 (2.7 to 5.5)	0.6 (0.3 to 1.0)	0.2 (0.1 to 0.3)	-9.4 (-18.8 to 11.9)	-43.6 (-49.4 to -30.3)
Meningococcal infection	12.6 (3.9 to 100.8)	1.9 (1.2 to 2.8)	1.5 (0.2 to 15.7)	642.8 (34.1 to 6,692.1)	362.8 (-16.5 to 4,131.9)
Other meningitis	18.0 (12.4 to 23.1)	5.1 (4.0 to 6.4)	0.8 (0.5 to 1.1)	78.1 (69.3 to 86.7)	11.0 (5.5 to 16.3)
Encephalitis	109.9 (56.8 to 183.7)	61.1 (53.8 to 69.7)	4.7 (3.2 to 6.6)	50.4 (42.6 to 59.3)	-6.3 (-11.2 to -0.7)
Diphtheria	--	--	--	--	--
Whooping cough	--	--	--	--	--
Tetanus	0.4 (0.4 to 0.4)	0.6 (0.2 to 0.8)	< 0.05 (< 0.05 to < 0.05)	1.5 (-21.0 to 31.4)	-36.8 (-50.8 to -18.1)
Measles	--	--	--	--	--
Varicella and herpes zoster	95.9 (75.0 to 123.1)	526.7 (414.5 to 661.1)	4.8 (2.8 to 7.7)	61.2 (53.3 to 68.9)	0.5 (-4.5 to 5.3)
**Neglected tropical diseases and malaria**	**5,705.6 (5,489.5 to 5,923.9)**	**2,560.3† (1,546.0 to 3,553.0)**	**51.8 (35.7 to 74.4)**	**25.1 (11.7 to 43.6)**	**-22.1 (-30.4 to -10.6)**
Malaria	58.4 (48.6 to 69.4)	126.8 (93.3 to 164.2)	0.6 (0.4 to 0.8)	-24.7 (-30.1 to -19.1)	-53.1 (-56.5 to -49.6)
Chagas disease	68.7 (58.2 to 80.9)	0.4 (0.2 to 0.6)	1.9 (1.2 to 3.0)	41.2 (33.2 to 49.9)	-12.0 (-17.0 to -6.6)
Leishmaniasis	21.7 (17.3 to 25.2)	5.1 (3.9 to 6.8)	1.1 (0.7 to 1.6)	32.9 (24.9 to 43.4)	-17.2 (-22.2 to -10.7)
Visceral leishmaniasis	0.6 (0.4 to 1.0)	2.4 (1.4 to 3.8)	< 0.05 (< 0.05 to 0.1)	-15.0 (-49.6 to 38.5)	-47.0 (-68.6 to -13.7)
Cutaneous and mucocutaneous leishmaniasis	18.1 (13.9 to 22.6)	2.7 (1.9 to 3.7)	1.0 (0.7 to 1.5)	35.7 (27.2 to 47.4)	-15.5 (-20.8 to -8.2)
African trypanosomiasis	< 0.05 (< 0.05 to < 0.05)	< 0.05 (< 0.05 to < 0.05)	< 0.05 (< 0.05 to < 0.05)	-70.3 (-96.0 to 125.2)	-81.5 (-97.5 to 40.3)
Schistosomiasis	434.1 (392.5 to 475.1)	94.9 (84.6 to 105.1)	3.0 (1.6 to 5.6)	-27.7 (-31.4 to -24.2)	-54.9 (-57.3 to -52.8)
Cysticercosis	24.0 (17.2 to 32.6)	--	3.3 (2.0 to 4.8)	29.5 (20.1 to 39.1)	-19.3 (-25.2 to -13.3)
Cystic echinococcosis	2.6 (1.4 to 5.3)	0.5 (0.2 to 1.3)	0.2 (0.1 to 0.5)	-19.9 (-70.1 to 108.8)	-50.1 (-81.4 to 30.1)
Lymphatic filariasis	58.3 (49.5 to 71.1)	15.1 (8.6 to 24.4)	1.9 (0.9 to 3.4)	-35.9 (-54.8 to -20.8)	-60.1 (-71.8 to -50.7)
Onchocerciasis	27.4 (18.4 to 41.0)	4.9 (2.0 to 9.8)	2.5 (1.5 to 4.5)	-8.0 (-26.7 to 19.3)	-42.6 (-54.3 to -25.6)
Trachoma	162.2 (110.6 to 225.4)	--	11.3 (7.4 to 16.5)	11.3 (0.6 to 22.7)	-30.7 (-37.3 to -23.5)
Dengue	23.3 (12.8 to 39.1)	388.9 (239.1 to 585.5)	3.3 (1.5 to 6.0)	148.6 (101.4 to 472.6)	54.9 (25.5 to 256.8)
Yellow fever	< 0.05 (< 0.05 to < 0.05)	0.1 (< 0.05 to 0.2)	< 0.05 (< 0.05 to < 0.05)	-17.2 (-50.0 to 32.4)	-48.4 (-68.8 to -17.5)
Rabies	< 0.05 (< 0.05 to < 0.05)	0.1 (< 0.05 to 0.2)	< 0.05 (< 0.05 to < 0.05)	27.8 (-42.3 to 103.5)	-20.4 (-64.0 to 26.8)
Intestinal nematode infections	4,693.7 (4,463.8 to 4,886.0)	--	7.9 (4.6 to 12.4)	9.6 (1.7 to 18.1)	-31.7 (-36.7 to -26.4)
Ascariasis	2,488.5 (2,198.1 to 2,849.9)	--	2.3 (1.2 to 3.8)	-8.8 (-20.2 to 3.5)	-43.2 (-50.3 to -35.5)

Cause	Prevalence in thousands (95% UI)	Incidence in thousands (95% UI)	YLDs in thousands (95% UI)		
	2016 counts	2016 counts	2016 counts	Percentage change in counts between 2006 and 2016	Percentage change in rates between 2006 and 2016
Trichuriasis	1,337.4 (1,218.1 to 1,472.5)	--	0.9 (0.5 to 1.5)	2.5 (-11.4 to 19.4)	-36.1 (-44.8 to -25.6)
Hookworm disease	1,357.3 (1,244.0 to 1,491.1)	--	4.7 (2.8 to 7.1)	23.8 (11.7 to 37.5)	-22.9 (-30.4 to -14.3)
Food-borne trematodiases	552.1 (375.7 to 720.1)	1,904.5 (877.6 to 2,890.4)	13.3 (4.0 to 28.5)	117.8 (77.9 to 181.5)	35.7 (10.9 to 75.4)
Leprosy	10.8 (10.5 to 11.2)	0.3 (0.3 to 0.4)	0.6 (0.4 to 0.8)	38.1 (31.9 to 45.0)	-14.0 (-17.8 to -9.7)
Ebola virus disease	< 0.05 (0.0 to < 0.05)	--	< 0.05 (0.0 to < 0.05)	--	--
Zika virus disease	0.3 (0.2 to 0.6)	18.7 (10.6 to 34.0)	< 0.05 (< 0.05 to < 0.05)	--	--
Guinea worm disease	< 0.05 (< 0.05 to < 0.05)	< 0.05 (< 0.05 to < 0.05)	< 0.05 (< 0.05 to < 0.05)	-99.4 (-99.7 to -99.1)	-99.7 (-99.8 to -99.5)
Other neglected tropical diseases	60.0 (58.2 to 61.7)	--	1.0 (0.7 to 1.4)	48.8 (40.0 to 57.0)	-7.3 (-12.8 to -2.2)
**Maternal disorders**	**--**	**--**	**--**	**--**	**--**
Maternal haemorrhage	--	--	--	--	--
Maternal sepsis and other pregnancy related infections	--	--	--	--	--
Maternal hypertensive disorders	--	--	--	--	--
Maternal obstructed labour and uterine rupture	--	--	--	--	--
Maternal abortion, miscarriage, and ectopic pregnancy	--	--	--	--	--
Other maternal disorders	--	--	--	--	--
**Neonatal disorders**	**63.3 (56.3 to 72.7)**	**--**	**3.7 (2.5 to 5.3)**	**77.7 (65.3 to 92.0)**	**10.7 (3.0 to 19.6)**
Neonatal preterm birth complications	57.7 (46.1 to 70.2)	--	3.0 (2.0 to 4.2)	69.2 (57.5 to 83.0)	5.4 (-1.9 to 14.0)
Neonatal encephalopathy due to birth asphyxia and trauma	0.3 (< 0.05 to 1.1)	--	0.1 (< 0.05 to 0.4)	158.7 (129.2 to 263.4)	61.1 (42.8 to 126.4)
Neonatal sepsis and other neonatal infections	1.1 (0.1 to 3.6)	--	0.5 (< 0.05 to 1.5)	135.2 (115.4 to 215.4)	46.6 (34.2 to 96.5)
Hemolytic disease and other neonatal jaundice	0.3 (0.2 to 0.4)	--	0.1 (0.1 to 0.1)	93.9 (67.0 to 115.1)	20.8 (4.0 to 34.0)
Other neonatal disorders	--	--	--	--	--
**Nutritional deficiencies**	**4,295.0 (4,231.9 to 4,345.0)**	**532.4† (406.8 to 659.8)**	**116.2 (81.2 to 158.8)**	**46.9 (45.0 to 48.6)**	**-8.5 (-9.6 to -7.4)**
Protein-energy malnutrition	--	--	--	--	--
Iodine deficiency	687.6 (614.4 to 767.7)	1.1 (0.8 to 1.4)	15.7 (10.9 to 21.6)	43.4 (39.6 to 47.2)	-10.6 (-13.1 to -8.3)
Vitamin A deficiency	513.0 (414.9 to 629.1)	531.3 (405.9 to 658.8)	0.3 (0.2 to 0.5)	55.0 (43.7 to 66.6)	-3.4 (-10.5 to 3.8)
Iron-deficiency anaemia	3,457.6 (3,304.2 to 3,593.2)	--	100.1 (69.7 to 136.3)	47.4 (45.5 to 49.3)	-8.1 (-9.3 to -7.0)
Other nutritional deficiencies	--	--	--	--	--
**Other communicable, maternal, neonatal, and nutritional diseases**	**10,498.0 (10,128.3 to 10,864.8)**	**1,111.2 (808.2 to 1,519.1)**	**10.6 (7.5 to 14.8)**	**58.4 (43.2 to 74.3)**	**-1.3 (-10.8 to 8.6)**
Sexually transmitted diseases excluding HIV	8,072.9 (7,450.1 to 8,924.3)	158.7 (136.7 to 182.7)	6.5 (4.5 to 9.2)	61.4 (53.4 to 70.4)	0.6 (-4.5 to 6.2)
Syphilis	36.3 (29.3 to 45.1)	1.6 (1.0 to 2.7)	4.6 (3.2 to 6.4)	64.6 (52.8 to 75.9)	2.5 (-4.8 to 9.6)
Chlamydial infection	< 0.05 (< 0.05 to < 0.05)	--	< 0.05 (< 0.05 to < 0.05)	89.6 (45.0 to 168.2)	18.1 (-9.7 to 67.1)
Gonococcal infection	< 0.05 (< 0.05 to < 0.05)	--	< 0.05 (< 0.05 to < 0.05)	108.0 (-13.5 to 444.9)	29.6 (-46.1 to 239.5)
Trichomoniasis	215.3 (183.1 to 250.2)	157.1 (135.3 to 181.1)	0.3 (0.1 to 0.6)	52.4 (46.0 to 59.3)	-5.1 (-9.0 to -0.7)
Genital herpes	7,723.7 (6,766.0 to 8,910.2)	--	1.7 (0.5 to 3.9)	55.0 (51.4 to 58.5)	-3.5 (-5.7 to -1.2)
Other sexually transmitted diseases	< 0.05 (< 0.05 to < 0.05)	--	< 0.05 (< 0.05 to < 0.05)	86.1 (42.3 to 163.3)	16.0 (-11.3 to 64.0)
Hepatitis	3,343.1 (2,965.1 to 3,734.3)	952.4 (659.2 to 1,354.9)	2.2 (1.3 to 3.7)	47.7 (-1.1 to 119.8)	-8.0 (-38.4 to 37.0)
Acute hepatitis A	10.5 (3.3 to 21.0)	136.7 (43.0 to 272.4)	0.4 (0.1 to 0.9)	29.2 (-61.1 to 290.1)	-19.5 (-75.7 to 143.0)
Hepatitis B	1,848.8 (1,508.8 to 2,216.7)	569.6 (310.2 to 946.2)	1.3 (0.7 to 2.5)	50.2 (-18.5 to 165.5)	-6.4 (-49.2 to 65.4)
Hepatitis C	1,467.0 (1,316.1 to 1,620.3)	28.7 (22.6 to 36.2)	< 0.05 (< 0.05 to 0.1)	46.4 (31.1 to 65.0)	-8.8 (-18.3 to 2.8)
Acute hepatitis E	16.7 (10.0 to 27.7)	217.4 (130.3 to 360.4)	0.5 (0.2 to 0.8)	59.8 (27.1 to 96.8)	-0.4 (-20.8 to 22.6)
Other infectious diseases	86.4 (83.4 to 89.3)	0.1 (0.1 to 0.2)	1.9 (1.3 to 2.6)	61.9 (56.0 to 67.6)	0.9 (-2.8 to 4.4)
**Non-communicable diseases**	**39,812.7 (39,804.4 to 39,827.4)**	**194,538.9† (174,958.4 to 217,985.4)**	**11,026.2 (8,417.7 to 13,946.7)**	**59.2 (58.0 to 60.3)**	**-0.8 (-1.5 to -0.1)**
**Neoplasms**	**1,895.7 (1,872.7 to 1,920.8)**	**881.4 (830.6 to 942.0)**	**246.0 (184.1 to 307.6)**	**61.8 (59.1 to 65.0)**	**0.8 (-0.9 to 2.8)**
Lip and oral cavity cancer	42.6 (41.3 to 44.0)	14.1 (13.7 to 14.6)	5.0 (3.7 to 6.4)	68.6 (58.6 to 78.6)	5.0 (-1.2 to 11.3)
Nasopharynx cancer	3.2 (3.0 to 3.4)	1.2 (1.1 to 1.2)	0.5 (0.4 to 0.7)	57.7 (46.5 to 68.8)	-1.7 (-8.7 to 5.2)
Other pharynx cancer	6.7 (6.4 to 7.0)	3.0 (2.8 to 3.1)	1.0 (0.7 to 1.3)	66.8 (53.3 to 82.5)	3.9 (-4.5 to 13.7)
Oesophageal cancer	23.2 (22.4 to 24.1)	19.8 (19.2 to 20.4)	4.6 (3.3 to 5.9)	47.0 (37.9 to 56.5)	-8.4 (-14.1 to -2.5)
Stomach cancer	112.9 (109.5 to 116.4)	66.2 (64.4 to 68.1)	15.6 (11.6 to 19.5)	57.9 (50.9 to 65.4)	-1.6 (-6.0 to 3.1)
Colon and rectum cancer	365.4 (352.9 to 380.9)	116.6 (112.6 to 121.3)	40.0 (30.2 to 50.3)	60.4 (53.6 to 67.5)	-0.1 (-4.3 to 4.4)
Liver cancer	31.6 (29.9 to 33.4)	34.4 (32.7 to 36.1)	7.3 (5.3 to 9.6)	92.5 (82.2 to 103.9)	19.9 (13.5 to 27.0)
Liver cancer due to hepatitis B	10.9 (8.3 to 13.5)	9.3 (7.1 to 11.6)	1.9 (1.3 to 2.7)	92.4 (74.6 to 113.1)	19.9 (8.8 to 32.8)

Cause	Prevalence in thousands (95% UI)	Incidence in thousands (95% UI)	YLDs in thousands (95% UI)		
	2016 counts	2016 counts	2016 counts	Percentage change in counts between 2006 and 2016	Percentage change in rates between 2006 and 2016
Liver cancer due to hepatitis C	16.4 (14.8 to 18.1)	13.0 (11.6 to 14.3)	2.8 (2.0 to 3.7)	91.0 (78.2 to 105.0)	19.0 (11.0 to 27.7)
Liver cancer due to alcohol use	6.5 (5.0 to 7.9)	5.8 (4.5 to 7.0)	1.3 (0.9 to 1.8)	96.4 (78.3 to 116.7)	22.4 (11.1 to 35.0)
Liver cancer due to other causes	7.6 (6.5 to 8.8)	6.3 (5.4 to 7.4)	1.4 (1.0 to 1.8)	92.3 (77.8 to 107.4)	19.8 (10.8 to 29.2)
Gallbladder and biliary tract cancer	14.0 (12.8 to 15.3)	15.6 (14.5 to 16.6)	3.2 (2.3 to 4.2)	55.6 (43.1 to 70.0)	-3.1 (-10.8 to 5.9)
Pancreatic cancer	28.2 (26.6 to 29.6)	33.1 (31.6 to 34.4)	6.1 (4.4 to 7.9)	72.0 (60.2 to 83.9)	7.1 (-0.2 to 14.6)
Larynx cancer	14.2 (13.8 to 14.7)	5.0 (4.9 to 5.2)	2.0 (1.5 to 2.5)	62.8 (52.4 to 73.9)	1.5 (-5.0 to 8.3)
Tracheal, bronchus, and lung cancer	140.3 (134.4 to 145.2)	107.7 (103.4 to 111.4)	23.4 (17.2 to 29.0)	77.2 (68.2 to 85.8)	10.4 (4.8 to 15.8)
Malignant skin melanoma	50.7 (39.7 to 54.7)	12.6 (10.0 to 13.6)	4.0 (2.8 to 5.2)	75.3 (61.3 to 88.9)	9.2 (0.5 to 17.7)
Non-melanoma skin cancer	103.5 (77.6 to 128.0)	131.6 (86.9 to 189.8)	3.5 (1.8 to 5.8)	22.5 (5.5 to 46.3)	-23.7 (-34.3 to -8.8)
Non-melanoma skin cancer (squamous-cell carcinoma)	84.2 (48.5 to 121.7)	73.0 (43.7 to 105.6)	3.4 (1.8 to 5.7)	22.3 (5.1 to 46.3)	-23.8 (-34.5 to -8.8)
Non-melanoma skin cancer (basal-cell carcinoma)	6.7 (3.3 to 12.4)	58.6 (31.0 to 107.5)	0.1 (< 0.05 to 0.1)	36.2 (18.5 to 56.1)	-15.1 (-26.2 to -2.7)
Breast cancer	230.8 (222.6 to 240.5)	54.3 (52.0 to 56.9)	25.9 (19.5 to 32.7)	44.1 (37.4 to 51.6)	-10.2 (-14.4 to -5.6)
Cervical cancer	19.6 (17.2 to 21.2)	6.2 (5.5 to 6.7)	2.3 (1.7 to 2.9)	34.3 (25.4 to 43.9)	-16.4 (-21.8 to -10.4)
Uterine cancer	44.9 (42.2 to 48.1)	11.3 (10.7 to 12.1)	4.2 (3.1 to 5.4)	56.4 (41.6 to 71.9)	-2.5 (-11.8 to 7.1)
Ovarian cancer	20.6 (19.3 to 21.6)	7.8 (7.4 to 8.2)	3.5 (2.5 to 4.5)	40.9 (26.5 to 55.7)	-12.2 (-21.2 to -3.0)
Prostate cancer	318.1 (279.4 to 363.9)	91.0 (80.9 to 103.1)	41.1 (29.7 to 51.8)	62.0 (53.4 to 73.4)	0.9 (-4.4 to 8.0)
Testicular cancer	1.2 (1.1 to 1.2)	0.3 (0.3 to 0.3)	0.1 (0.1 to 0.2)	91.5 (72.2 to 114.8)	19.3 (7.3 to 33.8)
Kidney cancer	37.5 (35.5 to 39.1)	12.1 (11.5 to 12.5)	3.8 (2.8 to 4.9)	63.8 (52.3 to 76.0)	2.1 (-5.1 to 9.7)
Bladder cancer	124.0 (118.4 to 129.8)	36.5 (34.9 to 38.2)	12.7 (9.5 to 16.2)	68.5 (60.1 to 77.6)	5.0 (-0.2 to 10.6)
Brain and nervous system cancer	13.0 (9.1 to 15.5)	6.7 (4.8 to 7.8)	2.1 (1.3 to 2.9)	84.2 (66.0 to 107.8)	14.7 (3.5 to 29.5)
Thyroid cancer	16.1 (15.5 to 17.0)	3.8 (3.6 to 4.0)	1.6 (1.2 to 2.1)	63.2 (55.1 to 73.8)	1.7 (-3.4 to 8.3)
Mesothelioma	2.5 (2.3 to 2.6)	1.8 (1.7 to 1.9)	0.6 (0.4 to 0.8)	70.8 (58.2 to 85.6)	6.4 (-1.4 to 15.6)
Hodgkin lymphoma	2.6 (2.3 to 2.7)	0.8 (0.7 to 0.8)	0.4 (0.3 to 0.5)	40.0 (30.8 to 49.3)	-12.8 (-18.5 to -7.0)
Non-Hodgkin's lymphoma	74.8 (65.5 to 78.7)	23.2 (20.4 to 24.4)	7.1 (5.2 to 9.2)	70.1 (59.4 to 80.8)	6.0 (-0.7 to 12.6)
Multiple myeloma	19.2 (15.2 to 21.8)	8.3 (6.7 to 9.5)	4.2 (2.9 to 5.4)	64.4 (50.8 to 79.7)	2.4 (-6.0 to 11.9)
Leukaemia	66.2 (63.1 to 69.3)	22.1 (21.0 to 23.0)	10.7 (7.8 to 13.7)	63.3 (54.1 to 73.1)	1.8 (-4.0 to 7.8)
Acute lymphoid leukaemia	2.6 (2.4 to 2.9)	0.9 (0.8 to 1.0)	0.4 (0.3 to 0.6)	88.4 (74.1 to 103.2)	17.4 (8.5 to 26.6)
Chronic lymphoid leukaemia	26.3 (24.1 to 28.6)	7.1 (6.5 to 7.8)	4.1 (3.0 to 5.3)	60.0 (45.6 to 76.1)	-0.3 (-9.3 to 9.8)
Acute myeloid leukaemia	5.7 (4.9 to 6.1)	4.8 (4.1 to 5.1)	1.2 (0.9 to 1.6)	74.8 (62.1 to 87.9)	8.9 (1.0 to 17.0)
Chronic myeloid leukaemia	4.1 (3.8 to 4.5)	1.7 (1.6 to 1.9)	0.6 (0.4 to 0.8)	38.3 (26.2 to 51.4)	-13.8 (-21.4 to -5.7)
Other leukaemia	27.5 (25.2 to 29.1)	7.5 (6.9 to 8.0)	4.4 (3.1 to 5.6)	65.4 (51.6 to 79.2)	3.1 (-5.6 to 11.7)
Other neoplasms	104.2 (95.4 to 111.3)	34.4 (31.6 to 36.5)	9.6 (7.1 to 12.3)	88.4 (73.7 to 103.2)	17.4 (8.2 to 26.6)
**Cardiovascular diseases**	**20,379.0 (20,012.9 to 20,682.7)**	**3,389.7† (3,112.2 to 3,729.9)**	**1,789.8 (1,341.2 to 2,263.9)**	**57.9 (52.6 to 63.2)**	**-1.6 (-4.9 to 1.7)**
Rheumatic heart disease	44.4 (38.9 to 49.9)	15.5 (13.8 to 17.4)	3.2 (2.3 to 4.4)	45.6 (38.2 to 53.2)	-9.3 (-13.9 to -4.5)
Ischaemic heart disease	7,426.2 (6,708.4 to 8,134.0)	1,731.5 (1,561.9 to 1,922.8)	339.7 (237.0 to 460.7)	63.9 (61.7 to 65.9)	2.1 (0.7 to 3.4)
Cerebrovascular disease	3,796.6 (3,545.1 to 4,036.0)	817.1‡ (745.2 to 889.1)	815.3 (567.1 to 1,042.1)	58.0 (46.0 to 69.2)	-1.5 (-9.0 to 5.4)
Ischaemic stroke	3,360.8 (2,861.5 to 3,870.7)	571.1‡ (503.9 to 641.0)	689.0 (476.0 to 893.0)	59.1 (44.8 to 73.0)	-0.8 (-9.8 to 7.8)
Hemorrhagic stroke	609.4 (504.2 to 718.1)	246.0‡ (217.7 to 274.0)	126.3 (88.3 to 165.2)	52.2 (40.0 to 65.5)	-5.2 (-12.8 to 3.1)
Hypertensive heart disease	1,427.2 (1,112.2 to 1,802.9)	--	108.4 (71.3 to 155.6)	60.6 (57.1 to 63.8)	0.1 (-2.1 to 2.1)
Cardiomyopathy and myocarditis	368.7 (323.0 to 430.3)	45.6† (34.0 to 55.6)	26.3 (17.4 to 39.1)	52.6 (47.1 to 58.4)	-4.9 (-8.4 to -1.3)
Myocarditis	11.2 (8.7 to 14.0)	45.6 (34.0 to 55.6)	0.5 (0.3 to 0.8)	62.4 (47.5 to 78.8)	1.2 (-8.1 to 11.4)
Alcoholic cardiomyopathy	20.8 (15.7 to 27.2)	--	1.6 (1.0 to 2.4)	82.4 (62.2 to 104.6)	13.7 (1.1 to 27.5)
Other cardiomyopathy	319.0 (244.6 to 414.7)	--	24.2 (16.0 to 36.0)	50.8 (45.0 to 56.8)	-6.1 (-9.7 to -2.3)
Atrial fibrillation and flutter	4,087.0 (3,574.5 to 4,654.4)	166.2 (135.6 to 201.6)	302.5 (208.3 to 416.2)	57.4 (55.3 to 59.4)	-2.0 (-3.2 to -0.7)
Peripheral vascular disease	7,815.4 (6,564.4 to 9,343.3)	573.9 (376.4 to 851.1)	41.2 (17.9 to 82.0)	50.3 (47.5 to 53.3)	-6.3 (-8.1 to -4.5)
Endocarditis	48.4 (36.7 to 62.2)	39.9 (32.8 to 47.7)	3.6 (2.3 to 5.3)	45.8 (38.0 to 53.3)	-9.2 (-14.0 to -4.5)
Other cardiovascular and circulatory diseases	2,975.2 (2,225.2 to 3,819.9)	--	149.5 (98.5 to 219.0)	47.6 (43.9 to 50.8)	-8.1 (-10.3 to -6.0)
**Chronic respiratory diseases**	**10,126.1 (9,921.9 to 10,351.4)**	**1,036.0† (938.1 to 1,142.9)**	**542.6 (475.7 to 609.5)**	**60.0 (55.7 to 63.9)**	**-0.3 (-3.0 to 2.1)**
Chronic obstructive pulmonary disease	8,336.0 (7,826.8 to 8,860.6)	502.7 (422.9 to 597.8)	423.1 (375.8 to 471.9)	59.5 (54.4 to 64.2)	-0.6 (-3.8 to 2.3)
Pneumoconiosis	25.5 (23.6 to 27.6)	1.4 (1.2 to 1.7)	3.6 (2.6 to 4.9)	70.0 (61.8 to 77.8)	5.9 (0.8 to 10.8)
Silicosis	8.2 (7.0 to 9.5)	0.8 (0.6 to 1.0)	1.2 (0.8 to 1.6)	76.3 (60.5 to 93.2)	9.8 (0.0 to 20.4)

Cause	Prevalence in thousands (95% UI)	Incidence in thousands (95% UI)	YLDs in thousands (95% UI)		
	2016 counts	2016 counts	2016 counts	Percentage change in counts between 2006 and 2016	Percentage change in rates between 2006 and 2016
Asbestosis	5.9 (5.3 to 6.5)	0.1 (0.1 to 0.2)	0.8 (0.6 to 1.1)	55.5 (46.1 to 65.6)	-3.1 (-9.0 to 3.2)
Coal workers pneumoconiosis	5.7 (5.0 to 6.6)	0.1 (0.1 to 0.1)	0.8 (0.5 to 1.1)	71.3 (52.2 to 92.5)	6.7 (-5.2 to 19.9)
Other pneumoconiosis	5.7 (4.8 to 6.8)	0.4 (0.2 to 0.6)	0.8 (0.6 to 1.2)	76.8 (62.9 to 94.0)	10.2 (1.5 to 20.9)
Asthma	2,599.1 (2,321.0 to 2,886.3)	509.5 (450.1 to 566.2)	90.7 (61.9 to 127.3)	59.6 (56.0 to 63.0)	-0.5 (-2.8 to 1.6)
Interstitial lung disease and pulmonary sarcoidosis	230.7 (201.1 to 264.4)	22.4 (19.1 to 26.5)	22.1 (14.9 to 31.2)	66.8 (61.6 to 72.0)	3.9 (0.7 to 7.2)
Other chronic respiratory diseases	--	--	3.1 (2.7 to 3.4)	93.4 (87.6 to 99.0)	20.5 (16.9 to 24.0)
**Cirrhosis and other chronic liver diseases**	**418.0 (387.2 to 450.7)**	**42.8 (37.7 to 48.1)**	**17.3 (12.0 to 24.1)**	**64.7 (58.3 to 70.9)**	**2.6 (-1.3 to 6.5)**
Cirrhosis and other chronic liver diseases due to hepatitis B	95.1 (82.4 to 108.2)	10.8 (9.0 to 13.0)	3.8 (2.5 to 5.5)	69.2 (60.0 to 78.6)	5.4 (-0.3 to 11.3)
Cirrhosis and other chronic liver diseases due to hepatitis C	95.7 (81.9 to 111.0)	10.4 (8.4 to 12.6)	4.2 (2.8 to 6.0)	65.5 (55.6 to 76.0)	3.1 (-3.0 to 9.7)
Cirrhosis and other chronic liver diseases due to alcohol use	129.8 (114.0 to 147.3)	12.1 (10.1 to 14.1)	5.5 (3.8 to 7.8)	63.9 (54.5 to 73.2)	2.1 (-3.7 to 7.9)
Cirrhosis and other chronic liver diseases due to other causes	97.5 (80.8 to 117.1)	9.6 (7.8 to 11.8)	3.8 (2.5 to 5.4)	60.5 (50.9 to 70.5)	0.0 (-6.0 to 6.2)
**Digestive diseases**	**4,230.7 (4,012.2 to 4,481.8)**	**3,597.1† (3,288.0 to 3,938.3)**	**117.8 (82.3 to 159.6)**	**65.8 (60.8 to 70.9)**	**3.3 (0.2 to 6.5)**
Peptic ulcer disease	1,249.7 (1,055.8 to 1,462.1)	146.9 (131.0 to 164.8)	25.7 (16.2 to 38.8)	63.6 (54.5 to 73.8)	1.9 (-3.8 to 8.3)
Gastritis and duodenitis	2,499.5 (1,916.7 to 3,183.9)	1,299.9 (1,139.4 to 1,467.1)	37.4 (24.3 to 54.7)	73.0 (66.0 to 80.2)	7.8 (3.4 to 12.3)
Appendicitis	7.5 (7.0 to 8.1)	202.7 (188.3 to 219.4)	2.0 (1.4 to 2.7)	49.2 (40.0 to 59.3)	-7.0 (-12.8 to -0.7)
Paralytic ileus and intestinal obstruction	4.6 (3.7 to 5.7)	141.8 (117.3 to 171.5)	1.3 (0.9 to 1.9)	63.6 (47.5 to 82.6)	1.9 (-8.1 to 13.8)
Inguinal, femoral, and abdominal hernia	491.8 (355.3 to 707.6)	743.3 (541.7 to 972.6)	23.8 (15.5 to 34.7)	62.5 (54.1 to 71.4)	1.2 (-4.0 to 6.8)
Inflammatory bowel disease	55.7 (47.9 to 63.8)	11.5 (9.4 to 13.8)	7.2 (5.0 to 10.0)	51.7 (44.1 to 59.2)	-5.5 (-10.2 to -0.8)
Vascular intestinal disorders	6.9 (5.4 to 8.9)	39.6 (31.9 to 49.3)	1.9 (1.2 to 2.7)	56.7 (36.1 to 79.8)	-2.4 (-15.2 to 12.0)
Gallbladder and biliary diseases	233.2 (180.7 to 291.3)	899.0 (724.5 to 1,100.0)	3.6 (2.3 to 5.1)	79.6 (63.7 to 96.5)	11.9 (2.0 to 22.5)
Pancreatitis	12.4 (10.9 to 14.2)	112.4 (98.9 to 127.2)	1.4 (1.0 to 2.0)	78.6 (61.3 to 98.5)	11.3 (0.5 to 23.7)
Other digestive diseases	--	--	13.6 (9.5 to 18.4)	64.2 (59.3 to 69.2)	2.3 (-0.7 to 5.5)
**Neurological disorders**	**13,530.7 (12,781.0 to 14,130.2)**	**3,441.5 (2,630.3 to 4,420.2)**	**1,465.8 (1,015.4 to 1,991.4)**	**59.8 (57.3 to 61.8)**	**-0.5 (-2.0 to 0.8)**
Alzheimer's disease and other dementias	7,601.5 (6,108.0 to 9,350.3)	1,308.2 (909.5 to 1,777.7)	1,217.9 (811.8 to 1,672.4)	59.7 (57.0 to 61.9)	-0.5 (-2.2 to 0.9)
Parkinson's disease	542.8 (423.9 to 689.2)	44.0 (27.6 to 65.7)	59.4 (38.4 to 86.0)	64.1 (58.9 to 68.8)	2.2 (-1.0 to 5.2)
Epilepsy	220.1 (175.1 to 264.1)	9.6 (6.3 to 13.6)	52.7 (36.1 to 72.0)	53.6 (30.9 to 81.1)	-4.3 (-18.4 to 12.8)
Multiple sclerosis	27.8 (25.0 to 30.9)	0.2 (0.2 to 0.3)	6.6 (4.6 to 8.5)	65.9 (52.9 to 79.8)	3.4 (-4.8 to 12.0)
Motor neuron disease	6.7 (5.5 to 8.2)	1.4 (1.0 to 1.7)	1.4 (1.0 to 2.0)	59.9 (54.2 to 65.3)	-0.4 (-3.9 to 3.0)
Migraine	1,899.2 (1,737.4 to 2,076.6)	146.1 (117.4 to 182.7)	96.2 (64.2 to 133.3)	60.9 (57.9 to 63.7)	0.2 (-1.6 to 2.0)
Tension-type headache	5,457.8 (3,883.8 to 7,368.7)	1,931.5 (1,248.8 to 2,842.6)	26.2 (16.6 to 39.0)	60.8 (56.9 to 65.1)	0.2 (-2.3 to 2.8)
Other neurological disorders	0.3 (0.2 to 0.5)	0.6 (0.3 to 0.9)	5.4 (3.7 to 7.4)	66.2 (63.5 to 68.6)	3.6 (1.9 to 5.0)
**Mental and substance use disorders**	**5,578.6 (5,484.1 to 5,665.1)**	**2,743.0† (2,358.0 to 3,155.0)**	**701.9 (523.9 to 906.8)**	**59.1 (57.7 to 60.5)**	**-0.8 (-1.7 to 0.0)**
Schizophrenia	35.9 (30.0 to 42.4)	--	19.6 (14.5 to 24.8)	69.4 (60.8 to 78.2)	5.6 (0.2 to 11.1)
Alcohol use disorders	212.3 (173.5 to 253.6)	73.3 (54.3 to 92.8)	18.7 (12.4 to 25.7)	73.1 (66.0 to 80.1)	7.9 (3.4 to 12.2)
Drug use disorders	151.1 (146.4 to 155.7)	8.5 (7.6 to 9.6)	38.7 (27.6 to 48.7)	63.0 (57.3 to 68.4)	1.6 (-2.0 to 4.9)
Opioid use disorders	82.1 (71.2 to 95.1)	--	29.0 (20.5 to 38.0)	65.0 (58.2 to 72.0)	2.8 (-1.4 to 7.2)
Cocaine use disorders	25.5 (22.3 to 28.9)	--	3.1 (1.9 to 4.4)	51.0 (39.5 to 63.6)	-5.9 (-13.1 to 1.9)
Amphetamine use disorders	6.4 (4.7 to 8.5)	2.0 (1.4 to 2.6)	0.8 (0.5 to 1.4)	48.5 (35.0 to 61.0)	-7.5 (-15.9 to 0.3)
Cannabis use disorders	18.1 (15.0 to 21.5)	--	0.5 (0.3 to 0.8)	56.5 (48.6 to 64.8)	-2.5 (-7.4 to 2.7)
Other drug use disorders	28.1 (25.1 to 31.8)	6.6 (5.8 to 7.4)	5.3 (3.7 to 7.1)	62.8 (54.8 to 71.1)	1.4 (-3.6 to 6.6)
Depressive disorders	2,419.4 (2,316.9 to 2,524.3)	2,532.0 (2,155.9 to 2,942.2)	362.9 (257.6 to 496.6)	58.1 (56.0 to 60.0)	-1.5 (-2.8 to -0.3)
Major depressive disorder	1,601.0 (1,369.8 to 1,838.7)	2,441.2 (2,062.2 to 2,843.1)	287.0 (197.5 to 398.2)	57.5 (55.1 to 59.6)	-1.8 (-3.4 to -0.5)
Dysthymia	876.7 (691.5 to 1,107.2)	90.8 (71.0 to 113.7)	75.9 (48.8 to 111.0)	60.1 (54.9 to 65.0)	-0.3 (-3.5 to 2.8)
Bipolar disorder	160.0 (129.7 to 198.5)	10.4 (7.7 to 13.4)	28.3 (17.2 to 41.2)	58.1 (52.3 to 63.6)	-1.5 (-5.1 to 1.9)
Anxiety disorders	1,510.1 (1,339.9 to 1,706.3)	103.0 (90.8 to 116.2)	127.0 (91.2 to 169.5)	55.7 (52.1 to 59.5)	-3.0 (-5.2 to -0.6)
Eating disorders	--	--	--	--	--
Anorexia nervosa	--	--	--	--	--
Bulimia nervosa	--	--	--	--	--
Autistic spectrum disorders	204.8 (195.7 to 212.0)	--	28.3 (19.6 to 39.0)	65.2 (61.8 to 68.7)	2.9 (0.8 to 5.1)
Autism	74.7 (64.0 to 87.5)	--	16.6 (11.0 to 23.7)	64.1 (59.1 to 69.3)	2.3 (-0.8 to 5.5)
Asperger syndrome and other autistic spectrum disorders	131.8 (109.5 to 158.7)	--	11.7 (7.9 to 16.6)	66.7 (62.8 to 70.4)	3.8 (1.4 to 6.2)

Cause	Prevalence in thousands (95% UI)	Incidence in thousands (95% UI)	YLDs in thousands (95% UI)		
	2016 counts	2016 counts	2016 counts	Percentage change in counts between 2006 and 2016	Percentage change in rates between 2006 and 2016
Attention-deficit/hyperactivity disorder	0.1 (0.1 to 0.2)	--	< 0.05 (< 0.05 to < 0.05)	66.3 (62.7 to 69.9)	3.6 (1.4 to 5.8)
Conduct disorder	--	--	--	--	--
Idiopathic developmental intellectual disability	141.8 (75.7 to 218.6)	--	6.8 (3.5 to 11.3)	49.7 (37.8 to 55.7)	-6.7 (-14.1 to -3.0)
Other mental and substance use disorders	1,089.3 (970.7 to 1,224.7)	15.8 (9.6 to 24.3)	71.6 (51.2 to 97.9)	61.8 (59.2 to 64.5)	0.8 (-0.8 to 2.5)
**Diabetes, urogenital, blood, and endocrine diseases**	**25,631.5 (25,413.0 to 25,887.5)**	**5,117.9† (4,555.2 to 5,775.0)**	**1,132.5 (813.1 to 1,491.4)**	**59.7 (57.0 to 62.5)**	**-0.5 (-2.2 to 1.2)**
Diabetes mellitus	6,334.7 (5,725.3 to 6,963.0)	88.3 (58.0 to 126.3)	510.1 (361.1 to 699.8)	57.9 (53.2 to 62.5)	-1.6 (-4.6 to 1.2)
Acute glomerulonephritis	1.5 (1.2 to 1.9)	23.0 (18.6 to 28.1)	0.1 (< 0.05 to 0.1)	58.4 (46.2 to 70.0)	-1.3 (-8.9 to 5.9)
Chronic kidney disease	13,877.0 (12,226.9 to 15,485.3)	673.4 (436.9 to 986.6)	360.7 (263.5 to 465.0)	60.6 (55.4 to 65.5)	0.0 (-3.2 to 3.1)
Chronic kidney disease due to diabetes mellitus	4,182.1 (3,034.5 to 5,505.2)	206.5 (122.8 to 322.9)	108.6 (72.6 to 157.4)	62.4 (55.5 to 69.4)	1.2 (-3.1 to 5.6)
Chronic kidney disease due to hypertension	3,403.8 (2,724.6 to 4,133.9)	81.9 (49.0 to 131.1)	128.6 (86.9 to 177.0)	57.4 (51.1 to 63.1)	-1.9 (-5.8 to 1.6)
Chronic kidney disease due to glomerulonephritis	3,158.2 (2,535.6 to 3,831.2)	233.2 (148.8 to 353.3)	43.5 (27.5 to 66.1)	57.0 (47.3 to 67.8)	-2.2 (-8.2 to 4.6)
Chronic kidney disease due to other causes	3,132.9 (2,204.5 to 4,222.3)	151.8 (84.7 to 247.7)	79.9 (50.3 to 118.6)	65.5 (57.2 to 74.3)	3.1 (-2.1 to 8.6)
Urinary diseases and male infertility	3,256.7 (2,920.0 to 3,805.9)	3,727.9† (3,250.5 to 4,277.5)	100.4 (64.3 to 146.6)	72.3 (70.6 to 73.9)	7.4 (6.3 to 8.4)
Interstitial nephritis and urinary tract infections	49.2 (42.0 to 58.4)	2,604.9 (2,195.1 to 3,107.0)	1.5 (0.9 to 2.2)	68.4 (59.4 to 77.1)	4.9 (-0.7 to 10.3)
Urolithiasis	33.4 (24.6 to 44.2)	859.0 (633.8 to 1,134.1)	2.2 (1.4 to 3.3)	84.0 (69.7 to 99.3)	14.6 (5.7 to 24.2)
Benign prostatic hyperplasia	3,121.0 (2,639.2 to 3,755.8)	263.9 (173.4 to 376.1)	96.1 (60.9 to 141.4)	72.2 (70.4 to 73.8)	7.3 (6.2 to 8.3)
Male infertility	--	--	--	--	--
Other urinary diseases	--	--	0.6 (0.4 to 0.8)	63.6 (54.9 to 72.9)	1.9 (-3.5 to 7.7)
Gynecological diseases	4,997.6 (4,687.2 to 5,492.9)	2.5† (1.8 to 3.5)	14.9 (7.3 to 26.4)	44.8 (42.5 to 47.0)	-9.8 (-11.2 to -8.4)
Uterine fibroids	121.2 (106.0 to 139.9)	2.5 (1.8 to 3.5)	0.8 (0.4 to 1.5)	56.3 (50.6 to 62.1)	-2.6 (-6.2 to 1.0)
Polycystic ovarian syndrome	--	--	--	--	--
Female infertility	--	--	--	--	--
Endometriosis	--	--	--	--	--
Genital prolapse	4,835.0 (4,244.7 to 5,477.1)	--	14.0 (6.9 to 25.2)	44.1 (41.8 to 46.4)	-10.2 (-11.7 to -8.8)
Premenstrual syndrome	--	--	--	--	--
Other gynecological diseases	--	--	--	--	--
Hemoglobinopathies and hemolytic anaemias	8,095.3 (8,025.9 to 8,171.1)	--	41.9 (28.7 to 58.2)	53.4 (49.8 to 56.8)	-4.5 (-6.7 to -2.3)
Thalassemias	< 0.05 (< 0.05 to < 0.05)	--	< 0.05 (< 0.05 to < 0.05)	46.0 (38.5 to 53.1)	-9.0 (-13.7 to -4.6)
Thalassaemias trait	1,455.2 (1,386.7 to 1,530.7)	--	24.2 (16.1 to 33.9)	67.5 (62.7 to 72.7)	4.4 (1.4 to 7.6)
Sickle cell disorders	< 0.05 (< 0.05 to < 0.05)	--	< 0.05 (< 0.05 to < 0.05)	43.5 (40.1 to 47.1)	-10.6 (-12.7 to -8.3)
Sickle cell trait	1,225.0 (1,119.8 to 1,369.5)	--	3.5 (2.4 to 4.9)	59.4 (54.1 to 65.5)	-0.7 (-4.0 to 3.1)
G6PD deficiency	989.6 (950.5 to 1,033.6)	--	0.1 (0.1 to 0.2)	56.5 (51.1 to 62.0)	-2.5 (-5.9 to 0.9)
G6PD trait	4,716.2 (4,626.0 to 4,819.0)	--	< 0.05 (< 0.05 to < 0.05)	54.7 (48.4 to 61.4)	-3.6 (-7.5 to 0.6)
Other hemoglobinopathies and hemolytic anaemias	434.2 (417.4 to 449.2)	--	14.1 (9.6 to 19.3)	32.9 (29.6 to 35.9)	-17.2 (-19.2 to -15.3)
Endocrine, metabolic, blood, and immune disorders	3,239.8 (2,580.8 to 4,041.5)	602.8 (442.6 to 800.1)	104.5 (69.5 to 155.8)	59.3 (52.3 to 66.8)	-0.7 (-5.1 to 3.9)
**Musculoskeletal disorders**	**18,269.4 (17,795.7 to 18,881.0)**	**6,900.3 (5,504.9 to 8,593.7)**	**1,768.7 (1,277.3 to 2,344.9)**	**58.1 (56.4 to 59.7)**	**-1.5 (-2.6 to -0.5)**
Rheumatoid arthritis	426.0 (379.7 to 473.7)	7.4 (5.7 to 9.6)	89.9 (62.9 to 115.6)	61.9 (57.8 to 65.9)	0.8 (-1.7 to 3.4)
Osteoarthritis	9,479.6 (8,956.2 to 10,021.0)	75.2 (58.2 to 93.8)	428.2 (302.2 to 582.8)	63.1 (61.5 to 64.8)	1.6 (0.6 to 2.7)
Low back and neck pain	10,134.6 (9,341.0 to 11,012.2)	4,460.4 (3,194.6 to 5,878.9)	1,085.6 (726.0 to 1,508.6)	59.1 (56.8 to 61.2)	-0.9 (-2.3 to 0.5)
Low back pain	7,747.4 (5,806.9 to 9,913.3)	3,736.5 (2,527.3 to 5,114.2)	768.3 (487.7 to 1,106.1)	59.0 (56.0 to 61.8)	-0.9 (-2.8 to 0.8)
Neck pain	3,516.9 (2,549.6 to 4,641.0)	724.0 (476.1 to 1,037.5)	317.3 (200.3 to 473.1)	59.1 (55.8 to 62.3)	-0.9 (-2.9 to 1.1)
Gout	872.6 (711.3 to 1,066.8)	124.8 (90.8 to 165.6)	24.7 (16.2 to 34.0)	63.7 (58.1 to 68.8)	2.0 (-1.5 to 5.2)
Other musculoskeletal disorders	1,852.7 (1,317.9 to 2,629.8)	2,232.5 (1,518.3 to 3,222.8)	140.3 (85.3 to 216.7)	36.2 (25.4 to 46.7)	-15.1 (-21.9 to -8.6)
**Other non-communicable diseases**	**38,878.6 (38,838.2 to 38,919.3)**	**167,389.2† (147,947.8 to 189,870.6)**	**3,243.9 (2,346.8 to 4,343.0)**	**59.7 (57.1 to 62.5)**	**-0.5 (-2.1 to 1.2)**
Congenital anomalies	329.6 (310.2 to 347.6)	--	34.9 (25.6 to 45.4)	57.0 (53.3 to 60.7)	-2.2 (-4.5 to 0.1)
Neural tube defects	23.2 (21.1 to 25.5)	--	6.5 (4.7 to 8.5)	58.1 (50.9 to 65.9)	-1.5 (-6.0 to 3.3)
Congenital heart anomalies	64.7 (58.2 to 71.8)	--	3.4 (1.4 to 5.9)	67.9 (60.5 to 75.2)	4.6 (0.0 to 9.1)
Orofacial clefts	20.7 (18.4 to 22.9)	--	0.2 (0.1 to 0.3)	52.4 (48.0 to 56.5)	-5.1 (-7.8 to -2.5)
Down's syndrome	--	--	--	--	--
Turner syndrome	2.0 (1.6 to 2.4)	--	< 0.05 (< 0.05 to < 0.05)	70.6 (66.9 to 74.5)	6.3 (4.0 to 8.7)
Klinefelter syndrome	0.2 (0.1 to 0.2)	--	< 0.05 (< 0.05 to < 0.05)	82.8 (77.2 to 89.0)	13.9 (10.4 to 17.8)

Cause	Prevalence in thousands (95% UI)	Incidence in thousands (95% UI)	YLDs in thousands (95% UI)		
	2016 counts	2016 counts	2016 counts	Percentage change in counts between 2006 and 2016	Percentage change in rates between 2006 and 2016
Other chromosomal abnormalities	< 0.05 (< 0.05 to < 0.05)	--	< 0.05 (< 0.05 to < 0.05)	86.3 (81.9 to 89.9)	16.1 (13.3 to 18.3)
Congenital musculoskeletal and limb anomalies	83.0 (76.6 to 89.4)	--	11.1 (7.8 to 14.8)	58.9 (51.9 to 66.0)	-1.0 (-5.3 to 3.4)
Urogenital congenital anomalies	18.8 (17.0 to 20.8)	--	0.3 (0.1 to 0.5)	67.2 (60.9 to 73.8)	4.2 (0.3 to 8.3)
Digestive congenital anomalies	59.5 (53.9 to 65.6)	--	2.4 (1.6 to 3.3)	59.1 (51.4 to 67.0)	-0.9 (-5.7 to 4.0)
Other congenital anomalies	132.3 (90.9 to 187.9)	--	11.1 (7.0 to 17.2)	50.8 (44.0 to 57.7)	-6.0 (-10.3 to -1.8)
Skin and subcutaneous diseases	23,844.5 (23,312.4 to 24,424.7)	109,090.1 (91,261.0 to 130,687.7)	309.6 (196.2 to 492.3)	60.6 (59.1 to 61.8)	0.1 (-0.9 to 0.8)
Dermatitis	2,052.1 (1,761.4 to 2,390.2)	2,517.0 (1,848.4 to 3,323.3)	59.5 (36.8 to 89.1)	56.6 (54.1 to 59.0)	-2.4 (-4.0 to -0.9)
Psoriasis	503.0 (460.8 to 552.5)	30.8 (25.8 to 36.0)	38.5 (27.9 to 49.7)	63.6 (59.1 to 68.3)	2.0 (-0.9 to 4.9)
Cellulitis	53.8 (45.7 to 64.7)	1,347.3 (1,165.2 to 1,569.3)	2.7 (1.7 to 3.9)	51.8 (44.0 to 59.9)	-5.4 (-10.3 to -0.4)
Pyoderma	379.2 (348.6 to 411.2)	10,088.6 (9,180.3 to 10,996.1)	1.9 (0.8 to 3.9)	68.2 (65.0 to 71.5)	4.8 (2.8 to 6.9)
Scabies	529.5 (370.8 to 728.4)	1,664.4 (1,174.6 to 2,314.2)	12.3 (6.5 to 21.0)	54.3 (51.3 to 56.9)	-3.9 (-5.8 to -2.2)
Fungal skin diseases	15,513.7 (13,025.9 to 18,309.2)	76,419.1 (58,743.1 to 97,748.5)	80.0 (32.3 to 170.6)	63.2 (62.6 to 63.8)	1.7 (1.3 to 2.0)
Viral skin diseases	508.2 (422.2 to 614.1)	779.0 (658.7 to 913.3)	14.0 (8.6 to 20.9)	58.5 (55.6 to 61.7)	-1.2 (-3.1 to 0.7)
Acne vulgaris	190.9 (185.1 to 196.6)	73.0 (70.3 to 76.1)	4.5 (3.1 to 6.4)	62.1 (55.5 to 68.9)	1.0 (-3.1 to 5.2)
Alopecia areata	36.4 (32.5 to 40.1)	59.7 (53.9 to 65.3)	1.1 (0.7 to 1.6)	50.3 (40.6 to 59.8)	-6.4 (-12.4 to -0.4)
Pruritus	760.6 (540.6 to 1,069.0)	577.3 (404.7 to 798.0)	7.4 (3.2 to 13.8)	62.9 (59.9 to 65.5)	1.5 (-0.4 to 3.1)
Urticaria	162.9 (112.5 to 224.1)	275.9 (190.5 to 374.1)	8.6 (5.1 to 13.6)	59.8 (54.1 to 65.0)	-0.5 (-4.0 to 2.8)
Decubitus ulcer	186.5 (141.2 to 238.5)	691.6 (519.9 to 910.0)	26.4 (17.7 to 37.8)	54.2 (44.5 to 62.9)	-3.9 (-10.0 to 1.5)
Other skin and subcutaneous diseases	10,390.7 (9,605.4 to 11,210.6)	14,566.6 (13,243.7 to 16,012.4)	52.6 (25.9 to 95.1)	64.6 (63.4 to 65.8)	2.6 (1.8 to 3.3)
Sense organ diseases	35,839.9 (35,507.1 to 36,164.4)	10,077.1† (9,191.8 to 10,966.4)	2,528.8 (1,853.7 to 3,353.3)	59.3 (56.0 to 62.9)	-0.7 (-2.8 to 1.5)
Glaucoma	265.5 (214.3 to 322.7)	--	25.2 (16.9 to 36.4)	64.9 (61.1 to 69.1)	2.7 (0.4 to 5.4)
Cataract	5,773.9 (4,834.1 to 6,814.8)	--	397.4 (283.0 to 543.1)	55.5 (53.4 to 57.5)	-3.1 (-4.4 to -1.9)
Macular degeneration	358.6 (289.0 to 446.8)	--	28.5 (19.5 to 40.0)	59.7 (55.4 to 64.1)	-0.5 (-3.2 to 2.3)
Refraction and accommodation disorders	18,091.9 (16,861.2 to 19,500.3)	--	282.9 (177.1 to 434.3)	56.5 (54.3 to 58.4)	-2.5 (-3.9 to -1.3)
Age-related and other hearing loss	35,574.4 (35,135.0 to 35,962.3)	--	1,568.4 (1,134.7 to 2,077.8)	60.4 (54.8 to 66.2)	-0.1 (-3.5 to 3.5)
Other vision loss	1,062.1 (817.3 to 1,330.2)	--	78.6 (53.6 to 109.1)	62.8 (59.9 to 66.0)	1.5 (-0.4 to 3.4)
Other sense organ diseases	5,044.3 (4,463.2 to 5,748.5)	10,077.1 (9,191.8 to 10,966.4)	147.9 (92.8 to 225.9)	61.7 (59.6 to 63.8)	0.7 (-0.5 to 2.1)
Oral disorders	24,913.0 (24,380.8 to 25,481.4)	48,222.0† (41,055.6 to 55,599.5)	370.6 (240.5 to 531.2)	61.7 (60.6 to 62.9)	0.8 (0.0 to 1.5)
Caries of deciduous teeth	--	--	--	--	--
Caries of permanent teeth	14,616.5 (12,820.9 to 16,594.0)	47,066.2 (39,839.6 to 54,466.7)	10.9 (4.8 to 20.9)	57.2 (55.2 to 59.1)	-2.1 (-3.3 to -0.9)
Periodontal disease	7,633.1 (5,725.5 to 9,577.5)	934.1 (497.8 to 1,468.7)	46.0 (17.4 to 95.2)	62.1 (60.8 to 63.3)	1.0 (0.2 to 1.8)
Edentulism and severe tooth loss	11,494.6 (9,660.1 to 13,547.5)	221.6 (128.8 to 349.3)	298.4 (198.2 to 425.2)	61.9 (60.5 to 63.4)	0.9 (-0.0 to 1.8)
Other oral disorders	576.1 (505.9 to 641.5)	--	15.3 (9.5 to 23.1)	59.9 (57.2 to 62.8)	-0.4 (-2.0 to 1.4)
**Injuries**	**29,999.8 (28,272.3 to 31,902.5)**	**9,862.1† (8,623.7 to 11,240.5)**	**1,131.3 (797.8 to 1,529.7)**	**65.0 (62.8 to 66.8)**	**2.8 (1.4 to 3.9)**
**Transport injuries**	**4,236.0 (3,980.5 to 4,538.8)**	**538.4 (442.8 to 640.4)**	**178.9 (121.0 to 252.0)**	**65.5 (64.2 to 66.8)**	**3.1 (2.3 to 4.0)**
Road injuries	3,513.6 (3,283.8 to 3,803.4)	418.3 (326.6 to 515.2)	148.2 (100.2 to 209.7)	68.4 (67.2 to 69.8)	5.0 (4.2 to 5.8)
Pedestrian road injuries	886.7 (763.8 to 1,033.2)	94.3 (67.9 to 125.4)	39.7 (26.9 to 56.2)	71.0 (69.4 to 72.8)	6.6 (5.5 to 7.7)
Cyclist road injuries	762.9 (642.6 to 878.1)	122.2 (87.8 to 160.4)	31.1 (20.8 to 44.3)	77.2 (75.6 to 79.2)	10.4 (9.4 to 11.6)
Motorcyclist road injuries	594.4 (509.6 to 686.0)	30.2 (19.7 to 44.9)	23.9 (16.1 to 33.8)	80.4 (79.0 to 81.7)	12.4 (11.5 to 13.2)
Motor vehicle road injuries	1,044.0 (879.2 to 1,200.8)	141.5 (104.8 to 181.2)	44.8 (30.4 to 62.9)	51.8 (50.1 to 53.5)	-5.4 (-6.5 to -4.4)
Other road injuries	225.6 (195.9 to 262.5)	30.2 (17.7 to 47.6)	8.8 (5.9 to 12.5)	94.8 (93.1 to 96.5)	21.4 (20.3 to 22.4)
Other transport injuries	722.4 (608.8 to 847.1)	120.1 (84.6 to 168.8)	30.7 (20.7 to 42.8)	52.6 (50.1 to 55.0)	-4.9 (-6.5 to -3.5)
**Unintentional injuries**	**24,591.7 (22,963.2 to 26,464.0)**	**9,120.7 (7,879.9 to 10,470.1)**	**916.6 (649.1 to 1,242.9)**	**65.2 (62.6 to 67.3)**	**2.9 (1.3 to 4.3)**
Falls	14,614.9 (13,092.2 to 16,436.4)	5,960.0 (4,776.0 to 7,270.2)	655.3 (466.2 to 882.4)	67.0 (63.5 to 69.7)	4.1 (1.9 to 5.8)
Drowning	155.1 (132.5 to 186.9)	35.8 (30.4 to 42.7)	6.9 (4.6 to 9.8)	33.9 (31.0 to 36.3)	-16.6 (-18.4 to -15.1)
Fire, heat, and hot substances	1,513.1 (1,343.9 to 1,703.7)	71.9 (57.9 to 86.8)	25.7 (15.1 to 40.6)	59.4 (57.6 to 60.9)	-0.7 (-1.8 to 0.3)
Poisonings	46.5 (40.5 to 52.9)	17.1 (11.9 to 24.4)	2.6 (1.8 to 3.7)	70.9 (65.7 to 76.4)	6.5 (3.3 to 9.9)
Exposure to mechanical forces	3,054.2 (2,667.0 to 3,582.7)	305.7 (242.6 to 386.9)	59.9 (39.6 to 86.2)	69.9 (68.4 to 71.1)	5.9 (4.9 to 6.6)
Unintentional firearm injuries	101.2 (85.3 to 119.6)	4.4 (2.6 to 7.0)	2.1 (1.3 to 3.1)	65.8 (64.7 to 66.9)	3.3 (2.6 to 4.0)
Unintentional suffocation	107.8 (89.7 to 126.2)	19.1 (13.0 to 28.0)	3.0 (1.9 to 4.4)	67.2 (64.9 to 69.4)	4.2 (2.8 to 5.6)
Other exposure to mechanical forces	2,845.2 (2,464.7 to 3,378.5)	282.2 (223.8 to 357.8)	54.8 (36.3 to 78.8)	70.2 (68.6 to 71.5)	6.1 (5.1 to 6.9)

Cause	Prevalence in thousands (95% UI)	Incidence in thousands (95% UI)	YLDs in thousands (95% UI)		
	2016 counts	2016 counts	2016 counts	Percentage change in counts between 2006 and 2016	Percentage change in rates between 2006 and 2016
Adverse effects of medical treatment	83.6 (59.9 to 111.8)	1,093.6 (854.8 to 1,386.1)	11.1 (6.8 to 17.9)	43.0 (34.4 to 51.5)	-10.9 (-16.2 to -5.6)
Animal contact	647.6 (585.3 to 718.3)	213.7 (159.4 to 283.9)	12.4 (8.6 to 17.3)	45.3 (43.0 to 47.2)	-9.5 (-10.9 to -8.2)
Venomous animal contact	230.4 (198.8 to 270.7)	93.8 (65.0 to 128.8)	6.5 (4.6 to 8.9)	47.3 (44.3 to 50.1)	-8.2 (-10.1 to -6.5)
Non-venomous animal contact	417.3 (365.3 to 468.8)	119.9 (84.7 to 164.1)	5.9 (3.8 to 8.9)	43.2 (40.8 to 45.0)	-10.8 (-12.3 to -9.7)
Foreign body	867.7 (760.5 to 989.4)	567.6 (476.2 to 688.2)	28.7 (19.1 to 41.5)	59.9 (58.0 to 61.8)	-0.4 (-1.6 to 0.8)
Pulmonary aspiration and foreign body in airway	445.1 (364.0 to 550.8)	382.9 (311.3 to 482.6)	16.0 (10.6 to 23.2)	62.1 (59.4 to 64.6)	1.0 (-0.7 to 2.6)
Foreign body in eyes	46.9 (35.8 to 61.8)	87.2 (48.9 to 152.5)	2.8 (1.8 to 4.2)	68.5 (66.1 to 71.9)	5.0 (3.5 to 7.1)
Foreign body in other body part	375.7 (314.9 to 455.5)	97.7 (67.2 to 136.9)	10.0 (6.6 to 14.5)	54.4 (52.4 to 56.2)	-3.8 (-5.0 to -2.7)
Environmental heat and cold exposure	1,026.5 (896.4 to 1,164.7)	186.2 (132.9 to 246.7)	37.1 (25.5 to 51.7)	61.5 (58.5 to 64.3)	0.7 (-1.3 to 2.4)
Other unintentional injuries	2,582.4 (2,236.9 to 2,962.5)	669.0 (464.6 to 933.6)	76.8 (52.0 to 109.0)	62.9 (61.4 to 64.3)	1.5 (0.6 to 2.4)
**Self-harm and interpersonal violence**	**1,559.1 (1,417.3 to 1,720.6)**	**197.3† (166.0 to 235.7)**	**31.2 (21.0 to 44.6)**	**52.1 (50.9 to 53.3)**	**-5.2 (-6.0 to -4.5)**
Self-harm	305.0 (257.5 to 365.0)	137.8 (113.6 to 171.6)	10.1 (6.6 to 14.4)	41.0 (38.8 to 43.0)	-12.2 (-13.5 to -10.9)
Self-harm by firearm	5.5 (4.7 to 7.0)	1.2 (0.5 to 2.6)	0.1 (0.1 to 0.2)	40.5 (37.6 to 43.3)	-12.5 (-14.3 to -10.7)
Self-harm by other specified means	299.4 (252.1 to 359.1)	136.6 (112.9 to 170.0)	9.9 (6.5 to 14.2)	41.0 (38.8 to 43.0)	-12.2 (-13.5 to -10.9)
Interpersonal violence	1,254.1 (1,122.1 to 1,411.5)	59.5† (42.0 to 83.0)	21.1 (14.3 to 30.2)	58.1 (57.1 to 59.0)	-1.5 (-2.1 to -0.9)
Assault by firearm	44.4 (37.7 to 50.8)	3.6 (1.9 to 6.1)	1.1 (0.7 to 1.6)	54.5 (52.9 to 56.2)	-3.7 (-4.7 to -2.6)
Assault by sharp object	161.1 (136.1 to 189.1)	13.7 (7.8 to 21.7)	3.2 (2.1 to 4.7)	55.1 (53.6 to 56.3)	-3.4 (-4.3 to -2.6)
Sexual violence	543.5 (445.0 to 659.0)	--	3.1 (2.0 to 4.5)	52.7 (50.8 to 54.6)	-4.9 (-6.0 to -3.7)
Assault by other means	505.2 (434.8 to 599.5)	42.2 (29.1 to 59.2)	13.8 (9.2 to 20.0)	60.3 (59.3 to 61.4)	-0.1 (-0.7 to 0.5)
**Forces of nature, conflict and terrorism, and executions and police conflict**	**156.6 (106.5 to 223.2)**	**5.7† (2.4 to 9.5)**	**4.6 (1.2 to 11.9)**	**108.0 (93.2 to 129.4)**	**29.6 (20.4 to 42.9)**
Forces of nature, conflict and terrorism, and state actor violence	50.5 (28.1 to 77.4)	1.7 (0.8 to 2.7)	1.7 (0.4 to 4.4)	135.4 (114.8 to 168.0)	46.7 (33.8 to 67.0)
Conflict and terrorism	90.7 (54.3 to 146.2)	--	2.7 (0.8 to 6.9)	91.5 (78.3 to 119.4)	19.3 (11.1 to 36.7)
Executions and police conflict	15.4 (5.4 to 23.9)	4.0 (1.0 to 7.7)	0.4 (0.1 to 1.3)	87.4 (46.4 to 129.3)	16.8 (-8.8 to 42.9)

We did not calculate incidence for certain NTDs and causes with only birth prevalence. Blank cells mean that no estimate is available.

† Incidence for aggregate causes represents the sum of the incidence of the causes below the aggregate.

‡ Incidence estimates for stroke represent first-ever stroke only.

^ Incidence of HIV/AIDS represents new infections of HIV only and does not include new infections of Tuberculosis in HIV positive cases.

SR Table 12: Global prevalence, incidence, and YLDs for 2016, percentage change of YLD counts and percentage change of YLD rates between 2006 and 2016 by cause, age 90-94 years.

Cause	Prevalence in thousands (95% UI)	Incidence in thousands (95% UI)	YLDs in thousands (95% UI)		
	2016 counts	2016 counts	2016 counts	Percentage change in counts between 2006 and 2016	Percentage change in rates between 2006 and 2016
**All causes**	**14,766.4 (14,761.6 to 14,778.5)**	**132,203.1† (119,155.4 to 148,346.4)**	**4,970.7 (3,755.7 to 6,260.5)**	**66.1 (65.0 to 67.2)**	**-0.8 (-1.4 to -0.1)**
**Communicable, maternal, neonatal, and nutritional disorders**	**7,344.3 (7,248.1 to 7,431.3)**	**45,425.0† (38,831.7 to 54,379.4)**	**109.1 (79.0 to 143.0)**	**56.4 (52.1 to 73.4)**	**-6.5 (-9.2 to 3.6)**
**HIV/AIDS and tuberculosis**	**2,367.2 (2,174.6 to 2,609.6)**	**19.6† (16.3 to 23.8)**	**8.6 (6.0 to 11.7)**	**40.5 (32.2 to 48.2)**	**-16.1 (-21.0 to -11.4)**
Tuberculosis	2,273.4 (2,002.2 to 2,601.1)	19.6† (16.3 to 23.8)	7.5 (5.2 to 10.3)	37.5 (32.7 to 42.7)	-17.9 (-20.7 to -14.8)
Drug-susceptible tuberculosis	27.2 (22.4 to 32.8)	19.0 (15.7 to 23.0)	7.2 (4.9 to 10.0)	37.6 (32.6 to 42.9)	-17.8 (-20.8 to -14.6)
Multidrug-resistant tuberculosis without extensive drug resistance	1.0 (0.8 to 1.2)	0.6 (0.5 to 0.8)	0.3 (0.2 to 0.4)	28.4 (10.5 to 50.3)	-23.3 (-34.0 to -10.2)
Extensively drug-resistant tuberculosis	0.1 (0.1 to 0.1)	< 0.05 (< 0.05 to 0.1)	< 0.05 (< 0.05 to < 0.05)	308.9 (256.8 to 374.1)	144.3 (113.2 to 183.3)
Latent tuberculosis infection	2,245.1 (1,973.4 to 2,570.7)	--	--	--	--
HIV/AIDS	10.9 (9.2 to 13.3)	--	1.1 (0.7 to 1.6)	65.1 (11.8 to 149.8)	-1.4 (-33.2 to 49.2)
Drug-susceptible HIV/AIDS - Tuberculosis	0.4 (0.2 to 0.5)	0.4 (0.3 to 0.5)	0.1 (0.1 to 0.2)	29.5 (18.7 to 39.7)	-22.6 (-29.1 to -16.5)
Multidrug-resistant HIV/AIDS - Tuberculosis without extensive drug resistance	< 0.05 (< 0.05 to < 0.05)	< 0.05 (< 0.05 to < 0.05)	< 0.05 (< 0.05 to < 0.05)	50.9 (6.6 to 95.9)	-9.8 (-36.3 to 17.0)
Extensively drug-resistant HIV/AIDS - Tuberculosis	< 0.05 (< 0.05 to < 0.05)	< 0.05 (< 0.05 to < 0.05)	< 0.05 (< 0.05 to < 0.05)	471.3 (266.0 to 717.0)	241.3 (118.7 to 388.1)
HIV/AIDS resulting in other diseases	10.5 (8.8 to 13.0)	--	1.0 (0.6 to 1.5)	71.2 (10.8 to 184.7)	2.3 (-33.8 to 70.1)
**Diarrhoea, lower respiratory infections, and other common infectious diseases**	**789.6 (751.7 to 824.5)**	**43,940.3† (37,390.6 to 52,844.1)**	**41.3 (29.5 to 57.1)**	**71.9 (65.4 to 123.2)**	**2.7 (-1.2 to 33.4)**
Diarrhoeal diseases	221.3 (197.7 to 244.6)	15,263.9 (13,580.3 to 16,934.1)	21.6 (15.3 to 29.0)	68.0 (64.2 to 71.8)	0.4 (-1.9 to 2.6)
Intestinal infectious diseases	0.5 (0.4 to 0.6)	10.1† (8.6 to 11.9)	0.1 (0.1 to 0.1)	32.1 (4.4 to 64.7)	-21.1 (-37.6 to -1.6)
Typhoid fever	0.5 (0.4 to 0.6)	7.8 (6.6 to 9.3)	0.1 (< 0.05 to 0.1)	34.7 (4.5 to 71.8)	-19.5 (-37.6 to 2.6)
Paratyphoid fever	0.1 (0.1 to 0.1)	2.3 (1.9 to 2.8)	< 0.05 (< 0.05 to < 0.05)	29.0 (6.1 to 58.1)	-22.9 (-36.6 to -5.5)
Other intestinal infectious diseases	--	--	< 0.05 (< 0.05 to < 0.05)	21.4 (-4.0 to 51.4)	-27.4 (-42.6 to -9.6)
Lower respiratory infections	98.0 (87.9 to 109.1)	4,258.6 (3,746.8 to 4,805.0)	5.2 (3.6 to 7.2)	86.1 (77.1 to 95.0)	11.2 (5.8 to 16.5)
Upper respiratory infections	322.2 (238.1 to 440.8)	24,121.5 (17,924.1 to 32,860.8)	7.2 (4.0 to 11.6)	65.0 (61.3 to 68.7)	-1.4 (-3.6 to 0.8)
Otitis media	87.8 (61.6 to 118.8)	70.0 (49.2 to 91.7)	2.2 (1.3 to 3.5)	57.5 (48.2 to 67.1)	-5.9 (-11.4 to -0.1)
Meningitis	22.6 (13.0 to 98.8)	3.8 (3.0 to 4.7)	1.7 (0.4 to 13.2)	232.0 (39.8 to 2,334.3)	98.4 (-16.5 to 1,354.4)
Pneumococcal meningitis	7.4 (5.6 to 9.2)	1.0 (0.7 to 1.4)	0.3 (0.2 to 0.5)	33.9 (28.3 to 42.0)	-20.0 (-23.3 to -15.1)
H influenzae type B meningitis	1.3 (0.9 to 1.7)	0.2 (0.1 to 0.4)	0.1 (< 0.05 to 0.1)	-5.2 (-14.9 to 16.5)	-43.4 (-49.1 to -30.4)
Meningococcal infection	8.0 (1.3 to 82.7)	0.8 (0.5 to 1.1)	1.1 (0.1 to 12.5)	1,482.6 (38.6 to 11,779.8)	845.5 (-17.2 to 6,997.7)
Other meningitis	5.9 (4.1 to 7.6)	1.8 (1.3 to 2.4)	0.3 (0.1 to 0.4)	81.8 (73.4 to 89.6)	8.6 (3.6 to 13.3)
Encephalitis	38.5 (19.6 to 64.5)	24.5 (21.5 to 28.0)	1.6 (1.1 to 2.2)	55.1 (46.6 to 64.9)	-7.3 (-12.4 to -1.5)
Diphtheria	--	--	--	--	--
Whooping cough	--	--	--	--	--
Tetanus	0.1 (0.1 to 0.1)	0.2 (0.1 to 0.3)	< 0.05 (< 0.05 to < 0.05)	6.5 (-16.5 to 38.8)	-36.4 (-50.1 to -17.1)
Measles	--	--	--	--	--
Varicella and herpes zoster	34.2 (27.5 to 43.1)	187.8 (147.9 to 237.8)	1.7 (1.0 to 2.6)	68.7 (60.7 to 77.1)	0.8 (-4.0 to 5.8)
**Neglected tropical diseases and malaria**	**1,790.3 (1,719.1 to 1,862.4)**	**987.9† (594.5 to 1,393.9)**	**17.5 (12.1 to 24.9)**	**34.5 (19.6 to 53.1)**	**-19.7 (-28.6 to -8.5)**
Malaria	14.6 (11.5 to 18.0)	30.5 (22.3 to 39.7)	0.1 (0.1 to 0.2)	-22.7 (-29.0 to -16.4)	-53.8 (-57.6 to -50.0)
Chagas disease	25.4 (21.2 to 30.3)	0.1 (0.1 to 0.2)	0.8 (0.4 to 1.5)	58.4 (49.8 to 67.3)	-5.4 (-10.5 to -0.1)
Leishmaniasis	6.5 (5.3 to 7.5)	1.3 (1.0 to 1.8)	0.3 (0.2 to 0.5)	49.4 (40.0 to 60.7)	-10.8 (-16.3 to -4.0)
Visceral leishmaniasis	0.1 (< 0.05 to 0.1)	0.4 (0.2 to 0.6)	< 0.05 (< 0.05 to < 0.05)	-39.0 (-72.9 to 18.1)	-63.6 (-83.8 to -29.4)
Cutaneous and mucocutaneous leishmaniasis	5.5 (4.3 to 6.9)	1.0 (0.7 to 1.3)	0.3 (0.2 to 0.5)	53.5 (44.1 to 66.1)	-8.3 (-13.9 to -0.8)
African trypanosomiasis	< 0.05 (< 0.05 to < 0.05)	< 0.05 (< 0.05 to < 0.05)	< 0.05 (< 0.05 to < 0.05)	-66.2 (-95.4 to 194.2)	-79.8 (-97.3 to 75.8)
Schistosomiasis	124.1 (109.1 to 139.6)	26.1 (22.4 to 29.9)	0.9 (0.5 to 1.6)	-24.6 (-28.7 to -20.4)	-55.0 (-57.4 to -52.4)
Cysticercosis	8.0 (5.4 to 12.2)	--	1.1 (0.6 to 1.7)	34.4 (24.3 to 44.4)	-19.7 (-25.7 to -13.8)
Cystic echinococcosis	0.7 (0.4 to 1.5)	0.1 (0.1 to 0.3)	0.1 (< 0.05 to 0.1)	-16.0 (-66.8 to 125.4)	-49.8 (-80.2 to 34.7)
Lymphatic filariasis	18.4 (15.1 to 23.8)	4.5 (2.6 to 7.1)	0.7 (0.4 to 1.3)	-35.6 (-55.0 to -21.8)	-61.5 (-73.1 to -53.3)
Onchocerciasis	7.2 (4.8 to 10.9)	1.4 (0.6 to 2.7)	0.7 (0.4 to 1.2)	-7.1 (-27.1 to 23.5)	-44.5 (-56.5 to -26.2)
Trachoma	51.5 (34.0 to 75.7)	--	3.6 (2.3 to 5.3)	18.1 (6.7 to 29.9)	-29.4 (-36.2 to -22.4)
Dengue	12.6 (6.9 to 21.2)	211.0 (130.5 to 316.4)	1.8 (0.8 to 3.2)	173.9 (122.5 to 517.3)	63.6 (32.9 to 268.8)
Yellow fever	< 0.05 (< 0.05 to < 0.05)	< 0.05 (< 0.05 to < 0.05)	< 0.05 (< 0.05 to < 0.05)	-16.6 (-48.6 to 34.1)	-50.2 (-69.3 to -19.9)
Rabies	< 0.05 (< 0.05 to < 0.05)	< 0.05 (< 0.05 to 0.1)	< 0.05 (< 0.05 to < 0.05)	139.0 (-33.3 to 324.8)	42.8 (-60.1 to 153.8)
Intestinal nematode infections	1,462.2 (1,392.2 to 1,524.2)	--	2.5 (1.5 to 3.9)	13.7 (5.9 to 22.2)	-32.1 (-36.8 to -27.0)
Ascariasis	778.6 (689.2 to 884.3)	--	0.7 (0.4 to 1.2)	-5.1 (-16.0 to 6.8)	-43.3 (-49.8 to -36.2)

Cause	Prevalence in thousands (95% UI)	Incidence in thousands (95% UI)	YLDs in thousands (95% UI)		
	2016 counts	2016 counts	2016 counts	Percentage change in counts between 2006 and 2016	Percentage change in rates between 2006 and 2016
Trichuriasis	424.1 (387.9 to 465.2)	--	0.3 (0.2 to 0.5)	9.3 (-6.8 to 27.8)	-34.7 (-44.3 to -23.6)
Hookworm disease	410.7 (377.6 to 449.6)	--	1.4 (0.9 to 2.2)	27.5 (15.0 to 40.2)	-23.9 (-31.3 to -16.2)
Food-borne trematodiases	185.5 (124.9 to 243.7)	706.8 (319.9 to 1,099.1)	4.4 (1.3 to 9.7)	121.9 (80.6 to 184.8)	32.6 (7.9 to 70.2)
Leprosy	3.6 (3.4 to 3.7)	0.1 (0.1 to 0.1)	0.2 (0.1 to 0.3)	53.1 (46.0 to 60.6)	-8.5 (-12.8 to -4.0)
Ebola virus disease	< 0.05 (0.0 to < 0.05)	--	< 0.05 (0.0 to < 0.05)	--	--
Zika virus disease	0.1 (0.1 to 0.2)	5.9 (3.3 to 10.6)	< 0.05 (< 0.05 to < 0.05)	--	--
Guinea worm disease	< 0.05 (< 0.05 to < 0.05)	< 0.05 (< 0.05 to < 0.05)	< 0.05 (< 0.05 to < 0.05)	-99.4 (-99.6 to -99.1)	-99.7 (-99.8 to -99.5)
Other neglected tropical diseases	18.5 (17.9 to 19.1)	--	0.3 (0.2 to 0.4)	57.7 (48.8 to 67.1)	-5.8 (-11.1 to -0.2)
**Maternal disorders**	**--**	**--**	**--**	**--**	**--**
Maternal haemorrhage	--	--	--	--	--
Maternal sepsis and other pregnancy related infections	--	--	--	--	--
Maternal hypertensive disorders	--	--	--	--	--
Maternal obstructed labour and uterine rupture	--	--	--	--	--
Maternal abortion, miscarriage, and ectopic pregnancy	--	--	--	--	--
Other maternal disorders	--	--	--	--	--
**Neonatal disorders**	**22.8 (20.2 to 26.3)**	**--**	**1.1 (0.7 to 1.5)**	**77.2 (62.9 to 92.8)**	**5.9 (-2.7 to 15.2)**
Neonatal preterm birth complications	20.8 (16.5 to 25.4)	--	1.0 (0.7 to 1.4)	73.8 (59.9 to 88.2)	3.8 (-4.5 to 12.4)
Neonatal encephalopathy due to birth asphyxia and trauma	< 0.05 (< 0.05 to 0.2)	--	< 0.05 (< 0.05 to 0.1)	193.2 (150.8 to 393.7)	75.2 (49.9 to 194.9)
Neonatal sepsis and other neonatal infections	0.1 (< 0.05 to 0.4)	--	< 0.05 (< 0.05 to 0.1)	173.9 (144.2 to 322.5)	63.6 (45.9 to 152.4)
Hemolytic disease and other neonatal jaundice	< 0.05 (< 0.05 to < 0.05)	--	< 0.05 (< 0.05 to < 0.05)	91.2 (49.2 to 129.8)	14.2 (-10.8 to 37.3)
Other neonatal disorders	--	--	--	--	--
**Nutritional deficiencies**	**1,409.0 (1,388.9 to 1,429.2)**	**146.2† (104.8 to 187.8)**	**36.9 (25.9 to 50.4)**	**55.5 (53.3 to 57.6)**	**-7.1 (-8.4 to -5.8)**
Protein-energy malnutrition	--	--	--	--	--
Iodine deficiency	246.5 (219.4 to 277.2)	0.3 (0.2 to 0.5)	5.6 (3.9 to 7.7)	53.3 (48.5 to 58.2)	-8.4 (-11.3 to -5.5)
Vitamin A deficiency	142.5 (107.8 to 182.0)	145.9 (104.4 to 187.5)	0.1 (0.1 to 0.1)	66.1 (54.5 to 77.5)	-0.8 (-7.7 to 6.0)
Iron-deficiency anaemia	1,124.5 (1,071.2 to 1,181.0)	--	31.3 (21.7 to 42.4)	55.9 (53.5 to 58.3)	-6.9 (-8.3 to -5.4)
Other nutritional deficiencies	--	--	--	--	--
**Other communicable, maternal, neonatal, and nutritional diseases**	**3,789.7 (3,648.9 to 3,935.4)**	**330.9 (232.1 to 458.0)**	**3.7 (2.6 to 5.0)**	**65.1 (52.4 to 78.5)**	**-1.3 (-8.9 to 6.7)**
Sexually transmitted diseases excluding HIV	3,008.4 (2,764.8 to 3,335.2)	36.2 (31.2 to 41.8)	2.4 (1.7 to 3.4)	64.2 (56.1 to 73.2)	-1.9 (-6.8 to 3.5)
Syphilis	14.2 (11.2 to 18.4)	0.6 (0.4 to 1.1)	1.7 (1.1 to 2.4)	65.3 (53.5 to 76.7)	-1.2 (-8.3 to 5.6)
Chlamydial infection	< 0.05 (< 0.05 to < 0.05)	--	< 0.05 (< 0.05 to < 0.05)	90.2 (31.5 to 189.2)	13.6 (-21.4 to 72.8)
Gonococcal infection	< 0.05 (< 0.05 to < 0.05)	--	< 0.05 (< 0.05 to < 0.05)	148.0 (-76.3 to 1,262.9)	48.2 (-85.9 to 714.3)
Trichomoniasis	52.1 (44.0 to 60.9)	35.6 (30.6 to 41.0)	0.1 (< 0.05 to 0.1)	62.5 (52.7 to 72.8)	-2.9 (-8.8 to 3.2)
Genital herpes	2,890.3 (2,534.3 to 3,330.2)	--	0.6 (0.2 to 1.4)	61.4 (57.2 to 65.7)	-3.6 (-6.1 to -1.0)
Other sexually transmitted diseases	< 0.05 (< 0.05 to < 0.05)	--	< 0.05 (< 0.05 to < 0.05)	86.6 (29.0 to 183.7)	11.5 (-22.9 to 69.5)
Hepatitis	1,099.5 (977.9 to 1,232.7)	294.7 (197.2 to 423.9)	0.7 (0.4 to 1.1)	64.2 (13.2 to 132.6)	-1.9 (-32.4 to 38.9)
Acute hepatitis A	3.3 (1.2 to 6.1)	43.0 (16.1 to 79.5)	0.1 (< 0.05 to 0.3)	45.7 (-49.4 to 253.0)	-13.0 (-69.8 to 110.9)
Hepatitis B	559.2 (451.8 to 675.9)	154.0 (79.7 to 262.6)	0.4 (0.2 to 0.7)	63.7 (-11.6 to 196.9)	-2.2 (-47.2 to 77.4)
Hepatitis C	530.1 (475.7 to 585.3)	8.8 (6.6 to 11.5)	< 0.05 (< 0.05 to < 0.05)	61.0 (43.8 to 81.4)	-3.8 (-14.1 to 8.4)
Acute hepatitis E	6.8 (3.7 to 12.5)	88.8 (48.3 to 162.1)	0.2 (0.1 to 0.4)	80.8 (43.9 to 123.7)	8.0 (-14.0 to 33.6)
Other infectious diseases	28.3 (27.2 to 29.6)	< 0.05 (< 0.05 to 0.1)	0.6 (0.4 to 0.8)	70.5 (64.5 to 76.5)	1.9 (-1.7 to 5.5)
**Non-communicable diseases**	**14,761.4 (14,757.3 to 14,769.5)**	**81,118.6† (70,401.8 to 94,544.7)**	**4,329.0 (3,281.3 to 5,424.1)**	**66.0 (64.8 to 67.2)**	**-0.8 (-1.6 to -0.1)**
**Neoplasms**	**903.6 (890.9 to 916.6)**	**446.1 (417.3 to 483.7)**	**127.5 (96.7 to 158.0)**	**73.8 (70.7 to 77.1)**	**3.9 (2.0 to 5.8)**
Lip and oral cavity cancer	20.0 (19.5 to 20.7)	7.4 (7.2 to 7.7)	2.5 (1.9 to 3.2)	77.7 (66.4 to 88.7)	6.2 (-0.6 to 12.7)
Nasopharynx cancer	1.2 (1.1 to 1.2)	0.5 (0.5 to 0.5)	0.2 (0.1 to 0.3)	67.1 (57.9 to 78.0)	-0.1 (-5.7 to 6.3)
Other pharynx cancer	2.3 (2.2 to 2.4)	1.1 (1.0 to 1.1)	0.4 (0.3 to 0.5)	76.7 (64.7 to 89.4)	5.5 (-1.6 to 13.1)
Oesophageal cancer	8.4 (8.2 to 8.7)	7.5 (7.4 to 7.8)	1.8 (1.3 to 2.2)	54.8 (45.9 to 64.5)	-7.5 (-12.8 to -1.7)
Stomach cancer	50.1 (48.6 to 51.6)	31.3 (30.5 to 32.2)	7.1 (5.4 to 8.8)	62.1 (54.4 to 69.8)	-3.2 (-7.8 to 1.4)
Colon and rectum cancer	181.8 (176.2 to 187.6)	65.2 (63.2 to 67.3)	21.8 (16.4 to 27.0)	69.1 (62.2 to 76.2)	1.0 (-3.1 to 5.3)
Liver cancer	10.8 (10.2 to 11.3)	12.8 (12.2 to 13.3)	2.7 (1.9 to 3.5)	96.0 (84.8 to 107.8)	17.1 (10.4 to 24.2)
Liver cancer due to hepatitis B	3.3 (2.5 to 4.3)	3.1 (2.3 to 4.0)	0.6 (0.4 to 0.9)	92.9 (72.8 to 114.4)	15.3 (3.2 to 28.1)

Cause	Prevalence in thousands (95% UI)	Incidence in thousands (95% UI)	YLDs in thousands (95% UI)		
	2016 counts	2016 counts	2016 counts	Percentage change in counts between 2006 and 2016	Percentage change in rates between 2006 and 2016
Liver cancer due to hepatitis C	6.6 (5.9 to 7.3)	5.6 (5.0 to 6.2)	1.2 (0.8 to 1.5)	96.7 (81.5 to 113.0)	17.5 (8.4 to 27.3)
Liver cancer due to alcohol use	2.0 (1.5 to 2.6)	1.9 (1.4 to 2.5)	0.4 (0.3 to 0.6)	101.5 (85.3 to 120.8)	20.4 (10.7 to 31.9)
Liver cancer due to other causes	2.3 (1.9 to 2.8)	2.1 (1.7 to 2.5)	0.5 (0.3 to 0.6)	93.5 (81.0 to 107.7)	15.6 (8.1 to 24.1)
Gallbladder and biliary tract cancer	7.2 (6.7 to 8.0)	8.3 (7.8 to 8.9)	1.7 (1.2 to 2.1)	63.2 (48.6 to 80.7)	-2.5 (-11.2 to 8.0)
Pancreatic cancer	13.3 (12.7 to 14.0)	16.1 (15.6 to 16.8)	2.9 (2.0 to 3.6)	82.0 (67.8 to 96.3)	8.7 (0.3 to 17.3)
Larynx cancer	4.8 (4.7 to 5.0)	1.9 (1.9 to 2.0)	0.7 (0.6 to 0.9)	72.5 (60.8 to 85.0)	3.1 (-3.9 to 10.6)
Tracheal, bronchus, and lung cancer	51.5 (49.1 to 53.3)	41.4 (39.6 to 42.8)	8.8 (6.5 to 10.9)	86.4 (76.6 to 95.3)	11.4 (5.5 to 16.7)
Malignant skin melanoma	25.3 (20.1 to 27.5)	7.2 (5.8 to 7.9)	2.2 (1.6 to 2.9)	96.1 (78.5 to 114.1)	17.2 (6.6 to 27.9)
Non-melanoma skin cancer	67.4 (48.8 to 87.6)	70.9 (44.1 to 107.4)	2.2 (1.1 to 3.7)	40.9 (16.1 to 71.7)	-15.8 (-30.7 to 2.6)
Non-melanoma skin cancer (squamous-cell carcinoma)	54.7 (31.3 to 84.0)	40.4 (23.7 to 62.1)	2.1 (1.1 to 3.7)	40.8 (15.9 to 71.9)	-15.9 (-30.7 to 2.7)
Non-melanoma skin cancer (basal-cell carcinoma)	3.5 (1.3 to 7.6)	30.5 (12.9 to 62.0)	< 0.05 (< 0.05 to 0.1)	45.4 (24.9 to 64.6)	-13.1 (-25.4 to -1.6)
Breast cancer	126.2 (121.8 to 131.6)	34.8 (33.5 to 36.4)	16.4 (12.5 to 20.2)	60.9 (52.7 to 69.4)	-3.9 (-8.8 to 1.2)
Cervical cancer	8.1 (7.4 to 8.6)	2.9 (2.6 to 3.0)	1.0 (0.8 to 1.3)	45.2 (32.4 to 58.9)	-13.2 (-20.9 to -5.1)
Uterine cancer	21.2 (20.0 to 22.5)	6.1 (5.8 to 6.5)	2.3 (1.7 to 2.8)	68.6 (51.6 to 88.0)	0.7 (-9.4 to 12.3)
Ovarian cancer	9.4 (8.9 to 9.8)	4.0 (3.8 to 4.1)	1.8 (1.3 to 2.2)	53.2 (38.0 to 69.2)	-8.5 (-17.6 to 1.1)
Prostate cancer	155.6 (134.9 to 174.2)	49.9 (43.4 to 54.9)	24.3 (17.6 to 30.9)	80.6 (71.5 to 94.5)	7.9 (2.4 to 16.2)
Testicular cancer	0.5 (0.5 to 0.5)	0.1 (0.1 to 0.2)	0.1 (0.1 to 0.1)	105.1 (85.0 to 129.2)	22.5 (10.5 to 36.9)
Kidney cancer	15.9 (15.1 to 16.6)	5.8 (5.6 to 6.0)	1.8 (1.3 to 2.2)	73.5 (60.6 to 88.2)	3.6 (-4.0 to 12.4)
Bladder cancer	59.6 (57.5 to 61.9)	20.1 (19.4 to 20.9)	6.7 (5.0 to 8.4)	79.4 (69.8 to 89.4)	7.2 (1.4 to 13.2)
Brain and nervous system cancer	4.2 (3.0 to 5.2)	2.4 (1.7 to 2.9)	0.7 (0.5 to 1.0)	95.0 (74.1 to 126.7)	16.5 (4.0 to 35.4)
Thyroid cancer	7.2 (7.0 to 7.6)	1.9 (1.9 to 2.0)	0.9 (0.6 to 1.1)	76.4 (63.4 to 91.2)	5.4 (-2.4 to 14.2)
Mesothelioma	0.9 (0.8 to 1.0)	0.7 (0.7 to 0.8)	0.2 (0.1 to 0.3)	84.8 (71.6 to 97.9)	10.4 (2.5 to 18.2)
Hodgkin lymphoma	0.8 (0.8 to 0.9)	0.3 (0.3 to 0.3)	0.1 (0.1 to 0.2)	54.2 (43.9 to 63.1)	-7.9 (-14.0 to -2.6)
Non-Hodgkin's lymphoma	32.1 (28.6 to 33.9)	11.2 (10.1 to 11.8)	3.4 (2.5 to 4.3)	88.4 (76.2 to 101.6)	12.6 (5.2 to 20.4)
Multiple myeloma	7.9 (6.5 to 9.0)	3.8 (3.2 to 4.4)	1.9 (1.3 to 2.4)	80.0 (64.5 to 97.7)	7.6 (-1.7 to 18.1)
Leukaemia	32.2 (30.7 to 33.8)	11.8 (11.3 to 12.3)	6.0 (4.3 to 7.6)	80.1 (69.7 to 91.5)	7.6 (1.4 to 14.4)
Acute lymphoid leukaemia	1.1 (1.0 to 1.1)	0.4 (0.4 to 0.4)	0.2 (0.1 to 0.2)	95.4 (82.0 to 109.0)	16.7 (8.7 to 24.9)
Chronic lymphoid leukaemia	13.6 (12.5 to 14.8)	4.2 (3.9 to 4.6)	2.5 (1.8 to 3.2)	77.1 (60.6 to 95.1)	5.8 (-4.1 to 16.6)
Acute myeloid leukaemia	2.3 (2.1 to 2.6)	2.1 (1.9 to 2.3)	0.5 (0.4 to 0.7)	91.1 (77.4 to 105.7)	14.2 (6.0 to 22.9)
Chronic myeloid leukaemia	2.1 (2.0 to 2.4)	1.0 (0.9 to 1.1)	0.3 (0.2 to 0.4)	54.9 (40.5 to 71.5)	-7.4 (-16.0 to 2.5)
Other leukaemia	13.0 (12.1 to 13.9)	4.1 (3.8 to 4.3)	2.4 (1.7 to 3.1)	84.1 (68.8 to 100.5)	10.0 (0.9 to 19.8)
Other neoplasms	50.2 (46.2 to 53.8)	18.6 (17.2 to 19.8)	5.1 (3.9 to 6.4)	101.9 (86.6 to 119.3)	20.6 (11.5 to 31.0)
**Cardiovascular diseases**	**7,965.3 (7,795.8 to 8,140.9)**	**1,549.0† (1,430.2 to 1,686.2)**	**711.3 (525.3 to 913.0)**	**59.7 (53.9 to 65.0)**	**-4.6 (-8.0 to -1.4)**
Rheumatic heart disease	12.8 (11.3 to 14.3)	10.9 (10.1 to 11.8)	0.9 (0.7 to 1.3)	63.1 (53.5 to 73.9)	-2.6 (-8.3 to 3.9)
Ischaemic heart disease	2,459.7 (2,161.3 to 2,769.3)	852.8 (783.2 to 935.0)	132.9 (92.5 to 182.4)	68.3 (65.6 to 70.9)	0.6 (-1.1 to 2.1)
Cerebrovascular disease	1,421.3 (1,333.3 to 1,509.6)	347.5‡ (311.4 to 386.6)	311.9 (217.3 to 399.3)	51.9 (39.1 to 63.5)	-9.2 (-16.9 to -2.3)
Ischaemic stroke	1,237.2 (1,014.4 to 1,446.0)	240.4‡ (207.8 to 275.0)	260.9 (178.7 to 342.5)	51.5 (36.2 to 65.8)	-9.5 (-18.6 to -0.9)
Hemorrhagic stroke	239.0 (195.7 to 285.9)	107.1‡ (91.7 to 122.6)	51.0 (36.0 to 66.8)	54.1 (40.4 to 67.3)	-8.0 (-16.1 to -0.0)
Hypertensive heart disease	678.2 (502.5 to 882.9)	--	50.9 (32.2 to 74.2)	69.4 (64.8 to 73.2)	1.2 (-1.5 to 3.5)
Cardiomyopathy and myocarditis	192.4 (161.9 to 236.1)	16.7† (12.5 to 20.5)	13.3 (8.6 to 19.8)	66.7 (59.8 to 74.0)	-0.4 (-4.5 to 4.0)
Myocarditis	4.1 (3.0 to 5.2)	16.7 (12.5 to 20.5)	0.2 (0.1 to 0.3)	68.6 (51.9 to 87.6)	0.7 (-9.3 to 12.1)
Alcoholic cardiomyopathy	2.1 (1.5 to 2.8)	--	0.2 (0.1 to 0.2)	60.1 (26.2 to 102.2)	-4.3 (-24.6 to 20.8)
Other cardiomyopathy	173.8 (125.3 to 232.7)	--	13.0 (8.3 to 19.3)	66.7 (59.6 to 74.2)	-0.4 (-4.6 to 4.0)
Atrial fibrillation and flutter	1,679.4 (1,475.8 to 1,900.1)	51.8 (40.6 to 65.2)	122.6 (84.2 to 167.8)	66.2 (64.0 to 68.4)	-0.7 (-2.0 to 0.6)
Peripheral vascular disease	3,532.6 (2,939.9 to 4,227.5)	249.3 (163.2 to 353.0)	18.4 (8.0 to 35.3)	57.5 (54.7 to 60.4)	-5.9 (-7.5 to -4.2)
Endocarditis	14.8 (11.0 to 19.4)	19.9 (17.1 to 23.0)	1.1 (0.7 to 1.6)	61.8 (51.5 to 72.1)	-3.4 (-9.5 to 2.8)
Other cardiovascular and circulatory diseases	1,172.3 (868.7 to 1,544.8)	--	59.3 (38.4 to 86.7)	62.5 (58.1 to 66.7)	-2.9 (-5.5 to -0.4)
**Chronic respiratory diseases**	**3,679.3 (3,584.4 to 3,770.2)**	**450.3† (405.4 to 500.4)**	**187.0 (164.6 to 210.0)**	**69.8 (65.5 to 74.4)**	**1.5 (-1.2 to 4.2)**
Chronic obstructive pulmonary disease	3,011.9 (2,805.1 to 3,248.4)	262.6 (227.7 to 302.9)	143.5 (126.6 to 161.3)	69.7 (64.0 to 75.2)	1.4 (-2.0 to 4.6)
Pneumoconiosis	6.7 (6.1 to 7.3)	0.6 (0.5 to 0.7)	0.9 (0.7 to 1.3)	74.0 (66.1 to 82.8)	4.0 (-0.7 to 9.2)
Silicosis	1.7 (1.4 to 2.0)	0.3 (0.2 to 0.4)	0.2 (0.1 to 0.3)	68.7 (48.7 to 90.0)	0.8 (-11.2 to 13.5)

Cause	Prevalence in thousands (95% UI)	Incidence in thousands (95% UI)	YLDs in thousands (95% UI)		
	2016 counts	2016 counts	2016 counts	Percentage change in counts between 2006 and 2016	Percentage change in rates between 2006 and 2016
Asbestosis	1.9<br>(1.7 to 2.1)	0.1<br>(< 0.05 to 0.1)	0.3<br>(0.2 to 0.4)	77.0<br>(66.8 to 89.7)	5.8<br>(-0.3 to 13.3)
Coal workers pneumoconiosis	1.4<br>(1.2 to 1.7)	< 0.05<br>(< 0.05 to 0.1)	0.2<br>(0.1 to 0.3)	69.1<br>(50.8 to 88.9)	1.0<br>(-9.9 to 12.8)
Other pneumoconiosis	1.7<br>(1.4 to 2.1)	0.2<br>(0.2 to 0.3)	0.2<br>(0.2 to 0.4)	80.0<br>(66.8 to 93.7)	7.6<br>(-0.3 to 15.7)
Asthma	928.4<br>(840.4 to 1,020.3)	176.8<br>(152.9 to 202.4)	32.0<br>(21.8 to 44.8)	67.8<br>(64.6 to 70.9)	0.2<br>(-1.6 to 2.1)
Interstitial lung disease and pulmonary sarcoidosis	99.0<br>(85.2 to 114.8)	10.3<br>(8.1 to 12.7)	9.3<br>(6.4 to 13.1)	75.4<br>(69.8 to 81.3)	4.8<br>(1.4 to 8.3)
Other chronic respiratory diseases	--	--	1.2<br>(1.1 to 1.4)	106.8<br>(100.3 to 113.1)	23.5<br>(19.7 to 27.3)
**Cirrhosis and other chronic liver diseases**	**157.8<br>(144.9 to 171.4)**	**21.1<br>(18.9 to 23.5)**	**6.7<br>(4.6 to 9.0)**	**75.0<br>(67.8 to 82.2)**	**4.5<br>(0.2 to 8.9)**
Cirrhosis and other chronic liver diseases due to hepatitis B	31.3<br>(26.0 to 36.8)	4.6<br>(3.7 to 5.6)	1.2<br>(0.8 to 1.8)	77.8<br>(66.1 to 90.0)	6.2<br>(-0.8 to 13.5)
Cirrhosis and other chronic liver diseases due to hepatitis C	38.0<br>(31.5 to 45.3)	5.1<br>(4.0 to 6.2)	1.7<br>(1.1 to 2.3)	74.4<br>(63.3 to 86.1)	4.2<br>(-2.5 to 11.2)
Cirrhosis and other chronic liver diseases due to alcohol use	48.6<br>(41.4 to 56.2)	6.3<br>(5.2 to 7.5)	2.3<br>(1.5 to 3.1)	76.6<br>(66.9 to 87.4)	5.5<br>(-0.3 to 12.0)
Cirrhosis and other chronic liver diseases due to other causes	39.9<br>(31.8 to 50.1)	5.1<br>(4.0 to 6.4)	1.5<br>(1.0 to 2.2)	71.0<br>(60.4 to 82.0)	2.2<br>(-4.2 to 8.7)
**Digestive diseases**	**1,507.8<br>(1,409.9 to 1,592.5)**	**1,298.8†<br>(1,193.3 to 1,412.9)**	**41.9<br>(29.5 to 56.0)**	**73.7<br>(68.3 to 79.1)**	**3.8<br>(0.6 to 7.0)**
Peptic ulcer disease	473.9<br>(401.5 to 553.3)	61.9<br>(55.1 to 69.6)	9.5<br>(5.9 to 14.2)	67.9<br>(57.2 to 77.8)	0.3<br>(-6.1 to 6.2)
Gastritis and duodenitis	856.4<br>(673.6 to 1,077.8)	480.3<br>(413.8 to 556.7)	13.2<br>(8.5 to 19.1)	81.9<br>(73.5 to 90.1)	8.7<br>(3.6 to 13.6)
Appendicitis	3.0<br>(2.8 to 3.3)	80.5<br>(73.9 to 88.0)	0.8<br>(0.5 to 1.1)	55.6<br>(47.3 to 65.7)	-7.1<br>(-12.0 to -1.0)
Paralytic ileus and intestinal obstruction	2.1<br>(1.7 to 2.6)	68.9<br>(57.9 to 81.4)	0.6<br>(0.4 to 0.8)	71.3<br>(49.5 to 95.9)	2.4<br>(-10.7 to 17.0)
Inguinal, femoral, and abdominal hernia	178.5<br>(119.4 to 279.7)	248.1<br>(184.2 to 322.2)	7.6<br>(4.8 to 10.8)	67.7<br>(58.7 to 76.6)	0.2<br>(-5.2 to 5.5)
Inflammatory bowel disease	17.2<br>(14.8 to 19.7)	3.4<br>(2.8 to 4.2)	2.2<br>(1.5 to 3.0)	67.7<br>(58.6 to 76.5)	0.2<br>(-5.2 to 5.5)
Vascular intestinal disorders	2.8<br>(2.2 to 3.5)	18.2<br>(15.1 to 22.3)	0.7<br>(0.5 to 1.0)	71.8<br>(46.0 to 98.9)	2.6<br>(-12.8 to 18.8)
Gallbladder and biliary diseases	77.4<br>(62.0 to 97.3)	295.3<br>(239.1 to 356.3)	1.1<br>(0.8 to 1.6)	88.9<br>(70.2 to 109.2)	12.8<br>(1.7 to 25.0)
Pancreatitis	4.6<br>(4.0 to 5.3)	42.1<br>(36.8 to 48.2)	0.5<br>(0.4 to 0.7)	83.7<br>(64.6 to 102.9)	9.8<br>(-1.7 to 21.2)
Other digestive diseases	--	--	5.5<br>(3.9 to 7.4)	76.4<br>(70.9 to 81.8)	5.4<br>(2.1 to 8.6)
**Neurological disorders**	**5,262.5<br>(5,000.6 to 5,546.7)**	**1,331.4<br>(1,022.2 to 1,756.1)**	**696.2<br>(484.4 to 925.6)**	**67.7<br>(65.2 to 69.7)**	**0.2<br>(-1.3 to 1.4)**
Alzheimer's disease and other dementias	3,830.7<br>(3,128.1 to 4,621.7)	761.1<br>(500.5 to 1,084.8)	605.3<br>(406.1 to 818.6)	67.5<br>(65.0 to 69.7)	0.1<br>(-1.4 to 1.4)
Parkinson's disease	188.0<br>(144.9 to 243.2)	15.1<br>(8.6 to 23.5)	20.2<br>(13.0 to 28.7)	72.6<br>(67.3 to 77.6)	3.1<br>(-0.1 to 6.1)
Epilepsy	85.5<br>(67.7 to 103.6)	3.9<br>(2.5 to 5.5)	19.6<br>(13.4 to 26.9)	64.7<br>(35.8 to 100.8)	-1.6<br>(-18.9 to 19.9)
Multiple sclerosis	11.7<br>(10.4 to 13.0)	0.1<br>(0.1 to 0.1)	2.7<br>(1.9 to 3.5)	79.4<br>(64.2 to 97.1)	7.2<br>(-1.9 to 17.8)
Motor neuron disease	2.2<br>(1.8 to 2.8)	0.4<br>(0.3 to 0.5)	0.5<br>(0.3 to 0.7)	77.2<br>(71.9 to 81.9)	5.8<br>(2.7 to 8.7)
Migraine	595.7<br>(540.1 to 657.3)	68.5<br>(53.5 to 85.7)	35.6<br>(24.2 to 49.3)	68.7<br>(65.4 to 71.8)	0.8<br>(-1.2 to 2.6)
Tension-type headache	1,476.6<br>(1,061.6 to 2,031.9)	482.1<br>(297.2 to 694.1)	10.6<br>(6.6 to 15.8)	68.4<br>(63.5 to 73.4)	0.6<br>(-2.3 to 3.6)
Other neurological disorders	0.1<br>(0.1 to 0.2)	0.2<br>(0.1 to 0.4)	1.6<br>(1.1 to 2.2)	83.6<br>(80.7 to 85.9)	9.7<br>(8.0 to 11.1)
**Mental and substance use disorders**	**1,990.3<br>(1,945.6 to 2,034.0)**	**1,030.1†<br>(854.1 to 1,260.0)**	**246.8<br>(181.2 to 320.6)**	**66.5<br>(64.8 to 68.2)**	**-0.5<br>(-1.5 to 0.5)**
Schizophrenia	9.2<br>(7.4 to 11.1)	--	4.9<br>(3.6 to 6.3)	82.0<br>(68.9 to 96.8)	8.8<br>(0.9 to 17.6)
Alcohol use disorders	83.5<br>(66.7 to 100.6)	30.2<br>(23.7 to 37.8)	7.2<br>(4.9 to 10.1)	78.0<br>(69.7 to 85.6)	6.3<br>(1.4 to 10.9)
Drug use disorders	55.4<br>(53.7 to 57.0)	4.2<br>(3.7 to 4.9)	13.2<br>(9.5 to 16.6)	67.8<br>(61.6 to 74.3)	0.3<br>(-3.4 to 4.1)
Opioid use disorders	25.5<br>(21.7 to 29.7)	--	8.8<br>(6.2 to 11.4)	68.5<br>(60.7 to 76.2)	0.7<br>(-4.0 to 5.3)
Cocaine use disorders	9.7<br>(8.5 to 11.0)	--	1.2<br>(0.7 to 1.6)	64.5<br>(48.8 to 81.9)	-1.7<br>(-11.1 to 8.7)
Amphetamine use disorders	4.8<br>(3.5 to 6.3)	1.3<br>(0.9 to 1.7)	0.6<br>(0.3 to 0.9)	53.3<br>(34.5 to 74.4)	-8.4<br>(-19.7 to 4.2)
Cannabis use disorders	5.3<br>(4.3 to 6.4)	--	0.1<br>(0.1 to 0.2)	66.8<br>(59.1 to 74.6)	-0.3<br>(-5.0 to 4.3)
Other drug use disorders	13.6<br>(12.1 to 15.5)	3.0<br>(2.6 to 3.4)	2.4<br>(1.7 to 3.2)	71.0<br>(61.8 to 80.5)	2.1<br>(-3.4 to 7.8)
Depressive disorders	870.6<br>(829.2 to 905.6)	961.1<br>(786.0 to 1,187.3)	131.2<br>(91.9 to 182.3)	65.0<br>(62.7 to 67.4)	-1.4<br>(-2.8 to -0.0)
Major depressive disorder	609.0<br>(508.1 to 732.0)	931.9<br>(760.0 to 1,157.5)	107.6<br>(73.5 to 152.2)	64.8<br>(62.1 to 67.4)	-1.5<br>(-3.2 to -0.0)
Dysthymia	276.5<br>(219.1 to 346.4)	29.2<br>(21.8 to 36.9)	23.6<br>(15.3 to 34.6)	66.0<br>(61.1 to 71.0)	-0.8<br>(-3.7 to 2.1)
Bipolar disorder	53.3<br>(42.9 to 66.8)	3.4<br>(2.4 to 5.0)	9.3<br>(5.7 to 13.8)	66.8<br>(60.0 to 73.9)	-0.4<br>(-4.4 to 3.9)
Anxiety disorders	515.6<br>(456.4 to 586.1)	22.9<br>(20.2 to 25.9)	42.7<br>(31.0 to 57.0)	65.1<br>(60.8 to 69.8)	-1.4<br>(-3.9 to 1.4)
Eating disorders	--	--	--	--	--
Anorexia nervosa	--	--	--	--	--
Bulimia nervosa	--	--	--	--	--
Autistic spectrum disorders	68.1<br>(65.2 to 70.9)	--	9.5<br>(6.6 to 13.1)	71.7<br>(67.3 to 75.7)	2.6<br>(-0.0 to 5.0)
Autism	26.3<br>(22.4 to 30.8)	--	5.8<br>(3.8 to 8.3)	71.5<br>(65.3 to 77.5)	2.4<br>(-1.2 to 6.1)
Asperger syndrome and other autistic spectrum disorders	42.4<br>(35.0 to 51.4)	--	3.7<br>(2.5 to 5.3)	72.0<br>(67.7 to 76.6)	2.7<br>(0.2 to 5.5)

Cause	Prevalence in thousands (95% UI)	Incidence in thousands (95% UI)	YLDs in thousands (95% UI)		
	2016 counts	2016 counts	2016 counts	Percentage change in counts between 2006 and 2016	Percentage change in rates between 2006 and 2016
Attention-deficit/hyperactivity disorder	< 0.05 (< 0.05 to < 0.05)	--	< 0.05 (< 0.05 to < 0.05)	90.7 (85.9 to 96.5)	14.0 (11.1 to 17.4)
Conduct disorder	--	--	--	--	--
Idiopathic developmental intellectual disability	37.1 (18.4 to 59.9)	--	1.9 (0.9 to 3.1)	57.2 (34.1 to 65.7)	-6.1 (-19.9 to -1.0)
Other mental and substance use disorders	415.6 (370.8 to 464.8)	8.2 (5.1 to 11.9)	26.9 (19.4 to 36.8)	68.6 (65.3 to 72.1)	0.7 (-1.2 to 2.8)
**Diabetes, urogenital, blood, and endocrine diseases**	**9,766.1 (9,656.4 to 9,859.5)**	**2,180.3† (1,953.8 to 2,437.8)**	**425.3 (307.8 to 553.5)**	**68.1 (65.0 to 71.3)**	**0.4 (-1.4 to 2.3)**
Diabetes mellitus	2,162.9 (1,931.7 to 2,387.9)	28.9 (17.9 to 42.0)	176.3 (124.9 to 243.1)	67.2 (61.9 to 72.6)	-0.1 (-3.3 to 3.1)
Acute glomerulonephritis	0.6 (0.5 to 0.8)	8.9 (6.9 to 11.3)	< 0.05 (< 0.05 to < 0.05)	66.7 (55.6 to 78.4)	-0.4 (-7.0 to 6.5)
Chronic kidney disease	5,994.4 (5,369.5 to 6,718.2)	399.3 (273.8 to 549.8)	150.9 (109.3 to 194.6)	68.8 (62.8 to 74.8)	0.9 (-2.8 to 4.4)
Chronic kidney disease due to diabetes mellitus	1,722.9 (1,254.5 to 2,265.5)	119.0 (74.1 to 182.0)	42.8 (27.6 to 60.5)	73.0 (64.8 to 81.1)	3.4 (-1.6 to 8.2)
Chronic kidney disease due to hypertension	1,554.5 (1,261.6 to 1,864.8)	39.2 (24.4 to 59.9)	60.7 (41.3 to 83.2)	68.2 (61.2 to 74.9)	0.5 (-3.7 to 4.5)
Chronic kidney disease due to glomerulonephritis	1,439.0 (1,170.4 to 1,726.5)	155.2 (102.2 to 223.5)	15.7 (10.0 to 23.5)	55.0 (44.0 to 65.2)	-7.4 (-14.0 to -1.3)
Chronic kidney disease due to other causes	1,278.0 (938.1 to 1,703.0)	85.9 (51.6 to 127.2)	31.7 (20.3 to 45.9)	71.9 (62.6 to 82.2)	2.7 (-2.9 to 8.9)
Urinary diseases and male infertility	946.3 (839.5 to 1,106.0)	1,473.2† (1,305.1 to 1,670.8)	28.7 (18.6 to 41.5)	74.9 (73.2 to 76.9)	4.5 (3.5 to 5.7)
Interstitial nephritis and urinary tract infections	21.6 (18.6 to 25.5)	1,158.8 (1,001.2 to 1,363.4)	0.6 (0.4 to 1.0)	82.3 (73.9 to 91.1)	8.9 (3.9 to 14.2)
Urolithiasis	9.5 (7.1 to 12.7)	245.3 (184.0 to 323.4)	0.6 (0.4 to 1.0)	81.8 (65.9 to 98.5)	8.6 (-0.9 to 18.6)
Benign prostatic hyperplasia	898.6 (746.1 to 1,084.0)	69.1 (41.9 to 103.9)	27.3 (17.4 to 39.7)	74.7 (73.0 to 76.6)	4.4 (3.3 to 5.5)
Male infertility	--	--	--	--	--
Other urinary diseases	--	--	0.2 (0.1 to 0.2)	67.2 (59.3 to 75.5)	-0.1 (-4.8 to 4.9)
Gynecological diseases	1,928.3 (1,803.5 to 2,118.8)	1.0† (0.7 to 1.5)	5.6 (2.8 to 9.9)	56.5 (53.5 to 59.5)	-6.5 (-8.3 to -4.7)
Uterine fibroids	36.3 (31.5 to 41.8)	1.0 (0.7 to 1.5)	0.2 (0.1 to 0.5)	64.6 (57.4 to 71.2)	-1.7 (-6.0 to 2.3)
Polycystic ovarian syndrome	--	--	--	--	--
Female infertility	--	--	--	--	--
Endometriosis	--	--	--	--	--
Genital prolapse	1,869.9 (1,647.5 to 2,116.3)	--	5.4 (2.6 to 9.5)	56.1 (53.2 to 59.2)	-6.7 (-8.5 to -4.9)
Premenstrual syndrome	--	--	--	--	--
Other gynecological diseases	--	--	--	--	--
Hemoglobinopathies and hemolytic anaemias	2,996.0 (2,971.5 to 3,023.1)	--	18.1 (12.3 to 25.2)	53.6 (49.0 to 58.5)	-8.2 (-11.0 to -5.3)
Thalassemias	< 0.05 (< 0.05 to < 0.05)	--	< 0.05 (< 0.05 to < 0.05)	32.9 (24.2 to 41.0)	-20.6 (-25.8 to -15.8)
Thalassaemias trait	499.8 (473.3 to 531.4)	--	8.6 (5.7 to 12.1)	73.6 (66.1 to 80.7)	3.7 (-0.7 to 8.0)
Sickle cell disorders	< 0.05 (< 0.05 to < 0.05)	--	< 0.05 (< 0.05 to < 0.05)	67.1 (62.5 to 72.1)	-0.2 (-2.9 to 2.8)
Sickle cell trait	411.2 (378.0 to 456.8)	--	1.2 (0.8 to 1.7)	72.1 (65.7 to 78.5)	2.8 (-1.0 to 6.7)
G6PD deficiency	322.1 (308.9 to 337.1)	--	0.1 (< 0.05 to 0.1)	62.2 (55.9 to 68.9)	-3.1 (-6.9 to 0.9)
G6PD trait	1,854.4 (1,816.1 to 1,897.0)	--	< 0.05 (< 0.05 to < 0.05)	61.7 (50.7 to 72.7)	-3.4 (-10.0 to 3.2)
Other hemoglobinopathies and hemolytic anaemias	157.2 (149.6 to 165.7)	--	8.3 (5.7 to 11.5)	35.5 (31.6 to 39.6)	-19.1 (-21.4 to -16.6)
Endocrine, metabolic, blood, and immune disorders	1,415.2 (1,147.6 to 1,742.8)	268.9 (196.8 to 352.5)	45.5 (29.6 to 65.1)	73.0 (65.0 to 80.5)	3.4 (-1.4 to 7.8)
**Musculoskeletal disorders**	**6,678.5 (6,542.9 to 6,844.9)**	**2,265.4 (1,782.0 to 2,878.9)**	**609.1 (448.4 to 789.0)**	**66.0 (64.2 to 67.6)**	**-0.8 (-1.9 to 0.1)**
Rheumatoid arthritis	163.0 (146.1 to 181.1)	3.1 (2.2 to 4.2)	33.9 (24.0 to 43.4)	72.5 (68.1 to 77.5)	3.1 (0.4 to 6.1)
Osteoarthritis	3,801.9 (3,601.8 to 4,011.4)	32.1 (25.5 to 39.1)	160.7 (114.2 to 218.3)	69.5 (68.3 to 70.8)	1.2 (0.6 to 2.0)
Low back and neck pain	3,331.8 (3,080.7 to 3,625.3)	1,402.4 (1,034.1 to 1,839.9)	349.5 (240.3 to 473.8)	65.9 (63.4 to 68.2)	-0.9 (-2.4 to 0.5)
Low back pain	2,601.0 (1,973.3 to 3,347.6)	1,199.0 (817.8 to 1,649.3)	249.8 (163.4 to 348.0)	64.7 (61.4 to 67.8)	-1.6 (-3.6 to 0.3)
Neck pain	1,120.8 (848.2 to 1,474.1)	203.4 (124.3 to 302.2)	99.7 (64.4 to 148.5)	69.1 (66.2 to 72.3)	1.0 (-0.7 to 2.9)
Gout	297.0 (236.2 to 369.2)	40.5 (27.8 to 54.4)	8.3 (5.4 to 11.6)	73.0 (66.4 to 79.8)	3.4 (-0.6 to 7.5)
Other musculoskeletal disorders	760.4 (547.2 to 1,094.7)	787.4 (503.4 to 1,176.4)	56.8 (35.1 to 88.0)	52.6 (42.0 to 63.5)	-8.8 (-15.2 to -2.3)
**Other non-communicable diseases**	**14,440.0 (14,422.0 to 14,461.8)**	**70,546.2† (60,123.4 to 84,117.6)**	**1,277.3 (922.5 to 1,706.6)**	**66.3 (63.2 to 69.4)**	**-0.6 (-2.5 to 1.2)**
Congenital anomalies	120.7 (114.4 to 126.6)	--	12.7 (9.4 to 16.4)	65.3 (60.9 to 69.6)	-1.2 (-3.9 to 1.3)
Neural tube defects	8.4 (7.7 to 9.2)	--	2.3 (1.7 to 3.1)	67.2 (57.5 to 76.4)	-0.1 (-5.9 to 5.4)
Congenital heart anomalies	25.9 (23.3 to 28.7)	--	1.4 (0.6 to 2.3)	77.1 (68.7 to 84.8)	5.8 (0.8 to 10.4)
Orofacial clefts	7.8 (6.9 to 8.6)	--	0.1 (0.1 to 0.1)	61.6 (56.5 to 66.2)	-3.5 (-6.5 to -0.7)
Down's syndrome	--	--	--	--	--
Turner syndrome	0.7 (0.6 to 0.9)	--	< 0.05 (< 0.05 to < 0.05)	73.3 (69.3 to 77.2)	3.5 (1.2 to 5.9)
Klinefelter syndrome	< 0.05 (< 0.05 to < 0.05)	--	< 0.05 (< 0.05 to < 0.05)	90.0 (83.4 to 97.2)	13.5 (9.6 to 17.9)

Cause	Prevalence in thousands (95% UI)	Incidence in thousands (95% UI)	YLDs in thousands (95% UI)		
	2016 counts	2016 counts	2016 counts	Percentage change in counts between 2006 and 2016	Percentage change in rates between 2006 and 2016
Other chromosomal abnormalities	< 0.05 (< 0.05 to < 0.05)	--	< 0.05 (< 0.05 to < 0.05)	96.0 (85.6 to 104.0)	17.1 (10.9 to 21.9)
Congenital musculoskeletal and limb anomalies	33.2 (30.7 to 35.6)	--	4.4 (3.1 to 5.8)	67.8 (59.8 to 75.6)	0.2 (-4.5 to 4.9)
Urogenital congenital anomalies	7.3 (6.6 to 8.1)	--	0.1 (0.1 to 0.2)	77.5 (69.5 to 85.3)	6.0 (1.2 to 10.7)
Digestive congenital anomalies	22.1 (20.0 to 24.4)	--	0.9 (0.6 to 1.2)	65.1 (56.2 to 74.3)	-1.4 (-6.7 to 4.2)
Other congenital anomalies	43.7 (29.9 to 62.1)	--	3.6 (2.3 to 5.6)	57.4 (49.3 to 65.0)	-6.0 (-10.8 to -1.4)
Skin and subcutaneous diseases	9,500.4 (9,221.4 to 9,803.5)	48,731.8 (38,890.8 to 61,412.5)	119.6 (76.3 to 188.3)	68.4 (66.7 to 70.1)	0.6 (-0.4 to 1.6)
Dermatitis	736.4 (639.1 to 850.1)	892.2 (647.9 to 1,178.7)	21.4 (13.2 to 33.0)	66.2 (63.4 to 69.3)	-0.7 (-2.4 to 1.1)
Psoriasis	146.8 (134.8 to 160.1)	8.3 (6.9 to 9.8)	11.0 (8.0 to 14.3)	72.6 (66.4 to 78.9)	3.1 (-0.6 to 6.9)
Cellulitis	26.2 (22.3 to 31.2)	652.4 (562.4 to 755.3)	1.3 (0.9 to 1.8)	65.7 (57.1 to 75.3)	-1.0 (-6.2 to 4.7)
Pyoderma	126.7 (116.5 to 137.8)	3,405.0 (3,112.4 to 3,736.1)	0.7 (0.3 to 1.3)	73.5 (68.8 to 78.1)	3.6 (0.8 to 6.4)
Scabies	190.6 (133.0 to 258.1)	599.0 (413.3 to 812.5)	4.4 (2.3 to 7.7)	58.6 (55.2 to 61.7)	-5.2 (-7.3 to -3.4)
Fungal skin diseases	6,764.8 (5,585.5 to 8,026.9)	36,709.4 (26,898.5 to 49,675.8)	34.5 (14.0 to 71.9)	68.8 (68.2 to 69.2)	0.8 (0.5 to 1.1)
Viral skin diseases	162.5 (134.7 to 201.0)	255.5 (211.4 to 308.8)	4.4 (2.8 to 6.7)	67.0 (63.0 to 71.4)	-0.2 (-2.6 to 2.4)
Acne vulgaris	50.6 (48.8 to 52.4)	16.9 (16.3 to 17.6)	1.2 (0.8 to 1.7)	70.4 (64.1 to 77.1)	1.8 (-2.0 to 5.8)
Alopecia areata	9.4 (8.4 to 10.6)	15.4 (13.8 to 17.3)	0.3 (0.2 to 0.4)	60.4 (46.2 to 75.9)	-4.2 (-12.6 to 5.1)
Pruritus	271.0 (187.4 to 377.6)	207.5 (145.6 to 278.3)	2.6 (1.2 to 5.1)	70.2 (66.9 to 73.3)	1.7 (-0.3 to 3.5)
Urticaria	48.2 (33.9 to 67.0)	82.8 (59.9 to 113.8)	2.5 (1.5 to 4.0)	67.1 (60.1 to 74.7)	-0.1 (-4.4 to 4.4)
Decubitus ulcer	112.4 (84.2 to 144.6)	419.4 (325.6 to 527.8)	15.7 (10.3 to 22.3)	66.1 (57.5 to 75.6)	-0.8 (-5.9 to 4.9)
Other skin and subcutaneous diseases	3,916.9 (3,642.8 to 4,204.4)	5,468.2 (4,977.6 to 6,009.4)	19.6 (9.6 to 34.4)	72.5 (70.8 to 74.2)	3.1 (2.1 to 4.1)
Sense organ diseases	13,226.2 (13,092.2 to 13,356.1)	3,691.5† (3,353.9 to 4,076.8)	1,012.8 (738.1 to 1,333.5)	65.8 (62.0 to 69.6)	-0.9 (-3.2 to 1.4)
Glaucoma	101.1 (80.1 to 126.3)	--	9.7 (6.7 to 13.9)	71.1 (66.5 to 75.8)	2.2 (-0.5 to 5.0)
Cataract	2,332.2 (1,916.7 to 2,815.5)	--	166.2 (116.3 to 227.1)	62.8 (60.9 to 64.8)	-2.7 (-3.9 to -1.5)
Macular degeneration	152.6 (120.7 to 188.7)	--	14.0 (9.7 to 19.9)	68.5 (63.3 to 74.2)	0.7 (-2.4 to 4.1)
Refraction and accommodation disorders	6,778.8 (6,249.8 to 7,353.1)	--	102.5 (63.8 to 155.3)	62.2 (60.4 to 63.9)	-3.1 (-4.2 to -2.1)
Age-related and other hearing loss	13,091.2 (12,906.7 to 13,262.2)	--	637.8 (460.7 to 834.0)	66.8 (60.8 to 72.9)	-0.3 (-3.9 to 3.3)
Other vision loss	454.5 (348.6 to 590.8)	--	34.1 (23.2 to 47.6)	67.8 (64.7 to 71.0)	0.2 (-1.6 to 2.2)
Other sense organ diseases	1,688.0 (1,470.1 to 1,928.5)	3,691.5 (3,353.9 to 4,076.8)	48.6 (31.2 to 71.5)	67.6 (64.1 to 72.1)	0.1 (-2.0 to 2.8)
Oral disorders	9,147.5 (8,948.4 to 9,319.8)	18,122.8† (15,337.5 to 20,856.8)	132.2 (85.4 to 189.3)	68.4 (67.0 to 69.8)	0.6 (-0.2 to 1.4)
Caries of deciduous teeth	--	--	--	--	--
Caries of permanent teeth	5,447.0 (4,730.2 to 6,203.2)	17,719.3 (15,026.5 to 20,496.1)	4.2 (1.8 to 7.7)	64.3 (62.1 to 66.8)	-1.8 (-3.1 to -0.4)
Periodontal disease	2,744.6 (2,102.1 to 3,515.2)	339.7 (142.1 to 558.9)	16.4 (6.2 to 34.4)	68.2 (66.9 to 69.6)	0.5 (-0.3 to 1.4)
Edentulism and severe tooth loss	4,156.6 (3,464.9 to 4,990.2)	63.9 (38.8 to 98.9)	106.6 (70.6 to 150.8)	68.7 (67.0 to 70.3)	0.8 (-0.2 to 1.8)
Other oral disorders	195.6 (172.8 to 219.8)	--	5.2 (3.2 to 7.7)	67.0 (63.4 to 70.3)	-0.2 (-2.4 to 1.7)
**Injuries**	**13,463.8 (12,607.8 to 14,384.1)**	**5,659.5† (5,089.8 to 6,290.6)**	**532.7 (375.9 to 728.2)**	**69.6 (67.2 to 71.7)**	**1.3 (-0.1 to 2.6)**
**Transport injuries**	**1,677.9 (1,574.0 to 1,796.4)**	**228.3 (188.8 to 273.1)**	**69.3 (46.4 to 99.4)**	**69.2 (68.0 to 70.4)**	**1.1 (0.3 to 1.8)**
Road injuries	1,369.6 (1,278.8 to 1,478.0)	170.4 (134.8 to 209.1)	56.4 (37.6 to 80.8)	70.8 (69.5 to 72.2)	2.1 (1.3 to 2.9)
Pedestrian road injuries	348.2 (301.4 to 400.6)	38.6 (28.5 to 51.8)	15.3 (10.3 to 21.9)	72.5 (71.0 to 74.1)	3.1 (2.2 to 4.0)
Cyclist road injuries	300.5 (254.8 to 344.9)	44.0 (31.1 to 59.1)	12.0 (7.9 to 17.3)	77.2 (75.6 to 78.9)	5.9 (4.9 to 6.9)
Motorcyclist road injuries	209.1 (179.4 to 242.5)	11.0 (7.1 to 16.0)	8.1 (5.4 to 11.6)	84.8 (83.4 to 86.2)	10.4 (9.6 to 11.2)
Motor vehicle road injuries	426.5 (365.7 to 489.4)	64.5 (48.6 to 82.3)	17.8 (12.0 to 25.3)	56.7 (55.0 to 58.4)	-6.4 (-7.4 to -5.4)
Other road injuries	85.2 (73.9 to 98.1)	12.4 (7.5 to 19.1)	3.2 (2.1 to 4.6)	96.3 (94.6 to 98.2)	17.3 (16.3 to 18.4)
Other transport injuries	308.3 (259.0 to 378.0)	57.9 (41.6 to 79.6)	13.0 (8.6 to 19.1)	62.6 (60.0 to 64.9)	-2.9 (-4.4 to -1.5)
**Unintentional injuries**	**11,350.2 (10,529.5 to 12,242.0)**	**5,330.5 (4,766.7 to 5,962.6)**	**450.7 (319.1 to 619.8)**	**70.0 (67.2 to 72.5)**	**1.6 (-0.1 to 3.0)**
Falls	7,198.4 (6,406.3 to 8,067.9)	3,438.0 (2,908.0 to 4,043.6)	336.3 (240.0 to 457.0)	71.8 (68.0 to 75.0)	2.6 (0.4 to 4.6)
Drowning	79.2 (66.0 to 99.3)	17.9 (15.3 to 21.5)	3.4 (2.3 to 4.9)	20.3 (16.6 to 23.4)	-28.1 (-30.3 to -26.3)
Fire, heat, and hot substances	628.6 (560.7 to 710.9)	44.5 (37.8 to 52.4)	10.7 (6.3 to 16.8)	60.5 (58.7 to 62.1)	-4.1 (-5.2 to -3.2)
Poisonings	18.7 (16.4 to 21.3)	8.2 (6.1 to 11.1)	1.0 (0.7 to 1.5)	76.7 (70.0 to 84.4)	5.5 (1.6 to 10.2)
Exposure to mechanical forces	1,208.2 (1,060.3 to 1,403.0)	202.1 (169.0 to 240.9)	24.3 (16.0 to 35.3)	72.8 (71.2 to 74.2)	3.2 (2.3 to 4.1)
Unintentional firearm injuries	42.1 (35.7 to 48.9)	3.7 (2.6 to 5.2)	0.8 (0.5 to 1.2)	70.8 (68.7 to 72.4)	2.0 (0.8 to 3.0)
Unintentional suffocation	52.3 (43.8 to 63.1)	17.0 (12.3 to 22.8)	1.5 (1.0 to 2.2)	72.2 (70.3 to 74.6)	2.9 (1.8 to 4.3)
Other exposure to mechanical forces	1,113.8 (967.5 to 1,312.2)	181.4 (151.6 to 218.2)	22.0 (14.4 to 31.9)	72.9 (71.2 to 74.3)	3.3 (2.3 to 4.2)

Cause	Prevalence in thousands (95% UI)	Incidence in thousands (95% UI)	YLDs in thousands (95% UI)		
	2016 counts	2016 counts	2016 counts	Percentage change in counts between 2006 and 2016	Percentage change in rates between 2006 and 2016
Adverse effects of medical treatment	48.8 (36.4 to 62.9)	639.2 (529.0 to 769.5)	6.5 (4.1 to 9.9)	60.0 (52.1 to 68.2)	-4.4 (-9.2 to 0.5)
Animal contact	242.9 (218.3 to 269.2)	92.1 (71.4 to 119.2)	4.5 (3.1 to 6.3)	54.8 (52.3 to 56.9)	-7.5 (-9.0 to -6.3)
Venomous animal contact	75.1 (64.6 to 88.0)	36.8 (27.2 to 50.0)	2.1 (1.5 to 3.0)	56.0 (52.7 to 59.2)	-6.8 (-8.8 to -4.9)
Non-venomous animal contact	167.8 (146.2 to 188.1)	55.3 (41.2 to 73.4)	2.4 (1.5 to 3.6)	53.8 (51.3 to 55.5)	-8.1 (-9.6 to -7.1)
Foreign body	420.7 (359.9 to 496.5)	413.5 (361.6 to 470.8)	14.5 (9.6 to 21.0)	66.2 (64.4 to 67.8)	-0.7 (-1.8 to 0.3)
Pulmonary aspiration and foreign body in airway	231.7 (185.4 to 298.4)	321.6 (274.3 to 369.3)	8.7 (5.7 to 12.7)	67.4 (65.0 to 69.7)	0.0 (-1.4 to 1.4)
Foreign body in eyes	20.2 (16.1 to 27.2)	32.4 (16.8 to 72.2)	1.2 (0.8 to 1.8)	75.5 (72.8 to 78.9)	4.9 (3.2 to 6.9)
Foreign body in other body part	168.8 (139.6 to 198.5)	59.5 (46.4 to 75.0)	4.6 (3.0 to 6.7)	61.9 (60.1 to 63.7)	-3.3 (-4.3 to -2.2)
Environmental heat and cold exposure	395.7 (352.2 to 445.1)	106.5 (84.8 to 133.5)	14.9 (10.3 to 20.7)	61.4 (58.9 to 63.5)	-3.6 (-5.1 to -2.3)
Other unintentional injuries	1,109.0 (959.5 to 1,275.4)	368.4 (268.3 to 480.8)	34.6 (23.1 to 49.4)	70.7 (69.1 to 72.0)	2.0 (1.0 to 2.8)
**Self-harm and interpersonal violence**	**495.8 (453.4 to 538.4)**	**97.5† (82.7 to 113.6)**	**11.2 (7.4 to 15.8)**	**52.6 (51.2 to 54.0)**	**-8.8 (-9.7 to -8.0)**
Self-harm	126.1 (105.7 to 146.3)	66.5 (54.4 to 80.0)	4.1 (2.7 to 5.8)	39.1 (37.1 to 41.2)	-16.9 (-18.1 to -15.6)
Self-harm by firearm	2.2 (1.8 to 2.8)	0.5 (0.2 to 1.1)	0.1 (< 0.05 to 0.1)	55.4 (51.2 to 59.5)	-7.2 (-9.7 to -4.7)
Self-harm by other specified means	123.8 (103.5 to 144.2)	66.0 (54.0 to 79.2)	4.0 (2.6 to 5.7)	39.0 (36.9 to 41.0)	-17.0 (-18.2 to -15.8)
Interpersonal violence	369.7 (334.9 to 413.6)	31.0† (22.9 to 40.0)	7.1 (4.7 to 10.1)	61.7 (60.5 to 62.8)	-3.4 (-4.1 to -2.7)
Assault by firearm	18.8 (16.1 to 21.7)	2.3 (1.4 to 3.5)	0.5 (0.3 to 0.7)	70.9 (69.4 to 72.4)	2.1 (1.2 to 3.0)
Assault by sharp object	58.4 (49.8 to 68.7)	7.8 (5.2 to 11.2)	1.2 (0.8 to 1.7)	59.2 (57.2 to 60.9)	-4.9 (-6.1 to -3.9)
Sexual violence	110.3 (90.6 to 133.3)	--	0.6 (0.4 to 0.9)	59.4 (57.4 to 61.3)	-4.8 (-6.0 to -3.6)
Assault by other means	182.2 (156.5 to 214.3)	20.9 (14.9 to 27.7)	4.9 (3.2 to 7.0)	61.8 (60.6 to 63.0)	-3.3 (-4.0 to -2.6)
**Forces of nature, conflict and terrorism, and executions and police conflict**	**50.1 (34.0 to 69.8)**	**3.2† (1.1 to 6.5)**	**1.5 (0.4 to 4.0)**	**103.6 (87.5 to 129.4)**	**21.6 (12.1 to 37.1)**
Forces of nature, conflict and terrorism, and state actor violence	19.1 (10.4 to 30.1)	0.4 (0.2 to 0.6)	0.6 (0.1 to 1.7)	138.0 (118.4 to 168.6)	42.2 (30.5 to 60.5)
Conflict and terrorism	26.0 (15.8 to 42.8)	--	0.8 (0.2 to 2.1)	87.9 (74.7 to 116.2)	12.3 (4.4 to 29.2)
Executions and police conflict	5.0 (1.7 to 8.2)	2.8 (0.8 to 6.1)	0.2 (< 0.05 to 0.5)	44.8 (5.9 to 110.2)	-13.5 (-36.8 to 25.6)

We did not calculate incidence for certain NTDs and causes with only birth prevalence. Blank cells mean that no estimate is available.

† Incidence for aggregate causes represents the sum of the incidence of the causes below the aggregate.

‡ Incidence estimates for stroke represent first-ever stroke only.

^ Incidence of HIV/AIDS represents new infections of HIV only and does not include new infections of Tuberculosis in HIV positive cases.

SR Table 12: Global prevalence, incidence, and YLDs for 2016, percentage change of YLD counts and percentage change of YLD rates between 2006 and 2016 by cause, age 95+ years.					
Cause	Prevalence in thousands (95% UI)	Incidence in thousands (95% UI)	YLDs in thousands (95% UI)		
	2016 counts	2016 counts	2016 counts	Percentage change in counts between 2006 and 2016	Percentage change in rates between 2006 and 2016
**All causes**	**3,297.9 (3,296.7 to 3,300.2)**	**32,291.1† (28,207.4 to 36,948.2)**	**1,114.4 (840.4 to 1,410.2)**	**55.1 (53.8 to 56.6)**	**-1.0 (-1.9 to -0.1)**
**Communicable, maternal, neonatal, and nutritional disorders**	**1,576.7 (1,553.9 to 1,603.4)**	**10,494.1† (8,625.0 to 12,908.5)**	**23.1 (16.8 to 31.9)**	**57.0 (50.6 to 97.9)**	**0.2 (-3.9 to 26.3)**
**HIV/AIDS and tuberculosis**	**457.1 (403.0 to 538.7)**	**3.9† (2.9 to 5.1)**	**1.6 (1.0 to 2.2)**	**48.4 (38.1 to 59.1)**	**-5.3 (-11.9 to 1.5)**
Tuberculosis	429.4 (350.1 to 536.0)	3.9† (2.9 to 5.1)	1.4 (0.9 to 1.9)	45.2 (39.2 to 52.7)	-7.3 (-11.2 to -2.5)
Drug-susceptible tuberculosis	4.8 (3.7 to 6.2)	3.7 (2.8 to 4.9)	1.3 (0.8 to 1.9)	45.2 (39.0 to 52.6)	-7.3 (-11.3 to -2.6)
Multidrug-resistant tuberculosis without extensive drug resistance	0.2 (0.1 to 0.2)	0.1 (0.1 to 0.1)	< 0.05 (< 0.05 to 0.1)	36.3 (15.5 to 64.6)	-13.0 (-26.3 to 5.0)
Extensively drug-resistant tuberculosis	< 0.05 (< 0.05 to < 0.05)	< 0.05 (< 0.05 to < 0.05)	< 0.05 (< 0.05 to < 0.05)	322.3 (260.6 to 407.3)	169.5 (130.1 to 223.7)
Latent tuberculosis infection	424.4 (344.6 to 530.3)	--	--	--	--
HIV/AIDS	2.1 (1.8 to 2.5)	--	0.2 (0.1 to 0.3)	72.5 (17.2 to 155.7)	10.1 (-25.2 to 63.1)
Drug-susceptible HIV/AIDS - Tuberculosis	0.1 (< 0.05 to 0.1)	0.1 (< 0.05 to 0.1)	< 0.05 (< 0.05 to < 0.05)	26.5 (16.1 to 36.9)	-19.2 (-25.9 to -12.7)
Multidrug-resistant HIV/AIDS - Tuberculosis without extensive drug resistance	< 0.05 (< 0.05 to < 0.05)	< 0.05 (< 0.05 to < 0.05)	< 0.05 (< 0.05 to < 0.05)	49.2 (3.9 to 102.9)	-4.8 (-33.7 to 29.5)
Extensively drug-resistant HIV/AIDS - Tuberculosis	< 0.05 (< 0.05 to < 0.05)	< 0.05 (< 0.05 to < 0.05)	< 0.05 (< 0.05 to < 0.05)	454.4 (244.0 to 722.2)	253.8 (119.5 to 424.7)
HIV/AIDS resulting in other diseases	2.0 (1.7 to 2.5)	--	0.2 (0.1 to 0.3)	80.6 (16.3 to 191.3)	15.3 (-25.8 to 85.9)
**Diarrhoea, lower respiratory infections, and other common infectious diseases**	**185.0 (171.3 to 202.2)**	**10,122.9† (8,270.7 to 12,546.7)**	**9.9 (6.9 to 15.9)**	**69.8 (58.3 to 174.7)**	**8.3 (1.0 to 75.3)**
Diarrhoeal diseases	51.0 (44.1 to 57.7)	3,506.6 (3,042.9 to 3,972.8)	5.0 (3.5 to 6.8)	64.0 (58.8 to 69.4)	4.7 (1.4 to 8.1)
Intestinal infectious diseases	0.1 (0.1 to 0.1)	1.8† (1.4 to 2.4)	< 0.05 (< 0.05 to < 0.05)	29.3 (2.0 to 66.1)	-17.5 (-34.9 to 6.0)
Typhoid fever	0.1 (0.1 to 0.1)	1.4 (1.1 to 1.9)	< 0.05 (< 0.05 to < 0.05)	32.8 (2.9 to 74.7)	-15.2 (-34.3 to 11.5)
Paratyphoid fever	< 0.05 (< 0.05 to < 0.05)	0.4 (0.3 to 0.5)	< 0.05 (< 0.05 to < 0.05)	23.8 (-1.5 to 55.5)	-21.0 (-37.1 to -0.8)
Other intestinal infectious diseases	--	--	< 0.05 (< 0.05 to < 0.05)	23.0 (-3.0 to 58.1)	-21.5 (-38.1 to 0.9)
Lower respiratory infections	25.8 (22.5 to 29.2)	1,115.0 (958.1 to 1,276.1)	1.4 (1.0 to 1.9)	72.8 (62.7 to 82.7)	10.2 (3.8 to 16.6)
Upper respiratory infections	72.7 (50.3 to 101.3)	5,440.4 (3,638.2 to 7,818.7)	1.6 (0.9 to 2.6)	51.6 (48.0 to 55.4)	-3.2 (-5.5 to -0.9)
Otitis media	19.3 (13.2 to 27.4)	10.4 (6.3 to 15.6)	0.5 (0.3 to 0.8)	51.1 (42.9 to 59.9)	-3.6 (-8.8 to 2.0)
Meningitis	6.7 (2.8 to 43.8)	0.9 (0.7 to 1.2)	0.6 (0.1 to 6.3)	519.2 (42.3 to 4,718.9)	295.2 (-9.2 to 2,975.1)
Pneumococcal meningitis	1.7 (1.2 to 2.1)	0.2 (0.1 to 0.4)	0.1 (< 0.05 to 0.1)	39.0 (33.4 to 45.7)	-11.3 (-14.8 to -7.0)
H influenzae type B meningitis	0.3 (0.2 to 0.4)	0.1 (< 0.05 to 0.1)	< 0.05 (< 0.05 to < 0.05)	1.6 (-9.0 to 19.8)	-35.1 (-42.0 to -23.6)
Meningococcal infection	3.5 (0.3 to 40.5)	0.2 (0.1 to 0.3)	0.5 (< 0.05 to 6.2)	3,128.5 (36.3 to 13,160.7)	1,960.2 (-13.0 to 8,362.0)
Other meningitis	1.2 (0.9 to 1.6)	0.4 (0.3 to 0.6)	0.1 (< 0.05 to 0.1)	78.6 (70.7 to 86.3)	13.9 (8.9 to 18.9)
Encephalitis	8.9 (4.6 to 14.8)	6.6 (5.3 to 7.9)	0.4 (0.3 to 0.5)	56.1 (45.9 to 66.9)	-0.4 (-6.9 to 6.5)
Diphtheria	--	--	--	--	--
Whooping cough	--	--	--	--	--
Tetanus	< 0.05 (< 0.05 to < 0.05)	0.1 (< 0.05 to 0.1)	< 0.05 (< 0.05 to < 0.05)	-2.3 (-26.2 to 34.3)	-37.6 (-52.9 to -14.3)
Measles	--	--	--	--	--
Varicella and herpes zoster	7.5 (5.1 to 10.7)	41.0 (27.5 to 58.8)	0.4 (0.2 to 0.6)	63.3 (54.2 to 72.5)	4.2 (-1.6 to 10.1)
**Neglected tropical diseases and malaria**	**369.9 (355.6 to 382.0)**	**277.2† (177.3 to 381.6)**	**3.5 (2.4 to 4.8)**	**37.6 (23.4 to 55.1)**	**-12.2 (-21.2 to -1.0)**
Malaria	0.6 (0.4 to 0.9)	10.1 (7.4 to 13.2)	< 0.05 (< 0.05 to < 0.05)	-7.3 (-20.2 to 5.0)	-40.9 (-49.1 to -33.0)
Chagas disease	7.6 (6.5 to 8.9)	< 0.05 (< 0.05 to 0.1)	0.3 (0.1 to 0.6)	54.9 (47.4 to 62.6)	-1.1 (-6.0 to 3.8)
Leishmaniasis	1.5 (1.2 to 1.8)	0.4 (0.3 to 0.6)	0.1 (< 0.05 to 0.1)	76.4 (62.9 to 91.0)	12.5 (3.9 to 21.9)
Visceral leishmaniasis	< 0.05 (< 0.05 to 0.1)	0.2 (0.1 to 0.3)	< 0.05 (< 0.05 to < 0.05)	-20.3 (-71.9 to 91.7)	-49.1 (-82.1 to 22.3)
Cutaneous and mucocutaneous leishmaniasis	1.3 (1.0 to 1.6)	0.2 (0.1 to 0.3)	0.1 (< 0.05 to 0.1)	84.0 (73.4 to 98.0)	17.4 (10.7 to 26.4)
African trypanosomiasis	< 0.05 (< 0.05 to < 0.05)	< 0.05 (< 0.05 to < 0.05)	< 0.05 (< 0.05 to < 0.05)	-61.4 (-96.4 to 228.2)	-75.4 (-97.7 to 109.5)
Schistosomiasis	23.9 (19.9 to 27.9)	4.7 (3.8 to 5.6)	0.2 (0.1 to 0.3)	-22.1 (-27.3 to -17.0)	-50.3 (-53.6 to -47.0)
Cysticercosis	1.9 (1.0 to 3.4)	--	0.2 (0.1 to 0.5)	41.4 (30.0 to 52.3)	-9.8 (-17.1 to -2.8)
Cystic echinococcosis	0.1 (0.1 to 0.3)	< 0.05 (< 0.05 to 0.1)	< 0.05 (< 0.05 to < 0.05)	-19.5 (-69.7 to 106.8)	-48.7 (-80.6 to 32.0)
Lymphatic filariasis	3.8 (3.0 to 5.1)	0.9 (0.5 to 1.4)	0.2 (0.1 to 0.3)	-34.3 (-53.8 to -19.5)	-58.1 (-70.5 to -48.6)
Onchocerciasis	1.3 (0.8 to 2.0)	0.3 (0.1 to 0.5)	0.1 (0.1 to 0.2)	-5.2 (-26.5 to 27.2)	-39.5 (-53.1 to -18.8)
Trachoma	11.0 (6.6 to 16.2)	--	0.8 (0.4 to 1.2)	26.3 (12.0 to 41.4)	-19.4 (-28.5 to -9.8)
Dengue	6.1 (3.4 to 10.2)	102.2 (63.6 to 152.6)	0.9 (0.4 to 1.6)	192.2 (139.3 to 562.6)	86.5 (52.7 to 322.8)
Yellow fever	< 0.05 (< 0.05 to < 0.05)	< 0.05 (< 0.05 to < 0.05)	< 0.05 (< 0.05 to < 0.05)	-9.8 (-44.2 to 50.1)	-42.4 (-64.4 to -4.2)
Rabies	< 0.05 (< 0.05 to < 0.05)	< 0.05 (< 0.05 to < 0.05)	< 0.05 (< 0.05 to < 0.05)	86.4 (-22.3 to 226.9)	18.9 (-50.4 to 108.6)
Intestinal nematode infections	304.8 (290.4 to 317.3)	--	0.5 (0.3 to 0.8)	14.3 (6.7 to 23.1)	-27.1 (-31.9 to -21.5)
Ascariasis	162.2 (144.4 to 184.1)	--	0.2 (0.1 to 0.3)	-4.4 (-15.2 to 8.2)	-39.0 (-45.9 to -30.9)

Cause	Prevalence in thousands (95% UI)	Incidence in thousands (95% UI)	YLDs in thousands (95% UI)		
	2016 counts	2016 counts	2016 counts	Percentage change in counts between 2006 and 2016	Percentage change in rates between 2006 and 2016
Trichuriasis	91.1 (83.1 to 99.8)	--	0.1 (< 0.05 to 0.1)	12.9 (-4.8 to 34.7)	-28.0 (-39.3 to -14.1)
Hookworm disease	82.5 (75.8 to 90.2)	--	0.3 (0.2 to 0.5)	28.3 (16.5 to 42.1)	-18.2 (-25.7 to -9.3)
Food-borne trematodiases	30.6 (20.8 to 38.0)	157.5 (70.3 to 248.1)	0.1 (< 0.05 to 0.2)	106.5 (74.3 to 147.1)	31.8 (11.2 to 57.7)
Leprosy	0.7 (0.7 to 0.8)	< 0.05 (< 0.05 to < 0.05)	< 0.05 (< 0.05 to 0.1)	65.5 (56.3 to 74.9)	5.6 (-0.2 to 11.6)
Ebola virus disease	< 0.05 (0.0 to < 0.05)	--	< 0.05 (0.0 to < 0.05)	--	--
Zika virus disease	< 0.05 (< 0.05 to < 0.05)	1.0 (0.5 to 1.9)	< 0.05 (< 0.05 to < 0.05)	--	--
Guinea worm disease	< 0.05 (< 0.05 to < 0.05)	< 0.05 (< 0.05 to < 0.05)	< 0.05 (< 0.05 to < 0.05)	-99.5 (-99.7 to -99.2)	-99.7 (-99.8 to -99.5)
Other neglected tropical diseases	3.8 (3.6 to 4.0)	--	0.1 (0.1 to 0.1)	76.4 (64.2 to 105.3)	12.6 (4.8 to 31.0)
**Maternal disorders**	**--**	**--**	**--**	**--**	**--**
Maternal haemorrhage	--	--	--	--	--
Maternal sepsis and other pregnancy related infections	--	--	--	--	--
Maternal hypertensive disorders	--	--	--	--	--
Maternal obstructed labour and uterine rupture	--	--	--	--	--
Maternal abortion, miscarriage, and ectopic pregnancy	--	--	--	--	--
Other maternal disorders	--	--	--	--	--
**Neonatal disorders**	**5.0 (4.5 to 5.8)**	**--**	**0.2 (0.1 to 0.3)**	**65.9 (51.8 to 81.2)**	**5.9 (-3.1 to 15.7)**
Neonatal preterm birth complications	4.6 (3.6 to 5.6)	--	0.2 (0.1 to 0.3)	65.5 (51.5 to 81.0)	5.6 (-3.3 to 15.5)
Neonatal encephalopathy due to birth asphyxia and trauma	< 0.05 (< 0.05 to < 0.05)	--	< 0.05 (< 0.05 to < 0.05)	188.8 (142.4 to 569.3)	84.3 (54.7 to 327.1)
Neonatal sepsis and other neonatal infections	< 0.05 (< 0.05 to < 0.05)	--	< 0.05 (< 0.05 to < 0.05)	189.4 (151.0 to 463.7)	84.7 (60.1 to 259.7)
Hemolytic disease and other neonatal jaundice	< 0.05 (< 0.05 to < 0.05)	--	< 0.05 (< 0.05 to < 0.05)	97.2 (24.8 to 189.7)	25.9 (-20.4 to 84.9)
Other neonatal disorders	--	--	--	--	--
**Nutritional deficiencies**	**289.3 (280.9 to 296.8)**	**26.1† (17.0 to 35.3)**	**7.2 (5.0 to 9.8)**	**52.9 (50.0 to 55.7)**	**-2.5 (-4.3 to -0.6)**
Protein-energy malnutrition	--	--	--	--	--
Iodine deficiency	52.5 (46.9 to 59.0)	0.1 (< 0.05 to 0.1)	1.2 (0.8 to 1.6)	43.9 (38.8 to 48.9)	-8.2 (-11.4 to -5.0)
Vitamin A deficiency	25.7 (17.8 to 33.9)	26.1 (17.0 to 35.2)	< 0.05 (< 0.05 to < 0.05)	75.9 (64.4 to 88.2)	12.3 (4.9 to 20.1)
Iron-deficiency anaemia	229.2 (214.3 to 250.4)	--	6.0 (4.1 to 8.2)	54.8 (51.5 to 58.2)	-1.2 (-3.3 to 0.9)
Other nutritional deficiencies	--	--	--	--	--
**Other communicable, maternal, neonatal, and nutritional diseases**	**846.4 (816.4 to 879.5)**	**64.1 (41.2 to 93.6)**	**0.8 (0.6 to 1.1)**	**59.6 (48.0 to 71.7)**	**1.9 (-5.6 to 9.5)**
Sexually transmitted diseases excluding HIV	684.5 (628.2 to 761.8)	2.9 (2.5 to 3.4)	0.6 (0.4 to 0.8)	57.0 (48.5 to 66.3)	0.2 (-5.2 to 6.1)
Syphilis	3.4 (2.6 to 4.8)	0.1 (0.1 to 0.3)	0.4 (0.2 to 0.6)	59.0 (47.0 to 70.1)	1.5 (-6.2 to 8.6)
Chlamydial infection	< 0.05 (< 0.05 to < 0.05)	--	< 0.05 (< 0.05 to < 0.05)	56.3 (2.6 to 172.3)	-0.2 (-34.5 to 73.8)
Gonococcal infection	< 0.05 (< 0.05 to < 0.05)	--	< 0.05 (< 0.05 to < 0.05)	155.1 (-97.8 to 3,353.1)	62.8 (-98.6 to 2,103.5)
Trichomoniasis	5.2 (4.4 to 6.2)	2.8 (2.4 to 3.2)	< 0.05 (< 0.05 to < 0.05)	56.0 (41.7 to 70.7)	-0.5 (-9.6 to 8.9)
Genital herpes	660.1 (579.1 to 760.4)	--	0.1 (< 0.05 to 0.3)	51.7 (47.8 to 55.6)	-3.2 (-5.7 to -0.7)
Other sexually transmitted diseases	--	--	< 0.05 (< 0.05 to < 0.05)	49.8 (-1.7 to 160.9)	-4.4 (-37.3 to 66.5)
Hepatitis	232.6 (206.5 to 261.4)	61.1 (38.3 to 90.6)	0.1 (0.1 to 0.2)	66.0 (18.5 to 132.7)	5.9 (-24.4 to 48.5)
Acute hepatitis A	0.6 (0.3 to 1.1)	8.2 (3.3 to 13.8)	< 0.05 (< 0.05 to < 0.05)	43.8 (-45.1 to 235.2)	-8.2 (-65.0 to 113.9)
Hepatitis B	111.4 (89.5 to 137.4)	28.9 (11.4 to 51.0)	0.1 (< 0.05 to 0.1)	65.2 (-9.4 to 192.4)	5.4 (-42.2 to 86.6)
Hepatitis C	118.9 (106.7 to 131.6)	1.8 (1.2 to 2.6)	< 0.05 (< 0.05 to < 0.05)	58.8 (41.3 to 79.5)	1.3 (-9.8 to 14.5)
Acute hepatitis E	1.7 (0.8 to 3.2)	22.3 (11.0 to 42.2)	< 0.05 (< 0.05 to 0.1)	81.9 (41.2 to 130.0)	16.1 (-9.9 to 46.8)
Other infectious diseases	6.0 (5.7 to 6.5)	< 0.05 (< 0.05 to < 0.05)	0.1 (0.1 to 0.2)	65.1 (59.2 to 70.8)	5.3 (1.6 to 9.0)
**Non-communicable diseases**	**3,296.7 (3,295.5 to 3,299.1)**	**19,945.8† (16,676.7 to 24,079.5)**	**935.8 (707.9 to 1,174.8)**	**55.4 (54.0 to 56.7)**	**-0.8 (-1.7 to 0.0)**
**Neoplasms**	**234.6 (230.6 to 238.4)**	**125.7 (116.3 to 138.0)**	**36.3 (27.3 to 44.8)**	**61.6 (58.5 to 64.7)**	**3.1 (1.2 to 5.1)**
Lip and oral cavity cancer	5.7 (5.5 to 5.9)	2.3 (2.2 to 2.4)	0.8 (0.6 to 1.0)	70.4 (59.1 to 82.4)	8.7 (1.5 to 16.4)
Nasopharynx cancer	0.3 (0.3 to 0.3)	0.1 (0.1 to 0.1)	0.1 (< 0.05 to 0.1)	62.9 (51.3 to 75.3)	3.9 (-3.5 to 11.9)
Other pharynx cancer	0.5 (0.4 to 0.5)	0.2 (0.2 to 0.2)	0.1 (0.1 to 0.1)	67.2 (57.2 to 77.8)	6.7 (0.3 to 13.5)
Oesophageal cancer	1.9 (1.8 to 2.0)	1.8 (1.7 to 1.8)	0.4 (0.3 to 0.5)	57.0 (47.8 to 66.5)	0.1 (-5.7 to 6.3)
Stomach cancer	14.3 (13.9 to 14.7)	9.3 (9.1 to 9.6)	2.1 (1.6 to 2.6)	64.3 (57.1 to 71.8)	4.9 (0.2 to 9.6)
Colon and rectum cancer	49.9 (48.4 to 51.5)	19.7 (19.2 to 20.3)	6.7 (5.1 to 8.2)	56.4 (50.4 to 62.2)	-0.2 (-4.0 to 3.5)
Liver cancer	2.5 (2.4 to 2.6)	3.0 (2.9 to 3.2)	0.6 (0.4 to 0.8)	93.1 (81.6 to 106.1)	23.2 (15.9 to 31.5)
Liver cancer due to hepatitis B	0.7 (0.4 to 1.0)	0.7 (0.4 to 0.9)	0.1 (0.1 to 0.2)	102.1 (83.4 to 126.3)	29.0 (17.1 to 44.4)

Cause	Prevalence in thousands (95% UI)	Incidence in thousands (95% UI)	YLDs in thousands (95% UI)		
	2016 counts	2016 counts	2016 counts	Percentage change in counts between 2006 and 2016	Percentage change in rates between 2006 and 2016
Liver cancer due to hepatitis C	1.7 (1.5 to 1.9)	1.5 (1.3 to 1.7)	0.3 (0.2 to 0.4)	90.2 (76.1 to 106.5)	21.4 (12.3 to 31.8)
Liver cancer due to alcohol use	0.4 (0.2 to 0.5)	0.4 (0.2 to 0.5)	0.1 (< 0.05 to 0.1)	89.5 (72.9 to 111.7)	20.9 (10.3 to 35.1)
Liver cancer due to other causes	0.5 (0.4 to 0.6)	0.5 (0.4 to 0.6)	0.1 (0.1 to 0.1)	93.0 (81.0 to 107.0)	23.2 (15.5 to 32.1)
Gallbladder and biliary tract cancer	2.2 (2.1 to 2.4)	2.6 (2.5 to 2.8)	0.5 (0.4 to 0.6)	60.4 (47.1 to 75.6)	2.3 (-6.2 to 12.1)
Pancreatic cancer	3.5 (3.3 to 3.6)	4.3 (4.2 to 4.4)	0.8 (0.5 to 1.0)	71.9 (59.3 to 85.9)	9.7 (1.7 to 18.6)
Larynx cancer	0.9 (0.9 to 0.9)	0.4 (0.4 to 0.4)	0.1 (0.1 to 0.2)	64.8 (54.9 to 75.5)	5.2 (-1.2 to 12.0)
Tracheal, bronchus, and lung cancer	12.2 (11.6 to 12.8)	10.1 (9.6 to 10.5)	2.1 (1.6 to 2.7)	88.9 (78.2 to 99.8)	20.5 (13.7 to 27.5)
Malignant skin melanoma	6.8 (5.3 to 8.0)	2.2 (1.7 to 2.6)	0.7 (0.5 to 0.9)	73.1 (58.9 to 87.9)	10.5 (1.4 to 19.9)
Non-melanoma skin cancer	25.0 (17.1 to 33.7)	21.0 (12.1 to 33.7)	0.8 (0.4 to 1.4)	34.8 (5.4 to 67.9)	-14.0 (-32.8 to 7.1)
Non-melanoma skin cancer (squamous-cell carcinoma)	20.2 (11.1 to 32.6)	12.3 (7.0 to 20.0)	0.8 (0.4 to 1.4)	34.8 (5.2 to 68.5)	-14.0 (-32.9 to 7.5)
Non-melanoma skin cancer (basal-cell carcinoma)	1.0 (0.3 to 2.6)	8.7 (2.8 to 19.2)	< 0.05 (< 0.05 to < 0.05)	27.9 (6.1 to 44.3)	-18.4 (-32.3 to -7.9)
Breast cancer	40.0 (38.6 to 41.5)	12.6 (12.1 to 13.1)	5.9 (4.5 to 7.2)	46.6 (39.1 to 53.7)	-6.4 (-11.2 to -1.9)
Cervical cancer	2.1 (1.9 to 2.2)	0.8 (0.8 to 0.9)	0.3 (0.2 to 0.4)	38.8 (25.6 to 51.9)	-11.4 (-19.9 to -3.1)
Uterine cancer	5.4 (5.1 to 5.8)	1.8 (1.7 to 1.9)	0.7 (0.5 to 0.8)	56.7 (41.6 to 71.1)	0.0 (-9.7 to 9.2)
Ovarian cancer	2.2 (2.2 to 2.3)	1.0 (1.0 to 1.1)	0.5 (0.3 to 0.6)	39.2 (25.6 to 54.6)	-11.2 (-19.9 to -1.3)
Prostate cancer	33.4 (28.3 to 35.8)	12.2 (10.4 to 13.0)	6.2 (4.5 to 7.8)	70.1 (63.3 to 79.4)	8.5 (4.2 to 14.5)
Testicular cancer	0.1 (0.1 to 0.1)	< 0.05 (< 0.05 to < 0.05)	< 0.05 (< 0.05 to < 0.05)	91.5 (74.3 to 111.6)	22.2 (11.2 to 35.1)
Kidney cancer	3.4 (3.3 to 3.5)	1.4 (1.3 to 1.4)	0.4 (0.3 to 0.5)	55.0 (43.2 to 67.3)	-1.1 (-8.6 to 6.7)
Bladder cancer	14.3 (13.9 to 14.7)	5.5 (5.3 to 5.6)	1.8 (1.3 to 2.2)	61.7 (54.7 to 69.4)	3.2 (-1.3 to 8.1)
Brain and nervous system cancer	0.8 (0.6 to 1.0)	0.5 (0.4 to 0.6)	0.2 (0.1 to 0.2)	85.0 (63.0 to 113.2)	18.0 (4.0 to 36.0)
Thyroid cancer	1.9 (1.8 to 2.0)	0.6 (0.5 to 0.6)	0.2 (0.2 to 0.3)	74.8 (58.2 to 92.5)	11.5 (1.0 to 22.9)
Mesothelioma	0.2 (0.2 to 0.2)	0.1 (0.1 to 0.2)	< 0.05 (< 0.05 to 0.1)	64.6 (56.6 to 72.5)	5.0 (-0.1 to 10.1)
Hodgkin lymphoma	0.2 (0.1 to 0.2)	0.1 (0.1 to 0.1)	< 0.05 (< 0.05 to < 0.05)	47.2 (37.1 to 55.3)	-6.1 (-12.5 to -0.9)
Non-Hodgkin's lymphoma	7.0 (6.5 to 7.3)	2.7 (2.6 to 2.9)	0.8 (0.6 to 1.0)	77.6 (66.1 to 90.5)	13.3 (6.0 to 21.5)
Multiple myeloma	1.5 (1.2 to 1.7)	0.8 (0.7 to 0.9)	0.4 (0.3 to 0.5)	64.8 (49.1 to 82.4)	5.1 (-4.9 to 16.4)
Leukaemia	7.9 (7.6 to 8.2)	3.2 (3.1 to 3.3)	1.7 (1.2 to 2.1)	60.5 (50.8 to 69.8)	2.4 (-3.8 to 8.3)
Acute lymphoid leukaemia	0.3 (0.3 to 0.3)	0.1 (0.1 to 0.1)	0.1 (< 0.05 to 0.1)	91.0 (79.3 to 103.2)	21.9 (14.4 to 29.7)
Chronic lymphoid leukaemia	3.4 (3.1 to 3.6)	1.2 (1.1 to 1.3)	0.7 (0.5 to 0.9)	53.6 (40.3 to 67.9)	-2.0 (-10.5 to 7.2)
Acute myeloid leukaemia	0.5 (0.5 to 0.6)	0.5 (0.5 to 0.5)	0.1 (0.1 to 0.2)	70.3 (58.8 to 82.4)	8.7 (1.3 to 16.4)
Chronic myeloid leukaemia	0.5 (0.5 to 0.6)	0.3 (0.2 to 0.3)	0.1 (0.1 to 0.1)	30.3 (17.2 to 45.6)	-16.8 (-25.2 to -7.1)
Other leukaemia	3.2 (3.1 to 3.4)	1.1 (1.1 to 1.2)	0.7 (0.5 to 0.9)	69.4 (55.5 to 84.0)	8.1 (-0.8 to 17.4)
Other neoplasms	12.7 (12.0 to 13.5)	5.2 (4.9 to 5.5)	1.4 (1.1 to 1.8)	81.4 (68.4 to 94.9)	15.8 (7.5 to 24.4)
**Cardiovascular diseases**	**1,845.2 (1,796.5 to 1,889.4)**	**419.9† (369.0 to 477.4)**	**169.6 (124.7 to 217.8)**	**45.4 (38.8 to 52.2)**	**-7.2 (-11.4 to -2.9)**
Rheumatic heart disease	2.4 (2.0 to 2.8)	4.2 (3.7 to 4.7)	0.2 (0.1 to 0.2)	67.9 (56.1 to 80.7)	7.2 (-0.4 to 15.3)
Ischaemic heart disease	463.9 (400.7 to 547.4)	243.2 (209.8 to 285.9)	30.6 (20.6 to 42.5)	55.4 (51.7 to 58.8)	-0.9 (-3.2 to 1.3)
Cerebrovascular disease	322.1 (294.0 to 349.3)	88.9‡ (77.2 to 101.4)	73.8 (50.8 to 97.1)	35.9 (22.4 to 50.8)	-13.3 (-21.9 to -3.8)
Ischaemic stroke	274.8 (214.3 to 337.8)	61.2‡ (51.0 to 72.2)	61.1 (40.6 to 82.2)	34.0 (17.7 to 50.6)	-14.5 (-24.9 to -3.9)
Hemorrhagic stroke	56.4 (44.1 to 71.9)	27.6‡ (22.8 to 32.4)	12.7 (8.7 to 17.0)	45.9 (29.4 to 63.7)	-6.9 (-17.4 to 4.5)
Hypertensive heart disease	181.5 (125.2 to 253.1)	--	13.6 (8.2 to 21.2)	61.1 (56.1 to 65.6)	2.8 (-0.4 to 5.7)
Cardiomyopathy and myocarditis	48.1 (37.1 to 62.8)	3.7† (2.6 to 4.7)	3.2 (1.9 to 5.1)	44.0 (37.2 to 51.3)	-8.1 (-12.5 to -3.4)
Myocarditis	0.9 (0.7 to 1.2)	3.7 (2.6 to 4.7)	< 0.05 (< 0.05 to 0.1)	56.3 (40.0 to 74.4)	-0.3 (-10.7 to 11.3)
Alcoholic cardiomyopathy	< 0.05 (< 0.05 to < 0.05)	--	< 0.05 (< 0.05 to < 0.05)	43.4 (-29.5 to 199.5)	-8.5 (-55.0 to 91.1)
Other cardiomyopathy	42.5 (26.3 to 62.4)	--	3.2 (1.8 to 5.1)	43.9 (36.9 to 51.3)	-8.2 (-12.6 to -3.4)
Atrial fibrillation and flutter	378.9 (333.1 to 429.2)	8.7 (5.2 to 13.2)	27.7 (19.1 to 37.8)	53.7 (51.7 to 55.8)	-1.9 (-3.2 to -0.6)
Peripheral vascular disease	925.9 (759.1 to 1,114.1)	64.8 (35.1 to 107.8)	5.1 (2.2 to 9.8)	47.4 (44.3 to 50.4)	-6.0 (-7.9 to -4.0)
Endocarditis	6.3 (4.2 to 8.9)	6.5 (5.0 to 8.2)	0.5 (0.3 to 0.7)	52.1 (43.1 to 61.0)	-3.0 (-8.7 to 2.7)
Other cardiovascular and circulatory diseases	292.0 (194.0 to 419.7)	--	14.9 (8.8 to 23.1)	48.6 (43.6 to 53.4)	-5.2 (-8.3 to -2.1)
**Chronic respiratory diseases**	**231.1 (216.9 to 246.3)**	**41.9† (33.6 to 51.0)**	**9.8 (6.8 to 13.3)**	**61.5 (58.6 to 64.7)**	**3.1 (1.2 to 5.1)**
Chronic obstructive pulmonary disease	--	--	--	--	--
Pneumoconiosis	1.2 (1.1 to 1.3)	0.2 (0.1 to 0.2)	0.2 (0.1 to 0.2)	80.4 (72.0 to 89.0)	15.1 (9.8 to 20.6)
Silicosis	0.2 (0.2 to 0.3)	0.1 (< 0.05 to 0.1)	< 0.05 (< 0.05 to < 0.05)	78.8 (55.5 to 102.9)	14.1 (-0.8 to 29.5)

Cause	Prevalence in thousands (95% UI)	Incidence in thousands (95% UI)	YLDs in thousands (95% UI)		
	2016 counts	2016 counts	2016 counts	Percentage change in counts between 2006 and 2016	Percentage change in rates between 2006 and 2016
Asbestosis	0.3<br>(0.3 to 0.4)	< 0.05<br>(< 0.05 to < 0.05)	< 0.05<br>(< 0.05 to 0.1)	79.5<br>(67.9 to 92.6)	14.6<br>(7.2 to 22.9)
Coal workers pneumoconiosis	0.2<br>(0.2 to 0.3)	< 0.05<br>(< 0.05 to < 0.05)	< 0.05<br>(< 0.05 to < 0.05)	71.4<br>(51.2 to 95.2)	9.4<br>(-3.5 to 24.6)
Other pneumoconiosis	0.4<br>(0.3 to 0.5)	0.1<br>(0.1 to 0.1)	0.1<br>(< 0.05 to 0.1)	87.8<br>(73.8 to 103.4)	19.8<br>(10.9 to 29.8)
Asthma	205.8<br>(180.0 to 236.6)	39.0<br>(30.9 to 48.0)	7.1<br>(4.7 to 10.1)	57.2<br>(54.0 to 60.6)	0.3<br>(-1.8 to 2.5)
Interstitial lung disease and pulmonary sarcoidosis	26.1<br>(21.5 to 31.5)	2.8<br>(2.0 to 3.6)	2.5<br>(1.7 to 3.6)	73.8<br>(68.0 to 79.8)	10.9<br>(7.2 to 14.7)
Other chronic respiratory diseases	--	--	< 0.05<br>(< 0.05 to < 0.05)	102.0<br>(95.5 to 108.6)	28.9<br>(24.8 to 33.1)
**Cirrhosis and other chronic liver diseases**	**50.6**<br>**(45.3 to 55.7)**	**7.3**<br>**(6.3 to 8.3)**	**2.1**<br>**(1.5 to 3.0)**	**66.0**<br>**(58.1 to 73.7)**	**5.9**<br>**(0.9 to 10.8)**
Cirrhosis and other chronic liver diseases due to hepatitis B	9.8<br>(7.5 to 12.1)	1.4<br>(1.0 to 1.8)	0.4<br>(0.2 to 0.6)	70.5<br>(59.1 to 81.9)	8.8<br>(1.5 to 16.1)
Cirrhosis and other chronic liver diseases due to hepatitis C	13.0<br>(10.2 to 16.3)	1.7<br>(1.2 to 2.2)	0.5<br>(0.3 to 0.8)	62.7<br>(51.9 to 73.3)	3.8<br>(-3.1 to 10.6)
Cirrhosis and other chronic liver diseases due to alcohol use	14.4<br>(11.5 to 17.3)	2.2<br>(1.7 to 2.7)	0.7<br>(0.5 to 1.0)	64.2<br>(54.2 to 73.6)	4.8<br>(-1.6 to 10.8)
Cirrhosis and other chronic liver diseases due to other causes	13.5<br>(9.8 to 17.8)	1.9<br>(1.4 to 2.6)	0.5<br>(0.3 to 0.8)	68.5<br>(57.4 to 79.2)	7.5<br>(0.5 to 14.3)
**Digestive diseases**	**363.2**<br>**(345.0 to 382.6)**	**298.1†**<br>**(263.3 to 339.9)**	**9.8**<br>**(6.8 to 13.4)**	**71.5**<br>**(64.5 to 78.1)**	**9.5**<br>**(5.0 to 13.7)**
Peptic ulcer disease	124.4<br>(102.7 to 149.2)	15.5<br>(13.5 to 17.9)	2.4<br>(1.4 to 3.7)	68.2<br>(56.2 to 80.2)	7.4<br>(-0.3 to 15.0)
Gastritis and duodenitis	199.3<br>(155.0 to 254.9)	110.0<br>(89.1 to 135.0)	2.9<br>(1.8 to 4.4)	86.7<br>(77.1 to 96.2)	19.1<br>(13.0 to 25.2)
Appendicitis	0.8<br>(0.7 to 0.8)	19.9<br>(18.1 to 22.0)	0.2<br>(0.1 to 0.3)	56.3<br>(46.4 to 65.7)	-0.3<br>(-6.6 to 5.7)
Paralytic ileus and intestinal obstruction	0.6<br>(0.5 to 0.7)	19.4<br>(15.5 to 23.7)	0.1<br>(0.1 to 0.2)	56.9<br>(35.2 to 79.0)	0.1<br>(-13.7 to 14.2)
Inguinal, femoral, and abdominal hernia	44.5<br>(23.5 to 78.5)	52.5<br>(35.1 to 74.8)	1.7<br>(1.1 to 2.6)	55.7<br>(45.0 to 66.3)	-0.6<br>(-7.5 to 6.1)
Inflammatory bowel disease	3.5<br>(2.9 to 4.1)	0.7<br>(0.5 to 0.9)	0.5<br>(0.3 to 0.6)	66.7<br>(57.4 to 77.1)	6.4<br>(0.4 to 13.0)
Vascular intestinal disorders	0.6<br>(0.4 to 0.7)	4.5<br>(3.6 to 5.8)	0.1<br>(0.1 to 0.2)	56.3<br>(32.5 to 83.1)	-0.3<br>(-15.4 to 16.8)
Gallbladder and biliary diseases	17.7<br>(14.0 to 23.0)	66.0<br>(51.3 to 86.3)	0.2<br>(0.2 to 0.4)	78.6<br>(59.7 to 98.4)	14.0<br>(1.9 to 26.6)
Pancreatitis	1.0<br>(0.8 to 1.3)	9.6<br>(7.8 to 11.8)	0.1<br>(0.1 to 0.2)	70.2<br>(48.4 to 92.1)	8.6<br>(-5.3 to 22.6)
Other digestive diseases	--	--	1.5<br>(1.0 to 2.0)	75.3<br>(68.1 to 82.0)	11.8<br>(7.3 to 16.2)
**Neurological disorders**	**1,305.9**<br>**(1,215.0 to 1,443.6)**	**384.3**<br>**(268.9 to 589.9)**	**191.5**<br>**(129.1 to 259.4)**	**57.4**<br>**(55.1 to 59.4)**	**0.4**<br>**(-1.1 to 1.7)**
Alzheimer's disease and other dementias	1,063.1<br>(838.1 to 1,373.5)	252.1<br>(150.4 to 440.7)	168.4<br>(110.7 to 233.7)	57.7<br>(55.2 to 59.9)	0.6<br>(-1.0 to 2.0)
Parkinson's disease	34.5<br>(25.9 to 45.8)	3.1<br>(1.6 to 5.9)	3.7<br>(2.4 to 5.5)	60.8<br>(55.0 to 65.8)	2.6<br>(-1.1 to 5.8)
Epilepsy	19.8<br>(15.6 to 23.9)	1.1<br>(0.6 to 1.8)	4.6<br>(3.1 to 6.3)	49.6<br>(24.9 to 82.4)	-4.5<br>(-20.3 to 16.4)
Multiple sclerosis	2.6<br>(2.3 to 3.0)	< 0.05<br>(< 0.05 to < 0.05)	0.6<br>(0.4 to 0.8)	57.6<br>(43.1 to 72.3)	0.5<br>(-8.7 to 10.0)
Motor neuron disease	0.4<br>(0.3 to 0.5)	0.1<br>(< 0.05 to 0.1)	0.1<br>(0.1 to 0.1)	62.3<br>(57.2 to 67.3)	3.6<br>(0.3 to 6.8)
Migraine	130.9<br>(113.2 to 152.0)	25.9<br>(16.1 to 38.9)	10.3<br>(6.8 to 14.6)	54.6<br>(51.5 to 57.8)	-1.4<br>(-3.3 to 0.7)
Tension-type headache	288.2<br>(208.4 to 395.5)	101.9<br>(62.6 to 167.0)	3.4<br>(2.1 to 5.2)	54.6<br>(50.7 to 59.0)	-1.3<br>(-3.8 to 1.5)
Other neurological disorders	< 0.05<br>(< 0.05 to 0.1)	0.1<br>(< 0.05 to 0.1)	0.4<br>(0.2 to 0.5)	61.2<br>(58.9 to 63.4)	2.9<br>(1.4 to 4.3)
**Mental and substance use disorders**	**432.4**<br>**(419.5 to 445.7)**	**241.6†**<br>**(184.1 to 313.8)**	**54.1**<br>**(39.8 to 71.6)**	**56.1**<br>**(54.2 to 58.0)**	**-0.4**<br>**(-1.6 to 0.8)**
Schizophrenia	1.4<br>(1.1 to 1.8)	--	0.8<br>(0.5 to 1.0)	75.6<br>(59.6 to 92.4)	12.0<br>(1.8 to 22.8)
Alcohol use disorders	24.1<br>(18.7 to 31.2)	8.4<br>(5.3 to 11.8)	2.1<br>(1.4 to 3.0)	70.5<br>(62.6 to 78.3)	8.8<br>(3.7 to 13.8)
Drug use disorders	12.9<br>(12.5 to 13.2)	1.2<br>(1.0 to 1.4)	2.9<br>(2.1 to 3.6)	60.4<br>(54.1 to 66.9)	2.4<br>(-1.6 to 6.5)
Opioid use disorders	4.9<br>(4.1 to 5.7)	--	1.7<br>(1.2 to 2.2)	65.8<br>(57.3 to 74.0)	5.8<br>(0.4 to 11.1)
Cocaine use disorders	2.1<br>(1.9 to 2.4)	--	0.2<br>(0.2 to 0.4)	54.1<br>(40.2 to 68.7)	-1.6<br>(-10.6 to 7.6)
Amphetamine use disorders	1.9<br>(1.3 to 2.6)	0.4<br>(0.3 to 0.6)	0.2<br>(0.1 to 0.3)	39.7<br>(21.6 to 58.0)	-10.8<br>(-22.4 to 0.8)
Cannabis use disorders	0.8<br>(0.6 to 1.0)	--	< 0.05<br>(< 0.05 to < 0.05)	61.0<br>(52.9 to 69.1)	2.7<br>(-2.5 to 7.9)
Other drug use disorders	4.0<br>(3.4 to 4.8)	0.8<br>(0.7 to 0.9)	0.7<br>(0.5 to 0.9)	57.3<br>(48.9 to 65.2)	0.4<br>(-5.0 to 5.5)
Depressive disorders	194.7<br>(181.2 to 207.7)	227.1<br>(170.3 to 299.5)	30.0<br>(20.4 to 43.3)	55.3<br>(52.3 to 57.9)	-0.9<br>(-2.8 to 0.8)
Major depressive disorder	144.1<br>(110.2 to 185.5)	221.5<br>(165.5 to 294.1)	25.5<br>(16.7 to 38.0)	55.0<br>(51.6 to 58.0)	-1.1<br>(-3.2 to 0.8)
Dysthymia	52.0<br>(38.8 to 67.6)	5.6<br>(3.4 to 7.9)	4.5<br>(2.8 to 6.7)	56.9<br>(51.6 to 62.0)	0.1<br>(-3.2 to 3.4)
Bipolar disorder	10.2<br>(7.9 to 13.2)	0.6<br>(0.3 to 1.1)	1.8<br>(1.1 to 2.8)	51.9<br>(44.8 to 60.2)	-3.1<br>(-7.6 to 2.2)
Anxiety disorders	96.8<br>(83.7 to 112.0)	1.7<br>(1.5 to 1.9)	8.0<br>(5.7 to 10.8)	51.8<br>(46.7 to 56.9)	-3.2<br>(-6.4 to 0.1)
Eating disorders	--	--	--	--	--
Anorexia nervosa	--	--	--	--	--
Bulimia nervosa	--	--	--	--	--
Autistic spectrum disorders	13.5<br>(12.8 to 14.0)	--	1.9<br>(1.3 to 2.6)	61.0<br>(56.9 to 65.2)	2.7<br>(0.1 to 5.4)
Autism	5.5<br>(4.7 to 6.4)	--	1.2<br>(0.8 to 1.7)	60.0<br>(53.9 to 66.5)	2.1<br>(-1.8 to 6.2)
Asperger syndrome and other autistic spectrum disorders	8.2<br>(6.8 to 9.9)	--	0.7<br>(0.5 to 1.0)	62.7<br>(58.5 to 67.4)	3.9<br>(1.1 to 6.8)

Cause	Prevalence in thousands (95% UI)	Incidence in thousands (95% UI)	YLDs in thousands (95% UI)		
	2016 counts	2016 counts	2016 counts	Percentage change in counts between 2006 and 2016	Percentage change in rates between 2006 and 2016
Attention-deficit/hyperactivity disorder	< 0.05 (< 0.05 to < 0.05)	--	< 0.05 (< 0.05 to < 0.05)	99.2 (91.9 to 106.5)	27.2 (22.4 to 31.7)
Conduct disorder	--	--	--	--	--
Idiopathic developmental intellectual disability	5.7 (2.7 to 9.8)	--	0.3 (0.2 to 0.5)	47.4 (20.1 to 57.5)	-6.0 (-23.4 to 0.5)
Other mental and substance use disorders	98.3 (86.7 to 111.7)	2.5 (1.3 to 4.6)	6.4 (4.5 to 8.8)	57.9 (54.8 to 60.8)	0.7 (-1.2 to 2.6)
**Diabetes, urogenital, blood, and endocrine diseases**	**2,250.0 (2,226.4 to 2,275.6)**	**627.1† (520.1 to 744.7)**	**99.3 (72.0 to 131.2)**	**60.1 (56.3 to 63.7)**	**2.2 (-0.2 to 4.4)**
Diabetes mellitus	432.5 (379.2 to 485.0)	6.8 (3.8 to 10.7)	36.2 (25.0 to 50.8)	61.8 (55.8 to 67.7)	3.2 (-0.6 to 7.0)
Acute glomerulonephritis	0.1 (0.1 to 0.2)	2.2 (1.6 to 3.0)	< 0.05 (< 0.05 to < 0.05)	69.2 (58.0 to 81.1)	8.0 (0.8 to 15.6)
Chronic kidney disease	1,622.3 (1,454.0 to 1,795.2)	152.9 (93.7 to 229.8)	40.9 (29.8 to 53.7)	60.2 (53.4 to 66.8)	2.2 (-2.1 to 6.5)
Chronic kidney disease due to diabetes mellitus	482.3 (327.5 to 647.2)	47.8 (26.8 to 78.7)	12.0 (7.4 to 17.6)	69.4 (60.7 to 79.6)	8.1 (2.6 to 14.6)
Chronic kidney disease due to hypertension	402.9 (320.9 to 504.3)	12.5 (6.2 to 21.7)	17.3 (11.7 to 24.0)	60.7 (53.0 to 68.9)	2.6 (-2.4 to 7.8)
Chronic kidney disease due to glomerulonephritis	421.3 (332.8 to 521.7)	62.6 (35.3 to 100.6)	3.8 (2.1 to 6.5)	38.8 (26.3 to 50.8)	-11.5 (-19.4 to -3.8)
Chronic kidney disease due to other causes	315.9 (205.6 to 450.0)	30.0 (15.5 to 50.0)	7.9 (4.5 to 12.2)	57.7 (47.2 to 68.5)	0.7 (-6.1 to 7.5)
Urinary diseases and male infertility	160.6 (142.2 to 187.7)	392.8† (320.0 to 479.9)	4.9 (3.2 to 7.1)	66.5 (64.8 to 68.2)	6.2 (5.2 to 7.3)
Interstitial nephritis and urinary tract infections	6.1 (4.9 to 7.7)	329.0 (257.8 to 414.7)	0.2 (0.1 to 0.3)	80.0 (71.7 to 90.5)	14.8 (9.6 to 21.6)
Urolithiasis	2.0 (1.4 to 2.7)	53.3 (37.6 to 71.6)	0.1 (0.1 to 0.2)	80.3 (66.0 to 94.8)	15.0 (5.9 to 24.3)
Benign prostatic hyperplasia	149.1 (119.2 to 181.5)	10.5 (6.0 to 17.9)	4.5 (2.9 to 6.7)	65.6 (63.9 to 67.3)	5.7 (4.6 to 6.7)
Male infertility	--	--	--	--	--
Other urinary diseases	--	--	< 0.05 (< 0.05 to < 0.05)	68.6 (61.1 to 76.8)	7.6 (2.8 to 12.8)
Gynecological diseases	374.9 (348.4 to 417.2)	0.2† (0.1 to 0.4)	1.1 (0.5 to 1.9)	41.0 (38.5 to 43.9)	-10.0 (-11.6 to -8.2)
Uterine fibroids	6.9 (5.7 to 8.2)	0.2 (0.1 to 0.4)	< 0.05 (< 0.05 to 0.1)	56.9 (49.1 to 65.2)	0.1 (-4.9 to 5.4)
Polycystic ovarian syndrome	--	--	--	--	--
Female infertility	--	--	--	--	--
Endometriosis	--	--	--	--	--
Genital prolapse	361.7 (312.5 to 416.6)	--	1.0 (0.5 to 1.8)	40.3 (37.6 to 43.3)	-10.4 (-12.2 to -8.6)
Premenstrual syndrome	--	--	--	--	--
Other gynecological diseases	--	--	--	--	--
Hemoglobinopathies and hemolytic anaemias	670.6 (665.4 to 676.1)	--	4.0 (2.7 to 5.8)	50.0 (42.2 to 57.7)	-4.3 (-9.3 to 0.6)
Thalassemias	< 0.05 (< 0.05 to < 0.05)	--	< 0.05 (< 0.05 to < 0.05)	39.0 (28.4 to 52.3)	-11.3 (-18.0 to -2.8)
Thalassaemias trait	107.7 (102.0 to 114.6)	--	1.7 (1.1 to 2.5)	77.4 (66.4 to 88.4)	13.2 (6.2 to 20.2)
Sickle cell disorders	< 0.05 (< 0.05 to < 0.05)	--	< 0.05 (< 0.05 to < 0.05)	47.6 (42.7 to 52.8)	-5.8 (-8.9 to -2.5)
Sickle cell trait	86.3 (80.0 to 95.0)	--	0.2 (0.1 to 0.3)	63.2 (55.6 to 70.8)	4.2 (-0.7 to 9.0)
G6PD deficiency	62.9 (60.4 to 65.7)	--	< 0.05 (< 0.05 to < 0.05)	59.8 (50.1 to 70.6)	1.9 (-4.2 to 8.9)
G6PD trait	437.2 (428.9 to 447.0)	--	< 0.05 (< 0.05 to < 0.05)	48.0 (33.3 to 62.9)	-5.5 (-14.9 to 4.0)
Other hemoglobinopathies and hemolytic anaemias	28.7 (27.0 to 31.0)	--	2.1 (1.3 to 3.1)	32.2 (25.4 to 39.2)	-15.6 (-20.0 to -11.2)
Endocrine, metabolic, blood, and immune disorders	373.8 (291.4 to 466.6)	72.2 (43.6 to 105.3)	12.1 (7.8 to 17.4)	58.2 (49.9 to 66.5)	0.9 (-4.4 to 6.2)
**Musculoskeletal disorders**	**1,477.2 (1,446.9 to 1,510.1)**	**456.9 (339.2 to 614.8)**	**127.6 (93.8 to 164.6)**	**55.6 (53.7 to 57.7)**	**-0.7 (-1.9 to 0.6)**
Rheumatoid arthritis	36.3 (32.4 to 40.6)	0.8 (0.5 to 1.1)	7.6 (5.3 to 9.8)	60.1 (55.1 to 64.4)	2.1 (-1.0 to 4.9)
Osteoarthritis	908.7 (863.7 to 957.4)	10.1 (7.5 to 13.4)	37.4 (26.7 to 50.9)	59.3 (57.6 to 61.4)	1.7 (0.6 to 3.0)
Low back and neck pain	664.7 (600.5 to 745.3)	268.5 (176.8 to 392.9)	67.3 (45.3 to 92.8)	53.9 (51.6 to 56.4)	-1.8 (-3.3 to -0.2)
Low back pain	525.7 (395.2 to 694.4)	234.5 (147.4 to 356.2)	49.6 (31.9 to 72.0)	52.1 (49.1 to 55.1)	-3.0 (-4.8 to -1.0)
Neck pain	199.7 (144.6 to 271.2)	34.0 (20.2 to 56.2)	17.8 (11.1 to 27.2)	59.1 (56.1 to 62.4)	1.6 (-0.4 to 3.6)
Gout	58.8 (45.4 to 73.6)	7.8 (4.9 to 11.8)	1.7 (1.1 to 2.4)	61.7 (54.8 to 68.6)	3.2 (-1.2 to 7.6)
Other musculoskeletal disorders	181.6 (126.8 to 263.7)	169.7 (100.9 to 276.8)	13.6 (8.2 to 21.3)	51.2 (40.2 to 62.4)	-3.5 (-10.5 to 3.6)
**Other non-communicable diseases**	**3,207.0 (3,197.6 to 3,215.0)**	**17,343.0† (13,986.0 to 21,429.5)**	**235.8 (170.5 to 319.3)**	**57.5 (55.9 to 59.3)**	**0.5 (-0.5 to 1.6)**
Congenital anomalies	26.3 (25.1 to 27.5)	--	2.8 (2.1 to 3.6)	53.7 (49.7 to 57.8)	-1.9 (-4.5 to 0.7)
Neural tube defects	1.9 (1.7 to 2.0)	--	0.5 (0.4 to 0.7)	57.0 (49.0 to 65.6)	0.2 (-4.9 to 5.7)
Congenital heart anomalies	6.1 (5.5 to 6.8)	--	0.3 (0.1 to 0.6)	65.3 (57.2 to 73.0)	5.5 (0.3 to 10.4)
Orofacial clefts	1.8 (1.6 to 2.0)	--	< 0.05 (< 0.05 to < 0.05)	58.4 (52.8 to 63.4)	1.1 (-2.5 to 4.2)
Down's syndrome	--	--	--	--	--
Turner syndrome	0.1 (0.1 to 0.2)	--	< 0.05 (< 0.05 to < 0.05)	50.0 (46.5 to 53.1)	-4.3 (-6.5 to -2.3)
Klinefelter syndrome	< 0.05 (< 0.05 to < 0.05)	--	< 0.05 (< 0.05 to < 0.05)	63.6 (57.6 to 69.6)	4.4 (0.6 to 8.2)

Cause	Prevalence in thousands (95% UI)	Incidence in thousands (95% UI)	YLDs in thousands (95% UI)		
	2016 counts	2016 counts	2016 counts	Percentage change in counts between 2006 and 2016	Percentage change in rates between 2006 and 2016
Other chromosomal abnormalities	< 0.05 (< 0.05 to < 0.05)	--	< 0.05 (< 0.05 to < 0.05)	107.1 (78.8 to 131.0)	32.1 (14.1 to 47.4)
Congenital musculoskeletal and limb anomalies	7.5 (6.9 to 8.0)	--	1.0 (0.7 to 1.3)	49.9 (43.0 to 57.5)	-4.3 (-8.7 to 0.5)
Urogenital congenital anomalies	1.6 (1.4 to 1.8)	--	< 0.05 (< 0.05 to < 0.05)	58.4 (51.4 to 65.4)	1.1 (-3.4 to 5.5)
Digestive congenital anomalies	4.9 (4.4 to 5.4)	--	0.2 (0.1 to 0.3)	53.5 (44.6 to 63.0)	-2.0 (-7.7 to 4.0)
Other congenital anomalies	8.8 (5.9 to 12.7)	--	0.8 (0.5 to 1.2)	51.7 (43.8 to 60.0)	-3.2 (-8.3 to 2.1)
Skin and subcutaneous diseases	2,221.6 (2,128.5 to 2,303.9)	12,497.3 (9,449.5 to 16,455.0)	28.1 (18.2 to 43.8)	56.5 (54.0 to 58.5)	-0.2 (-1.8 to 1.2)
Dermatitis	154.5 (133.2 to 181.0)	198.2 (142.5 to 267.0)	4.5 (2.8 to 6.9)	55.1 (52.2 to 57.9)	-1.0 (-2.9 to 0.8)
Psoriasis	24.9 (22.4 to 27.7)	1.7 (1.2 to 2.3)	1.9 (1.3 to 2.5)	56.9 (50.6 to 64.0)	0.1 (-3.9 to 4.6)
Cellulitis	6.9 (5.8 to 8.2)	171.8 (140.0 to 205.1)	0.3 (0.2 to 0.5)	56.1 (47.9 to 64.2)	-0.4 (-5.6 to 4.8)
Pyoderma	24.0 (22.0 to 26.2)	635.6 (570.1 to 709.4)	0.1 (0.1 to 0.2)	52.5 (48.1 to 56.8)	-2.7 (-5.5 to 0.0)
Scabies	44.4 (28.8 to 66.0)	139.5 (85.6 to 214.8)	1.0 (0.5 to 1.8)	69.2 (65.6 to 73.0)	8.0 (5.7 to 10.4)
Fungal skin diseases	1,684.7 (1,367.1 to 2,023.6)	9,947.2 (6,833.2 to 13,934.4)	8.6 (3.5 to 17.9)	56.9 (56.1 to 57.6)	0.1 (-0.4 to 0.6)
Viral skin diseases	32.5 (27.0 to 40.2)	50.4 (41.2 to 63.0)	0.9 (0.6 to 1.3)	54.6 (47.8 to 61.6)	-1.3 (-5.7 to 3.1)
Acne vulgaris	6.0 (5.7 to 6.3)	1.3 (1.3 to 1.4)	0.1 (0.1 to 0.2)	60.7 (54.5 to 67.2)	2.5 (-1.4 to 6.7)
Alopecia areata	1.4 (1.2 to 1.5)	2.2 (1.9 to 2.5)	< 0.05 (< 0.05 to 0.1)	52.6 (35.0 to 71.8)	-2.6 (-13.9 to 9.6)
Pruritus	62.0 (41.6 to 87.8)	45.8 (28.1 to 66.9)	0.6 (0.3 to 1.2)	61.1 (58.2 to 64.0)	2.8 (1.0 to 4.7)
Urticaria	9.4 (6.4 to 14.0)	16.2 (10.4 to 24.4)	0.5 (0.3 to 0.8)	57.5 (50.2 to 65.4)	0.5 (-4.2 to 5.5)
Decubitus ulcer	37.4 (27.0 to 49.6)	137.2 (97.5 to 180.8)	5.2 (3.3 to 7.6)	52.3 (43.0 to 62.1)	-2.8 (-8.7 to 3.4)
Other skin and subcutaneous diseases	843.5 (779.5 to 912.0)	1,150.2 (1,008.9 to 1,300.9)	4.2 (2.0 to 7.7)	59.0 (57.6 to 60.5)	1.5 (0.5 to 2.4)
Sense organ diseases	2,629.7 (2,601.0 to 2,657.0)	772.1† (680.5 to 876.0)	176.4 (128.4 to 236.8)	58.1 (55.9 to 60.4)	0.9 (-0.5 to 2.4)
Glaucoma	24.1 (18.4 to 30.2)	--	2.3 (1.5 to 3.5)	65.7 (61.3 to 70.5)	5.8 (2.9 to 8.8)
Cataract	603.8 (483.0 to 738.3)	--	44.9 (30.6 to 62.5)	59.1 (57.1 to 61.1)	1.5 (0.3 to 2.8)
Macular degeneration	38.6 (29.7 to 48.6)	--	3.8 (2.4 to 5.7)	52.2 (46.6 to 58.1)	-2.9 (-6.4 to 0.9)
Refraction and accommodation disorders	1,563.3 (1,400.2 to 1,756.2)	--	23.2 (14.2 to 36.4)	56.7 (54.7 to 58.5)	0.0 (-1.3 to 1.1)
Age-related and other hearing loss	2,093.2 (1,954.0 to 2,221.4)	--	83.7 (59.4 to 113.1)	57.2 (52.7 to 62.0)	0.3 (-2.5 to 3.4)
Other vision loss	124.7 (85.8 to 173.2)	--	9.5 (6.1 to 13.8)	65.5 (62.0 to 69.0)	5.6 (3.4 to 7.8)
Other sense organ diseases	312.4 (271.1 to 356.6)	772.1 (680.5 to 876.0)	9.0 (5.8 to 13.4)	57.1 (54.2 to 61.1)	0.2 (-1.6 to 2.8)
Oral disorders	2,002.5 (1,944.9 to 2,055.4)	4,073.7† (3,152.9 to 4,934.6)	28.6 (18.5 to 41.5)	55.6 (54.3 to 56.9)	-0.7 (-1.5 to 0.1)
Caries of deciduous teeth	--	--	--	--	--
Caries of permanent teeth	1,190.4 (997.8 to 1,401.7)	3,984.7 (3,072.7 to 4,849.7)	0.9 (0.4 to 1.8)	48.2 (45.5 to 50.7)	-5.4 (-7.1 to -3.8)
Periodontal disease	614.5 (430.6 to 816.8)	75.2 (26.3 to 131.4)	3.7 (1.3 to 7.9)	59.6 (58.3 to 61.0)	1.9 (1.0 to 2.8)
Edentulism and severe tooth loss	881.7 (725.0 to 1,085.9)	13.8 (7.0 to 23.7)	22.6 (15.0 to 32.1)	55.2 (53.7 to 56.9)	-0.9 (-1.9 to 0.1)
Other oral disorders	49.5 (39.8 to 60.1)	--	1.3 (0.8 to 1.9)	56.4 (52.8 to 60.2)	-0.2 (-2.5 to 2.2)
**Injuries**	**3,719.6 (3,453.7 to 3,987.1)**	**1,851.2† (1,575.2 to 2,180.9)**	**155.5 (109.3 to 215.5)**	**53.0 (49.3 to 56.8)**	**-2.4 (-4.7 to 0.0)**
**Transport injuries**	**411.9 (382.6 to 444.2)**	**61.9 (48.0 to 80.9)**	**17.1 (11.4 to 25.1)**	**61.3 (59.7 to 62.9)**	**2.9 (1.9 to 3.9)**
Road injuries	324.7 (302.1 to 355.4)	43.8 (31.8 to 60.7)	13.4 (8.9 to 19.6)	59.3 (57.6 to 61.1)	1.7 (0.6 to 2.8)
Pedestrian road injuries	85.8 (74.6 to 101.0)	9.7 (6.0 to 14.8)	3.8 (2.5 to 5.6)	61.9 (59.9 to 64.0)	3.3 (2.1 to 4.7)
Cyclist road injuries	68.3 (58.7 to 79.1)	9.3 (4.9 to 15.0)	2.7 (1.8 to 4.0)	62.2 (60.2 to 64.5)	3.5 (2.2 to 4.9)
Motorcyclist road injuries	44.9 (38.6 to 51.7)	2.9 (1.5 to 5.0)	1.7 (1.2 to 2.6)	76.5 (74.6 to 78.4)	12.6 (11.4 to 13.8)
Motor vehicle road injuries	105.7 (92.0 to 121.4)	18.5 (12.3 to 26.7)	4.4 (3.0 to 6.4)	46.6 (44.7 to 48.7)	-6.5 (-7.7 to -5.1)
Other road injuries	19.9 (17.4 to 22.8)	3.3 (1.7 to 5.9)	0.7 (0.5 to 1.1)	84.2 (81.9 to 87.3)	17.5 (16.1 to 19.5)
Other transport injuries	87.3 (70.5 to 104.9)	18.2 (12.0 to 26.2)	3.7 (2.4 to 5.5)	68.8 (66.0 to 71.5)	7.7 (5.9 to 9.5)
**Unintentional injuries**	**3,200.0 (2,943.7 to 3,457.7)**	**1,756.1 (1,484.0 to 2,086.9)**	**135.3 (95.2 to 185.8)**	**52.1 (47.9 to 56.3)**	**-3.0 (-5.6 to -0.2)**
Falls	2,080.4 (1,818.8 to 2,340.4)	1,089.8 (817.9 to 1,430.7)	101.9 (71.9 to 138.9)	51.2 (45.9 to 56.7)	-3.5 (-6.9 to -0.0)
Drowning	28.0 (22.8 to 34.2)	5.7 (4.6 to 7.2)	1.2 (0.8 to 1.7)	22.4 (18.7 to 25.9)	-21.9 (-24.2 to -19.7)
Fire, heat, and hot substances	169.4 (149.8 to 192.4)	16.9 (13.8 to 20.6)	3.0 (1.8 to 4.7)	48.0 (46.7 to 49.3)	-5.6 (-6.4 to -4.7)
Poisonings	4.6 (4.1 to 5.3)	2.7 (1.9 to 3.8)	0.2 (0.2 to 0.4)	62.9 (57.3 to 68.3)	4.0 (0.4 to 7.4)
Exposure to mechanical forces	300.2 (263.6 to 345.0)	74.0 (58.3 to 94.8)	6.5 (4.2 to 9.5)	57.5 (56.0 to 58.9)	0.5 (-0.5 to 1.4)
Unintentional firearm injuries	11.2 (9.4 to 13.0)	1.7 (1.0 to 2.6)	0.2 (0.1 to 0.3)	58.5 (56.6 to 60.6)	1.2 (-0.1 to 2.5)
Unintentional suffocation	17.6 (14.3 to 24.6)	7.0 (4.9 to 9.7)	0.5 (0.3 to 0.8)	60.2 (58.0 to 62.7)	2.2 (0.8 to 3.8)
Other exposure to mechanical forces	271.3 (235.3 to 316.5)	65.4 (51.0 to 84.7)	5.8 (3.8 to 8.4)	57.2 (55.7 to 58.7)	0.3 (-0.6 to 1.2)

Cause	Prevalence in thousands (95% UI)	Incidence in thousands (95% UI)	YLDs in thousands (95% UI)		
	2016 counts	2016 counts	2016 counts	Percentage change in counts between 2006 and 2016	Percentage change in rates between 2006 and 2016
Adverse effects of medical treatment	16.2 (11.6 to 22.2)	212.0 (161.7 to 275.5)	2.1 (1.3 to 3.4)	52.7 (42.1 to 63.4)	-2.6 (-9.3 to 4.2)
Animal contact	59.4 (53.6 to 65.7)	28.1 (20.8 to 37.4)	1.1 (0.8 to 1.6)	54.0 (51.9 to 55.9)	-1.8 (-3.1 to -0.5)
Venomous animal contact	16.1 (13.9 to 18.6)	10.6 (7.2 to 15.0)	0.5 (0.3 to 0.7)	57.1 (53.8 to 60.5)	0.3 (-1.9 to 2.4)
Non-venomous animal contact	43.3 (38.1 to 48.4)	17.5 (12.9 to 23.7)	0.6 (0.4 to 1.0)	51.6 (49.7 to 53.4)	-3.3 (-4.5 to -2.1)
Foreign body	145.5 (120.6 to 177.8)	174.3 (143.9 to 207.5)	5.2 (3.4 to 7.7)	63.8 (61.5 to 66.0)	4.5 (3.0 to 5.9)
Pulmonary aspiration and foreign body in airway	89.5 (68.0 to 123.6)	144.6 (116.7 to 174.8)	3.4 (2.2 to 5.1)	66.6 (63.2 to 69.5)	6.3 (4.2 to 8.2)
Foreign body in eyes	5.5 (4.4 to 7.8)	8.2 (3.3 to 23.7)	0.3 (0.2 to 0.5)	64.5 (61.6 to 67.7)	5.0 (3.1 to 7.0)
Foreign body in other body part	50.6 (40.9 to 59.7)	21.5 (14.5 to 32.2)	1.5 (1.0 to 2.2)	57.5 (55.9 to 59.0)	0.5 (-0.5 to 1.5)
Environmental heat and cold exposure	107.5 (94.5 to 127.0)	36.2 (25.1 to 51.5)	4.3 (2.9 to 6.1)	55.2 (52.8 to 57.3)	-1.0 (-2.5 to 0.4)
Other unintentional injuries	288.8 (247.3 to 337.7)	116.4 (79.9 to 169.7)	9.7 (6.5 to 13.9)	55.8 (53.9 to 58.1)	-0.6 (-1.8 to 0.9)
**Self-harm and interpersonal violence**	**99.8 (90.1 to 108.8)**	**32.6† (25.9 to 40.7)**	**2.7 (1.8 to 3.9)**	**47.2 (45.6 to 48.8)**	**-6.1 (-7.1 to -5.0)**
Self-harm	34.3 (28.9 to 40.6)	22.3 (16.6 to 28.8)	1.1 (0.7 to 1.6)	35.1 (32.6 to 37.7)	-13.8 (-15.4 to -12.1)
Self-harm by firearm	0.6 (0.5 to 0.7)	0.1 (< 0.05 to 0.3)	< 0.05 (< 0.05 to < 0.05)	61.7 (56.7 to 66.6)	3.2 (0.0 to 6.3)
Self-harm by other specified means	33.7 (28.4 to 40.0)	22.1 (16.4 to 28.6)	1.1 (0.7 to 1.6)	34.9 (32.4 to 37.4)	-13.9 (-15.5 to -12.3)
Interpersonal violence	65.5 (58.9 to 72.7)	10.3† (7.2 to 14.6)	1.6 (1.0 to 2.3)	57.2 (55.7 to 58.9)	0.3 (-0.6 to 1.4)
Assault by firearm	5.0 (4.3 to 5.7)	0.8 (0.4 to 1.5)	0.1 (0.1 to 0.2)	68.0 (66.0 to 70.3)	7.2 (5.9 to 8.7)
Assault by sharp object	13.6 (11.6 to 15.8)	2.7 (1.6 to 4.4)	0.3 (0.2 to 0.4)	57.1 (54.8 to 59.6)	0.3 (-1.2 to 1.9)
Sexual violence	5.3 (4.2 to 6.7)	--	< 0.05 (< 0.05 to < 0.05)	53.9 (51.4 to 55.9)	-1.8 (-3.4 to -0.5)
Assault by other means	41.6 (35.9 to 48.9)	6.8 (4.5 to 9.9)	1.1 (0.8 to 1.7)	56.1 (54.7 to 57.9)	-0.4 (-1.3 to 0.8)
**Forces of nature, conflict and terrorism, and executions and police conflict**	**13.2 (8.7 to 18.4)**	**0.5† (0.3 to 0.8)**	**0.4 (0.1 to 1.1)**	**88.6 (76.1 to 109.0)**	**20.4 (12.4 to 33.3)**
Forces of nature, conflict and terrorism, and state actor violence	5.0 (2.5 to 8.0)	0.2 (0.1 to 0.3)	0.2 (< 0.05 to 0.5)	131.1 (113.7 to 150.2)	47.5 (36.3 to 59.7)
Conflict and terrorism	7.0 (4.4 to 11.2)	--	0.2 (0.1 to 0.6)	62.1 (49.0 to 89.9)	3.5 (-4.9 to 21.2)
Executions and police conflict	1.2 (0.5 to 2.0)	0.3 (0.1 to 0.6)	< 0.05 (< 0.05 to 0.1)	23.9 (12.8 to 38.2)	-21.0 (-28.0 to -11.8)

We did not calculate incidence for certain NTDs and causes with only birth prevalence. Blank cells mean that no estimate is available.

† Incidence for aggregate causes represents the sum of the incidence of the causes below the aggregate.

‡ Incidence estimates for stroke represent first-ever stroke only.

^ Incidence of HIV/AIDS represents new infections of HIV only and does not include new infections of Tuberculosis in HIV positive cases.

SR Table 13a: Global prevalence and years lived with disability (YLDs) for 2016, percentage change of YLD counts and rates between 2006 and 2016 of anaemia by cause, ages 0-6 days				
Cause	Prevalence in thousands (95% uncertainty interval)	YLDs in thousands (95% uncertainty interval)		
	2016	2016	Percentage change in counts between 2006 and 2016	Percentage change in rates between 2006 and 2016
Iron-deficiency anaemia	305.6 (245.7 to 358.0)	10.9 (6.9 to 16.2)	-4.9% (-7.6% to -2.3%)	-4.4% (-7.1% to -1.8%)
Other infectious diseases	19.2 (15.5 to 22.5)	0.7 (0.4 to 1.0)	5.3% (1.3% to 9.0%)	5.8% (1.9% to 9.5%)
Endocrine, metabolic, blood, and immune disorders	14.1 (11.3 to 16.5)	0.5 (0.3 to 0.7)	-5.1% (-8.9% to -1.6%)	-4.6% (-8.4% to -1.1%)
Thalassaemias trait	15.1 (11.3 to 18.6)	0.5 (0.3 to 0.7)	-19.1% (-25.8% to -12.6%)	-18.6% (-25.4% to -12.1%)
Sickle cell trait	12.7 (10.3 to 15.4)	0.5 (0.3 to 0.7)	-5.8% (-10.9% to -0.8%)	-5.3% (-10.4% to -0.3%)
Other hemoglobinopathies and hemolytic anaemias	11.1 (9.0 to 13.0)	0.4 (0.2 to 0.6)	-8.5% (-12.5% to -4.7%)	-8.0% (-12.0% to -4.2%)
Sickle cell disorders	5.4 (4.5 to 7.0)	0.3 (0.2 to 0.4)	-0.4% (-8.2% to 7.2%)	0.1% (-7.7% to 7.8%)
Thalassemias	1.1 (0.9 to 1.4)	0.1 (<0.05 to 0.1)	-16.5% (-23.6% to -10.0%)	-16.0% (-23.2% to -9.5%)
G6PD deficiency	0.1 (0.1 to 0.1)	<0.05 (<0.05 to <0.05)	-8.3% (-11.6% to -4.8%)	-7.8% (-11.2% to -4.3%)
Chronic kidney disease due to other causes	<0.05 (<0.05 to <0.05)	<0.05 (<0.05 to <0.05)	-8.5% (-15.4% to -1.9%)	-8.0% (-14.9% to -1.4%)
Chronic kidney disease due to glomerulonephritis	<0.05 (<0.05 to <0.05)	<0.05 (<0.05 to <0.05)	-8.3% (-15.0% to -1.6%)	-7.9% (-14.6% to -1.0%)
G6PD trait	<0.05 (<0.05 to <0.05)	<0.05 (<0.05 to <0.05)	-15.0% (-19.2% to -10.6%)	-14.6% (-18.8% to -10.1%)
Chronic kidney disease due to diabetes mellitus	<0.05 (<0.05 to <0.05)	<0.05 (<0.05 to <0.05)	-7.7% (-15.1% to 0.5%)	-7.2% (-14.7% to 1.0%)
Chronic kidney disease due to hypertension	<0.05 (<0.05 to <0.05)	<0.05 (<0.05 to <0.05)	-10.4% (-18.3% to -2.7%)	-9.9% (-17.9% to -2.2%)
**All causes**	**384.4 (308.8 to 450.6)**	**13.8 (8.7 to 20.4)**	**-5.2% (-7.8% to -2.5%)**	**-4.7% (-7.3% to -2.0%)**

SR Table 13a: Global prevalence and years lived with disability (YLDs) for 2016, percentage change of YLD counts and rates between 2006 and 2016 of anaemia by cause, ages 7-27 days				
**Cause**	**Prevalence in thousands (95% uncertainty interval)**	**YLDs in thousands (95% uncertainty interval)**		
	**2016**	**2016**	**Percentage change in counts between 2006 and 2016**	**Percentage change in rates between 2006 and 2016**
Iron-deficiency anaemia	1,204.2 (1,055.1 to 1,334.1)	43.3 (28.1 to 62.3)	-2.5% (-5.2% to -0.0%)	-2.3% (-5.0% to 0.1%)
Other infectious diseases	75.8 (66.5 to 84.0)	2.7 (1.7 to 3.9)	7.7% (3.9% to 11.7%)	7.9% (4.1% to 11.9%)
Thalassaemias trait	69.5 (58.2 to 80.5)	2.3 (1.4 to 3.3)	-5.8% (-11.1% to -0.4%)	-5.6% (-11.0% to -0.2%)
Endocrine, metabolic, blood, and immune disorders	55.4 (48.6 to 61.4)	2.0 (1.3 to 2.9)	-2.5% (-6.4% to 0.9%)	-2.3% (-6.3% to 1.1%)
Sickle cell trait	50.4 (43.4 to 58.8)	1.8 (1.2 to 2.7)	2.5% (-2.1% to 7.2%)	2.6% (-1.9% to 7.4%)
Other hemoglobinopathies and hemolytic anaemias	43.8 (38.4 to 48.4)	1.5 (1.0 to 2.1)	-6.4% (-10.1% to -2.5%)	-6.2% (-9.9% to -2.4%)
Sickle cell disorders	18.2 (15.9 to 23.1)	1.0 (0.6 to 1.5)	2.8% (-3.8% to 9.5%)	3.0% (-3.6% to 9.7%)
Malaria	11.7 (8.5 to 15.4)	0.7 (0.4 to 1.1)	-36.2% (-46.3% to -23.7%)	-36.1% (-46.2% to -23.6%)
Thalassemias	4.2 (3.6 to 4.7)	0.2 (0.1 to 0.3)	-12.1% (-17.9% to -6.7%)	-11.9% (-17.7% to -6.6%)
G6PD deficiency	0.4 (0.4 to 0.5)	<0.05 (<0.05 to <0.05)	-6.9% (-10.5% to -3.9%)	-6.7% (-10.3% to -3.7%)
Chronic kidney disease due to other causes	<0.05 (<0.05 to <0.05)	<0.05 (<0.05 to <0.05)	-6.4% (-13.2% to 0.4%)	-6.2% (-13.0% to 0.6%)
Chronic kidney disease due to glomerulonephritis	<0.05 (<0.05 to <0.05)	<0.05 (<0.05 to <0.05)	-4.8% (-11.6% to 2.0%)	-4.6% (-11.5% to 2.1%)
G6PD trait	<0.05 (<0.05 to <0.05)	<0.05 (<0.05 to <0.05)	1.2% (-2.7% to 5.4%)	1.4% (-2.5% to 5.6%)
Chronic kidney disease due to diabetes mellitus	<0.05 (<0.05 to <0.05)	<0.05 (<0.05 to <0.05)	-5.4% (-13.2% to 3.3%)	-5.3% (-13.1% to 3.5%)
Chronic kidney disease due to hypertension	<0.05 (<0.05 to <0.05)	<0.05 (<0.05 to <0.05)	-9.2% (-16.8% to -1.3%)	-9.0% (-16.7% to -1.2%)
**All causes**	**1,533.6 (1,342.7 to 1,698.2)**	**55.4 (35.9 to 80.2)**	**-2.7% (-5.4% to -0.4%)**	**-2.5% (-5.2% to -0.2%)**

SR Table 13a: Global prevalence and years lived with disability (YLDs) for 2016, percentage change of YLD counts and rates between 2006 and 2016 of anaemia by cause, ages 28-364 days

Cause	Prevalence in thousands (95% uncertainty interval)	YLDs in thousands (95% uncertainty interval)		
	2016	2016	Percentage change in counts between 2006 and 2016	Percentage change in rates between 2006 and 2016
Iron-deficiency anaemia	42,772.2<br>(38,533.5 to 47,596.6)	1,455.7<br>(928.2 to 2,125.9)	0.2%<br>(-2.4% to 2.6%)	-0.7%<br>(-3.2% to 1.7%)
Other neglected tropical diseases	3,755.9<br>(3,414.9 to 4,134.5)	129.7<br>(83.4 to 190.0)	2.1%<br>(-0.8% to 4.9%)	1.3%<br>(-1.6% to 4.0%)
Thalassaemias trait	2,634.6<br>(2,231.4 to 3,125.8)	91.0<br>(56.0 to 136.4)	-2.4%<br>(-7.7% to 4.3%)	-3.2%<br>(-8.4% to 3.5%)
Other infectious diseases	2,694.4<br>(2,427.9 to 2,997.6)	90.9<br>(58.0 to 132.8)	12.6%<br>(9.0% to 16.3%)	11.7%<br>(8.1% to 15.3%)
Endocrine, metabolic, blood, and immune disorders	1,969.7<br>(1,775.4 to 2,191.4)	66.3<br>(42.4 to 97.3)	0.3%<br>(-2.7% to 3.3%)	-0.5%<br>(-3.5% to 2.4%)
Sickle cell trait	1,859.8<br>(1,607.3 to 2,162.6)	64.1<br>(41.3 to 95.8)	7.0%<br>(3.2% to 10.6%)	6.1%<br>(2.4% to 9.7%)
Other hemoglobinopathies and hemolytic anaemias	1,558.2<br>(1,410.0 to 1,728.8)	49.5<br>(31.7 to 72.5)	-5.3%<br>(-8.5% to -2.1%)	-6.0%<br>(-9.3% to -2.9%)
Malaria	587.6<br>(431.0 to 799.2)	33.7<br>(19.9 to 54.4)	-35.0%<br>(-45.2% to -24.5%)	-35.5%<br>(-45.6% to -25.1%)
Sickle cell disorders	323.8<br>(279.6 to 428.5)	21.8<br>(14.0 to 32.7)	8.2%<br>(1.9% to 14.4%)	7.3%<br>(1.0% to 13.5%)
HIV/AIDS resulting in other diseases	148.1<br>(138.4 to 158.1)	15.5<br>(11.1 to 20.4)	-22.4%<br>(-29.8% to -14.6%)	-23.0%<br>(-30.4% to -15.3%)
Hookworm disease	414.0<br>(361.5 to 473.7)	13.2<br>(8.3 to 19.7)	-6.2%<br>(-17.3% to 6.6%)	-7.0%<br>(-18.0% to 5.7%)
Thalassemias	65.4<br>(54.8 to 73.5)	4.8<br>(3.1 to 7.0)	-8.9%<br>(-14.4% to -3.9%)	-9.7%<br>(-15.1% to -4.7%)
Drug-susceptible HIV/AIDS - Tuberculosis	3.9<br>(2.7 to 5.3)	1.6<br>(0.9 to 2.4)	-56.2%<br>(-61.1% to -51.3%)	-56.6%<br>(-61.4% to -51.7%)
G6PD deficiency	20.8<br>(17.9 to 24.1)	1.0<br>(0.6 to 1.4)	-5.9%<br>(-14.7% to 2.2%)	-6.6%<br>(-15.4% to 1.4%)
Chronic kidney disease due to other causes	1.4<br>(0.9 to 2.0)	0.3<br>(0.2 to 0.6)	-2.8%<br>(-9.4% to 3.0%)	-3.6%<br>(-10.2% to 2.2%)
Chronic kidney disease due to glomerulonephritis	1.1<br>(0.7 to 1.5)	0.3<br>(0.1 to 0.4)	-1.4%<br>(-8.2% to 4.6%)	-2.2%<br>(-9.0% to 3.8%)
Multidrug-resistant HIV/AIDS - Tuberculosis without extensive drug resistance	0.1<br>(0.1 to 0.2)	<0.05<br>(<0.05 to 0.1)	-54.8%<br>(-65.2% to -43.1%)	-55.2%<br>(-65.5% to -43.6%)
Chronic kidney disease due to diabetes mellitus	0.1<br>(<0.05 to 0.1)	<0.05<br>(<0.05 to <0.05)	-1.3%<br>(-9.1% to 6.6%)	-2.1%<br>(-9.9% to 5.7%)
G6PD trait	0.2<br>(0.2 to 0.2)	<0.05<br>(<0.05 to <0.05)	3.0%<br>(-1.4% to 7.8%)	2.2%<br>(-2.2% to 6.9%)
Chronic kidney disease due to hypertension	<0.05<br>(<0.05 to <0.05)	<0.05<br>(<0.05 to <0.05)	-6.1%<br>(-13.6% to 0.9%)	-6.9%<br>(-14.3% to 0.0%)
Schistosomiasis	<0.05<br>(<0.05 to <0.05)	<0.05<br>(<0.05 to <0.05)	7.0%<br>(-1.9% to 16.7%)	6.2%<br>(-2.8% to 15.8%)
Extensively drug-resistant HIV/AIDS - Tuberculosis	<0.05<br>(<0.05 to <0.05)	<0.05<br>(<0.05 to <0.05)	109.3%<br>(70.0% to 155.5%)	107.6%<br>(68.6% to 153.4%)
**All causes**	**58,811.2**<br>**(52,889.6 to 65,426.2)**	**2,039.5**<br>**(1,309.4 to 2,980.6)**	**-0.5%**<br>**(-3.0% to 2.0%)**	**-1.3%**<br>**(-3.7% to 1.1%)**

SR Table 13a: Global prevalence and years lived with disability (YLDs) for 2016, percentage change of YLD counts and rates between 2006 and 2016 of anaemia by cause, ages 1-4 years				
**Cause**	**Prevalence in thousands (95% uncertainty interval)**	**YLDs in thousands (95% uncertainty interval)**		
	**2016**	**2016**	**Percentage change in counts between 2006 and 2016**	**Percentage change in rates between 2006 and 2016**
Iron-deficiency anaemia	154,787.1 (141,563.8 to 172,446.2)	4,961.5 (3,095.6 to 7,427.5)	0.1% (-4.3% to 4.7%)	-5.2% (-9.3% to -0.8%)
Other neglected tropical diseases	13,518.1 (12,450.5 to 14,909.0)	434.8 (271.5 to 650.2)	2.4% (-2.3% to 7.0%)	-3.0% (-7.4% to 1.4%)
Other infectious diseases	9,745.9 (8,917.1 to 10,849.4)	309.1 (192.3 to 461.7)	12.0% (6.8% to 18.0%)	6.1% (1.2% to 11.8%)
Thalassaemias trait	10,501.4 (8,287.1 to 13,524.5)	301.6 (171.5 to 495.6)	8.3% (-0.0% to 19.7%)	2.5% (-5.3% to 13.3%)
Malaria	4,689.2 (3,260.5 to 6,172.0)	231.0 (139.0 to 361.2)	-23.6% (-36.2% to -12.9%)	-27.6% (-39.6% to -17.5%)
Endocrine, metabolic, blood, and immune disorders	7,126.3 (6,519.5 to 7,936.4)	225.8 (142.2 to 338.7)	0.6% (-4.1% to 5.6%)	-4.7% (-9.2% to 0.0%)
Sickle cell trait	6,777.4 (5,921.3 to 7,924.5)	216.0 (134.9 to 321.3)	12.6% (6.7% to 19.2%)	6.7% (1.1% to 12.9%)
Other hemoglobinopathies and hemolytic anaemias	5,634.4 (5,174.4 to 6,243.6)	171.4 (107.1 to 253.9)	-3.2% (-8.8% to 3.8%)	-8.3% (-13.6% to -1.7%)
Sickle cell disorders	1,013.5 (895.9 to 1,269.2)	63.3 (40.8 to 94.8)	13.9% (6.0% to 22.5%)	7.9% (0.4% to 16.0%)
HIV/AIDS resulting in other diseases	606.8 (569.0 to 646.8)	62.1 (44.3 to 81.3)	-19.2% (-26.4% to -11.1%)	-23.4% (-30.3% to -15.8%)
Hookworm disease	1,544.0 (1,327.5 to 1,825.1)	43.5 (26.3 to 65.9)	-0.8% (-13.7% to 14.7%)	-6.0% (-18.2% to 8.6%)
Gastritis and duodenitis	962.0 (661.5 to 1,391.2)	24.5 (12.9 to 42.3)	17.6% (7.7% to 30.8%)	11.4% (2.0% to 23.9%)
Thalassemias	150.6 (112.3 to 174.9)	9.6 (5.6 to 14.7)	-3.2% (-11.7% to 4.8%)	-8.3% (-16.4% to -0.7%)
Drug-susceptible HIV/AIDS - Tuberculosis	16.3 (11.2 to 22.5)	6.6 (3.8 to 10.5)	-54.2% (-59.1% to -48.9%)	-56.6% (-61.3% to -51.6%)
G6PD deficiency	85.9 (71.5 to 106.5)	3.6 (2.1 to 5.8)	4.4% (-9.7% to 16.7%)	-1.1% (-14.4% to 10.6%)
Chronic kidney disease due to other causes	19.0 (11.1 to 30.7)	3.2 (1.5 to 6.2)	11.5% (2.5% to 18.9%)	5.6% (-2.9% to 12.7%)
Chronic kidney disease due to glomerulonephritis	13.5 (7.3 to 22.3)	2.3 (1.1 to 4.5)	12.4% (3.0% to 20.1%)	6.4% (-2.4% to 13.8%)
Schistosomiasis	6.6 (5.0 to 8.6)	0.4 (0.3 to 0.7)	-12.9% (-23.3% to -1.0%)	-17.5% (-27.4% to -6.2%)
Chronic kidney disease due to diabetes mellitus	1.1 (0.5 to 2.0)	0.2 (0.1 to 0.4)	13.5% (4.1% to 22.7%)	7.5% (-1.4% to 16.2%)
Multidrug-resistant HIV/AIDS - Tuberculosis without extensive drug resistance	0.5 (0.3 to 0.7)	0.2 (0.1 to 0.3)	-52.6% (-63.3% to -40.2%)	-55.1% (-65.2% to -43.3%)
Chronic kidney disease due to hypertension	0.3 (0.1 to 0.5)	0.1 (<0.05 to 0.1)	7.9% (-2.0% to 16.5%)	2.2% (-7.2% to 10.3%)
G6PD trait	0.6 (0.4 to 0.9)	<0.05 (<0.05 to 0.1)	12.0% (1.4% to 21.9%)	6.1% (-4.0% to 15.5%)
Extensively drug-resistant HIV/AIDS - Tuberculosis	<0.05 (<0.05 to <0.05)	<0.05 (<0.05 to <0.05)	150.2% (96.6% to 211.5%)	137.0% (86.2% to 195.0%)
**All causes**	**217,200.4 (197,837.8 to 243,074.4)**	**7,070.7 (4,408.1 to 10,631.5)**	**0.1% (-4.3% to 4.9%)**	**-5.2% (-9.3% to -0.7%)**

SR Table 13a: Global prevalence and years lived with disability (YLDs) for 2016, percentage change of YLD counts and rates between 2006 and 2016 of anaemia by cause, ages 5-9 years				
**Cause**	**Prevalence in thousands (95% uncertainty interval)**	**YLDs in thousands (95% uncertainty interval)**		
	**2016**	**2016**	**Percentage change in counts between 2006 and 2016**	**Percentage change in rates between 2006 and 2016**
Iron-deficiency anaemia	153,365.1 (142,685.4 to 164,250.8)	6,147.9 (3,986.2 to 9,111.0)	2.6% (0.3% to 5.6%)	-4.7% (-6.9% to -1.9%)
Other neglected tropical diseases	13,238.1 (12,322.8 to 14,154.6)	529.3 (344.3 to 781.4)	3.1% (-0.0% to 6.4%)	-4.3% (-7.1% to -1.1%)
Thalassaemias trait	11,037.5 (9,686.7 to 12,623.2)	385.3 (242.8 to 579.2)	-0.8% (-6.5% to 5.0%)	-7.9% (-13.2% to -2.5%)
Other infectious diseases	9,658.7 (8,986.7 to 10,343.7)	384.9 (249.2 to 566.0)	8.6% (5.4% to 12.2%)	0.8% (-2.1% to 4.2%)
Malaria	6,196.1 (4,523.2 to 7,970.4)	294.8 (175.8 to 473.2)	-21.2% (-30.6% to -12.7%)	-26.8% (-35.6% to -18.9%)
Endocrine, metabolic, blood, and immune disorders	7,058.7 (6,567.9 to 7,560.5)	280.5 (182.2 to 416.5)	2.8% (-0.4% to 6.3%)	-4.5% (-7.5% to -1.2%)
Sickle cell trait	6,892.3 (6,153.8 to 7,848.7)	273.8 (176.2 to 407.5)	4.3% (0.5% to 7.9%)	-3.2% (-6.7% to 0.2%)
Other hemoglobinopathies and hemolytic anaemias	5,574.8 (5,195.4 to 5,961.7)	222.2 (144.7 to 325.5)	3.1% (-0.7% to 7.7%)	-4.3% (-7.7% to 0.0%)
HIV/AIDS resulting in other diseases	885.1 (843.5 to 931.0)	115.7 (83.7 to 149.4)	-1.7% (-12.8% to 11.6%)	-8.7% (-19.0% to 3.6%)
Hookworm disease	2,184.2 (1,936.5 to 2,460.8)	80.3 (50.5 to 119.9)	-12.5% (-23.6% to 0.6%)	-18.8% (-29.0% to -6.6%)
Sickle cell disorders	886.6 (808.7 to 1,017.0)	64.1 (43.2 to 92.9)	15.5% (6.4% to 25.1%)	7.3% (-1.2% to 16.2%)
Gastritis and duodenitis	1,130.2 (809.8 to 1,533.4)	39.9 (23.3 to 63.9)	1.6% (-5.6% to 9.7%)	-5.6% (-12.3% to 1.9%)
Chronic kidney disease due to other causes	79.0 (49.6 to 117.6)	10.8 (5.9 to 18.3)	4.1% (-1.7% to 9.3%)	-3.3% (-8.7% to 1.5%)
Drug-susceptible HIV/AIDS - Tuberculosis	23.8 (16.4 to 33.1)	9.6 (5.6 to 15.4)	-27.3% (-36.8% to -17.7%)	-32.5% (-41.3% to -23.6%)
Chronic kidney disease due to glomerulonephritis	60.0 (34.5 to 91.5)	8.4 (4.3 to 14.2)	5.8% (0.4% to 10.5%)	-1.8% (-6.8% to 2.7%)
Peptic ulcer disease	139.3 (92.8 to 199.8)	8.0 (4.3 to 13.0)	-12.1% (-19.4% to -4.4%)	-18.4% (-25.1% to -11.2%)
Schistosomiasis	146.0 (117.4 to 179.0)	5.4 (3.2 to 8.2)	-28.3% (-37.1% to -18.4%)	-33.4% (-41.6% to -24.2%)
Thalassemias	63.4 (54.8 to 71.0)	4.3 (2.7 to 6.4)	-2.8% (-10.8% to 5.2%)	-9.7% (-17.1% to -2.3%)
G6PD deficiency	74.0 (66.1 to 82.1)	3.7 (2.3 to 5.3)	-7.9% (-14.9% to -0.7%)	-14.5% (-21.0% to -7.8%)
Chronic kidney disease due to diabetes mellitus	5.5 (2.8 to 9.4)	0.8 (0.4 to 1.5)	0.5% (-6.2% to 7.6%)	-6.7% (-12.9% to -0.1%)
Chronic kidney disease due to hypertension	2.2 (1.2 to 3.7)	0.3 (0.2 to 0.6)	1.1% (-5.1% to 7.2%)	-6.1% (-11.9% to -0.4%)
Multidrug-resistant HIV/AIDS - Tuberculosis without extensive drug resistance	0.6 (0.4 to 0.9)	0.3 (0.1 to 0.4)	-25.6% (-42.1% to -7.7%)	-30.9% (-46.2% to -14.3%)
G6PD trait	0.6 (0.5 to 0.7)	<0.05 (<0.05 to 0.1)	8.9% (3.9% to 14.5%)	1.2% (-3.5% to 6.3%)
Extensively drug-resistant HIV/AIDS - Tuberculosis	<0.05 (<0.05 to <0.05)	<0.05 (<0.05 to <0.05)	253.8% (187.1% to 338.1%)	228.6% (166.6% to 306.9%)
**All causes**	**218,702.1 (202,866.2 to 234,767.9)**	**8,870.4 (5,770.6 to 13,089.9)**	**1.6% (-0.8% to 4.6%)**	**-5.7% (-7.9% to -2.9%)**

SR Table 13a: Global prevalence and years lived with disability (YLDs) for 2016, percentage change of YLD counts and rates between 2006 and 2016 of anaemia by cause, ages 10-14 years				
**Cause**	**Prevalence in thousands (95% uncertainty interval)**	**YLDs in thousands (95% uncertainty interval)**		
	**2016**	**2016**	**Percentage change in counts between 2006 and 2016**	**Percentage change in rates between 2006 and 2016**
Iron-deficiency anaemia	97,982.7<br>(89,090.4 to 108,171.7)	3,720.6<br>(2,386.0 to 5,439.3)	-0.7%<br>(-3.7% to 2.4%)	-1.5%<br>(-4.5% to 1.5%)
Thalassaemias trait	6,581.0<br>(5,750.1 to 7,607.8)	224.2<br>(144.9 to 324.8)	-10.7%<br>(-16.4% to -4.8%)	-11.5%<br>(-17.1% to -5.5%)
Other hemoglobinopathies and hemolytic anaemias	4,838.0<br>(4,259.4 to 5,465.6)	187.0<br>(120.6 to 272.5)	2.1%<br>(-4.2% to 9.1%)	1.3%<br>(-5.0% to 8.2%)
Sickle cell trait	4,415.0<br>(3,877.4 to 5,112.7)	166.4<br>(107.7 to 249.3)	7.2%<br>(3.3% to 11.4%)	6.4%<br>(2.4% to 10.5%)
Malaria	2,787.1<br>(2,114.3 to 3,760.4)	129.8<br>(80.0 to 205.3)	-23.3%<br>(-32.8% to -10.5%)	-24.0%<br>(-33.3% to -11.2%)
Endocrine, metabolic, blood, and immune disorders	3,284.1<br>(2,951.4 to 3,669.3)	123.6<br>(79.0 to 182.0)	-0.7%<br>(-4.8% to 4.0%)	-1.5%<br>(-5.6% to 3.1%)
HIV/AIDS resulting in other diseases	632.5<br>(599.1 to 666.4)	115.9<br>(83.9 to 150.3)	37.3%<br>(18.4% to 59.7%)	36.2%<br>(17.4% to 58.4%)
Other infectious diseases	2,619.6<br>(2,314.1 to 2,954.9)	97.2<br>(62.4 to 141.1)	4.6%<br>(-0.5% to 9.7%)	3.7%<br>(-1.3% to 8.8%)
Other neglected tropical diseases	2,352.0<br>(2,073.0 to 2,649.3)	87.6<br>(55.8 to 127.4)	1.1%<br>(-3.8% to 6.6%)	0.3%<br>(-4.6% to 5.8%)
Hookworm disease	1,604.3<br>(1,396.5 to 1,835.3)	56.9<br>(36.6 to 83.9)	-11.3%<br>(-22.2% to 1.1%)	-12.0%<br>(-22.8% to 0.3%)
Gastritis and duodenitis	916.0<br>(704.2 to 1,205.8)	33.3<br>(20.1 to 52.0)	-12.0%<br>(-17.7% to -5.3%)	-12.8%<br>(-18.4% to -6.1%)
Sickle cell disorders	539.1<br>(494.1 to 599.7)	32.1<br>(21.2 to 47.6)	17.7%<br>(7.9% to 29.1%)	16.7%<br>(7.0% to 28.1%)
Peptic ulcer disease	474.3<br>(345.6 to 633.5)	19.8<br>(11.6 to 32.2)	-7.4%<br>(-14.1% to -0.5%)	-8.1%<br>(-14.8% to -1.3%)
Chronic kidney disease due to other causes	153.9<br>(99.7 to 226.3)	17.9<br>(9.6 to 29.7)	-1.2%<br>(-7.1% to 4.5%)	-2.0%<br>(-7.9% to 3.7%)
Schistosomiasis	473.8<br>(386.5 to 566.7)	17.4<br>(11.0 to 25.9)	-33.6%<br>(-39.8% to -27.9%)	-34.2%<br>(-40.3% to -28.5%)
Chronic kidney disease due to glomerulonephritis	135.3<br>(85.6 to 194.8)	15.7<br>(8.4 to 26.1)	-2.7%<br>(-8.4% to 2.3%)	-3.5%<br>(-9.2% to 1.4%)
Drug-susceptible HIV/AIDS - Tuberculosis	15.8<br>(10.7 to 22.2)	6.3<br>(3.6 to 10.1)	9.0%<br>(-5.5% to 23.6%)	8.1%<br>(-6.3% to 22.6%)
G6PD deficiency	38.1<br>(33.1 to 44.2)	2.1<br>(1.3 to 3.0)	1.2%<br>(-4.6% to 8.5%)	0.4%<br>(-5.4% to 7.6%)
Chronic kidney disease due to diabetes mellitus	13.4<br>(7.5 to 22.7)	1.7<br>(0.8 to 3.1)	-5.9%<br>(-12.4% to 0.5%)	-6.6%<br>(-13.1% to -0.3%)
Chronic kidney disease due to hypertension	7.7<br>(4.5 to 12.3)	1.0<br>(0.5 to 1.7)	-5.0%<br>(-10.9% to 0.9%)	-5.8%<br>(-11.6% to 0.0%)
Thalassemias	14.3<br>(12.0 to 16.8)	0.9<br>(0.6 to 1.4)	-9.8%<br>(-18.9% to -1.2%)	-10.5%<br>(-19.5% to -2.0%)
Multidrug-resistant HIV/AIDS - Tuberculosis without extensive drug resistance	0.4<br>(0.3 to 0.7)	0.2<br>(0.1 to 0.3)	21.0%<br>(-7.6% to 52.9%)	20.0%<br>(-8.4% to 51.6%)
Maternal haemorrhage	0.8<br>(0.5 to 1.3)	0.1<br>(<0.05 to 0.1)	-18.5%<br>(-23.7% to -13.7%)	-19.2%<br>(-24.4% to -14.4%)
G6PD trait	0.5<br>(0.4 to 0.5)	<0.05<br>(<0.05 to <0.05)	-2.1%<br>(-6.4% to 1.8%)	-2.9%<br>(-7.1% to 1.0%)
Extensively drug-resistant HIV/AIDS - Tuberculosis	<0.05<br>(<0.05 to <0.05)	<0.05<br>(<0.05 to <0.05)	396.8%<br>(300.8% to 506.5%)	392.7%<br>(297.5% to 501.5%)
**All causes**	**129,879.8**<br>**(117,967.0 to 143,789.1)**	**5,057.8**<br>**(3,263.0 to 7,335.2)**	**-1.1%**<br>**(-4.0% to 1.8%)**	**-2.0%**<br>**(-4.8% to 1.0%)**

SR Table 13a: Global prevalence and years lived with disability (YLDs) for 2016, percentage change of YLD counts and rates between 2006 and 2016 of anaemia by cause, ages 15-19 years				
**Cause**	**Prevalence in thousands (95% uncertainty interval)**	**YLDs in thousands (95% uncertainty interval)**		
	**2016**	**2016**	**Percentage change in counts between 2006 and 2016**	**Percentage change in rates between 2006 and 2016**
Iron-deficiency anaemia	107,466.9 (97,780.5 to 118,249.0)	2,477.5 (1,604.2 to 3,642.2)	1.8% (-1.5% to 5.4%)	7.7% (4.3% to 11.6%)
Thalassaemias trait	9,650.8 (8,356.5 to 11,228.0)	178.5 (111.2 to 275.3)	-19.2% (-26.3% to -12.1%)	-14.4% (-22.0% to -7.0%)
Sickle cell trait	5,353.2 (4,655.6 to 6,190.5)	119.2 (76.5 to 176.9)	11.4% (6.9% to 16.2%)	18.0% (13.1% to 23.0%)
Other hemoglobinopathies and hemolytic anaemias	5,418.6 (4,806.3 to 6,086.0)	101.3 (65.6 to 149.9)	19.2% (11.0% to 29.3%)	26.2% (17.5% to 36.9%)
Other gynecological diseases	2,853.2 (2,641.7 to 3,085.4)	85.6 (57.1 to 124.2)	1.2% (-3.1% to 5.8%)	7.1% (2.5% to 12.0%)
HIV/AIDS resulting in other diseases	738.6 (667.4 to 826.6)	81.0 (51.3 to 132.3)	44.3% (13.0% to 90.6%)	52.7% (19.6% to 101.7%)
Malaria	2,614.5 (2,131.1 to 3,259.1)	75.9 (47.1 to 115.3)	-23.6% (-31.3% to -14.1%)	-19.2% (-27.2% to -9.1%)
Endocrine, metabolic, blood, and immune disorders	3,635.9 (3,283.9 to 4,031.9)	70.4 (45.5 to 105.6)	4.5% (-0.4% to 9.7%)	10.6% (5.5% to 16.1%)
Hookworm disease	2,730.1 (2,376.5 to 3,157.4)	54.0 (33.6 to 81.6)	-6.4% (-19.6% to 9.2%)	-0.9% (-14.9% to 15.6%)
Other infectious diseases	2,923.0 (2,605.0 to 3,270.5)	49.2 (31.0 to 74.9)	8.9% (3.5% to 15.4%)	15.3% (9.6% to 22.1%)
Other neglected tropical diseases	2,582.7 (2,305.9 to 2,888.4)	43.3 (27.2 to 66.0)	8.3% (1.5% to 15.3%)	14.6% (7.5% to 22.0%)
Chronic kidney disease due to glomerulonephritis	488.6 (321.3 to 689.9)	40.7 (23.5 to 66.3)	2.0% (-4.4% to 7.6%)	8.0% (1.1% to 13.9%)
Chronic kidney disease due to other causes	464.3 (305.6 to 668.5)	37.0 (20.4 to 59.9)	4.4% (-2.6% to 10.7%)	10.5% (3.1% to 17.2%)
Schistosomiasis	1,294.0 (1,084.5 to 1,518.0)	28.1 (17.9 to 42.2)	-27.9% (-33.5% to -22.3%)	-23.7% (-29.6% to -17.8%)
Gastritis and duodenitis	1,382.9 (1,049.3 to 1,767.3)	27.2 (16.2 to 42.2)	-17.2% (-23.8% to -10.3%)	-12.3% (-19.4% to -5.1%)
Peptic ulcer disease	1,074.5 (785.4 to 1,479.8)	22.6 (12.9 to 37.2)	-8.7% (-15.6% to -1.3%)	-3.3% (-10.7% to 4.4%)
Sickle cell disorders	360.4 (332.3 to 394.6)	16.7 (11.0 to 25.2)	14.6% (2.5% to 26.8%)	21.3% (8.5% to 34.2%)
Drug-susceptible HIV/AIDS - Tuberculosis	31.4 (22.3 to 42.7)	12.5 (7.4 to 19.4)	-18.5% (-25.6% to -10.4%)	-13.8% (-21.3% to -5.1%)
Chronic kidney disease due to diabetes mellitus	75.3 (42.1 to 124.2)	8.3 (3.9 to 15.0)	0.4% (-7.7% to 7.7%)	6.2% (-2.3% to 14.0%)
Uterine fibroids	201.8 (154.3 to 258.2)	7.0 (4.0 to 11.5)	-5.5% (-12.5% to 2.1%)	0.1% (-7.4% to 8.1%)
Chronic kidney disease due to hypertension	41.9 (24.9 to 65.5)	4.7 (2.4 to 7.9)	0.6% (-6.5% to 7.5%)	6.5% (-1.1% to 13.7%)
Maternal haemorrhage	80.8 (57.0 to 111.9)	2.3 (1.3 to 3.7)	-21.8% (-32.4% to -10.7%)	-17.2% (-28.5% to -5.5%)
G6PD deficiency	57.0 (49.6 to 65.7)	1.9 (1.3 to 2.8)	7.2% (2.5% to 12.1%)	13.4% (8.5% to 18.6%)
Thalassemias	6.7 (5.5 to 7.7)	0.6 (0.4 to 0.8)	-11.6% (-20.4% to -3.8%)	-6.5% (-15.8% to 1.8%)
Multidrug-resistant HIV/AIDS - Tuberculosis without extensive drug resistance	0.6 (0.4 to 0.9)	0.3 (0.1 to 0.4)	-44.1% (-54.0% to -32.3%)	-40.8% (-51.3% to -28.3%)
G6PD trait	0.6 (0.5 to 0.7)	<0.05 (<0.05 to 0.1)	-8.5% (-14.0% to -2.4%)	-3.1% (-9.0% to 3.3%)
Extensively drug-resistant HIV/AIDS - Tuberculosis	<0.05 (<0.05 to <0.05)	<0.05 (<0.05 to <0.05)	78.4% (37.3% to 141.2%)	88.8% (45.4% to 155.3%)
**All causes**	**151,528.2 (137,702.7 to 167,102.8)**	**3,545.6 (2,329.2 to 5,233.3)**	**0.6% (-2.7% to 4.2%)**	**6.5% (3.0% to 10.3%)**

SR Table 13a: Global prevalence and years lived with disability (YLDs) for 2016, percentage change of YLD counts and rates between 2006 and 2016 of anaemia by cause, ages 20-24 years				
**Cause**	**Prevalence in thousands (95% uncertainty interval)**	**YLDs in thousands (95% uncertainty interval)**		
	**2016**	**2016**	**Percentage change in counts between 2006 and 2016**	**Percentage change in rates between 2006 and 2016**
Iron-deficiency anaemia	103,049.8 (90,945.7 to 115,758.9)	2,514.9 (1,636.2 to 3,738.0)	8.4% (3.9% to 13.2%)	3.4% (-0.9% to 7.9%)
HIV/AIDS resulting in other diseases	2,455.7 (2,257.7 to 2,693.6)	215.6 (152.8 to 288.7)	-8.6% (-18.4% to 6.8%)	-12.9% (-22.2% to 1.9%)
Thalassaemias trait	10,829.0 (9,158.6 to 12,713.8)	214.9 (136.2 to 325.1)	-2.3% (-10.9% to 7.5%)	-6.8% (-15.0% to 2.5%)
Sickle cell trait	5,108.2 (4,350.0 to 6,043.5)	116.9 (73.9 to 172.0)	17.6% (12.3% to 23.1%)	12.1% (7.1% to 17.5%)
Other gynecological diseases	3,267.1 (2,917.4 to 3,608.7)	98.6 (64.8 to 144.7)	8.3% (3.2% to 13.9%)	3.3% (-1.6% to 8.6%)
Other hemoglobinopathies and hemolytic anaemias	4,268.9 (3,658.4 to 4,949.5)	77.6 (49.7 to 116.1)	26.6% (17.4% to 37.4%)	20.8% (12.0% to 31.1%)
Chronic kidney disease due to glomerulonephritis	839.0 (557.8 to 1,157.9)	77.0 (43.7 to 120.0)	12.2% (4.8% to 19.5%)	7.0% (-0.1% to 14.0%)
Chronic kidney disease due to other causes	715.6 (453.1 to 995.1)	63.6 (34.7 to 100.0)	15.8% (7.6% to 24.4%)	10.5% (2.6% to 18.7%)
Endocrine, metabolic, blood, and immune disorders	3,197.4 (2,789.8 to 3,639.2)	62.5 (40.2 to 93.2)	10.4% (5.0% to 16.0%)	5.3% (0.1% to 10.7%)
Malaria	2,058.3 (1,678.6 to 2,522.8)	62.2 (38.4 to 94.6)	-24.5% (-31.9% to -15.3%)	-28.0% (-35.0% to -19.2%)
Hookworm disease	2,690.9 (2,304.9 to 3,127.9)	56.0 (35.0 to 84.9)	-1.4% (-14.5% to 12.7%)	-6.0% (-18.4% to 7.5%)
Other infectious diseases	2,378.2 (2,049.9 to 2,742.2)	39.5 (24.9 to 60.3)	12.8% (6.7% to 19.5%)	7.6% (1.8% to 14.0%)
Drug-susceptible HIV/AIDS - Tuberculosis	100.3 (71.1 to 137.0)	39.3 (23.2 to 61.3)	-37.1% (-41.6% to -32.5%)	-40.0% (-44.3% to -35.6%)
Peptic ulcer disease	1,699.0 (1,204.1 to 2,276.6)	37.4 (21.6 to 61.6)	11.9% (3.9% to 20.8%)	6.7% (-0.9% to 15.2%)
Gastritis and duodenitis	1,751.9 (1,329.0 to 2,238.2)	36.8 (22.3 to 59.0)	10.8% (2.8% to 19.6%)	5.7% (-2.0% to 14.1%)
Schistosomiasis	1,590.1 (1,345.9 to 1,870.8)	36.3 (23.0 to 55.4)	-26.0% (-30.7% to -21.0%)	-29.4% (-33.9% to -24.6%)
Other neglected tropical diseases	2,058.0 (1,775.9 to 2,358.3)	34.1 (21.5 to 52.4)	16.0% (9.6% to 23.6%)	10.6% (4.6% to 17.9%)
Chronic kidney disease due to diabetes mellitus	234.5 (139.8 to 358.9)	24.8 (12.9 to 42.2)	19.2% (10.9% to 27.9%)	13.7% (5.8% to 22.0%)
Uterine fibroids	573.6 (446.9 to 721.1)	20.8 (12.3 to 33.7)	5.6% (0.6% to 10.8%)	0.7% (-4.0% to 5.7%)
Chronic kidney disease due to hypertension	105.9 (66.0 to 159.5)	12.1 (6.5 to 20.5)	11.9% (5.0% to 19.3%)	6.7% (0.1% to 13.8%)
Sickle cell disorders	207.2 (190.5 to 228.0)	11.6 (7.8 to 16.8)	20.5% (8.5% to 33.0%)	14.9% (3.5% to 26.9%)
Maternal haemorrhage	279.0 (198.7 to 392.8)	7.1 (3.9 to 11.5)	-4.7% (-14.5% to 8.2%)	-9.1% (-18.5% to 3.2%)
G6PD deficiency	52.1 (44.2 to 61.3)	1.7 (1.1 to 2.4)	15.2% (10.0% to 20.6%)	9.9% (4.9% to 15.0%)
Multidrug-resistant HIV/AIDS - Tuberculosis without extensive drug resistance	2.2 (1.4 to 3.3)	0.9 (0.5 to 1.5)	-51.2% (-60.3% to -41.0%)	-53.5% (-62.1% to -43.7%)
Thalassemias	3.7 (2.9 to 4.4)	0.4 (0.2 to 0.5)	-1.8% (-9.7% to 7.6%)	-6.3% (-13.8% to 2.6%)
G6PD trait	0.7 (0.6 to 0.8)	<0.05 (<0.05 to 0.1)	10.4% (4.7% to 17.5%)	5.3% (-0.1% to 12.1%)
Extensively drug-resistant HIV/AIDS - Tuberculosis	<0.05 (<0.05 to <0.05)	<0.05 (<0.05 to <0.05)	79.4% (44.6% to 125.1%)	71.2% (37.9% to 114.7%)
**All causes**	**149,516.3 (131,283.0 to 168,240.8)**	**3,862.4 (2,553.0 to 5,651.8)**	**5.5% (1.4% to 9.6%)**	**0.6% (-3.3% to 4.5%)**

SR Table 13a: Global prevalence and years lived with disability (YLDs) for 2016, percentage change of YLD counts and rates between 2006 and 2016 of anaemia by cause, ages 25-29 years				
**Cause**	**Prevalence in thousands (95% uncertainty interval)**	**YLDs in thousands (95% uncertainty interval)**		
	**2016**	**2016**	**Percentage change in counts between 2006 and 2016**	**Percentage change in rates between 2006 and 2016**
Iron-deficiency anaemia	88,606.0<br>(78,161.5 to 101,225.4)	2,085.9<br>(1,344.4 to 3,083.1)	13.0%<br>(8.9% to 17.2%)	-4.8%<br>(-8.2% to -1.3%)
HIV/AIDS resulting in other diseases	4,408.0<br>(4,143.6 to 4,678.0)	420.2<br>(298.0 to 563.9)	-16.6%<br>(-28.6% to -5.0%)	-29.7%<br>(-39.9% to -20.0%)
Thalassaemias trait	11,489.4<br>(9,732.4 to 13,609.6)	214.4<br>(136.5 to 323.7)	30.5%<br>(19.2% to 41.5%)	9.9%<br>(0.5% to 19.2%)
Sickle cell trait	4,566.3<br>(3,877.5 to 5,426.5)	96.4<br>(61.8 to 145.1)	23.6%<br>(18.4% to 28.9%)	4.1%<br>(-0.3% to 8.6%)
Chronic kidney disease due to glomerulonephritis	1,044.1<br>(704.2 to 1,462.2)	95.3<br>(56.5 to 151.9)	26.0%<br>(16.0% to 35.2%)	6.2%<br>(-2.3% to 13.9%)
Other gynecological diseases	2,867.6<br>(2,578.1 to 3,198.8)	82.0<br>(52.7 to 119.7)	13.7%<br>(8.4% to 19.3%)	-4.3%<br>(-8.7% to 0.5%)
Chronic kidney disease due to other causes	870.1<br>(575.8 to 1,234.8)	76.7<br>(43.2 to 121.0)	25.9%<br>(15.9% to 35.4%)	6.1%<br>(-2.4% to 14.1%)
Drug-susceptible HIV/AIDS - Tuberculosis	156.6<br>(112.2 to 212.4)	60.9<br>(36.2 to 94.0)	-35.5%<br>(-39.7% to -30.9%)	-45.7%<br>(-49.2% to -41.8%)
Other hemoglobinopathies and hemolytic anaemias	3,569.1<br>(3,036.4 to 4,208.5)	55.9<br>(35.2 to 85.3)	25.5%<br>(18.5% to 34.1%)	5.7%<br>(-0.2% to 13.0%)
Uterine fibroids	1,471.6<br>(1,152.3 to 1,831.9)	52.4<br>(31.1 to 85.5)	17.9%<br>(14.0% to 22.0%)	-0.7%<br>(-4.0% to 2.7%)
Chronic kidney disease due to diabetes mellitus	530.0<br>(343.5 to 771.5)	52.2<br>(29.3 to 83.5)	40.1%<br>(29.0% to 51.8%)	18.0%<br>(8.6% to 27.8%)
Peptic ulcer disease	2,321.7<br>(1,645.4 to 3,082.7)	51.0<br>(29.6 to 82.0)	29.8%<br>(21.0% to 38.1%)	9.4%<br>(1.9% to 16.3%)
Hookworm disease	2,462.6<br>(2,094.7 to 2,897.3)	49.9<br>(30.5 to 77.1)	14.4%<br>(-0.9% to 33.4%)	-3.6%<br>(-16.5% to 12.3%)
Malaria	1,673.0<br>(1,385.8 to 2,029.0)	49.1<br>(31.1 to 72.7)	-21.3%<br>(-28.2% to -13.2%)	-33.7%<br>(-39.5% to -26.9%)
Endocrine, metabolic, blood, and immune disorders	2,715.8<br>(2,373.2 to 3,127.3)	48.9<br>(31.4 to 72.8)	16.8%<br>(11.1% to 22.9%)	-1.6%<br>(-6.4% to 3.5%)
Gastritis and duodenitis	2,189.9<br>(1,668.4 to 2,830.4)	46.9<br>(27.9 to 73.2)	32.6%<br>(24.2% to 40.9%)	11.7%<br>(4.6% to 18.7%)
Schistosomiasis	1,584.1<br>(1,324.7 to 1,877.9)	35.9<br>(22.6 to 53.5)	-20.6%<br>(-25.6% to -15.2%)	-33.1%<br>(-37.3% to -28.6%)
Other infectious diseases	1,996.2<br>(1,716.1 to 2,336.0)	30.0<br>(18.6 to 46.5)	14.2%<br>(7.9% to 20.5%)	-3.8%<br>(-9.1% to 1.5%)
Other neglected tropical diseases	1,717.0<br>(1,478.0 to 1,997.8)	26.0<br>(16.3 to 40.2)	18.9%<br>(12.2% to 26.4%)	0.2%<br>(-5.5% to 6.5%)
Chronic kidney disease due to hypertension	190.5<br>(121.6 to 286.5)	19.5<br>(10.9 to 32.5)	27.0%<br>(18.1% to 35.5%)	6.9%<br>(-0.5% to 14.2%)
Sickle cell disorders	124.3<br>(112.0 to 137.7)	9.6<br>(6.5 to 13.3)	32.1%<br>(19.2% to 45.3%)	11.3%<br>(0.4% to 22.4%)
Maternal haemorrhage	338.8<br>(239.5 to 474.5)	7.0<br>(3.9 to 11.5)	16.4%<br>(4.6% to 31.7%)	-2.0%<br>(-11.9% to 10.9%)
Multidrug-resistant HIV/AIDS - Tuberculosis without extensive drug resistance	3.9<br>(2.6 to 5.8)	1.6<br>(0.9 to 2.6)	-42.5%<br>(-53.5% to -29.8%)	-51.6%<br>(-60.8% to -40.9%)
G6PD deficiency	47.9<br>(40.4 to 57.9)	1.5<br>(0.9 to 2.2)	35.8%<br>(29.9% to 42.2%)	14.4%<br>(9.4% to 19.8%)
Thalassemias	2.7<br>(2.0 to 3.1)	0.3<br>(0.2 to 0.4)	21.7%<br>(10.7% to 33.7%)	2.5%<br>(-6.7% to 12.6%)
G6PD trait	0.7<br>(0.6 to 0.8)	<0.05<br>(<0.05 to 0.1)	26.9%<br>(20.5% to 33.0%)	6.9%<br>(1.5% to 12.0%)
Extensively drug-resistant HIV/AIDS - Tuberculosis	<0.05<br>(<0.05 to 0.1)	<0.05<br>(<0.05 to <0.05)	152.5%<br>(105.5% to 213.0%)	112.7%<br>(73.1% to 163.7%)
**All causes**	**136,948.0<br>(120,814.2 to 156,266.5)**	**3,669.4<br>(2,439.7 to 5,262.9)**	**8.9%<br>(3.4% to 13.4%)**	**-8.3%<br>(-12.9% to -4.4%)**

SR Table 13a: Global prevalence and years lived with disability (YLDs) for 2016, percentage change of YLD counts and rates between 2006 and 2016 of anaemia by cause, ages 30-34 years				
**Cause**	**Prevalence in thousands (95% uncertainty interval)**	**YLDs in thousands (95% uncertainty interval)**		
	**2016**	**2016**	**Percentage change in counts between 2006 and 2016**	**Percentage change in rates between 2006 and 2016**
Iron-deficiency anaemia	86,355.7 (75,807.4 to 98,633.7)	2,033.8 (1,294.4 to 3,003.7)	21.3% (17.1% to 25.5%)	7.5% (3.7% to 11.1%)
HIV/AIDS resulting in other diseases	5,373.9 (5,025.1 to 5,760.5)	531.5 (361.4 to 747.8)	-14.5% (-34.2% to 5.7%)	-24.2% (-41.7% to -6.4%)
Thalassaemias trait	12,173.8 (10,062.2 to 14,543.0)	247.7 (152.3 to 380.9)	-1.8% (-13.0% to 8.6%)	-13.1% (-22.9% to -3.9%)
Chronic kidney disease due to glomerulonephritis	1,294.4 (878.7 to 1,793.9)	108.7 (62.8 to 166.8)	26.4% (18.5% to 34.5%)	11.9% (4.9% to 19.2%)
Uterine fibroids	3,003.5 (2,461.5 to 3,582.2)	107.6 (63.0 to 169.7)	21.6% (18.0% to 25.6%)	7.6% (4.5% to 11.2%)
Chronic kidney disease due to other causes	1,262.8 (845.7 to 1,810.0)	103.3 (61.1 to 164.9)	28.1% (19.2% to 37.0%)	13.4% (5.6% to 21.4%)
Sickle cell trait	4,865.4 (4,077.2 to 5,752.6)	100.7 (63.5 to 150.3)	29.1% (23.8% to 34.9%)	14.3% (9.7% to 19.5%)
Chronic kidney disease due to diabetes mellitus	1,109.8 (731.0 to 1,572.0)	97.5 (58.0 to 153.8)	25.0% (15.2% to 33.7%)	10.7% (2.0% to 18.4%)
Peptic ulcer disease	3,704.2 (2,623.0 to 5,147.5)	90.1 (52.0 to 146.4)	8.8% (-0.9% to 18.9%)	-3.7% (-12.3% to 5.3%)
Gastritis and duodenitis	3,478.0 (2,602.2 to 4,534.8)	83.5 (47.1 to 133.1)	2.8% (-7.3% to 13.1%)	-8.9% (-17.9% to 0.1%)
Other gynecological diseases	2,685.8 (2,401.9 to 2,993.5)	74.5 (48.0 to 110.1)	21.0% (15.6% to 27.0%)	7.2% (2.3% to 12.4%)
Drug-susceptible HIV/AIDS - Tuberculosis	182.7 (130.2 to 250.5)	70.5 (43.0 to 108.4)	-25.7% (-30.4% to -21.4%)	-34.2% (-38.4% to -30.4%)
Other hemoglobinopathies and hemolytic anaemias	3,694.8 (3,152.3 to 4,333.6)	52.3 (32.4 to 82.5)	34.9% (28.1% to 42.6%)	19.5% (13.4% to 26.3%)
Hookworm disease	2,570.7 (2,163.7 to 3,022.2)	50.4 (30.7 to 77.2)	3.8% (-10.1% to 22.2%)	-8.1% (-20.4% to 8.2%)
Endocrine, metabolic, blood, and immune disorders	2,722.5 (2,370.8 to 3,135.7)	47.1 (29.9 to 70.7)	27.5% (21.6% to 33.7%)	13.0% (7.7% to 18.4%)
Malaria	1,546.7 (1,289.7 to 1,835.7)	44.4 (27.8 to 66.1)	-18.2% (-25.0% to -10.0%)	-27.5% (-33.6% to -20.3%)
Schistosomiasis	1,686.2 (1,413.8 to 1,966.9)	37.7 (23.5 to 56.5)	-18.3% (-23.8% to -13.0%)	-27.6% (-32.5% to -22.9%)
Chronic kidney disease due to hypertension	335.9 (228.8 to 472.2)	31.7 (18.9 to 50.5)	22.1% (13.5% to 31.6%)	8.2% (0.6% to 16.6%)
Other infectious diseases	2,044.8 (1,754.7 to 2,383.6)	29.5 (18.1 to 46.7)	23.1% (17.0% to 29.7%)	9.1% (3.6% to 14.8%)
Other neglected tropical diseases	1,728.6 (1,491.7 to 2,004.3)	24.8 (15.0 to 39.3)	24.9% (17.9% to 32.1%)	10.6% (4.4% to 17.0%)
Sickle cell disorders	81.4 (73.0 to 90.7)	9.0 (6.3 to 12.1)	48.4% (33.4% to 62.9%)	31.4% (18.2% to 44.2%)
Maternal haemorrhage	298.6 (211.3 to 427.7)	5.0 (2.8 to 8.4)	16.0% (0.8% to 33.2%)	2.7% (-10.7% to 17.9%)
Multidrug-resistant HIV/AIDS - Tuberculosis without extensive drug resistance	4.6 (3.0 to 6.8)	1.8 (1.1 to 3.1)	-33.8% (-45.9% to -20.5%)	-41.3% (-52.1% to -29.6%)
G6PD deficiency	51.4 (42.5 to 61.2)	1.6 (1.0 to 2.4)	29.9% (23.2% to 36.6%)	15.0% (9.1% to 20.9%)
Thalassemias	1.9 (1.4 to 2.3)	0.2 (0.1 to 0.3)	1.9% (-17.7% to 23.1%)	-9.8% (-27.1% to 9.0%)
G6PD trait	0.8 (0.6 to 0.9)	0.1 (<0.05 to 0.1)	12.5% (4.1% to 22.5%)	-0.3% (-7.8% to 8.5%)
Extensively drug-resistant HIV/AIDS - Tuberculosis	0.1 (<0.05 to 0.1)	<0.05 (<0.05 to <0.05)	214.5% (149.7% to 291.0%)	178.5% (121.2% to 246.2%)
**All causes**	**142,259.0 (124,468.6 to 163,586.4)**	**3,984.9 (2,660.6 to 5,729.0)**	**11.0% (2.2% to 17.0%)**	**-1.7% (-9.5% to 3.6%)**

SR Table 13a: Global prevalence and years lived with disability (YLDs) for 2016, percentage change of YLD counts and rates between 2006 and 2016 of anaemia by cause, ages 35-39 years				
**Cause**	**Prevalence in thousands (95% uncertainty interval)**	**YLDs in thousands (95% uncertainty interval)**		
	**2016**	**2016**	**Percentage change in counts between 2006 and 2016**	**Percentage change in rates between 2006 and 2016**
Iron-deficiency anaemia	75,861.4 (67,087.5 to 85,343.6)	1,793.7 (1,163.6 to 2,628.5)	12.8% (8.7% to 17.2%)	6.6% (2.7% to 10.8%)
HIV/AIDS resulting in other diseases	5,350.7 (5,019.4 to 5,698.5)	557.0 (370.6 to 809.9)	-16.1% (-34.5% to 11.6%)	-20.7% (-38.1% to 5.5%)
Thalassaemias trait	11,290.2 (9,511.4 to 13,297.5)	236.1 (146.1 to 354.7)	0.4% (-9.7% to 10.9%)	-5.1% (-14.6% to 4.9%)
Uterine fibroids	4,636.3 (3,938.6 to 5,355.2)	172.9 (104.7 to 266.6)	12.3% (8.7% to 15.6%)	6.1% (2.8% to 9.3%)
Chronic kidney disease due to diabetes mellitus	1,683.1 (1,169.9 to 2,314.3)	139.7 (83.9 to 215.8)	20.5% (12.0% to 28.3%)	13.9% (5.9% to 21.3%)
Peptic ulcer disease	4,333.7 (3,033.7 to 5,952.0)	116.0 (64.9 to 187.8)	17.4% (6.1% to 28.4%)	11.0% (0.3% to 21.4%)
Gastritis and duodenitis	4,305.3 (3,234.2 to 5,531.0)	112.6 (67.6 to 176.4)	11.4% (0.4% to 22.2%)	5.3% (-5.1% to 15.5%)
Chronic kidney disease due to other causes	1,377.7 (926.0 to 1,880.1)	105.8 (61.3 to 162.5)	26.8% (18.6% to 35.2%)	19.8% (12.1% to 27.8%)
Chronic kidney disease due to glomerulonephritis	1,271.9 (820.9 to 1,802.2)	97.8 (56.2 to 148.8)	25.3% (17.0% to 32.3%)	18.4% (10.6% to 25.0%)
Sickle cell trait	4,285.1 (3,615.5 to 5,038.3)	88.5 (56.0 to 134.2)	30.1% (24.8% to 35.4%)	23.0% (18.0% to 28.0%)
Other gynecological diseases	2,347.2 (2,112.2 to 2,593.3)	63.6 (41.8 to 93.0)	14.1% (8.4% to 19.7%)	7.9% (2.5% to 13.2%)
Drug-susceptible HIV/AIDS - Tuberculosis	163.0 (115.3 to 226.1)	62.4 (38.4 to 95.5)	-17.0% (-22.1% to -11.7%)	-21.5% (-26.4% to -16.6%)
Other hemoglobinopathies and hemolytic anaemias	3,262.7 (2,813.1 to 3,742.5)	44.0 (26.7 to 69.4)	23.6% (16.9% to 30.9%)	16.8% (10.5% to 23.8%)
Chronic kidney disease due to hypertension	474.1 (326.9 to 647.6)	41.3 (25.2 to 64.0)	19.0% (10.5% to 27.3%)	12.5% (4.5% to 20.3%)
Hookworm disease	2,254.6 (1,930.9 to 2,630.8)	41.0 (25.0 to 63.6)	3.9% (-11.4% to 21.3%)	-1.8% (-16.3% to 14.7%)
Endocrine, metabolic, blood, and immune disorders	2,395.5 (2,105.8 to 2,704.3)	41.0 (25.9 to 61.7)	19.5% (12.9% to 25.7%)	13.0% (6.7% to 18.8%)
Malaria	1,190.0 (1,004.2 to 1,402.2)	34.6 (21.6 to 51.9)	-19.0% (-25.9% to -11.4%)	-23.5% (-30.0% to -16.2%)
Schistosomiasis	1,519.1 (1,269.5 to 1,799.7)	32.1 (20.2 to 47.6)	-18.5% (-23.9% to -13.2%)	-22.9% (-28.1% to -17.9%)
Other infectious diseases	1,801.9 (1,563.8 to 2,058.6)	25.9 (15.6 to 40.9)	18.3% (12.0% to 25.8%)	11.8% (5.9% to 18.9%)
Other neglected tropical diseases	1,519.3 (1,324.9 to 1,722.4)	21.9 (13.2 to 34.5)	20.2% (13.0% to 28.1%)	13.6% (6.8% to 21.1%)
Sickle cell disorders	50.1 (44.8 to 55.6)	7.2 (5.1 to 9.6)	45.3% (32.0% to 59.1%)	37.3% (24.8% to 50.4%)
Maternal haemorrhage	137.9 (92.6 to 199.4)	2.0 (1.0 to 3.2)	20.9% (4.2% to 43.6%)	14.3% (-1.5% to 35.7%)
Multidrug-resistant HIV/AIDS - Tuberculosis without extensive drug resistance	4.1 (2.7 to 6.0)	1.7 (1.0 to 2.7)	-26.8% (-39.0% to -13.5%)	-30.8% (-42.3% to -18.2%)
G6PD deficiency	47.3 (39.9 to 56.5)	1.4 (0.9 to 2.0)	37.6% (30.5% to 44.7%)	30.1% (23.4% to 36.8%)
Thalassemias	1.4 (1.0 to 1.7)	0.1 (0.1 to 0.2)	4.0% (-14.7% to 30.6%)	-1.6% (-19.4% to 23.5%)
G6PD trait	0.7 (0.6 to 0.8)	<0.05 (<0.05 to 0.1)	6.1% (-0.0% to 13.0%)	0.3% (-5.5% to 6.9%)
Extensively drug-resistant HIV/AIDS - Tuberculosis	0.1 (<0.05 to 0.1)	<0.05 (<0.05 to <0.05)	300.8% (213.3% to 402.9%)	278.9% (196.1% to 375.4%)
**All causes**	**131,564.4 (116,309.8 to 148,105.1)**	**3,840.3 (2,583.0 to 5,505.4)**	**6.8% (-1.8% to 13.5%)**	**0.9% (-7.2% to 7.3%)**

SR Table 13a: Global prevalence and years lived with disability (YLDs) for 2016, percentage change of YLD counts and rates between 2006 and 2016 of anaemia by cause, ages 40-44 years				
**Cause**	**Prevalence in thousands (95% uncertainty interval)**	**YLDs in thousands (95% uncertainty interval)**		
	**2016**	**2016**	**Percentage change in counts between 2006 and 2016**	**Percentage change in rates between 2006 and 2016**
Iron-deficiency anaemia	73,083.6 (64,779.9 to 82,314.4)	1,710.9 (1,131.1 to 2,556.0)	12.4% (8.4% to 16.6%)	-1.3% (-4.8% to 2.4%)
HIV/AIDS resulting in other diseases	4,557.5 (4,065.3 to 5,102.4)	471.5 (299.0 to 716.4)	-12.2% (-33.6% to 22.2%)	-22.9% (-41.7% to 7.3%)
Thalassaemias trait	12,115.0 (10,241.4 to 14,236.6)	279.4 (177.7 to 420.2)	29.9% (17.7% to 42.8%)	14.1% (3.4% to 25.4%)
Uterine fibroids	5,411.6 (4,800.6 to 6,095.0)	207.6 (131.8 to 314.0)	12.5% (9.5% to 15.6%)	-1.1% (-3.8% to 1.5%)
Chronic kidney disease due to diabetes mellitus	2,621.6 (1,871.9 to 3,479.9)	191.4 (116.5 to 284.9)	31.0% (22.9% to 38.8%)	15.1% (7.9% to 21.9%)
Gastritis and duodenitis	6,223.1 (4,769.8 to 7,976.8)	184.3 (111.0 to 292.2)	44.6% (30.9% to 59.6%)	27.0% (15.0% to 40.2%)
Peptic ulcer disease	5,760.6 (4,107.5 to 7,856.1)	170.6 (97.8 to 275.0)	45.4% (32.8% to 58.1%)	27.7% (16.7% to 38.8%)
Chronic kidney disease due to other causes	1,461.2 (973.5 to 2,081.6)	97.6 (57.1 to 154.2)	28.4% (20.5% to 36.2%)	12.8% (5.9% to 19.7%)
Chronic kidney disease due to glomerulonephritis	1,309.3 (843.2 to 1,818.6)	88.1 (49.5 to 140.6)	27.2% (19.4% to 34.7%)	11.7% (4.8% to 18.3%)
Sickle cell trait	3,779.0 (3,221.3 to 4,389.1)	81.9 (52.4 to 123.7)	34.3% (28.6% to 39.9%)	17.9% (13.0% to 22.9%)
Other gynecological diseases	2,181.5 (1,974.0 to 2,399.7)	57.3 (38.5 to 84.2)	13.1% (8.1% to 18.7%)	-0.6% (-5.1% to 4.3%)
Chronic kidney disease due to hypertension	731.5 (527.6 to 1,007.3)	55.2 (33.5 to 84.5)	29.2% (19.8% to 38.4%)	13.4% (5.3% to 21.5%)
Drug-susceptible HIV/AIDS - Tuberculosis	127.5 (90.1 to 179.4)	48.6 (28.7 to 75.3)	-7.7% (-13.7% to -1.4%)	-18.9% (-24.2% to -13.4%)
Other hemoglobinopathies and hemolytic anaemias	3,297.2 (2,867.3 to 3,786.0)	40.6 (25.6 to 63.9)	28.1% (21.2% to 36.0%)	12.5% (6.5% to 19.4%)
Endocrine, metabolic, blood, and immune disorders	2,356.3 (2,084.9 to 2,661.4)	39.6 (25.7 to 59.5)	19.4% (13.4% to 25.5%)	4.9% (-0.4% to 10.3%)
Hookworm disease	2,184.1 (1,855.7 to 2,546.7)	38.5 (24.2 to 60.7)	19.3% (2.3% to 38.7%)	4.8% (-10.2% to 21.8%)
Malaria	967.2 (811.2 to 1,141.2)	29.6 (18.9 to 43.4)	-18.0% (-25.2% to -10.2%)	-28.0% (-34.3% to -21.1%)
Schistosomiasis	1,352.7 (1,132.4 to 1,602.6)	28.2 (17.7 to 42.2)	-18.7% (-24.5% to -13.1%)	-28.6% (-33.7% to -23.7%)
Other infectious diseases	1,803.9 (1,574.7 to 2,065.0)	24.2 (15.3 to 38.3)	25.9% (18.7% to 33.9%)	10.6% (4.3% to 17.6%)
Other neglected tropical diseases	1,510.3 (1,325.8 to 1,715.2)	20.4 (12.9 to 32.2)	25.4% (18.1% to 33.6%)	10.1% (3.7% to 17.3%)
Sickle cell disorders	29.9 (26.3 to 33.5)	4.9 (3.5 to 6.8)	53.9% (42.5% to 66.3%)	35.2% (25.1% to 46.0%)
G6PD deficiency	43.2 (36.4 to 50.9)	1.4 (0.9 to 2.0)	43.4% (36.8% to 50.0%)	25.9% (20.2% to 31.7%)
Multidrug-resistant HIV/AIDS - Tuberculosis without extensive drug resistance	3.3 (2.2 to 5.0)	1.4 (0.8 to 2.2)	-16.1% (-31.1% to 0.6%)	-26.4% (-39.5% to -11.6%)
Maternal haemorrhage	45.1 (32.0 to 60.8)	0.8 (0.5 to 1.3)	15.5% (1.9% to 32.2%)	1.4% (-10.5% to 16.1%)
Thalassemias	1.1 (0.8 to 1.4)	0.1 (0.1 to 0.2)	27.8% (-6.2% to 72.1%)	12.3% (-17.6% to 51.1%)
G6PD trait	0.7 (0.6 to 0.8)	0.1 (<0.05 to 0.1)	39.3% (30.2% to 49.9%)	22.4% (14.4% to 31.7%)
Extensively drug-resistant HIV/AIDS - Tuberculosis	<0.05 (<0.05 to 0.1)	<0.05 (<0.05 to <0.05)	282.9% (214.7% to 360.9%)	236.3% (176.4% to 304.8%)
**All causes**	**132,958.1 (117,331.8 to 150,563.0)**	**3,874.3 (2,624.2 to 5,567.6)**	**13.5% (3.6% to 20.7%)**	**-0.3% (-9.0% to 6.0%)**

SR Table 13a: Global prevalence and years lived with disability (YLDs) for 2016, percentage change of YLD counts and rates between 2006 and 2016 of anaemia by cause, ages 45-49 years				
**Cause**	**Prevalence in thousands (95% uncertainty interval)**	**YLDs in thousands (95% uncertainty interval)**		
	**2016**	**2016**	**Percentage change in counts between 2006 and 2016**	**Percentage change in rates between 2006 and 2016**
Iron-deficiency anaemia	62,779.0 (54,938.3 to 70,914.3)	1,419.5 (900.3 to 2,101.1)	17.8% (13.6% to 21.6%)	-5.6% (-8.9% to -2.5%)
HIV/AIDS resulting in other diseases	3,505.6 (3,059.9 to 4,046.3)	365.9 (224.7 to 554.7)	-11.0% (-30.6% to 23.0%)	-28.6% (-44.4% to -1.3%)
Thalassaemias trait	11,227.3 (9,341.7 to 13,208.8)	234.8 (143.9 to 363.6)	44.0% (31.8% to 56.1%)	15.5% (5.7% to 25.2%)
Chronic kidney disease due to diabetes mellitus	3,407.3 (2,465.1 to 4,503.3)	233.3 (149.3 to 340.6)	37.0% (28.9% to 44.6%)	9.9% (3.4% to 16.0%)
Gastritis and duodenitis	7,174.4 (5,377.9 to 9,482.7)	196.5 (113.8 to 308.6)	65.3% (50.6% to 79.6%)	32.5% (20.7% to 44.1%)
Uterine fibroids	5,006.7 (4,477.8 to 5,550.5)	188.7 (118.4 to 283.9)	17.9% (14.2% to 21.4%)	-5.4% (-8.4% to -2.7%)
Peptic ulcer disease	6,298.4 (4,512.6 to 8,587.2)	171.3 (97.1 to 270.5)	60.7% (47.5% to 73.0%)	28.8% (18.2% to 38.7%)
Chronic kidney disease due to other causes	1,490.6 (926.9 to 2,169.8)	91.4 (48.4 to 144.9)	30.8% (22.6% to 38.6%)	4.9% (-1.7% to 11.1%)
Chronic kidney disease due to glomerulonephritis	1,279.7 (814.6 to 1,840.8)	77.5 (43.1 to 124.5)	23.4% (14.1% to 32.3%)	-1.0% (-8.5% to 6.1%)
Chronic kidney disease due to hypertension	974.3 (689.4 to 1,337.1)	68.4 (42.0 to 104.0)	38.7% (28.4% to 49.7%)	11.2% (3.0% to 20.0%)
Sickle cell trait	3,114.4 (2,642.6 to 3,709.2)	64.5 (40.0 to 95.9)	32.8% (27.8% to 38.3%)	6.5% (2.4% to 10.9%)
Other gynecological diseases	1,837.8 (1,649.3 to 2,033.4)	44.7 (28.7 to 64.6)	18.9% (13.3% to 24.2%)	-4.6% (-9.2% to -0.4%)
Drug-susceptible HIV/AIDS - Tuberculosis	90.4 (63.4 to 124.6)	34.3 (20.0 to 52.7)	1.8% (-6.2% to 9.8%)	-18.4% (-24.7% to -12.0%)
Endocrine, metabolic, blood, and immune disorders	2,027.7 (1,772.2 to 2,292.8)	32.4 (20.5 to 49.2)	22.0% (15.5% to 28.4%)	-2.2% (-7.4% to 3.0%)
Other hemoglobinopathies and hemolytic anaemias	2,874.6 (2,488.7 to 3,273.1)	30.8 (18.3 to 48.3)	34.5% (26.8% to 43.3%)	7.8% (1.7% to 14.9%)
Hookworm disease	1,736.3 (1,466.1 to 2,039.5)	26.3 (15.7 to 40.8)	17.9% (1.2% to 38.7%)	-5.4% (-18.8% to 11.2%)
Malaria	694.7 (585.0 to 811.9)	20.5 (12.5 to 30.1)	-23.4% (-29.6% to -17.1%)	-38.6% (-43.6% to -33.5%)
Other infectious diseases	1,565.7 (1,357.9 to 1,780.8)	18.6 (10.9 to 29.6)	27.0% (19.5% to 34.4%)	1.8% (-4.2% to 7.7%)
Schistosomiasis	978.9 (830.1 to 1,129.8)	17.8 (10.8 to 26.7)	-30.1% (-35.1% to -25.4%)	-44.0% (-47.9% to -40.2%)
Other neglected tropical diseases	1,313.4 (1,146.2 to 1,490.0)	15.6 (9.1 to 24.6)	28.5% (20.2% to 36.6%)	3.0% (-3.6% to 9.6%)
Sickle cell disorders	16.0 (14.0 to 18.0)	2.6 (1.8 to 3.5)	53.7% (41.3% to 67.1%)	23.3% (13.3% to 34.0%)
G6PD deficiency	38.6 (32.3 to 45.6)	1.1 (0.7 to 1.6)	43.0% (36.7% to 49.9%)	14.6% (9.6% to 20.2%)
Multidrug-resistant HIV/AIDS - Tuberculosis without extensive drug resistance	2.5 (1.7 to 3.7)	1.0 (0.6 to 1.6)	-6.3% (-22.1% to 11.6%)	-24.9% (-37.5% to -10.5%)
Maternal haemorrhage	8.3 (5.3 to 12.5)	0.4 (0.2 to 0.7)	5.1% (-4.9% to 17.8%)	-15.7% (-23.8% to -5.6%)
Thalassemias	0.8 (0.6 to 0.9)	0.1 (<0.05 to 0.2)	28.8% (-41.8% to 195.6%)	3.3% (-53.3% to 137.1%)
G6PD trait	0.6 (0.5 to 0.7)	<0.05 (<0.05 to 0.1)	31.6% (20.0% to 44.0%)	5.5% (-3.7% to 15.4%)
Extensively drug-resistant HIV/AIDS - Tuberculosis	<0.05 (<0.05 to <0.05)	<0.05 (<0.05 to <0.05)	335.9% (264.1% to 423.7%)	249.6% (192.0% to 320.0%)
**All causes**	**119,444.1 (103,485.2 to 136,131.2)**	**3,358.1 (2,217.8 to 4,754.0)**	**20.3% (10.3% to 27.7%)**	**-3.6% (-11.6% to 2.4%)**

SR Table 13a: Global prevalence and years lived with disability (YLDs) for 2016, percentage change of YLD counts and rates between 2006 and 2016 of anaemia by cause, ages 50-54 years				
**Cause**	**Prevalence in thousands (95% uncertainty interval)**	**YLDs in thousands (95% uncertainty interval)**		
	**2016**	**2016**	**Percentage change in counts between 2006 and 2016**	**Percentage change in rates between 2006 and 2016**
Iron-deficiency anaemia	46,462.9 (40,610.8 to 53,182.0)	941.6 (596.9 to 1,439.1)	10.8% (6.7% to 14.8%)	-11.0% (-14.3% to -7.9%)
HIV/AIDS resulting in other diseases	2,467.2 (2,115.2 to 2,903.1)	243.9 (150.0 to 374.1)	2.3% (-26.6% to 56.8%)	-17.9% (-41.1% to 25.9%)
Chronic kidney disease due to diabetes mellitus	3,799.5 (2,692.2 to 5,032.2)	230.7 (143.8 to 339.8)	41.3% (33.8% to 49.2%)	13.5% (7.4% to 19.8%)
Gastritis and duodenitis	6,112.6 (4,692.6 to 8,087.0)	149.9 (88.0 to 234.5)	61.8% (46.9% to 76.9%)	29.9% (17.9% to 42.0%)
Thalassaemias trait	7,724.4 (6,339.7 to 9,249.1)	143.9 (88.0 to 228.7)	42.8% (29.3% to 54.7%)	14.7% (3.8% to 24.2%)
Peptic ulcer disease	5,035.1 (3,649.8 to 6,944.0)	127.5 (70.0 to 206.4)	61.0% (45.9% to 77.1%)	29.3% (17.1% to 42.2%)
Uterine fibroids	3,441.4 (3,064.3 to 3,853.1)	126.6 (79.6 to 199.3)	15.7% (11.5% to 19.7%)	-7.1% (-10.5% to -3.9%)
Chronic kidney disease due to other causes	1,613.4 (1,027.8 to 2,407.1)	90.0 (50.1 to 143.9)	38.9% (30.2% to 47.8%)	11.5% (4.6% to 18.6%)
Chronic kidney disease due to hypertension	1,174.1 (823.1 to 1,623.4)	75.8 (46.0 to 114.3)	46.5% (35.4% to 57.4%)	17.6% (8.7% to 26.4%)
Chronic kidney disease due to glomerulonephritis	1,262.9 (805.7 to 1,838.4)	67.9 (37.1 to 107.2)	31.5% (20.3% to 42.1%)	5.6% (-3.4% to 14.1%)
Sickle cell trait	2,369.1 (1,990.5 to 2,811.6)	44.8 (28.2 to 68.2)	36.6% (30.7% to 42.6%)	9.7% (4.9% to 14.5%)
Other gynecological diseases	1,244.1 (1,104.2 to 1,399.1)	27.3 (17.4 to 41.2)	13.8% (7.8% to 19.4%)	-8.6% (-13.4% to -4.1%)
Other hemoglobinopathies and hemolytic anaemias	2,318.3 (2,035.7 to 2,656.6)	24.2 (14.5 to 39.3)	21.1% (13.3% to 28.4%)	-2.8% (-9.0% to 3.1%)
Endocrine, metabolic, blood, and immune disorders	1,552.6 (1,365.4 to 1,772.8)	23.4 (14.6 to 36.3)	16.9% (10.5% to 22.9%)	-6.1% (-11.2% to -1.3%)
Drug-susceptible HIV/AIDS - Tuberculosis	56.1 (39.2 to 78.3)	21.1 (12.4 to 32.8)	7.4% (-1.5% to 16.4%)	-13.8% (-20.9% to -6.5%)
Hookworm disease	1,244.6 (1,053.7 to 1,468.3)	16.8 (10.3 to 27.8)	23.3% (6.2% to 43.7%)	-1.0% (-14.7% to 15.4%)
Malaria	477.0 (395.3 to 561.3)	14.4 (9.0 to 21.5)	-22.4% (-29.0% to -15.7%)	-37.7% (-43.0% to -32.3%)
Other infectious diseases	1,242.6 (1,093.8 to 1,422.8)	13.8 (8.2 to 22.5)	16.3% (9.1% to 24.0%)	-6.6% (-12.4% to -0.4%)
Schistosomiasis	731.1 (614.5 to 870.1)	12.1 (7.5 to 18.7)	-21.6% (-27.2% to -15.6%)	-37.1% (-41.5% to -32.3%)
Other neglected tropical diseases	1,054.7 (926.9 to 1,197.7)	11.2 (6.8 to 18.5)	16.1% (7.8% to 23.9%)	-6.8% (-13.5% to -0.5%)
Sickle cell disorders	8.3 (7.2 to 9.4)	1.3 (0.9 to 1.7)	59.2% (46.8% to 72.0%)	27.8% (17.9% to 38.1%)
G6PD deficiency	31.0 (25.7 to 36.9)	0.8 (0.5 to 1.2)	29.5% (20.4% to 37.5%)	4.0% (-3.3% to 10.4%)
Multidrug-resistant HIV/AIDS - Tuberculosis without extensive drug resistance	1.6 (1.1 to 2.4)	0.7 (0.4 to 1.1)	-0.2% (-17.0% to 17.1%)	-19.9% (-33.4% to -6.0%)
Thalassemias	0.4 (0.3 to 0.5)	<0.05 (<0.05 to 0.1)	31.0% (9.5% to 84.7%)	5.2% (-12.0% to 48.3%)
Maternal haemorrhage	0.4 (0.3 to 0.6)	<0.05 (<0.05 to 0.1)	20.8% (6.7% to 39.3%)	-3.0% (-14.3% to 11.9%)
G6PD trait	0.4 (0.3 to 0.5)	<0.05 (<0.05 to 0.1)	66.0% (46.1% to 91.7%)	33.3% (17.3% to 53.9%)
Extensively drug-resistant HIV/AIDS - Tuberculosis	<0.05 (<0.05 to <0.05)	<0.05 (<0.05 to <0.05)	399.2% (317.4% to 493.2%)	300.8% (235.2% to 376.3%)
**All causes**	**91,426.0 (78,931.9 to 105,124.7)**	**2,410.0 (1,597.2 to 3,487.3)**	**21.1% (11.4% to 27.6%)**	**-2.7% (-10.5% to 2.5%)**

SR Table 13a: Global prevalence and years lived with disability (YLDs) for 2016, percentage change of YLD counts and rates between 2006 and 2016 of anaemia by cause, ages 55-59 years				
**Cause**	**Prevalence in thousands (95% uncertainty interval)**	**YLDs in thousands (95% uncertainty interval)**		
	**2016**	**2016**	**Percentage change in counts between 2006 and 2016**	**Percentage change in rates between 2006 and 2016**
Iron-deficiency anaemia	47,157.0 (40,890.7 to 52,725.3)	987.0 (631.8 to 1,478.9)	33.4% (29.3% to 37.4%)	2.2% (-0.9% to 5.3%)
Chronic kidney disease due to diabetes mellitus	4,811.2 (3,383.5 to 6,445.2)	252.6 (153.2 to 378.4)	39.7% (32.2% to 46.3%)	7.0% (1.3% to 12.1%)
HIV/AIDS resulting in other diseases	1,628.5 (1,361.0 to 1,953.4)	155.9 (96.3 to 237.0)	16.2% (-17.7% to 76.8%)	-11.0% (-36.9% to 35.5%)
Gastritis and duodenitis	6,157.8 (4,703.5 to 7,917.7)	153.7 (89.6 to 238.4)	46.5% (32.4% to 59.8%)	12.2% (1.5% to 22.5%)
Thalassaemias trait	6,621.1 (5,394.7 to 7,820.5)	131.4 (82.6 to 204.7)	35.4% (23.7% to 45.7%)	3.7% (-5.2% to 11.6%)
Peptic ulcer disease	4,718.8 (3,316.8 to 6,481.8)	123.6 (70.7 to 203.7)	50.1% (35.1% to 64.6%)	15.0% (3.5% to 26.1%)
Uterine fibroids	2,769.4 (2,443.9 to 3,094.9)	97.1 (61.1 to 151.9)	36.5% (32.6% to 40.4%)	4.6% (1.6% to 7.6%)
Chronic kidney disease due to other causes	1,898.2 (1,192.6 to 2,831.3)	93.0 (53.1 to 154.7)	38.6% (30.6% to 47.3%)	6.2% (0.1% to 12.8%)
Chronic kidney disease due to hypertension	1,637.9 (1,180.0 to 2,273.7)	92.1 (57.0 to 142.0)	44.2% (33.6% to 54.5%)	10.5% (2.4% to 18.4%)
Chronic kidney disease due to glomerulonephritis	1,335.3 (820.1 to 1,970.4)	63.7 (35.0 to 104.4)	30.5% (18.2% to 42.3%)	-0.0% (-9.4% to 9.0%)
Sickle cell trait	2,088.3 (1,752.9 to 2,452.1)	40.8 (26.0 to 61.4)	39.5% (34.1% to 45.1%)	6.9% (2.7% to 11.2%)
Other hemoglobinopathies and hemolytic anaemias	2,540.2 (2,203.9 to 2,834.7)	30.9 (19.3 to 47.6)	39.4% (31.2% to 47.4%)	6.9% (0.5% to 13.0%)
Endocrine, metabolic, blood, and immune disorders	1,638.9 (1,427.8 to 1,830.9)	25.7 (16.2 to 38.8)	39.5% (32.4% to 46.3%)	6.9% (1.5% to 12.1%)
Other infectious diseases	1,350.6 (1,172.4 to 1,507.3)	16.3 (10.0 to 25.7)	35.4% (28.2% to 42.6%)	3.8% (-1.8% to 9.3%)
Hookworm disease	1,071.0 (913.1 to 1,247.9)	14.9 (9.0 to 23.6)	26.6% (9.2% to 45.5%)	-3.0% (-16.3% to 11.5%)
Other neglected tropical diseases	1,126.7 (984.8 to 1,250.8)	13.3 (8.0 to 21.2)	37.8% (29.3% to 46.2%)	5.6% (-0.9% to 12.0%)
Drug-susceptible HIV/AIDS - Tuberculosis	35.1 (24.5 to 48.6)	13.2 (7.8 to 20.4)	13.4% (3.3% to 24.2%)	-13.1% (-20.8% to -4.8%)
Malaria	403.8 (337.7 to 476.6)	11.7 (7.3 to 17.0)	-31.8% (-38.0% to -25.5%)	-47.7% (-52.5% to -42.9%)
Schistosomiasis	608.3 (502.5 to 714.7)	10.1 (6.2 to 15.4)	-32.3% (-37.3% to -27.6%)	-48.1% (-51.9% to -44.5%)
G6PD deficiency	27.3 (22.7 to 31.7)	0.8 (0.5 to 1.2)	29.1% (22.1% to 36.0%)	-1.1% (-6.4% to 4.2%)
Sickle cell disorders	4.1 (3.6 to 4.7)	0.7 (0.5 to 0.9)	65.6% (59.7% to 71.9%)	26.9% (22.4% to 31.7%)
Multidrug-resistant HIV/AIDS - Tuberculosis without extensive drug resistance	1.1 (0.7 to 1.6)	0.4 (0.2 to 0.7)	9.0% (-10.6% to 29.4%)	-16.5% (-31.5% to -0.9%)
G6PD trait	0.3 (0.2 to 0.4)	<0.05 (<0.05 to <0.05)	32.7% (14.5% to 57.7%)	1.7% (-12.3% to 20.8%)
Thalassemias	0.2 (0.1 to 0.2)	<0.05 (<0.05 to <0.05)	-3.7% (-30.4% to 14.3%)	-26.2% (-46.7% to -12.4%)
Extensively drug-resistant HIV/AIDS - Tuberculosis	<0.05 (<0.05 to <0.05)	<0.05 (<0.05 to <0.05)	337.0% (267.5% to 417.2%)	234.9% (181.6% to 296.3%)
**All causes**	**89,630.9 (76,159.1 to 102,615.7)**	**2,329.0 (1,544.5 to 3,429.7)**	**33.9% (26.1% to 39.8%)**	**2.6% (-3.3% to 7.1%)**

SR Table 13a: Global prevalence and years lived with disability (YLDs) for 2016, percentage change of YLD counts and rates between 2006 and 2016 of anaemia by cause, ages 60-64 years				
**Cause**	**Prevalence in thousands (95% uncertainty interval)**	**YLDs in thousands (95% uncertainty interval)**		
	**2016**	**2016**	**Percentage change in counts between 2006 and 2016**	**Percentage change in rates between 2006 and 2016**
Iron-deficiency anaemia	34,336.4<br>(31,135.3 to 37,565.5)	702.9<br>(457.4 to 1,009.5)	36.6%<br>(32.8% to 39.8%)	-9.4%<br>(-11.9% to -7.3%)
Chronic kidney disease due to diabetes mellitus	5,006.7<br>(3,690.6 to 6,444.4)	237.0<br>(153.9 to 342.6)	48.5%<br>(41.1% to 56.1%)	-1.5%<br>(-6.4% to 3.5%)
Gastritis and duodenitis	5,101.8<br>(3,965.2 to 6,554.6)	120.4<br>(74.3 to 180.3)	85.3%<br>(71.7% to 98.9%)	22.9%<br>(13.9% to 31.9%)
Chronic kidney disease due to hypertension	1,902.4<br>(1,392.8 to 2,628.8)	99.1<br>(63.6 to 148.7)	55.1%<br>(45.4% to 65.3%)	2.9%<br>(-3.6% to 9.7%)
HIV/AIDS resulting in other diseases	1,031.4<br>(872.4 to 1,238.5)	95.9<br>(59.9 to 145.3)	15.8%<br>(-16.9% to 72.5%)	-23.2%<br>(-44.9% to 14.4%)
Thalassaemias trait	4,938.7<br>(4,254.8 to 5,745.0)	91.9<br>(57.1 to 136.1)	59.8%<br>(49.1% to 69.9%)	6.0%<br>(-1.1% to 12.7%)
Peptic ulcer disease	3,639.9<br>(2,688.2 to 4,825.9)	90.3<br>(54.8 to 138.5)	77.9%<br>(63.7% to 93.8%)	18.0%<br>(8.6% to 28.5%)
Chronic kidney disease due to other causes	1,805.9<br>(1,163.4 to 2,697.7)	82.3<br>(46.1 to 132.8)	46.3%<br>(38.4% to 54.5%)	-3.0%<br>(-8.2% to 2.5%)
Other hemoglobinopathies and hemolytic anaemias	4,136.5<br>(3,764.5 to 4,507.1)	74.6<br>(48.8 to 107.7)	39.4%<br>(35.0% to 43.5%)	-7.6%<br>(-10.5% to -4.8%)
Chronic kidney disease due to glomerulonephritis	1,169.7<br>(735.1 to 1,754.2)	52.8<br>(29.5 to 88.5)	44.8%<br>(34.4% to 54.8%)	-4.0%<br>(-10.9% to 2.7%)
Endocrine, metabolic, blood, and immune disorders	2,486.0<br>(2,263.7 to 2,709.1)	46.5<br>(30.9 to 66.2)	42.5%<br>(37.3% to 47.3%)	-5.5%<br>(-8.9% to -2.3%)
Sickle cell trait	1,457.5<br>(1,282.5 to 1,678.3)	22.1<br>(14.0 to 32.7)	35.9%<br>(29.4% to 42.3%)	-9.9%<br>(-14.1% to -5.6%)
Other infectious diseases	823.3<br>(752.0 to 893.4)	11.6<br>(7.5 to 17.1)	42.0%<br>(35.0% to 49.6%)	-5.8%<br>(-10.4% to -0.8%)
Hookworm disease	757.1<br>(657.3 to 863.9)	10.4<br>(6.3 to 15.8)	32.2%<br>(13.8% to 52.9%)	-12.3%<br>(-24.5% to 1.4%)
Other neglected tropical diseases	695.9<br>(637.2 to 752.9)	9.2<br>(5.8 to 13.7)	44.9%<br>(36.1% to 53.5%)	-3.9%<br>(-9.7% to 1.8%)
Drug-susceptible HIV/AIDS - Tuberculosis	23.7<br>(16.3 to 33.2)	8.9<br>(5.1 to 13.9)	12.7%<br>(1.2% to 24.3%)	-25.2%<br>(-32.9% to -17.6%)
Malaria	268.4<br>(227.3 to 312.5)	8.1<br>(5.1 to 11.7)	-27.5%<br>(-33.7% to -21.2%)	-51.9%<br>(-56.0% to -47.8%)
Schistosomiasis	408.2<br>(342.4 to 472.5)	6.3<br>(3.9 to 9.3)	-27.5%<br>(-32.5% to -22.3%)	-51.9%<br>(-55.2% to -48.4%)
G6PD deficiency	20.5<br>(17.8 to 23.5)	0.6<br>(0.4 to 0.9)	49.2%<br>(44.0% to 54.6%)	-1.0%<br>(-4.5% to 2.5%)
Multidrug-resistant HIV/AIDS - Tuberculosis without extensive drug resistance	0.8<br>(0.5 to 1.2)	0.3<br>(0.2 to 0.5)	21.5%<br>(-1.9% to 45.3%)	-19.4%<br>(-35.0% to -3.6%)
Sickle cell disorders	1.8<br>(1.6 to 2.1)	0.3<br>(0.2 to 0.4)	71.2%<br>(65.4% to 77.1%)	13.5%<br>(9.7% to 17.5%)
G6PD trait	0.2<br>(0.2 to 0.2)	<0.05<br>(<0.05 to <0.05)	51.0%<br>(43.1% to 58.0%)	0.1%<br>(-5.1% to 4.8%)
Thalassemias	0.1<br>(0.1 to 0.1)	<0.05<br>(<0.05 to <0.05)	48.9%<br>(19.5% to 99.3%)	-1.3%<br>(-20.7% to 32.2%)
Extensively drug-resistant HIV/AIDS - Tuberculosis	<0.05<br>(<0.05 to <0.05)	<0.05<br>(<0.05 to <0.05)	495.1%<br>(399.5% to 609.0%)	294.7%<br>(231.3% to 370.3%)
**All causes**	**70,012.8<br>(62,218.0 to 78,289.3)**	**1,771.6<br>(1,207.9 to 2,507.2)**	**42.8%<br>(35.5% to 48.3%)**	**-5.3%<br>(-10.1% to -1.6%)**

SR Table 13a: Global prevalence and years lived with disability (YLDs) for 2016, percentage change of YLD counts and rates between 2006 and 2016 of anaemia by cause, ages 65-69 years				
**Cause**	**Prevalence in thousands (95% uncertainty interval)**	**YLDs in thousands (95% uncertainty interval)**		
	**2016**	**2016**	**Percentage change in counts between 2006 and 2016**	**Percentage change in rates between 2006 and 2016**
Iron-deficiency anaemia	26,488.7 (24,603.9 to 27,878.7)	614.8 (408.7 to 879.4)	21.1% (19.0% to 23.2%)	-8.6% (-10.2% to -7.0%)
Chronic kidney disease due to diabetes mellitus	6,434.9 (4,892.7 to 8,107.3)	284.7 (188.5 to 414.6)	36.5% (30.7% to 42.5%)	3.0% (-1.3% to 7.6%)
Gastritis and duodenitis	6,122.7 (4,944.9 to 7,502.1)	143.6 (89.8 to 212.4)	56.9% (48.1% to 65.4%)	18.4% (11.8% to 24.8%)
Chronic kidney disease due to hypertension	2,781.2 (2,073.4 to 3,686.6)	132.3 (84.5 to 193.7)	39.0% (31.3% to 46.4%)	4.9% (-0.9% to 10.5%)
Chronic kidney disease due to other causes	2,358.1 (1,550.6 to 3,389.3)	104.8 (60.4 to 168.2)	32.8% (26.0% to 39.7%)	0.2% (-4.9% to 5.4%)
Peptic ulcer disease	4,035.8 (3,063.1 to 5,355.5)	102.7 (64.0 to 157.3)	51.6% (40.1% to 63.3%)	14.4% (5.7% to 23.3%)
Thalassaemias trait	4,824.4 (4,294.3 to 5,309.4)	98.2 (62.6 to 144.0)	43.5% (37.4% to 49.7%)	8.3% (3.7% to 13.0%)
Chronic kidney disease due to glomerulonephritis	1,519.6 (990.8 to 2,200.8)	66.2 (37.9 to 105.9)	23.0% (12.4% to 33.2%)	-7.2% (-15.2% to 0.6%)
HIV/AIDS resulting in other diseases	639.8 (546.6 to 762.1)	58.7 (37.1 to 88.4)	10.8% (-21.8% to 66.4%)	-16.4% (-41.0% to 25.6%)
Other hemoglobinopathies and hemolytic anaemias	3,189.8 (2,976.2 to 3,339.6)	58.0 (38.0 to 83.7)	20.2% (16.8% to 23.1%)	-9.3% (-11.8% to -7.1%)
Endocrine, metabolic, blood, and immune disorders	1,914.1 (1,787.4 to 2,005.7)	36.5 (24.3 to 51.5)	21.3% (17.8% to 24.9%)	-8.4% (-11.1% to -5.8%)
Sickle cell trait	1,251.7 (1,121.5 to 1,405.7)	19.8 (12.8 to 28.9)	32.0% (27.5% to 36.5%)	-0.4% (-3.7% to 3.0%)
Hookworm disease	679.9 (606.2 to 756.9)	9.6 (5.9 to 14.5)	23.2% (8.1% to 40.9%)	-7.1% (-18.4% to 6.4%)
Other infectious diseases	633.9 (593.6 to 662.3)	9.5 (6.1 to 13.9)	23.2% (17.9% to 29.1%)	-7.1% (-11.0% to -2.6%)
Other neglected tropical diseases	517.4 (487.7 to 539.2)	7.5 (4.8 to 10.9)	29.9% (23.4% to 36.2%)	-2.0% (-6.9% to 2.8%)
Malaria	219.5 (188.3 to 252.1)	6.8 (4.4 to 9.7)	-27.1% (-32.7% to -21.6%)	-45.0% (-49.2% to -40.8%)
Drug-susceptible HIV/AIDS - Tuberculosis	16.3 (11.1 to 23.1)	6.1 (3.6 to 9.6)	12.8% (0.0% to 24.5%)	-14.8% (-24.5% to -6.0%)
Schistosomiasis	364.2 (319.0 to 409.5)	5.6 (3.6 to 8.1)	-31.1% (-35.6% to -26.4%)	-48.0% (-51.4% to -44.4%)
G6PD deficiency	17.4 (15.5 to 19.0)	0.6 (0.4 to 0.8)	30.8% (26.4% to 35.0%)	-1.3% (-4.6% to 1.9%)
Multidrug-resistant HIV/AIDS - Tuberculosis without extensive drug resistance	0.6 (0.4 to 0.8)	0.2 (0.1 to 0.4)	24.8% (0.4% to 48.0%)	-5.8% (-24.2% to 11.7%)
Sickle cell disorders	0.9 (0.8 to 1.0)	0.2 (0.1 to 0.2)	63.2% (58.0% to 68.6%)	23.2% (19.3% to 27.2%)
G6PD trait	0.2 (0.2 to 0.2)	<0.05 (<0.05 to <0.05)	44.4% (39.3% to 49.9%)	9.0% (5.1% to 13.1%)
Thalassemias	0.1 (<0.05 to 0.1)	<0.05 (<0.05 to <0.05)	26.0% (10.0% to 43.0%)	-4.9% (-17.0% to 7.9%)
Extensively drug-resistant HIV/AIDS - Tuberculosis	<0.05 (<0.05 to <0.05)	<0.05 (<0.05 to <0.05)	528.6% (433.2% to 638.0%)	374.4% (302.4% to 457.0%)
**All causes**	**64,011.0 (58,220.6 to 69,447.8)**	**1,766.0 (1,212.6 to 2,453.1)**	**29.6% (25.2% to 33.0%)**	**-2.2% (-5.5% to 0.4%)**

SR Table 13a: Global prevalence and years lived with disability (YLDs) for 2016, percentage change of YLD counts and rates between 2006 and 2016 of anaemia by cause, ages 70-74 years				
**Cause**	**Prevalence in thousands (95% uncertainty interval)**	**YLDs in thousands (95% uncertainty interval)**		
	**2016**	**2016**	**Percentage change in counts between 2006 and 2016**	**Percentage change in rates between 2006 and 2016**
Iron-deficiency anaemia	18,416.6 (17,487.2 to 19,254.5)	449.7 (303.6 to 643.1)	14.4% (12.7% to 16.0%)	-4.4% (-5.8% to -3.0%)
Chronic kidney disease due to diabetes mellitus	6,110.9 (4,727.1 to 7,555.8)	252.7 (170.0 to 360.3)	28.1% (23.4% to 32.6%)	7.1% (3.2% to 10.8%)
Chronic kidney disease due to hypertension	3,105.2 (2,355.0 to 3,964.4)	134.7 (86.1 to 195.8)	26.9% (21.1% to 32.2%)	6.0% (1.2% to 10.5%)
Gastritis and duodenitis	4,752.0 (3,779.8 to 5,861.4)	106.9 (69.3 to 157.0)	31.3% (25.1% to 37.2%)	9.7% (4.6% to 14.6%)
Chronic kidney disease due to other causes	2,423.4 (1,658.0 to 3,411.1)	103.2 (63.7 to 158.4)	25.3% (19.2% to 31.0%)	4.8% (-0.4% to 9.5%)
Peptic ulcer disease	2,906.0 (2,250.0 to 3,735.3)	73.3 (45.5 to 113.0)	28.6% (20.5% to 36.1%)	7.5% (0.7% to 13.8%)
Thalassaemias trait	3,407.1 (3,112.9 to 3,732.1)	72.1 (46.7 to 106.8)	28.9% (24.9% to 33.1%)	7.8% (4.4% to 11.3%)
Chronic kidney disease due to glomerulonephritis	1,503.8 (1,019.6 to 2,151.7)	63.9 (38.5 to 103.9)	14.9% (6.0% to 23.2%)	-4.0% (-11.4% to 3.0%)
Other hemoglobinopathies and hemolytic anaemias	2,227.9 (2,129.3 to 2,321.5)	39.5 (26.5 to 57.5)	11.0% (8.3% to 13.5%)	-7.3% (-9.5% to -5.1%)
HIV/AIDS resulting in other diseases	375.3 (324.8 to 438.5)	33.9 (21.7 to 50.0)	4.7% (-26.9% to 61.1%)	-12.5% (-38.9% to 34.6%)
Endocrine, metabolic, blood, and immune disorders	1,336.7 (1,278.0 to 1,393.4)	25.3 (17.1 to 36.0)	12.0% (8.8% to 15.3%)	-6.4% (-9.1% to -3.7%)
Sickle cell trait	931.5 (836.4 to 1,053.4)	14.6 (9.6 to 21.4)	31.6% (26.9% to 36.1%)	10.0% (6.1% to 13.8%)
Hookworm disease	499.2 (451.0 to 552.9)	6.6 (4.2 to 9.8)	12.6% (-1.4% to 28.7%)	-5.9% (-17.6% to 7.6%)
Other infectious diseases	442.8 (424.3 to 460.2)	6.5 (4.3 to 9.6)	15.5% (10.5% to 20.9%)	-3.5% (-7.6% to 1.1%)
Other neglected tropical diseases	356.4 (342.3 to 369.3)	5.1 (3.4 to 7.7)	19.8% (13.8% to 26.3%)	0.1% (-4.9% to 5.5%)
Malaria	156.1 (133.5 to 179.1)	5.0 (3.3 to 7.1)	-26.6% (-32.3% to -20.8%)	-38.6% (-43.4% to -33.8%)
Schistosomiasis	274.0 (240.4 to 308.9)	4.1 (2.7 to 6.0)	-33.0% (-36.9% to -28.8%)	-44.0% (-47.3% to -40.5%)
Drug-susceptible HIV/AIDS - Tuberculosis	10.8 (7.4 to 15.2)	4.0 (2.3 to 6.1)	4.3% (-5.8% to 14.2%)	-12.9% (-21.2% to -4.6%)
G6PD deficiency	13.0 (11.9 to 14.0)	0.4 (0.3 to 0.6)	20.4% (15.7% to 25.1%)	0.7% (-3.3% to 4.6%)
Multidrug-resistant HIV/AIDS - Tuberculosis without extensive drug resistance	0.3 (0.2 to 0.5)	0.1 (0.1 to 0.2)	10.2% (-10.4% to 30.2%)	-7.9% (-25.1% to 8.8%)
Sickle cell disorders	0.4 (0.3 to 0.4)	0.1 (<0.05 to 0.1)	39.0% (34.8% to 43.3%)	16.2% (12.7% to 19.8%)
G6PD trait	0.1 (0.1 to 0.2)	<0.05 (<0.05 to <0.05)	27.9% (23.6% to 32.9%)	6.9% (3.3% to 11.1%)
Thalassemias	<0.05 (<0.05 to <0.05)	<0.05 (<0.05 to <0.05)	6.2% (-4.9% to 17.9%)	-11.2% (-20.5% to -1.5%)
Extensively drug-resistant HIV/AIDS - Tuberculosis	<0.05 (<0.05 to <0.05)	<0.05 (<0.05 to <0.05)	430.9% (348.8% to 519.4%)	343.8% (275.1% to 417.7%)
**All causes**	**49,249.4 (45,498.9 to 52,905.0)**	**1,401.5 (1,003.0 to 1,918.2)**	**20.5% (17.7% to 23.1%)**	**0.7% (-1.7% to 2.9%)**

SR Table 13a: Global prevalence and years lived with disability (YLDs) for 2016, percentage change of YLD counts and rates between 2006 and 2016 of anaemia by cause, ages 75-79 years				
**Cause**	**Prevalence in thousands (95% uncertainty interval)**	**YLDs in thousands (95% uncertainty interval)**		
	**2016**	**2016**	**Percentage change in counts between 2006 and 2016**	**Percentage change in rates between 2006 and 2016**
Iron-deficiency anaemia	12,417.4<br>(11,899.1 to 12,991.8)	315.6<br>(214.3 to 443.4)	19.7%<br>(17.7% to 21.5%)	-3.7%<br>(-5.3% to -2.3%)
Chronic kidney disease due to diabetes mellitus	5,493.6<br>(4,166.4 to 6,889.4)	212.6<br>(142.2 to 298.6)	34.6%<br>(29.5% to 40.3%)	8.2%<br>(4.1% to 12.8%)
Chronic kidney disease due to hypertension	3,416.5<br>(2,573.7 to 4,407.9)	138.5<br>(88.7 to 196.0)	31.8%<br>(25.8% to 37.5%)	6.0%<br>(1.2% to 10.5%)
Chronic kidney disease due to other causes	2,691.3<br>(1,853.2 to 3,753.6)	107.0<br>(65.0 to 165.6)	30.3%<br>(23.2% to 37.6%)	4.8%<br>(-0.9% to 10.7%)
Gastritis and duodenitis	3,861.8<br>(3,081.6 to 4,785.1)	88.8<br>(55.9 to 128.2)	38.0%<br>(32.0% to 44.0%)	10.9%<br>(6.2% to 15.8%)
Chronic kidney disease due to glomerulonephritis	1,801.9<br>(1,256.3 to 2,533.0)	69.6<br>(41.3 to 104.8)	18.3%<br>(7.2% to 27.8%)	-4.9%<br>(-13.8% to 2.8%)
Peptic ulcer disease	2,343.5<br>(1,830.6 to 2,957.5)	60.3<br>(37.1 to 92.6)	36.2%<br>(28.1% to 44.9%)	9.5%<br>(3.0% to 16.5%)
Thalassaemias trait	2,572.3<br>(2,346.4 to 2,824.3)	56.9<br>(37.3 to 83.7)	33.3%<br>(29.1% to 37.8%)	7.2%<br>(3.8% to 10.8%)
Other hemoglobinopathies and hemolytic anaemias	1,520.5<br>(1,464.0 to 1,584.3)	27.1<br>(18.2 to 38.8)	13.1%<br>(9.9% to 15.9%)	-9.1%<br>(-11.6% to -6.8%)
HIV/AIDS resulting in other diseases	204.9<br>(175.8 to 245.0)	18.3<br>(11.5 to 27.3)	11.9%<br>(-21.4% to 70.1%)	-10.0%<br>(-36.8% to 36.8%)
Endocrine, metabolic, blood, and immune disorders	914.5<br>(879.7 to 953.6)	17.5<br>(11.8 to 24.8)	18.7%<br>(15.2% to 22.4%)	-4.5%<br>(-7.4% to -1.5%)
Sickle cell trait	670.9<br>(600.6 to 763.5)	10.7<br>(7.1 to 15.3)	40.0%<br>(35.8% to 44.7%)	12.6%<br>(9.2% to 16.3%)
Hookworm disease	355.6<br>(323.2 to 392.8)	4.7<br>(3.0 to 7.1)	19.8%<br>(5.9% to 36.8%)	-3.6%<br>(-14.8% to 10.0%)
Other infectious diseases	304.5<br>(294.0 to 317.1)	4.6<br>(3.1 to 6.6)	19.8%<br>(14.7% to 25.4%)	-3.7%<br>(-7.8% to 0.9%)
Other neglected tropical diseases	234.3<br>(227.3 to 242.2)	3.4<br>(2.3 to 5.0)	23.5%<br>(17.2% to 29.8%)	-0.7%<br>(-5.7% to 4.4%)
Schistosomiasis	184.7<br>(159.1 to 212.2)	2.9<br>(1.9 to 4.3)	-31.1%<br>(-35.5% to -26.7%)	-44.6%<br>(-48.1% to -41.1%)
Malaria	91.7<br>(77.1 to 106.6)	2.9<br>(2.0 to 4.2)	-25.7%<br>(-31.6% to -20.2%)	-40.3%<br>(-45.0% to -35.8%)
Drug-susceptible HIV/AIDS - Tuberculosis	6.1<br>(4.2 to 8.6)	2.2<br>(1.3 to 3.5)	-5.9%<br>(-14.3% to 3.1%)	-24.3%<br>(-31.1% to -17.1%)
G6PD deficiency	9.5<br>(8.8 to 10.3)	0.3<br>(0.2 to 0.5)	26.7%<br>(21.2% to 31.8%)	1.9%<br>(-2.5% to 5.9%)
Multidrug-resistant HIV/AIDS - Tuberculosis without extensive drug resistance	0.2<br>(0.1 to 0.3)	0.1<br>(<0.05 to 0.1)	15.1%<br>(-10.7% to 39.5%)	-7.5%<br>(-28.2% to 12.2%)
Sickle cell disorders	0.1<br>(0.1 to 0.2)	<0.05<br>(<0.05 to <0.05)	23.0%<br>(19.6% to 27.0%)	-1.1%<br>(-3.8% to 2.1%)
G6PD trait	0.1<br>(0.1 to 0.1)	<0.05<br>(<0.05 to <0.05)	18.3%<br>(6.4% to 29.0%)	-4.9%<br>(-14.5% to 3.8%)
Thalassemias	<0.05<br>(<0.05 to <0.05)	<0.05<br>(<0.05 to <0.05)	1.8%<br>(-9.1% to 10.3%)	-18.1%<br>(-26.9% to -11.3%)
Extensively drug-resistant HIV/AIDS - Tuberculosis	<0.05<br>(<0.05 to <0.05)	<0.05<br>(<0.05 to <0.05)	511.5%<br>(386.5% to 636.0%)	391.7%<br>(291.2% to 491.9%)
**All causes**	**39,095.9**<br>**(35,951.6 to 42,649.5)**	**1,144.0**<br>**(819.6 to 1,545.0)**	**26.7%**<br>**(23.5% to 29.8%)**	**1.9%**<br>**(-0.7% to 4.4%)**

SR Table 13a: Global prevalence and years lived with disability (YLDs) for 2016, percentage change of YLD counts and rates between 2006 and 2016 of anaemia by cause, ages 80-84				
**Cause**	**Prevalence in thousands (95% uncertainty interval)**	**YLDs in thousands (95% uncertainty interval)**		
	**2016**	**2016**	**Percentage change in counts between 2006 and 2016**	**Percentage change in rates between 2006 and 2016**
Iron-deficiency anaemia	7,066.9<br>(6,820.5 to 7,337.9)	201.9<br>(138.1 to 278.8)	29.4%<br>(27.5% to 31.2%)	-1.2%<br>(-2.6% to 0.2%)
Chronic kidney disease due to diabetes mellitus	3,766.1<br>(2,794.2 to 4,786.9)	148.7<br>(97.3 to 213.3)	40.5%<br>(34.6% to 46.3%)	7.2%<br>(2.8% to 11.7%)
Chronic kidney disease due to hypertension	3,160.6<br>(2,407.8 to 3,996.6)	130.2<br>(86.0 to 184.3)	36.8%<br>(30.6% to 42.4%)	4.4%<br>(-0.3% to 8.8%)
Chronic kidney disease due to other causes	2,333.1<br>(1,666.7 to 3,149.7)	94.3<br>(58.1 to 139.6)	33.6%<br>(26.3% to 41.0%)	2.0%<br>(-3.6% to 7.6%)
Gastritis and duodenitis	2,607.8<br>(2,104.3 to 3,206.5)	67.2<br>(43.8 to 98.2)	45.6%<br>(39.9% to 51.5%)	11.2%<br>(6.8% to 15.7%)
Chronic kidney disease due to glomerulonephritis	1,425.1<br>(1,023.9 to 1,918.0)	56.9<br>(35.4 to 86.4)	14.7%<br>(4.4% to 23.8%)	-12.4%<br>(-20.3% to -5.4%)
Peptic ulcer disease	1,598.3<br>(1,246.9 to 2,068.5)	44.3<br>(27.1 to 68.9)	41.8%<br>(33.6% to 49.8%)	8.3%<br>(2.0% to 14.4%)
Thalassaemias trait	1,710.4<br>(1,570.9 to 1,863.1)	40.9<br>(27.7 to 58.5)	38.6%<br>(34.3% to 42.9%)	5.9%<br>(2.6% to 9.1%)
Other hemoglobinopathies and hemolytic anaemias	869.7<br>(842.6 to 899.5)	17.6<br>(12.1 to 24.5)	32.5%<br>(29.5% to 35.3%)	1.2%<br>(-1.1% to 3.3%)
Endocrine, metabolic, blood, and immune disorders	523.9<br>(508.0 to 541.7)	10.6<br>(7.3 to 14.5)	35.5%<br>(31.6% to 39.5%)	3.5%<br>(0.5% to 6.5%)
HIV/AIDS resulting in other diseases	77.1<br>(64.0 to 95.8)	7.0<br>(4.2 to 10.9)	26.5%<br>(-16.5% to 115.3%)	-3.4%<br>(-36.3% to 64.4%)
Sickle cell trait	410.7<br>(367.0 to 471.1)	6.8<br>(4.6 to 9.9)	41.9%<br>(37.4% to 46.8%)	8.4%<br>(4.9% to 12.1%)
Hookworm disease	229.4<br>(209.0 to 251.8)	3.1<br>(2.1 to 4.6)	22.9%<br>(8.7% to 40.3%)	-6.1%<br>(-17.0% to 7.1%)
Other infectious diseases	175.5<br>(170.3 to 181.1)	2.8<br>(1.9 to 3.9)	35.8%<br>(29.5% to 42.4%)	3.7%<br>(-1.1% to 8.7%)
Other neglected tropical diseases	131.8<br>(128.6 to 135.4)	2.1<br>(1.4 to 2.9)	33.8%<br>(26.5% to 40.9%)	2.1%<br>(-3.4% to 7.6%)
Schistosomiasis	102.2<br>(88.5 to 117.2)	1.7<br>(1.1 to 2.5)	-33.0%<br>(-37.4% to -28.5%)	-48.8%<br>(-52.2% to -45.4%)
Malaria	38.9<br>(32.3 to 45.3)	1.3<br>(0.9 to 1.9)	-28.1%<br>(-33.5% to -22.6%)	-45.1%<br>(-49.2% to -40.9%)
Drug-susceptible HIV/AIDS - Tuberculosis	3.0<br>(2.0 to 4.2)	1.1<br>(0.6 to 1.7)	22.6%<br>(10.6% to 34.7%)	-6.4%<br>(-15.5% to 2.8%)
G6PD deficiency	6.0<br>(5.5 to 6.4)	0.2<br>(0.1 to 0.3)	31.1%<br>(26.8% to 35.8%)	0.1%<br>(-3.2% to 3.7%)
Multidrug-resistant HIV/AIDS - Tuberculosis without extensive drug resistance	0.1<br>(0.1 to 0.1)	<0.05<br>(<0.05 to 0.1)	45.0%<br>(6.8% to 83.2%)	10.7%<br>(-18.5% to 39.9%)
Sickle cell disorders	0.1<br>(0.1 to 0.1)	<0.05<br>(<0.05 to <0.05)	17.4%<br>(14.7% to 20.5%)	-10.4%<br>(-12.4% to -8.0%)
G6PD trait	0.1<br>(0.1 to 0.1)	<0.05<br>(<0.05 to <0.05)	28.0%<br>(22.4% to 33.1%)	-2.3%<br>(-6.5% to 1.7%)
Thalassemias	<0.05<br>(<0.05 to <0.05)	<0.05<br>(<0.05 to <0.05)	20.4%<br>(11.2% to 28.1%)	-8.1%<br>(-15.1% to -2.2%)
Extensively drug-resistant HIV/AIDS - Tuberculosis	<0.05<br>(<0.05 to <0.05)	<0.05<br>(<0.05 to <0.05)	800.0%<br>(563.0% to 1,047.8%)	587.2%<br>(406.2% to 776.4%)
**All causes**	**26,236.5<br>(24,062.1 to 28,508.1)**	**838.7<br>(589.0 to 1,127.4)**	**33.7%<br>(30.5% to 37.0%)**	**2.1%<br>(-0.4% to 4.6%)**

SR Table 13a: Global prevalence and years lived with disability (YLDs) for 2016, percentage change of YLD counts and rates between 2006 and 2016 of anaemia by cause, ages 85-89				
**Cause**	**Prevalence in thousands (95% uncertainty interval)**	**YLDs in thousands (95% uncertainty interval)**		
	**2016**	**2016**	**Percentage change in counts between 2006 and 2016**	**Percentage change in rates between 2006 and 2016**
Chronic kidney disease due to hypertension	2,468.7 (1,892.7 to 3,081.0)	101.6 (66.2 to 140.9)	58.2% (51.4% to 64.7%)	-1.4% (-5.7% to 2.6%)
Iron-deficiency anaemia	3,456.9 (3,303.5 to 3,592.5)	100.1 (69.6 to 136.2)	47.4% (45.5% to 49.3%)	-8.1% (-9.4% to -7.0%)
Chronic kidney disease due to diabetes mellitus	2,169.0 (1,551.6 to 2,862.6)	85.1 (55.3 to 126.9)	63.8% (55.9% to 71.7%)	2.0% (-2.9% to 7.0%)
Chronic kidney disease due to other causes	1,641.0 (1,162.1 to 2,180.8)	65.9 (39.8 to 97.1)	64.1% (55.4% to 73.5%)	2.2% (-3.2% to 8.1%)
Gastritis and duodenitis	1,419.5 (1,170.6 to 1,753.2)	37.3 (24.2 to 54.6)	73.1% (66.0% to 80.3%)	7.9% (3.4% to 12.4%)
Chronic kidney disease due to glomerulonephritis	890.2 (639.0 to 1,240.4)	36.3 (22.3 to 55.8)	56.9% (45.7% to 69.8%)	-2.2% (-9.2% to 5.8%)
Peptic ulcer disease	933.8 (734.8 to 1,191.2)	25.5 (16.1 to 38.5)	63.8% (54.6% to 74.0%)	2.0% (-3.7% to 8.4%)
Thalassaemias trait	939.4 (861.0 to 1,017.4)	24.2 (16.1 to 33.9)	67.5% (62.7% to 72.7%)	4.4% (1.3% to 7.6%)
Other hemoglobinopathies and hemolytic anaemias	427.3 (410.8 to 442.6)	8.7 (6.0 to 11.9)	49.2% (46.1% to 52.2%)	-7.0% (-9.0% to -5.1%)
Endocrine, metabolic, blood, and immune disorders	258.1 (248.2 to 267.2)	5.2 (3.6 to 7.1)	58.7% (54.0% to 63.6%)	-1.1% (-4.1% to 1.9%)
Sickle cell trait	209.7 (188.0 to 239.2)	3.5 (2.4 to 4.9)	59.4% (54.0% to 65.5%)	-0.7% (-4.0% to 3.1%)
HIV/AIDS resulting in other diseases	34.6 (28.9 to 43.0)	3.1 (1.9 to 4.8)	54.0% (0.9% to 155.5%)	-4.1% (-37.1% to 59.2%)
Hookworm disease	114.0 (104.1 to 124.6)	1.6 (1.1 to 2.3)	30.8% (15.1% to 48.1%)	-18.5% (-28.3% to -7.7%)
Other infectious diseases	86.4 (83.3 to 89.2)	1.3 (0.9 to 1.9)	52.4% (45.6% to 59.5%)	-5.1% (-9.3% to -0.6%)
Other neglected tropical diseases	60.0 (58.2 to 61.7)	0.9 (0.7 to 1.3)	48.0% (39.3% to 55.8%)	-7.8% (-13.2% to -2.9%)
Schistosomiasis	46.2 (40.0 to 52.6)	0.8 (0.5 to 1.2)	-33.6% (-38.2% to -29.1%)	-58.7% (-61.5% to -55.9%)
Malaria	15.7 (13.0 to 18.6)	0.5 (0.3 to 0.7)	-25.8% (-31.4% to -20.4%)	-53.8% (-57.3% to -50.4%)
Drug-susceptible HIV/AIDS - Tuberculosis	1.3 (0.9 to 1.8)	0.4 (0.3 to 0.7)	34.8% (22.5% to 46.9%)	-16.0% (-23.6% to -8.5%)
G6PD deficiency	3.0 (2.8 to 3.3)	0.1 (0.1 to 0.2)	57.0% (51.1% to 63.1%)	-2.2% (-5.8% to 1.6%)
Multidrug-resistant HIV/AIDS - Tuberculosis without extensive drug resistance	<0.05 (<0.05 to 0.1)	<0.05 (<0.05 to <0.05)	62.1% (17.9% to 108.1%)	1.0% (-26.5% to 29.6%)
Sickle cell disorders	<0.05 (<0.05 to <0.05)	<0.05 (<0.05 to <0.05)	43.5% (40.2% to 47.1%)	-10.6% (-12.6% to -8.3%)
G6PD trait	<0.05 (<0.05 to <0.05)	<0.05 (<0.05 to <0.05)	54.7% (48.4% to 61.4%)	-3.6% (-7.5% to 0.6%)
Thalassemias	<0.05 (<0.05 to <0.05)	<0.05 (<0.05 to <0.05)	46.0% (37.1% to 54.6%)	-9.0% (-14.6% to -3.7%)
Extensively drug-resistant HIV/AIDS - Tuberculosis	<0.05 (<0.05 to <0.05)	<0.05 (<0.05 to <0.05)	831.7% (568.0% to 1,117.1%)	480.5% (316.2% to 658.3%)
**All causes**	**15,174.9 (13,961.2 to 16,396.4)**	**502.3 (350.1 to 668.0)**	**58.3% (54.3% to 62.4%)**	**-1.4% (-3.9% to 1.2%)**

SR Table 13a: Global prevalence and years lived with disability (YLDs) for 2016, percentage change of YLD counts and rates between 2006 and 2016 of anaemia by cause, ages 90-94				
**Cause**	**Prevalence in thousands (95% uncertainty interval)**	**YLDs in thousands (95% uncertainty interval)**		
	**2016**	**2016**	**Percentage change in counts between 2006 and 2016**	**Percentage change in rates between 2006 and 2016**
Chronic kidney disease due to hypertension	1,212.1 (954.3 to 1,492.7)	50.3 (33.6 to 69.5)	69.3% (60.9% to 76.7%)	1.2% (-3.9% to 5.6%)
Chronic kidney disease due to diabetes mellitus	883.9 (649.1 to 1,172.7)	33.9 (21.2 to 48.8)	74.9% (65.9% to 83.7%)	4.5% (-0.9% to 9.8%)
Iron-deficiency anaemia	1,124.2 (1,070.9 to 1,180.7)	31.2 (21.7 to 42.3)	55.9% (53.5% to 58.3%)	-6.9% (-8.3% to -5.4%)
Chronic kidney disease due to other causes	678.8 (498.4 to 908.4)	27.2 (17.0 to 39.7)	69.6% (59.4% to 80.6%)	1.3% (-4.8% to 7.9%)
Chronic kidney disease due to glomerulonephritis	326.0 (232.3 to 442.1)	13.6 (8.6 to 20.4)	54.0% (41.6% to 65.6%)	-8.0% (-15.4% to -1.0%)
Gastritis and duodenitis	505.2 (418.9 to 607.8)	13.2 (8.5 to 19.0)	82.0% (73.5% to 90.1%)	8.7% (3.7% to 13.6%)
Peptic ulcer disease	352.3 (278.2 to 437.2)	9.4 (5.9 to 14.1)	67.9% (57.3% to 77.9%)	0.3% (-6.0% to 6.3%)
Thalassaemias trait	331.5 (299.1 to 366.2)	8.6 (5.7 to 12.1)	73.6% (66.1% to 80.7%)	3.7% (-0.7% to 7.9%)
Other hemoglobinopathies and hemolytic anaemias	140.7 (134.6 to 147.1)	2.8 (1.9 to 3.9)	59.1% (55.5% to 62.7%)	-5.0% (-7.1% to -2.8%)
Endocrine, metabolic, blood, and immune disorders	84.2 (80.7 to 87.9)	1.6 (1.1 to 2.3)	66.3% (61.1% to 71.8%)	-0.7% (-3.8% to 2.7%)
Sickle cell trait	73.1 (65.3 to 83.7)	1.2 (0.8 to 1.7)	72.1% (65.7% to 78.5%)	2.8% (-1.0% to 6.7%)
HIV/AIDS resulting in other diseases	10.4 (8.7 to 12.8)	0.9 (0.6 to 1.4)	70.5% (9.9% to 185.4%)	1.9% (-34.3% to 70.5%)
Hookworm disease	36.4 (33.3 to 39.7)	0.5 (0.3 to 0.8)	32.5% (16.8% to 49.7%)	-20.8% (-30.2% to -10.6%)
Other infectious diseases	28.3 (27.2 to 29.6)	0.4 (0.3 to 0.6)	58.7% (51.2% to 65.6%)	-5.2% (-9.7% to -1.1%)
Other neglected tropical diseases	18.5 (17.9 to 19.1)	0.3 (0.2 to 0.4)	55.6% (47.2% to 63.6%)	-7.1% (-12.1% to -2.2%)
Schistosomiasis	14.1 (11.7 to 16.5)	0.3 (0.2 to 0.4)	-27.9% (-32.8% to -22.7%)	-56.9% (-59.9% to -53.8%)
Malaria	4.1 (3.2 to 5.1)	0.1 (0.1 to 0.2)	-23.7% (-30.3% to -17.3%)	-54.4% (-58.4% to -50.6%)
Drug-susceptible HIV/AIDS - Tuberculosis	0.3 (0.2 to 0.5)	0.1 (0.1 to 0.2)	34.8% (23.1% to 46.5%)	-19.5% (-26.4% to -12.5%)
G6PD deficiency	1.0 (0.9 to 1.1)	<0.05 (<0.05 to 0.1)	61.9% (54.2% to 69.7%)	-3.3% (-7.9% to 1.4%)
Multidrug-resistant HIV/AIDS - Tuberculosis without extensive drug resistance	<0.05 (<0.05 to <0.05)	<0.05 (<0.05 to <0.05)	56.6% (12.1% to 102.6%)	-6.4% (-33.0% to 21.1%)
G6PD trait	<0.05 (<0.05 to <0.05)	<0.05 (<0.05 to <0.05)	61.7% (50.7% to 72.7%)	-3.4% (-10.0% to 3.2%)
Sickle cell disorders	<0.05 (<0.05 to <0.05)	<0.05 (<0.05 to <0.05)	67.1% (62.5% to 72.1%)	-0.2% (-2.9% to 2.8%)
Extensively drug-resistant HIV/AIDS - Tuberculosis	<0.05 (<0.05 to <0.05)	<0.05 (<0.05 to <0.05)	758.4% (503.6% to 1,048.3%)	412.8% (260.6% to 586.0%)
Thalassemias	<0.05 (<0.05 to <0.05)	<0.05 (<0.05 to <0.05)	31.8% (21.7% to 41.6%)	-21.2% (-27.3% to -15.4%)
**All causes**	**5,825.0 (5,320.7 to 6,331.1)**	**195.8 (136.9 to 260.2)**	**66.9% (61.8% to 71.8%)**	**-0.3% (-3.4% to 2.6%)**

SR Table 13a: Global prevalence and years lived with disability (YLDs) for 2016, percentage change of YLD counts and rates between 2006 and 2016 of anaemia by cause, ages 95+ years				
**Cause**	**Prevalence in thousands (95% uncertainty interval)**	**YLDs in thousands (95% uncertainty interval)**		
	**2016**	**2016**	**Percentage change in counts between 2006 and 2016**	**Percentage change in rates between 2006 and 2016**
Chronic kidney disease due to hypertension	310.2<br>(232.7 to 394.2)	13.9<br>(9.2 to 20.1)	62.6%<br>(54.0% to 71.7%)	3.8%<br>(-1.7% to 9.6%)
Chronic kidney disease due to diabetes mellitus	220.1<br>(147.5 to 297.0)	9.2<br>(5.4 to 14.0)	71.4%<br>(61.8% to 82.4%)	9.4%<br>(3.2% to 16.4%)
Chronic kidney disease due to other causes	148.4<br>(94.5 to 219.6)	6.2<br>(3.5 to 9.9)	55.5%<br>(43.8% to 67.7%)	-0.8%<br>(-8.2% to 7.0%)
Iron-deficiency anaemia	228.9<br>(214.1 to 250.1)	5.9<br>(4.1 to 8.2)	54.8%<br>(51.5% to 58.1%)	-1.2%<br>(-3.3% to 0.9%)
Chronic kidney disease due to glomerulonephritis	69.8<br>(42.9 to 108.2)	3.2<br>(1.8 to 5.5)	37.4%<br>(24.0% to 50.4%)	-12.3%<br>(-20.9% to -4.0%)
Gastritis and duodenitis	117.5<br>(94.3 to 146.6)	2.9<br>(1.8 to 4.4)	86.7%<br>(77.1% to 96.3%)	19.2%<br>(13.0% to 25.3%)
Peptic ulcer disease	91.1<br>(69.0 to 121.6)	2.3<br>(1.4 to 3.7)	68.3%<br>(56.3% to 80.3%)	7.4%<br>(-0.2% to 15.0%)
Thalassaemias trait	66.2<br>(57.5 to 77.9)	1.7<br>(1.1 to 2.5)	77.4%<br>(66.4% to 88.3%)	13.2%<br>(6.2% to 20.2%)
Other hemoglobinopathies and hemolytic anaemias	28.7<br>(27.0 to 31.0)	0.5<br>(0.4 to 0.7)	53.5%<br>(49.1% to 58.2%)	-2.0%<br>(-4.8% to 0.9%)
Endocrine, metabolic, blood, and immune disorders	17.4<br>(16.4 to 18.8)	0.3<br>(0.2 to 0.4)	59.8%<br>(54.0% to 65.1%)	2.0%<br>(-1.8% to 5.3%)
Sickle cell trait	14.7<br>(13.0 to 17.0)	0.2<br>(0.1 to 0.3)	63.2%<br>(55.7% to 70.8%)	4.2%<br>(-0.7% to 9.0%)
HIV/AIDS resulting in other diseases	2.0<br>(1.7 to 2.4)	0.2<br>(0.1 to 0.3)	80.5%<br>(15.8% to 193.2%)	15.2%<br>(-26.1% to 87.1%)
Hookworm disease	7.4<br>(6.6 to 8.2)	0.1<br>(0.1 to 0.1)	34.3%<br>(17.7% to 52.2%)	-14.3%<br>(-24.9% to -2.8%)
Other infectious diseases	6.0<br>(5.7 to 6.5)	0.1<br>(0.1 to 0.1)	59.0%<br>(51.1% to 66.3%)	1.5%<br>(-3.6% to 6.1%)
Other neglected tropical diseases	3.8<br>(3.6 to 4.0)	0.1<br>(<0.05 to 0.1)	64.7%<br>(56.0% to 73.6%)	5.1%<br>(-0.5% to 10.8%)
Schistosomiasis	2.8<br>(2.2 to 3.5)	0.1<br>(<0.05 to 0.1)	-27.0%<br>(-33.4% to -20.1%)	-53.4%<br>(-57.5% to -49.0%)
Drug-susceptible HIV/AIDS - Tuberculosis	0.1<br>(<0.05 to 0.1)	<0.05<br>(<0.05 to <0.05)	32.0%<br>(20.6% to 43.0%)	-15.7%<br>(-23.0% to -8.8%)
G6PD deficiency	0.2<br>(0.2 to 0.2)	<0.05<br>(<0.05 to <0.05)	59.8%<br>(50.1% to 70.6%)	2.0%<br>(-4.2% to 8.9%)
Multidrug-resistant HIV/AIDS - Tuberculosis without extensive drug resistance	<0.05<br>(<0.05 to <0.05)	<0.05<br>(<0.05 to <0.05)	54.6%<br>(9.6% to 109.6%)	-1.3%<br>(-30.0% to 33.8%)
G6PD trait	<0.05<br>(<0.05 to <0.05)	<0.05<br>(<0.05 to <0.05)	48.0%<br>(33.3% to 62.9%)	-5.6%<br>(-14.9% to 4.0%)
Sickle cell disorders	<0.05<br>(<0.05 to <0.05)	<0.05<br>(<0.05 to <0.05)	47.4%<br>(41.4% to 52.8%)	-5.9%<br>(-9.7% to -2.5%)
Extensively drug-resistant HIV/AIDS - Tuberculosis	<0.05<br>(<0.05 to <0.05)	<0.05<br>(<0.05 to <0.05)	699.0%<br>(428.5% to 1,035.8%)	409.8%<br>(237.3% to 624.8%)
Thalassemias	<0.05<br>(<0.05 to <0.05)	<0.05<br>(<0.05 to <0.05)	38.3%<br>(24.0% to 56.2%)	-11.8%<br>(-20.9% to -0.3%)
**All causes**	**1,335.2<br>(1,183.0 to 1,543.1)**	**47.0<br>(33.2 to 63.1)**	**61.9%<br>(55.6% to 68.3%)**	**3.3%<br>(-0.7% to 7.4%)**

SR Table 13b: Global prevalence and years lived with disability (YLDs) for 2016, percentage change of YLD counts and rates between 2006 and 2016 of developmental intellectual disability by cause, ages 0-6 days				
**Cause**	**Prevalence in thousands (95% uncertainty interval)**	**YLDs in thousands (95% uncertainty interval)**		
	**2016**	**2016**	**Percentage change in counts between 2006 and 2016**	**Percentage change in rates between 2006 and 2016**
Neonatal sepsis and other neonatal infections	8.7<br>(3.9 to 18.9)	4.4<br>(1.8 to 10.2)	-4.9%<br>(-8.3% to -1.4%)	-4.4%<br>(-7.8% to -0.9%)
Congenital heart anomalies	27.2<br>(21.9 to 33.2)	1.4<br>(0.6 to 2.5)	-1.6%<br>(-4.6% to 1.3%)	-1.0%<br>(-4.1% to 1.8%)
Autism	2.1<br>(1.3 to 3.1)	0.6<br>(0.3 to 1.0)	-0.4%<br>(-4.8% to 4.1%)	0.1%<br>(-4.3% to 4.6%)
Neonatal preterm birth complications	2.9<br>(2.0 to 4.0)	0.4<br>(0.3 to 0.7)	-16.1%<br>(-23.9% to -8.6%)	-15.7%<br>(-23.5% to -8.1%)
Other chromosomal abnormalities	4.4<br>(3.7 to 5.2)	0.4<br>(0.3 to 0.6)	5.0%<br>(1.4% to 9.0%)	5.6%<br>(2.0% to 9.6%)
Neural tube defects	1.0<br>(0.8 to 1.2)	0.3<br>(0.2 to 0.5)	3.3%<br>(-1.7% to 8.6%)	3.8%<br>(-1.2% to 9.1%)
Idiopathic developmental intellectual disability	5.2<br>(1.6 to 10.2)	0.2<br>(0.1 to 0.5)	-13.1%<br>(-18.2% to -7.0%)	-12.7%<br>(-17.7% to -6.5%)
Neonatal encephalopathy due to birth asphyxia and trauma	1.2<br>(0.7 to 1.7)	0.2<br>(0.1 to 0.3)	-12.6%<br>(-24.5% to -0.2%)	-12.1%<br>(-24.1% to 0.3%)
Down's syndrome	1.4<br>(1.2 to 1.7)	0.1<br>(0.1 to 0.2)	-1.0%<br>(-4.6% to 3.0%)	-0.4%<br>(-4.1% to 3.6%)
Haemolytic disease and other neonatal jaundice	<0.05<br>(<0.05 to 0.1)	<0.05<br>(<0.05 to <0.05)	-9.4%<br>(-21.3% to 1.0%)	-8.9%<br>(-20.8% to 1.5%)
Encephalitis	<0.05<br>(<0.05 to <0.05)	<0.05<br>(<0.05 to <0.05)	-5.0%<br>(-28.9% to 3.6%)	-4.5%<br>(-28.5% to 4.1%)
Klinefelter syndrome	0.4<br>(0.3 to 0.6)	<0.05<br>(<0.05 to <0.05)	1.3%<br>(-6.7% to 9.8%)	1.8%<br>(-6.2% to 10.4%)
Alcohol use disorders	0.1<br>(0.1 to 0.1)	<0.05<br>(<0.05 to <0.05)	0.3%<br>(-6.3% to 5.9%)	0.8%<br>(-5.8% to 6.5%)
Meningococcal infection	<0.05<br>(<0.05 to 0.1)	<0.05<br>(<0.05 to <0.05)	700.3%<br>(-19.2% to 3,358.4%)	704.5%<br>(-18.8% to 3,376.5%)
Pneumococcal meningitis	<0.05<br>(<0.05 to <0.05)	<0.05<br>(<0.05 to <0.05)	-11.2%<br>(-27.9% to -2.6%)	-10.7%<br>(-27.5% to -2.0%)
Digestive congenital anomalies	<0.05<br>(<0.05 to <0.05)	<0.05<br>(<0.05 to <0.05)	-5.8%<br>(-8.6% to -3.2%)	-5.3%<br>(-8.2% to -2.7%)
Other meningitis	<0.05<br>(<0.05 to <0.05)	<0.05<br>(<0.05 to <0.05)	-0.1%<br>(-18.7% to 8.6%)	0.4%<br>(-18.2% to 9.2%)
H influenzae type B meningitis	<0.05<br>(<0.05 to <0.05)	<0.05<br>(<0.05 to <0.05)	-17.7%<br>(-44.1% to -4.1%)	-17.3%<br>(-43.8% to -3.6%)
Tetanus	<0.05<br>(<0.05 to <0.05)	<0.05<br>(<0.05 to <0.05)	-16.7%<br>(-22.8% to -11.2%)	-16.2%<br>(-22.4% to -10.7%)
Malaria	<0.05<br>(<0.05 to <0.05)	<0.05<br>(<0.05 to <0.05)	18.4%<br>(6.4% to 28.5%)	19.0%<br>(7.0% to 29.2%)
**All causes**	**54.6<br>(46.2 to 66.4)**	**8.0<br>(5.0 to 13.7)**	**-4.3%<br>(-6.4% to -2.1%)**	**-3.8%<br>(-5.9% to -1.6%)**

SR Table 13b: Global prevalence and years lived with disability (YLDs) for 2016, percentage change of YLD counts and rates between 2006 and 2016 of developmental intellectual disability by cause, ages 7-27 days				
**Cause**	**Prevalence in thousands (95% uncertainty interval)**	**YLDs in thousands (95% uncertainty interval)**		
	**2016**	**2016**	**Percentage change in counts between 2006 and 2016**	**Percentage change in rates between 2006 and 2016**
Neonatal sepsis and other neonatal infections	25.1 (11.1 to 54.8)	12.5 (5.3 to 28.5)	-3.3% (-6.8% to 0.5%)	-3.2% (-6.6% to 0.7%)
Congenital heart anomalies	76.5 (61.5 to 93.3)	4.0 (1.7 to 7.0)	-1.5% (-4.3% to 1.4%)	-1.3% (-4.2% to 1.5%)
Other chromosomal abnormalities	15.4 (13.0 to 17.8)	2.4 (1.7 to 3.3)	-2.3% (-6.6% to 1.6%)	-2.2% (-6.5% to 1.8%)
Autism	6.3 (3.9 to 9.2)	1.7 (0.9 to 2.9)	-0.2% (-5.0% to 5.0%)	-0.1% (-4.8% to 5.2%)
Neonatal preterm birth complications	9.0 (6.2 to 12.3)	1.4 (0.8 to 2.2)	-15.9% (-24.1% to -8.0%)	-15.7% (-24.0% to -7.8%)
Neural tube defects	2.8 (2.2 to 3.6)	0.9 (0.6 to 1.4)	3.7% (-1.3% to 9.1%)	3.9% (-1.2% to 9.3%)
Idiopathic developmental intellectual disability	15.9 (5.1 to 31.0)	0.7 (0.2 to 1.5)	-13.0% (-18.0% to -7.0%)	-12.8% (-17.9% to -6.8%)
Neonatal encephalopathy due to birth asphyxia and trauma	3.7 (2.2 to 5.3)	0.5 (0.3 to 0.9)	-11.7% (-23.6% to 0.4%)	-11.5% (-23.4% to 0.6%)
Down's syndrome	4.1 (3.4 to 4.8)	0.4 (0.2 to 0.5)	-0.7% (-4.7% to 3.2%)	-0.5% (-4.5% to 3.4%)
Haemolytic disease and other neonatal jaundice	0.1 (0.1 to 0.2)	0.1 (<0.05 to 0.1)	-9.0% (-20.5% to 0.9%)	-8.8% (-20.4% to 1.1%)
Zika virus disease	0.1 (0.1 to 0.2)	0.1 (<0.05 to 0.1)	--	--
Klinefelter syndrome	1.3 (0.8 to 1.8)	<0.05 (<0.05 to <0.05)	2.0% (-7.0% to 12.0%)	2.2% (-6.8% to 12.2%)
Encephalitis	<0.05 (<0.05 to <0.05)	<0.05 (<0.05 to <0.05)	-5.7% (-31.5% to 4.4%)	-5.5% (-31.4% to 4.6%)
Alcohol use disorders	0.2 (0.2 to 0.3)	<0.05 (<0.05 to <0.05)	0.8% (-5.6% to 6.5%)	1.0% (-5.4% to 6.7%)
Tetanus	<0.05 (<0.05 to 0.1)	<0.05 (<0.05 to <0.05)	-16.9% (-42.2% to 18.7%)	-16.7% (-42.1% to 18.9%)
Digestive congenital anomalies	<0.05 (<0.05 to <0.05)	<0.05 (<0.05 to <0.05)	-5.1% (-8.0% to -2.4%)	-4.9% (-7.8% to -2.2%)
Pneumococcal meningitis	<0.05 (<0.05 to <0.05)	<0.05 (<0.05 to <0.05)	-24.1% (-34.3% to -12.9%)	-24.0% (-34.2% to -12.7%)
Other meningitis	<0.05 (<0.05 to <0.05)	<0.05 (<0.05 to <0.05)	24.8% (2.6% to 45.9%)	25.0% (2.8% to 46.2%)
H influenzae type B meningitis	<0.05 (<0.05 to <0.05)	<0.05 (<0.05 to <0.05)	-44.3% (-54.8% to -34.5%)	-44.2% (-54.7% to -34.4%)
Meningococcal infection	<0.05 (<0.05 to <0.05)	<0.05 (<0.05 to <0.05)	-7.0% (-17.3% to 2.6%)	-6.8% (-17.1% to 2.8%)
Malaria	<0.05 (<0.05 to <0.05)	<0.05 (<0.05 to <0.05)	-3.6% (-17.3% to 9.3%)	-3.5% (-17.2% to 9.5%)
**All causes**	**160.6 (136.3 to 195.6)**	**24.7 (16.0 to 41.1)**	**-3.6% (-5.5% to -1.3%)**	**-3.4% (-5.4% to -1.2%)**

SR Table 13b: Global prevalence and years lived with disability (YLDs) for 2016, percentage change of YLD counts and rates between 2006 and 2016 of developmental intellectual disability by cause, ages 28-364 days				
**Cause**	**Prevalence in thousands (95% uncertainty interval)**	**YLDs in thousands (95% uncertainty interval)**		
	**2016**	**2016**	**Percentage change in counts between 2006 and 2016**	**Percentage change in rates between 2006 and 2016**
Neonatal sepsis and other neonatal infections	301.0<br>(132.6 to 669.8)	145.4<br>(59.1 to 342.9)	5.6%<br>(0.2% to 12.5%)	4.8%<br>(-0.6% to 11.5%)
Other chromosomal abnormalities	249.0<br>(215.0 to 288.6)	47.8<br>(32.5 to 67.0)	-3.1%<br>(-6.7% to 0.6%)	-3.9%<br>(-7.5% to -0.3%)
Congenital heart anomalies	889.6<br>(728.5 to 1,092.1)	45.6<br>(19.9 to 78.1)	-1.3%<br>(-4.1% to 1.6%)	-2.1%<br>(-4.9% to 0.8%)
Autism	116.3<br>(77.5 to 164.5)	32.0<br>(17.6 to 52.1)	0.9%<br>(-3.5% to 5.0%)	0.1%<br>(-4.3% to 4.1%)
Neonatal preterm birth complications	156.9<br>(109.5 to 211.2)	24.1<br>(14.1 to 37.4)	-15.4%<br>(-23.5% to -7.9%)	-16.1%<br>(-24.2% to -8.7%)
Neural tube defects	49.4<br>(40.2 to 60.3)	18.3<br>(12.2 to 25.8)	3.4%<br>(-2.5% to 9.0%)	2.5%<br>(-3.3% to 8.1%)
Idiopathic developmental intellectual disability	335.3<br>(115.6 to 615.5)	14.2<br>(4.1 to 30.5)	-11.4%<br>(-16.2% to -7.1%)	-12.1%<br>(-16.9% to -7.9%)
Neonatal encephalopathy due to birth asphyxia and trauma	59.9<br>(36.2 to 87.8)	7.7<br>(3.9 to 13.4)	-5.4%<br>(-17.8% to 7.7%)	-6.2%<br>(-18.5% to 6.8%)
Down's syndrome	54.3<br>(46.1 to 63.8)	4.8<br>(3.2 to 6.9)	1.4%<br>(-2.2% to 5.1%)	0.6%<br>(-3.0% to 4.2%)
Haemolytic disease and other neonatal jaundice	3.1<br>(1.7 to 5.0)	1.3<br>(0.7 to 2.3)	-7.9%<br>(-18.5% to 1.5%)	-8.6%<br>(-19.2% to 0.7%)
Zika virus disease	2.2<br>(1.5 to 4.7)	1.3<br>(0.8 to 2.4)	--	--
Klinefelter syndrome	19.8<br>(12.9 to 27.5)	0.3<br>(0.1 to 0.5)	2.4%<br>(-7.0% to 11.7%)	1.6%<br>(-7.8% to 10.8%)
Pneumococcal meningitis	4.6<br>(3.2 to 6.5)	0.2<br>(0.1 to 0.3)	-18.5%<br>(-27.4% to -8.3%)	-19.2%<br>(-28.0% to -9.0%)
Other meningitis	4.7<br>(2.3 to 7.2)	0.2<br>(0.1 to 0.3)	31.1%<br>(9.8% to 51.0%)	30.1%<br>(8.9% to 49.8%)
Alcohol use disorders	3.7<br>(2.9 to 4.4)	0.2<br>(0.1 to 0.2)	2.6%<br>(-3.2% to 7.9%)	1.8%<br>(-4.0% to 7.1%)
H influenzae type B meningitis	2.6<br>(1.4 to 4.3)	0.1<br>(0.1 to 0.2)	-40.7%<br>(-50.2% to -31.6%)	-41.2%<br>(-50.7% to -32.1%)
Encephalitis	2.3<br>(0.9 to 4.4)	0.1<br>(<0.05 to 0.1)	-16.2%<br>(-22.5% to -9.3%)	-16.9%<br>(-23.2% to -10.1%)
Malaria	0.2<br>(0.1 to 0.3)	0.1<br>(<0.05 to 0.1)	-5.1%<br>(-16.4% to 6.3%)	-5.8%<br>(-17.1% to 5.5%)
Meningococcal infection	1.9<br>(0.8 to 3.2)	0.1<br>(<0.05 to 0.1)	-2.2%<br>(-11.7% to 7.7%)	-3.0%<br>(-12.4% to 6.9%)
Tetanus	1.2<br>(0.7 to 1.6)	<0.05<br>(<0.05 to 0.1)	-12.4%<br>(-42.7% to 32.8%)	-13.2%<br>(-43.2% to 31.7%)
Digestive congenital anomalies	0.7<br>(0.5 to 0.9)	<0.05<br>(<0.05 to 0.1)	-3.4%<br>(-5.9% to -1.0%)	-4.1%<br>(-6.6% to -1.8%)
**All causes**	**2,258.6<br>(1,919.1 to 2,719.5)**	**343.8<br>(236.7 to 556.0)**	**0.3%<br>(-2.6% to 3.3%)**	**-0.5%<br>(-3.4% to 2.5%)**

SR Table 13b: Global prevalence and years lived with disability (YLDs) for 2016, percentage change of YLD counts and rates between 2006 and 2016 of developmental intellectual disability by cause, ages 1-4 years				
**Cause**	**Prevalence in thousands (95% uncertainty interval)**	**YLDs in thousands (95% uncertainty interval)**		
	**2016**	**2016**	**Percentage change in counts between 2006 and 2016**	**Percentage change in rates between 2006 and 2016**
Neonatal sepsis and other neonatal infections	783.2 (321.9 to 1,793.0)	357.7 (130.0 to 865.5)	25.5% (16.5% to 38.8%)	18.9% (10.3% to 31.4%)
Autism	800.5 (620.5 to 1,003.4)	219.1 (131.9 to 336.3)	5.7% (2.4% to 9.1%)	0.1% (-3.0% to 3.3%)
Other chromosomal abnormalities	989.3 (866.0 to 1,136.6)	191.5 (130.9 to 265.9)	2.0% (-1.8% to 5.7%)	-3.4% (-7.0% to 0.1%)
Neonatal preterm birth complications	942.2 (666.4 to 1,242.7)	146.6 (86.7 to 223.8)	-9.9% (-17.5% to -2.1%)	-14.6% (-21.8% to -7.3%)
Idiopathic developmental intellectual disability	3,277.7 (1,484.6 to 5,212.9)	135.4 (50.9 to 255.7)	-2.5% (-6.9% to 0.5%)	-7.7% (-11.8% to -4.8%)
Congenital heart anomalies	2,177.2 (1,816.9 to 2,570.9)	111.2 (52.8 to 183.1)	2.8% (-0.4% to 5.7%)	-2.6% (-5.6% to 0.1%)
Neural tube defects	149.4 (116.3 to 190.0)	49.5 (31.9 to 72.4)	9.5% (5.2% to 14.4%)	3.7% (-0.3% to 8.4%)
Neonatal encephalopathy due to birth asphyxia and trauma	307.1 (173.2 to 485.4)	32.6 (10.7 to 76.4)	17.9% (-0.9% to 48.2%)	11.7% (-6.2% to 40.4%)
Down's syndrome	183.1 (159.5 to 210.7)	16.3 (10.9 to 23.1)	7.2% (4.0% to 10.3%)	1.6% (-1.5% to 4.5%)
Haemolytic disease and other neonatal jaundice	24.4 (14.0 to 36.6)	10.5 (5.6 to 16.9)	-3.8% (-13.7% to 5.4%)	-8.8% (-18.3% to -0.2%)
Pneumococcal meningitis	79.5 (58.4 to 104.3)	6.0 (3.4 to 9.2)	-6.8% (-18.9% to 4.2%)	-11.7% (-23.2% to -1.3%)
Iodine deficiency	17.3 (10.7 to 23.0)	6.0 (3.1 to 9.0)	-30.4% (-40.6% to -18.8%)	-34.1% (-43.8% to -23.1%)
Zika virus disease	9.5 (6.4 to 20.5)	5.6 (3.3 to 10.1)	--	--
Malaria	15.1 (8.4 to 22.0)	5.5 (2.8 to 8.7)	3.8% (-24.9% to 34.4%)	-1.7% (-28.9% to 27.3%)
Other meningitis	65.8 (36.8 to 98.9)	3.8 (2.1 to 6.0)	26.8% (9.2% to 44.6%)	20.1% (3.4% to 37.0%)
H influenzae type B meningitis	39.8 (24.7 to 59.0)	3.0 (1.7 to 4.7)	-29.4% (-39.9% to -19.2%)	-33.2% (-43.0% to -23.5%)
Encephalitis	60.5 (27.4 to 109.0)	2.9 (1.6 to 4.7)	-13.2% (-21.2% to -5.6%)	-17.7% (-25.3% to -10.6%)
Meningococcal infection	27.2 (14.3 to 43.2)	1.8 (1.0 to 2.9)	3.6% (-7.1% to 13.5%)	-1.9% (-12.0% to 7.5%)
Klinefelter syndrome	79.7 (51.9 to 111.0)	1.0 (0.5 to 2.0)	6.8% (-2.3% to 16.4%)	1.2% (-7.5% to 10.2%)
Alcohol use disorders	19.7 (17.0 to 21.8)	1.0 (0.6 to 1.4)	7.0% (2.5% to 11.0%)	1.4% (-2.9% to 5.1%)
Digestive congenital anomalies	6.0 (4.6 to 7.6)	0.3 (0.2 to 0.5)	1.3% (-0.9% to 3.5%)	-4.0% (-6.2% to -2.0%)
Tetanus	5.9 (3.7 to 8.1)	0.2 (0.1 to 0.3)	1.1% (-36.1% to 59.1%)	-4.2% (-39.5% to 50.7%)
**All causes**	**10,060.2 (8,065.7 to 12,271.8)**	**1,307.7 (937.1 to 1,897.6)**	**6.8% (3.5% to 11.6%)**	**1.2% (-2.0% to 5.7%)**

SR Table 13b: Global prevalence and years lived with disability (YLDs) for 2016, percentage change of YLD counts and rates between 2006 and 2016 of developmental intellectual disability by cause, ages 5-9 years				
**Cause**	**Prevalence in thousands (95% uncertainty interval)**	**YLDs in thousands (95% uncertainty interval)**		
	**2016**	**2016**	**Percentage change in counts between 2006 and 2016**	**Percentage change in rates between 2006 and 2016**
Idiopathic developmental intellectual disability	10,735.9 (6,519.2 to 15,306.6)	425.6 (196.6 to 737.0)	-0.2% (-4.4% to 2.7%)	-7.3% (-11.2% to -4.6%)
Autism	1,426.2 (1,165.3 to 1,731.4)	389.1 (245.6 to 589.1)	7.6% (4.4% to 10.6%)	-0.1% (-3.0% to 2.7%)
Neonatal sepsis and other neonatal infections	765.9 (264.7 to 1,843.6)	328.4 (98.5 to 837.8)	32.9% (22.2% to 50.0%)	23.4% (13.5% to 39.3%)
Neonatal preterm birth complications	1,678.2 (1,218.1 to 2,168.2)	316.9 (198.4 to 455.3)	4.4% (-7.9% to 16.6%)	-3.1% (-14.5% to 8.3%)
Other chromosomal abnormalities	1,109.6 (962.9 to 1,280.6)	188.2 (130.9 to 257.8)	7.7% (3.9% to 11.7%)	0.1% (-3.5% to 3.7%)
Congenital heart anomalies	1,692.8 (1,466.1 to 1,954.5)	101.4 (41.3 to 174.4)	5.9% (2.5% to 9.3%)	-1.7% (-4.8% to 1.5%)
Neural tube defects	171.4 (131.7 to 220.7)	57.6 (36.7 to 84.0)	9.9% (5.1% to 15.4%)	2.1% (-2.4% to 7.2%)
Neonatal encephalopathy due to birth asphyxia and trauma	445.9 (248.8 to 719.9)	54.2 (13.7 to 141.7)	32.4% (10.1% to 68.8%)	23.0% (2.3% to 56.8%)
Iodine deficiency	151.7 (103.7 to 196.9)	50.1 (29.6 to 74.1)	-29.7% (-36.8% to -21.4%)	-34.7% (-41.3% to -27.0%)
Malaria	64.8 (52.1 to 77.9)	24.8 (17.1 to 33.8)	14.4% (0.9% to 28.9%)	6.2% (-6.3% to 19.7%)
Haemolytic disease and other neonatal jaundice	55.1 (38.2 to 71.5)	23.8 (14.4 to 34.6)	6.3% (-3.4% to 13.7%)	-1.3% (-10.3% to 5.6%)
Down's syndrome	212.7 (185.8 to 245.7)	19.0 (12.7 to 26.9)	7.1% (4.0% to 10.3%)	-0.5% (-3.4% to 2.4%)
Pneumococcal meningitis	180.7 (137.4 to 233.0)	18.4 (11.1 to 27.7)	1.1% (-10.5% to 12.3%)	-6.1% (-16.9% to 4.3%)
Encephalitis	219.8 (113.2 to 372.4)	15.2 (9.1 to 22.5)	-6.3% (-17.2% to 3.9%)	-13.0% (-23.1% to -3.5%)
Other meningitis	134.1 (80.1 to 196.1)	11.4 (6.7 to 17.0)	27.1% (13.2% to 40.3%)	18.1% (5.1% to 30.3%)
H influenzae type B meningitis	82.2 (55.2 to 113.9)	9.0 (5.4 to 13.4)	-23.0% (-32.9% to -13.5%)	-28.5% (-37.7% to -19.6%)
Meningococcal infection	54.1 (30.8 to 81.7)	5.2 (3.2 to 7.9)	9.8% (1.4% to 16.9%)	2.0% (-5.9% to 8.6%)
Zika virus disease	4.6 (3.0 to 8.0)	2.7 (1.5 to 4.8)	--	--
Alcohol use disorders	28.1 (25.6 to 30.7)	1.5 (1.0 to 2.3)	10.3% (6.5% to 12.7%)	2.4% (-1.1% to 4.7%)
Klinefelter syndrome	93.0 (60.3 to 128.8)	1.2 (0.6 to 2.3)	8.2% (-1.2% to 18.2%)	0.5% (-8.2% to 9.8%)
Digestive congenital anomalies	9.2 (7.2 to 11.6)	0.5 (0.3 to 0.8)	3.6% (1.5% to 5.5%)	-3.8% (-5.7% to -2.0%)
Tetanus	8.3 (5.3 to 11.3)	0.3 (0.1 to 0.5)	4.6% (-32.3% to 62.1%)	-2.8% (-37.2% to 50.6%)
**All causes**	**19,324.3 (14,750.6 to 23,994.5)**	**2,044.5 (1,490.9 to 2,776.1)**	**7.7% (4.6% to 12.2%)**	**-0.0% (-2.8% to 4.2%)**

SR Table 13b: Global prevalence and years lived with disability (YLDs) for 2016, percentage change of YLD counts and rates between 2006 and 2016 of developmental intellectual disability by cause, ages 10-14 years				
**Cause**	**Prevalence in thousands (95% uncertainty interval)**	**YLDs in thousands (95% uncertainty interval)**		
	**2016**	**2016**	**Percentage change in counts between 2006 and 2016**	**Percentage change in rates between 2006 and 2016**
Idiopathic developmental intellectual disability	14,318.2 (8,889.3 to 19,655.1)	559.6 (260.8 to 960.6)	-4.9% (-8.4% to -2.5%)	-5.7% (-9.2% to -3.3%)
Autism	1,491.6 (1,254.5 to 1,785.8)	404.6 (261.4 to 608.5)	1.5% (-1.2% to 4.2%)	0.6% (-2.0% to 3.4%)
Neonatal preterm birth complications	1,770.0 (1,296.6 to 2,278.4)	361.1 (227.6 to 523.6)	3.0% (-10.7% to 18.3%)	2.1% (-11.4% to 17.3%)
Neonatal sepsis and other neonatal infections	663.2 (212.6 to 1,610.4)	273.7 (75.9 to 718.4)	26.0% (16.9% to 40.7%)	25.0% (15.9% to 39.6%)
Other chromosomal abnormalities	924.1 (794.9 to 1,073.8)	114.6 (79.7 to 154.6)	2.9% (-0.4% to 6.5%)	2.1% (-1.3% to 5.6%)
Iodine deficiency	328.2 (223.7 to 413.3)	107.8 (63.9 to 157.9)	-12.8% (-19.7% to -5.9%)	-13.5% (-20.3% to -6.6%)
Congenital heart anomalies	1,153.7 (1,008.8 to 1,308.8)	69.1 (28.8 to 118.7)	-2.9% (-6.0% to 0.3%)	-3.7% (-6.8% to -0.6%)
Neonatal encephalopathy due to birth asphyxia and trauma	437.0 (247.2 to 723.3)	59.5 (15.4 to 158.9)	26.4% (8.6% to 50.4%)	25.4% (7.7% to 49.1%)
Neural tube defects	153.4 (116.4 to 200.3)	52.3 (32.8 to 77.2)	5.4% (1.1% to 9.9%)	4.5% (0.3% to 9.0%)
Malaria	101.1 (81.8 to 121.6)	43.4 (29.1 to 58.9)	12.0% (0.5% to 25.2%)	11.0% (-0.3% to 24.2%)
Encephalitis	345.3 (191.7 to 571.3)	30.4 (19.3 to 43.7)	-13.4% (-21.2% to -6.0%)	-14.1% (-21.8% to -6.8%)
Haemolytic disease and other neonatal jaundice	63.0 (45.6 to 78.2)	28.0 (17.4 to 39.5)	3.8% (-3.0% to 9.4%)	2.9% (-3.8% to 8.5%)
Pneumococcal meningitis	211.7 (164.0 to 267.7)	24.1 (14.9 to 35.6)	0.7% (-9.7% to 11.7%)	-0.1% (-10.4% to 10.8%)
Down's syndrome	200.0 (175.0 to 231.1)	17.8 (12.0 to 25.2)	-0.6% (-3.8% to 2.4%)	-1.4% (-4.6% to 1.6%)
Other meningitis	149.1 (92.5 to 211.7)	14.7 (8.9 to 21.8)	22.0% (11.5% to 31.9%)	21.0% (10.6% to 30.8%)
H influenzae type B meningitis	89.5 (63.6 to 121.4)	11.3 (6.7 to 16.4)	-21.5% (-30.1% to -13.7%)	-22.1% (-30.7% to -14.4%)
Meningococcal infection	57.9 (34.2 to 86.0)	6.4 (3.9 to 9.6)	8.8% (1.8% to 14.4%)	7.9% (1.0% to 13.5%)
Alcohol use disorders	27.9 (25.6 to 30.2)	1.6 (1.0 to 2.3)	1.3% (-2.1% to 4.1%)	0.5% (-2.9% to 3.2%)
Klinefelter syndrome	84.1 (54.6 to 116.2)	1.1 (0.5 to 2.1)	2.9% (-5.2% to 12.8%)	2.1% (-6.0% to 11.8%)
Digestive congenital anomalies	12.2 (9.5 to 15.2)	0.6 (0.4 to 1.0)	-6.3% (-8.2% to -4.4%)	-7.1% (-9.0% to -5.1%)
Tetanus	8.1 (5.2 to 11.0)	0.3 (0.1 to 0.5)	8.3% (-23.6% to 50.2%)	7.4% (-24.2% to 49.0%)
Zika virus disease	0.2 (0.1 to 0.3)	0.1 (0.1 to 0.2)	--	--
**All causes**	**22,589.4 (17,173.4 to 27,942.0)**	**2,182.0 (1,592.4 to 2,917.8)**	**2.1% (-0.6% to 6.0%)**	**1.3% (-1.4% to 5.1%)**

SR Table 13b: Global prevalence and years lived with disability (YLDs) for 2016, percentage change of YLD counts and rates between 2006 and 2016 of developmental intellectual disability by cause, ages 15-19 years				
**Cause**	**Prevalence in thousands (95% uncertainty interval)**	**YLDs in thousands (95% uncertainty interval)**		
	**2016**	**2016**	**Percentage change in counts between 2006 and 2016**	**Percentage change in rates between 2006 and 2016**
Idiopathic developmental intellectual disability	13,913.4 (8,840.6 to 19,167.8)	539.9 (259.0 to 930.0)	-10.1% (-13.5% to -8.0%)	-4.9% (-8.4% to -2.6%)
Autism	1,417.7 (1,189.8 to 1,701.5)	381.7 (245.4 to 569.3)	-4.8% (-7.2% to -2.2%)	0.8% (-1.7% to 3.5%)
Neonatal preterm birth complications	1,689.6 (1,245.9 to 2,172.0)	342.1 (217.4 to 499.9)	-1.0% (-15.1% to 14.0%)	4.8% (-10.2% to 20.7%)
Neonatal sepsis and other neonatal infections	593.0 (199.0 to 1,429.7)	240.0 (67.0 to 631.0)	18.4% (10.1% to 30.5%)	25.3% (16.6% to 38.2%)
Iodine deficiency	360.0 (245.3 to 452.2)	117.2 (68.8 to 171.4)	-5.8% (-12.5% to 1.1%)	-0.3% (-7.4% to 7.0%)
Other chromosomal abnormalities	840.9 (716.3 to 980.7)	79.1 (53.2 to 110.7)	-5.2% (-8.3% to -1.9%)	0.4% (-2.9% to 3.9%)
Neonatal encephalopathy due to birth asphyxia and trauma	405.5 (232.1 to 664.4)	56.5 (15.7 to 148.7)	16.3% (2.3% to 34.3%)	23.1% (8.3% to 42.1%)
Congenital heart anomalies	894.8 (791.8 to 1,005.8)	53.4 (22.6 to 92.2)	-12.3% (-15.4% to -9.1%)	-7.1% (-10.5% to -3.8%)
Malaria	113.9 (89.1 to 136.8)	52.6 (34.3 to 71.3)	9.6% (-1.9% to 21.0%)	16.0% (3.9% to 28.1%)
Neural tube defects	140.6 (106.3 to 184.8)	48.6 (31.0 to 72.0)	1.0% (-3.5% to 5.6%)	6.9% (2.2% to 11.8%)
Encephalitis	433.3 (246.3 to 705.9)	44.8 (28.0 to 63.4)	-20.6% (-28.0% to -13.7%)	-15.9% (-23.8% to -8.7%)
Haemolytic disease and other neonatal jaundice	60.4 (43.8 to 74.8)	27.0 (16.9 to 37.8)	-0.9% (-7.5% to 4.1%)	4.9% (-2.1% to 10.2%)
Pneumococcal meningitis	221.1 (173.0 to 275.2)	25.5 (15.7 to 37.0)	-2.6% (-12.9% to 7.3%)	3.1% (-7.8% to 13.6%)
Down's syndrome	192.9 (168.6 to 222.8)	17.2 (11.7 to 24.2)	-7.3% (-10.2% to -4.1%)	-1.8% (-5.0% to 1.5%)
Other meningitis	151.7 (95.0 to 213.9)	15.4 (9.6 to 22.7)	15.6% (6.7% to 24.4%)	22.3% (12.9% to 31.6%)
H influenzae type B meningitis	87.3 (61.9 to 118.4)	11.4 (6.9 to 16.5)	-22.2% (-29.8% to -15.8%)	-17.6% (-25.7% to -10.8%)
Meningococcal infection	56.7 (33.6 to 84.0)	6.4 (3.9 to 9.6)	4.9% (-0.7% to 9.8%)	11.1% (5.1% to 16.2%)
Alcohol use disorders	26.8 (24.5 to 28.9)	1.5 (1.0 to 2.2)	-6.7% (-10.0% to -3.9%)	-1.3% (-4.7% to 1.8%)
Klinefelter syndrome	77.1 (50.0 to 106.5)	1.0 (0.5 to 1.9)	-2.4% (-10.8% to 6.8%)	3.4% (-5.6% to 13.1%)
Digestive congenital anomalies	12.0 (9.4 to 14.9)	0.6 (0.4 to 1.0)	-14.9% (-16.9% to -12.9%)	-10.0% (-12.0% to -7.8%)
Tetanus	7.7 (4.9 to 10.4)	0.2 (0.1 to 0.4)	12.9% (-29.1% to 76.1%)	19.5% (-25.0% to 86.4%)
**All causes**	**21,696.5 (16,377.0 to 26,840.1)**	**2,062.3 (1,499.0 to 2,746.6)**	**-3.2% (-5.5% to 0.2%)**	**2.5% (-0.0% to 6.1%)**

SR Table 13b: Global prevalence and years lived with disability (YLDs) for 2016, percentage change of YLD counts and rates between 2006 and 2016 of developmental intellectual disability by cause, ages 20-24 years				
**Cause**	**Prevalence in thousands (95% uncertainty interval)**	**YLDs in thousands (95% uncertainty interval)**		
	**2016**	**2016**	**Percentage change in counts between 2006 and 2016**	**Percentage change in rates between 2006 and 2016**
Idiopathic developmental intellectual disability	13,182.9 (8,414.5 to 18,141.6)	519.0 (251.3 to 888.7)	-2.2% (-5.7% to 0.1%)	-6.7% (-10.1% to -4.5%)
Autism	1,393.4 (1,171.5 to 1,677.9)	374.6 (241.4 to 557.0)	5.2% (2.5% to 8.1%)	0.3% (-2.2% to 3.1%)
Neonatal preterm birth complications	1,704.5 (1,258.3 to 2,193.2)	343.2 (217.1 to 501.6)	9.8% (-5.9% to 27.5%)	4.7% (-10.2% to 21.6%)
Neonatal sepsis and other neonatal infections	556.8 (195.9 to 1,325.7)	220.5 (64.9 to 578.0)	25.7% (16.1% to 39.5%)	19.9% (10.7% to 33.1%)
Iodine deficiency	349.2 (240.8 to 431.1)	113.2 (68.1 to 163.3)	-2.6% (-9.0% to 3.8%)	-7.1% (-13.2% to -0.9%)
Neonatal encephalopathy due to birth asphyxia and trauma	401.5 (235.8 to 647.3)	60.8 (19.6 to 150.9)	21.8% (9.6% to 36.5%)	16.2% (4.6% to 30.2%)
Encephalitis	516.9 (301.8 to 832.3)	57.3 (36.5 to 81.2)	-11.3% (-19.1% to -3.7%)	-15.4% (-22.9% to -8.2%)
Malaria	103.8 (79.9 to 124.9)	49.9 (32.8 to 67.2)	20.8% (8.6% to 32.4%)	15.2% (3.6% to 26.3%)
Congenital heart anomalies	820.9 (731.0 to 918.5)	49.0 (20.6 to 84.5)	2.6% (-0.9% to 6.1%)	-2.2% (-5.5% to 1.2%)
Neural tube defects	133.0 (99.7 to 176.3)	46.4 (29.3 to 69.2)	7.5% (3.2% to 12.3%)	2.5% (-1.6% to 7.1%)
Haemolytic disease and other neonatal jaundice	62.6 (46.2 to 76.3)	28.0 (17.6 to 38.9)	8.7% (1.5% to 14.5%)	3.7% (-3.2% to 9.2%)
Pneumococcal meningitis	225.9 (176.0 to 280.2)	26.5 (16.3 to 38.2)	1.1% (-8.9% to 10.2%)	-3.6% (-13.1% to 5.2%)
Other chromosomal abnormalities	283.9 (244.9 to 330.6)	25.5 (16.8 to 35.4)	2.3% (-1.9% to 6.5%)	-2.4% (-6.4% to 1.6%)
Down's syndrome	195.6 (170.6 to 225.1)	17.4 (11.7 to 24.6)	0.5% (-3.3% to 4.2%)	-4.1% (-7.8% to -0.6%)
Other meningitis	152.6 (96.7 to 213.8)	16.0 (9.8 to 23.8)	22.3% (12.7% to 31.3%)	16.6% (7.5% to 25.3%)
H influenzae type B meningitis	83.9 (60.9 to 112.3)	11.3 (6.9 to 16.3)	-17.3% (-24.6% to -11.3%)	-21.2% (-28.1% to -15.4%)
Meningococcal infection	55.4 (33.5 to 81.3)	6.5 (4.0 to 9.8)	8.0% (2.3% to 13.1%)	3.1% (-2.4% to 7.9%)
Alcohol use disorders	29.4 (27.4 to 31.2)	1.8 (1.2 to 2.6)	4.3% (-0.2% to 8.5%)	-0.5% (-4.8% to 3.5%)
Klinefelter syndrome	72.0 (44.7 to 99.6)	1.5 (0.7 to 2.8)	5.5% (-4.6% to 15.5%)	0.7% (-9.0% to 10.2%)
Digestive congenital anomalies	11.8 (9.3 to 14.7)	0.6 (0.3 to 1.0)	-1.1% (-3.1% to 1.0%)	-5.7% (-7.6% to -3.7%)
Tetanus	6.7 (4.2 to 9.1)	0.2 (0.1 to 0.4)	11.6% (-23.7% to 65.9%)	6.5% (-27.2% to 58.3%)
**All causes**	**20,342.7 (15,332.2 to 25,296.5)**	**1,969.2 (1,442.8 to 2,607.8)**	**5.4% (2.8% to 8.6%)**	**0.5% (-1.9% to 3.6%)**

SR Table 13b: Global prevalence and years lived with disability (YLDs) for 2016, percentage change of YLD counts and rates between 2006 and 2016 of developmental intellectual disability by cause, ages 25-29 years				
**Cause**	**Prevalence in thousands (95% uncertainty interval)**	**YLDs in thousands (95% uncertainty interval)**		
	**2016**	**2016**	**Percentage change in counts between 2006 and 2016**	**Percentage change in rates between 2006 and 2016**
Idiopathic developmental intellectual disability	12,277.1 (7,785.9 to 16,947.2)	487.6 (238.0 to 827.7)	9.8% (5.7% to 12.6%)	-7.5% (-11.0% to -5.2%)
Autism	1,371.9 (1,156.9 to 1,645.4)	367.7 (239.7 to 553.7)	18.8% (15.7% to 21.7%)	0.0% (-2.5% to 2.5%)
Neonatal preterm birth complications	1,701.8 (1,254.8 to 2,184.9)	343.5 (217.6 to 497.6)	23.8% (5.8% to 44.0%)	4.3% (-10.9% to 21.3%)
Neonatal sepsis and other neonatal infections	532.9 (197.4 to 1,279.9)	209.0 (62.2 to 542.3)	39.5% (28.7% to 59.1%)	17.5% (8.4% to 34.0%)
Iodine deficiency	316.7 (222.0 to 387.1)	102.3 (62.5 to 146.0)	5.2% (-2.0% to 12.5%)	-11.4% (-17.5% to -5.2%)
Encephalitis	587.8 (347.9 to 943.3)	67.8 (42.4 to 95.3)	3.7% (-5.3% to 12.4%)	-12.6% (-20.3% to -5.3%)
Neonatal encephalopathy due to birth asphyxia and trauma	398.3 (240.3 to 627.6)	64.3 (23.6 to 147.5)	35.2% (22.3% to 53.0%)	13.9% (3.1% to 28.9%)
Congenital heart anomalies	801.2 (712.6 to 892.7)	47.7 (19.9 to 82.2)	22.4% (18.3% to 26.9%)	3.1% (-0.3% to 6.9%)
Neural tube defects	127.3 (94.7 to 169.6)	44.8 (27.9 to 66.6)	18.5% (13.6% to 23.9%)	-0.2% (-4.3% to 4.4%)
Malaria	84.7 (64.1 to 102.5)	40.9 (26.6 to 55.3)	27.4% (13.6% to 39.8%)	7.3% (-4.3% to 17.8%)
Haemolytic disease and other neonatal jaundice	63.1 (46.7 to 76.4)	28.3 (17.6 to 39.5)	22.4% (14.0% to 28.4%)	3.1% (-3.9% to 8.1%)
Pneumococcal meningitis	225.7 (177.1 to 279.4)	26.5 (16.5 to 38.2)	8.0% (-2.3% to 17.9%)	-9.0% (-17.7% to -0.7%)
Down's syndrome	196.9 (171.5 to 225.6)	17.5 (11.7 to 24.6)	13.7% (9.2% to 18.2%)	-4.2% (-8.0% to -0.4%)
Other meningitis	151.5 (96.7 to 211.6)	16.2 (9.9 to 24.1)	31.9% (23.1% to 41.1%)	11.1% (3.7% to 18.9%)
H influenzae type B meningitis	79.7 (57.7 to 106.5)	11.0 (6.7 to 16.0)	-10.0% (-17.7% to -3.4%)	-24.2% (-30.6% to -18.7%)
Meningococcal infection	54.0 (33.4 to 78.3)	6.5 (4.0 to 9.7)	15.3% (7.2% to 22.7%)	-2.9% (-9.7% to 3.4%)
Alcohol use disorders	30.2 (28.4 to 31.9)	1.9 (1.2 to 2.7)	15.7% (10.6% to 20.2%)	-2.6% (-6.9% to 1.3%)
Klinefelter syndrome	63.0 (39.4 to 87.0)	1.3 (0.6 to 2.5)	15.9% (5.0% to 28.7%)	-2.4% (-11.5% to 8.4%)
Digestive congenital anomalies	13.8 (10.9 to 17.2)	0.7 (0.4 to 1.1)	17.0% (14.7% to 19.3%)	-1.4% (-3.4% to 0.5%)
Tetanus	6.1 (3.9 to 8.3)	0.2 (0.1 to 0.3)	19.9% (-16.8% to 71.9%)	1.0% (-29.9% to 44.8%)
Other chromosomal abnormalities	0.7 (0.7 to 0.8)	0.2 (0.1 to 0.2)	3.1% (-15.3% to 26.7%)	-13.2% (-28.6% to 6.7%)
**All causes**	**19,084.4 (14,385.6 to 23,663.1)**	**1,885.4 (1,396.3 to 2,487.2)**	**18.1% (15.3% to 22.0%)**	**-0.5% (-2.8% to 2.8%)**

SR Table 13b: Global prevalence and years lived with disability (YLDs) for 2016, percentage change of YLD counts and rates between 2006 and 2016 of developmental intellectual disability by cause, ages 30-34 years				
**Cause**	**Prevalence in thousands (95% uncertainty interval)**	**YLDs in thousands (95% uncertainty interval)**		
	**2016**	**2016**	**Percentage change in counts between 2006 and 2016**	**Percentage change in rates between 2006 and 2016**
Idiopathic developmental intellectual disability	10,216.1 (6,323.2 to 14,187.5)	405.7 (196.1 to 689.1)	4.9% (0.7% to 7.7%)	-7.1% (-10.8% to -4.6%)
Autism	1,209.1 (1,019.4 to 1,452.2)	322.2 (206.0 to 475.3)	12.7% (9.8% to 15.8%)	-0.2% (-2.7% to 2.5%)
Neonatal preterm birth complications	1,519.6 (1,117.9 to 1,948.9)	296.2 (188.0 to 430.0)	19.3% (1.6% to 39.4%)	5.7% (-10.0% to 23.5%)
Neonatal sepsis and other neonatal infections	472.2 (179.1 to 1,131.0)	184.1 (58.0 to 479.7)	39.3% (30.0% to 55.0%)	23.3% (15.1% to 37.3%)
Iodine deficiency	267.9 (187.3 to 328.2)	85.9 (51.9 to 123.0)	10.1% (1.9% to 17.2%)	-2.5% (-9.8% to 3.8%)
Encephalitis	575.6 (339.9 to 925.0)	67.7 (42.5 to 94.3)	-0.5% (-9.2% to 7.6%)	-11.9% (-19.6% to -4.7%)
Neonatal encephalopathy due to birth asphyxia and trauma	355.5 (215.8 to 555.2)	56.8 (21.6 to 127.5)	29.1% (17.2% to 45.5%)	14.4% (3.8% to 28.8%)
Neural tube defects	114.3 (84.3 to 152.8)	40.4 (24.8 to 60.1)	19.5% (15.3% to 24.1%)	5.9% (2.1% to 9.9%)
Congenital heart anomalies	678.9 (602.8 to 755.4)	40.2 (16.6 to 69.5)	11.7% (7.8% to 15.6%)	-1.0% (-4.5% to 2.4%)
Malaria	62.8 (48.4 to 75.8)	29.7 (19.2 to 39.7)	16.6% (5.0% to 27.6%)	3.3% (-7.0% to 13.0%)
Haemolytic disease and other neonatal jaundice	55.3 (40.4 to 67.1)	24.8 (15.4 to 34.6)	18.7% (10.4% to 24.6%)	5.2% (-2.2% to 10.3%)
Pneumococcal meningitis	213.7 (166.3 to 264.6)	24.4 (14.9 to 35.1)	8.5% (-0.8% to 17.5%)	-3.9% (-12.2% to 4.1%)
Down's syndrome	177.5 (154.7 to 203.0)	15.8 (10.5 to 22.3)	14.1% (9.6% to 18.9%)	1.0% (-2.9% to 5.3%)
Other meningitis	141.6 (89.3 to 196.9)	14.8 (8.9 to 22.1)	25.4% (16.2% to 34.4%)	11.0% (2.9% to 19.0%)
H influenzae type B meningitis	71.9 (52.0 to 95.9)	9.7 (5.9 to 14.5)	-9.9% (-16.5% to -3.9%)	-20.2% (-26.1% to -14.9%)
Meningococcal infection	49.9 (30.8 to 72.4)	5.9 (3.5 to 9.0)	15.1% (7.5% to 21.9%)	1.9% (-4.8% to 8.0%)
Alcohol use disorders	27.5 (25.7 to 29.2)	1.7 (1.1 to 2.4)	8.8% (2.9% to 13.8%)	-3.7% (-8.9% to 0.7%)
Klinefelter syndrome	47.0 (29.5 to 64.2)	1.0 (0.4 to 1.9)	13.7% (3.1% to 25.3%)	0.7% (-8.7% to 11.0%)
Digestive congenital anomalies	14.7 (11.6 to 18.3)	0.7 (0.4 to 1.2)	6.1% (3.7% to 8.6%)	-6.1% (-8.2% to -3.8%)
Tetanus	5.7 (3.6 to 7.7)	0.2 (0.1 to 0.3)	22.9% (-13.7% to 77.0%)	8.9% (-23.5% to 56.7%)
Other chromosomal abnormalities	0.2 (0.1 to 0.2)	0.1 (<0.05 to 0.1)	37.7% (-19.7% to 123.2%)	22.0% (-28.9% to 97.7%)
**All causes**	**16,277.2 (12,275.2 to 20,226.8)**	**1,627.9 (1,198.2 to 2,139.9)**	**14.1% (11.4% to 18.4%)**	**1.1% (-1.4% to 4.8%)**

SR Table 13b: Global prevalence and years lived with disability (YLDs) for 2016, percentage change of YLD counts and rates between 2006 and 2016 of developmental intellectual disability by cause, ages 35-39 years				
**Cause**	**Prevalence in thousands (95% uncertainty interval)**	**YLDs in thousands (95% uncertainty interval)**		
	**2016**	**2016**	**Percentage change in counts between 2006 and 2016**	**Percentage change in rates between 2006 and 2016**
Idiopathic developmental intellectual disability	8,287.4 (5,049.1 to 11,564.2)	330.7 (159.3 to 564.9)	-1.2% (-5.4% to 1.5%)	-6.6% (-10.6% to -4.0%)
Autism	1,046.4 (882.2 to 1,256.8)	277.0 (178.0 to 410.8)	5.2% (2.4% to 8.1%)	-0.5% (-3.2% to 2.2%)
Neonatal preterm birth complications	1,309.8 (959.8 to 1,683.1)	245.0 (154.7 to 354.7)	11.8% (-4.3% to 29.1%)	5.7% (-9.5% to 22.1%)
Neonatal sepsis and other neonatal infections	399.1 (155.7 to 946.4)	154.9 (50.2 to 398.0)	33.3% (24.6% to 47.0%)	26.0% (17.8% to 39.0%)
Iodine deficiency	213.5 (149.4 to 261.5)	67.9 (40.3 to 96.4)	9.9% (1.6% to 17.6%)	3.8% (-4.0% to 11.2%)
Encephalitis	543.6 (319.5 to 878.9)	62.7 (38.9 to 87.6)	-8.3% (-16.4% to -0.8%)	-13.4% (-21.0% to -6.3%)
Neonatal encephalopathy due to birth asphyxia and trauma	306.3 (186.2 to 474.0)	48.1 (19.0 to 106.1)	20.9% (9.9% to 35.4%)	14.3% (3.9% to 28.0%)
Neural tube defects	99.5 (73.2 to 133.5)	35.3 (22.0 to 52.9)	15.2% (11.1% to 20.1%)	8.9% (5.0% to 13.5%)
Congenital heart anomalies	579.1 (514.9 to 644.9)	34.2 (14.3 to 60.0)	2.3% (-1.7% to 6.0%)	-3.3% (-7.1% to 0.2%)
Malaria	46.5 (36.2 to 56.3)	21.7 (14.4 to 29.3)	13.8% (1.8% to 25.9%)	7.6% (-3.7% to 19.0%)
Pneumococcal meningitis	190.1 (148.8 to 237.3)	21.3 (13.1 to 31.2)	6.9% (-2.6% to 15.5%)	1.1% (-7.9% to 9.1%)
Haemolytic disease and other neonatal jaundice	46.6 (33.6 to 56.8)	20.8 (12.9 to 29.2)	12.8% (4.6% to 18.6%)	6.6% (-1.1% to 12.1%)
Down's syndrome	150.9 (131.9 to 173.4)	13.4 (9.0 to 19.0)	10.2% (6.2% to 14.6%)	4.2% (0.4% to 8.3%)
Other meningitis	126.0 (78.6 to 176.0)	12.9 (7.5 to 19.2)	17.1% (8.1% to 25.2%)	10.7% (2.2% to 18.4%)
H influenzae type B meningitis	61.6 (44.1 to 83.3)	8.2 (4.9 to 12.4)	-11.3% (-17.8% to -5.7%)	-16.1% (-22.3% to -10.9%)
Meningococcal infection	43.8 (27.1 to 63.4)	5.1 (3.0 to 7.8)	12.8% (5.1% to 19.8%)	6.6% (-0.7% to 13.3%)
Alcohol use disorders	24.7 (22.9 to 26.2)	1.5 (1.0 to 2.2)	0.2% (-5.4% to 5.2%)	-5.2% (-10.6% to -0.6%)
Digestive congenital anomalies	16.1 (12.7 to 20.1)	0.8 (0.5 to 1.3)	-3.7% (-6.2% to -1.1%)	-9.0% (-11.3% to -6.5%)
Klinefelter syndrome	31.5 (19.9 to 42.9)	0.6 (0.3 to 1.2)	6.9% (-5.2% to 20.0%)	1.0% (-10.4% to 13.4%)
Tetanus	5.1 (3.2 to 6.9)	0.2 (0.1 to 0.3)	22.7% (-13.3% to 77.2%)	16.0% (-18.1% to 67.5%)
Other chromosomal abnormalities	0.1 (0.1 to 0.1)	0.1 (<0.05 to 0.1)	37.1% (-20.1% to 137.6%)	29.6% (-24.5% to 124.6%)
**All causes**	**13,527.5 (10,215.0 to 16,815.6)**	**1,362.4 (1,000.6 to 1,785.0)**	**7.7% (5.0% to 11.8%)**	**1.8% (-0.8% to 5.7%)**

SR Table 13b: Global prevalence and years lived with disability (YLDs) for 2016, percentage change of YLD counts and rates between 2006 and 2016 of developmental intellectual disability by cause, ages 40-44 years				
**Cause**	**Prevalence in thousands (95% uncertainty interval)**	**YLDs in thousands (95% uncertainty interval)**		
	**2016**	**2016**	**Percentage change in counts between 2006 and 2016**	**Percentage change in rates between 2006 and 2016**
Idiopathic developmental intellectual disability	7,191.1<br>(4,325.7 to 10,078.4)	290.0<br>(140.7 to 496.2)	6.2%<br>(1.8% to 9.1%)	-6.7%<br>(-10.6% to -4.2%)
Autism	982.2<br>(827.9 to 1,180.0)	258.5<br>(165.2 to 384.9)	12.9%<br>(10.1% to 16.2%)	-0.9%<br>(-3.3% to 2.1%)
Neonatal preterm birth complications	1,194.0<br>(873.4 to 1,535.4)	215.6<br>(135.2 to 311.6)	20.3%<br>(3.2% to 40.0%)	5.6%<br>(-9.4% to 23.0%)
Neonatal sepsis and other neonatal infections	345.7<br>(137.1 to 810.7)	133.6<br>(44.9 to 346.0)	34.8%<br>(26.0% to 49.1%)	18.4%<br>(10.7% to 31.0%)
Encephalitis	559.0<br>(323.9 to 912.2)	61.8<br>(38.6 to 85.6)	-0.8%<br>(-9.8% to 7.9%)	-12.9%<br>(-20.8% to -5.2%)
Iodine deficiency	166.8<br>(117.4 to 203.8)	52.5<br>(31.5 to 74.4)	7.5%<br>(-0.1% to 15.4%)	-5.6%<br>(-12.3% to 1.4%)
Neonatal encephalopathy due to birth asphyxia and trauma	273.9<br>(168.3 to 417.8)	43.5<br>(17.9 to 92.3)	26.3%<br>(15.4% to 41.9%)	11.0%<br>(1.4% to 24.7%)
Congenital heart anomalies	566.1<br>(505.4 to 631.4)	33.3<br>(14.0 to 57.6)	14.9%<br>(10.3% to 19.6%)	1.0%<br>(-3.1% to 5.0%)
Neural tube defects	89.1<br>(65.4 to 119.6)	31.7<br>(19.9 to 47.4)	17.2%<br>(13.0% to 22.2%)	3.0%<br>(-0.8% to 7.3%)
Pneumococcal meningitis	167.9<br>(130.6 to 212.2)	18.7<br>(11.4 to 27.8)	10.4%<br>(1.3% to 19.0%)	-3.0%<br>(-11.0% to 4.5%)
Haemolytic disease and other neonatal jaundice	41.7<br>(29.9 to 50.9)	18.7<br>(11.6 to 26.1)	21.4%<br>(12.8% to 27.8%)	6.7%<br>(-1.0% to 12.3%)
Malaria	35.8<br>(27.4 to 43.5)	17.0<br>(11.0 to 22.8)	26.2%<br>(11.9% to 40.5%)	10.9%<br>(-1.7% to 23.4%)
Other meningitis	113.2<br>(71.4 to 158.1)	11.6<br>(6.8 to 17.0)	22.0%<br>(13.4% to 29.7%)	7.1%<br>(-0.4% to 13.9%)
Down's syndrome	129.2<br>(112.8 to 147.8)	11.5<br>(7.7 to 16.3)	13.0%<br>(7.9% to 18.2%)	-0.8%<br>(-5.2% to 3.8%)
H influenzae type B meningitis	52.7<br>(37.5 to 71.5)	7.0<br>(4.2 to 10.6)	-8.1%<br>(-15.4% to -2.1%)	-19.3%<br>(-25.7% to -14.0%)
Meningococcal infection	38.1<br>(23.4 to 55.4)	4.4<br>(2.6 to 6.8)	15.6%<br>(9.3% to 20.9%)	1.5%<br>(-4.0% to 6.2%)
Alcohol use disorders	24.1<br>(22.2 to 25.7)	1.5<br>(0.9 to 2.1)	5.1%<br>(-1.3% to 10.5%)	-7.7%<br>(-13.3% to -2.9%)
Digestive congenital anomalies	17.6<br>(13.9 to 21.9)	0.9<br>(0.5 to 1.4)	6.6%<br>(4.0% to 9.3%)	-6.3%<br>(-8.7% to -4.0%)
Klinefelter syndrome	20.8<br>(13.1 to 28.8)	0.4<br>(0.2 to 0.8)	10.5%<br>(1.2% to 21.0%)	-2.9%<br>(-11.1% to 6.3%)
Tetanus	4.4<br>(2.8 to 6.0)	0.1<br>(0.1 to 0.2)	21.5%<br>(-14.8% to 74.4%)	6.7%<br>(-25.2% to 53.2%)
Other chromosomal abnormalities	0.1<br>(0.1 to 0.1)	<0.05<br>(<0.05 to 0.1)	7.6%<br>(-43.3% to 102.2%)	-5.5%<br>(-50.2% to 77.6%)
**All causes**	**12,013.4**<br>**(9,074.8 to 14,910.8)**	**1,212.4**<br>**(893.4 to 1,584.8)**	**14.2%**<br>**(11.5% to 17.8%)**	**0.3%**<br>**(-2.0% to 3.4%)**

SR Table 13b: Global prevalence and years lived with disability (YLDs) for 2016, percentage change of YLD counts and rates between 2006 and 2016 of developmental intellectual disability by cause, ages 45-49 years				
**Cause**	**Prevalence in thousands (95% uncertainty interval)**	**YLDs in thousands (95% uncertainty interval)**		
	**2016**	**2016**	**Percentage change in counts between 2006 and 2016**	**Percentage change in rates between 2006 and 2016**
Idiopathic developmental intellectual disability	6,118.8 (3,627.6 to 8,621.6)	249.7 (121.2 to 424.1)	15.7% (11.6% to 18.6%)	-7.2% (-10.5% to -4.9%)
Autism	906.0 (764.0 to 1,085.7)	236.6 (152.2 to 351.0)	23.6% (20.1% to 27.3%)	-0.9% (-3.7% to 2.1%)
Neonatal preterm birth complications	1,069.2 (784.7 to 1,370.7)	185.5 (115.4 to 267.6)	32.3% (12.6% to 53.6%)	6.1% (-9.7% to 23.1%)
Neonatal sepsis and other neonatal infections	295.1 (120.5 to 689.2)	113.2 (38.5 to 293.4)	40.9% (31.3% to 57.3%)	13.0% (5.3% to 26.1%)
Encephalitis	556.3 (317.9 to 913.0)	59.9 (37.2 to 83.8)	12.7% (3.1% to 21.3%)	-9.6% (-17.4% to -2.7%)
Iodine deficiency	127.5 (90.7 to 155.9)	39.6 (24.2 to 56.0)	7.2% (-1.0% to 15.7%)	-14.0% (-20.7% to -7.2%)
Neonatal encephalopathy due to birth asphyxia and trauma	243.2 (151.9 to 364.6)	38.9 (16.4 to 80.7)	34.2% (22.2% to 50.5%)	7.6% (-2.0% to 20.7%)
Congenital heart anomalies	550.6 (492.3 to 614.5)	32.1 (13.3 to 55.2)	31.4% (26.1% to 36.9%)	5.4% (1.1% to 9.8%)
Neural tube defects	80.1 (58.8 to 107.6)	28.6 (17.8 to 42.5)	23.0% (19.1% to 27.5%)	-1.4% (-4.5% to 2.2%)
Haemolytic disease and other neonatal jaundice	36.4 (25.9 to 44.4)	16.4 (10.1 to 22.9)	32.6% (23.1% to 39.7%)	6.3% (-1.3% to 12.0%)
Pneumococcal meningitis	145.7 (113.4 to 184.9)	16.0 (9.7 to 23.8)	15.0% (5.9% to 23.5%)	-7.8% (-15.0% to -1.0%)
Malaria	26.3 (20.1 to 31.9)	12.5 (8.1 to 17.0)	31.4% (16.1% to 47.8%)	5.4% (-6.9% to 18.5%)
Other meningitis	100.9 (63.4 to 140.5)	10.2 (6.0 to 14.9)	30.5% (21.2% to 38.4%)	4.6% (-2.8% to 11.0%)
Down's syndrome	97.5 (84.1 to 112.0)	9.4 (6.4 to 13.2)	16.9% (11.4% to 21.9%)	-6.3% (-10.6% to -2.3%)
H influenzae type B meningitis	44.2 (31.0 to 60.7)	5.8 (3.4 to 9.0)	-4.1% (-12.3% to 2.6%)	-23.1% (-29.7% to -17.8%)
Meningococcal infection	32.8 (19.6 to 47.4)	3.8 (2.2 to 5.8)	18.5% (11.9% to 24.0%)	-4.9% (-10.3% to -0.5%)
Alcohol use disorders	23.0 (21.0 to 24.6)	1.4 (0.9 to 2.0)	11.6% (5.6% to 17.6%)	-10.5% (-15.3% to -5.7%)
Digestive congenital anomalies	18.2 (14.3 to 22.5)	0.9 (0.5 to 1.5)	20.8% (17.9% to 23.7%)	-3.1% (-5.5% to -0.8%)
Klinefelter syndrome	13.2 (8.4 to 18.3)	0.3 (0.1 to 0.5)	18.9% (8.3% to 31.0%)	-4.6% (-13.2% to 5.0%)
Tetanus	3.8 (2.4 to 5.2)	0.1 (0.1 to 0.2)	28.5% (-10.3% to 81.3%)	3.1% (-28.1% to 45.4%)
Other chromosomal abnormalities	<0.05 (<0.05 to <0.05)	<0.05 (<0.05 to <0.05)	18.1% (-41.8% to 147.2%)	-5.3% (-53.4% to 98.2%)
**All causes**	**10,488.9 (7,979.6 to 13,021.3)**	**1,060.8 (775.0 to 1,382.8)**	**23.7% (20.9% to 27.4%)**	**-0.8% (-3.1% to 2.1%)**

SR Table 13b: Global prevalence and years lived with disability (YLDs) for 2016, percentage change of YLD counts and rates between 2006 and 2016 of developmental intellectual disability by cause, ages 50-54 years				
**Cause**	**Prevalence in thousands (95% uncertainty interval)**	**YLDs in thousands (95% uncertainty interval)**		
	**2016**	**2016**	**Percentage change in counts between 2006 and 2016**	**Percentage change in rates between 2006 and 2016**
Autism	788.9 (664.7 to 945.1)	204.1 (129.9 to 301.8)	24.2% (20.7% to 27.9%)	-0.3% (-3.0% to 2.7%)
Idiopathic developmental intellectual disability	4,838.2 (2,802.0 to 6,867.4)	200.4 (97.2 to 342.3)	13.3% (8.2% to 16.2%)	-9.0% (-13.1% to -6.7%)
Neonatal preterm birth complications	898.7 (659.5 to 1,153.8)	146.7 (91.0 to 210.7)	33.0% (13.1% to 54.9%)	6.8% (-9.2% to 24.4%)
Neonatal sepsis and other neonatal infections	243.8 (104.0 to 561.8)	92.5 (32.8 to 236.9)	45.1% (34.1% to 62.3%)	16.5% (7.7% to 30.3%)
Encephalitis	504.4 (276.1 to 833.5)	53.1 (32.5 to 74.5)	13.6% (3.5% to 22.3%)	-8.8% (-16.9% to -1.8%)
Neonatal encephalopathy due to birth asphyxia and trauma	212.1 (134.1 to 316.3)	33.5 (14.9 to 68.1)	35.7% (23.0% to 54.3%)	8.9% (-1.3% to 23.9%)
Iodine deficiency	96.5 (68.3 to 117.0)	29.6 (18.2 to 41.7)	12.1% (2.6% to 21.6%)	-10.0% (-17.6% to -2.3%)
Congenital heart anomalies	498.8 (445.4 to 557.2)	28.9 (12.0 to 50.2)	30.1% (25.0% to 35.0%)	4.5% (0.4% to 8.4%)
Neural tube defects	70.4 (51.6 to 94.8)	25.2 (15.6 to 37.4)	25.8% (21.7% to 30.2%)	1.0% (-2.3% to 4.6%)
Haemolytic disease and other neonatal jaundice	29.7 (21.0 to 36.1)	13.4 (8.1 to 18.8)	30.8% (21.1% to 37.5%)	5.0% (-2.8% to 10.4%)
Pneumococcal meningitis	123.1 (94.2 to 158.2)	13.2 (7.7 to 19.6)	17.0% (8.0% to 24.7%)	-6.0% (-13.3% to 0.1%)
Down's syndrome	63.5 (54.1 to 74.2)	9.1 (6.2 to 12.4)	23.4% (19.3% to 27.0%)	-0.9% (-4.2% to 2.0%)
Malaria	18.3 (14.0 to 22.2)	8.6 (5.7 to 11.8)	23.2% (9.7% to 37.5%)	-1.1% (-11.9% to 10.4%)
Other meningitis	87.0 (53.4 to 121.4)	8.4 (4.8 to 12.5)	28.7% (19.7% to 36.4%)	3.3% (-3.9% to 9.5%)
H influenzae type B meningitis	36.0 (24.8 to 50.3)	4.5 (2.7 to 7.1)	-3.2% (-12.0% to 3.6%)	-22.3% (-29.4% to -16.8%)
Meningococcal infection	28.1 (16.7 to 40.8)	3.1 (1.8 to 4.7)	18.9% (11.8% to 24.6%)	-4.5% (-10.2% to 0.1%)
Alcohol use disorders	21.4 (19.3 to 23.1)	1.3 (0.8 to 1.9)	16.5% (9.1% to 23.3%)	-6.4% (-12.4% to -1.0%)
Digestive congenital anomalies	14.0 (11.0 to 17.4)	0.7 (0.4 to 1.1)	19.5% (16.4% to 22.5%)	-4.1% (-6.5% to -1.7%)
Klinefelter syndrome	8.0 (5.0 to 11.5)	0.1 (0.1 to 0.2)	23.9% (11.2% to 37.1%)	-0.5% (-10.7% to 10.1%)
Tetanus	3.3 (2.1 to 4.5)	0.1 (<0.05 to 0.2)	26.2% (-13.1% to 79.6%)	1.4% (-30.2% to 44.2%)
Other chromosomal abnormalities	<0.05 (<0.05 to <0.05)	<0.05 (<0.05 to <0.05)	70.8% (-30.6% to 308.4%)	37.1% (-44.3% to 227.9%)
**All causes**	**8,584.2 (6,542.8 to 10,634.4)**	**876.6 (640.0 to 1,140.3)**	**24.0% (21.3% to 28.0%)**	**-0.4% (-2.6% to 2.7%)**

SR Table 13b: Global prevalence and years lived with disability (YLDs) for 2016, percentage change of YLD counts and rates between 2006 and 2016 of developmental intellectual disability by cause, ages 55-59 years				
**Cause**	**Prevalence in thousands (95% uncertainty interval)**	**YLDs in thousands (95% uncertainty interval)**		
	**2016**	**2016**	**Percentage change in counts between 2006 and 2016**	**Percentage change in rates between 2006 and 2016**
Autism	644.5<br>(541.8 to 773.4)	165.0<br>(105.1 to 243.2)	28.4%<br>(24.9% to 32.3%)	-1.6%<br>(-4.3% to 1.4%)
Idiopathic developmental intellectual disability	3,595.2<br>(2,027.2 to 5,162.7)	152.4<br>(73.9 to 259.7)	19.8%<br>(14.3% to 23.1%)	-8.2%<br>(-12.4% to -5.7%)
Neonatal preterm birth complications	704.1<br>(514.1 to 905.2)	105.6<br>(65.1 to 151.0)	41.8%<br>(19.8% to 65.1%)	8.6%<br>(-8.2% to 26.5%)
Neonatal sepsis and other neonatal infections	183.5<br>(79.8 to 431.7)	68.7<br>(24.6 to 176.6)	53.7%<br>(43.0% to 68.9%)	17.8%<br>(9.6% to 29.4%)
Encephalitis	432.1<br>(222.2 to 734.0)	43.1<br>(26.2 to 61.5)	19.4%<br>(9.1% to 28.6%)	-8.5%<br>(-16.4% to -1.4%)
Neonatal encephalopathy due to birth asphyxia and trauma	175.2<br>(110.8 to 258.3)	27.1<br>(12.3 to 54.6)	40.9%<br>(27.9% to 58.9%)	8.0%<br>(-2.0% to 21.8%)
Congenital heart anomalies	420.8<br>(375.0 to 470.7)	24.2<br>(9.9 to 42.2)	32.7%<br>(27.6% to 38.1%)	1.7%<br>(-2.2% to 5.8%)
Neural tube defects	58.6<br>(42.9 to 78.9)	21.0<br>(13.1 to 31.1)	32.3%<br>(27.9% to 36.9%)	1.4%<br>(-2.0% to 4.9%)
Iodine deficiency	64.8<br>(46.3 to 77.9)	19.6<br>(11.9 to 27.4)	22.8%<br>(10.5% to 35.8%)	-5.9%<br>(-15.3% to 4.1%)
Haemolytic disease and other neonatal jaundice	22.7<br>(15.9 to 27.8)	10.3<br>(6.1 to 14.5)	39.0%<br>(29.0% to 45.9%)	6.5%<br>(-1.1% to 11.8%)
Pneumococcal meningitis	96.9<br>(73.6 to 125.7)	10.1<br>(5.9 to 15.2)	25.7%<br>(16.3% to 33.9%)	-3.7%<br>(-10.9% to 2.6%)
Down's syndrome	37.9<br>(31.9 to 44.1)	6.6<br>(4.5 to 9.0)	29.0%<br>(24.4% to 33.3%)	-1.1%<br>(-4.7% to 2.1%)
Other meningitis	71.1<br>(41.9 to 100.2)	6.4<br>(3.8 to 9.5)	35.9%<br>(26.6% to 44.3%)	4.1%<br>(-2.9% to 10.6%)
Malaria	12.2<br>(9.3 to 14.8)	5.8<br>(3.7 to 7.8)	25.5%<br>(11.1% to 40.5%)	-3.8%<br>(-14.9% to 7.7%)
H influenzae type B meningitis	27.8<br>(18.5 to 38.7)	3.3<br>(1.9 to 5.1)	3.9%<br>(-5.8% to 11.8%)	-20.4%<br>(-27.8% to -14.3%)
Meningococcal infection	22.9<br>(13.0 to 33.7)	2.4<br>(1.4 to 3.6)	26.6%<br>(18.2% to 33.7%)	-3.0%<br>(-9.4% to 2.5%)
Alcohol use disorders	18.4<br>(16.4 to 20.0)	1.1<br>(0.7 to 1.6)	21.3%<br>(13.7% to 28.0%)	-7.1%<br>(-12.8% to -1.9%)
Digestive congenital anomalies	13.2<br>(10.4 to 16.5)	0.7<br>(0.4 to 1.1)	25.8%<br>(22.5% to 29.1%)	-3.6%<br>(-6.2% to -1.1%)
Tetanus	2.8<br>(1.7 to 3.8)	0.1<br>(<0.05 to 0.2)	39.9%<br>(-3.3% to 97.6%)	7.2%<br>(-25.9% to 51.4%)
Klinefelter syndrome	4.3<br>(2.7 to 6.3)	0.1<br>(<0.05 to 0.1)	23.3%<br>(19.6% to 26.9%)	-5.5%<br>(-8.3% to -2.8%)
Other chromosomal abnormalities	<0.05<br>(<0.05 to <0.05)	<0.05<br>(<0.05 to <0.05)	63.2%<br>(-10.9% to 229.9%)	25.0%<br>(-31.7% to 152.8%)
**All causes**	**6,609.0**<br>**(5,020.2 to 8,189.0)**	**673.5**<br>**(494.4 to 873.3)**	**30.3%**<br>**(27.4% to 34.3%)**	**-0.1%**<br>**(-2.4% to 2.9%)**

SR Table 13b: Global prevalence and years lived with disability (YLDs) for 2016, percentage change of YLD counts and rates between 2006 and 2016 of developmental intellectual disability by cause, ages 60-64 years				
**Cause**	**Prevalence in thousands (95% uncertainty interval)**	**YLDs in thousands (95% uncertainty interval)**		
	**2016**	**2016**	**Percentage change in counts between 2006 and 2016**	**Percentage change in rates between 2006 and 2016**
Autism	541.6 (453.3 to 650.7)	136.9 (88.1 to 202.9)	48.5% (44.3% to 52.5%)	-1.5% (-4.3% to 1.2%)
Idiopathic developmental intellectual disability	2,699.5 (1,466.4 to 3,903.0)	118.3 (58.7 to 199.6)	36.2% (29.7% to 40.1%)	-9.7% (-14.0% to -7.1%)
Neonatal preterm birth complications	546.3 (394.3 to 707.5)	73.4 (45.1 to 105.5)	66.1% (40.7% to 94.9%)	10.1% (-6.7% to 29.3%)
Neonatal sepsis and other neonatal infections	133.1 (57.3 to 320.4)	49.2 (17.1 to 128.5)	77.1% (64.4% to 97.0%)	17.4% (9.0% to 30.7%)
Encephalitis	379.8 (183.4 to 662.0)	35.0 (21.1 to 49.5)	38.5% (27.0% to 49.6%)	-8.1% (-15.7% to -0.8%)
Neonatal encephalopathy due to birth asphyxia and trauma	142.7 (90.3 to 208.5)	21.7 (9.9 to 42.5)	67.1% (49.7% to 89.4%)	10.8% (-0.7% to 25.6%)
Congenital heart anomalies	379.9 (338.7 to 424.6)	21.7 (9.2 to 37.8)	59.8% (53.3% to 66.2%)	6.0% (1.7% to 10.2%)
Neural tube defects	48.8 (35.7 to 65.8)	17.5 (10.9 to 26.0)	46.6% (41.9% to 52.1%)	-2.7% (-5.9% to 0.9%)
Iodine deficiency	36.7 (26.6 to 42.7)	10.9 (7.0 to 15.1)	33.6% (17.3% to 50.7%)	-11.4% (-22.2% to -0.1%)
Haemolytic disease and other neonatal jaundice	17.3 (11.9 to 21.3)	8.0 (4.7 to 11.3)	63.3% (51.0% to 72.1%)	8.3% (0.2% to 14.1%)
Pneumococcal meningitis	72.2 (54.4 to 94.4)	7.3 (4.2 to 11.0)	38.6% (27.5% to 48.4%)	-8.1% (-15.4% to -1.5%)
Other meningitis	56.6 (32.2 to 81.3)	4.8 (2.8 to 7.2)	52.5% (42.4% to 61.9%)	1.1% (-5.5% to 7.4%)
Malaria	7.9 (5.8 to 9.7)	3.9 (2.5 to 5.3)	39.9% (22.2% to 59.5%)	-7.2% (-19.0% to 5.8%)
Down's syndrome	22.1 (18.2 to 26.0)	3.9 (2.6 to 5.3)	58.2% (52.4% to 63.9%)	4.9% (1.1% to 8.7%)
H influenzae type B meningitis	20.6 (13.1 to 29.3)	2.3 (1.4 to 3.6)	15.5% (2.1% to 26.7%)	-23.4% (-32.3% to -16.0%)
Meningococcal infection	17.9 (9.8 to 26.9)	1.8 (1.0 to 2.7)	42.2% (30.6% to 53.7%)	-5.7% (-13.4% to 2.0%)
Alcohol use disorders	15.6 (13.8 to 17.2)	1.0 (0.6 to 1.4)	44.6% (38.1% to 50.9%)	-4.1% (-8.4% to 0.1%)
Digestive congenital anomalies	12.4 (9.7 to 15.4)	0.6 (0.4 to 1.0)	49.4% (45.5% to 53.3%)	-0.9% (-3.5% to 1.7%)
Tetanus	2.4 (1.5 to 3.2)	0.1 (<0.05 to 0.1)	48.3% (-4.4% to 121.5%)	-1.6% (-36.6% to 46.9%)
Klinefelter syndrome	2.4 (1.5 to 3.5)	<0.05 (<0.05 to 0.1)	41.2% (38.6% to 44.2%)	-6.3% (-8.1% to -4.4%)
Other chromosomal abnormalities	<0.05 (<0.05 to <0.05)	<0.05 (<0.05 to <0.05)	22.1% (-56.5% to 234.3%)	-19.0% (-71.1% to 121.8%)
**All causes**	**5,155.7 (3,919.5 to 6,357.7)**	**518.4 (377.8 to 674.2)**	**49.8% (46.2% to 54.4%)**	**-0.6% (-3.0% to 2.4%)**

SR Table 13b: Global prevalence and years lived with disability (YLDs) for 2016, percentage change of YLD counts and rates between 2006 and 2016 of developmental intellectual disability by cause, ages 65-69 years				
Cause	Prevalence in thousands (95% uncertainty interval)	YLDs in thousands (95% uncertainty interval)		
	2016	2016	Percentage change in counts between 2006 and 2016	Percentage change in rates between 2006 and 2016
Autism	399.9<br>(335.2 to 481.1)	99.5<br>(63.6 to 148.1)	33.0%<br>(29.0% to 37.2%)	0.4%<br>(-2.7% to 3.5%)
Idiopathic developmental intellectual disability	1,698.2<br>(873.4 to 2,505.7)	78.5<br>(39.1 to 130.5)	21.2%<br>(16.5% to 24.9%)	-8.5%<br>(-12.1% to -5.7%)
Neonatal preterm birth complications	359.9<br>(260.9 to 470.4)	41.1<br>(25.4 to 59.2)	49.1%<br>(26.0% to 75.2%)	12.5%<br>(-4.9% to 32.3%)
Neonatal sepsis and other neonatal infections	80.0<br>(33.4 to 196.1)	28.6<br>(9.0 to 77.6)	52.7%<br>(40.2% to 76.0%)	15.3%<br>(5.8% to 32.9%)
Encephalitis	273.9<br>(119.8 to 494.3)	23.5<br>(13.6 to 32.9)	22.7%<br>(12.2% to 32.5%)	-7.4%<br>(-15.3% to 0.0%)
Congenital heart anomalies	296.9<br>(265.5 to 331.4)	16.8<br>(7.0 to 29.0)	42.4%<br>(36.7% to 47.9%)	7.5%<br>(3.1% to 11.6%)
Neural tube defects	36.3<br>(26.6 to 49.0)	13.0<br>(8.1 to 19.3)	28.7%<br>(24.8% to 32.7%)	-2.9%<br>(-5.8% to 0.2%)
Neonatal encephalopathy due to birth asphyxia and trauma	94.7<br>(59.6 to 138.6)	12.9<br>(5.9 to 24.8)	43.3%<br>(29.6% to 59.2%)	8.2%<br>(-2.2% to 20.1%)
Haemolytic disease and other neonatal jaundice	10.8<br>(7.3 to 13.3)	5.1<br>(2.9 to 7.1)	44.3%<br>(35.3% to 51.8%)	8.9%<br>(2.1% to 14.6%)
Pneumococcal meningitis	46.7<br>(34.2 to 62.3)	4.2<br>(2.5 to 6.2)	23.2%<br>(13.9% to 31.4%)	-7.0%<br>(-14.0% to -0.8%)
Iodine deficiency	13.5<br>(10.2 to 15.2)	4.2<br>(2.7 to 5.7)	15.6%<br>(1.9% to 30.2%)	-12.7%<br>(-23.1% to -1.8%)
Other meningitis	40.1<br>(21.6 to 58.9)	2.9<br>(1.7 to 4.4)	36.9%<br>(28.0% to 45.3%)	3.3%<br>(-3.4% to 9.6%)
Malaria	4.3<br>(3.1 to 5.2)	2.1<br>(1.4 to 2.9)	21.1%<br>(3.8% to 38.6%)	-8.6%<br>(-21.7% to 4.6%)
Down's syndrome	8.6<br>(7.0 to 10.4)	1.5<br>(1.0 to 2.1)	15.2%<br>(9.6% to 20.8%)	-13.1%<br>(-17.3% to -8.9%)
H influenzae type B meningitis	13.4<br>(8.0 to 19.4)	1.3<br>(0.8 to 1.9)	2.2%<br>(-11.2% to 13.1%)	-22.9%<br>(-33.0% to -14.7%)
Meningococcal infection	12.4<br>(6.2 to 19.5)	1.1<br>(0.6 to 1.7)	26.7%<br>(16.2% to 41.1%)	-4.4%<br>(-12.3% to 6.5%)
Alcohol use disorders	11.7<br>(10.2 to 13.1)	0.7<br>(0.5 to 1.1)	29.4%<br>(23.7% to 34.7%)	-2.4%<br>(-6.6% to 1.7%)
Digestive congenital anomalies	10.3<br>(8.1 to 12.9)	0.5<br>(0.3 to 0.8)	30.6%<br>(27.1% to 34.0%)	-1.4%<br>(-4.1% to 1.1%)
Tetanus	1.6<br>(1.0 to 2.2)	<0.05<br>(<0.05 to 0.1)	33.0%<br>(-12.8% to 92.8%)	0.4%<br>(-34.2% to 45.5%)
Klinefelter syndrome	1.2<br>(0.8 to 1.7)	<0.05<br>(<0.05 to <0.05)	30.0%<br>(27.2% to 33.1%)	-1.9%<br>(-4.0% to 0.5%)
Other chromosomal abnormalities	<0.05<br>(<0.05 to <0.05)	<0.05<br>(<0.05 to <0.05)	6.0%<br>(-72.3% to 334.9%)	-20.0%<br>(-79.1% to 228.2%)
**All causes**	**3,414.5<br>(2,606.8 to 4,215.3)**	**337.5<br>(248.5 to 438.9)**	**32.5%<br>(29.6% to 36.4%)**	**0.0%<br>(-2.2% to 2.9%)**

SR Table 13b: Global prevalence and years lived with disability (YLDs) for 2016, percentage change of YLD counts and rates between 2006 and 2016 of developmental intellectual disability by cause, ages 70-74 years				
**Cause**	**Prevalence in thousands (95% uncertainty interval)**	**YLDs in thousands (95% uncertainty interval)**		
	**2016**	**2016**	**Percentage change in counts between 2006 and 2016**	**Percentage change in rates between 2006 and 2016**
Autism	272.2<br>(228.3 to 328.1)	66.5<br>(43.6 to 98.0)	20.7%<br>(17.3% to 24.2%)	0.9%<br>(-2.0% to 3.8%)
Idiopathic developmental intellectual disability	978.0<br>(477.9 to 1,469.4)	48.0<br>(24.0 to 79.3)	9.8%<br>(5.6% to 12.9%)	-8.2%<br>(-11.7% to -5.6%)
Neonatal preterm birth complications	216.5<br>(154.1 to 288.6)	19.7<br>(12.0 to 27.8)	34.8%<br>(13.9% to 59.9%)	12.7%<br>(-4.8% to 33.7%)
Neonatal sepsis and other neonatal infections	42.5<br>(17.0 to 108.3)	14.4<br>(4.0 to 41.3)	42.0%<br>(31.7% to 62.7%)	18.7%<br>(10.1% to 36.0%)
Encephalitis	184.6<br>(70.4 to 343.6)	13.8<br>(8.0 to 19.8)	13.1%<br>(3.3% to 22.1%)	-5.5%<br>(-13.7% to 2.1%)
Congenital heart anomalies	214.3<br>(192.0 to 238.7)	11.9<br>(4.9 to 20.6)	26.5%<br>(21.7% to 31.2%)	5.8%<br>(1.7% to 9.6%)
Neural tube defects	25.4<br>(18.6 to 34.3)	9.1<br>(5.6 to 13.5)	19.3%<br>(15.4% to 23.3%)	-0.3%<br>(-3.5% to 3.1%)
Neonatal encephalopathy due to birth asphyxia and trauma	55.5<br>(33.8 to 82.1)	6.0<br>(2.7 to 11.6)	33.5%<br>(20.6% to 48.4%)	11.6%<br>(0.8% to 24.0%)
Haemolytic disease and other neonatal jaundice	5.6<br>(3.7 to 7.0)	2.7<br>(1.5 to 3.8)	34.1%<br>(26.1% to 40.6%)	12.1%<br>(5.4% to 17.5%)
Pneumococcal meningitis	28.8<br>(20.4 to 39.8)	2.1<br>(1.3 to 3.0)	15.8%<br>(7.8% to 23.9%)	-3.2%<br>(-9.9% to 3.5%)
Other meningitis	26.9<br>(13.6 to 40.1)	1.5<br>(0.9 to 2.3)	30.5%<br>(22.6% to 38.9%)	9.0%<br>(2.4% to 16.1%)
Iodine deficiency	3.1<br>(2.4 to 3.5)	1.1<br>(0.7 to 1.4)	10.9%<br>(8.4% to 12.8%)	-7.3%<br>(-9.4% to -5.7%)
Malaria	2.0<br>(1.5 to 2.4)	1.0<br>(0.7 to 1.4)	15.3%<br>(-0.9% to 30.8%)	-3.6%<br>(-17.2% to 9.3%)
H influenzae type B meningitis	8.3<br>(4.7 to 12.5)	0.6<br>(0.4 to 0.9)	-5.4%<br>(-18.7% to 6.1%)	-20.9%<br>(-32.1% to -11.3%)
Meningococcal infection	8.3<br>(3.7 to 13.3)	0.6<br>(0.3 to 1.1)	30.3%<br>(10.7% to 67.2%)	8.9%<br>(-7.5% to 39.8%)
Alcohol use disorders	7.5<br>(6.5 to 8.7)	0.5<br>(0.3 to 0.7)	15.4%<br>(10.5% to 19.9%)	-3.6%<br>(-7.6% to 0.2%)
Digestive congenital anomalies	7.6<br>(6.0 to 9.5)	0.4<br>(0.2 to 0.6)	17.3%<br>(14.1% to 20.4%)	-2.0%<br>(-4.6% to 0.6%)
Tetanus	1.1<br>(0.7 to 1.5)	<0.05<br>(<0.05 to 0.1)	25.6%<br>(-16.0% to 80.0%)	5.0%<br>(-29.8% to 50.4%)
Klinefelter syndrome	0.6<br>(0.3 to 0.8)	<0.05<br>(<0.05 to <0.05)	23.9%<br>(21.0% to 27.3%)	3.5%<br>(1.2% to 6.4%)
Other chromosomal abnormalities	<0.05<br>(<0.05 to <0.05)	<0.05<br>(<0.05 to <0.05)	28.1%<br>(-68.0% to 484.4%)	7.1%<br>(-73.2% to 388.4%)
**All causes**	**2,088.8**<br>**(1,608.6 to 2,566.2)**	**199.9**<br>**(146.5 to 259.5)**	**20.4%**<br>**(18.0% to 23.5%)**	**0.7%**<br>**(-1.3% to 3.2%)**

SR Table 13b: Global prevalence and years lived with disability (YLDs) for 2016, percentage change of YLD counts and rates between 2006 and 2016 of developmental intellectual disability by cause, ages 75-79 years				
**Cause**	**Prevalence in thousands (95% uncertainty interval)**	**YLDs in thousands (95% uncertainty interval)**		
	**2016**	**2016**	**Percentage change in counts between 2006 and 2016**	**Percentage change in rates between 2006 and 2016**
Autism	194.0 (162.2 to 234.1)	46.6 (30.4 to 68.6)	24.2% (20.3% to 28.3%)	-0.1% (-3.2% to 3.1%)
Idiopathic developmental intellectual disability	573.5 (265.7 to 887.4)	30.0 (15.0 to 48.3)	15.7% (10.7% to 19.1%)	-7.0% (-11.0% to -4.3%)
Congenital heart anomalies	165.0 (148.0 to 183.7)	9.0 (3.7 to 15.7)	28.8% (23.6% to 34.1%)	3.6% (-0.6% to 7.8%)
Neonatal preterm birth complications	133.1 (91.9 to 179.5)	8.4 (5.2 to 12.2)	34.1% (15.9% to 58.5%)	7.8% (-6.8% to 27.5%)
Encephalitis	130.3 (42.9 to 251.7)	8.1 (4.8 to 11.7)	21.8% (11.2% to 32.4%)	-2.0% (-10.6% to 6.5%)
Neonatal sepsis and other neonatal infections	22.6 (8.8 to 53.8)	7.1 (1.6 to 20.4)	54.3% (43.4% to 77.0%)	24.1% (15.3% to 42.3%)
Neural tube defects	18.5 (13.5 to 25.0)	6.6 (4.1 to 9.9)	23.4% (19.8% to 27.2%)	-0.8% (-3.6% to 2.3%)
Neonatal encephalopathy due to birth asphyxia and trauma	35.1 (20.9 to 52.4)	2.7 (1.2 to 5.4)	45.0% (25.2% to 60.5%)	16.6% (0.7% to 29.1%)
Haemolytic disease and other neonatal jaundice	2.7 (1.7 to 3.4)	1.4 (0.7 to 1.9)	45.0% (35.6% to 52.6%)	16.6% (9.1% to 22.7%)
Pneumococcal meningitis	17.6 (11.8 to 25.2)	1.0 (0.6 to 1.4)	19.6% (9.8% to 28.8%)	-3.9% (-11.7% to 3.6%)
Other meningitis	17.7 (8.4 to 27.0)	0.7 (0.4 to 1.1)	42.8% (34.2% to 52.4%)	14.8% (7.9% to 22.6%)
Malaria	0.8 (0.6 to 1.0)	0.4 (0.3 to 0.6)	33.5% (18.8% to 47.1%)	7.3% (-4.4% to 18.3%)
Meningococcal infection	5.9 (2.2 to 9.5)	0.4 (0.2 to 1.1)	92.5% (14.8% to 400.8%)	54.8% (-7.7% to 302.7%)
Alcohol use disorders	5.3 (4.5 to 6.2)	0.3 (0.2 to 0.5)	16.5% (11.8% to 20.9%)	-6.3% (-10.1% to -2.8%)
Digestive congenital anomalies	6.2 (4.9 to 7.8)	0.3 (0.2 to 0.5)	22.0% (18.4% to 25.4%)	-1.9% (-4.8% to 0.9%)
H influenzae type B meningitis	5.1 (2.7 to 7.9)	0.3 (0.2 to 0.4)	-2.8% (-18.5% to 12.5%)	-21.9% (-34.5% to -9.5%)
Iodine deficiency	0.4 (0.3 to 0.5)	0.1 (0.1 to 0.2)	20.3% (14.1% to 25.2%)	-3.2% (-8.3% to 0.7%)
Tetanus	0.7 (0.4 to 0.9)	<0.05 (<0.05 to <0.05)	33.2% (-8.4% to 95.5%)	7.1% (-26.4% to 57.2%)
Klinefelter syndrome	0.3 (0.2 to 0.4)	<0.05 (<0.05 to <0.05)	29.0% (25.5% to 32.9%)	3.7% (0.9% to 6.9%)
Other chromosomal abnormalities	<0.05 (<0.05 to <0.05)	<0.05 (<0.05 to <0.05)	26.9% (-35.4% to 139.5%)	2.0% (-48.0% to 92.6%)
**All causes**	**1,334.9 (1,033.5 to 1,629.7)**	**123.6 (90.8 to 161.8)**	**24.8% (22.4% to 28.2%)**	**0.4% (-1.5% to 3.1%)**

SR Table 13b: Global prevalence and years lived with disability (YLDs) for 2016, percentage change of YLD counts and rates between 2006 and 2016 of developmental intellectual disability by cause, ages 80-84				
**Cause**	**Prevalence in thousands (95% uncertainty interval)**	**YLDs in thousands (95% uncertainty interval)**		
	**2016**	**2016**	**Percentage change in counts between 2006 and 2016**	**Percentage change in rates between 2006 and 2016**
Autism	118.3 (98.5 to 143.0)	27.9 (18.3 to 41.1)	33.8% (29.8% to 38.4%)	2.1% (-0.9% to 5.7%)
Idiopathic developmental intellectual disability	354.8 (196.5 to 523.4)	16.3 (8.5 to 26.2)	24.9% (19.2% to 29.0%)	-4.6% (-9.0% to -1.5%)
Congenital heart anomalies	108.7 (97.5 to 121.0)	5.9 (2.4 to 10.2)	34.7% (29.3% to 40.5%)	2.8% (-1.3% to 7.2%)
Neural tube defects	11.1 (8.1 to 14.9)	4.0 (2.4 to 5.9)	29.7% (26.0% to 33.7%)	-1.0% (-3.8% to 2.1%)
Encephalitis	79.2 (22.3 to 157.3)	3.8 (2.2 to 5.7)	31.8% (20.8% to 41.9%)	0.6% (-7.7% to 8.4%)
Neonatal sepsis and other neonatal infections	5.6 (1.0 to 16.4)	2.3 (0.4 to 7.3)	70.9% (59.7% to 104.7%)	30.5% (21.9% to 56.3%)
Neonatal preterm birth complications	1.3 (0.5 to 2.1)	0.7 (0.2 to 1.2)	48.5% (9.1% to 95.2%)	13.4% (-16.7% to 49.0%)
Neonatal encephalopathy due to birth asphyxia and trauma	1.0 (0.2 to 2.7)	0.5 (0.1 to 1.4)	79.4% (53.4% to 121.2%)	37.0% (17.1% to 68.9%)
Haemolytic disease and other neonatal jaundice	0.9 (0.5 to 1.1)	0.4 (0.2 to 0.6)	55.8% (42.1% to 66.7%)	19.0% (8.5% to 27.3%)
Pneumococcal meningitis	10.3 (6.7 to 15.1)	0.4 (0.2 to 0.6)	20.2% (10.9% to 29.6%)	-8.2% (-15.3% to -1.1%)
Other meningitis	10.4 (4.8 to 16.1)	0.3 (0.2 to 0.5)	53.8% (44.0% to 64.9%)	17.4% (9.9% to 25.9%)
Meningococcal infection	3.7 (1.2 to 9.9)	0.3 (0.1 to 1.6)	203.5% (15.3% to 2,127.3%)	131.8% (-11.9% to 1,600.6%)
Alcohol use disorders	3.9 (3.3 to 4.6)	0.2 (0.2 to 0.4)	18.3% (11.8% to 23.9%)	-9.7% (-14.6% to -5.4%)
Digestive congenital anomalies	4.1 (3.2 to 5.1)	0.2 (0.1 to 0.3)	29.2% (25.3% to 32.9%)	-1.4% (-4.3% to 1.5%)
H influenzae type B meningitis	2.8 (1.4 to 4.4)	0.1 (0.1 to 0.2)	-4.4% (-21.7% to 13.7%)	-27.0% (-40.2% to -13.2%)
Malaria	0.2 (0.1 to 0.2)	0.1 (0.1 to 0.2)	50.7% (38.8% to 55.9%)	15.1% (6.0% to 19.1%)
Tetanus	0.4 (0.3 to 0.6)	<0.05 (<0.05 to <0.05)	45.1% (-3.5% to 121.3%)	10.8% (-26.3% to 69.0%)
Iodine deficiency	<0.05 (<0.05 to <0.05)	<0.05 (<0.05 to <0.05)	22.6% (10.6% to 32.2%)	-6.4% (-15.6% to 0.9%)
Klinefelter syndrome	0.1 (0.1 to 0.2)	<0.05 (<0.05 to <0.05)	43.2% (39.0% to 47.5%)	9.4% (6.1% to 12.6%)
Other chromosomal abnormalities	<0.05 (<0.05 to <0.05)	<0.05 (<0.05 to <0.05)	13.1% (11.4% to 15.0%)	-13.6% (-14.9% to -12.2%)
**All causes**	**716.7 (559.1 to 871.2)**	**63.4 (46.5 to 83.4)**	**32.8% (30.2% to 36.6%)**	**1.4% (-0.6% to 4.3%)**

SR Table 13b: Global prevalence and years lived with disability (YLDs) for 2016, percentage change of YLD counts and rates between 2006 and 2016 of developmental intellectual disability by cause, ages 85-89				
**Cause**	**Prevalence in thousands (95% uncertainty interval)**	**YLDs in thousands (95% uncertainty interval)**		
	**2016**	**2016**	**Percentage change in counts between 2006 and 2016**	**Percentage change in rates between 2006 and 2016**
Autism	62.1<br>(51.8 to 75.1)	14.4<br>(9.5 to 21.3)	64.1%<br>(58.6% to 70.0%)	2.3%<br>(-1.2% to 5.9%)
Idiopathic developmental intellectual disability	141.8<br>(75.6 to 218.6)	6.8<br>(3.5 to 11.3)	49.7%<br>(37.8% to 55.7%)	-6.7%<br>(-14.1% to -3.0%)
Congenital heart anomalies	64.1<br>(57.7 to 71.2)	3.4<br>(1.4 to 5.9)	67.9%<br>(60.5% to 75.2%)	4.6%<br>(-0.0% to 9.2%)
Neural tube defects	5.9<br>(4.3 to 8.0)	2.1<br>(1.3 to 3.2)	58.5%<br>(53.8% to 63.2%)	-1.2%<br>(-4.2% to 1.7%)
Encephalitis	40.1<br>(10.0 to 80.4)	1.5<br>(0.8 to 2.2)	61.0%<br>(47.6% to 73.2%)	0.3%<br>(-8.0% to 7.9%)
Neonatal sepsis and other neonatal infections	1.2<br>(0.2 to 3.9)	0.5<br>(0.1 to 1.7)	128.6%<br>(111.8% to 183.4%)	42.4%<br>(32.0% to 76.6%)
Meningococcal infection	2.0<br>(0.5 to 8.9)	0.2<br>(<0.05 to 1.8)	410.4%<br>(31.1% to 4,524.4%)	218.0%<br>(-18.3% to 2,781.3%)
Pneumococcal meningitis	4.9<br>(3.1 to 7.2)	0.2<br>(0.1 to 0.2)	32.7%<br>(22.6% to 43.8%)	-17.3%<br>(-23.6% to -10.4%)
Alcohol use disorders	2.1<br>(1.8 to 2.5)	0.1<br>(0.1 to 0.2)	52.2%<br>(41.2% to 58.1%)	-5.2%<br>(-12.1% to -1.5%)
Digestive congenital anomalies	2.5<br>(1.9 to 3.1)	0.1<br>(0.1 to 0.2)	60.2%<br>(55.4% to 65.0%)	-0.2%<br>(-3.2% to 2.8%)
Other meningitis	4.9<br>(2.2 to 7.7)	0.1<br>(0.1 to 0.2)	78.1%<br>(67.1% to 90.2%)	11.0%<br>(4.1% to 18.5%)
Neonatal encephalopathy due to birth asphyxia and trauma	0.2<br>(<0.05 to 0.6)	0.1<br>(<0.05 to 0.3)	139.1%<br>(92.7% to 225.8%)	49.0%<br>(20.0% to 103.0%)
Haemolytic disease and other neonatal jaundice	0.2<br>(0.1 to 0.2)	0.1<br>(<0.05 to 0.1)	93.0%<br>(64.2% to 115.8%)	20.3%<br>(2.3% to 34.4%)
H influenzae type B meningitis	1.2<br>(0.6 to 2.0)	<0.05<br>(<0.05 to 0.1)	2.8%<br>(-17.0% to 28.0%)	-35.9%<br>(-48.3% to -20.3%)
Neonatal preterm birth complications	0.1<br>(<0.05 to 0.1)	<0.05<br>(<0.05 to 0.1)	75.8%<br>(15.8% to 163.4%)	9.6%<br>(-27.9% to 64.1%)
Malaria	<0.05<br>(<0.05 to <0.05)	<0.05<br>(<0.05 to <0.05)	89.8%<br>(79.0% to 96.6%)	18.2%<br>(11.6% to 22.5%)
Tetanus	0.2<br>(0.1 to 0.2)	<0.05<br>(<0.05 to <0.05)	55.8%<br>(5.1% to 122.0%)	-2.9%<br>(-34.5% to 38.3%)
Klinefelter syndrome	<0.05<br>(<0.05 to 0.1)	<0.05<br>(<0.05 to <0.05)	82.8%<br>(77.2% to 89.0%)	13.9%<br>(10.4% to 17.8%)
Other chromosomal abnormalities	<0.05<br>(<0.05 to <0.05)	<0.05<br>(<0.05 to <0.05)	59.3%<br>(55.4% to 63.2%)	-0.7%<br>(-3.2% to 1.7%)
Iodine deficiency	<0.05<br>(<0.05 to <0.05)	<0.05<br>(<0.05 to <0.05)	66.8%<br>(39.2% to 82.0%)	3.9%<br>(-13.3% to 13.4%)
**All causes**	**333.6**<br>**(263.4 to 402.7)**	**29.7**<br>**(21.8 to 38.9)**	**61.8%**<br>**(58.1% to 68.2%)**	**0.8%**<br>**(-1.5% to 4.8%)**

SR Table 13b: Global prevalence and years lived with disability (YLDs) for 2016, percentage change of YLD counts and rates between 2006 and 2016 of developmental intellectual disability by cause, ages 90-94				
**Cause**	**Prevalence in thousands (95% uncertainty interval)**	**YLDs in thousands (95% uncertainty interval)**		
	**2016**	**2016**	**Percentage change in counts between 2006 and 2016**	**Percentage change in rates between 2006 and 2016**
Autism	21.9 (18.3 to 26.6)	5.0 (3.3 to 7.4)	71.4% (65.0% to 78.0%)	2.4% (-1.4% to 6.3%)
Idiopathic developmental intellectual disability	37.1 (18.4 to 59.9)	1.9 (0.9 to 3.1)	57.2% (34.1% to 65.7%)	-6.1% (-19.9% to -1.0%)
Congenital heart anomalies	25.7 (23.1 to 28.5)	1.3 (0.6 to 2.3)	77.1% (68.7% to 84.8%)	5.8% (0.8% to 10.4%)
Neural tube defects	2.2 (1.6 to 2.9)	0.8 (0.5 to 1.2)	67.6% (62.7% to 72.5%)	0.2% (-2.8% to 3.1%)
Encephalitis	13.0 (3.1 to 25.8)	0.4 (0.2 to 0.6)	62.3% (49.6% to 75.4%)	-3.0% (-10.6% to 4.8%)
Meningococcal infection	0.7 (0.2 to 5.5)	0.1 (<0.05 to 1.1)	657.3% (28.8% to 4,982.5%)	352.5% (-23.1% to 2,936.5%)
Neonatal sepsis and other neonatal infections	0.1 (<0.05 to 0.4)	0.1 (<0.05 to 0.2)	158.7% (134.2% to 218.7%)	54.5% (39.9% to 90.4%)
Digestive congenital anomalies	1.0 (0.8 to 1.3)	0.1 (<0.05 to 0.1)	69.0% (64.1% to 74.1%)	1.0% (-2.0% to 4.0%)
Alcohol use disorders	0.8 (0.6 to 0.9)	<0.05 (<0.05 to 0.1)	56.5% (27.5% to 64.8%)	-6.5% (-23.8% to -1.5%)
Pneumococcal meningitis	1.6 (1.0 to 2.3)	<0.05 (<0.05 to 0.1)	35.7% (25.5% to 46.6%)	-18.9% (-25.0% to -12.4%)
Other meningitis	1.5 (0.6 to 2.4)	<0.05 (<0.05 to 0.1)	79.8% (67.8% to 91.2%)	7.4% (0.2% to 14.2%)
Neonatal encephalopathy due to birth asphyxia and trauma	<0.05 (<0.05 to 0.1)	<0.05 (<0.05 to 0.1)	175.4% (67.1% to 375.5%)	64.5% (-0.2% to 184.1%)
H influenzae type B meningitis	0.4 (0.2 to 0.6)	<0.05 (<0.05 to <0.05)	3.1% (-17.9% to 32.3%)	-38.4% (-51.0% to -21.0%)
Haemolytic disease and other neonatal jaundice	<0.05 (<0.05 to <0.05)	<0.05 (<0.05 to <0.05)	95.8% (37.6% to 131.8%)	17.0% (-17.8% to 38.5%)
Tetanus	<0.05 (<0.05 to 0.1)	<0.05 (<0.05 to <0.05)	63.6% (15.8% to 136.3%)	-2.2% (-30.8% to 41.2%)
Malaria	<0.05 (<0.05 to <0.05)	<0.05 (<0.05 to <0.05)	101.0% (90.3% to 110.9%)	20.1% (13.7% to 26.0%)
Neonatal preterm birth complications	<0.05 (<0.05 to <0.05)	<0.05 (<0.05 to <0.05)	108.9% (12.9% to 206.2%)	24.8% (-32.5% to 82.9%)
Klinefelter syndrome	<0.05 (<0.05 to <0.05)	<0.05 (<0.05 to <0.05)	90.0% (83.4% to 97.3%)	13.5% (9.6% to 17.8%)
Other chromosomal abnormalities	<0.05 (<0.05 to <0.05)	<0.05 (<0.05 to <0.05)	91.2% (87.6% to 94.7%)	14.2% (12.1% to 16.3%)
Iodine deficiency	<0.05 (<0.05 to <0.05)	<0.05 (<0.05 to <0.05)	70.8% (44.9% to 97.2%)	2.0% (-13.4% to 17.8%)
**All causes**	**106.1 (85.1 to 126.8)**	**9.7 (7.1 to 12.7)**	**69.6% (65.0% to 82.4%)**	**1.3% (-1.4% to 9.0%)**

SR Table 13b: Global prevalence and years lived with disability (YLDs) for 2016, percentage change of YLD counts and rates between 2006 and 2016 of developmental intellectual disability by cause, ages 95+ years				
**Cause**	**Prevalence in thousands (95% uncertainty interval)**	**YLDs in thousands (95% uncertainty interval)**		
	**2016**	**2016**	**Percentage change in counts between 2006 and 2016**	**Percentage change in rates between 2006 and 2016**
Autism	4.6<br>(3.8 to 5.5)	1.0<br>(0.7 to 1.5)	59.9%<br>(53.4% to 66.8%)	2.0%<br>(-2.1% to 6.4%)
Congenital heart anomalies	6.1<br>(5.5 to 6.7)	0.3<br>(0.1 to 0.5)	65.3%<br>(57.2% to 73.1%)	5.5%<br>(0.3% to 10.4%)
Idiopathic developmental intellectual disability	5.7<br>(2.7 to 9.8)	0.3<br>(0.2 to 0.5)	47.4%<br>(20.1% to 57.4%)	-6.0%<br>(-23.4% to 0.5%)
Neural tube defects	0.5<br>(0.4 to 0.6)	0.2<br>(0.1 to 0.3)	57.5%<br>(52.5% to 63.0%)	0.5%<br>(-2.7% to 4.0%)
Encephalitis	2.7<br>(0.6 to 5.2)	0.1<br>(<0.05 to 0.1)	54.3%<br>(32.8% to 63.0%)	-1.5%<br>(-15.3% to 4.0%)
Meningococcal infection	0.2<br>(<0.05 to 1.6)	<0.05<br>(<0.05 to 0.3)	737.1%<br>(18.2% to 3,374.8%)	434.2%<br>(-24.6% to 2,117.4%)
Digestive congenital anomalies	0.2<br>(0.2 to 0.3)	<0.05<br>(<0.05 to <0.05)	58.4%<br>(53.2% to 63.7%)	1.1%<br>(-2.2% to 4.5%)
Alcohol use disorders	0.2<br>(0.1 to 0.2)	<0.05<br>(<0.05 to <0.05)	37.2%<br>(-8.4% to 47.9%)	-12.4%<br>(-41.5% to -5.6%)
Pneumococcal meningitis	0.3<br>(0.2 to 0.5)	<0.05<br>(<0.05 to <0.05)	35.1%<br>(22.3% to 46.4%)	-13.8%<br>(-22.0% to -6.6%)
Other meningitis	0.3<br>(0.1 to 0.5)	<0.05<br>(<0.05 to <0.05)	74.0%<br>(58.3% to 85.7%)	11.0%<br>(1.0% to 18.5%)
H influenzae type B meningitis	0.1<br>(<0.05 to 0.1)	<0.05<br>(<0.05 to <0.05)	-0.1%<br>(-21.4% to 28.8%)	-36.2%<br>(-49.8% to -17.8%)
Neonatal sepsis and other neonatal infections	<0.05<br>(<0.05 to <0.05)	<0.05<br>(<0.05 to <0.05)	149.0%<br>(124.2% to 223.1%)	58.9%<br>(43.0% to 106.2%)
Neonatal encephalopathy due to birth asphyxia and trauma	<0.05<br>(<0.05 to <0.05)	<0.05<br>(<0.05 to <0.05)	170.1%<br>(10.3% to 569.0%)	72.3%<br>(-29.6% to 326.9%)
Haemolytic disease and other neonatal jaundice	<0.05<br>(<0.05 to <0.05)	<0.05<br>(<0.05 to <0.05)	77.8%<br>(-12.2% to 139.1%)	13.5%<br>(-44.0% to 52.6%)
Tetanus	<0.05<br>(<0.05 to <0.05)	<0.05<br>(<0.05 to <0.05)	70.0%<br>(19.8% to 135.1%)	8.5%<br>(-23.6% to 50.0%)
Malaria	<0.05<br>(<0.05 to <0.05)	<0.05<br>(<0.05 to <0.05)	78.7%<br>(65.7% to 90.4%)	14.1%<br>(5.7% to 21.5%)
Neonatal preterm birth complications	<0.05<br>(<0.05 to <0.05)	<0.05<br>(<0.05 to <0.05)	108.1%<br>(-33.0% to 246.1%)	32.8%<br>(-57.2% to 120.9%)
Klinefelter syndrome	<0.05<br>(<0.05 to <0.05)	<0.05<br>(<0.05 to <0.05)	63.6%<br>(57.6% to 69.6%)	4.4%<br>(0.6% to 8.2%)
Other chromosomal abnormalities	<0.05<br>(<0.05 to <0.05)	<0.05<br>(<0.05 to <0.05)	84.5%<br>(77.7% to 90.0%)	17.7%<br>(13.4% to 21.3%)
Iodine deficiency	<0.05<br>(<0.05 to <0.05)	<0.05<br>(<0.05 to <0.05)	74.5%<br>(40.5% to 120.1%)	11.3%<br>(-10.3% to 40.5%)
**All causes**	**20.8<br>(17.0 to 24.5)**	**2.0<br>(1.4 to 2.6)**	**59.1%<br>(54.4% to 74.0%)**	**1.5%<br>(-1.5% to 11.0%)**

SR Table 13c: Global prevalence and years lived with disability (YLDs) for 2016, percentage change of YLD counts and rates between 2006 and 2016 of epilepsy by cause, ages 0-6 days				
**Cause**	**Prevalence in thousands (95% uncertainty interval)**	**YLDs in thousands (95% uncertainty interval)**		
	**2016**	**2016**	**Percentage change in counts between 2006 and 2016**	**Percentage change in rates between 2006 and 2016**
Neonatal sepsis and other neonatal infections	8.5<br>(3.6 to 18.5)	4.3<br>(1.8 to 10.1)	-4.9%<br>(-8.3% to -1.3%)	-4.4%<br>(-7.8% to -0.8%)
Neonatal preterm birth complications	0.9<br>(0.5 to 1.3)	0.4<br>(0.2 to 0.6)	-16.4%<br>(-25.5% to -7.5%)	-15.9%<br>(-25.1% to -7.1%)
Neonatal encephalopathy due to birth asphyxia and trauma	0.3<br>(0.2 to 0.5)	0.1<br>(0.1 to 0.2)	-14.0%<br>(-29.5% to 1.3%)	-13.5%<br>(-29.1% to 1.9%)
Zika virus disease	<0.05<br>(<0.05 to 0.1)	<0.05<br>(<0.05 to <0.05)	--	--
Epilepsy	0.1<br>(<0.05 to 0.1)	<0.05<br>(<0.05 to <0.05)	-7.6%<br>(-19.3% to 6.0%)	-7.1%<br>(-18.8% to 6.6%)
Haemolytic disease and other neonatal jaundice	<0.05<br>(<0.05 to 0.1)	<0.05<br>(<0.05 to <0.05)	-9.2%<br>(-20.5% to 0.7%)	-8.7%<br>(-20.1% to 1.2%)
Tetanus	<0.05<br>(<0.05 to <0.05)	<0.05<br>(<0.05 to <0.05)	-32.7%<br>(-42.7% to -22.9%)	-32.4%<br>(-42.4% to -22.5%)
Encephalitis	<0.05<br>(<0.05 to <0.05)	<0.05<br>(<0.05 to <0.05)	-2.8%<br>(-23.4% to 23.9%)	-2.3%<br>(-23.0% to 24.5%)
Pneumococcal meningitis	<0.05<br>(<0.05 to <0.05)	<0.05<br>(<0.05 to <0.05)	-4.7%<br>(-25.9% to 19.3%)	-4.2%<br>(-25.5% to 19.9%)
H influenzae type B meningitis	<0.05<br>(<0.05 to <0.05)	<0.05<br>(<0.05 to <0.05)	-2.2%<br>(-26.3% to 26.6%)	-1.7%<br>(-25.9% to 27.3%)
Other meningitis	<0.05<br>(<0.05 to <0.05)	<0.05<br>(<0.05 to <0.05)	26.7%<br>(-1.2% to 57.4%)	27.3%<br>(-0.7% to 58.2%)
Meningococcal infection	<0.05<br>(<0.05 to <0.05)	<0.05<br>(<0.05 to <0.05)	4.8%<br>(-17.3% to 27.4%)	5.4%<br>(-16.8% to 28.1%)
**All causes**	**9.8**<br>**(5.1 to 19.8)**	**4.9**<br>**(2.4 to 10.6)**	**-5.7%**<br>**(-8.8% to -2.7%)**	**-5.2%**<br>**(-8.3% to -2.2%)**

SR Table 13c: Global prevalence and years lived with disability (YLDs) for 2016, percentage change of YLD counts and rates between 2006 and 2016 of epilepsy by cause, ages 7-27 days				
**Cause**	**Prevalence in thousands (95% uncertainty interval)**	**YLDs in thousands (95% uncertainty interval)**		
	**2016**	**2016**	**Percentage change in counts between 2006 and 2016**	**Percentage change in rates between 2006 and 2016**
Neonatal sepsis and other neonatal infections	24.3<br>(10.5 to 53.8)	12.4<br>(5.3 to 28.5)	-3.3%<br>(-6.8% to 0.5%)	-3.2%<br>(-6.6% to 0.7%)
Neonatal preterm birth complications	2.8<br>(1.8 to 4.0)	1.1<br>(0.6 to 1.8)	-14.9%<br>(-24.6% to -5.4%)	-14.7%<br>(-24.5% to -5.2%)
Neonatal encephalopathy due to birth asphyxia and trauma	1.0<br>(0.6 to 1.7)	0.4<br>(0.2 to 0.7)	-11.8%<br>(-26.7% to 3.1%)	-11.6%<br>(-26.6% to 3.2%)
Epilepsy	0.8<br>(0.6 to 1.0)	0.3<br>(0.2 to 0.4)	-6.8%<br>(-18.5% to 6.9%)	-6.6%<br>(-18.4% to 7.1%)
Zika virus disease	0.1<br>(0.1 to 0.2)	0.1<br>(<0.05 to 0.1)	--	--
Haemolytic disease and other neonatal jaundice	0.2<br>(0.1 to 0.2)	0.1<br>(<0.05 to 0.1)	-6.9%<br>(-17.6% to 3.0%)	-6.7%<br>(-17.4% to 3.1%)
Tetanus	<0.05<br>(<0.05 to <0.05)	<0.05<br>(<0.05 to <0.05)	-32.0%<br>(-41.6% to -22.8%)	-31.9%<br>(-41.5% to -22.6%)
Malaria	<0.05<br>(<0.05 to <0.05)	<0.05<br>(<0.05 to <0.05)	-3.5%<br>(-17.3% to 9.5%)	-3.4%<br>(-17.2% to 9.7%)
Encephalitis	<0.05<br>(<0.05 to <0.05)	<0.05<br>(<0.05 to <0.05)	-5.0%<br>(-24.4% to 19.6%)	-4.8%<br>(-24.2% to 19.9%)
Pneumococcal meningitis	<0.05<br>(<0.05 to <0.05)	<0.05<br>(<0.05 to <0.05)	-3.0%<br>(-23.8% to 18.1%)	-2.8%<br>(-23.6% to 18.3%)
H influenzae type B meningitis	<0.05<br>(<0.05 to <0.05)	<0.05<br>(<0.05 to <0.05)	-1.8%<br>(-24.7% to 24.4%)	-1.6%<br>(-24.6% to 24.7%)
Other meningitis	<0.05<br>(<0.05 to <0.05)	<0.05<br>(<0.05 to <0.05)	27.0%<br>(2.0% to 52.9%)	27.2%<br>(2.2% to 53.2%)
Meningococcal infection	<0.05<br>(<0.05 to <0.05)	<0.05<br>(<0.05 to <0.05)	8.3%<br>(-14.5% to 36.0%)	8.5%<br>(-14.4% to 36.2%)
**All causes**	**29.1<br>(15.8 to 57.7)**	**14.4<br>(7.0 to 30.2)**	**-4.2%<br>(-7.4% to -1.0%)**	**-4.0%<br>(-7.2% to -0.8%)**

SR Table 13c: Global prevalence and years lived with disability (YLDs) for 2016, percentage change of YLD counts and rates between 2006 and 2016 of epilepsy by cause, ages 28-364 days				
**Cause**	**Prevalence in thousands (95% uncertainty interval)**	**YLDs in thousands (95% uncertainty interval)**		
	**2016**	**2016**	**Percentage change in counts between 2006 and 2016**	**Percentage change in rates between 2006 and 2016**
Neonatal sepsis and other neonatal infections	291.2<br>(122.4 to 659.8)	147.2<br>(59.8 to 347.0)	5.9%<br>(0.5% to 12.8%)	5.0%<br>(-0.4% to 11.9%)
Epilepsy	118.8<br>(91.8 to 150.9)	41.4<br>(26.7 to 59.2)	-2.5%<br>(-15.1% to 12.9%)	-3.3%<br>(-15.8% to 12.0%)
Neonatal preterm birth complications	102.0<br>(74.5 to 132.5)	36.2<br>(22.8 to 53.2)	-8.0%<br>(-20.7% to 6.6%)	-8.7%<br>(-21.3% to 5.7%)
Neonatal encephalopathy due to birth asphyxia and trauma	33.4<br>(18.7 to 55.7)	11.8<br>(5.8 to 20.9)	6.6%<br>(-6.1% to 21.5%)	5.7%<br>(-6.9% to 20.5%)
Haemolytic disease and other neonatal jaundice	8.5<br>(6.5 to 11.1)	3.0<br>(2.0 to 4.3)	2.2%<br>(-6.5% to 10.1%)	1.3%<br>(-7.3% to 9.2%)
Zika virus disease	1.9<br>(1.3 to 3.9)	1.3<br>(0.7 to 2.2)	--	--
Pneumococcal meningitis	1.5<br>(0.9 to 2.2)	0.3<br>(0.1 to 0.5)	-5.2%<br>(-17.1% to 8.3%)	-5.9%<br>(-17.8% to 7.4%)
H influenzae type B meningitis	1.1<br>(0.7 to 1.7)	0.2<br>(0.1 to 0.4)	-0.1%<br>(-17.5% to 19.2%)	-0.9%<br>(-18.2% to 18.2%)
Malaria	0.5<br>(0.3 to 0.8)	0.2<br>(0.1 to 0.3)	9.1%<br>(-3.2% to 21.7%)	8.2%<br>(-4.0% to 20.7%)
Other meningitis	1.0<br>(0.5 to 1.5)	0.2<br>(0.1 to 0.3)	39.5%<br>(19.5% to 61.1%)	38.4%<br>(18.5% to 59.7%)
Encephalitis	0.9<br>(0.6 to 1.4)	0.2<br>(0.1 to 0.3)	-6.5%<br>(-18.5% to 7.5%)	-7.3%<br>(-19.2% to 6.6%)
Meningococcal infection	0.4<br>(0.2 to 0.7)	0.1<br>(<0.05 to 0.1)	21.5%<br>(5.0% to 41.3%)	20.5%<br>(4.1% to 40.1%)
Tetanus	<0.05<br>(<0.05 to <0.05)	<0.05<br>(<0.05 to <0.05)	-20.2%<br>(-29.7% to -10.5%)	-20.8%<br>(-30.3% to -11.2%)
**All causes**	**561.3<br>(395.5 to 887.2)**	**241.9<br>(149.5 to 433.2)**	**2.6%<br>(-2.5% to 7.6%)**	**1.8%<br>(-3.3% to 6.7%)**

SR Table 13c: Global prevalence and years lived with disability (YLDs) for 2016, percentage change of YLD counts and rates between 2006 and 2016 of epilepsy by cause, ages 1-4 years				
**Cause**	**Prevalence in thousands (95% uncertainty interval)**	**YLDs in thousands (95% uncertainty interval)**		
	**2016**	**2016**	**Percentage change in counts between 2006 and 2016**	**Percentage change in rates between 2006 and 2016**
Epilepsy	1,568.9<br>(1,180.5 to 2,073.8)	545.0<br>(344.9 to 820.0)	8.2%<br>(-4.9% to 23.8%)	2.5%<br>(-9.9% to 17.2%)
Neonatal sepsis and other neonatal infections	722.7<br>(271.6 to 1,718.1)	355.5<br>(125.2 to 867.6)	26.0%<br>(16.9% to 39.6%)	19.3%<br>(10.7% to 32.2%)
Neonatal preterm birth complications	593.1<br>(433.2 to 776.4)	209.9<br>(130.1 to 312.1)	-1.7%<br>(-15.1% to 13.6%)	-6.9%<br>(-19.6% to 7.6%)
Neonatal encephalopathy due to birth asphyxia and trauma	118.0<br>(24.2 to 305.2)	42.4<br>(7.6 to 115.7)	32.8%<br>(13.4% to 86.2%)	25.8%<br>(7.4% to 76.4%)
Haemolytic disease and other neonatal jaundice	51.1<br>(41.0 to 63.7)	18.6<br>(12.2 to 26.5)	4.5%<br>(-1.6% to 10.6%)	-1.1%<br>(-6.8% to 4.8%)
Malaria	32.8<br>(23.1 to 43.8)	13.2<br>(7.9 to 20.2)	6.5%<br>(-8.5% to 22.1%)	0.9%<br>(-13.3% to 15.7%)
Pneumococcal meningitis	34.8<br>(28.1 to 42.3)	6.4<br>(4.2 to 9.4)	-2.6%<br>(-19.3% to 16.7%)	-7.7%<br>(-23.6% to 10.5%)
Encephalitis	32.3<br>(24.9 to 41.3)	5.7<br>(3.5 to 8.4)	-2.6%<br>(-16.0% to 12.4%)	-7.8%<br>(-20.5% to 6.5%)
H influenzae type B meningitis	24.8<br>(19.4 to 30.8)	4.6<br>(3.0 to 6.8)	-4.2%<br>(-23.4% to 20.8%)	-9.2%<br>(-27.5% to 14.4%)
Other meningitis	21.1<br>(16.5 to 26.1)	3.9<br>(2.4 to 5.8)	30.8%<br>(10.5% to 55.6%)	23.9%<br>(4.7% to 47.3%)
Meningococcal infection	8.7<br>(6.8 to 11.0)	1.7<br>(1.0 to 2.4)	14.7%<br>(1.4% to 28.4%)	8.7%<br>(-3.9% to 21.6%)
Zika virus disease	0.4<br>(0.2 to 0.6)	0.3<br>(0.1 to 0.4)	--	--
Cystic echinococcosis	0.4<br>(0.1 to 1.1)	0.1<br>(<0.05 to 0.2)	-40.8%<br>(-80.8% to 85.4%)	-43.9%<br>(-81.8% to 75.6%)
Food-borne trematodiases	0.3<br>(0.1 to 0.7)	<0.05<br>(<0.05 to 0.1)	-20.4%<br>(-26.1% to -14.8%)	-24.6%<br>(-30.0% to -19.3%)
Tetanus	<0.05<br>(<0.05 to <0.05)	<0.05<br>(<0.05 to <0.05)	-22.2%<br>(-32.7% to -12.5%)	-26.3%<br>(-36.3% to -17.1%)
**All causes**	**3,209.4<br>(2,531.5 to 4,277.6)**	**1,207.2<br>(861.1 to 1,775.9)**	**11.4%<br>(3.0% to 20.0%)**	**5.5%<br>(-2.4% to 13.7%)**

SR Table 13c: Global prevalence and years lived with disability (YLDs) for 2016, percentage change of YLD counts and rates between 2006 and 2016 of epilepsy by cause, ages 5-9 years				
**Cause**	**Prevalence in thousands (95% uncertainty interval)**	**YLDs in thousands (95% uncertainty interval)**		
	**2016**	**2016**	**Percentage change in counts between 2006 and 2016**	**Percentage change in rates between 2006 and 2016**
Epilepsy	2,406.8 (1,798.7 to 3,146.4)	827.9 (519.9 to 1,251.8)	10.7% (-2.8% to 26.6%)	2.8% (-9.7% to 17.6%)
Neonatal preterm birth complications	949.9 (691.4 to 1,231.4)	361.7 (223.7 to 527.6)	7.3% (-6.9% to 22.0%)	-0.3% (-13.6% to 13.3%)
Neonatal sepsis and other neonatal infections	667.7 (192.5 to 1,674.2)	312.4 (89.1 to 804.4)	33.1% (22.1% to 52.1%)	23.6% (13.4% to 41.3%)
Neonatal encephalopathy due to birth asphyxia and trauma	157.5 (28.4 to 440.4)	60.2 (9.8 to 174.8)	41.0% (18.8% to 95.1%)	30.9% (10.3% to 81.2%)
Malaria	122.6 (97.7 to 149.9)	51.5 (32.6 to 72.2)	14.7% (1.0% to 30.4%)	6.5% (-6.2% to 21.1%)
Haemolytic disease and other neonatal jaundice	80.4 (63.9 to 97.6)	31.4 (20.4 to 44.5)	8.2% (1.0% to 14.6%)	0.5% (-6.2% to 6.4%)
Encephalitis	72.0 (54.0 to 94.3)	12.4 (7.7 to 18.1)	-1.7% (-16.6% to 16.9%)	-8.7% (-22.6% to 8.6%)
Pneumococcal meningitis	44.8 (34.7 to 56.8)	8.1 (5.1 to 12.0)	-1.5% (-20.4% to 17.2%)	-8.5% (-26.1% to 8.9%)
H influenzae type B meningitis	29.0 (22.2 to 37.4)	5.4 (3.4 to 8.0)	-4.3% (-21.5% to 17.0%)	-11.1% (-27.1% to 8.6%)
Other meningitis	24.5 (18.9 to 30.8)	4.5 (2.9 to 6.6)	21.4% (2.9% to 43.0%)	12.7% (-4.5% to 32.8%)
Meningococcal infection	10.1 (7.7 to 13.3)	1.9 (1.2 to 2.8)	13.8% (2.5% to 25.1%)	5.7% (-4.8% to 16.2%)
Food-borne trematodiases	4.7 (1.3 to 11.2)	0.7 (0.2 to 1.7)	-18.7% (-42.3% to 15.0%)	-24.5% (-46.4% to 6.8%)
Cystic echinococcosis	0.6 (0.1 to 1.7)	0.1 (<0.05 to 0.4)	-43.5% (-82.3% to 67.6%)	-47.5% (-83.6% to 55.7%)
Cysticercosis	0.4 (0.1 to 0.8)	0.1 (<0.05 to 0.2)	-2.4% (-17.6% to 12.8%)	-9.3% (-23.5% to 4.7%)
Tetanus	<0.05 (<0.05 to <0.05)	<0.05 (<0.05 to <0.05)	-27.1% (-46.8% to -2.3%)	-32.3% (-50.6% to -9.3%)
**All causes**	**4,571.0 (3,674.1 to 5,781.8)**	**1,678.3 (1,243.9 to 2,364.3)**	**14.2% (5.7% to 23.1%)**	**6.1% (-1.9% to 14.4%)**

SR Table 13c: Global prevalence and years lived with disability (YLDs) for 2016, percentage change of YLD counts and rates between 2006 and 2016 of epilepsy by cause, ages 10-14 years				
**Cause**	**Prevalence in thousands (95% uncertainty interval)**	**YLDs in thousands (95% uncertainty interval)**		
	**2016**	**2016**	**Percentage change in counts between 2006 and 2016**	**Percentage change in rates between 2006 and 2016**
Epilepsy	2,271.1<br>(1,756.6 to 2,843.9)	768.5<br>(487.2 to 1,114.3)	0.6%<br>(-11.3% to 14.5%)	-0.2%<br>(-12.0% to 13.6%)
Neonatal preterm birth complications	983.3<br>(711.4 to 1,280.9)	380.3<br>(232.0 to 562.2)	2.4%<br>(-12.6% to 19.0%)	1.5%<br>(-13.3% to 18.0%)
Neonatal sepsis and other neonatal infections	573.8<br>(155.9 to 1,462.1)	261.1<br>(69.3 to 691.2)	25.8%<br>(16.7% to 41.4%)	24.7%<br>(15.7% to 40.3%)
Malaria	153.1<br>(127.8 to 179.5)	64.6<br>(42.1 to 87.0)	17.1%<br>(5.1% to 30.0%)	16.2%<br>(4.2% to 29.0%)
Neonatal encephalopathy due to birth asphyxia and trauma	157.9<br>(31.5 to 429.5)	61.5<br>(11.4 to 177.9)	29.0%<br>(13.6% to 60.0%)	27.9%<br>(12.7% to 58.7%)
Haemolytic disease and other neonatal jaundice	83.5<br>(66.8 to 99.4)	33.3<br>(21.8 to 46.4)	3.6%<br>(-2.7% to 9.0%)	2.8%<br>(-3.5% to 8.1%)
Encephalitis	82.3<br>(63.8 to 102.8)	14.1<br>(9.1 to 20.4)	-12.0%<br>(-23.4% to 1.2%)	-12.7%<br>(-24.0% to 0.4%)
Pneumococcal meningitis	34.6<br>(27.6 to 43.6)	6.3<br>(4.0 to 9.1)	-4.4%<br>(-20.2% to 14.1%)	-5.2%<br>(-20.9% to 13.2%)
H influenzae type B meningitis	19.2<br>(14.9 to 24.7)	3.6<br>(2.3 to 5.2)	-7.4%<br>(-19.3% to 5.5%)	-8.2%<br>(-19.9% to 4.6%)
Other meningitis	16.8<br>(13.3 to 20.6)	3.1<br>(2.0 to 4.3)	10.6%<br>(2.3% to 17.6%)	9.7%<br>(1.5% to 16.6%)
Cysticercosis	13.2<br>(6.4 to 22.3)	2.4<br>(1.1 to 4.3)	-9.7%<br>(-24.8% to 5.0%)	-10.4%<br>(-25.4% to 4.2%)
Food-borne trematodiases	14.4<br>(4.2 to 31.2)	2.2<br>(0.6 to 5.3)	-30.6%<br>(-48.1% to -8.4%)	-31.2%<br>(-48.5% to -9.1%)
Meningococcal infection	6.7<br>(5.1 to 8.6)	1.3<br>(0.8 to 1.9)	10.3%<br>(3.7% to 16.5%)	9.4%<br>(2.9% to 15.5%)
Cystic echinococcosis	0.6<br>(0.1 to 1.9)	0.1<br>(<0.05 to 0.4)	-42.5%<br>(-81.0% to 65.6%)	-43.0%<br>(-81.2% to 64.2%)
Tetanus	<0.05<br>(<0.05 to <0.05)	<0.05<br>(<0.05 to <0.05)	-49.0%<br>(-70.0% to -12.4%)	-49.4%<br>(-70.3% to -13.1%)
**All causes**	**4,410.5<br>(3,630.9 to 5,423.6)**	**1,602.3<br>(1,183.3 to 2,159.2)**	**5.8%<br>(-2.3% to 14.0%)**	**4.9%<br>(-3.1% to 13.1%)**

SR Table 13c: Global prevalence and years lived with disability (YLDs) for 2016, percentage change of YLD counts and rates between 2006 and 2016 of epilepsy by cause, ages 15-19 years				
**Cause**	**Prevalence in thousands (95% uncertainty interval)**	**YLDs in thousands (95% uncertainty interval)**		
	**2016**	**2016**	**Percentage change in counts between 2006 and 2016**	**Percentage change in rates between 2006 and 2016**
Epilepsy	2,136.2 (1,699.5 to 2,646.5)	707.9 (450.5 to 1,026.7)	-7.1% (-17.2% to 5.9%)	-1.7% (-12.3% to 12.1%)
Neonatal preterm birth complications	904.8 (645.6 to 1,187.3)	347.0 (214.9 to 513.6)	-1.4% (-16.6% to 14.6%)	4.3% (-11.7% to 21.3%)
Neonatal sepsis and other neonatal infections	511.8 (147.3 to 1,296.5)	229.7 (62.0 to 599.5)	18.4% (10.3% to 30.9%)	25.3% (16.7% to 38.6%)
Malaria	139.1 (115.7 to 160.9)	58.2 (39.3 to 78.8)	15.1% (4.5% to 25.8%)	21.8% (10.6% to 33.2%)
Neonatal encephalopathy due to birth asphyxia and trauma	146.7 (32.7 to 387.0)	56.6 (11.7 to 160.7)	16.1% (3.4% to 36.4%)	22.9% (9.5% to 44.4%)
Haemolytic disease and other neonatal jaundice	77.8 (62.8 to 91.9)	30.9 (20.0 to 43.3)	-1.2% (-7.4% to 3.7%)	4.6% (-2.0% to 9.8%)
Cysticercosis	82.2 (51.6 to 122.3)	14.2 (7.7 to 24.0)	-17.2% (-32.0% to -4.6%)	-12.3% (-28.0% to 0.9%)
Encephalitis	78.3 (62.7 to 97.7)	13.3 (8.9 to 18.4)	-17.9% (-28.7% to -6.1%)	-13.0% (-24.5% to -0.6%)
Pneumococcal meningitis	27.0 (21.4 to 33.3)	4.9 (3.2 to 7.1)	-5.0% (-18.4% to 9.5%)	0.5% (-13.7% to 15.9%)
Food-borne trematodiases	20.2 (6.4 to 40.3)	3.1 (0.8 to 6.8)	-48.4% (-59.7% to -32.3%)	-45.4% (-57.3% to -28.3%)
H influenzae type B meningitis	12.7 (9.9 to 16.1)	2.3 (1.5 to 3.5)	-8.1% (-15.5% to -1.1%)	-2.7% (-10.5% to 4.7%)
Other meningitis	11.8 (9.3 to 14.3)	2.1 (1.4 to 3.0)	3.5% (-3.9% to 9.3%)	9.6% (1.7% to 15.7%)
Meningococcal infection	4.5 (3.5 to 5.8)	0.8 (0.5 to 1.2)	6.1% (1.6% to 10.1%)	12.3% (7.5% to 16.5%)
Cystic echinococcosis	0.7 (0.1 to 2.0)	0.1 (<0.05 to 0.4)	-43.9% (-80.7% to 60.8%)	-40.6% (-79.6% to 70.2%)
Tetanus	<0.05 (<0.05 to <0.05)	<0.05 (<0.05 to <0.05)	-36.4% (-74.3% to 61.2%)	-32.6% (-72.8% to 70.7%)
**All causes**	**4,153.8 (3,440.4 to 5,037.5)**	**1,471.2 (1,073.8 to 1,968.9)**	**-1.2% (-8.6% to 6.5%)**	**4.6% (-3.3% to 12.7%)**

SR Table 13c: Global prevalence and years lived with disability (YLDs) for 2016, percentage change of YLD counts and rates between 2006 and 2016 of epilepsy by cause, ages 20-24 years				
**Cause**	**Prevalence in thousands (95% uncertainty interval)**	**YLDs in thousands (95% uncertainty interval)**		
	**2016**	**2016**	**Percentage change in counts between 2006 and 2016**	**Percentage change in rates between 2006 and 2016**
Epilepsy	2,050.8 (1,598.0 to 2,549.4)	668.9 (431.0 to 943.5)	-0.1% (-11.0% to 12.8%)	-4.7% (-15.1% to 7.6%)
Neonatal preterm birth complications	898.5 (631.6 to 1,185.0)	346.5 (212.7 to 517.3)	9.5% (-7.3% to 28.8%)	4.4% (-11.6% to 22.9%)
Neonatal sepsis and other neonatal infections	474.9 (149.7 to 1,191.6)	209.0 (60.2 to 549.5)	25.7% (16.0% to 40.0%)	19.9% (10.7% to 33.5%)
Neonatal encephalopathy due to birth asphyxia and trauma	159.8 (45.9 to 392.8)	61.7 (16.9 to 161.9)	22.6% (10.4% to 44.9%)	17.0% (5.3% to 38.3%)
Malaria	109.1 (91.5 to 127.0)	45.6 (30.6 to 61.2)	13.7% (1.4% to 25.4%)	8.4% (-3.2% to 19.6%)
Haemolytic disease and other neonatal jaundice	79.1 (63.7 to 92.8)	31.7 (20.7 to 44.2)	8.0% (1.5% to 13.4%)	3.0% (-3.2% to 8.2%)
Cysticercosis	180.3 (117.7 to 253.5)	30.5 (16.9 to 47.9)	-14.8% (-26.2% to -5.0%)	-18.8% (-29.6% to -9.4%)
Encephalitis	70.3 (57.4 to 86.1)	11.8 (7.7 to 16.7)	-7.5% (-19.3% to 5.6%)	-11.8% (-23.0% to 0.7%)
Pneumococcal meningitis	21.5 (17.1 to 26.5)	3.9 (2.5 to 5.5)	-1.0% (-12.5% to 11.2%)	-5.6% (-16.6% to 6.1%)
Food-borne trematodiases	25.1 (8.0 to 50.5)	3.8 (1.0 to 8.4)	-23.6% (-40.1% to 1.1%)	-27.1% (-42.9% to -3.5%)
Other meningitis	8.8 (6.9 to 11.0)	1.6 (1.0 to 2.3)	6.7% (0.0% to 11.8%)	1.8% (-4.6% to 6.7%)
H influenzae type B meningitis	8.5 (6.6 to 10.6)	1.6 (1.0 to 2.3)	-3.7% (-9.0% to 1.4%)	-8.1% (-13.2% to -3.3%)
Meningococcal infection	3.2 (2.5 to 4.1)	0.6 (0.4 to 0.9)	7.4% (3.1% to 11.4%)	2.4% (-1.6% to 6.3%)
Cystic echinococcosis	0.7 (0.1 to 2.1)	0.1 (<0.05 to 0.4)	-42.4% (-80.5% to 70.8%)	-45.0% (-81.4% to 62.9%)
Tetanus	<0.05 (<0.05 to <0.05)	<0.05 (<0.05 to <0.05)	-26.4% (-79.9% to 219.0%)	-29.8% (-80.8% to 204.3%)
**All causes**	**4,090.5 (3,400.9 to 4,961.3)**	**1,417.3 (1,044.2 to 1,888.8)**	**6.3% (-1.5% to 14.2%)**	**1.4% (-6.0% to 8.9%)**

SR Table 13c: Global prevalence and years lived with disability (YLDs) for 2016, percentage change of YLD counts and rates between 2006 and 2016 of epilepsy by cause, ages 25-29 years				
**Cause**	**Prevalence in thousands (95% uncertainty interval)**	**YLDs in thousands (95% uncertainty interval)**		
	**2016**	**2016**	**Percentage change in counts between 2006 and 2016**	**Percentage change in rates between 2006 and 2016**
Epilepsy	1,910.3 (1,524.2 to 2,328.8)	613.8 (400.2 to 861.9)	10.7% (-0.7% to 24.5%)	-6.7% (-16.3% to 4.9%)
Neonatal preterm birth complications	884.1 (624.3 to 1,174.4)	341.2 (204.8 to 505.3)	23.5% (3.2% to 45.8%)	4.0% (-13.1% to 22.8%)
Neonatal sepsis and other neonatal infections	451.7 (148.6 to 1,142.2)	196.6 (58.0 to 517.3)	39.6% (28.6% to 59.9%)	17.6% (8.3% to 34.7%)
Neonatal encephalopathy due to birth asphyxia and trauma	167.7 (56.4 to 388.2)	64.4 (21.0 to 157.3)	37.7% (22.8% to 66.4%)	16.0% (3.5% to 40.1%)
Cysticercosis	239.6 (155.6 to 339.4)	39.9 (22.3 to 62.5)	-8.4% (-19.9% to 1.4%)	-22.8% (-32.6% to -14.6%)
Malaria	84.9 (70.8 to 99.2)	35.2 (23.4 to 47.9)	16.6% (5.2% to 27.2%)	-1.8% (-11.4% to 7.1%)
Haemolytic disease and other neonatal jaundice	78.0 (63.1 to 91.6)	31.3 (20.1 to 43.4)	21.7% (14.3% to 27.5%)	2.5% (-3.7% to 7.4%)
Encephalitis	63.0 (51.2 to 75.7)	10.4 (6.7 to 14.5)	4.4% (-8.3% to 19.6%)	-12.0% (-22.8% to 0.8%)
Food-borne trematodiases	33.4 (10.7 to 66.8)	5.0 (1.4 to 11.0)	14.3% (-14.7% to 51.6%)	-3.7% (-28.2% to 27.7%)
Pneumococcal meningitis	17.7 (14.1 to 21.9)	3.1 (2.0 to 4.6)	7.1% (-5.0% to 19.0%)	-9.8% (-19.9% to 0.3%)
Other meningitis	7.1 (5.5 to 9.1)	1.3 (0.8 to 1.8)	14.1% (7.4% to 19.4%)	-3.9% (-9.5% to 0.6%)
H influenzae type B meningitis	6.0 (4.7 to 7.5)	1.1 (0.7 to 1.6)	6.5% (0.7% to 12.0%)	-10.3% (-15.2% to -5.6%)
Meningococcal infection	2.4 (1.9 to 3.1)	0.4 (0.3 to 0.6)	14.7% (10.1% to 19.0%)	-3.4% (-7.3% to 0.2%)
Cystic echinococcosis	0.7 (0.2 to 2.1)	0.1 (<0.05 to 0.4)	-40.8% (-79.0% to 74.7%)	-50.1% (-82.3% to 47.1%)
Tetanus	<0.05 (<0.05 to <0.05)	<0.05 (<0.05 to <0.05)	-24.2% (-83.0% to 316.1%)	-36.2% (-85.6% to 250.5%)
**All causes**	**3,946.4 (3,267.4 to 4,795.3)**	**1,343.9 (992.8 to 1,789.4)**	**18.1% (9.6% to 26.5%)**	**-0.5% (-7.7% to 6.6%)**

SR Table 13c: Global prevalence and years lived with disability (YLDs) for 2016, percentage change of YLD counts and rates between 2006 and 2016 of epilepsy by cause, ages 30-34 years				
**Cause**	**Prevalence in thousands (95% uncertainty interval)**	**YLDs in thousands (95% uncertainty interval)**		
	**2016**	**2016**	**Percentage change in counts between 2006 and 2016**	**Percentage change in rates between 2006 and 2016**
Epilepsy	1,605.1<br>(1,303.5 to 1,915.6)	509.3<br>(334.1 to 716.5)	8.0%<br>(-3.5% to 21.1%)	-4.4%<br>(-14.6% to 7.3%)
Neonatal preterm birth complications	754.2<br>(536.2 to 999.1)	289.1<br>(173.8 to 427.2)	19.3%<br>(-0.0% to 40.2%)	5.6%<br>(-11.4% to 24.2%)
Neonatal sepsis and other neonatal infections	399.0<br>(135.1 to 1,002.2)	172.8<br>(53.6 to 455.6)	39.9%<br>(30.1% to 55.3%)	23.9%<br>(15.2% to 37.5%)
Neonatal encephalopathy due to birth asphyxia and trauma	147.4<br>(52.0 to 333.5)	56.1<br>(18.4 to 135.8)	31.1%<br>(19.0% to 55.1%)	16.1%<br>(5.4% to 37.3%)
Cysticercosis	245.5<br>(162.3 to 349.0)	40.4<br>(22.5 to 62.9)	-13.9%<br>(-24.8% to -4.3%)	-23.7%<br>(-33.4% to -15.3%)
Malaria	66.7<br>(55.8 to 78.0)	27.5<br>(18.5 to 36.8)	21.7%<br>(8.7% to 36.3%)	7.8%<br>(-3.8% to 20.7%)
Haemolytic disease and other neonatal jaundice	68.0<br>(54.6 to 80.4)	27.2<br>(17.6 to 37.8)	18.7%<br>(11.2% to 24.6%)	5.1%<br>(-1.5% to 10.4%)
Encephalitis	52.3<br>(41.7 to 64.7)	8.7<br>(5.5 to 12.5)	0.3%<br>(-12.1% to 14.2%)	-11.2%<br>(-22.1% to 1.1%)
Food-borne trematodiases	25.1<br>(8.2 to 50.9)	3.8<br>(1.0 to 8.2)	-23.3%<br>(-42.5% to -2.5%)	-32.1%<br>(-49.1% to -13.6%)
Pneumococcal meningitis	14.5<br>(11.3 to 18.2)	2.6<br>(1.6 to 3.8)	12.4%<br>(5.2% to 19.7%)	-0.5%<br>(-6.9% to 6.0%)
Other meningitis	5.7<br>(4.4 to 7.4)	1.0<br>(0.6 to 1.5)	11.0%<br>(4.0% to 16.6%)	-1.7%<br>(-7.9% to 3.2%)
H influenzae type B meningitis	4.3<br>(3.3 to 5.5)	0.8<br>(0.5 to 1.2)	13.8%<br>(6.8% to 20.4%)	0.8%<br>(-5.4% to 6.6%)
Meningococcal infection	1.9<br>(1.4 to 2.4)	0.3<br>(0.2 to 0.5)	18.8%<br>(13.8% to 23.7%)	5.2%<br>(0.8% to 9.6%)
Cystic echinococcosis	0.7<br>(0.2 to 1.9)	0.1<br>(<0.05 to 0.4)	-40.6%<br>(-78.0% to 53.9%)	-47.4%<br>(-80.5% to 36.3%)
Tetanus	<0.05<br>(<0.05 to <0.05)	<0.05<br>(<0.05 to <0.05)	-14.4%<br>(-83.2% to 445.5%)	-24.2%<br>(-85.2% to 383.1%)
**All causes**	**3,390.4**<br>**(2,861.0 to 4,101.0)**	**1,139.6**<br>**(848.4 to 1,512.5)**	**15.0%**<br>**(6.6% to 23.5%)**	**1.9%**<br>**(-5.6% to 9.4%)**

SR Table 13c: Global prevalence and years lived with disability (YLDs) for 2016, percentage change of YLD counts and rates between 2006 and 2016 of epilepsy by cause, ages 35-39 years				
**Cause**	**Prevalence in thousands (95% uncertainty interval)**	**YLDs in thousands (95% uncertainty interval)**		
	**2016**	**2016**	**Percentage change in counts between 2006 and 2016**	**Percentage change in rates between 2006 and 2016**
Epilepsy	1,360.5 (1,116.4 to 1,631.7)	425.9 (281.9 to 596.1)	1.8% (-8.9% to 13.8%)	-3.8% (-13.8% to 7.6%)
Neonatal preterm birth complications	625.0 (441.3 to 823.4)	238.4 (144.3 to 352.0)	11.7% (-6.3% to 32.4%)	5.6% (-11.4% to 25.1%)
Neonatal sepsis and other neonatal infections	336.5 (117.8 to 838.5)	145.2 (46.7 to 379.0)	33.9% (24.9% to 47.7%)	26.6% (18.1% to 39.7%)
Neonatal encephalopathy due to birth asphyxia and trauma	126.3 (46.7 to 275.1)	47.8 (16.4 to 113.8)	22.5% (10.9% to 44.9%)	15.8% (4.8% to 37.0%)
Cysticercosis	238.1 (157.2 to 332.8)	38.6 (22.6 to 59.2)	-19.2% (-29.1% to -10.2%)	-23.6% (-33.0% to -15.1%)
Haemolytic disease and other neonatal jaundice	57.5 (46.2 to 68.1)	23.0 (14.8 to 31.8)	13.0% (5.8% to 18.6%)	6.8% (-0.0% to 12.1%)
Malaria	50.3 (41.9 to 58.8)	20.6 (13.9 to 27.9)	24.9% (11.7% to 40.4%)	18.1% (5.6% to 32.8%)
Encephalitis	45.5 (36.5 to 56.1)	7.5 (4.8 to 10.5)	-4.4% (-17.2% to 8.6%)	-9.7% (-21.8% to 2.7%)
Food-borne trematodiases	20.1 (6.5 to 40.9)	3.0 (0.9 to 6.6)	-39.5% (-56.4% to -20.3%)	-42.8% (-58.8% to -24.6%)
Pneumococcal meningitis	11.6 (9.0 to 14.7)	2.1 (1.3 to 3.1)	11.7% (4.3% to 18.5%)	5.6% (-1.4% to 12.1%)
Other meningitis	4.8 (3.6 to 6.2)	0.8 (0.5 to 1.2)	6.0% (-0.6% to 11.3%)	0.2% (-6.0% to 5.2%)
H influenzae type B meningitis	3.2 (2.5 to 4.0)	0.6 (0.4 to 0.8)	15.4% (7.9% to 22.4%)	9.1% (2.0% to 15.8%)
Meningococcal infection	1.5 (1.1 to 1.9)	0.3 (0.2 to 0.4)	18.3% (13.4% to 23.3%)	11.8% (7.2% to 16.6%)
Cystic echinococcosis	0.7 (0.2 to 1.8)	0.1 (<0.05 to 0.4)	-40.2% (-77.4% to 59.8%)	-43.5% (-78.7% to 51.1%)
Tetanus	<0.05 (<0.05 to <0.05)	<0.05 (<0.05 to <0.05)	-12.6% (-85.0% to 564.6%)	-17.4% (-85.8% to 528.3%)
**All causes**	**2,881.5 (2,449.5 to 3,496.2)**	**953.9 (709.5 to 1,267.1)**	**8.4% (0.7% to 16.5%)**	**2.4% (-4.8% to 10.1%)**

SR Table 13c: Global prevalence and years lived with disability (YLDs) for 2016, percentage change of YLD counts and rates between 2006 and 2016 of epilepsy by cause, ages 40-44 years				
**Cause**	**Prevalence in thousands (95% uncertainty interval)**	**YLDs in thousands (95% uncertainty interval)**		
	**2016**	**2016**	**Percentage change in counts between 2006 and 2016**	**Percentage change in rates between 2006 and 2016**
Epilepsy	1,278.1 (1,058.2 to 1,545.3)	393.2 (266.4 to 552.7)	7.4% (-3.7% to 19.9%)	-5.7% (-15.4% to 5.4%)
Neonatal preterm birth complications	551.5 (386.7 to 740.2)	209.7 (126.8 to 310.2)	21.1% (1.7% to 43.4%)	6.3% (-10.6% to 26.0%)
Neonatal sepsis and other neonatal infections	291.6 (104.0 to 721.2)	125.1 (41.4 to 328.6)	34.5% (25.4% to 49.3%)	18.1% (10.2% to 31.1%)
Neonatal encephalopathy due to birth asphyxia and trauma	115.7 (44.9 to 240.4)	43.5 (16.0 to 99.5)	27.8% (15.7% to 49.4%)	12.2% (1.7% to 31.2%)
Cysticercosis	248.7 (170.5 to 350.7)	39.6 (23.3 to 61.7)	-11.4% (-21.8% to -3.0%)	-22.2% (-31.3% to -14.8%)
Haemolytic disease and other neonatal jaundice	51.8 (41.2 to 61.2)	20.6 (13.4 to 28.4)	21.6% (14.0% to 27.6%)	6.8% (0.1% to 12.1%)
Malaria	36.8 (30.7 to 43.1)	15.1 (9.9 to 20.6)	23.8% (9.2% to 38.7%)	8.7% (-4.1% to 21.8%)
Encephalitis	45.0 (35.8 to 55.3)	7.2 (4.5 to 10.5)	2.7% (-9.9% to 16.4%)	-9.8% (-20.8% to 2.3%)
Food-borne trematodiases	22.2 (7.2 to 44.1)	3.3 (1.0 to 7.2)	-13.6% (-34.9% to 14.5%)	-24.1% (-42.8% to 0.5%)
Pneumococcal meningitis	9.7 (7.4 to 12.2)	1.7 (1.1 to 2.5)	12.2% (5.2% to 18.5%)	-1.4% (-7.6% to 4.1%)
Other meningitis	4.4 (3.4 to 5.7)	0.8 (0.5 to 1.1)	10.2% (3.6% to 15.1%)	-3.2% (-9.0% to 1.1%)
H influenzae type B meningitis	2.5 (1.9 to 3.2)	0.4 (0.3 to 0.6)	18.5% (10.9% to 25.8%)	4.0% (-2.6% to 10.5%)
Meningococcal infection	1.2 (0.9 to 1.6)	0.2 (0.1 to 0.3)	17.5% (12.9% to 21.9%)	3.2% (-0.8% to 7.1%)
Cystic echinococcosis	0.6 (0.1 to 1.5)	0.1 (<0.05 to 0.3)	-40.9% (-78.0% to 53.0%)	-48.1% (-80.7% to 34.4%)
Tetanus	<0.05 (<0.05 to <0.05)	<0.05 (<0.05 to <0.05)	-20.3% (-88.2% to 586.2%)	-30.0% (-89.6% to 502.7%)
**All causes**	**2,659.7 (2,262.6 to 3,211.9)**	**860.8 (651.5 to 1,140.5)**	**14.1% (6.1% to 22.4%)**	**0.2% (-6.8% to 7.5%)**

SR Table 13c: Global prevalence and years lived with disability (YLDs) for 2016, percentage change of YLD counts and rates between 2006 and 2016 of epilepsy by cause, ages 45-49 years				
**Cause**	**Prevalence in thousands (95% uncertainty interval)**	**YLDs in thousands (95% uncertainty interval)**		
	**2016**	**2016**	**Percentage change in counts between 2006 and 2016**	**Percentage change in rates between 2006 and 2016**
Epilepsy	1,237.3 (1,021.2 to 1,511.5)	373.5 (257.4 to 521.5)	16.7% (4.8% to 30.5%)	-6.4% (-16.0% to 4.6%)
Neonatal preterm birth complications	476.5 (330.4 to 641.2)	180.6 (106.5 to 270.2)	34.3% (10.9% to 59.2%)	7.7% (-11.1% to 27.7%)
Neonatal sepsis and other neonatal infections	248.7 (89.5 to 613.8)	106.2 (35.7 to 279.6)	40.5% (30.8% to 57.7%)	12.7% (4.9% to 26.4%)
Cysticercosis	261.7 (175.7 to 374.6)	41.0 (24.7 to 65.0)	-0.6% (-9.5% to 7.6%)	-20.3% (-27.5% to -13.7%)
Neonatal encephalopathy due to birth asphyxia and trauma	104.9 (42.5 to 208.8)	39.2 (15.2 to 86.8)	36.8% (24.4% to 61.1%)	9.7% (-0.2% to 29.2%)
Haemolytic disease and other neonatal jaundice	45.5 (35.9 to 53.8)	18.2 (11.6 to 25.3)	32.4% (23.9% to 38.8%)	6.1% (-0.6% to 11.3%)
Malaria	26.7 (22.3 to 31.2)	10.9 (7.1 to 14.9)	21.2% (6.7% to 36.3%)	-2.8% (-14.4% to 9.3%)
Encephalitis	46.7 (37.8 to 56.7)	7.3 (4.6 to 10.3)	11.9% (-1.1% to 26.4%)	-10.3% (-20.7% to 1.3%)
Food-borne trematodiases	24.1 (7.7 to 47.9)	3.6 (1.1 to 7.4)	28.7% (2.0% to 66.5%)	3.2% (-18.2% to 33.5%)
Pneumococcal meningitis	8.6 (6.7 to 10.6)	1.5 (0.9 to 2.1)	16.0% (9.8% to 21.1%)	-7.0% (-12.0% to -2.9%)
Other meningitis	4.3 (3.3 to 5.6)	0.7 (0.4 to 1.1)	17.2% (11.1% to 21.7%)	-6.0% (-10.9% to -2.4%)
H influenzae type B meningitis	2.1 (1.7 to 2.7)	0.4 (0.2 to 0.6)	24.2% (16.7% to 31.6%)	-0.4% (-6.4% to 5.5%)
Meningococcal infection	1.1 (0.8 to 1.4)	0.2 (0.1 to 0.3)	18.6% (14.1% to 22.7%)	-4.9% (-8.5% to -1.6%)
Cystic echinococcosis	0.5 (0.1 to 1.4)	0.1 (<0.05 to 0.3)	-42.5% (-79.4% to 54.2%)	-53.9% (-83.4% to 23.7%)
Tetanus	<0.05 (<0.05 to <0.05)	<0.05 (<0.05 to <0.05)	-13.5% (-90.8% to 872.6%)	-30.7% (-92.6% to 679.9%)
**All causes**	**2,488.9 (2,130.4 to 2,968.1)**	**783.3 (597.3 to 1,035.7)**	**23.5% (14.8% to 32.5%)**	**-1.0% (-8.0% to 6.3%)**

SR Table 13c: Global prevalence and years lived with disability (YLDs) for 2016, percentage change of YLD counts and rates between 2006 and 2016 of epilepsy by cause, ages 50-54 years				
**Cause**	**Prevalence in thousands (95% uncertainty interval)**	**YLDs in thousands (95% uncertainty interval)**		
	**2016**	**2016**	**Percentage change in counts between 2006 and 2016**	**Percentage change in rates between 2006 and 2016**
Epilepsy	1,168.8 (953.3 to 1,426.3)	345.8 (238.0 to 485.5)	18.1% (6.1% to 31.1%)	-5.2% (-14.8% to 5.2%)
Neonatal preterm birth complications	371.3 (253.4 to 506.4)	141.1 (83.9 to 215.0)	36.2% (12.3% to 62.1%)	9.4% (-9.9% to 30.2%)
Neonatal sepsis and other neonatal infections	204.6 (76.3 to 504.9)	86.9 (29.9 to 224.0)	45.6% (34.1% to 63.7%)	16.9% (7.7% to 31.4%)
Cysticercosis	258.6 (176.5 to 355.3)	39.8 (24.0 to 61.6)	-3.0% (-11.5% to 4.5%)	-22.1% (-28.9% to -16.1%)
Neonatal encephalopathy due to birth asphyxia and trauma	91.4 (39.6 to 177.5)	34.0 (13.5 to 73.9)	40.2% (26.0% to 69.4%)	12.6% (1.2% to 36.1%)
Haemolytic disease and other neonatal jaundice	37.3 (29.3 to 44.1)	14.9 (9.5 to 20.5)	31.2% (23.2% to 37.2%)	5.3% (-1.1% to 10.2%)
Malaria	19.3 (16.1 to 22.5)	7.9 (5.3 to 10.7)	23.1% (9.7% to 38.0%)	-1.1% (-11.9% to 10.8%)
Encephalitis	48.0 (39.0 to 59.7)	7.4 (4.8 to 10.6)	13.9% (2.0% to 27.7%)	-8.6% (-18.1% to 2.6%)
Food-borne trematodiases	19.0 (5.9 to 40.6)	2.8 (0.8 to 6.1)	8.4% (-14.2% to 41.7%)	-12.9% (-31.1% to 13.8%)
Pneumococcal meningitis	7.8 (6.2 to 9.7)	1.3 (0.9 to 1.9)	21.4% (16.4% to 25.3%)	-2.5% (-6.5% to 0.6%)
Other meningitis	4.0 (3.0 to 5.2)	0.7 (0.4 to 1.0)	18.5% (13.7% to 22.1%)	-4.9% (-8.7% to -1.9%)
H influenzae type B meningitis	1.9 (1.5 to 2.5)	0.3 (0.2 to 0.5)	30.6% (23.1% to 38.6%)	4.9% (-1.2% to 11.3%)
Meningococcal infection	1.0 (0.7 to 1.3)	0.2 (0.1 to 0.3)	22.2% (18.6% to 25.9%)	-1.9% (-4.8% to 1.1%)
Cystic echinococcosis	0.5 (0.1 to 1.3)	0.1 (<0.05 to 0.3)	-42.1% (-77.7% to 43.6%)	-53.5% (-82.1% to 15.3%)
Tetanus	<0.05 (<0.05 to <0.05)	<0.05 (<0.05 to <0.05)	12.3% (-89.5% to 1,647.6%)	-9.8% (-91.6% to 1,303.2%)
**All causes**	**2,233.7 (1,893.4 to 2,638.3)**	**683.2 (518.2 to 895.7)**	**24.1% (15.4% to 33.8%)**	**-0.3% (-7.4% to 7.4%)**

SR Table 13c: Global prevalence and years lived with disability (YLDs) for 2016, percentage change of YLD counts and rates between 2006 and 2016 of epilepsy by cause, ages 55-59 years				
**Cause**	**Prevalence in thousands (95% uncertainty interval)**	**YLDs in thousands (95% uncertainty interval)**		
	**2016**	**2016**	**Percentage change in counts between 2006 and 2016**	**Percentage change in rates between 2006 and 2016**
Epilepsy	1,055.2<br>(857.2 to 1,273.7)	306.9<br>(208.0 to 426.2)	26.6%<br>(13.5% to 41.5%)	-3.0%<br>(-13.1% to 8.5%)
Neonatal preterm birth complications	256.9<br>(173.6 to 354.6)	98.7<br>(56.3 to 150.7)	47.5%<br>(19.8% to 77.9%)	13.1%<br>(-8.2% to 36.4%)
Neonatal sepsis and other neonatal infections	151.9<br>(57.4 to 381.8)	64.2<br>(22.5 to 166.5)	54.3%<br>(43.3% to 70.0%)	18.3%<br>(9.8% to 30.3%)
Cysticercosis	233.5<br>(167.7 to 319.2)	35.6<br>(22.2 to 53.9)	7.5%<br>(-0.3% to 14.9%)	-17.6%<br>(-23.6% to -11.9%)
Neonatal encephalopathy due to birth asphyxia and trauma	73.5<br>(32.5 to 140.9)	27.3<br>(11.2 to 58.5)	45.3%<br>(31.6% to 68.1%)	11.3%<br>(0.9% to 28.8%)
Haemolytic disease and other neonatal jaundice	28.5<br>(22.1 to 33.7)	11.5<br>(7.3 to 15.9)	41.4%<br>(33.0% to 47.8%)	8.4%<br>(1.9% to 13.2%)
Encephalitis	48.2<br>(39.9 to 57.9)	7.3<br>(4.8 to 10.1)	23.7%<br>(10.9% to 37.8%)	-5.2%<br>(-15.0% to 5.6%)
Malaria	12.8<br>(10.6 to 15.0)	5.3<br>(3.5 to 7.2)	28.3%<br>(11.2% to 47.0%)	-1.6%<br>(-14.8% to 12.7%)
Food-borne trematodiases	13.1<br>(3.9 to 28.5)	1.9<br>(0.5 to 4.4)	12.3%<br>(-9.0% to 41.2%)	-13.9%<br>(-30.2% to 8.2%)
Pneumococcal meningitis	6.8<br>(5.4 to 8.4)	1.2<br>(0.7 to 1.6)	31.6%<br>(26.4% to 36.9%)	0.8%<br>(-3.1% to 4.9%)
Other meningitis	3.7<br>(2.8 to 4.7)	0.6<br>(0.4 to 0.9)	26.5%<br>(22.8% to 29.7%)	-3.0%<br>(-5.9% to -0.6%)
H influenzae type B meningitis	1.7<br>(1.4 to 2.2)	0.3<br>(0.2 to 0.4)	42.1%<br>(34.3% to 51.0%)	8.9%<br>(2.9% to 15.7%)
Meningococcal infection	0.9<br>(0.7 to 1.2)	0.2<br>(0.1 to 0.2)	29.3%<br>(26.0% to 32.9%)	-0.9%<br>(-3.5% to 1.8%)
Cystic echinococcosis	0.4<br>(0.1 to 1.1)	0.1<br>(<0.05 to 0.2)	-38.0%<br>(-77.5% to 60.7%)	-52.5%<br>(-82.7% to 23.2%)
Tetanus	<0.05<br>(<0.05 to <0.05)	<0.05<br>(<0.05 to <0.05)	23.7%<br>(-91.2% to 2,330.2%)	-5.2%<br>(-93.3% to 1,762.4%)
**All causes**	**1,887.2**<br>**(1,603.3 to 2,214.9)**	**561.0**<br>**(424.9 to 733.8)**	**32.1%**<br>**(22.3% to 42.8%)**	**1.3%**<br>**(-6.3% to 9.4%)**

SR Table 13c: Global prevalence and years lived with disability (YLDs) for 2016, percentage change of YLD counts and rates between 2006 and 2016 of epilepsy by cause, ages 60-64 years				
**Cause**	**Prevalence in thousands (95% uncertainty interval)**	**YLDs in thousands (95% uncertainty interval)**		
	**2016**	**2016**	**Percentage change in counts between 2006 and 2016**	**Percentage change in rates between 2006 and 2016**
Epilepsy	989.3 (805.6 to 1,200.4)	281.7 (189.3 to 396.9)	43.0% (27.9% to 60.3%)	-5.2% (-15.2% to 6.3%)
Neonatal preterm birth complications	165.5 (111.1 to 235.2)	64.8 (36.6 to 100.6)	75.0% (40.8% to 112.2%)	16.1% (-6.6% to 40.8%)
Neonatal sepsis and other neonatal infections	108.1 (40.0 to 277.1)	45.6 (15.4 to 120.5)	77.4% (64.2% to 99.0%)	17.7% (8.9% to 32.0%)
Cysticercosis	219.5 (160.0 to 305.5)	33.0 (20.2 to 50.2)	23.7% (15.2% to 32.3%)	-18.0% (-23.6% to -12.3%)
Neonatal encephalopathy due to birth asphyxia and trauma	57.2 (25.7 to 108.8)	21.4 (8.9 to 45.2)	77.1% (59.3% to 107.9%)	17.4% (5.6% to 37.9%)
Haemolytic disease and other neonatal jaundice	21.5 (16.4 to 25.5)	8.8 (5.4 to 12.2)	66.0% (56.6% to 73.7%)	10.1% (3.9% to 15.2%)
Encephalitis	48.9 (40.0 to 59.0)	7.3 (4.8 to 10.3)	39.9% (25.1% to 57.1%)	-7.2% (-17.0% to 4.2%)
Malaria	7.5 (6.2 to 8.8)	3.2 (2.1 to 4.3)	32.5% (14.6% to 51.4%)	-12.1% (-24.0% to 0.4%)
Food-borne trematodiases	11.8 (3.3 to 26.8)	1.7 (0.4 to 4.0)	58.3% (26.6% to 98.9%)	5.0% (-16.0% to 31.9%)
Pneumococcal meningitis	5.8 (4.6 to 7.2)	1.0 (0.6 to 1.4)	39.3% (34.5% to 44.0%)	-7.6% (-10.8% to -4.5%)
Other meningitis	3.5 (2.7 to 4.4)	0.6 (0.4 to 0.9)	37.4% (33.9% to 40.7%)	-8.9% (-11.2% to -6.7%)
H influenzae type B meningitis	1.6 (1.2 to 2.0)	0.3 (0.2 to 0.4)	51.2% (43.3% to 60.9%)	0.3% (-5.0% to 6.7%)
Meningococcal infection	0.8 (0.6 to 1.1)	0.1 (0.1 to 0.2)	37.6% (34.1% to 40.8%)	-8.7% (-11.0% to -6.6%)
Cystic echinococcosis	0.3 (0.1 to 1.0)	0.1 (<0.05 to 0.2)	-33.6% (-76.0% to 91.3%)	-56.0% (-84.1% to 26.9%)
Tetanus	<0.05 (<0.05 to <0.05)	<0.05 (<0.05 to <0.05)	66.1% (-90.2% to 3,636.5%)	10.2% (-93.5% to 2,378.5%)
**All causes**	**1,641.2 (1,391.0 to 1,947.5)**	**469.6 (349.1 to 618.0)**	**49.5% (38.0% to 61.9%)**	**-0.8% (-8.4% to 7.4%)**

SR Table 13c: Global prevalence and years lived with disability (YLDs) for 2016, percentage change of YLD counts and rates between 2006 and 2016 of epilepsy by cause, ages 65-69 years				
**Cause**	**Prevalence in thousands (95% uncertainty interval)**	**YLDs in thousands (95% uncertainty interval)**		
	**2016**	**2016**	**Percentage change in counts between 2006 and 2016**	**Percentage change in rates between 2006 and 2016**
Epilepsy	846.3<br>(688.3 to 1,024.8)	234.0<br>(159.0 to 326.5)	27.3%<br>(13.3% to 43.3%)	-3.9%<br>(-14.5% to 8.1%)
Neonatal preterm birth complications	82.8<br>(54.4 to 116.7)	33.1<br>(18.6 to 52.3)	61.1%<br>(28.9% to 97.2%)	21.6%<br>(-2.7% to 48.9%)
Cysticercosis	176.9<br>(126.7 to 246.0)	26.2<br>(15.7 to 40.9)	11.2%<br>(4.0% to 18.3%)	-16.1%<br>(-21.5% to -10.7%)
Neonatal sepsis and other neonatal infections	62.7<br>(21.1 to 169.5)	26.1<br>(7.9 to 72.2)	52.6%<br>(39.4% to 77.8%)	15.2%<br>(5.2% to 34.2%)
Neonatal encephalopathy due to birth asphyxia and trauma	30.1<br>(13.2 to 58.0)	11.5<br>(4.5 to 24.5)	50.8%<br>(37.0% to 76.1%)	13.8%<br>(3.4% to 32.9%)
Encephalitis	41.6<br>(34.8 to 49.9)	6.1<br>(4.1 to 8.5)	20.2%<br>(8.1% to 34.4%)	-9.3%<br>(-18.4% to 1.5%)
Haemolytic disease and other neonatal jaundice	12.9<br>(9.8 to 15.5)	5.4<br>(3.3 to 7.6)	45.9%<br>(38.8% to 52.7%)	10.1%<br>(4.7% to 15.2%)
Malaria	3.8<br>(3.2 to 4.5)	1.6<br>(1.1 to 2.2)	32.7%<br>(13.7% to 52.4%)	0.2%<br>(-14.2% to 15.1%)
Food-borne trematodiases	7.2<br>(2.0 to 17.0)	1.0<br>(0.3 to 2.4)	29.7%<br>(0.1% to 64.7%)	-2.1%<br>(-24.4% to 24.3%)
Pneumococcal meningitis	4.7<br>(3.7 to 5.6)	0.8<br>(0.5 to 1.1)	24.2%<br>(17.5% to 31.1%)	-6.3%<br>(-11.3% to -1.1%)
Other meningitis	3.0<br>(2.4 to 3.7)	0.5<br>(0.3 to 0.7)	23.6%<br>(20.6% to 26.3%)	-6.7%<br>(-9.0% to -4.6%)
H influenzae type B meningitis	1.3<br>(1.0 to 1.6)	0.2<br>(0.1 to 0.3)	35.6%<br>(28.5% to 43.4%)	2.3%<br>(-3.0% to 8.2%)
Meningococcal infection	0.7<br>(0.5 to 0.9)	0.1<br>(0.1 to 0.2)	24.2%<br>(21.4% to 26.9%)	-6.3%<br>(-8.4% to -4.3%)
Cystic echinococcosis	0.2<br>(0.1 to 0.7)	<0.05<br>(<0.05 to 0.1)	-39.9%<br>(-78.2% to 60.5%)	-54.7%<br>(-83.5% to 21.1%)
Tetanus	<0.05<br>(<0.05 to <0.05)	<0.05<br>(<0.05 to <0.05)	25.8%<br>(-90.6% to 2,320.5%)	-5.1%<br>(-92.9% to 1,726.8%)
**All causes**	**1,274.1**<br>**(1,075.8 to 1,492.1)**	**346.7**<br>**(252.0 to 457.5)**	**30.9%**<br>**(20.1% to 43.1%)**	**-1.2%**<br>**(-9.4% to 8.0%)**

SR Table 13c: Global prevalence and years lived with disability (YLDs) for 2016, percentage change of YLD counts and rates between 2006 and 2016 of epilepsy by cause, ages 70-74 years				
**Cause**	**Prevalence in thousands (95% uncertainty interval)**	**YLDs in thousands (95% uncertainty interval)**		
	**2016**	**2016**	**Percentage change in counts between 2006 and 2016**	**Percentage change in rates between 2006 and 2016**
Epilepsy	683.6<br>(555.2 to 821.2)	183.5<br>(123.7 to 255.3)	17.0%<br>(4.0% to 32.5%)	-2.2%<br>(-13.1% to 10.8%)
Cysticercosis	117.6<br>(84.6 to 162.4)	17.2<br>(10.2 to 25.9)	0.8%<br>(-6.0% to 7.5%)	-15.8%<br>(-21.4% to -10.2%)
Neonatal preterm birth complications	33.4<br>(20.7 to 48.6)	13.7<br>(7.5 to 21.9)	48.1%<br>(15.5% to 85.4%)	23.8%<br>(-3.4% to 54.9%)
Neonatal sepsis and other neonatal infections	31.4<br>(8.9 to 92.8)	13.0<br>(3.2 to 37.7)	42.7%<br>(31.9% to 67.3%)	19.3%<br>(10.2% to 39.9%)
Encephalitis	33.1<br>(27.9 to 39.3)	4.7<br>(3.2 to 6.6)	9.5%<br>(-1.4% to 21.7%)	-8.4%<br>(-17.6% to 1.7%)
Neonatal encephalopathy due to birth asphyxia and trauma	11.3<br>(4.3 to 23.7)	4.5<br>(1.5 to 10.1)	47.6%<br>(35.1% to 73.3%)	23.4%<br>(12.9% to 44.8%)
Haemolytic disease and other neonatal jaundice	6.5<br>(4.8 to 7.8)	2.7<br>(1.7 to 3.8)	36.1%<br>(28.9% to 42.3%)	13.7%<br>(7.7% to 18.9%)
Malaria	1.5<br>(1.3 to 1.9)	0.7<br>(0.5 to 0.9)	35.3%<br>(28.7% to 39.8%)	13.1%<br>(7.6% to 16.9%)
Pneumococcal meningitis	3.8<br>(2.9 to 4.7)	0.6<br>(0.4 to 0.9)	18.2%<br>(7.7% to 29.9%)	-1.2%<br>(-10.0% to 8.5%)
Food-borne trematodiases	4.1<br>(1.1 to 9.5)	0.6<br>(0.2 to 1.4)	6.0%<br>(-17.9% to 35.9%)	-11.4%<br>(-31.4% to 13.6%)
Other meningitis	2.4<br>(1.9 to 3.1)	0.4<br>(0.3 to 0.6)	16.1%<br>(13.2% to 18.8%)	-3.0%<br>(-5.3% to -0.7%)
H influenzae type B meningitis	1.0<br>(0.8 to 1.3)	0.2<br>(0.1 to 0.3)	28.6%<br>(22.3% to 36.5%)	7.5%<br>(2.2% to 14.1%)
Meningococcal infection	0.6<br>(0.4 to 0.7)	0.1<br>(0.1 to 0.1)	18.4%<br>(15.6% to 21.1%)	-1.0%<br>(-3.4% to 1.2%)
Cystic echinococcosis	0.2<br>(<0.05 to 0.4)	<0.05<br>(<0.05 to 0.1)	-42.7%<br>(-77.8% to 36.7%)	-52.1%<br>(-81.4% to 14.2%)
Tetanus	<0.05<br>(<0.05 to <0.05)	<0.05<br>(<0.05 to <0.05)	-20.8%<br>(-90.0% to 489.8%)	-33.8%<br>(-91.7% to 393.0%)
**All causes**	**930.5**<br>**(780.0 to 1,095.0)**	**241.9**<br>**(172.8 to 322.5)**	**18.7%**<br>**(8.0% to 30.9%)**	**-0.8%**<br>**(-9.7% to 9.4%)**

SR Table 13c: Global prevalence and years lived with disability (YLDs) for 2016, percentage change of YLD counts and rates between 2006 and 2016 of epilepsy by cause, ages 75-79 years				
**Cause**	**Prevalence in thousands (95% uncertainty interval)**	**YLDs in thousands (95% uncertainty interval)**		
	**2016**	**2016**	**Percentage change in counts between 2006 and 2016**	**Percentage change in rates between 2006 and 2016**
Epilepsy	563.2 (462.1 to 678.4)	146.1 (99.8 to 200.2)	21.6% (6.4% to 40.1%)	-2.2% (-14.4% to 12.6%)
Cysticercosis	76.9 (54.5 to 106.4)	11.0 (6.7 to 17.0)	6.7% (-0.6% to 14.0%)	-14.2% (-20.0% to -8.4%)
Neonatal sepsis and other neonatal infections	15.2 (3.4 to 43.1)	6.3 (1.2 to 18.6)	56.2% (44.3% to 87.5%)	25.6% (16.0% to 50.8%)
Encephalitis	27.8 (23.8 to 32.8)	3.9 (2.6 to 5.4)	17.6% (6.3% to 30.7%)	-5.5% (-14.5% to 5.1%)
Neonatal preterm birth complications	9.2 (5.4 to 14.1)	3.8 (2.0 to 6.5)	48.4% (11.4% to 91.3%)	19.3% (-10.4% to 53.8%)
Neonatal encephalopathy due to birth asphyxia and trauma	4.0 (1.0 to 9.4)	1.6 (0.4 to 4.2)	71.9% (52.2% to 102.5%)	38.3% (22.4% to 62.8%)
Haemolytic disease and other neonatal jaundice	3.0 (2.1 to 3.7)	1.3 (0.8 to 1.8)	47.7% (37.9% to 56.1%)	18.8% (10.9% to 25.5%)
Pneumococcal meningitis	3.2 (2.6 to 3.9)	0.5 (0.3 to 0.7)	24.4% (15.9% to 34.1%)	0.0% (-6.8% to 7.8%)
Food-borne trematodiases	2.5 (0.7 to 5.9)	0.4 (0.1 to 0.8)	18.2% (-11.7% to 52.4%)	-5.0% (-29.0% to 22.6%)
Other meningitis	2.0 (1.6 to 2.6)	0.3 (0.2 to 0.5)	23.1% (16.6% to 30.4%)	-1.0% (-6.2% to 4.9%)
Malaria	0.4 (0.3 to 0.4)	0.2 (0.1 to 0.2)	44.0% (38.1% to 48.8%)	15.8% (11.1% to 19.6%)
H influenzae type B meningitis	0.8 (0.7 to 1.0)	0.1 (0.1 to 0.2)	35.4% (29.6% to 42.1%)	8.9% (4.2% to 14.3%)
Meningococcal infection	0.5 (0.4 to 0.6)	0.1 (0.1 to 0.1)	23.9% (21.1% to 26.5%)	-0.4% (-2.6% to 1.7%)
Cystic echinococcosis	0.1 (<0.05 to 0.3)	<0.05 (<0.05 to 0.1)	-41.6% (-77.0% to 46.8%)	-53.1% (-81.5% to 18.0%)
Tetanus	<0.05 (<0.05 to <0.05)	<0.05 (<0.05 to <0.05)	-24.3% (-88.2% to 300.1%)	-39.1% (-90.5% to 221.7%)
**All causes**	**708.7 (596.5 to 833.5)**	**175.6 (124.1 to 235.2)**	**22.4% (9.1% to 38.0%)**	**-1.6% (-12.3% to 11.0%)**

SR Table 13c: Global prevalence and years lived with disability (YLDs) for 2016, percentage change of YLD counts and rates between 2006 and 2016 of epilepsy by cause, ages 80-84				
**Cause**	**Prevalence in thousands (95% uncertainty interval)**	**YLDs in thousands (95% uncertainty interval)**		
	**2016**	**2016**	**Percentage change in counts between 2006 and 2016**	**Percentage change in rates between 2006 and 2016**
Epilepsy	385.8 (311.8 to 464.1)	96.3 (66.7 to 132.5)	27.5% (10.2% to 48.7%)	-2.7% (-15.9% to 13.6%)
Cysticercosis	49.0 (34.2 to 68.3)	6.9 (4.0 to 10.4)	14.9% (6.7% to 23.0%)	-12.3% (-18.5% to -6.1%)
Encephalitis	19.1 (16.3 to 22.2)	2.6 (1.8 to 3.5)	25.5% (13.3% to 39.5%)	-4.2% (-13.5% to 6.5%)
Neonatal sepsis and other neonatal infections	4.8 (0.8 to 14.3)	2.0 (0.3 to 6.4)	73.2% (60.9% to 113.5%)	32.3% (22.9% to 63.0%)
Neonatal encephalopathy due to birth asphyxia and trauma	0.9 (0.1 to 2.5)	0.4 (<0.05 to 1.1)	83.6% (57.8% to 137.2%)	40.2% (20.5% to 81.1%)
Haemolytic disease and other neonatal jaundice	0.8 (0.6 to 1.1)	0.4 (0.2 to 0.5)	57.7% (42.8% to 69.9%)	20.4% (9.1% to 29.8%)
Pneumococcal meningitis	2.2 (1.8 to 2.8)	0.3 (0.2 to 0.5)	30.4% (20.1% to 41.5%)	-0.4% (-8.3% to 8.1%)
Neonatal preterm birth complications	0.6 (0.3 to 1.1)	0.2 (0.1 to 0.5)	40.3% (-7.7% to 104.3%)	7.1% (-29.5% to 56.0%)
Other meningitis	1.3 (1.0 to 1.7)	0.2 (0.1 to 0.3)	31.2% (23.6% to 38.1%)	0.2% (-5.7% to 5.5%)
Food-borne trematodiases	1.3 (0.4 to 2.9)	0.2 (<0.05 to 0.4)	28.7% (-6.7% to 69.6%)	-1.8% (-28.7% to 29.5%)
H influenzae type B meningitis	0.6 (0.4 to 0.7)	0.1 (0.1 to 0.1)	44.0% (37.3% to 51.9%)	10.0% (4.9% to 16.0%)
Meningococcal infection	0.3 (0.2 to 0.4)	0.1 (<0.05 to 0.1)	32.0% (29.0% to 34.7%)	0.8% (-1.5% to 2.9%)
Malaria	<0.05 (<0.05 to 0.1)	<0.05 (<0.05 to <0.05)	56.9% (50.1% to 64.4%)	19.8% (14.6% to 25.5%)
Cystic echinococcosis	0.1 (<0.05 to 0.1)	<0.05 (<0.05 to <0.05)	-38.5% (-75.9% to 60.3%)	-53.0% (-81.6% to 22.4%)
Tetanus	<0.05 (<0.05 to <0.05)	<0.05 (<0.05 to <0.05)	-11.8% (-85.6% to 448.3%)	-32.7% (-89.0% to 318.6%)
**All causes**	**466.7 (386.6 to 548.8)**	**109.6 (76.6 to 148.0)**	**27.4% (12.1% to 46.0%)**	**-2.7% (-14.4% to 11.5%)**

SR Table 13c: Global prevalence and years lived with disability (YLDs) for 2016, percentage change of YLD counts and rates between 2006 and 2016 of epilepsy by cause, ages 85-89				
**Cause**	**Prevalence in thousands (95% uncertainty interval)**	**YLDs in thousands (95% uncertainty interval)**		
	**2016**	**2016**	**Percentage change in counts between 2006 and 2016**	**Percentage change in rates between 2006 and 2016**
Epilepsy	220.1 (175.1 to 264.1)	52.7 (36.1 to 71.9)	53.6% (30.9% to 81.1%)	-4.3% (-18.4% to 12.8%)
Cysticercosis	24.0 (17.2 to 32.6)	3.3 (2.0 to 4.8)	29.5% (20.1% to 39.1%)	-19.3% (-25.2% to -13.3%)
Encephalitis	11.1 (9.5 to 12.9)	1.5 (1.0 to 2.0)	48.6% (33.9% to 65.7%)	-7.4% (-16.5% to 3.3%)
Neonatal sepsis and other neonatal infections	1.0 (0.1 to 3.3)	0.4 (<0.05 to 1.4)	135.0% (115.1% to 214.1%)	46.4% (34.0% to 95.7%)
Pneumococcal meningitis	1.3 (1.0 to 1.5)	0.2 (0.1 to 0.3)	51.1% (36.2% to 67.8%)	-5.8% (-15.1% to 4.5%)
Other meningitis	0.7 (0.6 to 0.9)	0.1 (0.1 to 0.2)	51.4% (43.6% to 58.7%)	-5.7% (-10.5% to -1.1%)
Food-borne trematodiases	0.5 (0.1 to 1.2)	0.1 (<0.05 to 0.2)	45.9% (1.3% to 98.0%)	-9.1% (-36.9% to 23.4%)
Neonatal encephalopathy due to birth asphyxia and trauma	0.2 (<0.05 to 0.6)	0.1 (<0.05 to 0.3)	153.6% (95.9% to 282.0%)	58.0% (22.0% to 138.0%)
Haemolytic disease and other neonatal jaundice	0.1 (0.1 to 0.2)	0.1 (<0.05 to 0.1)	91.5% (56.4% to 115.2%)	19.3% (-2.6% to 34.1%)
H influenzae type B meningitis	0.3 (0.2 to 0.4)	0.1 (<0.05 to 0.1)	63.2% (56.0% to 70.7%)	1.7% (-2.8% to 6.4%)
Meningococcal infection	0.2 (0.1 to 0.2)	<0.05 (<0.05 to <0.05)	55.5% (51.0% to 60.0%)	-3.1% (-5.9% to -0.3%)
Neonatal preterm birth complications	<0.05 (<0.05 to <0.05)	<0.05 (<0.05 to <0.05)	95.4% (21.7% to 174.7%)	21.8% (-24.2% to 71.1%)
Cystic echinococcosis	<0.05 (<0.05 to 0.1)	<0.05 (<0.05 to <0.05)	-26.6% (-73.0% to 96.1%)	-54.3% (-83.2% to 22.2%)
Malaria	<0.05 (<0.05 to <0.05)	<0.05 (<0.05 to <0.05)	76.4% (65.5% to 86.5%)	9.9% (3.1% to 16.2%)
Tetanus	<0.05 (<0.05 to <0.05)	<0.05 (<0.05 to <0.05)	2.7% (-83.5% to 796.3%)	-36.0% (-89.7% to 458.5%)
**All causes**	**259.6 (213.3 to 307.6)**	**58.4 (40.5 to 79.3)**	**52.3% (31.8% to 77.2%)**	**-5.1% (-17.9% to 10.4%)**

SR Table 13c: Global prevalence and years lived with disability (YLDs) for 2016, percentage change of YLD counts and rates between 2006 and 2016 of epilepsy by cause, ages 90-94				
**Cause**	**Prevalence in thousands (95% uncertainty interval)**	**YLDs in thousands (95% uncertainty interval)**		
	**2016**	**2016**	**Percentage change in counts between 2006 and 2016**	**Percentage change in rates between 2006 and 2016**
Epilepsy	85.5 (67.7 to 103.6)	19.6 (13.4 to 26.9)	64.7% (35.8% to 100.8%)	-1.6% (-18.9% to 19.9%)
Cysticercosis	8.0 (5.4 to 12.2)	1.1 (0.6 to 1.7)	34.4% (24.3% to 44.4%)	-19.7% (-25.7% to -13.8%)
Encephalitis	4.2 (3.6 to 5.0)	0.5 (0.4 to 0.7)	52.8% (38.5% to 69.5%)	-8.7% (-17.2% to 1.3%)
Pneumococcal meningitis	0.5 (0.4 to 0.6)	0.1 (<0.05 to 0.1)	57.3% (43.7% to 72.5%)	-6.0% (-14.1% to 3.0%)
Other meningitis	0.3 (0.2 to 0.3)	<0.05 (<0.05 to 0.1)	57.2% (45.7% to 68.6%)	-6.1% (-13.0% to 0.7%)
Neonatal sepsis and other neonatal infections	0.1 (<0.05 to 0.3)	<0.05 (<0.05 to 0.1)	173.7% (143.1% to 322.3%)	63.5% (45.3% to 152.3%)
H influenzae type B meningitis	0.1 (0.1 to 0.1)	<0.05 (<0.05 to <0.05)	68.9% (59.2% to 79.9%)	0.9% (-4.9% to 7.5%)
Food-borne trematodiases	0.1 (<0.05 to 0.3)	<0.05 (<0.05 to <0.05)	38.5% (-3.7% to 99.1%)	-17.3% (-42.4% to 18.9%)
Meningococcal infection	0.1 (0.1 to 0.1)	<0.05 (<0.05 to <0.05)	62.7% (57.0% to 67.6%)	-2.8% (-6.2% to 0.1%)
Neonatal encephalopathy due to birth asphyxia and trauma	<0.05 (<0.05 to 0.1)	<0.05 (<0.05 to <0.05)	186.2% (82.7% to 442.1%)	71.0% (9.1% to 223.9%)
Haemolytic disease and other neonatal jaundice	<0.05 (<0.05 to <0.05)	<0.05 (<0.05 to <0.05)	86.8% (15.5% to 128.2%)	11.6% (-31.0% to 36.3%)
Cystic echinococcosis	<0.05 (<0.05 to <0.05)	<0.05 (<0.05 to <0.05)	-23.7% (-72.7% to 113.4%)	-54.4% (-83.7% to 27.5%)
Neonatal preterm birth complications	<0.05 (<0.05 to <0.05)	<0.05 (<0.05 to <0.05)	117.4% (4.8% to 243.2%)	29.9% (-37.4% to 105.0%)
Malaria	<0.05 (<0.05 to <0.05)	<0.05 (<0.05 to <0.05)	98.6% (82.1% to 116.0%)	18.7% (8.8% to 29.0%)
Tetanus	<0.05 (<0.05 to <0.05)	<0.05 (<0.05 to <0.05)	19.7% (-85.6% to 1,262.1%)	-28.5% (-91.4% to 713.8%)
**All causes**	**98.9 (80.5 to 119.1)**	**21.4 (14.8 to 29.4)**	**62.6% (36.3% to 95.2%)**	**-2.8% (-18.5% to 16.6%)**

SR Table 13c: Global prevalence and years lived with disability (YLDs) for 2016, percentage change of YLD counts and rates between 2006 and 2016 of epilepsy by cause, ages 95+ years				
**Cause**	**Prevalence in thousands (95% uncertainty interval)**	**YLDs in thousands (95% uncertainty interval)**		
	**2016**	**2016**	**Percentage change in counts between 2006 and 2016**	**Percentage change in rates between 2006 and 2016**
Epilepsy	19.8 (15.6 to 23.9)	4.6 (3.1 to 6.3)	49.6% (24.9% to 82.4%)	-4.5% (-20.3% to 16.4%)
Cysticercosis	1.9 (1.0 to 3.4)	0.3 (0.1 to 0.5)	41.4% (30.0% to 52.3%)	-9.8% (-17.1% to -2.8%)
Encephalitis	1.1 (0.9 to 1.2)	0.1 (0.1 to 0.2)	52.0% (35.1% to 70.1%)	-3.0% (-13.8% to 8.6%)
Pneumococcal meningitis	0.1 (0.1 to 0.1)	<0.05 (<0.05 to <0.05)	56.6% (44.8% to 68.0%)	-0.1% (-7.6% to 7.2%)
Other meningitis	0.1 (<0.05 to 0.1)	<0.05 (<0.05 to <0.05)	59.9% (47.5% to 73.8%)	2.0% (-5.9% to 10.9%)
H influenzae type B meningitis	<0.05 (<0.05 to <0.05)	<0.05 (<0.05 to <0.05)	66.5% (58.0% to 76.4%)	6.2% (0.8% to 12.6%)
Meningococcal infection	<0.05 (<0.05 to <0.05)	<0.05 (<0.05 to <0.05)	63.4% (50.4% to 75.7%)	4.3% (-4.0% to 12.1%)
Neonatal encephalopathy due to birth asphyxia and trauma	<0.05 (<0.05 to <0.05)	<0.05 (<0.05 to <0.05)	176.1% (25.0% to 653.2%)	76.2% (-20.2% to 380.6%)
Neonatal sepsis and other neonatal infections	<0.05 (<0.05 to <0.05)	<0.05 (<0.05 to <0.05)	189.2% (150.3% to 460.6%)	84.5% (59.7% to 257.7%)
Cystic echinococcosis	<0.05 (<0.05 to <0.05)	<0.05 (<0.05 to <0.05)	-26.1% (-71.7% to 89.1%)	-52.8% (-81.9% to 20.7%)
Haemolytic disease and other neonatal jaundice	<0.05 (<0.05 to <0.05)	<0.05 (<0.05 to <0.05)	88.4% (-14.7% to 188.2%)	20.2% (-45.6% to 83.9%)
Neonatal preterm birth complications	<0.05 (<0.05 to <0.05)	<0.05 (<0.05 to <0.05)	118.2% (-18.1% to 279.7%)	39.2% (-47.7% to 142.3%)
Malaria	<0.05 (<0.05 to <0.05)	<0.05 (<0.05 to <0.05)	119.8% (95.4% to 150.8%)	40.3% (24.7% to 60.1%)
Tetanus	<0.05 (<0.05 to <0.05)	<0.05 (<0.05 to <0.05)	34.4% (-86.2% to 5,001.1%)	-14.2% (-91.2% to 3,155.2%)
**All causes**	**22.9 (18.7 to 27.7)**	**5.0 (3.4 to 6.9)**	**49.3% (26.4% to 78.9%)**	**-4.7% (-19.4% to 14.2%)**

SR Table 13d: Global prevalence and years lived with disability (YLDs) for 2016, percentage change of YLD counts and rates between 2006 and 2016 of Guillain-Barré syndrome by cause, ages 0-6 days				
**Cause**	**Prevalence in thousands (95% uncertainty interval)**	**YLDs in thousands (95% uncertainty interval)**		
	**2016**	**2016**	**Percentage change in counts between 2006 and 2016**	**Percentage change in rates between 2006 and 2016**
Zika virus disease	<0.05<br>(<0.05 to <0.05)	<0.05<br>(<0.05 to <0.05)	--	--
Upper respiratory infections	<0.05<br>(<0.05 to <0.05)	<0.05<br>(<0.05 to <0.05)	-0.6%<br>(-1.6% to 0.2%)	-0.1%<br>(-1.1% to 0.7%)
Lower respiratory infections	<0.05<br>(<0.05 to <0.05)	<0.05<br>(<0.05 to <0.05)	-0.6%<br>(-1.6% to 0.2%)	-0.1%<br>(-1.1% to 0.8%)
Diarrhoeal diseases	<0.05<br>(<0.05 to <0.05)	<0.05<br>(<0.05 to <0.05)	-0.6%<br>(-1.6% to 0.2%)	-0.1%<br>(-1.1% to 0.7%)
Other infectious diseases	<0.05<br>(<0.05 to <0.05)	<0.05<br>(<0.05 to <0.05)	-0.6%<br>(-1.7% to 0.2%)	-0.1%<br>(-1.1% to 0.8%)
**All causes**	**<0.05<br>(<0.05 to <0.05)**	**<0.05<br>(<0.05 to <0.05)**	**90.9%<br>(38.8% to 226.2%)**	**91.9%<br>(39.5% to 227.9%)**

SR Table 13d: Global prevalence and years lived with disability (YLDs) for 2016, percentage change of YLD counts and rates between 2006 and 2016 of Guillain-Barré syndrome by cause, ages 7-27 days				
**Cause**	**Prevalence in thousands (95% uncertainty interval)**	**YLDs in thousands (95% uncertainty interval)**		
	**2016**	**2016**	**Percentage change in counts between 2006 and 2016**	**Percentage change in rates between 2006 and 2016**
Upper respiratory infections	<0.05<br>(<0.05 to <0.05)	<0.05<br>(<0.05 to <0.05)	-0.3%<br>(-1.3% to 0.6%)	-0.1%<br>(-1.1% to 0.8%)
Zika virus disease	<0.05<br>(<0.05 to <0.05)	<0.05<br>(<0.05 to <0.05)	--	--
Lower respiratory infections	<0.05<br>(<0.05 to <0.05)	<0.05<br>(<0.05 to <0.05)	-0.3%<br>(-1.3% to 0.6%)	-0.1%<br>(-1.1% to 0.8%)
Diarrhoeal diseases	<0.05<br>(<0.05 to <0.05)	<0.05<br>(<0.05 to <0.05)	-0.3%<br>(-1.3% to 0.6%)	-0.1%<br>(-1.1% to 0.8%)
Other infectious diseases	<0.05<br>(<0.05 to <0.05)	<0.05<br>(<0.05 to <0.05)	-0.3%<br>(-1.3% to 0.6%)	-0.1%<br>(-1.1% to 0.8%)
**All causes**	**<0.05<br>(<0.05 to <0.05)**	**<0.05<br>(<0.05 to <0.05)**	**19.9%<br>(8.4% to 48.4%)**	**20.2%<br>(8.6% to 48.6%)**

SR Table 13d: Global prevalence and years lived with disability (YLDs) for 2016, percentage change of YLD counts and rates between 2006 and 2016 of Guillain-Barré syndrome by cause, ages 28-364 days				
**Cause**	**Prevalence in thousands (95% uncertainty interval)**	**YLDs in thousands (95% uncertainty interval)**		
	**2016**	**2016**	**Percentage change in counts between 2006 and 2016**	**Percentage change in rates between 2006 and 2016**
Upper respiratory infections	0.1<br>(0.1 to 0.2)	<0.05<br>(<0.05 to 0.1)	0.7%<br>(-0.4% to 1.6%)	-0.2%<br>(-1.2% to 0.8%)
Lower respiratory infections	0.1<br>(<0.05 to 0.1)	<0.05<br>(<0.05 to <0.05)	0.7%<br>(-0.4% to 1.6%)	-0.2%<br>(-1.2% to 0.8%)
Diarrhoeal diseases	<0.05<br>(<0.05 to 0.1)	<0.05<br>(<0.05 to <0.05)	0.7%<br>(-0.4% to 1.6%)	-0.1%<br>(-1.2% to 0.8%)
Other infectious diseases	<0.05<br>(<0.05 to 0.1)	<0.05<br>(<0.05 to <0.05)	0.7%<br>(-0.4% to 1.6%)	-0.2%<br>(-1.2% to 0.8%)
Zika virus disease	<0.05<br>(<0.05 to <0.05)	<0.05<br>(<0.05 to <0.05)	--	--
**All causes**	**0.3**<br>**(0.2 to 0.5)**	**0.1**<br>**(<0.05 to 0.1)**	**3.7%**<br>**(1.8% to 7.2%)**	**2.9%**<br>**(1.0% to 6.4%)**

SR Table 13d: Global prevalence and years lived with disability (YLDs) for 2016, percentage change of YLD counts and rates between 2006 and 2016 of Guillain-Barré syndrome by cause, ages 1-4 years				
**Cause**	**Prevalence in thousands (95% uncertainty interval)**	**YLDs in thousands (95% uncertainty interval)**		
	**2016**	**2016**	**Percentage change in counts between 2006 and 2016**	**Percentage change in rates between 2006 and 2016**
Other neurological disorders	1.4<br>(0.7 to 2.3)	0.4<br>(0.2 to 0.8)	5.2%<br>(3.5% to 6.5%)	-0.4%<br>(-1.9% to 0.9%)
Upper respiratory infections	1.2<br>(0.6 to 2.0)	0.3<br>(0.2 to 0.6)	5.2%<br>(3.5% to 6.5%)	-0.4%<br>(-1.9% to 0.9%)
Lower respiratory infections	0.4<br>(0.2 to 0.8)	0.1<br>(0.1 to 0.3)	5.2%<br>(3.5% to 6.5%)	-0.4%<br>(-1.9% to 0.9%)
Diarrhoeal diseases	0.4<br>(0.2 to 0.7)	0.1<br>(0.1 to 0.2)	5.2%<br>(3.6% to 6.5%)	-0.4%<br>(-1.9% to 0.9%)
Other infectious diseases	0.3<br>(0.1 to 0.5)	0.1<br>(<0.05 to 0.1)	5.2%<br>(3.5% to 6.5%)	-0.4%<br>(-1.9% to 0.9%)
Zika virus disease	<0.05<br>(<0.05 to 0.1)	<0.05<br>(<0.05 to <0.05)	--	--
**All causes**	**3.7<br>(2.0 to 6.0)**	**1.1<br>(0.5 to 2.0)**	**6.3%<br>(4.8% to 7.7%)**	**0.7%<br>(-0.7% to 2.0%)**

SR Table 13d: Global prevalence and years lived with disability (YLDs) for 2016, percentage change of YLD counts and rates between 2006 and 2016 of Guillain-Barré syndrome by cause, ages 5-9 years				
**Cause**	**Prevalence in thousands (95% uncertainty interval)**	**YLDs in thousands (95% uncertainty interval)**		
	**2016**	**2016**	**Percentage change in counts between 2006 and 2016**	**Percentage change in rates between 2006 and 2016**
Other neurological disorders	2.0<br>(0.9 to 3.9)	0.6<br>(0.3 to 1.2)	7.1%<br>(4.7% to 8.4%)	-0.5%<br>(-2.8% to 0.7%)
Upper respiratory infections	1.7<br>(0.8 to 3.1)	0.5<br>(0.2 to 1.0)	7.1%<br>(4.7% to 8.4%)	-0.5%<br>(-2.7% to 0.7%)
Lower respiratory infections	0.6<br>(0.3 to 1.2)	0.2<br>(0.1 to 0.4)	7.1%<br>(4.7% to 8.4%)	-0.5%<br>(-2.7% to 0.7%)
Diarrhoeal diseases	0.6<br>(0.3 to 1.1)	0.2<br>(0.1 to 0.3)	7.1%<br>(4.7% to 8.4%)	-0.5%<br>(-2.7% to 0.7%)
Other infectious diseases	0.4<br>(0.2 to 0.7)	0.1<br>(<0.05 to 0.2)	7.1%<br>(4.7% to 8.4%)	-0.5%<br>(-2.7% to 0.7%)
Zika virus disease	0.1<br>(<0.05 to 0.1)	<0.05<br>(<0.05 to <0.05)	--	--
**All causes**	**5.4<br>(2.7 to 9.6)**	**1.6<br>(0.7 to 3.1)**	**8.2%<br>(6.3% to 9.6%)**	**0.5%<br>(-1.2% to 1.8%)**

SR Table 13d: Global prevalence and years lived with disability (YLDs) for 2016, percentage change of YLD counts and rates between 2006 and 2016 of Guillain-Barré syndrome by cause, ages 10-14 years				
**Cause**	**Prevalence in thousands (95% uncertainty interval)**	**YLDs in thousands (95% uncertainty interval)**		
	**2016**	**2016**	**Percentage change in counts between 2006 and 2016**	**Percentage change in rates between 2006 and 2016**
Other neurological disorders	1.8 (0.8 to 3.5)	0.5 (0.2 to 1.1)	3.0% (0.9% to 4.2%)	2.1% (0.1% to 3.3%)
Upper respiratory infections	1.5 (0.7 to 2.8)	0.4 (0.2 to 0.9)	3.0% (0.9% to 4.2%)	2.1% (0.1% to 3.3%)
Lower respiratory infections	0.6 (0.2 to 1.1)	0.2 (0.1 to 0.4)	3.0% (0.9% to 4.2%)	2.1% (0.1% to 3.3%)
Diarrhoeal diseases	0.5 (0.2 to 1.0)	0.2 (0.1 to 0.3)	3.0% (0.9% to 4.2%)	2.1% (0.1% to 3.3%)
Other infectious diseases	0.3 (0.2 to 0.7)	0.1 (<0.05 to 0.2)	3.0% (0.9% to 4.2%)	2.1% (0.1% to 3.3%)
Zika virus disease	0.1 (<0.05 to 0.1)	<0.05 (<0.05 to <0.05)	--	--
**All causes**	**4.7 (2.3 to 8.6)**	**1.4 (0.6 to 2.9)**	**4.5% (3.0% to 6.1%)**	**3.6% (2.2% to 5.2%)**

SR Table 13d: Global prevalence and years lived with disability (YLDs) for 2016, percentage change of YLD counts and rates between 2006 and 2016 of Guillain-Barré syndrome by cause, ages 15-19 years				
**Cause**	**Prevalence in thousands (95% uncertainty interval)**	**YLDs in thousands (95% uncertainty interval)**		
	**2016**	**2016**	**Percentage change in counts between 2006 and 2016**	**Percentage change in rates between 2006 and 2016**
Other neurological disorders	1.4<br>(0.7 to 2.5)	0.4<br>(0.2 to 0.8)	-2.2%<br>(-3.6% to -1.0%)	3.5%<br>(2.0% to 4.8%)
Upper respiratory infections	1.2<br>(0.6 to 2.1)	0.4<br>(0.2 to 0.7)	-2.2%<br>(-3.6% to -1.0%)	3.5%<br>(2.0% to 4.8%)
Lower respiratory infections	0.4<br>(0.2 to 0.8)	0.1<br>(0.1 to 0.3)	-2.2%<br>(-3.6% to -1.0%)	3.5%<br>(2.0% to 4.8%)
Diarrhoeal diseases	0.4<br>(0.2 to 0.7)	0.1<br>(0.1 to 0.2)	-2.2%<br>(-3.6% to -1.0%)	3.5%<br>(2.0% to 4.8%)
Other infectious diseases	0.3<br>(0.1 to 0.5)	0.1<br>(<0.05 to 0.1)	-2.2%<br>(-3.6% to -1.0%)	3.5%<br>(2.0% to 4.8%)
Zika virus disease	0.1<br>(<0.05 to 0.1)	<0.05<br>(<0.05 to <0.05)	--	--
**All causes**	**3.8**<br>**(2.1 to 6.2)**	**1.1**<br>**(0.5 to 2.0)**	**-0.2%**<br>**(-1.7% to 1.8%)**	**5.6%**<br>**(4.0% to 7.7%)**

SR Table 13d: Global prevalence and years lived with disability (YLDs) for 2016, percentage change of YLD counts and rates between 2006 and 2016 of Guillain-Barré syndrome by cause, ages 20-24 years				
**Cause**	**Prevalence in thousands (95% uncertainty interval)**	**YLDs in thousands (95% uncertainty interval)**		
	**2016**	**2016**	**Percentage change in counts between 2006 and 2016**	**Percentage change in rates between 2006 and 2016**
Other neurological disorders	1.3<br>(0.7 to 2.4)	0.4<br>(0.2 to 0.7)	5.8%<br>(4.0% to 7.2%)	0.9%<br>(-0.8% to 2.2%)
Upper respiratory infections	1.1<br>(0.6 to 2.0)	0.3<br>(0.2 to 0.6)	5.8%<br>(4.0% to 7.2%)	0.9%<br>(-0.8% to 2.2%)
Lower respiratory infections	0.4<br>(0.2 to 0.8)	0.1<br>(<0.05 to 0.2)	5.7%<br>(4.0% to 7.2%)	0.9%<br>(-0.8% to 2.2%)
Diarrhoeal diseases	0.4<br>(0.2 to 0.7)	0.1<br>(0.1 to 0.2)	5.8%<br>(4.0% to 7.2%)	0.9%<br>(-0.8% to 2.2%)
Other infectious diseases	0.2<br>(0.1 to 0.5)	0.1<br>(<0.05 to 0.1)	5.8%<br>(4.0% to 7.2%)	0.9%<br>(-0.8% to 2.2%)
Zika virus disease	0.1<br>(<0.05 to 0.2)	<0.05<br>(<0.05 to <0.05)	--	--
**All causes**	**3.5<br>(2.1 to 6.0)**	**1.0<br>(0.5 to 1.9)**	**8.2%<br>(6.5% to 10.5%)**	**3.2%<br>(1.6% to 5.4%)**

SR Table 13d: Global prevalence and years lived with disability (YLDs) for 2016, percentage change of YLD counts and rates between 2006 and 2016 of Guillain-Barré syndrome by cause, ages 25-29 years				
**Cause**	**Prevalence in thousands (95% uncertainty interval)**	**YLDs in thousands (95% uncertainty interval)**		
	**2016**	**2016**	**Percentage change in counts between 2006 and 2016**	**Percentage change in rates between 2006 and 2016**
Other neurological disorders	1.4 (0.7 to 2.4)	0.4 (0.2 to 0.8)	18.0% (16.1% to 19.5%)	-0.6% (-2.2% to 0.7%)
Upper respiratory infections	1.2 (0.6 to 2.0)	0.4 (0.2 to 0.7)	18.0% (16.2% to 19.5%)	-0.6% (-2.2% to 0.7%)
Lower respiratory infections	0.4 (0.2 to 0.8)	0.1 (<0.05 to 0.3)	18.0% (16.2% to 19.5%)	-0.6% (-2.1% to 0.7%)
Diarrhoeal diseases	0.4 (0.2 to 0.7)	0.1 (0.1 to 0.2)	18.0% (16.2% to 19.5%)	-0.6% (-2.2% to 0.7%)
Other infectious diseases	0.3 (0.1 to 0.5)	0.1 (<0.05 to 0.1)	18.0% (16.2% to 19.5%)	-0.6% (-2.2% to 0.7%)
Zika virus disease	0.1 (<0.05 to 0.2)	<0.05 (<0.05 to 0.1)	--	--
**All causes**	**3.8 (2.1 to 6.2)**	**1.1 (0.6 to 2.1)**	**20.8% (18.9% to 23.1%)**	**1.7% (0.2% to 3.7%)**

SR Table 13d: Global prevalence and years lived with disability (YLDs) for 2016, percentage change of YLD counts and rates between 2006 and 2016 of Guillain-Barré syndrome by cause, ages 30-34 years				
**Cause**	**Prevalence in thousands (95% uncertainty interval)**	**YLDs in thousands (95% uncertainty interval)**		
	**2016**	**2016**	**Percentage change in counts between 2006 and 2016**	**Percentage change in rates between 2006 and 2016**
Other neurological disorders	1.4<br>(0.7 to 2.6)	0.4<br>(0.2 to 0.9)	15.4%<br>(13.5% to 17.0%)	2.2%<br>(0.5% to 3.6%)
Upper respiratory infections	1.2<br>(0.6 to 2.1)	0.4<br>(0.2 to 0.7)	15.4%<br>(13.6% to 17.0%)	2.2%<br>(0.6% to 3.6%)
Lower respiratory infections	0.4<br>(0.2 to 0.9)	0.1<br>(<0.05 to 0.3)	15.4%<br>(13.5% to 17.0%)	2.2%<br>(0.6% to 3.6%)
Diarrhoeal diseases	0.4<br>(0.2 to 0.8)	0.1<br>(0.1 to 0.2)	15.4%<br>(13.6% to 17.0%)	2.2%<br>(0.6% to 3.6%)
Other infectious diseases	0.3<br>(0.1 to 0.5)	0.1<br>(<0.05 to 0.2)	15.4%<br>(13.6% to 17.0%)	2.2%<br>(0.6% to 3.6%)
Zika virus disease	0.1<br>(<0.05 to 0.2)	<0.05<br>(<0.05 to 0.1)	--	--
**All causes**	**3.8**<br>**(2.1 to 6.8)**	**1.1**<br>**(0.5 to 2.2)**	**18.2%**<br>**(16.2% to 20.9%)**	**4.7%**<br>**(2.9% to 7.1%)**

SR Table 13d: Global prevalence and years lived with disability (YLDs) for 2016, percentage change of YLD counts and rates between 2006 and 2016 of Guillain-Barré syndrome by cause, ages 35-39 years				
**Cause**	**Prevalence in thousands (95% uncertainty interval)**	**YLDs in thousands (95% uncertainty interval)**		
	**2016**	**2016**	**Percentage change in counts between 2006 and 2016**	**Percentage change in rates between 2006 and 2016**
Other neurological disorders	1.4<br>(0.7 to 2.5)	0.4<br>(0.2 to 0.8)	9.5%<br>(7.3% to 11.2%)	3.5%<br>(1.5% to 5.1%)
Upper respiratory infections	1.2<br>(0.6 to 2.0)	0.4<br>(0.2 to 0.6)	9.5%<br>(7.4% to 11.2%)	3.5%<br>(1.5% to 5.1%)
Lower respiratory infections	0.4<br>(0.2 to 0.8)	0.1<br>(0.1 to 0.3)	9.5%<br>(7.3% to 11.2%)	3.5%<br>(1.5% to 5.1%)
Diarrhoeal diseases	0.4<br>(0.2 to 0.7)	0.1<br>(0.1 to 0.2)	9.5%<br>(7.4% to 11.2%)	3.5%<br>(1.5% to 5.1%)
Other infectious diseases	0.3<br>(0.1 to 0.5)	0.1<br>(<0.05 to 0.1)	9.5%<br>(7.4% to 11.2%)	3.5%<br>(1.5% to 5.1%)
Zika virus disease	0.1<br>(<0.05 to 0.2)	<0.05<br>(<0.05 to 0.1)	--	--
**All causes**	**3.8**<br>**(2.2 to 6.3)**	**1.1**<br>**(0.6 to 2.0)**	**12.0%**<br>**(10.1% to 14.3%)**	**5.9%**<br>**(4.1% to 8.1%)**

SR Table 13d: Global prevalence and years lived with disability (YLDs) for 2016, percentage change of YLD counts and rates between 2006 and 2016 of Guillain-Barré syndrome by cause, ages 40-44 years				
**Cause**	**Prevalence in thousands (95% uncertainty interval)**	**YLDs in thousands (95% uncertainty interval)**		
	**2016**	**2016**	**Percentage change in counts between 2006 and 2016**	**Percentage change in rates between 2006 and 2016**
Other neurological disorders	1.5<br>(0.7 to 2.8)	0.4<br>(0.2 to 0.9)	14.1%<br>(11.6% to 16.1%)	0.2%<br>(-2.0% to 2.0%)
Upper respiratory infections	1.3<br>(0.7 to 2.4)	0.4<br>(0.2 to 0.7)	14.1%<br>(11.6% to 16.1%)	0.2%<br>(-2.0% to 2.0%)
Lower respiratory infections	0.5<br>(0.2 to 1.0)	0.1<br>(0.1 to 0.3)	14.1%<br>(11.6% to 16.1%)	0.2%<br>(-2.0% to 2.0%)
Diarrhoeal diseases	0.4<br>(0.2 to 0.8)	0.1<br>(0.1 to 0.2)	14.1%<br>(11.6% to 16.1%)	0.2%<br>(-2.0% to 2.0%)
Other infectious diseases	0.3<br>(0.1 to 0.5)	0.1<br>(<0.05 to 0.2)	14.1%<br>(11.6% to 16.1%)	0.2%<br>(-2.0% to 2.0%)
Zika virus disease	0.1<br>(<0.05 to 0.1)	<0.05<br>(<0.05 to <0.05)	--	--
**All causes**	**4.0<br>(2.3 to 7.2)**	**1.2<br>(0.6 to 2.2)**	**16.3%<br>(14.1% to 18.6%)**	**2.1%<br>(0.2% to 4.1%)**

SR Table 13d: Global prevalence and years lived with disability (YLDs) for 2016, percentage change of YLD counts and rates between 2006 and 2016 of Guillain-Barré syndrome by cause, ages 45-49 years				
**Cause**	**Prevalence in thousands (95% uncertainty interval)**	**YLDs in thousands (95% uncertainty interval)**		
	**2016**	**2016**	**Percentage change in counts between 2006 and 2016**	**Percentage change in rates between 2006 and 2016**
Other neurological disorders	1.5<br>(0.7 to 2.6)	0.5<br>(0.2 to 0.8)	21.7%<br>(18.8% to 23.8%)	-2.4%<br>(-4.7% to -0.7%)
Upper respiratory infections	1.3<br>(0.7 to 2.2)	0.4<br>(0.2 to 0.7)	21.7%<br>(18.9% to 23.8%)	-2.4%<br>(-4.7% to -0.7%)
Lower respiratory infections	0.5<br>(0.2 to 0.9)	0.1<br>(0.1 to 0.3)	21.7%<br>(18.9% to 23.8%)	-2.4%<br>(-4.7% to -0.7%)
Diarrhoeal diseases	0.4<br>(0.2 to 0.8)	0.1<br>(0.1 to 0.2)	21.7%<br>(18.9% to 23.8%)	-2.4%<br>(-4.7% to -0.7%)
Other infectious diseases	0.3<br>(0.1 to 0.5)	0.1<br>(<0.05 to 0.2)	21.7%<br>(18.9% to 23.8%)	-2.4%<br>(-4.7% to -0.7%)
Zika virus disease	0.1<br>(<0.05 to 0.1)	<0.05<br>(<0.05 to <0.05)	--	--
**All causes**	**4.1**<br>**(2.2 to 6.5)**	**1.2**<br>**(0.6 to 2.1)**	**23.8%**<br>**(21.5% to 25.8%)**	**-0.7%**<br>**(-2.5% to 0.9%)**

SR Table 13d: Global prevalence and years lived with disability (YLDs) for 2016, percentage change of YLD counts and rates between 2006 and 2016 of Guillain-Barré syndrome by cause, ages 50-54 years				
**Cause**	**Prevalence in thousands (95% uncertainty interval)**	**YLDs in thousands (95% uncertainty interval)**		
	**2016**	**2016**	**Percentage change in counts between 2006 and 2016**	**Percentage change in rates between 2006 and 2016**
Other neurological disorders	1.6<br>(0.8 to 2.8)	0.5<br>(0.2 to 0.9)	25.0%<br>(22.6% to 26.7%)	0.4%<br>(-1.6% to 1.7%)
Upper respiratory infections	1.4<br>(0.7 to 2.4)	0.4<br>(0.2 to 0.7)	25.0%<br>(22.6% to 26.7%)	0.4%<br>(-1.6% to 1.7%)
Lower respiratory infections	0.5<br>(0.2 to 1.0)	0.1<br>(0.1 to 0.3)	25.0%<br>(22.6% to 26.7%)	0.4%<br>(-1.6% to 1.7%)
Diarrhoeal diseases	0.5<br>(0.2 to 0.8)	0.1<br>(0.1 to 0.3)	25.0%<br>(22.6% to 26.7%)	0.4%<br>(-1.6% to 1.7%)
Other infectious diseases	0.3<br>(0.2 to 0.5)	0.1<br>(<0.05 to 0.2)	25.0%<br>(22.6% to 26.7%)	0.4%<br>(-1.6% to 1.7%)
Zika virus disease	0.1<br>(<0.05 to 0.1)	<0.05<br>(<0.05 to <0.05)	--	--
**All causes**	**4.3<br>(2.3 to 7.1)**	**1.3<br>(0.6 to 2.3)**	**26.7%<br>(24.9% to 28.6%)**	**1.8%<br>(0.3% to 3.3%)**

SR Table 13d: Global prevalence and years lived with disability (YLDs) for 2016, percentage change of YLD counts and rates between 2006 and 2016 of Guillain-Barré syndrome by cause, ages 55-59 years				
**Cause**	**Prevalence in thousands (95% uncertainty interval)**	**YLDs in thousands (95% uncertainty interval)**		
	**2016**	**2016**	**Percentage change in counts between 2006 and 2016**	**Percentage change in rates between 2006 and 2016**
Other neurological disorders	1.6<br>(0.8 to 2.8)	0.5<br>(0.2 to 0.9)	32.1%<br>(29.4% to 34.1%)	1.2%<br>(-0.8% to 2.8%)
Upper respiratory infections	1.4<br>(0.8 to 2.3)	0.4<br>(0.2 to 0.7)	32.1%<br>(29.4% to 34.1%)	1.2%<br>(-0.8% to 2.8%)
Lower respiratory infections	0.5<br>(0.2 to 1.0)	0.1<br>(0.1 to 0.3)	32.1%<br>(29.4% to 34.1%)	1.3%<br>(-0.8% to 2.8%)
Diarrhoeal diseases	0.5<br>(0.2 to 0.8)	0.1<br>(0.1 to 0.3)	32.1%<br>(29.4% to 34.2%)	1.3%<br>(-0.8% to 2.8%)
Other infectious diseases	0.3<br>(0.2 to 0.5)	0.1<br>(<0.05 to 0.2)	32.1%<br>(29.4% to 34.2%)	1.2%<br>(-0.8% to 2.8%)
Zika virus disease	<0.05<br>(<0.05 to 0.1)	<0.05<br>(<0.05 to <0.05)	--	--
**All causes**	**4.3**<br>**(2.5 to 7.1)**	**1.3**<br>**(0.6 to 2.2)**	**33.5%**<br>**(31.1% to 35.5%)**	**2.3%**<br>**(0.5% to 3.8%)**

SR Table 13d: Global prevalence and years lived with disability (YLDs) for 2016, percentage change of YLD counts and rates between 2006 and 2016 of Guillain-Barré syndrome by cause, ages 60-64 years				
**Cause**	**Prevalence in thousands (95% uncertainty interval)**	**YLDs in thousands (95% uncertainty interval)**		
	**2016**	**2016**	**Percentage change in counts between 2006 and 2016**	**Percentage change in rates between 2006 and 2016**
Other neurological disorders	1.6<br>(0.8 to 2.9)	0.5<br>(0.2 to 0.9)	49.6%<br>(45.9% to 52.1%)	-0.8%<br>(-3.3% to 0.9%)
Upper respiratory infections	1.4<br>(0.7 to 2.4)	0.4<br>(0.2 to 0.8)	49.6%<br>(45.9% to 52.1%)	-0.8%<br>(-3.3% to 0.9%)
Lower respiratory infections	0.5<br>(0.2 to 1.0)	0.1<br>(0.1 to 0.3)	49.6%<br>(45.9% to 52.1%)	-0.8%<br>(-3.2% to 0.9%)
Diarrhoeal diseases	0.5<br>(0.2 to 0.9)	0.1<br>(0.1 to 0.3)	49.6%<br>(45.9% to 52.1%)	-0.8%<br>(-3.3% to 0.9%)
Other infectious diseases	0.3<br>(0.1 to 0.6)	0.1<br>(<0.05 to 0.2)	49.6%<br>(45.9% to 52.1%)	-0.8%<br>(-3.3% to 0.9%)
Zika virus disease	<0.05<br>(<0.05 to 0.1)	<0.05<br>(<0.05 to <0.05)	--	--
**All causes**	**4.2**<br>**(2.5 to 7.3)**	**1.3**<br>**(0.6 to 2.3)**	**50.8%**<br>**(47.5% to 53.3%)**	**0.0%**<br>**(-2.2% to 1.7%)**

SR Table 13d: Global prevalence and years lived with disability (YLDs) for 2016, percentage change of YLD counts and rates between 2006 and 2016 of Guillain-Barré syndrome by cause, ages 65-69 years				
**Cause**	**Prevalence in thousands (95% uncertainty interval)**	**YLDs in thousands (95% uncertainty interval)**		
	**2016**	**2016**	**Percentage change in counts between 2006 and 2016**	**Percentage change in rates between 2006 and 2016**
Other neurological disorders	1.3<br>(0.7 to 2.2)	0.4<br>(0.2 to 0.7)	31.1%<br>(26.9% to 33.7%)	-1.1%<br>(-4.2% to 0.9%)
Upper respiratory infections	1.1<br>(0.6 to 1.8)	0.3<br>(0.2 to 0.6)	31.1%<br>(26.8% to 33.7%)	-1.1%<br>(-4.3% to 0.9%)
Lower respiratory infections	0.4<br>(0.2 to 0.8)	0.1<br>(0.1 to 0.2)	31.1%<br>(26.8% to 33.7%)	-1.1%<br>(-4.3% to 0.9%)
Diarrhoeal diseases	0.4<br>(0.2 to 0.6)	0.1<br>(0.1 to 0.2)	31.1%<br>(26.9% to 33.7%)	-1.1%<br>(-4.2% to 0.9%)
Other infectious diseases	0.2<br>(0.1 to 0.4)	0.1<br>(<0.05 to 0.1)	31.1%<br>(26.8% to 33.7%)	-1.1%<br>(-4.3% to 0.9%)
Zika virus disease	<0.05<br>(<0.05 to <0.05)	<0.05<br>(<0.05 to <0.05)	--	--
**All causes**	**3.5<br>(2.1 to 5.6)**	**1.0<br>(0.6 to 1.8)**	**31.9%<br>(27.8% to 34.5%)**	**-0.5%<br>(-3.5% to 1.5%)**

SR Table 13d: Global prevalence and years lived with disability (YLDs) for 2016, percentage change of YLD counts and rates between 2006 and 2016 of Guillain-Barré syndrome by cause, ages 70-74 years				
**Cause**	**Prevalence in thousands (95% uncertainty interval)**	**YLDs in thousands (95% uncertainty interval)**		
	**2016**	**2016**	**Percentage change in counts between 2006 and 2016**	**Percentage change in rates between 2006 and 2016**
Other neurological disorders	1.0 (0.5 to 1.7)	0.3 (0.1 to 0.6)	20.2% (16.5% to 22.4%)	0.4% (-2.7% to 2.3%)
Upper respiratory infections	0.9 (0.5 to 1.4)	0.3 (0.1 to 0.5)	20.2% (16.5% to 22.4%)	0.4% (-2.6% to 2.3%)
Lower respiratory infections	0.3 (0.1 to 0.6)	0.1 (<0.05 to 0.2)	20.1% (16.5% to 22.5%)	0.4% (-2.6% to 2.3%)
Diarrhoeal diseases	0.3 (0.2 to 0.5)	0.1 (<0.05 to 0.2)	20.2% (16.5% to 22.5%)	0.4% (-2.6% to 2.3%)
Other infectious diseases	0.2 (0.1 to 0.3)	0.1 (<0.05 to 0.1)	20.2% (16.5% to 22.5%)	0.4% (-2.7% to 2.4%)
Zika virus disease	<0.05 (<0.05 to <0.05)	<0.05 (<0.05 to <0.05)	--	--
**All causes**	**2.7 (1.5 to 4.4)**	**0.8 (0.4 to 1.4)**	**20.7% (17.3% to 23.0%)**	**0.9% (-1.9% to 2.8%)**

SR Table 13d: Global prevalence and years lived with disability (YLDs) for 2016, percentage change of YLD counts and rates between 2006 and 2016 of Guillain-Barré syndrome by cause, ages 75-79 years				
**Cause**	**Prevalence in thousands (95% uncertainty interval)**	**YLDs in thousands (95% uncertainty interval)**		
	**2016**	**2016**	**Percentage change in counts between 2006 and 2016**	**Percentage change in rates between 2006 and 2016**
Other neurological disorders	0.8<br>(0.4 to 1.4)	0.2<br>(0.1 to 0.5)	23.9%<br>(20.1% to 26.2%)	-0.4%<br>(-3.4% to 1.5%)
Upper respiratory infections	0.7<br>(0.4 to 1.1)	0.2<br>(0.1 to 0.4)	23.9%<br>(20.1% to 26.2%)	-0.4%<br>(-3.4% to 1.5%)
Lower respiratory infections	0.3<br>(0.1 to 0.5)	0.1<br>(<0.05 to 0.1)	23.8%<br>(20.1% to 26.2%)	-0.4%<br>(-3.4% to 1.5%)
Diarrhoeal diseases	0.2<br>(0.1 to 0.4)	0.1<br>(<0.05 to 0.1)	23.9%<br>(20.1% to 26.2%)	-0.4%<br>(-3.4% to 1.5%)
Other infectious diseases	0.2<br>(0.1 to 0.3)	<0.05<br>(<0.05 to 0.1)	23.9%<br>(20.2% to 26.2%)	-0.4%<br>(-3.4% to 1.5%)
Zika virus disease	<0.05<br>(<0.05 to <0.05)	<0.05<br>(<0.05 to <0.05)	--	--
**All causes**	**2.2**<br>**(1.3 to 3.3)**	**0.7**<br>**(0.3 to 1.1)**	**24.4%**<br>**(20.9% to 26.6%)**	**-0.0%**<br>**(-2.8% to 1.8%)**

SR Table 13d: Global prevalence and years lived with disability (YLDs) for 2016, percentage change of YLD counts and rates between 2006 and 2016 of Guillain-Barré syndrome by cause, ages 80-84				
**Cause**	**Prevalence in thousands (95% uncertainty interval)**	**YLDs in thousands (95% uncertainty interval)**		
	**2016**	**2016**	**Percentage change in counts between 2006 and 2016**	**Percentage change in rates between 2006 and 2016**
Other neurological disorders	0.5<br>(0.3 to 1.0)	0.2<br>(0.1 to 0.3)	30.2%<br>(25.2% to 33.4%)	-0.6%<br>(-4.4% to 1.8%)
Upper respiratory infections	0.5<br>(0.2 to 0.8)	0.1<br>(0.1 to 0.3)	30.2%<br>(25.2% to 33.4%)	-0.6%<br>(-4.4% to 1.8%)
Lower respiratory infections	0.2<br>(0.1 to 0.3)	0.1<br>(<0.05 to 0.1)	30.1%<br>(25.2% to 33.4%)	-0.6%<br>(-4.4% to 1.8%)
Diarrhoeal diseases	0.2<br>(0.1 to 0.3)	<0.05<br>(<0.05 to 0.1)	30.2%<br>(25.2% to 33.4%)	-0.6%<br>(-4.4% to 1.8%)
Other infectious diseases	0.1<br>(0.1 to 0.2)	<0.05<br>(<0.05 to 0.1)	30.2%<br>(25.2% to 33.4%)	-0.6%<br>(-4.4% to 1.9%)
Zika virus disease	<0.05<br>(<0.05 to <0.05)	<0.05<br>(<0.05 to <0.05)	--	--
**All causes**	**1.5**<br>**(0.8 to 2.4)**	**0.4**<br>**(0.2 to 0.8)**	**30.6%**<br>**(25.7% to 33.8%)**	**-0.3%**<br>**(-4.0% to 2.1%)**

SR Table 13d: Global prevalence and years lived with disability (YLDs) for 2016, percentage change of YLD counts and rates between 2006 and 2016 of Guillain-Barré syndrome by cause, ages 85-89				
**Cause**	**Prevalence in thousands (95% uncertainty interval)**	**YLDs in thousands (95% uncertainty interval)**		
	**2016**	**2016**	**Percentage change in counts between 2006 and 2016**	**Percentage change in rates between 2006 and 2016**
Other neurological disorders	0.3<br>(0.2 to 0.5)	0.1<br>(<0.05 to 0.2)	62.4%<br>(56.1% to 66.3%)	1.2%<br>(-2.7% to 3.6%)
Upper respiratory infections	0.2<br>(0.1 to 0.4)	0.1<br>(<0.05 to 0.1)	62.4%<br>(56.1% to 66.3%)	1.2%<br>(-2.7% to 3.6%)
Lower respiratory infections	0.1<br>(<0.05 to 0.2)	<0.05<br>(<0.05 to 0.1)	62.4%<br>(56.1% to 66.3%)	1.2%<br>(-2.7% to 3.6%)
Diarrhoeal diseases	0.1<br>(<0.05 to 0.1)	<0.05<br>(<0.05 to <0.05)	62.4%<br>(56.1% to 66.3%)	1.2%<br>(-2.7% to 3.6%)
Other infectious diseases	0.1<br>(<0.05 to 0.1)	<0.05<br>(<0.05 to <0.05)	62.4%<br>(56.1% to 66.3%)	1.2%<br>(-2.7% to 3.6%)
Zika virus disease	<0.05<br>(<0.05 to <0.05)	<0.05<br>(<0.05 to <0.05)	--	--
**All causes**	**0.8**<br>**(0.5 to 1.2)**	**0.2**<br>**(0.1 to 0.4)**	**62.9%**<br>**(56.8% to 66.6%)**	**1.5%**<br>**(-2.3% to 3.8%)**

SR Table 13d: Global prevalence and years lived with disability (YLDs) for 2016, percentage change of YLD counts and rates between 2006 and 2016 of Guillain-Barré syndrome by cause, ages 90-94				
**Cause**	**Prevalence in thousands (95% uncertainty interval)**	**YLDs in thousands (95% uncertainty interval)**		
	**2016**	**2016**	**Percentage change in counts between 2006 and 2016**	**Percentage change in rates between 2006 and 2016**
Other neurological disorders	0.1<br>(0.1 to 0.2)	<0.05<br>(<0.05 to 0.1)	76.3%<br>(71.1% to 80.8%)	5.3%<br>(2.2% to 8.0%)
Upper respiratory infections	0.1<br>(0.1 to 0.2)	<0.05<br>(<0.05 to 0.1)	76.3%<br>(71.1% to 80.8%)	5.3%<br>(2.2% to 8.0%)
Lower respiratory infections	<0.05<br>(<0.05 to 0.1)	<0.05<br>(<0.05 to <0.05)	76.4%<br>(71.1% to 80.8%)	5.4%<br>(2.2% to 8.0%)
Diarrhoeal diseases	<0.05<br>(<0.05 to 0.1)	<0.05<br>(<0.05 to <0.05)	76.3%<br>(71.1% to 80.8%)	5.4%<br>(2.2% to 8.0%)
Other infectious diseases	<0.05<br>(<0.05 to <0.05)	<0.05<br>(<0.05 to <0.05)	76.3%<br>(71.1% to 80.8%)	5.3%<br>(2.2% to 8.0%)
Zika virus disease	<0.05<br>(<0.05 to <0.05)	<0.05<br>(<0.05 to <0.05)	--	--
**All causes**	**0.3<br>(0.2 to 0.5)**	**0.1<br>(<0.05 to 0.2)**	**76.7%<br>(71.6% to 81.2%)**	**5.6%<br>(2.5% to 8.3%)**

SR Table 13d: Global prevalence and years lived with disability (YLDs) for 2016, percentage change of YLD counts and rates between 2006 and 2016 of Guillain-Barré syndrome by cause, ages 95+ years				
**Cause**	**Prevalence in thousands (95% uncertainty interval)**	**YLDs in thousands (95% uncertainty interval)**		
	**2016**	**2016**	**Percentage change in counts between 2006 and 2016**	**Percentage change in rates between 2006 and 2016**
Other neurological disorders	<0.05 (<0.05 to 0.1)	<0.05 (<0.05 to <0.05)	79.0% (73.5% to 84.2%)	14.2% (10.7% to 17.5%)
Upper respiratory infections	<0.05 (<0.05 to 0.1)	<0.05 (<0.05 to <0.05)	79.0% (73.5% to 84.2%)	14.2% (10.7% to 17.6%)
Lower respiratory infections	<0.05 (<0.05 to <0.05)	<0.05 (<0.05 to <0.05)	79.0% (73.5% to 84.2%)	14.2% (10.7% to 17.6%)
Diarrhoeal diseases	<0.05 (<0.05 to <0.05)	<0.05 (<0.05 to <0.05)	79.0% (73.5% to 84.2%)	14.2% (10.7% to 17.5%)
Other infectious diseases	<0.05 (<0.05 to <0.05)	<0.05 (<0.05 to <0.05)	79.0% (73.5% to 84.2%)	14.2% (10.7% to 17.5%)
Zika virus disease	<0.05 (<0.05 to <0.05)	<0.05 (<0.05 to <0.05)	--	--
**All causes**	**0.1 (<0.05 to 0.2)**	**<0.05 (<0.05 to <0.05)**	**79.2% (73.9% to 84.6%)**	**14.4% (11.0% to 17.8%)**

SR Table 13e: Global prevalence and years lived with disability (YLDs) for 2016, percentage change of YLD counts and rates between 2006 and 2016 of hearing loss by cause, ages 0-6 days				
**Cause**	**Prevalence in thousands (95% uncertainty interval)**	**YLDs in thousands (95% uncertainty interval)**		
	**2016**	**2016**	**Percentage change in counts between 2006 and 2016**	**Percentage change in rates between 2006 and 2016**
Other congenital anomalies	12.7<br>(8.9 to 18.0)	1.3<br>(0.8 to 2.1)	-9.6%<br>(-14.1% to -5.0%)	-9.1%<br>(-13.6% to -4.5%)
Age-related and other hearing loss	5.6<br>(3.9 to 7.9)	0.6<br>(0.4 to 1.0)	-7.3%<br>(-12.6% to -1.7%)	-6.9%<br>(-12.2% to -1.2%)
Otitis media	0.6<br>(0.5 to 0.8)	<0.05<br>(<0.05 to <0.05)	-6.5%<br>(-10.1% to -2.5%)	-6.0%<br>(-9.6% to -2.0%)
Other meningitis	<0.05<br>(<0.05 to <0.05)	<0.05<br>(<0.05 to <0.05)	71.9%<br>(50.8% to 94.7%)	72.8%<br>(51.6% to 95.7%)
Pneumococcal meningitis	<0.05<br>(<0.05 to <0.05)	<0.05<br>(<0.05 to <0.05)	-31.8%<br>(-38.8% to -25.0%)	-31.4%<br>(-38.5% to -24.6%)
Meningococcal infection	<0.05<br>(<0.05 to <0.05)	<0.05<br>(<0.05 to <0.05)	1.1%<br>(-8.8% to 12.3%)	1.6%<br>(-8.3% to 12.8%)
H influenzae type B meningitis	<0.05<br>(<0.05 to <0.05)	<0.05<br>(<0.05 to <0.05)	-48.5%<br>(-55.7% to -40.1%)	-48.2%<br>(-55.4% to -39.8%)
**All causes**	**18.9<br>(13.4 to 26.7)**	**2.0<br>(1.2 to 3.1)**	**-8.9%<br>(-12.7% to -5.0%)**	**-8.4%<br>(-12.2% to -4.5%)**

SR Table 13e: Global prevalence and years lived with disability (YLDs) for 2016, percentage change of YLD counts and rates between 2006 and 2016 of hearing loss by cause, ages 7-27 days				
**Cause**	**Prevalence in thousands (95% uncertainty interval)**	**YLDs in thousands (95% uncertainty interval)**		
	**2016**	**2016**	**Percentage change in counts between 2006 and 2016**	**Percentage change in rates between 2006 and 2016**
Other congenital anomalies	37.6 (26.4 to 53.4)	4.0 (2.4 to 6.3)	-9.2% (-13.5% to -4.8%)	-9.0% (-13.3% to -4.6%)
Age-related and other hearing loss	11.3 (8.1 to 15.7)	1.5 (0.9 to 2.2)	-6.0% (-11.9% to -0.4%)	-5.8% (-11.7% to -0.2%)
Otitis media	7.4 (5.9 to 9.2)	0.3 (0.2 to 0.5)	-6.0% (-9.4% to -2.3%)	-5.8% (-9.3% to -2.1%)
Other meningitis	<0.05 (<0.05 to <0.05)	<0.05 (<0.05 to <0.05)	72.7% (52.6% to 95.9%)	73.0% (52.9% to 96.2%)
Pneumococcal meningitis	<0.05 (<0.05 to <0.05)	<0.05 (<0.05 to <0.05)	-31.4% (-38.4% to -24.3%)	-31.3% (-38.3% to -24.2%)
Meningococcal infection	<0.05 (<0.05 to <0.05)	<0.05 (<0.05 to <0.05)	1.4% (-7.9% to 12.4%)	1.6% (-7.8% to 12.6%)
H influenzae type B meningitis	<0.05 (<0.05 to <0.05)	<0.05 (<0.05 to <0.05)	-48.3% (-55.7% to -40.2%)	-48.2% (-55.6% to -40.1%)
**All causes**	**56.3 (41.5 to 76.4)**	**5.8 (3.6 to 8.7)**	**-8.2% (-11.5% to -4.7%)**	**-8.1% (-11.4% to -4.5%)**

SR Table 13e: Global prevalence and years lived with disability (YLDs) for 2016, percentage change of YLD counts and rates between 2006 and 2016 of hearing loss by cause, ages 28-364 days				
**Cause**	**Prevalence in thousands (95% uncertainty interval)**	**YLDs in thousands (95% uncertainty interval)**		
	**2016**	**2016**	**Percentage change in counts between 2006 and 2016**	**Percentage change in rates between 2006 and 2016**
Other congenital anomalies	598.1<br>(420.9 to 849.7)	62.7<br>(37.8 to 98.0)	-8.3%<br>(-13.0% to -3.6%)	-9.1%<br>(-13.7% to -4.4%)
Otitis media	608.7<br>(513.5 to 705.0)	36.9<br>(23.2 to 54.6)	-4.4%<br>(-8.3% to -0.1%)	-5.1%<br>(-9.1% to -0.9%)
Age-related and other hearing loss	146.0<br>(82.3 to 210.3)	21.0<br>(10.2 to 34.3)	-1.9%<br>(-10.0% to 6.4%)	-2.7%<br>(-10.7% to 5.5%)
Other meningitis	1.8<br>(0.5 to 2.5)	0.2<br>(<0.05 to 0.3)	73.4%<br>(52.8% to 97.0%)	72.0%<br>(51.5% to 95.4%)
Pneumococcal meningitis	1.7<br>(0.5 to 2.7)	0.2<br>(<0.05 to 0.4)	-30.5%<br>(-37.7% to -23.2%)	-31.1%<br>(-38.2% to -23.9%)
H influenzae type B meningitis	0.8<br>(0.2 to 1.4)	0.1<br>(<0.05 to 0.2)	-48.3%<br>(-55.2% to -39.9%)	-48.7%<br>(-55.6% to -40.4%)
Meningococcal infection	0.8<br>(0.2 to 1.3)	0.1<br>(<0.05 to 0.2)	1.5%<br>(-8.6% to 12.6%)	0.7%<br>(-9.4% to 11.7%)
**All causes**	**1,357.9<br>(1,126.9 to 1,647.1)**	**121.3<br>(79.3 to 171.4)**	**-6.1%<br>(-9.1% to -3.0%)**	**-6.9%<br>(-9.8% to -3.8%)**

SR Table 13e: Global prevalence and years lived with disability (YLDs) for 2016, percentage change of YLD counts and rates between 2006 and 2016 of hearing loss by cause, ages 1-4 years				
**Cause**	**Prevalence in thousands (95% uncertainty interval)**	**YLDs in thousands (95% uncertainty interval)**		
	**2016**	**2016**	**Percentage change in counts between 2006 and 2016**	**Percentage change in rates between 2006 and 2016**
Otitis media	8,212.7<br>(6,938.4 to 9,625.0)	426.5<br>(261.5 to 638.7)	1.4%<br>(-1.8% to 4.5%)	-3.9%<br>(-7.0% to -1.0%)
Other congenital anomalies	2,606.1<br>(1,834.1 to 3,702.7)	270.8<br>(165.1 to 420.7)	-4.0%<br>(-9.0% to 1.0%)	-9.0%<br>(-13.8% to -4.4%)
Age-related and other hearing loss	3,141.4<br>(2,045.8 to 4,306.5)	266.6<br>(146.1 to 411.2)	2.1%<br>(-3.1% to 7.5%)	-3.3%<br>(-8.2% to 1.8%)
Pneumococcal meningitis	20.9<br>(6.9 to 33.1)	1.9<br>(0.2 to 3.5)	-24.3%<br>(-31.4% to -17.0%)	-28.3%<br>(-35.0% to -21.4%)
Other meningitis	19.8<br>(6.9 to 28.4)	1.8<br>(0.2 to 3.1)	85.1%<br>(63.7% to 107.1%)	75.3%<br>(55.1% to 96.2%)
H influenzae type B meningitis	9.3<br>(3.1 to 14.8)	0.9<br>(0.1 to 1.6)	-45.8%<br>(-53.1% to -38.0%)	-48.6%<br>(-55.6% to -41.2%)
Meningococcal infection	8.7<br>(2.9 to 13.6)	0.8<br>(0.1 to 1.5)	7.8%<br>(-2.3% to 19.1%)	2.1%<br>(-7.5% to 12.8%)
**All causes**	**14,018.8<br>(12,015.6 to 16,090.2)**	**969.2<br>(653.6 to 1,357.1)**	**-0.0%<br>(-2.6% to 2.3%)**	**-5.3%<br>(-7.7% to -3.1%)**

SR Table 13e: Global prevalence and years lived with disability (YLDs) for 2016, percentage change of YLD counts and rates between 2006 and 2016 of hearing loss by cause, ages 5-9 years				
**Cause**	**Prevalence in thousands (95% uncertainty interval)**	**YLDs in thousands (95% uncertainty interval)**		
	**2016**	**2016**	**Percentage change in counts between 2006 and 2016**	**Percentage change in rates between 2006 and 2016**
Age-related and other hearing loss	12,024.2 (7,992.8 to 16,633.2)	663.1 (415.8 to 981.5)	3.4% (-0.6% to 7.4%)	-4.0% (-7.6% to -0.3%)
Otitis media	13,999.9 (11,307.5 to 17,410.7)	550.7 (329.7 to 866.1)	3.9% (0.5% to 6.9%)	-3.5% (-6.6% to -0.7%)
Other congenital anomalies	3,318.3 (2,345.3 to 4,695.9)	341.5 (208.5 to 535.6)	-3.2% (-8.4% to 1.8%)	-10.1% (-14.9% to -5.5%)
Pneumococcal meningitis	52.3 (21.8 to 79.8)	3.7 (0.5 to 6.7)	-22.7% (-29.4% to -15.3%)	-28.2% (-34.4% to -21.4%)
Other meningitis	46.4 (19.5 to 67.1)	3.2 (0.4 to 5.5)	85.4% (66.8% to 104.9%)	72.2% (54.9% to 90.3%)
H influenzae type B meningitis	21.0 (8.2 to 33.5)	1.5 (0.2 to 2.9)	-46.2% (-53.1% to -38.8%)	-50.1% (-56.5% to -43.2%)
Meningococcal infection	19.9 (8.0 to 30.3)	1.4 (0.2 to 2.6)	8.6% (-1.1% to 19.6%)	0.8% (-8.2% to 11.1%)
**All causes**	**29,482.0 (23,882.9 to 35,736.0)**	**1,565.2 (1,074.7 to 2,197.7)**	**2.0% (-0.4% to 4.2%)**	**-5.3% (-7.5% to -3.3%)**

SR Table 13e: Global prevalence and years lived with disability (YLDs) for 2016, percentage change of YLD counts and rates between 2006 and 2016 of hearing loss by cause, ages 10-14 years				
**Cause**	**Prevalence in thousands (95% uncertainty interval)**	**YLDs in thousands (95% uncertainty interval)**		
	**2016**	**2016**	**Percentage change in counts between 2006 and 2016**	**Percentage change in rates between 2006 and 2016**
Age-related and other hearing loss	16,310.2 (12,260.5 to 21,034.3)	838.5 (543.3 to 1,198.9)	-1.2% (-4.9% to 2.3%)	-2.0% (-5.7% to 1.5%)
Otitis media	10,987.5 (8,668.2 to 13,531.2)	384.6 (224.8 to 610.9)	-3.6% (-7.7% to -0.4%)	-4.3% (-8.5% to -1.2%)
Other congenital anomalies	3,166.6 (2,243.0 to 4,461.5)	323.1 (199.1 to 502.6)	-6.4% (-11.1% to -1.6%)	-7.1% (-11.8% to -2.4%)
Pneumococcal meningitis	73.4 (32.0 to 107.4)	4.7 (0.6 to 8.3)	-22.0% (-28.3% to -14.3%)	-22.7% (-28.9% to -15.0%)
Other meningitis	61.4 (26.1 to 85.6)	3.9 (0.5 to 6.7)	83.3% (66.8% to 101.4%)	81.7% (65.5% to 99.7%)
H influenzae type B meningitis	26.8 (11.1 to 42.2)	1.8 (0.2 to 3.3)	-46.1% (-52.8% to -38.8%)	-46.5% (-53.2% to -39.3%)
Meningococcal infection	25.5 (10.3 to 37.7)	1.7 (0.2 to 3.0)	8.6% (-0.0% to 19.3%)	7.7% (-0.8% to 18.3%)
**All causes**	**30,651.4 (25,736.3 to 36,155.0)**	**1,558.4 (1,066.5 to 2,158.9)**	**-2.9% (-5.4% to -0.8%)**	**-3.7% (-6.1% to -1.6%)**

SR Table 13e: Global prevalence and years lived with disability (YLDs) for 2016, percentage change of YLD counts and rates between 2006 and 2016 of hearing loss by cause, ages 15-19 years				
Cause	Prevalence in thousands (95% uncertainty interval)	YLDs in thousands (95% uncertainty interval)		
	2016	2016	Percentage change in counts between 2006 and 2016	Percentage change in rates between 2006 and 2016
Age-related and other hearing loss	19,607.8<br>(15,285.1 to 24,465.1)	939.0<br>(621.8 to 1,342.5)	-6.3%<br>(-9.9% to -3.1%)	-0.8%<br>(-4.7% to 2.6%)
Other congenital anomalies	3,026.2<br>(2,138.7 to 4,264.3)	304.1<br>(185.1 to 470.5)	-10.1%<br>(-14.6% to -5.5%)	-4.8%<br>(-9.6% to 0.1%)
Otitis media	8,901.3<br>(7,046.5 to 10,874.8)	289.4<br>(167.0 to 469.9)	-10.0%<br>(-13.7% to -6.6%)	-4.7%<br>(-8.7% to -1.1%)
Pneumococcal meningitis	88.6<br>(39.1 to 126.4)	5.2<br>(0.8 to 8.9)	-22.6%<br>(-28.3% to -15.2%)	-18.1%<br>(-24.1% to -10.2%)
Other meningitis	70.8<br>(30.8 to 97.0)	4.2<br>(0.6 to 7.0)	77.3%<br>(63.8% to 93.3%)	87.7%<br>(73.3% to 104.6%)
H influenzae type B meningitis	29.3<br>(12.8 to 45.1)	1.8<br>(0.3 to 3.3)	-47.0%<br>(-53.5% to -40.0%)	-43.9%<br>(-50.8% to -36.4%)
Meningococcal infection	28.4<br>(11.7 to 42.0)	1.8<br>(0.2 to 3.0)	6.1%<br>(-2.1% to 15.7%)	12.3%<br>(3.7% to 22.5%)
**All causes**	**31,752.5**<br>**(27,287.5 to 36,967.0)**	**1,545.5**<br>**(1,048.9 to 2,145.2)**	**-7.8%**<br>**(-10.2% to -5.7%)**	**-2.4%**<br>**(-4.9% to -0.1%)**

SR Table 13e: Global prevalence and years lived with disability (YLDs) for 2016, percentage change of YLD counts and rates between 2006 and 2016 of hearing loss by cause, ages 20-24 years				
**Cause**	**Prevalence in thousands (95% uncertainty interval)**	**YLDs in thousands (95% uncertainty interval)**		
	**2016**	**2016**	**Percentage change in counts between 2006 and 2016**	**Percentage change in rates between 2006 and 2016**
Age-related and other hearing loss	25,408.9<br>(18,796.3 to 32,528.2)	1,073.2<br>(715.0 to 1,553.5)	2.8%<br>(-1.0% to 6.4%)	-1.9%<br>(-5.6% to 1.5%)
Other congenital anomalies	2,979.1<br>(2,098.1 to 4,206.4)	295.4<br>(181.6 to 457.5)	-2.4%<br>(-7.1% to 2.4%)	-6.9%<br>(-11.4% to -2.3%)
Otitis media	7,481.9<br>(5,295.4 to 10,292.7)	222.4<br>(125.1 to 364.4)	-6.1%<br>(-10.3% to -1.6%)	-10.4%<br>(-14.5% to -6.2%)
Pneumococcal meningitis	103.9<br>(50.1 to 146.3)	5.5<br>(0.9 to 9.1)	-23.0%<br>(-29.0% to -16.3%)	-26.6%<br>(-32.2% to -20.2%)
Other meningitis	80.8<br>(39.5 to 110.2)	4.3<br>(0.7 to 7.1)	80.3%<br>(66.5% to 96.0%)	72.0%<br>(58.8% to 86.9%)
Meningococcal infection	32.6<br>(15.1 to 47.1)	1.8<br>(0.3 to 2.9)	4.6%<br>(-3.0% to 14.2%)	-0.2%<br>(-7.5% to 8.9%)
H influenzae type B meningitis	31.9<br>(15.4 to 47.0)	1.7<br>(0.3 to 3.0)	-48.0%<br>(-54.2% to -41.2%)	-50.4%<br>(-56.3% to -43.9%)
**All causes**	**36,119.1<br>(30,393.2 to 43,131.9)**	**1,604.2<br>(1,082.8 to 2,229.8)**	**0.4%<br>(-2.4% to 2.9%)**	**-4.2%<br>(-6.9% to -1.9%)**

SR Table 13e: Global prevalence and years lived with disability (YLDs) for 2016, percentage change of YLD counts and rates between 2006 and 2016 of hearing loss by cause, ages 25-29 years				
**Cause**	**Prevalence in thousands (95% uncertainty interval)**	**YLDs in thousands (95% uncertainty interval)**		
	**2016**	**2016**	**Percentage change in counts between 2006 and 2016**	**Percentage change in rates between 2006 and 2016**
Age-related and other hearing loss	37,276.9<br>(29,915.8 to 46,229.8)	1,291.9<br>(846.6 to 1,868.0)	14.9%<br>(11.0% to 18.6%)	-3.2%<br>(-6.5% to -0.1%)
Other congenital anomalies	2,959.8<br>(2,076.7 to 4,181.7)	289.1<br>(178.8 to 449.4)	8.7%<br>(3.6% to 13.9%)	-8.5%<br>(-12.7% to -4.0%)
Otitis media	5,196.8<br>(3,787.8 to 7,174.4)	153.9<br>(87.9 to 245.6)	4.4%<br>(-0.2% to 9.2%)	-12.1%<br>(-15.9% to -8.0%)
Pneumococcal meningitis	99.5<br>(50.1 to 138.0)	4.8<br>(0.8 to 8.1)	-18.4%<br>(-24.3% to -11.4%)	-31.2%<br>(-36.3% to -25.3%)
Other meningitis	75.6<br>(38.4 to 102.3)	3.6<br>(0.6 to 6.0)	88.0%<br>(73.1% to 103.4%)	58.3%<br>(45.8% to 71.4%)
Meningococcal infection	29.5<br>(14.2 to 42.5)	1.4<br>(0.3 to 2.4)	8.8%<br>(0.9% to 18.6%)	-8.3%<br>(-15.0% to -0.1%)
H influenzae type B meningitis	28.2<br>(14.2 to 41.3)	1.4<br>(0.2 to 2.4)	-46.1%<br>(-52.4% to -39.7%)	-54.6%<br>(-59.9% to -49.2%)
**All causes**	**45,666.3<br>(38,723.7 to 54,357.4)**	**1,746.2<br>(1,192.9 to 2,448.4)**	**12.7%<br>(9.6% to 15.4%)**	**-5.1%<br>(-7.7% to -2.8%)**

SR Table 13e: Global prevalence and years lived with disability (YLDs) for 2016, percentage change of YLD counts and rates between 2006 and 2016 of hearing loss by cause, ages 30-34 years				
**Cause**	**Prevalence in thousands (95% uncertainty interval)**	**YLDs in thousands (95% uncertainty interval)**		
	**2016**	**2016**	**Percentage change in counts between 2006 and 2016**	**Percentage change in rates between 2006 and 2016**
Age-related and other hearing loss	47,989.4 (38,545.3 to 60,363.6)	1,444.2 (936.2 to 2,104.5)	11.5% (7.7% to 15.1%)	-1.2% (-4.6% to 1.9%)
Other congenital anomalies	2,675.1 (1,880.3 to 3,785.8)	259.7 (161.0 to 399.4)	5.6% (0.9% to 10.8%)	-6.5% (-10.7% to -1.9%)
Otitis media	3,406.0 (2,289.1 to 4,864.8)	100.3 (55.8 to 165.5)	0.6% (-4.7% to 5.8%)	-10.9% (-15.6% to -6.3%)
Pneumococcal meningitis	91.8 (48.4 to 126.3)	4.1 (0.7 to 6.7)	-14.1% (-20.1% to -6.9%)	-23.9% (-29.2% to -17.5%)
Other meningitis	68.0 (36.5 to 91.8)	3.0 (0.5 to 5.1)	86.6% (73.8% to 101.1%)	65.2% (53.9% to 78.1%)
Meningococcal infection	26.0 (13.2 to 37.0)	1.2 (0.2 to 1.9)	12.7% (4.8% to 22.1%)	-0.2% (-7.2% to 8.1%)
H influenzae type B meningitis	24.4 (12.8 to 35.5)	1.1 (0.2 to 2.0)	-44.4% (-50.2% to -37.7%)	-50.7% (-55.9% to -44.9%)
**All causes**	**54,280.7 (44,708.1 to 65,719.7)**	**1,813.4 (1,231.6 to 2,568.0)**	**9.9% (6.9% to 12.7%)**	**-2.6% (-5.3% to -0.2%)**

SR Table 13e: Global prevalence and years lived with disability (YLDs) for 2016, percentage change of YLD counts and rates between 2006 and 2016 of hearing loss by cause, ages 35-39 years				
Cause	Prevalence in thousands (95% uncertainty interval)	YLDs in thousands (95% uncertainty interval)		
	2016	2016	Percentage change in counts between 2006 and 2016	Percentage change in rates between 2006 and 2016
Age-related and other hearing loss	59,845.0<br>(49,074.9 to 72,381.6)	1,527.6<br>(1,012.6 to 2,258.4)	6.0%<br>(2.9% to 8.7%)	0.2%<br>(-2.7% to 2.7%)
Other congenital anomalies	2,361.9<br>(1,661.7 to 3,336.1)	227.7<br>(141.5 to 351.3)	0.1%<br>(-4.4% to 5.3%)	-5.4%<br>(-9.6% to -0.4%)
Otitis media	2,936.6<br>(2,173.4 to 3,932.4)	86.1<br>(49.8 to 135.4)	-4.6%<br>(-9.1% to 0.2%)	-9.8%<br>(-14.1% to -5.2%)
Pneumococcal meningitis	77.4<br>(43.8 to 105.1)	3.1<br>(0.6 to 5.1)	-14.0%<br>(-19.8% to -6.8%)	-18.7%<br>(-24.2% to -11.9%)
Other meningitis	56.8<br>(32.2 to 75.6)	2.2<br>(0.5 to 3.7)	79.2%<br>(67.5% to 92.6%)	69.4%<br>(58.3% to 82.1%)
Meningococcal infection	21.4<br>(11.7 to 29.7)	0.8<br>(0.2 to 1.4)	12.9%<br>(5.6% to 22.4%)	6.7%<br>(-0.2% to 15.7%)
H influenzae type B meningitis	19.6<br>(10.9 to 28.2)	0.8<br>(0.2 to 1.4)	-45.0%<br>(-51.2% to -38.3%)	-48.0%<br>(-53.9% to -41.7%)
**All causes**	**65,318.8<br>(54,365.7 to 77,448.1)**	**1,848.4<br>(1,240.8 to 2,667.9)**	**4.7%<br>(2.1% to 6.9%)**	**-1.1%<br>(-3.5% to 1.1%)**

SR Table 13e: Global prevalence and years lived with disability (YLDs) for 2016, percentage change of YLD counts and rates between 2006 and 2016 of hearing loss by cause, ages 40-44 years				
**Cause**	**Prevalence in thousands (95% uncertainty interval)**	**YLDs in thousands (95% uncertainty interval)**		
	**2016**	**2016**	**Percentage change in counts between 2006 and 2016**	**Percentage change in rates between 2006 and 2016**
Age-related and other hearing loss	76,577.7<br>(61,565.5 to 93,710.6)	1,738.1<br>(1,133.7 to 2,609.1)	12.7%<br>(9.6% to 15.4%)	-1.0%<br>(-3.8% to 1.3%)
Other congenital anomalies	2,217.6<br>(1,556.5 to 3,116.9)	211.4<br>(133.1 to 323.8)	6.7%<br>(1.8% to 12.2%)	-6.3%<br>(-10.6% to -1.5%)
Otitis media	3,258.5<br>(2,189.1 to 4,578.3)	94.9<br>(53.8 to 160.5)	2.4%<br>(-2.5% to 7.6%)	-10.0%<br>(-14.3% to -5.5%)
Pneumococcal meningitis	66.1<br>(39.2 to 89.0)	2.4<br>(0.5 to 4.0)	-14.4%<br>(-20.0% to -7.0%)	-24.8%<br>(-29.7% to -18.4%)
Other meningitis	48.5<br>(28.9 to 64.8)	1.8<br>(0.4 to 2.9)	77.0%<br>(64.9% to 90.3%)	55.4%<br>(44.8% to 67.2%)
Meningococcal infection	17.7<br>(10.2 to 24.7)	0.6<br>(0.1 to 1.1)	10.1%<br>(3.0% to 19.4%)	-3.3%<br>(-9.6% to 4.9%)
H influenzae type B meningitis	16.0<br>(9.2 to 23.0)	0.6<br>(0.1 to 1.1)	-46.2%<br>(-52.0% to -40.2%)	-52.8%<br>(-57.8% to -47.5%)
**All causes**	**82,202.1<br>(67,058.3 to 99,255.0)**	**2,049.8<br>(1,360.1 to 2,984.5)**	**11.5%<br>(8.8% to 13.8%)**	**-2.1%<br>(-4.4% to -0.0%)**

SR Table 13e: Global prevalence and years lived with disability (YLDs) for 2016, percentage change of YLD counts and rates between 2006 and 2016 of hearing loss by cause, ages 45-49 years				
**Cause**	**Prevalence in thousands (95% uncertainty interval)**	**YLDs in thousands (95% uncertainty interval)**		
	**2016**	**2016**	**Percentage change in counts between 2006 and 2016**	**Percentage change in rates between 2006 and 2016**
Age-related and other hearing loss	97,763.0<br>(81,418.0 to 115,473.4)	2,189.5<br>(1,425.0 to 3,291.7)	22.4%<br>(18.9% to 25.3%)	-1.9%<br>(-4.7% to 0.5%)
Other congenital anomalies	2,062.4<br>(1,445.3 to 2,888.1)	193.6<br>(122.8 to 297.5)	17.0%<br>(11.5% to 23.4%)	-6.2%<br>(-10.6% to -1.1%)
Otitis media	3,950.0<br>(2,938.9 to 5,134.4)	114.4<br>(67.3 to 181.1)	12.7%<br>(7.6% to 18.0%)	-9.6%<br>(-13.8% to -5.4%)
Pneumococcal meningitis	59.7<br>(35.1 to 80.0)	2.2<br>(0.5 to 3.5)	-12.6%<br>(-18.1% to -5.9%)	-29.9%<br>(-34.3% to -24.5%)
Other meningitis	44.0<br>(26.2 to 58.1)	1.6<br>(0.4 to 2.5)	78.7%<br>(67.1% to 92.3%)	43.3%<br>(34.0% to 54.2%)
Meningococcal infection	15.4<br>(8.9 to 21.3)	0.6<br>(0.1 to 0.9)	8.7%<br>(1.3% to 17.1%)	-12.8%<br>(-18.7% to -6.1%)
H influenzae type B meningitis	13.8<br>(8.0 to 19.7)	0.5<br>(0.1 to 0.9)	-46.7%<br>(-52.3% to -40.6%)	-57.3%<br>(-61.8% to -52.3%)
**All causes**	**103,908.2**<br>**(87,224.5 to 121,504.6)**	**2,502.3**<br>**(1,661.8 to 3,670.6)**	**21.4%**<br>**(18.4% to 24.0%)**	**-2.6%**<br>**(-5.0% to -0.5%)**

SR Table 13e: Global prevalence and years lived with disability (YLDs) for 2016, percentage change of YLD counts and rates between 2006 and 2016 of hearing loss by cause, ages 50-54 years				
**Cause**	**Prevalence in thousands (95% uncertainty interval)**	**YLDs in thousands (95% uncertainty interval)**		
	**2016**	**2016**	**Percentage change in counts between 2006 and 2016**	**Percentage change in rates between 2006 and 2016**
Age-related and other hearing loss	119,143.0 (102,229.9 to 138,158.8)	2,661.4 (1,737.8 to 3,959.2)	22.0% (18.5% to 24.9%)	-2.0% (-4.8% to 0.3%)
Other congenital anomalies	1,795.3 (1,256.7 to 2,517.1)	166.4 (104.3 to 254.9)	17.5% (11.9% to 23.4%)	-5.7% (-10.1% to -0.9%)
Otitis media	4,114.6 (2,849.0 to 5,567.8)	118.3 (68.0 to 184.4)	12.3% (7.1% to 17.8%)	-9.8% (-14.0% to -5.4%)
Pneumococcal meningitis	54.2 (31.6 to 72.2)	1.9 (0.4 to 3.1)	-8.4% (-13.6% to -1.7%)	-26.5% (-30.7% to -21.0%)
Other meningitis	39.8 (23.6 to 52.4)	1.4 (0.3 to 2.2)	78.0% (67.3% to 90.9%)	43.0% (34.3% to 53.3%)
Meningococcal infection	13.8 (7.9 to 19.3)	0.5 (0.1 to 0.8)	11.8% (4.8% to 20.8%)	-10.2% (-15.9% to -3.0%)
H influenzae type B meningitis	12.0 (7.1 to 17.1)	0.4 (0.1 to 0.8)	-45.8% (-51.3% to -39.5%)	-56.5% (-60.9% to -51.5%)
**All causes**	**125,172.7 (107,940.2 to 144,066.3)**	**2,950.4 (1,930.2 to 4,338.3)**	**21.3% (18.2% to 23.8%)**	**-2.6% (-5.1% to -0.6%)**

SR Table 13e: Global prevalence and years lived with disability (YLDs) for 2016, percentage change of YLD counts and rates between 2006 and 2016 of hearing loss by cause, ages 55-59 years				
**Cause**	**Prevalence in thousands (95% uncertainty interval)**	**YLDs in thousands (95% uncertainty interval)**		
	**2016**	**2016**	**Percentage change in counts between 2006 and 2016**	**Percentage change in rates between 2006 and 2016**
Age-related and other hearing loss	137,270.3 (122,671.9 to 150,884.8)	3,057.7 (1,999.7 to 4,571.5)	28.7% (25.5% to 31.5%)	-1.4% (-3.8% to 0.8%)
Other congenital anomalies	1,478.4 (1,034.0 to 2,072.0)	135.8 (85.3 to 208.5)	22.2% (16.4% to 28.1%)	-6.3% (-10.8% to -1.8%)
Otitis media	3,077.7 (2,267.0 to 4,069.5)	87.7 (51.9 to 138.6)	17.4% (11.9% to 22.9%)	-10.0% (-14.2% to -5.8%)
Pneumococcal meningitis	47.7 (27.8 to 61.7)	1.7 (0.4 to 2.7)	-3.7% (-10.0% to 3.8%)	-26.2% (-31.0% to -20.4%)
Other meningitis	35.0 (20.5 to 45.5)	1.2 (0.3 to 1.9)	84.2% (72.9% to 96.7%)	41.2% (32.5% to 50.8%)
Meningococcal infection	12.1 (7.0 to 16.5)	0.4 (0.1 to 0.7)	16.3% (9.1% to 26.5%)	-10.9% (-16.4% to -3.1%)
H influenzae type B meningitis	10.2 (6.0 to 14.4)	0.4 (0.1 to 0.6)	-43.2% (-49.0% to -36.7%)	-56.5% (-60.9% to -51.5%)
**All causes**	**141,931.3 (127,223.5 to 155,647.8)**	**3,285.0 (2,151.5 to 4,852.5)**	**28.1% (25.1% to 30.7%)**	**-1.9% (-4.1% to 0.2%)**

SR Table 13e: Global prevalence and years lived with disability (YLDs) for 2016, percentage change of YLD counts and rates between 2006 and 2016 of hearing loss by cause, ages 60-64 years				
Cause	Prevalence in thousands (95% uncertainty interval)	YLDs in thousands (95% uncertainty interval)		
	2016	2016	Percentage change in counts between 2006 and 2016	Percentage change in rates between 2006 and 2016
Age-related and other hearing loss	151,702.8<br>(139,765.3 to 162,110.6)	3,514.3<br>(2,297.2 to 5,129.6)	46.2%<br>(42.1% to 49.6%)	-3.0%<br>(-5.7% to -0.8%)
Other congenital anomalies	1,249.0<br>(871.8 to 1,748.2)	113.0<br>(71.4 to 172.5)	34.8%<br>(29.1% to 41.3%)	-10.6%<br>(-14.4% to -6.3%)
Otitis media	2,109.1<br>(1,409.8 to 2,922.2)	59.6<br>(32.8 to 99.0)	32.6%<br>(26.5% to 39.5%)	-12.0%<br>(-16.1% to -7.4%)
Pneumococcal meningitis	41.6<br>(23.4 to 53.7)	1.5<br>(0.3 to 2.4)	-2.1%<br>(-8.4% to 5.3%)	-35.0%<br>(-39.3% to -30.2%)
Other meningitis	30.9<br>(17.5 to 39.8)	1.1<br>(0.2 to 1.8)	85.5%<br>(73.8% to 98.6%)	23.1%<br>(15.3% to 31.8%)
Meningococcal infection	10.5<br>(5.9 to 14.3)	0.4<br>(0.1 to 0.6)	18.3%<br>(10.5% to 28.1%)	-21.5%<br>(-26.7% to -15.1%)
H influenzae type B meningitis	8.7<br>(4.9 to 12.2)	0.3<br>(0.1 to 0.5)	-43.1%<br>(-48.6% to -36.8%)	-62.2%<br>(-65.9% to -58.1%)
**All causes**	**155,152.6**<br>**(143,200.7 to 165,592.4)**	**3,690.2**<br>**(2,433.1 to 5,365.9)**	**45.6%**<br>**(41.6% to 48.8%)**	**-3.4%**<br>**(-6.1% to -1.3%)**

SR Table 13e: Global prevalence and years lived with disability (YLDs) for 2016, percentage change of YLD counts and rates between 2006 and 2016 of hearing loss by cause, ages 65-69 years				
**Cause**	**Prevalence in thousands (95% uncertainty interval)**	**YLDs in thousands (95% uncertainty interval)**		
	**2016**	**2016**	**Percentage change in counts between 2006 and 2016**	**Percentage change in rates between 2006 and 2016**
Age-related and other hearing loss	140,789.6<br>(134,674.2 to 146,338.0)	3,575.2<br>(2,372.0 to 5,152.1)	27.9%<br>(24.6% to 31.3%)	-3.5%<br>(-6.0% to -0.9%)
Other congenital anomalies	911.8<br>(634.1 to 1,278.3)	81.5<br>(51.5 to 124.9)	19.6%<br>(14.0% to 25.7%)	-9.7%<br>(-14.0% to -5.1%)
Otitis media	1,266.2<br>(912.0 to 1,677.0)	35.3<br>(21.0 to 56.8)	18.3%<br>(12.6% to 24.5%)	-10.7%<br>(-15.0% to -6.0%)
Pneumococcal meningitis	33.9<br>(17.8 to 43.5)	1.3<br>(0.3 to 2.1)	-9.7%<br>(-15.2% to -3.3%)	-31.9%<br>(-36.0% to -27.0%)
Other meningitis	25.9<br>(13.8 to 32.9)	1.0<br>(0.2 to 1.6)	68.9%<br>(58.7% to 79.9%)	27.4%<br>(19.8% to 35.8%)
Meningococcal infection	8.7<br>(4.6 to 11.7)	0.3<br>(0.1 to 0.5)	8.9%<br>(2.8% to 16.1%)	-17.8%<br>(-22.4% to -12.4%)
H influenzae type B meningitis	6.9<br>(3.7 to 9.6)	0.3<br>(0.1 to 0.5)	-47.6%<br>(-52.7% to -42.0%)	-60.4%<br>(-64.3% to -56.2%)
**All causes**	**143,042.9<br>(136,910.1 to 148,685.3)**	**3,694.9<br>(2,480.1 to 5,305.6)**	**27.6%<br>(24.3% to 30.9%)**	**-3.7%<br>(-6.2% to -1.2%)**

SR Table 13e: Global prevalence and years lived with disability (YLDs) for 2016, percentage change of YLD counts and rates between 2006 and 2016 of hearing loss by cause, ages 70-74 years				
**Cause**	**Prevalence in thousands (95% uncertainty interval)**	**YLDs in thousands (95% uncertainty interval)**		
	**2016**	**2016**	**Percentage change in counts between 2006 and 2016**	**Percentage change in rates between 2006 and 2016**
Age-related and other hearing loss	116,630.9 (112,964.3 to 119,989.2)	3,414.8 (2,376.2 to 4,765.3)	17.2% (14.2% to 20.0%)	-2.1% (-4.6% to 0.3%)
Other congenital anomalies	631.2 (438.2 to 885.7)	55.5 (35.0 to 84.2)	11.5% (6.4% to 16.9%)	-6.8% (-11.0% to -2.3%)
Otitis media	764.9 (515.1 to 1,071.6)	21.1 (11.8 to 34.9)	8.9% (3.4% to 14.8%)	-9.0% (-13.6% to -4.0%)
Pneumococcal meningitis	26.9 (13.3 to 34.3)	1.2 (0.2 to 1.8)	-11.6% (-17.6% to -4.6%)	-26.1% (-31.1% to -20.2%)
Other meningitis	20.9 (10.4 to 26.5)	0.9 (0.2 to 1.4)	59.1% (49.0% to 69.9%)	33.0% (24.5% to 42.0%)
Meningococcal infection	7.0 (3.4 to 9.3)	0.3 (0.1 to 0.5)	6.9% (0.8% to 14.1%)	-10.6% (-15.7% to -4.6%)
H influenzae type B meningitis	5.4 (2.7 to 7.4)	0.2 (<0.05 to 0.4)	-48.5% (-53.5% to -43.0%)	-57.0% (-61.2% to -52.4%)
**All causes**	**118,087.2 (114,450.9 to 121,458.9)**	**3,494.0 (2,448.9 to 4,868.2)**	**17.0% (14.1% to 19.7%)**	**-2.2% (-4.6% to 0.1%)**

SR Table 13e: Global prevalence and years lived with disability (YLDs) for 2016, percentage change of YLD counts and rates between 2006 and 2016 of hearing loss by cause, ages 75-79 years				
**Cause**	**Prevalence in thousands (95% uncertainty interval)**	**YLDs in thousands (95% uncertainty interval)**		
	**2016**	**2016**	**Percentage change in counts between 2006 and 2016**	**Percentage change in rates between 2006 and 2016**
Age-related and other hearing loss	95,820.4<br>(93,893.6 to 97,633.7)	3,288.2<br>(2,328.3 to 4,450.3)	23.1%<br>(19.7% to 26.9%)	-1.0%<br>(-3.8% to 2.0%)
Other congenital anomalies	447.0<br>(309.1 to 628.3)	38.4<br>(24.4 to 58.3)	17.9%<br>(12.5% to 24.0%)	-5.2%<br>(-9.5% to -0.3%)
Otitis media	556.6<br>(402.3 to 730.5)	15.1<br>(9.1 to 23.8)	14.5%<br>(8.7% to 20.8%)	-7.9%<br>(-12.6% to -2.9%)
Pneumococcal meningitis	20.6<br>(9.4 to 25.9)	1.0<br>(0.2 to 1.5)	-5.8%<br>(-12.7% to 2.0%)	-24.2%<br>(-29.8% to -18.0%)
Other meningitis	16.2<br>(7.5 to 20.1)	0.8<br>(0.1 to 1.2)	65.8%<br>(55.0% to 77.3%)	33.3%<br>(24.6% to 42.6%)
Meningococcal infection	5.5<br>(2.5 to 7.1)	0.3<br>(<0.05 to 0.4)	12.0%<br>(5.8% to 18.7%)	-9.9%<br>(-14.9% to -4.5%)
H influenzae type B meningitis	4.0<br>(1.8 to 5.4)	0.2<br>(<0.05 to 0.3)	-44.9%<br>(-50.3% to -39.3%)	-55.7%<br>(-60.0% to -51.2%)
**All causes**	**96,870.3**<br>**(95,023.7 to 98,655.8)**	**3,343.9**<br>**(2,376.7 to 4,516.6)**	**23.0%**<br>**(19.6% to 26.7%)**	**-1.1%**<br>**(-3.8% to 1.9%)**

SR Table 13e: Global prevalence and years lived with disability (YLDs) for 2016, percentage change of YLD counts and rates between 2006 and 2016 of hearing loss by cause, ages 80-84				
**Cause**	**Prevalence in thousands (95% uncertainty interval)**	**YLDs in thousands (95% uncertainty interval)**		
	**2016**	**2016**	**Percentage change in counts between 2006 and 2016**	**Percentage change in rates between 2006 and 2016**
Age-related and other hearing loss	63,451.7 (62,326.0 to 64,381.4)	2,491.2 (1,783.3 to 3,312.2)	30.7% (26.3% to 34.8%)	-0.2% (-3.6% to 2.9%)
Other congenital anomalies	264.6 (182.6 to 371.9)	22.5 (14.4 to 34.4)	26.3% (20.5% to 32.6%)	-3.6% (-8.0% to 1.3%)
Otitis media	363.4 (249.1 to 516.9)	9.7 (5.5 to 16.3)	22.0% (15.8% to 28.8%)	-6.8% (-11.6% to -1.6%)
Pneumococcal meningitis	13.2 (5.8 to 16.6)	0.7 (0.1 to 1.1)	2.9% (-3.7% to 10.6%)	-21.4% (-26.5% to -15.6%)
Other meningitis	10.4 (4.6 to 12.8)	0.5 (0.1 to 0.8)	70.9% (59.8% to 82.6%)	30.5% (22.0% to 39.4%)
Meningococcal infection	3.3 (1.5 to 4.2)	0.2 (<0.05 to 0.3)	19.4% (12.2% to 27.0%)	-8.9% (-14.3% to -3.0%)
H influenzae type B meningitis	2.4 (1.0 to 3.2)	0.1 (<0.05 to 0.2)	-40.7% (-46.5% to -34.1%)	-54.7% (-59.2% to -49.7%)
**All causes**	**64,108.9 (62,995.5 to 64,999.0)**	**2,524.9 (1,810.3 to 3,345.7)**	**30.6% (26.3% to 34.6%)**	**-0.3% (-3.6% to 2.8%)**

SR Table 13e: Global prevalence and years lived with disability (YLDs) for 2016, percentage change of YLD counts and rates between 2006 and 2016 of hearing loss by cause, ages 85-89				
**Cause**	**Prevalence in thousands (95% uncertainty interval)**	**YLDs in thousands (95% uncertainty interval)**		
	**2016**	**2016**	**Percentage change in counts between 2006 and 2016**	**Percentage change in rates between 2006 and 2016**
Age-related and other hearing loss	35,574.4 (35,135.0 to 35,962.3)	1,568.4 (1,134.7 to 2,077.8)	60.4% (54.8% to 66.2%)	-0.1% (-3.5% to 3.5%)
Other congenital anomalies	132.3 (90.9 to 187.9)	11.1 (7.0 to 17.2)	50.8% (44.0% to 57.7%)	-6.0% (-10.3% to -1.7%)
Otitis media	214.9 (157.9 to 288.8)	5.6 (3.3 to 8.9)	49.0% (41.3% to 57.5%)	-7.2% (-12.0% to -1.9%)
Pneumococcal meningitis	7.0 (2.9 to 8.7)	0.4 (0.1 to 0.6)	17.6% (9.9% to 25.7%)	-26.7% (-31.5% to -21.7%)
Other meningitis	5.4 (2.3 to 6.6)	0.3 (<0.05 to 0.5)	88.0% (76.4% to 101.1%)	17.2% (9.9% to 25.3%)
Meningococcal infection	1.8 (0.8 to 2.2)	0.1 (<0.05 to 0.2)	38.8% (30.4% to 47.4%)	-13.5% (-18.7% to -8.2%)
H influenzae type B meningitis	1.2 (0.5 to 1.6)	0.1 (<0.05 to 0.1)	-33.0% (-39.1% to -26.2%)	-58.3% (-62.1% to -54.0%)
**All causes**	**35,936.9 (35,510.5 to 36,314.7)**	**1,586.0 (1,151.6 to 2,098.3)**	**60.2% (54.7% to 66.0%)**	**-0.2% (-3.6% to 3.4%)**

SR Table 13e: Global prevalence and years lived with disability (YLDs) for 2016, percentage change of YLD counts and rates between 2006 and 2016 of hearing loss by cause, ages 90-94				
Cause	Prevalence in thousands (95% uncertainty interval)	YLDs in thousands (95% uncertainty interval)		
	2016	2016	Percentage change in counts between 2006 and 2016	Percentage change in rates between 2006 and 2016
Age-related and other hearing loss	13,091.3<br>(12,906.7 to 13,262.2)	637.8<br>(460.7 to 834.0)	66.8%<br>(60.8% to 72.9%)	-0.3%<br>(-3.9% to 3.3%)
Other congenital anomalies	43.7<br>(29.9 to 62.1)	3.6<br>(2.3 to 5.6)	57.3%<br>(49.3% to 65.0%)	-6.0%<br>(-10.8% to -1.4%)
Otitis media	83.8<br>(57.1 to 114.4)	2.2<br>(1.2 to 3.4)	57.3%<br>(47.9% to 67.1%)	-6.0%<br>(-11.6% to -0.2%)
Pneumococcal meningitis	2.5<br>(1.0 to 3.1)	0.1<br>(<0.05 to 0.2)	26.0%<br>(18.3% to 34.6%)	-24.7%<br>(-29.3% to -19.6%)
Other meningitis	1.9<br>(0.8 to 2.2)	0.1<br>(<0.05 to 0.2)	90.3%<br>(78.2% to 102.6%)	13.7%<br>(6.5% to 21.1%)
Meningococcal infection	0.6<br>(0.3 to 0.8)	<0.05<br>(<0.05 to 0.1)	44.0%<br>(36.1% to 52.2%)	-13.9%<br>(-18.7% to -9.1%)
H influenzae type B meningitis	0.4<br>(0.2 to 0.5)	<0.05<br>(<0.05 to <0.05)	-28.8%<br>(-35.1% to -21.3%)	-57.5%<br>(-61.2% to -53.0%)
**All causes**	**13,224.2**<br>**(13,047.7 to 13,397.6)**	**643.9**<br>**(465.1 to 840.2)**	**66.7%**<br>**(60.8% to 72.8%)**	**-0.4%**<br>**(-4.0% to 3.2%)**

SR Table 13e: Global prevalence and years lived with disability (YLDs) for 2016, percentage change of YLD counts and rates between 2006 and 2016 of hearing loss by cause, ages 95+ years				
Cause	Prevalence in thousands (95% uncertainty interval)	YLDs in thousands (95% uncertainty interval)		
	2016	2016	Percentage change in counts between 2006 and 2016	Percentage change in rates between 2006 and 2016
Age-related and other hearing loss	2,093.2 (1,954.0 to 2,221.4)	83.7 (59.4 to 113.1)	57.2% (52.7% to 62.0%)	0.3% (-2.5% to 3.4%)
Other congenital anomalies	8.8 (5.9 to 12.7)	0.7 (0.5 to 1.1)	51.7% (43.8% to 60.0%)	-3.2% (-8.3% to 2.1%)
Otitis media	18.7 (12.6 to 26.7)	0.5 (0.3 to 0.8)	51.1% (42.8% to 60.0%)	-3.6% (-8.9% to 2.1%)
Pneumococcal meningitis	0.6 (0.2 to 0.7)	<0.05 (<0.05 to <0.05)	33.1% (25.7% to 41.4%)	-15.1% (-19.8% to -9.8%)
Other meningitis	0.4 (0.2 to 0.5)	<0.05 (<0.05 to <0.05)	87.0% (77.0% to 97.8%)	19.3% (13.0% to 26.2%)
Meningococcal infection	0.1 (0.1 to 0.2)	<0.05 (<0.05 to <0.05)	45.0% (38.2% to 52.5%)	-7.5% (-11.8% to -2.7%)
H influenzae type B meningitis	0.1 (<0.05 to 0.1)	<0.05 (<0.05 to <0.05)	-24.2% (-30.3% to -17.1%)	-51.6% (-55.5% to -47.1%)
**All causes**	**2,121.9 (1,983.5 to 2,252.2)**	**85.0 (60.4 to 114.8)**	**57.1% (52.7% to 61.8%)**	**0.3% (-2.6% to 3.2%)**

SR Table 13f: Global prevalence and years lived with disability (YLDs) for 2016, percentage change of YLD counts and rates between 2006 and 2016 of heart failure by cause, ages 0-6 days				
**Cause**	**Prevalence in thousands (95% uncertainty interval)**	**YLDs in thousands (95% uncertainty interval)**		
	**2016**	**2016**	**Percentage change in counts between 2006 and 2016**	**Percentage change in rates between 2006 and 2016**
Congenital heart anomalies	0.1 (<0.05 to 0.2)	<0.05 (<0.05 to 0.1)	-14.7% (-24.9% to -8.1%)	-14.3% (-24.5% to -7.6%)
Zika virus disease	<0.05 (<0.05 to 0.1)	<0.05 (<0.05 to <0.05)	--	--
Endocarditis	<0.05 (<0.05 to <0.05)	<0.05 (<0.05 to <0.05)	11.4% (8.4% to 14.9%)	12.0% (8.9% to 15.5%)
Endocrine, metabolic, blood, and immune disorders	<0.05 (<0.05 to <0.05)	<0.05 (<0.05 to <0.05)	1.0% (-0.8% to 2.8%)	1.6% (-0.3% to 3.3%)
Iron-deficiency anaemia	<0.05 (<0.05 to <0.05)	<0.05 (<0.05 to <0.05)	-2.0% (-15.6% to 13.1%)	-1.5% (-15.2% to 13.7%)
**All causes**	**0.2 (0.1 to 0.3)**	**0.1 (<0.05 to 0.1)**	**64.6% (25.0% to 238.8%)**	**65.5% (25.6% to 240.6%)**

SR Table 13f: Global prevalence and years lived with disability (YLDs) for 2016, percentage change of YLD counts and rates between 2006 and 2016 of heart failure by cause, ages 7-27 days				
**Cause**	**Prevalence in thousands (95% uncertainty interval)**	**YLDs in thousands (95% uncertainty interval)**		
	**2016**	**2016**	**Percentage change in counts between 2006 and 2016**	**Percentage change in rates between 2006 and 2016**
Congenital heart anomalies	0.5<br>(0.2 to 0.7)	0.1<br>(<0.05 to 0.2)	-13.3%<br>(-22.5% to -6.6%)	-13.1%<br>(-22.3% to -6.5%)
Zika virus disease	0.1<br>(0.1 to 0.3)	0.1<br>(<0.05 to 0.1)	--	--
Endocarditis	<0.05<br>(<0.05 to <0.05)	<0.05<br>(<0.05 to <0.05)	11.1%<br>(8.0% to 14.3%)	11.3%<br>(8.2% to 14.5%)
Endocrine, metabolic, blood, and immune disorders	<0.05<br>(<0.05 to <0.05)	<0.05<br>(<0.05 to <0.05)	2.2%<br>(0.7% to 3.5%)	2.3%<br>(0.9% to 3.7%)
Iron-deficiency anaemia	<0.05<br>(<0.05 to <0.05)	<0.05<br>(<0.05 to <0.05)	-1.5%<br>(-14.9% to 13.0%)	-1.3%<br>(-14.7% to 13.2%)
**All causes**	**0.6<br>(0.3 to 0.8)**	**0.2<br>(0.1 to 0.3)**	**65.7%<br>(26.5% to 223.7%)**	**66.0%<br>(26.7% to 224.2%)**

SR Table 13f: Global prevalence and years lived with disability (YLDs) for 2016, percentage change of YLD counts and rates between 2006 and 2016 of heart failure by cause, ages 28-364 days				
**Cause**	**Prevalence in thousands (95% uncertainty interval)**	**YLDs in thousands (95% uncertainty interval)**		
	**2016**	**2016**	**Percentage change in counts between 2006 and 2016**	**Percentage change in rates between 2006 and 2016**
Congenital heart anomalies	10.4<br>(6.9 to 13.5)	1.7<br>(0.9 to 2.7)	-5.9%<br>(-11.0% to -1.6%)	-6.6%<br>(-11.8% to -2.4%)
Zika virus disease	2.2<br>(1.5 to 4.7)	1.3<br>(0.8 to 2.3)	--	--
Endocarditis	0.3<br>(0.2 to 0.5)	<0.05<br>(<0.05 to <0.05)	1.7%<br>(-0.1% to 3.3%)	0.9%<br>(-0.9% to 2.5%)
Endocrine, metabolic, blood, and immune disorders	<0.05<br>(<0.05 to 0.1)	<0.05<br>(<0.05 to <0.05)	3.7%<br>(2.4% to 4.8%)	2.9%<br>(1.6% to 4.0%)
Chagas disease	<0.05<br>(<0.05 to <0.05)	<0.05<br>(<0.05 to <0.05)	-18.3%<br>(-23.4% to -11.5%)	-19.0%<br>(-24.0% to -12.2%)
Iron-deficiency anaemia	<0.05<br>(<0.05 to <0.05)	<0.05<br>(<0.05 to <0.05)	2.3%<br>(-9.1% to 14.6%)	1.5%<br>(-9.8% to 13.7%)
**All causes**	**13.0**<br>**(9.2 to 16.5)**	**3.1**<br>**(2.0 to 4.4)**	**63.0%**<br>**(30.2% to 144.5%)**	**61.7%**<br>**(29.2% to 142.5%)**

SR Table 13f: Global prevalence and years lived with disability (YLDs) for 2016, percentage change of YLD counts and rates between 2006 and 2016 of heart failure by cause, ages 1-4 years				
**Cause**	**Prevalence in thousands (95% uncertainty interval)**	**YLDs in thousands (95% uncertainty interval)**		
	**2016**	**2016**	**Percentage change in counts between 2006 and 2016**	**Percentage change in rates between 2006 and 2016**
Congenital heart anomalies	48.8 (36.7 to 60.9)	7.5 (4.6 to 10.7)	2.5% (0.3% to 4.6%)	-2.9% (-5.0% to -0.9%)
Other cardiomyopathy	45.2 (31.5 to 62.4)	4.1 (2.4 to 6.3)	8.0% (5.8% to 10.2%)	2.3% (0.2% to 4.4%)
Myocarditis	12.1 (8.5 to 16.7)	1.1 (0.6 to 1.7)	5.8% (4.2% to 7.4%)	0.2% (-1.3% to 1.8%)
Other cardiovascular and circulatory diseases	11.7 (8.4 to 15.8)	1.0 (0.6 to 1.6)	14.2% (12.3% to 16.1%)	8.2% (6.3% to 10.0%)
Endocarditis	6.2 (4.4 to 8.3)	0.6 (0.3 to 0.9)	10.5% (8.6% to 12.3%)	4.6% (2.8% to 6.4%)
Zika virus disease	0.4 (0.3 to 0.7)	0.3 (0.1 to 0.5)	--	--
Thalassemias	1.5 (1.1 to 2.0)	0.1 (0.1 to 0.2)	10.9% (6.5% to 15.2%)	5.1% (0.9% to 9.1%)
Endocrine, metabolic, blood, and immune disorders	1.1 (0.8 to 1.5)	0.1 (0.1 to 0.2)	4.4% (3.3% to 5.5%)	-1.1% (-2.2% to -0.1%)
Ischaemic heart disease	0.2 (0.1 to 0.4)	<0.05 (<0.05 to <0.05)	26.3% (15.0% to 34.2%)	19.7% (8.9% to 27.1%)
Hypertensive heart disease	0.1 (<0.05 to 0.1)	<0.05 (<0.05 to <0.05)	24.2% (18.6% to 30.4%)	17.7% (12.3% to 23.6%)
Other haemoglobinopathies and haemolytic anaemias	<0.05 (<0.05 to <0.05)	<0.05 (<0.05 to <0.05)	19.8% (14.6% to 25.0%)	13.5% (8.5% to 18.4%)
Iron-deficiency anaemia	<0.05 (<0.05 to <0.05)	<0.05 (<0.05 to <0.05)	8.3% (4.6% to 11.3%)	2.6% (-0.9% to 5.5%)
Rheumatic heart disease	<0.05 (<0.05 to <0.05)	<0.05 (<0.05 to <0.05)	15.1% (6.6% to 22.6%)	9.1% (1.0% to 16.1%)
Chagas disease	<0.05 (<0.05 to <0.05)	<0.05 (<0.05 to <0.05)	-21.9% (-26.6% to -17.6%)	-26.0% (-30.4% to -22.0%)
Interstitial lung disease and pulmonary sarcoidosis	<0.05 (<0.05 to <0.05)	<0.05 (<0.05 to <0.05)	9.5% (-2.0% to 22.8%)	3.7% (-7.2% to 16.4%)
Chronic obstructive pulmonary disease	<0.05 (<0.05 to <0.05)	<0.05 (<0.05 to <0.05)	10.7% (-0.6% to 24.2%)	4.8% (-5.8% to 17.6%)
**All causes**	**127.5 (103.0 to 156.7)**	**14.7 (9.5 to 21.1)**	**7.3% (5.5% to 9.6%)**	**1.7% (-0.1% to 3.8%)**

SR Table 13f: Global prevalence and years lived with disability (YLDs) for 2016, percentage change of YLD counts and rates between 2006 and 2016 of heart failure by cause, ages 5-9 years				
**Cause**	**Prevalence in thousands (95% uncertainty interval)**	**YLDs in thousands (95% uncertainty interval)**		
	**2016**	**2016**	**Percentage change in counts between 2006 and 2016**	**Percentage change in rates between 2006 and 2016**
Myocarditis	65.8<br>(43.5 to 95.3)	5.9<br>(3.4 to 9.8)	8.3%<br>(6.1% to 10.4%)	0.6%<br>(-1.4% to 2.6%)
Other cardiomyopathy	30.3<br>(20.1 to 43.8)	2.7<br>(1.6 to 4.5)	9.0%<br>(7.4% to 10.4%)	1.2%<br>(-0.3% to 2.5%)
Congenital heart anomalies	19.1<br>(13.1 to 26.2)	2.5<br>(1.5 to 3.9)	11.0%<br>(9.2% to 12.6%)	3.1%<br>(1.4% to 4.6%)
Other cardiovascular and circulatory diseases	14.3<br>(9.1 to 21.1)	1.3<br>(0.7 to 2.1)	16.6%<br>(14.3% to 18.6%)	8.3%<br>(6.1% to 10.1%)
Endocrine, metabolic, blood, and immune disorders	2.1<br>(1.4 to 2.9)	0.2<br>(0.1 to 0.3)	5.2%<br>(3.7% to 6.5%)	-2.3%<br>(-3.7% to -1.1%)
Ischaemic heart disease	0.2<br>(0.1 to 0.4)	<0.05<br>(<0.05 to <0.05)	25.8%<br>(17.3% to 33.4%)	16.8%<br>(9.0% to 23.9%)
Other haemoglobinopathies and haemolytic anaemias	0.1<br>(0.1 to 0.2)	<0.05<br>(<0.05 to <0.05)	10.8%<br>(7.0% to 14.2%)	2.9%<br>(-0.6% to 6.1%)
Iron-deficiency anaemia	0.1<br>(0.1 to 0.2)	<0.05<br>(<0.05 to <0.05)	7.5%<br>(4.2% to 10.6%)	-0.1%<br>(-3.2% to 2.7%)
Hypertensive heart disease	0.1<br>(<0.05 to 0.1)	<0.05<br>(<0.05 to <0.05)	24.2%<br>(18.4% to 30.8%)	15.3%<br>(9.9% to 21.5%)
Chagas disease	0.1<br>(<0.05 to 0.1)	<0.05<br>(<0.05 to <0.05)	-25.9%<br>(-30.4% to -22.3%)	-31.2%<br>(-35.4% to -27.8%)
Rheumatic heart disease	<0.05<br>(<0.05 to <0.05)	<0.05<br>(<0.05 to <0.05)	16.4%<br>(9.8% to 22.3%)	8.1%<br>(1.9% to 13.6%)
Interstitial lung disease and pulmonary sarcoidosis	<0.05<br>(<0.05 to <0.05)	<0.05<br>(<0.05 to <0.05)	12.0%<br>(-2.0% to 27.2%)	4.0%<br>(-8.9% to 18.2%)
Chronic obstructive pulmonary disease	<0.05<br>(<0.05 to <0.05)	<0.05<br>(<0.05 to <0.05)	6.9%<br>(-7.9% to 24.9%)	-0.7%<br>(-14.5% to 16.0%)
**All causes**	**132.2**<br>**(94.1 to 179.5)**	**12.6**<br>**(7.6 to 19.9)**	**9.7%**<br>**(8.5% to 11.0%)**	**1.9%**<br>**(0.8% to 3.1%)**

SR Table 13f: Global prevalence and years lived with disability (YLDs) for 2016, percentage change of YLD counts and rates between 2006 and 2016 of heart failure by cause, ages 10-14 years				
**Cause**	**Prevalence in thousands (95% uncertainty interval)**	**YLDs in thousands (95% uncertainty interval)**		
	**2016**	**2016**	**Percentage change in counts between 2006 and 2016**	**Percentage change in rates between 2006 and 2016**
Myocarditis	79.8<br>(55.3 to 108.4)	7.1<br>(4.1 to 11.4)	-2.0%<br>(-5.6% to 1.4%)	-2.8%<br>(-6.4% to 0.6%)
Other cardiomyopathy	44.5<br>(31.1 to 60.3)	4.0<br>(2.3 to 6.4)	0.6%<br>(-1.3% to 2.4%)	-0.2%<br>(-2.1% to 1.6%)
Congenital heart anomalies	13.5<br>(9.5 to 18.5)	1.8<br>(1.1 to 2.7)	-3.7%<br>(-5.5% to -2.1%)	-4.5%<br>(-6.3% to -2.9%)
Other cardiovascular and circulatory diseases	7.9<br>(5.0 to 11.4)	0.7<br>(0.4 to 1.2)	7.6%<br>(4.8% to 10.7%)	6.8%<br>(3.9% to 9.8%)
Endocarditis	7.1<br>(4.6 to 10.1)	0.6<br>(0.4 to 1.0)	7.6%<br>(5.2% to 10.1%)	6.7%<br>(4.3% to 9.2%)
Endocrine, metabolic, blood, and immune disorders	1.3<br>(0.9 to 1.8)	0.1<br>(0.1 to 0.2)	0.4%<br>(-1.1% to 2.2%)	-0.4%<br>(-2.0% to 1.4%)
Thalassemias	1.2<br>(0.8 to 1.6)	0.1<br>(0.1 to 0.2)	-6.8%<br>(-9.8% to -3.7%)	-7.5%<br>(-10.5% to -4.5%)
Chagas disease	0.4<br>(0.2 to 0.7)	<0.05<br>(<0.05 to <0.05)	-19.4%<br>(-24.1% to -16.0%)	-20.1%<br>(-24.7% to -16.7%)
Other haemoglobinopathies and haemolytic anaemias	0.2<br>(0.1 to 0.3)	<0.05<br>(<0.05 to <0.05)	-2.4%<br>(-5.0% to 0.4%)	-3.2%<br>(-5.8% to -0.5%)
Ischaemic heart disease	0.1<br>(<0.05 to 0.3)	<0.05<br>(<0.05 to <0.05)	24.9%<br>(15.0% to 31.7%)	23.9%<br>(14.1% to 30.7%)
Iron-deficiency anaemia	0.1<br>(0.1 to 0.1)	<0.05<br>(<0.05 to <0.05)	5.3%<br>(2.2% to 8.0%)	4.4%<br>(1.4% to 7.1%)
Hypertensive heart disease	0.1<br>(<0.05 to 0.1)	<0.05<br>(<0.05 to <0.05)	22.9%<br>(17.0% to 29.8%)	21.9%<br>(16.0% to 28.8%)
Rheumatic heart disease	<0.05<br>(<0.05 to <0.05)	<0.05<br>(<0.05 to <0.05)	13.4%<br>(6.9% to 21.9%)	12.4%<br>(6.0% to 20.9%)
Interstitial lung disease and pulmonary sarcoidosis	<0.05<br>(<0.05 to <0.05)	<0.05<br>(<0.05 to <0.05)	9.9%<br>(-7.4% to 30.2%)	9.0%<br>(-8.2% to 29.2%)
Chronic obstructive pulmonary disease	<0.05<br>(<0.05 to <0.05)	<0.05<br>(<0.05 to <0.05)	9.4%<br>(-6.3% to 28.0%)	8.5%<br>(-7.1% to 26.9%)
**All causes**	**156.2<br>(113.5 to 206.5)**	**14.5<br>(8.7 to 22.4)**	**-0.7%<br>(-2.6% to 1.2%)**	**-1.5%<br>(-3.4% to 0.4%)**

SR Table 13f: Global prevalence and years lived with disability (YLDs) for 2016, percentage change of YLD counts and rates between 2006 and 2016 of heart failure by cause, ages 15-19 years				
**Cause**	**Prevalence in thousands (95% uncertainty interval)**	**YLDs in thousands (95% uncertainty interval)**		
	**2016**	**2016**	**Percentage change in counts between 2006 and 2016**	**Percentage change in rates between 2006 and 2016**
Myocarditis	104.9<br>(80.1 to 134.9)	9.3<br>(5.8 to 14.5)	-9.6%<br>(-14.5% to -4.7%)	-4.3%<br>(-9.5% to 0.9%)
Other cardiomyopathy	92.1<br>(70.5 to 118.0)	8.2<br>(5.1 to 12.8)	-5.9%<br>(-9.7% to -2.0%)	-0.4%<br>(-4.4% to 3.7%)
Congenital heart anomalies	12.1<br>(9.3 to 15.4)	1.6<br>(1.0 to 2.3)	-17.6%<br>(-19.0% to -16.3%)	-12.8%<br>(-14.3% to -11.4%)
Other cardiovascular and circulatory diseases	15.7<br>(11.1 to 21.0)	1.4<br>(0.8 to 2.2)	3.5%<br>(0.3% to 6.6%)	9.6%<br>(6.2% to 12.9%)
Endocarditis	5.3<br>(3.8 to 7.1)	0.5<br>(0.3 to 0.7)	7.4%<br>(4.9% to 9.9%)	13.7%<br>(11.1% to 16.3%)
Endocrine, metabolic, blood, and immune disorders	1.5<br>(1.1 to 2.0)	0.1<br>(0.1 to 0.2)	-4.2%<br>(-5.7% to -2.7%)	1.4%<br>(-0.2% to 3.0%)
Thalassemias	1.1<br>(0.8 to 1.5)	0.1<br>(0.1 to 0.2)	-13.4%<br>(-16.0% to -10.7%)	-8.4%<br>(-11.1% to -5.5%)
Chagas disease	1.5<br>(0.9 to 2.5)	0.1<br>(<0.05 to 0.1)	-16.4%<br>(-26.8% to -6.5%)	-11.5%<br>(-22.5% to -1.1%)
Other haemoglobinopathies and haemolytic anaemias	0.9<br>(0.6 to 1.1)	0.1<br>(<0.05 to 0.1)	-5.4%<br>(-9.9% to -0.4%)	0.1%<br>(-4.6% to 5.4%)
Iron-deficiency anaemia	0.2<br>(0.2 to 0.3)	<0.05<br>(<0.05 to <0.05)	8.6%<br>(5.7% to 11.3%)	15.0%<br>(11.9% to 17.8%)
Ischaemic heart disease	0.1<br>(<0.05 to 0.3)	<0.05<br>(<0.05 to <0.05)	19.7%<br>(8.8% to 26.8%)	26.7%<br>(15.2% to 34.2%)
Hypertensive heart disease	0.1<br>(<0.05 to 0.1)	<0.05<br>(<0.05 to <0.05)	18.4%<br>(12.5% to 25.4%)	25.3%<br>(19.1% to 32.7%)
Rheumatic heart disease	<0.05<br>(<0.05 to <0.05)	<0.05<br>(<0.05 to <0.05)	8.7%<br>(0.7% to 18.7%)	15.0%<br>(6.5% to 25.6%)
Alcoholic cardiomyopathy	<0.05<br>(<0.05 to <0.05)	<0.05<br>(<0.05 to <0.05)	3.2%<br>(-8.6% to 15.7%)	9.3%<br>(-3.2% to 22.5%)
Chronic obstructive pulmonary disease	<0.05<br>(<0.05 to <0.05)	<0.05<br>(<0.05 to <0.05)	-1.8%<br>(-14.2% to 11.4%)	3.9%<br>(-9.2% to 17.9%)
Interstitial lung disease and pulmonary sarcoidosis	<0.05<br>(<0.05 to <0.05)	<0.05<br>(<0.05 to <0.05)	2.0%<br>(-13.8% to 19.2%)	8.0%<br>(-8.7% to 26.2%)
**All causes**	**235.5<br>(183.3 to 298.9)**	**21.4<br>(13.6 to 32.7)**	**-7.7%<br>(-10.3% to -5.2%)**	**-2.3%<br>(-5.1% to 0.4%)**

SR Table 13f: Global prevalence and years lived with disability (YLDs) for 2016, percentage change of YLD counts and rates between 2006 and 2016 of heart failure by cause, ages 20-24 years				
**Cause**	**Prevalence in thousands (95% uncertainty interval)**	**YLDs in thousands (95% uncertainty interval)**		
	**2016**	**2016**	**Percentage change in counts between 2006 and 2016**	**Percentage change in rates between 2006 and 2016**
Other cardiomyopathy	190.4 (147.9 to 241.8)	16.7 (10.4 to 25.0)	4.5% (-1.8% to 11.5%)	-0.4% (-6.4% to 6.3%)
Myocarditis	122.2 (94.5 to 155.1)	10.8 (6.7 to 16.5)	4.7% (-1.9% to 11.6%)	-0.2% (-6.4% to 6.5%)
Other cardiovascular and circulatory diseases	16.2 (11.6 to 21.4)	1.5 (0.9 to 2.3)	7.4% (4.8% to 9.8%)	2.5% (-0.0% to 4.7%)
Congenital heart anomalies	10.5 (8.2 to 13.4)	1.4 (0.9 to 2.0)	3.8% (2.4% to 5.2%)	-1.0% (-2.3% to 0.4%)
Endocarditis	14.9 (10.8 to 19.6)	1.3 (0.8 to 2.1)	9.7% (7.6% to 11.8%)	4.6% (2.7% to 6.6%)
Other haemoglobinopathies and haemolytic anaemias	3.2 (2.3 to 4.2)	0.3 (0.2 to 0.4)	6.5% (3.7% to 9.4%)	1.6% (-1.1% to 4.3%)
Endocrine, metabolic, blood, and immune disorders	2.6 (1.9 to 3.4)	0.2 (0.1 to 0.4)	4.1% (2.3% to 5.8%)	-0.7% (-2.4% to 0.9%)
Chagas disease	4.2 (2.4 to 6.7)	0.2 (0.1 to 0.4)	-15.7% (-29.4% to -0.5%)	-19.6% (-32.6% to -5.1%)
Iron-deficiency anaemia	0.3 (0.2 to 0.4)	<0.05 (<0.05 to <0.05)	16.4% (12.9% to 19.6%)	11.0% (7.7% to 14.1%)
Ischaemic heart disease	0.3 (0.1 to 0.5)	<0.05 (<0.05 to 0.1)	21.6% (16.0% to 26.8%)	16.0% (10.6% to 21.0%)
Hypertensive heart disease	0.2 (0.1 to 0.4)	<0.05 (<0.05 to <0.05)	17.7% (12.6% to 22.8%)	12.3% (7.4% to 17.2%)
Rheumatic heart disease	<0.05 (<0.05 to <0.05)	<0.05 (<0.05 to <0.05)	15.2% (11.3% to 19.5%)	9.8% (6.2% to 14.0%)
Alcoholic cardiomyopathy	<0.05 (<0.05 to <0.05)	<0.05 (<0.05 to <0.05)	8.3% (-1.3% to 18.3%)	3.3% (-5.8% to 12.8%)
Interstitial lung disease and pulmonary sarcoidosis	<0.05 (<0.05 to <0.05)	<0.05 (<0.05 to <0.05)	8.2% (-4.2% to 21.9%)	3.2% (-8.6% to 16.3%)
Chronic obstructive pulmonary disease	<0.05 (<0.05 to <0.05)	<0.05 (<0.05 to <0.05)	10.8% (-2.7% to 25.6%)	5.7% (-7.2% to 19.8%)
**All causes**	**365.2 (286.8 to 459.0)**	**32.5 (20.0 to 48.8)**	**4.7% (0.3% to 8.9%)**	**-0.1% (-4.3% to 3.9%)**

SR Table 13f: Global prevalence and years lived with disability (YLDs) for 2016, percentage change of YLD counts and rates between 2006 and 2016 of heart failure by cause, ages 25-29 years				
**Cause**	**Prevalence in thousands (95% uncertainty interval)**	**YLDs in thousands (95% uncertainty interval)**		
	**2016**	**2016**	**Percentage change in counts between 2006 and 2016**	**Percentage change in rates between 2006 and 2016**
Other cardiomyopathy	273.7 (201.1 to 357.3)	23.8 (14.0 to 36.8)	18.0% (10.7% to 25.8%)	-0.6% (-6.8% to 6.0%)
Myocarditis	195.8 (144.1 to 256.6)	17.2 (10.3 to 26.7)	24.3% (15.5% to 33.3%)	4.7% (-2.7% to 12.3%)
Endocarditis	30.9 (20.6 to 42.5)	2.8 (1.5 to 4.5)	17.5% (15.0% to 20.0%)	-1.0% (-3.2% to 1.1%)
Other cardiovascular and circulatory diseases	16.2 (10.7 to 22.5)	1.5 (0.8 to 2.4)	20.6% (17.9% to 23.1%)	1.6% (-0.7% to 3.7%)
Congenital heart anomalies	8.4 (6.4 to 10.8)	1.1 (0.7 to 1.7)	19.4% (17.7% to 21.2%)	0.6% (-0.8% to 2.1%)
Chagas disease	8.9 (5.3 to 14.0)	0.5 (0.2 to 0.8)	-10.8% (-22.0% to 2.6%)	-24.9% (-34.3% to -13.5%)
Endocrine, metabolic, blood, and immune disorders	2.8 (1.9 to 3.8)	0.3 (0.1 to 0.4)	15.9% (13.7% to 17.9%)	-2.4% (-4.2% to -0.7%)
Other haemoglobinopathies and haemolytic anaemias	2.7 (1.8 to 3.7)	0.2 (0.1 to 0.4)	25.2% (22.4% to 28.0%)	5.4% (3.1% to 7.8%)
Hypertensive heart disease	0.8 (0.4 to 1.3)	0.1 (<0.05 to 0.1)	20.5% (12.6% to 27.5%)	1.5% (-5.1% to 7.4%)
Ischaemic heart disease	0.7 (0.3 to 1.5)	0.1 (<0.05 to 0.1)	26.5% (19.9% to 32.0%)	6.5% (1.0% to 11.2%)
Iron-deficiency anaemia	0.2 (0.1 to 0.3)	<0.05 (<0.05 to <0.05)	28.0% (24.8% to 31.3%)	7.8% (5.1% to 10.6%)
Rheumatic heart disease	0.1 (0.1 to 0.2)	<0.05 (<0.05 to <0.05)	23.7% (19.5% to 27.0%)	4.2% (0.7% to 7.0%)
Thalassemias	0.1 (0.1 to 0.1)	<0.05 (<0.05 to <0.05)	15.0% (11.4% to 18.7%)	-3.1% (-6.2% to -0.0%)
Alcoholic cardiomyopathy	<0.05 (<0.05 to <0.05)	<0.05 (<0.05 to <0.05)	3.0% (-6.5% to 13.5%)	-13.3% (-21.2% to -4.4%)
Interstitial lung disease and pulmonary sarcoidosis	<0.05 (<0.05 to <0.05)	<0.05 (<0.05 to <0.05)	2.2% (-10.4% to 17.5%)	-13.9% (-24.5% to -1.0%)
Chronic obstructive pulmonary disease	<0.05 (<0.05 to <0.05)	<0.05 (<0.05 to <0.05)	3.2% (-10.2% to 18.7%)	-13.1% (-24.4% to 0.0%)
**All causes**	**541.2 (401.4 to 695.7)**	**47.4 (28.6 to 73.0)**	**19.9% (15.0% to 24.8%)**	**1.0% (-3.1% to 5.1%)**

SR Table 13f: Global prevalence and years lived with disability (YLDs) for 2016, percentage change of YLD counts and rates between 2006 and 2016 of heart failure by cause, ages 30-34 years

Cause	Prevalence in thousands (95% uncertainty interval)	YLDs in thousands (95% uncertainty interval)		
	2016	2016	Percentage change in counts between 2006 and 2016	Percentage change in rates between 2006 and 2016
Ischaemic heart disease	203.0 (155.6 to 254.9)	17.6 (10.8 to 26.2)	17.5% (9.5% to 27.3%)	4.1% (-3.0% to 12.7%)
Other cardiomyopathy	201.1 (152.5 to 253.1)	17.5 (10.7 to 26.3)	13.4% (6.9% to 20.8%)	0.4% (-5.3% to 7.0%)
Hypertensive heart disease	167.6 (119.2 to 221.1)	14.6 (8.7 to 22.8)	12.9% (4.3% to 21.7%)	0.0% (-7.6% to 7.8%)
Myocarditis	99.3 (74.3 to 125.4)	8.7 (5.3 to 13.4)	7.0% (-0.4% to 14.4%)	-5.2% (-11.8% to 1.4%)
Alcoholic cardiomyopathy	37.6 (28.4 to 47.1)	3.3 (2.0 to 5.0)	7.6% (-6.9% to 22.2%)	-4.7% (-17.6% to 8.2%)
Rheumatic heart disease	29.1 (21.7 to 38.2)	2.6 (1.5 to 4.0)	12.2% (6.4% to 17.9%)	-0.6% (-5.7% to 4.4%)
Chronic obstructive pulmonary disease	3.8 (2.6 to 5.2)	1.7 (1.1 to 2.7)	1.9% (-0.2% to 4.4%)	-9.7% (-11.6% to -7.5%)
Other cardiovascular and circulatory diseases	16.3 (10.2 to 23.9)	1.5 (0.8 to 2.4)	14.7% (11.7% to 17.6%)	1.6% (-1.0% to 4.1%)
Congenital heart anomalies	5.8 (4.5 to 7.2)	0.8 (0.5 to 1.1)	6.1% (4.6% to 7.4%)	-6.1% (-7.4% to -4.9%)
Chagas disease	14.6 (8.9 to 22.6)	0.8 (0.4 to 1.3)	-1.8% (-11.5% to 8.9%)	-13.0% (-21.6% to -3.5%)
Endocarditis	7.8 (5.0 to 11.5)	0.7 (0.4 to 1.1)	14.7% (12.2% to 17.1%)	1.5% (-0.7% to 3.7%)
Interstitial lung disease and pulmonary sarcoidosis	0.9 (0.6 to 1.2)	0.4 (0.2 to 0.6)	11.6% (9.8% to 13.4%)	-1.2% (-2.7% to 0.4%)
Other haemoglobinopathies and haemolytic anaemias	2.9 (1.9 to 4.3)	0.3 (0.1 to 0.4)	7.8% (5.0% to 10.3%)	-4.6% (-7.0% to -2.3%)
Endocrine, metabolic, blood, and immune disorders	2.3 (1.5 to 3.3)	0.2 (0.1 to 0.3)	10.7% (8.5% to 12.6%)	-2.0% (-3.9% to -0.3%)
Iron-deficiency anaemia	0.2 (0.1 to 0.2)	<0.05 (<0.05 to <0.05)	21.5% (17.5% to 25.5%)	7.6% (4.1% to 11.1%)
Iodine deficiency	<0.05 (<0.05 to <0.05)	<0.05 (<0.05 to <0.05)	36.6% (29.3% to 43.3%)	20.9% (14.5% to 26.9%)
Thalassemias	<0.05 (<0.05 to 0.1)	<0.05 (<0.05 to <0.05)	-9.6% (-14.0% to -4.8%)	-19.9% (-23.8% to -15.7%)
**All causes**	**792.2 (619.6 to 976.6)**	**70.7 (43.9 to 105.2)**	**12.5% (9.1% to 15.9%)**	**-0.3% (-3.4% to 2.6%)**

SR Table 13f: Global prevalence and years lived with disability (YLDs) for 2016, percentage change of YLD counts and rates between 2006 and 2016 of heart failure by cause, ages 35-39 years				
**Cause**	**Prevalence in thousands (95% uncertainty interval)**	**YLDs in thousands (95% uncertainty interval)**		
	**2016**	**2016**	**Percentage change in counts between 2006 and 2016**	**Percentage change in rates between 2006 and 2016**
Hypertensive heart disease	412.0 (305.8 to 547.1)	35.2 (21.5 to 53.6)	4.4% (-1.2% to 10.2%)	-1.3% (-6.6% to 4.2%)
Ischaemic heart disease	384.0 (293.0 to 504.0)	32.9 (19.8 to 49.2)	8.0% (2.1% to 14.1%)	2.1% (-3.5% to 7.9%)
Other cardiomyopathy	117.3 (86.0 to 155.4)	10.2 (6.0 to 15.5)	6.5% (0.6% to 12.8%)	0.7% (-4.9% to 6.6%)
Myocarditis	69.7 (51.1 to 93.0)	6.1 (3.6 to 9.3)	-0.4% (-7.4% to 6.8%)	-5.9% (-12.4% to 0.9%)
Rheumatic heart disease	64.8 (48.8 to 84.9)	5.6 (3.4 to 8.8)	5.0% (-6.1% to 16.3%)	-0.7% (-11.3% to 9.9%)
Chronic obstructive pulmonary disease	9.3 (6.4 to 13.3)	4.3 (2.5 to 6.5)	-9.3% (-11.6% to -7.0%)	-14.3% (-16.4% to -12.0%)
Alcoholic cardiomyopathy	47.8 (34.8 to 64.0)	4.2 (2.4 to 6.6)	4.2% (-8.4% to 19.3%)	-1.5% (-13.4% to 12.8%)
Other cardiovascular and circulatory diseases	14.6 (9.9 to 20.6)	1.3 (0.7 to 2.1)	6.9% (4.4% to 9.8%)	1.1% (-1.3% to 3.8%)
Chagas disease	19.9 (12.2 to 30.4)	1.0 (0.5 to 1.8)	2.2% (-7.3% to 12.0%)	-3.4% (-12.4% to 5.9%)
Interstitial lung disease and pulmonary sarcoidosis	1.7 (1.1 to 2.4)	0.8 (0.4 to 1.2)	3.2% (1.5% to 4.8%)	-2.5% (-4.0% to -0.9%)
Congenital heart anomalies	5.7 (4.3 to 7.4)	0.7 (0.5 to 1.1)	1.6% (-0.3% to 3.3%)	-4.0% (-5.7% to -2.4%)
Endocarditis	2.3 (1.6 to 3.2)	0.2 (0.1 to 0.3)	10.8% (8.0% to 13.8%)	4.7% (2.1% to 7.6%)
Endocrine, metabolic, blood, and immune disorders	2.3 (1.6 to 3.2)	0.2 (0.1 to 0.3)	3.8% (1.2% to 6.0%)	-1.9% (-4.3% to 0.2%)
Other haemoglobinopathies and haemolytic anaemias	2.3 (1.6 to 3.2)	0.2 (0.1 to 0.3)	-2.6% (-5.0% to -0.4%)	-8.0% (-10.2% to -5.8%)
Coal workers pneumoconiosis	0.1 (<0.05 to 0.1)	<0.05 (<0.05 to <0.05)	-29.8% (-33.2% to -26.5%)	-33.6% (-36.9% to -30.5%)
Other pneumoconiosis	0.1 (<0.05 to 0.1)	<0.05 (<0.05 to <0.05)	-20.9% (-24.0% to -17.6%)	-25.2% (-28.1% to -22.1%)
Iron-deficiency anaemia	0.1 (0.1 to 0.2)	<0.05 (<0.05 to <0.05)	15.9% (12.1% to 19.5%)	9.5% (6.0% to 12.9%)
Thalassemias	0.1 (<0.05 to 0.1)	<0.05 (<0.05 to <0.05)	-10.9% (-14.2% to -7.7%)	-15.8% (-18.9% to -12.7%)
**All causes**	**1,154.0 (893.3 to 1,496.5)**	**103.0 (62.6 to 153.2)**	**4.7% (1.8% to 7.7%)**	**-1.0% (-3.8% to 1.8%)**

SR Table 13f: Global prevalence and years lived with disability (YLDs) for 2016, percentage change of YLD counts and rates between 2006 and 2016 of heart failure by cause, ages 40-44 years				
**Cause**	**Prevalence in thousands (95% uncertainty interval)**	**YLDs in thousands (95% uncertainty interval)**		
	**2016**	**2016**	**Percentage change in counts between 2006 and 2016**	**Percentage change in rates between 2006 and 2016**
Hypertensive heart disease	646.6<br>(499.1 to 833.7)	54.8<br>(33.7 to 81.4)	15.6%<br>(10.6% to 21.0%)	1.5%<br>(-2.8% to 6.2%)
Ischaemic heart disease	640.8<br>(502.2 to 823.2)	54.4<br>(33.3 to 79.5)	17.3%<br>(12.4% to 22.4%)	3.0%<br>(-1.2% to 7.5%)
Chronic obstructive pulmonary disease	24.9<br>(18.1 to 33.7)	11.4<br>(7.1 to 16.9)	8.2%<br>(3.0% to 13.3%)	-4.9%<br>(-9.5% to -0.5%)
Other cardiomyopathy	119.3<br>(86.3 to 160.9)	10.3<br>(6.0 to 15.6)	12.4%<br>(6.0% to 18.7%)	-1.3%<br>(-6.9% to 4.2%)
Rheumatic heart disease	98.9<br>(79.0 to 123.4)	8.6<br>(5.3 to 12.5)	12.3%<br>(2.0% to 23.3%)	-1.4%<br>(-10.4% to 8.3%)
Myocarditis	66.9<br>(47.6 to 91.6)	5.9<br>(3.4 to 9.1)	15.3%<br>(9.0% to 22.0%)	1.3%<br>(-4.2% to 7.1%)
Alcoholic cardiomyopathy	55.8<br>(41.6 to 74.1)	4.9<br>(2.8 to 7.5)	2.0%<br>(-12.3% to 19.9%)	-10.4%<br>(-23.0% to 5.3%)
Interstitial lung disease and pulmonary sarcoidosis	3.5<br>(2.5 to 4.6)	1.6<br>(1.0 to 2.3)	8.6%<br>(7.1% to 10.1%)	-4.6%<br>(-6.0% to -3.3%)
Other cardiovascular and circulatory diseases	15.7<br>(10.7 to 22.2)	1.4<br>(0.8 to 2.2)	11.8%<br>(9.5% to 14.3%)	-1.8%<br>(-3.8% to 0.4%)
Chagas disease	23.9<br>(14.7 to 36.1)	1.2<br>(0.6 to 2.1)	-1.1%<br>(-11.1% to 9.5%)	-13.1%<br>(-21.9% to -3.8%)
Congenital heart anomalies	5.1<br>(3.9 to 6.5)	0.7<br>(0.4 to 1.0)	9.7%<br>(8.0% to 11.3%)	-3.7%<br>(-5.2% to -2.2%)
Endocarditis	7.3<br>(5.0 to 10.2)	0.7<br>(0.4 to 1.0)	13.8%<br>(8.9% to 18.5%)	-0.1%<br>(-4.4% to 4.1%)
Other haemoglobinopathies and haemolytic anaemias	3.1<br>(2.1 to 4.4)	0.3<br>(0.2 to 0.4)	11.5%<br>(9.2% to 13.7%)	-2.0%<br>(-4.1% to -0.2%)
Endocrine, metabolic, blood, and immune disorders	2.4<br>(1.7 to 3.4)	0.2<br>(0.1 to 0.3)	6.4%<br>(4.1% to 8.6%)	-6.6%<br>(-8.6% to -4.6%)
Other pneumoconiosis	<0.05<br>(<0.05 to 0.1)	<0.05<br>(<0.05 to <0.05)	2.1%<br>(-1.2% to 5.5%)	-10.3%<br>(-13.3% to -7.4%)
Iron-deficiency anaemia	0.1<br>(0.1 to 0.1)	<0.05<br>(<0.05 to <0.05)	20.2%<br>(17.2% to 23.3%)	5.6%<br>(2.9% to 8.3%)
Thalassemias	<0.05<br>(<0.05 to <0.05)	<0.05<br>(<0.05 to <0.05)	9.5%<br>(5.2% to 13.8%)	-3.8%<br>(-7.6% to -0.0%)
Iodine deficiency	<0.05<br>(<0.05 to <0.05)	<0.05<br>(<0.05 to <0.05)	32.8%<br>(25.8% to 39.2%)	16.7%<br>(10.5% to 22.3%)
**All causes**	**1,714.5**<br>**(1,359.7 to 2,183.1)**	**156.3**<br>**(97.2 to 228.3)**	**14.4%**<br>**(11.6% to 17.2%)**	**0.5%**<br>**(-2.0% to 3.0%)**

SR Table 13f: Global prevalence and years lived with disability (YLDs) for 2016, percentage change of YLD counts and rates between 2006 and 2016 of heart failure by cause, ages 45-49 years				
**Cause**	**Prevalence in thousands (95% uncertainty interval)**	**YLDs in thousands (95% uncertainty interval)**		
	**2016**	**2016**	**Percentage change in counts between 2006 and 2016**	**Percentage change in rates between 2006 and 2016**
Ischaemic heart disease	1,113.2 (920.3 to 1,345.2)	93.9 (62.2 to 134.5)	30.2% (25.9% to 34.7%)	4.4% (0.9% to 8.0%)
Hypertensive heart disease	997.0 (797.8 to 1,215.1)	83.9 (54.5 to 119.9)	29.6% (24.9% to 33.9%)	3.9% (0.2% to 7.4%)
Chronic obstructive pulmonary disease	83.6 (59.5 to 113.9)	36.6 (22.2 to 54.1)	25.0% (15.5% to 35.4%)	0.3% (-7.4% to 8.6%)
Other cardiomyopathy	191.2 (147.5 to 239.8)	16.3 (10.0 to 24.1)	19.7% (13.1% to 26.2%)	-4.0% (-9.3% to 1.2%)
Rheumatic heart disease	137.3 (110.8 to 166.1)	11.7 (7.6 to 16.6)	23.8% (14.5% to 33.7%)	-0.7% (-8.2% to 7.2%)
Alcoholic cardiomyopathy	126.0 (99.2 to 156.3)	10.8 (6.6 to 15.9)	5.9% (-3.9% to 17.5%)	-15.1% (-22.9% to -5.8%)
Myocarditis	70.8 (53.8 to 89.6)	6.2 (3.8 to 9.2)	29.3% (21.0% to 38.4%)	3.7% (-3.0% to 11.0%)
Other cardiovascular and circulatory diseases	34.8 (25.5 to 47.1)	3.1 (1.9 to 4.7)	23.3% (19.9% to 26.7%)	-1.1% (-3.9% to 1.6%)
Interstitial lung disease and pulmonary sarcoidosis	6.7 (4.8 to 8.9)	3.1 (1.9 to 4.6)	15.7% (13.8% to 17.6%)	-7.2% (-8.7% to -5.7%)
Chagas disease	28.4 (17.8 to 42.2)	1.4 (0.8 to 2.5)	3.8% (-5.3% to 14.8%)	-16.8% (-24.0% to -7.9%)
Endocarditis	14.2 (10.5 to 18.8)	1.3 (0.8 to 1.9)	18.7% (14.7% to 22.9%)	-4.8% (-8.0% to -1.4%)
Other haemoglobinopathies and haemolytic anaemias	6.8 (5.0 to 9.1)	0.6 (0.4 to 0.9)	32.8% (30.4% to 35.2%)	6.5% (4.6% to 8.4%)
Endocrine, metabolic, blood, and immune disorders	5.0 (3.7 to 6.6)	0.5 (0.3 to 0.7)	16.7% (13.4% to 19.8%)	-6.5% (-9.1% to -3.9%)
Congenital heart anomalies	3.1 (2.5 to 3.8)	0.4 (0.3 to 0.6)	15.0% (12.8% to 17.2%)	-7.8% (-9.5% to -6.0%)
G6PD deficiency	1.0 (0.7 to 1.4)	0.1 (0.1 to 0.1)	35.1% (32.2% to 38.4%)	8.4% (6.0% to 10.9%)
Coal workers pneumoconiosis	0.1 (0.1 to 0.1)	<0.05 (<0.05 to <0.05)	26.0% (20.1% to 32.2%)	1.0% (-3.7% to 6.0%)
Iron-deficiency anaemia	0.1 (0.1 to 0.2)	<0.05 (<0.05 to <0.05)	34.3% (30.8% to 37.7%)	7.7% (4.9% to 10.4%)
Thalassemias	<0.05 (<0.05 to <0.05)	<0.05 (<0.05 to <0.05)	16.9% (12.4% to 21.4%)	-6.3% (-9.9% to -2.6%)
Iodine deficiency	<0.05 (<0.05 to <0.05)	<0.05 (<0.05 to <0.05)	35.3% (28.3% to 41.9%)	8.5% (2.9% to 13.8%)
**All causes**	**2,819.4 (2,367.7 to 3,346.1)**	**270.0 (177.6 to 380.9)**	**26.6% (23.9% to 29.3%)**	**1.5% (-0.6% to 3.7%)**

SR Table 13f: Global prevalence and years lived with disability (YLDs) for 2016, percentage change of YLD counts and rates between 2006 and 2016 of heart failure by cause, ages 50-54 years				
Cause	Prevalence in thousands (95% uncertainty interval)	YLDs in thousands (95% uncertainty interval)		
	2016	2016	Percentage change in counts between 2006 and 2016	Percentage change in rates between 2006 and 2016
Ischaemic heart disease	1,510.6<br>(1,214.1 to 1,860.1)	126.3<br>(82.2 to 184.1)	30.0%<br>(26.4% to 33.7%)	4.3%<br>(1.5% to 7.4%)
Hypertensive heart disease	1,264.3<br>(981.5 to 1,582.1)	105.8<br>(68.6 to 153.6)	26.2%<br>(22.2% to 30.3%)	1.3%<br>(-1.9% to 4.6%)
Chronic obstructive pulmonary disease	209.4<br>(153.4 to 273.1)	88.1<br>(56.6 to 130.6)	17.8%<br>(11.4% to 24.6%)	-5.4%<br>(-10.5% to 0.1%)
Other cardiomyopathy	250.6<br>(186.0 to 323.4)	21.1<br>(13.0 to 31.0)	23.2%<br>(16.7% to 29.5%)	-1.1%<br>(-6.3% to 3.9%)
Alcoholic cardiomyopathy	175.6<br>(134.4 to 225.2)	14.9<br>(9.3 to 21.9)	20.0%<br>(9.3% to 31.3%)	-3.7%<br>(-12.2% to 5.4%)
Rheumatic heart disease	158.1<br>(118.9 to 198.6)	13.4<br>(8.4 to 19.6)	17.3%<br>(7.9% to 26.0%)	-5.8%<br>(-13.3% to 1.2%)
Interstitial lung disease and pulmonary sarcoidosis	11.1<br>(8.3 to 14.0)	5.1<br>(3.3 to 7.4)	19.5%<br>(16.8% to 22.1%)	-4.1%<br>(-6.2% to -2.0%)
Myocarditis	58.1<br>(42.7 to 76.4)	5.1<br>(3.0 to 7.7)	20.3%<br>(12.1% to 28.9%)	-3.4%<br>(-10.0% to 3.5%)
Other cardiovascular and circulatory diseases	57.4<br>(40.9 to 77.0)	5.1<br>(3.1 to 7.9)	23.1%<br>(15.8% to 30.0%)	-1.1%<br>(-7.0% to 4.4%)
Chagas disease	33.2<br>(21.1 to 48.8)	1.7<br>(0.9 to 2.8)	12.2%<br>(2.5% to 21.9%)	-9.9%<br>(-17.7% to -2.1%)
Endocarditis	18.4<br>(13.2 to 24.4)	1.6<br>(1.0 to 2.5)	23.2%<br>(19.4% to 27.1%)	-1.1%<br>(-4.2% to 2.1%)
Other haemoglobinopathies and haemolytic anaemias	10.9<br>(7.8 to 14.7)	1.0<br>(0.6 to 1.5)	27.5%<br>(25.0% to 29.7%)	2.4%<br>(0.4% to 4.1%)
Endocrine, metabolic, blood, and immune disorders	8.6<br>(6.2 to 11.4)	0.8<br>(0.5 to 1.2)	19.9%<br>(16.9% to 22.5%)	-3.7%<br>(-6.1% to -1.6%)
Congenital heart anomalies	2.6<br>(2.1 to 3.2)	0.4<br>(0.2 to 0.5)	18.3%<br>(16.2% to 20.3%)	-5.0%<br>(-6.7% to -3.4%)
Silicosis	0.4<br>(0.3 to 0.5)	0.2<br>(0.1 to 0.3)	7.9%<br>(3.8% to 11.8%)	-13.4%<br>(-16.6% to -10.2%)
G6PD deficiency	1.6<br>(1.1 to 2.2)	0.1<br>(0.1 to 0.2)	34.7%<br>(31.6% to 37.7%)	8.1%<br>(5.7% to 10.6%)
Coal workers pneumoconiosis	0.1<br>(0.1 to 0.1)	<0.05<br>(<0.05 to 0.1)	12.7%<br>(8.8% to 16.7%)	-9.5%<br>(-12.6% to -6.3%)
Asbestosis	0.1<br>(0.1 to 0.1)	<0.05<br>(<0.05 to <0.05)	19.1%<br>(17.0% to 21.3%)	-4.4%<br>(-6.0% to -2.6%)
Iron-deficiency anaemia	0.2<br>(0.1 to 0.2)	<0.05<br>(<0.05 to <0.05)	32.5%<br>(29.2% to 35.8%)	6.4%<br>(3.7% to 9.1%)
Thalassemias	<0.05<br>(<0.05 to 0.1)	<0.05<br>(<0.05 to <0.05)	-2.0%<br>(-6.8% to 3.1%)	-21.3%<br>(-25.2% to -17.2%)
Iodine deficiency	<0.05<br>(<0.05 to <0.05)	<0.05<br>(<0.05 to <0.05)	39.8%<br>(32.6% to 45.9%)	12.3%<br>(6.5% to 17.1%)
**All causes**	**3,771.2**<br>**(3,085.0 to 4,526.7)**	**390.8**<br>**(256.5 to 558.4)**	**24.3%**<br>**(21.8% to 26.8%)**	**-0.2%**<br>**(-2.2% to 1.8%)**

SR Table 13f: Global prevalence and years lived with disability (YLDs) for 2016, percentage change of YLD counts and rates between 2006 and 2016 of heart failure by cause, ages 55-59 years				
**Cause**	**Prevalence in thousands (95% uncertainty interval)**	**YLDs in thousands (95% uncertainty interval)**		
	**2016**	**2016**	**Percentage change in counts between 2006 and 2016**	**Percentage change in rates between 2006 and 2016**
Chronic obstructive pulmonary disease	373.9 (264.9 to 514.6)	153.2 (93.5 to 232.1)	25.2% (20.0% to 30.5%)	-4.0% (-8.1% to 0.0%)
Ischaemic heart disease	1,780.6 (1,377.3 to 2,205.3)	147.4 (95.3 to 216.9)	32.8% (29.2% to 36.4%)	1.8% (-1.0% to 4.6%)
Hypertensive heart disease	1,399.6 (1,081.8 to 1,790.0)	116.0 (74.1 to 171.3)	32.3% (28.3% to 36.1%)	1.4% (-1.7% to 4.3%)
Other cardiomyopathy	237.1 (177.5 to 309.9)	19.8 (12.3 to 29.9)	24.0% (18.1% to 29.5%)	-5.0% (-9.5% to -0.7%)
Rheumatic heart disease	186.0 (139.4 to 234.4)	15.6 (10.0 to 22.9)	25.4% (17.4% to 33.9%)	-3.9% (-10.0% to 2.6%)
Alcoholic cardiomyopathy	155.0 (117.7 to 199.6)	13.0 (8.1 to 19.7)	29.9% (16.6% to 43.7%)	-0.4% (-10.6% to 10.1%)
Interstitial lung disease and pulmonary sarcoidosis	15.8 (11.9 to 20.5)	7.3 (4.6 to 10.6)	22.3% (16.5% to 26.5%)	-6.2% (-10.7% to -3.0%)
Other cardiovascular and circulatory diseases	71.5 (50.9 to 98.9)	6.2 (3.7 to 9.7)	25.5% (17.9% to 33.4%)	-3.8% (-9.7% to 2.2%)
Myocarditis	55.8 (40.9 to 74.1)	4.9 (3.0 to 7.4)	19.0% (11.7% to 27.2%)	-8.8% (-14.4% to -2.5%)
Endocarditis	20.6 (14.8 to 28.2)	1.8 (1.1 to 2.9)	24.9% (18.9% to 30.7%)	-4.3% (-8.9% to 0.2%)
Chagas disease	35.4 (22.8 to 52.1)	1.8 (0.9 to 3.0)	19.4% (8.5% to 31.2%)	-8.5% (-16.8% to 0.5%)
Other haemoglobinopathies and haemolytic anaemias	12.8 (9.0 to 17.8)	1.2 (0.7 to 1.9)	25.3% (22.7% to 27.9%)	-4.0% (-6.0% to -2.0%)
Endocrine, metabolic, blood, and immune disorders	11.4 (8.3 to 15.5)	1.0 (0.6 to 1.6)	21.4% (18.1% to 24.8%)	-7.0% (-9.5% to -4.4%)
Congenital heart anomalies	2.3 (1.8 to 2.9)	0.3 (0.2 to 0.4)	17.1% (14.8% to 19.5%)	-10.2% (-12.0% to -8.4%)
G6PD deficiency	2.6 (1.9 to 3.6)	0.2 (0.1 to 0.4)	36.7% (33.0% to 40.6%)	4.7% (1.9% to 7.8%)
Coal workers pneumoconiosis	0.1 (0.1 to 0.2)	0.1 (<0.05 to 0.1)	13.0% (8.9% to 16.6%)	-13.4% (-16.5% to -10.6%)
Asbestosis	0.1 (0.1 to 0.1)	0.1 (<0.05 to 0.1)	18.1% (14.8% to 21.2%)	-9.5% (-12.0% to -7.1%)
Other pneumoconiosis	0.1 (<0.05 to 0.1)	<0.05 (<0.05 to <0.05)	14.8% (9.5% to 19.8%)	-12.0% (-16.1% to -8.2%)
Iron-deficiency anaemia	0.2 (0.1 to 0.3)	<0.05 (<0.05 to <0.05)	39.6% (36.0% to 43.1%)	7.0% (4.2% to 9.7%)
Iodine deficiency	<0.05 (<0.05 to <0.05)	<0.05 (<0.05 to <0.05)	48.4% (42.5% to 53.5%)	13.7% (9.2% to 17.6%)
Thalassemias	<0.05 (<0.05 to 0.1)	<0.05 (<0.05 to <0.05)	-20.2% (-24.5% to -15.6%)	-38.8% (-42.1% to -35.3%)
**All causes**	**4,361.0 (3,443.6 to 5,338.0)**	**489.8 (320.7 to 702.5)**	**29.0% (26.3% to 31.6%)**	**-1.1% (-3.2% to 0.9%)**

SR Table 13f: Global prevalence and years lived with disability (YLDs) for 2016, percentage change of YLD counts and rates between 2006 and 2016 of heart failure by cause, ages 60-64 years				
**Cause**	**Prevalence in thousands (95% uncertainty interval)**	**YLDs in thousands (95% uncertainty interval)**		
	**2016**	**2016**	**Percentage change in counts between 2006 and 2016**	**Percentage change in rates between 2006 and 2016**
Chronic obstructive pulmonary disease	764.1<br>(587.7 to 978.6)	307.3<br>(203.5 to 440.9)	46.8%<br>(42.0% to 51.5%)	-2.6%<br>(-5.8% to 0.5%)
Ischaemic heart disease	2,409.4<br>(1,944.1 to 2,924.6)	197.2<br>(131.2 to 282.8)	50.9%<br>(46.8% to 54.4%)	0.1%<br>(-2.6% to 2.4%)
Hypertensive heart disease	1,796.2<br>(1,394.0 to 2,276.0)	147.3<br>(95.3 to 214.4)	53.4%<br>(49.4% to 57.2%)	1.7%<br>(-0.9% to 4.3%)
Other cardiomyopathy	280.7<br>(204.5 to 370.6)	23.2<br>(14.5 to 36.3)	39.0%<br>(33.1% to 45.2%)	-7.8%<br>(-11.7% to -3.7%)
Rheumatic heart disease	240.9<br>(183.1 to 298.4)	19.9<br>(12.6 to 28.9)	49.0%<br>(40.8% to 57.9%)	-1.2%<br>(-6.6% to 4.8%)
Alcoholic cardiomyopathy	165.8<br>(124.1 to 216.7)	13.7<br>(8.6 to 21.1)	59.0%<br>(45.9% to 73.8%)	5.5%<br>(-3.2% to 15.3%)
Interstitial lung disease and pulmonary sarcoidosis	22.4<br>(17.3 to 27.6)	10.1<br>(6.7 to 14.4)	37.8%<br>(31.4% to 44.0%)	-8.6%<br>(-12.9% to -4.5%)
Other cardiovascular and circulatory diseases	115.1<br>(83.5 to 154.8)	9.7<br>(6.0 to 14.9)	43.9%<br>(35.0% to 53.8%)	-4.5%<br>(-10.5% to 2.0%)
Myocarditis	38.1<br>(27.3 to 51.2)	3.3<br>(2.0 to 5.3)	48.6%<br>(39.9% to 57.8%)	-1.4%<br>(-7.2% to 4.7%)
Endocarditis	27.9<br>(20.3 to 37.1)	2.5<br>(1.5 to 3.8)	38.6%<br>(30.8% to 47.0%)	-8.0%<br>(-13.3% to -2.5%)
Other haemoglobinopathies and haemolytic anaemias	20.3<br>(14.5 to 27.4)	1.8<br>(1.1 to 2.8)	45.2%<br>(39.6% to 50.8%)	-3.7%<br>(-7.4% to 0.0%)
Chagas disease	36.0<br>(23.4 to 52.2)	1.8<br>(1.0 to 3.0)	25.8%<br>(16.2% to 35.7%)	-16.6%<br>(-22.9% to -10.0%)
Endocrine, metabolic, blood, and immune disorders	16.7<br>(12.3 to 21.9)	1.5<br>(0.9 to 2.2)	35.9%<br>(30.8% to 41.2%)	-9.8%<br>(-13.3% to -6.4%)
Silicosis	0.7<br>(0.5 to 0.8)	0.3<br>(0.2 to 0.4)	30.5%<br>(26.2% to 35.3%)	-13.4%<br>(-16.3% to -10.3%)
Congenital heart anomalies	2.3<br>(1.9 to 2.8)	0.3<br>(0.2 to 0.4)	35.1%<br>(32.5% to 38.1%)	-10.4%<br>(-12.1% to -8.4%)
Coal workers pneumoconiosis	0.3<br>(0.2 to 0.4)	0.1<br>(0.1 to 0.2)	35.1%<br>(30.5% to 40.0%)	-10.4%<br>(-13.5% to -7.2%)
Asbestosis	0.2<br>(0.2 to 0.3)	0.1<br>(0.1 to 0.2)	32.3%<br>(28.1% to 36.2%)	-12.2%<br>(-15.0% to -9.7%)
Other pneumoconiosis	0.2<br>(0.1 to 0.2)	0.1<br>(0.1 to 0.1)	34.5%<br>(29.8% to 39.1%)	-10.8%<br>(-13.9% to -7.8%)
G6PD deficiency	0.7<br>(0.5 to 0.9)	0.1<br>(<0.05 to 0.1)	53.2%<br>(49.0% to 57.2%)	1.6%<br>(-1.1% to 4.3%)
Iron-deficiency anaemia	0.2<br>(0.2 to 0.3)	<0.05<br>(<0.05 to <0.05)	55.2%<br>(51.2% to 59.4%)	2.9%<br>(0.3% to 5.8%)
Iodine deficiency	<0.05<br>(<0.05 to <0.05)	<0.05<br>(<0.05 to <0.05)	52.7%<br>(47.4% to 58.0%)	1.3%<br>(-2.3% to 4.8%)
Thalassemias	<0.05<br>(<0.05 to <0.05)	<0.05<br>(<0.05 to <0.05)	6.9%<br>(0.8% to 13.9%)	-29.1%<br>(-33.1% to -24.5%)
**All causes**	**5,938.1**<br>**(4,925.3 to 7,001.4)**	**740.2**<br>**(512.2 to 1,028.0)**	**48.8%**<br>**(46.0% to 51.6%)**	**-1.3%**<br>**(-3.2% to 0.5%)**

SR Table 13f: Global prevalence and years lived with disability (YLDs) for 2016, percentage change of YLD counts and rates between 2006 and 2016 of heart failure by cause, ages 65-69 years				
**Cause**	**Prevalence in thousands (95% uncertainty interval)**	**YLDs in thousands (95% uncertainty interval)**		
	**2016**	**2016**	**Percentage change in counts between 2006 and 2016**	**Percentage change in rates between 2006 and 2016**
Chronic obstructive pulmonary disease	1,425.1 (1,099.6 to 1,786.0)	559.4 (385.0 to 741.1)	32.0% (28.9% to 35.1%)	-0.4% (-2.7% to 1.9%)
Ischaemic heart disease	3,359.9 (2,775.8 to 4,007.6)	270.8 (184.0 to 374.9)	26.9% (23.6% to 30.0%)	-4.2% (-6.7% to -1.9%)
Hypertensive heart disease	2,142.6 (1,681.2 to 2,682.4)	173.2 (112.7 to 248.4)	31.2% (28.2% to 34.3%)	-1.0% (-3.2% to 1.3%)
Other cardiomyopathy	361.7 (275.7 to 462.7)	29.4 (19.1 to 43.1)	30.1% (24.6% to 35.5%)	-1.8% (-6.0% to 2.3%)
Rheumatic heart disease	262.2 (209.3 to 312.0)	21.2 (13.7 to 30.0)	45.1% (37.3% to 53.0%)	9.5% (3.6% to 15.4%)
Other cardiovascular and circulatory diseases	198.7 (147.2 to 258.1)	16.2 (10.2 to 25.0)	28.8% (21.3% to 36.6%)	-2.8% (-8.4% to 3.1%)
Interstitial lung disease and pulmonary sarcoidosis	31.5 (22.7 to 40.8)	13.6 (8.7 to 19.8)	32.6% (25.8% to 40.4%)	0.1% (-5.0% to 5.9%)
Alcoholic cardiomyopathy	155.6 (119.4 to 199.0)	12.7 (8.2 to 18.6)	16.8% (6.4% to 28.7%)	-11.8% (-19.7% to -2.9%)
Myocarditis	42.6 (32.3 to 55.2)	3.6 (2.3 to 5.4)	30.6% (21.4% to 39.4%)	-1.4% (-8.4% to 5.2%)
Endocarditis	35.2 (26.2 to 45.6)	3.0 (1.9 to 4.7)	29.1% (18.5% to 40.2%)	-2.6% (-10.6% to 5.8%)
Other haemoglobinopathies and haemolytic anaemias	34.9 (25.6 to 46.0)	3.0 (1.8 to 4.7)	27.5% (16.9% to 38.2%)	-3.7% (-11.7% to 4.3%)
Endocrine, metabolic, blood, and immune disorders	32.2 (24.2 to 41.3)	2.7 (1.7 to 4.2)	30.5% (19.0% to 43.1%)	-1.5% (-10.2% to 8.0%)
Chagas disease	33.1 (21.8 to 47.2)	1.6 (0.9 to 2.6)	20.8% (12.2% to 30.2%)	-8.8% (-15.3% to -1.7%)
Silicosis	2.1 (1.7 to 2.6)	1.0 (0.7 to 1.4)	15.1% (10.5% to 19.8%)	-13.1% (-16.6% to -9.6%)
Congenital heart anomalies	2.3 (1.9 to 2.8)	0.3 (0.2 to 0.4)	30.3% (27.9% to 32.8%)	-1.7% (-3.5% to 0.2%)
G6PD deficiency	2.7 (1.9 to 3.5)	0.2 (0.1 to 0.4)	35.2% (28.9% to 41.3%)	2.0% (-2.7% to 6.7%)
Coal workers pneumoconiosis	0.5 (0.4 to 0.6)	0.2 (0.2 to 0.3)	31.6% (25.3% to 38.0%)	-0.7% (-5.4% to 4.2%)
Asbestosis	0.5 (0.4 to 0.6)	0.2 (0.1 to 0.3)	28.0% (25.0% to 30.6%)	-3.4% (-5.6% to -1.4%)
Other pneumoconiosis	0.3 (0.3 to 0.4)	0.2 (0.1 to 0.2)	22.2% (19.1% to 25.4%)	-7.7% (-10.1% to -5.4%)
Iron-deficiency anaemia	0.3 (0.2 to 0.4)	<0.05 (<0.05 to <0.05)	36.8% (32.0% to 41.7%)	3.2% (-0.4% to 7.0%)
Iodine deficiency	0.1 (<0.05 to 0.1)	<0.05 (<0.05 to <0.05)	32.8% (28.4% to 37.2%)	0.3% (-3.1% to 3.6%)
Thalassemias	<0.05 (<0.05 to <0.05)	<0.05 (<0.05 to <0.05)	9.6% (1.3% to 17.8%)	-17.3% (-23.5% to -11.1%)
**All causes**	**8,124.3 (6,978.1 to 9,474.2)**	**1,112.7 (805.8 to 1,462.9)**	**30.5% (28.6% to 32.4%)**	**-1.5% (-2.9% to -0.1%)**

SR Table 13f: Global prevalence and years lived with disability (YLDs) for 2016, percentage change of YLD counts and rates between 2006 and 2016 of heart failure by cause, ages 70-74 years				
**Cause**	**Prevalence in thousands (95% uncertainty interval)**	**YLDs in thousands (95% uncertainty interval)**		
	**2016**	**2016**	**Percentage change in counts between 2006 and 2016**	**Percentage change in rates between 2006 and 2016**
Chronic obstructive pulmonary disease	1,700.4<br>(1,356.9 to 2,104.1)	651.5<br>(477.7 to 820.0)	20.3%<br>(17.3% to 23.2%)	0.5%<br>(-1.9% to 3.0%)
Ischaemic heart disease	3,603.8<br>(2,925.1 to 4,406.2)	286.1<br>(191.4 to 405.5)	16.0%<br>(13.2% to 18.7%)	-3.0%<br>(-5.4% to -0.8%)
Hypertensive heart disease	2,133.7<br>(1,622.3 to 2,735.3)	169.8<br>(108.6 to 245.0)	18.1%<br>(15.2% to 20.9%)	-1.3%<br>(-3.7% to 1.0%)
Other cardiomyopathy	350.8<br>(247.4 to 472.1)	28.1<br>(17.8 to 41.4)	13.1%<br>(8.2% to 18.2%)	-5.5%<br>(-9.6% to -1.2%)
Other cardiovascular and circulatory diseases	279.9<br>(207.9 to 366.4)	22.4<br>(14.3 to 34.3)	13.9%<br>(8.6% to 19.3%)	-4.8%<br>(-9.2% to -0.3%)
Rheumatic heart disease	206.7<br>(162.7 to 249.2)	16.4<br>(10.4 to 23.4)	41.2%<br>(33.3% to 50.6%)	18.0%<br>(11.4% to 25.9%)
Interstitial lung disease and pulmonary sarcoidosis	33.9<br>(23.0 to 44.7)	13.9<br>(8.5 to 20.9)	24.3%<br>(17.2% to 32.7%)	3.9%<br>(-2.0% to 10.9%)
Alcoholic cardiomyopathy	100.6<br>(71.2 to 133.9)	8.1<br>(5.0 to 12.0)	9.3%<br>(0.1% to 20.1%)	-8.7%<br>(-16.3% to 0.4%)
Endocrine, metabolic, blood, and immune disorders	44.4<br>(33.7 to 56.7)	3.7<br>(2.3 to 5.6)	17.3%<br>(7.8% to 28.1%)	-2.0%<br>(-9.9% to 7.1%)
Myocarditis	42.0<br>(29.1 to 57.1)	3.5<br>(2.2 to 5.2)	13.6%<br>(5.4% to 21.4%)	-5.1%<br>(-11.9% to 1.5%)
Endocarditis	41.8<br>(31.2 to 53.8)	3.5<br>(2.2 to 5.4)	17.0%<br>(7.6% to 26.9%)	-2.2%<br>(-10.1% to 6.1%)
Other haemoglobinopathies and haemolytic anaemias	19.6<br>(14.6 to 25.8)	1.7<br>(1.0 to 2.6)	13.8%<br>(2.9% to 25.3%)	-4.9%<br>(-14.0% to 4.8%)
Chagas disease	29.0<br>(19.4 to 40.7)	1.4<br>(0.8 to 2.2)	17.5%<br>(9.6% to 26.5%)	-1.8%<br>(-8.4% to 5.7%)
Silicosis	1.8<br>(1.5 to 2.2)	0.8<br>(0.6 to 1.2)	4.7%<br>(-2.4% to 12.1%)	-12.5%<br>(-18.4% to -6.3%)
Coal workers pneumoconiosis	1.0<br>(0.8 to 1.2)	0.4<br>(0.3 to 0.6)	15.0%<br>(3.8% to 27.2%)	-3.9%<br>(-13.3% to 6.3%)
Asbestosis	0.7<br>(0.6 to 0.8)	0.3<br>(0.2 to 0.4)	20.2%<br>(18.2% to 22.2%)	0.5%<br>(-1.2% to 2.1%)
Other pneumoconiosis	0.5<br>(0.4 to 0.6)	0.2<br>(0.2 to 0.3)	15.4%<br>(12.6% to 18.2%)	-3.5%<br>(-5.9% to -1.2%)
G6PD deficiency	0.7<br>(0.5 to 1.0)	0.1<br>(<0.05 to 0.1)	18.2%<br>(15.1% to 21.6%)	-1.2%<br>(-3.8% to 1.6%)
Iron-deficiency anaemia	0.6<br>(0.5 to 0.8)	0.1<br>(<0.05 to 0.1)	25.6%<br>(21.4% to 29.8%)	4.9%<br>(1.5% to 8.5%)
Congenital heart anomalies	0.2<br>(0.1 to 0.2)	<0.05<br>(<0.05 to <0.05)	26.6%<br>(22.1% to 29.1%)	5.8%<br>(2.1% to 7.9%)
Iodine deficiency	0.1<br>(<0.05 to 0.1)	<0.05<br>(<0.05 to <0.05)	33.6%<br>(27.3% to 39.2%)	11.6%<br>(6.4% to 16.3%)
Thalassemias	<0.05<br>(<0.05 to <0.05)	<0.05<br>(<0.05 to <0.05)	3.7%<br>(-4.9% to 13.3%)	-13.3%<br>(-20.5% to -5.3%)
**All causes**	**8,592.2**<br>**(7,191.7 to 10,196.2)**	**1,212.0**<br>**(896.7 to 1,555.2)**	**18.8%**<br>**(17.1% to 20.5%)**	**-0.7%**<br>**(-2.2% to 0.7%)**

SR Table 13f: Global prevalence and years lived with disability (YLDs) for 2016, percentage change of YLD counts and rates between 2006 and 2016 of heart failure by cause, ages 75-79 years				
**Cause**	**Prevalence in thousands (95% uncertainty interval)**	**YLDs in thousands (95% uncertainty interval)**		
	**2016**	**2016**	**Percentage change in counts between 2006 and 2016**	**Percentage change in rates between 2006 and 2016**
Chronic obstructive pulmonary disease	1,638.0<br>(1,272.3 to 2,049.9)	613.9<br>(463.2 to 748.1)	26.0%<br>(22.6% to 29.4%)	1.3%<br>(-1.4% to 4.0%)
Ischaemic heart disease	3,836.7<br>(3,023.3 to 4,704.9)	299.9<br>(199.2 to 424.2)	20.6%<br>(17.0% to 23.8%)	-3.0%<br>(-5.9% to -0.5%)
Hypertensive heart disease	2,120.6<br>(1,572.9 to 2,749.4)	166.1<br>(107.1 to 246.4)	24.3%<br>(21.0% to 27.4%)	-0.1%<br>(-2.7% to 2.4%)
Other cardiomyopathy	350.2<br>(258.5 to 455.0)	27.5<br>(17.3 to 41.2)	10.6%<br>(5.5% to 15.8%)	-11.0%<br>(-15.1% to -6.9%)
Other cardiovascular and circulatory diseases	302.1<br>(220.2 to 403.7)	23.8<br>(15.0 to 36.2)	14.3%<br>(9.3% to 19.2%)	-8.1%<br>(-12.1% to -4.1%)
Interstitial lung disease and pulmonary sarcoidosis	33.7<br>(23.0 to 44.9)	13.5<br>(8.4 to 20.1)	23.7%<br>(16.1% to 31.5%)	-0.5%<br>(-6.6% to 5.7%)
Rheumatic heart disease	147.9<br>(123.6 to 170.3)	11.6<br>(7.9 to 15.9)	39.9%<br>(30.4% to 50.2%)	12.5%<br>(4.8% to 20.8%)
Alcoholic cardiomyopathy	81.6<br>(59.7 to 106.1)	6.4<br>(4.0 to 9.7)	21.8%<br>(8.7% to 36.5%)	-2.0%<br>(-12.6% to 9.8%)
Endocarditis	62.5<br>(45.9 to 83.1)	5.0<br>(3.1 to 7.7)	13.9%<br>(5.9% to 22.2%)	-8.4%<br>(-14.9% to -1.7%)
Endocrine, metabolic, blood, and immune disorders	40.3<br>(30.1 to 53.1)	3.3<br>(2.1 to 5.0)	12.2%<br>(3.5% to 21.6%)	-9.8%<br>(-16.8% to -2.2%)
Myocarditis	35.1<br>(25.2 to 46.4)	2.9<br>(1.8 to 4.4)	15.4%<br>(5.1% to 25.6%)	-7.2%<br>(-15.5% to 1.0%)
Chagas disease	24.2<br>(16.4 to 33.1)	1.1<br>(0.7 to 1.8)	16.2%<br>(9.2% to 23.7%)	-6.5%<br>(-12.2% to -0.5%)
Other haemoglobinopathies and haemolytic anaemias	12.0<br>(8.6 to 16.1)	1.0<br>(0.6 to 1.6)	15.1%<br>(3.7% to 26.3%)	-7.5%<br>(-16.6% to 1.6%)
Silicosis	1.1<br>(0.9 to 1.3)	0.5<br>(0.3 to 0.7)	13.8%<br>(6.0% to 21.9%)	-8.5%<br>(-14.7% to -2.0%)
Coal workers pneumoconiosis	0.9<br>(0.7 to 1.1)	0.4<br>(0.3 to 0.6)	18.5%<br>(6.6% to 31.9%)	-4.7%<br>(-14.3% to 6.1%)
Asbestosis	0.8<br>(0.6 to 0.9)	0.4<br>(0.2 to 0.5)	12.8%<br>(10.4% to 15.1%)	-9.3%<br>(-11.2% to -7.5%)
Other pneumoconiosis	0.5<br>(0.4 to 0.6)	0.2<br>(0.1 to 0.3)	23.8%<br>(21.1% to 26.7%)	-0.4%<br>(-2.6% to 1.9%)
G6PD deficiency	1.9<br>(1.3 to 2.6)	0.2<br>(0.1 to 0.3)	28.8%<br>(24.1% to 33.4%)	3.6%<br>(-0.2% to 7.3%)
Iron-deficiency anaemia	0.7<br>(0.5 to 0.9)	0.1<br>(<0.05 to 0.1)	24.4%<br>(11.4% to 38.7%)	0.0%<br>(-10.4% to 11.6%)
Congenital heart anomalies	0.1<br>(0.1 to 0.2)	<0.05<br>(<0.05 to <0.05)	28.9%<br>(23.9% to 31.7%)	3.6%<br>(-0.4% to 5.9%)
Iodine deficiency	<0.05<br>(<0.05 to <0.05)	<0.05<br>(<0.05 to <0.05)	34.6%<br>(30.0% to 38.4%)	8.3%<br>(4.5% to 11.3%)
Thalassemias	<0.05<br>(<0.05 to <0.05)	<0.05<br>(<0.05 to <0.05)	0.6%<br>(-8.1% to 10.0%)	-19.1%<br>(-26.1% to -11.5%)
**All causes**	**8,690.7<br>(7,049.3 to 10,485.7)**	**1,177.7<br>(868.7 to 1,487.6)**	**23.6%<br>(21.5% to 25.6%)**	**-0.6%<br>(-2.3% to 1.0%)**

SR Table 13f: Global prevalence and years lived with disability (YLDs) for 2016, percentage change of YLD counts and rates between 2006 and 2016 of heart failure by cause, ages 80-84				
**Cause**	**Prevalence in thousands (95% uncertainty interval)**	**YLDs in thousands (95% uncertainty interval)**		
	**2016**	**2016**	**Percentage change in counts between 2006 and 2016**	**Percentage change in rates between 2006 and 2016**
Chronic obstructive pulmonary disease	1,296.6<br>(988.8 to 1,639.3)	473.5<br>(385.7 to 546.3)	35.1%<br>(30.6% to 40.2%)	3.1%<br>(-0.3% to 7.1%)
Ischaemic heart disease	3,220.1<br>(2,624.1 to 3,870.8)	247.8<br>(166.8 to 342.4)	24.2%<br>(20.4% to 27.7%)	-5.2%<br>(-8.1% to -2.5%)
Hypertensive heart disease	1,821.4<br>(1,365.0 to 2,326.1)	140.3<br>(93.8 to 207.8)	31.4%<br>(28.2% to 34.9%)	0.3%<br>(-2.1% to 3.0%)
Other cardiomyopathy	333.8<br>(245.4 to 439.4)	25.8<br>(16.7 to 38.4)	17.9%<br>(12.2% to 23.7%)	-10.0%<br>(-14.3% to -5.6%)
Other cardiovascular and circulatory diseases	309.0<br>(229.8 to 407.4)	23.9<br>(15.0 to 35.7)	18.8%<br>(13.8% to 24.1%)	-9.3%<br>(-13.1% to -5.3%)
Interstitial lung disease and pulmonary sarcoidosis	28.4<br>(19.1 to 39.0)	11.0<br>(6.9 to 16.7)	32.7%<br>(25.2% to 41.5%)	1.3%<br>(-4.4% to 8.1%)
Rheumatic heart disease	84.0<br>(73.4 to 94.9)	6.5<br>(4.5 to 8.8)	32.5%<br>(24.6% to 41.5%)	1.2%<br>(-4.9% to 8.1%)
Endocarditis	46.9<br>(34.9 to 61.5)	3.7<br>(2.3 to 5.5)	20.2%<br>(11.4% to 28.5%)	-8.2%<br>(-14.9% to -1.9%)
Endocrine, metabolic, blood, and immune disorders	41.1<br>(30.9 to 53.6)	3.2<br>(2.0 to 4.8)	17.4%<br>(9.2% to 26.1%)	-10.4%<br>(-16.6% to -3.7%)
Alcoholic cardiomyopathy	40.9<br>(29.8 to 54.7)	3.2<br>(2.0 to 4.8)	16.0%<br>(3.0% to 31.0%)	-11.4%<br>(-21.4% to 0.1%)
Myocarditis	30.6<br>(22.1 to 40.7)	2.4<br>(1.5 to 3.7)	25.3%<br>(14.6% to 36.6%)	-4.3%<br>(-12.5% to 4.3%)
Other haemoglobinopathies and haemolytic anaemias	23.1<br>(17.1 to 30.8)	1.9<br>(1.2 to 2.8)	27.5%<br>(18.3% to 37.9%)	-2.6%<br>(-9.7% to 5.3%)
Chagas disease	18.3<br>(12.7 to 24.6)	0.9<br>(0.5 to 1.3)	26.6%<br>(18.7% to 35.4%)	-3.3%<br>(-9.4% to 3.4%)
Silicosis	1.1<br>(0.9 to 1.3)	0.5<br>(0.3 to 0.7)	34.5%<br>(23.6% to 46.0%)	2.7%<br>(-5.6% to 11.5%)
Asbestosis	0.7<br>(0.6 to 0.8)	0.3<br>(0.2 to 0.4)	15.9%<br>(10.7% to 21.4%)	-11.5%<br>(-15.5% to -7.3%)
Coal workers pneumoconiosis	0.6<br>(0.5 to 0.7)	0.3<br>(0.2 to 0.4)	23.5%<br>(11.3% to 36.1%)	-5.7%<br>(-15.0% to 3.9%)
Other pneumoconiosis	0.5<br>(0.4 to 0.6)	0.2<br>(0.2 to 0.3)	36.6%<br>(33.2% to 39.8%)	4.3%<br>(1.7% to 6.7%)
G6PD deficiency	1.0<br>(0.7 to 1.3)	0.1<br>(0.1 to 0.1)	31.9%<br>(27.6% to 36.1%)	0.7%<br>(-2.6% to 4.0%)
Iron-deficiency anaemia	0.7<br>(0.5 to 1.0)	0.1<br>(<0.05 to 0.1)	36.3%<br>(25.4% to 48.1%)	4.1%<br>(-4.3% to 13.1%)
Congenital heart anomalies	0.1<br>(<0.05 to 0.1)	<0.05<br>(<0.05 to <0.05)	34.1%<br>(27.9% to 37.4%)	2.4%<br>(-2.4% to 4.9%)
Iodine deficiency	<0.05<br>(<0.05 to <0.05)	<0.05<br>(<0.05 to <0.05)	29.9%<br>(21.9% to 37.1%)	-0.8%<br>(-6.9% to 4.7%)
Thalassemias	<0.05<br>(<0.05 to <0.05)	<0.05<br>(<0.05 to <0.05)	10.8%<br>(0.4% to 21.6%)	-15.4%<br>(-23.4% to -7.2%)
**All causes**	**7,298.8<br>(6,171.6 to 8,603.9)**	**945.5<br>(729.1 to 1,139.5)**	**30.3%<br>(27.9% to 32.8%)**	**-0.5%<br>(-2.4% to 1.4%)**

SR Table 13f: Global prevalence and years lived with disability (YLDs) for 2016, percentage change of YLD counts and rates between 2006 and 2016 of heart failure by cause, ages 85-89				
**Cause**	**Prevalence in thousands (95% uncertainty interval)**	**YLDs in thousands (95% uncertainty interval)**		
	**2016**	**2016**	**Percentage change in counts between 2006 and 2016**	**Percentage change in rates between 2006 and 2016**
Chronic obstructive pulmonary disease	775.2<br>(556.1 to 999.6)	276.6<br>(233.4 to 316.6)	59.8%<br>(53.5% to 65.8%)	-0.4%<br>(-4.4% to 3.3%)
Ischaemic heart disease	2,612.0<br>(2,183.9 to 3,133.7)	198.1<br>(136.6 to 274.0)	60.9%<br>(57.6% to 64.0%)	0.2%<br>(-1.8% to 2.2%)
Hypertensive heart disease	1,427.3<br>(1,112.2 to 1,802.9)	108.4<br>(71.3 to 155.6)	60.6%<br>(57.1% to 63.8%)	0.1%<br>(-2.1% to 2.1%)
Other cardiomyopathy	319.0<br>(244.6 to 414.7)	24.2<br>(16.0 to 36.0)	50.8%<br>(45.0% to 56.8%)	-6.1%<br>(-9.7% to -2.3%)
Other cardiovascular and circulatory diseases	290.7<br>(216.9 to 376.0)	22.1<br>(14.1 to 32.4)	45.2%<br>(40.6% to 49.8%)	-9.5%<br>(-12.4% to -6.7%)
Interstitial lung disease and pulmonary sarcoidosis	19.6<br>(13.7 to 26.5)	7.4<br>(4.7 to 10.8)	61.0%<br>(52.8% to 70.7%)	0.3%<br>(-4.8% to 6.4%)
Endocarditis	45.9<br>(34.4 to 59.9)	3.5<br>(2.2 to 5.2)	45.2%<br>(37.1% to 53.2%)	-9.5%<br>(-14.6% to -4.5%)
Rheumatic heart disease	38.7<br>(34.2 to 43.2)	3.0<br>(2.1 to 4.1)	47.1%<br>(39.1% to 55.2%)	-8.3%<br>(-13.3% to -3.3%)
Endocrine, metabolic, blood, and immune disorders	37.5<br>(28.2 to 48.3)	2.9<br>(1.8 to 4.2)	43.0%<br>(34.6% to 50.7%)	-10.9%<br>(-16.1% to -6.1%)
Alcoholic cardiomyopathy	20.8<br>(15.7 to 27.2)	1.6<br>(1.0 to 2.4)	82.4%<br>(62.2% to 104.6%)	13.6%<br>(1.1% to 27.5%)
Other haemoglobinopathies and haemolytic anaemias	6.9<br>(5.1 to 9.1)	0.5<br>(0.3 to 0.8)	59.4%<br>(43.2% to 76.9%)	-0.7%<br>(-10.8% to 10.2%)
Chagas disease	11.1<br>(7.8 to 14.7)	0.5<br>(0.3 to 0.8)	41.0%<br>(31.1% to 51.6%)	-12.2%<br>(-18.3% to -5.6%)
Silicosis	1.0<br>(0.8 to 1.2)	0.4<br>(0.3 to 0.6)	73.9%<br>(51.9% to 99.3%)	8.3%<br>(-5.3% to 24.2%)
Asbestosis	0.5<br>(0.4 to 0.6)	0.2<br>(0.2 to 0.3)	40.0%<br>(31.3% to 49.9%)	-12.7%<br>(-18.2% to -6.6%)
Coal workers pneumoconiosis	0.4<br>(0.3 to 0.5)	0.2<br>(0.1 to 0.2)	50.9%<br>(37.4% to 66.0%)	-6.0%<br>(-14.4% to 3.4%)
Other pneumoconiosis	0.3<br>(0.3 to 0.4)	0.2<br>(0.1 to 0.2)	67.6%<br>(64.4% to 71.1%)	4.4%<br>(2.4% to 6.6%)
Iron-deficiency anaemia	0.7<br>(0.5 to 0.9)	0.1<br>(<0.05 to 0.1)	61.9%<br>(43.6% to 83.4%)	0.9%<br>(-10.5% to 14.2%)
G6PD deficiency	0.1<br>(0.1 to 0.2)	<0.05<br>(<0.05 to <0.05)	52.0%<br>(43.2% to 61.3%)	-5.3%<br>(-10.8% to 0.5%)
Congenital heart anomalies	<0.05<br>(<0.05 to 0.1)	<0.05<br>(<0.05 to <0.05)	67.4%<br>(58.4% to 71.3%)	4.3%<br>(-1.3% to 6.7%)
Myocarditis	<0.05<br>(<0.05 to <0.05)	<0.05<br>(<0.05 to <0.05)	5.8%<br>(-4.4% to 16.2%)	-34.1%<br>(-40.4% to -27.6%)
Iodine deficiency	<0.05<br>(<0.05 to <0.05)	<0.05<br>(<0.05 to <0.05)	68.2%<br>(49.6% to 81.8%)	4.8%<br>(-6.8% to 13.3%)
Thalassemias	<0.05<br>(<0.05 to <0.05)	<0.05<br>(<0.05 to <0.05)	46.0%<br>(35.8% to 56.0%)	-9.1%<br>(-15.4% to -2.8%)
**All causes**	**5,607.8<br>(4,827.6 to 6,549.0)**	**649.7<br>(522.2 to 784.7)**	**59.2%<br>(56.1% to 62.0%)**	**-0.8%<br>(-2.8% to 0.9%)**

SR Table 13f: Global prevalence and years lived with disability (YLDs) for 2016, percentage change of YLD counts and rates between 2006 and 2016 of heart failure by cause, ages 90-94				
**Cause**	**Prevalence in thousands (95% uncertainty interval)**	**YLDs in thousands (95% uncertainty interval)**		
	**2016**	**2016**	**Percentage change in counts between 2006 and 2016**	**Percentage change in rates between 2006 and 2016**
Chronic obstructive pulmonary disease	279.2 (194.4 to 371.5)	97.9 (81.3 to 114.3)	70.1% (63.2% to 76.5%)	1.6% (-2.5% to 5.4%)
Ischaemic heart disease	1,212.8 (964.2 to 1,516.4)	90.9 (61.3 to 127.2)	67.6% (63.8% to 71.3%)	0.1% (-2.1% to 2.4%)
Hypertensive heart disease	678.2 (502.5 to 882.9)	50.9 (32.2 to 74.2)	69.4% (64.8% to 73.2%)	1.2% (-1.5% to 3.5%)
Other cardiomyopathy	173.7 (125.3 to 232.7)	13.0 (8.3 to 19.3)	66.7% (59.6% to 74.1%)	-0.4% (-4.6% to 4.0%)
Other cardiovascular and circulatory diseases	159.4 (115.8 to 213.8)	11.9 (7.6 to 17.7)	60.2% (54.7% to 66.0%)	-4.3% (-7.6% to -0.8%)
Interstitial lung disease and pulmonary sarcoidosis	8.6 (6.0 to 11.3)	3.1 (2.0 to 4.6)	76.7% (65.5% to 88.5%)	5.5% (-1.1% to 12.6%)
Endocrine, metabolic, blood, and immune disorders	19.7 (14.5 to 26.2)	1.5 (1.0 to 2.2)	57.7% (49.0% to 66.8%)	-5.8% (-11.0% to -0.3%)
Other haemoglobinopathies and haemolytic anaemias	16.6 (12.0 to 22.4)	1.2 (0.8 to 1.9)	59.8% (48.3% to 70.7%)	-4.6% (-11.4% to 2.0%)
Endocarditis	13.7 (10.0 to 18.4)	1.0 (0.7 to 1.6)	61.4% (50.3% to 72.1%)	-3.6% (-10.2% to 2.8%)
Rheumatic heart disease	11.9 (10.5 to 13.3)	0.9 (0.6 to 1.2)	63.6% (53.7% to 74.4%)	-2.3% (-8.2% to 4.2%)
Chagas disease	4.6 (3.3 to 6.0)	0.2 (0.1 to 0.3)	58.1% (47.7% to 68.5%)	-5.5% (-11.7% to 0.7%)
Alcoholic cardiomyopathy	2.1 (1.5 to 2.8)	0.2 (0.1 to 0.2)	60.2% (26.2% to 102.3%)	-4.3% (-24.6% to 20.8%)
Asbestosis	0.2 (0.2 to 0.3)	0.1 (0.1 to 0.1)	67.5% (54.7% to 81.8%)	0.1% (-7.6% to 8.6%)
Other pneumoconiosis	0.1 (0.1 to 0.2)	0.1 (<0.05 to 0.1)	72.4% (66.3% to 78.5%)	3.0% (-0.6% to 6.7%)
Coal workers pneumoconiosis	0.1 (0.1 to 0.1)	<0.05 (<0.05 to 0.1)	62.7% (48.7% to 80.2%)	-2.8% (-11.2% to 7.6%)
Iron-deficiency anaemia	0.3 (0.2 to 0.5)	<0.05 (<0.05 to <0.05)	64.8% (54.9% to 75.2%)	-1.5% (-7.4% to 4.7%)
G6PD deficiency	0.1 (0.1 to 0.1)	<0.05 (<0.05 to <0.05)	64.3% (59.8% to 68.7%)	-1.8% (-4.5% to 0.8%)
Congenital heart anomalies	<0.05 (<0.05 to <0.05)	<0.05 (<0.05 to <0.05)	76.3% (62.4% to 80.5%)	5.4% (-3.0% to 7.9%)
Myocarditis	<0.05 (<0.05 to <0.05)	<0.05 (<0.05 to <0.05)	36.7% (10.4% to 69.8%)	-18.3% (-34.0% to 1.4%)
Silicosis	<0.05 (<0.05 to <0.05)	<0.05 (<0.05 to <0.05)	58.3% (36.2% to 83.1%)	-5.4% (-18.6% to 9.4%)
Thalassemias	<0.05 (<0.05 to <0.05)	<0.05 (<0.05 to <0.05)	49.8% (40.1% to 60.3%)	-10.5% (-16.3% to -4.3%)
Iodine deficiency	<0.05 (<0.05 to <0.05)	<0.05 (<0.05 to <0.05)	71.7% (47.8% to 96.4%)	2.6% (-11.7% to 17.3%)
**All causes**	**2,581.2 (2,124.4 to 3,119.3)**	**273.0 (211.5 to 337.2)**	**68.4% (65.2% to 71.1%)**	**0.6% (-1.3% to 2.2%)**

SR Table 13f: Global prevalence and years lived with disability (YLDs) for 2016, percentage change of YLD counts and rates between 2006 and 2016 of heart failure by cause, ages 95+ years				
**Cause**	**Prevalence in thousands (95% uncertainty interval)**	**YLDs in thousands (95% uncertainty interval)**		
	**2016**	**2016**	**Percentage change in counts between 2006 and 2016**	**Percentage change in rates between 2006 and 2016**
Ischaemic heart disease	303.6 (228.4 to 394.6)	22.8 (14.9 to 32.8)	55.8% (51.0% to 60.1%)	-0.6% (-3.6% to 2.2%)
Hypertensive heart disease	181.5 (125.2 to 253.1)	13.6 (8.2 to 21.2)	61.1% (56.1% to 65.6%)	2.8% (-0.4% to 5.7%)
Other cardiovascular and circulatory diseases	44.8 (29.9 to 64.2)	3.4 (2.0 to 5.2)	46.5% (40.4% to 52.2%)	-6.5% (-10.4% to -2.9%)
Other cardiomyopathy	42.5 (26.3 to 62.4)	3.2 (1.8 to 5.1)	43.9% (36.9% to 51.3%)	-8.2% (-12.6% to -3.4%)
Interstitial lung disease and pulmonary sarcoidosis	2.1 (1.5 to 2.9)	0.8 (0.5 to 1.1)	68.2% (58.1% to 79.3%)	7.3% (0.9% to 14.4%)
Endocrine, metabolic, blood, and immune disorders	6.8 (4.6 to 9.7)	0.5 (0.3 to 0.8)	54.4% (46.5% to 61.9%)	-1.5% (-6.5% to 3.3%)
Endocarditis	6.0 (3.9 to 8.5)	0.4 (0.3 to 0.7)	51.8% (42.6% to 60.9%)	-3.1% (-9.0% to 2.7%)
Rheumatic heart disease	2.3 (1.9 to 2.7)	0.2 (0.1 to 0.2)	68.0% (56.0% to 81.4%)	7.2% (-0.5% to 15.7%)
Chagas disease	1.9 (1.6 to 2.3)	0.1 (0.1 to 0.1)	55.2% (46.4% to 63.5%)	-0.9% (-6.6% to 4.3%)
Iron-deficiency anaemia	0.3 (0.2 to 0.4)	<0.05 (<0.05 to <0.05)	52.0% (22.7% to 87.3%)	-3.0% (-21.7% to 19.5%)
Asbestosis	<0.05 (<0.05 to <0.05)	<0.05 (<0.05 to <0.05)	76.8% (65.9% to 87.6%)	12.8% (5.9% to 19.7%)
Congenital heart anomalies	<0.05 (<0.05 to <0.05)	<0.05 (<0.05 to <0.05)	64.9% (52.4% to 69.9%)	5.2% (-2.8% to 8.4%)
Alcoholic cardiomyopathy	<0.05 (<0.05 to <0.05)	<0.05 (<0.05 to <0.05)	43.4% (-29.5% to 199.5%)	-8.5% (-55.0% to 91.1%)
Other haemoglobinopathies and haemolytic anaemias	<0.05 (<0.05 to <0.05)	<0.05 (<0.05 to <0.05)	48.7% (-19.3% to 245.2%)	-5.1% (-48.5% to 120.3%)
Myocarditis	<0.05 (<0.05 to <0.05)	<0.05 (<0.05 to <0.05)	40.7% (-6.0% to 115.1%)	-10.2% (-40.0% to 37.3%)
Other pneumoconiosis	<0.05 (<0.05 to <0.05)	<0.05 (<0.05 to <0.05)	93.5% (48.6% to 160.8%)	23.5% (-5.2% to 66.4%)
Silicosis	<0.05 (<0.05 to <0.05)	<0.05 (<0.05 to <0.05)	55.6% (32.5% to 82.3%)	-0.7% (-15.5% to 16.4%)
Thalassemias	<0.05 (<0.05 to <0.05)	<0.05 (<0.05 to <0.05)	41.9% (31.8% to 53.6%)	-9.5% (-15.9% to -2.0%)
G6PD deficiency	<0.05 (<0.05 to <0.05)	<0.05 (<0.05 to <0.05)	49.5% (30.4% to 72.3%)	-4.6% (-16.8% to 9.9%)
Iodine deficiency	<0.05 (<0.05 to <0.05)	<0.05 (<0.05 to <0.05)	77.2% (32.3% to 138.7%)	13.1% (-15.6% to 52.3%)
**All causes**	**591.9 (457.6 to 752.9)**	**45.0 (29.4 to 64.6)**	**55.9% (52.9% to 58.9%)**	**-0.5% (-2.4% to 1.4%)**

SR Table 13g: Global prevalence and years lived with disability (YLDs) for 2016, percentage change of YLD counts and rates between 2006 and 2016 of infertility by cause, ages 0-6 days				
**Cause**	**Prevalence in thousands (95% uncertainty interval)**	**YLDs in thousands (95% uncertainty interval)**		
	**2016**	**2016**	**Percentage change in counts between 2006 and 2016**	**Percentage change in rates between 2006 and 2016**
Urogenital congenital anomalies	0.8<br>(0.5 to 1.1)	<0.05<br>(<0.05 to <0.05)	-5.5%<br>(-10.2% to -0.7%)	-5.0%<br>(-9.7% to -0.2%)
**All causes**	**0.8<br>(0.5 to 1.1)**	**<0.05<br>(<0.05 to <0.05)**	**-5.5%<br>(-10.2% to -0.7%)**	**-5.0%<br>(-9.7% to -0.2%)**

SR Table 13g: Global prevalence and years lived with disability (YLDs) for 2016, percentage change of YLD counts and rates between 2006 and 2016 of infertility by cause, ages 7-27 days				
**Cause**	**Prevalence in thousands (95% uncertainty interval)**	**YLDs in thousands (95% uncertainty interval)**		
	**2016**	**2016**	**Percentage change in counts between 2006 and 2016**	**Percentage change in rates between 2006 and 2016**
Urogenital congenital anomalies	2.3<br>(1.5 to 3.2)	<0.05<br>(<0.05 to 0.1)	-5.1%<br>(-10.0% to 0.3%)	-5.0%<br>(-9.8% to 0.5%)
**All causes**	**2.3<br>(1.5 to 3.2)**	**<0.05<br>(<0.05 to 0.1)**	**-5.1%<br>(-10.0% to 0.3%)**	**-5.0%<br>(-9.8% to 0.5%)**

SR Table 13g: Global prevalence and years lived with disability (YLDs) for 2016, percentage change of YLD counts and rates between 2006 and 2016 of infertility by cause, ages 28-364 days				
**Cause**	**Prevalence in thousands (95% uncertainty interval)**	**YLDs in thousands (95% uncertainty interval)**		
	**2016**	**2016**	**Percentage change in counts between 2006 and 2016**	**Percentage change in rates between 2006 and 2016**
Urogenital congenital anomalies	33.7<br>(22.1 to 47.6)	0.4<br>(0.2 to 0.9)	-4.2%<br>(-9.1% to 0.7%)	-5.0%<br>(-9.8% to -0.1%)
**All causes**	**33.7<br>(22.1 to 47.6)**	**0.4<br>(0.2 to 0.9)**	**-4.2%<br>(-9.1% to 0.7%)**	**-5.0%<br>(-9.8% to -0.1%)**

SR Table 13g: Global prevalence and years lived with disability (YLDs) for 2016, percentage change of YLD counts and rates between 2006 and 2016 of infertility by cause, ages 1-4 years				
Cause	Prevalence in thousands (95% uncertainty interval)	YLDs in thousands (95% uncertainty interval)		
	2016	2016	Percentage change in counts between 2006 and 2016	Percentage change in rates between 2006 and 2016
Urogenital congenital anomalies	114.5<br>(75.3 to 160.1)	1.5<br>(0.6 to 3.2)	0.2%<br>(-4.0% to 4.6%)	-5.1%<br>(-9.1% to -0.9%)
**All causes**	**114.5<br>(75.3 to 160.1)**	**1.5<br>(0.6 to 3.2)**	**0.2%<br>(-4.0% to 4.6%)**	**-5.1%<br>(-9.1% to -0.9%)**

SR Table 13g: Global prevalence and years lived with disability (YLDs) for 2016, percentage change of YLD counts and rates between 2006 and 2016 of infertility by cause, ages 5-9 years				
**Cause**	**Prevalence in thousands (95% uncertainty interval)**	**YLDs in thousands (95% uncertainty interval)**		
	**2016**	**2016**	**Percentage change in counts between 2006 and 2016**	**Percentage change in rates between 2006 and 2016**
Urogenital congenital anomalies	104.3<br>(69.3 to 144.3)	1.3<br>(0.5 to 2.9)	3.3%<br>(-0.7% to 8.0%)	-4.1%<br>(-7.8% to 0.3%)
**All causes**	**104.3<br>(69.3 to 144.3)**	**1.3<br>(0.5 to 2.9)**	**3.3%<br>(-0.7% to 8.0%)**	**-4.1%<br>(-7.8% to 0.3%)**

SR Table 13g: Global prevalence and years lived with disability (YLDs) for 2016, percentage change of YLD counts and rates between 2006 and 2016 of infertility by cause, ages 10-14 years				
**Cause**	**Prevalence in thousands (95% uncertainty interval)**	**YLDs in thousands (95% uncertainty interval)**		
	**2016**	**2016**	**Percentage change in counts between 2006 and 2016**	**Percentage change in rates between 2006 and 2016**
Urogenital congenital anomalies	74.8<br>(50.1 to 102.7)	1.0<br>(0.4 to 2.0)	-3.3%<br>(-5.4% to -1.1%)	-4.1%<br>(-6.2% to -2.0%)
Maternal sepsis and other pregnancy related infections	0.9<br>(0.6 to 1.4)	<0.05<br>(<0.05 to <0.05)	14.0%<br>(8.1% to 23.6%)	13.1%<br>(7.2% to 22.6%)
**All causes**	**75.7**<br>**(51.1 to 103.4)**	**1.0**<br>**(0.4 to 2.1)**	**-3.3%**<br>**(-5.3% to -1.1%)**	**-4.1%**<br>**(-6.1% to -1.9%)**

SR Table 13g: Global prevalence and years lived with disability (YLDs) for 2016, percentage change of YLD counts and rates between 2006 and 2016 of infertility by cause, ages 15-19 years				
**Cause**	**Prevalence in thousands (95% uncertainty interval)**	**YLDs in thousands (95% uncertainty interval)**		
	**2016**	**2016**	**Percentage change in counts between 2006 and 2016**	**Percentage change in rates between 2006 and 2016**
Male infertility	1,041.5<br>(628.3 to 1,600.0)	7.3<br>(2.7 to 16.2)	12.2%<br>(1.5% to 22.2%)	18.7%<br>(7.5% to 29.3%)
Female infertility	1,021.7<br>(505.1 to 1,775.6)	6.8<br>(2.2 to 15.4)	24.2%<br>(-29.0% to 74.8%)	31.4%<br>(-24.8% to 85.1%)
Turner syndrome	294.7<br>(257.7 to 336.1)	6.1<br>(3.1 to 9.9)	-6.3%<br>(-14.6% to 2.1%)	-0.8%<br>(-9.6% to 8.1%)
Other sexually transmitted diseases	384.7<br>(277.5 to 495.4)	2.5<br>(1.0 to 5.2)	-5.8%<br>(-11.7% to 0.1%)	-0.3%<br>(-6.6% to 6.0%)
Chlamydial infection	305.1<br>(234.5 to 376.1)	2.0<br>(0.8 to 4.2)	-0.6%<br>(-7.7% to 6.8%)	5.2%<br>(-2.3% to 13.1%)
Endometriosis	11.9<br>(7.5 to 17.8)	1.2<br>(0.7 to 2.0)	-17.4%<br>(-22.0% to -11.6%)	-12.6%<br>(-17.4% to -6.4%)
Polycystic ovarian syndrome	57.4<br>(30.8 to 96.3)	0.8<br>(0.3 to 1.7)	-4.3%<br>(-15.9% to 9.0%)	1.3%<br>(-11.0% to 15.4%)
Urogenital congenital anomalies	58.4<br>(39.3 to 79.8)	0.7<br>(0.3 to 1.6)	-9.8%<br>(-12.2% to -7.5%)	-4.5%<br>(-7.0% to -2.1%)
Gonococcal infection	72.8<br>(53.5 to 93.4)	0.5<br>(0.2 to 1.0)	-0.9%<br>(-12.2% to 11.7%)	4.9%<br>(-7.1% to 18.2%)
Maternal sepsis and other pregnancy related infections	12.6<br>(9.4 to 15.9)	0.1<br>(<0.05 to 0.1)	2.2%<br>(-17.8% to 28.5%)	8.2%<br>(-13.0% to 36.0%)
**All causes**	**3,260.8**<br>**(2,140.8 to 4,771.7)**	**28.0**<br>**(13.2 to 53.6)**	**4.3%**<br>**(-11.3% to 15.1%)**	**10.4%**<br>**(-6.1% to 21.8%)**

SR Table 13g: Global prevalence and years lived with disability (YLDs) for 2016, percentage change of YLD counts and rates between 2006 and 2016 of infertility by cause, ages 20-24 years				
Cause	Prevalence in thousands (95% uncertainty interval)	YLDs in thousands (95% uncertainty interval)		
	2016	2016	Percentage change in counts between 2006 and 2016	Percentage change in rates between 2006 and 2016
Female infertility	6,529.8<br>(3,772.0 to 9,869.6)	41.0<br>(14.8 to 93.6)	30.1%<br>(19.8% to 41.9%)	24.1%<br>(14.3% to 35.4%)
Male infertility	4,431.7<br>(2,994.6 to 6,293.7)	29.4<br>(10.9 to 62.3)	21.2%<br>(13.6% to 28.3%)	15.6%<br>(8.3% to 22.4%)
Other sexually transmitted diseases	1,827.0<br>(1,638.4 to 2,011.9)	11.1<br>(4.6 to 22.3)	6.5%<br>(2.4% to 10.0%)	1.6%<br>(-2.3% to 5.0%)
Chlamydial infection	981.8<br>(864.4 to 1,081.9)	6.0<br>(2.5 to 11.9)	9.1%<br>(4.5% to 13.7%)	4.1%<br>(-0.3% to 8.5%)
Turner syndrome	284.3<br>(248.6 to 323.3)	5.9<br>(3.0 to 9.6)	2.2%<br>(-7.1% to 11.9%)	-2.5%<br>(-11.4% to 6.7%)
Klinefelter syndrome	353.6<br>(305.8 to 407.4)	3.6<br>(1.7 to 6.9)	5.2%<br>(-0.4% to 10.7%)	0.3%<br>(-5.0% to 5.6%)
Endometriosis	33.0<br>(23.8 to 45.6)	3.4<br>(2.0 to 5.2)	-0.9%<br>(-4.7% to 3.7%)	-5.5%<br>(-9.1% to -1.1%)
Polycystic ovarian syndrome	226.7<br>(150.6 to 333.4)	2.6<br>(1.1 to 5.5)	5.0%<br>(-2.3% to 12.5%)	0.2%<br>(-6.8% to 7.3%)
Gonococcal infection	294.0<br>(261.5 to 327.6)	1.8<br>(0.7 to 3.6)	9.2%<br>(2.1% to 17.0%)	4.2%<br>(-2.6% to 11.6%)
Urogenital congenital anomalies	51.5<br>(34.9 to 69.7)	0.7<br>(0.3 to 1.4)	4.1%<br>(2.0% to 6.1%)	-0.7%<br>(-2.7% to 1.2%)
Maternal sepsis and other pregnancy related infections	55.1<br>(43.0 to 67.8)	0.3<br>(0.1 to 0.6)	3.6%<br>(-10.1% to 17.7%)	-1.1%<br>(-14.2% to 12.3%)
**All causes**	**15,068.4**<br>**(10,654.0 to 20,202.6)**	**105.7**<br>**(45.0 to 216.3)**	**18.4%**<br>**(11.5% to 24.2%)**	**12.9%**<br>**(6.3% to 18.5%)**

SR Table 13g: Global prevalence and years lived with disability (YLDs) for 2016, percentage change of YLD counts and rates between 2006 and 2016 of infertility by cause, ages 25-29 years				
**Cause**	**Prevalence in thousands (95% uncertainty interval)**	**YLDs in thousands (95% uncertainty interval)**		
	**2016**	**2016**	**Percentage change in counts between 2006 and 2016**	**Percentage change in rates between 2006 and 2016**
Female infertility	7,382.6 (3,316.0 to 13,874.7)	42.7 (12.2 to 113.4)	55.0% (42.0% to 80.1%)	30.5% (19.6% to 51.7%)
Male infertility	5,256.9 (3,526.7 to 7,573.8)	31.9 (11.7 to 71.7)	32.9% (25.5% to 39.0%)	11.9% (5.7% to 17.1%)
Other sexually transmitted diseases	4,226.3 (3,728.7 to 4,749.0)	24.1 (9.9 to 49.1)	21.1% (15.8% to 25.9%)	2.0% (-2.4% to 6.0%)
Chlamydial infection	1,812.7 (1,588.8 to 2,019.8)	10.4 (4.3 to 21.1)	22.0% (16.1% to 27.5%)	2.8% (-2.2% to 7.4%)
Turner syndrome	267.3 (232.3 to 303.5)	5.5 (2.8 to 8.9)	16.3% (5.5% to 26.7%)	-2.0% (-11.1% to 6.7%)
Gonococcal infection	642.3 (557.5 to 716.5)	3.7 (1.5 to 7.6)	22.3% (14.4% to 29.3%)	3.0% (-3.6% to 8.9%)
Endometriosis	34.5 (25.0 to 46.8)	3.6 (2.1 to 5.5)	21.9% (15.1% to 29.5%)	2.7% (-3.0% to 9.1%)
Polycystic ovarian syndrome	327.8 (212.2 to 483.8)	3.3 (1.3 to 7.2)	19.9% (11.9% to 28.3%)	1.0% (-5.7% to 8.1%)
Klinefelter syndrome	309.1 (267.6 to 355.7)	3.1 (1.4 to 6.0)	16.1% (9.8% to 23.0%)	-2.2% (-7.5% to 3.6%)
Urogenital congenital anomalies	47.0 (31.8 to 63.4)	0.6 (0.2 to 1.3)	20.9% (18.3% to 23.7%)	1.9% (-0.4% to 4.2%)
Maternal sepsis and other pregnancy related infections	98.8 (80.9 to 117.9)	0.5 (0.2 to 1.1)	14.3% (3.5% to 25.1%)	-3.7% (-12.8% to 5.4%)
**All causes**	**20,405.4 (14,010.6 to 29,483.7)**	**129.6 (52.9 to 278.0)**	**33.3% (26.8% to 39.2%)**	**12.3% (6.9% to 17.3%)**

SR Table 13g: Global prevalence and years lived with disability (YLDs) for 2016, percentage change of YLD counts and rates between 2006 and 2016 of infertility by cause, ages 30-34 years				
**Cause**	**Prevalence in thousands (95% uncertainty interval)**	**YLDs in thousands (95% uncertainty interval)**		
	**2016**	**2016**	**Percentage change in counts between 2006 and 2016**	**Percentage change in rates between 2006 and 2016**
Female infertility	7,124.9 (2,363.7 to 14,286.5)	39.3 (9.1 to 106.4)	46.8% (31.0% to 95.0%)	30.0% (16.0% to 72.7%)
Other sexually transmitted diseases	6,709.5 (5,751.4 to 7,678.4)	36.9 (15.2 to 78.1)	12.0% (7.5% to 17.3%)	-0.8% (-4.8% to 3.9%)
Male infertility	5,747.5 (3,794.3 to 8,283.2)	33.0 (12.5 to 72.6)	21.7% (15.8% to 26.8%)	7.8% (2.6% to 12.3%)
Chlamydial infection	2,586.1 (2,208.8 to 2,953.7)	14.3 (5.9 to 30.1)	18.4% (13.0% to 24.1%)	4.8% (0.1% to 9.9%)
Gonococcal infection	996.7 (837.0 to 1,143.3)	5.5 (2.3 to 11.6)	17.6% (11.0% to 24.8%)	4.2% (-1.7% to 10.5%)
Turner syndrome	220.2 (190.9 to 250.9)	4.5 (2.3 to 7.2)	14.4% (3.4% to 26.4%)	1.3% (-8.4% to 12.0%)
Polycystic ovarian syndrome	398.9 (259.5 to 574.2)	3.8 (1.5 to 8.3)	17.8% (10.6% to 26.0%)	4.3% (-2.1% to 11.6%)
Endometriosis	24.7 (18.1 to 34.0)	2.6 (1.5 to 4.0)	9.3% (5.4% to 14.2%)	-3.2% (-6.7% to 1.2%)
Klinefelter syndrome	230.8 (198.1 to 269.2)	2.3 (1.1 to 4.5)	13.6% (6.6% to 20.3%)	0.6% (-5.6% to 6.5%)
Maternal sepsis and other pregnancy related infections	126.9 (108.3 to 146.8)	0.6 (0.2 to 1.4)	19.1% (10.5% to 29.5%)	5.5% (-2.1% to 14.7%)
Urogenital congenital anomalies	38.4 (25.9 to 51.5)	0.5 (0.2 to 1.0)	13.1% (10.5% to 15.5%)	0.2% (-2.1% to 2.3%)
**All causes**	**24,204.6 (16,138.7 to 35,203.3)**	**143.5 (57.4 to 309.5)**	**23.4% (18.4% to 28.3%)**	**9.3% (4.8% to 13.6%)**

SR Table 13g: Global prevalence and years lived with disability (YLDs) for 2016, percentage change of YLD counts and rates between 2006 and 2016 of infertility by cause, ages 35-39 years				
**Cause**	**Prevalence in thousands (95% uncertainty interval)**	**YLDs in thousands (95% uncertainty interval)**		
	**2016**	**2016**	**Percentage change in counts between 2006 and 2016**	**Percentage change in rates between 2006 and 2016**
Other sexually transmitted diseases	8,569.9 (6,533.8 to 10,072.1)	46.2 (18.3 to 94.7)	2.5% (-0.9% to 6.1%)	-3.1% (-6.3% to 0.3%)
Male infertility	5,534.0 (3,396.4 to 8,685.7)	30.6 (11.3 to 64.4)	8.8% (3.5% to 13.3%)	2.9% (-2.1% to 7.1%)
Female infertility	5,312.6 (1,115.6 to 14,738.0)	27.9 (4.6 to 82.3)	25.8% (8.0% to 60.4%)	18.9% (2.1% to 51.6%)
Chlamydial infection	3,097.1 (2,345.5 to 3,676.1)	16.8 (6.8 to 33.9)	12.6% (8.5% to 17.3%)	6.4% (2.5% to 10.9%)
Gonococcal infection	1,183.2 (891.7 to 1,419.3)	6.4 (2.5 to 12.9)	10.6% (5.3% to 15.3%)	4.5% (-0.4% to 9.0%)
Polycystic ovarian syndrome	542.7 (345.9 to 777.4)	4.9 (1.9 to 10.2)	13.4% (6.4% to 22.6%)	7.2% (0.6% to 15.9%)
Turner syndrome	169.5 (147.1 to 193.8)	3.5 (1.8 to 5.6)	9.4% (-0.6% to 21.2%)	3.4% (-6.1% to 14.6%)
Endometriosis	23.4 (16.5 to 31.9)	2.4 (1.4 to 3.8)	-6.6% (-16.9% to 5.9%)	-11.7% (-21.4% to 0.1%)
Klinefelter syndrome	154.6 (133.1 to 181.3)	1.6 (0.7 to 3.0)	6.9% (0.4% to 13.9%)	1.1% (-5.1% to 7.7%)
Maternal sepsis and other pregnancy related infections	136.3 (118.4 to 154.9)	0.7 (0.3 to 1.4)	17.0% (8.5% to 25.6%)	10.6% (2.6% to 18.7%)
Urogenital congenital anomalies	31.4 (21.1 to 42.0)	0.4 (0.2 to 0.9)	3.2% (0.7% to 5.5%)	-2.4% (-4.8% to -0.3%)
**All causes**	**24,754.7 (15,240.8 to 39,347.8)**	**141.2 (53.1 to 293.8)**	**9.9% (4.9% to 14.3%)**	**3.9% (-0.9% to 8.1%)**

SR Table 13g: Global prevalence and years lived with disability (YLDs) for 2016, percentage change of YLD counts and rates between 2006 and 2016 of infertility by cause, ages 40-44 years				
**Cause**	**Prevalence in thousands (95% uncertainty interval)**	**YLDs in thousands (95% uncertainty interval)**		
	**2016**	**2016**	**Percentage change in counts between 2006 and 2016**	**Percentage change in rates between 2006 and 2016**
Other sexually transmitted diseases	10,530.5 (7,426.1 to 12,650.4)	56.2 (21.8 to 114.7)	13.9% (9.4% to 19.4%)	0.1% (-3.9% to 4.9%)
Male infertility	5,486.8 (3,676.0 to 7,917.3)	30.1 (11.1 to 65.4)	15.4% (9.2% to 20.8%)	1.3% (-4.1% to 6.1%)
Female infertility	3,887.0 (1,557.8 to 10,320.0)	20.9 (5.3 to 62.1)	35.5% (15.7% to 61.6%)	19.0% (1.7% to 42.0%)
Chlamydial infection	3,557.3 (2,581.1 to 4,286.1)	19.2 (7.3 to 40.2)	17.7% (11.9% to 23.5%)	3.4% (-1.7% to 8.5%)
Gonococcal infection	1,324.1 (981.1 to 1,591.5)	7.1 (2.7 to 14.5)	15.6% (8.8% to 21.2%)	1.5% (-4.5% to 6.5%)
Polycystic ovarian syndrome	438.4 (270.1 to 639.5)	3.9 (1.5 to 8.1)	20.3% (12.6% to 29.2%)	5.7% (-1.1% to 13.5%)
Endometriosis	31.0 (19.5 to 44.4)	3.1 (1.7 to 5.1)	15.4% (1.0% to 33.3%)	1.4% (-11.3% to 17.1%)
Turner syndrome	135.6 (118.0 to 154.7)	2.8 (1.4 to 4.5)	16.5% (4.4% to 29.4%)	2.3% (-8.3% to 13.6%)
Klinefelter syndrome	102.3 (86.3 to 119.8)	1.0 (0.5 to 2.0)	10.5% (3.5% to 17.9%)	-3.0% (-9.1% to 3.6%)
Maternal sepsis and other pregnancy related infections	135.4 (120.0 to 152.5)	0.7 (0.3 to 1.5)	12.6% (5.3% to 20.2%)	-1.1% (-7.5% to 5.6%)
Urogenital congenital anomalies	29.0 (19.4 to 38.7)	0.4 (0.1 to 0.8)	11.5% (8.5% to 14.5%)	-2.1% (-4.7% to 0.6%)
**All causes**	**25,657.4 (17,162.9 to 37,055.0)**	**145.2 (56.2 to 311.3)**	**17.7% (11.7% to 23.0%)**	**3.4% (-1.9% to 8.1%)**

SR Table 13g: Global prevalence and years lived with disability (YLDs) for 2016, percentage change of YLD counts and rates between 2006 and 2016 of infertility by cause, ages 45-49 years				
Cause	Prevalence in thousands (95% uncertainty interval)	YLDs in thousands (95% uncertainty interval)		
	2016	2016	Percentage change in counts between 2006 and 2016	Percentage change in rates between 2006 and 2016
Other sexually transmitted diseases	1,469.5<br>(857.2 to 2,234.3)	7.8<br>(2.7 to 18.0)	30.6%<br>(18.8% to 41.4%)	4.7%<br>(-4.7% to 13.4%)
Chlamydial infection	531.3<br>(318.3 to 795.5)	2.8<br>(1.0 to 6.2)	27.9%<br>(14.2% to 40.7%)	2.5%<br>(-8.4% to 12.8%)
Male infertility	545.9<br>(296.2 to 885.8)	2.8<br>(0.9 to 6.5)	23.0%<br>(9.6% to 34.1%)	-1.3%<br>(-12.1% to 7.5%)
Turner syndrome	104.5<br>(91.1 to 120.0)	2.2<br>(1.1 to 3.5)	25.1%<br>(12.1% to 39.2%)	0.3%<br>(-10.1% to 11.6%)
Female infertility	218.3<br>(127.6 to 369.0)	1.3<br>(0.5 to 2.8)	29.9%<br>(16.5% to 42.4%)	4.1%<br>(-6.6% to 14.2%)
Gonococcal infection	209.3<br>(126.3 to 314.2)	1.1<br>(0.4 to 2.4)	25.5%<br>(11.3% to 37.5%)	0.6%<br>(-10.7% to 10.2%)
Klinefelter syndrome	64.9<br>(54.3 to 76.5)	0.7<br>(0.3 to 1.2)	19.0%<br>(10.5% to 27.6%)	-4.6%<br>(-11.4% to 2.4%)
Maternal sepsis and other pregnancy related infections	127.5<br>(113.6 to 143.5)	0.6<br>(0.2 to 1.3)	11.1%<br>(4.0% to 18.7%)	-10.9%<br>(-16.6% to -4.8%)
Endometriosis	4.0<br>(2.0 to 6.5)	0.4<br>(0.2 to 0.8)	42.4%<br>(34.6% to 50.1%)	14.2%<br>(7.9% to 20.4%)
Urogenital congenital anomalies	26.8<br>(17.9 to 35.9)	0.3<br>(0.1 to 0.7)	23.6%<br>(20.1% to 27.0%)	-0.9%<br>(-3.7% to 1.9%)
Polycystic ovarian syndrome	4.5<br>(2.3 to 7.6)	<0.05<br>(<0.05 to 0.1)	22.9%<br>(7.4% to 41.9%)	-1.5%<br>(-13.9% to 13.8%)
**All causes**	**3,306.4**<br>**(2,152.1 to 4,899.9)**	**20.0**<br>**(8.3 to 41.6)**	**27.2%**<br>**(18.6% to 35.9%)**	**2.0%**<br>**(-4.9% to 9.0%)**

SR Table 13g: Global prevalence and years lived with disability (YLDs) for 2016, percentage change of YLD counts and rates between 2006 and 2016 of infertility by cause, ages 50-54 years				
**Cause**	**Prevalence in thousands (95% uncertainty interval)**	**YLDs in thousands (95% uncertainty interval)**		
	**2016**	**2016**	**Percentage change in counts between 2006 and 2016**	**Percentage change in rates between 2006 and 2016**
Maternal sepsis and other pregnancy related infections	118.7<br>(105.7 to 133.4)	0.6<br>(0.2 to 1.3)	17.6%<br>(8.9% to 25.5%)	-5.6%<br>(-12.6% to 0.8%)
Urogenital congenital anomalies	23.8<br>(15.9 to 31.9)	0.3<br>(0.1 to 0.6)	27.3%<br>(23.7% to 30.9%)	2.2%<br>(-0.7% to 5.1%)
**All causes**	**142.5<br>(127.6 to 158.9)**	**0.9<br>(0.4 to 1.8)**	**20.7%<br>(14.3% to 26.3%)**	**-3.1%<br>(-8.2% to 1.4%)**

SR Table 13g: Global prevalence and years lived with disability (YLDs) for 2016, percentage change of YLD counts and rates between 2006 and 2016 of infertility by cause, ages 55-59 years				
**Cause**	**Prevalence in thousands (95% uncertainty interval)**	**YLDs in thousands (95% uncertainty interval)**		
	**2016**	**2016**	**Percentage change in counts between 2006 and 2016**	**Percentage change in rates between 2006 and 2016**
Urogenital congenital anomalies	20.0<br>(13.4 to 26.9)	0.3<br>(0.1 to 0.5)	34.0%<br>(30.0% to 38.0%)	2.7%<br>(-0.4% to 5.7%)
**All causes**	**20.0<br>(13.4 to 26.9)**	**0.3<br>(0.1 to 0.5)**	**34.0%<br>(30.0% to 38.0%)**	**2.7%<br>(-0.4% to 5.7%)**

SR Table 13g: Global prevalence and years lived with disability (YLDs) for 2016, percentage change of YLD counts and rates between 2006 and 2016 of infertility by cause, ages 60-64 years				
**Cause**	**Prevalence in thousands (95% uncertainty interval)**	**YLDs in thousands (95% uncertainty interval)**		
	**2016**	**2016**	**Percentage change in counts between 2006 and 2016**	**Percentage change in rates between 2006 and 2016**
Urogenital congenital anomalies	17.4<br>(11.7 to 23.4)	0.2<br>(0.1 to 0.5)	59.4%<br>(54.8% to 64.3%)	5.7%<br>(2.6% to 9.0%)
**All causes**	**17.4**<br>**(11.7 to 23.4)**	**0.2**<br>**(0.1 to 0.5)**	**59.4%**<br>**(54.8% to 64.3%)**	**5.7%**<br>**(2.6% to 9.0%)**

SR Table 13g: Global prevalence and years lived with disability (YLDs) for 2016, percentage change of YLD counts and rates between 2006 and 2016 of infertility by cause, ages 65-69 years				
Cause	Prevalence in thousands (95% uncertainty interval)	YLDs in thousands (95% uncertainty interval)		
	2016	2016	Percentage change in counts between 2006 and 2016	Percentage change in rates between 2006 and 2016
Urogenital congenital anomalies	13.3 (8.9 to 17.9)	0.2 (0.1 to 0.4)	38.5% (33.9% to 42.8%)	4.5% (1.0% to 7.8%)
**All causes**	**13.3 (8.9 to 17.9)**	**0.2 (0.1 to 0.4)**	**38.5% (33.9% to 42.8%)**	**4.5% (1.0% to 7.8%)**

SR Table 13g: Global prevalence and years lived with disability (YLDs) for 2016, percentage change of YLD counts and rates between 2006 and 2016 of infertility by cause, ages 70-74 years				
**Cause**	**Prevalence in thousands (95% uncertainty interval)**	**YLDs in thousands (95% uncertainty interval)**		
	**2016**	**2016**	**Percentage change in counts between 2006 and 2016**	**Percentage change in rates between 2006 and 2016**
Urogenital congenital anomalies	9.4<br>(6.3 to 12.6)	0.1<br>(<0.05 to 0.3)	25.6%<br>(21.5% to 29.7%)	4.9%<br>(1.6% to 8.4%)
**All causes**	**9.4**<br>**(6.3 to 12.6)**	**0.1**<br>**(<0.05 to 0.3)**	**25.6%**<br>**(21.5% to 29.7%)**	**4.9%**<br>**(1.6% to 8.4%)**

SR Table 13g: Global prevalence and years lived with disability (YLDs) for 2016, percentage change of YLD counts and rates between 2006 and 2016 of infertility by cause, ages 75-79 years				
**Cause**	**Prevalence in thousands (95% uncertainty interval)**	**YLDs in thousands (95% uncertainty interval)**		
	**2016**	**2016**	**Percentage change in counts between 2006 and 2016**	**Percentage change in rates between 2006 and 2016**
Urogenital congenital anomalies	7.3<br>(4.9 to 9.8)	0.1<br>(<0.05 to 0.2)	28.5%<br>(24.2% to 33.2%)	3.3%<br>(-0.2% to 7.1%)
**All causes**	**7.3**<br>**(4.9 to 9.8)**	**0.1**<br>**(<0.05 to 0.2)**	**28.5%**<br>**(24.2% to 33.2%)**	**3.3%**<br>**(-0.2% to 7.1%)**

SR Table 13g: Global prevalence and years lived with disability (YLDs) for 2016, percentage change of YLD counts and rates between 2006 and 2016 of infertility by cause, ages 80-84				
**Cause**	**Prevalence in thousands (95% uncertainty interval)**	**YLDs in thousands (95% uncertainty interval)**		
	**2016**	**2016**	**Percentage change in counts between 2006 and 2016**	**Percentage change in rates between 2006 and 2016**
Urogenital congenital anomalies	4.6<br>(3.1 to 6.2)	0.1<br>(<0.05 to 0.1)	31.3%<br>(27.2% to 35.5%)	0.2%<br>(-2.9% to 3.5%)
**All causes**	**4.6<br>(3.1 to 6.2)**	**0.1<br>(<0.05 to 0.1)**	**31.3%<br>(27.2% to 35.5%)**	**0.2%<br>(-2.9% to 3.5%)**

SR Table 13g: Global prevalence and years lived with disability (YLDs) for 2016, percentage change of YLD counts and rates between 2006 and 2016 of infertility by cause, ages 85-89				
Cause	Prevalence in thousands (95% uncertainty interval)	YLDs in thousands (95% uncertainty interval)		
	2016	2016	Percentage change in counts between 2006 and 2016	Percentage change in rates between 2006 and 2016
Urogenital congenital anomalies	2.7<br>(1.8 to 3.6)	<0.05<br>(<0.05 to 0.1)	66.9%<br>(62.2% to 72.4%)	4.0%<br>(1.1% to 7.4%)
**All causes**	**2.7**<br>**(1.8 to 3.6)**	**<0.05**<br>**(<0.05 to 0.1)**	**66.9%**<br>**(62.2% to 72.4%)**	**4.0%**<br>**(1.1% to 7.4%)**

SR Table 13g: Global prevalence and years lived with disability (YLDs) for 2016, percentage change of YLD counts and rates between 2006 and 2016 of infertility by cause, ages 90-94				
**Cause**	**Prevalence in thousands (95% uncertainty interval)**	**YLDs in thousands (95% uncertainty interval)**		
	**2016**	**2016**	**Percentage change in counts between 2006 and 2016**	**Percentage change in rates between 2006 and 2016**
Urogenital congenital anomalies	1.0<br>(0.7 to 1.4)	<0.05<br>(<0.05 to <0.05)	77.8%<br>(72.9% to 82.8%)	6.2%<br>(3.3% to 9.2%)
**All causes**	**1.0**<br>**(0.7 to 1.4)**	**<0.05**<br>**(<0.05 to <0.05)**	**77.8%**<br>**(72.9% to 82.8%)**	**6.2%**<br>**(3.3% to 9.2%)**

SR Table 13g: Global prevalence and years lived with disability (YLDs) for 2016, percentage change of YLD counts and rates between 2006 and 2016 of infertility by cause, ages 95+ years				
**Cause**	**Prevalence in thousands (95% uncertainty interval)**	**YLDs in thousands (95% uncertainty interval)**		
	**2016**	**2016**	**Percentage change in counts between 2006 and 2016**	**Percentage change in rates between 2006 and 2016**
Urogenital congenital anomalies	0.2<br>(0.2 to 0.3)	<0.05<br>(<0.05 to <0.05)	58.4%<br>(54.6% to 62.5%)	1.1%<br>(-1.4% to 3.7%)
**All causes**	**0.2<br>(0.2 to 0.3)**	**<0.05<br>(<0.05 to <0.05)**	**58.4%<br>(54.6% to 62.5%)**	**1.1%<br>(-1.4% to 3.7%)**

SR Table 13h: Global prevalence and years lived with disability (YLDs) for 2016, percentage change of YLD counts and rates between 2006 and 2016 of pelvic inflammatory disease by cause, ages 10-14 years				
**Cause**	**Prevalence in thousands (95% uncertainty interval)**	**YLDs in thousands (95% uncertainty interval)**		
	**2016**	**2016**	**Percentage change in counts between 2006 and 2016**	**Percentage change in rates between 2006 and 2016**
Other sexually transmitted diseases	20.7<br>(15.0 to 28.1)	2.8<br>(1.7 to 4.4)	-0.1%<br>(-12.3% to 13.5%)	-0.9%<br>(-13.1% to 12.6%)
Chlamydial infection	12.2<br>(8.7 to 16.8)	1.7<br>(1.0 to 2.7)	2.8%<br>(0.3% to 5.8%)	2.0%<br>(-0.5% to 4.9%)
Gonococcal infection	3.2<br>(2.3 to 4.4)	0.4<br>(0.3 to 0.7)	4.2%<br>(1.6% to 7.1%)	3.3%<br>(0.8% to 6.2%)
**All causes**	**36.1**<br>**(26.8 to 48.5)**	**4.9**<br>**(3.0 to 7.7)**	**1.3%**<br>**(-6.2% to 9.5%)**	**0.4%**<br>**(-7.0% to 8.6%)**

SR Table 13h: Global prevalence and years lived with disability (YLDs) for 2016, percentage change of YLD counts and rates between 2006 and 2016 of pelvic inflammatory disease by cause, ages 15-19 years				
**Cause**	**Prevalence in thousands (95% uncertainty interval)**	**YLDs in thousands (95% uncertainty interval)**		
	**2016**	**2016**	**Percentage change in counts between 2006 and 2016**	**Percentage change in rates between 2006 and 2016**
Other sexually transmitted diseases	91.0<br>(66.8 to 122.3)	12.2<br>(7.4 to 19.1)	-8.3%<br>(-15.3% to 0.4%)	-3.0%<br>(-10.4% to 6.2%)
Chlamydial infection	48.7<br>(35.2 to 68.1)	6.5<br>(3.9 to 10.4)	-2.7%<br>(-14.4% to 9.6%)	3.0%<br>(-9.3% to 16.1%)
Gonococcal infection	15.4<br>(10.9 to 21.5)	2.1<br>(1.2 to 3.3)	-2.5%<br>(-14.8% to 10.7%)	3.2%<br>(-9.8% to 17.2%)
**All causes**	**155.1**<br>**(115.3 to 211.5)**	**20.8**<br>**(12.5 to 33.0)**	**-6.0%**<br>**(-11.7% to 0.3%)**	**-0.5%**<br>**(-6.6% to 6.2%)**

SR Table 13h: Global prevalence and years lived with disability (YLDs) for 2016, percentage change of YLD counts and rates between 2006 and 2016 of pelvic inflammatory disease by cause, ages 20-24 years				
**Cause**	**Prevalence in thousands (95% uncertainty interval)**	**YLDs in thousands (95% uncertainty interval)**		
	**2016**	**2016**	**Percentage change in counts between 2006 and 2016**	**Percentage change in rates between 2006 and 2016**
Other sexually transmitted diseases	207.0<br>(164.5 to 257.4)	27.4<br>(17.7 to 40.6)	0.3%<br>(-6.4% to 7.9%)	-4.3%<br>(-10.8% to 3.0%)
Chlamydial infection	90.8<br>(70.0 to 117.6)	12.1<br>(7.7 to 18.2)	3.1%<br>(-5.2% to 11.9%)	-1.7%<br>(-9.6% to 6.8%)
Gonococcal infection	34.3<br>(26.4 to 43.6)	4.6<br>(2.9 to 7.0)	3.3%<br>(-6.4% to 13.2%)	-1.4%<br>(-10.7% to 8.0%)
**All causes**	**332.1**<br>**(264.9 to 415.2)**	**44.2**<br>**(28.8 to 65.4)**	**1.3%**<br>**(-3.6% to 7.0%)**	**-3.3%**<br>**(-8.1% to 2.1%)**

SR Table 13h: Global prevalence and years lived with disability (YLDs) for 2016, percentage change of YLD counts and rates between 2006 and 2016 of pelvic inflammatory disease by cause, ages 25-29 years				
**Cause**	**Prevalence in thousands (95% uncertainty interval)**	**YLDs in thousands (95% uncertainty interval)**		
	**2016**	**2016**	**Percentage change in counts between 2006 and 2016**	**Percentage change in rates between 2006 and 2016**
Other sexually transmitted diseases	399.4<br>(299.6 to 537.0)	52.6<br>(33.3 to 81.3)	12.6%<br>(6.2% to 19.2%)	-5.2%<br>(-10.5% to 0.4%)
Chlamydial infection	126.8<br>(92.6 to 171.2)	16.8<br>(10.2 to 25.7)	13.2%<br>(4.1% to 22.8%)	-4.6%<br>(-12.3% to 3.5%)
Gonococcal infection	58.5<br>(42.6 to 79.7)	7.8<br>(4.7 to 12.1)	13.7%<br>(4.0% to 25.7%)	-4.2%<br>(-12.4% to 5.9%)
**All causes**	**584.8<br>(443.2 to 788.0)**	**77.1<br>(49.0 to 118.2)**	**12.8%<br>(8.0% to 18.0%)**	**-5.0%<br>(-9.1% to -0.6%)**

SR Table 13h: Global prevalence and years lived with disability (YLDs) for 2016, percentage change of YLD counts and rates between 2006 and 2016 of pelvic inflammatory disease by cause, ages 30-34 years				
**Cause**	**Prevalence in thousands (95% uncertainty interval)**	**YLDs in thousands (95% uncertainty interval)**		
	**2016**	**2016**	**Percentage change in counts between 2006 and 2016**	**Percentage change in rates between 2006 and 2016**
Other sexually transmitted diseases	491.5<br>(381.8 to 633.9)	64.1<br>(41.5 to 95.2)	5.5%<br>(0.6% to 11.1%)	-6.5%<br>(-10.9% to -1.6%)
Chlamydial infection	135.7<br>(101.7 to 181.0)	17.9<br>(10.8 to 27.2)	10.9%<br>(2.7% to 20.4%)	-1.8%<br>(-9.1% to 6.6%)
Gonococcal infection	63.8<br>(47.7 to 86.1)	8.5<br>(5.1 to 13.0)	11.0%<br>(1.8% to 21.6%)	-1.7%<br>(-9.8% to 7.7%)
**All causes**	**691.0**<br>**(537.4 to 886.6)**	**90.4**<br>**(58.5 to 136.2)**	**7.1%**<br>**(2.9% to 11.5%)**	**-5.2%**<br>**(-8.9% to -1.3%)**

SR Table 13h: Global prevalence and years lived with disability (YLDs) for 2016, percentage change of YLD counts and rates between 2006 and 2016 of pelvic inflammatory disease by cause, ages 35-39 years				
**Cause**	**Prevalence in thousands (95% uncertainty interval)**	**YLDs in thousands (95% uncertainty interval)**		
	**2016**	**2016**	**Percentage change in counts between 2006 and 2016**	**Percentage change in rates between 2006 and 2016**
Other sexually transmitted diseases	480.3<br>(369.6 to 612.8)	62.2<br>(39.3 to 90.4)	-5.8%<br>(-10.3% to -0.3%)	-10.9%<br>(-15.2% to -5.8%)
Chlamydial infection	142.6<br>(106.6 to 188.5)	18.7<br>(11.7 to 28.5)	3.1%<br>(-3.3% to 11.1%)	-2.6%<br>(-8.6% to 5.0%)
Gonococcal infection	53.0<br>(38.8 to 69.4)	7.0<br>(4.3 to 10.7)	2.0%<br>(-7.6% to 12.4%)	-3.6%<br>(-12.7% to 6.3%)
**All causes**	**675.9**<br>**(524.5 to 857.2)**	**88.0**<br>**(54.8 to 128.1)**	**-3.4%**<br>**(-7.1% to 1.0%)**	**-8.7%**<br>**(-12.2% to -4.6%)**

SR Table 13h: Global prevalence and years lived with disability (YLDs) for 2016, percentage change of YLD counts and rates between 2006 and 2016 of pelvic inflammatory disease by cause, ages 40-44 years				
**Cause**	**Prevalence in thousands (95% uncertainty interval)**	**YLDs in thousands (95% uncertainty interval)**		
	**2016**	**2016**	**Percentage change in counts between 2006 and 2016**	**Percentage change in rates between 2006 and 2016**
Other sexually transmitted diseases	448.4<br>(331.5 to 588.0)	57.8<br>(35.5 to 85.3)	5.9%<br>(0.4% to 12.2%)	-7.0%<br>(-11.8% to -1.5%)
Chlamydial infection	142.9<br>(102.5 to 190.1)	18.7<br>(11.0 to 28.8)	8.9%<br>(2.2% to 17.0%)	-4.4%<br>(-10.2% to 2.8%)
Gonococcal infection	39.4<br>(27.8 to 53.5)	5.2<br>(3.0 to 8.0)	8.7%<br>(-2.6% to 21.4%)	-4.5%<br>(-14.4% to 6.7%)
**All causes**	**630.7**<br>**(462.1 to 822.2)**	**81.6**<br>**(50.2 to 119.6)**	**6.7%**<br>**(2.0% to 11.8%)**	**-6.3%**<br>**(-10.4% to -1.8%)**

SR Table 13h: Global prevalence and years lived with disability (YLDs) for 2016, percentage change of YLD counts and rates between 2006 and 2016 of pelvic inflammatory disease by cause, ages 45-49 years				
**Cause**	**Prevalence in thousands (95% uncertainty interval)**	**YLDs in thousands (95% uncertainty interval)**		
	**2016**	**2016**	**Percentage change in counts between 2006 and 2016**	**Percentage change in rates between 2006 and 2016**
Other sexually transmitted diseases	304.9<br>(239.6 to 380.0)	39.1<br>(25.6 to 55.7)	20.1%<br>(13.0% to 27.9%)	-3.7%<br>(-9.4% to 2.5%)
Chlamydial infection	109.6<br>(83.2 to 140.5)	14.3<br>(9.0 to 21.0)	18.7%<br>(10.6% to 27.8%)	-4.8%<br>(-11.3% to 2.5%)
Gonococcal infection	21.4<br>(16.2 to 27.2)	2.9<br>(1.8 to 4.2)	18.1%<br>(5.5% to 31.0%)	-5.3%<br>(-15.4% to 5.1%)
**All causes**	**436.0**<br>**(339.4 to 541.7)**	**56.3**<br>**(36.4 to 81.2)**	**19.7%**<br>**(14.2% to 25.7%)**	**-4.0%**<br>**(-8.4% to 0.8%)**

SR Table 13h: Global prevalence and years lived with disability (YLDs) for 2016, percentage change of YLD counts and rates between 2006 and 2016 of pelvic inflammatory disease by cause, ages 50-54 years				
**Cause**	**Prevalence in thousands (95% uncertainty interval)**	**YLDs in thousands (95% uncertainty interval)**		
	**2016**	**2016**	**Percentage change in counts between 2006 and 2016**	**Percentage change in rates between 2006 and 2016**
Other sexually transmitted diseases	191.2 (136.1 to 262.9)	24.5 (14.9 to 37.9)	12.0% (4.4% to 20.5%)	-10.1% (-16.2% to -3.2%)
Chlamydial infection	63.0 (42.9 to 88.2)	8.2 (4.9 to 13.1)	14.9% (6.1% to 25.5%)	-7.7% (-14.8% to 0.8%)
Gonococcal infection	11.0 (7.4 to 15.3)	1.5 (0.9 to 2.3)	13.6% (9.6% to 18.1%)	-8.8% (-12.0% to -5.1%)
**All causes**	**265.1 (187.8 to 363.4)**	**34.2 (20.9 to 53.0)**	**12.7% (6.5% to 19.9%)**	**-9.5% (-14.5% to -3.7%)**

SR Table 13h: Global prevalence and years lived with disability (YLDs) for 2016, percentage change of YLD counts and rates between 2006 and 2016 of pelvic inflammatory disease by cause, ages 55-59 years				
**Cause**	**Prevalence in thousands (95% uncertainty interval)**	**YLDs in thousands (95% uncertainty interval)**		
	**2016**	**2016**	**Percentage change in counts between 2006 and 2016**	**Percentage change in rates between 2006 and 2016**
Other sexually transmitted diseases	132.7<br>(106.3 to 165.4)	16.9<br>(11.3 to 24.1)	24.2%<br>(15.7% to 33.7%)	-4.8%<br>(-11.4% to 2.5%)
Chlamydial infection	29.3<br>(22.9 to 36.7)	3.8<br>(2.4 to 5.6)	26.3%<br>(13.5% to 41.2%)	-3.2%<br>(-13.0% to 8.2%)
Gonococcal infection	6.1<br>(4.7 to 7.8)	0.8<br>(0.5 to 1.2)	24.2%<br>(21.3% to 27.2%)	-4.8%<br>(-7.1% to -2.5%)
**All causes**	**168.1<br>(134.0 to 209.6)**	**21.6<br>(14.3 to 30.9)**	**24.6%<br>(17.8% to 32.6%)**	**-4.5%<br>(-9.7% to 1.6%)**

SR Table 13h: Global prevalence and years lived with disability (YLDs) for 2016, percentage change of YLD counts and rates between 2006 and 2016 of pelvic inflammatory disease by cause, ages 60-64 years				
**Cause**	**Prevalence in thousands (95% uncertainty interval)**	**YLDs in thousands (95% uncertainty interval)**		
	**2016**	**2016**	**Percentage change in counts between 2006 and 2016**	**Percentage change in rates between 2006 and 2016**
Other sexually transmitted diseases	81.2<br>(62.4 to 104.4)	10.3<br>(6.5 to 15.2)	55.8%<br>(43.4% to 72.1%)	3.3%<br>(-4.9% to 14.1%)
Chlamydial infection	13.1<br>(9.8 to 17.3)	1.7<br>(1.1 to 2.7)	50.2%<br>(32.7% to 71.8%)	-0.4%<br>(-12.0% to 13.9%)
Gonococcal infection	3.1<br>(2.3 to 4.2)	0.4<br>(0.3 to 0.7)	50.7%<br>(45.6% to 57.0%)	-0.1%<br>(-3.5% to 4.1%)
**All causes**	**97.5<br>(75.0 to 125.4)**	**12.5<br>(7.8 to 18.4)**	**54.8%<br>(44.4% to 68.9%)**	**2.7%<br>(-4.2% to 12.1%)**

SR Table 13h: Global prevalence and years lived with disability (YLDs) for 2016, percentage change of YLD counts and rates between 2006 and 2016 of pelvic inflammatory disease by cause, ages 65-69 years				
**Cause**	**Prevalence in thousands (95% uncertainty interval)**	**YLDs in thousands (95% uncertainty interval)**		
	**2016**	**2016**	**Percentage change in counts between 2006 and 2016**	**Percentage change in rates between 2006 and 2016**
Other sexually transmitted diseases	22.5<br>(17.1 to 28.8)	2.9<br>(1.8 to 4.2)	34.8%<br>(22.6% to 50.4%)	1.8%<br>(-7.4% to 13.5%)
Chlamydial infection	3.4<br>(2.6 to 4.5)	0.5<br>(0.3 to 0.7)	30.0%<br>(25.5% to 35.7%)	-1.9%<br>(-5.3% to 2.4%)
Gonococcal infection	0.8<br>(0.6 to 1.1)	0.1<br>(0.1 to 0.2)	30.8%<br>(26.4% to 36.7%)	-1.3%<br>(-4.6% to 3.1%)
**All causes**	**26.7**<br>**(20.4 to 34.2)**	**3.5**<br>**(2.2 to 5.0)**	**34.0%**<br>**(23.6% to 47.5%)**	**1.1%**<br>**(-6.7% to 11.3%)**

SR Table 13h: Global prevalence and years lived with disability (YLDs) for 2016, percentage change of YLD counts and rates between 2006 and 2016 of pelvic inflammatory disease by cause, ages 70-74 years				
**Cause**	**Prevalence in thousands (95% uncertainty interval)**	**YLDs in thousands (95% uncertainty interval)**		
	**2016**	**2016**	**Percentage change in counts between 2006 and 2016**	**Percentage change in rates between 2006 and 2016**
Other sexually transmitted diseases	0.3 (0.2 to 0.3)	<0.05 (<0.05 to 0.1)	15.7% (11.1% to 21.8%)	-3.3% (-7.1% to 1.8%)
Chlamydial infection	<0.05 (<0.05 to <0.05)	<0.05 (<0.05 to <0.05)	14.8% (11.3% to 19.4%)	-4.1% (-7.0% to -0.2%)
Gonococcal infection	<0.05 (<0.05 to <0.05)	<0.05 (<0.05 to <0.05)	14.8% (11.3% to 19.5%)	-4.1% (-7.0% to -0.1%)
**All causes**	**0.3 (0.2 to 0.4)**	**<0.05 (<0.05 to 0.1)**	**15.6% (11.2% to 21.3%)**	**-3.4% (-7.0% to 1.4%)**

SR Table 13h: Global prevalence and years lived with disability (YLDs) for 2016, percentage change of YLD counts and rates between 2006 and 2016 of pelvic inflammatory disease by cause, ages 75-79 years				
**Cause**	**Prevalence in thousands (95% uncertainty interval)**	**YLDs in thousands (95% uncertainty interval)**		
	**2016**	**2016**	**Percentage change in counts between 2006 and 2016**	**Percentage change in rates between 2006 and 2016**
Other sexually transmitted diseases	<0.05 (<0.05 to <0.05)	<0.05 (<0.05 to <0.05)	19.5% (-43.4% to 65.1%)	-3.9% (-54.5% to 32.7%)
Chlamydial infection	<0.05 (<0.05 to <0.05)	<0.05 (<0.05 to <0.05)	20.5% (-35.2% to 72.5%)	-3.1% (-47.9% to 38.7%)
Gonococcal infection	<0.05 (<0.05 to <0.05)	<0.05 (<0.05 to <0.05)	18.7% (-40.6% to 59.1%)	-4.5% (-52.2% to 27.9%)
**All causes**	**<0.05 (<0.05 to <0.05)**	**<0.05 (<0.05 to <0.05)**	**19.6% (-42.3% to 66.0%)**	**-3.9% (-53.6% to 33.5%)**

SR Table 13h: Global prevalence and years lived with disability (YLDs) for 2016, percentage change of YLD counts and rates between 2006 and 2016 of pelvic inflammatory disease by cause, ages 80-84				
**Cause**	**Prevalence in thousands (95% uncertainty interval)**	**YLDs in thousands (95% uncertainty interval)**		
	**2016**	**2016**	**Percentage change in counts between 2006 and 2016**	**Percentage change in rates between 2006 and 2016**
Other sexually transmitted diseases	<0.05 (<0.05 to <0.05)	<0.05 (<0.05 to <0.05)	25.7% (-40.3% to 123.2%)	-4.0% (-54.4% to 70.4%)
Chlamydial infection	<0.05 (<0.05 to <0.05)	<0.05 (<0.05 to <0.05)	26.1% (-33.6% to 131.3%)	-3.7% (-49.3% to 76.6%)
Gonococcal infection	<0.05 (<0.05 to <0.05)	<0.05 (<0.05 to <0.05)	23.1% (-37.2% to 124.7%)	-6.0% (-52.0% to 71.6%)
**All causes**	**<0.05 (<0.05 to <0.05)**	**<0.05 (<0.05 to <0.05)**	**25.7% (-39.9% to 127.3%)**	**-4.0% (-54.1% to 73.5%)**

SR Table 13h: Global prevalence and years lived with disability (YLDs) for 2016, percentage change of YLD counts and rates between 2006 and 2016 of pelvic inflammatory disease by cause, ages 85-89				
**Cause**	**Prevalence in thousands (95% uncertainty interval)**	**YLDs in thousands (95% uncertainty interval)**		
	**2016**	**2016**	**Percentage change in counts between 2006 and 2016**	**Percentage change in rates between 2006 and 2016**
Other sexually transmitted diseases	<0.05<br>(<0.05 to <0.05)	<0.05<br>(<0.05 to <0.05)	--	--
Chlamydial infection	<0.05<br>(<0.05 to <0.05)	<0.05<br>(<0.05 to <0.05)	--	--
Gonococcal infection	<0.05<br>(<0.05 to <0.05)	<0.05<br>(<0.05 to <0.05)	--	--
**All causes**	**<0.05<br>(<0.05 to <0.05)**	**<0.05<br>(<0.05 to <0.05)**	**--**	**--**

SR Table 13h: Global prevalence and years lived with disability (YLDs) for 2016, percentage change of YLD counts and rates between 2006 and 2016 of pelvic inflammatory disease by cause, ages 90-94				
**Cause**	**Prevalence in thousands (95% uncertainty interval)**	**YLDs in thousands (95% uncertainty interval)**		
	**2016**	**2016**	**Percentage change in counts between 2006 and 2016**	**Percentage change in rates between 2006 and 2016**
Other sexually transmitted diseases	<0.05 (<0.05 to <0.05)	<0.05 (<0.05 to <0.05)	--	--
Chlamydial infection	<0.05 (<0.05 to <0.05)	<0.05 (<0.05 to <0.05)	--	--
Gonococcal infection	<0.05 (<0.05 to <0.05)	<0.05 (<0.05 to <0.05)	--	--
**All causes**	**<0.05 (<0.05 to <0.05)**	**<0.05 (<0.05 to <0.05)**	**--**	**--**

SR Table 13h: Global prevalence and years lived with disability (YLDs) for 2016, percentage change of YLD counts and rates between 2006 and 2016 of vision loss by cause, ages 0-6 days				
**Cause**	**Prevalence in thousands (95% uncertainty interval)**	**YLDs in thousands (95% uncertainty interval)**		
	**2016**	**2016**	**Percentage change in counts between 2006 and 2016**	**Percentage change in rates between 2006 and 2016**
Neonatal sepsis and other neonatal infections	8.3<br>(3.6 to 18.3)	4.3<br>(1.8 to 10.1)	-4.8%<br>(-8.2% to -1.2%)	-4.3%<br>(-7.7% to -0.7%)
Refraction and accommodation disorders	54.4<br>(49.1 to 59.7)	0.6<br>(0.3 to 1.1)	-6.4%<br>(-8.0% to -4.7%)	-5.9%<br>(-7.6% to -4.3%)
Near vision impairment	53.1<br>(47.9 to 58.2)	0.6<br>(0.3 to 1.0)	-6.1%<br>(-7.8% to -4.5%)	-5.6%<br>(-7.3% to -4.0%)
Other vision loss	9.5<br>(5.4 to 15.5)	0.5<br>(0.3 to 0.9)	-8.5%<br>(-14.0% to -4.1%)	-8.0%<br>(-13.5% to -3.6%)
Neonatal preterm birth complications	2.9<br>(2.1 to 3.9)	0.4<br>(0.2 to 0.5)	-1.7%<br>(-8.7% to 4.7%)	-1.2%<br>(-8.2% to 5.3%)
Neonatal encephalopathy due to birth asphyxia and trauma	0.1<br>(0.1 to 0.2)	<0.05<br>(<0.05 to 0.1)	-9.1%<br>(-19.7% to 0.4%)	-8.7%<br>(-19.3% to 0.9%)
[truncated: 511,281 more chars]
